
JOURNAL INFORMATION
==============================
NLM Title Abbreviation: Cancer Sci
Journal ID: Cancer Sci
NLM Journal ID: 101168776
Journal ID: CAS

Cancer Science

ISSN: 1347-9032
EISSN: 1349-7006
Publisher: Wiley

ARTICLE INFORMATION
==============================
PMCID: PMC12772631
PMID: 41493844
DOI: 10.1111/cas.70254
Article ID: CAS70254
Article version: 1
Subjects: Abstracts, Abstracts of the 84th Annual Meeting of the Japanese Cancer Association, Abstracts

Abstracts

Electronic publication date: 2026 Jan 6
Publication date: 2026 Jan
Volume: 117
Issue: Suppl 1
Issue title: Special Issue: Abstracts of the 84th Annual Meeting of the Japanese Cancer Association
First page: 1
Last page: 2148
Copyright: © 2026 The Author(s). Cancer Science published by John Wiley & Sons Australia, Ltd on behalf of the Japanese Cancer Association.
License: This is an open access article under the terms of the http://creativecommons.org/licenses/by/4.0/ License, which permits use, distribution and reproduction in any medium, provided the original work is properly cited.
License URL: https://creativecommons.org/licenses/by/4.0/

Figure count: 0
Table count: 0
Page count: 2231

source-schema-version-number: 2.0
cover-date: January 2026
details-of-publishers-convertor: Converter:WILEY_ML3GV2_TO_JATSPMC version:6.6.6 mode:remove_FC converted:06.01.2026

==============================

### The 84th Annual Meeting of the Japanese Cancer Association

International Cancer Meeting in Kanazawa

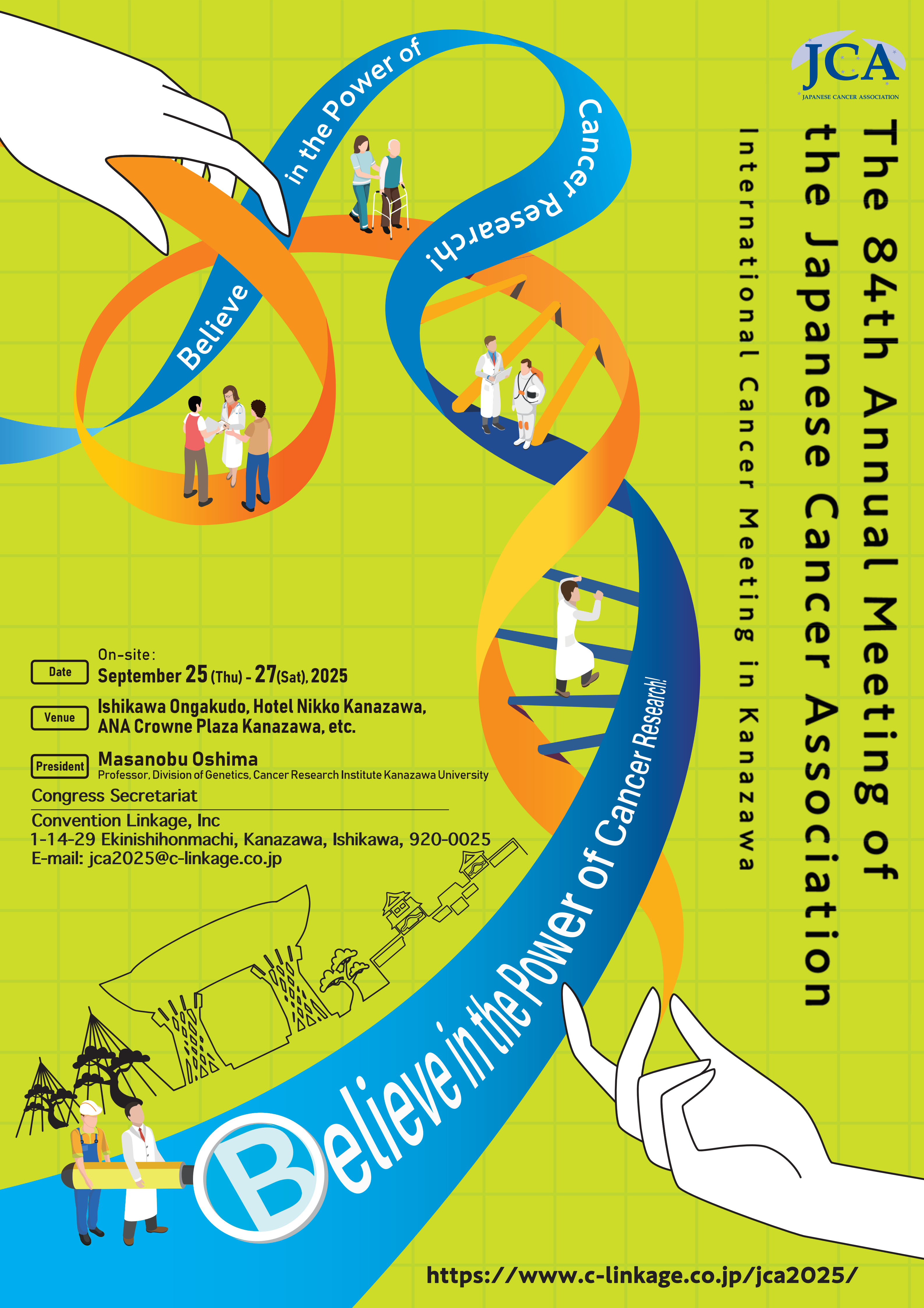

**Date** On-site:
September 25 (Thu) - 27 (Sat), 2025

**Venue** Ishikawa Ongakudo, Hotel Nikko Kanazawa, ANA Crowne Plaza Kanazawa, etc.

**President** Masanobu Oshima
Professor, Division of Genetics, Cancer Research Institute Kanazawa University

Congress Secretariat

Convention Linkage, Inc
1-14-29 Ekinishihonmachi, Kanazawa, Ishikawa, 920-0025
E-mail: jca2025@c-linkage.co.jp https://www.c-linkage.co.jp/jca2025/

### Program

The 84th Annual Meeting of the Japanese Cancer Association

### How to cite abstract in this supplement:

Citation Format:

[Authors of abstract]. [Title of abstract] (abstract). In: Abstracts of the 84th Annual Meeting of the Japanese Cancer Association; 2025 September 25–27; Kanazawa, Japan. Cancer Sci. 2026: 117 (Suppl 1); [pages]

Copyright

Editorial material and organization © 2026 Japanese Cancer Association. Copyright of individual abstracts remains with the authors. Permission is required for the use of any of the content included in this issue.

Disclaimer

This abstract book has been produced using author-supplied copy. Editing has been restricted to some corrections of spelling and style where appropriate. The publisher assumes no responsibility for any claims, instructions, methods or drug dosages contained in the abstracts. It is recommended that these are verified independently. The contents contained herein are correct at the time of printing and may be subject to change.

### CONTENTS

### September 25 (Thu)

**8:45 - 9:00** Opening:Opening
Room 1 / (Ishikawa Ongakudo 2F Concert Hall)
······ 1

**9:00 - 11:30** AACR1:Early Cancer Evolution and prevention -crossroad of genomics and biology-
Room 1 / (Ishikawa Ongakudo 2F Concert Hall)
AACR1-1 Somatic mosaicism and cancer ······ 2
AACR1-2 3D Genomic Analysis of Human Pancreatic Precancers ······ 3
AACR1-3 Comprehensive Insights into Environmental Carcinogenesis via DNA Adducts and Genome-wide Mutation Profiling ······ 4
AACR1-4 Deciphering Early Gastric Cancer: Genomic Mutations and Cellular Plasticity in Intestinal Metaplasia ······ 5

**9:00 - 11:30** YIA1:The Young Investigator Awards Lecture1
Room 2 / (Ishikawa Ongakudo 2F Hogaku Hall)
YIA1-1 Multifaceted interactions between cancer cells and glial cells in brain metastasis ······ 6
YIA1-2 Mechanism of cancer progression triggered by extracellular matrix stiffness ······ 7
YIA1-3 Elucidating the Developmental Mechanisms of Regulatory T Cells in the Tumor Microenvironment ······ 8
YIA1-4 Overcoming anti-cancer drug resistance by targeting YB-1 phosphorylation pathway ······ 9
YIA1-5 Establishment of novel 3D cell culture models of the fibrotic pancreatic cancer microenvironment using cell accumulation ······ 10
YIA1-6 Identification of novel negative regulators of anti-tumor T cell responses within the tumor microenv ironment ······ 11
YIA1-7 Elucidating the Mechanisms of Lymphomagenesis with Clonal Hematopoiesis ······ 12

**9:00 - 11:30** YIA2:The Young Investigator Awards Lecture2
Room 2 / (Ishikawa Ongakudo 2F Hogaku Hall)
YIA2-1 Genomic analysis of mature lymphoid neoplasms: Molecular mechanisms and clinical implications ······ 13
YIA2-2 Therapeutic targeting of syndecan-1 axis overcomes acquired resistance to KRAS-targeted therapy ······ 14
YIA2-3 Elucidation of the molecular mechanisms of HPV-associated oropharyngeal carcinoma caused by epigenetic abnormality ······ 15
YIA2-4 Sequencing-based Translational Research in Gynecologic Malignancies Through Comprehensive Molecular Analyses ······ 16

**9:00 - 11:30** S1:ORGANOIDS: Unlocking Deep Insights into Cancer Biology
Room 3 / (Hotel Nikko Kanazawa 4F Tsuru A)
S1-1 Cellular Plasticity Drives Malignant Progression of Inflammation-Associated Colorectal Cancer ······ 17
S1-2 Digestive organ tumors from precursor to intractable cancer ······ 18
S1-3 TBD ······ 19
S1-4 Identification of novel gastric cancer driver signals using mouse models, organoids, and multi-omics a nalysis ······ 20
S1-5 Multi-omics analysis of organoids and xenograft models for exploring epigenetic abnormalities in pancreatic cancer ······ 21
S1-6 Modeling Lung Cancer Heterogeneity Using Patient-Derived Organoids ······ 22

**9:00 - 11:30** S2:Large-scale clinico-genomic data analysis in cancer precision medicine
Room 4 / (Hotel Nikko Kanazawa 4F Tsuru B)
S2-1 Transforming Oncology Electronic Health Records into Meaningful Multinational Evidence ······ 23
S2-2 Epidemiological cancer research using health insurance claims data: An introduction to the LIFE Study ······ 24
S2-3 Understanding the impact of common germline variants on cancer progression using real-world data ······ 25
S2-4 Real-world clinical utility of comprehensive genomic profiling in advanced solid cancers ······ 26
S2-5 Current Status and Future Perspective of Precision Oncology in Advanced Solid Tumors ······ 27
S2-6 Large-scale analysis of the genetic and environmental factors in cancer ······ 28

### CONTENTS

### 9:00 - 11:30 IS1:Targeting vulnerability in mitotic cell cycle: emerging anti-cancer strategies
### Room 5 / (Hotel Nikko Kanazawa 4F Tsuru C)

IS1-1 Targeting replication stress and CDK dysregulation in cancer ········· 29
IS1-2 Targeting replication stress in Cancer with ATR and PARP Inhibition in SMARCA4-Deficient lung adenoc arcinoma ········· 30
IS1-3 Circular RNA SCAP promotes DNA replication in hepatocellular carcinoma cells ········· 31
IS1-4 Vulnerability of mitotic chromosome segregation in cancers ········· 32
IS1-5 Functional genetics with a hypomorphic CENP-C mutant reveals a regulatory system for chromosome con gression ········· 33
IS1-6 Development of WEE1 inhibition-based strategies to overcome resistance to molecularly targeted therapies in cancer ········· 34
IS1-7 Modeling cell renewal and dynamic transition of gastritis to cancer in 3D organoids ········· 35
IS1-8 The origin and consequences of aneuploidy in cancer ········· 36

### 9:00 - 11:30 OS1:The Contribution of Epidemiology to Cancer Control Policy - new approaches
### Room 6 / (Hotel Nikko Kanazawa 3F Kujaku A)

OS1-1 The High-Benefit Approach: A New Targeting Strategy in Precision Medicine ········· 37
OS1-2 Target trial emulation: an accessible framework for formal causal inference from observational dat abases ········· 38
OS1-3 Aggregating clinicogenomic data into C-CAT repository for utilization and incorporation ········· 39
OS1-4 Monitoring the quality of cancer control measures using government statistics and databases ········· 40
OS1-5 A new perspective on monitoring health inequalities to promote cancer control that leaves no one b ehind ········· 41
OS1-6 Epidemiology to cancer control policy -Need for policy impact assessments ········· 42

### 9:00 - 10:15 I-E12-1:The latest advancement in liquid biopsy
### Room 7 / (Hotel Nikko Kanazawa 3F Kujaku B)

E-1001 Bile liquid biopsy is a useful modality for molecular diagnosis of pancreaticobiliary cancer ········· 43
E-1002 Predictive potential of ctDNA dynamics for RECIST-based CT imaging outcomes ········· 44
E-1003 Comparative genomic profiling of cell-free and tumor-derived DNA in Japanese patients with metastatic b reast cancer ········· 45
E-1004 OTS-Select: A Target Mutation Selection Algorithm for ctDNA Monitoring using digital PCR ········· 46
E-1005 A digital PCR probe library for TP53 mutations in longitudinal ctDNA monitoring ········· 47
E-1006 Novel liquid biopsy for NSCLC using intratumoral exosome extraction and ultrasensitive digital assay ········· 48

### 9:00 - 10:15 I-E11-1:Tumor antigens/Antitumor immune response (1)
### Room 8 / (Hotel Nikko Kanazawa 3F Kujaku C)

E-1007 Biomodeling-Guided Shared Neoantigen Discovery via Comprehensive Tumor Profiling for Neoantigen Va ccines in Asia ········· 49
E-1008 Single-Cell and Spatial Profiling of Tumor-Infiltrating B Cells Reveals Distinct Tumor-Specific Clones in Ga stric Cancer ········· 50
E-1009 Combination of proton irradiation and immunotherapy promotes antitumor activities in pancreatic cancer model ········· 51
E-1010 Active aldehydes accelerate glycolysis/FAO imbalance and exhaustion of CD8+ T cells in tumor micro environment ········· 52
E-1011 THEMIS2 impairs antitumor activity of natural killer cells by suppressing activating NK receptor s ignaling ········· 53
E-1012 Exploring miRNAs Contributing to Resistance against Anti-CD40 Agonist Antibody Therapy in Mouse Pan creatic Cancer Cells ········· 54

### 9:00 - 10:15 I-J4-1:Oncogenes & tumor-suppressor genes (1)
### Room 9 / (Hotel Nikko Kanazawa 3F Lumiere)

J-1007 Detection and functional analysis of germline mutations associated with disease-free survival in colorectal cancer. ········· 55
J-1008 Mechanisms for anticancer effects of G-quadruplex ligands through translation inhibition ········· 56

### CONTENTS

J-1009 Humanized anti-CKAP4 antibody suppresses tumor growth and may regulate immune microenvironment of pancreatic cancer ········ 57
J-1010 MiR- 155- targeted IcosL controls tumor rejection ········ 58
J-1011 Single-cell transcriptomics reveal distinct TME remodeling in liver metastasis of pancreatic cancer upon K RAS inhibition ········ 59
J-1012 Calcineurin-mediated dephosphorylation stabilizes E2F1 and promotes cancer cell proliferation ········ 60

9:00 - 11:30 IS2:Spatial Single Cell Analysis for clinical implementation in terms of clinical development
Room 10 / (Art Hall)
IS2-1 Spatial transcriptomics reveals predictive insights for neoadjuvant chemotherapy of breast cancer ········ 61
IS2-2 Beyond Karyotype: Heterogeneity of Gene Regulatory Programs in Acute Myeloid Leukemia ········ 62
IS2-3 Structured Analysis of the Tumor Microenvironment Using SpatialKnifeY: A Cluster-Based Framework for Sp atial Omics Data ········ 63
IS2-4 Prediction of cell types from histopathological images using spatial single-cell transcriptome data ········ 64
IS2-5 Robust and interpretable prediction of cancer gene markers and cell types from spatial transc riptomics data ········ 65
IS2-6 Spatial transcriptomics reveals epithelial heterogeneity and microenvironmental divergence in gastric cancer subtypes ········ 66
IS2-7 Spatial Single-Cell Analysis through Collaboration by Clinician, Pathologist, and Researcher Enhance Precision Medicine ········ 67
IS2-8 Single-cell and spatially-resolved transcriptomic landscapes of large cell neuroendocrine carcinoma of the l ung ········ 68

9:00 - 11:30 AOSR1:Hepatocellular Carcinoma: The Cutting Edge
Room 11 / (ANA Crowne Plaza Hotel Kanazawa 3F Ohtori (West))
AOSR1-1 AI-driven characterization of hepatic fiber morphology to predict molecular alterations and future HCC r isk in MASLD ········ 69
AOSR1-2 Impact of the Microenvironment on the Development and Progression of Liver Cancer ········ 70
AOSR1-3 Novel Molecular and Immunological Subtyping for Therapeutic Innovation in Liver Cancer ········ 71
AOSR1-4 Utility of a novel tumor marker for the diagnosis of hepatocellular carcinoma based on the cancer stem c ell hypothesis ········ 72
AOSR1-5 Cutting-Edge Drug Therapies for Hepatocellular Carcinoma ········ 73

9:00 - 11:30 OS2:Innovative Cancer Treatment via Artificial Protein Engineering: Functional Domain Modification and Bispecific Antibody
Room 12 / (ANA Crowne Plaza Hotel Kanazawa 3F Ohtori (Central))
OS2-1 Local activation of immune cells and depletion of Tregs by tumor collagen-binding checkpoint immuno therapy ········ 74
OS2-2 Acceleration of antibody engineering pioneered by machine learning ········ 75
OS2-3 Biparatopic strategy for the optimization of antibody antagonists against TNFR2 ········ 76
OS2-4 Development and prodrugation of cancer therapeutic bispecific antibodies ········ 77
OS2-5 Advances and Challenges in T-Cell Bispecific Antibody Development ········ 78
OS2-6 Development shared neoantigen-targeting bispecific antibodies ········ 79

9:00 - 11:30 OS3:Epigenetics and precision medicine in digestive cancers
Room 13 / (ANA Crowne Plaza Hotel Kanazawa 3F Ohtori (East))
OS3-1 Diagnosis of Cancer Risk and Control of Distant Metastasis via Targeting Epigenetic Alterations ········ 80
OS3-2 Epigenomic alterations driven by environmental factors and the progression of gastric cancer ········ 81
OS3-3 Oncogenic transcriptomic/methylomic profile sustained in the liver after the eradication of hepatitis C virus. ········ 82
OS3-4 Targeting methylation-silenced genes as a source of synthetic lethality ········ 83
OS3-5 Comprehensive colorectal cancer stem cell transcriptomic signatures that affect outcomes possibly by epi genetic changes ········ 84

9:00 - 10:15 I-J17-1:Surgical intervention
Room 14 / (ANA Crowne Plaza Hotel Kanazawa 3F Zuiun 1)
J-1013 Clinicopathological features and treatment outcomes of esophageal carcinosarcoma ········ 85

### CONTENTS

J-1014 Impact of Preserving Azygos Vein Arch in Postoperative Pneumonitis for Thoracoscopic Esoph agectomy ........ 86
J-1015 Association of Clinical Frailty Scale with Surgical Outcome and Survival Outcome in Elderly Colon Cancer P atients ........ 87
J-1016 Development of an intraoperative margin assessment method for breast cancer using the a-Man probe: a m ulti-center study ........ 88
J-1017 Prognosis of Intrahepatic Cholangiocarcinoma Patients with Sarcopenic Obesity ........ 89
J-1018 Clinical significance of the Tumor Marker Gene Model before conversion surgery for advanced pancreatic c ancer ........ 90

9:00 - 10:15 II-J2-1:Colorectal cancer
Room 15 / (ANA Crowne Plaza Hotel Kanazawa 3F Zuiun 2)
J-1025 Contribution of cancer-associated fibroblasts in early colorectal cancer to cancer progression and morp hogenesis ........ 91
J-1026 The spatial transcriptomic landscape of the Ulcerative Colitis-Related Colorectal Cancer in mice ........ 92
J-1027 Analysis of combination strategy with KRASG12D inhibitor for KRASG12D mutant colorectal cancer patient derived-organoids ........ 93
J-1028 Multi-transcriptomic Analysis Reveals that EREG-driven TME Crosstalk Defines Anti-EGFR Response in Co lorectal Cancer ........ 94
J-1029 COX19, a gene identified by chromosome copy number analysis, is a novel driver gene for colorectal cancer. ........ 95
J-1030 Increased level of ribosome biogenesis induced by MEK inhibition as a potential therapeutic target for KR AS-mutant CRC ........ 96

9:00 - 10:15 I-E13-1:Molecular targeted therapy (1)
Room 16 / (11F Visage)
E-1025 Targeting EphA2/Ephexin4 signaling to disrupt cell division and enhance anticancer drug sensitivity ........ 97
E-1026 ATM inhibition potentiate anti-tumor effect of IFN-γ by inducing ferroptosis ........ 98
E-1027 Characterization of URST4 as a new promising biomarker and therapeutic target for breast cancer ........ 99
E-1028 PARP1 selective inhibitor saruparib exhibits distinct pharmacological properties compared to PAPP1/2 i nhibitor olaparib ........ 100
E-1029 Identification of Stromal-Derived Factors Linked to T-DXd Efficacy in HER2-Mutant Lung Cancer and HER 2-Low Breast Cancer ........ 101
E-1030 Clinical relevance of HER2 mutations in HER2-low metastatic breast cancer: real-world analysis of tras tuzumab deruxtecan ........ 102

10:15 - 11:30 I-J12-1:Pathological analysis
Room 7 / (Hotel Nikko Kanazawa 3F Kujaku B)
J-1001 Risk Factors and Prognostic Impact for Spread Through Air Spaces in Pulmonary Metastases from Colo rectal Cancer ........ 103
J-1002 Establishment of a novel antibody that shows positivity in cancer cells of infiltrating lobular car cinoma ........ 104
J-1003 Potential transdifferentiation from hepatocellular carcinoma to neuroendocrine carcinoma in hepatic MiN ENs ........ 105
J-1004 "Hepatocyte Islands": A Novel Histological Feature Associated with Prognosis in Intrahepatic C holangiocarcinoma ........ 106
J-1005 The spatial profiling of stroma and immune interaction in gastric cancer by RePROBE multiplexed imaging analysis ........ 107
J-1006 Concordance of claudin-18.2 positivity in biopsy and resected tissues in pancreatic ductal aden ocarcinoma ........ 108

10:15 - 11:30 I-E11-2:Tumor immune microenvironment/Tumor immune escape (1)
Room 8 / (Hotel Nikko Kanazawa 3F Kujaku C)
E-1013 Acrolein accelerates lipid peroxidation and ferroptosis process of CD8+ T cells in TME ........ 109
E-1014 Targeting DRP1 Promotes RT-Induced Antitumor Immunity via mtDNA-Mediated cGAS-STING Axis in KRA S-Mutated Cancer ........ 110
E-1015 Psychological Stress Promotes HCC Progression via Macrophage-Derived CXCL2 ........ 111

### CONTENTS

E-1016 Secretary IL-1Ra repressed tumor growth of oral squamous cell carcinoma cells via modulation of macr ophage polarization ........ 112
E-1017 Activated platelets adhere to CD8+ T cells via CD62p-PSGL-1 and inhibit their antigen specific tumor cell k illing ........ 113
E-1018 Metabolic environment shapes immune landscape of tumor microenvironment in CNS lymphoma ..... 114

### 10:15 - 11:30 I-E4-1:Oncogenes & tumor-suppressor genes (2)
### Room 9 / (Hotel Nikko Kanazawa 3F Lumiere)

E-1019 The Non-Senolytic Effects of Dasatinib and Quercetin (D+Q) on Cellular Senescence ........ 115
E-1020 Pan-RAS Inhibitors Show Preclinical Efficacy Against Oncogenic NRAS Variants in Melanoma ........ 116
E-1021 S100A2 mediates a survival signal downstream of RUNX3 and p53 in gastric carcinoma cells ........ 117
E-1022 Wnt-mediated transcriptional activation of Igf1 in intrahepatic cholangiocarcinoma ........ 118
E-1023 E3 ubiquitin ligase STUB1 suppresses IFNGR1 expression and accelerates the development of AML ... 119
E-1024 Distinct roles of NEDD8-conjugating enzymes in acute myeloid leukemia progression ........ 120

### 10:15 - 11:30 I-J17-2:Multimodal treatment in surgery
### Room 14 / (ANA Crowne Plaza Hotel Kanazawa 3F Zuiun 1)

J-1019 Prognostic significance of MUC1-C expression in lung squamous cell carcinoma ........ 121
J-1020 Organ-specific Prognostic Impact of Checkpoint Inhibitor Therapy in Patients with Metastatic Esophageal c ancer ........ 122
J-1021 HER2 overexpression is a biomarker of conversion surgery for initially unresectable EGJ aden ocarcinoma ........ 123
J-1022 Impact of surgical resection on outcomes in patients with BR1 hepatocellular carcinoma ........ 124
J-1023 Treatment outcomes of lower rectal cancer with TNT and evaluation about comprehensive analysis for total RNA sequencing ........ 125
J-1024 Long-term outcomes of perioperative chemotherapy with capecitabine plus oxaliplatin for locally advanced rectal cancer ........ 126

### 10:15 - 11:30 II-J2-2:Gastric cancer, eophageal cancer
### Room 15 / (ANA Crowne Plaza Hotel Kanazawa 3F Zuiun 2)

J-1031 Single Cell RNA- and ATAC-seq Revealed Aberrant Molecular Features of Intestinal Metaplasia and SPEM ........ 127
J-1032 Gene expression profile of organoids from residual esophageal squamous cell carcinoma after neoadjuvant chemotherapy ........ 128
J-1033 Clinicopathological and genomic features of extrachromosomal DNA in gastric cancer ........ 129
J-1034 Prognostic significance of c-JUN and its functional suppression by FBXW7 in gastrointestinal stromal tu mors ........ 130
J-1035 Tumor stroma-derived Thrombospondin-1 contributes to lung metastasis by modulating immune activity in gastric cancer ........ 131
J-1036 Risk of Recurrence and Use of Adjuvant Nivolumab After Esophagectomy: A Retrospective Review of 39 C ases ........ 132

### 10:15 - 11:30 I-J13-1:Signal transduction inhibitors
### Room 16 / (11F Visage)

J-1037 Proximity biotinylation based analysis of EGFR interacting protein changes induced by EGFR-targeted drugs ........ 133
J-1038 Loss of BMF enhances drug tolerant persister cell survival in ALK-positive lung cancer ........ 134
J-1039 Combination of avutometinib and MRTX1133 synergistically suppresses cell growth in KRASG12D-mutated pancreatic cancer ........ 135
J-1040 Involvement of aberrant GSK3β in malignant properties of pancreatic cancer with acquired resistance to gemcitabine. ........ 136
J-1041 CBP/p300, histone acetyltransferase, regulate PROTAC efficacy by acetylating histones ........ 137
J-1042 Mechanism of Hand-Foot Syndrome Induced by Cancer Chemotherapy and Development of Therapeutic A pproaches ........ 138

### 11:40 - 12:40 CS Associate Editors ’ Meeting:CS Associate Editors ’ Meeting
### Room 1 / (Ishikawa Ongakudo 2F Concert Hall)

### CONTENTS

…… 139

11:50 - 12:40 LS1:Janssen Pharmaceutical K.K. / Treatment strategies for multiple myeloma ~Considering the immune environment~
Room 3 / (Hotel Nikko Kanazawa 4F Tsuru A)
LS1 Treatment strategies for multiple myeloma ~ Considering the immune environment~ …… 140

11:50 - 12:40 LS2:Nippon Becton Dickinson Company, Ltd. / A novel flow cytometry for analyzing the immunosuppressive tumor microenvironment
Room 4 / (Hotel Nikko Kanazawa 4F Tsuru B)
LS2 A novel flow cytometry for analyzing the immunosuppressive tumor microenvironment …… 141

11:50 - 12:40 LS3:Twist Bioscience / Evolutions in Sequencing Technologies and Genomic Analysis Supporting Cancer Research
Room 5 / (Hotel Nikko Kanazawa 4F Tsuru C)
LS3-1 1) Genetic analysis of hematological malignancies by targeted sequencing using custom panels …… 142
LS3-2 2) Evolving Genomic Technologies and Their Potential …… 143

11:50 - 12:40 LS4:Abbott Japan LLC / Molecular Mechanisms of Laminin γ2 Monomer in Liver Cancer: Pathological Relevance and Future Perspectives
Room 6 / (Hotel Nikko Kanazawa 3F Kujaku A)
LS4 Molecular Mechanisms of Laminin γ2 Monomer in Liver Cancer: Pathological Relevance and Future Pers pectives …… 144

11:50 - 12:40 LS5:TOMY DIGITAL BIOLOGY CO., LTD. / Latest Perspective in Cancer Immunotherapy Research using Long-Read Sequencing
Room 7 / (Hotel Nikko Kanazawa 3F Kujaku B)
LS5-1 1) Transcriptome Analysis using PacBio Long-Read Sequencing …… 145
LS5-2 2) Cancer Immunotherapy Targeting Neoantigens: Discovery of New Therapeutic Targets using Long-Read S equencing …… 146

11:50 - 12:40 LS6:Cytek Japan Corporation / Multidimensional Analysis of Tumor Immunity Using Full-Spectrum Flow Cytometry
Room 8 / (Hotel Nikko Kanazawa 3F Kujaku C)
LS6 Diving Deep: Profiling Exhausted T Cells in the Tumor Microenvironment Using Full Spectrum Flow C ytometry …… 147

11:50 - 12:40 LS7:MSD K.K. / Role of Macrophages and Correlation with PD-L1 in Cancer Immunity
Room 9 / (Hotel Nikko Kanazawa 3F Lumiere)
LS7 Role of Macrophages and Correlation with PD-L1 in Cancer Immunity …… 148

11:50 - 12:40 LS8:AstraZeneca K.K. / Is Molecular-Targeted Therapy Necessary for Unresectable Stage III NSCLC? — Clinical Considerations from the LAURA Regimen
Room 11 / (ANA Crowne Plaza Hotel Kanazawa 3F Ohtori (West))
LS8 Is Molecular-Targeted Therapy Necessary for Unresectable Stage III NSCLC? — Clinical Considerations from the LAURA Regimen …… 149

11:50 - 12:40 LS9:CyberomiX Inc. / Clinical potential of Spatial Transcriptome Analysis for Cancer Genomics
Room 12 / (ANA Crowne Plaza Hotel Kanazawa 3F Ohtori (Central))
LS9 Clinical potential of Spatial Transcriptome Analysis for Cancer Genomics …… 150

### CONTENTS

**11:50 - 12:40 LS10:Eisai Co., Ltd. / The forefront of new drug discovery: Drug discovery innovation through collaboration between doctors and companies**
**Room 13 / (ANA Crowne Plaza Hotel Kanazawa 3F Ohtori (East))**

LS10 The future of biliary tract cancer treatment based on cancer genomic medicine -The discovery of FGFR2 fu
sion genes in Japan and the path to the launch of Tasurgratinib- ........ 151

**11:50 - 12:40 LS11:Leica Microsystems K.K. / Innovation in 3D Immune Analysis**
**Room 14 / (ANA Crowne Plaza Hotel Kanazawa 3F Zuiun 1)**

LS11-1 1) Modeling Tumor Microenvironment Factors Driving Cancer Malignancy ........ 152
LS11-2 2) Proposal of SpectraPlex, a Novel Technology of 3D Multiplex Imaging and AIVIA, an AI-Powered Image A
nalysis Software, for Cancer Immuno-Microenvironment Research ........ 153

**11:50 - 12:40 LS12:Oxford Nanopore Technologies / Unlock Transformative Cancer Insights: The power of multi-omics Oxford Nanopore sequencing.**
**Room 15 / (ANA Crowne Plaza Hotel Kanazawa 3F Zuiun 2)**

LS12-1 1) A compendium of human RNA structures and modifications ........ 154
LS12-2 2) Expanding the potential of multi-omics analysis: The latest advancements in Oxford Nanopore s
equencing ........ 155

**12:50 - 15:20 SS5:Exit-Oriented Drug Discovery Technology from Academia**
**Room 1 / (Ishikawa Ongakudo 2F Concert Hall)**

SS5-1 "Kanazawa Medicine of the World" - a story of Hajime Okamoto, S. pyogenes, RNA effect, and an
ti-cancer drug OK-432 ........ 156
SS5-2 Paradigm shift in antibody drug conjugates ........ 157
SS5-3 Clinical development of next-generation CAR-T technologies originated from academic institutions ........ 158
SS5-4 Transforming Undruggable Targets into Therapeutic Opportunities with xFOREST RNA-Targeted Discovery P
latform ........ 159
SS5-5 Targeted protein degradation by IAP-based PROTACs and anti-cancer drug discovery ........ 160
SS5-6 Overcoming the tumour barriers for drug and immune cell infiltration by protein-engineered new generation
CAR-T cells ........ 161
SS5-Special Remarks Special Remarks ........ 162

**12:50 - 14:50 SS3:Woman scientists in cancer research (WSCR)**
**Room 2 / (Ishikawa Ongakudo 2F Hogaku Hall)**

SS3-1 Metabolic Vulnerability Drives ETC Inhibitor Sensitivity in TTF-1 Negative Lung Cancer ........ 163
SS3-2 Coping Strategies of Glioblastoma Cells to Chromosomal Instability ........ 164
SS3-3 Application of EV sheets to realize ideal extracellular vesicle biomarker development in ovarian ca
ncer ........ 165
SS3-4 Roles of GPNMB in tumor initiation and tumor development of triple-negative breast cancer ........ 166
SS3-5 Gastric gland mucin regulates malignancy of various tumors showing gastric phenotype ........ 167
SS3-6 FADS2, fatty acid desaturase2 required for tumorigenesis in cholangiocarcinoma ........ 168

**12:50 - 15:20 JS1:Animal Models for Cancer Research - From the Mice to Comparative Oncology**
**Room 3 / (Hotel Nikko Kanazawa 4F Tsuru A)**

JS1-1 Genetic mouse and organoid models for intestinal cancer research ........ 169
JS1-2 Understanding the Rarity of Rare Cancers: Insights from Comparative Oncology ........ 170
JS1-3 The CD3L1 immunotherapeutic target: from discovery to companion animal model and human cl
inical trial ........ 171
JS1-4 Spontaneous Canine Osteosarcoma: A Translationally Relevant Patient Model for Humans ........ 172
JS1-6 Evaluation of podplanin as a therapeutic target and as an amoeboid invasion driver by comparative onco
logical approach ........ 173
JS1-5 Evaluation of Frozen Tissue in Zebrafish PDX Models for Bladder Cancer Research and Preclinical V
alidation ........ 174

### CONTENTS

### 12:50 - 15:20 OS4:Current Advances in Bone-Related Cancer Research
### Room 4 / (Hotel Nikko Kanazawa 4F Tsuru B)

OS4-1 Bone's unique traits driving the progression of bone metastasis ...... 175
OS4-2 The malignant transformation of endosteal skeletal stem cells into cancer-initiating cells ...... 176
OS4-3 The interaction between hematological malignancies and the bone marrow microenvironment through the C XCL12-CXCR4 axis ...... 177
OS4-4 Beyond defense: innate immune signaling in the pathogenesis of hematologic cancers ...... 178
OS4-5 Multi omics analysis of bone and soft tissue sarcomas ...... 179
OS4-6 Defenses and vulnerabilities specific to bone-related cancers ...... 180

### 12:50 - 15:20 IS3:Novel application of extracellular secretory vesicles (Evs) in cancer therapy
### Room 5 / (Hotel Nikko Kanazawa 4F Tsuru C)

IS3-1 Gene Editing-Based Cancer Therapy Using Acerola-Derived Nanovesicles ...... 181
IS3-2 Translation of extracellular vesicles from bench to bedside ...... 182
IS3-3 Spatiotemporal analysis of metastatic niche formation by breast cancer extracellular vesicles using tissue slice models ...... 183
IS3-4 Intercepting Extracellular Vesicle Signals to improve diagnosis and treatment of Endometrial Cancer ...... 184
IS3-5 Development of Bispecific Extracellular Vesicles for Cancer Immunotherapy ...... 185
IS3-6 Bioengineered iRGD exosomes antagonize the malignant properties enabled by EBV-miR-BART3-3p ...... 186

### 12:50 - 14:20 OS5:Lights and Shadows of Database Utilization in Oncology Research
### Room 6 / (Hotel Nikko Kanazawa 3F Kujaku A)

OS5-1 Development of Genome Editing Data Analysis Platform by Utilizing Public Databases ...... 187
OS5-2 Database analyses on colorectal cancer focusing on the gene coding estrogen receptor-β ...... 188
OS5-3 Targeting genomic fragile regions to uncover metabolic vulnerabilities: bridging prediction and va lidation ...... 189
OS5-4 Clinical impact of endogenous retrovirus reactivation in acute myeloid leukemia ~utilizing TCGA controlled d ataset~ ...... 190
OS5-5 An example of a side effect of data analysis: novel transcripts discovered unexpectedly from public data. ...... 191

### 12:50 - 14:05 I-E5-1:Signal transduction in cancer progression
### Room 7 / (Hotel Nikko Kanazawa 3F Kujaku B)

E-1037 Prohaptoglobin regulates Hippo pathway status and augments pancreatic cancer malignancy ...... 192
E-1038 Intercellular signaling factors drive communal endocrine therapy resistance in breast cancer ...... 193
E-1039 Cytoplasmic KRAS salvage KRAS-MAPK signal from oncogene overdose in Her2 amplified with KRAS-mutated colorectal cancer ...... 194
E-1040 HER2/HER3-regulated expression program of the integrated stress response in breast cancer cell lines ...... 195
E-1041 Discovery of NNC 05-2090, a small molecule antagonist of the Neuromedin U receptor 2 for glioma tre atment ...... 196
E-1042 Comprehensive understanding of BCR-ABL interactors in chronic myeloid leukemia by proximity-dependent biotinylation ...... 197

### 12:50 - 13:30 ME1:Science Translational Medicine
### Room 8 / (Hotel Nikko Kanazawa 3F Kujaku C)

ME1 Science Translational Medicine ...... 198

### 12:50 - 14:05 I-E3-1:Bacteria, Inflammation & Cancer
### Room 9 / (Hotel Nikko Kanazawa 3F Lumiere)

E-1049 Impact of tsRNA derived from Klebsiella pneumoniae on hepatocarcinogenesis ...... 199
E-1050 Functional analysis of Pak1 in monocyte-derived Langerhans cells during skin carcinogenesis ...... 200
E-1051 ETBF promotes cecal tumorigenesis in Ahr-/- mice by modification of gut microbiota ...... 201
E-1052 Extracellular vesicles derived from vaginal bacteria involves the progression of early-phase ovarian car cinoma ...... 202

### CONTENTS

E-1053 Clonal expansion in the bile duct associated with primary sclerosing cholangitis ···················· 203
E-1054 A potential role of fatty acid binding protein 5 in the progression of idiopathic pulmonary fibrosis ···· 204

12:50 - 15:20 SS6:Defeating cancer from a host perspective: cancer cachexia
Room 10 / (Art Hall)
SS6-1 Immuno-metabolic crosstalk in cancer cachexia ················································ 205
SS6-2 Muscle-cancer communication in cachexia: signals, pathways, and implications ······················ 206
SS6-3 RNA–derived extracellular modified nucleosides and their roles in cancer pathophysiology ······ 207
SS6-4 Iron-Dependent Redox Control of Adipose Remodeling in Cancer Cachexia ························· 208
SS6-5 Identification and application of angiocrine factors suppressing cancer cachexia ····················· 209
SS6-6 Nicotinamide Metabolism in Diagnostic and Therapeutic Approaches for Cancer-Associated Metabolic Abn ormalities ··························································································· 210

12:50 - 15:20 AOSR2:The forefront of lung cancer research
Room 11 / (ANA Crowne Plaza Hotel Kanazawa 3F Ohtori (West))
AOSR2-1 Unraveling the Genomic Landscape of Lung Adenocarcinoma: Risk, Evolution, and Emerging Therapeutic Targets ······························································································ 211
AOSR2-2 Current Topics in Small Cell Lung Cancer Research ··········································· 212
AOSR2-3 Targeted therapy-induced tumor evolution in non-small cell lung cancer ························· 213
AOSR2-4 Development of cancer immunotherapy against tumor microenvironment in lung cancer ·········· 214
AOSR2-5 Toward overcoming drug resistance and predicting efficacy of new antibody-based therapy for lung ca ncer ································································································ 215
AOSR2-6 Identification of drug resistance mechanisms using 3D co-culture models and CRISPR screening with pa tient derived cells ··················································································· 216

12:50 - 15:20 IS4:Breaking New Ground in Cancer Research with Advanced Proteomics
Room 12 / (ANA Crowne Plaza Hotel Kanazawa 3F Ohtori (Central))
IS4-1 Symposium Overview_Breaking New Ground in Cancer Research with Advanced Proteomics ·········· 217
IS4-2 Proteomics-guided Precision Oncology in Non-Smoking Lung Cancer ······························· 218
IS4-3 Phosphoproteome subtyping of gastric cancer reveals dynamic transformation with chemotherapy and guides targeted therapy ···················································································· 219
IS4-4 Multi-Omics Deconvolution of TNBC Reveals Subtype-Specific Therapeutic Vulnerabilities ············· 220
IS4-5 Federated deep learning enables cancer subtyping by proteomics ································· 221
IS4-6 Proteogenomics unlocks noncanonical cancer antigens for diagnostic and therapeutic development ····· 222
IS4-7 Application of ion mobility-assisted mass spectrometry for clinical immunopeptidomics ················ 223

12:50 - 15:20 OS6:Next-generation cancer research pioneered by designer cells and RNA science
Room 13 / (ANA Crowne Plaza Hotel Kanazawa 3F Ohtori (East))
OS6-1 Next-Generation In Vivo CAR-T Therapy Driven by RNA Engineering and LNP Optimization ··········· 224
OS6-2 Investigation of Immuno-Enhanced CAR-T Cells in Intractable Solid Tumor Models ··················· 225
OS6-3 Anti-cancer immune construction using iPS cell-derived NKT cells ································· 226
OS6-4 Anti-CCR8 antibody therapy promotes Dendritic Cell-driven antitumor immunity ····················· 227
OS6-5 Cancer stem cell plasticity in CRC chemoresistance and metastasis: A critical target for total kill th erapy. ·································································································· 228
OS6-6 Engineering mRNA for advanced cancer vaccines ··············································· 229
OS6-7 CXCR4 induces memory formation over exhaustion in CAR-T cells to achieve durable leukemia ta rgeting ······························································································· 230
OS6-8 Integrating Genome Editing and iPSC Technology for Cancer Immunotherapy: The Promise of Universal T C ells ··································································································· 231
OS6-9 Elucidation of the mechanisms of cancer malignancy through comprehensive RNA modification an alysis ································································································· 232
OS6-10 Application of Single-Molecule Electrical Measurement in Precision Medicine for Refractory Can cers ·································································································· 233

12:50 - 14:05 II-E7:Gynecologic cancer (1)
Room 14 / (ANA Crowne Plaza Hotel Kanazawa 3F Zuiun 1)

### CONTENTS

E-1079 HER2 targeted alpha therapy against trastuzumab resistant peritoneal metastasis of uterine serous carc inoma ........ 234
E-1080 FIH regulates adaptation of ovarian cancer cells to hypoxia through suppression of HIF2-Sp1 dependent g ene expression ........ 235
E-1081 Cancer Cell Properties in Uterine Serous Carcinoma with an Anti-Inflammatory Tumor Micro environment ........ 236
E-1082 Establishment and genomic characterization of patient-derived organoids from gastric-type cervical ade nocarcinoma ........ 237
E-1083 Osteopontin blockade elicits anti-tumor immunity in a mouse model of aggressive endometrial ca ncer ........ 238
E-1084 Site-specific miRNA alternation in fimbriae/fallopian tube/tumor of BRCA1 mutated high grade serous ov arian carcinoma ........ 239

### 12:50 - 14:05 I-J11-1:Tumor antigens/Antitumor immune response (2)
### Room 15 / (ANA Crowne Plaza Hotel Kanazawa 3F Zuiun 2)

J-1079 Anti-tumor immune responses triggered by abnormal spindle formation through the double-stranded RNA recognition pathway ........ 240
J-1080 Efficacy of PDPN-Targeted Photoimmunotherapy in Esophageal Cancer: Comparison with FAP-Targeted P hotoimmunotherapy ........ 241
J-1081 Clonal competition between CD8+ T cells with different TCR affinities modulates proliferation and diff erentiation ........ 242
J-1082 Oral administration of arginine enhances antitumor immunity and reduces tumor growth in a hepatocellular carcinoma model ........ 243
J-1083 ATR inhibition activates cGAS/STING-interferon signaling and promotes antitumor immunity in small-cell l ung cancer ........ 244
J-1084 Reprogramming the Tumor Immune Microenvironment in HPV+ Cervical Cancer and the Antitumor Effects by Therapeutic Vaccine ........ 245

### 12:50 - 14:05 I-J9-2:Metastasis & cancer microenvironment
### Room 16 / (11F Visage)

J-1097 Cancer cells invade lymph vessels by inducing EndMT in lymphatic endothelial cells ........ 246
J-1098 Development of a novel therapeutic approach for bone metastasis targeting lipids in the bone microe nvironment ........ 247
J-1099 The regulation of brain metastasis by the vascular endothelial FAK ........ 248
J-1100 Cancer-associated fibroblasts in "Immature" DR promote colorectal cancer cell behavior via type I procollagen processing ........ 249
J-1101 Host-derived factor induces premetastatic niche formation in liver. ........ 250
J-1102 Deciphering the contributions of cellular and exosomal heterogeneity to the multistep progression of cancer ........ 251

### 13:50 - 15:20 OS8:Frontier of cancer research driven by multi-modal extracellular matrix
### Room 8 / (Hotel Nikko Kanazawa 3F Kujaku C)

OS8-1 Basement membrane dynamics in epithelial tissue morphogenesis ........ 252
OS8-2 Viscous ECM: In Vitro Vascularized Cancer Stromal Tissues for Functional Cancer Personalized Me dicine ........ 253
OS8-3 Stiff ECM triggers activation of transcription factor ATF5 for cancer progression ........ 254
OS8-4 ERK-dependent translocation of ZO-1 regulates epithelial cell invasion ........ 255
OS8-5 Molecular Mechanisms of Therapeutic Resistance Driven by Dynamics of Tumor Vascular Matrix ........ 256
OS8-6 Stromal defense against cancer by the periosteum ........ 257

### 14:05 - 15:20 I-J5:Diversity of signal transduction pathways in cancer
### Room 7 / (Hotel Nikko Kanazawa 3F Kujaku B)

J-1055 CD73 as a Downstream Target of TGF-β Signaling in Tumor Immune Evasion ........ 258
J-1056 Unrevealing the mechanisms of the TGF-β-induced endothelial-mesenchymal transition (EndoMT) in t umor microenvironment ........ 259
J-1057 Regulation of KRAS signaling by lipid domain in the inner leaflet of cell membranes ........ 260

### CONTENTS

J-1058 Senescence-associated microRNA-3140-3p suppresses malignant pleural mesothelioma by targeting SLC 7A11 and ASF1B ········ 261
J-1059 The long noncoding RNA GSEC encodes a small peptide to promote colorectal tumorigenesis ········ 262
J-1060 Mitochondrial DNA mutation is associated with chemoresistance in esophageal squamous cell ca rcinoma ········ 263

14:05 - 15:20 I-E3-2:Viruses & Cancer
Room 9 / (Hotel Nikko Kanazawa 3F Lumiere)
E-1055 Exploration of the predictive marker for post HCV-SVR hepatocarcinogenesis based on DNA methylo me/transcriptome analysis ········ 264
E-1056 Oncolytic reovirus restores hepatic stellate cells from an activated status to a quiescent status ········ 265
E-1057 HDAC inhibitor SAHA suppresses the expression of viral microRNAs and induces apoptosis in EBV-infected cells ········ 266
E-1058 The type of HPV and the tissues it targets influence the level of malignancy. ········ 267
E-1059 Breaking Away from Low HPV Vaccination Rates: Reconstructing Cervical Cancer Prevention on Epide miological Surveys ········ 268
E-1060 The nucleolar protein, fibrillarin is essential for the production of Epstein-Barr virus ········ 269

14:05 - 15:20 II-J7:Gynecologic cancer (2)
Room 14 / (ANA Crowne Plaza Hotel Kanazawa 3F Zuiun 1)
J-1073 LRRK2 is a favorable prognostic factor inducing apoptosis in ovarian clear cell carcinoma ········ 270
J-1074 Human leucocyte antigen in gynecologic cancer before and after heavy-ion radiotherapy ········ 271
J-1075 Antibody-drug conjugate targeting CADM1/TSLC1 for treatment of early-stage endometrioid ca rcinoma ········ 272
J-1076 Exploration of new treatment for ovarian high-grade serous cancer with cyclin E1 amplification. ········ 273
J-1077 Significance of Molecular Residual Disease (MRD) in Gestational Trophoblastic Neoplasia ········ 274
J-1078 Unveiling the Th17-IL17 immune axis in ovarian clear cell carcinoma: Implications for immun otherapy ········ 275

14:05 - 15:20 I-J11-2:Tumor immune microenvironment/Tumor immune escape (2)
Room 15 / (ANA Crowne Plaza Hotel Kanazawa 3F Zuiun 2)
J-1085 The influence of astrocytes on the immune response of microglia in the microenvironment of metastatic b rain tumors ········ 276
J-1086 Myeloid heterogeneity in bone metastatic microenvironment ········ 277
J-1087 Caloric restriction ameliorates aging and tumor progression via tumor immunity ········ 278
J-1088 TIL Composition in Colorectal Cancer Liver Metastases: Comparison with Colorectal Cancer and Primary L iver Cancers ········ 279
J-1089 Inhibition of C5a-C5aR1 axis suppresses tumor progression with antitumor immunity in pancreatic ductal adenocarcinoma. ········ 280
J-1090 Identification of the mechanisms underlying cancer immune exclusion by extracellular matrix component a nalysis ········ 281

14:05 - 15:20 I-E9:Cellular plasticity & heterogeneity in metastasis
Room 16 / (11F Visage)
E-1091 Histamine-Producing Mast Cells Mediate Resistance to mTOR Inhibitors in Invasive Colorectal Ca ncer ········ 282
E-1092 Multi-Omics Insights into Mechanisms Driving Early Postoperative Recurrence in BCLC-0/A Hepatocellular Carcinoma ········ 283
E-1093 Novel Endothelial-Myeloma Cell Crosstalk Through CXCL10-CXCR3 Contributes to Chemotherapy Resi stance ········ 284
E-1094 The role of MAGT1 and TUSC3 in vasculogenic mimicry of human cancer cells in vitro ········ 285
E-1095 Single-Cell Spatial Analysis Reveals the Process of Sarcomatous change in Undifferentiated Pancreatic Ca ncer ········ 286
E-1096 Modeling PDAC intratumor heterogeneity and metastasis ········ 287

14:20 - 15:50 OS7:How Mechanical Forces Shape Cancer Cell Identity
Room 6 / (Hotel Nikko Kanazawa 3F Kujaku A)

### CONTENTS

OS7-1 Mechanism of nestin-mediated cellular softening in cancer cells ...... 288
OS7-2 Strategies to suppress cancer metastasis based on mechano-design ...... 289
OS7-3 Organelle Resilience and Its Regulation in Cell Fate Determination ...... 290
OS7-4 High-throughput profiling of the cell mechanics with unbiased transcriptional screening in cancer c ells ...... 291
OS7-5 Mechanical cues in tumor microenvironment: ECM-linked responses mediated by actin and p53 ...... 292

15:20 - 16:35 I-E2-1:Organoid/Animal model (1)
Room 3 / (Hotel Nikko Kanazawa 4F Tsuru A)
E-1031 Functional annotation of the PTEN gene variants of unknown significance (VUS) using endometrial cancer organoids ...... 293
E-1032 Novel molecular classification of large cell neuroendocrine carcinoma and its potential therapeutic impl ications ...... 294
E-1033 Helicobacter pylori CagA drives in situ gastric signet-ring cell carcinoma in the absence of E-cadherin ex pression ...... 295
E-1034 Tumor-Associated Fibrosis in Ovarian Cancer: Insights from a Highly Metastatic Xenograft Model ...... 296
E-1035 Loss of SWI/SNF subunits with PI3K/AKT pathway activation forms intraductal tubular papillary neoplasm in the pancreas ...... 297
E-1036 The IQGAP3-MYC axis maintains a stem-like population to drive gastric carcinogenesis ...... 298

15:20 - 16:35 I-J9-1:Metastasis & vascular biology
Room 4 / (Hotel Nikko Kanazawa 4F Tsuru B)
J-1043 Tumor endothelial cells form a drug-resistant niche in glioblastoma by regulating iron metabolism through ceruloplasmin ...... 299
J-1044 Ets2 transcription factor maintains vascular integrity by suppressing endothelial-mesenchymal t ransition ...... 300
J-1045 Multi-omics analyses using paired samples from newly diagnosed and recurrent glioblastomas during be vacizumab therapy ...... 301
J-1046 CROX (Cluster Regulation of RUNX) strategy-mediated suppression of lung metastasis in triple-negative br east cancer ...... 302
J-1047 LAT1 inhibition facilitated tumor vascular normalization through enhancing CTH expression. ...... 303
J-1048 Translocation renal cell carcinoma-on-a-chip to assess tumor microenvironmental factors regulating ang iogenesis ...... 304

15:20 - 16:35 I-J10-1:Extracellular vesicles/Exosome
Room 5 / (Hotel Nikko Kanazawa 4F Tsuru C)
J-1049 Paracrine adhesion signal by small extracellular vesicles promotes their clathrin-independent uptake by r ecipient cells ...... 305
J-1050 Expression and functional analysis of exo-miRNA in portal blood associated with liver metastasis of panc reatic cancer ...... 306
J-1051 Enzyme activity profiling and protease identification in extracellular vesicles for pancreatic cancer biom arkers ...... 307
J-1052 Differential immunosuppression effects of CAF's exosome depending on the desmoplastic patterns in col orectal cancer ...... 308
J-1053 Development of personalized therapy using cancer-derived exosomes for esophageal squamous cell car cinoma ...... 309
J-1054 Suppression of exosome secretion as an effect of cellular exposure to atmospheric dust in the promotion of lung cancer ...... 310

15:20 - 16:35 I-E5-2:Regulation of gene expression in cancer
Room 7 / (Hotel Nikko Kanazawa 3F Kujaku B)
E-1043 Hypoxia-related protein IREB2-268aa promotes gastric cancer progression through DHODH-induced fer roptosis defense ...... 311
E-1044 Identification of a novel lncRNA regulating Wnt pathway in pancreatic cancer ...... 312
E-1045 NEAT1-triggered collateral activity of CRISPR/Cas13 shows antileukemic activity ...... 313
E-1046 Regulation of structural diversity of miRNAs by IGF2BP3 in the malignant properties of early-stage lung a denocarcinoma ...... 314

### CONTENTS

E-1047 Association between tumor-specific alternative promoter usage and patient survival in lung ade nocarcinoma ........ 315
E-1048 Mechanistic insight into super-enhancer-mediated gene gating in cancer cells ........ 316

15:20 - 16:35 I-J3:Bacteria & Microbiome
Room 9 / (Hotel Nikko Kanazawa 3F Lumiere)
J-1061 The involvement of Fusobacterium nucleatum subspecies animalis in the pathogenesis of human esophageal adenocarcinoma ........ 317
J-1062 Butyrate and CBM588 Boost Oncolytic Adenovirus Immunotherapy via MHC-I Upregulation in Colorectal C ancer ........ 318
J-1063 Fusobacterium nucleatum-induced activation of the STING pathway contributes to esophageal cancer pro gression ........ 319
J-1064 IL-33 may contribute to sex-related pathology of inflammatory colorectal cancer with alterations in the gut microbiota ........ 320
J-1065 Oral administration of Enterococcus faecalis induces pancreatitis, which is a risk factor for cancer. ........ 321
J-1066 Influence of F. nucleatum, pks+ E. coli, and ETBF on the Tumor Microenvironment in Colorectal Cancer Liver Metastasis ........ 322

15:20 - 16:35 II-J5:Lung cancer
Room 11 / (ANA Crowne Plaza Hotel Kanazawa 3F Ohtori (West))
J-1067 Analysis of lung cancer stem cells reprogrammed by positively charged hydrogels ........ 323
J-1068 PDZ-binding kinase promotes cell migration and could be a promising therapeutic target in mes othelioma ........ 324
J-1069 Antibiotics as a negative factor for Chemoimmunotherapy for non-small cell lung cancer with low PD-L1 e xpression ........ 325
J-1070 Identification of a neuropeptide receptor as a promising therapeutic target for small cell lung ca ncer ........ 326
J-1071 CHK1 as a novel therapeutic target for pleural mesothelioma ........ 327
J-1072 Exploring molecular mechanisms of lung adenocarcinoma with interstitial pneumonia using spatial tran scriptomic analysis ........ 328

15:20 - 16:35 I-E11-3:Immune checkpoint inhibitors (1)
Room 12 / (ANA Crowne Plaza Hotel Kanazawa 3F Ohtori (Central))
E-1061 Withdrawn ........ 329
E-1062 Discovery of Novel Peptide Inhibitors Targeting the NKG2A–HLA-E Immune Checkpoint via RaPID s election ........ 330
E-1063 Low-Dose Radioimmunotherapy Using LDDS with Anti-CTLA-4 mAb for Lymph Node Metastasis ........ 331
E-1064 Intracranial myeloid cells increase combination immunotherapy against brain metastasis by activating CD4+ TFH cells ........ 332
E-1065 SIGLEC7 in NK cells act as an immune checkpoint receptor for VSIG4 ........ 333
E-1066 Identification of an immune-activating and tumor-suppressive cancer-associated fibroblast subtype in ga stric cancer ........ 334

15:20 - 16:35 I-E12-2:Cancer diagnosis & biomarkers
Room 13 / (ANA Crowne Plaza Hotel Kanazawa 3F Ohtori (East))
E-1067 Withdrawn ........ 335
E-1068 Withdrawn ........ 336
E-1069 Early detection of pancreatic cancer by comprehensive serummiRNA sequencing with machine learning: A multicenter study ........ 337
E-1070 Development of serum immunodiagnostics for very-early pancreatic cancer ........ 338
E-1071 Validation of extracellular vesicle protein biomarkers in multiple myeloma ........ 339
E-1072 Rapid Intraoperative Genetic Analysis of Adult-type Diffuse Gliomas Using a Microfluidic Real-Time PCR D evice ........ 340

15:20 - 16:35 II-E6:Frontiers in breast cancer
Room 14 / (ANA Crowne Plaza Hotel Kanazawa 3F Zuiun 1)
E-1073 Soluble TREM2 Drives Triple-Negative Breast Cancer Progression via TG2-AKT Pathway ........ 341

### CONTENTS

E-1074 Immediate Chemotherapy-Induced Cognitive Impairment (CICI) among Cancer Patients in Yogyakarta Region, Indonesia ........ 342
E-1075 Whole exome sequencing identifies Japanese familial breast cancer susceptibility gene ........ 343
E-1076 Single-cell analysis reveals a metastatic gene expression signature in a rare subpopulation of primary tumor cells ........ 344
E-1077 Novel Targets of CDK4/6 Inhibitors Identified Through Histopathology-Based AI Models and Spatial Transcriptomics ........ 345
E-1078 Integration of Single-Cell and Spatial Transcriptomics Reveals Treatment-Induced Clonal Reorganization in Breast Cancer ........ 346

15:20 - 16:35 I-E10-1:Cell death
Room 15 / (ANA Crowne Plaza Hotel Kanazawa 3F Zuiun 2)
E-1085 Sublethal disulfidoptosis contributes to TLR2-mediated pyroptosis induction in THP1 human leukemia cells ........ 347
E-1086 Necroptosis and MLKL in Cholangiocarcinoma: Tumor Promotion and Therapeutic Implications ........ 348
E-1087 Anticancer potential of Machilus thunbergii leaf extract and its component and proapoptotic effects ........ 349
E-1088 Z36 triggers a dose-dependent switch between cytoplasmic vacuolation death and apoptosis in colorectal cancer cells ........ 350
E-1089 Exploration of New Therapeutic Strategies for Peritoneal Metastasis of Gastric Cancer Based on Drug Relocalization ........ 351
E-1090 The role of mitophagy in the chemotherapy-induced senescence of cancer cells ........ 352

15:20 - 16:35 I-J7-1:Cancer genomics
Room 16 / (11F Visage)
J-1091 Genome profiling of central nervous system germ cell tumor by whole genome sequencing ........ 353
J-1092 Identification of novel driver genes in Wilms Tumor by whole-genome sequencing ........ 354
J-1093 Molecular evolutionary analysis revealed early-onset colorectal cancer-related driver cancer genes ........ 355
J-1094 Deciphering FGFR2-IIIb/-IIIc isoform switching in advanced gastric cancer through whole-transcriptome sequencing ........ 356
J-1095 Contribution of 15q15.1 deletion as a genomic alteration for brain metastasis development in non-small cell lung cancer ........ 357
J-1096 Segment specific variations in genetic alterations across the renal nephron ........ 358

15:30 - 16:10 SL1:Expanding Precision Oncology with Novel Therapeutics, Technologies and Clinical Trials Design
Room 1 / (Ishikawa Ongakudo 2F Concert Hall)
SL1 Expanding Precision Oncology with Novel Therapeutics, Technologies and Clinical Trials Design ........ 359

15:30 - 16:10 ME2:Nature Communications
Room 8 / (Hotel Nikko Kanazawa 3F Kujaku C)
ME2 Nature Communications ........ 360

16:45 - 17:30 I-P1-1:Animal models for cancer (1)
Room P / (Motenashi Dome)
P-1001 Characteristics of colon carcinogenesis in Mlh1 heterozygous mice after combined exposure to radiation and inflammation ........ 361
P-1002 Genotoxicity assessment of advanced materials using mouse liver organoids ........ 362
P-1003 Iron-catalyzed oxidative stress cancels cancer promotional effect of BRCA2 haploinsufficiency through ferroptosis ........ 363
P-1004 Histopathological and immunohistochemical analysis of antithyroid mechanism of VA-K-14 in rats ........ 364
P-1005 Withdrawn ........ 365
P-1006 Impacts of circadian rhythm disruption by time-restricted feeding and exercise on oxidative DNA damage and immune ........ 366
P-1007 Unveiling the therapeutic potential of alkylating PI polyamide in MET amplified tumors ........ 367

### CONTENTS

### 16:45 - 17:30 I-P2-1:Animal model (1)
### Room P / (Motenashi Dome)

P-1015 Effect of high-fat diet on colorectal cancer development ........ 368
P-1016 Suppression of intestinal adenoma and/or adenocarcinoma by loss of the TMEPAI gene ........ 369
P-1017 Novel PI3K-Driven Intrahepatic Cholangiocarcinoma Mouse Model and Synergistic Effects of PI3K and WEE1 Inhibition ........ 370
P-1018 Generation and phenotype analysis of a genome-edited mouse with a germline Apc variant in the 5' region ........ 371
P-1019 Possible roles of RECK in cancer immunity ........ 372
P-1020 Establishment of lung metastasis mice model for feline breast cancer and evaluation of its usefulness ........ 373
P-1021 Establishment of a highly metastatic pancreatic adenocarcinoma in Ogt-transgenic mice ........ 374
P-1022 Development of animal models for cancer cachexia and strategies to improve the quality of life in sarcopenia ........ 375

### 16:45 - 17:30 I-P2-3:Animal model (3)
### Room P / (Motenashi Dome)

P-1031 Evaluation of IL-6-targeted therapy in a mouse model of ovarian clear cell carcinoma ........ 376
P-1032 Establishment of a mouse intrahepatic cholangiocarcinoma cell line from Pten-null and Kras mutant mouse model ........ 377
P-1033 Mutational profiles of spontaneous and radiation-associated mammary carcinomas in a Brca1 haploinsufficiency rat model ........ 378
P-1034 The relationship between cellular senescence and invasive potential of cancer cells ........ 379
P-1035 Investigation of Size-Dependent Anti-Inflammatory and Antitumor Effects of Oxygen Nanobubble Water ........ 380
P-1036 Relationship Between Brain Weight and Cancer Mortality: Insights from a Comparative Oncological Analysis of Primates ........ 381
P-1037 Study of carcinogenesis by transplacental exposure to dimethylarsinic acid in F1 mice ........ 382
P-1038 Short-term exposure of chemical carcinogen determines the carcinogenic fate of upper tract urothelial carcinoma ........ 383

### 16:45 - 17:30 I-P3-2:Viral Oncology: Clinical & Applied
### Room P / (Motenashi Dome)

P-1045 HPV vaccine effectiveness against cervical cancer and precancer in Japan: a test-negative case-control study ........ 384
P-1046 Citrus limon (Linn) Burm. f. compounds as Potential HIV-1 Reverse Transcriptase Inhibitors ........ 385
P-1047 Persistence of serum antibody titers against HPV16/18/52/58 in vaccinated women in Japan ........ 386
P-1048 Lipid Droplet Accumulation is associated with Altered Gene Expression and HCC-Related Mutations in HBV-Infected Livers ........ 387
P-1049 Withdrawn ........ 388
P-1050 Disruption of HPV16 E6/E7 Genes Using All-in-One Adenovirus Vectors Expressing Eight Double-Nicking Guide RNAs ........ 389

### 16:45 - 17:30 I-P4-2:Oncogenes & tumor-suppressor genes (1)
### Room P / (Motenashi Dome)

P-1058 Myc upregulates Ggct, γ–glutamylcyclotransferase to promote development of p53–deficient osteosarcoma ........ 390
P-1059 Involvement of the Wnt pathway-related factors downregulation in anticancer activity of GGCT depletion ........ 391
P-1060 Glutaredoxin 2 has an oncogenic role in colon cancer ........ 392
P-1061 Amino acid transporter SLC38A1 is a tumour promoter and a potential therapeutic target for urothelial carcinoma ........ 393
P-1062 HIF1α Promotes Development of TFE3-Rearranged Renal Cell Carcinoma via a Metabolic Shift Toward Fatty Acid Synthesis ........ 394
P-1063 Impact of BTB and CNC homology 1 (BACH1) on cellular dynamics in triple negative breast cancer (TNBC) cells ........ 395

### CONTENTS

P-1064 Analysis of tumor progression signaling in TFE3-rearranged renal cell carcinoma induced by ARID2 def iciency ····· 396
P-1065 Modeling tumor-suppressive roles of ARID1A in early pancreatic tumorigenesis ····· 397
P-1066 Combination of a folate antimetabolite and a topoisomerase I inhibitor has synergy on ARID1A -mutated o varian cancer. ····· 398

### 16:45 - 17:30 I-P5-1:Extracellular/Intercellular signaling factors

### Room P / (Motenashi Dome)

P-1076 CD44 and EGFR may play a significant role in the cross talk between oral cancer stem cells and tumor mi croenvironment ····· 399
P-1077 The role of ER-localized enzymes in the pathway of cancer stem cell formation ····· 400
P-1078 The role of VEGFA and VEGFC in canine hemangiosarcoma ····· 401
P-1079 Analysis of Transcriptomic Changes in Extracellular Vesicles Under In Vitro Coculture Conditions Using an EV Tracer ····· 402
P-1080 Oleic acid-induced metastasis of KRAS/p53-mutant CRC relies on extracellular vesicles containing ICAM-1 and fibronectin ····· 403
P-1081 Development of a biotin proximity labeling method for cell surface proteins. ····· 404
P-1082 Elucidation of the cause of malignant of endometrial adenocarcinoma caused by decreased progesterone r eceptor expression ····· 405
P-1083 Effect of Clostridium butyricum TO-A Culture Supernatant and n-Butyric Acid on Gene Expression in HeLa S3 cells ····· 406
P-1084 Withdrawn ····· 407
P-1085 Exploring the role of mechanical forces in chromosomal instability during metastasis ····· 408

### 16:45 - 17:30 I-P9-1:Metastasis & EMT (1)

### Room P / (Motenashi Dome)

P-1095 TGF-&BetaR2 signaling regulates metastatic spreading by promoting invasive plasticity in nasopharyngeal carcinoma cells ····· 409
P-1096 Palmitic acid inhibits migration and invasion in human oral squamous cell carcinoma ····· 410
P-1097 HMGA1 is a poor prognostic marker and confers the aggressiveness of cholangiocarcinoma ····· 411
P-1098 The organogermanium compound THGP suppresses norepinephrine-induced EMT in the gastric cancer cell line HGC-27 cells ····· 412
P-1099 EMT-promoted cell migration depends on the non-canonical activation of EphA2 via FGFR1 ····· 413
P-1100 Efficacy and mechanism of action of Bergamottin for HB-EGF-induced EMT in esophageal Cancer ····· 414
P-1101 Oxaliplatin induces EMT via activation of KRAS/ERK pathway in KRAS-mutated colorectal cancer c ells ····· 415

### 16:45 - 17:30 I-P10-1:Extracellular vesicles/Exosome (1)

### Room P / (Motenashi Dome)

P-1109 Extracellular vesicles derived from mesenchymal stem cells inhibit tumor environment of triple negative b reast cancer ····· 416
P-1110 Comparison of Urine exosome and Plasma exosome in colorectal cancer ····· 417
P-1111 Development of breast cancer screening strategies by using extracellular vesicle in nipple disc harge ····· 418
P-1112 RNA-Seq of EVs-related genes analysis by t-SNE/PAM identifies molecular subtypes of bladder ca ncer ····· 419
P-1113 Cancer progression enhanced by sEVs released from gastric cancer cells during chemotherapy ····· 420

### 16:45 - 17:30 I-P11-1:Tumor antigens

### Room P / (Motenashi Dome)

P-1120 Neoantigen identification with NESSIE for personalized cancer vaccine ····· 421
P-1121 Detection of neoepitopes from cancer cell lines using targeted mass spectrometry ····· 422
P-1122 Bacterial infection induces transient melanoma dedifferentiation with attenuated antigenicity. ····· 423
P-1123 Frameshift Mutations as Sources of Neoantigens in Microsatellite-Stable Tumors ····· 424
P-1124 Withdrawn ····· 425
P-1125 Neoantigen Prediction in Gliomas Enhanced by WHO CNS5 Reclassification and Multi-Omics Pr ofiling ····· 426

### CONTENTS

### 16:45 - 17:30 I-P11-3:Antitumor immune response (2)
### Room P / (Motenashi Dome)

P-1134 Identification of a novel immune-related factor associated with ICB resistance in EGFR-mutated lung ade nocarcinoma ········ 427
P-1135 Effects of Anamorelin on Tumor Growth and Immune Modulation in Lung Cancer Cells ········ 428
P-1136 Research for immune response pathway related molecules following cancer radiation therapy ········ 429
P-1137 Targeting DHX9 as a therapeutic strategy in Human Gastric Cancer ········ 430
P-1138 CYLD regulates cGAS-STING pathway and metastasis in HNSCC ········ 431
P-1139 Cystatin A antitumor activity is mediated by Th1 lymphocytes and M1 macrophages in mouse models of p ancreatic cancer ········ 432
P-1140 Comparing the efficacy of neoadjuvant immunotherapy for locally advanced gastric cancer ········ 433
P-1141 Combination therapy of cGAMP and agonistic anti-CD40 antibody suppresses tumor growth via activating CD8+ T-cells. ········ 434
P-1142 Enhancement of anti-tumor immune responses by anti-CD155 antibody ········ 435

### 16:45 - 17:30 I-P11-5:Tumor immune microenvironment/Tumor immune escape (1)
### Room P / (Motenashi Dome)

P-1151 Elucidating immune-excluded microenvironment formation via integrative spatial and single-cell trans criptomics ········ 436
P-1152 EML4-ALK-rearrangement constructs a distinctive myeloid-cell-dominant immunosuppressive microe nvironment in lung cancer ········ 437
P-1153 CD63 Recruits Tumor-Suppressive Macrophages in Tumor Microenvironment and Suppresses Metastasis in Breast Cancer ········ 438
P-1154 Comprehensive NY-ESO and Immune Profiling across 150 Soft Tissue Sarcoma Cases ········ 439
P-1155 Single-cell profiling of neoantigen-reactive CD8+ T cells in sarcoma ········ 440
P-1156 Investigation of the indicator related to fibrotic tumor microenvironment in gastric cancer ········ 441
P-1157 DMC inhibits iCAF-mediated migration of esophageal squamous cell carcinoma cells via suppressing inf lammatory chemokines ········ 442
P-1158 The associations of nutritional indicators and immune cells in microenvironment in colorectal cancer pa tients ········ 443
P-1159 TNFR2 deficiency in M-MDSCs impairs immunosuppressive function in tumors ········ 444

### 16:45 - 17:30 I-P11-7:Tumor immune microenvironment/Tumor immune escape (3)
### Room P / (Motenashi Dome)

P-1168 Distribution analysis of cancer immune-related PUFAs using MSI in a mouse subcutaneous tumor model ········ 445
P-1169 Interaction of Notch1 mutation and PD-L1 modulates tumour-infiltrating CD8+ T cells in oral squamous cell carcinoma ········ 446
P-1170 Potentiation of PD-1 Blockade Efficacy via T Cell Activation Induced by Inhibition of Amino Acid Trans porter LAT1 ········ 447
P-1171 Effect of cancer cell-derived IL-6 on antitumor immune responses ········ 448
P-1172 The CCL17-CCR4 Axis Regulates TLS Formation and Anti-tumor Immunity with Prognostic Implications in PDAC ········ 449
P-1173 sTREM2-Bound Tumor Cell Debris Enhances Macrophage Phagocytosis in the TNBC Microe nvironment ········ 450
P-1174 Engineering a 3D Multilayered Tumor Model to Recapitulate Neutrophil Extracellular Trap-Coated Tumor M icroenvironment ········ 451
P-1175 Kasugamycin Derivatives: Modulating Chitinase Activity to Reprogram Immune Responses in the Tumor M icroenvironment ········ 452

### 16:45 - 17:30 I-P12-2:Liquid biopsy (2)
### Room P / (Motenashi Dome)

P-1184 Optimization of bile processing for liquid biopsy in cholangiocarcinoma focusing on ctDNA and protein st ability ········ 453
P-1185 Withdrawn ········ 454

### CONTENTS

P-1186 Exploring the Potential of Exosomal LAT1 as a Diagnostic Marker for Prostate Cancer Resistant to Standard Therapies ········ 455
P-1187 cfDNA-based mutational signature analysis reflects early carcinogenesis in esophageal squamous cell ca rcinoma ········ 456
P-1188 Utility of multi-body fluid integration analysis in liquid biopsy for pancreatic cancer. ········ 457
P-1189 Comparative Analysis of Extracellular Vesicle Surface Proteins Using Serum from Breast Cancer P atients ········ 458
P-1190 DNA extracted from peritoneal lavage fluid in Stage II/III colorectal cancer patients ········ 459

### 16:45 - 17:30 I-P13-1:Drug resistance (1)
### Room P / (Motenashi Dome)

P-1199 Signaling Pathway Analysis of PD-L1 Regulation in Drug-Resistant Lung Cancer Cells ········ 460
P-1200 Afatinib plus PEM and CBDCA overcome osimertinib resistance in EGFR-mutated NSCLC with high TSP-1 e xpression ········ 461
P-1201 Investigating the diversity of drug-tolerant persister cells in EGFR-Mutant NSCLC by single-cell c lones ········ 462
P-1202 CD74 antibody-drug conjugate eliminates targeted therapy-tolerant persisters in oncogenic kinase-driven l ung cancer ········ 463
P-1203 PLK1-driven cell cycle dynamics contribute to osimertinib resistance in EGFR-mutated non-small cell lung cancer ········ 464
P-1204 The role of METTL3 on osimertinib resistance in non-small cell lung cancer cells ········ 465
P-1205 Characterization of Drug-Tolerant Cells in EGFR-Mutant NSCLC Following Osimertinib Exposure ········ 466
P-1206 Suppression of drug resistance development based on cystine metabolism alteration in drug-tolerant per sister cells ········ 467
P-1207 AXL mediates intrinsic resistance to HER2 inhibitors via the SHC1BP-SHC1 pathway in HER2-aberrant ca ncers ········ 468

### 16:45 - 17:30 I-P13-3:Drug resistance (3)
### Room P / (Motenashi Dome)

P-1217 Analysis of lenvatinib resistance mechanisms to overcome its resistance in thyroid cancer ········ 469
P-1218 CYP17A1-HDAC6 Inhibitor Overcomes Temozolomide Resistance in Glioblastoma ········ 470
P-1219 Combinatorial drug strategies targeting histone deacetylases for the treatment of resistant gli oblastoma ········ 471
P-1220 AKT inhibition enhances the efficacy of androgen receptor-targeted therapy in mouse hormone-sensitive p rostate cancer ········ 472
P-1221 Emergence of Progenitor Cell-Like Features in a CD20 Antibody-Resistant Subpopulation of Relapsed Ma ture B-Cell Lymphoma ········ 473
P-1222 NSC-3852 synergistically enhances the cytotoxicity of olaparib via ROS-induced DNA damage. ········ 474
P-1223 DNA damage enhances tumor immunogenicity via the Src-STAT1 pathway ········ 475
P-1224 Targeting HSF4 enhanced the Efficacy of Cabozantinib and Immune Checkpoint Inhibitors in Renal Cell C arcinoma ········ 476
P-1225 Extracellular domain mutations alter sensitivity to small molecule FLT3 kinase inhibitors ········ 477

### 16:45 - 17:30 I-P14-2:Natural anticancer compounds (2)
### Room P / (Motenashi Dome)

P-1235 Flower extract of Kalanchoe pinnata potentiates Bax-activated apoptosis of cancer cells and cell c ycle arrest ········ 478
P-1236 Anti-tumor effect of the extracts from tea seed residue after oil extraction on gastrointestinal ca ncer. ········ 479
P-1237 Anticancer potentials of chaga and notoginseng against feline mammary tumor and canine hemangi osarcoma organoids ········ 480
P-1238 Propolis reduces the colorectal cancer stemness ········ 481
P-1239 D-allose-induced glycolytic inhibition associates with an interaction of D-allose 6-phosphate and P GI ········ 482
P-1240 Anti-tumor effects of diosmetin in cholangiocarcinoma cells via inhibition of cancer cell growth and mig ration ········ 483
P-1241 New Pyrazoline Derivatives as a Potential of Antiproliferative Against Breast Cancer ········ 484

### CONTENTS

P-1242 Regulation of Cancer Metabolism by Prodigiosin through Interaction with GLUT1 ·················· 485

### 16:45 - 17:30 I-P14-4:Synthetic anticancer compounds (2)
Room P / (Motenashi Dome)

P-1249 Preclinical Efficacy Assessment of Brincidofovir against Glioblastoma ······························ 486
P-1250 Antitumor Effects and Mechanisms of Benzaldehyde, a Fig-Derived Compound, in Human Mela noma Cells ························································································ 487
P-1251 Effects of siRNA on suppression of HPV16 E7 gene expression in cervical cancer ·················· 488
P-1252 Effects of the number of ethylene glycol units on the efficacy of novel complex I inhibitor 9bw ······· 489
P-1253 Single-molecule observation system to identify NCYM inhibitors ································ 490

### 16:45 - 17:30 I-P14-6:Anticancer drug sensitivity/Drug resistance (2)
Room P / (Motenashi Dome)

P-1264 Chemotherapy-induced inflammation in oral cancer tissues alters the tumor microenvironment via E-se lectin expression ········································································ 491
P-1265 Upregulation of ATF4 on LAT1 inhibition in pancreatic cancer cell lines ························· 492
P-1266 Exploring a Novel Treatment for Drug-Resistant Prostate Cancer Based on the Susceptibility to Cell Death ································································································ 493
P-1267 Withdrawn ··························································································· 494
P-1268 Decoding Spatiotemporal Complexity of the Tumor Microenvironment Driving Cancer Recurrence ···· 495
P-1269 Modeling Cellular Plasticity During Tumor Invasion and Drug Treatment in a Biomimetic 3D Pla tform ································································································ 496
P-1270 The Molecular Roles of Metformin: Potential to Reverse Docetaxel Resistance in Prostate Cancer C ells ································································································· 497
P-1271 Befotertinib restores chemosensitivity by inhibiting ABCB1-mediated drug efflux in resistant cancer cells ································································································ 498
P-1272 An epigenetic regulator drives regrowth of drug-tolerant persisters in colorectal cancer. ············· 499
P-1273 Fatty Acid Signaling Promotes EGFR-Dependent Metastasis and Cisplatin Resistance in Hyperlipi demia-associated HNSCC ··········································································· 500

### 16:45 - 17:30 I-P16-2:Radiosensitivity/Efficacy enhancement
Room P / (Motenashi Dome)

P-1279 Changes in Radiosensitivity During Neuronal Differentiation of Human iPS Cell-Derived Neural Progenitor Cells ································································································ 501
P-1280 SQAP, a Novel Radiation Sensitizer developed in Japan: From Veterinary Approval to Human Cancer The rapy Potential ······················································································ 502
P-1281 Effects of reoxygenation and fractionated X-ray irradiation on DNA damage and repair in osteosarcoma c ells ································································································· 503
P-1282 The early increase of long non-coding RNA SNHG12 expression after boron neutron capture the rapy ································································································ 504
P-1283 Fusion between Macrophages and Cancer Cells Contributes to Radiation-Induced Malignant Progression by Activating SIRPb ························································································ 505
P-1284 Investigation of IL27RA as a potential target for radiosensitization ······························ 506

### 16:45 - 17:30 I-P20-1:Immune Regulation & Tumor Microenvironment Dynamics in Cancer
Room P / (Motenashi Dome)

P-1293 A multi-task learning framework for TCR-antigen binding prediction guided by MHC-derived motif sign ature ································································································ 507
P-1294 Modeling Early Transcriptional Reprogramming of CD8+ T Cells in Melanoma Following Anti-CTLA-4 Im munotherapy ························································································ 508
P-1295 Mapping TLS Architecture Across Cancer Types: Key Roles of Dendritic Cells and Chemokines in Their Fo rmation ······························································································ 509
P-1296 Mutation-Driven Heterogeneity of Tumor Microenvironment: Insights into Cancer Immune Evasion Mec hanisms ······························································································ 510
P-1297 Elucidation of drug resistance mechanisms in multiple myeloma by single-cell meta-analysis ········ 511
P-1298 Machine Learning-Based Identification of Metastasis-Associated Genes in Normal Adjacent Tissue (NAT) ································································································· 512

### CONTENTS

P-1299 Development of a Transformer-based Cell Type Deconvolution Model ........ 513
P-1300 Evolutionary significance of endocytosis in CAF-TAM heterotypic nucleation: Insights from multi-agent si mulation ........ 514

16:45 - 17:30 I-P20-3:AI & Computational Modeling for Cancer Prognosis & Personalization
Room P / (Motenashi Dome)

P-1310 A study on the treatment of continuous variables in statistical risk models predicting cancer treatment ou tcomes ........ 515
P-1311 Virtual staining of fresh tissues with generative AI models ........ 516
P-1312 Development of a CNN-Cox Integrated Model for Predicting Multiple Drug Responses in Multiple Myeloma Treatment ........ 517
P-1313 Pharmacokinetic Evaluation of a Lymphatic Drug Delivery System(LDDS) Targeting Clinically Occult N0 L ymph Nodes ........ 518
P-1314 Culturing personalized tumor cells in computer for predictive cancer genome medicine ........ 519
P-1315 Comparative modeling of HER2 signaling dynamics in breast cancer cells and iPS cell-derived cardio myocytes ........ 520
P-1316 The integration of pathology images and multi-omics data to improve cancer prognosis prediction and ex planation ........ 521
P-1317 Generating Patient Documents from Electronic Health Records Using Generative Artificial Inte lligence ........ 522

16:45 - 17:30 II-P2-1:Colorectal cancer (1)
Room P / (Motenashi Dome)

P-1324 Withdrawn ........ 523
P-1325 ARID5A promotes colorectal cancer tumorigenesis via STAT3 and YAP activation ........ 524
P-1326 TRPV2 has an oncogenic role in colon cancer ........ 525
P-1327 Clinicopathological analyses of colorectal cancer with neuroendocrine differentiation ........ 526
P-1328 Functional and clinical significance of Anoctamin 5 in colon cancer ........ 527
P-1329 Regulation of colon cancer cell functions through LPA receptor signaling activation in cobalt chlori de-induced hypoxia ........ 528
P-1330 Association Between Iron Accumulation by Colorectal Cancer Drugs and the Synergistic Effect of Pter ostilbene ........ 529

16:45 - 17:30 II-P2-3:Gastric cancer
Room P / (Motenashi Dome)

P-1338 Withdrawn ........ 530
P-1339 Neutrophil extracellular traps contributes to peritoneal and liver metastasis of gastric cancer in diabetic c onditions ........ 531
P-1340 Selective dual inhibition of MET and VEGFR2 as a therapeutic strategy for gastric cancer with malignant a scites ........ 532
P-1341 Distinct modulation of gastric cancer cell function via LPA receptor signaling under CoCl2-induced and 1% oxygen hypoxia ........ 533
P-1342 Spatial Heterogeneity of CLDN18.2 Expression and Its Association with Clinicopathological Factors in Ga stric Cancer ........ 534
P-1343 Fascin1 as a potential therapeutic target for scirrhous gastric cancer ........ 535
P-1344 Spatial Transcriptomic Profiling Reveals Niche-Specific Interactions in Early Gastric Cancer Microen vironment ........ 536
P-1345 Identification of critical oncogenic genes and super enhancers by CRISPRi Screening ........ 537
P-1346 Withdrawn ........ 538

16:45 - 17:30 II-P3-1:Hepatocellular cancer
Room P / (Motenashi Dome)

P-1349 Investigation of Congestive Liver Microenvironment on Hepatocellular Carcinoma ........ 539
P-1350 Restoring Intestinal-LSEC Barriers Prevents Metabolic Dysfunction-Associated Steatohepatitis and Liver C ancer ........ 540
P-1351 Transmembrane Protein 206 is a candidate driver gene for hepatocellular carcinoma ........ 541
P-1352 Identification of a Novel Therapeutic Target Gene TONSL in Hepatocellular Carcinoma ........ 542

### CONTENTS

P-1353 Molecular biological significance of interleukin-11 in hepatocellular carcinoma ····· 543
P-1354 Withdrawn ····· 544
P-1355 Withdrawn ····· 545
P-1356 Withdrawn ····· 546

16:45 - 17:30 II-P3-3:Pancreatic cancer & endocrine tumor
Room P / (Motenashi Dome)
P-1364 p62 can be a useful for prognostic biomarker for the patients of pancreatic neuroendocrine tum ors. ····· 547
P-1365 Hypoxic Primary Culture Identified Proliferating CAFs in Pancreatic Cancer: Distinct Growth and Spatial F eatures ····· 548
P-1366 ECM1 and KRT6A Mediate Dexamethasone-Induced Tumor Progression and Chemoresistance in pancreatic cancer ····· 549
P-1367 Elucidation of the mechanism of pancreatic cancer malignant transformation by stabilization of modified p assenger miRNAs ····· 550
P-1368 RON kinase regulates HIF-1a-driven invasiveness in PDAC: Implications for targeted therapy ····· 551
P-1369 Rap1b regulates cell cycle progression and proliferation in pancreatic cancer cells ····· 552
P-1370 Glucose-Induced Suppression of the STING Pathway Impairs Innate Immunity via Autophagy in Pancreatic Cancer ····· 553

16:45 - 17:30 II-P4-2:Head & neck tumors (2)
Room P / (Motenashi Dome)
P-1380 MTDH as a cancer fitness gene driving metastasis in oral squamous cell carcinoma ····· 554
P-1381 Improving risk stratification for oropharyngeal cancer by evaluating initial diagnosis risk of distant met astasis ····· 555
P-1382 Withdrawn ····· 556
P-1383 Establishment of a Salivary Gland Tumorigenesis Model in salivary gland-specific EWSR1::ATF1 Transgenic Mice ····· 557
P-1384 PLAU as a Prognostic Biomarker Based on Transcriptomic Analysis of P. gingivalis-Infected Head and Neck Cancer Cells ····· 558
P-1385 Elucidation of epigenetic mechanisms underlying carcinogenesis in maxillary sinus carcinoma ····· 559
P-1386 Integrated analysis of immune cells and fibroblasts in head and neck squamous cell carcinoma ····· 560
P-1387 Expression of CD74 and macrophage migration inhibitory factor in nasopharyngeal carcinoma ····· 561
P-1388 In HNSCC, Trop2 is a marker of cancer stem cells and CD73 regulates metastasis. ····· 562

17:30 - 18:15 I-P1-2:Animal models for cancer (2)
Room P / (Motenashi Dome)
P-1008 LPO-caused cancer initiation to transform hepatocytes into GST-P(+) preneopastic single hepatocytes in rat livers ····· 563
P-1009 Early Gene Expression Alterations in DMH-Induced Rat Colon Carcinogenesis ····· 564
P-1010 Genomic mutation profiling of hepatocellular carcinoma induced by heavy-ion radiation in B6C3F1 mice ····· 565
P-1011 Mutational analysis of forestomach tumors induced by benzo[a]pyrene in wild-type and Pol&kappa ;-deficient mice ····· 566
P-1012 Gene mutation analysis of amitrole-induced mouse thyroid benign tumor cell lines ····· 567
P-1013 The mechanism of leukemia development after radiation exposure in Pax5 heterozygous mice ····· 568
P-1014 Metabolic signature of diethylnitrosamine-induced hepatocellular carcinoma development and sorafenib r esistance in rats ····· 569

17:30 - 18:15 I-P2-2:Animal model (2)
Room P / (Motenashi Dome)
P-1023 Pak1 Deficiency in CD11c-positive Cells Suppresses Skin Carcinogenesis ····· 570
P-1024 Analysis of systemic exacerbation mechanisms linked to abnormal brain histamine pathways in c ancer ····· 571
P-1025 The proto-oncogene WWP1 promotes pancreatic tumorigenesis via acinar-to-ductal metaplasia ····· 572
P-1026 CblQ367P cooperates with loss of Tet2 to drive a CNL-like disease via the NF-κB pathway ····· 573

### CONTENTS

P-1027 Antitumor effects and mechanisms of lenvatinib plus anti-PD-1 antibody in a novel endometrial cancer mo del. ........ 574
P-1028 Pathological and molecular analyses of a newly established cancer cachexia mouse model ........ 575
P-1029 Identification of genetic modifiers of Pak1 identified using a Japanese wild-derived inbred mouse strain, M SM/Ms ........ 576
P-1030 Liver-specific DICER1 syndrome model mice develop cystic liver tumors with defective primary c ilia ........ 577

17:30 - 18:15 I-P3-1:Viral Oncology: Basic
Room P / (Motenashi Dome)
P-1039 Characterization of high-risk HPVs through E6-associated chromosomal abnormalities ........ 578
P-1040 Elucidation of chromatin interaction between HPV-hybrid extrachromosomal DNA and host genome in head and neck cancer ........ 579
P-1041 Sustained expression of intact ift81 mRNA despite intron-oriented HTLV-1 integration in an ATL c ell line ED ........ 580
P-1042 The identification of a novel viral factor that contributes to EBV-driven transformation ........ 581
P-1043 Establishment and analysis of a novel post-SVR HCC model by manipulating the expression of Core J1 in m ouse liver ........ 582
P-1044 HPV18-related cancer model development and its application in the efficacy evaluation of a saRNA cancer vaccine ........ 583

17:30 - 18:15 I-P4-1:RAS-RB-E2F axis
Room P / (Motenashi Dome)
P-1051 Polo-like kinase 1 expression in colorectal cancer: association with RAS mutations ........ 584
P-1052 Enhancement of deregulated E2F1 activity enables induction of apoptosis in cancer cells via p53-in dependent pathways ........ 585
P-1053 WDR1 functions as a coactivator of E2F1 in the nucleus and enhances tumor suppressive E2F1 a ctivity ........ 586
P-1054 Deregulated E2F activity out of control by pRB discriminates cancer cells from normal growing c ells ........ 587
P-1055 Knockdown of Gene Y, Induced by Mutant KRAS, Inhibits Growth of Lung Cancer Cells ........ 588
P-1056 Functional analysis of BRAF V600E-induced protein kinase in colorectal cancer ........ 589
P-1057 Comprehensive and Rapid Identification of Driver Mutations via Single-Nucleotide Random Mutagenesis of Target Genes ........ 590

17:30 - 18:15 I-P4-3:Oncogenes & tumor-suppressor genes (2)
Room P / (Motenashi Dome)
P-1067 The role of TGF-β/Smad3/Smad4 in cancer immune evasion through suppression of STING ........ 591
P-1068 SPZ1-STING Axis Regulates Macrophage Polarization in the Lung Tumor Microenvironment ........ 592
P-1069 Targeting oxidative and immune signaling to suppress lymphovascular invasion in urothelial ca rcinoma ........ 593
P-1070 CD47-ISX Axis Triggers Inflammasome Activation, Modulates Macrophage Polarization, and Tumor-P romoting Microenvironment ........ 594
P-1071 Formaldehyde Exposure Activates the RUNX1-ISX Signaling Axis to Promote Oncogenic Activity in B-ALL ........ 595
P-1072 ISX Axis Drives Bone Marrow Remodeling, Macrophage Polarization, and Cancer Stemness in BC P-ALL ........ 596
P-1073 Functional characterization of an endogenous retroviral gene expressed in melanoma ........ 597
P-1074 The FAM72A-ETV4 Complex Mediates HBx Signaling to Promote Oncogenic Activities and Accelerate HCC Progression ........ 598
P-1075 The tumor suppressive function of MTSS1 and its potential mechanism ........ 599

17:30 - 18:15 I-P5-2:Regulation of gene expression
Room P / (Motenashi Dome)
P-1086 Epigenomic Regulation of Enhancers Characteristic of ER-Positive Breast Cancers ........ 600
P-1087 Inhibition of LRRC8A overcomes chemoresistance in the 3D spheroid model of breast cancer cells ........ 601

### CONTENTS

P-1088 YAP1-2α isoform, fosters the formation of LLPS-mediated super-enhancers that increase cancer cell malignancy ... 602
P-1089 Analysis of the expression mechanism and molecular function of an oncogenic ERK-inducible tetraspanin p rotein in cancer ... 603
P-1090 Liver cancer-specific repression of amino acid metabolism-related genes by the Wnt/β-catenin sig naling ... 604
P-1091 The Role of a Hormone-Independent Novel GREB1 Transcript ... 605
P-1092 Analysis of Expressional Alterations of HER2 and Associated Phenotypes in Breast Cancer Cell Li nes ... 606
P-1093 Investigating the Impact of KIF12 Motor Protein on Extracellular Vesicle Phenomenon ... 607
P-1094 Suppression of EWS-FLI1 function by competitive inhibition of its binding to enhancer elements in Ewing s arcoma cells ... 608

17:30 - 18:15 I-P9-2:Metastasis & EMT (2)
Room P / (Motenashi Dome)
P-1102 TGFβ1 induces Vasorin/slit-like2 protein and accelerates cellular migration in gastric cancer cells ... 609
P-1103 The role of INHBA in the EMT Induction of Oral squamous cell carcinoma ... 610
P-1104 RUNX3-MYC-p53 Axis: A Critical Regulatory Node of Cancer Metastasis and Therapeutic Targeting ... 611
P-1105 MRC2 promotes gastric cancer malignancy through SCD-1-mediated modulation of epithelial-mesenchymal transition ... 612
P-1106 Mapping Tumor Metastasis and EMT-Associated Immune Changes in HNSCC Through Spatial Transc riptomic Analysis ... 613
P-1107 Elucidation of how the immunosuppressive microenvironment promotes peritoneal dissemination and its t herapeutic targets ... 614
P-1108 MCE Suppresses Oral Cancer Metastasis by Inhibiting EMT through the β-Catenin/NOTCH1 pathways. ... 615

17:30 - 18:15 I-P10-2:Extracellular vesicles/Exosome (2)
Room P / (Motenashi Dome)
P-1114 Assessment of Surface PD-L1 Expression in Small Extracellular Vesicles using High-Speed Atomic Force M icroscopy ... 616
P-1115 FMR1-Mediated Selective Loading of Tumor-Promoting microRNAs into Extracellular Vesicles ... 617
P-1116 Ouabain Selectively Inhibits Extracellular Vesicle Secretion in Cancer Cells and Suppresses Their g rowth ... 618
P-1117 Extracellular vesicles-containing Regucalcin suppress tumor development by suppress M2-like m acrophage. ... 619
P-1118 Withdrawn ... 620
P-1119 Withdrawn ... 621

17:30 - 18:15 I-P11-2:Antitumor immune response (1)
Room P / (Motenashi Dome)
P-1126 The difference in the affinity of antigenic peptides affects the antitumor effect of T-cell adoptive immun otherapy ... 622
P-1127 High expression of HNF1A in cancer cells promotes anti-tumor immunity ... 623
P-1128 Anti-tumour effects of Naringin are due to the enhancement of the cross-presentation ability of antig en-presenting cells ... 624
P-1129 Identification of a Novel Immunoregulatory CD86high CD8 T Cell Subset in the Tumor Micro environment ... 625
P-1130 Differential Expression of Regulatory T cells Markers in lymphocytes of Oral Cancer Patients ... 626
P-1131 Antifolate pemetrexed activates innate immunity through STING regulation ... 627
P-1132 Peptide modulation overrides glycan synergy in gold nanoparticle-based vaccines for cancer immuno therapy ... 628
P-1133 Investigation of nivolumab-induced anti-tumor immune responses in a humanized NOG-Fc&gamm a;R-/-mouse model ... 629

### CONTENTS

### 17:30 - 18:15 I-P11-4:Antitumor immune response (3)
### Room P / (Motenashi Dome)

P-1143 Expression analysis of representative regulatory T-cell (Treg) targeting candidate molecules in renal cell c arcinoma ........ 630
P-1144 Establishment of murine NK cell line having cytotoxicity and capability for genetic modification ........ 631
P-1145 Method to know patient-wise tumor-immune state making model in detail by sensitivity from added IL2, etc. for cure ........ 632
P-1146 Potential Novel Mesothelioma Therapy by Targeting VISTA ........ 633
P-1147 Relationship between HLA allelic polymorphism and prognosis in pan-cancer with immune-associated mu tations ........ 634
P-1148 In vitro analysis of interaction between patient-derived organoids and tumor-specific T cells in renal cell c arcinoma ........ 635
P-1149 Immunological characteristics of hyper-differentiated melanoma cells ........ 636
P-1150 Expression of asialo-GM1 identifies the distinct subpopulation of NK cell lineage ........ 637

### 17:30 - 18:15 I-P11-6:Tumor immune microenvironment/Tumor immune escape (2)
### Room P / (Motenashi Dome)

P-1160 The Innate Immune System Activated by Temozolomide Predicts Response to Immune Checkpoint Inhi bitors in Glioblastoma ........ 638
P-1161 Effects of Photodynamic Therapy Using Novel Light-Sensitive Substances on Tumor-Associated Macro phages ........ 639
P-1162 Pan-cancer Immune Profiling Across Solid Cancers by Flow Cytometry ........ 640
P-1163 Restoring immunosuppressive tumor microenvironment by targeting HNF1beta in ovarian clear cellc arcinoma (OCCC) ........ 641
P-1164 Inhibition of TGFβ Type I Receptor Prevents M2 Polarization of TAMs and Suppresses Colorectal Ca ncer Progression ........ 642
P-1165 Prognostic Significance of PDGFC and its role in myCAF-iCAF interactions in Pancreatic Ductal Adeno carcinoma ........ 643
P-1166 Tumor microRNA induces regulatory T cells and affects their in vivo growth ........ 644
P-1167 RIG-I-Driven Neutrophil Activation Induces Anti-Tumor Efficacy and Mitigates Immune Checkpoint Therapy Resistance ........ 645

### 17:30 - 18:15 I-P12-1:Liquid biopsy (1)
### Room P / (Motenashi Dome)

P-1176 Circulating tumor DNA analysis based on CAPP-seq in patients with metastatic cutaneous angi osarcoma ........ 646
P-1177 Ultrasensitive measurement of HPV16 E6/E7 proteins in cervical cancer ........ 647
P-1178 Development of a machine learning model for ovarian cancer using methylation liquid biopsy ........ 648
P-1179 Ongoing Efforts for Long-Term Expansion of Circulating Tumor Cells (CTCs) ........ 649
P-1180 Potential role of circulating tumor cells and cell-free DNA as biomarkers for metastasis in oral squamous c ell carcinoma ........ 650
P-1181 Preliminary Use of a New Hybrid-Capture Panel for Liquid Biopsy of Bile cfDNA in Biliary Tract C ancer ........ 651
P-1182 Proteomic exploration of new pharmacodynamic biomarkers of tankyrase inhibitors ........ 652
P-1183 Serum exosomal BCYRN1 as a potential biomarker and therapeutic target in renal cell cancer ........ 653

### 17:30 - 18:15 I-P12-3:Genomic diagnostics / Panel sequencing
### Room P / (Motenashi Dome)

P-1191 Li-Fraumeni syndrome caused by a 3.6 kb deletion in the TP53 gene suggested by additional data from the NCC Oncopanel ........ 654
P-1192 Effects of a negative-pressure fixation method on nucleic acid preservation in FFPE tissue samples ........ 655
P-1193 Verification of the effectiveness of technology to improve the quality of library preparation for p anel testing ........ 656
P-1194 Detection of gene fusions in rare tumor cases ........ 657
P-1195 Comparison of genomic and clinical features of endometrial carcinoma between common and rare muta tions detected in POLE ........ 658

### CONTENTS

P-1196 Automated NGS and dPCR for Malignant Lymphoma in a Community Setting: Use of the Liverpool Lymphoid Network Panel ........ 659
P-1197 The benefit and limitation of Comprehensive Cancer Genome Panel Testing in Rural Areas ........ 660
P-1198 Novel pathogenic variants of HBOC that were not reported by a genetic test, BRACAnalysis covered by National Insurance ........ 661

### 17:30 - 18:15 I-P13-2:Drug resistance (2)
### Room P / (Motenashi Dome)

P-1208 Gene alteration of SKI gene affects ALK-TKI sensitivity in ALK-positive lung cancer ........ 662
P-1209 Analyses of ROS1-rearranged lung adenocarcinoma cell lines with acquired resistance to entrectinib and repotrectinib ........ 663
P-1210 STAT3 inhibition overcomes apoptotic resistance to KRAS-G12C inhibitors ........ 664
P-1211 Approaches to Elucidate Mechanisms of Resistance to KRAS Inhibitors ........ 665
P-1212 KRAS inhibitor tolerant cancer cells utilize glutamine metabolism for survival ........ 666
P-1213 Genome-wide CRISPR-Cas9 screen identifies genes involved in acquired KRAS G12C inhibitor resistance. ........ 667
P-1214 TDO2 plays a role in mediating Lenvatinib resistance in hepatocellular carcinoma. ........ 668
P-1215 Genome-wide CRISPR-Cas9 screen identifies genes conferring Lenvatinib resistance in hepatocellular carcinoma ........ 669
P-1216 Exploring the role of YAP in BRAF inhibitors resistance in Melanoma ........ 670

### 17:30 - 18:15 I-P14-1:Natural anticancer compounds (1)
### Room P / (Motenashi Dome)

P-1226 Harnessing Nature's Arsenal: Targeting Aggressive Cancers with Bioactive Natural Compounds ........ 671
P-1227 Withdrawn ........ 672
P-1228 Withdrawn ........ 673
P-1229 Withdrawn ........ 674
P-1230 Senna garrettiana extract inhibits hepatocellular carcinoma through antiproliferation and apoptosis induction ........ 675
P-1231 E413-YJ, a Flavonoid Phytochemical, Exhibits Anti-Leukemic Effects in Human AML Cells and Zebrafish Xenograft Models ........ 676
P-1232 Capsaicin: A Natural Therapeutic Agent Targeting Epstein-Barr Virus-Associated Epithelial Cancers ........ 677
P-1233 Withdrawn ........ 678
P-1234 Antitumor Effect of the Natural Compound Calebin A in Pancreatic Cance ........ 679

### 17:30 - 18:15 I-P14-3:Synthetic anticancer compounds (1)
### Room P / (Motenashi Dome)

P-1243 Inhibition of cell-intrinsic NF-κB activity and metastatic abilities of breast cancer by curcumin analogs ........ 680
P-1244 A potential indole compound for Glioblastoma therapy ........ 681
P-1245 ATF3 induces effects on Castration-resistant Prostate cancer and Olaparib-resistant cells ........ 682
P-1246 Withdrawn ........ 683
P-1247 Withdrawn ........ 684
P-1248 Antitumor effects of p300-targeting curcumin derivatives on TNBC patient-derived organoids ........ 685

### 17:30 - 18:15 I-P14-5:Anticancer drug sensitivity/Drug resistance (1)
### Room P / (Motenashi Dome)

P-1254 Investigation of survival mechanisms of doxorubicin tolerant persister cells in triple-negative breast cancer ........ 686
P-1255 Attenuation of SLC46A3 through methylation confers resistance to T-DM1 in gastric cancer N87 cells ........ 687
P-1256 Dissecting Chemotherapy Resistance in Gastric Cancer at Single-cell Resolution Using Orthotopic Syngeneic Mouse Model ........ 688
P-1257 Activated mitophagy endows hepatocellular carcinoma with resistance to lenvatinib ........ 689
P-1258 Identification of HIF-1+ M2-type macrophages as a chemoresistance niche component in ovarian clear cell carcinoma ........ 690

### CONTENTS

P-1259 Reprogramming pancreatic stellate cells toward a quiescent-like state via hTCF21 overexpression in vitro. ... 691
P-1260 Overcoming Chemoresistance in Glioblastoma through Cell Death Induced by Lysosomal Membrane Per meabilization ... 692
P-1261 Development of A3 Receptor Ligands as Modulators of the Multidrug Transporter ABCG2 ... 693
P-1262 Mechanism of therapy resistance mediated by the TFE3-lysosome axis in glioblastoma ... 694
P-1263 Identification of novel therapeutic targets for radioresistant OSCC focusing on tumor suppressor CYLD ex pression ... 695

17:30 - 18:15 I-P16-1:Radiation & particle therapy
Room P / (Motenashi Dome)
P-1274 GM-CSF mediates immune regulation after BNCT by activating M1 macrophages ... 696
P-1275 Evaluation of radiohalogenated anti-ADAM8 antibodies as theranostic agents for pancreatic cancer ... 697
P-1276 The radiosensitivity of tumors is predominantly determined by cancer cells, not stromal cells. ... 698
P-1277 Evaluation of Early and Late Effects of BNCT in an Immunocompetent Mouse Model of Pelvic Recurrent C olorectal Cancer ... 699
P-1278 Current BNCT & Precision BNCT based on Novel boron drug discovery strategies ... 700

17:30 - 18:15 I-P16-3:RI therapy/Others
Room P / (Motenashi Dome)
P-1285 CD154 change in peripheral blood after PRRT in NET patients ... 701
P-1286 Novel Carbonic Anhydrase IX targeting 64Cu- and 225Ac-PD-32766 are promising theranostic peptides for ccRCC and CRC ... 702
P-1287 Development of targeted alpha therapeutics aimed at eliminating drug resistant cancers ... 703
P-1288 PD-29875 is a promising radiotheranostic peptide targeting Claudin 18.2 for gastric and pancreatic can cers. ... 704
P-1289 Development of GPC3 antibody targeted delivery of isotope as a theranostic and diagnostic radionuclide p latform for HCC ... 705
P-1290 Changes in Mesorectal CT Values and Histological Effect By Preoperative Treatments for Rectal C ancer ... 706
P-1291 P53-independent bystander effects via secreted factors between carbon-ion irradiated tumor and unir radiated normal cells ... 707
P-1292 Assessing the potential of treating neuroblastoma by an isotopic compound ... 708

17:30 - 18:15 I-P20-2:Frontiers in RNA Structure & Spatial Transcriptomics
Room P / (Motenashi Dome)
P-1301 Augmented Transcript-Level Quantification Enhances Tumor scRNA-seq Analysis ... 709
P-1302 Optimal detection of allele-specific copy number alterations and haplotype swapping from short-read se quences data ... 710
P-1303 Identifying copy number alterations from RNA-Seq data and evaluating their confidence ... 711
P-1304 Unsupervised annotation of spatial transcriptomes based on vectorial information between tra nscripts ... 712
P-1305 Resolving cancer-associated isoform landscapes with epitranscriptomic signatures ... 713
P-1306 Comprehensive Analysis of RNA Modifications in Coding and Non-Coding Transcripts Using Nanopore Se quencing ... 714
P-1307 Mapping epigenomic states defining cancer cell states by integrating public single-cell datasets ... 715
P-1308 Mining spatial transcriptomics datasets using DeepSpaceDB ... 716
P-1309 Evaluation of copy number alteration detection tools using whole-genome sequence data ... 717

17:30 - 18:15 I-P20-4:Understanding Cancer Outcomes through Real-World & Multi-Factorial Analysis
Room P / (Motenashi Dome)
P-1318 Differences in the genome structure of Akkermansia muciniphila between elderly people in Ogimi Village a nd patients ... 718
P-1319 Study on the Impact of Co-mutations on Survival in Patients with Biliary Tract Cancer using real-world data in Japan ... 719
P-1320 Data Visualizer for Proteome: Software for Interactive Analysis of Olink Proteomic Data ... 720
P-1321 Multi-omics analysis of cancers utilizing alternative lengthening of telomeres ... 721

### CONTENTS

P-1322 Interrupt time series analysis for psychiatric diseases on cancer survivors using real world data in Japan ······ 722
P-1323 Prognostic value of perineural invasion in head and neck squamous cell carcinoma: A systematic review and meta-analysis ······ 723

17:30 - 18:15 II-P2-2:Colorectal cancer (2)
Room P / (Motenashi Dome)
P-1331 Functional analysis of Galectin-4 in colorectal cancer cells ······ 724
P-1332 Dual inhibition of OXPHOS and glycolysis enhances antitumor effects by blocking metabolic shift in col orectal cancer ······ 725
P-1333 Oncogenic role of EEPD1 via protein kinase B phosphorylation in colorectal cancer ······ 726
P-1334 Low-molecular-weight hyaluronan may cause vascular hypoplasia in the Immature-type stroma of colo rectal cancer (CRC) ······ 727
P-1335 Genome-wide DNA methylation profiling of colitis-associated and sporadic colorectal cancer using FFPE t issues ······ 728
P-1336 Synergistic Antitumor Effects of Aspirin and Metformin in Colorectal Cancer Organoids ······ 729
P-1337 Sex-Dependent Prognostic Significance of CD99 in Tumor Cells and Cancer-Associated Fibroblasts in Col orectal Cancer ······ 730

17:30 - 18:15 II-P2-4:Eophageal cancer
Room P / (Motenashi Dome)
P-1347 Survival and recurrence characteristics of PCR in esophageal cancer after neoadjuvant immunoche motherapy ······ 731
P-1348 Evaluation of Factors Influencing the Efficacy of Preoperative HIMAC Therapy in Esophageal Squamous Cell Carcinoma ······ 732

17:30 - 18:15 II-P3-2:Biliary tract cancer, pancreatic cancer
Room P / (Motenashi Dome)
P-1357 The Functional Role of the SWI/SNF Complex Subunit BRG1 in Biliary Carcinogenesis ······ 733
P-1358 Expression and functional analysis of PODXL2 in intrahepatic cholangiocarcinoma cell lines ······ 734
P-1359 Development of a Novel Cholangioscopy System for 5-ALA based Photodynamic Diagnosis of Cholan giocarcinoma ······ 735
P-1360 Hilar Cholangiocarcinoma Treated with Chemoimmunotherapy and Conversion Surgery ······ 736
P-1361 Prognostic Value of Fibrosis in Metastatic Lymph Nodes After NAC and Surgery for Resectable Pancreatic Cancer ······ 737
P-1362 The role of Actinin-4 and its phosphorylation in pancreatitis and pancreatic cancer ······ 738
P-1363 Surfaceome-Based therapeutic Target Discovery in Pancreatic Ductal Adenocarcinoma. ······ 739

17:30 - 18:15 II-P4-1:Head & neck tumors (1)
Room P / (Motenashi Dome)
P-1371 Therapeutic targeting of STAT3 with BBI608 induces apoptosis in oral squamous cell carcinoma ······ 740
P-1372 Withdrawn ······ 741
P-1373 Transcriptome Analysis Identifies a Prognostic Gene Signature and Therapeutic Target in Oral Squamous C ell Carcinoma ······ 742
P-1374 Analysis for antitumor effect of Src inhibitors and their combination with cisplatin in oral squamous cell c arcinoma ······ 743
P-1375 Identification of bone marrow stem cell-derived oral squamous cell carcinoma and its verification in an an imal model ······ 744
P-1376 Cytotoxic T cell density and PD-L1 expression as predictors of anti-PD1 therapy in recurrent OSCC ······ 745
P-1377 Establish of FAT1 knockout cell lines for functional analyses in oral squamous cell carcinoma. ······ 746
P-1378 Abnormal promoter methylation of smoking-related genes in oral squamous cell carcinoma and oral pre cancerous lesions ······ 747
P-1379 Effect of free fatty acids on the proliferation of oral squamous cell carcinoma: Mechanism of cell death in duced by FFAs ······ 748

### CONTENTS

### September 26 (Fri)

8:00 - 8:50 EL1:Frontiers in the clinical and basic research of KRAS mutant cancer
Room 3 / (Hotel Nikko Kanazawa 4F Tsuru A)
EL1 Clinical and Basic Research of KRAS-Mutant Cancers: History and future ······ 749

8:00 - 8:50 EL2:Elucidation of immune escape mechanisms and development of novel therapeutic strategies
Room 4 / (Hotel Nikko Kanazawa 4F Tsuru B)
EL2 Elucidation of immune escape mechanisms and development of novel therapeutic strategies ······ 750

8:00 - 8:50 EL3:Gut microbiota and cancer
Room 5 / (Hotel Nikko Kanazawa 4F Tsuru C)
EL3 Gut microbiota and cancer ······ 751

8:00 - 8:50 ML:Morning Lecture
Room 8 / (Hotel Nikko Kanazawa 3F Kujaku C)
ML How to write an engaging scientific paper in English ······ 752

8:00 - 8:50 EL4:AI is opening up new frontiers in cancer research and medicine
Room 11 / (ANA Crowne Plaza Hotel Kanazawa 3F Ohtori (West))
EL4 Research trends in medical AI and efforts toward clinical application ······ 753

8:00 - 8:50 EL5:Cancer Heterogeneity, Stem Cell Regulation, and Treatment Resistance
Room 12 / (ANA Crowne Plaza Hotel Kanazawa 3F Ohtori (Central))
EL5 Cancer Heterogeneity, Stem Cell Regulation, and Treatment Resistance ······ 754

9:00 - 11:30 AACR2:Spatio-temporal regulation of cancer phenotypes by cancer epigenetics
Room 1 / (Ishikawa Ongakudo 2F Concert Hall)
AACR2-1 Long Non-Coding RNAs: Illuminating Their Biological Functions and Potential as Novel Cancer Thera peutics ······ 755
AACR2-2 Role of histone modifications in transcription, differentiation and disease ······ 756
AACR2-3 Epimutation burden in stem cells precisely predicts gastric cancer risk ······ 757
AACR2-4 EPIGENETICS: A GATEKEEPER TO GENERATING EXTRACHROMOSOMAL DNA AMPLIFICATIONS & REA RRANGEMENTS ······ 758

9:00 - 10:40 YSA:Canser Sicience　Young Scientists Awards Lectures
Room 2 / (Ishikawa Ongakudo 2F Hogaku Hall)
YSA-1 Discovering cancer stem-like molecule, nuclear factor I X, using spatial transcriptome analysis in gastric ca ncer ······ 759
YSA-2 Circulating KRAS Mutations as Predictive Biomarkers for Targeted Therapy in Pancreatic Cancer ······ 760
YSA-3 Long noncoding RNA TUG1 promotes cisplatin resistance in ovarian cancer via upregulation of DNA pol ymerase eta ······ 761
YSA-4 Exploring the tumor microenvironment of colorectal cancer patients post renal transplantation by single-cell analysis ······ 762

9:00 - 11:30 S3:Frontiers in the clinical and basic research of KRAS mutant cancer
Room 3 / (Hotel Nikko Kanazawa 4F Tsuru A)
S3-1 Basic and clinical research on KRAS mutant lung cancer ······ 763
S3-2 Basic and clinical research on KRAS mutant pancreatic cancer ······ 764
S3-3 ASP3082, a First-in-Class KRAS G12D Degrader: Nonclinical Efficacy and Early Clinical Evidence ······ 765
S3-4 ASP3082, a First-in-Class KRAS G12D Degrader: Nonclinical Efficacy and Early Clinical Evidence ······ 766
S3-5 The mechanisms of reprogramming tumor immune microenvironment by KRAS inhibitor ······ 767

### CONTENTS

S3-6 Novel CRISPR-based treatment strategy for KRAS mutant cancer ········ 768
S3-7 KRAS-targeted therapy via splicing modulation using antisense oligonucleotides ········ 769

### 9:00 - 11:30 S4:Elucidation of immune escape mechanisms and development of novel therapeutic strategies

### Room 4 / (Hotel Nikko Kanazawa 4F Tsuru B)

S4-1 Regulatory mechanisms of tumor-infiltrating innate immune cells ········ 770
S4-2 TBD ········ 771
S4-3 Identifying the mechanism of acquired resistance against cancer immunotherapy targeting innate i mmunity ········ 772
S4-4 Mitochondrial Transfer in the Tumor Microenvironment ········ 773
S4-5 Single Cell Profiling of Tregs: Implications for Tumor Immunity and Cancer Therapy ········ 774
S4-6 Interplay Between Gut Microbiota and the Tumor Immune Microenvironment ········ 775

### 9:00 - 11:30 S5:Gut microbiota and cancer

### Room 5 / (Hotel Nikko Kanazawa 4F Tsuru C)

S5-1 Colibactin, a toxin produced by intestinal bacteria, is involved in colorectal carcinogenesis ········ 776
S5-2 Colorectal cancer risk in familial adenomatous polyposis (FAP): The Impact of the Gut Microbiome ········ 777
S5-3 Effect of Fusobacterium infection on ovarian cancer progression ········ 778
S5-4 Innate lymphoid cells in tumor immunity and therapeutic potential ········ 779
S5-5 An Enterococcus phage-derived lytic enzyme suppresses graft-versus-host disease ········ 780
S5-6 Development of a Catalog of Anticancer Drug Effects on the Human Gut Microbiome Across Cancer T ypes ········ 781

### 9:00 - 10:15 I-E1:DNA damage response & carcinogenesis

### Room 6 / (Hotel Nikko Kanazawa 3F Kujaku A)

E-2001 Identification of novel regulator of ATR inhibitor sensitivity and splicing vulnerability in prostate c ancer ········ 782
E-2002 Lung carcinogenicity of single-walled carbon nanotube and comparative analysis with multi-walled carbon nanotube in rats ········ 783
E-2003 Identifying carcinogenic chemical agents based on genomic signatures in cancers of unknown pr imary ········ 784
E-2004 OLA1 is phosphorylated by ATR and transported to centrosomes to promote DNA damage-induced cent rosome amplification ········ 785
E-2005 A DNA damage profiling of human blood cells using mass spectrometry with a chemical compound library of DNA adducts ········ 786
E-2006 Mutagenicity of 5-hydroxycytosine, an oxidized form of cytosine, in human cells ········ 787

### 9:00 - 10:15 I-E12-3:New cancer diagnostic technologies

### Room 7 / (Hotel Nikko Kanazawa 3F Kujaku B)

E-2007 Development of AI model for 3D polygon data generation toward the XR navigation of gynecological cancer surgery ········ 788
E-2008 Clinical Insights from Pre-Biopsy Germline NGS Testing in Gynecologic Oncology ········ 789
E-2009 Constructing a Deep Learning-Based Proteomic Classifier for Unknown Primary Squamous Cell C arcinoma ········ 790
E-2010 Development of a treatment decision-making support system for intraductal papillary mucinous n eoplasm ········ 791
E-2011 A prospective observational study of oxygen saturation endoscopic imaging and radiosensitivity in head and neck cancer ········ 792
E-2012 Withdrawn ········ 793

### 9:00 - 10:15 I-E10-2:Metabolism/Metabolome (1)

### Room 8 / (Hotel Nikko Kanazawa 3F Kujaku C)

E-2013 KEAP1 mutation in addition to KRAS-LKB1 mutation in NSCLC confers resistance to KRAS G12C inhibitor v ia anaplerosis ········ 794
E-2014 Suppressing glutaminase 1 expression synergizes the anti-tumor effects of metformin on cholangi ocarcinoma ········ 795

### CONTENTS

E-2015 Disruption of hepatocyte glutathione metabolism under breast cancer conditions: an integrated in vitro and in vivo study ··· 796
E-2016 Long chain fatty acid elongase 6 (ELOVL6) is a novel therapeutic target for breast cancer ··· 797
E-2017 Changes in glycosylation enzymes in mesothelial cells adjacent to adipose tissue and progression of ovarian cancer ··· 798
E-2018 High-Fat Diet inhibits ferroptosis and promotes cell proliferation of ovarian and endometrial cancer ··· 799

### 9:00 - 10:15 I-E19:Cancer Prevention & Epidemiology (1)
### Room 9 / (Hotel Nikko Kanazawa 3F Lumiere)

E-2019 Prefectural Success in Smoking Target Projections Correlates with Minimum Wage & Density ··· 800
E-2020 Withdrawn ··· 801
E-2021 Comparing Trends in Female Breast Cancer Incidence in Native Japanese and US Populations: An Age-Period-Cohort Analysis ··· 802
E-2022 Exploring the Role of Helicobacter pylori in Biliary Tract and Pancreatic Cancers ··· 803
E-2023 Insulin-like growth factor-binding protein 3 and incidence of pancreatic cancer in a nested case-control s tudy ··· 804
E-2024 Area-Level Socioeconomic Inequalities in Pancreatic Cancer Mortality in Japan: A Multilevel Ana lysis ··· 805

### 9:00 - 11:30 SP1:10th Anniversary of the Survivor-Scientist Program (SSP)
### Room 10 / (Art Hall)

SP1-1 TBD ··· 806
SP1-2 TBD ··· 807
SP1-3 TBD ··· 808
SP1-4 TBD ··· 809
SP1-5 TBD ··· 810
SP1-6 TBD ··· 811
SP1-Panelist ··· 812
SP1-Panelist ··· 813

### 9:00 - 11:30 JS2:AI is opening up new frontiers in cancer research and medicine.
### Room 11 / (ANA Crowne Plaza Hotel Kanazawa 3F Ohtori (West))

JS2-1 Data-Driven Analysis Using Cancer Histopathology ··· 814
JS2-2 Performance Comparison of Multiple Large Language Models for Cancer Gene Profiling Test Support System ··· 815
JS2-3 Cell State Analysis of Immune Cells in the Tumor Microenvironment with Deep Semi-NMF ··· 816
JS2-4 Integrating Transcriptomic Profiling and Machine Learning: A Prognostic Model for Infant Acute Myeloid L eukemia ··· 817
JS2-5 Rational design of de novo proteins using large datasets and AI ··· 818

### 9:00 - 11:30 JS3:Cancer Heterogeneity, Stem Cell Regulation, and Treatment Resistance
### Room 12 / (ANA Crowne Plaza Hotel Kanazawa 3F Ohtori (Central))

JS3-1 AI-based analysis of pathologic images incorporating tumor heterogeneity and the microenvironment in pa ncreatic cancer ··· 819
JS3-2 Breast cancer cells leverage bone marrow cell plasticity at the single-cell level to drive metastasis i nitiation ··· 820
JS3-3 Clinical and Biological Implications of Fusobacterium nucleatum in Esophageal Cancer ··· 821
JS3-4 Targeting tumor stromal components for therapeutic innovation in GI cancers with heterogeneous micro environments ··· 822
JS3-5 Application of Medium-Range Cell-Cell Interaction Analysis to Colorectal Cancer ··· 823
JS3-6 Targeting ECM-Secreted Matricellular Proteins to Overcome Immune Resistance in Aggressive Colorectal Ca ncer ··· 824

### 9:00 - 11:30 AOSR3:Urologic Cancer
### Room 13 / (ANA Crowne Plaza Hotel Kanazawa 3F Ohtori (East))

AOSR3-1 Development of precision medicine for kidney cancer ··· 825

### CONTENTS

AOSR3-2 Identification of Predictive Biomarkers for the Efficacy of Immune Checkpoint Inhibitors through Mult i-Omics Analysis ........ 826
AOSR3-3 Development of bladder cancer urine liquid biopsy technique using mutant proteins in extracellular ve sicles ........ 827
AOSR3-4 A new era in the treatment of advanced urothelial cancer pioneered by basic cancer research ........ 828
AOSR3-5 The latest status and issues of prostate cancer: especially from the perspective of HBCO ........ 829
AOSR3-6 Mechanisms Underlying Double Negative CRPC: Interplay Between FGF and CCL2 ........ 830

### 9:00 - 10:15 I-J14-1:Drug delivery system (1)
### Room 14 / (ANA Crowne Plaza Hotel Kanazawa 3F Zuiun 1)

J-2025 Enhancing anti-tumor effect by improving nanomedicine delivery with microbubbles and ultr asound ........ 831
J-2026 Therapy-oriented bioabsorbable nanofiber sheet toward location-specific targeted delivery of anti-cancer v esicles ........ 832
J-2027 Intraperitoneal administration of multilayer-structured anticancer nanoparticles prevents peritoneal dis semination ........ 833
J-2028 IL-7R-Targeted ADC Enables Non-Invasive Eradication of CNS-Infiltrated ALL via CSF-Mediated Pa thway ........ 834
J-2029 Mitochondria-targeted nanocapsules enhance photodynamic therapy efficacy by improving photosensitizer encapsulation ........ 835
J-2030 A multicenter phase II trial of CED of ACNU in children with DIPG: Comparison with other studies ........ 836

### 9:00 - 10:15 II-E3:Hepatobiliary & pancreatic cancers
### Room 15 / (ANA Crowne Plaza Hotel Kanazawa 3F Zuiun 2)

E-2031 Withdrawn ........ 837
E-2032 Withdrawn ........ 838
E-2033 Functional characterization of ATM variants of uncertain significance in patients with pancreatic c ancer ........ 839
E-2034 Integrative multi-omics profiling of patient-derived cell line to define the network pharmacology for Pre cision Oncology ........ 840
E-2035 HER2 Amplification in PBM-Associated Gallbladder Cancer ........ 841
E-2036 Diagnostic and Prognostic Utility of Bile-to-Serum exosomal miR-21-5p Ratio in Biliary Tract Ca ncer ........ 842

### 9:00 - 10:15 I-J2:Patient-derived experimental models
### Room 16 / (11F Visage)

J-2037 Organoid library analysis of human esophageal squamous cell carcinoma reveals features of chemotherapy resistance. ........ 843
J-2038 Establishment and characterization of novel patient-derived esophageal tumoroids with long-term cult ivability ........ 844
J-2039 Prediction of therapeutic Response Using ESCC Organoids and Recurrence Risk Assessment by MDR1 Ex pression Analysis ........ 845
J-2040 Organoid-Based Evaluation of Therapeutic Responses in Atypical Choroid Plexus Papilloma ........ 846
J-2041 TFRC inhibitor antibody drugs have anti-tumor effects on metastatic liver tumor derived from colorectal c ancer ........ 847
J-2042 Establishment of a high-risk AML cell line YCU-AML2 harboring KMT2A::MLLT3 fusion and MECOM ove rexpression ........ 848

### 10:15 - 11:30 I-J6:DNA damage response & genomic instability
### Room 6 / (Hotel Nikko Kanazawa 3F Kujaku A)

J-2001 Analysis of genomic instability and aging-inducing effects of glucocorticoids ........ 849
J-2002 Inactivation of an O2/Fe2+-dependent enzyme causes cell cycle retardation at early S phase under hypoxia ........ 850
J-2003 Mechanism of the Selective Cytotoxic Antitumor Effect Induced by the Novel CDK Inhibitor Aza lam4 ........ 851
J-2004 Cyclin E1 Overexpression Predicts Sensitivity to PLK1 Inhibition in Ovarian Cancer Cells ........ 852
J-2005 VCP contributes to DNA-PK-mediated repair of etoposide-induced DNA double-strand breaks ........ 853

### CONTENTS

J-2006 CENP-E Inhibition Induces cGAS-STING Activation to Enhance Cancer Immunotherapy ··············· 854

### 10:15 - 11:30 I-J12-2:Artificial intelligence based diagnostics
### Room 7 / (Hotel Nikko Kanazawa 3F Kujaku B)

J-2007 Development of a deep learning model "DWI-ResViT" to accelerate acquisition time for Diffusion Kurtosis Imaging. ··· 855

J-2008 Multimodal AI with CTC images and clinical information for postoperative prognosis prediction in colorectal cancer ··· 856

J-2009 Analysis of the Tumor Immune Microenvironment in Rectal Cancer Using Deep Learning-Based Imaging C ytometry ··· 857

J-2010 Radiomics-Based Difficulty-Aware Learning Algorithm for Robust Segmentation of Hard-to-Detect Lesions in PET/CT ··· 858

J-2011 New method for tumor cytology: AI analysis of white light scattering spectrum ··· 859

J-2012 AI-Powered Cytological Diagnosis Support System Using Multi-Focus Image-Based 3D Recognition ···· 860

### 10:15 - 11:30 I-J10-2:Metabolism/Metabolome (2)
### Room 8 / (Hotel Nikko Kanazawa 3F Kujaku C)

J-2013 Tumor Suppression Through Regular Exercise and PGC-1α-Driven Metabolic Changes in Hepato cellular Carcinoma ··· 861

J-2014 Withdrawn ··· 862

J-2015 miR-216b-5p Functions as a Tumor Suppressor via the PTBP1/TYMS Axis in Pancreatic Cancer ··· 863

J-2016 Identification of anoikis resistant-related molecules in breast cancer stem cell models ··· 864

J-2017 HSD17B4-mediated lipid metabolism is involved in breast cancer drug response ··· 865

J-2018 MUC1-C drives stemness by integrating metabolic reprogramming in triple-negative breast cancer ···· 866

### 10:15 - 11:30 I-J19:Cancer Prevention & Epidemiology (2)
### Room 9 / (Hotel Nikko Kanazawa 3F Lumiere)

J-2019 Cancer chemoprevention with DNA demethylating agents in oncovirus-associated leukemogenesis in mice. ··· 867

J-2020 Predicting colorectal cancer risk using genome-wide polygenic risk scores and lifestyle factors in a Japanese population ··· 868

J-2021 Association of plasma branched-chain amino acid levels with colorectal cancer risk in a nested case-control study ··· 869

J-2022 Impact of Alcohol Consumption-Associated Loci on Alcohol-Related Cancer Risk: An Assessment by Medi ation Analysis ··· 870

J-2023 Cardiovascular Disease Risk Among Childhood Leukemia Survivors: A retrospective matched cohort study ··· 871

J-2024 Cancer survival varied by cancer type, regardless of rarity/commonality ··· 872

### 10:15 - 11:30 I-E14-1:Drug delivery system (2)
### Room 14 / (ANA Crowne Plaza Hotel Kanazawa 3F Zuiun 1)

E-2025 Multifunctional Drug Delivery System to Inhibit the Proliferation of SW480 Cells ··· 873

E-2026 Withdrawn ··· 874

E-2027 Inhalable ivermectin loaded lipid polymer hybrid nanoparticles against lung cancer with enhanced anti cancer activity ··· 875

E-2028 Nanomedicine-Enhanced PROTAC Delivery for Improved Glioblastoma Therapy ··· 876

E-2029 Broader therapeutic window via tumor-selective delivery of astatine-211 in radioimmunotherapy ···· 877

E-2030 Nano-designed carbon monoxide donor suppresses tumor via modulation of microenvironment and met abolism ··· 878

### 10:15 - 11:30 II-J3-1:Hepatocellular cancer, biliary tract cancer
### Room 15 / (ANA Crowne Plaza Hotel Kanazawa 3F Zuiun 2)

J-2031 Evaluation of the Combined Effect of a Novel CompoundX and GC Therapy Using Patient-Derived Chol angiocarcinoma Organoids ··· 879

J-2032 Lipid profiling in metabolic dysfunction associated steatotic liver disease to elucidate hepatocarcinogenesis mechanisms ··· 880

J-2033 The impact of polyploidy on the pathogenesis of human intrahepatic cholangiocarcinoma ··· 881

### CONTENTS

J-2034 Functional difference of MYC and MYCN in liver cancer: role of HNF1B in combined hepatocell ular-cholangiocarcinoma ..... 882
J-2035 Transcription factor JUNB promotes EMT of hepatocarcinoma stem cells and induces distant me tastasis. ..... 883
J-2036 Withdrawn ..... 884

### 10:15 - 11:30 I-E2-2:Organoid/Animal model (2)
### Room 16 / (11F Visage)

E-2037 Elucidation of the mechanisms underlying drug resistance caused by heterogeneity of high-grade endo metrial cancer ..... 885
E-2038 Transcription differences in hemangiosarcoma vs. nodular hyperplasia using organoids&xenograft models reveal new targets ..... 886
E-2039 The Iqgap3–Kras axis orchestrates early gastric tumorigenesis ..... 887
E-2040 NCG-MHC-dKO Mice - an Ideal Model for the Evaluation of Bispecific T Cell Engagers and in vivo CAR-T T herapies ..... 888
E-2041 Preclinical evaluation of cancer immunotherapy targeting human macrophages in humanized mouse mod els ..... 889
E-2042 Meflin-positive cancer-associated fibroblasts exhibit anti-tumor effects in advanced ovarian cancer. ..... 890

### 10:40 - 11:30 IAL:JCA International Award Lecture
### Room 2 / (Ishikawa Ongakudo 2F Hogaku Hall)

IAL Updated perspectives on innate immune regulators in upper gastrointestinal cancers ..... 891

### 11:50 - 12:40 LS13:Team for advanced technical support and efficient research management in P-PROMOTE/ BINDS Activity Support Team for Collaboration Promotion Office / Innovative technologies or resources to support translational research in the field of drug development against cancer
### Room 1 / (Ishikawa Ongakudo 2F Concert Hall)

LS13-1 1) Advanced Technical Support by BINDS: New Initiatives to Accelerate Drug Discovery in Acad emia ..... 892
LS13-2 2) An approach to preclinical R&D from seed identification to POC establishment for antitumor efficacy in academia (Drug discovery initiatives in P-PROMOTE) ..... 893

### 11:50 - 12:40 LS14:CHUGAI PHARMACEUTICAL CO.,LTD. / Cell-free DNA Analysis in Malignant Lymphoma: Recent Findings and Clinical Applications
### Room 3 / (Hotel Nikko Kanazawa 4F Tsuru A)

LS14 Cell-free DNA Analysis in Malignant Lymphoma: Recent Findings and Clinical Applications ..... 894

### 11:50 - 12:40 LS15:Amgen K.K. / Transforming Small Cell Lung Cancer Treatment Ðurable survival benefit from bispecific antibody~
### Room 4 / (Hotel Nikko Kanazawa 4F Tsuru B)

LS15 Transforming Small Cell Lung Cancer Treatment ~Durable survival benefit from bispecific antibody~ ..... 895

### 11:50 - 12:40 LS16:SHIBUYA CORPORATION / Future perspective of next-generation CAR-T cell therapy and automated cell manufacturing technologies
### Room 5 / (Hotel Nikko Kanazawa 4F Tsuru C)

LS16 Future perspective of next-generation CAR-T cell therapy and automated cell manufacturing tech nologies ..... 896

### 11:50 - 12:40 LS17:Nippon Shinyaku Co., Ltd. / Current treatments for lymphoid neoplasms: The Impact of Pirtobrutinib on Treatment Strategies
### Room 6 / (Hotel Nikko Kanazawa 3F Kujaku A)

LS17 Current treatments for lymphoid neoplasms: The Impact of Pirtobrutinib on Treatment Strategies ..... 897

### CONTENTS

11:50 - 12:40 LS18:TAKARA BIO, Inc. / Technology Development for the expanding spatial analysis of cancers
Room 7 / (Hotel Nikko Kanazawa 3F Kujaku B)
LS18 Technology Development for the expanding spatial analysis of cancers ............................ 898

11:50 - 12:40 LS19:Kyowa Kirin Co., Ltd. / The Role of Gut Microbiota in the Era of Immune Checkpoint Inhibitor Chemotherapy Combination Therapy: Implications for Febrile Neutropenia Management with G-CSF
Room 8 / (Hotel Nikko Kanazawa 3F Kujaku C)
LS19 The Role of Gut Microbiota in the Era of Immune Checkpoint Inhibitor–Chemotherapy Combination Therapy: Implications for Febrile Neutropenia Management with G-CSF ............................ 899

11:50 - 12:40 LS20:10x Genomics / Pioneering cancer research: unlocking new frontiers with spatial multiomics
Room 9 / (Hotel Nikko Kanazawa 3F Lumiere)
LS20-1 1) Spatial analysis reveals origin of cancer ............................ 900
LS20-2 2) Revolutionizing cancer research through spatial transcriptomics with Visium HD & Xenium ........ 901

11:50 - 12:40 LS21:AstraZeneca K.K. / BTK inhibition in the treatment of CLL: past and future
Room 12 / (ANA Crowne Plaza Hotel Kanazawa 3F Ohtori (Central))
LS21 BTK inhibition in the treatment of CLL: past and future ............................ 902

11:50 - 12:40 LS22:Astellas Pharma Inc. / Treatment strategies for AML in the era of molecular targeted therapy
Room 13 / (ANA Crowne Plaza Hotel Kanazawa 3F Ohtori (East))
LS22 Treatment strategies for AML in the era of molecular targeted therapy ............................ 903

11:50 - 12:40 LS23:Shimadzu Corporation / Visualization of Drug Distribution in cancer tissue by Mass Spectrometry Imaging— Exploring the Future of Cancer Research in Comparison with Raman Spectroscopic Analysis and Comprehensive Genetic Analysis —
Room 14 / (ANA Crowne Plaza Hotel Kanazawa 3F Zuiun 1)
LS23 ............................ 904

11:50 - 12:40 LS24:TOHO Holdings Co., Ltd.／REPROCELL Inc. / Perspectives for Adoptive Cellular Immunotherapy of Cancer -TIL, TCR-T, CAR-T-
Room 15 / (ANA Crowne Plaza Hotel Kanazawa 3F Zuiun 2)
LS24 Perspectives for Adoptive Cellular Immunotherapy of Cancer -TIL, TCR-T, CAR-T- ............................ 905

12:50 - 15:20 SS4:AMED Symposium "Aiming for prevention and cure of cancer-Challenges and prospects for cancer research in AMED's third term"
Room 1 / (Ishikawa Ongakudo 2F Concert Hall)
SS4-1 Strategy for Controlling Metastatic Recurrence by Dormancy Induction ............................ 906
SS4-2 Controlling the origin of cancer clones for esophageal cancer prevention ............................ 907
SS4-3 Unequal Segregation of Extrachromosomal DNA as a Driver of Intratumoral Heterogeneity ............ 908
SS4-4 Development of an AI Model for Early Diagnosis of Pancreatic and Bile Duct Cancer via Cachexia-Related Metabolic Changes ............................ 909

12:50 - 15:20 SS4:AMED Symposium "Aiming for prevention and cure of cancer-Challenges and prospects for cancer research in AMED's third term"
Room 1 / (Ishikawa Ongakudo 2F Concert Hall)
SS4-5 Somatic Mosaicism and the Origins of Cancer ............................ 910
SS4-6 Development and Therapeutic Application of Next-Generation Nucleic Acid Drugs for Glioblastoma .... 911

### CONTENTS

SS4-7 Research and development aimed at eradicating refractory cancer ··· 912
SS4-8 From discovery to clinical implementation of blood biomarker for early diagnosis of pancreatic ca ncer ··· 913

### 12:50 - 15:20 SS2:Young Plenary Symposium
### Room 2 / (Ishikawa Ongakudo 2F Hogaku Hall)

SS2-1 Immune evasion through mitochondrial transfer in the tumor microenvironment ··· 914
SS2-2 Maintenance of R-loop structures by phosphorylated hTERT preserves genome integrity ··· 915
SS2-3 Pan-cancer comparative and integrative analyses of driver alterations using Japanese and international ge nomic databases ··· 916
SS2-4 Differentiation of Tregs into Th1-Tregs induced by tumor-associated macrophages promotes tumor growth ··· 917
SS2-5 Genetic architecture of alcohol consumption identified by genotype-stratified GWAS and impact on esop hageal cancer risk ··· 918
SS2-6 IL33-ILC2s: Novel lymphoneogenic pathway ··· 919
SS2-7 Evolutionary histories of breast cancer and related clones ··· 920

### 12:50 - 15:20 AOSR4:Recent advances in pediatic, AYA cancer research
### Room 3 / (Hotel Nikko Kanazawa 4F Tsuru A)

AOSR4-1 The Coding and Non-Coding Mutational Landscapes of Pediatric Acute Myeloid Leukemia ··· 921
AOSR4-2 Multi-omics analysis of pediatric mesenchymal liver tumors; UESL and MHL ··· 922
AOSR4-3 Targeting MLL-rearranged leukemia by MENIN inhibitors ··· 923
AOSR4-4 The Landscape of Clonal Hematopoiesis and Structural Variation in Aplastic Anemia ··· 924
AOSR4-5 Tracing the origin of pediatric cancer using single-cell whole genome sequencing ··· 925
AOSR4-6 Tracing the origins of childhood cancer through genome sequencing ··· 926

### 12:50 - 15:20 S6:Understanding the mechanism of cancer progression and metastasis orchestrated by stroma
### Room 4 / (Hotel Nikko Kanazawa 4F Tsuru B)

S6-1 CEP55 positive EVs Mediate Glucose-Driven Lung Metastasis via Fibroblast Reprogramming ··· 927
S6-2 Wnt-Induced hyaluronan expression promotes liver metastasis of gastric cancer ··· 928
S6-3 The epigenetic dysregulation of SETD2 in pancreatic cancer: from tumor cells to tumor microe nvironment ··· 929
S6-4 Identification of CD44v6 as a marker for precancerous lesions in pancreatic cancer ··· 930
S6-5 mRNA therapeutics for in situ cancer immunotherapy ··· 931
S6-6 Retinoid-Driven CAF Reprogramming for Pancreatic Cancer: MIKE-1 and APPLE Trials ··· 932

### 12:50 - 15:20 IS5:Epigenetic modifications: from mechanisms to cancer
### Room 5 / (Hotel Nikko Kanazawa 4F Tsuru C)

IS5-1 Lysine hypoacetylation-defined enhancers in Luminar breast cancers ··· 933
IS5-2 TERC interactome reveals its function beyond telomeres ··· 934
IS5-3 Epigenetic tumorigenesis by 3D interaction between external viral DNA and host genome ··· 935
IS5-4 Toward Enhancing Precision Medicine Across Diverse Bile Duct Cancers ··· 936
IS5-5 Molecular mechanisms of epigenetic regulation ··· 937
IS5-6 Reactivation of embryonic programs in cancer ··· 938
IS5-7 Protein N-terminal Acetylase Naa10: From Development to Cancer and Metabolism ··· 939

### 12:50 - 13:30 ME3:Nature Immunology
### Room 6 / (Hotel Nikko Kanazawa 3F Kujaku A)

ME3 Nature Immunology ··· 940

### 12:50 - 14:05 I-J14-2:Natural & Synthetic Anticancer Compounds
### Room 7 / (Hotel Nikko Kanazawa 3F Kujaku B)

J-2049 Identification of plumbagin as a novel GGCT inhibitor by cell-based screening using a luminescence probe L ISA-103 ··· 941

### CONTENTS

J-2050 Anti-Cancer Effects of Coffee Diterpenes, Kahweol Acetate and Cafestol, on Taxane-Resistant Pro state Cancer ···· 942
J-2051 Novel compounds inhibiting fibroblast-mediated pancreatic cancer stem-like cell growth ···· 943
J-2052 4-Octyl itaconate disrupts the antioxidant system in melanoma via glutathione metabolism dis orders. ···· 944
J-2053 Development of selective inhibitors of Na+/H+ exchanger 5 (NHE5) related in cancer metastasis ···· 945
J-2054 M4N as a Novel Therapeutic Approach for KDM6A-mutant Bladder Cancer ···· 946

12:50 - 15:20 SP2:Achievement to cancer control no one left behind -cancer research focusing on the health inequalities-
Room 8 / (Hotel Nikko Kanazawa 3F Kujaku C)
SP2-1 Approaches to Health Inequalities in Cancer Control Plans Abroad: A Review of Monitoring Indi cators ···· 947
SP2-2 Monitoring for health inequalities and socioeconomic inequalities in cancer prevention (smoking) ···· 948
SP2-3 Neighorhood-based inequalities in Human Papillomavirus vaccination ···· 949
SP2-4 Health Disparities Revealed Through Cancer Screening and Their Social Context ···· 950
SP2-5 Inequalities in Cancer Medicine: Survival, Treatment Gaps, and Underlying Factors ···· 951
SP2-6 The Current Landscape and Challenges of Financial Toxicity in Oncology ···· 952
SP2-7 Expectations for Evidence-Based Policymaking ···· 953
SP2-8 Unheard Realities in Cancer Control: Insights from Frontline Patient Support Practice ···· 954

12:50 - 14:05 I-E4-2:Oncogenes & tumor-suppressor genes (3)
Room 9 / (Hotel Nikko Kanazawa 3F Lumiere)
E-2055 Identification of a Novel Secretion Mechanism by Proximal-Dependent Interactomes for Endoplasmic Ret iculum Therapy ···· 955
E-2056 Pan-cancer analysis exploring the relationship between chromothripsis and global DNA hypom ethylation ···· 956
E-2057 The role of FOXO3 in the mechanism of malignant progression of gastric cancer ···· 957
E-2058 B4GALT3(Beta-1,4-Galactosyltransferase 3) Promotes Breast Cancer Cell Invasion via Integrin β 1-Targeted Glycosylation. ···· 958
E-2059 Identification of URST1 as a novel biomarker and therapeutic target for lung cancer. ···· 959
E-2060 A functional assessment can identify therapeutic targets without either any expressional changes or genetic mutations ···· 960

12:50 - 15:20 S7:Cancer metabolism - Bench to Bedside
Room 10 / (Art Hall)
S7-1 TBD ···· 961
S7-2 One-carbon metabolism-polyamine axis controls tumor immune microenvironment and potential metastatic n iche establishment ···· 962
S7-3 Systemic metabolic alterations in cancer cachexia revealed through multi-omics analysis ···· 963
S7-4 Guanine nucleotides drive ribosome biogenesis and glycolytic reprogramming in acute myeloid leukemia stem cells ···· 964
S7-5 Targeting RB1-SUCLA2 Deletion in Advanced Prostate Cancer ···· 965
S7-6 Chemical Medicine: Development of novel chemistry-based and enzyme-driven theranostics technologies for c ancer ···· 966

12:50 - 14:05 I-E20:Advances in Cancer Stratification & Therapeutic Strategies: From Pathophysiology to Clinical Applications
Room 11 / (ANA Crowne Plaza Hotel Kanazawa 3F Ohtori (West))
E-2067 Adunctin E Suppresses Lung Cancer Cells by Targeting HSP90AA1 ···· 967
E-2068 A Clinically Accessible Model for Predicting Induction Failure in pediatric acute myeloid leukemia ···· 968
E-2069 Stratifying Metastatic Breast Cancer by Systemic Symptoms: Biological Insights from Patient Clu sters ···· 969
E-2070 Computational Pathology with Topological Data Analysis ···· 970
E-2071 Spatial transcriptomic profiling to elucidate drug resistance via intercellular crosstalk in HGSOC ···· 971
E-2072 Design of T Cell Infusion Schedules Using a Mathematical Model of Adoptive Cell Therapy ···· 972

### CONTENTS

12:50 - 15:20 S8:Foundational and innovative technologies to bridge basic cancer research to drug development
Room 12 / (ANA Crowne Plaza Hotel Kanazawa 3F Ohtori (Central))
S8-1 Utilization of the key technologies for translating Academia's Seeds to Industries through the iD3 Booster ........ 973
S8-2 Parallel epitope and affinity evaluation of antibodies via mammalian cell display and sequencing ........ 974
S8-3 Next-Generation SNPD-siRNA Enables Allele-Specific Silencing of Mutant Genes ........ 975
S8-4 Drug discovery targeting KRAS mutants using targeted protein degradation ........ 976
S8-5 Development of Enzomenib: A promising MENIN-MLL interaction inhibitor for precision medicine in acute myeloid leukemia. ........ 977
S8-6 Development and Advancement of DXd ADC Platform ........ 978

12:50 - 15:20 IS6:Host Factors in EBV-induced Carcinogenesis
Room 13 / (ANA Crowne Plaza Hotel Kanazawa 3F Ohtori (East))
IS6-1 Host factors in carcinogenesis of nasopharyngeal carcinoma induced by Epstein-Barr virus ........ 979
IS6-2 Elucidating the mechanism of aberrant chromatin activation induced by the virus-host chromatin interactions ........ 980
IS6-3 Intravesicular microRNA in EVs from ENKTL Patients: Prognostic and CAEBV-Associated Signatures ........ 981
IS6-4 Host Context and Early Clinical Features of Pediatric Chronic Active EBV Infection ........ 982
IS6-5 Investigating the pathogenesis of CAEBV: our approaches today and future ........ 983
IS6-6 Distinct Immune Landscapes in Endemic and Sporadic Burkitt Lymphoma Reflect EBV Association ........ 984

12:50 - 14:05 II-J4:Head & neck tumors
Room 14 / (ANA Crowne Plaza Hotel Kanazawa 3F Zuiun 1)
J-2079 Influence of the Oral Microenvironment on the Progression of Head and Neck Squamous Cell Carcinoma ........ 985
J-2080 HPV E6 and E7 synergize with CBP haploinsufficiency to drive head and neck tumorigenesis ........ 986
J-2081 Depletion of nuclear protein X rescues the expression of malignancy-related genes in oral cancer cell lines ........ 987
J-2082 Oral microenvironment-associated gene signature reflects high malignancy and tumor microenvironment changes in HNSCC ........ 988
J-2083 Relationship between Self-care, Oral Environment, and Oral Mucositis during Chemoradiotherapy for Head and Neck Cancer ........ 989
J-2084 Potential involvement of myoblasts as a cellular origin of cancer-associated fibroblasts in tongue cancer ........ 990

12:50 - 14:05 I-J11-4:Tumor immune microenvironment/Tumor immune escape (3)
Room 15 / (ANA Crowne Plaza Hotel Kanazawa 3F Zuiun 2)
J-2091 Opposing roles of Semaphorin 4A and Semaphorin 6D in regulating antitumor immunity ........ 991
J-2092 Immune Suppression by CAF-Cancer Cell Close Interaction Revealed Through 3D Bioprinting ........ 992
J-2093 Stromal Cell Diversity in Angioimmunoblastic T-cell Lymphoma through Spatial Transcriptomics at Single Cell Resolution ........ 993
J-2094 Cancer-associated fibroblast-targeted near-infrared photoimmunotherapy induces tumor-specific memory T cells from TDLNs ........ 994
J-2095 The effect of age-related accumulation of MDSCs on the progression of metastatic liver tumors ........ 995
J-2096 Type 17 immunity promotes cancer immune evasion through the induction of inflammation ........ 996

12:50 - 14:05 I-E11-4:Antibody therapy/Immune cell therapy (CAR-T, T cell therapy) (1)
Room 16 / (11F Visage)
E-2091 An anti-CD44v10 antibody exerts antitumor activity in mouse xenograft models of oral squamous cell carcinomas ........ 997
E-2092 A cancer-specific anti-HER2 monoclonal antibody exhibits high reactivity in immunohistochemistry. ........ 998
E-2093 Development of a high-throughput platform for the rapid identification of neoantigens and their cognate TCRs ........ 999

### CONTENTS

E-2094 Prevention of NK cell-mediated reaction against HLA-KO cells by using membrane bound mAbs for inhi bitory NK receptors ........ 1000
E-2095 Development of an Antigen-Independent Dendritic Cell Therapy Using Tumor-Binding Antibodies Induced by Radiation Therapy ........ 1001
E-2096 The Runx3 R122C Variant induces Effector TEX prog Development and Improves CD8-T cell Anti-tumor I mmunity. ........ 1002

13:50 - 15:20 OS9:Lipid metabolism in caner biology
Room 6 / (Hotel Nikko Kanazawa 3F Kujaku A)
OS9-1 Adrenal Lipoma Formation Induced by Aberrant PIP3 Metabolism ........ 1003
OS9-2 Roles of the PLA2 family in cancer ........ 1004
OS9-3 Lysophosphatidic acid-mediated cancer progression and targeting of its receptor LPAR1 ........ 1005
OS9-4 Lipid Metabolism Shapes the Onco-Immune Landscape and Determines Immunotherapy Response in Hep atocellular Carcinoma ........ 1006
OS9-5 LGALS3BP Drives Tumorigenesis via sPLA2-X-Mediated EV Hydrolysis and TAM Uptake via Gal ectin-3 ........ 1007

14:05 - 15:20 I-E14-2:Cancer drug resistance & strategies to overcome it
Room 7 / (Hotel Nikko Kanazawa 3F Kujaku B)
E-2043 Role of miR-145-5p on cisplatin responsiveness in lung cancer cells ........ 1008
E-2044 Anti-Cholangiocarcinoma Activity of the Repurposed Antiemetic Drug Thiethylperazine ........ 1009
E-2045 Depletion of MLKL Augments Chemotherapy-Induced Apoptosis in Colorectal Cancer by Delaying TNFR-I E ndosomal Trafficking ........ 1010
E-2046 tRNA modifications drive glioma oncogenesis and are targets for therapy ........ 1011
E-2047 Pre-clinical efficacy evaluation of combined AKT inhibition and chemotherapy in mouse prostate cancer ........ 1012
E-2048 Targeting Telomeres by Lurbinectedin for ALT Cancer Therapy ........ 1013

14:05 - 15:20 I-J4-2:Oncogenes & tumor-suppressor genes (4)
Room 9 / (Hotel Nikko Kanazawa 3F Lumiere)
J-2061 Post-transcriptional regulation of the Hippo-YAP pathway ........ 1014
J-2062 Secretoglobin superfamily protein SCGB3A1 suppresses rhabdomyosarcoma development. ........ 1015
J-2063 GPR87 contributes to the malignant phenotype of esophageal squamous cell carcinoma by activating the A kt pathway. ........ 1016
J-2064 Pathogenic annotation of variants of unknown significance (VUS) based on real-world data and machine l earning tools ........ 1017
J-2065 ARID1A loss promotes proliferation of normal hepatocytes but suppresses inflammation-driven hepat ocarcinogenesis ........ 1018
J-2066 Novel candidate driver gene FIRRM (FIGNL1-interacting regulator of recombination and mitosis) in HCC ........ 1019

14:05 - 15:20 I-J20:Emerging Computational Approaches in Cancer Research: AI, Omics, & Precision Medicine
Room 11 / (ANA Crowne Plaza Hotel Kanazawa 3F Ohtori (West))
J-2067 Transcription Factor Binding Motif Analysis Method at the Single-Cell and Single-Gene Level Using Policy G radient ........ 1020
J-2068 Computational Identification of lncRNAs Related to Hematological Malignancies via the ab initio GO method ........ 1021
J-2069 Anti-cancer drug response prediction with quantum-classical hybrid machine learning with proper norm alization ........ 1022
J-2070 Spatial dynamics of the tumor microenvironment linked to emerging resistance in EGFR-mutated non-small cell lung cancer ........ 1023
J-2071 Application of SpatialKNifeY to Spatial Transcriptomics for Extracting Tissue Structure and Cataloging Mi croenvironment ........ 1024
J-2072 Reconstruction of cancer genome structure by personalized reference genome-based cancer genome ana lysis pipeline ........ 1025

### CONTENTS

14:05 - 15:20 II-E11:Advances in pathogenesis & treatment of hematological malignancies
Room 14 / (ANA Crowne Plaza Hotel Kanazawa 3F Zuiun 1)
E-2079 Specific Impact of Non-DNMT3A/TET2 Clonal Hematopoiesis on the Risk of Severe COVID-19 ······· 1026
E-2080 Genomic Landscape of Newly Diagnosed Adult Acute Lymphoblastic Leukemia ···················· 1027
E-2081 Cooperative Impact of Germline and Somatic DDX41 Mutations on Myeloid Pathogenesis ··········· 1028
E-2082 Engineered metabolic induction of excessive mitochondrial ROS as a promising therapeutic approach in a cute leukemia ······································································· 1029
E-2083 Restoring serotonin signaling to alleviate cytopenia in MDS as a novel therapeutic approach. ······· 1030
E-2084 Elucidating the unique pathogenesis of clonal hematopoiesis via single-cell multi-omics profiling. ··· 1031

14:05 - 15:20 I-J11-5:Tumor immune microenvironment/Tumor immune escape (4)
Room 15 / (ANA Crowne Plaza Hotel Kanazawa 3F Zuiun 2)
J-2097 Spatial Informatics and In Vivo Modeling Reveal Fibroblast-Mediated Immune Exclusion in Colorectal Can cer ·········································································· 1032
J-2098 Mechanisms of hepatocellular carcinoma recurrence after liver transplantation in the immunosuppressed s tate ·········································································· 1033
J-2099 Histamine H1 Receptor Antagonists Enhance Anti-PD-1 Therapy by Reducing Extracellular Matrix Prod uction in TNBC ································································ 1034
J-2100 Immunosuppressive mechanisms within the tumor microenvironment of Ewing's sarcoma mouse model ········································································ 1035
J-2101 The Role of Fusobacterium nucleatum in the Tumor Microenvironment of Oral Squamous Cell C arcinoma ······································································ 1036
J-2102 Immune and Molecular Profiling of NBNC HCC Based on Prior HBV Infection and Identification of Novel T herapeutic Targets ··························································· 1037

14:05 - 15:20 I-J11-6:Antibody therapy/Immune cell therapy (CAR-T, T cell therapy) (2)
Room 16 / (11F Visage)
J-2103 Therapeutic potential of a monoclonal antibody targeting the receptor tyrosine kinase EphB4 in breast ca ncer ·········································································· 1038
J-2104 A cancer-specific anti-podoplanin monoclonal antibody exerts antitumor activities in human tumor xeno graft models ································································· 1039
J-2105 Development of SRG system for selective regulation of CAR-T cell kinetics in vivo ················· 1040
J-2106 Antibody-Dependent Cellular Cytotoxicity Mediated by Anti-GD2 mAb in iPSC-Derived NKT Cells for Neu roblastoma ··································································· 1041
J-2107 Novel immunotherapy via induction of Tertiary Lymphoid Strcture ····························· 1042
J-2108 Development of novel adoptive T cell therapy that overcomes tumor heterogeneity with escape variant tu mor clones ································································· 1043

15:20 - 16:35 I-J8:Cancer epigenetics (3)
Room 5 / (Hotel Nikko Kanazawa 4F Tsuru C)
J-2043 Proton pump inhibitors intake is associated with DNA methylation ······························· 1044
J-2044 SMARCC1 Loss Impairs Differentiation and Enhances Self-Renewal in ASXL1-Mutant Hemat opoietic Cells ································································· 1045
J-2045 The pathophysiological role of Equilibrative Nucleoside Transporters (ENTs) in esophageal cancer ··· 1046
J-2046 Aberrant heterochromatin activation after oncovirus infection through H3K36me2 induction ········ 1047
J-2047 Dissecting the Mechanisms Underlying Cancer Cell Diversity Driven by Epigenomic Dysregulation ··· 1048
J-2048 Dynamics of mRNA modification in cancer microenvironment and EMT ························· 1049

15:20 - 16:35 I-J14-3:Mechanisms of Cancer Drug Resistance & Strategies to Overcome It
Room 7 / (Hotel Nikko Kanazawa 3F Kujaku B)
J-2055 Effect of acquisition of gemcitabine resistance in pancreatic cancer cells on chromosomal ins tability ····································································· 1050
J-2056 DCLK1 inhibition following gemcitabine decreases survival rate of pancreatic cancer cells escaped from s enescence ···································································· 1051
J-2057 PDAC cell treated by gemcitabine acquire chemoresistance by transition of nucleotide metabolism via TME. ································································· 1052

### CONTENTS

J-2058 Exploring status of Drug-Resistant Cancer Cells with Teton system ........ 1053
J-2059 Auranofin resensitizes ferroptosis-resistant lung cancer cells to ferroptosis inducers ........ 1054
J-2060 Elucidating Exosome-Mediated Mechanisms of Tumor Progression in Drug-Resistant Ovarian Ca ncer ........ 1055

15:20 - 16:35 I-E15:Evaluation & Prediction of Pharmacological Effects
Room 8 / (Hotel Nikko Kanazawa 3F Kujaku C)
E-2049 Pevonedistat response in Gastric Cancer: Proteomics for drug response prediction and biomarker discovery ........ 1056
E-2050 Risk Stratification and Therapeutic Suggestions for High-risk RUNX1::RUNX1T1-positive pAML p atients ........ 1057
E-2051 Association of ctDNA level with prognosis and efficacy in mCRC with anti-EGFR/anti-VEGF therapies: PAR ADIGM study ........ 1058
E-2052 Multicohort Retrospective Validation of a Predictive Biomarker for Irinotecan ........ 1059
E-2053 TLS predicts response and is associated with favorable outcomes in breast cancer patients rec eiving NAC ........ 1060
E-2054 3D Bone Marrow Niche: Scalable and Predictive Drug Screening Platform for Acute Myeloid Leukemia and Multiple Myeloma ........ 1061

15:20 - 16:35 I-E10-3:Telomere/Aging/Senescence/Immortalization/Cell differentiation/Others
Room 9 / (Hotel Nikko Kanazawa 3F Lumiere)
E-2061 HMMR Identifies a Transit-Amplifying Cell Population with TA-Specific Mitotic Behavior ........ 1062
E-2062 Osteocyte-like Differentiation of Osteosarcoma by Inorganic Phosphate treatment ........ 1063
E-2063 Selective targeting and disruption of the adaptive potential of tumor cells to the acidic pH microenv ironment ........ 1064
E-2064 Withdrawn ........ 1065
E-2065 Distinct Roles of Fanconi Anemia Pathway (FA) proteins on the Alternative Lengthening of Telomeres me chanism (ALT) ........ 1066
E-2066 A polyamine improves anti-tumor T cell function by promoting BiP chaperone activity in aged m ice ........ 1067

15:20 - 16:35 I-J11-3:Immune checkpoint inhibitors (2)
Room 12 / (ANA Crowne Plaza Hotel Kanazawa 3F Ohtori (Central))
J-2073 ROSE12 , FcγRs affinity-enhanced anti-CTLA-4 antibody, evokes tumor-selective immune response, activated by ATP in TME ........ 1068
J-2074 Activation of PD-1 antibody-bound progenitor-exhausted CD8+ T cells in lymph nodes after PD-1 blockade ........ 1069
J-2075 CLEC12A and CD200 mark tumor-reactive CD8+ T-cell clones in tumor-draining lymph nodes ........ 1070
J-2076 Risk factors & prognosis of immune checkpoint inhibitor-induced pneumonia in lung cancer with interstitial lung disease ........ 1071
J-2077 Investigation of the Activation Status and Significance of the cGAS-STING Pathway in Lung Squamous Cell C arcinoma ........ 1072
J-2078 PD-1 blockade and hematological malignancy exacerbation in lung cancer ........ 1073

15:20 - 16:35 I-E7-1:Advances in cancer research through genomic analysis
Room 13 / (ANA Crowne Plaza Hotel Kanazawa 3F Ohtori (East))
E-2073 Integrational analysis using somatic and germline alterations of lung cancer ........ 1074
E-2074 Differential immunogenic contributions of HLA-class I and -class II alleles to Lung Adenocarcinoma Dev elopment ........ 1075
E-2075 Intrapatient diversity of subclonal phenotypes in endometrial carcinomas ........ 1076
E-2076 Analysis of tumors with translocation breakpoints in the centromere using long read sequencing ........ 1077
E-2077 The genomic landscape of extrachromosomal DNA amplification in pediatric solid tumors ........ 1078
E-2078 Repertoire of Non-coding Drivers Identified by Whole-genome Sequencing of 913 Myeloid Neo plasms ........ 1079

15:20 - 16:35 II-J9:Urological Cancer
Room 14 / (ANA Crowne Plaza Hotel Kanazawa 3F Zuiun 1)

### CONTENTS

J-2085 Genomic landscape identifies therapeutic vulnerabilities in urachal carcinoma ······ 1080
J-2086 SLFN11 drives M1 macrophage activation and enhances response to immune checkpoint inhibitors in renal cell carcinoma ······ 1081
J-2087 Genome-wide CRISPR screening identifies a novel therapeutic agent to enhance radiation sensitivity in pr ostate cancer ······ 1082
J-2088 Phospholipid biosynthesis and oxidative stress interplay in the pathogenesis of renal cell carc inoma ······ 1083
J-2089 Molecular and clinical relevance of mitochondrial respiratory supercomplex in prostate cancer tumori genesis ······ 1084
J-2090 Whole-genome sequencing of 135 upper urinary tract urothelial carcinomas ······ 1085

### 15:20 - 16:35 I-E13-2:New molecular targeted therapy (1)
### Room 15 / (ANA Crowne Plaza Hotel Kanazawa 3F Zuiun 2)

E-2085 DOT1L inhibition reprograms innate immunity signaling to potentiate immunomodulatory drug responses in multiple myeloma ······ 1086
E-2086 A novel abiraterone derivative suppresses glioblastoma through FLG-mediated glioma stem cell d isruption ······ 1087
E-2087 Dysadherin Inhibition Suppresses Hepatocellular Carcinoma and Enhances Combination Therapy ······ 1088
E-2088 A bump & hole modification of IAP antagonist-IAP pair for a novel protein degradation sy stem ······ 1089
E-2089 Development of anti-metastatic drugs using synthetic short mRNA ······ 1090
E-2090 Withdrawn ······ 1091

### 15:30 - 16:10 SL2:Special Lecture2
### Room 1 / (Ishikawa Ongakudo 2F Concert Hall)

SL2 TBD ······ 1092

### 15:30 - 16:20 CS1:CHUGAI PHARMACEUTICAL CO., LTD. / Tumor Microenvironment Remodeling by VEGF Inhibition and Immune Checkpoint Blockade : Unraveling the Mechanisms of Synergistic Effects
### Room 2 / (Ishikawa Ongakudo 2F Hogaku Hall)

CS1 Tumor Microenvironment Remodeling by VEGF Inhibition and Immune Checkpoint Blockade : Unraveling the Mechanisms of Synergistic Effects ······ 1093

### 15:30 - 16:20 CS2:Integrated DNA Technologies / Comprehensive and Customizable IDT NGS Portfolio to Accelerate Cancer Research
### Room 3 / (Hotel Nikko Kanazawa 4F Tsuru A)

CS2 ······ 1094

### 15:30 - 16:10 ME4:Cancer Science
### Room 6 / (Hotel Nikko Kanazawa 3F Kujaku A)

ME4 Cancer Science ······ 1095

### 15:30 - 16:20 CS3:GeneBay, Inc. / Cancer Genome Analysis using Long Read Sequencing Technologies
### Room 11 / (ANA Crowne Plaza Hotel Kanazawa 3F Ohtori (West))

CS3-1 1) Introduction of Nanopore Sequencing: From Sample Prep to Data Analysis ······ 1096
CS3-2 2) Cancer Genome Informatic Analysis Pipeline for Nanopore Long Read Genome Sequencing ······ 1097
CS3-3 3) Landscape of Japanese Ovarian Cancer Genome ······ 1098

### 16:45 - 18:15 SSP:Survivor Scientist Program
### Room P / (Motenashi Dome)

SSP-1 ······ 1099
SSP-2 ······ 1100
SSP-3 ······ 1101
SSP-4 ······ 1102

### CONTENTS

SSP-5 ........ 1103
SSP-6 ........ 1104
SSP-7 ........ 1105
SSP-8 ........ 1106
SSP-9 ........ 1107
SSP-10 ........ 1108
SSP-11 ........ 1109
SSP-12 ........ 1110
SSP-13 ........ 1111
SSP-14 ........ 1112
SSP-15 ........ 1113
SSP-16 ........ 1114
SSP-17 ........ 1115

16:45 - 17:30 I-P1-3:Chemical carcinogens
Room P / (Motenashi Dome)

P-2001 Establishment of a chemical carcinogen-induced bladder cancer model using healthy dog bladder organoids ........ 1116
P-2002 Formation of the carcinogenic furan metabolite butenedial by hemin-induced degradation of alkadienals in fried foods ........ 1117
P-2003 Genotoxicity and mutational spectrum in lungs of gpt delta mice following inhalation exposure to heated t obacco products ........ 1118
P-2004 Comparative Gene Expression Analysis of Rat Lung Cancers Induced by Different Types of Carbon Nano tubes ........ 1119
P-2005 Structure-Dependent Hepatotoxicity and Carcinogenic Potential of PFASs in Immortalized Human Liver Ce lls ........ 1120
P-2006 Withdrawn ........ 1121
P-2007 Potential role of microplastics in oral cancer progression via oxidative stress ........ 1122
P-2008 Tamoxifen metabolites-induced DNA damage and cell proliferation in relation to endometrial ca rcinogenesis ........ 1123
P-2009 Dissecting the Carcinogenesis Mechanisms of Aristolochic Acid in Cholangiocarcinoma ........ 1124

16:45 - 17:30 I-P2-4:Organoid/Animal model (1)
Room P / (Motenashi Dome)

P-2019 Arid1a deficiency sensitizes pancreatic cancer to fatty acid synthase inhibition ........ 1125
P-2020 Establishment of Pancreatic Ductal Adenocarcinoma mouse model using phenotypically normal pancreatic ductal organoids ........ 1126
P-2021 Dual Targeting of CXCR7 Signaling and HDAC-Mediated Epigenetic Reprogramming to Disrupt the Desm oplasia in PDAC ........ 1127
P-2022 In vivo reprogramming unveils the epigenetic trigger of anaplastic transformation in thyroid ca ncer ........ 1128
P-2023 Orthotopic mouse model of distal bile duct cancer using tumor buds from genetically engineered bile duct organoids ........ 1129
P-2024 The Effect of Mint3 on Bladder Cancer Carcinogenesis and Lung Metastasis ........ 1130
P-2025 Withdrawn ........ 1131

16:45 - 17:30 I-P2-6:Organoid/animal model (3)
Room P / (Motenashi Dome)

P-2034 Lymphoproliferative Factors in the J-PDX Library: Role of Epstein-Barr Virus Infection and Rituximab Ef ficacy ........ 1132
P-2035 Considerations for Designing Drug Efficacy Studies Based on Tumor Characteristics in PDX Mo dels ........ 1133
P-2036 Evaluation of existing antibody drug efficacy based on survival time using PDX mouse models of hemat opoietic tumors ........ 1134
P-2037 Establishment of patient-derived xenograft and organoid models of pancreatic acinar cell car cinoma ........ 1135
P-2038 Evaluation of Tumor Characteristic Retention in TNBC-PDX Models ........ 1136

### CONTENTS

P-2039 Establishment of Two PDX Models from Organoids Derived from Circulating Tumor Cells of Prostate Cancer Patients ········ 1137
P-2040 Current status of human bone sarcoma cell line: publication and cell bank ········ 1138

16:45 - 17:30 I-P4-5:Oncogenes & tumor-suppressor genes (4)
Room P / (Motenashi Dome)
P-2050 Loss of ANK3 promotes cell proliferation, migration, and structural disruption in renal cell car cinoma ········ 1139
P-2051 GSTM3 Suppresses Autophagy via PINK1/Parkin Pathway to Promote Nasopharyngeal Carcinoma Prog ression ········ 1140
P-2052 Neuromedin U Promotes Tumor Progression and Fibrotic Remodeling in Lung Adenocarcinoma ········ 1141
P-2053 Allosteric SHP2 inhibitors inhibit non-canonical EphA2 activation in human lung adenocarcinoma cells ········ 1142
P-2054 Nuclear localization of insulin receptor substrate 1 (IRS1) correlates with estrogen signaling and CCA pr ogression ········ 1143
P-2055 DLL3 as a Common Molecular Marker in Neuroendocrine Carcinomas: Analysis in Esophageal N EC. ········ 1144
P-2056 Identification of therapeutic targets against double-negative prostate cancer ········ 1145
P-2057 The luciferase-based in vivo protein-protein interaction assay revealed that CHK1 promotes PP2A and PM E-1 interaction. ········ 1146
P-2058 Functional analysis of TSC22 family proteins in malignant transformation of glioblastoma ········ 1147

16:45 - 17:30 I-P5-3:Non-coding RNAs (1)
Room P / (Motenashi Dome)
P-2068 Upregulation of LINC02154 contributes to esophageal cancer progression by enhancing cell cy cle and EMT ········ 1148
P-2069 Novel strategy to cure cancer through inhibition of long non-coding RNA ········ 1149
P-2070 Androgen receptor-controlled microRNA-124-3p.2 suppresses prostate cancer progression via CCL2 inh ibition ········ 1150
P-2071 Investigation into the feasibility of treating lymph node metastasis with microRNA using lymphatic drug d elivery system ········ 1151
P-2072 CircESRP1 is highly expressed in HGSOC and promotes cell proliferation via the miR-182-5p/CDK1 pat hway. ········ 1152
P-2073 Characterization of colorectal cancer-associated microproteins translated from small ORFs within non-coding RNAs ········ 1153
P-2074 Malignant Alteration of Colorectal Cancer Cells Mediated by miR-4708 Expression ········ 1154
P-2075 miR-100-5p and miR-203a-3p Suppress Esophageal Squamous Cell Carcinoma Progression by Targeting F KBP5 ········ 1155
P-2076 Elucidation of the Mechanism of miR-20b in Regulating the Malignant Alteration of Colorectal C ancer Cells ········ 1156
P-2077 Momordica charantia Extract Combined with Doxorubicin via lncRNA Regulation Overcomes Resistance in Breast Cancer Cells ········ 1157

16:45 - 17:30 I-P8-1:DNA methylation
Room P / (Motenashi Dome)
P-2087 Epigenetic inactivation of DUOX1/DUOXA1 promotes tumor growth andmetastasis in nasopharyngeal ca rcinoma ········ 1158
P-2088 Collective Epigenetic Control in 14q32 miRNA Cluster of CRC: A Machine Learning Approach to Methy lation and Expression ········ 1159
P-2089 Characteristics of hypermethylated gastric intestinal metaplasia ········ 1160
P-2090 Analysis of somatic DNA variants and DNA methylation of tumor suppressor genes in colorectal cancer ········ 1161
P-2091 Concurrent CGI hypermethylation and non-CGI hypomethylation drive gastric cancer risk after H. pylori e radication ········ 1162
P-2092 Search for therapeutic target genes of synthetic lethality in CHFR-methylated cancer cells ········ 1163
P-2093 Defective chief cells in nodular gastritis in adults. ········ 1164

### CONTENTS

### 16:45 - 17:30 I-P8-3:Histone modification
### Room P / (Motenashi Dome)

P-2102 β-hydroxybutyrate-induced HDAC2 β-hydroxybutyrylation suppresses nasopharyngeal carcinoma malignancy ........ 1165
P-2103 Histone lactylation drives metabolic reprogramming in response to glucose deprivation in canine hema ngiosarcoma ........ 1166
P-2104 Molecular mechanisms of ATAD2 degradation under hypoxic conditions ........ 1167
P-2105 Epigenetic regulation of CD8+ T cell-dependent antitumor immunity by histone H3K27 methyltransferase Ezh2 ........ 1168
P-2106 HDAC5, an early osimertinib-responsive gene, is a novel target for drug resistance in EGFR-mutant lung c ancer cells. ........ 1169
P-2107 Withdrawn ........ 1170
P-2108 Targeting EHMT2 Overcomes 5 Fluorouracil Resistance in Colorectal Cancer ........ 1171

### 16:45 - 17:30 I-P9-4:Cell adhesion & invasion (2)
### Room P / (Motenashi Dome)

P-2117 Unravelling Cancer Evolution in a Gastric Cancer Mouse Model via SCNA Phylogeny from Single-Cell DNA Sequencing ........ 1172
P-2118 CCL5 promotes collective invasion of human skin squamaus carcinoma cells ........ 1173
P-2119 Elucidating the Mechanism of COMT-Mediated Suppression of Invasion and Metastasis in Ovarian Cancer ........ 1174
P-2120 Isoform-specific localization of p120-catenin in colorectal cancer cells is regulated by adaptins and related proteins ........ 1175
P-2121 ANKRD17 Promotes Pro-Survival Signaling to Enhance Invasion and Migration in Hepatocellular Carcinoma ........ 1176
P-2122 The Role of Extracellular Viscosity in Tumor Progression: From Single Cells to Spheroid Formation and In vasion ........ 1177
P-2123 Isoeugenol inhibits the growth, migration and invasion in different stages of colorectal cancer c ells ........ 1178
P-2124 Intracellular and extracellular functions of Adipsin (CFD) promote adipocyte maturation and tu mor invasion ........ 1179

### 16:45 - 17:30 I-P10-4:Metabolism/Metabolome (1)
### Room P / (Motenashi Dome)

P-2136 Plasma Lysophosphatidylcholine Levels Correlate with Prognosis and Immunotherapy Response in Squ amous Cell Carcinoma ........ 1180
P-2137 Tumor heterogeneity of unique BCAA metabolism regulates the aggressive nature in human triple-negative breast cancer ........ 1181
P-2138 Lysine acylation of plasma proteins as a potential biomarker for cancer cachexia ........ 1182
P-2139 Cancer-derived HMGB1 impairs cardiac mitochondria via mtDNA damage and CKMT2 suppr ession ........ 1183
P-2140 Cancer-derived Wnt ligand possibly inhibits adipogenesis via Wnt/β-catenin signaling pathway in pr eadipocytes. ........ 1184
P-2141 Metabolic reprogramming through enhanced cholesterol synthesis in CAFs of CMS4 colorectal c ancer. ........ 1185
P-2142 A novel organelle based nutrient sensing system in cancer cells ........ 1186

### 16:45 - 17:30 I-P10-6:Metabolism/Metabolome (3)
### Room P / (Motenashi Dome)

P-2151 Inhibition of PSMD14 by 7MeERT Suppresses Cancer Energy Metabolism via Regulation of PKM2 Ubiq uitination ........ 1187
P-2152 Targeting Carboxylation Pathway in RB1- SUCLA2 -Deficient Advanced Prostate Cancer ........ 1188
P-2153 Involvement of ER stress in the suppression of pancreatic cancer cell proliferation via FADS2 in hibition ........ 1189
P-2154 Mitochondrial one-carbon metabolic enzyme MTHFD1L is a novel molecular target for breast c ancer ........ 1190

### CONTENTS

P-2155 IDH1 upregulation contributes hepatoma cell survival via glutamine metabolism under the glucose-deprived condition. ........ 1191
P-2156 Preclinical evaluation of a NAD-targeting therapy for SCLC: potent antitumor activity and tolerable toxicities in mice. ........ 1192
P-2157 Analysis of G-body formation in quiescent cancer cells using a reversible cell model ........ 1193
P-2158 The Role of NNMT in Liver Metabolic Diseases and Its Therapeutic Applications ........ 1194
P-2159 Single-cell multiomics technology for the comprehensive analysis of transcriptomics and cancer driver mu tation ........ 1195
P-2160 Unveiling a Novel Role of Mesothelial Glycocalyx in Peritoneal Metastasis of Ovarian Cancer ........ 1196

### 16:45 - 17:30 I-P11-9:Tumor immune microenvironment/Tumor immune escape (5)
### Room P / (Motenashi Dome)

P-2169 Immunophenotypes of tumor microenvironment (TME) as prognostic factor for head and neck squamous c ell carcinoma (HNSCC) ........ 1197
P-2170 Epigenetic modifications of low blood miR-5193 as an immunotherapy target to PD-L1 in gastric and es ophageal cancer ........ 1198
P-2171 LncRNA NEAT1v1 confers resistance to immune checkpoint inhibitor therapy via CD24 ........ 1199
P-2172 T cell Repertoire of CML Patients Who Attempt Discontinuation of TKIs; the ISAC-TFR study ........ 1200
P-2173 The immunosuppressive role of CCR8+ Tregs in gastric cancer ........ 1201
P-2174 Analysis of tumor-infiltrating lymphocytes and their clinical significance in ovarian clear cell ca rcinoma ........ 1202
P-2175 Role of ANGPTL2 in multicellular network-mediated regulation of anti-tumor immunity in the tumor mic roenvironment ........ 1203
P-2176 Blockade of SIRPα enhances macrophage-dependent antitumor activity against head and neck squ amous cell carcinoma ........ 1204

### 16:45 - 17:30 I-P11-11:Immune checkpoint inhibitors (1)
### Room P / (Motenashi Dome)

P-2186 Identification of predictive peripheral blood biomarkers for NSCLC patient treated with anti-PD-1 antibody using PEA ........ 1205
P-2187 Exploratory study for the immunomodulatory effects of Juzentaihoto in preclinical cancer models ........ 1206
P-2188 Investigation of predictive biomarkers for immune checkpoint inhibitor efficacy in esophageal squamous c ell carcinoma ........ 1207
P-2189 Tissue-resident memory T cell infiltration predicts response to anti-PD-L1 therapy in extrahepatic chol angiocarcinoma ........ 1208
P-2190 CD80 is positively correlated with the efficacy of neoadjuvant PD-1 Ab combined with chemotherapy for e sophagus cancer ........ 1209
P-2191 Elucidation of the function of proline isomerase Pin1 on esophageal squamous cell carcinoma ........ 1210
P-2192 Development of personalized immunotherapy for metastatic renal cell carcinoma using integrative genomic analyses ........ 1211
P-2193 Immunotherapy outcomes and immune microenvironment profiles of patients with non-clear cell renal cell carcinoma ........ 1212

### 16:45 - 17:30 I-P11-13:Immune checkpoint inhibitors (3)
### Room P / (Motenashi Dome)

P-2203 Does LDDS inhibit lymph node metastasis and the development of distant tumors? Importance of LDDS de livered Docetaxel ........ 1213
P-2204 Impact of immediate inflammatory cytokines elevation after chemotherapy and pembrolizumab in advanced non-squamous NSCLC ........ 1214
P-2205 Evaluation of combination therapy with anti-PD-1 and anti-CD47 antibody in non-small cell lung cancer l iver metastasis ........ 1215
P-2206 Clinical Data and Mouse Model of NSCLC Brain Metastasis Reveal Efficacy of Anti-PD-1 + anti-CTLA-4 An tibody Therapy. ........ 1216
P-2207 CALLY Index as a Predictive Biomarker for ICI Plus Chemotherapy in Advanced HER2(-) Gastric Cancer: A First Report ........ 1217
P-2208 Evaluation of Chemoimmunotherapy for Metastatic Lymph Nodes via Treatment of Normal Ly mph Nodes ........ 1218

### CONTENTS

P-2209 Immune checkpoint inhibitor response and clinicopathological features of SMARCA4-deficient tumors ・・・ 1219
P-2210 The Impact of PGD2-CRTH2 Signaling on the Anti-Tumor Effects of Immune Checkpoint Inhibitors ・・・ 1220
P-2211 Enhanced efficacy of immune checkpoint inhibitors in combination with Japanese herbal medicine "TSUDOSAN" ・・・ 1221

### 16:45 - 17:30 I-P12-5:Pathological analysis & Biomarkers
Room P / (Motenashi Dome)

P-2221 Transgelin in Cancer Activated Fibroblasts Regulates Stromal Contractility and Correlates with Colon Cancer Progression ・・・ 1222
P-2222 Clinical significance of tertiary lymphoid structures in oral squamous cell carcinoma ・・・ 1223
P-2223 Role of zinc transporters in the immune microenvironment of the breast cancer ・・・ 1224
P-2224 Identification of urinary free N -glycan tumor marker having difucosylated type-1 structure ・・・ 1225
P-2225 Enhanced Specificity of 6-Color digital PCR with 3D Analysis for Multiplex Detection of Pancreatic Cancer Mutations ・・・ 1226
P-2226 Withdrawn ・・・ 1227
P-2227 Withdrawn ・・・ 1228
P-2228 Withdrawn ・・・ 1229
P-2229 Withdrawn ・・・ 1230

### 16:45 - 17:30 I-P13-5:Signal transduction inhibitor
Room P / (Motenashi Dome)

P-2239 USP8 Inhibition Induces Synthetic Lethality via FGFR2 Degradation in ARID1A-Deficient Ovarian Clear Cell Carcinoma ・・・ 1231
P-2240 Development of a novel covalent drug that allosterically inhibits the pro-oncogenic phosphatase SHP2 ・・・ 1232
P-2241 Lymphatic Drug Delivery System for Metastatic Lymph Nodes Using an HDAC/PI3K Dual Inhibitor ・・・ 1233
P-2242 DOT1L inhibition exerts anti-tumor effects through activating interferon signaling in breast cancer cells ・・・ 1234
P-2243 JAK inhibitors paradoxically activate an active JAK mutant, leading to discontinuation syndrome ・・・ 1235
P-2244 Development of novel anticancer agent targeting nucleolar stress pathway ・・・ 1236

### 16:45 - 17:30 I-P14-8:Combinational therapy/Adjuvant therapy
Room P / (Motenashi Dome)

P-2254 Amoxicillin, a β-lactam antibiotic, enhances cisplatin sensitivity in cancer cells affecting mitochondria. ・・・ 1237
P-2255 Effect of Synbiotics on Cancer Sarcopenia ・・・ 1238
P-2256 Targeting LIG1 and PARP for synthetic lethality-based treatment in prostate cancer ・・・ 1239
P-2257 Targeting DNA Repair Pathways in TNBC via Combined FAK and PARP Inhibition Independent of BRCA1 Status ・・・ 1240
P-2258 Targeting EGFR with Two DNA Intercalators to Inhibit Breast Cancer Metastasis ・・・ 1241
P-2259 Withdrawn ・・・ 1242
P-2260 Dimethoxyflavones potentiate the antitumor effects of microtubule polymerization inhibitors ・・・ 1243
P-2261 Shortening of the G2 phase avoids senescence and provides synergistic cytotoxicity with DNA replication stress inducers ・・・ 1244
P-2262 Development of novel combination cancer therapy with a catalytic inhibitor of PARP1 ・・・ 1245

### 16:45 - 17:30 I-P17-2:Multimodal treatment in surgery
Room P / (Motenashi Dome)

P-2272 Outcomes of Conversion Surgery After Combined Immunotherapy for cM1 and Recurrent Esophageal Cancer ・・・ 1246
P-2273 Outcome of conversion surgery for HER2-negative Stage IV gastric cancer ・・・ 1247
P-2274 Simultaneous colorectal and liver resection for colorectal cancer with synchronous liver metastasis ・・・ 1248
P-2275 Repeated R0 resection in cases of synchronous liver metastasis in colorectal cancer ・・・ 1249

### CONTENTS

P-2276 Conversion surgery for unresectable pancreatic ductal adenocarcinoma ........ 1250
P-2277 Conversion Surgery for unresectable pancreatic cancer ........ 1251
P-2278 The significance of neoadjuvant therapy for pancreatic cancer ........ 1252
P-2279 Indications and Outcomes of Conversion Surgery after Chemotherapy for Biliary Tract Cancer ........ 1253

### 16:45 - 17:30 I-P19-1:Cancer Prevention & Epidemiology (1)
### Room P / (Motenashi Dome)

P-2283 Efficacy of Caffeic acid in Coffee on Colon and Liver cancer cells ........ 1254
P-2284 Dinactin Exerts Anti-cancer Activity and Its Cellular Mechanism of Action ........ 1255
P-2285 Protective Effects of Lignosus rhinocerotis Extract Against Lipid Accumulation in Palmitic Acid-Induced H epG2 Cells ........ 1256
P-2286 Evaluation of the impact of health screening on mortality: A study using the Japanese Cancer Registry Da tabase ........ 1257
P-2287 Joinpoint analysis of age-standardized cancer mortality and comparison of annual percent change among prefectures ........ 1258

### 16:45 - 17:30 I-P19-3:Cancer Prevention & Epidemiology (3)
### Room P / (Motenashi Dome)

P-2294 Withdrawn ........ 1259
P-2295 Right versus left lung cancer: Findings from Chiba Prefecture Cancer Registry Database ........ 1260
P-2296 Null effects of diabetes mellitus on the prognosis of meningioma: A retrospective cohort study from a single center ........ 1261
P-2297 A cross-sectional study on post-marketing requirements for anticancer drugs approved in Japan from 2001 to 2024. ........ 1262
P-2298 A Novel Strategy to Bridge the Gap Between Cancer Screening and Treatment Research : Evaluation of a B ehavioral Model ........ 1263
P-2299 Withdrawn ........ 1264

### 16:45 - 17:30 II-P1:Brain tumor
### Room P / (Motenashi Dome)

P-2302 Spatial and genomic analyses for glioblastoma microenvironment with tertiary lymphoid stru ctures ........ 1265
P-2303 Effects of MTAP deletion on tumor survival and growth mechanisms in glioblastoma. ........ 1266
P-2304 Withdrawn ........ 1267
P-2305 Chromatin Modification Abnormalities by CHD7 and KMT2C Loss Promote Medulloblastoma Pr ogression ........ 1268
P-2306 Genetic analysis of H3K27-altered glioma utilizing the C-CAT data ........ 1269
P-2307 Molecular backgrounds of diffuse pediatric–type high-grade glioma, H3–wildtype and IDH –wildtype ........ 1270

### 16:45 - 17:30 II-P9-1:Prostate cancer (1)
### Room P / (Motenashi Dome)

P-2314 ASCT2/xCT Axis Promotes Castration-Resistant Prostate Cancer Progression viaMetabolic and Stress -Response Modulation ........ 1271
P-2315 Adipokines are involved in the proliferation, cell death, and biochemical recurrence of prostate c ancer ........ 1272
P-2316 Tumor-associated macrophages promote bladder cancer metastasis through the CCL20-CCR6 axis ... 1273
P-2317 CD169-Positive Macrophages and the Tumor Microenvironment in Regional Lymph Nodes of Prostate Ca ncer ........ 1274
P-2318 FABP4 promotes HFD-induced prostate cancer progression via inflammatory and metabolic pathways in T RAMP mice ........ 1275
P-2319 EndoMT in the tumor microenvironment promotes neuroendocrine differentiation of prostate cancer via G M-CSF signaling ........ 1276

### 16:45 - 17:30 II-P9-3:Urothelial cancer
### Room P / (Motenashi Dome)

### CONTENTS

P-2327 Elucidation of RNA modification function in upper tract urothelial carcinoma through multi-omics analysis ........ 1277
P-2328 PDGFR beta-STAT3 axis is involved in sphere-forming ability through regulating CD44 expression in bla dder cancer ........ 1278
P-2329 PRAME as a Prognostic Marker and Predictor of BCG Therapy Response in Bladder Cancer ........ 1279
P-2330 Therapeutic strategies targeting miR-424-5p-dependent CCNE1 amplification in cisplatin-resistant bladder cancer ........ 1280
P-2331 Tumor Microenvironment in Photodynamic Therapy for Upper Tract Urothelial Carcinoma ........ 1281
P-2332 Loss of wild-type p53 drives immune evasion in a novel urothelial organoid model harboring p53 missense mutation ........ 1282
P-2333 Improved diagnostic yield for non-muscle invasive bladder cancer using urinary cell-free DNA ........ 1283

### 16:45 - 17:30 II-P10:Skin tumors
### Room P / (Motenashi Dome)

P-2342 Analysis of the impact of a germline POLE mutation on voriconazole-associated cutaneous squamous cell c arcinoma ........ 1284
P-2343 NF-κB signaling is suppressed by diacylglycerol kinase γ in melanoma cells. ........ 1285
P-2344 Enhanced tumor endothelial marker 8 protein predicts poor prognosis in angiosarcoma. ........ 1286
P-2345 Treatment of advanced melanoma using IPS cell-derived myeloid cells overexpressing OX40 li gand ........ 1287

### 16:45 - 17:30 II-P11-2:Hematological malignancies (2)
### Room P / (Motenashi Dome)

P-2352 Response to valemetostat is linked to the induction of anti-HTLV-1 immune responses in adult T-cell leu kemia (ATL) ........ 1288
P-2353 Analysis of the mechanism by which vitamin K2 induces an integrated stress response in AML c ell lines. ........ 1289
P-2354 Impact of Clinico-genetic Factors on Prognosis After allogeneic HSCT in Acute Lymphoblastic L eukemia ........ 1290
P-2355 Differential regulatory T cell-like activity of novel paired HTLV-1 infected cell lines with and without CD13 expression ........ 1291
P-2356 CRISPR-based inducible knockout system in human blood cancer xenograft models ........ 1292
P-2357 Ambroxol induces myeloma cell death via inhibiting late stage of autophagy. ........ 1293

### 16:45 - 17:30 II-P13:Challenges in elucidating the nature of rare cancers & exploring new treatment options
### Room P / (Motenashi Dome)

P-2364 Development of hypoxically activated Magic Bullet having ultrahigh tumor-specificity ........ 1294
P-2365 Epigenetic suppression of GPX7 in pseudomyxoma peritonei may promote mucin production via enhanced PKA signaling ........ 1295
P-2366 Development of a Comprehensive Specimen Collection Timepoint Management System for Spatiotemporal Multi-omics Analysis ........ 1296
P-2367 Uncovering Prognostic Biomarkers for Enhanced Risk Stratification in Extraskeletal Myxoid Chondro sarcoma ........ 1297
P-2368 The effect of free fatty acids on the survival of malignant mesothelioma cells: Mechanism of cell death in duced by FFAs ........ 1298
P-2369 Pre-existing autoimmune disease as a risk factor for irAEs in cancer patients receiving immune checkpoint inhibitors ........ 1299
P-2370 Cross-Tumor Single-cell RNA-Seq Analysis Reveals Cancer-Specific Cellular and Molecular Fea tures ........ 1300
P-2371 Two neuroendocrine tumors of kidney with ghrelin expression ........ 1301

### 17:30 - 18:15 I-P1-4:Evaluation of Chemical & Radiation Carcinogenesis
### Room P / (Motenashi Dome)

P-2010 Leveraging mathematical models for extrapolation from laboratory animal data to human cancer risk ........ 1302

### CONTENTS

P-2011 Robustness of the four maker genes (Bax, Btg2, Ccng1, and Cdkn1a) for predicting genotoxicity of hepa tocarcinogens ······ 1303
P-2012 Direct visualization of clustered DNA damage using atomic force microscope in TK6 cells exposed on irr adiation. ······ 1304
P-2013 Effect of free fatty acid receptor signaling on X-ray-induced DNA damages in osteosarcoma cells ······ 1305
P-2014 Effect of radiation quality on Ptch1 allele deletion in radiation-induced mouse medulloblastoma ······ 1306
P-2015 Potential roles of chronic inflammation in radiation-induced mammary carcinogenesis ······ 1307
P-2016 A Tumor-Suppressive miRNA Induces Senescence via Targeting DNA Replication and Repair genes in Oral Cancer ······ 1308
P-2017 WDR82-Mediated Regulation of LncRNA Expression and R-loop Formation at Bidirectional Promoters in R esponse to UV Damage ······ 1309
P-2018 The vulnerability of NRF2-activated cancer cells ······ 1310

### 17:30 - 18:15 I-P2-5:Organoid/Animal model (2)
### Room P / (Motenashi Dome)

P-2026 A lung cancer patient-derived xenograft with the KRAS allelic imbalance showed a cancer stem cell pro perty ······ 1311
P-2027 Development and application of a 3D organotypic in vitro oral cancer model composed of patient-derived c ells ······ 1312
P-2028 Development of Predictive Biomarkers Using Multiple In Vitro Models: Pseudo-organoid and Monolayer C ultured Cells ······ 1313
P-2029 Establishment and Characterization of a Cancer Organoid from Esophageal SCC with Large Cytoplasmic V acuoles ······ 1314
P-2030 A model of peritoneal dissemination using co-culture of gastric cancer organoids and mesothelial cells ······ 1315
P-2031 Orthotopic Lung Cancer Model with Endoscopic Cell Delivery for Evaluating Tumor Environment and Drug Effects ······ 1316
P-2032 Bladder Cancer Xenografts in Mice and Zebrafish: Comparative Human Gene Expression In Vitro and In V ivo ······ 1317
P-2033 Proteogenomics of patient-derived cancer cachexia models: toward organ-targeted therapeutic s trategies ······ 1318

### 17:30 - 18:15 I-P4-4:Oncogenes & tumor-suppressor genes (3)
### Room P / (Motenashi Dome)

P-2041 Novel regulatory mechanisms for c-Myc oncoprotein degradation by the PP2A-B55α co mplex ······ 1319
P-2042 Identification of an NCYM-targeting compound that induces mitotic and apoptotic cell death ······ 1320
P-2043 Photochemical method inhibiting expression of c-Myc gene by using photo-crosslinkable antisense oli gonucleotides ······ 1321
P-2044 Functional analysis implicates oncogenic potential of SNIP1 in 1p34 risk locus in ovarian cancer. ······ 1322
P-2045 HSPD1 acts as an oncogenic driver in lung adenocarcinoma: Insights from proteomic analysis and func tional validation ······ 1323
P-2046 SPZ1 as an Oncogene: Its Role in Tumorigenesis and Stemness Regulation in NSCLC ······ 1324
P-2047 Downregulation of KRTAP2-3 suppresses proliferation and tumor formation in oral cancer cells ······ 1325
P-2048 Regulatory mechanism of BRCA1 ubiquitination and centrosomal localization by E3 activity of A urora A ······ 1326
P-2049 BARD1β increases the OLA1 polyubiquitination by Aurora A to promote centrosome mat uration ······ 1327

### 17:30 - 18:15 I-P4-6:Oncogenes & tumor-suppressor genes (5)
### Room P / (Motenashi Dome)

P-2059 Functional Characterization and Clinical Relevance of KCNB1 in Esophageal Cancer ······ 1328
P-2060 Characteristics of Gastric Cancer Revealed by Single-Cell RNA Sequencing ······ 1329
P-2061 Collaboration between Wnt Signaling and E-Cadherin Loss in the Development of Diffuse-Type Gastric Ca ncer ······ 1330
P-2062 Significance of CCNE1 Amplification in Lymph Node Metastasis of Gastric Cancer ······ 1331
P-2063 Investigating the role of ADAMTS6 in gastric cancer developing ······ 1332

### CONTENTS

P-2064 CKAP4 is highly expressed in malignant pulmonary mesothelioma and regulates AKT and tumor growth ···· 1333
P-2065 AHNAK2 promotes proliferation and survival of pancreatic cancer cells through enhanced p21 e xpression ···· 1334
P-2066 Elucidation of the function of mRNA nuclease Regnase-1 in renal cell carcinoma ···· 1335
P-2067 Development of Rapid Variant Assessment Method Using Zebrafish ···· 1336

17:30 - 18:15 I-P5-4:Non-coding RNAs (2)
Room P / (Motenashi Dome)
P-2078 Induction of APOBEC3B expression via the TGFβ-Smad pathway is regulated by lincNMR ···· 1337
P-2079 A high-throughput 3'UTR reporter screening identifies microRNAs targeting ASF1B in pancreatic aden ocarcinoma ···· 1338
P-2080 The Role of miR-224 in Hepatocellular Carcinoma: An Integrated In Vitro and In Vivo Analysis ···· 1339
P-2081 Withdrawn ···· 1340
P-2082 Withdrawn ···· 1341
P-2083 Tumor suppressive effect and potential as a molecular target of miR-224 in colorectal cancer ···· 1342
P-2084 circPPFIA1 induces cell growth in squamous cell carcinoma by modulating cancer-associated gene expr ession. ···· 1343
P-2085 The utility of plasma miR-1254 as chemosensitivity biomarker in esophageal squamous cell ca rcinoma ···· 1344
P-2086 Circular RNA circWDPCP_0026 and Its Regulatory Network in Cervical Cancer ···· 1345

17:30 - 18:15 I-P8-2:DNA methylation & others
Room P / (Motenashi Dome)
P-2094 Cell-fate reprogramming of gastric cells with tumorigenic aberrant epigenome by transient Yamanaka fa ctor expression ···· 1346
P-2095 Causal roles of DNA hypermethylation in better treatment response of the head and neck car cinoma ···· 1347
P-2096 Methylated HOXA1 with conventional tumor markers shows high diagnostic sensitivity for early hepat ocellular carcinoma ···· 1348
P-2097 Identification of the structural alterations causing epigenetic aberrations by long-read sequencing in mes othelioma ···· 1349
P-2098 The Impact of SLC25A22-Regulated Metabolic Pathways on the epigenetic regulation of NRXN1 in Gastric Cancer Progression ···· 1350
P-2099 Heterogeneity and biological implications of critical transcription factor in hepatoblastoma de velopment ···· 1351
P-2100 Improvement of prognostic marker assessment and application to patient stratification ···· 1352
P-2101 SEgene: A platform for analyzing cancer-related super-enhancers based on epigenetic correlation data and public database ···· 1353

17:30 - 18:15 I-P9-3:Cell adhesion & invasion (1)
Room P / (Motenashi Dome)
P-2109 Avasimibe's pan-cancer action: Blocking lipid accumulation and STAT1/3 signaling ···· 1354
P-2110 Angulin-1/LSR suppresses vasculogenic mimicry in human breast cancer isoform-specific manner ···· 1355
P-2111 Zyxin is mainly involved in persistent cell migration during migration and invasion. ···· 1356
P-2112 EVs of cancer cells induce FOXP3+ fibroblasts and facilitate tumor invasion via Wnt3-b-catenin p athway ···· 1357
P-2113 PTEN regulates MT1-MMP-mediated proMMP-2 activation in cancer cells. ···· 1358
P-2114 TFEC inhibits the motility of renal cell carcinoma through enhanced cell-cell adhesion by upregulation of K sp-cadherin ···· 1359
P-2115 Contribution of FAK to pS-EphA2-mediated cell migration ···· 1360
P-2116 SOX10 regulates melanoma metastasis through ITGA3 and EphA2. ···· 1361

17:30 - 18:15 I-P10-3:Telomere/Aging/Senescence/Immortalization
Room P / (Motenashi Dome)
P-2125 The crosstalk between NK cells and senescent fibroblasts ···· 1362

### CONTENTS

P-2126 Human Artificial Chromosome-based identification of an mTert suppression gene in pancreatic c ancer cells. ........ 1363
P-2127 The Role of Cellular Senescence in Fibrogenesis Phenotype ........ 1364
P-2128 SIN3A/SAP3-mediated hTERT upregulation in human oral squamous cell carcinoma cells. ........ 1365
P-2129 TERRA RNA regulates cellular senescence ........ 1366
P-2130 RNA acetylation enzyme NAT10 regulates telomerase activity ........ 1367
P-2131 The role of CREB1 in keratinocyte differentiation: Insights from a calcium-induced differentiation model ........ 1368
P-2132 Exploring the mechanisms regulating malignant micropapillary patterns of lung adenocarcinoma via epi genomic profiling ........ 1369
P-2133 Role of PDZD8 and MFN2 in MAM Remodeling During Neuroblastoma Differentiation ........ 1370
P-2134 Relation between MAM-associated proteins and differentiation in colorectal cancer cells ........ 1371
P-2135 Spatial Multi-omics Analysis Reveals Distinctive Molecular Events During Colorectal Adenoma-Carcinoma T ransition ........ 1372

### 17:30 - 18:15 I-P10-5:Metabolism/Metabolome (2)
### Room P / (Motenashi Dome)

P-2143 Combination therapeutic strategies targeting a glutamine transporter in colorectal cancer ........ 1373
P-2144 Functional linkage in mitochondrial electron transport, glycolysis, and AMPK signaling underlying cancer c ell survival ........ 1374
P-2145 Ferroptosis Overcomes AMG510 Resistance in KRASG12C /KEAP1-Mutant Lung Cancer via NRF2-Driven G lutathione Metabolism ........ 1375
P-2146 Analysis of cancer cachexia mechanisms using severely immune deficient mice ........ 1376
P-2147 Glutamine-polyamine metabolic axis promotes cell growth in cancer cells. ........ 1377
P-2148 Involvement of FUT8 in EMT and cancer stemness via behavior of HDAC in pancreatic cancer ........ 1378
P-2149 Withdrawn ........ 1379
P-2150 Effects of Synbiotics on Cancer Cachexia-Induced Tongue Atrophy ........ 1380

### 17:30 - 18:15 I-P11-8:Tumor immune microenvironment/Tumor immune escape (4)
### Room P / (Motenashi Dome)

P-2161 Evaluation of a Novel Dipeptide PD1 Inhibitor Across Three Syngeneic Models Reveals Broad Immune Mo dulatory Activity ........ 1381
P-2162 Etoposide-Induced tumor derived DAMPs upregulate Ccl2 and Ccl7 expression in macrophages ........ 1382
P-2163 Withdrawn ........ 1383
P-2164 Glutamine Deprivation Suppresses cGAS/TBK1 Signaling and Impairs Antitumor Immunity in KRAS-Mutant Lung Cancer ........ 1384
P-2165 SERBP1 Drives Immune Evasion via STING-Mediated Galectin-9 Upregulation in Pancreatic Can cer ........ 1385
P-2166 Complement c5: A Possible Linkage Molecule among diabetes and inflammation-mediated Progression of c holangiocarcinoma ........ 1386
P-2167 Withdrawn ........ 1387
P-2168 Single-cell RNA sequencing identifies immune evasion mechanisms in endometriosis resembling cancer ........ 1388

### 17:30 - 18:15 I-P11-10:Tumor immune microenvironment/Tumor immune escape (6)
### Room P / (Motenashi Dome)

P-2177 Carcinogenic mechanism of IFN-STAT1-dependent hepatocellular carcinoma in past HBV inf ection ........ 1389
P-2178 Tertiary Lymphoid Structures in CRC: Clinical Significance and Interaction with CAFs and the Immune Mi croenvironment ........ 1390
P-2179 Regulation of CD73 by Pyra-Metho-Carnil in Tumor Microenvironment and Its Link to Tumor-Associated M acrophage Maturation ........ 1391
P-2180 ICOS expression in the tumor immune microenvironment of lung adenocarcinoma ........ 1392
P-2181 Inhibition of TGF-β signals suppresses tumor formation by regulating multiple components of tumor microenvironments ........ 1393
P-2182 The impact of novel NLRC5-binding protein on anti-cancer immune response. ........ 1394
P-2183 Immunological assessment for right-sided colon cancer ........ 1395

### CONTENTS

P-2184 GSEA of FGF pathway signature in TME-evaluable specimens of ccRCC ..... 1396
P-2185 CD155 expression in pathological stage I lung adenocarcinoma ..... 1397

### 17:30 - 18:15 I-P11-12:Immune checkpoint inhibitors (2)
### Room P / (Motenashi Dome)

P-2194 Immune status and the efficacy of immune checkpoint inhibitors in non-small cell lung cancer patients with sarcopenia ..... 1398
P-2195 Development of an in vivo ICI evaluation system using humanized NOG-ΔMHC mice with colorectal c ancer-derived PDX ..... 1399
P-2196 Galectin-1 expression predicts the efficacy of anti-PD-1 therapy for non-small cell lung cancer p atients ..... 1400
P-2197 Identification and functional analysis of immune suppressor in liver cancer ..... 1401
P-2198 scRNA and scTCR sequencing of liver-infiltrating T cells and antigen search in fulminant hepatitis after ICI therapy ..... 1402
P-2199 Development of NK humanized mouse models for the in vivo evaluation of NK cell-based ther apeutics ..... 1403
P-2200 Withdrawn ..... 1404
P-2201 Withdrawn ..... 1405
P-2202 Immune Checkpoint Inhibitors for Advanced Triple Negative Breast Cancer Patients: A Bayesian Network M eta Analysis ..... 1406

### 17:30 - 18:15 I-P12-4:Diagnosis by tumor markers & biomarkers
### Room P / (Motenashi Dome)

P-2212 Prognostic Significance of EMT Markers in ARID1A-Deficient Colorectal Cancer: A Clinico pathological Study ..... 1407
P-2213 Withdrawn ..... 1408
P-2214 Combination of urinary free-glycan markers for the diagnosis of various malignant tumors ..... 1409
P-2215 Serum D-asparagine concentration adjusted for eGFR is a promising blood-based screening marker for ur othelial carcinoma ..... 1410
P-2216 Proteogenomics-based comprehensive profiling of autoantibody-bound novel cancer antigens ..... 1411
P-2217 Identification of candidate molecules for disease glycobiomarkers by data-driven analysis ..... 1412
P-2218 MicroRNA-Based Identification of Novel Basal-like Subtypes in Post-Treatment Pancreatic Cancer ..... 1413
P-2219 ADAR1 can be a effective biomarker for colorectal cancer patients with synchronous liver met astasis ..... 1414
P-2220 A novel index including age, sex, hTERT, and methylated RUNX3 is useful for predicting early g astric cancer ..... 1415

### 17:30 - 18:15 I-P13-4:Molecular targeted therapy
### Room P / (Motenashi Dome)

P-2230 Evaluation of the Effects of the NAMPT Inhibitor FK866 on Cell Proliferation in Colorectal Cancer Cells ..... 1416
P-2231 Targeting EGFR and STING Synergistically Enhances Pancreatic Cancer Therapy ..... 1417
P-2232 PVZB1077 Induces Mitochondrial Fragmentation and Cell Death in Nutrient-Stressed Pancreatic Cancer C ells ..... 1418
P-2233 Potential of Autotaxin-Targeted Therapy for Biliary Tract Cancer ..... 1419
P-2234 Targeting STAMBPL1 with a Novel Mushroom-Derived Peptide Sensitizes Cholangiocarcinoma to Apop tosis-Inducing Therapy ..... 1420
P-2235 Exploring Anti-Angiogenic Drug Repurposing as a Synergistic Approach with CDK4/6 Inhibitors in Chol angiocarcinoma ..... 1421
P-2236 TELO2 targeting therapy with ivermectin inhibits proliferation of malignant rhabdoid tumor cells ..... 1422
P-2237 The role of cetuximab in modulating calcium dynamics in oral cancer cells ..... 1423
P-2238 Rapid TKIs sensitivity profiling in CML cells using the opto-diagnostic FRET biosensor Pickles ..... 1424

### 17:30 - 18:15 I-P14-7:Anticancer drug & cell death
### Room P / (Motenashi Dome)

P-2245 Withdrawn ..... 1425

### CONTENTS

P-2246 Lysosome-dependent cell death mediates the cytotoxic effects of paroxetine in A549 lung adenocarcinoma
cells ........ 1426
P-2247 FUT8 Promotes TBK1 Stability via Autophagy to Enhance Lung Cancer Cell Survival ........ 1427
P-2248 Loperamide Enhances AMG-510 Efficacy via Autophagy-Mediated GPX4 Degradation in KRAS G12C-Mutant
Lung Cancer ........ 1428
P-2249 A Novel Eupatilin-Derived Multi-Kinase Inhibitor Enhances Therapeutic Efficacy in Triple-Negative Breast C
ancer ........ 1429
P-2250 Targeting of PTX3 impairs tumor metastasis by disruption of the tumor microenvironment in drug-tolerant
persister cells ........ 1430
P-2251 Withdrawn ........ 1431
P-2252 Tri–Synergy Metabo–Epi–Immune Therapy:Fe–Cu DAzymes &&be
ta;–Alanine for tumors ........ 1432
P-2253 Expression and roles of KIF18A in progression and survival of gemcitabine-resistance cholangiocarcinoma
cells ........ 1433

### 17:30 - 18:15 I-P17-1:Surgical intervention
### Room P / (Motenashi Dome)

P-2263 The emerging message from animal experiments on lymph node resection suggests what may happen in t
he real-world ........ 1434
P-2264 Efforts to Prevent Postoperative Complications in Esophageal Cancer Surgery to Improve Oncologic Out
comes ........ 1435
P-2265 Innovations and practice of laparoscopic gastric-tube reconstruction. ........ 1436
P-2266 Prognostic Significance of PLR and PNI in Remnant Gastric Cancer: A Multicenter Study ........ 1437
P-2267 Short-term Outcomes of Robot-assisted Left Hemicolectomy in Our Institution ........ 1438
P-2268 Surgical treatment using ICG fluorescence imaging for extrahepatic metastasis of hepatocellular
carcinoma ........ 1439
P-2269 A study of short-term outcomes of laparoscopic hepatectomy for four or more metastatic lesions ........ 1440
P-2270 Analysis of Perigallbladder Lymphatic Flow as a Pathway of Gallbladder Cancer Progression ........ 1441
P-2271 Severe complications in gynecological oncosurgery. ........ 1442

### 17:30 - 18:15 I-P18:Advances in Cell & Gene Therapy Technologies
### Room P / (Motenashi Dome)

P-2280 Development of Nucleic Acid Therapeutics Targeting the Warburg Effect ........ 1443
P-2281 Withdrawn ........ 1444
P-2282 Development of epigenome editing therapy for acute myeloid leukemia ........ 1445

### 17:30 - 18:15 I-P19-2:Cancer Prevention & Epidemiology (2)
### Room P / (Motenashi Dome)

P-2288 Cancer chemopreventive effect of Piper sarmentosum leaf extract on the early stage of liver and colon ca
rcinogenesis ........ 1446
P-2289 miR-101 mediates the inhibitory effects of active components from Antrodia cinnamomea mycelia on skin
cancer progression ........ 1447
P-2290 Growth Inhibitory Effect of Oxylipin on Human Lung Adenocarcinoma Cell Line PC-9 ........ 1448
P-2291 Withdrawn ........ 1449
P-2292 Withdrawn ........ 1450
P-2293 Prognostic value of HER2 discordance between primary and metastatic/recurrent breast cancer: a SR and
MA ........ 1451

### 17:30 - 18:15 I-P21:Palliative medicine
### Room P / (Motenashi Dome)

P-2300 Relationship Between Opioid Dose and Opioid-Induced Constipation in Cancer Patients: A Post Hoc Analysis
of OIC-J Study ........ 1452
P-2301 Investigation of the hypothalamic dysfunction in cancer cachexia aiming to discover novel druggable tar
gets ........ 1453

### 17:30 - 18:15 II-P8:Bone & soft tissue tumors
### Room P / (Motenashi Dome)

### CONTENTS

P-2308 Identification of sufficient conditions for osteosarcoma development in juvenile mice ··· 1454
P-2309 Identification of an antigen recognized by tumor-infiltrating lymphocyte in a patient with ost eosarcoma ··· 1455
P-2310 Megakaryoblastic leukemia 1 (MKL1) contributes to metastatic progression in osteosarcoma ··· 1456
P-2311 Molecular Pathology of the Tumor Microenvironment of Giant Cell Tumor of Bone by Spatial Transc riptomic Analysis ··· 1457
P-2312 Analysis of Tumor Microenvironment and Angiogenesis in Fibrosarcomatous Dermatofibrosarcoma Pro tuberans ··· 1458
P-2313 Immunohistochemical expression of SETD5 as a pathological factor for dedifferentiation and prognosis in liposarcoma ··· 1459

17:30 - 18:15 II-P9-2:Prostate cancer (2)
Room P / (Motenashi Dome)
P-2320 Exercise alters arginine metabolism in the tumor microenvironment and inhibits prostate cancer growth ··· 1460
P-2321 Association of PRC1.1 components in tumorigenesis of prostate cancer ··· 1461
P-2322 Elucidation of the tumor microenvironment that determines the chemotherapy sensitivity of bl adder cancer ··· 1462
P-2323 The critical role of the BIG3-PHB2 complex as a novel therapeutic target in prostate cancer ··· 1463
P-2324 Impact of androgen receptor signaling suppression on prostate cancer progression through KRAS activation ··· 1464
P-2325 The effects of microRNAs induced by luteolin on prostate cancer. ··· 1465
P-2326 GDF15 propeptide as a novel theranostic target in PSMA-negative and neuroendocrine prostate cancer ··· 1466

17:30 - 18:15 II-P9-4:Kidney/adrenal/testis cancer
Room P / (Motenashi Dome)
P-2334 PSMA-positive Tumo Vasculature in Renal Cell Carcinoma: An Angiogenic Phenotype Driven by the Mic roenvironment ··· 1467
P-2335 Alternative Lengthening of Telomeres in Chromophobe RCC: Prognostic Significance and Therapeutic Im plications ··· 1468
P-2336 FAXC regulates tumor progression via c-MET pathway in renal cell carcinoma ··· 1469
P-2337 YAP inhibits angiogenesis via suppression of HIF expression in clear cell renal cell carcinoma ··· 1470
P-2338 Investigation of the relationship between αE-catenin, migration ability, and intercellular adhesion in ccRCC ··· 1471
P-2339 Transcriptomic Features of Metabolically Classified Clear Cell Renal Cell Carcinoma Reveal Distinct Bio logical Pathways ··· 1472
P-2340 Elucidating the pathology of adrenocortical carcinoma using RNA-sequencing and exome seq uencing. ··· 1473
P-2341 Automated spatial analysis reveals proximity of HLA class-I positive tumor cells and CD8 positive cells in s eminoma. ··· 1474

17:30 - 18:15 II-P11-1:Hematological malignancies (1)
Room P / (Motenashi Dome)
P-2346 Withdrawn ··· 1475
P-2347 Investigation of the anti-cancer effects of Vitamin D3 (Rocaltrol) against primary effusion lym phoma (PEL) ··· 1476
P-2348 SREBF1 promotes the development of AML with poor prognosis by accelerating cholesterol me tabolism. ··· 1477
P-2349 Clinico-genomic characteristics of chromosome 7 aberrations in AML and MDS treated with stem cell tr ansplantation ··· 1478
P-2350 Serial gene panel testing is useful in the clinical management of hematopoietic neoplasms: insights from 1 ,500 analyses ··· 1479
P-2351 Distinctive Molecular Landscape of NUTM1-rearranged Infant Leukemias: Insights from Clinical and Animal Models ··· 1480

### CONTENTS

17:30 - 18:15 II-P12:Advances in Pediatric & AYA Cancers
Room P / (Motenashi Dome)
P-2358 Potential novel classification of pediatric osteosarcoma based on DNA methylation profiles and copy num ber aberrations ........ 1481
P-2359 Forkhead box O (FOXO) signal pathway activation in pulmonary metastasis-positive pediatric o steosarcoma ........ 1482
P-2360 The cellular and temporal landscape of neuroblastoma development ........ 1483
P-2361 Telomere maintenance abnormalities in high-risk neuroblastoma and their molecular subtypes ........ 1484
P-2362 Pharmacological blockade of BMI1 induces the p53 pathway, suppressing MYCN-amplified ne uroblastoma. ........ 1485
P-2363 Identification of a Synthetic Lethal Target in SMARCB1-Deficient Cancers ........ 1486

### September 27 (Sat)

8:00 - 8:50 EL6:Cellular senescence: From Guardians to Enablers of Cancer
Room 3 / (Hotel Nikko Kanazawa 4F Tsuru A)
EL6 Cellular Senescence at a Crossroads: Toward More Rigorous and Reproducible Research ........ 1487

8:00 - 8:50 EL7:Antigen-specific immunotherapy: cancer vaccine and cell therapy
Room 4 / (Hotel Nikko Kanazawa 4F Tsuru B)
EL7 TBD ........ 1488

8:00 - 8:50 EL8:Chemical Biology for Cancer Research
Room 5 / (Hotel Nikko Kanazawa 4F Tsuru C)
EL8 Life sciences and drug discovery based on chemical biology ........ 1489

8:00 - 8:50 EL9:The role of the vascular microenvironment in cancer development and progression
Room 11 / (ANA Crowne Plaza Hotel Kanazawa 3F Ohtori (West))
EL9 Tumor Blood Vessels Driving Cancer Progression and Metastasis: Cutting-Edge Research and Novel Therapeutic Strategies ........ 1490

8:00 - 8:50 EL10:Opening Up New Avenues for p53 Research
Room 12 / (ANA Crowne Plaza Hotel Kanazawa 3F Ohtori (Central))
EL10 New approach to targeting mutant p53 cancers ........ 1491

8:30 - 11:30 SS1:JCA-KCA Joint Symposium
Room 1 / (Ishikawa Ongakudo 2F Concert Hall)
SS1-1 Molecular Changes Underlying Cholangiocarcinoma Heterogeneity and their Clinical Implications ........ 1492
SS1-2 To Be or Not to Be: The Existentialism of PIK3CA Mutation in Lung Cancer ........ 1493
SS1-3 Precision Immunotherapy for Gastric Cancer: Insights from the Tumor Microenvironment ........ 1494
SS1-4 AACR Initiatives in Asia and Asia Pacific Regions ........ 1495
SS1-5 Trials for early detection of pancreatic cancer ........ 1496
SS1-6 Strategic drug development using in vitro and in vivo screening to overcome cancer heterogeneity ........ 1497
SS1-7 Never-smoker Lung Cancer in Asians: Epidemiology, Molecular Features, and Clinical Implications ........ 1498

9:00 - 11:30 SP3:JCA Work-Life-Balance Grand Conference - Achieving a well-being researcher life with JCA -
Room 2 / (Ishikawa Ongakudo 2F Hogaku Hall)
SP3-1 Back to the Heart of Cancer Research: Integrating Mission and Researcher Well-being ........ 1499
SP3-2 Mindset for Leading a Research Team: Balancing Personal and Team Well-being ........ 1500
SP3-3 Where is utopia for us scientists? ........ 1501
SP3-4 Considering work-life balance in basic medical research ........ 1502

### CONTENTS

### 9:00 - 11:30 S9:Cellular senescence: From Guardians to Enablers of Cancer
Room 3 / (Hotel Nikko Kanazawa 4F Tsuru A)

S9-1 Induction of Cellular Senescence and Ploidy Alterations in Cancer Evolution ........ 1503
S9-2 Plasma membrane damage-dependent senescence and its implications for cancer biology ........ 1504
S9-3 p53 loss enables breast cancer escape from CDK4/6 inhibitor-induced senescence ........ 1505
S9-4 A double-edged sword: cellular senescence in mammary gland development and breast cancer. ........ 1506
S9-5 Elucidation of ferroptosis resistance mechanisms in pancreatic cancer and senescent stromal cells ........ 1507

### 9:00 - 11:30 S10:Antigen-specific immunotherapy: cancer vaccine and cell therapy
Room 4 / (Hotel Nikko Kanazawa 4F Tsuru B)

S10-1 First in human study of allogeneic iPS-NKT cell infusion in patients with recurrent or advanced head and ne ck cancer ........ 1508
S10-2 Cytokine signaling to boost CAR-T cell therapy ........ 1509
S10-3 Development of novel cancer mRNA vaccine therapy using shared cancer antigens ........ 1510
S10-4 Engineering TCR-controlled fuzzy logic into CAR T cells enhances therapeutic specificity ........ 1511
S10-5 Targeting the cytosolic nucleic acid sensing pathway to drive anti-tumor immunity ........ 1512

### 9:00 - 11:30 JS4:Chemical Biology for Cancer Research
Room 5 / (Hotel Nikko Kanazawa 4F Tsuru C)

JS4-1 Synergistic Use of Functional Small Molecules and Proteins for Cancer Antigen Imaging ........ 1513
JS4-2 Therapeutic In Vivo Synthetic Chemistry ........ 1514
JS4-3 Identification of Redox and Druggable Vulnerabilities in Cancer ........ 1515
JS4-4 Design and evaluation of target-selective covalent inhibitors for cancer treatment ........ 1516
JS4-5 Precision Control of Cellular Functions via Photothermal Nanoheating Platforms ........ 1517

### 9:00 - 10:15 I-E8-1:Cancer epigenetics (1)
Room 6 / (Hotel Nikko Kanazawa 3F Kujaku A)

E-3001 Synthetic lethality using DNA methylation silencing ........ 1518
E-3002 Establishment of a single-cell epigenomic profiling pipeline ........ 1519
E-3003 Early-life epigenetic plasticity is shaped by dynamic chromatin accessibility changes and stem cell diversity ........ 1520
E-3004 Simultaneous single cell gene mutation and chromatin accessibility analysis of myeloid precursor lesions ........ 1521
E-3005 Non-coding RNA ELEANOR2-driven super-enhancer regulation of ESR1 with genomic ampli fication ........ 1522
E-3006 Oncogenic RAS activation drives transcriptional alterations through regulating chromatin dyn amics ........ 1523

### 9:00 - 10:15 I-J12-3:Liquid biopsy in cancer diagnosis
Room 7 / (Hotel Nikko Kanazawa 3F Kujaku B)

J-3001 GeMSort: Algorithm to Identify Germline Pathogenic Variants in Liquid Genomic Profiling - Insights from C -CAT Database ........ 1524
J-3002 cfDNA-Based Monitoring and MRD Prediction in BRAFV600E Mutant Colorectal Cancer: A Precision Med icine Biomarker Study ........ 1525
J-3003 Circulating tumor DNA detects minimal residual disease and predicts outcomes in esophageal cancer after esophagectomy ........ 1526
J-3004 Utility of Plasma and Urine ctDNA as Minimally Invasive Biomarkers in Advanced Esophageal Ca ncer ........ 1527
J-3005 Serial circulating tumor DNA analysis reveals clonal dynamics and prognostic alterations in high-risk neu roblastoma. ........ 1528
J-3006 TALnc1 (LINC01869) as a potential biomarker for liquid biopsy in colorectal cancer ........ 1529

### 9:00 - 11:30 IS7:UICC: Inequality and Cancer Outcome
Room 8 / (Hotel Nikko Kanazawa 3F Kujaku C)

### CONTENTS

IS7-2 Socioeconomic Inequalities and Cancer Outcomes at the Community Level: A Spatial Epidemiological Appr oach ........ 1530
IS7-3 Social disparities in cancer through the dual lens of place ........ 1531
IS7-4 Current situations of socioeconomic inequalities in cancer mortality in Japan ........ 1532
IS7-1 Underuse and overuse of medical cancer in cancer ........ 1533
IS7-5 Socioeconomic Disparities in Six Common Cancer Survival Rates in South Korea ........ 1534

9:00 - 10:15 I-E10-4:Cancer stem cells (1)
Room 9 / (Hotel Nikko Kanazawa 3F Lumiere)

E-3013 Metabolic vulnerability of ancestor-like cancer stem cells in radio-resistance ........ 1535
E-3014 G-CSF promotes tumorigenesis, metastasis, and macrophage recruitment via G-CSFR in CAF-driven breast c ancer. ........ 1536
E-3015 Formation of chemo-resistant gastric cancer microenvironment by ALDH1A3 through regulation of ext racellular matrix (ECM) ........ 1537
E-3016 Identification of MUC1-C as a Novel Oncogenic Regulator in Merkel Cell Carcinoma ........ 1538
E-3017 Perivascular niche generates glioblastoma stem cells via calcium ion-NFAT-c-Myc driven repr ogramming ........ 1539
E-3018 Lipid Droplet Profiling and Visualization of Lipid Metabolism in Ovarian Cancer by Fluorescence Lifetime I maging ........ 1540

9:00 - 11:30 SP4:Special Program4
Room 10 / (Art Hall)

SP4-reviewer1 ........ 1541
SP4-reviewer2 ........ 1542
SP4-1 Comprehensive molecular profiling of 84 cases of endometrial cancer. ........ 1543
SP4-2 DNA Damage Tolerance and Therapy Resistance in Polyploid Cancer Cells ........ 1544
SP4-3 Association between Green Tea Intake and bone strength and its sex differences -one example of S hika study- ........ 1545
SP4-4 The Innate Immune System Activated by Temozolomide Predicts Response to Immune Checkpoint Inhibitors in Glioblastoma ........ 1546
SP4-5 Identification of neural circuits responsible for abnormal social behavior in a mouse model of au tism ........ 1547
SP4-6 Investigation of the Mechanism of Radiosensitization by Bafilomycin ........ 1548
SP4-7 A study on changes in mindset and learning among medical students participating in a cancer genomic me dicine practicum ........ 1549

9:00 - 11:30 JS5:The role of the vascular microenvironment in cancer development and progression
Room 11 / (ANA Crowne Plaza Hotel Kanazawa 3F Ohtori (West))

JS5-1 How tumor cells cross endothelial barrier ........ 1550
JS5-2 Inflammaging of endothelial cells and cancer progression ........ 1551
JS5-3 The roles of endothelial-mesenchymal transition (EndoMT) in tumor progression and metastasis ........ 1552
JS5-4 Vascular encapsulation shields tumor clusters from immune cells in macrotrabecular-massive hepatocellular carcinoma ........ 1553
JS5-5 Endothelial heterogeneity in organogenesis and tissue repair ........ 1554
JS5-6 Trafficking inhibition to improve the vascular microenvironment in sarcoma ........ 1555

9:00 - 11:30 S11:Opening Up New Avenues for p53 Research
Room 12 / (ANA Crowne Plaza Hotel Kanazawa 3F Ohtori (Central))

S11-1 Cancer therapy based on co-targeting CKI&alpha and transcriptional kinases ........ 1556
S11-2 RUNX3-MYC-p53 Axis Governs Metastatic Switch in Gastric Cancer ........ 1557
S11-3 A tailored combination of a ROS inducer and a DDR inhibitor complements p53 loss, resulting in p53 syn thetic lethality ........ 1558
S11-4 The novel role of p53 in regulating macrophage iron metabolism for host defense ........ 1559
S11-5 Extrinsic induction of apoptosis and tumor suppression via the p53- Reprimo-Hippo-YAP-p73 pa thway ........ 1560
S11-6 Constitutive activation of hepatocyte p53 promotes liver carcinogenesis via connective tissue growth factor (CTGF). ........ 1561

### CONTENTS

### 9:00 - 11:30 AOSR5:The World of Hematologic Malignancy Research Unveiled by Emerging Technologies
### Room 13 / (ANA Crowne Plaza Hotel Kanazawa 3F Ohtori (East))

AOSR5-1 Liquid Biopsy-Based Monitoring of Disease Dynamics and Immune Landscape in Lymphoma ······· 1562
AOSR5-2 Uncovering Epigenetic Heterogeneity in Leukemia: Insights from Integrative Profiling ··········· 1563
AOSR5-3 Novel technology to understand and target aberrant RNA processing in cancer ················· 1564
AOSR5-4 Advances in SETBP1-Mutated Leukemia: Genomic, Epigenetic, and Therapeutic Insights ··········· 1565
AOSR5-5 Single-cell and spatial multi-omics reveals tumor heterogeneity and microenvironment diversity in T-cell lymphoma ······· 1566

### 9:00 - 10:15 I-J10-4:Cell culture system/Organoid
### Room 14 / (ANA Crowne Plaza Hotel Kanazawa 3F Zuiun 1)

J-3019 Creation of a new pancreatic cancer organoid model using tissue resident macrophages derived from human iPS cell ······· 1567
J-3020 The effect of oxygen in pancreatic organoid growth and anti-cancer drug responses revealed by l ive imaging ······· 1568
J-3021 Integrating Spatial Transcriptomics with Hypoxia-Based Organoid to Unveil Heterogeneity of Pancreatic C ancer ······· 1569
J-3022 Investigation of interactions between cancer cells and CAFs in desmoplastic reaction subtypes of colorectal cancer ······· 1570
J-3023 An Ex Vivo Lung Cancer Model Using Bioengineered Rat Lungs ······· 1571
J-3024 Development of a long-term primary culture model using prostate cancer needle biopsies: a new tool for t arget discovery ······· 1572

### 9:00 - 10:15 II-J3-2:Pancreatic cancer
### Room 15 / (ANA Crowne Plaza Hotel Kanazawa 3F Zuiun 2)

J-3031 NUAK2 inhibition improves macromolecular delivery in a 3D model of the fibrotic pancreatic tumor mic roenvironment ······· 1573
J-3032 Establishment of Precancerous Lesions of Pancreatic Cancer Organoids and Their Genomic and Trans criptomic Analysis ······· 1574
J-3033 Decoding the Mechanisms of Pancreatic Tumorigenesis through Spatial Analysis of Ultra-Early Resected P ancreatic Cancer ······· 1575
J-3034 Investigation of tumor mutation burden in de novo malignancies after liver/pancreas transplantation in our institution ······· 1576
J-3035 Autophagy inhibition enhances oncolytic virus-mediated antitumor immunity in pancreatic cancer via MHC-I upregulation ······· 1577
J-3036 Targeting Collagen Signaling via DDR1 Enhances Macromolecular Drug Delivery in a 3D Pancreatic Cancer Fibrosis Model ······· 1578

### 9:00 - 10:15 I-E7-2:Insights into cancer progression & pathogenesis based on genomic analysis
### Room 16 / (11F Visage)

E-3025 Novel molecular classification of colorectal cancer based on Wnt pathway alterations ······· 1579
E-3026 Multi-omics analyses of ELOC-mutated renal cell carcinoma identify the distinctive molecular fe atures ······· 1580
E-3027 DDX3Y is a synthetic dependency for therapy in high-grade B-cell lymphoma harbouring damaging DDX3X mutations. ······· 1581
E-3028 Whole genome landscape of asymptomatic HTLV-1 carriers with potential risk of ATL progr ession ······· 1582
E-3029 Genomic landscape of Japanese ovarian cancer: genomic features and driver mutations per histotype and m olecular subtype ······· 1583
E-3030 Evolution of Aneuploid Karyotypes during Malignant Transformation ······· 1584

### 10:15 - 11:30 I-E8-2:Cancer epigenetics (2)
### Room 6 / (Hotel Nikko Kanazawa 3F Kujaku A)

E-3007 The Role of Ezh2 of PCR2 in Driving Tumor-associated Macrophages toward Tumor Promoting Phenotypes in Liver Cancer Model ······· 1585
E-3008 Epigenetic modulation elicits an NK cell-mediated Immune Response in Urothelial Carcinoma ······· 1586

### CONTENTS

E-3009 Loss of SIRT1 drives a positive feedback loop pathway of H19-glycolysis-histone lactylation in gastric ca ncer ........ 1587
E-3010 Non canonical Polycomb group proteins epigenetically regulate tumorigenesis during lymphocyte deve lopment ........ 1588
E-3011 The DNMT inhibitor enhance the effects of EZH2 inhibitors through MYCN instability ........ 1589
E-3012 Regulation of H3K4me3 breadth and MYC expression by the SETD1B catalytic domain in MLL-rearranged leukemia ........ 1590

### 10:15 - 11:30 I-J12-4:Diagnosis by expression analysis
### Room 7 / (Hotel Nikko Kanazawa 3F Kujaku B)

J-3007 Heterogeneity of lung adenosquamous carcinoma visualized by histopathological images and spatial ana lysis using Xenium ........ 1591
J-3008 Unlocking the Past: A Comparative Study of Three Extraction Protocols for Proteomics of 30-Year Archival FFPE Samples ........ 1592
J-3009 Utilization of an Extracellular Vesicle Encapsulated RNA Panel as a Liquid Biopsy for the Diagnosis of Pan creatic Cancer ........ 1593
J-3010 Mass spectrometry-based exploration of glycan biomarkers for predicting cancer immunotherapy response ........ 1594
J-3011 Identification of Disease-Relevant Tumor Subpopulations in Pancreatic Cancer Microenvironment Using A bSeq Profiling ........ 1595
J-3012 Gene expression profiling of metaplastic breast carcinoma using spatial transcriptomics ........ 1596

### 10:15 - 11:30 I-J10-3:Cancer stem cells (2)
### Room 9 / (Hotel Nikko Kanazawa 3F Lumiere)

J-3013 Asymmetric cell division of ALDH1-positive cancer stem cells generates glycolytic metabolically diverse cell populations ........ 1597
J-3014 Expression of ALDH1 during serial transplantation of PDX for small bowel adenocarcinoma ........ 1598
J-3015 Regulation of γ-glutamylcyclotransferase (GGCT) expression and anti-proliferative effect by its kn ockdown ........ 1599
J-3016 Ferroptosis sensitivity in adipose-like differentiated cells derived from mesenchymal breast cancer cells. ........ 1600
J-3017 Contribution between breast cancer stem cells and cancer-associated fibroblasts in breast cancer formation. ........ 1601
J-3018 Development of Neuroblastoma Tumorigenesis Model from MYCN/ALK-Overexpressing Cranial Neural Cr est Cells ........ 1602

### 10:15 - 11:30 I-J16:Radiation therapy
### Room 14 / (ANA Crowne Plaza Hotel Kanazawa 3F Zuiun 1)

J-3025 Spatiotemporal metabolomic mapping of the brains of mouse glioma model treated with Boron Neutron C apture Therapy ........ 1603
J-3026 STING pathway activation enhances anti-tumor effect of photodynamic therapy through reactive oxygen s pecies production ........ 1604
J-3027 Nonclinical study of the Combined Therapeutic Approach of Bortezomib and Carbon-Ion Irra diation ........ 1605
J-3028 Radiation Therapy Enhancement Using Magnetosome@RGD via Lymphatic Drug Delivery System for Met astatic Lymph Nodes ........ 1606
J-3029 Radiotheranostics of pentadecanoic acid conjugated 111In/225Ac-RGD peptide ........ 1607
J-3030 Efficacy of the combination of ICG/Au/Resovist conjugated cell-permeable peptide and mild hyperthermia for oral cancer. ........ 1608

### 10:15 - 11:30 II-E1:Brain tumor
### Room 15 / (ANA Crowne Plaza Hotel Kanazawa 3F Zuiun 2)

E-3019 Dissecting p53 Regulation by Nuclear Pores in Glioblastoma: From Genomics to Nanoscale Ima ging ........ 1609
E-3020 Integrated Genomic and Transcriptomic Analysis Reveals Molecular Diversity and Novel Drivers in Japanese Medulloblastoma ........ 1610
E-3021 Identification of potentially novel therapeutic targets in supratentorial ependymoma ........ 1611

### CONTENTS

E-3022 Inhibition of Anti-apoptotic Bcl-2 Family Members Induces Synergistic Cell death under ER-Stress in Gli oblastoma ........ 1612
E-3023 Prediction of Malignant Transformation in IDH-mutant Glioma Using Pre- and Postoperative Tumor Vol umes ........ 1613
E-3024 Basic research and investigator-initiated trial with NS-REIC treatment for glioblastoma ........ 1614

10:15 - 11:30 I-J13-2:New molecular targeted therapy (2)
Room 16 / (11F Visage)
J-3037 Proteogenomic analysis to explore therapeutic target signaling pathways for advanced myxoid l iposarcoma ........ 1615
J-3038 Antibody-drug conjugate targeting glypican-1 shows an antitumor effect on gemcitabine-resistant panc reatic cancer ........ 1616
J-3039 Small molecule inhibitors targeting PSF modulate p53 signaling and suppress hormone therapy-resistant c ancer growth ........ 1617
J-3040 Anti-CSC Drug Discovery targeted for ER-stress Responsive Enzyme ........ 1618
J-3041 Development of therapeutic sustainable PPI inhibitor peptides targeting the BIG3-PHB2 complex in breast cancer ........ 1619
J-3042 Withdrawn ........ 1620

11:50 - 12:40 LS25:Otsuka Pharmaceutical Co., Ltd. - Medical Affairs / Mechanisms of T Cell Activation and Inhibition in the Tumor Microenvironment
Room 4 / (Hotel Nikko Kanazawa 4F Tsuru B)
LS25 Mechanisms of T Cell Activation and Inhibition in the Tumor Microenvironment ........ 1621

11:50 - 12:40 LS26:Janssen Pharmaceutical K.K. / Mastering Bispecific Antibody Novel therapeutic options for EGFR-Mutant NSCLC~
Room 6 / (Hotel Nikko Kanazawa 3F Kujaku A)
LS26 Mastering Bispecific Antibody ~Novel therapeutic options for EGFR-Mutant NSCLC~ ........ 1622

11:50 - 12:40 LS27:AbbVie GK / Real-world data of VEN+AZA therapy in Japanese patients with acute myeloid leukemia useful for clinical practice
Room 7 / (Hotel Nikko Kanazawa 3F Kujaku B)
LS27 Real-world data of VEN+AZA therapy in Japanese patients with acute myeloid leukemia useful for clinical pr actice ........ 1623

11:50 - 12:40 LS28:Bio-Rad Laboratories K.K. / The Forefront of Multiplex Digital PCR Analysis in Cancer Research
Room 8 / (Hotel Nikko Kanazawa 3F Kujaku C)
LS28-1 1) Basics of droplet digital PCR ........ 1624
LS28-2 2) Multiplex ddPCR analysis toward cancer genome diagnosis at a private hospital research in stitute ........ 1625

11:50 - 12:40 LS29:KONICA MINOLTA REALM, INC. / Comprehensive analysis using DNA/RNA twin panels and its development to cancer genomic medicine
Room 9 / (Hotel Nikko Kanazawa 3F Lumiere)
LS29 Comprehensive analysis using DNA/RNA twin panels and its development to cancer genomic med icine ........ 1626

11:50 - 12:40 LS30:DAIICHI SANKYO COMPANY, LIMITED / Pathogenesis of Malignant Lymphoma Explored by Single-Cell and Spatial Omics Analyses
Room 11 / (ANA Crowne Plaza Hotel Kanazawa 3F Ohtori (West))
LS30 Pathogenesis of Malignant Lymphoma Explored by Single-Cell and Spatial Omics Analyses ........ 1627

### CONTENTS

11:50 - 12:40 LS31:CyberomiX Inc. / The truth about the tumor microenvironment revealed by high-resolution spatial transcriptomics analysis
Room 12 / (ANA Crowne Plaza Hotel Kanazawa 3F Ohtori (Central))
LS31 The truth about the tumor microenvironment revealed by high-resolution spatial transcriptomics a nalysis ........ 1628

11:50 - 12:40 LS32:GlaxoSmithKline.K.K. / Fundamentals and Potential of Antibody-Drug Conjugates (ADCs) Learned through Belantamab mafodotin
Room 13 / (ANA Crowne Plaza Hotel Kanazawa 3F Ohtori (East))
LS32 Fundamentals and Potential of Antibody-Drug Conjugates (ADCs) Learned through Belantamab ma fodotin ........ 1629

11:50 - 12:40 LS33:Cosmo Bio Co., Ltd.／Proteintech Japan, Co., Ltd. /
Room 14 / (ANA Crowne Plaza Hotel Kanazawa 3F Zuiun 1)
LS33-1 1) Advancing Single-Cell Multi-Omics Analysis with Intracellular CITE-seq ........ 1630
LS33-2 2) Genotype-integrated single-cell transcriptomics reveals the pathogenesis of myeloid neoplasms ... 1631

11:50 - 12:40 LS34:Mediford Corporation / Translational Research contributing to cancer research by using PDX models PDX Models as a Platform for Drug Development and Translational Research
Room 15 / (ANA Crowne Plaza Hotel Kanazawa 3F Zuiun 2)
LS34 Translational Research contributing to cancer research by using PDX models PDX Models as a Platform for D rug Development and Translational Research ........ 1632

12:50 - 14:40 TYPL:The Tomizo Yoshida Prize Lecture
Room 1 / (Ishikawa Ongakudo 2F Concert Hall)
TYPL Fall down seven times, stand up eight with cancer epigenetics ........ 1633

12:50 - 14:40 MNPL:The Matayo Nagayo Prize Lecture
Room 1 / (Ishikawa Ongakudo 2F Concert Hall)
MNPL Development and implementation of oncolytic virus therapy using genetically engineered herpes viruses ........ 1634

12:50 - 13:20 JWAS:JCA Woman Scientists Awards
Room 2 / (Ishikawa Ongakudo 2F Hogaku Hall)
JWAS Molecular Pathogenesis of Malignant Lymphoma and Its Application to Precision Medicine ........ 1635

12:50 - 14:20 OS10:Cutting edge research on chronic inflammation and tumor microenvironment
Room 3 / (Hotel Nikko Kanazawa 4F Tsuru A)
OS10-1 Chronic Inflammation and Tumor Microenvironment Research: Past and Future. ........ 1636
OS10-2 Dual Functions of IL-11+ Fibroblasts: Cancer Promotion versus Colitis Protection in Colorectal D isease ........ 1637
OS10-3 Senescent cells regulate the progression of metabolic dysfunction-related steatohepatitis and hepatocellular carcinoma ........ 1638
OS10-4 TEAD Inhibition as a Therapeutic Strategy Targeting Hepatocellular Carcinoma and Its Fibrotic Micro environment ........ 1639
OS10-5 Interaction-activated transcriptomics dissects molecular crosstalk in cancer cell - microen vironment ........ 1640
OS10-6 Mesenchymal transition in glioblastoma driven by inflammatory cells ........ 1641

12:50 - 15:20 IS8:Triangular dynamics among iron, sulfur and oxygen in cancer: novel redox concepts toward cancer prevention and therapeutics
Room 4 / (Hotel Nikko Kanazawa 4F Tsuru B)

### CONTENTS

IS8-1 Pseudohypoxic Stabilization of HIF-1α by NRF2: Implications for Metabolic Reprogramming of Hepatoma Cells ........ 1642
IS8-2 NRF2-Driven Reductive Stress Impairs Anti-Tumor Immunity in the Tumor Microenvironment ........ 1643
IS8-3 Spatial transcriptomic morphometrics unveil renal carcinogenic shift via oxidative stress and BRCA1 haploinsufficiency ........ 1644
IS8-4 Hijacking Ferroptosis with Selenium: A Redox Therapeutic Avenue ........ 1645
IS8-5 A lactate-lipid peroxidation-acetate metabolic axis between TAMs and cancer cells fuels HCC metastasis ........ 1646
IS8-6 Oxidative stress adaptation and its role in cholangiocarcinogenesis: the EBF1-IRS1 axis in focus ........ 1647

### 12:50 - 14:05 I-E11-5:Cancer vaccines/Other immunotherapies (1)
### Room 5 / (Hotel Nikko Kanazawa 4F Tsuru C)

E-3031 Neo-DCVac in Combination with Immune Checkpoint Inhibitors for the Treatment of ICI-Resistant Advanced Lung Cancer ........ 1648
E-3032 EZH2 inhibitor combined with radiotherapy induces immune response by activating cGAS-STING pathway in prostate cancer ........ 1649
E-3033 Novel combination immunotherapy to enhance the efficacy of immune checkpoint inhibitors against hepatocellular carcinoma ........ 1650
E-3034 Development of a novel immunomodulation therapy for pancreatic ductal adenocarcinoma targeting complement factor B ........ 1651
E-3035 Near-infrared photoimmunotherapy using an affibody for EGFR-positive malignant brain tumor ........ 1652
E-3036 Anti-cancer cell therapy using CCL19-expressing mesenchymal stem cells exerts robust anti-tumor effect ........ 1653

### 12:50 - 13:30 ME5:Cancer Cell
### Room 6 / (Hotel Nikko Kanazawa 3F Kujaku A)

ME5 Cancer Cell ........ 1654

### 12:50 - 14:05 I-E6-1:Cell cycle & DNA damage response
### Room 7 / (Hotel Nikko Kanazawa 3F Kujaku B)

E-3043 Identification of Flavonols as PARP1 Inhibitors in Cholangiocarcinoma: Insights from Molecular Dynamics and Multi-Omics ........ 1655
E-3044 Targeting Motor Protein X to Disrupt Centrosome Clustering: A Therapeutic Strategy for Small Cell Lung Cancer (SCLC) ........ 1656
E-3045 Tissue-specificity in centriole duplication regulation by BRCA1-associated protein RACK1 ........ 1657
E-3046 A novel strategy for targeting chromosomal instability in cancer ........ 1658
E-3047 Dysregulation of supercoil dynamics associated with replication stress in cancer ........ 1659
E-3048 Synergism of Glutamine and the Calcium responsive NFATc1 transcription factor in pancreatic cancer progression ........ 1660

### 12:50 - 15:20 IS9:Elucidating the regulatory mechanisms involved in tumor tissue heterogeneity through advanced informatics technologies
### Room 8 / (Hotel Nikko Kanazawa 3F Kujaku C)

IS9-1 Deriving microenvironment associated with cell state distribution by transformer-based self supervised learning ........ 1661
IS9-2 Withdrawn ........ 1662
IS9-3 Geospatial Analysis of Liver Cancer Microenvironment for Comprehensive Insight and Drug Discovery ........ 1663
IS9-4 Intelligent Decoding of Spatial Biology ........ 1664
IS9-5 Spatial omics approaches to understand the evolution and therapeutic response of gastrointestinal cancers ........ 1665
IS9-6 The Multi-Layered Transcriptional Architecture of Glioblastoma Ecosystems ........ 1666

### 12:50 - 14:05 I-E10-5:Cell-to-cell interaction (1)
### Room 9 / (Hotel Nikko Kanazawa 3F Lumiere)

E-3055 Integrating spatial dissection of differentiation heterogeneity in immature-type gastric carcinoma ........ 1667

### CONTENTS

E-3056 A prospective study on the effect of preoperative rehabilitation on blood levels of a tumor-suppressor mi RNA ........ 1668
E-3057 Withdrawn ........ 1669
E-3058 Functional Characterization of Tumor-Educated Platelets in Hepatocellular Carcinoma Progr ession ........ 1670
E-3059 Cancer-associated lymphatic endothelial cells facilitate lymphatic metastasis: interactions with cancer or ganoids ........ 1671
E-3060 Exploring Mechanisms Driving Glioblastoma Invasion ........ 1672

### 12:50 - 15:20 OS11:Cancer Biology and Therapeutic Strategies in Cross-Organ Neuroendocrine Neoplasm
### Room 11 / (ANA Crowne Plaza Hotel Kanazawa 3F Ohtori (West))

OS11-1 Elucidation of the factors involved in the development of neuroendocrine neoplasm and its me tastasis ........ 1673
OS11-2 Patient-derived small cell lung cancer organoid library and elucidation of its biological dynamics ........ 1674
OS11-3 Treatment strategies for neuroendocrine carcinoma arising from head and neck, and gastr ointestinal area. ........ 1675
OS11-4 New treatment strategies for pancreatic neuroendocrine neoplasms (NEN) ........ 1676
OS11-5 Elucidation of Neuroendocrine Differentiation Mechanisms and Therapeutic Strategies for Pro state Cancer ........ 1677
OS11-6 Organoid analysis revealed differentiation plasticity in cervical neuroendocrine cancer ........ 1678

### 12:50 - 15:20 OS12:Frontier research on regulation of cancer progression deciphered by organelle dynamics
### Room 12 / (ANA Crowne Plaza Hotel Kanazawa 3F Ohtori (Central))

OS12-1 Tumor-driven mitochondrial "hijacking" suppresses antitumor immunity ........ 1679
OS12-2 Unraveling Cancer Progression Through Exosome-Mediated Intercellular Communication ........ 1680
OS12-3 Onset of glioblastoma by failure of alignment between ER and nucleus, and potential as a the rapeutic target. ........ 1681
OS12-4 Adaptation to the tumor microenvironment through ER-Golgi collaboration: A new therapeutic s trategy ........ 1682
OS12-5 Elucidation of novel nutrient sensing mechanisms through organelle dynamics ........ 1683
OS12-6 Targeting metabolic vulnerabilities to treat pancreatic ductal adenocarcinoma ........ 1684

### 12:50 - 15:20 AOSR7:Advances in Cancer Biology and Translational Research for Primary and Metastatic Brain Tumors
### Room 13 / (ANA Crowne Plaza Hotel Kanazawa 3F Ohtori (East))

AOSR7-1 Unraveling the complexity of glioblastoma invasion ........ 1685
AOSR7-2 Genomic heterogeneity within WNT medulloblastoma ........ 1686
AOSR7-3 Translational Research for Central Nervous System Lymphomas with paired Patient Tumors and PDX mo dels ........ 1687
AOSR7-4 Induction of cancer cell death by microglia and its regulatory mechanisms ........ 1688
AOSR7-5 Exploring the Therapeutic Potential of CAR-Engineered Immune Cells Using a Syngeneic GBM M odel ........ 1689
AOSR7-6 Extended Follow-Up of Phase 2 Trial on Survival Outcomes of Boron Neutron Capture Therapy in Recu rrent Glioblastoma ........ 1690

### 12:50 - 14:05 I-E13-3:Molecular targeted therapy (2)
### Room 14 / (ANA Crowne Plaza Hotel Kanazawa 3F Zuiun 1)

E-3061 The Oncogenic Impact of KRAS in Cholangiocarcinoma ........ 1691
E-3062 Resistance mechanism to KRAS Inhibitor in patient-derived xenograft with KRAS G12C mutation ........ 1692
E-3063 Perspectives on novel combination strategies for KRAS mutant lung cancer ........ 1693
E-3064 The inhibition of abnormal protein processing mechanisms suppresses the proliferation of drug-resistant c ancer ........ 1694
E-3065 Anti-tumor activity of orally available cyclic peptide LUNA18 through direct RAS inhibition ........ 1695
E-3066 Combination of RAS inhibitor LUNA18 and KRAS G12C inhibitors enhances anti-tumor effects by blocking MAPK reactivation. ........ 1696

### CONTENTS

### 12:50 - 14:05 I-J7-2:Clinical cancer genomics
### Room 15 / (ANA Crowne Plaza Hotel Kanazawa 3F Zuiun 2)

J-3055 Genetic architecture and clinical significance of clonal hematopoiesis among older Japanese in dividuals ........ 1697
J-3056 Clinical utility of detecting hereditary tumor-related pathogenic variants in ovarian cancer using adaptive s ampling ........ 1698
J-3057 Comprehensive HER2/ERBB2 landscape and its association with clinicopathology in bladder ca ncer ........ 1699
J-3058 An exploration of the advantages of whole-genome and transcriptome sequencing in cancer genomic med icine ........ 1700
J-3059 Therapeutic and diagnostic utility of clinical whole genome sequencing in cancer genome med icine. ........ 1701
J-3060 Whole-Genome Sequencing vs. Comprehensive Genomic Profiling in Solid Tumors: High Concordance and E xpanded Detection ........ 1702

### 12:50 - 14:05 I-J9-3:Metastasis & cell adhesion
### Room 16 / (11F Visage)

J-3067 Role of Claudin-11/MT1-MMP Axis in Small Cell Lung Cancer ........ 1703
J-3068 Laminin-γ2 fusion protein promotes metastatic potential in EGFR-wildtype non-small cell lung ca rcinoma ........ 1704
J-3069 Glycosylation of tetraspanin CD151 defines invasive phenotype of human breast cancer cells ........ 1705
J-3070 Gemcitabine-induced α6β4 integrin confers gemcitabine resistance to PDAC cells and promotes PDAC progression ........ 1706
J-3071 RSK/GSK3 mediated phosphorylation of FilGAP regulates chemotactic cancer invasion ........ 1707
J-3072 SUSD2 enhances tumor growth and metastasis by upregulating integrin-FAK signaling in pancreatic cancer cells ........ 1708

### 13:20 - 15:50 AOSR6:Ongoing development of targeted radionuclide therapy in Japan
### Room 2 / (Ishikawa Ongakudo 2F Hogaku Hall)

AOSR6-1 Development and Clinical Translation of Targeted Alpha Therapy using Astatine in Osaka ........ 1709
AOSR6-2 Development of Novel Alpha-Emitting Therapeutic Agents at Fukushima Medical University. ........ 1710
AOSR6-3 Development of 64Cu Radiotherapeutics Ongoing in Japan ........ 1711
AOSR6-4 Domestic production of Ac-225 using the experimental fast reactor Joyo ........ 1712
AOSR6-5 Production and supply of astatin-211 for targeted alpha-particle therapy ........ 1713

### 14:05 - 15:20 I-J11-7:Cancer vaccines/Other immunotherapies (2)
### Room 5 / (Hotel Nikko Kanazawa 4F Tsuru C)

J-3043 Novel Immune Drug Combination Induces Tumour Microenvironment Remodelling and Reduces the Dosage of Anti-PD-1 Antibody ........ 1714
J-3044 Dynamic analysis of long-term T cell immunity of TCR-T Vax, resulting in cure and recurrence suppression of solid tumors ........ 1715
J-3045 Anti-tumor effects of nucleic acid assembly triggered by miRNAs ........ 1716
J-3046 Intestinal bacterial metabolites enhance the efficacy of immune checkpoint inhibitor therapy for gastric ca ncer ........ 1717
J-3047 Enhancement of STING-targeting immune responses through co-treatment with the low molecular weight c ompound KIN1148 ........ 1718
J-3048 Cell fusion and immunostimulatory cytokines extremely impact on immunotherapeutic potential of oncolytic vaccinia virus ........ 1719

### 14:05 - 15:20 I-E11-6:Tumor immune microenvironment/Tumor immune escape (5)
### Room 6 / (Hotel Nikko Kanazawa 3F Kujaku A)

E-3037 Functional analysis of apCAF and mesothelial cells in colorectal cancer tumor microimmunity ........ 1720
E-3038 Immune cells derived from Tet2-mutant clonal haematopoiesis suppress liver metastasis of colorectal ca rcinoma ........ 1721
E-3039 PCSK9 from Mixed-Polarized Macrophages Induces CD8+ T Cell Exhaustion and Immune Res istance in DGC ........ 1722

### CONTENTS

E-3040 Secreted Peroxidasin Regulates Cancer Cell Immune Evasion by Modulating NK Cell-Mediated C ytotoxicity ........ 1723
E-3041 Molecular mechanisms of IL-23 overproduction by tumor-derived lactic acid ........ 1724
E-3042 The transcription factor MAFB defines the immunosuppressive nature of tumor-associated macrophages in pancreatic cancer ........ 1725

14:05 - 15:20 I-E6-2:Genomic instability
Room 7 / (Hotel Nikko Kanazawa 3F Kujaku B)
E-3049 UV-Associated Genome Instability is Increased by DNA Replication in Cells with CPD UV Photoproducts in Active Genes ........ 1726
E-3050 Molecular mechanism of centromeric HP1 enrichment ensuring mitotic fidelity ........ 1727
E-3051 Investigation of protein network involving ARID1A in SWI/SNF chromatin remodeler, and its function in g enome stability ........ 1728
E-3052 Chromosomal Instability Is Associated with cGAS-STING Activation in EGFR-TKI Refractory Non-Small-Cell Lung Cancer ........ 1729
E-3053 How Plk1 activity is regulated by conformational transition ........ 1730
E-3054 Sensitizing effects of inhibition of mitochondrial RNA polymerase on DNA damage-inducing ra diotherapy ........ 1731

14:05 - 15:20 I-J10-5:Cell-to-cell interaction (2)
Room 9 / (Hotel Nikko Kanazawa 3F Lumiere)
J-3049 Analysis of miRNA Expression Changes Induced by KrasG12D Mutant Cells in Pancreatic Ductal Progenitor Cells ........ 1732
J-3050 The role of tumor endothelial cells in tumor microenvironment in liver cancer ........ 1733
J-3051 Regulation of transforming growth factor-beta in cancer-associated fibroblasts in the hypoxic tumor mic roenvironment ........ 1734
J-3052 Functional distinctions between primary and metastatic cancer-associated fibroblasts in head and neck ca ncer ........ 1735
J-3053 Adipose-derived adipokines regulate cancer proliferation and metabolism, thereby determining cancer ma lignancy. ........ 1736
J-3054 Elucidation of the mechanism of tumor fibrosis through interorgan crosstalk ........ 1737

14:05 - 15:20 I-E13-4:Molecular targeted therapy (3)
Room 14 / (ANA Crowne Plaza Hotel Kanazawa 3F Zuiun 1)
E-3067 Osimertinib resistance via MET activation without amplification identified in a EGFR-mutant lung cancer s yngeneic model ........ 1738
E-3068 YAP-mediated regulation of HER3-driven adaptive resistance to RET inhibitors in RET-altered c ancer ........ 1739
E-3069 Dynamic Insights from MD Simulations Enable Prediction and Mechanistic Exploration of RET Inhibitor Re sistance ........ 1740
E-3070 Exploration of drug tolerance mechanisms in ALK-rearranged lung cancer using in vivo CRISPR s creening ........ 1741
E-3071 Patient-derived organoid-aided development of invasive cancer cells and angiogenesis dual targeting the rapeutic antibody ........ 1742
E-3072 A novel heavy-chain antibody-drug conjugate targeting CEACAM6 for cancer therapy ........ 1743

14:05 - 15:20 I-J18:Advances in Cell & Gene Therapy Technologies
Room 15 / (ANA Crowne Plaza Hotel Kanazawa 3F Zuiun 2)
J-3061 A transcriptomic model accurately predicting stem cell transplantation survival in pediatric acute myeloid l eukemia ........ 1744
J-3062 Exploring potential benefits of exploiting TCR machinery to CAR-T cells ........ 1745
J-3063 Disruption of the IGF2BP1–EWS::FLI1 Loop via ASO Therapy with Machine Learning-Optimized LNP Delivery in Ewing Sarcoma ........ 1746
J-3064 Mesenchymal stem cell (MSC)-based virotherapy for peritoneal metastasis in gastrointestinal ca ncers ........ 1747
J-3065 Development of innovative Triple-regulated oncolytic virus of effective and safer therapy of peritoneal ca rcinomatosis. ........ 1748

### CONTENTS

J-3066 Development of a sensor system to selectively induce cell death in chromosome 19 microRNA cluste r-positive tumors ........ 1749

14:05 - 15:20 I-J9-4:Metastasis models & EMT
Room 16 / (11F Visage)
J-3073 Colorectal cancer co-opts the wound healing program to form disseminated tumor cells during m etastasis ........ 1750
J-3074 Rho signaling is a critical driver of cancer stemness in metastatic colorectal cancer ........ 1751
J-3075 Epithelial and Mesenchymal Malignant Cells in Pleural Mesothelioma and Their Role in Intralesional Het erogeneity ........ 1752
J-3076 Impact of Increased Interstitial Pressure on Tumor Progression in Malignant Pleural Mesoth elioma ........ 1753
J-3077 Withdrawn ........ 1754
J-3078 Metastatic mechanism of pancreatic cancer based on the analysis of circulating tumor cells in portal vein b lood ........ 1755

14:20 - 15:50 OS13:New horizons of tumor microenvironment research via spatial single-cell analyses
Room 3 / (Hotel Nikko Kanazawa 4F Tsuru A)
OS13-1 Towards Multi-Omics Spatial Analysis to Reveal Cancer Heterogeneity ........ 1756
OS13-2 Unraveling the ecosystem of follicular lymphoma at Single-Cell and Spatial Resolution ........ 1757
OS13-3 Comprehensive analysis of the cancer microenvironment in MSI-H colorectal cancer ........ 1758
OS13-4 Understanding cellular networks in refractory cancers through spatial omics. ........ 1759

15:00 - 18:00 OPS:Public Open Seminar
Room 10 / (Art Hall)
Public Open Seminar ........ 1760
Public Open Seminar ........ 1761
Public Open Seminar ........ 1762
Public Open Seminar ........ 1763
Public Open Seminar ........ 1764

15:30 - 16:15 I-P3-3:Bacteria & Cancer
Room P / (Motenashi Dome)
P-3001 The association between tumoral pks+ Escherichia coli expression and oncological outcomes in colorectal cancer patients. ........ 1765
P-3002 Prognostic significance of Fusobacterium nucleatum abundance differs between right- and left-sided colon cancers. ........ 1766
P-3003 The effect of secretory DNA degrading enzymes of Prevotella intermedia on squamous cell car cinoma ........ 1767
P-3004 Glycosylation changes in macrophages induced by Fusobacterium within the endometriosis microen vironment ........ 1768
P-3005 Bacteria residing in the ovarian cancer microenvironment ........ 1769
P-3006 Withdrawn ........ 1770
P-3007 Withdrawn ........ 1771
P-3008 Inflammatory Signaling and RAGE in Pancreatic Cancer: A Potential Therapeutic Target ........ 1772
P-3009 The impacts of appendectomy for cancer development and disease control in patients with ulc erative colitis ........ 1773

15:30 - 16:15 I-P4-7:Cancer biology driven by TP53 abnormality
Room P / (Motenashi Dome)
P-3018 Cell Cycle-Related Gene Mutations and the Prognosis of Endometrial Cancer Patient: A Bayesian Network M eta-Analysis ........ 1774
P-3019 Crosstalk from Wnt signaling to p53 in multistep tumorigenesis ........ 1775
P-3020 TP53 Status as a Determinant of Response to Gemcitabine-Based Regimens in Pancreato-Biliary cancers ........ 1776
P-3021 Combined p53 Activation and Wnt Signaling Inhibition Synergistically Induce Cell Death in Colorectal Ca ncer Cells ........ 1777

### CONTENTS

P-3022 Identification of HSP40 members that stabilize conformational mutant p53 ······················ 1778
P-3023 Secreted PAD7 Shapes a Tumor-Suppressive Microenvironment by Inhibiting Lipid Metabolism in MASH -Driven Liver Cancer ························································ 1779
P-3024 The p53-PHLDA3 pathway promotes Liver cancer. ············································ 1780
P-3025 Mutant p53 Promotes Breast Cancer Malignancy through Transcriptional Regulation of the Mevalonate P athway ······························································· 1781
P-3026 p53 and Cellular Senescence in Cancer Cell Lines ········································ 1782

15:30 - 16:15 I-P5-6:Intracellular signal transduction (2)
Room P / (Motenashi Dome)
P-3036 RTK endocytois-dependent glycolysis regulation in ALK-expressing neuroblastoma ················ 1783
P-3037 Midkine promotes tumor growth and Cisplatin resistance through AKT pathway activation in small cell lung cancer ······························································· 1784
P-3038 The regulation and function of SAPK signaling pathways in the anticancer drug-induced genotoxic stress r esponses ······························································· 1785
P-3039 Significance of ULK1 in tolerance to EGFR TKI ············································ 1786
P-3040 Reciprocal regulation of ERK and MKP activities promotes mitogenic signaling and tumorigenesis ··· 1787
P-3041 Regulation of CTGF Protein Levels via AKT Signaling Activated by PDGF Receptor in Malignant Mesot helioma ······························································· 1788
P-3042 Taste 2 receptor-mediated signaling suppresses the malignancy of colorectalcancer cells ·········· 1789
P-3043 ERK mediated up-regulation of ErbB2/ErbB3 heterodimer in the LNCaP human prostate cancer c ells ······························································· 1790

15:30 - 16:15 I-P5-8:Intracellular signal transduction (4)
Room P / (Motenashi Dome)
P-3052 Functional Analysis of the Stress-Responsive MAP Kinase Pathway in Epithelial-Mesenchymal Transition ( EMT) ······························································· 1791
P-3053 Spatiotemporal regulation of KSR1 in RAS/MAPK signal propagation revealed by super-resolution micr oscopy ······························································· 1792
P-3054 HER2/HER3 signaling enhances activation of the integrated stress response in breast cancer cell lines ······························································· 1793
P-3055 ALPK2 Drives Cell Proliferation in Oral Squamous Cell Carcinoma ···························· 1794
P-3056 YAP reactivation drives ER stress-mediated dormancy under EGFR inhibition in oral squamous cell carc inoma ······························································· 1795
P-3057 Withdrawn ······························································· 1796
P-3058 Transcription-Dependent Autophagy Triggered by Nutrient Deprivation: Role of the IGF1R/PKC& beta;/CREB1 Axis ······························································· 1797

15:30 - 16:15 I-P6-2:DNA damage response
Room P / (Motenashi Dome)
P-3065 What is Lynch-like syndrome? Molecular pathology of MSI-H tumors. ·························· 1798
P-3066 The mechanisms of the recruitment of SLX4-XPF in the response to replication stress induced by lacO-LacI interaction ······························································· 1799
P-3067 Regulatory mechanism of BRCA1/BARD1 stability mediated by calcineurin. ···················· 1800
P-3068 Targeting the POLD4-PRIMPOL axis in non-small cell lung cancer ····························· 1801
P-3069 An ATR-PrimPol axis maintains chronic heterochromatin-associated replication stress tolerance in KRAS -driven cancer cell ······························································· 1802
P-3070 Functional analysis of ubiquitin ligase RFWD3 in DNA damage tolerance in human cells ··········· 1803
P-3071 Functional analysis of ARID1A in DNA repair ············································ 1804
P-3072 RNA helicase DHX8 promotes DNA damage response ········································ 1805
P-3073 Deletion of the deubiquitinating enzyme USP10 leads to prolonged DNA repair and chromosomal instability. ······························································· 1806

15:30 - 16:15 I-P7-1:Cancer genomics
Room P / (Motenashi Dome)
P-3081 Variation in Gene Expression and Methylation Profiling in Gingival and Tongue Cancers ··········· 1807

### CONTENTS

P-3082 Immunohistochemical and Molecular Evolutionary Characteristics of Jejunoileal Adenoca rcinoma ........ 1808
P-3083 Distinctive Immune Microenvironment in Pancreatic Cancer: Comparative Single-Cell Analysis with Gas trointestinal Cancers ........ 1809
P-3084 Chemotherapy resistance mechanisms of CF-based NAC in esophageal cancer ........ 1810
P-3085 Genomic and epigenomic analysis of lung large cell neuroendocrine carcinoma using a long read sequ encing technology ........ 1811
P-3086 Exploration of telomere maintenance mechanisms associated genetic abnormalities in 321 neuroblastomas (JCCG-JNBSG) ........ 1812
P-3087 Search for the conditions of library preparation aimed at obtaining optimized data using nanopore sequ ence ........ 1813
P-3088 Integrated analysis of Whole exome sequencing(WES) and Target capture sequencing ........ 1814
P-3089 Withdrawn ........ 1815

### 15:30 - 16:15 I-P7-3:Cancer genomic medicine
### Room P / (Motenashi Dome)

P-3096 Identification of Actionable Mutations and Prognostic factors using Cancer Genome Profiling in Biliary Tract Cancer ........ 1816
P-3097 Genomic analysis of meningiomas in Japan using C-CAT data ........ 1817
P-3098 Varporter software streamlines the evaluation of cancer gene panel test results ........ 1818
P-3099 Molecular and Cellular Profiling of Patient-Derived Hypopharyngeal Carcinoma Cell Lines Toward Clinical Application ........ 1819
P-3100 Clinical relevance of non-synonymous variants in BRCA2-related 6 cancer types across in vitro and in silico analysis ........ 1820
P-3101 Integrative Analysis of GATA3 Tissue Array and Genetic Variants Reveals Prognostic Implications in Uro thelial Carcinoma ........ 1821
P-3102 Withdrawn ........ 1822
P-3103 Significance of Pancreas Surveillance for HBOC Patients ........ 1823
P-3104 Sample Management System at TR Sample Management Office, National Cancer Center Hospital East. ........ 1824

### 15:30 - 16:15 I-P8-4:Histone modification & others
### Room P / (Motenashi Dome)

P-3110 MOA study of gut microbiome-derived propionate for colon cancer treatment ........ 1825
P-3111 Lacticaseibacillus rhamnosus is involved apoptosis in Renal cell carcinoma by targeting EHMT2 ........ 1826
P-3112 Lactobacillus reuteri Induces Apoptosis in HCC via SUV39H2 Modulation ........ 1827
P-3113 DUB-mediated KDM6 stabilization drives mitochondrial metabolism in lung adenocarcinoma ........ 1828
P-3114 The role of SYTL3 in restoring anti-tumor immunity in bladder cancer ........ 1829
P-3115 Characteristics of chromatin accessibility in gastric cancer cells with fetal phenotype ........ 1830
P-3116 Analysis of epitranscriptomic regulation of RNA demethylase ALKBH3 in pancreatic cancer ........ 1831
P-3117 Functional significance and epigenetic regulation of RXRG in head and neck squamous cell car cinoma ........ 1832

### 15:30 - 16:15 I-P9-5:Metastasis & vascular biology
### Room P / (Motenashi Dome)

P-3126 Proteinase-3 impairs anti-VEGF antibody efficacy via tumor angiogenesis in colorectal cancer ........ 1833
P-3127 The study of the inhibitory effects of IL-1R antagonists on pancreatic cancer invasion and ang iogenesis. ........ 1834
P-3128 The effects of beta-blockers on metastasis via their effects on vascular endothelial cells ........ 1835
P-3129 EphA2-mediated signaling is involved in the formation of vasculogenic mimicry structures constructed by cancer cells ........ 1836
P-3130 The angiostatic effects of clock gene inhibitors in pleural mesothelioma ........ 1837
P-3131 Cancer-associated mesothelial cells exhibit vascular differentiation potential in the ovarian tumor micr oenvironment ........ 1838
P-3132 Withdrawn ........ 1839
P-3133 FJX1 secreted by metastasis associated endothelial cells promote breast cancer cells stemness at the lung ........ 1840

### CONTENTS

P-3134 Squalene Epoxidase Drives Vascular Co-option and Astrocyte Reprogramming in Breast Cancer Brain Met astasis ........ 1841

### 15:30 - 16:15 I-P9-7:Metastasis models & circulating tumor cells
### Room P / (Motenashi Dome)

P-3142 Identification of RAS Mutations Using Circulating Tumor Cells, Circulating Tumor DNA, and Ex osomes ........ 1842
P-3143 Establishment of the detection and collection method of circulating tumor cells in sarcoma using a micr ofluidic device. ........ 1843
P-3144 A novel 9-gene classifier for predicting distant metastasis of soft-tissue sarcoma and multiple m alignancies ........ 1844
P-3145 A mitochondrial one-carbon metabolism promotes breast cancer tumorigenesis and lung met astasis ........ 1845
P-3146 Analysis of the biological property of peritoneal cancer cell clusters in gastric carcinoma ........ 1846
P-3147 Non-Target Organ Perturbation Enhances Pre-Metastatic Niche Conditioning in Experimental Metastasis M odels ........ 1847
P-3148 The autophagy-activated PTX3 glycosylation enhances HNSCC anoikis resistance and metastasis ........ 1848
P-3149 Analysis of Gene Regulatory Networks in Breast Cancer Dormancy ........ 1849
P-3150 Exploration of novel metastasis mechanisms using metastatic lung adenocarcinoma cell lines derived from CIEA-PDX ........ 1850

### 15:30 - 16:15 I-P10-8:Cell culture system/Organoid (2)
### Room P / (Motenashi Dome)

P-3160 NCYM promotes cholangiocarcinogenesis via activating of autophagy ........ 1851
P-3161 Investigation of an anti-tumor drug efficacy evaluation system using PDC ........ 1852
P-3162 NOTCH2NL is a context-dependent mediator of intestinal homeostasis in human ........ 1853
P-3163 Comparative Proteomic and Immunocytochemical Study of Squamous Components in 2D- and 3D-Cultured PDAC Cell Lines ........ 1854
P-3164 Interaction between breast cancer cells and tumor microenvironment in breast cancer organoids ........ 1855
P-3165 Targeting rare cancers: drug discovery using patient-derived organoids of small bowel adeno carcinoma. ........ 1856
P-3166 Activin Signaling Promotes Colon Cancer Cell Motility ........ 1857
P-3167 Clinical significance in vitro drug sensitivity profiling using pancreatic cancer organoids from EUS-FNA sa line flushes ........ 1858

### 15:30 - 16:15 I-P10-10:Cancer stem cells (2)
### Room P / (Motenashi Dome)

P-3176 Identification of pancreatic cancer stem cells and development of specific ablation therapy ........ 1859
P-3177 SLC14A1 regulates stemness and tumorigenicity of osteosarcoma stem cells ........ 1860
P-3178 Role of PLOD2 in regulating osteosarcoma stem cell properties ........ 1861
P-3179 Identification of a novel molecular regulator of chemoresistance in colorectal cancer ........ 1862
P-3180 Compound Screening Reveals a Key Signaling Pathway Involved in the Regulation of Colorectal Cancer St emness ........ 1863
P-3181 Development of novel targeted therapy for ovarian cancer stem cells using hydrogel ........ 1864
P-3182 CD80 Enhances Stemness and Environmental Adaptability in Rapidly Growing Oral Squamous Cell Carc inoma ........ 1865
P-3183 Depleting Anillin in 3D Culture Reveals Abnormal Cell Division of Enriched Triple-Negative Breast Cancer S tem-Like Cells ........ 1866
P-3184 Withdrawn ........ 1867

### 15:30 - 16:15 I-P10-12:Cell death (1)
### Room P / (Motenashi Dome)

P-3196 Induction of Ferroptosis by Dual Inhibition of GGCT and xCT Targeting Cysteine Metabolism in Gliob lastoma ........ 1868
P-3197 Regulation of cell death by tRNA modification enzyme, CDKAL1, in hepatocellular carcinoma ........ 1869
P-3198 The mechanism through which BACH1 inhibition induces iron-deficient cell death in head and neck cancer cells ........ 1870

### CONTENTS

P-3199 Analysis of JTC-801-induced cell death mechanisms in Osteosarcoma ···························· 1871
P-3200 Tolerance to Extracellular Acidic pH Facilitates Tumor Plasticity ································ 1872
P-3201 Effect of caprylic acid on colon cancer cells ············································ 1873
P-3202 Elucidating the role of MyD88 and its related factors in colorectal cancer progression ·············· 1874
P-3203 Elucidation of Cell Death Mechanisms Induced by NUDT5 Inhibition ··························· 1875
P-3204 Hybrid liposomes exhibit remarkable growth-inhibitory effects on esophageal cancer cells through apop tosis ·························································································· 1876

### 15:30 - 16:15 I-P11-14:Antibody therapy/Cancer vaccines (1)
### Room P / (Motenashi Dome)

P-3214 Enhancing T Cell-Mediated Tumor Killing via 4-1BB Agonism & CD3/Tumor Antigen Targeting to Improve Cancer Immunotherapy ··········································································· 1877
P-3215 Effects of EMT-inducing condition on the expression of cancer-associated membrane proteins ······ 1878
P-3216 The Role of Periostin Splicing Variants in Solid Tumors and Their Potential as Targets for Therapy ··· 1879
P-3217 Investigation of the mechanism of cytotoxicity of the anti-pan HLA class 2 mAb 4713 against ATL cells. ·························································································· 1880
P-3218 Trastuzumab deruxtecan resistance caused by increased expression of drug transporter inHER2-positive g astric cancer ·························································································· 1881
P-3219 The cancer-specific anti-human podoplanin antibody chLpMab-2f exerts antitumor effects on pleural mes othelioma. ·························································································· 1882
P-3220 Enhancing The Efficacy of T Cell-Dependent Bispecific Antibodies for Solid Tumors by Overcoming T Cell E xhaustion ·························································································· 1883
P-3221 Development of a Bispecific T-cell engaging antibody (BiTE) HLA-A2/Claspin Peptide Complex for Plat inum-Resistant Cancer ·························································································· 1884
P-3222 Analysis of cetuximab-induced EGFR interactome using proximity biotinylation AirID ·············· 1885

### 15:30 - 16:15 I-P11-16:Immune cell therapy (CAR-T, T cell therapy) (1)
### Room P / (Motenashi Dome)

P-3232 Vγ9Vδ2 T cell-mediated lung cancer cytotoxicity and NK cell-mediated mesothelioma cyt otoxicity ·························································································· 1886
P-3233 Withdrawn ·························································································· 1887
P-3234 Acquisition of glioblastoma-specific T cell receptors for TCR-T cell therapy ······················ 1888
P-3235 Short-term production of CAR-T cells using RetroNectin and G-Rex. ····························· 1889
P-3236 The Simple CAR-T Cell Manufacture: Simultaneous T cell activation and transduction in RetroNectin-coated G-Rex ·························································································· 1890
P-3237 Harnessing Antigen-Independent TNF-α Mechanisms to Overcome Tumor Heterogeneity in Adoptive T Cell Therapy ·························································································· 1891
P-3238 Development of a Novel T cell Activator using Rabbit anti-CD28 antibody to prevent T cell Exh austion ·························································································· 1892
P-3239 Withdrawn ·························································································· 1893

### 15:30 - 16:15 I-P12-6:Cancer diagnostic technologies (1)
### Room P / (Motenashi Dome)

P-3245 The Impact of Peroxiredoxin 3 on Diagnosis and Prognosis in Human Pancreatic Ductal Aden ocarcinoma ·························································································· 1894
P-3246 Identification of circulating miRNA markers using a rat pancreatic cancer model ·················· 1895
P-3247 Identification of genes involved in latent neck lymph node metastasis of early oral squamous cell carcinoma ·························································································· 1896
P-3248 Machine learning-integrated phenotyping predicts cisplatin sensitivity based on tumor gene expression p atterns ·························································································· 1897
P-3249 Search for breast cancer risk predictors in HBOC using comprehensive gene expression analysis ···· 1898
P-3250 Clinical significance of exosome-based microRNAs in urine of patients with gastric cancer and pancreatic c ancer ·························································································· 1899
P-3251 Investigation of the factors associated with high-grade fetal lung adenocarcinoma identified by gene net work analysis ·························································································· 1900
P-3252 Relationship between Desmoplastic Reaction (DR) Classification and Prognosis in Colorectal Ca ncer ·························································································· 1901

### CONTENTS

P-3253 HR eye & MMR eye: One-Day Assessment of DNA Repair-Defective Tumors Eligible for Targeted Therapy ..... 1902

### 15:30 - 16:15 I-P13-6:Engineered antibody
Room P / (Motenashi Dome)

P-3263 Understanding ADC Resistance using Preclinical Enhertu-induced Resistant Models ..... 1903
P-3264 CD44-Targeting Near-Infrared Photoimmunotherapy as a Potential Treatment for Mesothelioma ..... 1904
P-3265 Anti-CADM1/TSLC1 antibodies are potential therapeutic agents for cancer and pain ..... 1905
P-3266 Anti-tumor effect of nuclear transportable antibody-drug conjugate with triptolide via inhibition of RNA p olymerase II ..... 1906
P-3267 Safety assessment of trastuzumab emtansine (T-DM1) for use in companion dog patients ..... 1907
P-3268 Efficacy and Comparison of PPMX-T004b-PGAP-1, a New anti-CDH3 Antibody-Drug Conjugate (ADC), with Preceding ADC in Tumor ..... 1908
P-3269 Development of astatine-211 labeled anti-interleukin-7 receptor antibody for treating acute lymphoblastic leukemia ..... 1909
P-3270 Efficient internalization of EGFR-ADC by non-canonical endocytosis of EGFR ..... 1910
P-3271 Antitumor effect of anti-EpCAM antibody-drug conjugate against adenocarcinoma ..... 1911

### 15:30 - 16:15 I-P13-8:New molecular targeted therapy (2)
Room P / (Motenashi Dome)

P-3280 SLFN11 serves as a sensitivity biomarker for a novel ribonucleotide reductase inhibitor TAS1553 ..... 1912
P-3281 A novel PARG inhibitor MOD000582 exerts replication-independent anticancer effects by accumulating P ARylated proteins ..... 1913
P-3282 Decreased sensitivity of tumor cells to topoisomerase I inhibitor irinotecan under PARG defi ciency ..... 1914
P-3283 Anti-gastric cancer potential of the novel pyrrole-imidazole polyamide against SOX9 transcription factor ..... 1915
P-3284 Targeting of cyclin D3 by niclosamide in suppression of stomach cancer proliferation ..... 1916
P-3285 Prognostic significance of Claudin18.2 expression in patients with gastric cancer ..... 1917

### 15:30 - 16:15 I-P14-10:Anticancer drug
Room P / (Motenashi Dome)

P-3296 Effect of olaparib, a PARP inhibitor, on cell death induced by daunorubicin, an anthracycline an tibiotic ..... 1918
P-3297 Cell-death inducing mechanisms of the potential anti-cancer compound MO2455 in leukemia and lymp homa cells. ..... 1919
P-3298 Synergistic Effects of Microtubule Inhibitors and MK2 Inhibition in Cancer Treatment with Red uced Toxicity ..... 1920
P-3299 Synergistic effect on gastric cancer cells of combination treatment with platinum agents ..... 1921
P-3300 Novel prodrug targeting sulfotransferase for mesothelioma ..... 1922
P-3301 Effects of Anticancer Drugs on Mitochondrial Dynamics in Oral Cancer ..... 1923

### 15:30 - 16:15 I-P15:Evaluation & Prediction of Pharmacological Effects
Room P / (Motenashi Dome)

P-3307 Investigation of extraction methods suitable for quantification of antibody concentrations in tu mor tissue ..... 1924
P-3308 Automation and Optimization of a Three-Dimensional Blood Brain Barrier Model ..... 1925
P-3309 Integrated PK-PD/TD model-based prognostic prediction framework for FOLFIRINOX therapy in pancreatic cancer model mouse ..... 1926
P-3310 Withdrawn ..... 1927
P-3311 Withdrawn ..... 1928
P-3312 Phenotypic Classification and Drug Sensitivity Profiling of Pancreatic Cancer Cell Lines Based on Public D ata ..... 1929

### 15:30 - 16:15 II-P5-2:Lung cancer (2)
Room P / (Motenashi Dome)

P-3322 Design, synthesis, and biological evaluation of a novel 9-aminoacridine for lung cancer therapy ..... 1930

### CONTENTS

P-3323 Characteristics of CGP and RNA sequencing in EGFR-mutant lung cancer including cases of SCLC tran sformation ........ 1931
P-3324 Spatiotemporal analysis identifies EGFR-TKI-resistant tumor epithelial clusters. ........ 1932
P-3325 Clinical outcomes of osimertinib for advanced EGFR-mutated LUAD based on the 9th TNM classification: a multicenter study ........ 1933
P-3326 Impact of PD-L1 Expression on Outcomes in Advanced or Recurrent ALK-Positive NSCLC: A Multicenter R etrospective Study ........ 1934
P-3327 Multi-ion beam irradiation combined with KRASG12C inhibitors effectively destroys KRAS mutant lung ca ncer cells ........ 1935
P-3328 Clinical and prognostic significance of M2-like tumor-associated macrophages in malignant pleural mes othelioma ........ 1936
P-3329 Lung cancer secreting activin A recruited tumor associated macrophages to promote tumor gr owth ........ 1937
P-3330 SLC7A11 overexpression as a histological and prognostic marker in non-small cell lung cancer ........ 1938

### 15:30 - 16:15 II-P6-2:Tumor Immunity & Molecular Basis of Treatment Response in Breast Cancer Room P / (Motenashi Dome)

P-3339 Modeling the Role of Extracellular Matrix Stiffness and Alignment in Regulating Tumor Immune Inter actions ........ 1939
P-3340 Therapeutic strategy targeting FXYD3-positive ancestor-like cancer stem cells in triple-negative breast ca ncer ........ 1940
P-3341 Comparing Molecular Profile of Classical and Pleomorphic Invasive Lobular Carcinoma Using Spatial Tra nscriptome Analysis ........ 1941
P-3342 Low albumin-correlated systemic immunosuppression in metastatic breast cancer patients ........ 1942
P-3343 Mitochondrial BIG3-PHB2 complex as a potential therapeutic target in Triple-Negative Breast C ancer. ........ 1943
P-3344 The role of splicing variant Rac1/Rac1b in triple negative breast cancer ........ 1944
P-3345 The Relationship Between Hormone Levels, Lymphocyte Subsets, and Pathological Features in Triple -Negative Breast Cancer ........ 1945
P-3346 Functional analysis and drug discovery on RHBDL2, an intramembrane serine protease in TNBC ........ 1946

### 15:30 - 16:15 II-P6-4:Molecular Mechanisms of Breast Cancer Progression & Emerging Therapeutic Targets Room P / (Motenashi Dome)

P-3354 Biological characteristics of breast cancer subtypes based on tumor-infiltrating lymphocyte subclass and R NA Analysis ........ 1947
P-3355 Lysophosphatidic acid receptor 1 (LPAR1) is a poor prognostic factor associated with breast cancer pro gression ........ 1948
P-3356 Kallikrein Related Peptidase 11 as a potent prognostic factor in human breast cancer related to chemo therapy resistance ........ 1949
P-3357 Spatial Transcriptomic Characterization of the Tumor Microenvironment in Clinically Sequenced Breast C ancer Cases ........ 1950
P-3358 Analyses of the reactive oxygen species reduction abilities of gamma-glutamylcyclotransferase (GGCT) va riants. ........ 1951
P-3359 Aging and obesity contribute to breast cancer progression ........ 1952
P-3360 Analyses of the catalytic activity-independent promotion of cancer stemness by gamma-glutam ylcyclotransferase ........ 1953

### 15:30 - 16:15 II-P7-2:Ovarian Cancer: Molecular Mechanisms & Therapy Room P / (Motenashi Dome)

P-3369 Clinical impact and the potential therapeutic target of HIF2A in Ovarian clear cell carcinoma ........ 1954
P-3370 Association of cell adhesion related gene expression with chemoresistance in ascites derived spheroids of o varian cancer ........ 1955
P-3371 CRISPR-Cas9 screen identifies DHX38/PRP16 as an essential splicing factor for ovarian clear cell carcinoma tumorigenesis ........ 1956
P-3372 Exploring genes involved in anoikis resistancein ovarian cancer using the CRISPR/Cas9 libraries ........ 1957
P-3373 The molecular processes associated with the immune dynamics of macrophages in ovarian cancer ........ 1958

### CONTENTS

P-3374 Omental Tumor Microenvironment Abundant in IL-29 Is Crucial for Regression in Advanced Ov arian Cancer ..... 1959
P-3375 NGFR contributes to progression of ovarian cancer via promoting proliferation and invasion ..... 1960
P-3376 Intraperitoneal Administration of DNase1-expressing AAV Vector and Its Effect on Ovarian Cancer Diss emination ..... 1961

16:15 - 17:00 I-P3-4:Inflammation & Cancer
Room P / (Motenashi Dome)
P-3010 Genetic dissection of tumor-suppressive tissue microenvironment in Drosophila ..... 1962
P-3011 Impact of systemic inflammation on immunotherapy efficacy in PD-L1 high advanced non-small cell lung c ancer ..... 1963
P-3012 Red Blood Cell-Bound cell-free DNA Triggers Pro-Inflammatory Macrophage Responses in Cancer Cachexia ..... 1964
P-3013 Novel bioactive compounds and nanocarriers regulating macrophage diversity and malignant melanoma t herapeutic approach ..... 1965
P-3014 Over expression of SOX9 with inflammation-related DNA damage in cholangiocarcinoma induced by liver f luke ..... 1966
P-3015 Inhibition of NLRP3 inflammasome suppresses inflammation-related colon carcinogenesis ..... 1967
P-3016 Withdrawn ..... 1968
P-3017 Pentraxin 3 contributes to stromal cell senescence and hepatocellular carcinoma cell sorafenib r esistance ..... 1969

16:15 - 17:00 I-P5-5:Intracellular signal transduction (1)
Room P / (Motenashi Dome)
P-3027 p38-mediated non-canonical activation of Src facilitates tyrosine phosphorylation of TAB1 ..... 1970
P-3028 Analysis of Novel Genes Regulated by PRMT5 in Pancreatic Cancer Cells ..... 1971
P-3029 Traffic communications between the organelles by RNF213 ..... 1972
P-3030 Gallein, G protein βγ subunits inhibitor, suppresses the TGF-α-induced migration of HCC cells via inhibition of the JNK ..... 1973
P-3031 The role of GPRC6A, a nutrient-sensing receptor, in pancreatic cancer and cancer-associated ad ipocytes ..... 1974
P-3032 Withdrawn ..... 1975
P-3033 cGAS STING Pathway Related Proteins as Prognostic Marker in Cholangiocarcinoma Patients ..... 1976
P-3034 CULLIN3, as a master regulator of intestinal mucosa regeneration ..... 1977
P-3035 Analysis of tumor development through the increase of phosphorylated PKM2 in Adult T-cell leukem ia/lymphoma ..... 1978

16:15 - 17:00 I-P5-7:Intracellular signal transduction (3)
Room P / (Motenashi Dome)
P-3044 Non-canonical phosphorylation of EphA1 receptor tyrosine kinase ..... 1979
P-3045 Characterization of pseudogene-like EP400NL in gene expression ..... 1980
P-3046 Non-canonical phosphorylation of EphA2 in lung adenocarcinoma cells and lung squamous cell carcinoma cells ..... 1981
P-3047 Differences in the balance of canonical/non-canonical EGFR intracellular trafficking by different ligands ..... 1982
P-3048 Activation mechanism of RSK1 via the p38-MK2 stress response signaling pathway ..... 1983
P-3049 Dynamic movement of FGFR1-FRS2α complex observed at single-molecule level by High-Speed At omic Force Microscopy ..... 1984
P-3050 Analysis of the rapamycin and insulin-responsive mLST8 interactome using proximity bioti nylated AirID ..... 1985
P-3051 Single-molecule imaging enables high-resolution quantification of oncogenic KRAS activation and ther apeutic efficacy ..... 1986

16:15 - 17:00 I-P6-1:Cell cycle/Checkpoint
Room P / (Motenashi Dome)
P-3059 Conflicting cell cycle signal-mediated cytotoxicity in acute myeloid leukemia cells ..... 1987
P-3060 Ubiquitin-proteasome system-linked gene signature in prostate cancer ..... 1988

### CONTENTS

P-3061 DNA damage and cell cycle delay induced by 5-aminolevulinic acid in cancer cells: a potential therapeutic s trategy ······ 1989
P-3062 Novel Therapeutic Strategy for Neuroblastoma by Dual Chk1/Chk2 Inhibition ······ 1990
P-3063 LAT1 Inhibition-Induced Cell Cycle Arrest via p38 MAPK Mediated Cyclin D1 Downregulation in Cancer C ells ······ 1991
P-3064 Unveiling radiosensitization properties of PARP inhibitor talazoparib in cancer cells ······ 1992

### 16:15 - 17:00 I-P6-3:Genomic instability
### Room P / (Motenashi Dome)

P-3074 Chromosomal Instability in Patient-Derived Gliomas: Implications for Aneuploidy and Tumor Ev olution ······ 1993
P-3075 Cell fate of aneuploidy in cancer cells to facilitate adaptation overcoming aneuploid-mediated intracellular stress. ······ 1994
P-3076 PLK4 inhibition induces irreversible centrosome defect and DNA breakage in RB1-negative os teosarcoma ······ 1995
P-3077 SMARCA4 regulates SMARCAD1 expression for toleration of replication stress i n non-small cell lung cancer ······ 1996
P-3078 Molecular Basis of HP1-Dependent Activation of the Chromosomal Passenger Complex ······ 1997
P-3079 Effects of two active X chromosome on homologous recombination ······ 1998
P-3080 Inducing genomic instability: A novel therapeutic strategy for basal-like pancreatic cancer ······ 1999

### 16:15 - 17:00 I-P7-2:Insights into cancer pathogenesis based on genomic analysis
### Room P / (Motenashi Dome)

P-3090 Molecular characteristics of polyp lesions in Gastric Adenocarcinoma and Proximal Polyposis of the Sto mach (GAPPS) ······ 2000
P-3091 Whole-genome sequencing of osteosarcoma ······ 2001
P-3092 Molecular features of gastric metaplastic glands associated with carcinogenesis revealed by spatial tra nscriptomics ······ 2002
P-3093 Impact of TP53, KRAS, and APC Mutations on Neoadjuvant Chemotherapy Outcomes in Locally Advanced Rectal Cancer ······ 2003
P-3094 Molecular and cellular heterogeneities in CN-AML are driven by cell-type specific effects of somatic mut ations ······ 2004
P-3095 Highly chromosomal heterogeneity in parental sarcoma cells generating three phenotypically different tu mor cell lines ······ 2005

### 16:15 - 17:00 I-P7-4:Pathophysiology based on clonal evolution
### Room P / (Motenashi Dome)

P-3105 Characterization of Clonal Hematopoiesis in Liquid Biopsy Comprehensive Genomic Profiling Tests by C-CAT Repository Data ······ 2006
P-3106 Analysis of clonal evolution in endometrioid endometrial cancer ······ 2007
P-3107 Study on Acquisition Timing of KRAS Mutations in the Pancreatic Ductal Epithelium ······ 2008
P-3108 Genetic Insights into Clonal Evolution from Normal Prostate Epithelium to Prostate Cancer via H GPIN ······ 2009
P-3109 Splicing Junction Classifier for the Detection of Abnormal KEAP1-NRF2 System Activation ······ 2010

### 16:15 - 17:00 I-P8-5:Chromatin structure & others
### Room P / (Motenashi Dome)

P-3118 Integrated Analysis of Epigenome and Chromatin Structure in CAEBV ······ 2011
P-3119 Analysis of 3D chromatin architecture in PDAC ······ 2012
P-3120 Organization of the centromeric chromatin in cells entering mitosis ······ 2013
P-3121 Spatial Elucidation of the Transcriptional Regulatory Mechanisms of Differentiation Abnormalities in Lung Adenocarcinoma ······ 2014
P-3122 Elucidation of the Molecular Mechanisms of Key Epigenomic regulator in Hepatoblastoma Cells ······ 2015
P-3123 Effects of transient expression of Yamanaka factors to reprogram ARID1A-knockout gastric cells ······ 2016
P-3124 Breast CAF-derived exosomal miR-1290 promotes TNBC cell proliferation by targeting the TBC1D4-TSC1 a xis ······ 2017

### CONTENTS

P-3125 Increased m5C RNA Methylation of HDGF Induces Cell Growth and Proliferation in Human Colorectal Cancer ········ 2018

### 16:15 - 17:00 I-P9-6:Metastasis & extracellular matrix
### Room P / (Motenashi Dome)

P-3135 Matrix Stiffness Contributes to Tumor Progression in Intrahepatic Cholangiocarcinoma ········ 2019
P-3136 Elucidating the Mechanisms of Intercellular Interactions of Perineural Invasion in Pancreatic Cancer Microenvironment ········ 2020
P-3137 The ability of proliferation in a soft environment promotes lung metastasis of osteosarcoma ········ 2021
P-3138 Alternative splicing of FBLN2 generates a prometastatic tumor microenvironment in gastrointestinal cancers ········ 2022
P-3139 Hypoxia increased the hCXCL1 production from gastric cancer cells ········ 2023
P-3140 Shared morphological features between migrating neurons and invading glioblastoma in the adult brain ········ 2024
P-3141 The Role of Aligned Stromal Fibers-Induced Infiltrating Tumor Cells in Tumor Angiogenesis and Intravasation ········ 2025

### 16:15 - 17:00 I-P10-7:Cell culture system/Organoid (1)
### Room P / (Motenashi Dome)

P-3152 Spheroid Morphology of Lung Cancer Cell Lines Correlates with Oncological Profiles ········ 2026
P-3153 Glycolytic Metabolism of Pancreatic Cancer Cells in a Three-Dimensional Culture System ········ 2027
P-3154 Development of an Optimized Culture Medium Screening Method for Efficient Cancer Organoid (S-PDO) Establishment ········ 2028
P-3155 Establishment of a TME-Resembling In Vitro Assay System Using Freshly Isolated Human Tumoroids ········ 2029
P-3156 Immune Cell function and Effect of Immune Checkpoint Inhibitor in Freshly Dispersed Human Tumors ········ 2030
P-3157 Withdrawn ········ 2031
P-3158 Targeting BLU554-induced senescent cells to suppress pancreatic ductal adenocarcinoma ········ 2032
P-3159 Analysis of the interaction between tissue-resident macrophages and HrasG12V-expressing cells on mammary organoids ········ 2033

### 16:15 - 17:00 I-P10-9:Cancer stem cells (1)
### Room P / (Motenashi Dome)

P-3168 Elucidation of the mechanism of breast cancer progression by C1orf50, a gene of unknown function ········ 2034
P-3169 Fatty acid-binding protein 5 in the intestinal tumorigenesis accelerated by dietary stearic acid ········ 2035
P-3170 The stemness and cancer stemness of Jag2 in the small intestine ········ 2036
P-3171 Ovarian cancer-derived PDO models show different distribution patterns of cancer stem cell markers ········ 2037
P-3172 Tumor endothelial cell-derived Sfrp1 supports the maintenance of cancer stem cells via Wnt signaling ········ 2038
P-3173 p53-Phlda3 Axis Restrains Gastric Cancer Cell Plasticity by Blocking the PI3K-Akt Pathway ········ 2039
P-3174 Functional analysis of one-carbon metabolic enzyme SHMT2 in breast cancer ········ 2040
P-3175 Molecular characterization for a neuroblastoma-like tumorigenesis model from cranial neural crest cells ········ 2041

### 16:15 - 17:00 I-P10-11:Cell-to-cell interaction
### Room P / (Motenashi Dome)

P-3185 Biological significance of thrombospondin-1 in pancreatic cancer progression ········ 2042
P-3186 Identification of compounds that inhibit proliferation of tumor-promoting human breast cancer-associated fibroblasts ········ 2043
P-3187 PRSS3 from scirrhous gastric cancer, interact with cancer associated fibroblasts via PAR2/STC-1 axis ········ 2044
P-3188 Clinico-pathological significance of cancer-associated fibroblasts subtype, myCAF and apCAF, in gastric carcinomas ········ 2045
P-3189 Benchtop MALDI MS Imaging for Cancer Research: A New Approach Using HiPLEX-IHC Probes ········ 2046

### CONTENTS

P-3190 Mechanistic Elucidation of Connexin 43-Mediated Cancer Cell Extrusion from the Epithelial Mo nolayer ······ 2047
P-3191 Spatial transcriptomics uncovers the tumor microenvironment in a mouse model of diffuse gastric cancer ······ 2048
P-3192 Uncovering Gastric Cancer Heterogeneity with Histopathology and Spatial Transcriptomics ······ 2049
P-3193 SOX10low melanoma cells regulate the malignancies of SOX10high melanoma cells through secretion of I L-6. ······ 2050
P-3194 Effects of point mutations and nuclear localization of E-cadherin on entosis. ······ 2051
P-3195 Validation of a cancer-associated thrombosis model and early thrombosis detection in 4T1 breast canc er-bearing mice ······ 2052

### 16:15 - 17:00 I-P10-13:Cell death (2)
### Room P / (Motenashi Dome)

P-3205 The transcription factor SOX10 regulates melanoma growth through modulation of nuclear receptor RXR γ expression ······ 2053
P-3206 Analysis of ferroptotic cell death in mesothelioma cells ······ 2054
P-3207 Exploration of factor involved in vacuolar formation associated with cell death induced by CDK4/6 inhibitor abemaciclib. ······ 2055
P-3208 Elucidating compensatory metabolic changes induced by glycolytic inhibition in pancreatic ca ncer cell lines ······ 2056
P-3209 Role of Tubulin Acetylation in Paclitaxel Resistance in Lung Cancer ······ 2057
P-3210 Dual Targeting of mTOR and WNT Pathways Disrupts Mitosis and Induces Autophagic Cell Death in Neu roblastoma ······ 2058
P-3211 Withdrawn ······ 2059
P-3212 ACSL3 is an unfavorable prognostic marker and confers ferroptosis resistance in cholang iocarcinoma ······ 2060
P-3213 Yes associated protine dictates copper homeostasis through ATOX1 and confers to cuproptosis s ensitivity ······ 2061

### 16:15 - 17:00 I-P11-15:Antibody therapy/Cancer vaccines (2)
### Room P / (Motenashi Dome)

P-3223 Elucidating the pathophysiological role of monocyte and macrophage in antibody cancer the rapeutics ······ 2062
P-3224 Expanding the Application of Shark IgNAR Antibodies for Next-next-generation Cancer Antibody Thera peutics ······ 2063
P-3225 Research of a novel treatment for malignant pleural mesothelioma targeting Annexin A2 ······ 2064
P-3226 Targeting EphA2 and EGFR with Dual-Specific CrossMab Antibodies for Cholangiocarcinoma Th erapy ······ 2065
P-3227 Inhibiting EndoMT-Promoted Tumor Growth in Pancreatic Adenocarcinoma with a Humanized Anti-HSP90 alpha Antibody ······ 2066
P-3228 Trastuzumab Deruxtecan, HER2 targeting antibody-drug conjugates, exerted multiple anti-cancer activities against CCA. ······ 2067
P-3229 Possibility of different functional roles among the Fab arms in monoclonal antibody found by electron de nsity topography ······ 2068
P-3230 Establishment of the basis for the development of vaccine therapy for osteosarcoma ······ 2069
P-3231 Activation of Tumor-Specific CD8+ T Cells by Engineered Extracellular Vesicles ······ 2070

### 16:15 - 17:00 I-P11-17:Immune cell therapy (CAR-T, T cell therapy) (2)
### Room P / (Motenashi Dome)

P-3240 Withdrawn ······ 2071
P-3241 Withdrawn ······ 2072
P-3242 Oncolytic HSV enhances CAR T cell therapy by remodeling the tumor microenvironment in pancreatic ca ncer ······ 2073
P-3243 Combination therapy of T cell therapy and compound 433 overcomes tumor heterogeneity ······ 2074
P-3244 Replacement of the linker sequence of the self-activating CAR enhances in vitro cytotoxic activity. ······ 2075

### CONTENTS

### 16:15 - 17:00 I-P12-7:Cancer diagnostic technologies (2)
### Room P / (Motenashi Dome)

P-3254 Development of 67Ga-labeled PEGylated heterodivalent probes for imaging negative charges and integrins on cancer cells ····· 2076
P-3255 Withdrawn ····· 2077
P-3256 First-in-Human Phase 0 Study of Cu-PD32766, a CAIX-Targeting Peptide, in Patients with Clear Cell Renal Cell Carcinoma ····· 2078
P-3257 Validation of a Semi-automatic Artificial Intelligence Model for Pan-cancer Tumor Segmentation ····· 2079
P-3258 Assessment of Metabolic Features and Development of Inhibitory Therapy for GI Cancer via Hyperpolarized 13C-MRI ····· 2080
P-3259 AI-Driven Uterine Sarcoma Diagnosis: An Ensemble Approach with Radiomics Integration ····· 2081
P-3260 Development of EBUS image classification model in pulmonary lymph nodes using deep learning ····· 2082
P-3261 Withdrawn ····· 2083
P-3262 Withdrawn ····· 2084

### 16:15 - 17:00 I-P13-7:New molecular targeted therapy (1)
### Room P / (Motenashi Dome)

P-3272 Development of an Anti-IL-7R Antibody-PROTAC Conjugate for the Treatment of Acute Lymphoblastic Le ukemia ····· 2085
P-3273 TRIP13, a potential drug target for ATL pharmacotherapy ····· 2086
P-3274 Development of a cancer-specific cell death induction method using new synthetic mRNA ····· 2087
P-3275 Therapeutic efficacy and immune response of near-infrared photoimmunotherapy for lung cancer in ort hotopic murine model ····· 2088
P-3276 Near-Infrared Photoimmunotherapy Targeting CD98 for Cancer Therapy ····· 2089
P-3277 LPAR1 antagonist as a potential therapeutic agent for glioblastoma ····· 2090
P-3278 PI polyamide-TPP conjugate targeting m.A3243G inhibits the tumor growth of teratoma harboring the mu tation ····· 2091
P-3279 Development of surviving gene siRNA in the therapy of fibrotic tumors ····· 2092

### 16:15 - 17:00 I-P14-9:Drug delivery system
### Room P / (Motenashi Dome)

P-3286 Size-dependent malignant brain tumor targeting by polymeric nanoruler ····· 2093
P-3287 TXB-001: A novel DDS anthracycline with high tumor stromal penetration as a potential treatment for re fractory cancer ····· 2094
P-3288 Enhanced Anticancer Activity of LLAA peptide using self-assemble nanoparticles in high-metastasis cancer cells ····· 2095
P-3289 Intra-tumor Administration of Doxorubicin with a Guide-equipe Jet Injector, Actranza®, in K562-x enografted Mice ····· 2096
P-3290 Development of NK cell-targeting technology using antibody-modified lipid nanoparticles ····· 2097
P-3291 Transient transfection using 222nm Far UV-C irradiation ····· 2098
P-3292 Tumor-Type Specific Stromal Barriers in Antibody Delivery: Comparative 3D Spheroid Model ····· 2099
P-3293 Anti-tumor effects of folate receptor targeted paclitaxel releasing mesoporous silica nanoparticles ····· 2100
P-3294 Targeted co-delivery of Doxorubicin and H. perforata via aptamer-modified nanovesicles in colorectal ca ncer cells ····· 2101
P-3295 PLGA nanoparticles separately encapsulating ursolic acid and doxorubicin for improved cholangi ocarcinoma therapy ····· 2102

### 16:15 - 17:00 I-P14-11:Others
### Room P / (Motenashi Dome)

P-3302 Characterization of tumors of older breast cancer patients that show de novo resistance to endocrine the rapy ····· 2103
P-3303 The C11orf24 gene as a useful biomarker for predicting severe neutropenia in modified FOLFIRINOX for p ancreatic cancer ····· 2104
P-3304 Evaluation of Subclavian Vein Catheterization Under Ultrasound Guidance ····· 2105
P-3305 Photodynamic therapy with hybrid liposomes containing indocyanine green in mouse models of breast ca ncer ····· 2106

### CONTENTS

P-3306 Advanced mouse ascites carcinoma chemotherapy attempts to chase the localization of cancer s tem cells ........ 2107

16:15 - 17:00 II-P5-1:Lung cancer (1)
Room P / (Motenashi Dome)

P-3313 RBM10 Loss Modulates Splicing and Defines a Unique Transcriptomic Signature in EGFR-Mutant LUAD ........ 2108
P-3314 Investigating the impact of solid component-associated transcription factors on the lung adenocarcinoma m alignancy. ........ 2109
P-3315 PKM1 Expression in Neuroendocrine-Type SCLC Is Associated with Reduced T Cell Infiltration ........ 2110
P-3316 Role of brain abundant membrane attached signal protein 1 (BASP1) in large cell neuroendocrine carc inoma of the lung ........ 2111
P-3317 Establishment of intratumor heterogeneity cell model using lung cancer cell lines ........ 2112
P-3318 The role of phospholipase A2 in characteristics of pleural mesothelioma cells ........ 2113
P-3319 Promotion of cellular functions via LPA receptor-mediated signaling in lung cancer cells co-cultured with s tromal cells ........ 2114
P-3320 Regulation of cellular functions through activation of free fatty acid receptors (FFARs) in lung c ancer cells ........ 2115
P-3321 Roles of LPA receptor signaling in enhancing malignant potential of lung cancer cells under hypoxic con ditions ........ 2116

16:15 - 17:00 II-P6-1:Molecular Pathology of Prognostic & Progression Markers in Breast Cancer
Room P / (Motenashi Dome)

P-3331 Validation of PREDICT and development of postoperative prognostic models for Japanese breast cancer p atients ........ 2117
P-3332 Relationships between RS of Oncotype Dx and clinicopathological features in HR+/HER2- early breast ca ncer ........ 2118
P-3333 RUNX3 synergizes with RUNX1 to suppress MYC in breast cancer ........ 2119
P-3334 The clinical significance of MTX1 with genomic copy number amplification in breast cancer. ........ 2120
P-3335 The role of AMIGO2 in human breast cancer progression ........ 2121
P-3336 Tim1 is a poor prognostic factor associated with proliferation and migration in breast cancer. ........ 2122
P-3337 Does ABO blood type affect recurrence and prognosis in resected breast cancer? ........ 2123
P-3338 Analysis of gene expression and prognostic factors single-cell RNA sequencing (scRNA-seq) using in breast cancer(BC) ........ 2124

16:15 - 17:00 II-P6-3:Molecular Profiling & Immune microenviroment in breast cancer
Room P / (Motenashi Dome)

P-3347 Association Between E-cadherin Expression and Tumor-Infiltrating Lymphocytes in Invasive Lobular Car cinoma ........ 2125
P-3348 The influence of BRCA1/2 gene status on treatment strategies in patients with breast cancer ........ 2126
P-3349 Clinical Significance of FOXA1 Expression in Breast Cancer: A Retrospective Analysis of Cases Without Sy stemic Therapy ........ 2127
P-3350 Adipocytes induce CSC phenotype and tamoxifen resistance by activating PI3K/Akt/mTOR pathway in ER+ breast cancer cells ........ 2128
P-3351 EGFR/SRC/STAT3 signaling promotes radioresistance and cancer stemness by regulating hexokinase2 ex pression in TNBC cells ........ 2129
P-3352 Investigating the anticancer mechanism of Piperlongumine in Triple-negative breast cancer ........ 2130
P-3353 CDK4/6 Inhibitors retrial and therapy sequences after initial CDK4/6 Inhibitors in HR+, HER2- Advanced B reast Cancer ........ 2131

16:15 - 17:00 II-P7-1:Omics Analyses in Gynecologic Cancers
Room P / (Motenashi Dome)

P-3361 Investigation of Choriocarcinoma-Associated miRNAs Through Small RNA Sequencing ........ 2132
P-3362 Elucidation of Choriocarcinoma-Specific Genomic Profiles by Whole Exome Sequencing ........ 2133
P-3363 Identification of choriocarcinoma specific molecular profile by proteome analysis ........ 2134
P-3364 Impact of Copy Number Variations on EV-miRNA Profiles in Ovarian Cancer ........ 2135
P-3365 Investigating the malignant mechanism of gastric-type endocervical adenocarcinoma ........ 2136

### CONTENTS

P-3366 Genomic and molecular profiles of undifferentiated/dedifferentiated endometrial carcinomas ······· 2137
P-3367 Metabolomic and transcriptomic analyses identify metabolic alterations and immune suppression in ovarian cancer ······· 2138
P-3368 Landscape of genetic alterations associated with early-onset ovarian cancer development ······· 2139

16:15 - 17:00 II-P7-3:Molecular Mechanisms & Therapy in Gynecologic cancer
Room P / (Motenashi Dome)

P-3377 Expression of iron exporter ferroportin and its role in ovarian cancer ······· 2140
P-3378 Effect of p53 aggregation on the expression of 6-sulfo sialyl glycans in p53-mutated cancer ······· 2141
P-3379 Exploration of LRRN4 as a Marker for Normal Mesothelial Cells ······· 2142
P-3380 Immune microenvironment in uterine carcinosarcoma ······· 2143
P-3381 Histone methyltransferase SUV39H2 is associated with tumor growth and metastasis in uterine endometrial serous carcinoma ······· 2144
P-3382 Elucidation of the role of L1CAM-NF-κB pathway in human endometrial cancer ······· 2145
P-3383 Expression and Function of Malic Enzyme 1 in Endometrial Cancer ······· 2146
P-3384 Impact of Histological Type on MRI-Based Evaluation of Tumor Size and LVSI in Early-Stage Ce rvical Cancer ······· 2147
P-3385 Evaluation of zolbetuximab efficacy in gastric-type cervical adenocarcinoma using patient-derived organoid models ······· 2148

### Abstract

The 84th Annual Meeting of the Japanese Cancer Association

JCA-AACR Joint Symposia - Sponsored by
Princess Takamatsu Cancer Research Fund -
Special Lecture
Special Program
Special Symposia
International Sessions
Symposia
Open Symposia
Joint Symposia
Advances in Organ Site Research
Meet the Editor
Educational Lectures
Morning Lecture
The Tomizo Yoshida Prize Lecture
The Mataro Nagayo Prize Lecture
JCA International Award Lecture
JCA Women Scientists Awards
The Young Investigator Awards Lectures

Cancer Science Young Scientists Award Lectures Public Open Seminar

Survivor Scientist Program

Japanese Oral Session

English Oral Sessions

Poster Session

Luncheon Seminars

Coffee Break Seminars

Closing

Opening

Councilors' Meeting

General Assembly ／ Award Ceremony ／ Award Lectures

CS Associate Editors' Meeting

### Opening

### Opening

2025 / 9 / 25 (Thu) 8:45-9:00 Room 1/(Ishikawa Ongakudo 2F Concert Hall) Room 1

No Abstract

### [AACR1-1] AACR1
### Early Cancer Evolution and prevention -crossroad of genomics and biology-

2025 / 9 / 25 (Thu) 9:00-11:30 Room 1/(Ishikawa Ongakudo 2F Concert Hall) Room 1

[Chairpersons]Seishi Ogawa (Department of Pathology and Tumor Biology, Kyoto University)、Laura Wood (Johns Hopkins University)

Cancer population is shaped by multiple rounds of positive and negative selections, in which gene mutations accumulating over lifetime play central roles. Recent studies suggest that these processes are already on going in apparently normal tissues, where numerous independent "precancer clones" may evolve with aging to establish somatic mosaicism. Frequently influenced by various environmental insults and life-style cancer risks, somatic mosaicism is thought to be the source of cancer ancestors and its understanding is instrumental not only to shed light on cancer pathogenesis but also to device novel strategy of cancer prevention, two speakers will present the upfront findings on early cancer evolution and discuss how these knowledge could be used to improve cancer prevention and therapy.⊠The cancer population is shaped by multiple rounds of positive and negative selection, in which gene mutations accumulated over a lifetime play central roles. Recent studies suggest that these processes are already ongoing in apparently normal tissues, where numerous independent “precancer clones” may evolve with aging to establish somatic mosaicism. Frequently influenced by various environmental insults and lifestyle-related cancer risks, somatic mosaicism is thought to be the source of cancer ancestors that ultimately give rise to clinically diagnosed cancer. Understanding this phenomenon is instrumental not only in shedding light on cancer pathogenesis but also in devising novel strategies for cancer prevention. In this session, two speakers will present recent findings on early cancer evolution and discuss how this knowledge could be applied to improve cancer prevention and therapy.

---

### AACR1-1
### Somatic mosaicism and cancer

[Speaker]Seishi Ogawa (Department of Pathology and Tumor Biology, Kyoto University)

Recent studies have revealed frequent clonal expansions harboring cancer-associated mutations in histologically normal tissues, implicating these clones in cancer development. However, it remains unclear what additional driver events occur and in what sequence before such clones progress to malignancy. In this symposium, I will focus on somatic mosaicism in breast tissue and present a phylogenetic analysis of microdissected samples from breast cancers harboring der(1;16), a recurrent structural alteration found in ~20% of cases. We show that der(1;16) was acquired between early puberty and late adolescence, followed by the emergence of a common ancestor clone in the early 30s. Both malignant and non-malignant lineages diverged from this ancestor, and by the time of diagnosis, these clones had expanded to occupy a large area of the breast epithelium. Notably, multiple independent cancer founder clones frequently arose from non-malignant precursors, contributing to intratumour heterogeneity. These findings provide new insights into the temporal and spatial dynamics of somatic mosaicism and its role in breast cancer evolution.

[Disclosure of COI by Responsible researcher] Lead presenter is the Responsible researcher.
[Lead presenter: Seishi Ogawa] ■Advisor of the company and/or profit-making organization: エーザイ株式会社、Chordia Therapeutics Inc.、Montage Bio, INC. ■Profit of stock: 朝日ゲノミクス株式会社 ■Research expenses: エーザイ株式会社、Chordia Therapeutics Inc. ■Contributions: 日本新薬株式会社 ■Endowed chair: Chordia Therapeutics Inc.、公益社団法人鹿児島共済会 南風病院

▲TOP

### [AACR1-2] AACR1
### Early Cancer Evolution and prevention -crossroad of genomics and biology-

2025 / 9 / 25 (Thu) 9:00-11:30 Room 1/(Ishikawa Ongakudo 2F Concert Hall) Room 1

[Chairpersons]Seishi Ogawa (Department of Pathology and Tumor Biology, Kyoto University)、Laura Wood (Johns Hopkins University)

Cancer population is shaped by multiple rounds of positive and negative selections, in which gene mutations accumulating over lifetime play central roles. Recent studies suggest that these processes are already on going in apparently normal tissues, where numerous independent "precancer clones" may evolve with aging to establish somatic mosaicism. Frequently influenced by various environmental insults and life-style cancer risks, somatic mosaicism is thought to be the source of cancer ancestors and its understanding is instrumental not only to shed light on cancer pathogenesis but also to device novel strategy of cancer prevention, two speakers will present the upfront findings on early cancer evolution and discuss how these knowledge could be used to improve cancer prevention and therapy.⊠The cancer population is shaped by multiple rounds of positive and negative selection, in which gene mutations accumulated over a lifetime play central roles. Recent studies suggest that these processes are already ongoing in apparently normal tissues, where numerous independent “precancer clones” may evolve with aging to establish somatic mosaicism. Frequently influenced by various environmental insults and lifestyle-related cancer risks, somatic mosaicism is thought to be the source of cancer ancestors that ultimately give rise to clinically diagnosed cancer. Understanding this phenomenon is instrumental not only in shedding light on cancer pathogenesis but also in devising novel strategies for cancer prevention. In this session, two speakers will present recent findings on early cancer evolution and discuss how this knowledge could be applied to improve cancer prevention and therapy.

### AACR1-2
### 3D Genomic Analysis of Human Pancreatic Precancers

[Speaker]Laura Wood (Department of Pathology, Johns Hopkins University School of Medicine)

Pancreatic intraepithelial neoplasias (PanINs) are the most common precursors of pancreatic cancer, but their small size and inaccessibility in humans make them challenging to study. Here, we provide a microanatomical survey of human PanINs by analysing 46 large samples of grossly normal human pancreas with a machine-learning pipeline for quantitative 3D histological reconstruction at single-cell resolution. To elucidate genetic relationships between and within PanINs, we developed a workflow in which 3D modelling guides multi-region microdissection and targeted and whole-exome sequencing. From these samples, we calculated a mean burden of 13 PanINs per cm3 and extrapolated that the normal intact adult pancreas harbours hundreds of PanINs, almost all with oncogenic KRAS hotspot mutations. We found that most PanINs originate as independent clones with distinct somatic mutation profiles. The extensive multifocality and genetic heterogeneity of PanINs raises important questions about mechanisms that drive precancer initiation and confer differential progression risk in the human pancreas.

[Disclosure of COI by Responsible researcher] Lead presenter is the Responsible researcher.
[Lead presenter: Laura Wood] We have no financial relationships to disclose.

▲TOP

### [AACR1-3] AACR1
### Early Cancer Evolution and prevention -crossroad of genomics and biology-

2025 / 9 / 25 (Thu) 9:00-11:30 Room 1/(Ishikawa Ongakudo 2F Concert Hall) Room 1

[Chairpersons]Seishi Ogawa (Department of Pathology and Tumor Biology, Kyoto University)、Laura Wood (Johns Hopkins University)

Cancer population is shaped by multiple rounds of positive and negative selections, in which gene mutations accumulating over lifetime play central roles. Recent studies suggest that these processes are already on going in apparently normal tissues, where numerous independent "precancer clones" may evolve with aging to establish somatic mosaicism. Frequently influenced by various environmental insults and life-style cancer risks, somatic mosaicism is thought to be the source of cancer ancestors and its understanding is instrumental not only to shed light on cancer pathogenesis but also to device novel strategy of cancer prevention, two speakers will present the upfront findings on early cancer evolution and discuss how these knowledge could be used to improve cancer prevention and therapy.⊠The cancer population is shaped by multiple rounds of positive and negative selection, in which gene mutations accumulated over a lifetime play central roles. Recent studies suggest that these processes are already ongoing in apparently normal tissues, where numerous independent “precancer clones” may evolve with aging to establish somatic mosaicism. Frequently influenced by various environmental insults and lifestyle-related cancer risks, somatic mosaicism is thought to be the source of cancer ancestors that ultimately give rise to clinically diagnosed cancer. Understanding this phenomenon is instrumental not only in shedding light on cancer pathogenesis but also in devising novel strategies for cancer prevention. In this session, two speakers will present recent findings on early cancer evolution and discuss how this knowledge could be applied to improve cancer prevention and therapy.

---

### AACR1-3
### Comprehensive Insights into Environmental Carcinogenesis via DNA Adducts and Genome-wide Mutation Profiling

[Speaker]Yukari Totsuka (Dept. Environ. Health Sci., Hoshi Univ.)

Recent advances in large-scale genome analysis using next-generation sequencers have revealed patterns of somatic mutations (mutation signatures) that accumulate in various human tumors. Mutation signatures reflect exposure to various environmental factors that contribute to human cancer development. On the other hand, Humans are exposed to many chemicals in daily life. These chemicals covalently bind to nucleobases, forming DNA adducts and inducing mutations in the genome, and are therefore considered to be involved in cancer development as an early event. However, not all adducts contribute to human carcinogenesis to the same extent. Therefore, it is important to find adducts that contribute more to carcinogenesis to identify carcinogenic factors and develop cancer prevention strategies. For this purpose, we thought that the identification of "driver adducts" that define mutational signatures from a large number of adducts is a promising way to elucidate the relationship between environmental factors and human cancer. In this symposium, we will present our study on the comprehensive analysis of DNA adducts and genomic mutations.

[Disclosure of COI by Responsible researcher] Lead presenter is the Responsible researcher.
[Lead presenter: Yukari Totsuka] We have no financial relationships to disclose.

▲TOP

### [AACR1-4] AACR1
### Early Cancer Evolution and prevention -crossroad of genomics and biology-

2025 / 9 / 25 (Thu) 9:00-11:30 Room 1/(Ishikawa Ongakudo 2F Concert Hall) Room 1

[Chairpersons]Seishi Ogawa (Department of Pathology and Tumor Biology, Kyoto University)、Laura Wood (Johns Hopkins University)

Cancer population is shaped by multiple rounds of positive and negative selections, in which gene mutations accumulating over lifetime play central roles. Recent studies suggest that these processes are already on going in apparently normal tissues, where numerous independent "precancer clones" may evolve with aging to establish somatic mosaicism. Frequently influenced by various environmental insults and life-style cancer risks, somatic mosaicism is thought to be the source of cancer ancestors and its understanding is instrumental not only to shed light on cancer pathogenesis but also to device novel strategy of cancer prevention, two speakers will present the upfront findings on early cancer evolution and discuss how these knowledge could be used to improve cancer prevention and therapy.⊠The cancer population is shaped by multiple rounds of positive and negative selection, in which gene mutations accumulated over a lifetime play central roles. Recent studies suggest that these processes are already ongoing in apparently normal tissues, where numerous independent “precancer clones” may evolve with aging to establish somatic mosaicism. Frequently influenced by various environmental insults and lifestyle-related cancer risks, somatic mosaicism is thought to be the source of cancer ancestors that ultimately give rise to clinically diagnosed cancer. Understanding this phenomenon is instrumental not only in shedding light on cancer pathogenesis but also in devising novel strategies for cancer prevention. In this session, two speakers will present recent findings on early cancer evolution and discuss how this knowledge could be applied to improve cancer prevention and therapy.

### AACR1-4
### Deciphering Early Gastric Cancer: Genomic Mutations and Cellular Plasticity in Intestinal Metaplasia

[Speaker]Hoi Ning Helen Yan (Department of Pathology, The University of Hong Kong)

Gastric cancer (GC), a leading global cause of cancer-related mortality, often arises from intestinal metaplasia (IM), a precancerous condition driven by genomic and cellular changes. Whole-genome sequencing of 238 gastric gland microdissections from 30 individuals shows that normal gastric epithelium accumulates ~28 single-nucleotide variants yearly, with metaplastic glands in cancer patients exhibiting a 2.8-fold higher mutation burdens due to proliferation and oxidative damage. Recurrent trisomies, notably chromosome 20, and driver mutations in genes like ARID1A and CTNNB1 indicate early clonal selection. Organoid models of IM reveal lineage plasticity, with hybrid cells showing impaired differentiation and fetal reversion, facilitating neoplastic progression. Functionally, IMOs were FGF10 independent and showed downregulated FGFR2. Several IMOs exhibited a cell-matrix adhesion independent subpopulation with chromosome 20 gain but lacked key cancer driver mutations, potentially representing the earliest neoplastic precursor of IM-induced GC. These findings highlight the interplay of somatic mutations, copy-number variations, and cellular reprogramming in early GC.

[Disclosure of COI by Responsible researcher] Lead presenter is the Responsible researcher.
[Lead presenter: Hoi Ning Helen Yan] We have no financial relationships to disclose.

### [YIA1-1] YIA1
### The Young Investigator Awards Lecture1

2025 / 9 / 25 (Thu) 9:00-11:30 Room 2/(Ishikawa Ongakudo 2F Hogaku Hall) Room 2

[Chairperson]Shinya Tanaka (Hokkaido University,Fuculty of medicine)

### YIA1-1
### Multifaceted interactions between cancer cells and glial cells in brain metastasis

[Speaker]Kojiro Ishibashi (Cancer Res. Inst., Kanazawa Univ.) [Co-author]Eishu Hirata (Cancer Res. Inst., Kanazawa Univ.)

In cancer brain metastasis, the interactions between cancer cells and glial cells play a crucial role in the formation of brain metastases; however, there are few methods to stably analyze these events for a long period in vitro. Therefore, we have developed a stable culture method for prolonged observation of astrocytes and microglia, named MGS (mixed-glial culture on soft substrate). Using MGS, we found that Wnt5a secreted by astrocytes enhances the expression of mGluR1 in cancer cells, and that mGluR1 activates EGFR -ERK pathway, thereby enhancing the survival of brain metastatic cancer cells. Furthermore, in vivo experiments using LY456236, an inhibitor of mGluR1, revealed that mGluR1 signaling is also important for the progression of cancer brain metastasis in mice. Currently, we investigate the interaction between microglia and brain metastatic cancer cells and found that microglia directly interact with cancer cells and induce cancer cell death. These results suggest that, within the brain microenvironment, interactions with astrocytes are crucial for the progression of brain metastatic cancer cells, whereas interactions with microglia are crucial for their suppression.

[Disclosure of COI by Responsible researcher] Lead presenter is not the Responsible researcher.
[Lead presenter: Kojiro Ishibashi] We have no financial relationships to disclose.
[Principal Researcher: Eishu Hirata] We have no financial relationships to disclose.

▲TOP

### [YIA1-2] YIA1
### The Young Investigator Awards Lecture1

2025 / 9 / 25 (Thu) 9:00-11:30 Room 2/(Ishikawa Ongakudo 2F Hogaku Hall) Room 2

[Chairperson]Shinya Tanaka (Hokkaido University,Fuculty of medicine)

### YIA1-2
### Mechanism of cancer progression triggered by extracellular matrix stiffness

[Speaker]Seiichiro Ishihara (Cell Dynamics, Faculty Advanced Life Sci., Hokkaido Univ.) [Co-author]Atsushi Enomoto (Dept. Pathol., Nagoya Univ. Grad. Sch. Med.)、Akihiro Sakai (Dept. Pathol., Nagoya Univ. Grad. Sch. Med.)、Tadashi Iida (Dept. Pathol., Nagoya Univ. Grad. Sch. Med.)、Hisashi Haga (Cell Dynamics, Faculty Advanced Life Sci., Hokkaido Univ.)

Solid tumor tissues contain stiffer extracellular matrices (ECMs) than corresponding normal tissues. Cancer cells respond to the stiffness of ECMs and regulate their malignancy. However, the mechanism of cancer progression regulated by ECM stiffness is not well understood. We established novel methods to culture cells on ECMs with different stiffness. By using these methods, we found several transcription factors in cancer cells activated by stiff ECMs. Especially, activating transcription factor 5 (ATF5), which is highly expressed in cancer tissues than corresponding normal tissues, was localized to nuclei in pancreatic and lung cancer cells on stiff ECMs and promotes proliferation of the cells by suppressing the expression of early growth response 1 (***EGR1***). Furthermore, we revealed that mesenchymal stromal cells (MSCs) respond to ECM stiffness and differentiate to tumor-promoting cancer-associated fibroblasts (CAFs). The CAFs triggered proliferation of mammary cancer cells via secretion of prosaposin. These results show the critical role of ECM stiffness for progression of solid cancers.

[Disclosure of COI by Responsible researcher] Lead presenter is the Responsible researcher.
[Lead presenter: Seiichiro Ishihara] We have no financial relationships to disclose.

▲TOP

### [YIA1-3] YIA1
### The Young Investigator Awards Lecture1

2025 / 9 / 25 (Thu) 9:00-11:30 Room 2/(Ishikawa Ongakudo 2F Hogaku Hall) Room 2

[Chairperson]Shinya Tanaka (Hokkaido University,Fuculty of medicine)

### YIA1-3
### Elucidating the Developmental Mechanisms of Regulatory T Cells in the Tumor Microenvironment

[Speaker]Kota Itahashi (Unit Leader, Division of Cancer Immunotherapy, Exploratory Oncology Research and Clinical Trial Center, National Cancer Center) [Co-author]Hiroyoshi Nishikawa (Division of Cancer Immune Multicellular System Regulation, Center for Cancer Immunotherapy and Immunobiology (CCII) Graduate School of Medicine, Kyoto University ／ Division of Cancer Immunology, Research Institute／ Exploratory Oncology Research and Clinical Trial Center (EPOC), National Cancer Center ／ Department of Immunology, Nagoya University Graduate School of Medicine)

Regulatory T (Treg) cells play an important role in preserving self-tolerance and preventing autoimmunity. In contrast, Treg cells abundantly infiltrate the tumor microenvironment (TME) and foster tumor progression by dampening effective antitumor immune responses. The significance of Treg cells in the context of PD-1 blockade has recently been recognized, as they are implicated in therapeutic resistance and tumor hyperprogression. Although the differentiation process of $CD8^+$ T cells toward an exhausted state in the TME has been well investigated, the mechanisms underlying the differentiation of Treg cells into an activated phenotype in the TME remain poorly understood. We characterized the chromatin landscape and identified key transcription factor networks driving Treg-cell differentiation in human tumors using single-cell RNA-seq and single-cell ATAC-seq of Treg cells isolated from peripheral blood, tumor-draining lymph nodes, non-lymphoid tissues, and tumor tissues.

[Disclosure of COI by Responsible researcher] Lead presenter is not the Responsible researcher.
[Lead presenter: Kota Itahashi] We have no financial relationships to disclose.
[Principal Researcher: Hiroyoshi Nishikawa] We have no financial relationships to disclose.

### [YIA1-4] YIA1
### The Young Investigator Awards Lecture1

2025 / 9 / 25 (Thu) 9:00-11:30 Room 2/(Ishikawa Ongakudo 2F Hogaku Hall) Room 2

[Chairperson]Shinya Tanaka (Hokkaido University,Fuculty of medicine)

---

### YIA1-4
### Overcoming anti-cancer drug resistance by targeting YB-1 phosphorylation pathway

[Speaker]Tomohiro Shibata (Dept. Pharm., Yokohama City Univ. Sch. Med.)

Endocrine therapy significantly improves outcomes in patients with estrogen receptor alpha (ERα)-positive breast cancer, however, the development of resistance remains a major clinical challenge. Y-box binding protein 1 (YB-1), a multifunctional oncoprotein containing a cold shock domain, has been implicated in the regulation of genes associated with proliferation, survival, drug resistance, and DNA repair. Our previous studies have identified YB-1 as a key mediator of endocrine therapy resistance. We recently demonstrated that YB-1 expression is inversely correlated with ERα expression in breast cancer cells, both in vitro and in vivo. Moreover, inhibition of YB-1 phosphorylation effectively overcomes both acquired and intrinsic resistance to endocrine therapies and certain chemotherapeutic agents. These findings suggest that YB-1 plays a central role in endocrine resistance and may serve as a promising therapeutic target in endocrine-refractory and advanced-stage malignancies.

[Disclosure of COI by Responsible researcher] Lead presenter is the Responsible researcher.
[Lead presenter: Tomohiro Shibata] We have no financial relationships to disclose.

▲TOP

### [YIA1-5] YIA1
### The Young Investigator Awards Lecture1

2025 / 9 / 25 (Thu) 9:00-11:30 Room 2/(Ishikawa Ongakudo 2F Hogaku Hall) Room 2

[Chairperson]Shinya Tanaka (Hokkaido University,Fuculty of medicine)

### YIA1-5
### Establishment of novel 3D cell culture models of the fibrotic pancreatic cancer microenvironment using cell accumulation

[Speaker]Hiroyoshi Y. Tanaka (Faculty Med. Dent. Pharm. Sci., Okayama Univ.) [Co-author]Mitsunobu R. Kano (Faculty Interdisciplinary Sci. Engin. Health Syst., Okayama Univ.)

Pancreatic ductal adenocarcinoma (PDAC) is refractory to therapy, partly due to its dense, fibrotic microenvironment. The fibrotic stroma chiefly consists of pancreatic stellate cells (PSCs) and the extracellular matrix (ECM) that these cells excessively secrete. It poses a formidable barrier to drug delivery and regulates tumor cell behavior. Although the importance of targeting the fibrotic stroma in PDAC is widely recognized, effective targets remain elusive. A significant technical challenge is the lack of experimental models, especially ***in vitro***, that faithfully recapitulate the fibrotic microenvironment. Here, we overcome this challenge by applying the cell accumulation technique to develop novel 3D cell culture models of the fibrotic PDAC microenvironment. Specifically, by culturing patient PSCs in 3D, we recapitulate fibrotic tissue ***thickness*** (the distance from a tumor vessel to the closest tumor nest) and correlate ECM structure with drug delivery efficiency. Building on this model, we add tumor cells to establish a 3D coculture system that mimics the ***proportion*** of fibrotic tissue within PDAC, and apply this model to study the mechanisms driving the fibrotic phenotype.

[Disclosure of COI by Responsible researcher] Lead presenter is the Responsible researcher.
[Lead presenter: Hiroyoshi Y. Tanaka] ■Research funds from company: 科研費、ハーモニック伊藤財団、ウエスコ学術振興財団、薬学研究奨励財団、稲盛財団

▲TOP

### [YIA1-6] YIA1
### The Young Investigator Awards Lecture1

2025 / 9 / 25 (Thu) 9:00-11:30 Room 2/(Ishikawa Ongakudo 2F Hogaku Hall) Room 2

[Chairperson]Shinya Tanaka (Hokkaido University,Fuculty of medicine)

### YIA1-6
### Identification of novel negative regulators of anti-tumor T cell responses within the tumor microenvironment

[Speaker]Sana Hibino (Div. of Cancer Immunol., Natl. Cancer Ctr. Res. Inst.)

To overcome therapeutic resistance to cancer immunotherapy, it is critical to gain molecular insights into how effector T cells – the principal mediators of therapeutic response – are functionally regulated within the tumor microenvironment (TME). My research has focused on dissecting immunosuppressive mechanisms within the TME from multiple perspectives, leading to the identification of three novel negative regulators of anti-tumor T cell responses; 1) Nr4a factors, critical transcription factors governing the immunosuppressive function of regulatory T cells (Tregs) (Hibino ***et al., Cancer Res.*** 2018), 2) Spermidine, an oncometabolite with T cell-suppressive activity released by dying tumor cells (Hibino ***et al., Proc Natl Acad Sci U S A.*** 2023), 3) Molecular X, highly expressed in the specific subset of intratumoral T cells associated with non-responsiveness to immune checkpoint blockade therapy (***unpublished***). These findings may open new avenues for further breakthroughs in cancer immunotherapy.

[Disclosure of COI by Responsible researcher] Lead presenter is the Responsible researcher.
[Lead presenter: Sana Hibino] ■Research funds from company: 公益財団法人小林がん学術振興会、公益財団法人がん研究振興財団

▲TOP

### [YIA1-7] YIA1
### The Young Investigator Awards Lecture1

2025 / 9 / 25 (Thu) 9:00-11:30 Room 2/(Ishikawa Ongakudo 2F Hogaku Hall) Room 2

[Chairperson]Shinya Tanaka (Hokkaido University,Fuculty of medicine)

### YIA1-7
### Elucidating the Mechanisms of Lymphomagenesis with Clonal Hematopoiesis

[Speaker]Manabu Fujisawa (Department of Hematology, Institute of Medicine, University of Tsukuba／British Columbia Cancer Center, Department of Lymphoid Cancer Research)

血管免疫芽球性T細胞リンパ腫（AITL）は、T濾胞ヘルパー細胞様の表現型・機能を有する腫瘍性T細胞と、多彩な炎症細胞浸潤を特徴とする稀少T細胞リンパ腫である。造血幹細胞が***TET2***変異を獲得して加齢性クローン性造血（ACH）となり、さらにG17V ***RHOA***変異を得て腫瘍化するという多段階発がん説が提唱されている。AITL組織内のB細胞にもTET2変異が検出されており、変異炎症細胞が腫瘍支持ニッチとして機能する可能性がある。全血液細胞で***Tet2***欠損を導入したマウスは約1年でAITLを自然発症したが、T細胞特異的欠損マウスでは発症せず、変異炎症細胞の関与が示唆された。シングルセル発現解析では、暗帯・明帯の両特徴を示す異常胚中心B（GCB）細胞が増加し、体細胞変異を獲得してクローン進化していた。リガンドーレセプター解析でGCB細胞と腫瘍細胞間のCD40ーCD40LG相互作用を同定し、阻害抗体投与によりマウスの生存期間が延長した。以上から、***TET2***変異ACH由来GCB細胞はCD40ーCD40LGを介して腫瘍細胞を促進し、両者の相互作用が新規治療標的となり得ることが示唆された。

[Disclosure of COI by Responsible researcher] Lead presenter is the Responsible researcher.
[Lead presenter: Manabu Fujisawa] ■Research funds from company: 日本新薬

▲TOP

### [YIA2-1] YIA2
### The Young Investigator Awards Lecture2

2025 / 9 / 25 (Thu) 9:00-11:30 Room 2/(Ishikawa Ongakudo 2F Hogaku Hall) Room 2

[Chairperson]Toshihiko Doi (National Cancer Center Hospital East)

### YIA2-1
### Genomic analysis of mature lymphoid neoplasms: Molecular mechanisms and clinical implications

[Speaker]Yasunori Kogure (Div. Molecul. Oncol., Natl. Cancer Ctr. Res. Inst.) [Co-author]Keisuke Kataoka (Div. Molecul. Oncol., Natl. Cancer Ctr. Res. Inst.／Div. Hematol., Dept. Med., Keio Univ. Sch. Med.)

Mature lymphoid neoplasms comprise diverse disease entities, and despite advances in genomic analysis, their genetic basis and clinical implications remain elusive. We conducted genomic analyses of these diseases, focusing on adult T-cell leukemia/lymphoma (ATLL) and relapsed/refractory multiple myeloma (RRMM), yielding important insights into disease biology and clinical application.In ATLL, high-depth whole-genome sequencing (WGS) identified recurrent long isoform-specific alterations of CIC gene. We also discovered recurrent structural variations causing C-terminal truncation of c-Rel. In total, we discovered 56 driver genes, advancing our understanding of the genetic landscape of ATLL and underscoring the role of WGS in cancer research.In RRMM, analysis of circulating tumor DNA (ctDNA) and bone marrow plasma cells revealed that 59.2% of TP53 mutations were detected exclusively in ctDNA, highlighting its ability to detect subclones. Patients with KRAS mutations detected in ctDNA had significantly worse outcomes, leading to the development of a novel ctDNA-based prognostic model. These findings demonstrate the clinical importance of ctDNA analysis in RRMM.

[Disclosure of COI by Responsible researcher] Lead presenter is not the Responsible researcher.
[Lead presenter: Yasunori Kogure] ■Lecture fee: 武田薬品工業
[Principal Researcher: Keisuke Kataoka] ■Research funds from company: 公的研究費（JSPS, AMED, JST）、企業との契約研究（武田薬品工業）、その他（民間財団：第一三共生命科学研究振興財団[第一三共株式会社]、武田科学振興財団[武田薬品工業株式会社]、学会助成：日本血液学会）　■Profit of stock: 朝日ゲノミクス株式会社　■Lecture fee: 大塚製薬株式会社、Meiji Seika ファルマ株式会社、アッヴィ合同会社　■Research expenses: Meiji Seika ファルマ株式会社　■Contributions: エーザイ株式会社、協和キリン株式会社、中外製薬株式会社、大塚製薬株式会社、JCRファーマ株式会社、旭化成ファーマ株式会社

### [YIA2-2] YIA2
### The Young Investigator Awards Lecture2

2025 / 9 / 25 (Thu) 9:00-11:30 Room 2/(Ishikawa Ongakudo 2F Hogaku Hall) Room 2

[Chairperson]Toshihiko Doi (National Cancer Center Hospital East)

### YIA2-2
### Therapeutic targeting of syndecan-1 axis overcomes acquired resistance to KRAS-targeted therapy

[Speaker]Mitsunobu Takeda (Department of Gastroenterological Surgery, The University of Osaka) [Co-author]Wantong Yao (The University of Texas MD Anderson Cancer Center)、Haoqiang Ying (The University of Texas MD Anderson Cancer Center)、Yuki Sekido (Department of Gastroenterological Surgery, The University of Osaka)、Tsuyoshi Hata (Department of Gastroenterological Surgery, The University of Osaka)、Atsushi Hamabe (Department of Gastroenterological Surgery, The University of Osaka)、Takayuki Ogino (Department of Gastroenterological Surgery, The University of Osaka)、Norikatsu Miyoshi (Department of Gastroenterological Surgery, The University of Osaka)、Mamoru Uemura (Department of Gastroenterological Surgery, The University of Osaka)、Hirofumi Yamamoto (Department of Gastroenterological Surgery, The University of Osaka)、Yuichiro Doki (Department of Gastroenterological Surgery, The University of Osaka)、Hidetoshi Eguchi (Department of Gastroenterological Surgery, The University of Osaka)

KRAS変異を有する悪性腫瘍に対する標的治療薬は臨床応用が進む一方で、治療効果は一過性であり、獲得耐性の出現が大きな課題となっている。我々はこれまでに、KRAS活性によって細胞膜に局在するシンデカン-1（SDC1）が膵癌の進展に関与することを報告した。本研究では、KRAS阻害によって一時的に消失するSDC1の膜発現が、耐性腫瘍細胞においてYAP1活性化により再び誘導され、マクロピノサイトーシスや複数の受容体型チロシンキナーゼ（RTK）を介して腫瘍の維持やKRAS非依存性の獲得に寄与することを明らかにした。さらに、YAP1またはSDC1の阻害はRTK活性を抑制し、膵癌および大腸癌モデルにおける耐性腫瘍の増殖を著しく抑制した。YAP1に対するアンチセンスオリゴヌクレオチドはKRAS阻害薬との併用で、患者由来腫瘍モデルにおいて強力な治療効果を示した。YAP1-SDC1軸は、KRAS標的治療に対する獲得耐性を克服する新たな治療戦略の標的となる可能性がある。

[Disclosure of COI by Responsible researcher] Lead presenter is the Responsible researcher.
[Lead presenter: Mitsunobu Takeda] ■Research funds from company: 上原記念生命科学財団, 新谷医学国際交流財団, 細胞科学研究財団

### [YIA2-3] YIA2
### The Young Investigator Awards Lecture2

2025 / 9 / 25 (Thu) 9:00-11:30 Room 2/(Ishikawa Ongakudo 2F Hogaku Hall) Room 2

[Chairperson]Toshihiko Doi (National Cancer Center Hospital East)

---

### YIA2-3
### Elucidation of the molecular mechanisms of HPV-associated oropharyngeal carcinoma caused by epigenetic abnormality

[Speaker]Takuya Nakagawa (Health & Disease Omics Ctr., Chiba Univ. ／Dept Mol Oncol, Grad Sch Med, Chiba Univ. ／Dept Oto, Grad Sch Med, Chiba Univ.)

HPV-associated oropharyngeal squamous cell carcinoma (OPSCC) generally shows better prognosis than HPV-negative cases, but ~30% have poor outcomes. Lack of molecular mechanisms and markers hampers stratification and de-escalation strategies. We performed Infinium 450k DNA methylation profiling of 89 OPSCC cases with targeted exon sequencing and integrated these with 81 TCGA data. Unsupervised clustering analysis based on methylation patterns subdivided HPV-associated OPSCC cases into two distinct epigenetic subgroups with significantly different prognoses (P=0.0006). A panel of five methylation marker genes accurately distinguished these subgroups (AUC 0.969), while overall mutation profiles were similar between them. Furthermore, we recently found that in ~30% of cases, the HPV genome and adjacent human sequences form an extrachromosomal circular structure, termed hybrid ecDNA. By integrating epigenomic and ecDNA analyses, we elucidated its oncogenic mechanism and demonstrated therapeutic potential. The poor-prognosis methylation subgroup might be linked to these mechanisms, and integrated analyses could advance both prognostic stratification and novel treatment development.

[Disclosure of COI by Responsible researcher] Lead presenter is the Responsible researcher.
[Lead presenter: Takuya Nakagawa] We have no financial relationships to disclose.

▲TOP

### [YIA2-4] YIA2
### The Young Investigator Awards Lecture2

2025 / 9 / 25 (Thu) 9:00-11:30 Room 2/(Ishikawa Ongakudo 2F Hogaku Hall) Room 2

[Chairperson]Toshihiko Doi (National Cancer Center Hospital East)

### YIA2-4
### Sequencing-based Translational Research in Gynecologic Malignancies Through Comprehensive Molecular Analyses

[Speaker]Kosuke Yoshida (Dept. OBGYN, Nagoya Univ.)

Next-generation sequencing (NGS) enables comprehensive genomic and transcriptomic analysis, offering key insights into cancer biology. We conducted NGS-based molecular analyses to elucidate the pathology of gynecologic malignancies. In uterine leiomyosarcoma, transcriptome and pathway analysis highlighted activation of cell cycle-related kinases, and inhibitors of PLK1 and CHEK1 showed potent antitumor effects in vivo. Moreover, microRNA profiling revealed miR-10b-5p as a tumor suppressor candidate in uterine leiomyosarcoma. In malignant transformation of mature cystic teratoma, miRNA-seq identified disease-specific miRNAs, and spatial transcriptomics revealed KLF5 overexpression in cancer cells, which may contribute to the development of the disease. Furthermore, miRNA-seq was also applied to extracellular vesicle (EV) research. Our EV studies introduced a cellulose nanofiber (EV) sheet, which enables the evaluation of small-volume ascites diversity, and suggested its potential to contribute to the diagnosis and elucidation of the pathogenesis of ovarian cancer. These findings provide novel molecular insights and potential therapeutic targets in rare gynecologic cancers.

[Disclosure of COI by Responsible researcher] Lead presenter is the Responsible researcher.
[Lead presenter: Kosuke Yoshida] We have no financial relationships to disclose.

### [S1-1] S1
### ORGANOIDS: Unlocking Deep Insights into Cancer Biology

2025 / 9 / 25 (Thu) 9:00-11:30 Room 3/(Hotel Nikko Kanazawa 4F Tsuru A) Room 3

[Chairpersons]Haruna Takeda (National Cancer Center, Research Institute)、Toshiro Sato (Department of Medical Chemistry, Keio University School of Medicine )

Organoids can recapitulate the in vivo characteristics of cells and significantly contribute to elucidating the molecular mechanisms involved in the development and progression of cancer. Cancer progression is influenced by diverse factors in the microenvironment, such as inflammation, immune reaction, extracellular matrix remodeling, energy metabolism, and hypoxia. However, most of the molecular mechanisms promoting malignant progression remain unknown. Functional analysis using state-of-the-art technologies such as imaging, omics analysis, and novel devices in organoid culture systems has made it possible to analyze fundamental processes that take place in the microenvironment. Also, manipulating gene functions utilizing various genetic approaches in organoids has uncovered many novel gene functions that regulate differentiation, cell proliferation, and drug responses. In addition, constructing a library of patient-derived organoids and the stratification of cancers according to their responsiveness to cancer drugs or niche factors promote personalized medicine. Such analyses have provided new insights regarding cancer stem cells, cell plasticity, and the interactions between cancer cells and the immune system. These efforts will lead to identifying new therapeutic targets and establishing new therapies to cure malignant cancers for which no treatment is available. In this symposium, we will discuss the cutting edge of organoid research and the future direction.

---

### S1-1
### Cellular Plasticity Drives Malignant Progression of Inflammation-Associated Colorectal Cancer

[Speaker]Haruna Takeda (Natl. Cancer Ctr. Res. Inst.)

To elucidate the mechanisms underlying the malignant progression of inflammation-associated colorectal cancer (CRC), we performed in vivo screening using the Sleeping Beauty (SB) transposon system in mouse models of colitis. This approach identified ***Trp53*** and ***Cdkn2a*** as genes specifically involved in the development of inflammation-driven CRC. Detailed analyses using colonic organoids revealed distinct roles for inflammatory cytokines: TNF$\alpha$ activates cellular senescence pathways, Activin promotes cell motility, and IFN$\gamma$ triggers immune response pathways. The actions of these cytokines intricately interact to shape a complex tumor microenvironment, thereby promoting CRC progression. These cytokine-induced changes in cellular state are also accompanied by genome-wide epigenetic alterations. Our findings indicate that malignant progression in CRC, which cannot be fully accounted for by genetic mutations alone, is driven by inflammation-induced changes in cellular plasticity. Future studies will aim to identify epithelial-stromal interactions specific to inflammation-associated CRC, with the ultimate goal of developing novel therapeutic strategies.

[Disclosure of COI by Responsible researcher] Lead presenter is the Responsible researcher.
[Lead presenter: Haruna Takeda] We have no financial relationships to disclose.

▲TOP

### [S1-2] S1
### ORGANOIDS: Unlocking Deep Insights into Cancer Biology

2025 / 9 / 25 (Thu) 9:00-11:30 Room 3/(Hotel Nikko Kanazawa 4F Tsuru A) Room 3

[Chairpersons]Haruna Takeda (National Cancer Center, Research Institute)、Toshiro Sato (Department of Medical Chemistry, Keio University School of Medicine )

Organoids can recapitulate the in vivo characteristics of cells and significantly contribute to elucidating the molecular mechanisms involved in the development and progression of cancer. Cancer progression is influenced by diverse factors in the microenvironment, such as inflammation, immune reaction, extracellular matrix remodeling, energy metabolism, and hypoxia. However, most of the molecular mechanisms promoting malignant progression remain unknown. Functional analysis using state-of-the-art technologies such as imaging, omics analysis, and novel devices in organoid culture systems has made it possible to analyze fundamental processes that take place in the microenvironment. Also, manipulating gene functions utilizing various genetic approaches in organoids has uncovered many novel gene functions that regulate differentiation, cell proliferation, and drug responses. In addition, constructing a library of patient-derived organoids and the stratification of cancers according to their responsiveness to cancer drugs or niche factors promote personalized medicine. Such analyses have provided new insights regarding cancer stem cells, cell plasticity, and the interactions between cancer cells and the immune system. These efforts will lead to identifying new therapeutic targets and establishing new therapies to cure malignant cancers for which no treatment is available. In this symposium, we will discuss the cutting edge of organoid research and the future direction.

---

### S1-2
### Digestive organ tumors from precursor to intractable cancer

[Speaker]Hiroshi Seno (Dept. Gastroenterol & Hepatol, Kyoto Univ. Grad. Sch. Med.) [Co-author]Munemasa Nagao (Dept. Gastroenterol & Hepatol, Kyoto Univ. Grad. Sch. Med.)、Munenori Kawai (Dept. Gastroenterol & Hepatol, Kyoto Univ. Grad. Sch. Med.)、Go Yamakawa (Dept. Gastroenterol & Hepatol, Kyoto Univ. Grad. Sch. Med.)、Yuki Nakanishi (Dept. Gastroenterol & Hepatol, Kyoto Univ. Grad. Sch. Med.)、Akihisa Fukuda (Dept. Gastroenterol & Hepatol, Kyoto Univ. Grad. Sch. Med.)

Accumulating evidence has revealed developmental process of digestive organ tumors from precursor lesions to advanced and intractable cancers. In the case of pancreatic cancer, mouse and human studies indicated that both pancreatic ductal and acinar cells have potential to form precursor lesions (e.g., PanIN, IPMN and ITPN). As well, corresponding precursors are known in the biliary tract (e.g, BilIN, IPNB, etc.). In the gastrointestinal tract, colorectal cancers develop mainly through two different molecular pathways; the conventional and the serrated pathways (i.e., sessile serrated lesion and adenoma). Recent bulk or single-cell transcriptomics have dissected the roles of genetic and epigenetic alteration. We have reported that loss of SWI/SNF complex members in these precursor lesions and subsequent invasive cancers using genetically-engineered mice and human patient-derived organoids. In this session, I would like to introduce several models to mimick digestive organ cancer development from precursors to intractable cancers and discuss potential of those models for understanding cancer biology and therapeutics.

[Disclosure of COI by Responsible researcher] Lead presenter is the Responsible researcher.
[Lead presenter: Hiroshi Seno] We have no financial relationships to disclose.

### [S1-3] S1
### ORGANOIDS: Unlocking Deep Insights into Cancer Biology

2025 / 9 / 25 (Thu) 9:00-11:30 Room 3/(Hotel Nikko Kanazawa 4F Tsuru A) Room 3

[Chairpersons]Haruna Takeda (National Cancer Center, Research Institute)、Toshiro Sato (Department of Medical Chemistry, Keio University School of Medicine )

Organoids can recapitulate the in vivo characteristics of cells and significantly contribute to elucidating the molecular mechanisms involved in the development and progression of cancer. Cancer progression is influenced by diverse factors in the microenvironment, such as inflammation, immune reaction, extracellular matrix remodeling, energy metabolism, and hypoxia. However, most of the molecular mechanisms promoting malignant progression remain unknown. Functional analysis using state-of-the-art technologies such as imaging, omics analysis, and novel devices in organoid culture systems has made it possible to analyze fundamental processes that take place in the microenvironment. Also, manipulating gene functions utilizing various genetic approaches in organoids has uncovered many novel gene functions that regulate differentiation, cell proliferation, and drug responses. In addition, constructing a library of patient-derived organoids and the stratification of cancers according to their responsiveness to cancer drugs or niche factors promote personalized medicine. Such analyses have provided new insights regarding cancer stem cells, cell plasticity, and the interactions between cancer cells and the immune system. These efforts will lead to identifying new therapeutic targets and establishing new therapies to cure malignant cancers for which no treatment is available. In this symposium, we will discuss the cutting edge of organoid research and the future direction.

---

### S1-3
### TBD

[Speaker]Toshiro Sato (Department of Medical Chemistry, Keio University School of Medicine)

No Abstract

### [S1-4] S1
### ORGANOIDS: Unlocking Deep Insights into Cancer Biology

2025 / 9 / 25 (Thu) 9:00-11:30 Room 3/(Hotel Nikko Kanazawa 4F Tsuru A) Room 3

[Chairpersons]Haruna Takeda (National Cancer Center, Research Institute)、Toshiro Sato (Department of Medical Chemistry, Keio University School of Medicine )

Organoids can recapitulate the in vivo characteristics of cells and significantly contribute to elucidating the molecular mechanisms involved in the development and progression of cancer. Cancer progression is influenced by diverse factors in the microenvironment, such as inflammation, immune reaction, extracellular matrix remodeling, energy metabolism, and hypoxia. However, most of the molecular mechanisms promoting malignant progression remain unknown. Functional analysis using state-of-the-art technologies such as imaging, omics analysis, and novel devices in organoid culture systems has made it possible to analyze fundamental processes that take place in the microenvironment. Also, manipulating gene functions utilizing various genetic approaches in organoids has uncovered many novel gene functions that regulate differentiation, cell proliferation, and drug responses. In addition, constructing a library of patient-derived organoids and the stratification of cancers according to their responsiveness to cancer drugs or niche factors promote personalized medicine. Such analyses have provided new insights regarding cancer stem cells, cell plasticity, and the interactions between cancer cells and the immune system. These efforts will lead to identifying new therapeutic targets and establishing new therapies to cure malignant cancers for which no treatment is available. In this symposium, we will discuss the cutting edge of organoid research and the future direction.

---

### S1-4
### Identification of novel gastric cancer driver signals using mouse models, organoids, and multi-omics analysis

[Speaker]Yoku Hayakawa (Department of Gastroenterology, The University of Tokyo) [Co-author]Katsuyuki Oura (Department of Gastroenterology, The University of Tokyo)、Shintaro Shinohara (Department of Gastroenterology, The University of Tokyo)、Masahiro Hata (Department of Gastroenterology, The University of Tokyo)、Junya Arai (Department of Gastroenterology, The University of Tokyo)、Hiroaki Tateno (The National Institute of Advanced Industrial Science and Technology)、Mitsuhiro Fujishiro (Department of Gastroenterology, The University of Tokyo)

To elucidate mechanisms of gastric tumorigenesis, we employed genetically engineered mouse models, organoids, and multi-omics analysis. We first investigated R-spondin 3 (Rspo3), a regulator of gastric stem cell dynamics, and its receptors. Rspo3 overexpression promoted differentiation into neck and chief cells, while Lgr4 knockout (KO) blocked chief cell differentiation and induced metaplasia. Sdc4, a co-receptor, partially compensated for Rspo3 activity. Lgr4 and Sdc4 double KO led to pit cell expansion and disrupted gland homeostasis, highlighting receptor-specific roles in lineage allocation and tumor risk. Separately, we found that MUC6 KO gastric tumors showed increased mannose expression on clusterin, a glycoprotein downstream of Kras signaling. Clusterin and mannose accumulation was also observed in Kras-mutant tumors and correlated with marked infiltration of mannose receptor+ tumor-associated macrophages (TAMs). Inhibition of the clusterin-mannose axis suppressed TAM activation and tumor progression. These findings reveal epithelial-stromal-immune interactions driving gastric cancer and suggest new therapeutic targets.

[Disclosure of COI by Responsible researcher] Lead presenter is the Responsible researcher.
[Lead presenter: Yoku Hayakawa] We have no financial relationships to disclose.

### [S1-5] S1
### ORGANOIDS: Unlocking Deep Insights into Cancer Biology

2025 / 9 / 25 (Thu) 9:00-11:30 Room 3/(Hotel Nikko Kanazawa 4F Tsuru A) Room 3

[Chairpersons]Haruna Takeda (National Cancer Center, Research Institute)、Toshiro Sato (Department of Medical Chemistry, Keio University School of Medicine )

Organoids can recapitulate the in vivo characteristics of cells and significantly contribute to elucidating the molecular mechanisms involved in the development and progression of cancer. Cancer progression is influenced by diverse factors in the microenvironment, such as inflammation, immune reaction, extracellular matrix remodeling, energy metabolism, and hypoxia. However, most of the molecular mechanisms promoting malignant progression remain unknown. Functional analysis using state-of-the-art technologies such as imaging, omics analysis, and novel devices in organoid culture systems has made it possible to analyze fundamental processes that take place in the microenvironment. Also, manipulating gene functions utilizing various genetic approaches in organoids has uncovered many novel gene functions that regulate differentiation, cell proliferation, and drug responses. In addition, constructing a library of patient-derived organoids and the stratification of cancers according to their responsiveness to cancer drugs or niche factors promote personalized medicine. Such analyses have provided new insights regarding cancer stem cells, cell plasticity, and the interactions between cancer cells and the immune system. These efforts will lead to identifying new therapeutic targets and establishing new therapies to cure malignant cancers for which no treatment is available. In this symposium, we will discuss the cutting edge of organoid research and the future direction.

---

### S1-5
### Multi-omics analysis of organoids and xenograft models for exploring epigenetic abnormalities in pancreatic cancer

[Speaker]Takaaki Furukawa (Division of Cancer Epigenomics, Cancer Institute, JFCR／Division of Hepato-Biliary-Pancreatic Medicine, Cancer Institute Hospital of JFCR) [Co-author]Kenichi Miyata (Division of Cancer Epigenomics, Cancer Institute, JFCR)、Chikako Shibata (Division of Cancer Epigenomics, Cancer Institute, JFCR)、Kohei Kumegawa (Cancer cell diversity project, NEXT-Ganken Program, JFCR)、Miwa Tanaka (Division of Cancer Epigenomics, Cancer Institute, JFCR)、Takafumi Mie (Division of Hepato-Biliary-Pancreatic Medicine, Cancer Institute Hospital of JFCR)、Takeshi Okamoto (Division of Hepato-Biliary-Pancreatic Medicine, Cancer Institute Hospital of JFCR)、Tsuyoshi Takeda (Division of Hepato-Biliary-Pancreatic Medicine, Cancer Institute Hospital of JFCR)、Takashi Sasaki (Division of Hepato-Biliary-Pancreatic Medicine, Cancer Institute Hospital of JFCR)、Masato Ozaka (Division of Hepato-Biliary-Pancreatic Medicine, Cancer Institute Hospital of JFCR)、Naoki Sasahira (Division of Hepato-Biliary-Pancreatic Medicine, Cancer Institute Hospital of JFCR)、Tetsuo Noda (Director's room, Cancer Institute, JFCR)、Reo Maruyama (Division of Cancer Epigenomics, Cancer Institute, JFCR／Cancer cell diversity project, NEXT-Ganken Program, JFCR)

Epigenetic abnormalities are thought to underlie metastasis in pancreatic cancer (PC). Omics analysis in PC has been challenging due to difficulty in obtaining sufficient tissue. Organoid culture techniques have enabled propagation of cancer cells while preserving genetic traits of parental tumors. In this study, we established 18 patient-derived-organoids (PDOs) from PC (pancreas/malignant fluids: 12/6) and performed single-cell ATAC-sequencing (scATAC-seq), generating chromatin accessibility profiles from 42,861 cells. Subsequent analysis revealed increased motif scores for basic leucine zipper (bZIP) transcription factors in ascites-derived PDOs. Moreover, heterogeneity in bZIP motif scores was observed within each PDO at the single-cell level. To investigate bZIP motif activity in vivo, we established xenograft models with peritoneal metastases by orthotopic transplantation of ascites-derived PDOs. We performed scATAC-seq on both primary and peritoneal tumors, revealing heterogeneously elevated bZIP motif scores in the peritoneal tumor. We also conducted snRNA-seq and integrated it with scATAC-seq data to assess potential regulatory roles of bZIP transcription factors.

[Disclosure of COI by Responsible researcher] Lead presenter is not the Responsible researcher.
[Lead presenter: Takaaki Furukawa] We have no financial relationships to disclose.
[Principal Researcher: Reo Maruyama] We have no financial relationships to disclose.

### [S1-6] S1
### ORGANOIDS: Unlocking Deep Insights into Cancer Biology

2025 / 9 / 25 (Thu) 9:00-11:30 Room 3/(Hotel Nikko Kanazawa 4F Tsuru A) Room 3

[Chairpersons]Haruna Takeda (National Cancer Center, Research Institute)、Toshiro Sato (Department of Medical Chemistry, Keio University School of Medicine )

Organoids can recapitulate the in vivo characteristics of cells and significantly contribute to elucidating the molecular mechanisms involved in the development and progression of cancer. Cancer progression is influenced by diverse factors in the microenvironment, such as inflammation, immune reaction, extracellular matrix remodeling, energy metabolism, and hypoxia. However, most of the molecular mechanisms promoting malignant progression remain unknown. Functional analysis using state-of-the-art technologies such as imaging, omics analysis, and novel devices in organoid culture systems has made it possible to analyze fundamental processes that take place in the microenvironment. Also, manipulating gene functions utilizing various genetic approaches in organoids has uncovered many novel gene functions that regulate differentiation, cell proliferation, and drug responses. In addition, constructing a library of patient-derived organoids and the stratification of cancers according to their responsiveness to cancer drugs or niche factors promote personalized medicine. Such analyses have provided new insights regarding cancer stem cells, cell plasticity, and the interactions between cancer cells and the immune system. These efforts will lead to identifying new therapeutic targets and establishing new therapies to cure malignant cancers for which no treatment is available. In this symposium, we will discuss the cutting edge of organoid research and the future direction.

---

### S1-6
### Modeling Lung Cancer Heterogeneity Using Patient-Derived Organoids

[Speaker]Hiroyuki Yasuda (Keio University, School of Medicine, Department of Pulmonary Medicine)

Lung cancer is the leading cause of cancer-related deaths and is known for its poor prognosis. Despite advances in treatment, disease progression continues to drive high mortality. Lung cancer is highly heterogeneous, including adenocarcinoma, squamous cell carcinoma, and small cell carcinoma, and is further classified by driver gene alterations. Even within the same histologic and genetic subtypes, tumors vary significantly in behavior and treatment response. To improve therapy, a deeper molecular understanding using clinically relevant models is crucial. We have established a patient-derived lung cancer organoid library from diverse clinical specimens to investigate the molecular landscape of lung cancer. In this symposium, I will present our latest findings based on studies using these organoids.

[Disclosure of COI by Responsible researcher] Lead presenter is the Responsible researcher.
[Lead presenter: Hiroyuki Yasuda] We have no financial relationships to disclose.

### [S2-1] S2
### Large-scale clinico-genomic data analysis in cancer precision medicine

2025 / 9 / 25 (Thu) 9:00-11:30 Room 4/(Hotel Nikko Kanazawa 4F Tsuru B) Room 4

[Chairpersons]Keisuke Kataoka (Division of Hematology, Department of Medicine, Keio University School of Medicine)、Takashi Kohno (Division of Genome Biology, National Cancer Center, Research Institute)

Next-generation sequencing (NGS) technology has significantly deepened our understanding of cancer biology and genomics, transforming the landscape of cancer research and medicine. NGS-based studies have uncovered numerous novel driver alterations, mapping a comprehensive landscape of these genetic changes across human cancers. More recently, large-scale clinico-genomic studies have highlighted the clinical implications of these driver alterations, impacting not only treatment decisions but also diagnostics and prognostic predictions. In this session, we will present recent advancements in large-scale cancer studies, with a focus on real-world clinico-genomic research. Additionally, we will emphasize the clinical significance of genetic alterations, which paves the way for new horizons in both clinical and fundamental oncology.

---

### S2-1
### Transforming Oncology Electronic Health Records into Meaningful Multinational Evidence

[Speaker]Blythe Adamson (Flatiron Health, Inc.)

The integration of real-world data (RWD) from electronic health records (EHRs) is transforming oncology research and regulatory science worldwide. In the US, EHR-derived RWD has already supported regulatory submissions, drug development, real-world evidence generation and comparative effectiveness studies, demonstrating the value of robust, longitudinal data for both research and clinical care. Recent advances leverage machine learning and large language models to automate extraction of key clinical endpoints such as performance status and biomarker results at scale, enhancing both efficiency and depth of clinical insight. Robust quality assurance processes, including iterative validation and harmonization to global standards (e.g., RxNorm, LOINC), ensure traceability and auditability back to the source. The complexity of harmonizing structured and unstructured data across diverse healthcare systems, including Japan, presents unique challenges and opportunities. A tailored approach for Japan, with local language abstraction and compliance with local regulations, will allow us to learn from the experiences of people with cancer in Japan and benefit patients around the world.

[Disclosure of COI by Responsible researcher] Lead presenter is the Responsible researcher.
[Lead presenter: Blythe Adamson] We have no financial relationships to disclose.

▲TOP

### [S2-2] S2
### Large-scale clinico-genomic data analysis in cancer precision medicine

2025 / 9 / 25 (Thu) 9:00-11:30 Room 4/(Hotel Nikko Kanazawa 4F Tsuru B) Room 4

[Chairpersons]Keisuke Kataoka (Division of Hematology, Department of Medicine, Keio University School of Medicine)、Takashi Kohno (Division of Genome Biology, National Cancer Center, Research Institute)

Next-generation sequencing (NGS) technology has significantly deepened our understanding of cancer biology and genomics, transforming the landscape of cancer research and medicine. NGS-based studies have uncovered numerous novel driver alterations, mapping a comprehensive landscape of these genetic changes across human cancers. More recently, large-scale clinico-genomic studies have highlighted the clinical implications of these driver alterations, impacting not only treatment decisions but also diagnostics and prognostic predictions. In this session, we will present recent advancements in large-scale cancer studies, with a focus on real-world clinico-genomic research. Additionally, we will emphasize the clinical significance of genetic alterations, which paves the way for new horizons in both clinical and fundamental oncology.

---

### S2-2
### Epidemiological cancer research using health insurance claims data: An introduction to the LIFE Study

[Speaker]Kengo Kawaguchi (Dept. Health Care Admin. & Mgmt., Kyushu Univ.／Dept. Ortho. Surg., Grad. Sch. Med. Sci., Kyushu Univ.) [Co-author]Megumi Maeda (Dept. Health Care Admin. & Mgmt., Kyushu Univ.)、Yasuharu Nakashima (Dept. Ortho. Surg., Grad. Sch. Med. Sci., Kyushu Univ.)、Haruhisa Fukuda (Dept. Health Care Admin. & Mgmt., Kyushu Univ.)

Large-scale health insurance data have useful applications in advanced cancer research. The Longevity Improvement & Fair Evidence (LIFE) Study integrates medical claims, long-term care claims, and administrative data from over 4 million insurance enrollees in 30 municipalities across Japan, with potential follow-up for up to 8 years. Using claims data from 14 LIFE Study municipalities, we analyzed depression and anxiety incidence in 22,863 cancer patients diagnosed between April 2018 and March 2021. Depression incidence (3.36 per 1,000 person-months) peaked 2 months post-diagnosis, while anxiety (3.11 per 1,000 person-months) peaked at the time of diagnosis. A secondary peak for depression occurred 10 months post-diagnosis. Higher risks were observed in women and chemotherapy recipients. Pancreatic cancer had the highest incidence rates. This study highlights the critical periods for mental health interventions and emphasizes timely psychological support in cancer care. The LIFE Study's extensive and detailed data provide unique insights into real-world health outcomes, demonstrating its potential to guide effective public health policies and personalized medicine.

[Disclosure of COI by Responsible researcher] Lead presenter is not the Responsible researcher.
[Lead presenter: Kengo Kawaguchi] We have no financial relationships to disclose.
[Principal Researcher: Haruhisa Fukuda] We have no financial relationships to disclose.

### [S2-3] S2
### Large-scale clinico-genomic data analysis in cancer precision medicine

2025 / 9 / 25 (Thu) 9:00-11:30 Room 4/(Hotel Nikko Kanazawa 4F Tsuru B) Room 4

[Chairpersons]Keisuke Kataoka (Division of Hematology, Department of Medicine, Keio University School of Medicine)、Takashi Kohno (Division of Genome Biology, National Cancer Center, Research Institute)

Next-generation sequencing (NGS) technology has significantly deepened our understanding of cancer biology and genomics, transforming the landscape of cancer research and medicine. NGS-based studies have uncovered numerous novel driver alterations, mapping a comprehensive landscape of these genetic changes across human cancers. More recently, large-scale clinico-genomic studies have highlighted the clinical implications of these driver alterations, impacting not only treatment decisions but also diagnostics and prognostic predictions. In this session, we will present recent advancements in large-scale cancer studies, with a focus on real-world clinico-genomic research. Additionally, we will emphasize the clinical significance of genetic alterations, which paves the way for new horizons in both clinical and fundamental oncology.

---

### S2-3
### Understanding the impact of common germline variants on cancer progression using real-world data

[Speaker]Jian Carrot-Zhang (Memorial Sloan Kettering Cancer Center)

Germline risk variants modulate cancer evolution trajectories, yet for common, population-level variants, their impact on the tumor mutational landscape and clinical outcome remains largely unexplored. Leveraging two, independent clinical sequencing cohorts (MSK-IMPACT and DFCI-Profile), we imputed genome-wide, common variants from off-target reads and integrated germline, somatic and clinical data from a total of 91,275 cancer patients, enabling a comprehensive characterization of germline-somatic interactions and their influence on patient survival. Through genome-wide association study (GWAS) comparing driver alteration status within cancer patients at pan-cancer and cancer type-specific levels, we identify and validate several novel, germline determinants of tumor aggressiveness. Furthermore, our multi-ancestry analysis demonstrates that germline variants associated with genetic ancestry can contribute to somatic driver differences between populations. Our study highlights the power of large-scale, real-world data integration in refining our understanding of cancer biology, and motivates further investigation to improve risk assessment and treatment strategies.

[Disclosure of COI by Responsible researcher] Lead presenter is the Responsible researcher.
[Lead presenter: Jian Carrot-Zhang] We have no financial relationships to disclose.

▲TOP

### [S2-4] S2
### Large-scale clinico-genomic data analysis in cancer precision medicine

2025 / 9 / 25 (Thu) 9:00-11:30 Room 4/(Hotel Nikko Kanazawa 4F Tsuru B) Room 4

[Chairpersons]Keisuke Kataoka (Division of Hematology, Department of Medicine, Keio University School of Medicine)、Takashi Kohno (Division of Genome Biology, National Cancer Center, Research Institute)

Next-generation sequencing (NGS) technology has significantly deepened our understanding of cancer biology and genomics, transforming the landscape of cancer research and medicine. NGS-based studies have uncovered numerous novel driver alterations, mapping a comprehensive landscape of these genetic changes across human cancers. More recently, large-scale clinico-genomic studies have highlighted the clinical implications of these driver alterations, impacting not only treatment decisions but also diagnostics and prognostic predictions. In this session, we will present recent advancements in large-scale cancer studies, with a focus on real-world clinico-genomic research. Additionally, we will emphasize the clinical significance of genetic alterations, which paves the way for new horizons in both clinical and fundamental oncology.

### S2-4
### Real-world clinical utility of comprehensive genomic profiling in advanced solid cancers

[Speaker]Yuki Saito (Div. Mol. Oncol., Natl. Cancer Ctr. Res. Inst.／Div. Gastroenterol. Hepatol., Dept. Int. Med., Keio Univ. Sch. Med.) [Co-author]Sara Horie (Div. Mol. Oncol., Natl. Cancer Ctr. Res. Inst.／Div. Gastroenterol. Hepatol., Dept. Int. Med., Keio Univ. Sch. Med.)、Yasunori Kogure (Div. Mol. Oncol., Natl. Cancer Ctr. Res. Inst.)、Kota Mizuno (Div. Mol. Oncol., Natl. Cancer Ctr. Res. Inst.／Div. Hematol., Dept. Med., Keio Univ. Sch. Med.)、Yuta Ito (Div. Mol. Oncol., Natl. Cancer Ctr. Res. Inst.／Dept. Hematol., Natl. Cancer Ctr. Hosp.)、Yosuke Mizukami (Div. Mol. Oncol., Natl. Cancer Ctr. Res. Inst.／Div. Gastroenterol. Hepatol., Dept. Int. Med., Keio Univ. Sch. Med.)、Haryoon Kim (Div. Mol. Oncol., Natl. Cancer Ctr. Res. Inst.／Div. Hematol., Dept. Med., Keio Univ. Sch. Med.)、Zen Tamura (Div. Mol. Oncol., Natl. Cancer Ctr. Res. Inst.／Div. Hematol., Dept. Med., Keio Univ. Sch. Med.)、Junji Koya (Div. Mol. Oncol., Natl. Cancer Ctr. Res. Inst.／Div. Hematol., Dept. Med., Keio Univ. Sch. Med.)、Takeru Funakoshi (Dept. Dermatol., Keio Univ. Sch. Med.)、Kenro Hirata (Div. Gastroenterol. Hepatol., Dept. Int. Med., Keio Univ. Sch. Med.／Keio Cancer Center, Keio Univ. Sch. Med., Tokyo, Japan.)、Keisuke Kataoka (Div. Mol. Oncol., Natl. Cancer Ctr. Res. Inst.／Div. Hematol., Dept. Med., Keio Univ. Sch. Med.)

Comprehensive genomic profiling (CGP) is crucial in precision oncology, yet its real-world utility remains unclear. Through analyzing 54,185 advanced solid tumor cases receiving CGP with FoundationOne CDx from the Japanese nationwide C-CAT database, we demonstrate the prognostic value of CGP-guided clinical evidence level classification, with level A (16.6%) and B (8.1%) alterations associated with better prognosis. Only 8.0% of cases received CGP-guided approved or experimental targeted therapies (clinical trials or off-label drug use). There were substantial differences in this proportion across tumor types, with the highest in thyroid and lung cancers and the lowest in pancreatic and liver cancers. This proportion nearly doubled from 5.5% in 2019-2020 to 10.0% in 2023-2024, primarily due to the approval of new targeted therapies. Tumor-agnostic biomarker analyses reveal that tumor mutation burden (TMB) ≥ 20 mutations/Mb predicts better survival and objective response rate, regardless of microsatellite instability status, in TMB-high cases receiving pembrolizumab. Our findings reveal where CGP offers greater benefit, providing valuable information for individualized treatment.

[Disclosure of COI by Responsible researcher] Lead presenter is not the Responsible researcher.
[Lead presenter: Yuki Saito] ■Research funds from company: 文部科学省（科研費）、小林がん学術振興会、武田科学振興財団
[Principal Researcher: Keisuke Kataoka] ■Research funds from company: AMED、科学技術振興機構、上原記念生命科学財団
■Profit of stock: 朝日ゲノミクス株式会社　■Lecture fee: 大塚製薬株式会社、Meiji Seika ファルマ株式会社、アッヴィ合同会社
■Research expenses: Meiji Seika ファルマ株式会社　■Contributions: エーザイ株式会社、協和キリン株式会社、中外製薬株式会社、大塚製薬株式会社、JCRファーマ株式会社、旭化成ファーマ株式会社

### [S2-5] S2
### Large-scale clinico-genomic data analysis in cancer precision medicine

2025 / 9 / 25 (Thu) 9:00-11:30 Room 4/(Hotel Nikko Kanazawa 4F Tsuru B) Room 4

[Chairpersons]Keisuke Kataoka (Division of Hematology, Department of Medicine, Keio University School of Medicine)、Takashi Kohno (Division of Genome Biology, National Cancer Center, Research Institute)

Next-generation sequencing (NGS) technology has significantly deepened our understanding of cancer biology and genomics, transforming the landscape of cancer research and medicine. NGS-based studies have uncovered numerous novel driver alterations, mapping a comprehensive landscape of these genetic changes across human cancers. More recently, large-scale clinico-genomic studies have highlighted the clinical implications of these driver alterations, impacting not only treatment decisions but also diagnostics and prognostic predictions. In this session, we will present recent advancements in large-scale cancer studies, with a focus on real-world clinico-genomic research. Additionally, we will emphasize the clinical significance of genetic alterations, which paves the way for new horizons in both clinical and fundamental oncology.

---

### S2-5
### Current Status and Future Perspective of Precision Oncology in Advanced Solid Tumors

[Speaker]Tadayoshi Hashimoto (TR Support Office, NCCHE／Dept. GI Oncology, NCCHE) [Co-author]Naoko Iida (TR Support Office, NCCHE)、Hiroshi Ozaki (TR Support Office, NCCHE)、Masataka Amisaki (TR Support Office, NCCHE)、Taro Shibuki (TR Support Office, NCCHE)、Mitsuho Imai (TR Support Office, NCCHE)、Takao Fujisawa (TR Support Office, NCCHE)、Yoshiaki Nakamura (TR Support Office, NCCHE)、Hideaki Bando (TR Support Office, NCCHE／Dept. GI Oncology, NCCHE)、Takayuki Yoshino (Dept. GI Oncology, NCCHE)

The evolution of precision oncology has been dramatically accelerated by large-scale molecular profiling initiatives. The SCRUM-Japan MONSTAR-SCREEN consortium represents a pioneering nationwide effort that employs artificial intelligence-driven multiomics analyses for patients with advanced malignancies. Since its inception in 2015, our project has enrolled over 24,000 patients. Among 16,144 patients with advanced solid tumors in MONSTAR-SCREEN projects, 5.0% were successfully matched to targeted clinical trials. These matched patients demonstrated encouraging clinical outcomes, with a 29.2% objective response rate and 14.8-month median survival. Importantly, patients receiving matched therapy showed significantly improved overall survival compared to those who did not (hazard ratio 0.77; 95% confidence interval, 0.71-0.83). The MONSTAR-SCREEN-3 trial represents our comprehensive molecular profiling effort, incorporating liquid biopsy, bulk- and spatial-omics for tissue samples, and microbiome characterization. With a target enrollment of 3,200 patients, this multi-dimensional approach aims to provide unprecedented insights into tumor biology and treatment response.

[Disclosure of COI by Responsible researcher] Lead presenter is the Responsible researcher.
[Lead presenter: Tadayoshi Hashimoto] We have no financial relationships to disclose.

### [S2-6] S2
### Large-scale clinico-genomic data analysis in cancer precision medicine

2025 / 9 / 25 (Thu) 9:00-11:30 Room 4/(Hotel Nikko Kanazawa 4F Tsuru B) Room 4

[Chairpersons]Keisuke Kataoka (Division of Hematology, Department of Medicine, Keio University School of Medicine)、Takashi Kohno (Division of Genome Biology, National Cancer Center, Research Institute)

Next-generation sequencing (NGS) technology has significantly deepened our understanding of cancer biology and genomics, transforming the landscape of cancer research and medicine. NGS-based studies have uncovered numerous novel driver alterations, mapping a comprehensive landscape of these genetic changes across human cancers. More recently, large-scale clinico-genomic studies have highlighted the clinical implications of these driver alterations, impacting not only treatment decisions but also diagnostics and prognostic predictions. In this session, we will present recent advancements in large-scale cancer studies, with a focus on real-world clinico-genomic research. Additionally, we will emphasize the clinical significance of genetic alterations, which paves the way for new horizons in both clinical and fundamental oncology.

---

### S2-6
### Large-scale analysis of the genetic and environmental factors in cancer

[Speaker]Yukihide Momozawa (Laboratory for Genotyping Development, IMS, RIKEN)

The development of cancer is influenced by both genetic and environmental factors. We considered that a more efficacious strategy would be to initially characterize genetic risk, followed by a combination analysis of both factors. The present study has focused on pathogenic variants of 27 hereditary cancer genes, and 120,000 samples of 23 cancer types have been analyzed. We have reported multiple novel associations. For instance, pathogenic variants in BRCA1 and BRCA2 increased disease risk of various cancer types, including biliary tract cancer, esophageal cancer, and gastric cancer, with a higher prevalence observed in East Asian populations (JAMA Oncol 2022). Subsequently, we proceeded to the combination analysis of pathogenic variants and Helicobacter pylori on gastric cancer in 1433 patients and 5997 controls provided by the HERPACC. We identified that H. pylori infection was associated with an elevated risk of pathogenic variants in homologous-recombination genes (New Engl J Med 2023). The present study has expanded the analysis to encompass additional cancer types and environmental factors. The latest results will be presented.

[Disclosure of COI by Responsible researcher] Lead presenter is the Responsible researcher.
[Lead presenter: Yukihide Momozawa] We have no financial relationships to disclose.

### [IS1-1] IS1
### Targeting vulnerability in mitotic cell cycle: emerging anti-cancer strategies

2025 / 9 / 25 (Thu) 9:00-11:30 Room 5/(Hotel Nikko Kanazawa 4F Tsuru C) Room 5

[Chairpersons]Toru Hirota (The Cancer Institute of the JFCR)、Hongtao Yu (Westlake University)

Cell division is the basis of cell proliferation and has long been expected to provide promising targets for cancer therapy. A number of agents that inhibit microtubule dynamics, mitotic kinases and motors have therefore been clinically tested; however, their efficacy has been limited in practice, primarily because mitosis is a fundamental process for all cells and its disruption damages normal proliferating cells. How can we circumvent this narrow therapeutic window and find out means to selectively suppress the proliferation of cancer cells? One potential approach involves the identification of cancer-specific vulnerabilities, by figuring out the differences in the regulation of mitotic cell cycle between cancer and normal cells. In this symposium, we will highlight recent studies devising unique approaches on the basis of cancer-specific cellular responses. Recent progress in the understanding of cell cycle regulation and in technologies including CRISPR screening has allowed us to identify cancer-specific vulnerabilities in the regulation of cell division or chromosome dynamics, thereby revealed possible intervening points. Although we are still in early phases of the development, deep understandings of these processes in both physiological and pathological contexts should pave the way for innovative anti-cancer therapeutics targeting the mitotic cell cycle.

---

### IS1-1
### Targeting replication stress and CDK dysregulation in cancer

[Speaker]Mathew Jones (Frazer Institute, Univ. of Queensland／Sch. of Chemistry & Molecular Biosci, The Univ. of Queensland) [Co-author]Alexis Bonfim-Melo (Frazer Institute, Univ. of Queensland)、Rocco Ferguson (Frazer Institute, Univ. of Queensland)、Subash Rai (Frazer Institute, Univ. of Queensland)、David Cullen (Frazer Institute, Univ. of Queensland)、Beier Fu (Frazer Institute, Univ. of Queensland)

The dysregulation of cyclin-dependent kinases (CDKs) is a major driver of various cancer types. The overexpression of Cyclin E and the inactivation of CDK inhibitors result in premature cell cycle entry and increased DNA replication-associated damage, known as replication stress. To target replication stress in these cancers, we are building a systems-level understanding of the dependencies within the S-phase kinase network. Additionally, we are developing a functional genomics assay (DNAscent) that leverages long-read nanopore sequencing to assess the dynamic properties of DNA replication and define "replication stress signatures." Our long-term objective is to establish prognostic biomarkers that inform the clinical use of S-phase kinase inhibitors, such as Wee1, PKMYT1, Cdc7, and CDK2.

[Disclosure of COI by Responsible researcher] Lead presenter is the Responsible researcher.
[Lead presenter: Mathew Jones] ■Research funds from company: Schrodinger Inc.　■Research expenses: Schrodinger Inc.

▲TOP

### [IS1-2] IS1
### Targeting vulnerability in mitotic cell cycle: emerging anti-cancer strategies

2025 / 9 / 25 (Thu) 9:00-11:30 Room 5/(Hotel Nikko Kanazawa 4F Tsuru C) Room 5

[Chairpersons]Toru Hirota (The Cancer Institute of the JFCR)、Hongtao Yu (Westlake University)

Cell division is the basis of cell proliferation and has long been expected to provide promising targets for cancer therapy. A number of agents that inhibit microtubule dynamics, mitotic kinases and motors have therefore been clinically tested; however, their efficacy has been limited in practice, primarily because mitosis is a fundamental process for all cells and its disruption damages normal proliferating cells. How can we circumvent this narrow therapeutic window and find out means to selectively suppress the proliferation of cancer cells? One potential approach involves the identification of cancer-specific vulnerabilities, by figuring out the differences in the regulation of mitotic cell cycle between cancer and normal cells. In this symposium, we will highlight recent studies devising unique approaches on the basis of cancer-specific cellular responses. Recent progress in the understanding of cell cycle regulation and in technologies including CRISPR screening has allowed us to identify cancer-specific vulnerabilities in the regulation of cell division or chromosome dynamics, thereby revealed possible intervening points. Although we are still in early phases of the development, deep understandings of these processes in both physiological and pathological contexts should pave the way for innovative anti-cancer therapeutics targeting the mitotic cell cycle.

---

### IS1-2
### Targeting replication stress in Cancer with ATR and PARP Inhibition in SMARCA4-Deficient lung adenocarcinoma

[Speaker]Bunsyo Shiotani (Dept. Genome Stress Signaling,, Inst. Med. Sci., Tokyo Med. Univ.)

DNA replication stress is a hallmark of cancer. ATR kinase coordinates the response by stabilizing stalled replication forks and activating cell cycle checkpoints, allowing cancer cells to survive otherwise lethal damage. Thus, ATR inhibitors (ATRi) are promising therapeutic agents. However, predictive biomarkers and effective combination strategies remain to be defined. Here, we investigated genetic determinants and drug combinations that enhance ATRi sensitivity in lung adenocarcinoma cells deficient in SWI/SNF complex subunits (SMARCA4, ARID1A, PBRM1). SMARCA4-deficient cells showed marked synergistic cytotoxicity upon combined treatment with ATRi and PARP inhibitor (PARPi). PARPi increased H3K9me2, a marker of facultative heterochromatin, thereby enhancing replication stress and ATR dependency. Moreover, the combined treatment in SMARCA4-deficient cells triggered replication catastrophe via Mre11- and Mus81-mediated DNA damage. In a xenograft model, intermittent ATRi and continuous PARPi combined treatment significantly suppressed tumor growth. These findings highlight ATR/PARP co-inhibition as a promising strategy for SMARCA4-deficient lung cancer.

[Disclosure of COI by Responsible researcher] Lead presenter is the Responsible researcher.
[Lead presenter: Bunsyo Shiotani] ■Research funds from company: Merck KGaA

### [IS1-3] IS1
### Targeting vulnerability in mitotic cell cycle: emerging anti-cancer strategies

2025 / 9 / 25 (Thu) 9:00-11:30 Room 5/(Hotel Nikko Kanazawa 4F Tsuru C) Room 5

[Chairpersons]Toru Hirota (The Cancer Institute of the JFCR)、Hongtao Yu (Westlake University)

Cell division is the basis of cell proliferation and has long been expected to provide promising targets for cancer therapy. A number of agents that inhibit microtubule dynamics, mitotic kinases and motors have therefore been clinically tested; however, their efficacy has been limited in practice, primarily because mitosis is a fundamental process for all cells and its disruption damages normal proliferating cells. How can we circumvent this narrow therapeutic window and find out means to selectively suppress the proliferation of cancer cells? One potential approach involves the identification of cancer-specific vulnerabilities, by figuring out the differences in the regulation of mitotic cell cycle between cancer and normal cells. In this symposium, we will highlight recent studies devising unique approaches on the basis of cancer-specific cellular responses. Recent progress in the understanding of cell cycle regulation and in technologies including CRISPR screening has allowed us to identify cancer-specific vulnerabilities in the regulation of cell division or chromosome dynamics, thereby revealed possible intervening points. Although we are still in early phases of the development, deep understandings of these processes in both physiological and pathological contexts should pave the way for innovative anti-cancer therapeutics targeting the mitotic cell cycle.

---

### IS1-3
### Circular RNA SCAP promotes DNA replication in hepatocellular carcinoma cells

[Speaker]Chaiyaboot Ariyachet (Dept. of Biochem., Faculty of Med., Chulalongkorn Univ.) [Co-author]Archittapon Nokkeaw (Dept. of Biochem., Faculty of Med., Chulalongkorn Univ.)

Hepatocellular carcinoma (HCC) remains a leading cause of cancer mortality, with limited therapeutic options. Circular RNAs (circRNAs) have emerged as crucial regulators in cancer biology. We identified circSCAP as significantly upregulated in HCC tissues, suggesting its oncogenic role. Knockdown of circSCAP in HCC cells impaired proliferation, migration, and colony formation, indicating its importance in tumor progression. Transcriptomic analysis (RNA-seq) of circSCAP-knockdown HCC cells revealed a significant downregulation of genes associated with DNA replication. Mechanistically, we found that circSCAP functions as a molecular sponge for microRNA let-7c-5p. Inhibition of let-7c-5p reversed the impairment of DNA replication observed in circSCAP-knockdown HCC cells. Furthermore, we confirmed that let-7c-5p directly regulates genes essential for DNA replication using dual luciferase assays. These findings demonstrate that circSCAP promotes HCC progression by suppressing let-7c-5p, thereby facilitating DNA replication. Thus, circSCAP represents a novel oncogenic circRNA in HCC and a potential therapeutic target for future interventions.

[Disclosure of COI by Responsible researcher] Lead presenter is the Responsible researcher.
[Lead presenter: Chaiyaboot Ariyachet] We have no financial relationships to disclose.

▲TOP

### [IS1-4] IS1
### Targeting vulnerability in mitotic cell cycle: emerging anti-cancer strategies

2025 / 9 / 25 (Thu) 9:00-11:30 Room 5/(Hotel Nikko Kanazawa 4F Tsuru C) Room 5

[Chairpersons]Toru Hirota (The Cancer Institute of the JFCR)、Hongtao Yu (Westlake University)

Cell division is the basis of cell proliferation and has long been expected to provide promising targets for cancer therapy. A number of agents that inhibit microtubule dynamics, mitotic kinases and motors have therefore been clinically tested; however, their efficacy has been limited in practice, primarily because mitosis is a fundamental process for all cells and its disruption damages normal proliferating cells. How can we circumvent this narrow therapeutic window and find out means to selectively suppress the proliferation of cancer cells? One potential approach involves the identification of cancer-specific vulnerabilities, by figuring out the differences in the regulation of mitotic cell cycle between cancer and normal cells. In this symposium, we will highlight recent studies devising unique approaches on the basis of cancer-specific cellular responses. Recent progress in the understanding of cell cycle regulation and in technologies including CRISPR screening has allowed us to identify cancer-specific vulnerabilities in the regulation of cell division or chromosome dynamics, thereby revealed possible intervening points. Although we are still in early phases of the development, deep understandings of these processes in both physiological and pathological contexts should pave the way for innovative anti-cancer therapeutics targeting the mitotic cell cycle.

---

### IS1-4
### Vulnerability of mitotic chromosome segregation in cancers

[Speaker]Nana Kamakura (JFCR, Cancer Inst., Div. Exp. Pathol.／Dept. JFCR., Grad. Sch., Inst of Sci Tokyo) [Co-author]Toru Hirota (JFCR, Cancer Inst., Div. Exp. Pathol.／Dept. JFCR., Grad. Sch., Inst of Sci Tokyo)

Defects in kinetochore-microtubule attachments cause chromosome mis-segregation and chromosomal instability. Two mitotic kinases, Aurora B and Polo-like kinase1(Plk1) have been implicated in regulating the attachments. Aurora B is a well-known destabilizer, and Aurora B activity is reduced in many types of cancer cells. By contrast, Plk1 is a stabilizer, but how Plk1 is involved in cancer is largely unknown. Here, we found that cancer cells are vulnerable to both Plk1 activation and inactivation. To investigate what accounts the sensitivity to Plk1 perturbation, we focused on protein phosphatase PP2A, which is responsible for stabilizing microtubule attachments. We found that mitotic defect in perturbed Plk1 activity is associated with altered expression levels of a PP2A subunit, suggesting that sufficient levels of PP2A confers the resilience to Plk1 perturbation. To obtain a comprehensive view of the resilient regulatory system of Plk1, CRISPR screening is also underway. These findings identify the cancer-specific vulnerability in mitosis and therefore provide a target for emerging drug-development strategy to intervene cell division in cancers.

[Disclosure of COI by Responsible researcher] Lead presenter is not the Responsible researcher.
[Lead presenter: Nana Kamakura] We have no financial relationships to disclose.
[Principal Researcher: Toru Hirota] We have no financial relationships to disclose.

### [IS1-5] IS1
### Targeting vulnerability in mitotic cell cycle: emerging anti-cancer strategies

2025 / 9 / 25 (Thu) 9:00-11:30 Room 5/(Hotel Nikko Kanazawa 4F Tsuru C) Room 5

[Chairpersons]Toru Hirota (The Cancer Institute of the JFCR)、Hongtao Yu (Westlake University)

Cell division is the basis of cell proliferation and has long been expected to provide promising targets for cancer therapy. A number of agents that inhibit microtubule dynamics, mitotic kinases and motors have therefore been clinically tested; however, their efficacy has been limited in practice, primarily because mitosis is a fundamental process for all cells and its disruption damages normal proliferating cells. How can we circumvent this narrow therapeutic window and find out means to selectively suppress the proliferation of cancer cells? One potential approach involves the identification of cancer-specific vulnerabilities, by figuring out the differences in the regulation of mitotic cell cycle between cancer and normal cells. In this symposium, we will highlight recent studies devising unique approaches on the basis of cancer-specific cellular responses. Recent progress in the understanding of cell cycle regulation and in technologies including CRISPR screening has allowed us to identify cancer-specific vulnerabilities in the regulation of cell division or chromosome dynamics, thereby revealed possible intervening points. Although we are still in early phases of the development, deep understandings of these processes in both physiological and pathological contexts should pave the way for innovative anti-cancer therapeutics targeting the mitotic cell cycle.

---

### IS1-5
### Functional genetics with a hypomorphic CENP-C mutant reveals a regulatory system for chromosome congression

[Speaker]Tatsuo Fukagawa (FBS, Univ. Osaka)

Chromosome congression is a key process that acts to align chromosomes at the spindle equator via kinetochore-microtubule interactions, with defects in chromosome alignment leading to chromosomal instability. However, defining the mechanisms that underlie chromosome congression is limited due to the multiple factors that act in parallel to regulate chromosome movement. Here, we conducted a genome-wide Cas9-based functional genetics screen using a hypomorphic CENP-C mutant that affects its kinetochore interactions. Our analysis identified several genes, whose knockout resulted in synthetic lethality with the CENP-C mutant. Further analysis revealed that the synthetic defect was due to a reduction in some kinetochore functions in the CENP-C mutant. Our analysis allowed us to dissect parallel molecular mechanisms for chromosome congression and identify sensitivities and biomarkers for cancer therapy.

[Disclosure of COI by Responsible researcher] Lead presenter is the Responsible researcher.
[Lead presenter: Tatsuo Fukagawa] We have no financial relationships to disclose.

### [IS1-6] IS1
### Targeting vulnerability in mitotic cell cycle: emerging anti-cancer strategies

2025 / 9 / 25 (Thu) 9:00-11:30 Room 5/(Hotel Nikko Kanazawa 4F Tsuru C) Room 5

[Chairpersons]Toru Hirota (The Cancer Institute of the JFCR)、Hongtao Yu (Westlake University)

Cell division is the basis of cell proliferation and has long been expected to provide promising targets for cancer therapy. A number of agents that inhibit microtubule dynamics, mitotic kinases and motors have therefore been clinically tested; however, their efficacy has been limited in practice, primarily because mitosis is a fundamental process for all cells and its disruption damages normal proliferating cells. How can we circumvent this narrow therapeutic window and find out means to selectively suppress the proliferation of cancer cells? One potential approach involves the identification of cancer-specific vulnerabilities, by figuring out the differences in the regulation of mitotic cell cycle between cancer and normal cells. In this symposium, we will highlight recent studies devising unique approaches on the basis of cancer-specific cellular responses. Recent progress in the understanding of cell cycle regulation and in technologies including CRISPR screening has allowed us to identify cancer-specific vulnerabilities in the regulation of cell division or chromosome dynamics, thereby revealed possible intervening points. Although we are still in early phases of the development, deep understandings of these processes in both physiological and pathological contexts should pave the way for innovative anti-cancer therapeutics targeting the mitotic cell cycle.

---

### IS1-6
### Development of WEE1 inhibition-based strategies to overcome resistance to molecularly targeted therapies in cancer

[Speaker]Koji Fukuda (Cancer. Inst., Kanazawa Univ.／Nano. Inst., Kanazawa Univ.) [Co-author]Shinji Takeuchi (Nano. Inst., Kanazawa Univ.／Dept. Med. Oncol., Kanazawa Univ. Hosp.)、Shigeki Nanjo (Dept. Respir. Med., Inst. Med., Pharm., Health Sci., Kanazawa Univ.)、Hiroaki Taniguchi (Cancer. Inst., Kanazawa Univ.／Dept. Med. Oncol., Kanazawa Univ. Hosp.)、Seiji Yano (Dept. Respir. Med., Inst. Med., Pharm., Health Sci., Kanazawa Univ.)

**Background:**
Cell cycle-targeted therapies exploit vulnerabilities in checkpoint mechanisms. WEE1 inhibitors disrupt DNA damage responses (DDR) and the G2/M checkpoint, inducing selective cytotoxicity in cancer cells. We previously demonstrated that WEE1 inhibition enhances the efficacy of KRAS inhibitors in ***KRAS***-mutant lung cancer. Here, we investigated its ability to overcome intrinsic and acquired resistance to molecularly targeted therapies in lung and pancreatic cancers.
**Results:**
1. In ***KRAS***-mutant lung cancer, the combination of KRAS inhibitors and WEE1 inhibition enhanced apoptosis and overcame intrinsic resistance to KRAS-G12C inhibitors.
2. In ***KRAS***-mutant pancreatic cancer, WEE1 inhibition also enhanced apoptosis in intrinsic and acquired resistance to KRAS-G12D inhibitors.
3. In ***EGFR***-mutant lung cancer CNS models, WEE1 inhibition overcame ***ARID1A*** mutation-induced osimertinib resistance.
**Conclusion:**
Our findings suggest that targeting DDR defects via WEE1 inhibition may broadly overcome resistance to KRAS and EGFR inhibitors, supporting further clinical development of WEE1-based combination therapies for drug-resistant cancers.

[Disclosure of COI by Responsible researcher] Lead presenter is the Responsible researcher.
[Lead presenter: Koji Fukuda] We have no financial relationships to disclose.

▲TOP

### [IS1-7] IS1
### Targeting vulnerability in mitotic cell cycle: emerging anti-cancer strategies

2025 / 9 / 25 (Thu) 9:00-11:30 Room 5/(Hotel Nikko Kanazawa 4F Tsuru C) Room 5

[Chairpersons]Toru Hirota (The Cancer Institute of the JFCR)、Hongtao Yu (Westlake University)

Cell division is the basis of cell proliferation and has long been expected to provide promising targets for cancer therapy. A number of agents that inhibit microtubule dynamics, mitotic kinases and motors have therefore been clinically tested; however, their efficacy has been limited in practice, primarily because mitosis is a fundamental process for all cells and its disruption damages normal proliferating cells. How can we circumvent this narrow therapeutic window and find out means to selectively suppress the proliferation of cancer cells? One potential approach involves the identification of cancer-specific vulnerabilities, by figuring out the differences in the regulation of mitotic cell cycle between cancer and normal cells. In this symposium, we will highlight recent studies devising unique approaches on the basis of cancer-specific cellular responses. Recent progress in the understanding of cell cycle regulation and in technologies including CRISPR screening has allowed us to identify cancer-specific vulnerabilities in the regulation of cell division or chromosome dynamics, thereby revealed possible intervening points. Although we are still in early phases of the development, deep understandings of these processes in both physiological and pathological contexts should pave the way for innovative anti-cancer therapeutics targeting the mitotic cell cycle.

---

### IS1-7
### Modeling cell renewal and dynamic transition of gastritis to cancer in 3D organoids

[Speaker]Xuebiao Yao (School of Life Science, University of Science & Technology of China)

No Abstract

▲TOP

### [IS1-8] IS1
### Targeting vulnerability in mitotic cell cycle: emerging anti-cancer strategies

2025 / 9 / 25 (Thu) 9:00-11:30 Room 5/(Hotel Nikko Kanazawa 4F Tsuru C) Room 5

[Chairpersons]Toru Hirota (The Cancer Institute of the JFCR)、Hongtao Yu (Westlake University)

Cell division is the basis of cell proliferation and has long been expected to provide promising targets for cancer therapy. A number of agents that inhibit microtubule dynamics, mitotic kinases and motors have therefore been clinically tested; however, their efficacy has been limited in practice, primarily because mitosis is a fundamental process for all cells and its disruption damages normal proliferating cells. How can we circumvent this narrow therapeutic window and find out means to selectively suppress the proliferation of cancer cells? One potential approach involves the identification of cancer-specific vulnerabilities, by figuring out the differences in the regulation of mitotic cell cycle between cancer and normal cells. In this symposium, we will highlight recent studies devising unique approaches on the basis of cancer-specific cellular responses. Recent progress in the understanding of cell cycle regulation and in technologies including CRISPR screening has allowed us to identify cancer-specific vulnerabilities in the regulation of cell division or chromosome dynamics, thereby revealed possible intervening points. Although we are still in early phases of the development, deep understandings of these processes in both physiological and pathological contexts should pave the way for innovative anti-cancer therapeutics targeting the mitotic cell cycle.

---

### IS1-8
### The origin and consequences of aneuploidy in cancer

[Speaker]Hongtao Yu (School of Life Sciences, Westlake University, Hangzhou, China)

Aneuploidy is present in about 90% human solid tumors. Certain tumors remain addicted to aneuploidy. Paradoxically, artificially induced aneuploidy in normal cells elicits cellular stresses and decreases cell fitness. How aneuploidy initially emerges during tumorigenesis is thus unclear. Using human embryonic stem cells (ESCs) as a model, we show that aneuploid ESCs form immature teratomas with an enrichment of mesenchymal tissues. Specifically, chromosome 8 (chr8) gain, a prevalent form of aneuploidy in human cancers, promotes the expansion of mesenchymal stem cells (MSCs) in teratomas and MSC proliferation in vitro. We further show that human embryonal rhabdomyosarcomas, tumors of mesenchymal origin, lack dominant driver mutations but frequently harbor chr8 gain. Our study suggests a plausible mechanism for aneuploidy emergence during tumorigenesis, links specific aneuploidy to the mesenchymal lineage, and paves the way for identifying vulnerabilities of aneuploid MSCs, which may have important roles in tumorigenesis.

[Disclosure of COI by Responsible researcher] Lead presenter is the Responsible researcher.
[Lead presenter: Hongtao Yu] We have no financial relationships to disclose.

### [OS1-1] OS1
### The Contribution of Epidemiology to Cancer Control Policy - new approaches

2025 / 9 / 25 (Thu) 9:00-11:30 Room 6/(Hotel Nikko Kanazawa 3F Kujaku A) Room 6

[Chairpersons]Manami Inoue (National Cancer Center Institute for Cancer Control)、Keitaro Matsuo (Aichi Cancer Center)

National cancer control measures must be implemented with evidence-based approaches. Epidemiology has played various roles in cancer control policy decision-making. New research techniques utilizing large-scale databases, such as electronic medical records and government statistics, have been developed in recent years. This symposium will provide an opportunity to deepen understanding of new research approaches that have attracted attention in recent years and to consider epidemiological research that is expected to contribute to cancer control policy in the future.

---

### OS1-1
### The High-Benefit Approach: A New Targeting Strategy in Precision Medicine

[Speaker]Kosuke Inoue (Kyoto University)

In medicine, treatment decisions are often guided by the high-risk approach, which targets individuals at elevated risk of disease or mortality. However, it remains unclear whether those at highest risk benefit most from the treatment. To address this issue, we previously developed a novel strategy in personalized medicine called the high-benefit approach. This approach leverages machine learning techniques applied to data from randomized controlled trials and observational studies to identify populations most likely to experience substantial benefits from treatment. Rather than focusing solely on risk, the high-benefit approach targets individuals based on their potential for treatment benefit.

The high-benefit approach not only enhances the efficiency of resource allocation but also has the potential to reduce health disparities by identifying individuals who may receive limited benefit from standard treatments and guiding them toward alternative, more effective options. This presentation introduces the overall concept of the high-benefit approach and explores its potential applications in the healthcare literature to advance precision medicine and public health.

[Disclosure of COI by Responsible researcher] Lead presenter is the Responsible researcher.
[Lead presenter: Kosuke Inoue] We have no financial relationships to disclose.

▲TOP

### [OS1-2] OS1
### The Contribution of Epidemiology to Cancer Control Policy - new approaches

2025 / 9 / 25 (Thu) 9:00-11:30 Room 6/(Hotel Nikko Kanazawa 3F Kujaku A) Room 6

[Chairpersons]Manami Inoue (National Cancer Center Institute for Cancer Control)、Keitaro Matsuo (Aichi Cancer Center)

National cancer control measures must be implemented with evidence-based approaches. Epidemiology has played various roles in cancer control policy decision-making. New research techniques utilizing large-scale databases, such as electronic medical records and government statistics, have been developed in recent years. This symposium will provide an opportunity to deepen understanding of new research approaches that have attracted attention in recent years and to consider epidemiological research that is expected to contribute to cancer control policy in the future.

---

### OS1-2
### Target trial emulation: an accessible framework for formal causal inference from observational databases

[Speaker]Tomohiro Shinozaki (Dpt. Biostat., Sch. Public Health, Univ. Tokyo)

With the development of large-scale real-world databases collected in routine clinical practice, a foundation is being established across various fields, including cancer research, for revisiting research questions grounded in everyday medical care. However, real-world data alone do not necessarily generate real-world evidence. This talk introduces the framework of ***target trial emulation*** through applied examples using real-world databases. Unlike traditional approaches that focus on adjusting for confounding biases inherent to observational studies, this framework centers on structuring observational data to emulate a hypothetical intervention trial, known as the target trial. By sidestepping complex mathematical formulation, target trial emulation promotes the practical implementation of formal statistical causal inference, enabling (1) the natural avoidance of design-specific pitfalls in observational studies and (2) improved interpretability and transparency of study findings. As a potential common language for clinicians, epidemiologists, and data scientists, this framework may offer a breakthrough in addressing real-world research questions.

[Disclosure of COI by Responsible researcher] Lead presenter is the Responsible researcher.
[Lead presenter: Tomohiro Shinozaki] ■Advisor of the company and/or profit-making organization: （株 ）博報堂ＤＹホールディングス　■Lecture fee: SAS Institute Japan 株式会社

▲TOP

### [OS1-3] OS1
### The Contribution of Epidemiology to Cancer Control Policy - new approaches

2025 / 9 / 25 (Thu) 9:00-11:30 Room 6/(Hotel Nikko Kanazawa 3F Kujaku A) Room 6

[Chairpersons]Manami Inoue (National Cancer Center Institute for Cancer Control)、Keitaro Matsuo (Aichi Cancer Center)

National cancer control measures must be implemented with evidence-based approaches. Epidemiology has played various roles in cancer control policy decision-making. New research techniques utilizing large-scale databases, such as electronic medical records and government statistics, have been developed in recent years. This symposium will provide an opportunity to deepen understanding of new research approaches that have attracted attention in recent years and to consider epidemiological research that is expected to contribute to cancer control policy in the future.

---

### OS1-3
### Aggregating clinicogenomic data into C-CAT repository for utilization and incorporation

[Speaker]Yusuke Okuma (Sec of Liaison for Cancer Genomic Med Hosps)

Cancer genomic medicine based on comprehensive genomic profiling is the fundamental system for pursuing precision oncology, and has been implemented in Japan since June 2019 under the national health insurance system. C-CAT is currently aggregating the clinicogenomic data to improve the quality of genomic medicine by expanding treatment options through the promotion of clinical trials, contributing to the development of treatment, and providing key strategies for cancer control planning. To expand the data and facilitate the data input to reduce the physician workload, we need to overcome the challenge elaborating data between platforms to increase the value and advance the digital transformation for oncology. As the C-CAT portal becomes the connecting or transit gateway to connect platforms, such as electronic health data, the individual oncologists will gain direct access to multimodality datasets for practical purposes, and this will provide the clinical support from central experts. For the future development of this framework, the advances in information technology and international collaboration will be essential to overcome the problems and promote cancer genomic medicine.

[Disclosure of COI by Responsible researcher] Lead presenter is the Responsible researcher.
[Lead presenter: Yusuke Okuma] ■Lecture fee: アストラゼネカ株式会社、イーライリリー株式会社、エーザイ株式会社、中外製薬株式会社　■Research expenses: Summit Therapeutics、MSD

### [OS1-4] OS1
### The Contribution of Epidemiology to Cancer Control Policy - new approaches

2025 / 9 / 25 (Thu) 9:00-11:30 Room 6/(Hotel Nikko Kanazawa 3F Kujaku A) Room 6

[Chairpersons]Manami Inoue (National Cancer Center Institute for Cancer Control)、Keitaro Matsuo (Aichi Cancer Center)

National cancer control measures must be implemented with evidence-based approaches. Epidemiology has played various roles in cancer control policy decision-making. New research techniques utilizing large-scale databases, such as electronic medical records and government statistics, have been developed in recent years. This symposium will provide an opportunity to deepen understanding of new research approaches that have attracted attention in recent years and to consider epidemiological research that is expected to contribute to cancer control policy in the future.

---

### OS1-4
### Monitoring the quality of cancer control measures using government statistics and databases

[Speaker]Tomohiro Matsuda (Ctr. for Cancer Registries, NCC ICC)

Following the enforcement of the Cancer Registration Act, Japan has been rapidly improving its cancer statistics. A legally based data infrastructure is vital to quantitatively understand cancer incidence, diagnosis, treatment, and prognosis, and to evaluate cancer control efforts. As the national registry center, we work to visualize regional disparities, track time trends, and examine links between factors such as incidence risk, screening participation, patient centralization, and standard treatment implementation. We are also exploring the integration of multiple public databases, anticipating future legal revisions. However, compiling data into reports alone is not sufficient. Cancer statistics must be transformed into outcome indicators, while cancer control policies should be evaluated as processes-including budget, staffing, and implementation status. These must be integrated and continuously monitored at both national and regional levels. The International Cancer Benchmarking Partnership (ICBP), scores cancer policies and shows a positive correlation with survival. Stronger evidence generation and policy analysis are needed to align with international standards.

[Disclosure of COI by Responsible researcher] Lead presenter is the Responsible researcher.
[Lead presenter: Tomohiro Matsuda] We have no financial relationships to disclose.

▲TOP

### [OS1-5] OS1
### The Contribution of Epidemiology to Cancer Control Policy - new approaches

2025 / 9 / 25 (Thu) 9:00-11:30 Room 6/(Hotel Nikko Kanazawa 3F Kujaku A) Room 6

[Chairpersons]Manami Inoue (National Cancer Center Institute for Cancer Control)、Keitaro Matsuo (Aichi Cancer Center)

National cancer control measures must be implemented with evidence-based approaches. Epidemiology has played various roles in cancer control policy decision-making. New research techniques utilizing large-scale databases, such as electronic medical records and government statistics, have been developed in recent years. This symposium will provide an opportunity to deepen understanding of new research approaches that have attracted attention in recent years and to consider epidemiological research that is expected to contribute to cancer control policy in the future.

---

### OS1-5
### A new perspective on monitoring health inequalities to promote cancer control that leaves no one behind

[Speaker]Yuri Ito (Dept. of Med. Stats, Med., Osaka Med. & Pharm Univ.)

In Japan, the government has set "Overcome cancer with all citizens, promote cancer control that leaves no one behind" as the target of the 4th National Cancer Plan, focusing on tackling socioeconomic inequalities in the cancer control continuum. However, monitoring inequalities in all aspects of the continuum is challenging. We started monitoring inequalities in indicators in prevention, screening, treatment and survivorship, based on the Logic Model which was implemented after the 4th Cancer Plan. We can monitor some indicators at prefecture-level as regional inequalities. In order to understand regional differences in indicators, we need further examination using more detailed data from smaller geographical units. Using small geographical units, allows us to monitor area-level socio economic inequalities, and also consider issues such as accessibility to care, urban-rurality, walkability, and social isolation as social environmental factors in cancer indicators. Understanding the factors and mechanisms of regional differences in multiple indicators on the Logic Model route from output to outcome indicators enables us to tackle inequalities in cancer outcome in Japan.

[Disclosure of COI by Responsible researcher] Lead presenter is the Responsible researcher.
[Lead presenter: Yuri Ito] We have no financial relationships to disclose.

▲TOP

### [OS1-6] OS1
### The Contribution of Epidemiology to Cancer Control Policy - new approaches

2025 / 9 / 25 (Thu) 9:00-11:30 Room 6/(Hotel Nikko Kanazawa 3F Kujaku A) Room 6

[Chairpersons]Manami Inoue (National Cancer Center Institute for Cancer Control)、Keitaro Matsuo (Aichi Cancer Center)

National cancer control measures must be implemented with evidence-based approaches. Epidemiology has played various roles in cancer control policy decision-making. New research techniques utilizing large-scale databases, such as electronic medical records and government statistics, have been developed in recent years. This symposium will provide an opportunity to deepen understanding of new research approaches that have attracted attention in recent years and to consider epidemiological research that is expected to contribute to cancer control policy in the future.

---

### OS1-6
### Epidemiology to cancer control policy -Need for policy impact assessments

[Speaker]Kota Katanoda (Div. Data Sci. Natl. Can. Ctr. Inst. Can. Contr.)

In cancer control, scientific evidence is crucial not only for building policies but also for evaluating past or ongoing policies. In many high-income countries, policy evaluation, or more generally, health technology assessment (HTA), is one of the major research fields, closely connected to administrative activities. Policy assessment or evaluation requires diverse expertise and methodologies, including descriptive epidemiology, biostatistics, simulation modeling, health economics, and behavioral/social sciences. There are two time perspectives: retrospective and prospective, both involving hypothetical scenarios. I am going to present several examples of policy evaluation research and activities in Japan, ranging from tobacco, hepatitis, human papillomavirus, Helicobacter pylori, and cancer screening. One of the features of these topics is their high population impact. In other words, Japanese policy impact assessments are still limited to quantitative approaches. Internationally, many qualitative or narrative approaches have been used in policy assessments. I am going to touch on those aspects as well. I hope this symposium will help boost this new research field.

[Disclosure of COI by Responsible researcher] Lead presenter is the Responsible researcher.
[Lead presenter: Kota Katanoda] We have no financial relationships to disclose.

### [E-1001] I-E12-1
### The latest advancement in liquid biopsy

2025 / 9 / 25 (Thu) 9:00-10:15 Room 7/(Hotel Nikko Kanazawa 3F Kujaku B) Room 7

[Chairperson]Shinji Kohsaka (Div. of Cell. Sig., NCCRI.)

---

### E-1001
### Bile liquid biopsy is a useful modality for molecular diagnosis of pancreaticobiliary cancer

[Speaker]Shuichi Aoki (Dept. Surg., Tohoku Univ.) [Co-author]Shin Ito (Div. Mol. & Cell. Oncol., Miyagi Cancer Ctr. Res. Inst.)、Mitsuhiro Shimura (Dept. Surg., Tohoku Univ.)、Daisuke Douchi (Dept. Surg., Tohoku Univ.)、Akihiro Yamamura (Dept. Surg., Tohoku Univ.)、Hideaki Karasawa (Dept. Surg., Tohoku Univ.)、Masamichi Mizuma (Dept. Surg., Tohoku Univ.)、Shinobu Ohnuma (Dept. Surg., Tohoku Univ.)、Jun Yasuda (Div. Mol. & Cell. Oncol., Miyagi Cancer Ctr. Res. Inst.)、Michiaki Unno (Dept. Surg., Tohoku Univ.)

Objective: A liquid biopsy using circulating tumor DNA (ctDNA) in the blood is a valuable tool. However, a low positivity rate has been reported in pancreaticobiliary cancers.Methods: We examined the utility of bile-derived ctDNA in 13 cases diagnosed with pancreaticobiliary cancer, who underwent surgical resection.Results: Upon extracting ctDNA from both bile and blood, the amount of bile-ctDNA was significantly greater than blood-ctDNA, with the capability to extract longer fragment DNA (both $p<0.001$). The quantity of bile-ctDNA did not correlate with blood-ctDNA levels, nor was there a correlation with disease stage. Whole-exome sequencing of resected specimens revealed actionable genomic mutations and ddPCR confirmed identical genomic mutations from bile-ctDNA. Additionally, in a genomic analysis of 22 paired samples, the detection rate of driver mutations was superior in bile-ctDNA (59.0%), compared to those in blood-ctDNA (16.7%).Conclusion: Bile allows for the extraction of sufficient quantities and quality of ctDNA derived from cancer cells, regardless of disease stage. Bile-ctDNA is a more useful genomic analysis tool compared to blood-ctDNA.

[Disclosure of COI by Responsible researcher] Lead presenter is the Responsible researcher.
[Lead presenter: Shuichi Aoki] We have no financial relationships to disclose.

### [E-1002] I-E12-1
### The latest advancement in liquid biopsy

2025 / 9 / 25 (Thu) 9:00-10:15 Room 7/(Hotel Nikko Kanazawa 3F Kujaku B) Room 7

[Chairperson]Shinji Kohsaka (Div. of Cell. Sig., NCCRI.)

---

### E-1002
### Predictive potential of ctDNA dynamics for RECIST-based CT imaging outcomes

[Speaker]Minit Jain (Dept. of Biomed. R&D, Iwate Med. Univ. Inst. Biomed. Sci.) [Co-author]Akiko Yashima-Abo (Dept. of Biomed. R&D, Iwate Med. Univ. Inst. Biomed. Sci.)、Hayato Hiraki (Dept. of Biomed. R&D, Iwate Med. Univ. Inst. Biomed. Sci.)、Shinji Tamada (Dept. of Biomed. R&D, Iwate Med. Univ. Inst. Biomed. Sci.)、Taiga Sasaki (Dept. of Biomed. R&D, Iwate Med. Univ. Inst. Biomed. Sci.)、Akio Tamura (Dept. of Radiology Iwate Med. Univ. School of Med.)、Takeshi Iwaya (Dept. Clin. Oncology Iwate Med. Univ. School of Med.)、Satoshi Nishizuka (Dept. of Biomed. R&D, Iwate Med. Univ. Inst. Biomed. Sci.)

Variant allele frequency (VAF) in ctDNA represents a potentially new class of biomarker for assessing physical tumor burden. We hypothesized that the linear rate of change (LRoC) in ctDNA VAF% could help predict RECIST-based CT outcomes. In this study, we aimed to find corroborative evidence that ctDNA change may precede CT. We analyzed plasma samples from our pan-cancer MORIOKA study cohort (n=305), collected at periodic timepoints during treatment (n=1,521). The LRoC at post regimen was compared with CT categorized using RECIST-based criteria. First, we set a time window of ≤45 days between post regimen ctDNA and CT, and found that 41.8% of LRoC correctly predicted PD, while 44.8% of those did for PR. Next, we assessed LRoC performance for individual regimens. Here, 40% of LRoC correctly predicted PD while 60% of those did for PR. By regimen type, LRoC of chemotherapy (n=80) correctly predicted 33.3% of PD and 66.7% of PR; and those of immune-checkpoint inhibitors (ICIs, n=36) did 56.5% of PD and 83.3% of PR. These findings suggest that periodic ctDNA monitoring could assist in predicting CT outcomes, particularly for ICI-based regimens.

[Disclosure of COI by Responsible researcher] Lead presenter is not the Responsible researcher.
[Lead presenter: Minit Jain] ■ employment by a company related to the presentation: Quantdatect, Inc.
[Principal Researcher: Satoshi S. Nishizuka] ■employment by a company related to the presentation: Quantdetect, Inc. ■Advisor of the company and/or profit-making organization: Quantdetect, Inc. ■Profit of stock: Quantdetect, Inc. ■Research expenses: Quantdetect, Rosche, Taiho, Boerringer ■Representative of organization for clinical study receiving research expenses from company: Quantdetect, Rosche, Taiho, Boerringer

### [E-1003] I-E12-1
### The latest advancement in liquid biopsy

2025 / 9 / 25 (Thu) 9:00-10:15 Room 7/(Hotel Nikko Kanazawa 3F Kujaku B) Room 7

[Chairperson]Shinji Kohsaka (Div. of Cell. Sig., NCCRI.)

---

### E-1003
### Comparative genomic profiling of cell-free and tumor-derived DNA in Japanese patients with metastatic breast cancer

[Speaker]Yuri Fujimoto (Department of Breast Surgery Kyoto University Graduate school of medicine) [Co-author]Masahiro Kawashima (Department of Breast Surgery Kyoto University Graduate school of medicine)、Ai Yamaguchi (Department of Breast Surgery Kyoto University Graduate school of medicine)、Shun Kawaguchi (Department of Pathology and Tumor Biology Kyoto University)、Tomomi Nishimura (Department of Breast Surgery Kyoto University Graduate school of medicine／Department of Pathology and Tumor Biology Kyoto University／Department of Surgery, Japanese Red Cross Fukui Hospital)、Masakazu Toi (Tokyo Metropolitan Cancer Center Komagome Hospital Tokyo Metropolitan Hospital Organization)、Seishi Ogawa (Department of Pathology and Tumor Biology Kyoto University)、Norikazu Masuda (Department of Breast Surgery Kyoto University Graduate school of medicine)

Background: The utility of cfDNA analysis in Japanese metastatic breast cancer (MBC) and its genomic landscape remain unclear. This study assessed the performance of Guardant360 (G360) in detecting pathogenic variants (PVs) and changes over time using tissue DNA (tDNA) profiling. Methods: We analyzed 10 archived and 29 prospective plasma samples from 39 MBC patients using G360. Serial biopsies from primary/metastatic sites were profiled with an in-house tDNA NGS panel. Detection rates and patterns of PVs and actionable PVs (ERBB2, PIK3CA, BRCA1/2, AKT1, ESR1, PTEN) were compared between cfDNA and tDNA. Results: In 29 prospective cfDNA samples, PVs and actionable PVs were found in 86.2% and 51.7%, similar to 10 archived samples (80%, 60%). tDNA profiling succeeded in 23 cases; concordance with cfDNA was 96.5%. MYC amplification was more frequent in tDNA, ESR1 and TP53 in cfDNA. Among 19 patients with serial profiling, luminal subtype had the most actionable PVs (82.4% at initial biopsy). Conclusion: This study presents longitudinal genomic data in Japanese MBC. cfDNA and tDNA showed high concordance with gene level differences. G360 effectively detected actionable PVs.

[Disclosure of COI by Responsible researcher] Lead presenter is not the Responsible researcher.
[Lead presenter: Yuri Fujimoto] We have no financial relationships to disclose.
[Principal Researcher: Mashiro Kawashima] ■Research funds from company: Guardant Health Japan

▲TOP

### [E-1004] I-E12-1
### The latest advancement in liquid biopsy

2025 / 9 / 25 (Thu) 9:00-10:15 Room 7/(Hotel Nikko Kanazawa 3F Kujaku B) Room 7

[Chairperson]Shinji Kohsaka (Div. of Cell. Sig., NCCRI.)

---

### E-1004
### OTS-Select: A Target Mutation Selection Algorithm for ctDNA Monitoring using digital PCR

[Speaker]Hayato Hiraki (Div. Biomed. Res. & Development, Iwate Med. Univ.) [Co-author]Akiko Abo (Div. Biomed. Res. & Development, Iwate Med. Univ.)、Takeshi Iwaya (Dept. Clin. Oncology, Iwate Med. Univ.)、Satoshi Nishizuka (Div. Biomed. Res. & Development, Iwate Med. Univ.)

Circulating tumor DNA (ctDNA) monitoring is crucial for predicting and detecting tumor relapse during post-treatment surveillance. As ctDNA variant allele frequencies (VAFs) are often below 1%, digital PCR (dPCR) remains the most effective method. A key challenge in this context is selecting specific primers and probes for patient tumor-specific mutations. To address this, we developed OTS-Select, an algorithm designed to select target mutations considering germline mutations and sequencing errors from next generation sequencing data for ctDNA monitoring. OTS-Select evaluates sequencing quality, VAFs, mutation classification from dbSNP and ClinVar. It ranks candidate mutations into categories S, A, B, C, D, and E. Among 511 mutations from 375 patients, ctDNA of patient tumor-specific mutations was longitudinally monitored for up to 759 days. The success rates of ctDNA monitoring by OTS-Select were as follows: S, 95%; A, 84%; B, 81%; C, 35%; D, 25%; and E, 0%. We also assessed the clinical validity of patient tumor-specific mutations as individualized tumor markers, confirming the potential of OTS-Select to enable ctDNA monitoring using dPCR.

[Disclosure of COI by Responsible researcher] Lead presenter is not the Responsible researcher.
[Lead presenter: Hayato Hiraki] ■Research funds from company: 株式会社LSIメディエンス　■Advisor of the company and/or profit-making organization: 株式会社クオントディテクト　■Presents or other payment: 株式会社クオントディテクト
[Principal Researcher: Satoshi S. Nishizuka] ■Research funds from company: 株式会社クオントディテクト、日本ベーリンガーインゲルハイム株式会社、大鵬薬品工業株式会社、株式会社LSIメディエンス、株式会社キアゲン、ロシュ・ダイアグノスティックス株式会社　■employment by a company related to the presentation: 株式会社クオントディテクト　■Advisor of the company and/or profit-making organization: 株式会社日立ハイテク　■Profit of stock: 株式会社クオントディテクト　■Lecture fee: 株式会社フィンガルリンク、MSD株式会社、日本化薬株式会社、岩手県、Thermo Fisher Scientific社

### [E-1005] I-E12-1
### The latest advancement in liquid biopsy

2025 / 9 / 25 (Thu) 9:00-10:15 Room 7/(Hotel Nikko Kanazawa 3F Kujaku B) Room 7

[Chairperson]Shinji Kohsaka (Div. of Cell. Sig., NCCRI.)

---

### E-1005
### A digital PCR probe library for TP53 mutations in longitudinal ctDNA monitoring

[Speaker]Satoshi Nishizuka (Iwate Med. Univ. Inst. Biomed. Sci. Div. Biomed. R&D.) [Co-author]Hayato Hiraki (Iwate Med. Univ. Inst. Biomed. Sci. Div. Biomed. R&D.)、Minit Jain (Iwate Med. Univ. Inst. Biomed. Sci. Div. Biomed. R&D.)、Akiko Abo (Iwate Med. Univ. Inst. Biomed. Sci. Div. Biomed. R&D.)、Takeshi Iwaya (Iwate Med. Univ. Dept. Clin. Oncol.)

Although ***TP53*** mutations are highly prevalent in human cancer, they have yet to lead to significant improvements in patient care. Among > 1,500 ***TP53*** mutations identified within the DNA-binding domain coding region, about 10% of recurrent mutations account for >70% of all reported mutations. This suggests that limited numbers of digital PCR probes can enable immediate, sensitive, and individualized tumor-informed ctDNA monitoring in the majority of patients with ***TP53*** mutations. Here, we developed a uniquely designed digital PCR probe library targeting >200 ***TP53*** mutations, to quantify ***TP53*** mutations as ctDNA. In our advanced pan-cancer cohort, ***TP53*** mutations were detected in 59% (178/303) cases with the highest frequencies observed in uterine (76%, 19/25), ovarian (75%, 9/12), and colorectal (73%, 38/52) cancers. Moreover, overall 110 cases with ***TP53*** mutations demonstrated at least one of the following clinical validity during longitudinal ctDNA monitoring: early relapse prediction; treatment efficacy evaluation; and no relapse corroboration. These results suggest that digital PCR probe library for ***TP53*** mutations contribute to longitudinal ctDNA monitoring during cancer therapy.

[Disclosure of COI by Responsible researcher] Lead presenter is the Responsible researcher.
[Lead presenter: Satoshi Nishizuka] ■Research funds from company: クオントディテクト、ロシュ、大鵬、ベーリンガー　■employment by a company related to the presentation: クオントディテクト　■Advisor of the company and/or profit-making organization: クオントディテクト　■Profit of stock: クオントディテクト　■Research expenses: クオントディテクト、ロシュ、大鵬、ベーリンガー

### [E-1006] I-E12-1
### The latest advancement in liquid biopsy

2025 / 9 / 25 (Thu) 9:00-10:15 Room 7/(Hotel Nikko Kanazawa 3F Kujaku B) Room 7

[Chairperson]Shinji Kohsaka (Div. of Cell. Sig., NCCRI.)

---

### E-1006
### Novel liquid biopsy for NSCLC using intratumoral exosome extraction and ultrasensitive digital assay

[Speaker]Taketo Kato (Department of Thoracic Surgery, Nagoya University) [Co-author]Heng Huang (Department of Thoracic Surgery, Nagoya University)、Taiki Ryo (Department of Thoracic Surgery, Nagoya University)、Yoshito Imamura (Department of Thoracic Surgery, Nagoya University)、Yuji Nomata (Department of Thoracic Surgery, Nagoya University)、Hiroki Watanabe (Department of Thoracic Surgery, Nagoya University)、Yuta Kawasumi (Department of Thoracic Surgery, Nagoya University)、Keita Nakanishi (Department of Thoracic Surgery, Nagoya University)、Yuka Kadomatsu (Department of Thoracic Surgery, Nagoya University)、Harushi Ueno (Department of Thoracic Surgery, Nagoya University)、Shota Nakamura (Department of Thoracic Surgery, Nagoya University)、Tetsuya Mizuno (Department of Thoracic Surgery, Nagoya University)、Toyofumi Yoshikawa (Department of Thoracic Surgery, Nagoya University)

Introduction: Lung cancer is the leading cause of cancer deaths. Although survival for advanced cases is poor, early detection improves outcomes. We developed a liquid biopsy for early NSCLC detection using intratumoral EV extraction and an ultrasensitive digital EV assay (UDA), achieving higher sensitivity than ELISA. Methods: EVs were extracted from four lung adenocarcinoma/squamous cell carcinoma and four matched normal specimens, plus two lung cancer cell lines. Proteomic analysis with 10-plex TMT labeling was conducted. TCGA and HPA data were integrated to identify lung cancer-specific EV surface proteins. Plasma UDA was performed. Results: Proteomic analysis identified 1,653 proteins. PCA separated lung cancer from normal tissues. Cross-referencing with TCGA and HPA identified GLUT1 as a candidate marker. UDA targeting GLUT1 showed an AUC of 0.776, improving to 0.860 with CEA and CYFRA. In stage I cases, UDA achieved an AUC of 0.833, rising to 0.883 when combined. Conclusion: We developed a novel, highly sensitive liquid biopsy targeting low-abundance EV proteins, supporting early detection and precision medicine in lung cancer.

[Disclosure of COI by Responsible researcher] Lead presenter is the Responsible researcher.
[Lead presenter: Taketo Kato] We have no financial relationships to disclose.

### [E-1007] I-E11-1
### Tumor antigens/Antitumor immune response (1)

2025 / 9 / 25 (Thu) 9:00-10:15 Room 8/(Hotel Nikko Kanazawa 3F Kujaku C) Room 8

[Chairperson]Tetsuya Nakatsura (Div. Cancer Immunothera,, EPOC, NCC)

### E-1007
### Biomodeling-Guided Shared Neoantigen Discovery via Comprehensive Tumor Profiling for Neoantigen Vaccines in Asia

[Speaker]Diem TP Tran (Dept. of Oncology) [Co-author]Bui Que Tran Nguyen (Dept. of Oncology)、Thi Mong Quynh Pham (Dept. of Oncology)、Le Son Tran (Dept. of Oncology)

Personalized neoantigen vaccines are effective but costly and slow. We developed an off-the-shelf shared neoantigen panel tailored to Asian HLA-I diversity for colorectal (CRC) and lung cancer (LC). Mining TCGA and 20000 Vietnamese/Southeast Asian genomes, we identified recurrent tumor mutations and prevalent HLAI alleles. Candidate binding was predicted with NetMHCpan 4.1 and immunogenicity validated via Fastneo, a high throughput assay using CD80 expressing artificial APCs. In 55 CRC and 50 LC patients, our 67 peptide panel covered 44 % and 30 %, respectively. Of 47 HLA-A*11:01 restricted candidates (65.9 %), 31 peptides activated T cells in 10 healthy donors; KRAS G13D was most immunogenic (5/10 donors), while KRAS G12D responses varied by HLA/TCR context. Among 10 patients with matched PBMCs, two harbored panel mutations; TP53–R273H and TP53–V157F elicited T cell responses, with TP53–R273H driving cytotoxic and memory $CD8^{+}$ T cells marked by GZMA, PRF1, and NKG7 expression. This panel supports rapid, scalable neoantigen vaccine development for Asian CRC and LC populations.

[Disclosure of COI by Responsible researcher] Lead presenter is the Responsible researcher.
[Lead presenter: Diem TP Tran] We have no financial relationships to disclose.

▲TOP

### [E-1008] I-E11-1
### Tumor antigens/Antitumor immune response (1)

2025 / 9 / 25 (Thu) 9:00-10:15 Room 8/(Hotel Nikko Kanazawa 3F Kujaku C) Room 8

[Chairperson]Tetsuya Nakatsura (Div. Cancer Immunothera,, EPOC, NCC)

### E-1008
### Single-Cell and Spatial Profiling of Tumor-Infiltrating B Cells Reveals Distinct Tumor-Specific Clones in Gastric Cancer

[Speaker]Yichen Jiang (Dept. Prev. Med., Grad. Sch. Med., The Univ. Tokyo) [Co-author]Ayumu Tsubosaka (Dept. Prev. Med., Grad. Sch. Med., The Univ. Tokyo)、Kyohei Sano (Dept. Prev. Med., Grad. Sch. Med., The Univ. Tokyo)、Daisuke Komura (Dept. Prev. Med., Grad. Sch. Med., The Univ. Tokyo)、Miwako Kakiuchi (Dept. Prev. Med., Grad. Sch. Med., The Univ. Tokyo)、Hiroto Katoh (Div. Pathol., EPOC, Natl. Cancer Ctr.)、Shumpei Ishikawa (Dept. Prev. Med., Grad. Sch. Med., The Univ. Tokyo／Div. Pathol., EPOC, Natl. Cancer Ctr.)

Gastric cancer remains a leading malignancy in East Asia, particularly in China and Japan. The frequent presence of tertiary lymphoid structures (TLS) and plasma cell infiltration in tumors suggests a key role for B cells in antitumor immunity. While TLSs are linked to favorable prognosis, the functions of tumor-infiltrating plasma cells remain poorly understood.To investigate this, we performed single-cell B cell receptor (BCR) sequencing to analyze the clonal diversity and expansion of B cells in gastric cancer. In parallel, spatial transcriptomics was used to map the localization, organization, and microenvironmental interactions of B cells at single-cell resolution. This spatial profiling revealed functional immune niches, highlighting coordinated B cell activity in regions enriched for antigen presentation and immune signaling.Our integrative analysis reveals the spatial heterogeneity and immune architecture of B cell responses in gastric cancer, offering insights into tumor-associated antigen discovery and informing antibody-based therapies and cancer vaccine development.

[Disclosure of COI by Responsible researcher] Lead presenter is not the Responsible researcher.
[Lead presenter: Yichen Jiang] We have no financial relationships to disclose.
[Principal Researcher: Shumpei Ishikawa] ■Research funds from company: AMED、日本学術振興会　■Advisor of the company and/or profit-making organization: 新日本科学株式会社　■Research expenses: Chordis therapeutics株式会社、大鵬薬品工業株式会社、ロート製薬株式会社、アステラス製薬株式会社

▲TOP

### [E-1009] I-E11-1
### Tumor antigens/Antitumor immune response (1)

2025 / 9 / 25 (Thu) 9:00-10:15 Room 8/(Hotel Nikko Kanazawa 3F Kujaku C) Room 8

[Chairperson]Tetsuya Nakatsura (Div. Cancer Immunothera,, EPOC, NCC)

---

### E-1009
### Combination of proton irradiation and immunotherapy promotes antitumor activities in pancreatic cancer model

[Speaker]Ho Yagi (Information-Based Medicine Development, Kanazawa University, Kanazawa, Japan) [Co-author]Alessandro Nasti (Information-Based Medicine Development, Kanazawa University, Kanazawa, Japan)、Akihiro Seki (Department of Gastroenterology, Kanazawa University Hospital, Kanazawa, Japan)、Yoshio Sakai (Department of Gastroenterology, Kanazawa University Hospital, Kanazawa, Japan)、Kosuke Satomura (System biology, Kanazawa University, Kanazawa, Japan)、Shingo Inagaki (System biology, Kanazawa University, Kanazawa, Japan)、Kyo Kume (Proton Medical Research Division, Research & Development Department, WERC, Tsuruga, Japan)、Munetoshi Maeda (Proton Medical Research Division, Research & Development Department, WERC, Tsuruga, Japan)、Hiroyasu Tamamura (Proton Therapy Center, Fukui Prefectural Hospital, Fukui, Japan)、Makoto Sasaki (Proton Therapy Center, Fukui Prefectural Hospital, Fukui, Japan)、Kazutaka Yamamoto (Proton Therapy Center, Fukui Prefectural Hospital, Fukui, Japan)、Taro Yamashita (Department of Gastroenterology, Kanazawa University Hospital, Kanazawa, Japan／System biology, Kanazawa University, Kanazawa, Japan)、Shuichi Kaneko (Information-Based Medicine Development, Kanazawa University, Kanazawa, Japan／Department of Gastroenterology, Kanazawa University Hospital, Kanazawa, Japan／System biology, Kanazawa University, Kanazawa, Japan)

Proton therapy allows precision treatment (Tx) of cancer by avoiding irradiation to healthy tissues. We combined proton irradiation (PI) with anti-PD-L1 antibody (aPD-L1), and examined the effects on a pancreatic cancer model.Mouse pancreatic cancer cell line mT3-2D (CSH Laboratory) received PI at doses of 0, 2, 8 Gy, we confirmed that PI reduced the colony forming ability of cells. DNA microarray analysis of irradiated mT3-2D cells confirmed differential expression of immune response-related genes, cell cycle, and DNA damage-related genes.Next, a murine model was established by injecting mT3-2D subcutaneously into the leg of C57Bl6J mice, followed by PI and aPD-L1 Tx. Tumor growth was significantly inhibited by combination Tx. We confirmed by DNA microarray analysis, 697 upregulated genes in treated tumors, related to CD4+ T cells activity, Effector CD8+ T cells, DCs and NK cells pathways. Whereas 1285 downregulated genes were related to pluripotency, survival, proliferation, and migration.PI and aPD-L1 combination Tx promoted antitumor effects in a murine model of pancreatic cancer, inducing sustained immune response and decreasing tumor cell proliferation capability.

[Disclosure of COI by Responsible researcher] Lead presenter is not the Responsible researcher.
[Lead presenter: Ho Yagi] We have no financial relationships to disclose.
[Principal Researcher: Alessandro Nasti] We have no financial relationships to disclose.

▲TOP

### [E-1010] I-E11-1
### Tumor antigens/Antitumor immune response (1)

2025 / 9 / 25 (Thu) 9:00-10:15 Room 8/(Hotel Nikko Kanazawa 3F Kujaku C) Room 8

[Chairperson]Tetsuya Nakatsura (Div. Cancer Immunothera,, EPOC, NCC)

---

### E-1010
### Active aldehydes accelerate glycolysis/FAO imbalance and exhaustion of CD8+ T cells in tumor microenvironment

[Speaker]Koji Kitaoka (Kyoto Univ. Medical Sch. CCII) [Co-author]Yasuharu Haku (Kyoto Univ. Medical Sch. CCII)、Koki Ichimaru (Kyoto Univ. Medical Sch. CCII)、Tomoko Hirano (Kyoto Univ. Medical Sch. CCII)、Tomonori Yaguchi (Kyoto Univ. Medical Sch. CCII)、Tasuku Honjo (Kyoto Univ. Medical Sch. CCII)、Kenji Chamoto (Kyoto Univ. Medical Sch. CCII)

PD-1 is a checkpoint for T cells to avoid overactivation-induced cell death by inhibiting glycolysis and enhancing FAO through inactivation of the RAS pathway. Nevertheless, PD-1high terminally exhausted CD8+ T cells in TME rely on glycolysis and carry impaired mitochondrial metabolism. Here, we clarified how glycolysis and FAO balance are regulated during exhaustion in PD-1+ CD8+ T cells.Living PD1+ CD8+ T cells in TME were divided into three populations based on mitochondrial (mito) status; D1: Mito masslow potentiallow, D2: massint potentialint, D3: masshigh potentialhigh. Single-cell RNA sequencing analysis revealed a differentiation trajectory of living PD1+ CD8+ T cells from D1 to D3, corresponding to the depth of their exhaustion, cell division, and glycolysis/FAO imbalance. We demonstrated that active aldehydes, final products of lipid peroxidation, gradually accumulated from D1 to D3. Phosphoproteomics analysis showed that active aldehydes activate the phosphorylation of a PD-1 signal target, RAS pathway, in one hour . Furthermore, active aldehydes elevated glycolysis and attenuated FAO activity to promote T cell proliferation and hasten the transition from D1-D3.

[Disclosure of COI by Responsible researcher] Lead presenter is not the Responsible researcher.
[Lead presenter: Koji Kitaoka] We have no financial relationships to disclose.
[Principal Researcher: Kenji Chamoto] ■Research expenses: Imel Therapeutics, Inc. and Sysmex Corporation ■Contributions: UNIQLO Co., Ltd. ■Endowed chair: Meiji Holdings Co., Ltd. and Meiji Seika Pharma Co., Ltd.

### [E-1011] I-E11-1
### Tumor antigens/Antitumor immune response (1)

2025 / 9 / 25 (Thu) 9:00-10:15 Room 8/(Hotel Nikko Kanazawa 3F Kujaku C) Room 8

[Chairperson]Tetsuya Nakatsura (Div. Cancer Immunothera,, EPOC, NCC)

---

### E-1011
### THEMIS2 impairs antitumor activity of natural killer cells by suppressing activating NK receptor signaling

[Speaker]Tsukasa Nabekura (Aichi Cancer Center Research Institute, Division of Immune Response／Nagoya University Graduate School of Medicine／Life Science Center for Survival Dynamics, University of Tsukuba／R&D Center for Innovative Drug Discovery, University of Tsukuba) [Co-author]Elfira A. Deborah (Department of Immunology, Institute of Medicine, University of Tsukuba／Doctoral Program in Medical Science, University of Tsukuba)、Akira Shibuya (Life Science Center for Survival Dynamics, University of Tsukuba／R&D Center for Innovative Drug Discovery, University of Tsukuba／Department of Immunology, Institute of Medicine, University of Tsukuba／Doctoral Program in Medical Science, University of Tsukuba)

Natural killer (NK) cells are cytotoxic innate lymphocytes that play a critical role in antitumor immunity. NK cells recognize target cells by using a repertoire of activating NK receptors and exert the effector functions. Although the magnitude of activation signals through activating NK receptors controls NK cell function, it has not been fully understood how these activating signals are modulated in NK cells. In this study, we found that a scaffold protein, THEMIS2, inhibits activating NK receptor signaling. Overexpression of THEMIS2 attenuated the effector function of human NK cells, whereas knockdown of THEMIS2 enhanced it. Mechanistically, THEMIS2 binds to GRB2 and phosphorylated SHP-1 and SHP-2 at the proximity of activating NK receptors DNAM-1 and NKG2D. Knockdown of THEMIS2 in primary human NK cells promoted the effector functions. Furthermore, Themis2-deficient mice showed low metastatic burden in an NK cell-dependent manner. These findings demonstrate that THEMIS2 has an inhibitory role in the antitumor activity of NK cells, suggesting that THEMIS2 might be a potential therapeutic target for NK cell-mediated cancer immunotherapy.

[Disclosure of COI by Responsible researcher] Lead presenter is the Responsible researcher.
[Lead presenter: Tsukasa Nabekura] We have no financial relationships to disclose.

▲TOP

### [E-1012] I-E11-1
### Tumor antigens/Antitumor immune response (1)

2025 / 9 / 25 (Thu) 9:00-10:15 Room 8/(Hotel Nikko Kanazawa 3F Kujaku C) Room 8

[Chairperson]Tetsuya Nakatsura (Div. Cancer Immunothera,, EPOC, NCC)

---

### E-1012
### Exploring miRNAs Contributing to Resistance against Anti-CD40 Agonist Antibody Therapy in Mouse Pancreatic Cancer Cells

[Speaker]Yukiko Asakawa (Division of Phamacotherapeutics, Keio Univ. Faculty of Pharmacy) [Co-author]Juntaro Matsuzaki (Res. Ctr. for Drug Discovery, Keio Univ. Faculty of Pharmacy)、Taisuke Koreeda (Division of Phamacotherapeutics, Keio Univ. Faculty of Pharmacy)、Rina Shibagaki (Division of Phamacotherapeutics, Keio Univ. Faculty of Pharmacy)、Yuzhi Tan (Division of Phamacotherapeutics, Keio Univ. Faculty of Pharmacy)、Kazuki Oshima (Division of Phamacotherapeutics, Keio Univ. Faculty of Pharmacy)、Chihiro Oikawa (Division of Phamacotherapeutics, Keio Univ. Faculty of Pharmacy)、Marina Nakamura (Division of Phamacotherapeutics, Keio Univ. Faculty of Pharmacy)、Kako Imamoto (Division of Phamacotherapeutics, Keio Univ. Faculty of Pharmacy)、Yoshimasa Saito (Division of Phamacotherapeutics, Keio Univ. Faculty of Pharmacy)

Pancreatic cancer is characterized by an immunosuppressive tumor microenvironment (TME), contributing to high mortality and poor immunotherapy outcomes. CD40 agonist antibody (CD40a) enhances antigen-presenting cell activation, showing partial therapeutic efficacy. However, therapeutic responses are variable, highlighting the need for predictive biomarkers and resistance mechanisms. Tumor-derived miRNAs modulate the TME, but their role in CD40a resistance is unclear. We used two KPCY cell lines, KPCY2838c3 (high T-cell infiltration) and KPCY6419c5 (low T-cell infiltration), derived from the KPCY mouse model, which has Kras G12D and Tp53 R172H mutations. All miRNAs overexpressed in KPCY6419c5 identified by miRNA-seq were introduced into KPCY2838c3 cells by creating a pooled miRNA library. Amplicon sequencing of xenograft tumors under CD40a therapy revealed miRNAs conferring resistance. Notably, we identified miR-10a, which explained the resistance to CD40a. miR-10a overexpression reduced CD8+ T-cell infiltration, fostering an immunosuppressive TME. These results suggest miR-10a is a key regulator of TME, and targeting it could enhance CD40a-based immunotherapy in pancreatic cancer.

[Disclosure of COI by Responsible researcher] Lead presenter is not the Responsible researcher.
[Lead presenter: Yukiko Asakawa] We have no financial relationships to disclose.
[Principal Researcher: Juntaro Matsuzaki] ■Research funds from company: ISM株式会社、レグリア株式会社、フェムトディプロイメンツ株式会社

▲TOP

### [J-1007] I-J4-1
### Oncogenes & tumor-suppressor genes (1)

2025 / 9 / 25 (Thu) 9:00-10:15 Room 9/(Hotel Nikko Kanazawa 3F Lumiere) Room 9

[Chairperson]Yoji Andrew Minamishima (Dept. Biochem., Gunma Univ. Grad. Sch. Med.)

### J-1007
### Detection and functional analysis of germline mutations associated with disease-free survival in colorectal cancer.

[Speaker]Masaki Ohira (NCC Div. Transl. Inform.／Front. Sci. Dept. Integr. Biosci. Univ. Tokyo) [Co-author]Yoshiaki Nakamura (NCCE Dept. GI Oncology)、Masao Nagasaki (Div. Biomed. Info. Analysis Med. Inst. Bioreg. Kyushu Univ.／Ctr. for Genomic Med. Grad. Sch. Med. Kyoto Univ.)、Tadayoshi Hashimoto (NCCE Dept. GI Oncology)、Jun Watanabe (Dept. Colorectal Surg. Kansai Med. Univ.)、Saori Mishima (NCCE Dept. GI Oncology)、Hiromichi Ebi (Div. Mol. Therap. Aichi Cancer Ctr. Res. Inst.)、Hideaki Bando (NCCE Dept. GI Oncology)、Ichiro Takemasa (Dept. Surg. Surg. Oncology & Sci. Sapporo Med. Univ.)、Takeshi Kato (Dept. Surg. NHO Osaka Natl. Hosp.)、Daisuke Kotani (NCCE Dept. GI Oncology)、Eiji Oki (Dept. Surg. & Sci. Grad. Sch. Med. Sci. Kyushu Univ.)、Takayuki Yoshino (NCCE Dept. GI Oncology)、Katsuya Tsuchihara (NCC Div. Transl. Inform.／Front. Sci. Dept. Integr. Biosci. Univ. Tokyo)、Riu Yamashita (NCC Div. Transl. Inform.／Front. Sci., Dept. Comput. Biol. & Med. Sci. Univ. Tokyo)

Germline mutations influence clinical outcomes across various cancer types. In this study, we analyzed germline single nucleotide variants (germline SNVs) derived from whole-exome sequencing data in the CIRCULATE-Japan study, focusing specifically on colorectal cancer (CRC).
Among 35,293,493 loci across 3,485 patients, we identified 191 germline SNVs significantly associated with disease-free survival (DFS) (Log-rank test: $P < 1 \times 10^{-5}$). The most frequently observed variant was rs4418597 in the ***RASSF5*** gene (allele frequency = 0.213). The rs4418597 variant is located in a region that is intronic or causes a missense mutation, depending on the transcript isoform. Its allele frequency was nearly identical to that observed in healthy Japanese individuals from the ToMMo 60K cohort (allele frequency = 0.214), indicating no enrichment in CRC patients. According to the GTEx database, expression levels of ***RASSF5*** were reduced, while those of ***EIF2D*** were increased in both heterozygous and homozygous rs4418597 carriers in specific tissues. These findings suggest that our approach can identify germline mutations that serve as potential prognostic biomarkers for predicting DFS in CRC.

[Disclosure of COI by Responsible researcher] Lead presenter is not the Responsible researcher.
[Lead presenter: Masaki Ohira] We have no financial relationships to disclose.
[Principal Researcher: Riu Yamashita] We have no financial relationships to disclose.

▲TOP

### [J-1008] I-J4-1
### Oncogenes & tumor-suppressor genes (1)

2025 / 9 / 25 (Thu) 9:00-10:15 Room 9/(Hotel Nikko Kanazawa 3F Lumiere) Room 9

[Chairperson]Yoji Andrew Minamishima (Dept. Biochem., Gunma Univ. Grad. Sch. Med.)

### J-1008
### Mechanisms for anticancer effects of G-quadruplex ligands through translation inhibition

[Speaker]Sachiko Okabe (Div. Mol. Biother., JFCR Cancer Chemother. Ctr.) [Co-author]Risa Ito (Div. Mol. Biother., JFCR Cancer Chemother. Ctr.／Grad. Sch. Front. Sci., Univ. Tokyo)、Yuichi Shichino (RNA Systems Biochem. Lab., RIKEN PRI)、Shintaro Iwasaki (Grad. Sch. Front. Sci., Univ. Tokyo／RNA Systems Biochem. Lab., RIKEN PRI)、Kazuo Shinya (Biomed. Info. Res. Ctr., Natl. Inst. AIST.)、Kazuo Nagasawa (Biomed. Fac. Technol., Tokyo Univ. Agric. Technol.)、Hiroyuki Seimiya (Div. Mol. Biother., JFCR Cancer Chemother. Ctr.／Grad. Sch. Front. Sci., Univ. Tokyo)

G-quadruplex (G4) is a higher-order structure formed in G-rich sequences. We previously reported that G4-stabilizing compounds (G4 ligands) exert anticancer effects through inhibiting translation. However, its precise mechanism remains elusive. Here we show that G4 ligands modulate the translation of the specific mRNAs essential for cancer cell proliferation. To identify critical targets whose translation inhibition causes the anticancer effect, we performed a ribosome profiling of pancreatic cancer MIA-PaCa2 (***KRAS*** mutated) and NOR-P1 (***KRAS*** wild-type) cells treated with G4 ligands, telomestatin or Phen-DC3. As a result, mRNAs whose translation efficiency was reduced by G4 ligands had more G4-forming sequences in the 5'-untranslated regions as compared with unaffected mRNAs. Gene ontology analysis revealed that the target genes are related to MAP kinase (MAPK) pathway. While G4 ligands reduced those proteins in both cell lines, siRNA-mediated knockdown of them reduced MIA-PaCa2 but not NOR-P1 cell proliferation. These results indicate that G4 ligands target MAPK pathway at the translational level, implicated as a therapeutic strategy for intractable KRAS-driven pancreatic cancer.

[Disclosure of COI by Responsible researcher] Lead presenter is not the Responsible researcher.
[Lead presenter: Sachiko Okabe] We have no financial relationships to disclose.
[Principal Researcher: Hiroyuki Seimiya] ■Research funds from company: AMED次世代がん、JSPS科研費、日本財団、武田科学振興財団、富士フイルムメディカル　■Contributions: 日本財団、武田科学振興財団、富士フイルムメディカル

▲TOP

### [J-1009] I-J4-1
### Oncogenes & tumor-suppressor genes (1)

2025 / 9 / 25 (Thu) 9:00-10:15 Room 9/(Hotel Nikko Kanazawa 3F Lumiere) Room 9

[Chairperson]Yoji Andrew Minamishima (Dept. Biochem., Gunma Univ. Grad. Sch. Med.)

---

### J-1009
### Humanized anti-CKAP4 antibody suppresses tumor growth and may regulate immune microenvironment of pancreatic cancer

[Speaker]Ryota Sada (Med. Edu. Ctr., Faculty of Med., Osaka Univ. ／Signal Transduction & Biol. Response, CiDER, Osaka Univ.／Inst. for Open & Transdisciplinary Res. Initiatives, Osaka Univ.) [Co-author]Shinji Matsumoto (Signal Transduction & Biol. Response, CiDER, Osaka Univ.／Dept. of Oral. Physiol., Grad. Sch. of Dent., Tokushima Univ.)、Akikazu Harada (Signal Transduction & Biol. Response, CiDER, Osaka Univ.／Inst. for Open & Transdisciplinary Res. Initiatives, Osaka Univ.)、Hirokazu Kimura (Dept. of MolBio. Biochem., Grad. Sch. of Med., Osaka Univ.)、Akira Kikuchi (Signal Transduction & Biol. Response, CiDER, Osaka Univ.／Inst. for Open & Transdisciplinary Res. Initiatives, Osaka Univ.)

CKAP4 is a cell surface receptor for DKK1. The DKK1-CKAP4 pathway is activated in various cancers to promote tumor growth. Thus, DKK1-CKAP4 pathway has been expected to be promised molecular target of cancer therapy. We have previously developed mouse anti-CKAP4 monoclonal antibody 3F11-2B10, which inhibited DKK1-CKAP4 pathway in vitro and in vivo. From the 3F11-2B10 nucleotide sequence, a humanized anti-CKAP4 antibody Hv1Lt1 was newly established. The binding affinity of Hv1Lt1 for CKAP4 was superior to that of original 3F11-2B10. Hv1Lt1 inhibited DKK1 binding to CKAP4, AKT activity, and sphere formation of pancreatic cancer cells. Hv1Lt1 also suppressed xenograft tumor growth subcutaneously implanted into immunodeficient mice. Moreover, Hv1Lt1 inhibited tumor growth of syngeneic pancreatic tumor models using immunocompetent mice. Interestingly, cytotoxic T cells infiltration into tumor microenvironment was enhanced by Hv1Lt1. FACS analysis of resected tumors revealed that some immune cells express CKAP4 receptors and DKK1 may regulated immune microenvironment via CKAP4. These results suggest that our Hv1Lt1 antibody may represents a promising anti-cancer therapeutic.

[Disclosure of COI by Responsible researcher] Lead presenter is not the Responsible researcher.
[Lead presenter: Ryota Sada] We have no financial relationships to disclose.
[Principal Researcher: Akira Kikuchi] We have no financial relationships to disclose.

▲TOP

### [J-1010] I-J4-1
### Oncogenes & tumor-suppressor genes (1)

2025 / 9 / 25 (Thu) 9:00-10:15 Room 9/(Hotel Nikko Kanazawa 3F Lumiere) Room 9

[Chairperson]Yoji Andrew Minamishima (Dept. Biochem., Gunma Univ. Grad. Sch. Med.)

---

### J-1010
### MiR- 155- targeted IcosL controls tumor rejection

[Speaker]Hajime Otsu (Kyushu University Beppu Hospital) [Co-author]Esmerina Tili (Ohio State University)、Qingjiang Hu (Kyushu University Beppu Hospital)、Yuki Ando (Kyushu University Beppu Hospital)、Yusuke Yonemura (Kyushu University Beppu Hospital)、Carlo M. Croce (Ohio State University)、Koshi Mimori (Kyushu University Beppu Hospital)

Aim: To investigate how miR-155 affects anti-tumor immunity via regulation of IcosL, and whether restoring IcosL expression enhances tumor rejection. Method: Using Eμ-miR-155 and Cre-Tet-OFF mouse models, we evaluated the effect of miR-155 on IcosL expression in B cells and tumor immune response. IcosL expression was also restored in MC38 murine colon cancer cells via cDNA overexpression, and tumor growth and T-cell infiltration were analyzed in syngeneic C57BL/6 mice. Result: Overexpression of miR-155 resulted in the loss of IcosL in malignant B cells and suppressed CD8+ T-cell infiltration. Turning miR-155 OFF led to tumor regression and the formation of immunological synapses between IcosL+ B cells and Icos+ T cells. In MC38 models, IcosL expression reduced tumor size and increased intratumoral CD8+ T-cell infiltration. Conclusion: miR-155 promotes immune evasion by suppressing IcosL, a key regulator of T-cell-mediated tumor rejection. Targeting the miR-155-IcosL axis may serve as a novel therapeutic approach to enhance anti-tumor immunity in both hematologic and solid tumors.This work was published in PNAS, Volume 121, in 2024.

[Disclosure of COI by Responsible researcher] Lead presenter is not the Responsible researcher.
[Lead presenter: Hajime Otsu] We have no financial relationships to disclose.
[Principal Researcher: Koshi Mimori] We have no financial relationships to disclose.

### [J-1011] I-J4-1
### Oncogenes & tumor-suppressor genes (1)

2025 / 9 / 25 (Thu) 9:00-10:15 Room 9/(Hotel Nikko Kanazawa 3F Lumiere) Room 9

[Chairperson]Yoji Andrew Minamishima (Dept. Biochem., Gunma Univ. Grad. Sch. Med.)

---

### J-1011
### Single-cell transcriptomics reveal distinct TME remodeling in liver metastasis of pancreatic cancer upon KRAS inhibition

[Speaker]Yuuya Shiraishi (Department of Gastroenterology, The University of Tokyo) [Co-author]Keisuke Yamamoto (Department of Gastroenterology, The University of Tokyo)、Miwako Kakiuchi (Department of Preventive Medicine, The University of Tokyo)、Ayumu Tsubosaka (Department of Preventive Medicine, The University of Tokyo)、Kyohei Sano (Department of Preventive Medicine, The University of Tokyo)、Hirotaka Inoue (Department of Preventive Medicine, The University of Tokyo)、Keiko Nakamura (Department of Gastroenterology, The University of Tokyo)、Dosuke Iwadate (Department of Gastroenterology, The University of Tokyo)、Daisuke Komura (Department of Preventive Medicine, The University of Tokyo)、Shumpei Ishikawa (Department of Preventive Medicine, The University of Tokyo／National Cancer Center Exploratory Oncology Research & Clinical Trial Center)、Mitsuhiro Fujishiro (Department of Gastroenterology, The University of Tokyo)

Pancreatic ductal adenocarcinoma (PDAC) is a lethal malignancy characterized by extensive stromal expansion in primary tumors, driven by oncogenic KRAS. This unique tumor microenvironment (TME) contributes to resistance against therapies, including immune checkpoint inhibitors and KRAS-targeted agents. Although liver metastases (LM) are clinically important, how oncogenic KRAS influences the TME in LM and whether it differs from the primary TME remain unclear. In this study, we utilized a PDAC mouse model to analyze TME changes following acute ablation of oncogenic KRAS in both primary tumors and LM. KRAS extinction induced tumor regression and boosted anti-tumor immune responses, which were more pronounced in LM. Comprehensive analyses using flow cytometry, single-cell RNA sequencing, and spatial transcriptomics revealed distinct immune cell infiltration patterns in LM. These results suggest that immune suppression mechanisms vary between primary and metastatic sites, highlighting the importance of considering site-specific immune landscapes when designing combination therapies for metastatic PDAC.

[Disclosure of COI by Responsible researcher] Lead presenter is not the Responsible researcher.
[Lead presenter: Yuuya Shiraishi] We have no financial relationships to disclose.
[Principal Researcher: Keisuke Yamamoto] We have no financial relationships to disclose.

### [J-1012] I-J4-1
### Oncogenes & tumor-suppressor genes (1)

2025 / 9 / 25 (Thu) 9:00-10:15 Room 9/(Hotel Nikko Kanazawa 3F Lumiere) Room 9

[Chairperson]Yoji Andrew Minamishima (Dept. Biochem., Gunma Univ. Grad. Sch. Med.)

---

### J-1012
### Calcineurin-mediated dephosphorylation stabilizes E2F1 and promotes cancer cell proliferation

[Speaker]Makoto Habara (Lab. of Veterinary Physiol. & Biochem., Yamaguchi Univ.) [Co-author]Yuki Sato (Dept. of Mol. Biol., Grad. Sch. of Med., Nagoya Univ.)、Shunsuke Hanaki (Lab. of Veterinary Physiol. & Biochem., Yamaguchi Univ.)、Takahiro Masaki (Lab. of Veterinary Physiol. & Biochem., Yamaguchi Univ.)、Haruki Tomiyasu (Lab. of Veterinary Physiol. & Biochem., Yamaguchi Univ.)、Yosei Miki (Lab. of Veterinary Physiol. & Biochem., Yamaguchi Univ.)、Midori Shimada (Dept. of Mol. Biol., Grad. Sch. of Med., Nagoya Univ.)

The transcription factor E2F1 plays a key role in regulating the cell cycle and promoting cell proliferation, and its overexpression is linked to tumor progression. Understanding the molecular mechanisms governing E2F1 degradation is therefore critical for cancer therapy development. We identified that the tumor suppressor FBXW7, as part of a ubiquitin ligase complex, recognizes E2F1 phosphorylated at Ser403, promoting its polyubiquitination and proteasomal degradation. In contrast, calcineurin–a $Ca^{2+}$-dependent serine/threonine phosphatase–dephosphorylates E2F1 at Ser403, thereby preventing FBXW7 binding and stabilizing E2F1. Pharmacological modulation of intracellular calcium levels revealed that calcium channel blockers suppressed E2F1 protein and its target gene expression, while treatment with the calcium ionophore ionomycin enhanced E2F1 expression. Importantly, administration of the calcineurin inhibitor FK506 reduced tumor growth in a xenograft model, along with decreased E2F1 expression. These findings highlight a critical regulatory axis between FBXW7 and calcineurin in regulating E2F1 stability and suggest that disruption of this balance contributes to carcinogenesis.

[Disclosure of COI by Responsible researcher] Lead presenter is not the Responsible researcher.
[Lead presenter: Makoto Habara] We have no financial relationships to disclose.
[Principal Researcher: Midori Shimada] We have no financial relationships to disclose.

▲TOP

### [IS2-1] IS2
### Spatial Single Cell Analysis for clinical implementation in terms of clinical development

2025 / 9 / 25 (Thu) 9:00-11:30 Room 10/(Art Hall) Room 10

[Chairpersons]Mitsuho Imai (National Cancer Center Hospital East)、Woong-Yang Park (Samsung Genome Institute)

Recent advancements in spatial single-cell analysis have enabled high-resolution mapping of cellular organization and interactions within tissues. These technologies provide unprecedented insights into the cellular heterogeneity and spatial architecture of various biological systems, offering new perspectives on disease mechanisms, tumor microenvironments, and immune responses. By integrating spatial transcriptomics, proteomics, and machine learning-based analysis, researchers can now explore complex cellular ecosystems with greater precision, driving innovations in biomarker discovery and therapeutic development.
This session will bring together leading experts to discuss the latest advancements, applications, and challenges in spatial single-cell analysis. By examining cutting-edge technologies and their translational potential, we aim to foster interdisciplinary discussions on how these methods can bridge the gap between fundamental research and clinical practice. The session will highlight key developments in data integration, computational modeling, and clinical validation, providing insights into how spatial single-cell analysis can enhance disease diagnosis, treatment strategies, and precision medicine approaches.
Through this exchange, we hope to identify opportunities for collaboration and innovation, ultimately contributing to the broader implementation of spatial single-cell technologies in biomedical research and clinical applications.

---

### IS2-1
### Spatial transcriptomics reveals predictive insights for neoadjuvant chemotherapy of breast cancer

[Speaker]Woong-Yang Park (Translational Genomics Center, Samsung Medical Center, Sungkyunkwan University, Seoul, Korea)

Spatial transcriptomics (ST) offers detailed insights into tumor biology, enabling precise predictions of treatment outcomes. We performed spatial transcriptome analysis on breast cancer patients treated with neoadjuvant chemotherapy (NAC), mapping intratumoral heterogeneity and immune microenvironment interactions associated with therapeutic responses. ST analysis identified distinct spatial gene expression signatures linked to pathological complete response (pCR) and residual disease. Specifically, enhanced immune infiltration and inflammatory signaling pathways spatially co-localized with tumor regression areas, whereas immune-exclusion phenotypes correlated with resistance. This spatially resolved data highlights critical immune-related biomarkers, demonstrating ST's clinical utility for predicting NAC efficacy, guiding personalized treatment strategies in breast cancer.

[Disclosure of COI by Responsible researcher] Lead presenter is the Responsible researcher.
[Lead presenter: Woong-Yang Park] ■ employment by a company related to the presentation: GxD Inc. and Geninus Inc.
■Advisor of the company and/or profit-making organization: GxD Inc. and Geninus Inc. ■Profit of stock: Geninus Inc.

▲TOP

### [IS2-2] IS2
### Spatial Single Cell Analysis for clinical implementation in terms of clinical development

2025 / 9 / 25 (Thu) 9:00-11:30 Room 10/(Art Hall) Room 10

[Chairpersons]Mitsuho Imai (National Cancer Center Hospital East)、Woong-Yang Park (Samsung Genome Institute)

Recent advancements in spatial single-cell analysis have enabled high-resolution mapping of cellular organization and interactions within tissues. These technologies provide unprecedented insights into the cellular heterogeneity and spatial architecture of various biological systems, offering new perspectives on disease mechanisms, tumor microenvironments, and immune responses. By integrating spatial transcriptomics, proteomics, and machine learning-based analysis, researchers can now explore complex cellular ecosystems with greater precision, driving innovations in biomarker discovery and therapeutic development.
This session will bring together leading experts to discuss the latest advancements, applications, and challenges in spatial single-cell analysis. By examining cutting-edge technologies and their translational potential, we aim to foster interdisciplinary discussions on how these methods can bridge the gap between fundamental research and clinical practice. The session will highlight key developments in data integration, computational modeling, and clinical validation, providing insights into how spatial single-cell analysis can enhance disease diagnosis, treatment strategies, and precision medicine approaches.
Through this exchange, we hope to identify opportunities for collaboration and innovation, ultimately contributing to the broader implementation of spatial single-cell technologies in biomedical research and clinical applications.

---

### IS2-2
### Beyond Karyotype: Heterogeneity of Gene Regulatory Programs in Acute Myeloid Leukemia

[Speaker]Chung-chau Hon (RIKEN, Center for Integrative Medical Sciences, Japan) [Co-author]Manli Yang (RIKEN, Center for Integrative Medical Sciences, Japan)、Soren Lehmann (Karolinska institutet, Department of Haematology, Sweden)、Andreas Lennartsson (Karolinska institutet, Department of Haematology, Sweden)、Chung-Chau Hon (RIKEN, Center for Integrative Medical Sciences, Japan)

Cytogenetically normal AML (CN-AML) lacks recurrent karyotypic lesions yet shows broad heterogeneity driven by recurrent somatic mutations (e.g., NPM1, RUNX1). We profiled bone marrow mononuclear cells from CN-AML using 5' RNA-seq at bulk (CAGE, n=88) and single-cell (n=16) resolution. Both capture transcription start sites and reveal transcribed cis-regulatory elements (tCREs) in promoters and enhancers. Focusing on tCRE activity rather than gene counts, we defined four molecular subtypes with distinct cell-type composition, clinical outcomes, and mutation spectra. To map cell-type-specific mutation effects, we compared tCRE profiles of mutant- versus wild-type allele cells in single-cell data and used these signals to validate a bulk deconvolution that yielded differentially active tCREs for 10 major mutations. We then organized tCREs into 62 co-activity modules spanning identity modules and activity modules (e.g., DNA methylation, interferon response). Several modules were enriched for mutation-associated tCREs and linked to survival. Finally, we integrated modules with core TFs, epigenetic regulators, and high-AML-score targets to outline the CN-AML regulatory network.

[Disclosure of COI by Responsible researcher] Lead presenter is the Responsible researcher.
[Lead presenter: Chung-chau Hon] We have no financial relationships to disclose.

▲TOP

### [IS2-3] IS2
### Spatial Single Cell Analysis for clinical implementation in terms of clinical development

2025 / 9 / 25 (Thu) 9:00-11:30 Room 10/(Art Hall) Room 10

[Chairpersons]Mitsuho Imai (National Cancer Center Hospital East)、Woong-Yang Park (Samsung Genome Institute)

Recent advancements in spatial single-cell analysis have enabled high-resolution mapping of cellular organization and interactions within tissues. These technologies provide unprecedented insights into the cellular heterogeneity and spatial architecture of various biological systems, offering new perspectives on disease mechanisms, tumor microenvironments, and immune responses. By integrating spatial transcriptomics, proteomics, and machine learning-based analysis, researchers can now explore complex cellular ecosystems with greater precision, driving innovations in biomarker discovery and therapeutic development.
This session will bring together leading experts to discuss the latest advancements, applications, and challenges in spatial single-cell analysis. By examining cutting-edge technologies and their translational potential, we aim to foster interdisciplinary discussions on how these methods can bridge the gap between fundamental research and clinical practice. The session will highlight key developments in data integration, computational modeling, and clinical validation, providing insights into how spatial single-cell analysis can enhance disease diagnosis, treatment strategies, and precision medicine approaches.
Through this exchange, we hope to identify opportunities for collaboration and innovation, ultimately contributing to the broader implementation of spatial single-cell technologies in biomedical research and clinical applications.

---

### IS2-3
### Structured Analysis of the Tumor Microenvironment Using SpatialKnifeY: A Cluster-Based Framework for Spatial Omics Data

[Speaker]Riu Yamashita (National Cancer Center, EPOC, TI／CBMS, Grad. Sch. of Frontier Sci. Univ. of Tokyo) [Co-author]Shunsuke Sakai (National Cancer Center, EPOC, TI／Integrated Biosciences, Grad. Sch. of Frontier Sci. Univ. of Tokyo)、Shunichiro Kageyama (National Cancer Center, EPOC, TI)、Rhosuke Nomura (National Cancer Center, EPOC, TI／CBMS, Grad. Sch. of Frontier Sci. Univ. of Tokyo)

Single-cell spatial omics technologies are increasingly used in cancer research because they allow visualization of gene expression directly on tissue sections. However, standardized analytical methods are still lacking, and spatial information is often underutilized in downstream analysis.To address this, we propose an analytical framework using SpatialKnifeY (SKNY), a tool we developed to analyze the tumor microenvironment. SKNY extracts cancer cell clusters from spatial omics datasets (e.g., Xenium) based on known cancer marker gene expression. Once clusters are identified, SKNY defines tumor boundaries and classifies regions as intra- or peri-tumoral, enabling structured microenvironment analysis.We applied SKNY to breast cancer Xenium data. The tool identified nine distinct cancer cell clusters, successfully separating ductal carcinoma in situ (DCIS) from invasive ductal carcinoma (IDC). In addition, trajectory analysis revealed lineage relationships among the clusters. These results suggest that analyzing tumors at the cluster level, rather than at the single-cell level, may provide new insights into the spatial organization and biological behavior of cancer.

[Disclosure of COI by Responsible researcher] Lead presenter is the Responsible researcher.
[Lead presenter: Riu Yamashita] We have no financial relationships to disclose.

### [IS2-4] IS2
### Spatial Single Cell Analysis for clinical implementation in terms of clinical development

2025 / 9 / 25 (Thu) 9:00-11:30 Room 10/(Art Hall) Room 10

[Chairpersons]Mitsuho Imai (National Cancer Center Hospital East)、Woong-Yang Park (Samsung Genome Institute)

Recent advancements in spatial single-cell analysis have enabled high-resolution mapping of cellular organization and interactions within tissues. These technologies provide unprecedented insights into the cellular heterogeneity and spatial architecture of various biological systems, offering new perspectives on disease mechanisms, tumor microenvironments, and immune responses. By integrating spatial transcriptomics, proteomics, and machine learning-based analysis, researchers can now explore complex cellular ecosystems with greater precision, driving innovations in biomarker discovery and therapeutic development.
This session will bring together leading experts to discuss the latest advancements, applications, and challenges in spatial single-cell analysis. By examining cutting-edge technologies and their translational potential, we aim to foster interdisciplinary discussions on how these methods can bridge the gap between fundamental research and clinical practice. The session will highlight key developments in data integration, computational modeling, and clinical validation, providing insights into how spatial single-cell analysis can enhance disease diagnosis, treatment strategies, and precision medicine approaches.
Through this exchange, we hope to identify opportunities for collaboration and innovation, ultimately contributing to the broader implementation of spatial single-cell technologies in biomedical research and clinical applications.

---

### IS2-4
### Prediction of cell types from histopathological images using spatial single-cell transcriptome data

[Speaker]Sanghyuk Lee (Dept. of Life Science, Ewha Womans University) [Co-author]Min J. Kang (Dept. of Life Science, Ewha Womans University)、Ji-Hyun Park (Dept. of Life Science, Ewha Womans University)、Minkyung Kim (Dept. of Life Science, Ewha Womans University)、Woong-Yang Park (GxD Inc., Kashiwa)

Histopathology has long relied on visual interpretation of H&E-stained slides by pathologists, where accurate identification of diverse cell types remains subjective and challenging. Recent deep learning methods, including vision transformer (ViT)-based foundation models, have shown promise in predicting clinical features. However, directly predicting individual cell types from histologial images remains an unmet challenge, despite its significant potential to advance tumor pathology and guide treatment decisions. Here, we present a ViT-based AI model trained to predict cell types directly from histology images using annotations inferred from spatial single-cell transcriptomics. Our model integrates pretrained pathology foundation models as encoders and achieved an AUROC of 0.72 in distinguishing 17 distinct cell types. We also developed methods to infer clinically relevant features of the tumor microenvironment - such as tumor subtypes and tumor-infiltrating lymphocytes (TILs) - leveraging the spatially resolved cell-type annotations. This approach offers a strong potential to enhance tumor biology insights and enable AI-driven precision pathology from standard H&E slides.

[Disclosure of COI by Responsible researcher] Lead presenter is the Responsible researcher.
[Lead presenter: Sanghyuk Lee] ■Advisor of the company and/or profit-making organization: Geninus Inc., Korea

▲TOP

### [IS2-5] IS2
### Spatial Single Cell Analysis for clinical implementation in terms of clinical development

2025 / 9 / 25 (Thu) 9:00-11:30 Room 10/(Art Hall) Room 10

[Chairpersons]Mitsuho Imai (National Cancer Center Hospital East)、Woong-Yang Park (Samsung Genome Institute)

Recent advancements in spatial single-cell analysis have enabled high-resolution mapping of cellular organization and interactions within tissues. These technologies provide unprecedented insights into the cellular heterogeneity and spatial architecture of various biological systems, offering new perspectives on disease mechanisms, tumor microenvironments, and immune responses. By integrating spatial transcriptomics, proteomics, and machine learning-based analysis, researchers can now explore complex cellular ecosystems with greater precision, driving innovations in biomarker discovery and therapeutic development.
This session will bring together leading experts to discuss the latest advancements, applications, and challenges in spatial single-cell analysis. By examining cutting-edge technologies and their translational potential, we aim to foster interdisciplinary discussions on how these methods can bridge the gap between fundamental research and clinical practice. The session will highlight key developments in data integration, computational modeling, and clinical validation, providing insights into how spatial single-cell analysis can enhance disease diagnosis, treatment strategies, and precision medicine approaches.
Through this exchange, we hope to identify opportunities for collaboration and innovation, ultimately contributing to the broader implementation of spatial single-cell technologies in biomedical research and clinical applications.

---

### IS2-5
### Robust and interpretable prediction of cancer gene markers and cell types from spatial transcriptomics data

[Speaker]Quan Nguyen (QIMR Berghofer Medical Research Institute, Infection and Immunology Program) [Co-author]Xiao Tan (QIMR Berghofer Medical Research Institute, Infection and Immunology Program)、Albert Xiong (QIMR Berghofer Medical Research Institute, Infection and Immunology Program)、Prakrithi Pavithra (QIMR Berghofer Medical Research Institute, Infection and Immunology Program)

For over a century, cancer patient tissue biopsies are stained by Haematoxylin and Eosin (H&E) for pathologists to examine using light microscopy. We developed STimage as a comprehensive suite of models for predicting gene expression and classifying tissue regions and cell types. For robustness, STimage predicts gene expression based on parameter distributions rather than fixed data points and estimates uncertainty from the data and the model ans enable model interpretability. Using diverse datasets from three cancers, we assessed the model's performance on in-distribution and out-of-distribution samples, across platforms, data types, and sample preservation methods. Further, we implemented an ensemble approach, incorporating pre-trained foundation models, to improve performance and reliability, especially in cases with small training datasets. Finally, we showed that using STimage-predicted values based solely on imaging input, we could stratify patient survival groups. Overall, STimage enables the prediction of molecular and cellular information from histopathological images, opening a new direction to advance digital pathology applications.

[Disclosure of COI by Responsible researcher] Lead presenter is the Responsible researcher.
[Lead presenter: Quan Nguyen] We have no financial relationships to disclose.

▲TOP

### [IS2-6] IS2
### Spatial Single Cell Analysis for clinical implementation in terms of clinical development

2025 / 9 / 25 (Thu) 9:00-11:30 Room 10/(Art Hall) Room 10

[Chairpersons]Mitsuho Imai (National Cancer Center Hospital East)、Woong-Yang Park (Samsung Genome Institute)

Recent advancements in spatial single-cell analysis have enabled high-resolution mapping of cellular organization and interactions within tissues. These technologies provide unprecedented insights into the cellular heterogeneity and spatial architecture of various biological systems, offering new perspectives on disease mechanisms, tumor microenvironments, and immune responses. By integrating spatial transcriptomics, proteomics, and machine learning-based analysis, researchers can now explore complex cellular ecosystems with greater precision, driving innovations in biomarker discovery and therapeutic development.
This session will bring together leading experts to discuss the latest advancements, applications, and challenges in spatial single-cell analysis. By examining cutting-edge technologies and their translational potential, we aim to foster interdisciplinary discussions on how these methods can bridge the gap between fundamental research and clinical practice. The session will highlight key developments in data integration, computational modeling, and clinical validation, providing insights into how spatial single-cell analysis can enhance disease diagnosis, treatment strategies, and precision medicine approaches.
Through this exchange, we hope to identify opportunities for collaboration and innovation, ultimately contributing to the broader implementation of spatial single-cell technologies in biomedical research and clinical applications.

---

### IS2-6
### Spatial transcriptomics reveals epithelial heterogeneity and microenvironmental divergence in gastric cancer subtypes

[Speaker]Ayumu Tsubosaka (Dept. Prev. Med., Grad. Sch. Med., The Univ. Tokyo) [Co-author]Kyohei Sano (Dept. Prev. Med., Grad. Sch. Med., The Univ. Tokyo)、Miwako Kakiuchi (Dept. Prev. Med., Grad. Sch. Med., The Univ. Tokyo)、Daisuke Komura (Dept. Prev. Med., Grad. Sch. Med., The Univ. Tokyo)、Hiroto Katoh (Div. Pathol., EPOC, Natl. Cancer Ctr.)、Tetsuo Ushiku (Dept. Pathol., Med., Grad. Sch. Med., The Univ. Tokyo)、Shumpei Ishikawa (Dept. Prev. Med., Grad. Sch. Med., The Univ. Tokyo／Div. Pathol., EPOC, Natl. Cancer Ctr.)

Gastric cancer includes histologically and molecularly distinct subtypes, such as diffuse-type and intestinal-type tumors, each showing unique architecture and clinical behavior. However, the spatial organization of epithelial programs and their interaction with the tumor microenvironment (TME) remain elusive.We analyzed gastric cancer tissues from 43 patients, comprising over 7 million cells, via single-cell spatial transcriptomic analysis. This was integrated with single-cell RNA and whole genome sequencing, enabling multi-omics analysis of epithelial diversity and tumor microenvironment (TME) features. To characterize epithelial variation, we developed "diffuse" and "intestinal" scores based on epithelial transcriptional profiles. These scores enabled spatial quantification of epithelial heterogeneity across tumor subregions.We then evaluated TME patterns in relation to these scores. In regions with higher diffuse scores, we observed interferon signaling, elevated immunosuppressive scores, and mechanically stressed cancer-associated fibroblasts, suggesting a microenvironmental state potentially associated with immune evasion and adverse outcomes.

[Disclosure of COI by Responsible researcher] Lead presenter is not the Responsible researcher.
[Lead presenter: Ayumu Tsubosaka] We have no financial relationships to disclose.
[Principal Researcher: Shumpei Ishikawa] ■Research funds from company: 日本医療研究開発機構、日本学術振興会　■Advisor of the company and/or profit-making organization: 新日本科学株式会社　■Research expenses: Chordis therapeutics株式会社、大鵬薬品工業株式会社、ロート製薬株式会社、アステラス製薬株式会社

International Sessions

### [IS2-7] IS2
### Spatial Single Cell Analysis for clinical implementation in terms of clinical development

2025 / 9 / 25 (Thu) 9:00-11:30 Room 10/(Art Hall) Room 10

[Chairpersons]Mitsuho Imai (National Cancer Center Hospital East)、Woong-Yang Park (Samsung Genome Institute)

Recent advancements in spatial single-cell analysis have enabled high-resolution mapping of cellular organization and interactions within tissues. These technologies provide unprecedented insights into the cellular heterogeneity and spatial architecture of various biological systems, offering new perspectives on disease mechanisms, tumor microenvironments, and immune responses. By integrating spatial transcriptomics, proteomics, and machine learning-based analysis, researchers can now explore complex cellular ecosystems with greater precision, driving innovations in biomarker discovery and therapeutic development.
This session will bring together leading experts to discuss the latest advancements, applications, and challenges in spatial single-cell analysis. By examining cutting-edge technologies and their translational potential, we aim to foster interdisciplinary discussions on how these methods can bridge the gap between fundamental research and clinical practice. The session will highlight key developments in data integration, computational modeling, and clinical validation, providing insights into how spatial single-cell analysis can enhance disease diagnosis, treatment strategies, and precision medicine approaches.
Through this exchange, we hope to identify opportunities for collaboration and innovation, ultimately contributing to the broader implementation of spatial single-cell technologies in biomedical research and clinical applications.

---

### IS2-7
### Spatial Single-Cell Analysis through Collaboration by Clinician, Pathologist, and Researcher Enhance Precision Medicine

[Speaker]Kenya Kobayashi (Dept of Otolaryngology, H&N surgery, The University of Tokyo) [Co-author]Suguru Miyata (Div of Cell Therapy, Chiba Cancer Ctr, Research Inst.／Dept of Otolaryngology, H&N surgery, Chiba University.)、Koichi Saeki (Div of Integrated Research, The Tokyo University of Science.)、Taisuke Mori (Dept of Pathology, National Cancer Center Hosp.)、Katsushige Kawase (Div of Cell Therapy, Chiba Cancer Ctr, Research Inst.／Dept of Otolaryngology, H&N surgery, Chiba University.)、Ichiro Fukumoto (Div of Cell Therapy, Chiba Cancer Ctr, Research Inst.／Dept of Otolaryngology, H&N surgery, Chiba University.)、Toyoyuki Hanazawa (Dept of Otolaryngology, H&N surgery, Chiba University.)、Yuki Saito (Dept of Otolaryngology, H&N surgery, The University of Tokyo)、Kenji Kondo (Dept of Otolaryngology, H&N surgery, The University of Tokyo)、Hiroshi Haeno (Div of Integrated Research, The Tokyo University of Science.)、Mizuo Ando (Dept of Otolaryngology, H&N surgery, Okayama University.)、Masahito Kawazu (Div of Cell Therapy, Chiba Cancer Ctr, Research Inst.)

Spatial transcriptomics enables high-resolution mapping of cellular interactions. Identifying relevant clinicopathological questions is the first step in translating this technology into clinical practice, requiring collaboration between clinicians, pathologists, and researchers. We applied this technology to MYB rearrangement-derived adenoid cystic carcinoma, which consists of two distinct populations: epithelial and myoepithelial cells. Tumor malignancy depends on the distribution of these cells, with high-grade transformation (HGT) occurring in some cases. Key findings include: 1) NOTCH was highly expressed in epithelial cells, while NOTCH ligand genes were expressed in myoepithelial cells. The spatial arrangement of these populations is crucial for paracrine signaling. 2) High-grade tumors, lacking myoepithelial cells, exhibited elevated NOTCH and MYB expression and activation of ligand-independent Notch signaling. 3) Copy number abnormalities were linked to HGT, with clear boundaries. 4) Distinct gene expression was observed within the same section, indicating intratumoral heterogeneity. These findings offer valuable insights for ongoing clinical trials targeting MYB.

[Disclosure of COI by Responsible researcher] Lead presenter is the Responsible researcher.
[Lead presenter: Kenya Kobayashi] We have no financial relationships to disclose.

▲TOP

### [IS2-8] IS2
### Spatial Single Cell Analysis for clinical implementation in terms of clinical development

2025 / 9 / 25 (Thu) 9:00-11:30 Room 10/(Art Hall) Room 10

[Chairpersons]Mitsuho Imai (National Cancer Center Hospital East)、Woong-Yang Park (Samsung Genome Institute)

Recent advancements in spatial single-cell analysis have enabled high-resolution mapping of cellular organization and interactions within tissues. These technologies provide unprecedented insights into the cellular heterogeneity and spatial architecture of various biological systems, offering new perspectives on disease mechanisms, tumor microenvironments, and immune responses. By integrating spatial transcriptomics, proteomics, and machine learning-based analysis, researchers can now explore complex cellular ecosystems with greater precision, driving innovations in biomarker discovery and therapeutic development.
This session will bring together leading experts to discuss the latest advancements, applications, and challenges in spatial single-cell analysis. By examining cutting-edge technologies and their translational potential, we aim to foster interdisciplinary discussions on how these methods can bridge the gap between fundamental research and clinical practice. The session will highlight key developments in data integration, computational modeling, and clinical validation, providing insights into how spatial single-cell analysis can enhance disease diagnosis, treatment strategies, and precision medicine approaches.
Through this exchange, we hope to identify opportunities for collaboration and innovation, ultimately contributing to the broader implementation of spatial single-cell technologies in biomedical research and clinical applications.

---

### IS2-8
### Single-cell and spatially-resolved transcriptomic landscapes of large cell neuroendocrine carcinoma of the lung

[Speaker]Ayako Suzuki (Grad. Sch. of Front. Sci., Univ. Tokyo) [Co-author]Yoshiki Otsuka (Grad. Sch. of Front. Sci., Univ. Tokyo)、Megumi Tateishi (Grad. Sch. of Front. Sci., Univ. Tokyo)、Junko Zenkoh (Grad. Sch. of Front. Sci., Univ. Tokyo)、Ryota Matsuoka (Dept. Diag. Pathol., Fac. Med., Univ. Tsukuba)、Daisuke Matsubara (Dept. Diag. Pathol., Fac. Med., Univ. Tsukuba)、Yutaka Suzuki (Grad. Sch. of Front. Sci., Univ. Tokyo)

Large cell neuroendocrine carcinomas (LCNECs) have recently been suggested to be classified into four subtypes according to expression patterns of lineage-associated transcription factors, ***ASCL1***, ***NEUROD1***, ***POU2F3*** and ***YAP1***, in similar to small cell lung carcinomas. However, there are less therapeutic strategies based on molecular basis, which cause therapeutic difficulties and poor prognoses. In this study, we performed spatial transcriptome Xenium for 34 high-grade neuroendocrine carcinomas to understand diverse lineage differentiation of LCNEC cells for precise stratification of LCNEC cases and development of novel therapeutic strategies for them. According to the clustering results, tumor cells were roughly classified into three types with high expression levels of ***ASCL1***, ***ASCL2*** or ***YAP1***. Basically, NE and non-NE genes would be mutual exclusively expressed in individual cells. However, we found that tumor cells in some LCNEC cases showed both NE and non-NE marker expressions. These double-positive or both highly-nested features might transition in both directions and could potentially controlled epigenetically/transcriptionally intervention for tumor tissues.

[Disclosure of COI by Responsible researcher] Lead presenter is the Responsible researcher.
[Lead presenter: Ayako Suzuki] We have no financial relationships to disclose.

International Sessions

### [AOSR1-1] AOSR1
### Hepatocellular Carcinoma: The Cutting Edge

2025 / 9 / 25 (Thu) 9:00-11:30 Room 11/(ANA Crowne Plaza Hotel Kanazawa 3F Ohtori (West)) Room 11

[Chairpersons]Taro Yamashita (Kanazawa University School of Medicine)、Tatsuhiro Shibata (Laboratory of Molecular Medicine, The Institute of Medical Science, The University of Tokyo)

Hepatocellular carcinoma is the third leading cause of cancer death in the world, and its incidence rates are still globally increasing. Although the main etiology of hepatocellular carcinoma has been chronic viral hepatitis and cirrhosis in which continuous inflammation and hepatocytes regeneration occur, current epidemiological evidence suggests the emergence of hepatocellular carcinoma without hepatitis virus infection typically associated with steatotic liver diseases worldwide. Recent advancement of molecular biology has enabled the in-depth analysis of hepatocellular carcinoma pathobiology from the aspects of genome abnormality, impairment of host immune responses, and cancer stem cells as culprits of patients' poor prognosis. Although these research efforts have resulted in the development of novel diagnostics and therapeutics widely used in clinical practice, the patients'responses to certain treatment options widely varied case by case, which indicates the need for novel biomarkers and therapeutic target identification for the establishment of precision medicine in hepatocellular carcinoma patients. This symposium focuses on the current cutting-edge research of hepatocellular carcinoma from five experts' presentations to have fruitful discussions, to pave the way for the elimination of hepatocellular carcinoma in the near future.

---

### AOSR1-1
### AI-driven characterization of hepatic fiber morphology to predict molecular alterations and future HCC risk in MASLD

[Speaker]Hayato Nakagawa (Department of Gastroenterology and Hepatology, Mie University)

MASLD is a common liver disease that can progress to cirrhosis and hepatocellular carcinoma (HCC), but the mechanisms remain unclear. We conducted an integrative analysis using AI-based digital pathology and bulk transcriptomics on 94 liver biopsy samples to stratify MASLD patients and uncover key pathogenic features. FibroNest identified 327 collagen fiber phenotypes, summarized into eight principal components (FibroPC1-8). Among them, FibroPC4, characterized by reticular fibers, was associated with a transcriptomic signature predictive of future HCC. Spatial single-cell transcriptomics revealed that FibroPC4-rich areas contained hepatic stellate cells (HSCs) with HCC-promoting phenotypes adjacent to senescent periportal endothelial cells (ECs). Further analysis suggested that IGFBP-7 secreted from senescent ECs induces this tumor-promoting transformation in HSCs. In vitro experiments confirmed that IGFBP-7 reprograms HSCs into an HCC-promoting phenotype. Thus, morphological fiber phenotyping combined with spatial transcriptomics provides novel insights into the tumorigenic microenvironment in MASLD and may help identify high-risk patients for HCC.

[Disclosure of COI by Responsible researcher] Lead presenter is the Responsible researcher.
[Lead presenter: Hayato Nakagawa] We have no financial relationships to disclose.

### [AOSR1-2] AOSR1
### Hepatocellular Carcinoma: The Cutting Edge

2025 / 9 / 25 (Thu) 9:00-11:30 Room 11/(ANA Crowne Plaza Hotel Kanazawa 3F Ohtori (West)) Room 11

[Chairpersons]Taro Yamashita (Kanazawa University School of Medicine)、Tatsuhiro Shibata (Laboratory of Molecular Medicine, The Institute of Medical Science, The University of Tokyo)

Hepatocellular carcinoma is the third leading cause of cancer death in the world, and its incidence rates are still globally increasing. Although the main etiology of hepatocellular carcinoma has been chronic viral hepatitis and cirrhosis in which continuous inflammation and hepatocytes regeneration occur, current epidemiological evidence suggests the emergence of hepatocellular carcinoma without hepatitis virus infection typically associated with steatotic liver diseases worldwide. Recent advancement of molecular biology has enabled the in-depth analysis of hepatocellular carcinoma pathobiology from the aspects of genome abnormality, impairment of host immune responses, and cancer stem cells as culprits of patients' poor prognosis. Although these research efforts have resulted in the development of novel diagnostics and therapeutics widely used in clinical practice, the patients'responses to certain treatment options widely varied case by case, which indicates the need for novel biomarkers and therapeutic target identification for the establishment of precision medicine in hepatocellular carcinoma patients. This symposium focuses on the current cutting-edge research of hepatocellular carcinoma from five experts' presentations to have fruitful discussions, to pave the way for the elimination of hepatocellular carcinoma in the near future.

---

### AOSR1-2
### Impact of the Microenvironment on the Development and Progression of Liver Cancer

[Speaker]Hayato Hikita (Dept. Gastroenterol. & Hepatology, Grad. Sch. Med., The Univ. Osaka)

Liver cancer arises within a distinctive hepatic microenvironment shaped by chronic injury, inflammation, fibrosis, and regeneration. This environment, comprising non-parenchymal cells such as hepatic stellate cells, sinusoidal endothelial cells, macrophages, and lymphocytes, plays a crucial role not only in tumor progression but also in early tumor development. Rather than being passive, the microenvironment actively influences malignancy emergence through complex cellular and molecular interactions.As cancer advances, further alterations in the microenvironment promote proliferation, angiogenesis, immune modulation, and therapeutic resistance. Immune cells are integral to this network, engaging in continuous cross-talk with stromal and tumor cells, thereby affecting disease behavior.In this presentation, I will introduce recent research findings that enhance our understanding of how the hepatic microenvironment influences both the development and progression of liver cancer. I hope these insights contribute to a deeper comprehension of the microenvironment's role in liver cancer.

[Disclosure of COI by Responsible researcher] Lead presenter is the Responsible researcher.
[Lead presenter: Hayato Hikita] ■Lecture fee: Chugai Pharmaceutical Co., Gilead Sciences ■Research expenses: Chugai Pharmaceutical Co., Gilead Sciences, Janssen Pharmaceutical K.K., Ohara Pharmaceutical Co., AstraZeneca K.K., Fujifilm Medical Co., J-Pharma Co., Shionogi& Co., Bristol Myers Squibb, Nitto Denko Co. ■Contributions: Chugai Pharmaceutical Co., Otsuka Pharmaceutical Co., Aska Pharmaceutical Co., Eisai Co., AbbVie, Sumitomo Pharma Co., Mitsubishi Tanabe Pharma Co.

### [AOSR1-3] AOSR1
### Hepatocellular Carcinoma: The Cutting Edge

2025 / 9 / 25 (Thu) 9:00-11:30 Room 11/(ANA Crowne Plaza Hotel Kanazawa 3F Ohtori (West)) Room 11

[Chairpersons]Taro Yamashita (Kanazawa University School of Medicine)、Tatsuhiro Shibata (Laboratory of Molecular Medicine, The Institute of Medical Science, The University of Tokyo)

Hepatocellular carcinoma is the third leading cause of cancer death in the world, and its incidence rates are still globally increasing. Although the main etiology of hepatocellular carcinoma has been chronic viral hepatitis and cirrhosis in which continuous inflammation and hepatocytes regeneration occur, current epidemiological evidence suggests the emergence of hepatocellular carcinoma without hepatitis virus infection typically associated with steatotic liver diseases worldwide. Recent advancement of molecular biology has enabled the in-depth analysis of hepatocellular carcinoma pathobiology from the aspects of genome abnormality, impairment of host immune responses, and cancer stem cells as culprits of patients' poor prognosis. Although these research efforts have resulted in the development of novel diagnostics and therapeutics widely used in clinical practice, the patients'responses to certain treatment options widely varied case by case, which indicates the need for novel biomarkers and therapeutic target identification for the establishment of precision medicine in hepatocellular carcinoma patients. This symposium focuses on the current cutting-edge research of hepatocellular carcinoma from five experts' presentations to have fruitful discussions, to pave the way for the elimination of hepatocellular carcinoma in the near future.

### AOSR1-3
### Novel Molecular and Immunological Subtyping for Therapeutic Innovation in Liver Cancer

[Speaker]Shinji Tanaka (Dept. Mol. Oncol., Inst. Sci. Tokyo)

Heterogeneity is key in malignancies, driving treatment resistance and recurrence. Heterogeneous HCC background necessitates personalized medicine for significant pathological diversity based on stratification. Prior molecular immunological analysis of our surgical specimens identified 3 HCC subtypes (EBioMedicine 1999, Clarivate HiCi Paper). Subsequent clinical reports highlighted a significant correlation between these subtypes and immunotherapy efficacy. Conventional bulk data analysis, with mixed cancer and stromal cells, obscured if subtype characteristics reflected cancer cell properties. Single-cell RNA-seq and clustering of individual cancer cells revealed 5 distinct clusters. Pseudo-bulk analysis, reconstructing bulk data from single-cell data, validated the 3 original subtypes, confirming the intrinsic properties of the cancer cells. Distinct immune landscape understanding enables targeted treatments and predictive biomarkers, improving HCC outcomes. Current efforts focus on characterizing each subtype's immune microenvironment (e.g., T-cell exclusion, DC paucity, Treg enrichment) to develop subtype-specific strategies (JHEP Rep 2024, Hepatology 2025, Cell Rep Med 2025).

[Disclosure of COI by Responsible researcher] Lead presenter is the Responsible researcher.
[Lead presenter: Shinji Tanaka] ■Lecture fee: アストラゼネカ

▲TOP

### [AOSR1-4] AOSR1
### Hepatocellular Carcinoma: The Cutting Edge

2025 / 9 / 25 (Thu) 9:00-11:30 Room 11/(ANA Crowne Plaza Hotel Kanazawa 3F Ohtori (West)) Room 11

[Chairpersons]Taro Yamashita (Kanazawa University School of Medicine)、Tatsuhiro Shibata (Laboratory of Molecular Medicine, The Institute of Medical Science, The University of Tokyo)

Hepatocellular carcinoma is the third leading cause of cancer death in the world, and its incidence rates are still globally increasing. Although the main etiology of hepatocellular carcinoma has been chronic viral hepatitis and cirrhosis in which continuous inflammation and hepatocytes regeneration occur, current epidemiological evidence suggests the emergence of hepatocellular carcinoma without hepatitis virus infection typically associated with steatotic liver diseases worldwide. Recent advancement of molecular biology has enabled the in-depth analysis of hepatocellular carcinoma pathobiology from the aspects of genome abnormality, impairment of host immune responses, and cancer stem cells as culprits of patients' poor prognosis. Although these research efforts have resulted in the development of novel diagnostics and therapeutics widely used in clinical practice, the patients'responses to certain treatment options widely varied case by case, which indicates the need for novel biomarkers and therapeutic target identification for the establishment of precision medicine in hepatocellular carcinoma patients. This symposium focuses on the current cutting-edge research of hepatocellular carcinoma from five experts' presentations to have fruitful discussions, to pave the way for the elimination of hepatocellular carcinoma in the near future.

---

### AOSR1-4
### Utility of a novel tumor marker for the diagnosis of hepatocellular carcinoma based on the cancer stem cell hypothesis

[Speaker]Taro Yamashita (Dept. Gastroenterology, Kanazawa Univ., Sch. Med.)

Cancer stem cell hypothesis is that a subset of cells with stem cell features is indispensable for the maintenance of tumors. Advances in cancer stem cells have been achieved in solid tumors including hepatocellular carcinoma. Increasing evidence suggests the importance of liver cancer stem cells for tumor growth, metastasis, and chemo/radiation resistance in hepatocellular carcinoma, but the application of the liver cancer stem cells concept for the clinical diagnosis and treatment of hepatocellular carcinoma has not yet been achieved to the initially expected. Furthermore, the heterogeneity and plasticity of liver cancer stem cells has recently been noted and might be related to drug resistance and the rapid growth and/or metastasis of the tumor after treatment. Here, we introduce our recent advancement in liver cancer stem cells research and discuss the clinical implications using a novel tumor marker laminin gamma 2 monomer, which may lead to the development of improved diagnostics and treatment in hepatocellular carcinoma.

[Disclosure of COI by Responsible researcher] Lead presenter is the Responsible researcher.
[Lead presenter: Taro Yamashita] ■Research expenses: アボットジャパン合同会社

▲TOP

### [AOSR1-5] AOSR1
### Hepatocellular Carcinoma: The Cutting Edge

2025 / 9 / 25 (Thu) 9:00-11:30 Room 11/(ANA Crowne Plaza Hotel Kanazawa 3F Ohtori (West)) Room 11

[Chairpersons]Taro Yamashita (Kanazawa University School of Medicine)、Tatsuhiro Shibata (Laboratory of Molecular Medicine, The Institute of Medical Science, The University of Tokyo)

Hepatocellular carcinoma is the third leading cause of cancer death in the world, and its incidence rates are still globally increasing. Although the main etiology of hepatocellular carcinoma has been chronic viral hepatitis and cirrhosis in which continuous inflammation and hepatocytes regeneration occur, current epidemiological evidence suggests the emergence of hepatocellular carcinoma without hepatitis virus infection typically associated with steatotic liver diseases worldwide. Recent advancement of molecular biology has enabled the in-depth analysis of hepatocellular carcinoma pathobiology from the aspects of genome abnormality, impairment of host immune responses, and cancer stem cells as culprits of patients' poor prognosis. Although these research efforts have resulted in the development of novel diagnostics and therapeutics widely used in clinical practice, the patients'responses to certain treatment options widely varied case by case, which indicates the need for novel biomarkers and therapeutic target identification for the establishment of precision medicine in hepatocellular carcinoma patients. This symposium focuses on the current cutting-edge research of hepatocellular carcinoma from five experts' presentations to have fruitful discussions, to pave the way for the elimination of hepatocellular carcinoma in the near future.

---

### AOSR1-5
### Cutting-Edge Drug Therapies for Hepatocellular Carcinoma

[Speaker]Masatoshi Kudo (Dept. Gastroenterol. & Hepatol., Kindai Univ. Fac. Med.)

Recent progress in systemic therapy for hepatocellular carcinoma (HCC) has been remarkable. First-line options now include atezolizumab + bevacizumab (IO + anti-VEGF), durvalumab + tremelimumab, and nivolumab + ipilimumab (IO + IO). Selecting among these is a key clinical challenge. Patient selection is essential. Those likely to tolerate grade 3-4 immune-related adverse events (irAEs)-younger patients with good performance status, liver function, and no active cardiovascular disease-may benefit from IO + IO regimens. For others, IO + anti-VEGF is safer. Patients with high tumor burden may respond better to IO + IO, especially via CTLA-4-mediated Treg depletion. AFP and PIVKA-II should be checked at week 3; anti-VEGF can be added from week 4 if needed.As second-line treatment, lenvatinib or IO-based regimens are options after progression on atezolizumab + bevacizumab. Lenvatinib is often preferred, but IO + IO may offer benefit in selected cases. In intermediate-stage HCC, the combination of atezolizumab + bevacizumab with TACE appears most effective. Trials like IMPACT may validate on-demand TACE in stable disease, potentially establishing new global evidence from Japan.

[Disclosure of COI by Responsible researcher] Lead presenter is the Responsible researcher.
[Lead presenter: Masatoshi Kudo] ■Lecture fee: 中外製薬(株)、アストラゼネカ(株)、エーザイ(株)、日本イーライリリー(株) ■Contributions: 大塚製薬(株)、大鵬薬品工業(株)、中外製薬(株)、GEヘルスケアファーマ(株)、エーザイ(株) ■Fees of testimony judgement comment etc.: 中外製薬(株)、エーザイ(株)、アストラゼネカ(株)

### [OS2-1] OS2

### Innovative Cancer Treatment via Artificial Protein Engineering: Functional Domain Modification and Bispecific Antibody

2025 / 9 / 25 (Thu) 9:00-11:30 Room 12/(ANA Crowne Plaza Hotel Kanazawa 3F Ohtori (Central)) Room 12

[Chairpersons]Masahiro Yasunaga (National Cancer Center)、Jun Ishihara (Imperial College London)

本シンポジウムは、人工タンパク質技術と抗体改変を活用した革新的ながん治療をテーマに、最先端の研究成果とその臨床応用の可能性について議論します。抗体やタンパク質への機能性ドメインの改変や次世代設計技術により、特異性と治療効果を高める新たなアプローチや、タンパク質構造を再設計することでがん細胞を選択的に標的化する戦略を紹介します。また、進展が著しい二重特異性抗体の基礎研究と臨床応用に焦点を当て、複雑ながん病態に対応する多面的治療法の可能性を探ります。医療と工学の連携により、基礎研究者と臨床開発者が集い、次世代がん治療技術における課題や将来の展望を共有する場となります。参加者は、タンパク質工学を基盤とした医工薬連携やがん治療の知識を深め、実践的な洞察と医療技術革新への理解を得ることが期待されます。

---

### OS2-1

### Local activation of immune cells and depletion of Tregs by tumor collagen-binding checkpoint immunotherapy

[Speaker]Jun Ishihara (Imperial)

Our research developed a novel approach using tumour collagen-binding domain (CBD) fusion with checkpoint inhibitors for targeted delivery. While Ipilimumab depletes regulatory T cells (Tregs) in vitro through ADCC, it fails to do so in vivo due to poor tumour penetration.The engineered CBD-anti-CTLA4 construct accumulates specifically in tumour stroma by binding exposed collagen, significantly reducing systemic toxicity while enhancing anti-tumour efficacy across multiple cancer types including breast cancer, glioblastoma, colon cancer, and melanoma.Unlike conventional antibodies, CBD-anti-CTLA4 infiltrates deeply into tumour tissues, enabling efficient Treg depletion previously unachievable with standard anti-CTLA4 therapy. This has led to complete tumour regression in previously unresponsive models.We've developed a humanized version that demonstrated safety in preclinical studies and confirmed CBD technology works with other Treg-depleting antibodies across multiple cancer types. Testing in large animals is ongoing.This approach represents a significant advancement for treating immunologically cold tumours that don't respond to conventional immunotherapy.

[Disclosure of COI by Responsible researcher] Lead presenter is the Responsible researcher.
[Lead presenter: Jun Ishihara] ■Research funds from company: ICVI open grant, Sarcoma UK, Prostate cancer UK, Brain tumour charity　■Profit of stock: ArrowImmune and HeioThera　■Research expenses: Ono pharma

### [OS2-2] OS2
### Innovative Cancer Treatment via Artificial Protein Engineering: Functional Domain Modification and Bispecific Antibody

2025 / 9 / 25 (Thu) 9:00-11:30 Room 12/(ANA Crowne Plaza Hotel Kanazawa 3F Ohtori (Central)) Room 12

[Chairpersons]Masahiro Yasunaga (National Cancer Center)、Jun Ishihara (Imperial College London)

本シンポジウムは、人工タンパク質技術と抗体改変を活用した革新的ながん治療をテーマに、最先端の研究成果とその臨床応用の可能性について議論します。抗体やタンパク質への機能性ドメインの改変や次世代設計技術により、特異性と治療効果を高める新たなアプローチや、タンパク質構造を再設計することでがん細胞を選択的に標的化する戦略を紹介します。また、進展が著しい二重特異性抗体の基礎研究と臨床応用に焦点を当て、複雑ながん病態に対応する多面的治療法の可能性を探ります。医療と工学の連携により、基礎研究者と臨床開発者が集い、次世代がん治療技術における課題や将来の展望を共有する場となります。参加者は、タンパク質工学を基盤とした医工薬連携やがん治療の知識を深め、実践的な洞察と医療技術革新への理解を得ることが期待されます。

---

### OS2-2
### Acceleration of antibody engineering pioneered by machine learning

[Speaker]Mitsuo Umetsu (Dep. Biomol. Eng., Grad. Sch. Eng., Tohoku Univ.／RevolKa Ltd.)

The growing wealth of protein sequence and structure data has enabled highly accurate predictions of protein tertiary structures, notably through AlphaFold. However, However, functional prediction remains comparatively imprecise, and obtaining proteins with desired functions still requires numerous experimental steps. In antibody design, the simultaneous optimization of multiple physicochemical and pharmacological properties is essential. This process is complicated by trade-offs between properties: for example, the inverse correlation between target affinity and structural stability. A key advantage of machine learning lies in its ability to simultaneously optimize multiple parameters, significantly reducing the number of iterative experiments required to improve these properties. Here, I demonstrate how machine learning can be utilized to enhance the success rate of developing target antibodies, and further explore its potential applications to bispecific antibody design.

[Disclosure of COI by Responsible researcher] Lead presenter is the Responsible researcher.
[Lead presenter: Mitsuo Umetsu] ■Research funds from company: レボルカ　■ employment by a company related to the presentation: レボルカ　■Profit of stock: レボルカ

### [OS2-3] OS2
### Innovative Cancer Treatment via Artificial Protein Engineering: Functional Domain Modification and Bispecific Antibody

2025 / 9 / 25 (Thu) 9:00-11:30 Room 12/(ANA Crowne Plaza Hotel Kanazawa 3F Ohtori (Central)) Room 12

[Chairpersons]Masahiro Yasunaga (National Cancer Center)、Jun Ishihara (Imperial College London)

本シンポジウムは、人工タンパク質技術と抗体改変を活用した革新的ながん治療をテーマに、最先端の研究成果とその臨床応用の可能性について議論します。抗体やタンパク質への機能性ドメインの改変や次世代設計技術により、特異性と治療効果を高める新たなアプローチや、タンパク質構造を再設計することでがん細胞を選択的に標的化する戦略を紹介します。また、進展が著しい二重特異性抗体の基礎研究と臨床応用に焦点を当て、複雑ながん病態に対応する多面的治療法の可能性を探ります。医療と工学の連携により、基礎研究者と臨床開発者が集い、次世代がん治療技術における課題や将来の展望を共有する場となります。参加者は、タンパク質工学を基盤とした医工薬連携やがん治療の知識を深め、実践的な洞察と医療技術革新への理解を得ることが期待されます。

---

### OS2-3
### Biparatopic strategy for the optimization of antibody antagonists against TNFR2

[Speaker]Hiroki Akiba (Grad Sch. of Pharm. Sci., Kyoto Univ.)

Biparatopic antibodies (BpAbs) bind two distinct epitopes on the same target and form diverse types of antigen–antibody complexes. Major classification is ***cis***- and ***trans***-binding to the antigen, resulting in the formation of 1:1 and multimeric complexes. Thus, various mechanisms of action are expected through binding. While ***trans***-binding BpAbs like Zanidatamab (anti-HER2, FDA-approved 2024) drive receptor clustering for cell internalization or agonism, our strategy leverages ***cis***-binding to avoid unwanted activation. Applied to TNFR2, an immuno-oncology target enriched mainly on regulatory T cells, our ***cis***-binding BpAbs block signaling without inducing receptor clustering or activation. This overcomes a major hurdle in TNFR2 antagonist development, where bivalent formats risk agonistic effects. We present a rational design platform for ***cis***-binding BpAbs with strong potential for safe and effective cancer immunotherapy.

[Disclosure of COI by Responsible researcher] Lead presenter is the Responsible researcher.
[Lead presenter: Hiroki Akiba] We have no financial relationships to disclose.

### [OS2-4] OS2
### Innovative Cancer Treatment via Artificial Protein Engineering: Functional Domain Modification and Bispecific Antibody

2025 / 9 / 25 (Thu) 9:00-11:30 Room 12/(ANA Crowne Plaza Hotel Kanazawa 3F Ohtori (Central)) Room 12

[Chairpersons]Masahiro Yasunaga (National Cancer Center)、Jun Ishihara (Imperial College London)

本シンポジウムは、人工タンパク質技術と抗体改変を活用した革新的ながん治療をテーマに、最先端の研究成果とその臨床応用の可能性について議論します。抗体やタンパク質への機能性ドメインの改変や次世代設計技術により、特異性と治療効果を高める新たなアプローチや、タンパク質構造を再設計することでがん細胞を選択的に標的化する戦略を紹介します。また、進展が著しい二重特異性抗体の基礎研究と臨床応用に焦点を当て、複雑ながん病態に対応する多面的治療法の可能性を探ります。医療と工学の連携により、基礎研究者と臨床開発者が集い、次世代がん治療技術における課題や将来の展望を共有する場となります。参加者は、タンパク質工学を基盤とした医工薬連携やがん治療の知識を深め、実践的な洞察と医療技術革新への理解を得ることが期待されます。

---

### OS2-4
### Development and prodrugation of cancer therapeutic bispecific antibodies

[Speaker]Ryutaro Asano (Grad. Sch. of Eng., Tokyo Univ. of Agric. & Tech.)

We have developed T cell-engaging bispecific antibodies targeting CD3 and EGFR. We found that rearranging the domains significantly improves the effects bispecific antibodies, and recently elucidated this mechanism through structural analysis using cryo-electron microscopy. However, T cell-engaging bispecific antibodies have on-target toxicities because no highly specific cancer antigen has been identified. In addition, the depletion or exhaustion of T cells in the tumor tissue is a problem for T cell-engaging bispecific antibodies. To reduce on-target toxicity, prodrugation is a recent development trend. For example, the antigen binding site of the antibody could be masked prior to delivery to the tumor to reduce on-target toxicity, and then unmasked after delivery to the tumor. In this case, recognition sequences of proteases that are upregulated around the tumor are often used as an unmasking strategy. We have worked on the development of universal mask sequences and the use of T cell activating factors as mask sequences to reduce side effects and activate T cells around the tumor. In this presentation we would like to highlight our progress.

[Disclosure of COI by Responsible researcher] Lead presenter is the Responsible researcher.
[Lead presenter: Ryutaro Asano] ■Research funds from company: 日本学術振興会

### [OS2-5] OS2

### Innovative Cancer Treatment via Artificial Protein Engineering: Functional Domain Modification and Bispecific Antibody

2025 / 9 / 25 (Thu) 9:00-11:30 Room 12/(ANA Crowne Plaza Hotel Kanazawa 3F Ohtori (Central)) Room 12

[Chairpersons]Masahiro Yasunaga (National Cancer Center)、Jun Ishihara (Imperial College London)

本シンポジウムは、人工タンパク質技術と抗体改変を活用した革新的ながん治療をテーマに、最先端の研究成果とその臨床応用の可能性について議論します。抗体やタンパク質への機能性ドメインの改変や次世代設計技術により、特異性と治療効果を高める新たなアプローチや、タンパク質構造を再設計することでがん細胞を選択的に標的化する戦略を紹介します。また、進展が著しい二重特異性抗体の基礎研究と臨床応用に焦点を当て、複雑ながん病態に対応する多面的治療法の可能性を探ります。医療と工学の連携により、基礎研究者と臨床開発者が集い、次世代がん治療技術における課題や将来の展望を共有する場となります。参加者は、タンパク質工学を基盤とした医工薬連携やがん治療の知識を深め、実践的な洞察と医療技術革新への理解を得ることが期待されます。

---

### OS2-5

### Advances and Challenges in T-Cell Bispecific Antibody Development

[Speaker]Takahiro Ishiguro (CHUGAI PHARMACEUTICAL CO., LTD.)

T-cell bispecific antibodies (TCBs) bridge CD3 on T cells and tumor antigens, activating T cells in an MHC-independent manner to exert potent antitumor effects. This concept, dating back to the 1980s, revolutionized hematologic malignancy treatment with the success of blinatumomab targeting CD19. Recently, TCBs targeting DLL3 or HLA/gp100 have shown clinical efficacy in solid tumors, intensifying development efforts. However, challenges remain. One major hurdle is on-target off-tumor toxicity due to low-level antigen expression in normal tissues, which narrows the therapeutic window. Cytokine release syndrome (CRS), though increasingly manageable via step-up dosing and IL-6 receptor blockade, remains a key clinical issue. Moreover, intratumoral antigen heterogeneity and immunosuppressive tumor microenvironments can limit efficacy. To overcome these barriers, next-generation TCBs are under active development, including avidity-based targeting, tumor-selective binding, and co-stimulatory strategies to prevent T-cell exhaustion. This presentation will overview TCB mechanisms, clinical progress in solid tumors, and emerging technologies addressing current limitations.

[Disclosure of COI by Responsible researcher] Lead presenter is the Responsible researcher.
[Lead presenter: Takahiro Ishiguro] We have no financial relationships to disclose.

### [OS2-6] OS2
### Innovative Cancer Treatment via Artificial Protein Engineering: Functional Domain Modification and Bispecific Antibody

2025 / 9 / 25 (Thu) 9:00-11:30 Room 12/(ANA Crowne Plaza Hotel Kanazawa 3F Ohtori (Central)) Room 12

[Chairpersons]Masahiro Yasunaga (National Cancer Center)、Jun Ishihara (Imperial College London)

本シンポジウムは、人工タンパク質技術と抗体改変を活用した革新的ながん治療をテーマに、最先端の研究成果とその臨床応用の可能性について議論します。抗体やタンパク質への機能性ドメインの改変や次世代設計技術により、特異性と治療効果を高める新たなアプローチや、タンパク質構造を再設計することでがん細胞を選択的に標的化する戦略を紹介します。また、進展が著しい二重特異性抗体の基礎研究と臨床応用に焦点を当て、複雑ながん病態に対応する多面的治療法の可能性を探ります。医療と工学の連携により、基礎研究者と臨床開発者が集い、次世代がん治療技術における課題や将来の展望を共有する場となります。参加者は、タンパク質工学を基盤とした医工薬連携やがん治療の知識を深め、実践的な洞察と医療技術革新への理解を得ることが期待されます。

---

### OS2-6
### Development shared neoantigen-targeting bispecific antibodies

[Speaker]Kazuma Kiyotani (Lab. Immunogenomics, National Institute of Biomedical Innovation, Health and Nutrition) [Co-author]Xiaojing Wu (Lab. Immunogenomics, National Institute of Biomedical Innovation, Health and Nutrition)、Yusuke Nakamura (Lab. Immunogenomics, National Institute of Biomedical Innovation, Health and Nutrition)

Neoantigens are cancer-specific antigens derived from somatic gene alterations in cancer cells and are presented on the cell surface by HLA molecules. These neoantigen-HLA complexes are potential targets for cancer immunotherapy. In our previous research, we identified several shared neoantigens from gene alterations found in multiple cancer patients. In this study, we aimed to identify TCR-like antibodies that specifically recognize shared neoantigens presented on HLA, including KRAS and APC frameshift neoantigens, and evaluate their potential as therapeutic bispecific antibodies (BsAbs). Through phage display screening, we isolated single-chain variable fragments (scFvs) with high specificity for mutant neoantigen-HLA complexes. We then developed BsAbs incorporating these scFvs and confirmed their ability to bind both neoantigen-HLA complexes and CD3 molecules. Furthermore, these BsAbs effectively activated T cells and demonstrated cytotoxicity against cancer cells expressing the neoantigens in vitro. This study represents a key step forward in the development of immunotherapeutic BsAbs targeting shared neoantigens.

[Disclosure of COI by Responsible researcher] Lead presenter is the Responsible researcher.
[Lead presenter: Kazuma Kiyotani] ■Advisor of the company and/or profit-making organization: 株式会社Cancer Precision Medicine

▲TOP

### [OS3-1] OS3
### Epigenetics and precision medicine in digestive cancers

2025 / 9 / 25 (Thu) 9:00-11:30 Room 13/(ANA Crowne Plaza Hotel Kanazawa 3F Ohtori (East)) Room 13

[Chairpersons]Makoto Taketo (Med. Res. Inst. Kitano Hospital (MIC, Kyoto Univ. Grad. Sch. Med.)、Masahiro Maeda (Gastrointestinal Surgery, Graduate School of Medicine, Kyoto University)

Cancer exhibits an unstable, disorganized epigenome, often but not always caused by genetic alterations and driven by epigenetic alterations through aging, injury, or chronic infection in normal tissues. Precision medicine aims to tailor disease prevention and treatment based on individual characteristics and molecular data, including genomic and epigenomic profiles. This symposium highlights the transformative potential of cancer epigenetics in precision medicine.
Co-chaired by Dr. Taketo and Dr. Maeda (Kyoto University), the session features leading-edge presentations by emerging Japanese researchers. Dr. Maeda will provide an overview of cancer epigenetics and discuss the epigenetic field for cancerization and reprogramming during progression. Dr. Usui will explore tumor microenvironment-induced epigenetic changes in gastric cancer. Dr. Takai will present on hepatitis C virus-driven epigenetic alterations and their role in liver cancer development. Dr. Takeshima will address drug discovery targeting synthetic lethality through DNA methylation-mediated gene silencing. Finally, Dr. Taketo will discuss potential epigenetic targets revealed by transcriptomic profiling of colorectal cancer stem cells.
This session is distinct and does not overlap with other symposia at this meeting, including the JCA-JSGC and JCA-AACR joint sessions.

---

### OS3-1
### Diagnosis of Cancer Risk and Control of Distant Metastasis via Targeting Epigenetic Alterations

[Speaker]Masahiro Maeda (Dept. of GI Surgery, Kyoto Univ.) [Co-author]Takashi Tsukamoto (Johns Hopkins Drug Discovery, Johns Hopkins Univ., MD, USA.)、Barbara Slusher (Johns Hopkins Drug Discovery, Johns Hopkins Univ., MD, USA.)、Harumi Yamada (Dept. of Epigenomics, Inst. for Advanced Life Sciences, Hoshi Univ.)、Kazutaka Obama (Dept. of GI Surgery, Kyoto Univ.)、Andrew Feinberg (Dept. of Med., Johns Hopkins Univ., MD, USA.)、Toshikazu Ushijima (Dept. of Epigenomics, Inst. for Advanced Life Sciences, Hoshi Univ.)

Cancer development and progression are driven by the accumulation of genetic and epigenetic alterations. Particularly, epigenetic changes form a field defect for cancerization, correlating with cancer risk and have also been highlighted as metastatic drivers. I have addressed two critical challenges in cancer treatment by focusing on epigenetic mechanisms.
For the early detection of gastric cancer (GC), primarily caused by Helicobacter pylori-induced chronic inflammation, we demonstrated an epigenetic field defect associated with GC risk, developed novel DNA methylation markers for risk prediction, and initiated a multi-center prospective study, recently published [Yamada et. al., Gut, 2025].
Additionally, distant metastasis, a major cause of cancer-related mortality, can arise after curative surgery. In pancreatic cancer, genome-wide chromatin reorganization coupled with metabolic alterations promotes metastatic progression. To tackle this, I developed a high-throughput screening assay based on HTRF technology to measure cellular heterochromatin levels, leading to the discovery of novel inhibitors with improved specificity and potency compared to a conventional agent.

[Disclosure of COI by Responsible researcher] Lead presenter is not the Responsible researcher.
[Lead presenter: Masahiro Maeda] We have no financial relationships to disclose.
[Principal Researcher: Toshikazu Ushijima] ■Research funds from company: AMED　■Research expenses: シスメックス

### [OS3-2] OS3
### Epigenetics and precision medicine in digestive cancers

2025 / 9 / 25 (Thu) 9:00-11:30 Room 13/(ANA Crowne Plaza Hotel Kanazawa 3F Ohtori (East)) Room 13

[Chairpersons]Makoto Taketo (Med. Res. Inst. Kitano Hospital (MIC, Kyoto Univ. Grad. Sch. Med.)、Masahiro Maeda (Gastrointestinal Surgery, Graduate School of Medicine, Kyoto University)

Cancer exhibits an unstable, disorganized epigenome, often but not always caused by genetic alterations and driven by epigenetic alterations through aging, injury, or chronic infection in normal tissues. Precision medicine aims to tailor disease prevention and treatment based on individual characteristics and molecular data, including genomic and epigenomic profiles. This symposium highlights the transformative potential of cancer epigenetics in precision medicine.
Co-chaired by Dr. Taketo and Dr. Maeda (Kyoto University), the session features leading-edge presentations by emerging Japanese researchers. Dr. Maeda will provide an overview of cancer epigenetics and discuss the epigenetic field for cancerization and reprogramming during progression. Dr. Usui will explore tumor microenvironment-induced epigenetic changes in gastric cancer. Dr. Takai will present on hepatitis C virus-driven epigenetic alterations and their role in liver cancer development. Dr. Takeshima will address drug discovery targeting synthetic lethality through DNA methylation-mediated gene silencing. Finally, Dr. Taketo will discuss potential epigenetic targets revealed by transcriptomic profiling of colorectal cancer stem cells.
This session is distinct and does not overlap with other symposia at this meeting, including the JCA-JSGC and JCA-AACR joint sessions.

---

### OS3-2
### Epigenomic alterations driven by environmental factors and the progression of gastric cancer

[Speaker]Genki Usui (Dept. Quant. Biomed., Univ. Zurich／Inst. Mol. Health Sci., ETH Zurich／Dept. Mol. Oncol., Grad. Sch. Med., Chiba Univ.)
[Co-author]Keisuke Matsusaka (Dept. Mol. Oncol., Grad. Sch. Med., Chiba Univ.／Dept. Pathol., Chiba Univ. Hosp.)、Tomohiro Shinozaki (Dept. Info. & Comp. Technol., Fac. Eng., Tokyo Univ. of Sci.)、Masaki Fukuyo (Dept. Mol. Oncol., Grad. Sch. Med., Chiba Univ.)、Bahityar Rahmutulla (Dept. Mol. Oncol., Grad. Sch. Med., Chiba Univ.)、Motoaki Seki (Dept. Mol. Oncol., Grad. Sch. Med., Chiba Univ.)、Tomoka Okada (Dept. Mol. Oncol., Grad. Sch. Med., Chiba Univ.)、Eiji Sakai (Dept. Gastroenterol., NTT Med. Ctr. Tokyo)、Hiroyuki Abe (Dept. Path., Grad. Sch. Med., The Univ. Tokyo)、Toshiaki Gunji (Ctr. Prev. Med., NTT Med. Ctr. Tokyo)、Nobuyuki Matsuhashi (Dept. Gastroenterol., NTT Med. Ctr. Tokyo)、Teppei Morikawa (Dept. Diagn. Pathol., NTT Med. Ctr. Tokyo)、Tetsuo Ushiku (Dept. Path., Grad. Sch. Med., The Univ. Tokyo)、Khay G. Yeoh (Dept. Med., Yong Loo Lin Sch. Medi., NUS／Dept. Gastroenterol. & Hepatol., NUHS)、Patrick Tan (Cancer & Stem Cell Biology Program, Duke-NUS／Genome Inst. Singapore／Cancer Sci. Inst. Singapore)、Atsushi Kaneda (Dept. Mol. Oncol., Grad. Sch. Med., Chiba Univ.／Health & Dis. Omics Ctr., Chiba Univ.)

Gastric cancer remains a major cause of cancer mortality, and its development is influenced by environmental exposures and epigenetic changes. We examined how ***Helicobacter pylori*** (***HP***) infection, smoking, and alcohol consumption influence DNA methylation in gastric mucosa, and whether such epigenetic changes can predict primary gastric neoplasia (GN) using longitudinally monitored cohorts in Japan and Singapore. High levels of aberrant DNA methylation in gastric mucosa predicted elevated GN risk and a shorter time to GN onset. Methylation levels correlated with ***HP*** infection, lifestyles, and morphological alterations, yet remained an independent GN risk factor in multivariate analyses. Pro-carcinogenic epigenetic alterations initiated by ***HP*** exposure were amplified by unfavorable but modifiable lifestyle choices. Integration of methylation levels with clinical factors significantly improved risk prediction, providing greater resolution in GN risk stratification. These findings underscore the role of environmentally induced epigenetic alterations in gastric carcinogenesis and the value of integrating clinical and epigenetic data for early detection and prevention of gastric cancer.

[Disclosure of COI by Responsible researcher] Lead presenter is not the Responsible researcher.
[Lead presenter: Genki Usui] ■Research expenses: アステラス製薬　■Contributions: 中外創薬科学財団、上原記念生命科学財団
[Principal Researcher: Tomohiro Shinozaki] ■Research expenses: 株式会社エスアールエル、株式会社スクラム　■Contributions: 武田科学振興財団、タムス岡本記念財団

▲TOP

### [OS3-3] OS3
### Epigenetics and precision medicine in digestive cancers

2025 / 9 / 25 (Thu) 9:00-11:30 Room 13/(ANA Crowne Plaza Hotel Kanazawa 3F Ohtori (East)) Room 13

[Chairpersons]Makoto Taketo (Med. Res. Inst. Kitano Hospital (MIC, Kyoto Univ. Grad. Sch. Med.)、Masahiro Maeda (Gastrointestinal Surgery, Graduate School of Medicine, Kyoto University)

Cancer exhibits an unstable, disorganized epigenome, often but not always caused by genetic alterations and driven by epigenetic alterations through aging, injury, or chronic infection in normal tissues. Precision medicine aims to tailor disease prevention and treatment based on individual characteristics and molecular data, including genomic and epigenomic profiles. This symposium highlights the transformative potential of cancer epigenetics in precision medicine.
Co-chaired by Dr. Taketo and Dr. Maeda (Kyoto University), the session features leading-edge presentations by emerging Japanese researchers. Dr. Maeda will provide an overview of cancer epigenetics and discuss the epigenetic field for cancerization and reprogramming during progression. Dr. Usui will explore tumor microenvironment-induced epigenetic changes in gastric cancer. Dr. Takai will present on hepatitis C virus-driven epigenetic alterations and their role in liver cancer development. Dr. Takeshima will address drug discovery targeting synthetic lethality through DNA methylation-mediated gene silencing. Finally, Dr. Taketo will discuss potential epigenetic targets revealed by transcriptomic profiling of colorectal cancer stem cells.
This session is distinct and does not overlap with other symposia at this meeting, including the JCA-JSGC and JCA-AACR joint sessions.

---

### OS3-3
### Oncogenic transcriptomic/methylomic profile sustained in the liver after the eradication of hepatitis C virus.

[Speaker]Atsushi Takai (Dept. Gastroenterol. Hepatol., Kyoto Univ., Sch. Med.) [Co-author]Masako Mishima (Dept. Gastroenterol. Hepatol., Kyoto Univ., Sch. Med.)、Haruhiko Takeda (Dept. Gastroenterol. Hepatol., Kyoto Univ., Sch. Med.)、Hiroshi Seno (Dept. Gastroenterol. Hepatol., Kyoto Univ., Sch. Med.)

The molecular mechanisms of hepatocarcinogenesis after hepatitis C virus (HCV) eradication remains unclear. To explore the molecular profile associated with hepatocellular carcinoma (HCC) development after sustained viral response (SVR), we performed the comprehensive analysis of transcriptome and methylome using the tissues of normal livers, HCV-positive livers and post-SVR livers. The expression changes of carcinogenesis-associated molecules, such as genes related to ERK signaling and inositol phosphate metabolism, were commonly observed in the liver tissues of HCV-positive and post-SVR in the integrated analysis of transcriptome and methylome. In addition, the elevated expression of CYR61, one of the YAP/TAZ target molecules, and PIK3CG, a PI3K/AKT pathway-related molecule, in the serum immediately after anti-HCV therapy was significantly associated with the early development of HCC. The current data suggested that the oncogenic gene expression alteration and DNA methylation changes induced by HCV infection is related to hepatocarcinogenesis and the molecules whose abnormal expression levels persist after HCV clearance could be a potential biomarker predicting post-SVR HCC.

[Disclosure of COI by Responsible researcher] Lead presenter is the Responsible researcher.
[Lead presenter: Atsushi Takai] We have no financial relationships to disclose.

### [OS3-4] OS3
### Epigenetics and precision medicine in digestive cancers

2025 / 9 / 25 (Thu) 9:00-11:30 Room 13/(ANA Crowne Plaza Hotel Kanazawa 3F Ohtori (East)) Room 13

[Chairpersons]Makoto Taketo (Med. Res. Inst. Kitano Hospital (MIC, Kyoto Univ. Grad. Sch. Med.)、Masahiro Maeda (Gastrointestinal Surgery, Graduate School of Medicine, Kyoto University)

Cancer exhibits an unstable, disorganized epigenome, often but not always caused by genetic alterations and driven by epigenetic alterations through aging, injury, or chronic infection in normal tissues. Precision medicine aims to tailor disease prevention and treatment based on individual characteristics and molecular data, including genomic and epigenomic profiles. This symposium highlights the transformative potential of cancer epigenetics in precision medicine.
Co-chaired by Dr. Taketo and Dr. Maeda (Kyoto University), the session features leading-edge presentations by emerging Japanese researchers. Dr. Maeda will provide an overview of cancer epigenetics and discuss the epigenetic field for cancerization and reprogramming during progression. Dr. Usui will explore tumor microenvironment-induced epigenetic changes in gastric cancer. Dr. Takai will present on hepatitis C virus-driven epigenetic alterations and their role in liver cancer development. Dr. Takeshima will address drug discovery targeting synthetic lethality through DNA methylation-mediated gene silencing. Finally, Dr. Taketo will discuss potential epigenetic targets revealed by transcriptomic profiling of colorectal cancer stem cells.
This session is distinct and does not overlap with other symposia at this meeting, including the JCA-JSGC and JCA-AACR joint sessions.

---

### OS3-4
### Targeting methylation-silenced genes as a source of synthetic lethality

[Speaker]Hideyuki Takeshima (Dept. of Epigenomics, Inst. Adv. Life Sci, Hoshi Univ.) [Co-author]Yui Ohashi (Dept. of Epigenomics, Inst. Adv. Life Sci, Hoshi Univ.)、Toshikazu Ushijima (Dept. of Epigenomics, Inst. Adv. Life Sci, Hoshi Univ.)

Methylation-silencing is frequently present in many types of cancers, and can be a target for synthetic lethality. So far, BRCA1 methylation with a PARP inhibitor is known as a targeting synthetic lethality [Kawachi et al., Breast Cancer Res Treat, 181:323-329, 2020]. Here, we aimed to identify novel synthetic lethal combinations by focusing on SMARCA1 methylation. Therapeutic target genes were searched using SMARCA1 knockout (KO) cells and their normal counterpart cells by shRNA library screening or the sgRNA screening database [DepMap]. Several candidate target genes, such as the dynein-related gene, DYNC1H1, and pyrimidine synthesis-related genes, DHODH and UMPS, were able to be identified. The growth suppression effect of DYNC1H1 knockdown was verified using SMARCA1 KO cells and its wild-type counterparts. Cell growth was reduced to 42.8±2.8% of mock-treated cells in SMARCA1 KO cells , whereas 104±3.6% in wild-type cells. These results suggested that methylation-silenced genes might be a source of synthetic lethality.

[Disclosure of COI by Responsible researcher] Lead presenter is not the Responsible researcher.
[Lead presenter: Hideyuki Takeshima] ■Research funds from company: 科研費、AMED次世代がん
[Principal Researcher: Toshikazu Ushijima] ■Research funds from company: AMED次世代がん

### [OS3-5] OS3
### Epigenetics and precision medicine in digestive cancers

2025 / 9 / 25 (Thu) 9:00-11:30 Room 13/(ANA Crowne Plaza Hotel Kanazawa 3F Ohtori (East)) Room 13

[Chairpersons]Makoto Taketo (Med. Res. Inst. Kitano Hospital (MIC, Kyoto Univ. Grad. Sch. Med.)、Masahiro Maeda (Gastrointestinal Surgery, Graduate School of Medicine, Kyoto University)

Cancer exhibits an unstable, disorganized epigenome, often but not always caused by genetic alterations and driven by epigenetic alterations through aging, injury, or chronic infection in normal tissues. Precision medicine aims to tailor disease prevention and treatment based on individual characteristics and molecular data, including genomic and epigenomic profiles. This symposium highlights the transformative potential of cancer epigenetics in precision medicine.
Co-chaired by Dr. Taketo and Dr. Maeda (Kyoto University), the session features leading-edge presentations by emerging Japanese researchers. Dr. Maeda will provide an overview of cancer epigenetics and discuss the epigenetic field for cancerization and reprogramming during progression. Dr. Usui will explore tumor microenvironment-induced epigenetic changes in gastric cancer. Dr. Takai will present on hepatitis C virus-driven epigenetic alterations and their role in liver cancer development. Dr. Takeshima will address drug discovery targeting synthetic lethality through DNA methylation-mediated gene silencing. Finally, Dr. Taketo will discuss potential epigenetic targets revealed by transcriptomic profiling of colorectal cancer stem cells.
This session is distinct and does not overlap with other symposia at this meeting, including the JCA-JSGC and JCA-AACR joint sessions.

---

### OS3-5
### Comprehensive colorectal cancer stem cell transcriptomic signatures that affect outcomes possibly by epigenetic changes

[Speaker]Makoto M. Taketo (Cancer Ther., Med. Innov. Ctr., Kyoto Univ. Grad. Sch. Med.／Medical Research Institute KITANO HOSPITAL, PIIF Tazuke-Kofu-kai, Osaka)

While genomics of colorectal cancer (CRC) have made remarkable advances these decades, our knowledge remains limited regarding how much epigenetic changes affect its progression and patient prognosis. To overcome such limitations, we have added several new spins to our study design: Use CRC-SC spheroids that form a clonal single cell population. Compare their transcriptome with that of normal colonic epithelial SC focusing on relatively under- or over-expressed genes. Assess these candidate genes for patient outcomes using large public databases, bundling several associated genes as a signature. And, integrate subtype signatures thus obtained into a comprehensive signature (GCS). Finally, develop and validate the GCS scores by a novel mouse model where CRC-SC spheroids are transplanted orthotopically, which can show metastasis to the liver and lungs. As a result, we have identified 5 signatures that are consisted of 19 genes whose expression appears to be affected by epigenetic mechanisms with few protein-structural changes. These data underscore the importance of epigenetic mechanisms in CRC progression and may offer novel targets for therapeutic interventions.

[Disclosure of COI by Responsible researcher] Lead presenter is the Responsible researcher.
[Lead presenter: Taketo Makoto] ■Profit of stock: KyoDiagnostics社　（未公開）

▲TOP

### [J-1013] I-J17-1
### Surgical intervention

2025 / 9 / 25 (Thu) 9:00-10:15 Room 14/(ANA Crowne Plaza Hotel Kanazawa 3F Zuiun 1) Room 14

[Chairperson]Akinobu Taketomi (Dept. of Gastroenterological Surgery I, Hokkaido Univ.)

---

### J-1013
### Clinicopathological features and treatment outcomes of esophageal carcinosarcoma

[Speaker]Takashi Kato (The Cancer Institute Hospital of JFCR) [Co-author]Akihiko Okamura (The Cancer Institute Hospital of JFCR)、Naoki Takahashi (The Cancer Institute Hospital of JFCR)、Hiroki Ishida (The Cancer Institute Hospital of JFCR)、Masayoshi Terayama (The Cancer Institute Hospital of JFCR)、Jun Kanamori (The Cancer Institute Hospital of JFCR)、Yu Imamura (The Cancer Institute Hospital of JFCR)、Masayuki Watanabe (The Cancer Institute Hospital of JFCR)

Background: Esophageal carcinosarcoma is a rare tumor with both carcinoma and sarcoma components, often associated with vascular invasion and lymph node metastasis. Due to its rarity, no standard treatment has been established.Material and Method: We retrospectively analyzed the clinicopathological features and outcomes of 16 patients who underwent esophagectomy for esophageal carcinosarcoma at our institution from 2005 to 2023.Result: Median age was 67 years(range: 27-83), with a male-to-female ratio of 13:3. Tumor staging was pT1/2/3/4 in 9/2/4/1 cases, with submucosal invasion (SM2/SM3) seen in most pT1 cases. Lymphatic invasion occurred in 56.3%, and lymph node metastasis in 50%. The 3-year overall survival rate was 73%, and recurrence-free survival was 60%. Recurrence occurred in 4 patients, all with lymph node metastasis. These patients had significantly worse 3-year overall (73% vs. 88%, P=0.04) and recurrence-free survival (38% vs. 88%, P=0.05) compared to those without metastasis.Conclusion: Patients with lymph node metastasis have a poor prognosis, and it is necessary to develop treatments for the cases in the future.

[Disclosure of COI by Responsible researcher] Lead presenter is the Responsible researcher.
[Lead presenter: Takashi Kato] We have no financial relationships to disclose.

### [J-1014] I-J17-1
### Surgical intervention

2025 / 9 / 25 (Thu) 9:00-10:15 Room 14/(ANA Crowne Plaza Hotel Kanazawa 3F Zuiun 1) Room 14

[Chairperson]Akinobu Taketomi (Dept. of Gastroenterological Surgery I, Hokkaido Univ.)

---

### J-1014
### Impact of Preserving Azygos Vein Arch in Postoperative Pneumonitis for Thoracoscopic Esophagectomy

[Speaker]Tomonori Nakanoko (Surgery and Science, Kyushu University) [Co-author]Keita Natsugoe (Surgery and Science, Kyushu University)、Yuki Shin (Surgery and Science, Kyushu University)、Tetsuro Kawazoe (Surgery and Science, Kyushu University)、Yasuo Tsuda (Surgery and Science, Kyushu University)、Tomoya Harima (Surgery and Science, Kyushu University)、Sho Nambara (Surgery and Science, Kyushu University)、Koji Ando (Surgery and Science, Kyushu University)、Eiji Oki (Surgery and Science, Kyushu University)、Tomoharu Yoshizumi (Surgery and Science, Kyushu University)

Background: We have been preserving the right bronchial artery running ventral to the azygos vein arch together with the azygos vein arch in thoracoscopic sub-total esophagectomy. Purpose: To clarify the effect of preserving the right bronchial artery on the prevention of postoperative pneumonia. Patients and Methods: To investigate the effect of preservation of the arch of the azygos vein on prevention of postoperative pneumonia in 100 patients who underwent thoracoscopic subtotal esophagectomy for thoracic esophageal cancer between April 2015 and December 2021. Results: Forty-eight patients were treated with preservation of the azygos arch, and 52 patients were treated with resection. The number of superior mediastinal lymph nodes dissected was 5 vs. 6.5 ($P<0.05$), which was lower in the preserved cases. Postoperative pneumonia occurred in 2/48 (4%) vs. 10/52 (19%), and was less common in cases with preserved case ($P<0.05$). Conclusion: Preservation of the right bronchial artery with preservation of the azygos vein arch may reduce the incidence of postoperative pneumonia in thoracoscopic sub-total esophagectomy for thoracic esophageal cancer.

[Disclosure of COI by Responsible researcher] Lead presenter is the Responsible researcher.
[Lead presenter: Tomonori Nakanoko] We have no financial relationships to disclose.

▲TOP

### [J-1015] I-J17-1
### Surgical intervention

2025 / 9 / 25 (Thu) 9:00-10:15 Room 14/(ANA Crowne Plaza Hotel Kanazawa 3F Zuiun 1) Room 14

[Chairperson]Akinobu Taketomi (Dept. of Gastroenterological Surgery I, Hokkaido Univ.)

---

### J-1015
### Association of Clinical Frailty Scale with Surgical Outcome and Survival Outcome in Elderly Colon Cancer Patients

[Speaker]Takashi Tamura (Department of Surgery, Nara Medical University) [Co-author]Fumikazu Koyama (Department of Surgery, Nara Medical University／Department of Endoscopy, Nara Medical University Hospital)、Yosuke Iwasa (Department of Surgery, Nara Medical University／Department of Endoscopy, Nara Medical University Hospital)、Tadataka Takagi (Department of Surgery, Nara Medical University)、Kosuke Fujimoto (Department of Surgery, Nara Medical University)、Goki Ejiri (Department of Surgery, Nara Medical University)、Chihiro Yoshikawa (Department of Surgery, Nara Medical University)、Masayuki Sho (Department of Surgery, Nara Medical University)

PurposeThis study examined the impact of the Clinical Frailty Scale (CFS) on surgical outcomes and survival rates in elderly colon cancer patients.MethodsData from 93 stage II/III colon cancer patients (≧80 years old) who underwent radical resection from 2008 to 2022 were analyzed retrospectively. Patients were classified as Low-Frail (CFS 4≦, n=35) or High-Frail (CFS ≧5, n=58). Postoperative complications, discharge status, and survival outcomes were assessed.ResultsComplications occurred in 44.1% of patients. High frailty was significantly correlated with the occurrence of complications, with contributing factors including cardiac comorbidity, a low Prognostic Nutritional Index, and a high Glasgow Prognostic Score. All Low-Frail patients were discharged home, while 20% of High-Frail patients required facility transfer. Three-year survival rates were 92.8% for Low-Frail versus 76.0% for High-Frail.ConclusionElderly colon cancer patients with high frailty show significantly increased complication rates. Comprehensive risk assessment and individualized strategies need to be incorporated into treatment approaches to improve clinical outcomes in this vulnerable population.

[Disclosure of COI by Responsible researcher] Lead presenter is the Responsible researcher.
[Lead presenter: Takashi Tamura] We have no financial relationships to disclose.

### [J-1016] I-J17-1
### Surgical intervention

2025 / 9 / 25 (Thu) 9:00-10:15 Room 14/(ANA Crowne Plaza Hotel Kanazawa 3F Zuiun 1) Room 14

[Chairperson]Akinobu Taketomi (Dept. of Gastroenterological Surgery I, Hokkaido Univ.)

---

### J-1016
### Development of an intraoperative margin assessment method for breast cancer using the a-Man probe: a multi-center study

[Speaker]Takaaki Masuda (Breast Surg Oncol, Dept of Surg, Kochi Med School) [Co-author]Hiroki Ueo (Ueo Breast Cancer Hosp)、Hiroaki Ueo (Ueo Breast Cancer Hosp)、Takako Doi (Breast Cancer Ctr, Shonan Memorial Hosp)、Miki Yamaguchi (Dept of Breast Surg, JCHO Kurume General Hosp)、Sadako Akashi (Dept of Breast Surg, Tokyo Women's Med Univ Hosp)、Tomoko Takamaru (Dept of Breast Surg, SHOWA Univ Koto Toyosu Hosp)、Hitoshi Tsuda (Dept of Path, Chiba Med Ctr)、Takuya Moriya (Kawasaki Med School)、Rin Yamaguchi (Dept of Diagnostic Path／Breast ctr, Nagasaki Univ Hosp)、Yuji Kozuka (Dept of Path, Mie Univ Hosp)、Takeshi Sasaki (Cancer Ctr, Keio Univ School of Med)、Kyohei Fujita (Dept of Biomed Informatics, The Univ of Tokyo)、Yasuteru Urano (Grad Sch of Pharm Sci, The Univ of Tokyo)、Koshi Mimori (Dept of Surg, Kyushu Univ Beppu Hosp)

Background: Accurate surgical margin assessment is crucial for successful breast-conserving surgery (BCS) in breast cancer (BC). This study evaluated the a-Man probe, targeting a-mannosidase 2C1 (MAN2C1), for intraoperative margin assessment.Methods: MAN2C1 expression was analyzed using public databases (TN plot and HUMAN PROTEIN ATLAS). Fluorescence intensity (FI) was measured from 274 resected BC and benign samples after mixing with the a-Man probe. Definitive positive and negative thresholds for change in FI at 5 and 20 minutes (5-min dFI, 20-min dFI) were set via ROC analysis to maintain 3% false-positive and negative rates.Results: MAN2C1 was overexpressed in BC tissues compared to normal tissues ($p<0.05$). Using the 5-min and 20-min dFI thresholds, positivity rates were 24.1% and 28.1%, uncertainty rates were 53.6% and 38.3%, and negativity rates were 22.3% and 33.6%, respectively. Positive predictive values were 93.9% (5-min dFI) and 94.8% (20-min dFI), with negative predictive values of 95.1% and 96.7%, respectively.Conclusion: The a-Man fluorescent probe may provide a practical option for intraoperative surgical margin assessment during BCS in BC.

[Disclosure of COI by Responsible researcher] Lead presenter is the Responsible researcher.
[Lead presenter: Takaaki Masuda] We have no financial relationships to disclose.

▲TOP

### [J-1017] I-J17-1
### Surgical intervention

2025 / 9 / 25 (Thu) 9:00-10:15 Room 14/(ANA Crowne Plaza Hotel Kanazawa 3F Zuiun 1) Room 14

[Chairperson]Akinobu Taketomi (Dept. of Gastroenterological Surgery I, Hokkaido Univ.)

### J-1017
### Prognosis of Intrahepatic Cholangiocarcinoma Patients with Sarcopenic Obesity

[Speaker]Kyohei Yugawa (Department of Surgery and Science, Kyushu University) [Co-author]Shinji Itoh (Department of Surgery and Science, Kyushu University)、Takeo Toshima (Department of Surgery and Science, Kyushu University)、Takashi Motomura (Department of Surgery and Science, Kyushu University)、Yuriko Tsutsui (Department of Surgery and Science, Kyushu University)、Yuki Nakayama (Department of Surgery and Science, Kyushu University)、Tomoharu Yoshizumi (Department of Surgery and Science, Kyushu University)

Background: Obesity and sarcopenia are major physical changes associated with poor prognosis in patients with intrahepatic cholangiocarcinoma (ICC).Methods: This study included 93 patients who underwent liver resection for ICC between 2000 and 2022. Obesity was defined as a BMI of 25 kg/$m^2$ or higher. Skeletal muscle mass was assessed using the iliacus muscle area at the L3 level.Results: Obesity was present in 28 of 93 patients (30.1%) and 46 patients (49.5%) were classified as sarcopenia group. Patients with sarcopenic obesity had significantly worse outcomes in terms of overall survival and disease-free survival ($p<0.05$). BMI and tumor FDG uptake showed a significant correlation ($r=0.38$, $p<0.0001$), suggesting an association between obesity and cancer associated metabolism. Additionally, sarcopenia was significantly associated with various nutritional inflammation markers (PNI and CAR), potentially reflecting a state of malnutrition. Patients with sarcopenic obesity had extremely poor prognosis ($p<0.0001$).Conclusion: The consideration of both the cancer metabolism-enhancing state induced by obesity and the malnutrition-immunodeficiency state caused by sarcopenia are required.

[Disclosure of COI by Responsible researcher] Lead presenter is not the Responsible researcher.
[Lead presenter: Kyohei Yugawa] We have no financial relationships to disclose.
[Principal Researcher: Shinji Itoh] We have no financial relationships to disclose.

▲TOP

### [J-1018] I-J17-1
### Surgical intervention

2025 / 9 / 25 (Thu) 9:00-10:15 Room 14/(ANA Crowne Plaza Hotel Kanazawa 3F Zuiun 1) Room 14

[Chairperson]Akinobu Taketomi (Dept. of Gastroenterological Surgery I, Hokkaido Univ.)

---

### J-1018
### Clinical significance of the Tumor Marker Gene Model before conversion surgery for advanced pancreatic cancer

[Speaker]Haruyoshi Tanaka (Nagoya University Hospital, Depertment of Surgery) [Co-author]Masamichi Hayashi (Nagoya University Hospital, Depertment of Surgery)、Shinichi Umeda (Nagoya University Hospital, Depertment of Surgery)、Dai Shimizu (Nagoya University Hospital, Depertment of Surgery)、Taisuke Baba (Nagoya University Hospital, Depertment of Surgery)、Masaki Sunagawa (Nagoya University Hospital, Depertment of Surgery)、Norifumi Hattori (Nagoya University Hospital, Depertment of Surgery)、Toshio Kokuryo (Nagoya University Hospital, Depertment of Surgery)、Mitsuro Kanda (Nagoya University Hospital, Depertment of Surgery)

Background: Induction therapy for advanced pancreatic cancer and drawing a roadmap to conversion surgery (CS) require a comprehensive individualized treatment strategy that includes genetic and clinical information. In addition, the significance of induction therapy for UR-LA pancreatic cancer and the significance of CA19-9 and DUPAN-2 normalization for determining CS indication are controversial.
Methods: We genotyped 89 UR pancreatic cancer cases for which germline DNA could be obtained during 2012-2021 using Taqman PCR for FUT2/3 genotyping and developed a tumor marker gene model (TMGM).
Results: We do not hesitate to perform combined arteriovenous and portal vein resection using the Mesenteric approach and the Anthron bypass technique, aiming for R0 resection at the time of CS. Genotyping was possible in 88 patients (FUT2-null/FUT-intact/FUT3-null = 9/73/6). Compared to a single cutoff model, TMGM had better prognostic power for OS ($P < 0.001$, $HR = 4.47$).
Conclusions: Normalization of CA19-9 and DUPAN-2 after treatment is one of the important biomarkers during CS, and its use becomes more valuable when genotype variation is considered.

[Disclosure of COI by Responsible researcher] Lead presenter is the Responsible researcher.
[Lead presenter: Haruyoshi Tanaka] We have no financial relationships to disclose.

### [J-1025] II-J2-1
### Colorectal cancer

2025 / 9 / 25 (Thu) 9:00-10:15 Room 15/(ANA Crowne Plaza Hotel Kanazawa 3F Zuiun 2) Room 15

[Chairperson]Hideki Ueno (Dept. Surg., National Defense Medical College)

### J-1025
### Contribution of cancer-associated fibroblasts in early colorectal cancer to cancer progression and morphogenesis

[Speaker]Shuichi Tsukamoto (Dept. Pathol. Clin. Lab., Nat. Ca. Cen. Hosp. East／Div. Mol. Genomic Pathol., Dept. Pathol., Kobe Univ.) [Co-author]Takayuki Kodama (Div. Mol. Genomic Pathol., Dept. Pathol., Kobe Univ.)、Mari Nishio (Dept. Diagnostic Pathol., Kakogawa Central City Hosp.)、Manabu Shhigeoka (Div. Mol. Genomic Pathol., Dept. Pathol., Kobe Univ.)、Yuichiro Koma (Div. Mol. Genomic Pathol., Dept. Pathol., Kobe Univ.)、Masafumi Horie (Div. Mol. Genomic Pathol., Dept. Pathol., Kobe Univ.)

Cancer-associated fibroblasts (CAFs) are major components of cancerous stroma and play important roles in cancer progression. Recently, we reported that pericryptal fibroblasts (PCFs) are major sources of early-stage CAFs of colorectal carcinoma (CRC) expressing podoplanin (PDPN), a lymphatic vessel marker. Single-cell RNA sequence (scRNA-seq) analysis revealed that PDPN-expressing CAFs are polarized to inflammatory CAFs (iCAFs) that produce various cytokines and chemokines, which indicates these CAFs facilitate cancer cell survival, proliferation, or invasion. Once CRC invades submucosa, vigorous increase of myofibroblastic CAFs (myCAFs) called desmoplastic reaction (DR) emerged. In contrast to the early-stage CAFs we mentioned above, myCAFs are generally poor in iCAFs signatures. We now target this dynamic transition of CAFs´ features in CRC stroma. We found that at least a part of myCAFs in CRC seemed to originate from early-stage CAFs expressing PDPN and gene X, a candidate of another early-stage CAFs´ marker. Collectively, early-stage CAFs emerge at the beginning of carcinogenesis and arrange tumor microenvironment that is advantageous for cancer progression.

[Disclosure of COI by Responsible researcher] Lead presenter is the Responsible researcher.
[Lead presenter: Shuichi Tsukamoto] We have no financial relationships to disclose.

### [J-1026] II-J2-1
### Colorectal cancer

2025 / 9 / 25 (Thu) 9:00-10:15 Room 15/(ANA Crowne Plaza Hotel Kanazawa 3F Zuiun 2) Room 15

[Chairperson]Hideki Ueno (Dept. Surg., National Defense Medical College)

---

### J-1026
### The spatial transcriptomic landscape of the Ulcerative Colitis-Related Colorectal Cancer in mice

[Speaker]Yukina Kusunoki (Dept. of Gastroenterological Surgery, Osaka Metropolitan Univ. ／Dept. of Pathophysiology, Osaka Metropolitan Univ.) [Co-author]Tomonori Kamiya (Dept. of Pathophysiology, Osaka Metropolitan Univ.)、Kanae Echizen (Dept. of Pathophysiology, Osaka Metropolitan Univ.)、Shugo Suzuki (Dept. of Molecular Pathology, Osaka Metropolitan Univ.)、Yoshiki Nonaka (Dept. of Pathophysiology, Osaka Metropolitan Univ.)、Yu Muta (Dept. of Gastroenterology and Hepatology, Kyoto Univ.)、Hiroaki Kasashima (Dept. of Gastroenterological Surgery, Osaka Metropolitan Univ.)、Tsuyoshi Nishiyama (Dept. of Gastroenterological Surgery, Osaka Metropolitan Univ.)、Masanobu Oshima (Cancer Research Inst., Kanazawa Univ.)、Kiyoshi Maeda (Dept. of Gastroenterological Surgery, Osaka Metropolitan Univ.)、Naoko Ohtani (Dept. of Pathophysiology, Osaka Metropolitan Univ.)

Ulcerative colitis(UC) is a chronic inflammatory disease that have an increased risk of colorectal cancer(CRC). In this study, we use spatial transcriptomics to elucidate the tumorigenesis process of UC-associated CRC(UC-CRC). To mimic the process of UC-CRC development as it occurs clinically, DSS was treated in mice to create the colitis before introducing oncogenic mutations in Tgfbr2, Trp53, and Fbxw7 genes in genetically engineered mice(Sakai et al. Cancer Res 2018). We found the dysplasia lesions in 7 mice among the 9 mice 8 weeks after DSS treatment. Spatial transcriptomic analysis was performed on two mice that had dysplasia lesions. We verified the data to evaluate the inter-cellular networks. The analysis demonstrated that clusters included dysplasia lesions interacted with those cells containing fibroblasts and immune cells. Further, we analyzed publicly available single-cell data of human CRC and examined the expression of factors detected by the mouse model analysis. Spatial transcriptome analysis has been suggested to be useful for capturing the characteristics of geographical inter-cellular networks and identifying novel potential molecular targets.

[Disclosure of COI by Responsible researcher] Lead presenter is not the Responsible researcher.
[Lead presenter: Yukina Kusunoki] ■Research funds from company: 科研費特別研究員奨励費351万円、金沢大学がん進展制御研究所共同研究25万円
[Principal Researcher: Naoko Ohtani] ■Research expenses: 武田科学振興財団（2023年900万円）

▲TOP

### [J-1027] II-J2-1
### Colorectal cancer

2025 / 9 / 25 (Thu) 9:00-10:15 Room 15/(ANA Crowne Plaza Hotel Kanazawa 3F Zuiun 2) Room 15

[Chairperson]Hideki Ueno (Dept. Surg., National Defense Medical College)

### J-1027
### Analysis of combination strategy with KRASG12D inhibitor for KRASG12D mutant colorectal cancer patient derived-organoids

[Speaker]Hiroshi Saito (Division of Genetics, Cancer Research Institute, Kanazawa University) [Co-author]Mizuho Nakayama (Division of Genetics, Cancer Research Institute, Kanazawa University／WPINano-Life Science Institute (Nano-LSI), Kanazawa University)、Hiroko Oshima (Division of Genetics, Cancer Research Institute, Kanazawa University／WPINano-Life Science Institute (Nano-LSI), Kanazawa University)、Kenta Doden (Department of Gastrointestinal Surgery, Kanazawa University)、Yusuke Sakimura (Department of Gastrointestinal Surgery, Kanazawa University)、Kengo Hayashi (Department of Gastrointestinal Surgery, Kanazawa University)、Saki Hayashi (Department of Gastrointestinal Surgery, Kanazawa University)、Ryota Matsui (Department of Gastrointestinal Surgery, Kanazawa University)、Hiroto Saito (Department of Gastrointestinal Surgery, Kanazawa University)、Toshikatsu Tsuji (Department of Gastrointestinal Surgery, Kanazawa University)、Daisuke Yamamoto (Department of Gastrointestinal Surgery, Kanazawa University)、Hideki Moriyama (Department of Gastrointestinal Surgery, Kanazawa University)、Jun Kinoshita (Department of Gastrointestinal Surgery, Kanazawa University)、Noriyuki Inaki (Department of Gastrointestinal Surgery, Kanazawa University)、Masanobu Oshima (Division of Genetics, Cancer Research Institute, Kanazawa University／WPINano-Life Science Institute (Nano-LSI), Kanazawa University)

Background: The KRASG12D inhibitor, MRTX1133 has recently been developed, however, its efficacy in KRAS mutated colorectal cancer appears to be limited, due to feedback mechanisms. In this study, we evaluated the effectiveness of combination therapy using KRASG12D-mutated patient-derived organoids.Methods: We established organoids from liver metastases of KRASG12D-mutated colorectal cancer patient. Treatment experiments were conducted using an EGFR inhibitor, KRASG12D inhibitor, MEK inhibitor, and their combinations. Results: Compared to monotherapy treatment, combination of KRASG12D inh./ MEK inh., or EGFR inh./KRASG12D inh. treatments showed synergy effects. Furthermore, a triple combination of EGFR inh./KRASG12D inh./MEK inh showed a greater synergistic effects on organoid survival and ERK activation. Conclusions: For KRASG12D-mutated colon cancer, combination is potentially more effective than monotherapy with KRASG12D inhibitor. Future studies are needed to elucidate the tumor suppression mechanism involved in drug combination treatment.

[Disclosure of COI by Responsible researcher] Lead presenter is not the Responsible researcher.
[Lead presenter: Hiroshi Saito] We have no financial relationships to disclose.
[Principal Researcher: Masanobu Oshima] We have no financial relationships to disclose.

### [J-1028] II-J2-1
### Colorectal cancer

2025 / 9 / 25 (Thu) 9:00-10:15 Room 15/(ANA Crowne Plaza Hotel Kanazawa 3F Zuiun 2) Room 15

[Chairperson]Hideki Ueno (Dept. Surg., National Defense Medical College)

---

### J-1028
### Multi-transcriptomic Analysis Reveals that EREG-driven TME Crosstalk Defines Anti-EGFR Response in Colorectal Cancer

[Speaker]Atsuki Taniguchi (Dept. Gastroenterological Surg., Okayama Univ. Grad. Sch. Med. Dent. Pharm.) [Co-author]Shunsuke Kagawa (Dept. Gastroenterological Surg., Okayama Univ. Grad. Sch. Med. Dent. Pharm.)、Nobuhiko Kanaya (Dept. Gastroenterological Surg., Okayama Univ. Grad. Sch. Med. Dent. Pharm.)、Yoshihiko Kakiuchi (Dept. Gastroenterological Surg., Okayama Univ. Grad. Sch. Med. Dent. Pharm.)、Satoru Kikuchi (Dept. Gastroenterological Surg., Okayama Univ. Grad. Sch. Med. Dent. Pharm.)、Shinji Kuroda (Dept. Gastroenterological Surg., Okayama Univ. Grad. Sch. Med. Dent. Pharm.)、Hiroshi Tazawa (Dept. Gastroenterological Surg., Okayama Univ. Grad. Sch. Med. Dent. Pharm.)、Toshiyoshi Fujiwara (Dept. Gastroenterological Surg., Okayama Univ. Grad. Sch. Med. Dent. Pharm.)

Background: Colorectal cancer (CRC) sidedness impacts anti-EGFR therapy response, but the biological basis for this difference remains unclear. We explored factors associated with anti-EGFR therapy sensitivity (anti-EGFR eligibility) in terms of tumor microenvironment (TME) heterogeneity using multi-omics. Methods: We integrated multiple single-cell RNA-seq data from CRC tumors and normal tissues, focusing on anti-EGFR eligibility. Cancer cells, cancer-associated fibroblasts (CAFs), tumor-associated macrophages (TAMs), and intercellular communication were analyzed. The obtained findings were further validated by bulk RNA-seq and spatial transcriptomics. Results: anti-EGFR eligibility stratified cancer cells more explicitly than sidedness. Eligible tumors showed higher Epiregulin (EREG) expression. We identified an "EREG/EGFR/CSF axis" specific to eligible CRC. Spatial analysis confirmed localized EREG expression and adjacent EGFR pathway activity near cancer cells specifically in eligible tumors. Conclusion: An EREG-driven TME network underlies anti-EGFR sensitivity. This provides mechanistic insights underlying "sidedness" for patient stratification.

[Disclosure of COI by Responsible researcher] Lead presenter is the Responsible researcher.
[Lead presenter: Atsuki Taniguchi] We have no financial relationships to disclose.

▲TOP

### [J-1029] II-J2-1
### Colorectal cancer

2025 / 9 / 25 (Thu) 9:00-10:15 Room 15/(ANA Crowne Plaza Hotel Kanazawa 3F Zuiun 2) Room 15

[Chairperson]Hideki Ueno (Dept. Surg., National Defense Medical College)

---

### J-1029
### COX19, a gene identified by chromosome copy number analysis, is a novel driver gene for colorectal cancer.

[Speaker]Koto Kawata (Dept of Surg Kyushu Univ Beppu Hosp.／Dept of Surg and Sci,Grad Sch of MedSci KyushuUniv.) [Co-author]Takaaki Masuda (Dept of Surg Kochi Univ Sch of Med, Dept ofBreastOncology)、Hajime Otsu (Dept of Surg Kyushu Univ Beppu Hosp.／Dept of Surg and Sci,Grad Sch of MedSci KyushuUniv.)、Akinori Tsujimoto (Dept of Surg Kyushu Univ Beppu Hosp.)、Kazuki Omachi (Dept of Surg Kyushu Univ Beppu Hosp.)、Shinsaku Itoyama (Dept of Surg Kyushu Univ Beppu Hosp.)、Takashi Ofuchi (Dept of Surg Kyushu Univ Beppu Hosp.)、Tomohiko Ikehara (Dept of Surg Kyushu Univ Beppu Hosp.)、Shohei Shibuta (Dept of Surg Kyushu Univ Beppu Hosp.／Dept of Surg and Sci,Grad Sch of MedSci KyushuUniv.)、Kosuke Hirose (Dept of Surg Kyushu Univ Beppu Hosp.／Dept of Surg and Sci,Grad Sch of MedSci KyushuUniv.)、Yuki Ando (Dept of Surg Kyushu Univ Beppu Hosp.／Dept of Surg and Sci,Grad Sch of MedSci KyushuUniv.)、Qingjiang Hu (Dept of Surg Kyushu Univ Beppu Hosp.／Dept of Surg and Sci,Grad Sch of MedSci KyushuUniv.)、Yusuke Yonemura (Dept of Surg Kyushu Univ Beppu Hosp.／Dept of Surg and Sci,Grad Sch of MedSci KyushuUniv.)、Tomoharu Yoshizumi (Dept of Surg and Sci,Grad Sch of MedSci KyushuUniv.)、Koshi Mimori (Dept of Surg Kyushu Univ Beppu Hosp.／Dept of Surg and Sci,Grad Sch of MedSci KyushuUniv.)

Background: Amplification of chromosome 7p in CRC likely has a driver gene (Cancer Res 2006). In this study, we identified COX19 on 7p as a driver gene.Materials and Methods: COX19 expression, survival analysis, and GSEA were performed using TCGA, immunohistochemical staining (IHC), and single-cell analysis. In vitro proliferation assays and Western blotting were performed using COX19 knockout and overexpression CRC cell lines.Results: COX19 mRNA expression was significantly upregulated in CRC tissues ($p<0.01$), and overall survival was shortened in the high-expression group ($p<0.01$). Multivariate Cox regression confirmed COX19 as an independent poor prognostic factor ($p=0.02$); IHC and single-cell analysis showed high COX19 expression in tumor cells ($p<0.01$); knockout of COX19 inhibited proliferation, while overexpression promoted it; GSEA showed a negative correlation between COX19 and apoptosis-related genes.Conclusion: COX19 contributes to mitochondrial respiratory chain complex IV formation (Nat. Commun. 2022). Our findings suggest that overexpression of COX19 in CRC promotes tumor growth by enhancing ATP synthesis (Int J Mol Sci 2020) and inhibiting apoptosis.

[Disclosure of COI by Responsible researcher] Lead presenter is not the Responsible researcher.
[Lead presenter: Koto Kawata] We have no financial relationships to disclose.
[Principal Researcher: Koshi Mimori] We have no financial relationships to disclose.

▲TOP

### [J-1030] II-J2-1
### Colorectal cancer

2025 / 9 / 25 (Thu) 9:00-10:15 Room 15/(ANA Crowne Plaza Hotel Kanazawa 3F Zuiun 2) Room 15

[Chairperson]Hideki Ueno (Dept. Surg., National Defense Medical College)

---

### J-1030
### Increased level of ribosome biogenesis induced by MEK inhibition as a potential therapeutic target for KRAS-mutant CRC

[Speaker]Satoshi Nagayama (Dept. Surg., Uji-Tokusyukai Medical Center／Dept. Cell Biol., Cancer Institute, JFCR) [Co-author]Mizuho Sakahara (Dept. Cell Biol., Cancer Institute, JFCR)、Jun Adachi (Lab. Proteomics for Drug Discovery, NIBIOHN)、Yuichi Abe (Lab. Proteomics for Drug Discovery, NIBIOHN)、Ryoji Yao (Dept. Cell Biol., Cancer Institute, JFCR)

KRAS signaling represents a promising target for cancer treatment. However, KRAS-targeting therapeutic agents have shown limited effectiveness against colorectal cancer (CRC). Using mass spectrometry-based phospho-proteomics analysis, we previously showed that MEK inhibitor elevated the ribosome biogenesis, and its perturbation synergistically suppressed tumor growth of KRAS-mutant CRC organoids. In this study, we analyzed their expression profile and found that MEK inhibitor induced a cellular state resembling the pluripotent naive ground state of embryonic stem cells. These cells are characterized by elevated ribosome biogenesis and Myc expression but low global protein synthesis rate. Furthermore, these cells were vulnerable to the inhibition of ribosome biogenesis, and RNA polymerase I inhibition exerted synergistic anti-tumor effects with trametinib in an autochthonous mouse model of intestinal tumors. CRC cells have been shown to enter a pluripotent stem-like state by exploiting the cellular program of early embryogenesis and become tolerant to KRAS inhibition. Enhanced ribosome biogenesis is a novel, potential therapeutic target to overcome the tolerance to KRAS inhibition.

[Disclosure of COI by Responsible researcher] Lead presenter is not the Responsible researcher.
[Lead presenter: Satoshi Nagayama] We have no financial relationships to disclose.
[Principal Researcher: Ryoji Yao] We have no financial relationships to disclose.

English Oral Session

### [E-1025] I-E13-1
### Molecular targeted therapy (1)

2025 / 9 / 25 (Thu) 9:00-10:15 Room 16/(11F Visage) Room 16

[Chairperson]Masahiko Tanabe (Dept. Breast & Endocrine Surg., Tokyo Univ. Hosp.)

---

### E-1025
### Targeting EphA2/Ephexin4 signaling to disrupt cell division and enhance anticancer drug sensitivity

[Speaker]Yuji Nakayama (Kyoto Pharm. Univ., Lab. of Biochem. and Mol. Biol.) [Co-author]Ryuji Yasutake (Kyoto Pharm. Univ., Lab. of Biochem. and Mol. Biol.)、Yuichiro Kaibori (Kyoto Pharm. Univ., Lab. of Biochem. and Mol. Biol.)、Ryuzaburo Yuki (Kyoto Pharm. Univ., Lab. of Biochem. and Mol. Biol.)

Targeting cell division abnormalities is a promising strategy for cancer chemotherapy. This study reveals EphA2/Ephexin4's role in cell division and their involvement in drug sensitivity. Silencing either EphA2 or Ephexin4 induced abnormal cell division, including chromosome misalignment, reversed by protein re-expression. We found that EphA2 is phosphorylated at Ser897 by the CDK1/MEK/ERK/RSK pathway, and Ephexin4 at Ser41 downstream of CDK1, both of which phosphorylations were crucial for normal cell division. ERK inhibitor-mediated EphA2 phosphorylation inhibition or Ephexin4 knockdown led to RhoG loss from the cortex. Interestingly, active RhoG-G12V remained localized to the cortex despite Ephexin4 knockdown, suggesting that EphA2/Ephexin4 regulates RhoG activity and localization, and thereby regulating cell division. Furthermore, Ephexin4 knockdown worsened vincristine-induced chromosome misalignment, rescued by wild-type Ephexin4, but not unphosphorylated mutant, re-expression. These findings suggest that targeting the EphA2/Ephexin4 axis, in combination with mitosis-targeting agents, could be a promising strategy for novel cancer chemotherapies.

[Disclosure of COI by Responsible researcher] Lead presenter is the Responsible researcher.
[Lead presenter: Yuji Nakayama] We have no financial relationships to disclose.

### [E-1026] I-E13-1
### Molecular targeted therapy (1)

2025 / 9 / 25 (Thu) 9:00-10:15 Room 16/(11F Visage) Room 16

[Chairperson]Masahiko Tanabe (Dept. Breast & Endocrine Surg., Tokyo Univ. Hosp.)

### E-1026
### ATM inhibition potentiate anti-tumor effect of IFN-γ by inducing ferroptosis

[Speaker]MUHAMMAD IRSHAD FAROOQ (Inst. of Natural Med., Univ. of Toyama) [Co-author]Sisca Ucche (Inst. of Natural Med., Univ. of Toyama／Faculty of Pharm., Univ. Gadjah Mada)、Sana Jabbar (Inst. of Natural Med., Univ. of Toyama)、Ka He (Inst. of Natural Med., Univ. of Toyama)、Suthasinee Seephan (Inst. of Natural Med., Univ. of Toyama)、Yoshihiro Hayakawa (Inst. of Natural Med., Univ. of Toyama)

Ataxia telangiectasia mutated (ATM) is a kinase involved in the DNA damage response and activated by DNA double strand breaks (DSBs). Dysregulation of the ATM pathway is commonly found in various cancers and contributes to resistance against chemotherapies. ATM is also involved in regulating cancer cell responsiveness to immunotherapy and resistance to IFN-γ. In this study, we investigated whether ATM can be a therapeutic target to enhance cancer cell sensitivity to IFN-γ. In IFN-γ-resistant human cancer cell lines, ATM inhibitor (ATMi) significantly restored the cytotoxic effect of IFN-γ in the resistance cell lines along with the increased expression of γ-H2AX. In order to understand how ATMi and IFN-γ collaboratively exert an anti-tumor effect, we explored the cell death pathway involved and found that the ferroptosis inhibitor significantly blocked the cytotoxic effect of that combination. These findings suggest that ATM inhibition potentiate the anti-tumor effect of IFN-γ by inducing ferroptosis, therefore, targeting of the ATM pathway may provide a novel strategy for improving cancer immunotherapy efficacy.

[Disclosure of COI by Responsible researcher] Lead presenter is not the Responsible researcher.
[Lead presenter: MUHAMMAD IRSHAD FAROOQ] We have no financial relationships to disclose.
[Principal Researcher: Yoshihiro Hayakawa] We have no financial relationships to disclose.

▲TOP

### [E-1027] I-E13-1
### Molecular targeted therapy (1)

2025 / 9 / 25 (Thu) 9:00-10:15 Room 16/(11F Visage) Room 16

[Chairperson]Masahiko Tanabe (Dept. Breast & Endocrine Surg., Tokyo Univ. Hosp.)

---

### E-1027
### Characterization of URST4 as a new promising biomarker and therapeutic target for breast cancer

[Speaker]Hoa T. Nguyen (Dept. Med. Oncol. & Cancer Ctr., Shiga Univ. of Med. Sci.／Ctr. Advanced Med. against Cancer, Shiga Univ. of Med. Sci.) [Co-author]Atsushi Takano (Dept. Med. Oncol. & Cancer Ctr., Shiga Univ. of Med. Sci.／Ctr. Antibody and Vaccine Ther, Inst. Med. Sci., UTokyo／Ctr. Advanced Med. against Cancer, Shiga Univ. of Med. Sci.)、Yohei Miyagi (Mol. Pathol. & Genet. Div., Kanagawa Cancer Ctr.)、Yataro Daigo (Dept. Med. Oncol. & Cancer Ctr., Shiga Univ. of Med. Sci.／Ctr. Antibody and Vaccine Ther, Inst. Med. Sci., UTokyo／Ctr. Advanced Med. against Cancer, Shiga Univ. of Med. Sci.)

This study investigated URST4 (up-regulated in solid tumor 4) as a promising biomarker and therapeutic target for breast cancer. Real-time qPCR, western blotting, and immunocytochemistry showed overexpression of URST4 in breast cancer cell lines and clinical breast cancer tissues compared to normal epithelial breast cell lines and normal breast tissues. Immunohistochemistry using tissue microarrays demonstrated that URST4 was expressed in 72.3% (188/260) of breast cancer tissues but was scarcely expressed in normal breast tissues. URST4 expressions were significantly correlated with poor prognosis for breast cancer patients ($P = 0.0105$). Gene knock-down assays using siRNAs against URST4 resulted in significant cell growth suppression through cell cycle arrest at G2/M, mitotic program disruption, and/or senescence formation. Based on the pathway relationship of URST4 to PLK1, we attempted combined inhibition assays using siRNAs for URST4 and PLK1 inhibitors. The results demonstrated the superior anti-tumor activity of the combined inhibition compared to the single inhibitor treatment. URST4 could be a potential biomarker and therapeutic target for breast cancer.

[Disclosure of COI by Responsible researcher] Lead presenter is not the Responsible researcher.
[Lead presenter: Hoa T. Nguyen] We have no financial relationships to disclose.
[Principal Researcher: Yataro Daigo] We have no financial relationships to disclose.

▲TOP

### [E-1028] I-E13-1
### Molecular targeted therapy (1)

2025 / 9 / 25 (Thu) 9:00-10:15 Room 16/(11F Visage) Room 16

[Chairperson]Masahiko Tanabe (Dept. Breast & Endocrine Surg., Tokyo Univ. Hosp.)

---

### E-1028
### PARP1 selective inhibitor saruparib exhibits distinct pharmacological properties compared to PAPP1/2 inhibitor olaparib

[Speaker]Shiho Nakano (Ehime University of Medicine Department of Obstetrics and Gynecology／Ehime University of Medicine Department of Biochemistry and Molecular Genetics) [Co-author]Erina Kusakabe (Breast Center, Ehime University Hospital／Ehime University of Medicine Department of Biochemistry and Molecular Genetics)、Sota Tate (Ehime University of Medicine Department of Biochemistry and Molecular Genetics／Proteo-Science Center, Ehime University)、Takashi Sugiyama (Ehime University of Medicine Department of Obstetrics and Gynecology)、Junko Murai (Ehime University of Medicine Department of Biochemistry and Molecular Genetics／Proteo-Science Center, Ehime University)

PARP inhibitors (PARPis) have shown efficacy in homologous recombination repair (HRR)-deficient tumors, including BRCA-mutant tumors. First-generation PARPis, such as olaparib, target PARP1 and PARP2; however, PARP2 targeting can lead to severe anemia. This led to the development of PARP1-selective inhibitors like saruparib. In this study, we compared olaparib and saruparib. Saruparib exhibited 5-10 times greater PARylation inhibition than olaparib. As a single agent, saruparib showed little cytotoxicity in wild-type and DNA repair-deficient mutants, except for BRCA-deficient cells, while olaparib displayed dose-dependent cytotoxicity in both wild-type and other mutant cells. The presence of SLFN11, a sensitizer of replication stress, enhanced sensitivity to olaparib but not to saruparib. However, combining saruparib with the alkylating agent MMS drastically increased its cytotoxicity in the low nanomolar range, while olaparib required the low micromolar range for synergy. Both drugs trapped PARP1 under MMS treatment, but with distinct kinetics. We conclude that saruparib act through a different mechanism compared to olaparib, particularly as a single agent.

[Disclosure of COI by Responsible researcher] Lead presenter is not the Responsible researcher.
[Lead presenter: Shiho Nakano] We have no financial relationships to disclose.
[Principal Researcher: Junko Murai] ■Lecture fee: 武田薬品工業、ファイザー

### [E-1029] I-E13-1
### Molecular targeted therapy (1)

2025 / 9 / 25 (Thu) 9:00-10:15 Room 16/(11F Visage) Room 16

[Chairperson]Masahiko Tanabe (Dept. Breast & Endocrine Surg., Tokyo Univ. Hosp.)

---

### E-1029
### Identification of Stromal-Derived Factors Linked to T-DXd Efficacy in HER2-Mutant Lung Cancer and HER2-Low Breast Cancer

[Speaker]Akari Shiraishi (Dept. CBMS, Grad. Sch. Front. Sci., Univ. of Tokyo／Div. Experiment. Chemother., Cancer Chemother. Ctr., JFCR) [Co-author]Tomoko Ohhara (Div. Experiment. Chemother., Cancer Chemother. Ctr., JFCR)、Ryohei Katayama (Dept. CBMS, Grad. Sch. Front. Sci., Univ. of Tokyo／Div. Experiment. Chemother., Cancer Chemother. Ctr., JFCR)

Trastuzumab deruxtecan (T-DXd), an anti-HER2 antibody-drug conjugate, is approved for HER2-positive cancers. However, HER2 status alone does not fully explain the T-DXd efficacy, suggesting additional contributing factors. In this study, HER2-positive lung cancer cells and CRISPR/Cas9-engineered HER2-low breast cancer cells were evaluated for T-DXd sensitivity. Although these cells were resistant to T-DXd in 2D monoculture, they showed tumor regression in a xenograft mouse model after a single dose of T-DXd treatment. This contrast implied that the tumor microenvironment (TME) contributes to T-DXd efficacy. To explore the mechanisms behind this difference, we used a 3D co-culture system with fibroblasts (NHDF), layered with extracellular matrix to create about a 30-layer 3D structure. We compared three conditions: 3D-on (cancer cells on layered NHDF), 3D-out (cancer cells outside a chamber of the layers); and 2D monoculture. T-DXd-resistant cancer cells in 2D became sensitive under 3D-on conditions. Further analysis identified multiple stromal-derived factors locally modulate T-DXd sensitivity, suggesting that TME profiling may contribute to patient stratification.

[Disclosure of COI by Responsible researcher] Lead presenter is not the Responsible researcher.
[Lead presenter: Akari Shiraishi] We have no financial relationships to disclose.
[Principal Researcher: Ryohei Katayama] ■Research funds from company: 中外製薬、TOPPAN、UBE、BML　■Patent fee: 栄研化学

### [E-1030] I-E13-1
### Molecular targeted therapy (1)

2025 / 9 / 25 (Thu) 9:00-10:15 Room 16/(11F Visage) Room 16

[Chairperson]Masahiko Tanabe (Dept. Breast & Endocrine Surg., Tokyo Univ. Hosp.)

---

### E-1030
### Clinical relevance of HER2 mutations in HER2-low metastatic breast cancer: real-world analysis of trastuzumab deruxtecan

[Speaker]Yoshiya Horimoto (Tokyo Med. Univ. Dept. Breast Surg. Oncol.／Juntendo Univ. Dept. Breast Oncol.／Juntendo Univ. Dept. Human Path.) [Co-author]Masanori Oshi (Yokohama City Univ. Grad. Sch. Med. Dept. Gastroenterological Surg.)、Akimitsu Yamada (Yokohama City Univ. Grad. Sch. Med. Dept. Gastroenterological Surg.)、Masako Muguruma (Tokyo Med. Univ. Dept. Breast Surg. Oncol.)、Takahiko Kawate (Tokyo Med. Univ. Dept. Breast Surg. Oncol.)、Fumi Murakami (Juntendo Univ. Dept. Breast Oncol.)、Takuo Hayashi (Juntendo Univ. Dept. Human Path.)、Takashi Ishikawa (Tokyo Med. Univ. Dept. Breast Surg. Oncol.)、Junichiro Watanabe (Juntendo Univ. Dept. Breast Oncol.)、Goro Kutomi (Juntendo Univ. Dept. Breast Oncol.)

Background: In solid tumors with ***HER2*** mutations, trastuzumab deruxtecan (T-DXd), a HER2-targeted antibody-drug conjugate, has recently demonstrated clinical efficacy, thus attention has shifted not only to HER2 expression levels but also to ***HER2*** mutations as potential therapeutic targets. However, the impact of ***HER2*** mutations on the efficacy of T-DXd in metastatic breast cancer (MBC) remains unclear. Methods: We retrospectively analyzed 25 women with HER2-low MBC treated with T-DXd, assessing four ***HER2*** kinase domain mutations (L755S, D769Y, V777L, V842I) by droplet digital PCR and their associations with time-to-treatment discontinuation (TTD). Results: The frequencies of the four ***HER2*** mutations were: L755S (64%), D769Y (32%), V777L (8%), and V842I (92%). Mutation prevalence varied considerably by site. Patients with the L755S mutation had a shorter median TTD than wild-type (32 vs. 41 weeks), suggesting reduced sensitivity to T-DXd. In contrast, D769Y status had no clear impact on treatment duration, with similar median TTD (38 vs. 36 weeks). Conclusions: Specific ***HER2*** mutations may influence the response to T-DXd in HER2-low MBC.

[Disclosure of COI by Responsible researcher] Lead presenter is the Responsible researcher.
[Lead presenter: Yoshiya Horimoto] We have no financial relationships to disclose.

▲TOP

### [J-1001] I-J12-1
### Pathological analysis

2025 / 9 / 25 (Thu) 10:15-11:30 Room 7/(Hotel Nikko Kanazawa 3F Kujaku B) Room 7

[Chairperson]Etsuko Kiyokawa (Dept. Oncol. Pathol., Kanazawa Med. Univ.)

---

### J-1001
### Risk Factors and Prognostic Impact for Spread Through Air Spaces in Pulmonary Metastases from Colorectal Cancer

[Speaker]Taketo Nakai (Department of Thoracic Surg, Keio Univ.) [Co-author]Satoru Morita (Department of Surg, Keio Univ.)、Yutaka Kurebayashi (Department of Path, Keio Univ.)、Takahiro Suzuki (Department of Thoracic Surg, Keio Univ.)、Yu Okubo (Department of Thoracic Surg, Keio Univ.)、Shigeki Suzuki (Department of Thoracic Surg, Keio Univ.)、Kyohei Masai (Department of Thoracic Surg, Keio Univ.)、Kaoru Kaseda (Department of Thoracic Surg, Keio Univ.)、Yusuke Tomita (Department of Surg, Keio Univ.)、Yusaku Shogen (Department of Surg, Keio Univ.)、Hirochika Kato (Department of Surg, Keio Univ.)、Masayoshi Monno (Department of Surg, Keio Univ.)、Kohei Shigeta (Department of Surg, Keio Univ.)、Koji Okabayashi (Department of Surg, Keio Univ.)、Yuko Kitagawa (Department of Surg, Keio Univ.)、Keisuke Asakura (Department of Thoracic Surg, Keio Univ.)

Objectives: Spread through air spaces (STAS) is one of the invasion forms associated with poor prognosis in primary lung cancer. However, little is known about STAS in pulmonary metastases from colorectal cancer (PM-CRC). This study aimed to evaluate the prognostic impact of STAS in PM-CRC and to assess the risk factors of STAS with clinicopathological characteristics.Methods: A total of 120 patients who underwent complete resection for PM-CRC between 2014 and 2023 at our institution were retrospectively analyzed.Results: Histopathological examination confirmed STAS in 34% (n=41) of the patients. Patients with STAS showed significantly worse recurrence-free survival (RFS) (5-year RFS: 19% vs. 52%, p=0.003) than those without. Multivariate analysis revealed both PM size (p=0.03) and STAS (p=0.04) were independent predictors of poor RFS. Intrapulmonary recurrence was more frequent in the STAS-positive group (51% vs. 33%, p=0.07). Tumor budding in the primary CRC was significantly associated with STAS (OR=6.3, 95%CI 1.1-29.6, p=0.01).Conclusion: STAS in PM from CRC is an independent poor prognostic factor, and tumor budding in the primary lesion may be a risk factor for STAS.

[Disclosure of COI by Responsible researcher] Lead presenter is not the Responsible researcher.
[Lead presenter: Taketo Nakai] We have no financial relationships to disclose.
[Principal Researcher: Satoru Morita] We have no financial relationships to disclose.

▲TOP

### [J-1002] I-J12-1
### Pathological analysis

2025 / 9 / 25 (Thu) 10:15-11:30 Room 7/(Hotel Nikko Kanazawa 3F Kujaku B) Room 7

[Chairperson]Etsuko Kiyokawa (Dept. Oncol. Pathol., Kanazawa Med. Univ.)

---

### J-1002
### Establishment of a novel antibody that shows positivity in cancer cells of infiltrating lobular carcinoma

[Speaker]Natsuko Mizutani (Kyorin Univ. Faculty of Health Sciences. Dept. of Med. tech.) [Co-author]Yoshiya Horimoto (Dept of Imunol. diag. Grad. School. of Med. Juntendo)、Kazunori Kajino (Dept of Imunol. diag. Grad. School. of Med. Juntendo)、Ikumi Sakai (Dept of Imunol. diag. Grad. School. of Med. Juntendo)、Yasuhiko Ito (Dept of Imunol. diag.Grad. Sch. of Med. Juntendo)、Hiromichi Turui (Dept of Imunol. diag.Grad. Sch. of Med. Juntendo)、Takeshi Hirano (Dept of Imunol. diag.Grad. Sch. of Med. Juntendo／Dept of Latic acid bacteria. Grad. School. of Med. Juntendo)、Ryo Hatano (Dept of Therapy Develop. Grad. School. of Med. Juntendo)、Yuuka Tanno (Dept of Imunol. diag.Grad. Sch. of Med. Juntendo)、Masaaki Abe (Dept of Imunol. diag.Grad. Sch. of Med. Juntendo)、Hideo Yagita (Dept of Imunol. diag.Grad. Sch. of Med. Juntendo)、Shuji Matsuoka (Dept of Imunol. diag.Grad. Sch. of Med. Juntendo)

Lobular carcinoma of the mammary grand often grows and invaders while retaining most of the preexisting mammary gland structure. In addition to histopathological diagnosis, negative immunohistochemical staining for E-cadherin is used to confirm lobular carcinoma diagnosis. Lobular carcinoma often metastasizes to other organs at an early stage, and when the lesion is located outside the mammary gland, it is often difficult to distinguish from adenocarcinomas originating from other organs.Here, we sought to develop a new antibody that is specific for lobular carcinoma cells for use in confirming preoperative diagnosis.To this method, we immunized 8-week-old BALB/c mice with three cultured mammary lobular carcinoma cell lines every 2 weeks alternately for 3 months. Three days after the final immunization, P3U1 cells were fusedwith spleen cells from immunized mice using the PEG method. Afterwards, several effective antibodies were obtained by screening. Some of these antibodies could be immunostained on formalin-fixed paraffin sections.

[Disclosure of COI by Responsible researcher] Lead presenter is the Responsible researcher.
[Lead presenter: Natsuko Mizutani] ■Research funds from company: 高橋産業財団

▲TOP

### [J-1003] I-J12-1
### Pathological analysis

2025 / 9 / 25 (Thu) 10:15-11:30 Room 7/(Hotel Nikko Kanazawa 3F Kujaku B) Room 7

[Chairperson]Etsuko Kiyokawa (Dept. Oncol. Pathol., Kanazawa Med. Univ.)

---

### J-1003
### Potential transdifferentiation from hepatocellular carcinoma to neuroendocrine carcinoma in hepatic MiNENs

[Speaker]Sumie Ohni (Dept. of Path. & Microbiol., Nihon Univ. Sch. of Med.) [Co-author]Yoko Nakanishi (Dept. of Path. & Microbiol., Nihon Univ. Sch. of Med.)、Yukari Hirotani (Dept. of Path. & Microbiol., Nihon Univ. Sch. of Med.)、Ryosuke Toyonaka (Dept. of Surg., Nihon Univ. Sch. of Med.)、Osamu Aramaki (Dept. of Surg., Nihon Univ. Sch. of Med.)、Yukiyasu Okamura (Dept. of Surg., Nihon Univ. Sch. of Med.)、Shinobu Masuda (Dept. of Path. & Microbiol., Nihon Univ. Sch. of Med.)、Makoto Makishima (Dept. of Biomed. Sci., Nihon Univ. Sch. of Med.)、Mariko Esumi (Dept. of Biomed. Sci., Nihon Univ. Sch. of Med.)

Hepatic mixed neuroendocrine-non-neuroendocrine neoplasm (MiNEN) is too rare to be well investigated. We phenotypically characterized multiple cancer components of mixed hepatocellular carcinoma-neuroendocrine carcinoma (mixed HCC-NEC) by the immunohistochemistry for differentiation markers (HepPar1, INSM1, etc.) and cancer-related markers (TERT, Ki-67, p53). We demonstrate here two new findings; (1) peculiar and heterogeneous HCC in HCC components; HepPar1(-) HCC cells increased in HCC components with and without histological changes. The histologically changed HCC occasionally showed HepPar1(-)/INSM1(+) and HepPar1(+)/INSM1(+), and a part of them was NEC-like histologically and in terms of Ki-67-positive rate. (2) various positive rate of three cancer biomarkers in individual cancer components but the close correlation of the positive rate pattern in HCC and NEC of each case. It suggests the cancer-characteristic similarity in HCC and NEC. These results indicate the potential transdifferentiation from HCC to NEC in the evolution of mixed HCC-NEC.

[Disclosure of COI by Responsible researcher] Lead presenter is not the Responsible researcher.
[Lead presenter: Sumie Ohni] We have no financial relationships to disclose.
[Principal Researcher: Mariko Esumi] We have no financial relationships to disclose.

▲TOP

### [J-1004] I-J12-1
### Pathological analysis

2025 / 9 / 25 (Thu) 10:15-11:30 Room 7/(Hotel Nikko Kanazawa 3F Kujaku B) Room 7

[Chairperson]Etsuko Kiyokawa (Dept. Oncol. Pathol., Kanazawa Med. Univ.)

---

### J-1004
### "Hepatocyte Islands": A Novel Histological Feature Associated with Prognosis in Intrahepatic Cholangiocarcinoma

[Speaker]Naoto Kubota (Tochigi Cancer Center, Research Institute, Division of Molecular Pathology／Tochigi Cancer Center, Pathology) [Co-author]Ken Yamazaki (Tochigi Cancer Center, Research Institute, Division of Molecular Pathology)、Makoto Abe (Tochigi Cancer Center, Research Institute, Division of Molecular Pathology／Tochigi Cancer Center, Pathology)、Eisuke Miura (Tochigi Cancer Center, Research Institute, Division of Molecular Pathology／Tochigi Cancer Center, Pathology)、Masatsugu Ishii (Tochigi Cancer Center, Research Institute, Division of Molecular Pathology／Tochigi Cancer Center, Hepatobiliary and Pancreatic Surgery)、Hirofumi Shirakawa (Tochigi Cancer Center, Research Institute, Division of Molecular Pathology／Tochigi Cancer Center, Hepatobiliary and Pancreatic Surgery)、Kisho Mihara (Tochigi Cancer Center, Hepatobiliary and Pancreatic Surgery)、Moriaki Tomikawa (Tochigi Cancer Center, Hepatobiliary and Pancreatic Surgery)、Iwao Ozawa (Tochigi Cancer Center, Hepatobiliary and Pancreatic Surgery)、Shoichi Hishinuma (Tochigi Cancer Center, Hepatobiliary and Pancreatic Surgery)、Hidenori Ojima (Tochigi Cancer Center, Research Institute, Division of Molecular Pathology／Tochigi Cancer Center, Pathology)

Background: In intrahepatic cholangiocarcinoma (iCCA), non-neoplastic hepatocytes are often entrapped at the tumor margin. We termed this phenomenon "Hepatocyte Island (HepIS)", and investigated its clinicopathological significance. Methods: Forty-four surgically resected iCCA cases from Tochigi Cancer Center were retrospectively analyzed. HepIS was identified by Hep-par 1 immunostaining, and the maximum distance from the tumor edge to the deepest entrapped hepatocytes (HepIS-length) was measured. Using receiver operating characteristic analysis, an optimal HepIS-length cutoff was determined, classifying cases into HepIS-positive and -negative groups.Results: HepIS was observed in 22 cases. The mean HepIS-length was 0.77 mm (range: 0-5.0 mm). Hazard ratios for overall survival (OS) and recurrence-free survival (RFS) were 1.67 [95% CI: 1.24-2.26] and 1.76 [95% CI: 1.29-2.39], respectively. With a 0.5 mm cutoff, the HepIS-positive group had significantly poorer outcomes (OS: $p = 0.005$; RFS: $p = 0.002$), and similar results were observed even limited to mass-forming type iCCA.Conclusion: HepIS is a novel histopathological feature associated with recurrence and survival in iCCA.

[Disclosure of COI by Responsible researcher] Lead presenter is not the Responsible researcher.
[Lead presenter: Naoto Kubota] We have no financial relationships to disclose.
[Principal Researcher: Ojima Hidenori] We have no financial relationships to disclose.

### [J-1005] I-J12-1
### Pathological analysis

2025 / 9 / 25 (Thu) 10:15-11:30 Room 7/(Hotel Nikko Kanazawa 3F Kujaku B) Room 7

[Chairperson]Etsuko Kiyokawa (Dept. Oncol. Pathol., Kanazawa Med. Univ.)

---

### J-1005
### The spatial profiling of stroma and immune interaction in gastric cancer by RePROBE multiplexed imaging analysis

[Speaker]Takashi Semba (Division of Carcinogenesis, Cancer Institute, JFCR) [Co-author]Huaitao(JFCR) Wang (Division of Carcinogenesis, Cancer Institute, JFCR)、Atsuko Yonemura (Division of Carcinogenesis, Cancer Institute, JFCR)、Yilin(JFCR) Tong (Division of Carcinogenesis, Cancer Institute, JFCR)、Takatsugu Ishimoto (Division of Carcinogenesis, Cancer Institute, JFCR)

A tumor microenvironment (TME) drives tumor progression, and emerging evidence suggests that spatial cellular organization modulates the functional status of cells in the TME. Gastric cancer (GC), especially diffuse-type GC, is characterized by a stroma-rich TME and poor prognosis. However, the spatial organization of GC TME is not fully understood. In this study, we developed a simple and versatile workflow for fluorescent multiplexed immunohistochemistry named RePROBE (Repetitive Primary antibody Replacement and Overlaying images from a Broad range of fluorescence imaging Equipment), enabling more than 30-plex whole-slide single-cell resolution imaging. Spatial analysis revealed few lymphocyte-network formations in diffuse-type GC, which is associated with dysfunctional T cell status. Further analysis, including cell proximity, graph-based cell-cell communication, and cellular neighborhood, showed distinct immune and stromal profiles between diffuse-type and intestinal-type GC. This study demonstrates the flexibility of our RePROBE method and shows the impact of the histological subtype-specific spatial organization of TME on the cellular status and phenotypes in GC.

[Disclosure of COI by Responsible researcher] Lead presenter is not the Responsible researcher.
[Lead presenter: Takashi Semba] We have no financial relationships to disclose.
[Principal Researcher: Takatsugu Ishimoto] We have no financial relationships to disclose.

### [J-1006] I-J12-1
### Pathological analysis

2025 / 9 / 25 (Thu) 10:15-11:30 Room 7/(Hotel Nikko Kanazawa 3F Kujaku B) Room 7

[Chairperson]Etsuko Kiyokawa (Dept. Oncol. Pathol., Kanazawa Med. Univ.)

---

### J-1006
### Concordance of claudin-18.2 positivity in biopsy and resected tissues in pancreatic ductal adenocarcinoma

[Speaker]Daisuke Kyuno (Dept. Pathol., Sapporo Med. Univ., Sch. Med.／Dept. Surg., Sapporo Med. Univ.) [Co-author]Kazuhiko Yanazume (Dept. Pathol., Sapporo Med. Univ., Sch. Med.)、Akira Saito (Dept. Pathol., Sapporo Med. Univ., Sch. Med.)、Yusuke Ono (Dept. Pathol., Sapporo Med. Univ., Sch. Med.)、Tatsuya Ito (Dept. Surg., Sapporo Med. Univ.)、Masafumi Imamura (Dept. Surg., Sapporo Med. Univ.)、Makoto Osanai (Dept. Pathol., Sapporo Med. Univ., Sch. Med.)

[Background] Zolbetuximab, which specifically targets claudin-18.2 (CLDN18.2), is a potential treatment for pancreatic ductal adenocarcinoma (PDAC). Accurate assessment of CLDN18.2 positivity in biopsy specimens is essential for zolbetuximab treatment planning. This study aimed to compare the concordance of CLDN18.2 staining between biopsy and resected PDAC specimens. [Methods] Specimens from 222 patients diagnosed with pancreatic cancer were analyzed in this study. Immunohistochemistry was performed using an anti-cldn18 antibody (clone 43-14A).[Results] The prevalence of cldn18.2 positivity (moderate-to-strong staining $\geq$ 75%) was 9.5%. The concordance rate of cldn18.2 positivity between these specimens was 92.5% (P<0.001). No significant correlation was observed between concordance and any clinicopathological feature. The sensitivity was 54.6%, and five unmatched cases were negative in the biopsied specimen and positive in the resected specimen for cldn18.2 positivity.[Conclusion] Concordance of CLDN18.2 positivity was achieved between biopsy and resected specimens. Increasing the sensitivity is needed to avoid losing the chance of Zolbetuximab treatment for patients with PDAC.

[Disclosure of COI by Responsible researcher] Lead presenter is the Responsible researcher.
[Lead presenter: Daisuke Kyuno] We have no financial relationships to disclose.

### [E-1013] I-E11-2
### Tumor immune microenvironment/Tumor immune escape (1)

2025 / 9 / 25 (Thu) 10:15-11:30 Room 8/(Hotel Nikko Kanazawa 3F Kujaku C) Room 8

[Chairperson]Yoshihiro Hayakawa (Inst. Nat. Med., Univ. Toyama)

---

### E-1013
### Acrolein accelerates lipid peroxidation and ferroptosis process of CD8$^+$ T cells in TME

[Speaker]Koki Ichimaru (Kyoto University CCII) [Co-author]Koji Kitaoka (Kyoto University CCII)、Yasuharu Haku (Kyoto University CCII)、Tomonori Yaguchi (Kyoto University CCII)、Tasuku Honjo (Kyoto University CCII)、Kenji Chamoto (Kyoto University CCII)

Ferroptosis is known to be caused by lipid peroxidation (LPO). We have previously shown that mitochondrial dysfunction increases LPO in CD8$^+$ T cells in the TME, and that a final product of LPO, acrolein (ACR) , leads to a vicious cycle where ACR impaired mitochondrial function. However, it remains unclear how ACR is related to ferroptosis. We classified CD8$^+$ PD-1$^+$ T cells in MC38 into D1-D4 population based on the state of mitochondria (mito). D1: mito potential$^{low}$ mass$^{low}$, D2: mito potential$^{int}$ mass$^{int}$, D3: mito potential$^{high}$ mass$^{high}$, and D4: mito potential$^{low}$ mass$^{high}$. D4 indicated Annexin$^+$ dying cells and was positive for ferroptosis markers. LPO gradually increased from D1 to D3 in proportion to the accumulation of ACR. Upon the repetitive TCR stimulation in vitro, naive CD8$^+$ T cells differentiated from D1 to D3 with increased exhaustion markers. In the presence of ACR, the differentiation and exhaustion were accelerated. Furthermore, the administration of ACR increased the growth of MC38 in mice, which was canceled by a LPO inhibitor, Vitamin E. These results suggest that ACR accumulation accelerates LPO in CD8$^+$ T cells and induces their ferroptosis in TME.

[Disclosure of COI by Responsible researcher] Lead presenter is not the Responsible researcher.
[Lead presenter: Koki Ichimaru] We have no financial relationships to disclose.
[Principal Researcher: Kenji Chamoto] ■Research funds from company: イメル創薬、シスメックス株式会社　■Contributions: ユニクロ　■Endowed chair: 明治ホールディングズ、Meiji Seika

▲TOP

### [E-1014] I-E11-2
### Tumor immune microenvironment/Tumor immune escape (1)

2025 / 9 / 25 (Thu) 10:15-11:30 Room 8/(Hotel Nikko Kanazawa 3F Kujaku C) Room 8

[Chairperson]Yoshihiro Hayakawa (Inst. Nat. Med., Univ. Toyama)

### E-1014
### Targeting DRP1 Promotes RT-Induced Antitumor Immunity via mtDNA-Mediated cGAS-STING Axis in KRAS-Mutated Cancer

[Speaker]Kevin Chih-Yang Huang (China Medical University／China Medical University Hospital) [Co-author]Hsin-Yu Chang (China Medical University／National Yang Ming Chiao Tung University)、Wei-Ze Hong (China Medical University／China Medical University Hospital)、Jhen-Yu Chen (China Medical University／China Medical University Hospital)、Yuan-Yao Tsai (China Medical University／China Medical University Hospital)、Tao-Wei Ke (China Medical University／China Medical University Hospital)、Chi-Hsien Huang (China Medical University／China Medical University Hospital)、Yi-Wen Jiang (China Medical University)、K. S. Clifford Chao (China Medical University／China Medical University Hospital)

Oncogenic KRAS causes the immunosuppressive tumor microenvironment (TME) to decrease the therapeutic response to radiotherapy and is associated with poor outcomes. In this study, we observed that constitutive DRP1 phosphorylation by oncogenic KRAS resulted in a decrease in mitochondrial DNA (mtDNA) content, leading to attenuated radiotherapy-induced cGAS/STING-driven type I IFN secretion. Targeting DRP1 phosphorylation enhanced cytosolic mtDNA release to promote cGAS/STING activation in response to radiotherapy. Disruption of cGAS-STING1 signaling limited the immune-enhancing effect of DRP1 targeting on type I IFN production and T-cell killing ability. Notably, the immune-enhancing effects of DRP1 inhibition were impaired by IFN receptor blockade. Moreover, dual targeting of DRP1 and MEK significantly enhanced the therapeutic response to RT in a KRAS-mutated CRC model. These findings reveal that DRP1 phosphorylation-mediated mtDNA decrease is a suppressor of antitumor immunity, suggesting that targeting DRP1 could be used to increase tumor immunogenicity and improve responsiveness to radiotherapy.

[Disclosure of COI by Responsible researcher] Lead presenter is the Responsible researcher.
[Lead presenter: Kevin Chih-Yang Huang] ■ employment by a company related to the presentation: China Medical University

▲TOP

### [E-1015] I-E11-2
### Tumor immune microenvironment/Tumor immune escape (1)

2025 / 9 / 25 (Thu) 10:15-11:30 Room 8/(Hotel Nikko Kanazawa 3F Kujaku C) Room 8

[Chairperson]Yoshihiro Hayakawa (Inst. Nat. Med., Univ. Toyama)

---

### E-1015
### Psychological Stress Promotes HCC Progression via Macrophage-Derived CXCL2

[Speaker]Keita Ogawa (Depart. of Medical Immunology, Grad. Sch. of Med, Chiba Univ.／Depart. of Gastroenterology, Grad. Sch. of Med, Chiba Univ.) [Co-author]Hongxuan Wang (Depart. of Medical Immunology, Grad. Sch. of Med, Chiba Univ.)、Ko Ozaki (Depart. of Medical Immunology, Grad. Sch. of Med, Chiba Univ.)、Katsuhiro Nishimura (Depart. of Medical Immunology, Grad. Sch. of Med, Chiba Univ.)、Takahiro Aoki (Depart. of Medical Immunology, Grad. Sch. of Med, Chiba Univ.)、Mariko Takami (Depart. of Medical Immunology, Grad. Sch. of Med, Chiba Univ.)、Shinichiro Motohashi (Depart. of Medical Immunology, Grad. Sch. of Med, Chiba Univ.)

Background: Mental stress contributes to tumor progression, including hepatocellular carcinoma (HCC), but the mechanism remains unclear. This study investigated how stress alters the HCC tumor microenvironment. Methods: HCC-bearing mice were subjected to restraint stress. Tumor-infiltrating immune cells were analyzed by flow cytometry. RNA was extracted from tumors and neutrophils for transcriptome analysis. Public scRNA-seq data were reanalyzed to identify chemokine-expressing cells. In vitro, macrophages were treated with stress-hormones to assess chemokine expression. Results: Stress enhanced tumor growth, neutrophil infiltration, and ***Cxcl2*** expression. Neutrophils from stressed mice upregulated immunosuppressive genes. Neutrophil depletion suppressed stress-induced tumor growth. scRNA-seq revealed that macrophages expressed neutrophil-recruiting chemokines. Noradrenaline induced CXCL2 via ADRB2 receptors in macrophages. Tumor macrophages expressed higher ***Cxcl2*** than those from other tissues. Conclusion: Stress-induced noradrenaline may enhance HCC growth by increasing CXCL2 production in tumor macrophages. Targeting stress-related pathways may offer new therapeutic strategies.

[Disclosure of COI by Responsible researcher] Lead presenter is not the Responsible researcher.
[Lead presenter: Ogawa Keita] We have no financial relationships to disclose.
[Principal Researcher: Shinichiro Motohashi] We have no financial relationships to disclose.

### [E-1016] I-E11-2
### Tumor immune microenvironment/Tumor immune escape (1)

2025 / 9 / 25 (Thu) 10:15-11:30 Room 8/(Hotel Nikko Kanazawa 3F Kujaku C) Room 8

[Chairperson]Yoshihiro Hayakawa (Inst. Nat. Med., Univ. Toyama)

### E-1016
### Secretary IL-1Ra repressed tumor growth of oral squamous cell carcinoma cells via modulation of macrophage polarization

[Speaker]Ya-Wen Chen (National Institute of Cancer Research, NHRI, Miaoli, Taiwan) [Co-author]Jin-En Pong (National Institute of Cancer Research, NHRI, Miaoli, Taiwan／Institute of Analytic and Environmental Sciences, NTHU, Hsinchu, Taiwan)、Yu-Lin Chen (National Institute of Cancer Research, NHRI, Miaoli, Taiwan)、Ssu-Han Wang (National Institute of Cancer Research, NHRI, Miaoli, Taiwan)、Shine-Gwo Shiah (National Institute of Cancer Research, NHRI, Miaoli, Taiwan)、Shih Sheng Jiang (National Institute of Cancer Research, NHRI, Miaoli, Taiwan)、Ko-Jiunn Liu (National Institute of Cancer Research, NHRI, Tainan, Taiwan)、Hsiu-Chuan Chou (Institute of Analytic and Environmental Sciences, NTHU, Hsinchu, Taiwan)

Clinical data have revealed that low expression of Interleukin-1 Receptor Antagonist (IL-1Ra), encoded by IL1RN, in oral squamous cell carcinoma (OSCC) tissues when compared to nontumor parts. IL-1Ra exists in two main forms: the secreted form (secIL-1Ra) and the intracellular form (icIL-1Ra). In vivo studies demonstrated that OSCC cells, LN1-1, overexpressing secIL-1Ra formed smaller tumors compared to those overexpressing icIL-1Ra or vector control. Interestingly, this effect was not observed in in vitro assays. Tumor-associated immune cell infiltration prediction showed a strong correlation between IL1RN expression and macrophage infiltration. Next, we differentiated THP-1 cells into macrophages and treated with IL-1Ra, followed by M1 and M2 polarization. Assessments via qPCR, western blot, and flow cytometry revealed that IL-1Ra stimulation led to increased expression of M1 markers, such as CD86 and HLA-DR, and decreased expression of M2 markers, including Arginase-1. Our data implied that IL-1Ra inhibits OSCC tumor progression by regulation of macrophage polarization.

[Disclosure of COI by Responsible researcher] Lead presenter is the Responsible researcher.
[Lead presenter: Ya-Wen Chen] We have no financial relationships to disclose.

### [E-1017] I-E11-2
### Tumor immune microenvironment/Tumor immune escape (1)

2025 / 9 / 25 (Thu) 10:15-11:30 Room 8/(Hotel Nikko Kanazawa 3F Kujaku C) Room 8

[Chairperson]Yoshihiro Hayakawa (Inst. Nat. Med., Univ. Toyama)

### E-1017
### Activated platelets adhere to CD8+ T cells via CD62p-PSGL-1 and inhibit their antigen specific tumor cell killing

[Speaker]Sae Nishiguchi (Div. Exp. Chemother., Cancer Chemother. Ctr., JFCR／Dept. CBMS, Grad. Sch. Front. Sci., The Univ. of Tokyo) [Co-author]Masaru Yokomura (Div. Exp. Chemother., Cancer Chemother. Ctr., JFCR)、Seiji Nagano (Lab. Immunol., LiMe, Kyoto Univ.)、Hiroshi Kawamoto (Lab. Immunol., LiMe, Kyoto Univ.)、Satoshi Takagi (Div. Exp. Chemother., Cancer Chemother. Ctr., JFCR)、Ryohei Katayama (Div. Exp. Chemother., Cancer Chemother. Ctr., JFCR／Dept. CBMS, Grad. Sch. Front. Sci., The Univ. of Tokyo)

In the tumor microenvironment, platelets (PLTs) promote cancer malignancy through the release of soluble factors, such as EGF and PDGF, as well as direct interactions with cancer cells. PLTs also facilitate immune evasion by suppressing cytotoxic T lymphocytes (CTLs), which play an essential role in anti-tumor immunity. However, the direct mechanisms of PLT-CTL interaction remain insufficiently understood. In this study, we evaluated the direct effect of PLTs on the antigen-specific cancer-killing activity of $CD8^+$ T cells. To assess this, we used a co-culture system of cancer cells and $CD8^+$ T cells (CTL3-3) derived from iPS cells, which specifically recognize the WT1 antigen presented on the HLA of cancer cells. When PLTs were added to this co-culture system, killing activity of CTL3-3 to cancer cells were suppressed. Evaluation using FCM analysis revealed that activated PLTs, expressing CD62p, adhered to CTL3-3 through PSGL-1. Moreover, PLTs inhibited the killing activity of CTL3-3 via CD62p-PSGL-1 axis without affecting the proliferation of CTL3-3. In conclusion, activated PLTs directly suppress antigen-specific cancer-killing activity of $CD8^+$ T cells.

[Disclosure of COI by Responsible researcher] Lead presenter is not the Responsible researcher.
[Lead presenter: Sae Nishiguchi] We have no financial relationships to disclose.
[Principal Researcher: Ryohei Katayama] ■Patent fee: 栄研化学　■Research expenses: 中外製薬、TOPPAN、UBE、BML

▲TOP

### [E-1018] I-E11-2
### Tumor immune microenvironment/Tumor immune escape (1)

2025 / 9 / 25 (Thu) 10:15-11:30 Room 8/(Hotel Nikko Kanazawa 3F Kujaku C) Room 8

[Chairperson]Yoshihiro Hayakawa (Inst. Nat. Med., Univ. Toyama)

---

### E-1018
### Metabolic environment shapes immune landscape of tumor microenvironment in CNS lymphoma

[Speaker]Haryoon Kim (Div. Mol. Oncol., Natl. Cancer Ctr. Res. Inst., Tokyo, Japan.／Div. Hematol., Dept. Med., Keio Univ. Sch. Med., Tokyo, Japan.) [Co-author]Junji Koya (Div. Mol. Oncol., Natl. Cancer Ctr. Res. Inst., Tokyo, Japan.／Div. Hematol., Dept. Med., Keio Univ. Sch. Med., Tokyo, Japan.)、Nobuyoshi Sasaki (Dept. Neurosurg., Kyorin Univ. Fac. Med., Tokyo, Japan.)、Kota Mizuno (Div. Mol. Oncol., Natl. Cancer Ctr. Res. Inst., Tokyo, Japan.／Div. Hematol., Dept. Med., Keio Univ. Sch. Med., Tokyo, Japan.)、Yuki Saito (Div. Mol. Oncol., Natl. Cancer Ctr. Res. Inst., Tokyo, Japan.)、Mitsuhiro Yuasa (Div. Mol. Oncol., Natl. Cancer Ctr. Res. Inst., Tokyo, Japan.)、Yasunori Kogure (Div. Mol. Oncol., Natl. Cancer Ctr. Res. Inst., Tokyo, Japan.)、Masamichi Takahashi (Dept. Neurosurg., Tokai Univ. Sch. Med., Isehara, Japan.／Dept. Neurosurg. & Neuro-Oncol., Natl. Cancer Ctr. Hosp., Tokyo, Japan.)、Yoshitaka Narita (Dept. Neurosurg. & Neuro-Oncol., Natl. Cancer Ctr. Hosp., Tokyo, Japan.)、Tomoyoshi Soga (Inst. Adv. Biosci., Keio Univ., Yamagata, Japan.)、Akiko Maeshima (Dept. Diag. Path, Natl. Cancer Ctr. Hosp., Tokyo, Japan.)、Daisuke Ennishi (Ctr. Comprehensive Genomic Med., Okayama Univ. Hosp., Okayama, Japan.)、Daisuke Kaji (Dept. Hematol., Toranomon Hosp., Tokyo, Japan.)、Fumihito Tajima (Dept. Hematol. & Oncol., Yonago Med. Ctr., Yonago, Japan.)、Motoo Nagane (Dept. Neurosurg., Kyorin Univ. Fac. Med., Tokyo, Japan.)、Keisuke Kataoka (Div. Mol. Oncol., Natl. Cancer Ctr. Res. Inst., Tokyo, Japan.／Div. Hematol., Dept. Med., Keio Univ. Sch. Med., Tokyo, Japan.)

Although substantial heterogeneity exists in diffuse large B-cell lymphomas (DLBCL), the effect of tissue of origin on tumor microenvironment (TME) remains largely unknown. To address this, we performed single-cell multi-omics profiling, including transcriptomics, surface proteomics, and TCR/BCR repertoire analyses, on nearly 200,000 cells from tumors from 18 DLBCL and 16 central nervous system lymphoma (CNSL). CNSL showed dynamic changes of TME, including more pronounced CD8+ T cell exhaustion and clonal expansion, increased mature NK cells and macrophages, and decreased CD4+ T cells, compared with DLBCL. To confirm the effect of tissue of origin, we developed DLBCL and CNSL mouse models, which successfully recapitulated human TME. In the CNSL model, CD8+ T cell depletion shortened survival, whereas 4-1BB agonist prolonged survival, suggesting the anti-tumor role of CD8+ T cells. Metabolomic profiling of mouse tumors revealed enhanced glycolysis and increased glutarate and lactate levels in CNSL. In vitro exposure to glutarate or lactate induced CD8+ T cell exhaustion. These findings highlight the role of metabolomic environment to shape TME and its therapeutic potential in CNSL.

[Disclosure of COI by Responsible researcher] Lead presenter is not the Responsible researcher.
[Lead presenter: Haryoon Kim] ■Research funds from company: 公的研究費のみ（AMED、学術振興会）
[Principal Researcher: Keisuke Kataoka] ■Research funds from company: 公的研究費のみ（AMED、学術振興会） ■Profit of stock: 朝日ゲノミクス株式会社 ■Lecture fee: 大塚製薬株式会社、Meiji Seika ファルマ株式会社、アッヴィ合同会社 ■Research expenses: Meiji Seika ファルマ株式会社 ■Contributions: エーザイ株式会社、協和キリン株式会社、中外製薬株式会社、大塚製薬株式会社、JCRファーマ株式会社、旭化成ファーマ株式会社

English Oral Session

### [E-1019] I-E4-1
### Oncogenes & tumor-suppressor genes (2)

2025 / 9 / 25 (Thu) 10:15-11:30 Room 9/(Hotel Nikko Kanazawa 3F Lumiere) Room 9

[Chairperson]Shunsuke Kitajima (Cancer Immunotherapy Development, CPM Center, JFCR)

---

### E-1019
### The Non-Senolytic Effects of Dasatinib and Quercetin (D+Q) on Cellular Senescence

[Speaker]Koyu Ito (Dept. of Biochem. & Mol. Biol., Shiga Univ. of Med. Sci.／Res. Inst. for Microbial Diseases, Univ. of Osaka) [Co-author]Masahiro Wakita (Res. Inst. for Microbial Diseases, Univ. of Osaka)、Birte Jung (Res. Inst. for Microbial Diseases, Univ. of Osaka)、Shimpei Kawamoto (Res. Inst. for Microbial Diseases, Univ. of Osaka)、Yuuki Ohara (Nagoya Univ. Grad. Sch. of Med.)、Yumiko Okumura (Res. Inst. for Microbial Diseases, Univ. of Osaka)、Sosuke Nakano (Res. Inst. for Microbial Diseases, Univ. of Osaka)、Yasutoshi Agata (Dept. of Biochem. & Mol. Biol., Shiga Univ. of Med. Sci.)、Shinya Toyokuni (Nagoya Univ. Grad. Sch. of Med.)、Eiji Hara (Res. Inst. for Microbial Diseases, Univ. of Osaka／Immunol. Front. Res. Center, Univ. of Osaka)

Cellular senescence is a state of irreversible cell cycle arrest that can be induced by various potentially oncogenic stimuli, thereby serving as a critical tumor suppression mechanism. Paradoxically, however, excessive accumulation of senescent cells promotes tumorigenesis through the senescence-associated secretory phenotype (SASP), which creates a pro-inflammatory microenvironment. Consequently, the development of senolytic approaches to eliminate senescent cells has become an active research area worldwide. Among the most extensively studied senolytic strategies is the combination therapy of Dasatinib and Quercetin (D+Q). However, contrary to previous reports, our studies demonstrate that D+Q exhibits minimal senolytic activity across various senescent cell types in culture. Instead, we found that D+Q significantly prevents the onset of cellular senescence. While inhibiting the onset of cellular senescence could benefit health by preventing SASP, it also risks tumorigenesis by compromising a critical tumor suppression mechanism. Here, we present evidence supporting this risk of D+Q in cultured cells and mouse models, and elucidate the underlying molecular mechanisms.

[Disclosure of COI by Responsible researcher] Lead presenter is not the Responsible researcher.
[Lead presenter: Koyu Ito] We have no financial relationships to disclose.
[Principal Researcher: Eiji Hara] We have no financial relationships to disclose.

▲TOP

### [E-1020] I-E4-1
### Oncogenes & tumor-suppressor genes (2)

2025 / 9 / 25 (Thu) 10:15-11:30 Room 9/(Hotel Nikko Kanazawa 3F Lumiere) Room 9

[Chairperson]Shunsuke Kitajima (Cancer Immunotherapy Development, CPM Center, JFCR)

---

### E-1020
### Pan-RAS Inhibitors Show Preclinical Efficacy Against Oncogenic NRAS Variants in Melanoma

[Speaker]Sai Fung Yeung (School of Biomedical Sciences, Chinese University of Hong Kong) [Co-author]July Xi Chen (School of Biomedical Sciences, Chinese University of Hong Kong)、Mingo Ming Ho Yuen (Department of Obstetrics Gynaecology, The University of Hong Kong)、William Chi Shing Cho (Department of Clinical Oncology, The Queen Elizabeth Hospital)、Cherie Tze Yau Law (School of Biomedical Sciences, Chinese University of Hong Kong)、Stephen Kwok Wing Tsui (School of Biomedical Sciences, Chinese University of Hong Kong)

BackgroundMelanoma caused ~60,000 deaths in 2022, with NRAS mutations (20-30% of cases) driving aggressive disease and poor response to immune checkpoint blockade (ICB). Current therapies targeting downstream MAPK/PI3K pathways show limited efficacy, underscoring the need for NRAS-directed strategies. Recent breakthroughs in KRAS inhibition highlight therapeutic opportunities for NRAS-mutant melanoma.Methods Using GENIE v15.0, we characterized NRAS mutation profiles and evaluated functional/drug sensitivity effects of Q61L/K/R variants via spheroid formation, growth assays, and Western blotting. Saturation mutagenesis assessed oncogenic activity and inhibitor sensitivity across NRAS hotspots.Results: NRAS mutations concentrated in codons 12,13,59,60,61 (90% at Q61). A59V, Q61L, and Q61R displayed highest oncogenicity. Clinically prevalent Q61K/L/R mutations showed enhanced sensitivity to pan-RAS inhibitors RMC-7977, RMC-6236, and ADT-001, but resistance to G12 inhibitors sotorasib/adagrasib.ConclusionOur findings demonstrate broad sensitivity of NRAS mutants to pan-RAS inhibitors, positioning RMC-7977 and RMC-6236 as promising therapeutic candidates for this high-risk population.

[Disclosure of COI by Responsible researcher] Lead presenter is not the Responsible researcher.
[Lead presenter: Sai Fung Yeung] We have no financial relationships to disclose.
[Principal Researcher: Stephen Kwok Wing Tsui] We have no financial relationships to disclose.

▲TOP

### [E-1021] I-E4-1
### Oncogenes & tumor-suppressor genes (2)

2025 / 9 / 25 (Thu) 10:15-11:30 Room 9/(Hotel Nikko Kanazawa 3F Lumiere) Room 9

[Chairperson]Shunsuke Kitajima (Cancer Immunotherapy Development, CPM Center, JFCR)

---

### E-1021
### S100A2 mediates a survival signal downstream of RUNX3 and p53 in gastric carcinoma cells

[Speaker]Sarah Momtazkari (Cancer Research Institute, Kanazawa Univ.) [Co-author]Jason K. Koo (Cancer Science Institute of Singapore, Natl. Univ. of Singapore)、Shangying Chen (Dept. of Biochem., Sch. of Med., Natl. Univ. of Singapore)、Kenneth HK. Ban (Dept. of Biochem., Sch. of Med., Natl. Univ. of Singapore)、Chiaki Takahashi (Cancer Research Institute, Kanazawa Univ.／Inst. for Frontier Sci. Initiative, Kanazawa Univ.)、Masaharu Hazawa (Inst. for Frontier Sci. Initiative, Kanazawa Univ.／WPI Nano Life Science Inst., Kanazawa Univ.)、Yoshiaki Ito (Cancer Science Institute of Singapore, Natl. Univ. of Singapore)、Dominic C. Voon (Cancer Research Institute, Kanazawa Univ.／Inst. for Frontier Sci. Initiative, Kanazawa Univ.)

RUNX3 and p53 are key tumor suppressors frequently inactivated in gastric cancer through epigenetic silencing and mutation, respectively. Here, the EF-hand protein S100A2 was identified as a cooperative transcriptional target of RUNX3 and p53, most evidently during p53-mediated DNA damage response. Functionally, S100A2 enhanced adaptation to genotoxic stress, promoting proliferation in gastric carcinoma cells. Notably, S100A2 deficiency elevated reactive oxygen species (ROS) levels, indicative of altered OXPHOS and redox homeostasis, leading to oxidative DNA damage. Moreover, RNAi depletion of S100A2 altered cell cycle dynamics and sensitized cells to TGF-$\beta$-induced apoptosis, further implicating a role in stress adaptation. Though cooperatively regulated by two major tumor suppressor, S100A2 appeared to serve a protective role against genotoxic conditions. This paradoxical function identifies S100A2 as a therapeutic target in specific contexts, warranting further investigation into its nuanced role in tumor progression.

[Disclosure of COI by Responsible researcher] Lead presenter is not the Responsible researcher.
[Lead presenter: Sarah Momtazkari] We have no financial relationships to disclose.
[Principal Researcher: Dominic C. Voon] We have no financial relationships to disclose.

### [E-1022] I-E4-1
### Oncogenes & tumor-suppressor genes (2)

2025 / 9 / 25 (Thu) 10:15-11:30 Room 9/(Hotel Nikko Kanazawa 3F Lumiere) Room 9

[Chairperson]Shunsuke Kitajima (Cancer Immunotherapy Development, CPM Center, JFCR)

---

### E-1022
### Wnt-mediated transcriptional activation of ***Igf1*** in intrahepatic cholangiocarcinoma

[Speaker]Meihui Zuo (Grad. Sch. of Med., The Univ. of Tokyo) [Co-author]Kiyoshi Yamaguchi (Inst. of Med. Sci., The Univ. of Tokyo)、Saya Nakagawa (Inst. of Med. Sci., The Univ. of Tokyo)、Kiyoko Takane (Inst. of Med. Sci., The Univ. of Tokyo)、Yoichi Furukawa (Inst. of Med. Sci., The Univ. of Tokyo)

Although aberrant activation of Wnt/β-catenin signaling is a key oncogenic mechanism in a wide range of human cancers, its role in intrahepatic cholangiocarcinoma (ICC) remains poorly understood. We generated AKPP-β-catenin-Δexon3 cells with a deletion of ***Ctnnb1*** exon 3 from mouse ICC (AKPP) cells using the CRISPR-Cas9 system. RNA-seq comparison of AKPP-β-catenin-Δexon3 and parental AKPP cells identified 130 upregulated genes in the ***Ctnnb1***-mutant cells. Among them, we focused on ***Igf1*** due to its marked upregulation. To identify the regulatory region of ***Igf1***, we used ChIP-seq and found two candidate regions in intron 2. Reporter assays demonstrated that one of them, ***Igf1***-2, has enhancer activity, and knockdown of ***Ctnnb1*** or ***Tcf7l2*** significantly reduced reporter activity and ***Igf1*** expression. Furthermore, ChIP-qPCR with anti-β-catenin antibody confirmed interaction between β-catenin and ***Igf1***-2. These data suggest that ***Igf1*** is a direct transcriptional target of Wnt/β-catenin signaling in ICC and highlight a novel regulatory axis potentially contributing to ICC tumorigenesis.

[Disclosure of COI by Responsible researcher] Lead presenter is not the Responsible researcher.
[Lead presenter: Meihui Zuo] We have no financial relationships to disclose.
[Principal Researcher: Yoichi Furukawa] We have no financial relationships to disclose.

### [E-1023] I-E4-1
### Oncogenes & tumor-suppressor genes (2)

2025 / 9 / 25 (Thu) 10:15-11:30 Room 9/(Hotel Nikko Kanazawa 3F Lumiere) Room 9

[Chairperson]Shunsuke Kitajima (Cancer Immunotherapy Development, CPM Center, JFCR)

---

### E-1023
### E3 ubiquitin ligase STUB1 suppresses IFNGR1 expression and accelerates the development of AML

[Speaker]Yangying Hao (Division of Molecular Oncology, The University of Tokyo) [Co-author]Keita Yamamoto (Division of Molecular Oncology, The University of Tokyo)、Taishi Yonezawa (Division of Molecular Oncology, The University of Tokyo)、Takuya Tomita (Division of Protein Metabolism, IMSUT)、Yasushi Saeki (Division of Protein Metabolism, IMSUT)、Toshio Kitamura (Division of Molecular Pharmacology of Malignant Diseases, UTokyo／Foundation for Biomedical Research and Innovation at Kobe)、Susumu Goyama (Division of Molecular Oncology, The University of Tokyo)

STUB1 is an E3 ubiquitin ligase that targets misfolded proteins and IFNGR1 towards proteasomal degradation. Through its TPR domain, STUB1 interacts with heat shock proteins such as HSP70 and HSP90, forming a chaperone-dependent degradation complex. In this study, we found that HSP70 promotes, whereas HSP90 inhibits STUB1-induced ubiquitination. Using a Split-TurboID system, we profiled proteins interacting with the STUB1-HSP70 complex and identified several potential substrates, including components of the innate immune pathway. STUB1 depletion upregulated IFNGR1 in human leukemia cell lines (NOMO1, HEL, K562, etc.) and mouse myeloid leukemia cells driven by MLL-AF9 or ERG/Trp53 deficiency. Furthermore, STUB1 depletion suppressed MLL-AF9-driven leukemogenesis, particularly ***in vivo***. Additionally, we discovered several compounds that inhibit STUB1 function. Our findings establish STUB1 as a checkpoint E3 ligase regulating interferon-gamma signaling and myeloid leukemogenesis. Targeting STUB1-HSP70 may represent a novel therapeutic strategy for myeloid leukemia.

[Disclosure of COI by Responsible researcher] Lead presenter is not the Responsible researcher.
[Lead presenter: Yangying Hao] We have no financial relationships to disclose.
[Principal Researcher: Keita Yamamoto] We have no financial relationships to disclose.

▲TOP

### [E-1024] I-E4-1
### Oncogenes & tumor-suppressor genes (2)

2025 / 9 / 25 (Thu) 10:15-11:30 Room 9/(Hotel Nikko Kanazawa 3F Lumiere) Room 9

[Chairperson]Shunsuke Kitajima (Cancer Immunotherapy Development, CPM Center, JFCR)

---

### E-1024
### Distinct roles of NEDD8-conjugating enzymes in acute myeloid leukemia progression

[Speaker]Takeshi Ueda (Dept. of Biochem., Kindai Univ. Faculty of Med.) [Co-author]Suman Dash (Columbia Univ. Irving Med. Ctr.)、Ryoichi Sugisawa (Dept. of Biochem., Kindai Univ. Faculty of Med.)、Akiyoshi Komuro (Dept. of Biochem., Kindai Univ. Faculty of Med.)、Hitoshi Okada (Dept. of Biochem., Kindai Univ. Faculty of Med.)

Neddylation, a post-translational modification by the ubiquitin-like protein NEDD8, is mediated by a cascade of E1, E2, and E3 enzymes. Among these, the E2 enzymes UBE2F and UBE2M (also known as NEDD8-conjugating enzymes) transfer NEDD8 to substrates in cooperation with E3 ligases. E1 inhibitors have been explored in cancers, including myeloid malignancies, and E2 enzymes are generally known for their oncogenic roles in solid tumors. However, through CRISPR screening, we found that loss of UBE2F function induced cytokine-independent growth and ***in vivo*** expansion of a myeloid precursor cell line, and enhanced the clonogenic potential of bone marrow cells expressing a leukemia oncogene. UBE2F deficiency also abolished neddylation of a Cullin, whose loss similarly induced cytokine independence. Furthermore, UBE2M promoted AML cell line growth, whereas UBE2F suppressed it. Notably, expression levels of UBE2F, UBE2M, and the Cullin correlate with drug sensitivity to an E1 inhibitor, specifically in myeloid cell lines. These findings underscore the tumor-suppressive role of UBE2F in AML and the context-dependent impact of neddylation in cancer.

[Disclosure of COI by Responsible researcher] Lead presenter is the Responsible researcher.
[Lead presenter: Takeshi Ueda] We have no financial relationships to disclose.

▲TOP

### [J-1019] I-J17-2
### Multimodal treatment in surgery

2025 / 9 / 25 (Thu) 10:15-11:30 Room 14/(ANA Crowne Plaza Hotel Kanazawa 3F Zuiun 1) Room 14

[Chairperson]Hirotoshi Kikuchi (2nd Dept. Surg., Hamamatsu Univ. Shc. Med.)

---

### J-1019
### Prognostic significance of MUC1-C expression in lung squamous cell carcinoma

[Speaker]Giacomo Bassi (Graduate School of Medical Sciences, Kyushu University) [Co-author]Tomoyoshi Takenaka (Department of Thoracic Surgery, Kyushu University Hospital)、Kazuki Takada (Department of Thoracic Surgery, Kyushu University Hospital)、Taichi Matsubara (Department of Thoracic Surgery, Kyushu University Hospital)、Fumihiko Kinoshita (Department of Thoracic Surgery, Kyushu University Hospital)、Hiroki Funakoshi (Graduate School of Medical Sciences, Kyushu University)、Takayuki Tokunaga (Graduate School of Medical Sciences, Kyushu University)、Yuya Ono (Graduate School of Medical Sciences, Kyushu University)、Tomoharu Yoshizumi (Graduate School of Medical Sciences, Kyushu University)

Background: MUC1-C is a mucin associated with inflammatory response and cell proliferation. MUC1-C overexpression was reported to be correlated with poor prognosis and immune evasion in various cancers. However, the role of MUC1-C in lung squamous cell carcinoma (LUSC) remains poorly understood.Methods: 199 tissue specimens were collected from the patients who underwent surgical resection for LUSC. IHC staining was conducted to evaluate MUC1-C protein and tumor infiltrating immune cells expression. Results: Low MUC1-C expression was observed in 105 and high in 94 patients, and it was correlated to smoking history ($p = 0.029$). MUC1-C was associated with higher FOXP3+ cell count ($p < 0.001$). No correlation was observed between MUC1-C and PD-L1 expression. Recurrence-free survival (RFS) and overall survival were significantly worse in patients with high MUC1-C expression ($p=0.013$ and $p=0.021$). Multivariate analysis showed that high MUC1-C expression was an independent risk factor for RFS (HR 1.52, 95% CI 1.01-2.30, $p = 0.047$).Conclusion: MUC1-C overexpression in LUSC correlates with poor prognosis and it may influence immune cell populations within the tumor microenvironment.

[Disclosure of COI by Responsible researcher] Lead presenter is not the Responsible researcher.
[Lead presenter: Giacomo Bassi] We have no financial relationships to disclose.
[Principal Researcher: Tomoyoshi Takenaka] We have no financial relationships to disclose.

### [J-1020] I-J17-2
### Multimodal treatment in surgery

2025 / 9 / 25 (Thu) 10:15-11:30 Room 14/(ANA Crowne Plaza Hotel Kanazawa 3F Zuiun 1) Room 14

[Chairperson]Hirotoshi Kikuchi (2nd Dept. Surg., Hamamatsu Univ. Shc. Med.)

---

### J-1020
### Organ-specific Prognostic Impact of Checkpoint Inhibitor Therapy in Patients with Metastatic Esophageal cancer

[Speaker]Kazuto Harada (Dept of GE Surg, Kumamoto Univ) [Co-author]Keisuke Kosumi (Dept of GE Surg, Kumamoto Univ)、Hiroki Tsubakihara (Dept of GE Surg, Kumamoto Univ)、Chihiro Matsumoto (Dept of GE Surg, Kumamoto Univ)、Kohei Yamashita (Dept of GE Surg, Kumamoto Univ)、Kojiro Eto (Dept of GE Surg, Kumamoto Univ)、Satoshi Ida (Dept of GE Surg, Kumamoto Univ)、Kota Arima (Dept of GE Surg, Kumamoto Univ)、Yukiharu Hiyoshi (Dept of GE Surg, Kumamoto Univ)、Yuji Miyamoto (Dept of GE Surg, Kumamoto Univ)、Masaaki Iwatsuki (Dept of GE Surg, Kumamoto Univ)

Purpose: Immune checkpoint inhibitors (ICIs) have become a first-line treatment for esophageal squamous cell carcinoma (ESCC) . This study evaluated the prognostic impact of metastatic sites in ESCC patients treated with first-line ICI plus FP therapy.Methods: We retrospectively analyzed 75 patients with ESCC who received ICI+FP between Dec 2021 and Oct 2024. Prognosis was compared by metastatic organ.Results: Median age was 66, with 60 males. Thirty-five had unresectable disease; 40 had recurrence. Tumor sites included Ce (n=6), Ut (n=9), Mt (n=41), and Lt (n=19). Best response: CR 14, PR 31, SD 14, PD 12. Metastatic sites (overlapping): lymph nodes (n=55), lung (n=13), liver (n=12), bone (n=10), local (n=7), others (n=10). Overall median survival time (MST) was 605 days; 2-year survival rate was 45%. By site: lymph node and lung MST 734 days, 56%; liver 284 days, 0%; bone 171 days, 0%; others 277 days, 0%. Liver or bone metastasis correlated with worse outcomes (MST 272 vs 899 days; 0% vs 57%, $p<0.01$).Conclusion: ICI+FP therapy showed favorable real-world efficacy, but liver and bone metastases predicted poor prognosis, indicating the need for tailored strategies.

[Disclosure of COI by Responsible researcher] Lead presenter is the Responsible researcher.
[Lead presenter: Kazuto Harada] We have no financial relationships to disclose.

▲TOP

### [J-1021] I-J17-2
### Multimodal treatment in surgery

2025 / 9 / 25 (Thu) 10:15-11:30 Room 14/(ANA Crowne Plaza Hotel Kanazawa 3F Zuiun 1) Room 14

[Chairperson]Hirotoshi Kikuchi (2nd Dept. Surg., Hamamatsu Univ. Shc. Med.)

---

### J-1021
### HER2 overexpression is a biomarker of conversion surgery for initially unresectable EGJ adenocarcinoma

[Speaker]Yu Imamura (Department of Gastroenterological Surgery, The Cancer Institute Hospital of JFCR) [Co-author]Souya Nunobe (Department of Gastroenterological Surgery, The Cancer Institute Hospital of JFCR)、Naoki Takahashi (Department of Gastroenterological Surgery, The Cancer Institute Hospital of JFCR)、Masayoshi Terayama (Department of Gastroenterological Surgery, The Cancer Institute Hospital of JFCR)、Hiroki Ishida (Department of Gastroenterological Surgery, The Cancer Institute Hospital of JFCR)、Takashi Kato (Department of Gastroenterological Surgery, The Cancer Institute Hospital of JFCR)、Akihiko Okamura (Department of Gastroenterological Surgery, The Cancer Institute Hospital of JFCR)、Jun Kanamori (Department of Gastroenterological Surgery, The Cancer Institute Hospital of JFCR)、Masayuki Watanabe (Department of Gastroenterological Surgery, The Cancer Institute Hospital of JFCR)

Background: The indications for conversion therapy in EGJ adenocarcinoma remain unclear.Methods: Initially unresectable Siewert type I-II EGJ adenocarcinoma at our institution between 2012 and 2022 were enrolled (N=71). Results: Eleven cases (15.5 %) underwent conversion surgery, while 56 cases remained unresected ( 4 cases were transhiatal resections ). Unresectable factors were non-regional lymph node metastases (LNM) in 8 patients (#16: 5 cases, #106pre: 1, #104L: 1, #8p: 1), bilobar liver metastases in 2, and CY1 in one. Trastuzumab-containing regimens were used in 5 of the 8 patients with non-regional LNM. The median interval from initial chemotherapy to surgery was 7.1 months (range, 2.9-30). 3yr-overall survival (OS) was significantly higher in the conversion vs. unresected group (81.8 % vs. 10.9 %, P<0.0001) . Notably, all five HER2-positive patients who underwent conversion surgery remain alive at a mean postoperative follow-up of 38.5 months (range, 9-80).Conclusions: In patients with initially unresectable EGJ adenocarcinoma, conversion surgery after a favorable response to trastuzumab-based chemotherapy-especially may markedly improve prognosis.

[Disclosure of COI by Responsible researcher] Lead presenter is the Responsible researcher.
[Lead presenter: Yu Imamura] We have no financial relationships to disclose.

### [J-1022] I-J17-2
### Multimodal treatment in surgery

2025 / 9 / 25 (Thu) 10:15-11:30 Room 14/(ANA Crowne Plaza Hotel Kanazawa 3F Zuiun 1) Room 14

[Chairperson]Hirotoshi Kikuchi (2nd Dept. Surg., Hamamatsu Univ. Shc. Med.)

---

### J-1022
### Impact of surgical resection on outcomes in patients with BR1 hepatocellular carcinoma

[Speaker]Koichiro Haruki (Department of Surgery, The Jikei University School of Medicine) [Co-author]Kenei Furukawa (Department of Surgery, The Jikei University School of Medicine)、Mitsuru Yanagaki (Department of Surgery, The Jikei University School of Medicine)、Tomohiko Taniai (Department of Surgery, The Jikei University School of Medicine)、Munetoshi Akaoka (Department of Surgery, The Jikei University School of Medicine)、Yoshihiro Shirai (Department of Surgery, The Jikei University School of Medicine)、Yoshiaki Tanji (Department of Surgery, The Jikei University School of Medicine)、Michinori Matsumoto (Department of Surgery, The Jikei University School of Medicine)、Yuto Yamahata (Department of Surgery, The Jikei University School of Medicine)、Takeshi Gocho (Department of Surgery, The Jikei University School of Medicine)、Toru Ikegami (Department of Surgery, The Jikei University School of Medicine)

Background: With advances in systemic therapy for hepatocellular carcinoma (HCC), treatment strategies for BR1 cases defined by the Expert Consensus 2023 needs reconsideration.Methods: We analyzed 325 patients who underwent primary hepatic resection for HCC (2008 to 2023) to evaluate the prognostic impact of factors defining BR1 classification and treatment strategies for both primary and recurrent HCC. Results: R/BR1/BR2 classifications comprised 279/31/15 cases with 5-year survival rates of 82%/62%/50%. BR1 defining factors: tumor size (65%), tumor number (13%), vascular invasion (16%), extrahepatic disease (13%). Only extrahepatic disease showed significant survival differences (p=0.03). For multiple tumors, RFA combined with resection in 3 cases. Median survival: resection only (7.7 years), resection+RFA/TACE (3.2 years), resection+systemic therapy (0.7 years). Recurrence treatments: resection (11%), RFA (11%), systemic/radiotherapy (17%), TACE (50%), with TACE showing worse survival.Conclusions: For multiple tumors, resection+RFA appears acceptable, with locoregional approaches improving survival. For recurrent tumors, systemic therapy may offer better outcomes than TACE.

[Disclosure of COI by Responsible researcher] Lead presenter is the Responsible researcher.
[Lead presenter: Koichiro Haruki] We have no financial relationships to disclose.

▲TOP

### [J-1023] I-J17-2
### Multimodal treatment in surgery

2025 / 9 / 25 (Thu) 10:15-11:30 Room 14/(ANA Crowne Plaza Hotel Kanazawa 3F Zuiun 1) Room 14

[Chairperson]Hirotoshi Kikuchi (2nd Dept. Surg., Hamamatsu Univ. Shc. Med.)

---

### J-1023
### Treatment outcomes of lower rectal cancer with TNT and evaluation about comprehensive analysis for total RNA sequencing

[Speaker]Tadanobu Shimura (Dept of GIP Surg, Mie Univ) [Co-author]Takahito Kitajima (Dept of GIP Surg, Mie Univ／Dept of Genomic medicine, Mie Univ Hosp)、Ruiya Ma (Dept of GIP Surg, Mie Univ)、Yoshinaga Okugawa (Dept of GIP Surg, Mie Univ／Dept of Genomic medicine, Mie Univ Hosp)、Yuji Toiyama (Dept of GIP Surg, Mie Univ)

[Background and aims]: Accumulating evidence show that total neoadjuvant therapy (TNT) has been shown to improve oncological outcome and enable non-operative management (NOM) for advanced lower rectal cancer patients. Herein we assessed the clinical outcomes of TNT, and we investigated the intermediate results for comprehensive analysis of total RNA sequencing (seq) using pre-treatment biopsy specimens.
[Results] We assessed 91 patients who completed TNT from Jan.2018 to Dec.2023. During a median follow-up of 36 months (7-69 months), NOM was achieved in 36.6% among candidate patients. The recurrence free survival rates at 1 and 3 years were 100% and 91%. Furthermore, our total RNA seq using clinical complete-remission (cCR) cases and non-responder (NR) cases (n=3, cStageIIa, respectively) revealed that expression of 653 targets was significantly dysregulated ($p<0.05$) between cCR and NR groups. Then the gene ontology enrichment analysis showed that immune response related terms obviously differed between groups.
[Conclusion]: Our TNT regimen showed favorable clinical outcomes, and we expect to further develop our molecular based approach to detect cases with aggressive phenotypes.

[Disclosure of COI by Responsible researcher] Lead presenter is the Responsible researcher.
[Lead presenter: Tadanobu Shimura] We have no financial relationships to disclose.

### [J-1024] I-J17-2
### Multimodal treatment in surgery

2025 / 9 / 25 (Thu) 10:15-11:30 Room 14/(ANA Crowne Plaza Hotel Kanazawa 3F Zuiun 1) Room 14

[Chairperson]Hirotoshi Kikuchi (2nd Dept. Surg., Hamamatsu Univ. Shc. Med.)

---

### J-1024
### Long-term outcomes of perioperative chemotherapy with capecitabine plus oxaliplatin for locally advanced rectal cancer

[Speaker]Takayoshi Kishida (Dept of Surg, Nagoya Univ Grad Sch of Med) [Co-author]Shinichi Umeda (Dept of Surg, Nagoya Univ Grad Sch of Med)、Dai Shimizu (Dept of Surg, Nagoya Univ Grad Sch of Med)、Norifumi Hattori (Dept of Surg, Nagoya Univ Grad Sch of Med)、Masamichi Hayashi (Dept of Surg, Nagoya Univ Grad Sch of Med)、Mitsuro Kanda (Dept of Surg, Nagoya Univ Grad Sch of Med)

Objective. The aim of this analysis was to evaluate two multicenter clinical phase II trials of pre-and postoperative chemotherapy (CORONAI) and postoperative chemotherapy (CORONAII) with capecitabine plus oxaliplatin (CAPOX) for locally advanced rectal cancer. Methods. Patients received 4cycles of CAPOX therapy before and after curative surgical resection in CORONAI study and 8cycles after surgery in CORONAII study. A total of 81 patients (CORONAI,41;CORONAII,40) were enrolled between June 2012 and November 2015. We compared tumor characteristics, R0 resection rate, postoperative complication and postoperative outcomes between two trials. Results. CONONAI study included more advanced cases compared to CORONAII study (cT4b:27vs.3%;p<0.01,cN positive:76vs.47%;p=0.012). The proportion of R0 resection and postoperative complications (Clavien-Dindo classification Grade 2 and higher) were 90% and 45% in CORONA I, respectively, compared with 97% and 32% in CORONA II. Five-year RFS and OS rates were 76% and 80% in CORONAI and 73% and 91% in CORONAII.Conclusions. The CAPOX therapy could be feasible as perioperative and postoperative adjuvant setting for the locally advanced rectal cancer.

[Disclosure of COI by Responsible researcher] Lead presenter is not the Responsible researcher.
[Lead presenter: Takayoshi Kishida] We have no financial relationships to disclose.
[Principal Researcher: Mitsuro Kanda] We have no financial relationships to disclose.

### [J-1031] II-J2-2
### Gastric cancer, eophageal cancer

2025 / 9 / 25 (Thu) 10:15-11:30 Room 15/(ANA Crowne Plaza Hotel Kanazawa 3F Zuiun 2) Room 15

[Chairperson]Kazuo Yasumoto (Dept. Med. Oncol., Kanazawa Med. Univ., Sch. Med.)

---

### J-1031
### Single Cell RNA- and ATAC-seq Revealed Aberrant Molecular Features of Intestinal Metaplasia and SPEM

[Speaker]Chihiro Takeuchi (Dept. of Epigenomics, Hoshi Univ.／Dept. of Gastroenterology, The Univ. of Tokyo Hosp.) [Co-author]Yuyu Liu (Dept. of Epigenomics, Hoshi Univ.)、Hideyuki Takeshima (Dept. of Epigenomics, Hoshi Univ.)、Mitsuhiro Fujishiro (Dept. of Gastroenterology, The Univ. of Tokyo Hosp.)、Toshikazu Ushijima (Dept. of Epigenomics, Hoshi Univ.)

Intestinal metaplasia (IM) is a premalignant lesion that shares morphological features with the small intestine, but it remains unclear whether IM cells retain the molecular features of gastric cells at the single-cell level and whether they have additional ones. We performed single-cell RNA- and ATAC-seq on gastric mucosa with IM and small intestinal mucosa. IM cells exhibited gene expression patterns similar to small intestinal cells but retained gastric gene expression at the single-cell level. A fraction of IM cells showed distinct gene expression patterns from gastric and small intestinal cells, such as high expression levels of ***REG4*** and ***BBOX1*** genes. IM cells also had open chromatin at master regulator genes of both the stomach (***SOX2***) and the small intestine (***CDX2*** and ***PDX1***), which was considered to contribute to the hybrid gene expression in IM cells. In a mouse model, SPEM also had open chromatin at both gastric and intestinal regulator genes. These findings indicate that IM cells had hybrid gene expression and chromatin features at the single-cell level, and possess gene expression not present in either the stomach or small intestine, which may be involved in carcinogenesis.

[Disclosure of COI by Responsible researcher] Lead presenter is not the Responsible researcher.
[Lead presenter: Chihiro Takeuchi] We have no financial relationships to disclose.
[Principal Researcher: Toshikazu Ushijima] We have no financial relationships to disclose.

### [J-1032] II-J2-2
### Gastric cancer, eophageal cancer

2025 / 9 / 25 (Thu) 10:15-11:30 Room 15/(ANA Crowne Plaza Hotel Kanazawa 3F Zuiun 2) Room 15

[Chairperson]Kazuo Yasumoto (Dept. Med. Oncol., Kanazawa Med. Univ., Sch. Med.)

### J-1032
### Gene expression profile of organoids from residual esophageal squamous cell carcinoma after neoadjuvant chemotherapy

[Speaker]Yoshiyuki Tsukamoto (Dept. Mol. Path., Facult. Med., Oita Univ.) [Co-author]Shusaku Kurogi (Dept. Mol. Path., Facult. Med., Oita Univ.)、Tomotaka Shibata (Dept. Gastroenterol. and Pediatr. Surg., Facult. Med., Oita Univ.)、Shoichi Fumoto (Dept., Surg., Oita Nakamura Hosp.)、Yuka Hirashita (Dept. Gastroenterol., Facult. Med., Oita Univ.)、Chisato Nakada (Dept. Mol. Path., Facult. Med., Oita Univ.)、Masafumi Inomata (Dept. Gastroenterol. and Pediatr. Surg., Facult. Med., Oita Univ.)、Masatsugu Moriyama (Dept. Mol. Path., Facult. Med., Oita Univ.)、Naoki Hijiya (Dept. Mol. Path., Facult. Med., Oita Univ.)

Neoadjuvant chemotherapy (NAC) followed by surgery is a standard form of management for advanced esophageal squamous cell carcinoma (ESCC). While patients who achieve a complete response show favorable outcomes, those who do not respond well have a poor prognosis. To investigate the mechanism of NAC resistance, we established organoids from post-NAC residual (postNAC-Os; 10 lines) and NAC-naive (25 lines) tissues. When capability of 10 or more passages was defined as success, the success rate was 22%. Gene expression profiles were generated and subjected to gene set enrichment analysis (GSEA). GSEA showed that the hallmark gene set "HYPOXIA" was enriched in postNAC-Os (NES=2.31, FDR<0.001), suggestive of hypoxic adaptation despite normoxia in ***in vitro*** culture. In contrast, gene sets for MYC, G2M and E2F were downregulated in postNAC-Os. Furthermore, paired organoids derived from a single patient before and after NAC demonstrated that the postNAC-Os had higher tumorigenicity and enhanced expression of hypoxia-related genes (Fuchino et al., ***Human Cell*** 2024). Thus, our data suggest that intrinsic activation of the hypoxia response might be a therapeutic target for NAC-resistant ESCCs.

[Disclosure of COI by Responsible researcher] Lead presenter is the Responsible researcher.
[Lead presenter: Yoshiyuki Tsukamoto] We have no financial relationships to disclose.

### [J-1033] II-J2-2
### Gastric cancer, eophageal cancer

2025 / 9 / 25 (Thu) 10:15-11:30 Room 15/(ANA Crowne Plaza Hotel Kanazawa 3F Zuiun 2) Room 15

[Chairperson]Kazuo Yasumoto (Dept. Med. Oncol., Kanazawa Med. Univ., Sch. Med.)

---

### J-1033
### Clinicopathological and genomic features of extrachromosomal DNA in gastric cancer

[Speaker]Yukio Hokazono (Div. of Cancer Genomics, Natl. Cancer Ctr. Res. Inst.／Dept. of Pathology, The Univ. of Tokyo) [Co-author]Mihoko Adachi (Div. of Cancer Genomics, Natl. Cancer Ctr. Res. Inst.／Lab. of Mol. Med., The Univ. of Tokyo)、Natsuko Hama (Div. of Cancer Genomics, Natl. Cancer Ctr. Res. Inst.)、Yasushi Totoki (Div. of Cancer Genomics, Natl. Cancer Ctr. Res. Inst.／Dept. of Cancer Genome Informatics, Osaka Univ.)、Hiromi Nakamura (Div. of Cancer Genomics, Natl. Cancer Ctr. Res. Inst.)、Yasuhito Arai (Div. of Cancer Genomics, Natl. Cancer Ctr. Res. Inst.)、Akihiko Fukagawa (Div. of Cancer Genomics, Natl. Cancer Ctr. Res. Inst.／Dept. of Pathology, The Univ. of Tokyo)、Shinichi Yachida (Dept. of Cancer Genome Informatics, Osaka Univ.)、Hirofumi Rokutan (Tokyo Met. Hosp. and Inst. of Gerontol.)、Tetsuo Ushiku (Dept. of Pathology, The Univ. of Tokyo)、Tatsuhiro Shibata (Div. of Cancer Genomics, Natl. Cancer Ctr. Res. Inst.／Lab. of Mol. Med., The Univ. of Tokyo)

Background: Extrachromosomal DNA (ecDNA) is a common cause of oncogene amplification. It was reported that ecDNA is associated with cancer progression and specific genomic situations. However, in gastric cancer, the clinicopathological characteristics and genomic mechanism of ecDNA remain poorly understood. Method: Using WGS data of 81 Japanese gastric cancer samples, we performed computational analysis to detect ecDNA by AmpliconArchitect. Results: First, we revealed that the frequency of ecDNA is comparable between cancer stages and has a modest impact on prognosis. Second, several immunomodulatory genes were amplified on ecDNA, and ecDNA with immunomodulatory genes contributed to suppression of cytotoxic T cell immune response. Third, we confirmed that ecDNA-positive cases have more frequently ***TP53*** mutation and MMR-p than ecDNA-negative cases. Finally, in ecDNA-positive cases, SBS signature 17 was more frequently observed, indicating that oxidative stress due to gastric acid was associated with ecDNA genesis in gastric cancer. Conclusion: These results give us detailed information about the clinicopathological and genomic features of ecDNA in gastric cancer.

[Disclosure of COI by Responsible researcher] Lead presenter is not the Responsible researcher.
[Lead presenter: Yukio Hokazono] We have no financial relationships to disclose.
[Principal Researcher: Tatsuhiro Shibata] We have no financial relationships to disclose.

### [J-1034] II-J2-2
### Gastric cancer, eophageal cancer

2025 / 9 / 25 (Thu) 10:15-11:30 Room 15/(ANA Crowne Plaza Hotel Kanazawa 3F Zuiun 2) Room 15

[Chairperson]Kazuo Yasumoto (Dept. Med. Oncol., Kanazawa Med. Univ., Sch. Med.)

---

### J-1034
### Prognostic significance of c-JUN and its functional suppression by FBXW7 in gastrointestinal stromal tumors

[Speaker]Kohei Yamashita (Dept. of Gastroenterol. Surg., Grad. Sch. Med. Sci., Kumamoto Univ.) [Co-author]Xiyu Wu (Dept. of Gastroenterol. Surg., Grad. Sch. Med. Sci., Kumamoto Univ.)、Weiliyun Zhang (Dept. of Gastroenterol. Surg., Grad. Sch. Med. Sci., Kumamoto Univ.)、Hiroki Tsubakihara (Dept. of Gastroenterol. Surg., Grad. Sch. Med. Sci., Kumamoto Univ.)、Chihiro Matsumoto (Dept. of Gastroenterol. Surg., Grad. Sch. Med. Sci., Kumamoto Univ.)、Keisuke Kosumi (Dept. of Gastroenterol. Surg., Grad. Sch. Med. Sci., Kumamoto Univ.)、Kojiro Eto (Dept. of Gastroenterol. Surg., Grad. Sch. Med. Sci., Kumamoto Univ.)、Kazuto Harada (Dept. of Gastroenterol. Surg., Grad. Sch. Med. Sci., Kumamoto Univ.)、Satoshi Ida (Dept. of Gastroenterol. Surg., Grad. Sch. Med. Sci., Kumamoto Univ.)、Yuji Miyamoto (Dept. of Gastroenterol. Surg., Grad. Sch. Med. Sci., Kumamoto Univ.)、Masaaki Iwatsuki (Dept. of Gastroenterol. Surg., Grad. Sch. Med. Sci., Kumamoto Univ.)

Background: c-Jun, a key transcriptional activator, functions by promoting cell survival in various cancers. This study aimed to evaluate its prognostic significance in gastrointestinal stromal tumors (GISTs) and to elucidate its regulation by FBXW7. Methods: c-Jun expression was assessed in 167 GIST cases using immunohistochemistry and analyzed in relation to clinicopathological features. The FBXW7-c-Jun regulatory mechanism was examined through in vitro and in vivo experiments. Results: High c-Jun expression was observed in 47 cases (28.1%) and was significantly associated with poorer overall and recurrence-free survival compared to low expression cases (n = 120, 71.9%; both $p < 0.001$). Multivariate analysis identified c-Jun as an independent prognostic factor for 10-year overall and recurrence-free survival (both $p < 0.001$). FBXW7 directly targeted c-Jun to suppress GIST cell proliferation, invasion, and migration. FBXW7 knockdown-induced c-Jun upregulation was confirmed in xenograft and metastasis models. Additionally, c-Jun was upregulated in post-imatinib tumor samples. Conclusions: c-Jun is a potential prognostic biomarker and FBXW7 regulates its oncogenic roles in GIST.

[Disclosure of COI by Responsible researcher] Lead presenter is not the Responsible researcher.
[Lead presenter: Kohei Yamashita] ■Endowed chair: 熊本大学消化器外科消化器癌先端治療開発学寄附講座
[Principal Researcher: Masaaki Iwatsuki] We have no financial relationships to disclose.

### [J-1035] II-J2-2
### Gastric cancer, eophageal cancer

2025 / 9 / 25 (Thu) 10:15-11:30 Room 15/(ANA Crowne Plaza Hotel Kanazawa 3F Zuiun 2) Room 15

[Chairperson]Kazuo Yasumoto (Dept. Med. Oncol., Kanazawa Med. Univ., Sch. Med.)

---

### J-1035
### Tumor stroma-derived Thrombospondin-1 contributes to lung metastasis by modulating immune activity in gastric cancer

[Speaker]Naoki Aoyama (Kyoto Univ. Dept. of Gastroenterology & Hepatology) [Co-author]Yuki Nakanishi (Kyoto Univ. Dept. of Gastroenterology & Hepatology)、Kento Yasumura (Kyoto Univ. Dept. of Gastroenterology & Hepatology)、Ryo Yokota (Kyoto Univ. Dept. of Gastroenterology & Hepatology)、Munehiro Ikeda (Kyoto Univ. Dept. of Gastroenterology & Hepatology)、Yoko Masui (Kyoto Univ. Dept. of Gastroenterology & Hepatology)、Jiayu Chen (Kyoto Univ. Dept. of Gastroenterology & Hepatology)、Kosuke Iwane (Kyoto Univ. Dept. of Gastroenterology & Hepatology)、Mayuki Omatsu (Kyoto Univ. Dept. of Gastroenterology & Hepatology)、Yu Muta (Kyoto Univ. Dept. of Gastroenterology & Hepatology)、Hiroaki Kasashima (Osaka Metropolitan Univ. Dept. of Surg.)、Akihisa Fukuda (Kyoto Univ. Dept. of Gastroenterology & Hepatology)、Hiroshi Seno (Kyoto Univ. Dept. of Gastroenterology & Hepatology)

Background: Gastric cancer often metastasizes to the lung at advanced stages, but the role of stromal molecules remains unclear. We previously identified thrombospondin-1 (THBS1) as a key regulator of the tumor immune microenvironment in colorectal cancer. Methods: We examined THBS1 expression and its link to immune phenotypes via bioinformatics and histology. Mouse gastric cancer cells were injected subcutaneously or intravenously into wild-type and Thbs1 knockout (Thbs1-/-) mice. Tumors were analyzed by immunohistochemistry and RNA sequencing. Results: THBS1 was highly expressed in cancer-associated fibroblasts and monocyte-like cells, correlating with poor prognosis. THBS1 inhibition suppressed lung metastasis and prolonged survival. CD8+ T cell infiltration increased in both primary tumors and lung metastases in Thbs1-/- mice. Monocyte/macrophage-specific THBS1 knockout also reduced lung metastasis, suggesting monocyte-like cells as a main THBS1 source. RNA-seq showed enhanced immune activity in Thbs1-/- tumors. Conclusion: Monocyte-like cell-derived THBS1 promotes lung metastasis of gastric cancer by suppressing antitumor immunity. Targeting THBS1 may offer a new strategy.

[Disclosure of COI by Responsible researcher] Lead presenter is not the Responsible researcher.
[Lead presenter: Naoki Aoyama] We have no financial relationships to disclose.
[Principal Researcher: Yuki Nakanishi] We have no financial relationships to disclose.

▲TOP

### [J-1036] II-J2-2
### Gastric cancer, eophageal cancer

2025 / 9 / 25 (Thu) 10:15-11:30 Room 15/(ANA Crowne Plaza Hotel Kanazawa 3F Zuiun 2) Room 15

[Chairperson]Kazuo Yasumoto (Dept. Med. Oncol., Kanazawa Med. Univ., Sch. Med.)

---

### J-1036
### Risk of Recurrence and Use of Adjuvant Nivolumab After Esophagectomy: A Retrospective Review of 39 Cases

[Speaker]Yuma Obata (Dept. of Surg., Nagoya Univ. Hosp.) [Co-author]Dai Shimizu (Dept. of Surg., Nagoya Univ. Hosp.)、Mitsuro Kanda (Dept. of Surg., Nagoya Univ. Hosp.)、Masamichi Hayashi (Dept. of Surg., Nagoya Univ. Hosp.)、Norifumi Hattori (Dept. of Surg., Nagoya Univ. Hosp.)、Shinichi Umeda (Dept. of Surg., Nagoya Univ. Hosp.)

CheckMate 577 has made adjuvant nivolumab (adj-Nivo) available option for esophageal squamous cell carcinoma (ESCC). However, its efficacy and safety in patients who underwent neoadjuvant chemotherapy (NAC) remains lacking.We analyzed 332 ESCC patients who underwent curative resection after NAC between 2006 and 2020. 97 patients experienced recurrence (Rec), and 102 patients remained Rec-free for over 5-year. Rec risk factors were analyzed, and clinical outcomes of 39 patients who received adj-Nivo were evaluated.Fisher's exact test identified ly, v, tumor size, pT, pN, pStage and preoperative SCC as significant predictors of Rec. However, logistic regression analysis did not identify any independent significant predictors of Rec. Among the patients who received adj-Nivo, 9 completed the therapy, 10 discontinued due to recurrence, 13 due to irAEs, and 1 due to performance status decline. irAEs occurred in 14 patients, and hepatic disorder was the most frequently observed (n=11). Patients who experienced irAEs tended to have better prognoses.In real-world clinical practice, careful patient selection based on a risk-benefit assessment is essential when considering adj-Nivo.

[Disclosure of COI by Responsible researcher] Lead presenter is not the Responsible researcher.
[Lead presenter: Yuma Obata] We have no financial relationships to disclose.
[Principal Researcher: Mitsuro Kanda] We have no financial relationships to disclose.

### [J-1037] I-J13-1
### Signal transduction inhibitors

2025 / 9 / 25 (Thu) 10:15-11:30 Room 16/(11F Visage) Room 16

[Chairperson]Kazuko Sakai (Dept. of Genome Biol., Kindai Univ. Faculty of Med.)

### J-1037
### Proximity biotinylation based analysis of EGFR interacting protein changes induced by EGFR-targeted drugs

[Speaker]Kohdai Yamada (PROS, Ehime University) [Co-author]Tatsuya Sawasaki (PROS, Ehime University)

EGFR is a critical signaling mediator in many epithelial cancers. Abnormalities in EGFR cause cells to become cancerous, so that EGFR have long been considered an important cancer drug target. EGFR-targeted tyrosine kinase inhibitors (TKIs) exert their effects by inhibiting EGFR phosphorylation. However, whether intracellular PPI (Protein-protein interaction) changes caused by EGFR TKIs differ between TKIs and cell types has not been tested in depth. Furthermore, in the extracellular domain of the EGFR, TKI-induced changes of PPIs have not been analysed at all. In this study, we demonstrate a new approach using a proximity biotinylation enzyme AirID fusion EGFR or anti-EGFR fragment of antigen (Fab) to analyse proximity PPI changes of EGFR under extracellular and intracellular conditions. Using these approaches, we succeeded in identifying dynamic EGFR interactiong proteins changes caused by Gefitinib or Osimertinib extracellular and intracellular. These results indicate that AirID provides a useful PPI analysis of the EGFR caused by TKI responses on the plasma membrane and provide important information for future TKI development.

[Disclosure of COI by Responsible researcher] Lead presenter is the Responsible researcher.
[Lead presenter: Kohdai Yamada] We have no financial relationships to disclose.

▲TOP

### [J-1038] I-J13-1
### Signal transduction inhibitors

2025 / 9 / 25 (Thu) 10:15-11:30 Room 16/(11F Visage) Room 16

[Chairperson]Kazuko Sakai (Dept. of Genome Biol., Kindai Univ. Faculty of Med.)

### J-1038
### Loss of BMF enhances drug tolerant persister cell survival in ALK-positive lung cancer

[Speaker]Takahiro Utsumi (Div. Exp. Chemother., Cancer Chemother. Ctr., JFCR／Dep. Respiratory Med., Grad. Sch. Med. Sci., Kyushu Univ.)
[Co-author]Hiroto Azuma (Dept. CBMS, Grad. Sch. Front. Sci., Univ. of Tokyo)、Ken Uchibori (Dept. Thoracic Med. Oncology, Cancer Inst. Hosp., JFCR)、Makoto Nishio (Dept. Thoracic Med. Oncology, Cancer Inst. Hosp., JFCR)、Isamu Okamoto (Dep. Respiratory Med., Grad. Sch. Med. Sci., Kyushu Univ.)、Ryohei Katayama (Div. Exp. Chemother., Cancer Chemother. Ctr., JFCR／Dept. CBMS, Grad. Sch. Front. Sci., Univ. of Tokyo)

ALK-positive non-small cell lung cancer (NSCLC) responds well to ALK inhibitors (ALKi), but acquired resistance often causes relapse, with some cases recurring early. While drug-tolerant persister (DTP) cells are implicated in resistance, mechanisms of DTP survival remain unclear. We conducted a genome-wide CRISPR/Cas9 screen using ALKi-sensitive, patient-derived cells and identified loss of BMF (Bcl-2 modifying factor) as a top candidate associated with increased DTP cell populations. BMF promotes apoptosis by antagonizing anti-apoptotic proteins. We found that BMF expression was transcriptionally increased within 24 hours of ALKi treatment or dual ERK and PI3K inhibition. In contrast, BMF depletion reduced apoptosis and increased DTP cell populations under ALKi exposure. Notably, inhibition of MCL-1 restored impaired apoptosis and effectively eliminated cancer cells lacking BMF. These findings suggest that BMF plays a critical role in ALKi-induced apoptosis, and that its loss promotes DTP cell survival, thereby reducing therapeutic efficacy. Combining MCL-1 inhibitors with ALKi may overcome this resistance and provide a potential therapeutic strategy for ALK-positive NSCLC.

[Disclosure of COI by Responsible researcher] Lead presenter is not the Responsible researcher.
[Lead presenter: Takahiro Utsumi] We have no financial relationships to disclose.
[Principal Researcher: Ryohei Katayama] ■Research funds from company: 中外製薬、TOPPAN、UBE、日本化薬　■Patent fee: 栄研化学

### [J-1039] I-J13-1
### Signal transduction inhibitors

2025 / 9 / 25 (Thu) 10:15-11:30 Room 16/(11F Visage) Room 16

[Chairperson]Kazuko Sakai (Dept. of Genome Biol., Kindai Univ. Faculty of Med.)

---

### J-1039
### Combination of avutometinib and MRTX1133 synergistically suppresses cell growth in KRAS$^{G12D}$-mutated pancreatic cancer

[Speaker]Mano Horinaka (Dept. Drug Discov. Med., Kyoto Pref. Univ. Med.) [Co-author]Ema Toyokuni (Dept. Drug Discov. Med., Kyoto Pref. Univ. Med.／Dept. Endocrinol. Metab., Kyoto Pref. Univ. Med.)、Akihiro Yoshimura (Dept. Drug Discov. Med., Kyoto Pref. Univ. Med.／Dept. Pulm. Med., Kyoto Pref. Univ. Med.)、Emi Nishimoto (Dept. Drug Discov. Med., Kyoto Pref. Univ. Med.)、Michiaki Fukui (Dept. Endocrinol. Metab., Kyoto Pref. Univ. Med.)、Toshiyuki Sakai (Dept. Drug Discov. Med., Kyoto Pref. Univ. Med.)

Pancreatic ductal adenocarcinoma (PDAC) is a lethal malignancy with a poor prognosis, and over 90% of PDAC cases involve activating mutations in the oncogene KRAS. Among several molecular-targeted drugs, avutometinib (CH5126766/RO5126766/VS-6766), a novel dual RAF/MEK clamp, shows promise for patients with KRAS-mutated cancers. However, its efficacy as a single agent remains insufficient, highlighting the need for more effective treatment strategies. In this study, we found that combining avutometinib with the KRAS$^{G12D}$ inhibitor MRTX1133 demonstrated synergistic inhibitory effects on cell growth against pancreatic cancer cells with KRAS$^{G12D}$ mutation. We further investigated the efficacy of this combination therapy in ***in vitro*** and ***in vivo*** experiments. The combination therapy upregulated BIM, downregulated survivin, and induced apoptosis in pancreatic cancer cells with the KRAS$^{G12D}$ mutation. In ***in vivo*** experiments, the combination therapy markedly delayed tumor growth compared to either therapy alone. Therefore, the combination of avutometinib and MRTX1133 may represent a promising therapeutic approach for KRAS$^{G12D}$-mutated pancreatic cancer.

[Disclosure of COI by Responsible researcher] Lead presenter is the Responsible researcher.
[Lead presenter: Mano Horinaka] ■Research funds from company: ENT M Dr.浅野登＆暉子基金医学基礎研究助成事業

▲TOP

### [J-1040] I-J13-1
### Signal transduction inhibitors

2025 / 9 / 25 (Thu) 10:15-11:30 Room 16/(11F Visage) Room 16

[Chairperson]Kazuko Sakai (Dept. of Genome Biol., Kindai Univ. Faculty of Med.)

---

### J-1040
### Involvement of aberrant GSK3β in malignant properties of pancreatic cancer with acquired resistance to gemcitabine.

[Speaker]Takahiro Domoto (Div. Innov. Cancer Cont. Res., Cancer Res. Inst., Kanazawa Univ.) [Co-author]Satoshi Takenaka (Dept. Surg., Toyama City Hosp.)、Tomoharu Miyashita (Dept. Surg., Toyama City Hosp.)、Toshinari Minamoto (Dept. Mol. Cell. Pathol., Kanazawa Univ.／JCHO Kanazawa Hosp.)、Hiroaki Taniguchi (Div. Innov. Cancer Cont. Res., Cancer Res. Inst., Kanazawa Univ.)

Pancreatic cancer (PC) is characterized by high invasive capacity and therapy resistance, particularly acquired resistance to gemcitabine (GEM). These pathological properties presumably share cancer stemness and represent major causes of therapy failure. In this study, we investigated the potential link between acquired chemoresistance, invasion, and stemness in GEM-resistant PC cells and explored the underlying shared mechanisms, focusing on the tumor-promoting role of glycogen synthase kinase (GSK)3β. Compared to the parental PC cells, GEM-resistant clones showed higher invasive capacity and increased sphere formation, both associated with STAT3 activation. GSK3β inhibition synergistically enhanced the effect of GEM, and reduced stemness and invasion via STAT3 inactivation. ***In vivo***, administration of GSK3β inhibitor substantially enhanced GEM efficacy against the orthotopic xenografts of the resistant clone and its peritoneal metastases in mice. These findings suggest that GSK3β connects invasion and stemness through the STAT3-mediated pathway in GEM-resistant PC, underscoring its potential as a therapeutic target.

[Disclosure of COI by Responsible researcher] Lead presenter is the Responsible researcher.
[Lead presenter: Takahiro Domoto] We have no financial relationships to disclose.

▲TOP

### [J-1041] I-J13-1
### Signal transduction inhibitors

2025 / 9 / 25 (Thu) 10:15-11:30 Room 16/(11F Visage) Room 16

[Chairperson]Kazuko Sakai (Dept. of Genome Biol., Kindai Univ. Faculty of Med.)

---

### J-1041
### CBP/p300, histone acetyltransferase, regulate PROTAC efficacy by acetylating histones

[Speaker]Rikuto Honda (Graduate School of Pharmaceutical Sciences, Hoshi University／ Institute for Advanced Life Sciences, Hoshi University)
[Co-author]Fumiaki Ohtake (Graduate School of Pharmaceutical Sciences, Hoshi University／ Institute for Advanced Life Sciences, Hoshi University)

CBP/p300 promotes gene expression by acetylating histones and facilitating the binding of bromodomain (BD)-containing transcription factors (TFs) to acetylated histones. Inhibition of BD-TFs like BRD4 and SMARCA2 induces apoptosis. PROTAC which have two different ligands for a specific ubiquitin E3 ligase and for a target protein degrades target proteins by harnessing the ubiquitin-proteasome system. However, the mechanism of PROTAC is not fully understood. In this study, we investigated the role of CBP/p300 in modulating PROTAC efficacy. We found that CBP/p300 inhibition enhances PROTAC-mediated degradation of BRD4, BRD9, and SMARCA2, leading to increased cancer cell death. RNA-seq analysis showed that co-treatment significantly enhanced the downregulation of oncogenes and upregulation of p21. Furthermore, CBP-deficient cells showed stronger PROTAC efficacy than wild-type cells. Finally, in a mouse xenograft model, combination treatment significantly reduced tumor volume compared to monotherapies. In conclusion, we revealed CBP/p300 regulate activity of a wide range of PROTACs and identified CBP/p300 as a potential biomarker for cancer treatment by PROTACs.

[Disclosure of COI by Responsible researcher] Lead presenter is the Responsible researcher.
[Lead presenter: Rikuto Honda] We have no financial relationships to disclose.

▲TOP

### [J-1042] I-J13-1
### Signal transduction inhibitors

2025 / 9 / 25 (Thu) 10:15-11:30 Room 16/(11F Visage) Room 16

[Chairperson]Kazuko Sakai (Dept. of Genome Biol., Kindai Univ. Faculty of Med.)

---

### J-1042
### Mechanism of Hand-Foot Syndrome Induced by Cancer Chemotherapy and Development of Therapeutic Approaches

[Speaker]Sachiko Tanaka (Tokyo Univ. Pharm. & Life Sci. Dept. Clin. Pharmacol.) [Co-author]Jun Utsumi (Tokyo Univ. Pharm. & Life Sci. Dept. Clin. Pharmacol.)、Hisanori Shimizu (Cancer Inst. Hosp. JFCR. Dept. Pharmacy)、Tsuyoshi Aoyama (Cancer Inst. Hosp. JFCR. Dept. Pharmacy)、Hiroaki Maegouchi (Tokyo Univ. Pharm. & Life Sci. Dept. Clin. Pharmacol.)、Miu Hosoda (Tokyo Univ. Pharm. & Life Sci. Dept. Clin. Pharmacol.)、Karen Itoh (Tokyo Univ. Pharm. & Life Sci. Dept. Clin. Pharmacol.)、Kenji Onda (Tokyo Univ. Pharm. & Life Sci. Dept. Clin. Pharmacol.)、Takashi Yokokawa (Tokyo Univ. Pharm. & Life Sci. Dept. Clin. Pharmacol.)、Kenichi Suzuki (Tokyo Univ. Pharm. & Life Sci. Dept. Clin. Pharmacol.)

Hand-foot syndrome/hand-foot skin reaction (HFS/HFSR) is a common serious side effect of anti-cancer drugs, occurring in 60 to 80% of cases, however, its mechanisms remain unknown. This study focused on the transcriptome analysis in normal skin keratinocytes. The cell proliferation was suppressed to 40% when treated with capecitabine (CP, 2mM) or regorafenib (REG, 20μM). The top 500 downregulated genes were analyzed with Gene Ontology-Biological Process (Metascape), revealing that epidermis development (GO:0008544) ranked highest for both drugs. This is not only anti-cancer effect but also correlates with the side effects of impairing skin development. Among GO-BP(GO:0008544) related genes, 54 were for CP, 41 for REG, with 15 cornified envelope related genes shared between both, were identified as potential targets of epidermal barrier dysfunction of HFS/HFSR pathology. Drug screening revealed that Kampo medicines, Jumi-Haidoku-To (JHT) and Shofu-San (SFS), counteract relevant GO-BP and upregulate 13 of 15 genes. These results suggest repair and maintenance of the epidermal envelope as a novel option for HFS/HFSR topical treatment with JHT, SFS and their shared herbal components.

[Disclosure of COI by Responsible researcher] Lead presenter is the Responsible researcher.
[Lead presenter: Sachiko Tanaka] ■Research funds from company: ティア・リサーチ・コンサルティング合同会社

▲TOP

### CS Associate Editors' Meeting

CS Associate Editors' Meeting

2025 / 9 / 25 (Thu) 11:40-12:40 Room 1/(Ishikawa Ongakudo 2F Concert Hall) Room 1

No Abstract

### [LS1] LS1

### Janssen Pharmaceutical K.K. / Treatment strategies for multiple myeloma ~ Considering the immune environment~

2025 / 9 / 25 (Thu) 11:50-12:40 Room 3/(Hotel Nikko Kanazawa 4F Tsuru A) Room 3
[]Janssen Pharmaceutical K.K.

[Chairperson]Hiroyuki Takamatsu (Department of Hematology/Professor, Faculty of Transdisciplinary Sciences for Innovation, Institute of Transdisciplinary Sciences for Innovation, Kanazawa University)

---

### LS1

### Treatment strategies for multiple myeloma ~ Considering the immune environment~

[Speaker]Hideto Tamura (Division of Diabetes, Endocrinology and Hematology, Department of Internal Medicine, Dokkyo Medical University Saitama Medical Center)

No Abstract

▲TOP

### [LS2] LS2

### Nippon Becton Dickinson Company, Ltd. / A novel flow cytometry for analyzing the immunosuppressive tumor microenvironment

2025 / 9 / 25 (Thu) 11:50-12:40 Room 4/(Hotel Nikko Kanazawa 4F Tsuru B) Room 4
[]Nippon Becton Dickinson Company, Ltd.

[Chairperson]Osamu Takeuchi (Department of Medical Chemistry, Graduate School of Medicine, Kyoto University)

---

### LS2

### A novel flow cytometry for analyzing the immunosuppressive tumor microenvironment

[Speaker]Hiroyoshi Nishikawa (Division of Cancer Immunology, Research Institute ／ EPOC, National Cancer Center ／ Division of Cancer Immune Multicellular System Regulation, Center for Cancer Immunotherapy and Immunobiology, Kyoto University Graduate School of Medicine ／ Department of Immunology, Nagoya University Graduate School of Medicine)

No Abstract

### [LS3-1] LS3

### Twist Bioscience / Evolutions in Sequencing Technologies and Genomic Analysis Supporting Cancer Research

2025 / 9 / 25 (Thu) 11:50-12:40 Room 5/(Hotel Nikko Kanazawa 4F Tsuru C) Room 5
[]Twist Bioscience

[Chairperson]Motohiro Kato (Department of Prdiatrics, The University of Tokyo Hospital)

---

### LS3-1
### 1) Genetic analysis of hematological malignancies by targeted sequencing using custom panels

[Speaker]Kenichi Yoshida (Division of Cancer Evolution, National Cancer Center Japan)

No Abstract

▲TOP

### [LS3-2] LS3

### Twist Bioscience / Evolutions in Sequencing Technologies and Genomic Analysis Supporting Cancer Research

2025 / 9 / 25 (Thu) 11:50-12:40 Room 5/(Hotel Nikko Kanazawa 4F Tsuru C) Room 5
[]Twist Bioscience

[Chairperson]Motohiro Kato (Department of Prdiatrics, The University of Tokyo Hospital)

---

### LS3-2
### 2) Evolving Genomic Technologies and Their Potential

[Speaker]Motohiro Kato (Department of Prdiatrics, The University of Tokyo Hospital)

No Abstract

▲TOP

### [LS4] LS4

### Abbott Japan LLC / Molecular Mechanisms of Laminin γ2 Monomer in Liver Cancer: Pathological Relevance and Future Perspectives

2025 / 9 / 25 (Thu) 11:50-12:40 Room 6/(Hotel Nikko Kanazawa 3F Kujaku A) Room 6
[]Abbott Japan LLC

[Chairperson]Taro Yamashita (Department of Gastroenterology, Kanazawa University Graduate School of Medical Sciences)

---

### LS4

### Molecular Mechanisms of Laminin γ2 Monomer in Liver Cancer: Pathological Relevance and Future Perspectives

[Speaker]Kouki Nio (Innovative Clinical Research Center, Kanazawa University Hospital)

No Abstract

▲TOP

### [LS5-1] LS5

### TOMY DIGITAL BIOLOGY CO., LTD. / Latest Perspective in Cancer Immunotherapy Research using Long-Read Sequencing

2025 / 9 / 25 (Thu) 11:50-12:40 Room 7/(Hotel Nikko Kanazawa 3F Kujaku B) Room 7
[]TOMY DIGITAL BIOLOGY CO., LTD.

[Chairperson]Kotoe Katayama (Laboratory of Sequence Analysis, Human Genome Center, Institute of Medical Science, University of Tokyo)

---

### LS5-1
### 1) Transcriptome Analysis using PacBio Long-Read Sequencing

[Speaker]Miwako Kitazume (TOMY Digital Biology Co., Ltd.)

No Abstract

### [LS5-2] LS5

### TOMY DIGITAL BIOLOGY CO., LTD. / Latest Perspective in Cancer Immunotherapy Research using Long-Read Sequencing

2025 / 9 / 25 (Thu) 11:50-12:40 Room 7/(Hotel Nikko Kanazawa 3F Kujaku B) Room 7
[]TOMY DIGITAL BIOLOGY CO., LTD.

[Chairperson]Kotoe Katayama (Laboratory of Sequence Analysis, Human Genome Center, Institute of Medical Science, University of Tokyo)

---

### LS5-2

### 2) Cancer Immunotherapy Targeting Neoantigens: Discovery of New Therapeutic Targets using Long-Read Sequencing

[Speaker]Kazuma Kiyotani (Laboratory of Immunogenomics, Center for Intractable Diseases and ImmunoGenomics, National Institutes of Biomedical Innovation, Health and Nutrition (NIBN).)

No Abstract

### [LS6] LS6

### Cytek Japan Corporation / Multidimensional Analysis of Tumor Immunity Using Full-Spectrum Flow Cytometry

2025 / 9 / 25 (Thu) 11:50-12:40 Room 8/(Hotel Nikko Kanazawa 3F Kujaku C) Room 8
[]Cytek Japan Corporation

[Chairperson]Edmond Chua (Cytek Biosciences)

---

### LS6

### Diving Deep: Profiling Exhausted T Cells in the Tumor Microenvironment Using Full Spectrum Flow Cytometry

[Speaker]Samantha Ho (Merck Sharp & Dohme (MSD), Singapore)

No Abstract

### [LS7] LS7
### MSD K.K. / Role of Macrophages and Correlation with PD-L1 in Cancer Immunity

2025 / 9 / 25 (Thu) 11:50-12:40 Room 9/(Hotel Nikko Kanazawa 3F Lumiere) Room 9
[]MSD K.K.

[Chairperson]Yasuhiko Nishioka (Department of Respiratory Medicine and Rheumatology, Graduate School of Biomedical Sciences, Tokushima University)

---

### LS7
### Role of Macrophages and Correlation with PD-L1 in Cancer Immunity

[Speaker]Yoshihiro Komohara (Department of Cell Pathology, Graduate School of Medical Science, Kumamoto University)

No Abstract

▲TOP

### [LS8] LS8

### AstraZeneca K.K. / Is Molecular-Targeted Therapy Necessary for Unresectable Stage III NSCLC? — Clinical Considerations from the LAURA Regimen

2025 / 9 / 25 (Thu) 11:50-12:40 Room 11/(ANA Crowne Plaza Hotel Kanazawa 3F Ohtori (West)) Room 11
[]AstraZeneca K.K.

[Chairperson]Shigeki Nanjo (Department of Respiratory Medicine, Kanazawa University Hospital )

---

### LS8

### Is Molecular-Targeted Therapy Necessary for Unresectable Stage III NSCLC? — Clinical Considerations from the LAURA Regimen

[Speaker]Akito Hata (Division of Thoracic Oncology, Kobe Minimally Invasive Cancer Center)

No Abstract

### [LS9] LS9
### CyberomiX Inc. / Clinical potential of Spatial Transcriptome Analysis for Cancer Genomics

2025 / 9 / 25 (Thu) 11:50-12:40 Room 12/(ANA Crowne Plaza Hotel Kanazawa 3F Ohtori (Central)) Room 12
[]CyberomiX Inc.

[Chairperson]Akira Watanabe (CyberomiX Inc.)

---

### LS9
### Clinical potential of Spatial Transcriptome Analysis for Cancer Genomics

[Speaker]Hiroshi Nishihara (Keio University)

No Abstract

### [LS10] LS10

### Eisai Co., Ltd. / The forefront of new drug discovery: Drug discovery innovation through collaboration between doctors and companies

2025 / 9 / 25 (Thu) 11:50-12:40 Room 13/(ANA Crowne Plaza Hotel Kanazawa 3F Ohtori (East)) Room 13
[]Eisai Co., Ltd.

[Chairperson]Takashi Seto (NHO Kyushu Cancer Center, Thoracic Oncology)

---

### LS10

### The future of biliary tract cancer treatment based on cancer genomic medicine -The discovery of FGFR2 fusion genes in Japan and the path to the launch of Tasurgratinib-

[Speaker]Tatsuhiro Shibata (Laboratory of Molecular Medicine, The Institute of Medical Science, The University of Tokyo ／ Division of Cancer Genomics, National Cancer Center Research Institute)

No Abstract

Luncheon Seminars

### [LS11-1] LS11
### Leica Microsystems K.K. / Innovation in 3D Immune Analysis

2025 / 9 / 25 (Thu) 11:50-12:40 Room 14/(ANA Crowne Plaza Hotel Kanazawa 3F Zuiun 1) Room 14
[]Leica Microsystems K.K.

[Chairperson]Shintaro Tanaka (Leica Microsystems K.K.)

---

### LS11-1
### 1) Modeling Tumor Microenvironment Factors Driving Cancer Malignancy

[Speaker]Hiroko OSHIMA (Cancer Research Institute, Kanazawa University)

No Abstract

### [LS11-2] LS11
### Leica Microsystems K.K. / Innovation in 3D Immune Analysis

2025 / 9 / 25 (Thu) 11:50-12:40 Room 14/(ANA Crowne Plaza Hotel Kanazawa 3F Zuiun 1) Room 14
[]Leica Microsystems K.K.

[Chairperson]Shintaro Tanaka (Leica Microsystems K.K.)

---

### LS11-2
### 2) Proposal of SpectraPlex, a Novel Technology of 3D Multiplex Imaging and AIVIA, an AI-Powered Image Analysis Software, for Cancer Immuno-Microenvironment Research

[Speaker]Toshiyuki Hatano (Leica Microsystems K.K.)

No Abstract

### [LS12-1] LS12

### Oxford Nanopore Technologies / Unlock Transformative Cancer Insights: The power of multi-omics Oxford Nanopore sequencing.

2025 / 9 / 25 (Thu) 11:50-12:40 Room 15/(ANA Crowne Plaza Hotel Kanazawa 3F Zuiun 2) Room 15
[]Oxford Nanopore Technologies

[Chairperson]Ken Osaki (Oxford Nanopore Technologies)

---

### LS12-1
### 1) A compendium of human RNA structures and modifications

[Speaker]Yasuhiro Murakawa (Kyoto University)

No Abstract

▲TOP

### [LS12-2] LS12

### Oxford Nanopore Technologies / Unlock Transformative Cancer Insights: The power of multi-omics Oxford Nanopore sequencing.

2025 / 9 / 25 (Thu) 11:50-12:40 Room 15/(ANA Crowne Plaza Hotel Kanazawa 3F Zuiun 2) Room 15
[]Oxford Nanopore Technologies

[Chairperson]Ken Osaki (Oxford Nanopore Technologies)

---

### LS12-2

### 2) Expanding the potential of multi-omics analysis: The latest advancements in Oxford Nanopore sequencing

[Speaker]Rie Yamashige (Oxford Nanopore Technologies)

No Abstract

### [SS5-1] SS5
### Exit-Oriented Drug Discovery Technology from Academia

2025 / 9 / 25 (Thu) 12:50-15:20 Room 1/(Ishikawa Ongakudo 2F Concert Hall) Room 1

[Chairpersons]Akira Yokoi (Eisai Co., Ltd.)、Katsuya Tsuchihara (Exploratory Oncology Research and Clinical Trial Center, National Cancer Center Japan)

Professor Hajime Okamoto served as the President of the 28th Annual Meeting of the Japanese Cancer Association (1969) and was the founding Director of the Cancer Research Institute at Kanazawa University. He initiated the development of the immunostimulatory anticancer agent OK-432 (Picibanil) through his discovery of the anticancer effects of streptococcal bacteria. In commemoration of Professor Okamoto's achievements, this symposium will focus on academia-driven drug discovery based on novel modalities. Researchers who are advancing the development of anticancer agents through unique academic discoveries and technologies-leveraging clear exit strategies via industry-academia collaborations or academic spin-off ventures-will present their work. The symposium also aims to pave the way for a new era of academia-led drug discovery.

---

### SS5-1
### "Kanazawa Medicine of the World" - a story of Hajime Okamoto, ***S. pyogenes***, RNA effect, and anti-cancer drug OK-432

[Speaker]Hiroshi Yamamoto (President, Komatsu Univ.)

An Inspection Party from GHQ witnessed, in Kanazawa in 1947, two research teams working as if war had not occurred, and purportedly declared "Japan will revive". One team, led by Professor Masaru Kuru, clarified human nociceptive pathways. The other, led by Professor Hajime Okamoto, discovered that RNA can induce ***S. pyogenes*** production of hemolysin. This finding was the first demonstration of biologic activity of RNA, being reported in 1939, 5 years earlier than Avery's description about the genic nature of DNA. There are similarities between the two. First, Okamoto employed ***S. pyogenes*** and Avery ***S. pneumoniae***, respectively, throughout their lifetimes. Secondly, both focused on the host defense system. Avery identified CRP in ***S. pneumoniae***-infected animals. In the 1960's, Okamoto developed the anti-cancer drug "OK-432" from ***S. pyogenes***. He received the Japan Academy Prize in 1957, founded Kanazawa University Cancer Research Institute in 1967. In his address as Dean of School of Medicine in 1962, he said; "this school should attract talent to establish Kanazawa Medicine of the World". It must be said that it was Hajime Okamoto himself who most contributed to this aspiration.

[Disclosure of COI by Responsible researcher] Lead presenter is the Responsible researcher.
[Lead presenter: Hiroshi Yamamoto] We have no financial relationships to disclose.

▲TOP

### [SS5-2] SS5
### Exit-Oriented Drug Discovery Technology from Academia

2025 / 9 / 25 (Thu) 12:50-15:20 Room 1/(Ishikawa Ongakudo 2F Concert Hall) Room 1

[Chairpersons]Akira Yokoi (Eisai Co., Ltd.)、Katsuya Tsuchihara (Exploratory Oncology Research and Clinical Trial Center, National Cancer Center Japan)

Professor Hajime Okamoto served as the President of the 28th Annual Meeting of the Japanese Cancer Association (1969) and was the founding Director of the Cancer Research Institute at Kanazawa University. He initiated the development of the immunostimulatory anticancer agent OK-432 (Picibanil) through his discovery of the anticancer effects of streptococcal bacteria. In commemoration of Professor Okamoto's achievements, this symposium will focus on academia-driven drug discovery based on novel modalities. Researchers who are advancing the development of anticancer agents through unique academic discoveries and technologies-leveraging clear exit strategies via industry-academia collaborations or academic spin-off ventures-will present their work. The symposium also aims to pave the way for a new era of academia-led drug discovery.

---

### SS5-2
### Paradigm shift in antibody drug conjugates

[Speaker]Yasuiro Matsumura (RIN Institute Inc.)

Currently, 14 types of ADCs have been approved by the FDA, and more than 80 types are in clinical development or preclinical stages, making ADCs a major modality in cancer treatment. On the other hand, membrane proteins of epithelial cancer cells are also expressed to some extent in normal epithelial stem cells, causing adverse events such as interstitial pneumonia.We have developed an antibody-drug conjugate (ADC) that specifically binds to insoluble fibrin present in the tumor stroma, contains a linker that is selectively cleaved by plasmin, uniformly distributes anticancer agents in tumor tissue, and does not cause toxicity in normal tissue. We named this therapy Cancer Stromal Targeting (CAST) therapy. Meanwhile, both basic and clinical data have demonstrated that the antitumor effect of ADCs does not depend on internalization into cancer cells. ADC remaining in the cancer stroma releases its payload through cancer stroma enzymes, attacking the cancer from outside the cancer cells, representing a drastic change.In this presentation, I will focus on CAST therapy and discuss issues faced by venture companies in Japan through my experience in antibody drug development.

[Disclosure of COI by Responsible researcher] Lead presenter is the Responsible researcher.
[Lead presenter: Yasuiro Matsumura] ■ employment by a company related to the presentation: 株式会社凜研究所　■Profit of stock: 15%　■Research expenses: AMED革新がん事業

▲TOP

### [SS5-3] SS5
### Exit-Oriented Drug Discovery Technology from Academia

2025 / 9 / 25 (Thu) 12:50-15:20 Room 1/(Ishikawa Ongakudo 2F Concert Hall) Room 1

[Chairpersons]Akira Yokoi (Eisai Co., Ltd.)、Katsuya Tsuchihara (Exploratory Oncology Research and Clinical Trial Center, National Cancer Center Japan)

Professor Hajime Okamoto served as the President of the 28th Annual Meeting of the Japanese Cancer Association (1969) and was the founding Director of the Cancer Research Institute at Kanazawa University. He initiated the development of the immunostimulatory anticancer agent OK-432 (Picibanil) through his discovery of the anticancer effects of streptococcal bacteria. In commemoration of Professor Okamoto's achievements, this symposium will focus on academia-driven drug discovery based on novel modalities. Researchers who are advancing the development of anticancer agents through unique academic discoveries and technologies-leveraging clear exit strategies via industry-academia collaborations or academic spin-off ventures-will present their work. The symposium also aims to pave the way for a new era of academia-led drug discovery.

---

### SS5-3
### Clinical development of next-generation CAR-T technologies originated from academic institutions

[Speaker]Koji Tamada (Yamaguchi University Graduate School of Medicine)

Genetically engineered immune cell therapies are progressing worldwide and anticipated as the next-generation cancer immunotherapy. CAR-T cells are among the most advanced modalities, which have demonstrated an excellent effect on hematopoietic malignancies such as B-cell acute lymphocytic leukemia and multiple myeloma, leading to clinical approval in many countries including Japan. Current CAR-T cell therapy, however, harbors many hurdles to be overcome, including a necessity of target molecules specifically expressed on cancer cells, occurrence of severe adverse events, and tumor relapse after remission. Another major challenge is that CAR-T cell therapy has not yet demonstrated sufficient therapeutic efficacy against solid tumors. We have been engaged in the development of novel CAR-T cells that overcome these hurdles by adding various technological improvements to CAR structure, and some of these have been evaluated in clinical trials. In this lecture, representative examples of these next-generation CAR-T technologies will be introduced.

[Disclosure of COI by Responsible researcher] Lead presenter is the Responsible researcher.
[Lead presenter: Koji Tamada] ■ employment by a company related to the presentation: ノイルイミューン・バイオテック株式会社
■Profit of stock: ノイルイミューン・バイオテック株式会社

### [SS5-4] SS5
### Exit-Oriented Drug Discovery Technology from Academia

2025 / 9 / 25 (Thu) 12:50-15:20 Room 1/(Ishikawa Ongakudo 2F Concert Hall) Room 1

[Chairpersons]Akira Yokoi (Eisai Co., Ltd.)、Katsuya Tsuchihara (Exploratory Oncology Research and Clinical Trial Center, National Cancer Center Japan)

Professor Hajime Okamoto served as the President of the 28th Annual Meeting of the Japanese Cancer Association (1969) and was the founding Director of the Cancer Research Institute at Kanazawa University. He initiated the development of the immunostimulatory anticancer agent OK-432 (Picibanil) through his discovery of the anticancer effects of streptococcal bacteria. In commemoration of Professor Okamoto's achievements, this symposium will focus on academia-driven drug discovery based on novel modalities. Researchers who are advancing the development of anticancer agents through unique academic discoveries and technologies-leveraging clear exit strategies via industry-academia collaborations or academic spin-off ventures-will present their work. The symposium also aims to pave the way for a new era of academia-led drug discovery.

---

### SS5-4
### Transforming Undruggable Targets into Therapeutic Opportunities with xFOREST RNA-Targeted Discovery Platform

[Speaker]Shunnichi Kashida (xFOREST Therapeutics)

RNA-targeted small molecule (rSM) drug discovery is emerging as a promising alternative to protein-targeted approaches. The approval of Evrysdi, the first rSM drug, as a blockbuster therapy for SMA highlighted the potential of this field. rSM discovery is now advancing into oncology. Rgenta is conducting a Phase 1 trial of RGT-61159, targeting c-MYB mRNA decay (NCT06462183). However, many candidates are identified via phenotypic screens without validated RNA-binding mechanisms. Known RNA-binding compounds often suffer from low selectivity, weak affinity, and cytotoxicity. xFOREST Therapeutics addresses these challenges with its proprietary FOREST technology platform. MatrixFOREST enables high-throughput biochemical profiling of thousands of RNA motifs and 100,000 compounds, generating base-resolution interaction data for hit discovery and SAR acquisition. SpliceVerse allows multiplexed splicing modulator evaluation with nucleotide-level MoA profiles. In addition to collaborations with major pharmaceutical companies, xFOREST is advancing in-house lead discovery programs, including oncology targets, to pioneer next-generation rSM therapeutics.

[Disclosure of COI by Responsible researcher] Lead presenter is the Responsible researcher.
[Lead presenter: Shunnichi Kashida] ■ employment by a company related to the presentation: 株式会社イクスフォレストセラピューティクス

### [SS5-5] SS5
### Exit-Oriented Drug Discovery Technology from Academia

2025 / 9 / 25 (Thu) 12:50-15:20 Room 1/(Ishikawa Ongakudo 2F Concert Hall) Room 1

[Chairpersons]Akira Yokoi (Eisai Co., Ltd.)、Katsuya Tsuchihara (Exploratory Oncology Research and Clinical Trial Center, National Cancer Center Japan)

Professor Hajime Okamoto served as the President of the 28th Annual Meeting of the Japanese Cancer Association (1969) and was the founding Director of the Cancer Research Institute at Kanazawa University. He initiated the development of the immunostimulatory anticancer agent OK-432 (Picibanil) through his discovery of the anticancer effects of streptococcal bacteria. In commemoration of Professor Okamoto's achievements, this symposium will focus on academia-driven drug discovery based on novel modalities. Researchers who are advancing the development of anticancer agents through unique academic discoveries and technologies-leveraging clear exit strategies via industry-academia collaborations or academic spin-off ventures-will present their work. The symposium also aims to pave the way for a new era of academia-led drug discovery.

---

### SS5-5
### Targeted protein degradation by IAP-based PROTACs and anti-cancer drug discovery

[Speaker]Mikihiko Naito (TPD, Grad. Sch. Pharma. Sci., Univ. Tokyo)

Targeted protein degradation by PROTACs is a novel modality for drug development. We have been developed IAP-based PROTACs, named SNIPERs, to induce degradation of various oncogenic proteins, including estrogen receptors, androgen receptors, BCR-ABL and so on. SNIPERs recruit IAP ubiquitin ligases to target proteins and induce poly-ubiquitylation and degradation. The first-generation SNIPERs contain bestatin as an IAP binder module and recruit cIAP1 for degradation. In the second-generation SNIPERs, we employed pan-IAP antagonists to recruit several IAP family members, such as XIAP, cIAP1 and Apollon, and they degrade target proteins at nM concentrations. Recently we have successfully developed the third-generation SNIPERs that specifically recruit Livin for target degradation. Since Livin is not expressed in adult normal human tissues while abundantly expressed in some melanomas, Livin-SNIPERs could be a promising therapeutic modality against Livin-positive melanomas.

[Disclosure of COI by Responsible researcher] Lead presenter is the Responsible researcher.
[Lead presenter: Mikihiko Naito] ■Endowed chair: 社会連携講座：エーザイ株式会社

▲TOP

### [SS5-6] SS5
### Exit-Oriented Drug Discovery Technology from Academia

2025 / 9 / 25 (Thu) 12:50-15:20 Room 1/(Ishikawa Ongakudo 2F Concert Hall) Room 1

[Chairpersons]Akira Yokoi (Eisai Co., Ltd.)、Katsuya Tsuchihara (Exploratory Oncology Research and Clinical Trial Center, National Cancer Center Japan)

Professor Hajime Okamoto served as the President of the 28th Annual Meeting of the Japanese Cancer Association (1969) and was the founding Director of the Cancer Research Institute at Kanazawa University. He initiated the development of the immunostimulatory anticancer agent OK-432 (Picibanil) through his discovery of the anticancer effects of streptococcal bacteria. In commemoration of Professor Okamoto's achievements, this symposium will focus on academia-driven drug discovery based on novel modalities. Researchers who are advancing the development of anticancer agents through unique academic discoveries and technologies-leveraging clear exit strategies via industry-academia collaborations or academic spin-off ventures-will present their work. The symposium also aims to pave the way for a new era of academia-led drug discovery.

---

### SS5-6
### Overcoming the tumour barriers for drug and immune cell infiltration by protein-engineered new generation CAR-T cells

[Speaker]Jun Ishihara (Imperial ／NCC EPOC)

Metastatic castration-resistant prostate cancer (mCRPC) is a currently incurable, immunologically cold tumor. STEAP1 is a promising target expressed in 85% of mCRPC patients. CAR-T cell therapy is still not effective against solid cancers. IL-12 may address these issues. IL-12-producing CAR-T cells showed responses in patients but were limited by toxicity. IL-12 produced locally by CAR-T remains toxic due to IL12 diffusion into blood. Objectives: We localise IL-12 by harnessing the collagen-binding domain (CBD) of von Willebrand factor to turn tumour collagens into a drug reservoir. We developed STEAP1-targeting CAR-T that express CBD fused IL-12. Results: CBD fusion to IL-12 increased the concentration of IL-12 in tumors and reduced its systemic concentration following secretion from the CAR-T. This turned the TME from cold to hot. CBD-IL-12 CAR-T did not increase organ damage. CBD-IL-12 CAR-T increased tumor-infiltrating T/NK/dendritic cells. CBD-IL-12 CAR-T eradicated prostate cancers and induced epitope spreading. CBD fusion to promising yet toxic immunotherapies may improve their therapeutic window as payload of engineered immune cells to overcome solid tumors.

[Disclosure of COI by Responsible researcher] Lead presenter is the Responsible researcher.
[Lead presenter: Jun Ishihara] ■Research funds from company: ICVI open grant, Sarcoma UK, Prostate cancer UK, Brain tumour ■Profit of stock: ArrowImmune and HeioThera　■Research expenses: Ono pharma

▲TOP

### [SS5-Special Remarks] SS5
### Exit-Oriented Drug Discovery Technology from Academia

2025 / 9 / 25 (Thu) 12:50-15:20 Room 1/(Ishikawa Ongakudo 2F Concert Hall) Room 1

[Chairpersons]Akira Yokoi (Eisai Co., Ltd.)、Katsuya Tsuchihara (Exploratory Oncology Research and Clinical Trial Center, National Cancer Center Japan)

Professor Hajime Okamoto served as the President of the 28th Annual Meeting of the Japanese Cancer Association (1969) and was the founding Director of the Cancer Research Institute at Kanazawa University. He initiated the development of the immunostimulatory anticancer agent OK-432 (Picibanil) through his discovery of the anticancer effects of streptococcal bacteria. In commemoration of Professor Okamoto's achievements, this symposium will focus on academia-driven drug discovery based on novel modalities. Researchers who are advancing the development of anticancer agents through unique academic discoveries and technologies-leveraging clear exit strategies via industry-academia collaborations or academic spin-off ventures-will present their work. The symposium also aims to pave the way for a new era of academia-led drug discovery.

---

### SS5-Special Remarks
### Special Remarks

[Speaker]Hiroshi Okamoto (Professor Emeritus, Graduate School of Medicine Medical Sciences, Tohoku University)

No Abstract

### [SS3-1] SS3
### Woman scientists in cancer research (WSCR)

2025 / 9 / 25 (Thu) 12:50-14:50 Room 2/(Ishikawa Ongakudo 2F Hogaku Hall) Room 2

[Chairpersons]Hiroyuki Seimiya (Div. Mol. Biother., JFCR Cancer Chemother. Ctr.)、Ai Kohtani (Research Institute of Microbial Diseases, The University of Osaka)

Welcome to the 11th Women Scientists in Cancer Research (WSCR) Symposium. This symposium was established in JCA Annual Meeting in 2014 to encourage further advancement and career development of women researchers, especially recently, early- and mid-career scientists. From among numerous applicants who submitted presentation abstracts along with their career histories and achievement records, the six most excellent speakers were selected through a rigorous review process. At the symposium, these speakers will present their cutting-edge research, and based on the scientific content of their talks, the recipient of the WSCR Symposium Award will be chosen by JCA Committee of Women Scientists and members of Organizing Committee of this year's Annual Meeting. Scientifically, this session offers an exciting lineup, covering topics such as metabolic vulnerabilities in cancer, adaptive strategies to chromosomal instability, novel regulators of triple-negative breast cancer, diagnostic devices utilizing extracellular vesicles, and new diagnostic markers and therapeutic targets. Needless to say, there is absolutely no restriction on the gender of attendees. We look forward to your participation and active discussions.

---

### SS3-1
### Metabolic Vulnerability Drives ETC Inhibitor Sensitivity in TTF-1 Negative Lung Cancer

[Speaker]Aya Shiba (Diag Pathol., Inst. of Med., Univ. Tsukuba) [Co-author]Kanon Hirai (Diag Pathol., Inst. of Med., Univ. Tsukuba)、Daisuke Matsubara (Diag Pathol., Inst. of Med., Univ. Tsukuba) [Panelist]Naoko Otani (Graduate School of Medicine, Osaka Metropolitan University)、Chiaki Takahashi (Division of Oncology and Molecular Biology, Kanazawa University, Cancer Research Institute)、Takashi Kohno (Division of Genome Biology, National Cancer Center, Research Institute)、Yasuyuki Fujita (Kyoto University Graduate School of Medicine)、Yukari Totsuka (Environmental Health Sci., Hoshi Univ.)、Yasuhito Nannya (Dep. Hematol&Oncol Research Hospital IMSUT)、Etsuko Kiyokawa (Dept. Oncol. Pathol., Kanazawa Med. Univ.)、Noriko Gotoh (Cancer Research Institute, Kanazawa University)

Metabolic rewiring drives targeted therapy resistance in non-small cell lung cancer (NSCLC). The mitochondrial electron transport chain (ETC) is crucial for cancer cell bioenergetics, representing a potential therapeutic target. We explored ETC inhibitors (ETCi) in 41 lung adenocarcinoma cell lines, and found TTF-1 negative and poorly differentiated cells exhibited significantly higher ETCi sensitivity. Sensitive cells displayed higher proliferation rates, low mitochondrial spare respiratory capacity, perinuclear mitochondrial localization, and elevated ROS production and SOD1 expression, suggesting metabolic stress and dependence on antioxidant systems. Notably, ETCi sensitivity did not correlate with typical cytotoxic drugs sensitivity. These findings indicate that high proliferation and compromised mitochondrial reserve render the cells vulnerable to ETCi-induced energy depletion and oxidative stress. Ki67 expression and mitochondrial distribution patterns emerge as potential predictive biomarkers for ETCi sensitivity. These findings provide a basis for the development of novel ETCi-based therapies for specific NSCLC subtypes, with potential for improved patient outcomes.

[Disclosure of COI by Responsible researcher] Lead presenter is the Responsible researcher.
[Lead presenter: Aya Shiba] We have no financial relationships to disclose.

### [SS3-2] SS3
### Woman scientists in cancer research (WSCR)

2025 / 9 / 25 (Thu) 12:50-14:50 Room 2/(Ishikawa Ongakudo 2F Hogaku Hall) Room 2

[Chairpersons]Hiroyuki Seimiya (Div. Mol. Biother., JFCR Cancer Chemother. Ctr.)、Ai Kohtani (Research Institute of Microbial Diseases, The University of Osaka)

Welcome to the 11th Women Scientists in Cancer Research (WSCR) Symposium. This symposium was established in JCA Annual Meeting in 2014 to encourage further advancement and career development of women researchers, especially recently, early- and mid-career scientists. From among numerous applicants who submitted presentation abstracts along with their career histories and achievement records, the six most excellent speakers were selected through a rigorous review process. At the symposium, these speakers will present their cutting-edge research, and based on the scientific content of their talks, the recipient of the WSCR Symposium Award will be chosen by JCA Committee of Women Scientists and members of Organizing Committee of this year’s Annual Meeting. Scientifically, this session offers an exciting lineup, covering topics such as metabolic vulnerabilities in cancer, adaptive strategies to chromosomal instability, novel regulators of triple-negative breast cancer, diagnostic devices utilizing extracellular vesicles, and new diagnostic markers and therapeutic targets. Needless to say, there is absolutely no restriction on the gender of attendees. We look forward to your participation and active discussions.

---

### SS3-2
### Coping Strategies of Glioblastoma Cells to Chromosomal Instability

[Speaker]Minji Jo (Div. Exp. Pathol., Cancer Inst., JFCR) [Co-author]Oltea Sampetrean (Human Biology-Microbiome-Quantum Res. Ctr., Keio Univ.)、Tetsuya Negoto (Dept. Neurosurg., Sch. Med., Kurume Univ.)、Utako Kato (Div. Exp. Pathol., Cancer Inst., JFCR)、Hideyuki Saya (Oncology Innovation Ctr., Fujita Health Univ.)、Toru Hirota (Div. Exp. Pathol., Cancer Inst., JFCR) [Panelist]Naoko Otani (Graduate School of Medicine, Osaka Metropolitan University)、Chiaki Takahashi (Division of Oncology and Molecular Biology, Kanazawa University, Cancer Research Institute)、Takashi Kohno (Division of Genome Biology, National Cancer Center, Research Institute)、Yasuyuki Fujita (Kyoto University Graduate School of Medicine)、Yukari Totsuka (Environmental Health Sci., Hoshi Univ.)、Yasuhito Nannya (Dep. Hematol&Oncol Research Hospital IMSUT)、Etsuko Kiyokawa (Dept. Oncol. Pathol., Kanazawa Med. Univ.)、Noriko Gotoh (Cancer Research Institute, Kanazawa University)

Glioblastoma (GBM), the most aggressive brain tumor, exhibits genomic and chromosomal abnormalities due to chromosomal instability (CIN). While CIN promotes karyotypic diversity and drives cancer evolution, it also induces chromosomal stress, deteriorating cellular fitness. Yet, how GBM cells proliferate along with CIN-associated stress remain unclear. To explore this, we used mouse glioma stem cells that aggressively proliferate with high levels of CIN. In vitro evolution experiments and orthotopic implantation revealed that single-cell-derived clones, with varying degrees of CIN, underwent unique karyotypic changes and developed distinct malignant features. Furthermore, transcriptome profiling indicated that metabolic reprogramming is a strategy to overcome any levels of CIN-associated stress. In contrast, high CIN clones that lacked metabolic adaptations showed enhanced growth signaling and distinct interactions with immune cells, shaping a tumor-prone microenvironment. Our findings highlight diverse strategies GBM cells employ to cope with chromosomal stress. Understanding these coping strategies provides insights into evolution and thus possible therapeutic approaches of GBM.

[Disclosure of COI by Responsible researcher] Lead presenter is not the Responsible researcher.
[Lead presenter: Minji Jo] We have no financial relationships to disclose.
[Principal Researcher: Toru Hirota] We have no financial relationships to disclose.

### [SS3-3] SS3
### Woman scientists in cancer research (WSCR)

2025 / 9 / 25 (Thu) 12:50-14:50 Room 2/(Ishikawa Ongakudo 2F Hogaku Hall) Room 2

[Chairpersons]Hiroyuki Seimiya (Div. Mol. Biother., JFCR Cancer Chemother. Ctr.)、Ai Kohtani (Research Institute of Microbial Diseases, The University of Osaka)

Welcome to the 11th Women Scientists in Cancer Research (WSCR) Symposium. This symposium was established in JCA Annual Meeting in 2014 to encourage further advancement and career development of women researchers, especially recently, early- and mid-career scientists. From among numerous applicants who submitted presentation abstracts along with their career histories and achievement records, the six most excellent speakers were selected through a rigorous review process. At the symposium, these speakers will present their cutting-edge research, and based on the scientific content of their talks, the recipient of the WSCR Symposium Award will be chosen by JCA Committee of Women Scientists and members of Organizing Committee of this year's Annual Meeting. Scientifically, this session offers an exciting lineup, covering topics such as metabolic vulnerabilities in cancer, adaptive strategies to chromosomal instability, novel regulators of triple-negative breast cancer, diagnostic devices utilizing extracellular vesicles, and new diagnostic markers and therapeutic targets. Needless to say, there is absolutely no restriction on the gender of attendees. We look forward to your participation and active discussions.

---

### SS3-3
### Application of EV sheets to realize ideal extracellular vesicle biomarker development in ovarian cancer

[Speaker]Yukari Nagao (Dept. Obst. & Gynecol., Nagoya Univ. Grad. Sch. of Med.) [Co-author]Akira Yokoi (Dept. Obst. & Gynecol., Nagoya Univ. Grad. Sch. of Med.／Inst. Adv. Res., Nagoya Univ.)、Kosuke Yoshida (Dept. Obst. & Gynecol., Nagoya Univ. Grad. Sch. of Med.／Inst. Adv. Res., Nagoya Univ.)、Masami Kitagawa (Dept. Obst. & Gynecol., Nagoya Univ. Grad. Sch. of Med.)、Eri Inami (Dept. Obst. & Gynecol., Nagoya Univ. Grad. Sch. of Med.)、Kazuhiro Suzuki (Dept. Obst. & Gynecol., Nagoya Univ. Grad. Sch. of Med.)、Masato Yoshihara (Dept. Obst. & Gynecol., Nagoya Univ. Grad. Sch. of Med.)、Satoshi Tamauchi (Dept. Obst. & Gynecol., Nagoya Univ. Grad. Sch. of Med.)、Nobuhisa Yoshikawa (Dept. Obst. & Gynecol., Nagoya Univ. Grad. Sch. of Med.)、Kaoru Niimi (Dept. Obst. & Gynecol., Nagoya Univ. Grad. Sch. of Med.)、Takao Yasui (Life Sci. and Tech., Inst. of Sci. Tokyo)、Hiroaki Kajiyama (Dept. Obst. & Gynecol., Nagoya Univ. Grad. Sch. of Med.) [Panelist]Naoko Otani (Graduate School of Medicine, Osaka Metropolitan University)、Chiaki Takahashi (Division of Oncology and Molecular Biology, Kanazawa University, Cancer Research Institute)、Takashi Kohno (Division of Genome Biology, National Cancer Center, Research Institute)、Yasuyuki Fujita (Kyoto University Graduate School of Medicine)、Yukari Totsuka (Environmental Health Sci., Hoshi Univ.)、Yasuhito Nannya (Dep. Hematol&Oncol Research Hospital IMSUT)、Etsuko Kiyokawa (Dept. Oncol. Pathol., Kanazawa Med. Univ.)、Noriko Gotoh (Cancer Research Institute, Kanazawa University)

Objective: Extracellular vesicle (EV)-miRNA has attracted attention as a biomarker. This study aimed to establish novel biomarker strategies in ovarian cancer by applying EV sheets, which enable the isolation of EVs easily and efficiently.
Methods: EVs on multiple organ surfaces were collected intraoperatively from ovarian tumor patients by applying EV sheet attaching method, and EVs in serum, urine, and ascites were collected with soaking method. A total of 753 EV samples from 185 patients were analyzed by small RNA-seq and qRT-PCR.
Results: Based on miRNA profiles of tissues and the EVs of tumor surface and fimbriae, miR-200a-3p, miR-200c-3p, miR-425-5p, and miR-429 were highly expressed in cancer patients. They were also upregulated in the EVs from serum, urine, and ascites by qPCR, and AUC values were 0.85, 0.75, and 0.96, respectively. Moreover, EV-miRNA profiles of organ surface differed by sampling locations, which can reflect patients' clinical features, and the expressions of candidate EV-miRNAs could be validated by qPCR.
Conclusions: EV-miRNAs from EV sheets could be novel biomarkers for ovarian cancer diagnosis, staging, and recurrence, leading to new clinical strategies.

[Disclosure of COI by Responsible researcher] Lead presenter is not the Responsible researcher.
[Lead presenter: Yukari Nagao] We have no financial relationships to disclose.
[Principal Researcher: Akira Yokoi] We have no financial relationships to disclose.

▲TOP

### [SS3-4] SS3
### Woman scientists in cancer research (WSCR)

2025 / 9 / 25 (Thu) 12:50-14:50 Room 2/(Ishikawa Ongakudo 2F Hogaku Hall) Room 2

[Chairpersons]Hiroyuki Seimiya (Div. Mol. Biother., JFCR Cancer Chemother. Ctr.)、Ai Kohtani (Research Institute of Microbial Diseases, The University of Osaka)

Welcome to the 11th Women Scientists in Cancer Research (WSCR) Symposium. This symposium was established in JCA Annual Meeting in 2014 to encourage further advancement and career development of women researchers, especially recently, early- and mid-career scientists. From among numerous applicants who submitted presentation abstracts along with their career histories and achievement records, the six most excellent speakers were selected through a rigorous review process. At the symposium, these speakers will present their cutting-edge research, and based on the scientific content of their talks, the recipient of the WSCR Symposium Award will be chosen by JCA Committee of Women Scientists and members of Organizing Committee of this year's Annual Meeting. Scientifically, this session offers an exciting lineup, covering topics such as metabolic vulnerabilities in cancer, adaptive strategies to chromosomal instability, novel regulators of triple-negative breast cancer, diagnostic devices utilizing extracellular vesicles, and new diagnostic markers and therapeutic targets. Needless to say, there is absolutely no restriction on the gender of attendees. We look forward to your participation and active discussions.

---

### SS3-4
### Roles of GPNMB in tumor initiation and tumor development of triple-negative breast cancer

[Speaker]Yukari Okita (Dept. Exp. Path., Inst. Med., Univ. Tsukuba) [Co-author]Mitsuyasu Kato (Dept. Exp. Path., Inst. Med., Univ. Tsukuba)
[Panelist]Naoko Otani (Graduate School of Medicine, Osaka Metropolitan University)、Chiaki Takahashi (Division of Oncology and Molecular Biology, Kanazawa University, Cancer Research Institute)、Takashi Kohno (Division of Genome Biology, National Cancer Center, Research Institute)、Yasuyuki Fujita (Kyoto University Graduate School of Medicine)、Yukari Totsuka (Environmental Health Sci., Hoshi Univ.)、Yasuhito Nannya (Dep. Hematol&Oncol Research Hospital IMSUT)、Etsuko Kiyokawa (Dept. Oncol. Pathol., Kanazawa Med. Univ.)、Noriko Gotoh (Cancer Research Institute, Kanazawa University)

Breast cancer is recognized as a heterogeneous disease. Molecular subtypes are classified based on the expression of specific markers: estrogen receptor (ER), progesterone receptor (PR), and human epidermal growth factor receptor 2 (HER2). One such subtype is known as triple-negative breast cancer (TNBC), characterized by the lack of ER, PR, and HER2 expression. TNBC is the most aggressive subtype of breast cancer; therefore, further molecular characterization of TNBC is urgently needed to support the development of novel targeted therapies. Glycoprotein nonmetastatic melanoma protein B (GPNMB) is a type I transmembrane protein that is highly expressed in various cancers, including TNBC. Our previous studies have demonstrated the critical roles of GPNMB in induction of tumorigenesis, cellular motility, and stem-like properties through the induction of epithelial-mesenchymal transition (EMT). In this presentation, we would like to introduce the key motifs and the domain of GPNMB involved in its tumorigenic functions. Furthermore, we will discuss the crosstalk between GPNMB and receptor tyrosine kinase (RTK) signaling pathway.

[Disclosure of COI by Responsible researcher] Lead presenter is the Responsible researcher.
[Lead presenter: Yukari Okita] We have no financial relationships to disclose.

### [SS3-5] SS3
### Woman scientists in cancer research (WSCR)

2025 / 9 / 25 (Thu) 12:50-14:50 Room 2/(Ishikawa Ongakudo 2F Hogaku Hall) Room 2

[Chairpersons]Hiroyuki Seimiya (Div. Mol. Biother., JFCR Cancer Chemother. Ctr.)、Ai Kohtani (Research Institute of Microbial Diseases, The University of Osaka)

Welcome to the 11th Women Scientists in Cancer Research (WSCR) Symposium. This symposium was established in JCA Annual Meeting in 2014 to encourage further advancement and career development of women researchers, especially recently, early- and mid-career scientists. From among numerous applicants who submitted presentation abstracts along with their career histories and achievement records, the six most excellent speakers were selected through a rigorous review process. At the symposium, these speakers will present their cutting-edge research, and based on the scientific content of their talks, the recipient of the WSCR Symposium Award will be chosen by JCA Committee of Women Scientists and members of Organizing Committee of this year's Annual Meeting. Scientifically, this session offers an exciting lineup, covering topics such as metabolic vulnerabilities in cancer, adaptive strategies to chromosomal instability, novel regulators of triple-negative breast cancer, diagnostic devices utilizing extracellular vesicles, and new diagnostic markers and therapeutic targets. Needless to say, there is absolutely no restriction on the gender of attendees. We look forward to your participation and active discussions.

---

### SS3-5
### Gastric gland mucin regulates malignancy of various tumors showing gastric phenotype

[Speaker]Chifumi Fujii (Shinshu Univ., Sch. Med.／Dept. Biotech., Inst. Biomed. Sci., RCSI., Shinshu Univ.) [Panelist]Naoko Otani (Graduate School of Medicine, Osaka Metropolitan University)、Chiaki Takahashi (Division of Oncology and Molecular Biology, Kanazawa University, Cancer Research Institute)、Takashi Kohno (Division of Genome Biology, National Cancer Center, Research Institute)、Yasuyuki Fujita (Kyoto University Graduate School of Medicine)、Yukari Totsuka (Environmental Health Sci., Hoshi Univ.)、Yasuhito Nannya (Dep. Hematol&Oncol Research Hospital IMSUT)、Etsuko Kiyokawa (Dept. Oncol. Pathol., Kanazawa Med. Univ.)、Noriko Gotoh (Cancer Research Institute, Kanazawa University)

Gastric gland mucin is a molecular complex composed of MUC6, α1,4-linked ***N***-acetylglucosamine residues (αGlcNAc) bound to MUC6, and TFF2. Because loss of αGlcNAc on MUC6 in differentiated-type gastric adenocarcinoma patients is associated with worse prognosis, we studied the effect of αGlcNAc expression on malignant phenotypes in differentiated-type gastric cancer cells and revealed MUC1 is a key molecule underlining the molecular mechanisms. We also examined the association of αGlcNAc and MUC6 on pancreatic cancer malignancy and showed that αGlcNAc expression decreased early stage of the tumorigenesis, followed by reduced MUC6 expression and that lower ***A4GNT*** or ***MUC6*** mRNA levels correlated with poor prognosis. Ectopic expression of MUC6 in pancreatic cancer cells suppressed malignancy, with α4GnT co-expression enhancing this effect by MUC6-TFF2 complex. In invasive mucinous lung adenocarcinoma, MUC6-positive patients showed significantly favorable survival. Expression of MUC6 in A549 cells suppressed malignant phenotypes. These results suggest that αGlcNAc, MUC6, and TFF2 are potential novel diagnostic markers for the gastric, pancreatic and lung cancers showing gastric phenotype.

[Disclosure of COI by Responsible researcher] Lead presenter is the Responsible researcher.
[Lead presenter: Chifumi Fujii] We have no financial relationships to disclose.

▲TOP

### [SS3-6] SS3
### Woman scientists in cancer research (WSCR)

2025 / 9 / 25 (Thu) 12:50-14:50 Room 2/(Ishikawa Ongakudo 2F Hogaku Hall) Room 2

[Chairpersons]Hiroyuki Seimiya (Div. Mol. Biother., JFCR Cancer Chemother. Ctr.)、Ai Kohtani (Research Institute of Microbial Diseases, The University of Osaka)

Welcome to the 11th Women Scientists in Cancer Research (WSCR) Symposium. This symposium was established in JCA Annual Meeting in 2014 to encourage further advancement and career development of women researchers, especially recently, early- and mid-career scientists. From among numerous applicants who submitted presentation abstracts along with their career histories and achievement records, the six most excellent speakers were selected through a rigorous review process. At the symposium, these speakers will present their cutting-edge research, and based on the scientific content of their talks, the recipient of the WSCR Symposium Award will be chosen by JCA Committee of Women Scientists and members of Organizing Committee of this year's Annual Meeting. Scientifically, this session offers an exciting lineup, covering topics such as metabolic vulnerabilities in cancer, adaptive strategies to chromosomal instability, novel regulators of triple-negative breast cancer, diagnostic devices utilizing extracellular vesicles, and new diagnostic markers and therapeutic targets. Needless to say, there is absolutely no restriction on the gender of attendees. We look forward to your participation and active discussions.

---

### SS3-6
### FADS2, fatty acid desaturase2 required for tumorigenesis in cholangiocarcinoma

[Speaker]Haruna Fujimori (Div. Cancer Stem Cell) [Co-author]Kohsei Hasegawa (Div. Cancer Stem Cell)、Kohta Nakatani (Medical Institute of Bioregulation, Kyushu Univ.)、Masatomo Takahashi (Medical Institute of Bioregulation, Kyushu Univ.)、Yoshihiro Izumi (Medical Institute of Bioregulation, Kyushu Univ.)、Takeshi Bamba (Medical Institute of Bioregulation, Kyushu Univ.)、Rie Takahashi (Div. Cancer Stem Cell)、Mai Mochizuki (Div. Cancer Stem Cell)、Kazunori Yamaguchi (Div. Mol. & Cell. Oncol., Miyagi Cancer Ctr. Res. Inst.)、Jun Yasuda (Div. Mol. & Cell. Oncol., Miyagi Cancer Ctr. Res. Inst.)、Naoki Asano (Div. Cancer Stem Cell)、Keiichi Tamai (Div. Cancer Stem Cell) [Panelist]Naoko Otani (Graduate School of Medicine, Osaka Metropolitan University)、Chiaki Takahashi (Division of Oncology and Molecular Biology, Kanazawa University, Cancer Research Institute)、Takashi Kohno (Division of Genome Biology, National Cancer Center, Research Institute)、Yasuyuki Fujita (Kyoto University Graduate School of Medicine)、Yukari Totsuka (Environmental Health Sci., Hoshi Univ.)、Yasuhito Nannya (Dep. Hematol&Oncol Research Hospital IMSUT)、Etsuko Kiyokawa (Dept. Oncol. Pathol., Kanazawa Med. Univ.)、Noriko Gotoh (Cancer Research Institute, Kanazawa University)

Cholangiocarcinoma has limited treatment options. To identify a novel therapeutic target, we screened novel genes that play an important role in tumorigenesis. Using a xenograft mouse model, we found that fatty acid desaturase 2 (FADS2) is required for tumorigenesis in cholangiocarcinoma. ***in situ*** hybridization using clinical specimens showed that FADS2 was expressed in cancer cells, but not in the stromal cells. FADS2 depletion in cholangiocarcinoma cells suppressed cell migration and sphere formation. Furthermore, since FADS2 is known as a fatty acid desaturase, we conducted lipidomics analysis to investigate alterations in lipid species and found that triglycerides, which are used as an energy source, were significantly decreased by FADS2 depletion, while fatty acid composition was not altered. Mitochondrial oxygen consumption was also reduced by FADS2 depletion. These results suggest that FADS2 depletion leads to a decrease in energy sources including triglycerides, a subsequent reduction in oxygen consumption to produce ATP, and suppression of cell proliferation and migration. FADS2 is expected to be a novel therapeutic target for further investigation.

[Disclosure of COI by Responsible researcher] Lead presenter is not the Responsible researcher.
[Lead presenter: Haruna Fujimori] We have no financial relationships to disclose.
[Principal Researcher: Keiichi Tamai] We have no financial relationships to disclose.

### [JS1-1] JS1
### Animal Models for Cancer Research - From the Mice to Comparative Oncology

2025 / 9 / 25 (Thu) 12:50-15:20 Room 3/(Hotel Nikko Kanazawa 4F Tsuru A) Room 3

[Chairpersons]Masanobu Oshima (Division of Genetics, Cancer Research Institute, Kanazawa University)、Takayuki Nakagawa (Laboratory of Veterinary Surgery, Graduate School of Agricultural and Life sciences, The University of Tokyo)、Amy K LeBlanc (Senior Scientist and Director, Comparative Oncology Program, National Institutes of Health)

Animal models are essential tools for cancer research. To date, attempts have been made to develop animal models that faithfully recapitulate the molecular etiology and pathophysiology of human cancer, such as syngeneic/xenogeneic tumor-bearing mouse models, patient-derived xenografted mouse models, humanized mouse models and genetically modified mouse models. Furthermore, over the past two decades, the NIH/NIC in the USA has led the development of novel animal models using naturally occurring tumors in pet dogs, known as the “Comparative Oncology Project”, and has successfully accumulated critical knowledge through the biological studies including genome and informatics analysis. Moreover, the canine tumor models are now used to evaluate immunotherapy for rare cancers as the “Cancer Moonshot Project” in the United States, which is being promoted around the world. The aim of this symposium is to provide the basics from the cutting-edge mouse models to naturally occurring canine tumor models, as well as their practical application in future research. These approaches will greatly contribute to the innovative cancer research and therapeutic strategy.

---

### JS1-1
### Genetic mouse and organoid models for intestinal cancer research

[Speaker]Masanobu Oshima (Div. Genetics, Cancer Res. Inst., Kanazawa Univ.／Nano LSI, Kanazawa Univ.) [Co-author]Mizuho Nakayama (Div. Genetics, Cancer Res. Inst., Kanazawa Univ.／Nano LSI, Kanazawa Univ.)、Hiroko Oshima (Div. Genetics, Cancer Res. Inst., Kanazawa Univ.／Nano LSI, Kanazawa Univ.)、Yukiko T. Matsunaga (IIS, UTokyo)

Accumulation of driver mutations promotes the malignant progression of cancer, as the well-known concept of multistep tumorigenesis. However, the precise mechanisms underlying malignancy driven by genetic alterations remain incompletely understood. Here, we constructed genetically engineered mouse models carrying various combinations of driver mutations in the intestinal epithelia. Comprehensive pathological analyses revealed that concurrent mutations in ***Apc***, ***Kras***, and ***Tgfbr2*** are responsible for intravasation and liver metastasis. We subsequently established tumor-derived organoids from mouse models ranging from benign to highly malignant lesions and evaluated their metastatic capacity. Interestingly, when non-metastatic tumor cells disseminated to the liver in clusters together with highly metastatic cells, they formed chimeric metastatic tumors through a polyclonal metastasis mechanism. Moreover, microdevice-based imaging uncovered a unique mode of collective intravasation by tumor cell clusters. Collectively, our mouse models, organoid systems, and microdevice imaging techniques provide a powerful platform for dissecting the mechanisms of cancer progression and metastasis.

[Disclosure of COI by Responsible researcher] Lead presenter is the Responsible researcher.
[Lead presenter: Masanobu Oshima] ■Research funds from company: 科研費、AMED

▲TOP

### [JS1-2] JS1
### Animal Models for Cancer Research - From the Mice to Comparative Oncology

2025 / 9 / 25 (Thu) 12:50-15:20 Room 3/(Hotel Nikko Kanazawa 4F Tsuru A) Room 3

[Chairpersons]Masanobu Oshima (Division of Genetics, Cancer Research Institute, Kanazawa University)、Takayuki Nakagawa (Laboratory of Veterinary Surgery, Graduate School of Agricultural and Life sciences, The University of Tokyo)、Amy K LeBlanc (Senior Scientist and Director, Comparative Oncology Program, National Institutes of Health)

Animal models are essential tools for cancer research. To date, attempts have been made to develop animal models that faithfully recapitulate the molecular etiology and pathophysiology of human cancer, such as syngeneic/xenogeneic tumor-bearing mouse models, patient-derived xenografted mouse models, humanized mouse models and genetically modified mouse models. Furthermore, over the past two decades, the NIH/NIC in the USA has led the development of novel animal models using naturally occurring tumors in pet dogs, known as the “Comparative Oncology Project”, and has successfully accumulated critical knowledge through the biological studies including genome and informatics analysis. Moreover, the canine tumor models are now used to evaluate immunotherapy for rare cancers as the “Cancer Moonshot Project” in the United States, which is being promoted around the world. The aim of this symposium is to provide the basics from the cutting-edge mouse models to naturally occurring canine tumor models, as well as their practical application in future research. These approaches will greatly contribute to the innovative cancer research and therapeutic strategy.

### JS1-2
### Understanding the Rarity of Rare Cancers: Insights from Comparative Oncology

[Speaker]Tadashi Kondo (Division of Rare Cancer Research, National Cancer Center)

Laboratory animals have long served as key models in human cancer research. In this era of omics-driven discovery and rapid oncology drug development, comparative oncology offers new opportunities to advance cancer research. This field explores both similarities and differences between humans and animals, leading to new insights. One such question is: Why are rare cancers rare? In humans, variation in cancer incidence across organs is thought to reflect the number of proliferative stem cells. A common hypothesis suggests that cancer risk increases with the number of such cells. This may explain the rarity of some cancers in humans. However, this idea does not apply across species. For example, cancer frequency does not scale with body size. Moreover, in canines, osteosarcoma and hemangiosarcoma are common, while they are rare in humans. Conversely, lung cancer is common in humans but rare in canines. These examples show how comparative oncology can help address fundamental questions among cancers. However, research infrastructure for studying cancer in veterinary species remains underdeveloped. This presentation will outline current efforts and challenges in this field.

[Disclosure of COI by Responsible researcher] Lead presenter is the Responsible researcher.
[Lead presenter: Tadashi Kondo] ■Research expenses: 日本ゼオン株式会社

### [JS1-3] JS1
### Animal Models for Cancer Research - From the Mice to Comparative Oncology

2025 / 9 / 25 (Thu) 12:50-15:20 Room 3/(Hotel Nikko Kanazawa 4F Tsuru A) Room 3

[Chairpersons]Masanobu Oshima (Division of Genetics, Cancer Research Institute, Kanazawa University)、Takayuki Nakagawa (Laboratory of Veterinary Surgery, Graduate School of Agricultural and Life sciences, The University of Tokyo)、Amy K LeBlanc (Senior Scientist and Director, Comparative Oncology Program, National Institutes of Health)

Animal models are essential tools for cancer research. To date, attempts have been made to develop animal models that faithfully recapitulate the molecular etiology and pathophysiology of human cancer, such as syngeneic/xenogeneic tumor-bearing mouse models, patient-derived xenografted mouse models, humanized mouse models and genetically modified mouse models. Furthermore, over the past two decades, the NIH/NIC in the USA has led the development of novel animal models using naturally occurring tumors in pet dogs, known as the “Comparative Oncology Project”, and has successfully accumulated critical knowledge through the biological studies including genome and informatics analysis. Moreover, the canine tumor models are now used to evaluate immunotherapy for rare cancers as the “Cancer Moonshot Project” in the United States, which is being promoted around the world. The aim of this symposium is to provide the basics from the cutting-edge mouse models to naturally occurring canine tumor models, as well as their practical application in future research. These approaches will greatly contribute to the innovative cancer research and therapeutic strategy.

---

### JS1-3
### The CD3L1 immunotherapeutic target: from discovery to companion animal model and human clinical trial

[Speaker]Jie Xu (Fudan University)

No Abstract

▲TOP

### [JS1-4] JS1
### Animal Models for Cancer Research - From the Mice to Comparative Oncology

2025 / 9 / 25 (Thu) 12:50-15:20 Room 3/(Hotel Nikko Kanazawa 4F Tsuru A) Room 3

[Chairpersons]Masanobu Oshima (Division of Genetics, Cancer Research Institute, Kanazawa University)、Takayuki Nakagawa (Laboratory of Veterinary Surgery, Graduate School of Agricultural and Life sciences, The University of Tokyo)、Amy K LeBlanc (Senior Scientist and Director, Comparative Oncology Program, National Institutes of Health)

Animal models are essential tools for cancer research. To date, attempts have been made to develop animal models that faithfully recapitulate the molecular etiology and pathophysiology of human cancer, such as syngeneic/xenogeneic tumor-bearing mouse models, patient-derived xenografted mouse models, humanized mouse models and genetically modified mouse models. Furthermore, over the past two decades, the NIH/NIC in the USA has led the development of novel animal models using naturally occurring tumors in pet dogs, known as the “Comparative Oncology Project”, and has successfully accumulated critical knowledge through the biological studies including genome and informatics analysis. Moreover, the canine tumor models are now used to evaluate immunotherapy for rare cancers as the “Cancer Moonshot Project” in the United States, which is being promoted around the world. The aim of this symposium is to provide the basics from the cutting-edge mouse models to naturally occurring canine tumor models, as well as their practical application in future research. These approaches will greatly contribute to the innovative cancer research and therapeutic strategy.

---

### JS1-4
### Spontaneous Canine Osteosarcoma: A Translationally Relevant Patient Model for Humans

[Speaker]Amy K. LeBlanc (Comparative Oncology Program, Center for Cancer Research, NCI, NIH) [Co-author]Anjali Garg (Comparative Oncology Program, Center for Cancer Research, NCI, NIH)、Jessica A. Beck (Comparative Oncology Program, Center for Cancer Research, NCI, NIH)、Christina Mazcko (Comparative Oncology Program, Center for Cancer Research, NCI, NIH)

Canine osteosarcoma shares many features with human osteosarcoma and is an important preclinical model with the potential to inform pediatric oncology. However, key gaps persist in our understanding of the histologic, genomic, and transcriptomic features of canine osteosarcoma. Under the NCI's Decoding the Osteosarcoma Genome of the Dog (DOG2) project, the COP is addressing these gaps through a comprehensive assessment of outcome-linked tissue samples collected through the efforts of the Comparative Oncology Trials Consortium (COTC). Canine osteosarcomas demonstrate a similar metastatic progression to humans with heterogeneous tumors displaying multiple histologic subtypes, and formation of a complex TME with intra- and peri-tumoral lymphocytic aggregates, diffuse macrophage infiltration, and accumulation of tumor-derived osteoid and collagenous tumor matrix. The morphologic characterization of these samples facilitated the development of an AI predictive model based on histologic subtypes and reinforced the identification of transcriptional signatures of primary tumors highlighting a role for immune infiltrates and tumor stroma in overall survival and disease-free progression.

[Disclosure of COI by Responsible researcher] Lead presenter is the Responsible researcher.
[Lead presenter: Amy K. LeBlanc] We have no financial relationships to disclose.

▲TOP

### [JS1-6] JS1
### Animal Models for Cancer Research - From the Mice to Comparative Oncology

2025 / 9 / 25 (Thu) 12:50-15:20 Room 3/(Hotel Nikko Kanazawa 4F Tsuru A) Room 3

[Chairpersons]Masanobu Oshima (Division of Genetics, Cancer Research Institute, Kanazawa University)、Takayuki Nakagawa (Laboratory of Veterinary Surgery, Graduate School of Agricultural and Life sciences, The University of Tokyo)、Amy K LeBlanc (Senior Scientist and Director, Comparative Oncology Program, National Institutes of Health)

Animal models are essential tools for cancer research. To date, attempts have been made to develop animal models that faithfully recapitulate the molecular etiology and pathophysiology of human cancer, such as syngeneic/xenogeneic tumor-bearing mouse models, patient-derived xenografted mouse models, humanized mouse models and genetically modified mouse models. Furthermore, over the past two decades, the NIH/NIC in the USA has led the development of novel animal models using naturally occurring tumors in pet dogs, known as the “Comparative Oncology Project”, and has successfully accumulated critical knowledge through the biological studies including genome and informatics analysis. Moreover, the canine tumor models are now used to evaluate immunotherapy for rare cancers as the “Cancer Moonshot Project” in the United States, which is being promoted around the world. The aim of this symposium is to provide the basics from the cutting-edge mouse models to naturally occurring canine tumor models, as well as their practical application in future research. These approaches will greatly contribute to the innovative cancer research and therapeutic strategy.

### JS1-6
### Evaluation of podplanin as a therapeutic target and as an amoeboid invasion driver by comparative oncological approach

[Speaker]Takayuki Nakagawa (Lab. Vet. Surg., Grad. Sch. of Agri., Univ. of Tokyo) [Co-author]Daiki Kato (Lab. Vet. Surg., Grad. Sch. of Agri., Univ. of Tokyo)

There is a lack of ideal animal models that possess a naturally occurring tumor microenvironment and enable long-term observation of clinical behavior, thereby closely mimicking human cancer patients. This represents a significant barrier to the research and development of novel therapies. However, client-owned pet dogs develop naturally occurring cancers through exposure to similar carcinogenic factors as humans, receive veterinary care, and exhibit long-term clinical behavior comparable to that seen in human patients, making them a valuable animal model.We have revealed that podoplanin (PDPN) overexpression in tumor tissues is associated with poor prognosis in dogs with mucosal melanoma. Moreover, overexpressed PDPN promoted proliferation and aggressive amoeboid invasion in both canine and human mucosal melanoma. Furthermore, an anti-dog PDPN antibody demonstrated clinical feasibility in canine patients with naturally occurring mucosal melanoma. Overall, these findings demonstrate the cross-species conservation of PDPN-driven malignant transformation in mucosal melanoma and highlight the utility of naturally occurring canine cancer models.

[Disclosure of COI by Responsible researcher] Lead presenter is the Responsible researcher.
[Lead presenter: Takayuki Nakagawa] We have no financial relationships to disclose.

▲TOP

### [JS1-5] JS1
### Animal Models for Cancer Research - From the Mice to Comparative Oncology

2025 / 9 / 25 (Thu) 12:50-15:20 Room 3/(Hotel Nikko Kanazawa 4F Tsuru A) Room 3

[Chairpersons]Masanobu Oshima (Division of Genetics, Cancer Research Institute, Kanazawa University)、Takayuki Nakagawa (Laboratory of Veterinary Surgery, Graduate School of Agricultural and Life sciences, The University of Tokyo)、Amy K LeBlanc (Senior Scientist and Director, Comparative Oncology Program, National Institutes of Health)

Animal models are essential tools for cancer research. To date, attempts have been made to develop animal models that faithfully recapitulate the molecular etiology and pathophysiology of human cancer, such as syngeneic/xenogeneic tumor-bearing mouse models, patient-derived xenografted mouse models, humanized mouse models and genetically modified mouse models. Furthermore, over the past two decades, the NIH/NIC in the USA has led the development of novel animal models using naturally occurring tumors in pet dogs, known as the “Comparative Oncology Project”, and has successfully accumulated critical knowledge through the biological studies including genome and informatics analysis. Moreover, the canine tumor models are now used to evaluate immunotherapy for rare cancers as the “Cancer Moonshot Project” in the United States, which is being promoted around the world. The aim of this symposium is to provide the basics from the cutting-edge mouse models to naturally occurring canine tumor models, as well as their practical application in future research. These approaches will greatly contribute to the innovative cancer research and therapeutic strategy.

---

### JS1-5
### Evaluation of Frozen Tissue in Zebrafish PDX Models for Bladder Cancer Research and Preclinical Validation

[Speaker]Xin Bao (Department of Nephro-Urologic Surgery and Andrology, Mie University／Center for medical zebrafish research, Mie University) [Co-author]Yusuke Sugino (Department of Nephro-Urologic Surgery and Andrology, Mie University／Center for medical zebrafish research, Mie University)、Takumi Kageyama (Department of Nephro-Urologic Surgery and Andrology, Mie University)、Sho Sekito (Department of Nephro-Urologic Surgery and Andrology, Mie University)、Shiori Miyachi (Department of Nephro-Urologic Surgery and Andrology, Mie University)、Takeshi Sasaki (Department of Nephro-Urologic Surgery and Andrology, Mie University)、Toshio Tanaka (Center for medical zebrafish research, Mie University／Department of Systems Pharmacology, Mie University Graduate School of Medicine)、Takahiro Inoue (Department of Nephro-Urologic Surgery and Andrology, Mie University／Center for medical zebrafish research, Mie University)

**Objectives:** This study compared fresh vs frozen bladder tumor tissues in zebrafish patient-derived xenografts(zPDX) models to assess frozen tissue reliability. **Methods:** Bladder cancer tissues derived from mouse patient-derived xenografts (mPDX)models (mieUC5/9) and patient sample (mieUC17) were divided into fresh and frozen (cryopreserved for 1 week to 6 months) groups. Further, isolated cells were fluorescently labeled and injected into the zebrafish larvae. The control group was injected with cancer cells only, while the CDDP group was co-injected with cancer cells and 400 µM cisplatin. Relative tumor area were calculated at 1 and 2 days post-injection(dpi). **Results:** The frozen tissue groups achieved a transplantation success rate comparable to that of fresh tissues. Tumor proliferation (1/2 dpi relative area) in frozen groups showed minor reductions but no statistical significance. Additionally, mieUC17 outcomes indicated a cisplatin-resistant phenotype, which was consistent with clinical observations.**Conclusion:** Frozen tissues could be reliably applied in zPDX establishment, enabling multicenter collaborations for high-throughput CDDP resistance drug screening.

[Disclosure of COI by Responsible researcher] Lead presenter is not the Responsible researcher.
[Lead presenter: Xin Bao] ■Research funds from company: 三重大学　■Research expenses: 三重大学　■Representative of organization for clinical study receiving research expenses from company: 国立大学法人三重大学
[Principal Researcher: Takahiro Inoue] ■Research funds from company: 三重大学　■Research expenses: 三重大学
■Representative of organization for clinical study receiving research expenses from company: 国立大学法人三重大学

▲TOP

### [OS4-1] OS4
### Current Advances in Bone-Related Cancer Research

2025 / 9 / 25 (Thu) 12:50-15:20 Room 4/(Hotel Nikko Kanazawa 4F Tsuru B) Room 4

[Chairpersons]Kazuo Okamoto (Division of Immune Environment Dynamics, Cancer Research Institute, Kanazawa University)、Hiroshi Takayanagi (Department of Immunology Graduate School of Medicine and Faculty of Medicine, The University of Tokyo)

Bone tissue is a favorite site of tumor metastasis and is also the site where osteosarcoma and multiple myeloma develop. Bone has a unique assembly of cells, including osteoclasts, osteoblasts, chondrocytes and periosteal cells. In recent years, various mesenchymal stem cells involved in skeletal development have been identified, leading to new advances in the study of multicellular networks in bone. In addition, bone marrow provides the specialized microenvironment essential for the maintenance and differentiation of hematopoietic stem cells and the progeny. This symposium will be held as an interdisciplinary session by cancer researchers and experts in bone biology, focusing on cancers associated with the bone marrow environment, such as bone metastasis, osteosarcoma, cancer-related bone invasion and hematopoietic malignancies. We aim to deepen our understanding of the unique features of cancers associated with bone tissue and to discuss the prospects for innovative treatment strategies.

---

### OS4-1
### Bone's unique traits driving the progression of bone metastasis

[Speaker]Kazuo Okamoto (Div. of Immune Environment Dynamics, CRI, Kanazawa Univ／Immune Network Research Unit, InFiniti, Kanazawa Univ)

Bone possesses various properties that make it a fertile soil for tumor metastasis and progression. Aberrant expression of the TNF family cytokine RANKL by metastatic tumor cells causes pathological bone resorption, leading to skeletal tumor progression. The "vicious cycle" of tumor cells, osteoblasts and osteoclasts is a central mechanism underlying bone metastasis. Notably, RANKL contributes to bone metastasis not only by promoting the vicious cycle but also through osteoclast-independent mechanisms. We previously revealed that bone-derived soluble RANKL promotes bone metastasis by exerting chemotactic activity on tumor cells. Beyond soluble RANKL, bone-enriched chemokines such as CXCL12 also enhance cancer cell bone tropism. Moreover, since the bone marrow harbors hematopoietic stem cells and immune progenitor cells, disruption of the bone marrow by tumor burden can affect the immune system through the hematopoietic alterations. Deciphering the unique tumor microenvironment formed by the multicellular network of immune, bone and tumor cells would provide a more comprehensive understanding of the pathogenesis of bone metastases and guide the development of cancer immunotherapy.

[Disclosure of COI by Responsible researcher] Lead presenter is the Responsible researcher.
[Lead presenter: Kazuo Okamoto] ■Research funds from company: 日本学術振興会、科学技術振興機構　■Contributions: 公益財団法人アステラス病態代謝研究会

▲TOP

### [OS4-2] OS4
### Current Advances in Bone-Related Cancer Research

2025 / 9 / 25 (Thu) 12:50-15:20 Room 4/(Hotel Nikko Kanazawa 4F Tsuru B) Room 4

[Chairpersons]Kazuo Okamoto (Division of Immune Environment Dynamics, Cancer Research Institute, Kanazawa University)、Hiroshi Takayanagi (Department of Immunology Graduate School of Medicine and Faculty of Medicine, The University of Tokyo)

Bone tissue is a favorite site of tumor metastasis and is also the site where osteosarcoma and multiple myeloma develop. Bone has a unique assembly of cells, including osteoclasts, osteoblasts, chondrocytes and periosteal cells. In recent years, various mesenchymal stem cells involved in skeletal development have been identified, leading to new advances in the study of multicellular networks in bone. In addition, bone marrow provides the specialized microenvironment essential for the maintenance and differentiation of hematopoietic stem cells and the progeny. This symposium will be held as an interdisciplinary session by cancer researchers and experts in bone biology, focusing on cancers associated with the bone marrow environment, such as bone metastasis, osteosarcoma, cancer-related bone invasion and hematopoietic malignancies. We aim to deepen our understanding of the unique features of cancers associated with bone tissue and to discuss the prospects for innovative treatment strategies.

### OS4-2
### The malignant transformation of endosteal skeletal stem cells into cancer-initiating cells

[Speaker]Yuki Matsushita (Dept. Skeletal Dev. Regen. Biol., Nagasaki Univ.)

Bone provides mechanical support and enables locomotion, as well as serving as the primary site of hematopoiesis in mammals. Within the bone marrow, skeletal, hematopoietic, and vascular cells form a dynamic network known as the bone marrow niche, which is essential for tissue homeostasis. Although skeletal stem cells (SSCs) have long been assumed to exist in the bone marrow and are thought to support bone growth, regeneration, and maintenance, their precise localization has remained unclear.Here, we identified a novel SSC population in young bone marrow using single-cell transcriptomics and ***in vivo*** lineage tracing. We termed these cells endosteal stem cells (E-SSCs) and showed that they contribute to bone formation and repair. Conditional deletion of the tumor suppressor gene ***Trp53*** in E-SSCs caused abnormal expansion and the development of aggressive osteosarcomas. Δp53-E-SSCs exhibited enhanced self-renewal ***ex vivo,*** and transplantation into immunodeficient mouse bone marrow led to tumor formation ***in vivo.***These findings demonstrate that E-SSCs are essential for bone homeostasis and regeneration, and may act as a cell of origin for osteosarcoma under oncogenic stress.

[Disclosure of COI by Responsible researcher] Lead presenter is the Responsible researcher.
[Lead presenter: Yuki Matsushita] We have no financial relationships to disclose.

### [OS4-3] OS4
### Current Advances in Bone-Related Cancer Research

2025 / 9 / 25 (Thu) 12:50-15:20 Room 4/(Hotel Nikko Kanazawa 4F Tsuru B) Room 4

[Chairpersons]Kazuo Okamoto (Division of Immune Environment Dynamics, Cancer Research Institute, Kanazawa University)、Hiroshi Takayanagi (Department of Immunology Graduate School of Medicine and Faculty of Medicine, The University of Tokyo)

Bone tissue is a favorite site of tumor metastasis and is also the site where osteosarcoma and multiple myeloma develop. Bone has a unique assembly of cells, including osteoclasts, osteoblasts, chondrocytes and periosteal cells. In recent years, various mesenchymal stem cells involved in skeletal development have been identified, leading to new advances in the study of multicellular networks in bone. In addition, bone marrow provides the specialized microenvironment essential for the maintenance and differentiation of hematopoietic stem cells and the progeny. This symposium will be held as an interdisciplinary session by cancer researchers and experts in bone biology, focusing on cancers associated with the bone marrow environment, such as bone metastasis, osteosarcoma, cancer-related bone invasion and hematopoietic malignancies. We aim to deepen our understanding of the unique features of cancers associated with bone tissue and to discuss the prospects for innovative treatment strategies.

---

### OS4-3
### The interaction between hematological malignancies and the bone marrow microenvironment through the CXCL12-CXCR4 axis

[Speaker]Kazunari Aoki (LiMe, Kyoto University)

Acute leukemia cells and multiple myeloma cells are maintained within specialized bone marrow microenvironments known as bone marrow niches. Recent studies have identified the major cellular components of these niches as mesenchymal stem cells, known as CXCL12-abundant reticular (CAR)/LEPR$^{+}$ cells, and vascular endothelial cells. These niche cells are the primary source of CXCL12 in the bone marrow and play a critical role in retaining these malignant cells within the marrow, thereby supporting their survival and proliferation.
Acute leukemia cells and multiple myeloma cells express CXCR4, the physiological receptor for CXCL12. Although the regulatory mechanisms of CXCR4 expression had not been fully elucidated, we discovered that in T-cell acute lymphoblastic leukemia, the canonical BRG1/BRM-associated factor complex regulates the chromatin accessibility at RUNX1-binding sites, thereby controlling CXCR4 expression.
In the future, elucidating the disease-specific regulatory mechanisms of the CXCL12-CXCR4 axis may lead to the development of novel therapies that target the interaction between malignant cells and the bone marrow microenvironment, with reduced adverse effects.

[Disclosure of COI by Responsible researcher] Lead presenter is the Responsible researcher.
[Lead presenter: Kazunari Aoki] We have no financial relationships to disclose.

### [OS4-4] OS4
### Current Advances in Bone-Related Cancer Research

2025 / 9 / 25 (Thu) 12:50-15:20 Room 4/(Hotel Nikko Kanazawa 4F Tsuru B) Room 4

[Chairpersons]Kazuo Okamoto (Division of Immune Environment Dynamics, Cancer Research Institute, Kanazawa University)、Hiroshi Takayanagi (Department of Immunology Graduate School of Medicine and Faculty of Medicine, The University of Tokyo)

Bone tissue is a favorite site of tumor metastasis and is also the site where osteosarcoma and multiple myeloma develop. Bone has a unique assembly of cells, including osteoclasts, osteoblasts, chondrocytes and periosteal cells. In recent years, various mesenchymal stem cells involved in skeletal development have been identified, leading to new advances in the study of multicellular networks in bone. In addition, bone marrow provides the specialized microenvironment essential for the maintenance and differentiation of hematopoietic stem cells and the progeny. This symposium will be held as an interdisciplinary session by cancer researchers and experts in bone biology, focusing on cancers associated with the bone marrow environment, such as bone metastasis, osteosarcoma, cancer-related bone invasion and hematopoietic malignancies. We aim to deepen our understanding of the unique features of cancers associated with bone tissue and to discuss the prospects for innovative treatment strategies.

---

### OS4-4
### Beyond defense: innate immune signaling in the pathogenesis of hematologic cancers

[Speaker]Tomoya Muto (National Cancer Center Research Institute, Division of Cancer RNA Research)

Hematologic malignancies such as myelodysplastic syndromes and acute myeloid leukemia are rising with the aging population. These diseases often arise from clonal hematopoiesis, where driver mutations in hematopoietic stem cells trigger clonal expansion and subsequent mutations drive malignant transformation. Innate immunity, initially a defense against pathogens, is now recognized as a key contributor to hematologic cancer development. A central player is the E3 ubiquitin ligase TRAF6, which forms K63-linked polyubiquitin chains acting independently of protein degradation. These modifications promote TRAF6 ubiquitination and activate downstream signals like NF-κB, initiating inflammatory responses. Our recent experimental findings show that in hematologic malignancies, TRAF6 drives leukemogenesis not only via inflammation but also through mechanisms involving metabolism, transcriptional regulation, RNA splicing, and replication stress. These findings highlight the diverse, context-dependent roles of innate immune signaling-particularly TRAF6-mediated pathways-in disease progression. In this symposium, I will present our latest experimental data illustrating these mechanisms.

[Disclosure of COI by Responsible researcher] Lead presenter is the Responsible researcher.
[Lead presenter: Tomoya Muto] We have no financial relationships to disclose.

### [OS4-5] OS4
### Current Advances in Bone-Related Cancer Research

2025 / 9 / 25 (Thu) 12:50-15:20 Room 4/(Hotel Nikko Kanazawa 4F Tsuru B) Room 4

[Chairpersons]Kazuo Okamoto (Division of Immune Environment Dynamics, Cancer Research Institute, Kanazawa University)、Hiroshi Takayanagi (Department of Immunology Graduate School of Medicine and Faculty of Medicine, The University of Tokyo)

Bone tissue is a favorite site of tumor metastasis and is also the site where osteosarcoma and multiple myeloma develop. Bone has a unique assembly of cells, including osteoclasts, osteoblasts, chondrocytes and periosteal cells. In recent years, various mesenchymal stem cells involved in skeletal development have been identified, leading to new advances in the study of multicellular networks in bone. In addition, bone marrow provides the specialized microenvironment essential for the maintenance and differentiation of hematopoietic stem cells and the progeny. This symposium will be held as an interdisciplinary session by cancer researchers and experts in bone biology, focusing on cancers associated with the bone marrow environment, such as bone metastasis, osteosarcoma, cancer-related bone invasion and hematopoietic malignancies. We aim to deepen our understanding of the unique features of cancers associated with bone tissue and to discuss the prospects for innovative treatment strategies.

---

### OS4-5
### Multi omics analysis of bone and soft tissue sarcomas

[Speaker]Koichi Matsuda (GSFS Universigy of Tokyo)

Bone and soft tissue sarcoma (BSTs) is a rare cancer showing remarkable diversity. Through the collaboration of 40 institutes, we conducted whole exome and RNA sequencing of dedifferentiated liposarcoma and tenosynovial giant cell tumor, and revealed CTDSP1/2-DNM3OS fusion genes (8%) and CBL mutation (35%) that was associated with poor prognosis and JAK2 activation, respectively. Whole genome and transcriptome analysis of 594 BSTs across multiple subtypes, those are classified into 2 categories: translocation-related sarcomas (TRS) and complex-karyotype sarcomas (CKS) without known fusion. Both the tumor mutation burden and number of structural variants were higher in CKS than in TRS. Somatic mutations in cancer-related gene, such as TP53, KMT2C, MUC16 and ATRX, as well as in cis-regulatory element of TERT were detected as recurrent, most of which was more frequent in CKS than in TRS. In addition, proteogenomic analysis including WES, RNA-seq, and total proteome of myxoid liposarcoma identified differentially expressed proteins that were associated with poor prognosis. Our study provides a biological insight and suggests a novel therapeutic strategy for BSTs.

[Disclosure of COI by Responsible researcher] Lead presenter is the Responsible researcher.
[Lead presenter: Koichi Matsuda] We have no financial relationships to disclose.

▲TOP

### [OS4-6] OS4
### Current Advances in Bone-Related Cancer Research

2025 / 9 / 25 (Thu) 12:50-15:20 Room 4/(Hotel Nikko Kanazawa 4F Tsuru B) Room 4

[Chairpersons]Kazuo Okamoto (Division of Immune Environment Dynamics, Cancer Research Institute, Kanazawa University)、Hiroshi Takayanagi (Department of Immunology Graduate School of Medicine and Faculty of Medicine, The University of Tokyo)

Bone tissue is a favorite site of tumor metastasis and is also the site where osteosarcoma and multiple myeloma develop. Bone has a unique assembly of cells, including osteoclasts, osteoblasts, chondrocytes and periosteal cells. In recent years, various mesenchymal stem cells involved in skeletal development have been identified, leading to new advances in the study of multicellular networks in bone. In addition, bone marrow provides the specialized microenvironment essential for the maintenance and differentiation of hematopoietic stem cells and the progeny. This symposium will be held as an interdisciplinary session by cancer researchers and experts in bone biology, focusing on cancers associated with the bone marrow environment, such as bone metastasis, osteosarcoma, cancer-related bone invasion and hematopoietic malignancies. We aim to deepen our understanding of the unique features of cancers associated with bone tissue and to discuss the prospects for innovative treatment strategies.

---

### OS4-6
### Defenses and vulnerabilities specific to bone-related cancers

[Speaker]Hiroshi Takayanagi (Dept. of Immunol., Grad. Sch. of Med., Univ. Tokyo)

Bone is a unique site in cancer biology, where invasion or metastasis severely worsens prognosis, suggesting specialized defense mechanisms. The limited effect of immune checkpoint inhibitors in bone metastasis implies unique immune evasion strategies in the bone microenvironment. Cancer spreads via both metastasis and direct invasion, with bone invasion being particularly harmful. We studied the mechanism of periosteal thickening (periosteal reaction) in head and neck squamous cell carcinoma and its mouse model. When tumors approach bone, Timp1 expression inhibits MMP-mediated matrix degradation, causing ECM accumulation and periosteal cell proliferation. In mice, bone invasion does not occur unless the periosteum is damaged. However, in Timp1-deficient mice, invasion happens even without damage, indicating a protective stromal role. We also identified unique immune cell populations associated with immunosuppression in bone metastasis. This study reveals the distinctive defense mechanisms and vulnerabilities of bone in cancer, providing insights into potential therapeutic targets for bone-invasive cancers.

[Disclosure of COI by Responsible researcher] Lead presenter is the Responsible researcher.
[Lead presenter: Hiroshi Takayanagi] We have no financial relationships to disclose.

### [IS3-1] IS3
### Novel application of extracellular secretory vesicles (Evs) in cancer therapy

2025 / 9 / 25 (Thu) 12:50-15:20 Room 5/(Hotel Nikko Kanazawa 4F Tsuru C) Room 5

[Chairpersons]Masahiko Kuroda (Department of Molecular Pathology, Tokyo Medical University)、Andreas Moeller (University of Hong Kong)

Translational Research (TR) is crucial to the development of cancer therapy, as it enables medical discoveries and new technologies to be applied clinically. This session will focus on extracellular vesicles (EVs, exosomes), which are attracting attention as a new modality. EVs transport various cellular constituents such as miRNA, mRNA, DNA, lipids, and proteins over long distances and affect many physiological and pathological states including cancer microenvironment. Interestingly, vesicles with EVs-like activities like those in humans have been identified in plants and bacteria. Various clinical applications of these plant-derived EVs are also in progress. EVs also play a crucial role in intracellular signal transduction in cancer therapy, making them promising candidates for therapeutic agents, drug delivery systems（DDS）, and cancer biomarkers for Lquid biopsy. Therefore, in this session, we would like to focus on various types of EVs and introduce clinical applications of EVs in cancer TR research. We hope that this session will lead to suggestions for new cancer therapy.

---

### IS3-1
### Gene Editing-Based Cancer Therapy Using Acerola-Derived Nanovesicles

[Speaker]Masahiko Kuroda (Dep. Mol. Pathol., Tokyo Med. Univ.)

We previously focused on extracellular vesicles (EVs) as DDS and showed that engineered EVs targeting EGFR can selectively deliver tumor-suppressive miRNAs to cancer cells. However, EVs from animal cells face challenges like immunogenicity and high production costs, limiting clinical use. To address this, we turned to plant-derived EV-like nanovesicles and successfully isolated stable particles from acerola (Acerola-derived Exosome-Like Nanovesicles: AELNs). AELNs can form complexes with nucleic acid therapeutics and Cas proteins, are suitable for oral administration, and offer potential as next-generation DDS. In this study, we are developing a novel RNA-targeted therapeutic system, "K-CRASH," which uses the RNA endonuclease Cas13a and crRNAs that specifically recognize KRAS mutant mRNAs. By altering crRNA sequences, this system can flexibly target various mutations such as G12D and G12C. Since Cas13a acts on RNA without affecting the genome, it offers a safer and potentially faster-acting therapeutic option. Combining the safety and delivery benefits of AELNs with the precision of Cas13a, this strategy holds promise for treating therapy-resistant KRAS-mutant cancers.

[Disclosure of COI by Responsible researcher] Lead presenter is the Responsible researcher.
[Lead presenter: Kuroda Masahiko] We have no financial relationships to disclose.

▲TOP

### [IS3-2] IS3
### Novel application of extracellular secretory vesicles (Evs) in cancer therapy

2025 / 9 / 25 (Thu) 12:50-15:20 Room 5/(Hotel Nikko Kanazawa 4F Tsuru C) Room 5

[Chairpersons]Masahiko Kuroda (Department of Molecular Pathology, Tokyo Medical University)、Andreas Moeller (University of Hong Kong)

Translational Research (TR) is crucial to the development of cancer therapy, as it enables medical discoveries and new technologies to be applied clinically. This session will focus on extracellular vesicles (EVs, exosomes), which are attracting attention as a new modality. EVs transport various cellular constituents such as miRNA, mRNA, DNA, lipids, and proteins over long distances and affect many physiological and pathological states including cancer microenvironment. Interestingly, vesicles with EVs-like activities like those in humans have been identified in plants and bacteria. Various clinical applications of these plant-derived EVs are also in progress. EVs also play a crucial role in intracellular signal transduction in cancer therapy, making them promising candidates for therapeutic agents, drug delivery systems（DDS）, and cancer biomarkers for Lquid biopsy. Therefore, in this session, we would like to focus on various types of EVs and introduce clinical applications of EVs in cancer TR research. We hope that this session will lead to suggestions for new cancer therapy.

### IS3-2
### Translation of extracellular vesicles from bench to bedside

[Speaker]Andreas Moeller (Faculty of Science, The Chinese University of Hong Kong)

Extracellular vesicles (EVs) present a huge, yet largely untapped resource for disease diagnostics and therapeutics. While significant advancements have been made in bench-based investigations, these have largely not yet been successfully translated to clinical or commercial applications. In the context of cancer, the evaluation of EV cargo composition changes allowed us to develop highly sensitive, liquid biopsy diagnostic and prognostic biomarkers. In this presentation, the translational work, including the validation in retrospective patient cohorts and prospective trials will be discussed. Some of the technical and logistic challenges faced when transitioning bench methods to bedside applications, and their implementation into a hospital setting, will be highlighted.

[Disclosure of COI by Responsible researcher] Lead presenter is the Responsible researcher.
[Lead presenter: Andreas Moeller] We have no financial relationships to disclose.

### [IS3-3] IS3
### Novel application of extracellular secretory vesicles (Evs) in cancer therapy

2025 / 9 / 25 (Thu) 12:50-15:20 Room 5/(Hotel Nikko Kanazawa 4F Tsuru C) Room 5

[Chairpersons]Masahiko Kuroda (Department of Molecular Pathology, Tokyo Medical University)、Andreas Moeller (University of Hong Kong)

Translational Research (TR) is crucial to the development of cancer therapy, as it enables medical discoveries and new technologies to be applied clinically. This session will focus on extracellular vesicles (EVs, exosomes), which are attracting attention as a new modality. EVs transport various cellular constituents such as miRNA, mRNA, DNA, lipids, and proteins over long distances and affect many physiological and pathological states including cancer microenvironment. Interestingly, vesicles with EVs-like activities like those in humans have been identified in plants and bacteria. Various clinical applications of these plant-derived EVs are also in progress. EVs also play a crucial role in intracellular signal transduction in cancer therapy, making them promising candidates for therapeutic agents, drug delivery systems（DDS）, and cancer biomarkers for Lquid biopsy. Therefore, in this session, we would like to focus on various types of EVs and introduce clinical applications of EVs in cancer TR research. We hope that this session will lead to suggestions for new cancer therapy.

---

### IS3-3
### Spatiotemporal analysis of metastatic niche formation by breast cancer extracellular vesicles using tissue slice models

[Speaker]Nao Nishida-Aoki (Waseda Institute for Advanced Study, Waseda Univ.)

Metastatic colonization requires tumor cells to actively reprogram the local microenvironment into a tumor-supportive niche. Extracellular vesicles (EVs), small membrane-bound vesicles secreted by cancer cells, are key mediators of this early remodeling process, yet their spatiotemporal dynamics in distant organs remain poorly understood. To investigate the roles of breast cancer-derived EV dynamics in metastatic niche formation, we established ***ex vivo*** breast cancer metastatic models based on organotypic tissue slice cultures of mouse brain and lungs. By employing HaloTag-labeled EVs and two-photon microscopy, we achieved real-time visualization of EV secretion within native tissue architecture. We observed distinct spatiotemporal patterns of EV localization and organ-specific uptake by stromal and immune cell subsets, suggesting selective EV targeting contributes to organotropic metastasis. Our platform provides a physiologically relevant approach to dissect cancer-host cell interactions mediated by EVs and may contribute to the identification of therapeutic strategies targeting early metastatic niche formation.

[Disclosure of COI by Responsible researcher] Lead presenter is the Responsible researcher.
[Lead presenter: Nao Nishida] We have no financial relationships to disclose.

### [IS3-4] IS3
### Novel application of extracellular secretory vesicles (Evs) in cancer therapy

2025 / 9 / 25 (Thu) 12:50-15:20 Room 5/(Hotel Nikko Kanazawa 4F Tsuru C) Room 5

[Chairpersons]Masahiko Kuroda (Department of Molecular Pathology, Tokyo Medical University)、Andreas Moeller (University of Hong Kong)

Translational Research (TR) is crucial to the development of cancer therapy, as it enables medical discoveries and new technologies to be applied clinically. This session will focus on extracellular vesicles (EVs, exosomes), which are attracting attention as a new modality. EVs transport various cellular constituents such as miRNA, mRNA, DNA, lipids, and proteins over long distances and affect many physiological and pathological states including cancer microenvironment. Interestingly, vesicles with EVs-like activities like those in humans have been identified in plants and bacteria. Various clinical applications of these plant-derived EVs are also in progress. EVs also play a crucial role in intracellular signal transduction in cancer therapy, making them promising candidates for therapeutic agents, drug delivery systems（DDS）, and cancer biomarkers for Lquid biopsy. Therefore, in this session, we would like to focus on various types of EVs and introduce clinical applications of EVs in cancer TR research. We hope that this session will lead to suggestions for new cancer therapy.

---

### IS3-4
### Intercepting Extracellular Vesicle Signals to improve diagnosis and treatment of Endometrial Cancer

[Speaker]Cherie Blenkiron (FMHS, The University of Auckland, Auckland, New Zealand) [Co-author]Anastasiia Artuyants (FMHS, The University of Auckland, Auckland, New Zealand)、Zi Yin Enid He (FMHS, The University of Auckland, Auckland, New Zealand)、Sophia Bebelman (FMHS, The University of Auckland, Auckland, New Zealand)、Claire Henry (Department of Surgery, University of Otago, Wellington, New Zealand)、Sandra Fitzgerald (FMHS, The University of Auckland, Auckland, New Zealand)

Globally, the incidence of endometrial cancer, arising from the lining of the uterus, is increasing, in parallel with the co-morbidity of metabolic disease. New Zealand, in the Pacific, has one of the highest rates of metabolic disease, escalating diagnoses of endometrial cancer.Our research aims to understand the biology of endometrial cancer, and to exploit this knowledge to improve diagnosis and treatment selection. First, we have explored the effect of hyperglycemia 3D cultured cell-lines and their production of extracellular vesicles (EV), finding alterations in EV release and cargo. EVs had functional effects on cell behaviour such as adhesion to matrix and colony formation.Second, we create biomarker tests for minimally invasive diagnosis including the analysis of microbiomes, mutations and methylation in swabs. We are also developing a blood-based targeted EV proteomics panel designed to separate endometrial cancer subtypes for prognostication. Our goal is to develop sensitive, affordable and acceptable approaches to test for endometrial cancer. Additionally, understanding the effects of metabolic conditions on cancer initiation may identify new preventative approaches.

[Disclosure of COI by Responsible researcher] Lead presenter is the Responsible researcher.
[Lead presenter: Cherie Blenkiron] We have no financial relationships to disclose.

▲TOP

### [IS3-5] IS3
### Novel application of extracellular secretory vesicles (Evs) in cancer therapy

2025 / 9 / 25 (Thu) 12:50-15:20 Room 5/(Hotel Nikko Kanazawa 4F Tsuru C) Room 5

[Chairpersons]Masahiko Kuroda (Department of Molecular Pathology, Tokyo Medical University)、Andreas Moeller (University of Hong Kong)

Translational Research (TR) is crucial to the development of cancer therapy, as it enables medical discoveries and new technologies to be applied clinically. This session will focus on extracellular vesicles (EVs, exosomes), which are attracting attention as a new modality. EVs transport various cellular constituents such as miRNA, mRNA, DNA, lipids, and proteins over long distances and affect many physiological and pathological states including cancer microenvironment. Interestingly, vesicles with EVs-like activities like those in humans have been identified in plants and bacteria. Various clinical applications of these plant-derived EVs are also in progress. EVs also play a crucial role in intracellular signal transduction in cancer therapy, making them promising candidates for therapeutic agents, drug delivery systems（DDS）, and cancer biomarkers for Lquid biopsy. Therefore, in this session, we would like to focus on various types of EVs and introduce clinical applications of EVs in cancer TR research. We hope that this session will lead to suggestions for new cancer therapy.

---

### IS3-5
### Development of Bispecific Extracellular Vesicles for Cancer Immunotherapy

[Speaker]Minh Le (Dept. Pharm, National University of Singapore) [Co-author]Phuong Nguyen (Dept. Pharm, National University of Singapore)、Lan Tran (Dept. Pharm, National University of Singapore)、Migara Jayasinghe (Dept. Pharm, National University of Singapore)

We have developed a biocompatible and efficient bispecific extracellular vesicle (BEV) platform using natural EVs from human red blood cells. BEVs are surface-functionalized with antibodies targeting CD3 on T cells and tumor antigens on cancer cells, enabling localized immune activation. BEVs activate T cells as shown by increased pro-inflammatory cytokine secretion and upregulation of activation markers. BEVs induce stronger T cell responses than equivalent doses of free antibodies including bispecific antibodies, suggesting enhanced potency at lower therapeutic concentrations and reduced risk of toxicity.
BEVs targeting PD-L1 or Claudin-18.2 recruit activated T cells to tumor sites, significantly reduce tumor burden, and prolong survival in mouse models of immunosuppressive lung and pancreatic cancer, respectively. Importantly, systemic administration of BEVs demonstrates a favorable safety profile in non-human primates, with no evidence of cytokine release syndrome. Our engineering approach is rapid, stable, scalable and adaptable to multiple tumor antigens. This establishes a versatile EV-based immunotherapy platform with significant potential for clinical translation.

[Disclosure of COI by Responsible researcher] Lead presenter is the Responsible researcher.
[Lead presenter: Minh Le] ■Research funds from company: CARMINE THERAPEUTICS PTE LTD

### [IS3-6] IS3
### Novel application of extracellular secretory vesicles (Evs) in cancer therapy

2025 / 9 / 25 (Thu) 12:50-15:20 Room 5/(Hotel Nikko Kanazawa 4F Tsuru C) Room 5

[Chairpersons]Masahiko Kuroda (Department of Molecular Pathology, Tokyo Medical University)、Andreas Moeller (University of Hong Kong)

Translational Research (TR) is crucial to the development of cancer therapy, as it enables medical discoveries and new technologies to be applied clinically. This session will focus on extracellular vesicles (EVs, exosomes), which are attracting attention as a new modality. EVs transport various cellular constituents such as miRNA, mRNA, DNA, lipids, and proteins over long distances and affect many physiological and pathological states including cancer microenvironment. Interestingly, vesicles with EVs-like activities like those in humans have been identified in plants and bacteria. Various clinical applications of these plant-derived EVs are also in progress. EVs also play a crucial role in intracellular signal transduction in cancer therapy, making them promising candidates for therapeutic agents, drug delivery systems（DDS）, and cancer biomarkers for Lquid biopsy. Therefore, in this session, we would like to focus on various types of EVs and introduce clinical applications of EVs in cancer TR research. We hope that this session will lead to suggestions for new cancer therapy.

---

### IS3-6
### Bioengineered iRGD exosomes antagonize the malignant properties enabled by EBV-miR-BART3-3p

[Speaker]Qianqing Fan (Dept. of Med. Oncology, Cancer Hosp. of Guangxi Med. Univ.) [Co-author]Qiuyun Li (Dept. of Med. Oncology, Cancer Hosp. of Guangxi Med. Univ.)、Lihong Huang (Dept. of Otolaryngology & Head and Neck, People's Hosp. of Guangxi)、Yuping Liu (Dept. of Med. Oncology, Cancer Hosp. of Guangxi Med. Univ.)、Binli Cai (Dept. of Med. Oncology, Cancer Hosp. of Guangxi Med. Univ.)、Yong Chen (Dept. of Med. Oncology, Cancer Hosp. of Guangxi Med. Univ.)、Zhihui Liu (Dept. of Med. Oncology, Cancer Hosp. of Guangxi Med. Univ.)、Jiaxiang Ye (Dept. of Med. Oncology, Cancer Hosp. of Guangxi Med. Univ.)、Jiazhang Wei (Dept. of Otolaryngology & Head and Neck, People's Hosp. of Guangxi)、Jinyan Zhang (Dept. of Med. Oncology, Cancer Hosp. of Guangxi Med. Univ.)

Nasopharyngeal carcinoma (NPC) is a head and neck cancer closely related to Epstein-Barr virus (EBV) infection. EBV-encoded microRNAs modulate malignant phenotypes of NPC cells by inactivating various tumor suppressor genes. In the present study, bioinformatics analysis shows that of all the EBV-encoded miRNAs, EBV-miR-BART3-3p is abundantly expressed and associated with poor clinical outcomes in NPC. In vitro experiments demonstrated that EBV-miR-BART3-3p promoted proliferation and anti-apoptotic abilities in a Caspase 3-dependent manner by downregulating ***RASSF1*** in NPC cells. Bioengineered iRGD exosomes-mediated targeted delivery of the antagomiRs for EBV-miR-BART3-3p effectively reversed the malignant characteristics by rescuing ***RASSF1*** expression. These results indicate that development of the bioengineered exosomes carrying antagomiRs for EBV-miRNAs is a feasible strategy to treat EBV-relevant NPC.

[Disclosure of COI by Responsible researcher] Lead presenter is not the Responsible researcher.
[Lead presenter: Qianqing Fan] We have no financial relationships to disclose.
[Principal Researcher: Qianqing Fan] We have no financial relationships to disclose.

▲TOP

### [OS5-1] OS5
### Lights and Shadows of Database Utilization in Oncology Research

2025 / 9 / 25 (Thu) 12:50-14:20 Room 6/(Hotel Nikko Kanazawa 3F Kujaku A) Room 6

[Chairpersons]Shoichiro Tange (Department of Medical Genome Sciences, Sapporo Medical University)、Jun Nakayama (Osaka International Cancer Institute)

This year marks the 20th anniversary of TCGA, a project to collect clinical specimens primarily from the National Cancer Institute (NCI) and other institutions. While this database continues to be a useful source of information for cancer researchers, it does not fully reflect the progress of analysis methods in later years, and the possibility exists that hidden information may be buried. In addition, the lack of sharing of analysis methods and how-tos has led to misinterpretations due to wrong analysis methods and missing data in TCGA, resulting in erroneous research results. In this session, we will discuss the latest findings with researchers who aim to comprehensively reanalyze the information contained in TCGA and other public databases to find new horizons in cancer research. By sharing information gaps and analysis pitfalls in the databases, we hope to establish more robust analysis strategies, leading to the next generation of cancer research and the development of cancer treatments.

---

### OS5-1
### Development of Genome Editing Data Analysis Platform by Utilizing Public Databases

[Speaker]Hidemasa Bono (Genome Informatics, Grad. Sch. Integ. Sci. Hiroshima Univ.／Genome Editing Innov. Ctr. Hiroshima Univ.)

Cancer research is flooded with data, not only from Sequence Read Archive (SRA) but also from The Cancer Genome Atlas (TCGA) project. Although many studies have been conducted using such data, digital transformation (Bio-DX) in the life science field that fully utilizes such data is still in its infancy. Therefore, it is important to develop fundamental technologies for data utilization. We have developed a technology to utilize not only the transcriptome by RNA sequencing (RNA-seq) from public databases but also all the literature data (what we call bibliome) from Pubmed and PMC in order to find genes that can be targets for genome editing. In addition to individual genes, we have developed an integrated gene expression analysis environment based on pathway diagrams, called QPX (Quest for Pathways with eXpression), which will also be presented.

[Disclosure of COI by Responsible researcher] Lead presenter is the Responsible researcher.
[Lead presenter: Hidemasa Bono] We have no financial relationships to disclose.

### [OS5-2] OS5
### Lights and Shadows of Database Utilization in Oncology Research

2025 / 9 / 25 (Thu) 12:50-14:20 Room 6/(Hotel Nikko Kanazawa 3F Kujaku A) Room 6

[Chairpersons]Shoichiro Tange (Department of Medical Genome Sciences, Sapporo Medical University)、Jun Nakayama (Osaka International Cancer Institute)

This year marks the 20th anniversary of TCGA, a project to collect clinical specimens primarily from the National Cancer Institute (NCI) and other institutions. While this database continues to be a useful source of information for cancer researchers, it does not fully reflect the progress of analysis methods in later years, and the possibility exists that hidden information may be buried. In addition, the lack of sharing of analysis methods and how-tos has led to misinterpretations due to wrong analysis methods and missing data in TCGA, resulting in erroneous research results. In this session, we will discuss the latest findings with researchers who aim to comprehensively reanalyze the information contained in TCGA and other public databases to find new horizons in cancer research. By sharing information gaps and analysis pitfalls in the databases, we hope to establish more robust analysis strategies, leading to the next generation of cancer research and the development of cancer treatments.

### OS5-2
### Database analyses on colorectal cancer focusing on the gene coding estrogen receptor-β

[Speaker]Naoko Honma (Dept. Surg. Pathol., Toho Univ., Fucult. Med.) [Co-author]Takashi Nishina (Dept. Biochem., Toho Univ., Fucult. Med.)、Shoichiro Tange (Med. Genome Sci., Dept. Genomic Preventive Med., Sapporo Med. Univ.)

Estrogens and estrogen receptor-β (ER-β) have been proposed to play a suppressive role in colorectal cancer (CRC), although some controversies remain. Investigating the relationship between estrogens and CRC is complex, as aging and sex influence not only serum estrogen levels but also CRC characteristics, such as tumor locus or microsatellite instability (MSI). In a previous study, we examined estrogen levels and ER-β expression in paired cancerous and non-cancerous colon tissues from postmenopausal women. Our findings suggested that the estrogen-ER-β pathway plays a suppressive role in left-sided/microsatellite-stable tumors in younger women, but not in right-sided/MSI tumors in older women. In this study, we conducted a comprehensive analysis of the relationship between ESR2 (the gene encoding ER-β) and various factors in CRC using the TCGA database. Our results indicate that high ESR2 expression is correlated with right-sided, MSI, or BRAF-mutated tumors, suggesting a link to sporadic mismatch repair-deficient tumors, which are typically found in older women. These database analyses support the heterogeneous role of ER-β in CRC, as suggested in our previous study.

[Disclosure of COI by Responsible researcher] Lead presenter is the Responsible researcher.
[Lead presenter: Naoko Honma] We have no financial relationships to disclose.

### [OS5-3] OS5
### Lights and Shadows of Database Utilization in Oncology Research

2025 / 9 / 25 (Thu) 12:50-14:20 Room 6/(Hotel Nikko Kanazawa 3F Kujaku A) Room 6

[Chairpersons]Shoichiro Tange (Department of Medical Genome Sciences, Sapporo Medical University)、Jun Nakayama (Osaka International Cancer Institute)

This year marks the 20th anniversary of TCGA, a project to collect clinical specimens primarily from the National Cancer Institute (NCI) and other institutions. While this database continues to be a useful source of information for cancer researchers, it does not fully reflect the progress of analysis methods in later years, and the possibility exists that hidden information may be buried. In addition, the lack of sharing of analysis methods and how-tos has led to misinterpretations due to wrong analysis methods and missing data in TCGA, resulting in erroneous research results. In this session, we will discuss the latest findings with researchers who aim to comprehensively reanalyze the information contained in TCGA and other public databases to find new horizons in cancer research. By sharing information gaps and analysis pitfalls in the databases, we hope to establish more robust analysis strategies, leading to the next generation of cancer research and the development of cancer treatments.

---

### OS5-3
### Targeting genomic fragile regions to uncover metabolic vulnerabilities: bridging prediction and validation

[Speaker]Susumu Kohno (Div. Oncol. Mol. Biol., Cancer Res. Insti., Kanazawa Univ.) [Co-author]Chiaki Takahashi (Div. Oncol. Mol. Biol., Cancer Res. Insti., Kanazawa Univ.)

Common fragile sites (CFSs) are specific genomic regions prone to breakage, frequently observed in cancer cells. These regions often serve as hotspots for chromosomal rearrangements and deletions. Notably, many CFSs harbor tumor suppressor genes, and their deletion can result in the simultaneous loss of neighboring functional genes, leading to newly acquired vulnerabilities in tumors. Among these, metabolic enzymes are of particular interest, as their loss may create tumor-specific metabolic dependencies that can be therapeutically exploited. Deletion of such enzymes induces metabolic remodeling, enabling tumor cells to survive under altered conditions. By focusing on the physical proximity of tumor suppressor genes and metabolic enzymes within CFSs, we identified potential co-deleted gene pairs. In this study, we discovered that RB1 and SUCLA2 are frequently co-deleted in advanced prostate cancer. We further developed a novel compound targeting SUCLA2 deficiency, which leverages the upregulation of the anaplerotic pathway induced by SUCLA2 loss.

[Disclosure of COI by Responsible researcher] Lead presenter is not the Responsible researcher.
[Lead presenter: Susumu Kohno] We have no financial relationships to disclose.
[Principal Researcher: Chiaki Takahashi] We have no financial relationships to disclose.

### [OS5-4] OS5
### Lights and Shadows of Database Utilization in Oncology Research

2025 / 9 / 25 (Thu) 12:50-14:20 Room 6/(Hotel Nikko Kanazawa 3F Kujaku A) Room 6

[Chairpersons]Shoichiro Tange (Department of Medical Genome Sciences, Sapporo Medical University)、Jun Nakayama (Osaka International Cancer Institute)

This year marks the 20th anniversary of TCGA, a project to collect clinical specimens primarily from the National Cancer Institute (NCI) and other institutions. While this database continues to be a useful source of information for cancer researchers, it does not fully reflect the progress of analysis methods in later years, and the possibility exists that hidden information may be buried. In addition, the lack of sharing of analysis methods and how-tos has led to misinterpretations due to wrong analysis methods and missing data in TCGA, resulting in erroneous research results. In this session, we will discuss the latest findings with researchers who aim to comprehensively reanalyze the information contained in TCGA and other public databases to find new horizons in cancer research. By sharing information gaps and analysis pitfalls in the databases, we hope to establish more robust analysis strategies, leading to the next generation of cancer research and the development of cancer treatments.

### OS5-4
### Clinical impact of endogenous retrovirus reactivation in acute myeloid leukemia ~utilizing TCGA controlled dataset~

[Speaker]Ryo Yanagiya (Dept. Hematol. Oncol., Saga Univ.／Dept. Reg. Infect. Cancer, RIMD, The Univ. Osaka) [Co-author]Ai Kotani (Dept. Reg. Infect. Cancer, RIMD, The Univ. Osaka)、So Nakagawa (Dept. Mol. Life Sci., Tokai Univ.)

The Cancer Genome Atlas (TCGA) provides us various clinical and biosample-derived data of human malignancies. Furthermore, obtaining ethical approval from National Institutes of Health (NIH) allows us to access raw sequence data (controlled data) and analyze whole-transcriptome combined with clinical information. Herein I demonstrate an investigation of endogenous viral element (EVEs) expression profile in acute myelocytic leukemia (AML) and their impact to clinical outcomes.
Transcriptomic data of 151 AML cases are available in TCGA. Via re-mapping using EVE dataset, total of fourteen viral species were annotated in differentially expressed EVEs. Among 90 transplantation-eligible AML cases, significant prolongation of event-free survival was observed in cases with higher expression of human endogenous retrovirus type K9 (HERVK9)-derived elements, and multivariate analysis indicated that HERVK9 expression is a novel prognostic predictor independent of conventional risk stratifications.
Considering expensiveness of high-throuput experiments, the cost-effectiveness of utilizing deposited data should not be despised despite their various limitations because of retrospectivity.

[Disclosure of COI by Responsible researcher] Lead presenter is not the Responsible researcher.
[Lead presenter: Ryo Yanagiya] ■Research expenses: 大原薬品工業株式会社、ペルセウスプロテオミクス社　■Endowed chair: 大原薬品工業株式会社
[Principal Researcher: Nakagawa So] We have no financial relationships to disclose.

▲TOP

### [OS5-5] OS5
### Lights and Shadows of Database Utilization in Oncology Research

2025 / 9 / 25 (Thu) 12:50-14:20 Room 6/(Hotel Nikko Kanazawa 3F Kujaku A) Room 6

[Chairpersons]Shoichiro Tange (Department of Medical Genome Sciences, Sapporo Medical University)、Jun Nakayama (Osaka International Cancer Institute)

This year marks the 20th anniversary of TCGA, a project to collect clinical specimens primarily from the National Cancer Institute (NCI) and other institutions. While this database continues to be a useful source of information for cancer researchers, it does not fully reflect the progress of analysis methods in later years, and the possibility exists that hidden information may be buried. In addition, the lack of sharing of analysis methods and how-tos has led to misinterpretations due to wrong analysis methods and missing data in TCGA, resulting in erroneous research results. In this session, we will discuss the latest findings with researchers who aim to comprehensively reanalyze the information contained in TCGA and other public databases to find new horizons in cancer research. By sharing information gaps and analysis pitfalls in the databases, we hope to establish more robust analysis strategies, leading to the next generation of cancer research and the development of cancer treatments.

### OS5-5
### An example of a side effect of data analysis: novel transcripts discovered unexpectedly from public data.

[Speaker]Shoichiro Tange (Med. Genome Sci., Dept. Genomic Preventive Med., Sapporo Med. Univ.) [Co-author]Masashi Idogawa (Med. Genome Sci., Dept. Genomic Preventive Med., Sapporo Med. Univ.)、Issei Imoto (Res. Inst., Aichi Cancer Center)、Takashi Tokino (Med. Genome Sci., Dept. Genomic Preventive Med., Sapporo Med. Univ.／Sapporo Univ. of Health Sci.)

We screened for potential prognostic markers across cancer types based on the transcriptome data from The Cancer Genome Atlas (TCGA). Analysis of RNA-Seq data from 22 tumor samples revealed a gene ***Z***, common to seven tumor types, whose elevated expression correlated with shorter overall survival. This gene is exclusively expressed in skeletal muscle cells. Of note, in TCGA breast cancer specimens, higher expression was found in more aggressive triple negative breast cancers. The upregulation of ***Z*** was also confirmed in qRT-PCR analysis using breast cancer cell lines. On the other hand, we could not confirm the presence of ***Z*** protein from Western blot experiments. Analysis using long-read transcriptome sequencing data suggested that the transcript with this gene sequence also contained ***X***- and ***Y***-derived sequences located upstream of ***Z***, suggesting that it is a read-through product consisting of multiple genes. Based on these data, we hypothesized that although ***Z*** is not directly involved in tumor malignancy, it may be a marker of poor prognosis in some cancer types. We are currently in the process of analyzing the detailed mechanism by which this transcript is produced.

[Disclosure of COI by Responsible researcher] Lead presenter is the Responsible researcher.
[Lead presenter: Shoichiro Tange] We have no financial relationships to disclose.

### [E-1037] I-E5-1
### Signal transduction in cancer progression

2025 / 9 / 25 (Thu) 12:50-14:05 Room 7/(Hotel Nikko Kanazawa 3F Kujaku B) Room 7

[Chairperson]Hiromu Suzuki (Div. Mol. Biol., Sapporo Med. Univ., Sch. Med.)

### E-1037
### Prohaptoglobin regulates Hippo pathway status and augments pancreatic cancer malignancy

[Speaker]Daisuke Sakon (Mol. Biochem. & Clin. Invest., Univ. of Osaka. Med) [Co-author]Jumpei Kondo (Mol. Biochem. & Clin. Invest., Univ. of Osaka. Med)、Honoka Nakayama (Mol. Biochem. & Clin. Invest., Univ. of Osaka. Med)、Muya Matsumoto (Mol. Biochem. & Clin. Invest., Univ. of Osaka. Med)、Ayusa Kuroda (Mol. Biochem. & Clin. Invest., Univ. of Osaka. Med)、Ayumu Hayashibara (Mol. Biochem. & Clin. Invest., Univ. of Osaka. Med)、Shinji Takamatsu (Mol. Biochem. & Clin. Invest., Univ. of Osaka. Med)、Eiji Miyoshi (Mol. Biochem. & Clin. Invest., Univ. of Osaka. Med)

An acute-phase protein haptoglobin (Hp) is produced in the liver and prevents hemoglobin (Hb)-mediated damages by scavenging Hb. Recent reports highlights Prohaptoglobin (proHp), a precursor of haptoglobin, as a novel biomarker detected at high levels in the serum of patients with pancreatic cancer. Although reports have suggested functions of proHp distinct from that of mature Hp, the role for proHp in pancreatic cancer microenvironment remains unclear.In this study, we investigated the function of proHp in pancreatic cancer using PSN-1 cells which produce proHp. Wild-type (WT) PSN-1 cells exhibited significantly higher proliferative capacity compared to proHp knockouts (KO) in vitro. In subcutaneous transplantation model, PSN-1 WT cells yielded larger xenograft tumors than KO cells. RNAseq analysis revealed that YAP-mediated gene expression is activated in WT cells. Alteration in Hippo pathway status in proHp KO is supported by suppressed phosphorylation and upregulated translocation of YAP by proHp.Our findings suggest the pro-tumorigenic role of proHp, which may enhance pancreatic cancer malignancy through modulation of the Hippo pathway.

[Disclosure of COI by Responsible researcher] Lead presenter is not the Responsible researcher.
[Lead presenter: Daisuke Sakon] We have no financial relationships to disclose.
[Principal Researcher: Eiji Miyoshi] We have no financial relationships to disclose.

### [E-1038] I-E5-1
### Signal transduction in cancer progression

2025 / 9 / 25 (Thu) 12:50-14:05 Room 7/(Hotel Nikko Kanazawa 3F Kujaku B) Room 7

[Chairperson]Hiromu Suzuki (Div. Mol. Biol., Sapporo Med. Univ., Sch. Med.)

---

### E-1038
### Intercellular signaling factors drive communal endocrine therapy resistance in breast cancer

[Speaker]Yuji Kumagai (PHS and HB Divs, Fred Hutch Cancer Center) [Co-author]Brad Krajina (PHS and HB Divs, Fred Hutch Cancer Center)、Kevin Cheung (PHS and HB Divs, Fred Hutch Cancer Center)

Cancer cells engage with their neighbors via a variety of communication modes. How intercellular communication promotes therapy resistance remains poorly understood. Major challenges include identifying the signaling factors involved and validating their intercellular functions. In this work, we developed two complementary platforms: STEALTH (SPAAC Targeted Enrichment of Accumulated and Locally Transmitted Hormones and growth factors), to interrogate intercellular signals from organotypic models, and MISTIQ (Measuring Intercellular Signaling Tumor Interactions Quantitatively), to quantify the impact of intercellular interactions between distinct tumor clones. Applying these technologies to estrogen receptor-positive (ER+) breast cancer models, we found that ER+ breast tumor cells accumulate the EGFR ligand amphiregulin (AREG) within intercellular spaces. We further revealed that AREG diffuses through the tumor cell population and that the presence of only 10% AREG-expressing cells is sufficient to induce communal resistance to selective estrogen receptor degrader. These studies reveal that breast cancer cells form an intercellular ecosystem driving communal therapy resistance.

[Disclosure of COI by Responsible researcher] Lead presenter is the Responsible researcher.
[Lead presenter: Yuji Kumagai] We have no financial relationships to disclose.

### [E-1039] I-E5-1
### Signal transduction in cancer progression

2025 / 9 / 25 (Thu) 12:50-14:05 Room 7/(Hotel Nikko Kanazawa 3F Kujaku B) Room 7

[Chairperson]Hiromu Suzuki (Div. Mol. Biol., Sapporo Med. Univ., Sch. Med.)

---

### E-1039
### Cytoplasmic KRAS salvage KRAS-MAPK signal from oncogene overdose in Her2 amplified with KRAS-mutated colorectal cancer

[Speaker]Kohei Maruyama (Div. Exp. Chemother., Cancer Chemother. Ctr., JFCR) [Co-author]Tomoko Ohhara (Div. Exp. Chemother., Cancer Chemother. Ctr., JFCR)、Yosuke Seto (Div. Exp. Chemother., Cancer Chemother. Ctr., JFCR)、Satoshi Nagayama (Dept. Surg., Grad. Sch. Med, Kyoto Univ／Dept. Surg., Uji-Tokushukai Med Ctr)、Ryohei Katayama (Div. Exp. Chemother., Cancer Chemother. Ctr., JFCR／Dept. CBMS, Grad. Sch. Front. Sci., The Univ. of Tokyo)

Current studies highlighted cell growth and survival of KRAS-mutated colorectal cancer (KRAS-CRC) frequently depends on EGFR-KRAS-MAPK signaling in KRAS inhibition. We recently reported a cell with Her2 amplification (Her2amp) and PIK3CA minor mutation positive KRAS-CRC cells showed high PI3K pathway dependency. Notably, KRAS inhibition of this PDCX tends to be more growth than vehicle regardless of harboring KRAS-G12C mutation. However, the underlying mechanisms that conferred high PI3K dependency and low KRAS-MAPK dependency in the presence of concomitant KRAS and Her2 aberration remain unclear. In this study, we examined the regulating mechanisms between Her2-PI3K and KRAS-MAPK pathway in Her2amp with KRAS-CRC, by focusing on oncogene overdose. We discovered that Her2 knockout suppressed Her2-PI3K signal and activated KRAS-MAPK dependency with alteration of KRAS localization from cytoplasm to plasm membrane. These results suggest that Her2amp may maintain the cytoplasmic KRAS localization and low dependence on KRAS-MAPK pathway to salvage oncogene overdose. Furthermore, KRAS localization may regulate the balance between MAPK and PI3K signal intensity even in KRAS mutant cells.

[Disclosure of COI by Responsible researcher] Lead presenter is not the Responsible researcher.
[Lead presenter: Kohei Maruyama] We have no financial relationships to disclose.
[Principal Researcher: Ryohei Katayama] ■Patent fee: 栄研化学　■Research expenses: 中外製薬、TOPPAN、UBE、BML

▲TOP

### [E-1040] I-E5-1
### Signal transduction in cancer progression

2025 / 9 / 25 (Thu) 12:50-14:05 Room 7/(Hotel Nikko Kanazawa 3F Kujaku B) Room 7

[Chairperson]Hiromu Suzuki (Div. Mol. Biol., Sapporo Med. Univ., Sch. Med.)

---

### E-1040
### HER2/HER3-regulated expression program of the integrated stress response in breast cancer cell lines

[Speaker]Yuka Okamoto (Lab. Genome Tech., Human Genome Ctr., IMSUT ／Div. Genome Res., Cancer Chemother. Ctr. JFCR) [Co-author]Miku Otsuka (Div. Genome Res., Cancer Chemother. Ctr. JFCR／CBMS, Grad. School Frontier Sciences, the University of Tokyo)、Hitomi Shirahama (Div. Genome Res., Cancer Chemother. Ctr. JFCR)、Akihiro Tomida (Div. Genome Res., Cancer Chemother. Ctr. JFCR)

Tumor cells activate adaptive responses like the integrated stress response (ISR) to survive and proliferate under unfavorable conditions of malnutrition or hypoxia. Previous studies have shown involvement of oncogenic signals such as activating mutations in BRAF, EGFR, and PI3K in modulation of the ISR. We have also found that the induction of ATF4, a crucial transcription factor in the ISR, was enhanced by HER2/HER3 signaling. In this study we conducted transcriptome analysis in HER2-amplified breast cancer SKBR3 cells treated with chemical stressors (a glycosylation inhibitor tunicamycin; TM or a histidyl-tRNA synthetase inhibitor L-histidinol; His) in the presence or absence of HER3 ligand (heregulin; HRG). From hierarchical clustering for differentially expressed genes, a set of genes whose expression was increased by co-treatment of HRG compared to treatment with TM or His alone. Gene ontology analysis for this gene set revealed that stress-responsive genes including ATF4-targeted genes were significantly enriched. These results indicate that HER2/HER3 signal can boost the ISR gene expression programs.

[Disclosure of COI by Responsible researcher] Lead presenter is not the Responsible researcher.
[Lead presenter: Yuka Okamoto] We have no financial relationships to disclose.
[Principal Researcher: Akihiro Tomida] We have no financial relationships to disclose.

### [E-1041] I-E5-1
### Signal transduction in cancer progression

2025 / 9 / 25 (Thu) 12:50-14:05 Room 7/(Hotel Nikko Kanazawa 3F Kujaku B) Room 7

[Chairperson]Hiromu Suzuki (Div. Mol. Biol., Sapporo Med. Univ., Sch. Med.)

---

### E-1041
### Discovery of NNC 05-2090, a small molecule antagonist of the Neuromedin U receptor 2 for glioma treatment

[Speaker]Yuna Roh (Korea Research Institute of Bioscience and Biotechnology (KRIBB)／University of Science and Technology (UST) [Co-author]Jang S. Kim (Korea Research Institute of Bioscience and Biotechnology (KRIBB))、Tae S. Han (Korea Research Institute of Bioscience and Biotechnology (KRIBB)／University of Science and Technology (UST))、Tae S. Son (Korea Research Institute of Bioscience and Biotechnology (KRIBB)／University of Science and Technology (UST))、Sa R. Kim (Korea Research Institute of Bioscience and Biotechnology (KRIBB)／University of Science and Technology (UST))、Na Y. Gu (Korea Research Institute of Bioscience and Biotechnology (KRIBB))

Gliomas account for 30% of brain and CNS tumors and 80% of malignant brain tumors, yet their pathogenesis remains unclear. Temozolomide is the standard chemotherapy, but its clinical efficacy is often limited by recurrence and resistance. In this study, we identify Neuromedin U receptor 2 (NMUR2) as a novel therapeutic target in glioma and aim to discover NMUR2-specific inhibitors. NMUR2 is significantly overexpressed in glioma tissues, and its overexpression enhances cell proliferation and migration, while knockdown suppresses these processes. Transcriptomic analysis revealed that NMUR2 knockdown leads to downregulation of cell cycle related genes, potentially mediated by STAT5. To identify NMUR2-specific antagonists, we conducted cell-based screening of 6,331 repurposing drugs and discovered NNC 05-2090. Treatment with NNC 05-2090 impaired cell cycle progression in glioma cells via NMUR2-dependent signaling. Moreover, combining NNC 05-2090 with temozolomide demonstrated a synergistic effect both in vitro and in vivo. These findings suggest that NMUR2 is a viable target for glioma therapy, and NNC 05-2090 may enhance therapeutic outcomes when used in combination with temozolomide.

[Disclosure of COI by Responsible researcher] Lead presenter is not the Responsible researcher.
[Lead presenter: Yuna Roh] We have no financial relationships to disclose.
[Principal Researcher: Tae S. Han] We have no financial relationships to disclose.

### [E-1042] I-E5-1
### Signal transduction in cancer progression

2025 / 9 / 25 (Thu) 12:50-14:05 Room 7/(Hotel Nikko Kanazawa 3F Kujaku B) Room 7

[Chairperson]Hiromu Suzuki (Div. Mol. Biol., Sapporo Med. Univ., Sch. Med.)

### E-1042
### Comprehensive understanding of BCR-ABL interactors in chronic myeloid leukemia by proximity-dependent biotinylation

[Speaker]Takanori Nakamura (Proteo-Science Center, Ehime University) [Co-author]Mikihiko Naito (Graduate School of Pharmaceutical Sciences, The University of Tokyo)、Junya Masumoto (Proteo-Science Center, Ehime University)、Tatsuya Sawasaki (Proteo-Science Center, Ehime University)

Chronic myeloid leukemia (CML) is a hematological cancer caused by a reciprocal translocation between chromosomes 9 and 22 resulting in a constitutively active BCR-ABL protein, a fusion of BCR and tyrosine-kinase ABL. Tyrosine kinase inhibitors (TKI) have revolutionized the treatment of CML, while continued TKI treatment causes relapse because of resistance emerging. Therefore, to develop a new drug effective for TKI-resistant CML cells, a comprehensive understanding of BCR-ABL signal pathway by identifying BCR-ABL-associating proteins must be clarified. However, the databases of BCR-ABL-interacting proteins are not established enough, and therefore BCR-ABL-driven pathological signal transduction remains unclear.Here we performed proximity-dependent biotinylation for BCR-ABL-binding proteins and their detection by mass spectrometry. The interactome analysis identifed not only already-known BCR-ABL interactors such as SHIP2, but also several novel BCR-ABL interactors. Next, genetically depleting each of them, we found that protein-X serves as a key regulator for BCR-ABL signaling pathway, as the kinase activity of BCR-ABL was substantially reduced by the loss of protein-X.

[Disclosure of COI by Responsible researcher] Lead presenter is not the Responsible researcher.
[Lead presenter: Takanori Nakamura] ■Research funds from company: 高松宮妃癌研究基金 研究助成
[Principal Researcher: Tatsuya Sawasaki] ■Advisor of the company and/or profit-making organization: 株式会社セルフリーサイエンス

▲TOP

### [ME1] ME1
### Science Translational Medicine

2025 / 9 / 25 (Thu) 12:50-13:30 Room 8/(Hotel Nikko Kanazawa 3F Kujaku C) Room 8

[Chairperson]Hiroyoshi Nishikawa (Division of Cancer Immunology, Research Institute, National Cancer Center)

### ME1
### Science Translational Medicine

[Speaker]Orla Smith (AAAS／Science Translational Medicine)

No Abstract

### [E-1049] I-E3-1
### Bacteria, Inflammation & Cancer

2025 / 9 / 25 (Thu) 12:50-14:05 Room 9/(Hotel Nikko Kanazawa 3F Lumiere) Room 9

[Chairperson]Naoko Kamiya (Div. Microbial Oncol., Inst. Genetic Med., Hokkaido Univ.)

---

### E-1049
### Impact of tsRNA derived from ***Klebsiella pneumoniae*** on hepatocarcinogenesis

[Speaker]Kazuki Oshima (Division of Pharmacotherapeutics, Keio Univ. Faculty of Pharmacy) [Co-author]Juntaro Matsuzaki (Res. Ctr. for Drug Discovery, Keio Univ. Faculty of Pharmacy)、Jun(NCC) Nakayama (Dept. Oncogenesis & Growth Reg., Osaka)、Yusuke Yamamoto (Lab. Integr. Oncol. Natl. Cancer Ctr. Res. Inst.)、Hitoshi Tsugawa (Transkingdom Signaling Research Unit, Tokai Univ.)、Ken Kato (Natl. Cancer Ctr. Hosp.)、Takahiro Ochiya (Dept. Mol. Cell. Med., Tokyo Med. Univ.)、Yoshimasa Saito (Division of Pharmacotherapeutics, Keio Univ. Faculty of Pharmacy)

***Klebsiella pneumoniae*** (***K. pneumoniae***) has been reported to promote hepatocellular carcinoma (HCC) and is attracting increasing attention (Wang et al., ***Nat Microbiol*** 10, 2025). Recent RNA sequencing studies have identified circulating RNA fragments not derived from the human genome. Since bacterial RNAs are enclosed in bacterial extracellular vesicles (bEVs), we examined whether ***K. pneumoniae*** RNA exists in human blood and is linked to HCC. Two transfer RNA-derived small RNAs (tsRNAs) from ***K. pneumoniae*** were identified in HCC patients and also detected in bEVs purified from ***K. pneumoniae*** culture supernatants (Kp-bEVs). Tail vein injection of Kp-bEVs into STAM mice, a model of HCC after metabolic dysfunction-associated steatohepatitis (MASH), increased liver tumors and elevated IL-1β, IL-6, and Cyclin D3 (CCND3). Transfection of these tsRNAs into hepatic stellate cells (LX-2) also upregulated these markers, while CCND3 knockdown reduced IL-1β and IL-6. Since IL-1β and IL-6 contribute to HCC progression, our findings suggest that ***K. pneumoniae***-derived tsRNAs may promote liver carcinogenesis through inflammatory and oncogenic pathways.

[Disclosure of COI by Responsible researcher] Lead presenter is not the Responsible researcher.
[Lead presenter: Kazuki Oshima] We have no financial relationships to disclose.
[Principal Researcher: Juntaro Matsuzaki] ■Research funds from company: ISM株式会社、レグリア株式会社、フェムトディプロイメンツ株式会社

▲TOP

### [E-1050] I-E3-1
### Bacteria, Inflammation & Cancer

2025 / 9 / 25 (Thu) 12:50-14:05 Room 9/(Hotel Nikko Kanazawa 3F Lumiere) Room 9

[Chairperson]Naoko Kamiya (Div. Microbial Oncol., Inst. Genetic Med., Hokkaido Univ.)

### E-1050
### Functional analysis of ***Pak1*** in monocyte-derived Langerhans cells during skin carcinogenesis

[Speaker]Megumi Saito (Div. of Exp. Anim. Res., Chiba Cancer Center Res. Inst.) [Co-author]Kazuhiro Okumura (Div. of Exp. Anim. Res., Chiba Cancer Center Res. Inst.)、Sora Tanaka (Div. of Exp. Anim. Res., Chiba Cancer Center Res. Inst.／Grad. Sch. Of Med. & Pharm. Sci. Chiba Univ.)、Keisuke Otoyama (Div. of Exp. Anim. Res., Chiba Cancer Center Res. Inst.／Grad. Sch. Of Med. & Pharm. Sci. Chiba Univ.)、Yuichi Wakabayashi (Div. of Exp. Anim. Res., Chiba Cancer Center Res. Inst.)

The tissue inflammatory response plays an important role in the tumor microenvironment, which regulates cancer susceptibility. In particular, chronic tissue inflammation, is a major factor in cancer development. Recently, it has been shown to be monocyte-derived Langerhans cells (moLCs) are implicated in anti-inflammatory responses, they attract attention. On the other hand, we have previously identified ***Pak1*** as a key gene that significantly influences cancer susceptibility. Furthermore, we have found that ***Pak1*** is strongly expressed in Cd11c-positive cells. And Cd11c-positive cell-specific ***Pak1*** conditional knockout (cKO) mice acquired a strong resistance to carcinogenesis and showed the regulated inflammatory responses. Further analysis revealed an increase in Langerhans cells (LCs) marker Langerin (CD207) and anti-inflammatory cytokine Tgf-β in cKO mice. Based on these findings, we have prepared bone marrow cells (BMCs) from cKO or control mice, and cultured in the presence of GM-CSF and TGF-β1. Under these experimental conditions, we have found that BMCs prepared from cKO mice efficiently differentiate into CD207-positive cells in response to GM-CSF and TGF-β1.

[Disclosure of COI by Responsible researcher] Lead presenter is not the Responsible researcher.
[Lead presenter: Megumi Saito] We have no financial relationships to disclose.
[Principal Researcher: Yuichi Wakabayashi] We have no financial relationships to disclose.

▲TOP

### [E-1051] I-E3-1
### Bacteria, Inflammation & Cancer

2025 / 9 / 25 (Thu) 12:50-14:05 Room 9/(Hotel Nikko Kanazawa 3F Lumiere) Room 9

[Chairperson]Naoko Kamiya (Div. Microbial Oncol., Inst. Genetic Med., Hokkaido Univ.)

### E-1051
### ETBF promotes cecal tumorigenesis in Ahr-/- mice by modification of gut microbiota

[Speaker]Hisanori Matoba (Dept. of Mol. Pathol., Shinshu Univ., Sch. of Med.) [Co-author]Chifumi Fujii (Shinshu Univ., Sch. of Med.／Div. Biotech., Inst. Biomed. Sci., RCSI., Shinshu Univ.)、Masatomo Kawakubo (Dept. of Mol. Pathol., Shinshu Univ., Sch. of Med.)、Tatsuya Natori (Dept. of Lab. Med., Shinshu Univ. Hosp.)、Hitomi Imamura (Dept. of Mol. Pathol., Shinshu Univ., Sch. of Med.／Dept. of Lab. Med., Shinshu Univ. Hosp.)、Masaya Takamoto (Nagano Univ. of Health and Med.)、Shunichiro Taniguchi (Adv. Cancer Med., Grad. Sch. of Kagoshima Univ.)、Jun(Azumi Hosp.) Nakayama (Dept. of Pathol., North Alps Medical Center Azumi Hosp.)

The aryl hydrocarbon receptor (Ahr) is a transcription factor known as a dioxin receptor. Recently, it was revealed that all Ahr-/- mice developed cecal tumors by 10 weeks old in another facility, but germ-free Ahr-/- mice showed no tumor development, suggesting the importance of gut microbiota-driven inflammation. When we studied tumorigenesis in Ahr-/- mice in our facility, the tumor incidence decreased dramatically to 35.7% (10/28) by 50 weeks old, likely due to different gut microbiota between the original and our facilities. Enterotoxigenic Bacteroides fragilis (ETBF) is a human colonic commensal implicated in colorectal carcinogenesis. We attempted to promote cecal tumorigenesis in Ahr-/- mice by ETBF administration. Ahr-/- mice without ETBF showed no tumor development (0/56). This was a significant reduction compared to our previous study mentioned above. 13% (11/85) of Ahr-/- mice with ETBF showed cecal tumorigenesis. Analysis of gut microbiota revealed that microbiota changed between our previous and present study, and that ETBF administration also altered the microbiota, suggesting that these changes were responsible for above decrease and increase in tumor development.

[Disclosure of COI by Responsible researcher] Lead presenter is the Responsible researcher.
[Lead presenter: Hisanori Matoba] We have no financial relationships to disclose.

▲TOP

### [E-1052] I-E3-1
### Bacteria, Inflammation & Cancer

2025 / 9 / 25 (Thu) 12:50-14:05 Room 9/(Hotel Nikko Kanazawa 3F Lumiere) Room 9

[Chairperson]Naoko Kamiya (Div. Microbial Oncol., Inst. Genetic Med., Hokkaido Univ.)

---

### E-1052
### Extracellular vesicles derived from vaginal bacteria involves the progression of early-phase ovarian carcinoma

[Speaker]Eri Inami (Nagoya Univ. Med. Gynecol.) [Co-author]Akira Yokoi (Nagoya Univ. Med. Gynecol.)、Kosuke Yoshida (Nagoya Univ. Med. Gynecol.)、Masami Kitagawa (Nagoya Univ. Med. Gynecol.)、Yukari Nagao (Nagoya Univ. Med. Gynecol.)、Hiroaki Kajiyama (Nagoya Univ. Med. Gynecol.)

Recent understanding of ovarian high-grade serous carcinoma (HGSC) has revealed it arises from fallopian tube epithelial cells (FTE cells), but the detail mechanisms are unknown. Extracellular vesicles (EVs) play a crucial role in cell-cell communication, and residential bacteria in humans also secrete EVs (BEVs). This study aimed to elucidate how BEVs contribute to the progression of ovarian cancer malignancy. Compared to control vaginal BEVs, specific bacteria derived EVs promoted cell motility, and anoikic ability of p53 activated FTE cells ($p<0.05$). Treatment of BEVs in mouse vaginas resulted in efficient BEV uptakes in FTE cells. BEV on fallopian tubes were directly collected from fifteen ovarian tumor patients by using cellulose nanofiber-based EV sheets and small RNA-seq for bacteria references revealed different profiles of FT-EVs in HGSC. mRNA-seq of FTE cells treated with BEVs showed significantly altered gene expression, and IPA/GSEA analysis identified cancer-related pathways. These results provide the unknown mechanism for progression of ovarian cancer due to specific BEV exposure and identify target genes in FTE cells as new diagnostic and therapeutic targets.

[Disclosure of COI by Responsible researcher] Lead presenter is not the Responsible researcher.
[Lead presenter: Eri Inami] ■Research funds from company: 科研費　■Contributions: 内藤記念科学財団
[Principal Researcher: Akira Yokoi] ■Research funds from company: 科研費、AMED

### [E-1053] I-E3-1
### Bacteria, Inflammation & Cancer

2025 / 9 / 25 (Thu) 12:50-14:05 Room 9/(Hotel Nikko Kanazawa 3F Lumiere) Room 9

[Chairperson]Naoko Kamiya (Div. Microbial Oncol., Inst. Genetic Med., Hokkaido Univ.)

---

### E-1053
### Clonal expansion in the bile duct associated with primary sclerosing cholangitis

[Speaker]Hirona Maeda (Dept. Path. & Tumor Biol., Kyoto Univ.／Inst., for the Advanced Study of Human Biol., Kyoto Univ.) [Co-author]Nobuyuki Kakiuchi (Dept. Path. & Tumor Biol., Kyoto Univ.／Hakubi Ctr. for Advanced Res.／Dept. Gastroenterology & Hepatology, Kyoto Univ.)、Takashi Ito (Dept. Hepato-Biliary-Pancreatic Surg. & Transplantation Dept., Kyoto Univ.)、Eri Ogawa (Dept. Pediatric Surg. , Kyoto Univ.)、Masahiro Shiokawa (Dept. Gastroenterology & Hepatology, Kyoto Univ.)、Norimitsu Uza (Dept. Gastroenterology & Hepatology, Kyoto Univ.)、Koichi Watanabe (Dept. Gynecology & Obstetrics, Kyoto Univ.)、Yasuhito Nannya (Dept. Hematol., Inst. of Med. Sci., Tokyo Univ.)、Yuzo Kodama (Div. Gastroenterology, Dept. Internal Med., Kobe Univ.)、Tatsuki Kataoka (Dept. Pathol., Iwate Med. Univ.)、Etsuro Hatano (Dept. Hepato-Biliary-Pancreatic Surg. & Transplantation Dept., Kyoto Univ.)、Seishi Ogawa (Dept. Path. & Tumor Biol., Kyoto Univ.／Inst., for the Advanced Study of Human Biol., Kyoto Univ.)

Chronic inflammation has been implicated in the development of cancer and more recently, in the evolution of clones carrying cancer-related mutations. Primary sclerosing cholangitis (PSC) is a chronic inflammation of the bile duct and associated with a markedly increased risk of cholangiocarcinoma. However, clones in PSC-affected bile ducts have not been investigated. In this study, we analyzed 463 samples obtained from 9 PSC patients using laser-capture microdissection for somatic mutations and copy number alterations (CNAs) using whole exome sequencing. Somatic mutations and CNAs were detected in all (100%) and 141 samples (30.4%) each, with a median of 22 mutations per exome, while CNAs were found in 7 (78%) cases. A total of 79 clones were detected regardless of the presence or absence of BilIN and expanded up to a maximum of 18 mm in length. Expanded clones acquired mutations in driver genes commonly found in cancer such as ***ARID1A*** and ***KRAS***, along with CNAs, which could be extensive. Our results suggest clonal expansion in the bile duct of PSC patients is common and accompanied by known cancer driver mutations, suggesting their role in carcinogenesis associated with PSC.

[Disclosure of COI by Responsible researcher] Lead presenter is not the Responsible researcher.
[Lead presenter: Hirona Maeda] We have no financial relationships to disclose.
[Principal Researcher: Seishi Ogawa] ■Advisor of the company and/or profit-making organization: エーザイ株式会社, Chordia Therapeutics Inc., Montage Bio, INC.　■Profit of stock: 朝日ゲノミクス株式会社　■Lecture fee: 公益財団法人三菱財団 公益財団法人中谷医工計測技術振興財団　■Research expenses: エーザイ株式会社, Chordia Therapeutics Inc.　■Contributions: 日本新薬株式会社　■Endowed chair: Chordia Therapeutics Inc., 公益社団法人鹿児島共済会 南風病院

▲TOP

### [E-1054] I-E3-1
### Bacteria, Inflammation & Cancer

2025 / 9 / 25 (Thu) 12:50-14:05 Room 9/(Hotel Nikko Kanazawa 3F Lumiere) Room 9

[Chairperson]Naoko Kamiya (Div. Microbial Oncol., Inst. Genetic Med., Hokkaido Univ.)

---

### E-1054
### A potential role of fatty acid binding protein 5 in the progression of idiopathic pulmonary fibrosis

[Speaker]Hiroto Hatano (Dept. Clin. Coord., Natl. Inst. Global Health and Med., JIHS.) [Co-author]Kazuaki Nakata (Dept. Clin. Coord., Natl. Inst. Global Health and Med., JIHS.)、Yuki I. Kawamura (Dept. Clin. Coord., Natl. Inst. Global Health and Med., JIHS.)

Idiopathic pulmonary fibrosis (IPF) is a progressive lung disease recognized as a precancerous condition leading to lung cancer. Dysregulated lipid metabolism has been implicated in fibrosis and tumorigenesis. Fatty acid-binding protein 5 (FABP5), involved in intracellular fatty acid transport, is normally expressed in alveolar type 2 (AT2) cells, but its role in IPF remains unclear. In this study, immunohistochemistry of IPF lung tissues demonstrated loss of FABP5 in AT2 cells within fibrotic areas. Secondary analysis of publicly available single-cell RNA sequencing datasets also identified an AT2 subset lacking FABP5 expression in IPF lungs. In a murine model, FABP5 expression in AT2 cells was reduced by day 10 after bleomycin-induced injury, and fibrosis was more severe in FABP5-deficient mice. On day 4, Ki67-positive AT2 cells were more frequent in wild-type mice, while FABP5 deficiency tended to reduce AT2 proliferation. These findings suggest that FABP5 loss may impair AT2 regenerative capacity, contributing to fibrotic progression and possibly carcinogenesis.

[Disclosure of COI by Responsible researcher] Lead presenter is not the Responsible researcher.
[Lead presenter: Hiroto Hatano] We have no financial relationships to disclose.
[Principal Researcher: Yuki Kawamura] We have no financial relationships to disclose.

### [SS6-1] SS6
### Defeating cancer from a host perspective: cancer cachexia

2025 / 9 / 25 (Thu) 12:50-15:20 Room 10/(Art Hall) Room 10

[Chairpersons]Takeshi Suzuki (Cancer Research Intitute, Kanazawa University)、Shinpei Kawaoka (Institute of Development, Aging and Cancer, Tohoku University (Institute for Life and Medical Sciences, Kyoto University)

Cancer cachexia has long been viewed as a terminal condition marked by unintentional weight loss and organ dysfunction, especially skeletal muscle wasting. Although weight loss correlates strongly with poor clinical outcomes, it remains unclear whether reversing weight loss alone is sufficient to improve patient survival, due to the absence of key drugs. Recent discoveries urge us to broaden our view beyond traditional cachexia symptoms, highlighting the importance of systemic changes in organs and cells that may impair treatment outcomes even in the absence of weight loss. There is also growing interest in detecting host physiological changes at earlier stages, prior to overt cachexia, to enable timely interventions. These insights collectively challenge the current definition of cancer cachexia and call for its revision to reflect both clinical needs and biological complexity. A more comprehensive and operational definition may facilitate earlier diagnosis, better therapeutic targeting, and ultimately enhanced responses to cancer treatment. In this international symposium, leading researchers from Japan and around the world will gather to critically examine traditional cachexia concepts and discuss new biological and clinical frameworks that integrate early systemic alterations. Through this dialogue, we aim to lay the groundwork for redefining cachexia as a modifiable and treatable component of cancer care.

---

### SS6-1
### Immuno-metabolic crosstalk in cancer cachexia

[Speaker]Shinpei Kawaoka (Institute of Development, Aging and Cancer, Tohoku University／Insitute for Life and Medical Sciences, Kyoto University)

Cancer cachexia is a complex and multifaceted syndrome that profoundly compromises the health, therapeutic response, and socioeconomic well-being of cancer patients. Conventionally, it is defined by late-stage manifestations such as significant weight loss and sarcopenia. However, these macro-symptoms often emerge too late to allow effective intervention. To improve clinical outcomes, it is critical to elucidate the earlier, more subtle events that precede overt cachexia. Recent studies, including our own, highlight that cancer induces far-reaching systemic changes, even at early, localized stages. These include disruptions in hepatic metabolic function and immune homeostasis, which can occur in the absence of detectable distant metastases. Such evidence challenges the conventional notion of cancer cachexia as a late-stage complication and instead supports its reconceptualization as a progressive systemic syndrome initiated by the cancer-host interaction. In my presentation, I will summarize our recent findings with particular focus on immuno-metabolic crosstalk in cancer cachexia.

[Disclosure of COI by Responsible researcher] Lead presenter is the Responsible researcher.
[Lead presenter: Shinpei Kawaoka] We have no financial relationships to disclose.

▲TOP

### [SS6-2] SS6
### Defeating cancer from a host perspective: cancer cachexia

2025 / 9 / 25 (Thu) 12:50-15:20 Room 10/(Art Hall) Room 10

[Chairpersons]Takeshi Suzuki (Cancer Research Intitute, Kanazawa University)、Shinpei Kawaoka (Institute of Development, Aging and Cancer, Tohoku University (Institute for Life and Medical Sciences, Kyoto University)

Cancer cachexia has long been viewed as a terminal condition marked by unintentional weight loss and organ dysfunction, especially skeletal muscle wasting. Although weight loss correlates strongly with poor clinical outcomes, it remains unclear whether reversing weight loss alone is sufficient to improve patient survival, due to the absence of key drugs. Recent discoveries urge us to broaden our view beyond traditional cachexia symptoms, highlighting the importance of systemic changes in organs and cells that may impair treatment outcomes even in the absence of weight loss. There is also growing interest in detecting host physiological changes at earlier stages, prior to overt cachexia, to enable timely interventions. These insights collectively challenge the current definition of cancer cachexia and call for its revision to reflect both clinical needs and biological complexity. A more comprehensive and operational definition may facilitate earlier diagnosis, better therapeutic targeting, and ultimately enhanced responses to cancer treatment. In this international symposium, leading researchers from Japan and around the world will gather to critically examine traditional cachexia concepts and discuss new biological and clinical frameworks that integrate early systemic alterations. Through this dialogue, we aim to lay the groundwork for redefining cachexia as a modifiable and treatable component of cancer care.

---

### SS6-2
### Muscle-cancer communication in cachexia: signals, pathways, and implications

[Speaker]Hong-Wen Tang (Cancer and Stem Cell Biology Program, Duke-NUS Medical School, Singapore) [Co-author]Kah Yong Goh (Cancer and Stem Cell Biology Program, Duke-NUS Medical School, Singapore)、Wen XIng Lee (Cancer and Stem Cell Biology Program, Duke-NUS Medical School, Singapore)、Andrew Leidal (Department of Pathology, University of California San Francisco, USA)、Sze Mun Choy (Cancer and Stem Cell Biology Program, Duke-NUS Medical School, Singapore)、Gopal Krishnan Priyadarshini (Cancer and Stem Cell Biology Program, Duke-NUS Medical School, Singapore)、Nathan Harmston (Molecular Biosciences Division, Cardiff School of Biosciences, Cardiff University, UK)、Jayanta Debnath (Department of Pathology, University of California San Francisco, USA)

Cancer cachexia is a debilitating syndrome marked by skeletal muscle wasting and poor clinical outcomes. Emerging evidence identifies extracellular vesicles (EVs) as critical mediators of muscle-cancer communication. Tumor-derived EVs, enriched with microRNAs, proteins, and metabolites, can activate proteasomal and autophagic pathways in muscle, driving catabolism. In cachexia, oncogenic signaling alters EV biogenesis and cargo composition, skewing the crosstalk toward both muscle loss and tumor progression. Therapeutic strategies aimed at blocking tumor-derived EV uptake or restoring protective muscle-derived EV populations show promise, while EV-associated biomarkers may enable earlier detection and monitoring. This talk will synthesize current insights into EV-mediated muscle-cancer interactions, highlight underlying pathways, and discuss translational approaches to mitigate cachexia and improve patient outcomes.

[Disclosure of COI by Responsible researcher] Lead presenter is the Responsible researcher.
[Lead presenter: Hong-Wen Tang] We have no financial relationships to disclose.

### [SS6-3] SS6
### Defeating cancer from a host perspective: cancer cachexia

2025 / 9 / 25 (Thu) 12:50-15:20 Room 10/(Art Hall) Room 10

[Chairpersons]Takeshi Suzuki (Cancer Research Intitute, Kanazawa University)、Shinpei Kawaoka (Institute of Development, Aging and Cancer, Tohoku University (Institute for Life and Medical Sciences, Kyoto University)

Cancer cachexia has long been viewed as a terminal condition marked by unintentional weight loss and organ dysfunction, especially skeletal muscle wasting. Although weight loss correlates strongly with poor clinical outcomes, it remains unclear whether reversing weight loss alone is sufficient to improve patient survival, due to the absence of key drugs. Recent discoveries urge us to broaden our view beyond traditional cachexia symptoms, highlighting the importance of systemic changes in organs and cells that may impair treatment outcomes even in the absence of weight loss. There is also growing interest in detecting host physiological changes at earlier stages, prior to overt cachexia, to enable timely interventions. These insights collectively challenge the current definition of cancer cachexia and call for its revision to reflect both clinical needs and biological complexity. A more comprehensive and operational definition may facilitate earlier diagnosis, better therapeutic targeting, and ultimately enhanced responses to cancer treatment. In this international symposium, leading researchers from Japan and around the world will gather to critically examine traditional cachexia concepts and discuss new biological and clinical frameworks that integrate early systemic alterations. Through this dialogue, we aim to lay the groundwork for redefining cachexia as a modifiable and treatable component of cancer care.

---

### SS6-3
### RNA–derived extracellular modified nucleosides and their roles in cancer pathophysiology

[Speaker]Akiko Ogawa (Dept. Pharmacology. Tohoku Univ.)

Over 170 chemical modifications decorate cellular RNA and govern post-transcriptional gene expression. Defects in RNA modifications cause diverse disorders, including cancer or diabetes. Yet the metabolic fate of modified RNA is largely unknown. Using quantitative mass spectrometry, we found that modified RNAs are catabolized into modified nucleosides, that are actively secreted into extracellular fluids. The levels of these metabolites change dynamically in response to cellular stress and can act as signaling molecules. For example, N6-methyladenosine (m6A) potently activates G-protein-coupled receptor, triggering immune responses. Beyond receptor-mediated signaling, RNA metabolites track the course of cancer treatment, making them useful prognostic biomarkers. This talk will review the extracellular biology of RNA modification-derived metabolites and highlight their emerging roles in cancer pathophysiology.

[Disclosure of COI by Responsible researcher] Lead presenter is the Responsible researcher.
[Lead presenter: Akiko Ogawa] We have no financial relationships to disclose.

▲TOP

### [SS6-4] SS6
### Defeating cancer from a host perspective: cancer cachexia

2025 / 9 / 25 (Thu) 12:50-15:20 Room 10/(Art Hall) Room 10

[Chairpersons]Takeshi Suzuki (Cancer Research Intitute, Kanazawa University)、Shinpei Kawaoka (Institute of Development, Aging and Cancer, Tohoku University (Institute for Life and Medical Sciences, Kyoto University)

Cancer cachexia has long been viewed as a terminal condition marked by unintentional weight loss and organ dysfunction, especially skeletal muscle wasting. Although weight loss correlates strongly with poor clinical outcomes, it remains unclear whether reversing weight loss alone is sufficient to improve patient survival, due to the absence of key drugs. Recent discoveries urge us to broaden our view beyond traditional cachexia symptoms, highlighting the importance of systemic changes in organs and cells that may impair treatment outcomes even in the absence of weight loss. There is also growing interest in detecting host physiological changes at earlier stages, prior to overt cachexia, to enable timely interventions. These insights collectively challenge the current definition of cancer cachexia and call for its revision to reflect both clinical needs and biological complexity. A more comprehensive and operational definition may facilitate earlier diagnosis, better therapeutic targeting, and ultimately enhanced responses to cancer treatment. In this international symposium, leading researchers from Japan and around the world will gather to critically examine traditional cachexia concepts and discuss new biological and clinical frameworks that integrate early systemic alterations. Through this dialogue, we aim to lay the groundwork for redefining cachexia as a modifiable and treatable component of cancer care.

---

### SS6-4
### Iron-Dependent Redox Control of Adipose Remodeling in Cancer Cachexia

[Speaker]Christine Chio (Institute for Cancer Genetics, Department of Genetics & Development, Columbia University)

Cancer cachexia is a life-threatening syndrome marked by progressive weight loss, adipose tissue browning, and metabolic dysfunction, particularly prevalent in pancreatic ductal adenocarcinoma (PDA). In this talk, I will present our recent work uncovering an iron-regulated signaling axis that drives adipose remodeling in the context of cancer. We identify methionine sulfoxide reductase A (MSRA) as a critical mediator of beta-3-adrenergic-induced adipose browning, whose enzymatic activity is modulated through iron-induced multimerization. Genetic deletion of MsrA disrupts adipose thermogenesis, mitigates weight loss, and improves survival in mouse models of PDA. These findings define a novel methionine-based redox switch linking iron metabolism to systemic energy balance and reveal new avenues for therapeutic intervention in cancer cachexia.

[Disclosure of COI by Responsible researcher] Lead presenter is the Responsible researcher.
[Lead presenter: Christine Chio] We have no financial relationships to disclose.

▲TOP

### [SS6-5] SS6
### Defeating cancer from a host perspective: cancer cachexia

2025 / 9 / 25 (Thu) 12:50-15:20 Room 10/(Art Hall) Room 10

[Chairpersons]Takeshi Suzuki (Cancer Research Intitute, Kanazawa University)、Shinpei Kawaoka (Institute of Development, Aging and Cancer, Tohoku University (Institute for Life and Medical Sciences, Kyoto University)

Cancer cachexia has long been viewed as a terminal condition marked by unintentional weight loss and organ dysfunction, especially skeletal muscle wasting. Although weight loss correlates strongly with poor clinical outcomes, it remains unclear whether reversing weight loss alone is sufficient to improve patient survival, due to the absence of key drugs. Recent discoveries urge us to broaden our view beyond traditional cachexia symptoms, highlighting the importance of systemic changes in organs and cells that may impair treatment outcomes even in the absence of weight loss. There is also growing interest in detecting host physiological changes at earlier stages, prior to overt cachexia, to enable timely interventions. These insights collectively challenge the current definition of cancer cachexia and call for its revision to reflect both clinical needs and biological complexity. A more comprehensive and operational definition may facilitate earlier diagnosis, better therapeutic targeting, and ultimately enhanced responses to cancer treatment. In this international symposium, leading researchers from Japan and around the world will gather to critically examine traditional cachexia concepts and discuss new biological and clinical frameworks that integrate early systemic alterations. Through this dialogue, we aim to lay the groundwork for redefining cachexia as a modifiable and treatable component of cancer care.

---

### SS6-5
### Identification and application of angiocrine factors suppressing cancer cachexia

[Speaker]Nobuyuki Takakura (Dept. of Signal Transduction, RIMD, The University of Osaka)

The onset of cancer cachexia is closely linked to poor prognosis in cancer patients due to the loss of treatment opportunities. While tumor-derived inflammatory cytokines have been reported to promote muscle atrophy, lipolysis, and anorexia, the direct molecular mechanisms remain unclear. We found that intestinal TNF alpha, induced by gut microbiota dysbiosis, disrupts villous capillaries and causes mucosal injury. This inflammation also induces hepatic TNF alpha expression, leading to damage of sinusoidal vasculature. These findings suggest that inflammatory cytokines may impair capillaries in distant organs, potentially contributing to cachexia. We hypothesize that preventing systemic capillary damage may suppress cachexia development, even in cancer. Previously, we showed that the receptor tyrosine kinase Tie2 and the GPCR APJ, both expressed in endothelial cells, form a signaling axis critical for vascular stability. In this session, we present our recent findings that apelin, an APJ ligand secreted by endothelial cells, acts as an angiocrine factor that can suppress cancer cachexia.

[Disclosure of COI by Responsible researcher] Lead presenter is the Responsible researcher.
[Lead presenter: Nobuyuki Takakura] ■Advisor of the company and/or profit-making organization: ジーン・ステム（株）（ベンチャー企業）、丸善製薬（株）、（株）MTG, ニッタ（株）、ニッポー（株）、資生堂（株） ■Profit of stock: ジーン・ステム（株）、リバスキュラーバイオ（株）

Special Symposia

### [SS6-6] SS6
### Defeating cancer from a host perspective: cancer cachexia

2025 / 9 / 25 (Thu) 12:50-15:20 Room 10/(Art Hall) Room 10

[Chairpersons]Takeshi Suzuki (Cancer Research Intitute, Kanazawa University)、Shinpei Kawaoka (Institute of Development, Aging and Cancer, Tohoku University (Institute for Life and Medical Sciences, Kyoto University)

Cancer cachexia has long been viewed as a terminal condition marked by unintentional weight loss and organ dysfunction, especially skeletal muscle wasting. Although weight loss correlates strongly with poor clinical outcomes, it remains unclear whether reversing weight loss alone is sufficient to improve patient survival, due to the absence of key drugs. Recent discoveries urge us to broaden our view beyond traditional cachexia symptoms, highlighting the importance of systemic changes in organs and cells that may impair treatment outcomes even in the absence of weight loss. There is also growing interest in detecting host physiological changes at earlier stages, prior to overt cachexia, to enable timely interventions. These insights collectively challenge the current definition of cancer cachexia and call for its revision to reflect both clinical needs and biological complexity. A more comprehensive and operational definition may facilitate earlier diagnosis, better therapeutic targeting, and ultimately enhanced responses to cancer treatment. In this international symposium, leading researchers from Japan and around the world will gather to critically examine traditional cachexia concepts and discuss new biological and clinical frameworks that integrate early systemic alterations. Through this dialogue, we aim to lay the groundwork for redefining cachexia as a modifiable and treatable component of cancer care.

---

### SS6-6
### Nicotinamide Metabolism in Diagnostic and Therapeutic Approaches for Cancer-Associated Metabolic Abnormalities

[Speaker]Atsushi Hirao (Can Res Inst／WPI-Nano LSI, Kanazawa Univ)

Nicotinamide adenine dinucleotide (NAD+) functions not only as a coenzyme for dehydrogenases but also as a substrate for NAD+-consuming enzymes, playing key roles in energy metabolism and epigenetic regulation. Dysregulations in NAD+ metabolism are associated with age-related, lifestyle-related, and cancer-associated diseases. Nicotinamide, a central precursor in the NAD+ salvage pathway, is also converted by nicotinamide-N-methyltransferase (NNMT) into 1-methylnicotinamide (1-MNA), thereby affecting NAD+ availability. Thus, monitoring NAD+ metabolic states in vivo may offer novel insights into disease mechanisms and support diagnostic innovations. We are developing supramolecular biosensors of 1-MNA (Commun. Chem. 2020; Anal. Chem. 2024) to detect NAD+ metabolic abnormalities. In addition to metabolomic analyses in animal models, we have conducted clinical studies to establish a non-invasive diagnostic methodology for human metabolic disorders. This presentation will highlight our diagnostic strategy targeting NAD+ metabolism, with a focus on NNMT and 1-MNA, and present progress in clinical applications.

[Disclosure of COI by Responsible researcher] Lead presenter is the Responsible researcher.
[Lead presenter: Atsushi Hirao] ■Research funds from company: AMED

### [AOSR2-1] AOSR2
### The forefront of lung cancer research

2025 / 9 / 25 (Thu) 12:50-15:20 Room 11/(ANA Crowne Plaza Hotel Kanazawa 3F Ohtori (West)) Room 11

[Chairpersons]Seiji Yano (Kanazawa University)、Hideko Isozaki (Kanazawa University Cancer Research Institute)

Research on lung cancer is progressing rapidly. Recent advances in whole-genome analysis technology are beginning to elucidate the entire picture of genomic abnormalities in lung cancer. Furthermore, with the advent of immune checkpoint inhibitors, molecular target drugs, antibody-drug conjugates, and bispecific antibodies, the perioperative treatment of lung cancer and the treatment of advanced stage cancers are undergoing dramatic changes. There is a feeling that bispecific antibodies will lead to a paradigm shift in the treatment of small cell lung cancer, where no major changes have been seen in treatment. In this symposium, we would like to share cutting-edge information on research into lung cancer. We are looking forward to many participants.

---

### AOSR2-1
### Unraveling the Genomic Landscape of Lung Adenocarcinoma: Risk, Evolution, and Emerging Therapeutic Targets

[Speaker]Takashi Kohno (Div Genome Biol, Natl Cancer Ctr Res Inst／C-CAT, Natl Cancer Ctr) [Co-author]Takashi Nakaoku (Div Genome Biol, Natl Cancer Ctr Res Inst)、Kouya Shiraishi (Div Genome Biol, Natl Cancer Ctr Res Inst)

Comprehensive genomic profiling has elucidated critical aspects of lung cancer, including genetic risk factors, somatic alterations, oncogenic mechanisms, and therapeutic vulnerabilities. In lung adenocarcinoma (LADC) of Asian, EGFR mutations are frequently observed. Polymorphisms in TERT and HLA class II genes are significant risk factors. According to a six-country Asian study, individuals in the highest polygenic risk score tier face an 8.6-fold increased risk of EGFR-mutant LADC (Blechter, JTO, 2025). Adenocarcinoma in situ typically harbors driver mutations and evolve into invasive cancer through acquisition of TP53 mutations, genome-wide hypomethylation, and copy number alterations. Spatial transcriptomic analysis has further revealed dynamic tumor-immune interactions during invasion (Haga, Nat Com, 2023). A major unmet clinical need remains for patients with oncogene-negative LADC, who are not candidates for current targeted therapies. Through whole-genome and RNA sequencing of LADCs, we have identified promising therapeutic targets, including oncogene amplifications and mutations in SMARCA4 and RNA splicing genes, offering new avenues for precision lung cancer medicine.

[Disclosure of COI by Responsible researcher] Lead presenter is the Responsible researcher.
[Lead presenter: Takashi Kohno] ■Patent fee: 理研Genesis, Amoy, Foundation Medicine, ThermoFisher Scientific　■Lecture fee: 日本イーライリリー　■Research expenses: 中外製薬株式会社, シスメックス株式会社

### [AOSR2-2] AOSR2
### The forefront of lung cancer research

2025 / 9 / 25 (Thu) 12:50-15:20 Room 11/(ANA Crowne Plaza Hotel Kanazawa 3F Ohtori (West)) Room 11

[Chairpersons]Seiji Yano (Kanazawa University)、Hideko Isozaki (Kanazawa University Cancer Research Institute)

Research on lung cancer is progressing rapidly. Recent advances in whole-genome analysis technology are beginning to elucidate the entire picture of genomic abnormalities in lung cancer. Furthermore, with the advent of immune checkpoint inhibitors, molecular target drugs, antibody-drug conjugates, and bispecific antibodies, the perioperative treatment of lung cancer and the treatment of advanced stage cancers are undergoing dramatic changes. There is a feeling that bispecific antibodies will lead to a paradigm shift in the treatment of small cell lung cancer, where no major changes have been seen in treatment. In this symposium, we would like to share cutting-edge information on research into lung cancer. We are looking forward to many participants.

---

### AOSR2-2
### Current Topics in Small Cell Lung Cancer Research

[Speaker]Masafumi Horie (Div. Mol. Genomic Pathol. Kobe Univ. Grad. Sch. Med.)

Small cell lung cancer (SCLC) is a highly aggressive neuroendocrine carcinoma with a poor prognosis and limited treatment options. Recent advances in molecular profiling have revealed the heterogeneity of SCLC, leading to a refined classification based on transcriptional regulators such as ASCL1, NEUROD1, POU2F3, and YAP1. This has opened new avenues for targeted therapies and biomarker development. In addition, the emergence of novel technologies - including single-cell RNA sequencing, spatial transcriptomics, and epigenomic profiling - has enabled deeper insights into tumor biology and the tumor microenvironment. Current research also explores the role of non-coding RNAs, immune modulation, and lineage plasticity in treatment resistance. In this talk, we highlight recent findings and discuss their potential clinical implications in the era of precision oncology.

[Disclosure of COI by Responsible researcher] Lead presenter is the Responsible researcher.
[Lead presenter: Masafumi Horie] We have no financial relationships to disclose.

### [AOSR2-3] AOSR2
### The forefront of lung cancer research

2025 / 9 / 25 (Thu) 12:50-15:20 Room 11/(ANA Crowne Plaza Hotel Kanazawa 3F Ohtori (West)) Room 11

[Chairpersons]Seiji Yano (Kanazawa University)、Hideko Isozaki (Kanazawa University Cancer Research Institute)

Research on lung cancer is progressing rapidly. Recent advances in whole-genome analysis technology are beginning to elucidate the entire picture of genomic abnormalities in lung cancer. Furthermore, with the advent of immune checkpoint inhibitors, molecular target drugs, antibody-drug conjugates, and bispecific antibodies, the perioperative treatment of lung cancer and the treatment of advanced stage cancers are undergoing dramatic changes. There is a feeling that bispecific antibodies will lead to a paradigm shift in the treatment of small cell lung cancer, where no major changes have been seen in treatment. In this symposium, we would like to share cutting-edge information on research into lung cancer. We are looking forward to many participants.

---

### AOSR2-3
### Targeted therapy-induced tumor evolution in non-small cell lung cancer

[Speaker]Hideko Isozaki (Genome Biology, Cancer Research Institute, Kanazawa Univ.)

Acquired drug resistance to anticancer targeted therapies remains an unsolved clinical problem. Although many drivers of acquired drug resistance have been identified, the underlying molecular mechanisms shaping tumor evolution during treatment are incompletely understood. Genomic profiling of patient tumors has implicated cytidine deaminases APOBEC in tumor evolution; however, their role during therapy and the development of acquired drug resistance is undefined. Recently, we reported that lung cancer targeted therapies commonly used in the clinic can induce APOBEC3A (A3A), leading to sustained mutagenesis in drug-tolerant persister cells (DTPs). Deletion of A3A reduces APOBEC mutations and structural variations in DTPs and delays the development of drug resistance. APOBEC mutational signatures are enriched in tumors from patients with lung cancer who progressed after extended responses to targeted therapies. This study shows that induction of A3A in response to targeted therapies drives evolution of DTPs, suggesting that suppression of A3A may represent a potential therapeutic strategy in preventing or delaying acquired resistance to lung cancer targeted therapy.

[Disclosure of COI by Responsible researcher] Lead presenter is the Responsible researcher.
[Lead presenter: Hideko Isozaki] We have no financial relationships to disclose.

▲TOP

### [AOSR2-4] AOSR2
### The forefront of lung cancer research

2025 / 9 / 25 (Thu) 12:50-15:20 Room 11/(ANA Crowne Plaza Hotel Kanazawa 3F Ohtori (West)) Room 11

[Chairpersons]Seiji Yano (Kanazawa University)、Hideko Isozaki (Kanazawa University Cancer Research Institute)

Research on lung cancer is progressing rapidly. Recent advances in whole-genome analysis technology are beginning to elucidate the entire picture of genomic abnormalities in lung cancer. Furthermore, with the advent of immune checkpoint inhibitors, molecular target drugs, antibody-drug conjugates, and bispecific antibodies, the perioperative treatment of lung cancer and the treatment of advanced stage cancers are undergoing dramatic changes. There is a feeling that bispecific antibodies will lead to a paradigm shift in the treatment of small cell lung cancer, where no major changes have been seen in treatment. In this symposium, we would like to share cutting-edge information on research into lung cancer. We are looking forward to many participants.

### AOSR2-4
### Development of cancer immunotherapy against tumor microenvironment in lung cancer

[Speaker]Yasuhiko Nishioka (Dept. Respiratory Medicine and Rheumatology, Grad. Sch., Tokushima Univ.) [Co-author]Atsushi Mitsuhashi (Dept. Respiratory Medicine and Rheumatology, Grad. Sch., Tokushima Univ.)、Mika K. Kaneko (Dept. Antibody Drug Development, Tohoku University Grad. Sch of Med.)、Yukinari Kato (Dept. Antibody Drug Development, Tohoku University Grad. Sch of Med.)

Immune checkpoint inhibitors (ICIs) with anti-PD-1/PD-L1 antibodies have shown the durable clinical effects for lung cancer as a standard therapy. However, a substantial percentage of patients still do not respond to ICI therapy, and the resistance phenomena are experienced in the clinical setting. We have studied fibrocytes in tumor microenvironment, and reported that fibrocytes have ability to promote tumor progression and mediate drug resistance. To develop the novel targeted therapy, we focused the novel cell surface molecule expressing on fibrocytes, and generated the specific antibody. Treatment with the antibody enhanced the antitumor effects mediated by ICIs in mice. These findings suggest that targeted therapy to fibrocytes might be useful to overcome the drug resistance of ICIs.

[Disclosure of COI by Responsible researcher] Lead presenter is the Responsible researcher.
[Lead presenter: Yasuhiko Nishioka] ■Lecture fee: 日本ベーリンガーインゲルハイム株式会社 ■Research expenses: SANSHO株式会社、株式会社ティムス ■Contributions: 中外製薬株式会社、大鵬薬品工業株式会社、旭化成ファーマ株式会社、日本イーライリリー株式会社

▲TOP

### [AOSR2-5] AOSR2
### The forefront of lung cancer research

2025 / 9 / 25 (Thu) 12:50-15:20 Room 11/(ANA Crowne Plaza Hotel Kanazawa 3F Ohtori (West)) Room 11

[Chairpersons]Seiji Yano (Kanazawa University)、Hideko Isozaki (Kanazawa University Cancer Research Institute)

Research on lung cancer is progressing rapidly. Recent advances in whole-genome analysis technology are beginning to elucidate the entire picture of genomic abnormalities in lung cancer. Furthermore, with the advent of immune checkpoint inhibitors, molecular target drugs, antibody-drug conjugates, and bispecific antibodies, the perioperative treatment of lung cancer and the treatment of advanced stage cancers are undergoing dramatic changes. There is a feeling that bispecific antibodies will lead to a paradigm shift in the treatment of small cell lung cancer, where no major changes have been seen in treatment. In this symposium, we would like to share cutting-edge information on research into lung cancer. We are looking forward to many participants.

---

### AOSR2-5
### Toward overcoming drug resistance and predicting efficacy of new antibody-based therapy for lung cancer

[Speaker]Shigeki Nanjo (Department of Respiratory Medicine, Kanazawa University Hospital, Kanazawa, Japan) [Co-author]Seiji Yano (Department of Respiratory Medicine, Kanazawa University Hospital, Kanazawa, Japan)

As a new antibody-based therapy, the anti-Her2 antibody-drug conjugate (ADC), Trastuzumab Deruxtecan (T-DXd), has been developed for lung cancer treatment, and anti-MET antibody ADCs have demonstrated promising efficacy in clinical trials. However, drug resistance remains a critical issue in these antibody therapies, and biomarkers for predicting efficacy remain unclear.We established in vivo T-DXd-resistant HER2-amplified lung cancer cell line (Calu3 AR) . In this resistant cell line, HER2 expression was maintained, however, the expression of ABC transporters was elevated, and knocking down these genes or pharmacologically inhibition restored sensitivity to T-DXd.Additionally, in a Phase 2 trial of anti-MET antibody ADC, efficacy was demonstrated in cases with high MET protein expression; however, accurate determination via IHC in small biopsy samples is challenging. Then, we are developing a PET imaging probe using a MET-specific cyclic peptide grafted to ubiquitin (aMD4-Ub), labeled with a radioactive isotope, to accurately evaluate MET expression in human cancer.

[Disclosure of COI by Responsible researcher] Lead presenter is the Responsible researcher.
[Lead presenter: Shigeki Nanjo] ■Research funds from company: 23K27609、23K06648　■Lecture fee: AstraZeneca

▲TOP

### [AOSR2-6] AOSR2
### The forefront of lung cancer research

2025 / 9 / 25 (Thu) 12:50-15:20 Room 11/(ANA Crowne Plaza Hotel Kanazawa 3F Ohtori (West)) Room 11

[Chairpersons]Seiji Yano (Kanazawa University)、Hideko Isozaki (Kanazawa University Cancer Research Institute)

Research on lung cancer is progressing rapidly. Recent advances in whole-genome analysis technology are beginning to elucidate the entire picture of genomic abnormalities in lung cancer. Furthermore, with the advent of immune checkpoint inhibitors, molecular target drugs, antibody-drug conjugates, and bispecific antibodies, the perioperative treatment of lung cancer and the treatment of advanced stage cancers are undergoing dramatic changes. There is a feeling that bispecific antibodies will lead to a paradigm shift in the treatment of small cell lung cancer, where no major changes have been seen in treatment. In this symposium, we would like to share cutting-edge information on research into lung cancer. We are looking forward to many participants.

---

### AOSR2-6
### Identification of drug resistance mechanisms using 3D co-culture models and CRISPR screening with patient derived cells

[Speaker]Ryohei Katayama (Div. Exp. Chemother., Cancer Chemother. Ctr., JFCR／Dept. CBMS, Grad. Sch. Front. Sci., The Univ. of Tokyo) [Co-author]Ken Uchibori (Div. Exp. Chemother., Cancer Chemother. Ctr., JFCR／Dept. Thoracic Med. Oncology, Cancer Inst. Hosp.,)、Makoto Nishio (Dept. Thoracic Med. Oncology, Cancer Inst. Hosp.,)

Recent advances in drug therapy have largely improved the prognosis of advanced cancer patients, however, recurrence due to acquired or primary resistance make it difficult to cure the advanced cancer. We have focused on elucidating the mechanisms of drug resistance in lung cancer using clinical samples obtained from patients before and/or after anticancer drug treatment, as well as established cancer cell lines. Although a number of drug resistance mechanisms have been identified, there is considerable number of cases whose resistance mechanisms uncovered. To identify those unknown resistance mechanisms, we have performed 2 strategies, (1) genome-wide CRISPR screening using patient-derived cancer cell lines, and (2) apply clinical samples to our original layered three-dimensional co-culture system that combines extracellular matrix, fibroblasts, vascular cells, and patient-derived tumor cells. In this presentation, we'd like to present our latest findings on drug resistance mechanisms and overcoming strategy using this 3D co-culture models and patient-derived samples, mainly focusing on ***NF2*** loss mediated resistance and stromal cell derived factor dependent resistance.

[Disclosure of COI by Responsible researcher] Lead presenter is the Responsible researcher.
[Lead presenter: Ryohei Katayama] ■Patent fee: Eiken Chemical　■Research expenses: Chugai, TOPPAN, UBE

### [IS4-1] IS4
### Breaking New Ground in Cancer Research with Advanced Proteomics

2025 / 9 / 25 (Thu) 12:50-15:20 Room 12/(ANA Crowne Plaza Hotel Kanazawa 3F Ohtori (Central)) Room 12

[Chairpersons]Shungo Adachi (Department of Proteomics, National Cancer Center Research institute)、Yu-Ju Chen (Institute of Chemistry, Academia Sinica)

In advancing our understanding and treatment of cancer, it is essential to analyze not only genes but also the quantity and activity of functional proteins. Traditional approaches have primarily focused on individual proteins; however, recent advances in mass spectrometry now enable comprehensive proteomic analysis of over 10,000 proteins simultaneously. This breakthrough allows for in-depth investigation of cancer-related protein expression, cancer-specific mutations, and post-translational modifications, thereby enhancing our understanding of cancer biology, diagnosis, and therapeutic development. This symposium will highlight how state-of-the-art proteomics technologies are being utilized in cancer research around the world. It will focus on emerging challenges such as single-cell proteomics and the integration of proteomics with genomics, while also exploring their potential applications in clinical research.

---

### IS4-1
### Symposium Overview_Breaking New Ground in Cancer Research with Advanced Proteomics

[Speaker]Shungo Adachi (Dept. of Proteomics, NCCRI)

This overview introduces the theme and objectives of the symposium titled "Breaking New Ground in Cancer Research with Advanced Proteomics." While genomics and transcriptomics have laid the foundation for many breakthroughs in cancer biology, protein-level information is essential for understanding the functional landscape of tumors. With recent advances in mass spectrometry, researchers can now simultaneously quantify and characterize over 10,000 proteins, including those bearing cancer-specific mutations and post-translational modifications. This symposium will highlight the transformative impact of proteomics on cancer research-from unraveling disease mechanisms to identifying diagnostic markers and therapeutic targets. Key topics include single-cell proteomics, the integration of multi-omics data, and emerging clinical applications. By featuring global leaders in proteomics and oncology, the symposium will offer a comprehensive perspective on how cutting-edge technologies are reshaping the future of cancer research and precision medicine.

[Disclosure of COI by Responsible researcher] Lead presenter is the Responsible researcher.
[Lead presenter: Adachi Shungo] We have no financial relationships to disclose.

### [IS4-2] IS4
### Breaking New Ground in Cancer Research with Advanced Proteomics

2025 / 9 / 25 (Thu) 12:50-15:20 Room 12/(ANA Crowne Plaza Hotel Kanazawa 3F Ohtori (Central)) Room 12

[Chairpersons]Shungo Adachi (Department of Proteomics, National Cancer Center Research institute)、Yu-Ju Chen (Institute of Chemistry, Academia Sinica)

In advancing our understanding and treatment of cancer, it is essential to analyze not only genes but also the quantity and activity of functional proteins. Traditional approaches have primarily focused on individual proteins; however, recent advances in mass spectrometry now enable comprehensive proteomic analysis of over 10,000 proteins simultaneously. This breakthrough allows for in-depth investigation of cancer-related protein expression, cancer-specific mutations, and post-translational modifications, thereby enhancing our understanding of cancer biology, diagnosis, and therapeutic development. This symposium will highlight how state-of-the-art proteomics technologies are being utilized in cancer research around the world. It will focus on emerging challenges such as single-cell proteomics and the integration of proteomics with genomics, while also exploring their potential applications in clinical research.

---

### IS4-2
### Proteomics-guided Precision Oncology in Non-Smoking Lung Cancer

[Speaker]YuJu Chen (Institute of Chemistry, Academia Sinica)

Personalized proteomics is emerging as a transformative strategy in precision oncology, enabling the discovery of disease biomarkers, molecular subtypes, and druggable pathways tailored to individual patients. By integrating proteomics with genomics, we previously revealed a complex etiology and pathogenesis of early-stage non-smoking non-small-cell lung cancer (NSCLC). Proteomics-informed classification identified a high risk " late-like " subtype with early recurrence at stage I. This prospective study inspired development a few proteomics-based strategies to improve early-stage NSCLC management. Furthermore, our recent study of a comprehensive proteogenomic analysis of 406 lung adenocarcinoma patients across diverse geographic and demographic backgrounds also reveal the differential and sex-specific effects of endogenous mutagens and environmental carcinogens, and pathobiology of early-stage tumors with "late-like" characteristics. These findings highlights how innovations in proteomic technologies are paving the way for personalized and actionable insights in cancer care.

[Disclosure of COI by Responsible researcher] Lead presenter is the Responsible researcher.
[Lead presenter: YuJu Chen] We have no financial relationships to disclose.

▲TOP

### [IS4-3] IS4
### Breaking New Ground in Cancer Research with Advanced Proteomics

2025 / 9 / 25 (Thu) 12:50-15:20 Room 12/(ANA Crowne Plaza Hotel Kanazawa 3F Ohtori (Central)) Room 12

[Chairpersons]Shungo Adachi (Department of Proteomics, National Cancer Center Research institute)、Yu-Ju Chen (Institute of Chemistry, Academia Sinica)

In advancing our understanding and treatment of cancer, it is essential to analyze not only genes but also the quantity and activity of functional proteins. Traditional approaches have primarily focused on individual proteins; however, recent advances in mass spectrometry now enable comprehensive proteomic analysis of over 10,000 proteins simultaneously. This breakthrough allows for in-depth investigation of cancer-related protein expression, cancer-specific mutations, and post-translational modifications, thereby enhancing our understanding of cancer biology, diagnosis, and therapeutic development. This symposium will highlight how state-of-the-art proteomics technologies are being utilized in cancer research around the world. It will focus on emerging challenges such as single-cell proteomics and the integration of proteomics with genomics, while also exploring their potential applications in clinical research.

---

### IS4-3
### Phosphoproteome subtyping of gastric cancer reveals dynamic transformation with chemotherapy and guides targeted therapy

[Speaker]Jun Adachi (Lab. Proteomics for Drug Discovery, NIBN) [Co-author]Hirokazu Shoji (Dept. Gastrointestinal Medical Oncology, NCCH)、Hidekazu Hirano (Dept. Gastrointestinal Medical Oncology, NCCH)、Yosui Nojima (Center. Mathematical Modeling and Data Science, Osaka University)、Daigo Gunji (Lab. Proteomics for Drug Discovery, NIBN／Dept. Surgery, Graduate School of Medicine, Kyoto University)、Akina Shinkura (Lab. Proteomics for Drug Discovery, NIBN／Dept. Surgery, Graduate School of Medicine, Kyoto University)、Satoshi Muraoka (Lab. Proteomics for Drug Discovery, NIBN)、Yuichi Abe (Lab. Proteomics for Drug Discovery, NIBN／iGCORE, Gifu University)、Takeshi Tomonaga (Lab. Proteomics for Drug Discovery, NIBN)、Kenji Mizugichi (Lab. Bioinformatics, NIBN／ Institute for Protein Research, Osaka University)、Kazutaka Obama (Dept. Surgery, Graduate School of Medicine, Kyoto University)、Kazufumi Honda (Dept. Bioregulation, Graduate School of Medicine, NMS)、Yutaka Saito (Endoscopy Division, NCCH)、Takaki Yoshikawa (Dept. Gastric Surgery, NCCH)、Ken Kato (Dept. Gastrointestinal Medical Oncology, NCCH)、Narikazu Boku (Dept. Gastrointestinal Medical Oncology, NCCH／Dept. Medical Oncology, IMSUT Hospital, University of Tokyo)

Only a few molecular targeted agents are effective for advanced gastric cancer (AGC), thus, it is imperative to establish a new concept of precision medicine for AGC.
We collected endoscopic biopsy specimens from 84 chemo-naive gastric cancer patients as well as 9 AGC patients receiving 2nd line therapy. Each specimen was immediately snap frozen in liquid nitrogen within 20 s after collection. Proteome and phosphoproteome analysis were performed by multiplex analysis using TMT reagent.
Phosphoproteomics quantified an average of 21000 phosphorylation sites and clearly divided gastric cancer into proliferative subtype, EMT subtype, and OxPhos subtype. Subsequent analysis using serially collected gastric cancer specimens unveiled the dynamic transitions, ultimately resulting in the acquisition of the EMT subtype. We investigated elaborate intracellular signaling related to EMT in gastric cancer and validate therapeutic approaches targeting AXL in vitro and in vivo.
Our proof-of-concept of phosphoproteome analysis of fresh-frozen endoscopic biopsy specimens is capable of not only defining cancer subtyping but also capturing dynamic cancer signaling changes in response to therapy.

[Disclosure of COI by Responsible researcher] Lead presenter is the Responsible researcher.
[Lead presenter: Jun Adachi] We have no financial relationships to disclose.

### [IS4-4] IS4
### Breaking New Ground in Cancer Research with Advanced Proteomics

2025 / 9 / 25 (Thu) 12:50-15:20 Room 12/(ANA Crowne Plaza Hotel Kanazawa 3F Ohtori (Central)) Room 12

[Chairpersons]Shungo Adachi (Department of Proteomics, National Cancer Center Research institute)、Yu-Ju Chen (Institute of Chemistry, Academia Sinica)

In advancing our understanding and treatment of cancer, it is essential to analyze not only genes but also the quantity and activity of functional proteins. Traditional approaches have primarily focused on individual proteins; however, recent advances in mass spectrometry now enable comprehensive proteomic analysis of over 10,000 proteins simultaneously. This breakthrough allows for in-depth investigation of cancer-related protein expression, cancer-specific mutations, and post-translational modifications, thereby enhancing our understanding of cancer biology, diagnosis, and therapeutic development. This symposium will highlight how state-of-the-art proteomics technologies are being utilized in cancer research around the world. It will focus on emerging challenges such as single-cell proteomics and the integration of proteomics with genomics, while also exploring their potential applications in clinical research.

---

### IS4-4
### Multi-Omics Deconvolution of TNBC Reveals Subtype-Specific Therapeutic Vulnerabilities

[Speaker]Min-Sik Kim (Department of New Biology, DGIST)

Triple-negative breast cancer (TNBC) is known for its high heterogeneity and lack of targeted treatment options due to the absence of hormone receptors and HER2 amplification. To better understand this complexity, we performed a large-scale proteomic and phosphoproteomic analysis of 351 breast cancer samples including 68 TNBC cases. These data were integrated with publicly available CPTAC and TCGA proteogenomic datasets. Through supervised clustering based on global and phosphoproteomic profiles, breast cancers were grouped into four major subtypes: Luminal A, Luminal B, HER2-enriched, and Basal-like. While most TNBC were classified under the Basal group, we observed considerable molecular diversity within TNBC itself. Focusing on TNBC cases, we conducted further subtype analysis using non-negative matrix factorization, which revealed three distinct TNBC subtypes: EMT-like, Basal-proliferative, and Immune-enriched.This study systematically dissects the molecular complexity of TNBC and proposes subtype-specific biomarkers and signaling pathways, offering a critical foundation for developing precision therapies tailored to each molecular phenotype.

[Disclosure of COI by Responsible researcher] Lead presenter is the Responsible researcher.
[Lead presenter: Min-Sik Kim] We have no financial relationships to disclose.

▲TOP

### [IS4-5] IS4
### Breaking New Ground in Cancer Research with Advanced Proteomics

2025 / 9 / 25 (Thu) 12:50-15:20 Room 12/(ANA Crowne Plaza Hotel Kanazawa 3F Ohtori (Central)) Room 12

[Chairpersons]Shungo Adachi (Department of Proteomics, National Cancer Center Research institute)、Yu-Ju Chen (Institute of Chemistry, Academia Sinica)

In advancing our understanding and treatment of cancer, it is essential to analyze not only genes but also the quantity and activity of functional proteins. Traditional approaches have primarily focused on individual proteins; however, recent advances in mass spectrometry now enable comprehensive proteomic analysis of over 10,000 proteins simultaneously. This breakthrough allows for in-depth investigation of cancer-related protein expression, cancer-specific mutations, and post-translational modifications, thereby enhancing our understanding of cancer biology, diagnosis, and therapeutic development. This symposium will highlight how state-of-the-art proteomics technologies are being utilized in cancer research around the world. It will focus on emerging challenges such as single-cell proteomics and the integration of proteomics with genomics, while also exploring their potential applications in clinical research.

### IS4-5
### Federated deep learning enables cancer subtyping by proteomics

[Speaker]Qing Zhong (Children's Medical Research Institute, The University of Sydney)

Artificial intelligence applications in biomedicine face major challenges from data privacy requirements. To address this issue for clinically annotated tissue proteomic data, we developed a Federated Deep Learning (FDL) approach (ProCanFDL), training local models on simulated sites containing data from a pan-cancer cohort (n=1,260) and 29 cohorts held behind private firewalls (n=6,265), representing 19,930 replicate data-independent acquisition mass spectrometry (DIA-MS) runs. Local parameter updates were aggregated to build the global model, achieving a 43% performance gain on the hold-out test set (n=625) in 14 cancer subtyping tasks compared to local models, and matching centralized model performance. The approach's generalizability was demonstrated by retraining the global model with data from two external DIA-MS cohorts (n=55) and eight acquired by tandem mass tag (TMT) proteomics (n=832). ProCanFDL presents a solution for internationally collaborative machine learning initiatives using proteomic data, e.g., for discovering predictive biomarkers or treatment targets, while maintaining data privacy.

[Disclosure of COI by Responsible researcher] Lead presenter is the Responsible researcher.
[Lead presenter: Qing Zhong] We have no financial relationships to disclose.

### [IS4-6] IS4
### Breaking New Ground in Cancer Research with Advanced Proteomics

2025 / 9 / 25 (Thu) 12:50-15:20 Room 12/(ANA Crowne Plaza Hotel Kanazawa 3F Ohtori (Central)) Room 12

[Chairpersons]Shungo Adachi (Department of Proteomics, National Cancer Center Research institute)、Yu-Ju Chen (Institute of Chemistry, Academia Sinica)

In advancing our understanding and treatment of cancer, it is essential to analyze not only genes but also the quantity and activity of functional proteins. Traditional approaches have primarily focused on individual proteins; however, recent advances in mass spectrometry now enable comprehensive proteomic analysis of over 10,000 proteins simultaneously. This breakthrough allows for in-depth investigation of cancer-related protein expression, cancer-specific mutations, and post-translational modifications, thereby enhancing our understanding of cancer biology, diagnosis, and therapeutic development. This symposium will highlight how state-of-the-art proteomics technologies are being utilized in cancer research around the world. It will focus on emerging challenges such as single-cell proteomics and the integration of proteomics with genomics, while also exploring their potential applications in clinical research.

### IS4-6
### Proteogenomics unlocks noncanonical cancer antigens for diagnostic and therapeutic development

[Speaker]Ayumu Taguchi (Nagoya City Univ. Grad. Sch. Med. Sci. Dept. Mol. Oncol.) [Co-author]Hisanori Isomura (Nagoya City Univ. Grad. Sch. Med. Sci. Dept. Mol. Oncol.)、Yongwoon Han (Nagoya City Univ. Grad. Sch. Med. Sci. Dept. Mol. Oncol.)

Recent studies have demonstrated that microproteins can be translated from RNAs previously annotated as noncoding, such as circRNAs and lncRNAs. Alternative initiation at non-AUG codons can also give rise to entirely novel proteins from canonical mRNAs. These noncanonical proteins are thus often tumor-specific and immunogenic, highlighting their potential as diagnostic biomarkers and immunotherapeutic targets. We developed a proteogenomic pipeline for identifying noncanonical proteins, along with a targeted proteomics workflow for validation. Applied to colorectal cancer plasma autoantibody profiling, this approach identified nearly 400 novel noncanonical antigens, a subset of which were cancer-specific and showed promise as diagnostic biomarkers. Extension of the pipeline to the cell surface proteome of pancreatic cancer PDXs enabled the identification of transmembrane domain-containing noncanonical proteins. One of these proteins was validated by targeted proteomics and is currently undergoing antibody development. This strategy is now being expanded across multiple tumor types and diverse proteomic datasets to facilitate novel approaches in cancer diagnostics and immunotherapy.

[Disclosure of COI by Responsible researcher] Lead presenter is the Responsible researcher.
[Lead presenter: Ayumu Taguchi] ■Research funds from company: 科研費　挑戦的研究（萌芽）、AMED　次世代がん医療加速化研究事業　■Research expenses: 小野薬品工業株式会社

### [IS4-7] IS4
### Breaking New Ground in Cancer Research with Advanced Proteomics

2025 / 9 / 25 (Thu) 12:50-15:20 Room 12/(ANA Crowne Plaza Hotel Kanazawa 3F Ohtori (Central)) Room 12

[Chairpersons]Shungo Adachi (Department of Proteomics, National Cancer Center Research institute)、Yu-Ju Chen (Institute of Chemistry, Academia Sinica)

In advancing our understanding and treatment of cancer, it is essential to analyze not only genes but also the quantity and activity of functional proteins. Traditional approaches have primarily focused on individual proteins; however, recent advances in mass spectrometry now enable comprehensive proteomic analysis of over 10,000 proteins simultaneously. This breakthrough allows for in-depth investigation of cancer-related protein expression, cancer-specific mutations, and post-translational modifications, thereby enhancing our understanding of cancer biology, diagnosis, and therapeutic development. This symposium will highlight how state-of-the-art proteomics technologies are being utilized in cancer research around the world. It will focus on emerging challenges such as single-cell proteomics and the integration of proteomics with genomics, while also exploring their potential applications in clinical research.

---

### IS4-7
### Application of ion mobility-assisted mass spectrometry for clinical immunopeptidomics

[Speaker]Yuriko Minegishi (Dvi. Analytical Biochem., CPM Ctr, JFCR) [Co-author]Norio Tanaka (Dvi. Cancer Genomics, CPM Ctr, JFCR)、Yoshimi Haga (Dvi. Analytical Biochem., CPM Ctr, JFCR)、Ryo Yanagiya (Faculty of Med., Saga Univ.)、Noriko Iwamoto (SHIMADZU Corp.)、Takashi Shimada (SHIMADZU Corp.)、So Nakagawa (Tokai Univ. School of Med.)、Kazuma Kiyotani (Lab. Immunogenomics, CiDIG, NIBIOHN)、Seiichi Mori (Dvi. Cancer Genomics, CPM Ctr, JFCR)、Koji Ueda (Dvi. Analytical Biochem., CPM Ctr, JFCR)

Identifying the presented antigens (a.k.a. immunopeptides) by mass spectrometry (MS), called immunopeptidomics, can gain insights for cancer immunotherapy, such as adoptive T cell therapy and vaccines. Since it is generally required more than 1000 mg of tissue to identify more than 1K peptides, the analysis from limited amount of clinical sample has been unrealistic. We previously reported that the differential ion mobility (IM)-assisted MS allowed sufficient identification of immunopeptides, including oncogenic KRAS neoepitope, from around 40 mg of tissue. Furthermore, assuming the sample size of endoscopic biopsy (EB), another type of IM, called trapped-IM in timsTOF-ULTRA2 mass spectrometer (Bruker) was newly introduced. Under optimized methods, when samples analyzed for 2 and 1 million of HCT116 cells equivalent, 6915 and 4570 peptides with 7 and 4 neoepitopes were identified, respectively. From 16.2 and 9.1 mg of colon cancer EBs, 14086 and 10545 peptides were successfully identified, respectively. The IM-assisted MS can be a powerful strategy for clinical immunopeptidomics to identify the patient-specific and actually-presented neoepitopes for better precision immunotherapy.

[Disclosure of COI by Responsible researcher] Lead presenter is not the Responsible researcher.
[Lead presenter: Yuriko Minegishi] We have no financial relationships to disclose.
[Principal Researcher: Koji Ueda] ■Research funds from company: 株式会社島津製作所

▲TOP

### [OS6-1] OS6
### Next-generation cancer research pioneered by designer cells and RNA science

2025 / 9 / 25 (Thu) 12:50-15:20 Room 13/(ANA Crowne Plaza Hotel Kanazawa 3F Ohtori (East)) Room 13

[Chairpersons]Tomoaki Hara (Department of Medical Data Science, Center of Medical Innovation and Translational Research, Osaka University Graduate School of Medicine)、Yukimi Sakoda (Department of Immunology, Yamaguchi University Graduate School of Medicine)

キメラ抗原受容体T（CAR-T）細胞療法は、造血器腫瘍で顕著な成果を挙げていますが、固形がんでは依然として多くの課題が残り、さらなる革新が求められています。特に、がん幹細胞や線維芽細胞が豊富ながん微小環境、固形がん特有の抗原変化や喪失の多様性といった問題が、現行治療法の限界を浮き彫りにしています。これらの課題を克服するには、従来のCAR-T療法を超える新しい治療戦略が必要です。本企画では、精密なゲノム情報や最先端の核酸技術を活用し、「RNA科学が切り開く次世代デザイナー細胞療法」の可能性を探求します。RNA修飾の正確な測定やプロファイリング技術の進展により、がん治療の精密医療の実現だけでなく、核酸医薬の新たな可能性を拡大します。具体的には、抗原多様性に対応するエピトープ拡大、免疫記憶の制御、T細胞以外を対象とした治療戦略などを検討し、ヒト病態に忠実な革新的モデルを活用した評価を行います。また、若手研究者による画期的な技術開発を促進し、次世代治療法の創出を目指した革新的な研究を支援します。

---

### OS6-1
### Next-Generation In Vivo CAR-T Therapy Driven by RNA Engineering and LNP Optimization

[Speaker]Sikun Meng (Osaka Univ Grad Sch Med, CoMIT) [Co-author]Tomoaki Hara (Osaka Univ Grad Sch Med, CoMIT)、Shotaro Tatekawa (Osaka Univ Grad Sch Med, Rad Oncol)、Tetsuya Sato (Saitama Med Univ)、Yoshiko Saito (Osaka Univ Grad Sch Med, CoMIT)、Yasuko Arao (Osaka Univ Grad Sch Med, CoMIT)、Taroh Satoh (Osaka Univ Hosp Cancer Gen Med Center)、Hidetoshi Eguchi (Osaka Univ Grad Sch Med, Gastro Surg)、Kazuhiko Ogawa (Osaka Univ Grad Sch Med, Rad Oncol)、Yutaka Miura (Inst Sci Tokyo)、Hideshi Ishii (Osaka Univ Grad Sch Med, CoMIT)

Chimeric antigen receptor (CAR)-T therapy is effective for hematologic cancers but limited in solid tumors due to the suppressive tumor microenvironment (TME). Fibroblast activation protein (FAP) is broadly expressed by cancer-associated fibroblasts in solid tumors, offering a selective target. We developed an mRNA-based CAR-T strategy delivering FAP-targeting CAR via lipid nanoparticles (LNPs), enabling transient CAR expression in vivo. In colorectal and breast cancer models, combination with chemotherapy and immune checkpoint inhibitors led to tumor eradication and long-term immunity, mediated by tissue-resident memory T cells.To further improve efficacy, we incorporated m6A modifications to enhance mRNA translation and optimized the LNP formulation using polybetaine. In parallel, we explored the applicability of this platform to cancer stem cell markers, including EpCAM, LGR5, and CD133, in the MC38 model. Notably, LGR5-targeted CAR-T induced strong tumor suppression with complete regression in one case. This flexible, safer CAR-T strategy may overcome barriers in solid tumors and broaden RNA-based cancer immunotherapy.

[Disclosure of COI by Responsible researcher] Lead presenter is not the Responsible researcher.
[Lead presenter: Sikun Meng] ■Endowed chair: IDEA Consultants, INC. Kyowakai Medical Corporation. KINSHUKAI GROUP. HIROTSU BIO SCIENCE INC. UNITECH Inc.
[Principal Researcher: Hideshi Ishii] ■Endowed chair: IDEA Consultants, INC. Kyowakai Medical Corporation. KINSHUKAI GROUP. HIROTSU BIO SCIENCE INC. UNITECH Inc.

▲TOP

### [OS6-2] OS6
### Next-generation cancer research pioneered by designer cells and RNA science

2025 / 9 / 25 (Thu) 12:50-15:20 Room 13/(ANA Crowne Plaza Hotel Kanazawa 3F Ohtori (East)) Room 13

[Chairpersons]Tomoaki Hara (Department of Medical Data Science, Center of Medical Innovation and Translational Research, Osaka University Graduate School of Medicine)、Yukimi Sakoda (Department of Immunology, Yamaguchi University Graduate School of Medicine)

キメラ抗原受容体T（CAR-T）細胞療法は、造血器腫瘍で顕著な成果を挙げていますが、固形がんでは依然として多くの課題が残り、さらなる革新が求められています。特に、がん幹細胞や線維芽細胞が豊富ながん微小環境、固形がん特有の抗原変化や喪失の多様性といった問題が、現行治療法の限界を浮き彫りにしています。これらの課題を克服するには、従来のCAR-T療法を超える新しい治療戦略が必要です。本企画では、精密なゲノム情報や最先端の核酸技術を活用し、「RNA科学が切り開く次世代デザイナー細胞療法」の可能性を探求します。RNA修飾の正確な測定やプロファイリング技術の進展により、がん治療の精密医療の実現だけでなく、核酸医薬の新たな可能性を拡大します。具体的には、抗原多様性に対応するエピトープ拡大、免疫記憶の制御、T細胞以外を対象とした治療戦略などを検討し、ヒト病態に忠実な革新的モデルを活用した評価を行います。また、若手研究者による画期的な技術開発を促進し、次世代治療法の創出を目指した革新的な研究を支援します。

### OS6-2
### Investigation of Immuno-Enhanced CAR-T Cells in Intractable Solid Tumor Models

[Speaker]Keisuke Ohta (Dept of Immunology,Yamaguchi Univ.／Dept of Gastroenterological Surgery) [Co-author]Yukimi Sakoda (Dept of Immunology,Yamaguchi Univ.)、Keishi Adachi (Dept of Immunology,Yamaguchi Univ.)、Taro Shinozaki (Dept of Pulmonary Medicine, Keio Univ.)、Masao Nakajima (Dept of Gastroenterological Surgery)、Hiroyuki Yasuda (Dept of Pulmonary Medicine, Keio Univ.)、Hiroaki Nagano (Dept of Gastroenterological Surgery)、Koji Tamada (Dept of Immunology,Yamaguchi Univ.)

Cancer immunotherapy using immune checkpoint inhibitors and its combination with other anti-cancer therapies have emerged as a new standard of care, due to the encouraging therapeutic effects in various solid cancers. Nonetheless, glioblastoma and pancreatic cancer remain resistant to immunotherapy and represent intractable cancers with the poorest prognosis. We investigated therapeutic effects of next-generation CAR-T cells producing IL-7 and CCL19 (7x19 CAR-T) in these intractable cancers. The therapeutic efficacy of 7x19 CAR-T was evaluated using an EGFRvIII-positive glioblastoma model or HER2-positive pancreatic cancer organoid (PCO) model. In the PCO model, CAR-T cells are generated from the same patient's PBMC. Anti-EGFRvIII 7x19 CAR-T induced complete rejection of EGFRvIII-positive glioblastoma with massive T cell infiltration and tumor cell death in the tumor tissues, and consequently prolonged mouse survival. Anti-HER2 7x19 CAR-T demonstrated potent cytotoxic activity against autologous HER2-positive PCO along with prolonged mouse survival. Our results suggest that 7x19 CAR-T could become a therapeutic option for glioblastoma and pancreatic cancer.

[Disclosure of COI by Responsible researcher] Lead presenter is not the Responsible researcher.
[Lead presenter: Keisuke Ohta] We have no financial relationships to disclose.
[Principal Researcher: Koji Tamada] ■Research funds from company: ノイルイミューンバイオテック　■employment by a company related to the presentation: ノイルイミューンバイオテック　■Profit of stock: ノイルイミューンバイオテック　■Research expenses: ノイルイミューンバイオテック

▲TOP

### [OS6-3] OS6
### Next-generation cancer research pioneered by designer cells and RNA science

2025 / 9 / 25 (Thu) 12:50-15:20 Room 13/(ANA Crowne Plaza Hotel Kanazawa 3F Ohtori (East)) Room 13

[Chairpersons]Tomoaki Hara (Department of Medical Data Science, Center of Medical Innovation and Translational Research, Osaka University Graduate School of Medicine)、Yukimi Sakoda (Department of Immunology, Yamaguchi University Graduate School of Medicine)

キメラ抗原受容体T（CAR-T）細胞療法は、造血器腫瘍で顕著な成果を挙げていますが、固形がんでは依然として多くの課題が残り、さらなる革新が求められています。特に、がん幹細胞や線維芽細胞が豊富ながん微小環境、固形がん特有の抗原変化や喪失の多様性といった問題が、現行治療法の限界を浮き彫りにしています。これらの課題を克服するには、従来のCAR-T療法を超える新しい治療戦略が必要です。本企画では、精密なゲノム情報や最先端の核酸技術を活用し、「RNA科学が切り開く次世代デザイナー細胞療法」の可能性を探求します。RNA修飾の正確な測定やプロファイリング技術の進展により、がん治療の精密医療の実現だけでなく、核酸医薬の新たな可能性を拡大します。具体的には、抗原多様性に対応するエピトープ拡大、免疫記憶の制御、T細胞以外を対象とした治療戦略などを検討し、ヒト病態に忠実な革新的モデルを活用した評価を行います。また、若手研究者による画期的な技術開発を促進し、次世代治療法の創出を目指した革新的な研究を支援します。

### OS6-3
### Anti-cancer immune construction using iPS cell-derived NKT cells

[Speaker]Takahiro Aoki (Dept. Med. Immunol., Chiba Univ.／Lab. Develop. Genetics, RIKEN IMS) [Co-author]Shinichiro Motohashi (Dept. Med. Immunol., Chiba Univ.)、Haruhiko Koseki (Lab. Develop. Genetics, RIKEN IMS)

At Chiba University, we have developed cancer immunotherapies targeting invariant NKT (iNKT) cells. However, since the population of autologous iNKT cells varies among patients, achieving their stable expansion of these cells is challenging. To address this issue, we generated allogeneic iPS cell-derived NKT (iPS-NKT) cells from iNKT cells sourced from healthy donors. We initiated a phase I clinical trial to evaluate the safety of these novel artificial immune cells. Importantly, activation by the invariant NKT cell ligand αGalCer is essential for iNKT cells to effectively induce tumor-specific T cells and elicit an anti-cancer response. Consequently, we conducted a preclinical study to investigate the combination therapy of iPS-NKT cells with αGalCer-pulsed antigen-presenting cells, assessing the adjuvant capacity of iPS-NKT cells to stimulate tumor-specific T cells. In a humanized patient-derived xenograft murine model, this combination therapy resulted in tumor suppression by inducing tumor-reactive memory-phenotype T cells. Building on these findings, we are advancing the development of iPS-NKT cell therapy to promote host anti-cancer immunity.

[Disclosure of COI by Responsible researcher] Lead presenter is not the Responsible researcher.
[Lead presenter: Takahiro Aoki] We have no financial relationships to disclose.
[Principal Researcher: Haruhiko Koseki] ■Research funds from company: ブライトパスバイオ社

### [OS6-4] OS6
### Next-generation cancer research pioneered by designer cells and RNA science

2025 / 9 / 25 (Thu) 12:50-15:20 Room 13/(ANA Crowne Plaza Hotel Kanazawa 3F Ohtori (East)) Room 13

[Chairpersons]Tomoaki Hara (Department of Medical Data Science, Center of Medical Innovation and Translational Research, Osaka University Graduate School of Medicine)、Yukimi Sakoda (Department of Immunology, Yamaguchi University Graduate School of Medicine)

キメラ抗原受容体T（CAR-T）細胞療法は、造血器腫瘍で顕著な成果を挙げていますが、固形がんでは依然として多くの課題が残り、さらなる革新が求められています。特に、がん幹細胞や線維芽細胞が豊富ながん微小環境、固形がん特有の抗原変化や喪失の多様性といった問題が、現行治療法の限界を浮き彫りにしています。これらの課題を克服するには、従来のCAR-T療法を超える新しい治療戦略が必要です。本企画では、精密なゲノム情報や最先端の核酸技術を活用し、「RNA科学が切り開く次世代デザイナー細胞療法」の可能性を探求します。RNA修飾の正確な測定やプロファイリング技術の進展により、がん治療の精密医療の実現だけでなく、核酸医薬の新たな可能性を拡大します。具体的には、抗原多様性に対応するエピトープ拡大、免疫記憶の制御、T細胞以外を対象とした治療戦略などを検討し、ヒト病態に忠実な革新的モデルを活用した評価を行います。また、若手研究者による画期的な技術開発を促進し、次世代治療法の創出を目指した革新的な研究を支援します。

---

### OS6-4
### Anti-CCR8 antibody therapy promotes Dendritic Cell-driven antitumor immunity

[Speaker]Masaki Hagiwara (Dept. Frontier Res. Tumor Immunol, Grad. Sch. Med, Osaka Univ.／Drug discovery Res. Div, Shionogi & Co., Ltd.,／Dept. Experimental Immunol, Immunol. Frontier Res. Ctr, Osaka Univ.) [Co-author]Azumi Ueyama (Dept. Clin. Res. Tumor Immunol, Grad. Sch. Med, Osaka Univ.)、Yamami Nakamura (Dept. Frontier Res. Tumor Immunol, Grad. Sch. Med, Osaka Univ.／Dept. Experimental Immunol, Immunol. Frontier Res. Ctr, Osaka Univ.)、Shimon Sakaguchi (Dept. Experimental Immunol, Immunol. Frontier Res. Ctr, Osaka Univ.)、Naganari Ohkura (Dept. Frontier Res. Tumor Immunol, Grad. Sch. Med, Osaka Univ.)

The chemokine receptor CCR8 is selectively expressed on tumor-infiltrating regulatory T cells, making it a promising target for enhancing anti-tumor immunity via selective Treg depletion. While changes in T cell dynamics following anti-CCR8 antibody treatment have been reported, the role of antigen-presenting cells, especially dendritic cells (DCs), remains unclear. To address this, we investigated the contribution of DCs in anti-tumor immunity following CCR8+ Treg depletion. Single-cell RNA sequencing of tumor T cells and DCs revealed that a subset of activated DCs strongly interacted with effector T cells in the anti-CCR8-treated group. Spatial transcriptomics further showed that the region surrounding the tumors, which expanded after treatment, was enriched with immune cells, including activated DCs. Moreover, DCs capturing tumor antigens migrated to the draining lymph nodes, where they became activated. Correspondingly, T cells in the lymph nodes displayed an increase in tumor-specific CD8+ T cells. These findings suggest that depletion of CCR8+ Tregs enhances anti-tumor immunity by activating DCs, contributing to the therapeutic efficacy of anti-CCR8 antibodies.

[Disclosure of COI by Responsible researcher] Lead presenter is not the Responsible researcher.
[Lead presenter: Masaki Hagiwara] ■ employment by a company related to the presentation: 塩野義製薬株式会社
[Principal Researcher: Naganari Ohkura] ■Endowed chair: 塩野義製薬株式会社

▲TOP

### [OS6-5] OS6
### Next-generation cancer research pioneered by designer cells and RNA science

2025 / 9 / 25 (Thu) 12:50-15:20 Room 13/(ANA Crowne Plaza Hotel Kanazawa 3F Ohtori (East)) Room 13

[Chairpersons]Tomoaki Hara (Department of Medical Data Science, Center of Medical Innovation and Translational Research, Osaka University Graduate School of Medicine)、Yukimi Sakoda (Department of Immunology, Yamaguchi University Graduate School of Medicine)

キメラ抗原受容体T（CAR-T）細胞療法は、造血器腫瘍で顕著な成果を挙げていますが、固形がんでは依然として多くの課題が残り、さらなる革新が求められています。特に、がん幹細胞や線維芽細胞が豊富ながん微小環境、固形がん特有の抗原変化や喪失の多様性といった問題が、現行治療法の限界を浮き彫りにしています。これらの課題を克服するには、従来のCAR-T療法を超える新しい治療戦略が必要です。本企画では、精密なゲノム情報や最先端の核酸技術を活用し、「RNA科学が切り開く次世代デザイナー細胞療法」の可能性を探求します。RNA修飾の正確な測定やプロファイリング技術の進展により、がん治療の精密医療の実現だけでなく、核酸医薬の新たな可能性を拡大します。具体的には、抗原多様性に対応するエピトープ拡大、免疫記憶の制御、T細胞以外を対象とした治療戦略などを検討し、ヒト病態に忠実な革新的モデルを活用した評価を行います。また、若手研究者による画期的な技術開発を促進し、次世代治療法の創出を目指した革新的な研究を支援します。

---

### OS6-5
### Cancer stem cell plasticity in CRC chemoresistance and metastasis: A critical target for total kill therapy.

[Speaker]Norikatsu Miyoshi (Dept. Gastroenterol. Surg., The Univ. Osaka／Dept. Innov. Oncol. Regn. Med. Osaka Int'l Cancer Inst.) [Co-author]Shiki Fujino (Dept. Gastroenterol. Surg., The Univ. Osaka／Dept. Innov. Oncol. Regn. Med. Osaka Int'l Cancer Inst.／Dept. Surgery, Itami Hosp.)、Mitsunobu Takeda (Dept. Gastroenterol. Surg., The Univ. Osaka)、Yuki Sekido (Dept. Gastroenterol. Surg., The Univ. Osaka)、Tsuyoshi Hata (Dept. Gastroenterol. Surg., The Univ. Osaka)、Atsushi Hamabe (Dept. Gastroenterol. Surg., The Univ. Osaka)、Takayuki Ogino (Dept. Gastroenterol. Surg., The Univ. Osaka)、Mamoru Uemura (Dept. Gastroenterol. Surg., The Univ. Osaka)、Hirofumi Yamamoto (Dept. Gastroenterol. Surg., The Univ. Osaka)、Yuichiro Doki (Dept. Gastroenterol. Surg., The Univ. Osaka)、Hidetoshi Eguchi (Dept. Gastroenterol. Surg., The Univ. Osaka)

Cellular heterogeneity, regulatory, and epigenetic factors are critical to cancer biology and behavior, particularly regarding treatment resistance. This study aimed to identify CRC treatment resistance mechanisms, focusing on typical stem cells that induce cellular heterogeneity. We performed gene expression profiling of CRC patients along with drug susceptibility assays utilizing patient-derived organoids (PDOs). Despite their small number, POU5F1-positive single cells generated heterogeneous populations. Single-cell analysis revealed that heterogeneous populations, including cancer stem cells and persister cells, were derived from POU5F1-positive cells after anti-cancer drug administration. This study unveils a novel mechanism of CRC treatment resistance and highlights the potential of POU5F1-positive cells. Building upon these findings, we aim to develop chimeric antigen receptor (CAR)-T cell therapy targeting these chemotherapy-resistant persister cells to achieve total kill therapy for colorectal cancer. The mechanisms elucidated in this study are expected to contribute to the development of new immunotherapies and the fight against intractable colorectal cancer.

[Disclosure of COI by Responsible researcher] Lead presenter is the Responsible researcher.
[Lead presenter: Norikatsu Miyoshi] ■Research expenses: 科研費

### [OS6-6] OS6
### Next-generation cancer research pioneered by designer cells and RNA science

2025 / 9 / 25 (Thu) 12:50-15:20 Room 13/(ANA Crowne Plaza Hotel Kanazawa 3F Ohtori (East)) Room 13

[Chairpersons]Tomoaki Hara (Department of Medical Data Science, Center of Medical Innovation and Translational Research, Osaka University Graduate School of Medicine)、Yukimi Sakoda (Department of Immunology, Yamaguchi University Graduate School of Medicine)

キメラ抗原受容体T（CAR-T）細胞療法は、造血器腫瘍で顕著な成果を挙げていますが、固形がんでは依然として多くの課題が残り、さらなる革新が求められています。特に、がん幹細胞や線維芽細胞が豊富ながん微小環境、固形がん特有の抗原変化や喪失の多様性といった問題が、現行治療法の限界を浮き彫りにしています。これらの課題を克服するには、従来のCAR-T療法を超える新しい治療戦略が必要です。本企画では、精密なゲノム情報や最先端の核酸技術を活用し、「RNA科学が切り開く次世代デザイナー細胞療法」の可能性を探求します。RNA修飾の正確な測定やプロファイリング技術の進展により、がん治療の精密医療の実現だけでなく、核酸医薬の新たな可能性を拡大します。具体的には、抗原多様性に対応するエピトープ拡大、免疫記憶の制御、T細胞以外を対象とした治療戦略などを検討し、ヒト病態に忠実な革新的モデルを活用した評価を行います。また、若手研究者による画期的な技術開発を促進し、次世代治療法の創出を目指した革新的な研究を支援します。

---

### OS6-6
### Engineering mRNA for advanced cancer vaccines

[Speaker]Satoshi Uchida (Science Tokyo／iCONM)

COVID-19 mRNA vaccines have demonstrated their utility against infectious diseases in humans, prompting research into mRNA cancer vaccines. However, the efficacy remains limited in cancer vaccines. To enhance mRNA vaccine performance, we employ our original mRNA engineering technology, which functionalizes mRNA via hybridization with complementary RNAs conjugated to functional moieties. This approach has been used to enhance adjuvanticity and enable spleen targeting. For adjuvanticity, immunostimulatory double-stranded RNA targeting RIG-I, a cytosolic innate immune receptor, was hybridized to mRNA. This formulation improved cytotoxic T cell responses when delivered using anionic lipoplexes, ionizable lipid nanoparticles, and polymeric micelles coated with poly(ethylene glycol) (PEG). This strategy effectively reduced tumor size in mouse models targeting both a model antigen and a tumor-associated antigen. For spleen targeting, PEG density on polycation/mRNA polyplexes was controlled by hybridizing mRNA with PEGylated RNA oligonucleotides, enabling selective spleen accumulation and cellular immunity induction.

[Disclosure of COI by Responsible researcher] Lead presenter is the Responsible researcher.
[Lead presenter: Satoshi Uchida] ■ employment by a company related to the presentation: Crafton Biotechnology株式会社
■Profit of stock: Crafton Biotechnology株式会社

### [OS6-7] OS6
### Next-generation cancer research pioneered by designer cells and RNA science

2025 / 9 / 25 (Thu) 12:50-15:20 Room 13/(ANA Crowne Plaza Hotel Kanazawa 3F Ohtori (East)) Room 13

[Chairpersons]Tomoaki Hara (Department of Medical Data Science, Center of Medical Innovation and Translational Research, Osaka University Graduate School of Medicine)、Yukimi Sakoda (Department of Immunology, Yamaguchi University Graduate School of Medicine)

キメラ抗原受容体T（CAR-T）細胞療法は、造血器腫瘍で顕著な成果を挙げていますが、固形がんでは依然として多くの課題が残り、さらなる革新が求められています。特に、がん幹細胞や線維芽細胞が豊富ながん微小環境、固形がん特有の抗原変化や喪失の多様性といった問題が、現行治療法の限界を浮き彫りにしています。これらの課題を克服するには、従来のCAR-T療法を超える新しい治療戦略が必要です。本企画では、精密なゲノム情報や最先端の核酸技術を活用し、「RNA科学が切り開く次世代デザイナー細胞療法」の可能性を探求します。RNA修飾の正確な測定やプロファイリング技術の進展により、がん治療の精密医療の実現だけでなく、核酸医薬の新たな可能性を拡大します。具体的には、抗原多様性に対応するエピトープ拡大、免疫記憶の制御、T細胞以外を対象とした治療戦略などを検討し、ヒト病態に忠実な革新的モデルを活用した評価を行います。また、若手研究者による画期的な技術開発を促進し、次世代治療法の創出を目指した革新的な研究を支援します。

---

### OS6-7
### CXCR4 induces memory formation over exhaustion in CAR-T cells to achieve durable leukemia targeting

[Speaker]Minggao Liang (Dept. Comp. Path., ISCT, Sch. Med. Dent. Res.／Lab. Hum. Disease Models., RIKEN, RIKEN IMS,) [Co-author]Ari Itoh (Lab. Hum. Disease Models., RIKEN, RIKEN IMS,／Dept. Hyg. Pub. Health, Nippon Med. Sch., Grad. Sch. Med.)、Akiko Kaneko (Lab. Hum. Disease Models., RIKEN, RIKEN IMS,)、Chen Bibi (Lab. Hum. Disease Models., RIKEN, RIKEN IMS,)、Mariko Tomizawa (Lab. Hum. Disease Models., RIKEN, RIKEN IMS,)、Saera Fujiki (Lab. Hum. Disease Models., RIKEN, RIKEN IMS,)、Emi Kanamaru (Lab. Hum. Disease Models., RIKEN, RIKEN IMS,)、Mari Hashimoto (Lab. Hum. Disease Models., RIKEN, RIKEN IMS,)、Hiroshi Kajita (Lab. Hum. Disease Models., RIKEN, RIKEN IMS,)、Kaori Sato (Lab. Hum. Disease Models., RIKEN, RIKEN IMS,)、Jonathan Moody (Lab. Genome. Info. Ana., RIKEN, RIKEN IMS)、Yoshinari Ando (Lab. Transcriptome Tech., RIKEN, RIKEN IMS)、Michiel Dehoon (App. Comp. Genomics Team, RIKEN, RIKEN IMS,)、Leonard Schultz (The Jackson Laboratory)、Yoriko Saito (Lab. Hum. Disease Models., RIKEN, RIKEN IMS,)、Fumihiko Ishikawa (Dept. Comp. Path., ISCT, Sch. Med. Dent. Res.／Lab. Hum. Disease Models., RIKEN, RIKEN IMS,)

Chimeric antigen receptor (CAR)-T cell therapy has improved outcomes in B-cell malignancies, but its success in AML remains limited. Treatment efficacy depends on the acquisition of memory T cell states, while exhaustion contributes to relapse. In AML patients who achieved remission following cord blood (CB) transplantation, we observed an enrichment of CD4+ memory T cells with high CXCR4 expression. Based on this, we engineered CAR-T cells expressing CXCR4 and targeting CD25, a molecule enriched in AML-initiating cells and low potential for on-target off-tumor toxicity. Using single-cell transcriptomic and proteomic profiling, CXCR4 enhances CAR-T memory, reduces exhaustion, and sustains anti-leukemic activity in AML PDX models. Similar benefits were observed when targeting an alternate AML-associated antigen, CD96.Our results suggest that CXCR4 enhances CAR-T cell persistence and memory differentiation. Engineering of CXCR4 expression may improve the durability and efficacy of CAR-T cell therapies in AML and potentially other cancers where long-term T cell function is essential.

[Disclosure of COI by Responsible researcher] Lead presenter is not the Responsible researcher.
[Lead presenter: Minggao Liang] We have no financial relationships to disclose.
[Principal Researcher: Fumihiko Ishikawa] We have no financial relationships to disclose.

### [OS6-8] OS6
### Next-generation cancer research pioneered by designer cells and RNA science

2025 / 9 / 25 (Thu) 12:50-15:20 Room 13/(ANA Crowne Plaza Hotel Kanazawa 3F Ohtori (East)) Room 13

[Chairpersons]Tomoaki Hara (Department of Medical Data Science, Center of Medical Innovation and Translational Research, Osaka University Graduate School of Medicine)、Yukimi Sakoda (Department of Immunology, Yamaguchi University Graduate School of Medicine)

キメラ抗原受容体T（CAR-T）細胞療法は、造血器腫瘍で顕著な成果を挙げていますが、固形がんでは依然として多くの課題が残り、さらなる革新が求められています。特に、がん幹細胞や線維芽細胞が豊富ながん微小環境、固形がん特有の抗原変化や喪失の多様性といった問題が、現行治療法の限界を浮き彫りにしています。これらの課題を克服するには、従来のCAR-T療法を超える新しい治療戦略が必要です。本企画では、精密なゲノム情報や最先端の核酸技術を活用し、「RNA科学が切り開く次世代デザイナー細胞療法」の可能性を探求します。RNA修飾の正確な測定やプロファイリング技術の進展により、がん治療の精密医療の実現だけでなく、核酸医薬の新たな可能性を拡大します。具体的には、抗原多様性に対応するエピトープ拡大、免疫記憶の制御、T細胞以外を対象とした治療戦略などを検討し、ヒト病態に忠実な革新的モデルを活用した評価を行います。また、若手研究者による画期的な技術開発を促進し、次世代治療法の創出を目指した革新的な研究を支援します。

---

### OS6-8
### Integrating Genome Editing and iPSC Technology for Cancer Immunotherapy: The Promise of Universal T Cells

[Speaker]Bo(Kyoto Univ.) Wang (Kaneko Lab. CiRA. Kyoto University)

Adoptive immunotherapy with engineered T cells has transformed cancer treatment, especially through chimeric antigen receptor (CAR) T cells. However, reliance on autologous T cells poses major challenges, including time-consuming manufacturing, variable quality, and patient-specific limitations. Allogeneic T cells from healthy donors present a promising alternative, but require extensive gene editing to prevent graft-versus-host disease (GVHD) and immune rejection. This multiplex editing often leads to low cell yields, genotoxicity, and T cell exhaustion due to prolonged ex vivo culture.Induced pluripotent stem cells (iPSCs) offer a compelling solution. They possess unlimited proliferative capacity, allow precise genetic modification, and can differentiate into functional T cells. In this talk, I will present our recent progress in generating universal T cells from iPSCs. I will focus on engineering immune-compatible iPS cells and strategies to overcome immunological barriers, paving the way for next-generation allogeneic T cell therapies in cancer immunotherapy.

[Disclosure of COI by Responsible researcher] Lead presenter is the Responsible researcher.
[Lead presenter: Bo Wang] ■Research expenses: iPSアカデミアジャパン株式会社

▲TOP

### [OS6-9] OS6
### Next-generation cancer research pioneered by designer cells and RNA science

2025 / 9 / 25 (Thu) 12:50-15:20 Room 13/(ANA Crowne Plaza Hotel Kanazawa 3F Ohtori (East)) Room 13

[Chairpersons]Tomoaki Hara (Department of Medical Data Science, Center of Medical Innovation and Translational Research, Osaka University Graduate School of Medicine)、Yukimi Sakoda (Department of Immunology, Yamaguchi University Graduate School of Medicine)

キメラ抗原受容体T（CAR-T）細胞療法は、造血器腫瘍で顕著な成果を挙げていますが、固形がんでは依然として多くの課題が残り、さらなる革新が求められています。特に、がん幹細胞や線維芽細胞が豊富ながん微小環境、固形がん特有の抗原変化や喪失の多様性といった問題が、現行治療法の限界を浮き彫りにしています。これらの課題を克服するには、従来のCAR-T療法を超える新しい治療戦略が必要です。本企画では、精密なゲノム情報や最先端の核酸技術を活用し、「RNA科学が切り開く次世代デザイナー細胞療法」の可能性を探求します。RNA修飾の正確な測定やプロファイリング技術の進展により、がん治療の精密医療の実現だけでなく、核酸医薬の新たな可能性を拡大します。具体的には、抗原多様性に対応するエピトープ拡大、免疫記憶の制御、T細胞以外を対象とした治療戦略などを検討し、ヒト病態に忠実な革新的モデルを活用した評価を行います。また、若手研究者による画期的な技術開発を促進し、次世代治療法の創出を目指した革新的な研究を支援します。

---

### OS6-9
### Elucidation of the mechanisms of cancer malignancy through comprehensive RNA modification analysis

[Speaker]Masamitsu Konno (National Institute of Advanced Industrial Science and Technology)

RNA undergoes approximately 200 types of modifications that control its fate. In particular, RNA modifications control various life phenomena such as stem cell maintenance, biological clocks, development, and immune control, and it has been reported that RNA modification failure contributes to the onset and progression of diseases. In recent years, it has become clear that mRNA and microRNA modifications control life phenomena by affecting splicing control, stability, and processing.Our research group is investigating changes in RNA modifications and their functions in gastrointestinal cancers. As a result, we have identified specific modification types with altered modification rates in pancreatic cancer compared to healthy individuals. We found that multiple types of modification changes in pancreatic cancer promote tumor proliferation and enhanced invasive metastasis, contributing to increased malignancy. In this presentation, I will discuss the state-of-the-art in Elucidation of the mechanisms of cancer malignancy through comprehensive RNA modification analysis and the medical application of the comprehensive RNA modification information.

[Disclosure of COI by Responsible researcher] Lead presenter is the Responsible researcher.
[Lead presenter: Masamitsu Konno] We have no financial relationships to disclose.

▲TOP

### [OS6-10] OS6
### Next-generation cancer research pioneered by designer cells and RNA science

2025 / 9 / 25 (Thu) 12:50-15:20 Room 13/(ANA Crowne Plaza Hotel Kanazawa 3F Ohtori (East)) Room 13

[Chairpersons]Tomoaki Hara (Department of Medical Data Science, Center of Medical Innovation and Translational Research, Osaka University Graduate School of Medicine)、Yukimi Sakoda (Department of Immunology, Yamaguchi University Graduate School of Medicine)

キメラ抗原受容体T（CAR-T）細胞療法は、造血器腫瘍で顕著な成果を挙げていますが、固形がんでは依然として多くの課題が残り、さらなる革新が求められています。特に、がん幹細胞や線維芽細胞が豊富ながん微小環境、固形がん特有の抗原変化や喪失の多様性といった問題が、現行治療法の限界を浮き彫りにしています。これらの課題を克服するには、従来のCAR-T療法を超える新しい治療戦略が必要です。本企画では、精密なゲノム情報や最先端の核酸技術を活用し、「RNA科学が切り開く次世代デザイナー細胞療法」の可能性を探求します。RNA修飾の正確な測定やプロファイリング技術の進展により、がん治療の精密医療の実現だけでなく、核酸医薬の新たな可能性を拡大します。具体的には、抗原多様性に対応するエピトープ拡大、免疫記憶の制御、T細胞以外を対象とした治療戦略などを検討し、ヒト病態に忠実な革新的モデルを活用した評価を行います。また、若手研究者による画期的な技術開発を促進し、次世代治療法の創出を目指した革新的な研究を支援します。

### OS6-10
### Application of Single-Molecule Electrical Measurement in Precision Medicine for Refractory Cancers

[Speaker]Takahito Ohshiro (Sanken, Osaka Univ) [Co-author]Yuki Komoto (Sanken, Osaka Univ)、Masateru Taniguchi (Sanken, Osaka Univ)、Hideshi Ishii (Med, Osaka Univ)

Refractory cancers are a challenge in precision medicine, as conventional treatments often fail. A promising approach is single-molecule electrical current measurement with nanodevices. This technology enables sensitive detection of biomolecules such as DNA, RNA, and peptides at the single-molecule level, offering potential for identifying new biomarkers and therapeutic targets.Single-molecule measurements allow precise analysis of RNA molecules, including base types and modifications. This can reveal insights into cancer progression and drug resistance. Monitoring treatment effects in real-time improves personalized medicine, ensuring more effective treatments. Recent studies have demonstrated success in lung, breast, and colorectal cancers, using nanogap electrodes to detect RNA in plasma and tissue samples.Combined with machine learning, this technology enhances diagnostic accuracy and predicts patient outcomes. In conclusion, single-molecule electrical measurement is a powerful tool for detecting biomarkers in refractory cancers. It has the potential to advance personalized medicine and provide new treatment options.

[Disclosure of COI by Responsible researcher] Lead presenter is not the Responsible researcher.
[Lead presenter: Takahito Ohshiro] We have no financial relationships to disclose.
[Principal Researcher: Hideshi Ishii] We have no financial relationships to disclose.

▲TOP

### [E-1079] II-E7
### Gynecologic cancer (1)

2025 / 9 / 25 (Thu) 12:50-14:05 Room 14/(ANA Crowne Plaza Hotel Kanazawa 3F Zuiun 1) Room 14

[Chairperson]Satoru Kyo (Dept.OB/GYN., Shimane Univ. Fac. Med.)

---

### E-1079
### HER2 targeted alpha therapy against trastuzumab resistant peritoneal metastasis of uterine serous carcinoma

[Speaker]Huizi K. Li (Dept. of Charged Particle Therapy Res., QST) [Co-author]Mayuka Anko (Dept. of Obstetrics and Gynecology, Keio Univ. Sch. of Med.)、Sumitaka Hasegawa (Dept. of Charged Particle Therapy Res., QST)

Uterine serous carcinoma (USC) is a rare aggressive type 2 endometrial carcinoma with dismal prognosis. Peritoneal metastasis (PM) is often observed even at early stage in USC. HER2 is a therapeutic target of USC and trastuzumab, the anti-HER2 antibody, has shown benefit in the treatment of HER2 positive USC. However, the acquired resistance of trastuzumab also has been challenges to overcome for HER2 positive USC.Targeted alpha therapy (TAT) is useful not only for local tumor but also metastasis. Having the target specificity of antibody and high cell cytotoxicity and short range of alpha-particle, TAT can effectively control targeted tumor with minimal toxicity to untargeted normal tissues. Astatine 211 ($^{211}$ At) is one of the attractive alpha-emitter. We have performed experimental TAT using $^{211}$At-trastuzumab to preclinical mouse model with trastuzumab resistant PM of HER2 positive USC in order to investigate the therapeutic efficacy and toxicity. We will discuss the potential of TAT as a novel therapeutic option for drug resistant PM of HER2 positive USC.

[Disclosure of COI by Responsible researcher] Lead presenter is not the Responsible researcher.
[Lead presenter: Huizi K. Li] We have no financial relationships to disclose.
[Principal Researcher: Sumitaka Hasegawa] We have no financial relationships to disclose.

▲TOP

### [E-1080] II-E7
### Gynecologic cancer (1)

2025 / 9 / 25 (Thu) 12:50-14:05 Room 14/(ANA Crowne Plaza Hotel Kanazawa 3F Zuiun 1) Room 14

[Chairperson]Satoru Kyo (Dept.OB/GYN., Shimane Univ. Fac. Med.)

---

### E-1080
### FIH regulates adaptation of ovarian cancer cells to hypoxia through suppression of HIF2-Sp1 dependent gene expression

[Speaker]Shiro Koizume (Kanagawa Cancer Ctr Res Inst) [Co-author]Tomoko Takahashi (Kanagawa Cancer Ctr Res Inst)、Etsuko Miyagi (Yokohama City Univ OBGY)、Yohei Miyagi (Kanagawa Cancer Ctr Res Inst)

Ovarian clear cell carcinoma (CCC) is a lethal and poor prognostic gynecological malignancy. Factor inhibiting HIF1 (FIH) inhibits hypoxia inducible factor (HIF)-arylhydrocarbon receptor nuclear translocator (ARNT) complex-driven gene expression required for adaptation of cancer cells to hypoxia. Thus, FIH negatively regulate adaptive response of cancer cells to hypoxia. We have shown that HIF2-Sp1 interaction-driven transcription is important for adaptation of CCC cells to hypoxia through inhibition of apoptosis and induction of epithelial-mesenchymal transition. However, relative frequency of this unusual protein-protein interaction over HIF-ARNT interaction in CCC cells and effect of FIH on HIF2-Sp1-driven transcription have been unclear. Here, we show by proximity ligation assay that HIF2-Sp1 interaction is frequent as HIF2-ARNT interaction in CCC cells. We further showed that both PAS and C-terminal activation (CTA) domains of HIF2 can be involved in HIF2-Sp1 interaction. FIH can suppress HIF2-Sp1-driven gene expression by interfering association between CTA domain and Sp1. Our data raise the possibility that FIH contributes to CCC progression through HIF2-Sp1 interaction.

[Disclosure of COI by Responsible researcher] Lead presenter is the Responsible researcher.
[Lead presenter: Shiro Koizume] We have no financial relationships to disclose.

### [E-1081] II-E7
### Gynecologic cancer (1)

2025 / 9 / 25 (Thu) 12:50-14:05 Room 14/(ANA Crowne Plaza Hotel Kanazawa 3F Zuiun 1) Room 14

[Chairperson]Satoru Kyo (Dept.OB/GYN., Shimane Univ. Fac. Med.)

---

### E-1081
### Cancer Cell Properties in Uterine Serous Carcinoma with an Anti-Inflammatory Tumor Microenvironment

[Speaker]Shunpei Satoh (Saitama Cancer Ctr. Res. Inst. for Clin. Oncol.) [Co-author]Miki Ohira (Saitama Cancer Ctr. Res. Inst. for Clin. Oncol.)、Takehiko Kamijo (Saitama Cancer Ctr. Res. Inst. for Clin. Oncol.)

The inflammatory tumor microenvironment (TME) influences the efficacy of immune checkpoint blockade, which has been approved for the treatment of advanced endometrial cancers. However, the comprehensive TME landscape and the TME-associated cancer cell properties remain poorly understood in uterine serous carcinoma (USC). In this study, we deconvolved the transcriptomic profiles of 138 USC samples from The Cancer Genome Atlas (TCGA) and predicted their inflammatory TME signatures. Among them, anti-inflammatory (AI) tumors exhibited elevated expression of genes involved in WNT signaling pathway, along with increased β-catenin protein levels. Consistently, analysis of published sc/snRNA-seq data from 14 USC samples revealed an $LGR5^{high}$/$TCF7L1^{high}$ cluster within $MHC^{low}$ cancer cell populations. TCGA Infinium array data showed increased CpG methylation of hypoxia-responsive elements, a hallmark of immunotolerance, in AI tumors. Furthermore, we identified a positive correlation between high ***LGR5*** expression and hypomethylation of an intron 1 locus. These findings underscore distinct transcriptomic and epigenetic properties of AI USC cells, offering new insights for precision medicine.

[Disclosure of COI by Responsible researcher] Lead presenter is the Responsible researcher.
[Lead presenter: Shunpei Satoh] We have no financial relationships to disclose.

English Oral Session

### [E-1082] II-E7
### Gynecologic cancer (1)

2025 / 9 / 25 (Thu) 12:50-14:05 Room 14/(ANA Crowne Plaza Hotel Kanazawa 3F Zuiun 1) Room 14

[Chairperson]Satoru Kyo (Dept.OB/GYN., Shimane Univ. Fac. Med.)

---

### E-1082
### Establishment and genomic characterization of patient-derived organoids from gastric-type cervical adenocarcinoma

[Speaker]Kaede Katagiri (Department of Obstetrics and Gynecology, Keio University School of Medicine) [Co-author]Masaki Sugawara (Department of Obstetrics and Gynecology, Keio University School of Medicine)、Hiroshi Nishio (Department of Obstetrics and Gynecology, Keio University School of Medicine)、Ayase Futatsugi (Department of Obstetrics and Gynecology, Keio University School of Medicine／Clinical Physiology and Therapeutics, Keio University Faculty of Pharmacy)、Yuki Katoh (Department of Obstetrics and Gynecology, Keio University School of Medicine／Division of Anatomical Science, Department of Functional Morphology, Nihon University)、Takashi Iwata (Department of Obstetrics and Gynecology, Keio University School of Medicine)、Maiko Matsushita (Clinical Physiology and Therapeutics, Keio University Faculty of Pharmacy)、Wataru Yamagami (Department of Obstetrics and Gynecology, Keio University School of Medicine)

Gastric-type cervical adenocarcinoma (GAS) is a highly aggressive subtype of cervical cancer with poor prognosis and resistance to conventional therapies. Due to its rarity, few research models exist, limiting the understanding of its molecular features. In this study, we established and characterized patient-derived organoids from advanced-stage GAS. Fresh tumor tissues were obtained from GAS patients and cultured using a standard organoid protocol. The resulting organoids showed stable growth and preserved key histological and genetic traits of the original tumors. Genomic analysis revealed that they retained copy number variations associated with tumor survival. Interestingly, organoids derived from samples collected at different time points from the same patient exhibited distinct genomic profiles. These findings demonstrate that GAS organoids closely resemble the parental tumors and offer a promising tool for biological research and drug screening. This study provides a valuable platform for further investigation of GAS, contributing to improved understanding and potential therapeutic development.

[Disclosure of COI by Responsible researcher] Lead presenter is not the Responsible researcher.
[Lead presenter: Kaede Katagiri] We have no financial relationships to disclose.
[Principal Researcher: Hiroshi Nishio] We have no financial relationships to disclose.

▲TOP

### [E-1083] II-E7
### Gynecologic cancer (1)

2025 / 9 / 25 (Thu) 12:50-14:05 Room 14/(ANA Crowne Plaza Hotel Kanazawa 3F Zuiun 1) Room 14

[Chairperson]Satoru Kyo (Dept.OB/GYN., Shimane Univ. Fac. Med.)

---

### E-1083
### Osteopontin blockade elicits anti-tumor immunity in a mouse model of aggressive endometrial cancer

[Speaker]Yuko Arino (Dept. of Immunol., Yokohama City Univ.／Dept. of Obstet. & Gynecol., Yokohama City Univ.) [Co-author]Tatsuma Ban (Dept. of Immunol., Yokohama City Univ.)、Mone Watanabe (Dept. of Immunol., Yokohama City Univ.)、Haruto Furuta (Dept. of Immunol., Yokohama City Univ.)、Erika Muraoka (Dept. of Mol. Path., Yokohama City Univ.)、Yukiko Sasahara (Dept. of Mol. Path., Yokohama City Univ.)、Satoshi Fujii (Dept. of Mol. Path., Yokohama City Univ.)、Takiko Daikoku (Res. Ctr. for Exp. Modeling of Human Disease, Kanazawa Univ.)、Shigeyuki Kon (Faculty of Pharm. Sci., Fukuyama Univ.)、Etsuko Miyagi (Dept. of Obstet. & Gynecol., Yokohama City Univ.)、Tomohiko Tamura (Dept. of Immunol., Yokohama City Univ.)

Tumor-associated macrophages (TAMs) promote the progression of various cancers. However, the role of TAMs in the aggressive histological type of endometrial cancer (aggressive EC) remains unknown. Here, we found that a high proportion of M2-like TAMs positively correlated with the poor prognosis of patients with aggressive EC using the TCGA database. Macrophage depletion in an aggressive EC orthotopic mouse model with CSF1R inhibitor decreased cell number and CD163 expression of TAMs and reduced tumor burden, possibly by activating $CD8^+$ T cells. We identified genes highly expressed in aggressive EC TAMs compared to normal endometrium macrophages in both humans and mice. Among them, osteopontin (OPN), encoded by ***SPP1***, was selectively expressed in an M2-like subpopulation in TAMs, and its expression was associated with poor prognosis. Notably, the combination therapy of an immune checkpoint inhibitor and the OPN neutralization monoclonal antibody suppressed tumor progression in the mouse model, probably by increasing tissue-resident memory T cells, known to have an effector function. Thus, targeting OPN has the potential to be an effective strategy in aggressive EC.

[Disclosure of COI by Responsible researcher] Lead presenter is not the Responsible researcher.
[Lead presenter: Yuko Arino] We have no financial relationships to disclose.
[Principal Researcher: Tomohiko Tamura] We have no financial relationships to disclose.

▲TOP

### [E-1084] II-E7
### Gynecologic cancer (1)

2025 / 9 / 25 (Thu) 12:50-14:05 Room 14/(ANA Crowne Plaza Hotel Kanazawa 3F Zuiun 1) Room 14

[Chairperson]Satoru Kyo (Dept.OB/GYN., Shimane Univ. Fac. Med.)

---

### E-1084
### Site-specific miRNA alternation in fimbriae/fallopian tube/tumor of BRCA1 mutated high grade serous ovarian carcinoma

[Speaker]Yurika Yamada (Dept. of Obst. & Gyne. Nagoya Univ. Grad. Sch. of Med.) [Co-author]Akira Yokoi (Dept. of Obst. & Gyne. Nagoya Univ. Grad. Sch. of Med.)、Kosuke Yoshida (Dept. of Obst. & Gyne. Nagoya Univ. Grad. Sch. of Med.)、Hiroaki Yamada (Dept. of Obst. & Gyne. Nagoya Univ. Grad. Sch. of Med.)、Kazuhiro Suzuki (Dept. of Obst. & Gyne. Nagoya Univ. Grad. Sch. of Med.)、Eri Inami (Dept. of Obst. & Gyne. Nagoya Univ. Grad. Sch. of Med.)、Masami Kitagawa (Dept. of Obst. & Gyne. Nagoya Univ. Grad. Sch. of Med.)、Nobuhisa Yoshikawa (Dept. of Obst. & Gyne. Nagoya Univ. Grad. Sch. of Med.)、Kaoru Niimi (Dept. of Obst. & Gyne. Nagoya Univ. Grad. Sch. of Med.)、Hiroaki Kajiyama (Dept. of Obst. & Gyne. Nagoya Univ. Grad. Sch. of Med.)

The fimbrial epithelium, is widely recognized as a precursor lesion of high-grade serous carcinoma (HGSC). Although BRCA1 mutations have been implicated in the pathogenesis of these malignancies, molecular mechanism at fimbriae following the mutation occurrence remain poorly characterized. This study aims to investigate the impact of BRCA1 mutations on miRNA expression profiles in HGSC patients. We conducted miRNA sequencing on 36 specimens from fallopian tube, fimbriae, and tumor tissues obtained from HGSC patients with BRCA1mt/wt, and cancer-free BRCA1mt carriers. A total of 306 miRNAs were identified, and the miRNA profiles were clearly distinct between tumor and non-tumor tissues. The global effect of BRCA1 status across all samples was not identified, but especially in fallopian tube or fimbriae, the fimbriae exhibited substantial BRCA1-associated miRNA alterations than fallopian tubes. Three miRNAs were consistently associated with BRCA1mt status in the fimbriae, indicating their potential as early molecular markers. These findings suggest that BRCA1mt may induce site-specific miRNA regulation, and highlight candidate miRNAs as key factors for early-phase carcinogenesis.

[Disclosure of COI by Responsible researcher] Lead presenter is not the Responsible researcher.
[Lead presenter: Yurika Yamada] We have no financial relationships to disclose.
[Principal Researcher: Akira Yokoi] We have no financial relationships to disclose.

▲TOP

### [J-1079] I-J11-1
### Tumor antigens/Antitumor immune response (2)

2025 / 9 / 25 (Thu) 12:50-14:05 Room 15/(ANA Crowne Plaza Hotel Kanazawa 3F Zuiun 2) Room 15

[Chairperson]Ken-ichiro Seino (Immunobiology, IGM, Hokkaido Univ)

---

### J-1079
### Anti-tumor immune responses triggered by abnormal spindle formation through the double-stranded RNA recognition pathway

[Speaker]Nobunari Sasaki (JFCR CPM Center Cancer Immunotherapy Development) [Co-author]Mizuki Homme (JFCR CPM Center Cancer Immunotherapy Development)、Shunsuke Kitajima (JFCR CPM Center Cancer Immunotherapy Development)

The cytoplasmic double-stranded RNA (dsRNA) sensing pathway, which is crucial for antiviral defense, triggers anti-tumor immunity by detecting aberrant dsRNA in cancer cells. However, the mechanisms by which dsRNA-forming transcripts are regulated during cancer therapy remain unclear. Here, we show that pharmacologic induction of micronuclei following abnormal spindle formation leads to cytoplasmic dsRNA accumulation in cancer cells, activating the MAVS-mediated dsRNA sensing pathway and subsequently enhancing immune cell migration and antigen-presenting machinery. dsRNA-sequence analysis shows that these transcripts primarily originate from non-exonic regions, particularly those adjacent to genes. Moreover, dsRNA-forming transcripts are significantly enriched in the micronucleus fraction. Pulsed inhibition of MPS1, a master regulator of the spindle assembly checkpoint, efficiently induces micronuclei and enhances dsRNA-sensing-driven anti-tumor immunity via cytotoxic lymphocyte activation in vivo. These findings uncover a mechanism by which dsRNA sensing pathway boosts immune responses, offering a strategy to improve the efficacy of cancer therapies targeting genomic instability.

[Disclosure of COI by Responsible researcher] Lead presenter is not the Responsible researcher.
[Lead presenter: Nobunari Sasaki] We have no financial relationships to disclose.
[Principal Researcher: Shunsuke Kitajima] ■Research funds from company: 日本ベーリンガーインゲルハイム

### [J-1080] I-J11-1
### Tumor antigens/Antitumor immune response (2)

2025 / 9 / 25 (Thu) 12:50-14:05 Room 15/(ANA Crowne Plaza Hotel Kanazawa 3F Zuiun 2) Room 15

[Chairperson]Ken-ichiro Seino (Immunobiology, IGM, Hokkaido Univ)

---

### J-1080
### Efficacy of PDPN-Targeted Photoimmunotherapy in Esophageal Cancer: Comparison with FAP-Targeted Photoimmunotherapy

[Speaker]Tasuku Matsumoto (Dept. of Gastroenterological Surg., Grad. Sch. of Med., Okayama Univ.) [Co-author]Kazuhiro Noma (Dept. of Gastroenterological Surg., Grad. Sch. of Med., Okayama Univ.)、Yohei Mizusawa (Dept. of Gastroenterological Surg., Grad. Sch. of Med., Okayama Univ.)、Akito Shimizu (Dept. of Gastroenterological Surg., Grad. Sch. of Med., Okayama Univ.)、Seitaro Nishimura (Dept. of Gastroenterological Surg., Grad. Sch. of Med., Okayama Univ.)、Yasushige Takeda (Dept. of Gastroenterological Surg., Grad. Sch. of Med., Okayama Univ.)、Tatsuya Takahashi (Dept. of Gastroenterological Surg., Grad. Sch. of Med., Okayama Univ.)、Hijiri Matsumoto (Dept. of Gastroenterological Surg., Grad. Sch. of Med., Okayama Univ.)、Tomoyoshi Kunitomo (Dept. of Gastroenterological Surg., Grad. Sch. of Med., Okayama Univ.)、Hajime Kashima (Dept. of Gastroenterological Surg., Grad. Sch. of Med., Okayama Univ.)、Satoru Kikuchi (Dept. of Gastroenterological Surg., Grad. Sch. of Med., Okayama Univ.)、Toshiaki Ohara (Dept. of Gastroenterological Surg., Grad. Sch. of Med., Okayama Univ.)、Shunsuke Tanabe (Dept. of Gastroenterological Surg., Grad. Sch. of Med., Okayama Univ.)、Hiroshi Tazawa (Dept. of Gastroenterological Surg., Grad. Sch. of Med., Okayama Univ.)、Hisataka Kobayashi (Mol. Imaging, Cancer Res. Ctr., Natl. Cancer Inst., NIH, USA)、Toshiyoshi Fujiwara (Dept. of Gastroenterological Surg., Grad. Sch. of Med., Okayama Univ.)

[Objective]PDPN is highly expressed in cancer cells and CAFs in ESCC, promoting tumor progression and immunosuppression. We previously showed that CAF-targeted PIT improves the TME and boosts immunity. As PDPN exists in both, PDPNPIT may eliminate tumor cells and modulate the TME. This study evaluated its antitumor and immune effects vs. FAPPIT.[Methods]PDPN expression and prognosis were analyzed by IHC in 91 ESCC cases. SCC VII, a PDPN-high line, was used, and PDPN induction after CAF coculture was assessed by FACS. DAMPs after PDPNPIT or FAPPIT were measured by ELISA. In vivo, a tumor model with SCC VII and MEFs was used to assess tumor shrinkage, CD8+ T cell infiltration, and DC activation. Memory T cells are under analysis. [Results]High PDPN in cancer cells and CAFs was linked to poor prognosis, and PDPN-high CAFs were an independent factor (HR=2.314, p=0.017). Coculture increased PDPN in SCC VII. PDPNPIT induced more DAMPs than FAPPIT. Though preliminary, in vivo data suggest that PDPNPIT promotes stronger tumor shrinkage and immune activation.[Conclusion]PDPNPIT may be more effective than FAPPIT in inducing tumor regression and antitumor immunity.

[Disclosure of COI by Responsible researcher] Lead presenter is not the Responsible researcher.
[Lead presenter: Tasuku Matsumoto] We have no financial relationships to disclose.
[Principal Researcher: Kazuhiro Noma] We have no financial relationships to disclose.

▲TOP

### [J-1081] I-J11-1
### Tumor antigens/Antitumor immune response (2)

2025 / 9 / 25 (Thu) 12:50-14:05 Room 15/(ANA Crowne Plaza Hotel Kanazawa 3F Zuiun 2) Room 15

[Chairperson]Ken-ichiro Seino (Immunobiology, IGM, Hokkaido Univ)

---

### J-1081

### Clonal competition between CD8$^{+}$ T cells with different TCR affinities modulates proliferation and differentiation

[Speaker]Masaki Kurosu (Tokyo University of Science) [Co-author]Mikiya Tsunoda (Tokyo University of Science)、Haru Ogiwara (Tokyo University of Science)、Kouji Matsushima (Tokyo University of Science)、Satoshi Ueha (Tokyo University of Science)

Cocktail TCR-T cell therapy, utilizing multiple tumor-reactive TCRs, offers a promising strategy against tumor immune escape. However, competition among co-transferred T-cell clones can affect therapeutic efficacy by influencing proliferation and differentiation. The mechanisms behind such clonal competition are poorly understood. In this study, we established an in vitro model to examine. BMDCs were pulsed with melanoma-associated antigen hgp100 and/or OVA peptides and co-cultured with Pmel-1 and OT-1 CD8$^{+}$ T-cell clones at different ratios. T-cell responses were analyzed by flow cytometry, focusing on activation (CD69), degranulation (CD107a), exhaustion (PD-1), and proliferation. While CD69 and CD107a expression remained unaffected by clonal competition, proliferation of Pmel-1 was suppressed as the proportion of OT-1 increased. PD-1 expression was lower in proliferating Pmel-1 cells. Additionally, memory T cells suppressed the proliferation of naive T cells. These results indicate that clonal competition mainly impacts proliferation, requiring stable antigen-presenting cell T cell interactions. Our findings support optimizing transfer timing to improve cocktail TCR-T therapies.

[Disclosure of COI by Responsible researcher] Lead presenter is not the Responsible researcher.
[Lead presenter: Masaki Kurosu] ■Research funds from company: JST 次世代研究者挑戦的研究プログラム JPMJSP2151
[Principal Researcher: Satoshi Ueha] ■Advisor of the company and/or profit-making organization: イムノジェネテクス株式会社
■Profit of stock: イムノジェネテクス株式会社

### [J-1082] I-J11-1
### Tumor antigens/Antitumor immune response (2)

2025 / 9 / 25 (Thu) 12:50-14:05 Room 15/(ANA Crowne Plaza Hotel Kanazawa 3F Zuiun 2) Room 15

[Chairperson]Ken-ichiro Seino (Immunobiology, IGM, Hokkaido Univ)

---

### J-1082
### Oral administration of arginine enhances antitumor immunity and reduces tumor growth in a hepatocellular carcinoma model

[Speaker]Hikaru Sekikawa (Dept. Biomed. Eng., Sci. & Eng., Toyo Univ.) [Co-author]Kaito Nakazato (Dept. Biomed. Eng., Sci. & Eng., Toyo Univ.)、Shunsuke Shichi (Dept. Gastroenterological Surg.I Hokkaido Univ. Grad. Sch. Med.)、Saori Kimura (Dept. Gastroenterological Surg.I Hokkaido Univ. Grad. Sch. Med.)、Hiroki Nakamoto (Dept. Gastroenterological Surg.I Hokkaido Univ. Grad. Sch. Med.)、Chisato Shirakawa (Dept. Gastroenterological Surg.I Hokkaido Univ. Grad. Sch. Med.)、Akinobu Taketomi (Dept. Gastroenterological Surg.I Hokkaido Univ. Grad. Sch. Med.)、Junya Ohtake (Res. Facility Ctr., Asaka, Toyo Univ.／Res. Ctr., Biomed. Eng., Toyo. Univ.)、Hidemitsu Kitamura (Dept. Biomed. Eng., Sci. & Eng., Toyo Univ.／Res. Facility Ctr., Asaka, Toyo Univ.／Res. Ctr., Biomed. Eng., Toyo. Univ.／Dept. Biomed. Eng., Life Sci., Toyo Univ.)

Hepatocellular carcinoma (HCC) remains a malignancy with a poor prognosis, and the development of novel therapeutic strategies is urgently needed. In this study, we investigated the effects of oral arginine administration on antitumor immune responses and tumorigenesis in a liver cancer model.A liver cancer model was established by intrasplenic injection of Hepa1-6 hepatoma cells into wild-type C57BL/6 mice. Mice received oral administration of either PBS or an arginine solution starting on the day of tumor cell injection for 14 days. Tumor burden in the liver and immune cell infiltration were evaluated by HE staining and immunohistochemistry. The oral administration of arginine significantly suppressed tumorigenesis in the liver. Arginine intake also enhanced the infiltration of $CD4^+$ T cells, $CD8^+$ T cells, and $CD11c^+$ dendritic cells within the tumor microenvironment. These findings suggest that oral arginine administration enhances host antitumor immune responses, thereby exerting antitumor effects in vivo. These results indicate that oral arginine supplementation may serve as a novel and promising therapeutic strategy for the treatment of HCC.

[Disclosure of COI by Responsible researcher] Lead presenter is not the Responsible researcher.
[Lead presenter: Hikaru Sekikawa] We have no financial relationships to disclose.
[Principal Researcher: Hidemitsu Kitamura] ■Contributions: テルモ生命科学振興財団

▲TOP

### [J-1083] I-J11-1
### Tumor antigens/Antitumor immune response (2)

2025 / 9 / 25 (Thu) 12:50-14:05 Room 15/(ANA Crowne Plaza Hotel Kanazawa 3F Zuiun 2) Room 15

[Chairperson]Ken-ichiro Seino (Immunobiology, IGM, Hokkaido Univ)

---

### J-1083
### ATR inhibition activates cGAS/STING-interferon signaling and promotes antitumor immunity in small-cell lung cancer

[Speaker]Hirokazu Taniguchi (Cli. Oncol. Ctr. Nagasaki Univ. Hosp.／Dept. Respiratory. Med. Nagasaki Univ. Hosp.) [Co-author]Kazumasa Akagi (Cli. Oncol. Ctr. Nagasaki Univ. Hosp.／Dept. Respiratory. Med. Nagasaki Univ. Hosp.)、Hiromi Tomono (Dept. Respiratory. Med. Nagasaki Univ. Hosp.)、Noritaka Honda (Dept. Respiratory. Med. Nagasaki Univ. Hosp.)、Yosuke Dotsu (Dept. Respiratory. Med. Nagasaki Univ. Hosp.)、Midori Matsuo (Dept. Respiratory. Med. Nagasaki Univ. Hosp.／Cli. Res. Ctr. Nagasaki Univ. Hosp.)、Shinnosuke Takemoto (Dept. Respiratory. Med. Nagasaki Univ. Hosp.)、Hiroshi Mukae (Dept. Respiratory. Med. Nagasaki Univ. Hosp.)

Small-cell lung cancer (SCLC) has poor prognosis and mostly experience only transient benefits from combined immune checkpoint blockade (ICB) and platinum-etoposide. In this study, we demonstrate that inhibition of ataxia telangiectasia and rad3 related (ATR), the primary replication stress response activator, induces DNA damage-mediated micronuclei formation in SCLC cells. ATR inhibition in SCLC activates the stimulator of interferon genes (STING)-mediated interferon signaling, recruits T cells, and augments the antitumor immune response of programmed death-ligand 1 (PD-L1) blockade in mouse models. We demonstrate that combined ATR and PD-L1 inhibition causes improved antitumor response than PD-L1 alone as the second-line treatment in SCLC mouse model. This study shows that targeting ATR up-regulates major histocompatibility class I expression in preclinical models. Inhibition of ATR represents a transformative vulnerability and is a complementary strategy to induce STING-interferon signaling-mediated immunogenicity in SCLC.

[Disclosure of COI by Responsible researcher] Lead presenter is the Responsible researcher.
[Lead presenter: Hirokazu Taniguchi] We have no financial relationships to disclose.

### [J-1084] I-J11-1
### Tumor antigens/Antitumor immune response (2)

2025 / 9 / 25 (Thu) 12:50-14:05 Room 15/(ANA Crowne Plaza Hotel Kanazawa 3F Zuiun 2) Room 15

[Chairperson]Ken-ichiro Seino (Immunobiology, IGM, Hokkaido Univ)

### J-1084
### Reprogramming the Tumor Immune Microenvironment in HPV+ Cervical Cancer and the Antitumor Effects by Therapeutic Vaccine

[Speaker]Kanako Shimizu (Lab for Immunotherapy, RIKEN-IMS) [Co-author]An Sanpei (Lab for Immunotherapy, RIKEN-IMS)、Yuhya Hirahara (Dept. of Obstetrics and Gynecology, Yokohama City University Hospital)、Satoru Yamasaki (Lab for Immunotherapy, RIKEN-IMS)、Shinichiro Fujii (Lab for Immunotherapy, RIKEN-IMS／aAVC Drug translational Unit)

Human papillomavirus (HPV) infection is a major cause of cervical cancer, found in over 95% of cases. While HPV antigens are promising targets for immunotherapy, checkpoint inhibitors alone have shown limited efficacy. We first evaluated the immune responses in cervical cancer (CC) patients. The frequency and function of NK/NKT cells in blood were comparable to those in healthy individuals. However, tumor and tumor margins showed infiltration of TIM-$3^+$/LAG-$3^+$ $CD4^+$ and $CD8^+$ T cells, as well as NK cells, indicating that CC exhibits a "hot tumor" phenotype. We then developed a novel therapeutic cellular vaccine, aAVC-HPV, designed to stimulate both innate and adaptive immunity. In CC mouse models, aAVC-HPV induced strong NK/NKT cell responses and generated E6/E7-specific $CD8^+$ T cells. Antitumor efficacy was demonstrated in B16 lung metastasis and TC-1 subcutaneous tumor models, with significant tumor inhibition and increased infiltration of NK and $CD8^+$ T cells. These findings suggest that aAVC-HPV is a promising therapeutic strategy for HPV-positive cervical cancer.

[Disclosure of COI by Responsible researcher] Lead presenter is not the Responsible researcher.
[Lead presenter: Kanako Shimizu] We have no financial relationships to disclose.
[Principal Researcher: Shinichiro Fujii] We have no financial relationships to disclose.

### [J-1097] I-J9-2
### Metastasis & cancer microenvironment

2025 / 9 / 25 (Thu) 12:50-14:05 Room 16/(11F Visage) Room 16

[Chairperson]Tetsturo Watabe (Dept. Biochem., Grad. Sch. Med. Dent. Sci., Inst. Sci. Tokyo)

---

### J-1097
### Cancer cells invade lymph vessels by inducing EndMT in lymphatic endothelial cells

[Speaker]Shunsuke Kon (RIBS. Tokyo Univ. Sci.) [Co-author]Hancheng Lin (RIBS. Tokyo Univ. Sci.)、Yukari Yamazaki (RIBS. Tokyo Univ. Sci.)、Kosuke Terao (RIBS. Tokyo Univ. Sci.)、Yuta Sakae (RIBS. Tokyo Univ. Sci.)

Cancer cells disseminate from the primary site to distant organs through blood vessels or lymphatic vessels. While the mechanical basis of hematogenous metastasis have been extensively studied, little is known about the underlying processes governing lymphatic metastasis, particularly lymphovascular invasion.In this study, we utilized a genetically engineered mouse model in which cancer cells exhibit a high propensity for lymphatic metastasis. We first observed that lymphatic structures become progressively diminished within neoplastic nevi as tumors expand in tissues. Subsequent scRNA-seq analysis revealed a marked alteration in the expression of genes related to endothelial-to-mesenchymal transition (EndMT) in lymphatic endothelial cells. Furthermore, immunofluorescence analysis demonstrated robust upregulation of EndMT markers in lymphatic endothelial cells close to cancer cells. These finding were corroborated by co-culture experiments using cancer cells and EndMT reporter cell lines. Taken together, our results suggest that cancer cells actively induce EndMT in lymphatic endothelial cells, thereby endowing cancer cells with a lymphatic invasion advantage.

[Disclosure of COI by Responsible researcher] Lead presenter is the Responsible researcher.
[Lead presenter: Shunsuke Kon] We have no financial relationships to disclose.

▲TOP

### [J-1098] I-J9-2
### Metastasis & cancer microenvironment

2025 / 9 / 25 (Thu) 12:50-14:05 Room 16/(11F Visage) Room 16

[Chairperson]Tetsturo Watabe (Dept. Biochem., Grad. Sch. Med. Dent. Sci., Inst. Sci. Tokyo)

---

### J-1098
### Development of a novel therapeutic approach for bone metastasis targeting lipids in the bone microenvironment

[Speaker]Soichiro Sasaki (Inst. of Nat. Med., Univ. of Toyama) [Co-author]Yoshihiro Hayakawa (Inst. of Nat. Med., Univ. of Toyama)

Bone metastasis is a common occurrence in cancer patients. Traditional treatments often have limited success, making it essential to explore the mechanisms of cancer progression in bone metastasis to develop more effective therapies. Within the bone microenvironment, mesenchymal and bone marrow-derived cells interact with cancer cells during bone metastasis. However, the comprehensive functions of these cells in the progression of bone metastasis have not yet been elucidated. To investigate this, tumor growth in bone marrow was confirmed in mice. We observed a significant increase in breast cancer cell proliferation in the bone marrow of mice depleted of the CD20-positive immature B cell population. Additionally, CD20-negative B cells derived conditioned medium promoted tumor growth in an anchorage-independent growth environment. Furthermore, lipid-related receptor molecules of tumor cells were upregulated under this condition, and the addition of exogenous lipids promoted cell growth. Finally, statin treatment inhibited the growth of bone metastatic tumors in mice. In conclusion, therapies targeting lipids could be promising for treating bone metastases.

[Disclosure of COI by Responsible researcher] Lead presenter is the Responsible researcher.
[Lead presenter: Soichiro Sasaki] We have no financial relationships to disclose.

### [J-1099] I-J9-2
### Metastasis & cancer microenvironment

2025 / 9 / 25 (Thu) 12:50-14:05 Room 16/(11F Visage) Room 16

[Chairperson]Tetsturo Watabe (Dept. Biochem., Grad. Sch. Med. Dent. Sci., Inst. Sci. Tokyo)

---

### J-1099
### The regulation of brain metastasis by the vascular endothelial FAK

[Speaker]Shoko Noda (Graduate School of Medicine, The University of Tokyo) [Co-author]Atsu Aiba (Graduate School of Medicine, The University of Tokyo)

In cancer metastasis, the cell adhesion molecules of vascular endothelial cells (ECs), such as VE-cadherin and PECAM-1, degrade in the response to the signals from the tumor, and it facilitates the invasion of cancer cells. However, brain ECs (BECs) maintain a very strong cell adhesion called blood-brain barrier (BBB), and how cancer cells invade the strong barrier of the BECs has not been fully elucidated. In this study, we generated EC-specific focal adhesion kinase (FAK) knockout (cKO) mice and analyzed the regulation of cell adhesion and barrier function of BECs against brain metastatic (BrM) cell lines.
FAK-cKO exacerbated the brain metastasis of the BrM cells, although FAK-cKO mice showed no difference in the expression of adhesion molecules or vascular permeability of the BBB compared to the control mice. Co-culture with BrM cells resulted in the internalization of PECAM-1 in primary BECs from FAK-cKO mice, but not in BECs from control mice. The susceptibility of BECs from FAK-cKO mice to BrM cell lines suggests a role of vascular FAK in maintaining the BBB barrier against the cancer metastasis.

[Disclosure of COI by Responsible researcher] Lead presenter is the Responsible researcher.
[Lead presenter: Shoko Noda] ■Research funds from company: 日本学術振興会 科学研究費助成事業

Japanese Oral Session

### [J-1100] I-J9-2
### Metastasis & cancer microenvironment

2025 / 9 / 25 (Thu) 12:50-14:05 Room 16/(11F Visage) Room 16

[Chairperson]Tetsturo Watabe (Dept. Biochem., Grad. Sch. Med. Dent. Sci., Inst. Sci. Tokyo)

---

### J-1100
### Cancer-associated fibroblasts in "Immature" DR promote colorectal cancer cell behavior via type I procollagen processing

[Speaker]Yuki Sota (Department of Surgery, National Defense Medical College) [Co-author]Satsuki Mochizuki (Department of Surgery, National Defense Medical College)、Koji Moriya (Department of Surgery, National Defense Medical College)、Toshiaki Tanaka (Department of Life Science and Technology, Institute of Science Tokyo)、Kenta Kikuya (Department of Surgery, National Defense Medical College)、Keita Tashiro (Department of Surgery, National Defense Medical College)、Masato Yamadera (Department of Surgery, National Defense Medical College)、Koichi Okamoto (Department of Surgery, National Defense Medical College)、Yoshiki Kajiwara (Department of Surgery, National Defense Medical College)、Yoji Kishi (Department of Surgery, National Defense Medical College)、Yasunori Okada (Department of Pathophysiology for Locomotive and Neoplastic Diseases, Juntendo University)、Hideki Ueno (Department of Surgery, National Defense Medical College)

Desmoplastic reaction (DR) classification with the Mature, Intermediate and Immature categories is a novel prognostic indicator in malignancies. We examined collagen production in cancer-associated fibroblasts (CAFs) by the immunoblotting assay using collagen-hybridizing peptides (Bin*d*COL), which can detect type I, II, III, IV, and V collagens, and found decreased production of type I collagen and increased secretion of type I procollagen in CAFs derived from Immature stroma (CAFs$^{Immature}$) compared to Mature stroma (CAFs$^{Mature}$). Migration and proliferation of CRC cells stimulated with conditioned media of CAFs$^{Immature}$ were inhibited by addition of recombinant type I collagen. Among procollagen N-proteinases (ADAMTS2, 3 and 14), ADAMTS3 expression was significantly lower in CAFs$^{Immature}$ than in CAFs$^{Mature}$. Overexpression of ADAMTS3 in CAFs$^{Immature}$ enhanced type I collagen production and inhibited CRC cell migration and proliferation. These data suggest that suppression of ADAMTS3-mediated processing of type I procollagen in CAFs$^{Immature}$ may be implicated in enhanced tumor cell migration/proliferation in CRC patients with the Immature DR pattern, causing their unfavorable prognosis.

[Disclosure of COI by Responsible researcher] Lead presenter is not the Responsible researcher.
[Lead presenter: Yuki Sota] We have no financial relationships to disclose.
[Principal Researcher: Satsuki Mochizuki] We have no financial relationships to disclose.

Japanese Oral Session

### [J-1101] I-J9-2
### Metastasis & cancer microenvironment

2025 / 9 / 25 (Thu) 12:50-14:05 Room 16/(11F Visage) Room 16

[Chairperson]Tetsturo Watabe (Dept. Biochem., Grad. Sch. Med. Dent. Sci., Inst. Sci. Tokyo)

---

### J-1101
### Host-derived factor induces premetastatic niche formation in liver.

[Speaker]Atsuko Deguchi (Inst. of Adv. Biomed. Engineering Sci., Tokyo Women's Med. Univ.) [Co-author]Masataka Kikuchi (Dept. of Mol. Genetics., Brain Res. Inst., Niigata Univ.)、Akihiro Nakaya (Life Sci. Data Res. Ctr., The Univ. of Tokyo)、Yutaka Suzuki (Life Sci. Data Res. Ctr., The Univ. of Tokyo)、Tsuneyasu Kaisho (Dept. of Immunol., Wakayama Med. Univ.)、Hiroaki Honda (Inst. of Lab. Animals, Tokyo Women's Med. Univ.)、Hiroko Oshima (Div. of Genetics, Cancer Res. Inst., Kanazawa Univ.)、Masanobu Oshima (Div. of Genetics, Cancer Res. Inst., Kanazawa Univ.)、Hiroki Ochiai (Teikyo Univ. Hosp.)、Kashiro Ayumi (Dept. of Bioregulation, Grad Sch. of Med., Nippon Med. Univ.)、Kazufumi Honda (Dept. of Bioregulation, Grad Sch. of Med., Nippon Med. Univ.)、Yoshiro Maru (Future Robotics Org., Waseda Univ.)

The inflammation-mediated tumor microenvironment at primary tumor site is involved in the progression of cancer. It has been found that a premetastatic niche similar to tumor microenvironment is formed in distant organs prior to metastasis, which promotes metastasis. We previously reported that S100A8, a TLR4 endogenous ligand, induces the recruitment of MDSCs to lungs at premetastatic phase. As similar to pulmonary premetastatic niche formation, MDSCs were recruited to liver at the premetastatic phase. Metastatic tumors tend to metastasize to favorable organs. We then identified a host-derived factor which was up-regulated in the liver at a premetastatic phase by using mouse orthotopic transplantation models, and generated genetically engineered mice to examine the underlying mechanisms using single-cell transcriptome analysis. To evaluate the clinical relevance, we investigated that plasma level of the factor in colorectal cancer patients. The plasma levels of host-derived factor increased as colorectal tumors became more malignant. In addition, the plasma levels significantly increased in colorectal cancer patients with distant metastasis to liver, but not to other organs.

[Disclosure of COI by Responsible researcher] Lead presenter is the Responsible researcher.
[Lead presenter: Atsuko Deguchi] ■Research funds from company: 科学研究費助成事業基盤研究C, AMED, アステラス病態代謝研究会研究助成, 内藤記念科学振興財団一般助成

### [J-1102] I-J9-2
### Metastasis & cancer microenvironment

2025 / 9 / 25 (Thu) 12:50-14:05 Room 16/(11F Visage) Room 16

[Chairperson]Tetsturo Watabe (Dept. Biochem., Grad. Sch. Med. Dent. Sci., Inst. Sci. Tokyo)

---

### J-1102
### Deciphering the contributions of cellular and exosomal heterogeneity to the multistep progression of cancer

[Speaker]Yuto Hamazaki (RCAST, UTokyo) [Co-author]Ayuko Hoshino (RCAST, UTokyo)

Exosomes derived from cancer cells, which are 30-150 nm in diameter and enclosed by a lipid bilayer, contribute to cancer progression. Tumor-derived exosomes are taken up by distant organs and modify the microenvironment to create favorable sites for metastasis. We have previously reported that the adhesion protein pair, integrin $\alpha_6$ and integrin $\beta_4$ (ITG$\alpha_6\beta_4$) on exosomes functions as a "zip code", directing ITG$\alpha_6\beta_4^+$ exosomes to the lung.
In recent years, advancements in technology have enabled the analysis of single exosomes. Using single-exosome analysis, we revealed that lung-tropic cancer cell-derived exosomes are heterogeneous, with only 1% having ITG$\alpha_6\beta_4$. Furthermore, the production of ITG$\alpha_6\beta_4^+$ exosomes from a single cell varies within the cell population. We discovered that, in addition to differences in exosome secretion ability, tumor growth at the primary site and adhesion to distant organs also vary among cells. In this research, we aim to decipher how individual cells secrete different amount of ITG$\alpha_6\beta_4^+$ exosomes and how these exosomes contribute to each stage of cancer progression.

[Disclosure of COI by Responsible researcher] Lead presenter is not the Responsible researcher.
[Lead presenter: Yuto Hamazaki] We have no financial relationships to disclose.
[Principal Researcher: Ayuko Hoshino] We have no financial relationships to disclose.

▲TOP

### [OS8-1] OS8
### Frontier of cancer research driven by multi-modal extracellular matrix

2025 / 9 / 25 (Thu) 13:50-15:20 Room 8/(Hotel Nikko Kanazawa 3F Kujaku C) Room 8

[Chairpersons]Hiroyasu Kidoya (Department of Integrative Vascular Biology, Faculty of Medical Sciences, University of Fukui)、Seiichiro Ishihara (Hokkaido University)

近年の解析技術の進歩により、細胞外基質（ECM）は従来考えられていたよりも遥かに動的な構造物であり、シグナル分子・高次構造・粘弾性などのマルチモーダルな作用を細胞に与えることで複雑な生命現象を支えていることが明らかになってきた。このような多細胞システムへのECMの作用は、がんの発症・進展においても大きな役割を果たしていると考えられる。しかしながら、これまでのがん研究はシングルセルオミクスなど細胞間相互作用の解析に集中しており、ECMは「単なる静的な足場」として軽視されてきた。本シンポジウムでは、ゲノムに明示的に書き込むことが困難な「作用場」の時空間情報としてECMを見直し、ECMを考慮せずに細胞中心で構築されてきたがん研究の体系に一石を投じる。講演者には、細胞・組織・生体の各階層でのがん研究者に加えてECMの生化学者や高分子化学者を交え、ECMの基礎からがん治療への可能性まで幅広く議論する。

### OS8-1
### Basement membrane dynamics in epithelial tissue morphogenesis

[Speaker]Hironobu Fujiwara (RIKEN, BDR)

Precisely regulated remodelling of the basement membrane (BM) plays essential roles not only in normal tissue morphogenesis but also in pathological processes such as in cancer. However, BMs molecular and tissue-level dynamics, as well as their underlying mechanisms and functional significance, remain poorly understood due to limited availability of visualization tools. To address this, we have recently developed mouse lines in which the endogenous core BM proteins are fluorescently tagged, enabling live imaging of BM dynamics in intact tissues. Using developing hair follicles as a model, we demonstrate that spatially regulated BM turnover rates control BM expansion, cell displacement, and the orientation of cell division, collectively driving anisotropic organ elongation. Furthermore, by performing targeted BM ablation, we investigate how tension gradients within the BM contribute to tissue mechanics and morphogenesis. In this study, I will discuss how dynamic interactions between cells and the BM coordinate tissue morphogenesis, which can also inform future strategies to elucidate and manipulate BM remodelling in cancer.

[Disclosure of COI by Responsible researcher] Lead presenter is the Responsible researcher.
[Lead presenter: Hironobu Fujiwara] We have no financial relationships to disclose.

### [OS8-2] OS8
### Frontier of cancer research driven by multi-modal extracellular matrix

2025 / 9 / 25 (Thu) 13:50-15:20 Room 8/(Hotel Nikko Kanazawa 3F Kujaku C) Room 8

[Chairpersons]Hiroyasu Kidoya (Department of Integrative Vascular Biology, Faculty of Medical Sciences, University of Fukui)、Seiichiro Ishihara (Hokkaido University)

近年の解析技術の進歩により、細胞外基質（ECM）は従来考えられていたよりも遥かに動的な構造物であり、シグナル分子・高次構造・粘弾性などのマルチモーダルな作用を細胞に与えることで複雑な生命現象を支えていることが明らかになってきた。このような多細胞システムへのECMの作用は、がんの発症・進展においても大きな役割を果たしていると考えられる。しかしながら、これまでのがん研究はシングルセルオミクスなど細胞間相互作用の解析に集中しており、ECMは「単なる静的な足場」として軽視されてきた。本シンポジウムでは、ゲノムに明示的に書き込むことが困難な「作用場」の時空間情報としてECMを見直し、ECMを考慮せずに細胞中心で構築されてきたがん研究の体系に一石を投じる。講演者には、細胞・組織・生体の各階層でのがん研究者に加えてECMの生化学者や高分子化学者を交え、ECMの基礎からがん治療への可能性まで幅広く議論する。

---

### OS8-2
### Viscous ECM: In Vitro Vascularized Cancer Stromal Tissues for Functional Cancer Personalized Medicine

[Speaker]Michiya Matsusaki (Dept. Appl. Chem., Grad. Sch. Eng., The Univ. of Osaka)

The development of high-throughput anticancer drug screening methods using patient-derived cancer cell (PDC) lines that maintain their original characteristics in an in vitro three-dimensional (3D) culture system possess a significant challenge to achieving personalized cancer medicine. Because stromal tissue plays a critical role in the composition and maintenance of the cancer microenvironment, in vitro 3D-culture using reconstructed stromal tissues has attracted considerable attention. Here, a simple and unique in vitro 3D-culture method using viscous extracellular matrix consisting of heparin and collagen together with fibroblasts and endothelial cells to fabricate vascularized 3D-stromal tissues for in vitro culture of PDCs is reported. The co-treatment with bevacizumab and 5-fluorouracil significantly reduced the survival rate of 3D-cultured PDCs to 30%. Surprisingly, drug evaluation using eight PDC lines with the 3D-culture method resulted in a drug efficacy concordance rate of 75% with clinical outcomes. The model is expected to be applicable to in vitro throughput drug screening for the development of personalized cancer medicine.

[Disclosure of COI by Responsible researcher] Lead presenter is the Responsible researcher.
[Lead presenter: Michiya Matsusaki] ■Endowed chair: TOPPANホールディングス株式会社

▲TOP

### [OS8-3] OS8
### Frontier of cancer research driven by multi-modal extracellular matrix

2025 / 9 / 25 (Thu) 13:50-15:20 Room 8/(Hotel Nikko Kanazawa 3F Kujaku C) Room 8

[Chairpersons]Hiroyasu Kidoya (Department of Integrative Vascular Biology, Faculty of Medical Sciences, University of Fukui)、Seiichiro Ishihara (Hokkaido University)

近年の解析技術の進歩により、細胞外基質（ECM）は従来考えられていたよりも遥かに動的な構造物であり、シグナル分子・高次構造・粘弾性などのマルチモーダルな作用を細胞に与えることで複雑な生命現象を支えていることが明らかになってきた。このような多細胞システムへのECMの作用は、がんの発症・進展においても大きな役割を果たしていると考えられる。しかしながら、これまでのがん研究はシングルセルオミクスなど細胞間相互作用の解析に集中しており、ECMは「単なる静的な足場」として軽視されてきた。本シンポジウムでは、ゲノムに明示的に書き込むことが困難な「作用場」の時空間情報としてECMを見直し、ECMを考慮せずに細胞中心で構築されてきたがん研究の体系に一石を投じる。講演者には、細胞・組織・生体の各階層でのがん研究者に加えてECMの生化学者や高分子化学者を交え、ECMの基礎からがん治療への可能性まで幅広く議論する。

---

### OS8-3
### Stiff ECM triggers activation of transcription factor ATF5 for cancer progression

[Speaker]Seiichiro Ishihara (Cell Dynamics, Faculty Advanced Life Sci., Hokkaido Univ.) [Co-author]Atsushi Enomoto (Dept. Path., Nagoya Univ. Grad. Sch. Med.)、Akihiro Sakai (Dept. Path., Nagoya Univ. Grad. Sch. Med.)、Tadashi Iida (Dept. Path., Nagoya Univ. Grad. Sch. Med.)、Hisashi Haga (Cell Dynamics, Faculty Advanced Life Sci., Hokkaido Univ.)

Solid tumor tissues contain stiffer extracellular matrices (ECMs) than corresponding normal tissues. Cancer cells respond to the stiffness of ECMs and regulate their malignancy. However, the transcription factors that specifically express in cancer cells and respond to ECM stiffness for cancer progression is poorly identified. In this study, we revealed that activating transcription factor 5 (ATF5), which is highly expressed in cancer tissues than corresponding normal tissues, is activated by stiff ECMs and promotes cancer progression. We found that ATF5 is localized at nuclei in cancer cells on a stiff ECM compared with that on a soft ECM. ATF5 knockdown in cancer cells suppressed proliferation in vitro and in vivo. On a stiff ECM, ATF5 suppressed the expression of early growth response 1 (EGR1), and as a result, promoted growth of cancer cells. ATF5 activation by stiff ECMs was regulated by the JAK-MYC pathway. Furthermore, immunohistochemistry showed that ATF5 is highly expressed and activated in pancreatic cancer tissues, especially collagen-rich (mechanically stiff) region. These results show a critical role of ATF5 for responding to ECM stiffness in cancer cells.

[Disclosure of COI by Responsible researcher] Lead presenter is the Responsible researcher.
[Lead presenter: Seiichiro Ishihara] We have no financial relationships to disclose.

### [OS8-4] OS8
### Frontier of cancer research driven by multi-modal extracellular matrix

2025 / 9 / 25 (Thu) 13:50-15:20 Room 8/(Hotel Nikko Kanazawa 3F Kujaku C) Room 8

[Chairpersons]Hiroyasu Kidoya (Department of Integrative Vascular Biology, Faculty of Medical Sciences, University of Fukui)、Seiichiro Ishihara (Hokkaido University)

近年の解析技術の進歩により、細胞外基質（ECM）は従来考えられていたよりも遥かに動的な構造物であり、シグナル分子・高次構造・粘弾性などのマルチモーダルな作用を細胞に与えることで複雑な生命現象を支えていることが明らかになってきた。このような多細胞システムへのECMの作用は、がんの発症・進展においても大きな役割を果たしていると考えられる。しかしながら、これまでのがん研究はシングルセルオミクスなど細胞間相互作用の解析に集中しており、ECMは「単なる静的な足場」として軽視されてきた。本シンポジウムでは、ゲノムに明示的に書き込むことが困難な「作用場」の時空間情報としてECMを見直し、ECMを考慮せずに細胞中心で構築されてきたがん研究の体系に一石を投じる。講演者には、細胞・組織・生体の各階層でのがん研究者に加えてECMの生化学者や高分子化学者を交え、ECMの基礎からがん治療への可能性まで幅広く議論する。

---

### OS8-4
### ERK-dependent translocation of ZO-1 regulates epithelial cell invasion

[Speaker]Sayuki Hirano (Grad. Sch. of Biost., Kyoto Univ.) [Co-author]Yohei Kondo (One Med. Transdiscipl. Life sci-Med. co-cr Platf. (TLiMP), Nagoya Univ.)、Naoto Ueno (Natl. Insts. of Nat. Scis. (NINS))、Kazuhiro Aoki (Grad. Sch. of Biost., Kyoto Univ.)

Collective cell migration is a coordinated form of cell movement that plays crucial role in cancer invasion. While both cell-cell and cell-ECM adhesions are essential for this process, the mechanisms that integrate these adhesions remain largely unclear. To address this question, we focused on ZO-1, a scaffold protein of tight junctions. In non-migrating cells, ZO-1 is localized at apical junctions. Upon initiation of collective migration, however, ZO-1 translocated to the basal cell surface, where it formed invadosome. Using a ZO-1 mutant lacking the actin-binding region (ZO-1ΔABR), we found that ZO-1 interaction with actin is essential for its localization to invadosome. Functional analysis demonstrated that ZO-1ΔABR-expressing cells exhibited reduced traction force generation and lower ECM degradation compared to wild-type ZO-1-expressing cells. Furthermore, we found that ERK activity, which propagates as waves during collective migration, is required for ZO-1 translocation to invadosome. Altogether, our study uncovers a role for ZO-1 in regulating epithelial cell invasion by dynamically translocating between apical junctions and invadosome in an ERK-dependent manner.

[Disclosure of COI by Responsible researcher] Lead presenter is the Responsible researcher.
[Lead presenter: Sayuki Hirano] We have no financial relationships to disclose.

### [OS8-5] OS8
### Frontier of cancer research driven by multi-modal extracellular matrix

2025 / 9 / 25 (Thu) 13:50-15:20 Room 8/(Hotel Nikko Kanazawa 3F Kujaku C) Room 8

[Chairpersons]Hiroyasu Kidoya (Department of Integrative Vascular Biology, Faculty of Medical Sciences, University of Fukui)、Seiichiro Ishihara (Hokkaido University)

近年の解析技術の進歩により、細胞外基質（ECM）は従来考えられていたよりも遥かに動的な構造物であり、シグナル分子・高次構造・粘弾性などのマルチモーダルな作用を細胞に与えることで複雑な生命現象を支えていることが明らかになってきた。このような多細胞システムへのECMの作用は、がんの発症・進展においても大きな役割を果たしていると考えられる。しかしながら、これまでのがん研究はシングルセルオミクスなど細胞間相互作用の解析に集中しており、ECMは「単なる静的な足場」として軽視されてきた。本シンポジウムでは、ゲノムに明示的に書き込むことが困難な「作用場」の時空間情報としてECMを見直し、ECMを考慮せずに細胞中心で構築されてきたがん研究の体系に一石を投じる。講演者には、細胞・組織・生体の各階層でのがん研究者に加えてECMの生化学者や高分子化学者を交え、ECMの基礎からがん治療への可能性まで幅広く議論する。

---

### OS8-5
### Molecular Mechanisms of Therapeutic Resistance Driven by Dynamics of Tumor Vascular Matrix

[Speaker]Hiroyasu Kidoya (Dept. Integrative Vascular Biol., Univ. Fukui, Sch. Med.) [Co-author]Kazuhiro Takara (Dept. Integrative Vascular Biol., Univ. Fukui, Sch. Med.)

Angiogenesis inhibitors are standard solid tumor therapies, but treatment resistance and rapid tumor progression after discontinuation pose clinical challenges. Previous studies focused on tumor vasculature and cells, but vascular matrix components' influence remains insufficiently investigated. This study examined anti-angiogenic resistance and post-discontinuation progression, investigating dynamic vascular matrix changes and effects on surrounding cells. Using imaging mice expressing fluorescent proteins in collagen type IV and vascular endothelial cells, we employed two-photon microscopy for high-resolution in vivo imaging of temporal tumor vascular matrix changes. Results revealed tubular collagen type IV structures remained after vascular endothelial cell regression, altering morphology while affecting surrounding cells. Examining associations between this extracellular matrix and treatment resistance/tumor progression showed evidence suggesting interstitial cell activation. This study suggests dynamic tumor vascular matrix changes contribute to anti-angiogenic resistance acquisition and post-treatment progression, providing foundation for new matrix-targeting therapies.

[Disclosure of COI by Responsible researcher] Lead presenter is the Responsible researcher.
[Lead presenter: Hiroyasu Kidoya] We have no financial relationships to disclose.

▲TOP

### [OS8-6] OS8
### Frontier of cancer research driven by multi-modal extracellular matrix

2025 / 9 / 25 (Thu) 13:50-15:20 Room 8/(Hotel Nikko Kanazawa 3F Kujaku C) Room 8

[Chairpersons]Hiroyasu Kidoya (Department of Integrative Vascular Biology, Faculty of Medical Sciences, University of Fukui)、Seiichiro Ishihara (Hokkaido University)

近年の解析技術の進歩により、細胞外基質（ECM）は従来考えられていたよりも遥かに動的な構造物であり、シグナル分子・高次構造・粘弾性などのマルチモーダルな作用を細胞に与えることで複雑な生命現象を支えていることが明らかになってきた。このような多細胞システムへのECMの作用は、がんの発症・進展においても大きな役割を果たしていると考えられる。しかしながら、これまでのがん研究はシングルセルオミクスなど細胞間相互作用の解析に集中しており、ECMは「単なる静的な足場」として軽視されてきた。本シンポジウムでは、ゲノムに明示的に書き込むことが困難な「作用場」の時空間情報としてECMを見直し、ECMを考慮せずに細胞中心で構築されてきたがん研究の体系に一石を投じる。講演者には、細胞・組織・生体の各階層でのがん研究者に加えてECMの生化学者や高分子化学者を交え、ECMの基礎からがん治療への可能性まで幅広く議論する。

---

### OS8-6
### Stromal defense against cancer by the periosteum

[Speaker]Masayuki Tsukasaki (Dep. Biochem. Dent. Showa Med. Univ.)

Cancer-bone interactions have been investigated primarily in the context of bone metastasis. However, hematogenous spread is not the only route by which cancer cells enter the bone. Certain cancer types, including oral cancer, affect the bone in a different way: the primary tumor directly invades adjacent bones. This process is called cancer bone invasion which significantly worsens prognosis and quality of life, but little is known about mechanisms of localized bone invasion by primary tumors. Here, I discuss our recent findings that periosteal cells produce Timp1 which inactivates matrix-degrading proteases, promoting periosteal thickening to inhibit cancer invasion. Our findings demonstrate that the periosteum acts as a functional stromal barrier against tumour progression, representing a unique example of anti-tumor machinery mediated by the non-immune system.

[Disclosure of COI by Responsible researcher] Lead presenter is the Responsible researcher.
[Lead presenter: Masayuki Tsukasaki] We have no financial relationships to disclose.

### [J-1055] I-J5
### Diversity of signal transduction pathways in cancer

2025 / 9 / 25 (Thu) 14:05-15:20 Room 7/(Hotel Nikko Kanazawa 3F Kujaku B) Room 7

[Chairperson]Kenichi Suzuki (iGCORE, Gifu Univ./NCCRI)

### J-1055
### CD73 as a Downstream Target of TGF-β Signaling in Tumor Immune Evasion

[Speaker]Etsu Tashiro (Showa Pharmaceutical University, Laboratory of Biochemistry／RIKEN Center for Integrative Medical Sciences) [Co-author]Naoko Nakano (Showa Pharmaceutical University, Laboratory of Biochemistry)、Daizo Koinuma (Dept. Path., Grad. Sch. Med., The Univ. of Tokyo)、Kohei Miyazono (Dept. Applied Path., Grad. Sch. Med., The Univ. of Tokyo／RIKEN Center for Integrative Medical Sciences)、Susumu Itoh (Showa Pharmaceutical University, Laboratory of Biochemistry)

TGF-β signaling has a dichotomous role in cancer progression. To examine the role of TGF-β signaling in the cancer progression, we performed RNA-seq analysis using total RNA from intestinal epithelium in small intestine-specific Smad2/Smad3 DKO mice. As a result, we found that mRNA of CD73 involved in tumor immune evasion was decreased in the small intestine of Smad2/Smad3 DKO mice. This result supposed that the expression of the CD73 gene might transcriptionally be regulated by TGF-β signaling.Here, we demonstrated that the expression of CD73 mRNA and CD73 protein located on the plasma membrane were increased in A549 cells after TGF-β stimulation. To understand the mechanism by which TGF-β induced upregulation of CD73, we performed luciferase reported assays. As a result, the transcriptional activity of the CD73 enhancer region located within the intron 1 of the CD73 gene was enhanced by TGF-β stimulation. Furthermore, we identified two Smad binding elements (SBEs), and the TGF-β-induced transcriptional activation was abolished in SBE mutants. We are currently investigating how Smad and other transcription factor(s) coordinately regulates the transcript of the CD73 gene in detail.

[Disclosure of COI by Responsible researcher] Lead presenter is the Responsible researcher.
[Lead presenter: Etsu Tashiro] We have no financial relationships to disclose.

### [J-1056] I-J5
### Diversity of signal transduction pathways in cancer

2025 / 9 / 25 (Thu) 14:05-15:20 Room 7/(Hotel Nikko Kanazawa 3F Kujaku B) Room 7

[Chairperson]Kenichi Suzuki (iGCORE, Gifu Univ./NCCRI)

---

### J-1056
### Unrevealing the mechanisms of the TGF-β-induced endothelial-mesenchymal transition (EndoMT) in tumor microenvironment

[Speaker]Mizuki Tanaka (Dept. Biochem, Grad. Sc Med. & Dent., Science Tokyo.) [Co-author]Kazuki Takahashi (Dept. Biochem, Grad. Sc Med. & Dent., Science Tokyo.)、Miho Kobayashi (Dept. Biochem, Grad. Sc Med. & Dent., Science Tokyo.)、Katarzyna A. Inoue (Dept. Biochem, Grad. Sc Med. & Dent., Science Tokyo.)、Yukiko T. Matsunaga (IIS, UTokyo.)、Tetsuro Watabe (Dept. Biochem, Grad. Sc Med. & Dent., Science Tokyo.)

Transforming growth factor-β (TGF-β) induces endothelial-mesenchymal transition (EndoMT) during which endothelial cells transdifferentiate to mesenchymal cells leading to tumor progression and metastasis. Cells undergoing EndoMT differentiate through a spectrum of intermediate phases called partial EndoMT, in which they exhibit both endothelial/mesenchymal phenotypes. However, detailed analysis of partial EndoMT has been hampered by the lack of specific markers. In this study, we developed EndoMT reporter endothelial cells (EMRECs), allowing real-time observation of stepwise changes in EndoMT and facilitating the visualization and analysis of this process. RNA sequencing of multiple EndoMT stages identified CD40 as a marker specifically upregulated during partial EndoMT. To investigate the role of CD40 in partial EndoMT, we suppressed its expression in TGF-β-stimulated EMRECs and found that TGF-β-induced EndoMT was more enhanced upon downregulation of CD40 expression, indicating that CD40 suppresses the transition from partial to full EndoMT stages. These findings may lead to the development of new therapeutic strategies targeting EndoMT-driven cancer progression and metastasis.

[Disclosure of COI by Responsible researcher] Lead presenter is not the Responsible researcher.
[Lead presenter: Mizuki Tanaka] We have no financial relationships to disclose.
[Principal Researcher: Tetsuro Watabe] We have no financial relationships to disclose.

### [J-1057] I-J5
### Diversity of signal transduction pathways in cancer

2025 / 9 / 25 (Thu) 14:05-15:20 Room 7/(Hotel Nikko Kanazawa 3F Kujaku B) Room 7

[Chairperson]Kenichi Suzuki (iGCORE, Gifu Univ./NCCRI)

---

### J-1057
### Regulation of KRAS signaling by lipid domain in the inner leaflet of cell membranes

[Speaker]Toshiki Mori (UGSAS, Gifu Univ. ／Natl. Cancer Ctr. Res. Inst.) [Co-author]Koichiro M. Hirosawa (iGCORE, Gifu Univ.)、Rinshi S. Kasai (Natl. Cancer Ctr. Res. Inst.)、Tomohiko Taguchi (Grad. Sch. Life. Sci., Tohoku Univ.)、Yasunari Yokota (Dept. Eng., Gifu Univ.)、Eiji Yamamoto (Dept. Sys. Des. Eng., Keio Univ.)、Kenichi Suzuki (UGSAS, Gifu Univ. ／Natl. Cancer Ctr. Res. Inst.／iGCORE, Gifu Univ.)

KRAS is a pivotal small G protein localized to the inner leaflet of the plasma membrane (PM) via a farnesyl-cysteine-methyl-ester anchor. Previous studies suggested that KRAS interacts with phosphatidylserine in the inner leaflet of the PM to form nanoclusters essential for downstream signal transduction (Science, 2015; Cell, 2017). However, how lipid domains regulate KRAS signaling in the PM of living cells remains poorly understood. To address this issue, we performed simultaneous dual-color super-resolution movie observation to visualize KRAS and lipid domains in living cells. Our results revealed that KRAS dynamically translocates between distinct lipid domains. Furthermore, simultaneous dual-color super-resolution imaging of lipid domains combined with single-molecule tracking of BRAF, a key effector of KRAS, demonstrated that BRAF is exclusively recruited to specific lipid domains. We further investigated how these membrane lipid domains influence KRAS orientation and the free energy landscape of the KRAS-BRAF complex using molecular dynamics simulation.

[Disclosure of COI by Responsible researcher] Lead presenter is not the Responsible researcher.
[Lead presenter: Toshiki Mori] We have no financial relationships to disclose.
[Principal Researcher: Kenichi Suzuki] We have no financial relationships to disclose.

### [J-1058] I-J5
### Diversity of signal transduction pathways in cancer

2025 / 9 / 25 (Thu) 14:05-15:20 Room 7/(Hotel Nikko Kanazawa 3F Kujaku B) Room 7

[Chairperson]Kenichi Suzuki (iGCORE, Gifu Univ./NCCRI)

---

### J-1058
### Senescence-associated microRNA-3140-3p suppresses malignant pleural mesothelioma by targeting SLC7A11 and ASF1B

[Speaker]Yuki Yamamoto (Cell. & Mol. Biol., Grad. Sch. Biomed. & Health Sci., Hiroshima Univ.) [Co-author]Kimiyoshi Yano (Cell. & Mol. Biol., Grad. Sch. Biomed. & Health Sci., Hiroshima Univ.)、Ryouu Takahashi (Cell. & Mol. Biol., Grad. Sch. Biomed. & Health Sci., Hiroshima Univ.)、Hidetoshi Tahara (Cell. & Mol. Biol., Grad. Sch. Biomed. & Health Sci., Hiroshima Univ.)

While microRNAs (miRNAs) are known to play critical roles in the regulation of cellular senescence and tumor development, the roles of individual miRNAs in these processes are not fully understood. In this study, we identified 349 senescence-associated miRNAs (SA-miRNAs) using high-throughput functional screening with an miRNA library (2028 miRNAs). Among these miRNAs, we identified miRNA 3140-3p as a novel SA-miRNA that induced cellular senescence through p53/p21 activation and stronger tumor suppression phenotypes than those of other well-known tumor suppressive miRNAs, such as miR-34a-5p and 22-3p. Integrated analysis with RNA-seq and proteomics also revealed that miR-3140-3p exerts tumor suppressive functions by directly targeting SLC7A11 and ASF1B which are highly expressed in multiple types of tumors including MPM. The findings contribute to our understanding of the miRNA profiles associated with senescence induction and provide evidence that "Aging switch" with tumor suppressive SA-miRNAs could represent promising drug candidates for cancer therapy.

[Disclosure of COI by Responsible researcher] Lead presenter is not the Responsible researcher.
[Lead presenter: Yuki Yamamoto] We have no financial relationships to disclose.
[Principal Researcher: Hidetoshi Tahara] ■Research funds from company: 株式会社PURMX Therapeutics　■Advisor of the company and/or profit-making organization: 株式会社PURMX Therapeutics　■Profit of stock: 株式会社PURMX Therapeutics

▲TOP

### [J-1059] I-J5
### Diversity of signal transduction pathways in cancer

2025 / 9 / 25 (Thu) 14:05-15:20 Room 7/(Hotel Nikko Kanazawa 3F Kujaku B) Room 7

[Chairperson]Kenichi Suzuki (iGCORE, Gifu Univ./NCCRI)

---

### J-1059
### The long noncoding RNA GSEC encodes a small peptide to promote colorectal tumorigenesis

[Speaker]Yoshihiro Kawasaki (Near InfraRed Photo-Immunotherapy Res. Inst., Kansai Medical University／IQB, The University of Tokyo) [Co-author]Tomoatsu Hayashi (IQB, The University of Tokyo)、Tetsu Akiyama (IQB, The University of Tokyo)、Eisaku Kondo (Near InfraRed Photo-Immunotherapy Res. Inst., Kansai Medical University)

Long non-coding RNAs (lncRNAs) are frequently dysregulated in multiple human cancers. We have recently reported that lncRNA GSEC is required for the motility of colorectal cancer cells by impairing DHX36 RNA helicase activity. However, the function of GSEC in colorectal tumorigenesis is still unclear. Here we show that GSEC is upregulated in epithelial cells of colorectal cancer and is required for the tumorigenicity of colorectal cancer cells. We also demonstrate that GSEC encodes a small peptide, termed GSEC-pep, and GSEC-pep promotes the growth of colorectal cancer cells through the E2F/Rb pathway. Furthermore, we show that exogenously expressed GSEC-pep is predominantly localized in the nucleolus and knockdown of GSEC leads to a reduction in nucleolar number in colorectal cancer cells. These findings suggest that GSEC-pep has a critical role in cancer cell proliferation and might be a promising molecular target for the treatment of colorectal cancer.

[Disclosure of COI by Responsible researcher] Lead presenter is the Responsible researcher.
[Lead presenter: Yoshihiro Kawasaki] We have no financial relationships to disclose.

### [J-1060] I-J5
### Diversity of signal transduction pathways in cancer

2025 / 9 / 25 (Thu) 14:05-15:20 Room 7/(Hotel Nikko Kanazawa 3F Kujaku B) Room 7

[Chairperson]Kenichi Suzuki (iGCORE, Gifu Univ./NCCRI)

---

### J-1060
### Mitochondrial DNA mutation is associated with chemoresistance in esophageal squamous cell carcinoma

[Speaker]Koji Tanaka (Department of Gastroenterological Surgery, Graduate School of Medicine, Osaka University) [Co-author]Takashi Harino (Department of Upper Gastrointestinal Surgery, Kansai Medical University Hospital)、Takaomi Hagi (Department of Gastroenterological Surgery, Graduate School of Medicine, Osaka University)、Kota Momose (Department of Gastroenterological Surgery, Graduate School of Medicine, Osaka University)、Kotaro Yamashita (Department of Gastroenterological Surgery, Graduate School of Medicine, Osaka University)、Takuro Saito (Department of Gastroenterological Surgery, Graduate School of Medicine, Osaka University)、Tomoki Makino (Department of Gastroenterological Surgery, Graduate School of Medicine, Osaka University)、Tsuyoshi Takahashi (Department of Gastroenterological Surgery, Graduate School of Medicine, Osaka University)、Yukinori Kurokawa (Department of Gastroenterological Surgery, Graduate School of Medicine, Osaka University)、Kiyokazu Nakajima (Department of Gastroenterological Surgery, Graduate School of Medicine, Osaka University)、Hidetoshi Eguchi (Department of Gastroenterological Surgery, Graduate School of Medicine, Osaka University)、Yuichiro Doki (Department of Gastroenterological Surgery, Graduate School of Medicine, Osaka University)

PurposeStandard treatment for esophageal squamous cell carcinoma (ESCC) includes neoadjuvant chemotherapy followed by surgery. However, some patients show chemoresistance, resulting in poor prognosis. We focused on mitochondrial DNA (mtDNA) to examine its role in resistance.Methods and ResultsCDDP-resistant sublines were established from TE11 and TE8 ESCC cell lines. mtDNA sequencing showed that somatic mutations induced by chemotherapy were enriched in the D-loop region. Resistant TE11-R cells had decreased mtDNA copy number.In 96 ESCC patients, D-loop mutations were analyzed in cancer cells from post-chemotherapy resection specimens. HVS1 mutations correlated with chemoresistance ($p=0.03$). Patients with>14 HVS1 mutations had more resistant cases ($p=0.002$) and worse recurrence-free survival ($p=0.03$). This group also had significantly lower mtDNA copy numbers.ConclusionD-loop mutations, especially in HVS1, are linked to chemoresistance and poor prognosis in ESCC. HVS1 may promote resistance via mtDNA copy number reduction, suggesting a potential therapeutic target.

[Disclosure of COI by Responsible researcher] Lead presenter is the Responsible researcher.
[Lead presenter: Koji Tanaka] We have no financial relationships to disclose.

▲TOP

### [E-1055] I-E3-2
### Viruses & Cancer

2025 / 9 / 25 (Thu) 14:05-15:20 Room 9/(Hotel Nikko Kanazawa 3F Lumiere) Room 9

[Chairperson]Jun-ichirou Yasunaga (Dept. Hematol., Fac. Life Sci., Kumamoto Univ.)

---

### E-1055
### Exploration of the predictive marker for post HCV-SVR hepatocarcinogenesis based on DNA methylome/transcriptome analysis

[Speaker]Masako Mishima (Kyoto Univ.) [Co-author]Haruhiko Takeda (Kyoto Univ.)、Haruka Amino (Kyoto Univ.)、Yosuke Fujii (Kyoto Univ.)、Takahiro Shimizu (Kobe City Medical Center West Hospital)、Atsushi Takai (Kyoto Univ.)、Hiroshi Seno (Kyoto Univ.)

We previously reported the gene expression changes related with hepatocarcinogenesis persist in the liver tissue even after the eradication of hepatitis C virus (HCV). We aimed to identify the molecules that can predict hepatocarcinogenesis after sustained viral response (SVR) by integrated analysis of DNA methylome/transcriptome using non-cancerous liver samples of 1) normal 2) HCV positive and 3) SVR cases. We also explored candidate molecules predicting early carcinogenesis after SVR. As a result, 36 genes with altered DNA methylation and expression changes were commonly identified in HCV positive and SVR groups. Among these genes, the expression of PIK3CG, which is related to inositol phosphate metabolism, was significantly increased in the serum collected at the time of HCV treatment in the cases that developed liver cancer within 3 years of SVR compared with the cases that developed cancer more than 3 years after SVR. Our data suggested that gene expression changes with altered DNA methylation were sustained in the liver tissue after HCV elimination, and PIK3CG upregulation at the time of HCV treatment could be a predictive marker for early hepatocarcinogenesis after SVR.

[Disclosure of COI by Responsible researcher] Lead presenter is not the Responsible researcher.
[Lead presenter: Masako Mishima] We have no financial relationships to disclose.
[Principal Researcher: Atsushi Takai] We have no financial relationships to disclose.

### [E-1056] I-E3-2
### Viruses & Cancer

2025 / 9 / 25 (Thu) 14:05-15:20 Room 9/(Hotel Nikko Kanazawa 3F Lumiere) Room 9

[Chairperson]Jun-ichirou Yasunaga (Dept. Hematol., Fac. Life Sci., Kumamoto Univ.)

---

### E-1056
### Oncolytic reovirus restores hepatic stellate cells from an activated status to a quiescent status

[Speaker]Ikuho Ishigami (Grad. Sch. of Pharm. Sci., Osaka Univ.) [Co-author]Shunsuke Inoue (Grad. Sch. of Pharm. Sci., Osaka Univ.)、Yuki Kibe (Grad. Sch. of Pharm. Sci., Osaka Univ.)、Kanako Nakagawa (Grad. Sch. of Pharm. Sci., Osaka Univ.)、Hiroyuki Mizuguchi (Grad. Sch. of Pharm. Sci., Osaka Univ.／NIBIOHN／OTRI, Osaka Univ.／MEI center, Osaka Univ.)

Fibrosis, often caused by chronic inflammation and characterized by hyperaccumulation of extracellular matrix (ECM), results in severe organ failure and carcinogenesis. In liver fibrosis, activated hepatic stellate cells (HSCs) are the main producers of ECM. Recently, several studies revealed that activated HSCs showed plasticity which enables them to regulate a range of physiologic and pathologic responses of the liver.Here, we showed that mammalian orthoreovirus (reovirus), an oncolytic virus containing a 10-segmented double-stranded RNA genome, recovered expression profiles and functions of hepatic stellate cells from an activated state to a quiescent state. First, we examined the anti-fibrotic effects of reovirus on human hepatic stellate cell line, LX-2 cells. Reovirus significantly reduced the several fibrosis marker expressions without inducing apparent cytotoxicity. RNA-sequencing analysis suggested that the reovirus treatment restored the properties of LX-2 cells from an activated status to a quiescent status. The functions of LX-2 cells, including cell migration and lipid droplet storage, were also recovered by reovirus treatment.

[Disclosure of COI by Responsible researcher] Lead presenter is not the Responsible researcher.
[Lead presenter: Ikuho Ishigami] We have no financial relationships to disclose.
[Principal Researcher: Hiroyuki Mizuguchi] We have no financial relationships to disclose.

▲TOP

### [E-1057] I-E3-2
### Viruses & Cancer

2025 / 9 / 25 (Thu) 14:05-15:20 Room 9/(Hotel Nikko Kanazawa 3F Lumiere) Room 9

[Chairperson]Jun-ichirou Yasunaga (Dept. Hematol., Fac. Life Sci., Kumamoto Univ.)

---

### E-1057
### HDAC inhibitor SAHA suppresses the expression of viral microRNAs and induces apoptosis in EBV-infected cells

[Speaker]Hisashi Iizasa (Dept. Microbiol., Faclt Med., Shimane Univ.) [Co-author]Yuxin Liu (Dept. Microbiol., Faclt Med., Shimane Univ. ／UPMC Hillman Cancer Center, Univ. Pittsburgh.)、Shunpei Okada (Dept. Microbiol., Faclt Med., Shimane Univ.)

Epstein-Barr virus (EBV) has been associated with a variety of cancers, including Burkitt's lymphoma, natural killer/T lymphoma, and EBV-associated gastric cancer. However, drugs targeting EBV-positive tumors remain to be developed. Noncoding RNA BamHI A Rightward Transcript (BART) intron-encoded microRNAs (miRNAs) are expressed in all EBV-positive tumors and suppress lytic infection and host cell apoptosis. Suberoylanilide hydroxamic acid (SAHA), a histone deacetylase inhibitor, was identified as a repressor of BART promoter activity and miRNA transcription. SAHA significantly inhibited cell proliferation of EBV-positive cells in a p53-independent manner. It induced cytotoxicity in EBV-positive cells with lytic infection and also enhanced cell death in lytic-defective EBV-infected cells. SAHA reduced BART gene expression in EBV-positive cells by 85%, upregulated proapoptotic factors targeted by BART miRNA, and activated caspase-3. These results indicate that SAHA promotes cell death by suppressing BART miRNA transcription and activating the apoptotic program.

[Disclosure of COI by Responsible researcher] Lead presenter is the Responsible researcher.
[Lead presenter: Hisashi Iizasa] ■Research funds from company: 科学研究費

▲TOP

### [E-1058] I-E3-2
### Viruses & Cancer

2025 / 9 / 25 (Thu) 14:05-15:20 Room 9/(Hotel Nikko Kanazawa 3F Lumiere) Room 9

[Chairperson]Jun-ichirou Yasunaga (Dept. Hematol., Fac. Life Sci., Kumamoto Univ.)

---

### E-1058
### The type of HPV and the tissues it targets influence the level of malignancy.

[Speaker]Toshiyuki Sasagawa (Kanazawa Medical University, Department of Obstetrics and Gynecology) [Co-author]Mitsuaki Okodo (Kyorin University, Department of Medical Technology)、Kaori Okayama (Gumma Paz University, Health Science)、Jinichi Sakamoto (Kanazawa Medical University, Department of Obstetrics and Gynecology)、Takeo Shibata (Kanazawa Medical University, Department of Obstetrics and Gynecology)、Masahiro Takakura (Kanazawa Medical University, Department of Obstetrics and Gynecology)

To examine HPV type and its target tissue, 693 eligible women visiting for cervical cancer screening and colposcopy were analyzed. All with abnormal Pap results undertook a histological diagnosis under the colposcopy. HPV typing was performed using cervical cell samples and the tissue-microdissection procedure. Among them, 59.7% had a single lesion, but 40.3% had multiple lesions, including cervicitis/vaginitis, CIN, VaIN, CxCa, and VagCa. Multiple HPV types were detected in 50.6%, a single HPV type in 42.8%, and negative in 6.6% in 1034 lesions, suggesting that more than half were infected with HPV in the cervix and vagina. As a single HPV type, HPV16, 31, 52, 58, 67 (alpha-9), HPV18, 39 (alpha-7), HPV69, 82 (alpha-5) were identified in CxCa, and HPV45, 68 (alpha-7), 53 (alpha-6) were in VagCa. HPV16 and 18 were more frequently identified in CIN3 than in CIN1, while HPV31, 39, 52, and 58 were almost equivalent between CIN1 and CIN3. HPV16 and 18 can induce cancer directly from CIN2/3 located in the squamocolumnar junction (SCJ) or the gland. The other oncogenic types infect the SCJ and the ectocervix. HPV53, 67, 69, and 82 are new oncogenic types.

[Disclosure of COI by Responsible researcher] Lead presenter is the Responsible researcher.
[Lead presenter: Toshiyuki Sasagawa] We have no financial relationships to disclose.

▲TOP

### [E-1059] I-E3-2
### Viruses & Cancer

2025 / 9 / 25 (Thu) 14:05-15:20 Room 9/(Hotel Nikko Kanazawa 3F Lumiere) Room 9

[Chairperson]Jun-ichirou Yasunaga (Dept. Hematol., Fac. Life Sci., Kumamoto Univ.)

---

### E-1059
### Breaking Away from Low HPV Vaccination Rates: Reconstructing Cervical Cancer Prevention on Epidemiological Surveys

[Speaker]Ryo Konno (Dept. Obstet and Gynecol, Jichi Med Univ Saitama Med Center)

Japan's high cervical cancer incidence requires improved HPV vaccination rates hindered by past recommendation pauses. This study uses extensive data to analyze the impact on prevention and propose solutions. Objectives: Assess HPV vaccine effectiveness (Japan Cancer Society-DB). Examine governmental intervention's impact on vaccination/disease rates. Clarify public/local understanding and intentions. Analyze information sharing and post-vaccination consultations. Project hesitancy's long-term impact (vs. Lancet Public Health 2020). Evaluate program effectiveness. Propose dissemination strategies and a registry framework. Recommend provider responses. Methods: Population-based epidemiology (330k), longitudinal HPV evaluation, hesitancy modeling, awareness surveys, intention data, consultation reviews. Outcomes: Quantify vaccine effectiveness; stagnation impact; public awareness; collaboration; assess consultations. Project incidence; outline dissemination; indicate registry direction. Conclusion: This analysis proposes evidence-based countermeasures to improve Japan's low HPV vaccination rates and reduce cervical cancer incidence, aiming to enhance public health and inform policy.

[Disclosure of COI by Responsible researcher] Lead presenter is the Responsible researcher.
[Lead presenter: Ryo Konno] ■Lecture fee: MSD　■Contributions: 中外製薬

▲TOP

### [E-1060] I-E3-2
### Viruses & Cancer

2025 / 9 / 25 (Thu) 14:05-15:20 Room 9/(Hotel Nikko Kanazawa 3F Lumiere) Room 9

[Chairperson]Jun-ichirou Yasunaga (Dept. Hematol., Fac. Life Sci., Kumamoto Univ.)

---

### E-1060
### The nucleolar protein, fibrillarin is essential for the production of Epstein-Barr virus

[Speaker]Atsuko Sugimoto (Dept. Virol., Nagoya Univ. Grad. Sch. Med.) [Co-author]Yuta Onuma (Dept. Virol., Nagoya Univ. Grad. Sch. Med.)、Tomoharu Ueta (Dept. Virol., Nagoya Univ. Sch. Med.)、Emi S. Mishiro (WPI-ITbM Mol. Structure Ctr., Nagoya Univ.)、Yuichi Abe (Immunoproteomics lab., iGCORE, Gifu Univ)、Yoshitaka Sato (Dept. Virol., Nagoya Univ. Grad. Sch. Med.)、Takayuki Murata (Dept. Virol., Fujita Health Univ. Sch. Med.)、Hiroshi Kimura (Dept. Virol., Nagoya Univ. Grad. Sch. Med.)

Epstein-Barr virus (EBV) is known as the human tumor virus. In EBV viral productive infection, called lytic infection, EBV expresses its genes in a well-coordinated manner, amplifies its viral genome in the nuclear replication compartments, and produces progeny virions. Recent studies reported that EBV lytic replication is also important for EBV-mediated oncogenesis, regulating oncogenic pathways and producing viral infectious particles. In our previous report, we performed electron microscopic analysis of primary B cells infected with wild-type EBV, that indicated that the nuclei were enlarged. We identified that this EBV-induced nucleolar enlargement was caused by the induction of IMPDH2, which was essential for B cell growth transformation by EBV (Sugimoto et al., 2023). Here, we demonstrated that enlarged nucleoli in EBV-infected cells regulated EBV lytic replication. Some EBV lytic gene products were located in nucleoli and interacted with one of the nucleolar proteins, fibrillarin. Knocking down of fibrillarin reduced the formation of the EBV replication compartment. Therefore, these results suggest that EBV regulates nucleoli for their transformation and lytic replication.

[Disclosure of COI by Responsible researcher] Lead presenter is not the Responsible researcher.
[Lead presenter: Atsuko Sugimoto] We have no financial relationships to disclose.
[Principal Researcher: Hiroshi Kimura] We have no financial relationships to disclose.

### [J-1073] II-J7
### Gynecologic cancer (2)

2025 / 9 / 25 (Thu) 14:05-15:20 Room 14/(ANA Crowne Plaza Hotel Kanazawa 3F Zuiun 1) Room 14

[Chairperson]Kiyoko Kato (Dept.Ob＆Gy ,Kyushu Univ. Sch..Med.)

---

### J-1073
### LRRK2 is a favorable prognostic factor inducing apoptosis in ovarian clear cell carcinoma

[Speaker]Toshihide Matsumoto (Department of Pathology, School of Allied Health Sciences, Kitasato University) [Co-author]Momoko Kusabuka (Department of Pathology, School of Allied Health Sciences, Kitasato University)、Yuka Ito (Department of Pathology, School of Allied Health Sciences, Kitasato University)、Ryo Konno (Department of Applied Genomics, Kazusa DNA Research Institute)、Yusuke Kawashima (Department of Applied Genomics, Kazusa DNA Research Institute)、Kazuyoshi Kato (Department of Gynecology, School of Medicine, Kitasato University)、Makoto Saegusa (Department of Pathology, School of Medicine, Kitasato University)、Hiroyuki Takahashi (Department of Pathology, School of Allied Health Sciences, Kitasato University)

<**Background**> Ovarian clear cell carcinoma (OCCC), often arising from endometriosis, is more common in Japan than in Western countries. LRRK2, a gene associated with Parkinson´s disease, has been observed in solid tumors, but its role in OCCC is unclear. This study investigated LRRK2 expression and clinical relevance in OCCC. <**Results**> LRRK2 showed cytoplasmic expression in OCCC cells and was strongly positive in 38 cases (46.6%). High LRRK2 expression correlated with metastasis, peritoneal spread, and better prognosis in OCCC. LRRK2 mRNA and protein levels were elevated without gene mutations. LRRK2 was highly expressed in tumor-adjacent endometriosis but low in benign cases. LRRK2-overexpression promoted apoptosis and cleaved caspase-3, while knockdown reduced apoptosis and CDDP sensitivity. TUNEL-positive cells also decreased with knockdown. Proteomics showed reduced apoptosis pathways in knockdown cells. IL-6 and pStat3 were elevated in LRRK2-high tumors, and IL-6 induced LRRK2/pStat3 expression dose-dependently. <**Conclusion**> LRRK2 is upregulated in OCCC via IL-6/Stat3 signaling and enhances apoptosis. It may serve as a favorable prognostic biomarker and therapeutic target.

[Disclosure of COI by Responsible researcher] Lead presenter is the Responsible researcher.
[Lead presenter: Toshihide Matsumoto] ■Research funds from company: 株式会社デ・ウエスタン・セラピテクス研究所

### [J-1074] II-J7
### Gynecologic cancer (2)

2025 / 9 / 25 (Thu) 14:05-15:20 Room 14/(ANA Crowne Plaza Hotel Kanazawa 3F Zuiun 1) Room 14

[Chairperson]Kiyoko Kato (Dept.Ob＆Gy ,Kyushu Univ. Sch..Med.)

---

### J-1074
### Human leucocyte antigen in gynecologic cancer before and after heavy-ion radiotherapy

[Speaker]Sumitaka Hasegawa (QST) [Co-author]Nakako Nakajima (QST)

Human leukocyte antigen (HLA) is a major histocompatibility complex (MHC) class I molecule in human and is critical for anti-tumor immunity in the host. To investigate the impact of heavy-ion irradiation on anti-tumor immune responses in gynecologic cancers, we conducted immunohistochemical analyses to evaluate protein expression of HLA-I on cell surface of clinical samples from the patients of uterine cervical adeno/adenocarcinoma (UCAA) that underwent heavy-ion radiotherapy (HIRT). Of the 55 UCAA patients, 31 who underwent pre-HIRT and post-12 Gy-HIRT tumor biopsies were used for this study. All clinical samples were obtained from a single center. Of 31 samples examined, 93% of the samples were positive for HPV. Pre-HIRT HLA-I positive (over 50% of cells) was 4 and partial positive (5-50% of cells) was 13 samples. In contrast, post-HIRT HLA-I positive was 14 and partial positive was 10 samples. This study demonstrated that HIRT can restore immune recognition in UCAA cells, warranting favorable combination with immunotherapy. This abstract is an encore abstract.

[Disclosure of COI by Responsible researcher] Lead presenter is the Responsible researcher.
[Lead presenter: Sumitaka Hasegawa] We have no financial relationships to disclose.

▲TOP

### [J-1075] II-J7
### Gynecologic cancer (2)

2025 / 9 / 25 (Thu) 14:05-15:20 Room 14/(ANA Crowne Plaza Hotel Kanazawa 3F Zuiun 1) Room 14

[Chairperson]Kiyoko Kato (Dept.Ob＆Gy ,Kyushu Univ. Sch..Med.)

---

### J-1075
### Antibody-drug conjugate targeting CADM1/TSLC1 for treatment of early-stage endometrioid carcinoma

[Speaker]Man Hagiyama (Dept. Pathol., Fac. Med., Kindai Univ.) [Co-author]Azusa Yoneshige (Dept. Pathol., Fac. Med., Kindai Univ.)、Fuka Takeuchi (Dept. Pathol., Fac. Med., Kindai Univ.)、Akihiko Ito (Dept. Pathol., Fac. Med., Kindai Univ.)

Cell adhesion molecule 1 (CADM1), an IgCAM member, is expressed in endometrial glandular cells highly in the proliferative phase but lowly in the secretory phase. Previously, we generated a humanized anti-CADM1 ectodomain antibody conjugated with monomethyl auristatin E (h3E1-MMAE ADC) for mesothelioma treatment. The present study aimed at probing whether this ADC could be useful for the treatment of endometrial neoplasm. Firstly, immunohistochemistry for CADM1 was conducted on proliferative-phase endometrium (n = 13), endometrial hyperplasia (n = 35), and endometrioid carcinoma at various stages (n = 166). CADM1 protein level was highest in atypical endometrial hyperplasia and endometrioid carcinoma confined within the endometrium and decreased stepwise as the carcinoma stage progressed. Next, h3E1-MMAE ADC was examined for its cytotoxicity in vitro using human endometrial adenocarcinoma cell lines expressing CADM1; HEC-1B, HEC-50B, JHUM-3, and OMC-2. The ADC killed these cells in a dose-dependent manner, with IC50 = 12.02 nM for HEC-1B, 2.04 nM for HEC-50B. Collectively, h3E1-MMAE ADC could potentially be an option in noninvasive treatment for early-stage endometrioid carcinoma.

[Disclosure of COI by Responsible researcher] Lead presenter is not the Responsible researcher.
[Lead presenter: Man Hagiyama] We have no financial relationships to disclose.
[Principal Researcher: Akihiko Ito] We have no financial relationships to disclose.

▲TOP

### [J-1076] II-J7
### Gynecologic cancer (2)

2025 / 9 / 25 (Thu) 14:05-15:20 Room 14/(ANA Crowne Plaza Hotel Kanazawa 3F Zuiun 1) Room 14

[Chairperson]Kiyoko Kato (Dept.Ob＆Gy ,Kyushu Univ. Sch..Med.)

### J-1076
### Exploration of new treatment for ovarian high-grade serous cancer with ***cyclin E1*** amplification.

[Speaker]Mio Takahashi (Dept. of Obstetrics & Gynecology, Keio University School of Medicine) [Co-author]Tatsuyuki Chiyoda (Dept. of Obstetrics & Gynecology, Keio University School of Medicine)、Yumiko Kimura (Dept. of Obstetrics & Gynecology, Keio University School of Medicine)、Daisuke Ochiai (Dept. of Obstetrics & Gynecology, Keio University School of Medicine)、Mitsuyo Jisaka (Dept. of Obstetrics & Gynecology, Keio University School of Medicine)、Tomomi Sakamaki (Dept. of Obstetrics & Gynecology, Keio University School of Medicine)、Shinya Oki (Dept. of Obstetrics & Gynecology, Keio University School of Medicine)、Tomoko Yoshihama (Dept. of Obstetrics & Gynecology, Keio University School of Medicine)、Kensuke Sakai (Dept. of Obstetrics & Gynecology, Keio University School of Medicine)、Aki Ookubo (JKiC)、Manabu Itoh (JKiC)、Wataru Yamagami (Dept. of Obstetrics & Gynecology, Keio University School of Medicine)

BACKGROUND
Ovarian high-grade serous cancer (HGSC), especially homologous recombination proficient (HRP) tumor has a poor prognosis. We investigated therapeutic options for HGSCs with ***cyclin E1*** (***CCNE1***) amplification, a major genomic feature of HRP, using patient-derived organoids.
METHODS
High-throughput drug screening for ***CCNE1***-amplified HGSC organoids was performed using a library of 4,560 compounds, and several drugs were selected as candidates. The expression of molecules related to the therapeutic targets of the candidates was examined by immunohistochemistry (IHC) in excised tumor specimens. Animal experiment was conducted to verify efficacy and safety in vivo.
RESULTS
PLK1 inhibitors were selected as drug candidates. They showed dose-dependent anti-proliferative activity in HRP organoids. In IHC, expression of PLK1 and CCNE1 were correlated, and PLK1-positive group had significantly worse overall survival than the negative group ($p<0.05$). In the animal experiment, the diameters of the implanted organoids was significantly smaller in the treatment group than in the control group ($p<0.01$).
CONCLUSIONS
PLK1 inhibitors can be a therapeutic strategy for ***CCNE1***-amplified HGSC.

[Disclosure of COI by Responsible researcher] Lead presenter is not the Responsible researcher.
[Lead presenter: Mio Takahashi] We have no financial relationships to disclose.
[Principal Researcher: Tatsuyuki Chiyoda] ■Research expenses: 武田薬品工業株式会社

▲TOP

### [J-1077] II-J7
### Gynecologic cancer (2)

2025 / 9 / 25 (Thu) 14:05-15:20 Room 14/(ANA Crowne Plaza Hotel Kanazawa 3F Zuiun 1) Room 14

[Chairperson]Kiyoko Kato (Dept.Ob＆Gy ,Kyushu Univ. Sch..Med.)

---

### J-1077
### Significance of Molecular Residual Disease (MRD) in Gestational Trophoblastic Neoplasia

[Speaker]Hirokazu Usui (Chiba Cancer Center Dept. Gynecology ／Chiba Univ. Grad. Sch. Med. Dept. Obstetrics and Gynecology ／Chiba Univ. Hosp. Dept. Obstetrics and Gynecology) [Co-author]Eri Katayama (Chiba Univ. Hosp. Dept. Obstetrics and Gynecology)、Natsuko Nakamura (Chiba Univ. Hosp. Dept. Obstetrics and Gynecology)、Nozomi Sakai (Chiba Univ. Hosp. Dept. Obstetrics and Gynecology)、Satoyo Otsuka (Chiba Univ. Hosp. Dept. Obstetrics and Gynecology)、Yuji Habu (Chiba Univ. Hosp. Dept. Obstetrics and Gynecology)、Rie Okuya (Chiba Univ. Hosp. Dept. Obstetrics and Gynecology)、Kyoko Nishikimi (Chiba Univ. Hosp. Dept. Obstetrics and Gynecology)、Shinichi Tate (Chiba Univ. Grad. Sch. Med. Dept. Obstetrics and Gynecology ／Chiba Univ. Hosp. Dept. Obstetrics and Gynecology)、Kaori Koga (Chiba Univ. Grad. Sch. Med. Dept. Obstetrics and Gynecology ／Chiba Univ. Hosp. Dept. Obstetrics and Gynecology)

BackgroundHuman chorionic gonadotropin (hCG) serves as a valuable serum marker for treatment response assessment in gestational trophoblastic neoplasia (GTN), though additional treatment remains necessary even when hCG levels fall below detection limits. Recent studies have demonstrated molecular residual disease (MRD) assessment utility in predicting recurrence. We aimed to develop a ctDNA-based SNP detection system for GTN management.MethodsWe selected SNP loci with ~0.5 minor allele frequencies in Japanese population and designed HRM primers. Control genomes validated primers for SNP genotype distinction. Amplicon sequencing quantified ctDNA compared to serum hCG levels. STR analysis was conducted in placental site trophoblastic tumor (n=1) and choriocarcinoma (n=3).ResultsMRD evaluation was performed on 10 ctDNA samples from four patients. Tumor-derived SNPs were detected in six samples from three patients. Four samples showed no detection when hCG levels exceeded cutoff threshold.ConclusionsWe established a ctDNA detection system for GTN that suggests ctDNA analysis does not demonstrate superior sensitivity compared to serum hCG in GTN monitoring.

[Disclosure of COI by Responsible researcher] Lead presenter is the Responsible researcher.
[Lead presenter: Hirokazu Usui] ■Research funds from company: JSPS KAKENHI 22H03220 and 23K24479

▲TOP

### [J-1078] II-J7
### Gynecologic cancer (2)

2025 / 9 / 25 (Thu) 14:05-15:20 Room 14/(ANA Crowne Plaza Hotel Kanazawa 3F Zuiun 1) Room 14

[Chairperson]Kiyoko Kato (Dept.Ob＆Gy ,Kyushu Univ. Sch..Med.)

---

### J-1078
### Unveiling the Th17-IL17 immune axis in ovarian clear cell carcinoma: Implications for immunotherapy

[Speaker]Kosuke Murakami (Department of Obstetrics and Gynecology, Kindai University Faculty of Medicine) [Co-author]Shiki Takamura (Laboratory for Immunological Memory, RIKEN Center for Integrative Medical Sciences)、Chiho Miyagawa (Department of Obstetrics and Gynecology, Kindai University Faculty of Medicine)、Shiro Takamatsu (Department of Obstetrics and Gynecology, Kindai University Faculty of Medicine)、Yoko Kashima (Department of Immunology, Kindai University Faculty of Medicine)、Yukari Kobayashi (Department of Immunology, Kindai University Faculty of Medicine)、Koji Nagaoka (Department of Immunology, Kindai University Faculty of Medicine)、Kazuhiro Kakimi (Department of Immunology, Kindai University Faculty of Medicine)、Noriomi Matsumura (Department of Obstetrics and Gynecology, Kindai University Faculty of Medicine)

Ovarian clear cell carcinoma (OCCC) is suggested to be responsive to immune checkpoint inhibitors (ICIs), although the mechanisms remain largely unknown. In this study, we analyzed the tumor immune microenvironment of OCCC using human samples and mouse models to investigate its association with ICI efficacy. RNA sequencing of OCCC cases from our institution and the JGOG3025-TR1 cohort revealed that Th17-related gene expression was enriched and positively correlated with the OCCC-specific gene signature. In the JGOG3025 cohort, a subset of OCCC exhibited an IL17-high, immune-hot phenotype independent of established biomarkers such as dMMR, MSI-high, and TMB-high. In vitro, IL17 stimulation of murine OCCC cells promoted tumor growth and increased immunogenic cytokine secretion. In vivo, IL17 administration increased infiltration of exhausted CD8+ and CD4+ T cells, and upregulated NF-kB expression. Furthermore, adoptive Th17 cell transfer elevated systemic IL17 levels, and anti-PD-L1 treatment in this context significantly prolonged survival. These findings suggest that the Th17-IL17 signature may characterize OCCC and enhance ICI efficacy.

[Disclosure of COI by Responsible researcher] Lead presenter is not the Responsible researcher.
[Lead presenter: Kosuke Murakami] ■Research funds from company: 野澤記念研究助成金
[Principal Researcher: Noriomi Matsumura] ■Advisor of the company and/or profit-making organization: タカラバイオ　■Lecture fee: 武田薬品工業、アストラゼネカ

### [J-1085] I-J11-2
### Tumor immune microenvironment/Tumor immune escape (2)

2025 / 9 / 25 (Thu) 14:05-15:20 Room 15/(ANA Crowne Plaza Hotel Kanazawa 3F Zuiun 2) Room 15

[Chairperson]Yasuyuki Saito (Dept. Immunol., Fac. Med., Shimane Univ.)

---

### J-1085
### The influence of astrocytes on the immune response of microglia in the microenvironment of metastatic brain tumors

[Speaker]Hiromi Satoi (Clinical Pharmacology and Pharmacometrics, Pharmaceuti. Sci., Chiba Univ.) [Co-author]Keitaro Sato (Clinical Pharmacology and Pharmacometrics, Pharmaceuti. Sci., Chiba Univ.)、Haruka Takemoto (Clinical Pharmacology and Pharmacometrics, Pharmaceuti. Sci., Chiba Univ.)、Mao Watanabe (Clinical Pharmacology and Pharmacometrics, Pharmaceuti. Sci., Chiba Univ.)、Yui Iida (Clinical Pharmacology and Pharmacometrics, Pharmaceuti. Sci., Chiba Univ.)、Iori Kojima (Neurological Surgery, Medicine, Chiba Univ.)、Yoshinori Higuchi (Neurological Surgery, Medicine, Chiba Univ.)、Akihiro Hisaka (Clinical Pharmacology and Pharmacometrics, Pharmaceuti. Sci., Chiba Univ.)

Although astrocytes are known to be involved in the progression of brain metastasis (BrM), their precise role in the formation of the immune microenvironment is not yet fully understood. We found that astrocytes that have received 2'3'-cyclic GMP-AMP (cGAMP), a second messenger of the innate immune response, from cancer cells secrete CCL5, a chemokine that regulates the immune response. Using a mouse melanoma-BrM model and in vitro assays, we found that microglia express the CCL5 receptor CCR5 and that CCL5 promotes M2-microglia migration. Furthermore, when M1 microglia treated with CCL5 were co-cultured with melanoma cells, the cytotoxic effects of M1 microglia were reduced and the survival of the melanoma cells was restored. Importantly, we observed CCR5-expressing cells in areas of human BrM tumor samples with highly microglia infiltration, which supports the clinical relevance of our findings. These results suggest that CCL5 derived from astrocytes contributes to the formation of an immunosuppressive microenvironment via CCR5-expressing microglia and promotes tumor progression in the BrM. Targeting the CCL5-CCR5 axis may provide a new therapeutic strategy for brain metastasis.

[Disclosure of COI by Responsible researcher] Lead presenter is the Responsible researcher.
[Lead presenter: Hiromi Satoi] ■Research funds from company: 科学研究費　基盤研究C

### [J-1086] I-J11-2
### Tumor immune microenvironment/Tumor immune escape (2)

2025 / 9 / 25 (Thu) 14:05-15:20 Room 15/(ANA Crowne Plaza Hotel Kanazawa 3F Zuiun 2) Room 15

[Chairperson]Yasuyuki Saito (Dept. Immunol., Fac. Med., Shimane Univ.)

---

### J-1086
### Myeloid heterogeneity in bone metastatic microenvironment

[Speaker]Kyoko Hashimoto (Dept. of Immunol., Grad. Sch. of Med., Univ. Tokyo) [Co-author]Kazuo Okamoto (Div. of Immune Environment Dynamics, CRI, Kanazawa Univ.)、Hiroshi Takayanagi (Dept. of Immunol., Grad. Sch. of Med., Univ. Tokyo)

Objective: Bone metastases are common in patients with certain solid tumors such as breast cancer, and significantly impact their quality of life and prognosis. While the bone marrow is the primary lymphoid tissue harboring a variety of immune cells, the immune microenvironment in bone metastases remains poorly understood. This study aimed to characterize the immune landscape in bone metastases.Methods: Utilizing the E0771 mouse breast cancer model, bone metastases and orthotopic tumor tissue were analyzed by immunohistochemistry and flow cytometry. CD11b-positive cells from both sites were isolated and analyzed by single-cell RNA sequencing.Results: Bone metastases exhibited significantly fewer lymphocytes, whereas CD11b-positive cells were enriched in bone metastases. Single-cell RNA sequencing revealed diverse myeloid cells, including neutrophils, monocytes, macrophages and osteoclasts, with distinct gene expression profile from normal bone marrow and orthotopic tumor.Conclusion: This study demonstrates a unique myeloid-dominant immune microenvironment in bone metastases. Targeting these myeloid populations may offer a novel therapeutic strategy for treating bone metastases.

[Disclosure of COI by Responsible researcher] Lead presenter is not the Responsible researcher.
[Lead presenter: Kyoko Hashimoto] ■Contributions: 武田科学振興財団　医学系研究助成
[Principal Researcher: Hiroshi Takayanagi] ■Lecture fee: エーザイ、ギリアド　■Research expenses: 櫻泉株式会社
■Contributions: 公益財団法人　セコム科学技術振興財団

▲TOP

### [J-1087] I-J11-2
### Tumor immune microenvironment/Tumor immune escape (2)

2025 / 9 / 25 (Thu) 14:05-15:20 Room 15/(ANA Crowne Plaza Hotel Kanazawa 3F Zuiun 2) Room 15

[Chairperson]Yasuyuki Saito (Dept. Immunol., Fac. Med., Shimane Univ.)

---

### J-1087
### Caloric restriction ameliorates aging and tumor progression via tumor immunity

[Speaker]Taichi Horino (Dept. of Molecular Genetics, Kumamoto Univ.／Dept. of Gastroenterological Surgery, Kumamoto Univ.) [Co-author]Haruki Horiguchi (Dept. of Molecular Genetics, Kumamoto Univ.)、Yuji Miyamoto (Dept. of Gastroenterological Surgery, Kumamoto Univ.)、Masaaki Iwatsuki (Dept. of Gastroenterological Surgery, Kumamoto Univ.)、Yuichi Oike (Dept. of Molecular Genetics, Kumamoto Univ.)

Caloric restriction (CR) without malnutrition reportedly extends lifespan and mitigates aging. Moreover, CR has been suggested to reduce cancer incidence and inhibit tumor growth, although the precise mechanisms remain unclear. In this study, we established a syngeneic tumor model using C57BL/6N male mice to investigate the tumor-suppressive effects of CR. Mice were randomized into ad libitum (AL) and CR groups (30% calorie reduction). CR significantly suppressed tumor growth in aged mice, modestly affecting younger mice. Additionally, intratumoral CD4+ and CD8+ T cells were notably increased in the CR group compared to the AL group. CD8+ T cell depletion reversed the tumor-suppressive effects observed under CR, highlighting the crucial role of CD8+ T cells in mediating this response. Furthermore, anti-PD-1 antibody treatment exhibited equivalent tumor-suppressive efficacy in both dietary conditions. These results suggest that CR enhances antitumor immunity, particularly via CD8+ T cell activity, contributing significantly to its tumor-suppressive effects. Further comprehensive analyses are underway to clarify the immunological mechanisms underlying CR-induced tumor suppression.

[Disclosure of COI by Responsible researcher] Lead presenter is not the Responsible researcher.
[Lead presenter: Taichi Horino] We have no financial relationships to disclose.
[Principal Researcher: Yuichi Oike] We have no financial relationships to disclose.

▲TOP

### [J-1088] I-J11-2
### Tumor immune microenvironment/Tumor immune escape (2)

2025 / 9 / 25 (Thu) 14:05-15:20 Room 15/(ANA Crowne Plaza Hotel Kanazawa 3F Zuiun 2) Room 15

[Chairperson]Yasuyuki Saito (Dept. Immunol., Fac. Med., Shimane Univ.)

---

### J-1088
### TIL Composition in Colorectal Cancer Liver Metastases: Comparison with Colorectal Cancer and Primary Liver Cancers

[Speaker]Shu Aoyama (Department of Gastroenterological Surgery, the University of Osaka ／Department of Clinical Tumor Immunology, the University of Osaka) [Co-author]Takehiro Noda (Department of Gastroenterological Surgery, the University of Osaka)、Azumi Ueyama (Shionogi & Co., Ltd.)、Hirofumi Akita (Department of Gastroenterological Surgery, the University of Osaka)、Yosuke Mukai (Department of Gastroenterological Surgery, the University of Osaka)、Kazuki Sasaki (Department of Gastroenterological Surgery, the University of Osaka)、Shinichiro Hasegawa (Department of Gastroenterological Surgery, the University of Osaka)、Daisaku Yamada (Department of Gastroenterological Surgery, the University of Osaka)、Yoshito Tomimaru (Department of Gastroenterological Surgery, the University of Osaka)、Hidenori Takahashi (Department of Gastroenterological Surgery, the University of Osaka)、Shogo Kobayashi (Department of Gastroenterological Surgery, the University of Osaka)、Mamoru Uemura (Department of Gastroenterological Surgery, the University of Osaka)、Takuro Saito (Department of Gastroenterological Surgery, the University of Osaka ／Department of Clinical Tumor Immunology, the University of Osaka)、Hisashi Wada (Department of Gastroenterological Surgery, the University of Osaka ／Department of Clinical Tumor Immunology, the University of Osaka)、Yuichiro Doki (Department of Gastroenterological Surgery, the University of Osaka)、Hidetoshi Eguchi (Department of Gastroenterological Surgery, the University of Osaka)

Background: Tumor-infiltrating lymphocytes (TILs) play key roles in tumor microenvironment and influence cancer progression. However, the characteristics in colorectal cancer liver metastases (CRLM) remain unclear. Methods: TILs were analyzed by flow cytometry using fresh tumor tissues from colorectal cancer (CRC), CRLM, hepatocellular carcinoma (HCC), and intrahepatic cholangiocarcinoma (ICC) cases resected between April 2021 and December 2024.Results: We analyzed 17 CRLM, 55 CRC, 40 HCC, and 2 ICC cases. Uniform manifold approximation and projection of TIL subsets demonstrated that CRLM and primary liver cancers (HCC and ICC) formed a shared cluster, separate from CRC. Compared to CRC, liver tumors (CRLM, HCC/ICC) exhibited higher proportions of $CD8^{+}$ T cells, lower frequencies of Tim-$3^{+}$ $CD8^{+}$ T cells, and fewer regulatory T cells (all $p^{+}0.001$). These patterns were consistent in CRLM vs. CRC and HCC vs. CRC. In HCC, $CD8^{+}$ T-cell exhaustion markers (PD-1, Tim-3) increased with tumor size (r = 0.41, 0.46), but not in CRLM.Conclusion: CRLM resembled primary liver cancers in TIL composition rather than CRC, but the trajectory of T-cell exhaustion may differ from HCC.

[Disclosure of COI by Responsible researcher] Lead presenter is the Responsible researcher.
[Lead presenter: Shu Aoyama] ■Endowed chair: 塩野義製薬株式会社

▲TOP

### [J-1089] I-J11-2
### Tumor immune microenvironment/Tumor immune escape (2)

2025 / 9 / 25 (Thu) 14:05-15:20 Room 15/(ANA Crowne Plaza Hotel Kanazawa 3F Zuiun 2) Room 15

[Chairperson]Yasuyuki Saito (Dept. Immunol., Fac. Med., Shimane Univ.)

---

### J-1089
### Inhibition of C5a-C5aR1 axis suppresses tumor progression with antitumor immunity in pancreatic ductal adenocarcinoma.

[Speaker]Shigetsugu Takano (Dept. General Surg., Sch., Med., Chiba Univ.) [Co-author]Ryotaro Eto (Dept. General Surg., Sch., Med., Chiba Univ.)、Daren Zhou (Dept. General Surg., Sch., Med., Chiba Univ.)、Kensuke Suzuki (Dept. General Surg., Sch., Med., Chiba Univ.)、Takanori Konishi (Dept. General Surg., Sch., Med., Chiba Univ.)、Masayuki Ohtsuka (Dept. General Surg., Sch., Med., Chiba Univ.)

Complement factors regulate tumor immunity in the tumor microenvironment (TME). We investigated the functions of complement 5a (C5a) and its receptor, C5aR1 in forming the C5a-C5aR1 signaling axis in the immune TME of pancreatic ductal adenocarcinoma (PDAC). C5a and C5aR1 were assessed in cancer cell cytoplasmic (c-) and stromal (s-) expressions in resected PDAC tissues. In vitro assays were conducted to examine endogenous C5aR1 functions in PDAC cells, and orthotopic transplantation was performed in a preclinical study. In IHC, High C5a-C5aR1 c-axis was correlated with poor prognosis and High C5a-C5aR1 s-axis was associated with a decrease in $CD8^+$ T cells and an increase in $CD11b^+$ MDSCs. C5aR1 knockdown and CCX168, the specific C5aR1 inhibitor, impaired proliferation and enhanced gemcitabine sensitivity by increasing apoptosis. The combination of CCX168 and GnP demonstrated a significant reduction in tumor volume. The number of $CD8^+$ T cells was significantly increased in CCX168-treated groups, whereas CCX168 treatment resulted in a decrease in MDSCs. CCX168 appears to modulate antitumor immunity, thereby warranting future complement-based immunomodulation therapies for PDAC.

[Disclosure of COI by Responsible researcher] Lead presenter is the Responsible researcher.
[Lead presenter: Shigetsugu Takano] ■Research expenses: デンカ株式会社

▲TOP

### [J-1090] I-J11-2
### Tumor immune microenvironment/Tumor immune escape (2)

2025 / 9 / 25 (Thu) 14:05-15:20 Room 15/(ANA Crowne Plaza Hotel Kanazawa 3F Zuiun 2) Room 15

[Chairperson]Yasuyuki Saito (Dept. Immunol., Fac. Med., Shimane Univ.)

---

### J-1090
### Identification of the mechanisms underlying cancer immune exclusion by extracellular matrix component analysis

[Speaker]Atsushi Mitsuhashi (Department of Respiratory Medicine and Rheumatology, Tokushima University) [Co-author]Aito Yoshida (Department of Respiratory Medicine and Rheumatology, Tokushima University)、Hirokazu Ogino (Department of Respiratory Medicine and Rheumatology, Tokushima University)、Ryohiko Ozaki (Department of Respiratory Medicine and Rheumatology, Tokushima University)、Yuki Tsukazaki (Department of Respiratory Medicine and Rheumatology, Tokushima University)、Yutaka Morita (Department of Respiratory Medicine and Rheumatology, Tokushima University)、Bayarmaa Agarzandan (Department of Respiratory Medicine and Rheumatology, Tokushima University)、Rikako Matsumoto (Department of Respiratory Medicine and Rheumatology, Tokushima University)、Hiroshi Nokihara (Center Hospital of National Center for Global Health and Medicine)、Masaki Hanibuchi (Departments of Community Medicine for Respirology, Tokushima University)、Yasuhiko Nishioka (Department of Respiratory Medicine and Rheumatology, Tokushima University)

Tumor immune exclusion is known to induce the resistance to immune checkpoint inhibitors (ICIs), in which tumor stromal cells and extracellular matrix (ECM) inhibited the lymphocyte infiltration. However, the key ECM components forming this barrier have been still unknown. In this study, we analyzed ECM components in decellularized ICI-resistant tumors to investigate the mechanisms of immune exclusion. In mouse subcutaneous tumor mode, ICIs-resistant tumor tissue showed a decrease in CD8 T-cell infiltration and a high-density ECM structure. Mass spectrometry analysis of decellularized tumor tissue identified several ECM components upregulated in ICI-resistant tissues. Among them, we focused on the collagen VI as a key ECM component forming immune exclusion. The transfection of collagen VI into the lung cancer cell induced the resistance to anti-PD-L1 antibody in vivo. Furthermore, high collagen VI expression was strongly associated with poor postoperative prognosis and pathological features as immune exclusion in surgically resected lung adenocarcinoma cases. These findings suggest that collagen VI play the pivotal roles in tumor immune exclusion and ICI resistance.

[Disclosure of COI by Responsible researcher] Lead presenter is the Responsible researcher.
[Lead presenter: Atsushi Mitsuhashi] ■Research expenses: 大鵬薬品工業

### [E-1091] I-E9
### Cellular plasticity & heterogeneity in metastasis

2025 / 9 / 25 (Thu) 14:05-15:20 Room 16/(11F Visage) Room 16

[Chairperson]Tsunaki Hongu (Div. Cancer Cell Biol., Cancer. Res. Inst., Kanazawa Univ.)

---

### E-1091
### Histamine-Producing Mast Cells Mediate Resistance to mTOR Inhibitors in Invasive Colorectal Cancer

[Speaker]Yuwen Liu (Div. Pathophysiol., Aichi Cancer Ctr. Res. Inst.／Div. Cancer Physiol., Nagoya Univ. Grad. Sch. Med.) [Co-author]Teruaki Fujishita (Div. Pathophysiol., Aichi Cancer Ctr. Res. Inst.)、Yasushi Kojima (Div. Pathophysiol., Aichi Cancer Ctr. Res. Inst.)、Rie Kajino (Div. Pathophysiol., Aichi Cancer Ctr. Res. Inst.)、Emi Mishiro (Inst. Trans.Bio-Mol., Nagoya Univ.)、Tomoyoshi Soga (Inst. Adv. Bios. Keio Univ.)、Makoto M. Taketo (Pers. Canc. Ther., MIC, Kyoto Univ. Grad. Sch. Med.)、Masahiro Aoki (Div. Pathophysiol., Aichi Cancer Ctr. Res. Inst.／Div. Cancer Physiol., Nagoya Univ. Grad. Sch. Med.)

The mTOR pathway regulates cell growth and proliferation by controlling cellular metabolism, including protein and lipid synthesis. Although this pathway is frequently activated in various cancers, clinical responses to mTOR inhibitors are limited. We previously demonstrated that mTOR inhibitors suppress benign intestinal adenomas in ***Apc*** mutant mice but fail against invasive adenocarcinomas in ***Apc/Smad4*** mice. To elucidate the mechanisms underlying this resistance, we conducted CE/MS-based metabolomic analysis. Invasive intestinal adenocarcinomas resistant to mTOR inhibitors exhibited elevated histamine levels, and co-treatment with histamine inhibitors significantly suppressed invasion. We identified HDC-positive mast cells accumulating at tumor invasion fronts as the primary source of histamine. Additionally, mTOR inhibitor treatment stimulated the secretion of several cytokines capable of activating mast cells. These findings indicate that invasive intestinal adenocarcinomas resist mTOR inhibitors via histamine derived from the tumor microenvironment.

[Disclosure of COI by Responsible researcher] Lead presenter is not the Responsible researcher.
[Lead presenter: Yuwen Liu] We have no financial relationships to disclose.
[Principal Researcher: Masahiro Aoki] ■Research funds from company: 科研費　基盤研究B

### [E-1092] I-E9
### Cellular plasticity & heterogeneity in metastasis

2025 / 9 / 25 (Thu) 14:05-15:20 Room 16/(11F Visage) Room 16

[Chairperson]Tsunaki Hongu (Div. Cancer Cell Biol., Cancer. Res. Inst., Kanazawa Univ.)

---

### E-1092
### Multi-Omics Insights into Mechanisms Driving Early Postoperative Recurrence in BCLC-0/A Hepatocellular Carcinoma

[Speaker]JunTao Huang (Department of Hepatobiliary Surgery, Guangxi Medical University Cancer Hospital) [Co-author]Rui Song (Department of Hepatobiliary Surgery, Guangxi Medical University Cancer Hospital)、Jie Zhang (Department of Hepatobiliary Surgery, Guangxi Medical University Cancer Hospital)、MingJun Li (Department of Hepatobiliary Surgery, Guangxi Medical University Cancer Hospital)、BangDe Xiang (Department of Hepatobiliary Surgery, Guangxi Medical University Cancer Hospital)

Postoperative recurrence critically determines prognosis in hepatocellular carcinoma (HCC), particularly for BCLC-0/A patients.While GCNT3 participates in tumor progression, its role in early recurrence remains undefined.Our multicenter study, involving data from 522 patients, demonstrated that patients with early recurrence exhibited significantly shorter overall survival (OS) compared to those in the non-recurrence group ($P<0.001$).Multi-platform analysis (RNA-seq/CyTOF/scRNA-seq) revealed recurrent tumors exhibit immunosuppressive microenvironment with macrophage infiltration ($P<0.01$ ), CD8+ T cell depletion ($p<0.01$), and GCNT3 upregulation (log2FC>0.04 ,$P<0.05$ ). Clinical validation (IHC cohort/KM Plotter database) confirmed GCNT3's prognostic value ($P<0.001$).Functional studies demonstrated GCNT3 promotes proliferation (CCK8/Transwell/Wound Healing assay/Colony-formation assay /Subcutaneous transplantation tumor), metastasis (lung nodules:$P<0.01$).Mechanistically, GCNT3 transcriptionally activates MUC4 , driving glycometabolic reprogramming and immune evasion.This work establishes GCNT3-MUC4 axis as a therapeutic target for early HCC recurrence.

[Disclosure of COI by Responsible researcher] Lead presenter is the Responsible researcher.
[Lead presenter: JunTao Huang] We have no financial relationships to disclose.

▲TOP

### [E-1093] I-E9
### Cellular plasticity & heterogeneity in metastasis

2025 / 9 / 25 (Thu) 14:05-15:20 Room 16/(11F Visage) Room 16

[Chairperson]Tsunaki Hongu (Div. Cancer Cell Biol., Cancer. Res. Inst., Kanazawa Univ.)

---

### E-1093
### Novel Endothelial-Myeloma Cell Crosstalk Through CXCL10-CXCR3 Contributes to Chemotherapy Resistance

[Speaker]Kensuke Hachiya (Department of Signal Transduction, RIMD, Osaka University) [Co-author]Fumitaka Muramatsu (Department of Signal Transduction, RIMD, Osaka University)、Kinnosuke Matsumoto (Department of Signal Transduction, RIMD, Osaka University)、Yoshimi Noda (Department of Signal Transduction, RIMD, Osaka University)、Nobuyuki Takakura (Department of Signal Transduction, RIMD, Osaka University)

Multiple myeloma (MM) actively induces abundant angiogenesis, but the biological significance of proliferated endothelial cells (ECs) remains largely unclear. In the Vk*MYC transplantable model, an allogeneic MM model, the bone marrow (BM) vessel structures changed to exhibit irregular arrangement and variable caliber, and the BMECs underwent morphological and functional transformations. After chemotherapy, residual myeloma cells were located near BM blood vessels. Using in vivo imaging with two-photon excitation microscopy, we discovered that myeloma cells exhibit vascular endothelial cell-dependent behavior. Therefore, we focused on the distance between myeloma and endothelial cells. MMECs exhibited high expression of CXCL10, while myeloma cells showed elevated expression of CXCR3. In vitro, myeloma cells migrated via the CXCL10-CXCR3 axis, and anti-CXCL10 antibodies blocked this chemotaxis. EC-specific CXCL10 knockout mice showed an improvement in survival duration. Furthermore, combination chemotherapy with CXCL10-neutralizing antibodies prolonged survival in mice.

[Disclosure of COI by Responsible researcher] Lead presenter is the Responsible researcher.
[Lead presenter: Kensuke Hachiya] ■Research funds from company: 科研費(23K14563・20H05698)

### [E-1094] I-E9
### Cellular plasticity & heterogeneity in metastasis

2025 / 9 / 25 (Thu) 14:05-15:20 Room 16/(11F Visage) Room 16

[Chairperson]Tsunaki Hongu (Div. Cancer Cell Biol., Cancer. Res. Inst., Kanazawa Univ.)

---

### E-1094
### The role of MAGT1 and TUSC3 in vasculogenic mimicry of human cancer cells ***in vitro***

[Speaker]Ryota Kawahara (Faculty of Science and Technology, Keio University) [Co-author]Siro Simizu (Faculty of Science and Technology, Keio University)

Vasculogenic mimicry (VM) is a phenomenon in which tumor cells themselves form vascular-like luminal structures. Since VM has been reported to correlate with poor prognosis of cancer patients, further analysis of the mechanism is warranted. Here, we focused on MAGT1 and TUSC3 which are subunits of the oligosaccharyltransferase complex, and aimed to demonstrate the role of these proteins in VM formation. In this study, we used two types of human cancer cell lines; fibrosarcoma HT1080 and lung adenocarcinoma A549. HT1080 is a VM-positive cell enough to easily constitute VM networks on Matrigel, but A549 does not have the ability. Using CRISPR/Cas9 system, we deleted ***MAGT1*** gene and evaluated VM formation capability ***in vitro***. As a result, knockout (KO) of MAGT1 induced significantly earlier VM formation compared with the parental HT1080 cells. Importantly, KO of MAGT1 also induced VM formation in VM-negative A549 cells. Consistent with this, KO of TUSC3 significantly promotes VM formation both in HT1080 and A549 cells. Our results indicate that MAGT1 and TUSC3 suppress VM formation of human cancer cells and imply the significance of glycosylation in VM.

[Disclosure of COI by Responsible researcher] Lead presenter is not the Responsible researcher.
[Lead presenter: Ryota Kawahara] We have no financial relationships to disclose.
[Principal Researcher: Siro Simizu] We have no financial relationships to disclose.

### [E-1095] I-E9
### Cellular plasticity & heterogeneity in metastasis

2025 / 9 / 25 (Thu) 14:05-15:20 Room 16/(11F Visage) Room 16

[Chairperson]Tsunaki Hongu (Div. Cancer Cell Biol., Cancer. Res. Inst., Kanazawa Univ.)

---

### E-1095
### Single-Cell Spatial Analysis Reveals the Process of Sarcomatous change in Undifferentiated Pancreatic Cancer

[Speaker]Hajime Kamiya (Dept of Hepatobiliary and Pancreatic Surgery, NCCHE) [Co-author]Shunsuke A. Sakai (Division of Translational Informatics, EPOC, NCC)、Shunichiro Kageyama (Division of Translational Informatics, EPOC, NCC)、Riu Yamashita (Division of Translational Informatics, EPOC, NCC)、Hiroto Katoh (Division of Pathology, NCC)、Motohiro Kojima (Division of Pathology, NCC／Department of Pathology, Kyoto Prefectural University of Medicine,)、Yutaka Suzuki (Dept. of Computational Biology and Medical Sciences, Univ. of Tokyo)、Masashi Kudo (Dept of Hepatobiliary and Pancreatic Surgery, NCCHE)、Motokazu Sugimoto (Dept of Hepatobiliary and Pancreatic Surgery, NCCHE)、Shin Kobayashi (Dept of Hepatobiliary and Pancreatic Surgery, NCCHE)、Naoto Gotohda (Dept of Hepatobiliary and Pancreatic Surgery, NCCHE)

Undifferentiated pancreatic cancer (UPC) has significantly poorer prognosis compared to ordinary pancreatic cancer (OPC), yet mechanisms driving its characteristic histology and aggressive clinical behavior remain unclear. This study aimed to identify molecular mechanism responsible for histological change from OPC to UPC. Among 832 surgically treated pancreatic cancer patients, 35 (4.2%) exhibited histological undifferentiated features, and showed significantly worse outcomes than OPC (P = 0.013). To investigate undifferentiated histology, we performed single cell spatial analysis using Xenium 5K and PhenoCycler with 61 antibodies on tissues from eight cases clearly showing OPC-to-UPC histological alteration. This analysis revealed sequential cellular mesenchymal differentiation from PanKRT-positive OPC cells, through a PanKRT and Vimentin dual-positive intermediate, to exclusively Vimentin-positive UPC cells. Early-stage transition prominently involved fatty acid metabolism, while TGFB signaling dominated later stages. Single cell spatial analysis revealed sequential transition steps, indicating potential mechanisms and therapeutic targets for UPC.

[Disclosure of COI by Responsible researcher] Lead presenter is not the Responsible researcher.
[Lead presenter: Hajime Kamiya] We have no financial relationships to disclose.
[Principal Researcher: Motohiro Kojima] We have no financial relationships to disclose.

▲TOP

### [E-1096] I-E9
### Cellular plasticity & heterogeneity in metastasis

2025 / 9 / 25 (Thu) 14:05-15:20 Room 16/(11F Visage) Room 16

[Chairperson]Tsunaki Hongu (Div. Cancer Cell Biol., Cancer. Res. Inst., Kanazawa Univ.)

### E-1096
### Modeling PDAC intratumor heterogeneity and metastasis

[Speaker]Shigekazu Murakami (Georgetown University, Lombardi Comprehensive Cancer Center, Departement of Oncology／Miyagi Cancer Center Research Institute, Division of Cancer Stem Cell) [Co-author]Benjamin Colonemeric (Georgetown University, Lombardi Comprehensive Cancer Center, Departement of Oncology)、Haruna Fujimori (Miyagi Cancer Center Research Institute, Division of Cancer Stem Cell)、Yuka Unno (Miyagi Cancer Center Research Institute, Division of Cancer Stem Cell)、Jun Yasuda (Miyagi Cancer Center Research Institute, Division of Cancer Stem Cell)、Naoki Asano (Miyagi Cancer Center Research Institute, Division of Cancer Stem Cell)、Chunling Yi (Georgetown University, Lombardi Comprehensive Cancer Center, Departement of Oncology)

Tumor cell plasticity, defined as progressive phenotypic and functional changes in cancer cells during tumor progression, arises from genetic instability or epigenetic alterations induced by a heterogeneous tumor microenvironment (TME). Genetic and epigenetic reprogramming involve coordinated changes in the signaling network and global transcriptome. To identify metastasis-driving pathways in pancreatic ductal adenocarcinoma (PDAC), we developed spontaneous metastasis mouse models exhibiting lung and liver macro-metastases at varying frequencies after primary tumor resection. We activated signaling pathways in PDAC cell lines by introducing pathway regulators previously linked to PDAC pathogenesis and EMT, and compared their ability to seed primary tumors and metastases via co-injections of each cell lines. Unexpectedly, EMT-inducing pathways did not enhance metastasis. Instead, we found clonal variation within primary tumors and metastatic nodules. Our findings indicate that signaling pathways play context-dependent roles in promoting or suppressing metastasis.

[Disclosure of COI by Responsible researcher] Lead presenter is the Responsible researcher.
[Lead presenter: Shigekazu Murakami] We have no financial relationships to disclose.

▲TOP

### [OS7-1] OS7
### How Mechanical Forces Shape Cancer Cell Identity

2025 / 9 / 25 (Thu) 14:20-15:50 Room 6/(Hotel Nikko Kanazawa 3F Kujaku A) Room 6

[Chairpersons]Naoko Hattori (Institute for Molecular and Cellular Regulation, Gunma University)、Yusuke Yamamoto (Laboratory of Integrative Oncology, National Cancer Center Research Institute)

Technological innovations in comprehensive DNA, RNA, and protein analyses, as well as spatial omics, have led to significant progress in cancer research. However, regarding the "mechanical properties, " such as flexibility of cancer cells, although their association with invasion and metastasis has been postulated, their molecular mechanisms and effects on gene expression regulatory mechanisms remain to be elucidated. To address this issue, innovative research strategies that integrate medicine, biology, and engineering, combining conventional molecular biological approaches with engineering methods, such as mechanical measurements, are imperative. This symposium aims to bring together emerging and established researchers from mechanical engineering, mechanobiology, imaging, and cell biology to explore fate determination and regulation of cancer cells through the lens of cellular flexibility.

---

### OS7-1
### Mechanism of nestin-mediated cellular softening in cancer cells

[Speaker]Chikashi Nakamura (Cell. Mol. Biotechnol. Res. Inst., AIST／Dept. Biotechnol. & Life Sci., TUAT) [Co-author]Ayana Yamagishi (Cell. Mol. Biotechnol. Res. Inst., AIST／Dept. Biotechnol. & Life Sci., TUAT)

Nestin, an intermediate filament (IF), is highly expressed in malignant cancer cells. In these cells, nestin copolymerizes with vimentin, which interacts with actin filaments through its C terminal tail. The 170 kDa tail domain of nestin inhibits the binding between vimentin and actin, suggesting that nestin modulates cytoskeletal architecture and mechanical properties. To evaluate cytoskeletal mobility, we examined the highly metastatic mouse breast cancer cell line FP10SC2 and its nestin-knockout (NKO) counterpart using anti-vimentin antibody-functionalized nanoneedles and AFM. The nanoneedles are capable of penetrating the plasma membrane and directly interacting with the cytoskeleton in living cells. During retraction of the needle, a distinct peak force corresponding to the rupture of antibody-vimentin interactions can be detected. By analyzing the slope of the force distance curve, we found that FP10SC2 cells exhibited significantly greater cytoskeletal mobility than NKO cells. These findings suggest that nestin enhances cytoskeletal flexibility by modulating vimentin-actin interactions, thereby reducing cellular stiffness and potentially promoting metastatic behavior.

[Disclosure of COI by Responsible researcher] Lead presenter is the Responsible researcher.
[Lead presenter: Chikashi Nakamura] We have no financial relationships to disclose.

### [OS7-2] OS7
### How Mechanical Forces Shape Cancer Cell Identity

2025 / 9 / 25 (Thu) 14:20-15:50 Room 6/(Hotel Nikko Kanazawa 3F Kujaku A) Room 6

[Chairpersons]Naoko Hattori (Institute for Molecular and Cellular Regulation, Gunma University)、Yusuke Yamamoto (Laboratory of Integrative Oncology, National Cancer Center Research Institute)

Technological innovations in comprehensive DNA, RNA, and protein analyses, as well as spatial omics, have led to significant progress in cancer research. However, regarding the "mechanical properties, " such as flexibility of cancer cells, although their association with invasion and metastasis has been postulated, their molecular mechanisms and effects on gene expression regulatory mechanisms remain to be elucidated. To address this issue, innovative research strategies that integrate medicine, biology, and engineering, combining conventional molecular biological approaches with engineering methods, such as mechanical measurements, are imperative. This symposium aims to bring together emerging and established researchers from mechanical engineering, mechanobiology, imaging, and cell biology to explore fate determination and regulation of cancer cells through the lens of cellular flexibility.

---

### OS7-2
### Strategies to suppress cancer metastasis based on mechano-design

[Speaker]Daisuke Yoshino (Div. Adv. Appl. Phys., Inst. Eng., TUAT／Dept. Biomed. Eng., Grad. Sch., TUAT) [Co-author]Norino Uenaka (Dept. Biomed. Eng., Grad. Sch., TUAT)

Cells in the living body are constantly exposed to mechanical forces, including pressure, which are sensed at the cell membrane and transduced into biochemical signals. Cancer cells are highly affected by the pressure of their environment, as they continue to proliferate even in crowded spaces. However, few studies have investigated the effects of mechanical factors on cancer cells. This study examines the mechanisms by which hydrostatic pressure affects pancreatic cancer cells, with a focus on nuclear dynamics. MIA PaCa-2 cells were exposed to sustained pressure for 24 hours, nuclear stiffness was measured, and localization and expression of relevant proteins were analyzed. Hydrostatic pressure altered H3K9me3 localization, resulting in punctate aggregation of Lamin A/C. Furthermore, cell nuclei were softened due to the reduced ability of Lamin A/C to maintain nuclear structure. Our results suggest that pressure contributes to the invasive ability of pancreatic cancer cells, as invasion is associated with nuclear stiffness. This study provides useful knowledge to develop a novel method to control the invasion of pancreatic cancer cells by pressure and/or through its mechanisms.

[Disclosure of COI by Responsible researcher] Lead presenter is the Responsible researcher.
[Lead presenter: Daisuke Yoshino] ■Research funds from company: 中谷財団、AMED

▲TOP

### [OS7-3] OS7
### How Mechanical Forces Shape Cancer Cell Identity

2025 / 9 / 25 (Thu) 14:20-15:50 Room 6/(Hotel Nikko Kanazawa 3F Kujaku A) Room 6

[Chairpersons]Naoko Hattori (Institute for Molecular and Cellular Regulation, Gunma University)、Yusuke Yamamoto (Laboratory of Integrative Oncology, National Cancer Center Research Institute)

Technological innovations in comprehensive DNA, RNA, and protein analyses, as well as spatial omics, have led to significant progress in cancer research. However, regarding the "mechanical properties, " such as flexibility of cancer cells, although their association with invasion and metastasis has been postulated, their molecular mechanisms and effects on gene expression regulatory mechanisms remain to be elucidated. To address this issue, innovative research strategies that integrate medicine, biology, and engineering, combining conventional molecular biological approaches with engineering methods, such as mechanical measurements, are imperative. This symposium aims to bring together emerging and established researchers from mechanical engineering, mechanobiology, imaging, and cell biology to explore fate determination and regulation of cancer cells through the lens of cellular flexibility.

---

### OS7-3
### Organelle Resilience and Its Regulation in Cell Fate Determination

[Speaker]Takafumi Miyamoto (Center for Cyber Med Res. Univ. Tsukuba)

Cells adapt to environmental stress to maintain homeostasis. Drug-tolerant persister cells (DTPs) are a transiently resistant subpopulation that can survive anticancer drugs without permanent genetic changes, later resuming growth and contributing to relapse. Although any cancer cell may stochastically acquire this state, the underlying mechanisms remain unclear.This study focuses on the early phase of drug tolerance induced by molecularly targeted therapies, with particular attention to dynamic changes in organelle morphology. We introduce live-cell imaging techniques for tracking mitochondrial and lysosomal remodeling and describe methods for experimentally modulating organelle structure. These approaches reveal how organelle resilience may regulate cell fate and support reversible drug-tolerant states.

[Disclosure of COI by Responsible researcher] Lead presenter is the Responsible researcher.
[Lead presenter: Takafumi Miyamoto] We have no financial relationships to disclose.

### [OS7-4] OS7
### How Mechanical Forces Shape Cancer Cell Identity

2025 / 9 / 25 (Thu) 14:20-15:50 Room 6/(Hotel Nikko Kanazawa 3F Kujaku A) Room 6

[Chairpersons]Naoko Hattori (Institute for Molecular and Cellular Regulation, Gunma University)、Yusuke Yamamoto (Laboratory of Integrative Oncology, National Cancer Center Research Institute)

Technological innovations in comprehensive DNA, RNA, and protein analyses, as well as spatial omics, have led to significant progress in cancer research. However, regarding the "mechanical properties, " such as flexibility of cancer cells, although their association with invasion and metastasis has been postulated, their molecular mechanisms and effects on gene expression regulatory mechanisms remain to be elucidated. To address this issue, innovative research strategies that integrate medicine, biology, and engineering, combining conventional molecular biological approaches with engineering methods, such as mechanical measurements, are imperative. This symposium aims to bring together emerging and established researchers from mechanical engineering, mechanobiology, imaging, and cell biology to explore fate determination and regulation of cancer cells through the lens of cellular flexibility.

---

### OS7-4
### High-throughput profiling of the cell mechanics with unbiased transcriptional screening in cancer cells

[Speaker]Akifumi Shiomi (LiMe, Kyoto Univ.,／Hakubi, Kyoto Univ.,／UCLA, Bioeng.,) [Co-author]Taikopaul Kaneko (LiMe, Kyoto Univ.,)、Kaori Nishikawa (LiMe, Kyoto Univ.,)、Dino Dicarlo (UCLA, Bioeng.,)、Hirofumi Shintaku (LiMe, Kyoto Univ.,)

Mechanical properties of cells are closely linked to their physiological states in various biological contexts, including cell differentiation, aging, and cancer malignancy. However, the molecular cascades underlying transitions in these mechanical properties remain largely unexplored because of the lack of tools that effectively probe the relationship between cell mechanics and molecular regulation. Here, we present a method that integrates the profiling of cell mechanics, such as cell surface tension, with unbiased transcriptomic analysis of thousands of single cells. This method, named ELASTomics, enables multimodal analysis by combining single cell RNA sequencing with cell surface tension–dependent labeling using DNA–tagged dextran (DTD) introduced via nanopore electroporation. To demonstrate the utility of ELASTomics, we applied it to four cell lines with varying malignancy levels and identified candidate genes that may contribute to decrease cell surface tension in cancer cells. In addition, we introduce MECHANOmics, a complementary method that integrates single cell transcriptomics with measurements of cell deformability.

[Disclosure of COI by Responsible researcher] Lead presenter is not the Responsible researcher.
[Lead presenter: Akifumi Shiomi] We have no financial relationships to disclose.
[Principal Researcher: Hirofumi Shintaku] ■Research funds from company: 中島記念国際交流財団、公益財団法人JKA
■Contributions: 株式会社アナナスジャパン　■Presents or other payment: 中谷財団(中谷賞貢献賞)

### [OS7-5] OS7
### How Mechanical Forces Shape Cancer Cell Identity

2025 / 9 / 25 (Thu) 14:20-15:50 Room 6/(Hotel Nikko Kanazawa 3F Kujaku A) Room 6

[Chairpersons]Naoko Hattori (Institute for Molecular and Cellular Regulation, Gunma University)、Yusuke Yamamoto (Laboratory of Integrative Oncology, National Cancer Center Research Institute)

Technological innovations in comprehensive DNA, RNA, and protein analyses, as well as spatial omics, have led to significant progress in cancer research. However, regarding the "mechanical properties, " such as flexibility of cancer cells, although their association with invasion and metastasis has been postulated, their molecular mechanisms and effects on gene expression regulatory mechanisms remain to be elucidated. To address this issue, innovative research strategies that integrate medicine, biology, and engineering, combining conventional molecular biological approaches with engineering methods, such as mechanical measurements, are imperative. This symposium aims to bring together emerging and established researchers from mechanical engineering, mechanobiology, imaging, and cell biology to explore fate determination and regulation of cancer cells through the lens of cellular flexibility.

---

### OS7-5
### Mechanical cues in tumor microenvironment: ECM-linked responses mediated by actin and p53

[Speaker]Keiko Kawauchi (FIRST, Konan Univ.) [Co-author]Toshiyuki Goto (FIRST, Konan Univ.／GSSTI, Kobe Univ.)、Hideaki Fujita (RIRBM, Hiroshima Univ.)、Madoka Suzuki (IPR, Univ. Osaka)、Hiroaki Machiyama (Dept. Immunol., TMU)、Hiroaki Hirata (Dept. Appl. Biosci., KIT)、Shinichi Ishiwata (Dept. Phys., Waseda Univ)、Yosky Kataoka (GSSTI, Kobe Univ.)、Daisuke Miyoshi (FIRST, Konan Univ.)

Mechanical stresses in tumors - such as elevated interstitial pressure and matrix stiffening - are known to influence cellular architecture and gene expression through actin-dependent mechanotransduction. Using an in vitro system that allows independent control of tensile force, substrate stiffness, and hydrostatic pressure, we investigated how fibroblasts and cancer cells respond to these mechanical cues. Tensile force applied to fibroblasts promoted stress fiber formation and phosphorylation of FAK and p130Cas, along with $\beta$-catenin phosphorylation at a site required for its nuclear translocation linked to the upregulation of extracellular matrix-related genes. In contrast, cancer cells exposed to increased stiffness and pressure exhibited actin reorganization and transcriptional changes that appeared to be p53-dependent. Ongoing in vivo studies aim to further elucidate the role of these mechanotransduction pathways in tumor-associated mechanical environments.

[Disclosure of COI by Responsible researcher] Lead presenter is the Responsible researcher.
[Lead presenter: Keiko Kawauchi] We have no financial relationships to disclose.

### [E-1031] I-E2-1
### Organoid/Animal model (1)

2025 / 9 / 25 (Thu) 15:20-16:35 Room 3/(Hotel Nikko Kanazawa 4F Tsuru A) Room 3

[Chairperson]Ryoji Yao (Dept. Cell Biology., Cancer Institute, JFCR)

---

### E-1031
### Functional annotation of the PTEN gene variants of unknown significance (VUS) using endometrial cancer organoids

[Speaker]Yoshitaka Hippo (Lab. Precis. Tumor Model. Syst., Chiba Cancer Ctr. Res. Inst.) [Co-author]Yoshiaki Maru (Lab. Precis. Tumor Model. Syst., Chiba Cancer Ctr. Res. Inst.)、Shingo Kato (Div. Cancer Genome Diag,, Yokohama City Univ. Hosp.)、Jo Nishino (Div. Bioinfo., Natl. Cancer Ctr. Res. Inst.)、Mamoru Kato (Div. Bioinfo., Natl. Cancer Ctr. Res. Inst.)

PTEN is a tumor suppressor gene whose inactivation promotes oncogenesis predominantly by inducing Akt phosphorylation. Its somatic mutations are frequent in a variety of malignancies, while germline mutations are responsible for Cowden syndrome, a hereditary disorder predisposed to benign tumors in colon and uterus. Among more than 2000 variants in the PTEN gene, the majority remain as variants of unknown significance (VUS), because their pathogenicity has mostly been inferred from sequence. Since Akt phosphorylation by PTEN inactivation is commonly observed in endometrioid type endometrial carcinoma (EC), we first used its PDO to evaluate the ability of VUS to suppress Akt phosphorylation. While known pathogenic mutations can be successfully introduced, the wild-type was never stably introduced into the PDO. We then tested PDO of undifferentiated EC that lacks the PTEN gene but is not addicted to PTEN loss, which allowed us to introduce the wildtype and thereby evaluating the impact of 80 VUS. Interestingly, there were several cases in which the results differed from those predicted by conventional in silico approach, reaffirmed the importance of functional validation in vitro.

[Disclosure of COI by Responsible researcher] Lead presenter is the Responsible researcher.
[Lead presenter: Yoshitaka Hippo] ■Research funds from company: AMED、日本学術振興会　■Research expenses: NECソリューションイノベータ、ワールドインテック

▲TOP

### [E-1032] I-E2-1
### Organoid/Animal model (1)

2025 / 9 / 25 (Thu) 15:20-16:35 Room 3/(Hotel Nikko Kanazawa 4F Tsuru A) Room 3

[Chairperson]Ryoji Yao (Dept. Cell Biology., Cancer Institute, JFCR)

---

### E-1032
### Novel molecular classification of large cell neuroendocrine carcinoma and its potential therapeutic implications

[Speaker]Takuya Fukazawa (Department of General Surgery, Kawasaki Medical School) [Co-author]Etsuko Yokota (Department of General Surgery, Kawasaki Medical School)、Miki Iwai (General Medical Center Research Unit, Kawasaki Medical School)、Takuro Yukawa (Department of General Surgery, Kawasaki Medical School)、Yoshio Naomoto (Department of General Surgery, Kawasaki Medical School)、Nagio Takigawa (General Medical Center Research Unit, Kawasaki Medical School／Department of General Internal Medicine 4, Kawasaki Medical School)、Yasumasa Monobe (Okayama Medical Laboratories CO., Ltd)、Minoru Haisa (Kawasaki Medical School General Medical)、Tomoki Yamatsuji (Department of General Surgery, Kawasaki Medical School)

Pulmonary large-cell neuroendocrine carcinoma (LCNEC) is a high-grade neuroendocrine malignancy, accounting about 1%-3% of all lung cancers. Despite numerous studies, an optimal treatment strategy for advanced LCNEC remains undefined. Recent genomic analyses have identified two molecular LCNEC subgroups: S-LCNEC, harboring ***RB1*** and ***TP53*** mutations, and N-LCNEC, lacking ***RB1*** loss but carrying ***STK11***, ***KEAP1***, or ***CDKN2A*** mutations with ***TP53*** mutations. Drug sensitivity testing using patient-derived LCNEC organoids revealed challenges in selecting effective drugs for each subclass. However, a microtubule disassembly inhibitor, docetaxel, and Aurora A kinase inhibitors, alisertib/LY3295668, showed antitumor effects against S-LCNEC and N-LCNEC with MYC amplification. Furthermore, we investigated whether the novel SCLC classification (NAPI) applies to LCNEC and found ASCL1-expressing LCNEC might be sensitive to BCL2 inhibitors, ABT-263/ABT-737 (Yokota et al. ***Cancer Lett.*** 2024). These results highlight the importance of molecular subtyping of LCNEC and the use of organoid models in understanding the pathogenesis of this rare cancer and developing effective treatments.

[Disclosure of COI by Responsible researcher] Lead presenter is the Responsible researcher.
[Lead presenter: Takuya Fukazawa] We have no financial relationships to disclose.

### [E-1033] I-E2-1
### Organoid/Animal model (1)

2025 / 9 / 25 (Thu) 15:20-16:35 Room 3/(Hotel Nikko Kanazawa 4F Tsuru A) Room 3

[Chairperson]Ryoji Yao (Dept. Cell Biology., Cancer Institute, JFCR)

---

### E-1033
### ***Helicobacter pylori*** CagA drives ***in situ*** gastric signet-ring cell carcinoma in the absence of E-cadherin expression

[Speaker]Ippei Kikuchi (Laboratory of Microbial Carcinogenesis, Institute of Microbial Chemistry (BIKAKEN) [Co-author]Shunichi Ohba (Numazu Branch, Institute of Microbial Chemistry (BIKAKEN))、Kyohei Kurosawa (Numazu Branch, Institute of Microbial Chemistry (BIKAKEN))、Tomokazu Ohishi (Laboratory of Oncology, Institute of Microbial Chemistry (BIKAKEN))、Naoko Kamiya (Institute for Genetic Medicine, Hokkaido University)、Masanori Hatakeyama (Laboratory of Microbial Carcinogenesis, Institute of Microbial Chemistry (BIKAKEN)／Institute for Genetic Medicine, Hokkaido University)

Signet-ring cell carcinoma (SRCC) is a distinct histological subtype of diffuse-type gastric cancer (DGC), characterized by tumor cells with prominent cytoplasmic mucin and an eccentrically positioned nucleus. While most cases of DGC are linked to ***Helicobacter pylori*** (***H. pylori***) infection, the mechanisms by which ***H. pylori*** contributes to the development of DGC remain largely unknown. Here, we developed a genetically engineered mouse (***cagA-LSL***; ***Cdh1-flox***; ***Cldn18-CreERT2***) that enables stomach epithelial-specific expression of the ***H. pylori*** CagA oncoprotein alongside the loss of the ***Cdh1***/E-cadherin tumor suppressor gene. Remarkably, CagA induction combined with E-cadherin loss led to extensive ***in situ*** SRCC formation in the gastric corpus within two months. Gastric organoid analysis revealed that while E-cadherin loss alone resulted in epithelial cell extrusion toward the luminal side, CagA expression together with E-cadherin loss caused extensive extrusion of epithelial cells toward the basal side of the epithelial monolayer. These findings highlight the role of CagA in SRCC formation, as well as basal invasion and spread, which contribute to the aggressive features of DGC.

[Disclosure of COI by Responsible researcher] Lead presenter is not the Responsible researcher.
[Lead presenter: Ippei Kikuchi] We have no financial relationships to disclose.
[Principal Researcher: Masanori Hatakeyama] We have no financial relationships to disclose.

▲TOP

### [E-1034] I-E2-1
### Organoid/Animal model (1)

2025 / 9 / 25 (Thu) 15:20-16:35 Room 3/(Hotel Nikko Kanazawa 4F Tsuru A) Room 3

[Chairperson]Ryoji Yao (Dept. Cell Biology., Cancer Institute, JFCR)

---

### E-1034
### Tumor-Associated Fibrosis in Ovarian Cancer: Insights from a Highly Metastatic Xenograft Model

[Speaker]Hiroki Fujimoto (Dept. of Obstet. & Gynecol., Aichi Med. Univ. Sch. of Med.／Dept. of Obstet. & Gynecol.,Grad. Sch. of Med., Nagoya Univ.) [Co-author]Marina Yoshikawa (Dept. of Obstet. & Gynecol.,Grad. Sch. of Med., Nagoya Univ.)、Emiri Miyamoto (Dept. of Obstet. & Gynecol.,Grad. Sch. of Med., Nagoya Univ.)、Kazumasa Mogi (Dept. of Obstet. & Gynecol.,Grad. Sch. of Med., Nagoya Univ.)、Atsushi Kunishima (Dept. of Obstet. & Gynecol.,Grad. Sch. of Med., Nagoya Univ.)、Kaname Uno (Dept. of Obstet. & Gynecol.,Grad. Sch. of Med., Nagoya Univ.)、Shohei Iyoshi (Dept. of Obstet. & Gynecol.,Grad. Sch. of Med., Nagoya Univ.)、Masato Yoshihara (Dept. of Obstet. & Gynecol.,Grad. Sch. of Med., Nagoya Univ.)、Hiroaki Kajiyama (Dept. of Obstet. & Gynecol.,Grad. Sch. of Med., Nagoya Univ.)

Ovarian cancer (OvCa) is often diagnosed at advanced stages with peritoneal dissemination and stromal remodeling. Tumor-associated fibrosis (TAF), a fibrosis-rich stroma, has been implicated in metastasis and chemoresistance. To investigate its role, we developed a highly metastatic OvCa cell line, OV90-IP4, through serial intraperitoneal transplantation in nude mice. OV90-IP4 xenografts exhibited increased fibrosis and malignancy compared to originall cells, despite similar cellular functions in vitro. Co-culture spheroid assays with mesothelial cells showed enhanced carboplatin resistance, suggesting stromal involvement in chemoresistance. Transcriptomic and proteomic analyses of xenograft tumors identified elevated host-derived ECM proteins, including proteoglycans VCAN, NID1/2, and DCN, in OV90-IP4. These ECM-enriched profiles were associated with poor prognosis in public OvCa datasets. Collectively, our findings highlight the critical role of mesothelial-derived CAFs and fibrotic stroma in OvCa progression and resistance. The OV90-IP4 model provides a valuable bench to bedside model to study tumor-stroma interactions and explore novel therapeutic targets.

[Disclosure of COI by Responsible researcher] Lead presenter is not the Responsible researcher.
[Lead presenter: Hiroki Fujimoto] We have no financial relationships to disclose.
[Principal Researcher: Masato Yoshihara] We have no financial relationships to disclose.

### [E-1035] I-E2-1
### Organoid/Animal model (1)

2025 / 9 / 25 (Thu) 15:20-16:35 Room 3/(Hotel Nikko Kanazawa 4F Tsuru A) Room 3

[Chairperson]Ryoji Yao (Dept. Cell Biology., Cancer Institute, JFCR)

---

### E-1035
### Loss of SWI/SNF subunits with PI3K/AKT pathway activation forms intraductal tubular papillary neoplasm in the pancreas

[Speaker]Kei Iimori (Dept. of Gastroenterol. & Hepatol., Kyoto Univ.) [Co-author]Akihisa Fukuda (Dept. of Gastroenterol. & Hepatol., Kyoto Univ.)、Munemasa Nagao (Dept. of Gastroenterol. & Hepatol., Kyoto Univ.)、Takahisa Maruno (Dept. of Gastroenterol. & Hepatol., Kyoto Univ.)、Yuki Nakanishi (Dept. of Gastroenterol. & Hepatol., Kyoto Univ.)、Yuichi Fukunaga (Sumitomo Pharma Cancer Drug Discovery Res. Unit.)、Takayuki Anazawa (Dept. of Hepatobiliary & Pancreatic Surg., Kyoto Univ.)、Kazuyuki Nagai (Dept. of Hepatobiliary & Pancreatic Surg., Kyoto Univ.)、Etsuro Hatano (Dept. of Hepatobiliary & Pancreatic Surg., Kyoto Univ.)、Kosuke Minaga (Dept. of Gastroenterol., Kindai Univ.)、Katsutoshi Kuriyama (Dept. of Gastroenterol., Kyoto Med. Ctr.)、Shujiro Yazumi (Dept. of Gastroenterol., Kitano Hosp.)、Osamu Araki (Dept. of Gastroenterol., Kyoto Katsura Hosp.)、Yuki Yamauchi (Dept. of Gastroenterol., Hyogo Pref. Amagasaki Gen. Med. Ctr.)、Hiroshi Seno (Dept. of Gastroenterol. & Hepatol., Kyoto Univ.)

Intraductal tubular papillary neoplasm (ITPN) is a rare precursor of pancreatic cancer. The pathogenesis of ITPN remains unclear. We focused on the SWI/SNF complex subunits and PI3K/AKT pathway, frequently mutated in human ITPNs. We generated mice with pancreatic duct cell-specific knockouts of Pten and either Arid1a or Brg1 (HPA and HPB mice, respectively). These mice developed ITPN-like tumors, some of which progressed to invasive cancer. Gene expression analysis showed YAP/TAZ activation in HPA and HPB mice, confirmed by increased TAZ expression. ITPN organoids from HPB mice formed invasive cancer in xenografts, showing high TAZ expression. Moreover, YAP/TAZ inhibitor suppressed ITPN formation in HPA and HPB mice. Immunoprecipitation revealed that the SWI/SNF complex was bound to YAP/TAZ in control mice, but was dissociated in HPA mice, suggesting its role in YAP/TAZ suppression. The SWI/SNF complex subunits expression was reduced in 50% of human ITPNs (n = 12) and inversely correlated with TAZ expression. Therefore, concomitant PI3K/AKT activation and Arid1a/Brg1 loss drive ITPN formation via YAP/TAZ activation, highlighting YAP/TAZ inhibitors as potential treatments for ITPN.

[Disclosure of COI by Responsible researcher] Lead presenter is not the Responsible researcher.
[Lead presenter: Kei Iimori] We have no financial relationships to disclose.
[Principal Researcher: Akihisa Fukuda] We have no financial relationships to disclose.

▲TOP

### [E-1036] I-E2-1
### Organoid/Animal model (1)

2025 / 9 / 25 (Thu) 15:20-16:35 Room 3/(Hotel Nikko Kanazawa 4F Tsuru A) Room 3

[Chairperson]Ryoji Yao (Dept. Cell Biology., Cancer Institute, JFCR)

---

### E-1036
### The IQGAP3-MYC axis maintains a stem-like population to drive gastric carcinogenesis

[Speaker]Junichi Matsuo (Cancer Sci. Inst. of Singapore, Natl. Univ. of Singapore) [Co-author]Takaomi Sanda (Cancer Sci. Inst. of Singapore, Natl. Univ. of Singapore／Nagoya City Univ. Grad. Sch. of Med. Sci.)、Mitsuhiro Shimura (Cancer Sci. Inst. of Singapore, Natl. Univ. of Singapore／Dept. of Surg., Tohoku Univ. Grad. Sch. of Med.)、Jungwon Lee (Cancer Sci. Inst. of Singapore, Natl. Univ. of Singapore)、Nawaphat Jangphattananont (Cancer Sci. Inst. of Singapore, Natl. Univ. of Singapore)、Wei Peng Yong (Cancer Sci. Inst. of Singapore, Natl. Univ. of Singapore／Dept. Hematology-Oncology, Natl. Univ. Cancer Inst.／NUS Ctr. for Cancer Res., Natl. Univ. of Singapore)、Linda S. H. Chuang (Cancer Sci. Inst. of Singapore, Natl. Univ. of Singapore)、Yoshiaki Ito (Cancer Sci. Inst. of Singapore, Natl. Univ. of Singapore／NUS Ctr. for Cancer Res., Natl. Univ. of Singapore)

Adult stem cells are functionally critical for tissue repair. Mechanistic parallels between repair and carcinogenesis imply that deregulated repair mechanisms may initiate cancer. Stem cell marker IQGAP3 is crucial for homeostasis in the mouse stomach. Upon tissue injury, IQGAP3 expression was rapidly elevated in the isthmus and chief cells, along with the activation of multiple oncogenic pathways. IQGAP3 conditional knockout mice exhibited defective tissue repair. The severe reduction of MYC ser62 phosphorylation in IQGAP3 deficient mice indicates that IQGAP3 enhanced MYC activity to drive chief cell dedifferentiation, stem cell proliferation, and subsequent tissue regeneration. We next investigated the IQGAP3-MYC axis in early gastric cancer. IQGAP3 ablation in metaplasia and dysplasia mouse models impaired metaplastic glands or dysplastic tissue development, with the tissues presenting downregulation of phosphorylated MYC as well as reduced CD44- and AQP5-expressing stem-like populations. Lastly, we demonstrated that IQGAP3 interacts with MYC to initiate and maintain phenotypic plasticity in early carcinogenesis. These findings pave the way to early gastric cancer intervention.

[Disclosure of COI by Responsible researcher] Lead presenter is not the Responsible researcher.
[Lead presenter: Junichi Matsuo] We have no financial relationships to disclose.
[Principal Researcher: Yoshiaki Ito ] We have no financial relationships to disclose.

### [J-1043] I-J9-1
### Metastasis & vascular biology

2025 / 9 / 25 (Thu) 15:20-16:35 Room 4/(Hotel Nikko Kanazawa 4F Tsuru B) Room 4

[Chairperson]Kyoko Hida (Vasc.Biol.Mol.Pathol., Dent Med. Hokkaido Univ.)

---

### J-1043
### Tumor endothelial cells form a drug-resistant niche in glioblastoma by regulating iron metabolism through ceruloplasmin

[Speaker]Fumitaka Muramatsu (the Univ. of Osaka, RIMD, Signal transduction) [Co-author]Hiroyasu Kidoya (Univ. of Fukui, Faculty of Medical Sciences)、Kinnosuke Matsumoto (the Univ. of Osaka, RIMD, Signal transduction)、Kensuke Hachiya (the Univ. of Osaka, RIMD, Signal transduction)、Nobuyuki Takakura (the Univ. of Osaka, RIMD, Signal transduction)

We used two-photon microscopy to investigate the dynamic relationship between tumor cells and vasculature in glioblastoma, which is largely resistant to current anti-angiogenic therapies. Our analysis revealed that tumor blood vessels play a central role in establishing a microenvironmental niche that confers chemoresistance.
Mgmt promoter methylation is a well-known epigenetic marker of drug resistance in glioblastoma. We searched for endothelial–derived factors that induce Mgmt and identified ceruloplasmin. In a co-culture system of glioblastoma and brain endothelial cells, we confirmed that ceruloplasmin enhances iron uptake in tumor cells. Internalized iron served as a Tet3, DNA demethylase, cofactor to control Mgmt promoter methylation.
Next, we generated endothelial cell–specific ceruloplasmin knockout mice. In these mice, the formation of vascular niches was suppressed, resulting in enhanced temozolomide efficacy. Our findings demonstrate that ceruloplasmin–mediated iron regulation by tumor endothelial cells promotes chemoresistance in glioblastoma. Targeting this mechanism may offer a novel strategy to improve the therapeutic response by disrupting the vascular niche.

[Disclosure of COI by Responsible researcher] Lead presenter is the Responsible researcher.
[Lead presenter: Fumitaka Muramatsu] We have no financial relationships to disclose.

▲TOP

### [J-1044] I-J9-1
### Metastasis & vascular biology

2025 / 9 / 25 (Thu) 15:20-16:35 Room 4/(Hotel Nikko Kanazawa 4F Tsuru B) Room 4

[Chairperson]Kyoko Hida (Vasc.Biol.Mol.Pathol., Dent Med. Hokkaido Univ.)

---

### J-1044
### Ets2 transcription factor maintains vascular integrity by suppressing endothelial-mesenchymal transition

[Speaker]Yasuhiro Yoshimatsu (Div. Pharm.,Grad. Sch. Med. Dent., Niigata Univ.) [Co-author]Kentaro Maeda (Lab. Oncol., Sch. Life Sci., Tokyo Univ. Pharma. Life Sci.)、Yusuke Watanabe (Dept. Mol. Physiol., NCVC)、Tomohiro Shiiya (Div. Pharm.,Grad. Sch. Med. Dent., Niigata Univ.)、Shiori Kimuro (Dept. Biochem., Grad. Sch. Med. Dent., Inst. Sci.Tokyo)、Hiroyuki Sugimoto (Dept. Biochem., Sch. Med., Dokkyo Med.Univ.)、Masahito Ikawa (Dept. Exp. Genome Res., RIMD, Osaka Univ.)、Robert G. Oshima (Sanford Burnham Prebys Med. Discov. Inst)、Osamu Nakagawa (Dept. Mol. Physiol., NCVC)、Tetsuro Watabe (Lab. Oncol., Sch. Life Sci., Tokyo Univ. Pharma. Life Sci.／Dept. Biochem., Grad. Sch. Med. Dent., Inst. Sci.Tokyo)、Masanori Hirashima (Div. Pharm.,Grad. Sch. Med. Dent., Niigata Univ.)

Endothelial-mesenchymal transition (EndoMT) increases vascular permeability by inducing partial disruption of vascular integrity and has been implicated in exacerbating inflammation and generating some cancer-associated fibroblasts. We have previously shown transcription factors such as Snail and MRTFA induce EndoMT, but the transcriptional network regulating EndoMT remains largely unknown. In this study, we investigated the involvement of Ets family transcription factors, which play crucial roles in vascular development, in EndoMT. First, we found expression of Ets2 was down-regulated during TGF-$\beta$-induced EndoMT. When Ets2 was knocked down by siRNA, increased expression of mesenchymal cell markers such as SM22$\alpha$ was observed, suggesting that EndoMT is induced by suppression of Ets2 expression. Next, we generated endothelial cell-specific conditional knockout mice of Ets2 to elucidate the role of Ets2 in vascular formation. During our examination of blood and lymphatic vessel development, the presence of erythrocytes was observed in some lymphatic vessels, suggesting that blood vessels misconnect to lymphatic vessels and Ets2 contributes to the maintenance of vascular integrity.

[Disclosure of COI by Responsible researcher] Lead presenter is the Responsible researcher.
[Lead presenter: Yasuhiro Yoshimatsu] We have no financial relationships to disclose.

### [J-1045] I-J9-1
### Metastasis & vascular biology

2025 / 9 / 25 (Thu) 15:20-16:35 Room 4/(Hotel Nikko Kanazawa 4F Tsuru B) Room 4

[Chairperson]Kyoko Hida (Vasc.Biol.Mol.Pathol., Dent Med. Hokkaido Univ.)

---

### J-1045
### Multi-omics analyses using paired samples from newly diagnosed and recurrent glioblastomas during bevacizumab therapy

[Speaker]Toshihide Tanaka (Department of Neurosurgery, Jikei University School of Medicine／Department of Neurosurgery, Jikei University School of Medicine Kashiwa Hospital) [Co-author]Ai Iwauchi (Department of Pathology, Jikei University School of Medicine)、Nei Fukasawa (Department of Pathology, Jikei University School of Medicine)、Jun Takei (Department of Neurosurgery, Jikei University School of Medicine)、Akihiko Teshigawara (Department of Neurosurgery, Jikei University School of Medicine Kashiwa Hospital)、Kyoichi Tomoto (Department of Neurosurgery, Jikei University School of Medicine／Department of Neurosurgery, Jikei University School of Medicine Kashiwa Hospital)、Youei Yamamoto (Department of Neurosurgery, Jikei University School of Medicine Daisan Hospital)、Yasuharu Akasaki (Department of Neurosurgery, Jikei University School of Medicine)、Masayuki Shimoda (Department of Pathology, Jikei University School of Medicine)、Yuichi Murayama (Department of Neurosurgery, Jikei University School of Medicine)

**Background;** We explored multi-omics analyses using surgical samples obtained from patients received preoperative neoadjuvant bevacizumab therapy (neoBev) for newly diagnosed glioblastoma (nGBM) and its recurrence (rGBM).

**Materials and Methods;** Of all eight surgical samples from two cases treated by neoBev and two cases without preoperative Bev therapy (naiveBev) at nGBM and rGBM were analysed.

**Results;** Compared with naiveBev, expression levels of VEGF, HIF-1 alpha, FOXM1 in neoBev were downregulated, whereas CD163 was upregulated. By comparison between long and short overall survival (OS), Foxp3/CD8 ratio and FOXM1 were decreased in long OS. FOXM1 was significantly elevated in naiveBev with long OS.

**Discussions and Conclusions;** Analyses by space multi-omics provided novel findings correlated with specific gene expressions and anatomical location, which might be quite useful to elucidate the mechanism of effectiveness and resistance of targeted therapies including antiangiogenic or immunotherapies. But problems were as follows; 1) interpretation of huge amount of information, 2) requirement of skillful bioinformaticians, 3) cost-effectiveness. These problems should be solved.

[Disclosure of COI by Responsible researcher] Lead presenter is the Responsible researcher.
[Lead presenter: Toshihide Tanaka] We have no financial relationships to disclose.

▲TOP

### [J-1046] I-J9-1
### Metastasis & vascular biology

2025 / 9 / 25 (Thu) 15:20-16:35 Room 4/(Hotel Nikko Kanazawa 4F Tsuru B) Room 4

[Chairperson]Kyoko Hida (Vasc.Biol.Mol.Pathol., Dent Med. Hokkaido Univ.)

---

### J-1046
### CROX (Cluster Regulation of RUNX) strategy-mediated suppression of lung metastasis in triple-negative breast cancer

[Speaker]Tatsuya Masuda (Dept. Mol. Carci., Chiba Cancer Ctr.) [Co-author]Takayoshi Watanabe (Dept. Mol. Carci., Chiba Cancer Ctr.)、Feng Cao (Dept. Mol. Carci., Chiba Cancer Ctr.)、Toshinori Ozaki (Dept. Mol. Carci., Chiba Cancer Ctr.)、Rikiya Nakamura (Dept. Breast Surg., Chiba Cancer Ctr. Hosp.)、Hiroshi Sugiyama (Dept. Chem., Grad. Sch. Sci., Kyoto Univ.)、Yasuhiko Kamikubo (Dept. Mol. Carci., Chiba Cancer Ctr.)

Triple-negative breast cancer (TNBC) is defined as a lack of the expression of ER, PgR and HER2. TNBC highly metastasizes to lung and bone. Previously, we have demonstrated that RUNX family serves as an important oncogenic driver, and thereby postulated a novel anti-cancer strategy termed CROX (Cluster Regulation of RUNX). The aim of the present study is to ask whether CROX-based our novel RUNX inhibitor termed Chb-M' could reduce a metastatic potential of TNBC. To this end, we performed a cell adhesion assay. Cultured TNBC cells and pulmonary endothelial cells were treated with Chb-M' and then co-cultured. After washing in PBS, Chb-M'-dependent decrease in number of attached TNBC cells was observed. To elucidate the molecular mechanism(s) how Chb-M' could suppress lung metastasis of TNBC, we searched for RUNX family-target genes under these experimental conditions. Based on our results, cell adhesion-related ***ICAM1*** and ***ITGB2*** genes were identified from the endothelial and TNBC cells, respectively. Thus, it is likely that Chb-M'-mediated down-regulation of its ICAM1/ITGB2-dependent adhesion potential could attenuate lung metastasis of TNBC.

[Disclosure of COI by Responsible researcher] Lead presenter is not the Responsible researcher.
[Lead presenter: Tatsuya Masuda] We have no financial relationships to disclose.
[Principal Researcher: Yasuhiko Kamikubo] We have no financial relationships to disclose.

### [J-1047] I-J9-1
### Metastasis & vascular biology

2025 / 9 / 25 (Thu) 15:20-16:35 Room 4/(Hotel Nikko Kanazawa 4F Tsuru B) Room 4

[Chairperson]Kyoko Hida (Vasc.Biol.Mol.Pathol., Dent Med. Hokkaido Univ.)

---

### J-1047
### LAT1 inhibition facilitated tumor vascular normalization through enhancing CTH expression.

[Speaker]Junichi Suehiro (Department of Pharmacology and Toxicology, Kyorin University School of Medicine) [Co-author]Hiroyuki Sakurai (Department of Pharmacology and Toxicology, Kyorin University School of Medicine)

System L amino acid transporter LAT1 plays an important role in supplying essential neutral amino acids and has been implicated in cancer cell proliferation. However, its role in tumor vasculature remained poorly understood. Analysis of endothelial-specific LAT1 conditional knockout mice revealed that lack of LAT1 function in endothelial cells caused intracellular tryptophan deficiency and subsequent cystathionine γ lyase (CTH) induction, which promoted hydrogen sulfide ($H_2S$) production and contributed to mesenchymal cell recruitment and vascular normalization in B16F10 or Ex3-LLC solid tumor bearing mice. LAT1 inhibition also enhanced anti-cancer drug delivery and attenuated pre-metastatic niche formation and metastatic colonization in the lung by suppressing inflammation in endothelial cells. These findings indicated that the inhibition of LAT1 facilitated vascular normalization and improved tumor microenvironment, which could be beneficial in cancer treatment. Because we found that 10-25% of tumor endothelial cells expressed LAT1 in several human cancer specimens, it is likely that LAT1 in the tumor vasculature may play a role in human cancers in a similar manner.

[Disclosure of COI by Responsible researcher] Lead presenter is not the Responsible researcher.
[Lead presenter: Junichi Suehiro] We have no financial relationships to disclose.
[Principal Researcher: Hiroyuki Sakurai] We have no financial relationships to disclose.

▲TOP

### [J-1048] I-J9-1
### Metastasis & vascular biology

2025 / 9 / 25 (Thu) 15:20-16:35 Room 4/(Hotel Nikko Kanazawa 4F Tsuru B) Room 4

[Chairperson]Kyoko Hida (Vasc.Biol.Mol.Pathol., Dent Med. Hokkaido Univ.)

---

### J-1048
### Translocation renal cell carcinoma-on-a-chip to assess tumor microenvironmental factors regulating angiogenesis

[Speaker]Atsuya Kitada (Kyoto Univ., Grad. Sch. of Eng.) [Co-author]Kazuya Fujimoto (Kyoto Univ., Grad. Sch. of Eng.)、Miwa Tanaka (JFCR, The Cancer Inst.)、Masaya Baba (Kumamoto Univ. Hosp.)、Takuro Nakamura (Tokyo Med. Univ., Inst. of Med. Sci.)、Ryuji Yokokawa (Kyoto Univ., Grad. Sch. of Eng.)

Translocation renal cell carcinoma (tRCC), driven by ***TFE3*** fusion genes, is a highly vascularized tumor, yet its angiogenic mechanisms remain poorly understood. In contrast, alveolar soft part sarcoma (ASPS), another ***TFE3*** fusion-driven tumor, has been investigated using tumor-on-a-chip (ToC) models based on microfluidic technology. ASPS ToC revealed the role of pericytes in angiogenic microenvironments and demonstrated that the loss of vesicle trafficking via Rab27a and Sytl2 reduced the angiogenesis. Based on these findings, we developed a microfluidic tRCC model, incorporating tRCC cells, pericytes, and endothelial cells to investigate tRCC angiogenic dynamics. This model enabled visualization of angiogenesis and revealed that blood vessel morphology is influenced by microenvironmental conditions. It further validated the essential role of pericytes in tRCC vessel formation and evaluated how the loss of Rab27a and Sytl2 affects angiogenesis. This model serves as a tool to assess the potential factors influencing angiogenesis in the tRCC microenvironment.

[Disclosure of COI by Responsible researcher] Lead presenter is not the Responsible researcher.
[Lead presenter: Atsuya Kitada] We have no financial relationships to disclose.
[Principal Researcher: Ryuji Yokokawa] ■employment by a company related to the presentation: 株式会社フィジオスバイオテック

### [J-1049] I-J10-1
### Extracellular vesicles/Exosome

2025 / 9 / 25 (Thu) 15:20-16:35 Room 5/(Hotel Nikko Kanazawa 4F Tsuru C) Room 5

[Chairperson]Shiro Koizume (Kanagawa Cancer Center Res Inst)

---

### J-1049
### Paracrine adhesion signal by small extracellular vesicles promotes their clathrin-independent uptake by recipient cells

[Speaker]Koichiro M. Hirosawa (iGCORE, Gifu univ.) [Co-author]Yusuke Sato (Dept. Chemistry, Grad. Sch. Sci., Tohoku Univ.)、Rinshi S. Kasai (Natl. Cancer Ctr Res. Inst.)、Eriko Yamaguchi (iGCORE, Gifu univ.)、Naoko Komura (iGCORE, Gifu univ.)、Hiromune Ando (iGCORE, Gifu univ.／Integrated Cell-Material Sciences, Kyoto Univ.／Innovation Res. Cent. Quantum Med., Gifu Univ.)、Ayuko Hoshino (Res. Ctr. for Adv. Sci. and Tech., Tokyo Univ.／Inamori Res. Inst. for Sci.)、Yasunari Yokota (Faculty of Eng., Gifu Univ.)、Kenichi Suzuki (iGCORE, Gifu univ.／Natl. Cancer Ctr Res. Inst.／Integrated Cell-Material Sciences, Kyoto Univ.／Innovation Res. Cent. Quantum Med., Gifu Univ.)

Tumor-derived small extracellular vesicles (sEVs) play a crucial role in cancer metastasis by facilitating the transfer of molecules between tumor cells and recipient cells. These sEVs mediate intercellular communication through distinct internalization mechanisms. Using single-molecule imaging and super-resolution microscopy, we observed that all sEV subtypes undergo clathrin-independent endocytosis via galectin-3 and LAMP-2C. Notably, specific subtypes that recruited raft markers are internalized through caveolae. Across all subtypes, we detected the accumulation of integrin β1 and talin-1 in recipient cell membranes. Significantly, we discovered that paracrine (not autocrine) sEV binding triggered calcium mobilization through Src family kinases and phospholipase Cγ, subsequently activating calcineurin-dynamin pathways that direct sEVs toward recycling pathways. This calcium-dependent regulation represents a novel mechanism of sEV internalization with potential therapeutic implications. Our findings have significant implications for understanding intercellular communication within tumor microenvironments. Reference: Hirosawa et al. ***Nature Communications***, 16, 2419 (2025)

[Disclosure of COI by Responsible researcher] Lead presenter is not the Responsible researcher.
[Lead presenter: Koichiro M. Hirosawa] We have no financial relationships to disclose.
[Principal Researcher: Kenichi Suzuki] We have no financial relationships to disclose.

▲TOP

### [J-1050] I-J10-1
### Extracellular vesicles/Exosome

2025 / 9 / 25 (Thu) 15:20-16:35 Room 5/(Hotel Nikko Kanazawa 4F Tsuru C) Room 5

[Chairperson]Shiro Koizume (Kanagawa Cancer Center Res Inst)

---

### J-1050
### Expression and functional analysis of exo-miRNA in portal blood associated with liver metastasis of pancreatic cancer

[Speaker]Satoru Miyahara (Dept. of Gastroenterol. Surg., Grad. Sch. of Med., Osaka Univ.) [Co-author]Hidenori Takahashi (Dept. of Gastroenterol. Surg., Grad. Sch. of Med., Osaka Univ.)、Hirofumi Akita (Dept. of Gastroenterol. Surg., Grad. Sch. of Med., Osaka Univ.)、Yoshito Tomimaru (Dept. of Gastroenterol. Surg., Grad. Sch. of Med., Osaka Univ.)、Kazuki Sasaki (Dept. of Gastroenterol. Surg., Grad. Sch. of Med., Osaka Univ.)、Shinichiro Hasegawa (Dept. of Gastroenterol. Surg., Grad. Sch. of Med., Osaka Univ.)、Daisaku Yamada (Dept. of Gastroenterol. Surg., Grad. Sch. of Med., Osaka Univ.)、Takehiro Noda (Dept. of Gastroenterol. Surg., Grad. Sch. of Med., Osaka Univ.)、Shogo Kobayashi (Dept. of Gastroenterol. Surg., Grad. Sch. of Med., Osaka Univ.)、Yuichiro Doki (Dept. of Gastroenterol. Surg., Grad. Sch. of Med., Osaka Univ.)、Hidetoshi Eguchi (Dept. of Gastroenterol. Surg., Grad. Sch. of Med., Osaka Univ.)

**Background:** In pancreatic cancer (PC), portal vein blood (PVB) has demonstrated greater potential than peripheral blood (PB) as a source for liquid biopsy. This study aimed to identify exosomal microRNAs (exo-miRNAs) in PVB associated with liver metastasis and to elucidate the underlying mechanisms.**Method:** miRNA microarray analyses were performed on exosomes isolated from PVB and PB samples of patients with liver recurrence of PC. The functions of miRNAs exclusively identified in PVB were investigated for their role in promoting liver metastasis.**Result:** Among the exo-miRNAs in PVB, miR-4800-3p was identified as a notable candidate, not detected in PB analysis. Overexpression of miR-4800-3p enhanced the malignant potential of PC cells and promoted liver metastasis in a mouse model. RNA-sequencing identified PLAA, involved in ubiquitin-mediated protein degradation, as a target of miR-4800-3p. Clinical samples of liver metastases exhibited significant influence of miR-4800-3p downstream molecules, including PLAA.**Conclusion:** Our findings highlight the importance of exo-miRNAs in PVB as a valuable liquid biopsy source for understanding the mechanisms of liver metastasis in PC.

[Disclosure of COI by Responsible researcher] Lead presenter is not the Responsible researcher.
[Lead presenter: Satoru Miyahara] We have no financial relationships to disclose.
[Principal Researcher: Hidetoshi Eguchi] We have no financial relationships to disclose.

▲TOP

### [J-1051] I-J10-1
### Extracellular vesicles/Exosome

2025 / 9 / 25 (Thu) 15:20-16:35 Room 5/(Hotel Nikko Kanazawa 4F Tsuru C) Room 5

[Chairperson]Shiro Koizume (Kanagawa Cancer Center Res Inst)

### J-1051
### Enzyme activity profiling and protease identification in extracellular vesicles for pancreatic cancer biomarkers

[Speaker]Yusuke Yoshioka (Dept. Mol. Cell. Med., Inst. Med. Sci, Tokyo Med. Univ.) [Co-author]Ryosuke Kojima (Grad. Sch. Med., Univ. Tokyo.)、Sei Furutsuki (Grad. Sch. Pharm. Sci., Univ. Tokyo)、Kazumasa Nagai (Dept. Gastroenterology & Hepatology, Tokyo Med. Univ.)、Tadahaya Mizuno (Grad. Sch. Pharm. Sci., Univ. Tokyo)、Winnie Wong (Grad. Sch. Med., Univ. Tokyo.)、Reiko Tsuchiya (Grad. Sch. Med., Univ. Tokyo.)、Takao Itoi (Dept. Gastroenterology & Hepatology, Tokyo Med. Univ.)、Yasuteru Urano (Grad. Sch. Med., Univ. Tokyo.／Grad. Sch. Pharm. Sci., Univ. Tokyo)、Takahiro Ochiya (Dept. Mol. Cell. Med., Inst. Med. Sci, Tokyo Med. Univ.)

Pancreatic ductal adenocarcinoma (PDAC) is often diagnosed late, highlighting the need for novel modalities to improve disease characterization. We previously reported at this conference that enzyme activity in serum-derived extracellular vesicles (EVs) could serve as a useful modality, using a unique fluorescent probe library. We identified specific probes that distinguished PDAC from diabetic patients, a high-risk group, and found that EV-based measurements outperformed direct serum analysis in diagnostic accuracy. In this study, we extended our panel by identifying novel fluorescent probes whose activity was significantly decreased in PDAC, yielding a probe set covering both increased and decreased activity. We are also identifying candidate enzymes responsible for both increased and decreased probe cleavage using the Diced Electrophoresis Gel (DEG) assay-a method combining 2D electrophoresis, gel fractionation into a 384-well plate, enzymatic activity screening, and peptide mass fingerprinting. These findings highlight EV-associated enzyme activity profiling as a promising and unconventional modality for cancer research and potential diagnostics.

[Disclosure of COI by Responsible researcher] Lead presenter is the Responsible researcher.
[Lead presenter: Yusuke Yoshioka] ■Research funds from company: 公的資金AMED　■Research expenses: キヤノン株式会社、堀場製作所

▲TOP

### [J-1052] I-J10-1
### Extracellular vesicles/Exosome

2025 / 9 / 25 (Thu) 15:20-16:35 Room 5/(Hotel Nikko Kanazawa 4F Tsuru C) Room 5

[Chairperson]Shiro Koizume (Kanagawa Cancer Center Res Inst)

---

### J-1052
### Differential immunosuppression effects of CAF's exosome depending on the desmoplastic patterns in colorectal cancer

[Speaker]Keita Tashiro (Department of surgery, National Defense Medical College) [Co-author]Koichi Okamoto (Department of surgery, National Defense Medical College)、Satsuki Mochizuki (Department of surgery, National Defense Medical College)、Yoshiki Kajiwara (Department of surgery, National Defense Medical College)、Masato Yamadera (Department of surgery, National Defense Medical College)、Koji Moriya (Department of surgery, National Defense Medical College)、Kenta Kikuya (Department of surgery, National Defense Medical College)、Mayu Tashiro (Department of surgery, National Defense Medical College)、Hideki Ueno (Department of surgery, National Defense Medical College)

**Background**
Desmoplastic reaction (DR) classification, having 3 categories of Mature, Intermediate, and Immature, is a novel prognosticator in colorectal cancer (CRC). Our previous proteomics on exosome (Ex) from cancer-associated fibroblast (CAF) showed that CAF derived from Immature stroma (Immature-CAF), reportedly causes unfavorable prognosis, contained some proteins associated with immunosuppression.
**Methods**
Ex from CAF culture supernatant was collected by ultracentrifugation. CD8+ T cell (CTL) was isolated from peripheral blood of a healthy donor. The difference in granzyme A/B and perforin produced by CTL educated with or without Ex derived from CAF was compared between Mature and Immature CAFs. These CTL were then cocultured with CRC cell and apoptosis was assessed.
**Results**
The increasing ratio in granzyme A and perforin by adding Ex was lower in the Immature group (0.97 and 1.16) than Mature group (1.31 and 1.46) ($p=0.005$ and 0.041, respectively). Granzyme B showed a similar trend. The expression of cleaved caspase 3 was lower in the Immature group (cleaved caspase 3/GAPDH: 0.02) than the Mature one (0.81, $p=0.03$).
**Conclusion**
Immature-CAF Ex may induce potent immunosuppression.

[Disclosure of COI by Responsible researcher] Lead presenter is not the Responsible researcher.
[Lead presenter: Keita Tashiro] We have no financial relationships to disclose.
[Principal Researcher: Hideki Ueno] We have no financial relationships to disclose.

▲TOP

### [J-1053] I-J10-1
### Extracellular vesicles/Exosome

2025 / 9 / 25 (Thu) 15:20-16:35 Room 5/(Hotel Nikko Kanazawa 4F Tsuru C) Room 5

[Chairperson]Shiro Koizume (Kanagawa Cancer Center Res Inst)

---

### J-1053
### Development of personalized therapy using cancer-derived exosomes for esophageal squamous cell carcinoma

[Speaker]Yasunori Matsumoto (Dept. Frontier Surg. Chiba Univ. Grad. Sch. Med.) [Co-author]Takeshi Toyozumi (Dept. Frontier Surg. Chiba Univ. Grad. Sch. Med.)、Nobufumi Sekino (Dept. Frontier Surg. Chiba Univ. Grad. Sch. Med.)、Tadashi Shiraishi (Dept. Frontier Surg. Chiba Univ. Grad. Sch. Med.)、Koichiro Okada (Dept. Frontier Surg. Chiba Univ. Grad. Sch. Med.)、Kazuya Kinoshita (Dept. Frontier Surg. Chiba Univ. Grad. Sch. Med.)、Tenshi Makiyama (Dept. Frontier Surg. Chiba Univ. Grad. Sch. Med.)、Yuri Nishioka (Dept. Frontier Surg. Chiba Univ. Grad. Sch. Med.)、Masanari Yamada (Dept. Frontier Surg. Chiba Univ. Grad. Sch. Med.)、Akane Morimoto (Dept. Frontier Surg. Chiba Univ. Grad. Sch. Med.)、Hisahiro Matsubara (Dept. Frontier Surg. Chiba Univ. Grad. Sch. Med.)

Background: Exosomes have attracted attention in recent years as a drug delivery system because of their high biocompatibility and blood stability. In this study, we investigate the usefulness of exosomes as a novel therapy for Esophageal squamous cell carcinoma (ESCC).
Method: We analyzed the function (proliferation, migration, invasion) of miR-148a and miR-185, tumor suppressive microRNAs involved in therapeutic resistance selected from comprehensive analysis, using ESCC cell lines (TE2, T.Tn, KYSE-960). Establishment of a pipeline to establish patient derived tumor xenograft (PDX) from resected cancer specimens and cell lines and extraction of tumor-derived exosomes for clinical application.
Results: Overexpression of miR-148a and miR-185, respectively, suppressed proliferative capacity, cell migration, and invasive ability in vitro. miR-185 also regulated apoptosis after cisplatin treatment. The addition of fluorescently labeled exosomes suggested that exosomes derived from allogeneic cells have higher uptake than those from heterogeneous cells.
Conclusion: Tumor-derived exosomes encapsulating tumor-suppressive miRs could be a novel therapeutic approach.

[Disclosure of COI by Responsible researcher] Lead presenter is the Responsible researcher.
[Lead presenter: Yasunori Matsumoto] We have no financial relationships to disclose.

### [J-1054] I-J10-1
### Extracellular vesicles/Exosome

2025 / 9 / 25 (Thu) 15:20-16:35 Room 5/(Hotel Nikko Kanazawa 4F Tsuru C) Room 5

[Chairperson]Shiro Koizume (Kanagawa Cancer Center Res Inst)

---

### J-1054
### Suppression of exosome secretion as an effect of cellular exposure to atmospheric dust in the promotion of lung cancer

[Speaker]Daisuke Onoshima (Aichi Cancer Ctr. Res. Inst.) [Co-author]Chitose Oneyama (Aichi Cancer Ctr. Res. Inst.)

PM2.5 acts as a tumor promoter for lung cancer. The changes in lung cells induced by air pollution are particularly important for the study of formation models leading to malignant tumors. In this study, exosome secretion and intracellular ROS levels were measured by cellular exposure to atmospheric dust in analyzing the process of tumor promotion. Urban air dust 1648a collected in the St Louis area of Missouri, USA, was used to measure exosome secretion following administration to lung cancer cells A549. Luciferase Nano-Luc luminescence measurements were used for the secretion experiments. ROS was detected using a cell-permeable fluorescent probe and used as an analytical indicator of oxidative stress. Atmospheric dust components were analyzed by SEM-EDS. Exosome secretion was reduced by approximately 20% with the use of 10 μg/mL of atmospheric dust. Intracellular ROS levels increased with the administration of atmospheric dust. The dust contained transition metals including Fe. By using polystyrene latex particles as a negative control, the correlation between the suppression of exosome secretion and oxidative stress was successfully analyzed.

[Disclosure of COI by Responsible researcher] Lead presenter is the Responsible researcher.
[Lead presenter: Daisuke Onoshima] We have no financial relationships to disclose.

### [E-1043] I-E5-2
### Regulation of gene expression in cancer

2025 / 9 / 25 (Thu) 15:20-16:35 Room 7/(Hotel Nikko Kanazawa 3F Kujaku B) Room 7

[Chairperson]Hidetoshi Tahara (Cell.Mol.Biol., Hiroshima univ)

---

### E-1043
### Hypoxia-related protein IREB2-268aa promotes gastric cancer progression through DHODH-induced ferroptosis defense

[Speaker]ZAI LUO (Department of Gastrointestinal Surgery, Shanghai General Hospital) [Co-author]Pengshan Zhang (Department of Gastrointestinal Surgery, Shanghai General Hospital)、Renchao Zhang (Department of Gastrointestinal Surgery, Shanghai General Hospital)、Yuan Fang (Department of Gastrointestinal Surgery, Shanghai General Hospital)、Zhengjun Qiu (Department of Gastrointestinal Surgery, Shanghai General Hospital)、Chen Huang (Department of Gastrointestinal Surgery, Shanghai General Hospital)

Emerging evidence suggests the potential of circular RNA to encode proteins. However, their roles in ferroptosis and gastric cancer (GC) progression remain largely unclear. We aimed to identify protein encoded by circRNA involved in GC progression. The translatability of circRNA was verified by employing multiple techniques, including polysome profiling, custom antibody, and mass spectrometry. The mechanism and function of the IREB2-268aa/DHODH signaling axis were extensively determined in vitro and in vivo. Here, we identified that IREB2-268aa encoded by circIREB2 was notably elevated in GC cells and promoted GC cell progression by DHODH induced ferroptosis defense. Mechanistically, IREB2-268aa, positively regulated by DDX3X, reinforces ferroptosis resistance by directly binds to and stabilizes DHODH, reducing CoQ to CoQH2 as well as detoxifying lipid peroxides in mitochondria. Furthermore, IREB2-268aa inhibits HERC4 and DHODH binding, by suppressing the recruitment of E3 ubiquitin ligase HERC4 and polyubiquitination degradation of DHODH at K306 site, which maintains DHODH protein stability. Overall, we identify IREB2-268aa as a promising therapeutic strategy for GC patients.

[Disclosure of COI by Responsible researcher] Lead presenter is the Responsible researcher.
[Lead presenter: ZAI LUO] We have no financial relationships to disclose.

▲TOP

### [E-1044] I-E5-2
### Regulation of gene expression in cancer

2025 / 9 / 25 (Thu) 15:20-16:35 Room 7/(Hotel Nikko Kanazawa 3F Kujaku B) Room 7

[Chairperson]Hidetoshi Tahara (Cell.Mol.Biol., Hiroshima univ)

---

### E-1044
### Identification of a novel lncRNA regulating Wnt pathway in pancreatic cancer

[Speaker]Keiko Shinjo (Div. of Cancer Biol., Nagoya Uni. Grad. Sch. of Med.) [Co-author]Xingxing Wang (Div. of Cancer Biol., Nagoya Uni. Grad. Sch. of Med.)、Kohei Kumegawa (Cancer Cell Diversity Project, NEXT-Ganken Program, JFCR)、Reo Maruyama (Cancer Cell Diversity Project, NEXT-Ganken Program, JFCR)、Shinji Mii (Molecular Path., Sch. of Med., Hiroshima Univ.)、Tatsunori Nishimura (Div. of Cancer Biol., Nagoya Uni. Grad. Sch. of Med.)、Miho Suzuki (Div. of Cancer Biol., Nagoya Uni. Grad. Sch. of Med.)、Atsushi Enomoto (Dept. of Path., Nagoya Uni. Grad. Sch. of Med.)、Yutaka Kondo (Div. of Cancer Biol., Nagoya Uni. Grad. Sch. of Med.)

Long non-coding RNAs (lncRNAs) are non-coding RNAs longer than 200 nucleotides that play essential roles in transcriptional regulation by interacting with various proteins. Through single-cell analysis of pancreatic tissue from a pancreatic ductal adenocarcinoma (PDAC) mouse model, we identified lncA, which is involved in tumor aggressiveness. In PDAC cell lines, lncA was highly expressed in mesenchymal-type cells. RNA fluorescence in situ hybridization (RNA-FISH) revealed that the localization of lncA varied depending on cellular conditions. When cells were tightly attached to each other, lncA was localized at the cell membrane, whereas during invasion, it translocated into the cytoplasm. Interestingly, a similar localization pattern was observed in both human and mouse PDAC tissues. Knockdown of lncA reduced β-catenin protein levels and inhibited the epithelial-mesenchymal transition phenotype. Furthermore, RNA immunoprecipitation suggested that lncA interact with β-catenin protein.Mutations in the β-catenin pathway are rare in PDAC; however, the overexpression of lncA may represent an alternative mechanism for dysregulating the β-catenin pathway in this tumor.

[Disclosure of COI by Responsible researcher] Lead presenter is not the Responsible researcher.
[Lead presenter: Keiko Shinjo] We have no financial relationships to disclose.
[Principal Researcher: Xingxing Wang] ■Research expenses: NANO MRNA社

### [E-1045] I-E5-2
### Regulation of gene expression in cancer

2025 / 9 / 25 (Thu) 15:20-16:35 Room 7/(Hotel Nikko Kanazawa 3F Kujaku B) Room 7

[Chairperson]Hidetoshi Tahara (Cell.Mol.Biol., Hiroshima univ)

---

### E-1045
### ***NEAT1***-triggered collateral activity of CRISPR/Cas13 shows antileukemic activity

[Speaker]Ruka Shimura (Division of Molecular Oncology, The University of Tokyo) [Co-author]Keita Yamamoto (Division of Molecular Oncology, The University of Tokyo)、Tomohiro Yabushita (Division of Molecular Oncology, The University of Tokyo)、Wenyu Zhang (Division of Molecular Oncology, The University of Tokyo)、Kohei Iida (Division of Molecular Oncology, The University of Tokyo)、Kaito Mimura (Division of Cancer Evolution, National Cancer Center Research Institute)、Toshio Kitamura (Division of Molecular Pharmacology of Malignant Diseases, UTokyo／Foundation for Biomedical Research and Innovation at Kobe)、Susumu Goyama (Division of Molecular Oncology, The University of Tokyo)

***NEAT1*** is a long non-coding RNA that is highly expressed in many cancers, including leukemia. In this study, we found ***NEAT1***-targeting CRISPR/Cas13 induced strong growth inhibition in human leukemia cells. The antileukemic effect of ***NEAT1***-targeting CRISPR/Cas13 was also confirmed ***in vivo*** by the xenograft assays. However, neither ***NEAT1*** KD with CRISPRi nor ***NEAT1*** KO with CRISPR/Cas9 showed growth inhibitory effects, indicating ***NEAT1*** itself is dispensable for leukemic proliferation. Subsequent RNA-seq and RIP-seq analyses revealed ***NEAT1***-targeting CRISPR/Cas13 cleaved both ***NEAT1*** and bystander RNAs surrounding ***NEAT1***, resulting in a proliferation defect. Importantly, this CRISPR/Cas13-mediated collateral transcriptome destruction was induced only by the highly expressed RNA such as ***NEAT1***. Furthermore, leukemia cells harboring SNP at ***NEAT1*** sequences were insensitive to growth inhibition, suggesting leukemia-specific passenger mutations in ***NEAT1*** may be selective therapeutic targets. We propose the collateral activity of CRISPR/Cas13 can be exploited for leukemia therapy to target marker RNAs and passenger mutations that are not considered therapeutic targets by the current treatment options.

[Disclosure of COI by Responsible researcher] Lead presenter is not the Responsible researcher.
[Lead presenter: Ruka Shimura] We have no financial relationships to disclose.
[Principal Researcher: Keita Yamamoto] We have no financial relationships to disclose.

### [E-1046] I-E5-2
### Regulation of gene expression in cancer

2025 / 9 / 25 (Thu) 15:20-16:35 Room 7/(Hotel Nikko Kanazawa 3F Kujaku B) Room 7

[Chairperson]Hidetoshi Tahara (Cell.Mol.Biol., Hiroshima univ)

---

### E-1046
### Regulation of structural diversity of miRNAs by IGF2BP3 in the malignant properties of early-stage lung adenocarcinoma

[Speaker]Naoto Tsuchiya (Lab. Mol. Carcinogenesis, Natl. Cancer Ctr. Res. Inst.) [Co-author]Yuko Fujiwara (Lab. Mol. Carcinogenesis, Natl. Cancer Ctr. Res. Inst.)、Ryouu Takahashi (Dep. Cell. and Mol. Biol., Hiroshima Univ.)、Motonobu Saito (Dep. Gastro. Tract Surg., Fukushima Med. Univ.)、Hidetoshi Tahara (Dep. Cell. and Mol. Biol., Hiroshima Univ.)、Kouya Shiraishi (Div. Genome Biol., Natl. Cancer Ctr. Res. Inst.)、Takashi Kohno (Div. Genome Biol., Natl. Cancer Ctr. Res. Inst.)、Akiteru Goto (Dep. Cell. And Org. Patho., Akita Univ.)

Abnormalities in microRNAs (miRNAs) are implicated in the pathogenesis of cancers. We have demonstrated that the oncofetal protein IGF2BP3 regulates the biogenesis of 3'-isomiRs, inducing substantial alterations in the cellular structural configuration of miRNAs, thereby contributing to the establishment of malignant gene expression profiles. We developed a metric, the D-score, which reflects the predominant production of the 3'-isomiR miR-21-5p+C. This score effectively distinguishes early-stage lung adenocarcinoma (LUAD) cases with a low risk of recurrence from those with a high risk. We found that IGF2BP3 forms a complex with Drosha in the nucleus and participates in the synthesis of 3'-isomiRs. IGF2BP3 also contributes to the biogenesis of the other 3'-isomiRs, which regulate p53, TGF-β, and TNF pathways in LUAD. Notably, the intracellular abundance of 3'-long isomiRs is significantly elevated in malignant LUAD, implying that structural diversification of miRNAs plays a critical role in the acquisition of malignant phenotypes. This principle offers a foundation for developing diagnostic biomarkers with broad clinical applicability in early-stage LUAD.

[Disclosure of COI by Responsible researcher] Lead presenter is the Responsible researcher.
[Lead presenter: Naoto Tsuchiya] We have no financial relationships to disclose.

▲TOP

[E-1047] I-E5-2

### Regulation of gene expression in cancer

2025 / 9 / 25 (Thu) 15:20-16:35 Room 7/(Hotel Nikko Kanazawa 3F Kujaku B) Room 7

[Chairperson]Hidetoshi Tahara (Cell.Mol.Biol., Hiroshima univ)

---

E-1047

### Association between tumor-specific alternative promoter usage and patient survival in lung adenocarcinoma

[Speaker]Ken Asada (Cancer Transl. Res. Team, RIKEN Ctr. for AIP project／Div. Medical AI Res. Dev., Natl. Cancer Ctr. Res. Inst.) [Co-author]Amina Bolatkan (Cancer Transl. Res. Team, RIKEN Ctr. for AIP project／Div. Medical AI Res. Dev., Natl. Cancer Ctr. Res. Inst.)、Kouya Shiraishi (Div. Genome Biol., Natl. Cancer Ctr. Res. Inst.)、Hidehito Horinouchi (Dept. Thoracic Oncol., Natl. Cancer Ctr., Hosp.)、Yukihiro Yoshida (Dept. Thoracic Surg., Natl. Cancer Ctr., Hosp.)、Masami Mukai (Div. Medical Info., Natl. Cancer Ctr., Hosp. Dept)、Yasushi Yatabe (Dept. Diagnostic Pathol., Natl. Cancer Ctr. Hosp.)、Takashi Kohno (Div. Genome Biol., Natl. Cancer Ctr. Res. Inst.)、Ryuji Hamamoto (Cancer Transl. Res. Team, RIKEN Ctr. for AIP project／Div. Medical AI Res. Dev., Natl. Cancer Ctr. Res. Inst.／NCC Cancer Sci., Grad. Sch., Inst. Sci. Tokyo)

It has been estimated that more than 50% of human genes are regulated by multiple promoters. Dysregulation of promoter usages is associated with tumor proliferarion, metastasis, and drug resistant. In this study, we analyzed alternative promoter usage to examine isoform-specific gene expression in Japanese lung adenocarcinoma (LUAD). Histone deacetylase 9 (HDAC9) has several promoters, of which eight were uniquely identified in our study. Notably, promoter 8 (P8) was exclusively active in tumor samples compared to normal adjucent to tumor samples. Additionally, higher activity of P8 in HDAC9 was associated with advers outcomes in LUAD, highliging its potential role as a prognostic biomarker. Mechanistically, we identidied that patients with KRAS mutation had a high P8 activity compared with no driver mutation and EGFR mutation. Hypomethylation was found in P8, while hypermethylation was observed in the inactive promoter regions. Furthermore, H3K27 histone acetylation was significantly enriched in the P8 regions. Collectively, genetic and epigenetic regulations are one of the mechanisms of isoform expression directed by distinct promoter and enhancer usages in LUAD.

[Disclosure of COI by Responsible researcher] Lead presenter is not the Responsible researcher.
[Lead presenter: Ken Asada] We have no financial relationships to disclose.
[Principal Researcher: Ryuji Hamamoto] We have no financial relationships to disclose.

▲TOP

### [E-1048] I-E5-2
### Regulation of gene expression in cancer

2025 / 9 / 25 (Thu) 15:20-16:35 Room 7/(Hotel Nikko Kanazawa 3F Kujaku B) Room 7

[Chairperson]Hidetoshi Tahara (Cell.Mol.Biol., Hiroshima univ)

---

### E-1048
### Mechanistic insight into super-enhancer-mediated gene gating in cancer cells

[Speaker]Dini K. Ikliptikawati (Kanazawa Univ. Infiniti) [Co-author]Yuta Kozuka (Kanazawa Univ. LST)、Richard Wong (Kanazawa Univ. Infiniti)、Masaharu Hazawa (Kanazawa Univ. Infiniti)

Super-enhancers (SEs) are expansive regulatory elements densely occupied by master transcription factors and coactivators like Mediator and BRD4, driving robust gene expression programs. In cancer, SEs often localize near nuclear pores, promoting oncogene activation by enhancing mRNA export. Although RNA-binding proteins (RBPs) are key regulators of RNA processing and export, their roles within SE condensates remain largely unexplored. Given that SEs form phase-separated condensates that serve as selective compartments, we identify RBPs that coordinate RNA metabolism within SEs at the nuclear periphery. Using an RNA-centric approach with oligo(dT) enrichment and quantitative mass spectrometry, we profiled RBPs in the presence or absence of the pan-BET degrader ARV-771. Among 79 high-confidence RBPs identified across cancer cell lines, 27 (34.2%) were SE-dependent. Functional annotation revealed their involvement across the mRNA life cycle, including transcription, splicing, processing, export, translation, and decay. Notably, SE-enriched RBPs often showed dual roles in transcriptional control and splicing, linking SE activity to integrated post-transcriptional regulation.

[Disclosure of COI by Responsible researcher] Lead presenter is not the Responsible researcher.
[Lead presenter: Dini K. Ikliptikawati] We have no financial relationships to disclose.
[Principal Researcher: Masaharu Hazawa] We have no financial relationships to disclose.

▲TOP

### [J-1061] I-J3
### Bacteria & Microbiome

2025 / 9 / 25 (Thu) 15:20-16:35 Room 9/(Hotel Nikko Kanazawa 3F Lumiere) Room 9

[Chairperson]Kentaro Inamura (Tumor Pathol., Jichi Med. Univ.)

### J-1061
### The involvement of Fusobacterium nucleatum subspecies animalis in the pathogenesis of human esophageal adenocarcinoma

[Speaker]Shintaro Okumura (Dept. Surg., Faculty Med. Kyoto Univ.) [Co-author]Taku Kitano (Dept. Surg., Faculty Med. Kyoto Univ.／Ploidy Path. Lab., Grad. Sch. Frontier Biosci, Osaka Univ.)、Tomonori Matsumoto (Ploidy Path. Lab., Grad. Sch. Frontier Biosci, Osaka Univ.)、Shigeru Tsunoda (Dept. Surg., Faculty Med. Kyoto Univ.)、Tatsuto Nishigori (Dept. Surg., Faculty Med. Kyoto Univ.)、Kazutaka Obama (Dept. Surg., Faculty Med. Kyoto Univ.)、Eiji Hara (Dept. Mol. Biol., Res. Inst. Microbial Diseases, Osaka Univ.)

Background: While bacterial involvement in the pathophysiology of the esophageal adenocarcinoma (EAC) was suggested, the details remain unclear. We aimed to identify bacteria associated with the pathogenesis of EAC and to investigate their potential role.Methods: Microbiota in EAC tissues was explored by 16S-rRNA sequencing. The impact of intra-tumoral bacteria on tumor microenvironment was histologically evaluated. The influence of pathological bacteria on EAC was examined by the treatment of OE33 cell line with bacterial culture supernatants.Results: Oral pathogenic bacteria were detected in EAC patients and was associated with poor prognosis. Intra-tumoral infiltration of oral pathogenic bacteria was accompanied with local neutrophil infiltration. Among the oral pathogenic bacteria, ***Fusobacterium nucleatum animalis*** was detected most frequently in the EAC tissue. Its culture supernatant induced ROS-mediated DNA damage in EAC cells. Conclusion: Intra-tumoral infiltration of oral pathogenic bacteria has a profound impact on the tumor microenvironment of EAC. ***F. nucleatum animalis***, the predominant oral pathogenic bacteria in the EAC tissue induced DNA damage in cancer cells.

[Disclosure of COI by Responsible researcher] Lead presenter is the Responsible researcher.
[Lead presenter: Okumura Shintaro] We have no financial relationships to disclose.

▲TOP

### [J-1062] I-J3
### Bacteria & Microbiome

2025 / 9 / 25 (Thu) 15:20-16:35 Room 9/(Hotel Nikko Kanazawa 3F Lumiere) Room 9

[Chairperson]Kentaro Inamura (Tumor Pathol., Jichi Med. Univ.)

---

### J-1062
### Butyrate and CBM588 Boost Oncolytic Adenovirus Immunotherapy via MHC-I Upregulation in Colorectal Cancer

[Speaker]Tetsuya Katayama (Department of Gastroenterological Surgery, Okayama University Graduate School of Medicine) [Co-author]Shinji Kuroda (Department of Gastroenterological Surgery, Okayama University Graduate School of Medicine)、Masaki Sakamoto (Department of Gastroenterological Surgery, Okayama University Graduate School of Medicine)、Hitoshi Minagi (Department of Gastroenterological Surgery, Okayama University Graduate School of Medicine)、Makoto Matsumoto (Department of Gastroenterological Surgery, Okayama University Graduate School of Medicine)、Eri Takeda (Department of Gastroenterological Surgery, Okayama University Graduate School of Medicine)、Yu Mikane (Department of Gastroenterological Surgery, Okayama University Graduate School of Medicine)、Masashi Hashimoto (Department of Gastroenterological Surgery, Okayama University Graduate School of Medicine)、Nobuhiko Kanaya (Department of Gastroenterological Surgery, Okayama University Graduate School of Medicine)、Yoshihiko Kakiuchi (Department of Gastroenterological Surgery, Okayama University Graduate School of Medicine)、Satoru Kikuchi (Department of Gastroenterological Surgery, Okayama University Graduate School of Medicine)、Shunsuke Kagawa (Department of Gastroenterological Surgery, Okayama University Graduate School of Medicine)、Hiroshi Tazawa (Department of Gastroenterological Surgery, Okayama University Graduate School of Medicine)、Yasuo Urata (Oncolys BioPharma, Inc., Tokyo, Japan)、Toshiyoshi Fujiwara (Department of Gastroenterological Surgery, Okayama University Graduate School of Medicine)

Butyrate, a short-chain fatty acid from gut microbiota, plays a key role in modulating antitumor immunity and enhancing therapy. OBP-702 is a telomerase-specific oncolytic adenovirus expressing p53, known to boost antitumor responses. We investigated the synergy between OBP-702 and butyrate in colorectal cancer (CRC). In vitro, the combination increased cytotoxicity in human and murine CRC cell lines. In vivo, it significantly reduced tumor growth in HCT116 and CT26 models versus each monotherapy. Mechanistically, butyrate upregulated the coxsackie and adenovirus receptor (CAR) and integrins, enhancing viral infection. It also activated the cGAS-STING pathway, increasing MHC-I and CXCL10 and promoting CD8 T cell infiltration. These effects were confirmed in an orthotopic CRC model with liver metastasis, which showed reduced tumor burden and better survival. Notably, replacing butyrate with the butyrate-producing bacterium CBM588 resulted in similar antitumor synergy with increased dendritic cell and CD8 T cell infiltration. This strategy offers a promising approach for CRC immunotherapy.

[Disclosure of COI by Responsible researcher] Lead presenter is not the Responsible researcher.
[Lead presenter: Tetsuya Katayama] We have no financial relationships to disclose.
[Principal Researcher: Shinji Kuroda, Masaki Sakamoto] We have no financial relationships to disclose.

▲TOP

### [J-1063] I-J3
### Bacteria & Microbiome

2025 / 9 / 25 (Thu) 15:20-16:35 Room 9/(Hotel Nikko Kanazawa 3F Lumiere) Room 9

[Chairperson]Kentaro Inamura (Tumor Pathol., Jichi Med. Univ.)

### J-1063
### ***Fusobacterium nucleatum***-induced activation of the STING pathway contributes to esophageal cancer progression

[Speaker]Shotaro Nakajima (Dept. Multidisciplinary Treatment of Cancer and Regional Medical Support, FMU／Dept. Gastrointestinal Tract Surgery, FMU) [Co-author]Saito Katsuharu (Dept. Gastrointestinal Tract Surgery, FMU)、Fukai Satoshi (Dept. Gastrointestinal Tract Surgery, FMU)、Okayama Hirokazu (Dept. Gastrointestinal Tract Surgery, FMU)、Motonobu Saito (Dept. Gastrointestinal Tract Surgery, FMU)、Mimura Kosaku (Dept. Gastrointestinal Tract Surgery, FMU／Dept. Blood Transfusion and Transplantation Immunology, FMU)、Nirei Azuma (Dept. Gastrointestinal Tract Surgery, FMU)、Kikuchi Tomohiro (Dept. Gastrointestinal Tract Surgery, FMU)、Hanayama Hiroyuki (Dept. Gastrointestinal Tract Surgery, FMU)、Zenichiro Saze (Dept. Gastrointestinal Tract Surgery, FMU)、Momma Tomoyuki (Dept. Gastrointestinal Tract Surgery, FMU)、Nishiyama Kyoko (Dept. Microbiology, FMU)、Suzutani Tatsuo (Dept. Microbiology, FMU)、Koji Kono (Dept. Multidisciplinary Treatment of Cancer and Regional Medical Support, FMU／Dept. Gastrointestinal Tract Surgery, FMU)

Intratumoral ***Fusobacterium nucleatum*** (***Fn***) infection is associated with poor prognosis in esophageal cancer (EC) by influencing the tumor microenvironment (TME). The tumor cell-intrinsic cyclic GMP-AMP synthase (cGAS)/stimulator of interferon genes (STING) pathway plays an important role in regulating immune cell activation in the TME. This study investigated the impact of ***Fn*** infection on the activation of the tumor cell-intrinsic cGAS/STING pathway and its role in EC progression. Analysis of an EC patient cohort and ***in vitro*** co-culture experiments with EC cell lines and ***Fn*** revealed that high STING expression correlates with worse prognosis in ***Fn***-infected EC patients. ***Fn*** exposure significantly activated the STING pathway in EC cells, as shown by RNA-seq analysis, which indicated enhanced cytokine- and chemokine-related signaling. Specifically, ***Fn*** infection upregulated ***CCL20***, ***CXCL10***, and ***CSF2***, whose expression was linked to poor prognosis based on TCGA data analysis. These findings suggest that ***Fn***-induced STING activation and subsequent inflammatory cytokine and chemokine expression may contribute to EC progression and poor clinical outcomes.

[Disclosure of COI by Responsible researcher] Lead presenter is the Responsible researcher.
[Lead presenter: Nakajima Shotaro] ■Endowed chair: 医療法人社団 恵周会 白河病院

### [J-1064] I-J3
### Bacteria & Microbiome

2025 / 9 / 25 (Thu) 15:20-16:35 Room 9/(Hotel Nikko Kanazawa 3F Lumiere) Room 9

[Chairperson]Kentaro Inamura (Tumor Pathol., Jichi Med. Univ.)

---

### J-1064
### IL-33 may contribute to sex-related pathology of inflammatory colorectal cancer with alterations in the gut microbiota

[Speaker]Miho Akimoto (Dept. of Biochem., Teikyo Univ. Sch. of Med.) [Co-author]Noriyuki Okudaira (Dept. of Biochem., Teikyo Univ. Sch. of Med.)、Mimi Adachi (Dept. of Biochem., Teikyo Univ. Sch. of Med.)

**Background:** A mouse model of inflammatory colorectal cancer (CRC) shows sex differences in pathology. In the same model, a proinflammatory cytokine IL-33 contributes to malignant progression by regulating gut microbiota. Our recent findings suggest that IL-33 affect sex-specific gut microbiota, leading to sex-related CRC pathology. **Purpose:** Here, we investigated whether IL-33 is involved in sex-related CRC pathology using a mouse model. **Methods:** Male and female wild-type (WT) or IL-33 knockout (KO) mice were treated with azoxymethane and dextran sodium sulfate. Body weight and disease-associated index (DAI) were monitored, and colonic polyps were assessed. Fecal DNA was analyzed by 16S rRNA amplicon sequencing. **Results:** In WT mice, males showed greater weight loss, higher DAI, and more numerous and larger polyps than females, whereas these differences disappeared in KO mice. Gut bacteria associated with pathology and influenced by IL-33 were identified. **Conclusion:** IL-33, together with specific gut bacteria, may contribute to sex differences in inflammatory CRC pathology. Our ongoing spatial transcriptome analysis will explore host-microbiota interactions and molecular mechanisms.

[Disclosure of COI by Responsible researcher] Lead presenter is the Responsible researcher.
[Lead presenter: Miho Akimoto] ■Research funds from company: 科研費

### [J-1065] I-J3
### Bacteria & Microbiome

2025 / 9 / 25 (Thu) 15:20-16:35 Room 9/(Hotel Nikko Kanazawa 3F Lumiere) Room 9

[Chairperson]Kentaro Inamura (Tumor Pathol., Jichi Med. Univ.)

---

### J-1065
### Oral administration of Enterococcus faecalis induces pancreatitis, which is a risk factor for cancer.

[Speaker]Munefumi Shimosaka (Dept. of Mol. Biochem. and Clin. Investigation, Osaka Univ) [Co-author]Yudai Ishida (Dept. of Mol. Biochem. and Clin. Investigation, Osaka Univ)、Jumpei Kondo (Dept. of Mol. Biochem. and Clin. Investigation, Osaka Univ)、Asuka Ogata (Dept. of Mol. Biochem. and Clin. Investigation, Osaka Univ)、Kaho Nishikori (Dept. of Mol. Biochem. and Clin. Investigation, Osaka Univ)、Shinji Takamatsu (Dept. of Mol. Biochem. and Clin. Investigation, Osaka Univ)、Hirofumi Akita (Dept. of Gastroenterological Surg., Osaka Univ)、Hidetoshi Eguchi (Dept. of Gastroenterological Surg., Osaka Univ)、Eiji Miyoshi (Dept. of Mol. Biochem. and Clin. Investigation, Osaka Univ)

Recent literature supports intestinal microbiota play important roles in carcinogenesis. We have previously reported that Enterococcus faecalis (E. faecalis) was detected in pancreatic juices of many pancreatic cancer patients as well as certain cases of the region in subclinical chronic pancreatitis surrounding pancreatic cancer. Furthermore, we also reported E. faecalis can survive in pancreatic juice and invade pancreatic ductal cells, leading to chronic inflammation. However, it remains unknown at all whether E. faecalis causes pancreatitis. In this study, E. faecalis was orally administered to mice to determine whether the bacteria migrate to the pancreas and induce chronic pancreatitis.We found that long-term oral administration of E. faecalis resulted in the development of chronic pancreatitis as well as an increase in the expression of inflammatory cytokines. Administration of fluorescently labeled E. faecalis for a brief period induced the migration of the bacteria into the pancreas tissue. These results suggest that E. faecalis present in pancreatic juice could be one of the causes of chronically latent pancreatitis which may lead to pancreatic cancer.

[Disclosure of COI by Responsible researcher] Lead presenter is the Responsible researcher.
[Lead presenter: Munefumi Shimosaka] We have no financial relationships to disclose.

### [J-1066] I-J3
### Bacteria & Microbiome

2025 / 9 / 25 (Thu) 15:20-16:35 Room 9/(Hotel Nikko Kanazawa 3F Lumiere) Room 9

[Chairperson]Kentaro Inamura (Tumor Pathol., Jichi Med. Univ.)

---

### J-1066
### Influence of ***F. nucleatum***, pks+ ***E. coli***, and ETBF on the Tumor Microenvironment in Colorectal Cancer Liver Metastasis

[Speaker]Rumiko Saito (JFCR) [Co-author]Kentaro Inamura (JFCR／Jichi Medical University)、Yasuyuki Shigematu (JFCR)、Shunji Takahashi (JFCR)、Yuji Miura (JFCR)

Microbial influences on tumor development and progression have attracted attention. ***Fusobacterium nucleatum***, ***Escherichia coli*** carrying the polyketide synthetase gene (pks+ ***E. coli***), and enterotoxigenic ***Bacteroides fragilis*** (ETBF) have been implicated in colorectal cancer (CRC) incidence and progression. We investigated these bacterial species in CRC liver metastases. DNA was detected and quantified by droplet digital PCR. Immune cell densities in tumor-immune microenvironment (TIME) were assessed by immunohistochemistry and digital image analysis using tissue microarrays. Systemic inflammation was assessed using serum CRP levels. ***F. nucleatum*** was associated with increased systemic inflammation and immune suppression in TIME. Higher levels of pks+ ***E. coli*** correlated with decreased systemic inflammation and the TIME modulation. ETBF was detected in almost all cases and was associated with the altered TIME. These bacterial species influence the TIME through diverse mechanisms and could be potential diagnostic and therapeutic targets in CRC liver metastases. In addition, we are analyzing the interplay among these bacterial species and will present these results.

[Disclosure of COI by Responsible researcher] Lead presenter is not the Responsible researcher.
[Lead presenter: Rumiko Saito] We have no financial relationships to disclose.
[Principal Researcher: Kentaro Inamura] We have no financial relationships to disclose.

▲TOP

### [J-1067] II-J5
### Lung cancer

2025 / 9 / 25 (Thu) 15:20-16:35 Room 11/(ANA Crowne Plaza Hotel Kanazawa 3F Ohtori (West)) Room 11

[Chairperson]Hidenori Kitai (Dept. of Respir Med., Fac. of Med., Hokkaido Univ.)

---

### J-1067
### Analysis of lung cancer stem cells reprogrammed by positively charged hydrogels

[Speaker]Shinya Otsuka (Department of Cancer Pathology, Faculty of Medicine, Hokkaido University／Department of Thoracic Surgery, Hokkaido University Hospital) [Co-author]Masumi Tsuda (Department of Cancer Pathology, Faculty of Medicine, Hokkaido University／Institute for Chemical Reaction Design and Discovery (WPI-ICReDD), Hokkaido University)、Lei Wang (Department of Cancer Pathology, Faculty of Medicine, Hokkaido University／Institute for Chemical Reaction Design and Discovery (WPI-ICReDD), Hokkaido University)、Yoshitaka Oda (Department of Cancer Pathology, Faculty of Medicine, Hokkaido University)、Tatsuya Kato (Department of Thoracic Surgery, Hokkaido University Hospital)、Shinya Tanaka (Department of Cancer Pathology, Faculty of Medicine, Hokkaido University／Institute for Chemical Reaction Design and Discovery (WPI-ICReDD), Hokkaido University／Department of Surgical Pathology, Hokkaido University Hospital)

The treatment for lung cancer including molecular targeted therapy has rapidly advanced; however, the acquisition of drug resistance associated with these treatments remains an unsolved issue, where cancer stem cells are reported to be involved. Recently, we have shown that tumor cells cultured on hydrogels can be induced stemness in a short period of time as HARP phenomenon. However, research on lung cancer using this approach is limited, and studies utilizing clinical specimens have not been conducted.
In this study, lung cancer cell lines (A549, NCI-H1975, EBC-1) cultured on several hydrogels increased expression of stem cell markers (***Sox-2***, ***Nanog***, ***Oct3/4***) confirmed by RT-PCR. Notably, this effect was particularly pronounced in positively charged hydrogels. Furthermore, Western blotting and RNA sequencing were employed to elucidate the factors which underly molecular mechanisms for the acquisition of stemness. Additionally, we are trying to establish patient-derived xenograft models from surgically resected lung cancer specimens and cultivate tumor cells originated from them on hydrogels to validate the induction of stemness, ultimately aiming to overcome drug resistance.

[Disclosure of COI by Responsible researcher] Lead presenter is not the Responsible researcher.
[Lead presenter: Shinya Otsuka] We have no financial relationships to disclose.
[Principal Researcher: Shinya Tanaka] We have no financial relationships to disclose.

### [J-1068] II-J5
### Lung cancer

2025 / 9 / 25 (Thu) 15:20-16:35 Room 11/(ANA Crowne Plaza Hotel Kanazawa 3F Ohtori (West)) Room 11

[Chairperson]Hidenori Kitai (Dept. of Respir Med., Fac. of Med., Hokkaido Univ.)

---

### J-1068
### PDZ-binding kinase promotes cell migration and could be a promising therapeutic target in mesothelioma

[Speaker]Kazumi Hori (Dept. of Respiratory Med. Nagoya Univ. Grad. Sch. of Med.) [Co-author]Ichidai Tanaka (Dept. of Respiratory Med. Nagoya Univ. Grad. Sch. of Med.)、Tatsuhiro Sato (Div. of Cancer Biol, Aichi Cancer Ctr. Res. Inst.)、Mika Sato (Dept. of Respiratory Med. Nagoya Univ. Grad. Sch. of Med.)、Yuta Kodama (Dept. of Respiratory Med. Nagoya Univ. Grad. Sch. of Med.)、Hideyuki Itoigawa (Dept. of Respiratory Med. Nagoya Univ. Grad. Sch. of Med.)、Yuichi Abe (Div. of Mol. Diagnostics, Aichi Cancer Ctr Res. Inst.)、Taketo Kato (Dept. of Thoracic Surg., Nagoya Univ. Grad. Sch. of Med.)、Ayumu Taguchi (Div. of Mol. Diagnostics, Aichi Cancer Ctr Res. Inst.／Div. of Advanced Cancer Diagnostics, Nagoya Univ.)、Mitsuo Sato (Div. of Host Defense Sciences, Nagoya Univ.)、Yoshitaka Sekido (Div. of Cancer Biol, Aichi Cancer Ctr. Res. Inst.／Dept. of Mol. and Cellular Oncology, Nagoya Univ.)、Toyofumi Yoshikawa (Dept. of Thoracic Surg., Nagoya Univ. Grad. Sch. of Med.)、Makoto Ishii (Dept. of Respiratory Med. Nagoya Univ. Grad. Sch. of Med.)

Mesothelioma is an aggressive neoplasms with poor prognosis. We previously found that oxytocin receptors (OXTR) are highly expressed in mesothelioma and that OXTR knockdown reduces proliferation in OXTR-high cells. Here, we performed proteomic profiling of OXTR-high mesothelioma cells and identified PDZ-binding kinase (PBK) as a downstream effector. PBK knockdown suppressed proliferation, migration, colony formation, and reduced Akt/MAPK phosphorylation. While OXTR overexpression in low-PBK H2452 cells enhanced proliferation but not migration, PBK overexpression promoted both. Immunohistochemistry of patient samples showed high PBK expression correlated with poor survival (OS: P=0.0031, HR 3.339; RFS: P=0.0024, HR 3.355). TCGA analysis confirmed PBK mRNA as an independent predictor of OS. PBK is a key driver of mesothelioma aggressiveness and a promising therapeutic target and biomarker.

[Disclosure of COI by Responsible researcher] Lead presenter is not the Responsible researcher.
[Lead presenter: Kazumi Hori] ■Research funds from company: AMED革新的がん医療実用化研究事業
[Principal Researcher: Tatsuhiro Sato] ■Research funds from company: 基盤研究B　■Lecture fee: Astrazeneca 中外製薬

▲TOP

### [J-1069] II-J5
### Lung cancer

2025 / 9 / 25 (Thu) 15:20-16:35 Room 11/(ANA Crowne Plaza Hotel Kanazawa 3F Ohtori (West)) Room 11

[Chairperson]Hidenori Kitai (Dept. of Respir Med., Fac. of Med., Hokkaido Univ.)

---

### J-1069
### Antibiotics as a negative factor for Chemoimmunotherapy for non-small cell lung cancer with low PD-L1 expression

[Speaker]Tae Hata (Department of Pulmonary Medicine, Kyoto Prefectural University of Medicine) [Co-author]Tadaaki Yamada (Department of Pulmonary Medicine, Kyoto Prefectural University of Medicine)、Yasuhiro Goto (Department of Respiratory Medicine, Fujita Health University School of Medicine)、Akihiko Amano (Department of Respiratory Medicine, Kurashiki Central Hospital)、Takashi Kijima (Department of Respiratory Medicine and Hematology, Hyogo Medical University)、Satoshi Watanabe (Department of Respiratory Medicine and Infectious Diseases, Niigata University)、Naoki Furuya (Division of Respiratory Medicine, St. Marianna University School of Medicine)、Tomohiro Oba (Department of Respiratory Medicine, Saitama Red Cross Hospital)、Tatsuki Ikoma (Department of Thoracic Oncology, Kansai Medical University)、Akira Nakao (Department of Respiratory Medicine, Fukuoka University Hospital)、Keiko Tanimura (Department of Medical Oncology, Fukuchiyama City Hospital)、Hirokazu Taniguchi (Department of Respiratory Medicine, Nagasaki University)、Akihiro Yoshimura (Department of Respiratory Medicine, Japanese Red Cross Kyoto Daini Hospital)、Tomoya Fukui (Department of Respiratory Medicine, Shonan Kamakura General Hospital)、Daiki Murata (Division of Respirology, Kurume University School of Medicine)、Koichi Takayama (Department of Pulmonary Medicine, Kyoto Prefectural University of Medicine)

Objective: Alterations in the gut microbiota have been reported to reduce the efficacy of immunotherapy. However, predictive factors for the efficacy of chemoimmunotherapy (Chemo-IO) in non-small cell lung cancer (NSCLC) remain unclear. This study aimed to clarify the impact of prior antimicrobial treatment (ATB) on the efficacy of Chemo-IO.Methods: Patients with advanced NSCLC with low PD-L1 expression who received Chemo-IO or chemotherapy (Chemo) at 19 institutions were retrospectively examined.Results: Of 851 patients, 126 of whom received ATB within 1 month prior to treatment. Median overall survival (OS) and progression-free survival (PFS) in patients without prior ATB were longer in the Chemo-IO group than in the Chemo group (OS, $P = 0.02$: PFS, $P < 0.001$), while median OS and PFS for patients with prior ATB were not significantly different between the two groups (OS, $P = 0.24$: PFS, $P = 0.09$). In multivariate analysis, the history of ATB was independently associated with shorter OS in the Chemo-IO group ($P = 0.02$). The incidence of grade >=3 adverse events (AEs) increased in the ATB group.Conclusions: ATB attenuates the efficacy of ICI and increases AEs.

[Disclosure of COI by Responsible researcher] Lead presenter is not the Responsible researcher.
[Lead presenter: Tae Hata] We have no financial relationships to disclose.
[Principal Researcher: Tadaaki Yamada] ■Lecture fee: イーライリリー株式会社、中外製薬株式会社　■Research expenses: 小野薬品工業株式会社、武田薬品株式会社、ヤンセンファーマ株式会社

▲TOP

### [J-1070] II-J5
### Lung cancer

2025 / 9 / 25 (Thu) 15:20-16:35 Room 11/(ANA Crowne Plaza Hotel Kanazawa 3F Ohtori (West)) Room 11

[Chairperson]Hidenori Kitai (Dept. of Respir Med., Fac. of Med., Hokkaido Univ.)

---

### J-1070
### Identification of a neuropeptide receptor as a promising therapeutic target for small cell lung cancer

[Speaker]Masakatsu Tokunaga (Department of Respiratory Medicine, The University of Tokyo) [Co-author]Natsuki Nakagawa (Department of Respiratory Medicine, The University of Tokyo)、Mirei Ka (Next-Generation Precision Medicine Development Laboratory, The University of Tokyo)、Yuriko Sugiura (Department of Respiratory Medicine, The University of Tokyo)、Takahiro Iida (Department of Thoracic Surgery, The University of Tokyo)、Hiroaki Ikushima (Department of Respiratory Medicine, The University of Tokyo)、Takahiro Ando (Department of Respiratory Medicine, The University of Tokyo)、Kousuke Watanabe (Next-Generation Precision Medicine Development Laboratory, The University of Tokyo)、Akiko Kunita (Next-Generation Precision Medicine Development Laboratory, The University of Tokyo)、Hidenori Kage (Department of Respiratory Medicine, The University of Tokyo)、Masanori Kawakami (Department of Respiratory Medicine, The University of Tokyo)

Small cell lung cancer (SCLC) has a poor prognosis and limited treatment options. Most of these subtypes are neuroendocrine (NE)-type tumors. Since neural signaling has recently been noted to have an impact on cancer etiology, we hypothesized that some neurotransmitters/receptors regulate SCLC biology. A drug library screening of 560 neural-signaling-targeting compounds identified neuropeptide receptor X as a potential target for NE-type SCLC. Growth inhibition by the receptor X inhibitor correlated with receptor X expression. In silico analysis showed that receptor X expression was elevated in NE-type SCLC cells. Genetic knockdown or knockout of receptor X suppressed cell proliferation and induced apoptosis in NE-type SCLC cell lines, while ligand stimulation promoted cell growth. Phosphoprotein analysis implicated MAPK and PI3K-Akt pathways in receptor X signaling. A receptor X inhibitor suppressed tumor growth in vivo in an NE-type SCLC xenograft model. These findings highlight neural signaling as a novel therapeutic target in SCLC.

[Disclosure of COI by Responsible researcher] Lead presenter is not the Responsible researcher.
[Lead presenter: Masakatsu Tokunaga] We have no financial relationships to disclose.
[Principal Researcher: Masanori Kawakami] We have no financial relationships to disclose.

▲TOP

### [J-1071] II-J5
### Lung cancer

2025 / 9 / 25 (Thu) 15:20-16:35 Room 11/(ANA Crowne Plaza Hotel Kanazawa 3F Ohtori (West)) Room 11

[Chairperson]Hidenori Kitai (Dept. of Respir Med., Fac. of Med., Hokkaido Univ.)

---

### J-1071
### CHK1 as a novel therapeutic target for pleural mesothelioma

[Speaker]Kazumasa Akagi (Dept. Respiratory Med., Nagasaki Univ. Hosp.／Clin. Oncology Ctr., Nagasaki Univ. Hosp.) [Co-author]Hirokazu Taniguchi (Dept. Respiratory Med., Nagasaki Univ. Hosp.／Clin. Oncology Ctr., Nagasaki Univ. Hosp.)、Hiromi Tomono (Dept. Respiratory Med., Nagasaki Univ. Hosp.)、Noritaka Honda (Dept. Respiratory Med., Nagasaki Univ. Hosp.)、Yosuke Dotsu (Dept. Respiratory Med., Nagasaki Univ. Hosp.)、Midori Matsuo (Dept. Respiratory Med., Nagasaki Univ. Hosp.)、Shinnosuke Takemoto (Dept. Respiratory Med., Nagasaki Univ. Hosp.)、Yasuhiko Nakao (Dept. Gastroenterology & Hepatology, Nagasaki Univ. Hosp.)、Seiji Yano (Dept. Respiratory Med., Kanazawa Univ. Hosp.／Nano Life Sci. Inst., Kanazawa Univ.)、Kazuto Ashizawa (Clin. Oncology Ctr., Nagasaki Univ. Hosp.)、Hiroshi Mukae (Dept. Respiratory Med., Nagasaki Univ. Hosp.)

Background Pleural mesothelioma (PM) is a rare thoracic malignant tumor with poor prognosis and development of therapeutic strategy with novel mechanisms is desired. In this study, we focused on CHK1 as a candidate for novel therapeutic target. Method The anti-tumor effects of CHK1 inhibitor (CHKi) were evaluated by MTT assay and annexin V assay. The mRNA and protein expression were evaluated by qRT-PCR and Western blotting, respectively. The anti-tumor effects of CHK1i in vivo were evaluated with the pleural carcinomatosis model established by injecting the mouse PM cells into the intrapleural space of Balb/c mouse. Results CHK1i had anti-tumor effect and induced apoptosis. CHK1i activated STING pathway and induced mRNA expression of type I and II interferons, suggesting CHK1i activated anti-tumor immune response. PD-L1 expression was also increased by CHK1i. Furthermore, CHK1i combined with anti-PD-L1 antibody significantly prolonged overall survival in the pleural carcinomatosis model. Conclusion The CHK1i showed immunostimulatory effect on PM in addition to direct anti-tumor effect. Furthermore, the therapeutic benefit was enhanced when combined with anti-PD-L1 antibody.

[Disclosure of COI by Responsible researcher] Lead presenter is not the Responsible researcher.
[Lead presenter: Kazumasa Akagi] We have no financial relationships to disclose.
[Principal Researcher: Hirokazu Taniguchi] We have no financial relationships to disclose.

### [J-1072] II-J5
### Lung cancer

2025 / 9 / 25 (Thu) 15:20-16:35 Room 11/(ANA Crowne Plaza Hotel Kanazawa 3F Ohtori (West)) Room 11

[Chairperson]Hidenori Kitai (Dept. of Respir Med., Fac. of Med., Hokkaido Univ.)

---

### J-1072
### Exploring molecular mechanisms of lung adenocarcinoma with interstitial pneumonia using spatial transcriptomic analysis

[Speaker]Akifumi Mochizuki (Dept. Resp. Med., Inst Sci Tokyo.／Div. Genome Biol., Natl. Cancer Ctr. Res. Inst.) [Co-author]Kouya Shiraishi (Div. Genome Biol., Natl. Cancer Ctr. Res. Inst.)、Takayuki Honda (Dept. Resp. Med., Inst Sci Tokyo.)、Ayako Suzuki (CBMS, Grad. Sch. Front. Sci., Univ. Tokyo)、Syuzo Kaneko (Div. Medical AI Res. Dev., Natl. Cancer Ctr. Res. Inst.)、Yukihiro Yoshida (Dept. Thoracic Surg., Natl. Cancer Ctr. Hosp.)、Genichiro Ishii (Dept. Pathol. Clin. Lab. National Cancer Center Hospital East)、Issei Imoto (Aichi Cancer Ctr. Res. Inst.)、Ryuji Hamamoto (Div. Medical AI Res. Dev., Natl. Cancer Ctr. Res. Inst.)、Yasushi Yatabe (Dept. Diagnostic Path., Natl. Cancer Ctr. Hosp.)、Yutaka Suzuki (CBMS, Grad. Sch. Front. Sci., Univ. Tokyo)、Takashi Kohno (Div. Genome Biol., Natl. Cancer Ctr. Res. Inst.)

Interstitial pneumonia increases lung cancer risk, yet underlying carcinogenic mechanisms remain poorly understood. We conducted comprehensive whole genome and RNA sequencing of 44 lung adenocarcinomas (LADCs) associated with usual interstitial pneumonia (UIP) compared to 216 UIP-negative LADCs. UIP-positive LADCs showed distinctive features: low mutation burden, high frequency of NKX2-1 genomic aberrations and abundant myofibroblastic cancer-associated fibroblasts (myCAFs). NKX2-1 genomic alterations (41%) created two distinct profiles: NKX2-1-deficient tumors had gastrointestinal differentiation, ERBB2 amplification with super-enhancer formation, and "cold" immune microenvironment; NKX2-1-proficient tumors showed "hot" immune characteristics. Spatial transcriptomics using Visium and Xenium in situ platform (lung/5K panel) revealed myCAFs expressing WNT5A or CTHRC1 potentially originated from UIP-associated myofibroblasts. Findings propose a dual model of UIP-associated lung carcinogenesis, offering targeted therapeutic potential based on NKX2-1 status.

[Disclosure of COI by Responsible researcher] Lead presenter is not the Responsible researcher.
[Lead presenter: Mochizuki Akifumi] We have no financial relationships to disclose.
[Principal Researcher: Takashi Kohno] ■Patent fee: Thermo Fisher Scientific、理研Genesis、Amoy、Foundation Medicine ■Lecture fee: 日本イーライリリー ■Manuscript fee: なし ■Research expenses: シスメックス 、中外製薬、コニカミノルタ、ガーダントヘルス、日本イーライリリー、ユーロフィンクリニカルジェネティックス

### [E-1061] I-E11-3
### Immune checkpoint inhibitors (1)

2025 / 9 / 25 (Thu) 15:20-16:35 Room 12/(ANA Crowne Plaza Hotel Kanazawa 3F Ohtori (Central)) Room 12

[Chairperson]Kenji Chamoto (Dept. Cancer Immunol. PDT, Kyoto Univ. Grad. Sch. Med.)

---

### E-1061
### Withdrawn

No Abstract

### [E-1062] I-E11-3
### Immune checkpoint inhibitors (1)

2025 / 9 / 25 (Thu) 15:20-16:35 Room 12/(ANA Crowne Plaza Hotel Kanazawa 3F Ohtori (Central)) Room 12

[Chairperson]Kenji Chamoto (Dept. Cancer Immunol. PDT, Kyoto Univ. Grad. Sch. Med.)

### E-1062
### Discovery of Novel Peptide Inhibitors Targeting the NKG2A–HLA-E Immune Checkpoint via RaPID selection

[Speaker]Nicholas Loh (Grad. Sch. Sci., UTokyo／JFCR) [Co-author]Haruo Aikawa (Grad. Sch. Sci., UTokyo)、Naohiro Terasaka (Grad. Sch. Sci., UTokyo)、Yoko Yoshida (JFCR)、Sho Isoyama (JFCR)、Shingo Dan (JFCR)、Hiroaki Suga (Grad. Sch. Sci., UTokyo)

The NKG2A–HLA-E immune checkpoint interaction is formed between NKG2A on NK and T cells, and the MHC Class I receptor HLA-E. Cancer cells upregulate the expression of HLA-E to escape from immune cells via this interaction. To inhibit this, NKG2A was selected as the target for the development of a peptide drug.Screening of a large peptide library against NKG2A led to the discovery of multiple potential inhibitors. However, NKG2C, an almost identical protein to NKG2A and is known to bind to HLA-E to stimulate a response in adaptive NK cells. This emphasizes the need for selective binders towards NKG2A. By including NKG2C in the screening process and collecting the peptides that bind only to NKG2A, hit peptides of $K_D$ <5 nM selective towards NKG2A were obtained.To evaluate the bioactivity of the hit peptides, a killing assay was designed using NK92MI as effector cells. With the insertion of the [HLA-ligand] – [β2m] – [HLA-E heavy chain] single chain trimer gene via lentiviral transduction into K562 and Daudi cells, the interaction was emulated for the evaluation of the hit peptides.Due to potential patent applications, the specifics of the peptides will be redacted.

[Disclosure of COI by Responsible researcher] Lead presenter is the Responsible researcher.
[Lead presenter: Nicholas Loh] We have no financial relationships to disclose.

### [E-1063] I-E11-3
### Immune checkpoint inhibitors (1)

2025 / 9 / 25 (Thu) 15:20-16:35 Room 12/(ANA Crowne Plaza Hotel Kanazawa 3F Ohtori (Central)) Room 12

[Chairperson]Kenji Chamoto (Dept. Cancer Immunol. PDT, Kyoto Univ. Grad. Sch. Med.)

### E-1063
### Low-Dose Radioimmunotherapy Using LDDS with Anti-CTLA-4 mAb for Lymph Node Metastasis

[Speaker]Wilda Septilah Aulia (Graduate school of dentistry, Tohoku University) [Co-author]Wilda S. Aulia (Graduate school of dentistry, Tohoku University)、Ariunbuyan Sukhbaatar (Graduate school of dentistry, Tohoku University／Laboratory of Biomedical engineering for cancer, Tohoku University)、Shiro Mori (Graduate school of dentistry, Tohoku University)、Tsuyoshi Sugiura (Graduate school of dentistry, Tohoku University)、Tetsuya Kodama (Laboratory of Biomedical engineering for cancer, Tohoku University)

Anti-CTLA-4 monoclonal antibody (mAb) therapy represents a promising strategy for treating lymph node metastases and advanced tumors. However, dose escalation of anti-CTLA-4 mAb has been associated with a significant increase in immune-related adverse events (irAEs). To address this limitation, recent attention has focused on combining low-dose radiotherapy with anti-CTLA-4 mAb delivered via a locoregional drug delivery system (LDDS). LDDS offers improved pharmacokinetics by enabling direct administration to the target site. Additionally, previous studies have shown that low-dose radiotherapy can enhance immune activation both locally and systemically. In this study, we investigated the therapeutic efficacy of combining 2 Gy radiotherapy with anti-CTLA-4 mAb (1 mg/kg) administered through LDDS to tumor-bearing lymph nodes. The combination treatment significantly suppressed tumor progression. Notably, this approach also prevented the onset of irAEs, suggesting a safer and effective alternative to systemic administration. These findings support the potential of LDDS-based low-dose radioimmunotherapy as a novel therapeutic strategy for metastatic cancers with reduced toxicity.

[Disclosure of COI by Responsible researcher] Lead presenter is the Responsible researcher.
[Lead presenter: Wilda Septilah Aulia] We have no financial relationships to disclose.

### [E-1064] I-E11-3
### Immune checkpoint inhibitors (1)

2025 / 9 / 25 (Thu) 15:20-16:35 Room 12/(ANA Crowne Plaza Hotel Kanazawa 3F Ohtori (Central)) Room 12

[Chairperson]Kenji Chamoto (Dept. Cancer Immunol. PDT, Kyoto Univ. Grad. Sch. Med.)

---

### E-1064
### Intracranial myeloid cells increase combination immunotherapy against brain metastasis by activating CD4+ TFH cells

[Speaker]Ryo Omae (Dept. of Tumor Microenvironment, Okayama Univ.／Dept. of Neurological Surg., Okayama Univ.) [Co-author]Toshifumi Ninomiya (Dept. of Tumor Microenvironment, Okayama Univ.／Dept. of Respiratory Med., Kyushu Univ.)、Naoya Kemmotsu (Dept. of Tumor Microenvironment, Okayama Univ.／Dept. of Neurological Surg., Okayama Univ.)、Masaki Magari (Dept. of Applied Cell Biol., Okayama Univ.)、Ai Miyamoto (Dept. of Medical Protein Engineering, Okayama Univ.)、Youki Ueda (Dept. of Tumor Microenvironment, Okayama Univ.)、Takamasa Ishino (Dept. of Tumor Microenvironment, Okayama Univ.)、Joji Nagasaki (Dept. of Tumor Microenvironment, Okayama Univ.)、Tomohiro Fujiwara (Dept. of Orthopaedic Surg., Okayama Univ.)、Shota Tanaka (Dept. of Neurological Surg., Okayama Univ.)、Shinichi Toyooka (Dept. of Thoracic, Breast and Endocrinological Surg., Okayama Univ.)、Isamu Okamoto (Dept. of Respiratory Med., Kyushu Univ.)、Yosuke Togashi (Dept. of Tumor Microenvironment, Okayama Univ.／Dept of Allergy and Respiratory Medicine, Okayama Univ. Hosp.／Kindai Univ. Faculty of Medicine)

Combination immunotherapy with an anti-CTLA-4 monoclonal antibody (mAb) has been reported to demonstrate potent antitumor effects against brain metastasis. We analyzed murine brain metastasis models, revealing that CD4+ T follicular helper (TFH) cells express high levels of CTLA-4 in the intracranial tumor microenvironment (TME). CTLA-4 blockade induced the secretion of IL-21 from intracranial TFH cells, which in turn activated CD8+ T cells, leading to antitumor effects. In contrast, the loss of TFH cells reduced the additive effect of anti-CTLA-4 mAb with anti-PD-1 mAb. Furthermore, single-cell RNA sequencing revealed that myeloid cells, particularly CD80hiCD206lo proinflammatory M1-like macrophages, which are abundant in the intracranial TME, produce B cell-activating factor (BAFF) and a proliferation-inducing ligand (APRIL), leading to the infiltration and activation of both B cells and TFH cells. These findings provide deep insights into the intracranial TME, supporting the potential of combination therapy with CTLA-4 blockade for brain metastasis.

[Disclosure of COI by Responsible researcher] Lead presenter is not the Responsible researcher.
[Lead presenter: Ryo Omae] ■Research funds from company: JP22K1945904・JP22K20824・JP23K14594（日本医療研究開発機構（AMED-CREST JP22gm1810002s0101）、科学技術振興機構（JPMJFR2049）、武田科学振興財団、第一三共生命科学財団、日本呼吸器学会、ウェスコ財団、上原記念生命科学財団、千里生命科学財団、未来プロジェクトがん研究助成金、興和生命科学財団、日本臨床薬理学会研究財団、中外製薬革新的薬物発見科学財団、内藤記念財団、安田医療財団、日本新薬公的研究助成金
[Principal Researcher: Yosuke Togashi] ■Research funds from company: JP22K1945904・JP22K20824・JP23K14594（日本医療研究開発機構（AMED-CREST JP22gm1810002s0101）、科学技術振興機構（JPMJFR2049）、武田科学振興財団、第一三共生命科学財団、日本呼吸器学会、ウェスコ財団、上原記念生命科学財団、千里生命科学財団、未来プロジェクトがん研究助成金、興和生命科学財団、日本臨床薬理学会研究財団、中外製薬革新的薬物発見科学財団、内藤記念財団、安田医療財団、日本新薬公的研究助成金　■Lecture fee: 中外製薬、エーザイ、アストラゼネカ、ブリストルマイヤーズスクイブ、MSD　■Research expenses: ヤンセンファーマ、アストラゼネカ、 WJOG、大鵬薬品工業、武田薬品工業　■Contributions: （公財）小野薬品 がん・免疫・神経研究財団、（公財）小林がん学術振興会、（公財）武田科学振興財団、（公財）アステラス病態代謝研究会、（公財）上原記念生命科学財団

### [E-1065] I-E11-3
### Immune checkpoint inhibitors (1)

2025 / 9 / 25 (Thu) 15:20-16:35 Room 12/(ANA Crowne Plaza Hotel Kanazawa 3F Ohtori (Central)) Room 12

[Chairperson]Kenji Chamoto (Dept. Cancer Immunol. PDT, Kyoto Univ. Grad. Sch. Med.)

---

### E-1065
### SIGLEC7 in NK cells act as an immune checkpoint receptor for VSIG4

[Speaker]Yutaka Kasai (Dept. Mol. Biol., Inst. Adv. Med. Sci., Nippon Med. Sch.) [Co-author]Takeshi Ito (Sect. Med. Oncol., Dept. Intern. Med., Yale Sch. Med.／Div. Mol. Pathol., Inst. Med. Sci., Univ. Tokyo)、Yoshinori Murakami (Dept. Mol. Biol., Inst. Adv. Med. Sci., Nippon Med. Sch.)

Immune checkpoint inhibitors (ICIs) have been approved for the treatment of various types of cancers, but only a limited number of cases respond to current ICIs. To identify novel targets for ICIs, we employed the interaction-screening within approximately 300 immunoglobulin superfamily (IgSF) proteins, which includes most of the known immune checkpoint molecules. Amplified luminescence proximity homogeneous assay (ALPHA) identified the novel interaction between VSIG4 (V-set and immunoglobulin-containing 4) and SIGLEC7 (Sialic acid-binding Ig-like lectin 7). VSIG4 has been considered as a ligand for an unknown immune checkpoint receptor, whereas SIGLEC7 is an inhibitory receptor expressed predominantly on NK cells. Co-culturing VSIG4-expressing cancer cells with NK cells inhibited NK cell activity, as indicated by reduced cytokine secretion and cytotoxicity. Moreover, the blockade of the VSIG4-SIGLEC7 interaction using anti-VSIG4 or anti-SIGLEC7 antibodies restored NK cell activity. Taken together, SIGLEC7 would function as an inhibitory receptor for VSIG4 and targeting the VSIG4-SIGLEC7 interaction represents a potential therapeutic approach for cancer treatment.

[Disclosure of COI by Responsible researcher] Lead presenter is not the Responsible researcher.
[Lead presenter: Yutaka Kasai] We have no financial relationships to disclose.
[Principal Researcher: Takeshi Ito, Yoshinori Murakami] We have no financial relationships to disclose.

### [E-1066] I-E11-3
### Immune checkpoint inhibitors (1)

2025 / 9 / 25 (Thu) 15:20-16:35 Room 12/(ANA Crowne Plaza Hotel Kanazawa 3F Ohtori (Central)) Room 12

[Chairperson]Kenji Chamoto (Dept. Cancer Immunol. PDT, Kyoto Univ. Grad. Sch. Med.)

### E-1066
### Identification of an immune-activating and tumor-suppressive cancer-associated fibroblast subtype in gastric cancer

[Speaker]Huaitao(Kumamoto Univ.) Wang (Department of Gastroenterological Surgery, Kumamoto University) [Co-author]Takashi Semba (Cancer Institute, Japanese Foundation for Cancer Research)、Masaya Yamazaki (Cancer Institute, Japanese Foundation for Cancer Research)、Atsuko Yonemura (Cancer Institute, Japanese Foundation for Cancer Research)、Takatsugu Ishimoto (Cancer Institute, Japanese Foundation for Cancer Research)

The tumor microenvironment (TME), particularly cancer-associated fibroblasts (CAFs), drives progression and therapy resistance in advanced gastric cancer (GC). While protumorigenic CAFs are well-studied, tumor-suppressive CAFs remain poorly characterized. We analyzed two scRNA-seq datasets from 27 GC patients to identify antitumor genes in CAF subclusters. Validations included multiplex IHC, Transwell assays, qPCR, ELISA, and in vivo models. scRNA-seq identified RARRES2(Chemerin) as a hallmark gene in tumor-suppressive CAFs. Chemerin+ CAFs spatially colocalized with CD8+ T cells in GC tissues, correlating with enhanced lymphocyte infiltration. Recombinant Chemerin promoted CD8+ T cell migration in vitro. Tumor cells suppressed Chemerin in CAFs via the Jag1-Notch2-HDAC5 axis, validated in co-culture models. CAF-specific RARRES2 overexpression reduced tumor growth and amplified CD8+ T cell recruitment in vivo. This study identifies Chemerin+ CAFs as stromal regulators of antitumor immunity via CD8+ T cell recruitment. Tumor-derived Jag1-Notch2-HDAC5 signaling epigenetically silences Chemerin, highlighting cancer-stromal crosstalk that subverts antitumor functions.

[Disclosure of COI by Responsible researcher] Lead presenter is not the Responsible researcher.
[Lead presenter: Huaitao Wang] We have no financial relationships to disclose.
[Principal Researcher: Takatsugu Ishimoto] We have no financial relationships to disclose.

### [E-1067] I-E12-2
### Cancer diagnosis & biomarkers

2025 / 9 / 25 (Thu) 15:20-16:35 Room 13/(ANA Crowne Plaza Hotel Kanazawa 3F Ohtori (East)) Room 13

[Chairperson]Kohei Taniguchi (Div. Trans. Res., Osaka Med. Pharm. Univ.)

---

### E-1067
### Withdrawn

No Abstract

▲TOP

### [E-1068] I-E12-2
### Cancer diagnosis & biomarkers

2025 / 9 / 25 (Thu) 15:20-16:35 Room 13/(ANA Crowne Plaza Hotel Kanazawa 3F Ohtori (East)) Room 13

[Chairperson]Kohei Taniguchi (Div. Trans. Res., Osaka Med. Pharm. Univ.)

---

### E-1068
### Withdrawn

No Abstract

▲TOP

### [E-1069] I-E12-2
### Cancer diagnosis & biomarkers

2025 / 9 / 25 (Thu) 15:20-16:35 Room 13/(ANA Crowne Plaza Hotel Kanazawa 3F Ohtori (East)) Room 13

[Chairperson]Kohei Taniguchi (Div. Trans. Res., Osaka Med. Pharm. Univ.)

---

### E-1069
### Early detection of pancreatic cancer by comprehensive serummiRNA sequencing with machine learning: A multicenter study

[Speaker]Munenori Kawai (Gastroenterology and Hepatology, Kyoto University Graduate School of Medicine) [Co-author]Akihisa Fukuda (Gastroenterology and Hepatology, Kyoto University Graduate School of Medicine)、Kosuke Minaga (Department of Gastroenterology and Hepatology, Kindai University Faculty of Medicine)、Masanori Asada (Department of Gastroenterology and Hepatology, Osaka Red Cross Hospital)、Atsushi Umemura (Department of Pharmacology, Kyoto Prefectural University of Medicine)、Yoshito Uenoyama (Gastroenterology and Hepatology, Japanese Red Cross Wakayama Medical Center)、Tomonori Masuda (Department of Gastroenterology and Hepatology, Otsu Red Cross Hospital)、Toshihiro Morita (Department of Gastroenterology and Hepatology, Kitano Hospital)、Ryuki Minami (Department of Gastroenterology, Tenri Hospital)、Saiko Marui (Department of Gastroenterology and Hepatology, Shiga General Hospital)、Yuki Yamauchi (Department of Gastroenterology, Hyogo Prefectural Amagasaki General Medical Center)、Yoshitaka Nakai (Department of Gastroenterology and Hepatology, Kyoto Katsura Hospital)、Yutaka Takada (Department of Gastroenterology and Hepatology, Kobe City Nishi-Kobe Medical Center)、Kei Iimori (Gastroenterology and Hepatology, Kyoto University Graduate School of Medicine)、Hiroshi Seno (Gastroenterology and Hepatology, Kyoto University Graduate School of Medicine)

Early-stage diagnosis of pancreatic cancer is difficult because of nonspecific symptoms and lack of available biomarkers. We performed comprehensive serum miRNA sequencing of 212 pancreatic cancer patient samples from 14 hospitals and 213 non-cancerous healthy control samples. We randomly classified the pancreatic cancer and control samples into the two cohorts: a training cohort (N = 185) and a validation cohort (N = 240). We created ensemble models that combined automated machine learning with 100 highly expressed miRNAs and their combination with CA19-9 and validated the performance of the models in the independent validation cohort. The diagnostic model with the combination of the 100 highly expressed miRNAs and CA19-9 could discriminate pancreatic cancer from non-cancer healthy control with high accuracy (area under the curve (AUC), 0.99). We validated high accuracy in an independent asymptomatic early-stage (stage 0-I) pancreatic cancer cohort (AUC, 0.97) and CA19-9 negative pancreatic cancer cohort (AUC, 0.97). We demonstrate that the 100 highly expressed miRNAs and their combination with CA19-9 could be biomarkers for the specific and early detection of pancreatic cancer.

[Disclosure of COI by Responsible researcher] Lead presenter is not the Responsible researcher.
[Lead presenter: Munenori Kawai] We have no financial relationships to disclose.
[Principal Researcher: Akihisa Fukuda] ■Research funds from company: ARKRAY, Inc

▲TOP

### [E-1070] I-E12-2
### Cancer diagnosis & biomarkers

2025 / 9 / 25 (Thu) 15:20-16:35 Room 13/(ANA Crowne Plaza Hotel Kanazawa 3F Ohtori (East)) Room 13

[Chairperson]Kohei Taniguchi (Div. Trans. Res., Osaka Med. Pharm. Univ.)

---

### E-1070
### Development of serum immunodiagnostics for very-early pancreatic cancer

[Speaker]Naohiko Koshikawa (Inst of Science Tokyo, Life Sciences and Technology) [Co-author]Shinya Sato (Kanagawa Cancer Cent Res Inst)、Masatoshi Nakagawa (Abbott Japan Res and Dev)、Kouki Nio (Grad Sch Med Sci, Kanazawa Univ)、Toru Yoshimura (Abbott Japan Res and Dev)、Makoto Ueno (Gastroentero Kanagawa Cancer Cent Hospital)、Taro Yamashita (Grad Sch Med Sci, Kanazawa Univ)

We have reported a novel molecular mechanism in which MT1-MMP cleaves the ligand-binding domain of EphA2, resulting in the loss of its tumor-suppressive function while promoting tumorigenic signaling (Cancer Res, 2015). This cleavage generates an N-terminal EphA2 fragment (EphA2-NF), which is released from the cell membrane and serves as a cancer-specific post-translational biomarker.To quantify serum EphA2-NF, we established a fully automated chemiluminescent immunoassay using an EphA2-NF-specific antibody. Analysis of serum from over 500 pancreatic cancer (PC) patients revealed that EphA2-NF is detectable in ~60% of early-stage (Stage I/II) cases. Furthermore, pathological analysis of resected intraductal papillary mucinous neoplasm (IPMN) tissues, precursors of PC, showed that all 19 cases diagnosed post-resection as pancreatic cystic adenocarcinoma exhibited loss of the EphA2 N-terminus (Cancer Res Comm, 2023).These findings suggest that EphA2 processing plays a role in PC development and that serum EphA2-NF is a promising biomarker for early-stage PC.

[Disclosure of COI by Responsible researcher] Lead presenter is the Responsible researcher.
[Lead presenter: Naohiko Koshikawa] ■Research expenses: Abbott Laboratories

### [E-1071] I-E12-2
### Cancer diagnosis & biomarkers

2025 / 9 / 25 (Thu) 15:20-16:35 Room 13/(ANA Crowne Plaza Hotel Kanazawa 3F Ohtori (East)) Room 13

[Chairperson]Kohei Taniguchi (Div. Trans. Res., Osaka Med. Pharm. Univ.)

---

### E-1071
### Validation of extracellular vesicle protein biomarkers in multiple myeloma

[Speaker]Yuko Shirouchi (Division of Analytical Biochemistry, CPM Center, JFCR／Department of Hematology Oncology, Cancer Institute Hospital, JFCR) [Co-author]Yoshimi Haga (Division of Analytical Biochemistry, CPM Center, JFCR)、Yuriko Minegishi (Division of Analytical Biochemistry, CPM Center, JFCR)、Kiminori Hori (Division of Analytical Biochemistry, CPM Center, JFCR)、Yuko Mishima (Department of Hematology Oncology, Cancer Institute Hospital, JFCR／Division of Clinical Research, the Cancer Chemotherapy Center, JFCR)、Dai Maruyama (Department of Hematology Oncology, Cancer Institute Hospital, JFCR)、Koji Ueda (Division of Analytical Biochemistry, CPM Center, JFCR)

Multiple myeloma (MM) is an incurable plasma cell neoplasm characterized by frequent relapses. Thus, developing biomarkers for early diagnosis, risk stratification, and disease monitoring is crucial. We previously identified 8839 proteins through comprehensive proteomic analysis of bone marrow-derived extracellular vesicles (EVs), from which 16 were selected for further evaluation in serum-derived EVs. Using targeted proteomic analysis, we identified serum EV-BCMA, IL5RA, and LSR as potential diagnostic biomarkers and serum EV-BCMA and IL5RA as potential prognostic biomarkers in untreated MM. In this study, we aimed to assess their potential for disease monitoring through longitudinal serum sampling and validate these findings in an independent cohort. Sera from 44 MM patients and 30 healthy individuals were collected. EVs were isolated and analyzed using targeted proteomics with mass spectrometric absolute quantification of the candidate proteins. We evaluated diagnostic accuracy, prognostic value for PFS, and feasibility of relapse detection, supporting the potential of the serum EV proteins as diagnostic and prognostic biomarkers and their application for disease monitoring.

[Disclosure of COI by Responsible researcher] Lead presenter is not the Responsible researcher.
[Lead presenter: Yuko Shirouchi] We have no financial relationships to disclose.
[Principal Researcher: Koji Ueda] We have no financial relationships to disclose.

▲TOP

### [E-1072] I-E12-2
### Cancer diagnosis & biomarkers

2025 / 9 / 25 (Thu) 15:20-16:35 Room 13/(ANA Crowne Plaza Hotel Kanazawa 3F Ohtori (East)) Room 13

[Chairperson]Kohei Taniguchi (Div. Trans. Res., Osaka Med. Pharm. Univ.)

---

### E-1072
### Rapid Intraoperative Genetic Analysis of Adult-type Diffuse Gliomas Using a Microfluidic Real-Time PCR Device

[Speaker]Fumiharu Ohka (Dept. of Neurosurgery, Nagoya Univ.) [Co-author]Sachi Maeda (Dept. of Neurosurgery, Nagoya Univ.)、Kazuya Motomura (Dept. of Neurosurgery, Nagoya Univ.)、Kosuke Aoki (Dept. of Neurosurgery, Nagoya Univ.)、Shoichi Deguchi (Dept. of Neurosurgery, Nagoya Univ.)、Yoshiki Shiba (Dept. of Neurosurgery, Nagoya Univ.)、Junya Yamaguchi (Dept. of Neurosurgery, Nagoya Univ.)、Keisuke Kimura (Dept. of Neurosurgery, Nagoya Univ.)、Yuhei Takido (Dept. of Neurosurgery, Nagoya Univ.)、Ryo Yamamoto (Dept. of Neurosurgery, Nagoya Univ.)、Akihiro Nakamura (Dept. of Neurosurgery, Nagoya Univ.)、Keiko Shinjo (Div. of Cancer Biology, Nagoya Univ.)、Yutaka Kondo (Div. of Cancer Biology, Nagoya Univ.)、Kennosuke Karube (Dept. of Pathology and Laboratory Medicine, Nagoya Univ.)、Ryuta Saito (Dept. of Neurosurgery, Nagoya Univ.)

In adult-type diffuse gliomas, IDH and ***TERT*** promoter mutations play pivotal roles in the molecular classification. This study aimed to develop a rapid genotyping system using GeneSoC®, a real-time PCR platform with microfluidic thermal cycling capable of completing 50-cycles of PCR within 20 min.
Using frozen tumor tissues from 67 patients, this system demonstrated a detection limit of at least 5% variant allele frequency for the ***IDH1*** R132H and ***TERT*** promoter C228T/C250T mutations. Subsequently, intraoperative testing was performed in 111 cases using this system. The sensitivity and specificity of ***IDH1*** R132H mutation were 0.983 and 0.979, respectively, whereas, those of ***TERT*** promoter C228T/C250T mutation were 1.000 and 1.000, respectively. These mutations were detected intraoperatively within approximately 25 min after collection of tumor tissue. Furthermore, this assay identified tumor boundaries in an IDH-mutated glioma case, where ***IDH1*** R132H mutations could not be detected.
The GeneSoC®-based rapid genotyping system may be effective not only for the intraoperative diagnosis of diffuse glioma but also for the detection of tumor boundaries.

[Disclosure of COI by Responsible researcher] Lead presenter is the Responsible researcher.
[Lead presenter: Fumiharu Ohka] We have no financial relationships to disclose.

▲TOP

### [E-1073] II-E6
### Frontiers in breast cancer

2025 / 9 / 25 (Thu) 15:20-16:35 Room 14/(ANA Crowne Plaza Hotel Kanazawa 3F Zuiun 1) Room 14

[Chairperson]Mamoru Takada (Dept. General Surg.,Chiba Univ.,Sch., Med.)

### E-1073
### Soluble TREM2 Drives Triple-Negative Breast Cancer Progression via TG2-AKT Pathway

[Speaker]Yu Tian (School of Life Sciences, Jiangsu University, Zhenjiang, China／International Genome Center, Jiangsu University, Zhenjiang, China) [Co-author]Yan Wu (School of Medicine, Jiangsu University, Zhenjiang, China)、JIng Jin (School of Medicine, Jiangsu University, Zhenjiang, China)、Zhaoliang Su (School of Life Sciences, Jiangsu University, Zhenjiang, China／International Genome Center, Jiangsu University, Zhenjiang, China)

Triggering receptor expressed on myeloid cells 2 (TREM2) plays a key role in immune regulation, particularly within tumor-associated macrophages (TAMs). In triple-negative breast cancer (TNBC), $TREM2^{+}$ TAMs have been shown to modulate the tumor microenvironment, but the role of its soluble form (sTREM2), produced through proteolytic cleavage, remains unclear. In this study, we investigated the effects of sTREM2 on TNBC progression. ***In vitro***, treatment of TNBC cells with recombinant sTREM2 or sTREM2-containing culture supernatant significantly enhanced cell proliferation, invasion, and migration. These effects were further confirmed by the use of TREM2-neutralizing antibodies, which abrogated sTREM2's tumor-promoting activities. In vivo, peri-tumoral injections of recombinant sTREM2 led to a notable acceleration of tumor growth in mouse models. Mechanistically, we found that sTREM2's effects were mediated through activation of the AKT signaling pathway. Collectively, our findings suggest that sTREM2 drives TNBC progression by enhancing critical tumor cell functions, positioning it as a potential therapeutic target for TNBC treatment.

[Disclosure of COI by Responsible researcher] Lead presenter is the Responsible researcher.
[Lead presenter: Yu Tian] We have no financial relationships to disclose.

▲TOP

### [E-1074] II-E6
### Frontiers in breast cancer

2025 / 9 / 25 (Thu) 15:20-16:35 Room 14/(ANA Crowne Plaza Hotel Kanazawa 3F Zuiun 1) Room 14

[Chairperson]Mamoru Takada (Dept. General Surg.,Chiba Univ.,Sch., Med.)

---

### E-1074
### Immediate Chemotherapy-Induced Cognitive Impairment (CICI) among Cancer Patients in Yogyakarta Region, Indonesia

[Speaker]Dwi Kartika Rukmi (Nursing Department, Universitas Jenderal Achmad Yani Yogyakarta, Sleman, Indonesia) [Co-author]Ike Wuri Winahyu Sari (Nursing Department, Universitas Jenderal Achmad Yani Yogyakarta, Sleman, Indonesia)、Francisca Romana Sri Supadmi (Nursing Department, Universitas Jenderal Achmad Yani Yogyakarta, Sleman, Indonesia)

The study aims to assess immediate cognitive impairment post-chemotherapy to enhance knowledge about its impact. A pre-posttest design was conducted on patients aged 18 or older without brain metastases, prior stroke, and allergic history at a regional hospital in Yogyakarta from May to October 2023. Patients' cognitive function was measured using the Indonesian Montreal Cognitive Assessment (MoCA-Ina) 30 minutes before and after chemotherapy. Of 90 patients (median age 54 yrs) who had breast cancer (60%) and neoadjuvant chemotherapy (53,35%) through an intravenous route (95.6%), MoCA-Ina tests showed a significant cognitive decline (p=0.33) post-chemotherapy compared to baseline. Visuospatial (p=.028) and attentional abilities (p=.011) are the most affected functions among 51% of patients who experienced cognitive decline. Simple linear regression found that education level (B=.878, t= 3.12, p=.002) and baseline cognitive state (B=.639, t=.666, p=<.001) were strongly correlated with their cognitive impairment. Further research is needed to understand the direct functional effects of this immediate condition.

[Disclosure of COI by Responsible researcher] Lead presenter is the Responsible researcher.
[Lead presenter: Dwi Kartika Rukmi] We have no financial relationships to disclose.

### [E-1075] II-E6
### Frontiers in breast cancer

2025 / 9 / 25 (Thu) 15:20-16:35 Room 14/(ANA Crowne Plaza Hotel Kanazawa 3F Zuiun 1) Room 14

[Chairperson]Mamoru Takada (Dept. General Surg.,Chiba Univ.,Sch., Med.)

### E-1075
### Whole exome sequencing identifies Japanese familial breast cancer susceptibility gene

[Speaker]Ili S. Abdullah (Lab. of Biofunctional Mol. Med., Natl. Inst. of Biomed. Innovation) [Co-author]Yosuke Matsushita (Lab. of Biofunctional Mol. Med., Natl. Inst. of Biomed. Innovation)、Yasuo Miyoshi (Dept. of Breast and Endocrine Surg., Hyogo College of Med)、Mitsunori Sasa (Dept. Breast Surg. Natl. Hosp. Org. Shikoku Cancer Ctr.)、Toyomasa Katagiri (Lab. of Biofunctional Mol. Med., Natl. Inst. of Biomed. Innovation)

The genetic basis and molecular mechanisms underlying most non-BRCA familial breast cancers remain largely unclear. To identify novel susceptibility genes, we performed whole-exome sequencing in 24 high-risk Japanese breast cancer families. Variants were filtered based on in silico pathogenicity predictions, minor allele frequency (< 0.01 or unknown), and co-segregation with disease status. Six candidate genes were identified in families without known mutations in ***BRCA1***, ***BRCA2***, or ***TP53***. Among them, we focused on Gene-A, whose mRNA expression was significantly reduced in breast tumors compared to adjacent normal tissues. Hypermethylation near its transcription start site was observed in tumor samples. Functional analysis revealed that the mutant Gene-A protein failed to interact with its binding partner, while the wild-type protein retained this interaction and inhibited the partner's nuclear translocation, suppressing breast cancer cell proliferation. Ongoing CRISPR-Cas9 knock-in cell line analysis will further clarify its role. These findings suggest Gene-A as a novel tumor suppressor and potential susceptibility gene for familial breast cancer.

[Disclosure of COI by Responsible researcher] Lead presenter is not the Responsible researcher.
[Lead presenter: Ili S. Abdullah] We have no financial relationships to disclose.
[Principal Researcher: Ili S. Abdullah] ■Research funds from company: Oncotherapy Science Co., Ltd ■Advisor of the company and/or profit-making organization: Oncotherapy Science Co., Ltd ■Contributions: Tokushima Breast Care Clinic

### [E-1076] II-E6
### Frontiers in breast cancer

2025 / 9 / 25 (Thu) 15:20-16:35 Room 14/(ANA Crowne Plaza Hotel Kanazawa 3F Zuiun 1) Room 14

[Chairperson]Mamoru Takada (Dept. General Surg.,Chiba Univ.,Sch., Med.)

---

### E-1076
### Single-cell analysis reveals a metastatic gene expression signature in a rare subpopulation of primary tumor cells

[Speaker]Marta Prietovila (Research Center for Drug Discovery, Faculty of Pharmacy, Keio University.／Division of Molecular and Cellular Medicine, Tokyo Medical University.) [Co-author]Jun(NCC) Nakayama (Division of molecular and cellular medicine, National Cancer Research Institute.)、Wataru Usuba (Department of Urology, St Marianna University.)、Yasuyuki Kojima (Department of Urology, St Marianna University.)、Sho Shiino (Department of Breast Surgery, National Cancer Center Hospital.)、Masayuki Yoshida (Department of Urology, St Marianna University.)、Juntaro Matsuzaki (Research Center for Drug Discovery, Faculty of Pharmacy, Keio University.)、Takahiro Ochiya (Division of Molecular and Cellular Medicine, Tokyo Medical University.)、Yusuke Yamamoto (Division of molecular and cellular medicine, National Cancer Research Institute.)

Despite the continuous progress in breast cancer (BC) treatment, metastasis remains the leading cause of cancer-related mortality. However, its development, especially in initial stages, remains unclear due to traditional molecular studies relying on bulk analysis, hindering the identification of individual cells with metastatic characteristics. To address this issue, we performed single-cell RNA sequencing in paired samples of primary (P) and metastatic (M) nodules from luminal A BC tumors.Single-cell analysis unraveled the complexity of tumor composition. Clustering analysis showed a preference for M-cells to cluster together and separate from P-cells. Through computational analyses, we established an M-cell gene expression profile, which was conserved at the protein level in tissue samples. Importantly, we identified a rare subset of P-cells sharing the M-profile and clustered with M-cells. Using the parental MCF7 cell line and its highly metastatic variant (BM22), we manipulated gene expression (downregulation/overexpression) to assess their metastatic capacity. These findings will shed light on the early steps of metastasis initiation to prevent metastatic spread in patients.

[Disclosure of COI by Responsible researcher] Lead presenter is the Responsible researcher.
[Lead presenter: Marta Prietovila] We have no financial relationships to disclose.

### [E-1077] II-E6
### Frontiers in breast cancer

2025 / 9 / 25 (Thu) 15:20-16:35 Room 14/(ANA Crowne Plaza Hotel Kanazawa 3F Zuiun 1) Room 14

[Chairperson]Mamoru Takada (Dept. General Surg.,Chiba Univ.,Sch., Med.)

---

### E-1077
### Novel Targets of CDK4/6 Inhibitors Identified Through Histopathology-Based AI Models and Spatial Transcriptomics

[Speaker]Maki Tanioka (Okayama University Hospital, Clinical Oncology Center) [Co-author]Taiyo Nakayama (Okayama University Faculty of Medicine)、Tomoki Marutani (Okayama University Faculty of Engineer)、Yuto Niki (Okayama University Faculty of Engineer)、Akihiro Nakaya (Tokyo Univ)、Zehao Li (Kumamoto Univ)、Kenichi Morooka (Kumamoto Univ)、Tadahiko Shien (Okayama University Hospital Breast and Endocrine Surgery)

Background: Cyclin-dependent kinase (CDK) 4/6 inhibitors have prolonged survival in patients with estrogen receptor-positive (ER+) metastatic breast cancer. However, subsequent studies analyzing bulk tumors and their microenvironment failed to identify actionable targets. Methods: We analyzed 511 H&E-stained slides from 178 breast cancer patients treated with CDK4/6 inhibitors plus endocrine therapy. Using GigaPath (Xu, Nature, 2024), we developed a deep learning model to predict progression-free survival (PFS). Grad-CAM was applied for spatial interpretation. Spatial transcriptomics (Visium v2) of 12 samples compared gene expression in cancer vs. stroma using a custom tile classifier. For 7 paired pre/post-treatment tissues, Visium HD was used, mapping gene expression within 30 um of Stardist-identified nuclei.Results: A GigaPath fine-tuned model on 330 samples (PFS -6 or 15- months) achieved cross-validated AUC 0.75. MAPK pathway genes were associated with sensitivity, while PI3K pathway and CAF-related genes correlated with resistance.Conclusion: Tissue morphology correlates with CDK4/6 inhibitor response. MAPK and CAF-associated pathways may represent novel therapeutic targets.

[Disclosure of COI by Responsible researcher] Lead presenter is the Responsible researcher.
[Lead presenter: Maki Tanioka] ■Research funds from company: 科研費国際共同研究強化B　■Research expenses: セルトリオン、ファイザー

▲TOP

### [E-1078] II-E6
### Frontiers in breast cancer

2025 / 9 / 25 (Thu) 15:20-16:35 Room 14/(ANA Crowne Plaza Hotel Kanazawa 3F Zuiun 1) Room 14

[Chairperson]Mamoru Takada (Dept. General Surg.,Chiba Univ.,Sch., Med.)

---

### E-1078
### Integration of Single-Cell and Spatial Transcriptomics Reveals Treatment-Induced Clonal Reorganization in Breast Cancer

[Speaker]Kazutaka Otsuji (NEXT-Ganken Program, JFCR) [Co-author]Tomo Osako (Div. of Pathol., Cancer Inst., JFCR)、Yoko Takahashi (Univ. of Hawai'i Cancer Center／Breast Oncology Ctr., Cancer Institute Hosp., JFCR)、Takayuki Kobayashi (Breast Oncology Ctr., Cancer Institute Hosp., JFCR)、Toshimi Takano (Breast Oncology Ctr., Cancer Institute Hosp., JFCR)、Chikako Shibata (Div.of Cancer Epigenomics, Cancer Inst., JFCR)、Sumito Saeki (Div.of Cancer Epigenomics, Cancer Inst., JFCR)、Asumi Iesato (NEXT-Ganken Program, JFCR)、Tetsuo Noda (Director's room, Cancer Inst., JFCR)、Kengo Takeuchi (Div. of Pathol., Cancer Inst., JFCR)、Takayuki Ueno (Breast Oncology Ctr., Cancer Institute Hosp., JFCR)、Reo Maruyama (NEXT-Ganken Program, JFCR／Div.of Cancer Epigenomics, Cancer Inst., JFCR)

Background:Although scRNA-seq has advanced our understanding of tumor heterogeneity, it lacks spatial context. Recent spatial transcriptomic technologies preserve cellular localization and allow exploration of how genomic heterogeneity relates to tumor architecture.Methods:We analyzed pre-treatment biopsy and post-treatment surgical samples from a lactating female with Stage IV breast cancer. scRNA-seq was performed to infer single-cell copy number variations. Xenium platform provided spatial transcriptomic data, integrated with the scRNA-seq results.Results:scRNA-seq showed that pre-treatment cancer cells displayed diverse copy number patterns, whereas post-treatment cells fell into two profiles, one pre-existing and one likely acquired during therapy. When spatially mapped, clones diffusely distributed in the biopsy became regionally segregated in the surgical specimen, suggesting treatment-induced reorganization.Conclusion:This study demonstrates that spatially resolved genomic profiling can reveal treatment-induced clonal evolution and tumor reorganization, offering insights that may improve patient stratification, guide targeted therapies, and facilitate biomarker discovery.

[Disclosure of COI by Responsible researcher] Lead presenter is the Responsible researcher.
[Lead presenter: Kazutaka Otsuji] We have no financial relationships to disclose.

### [E-1085] I-E10-1
### Cell death

2025 / 9 / 25 (Thu) 15:20-16:35 Room 15/(ANA Crowne Plaza Hotel Kanazawa 3F Zuiun 2) Room 15

[Chairperson]Tsuyoshi Osawa (RCAST, University of Tokyo)

---

### E-1085
### Sublethal disulfidoptosis contributes to TLR2-mediated pyroptosis induction in THP1 human leukemia cells

[Speaker]Kohsuke Tsuchiya (Immunol. Cancer Res. Inst., Kanazawa Univ.) [Co-author]Takashi Suda (Immunol. Cancer Res. Inst., Kanazawa Univ.)

Pyroptosis is inflammatory regulated necrosis mediated by the GSDM family. Its induction in cancer cells can trigger anti-tumor immunity. Inflammasomes activate caspase-1, which drives pyroptosis by cleaving GSDMD. Here, we show that bacterial lipopeptides (BLPs), known as TLR2 ligands, induce GSDMD-dependent pyroptosis in THP1 cells, and the mechanism was elucidated. Knockout of NLRP3, ASC, or caspase-1 abolished GSDMD cleavage and pyroptosis, indicating involvement of the NLRP3 inflammasome. KCl addition suppressed pyroptosis, suggesting $K^+$ efflux is required for inflammasome activation. We speculated that $K^+$ efflux results from sublethal membrane damage by some cell death mechanism. Among cell death inhibitors tested, DTT that blocks disulfidoptosis, necrotic cell death by excessive disulfide bonding in the cytoskeleton, reduced pyroptosis. BLPs caused NADPH depletion and $\beta$-actin disulfide formation, hallmarks of disulfidoptosis. These results suggest that disulfidoptosis helps BLP-induced NLRP3 activation and pyroptosis in THP1 cells. This is the first report linking disulfidoptosis to innate immune signaling and may guide strategies to induce pyroptosis in leukemia cells.

[Disclosure of COI by Responsible researcher] Lead presenter is the Responsible researcher.
[Lead presenter: Kohsuke Tsuchiya] We have no financial relationships to disclose.

### [E-1086] I-E10-1
### Cell death

2025 / 9 / 25 (Thu) 15:20-16:35 Room 15/(ANA Crowne Plaza Hotel Kanazawa 3F Zuiun 2) Room 15

[Chairperson]Tsuyoshi Osawa (RCAST, University of Tokyo)

---

### E-1086
### Necroptosis and MLKL in Cholangiocarcinoma: Tumor Promotion and Therapeutic Implications

[Speaker]Siriporn Jitkaew (Faculty of Allied Health Sciences, Chulalongkorn University, Thailand) [Co-author]Nattaya Duangthim (Faculty of Allied Health Sciences, Chulalongkorn University, Thailand)、Thanpisit Lomphithak (Faculty of Allied Health Sciences, Chulalongkorn University, Thailand)、Sasiprapa Sonkaew (Faculty of Allied Health Sciences, Chulalongkorn University, Thailand)、Perawatt Akara-Amornthum (Faculty of Allied Health Sciences, Chulalongkorn University, Thailand)、Rutaiwan Tohtong (Faculty of Science, Mahidol University, Thailand)、Choopet Nitsakulthong (Chulalongkorn University Laboratory Animal Center, Thailand)、Jeeraprapha Duangbupha (Chulalongkorn University Laboratory Animal Center, Thailand)、Poorichaya Somparn (Faculty of Medicine, Chulalongkorn University, Thailand)、Keigo Murakami (Tohoku University School of Medicine, Japan)、Masatoshi Hashimoto (Tohoku University School of Medicine, Japan)、Hajime Usubuchi (Tohoku University School of Medicine, Japan)、Erina Iwabuchi (Tohoku University School of Medicine, Japan)、Michiaki Unno (Tohoku University School of Medicine, Japan)、Zhenyu Cai (School of Medicine, Tongji University, China)、Hironobu Sasano (Tohoku University School of Medicine, Japan)

Necroptosis, a regulated form of necrosis, is gaining interest as a cancer therapeutic strategy, though its role in tumorigenesis remains debated. We investigated the clinical and functional relevance of necroptosis and MLKL in cholangiocarcinoma (CCA). In 88 CCA patients, immunohistochemistry revealed that high MLKL expression was associated with shorter survival and an unfavorable immune profile, while phosphorylated MLKL (pMLKL) correlated with increased CD8 T-cell infiltration, PD-L1 expression, and improved prognosis. In vitro, MLKL knockdown reduced proliferation, migration, and EMT; these effects were confirmed in vivo. Our findings suggest a dual role of necroptosis in CCA, with MLKL promoting tumor progression, while activated necroptosis enhances anti-tumor immunity supporting the potential of necroptosis-based therapeutic strategies.

[Disclosure of COI by Responsible researcher] Lead presenter is the Responsible researcher.
[Lead presenter: Siriporn Jitkaew] We have no financial relationships to disclose.

▲TOP

### [E-1087] I-E10-1
### Cell death

2025 / 9 / 25 (Thu) 15:20-16:35 Room 15/(ANA Crowne Plaza Hotel Kanazawa 3F Zuiun 2) Room 15

[Chairperson]Tsuyoshi Osawa (RCAST, University of Tokyo)

---

### E-1087
### Anticancer potential of ***Machilus thunbergii*** leaf extract and its component and proapoptotic effects

[Speaker]Abdullah Aljwanieh (Div. Biometab. Chem., Univ. the Ryukyus Facult. Med.) [Co-author]Nanae Harashima (Div. Biometab. Chem., Univ. the Ryukyus Facult. Med.)

In recent years, there has been interest in exploring the chemopreventive potential of phytochemicals. Various herbal derived ingredients such as alkaloids, polyphenols, flavonoids, terpenoids, and polysaccharides have been used against various cancers and infectious diseases with minimal side effects. We reported that natural plant crude extract, such as ***Machilus thunbergii*** leaf extract (MTLE), activated the expression of pro-apoptotic molecules and downregulated anti-apoptotic molecules in HeLa cells at last year's meeting. So there, the aim of this study is to further investigate the mechanism of MTLE and the anticancer effects of rutin, which is reported to be one of the components of ***Machilus thunbergii***. Rutin is a member of flavonoids contained in buckwheat and celery. Both MTLE and rutin induced apoptosis via the intrinsic pathway by activating Bax, Bim, and cleaved-caspase-9/3 and inactivating Bcl-2 and c-Myc in cancer cells. Moreover, MTLE induced S-phase cell cycle arrest by reduction in CDK2 and cyclin A2. These results suggest the potential of crude MTLE and one of the components, rutin, as a candidate for a therapeutic agent.

[Disclosure of COI by Responsible researcher] Lead presenter is not the Responsible researcher.
[Lead presenter: Abdullah Aljwanieh] We have no financial relationships to disclose.
[Principal Researcher: Nanae Harashima] We have no financial relationships to disclose.

▲TOP

### [E-1088] I-E10-1
### Cell death

2025 / 9 / 25 (Thu) 15:20-16:35 Room 15/(ANA Crowne Plaza Hotel Kanazawa 3F Zuiun 2) Room 15

[Chairperson]Tsuyoshi Osawa (RCAST, University of Tokyo)

### E-1088
### Z36 triggers a dose-dependent switch between cytoplasmic vacuolation death and apoptosis in colorectal cancer cells

[Speaker]Mati Ur Rehman (Dept. of Biol. & Biosci. Aga Khan Univ.) [Co-author]Almuayyad Gajani (Dept. of Biol. & Biosci. Aga Khan Univ.)、Mahwish Fatima (Dept. of Biol. & Biosci. Aga Khan Univ.)、Paras Jawaid (Dept. of Biol. & Biosci. Aga Khan Univ.／Ctr. for Oncological Res. in Surg. Aga Khan Univ.)、Arooj Shafiq (Dept. of Biol. & Biosci. Aga Khan Univ.)、Azhar Hussain (Dept. of Biol. & Biosci. Aga Khan Univ.)

Colorectal cancer remains the third leading cause of cancer-related deaths worldwide. Apoptosis resistance is a significant challenge in cancer therapy, contributing to poor prognosis and treatment failure. In this study, we investigated the effects of Z36, a novel BCL-XL inhibitor, on human colorectal cancer cell lines HCT-116 and HCT-15. Z36 treatment led to a dose- and time-dependent decrease in cell viability, with significant effects observed at concentrations as low as 2.5 µM. Notably, no DNA fragmentation was evident at doses up to 5 µM. Morphological analysis revealed cytoplasmic vacuolation-type death at 5 µM, whereas higher doses (10 µM) induced classical apoptotic features, including DNA fragmentation and Annexin V-FITC-PI positive changes. Giemsa staining confirmed these distinct cell death morphologies, and Western blot analysis highlighted modulation of key cell death-related proteins. Inducing non-apoptotic cell death, such as vacuolation, at lower doses may help overcome apoptosis resistance in colorectal cancer. These findings suggest that Z36 may overcome apoptosis resistance by promoting alternative cell death pathways in colorectal cancer.

[Disclosure of COI by Responsible researcher] Lead presenter is the Responsible researcher.
[Lead presenter: Mati Ur Rehman] We have no financial relationships to disclose.

### [E-1089] I-E10-1
### Cell death

2025 / 9 / 25 (Thu) 15:20-16:35 Room 15/(ANA Crowne Plaza Hotel Kanazawa 3F Zuiun 2) Room 15

[Chairperson]Tsuyoshi Osawa (RCAST, University of Tokyo)

---

### E-1089
### Exploration of New Therapeutic Strategies for Peritoneal Metastasis of Gastric Cancer Based on Drug Relocalization

[Speaker]Xiaofang Che (Med Onco Dept. 1st Hospital of CMU) [Co-author]Bowen Bao (Med Onco Dept. 1st Hospital of CMU)、Wenshi Li (Med Onco Dept. 1st Hospital of CMU)、Xiujuan Qu (Med Onco Dept. 1st Hospital of CMU)、Yunpeng Liu (Med Onco Dept. 1st Hospital of CMU)

Peritoneal dissemination (PD) remains a critical clinical challenge in gastric cancer (GC) due to the lack of effective therapeutic interventions. Targeting free gastric cancer cells (FGCCs) that detach from primary lesions and survive in the peritoneal cavity represents a pivotal strategy for impeding PD. This study identified disulfiram (DSF), an FDA-approved alcohol addiction treatment drug, as a promising anti-PD agent through systematic drug repositioning analysis. Notably, DSF in combination with copper gluconate (DSF-$Cu^{2+}$) exhibited selective cytotoxicity against anchorage-independent growth (AIG) GC cells, a key survival feature of FGCCs in peritoneal fluid. Mechanistic investigations revealed that DSF-$Cu^{2+}$ induces ferroptosis in AIG GC cells by upregulating HO-1 via increasing Nrf2 stability. Furthermore, to overcome the pharmacokinetic limitations of conventional DSF formulations, we developed a nanoparticle-based delivery system that demonstrated superior PM suppression efficacy in GC PM models. These findings not only elucidate the molecular mechanism underlying DSF-mediated ferroptosis in FGCCs, but also provide a novel therapeutic strategy for clinical PD treatment.

[Disclosure of COI by Responsible researcher] Lead presenter is the Responsible researcher.
[Lead presenter: Xiaofang Che] We have no financial relationships to disclose.

▲TOP

### [E-1090] I-E10-1
### Cell death

2025 / 9 / 25 (Thu) 15:20-16:35 Room 15/(ANA Crowne Plaza Hotel Kanazawa 3F Zuiun 2) Room 15

[Chairperson]Tsuyoshi Osawa (RCAST, University of Tokyo)

---

### E-1090
### The role of mitophagy in the chemotherapy-induced senescence of cancer cells

[Speaker]Chen Yan (Dept. of Stem Cell Biol., ABDI, Nagasaki Univ.) [Co-author]Taosheng Li (Dept. of Stem Cell Biol., ABDI, Nagasaki Univ.)

Therapy-induced senescence (TIS) is a state of cellular senescence that occurs in cancer cells in response to anticancer treatments. TIS has been proposed as a contributing factor to therapy resistance and tumor relapse. Mitophagy, a selective form of autophagy that removes damaged mitochondria, plays a critical role in maintaining cellular homeostasis and regulating cell survival. In this study, we investigated how mitophagy is involved in therapy-induced senescence of cancer cells. To induce senescence, HCT116 colorectal cancer cells were treated with doxorubicin (DOX) for 7days. DOX treatment resulted in the emergence of senescence-associated features, including enlarged cell body, cell cycle arrest, increased SA-$\beta$-galactosidase activity, and upregulation of canonical senescence markers. Compared to untreated cells, senescent cells exhibited increased co-localization of mitochondria with autophagosomes, along with elevated expression of mitophagy-related genes, suggesting enhanced mitophagy in these senescent cells. Further mechanistic studies are warranted to elucidate the functional role of mitophagy in regulating the senescence of cancer cells.

[Disclosure of COI by Responsible researcher] Lead presenter is the Responsible researcher.
[Lead presenter: Chen Yan] We have no financial relationships to disclose.

▲TOP

### [J-1091] I-J7-1
### Cancer genomics

2025 / 9 / 25 (Thu) 15:20-16:35 Room 16/(11F Visage) Room 16

[Chairperson]Yoshinori Murakami (Dept. Mol. Biol., Inst. for Adv. Med. Sci., Nippon Med. Sch.)

---

### J-1091
### Genome profiling of central nervous system germ cell tumor by whole genome sequencing

[Speaker]Shota Kato (Dept. Pediatrics, UTokyo, Tokyo, Japan) [Co-author]Hirokazu Takami (Dept. Neurosurgery, UTokyo, Tokyo, Japan)、Yuki Yamagishi (Dept. Neurosurgery, Kyorin Univ., Tokyo, Japan)、Kentaro Watanabe (Dept. Pediatrics, UTokyo, Tokyo, Japan)、Junko Hirato (Dept. Pathology, Public Tomioka General Hosp., Gunma, Japan)、Hajime Okita (Div. Diagnostic Pathology, Keio Univ., Tokyo, Japan)、Takako Yoshioka (Dept. Pathology, NCCHD, Tokyo, Japan)、Tomonari Suzuki (Dept. Neuro-Oncology／Neurosurgery, Saitama Medical Univ. International Medical Ctr., Saitama, Japan)、Tatsuhiro Shibata (Div. Cancer Genomics, Natl. Cancer Ctr., Tokyo, Japan)、Kenichi Yoshida (Div. Cancer Evolution, Natl. Cancer Ctr., Tokyo, Japan)、Yuichi Shiraishi (Div. Genome Analysis Platform Development, Natl. Cancer Ctr., Tokyo, Japan)、Ryo Nishikawa (Dept. Neuro-Oncology／Neurosurgery, Saitama Medical Univ. International Medical Ctr., Saitama, Japan)、Toshihiro Kumabe (Dept. Neurosurgery, Kitasato Univ., Kanagawa, Japan)、Motohiro Kato (Dept. Pediatrics, UTokyo, Tokyo, Japan)、Koichi Ichimura (Dept. Neurosurgery, Kyorin Univ., Tokyo, Japan／Dept. Pathology, Kyorin Univ., Tokyo, Japan)

Central nervous system germ cell tumors (CNS GCTs) are driven by somatic alterations involving RAS/MAPK and/or PI3K/Akt/mTOR pathways. However, conventional analyses have identified driver mutations in only 70% and 30% of patients with germinoma and non-germinomatous GCTs (NGGCTs), respectively. This study conducted whole genome sequencing (WGS) on 71 patients with CNS GCTs from JCCG and iGCT consortium (37 with germinoma and 34 with NGGCTs), and identified somatic driver alterations involving RAS/MAPK or PI3K/Akt/mTOR pathways in 84% of the patients with germinoma and 62% with NGGCTs. The most frequently altered genes were ***KIT*** (54%) in germinomas and ***USP28*** (21%) in NGGCTs. Among patients with driver alterations, 17% harbored only alterations involving non-coding regions, such as structural variations (SVs) and copy number variations (CNVs). Three of four patients with germline chromosomal abnormalities (two with Down syndrome and two with Klinefelter syndrome) had no somatic driver alterations. WGS identified driver alterations of CNS GCTs with higher sensitivity compared to previous studies, highlighting the importance of comprehensive genomic profiling including SVs and CNVs.

[Disclosure of COI by Responsible researcher] Lead presenter is not the Responsible researcher.
[Lead presenter: Shota Kato] We have no financial relationships to disclose.
[Principal Researcher: Koichi Ichimura] ■Research expenses: 第一三共株式会社、理研ジェネシス株式会社　■Endowed chair: イドルシアファーマシューティカルジャパン株式会社

▲TOP

### [J-1092] I-J7-1
### Cancer genomics

2025 / 9 / 25 (Thu) 15:20-16:35 Room 16/(11F Visage) Room 16

[Chairperson]Yoshinori Murakami (Dept. Mol. Biol., Inst. for Adv. Med. Sci., Nippon Med. Sch.)

### J-1092
### Identification of novel driver genes in Wilms Tumor by whole-genome sequencing

[Speaker]Yusuke Tsumura (Division of Cancer Evolution, National Cancer Center) [Co-author]Kaito Mimura (Division of Cancer Evolution, National Cancer Center)、Ai Okada (Division of Genome Analysis Platform Development, National Cancer Center)、Kentaro Watanabe (Department of Pediatrics, Graduate School of Medicine, The Tokyo Univ.)、Miho Kato (Department of Childhood Cancer Data Management, Childhood Cancer Center, NCCHD)、Takako Yoshioka (Department of Pathology, NCCHD)、Takaharu Oue (Department of Pediatric Surgery, Hyogo College of Medicine)、Shuichiro Uehara (Division of Pediatric Surgery, Nihon University Itabashi Hospital)、Hajime Okita (Division of Diagnostic Pathology, Keio University School of Medicine)、Tatsuro Tajiri (Department of Pediatric Surgery, Kyushu University)、Akihiro Yoneda (Division of Surgery, NCCHD)、Seiya Imoto (Human Genome Center, The Institute of Medical Science)、Eiso Hiyama (Natural Science Center for Basic Research and Development, Hiroshima University)、Yuichi Shiraishi (Division of Genome Analysis Platform Development, National Cancer Center)、Motohiro Kato (Department of Pediatrics, Graduate School of Medicine, The Tokyo Univ.)、Kenichi Yoshida (Division of Cancer Evolution, National Cancer Center)

Wilms tumor (WT), a pediatric kidney tumor, has been reported to have diverse driver genes. The most frequently mutated genes include ***WT1***, ***CTNNB1***, and ***AMER1*** in the Wnt/β-catenin signaling pathway (~20%), and many other rare driver genes (~40 genes) are associated with other pathways including miRNA biogenesis and epigenetic modification. To further investigate the pathogenesis of WT, we analyzed a total of 407 WTs, by integrating whole-genome sequencing (WGS) data from 68 Japanese WT samples collected by the Observational Study of Childhood, Adolescent, and Young Adult Solid Tumors by the Japan Children's Cancer Group, and whole-exome sequencing/WGS data from the publicly available datasets. dN/dS analysis identified ***KDM5C***, a histone modifier, besides 22 known driver genes. Three additional genes were also considered as new drivers based on the distribution and type of mutations, including ***MED12***, with hotspot mutations found in uterine leiomyomas. While the frequency was low (~2%), the candidate novel driver genes were components of pathways related to WT, suggesting their biological relevance. Our findings contribute to further understand the pathogenesis of WT.

[Disclosure of COI by Responsible researcher] Lead presenter is not the Responsible researcher.
[Lead presenter: Yusuke Tsumura] We have no financial relationships to disclose.
[Principal Researcher: Kenichi Yoshida] ■Contributions: 公益財団法人持田記念医学薬学振興財団 公益財団法人内藤記念科学振興財団 公益財団法人第一三共生命科学研究振興財団 公益財団法人住友財団 公益財団法人安田記念医学財団 公益財団法人アステラス病態代謝研究会 公益財団法人MSD生命科学財団 一般社団法人日本血液学会 公益財団法人小児医学研究振興財団 公益財団法人上原記念生命科学財団 公益財団法人高松宮妃癌研究基金 公益信託日本白血病研究基金 公益財団法人小林がん学術振興会 公益財団法人武田科学振興財団 公益財団法人大和証券財団 公益財団法人ＳＧＨ財団 公益財団法人川野小児医学奨学財団 認定NPO法人ゴールドリボン・ネットワーク 公益財団法人先進医薬研究振興財団 公益財団法人テルモ生命科学振興財団 公益財団法人小野薬品がん・免疫・神経研究財団 公益財団法人細胞科学研究財団

### [J-1093] I-J7-1
### Cancer genomics

2025 / 9 / 25 (Thu) 15:20-16:35 Room 16/(11F Visage) Room 16

[Chairperson]Yoshinori Murakami (Dept. Mol. Biol., Inst. for Adv. Med. Sci., Nippon Med. Sch.)

---

### J-1093
### Molecular evolutionary analysis revealed early-onset colorectal cancer-related driver cancer genes

[Speaker]Yosuke Seto (Div. Exp. Chemother. Cancer Chemother. Ctr. JFCR) [Co-author]Satoshi Nagayama (Dept. Surg. Grad. Sch. Med. Kyoto Univ.／Dept. Surg. Uji-Tokushukai Med. Ctr.)、Ryohei Katayama (Div. Exp. Chemother. Cancer Chemother. Ctr. JFCR)

Colorectal cancer (CRC) is the second leading cause of cancer death in the world. The developmental process of CRC is known as multistep carcinogenesis, explained by tumor initiation, tumor promotion, malignant conversion and tumor progression, caused by mutations occurring on tumor suppressor and driver genes over decades. Recently, the increasing incidence rate of colorectal cancer in younger people has become a problem in the world as well as in Japan. However, the molecular characteristics of early–onset colorectal cancer (EOCRC) have not been fully understood. In this study, we developed a new method based on evolutionary theory to identify EOCRC–related cancer driver genes, named SPEC (Selection Pressure Estimation for Cohort) using sequencing data from EOCRC and elderly CRC patients' tumor. SPEC identified that NF1, RAF1 and SMAD4 were under positive selection in EOCRC patients and EOCRC–specific hotspot mutations on SMAD4. Additionally, several genes encoding G protein–coupled receptor and scaffolding protein were identified as positively selected genes. These results suggest that SPEC is a powerful tool for identifying genes involved in in cancer development.

[Disclosure of COI by Responsible researcher] Lead presenter is not the Responsible researcher.
[Lead presenter: Yosuke Seto] We have no financial relationships to disclose.
[Principal Researcher: Ryohei Katayama] ■Research funds from company: 中外製薬、TOPPAN、UBE、BML　■Patent fee: 栄研化学

### [J-1094] I-J7-1
### Cancer genomics

2025 / 9 / 25 (Thu) 15:20-16:35 Room 16/(11F Visage) Room 16

[Chairperson]Yoshinori Murakami (Dept. Mol. Biol., Inst. for Adv. Med. Sci., Nippon Med. Sch.)

---

### J-1094
### Deciphering FGFR2-IIIb/-IIIc isoform switching in advanced gastric cancer through whole-transcriptome sequencing

[Speaker]Naoko Iida (TRS, Natl. Cancer Ctr. East) [Co-author]Tadayoshi Hashimoto (TRS, Natl. Cancer Ctr. East／Gastro & GI Oncol. Dept., Natl. Cancer Ctr. East)、Shigenori Kadowaki (Clin. Oncol. Dept. Aichi Cancer Ctr. Hosp.)、Akitaka Makiyama (Gifu Univ. Hosp. Cancer Ctr.／COMIT, Gifu Univ.)、Nozomu Machida (Gastroenterol. Dept. Kanagawa Cancer Ctr.)、Naoki Takahashi (Gastroenterol. Dept. Saitama Cancer Cntr.)、Shogen Boku (Clin. Oncol. Dept. Kyushu Med. Univ. Hosp.)、Toshihiro Kudo (Med. Oncology Dept., Osaka Intl. Cancer Inst.)、Eiji Oki (Surgery & Science Dept., Kyushu Univ. Med. Sci.)、Taro Shibuki (HB&P Oncology Dept. Natl. Cancer Ctr. East)、Mitsuho Imai (TRS, Natl. Cancer Ctr. East)、Takao Fujisawa (TRS, Natl. Cancer Ctr. East／H&N Medical Oncology Dep., Natl. Cancer Ctr. East)、Yoshiaki Nakamura (TRS, Natl. Cancer Ctr. East／Gastro & GI Oncol. Dept., Natl. Cancer Ctr. East)、Hideaki Bando (TRS, Natl. Cancer Ctr. East／Gastro & GI Oncol. Dept., Natl. Cancer Ctr. East)、Takayuki Yoshino (Gastro & GI Oncol. Dept., Natl. Cancer Ctr. East)

FGFR2-IIIb alternative spliced isoform of FGFR2 is currently a promising therapeutic target for advanced gastric or gastroesophageal junction cancer (GC/GEJC). Abnormal switching of FGFR2-IIIb to IIIc has been implicated in various malignancies. However, systematic differentiation of FGFR2 isoform and elucidating the role remain challenging. In this study, we evaluated the FGFR2 isoform switching utilizing the transcriptome data of GC/GEJC (n= 272) through a large-scale cancer genome sequencing project, SCRUM-Japan MONSTAR-SCREEN-2 (UMIN000043899). We observed that 10% of samples showed a high IIIc ratio of greater than 0.5. The high IIIc group was associated with significantly shorter overall survival than the low IIIc groups (IIIc ratio 0.29; hazard ratio 1.98; log-rank P=0.002). Gene set enrichment analysis showed an enrichment of myogenesis-related genes such as ***MYH11*** and ***CDKN1A***, as well as epithelial-mesenchymal transition-associated genes such as ***MLK***, in the IIIc-high group, which may have crucial roles in cancer metastasis. The quantifying of FGFR2 isoforms using transcriptome data might be useful in predicting prognosis and developing effective FGFR2-targeted therapy.

[Disclosure of COI by Responsible researcher] Lead presenter is not the Responsible researcher.
[Lead presenter: Naoko Iida] We have no financial relationships to disclose.
[Principal Researcher: Tadayoshi Hashimoto] We have no financial relationships to disclose.

▲TOP

### [J-1095] I-J7-1
### Cancer genomics

2025 / 9 / 25 (Thu) 15:20-16:35 Room 16/(11F Visage) Room 16

[Chairperson]Yoshinori Murakami (Dept. Mol. Biol., Inst. for Adv. Med. Sci., Nippon Med. Sch.)

---

### J-1095
### Contribution of 15q15.1 deletion as a genomic alteration for brain metastasis development in non-small cell lung cancer

[Speaker]Jun Miyakoshi (Division of Genome Biology, National Cancer Center Research Institute／Department of Respiratory Medicine, Chiba University Graduate School of Medicine／Department of Thoracic Oncology, National Cancer Center Hospital) [Co-author]Kouya Shiraishi (Division of Genome Biology, National Cancer Center Research Institute)、Akifumi Mochizuki (Division of Genome Biology, National Cancer Center Research Institute)、Akiko Tateishi (Division of Genome Biology, National Cancer Center Research Institute／Department of Thoracic Oncology, National Cancer Center Hospital)、Yukiko Shimoda (Division of Genome Biology, National Cancer Center Research Institute／Department of Thoracic Oncology, National Cancer Center Hospital)、Masahiro Torasawa (Division of Genome Biology, National Cancer Center Research Institute／Department of Thoracic Oncology, National Cancer Center Hospital)、Makoto Ohno (Department of Neurosurgery and Neuro-Oncology, National Cancer Center Hospital)、Yukihiro Yoshida (Department of Thoracic Surgery, National Cancer Center Hospital)、Tatsuya Yoshida (Department of Thoracic Oncology, National Cancer Center Hospital)、Yasushi Yatabe (Department of Diagnostic Pathology, National Cancer Center Hospital)、Yasushi Goto (Department of Thoracic Oncology, National Cancer Center Hospital)、Yoshitaka Narita (Department of Neurosurgery and Neuro-Oncology, National Cancer Center Hospital)、Shunichi Watanabe (Department of Thoracic Surgery, National Cancer Center Hospital)、Ryuji Hamamoto (Division of Medical AI Research and Development, NCC Research Institute／Cancer Translational Research Team, RIKEN Center for Advanced Intelligence Project)、Takashi Kohno (Division of Genome Biology, National Cancer Center Research Institute)

Brain metastasis (BM) is a common and fatal complication in advanced NSCLC. While genomic drivers of BM have been studied in Caucasians, data in Asian populations remain limited. We analyzed three independent cohorts of resected NSCLC tumors: (1) 183 WGS samples from primary tumors with postoperative brain imaging follow-up, including 26 samples with future BM relapse (discovery cohort); (2) 929 WES samples with similar follow-up, including 225 with BM relapse (validation cohort); and (3) 31 WGS samples, including 7 samples from BM and 12 matched primary–BM pairs (BM cohort) (IRB number 2005-109). Comprehensive analysis of the discovery cohort revealed that 15q15.1 deletion was significantly enriched in tumors with BM relapse compared to those without, whereas there was no enrichment of specific SNVs/IDs and structural variants. This enrichment of 15q15.1 deletion was also observed in the validation cohort. In the BM cohort, this deletion was more frequent in BM than in matched primary tumors, whereas previously known driver alterations of BM were shared as trunk clones. 15q15.1 deletion may contribute to BM development and serve as a potential biomarker for future BM relapse.

[Disclosure of COI by Responsible researcher] Lead presenter is not the Responsible researcher.
[Lead presenter: Jun Miyakoshi] We have no financial relationships to disclose.
[Principal Researcher: Takashi Kohno] ■Research funds from company: AMED　■Patent fee: 理研Genesis、Amoy、Foundation Medicine、サーモフィッシャーサイエンティフィック　■Lecture fee: 日本イーライリリー　■Research expenses: 中外製薬、シスメックス、コニカミノルタ、ガーダントヘルス、イーライリリージャパン、ユーロフィンクリニカルジェネティクス

▲TOP

### [J-1096] I-J7-1
### Cancer genomics

2025 / 9 / 25 (Thu) 15:20-16:35 Room 16/(11F Visage) Room 16

[Chairperson]Yoshinori Murakami (Dept. Mol. Biol., Inst. for Adv. Med. Sci., Nippon Med. Sch.)

---

### J-1096
### Segment specific variations in genetic alterations across the renal nephron

[Speaker]Kosuke Ieiri (Department of Pathology and Tumor Biology, Kyoto University) [Co-author]Nobuyuki Kakiuchi (The Hakubi Center for Advanced Research, Kyoto University)、Tomonori Hirano (Department of Pathology and Tumor Biology, Kyoto University)、Koichi Watanabe (Department of Pathology and Tumor Biology, Kyoto University)、Hirona Maeda (Department of Pathology and Tumor Biology, Kyoto University)、Shun Kawaguchi (Department of Pathology and Tumor Biology, Kyoto University)、Yoshikage Inoue (Department of Pathology and Tumor Biology, Kyoto University)、Hiroko Tanaka (M&D Data Science Center, Tokyo Medical and Dental University)、Satoru Miyano (M&D Data Science Center, Tokyo Medical and Dental University)、Dai Takamatsu (Department of Urology, Graduate School of Medical Science, Kyushu University)、Shunsuke Goto (Department of Urology, Graduate School of Medical Science, Kyushu University)、Takashi Matsumoto (Department of Urology, Graduate School of Medical Science, Kyushu University)、Masaki Shiota (Department of Urology, Graduate School of Medical Science, Kyushu University)、Masatoshi Eto (Department of Urology, Graduate School of Medical Science, Kyushu University)、Seishi Ogawa (Department of Pathology and Tumor Biology, Kyoto University)

Renal cell carcinoma (RCC) subtypes are thought to originate from distinct nephron segments, suggesting that mutational background in normal nephron may also differ by segment. To investigate this, we sampled and sequenced five nephron segments: proximal convoluted and straight tubules (PCT/PST), distal straight and convoluted tubules (DST/DCT), and collecting ducts (CD). The number of mutations per cell per year was highest in PST (101.1), followed by PCT (57.9), DST (25.9), DCT (26.8), and CD (18.0). Three de novo mutational signatures were identified: Signature A (corresponding to SBS40b/40c), Signature B (corresponding to SBS1/5/40a), and Signature C (corresponding to SBS12). Signature A was enriched in PST and accounted for its high burden. Signature C was frequently observed in the proximal tubule, particularly enriched in PCT. These results demonstrate segment-specific differences in mutation accumulation and the underlying mutational processes. The enrichment of SBS12 in PCT, along with its strong strand bias, suggests the involvement of an exogenous mutagen, possibly related to substances reabsorbed or metabolized in PCT.

[Disclosure of COI by Responsible researcher] Lead presenter is not the Responsible researcher.
[Lead presenter: Kosuke Ieiri] ■Endowed chair: 公益社団法人鹿児島共済会 南風病院
[Principal Researcher: Seishi Ogawa] ■Advisor of the company and/or profit-making organization: エーザイ株式会社 Chordia Therapeutics Inc. Montage Bio, INC. ■Profit of stock: 朝日ゲノミクス株式会社 ■Research expenses: エーザイ株式会社 Chordia Therapeutics Inc. ■Contributions: 日本新薬株式会社 ■Endowed chair: Chordia Therapeutics Inc. 公益社団法人鹿児島共済会 南風病院

### [SL1] SL1
### Expanding Precision Oncology with Novel Therapeutics, Technologies and Clinical Trials Design

2025 / 9 / 25 (Thu) 15:30-16:10 Room 1/(Ishikawa Ongakudo 2F Concert Hall) Room 1

[Chairperson]Tomokazu Yosizaki (Kanazawa University, Otolaryngology -Head and Neck Surgery)

### SL1
### Expanding Precision Oncology with Novel Therapeutics, Technologies and Clinical Trials Design

[Speaker]Lillian L. Siu (Princess Margaret Cancer Centre, Toronto, Canada)

The clinical utility of precision oncology through molecular characterization of tumors has been demonstrated in many malignancies. This presentation will explore ways to expand the current scope of precision oncology. From the therapeutics perspective, there are growing interests in drugging previously deemed undruggable targets through different types of chemical inhibitors and protein degraders. The challenges and opportunities in developing emerging therapeutic classes will be discussed. From the technology perspective, expansion beyond next generation sequencing panels with whole genome transcriptome sequencing, nanotechnologies, functional drug testing, and artificial intelligence, are being increasingly applied for target identification. Liquid biopsies to track specific tumor clones are of great interest to enable non-invasive monitoring of response or resistance to treatment, and the identification of molecular residual disease. Lastly, from the perspective of clinical trial designs, innovative neural network-based models are being developed to create comprehensive multimodal predictive biomarker profiles, learning from both patient-intrinsic and extrinsic data.

[Disclosure of COI by Responsible researcher] Lead presenter is the Responsible researcher.
[Lead presenter: Lillian L. Siu] ■Advisor of the company and/or profit-making organization: Voronoi ■Endowed chair: BMO Chair in Precision Genomics ■Representative of organization for clinical study receiving research expenses from company: Clinical trials support for institution: Merck, Novartis, Bristol-Myers Squibb, Pfizer/SeaGen, Boerhinger-Ingelheim, GlaxoSmithKline, Roche/Genentech, AstraZeneca/Medimmune, Bayer, Abbvie, Amgen, EMD Serono, Daiichi Sankyo, Gilead, Marengo, Incyte, LegoChem, Loxo/Eli Lilly, Medicenna, Takara

### [ME2] ME2
### Nature Communications

2025 / 9 / 25 (Thu) 15:30-16:10 Room 8/(Hotel Nikko Kanazawa 3F Kujaku C) Room 8

[Chairperson]Masahiro Sonoshita (Hokkaido University)

---

### ME2
### Nature Communications

[Speaker]Laura Sanchez Burgos (Springer Nature, Nature Communications)

No Abstract

### [P-1001] I-P1-1
### Animal models for cancer (1)

2025 / 9 / 25 (Thu) 16:45-17:30 Room P/(Motenashi Dome) Room P

---

### P-1001
### Characteristics of colon carcinogenesis in ***Mlh1*** heterozygous mice after combined exposure to radiation and inflammation

[Speaker]Takamitsu Morioka (Dept. Rad. Effects. Res., NIRS, QST／Quantum Cancer Sci. Team, iQLS, QST) [Co-author]Yi Shang (Dept. Rad. Effects. Res., NIRS, QST／Quantum Cancer Sci. Team, iQLS, QST)、Kazuhiro Daino (Dept. Rad. Effects. Res., NIRS, QST／Quantum Cancer Sci. Team, iQLS, QST)、Atsuko Ishikawa (Dept. Rad. Effects. Res., NIRS, QST／Quantum Cancer Sci. Team, iQLS, QST)、Chizuru Tsuruoka (Dept. Rad. Effects. Res., NIRS, QST／Quantum Cancer Sci. Team, iQLS, QST)、Masaaki Sunaoshi (Dept. Rad. Effects. Res., NIRS, QST／Quantum Cancer Sci. Team, iQLS, QST)、Tatsuhiko Imaoka (Dept. Rad. Effects. Res., NIRS, QST／Quantum Cancer Sci. Team, iQLS, QST)、Shizuko Kakinuma (Dept. Rad. Effects. Res., NIRS, QST／IES)

Both genetic and environmental factors play critical roles in colon tumorigenesis. The mutL homolog 1 (MLH1) gene, one of the DNA mismatch repair genes, is known as the causal gene of hereditary nonpolyposis colorectal cancer (HNPCC). Ionizing radiation and inflammation are also known as risk factors for colorectal cancer. However, little is known about the interaction of these factors. Hence, we examined the combined effects of radiation and inflammation on colon carcinogenesis using ***Mlh1*** heterozygous mice. Male and female 2- and 7-week-old ***Mlh1***$^{+/-}$ and ***Mlh1***$^{+/+}$ mice were irradiated with X-rays at a dose of 2 Gy. Then, they were treated with 1% dextran sodium sulfate (DSS) or vehicle in drinking water for 7 days to induce colitis. Colons were examined for tumors at 25 and 50 weeks of age. The tumor number after combined exposure to radiation followed by DSS was higher than that after a single exposure and in untreated mice. In addition, the tumor number was higher at 50 weeks of age compared to 25 weeks of age. In conclusion, combined exposure to radiation followed by mild inflammation enhances colon tumorigenesis in ***Mlh1*** heterozygous mice.

[Disclosure of COI by Responsible researcher] Lead presenter is the Responsible researcher.
[Lead presenter: Takamitsu Morioka] We have no financial relationships to disclose.

▲TOP

### [P-1002] I-P1-1
### Animal models for cancer (1)

2025 / 9 / 25 (Thu) 16:45-17:30 Room P/(Motenashi Dome) Room P

---

### P-1002
### Genotoxicity assessment of advanced materials using mouse liver organoids

[Speaker]Rikako Ishigamori (Environmental Health Sci., Hoshi Univ.) [Co-author]Akiko Ohno (Div. of Genome Safety Sci., Natl. Inst. of Health Sci.)、Yukari Totsuka (Environmental Health Sci., Hoshi Univ.)

Advanced materials are commonly used in various applications in industrial, medical, and cosmetic fields because of their useful properties. It is likely that these materials can be released into the environment and then humans can be exposed to them, thus there is an urgent need for their risk assessment. In the present study, we examined cellular uptake, cytotoxicity and micronucleus tests as a genotoxicity evaluation of advanced materials, such as mesoporous silica, graphene oxide and nano silica, using mouse liver organoids. To evaluate these advanced materials under more biologically relevant conditions, we used mouse liver progenitor organoids. Through confocal laser microscopy and flow cytometry analysis on cellular uptake of nanosilica, we confirmed that nanosilica is incorporated into organoids. When all advanced nanomaterials used in the present study were exposed to mouse hepatic organoids, cytotoxicity and genotoxicity were increased dose-dependently compared to a solvent control.

[Disclosure of COI by Responsible researcher] Lead presenter is not the Responsible researcher.
[Lead presenter: Rikako Ishigamori] We have no financial relationships to disclose.
[Principal Researcher: Yukari Totsuka] We have no financial relationships to disclose.

### [P-1003] I-P1-1
### Animal models for cancer (1)

2025 / 9 / 25 (Thu) 16:45-17:30 Room P/(Motenashi Dome) Room P

### P-1003
### Iron-catalyzed oxidative stress cancels cancer promotional effect of BRCA2 haploinsufficiency through ferroptosis

[Speaker]Yuki Maeda (Dept. Pathol. Biol. Responses., Nagoya. Univ., Grad. Sch. Med.) [Co-author]Tomoji Mashimo (Anim. Genet., Univ. of Tokyo., Inst. Med. Sci.)、Shinya Toyokuni (Dept. Pathol. Biol. Responses., Nagoya. Univ., Grad. Sch. Med.)

Pathogenic variants in ***BRCA2*** are risks for various cancers. Genomic instability due to insufficient homologous recombination is thought as responsible for carcinogenesis. However, molecular sequences how BRCA2 insufficiency leads to carcinogenesis remains unelucidated. We here applied iron-induced renal carcinogenesis to a rat heterozygous mutation model of ***Brca2*** (mutant ;MUT). Rat MUT model showed no promotional effect of renal carcinogenesis induced by ferric nitrilotriacetate (Fe-NTA). Whereas acute-phase analysis of the kidney after single or 1-week Fe-NTA administration to MUT showed suppressed lipid peroxidation, consistent with ferroptosis-resistance, ferroptosis and regeneration of tubular cells were coexistent with higher cytoplasmic catalytic Fe(II) levels in the subacute phase of MUT after 3-week Fe-NTA administration. In conclusion, iron-dependent oxidative stress plays double-edged roles either for cell death or proliferation in carcinogenesis and its biological consequences are distinct between ***BRCA2*** and ***BRCA1*** haploinsufficiency. Our results suggest that iron-catalyzed oxidative stress is not a major driving force of carcinogenesis in ***BRCA2*** pathogenic variants.

[Disclosure of COI by Responsible researcher] Lead presenter is not the Responsible researcher.
[Lead presenter: Yuki Maeda] We have no financial relationships to disclose.
[Principal Researcher: Shinya Toyokuni] We have no financial relationships to disclose.

### [P-1004] I-P1-1
### Animal models for cancer (1)

2025 / 9 / 25 (Thu) 16:45-17:30 Room P/(Motenashi Dome) Room P

### P-1004
### Histopathological and immunohistochemical analysis of antithyroid mechanism of VA-K-14 in rats

[Speaker]Hirotoshi Akane (Division of Pathology, National Institute of Health Sciences) [Co-author]Kohei Matsushita (Division of Pathology, National Institute of Health Sciences)、Mizuho Uneyama (Division of Pathology, National Institute of Health Sciences)、Tomomi Morikawa (Division of Pathology, National Institute of Health Sciences)、Shinji Takasu (Division of Pathology, National Institute of Health Sciences)、Kumiko Ogawa (Division of Pathology, National Institute of Health Sciences／Department of Toxicology, Hoshi University)、Takeshi Toyoda (Division of Pathology, National Institute of Health Sciences)

Changes in blood thyroid hormone levels are closely associated with nongenotoxic mechanism of thyroid carcinogenesis. We have recently reported a method to detect antithyroid effects of chemicals and estimate their mechanisms by histopathological and immunohistochemical analyses in rats. Here, we examine the antithyroid effects of VA-K-14, a TSH receptor antagonist ***in vitro***, in rats by our method. VA-K-14 was administered by gavage to 6-week-old male SD rats at doses of 1-100 mg/kg for 28 days. Despite no changes in serum T3 or TSH, decreased serum T4 and follicular cell hypertrophy in the thyroid were detected at 100 mg/kg, demonstrating the antithyroid effect of VA-K-14 in rats. Immunohistochemical analysis showed no obvious change in T3 or T4 in the thyroid and increased TSH+ area in the anterior pituitary gland, which were not consistent with immunostaining profiles of thyroid peroxidase and iodine uptake inhibitors, or TSH synthesis inhibitors, respectively. In contrast, UGT1A6+ area in the liver was significantly increased at 3 mg/kg and higher. These results indicated that antithyroid effects of VA-K-14 in rats is caused by thyroid hormone metabolism promotion in the liver.

[Disclosure of COI by Responsible researcher] Lead presenter is not the Responsible researcher.
[Lead presenter: Hirotoshi Akane] We have no financial relationships to disclose.
[Principal Researcher: Takeshi Toyoda] We have no financial relationships to disclose.

▲TOP

### [P-1005] I-P1-1
### Animal models for cancer (1)

2025 / 9 / 25 (Thu) 16:45-17:30 Room P/(Motenashi Dome) Room P

### P-1005
### Withdrawn

No Abstract

[P-1006] I-P1-1
### Animal models for cancer (1)

2025 / 9 / 25 (Thu) 16:45-17:30 Room P/(Motenashi Dome) Room P

P-1006
### Impacts of circadian rhythm disruption by time-restricted feeding and exercise on oxidative DNA damage and immune

[Speaker]Yunshan Li (Dept. Environ. Oncol., Univ. Occup. Environ. Health. Japan) [Co-author]Hiroaki Fujihara (Dept. Physiol., Univ. Occup. Environ. Health. Japan)、Koichi Fujisawa (Dept. Environ. Oncol., Univ. Occup. Environ. Health. Japan)、Kazuaki Kawai (Dept. Environ. Oncol., Univ. Occup. Environ. Health. Japan)

Circadian rhythm disruption can dysregulate cellular activity and metabolism, leading to various health problems, including sleep disorders, cardiovascular diseases, and even cancer. Recently, the complex relationship between circadian rhythm, oxidative stress, and immune function has garnered increasing attention. The aim of this study is investigating the impacts of circadian rhythm disorder by controlling the timing of feeding and running wheel activity on oxidative DNA damage and immune responses in 8-week-old mice for 14 days. Body weight, daytime running wheel activity, serum interleukin-6 levels, urinary 8-hydroxy-2´-deoxyguanosine levels, and nuclear DNA (liver, lung, testes, and pancreas) were significantly increased in the night-restricted group compared with the control group. In additionally, the mice in the night-restricted group indicated anxiety-like behavior. These results suggest that circadian rhythm disruption induced by abnormal dietary timing can lead to obesity, mental state dysregulation, oxidative DNA damage, and immune function variations in mice. This oxidative DNA damage may contribute to the initiation and increased risk of cancer.

[Disclosure of COI by Responsible researcher] Lead presenter is the Responsible researcher.
[Lead presenter: Yunshan Li] ■Research funds from company: 科研費　基盤研究C

### [P-1007] I-P1-1
### Animal models for cancer (1)

2025 / 9 / 25 (Thu) 16:45-17:30 Room P/(Motenashi Dome) Room P

---

### P-1007
### Unveiling the therapeutic potential of alkylating PI polyamide in ***MET*** amplified tumors

[Speaker]Zhilin Feng (Div. of Innovative Cancer Therap., Chiba Cancer Ctr. Res. Inst.／Chiba Univ. Grad. Sch. of Med. & Pharm. Sci.) [Co-author]Hiroyuki Yoda (Div. of Innovative Cancer Therap., Chiba Cancer Ctr. Res. Inst.)、Yoshiaki Maru (Div. of Mol. Carcinogenesis, Chiba Cancer Ctr. Res. Inst.)、Atsushi Takatori (Div. of Innovative Cancer Therap., Chiba Cancer Ctr. Res. Inst.)

In general, aberrant amplification and overexpression of proto-oncogene ***MET*** are tightly linked to poor survival and prognosis of cancer patients. Currently, the conventional MET inhibitors face the challenges of drug resistance and/or lack of specificity. To overcome these issues, the development of a gene-level-inhibitory strategy against ***MET*** amplification is urgently required. Previously, our group reported an alkylating pyrrole-imidazole polyamide (CCC-002) targeting the amplified ***MYCN*** in neuroblastoma cells. During the subsequent study, we also found out two CCC-002-binding sites within ***MET***. Therefore, we sought to ask whether CCC-002 directly targets the ***MET*** gene for the treatment of ***MET***-amplified (***MET***-amp) tumors. In the present study, we have demonstrated that CCC-002 downregulated MET expression in ***MET***-amp cell lines (MKN45 and NCI-H1993) and patient-derived organoids. Besides, we also found that the relative copy number of ***MET*** was significantly reduced in MKN45 and NCI-H1993 cells exposed to CCC-002. Together, our present results suggest that CCC-002 has a potential to target ***MET*** amplification.

[Disclosure of COI by Responsible researcher] Lead presenter is not the Responsible researcher.
[Lead presenter: Zhilin Feng] We have no financial relationships to disclose.
[Principal Researcher: Atsushi Takatori] ■Research funds from company: JSPS

### [P-1015] I-P2-1
### Animal model (1)

2025 / 9 / 25 (Thu) 16:45-17:30 Room P/(Motenashi Dome) Room P

---

### P-1015
### Effect of high-fat diet on colorectal cancer development

[Speaker]Takafumi Hanashima (Lab. Mol. Genetics., Nat. Cancer Ctr. Res. Inst.／Dept. Pharmacolo., Hoshi Univ.) [Co-author]Haruna Takeda (Lab. Mol. Genetics., Nat. Cancer Ctr. Res. Inst.)、Minoru Narita (Dept. Pharmacolo., Hoshi Univ.)

It has been reported that the high-fat diet (HFD) increases the risk of colorectal cancer (CRC) development. To elucidate the molecular mechanisms underlying HFD-associated CRC, we performed in vivo Sleeping Beauty transposon screening in mice carrying a KrasG12D mutation. HFD-fed mice showed increased body weight, increased blood glucose levels, and increased number of tumors in the GI tract compared to control mice. The data showed that HFD accelerated tumor development in our mouse model. We used tumor genomes to determine transposon insertion sites, identify tumor-related genes, and found that the AMPK pathway genes are frequently mutated in tumors of HFD mice. We have administered drugs targeting AMPK to the HFD mice and analyzed their effects on blood glucose levels and the number of tumors. In addition, we have performed single-cell RNA-seq analysis on these tumor tissues to evaluate the effects of the drug on the cancer microenvironment. Our study will clarify the impact of HFD intake on CRC and lead to the development of novel therapeutic targets.

[Disclosure of COI by Responsible researcher] Lead presenter is not the Responsible researcher.
[Lead presenter: Takafumi Hanashima] We have no financial relationships to disclose.
[Principal Researcher: Haruna Takeda] We have no financial relationships to disclose.

▲TOP

### [P-1016] I-P2-1
### Animal model (1)

2025 / 9 / 25 (Thu) 16:45-17:30 Room P/(Motenashi Dome) Room P

---

### P-1016
### Suppression of intestinal adenoma and/or adenocarcinoma by loss of the TMEPAI gene

[Speaker]Naoko Nakano (Lab. Biol., Showa Pharm. Univ.) [Co-author]Fumiko Itoh (Lab. Stem Cell Reg., Tokyo Univ. Pharm.)、Makoto M. Taketo (Med. Res. Inst. KITANO HOSP.)、Mitsuyasu Kato (Lab. Exp. Path., Univ. Tsukuba.)、Susumu Itoh (Lab. Biol., Showa Pharm. Univ.)

We have previously found that the TMEPAI family, including TMEPAI and C18ORF1, inhibits TGF-β signaling by sequestering Smad2/3. Analysis using the TNMplot database revealed that TMEPAI expression is elevated in various tumors, including human colon cancer. To investigate the role of the TMEPAI family in intestinal tumorigenesis, we generated $Apc^{\Delta 716/+}$mice lacking TMEPAI and/or C18ORF1 genes. Loss of the TMEPAI gene markedly reduced both the number and size of adenomas in $Apc^{\Delta 716/+}$ mice. Consistently, in the AOM/DSS tumor model, mice lacking the TMEPAI gene developed fewer colorectal adenocarcinomas compared to wild-type controls. To further elucidate the molecular mechanisms by which TMEPAI deficiency suppresses tumorigenesis, we utilized $Apc^{F/F}$/Villin-CreER mice in the presence or absence of the TMEPAI gene. A single injection of tamoxifen in $Apc^{F/F}$/Villin-CreER mice promoted the migration of Olfm4- and Ki67-positive intestinal cells along the villi, whereas this effect was attenuated in mice lacking the TMEPAI gene. These findings suggest that TMEPAI deficiency may enhance the tumor-suppressive effects of TGF-β signaling, thereby contributing to the suppression of tumor growth.

[Disclosure of COI by Responsible researcher] Lead presenter is not the Responsible researcher.
[Lead presenter: Naoko Nakano] We have no financial relationships to disclose.
[Principal Researcher: Susumu Itoh] We have no financial relationships to disclose.

▲TOP

### [P-1017] I-P2-1
### Animal model (1)

2025 / 9 / 25 (Thu) 16:45-17:30 Room P/(Motenashi Dome) Room P

---

### P-1017
### Novel PI3K-Driven Intrahepatic Cholangiocarcinoma Mouse Model and Synergistic Effects of PI3K and WEE1 Inhibition

[Speaker]Hiromi Tsuchiya (Dept. of Gastroenterology, Yokohama City University Medical Ctr.) [Co-author]Makoto Sugimori (Dept. of Gastroenterology, Yokohama City University Medical Ctr./Divi. of Cancer Genome, Yokohama City University Medical Ctr.)、Yugo Ishino (Dept. of Gastroenterology, Yokohama City University Medical Ctr.)、Shotaro Tsunoda (Dept. of Gastroenterology, Yokohama City University Medical Ctr.)、Akihiro Funaoka (Dept. of Gastroenterology, Yokohama City University Medical Ctr.)、Kazuki Endo (Dept. of Gastroenterology, Yokohama City University Medical Ctr.)、Ritsuko Oishi (Dept. of Gastroenterology, Yokohama City University Medical Ctr.)、Yuichi Suzuki (Dept. of Gastroenterology, Yokohama City University Medical Ctr.)、Yushi Kanemaru (Dept. of Gastroenterology, Yokohama City University Graduate School of Med.)、Yoshimasa Suzuki (Dept. of Gastroenterology, Yokohama City University Graduate School of Med.)、Sho Onodera (Dept. of Gastroenterology, Yokohama City University Graduate School of Med.)、Haruo Miwa (Dept. of Gastroenterology, Yokohama City University Medical Ctr.)、Akito Nozaki (Dept. of Gastroenterology, Yokohama City University Medical Ctr.)、Manabu Morimoto (Dept. of Gastroenterology, Yokohama City University Medical Ctr.)、Shin Maeda (Dept. of Gastroenterology, Yokohama City University Graduate School of Med.)

Aim:Cholangiocarcinoma (CCA) is a lethal malignancy with limited treatment options. About 7–12% of cases involve PI3K pathway activation, indicating potential for targeted therapy. This study evaluated PI3K inhibition in a novel PI3K-activated CCA mouse model.Methods:***Foxa3-Cre*** mice were crossed with ***Rosa26-LSL-Pik3ca***$^{H1047R}$, ***PTEN flox***, and ***TP53 flox*** mice to generate the model. Tumor-derived organoids and intrahepatic CCA cell lines (HuH28, HuCCT1) were treated with alpelisib (PI3K inhibitor) and adavosertib (WEE1 inhibitor).Results: Mice harboring PI3K activation and TP53 loss developed intrahepatic CCA at 4–5 months. Alpelisib suppressed proliferation in ***PIK3CA***-mutant HuH28 cells and mouse organoids. Combination with adavosertib, a WEE1 inhibitor that disrupts the G2/M checkpoint and is particularly effective in TP53-deficient tumors, induced synergistic growth inhibition. Combination treatment more strongly inhibited CDK1 phosphorylation and increased γ–H2AX, indicating enhanced DNA damage and checkpoint disruption.Conclusion: PI3K pathway activation with TP53 loss promotes cholangiocarcinogenesis, and dual inhibition of PI3K and WEE1 could be a promising treatment approach.

[Disclosure of COI by Responsible researcher] Lead presenter is not the Responsible researcher.
[Lead presenter: Hiromi Tsuchiya] We have no financial relationships to disclose.
[Principal Researcher: Shin Maeda] We have no financial relationships to disclose.

[P-1018] I-P2-1
Animal model (1)

2025 / 9 / 25 (Thu) 16:45-17:30 Room P/(Motenashi Dome) Room P

P-1018

### Generation and phenotype analysis of a genome-edited mouse with a germline *Apc* variant in the 5' region

[Speaker]Teruaki Hirota (Joint Graduate School of Veterinary Sci., Gifu Univ.／Lab. of Veterinary Path., Gifu Univ.／JSPS Research Fellowship for Young Scientist DC1) [Co-author]Shingo Miyawaki (Lab. of Veterunary Surg., Gifu Univ.／Center for One Medicine Innovative Translational Research (COMIT), Gifu Univ.)、Hiroki Sakai (Joint Graduate School of Veterinary Sci., Gifu Univ.／Lab. of Veterinary Path., Gifu Univ.／Center for One Medicine Innovative Translational Research (COMIT), Gifu Univ.)、Akihiro Hirata (Joint Graduate School of Veterinary Sci., Gifu Univ.／Lab. of Veterinary Path., Gifu Univ.／Center for One Medicine Innovative Translational Research (COMIT), Gifu Univ.)

[Background] Germline ***APC*** variants cause familial adenomatous polyposis (FAP), an autosomal dominant disorder characterized by numerous colorectal adenomatous polyps. Recently, we identified a canine counterpart of FAP that is caused by a germline ***APC*** variant at codons 154 and 155 (c.[462_463delinsTT]). Compared to patients with typical FAP, the affected dog developed fewer tumors overall but showed a markedly higher incidence of gastric tumors.

[Methods] We generated a mouse model harboring the same ***Apc*** variant as the affected dogs, ***Apc***$^{\Delta155/+}$ mice, using the CRISPR-Cas9 system and analyzed the phenotypes at 8, 12, 16, and 20 weeks of age.

[Results] ***Apc***Δ155/+ mice developed more tumors in the stomach and proximal small bowel than in the colorectum. The number of tumors in these regions increased significantly with age.

[Discussion] Compared to ***Apc***$^{Min/+}$ mice harboring the germline variant at codon 850, ***Apc***$^{\Delta155/+}$ mice developed a larger number of gastric tumors, but the total number of gastrointestinal tumors was smaller. Therefore, the germline variant in the 5' region results in a smaller number of tumors and predilection for the stomach in the gastrointestinal tract.

[Disclosure of COI by Responsible researcher] Lead presenter is not the Responsible researcher.
[Lead presenter: Teruaki Hirota] We have no financial relationships to disclose.
[Principal Researcher: Akihiro Hirata] We have no financial relationships to disclose.

▲TOP

### [P-1019] I-P2-1
### Animal model (1)

2025 / 9 / 25 (Thu) 16:45-17:30 Room P/(Motenashi Dome) Room P

### P-1019
### Possible roles of RECK in cancer immunity

[Speaker]Tomoko Matsuzaki (Institute of Laboratory Animals, Graduate School of Medicine, Kyoto University) [Co-author]Makoto Noda (Graduate School of Medicine, Kyoto University)

RECK is known to regulate several metalloproteinases, but we recently found that it also affects plasma TGF-β levels probably by promoting the formation of robust fibrillin microfibrils that serve as a tissue reservoir for TGF-β precursor. We also found that RECK can suppress tumor growth via both cell-autonomous and non-cell-autonomous mechanisms; the latter may involve immunological suppression. We therefore attempt to further elucidate these mechanisms through two approaches, <1> characterizing mice carrying ***Reck*** mutations in hematopoietic lineages, and <2> transcriptome analyses of tumor cells with and without RECK expression, focusing on cell surface molecules. New findings will be presented in the meeting.

[Disclosure of COI by Responsible researcher] Lead presenter is the Responsible researcher.
[Lead presenter: Tomoko Matsuzaki] ■Research funds from company: 科研費　基盤C

### [P-1020] I-P2-1
### Animal model (1)

2025 / 9 / 25 (Thu) 16:45-17:30 Room P/(Motenashi Dome) Room P

---

### P-1020
### Establishment of lung metastasis mice model for feline breast cancer and evaluation of its usefulness

[Speaker]Haru Yamamoto (Lab. of Vet. Pharm., Tokyo Univ. of Agri & Tech.) [Co-author]Kazuaki Sasaki (Lab. of Vet. Pharm., Tokyo Univ. of Agri & Tech.)

**Introdultion**Feline breast cancer (FBC) is 3rd common cancer in feline tumors. Even if in early stage of disease, many cases had already had metastasis to another region and poor prognosis. Therefore, hematogenous metastasis to the lungs has been often observed.
**Purpose**We established lung metastasis mouse model for FBC and evaluated its usefulness as in vivo model.
**Method**Using FBC tissue, tumor cells (Ori.-FBC cells) was extracted and cultured. After 4 weeks of tail vein administration to mice, lungs were extracted and pathological changes were observed. Tumor cells from metastatic lung tissue (Meta-FBC cells) were cultured, and marker expression was confirmed.
**Result**Ori.-FBC cells could be cultured and extracted lung tissue had multiple metastatic regions. Marker expression corresponded between original tissue and metastatic lung tissue. Meta.-FBC cells were exhibited mesenchymal morphology and IF showed consistent marker expression.
**Conclusion**Through this study, lung metastatic mouse model was established, and we succeeded new experimental model which can be a novel experimental model of hematogenous pulmonary metastasis

[Disclosure of COI by Responsible researcher] Lead presenter is not the Responsible researcher.
[Lead presenter: Haru Yamamoto] We have no financial relationships to disclose.
[Principal Researcher: Kazuaki Sasaki] We have no financial relationships to disclose.

▲TOP

### [P-1021] I-P2-1
### Animal model (1)

2025 / 9 / 25 (Thu) 16:45-17:30 Room P/(Motenashi Dome) Room P

---

### P-1021
### Establishment of a highly metastatic pancreatic adenocarcinoma in ***Ogt***-transgenic mice

[Speaker]Kazumasa Moriwaki (Dept. Pharmacol. Fac. Med., Osaka Med. Pharmaceut. Univ.)

***O***-GlcNAcylation is a post-translational modification regulating dynamic intracellular signaling by ***O***-GlcNAc transferase (OGT) that add the modification. In cancer cells, ***O***-GlcNAcylation is generally elevated because of increased glucose uptake, which promotes cancer progression. To examine the role of tumor microenvironment (TME) ***O***-GlcNAcylation in cancer progression, we generated ***Ogt*** transgenic (***Ogt***-Tg) mice and previously reported that elevated ***O***-GlcNAcylation in the TME promotes B16 tumor growth. This time, we show augmented TME OGT promotes cancer engraftment in a syngeneic orthotopic murine model of Panc02 pancreatic adenocarcinoma. Upon orthotopic inoculation, a small number of the cells did not survive in WT mice but did survive in ***Ogt***-Tg mice. Using ***Ogt***-Tg mice, serial transplantations were performed in the orthotopic transplantation model to establish highly malignant Panc02 sublines. One of them showed high metastatic potential ***in vitro*** and ***in vivo***. These sublines were screened for key molecules that promote cancer progression. RNA-sequencing revealed elevated expression levels of distinct molecules in these sublines. Some of these molecules are presented here.

[Disclosure of COI by Responsible researcher] Lead presenter is the Responsible researcher.
[Lead presenter: Kazumasa Moriwaki] We have no financial relationships to disclose.

### [P-1022] I-P2-1
### Animal model (1)

2025 / 9 / 25 (Thu) 16:45-17:30 Room P/(Motenashi Dome) Room P

---

### P-1022
### Development of animal models for cancer cachexia and strategies to improve the quality of life in sarcopenia

[Speaker]Junichi Tanuma (Div. Oral Pathol., Niigata Univ. Grad. Sch. Med. & Dent. Sci.) [Co-author]Satoshi Maruyama (Div. Oral Pathol., Niigata Univ. Grad. Sch. Med. & Dent. Sci.／Oral Pathol. Sect., Dept. Surg. Pathol., Niigata Univ. Hosp.)、Tatsuya Abe (Div. Oral Pathol., Niigata Univ. Grad. Sch. Med. & Dent. Sci.)、Kenta Haga (Div. Oral Pathol., Niigata Univ. Grad. Sch. Med. & Dent. Sci.)、Alex Kesuma (Div. Oral Anatomy., Niigata Univ. Grad. Sch. Med. & Dent. Sci.)、Maiko Kawasaki (Div. Oral Anatomy., Niigata Univ. Grad. Sch. Med. & Dent. Sci.)、Manabu Yamazaki (Div. Oral Pathol., Niigata Univ. Grad. Sch. Med. & Dent. Sci.)

Cancer cachexia is a condition characterized by anorexia and weight loss and is associated with many diseases, including oral cancer, while sarcopenia is similarly marked by muscle weakness due to skeletal muscle atrophy, and bone resorption. However, there is no animal model to elucidate this cancer cachexia and sarcopenia, so we report here that we have succeeded in creating a rat model. We also report data (blood tests, antibody tests, histopathology, μCT) on this model in comparison with human cachexia and sarcopenia, as well as on treatment with Chinese herbal medicine to improve the quality of life of these model rats.

[Disclosure of COI by Responsible researcher] Lead presenter is the Responsible researcher.
[Lead presenter: Junichi Tanuma] ■Research funds from company: 株式会社ツムラ

### [P-1031] I-P2-3
### Animal model (3)

2025 / 9 / 25 (Thu) 16:45-17:30 Room P/(Motenashi Dome) Room P

### P-1031
### Evaluation of IL-6-targeted therapy in a mouse model of ovarian clear cell carcinoma

[Speaker]Chiho Miyagawa (Kindai Univ., Faculty of Med., Gynecol. & Obstet.／Tondabayashi Hosp.) [Co-author]Kosuke Murakami (Kindai Univ., Faculty of Med., Gynecol. & Obstet.)、Shiki Takamura (RIKEN, Lab of Immunol. memory)、Yoko Kashima (Kindai Univ., Faculty of Med., Gynecol. & Obstet.)、Yukari Kobayashi (Kindai Univ., Faculty of Med., Gynecol.)、Koji Nagaoka (Kindai Univ., Faculty of Med., Gynecol.)、Kazuhiro Kakimi (Kindai Univ., Faculty of Med., Gynecol.)、Noriomi Matsumura (Kindai Univ., Faculty of Med., Gynecol. & Obstet.)

Objective:Recent studies suggest immune checkpoint inhibitors (ICIs) are effective against ovarian clear cell carcinoma. We previously established a C57BL/6-based conditional Arid1a/Pik3ca co-mutated mouse model producing high IL-6 levels and derived cell lines. We evaluated IL-6-targeted therapy using this model. Methods:IL-6 knockdown (KD) cell lines were generated via shRNA. Their proliferation and subcutaneous tumor growth in mice were compared with parental lines. The effects of anti-IL-6, anti-PD-L1, and anti-CTLA-4 antibodies were assessed in a carcinomatous peritonitis model.Results:In vitro proliferation was similar between IL-6KD and parental lines. However, IL-6KD tumors showed significantly reduced growth in both C57BL/6 and nude mice ($P<0.0001$, $P=0.002$). RNA-seq revealed up-regulation of apoptosis-related and down-regulation of G2/M checkpoint genes ($P=0.0004$,$P=0.02$). In the peritonitis model, anti-IL-6 significantly prolonged survival ($P=0.004$), and combination with triple antibody therapy further improved survival ($P=0.0009$).Conclusion:IL-6-targeted therapy shows antitumor effects via non-immune mechanisms, and combination with ICIs may enhance efficacy.

[Disclosure of COI by Responsible researcher] Lead presenter is the Responsible researcher.
[Lead presenter: Chiho Miyagawa] We have no financial relationships to disclose.

### [P-1032] I-P2-3
### Animal model (3)

2025 / 9 / 25 (Thu) 16:45-17:30 Room P/(Motenashi Dome) Room P

---

### P-1032
### Establishment of a mouse intrahepatic cholangiocarcinoma cell line from Pten-null and Kras mutant mouse model

[Speaker]Takahiro Oshina (Division of Clinical Genome Research, IMSUT／Department of Urology, Graduate School of Medicine, Tokyo Univ.)
[Co-author]Kiyoko Takane (Division of Clinical Genome Research, IMSUT)、Hideaki Ijichi (Department of Gastroenterology, Graduate School of Medicine, Tokyo univ.)、Satoru Taguchi (Department of Urology, Graduate School of Medicine, Tokyo Univ.)、Haruki Kume (Department of Urology, Graduate School of Medicine, Tokyo Univ.)、Kiyoshi Yamaguchi (Division of Clinical Genome Research, IMSUT)、Yoichi Furukawa (Division of Clinical Genome Research, IMSUT)

Intrahepatic cholangiocarcinoma (ICC) is the second frequent primary liver cancer, and its prognosis remains poor. Although the standard chemotherapy regimen of gemcitabine and cisplatin for advanced and/or recurrent ICC shows a median overall survival of approximately 11 months, the efficacy is limited and unsatisfactory. Therefore, the development of new therapeutic strategies for ICC is needed. We previously generated AKPP mouse, a containing ***Kras*** activation and homozygous ***Pten*** deletion in liver progenitor cells and showed that the mice exclusively developed cholangiocarcinoma. In this study, we successfully established a murine cell line, AKPP, from the tumors. We corroborated that the cells carried ***Kras*** G12D mutation by genetic analysis and increased phosphorylation of Erk and Akt and loss of Pten expression by western blot analysis. Notably, the AKPP cells showed in vivo tumor formation in the syngeneic C57BL/6 mice. In addition, treatment of the cells with Kras and Pi3k inhibitors suppressed their proliferation. These data suggest that this line will be useful for the preclinical studies of anticancer drugs including immuno-checkpoint inhibitors.

[Disclosure of COI by Responsible researcher] Lead presenter is not the Responsible researcher.
[Lead presenter: Takahiro Oshina] We have no financial relationships to disclose.
[Principal Researcher: Yoichi Furukawa] We have no financial relationships to disclose.

▲TOP

### [P-1033] I-P2-3
### Animal model (3)

2025 / 9 / 25 (Thu) 16:45-17:30 Room P/(Motenashi Dome) Room P

---

### P-1033
### Mutational profiles of spontaneous and radiation-associated mammary carcinomas in a Brca1 haploinsufficiency rat model

[Speaker]Yuzuki Nakamura (Inst. for Radiol. Sci., QST／Tokyo Metropolitan Univ.／JSPS Research Fellow, DC) [Co-author]Kazuhiro Daino (Inst. for Radiol. Sci., QST／Inst. for Quantum Sci., QST)、Atsuko Ishikawa (Inst. for Radiol. Sci., QST)、Shizuko Kakinuma (Inst. for Radiol. Sci., QST／Inst. for Environ. Sci.)、Yukiko Nishimura-Yano (Inst. for Radiol. Sci., QST)、Kento Nagata (Inst. for Radiol. Sci., QST／Inst. for Quantum Sci., QST)、Masaru Takabatake (Inst. for Radiol. Sci., QST／Tokyo Metropolitan Univ.)、Kazumasa Inoue (Tokyo Metropolitan Univ.)、Tatsuhiko Imaoka (Inst. for Radiol. Sci., QST／Tokyo Metropolitan Univ.／Inst. for Quantum Sci., QST)

Carriers of a ***BRCA1*** pathogenic variant have a high incidence of breast cancer. Recently, BRCA1 haploinsufficiency has been reported to contribute to the early phase of BRCA1-associated carcinogenesis. ***Brca1***$^{L63X/+}$ rat (MUT) exhibits a higher incidence of mammary carcinoma than wild-type rats (WT) under DNA damage induced by ionizing radiation. No inactivation of the wild-type Brca1 allele has been observed in mammary carcinomas of MUT, suggesting that this model recapitulates the Brca1 haploinsufficiency carcinogenesis. Here, we performed whole-exome sequencing of carcinomas in untreated or irradiated MUT and WT, to clarify the mechanism of carcinogenesis based on Brca1 haploinsufficiency. There were no significant differences between MUT and WT in the number of somatic mutations in either spontaneous or radiation-associated carcinomas. In the irradiated group, cancer-driver mutations by single nucleotide variants (SNVs) or insertions/deletions (InDels) were significantly fewer in carcinomas of MUT than WT. Thus, Brca1 haploinsufficiency may promote carcinogenesis through mechanisms other than SNVs and InDels, such as structural variants or tissue microenvironment changes.

[Disclosure of COI by Responsible researcher] Lead presenter is not the Responsible researcher.
[Lead presenter: Yuzuki Nakamura] We have no financial relationships to disclose.
[Principal Researcher: Tatsuhiko Imaoka] We have no financial relationships to disclose.

### [P-1034] I-P2-3
### Animal model (3)

2025 / 9 / 25 (Thu) 16:45-17:30 Room P/(Motenashi Dome) Room P

### P-1034
### The relationship between cellular senescence and invasive potential of cancer cells

[Speaker]Heewon Seo (Tokyo Univ. of Sci. Graduate School of Biological Sci.) [Co-author]Shunsuke Kon (Tokyo Univ. of Sci. Research institute for Biomedical Sciences)

Cellular senescence, a stable state of cell-cycle arrest, has traditionally been considered a tumor-suppressive mechanism that prevents uncontrolled proliferation of cancer cells. However, recent studies have demonstrated a paradoxical role of senescence in cancer progression. For instance, the senescence-associated secretory phenotype (SASP), characterized by the secretion of pro-inflammatory factors, has been shown to promote tumorigenesis. Moreover, the impact of senescence induction on the invasiveness and malignant potential of cancer cells remains poorly understood. In this study, using an intestinal de novo cancer mouse model, we found that increased invasiveness and malignancy of cancer cells are accompanied by elevated levels of senescence. This observation was further supoorted by in vitro invasion assays. Additionally, single-cell RNA sequencing (scRNA-seq) analysis revealed a significant correlation between cellular senescence markers and the expression of invasion-related genes. Taken together, our findings suggest that cellular senescence may play a promotive role in enhancing the invasive and malignant characteristics of cancer cells.

[Disclosure of COI by Responsible researcher] Lead presenter is not the Responsible researcher.
[Lead presenter: Heewon Seo] We have no financial relationships to disclose.
[Principal Researcher: Shunsuke Kon] We have no financial relationships to disclose.

▲TOP

### [P-1035] I-P2-3
### Animal model (3)

2025 / 9 / 25 (Thu) 16:45-17:30 Room P/(Motenashi Dome) Room P

---

### P-1035
### Investigation of Size-Dependent Anti-Inflammatory and Antitumor Effects of Oxygen Nanobubble Water

[Speaker]Haruka Okami (Initiative for Advanced Res., Gunma Univ.) [Co-author]Gendensuren Dorjkhorloo (Dept. of General Surg. Gastroenterological Surg., Gunma Univ. Grad. Sch.)、Nobutoshi Mutsuki (Inc., Sigma Tech.)、Dai Yamanouchi (Dept. of Vascular Surg., Fujita Health Univ.)、Takehiko Yokobori (Initiative for Advanced Res., Gunma Univ.)、Eri Miyata (Dept. of General Surg. Gastroenterological Surg., Gunma Univ. Grad. Sch.)、Chika Katayama (Dept. of General Surg. Gastroenterological Surg., Gunma Univ. Grad. Sch.)、Chika Komine (Dept. of General Surg. Gastroenterological Surg., Gunma Univ. Grad. Sch.)、Takuhisa Okada (Dept. of General Surg. Gastroenterological Surg., Gunma Univ. Grad. Sch.)、Akihiko Sano (Dept. of General Surg. Gastroenterological Surg., Gunma Univ. Grad. Sch.)、Takuya Shiraishi (Dept. of General Surg. Gastroenterological Surg., Gunma Univ. Grad. Sch.)、Makoto Sakai (Dept. of General Surg. Gastroenterological Surg., Gunma Univ. Grad. Sch.)、Ken Shirabe (Dept. of General Surg. Gastroenterological Surg., Gunma Univ. Grad. Sch.)、Hiroshi Saeki (Dept. of General Surg. Gastroenterological Surg., Gunma Univ. Grad. Sch.)

Background: The incidence of ulcerative colitis and colitis-associated colorectal cancer is rising. Chronic inflammation induces a hypoxic tumor microenvironment, enhancing hypoxia-inducible factor-1α (HIF-1α) accumulation and tumor progression. We previously showed that nano-bubble oxygenated water ($NBO_2$) suppressed HIF-1α and reduced radioresistance. This study examined the effects of 10 nm and 100 nm $NBO_2$ in a murine colitis-associated cancer model.Methods: Mice were treated with azoxymethane and dextran sodium sulfate, and given distilled water (DW), 10 nm, or 100 nm $NBO_2$. Disease activity index (DAI) and histology (HIF-1α, Ki67, CD8, CD68, FOXP3, goblet cells) were evaluated.Results: The 10 nm $NBO_2$ group showed lower DAI, more goblet cells, improved mucosa, fewer tumors and Ki67+ cells, and reduced CD68+ macrophages vs DW. No significant effects were observed with 100 nm $NBO_2$.Conclusion: 10 nm $NBO_2$ suppressed inflammation and tumor formation, suggesting potential for inflammation-related cancer therapy.

[Disclosure of COI by Responsible researcher] Lead presenter is not the Responsible researcher.
[Lead presenter: Hruka Okami] We have no financial relationships to disclose.
[Principal Researcher: Takehiko Yokobori] ■Research funds from company: 株式会社シグマテクノロジー

▲TOP

### [P-1036] I-P2-3
### Animal model (3)

2025 / 9 / 25 (Thu) 16:45-17:30 Room P/(Motenashi Dome) Room P

---

### P-1036
### Relationship Between Brain Weight and Cancer Mortality: Insights from a Comparative Oncological Analysis of Primates

[Speaker]Yusuke Suenaga (Lab. Evo. Oncol. Chiba Cancer Ctr. Res. Ins.) [Co-author]Kazuma Nakatani (Lab. Evo. Oncol. Chiba Cancer Ctr. Res. Ins.)、Daisuke Muto (Lab. Evo. Oncol. Chiba Cancer Ctr. Res. Ins.)、Akiko Endo (Lab. Evo. Oncol. Chiba Cancer Ctr. Res. Ins.)、Shino Okamoto (Lab. Evo. Oncol. Chiba Cancer Ctr. Res. Ins.)、Ummi M. Zulfin (Lab. Evo. Oncol. Chiba Cancer Ctr. Res. Ins.)、Yuta Shintaku (Japan Monkey Centre)、Koshiro Watanuki (Japan Monkey Centre)

Cancer mortality is markedly higher in humans than in great apes; however, comprehensive data across primates remain limited. Thus, it is uncertain whether elevated cancer mortality in humans reflects an ancestral or derived trait. Here, we examined tumor incidence using necropsy records from 6,852 individuals across 128 primate species maintained at the Japan Monkey Centre over 65 years (1959-2024). No species exhibited mortality rates comparable to those of humans, suggesting heightened cancer mortality is a derived trait. Tumor incidence was significantly greater in Strepsirrhini than in Haplorhini. Among closely related taxa, Taiwanese macaques showed higher tumor incidence than rhesus macaques, and Yakushima macaques (***Macaca fuscata yakui***) higher than mainland Japanese macaques (***Macaca fuscata fuscata***). In both cases, elevated tumor incidence correlated with lower brain-to-body weight ratios, with no associations found for other organs. These findings suggest a possible "island effect," where island primates exhibit greater tumor susceptibility and reduced brain size. We further explore the potential role of effective population size in shaping these traits.

[Disclosure of COI by Responsible researcher] Lead presenter is the Responsible researcher.
[Lead presenter: Yusuke Suenaga] We have no financial relationships to disclose.

Poster Session

### [P-1037] I-P2-3
### Animal model (3)

2025 / 9 / 25 (Thu) 16:45-17:30 Room P/(Motenashi Dome) Room P

---

### P-1037
### Study of carcinogenesis by transplacental exposure to dimethylarsinic acid in F1 mice

[Speaker]Masaki Fujioka (Dept. Mol. Pathol., Osaka Metro. Univ. Grad. Sch. Med.) [Co-author]Shugo Suzuki (Dept. Mol. Pathol., Osaka Metro. Univ. Grad. Sch. Med.)、Min Gi (Dept. Env. Risk Assess., Osaka Metro. Univ. Grad. Sch. Med.)、Arpamas Vachiraarunwong (Dept. Env. Risk Assess., Osaka Metro. Univ. Grad. Sch. Med.)、Runjie Guo (Dept. Env. Risk Assess., Osaka Metro. Univ. Grad. Sch. Med.)、Yurina Kawamura (Dept. Env. Risk Assess., Osaka Metro. Univ. Grad. Sch. Med.)、Yuji Oishi (Dept. Mol. Pathol., Osaka Metro. Univ. Grad. Sch. Med.)、Anna Kakehashi (Dept. Mol. Pathol., Osaka Metro. Univ. Grad. Sch. Med.)、Hideki Wanibuchi (Dept. Env. Risk Assess., Osaka Metro. Univ. Grad. Sch. Med.)

Accumulating evidence indicates that prenatal exposure to inorganic arsenic, a human carcinogen, can subsequently cause cancer in adulthood in mice. In our previous study, transplacental exposure to DMA, an organic arsenic compound major metabolite of inorganic arsenic, occurred in lung adenocarcinoma in male F1 mice. Moreover, our reports show that altered histone modifications are essential to lung carcinogenesis by DMA transplacental exposure. However, little is known about the hepatocarcinogenic risk and its mechanism of transplacental DMA exposure. This study aims to determine the hepatocarcinogenic effects of transplacental DMA exposure in CD1 mice. Pregnant CD1 mice were given 0 or 200 ppm DMA in drinking water from gestation day 8 to 18. The offspring (F1) were observed for up to 84 weeks of age. In male offspring, there is a significantly increased incidence of liver tumors (hepatocellular adenoma and carcinoma) in the transplacental DMA exposure group. Conversely, there were no increases in the tumor incidence in the female offspring. These results show that transplacental exposure of CD1 mice to DMA through drinking water induces hepatocarcinogenesis in male offspring.

[Disclosure of COI by Responsible researcher] Lead presenter is not the Responsible researcher.
[Lead presenter: Masaki Fujioka] We have no financial relationships to disclose.
[Principal Researcher: Hideki Wanibuchi] We have no financial relationships to disclose.

[P-1038] I-P2-3
Animal model (3)

2025 / 9 / 25 (Thu) 16:45-17:30 Room P/(Motenashi Dome) Room P

P-1038
### Short-term exposure of chemical carcinogen determines the carcinogenic fate of upper tract urothelial carcinoma

[Speaker]Yuki Horibe (Department of Urology, The University of Osaka, Medicine) [Co-author]Atsunari Kawashima (Department of Urology, The University of Osaka, Medicine)、Kentaro Jingushi (Molecular and Cellular Physiology, The University of Osaka, Pharmaceutical Sciences)、Kansuke Kido (Department of Pathology, The University of Osaka, Medicine)、Hiromu Horitani (Department of Urology, The University of Osaka, Medicine)、Shunsuke Inoguchi (Department of Urology, The University of Osaka, Medicine)、Nesrine Sassi (Department of Urology, The University of Osaka, Medicine)、Akinaru Yamamoto (Department of Urology, The University of Osaka, Medicine)、Toshihiro Uemura (Department of Urology, The University of Osaka, Medicine)、Yu Ishizuya (Department of Urology, The University of Osaka, Medicine)、Takuji Hayashi (Department of Urology, The University of Osaka, Medicine)、Yoshiyuki Yamamoto (Department of Urology, The University of Osaka, Medicine)、Taigo Kato (Department of Urology, The University of Osaka, Medicine)、Koji Hatano (Department of Urology, The University of Osaka, Medicine)、Satoshi Nojima (Department of Pathology, The University of Osaka, Medicine)、Norio Nonomura (Department of Urology, The University of Osaka, Medicine)

Objective: We previously reported a mouse model with 18 weeks of N-Butyl-N-(4-hydroxybutyl) nitrosamine (BBN) administration of upper tract urothelial carcinoma (UTUC). We aimed to elucidate the timing of carcinogenesis by varying the duration of BBN exposure.Methods: Six-week-old female Balb/c mice were given 0.05% BBN for 4 or 8 weeks, followed by tap water until 18 weeks (n=16/group). To assess early carcinogenesis, other mice were sacrificed after BBN administration for 4 or 8 weeks (n=6/group). UTUC was evaluated by histopathological findings.Results: At 4 weeks of BBN administration, only dysplasia was observed. In contrast, 83% of mice developed UTUC after 8 weeks BBN administration. After switching to tap water, both 4 weeks and 8 weeks BBN administration groups showed a high incidence of UTUC by week 18 (93%, 100%). Immunostaining showed significantly more p53-positive cells in the 8 weeks BBN administration group than 4 weeks (0.4% vs 12%, p=0.02) and no difference of Ki-67 positive cells between both groups (11.9% vs 13%, p=0.80).Conclusion: While UTUC did not develop after 4 weeks of BBN administration, extended observation revealed a high incidence of UTUC by 18 weeks.

[Disclosure of COI by Responsible researcher] Lead presenter is the Responsible researcher.
[Lead presenter: Yuki Horibe] We have no financial relationships to disclose.

### [P-1045] I-P3-2
### Viral Oncology: Clinical & Applied

2025 / 9 / 25 (Thu) 16:45-17:30 Room P/(Motenashi Dome) Room P

---

### P-1045
### HPV vaccine effectiveness against cervical cancer and precancer in Japan: a test-negative case-control study

[Speaker]Mamiko Onuki (Showa Med. Univ., Dept. Obstet. Gynecol.) [Co-author]Iwao Kukimoto (Natl. Inst. Infect. Dis., Pathogen Genomics Ctr.)、Fumiaki Takahashi (Iwate Med. Univ., Div. Med. Engineering, Dept. Information Sci.,)、Takashi Iwata (Keio Univ., Dept. Obstet. Gynecol.)、Hideaki Yahata (Kyusyu Univ., Dept. Obstet. Gynecol.)、Kazuya Ariyoshi (Kyushu Cancer Ctr., Gynecol. Service)、Kazuhiro Takehara (Shikoku Cancer Ctr., Dept. Gynecol. Oncol.)、Koji Horie (Saitama Cancer Ctr., Dept. Gynecol.)、Haruya Saji (Kanagawa Cancer Ctr., Dept. Gynecol,)、Satoshi Yamaguchi (Hyogo Cancer Ctr., Dept. Gynecol. Oncol.,)、Hiroyuki Kanao (Cancer Inst. Hosp., Dept. Gynecol.)、Yuji Takei (Jichi Med. Univ., Dept. Obstet. Gynecol.)、Hiroyuki Yoshikawa (Tsukuba Univ., Dept. Obstet. Gynecol.)、Nobuo Yaegashi (Tohoku Univ., Dept. Obstet. Gynecol.)、Koji Matsumoto (Showa Med. Univ., Dept. Obstet. Gynecol.)

To evaluate the population-level effectiveness of human papillomavirus (HPV) vaccination, 8065 women (<40 years) newly diagnosed with invasive cervical cancer (ICC) or precancer (CIN2-3/AIS) in 24 hospitals throughout Japan were registered with HPV genotyping results and vaccination status between 2012 and 2024. Using a test-negative, case-control design, the vaccine effectiveness (VE) was calculated as the percentage reduction of HPV16/18 (vaccine types)-positive CIN2-3/AIS/ICC in the vaccinated group compared with the unvaccinated group. VE was 100% in women vaccinated before their first sexual intercourse. Accordingly, VE was strongly associated with age at the first vaccination ($P<0.0001$; 12-15 years, 96%; 16-18 years, 87%; 19-22 years, 38%; and >22 years, 18%). Additionally, the 2-valent vaccine had a higher VE than the 4-valent vaccine (70% vs. 30%, $P=0.001$), but VE was not correlated with vaccine doses ($P=0.59$). A VE ≧ 80% and a herd effect were observed in the 1994-1999 birth cohorts that were highly vaccinated at a younger age. These results indicate the real-world effectiveness of 2- or 4-valent HPV vaccines after the introduction of the National Immunization Program.

[Disclosure of COI by Responsible researcher] Lead presenter is not the Responsible researcher.
[Lead presenter: Mamiko Onuki] ■Research funds from company: 科研費基盤(C)
[Principal Researcher: Koji Matsumoto] ■Research funds from company: AMED　■Research expenses: カシオ計算機

▲TOP

### [P-1046] I-P3-2
### Viral Oncology: Clinical & Applied

2025 / 9 / 25 (Thu) 16:45-17:30 Room P/(Motenashi Dome) Room P

---

### P-1046
### Citrus limon (Linn) Burm. f. compounds as Potential HIV-1 Reverse Transcriptase Inhibitors

[Speaker]Shela Salsabila (Faculty of Pharmacy, Universitas Padjadjaran, Indonesia) [Co-author]Teuku A.S. Haq (Faculty of Pharmacy, Universitas Padjadjaran, Indonesia)、Btari K. Nyratri (Faculty of Pharmacy, Universitas Padjadjaran, Indonesia)、Ira Afridona (Faculty of Pharmacy, Universitas Padjadjaran, Indonesia)、Reneta A.M. Junus (Faculty of Pharmacy, Universitas Padjadjaran, Indonesia)、Bagus Adhinagoro (Faculty of Pharmacy, Universitas Padjadjaran, Indonesia)、Muchtaridi Muchtaridi (Faculty of Pharmacy, Universitas Padjadjaran, Indonesia／Research Collaboration Centre for Radiopharmaceuticals Theranostic, BRIN, Indonesia)

The complex relationship between Human Immunodeficiency Virus (HIV) infection and cancer development has emerged as a critical area of investigation. Studies have underscored the role of chronic inflammation and immune system suppression in the development of cancer in individuals infected with HIV, leading to the identification of novel therapeutic targets. This study investigates the potential anti-HIV properties of bioactive compounds isolated from Citrus limon (Linn) Burm. f. through comprehensive in silico approaches. ADMET predictions indicated favorable pharmacokinetic profiles. QSAR analysis identified key structural features responsible for anti-HIV activity. Molecular docking analyses revealed significant binding affinities between citrus compounds and reverse transcriptase HIV type 1 (HIV-1 RT) enzymes, with binding energies ranging from -5.50 to -8.84 kcal/mol. Molecular dynamics simulations over 50 ns demonstrated stable ligand-protein complexes. This n silico investigation provides compelling evidence for the potential development of Citrus limon compounds as novel anti-HIV therapeutic agents, warranting further in vitro and in vivo validation studies.

[Disclosure of COI by Responsible researcher] Lead presenter is the Responsible researcher.
[Lead presenter: Shela Salsabila] We have no financial relationships to disclose.

### [P-1047] I-P3-2
### Viral Oncology: Clinical & Applied

2025 / 9 / 25 (Thu) 16:45-17:30 Room P/(Motenashi Dome) Room P

---

### P-1047
### Persistence of serum antibody titers against HPV16/18/52/58 in vaccinated women in Japan

[Speaker]Iwao Kukimoto (Natl. Inst. Infect. Dis., Pathogen Genomics Ctr.) [Co-author]Seiichiro Mori (Natl. Inst. Infect. Dis., Pathogen Genomics Ctr.)、Yoshiyuki Ishii (Natl. Inst. Infect. Dis., Pathogen Genomics Ctr.)、Mamiko Onuki (Showa Med. Univ., Dept. Obstet. Gynecol.)、Hideaki Yahata (Kyusyu Univ., Dept. Obstet. Gynecol.)、Takashi Iwata (Keio Univ., Dept. Obstet. Gynecol.)、Kazuhiro Takehara (Shikoku Cancer Ctr., Dept. Gynecol.)、Hidemichi Watari (Hokkaido Univ., Dept. Obstet. Gynecol.)、Wataru Kudaka (Ryukyu Univ., Dept. Obstet. Gynecol.)、Satoshi Yamaguchi (Hyogo Cancer Ctr., Dept. Gynecol.)、Noriomi Matsumura (Kindai Univ., Dept. Obstet. Gynecol.)、Takeshi Motohara (Kumamoto Univ., Dept. Obstet. Gynecol.)、Kazuya Ariyoshi (Kyushu Cancer Ctr., Gynecol. Service)、Muneaki Shimada (Tohoku Univ., Dept. Obstet. Gynecol.)、Naotake Tsuda (Kurume Univ., Dept. Obstet. Gynecol.)、Koji Matsumoto (Showa Med. Univ., Dept. Obstet. Gynecol.)

[Objective] We aimed to evaluate serum antibody levels against HPV16/18/52/58 among Japanese women who received HPV vaccination to examine the durability of vaccine effectiveness.[Methods] Serum specimens were collected from HPV vaccinees diagnosed with cervical intraepithelial neoplasia (CIN) and vaccinated healthy controls. HPV16/18/52/58 IgG antibody titers were measured by ELISA using corresponding virus-like particles and international standard sera.[Results] The mean age at vaccination was 16.6 years, and the mean interval between vaccination and blood collection was 10.1 years. Much higher levels of HPV16/18 antibody titers than observed in natural infection were detected in the sera from the vaccinees, indicating persistence of HPV16/18 antibody titers. The bivalent vaccine induced higher antibody titers than the 4/9-valent vaccines. There was no significant difference in HPV16/18 antibody titers between CIN and healthy controls. The 9-valent vaccine induced high levels of HPV52/58 antibody titers that were not seen with the bivalent or 4-valent vaccines.[Conclusion] Serum HPV16/18 antibodies persist at high levels for about 10 years after HPV vaccination.

[Disclosure of COI by Responsible researcher] Lead presenter is the Responsible researcher.
[Lead presenter: Iwao Kukimoto] We have no financial relationships to disclose.

### [P-1048] I-P3-2
### Viral Oncology: Clinical & Applied

2025 / 9 / 25 (Thu) 16:45-17:30 Room P/(Motenashi Dome) Room P

---

### P-1048
### Lipid Droplet Accumulation is associated with Altered Gene Expression and HCC-Related Mutations in HBV-Infected Livers

[Speaker]Kousho Wakae (Dept. Virology II, NIID, JIHS) [Co-author]Ryuta Ueda (Dept. Virology II, NIID, JIHS)、Haruyo Aoyagi (Dept. Virology II, NIID, JIHS)、Akihito Tsubota (Res. Ctr. for Med. Sci., Jikei Univ. Sch. of Med.)、Kazuyoshi Hosomichi (Tokyo Univ. of Pharm. and Life Sci.)、Kento Fukano (Dept. Virology II, NIID, JIHS／Ctr. for Clin. Sci. NCGM, JIHS)、Naganori Nao (Intl. Inst. for Zoonosis Control, Hokkaido Univ.／One Health Res. Ctr. Hokkaido Univ.)、Hiroko Ijima (Dep.of Gastroenterol., Hyogo Med. Univ.)、Masanori Isogawa (Dept. Virology II, NIID, JIHS)、Masamichi Muramatsu (Dept. of Infectious Disease Res., FBRI)、Hideki Aizaki (Dept. Virology II, NIID, JIHS)

Hepatitis B virus (HBV)-associated carcinogenesis involves multiple molecular events, including altered gene expression, viral integration into the host genome, and host gene mutations. While HBV gene expression reportedly alter host subcellular organelles in cellular and mice models, its impact on human livers remains incompletely understood. In this study, we utilized transmission electron microscopy to analyze 10 non-tumorous liver tissues adjacent to chronic hepatitis B-induced hepatocellular carcinoma (HBV-HCC), and 27 from chronic hepatitis B (CHB) without a history of HCC. The analysis revealed ultrastructural abnormalities in both HBV-HCC adjacent and CHB tissues, and RT-PCR revealed association of lipid droplet (LD) accumulation in tumor adjacent tissues and HBs/HBx mRNA expressions. Transcriptomic and whole exome sequencing analysis revealed association of LD with enhanced inflammatory gene expression and enriched HCC-associated host gene mutations, respectively. Overall, the results highlight the association of LD accumulation with HBs/HBx expression, inflammation, and HCC-associated host gene mutations.

[Disclosure of COI by Responsible researcher] Lead presenter is the Responsible researcher.
[Lead presenter: Kousho Wakae] We have no financial relationships to disclose.

### [P-1049] I-P3-2
### Viral Oncology: Clinical & Applied

2025 / 9 / 25 (Thu) 16:45-17:30 Room P/(Motenashi Dome) Room P

---

### P-1049
### Withdrawn

No Abstract

### [P-1050] I-P3-2
### Viral Oncology: Clinical & Applied

2025 / 9 / 25 (Thu) 16:45-17:30 Room P/(Motenashi Dome) Room P

---

### P-1050
### Disruption of HPV16 E6/E7 Genes Using All-in-One Adenovirus Vectors Expressing Eight Double-Nicking Guide RNAs

[Speaker]Tohru Kiyono (Sasaki Inst., Dep Cancer Cell Res.／Nat. Cancer Ctr, EPOC, Div. Collab. Res. and Dev.) [Co-author]Tomomi Nakahara (Nat. Cancer Ctr. Res. Inst.／Osaka Univ., CAMaD)、Satomi Aoyama (Nat. Cancer Ctr. Res. Inst.／Hokkaido Cancer Ctr., Div. Gynecol. Oncol)、Kiyoshi Yamaguchi (Univ Tokyo, Inst. Med. Sci., Div. Clin. Genome Res.)、Yoichi Furukawa (Univ Tokyo, Inst. Med. Sci., Div. Clin. Genome Res.)、Mariko Nakamura (Juntendo Univ., Ctr. Biomed. Res. Resources)、Megumi Yamaji (Juntendo Univ., Ctr. Biomed. Res. Resources)、Tadashi Okada (Okayama Gakuin Univ., Dep. Food Management)、Hirotaka Tabata (Toyama Prefectural Univ., Dep. Pharmaceutical Engineering)、Ryoko Fuse (Inst. Microbial Chemistry, Lab. Virol.)、Eigo Shimizu (Univ. Tokyo, Inst. Med. Sci., HGC, Div. Health Med. Intelligence)、Rika Kasajima (Univ. Tokyo, Inst. Med. Sci., HGC, Div. Health Med. Intelligence／Kanagawa Cancer Ctr. Res. Inst., Mol. Pathol. Genet. Div.)、Seiya Imoto (Univ. Tokyo, Inst. Med. Sci., HGC, Div. Health Med. Intelligence)、Iwao Kukimoto (Nat. Inst. Infectious Dis., Pathogen Genomics Ctr.)、Izumu Saito (Juntendo Univ. Grad. Sch. Med.)、Tomoko Nakanishi (Juntendo Univ., Ctr. Biomed. Res. Resources)

Human papillomavirus (HPV) is a prime target for genome-editing therapy as its E6 and E7 oncogenes are crucial for cancer development and maintenance. A key challenge in CRISPR/Cas9 therapy is off-target effects. This study utilized a double-nicking technique to introduce DNA breaks in the E6 and E7 regions of HPV16. From 146 gRNA candidates, 16 double-nicking pairs were selected. Multiple combinations of double-nicking (DN)-gRNA pairs were delivered to HPV16-positive cells via lentiviruses, followed by Cas9 nickase (Cas9n) expression. Combinations of 3-4 DN-gRNA pairs effectively killed HPV16-positive cells while sparing HPV-negative cells. Off-target effects were reduced by nearly three orders of magnitude. An "all-in-one" adenovirus (AdV) system expressing four gRNA pairs and Cas9n showed promise in inhibiting tumor growth in HPV16-positive cancer models, demonstrating its potential as a safe and effective treatment for HPV-induced tumors.

[Disclosure of COI by Responsible researcher] Lead presenter is the Responsible researcher.
[Lead presenter: Tohru Kiyono] We have no financial relationships to disclose.

[P-1058] I-P4-2
Oncogenes & tumor-suppressor genes (1)

2025 / 9 / 25 (Thu) 16:45-17:30 Room P/(Motenashi Dome) Room P

P-1058

### Myc upregulates Ggct, γ–glutamylcyclotransferase to promote development of p53–deficient osteosarcoma

[Speaker]Tomoya Ueno (Dept. of Mol. Tumor Biol., Nagasaki Univ.) [Co-author]Shohei Otani (Dept. of Mol. Tumor Biol., Nagasaki Univ.)、Hiromi Ii (Lab. Of Clin. Oncol., Kyoto Pharm. Univ.)、Susumu Kageyama (Dept. of Urol., Shiga Univ. of Med. Sci.)、Susumu Nakata (Lab. Of Clin. Oncol., Kyoto Pharm. Univ.)、Kosei Ito (Dept. of Mol. Tumor Biol., Nagasaki Univ.)

We have previously reported that dysregulation of ***c-Myc (Myc)***, which follows ***p53*** deficiency, is the core mechanism underlying the development of osteosarcoma (OS) in both humans and mice. However, little is known about which genes are targeted by Myc to promote osteosarcomagenesis.

In this study, we found that Myc directly upregulates Ggct in OS cells derived from a mouse model of human OS, ***Osx***-Cre; ***p53***$^{fl/fl}$ mice (***OS*** mice). Ggct is a component enzyme of the γ–glutamyl cycle essential for homeostasis of glutathione, which has antioxidant properties. In a ***Ggct***$^{-/-}$ ***OS*** mouse line we generated, lifespan was extended and OS incidence was reduced. Similarly, in human OS, high expression levels of ***MYC*** and ***GGCT*** are associated with poor prognosis. The identification of Ggct as a Myc target gene reveals a critical axis in OS development and highlights the potential target for therapeutic intervention.

[Disclosure of COI by Responsible researcher] Lead presenter is not the Responsible researcher.
[Lead presenter: Tomoya Ueno] We have no financial relationships to disclose.
[Principal Researcher: Kosei Ito] We have no financial relationships to disclose.

▲TOP

### [P-1059] I-P4-2
### Oncogenes & tumor-suppressor genes (1)

2025 / 9 / 25 (Thu) 16:45-17:30 Room P/(Motenashi Dome) Room P

---

### P-1059
### Involvement of the Wnt pathway-related factors downregulation in anticancer activity of GGCT depletion

[Speaker]Hiromi Ii (Laboratory of Clinical Oncology, Kyoto Pharmaceutical University) [Co-author]Kozue Nose (Laboratory of Clinical Oncology, Kyoto Pharmaceutical University)、Masaya Mori (Laboratory of Clinical Oncology, Kyoto Pharmaceutical University)、Yuhi Sone (Laboratory of Clinical Oncology, Kyoto Pharmaceutical University)、Ryota Ito (Laboratory of Clinical Oncology, Kyoto Pharmaceutical University)、Kosei Ito (Dept of Molecular Tumor Biology, Nagasaki University)、Susumu Nakata (Laboratory of Clinical Oncology, Kyoto Pharmaceutical University)

Aims: $\gamma$-Glutamylcyclotransferase (GGCT) is upregulated in various cancers, including colorectal cancer (CRC). In this study, we analyzed the antiproliferative activity of GGCT knockdown (KD) in human CRC cell lines and knockout in the $Apc^{Min/+}$ mice, and also investigated the involvement of downregulation of Wnt pathway-related factors to elucidate the underlying mechanism.
Results & Conclusion GGCT-KD inhibited proliferation and induced cell cycle arrest in the G0/G1 phase of multiple human CRC cell lines with p21 upregulation. The total expression levels and nuclear accumulation of $\beta$-catenin together with the downstream related factors including Survivin, cMyc, OCT4, and cyclin D1 were downregulated by GGCT-KD in the CRC cells. Among them, overexpression of Survivin restored the antiproliferative effect of GGCT-KD and reduced cell death. Furthermore, the simultaneous Ggct knockout prolonged overall survival of the $Apc^{Min/+}$ mice. These results suggest that GGCT depletion inhibits the growth of CRC cells, indicating that GGCT may be a novel target molecule for CRC treatment.

[Disclosure of COI by Responsible researcher] Lead presenter is not the Responsible researcher.
[Lead presenter: Hiromi Ii] We have no financial relationships to disclose.
[Principal Researcher: Susumu Nakata] We have no financial relationships to disclose.

### [P-1060] I-P4-2
### Oncogenes & tumor-suppressor genes (1)

2025 / 9 / 25 (Thu) 16:45-17:30 Room P/(Motenashi Dome) Room P

---

### P-1060
### Glutaredoxin 2 has an oncogenic role in colon cancer

[Speaker]Yuta Marunaka (Div. of Digestive Surg, Kyoto Pref. Univ. of Med.) [Co-author]Hiroki Shimizu (Div. of Digestive Surg, Kyoto Pref. Univ. of Med.)、Kento Kurashima (Div. of Digestive Surg, Kyoto Pref. Univ. of Med.)、Hiroyuki Inoue (Div. of Digestive Surg, Kyoto Pref. Univ. of Med.)、Takayuki Miyoshi (Div. of Digestive Surg, Kyoto Pref. Univ. of Med.)、Jun Kiuchi (Div. of Digestive Surg, Kyoto Pref. Univ. of Med.)、Taisuke Imamura (Div. of Digestive Surg, Kyoto Pref. Univ. of Med.)、Kenji Nanishi (Div. of Digestive Surg, Kyoto Pref. Univ. of Med.)、Tomohiro Arita (Div. of Digestive Surg, Kyoto Pref. Univ. of Med.)、Toshiyuki Kosuga (Div. of Digestive Surg, Kyoto Pref. Univ. of Med.)、Yusuke Yamamoto (Div. of Digestive Surg, Kyoto Pref. Univ. of Med.)、Hirotaka Konishi (Div. of Digestive Surg, Kyoto Pref. Univ. of Med.)、Ryo Morimura (Div. of Digestive Surg, Kyoto Pref. Univ. of Med.)、Hitoshi Fujiwara (Div. of Digestive Surg, Kyoto Pref. Univ. of Med.)、Atsushi Shiozaki (Div. of Digestive Surg, Kyoto Pref. Univ. of Med.)

Background: Glutaredoxin 2 (Grx2) is a member of the glutaredoxin family which maintains thiol homeostasis. We investigated the association between Grx2 expression and tumor progression in colon cancer.Methods: 1) Grx2-knockdown experiments were performed using two human colon cancer cell lines. 2) mRNA Microarray analysis was performed to assess changes in gene profiles. 3) Samples from 141 patients with colon cancer were subjected to immunohistochemistry for Grx2, and the Grx2 expression prognostic value was analyzed.Results: 1) Grx2-knockdown suppressed cell proliferation with G0/G1 arrest, induced apoptosis, and cell migration inhibition. 2) mRNA Microarray analysis revealed that Grx2 gene expression was related to the signaling pathway of G1/S cell cycle checkpoint regulation. 3) In survival analysis, high Grx2 expression was associated with poor prognosis, and multivariate analysis identified Grx2 expression as an independent prognostic factor with colon cancer.Conclusion: Grx2 plays an oncogenic role in colon cancer cells and also affects the prognosis of patients with colon cancer. Grx2 may be a novel prognostic biomarker and therapeutic target for colon cancer.

[Disclosure of COI by Responsible researcher] Lead presenter is not the Responsible researcher.
[Lead presenter: Yuta Marunaka] We have no financial relationships to disclose.
[Principal Researcher: Hiroki Shimizu] We have no financial relationships to disclose.

▲TOP

### [P-1061] I-P4-2
### Oncogenes & tumor-suppressor genes (1)

2025 / 9 / 25 (Thu) 16:45-17:30 Room P/(Motenashi Dome) Room P

---

P-1061

### Amino acid transporter ***SLC38A1*** is a tumour promoter and a potential therapeutic target for urothelial carcinoma

[Speaker]Nesrine Sassi (Dep. of Urology, UO) [Co-author]Atsunari Kawashima (Dep. of Urology, UO)、Kentaro Jingushi (Lab. of Mol Cel Phy, UO)、Hiroumi Hiritane (Dep. of Urology, UO)、Shunsuke Inoguchi (Dep. of Urology, UO)、Yuki Horibe (Dep. of Urology, UO)、Masaru Tani (Dep. of Urology, UO)、Yutong Liu (Dep. of Urology, UO)、Yoshiyuki Yamamoto (Dep. of Urology, UO)、Yu Ishizuya (Dep. of Urology, UO)、Takuji Hayashi (Dep. of Urology, UO)、Taigo Kato (Dep. of Urology, UO)、Koji Hatano (Dep. of Urology, UO)、Norio Nonomura (Dep. of Urology, UO)

Cancer cells require efficient amino acid acquisition to support their survival. ***SLC38A1***, an amino acid transporter proved to be involved in different types of cancer, remains poorly explored in urothelial carcinoma (UC). In house data (n=96 UC tissues, n=79 adjacent/normal tissues) and public data were analyzed by RNA sequencing to assess SLC38 family mRNA expression. To further explore the biological significance of ***SLC38A1***, loss of function studies using short interfering RNAs in TCCSUP and UMUC-3 cells were confirmed. Functional assays (proliferation, wound healing, invasion) and PI3K/AKT/mTOR pathway analysis were performed. ***SLC38A1*** was significantly upregulated in UC tissues (p = 0.0004, p = 0.00005) compared to normal in all cohorts. ***SLC38A1*** knockdown in UMUC-3 and TCCSUP cells reduced proliferation (p = 0.0001, p = 0.01), invasion (p = 0.01, p = 0.04), and migration (p = 0.01, p = 0.0009), respectively. Western blotting analysis revealed that phosphorylated forms of PI3K/AKT/mTOR were significantly reduced following ***SLC38A1*** knockdown and IGF-1 treatment. ***SLC38A1*** is highly expressed in UC tissues, modulates cell behavior, and may serve as a potential target in UC patients.

[Disclosure of COI by Responsible researcher] Lead presenter is not the Responsible researcher.
[Lead presenter: Nesrine Sassi] We have no financial relationships to disclose.
[Principal Researcher: Atsunari Kawashima] We have no financial relationships to disclose.

▲TOP

### [P-1062] I-P4-2
### Oncogenes & tumor-suppressor genes (1)

2025 / 9 / 25 (Thu) 16:45-17:30 Room P/(Motenashi Dome) Room P

---

### P-1062
### HIF1α Promotes Development of TFE3-Rearranged Renal Cell Carcinoma via a Metabolic Shift Toward Fatty Acid Synthesis

[Speaker]Reio Kudaka (Dep. of Urol. Grad. Sch. of Med. Sci. Kumamoto Univ.) [Co-author]Hidekazu Nishizawa (Dep. of Urol. Grad. Sch. of Med. Sci. Kumamoto Univ.)、Shintaro Funasaki (Div. of Mol. and Vascular Biol. IRDA, Kumamoto Univ.)、Wenjuan Ma (Cambridge Stem Cell Inst. University of Cambridge)、Shoichiro Kuroda (Dep. of Urol. Grad. Sch. of Med. Sci. Kumamoto Univ.)、Yoshiaki Kubota (Dep. Of Anat. Sch. of Med. Keio Univ.)、Yuichiro Arima (Developmental Cardiology, IRCMS, Kumamoto Univ.)、Mitsuko Furuya (Dep. of Surgi. Path. Hokkaido Univ. Hosp.)、Yorifumi Satou (Joint Res. Cent. for Human Retro. Infect. Kumamoto Univ.)、Hisashi Hasumi (Dep. of Urol. Yokohama City Univ. Grad. School of Med.)、Ryosuke Jikuya (Dep. of Urol. Yokohama City Univ. Grad. School of Med.)、Yuichi Oike (Mol. Genet. Grad. Sch. of Med. Sci. Kumamoto Univ.)、Yasuhito Tanaka (Gastroent. and Hepatol. Grad. Sch. of Med. Sci. Kumamoto Univ.)、Toshio Suda (State Key Lab. of Hematol. and Health)、Masaya Baba (Dep. of Urol. Grad. Sch. of Med. Sci. Kumamoto Univ.)、Tomomi Kamba (Dep. of Urol. Grad. Sch. of Med. Sci. Kumamoto Univ.)

Tumor development often requires adaptation to a high metabolic state; however, mechanisms driving such changes in TFE3-rearranged renal cell carcinoma (TFE3-RCC) are not fully understood. TFE3-RCC, a rare renal cancer subtype, is defined by chimeric proteins involving the transcription factor TFE3. In this study, using cell lines and genetically engineered mice, we showed that PRCC-TFE3 expression induced a hypoxia-related signature by transcriptionally upregulating HIF1α and HIF2α. HIF1α activation promoted glycolysis, boosting ATP production and providing substrates for the TCA cycle while supporting oxidative phosphorylation. Crossing TFE3-RCC model mice with Hif1α and/or Hif2α knockout mice revealed that HIF1α, but not HIF2α, is essential for tumor development. RNA-seq and metabolomic analyses of kidney tissues demonstrated an inverse correlation between ketone body production and tumorigenesis, while de novo lipid synthesis was increased through a HIF1α/SREBP1-dependent mechanism. These results suggest that PRCC-TFE3 enhances tumor development in TFE3-RCC by driving a metabolic shift via the HIF1α/SREBP1 axis.

[Disclosure of COI by Responsible researcher] Lead presenter is not the Responsible researcher.
[Lead presenter: Reio Kudaka] We have no financial relationships to disclose.
[Principal Researcher: Masaya Baba] We have no financial relationships to disclose.

### [P-1063] I-P4-2
### Oncogenes & tumor-suppressor genes (1)

2025 / 9 / 25 (Thu) 16:45-17:30 Room P/(Motenashi Dome) Room P

---

### P-1063
### Impact of BTB and CNC homology 1 (BACH1) on cellular dynamics in triple negative breast cancer (TNBC) cells

[Speaker]Jingyao Pang (Dept. of Biochem., Tohoku Univ. Grad. Sch. Med.) [Co-author]Hironari Nishizawa (Dept. of Biochem., Tohoku Univ. Grad. Sch. Med.)、Mitsuyo Matsumoto (Dept. of Biochem., Tohoku Univ. Grad. Sch. Med.／IMRAM Tohoku Univ.)、Akihiko Muto (Dept. of Biochem., Tohoku Univ. Grad. Sch. Med.)、Kazuki Nakamura (Dept. of Biochem., Tohoku Univ. Grad. Sch. Med.／Dept. of Otolaryngology-HNS, Tohoku Univ. Grad. Sch. Med.)、Takumi Akiyama (IMRAM Tohoku Univ.)、Takeshi Kurasawa (Dept. of Biochem., Tohoku Univ. Grad. Sch. Med.)、Kazuhiko Igarashi (Dept. of Biochem., Tohoku Univ. Grad. Sch. Med.／Ctr. for Reg. Epi. & Dis., Tohoku Univ. Grad. Sch. Med.)

BACH1 promotes metastasis of various cancers, including breast cancer, and enhances ferroptosis by repressing the expression of iron metabolism-related genes. However, the role of iron metabolism in TNBC remains unclear. Using the MDA-MB-231 cell line to investigate the effects of BACH1 overexpression on iron metabolism, ferroptosis, and EMT.Lentiviral vectors transduction was used to overexpress wild-type mouse BACH1 (OE-BACH1$^{wt}$) and mutant BACH1 (OE-BACH1$^{mt}$) whose multiple serine residues, targets of phosphorylation by TBK1, were substituted with alanine.Both OE-BACH1$^{wt}$ and OE-BACH1$^{mt}$ suppressed FTH1 transcription but increased FTH1 protein levels. IRP2 protein was also upregulated. NCOA4 and p62 proteins were reduced, suggesting a suppression of ferritinophagy. Total intracellular iron content was increased. OE-BACH1$^{wt}$ and OE-BACH1$^{mt}$ decreased E-cadherin, but only the former reduced TBK1 protein. Compared to OE-BACH1$^{wt}$, OE-BACH1$^{mt}$ resulted in stronger cell proliferation inhibition and heightened ferroptosis sensitivity.BACH1 may play a key role in regulating iron metabolism, ferroptosis and EMT in TNBC cells. Its phosphorylation may coordinate the balance of these outputs.

[Disclosure of COI by Responsible researcher] Lead presenter is not the Responsible researcher.
[Lead presenter: Jingyao Pang] We have no financial relationships to disclose.
[Principal Researcher: Kazuhiko Igrashi] We have no financial relationships to disclose.

### [P-1064] I-P4-2
### Oncogenes & tumor-suppressor genes (1)

2025 / 9 / 25 (Thu) 16:45-17:30 Room P/(Motenashi Dome) Room P

---

### P-1064
### Analysis of tumor progression signaling in TFE3-rearranged renal cell carcinoma induced by ARID2 deficiency

[Speaker]Shoichiro Kuroda (Dep. of Urol, Grad. Sch. of Med. Sci. Kumamoto Univ.) [Co-author]Jinglong Tang (Dep. of Urol, Grad. Sch. of Med. Sci. Kumamoto Univ.)、Hidekazu Nishizawa (Dep. of Urol, Grad. Sch. of Med. Sci. Kumamoto Univ.)、Shintaro Funasaki (Div. of Mol. and Vascular Biol. IRDA, Kumamoto Univ.)、Takanobu Motoshima (Dep. of Urol, Grad. Sch. of Med. Sci. Kumamoto Univ.)、Yuichiro Arima (Developmental Cardiology, IRCMS, Kumamoto Univ.)、Yorifumi Satou (Joint Res. Cent. for Human Retro. Infect. Kumamoto Univ.)、Hisashi Hasumi (Dep. of Urol. Yokohama City Univ. Grad. School of Med.)、Ryosuke Jikuya (Dep. of Urol. Yokohama City Univ. Grad. School of Med.)、Yuichi Oike (Mol. Genet. Grad. Sch. of Med. Sci. Kumamoto Univ.)、Yasuhito Tanaka (Gastroent. and Hepatol. Grad. Sch. of Med. Sci. Kumamoto Univ.)、Masaya Baba (Dep. of Urol, Grad. Sch. of Med. Sci. Kumamoto Univ.)、Tomomi Kamba (Dep. of Urol, Grad. Sch. of Med. Sci. Kumamoto Univ.)

TFE3-rearranged renal cell carcinoma (TFE3-RCC) is a subtype of RCC characterized by chromosomal rearrangements involving Xp11.2, producing TFE3 fusion proteins that function as aberrant transcription factors driving tumorigenesis. Recent studies suggest additional driver genes may contribute to TFE3-RCC. We focused on ARID2, a subunit of the SWI/SNF chromatin remodeling complex, known to be mutated in various cancers. In this study, we investigated the role of ARID2 deficiency in TFE3-RCC using CRISPR-Cas9-mediated knockout in the TFE3-RCC cell line UOK124. Functional assays including proliferation, wound healing, and colony formation were conducted, along with in vivo xenograft models. We also performed RNA sequencing and chromatin immunoprecipitation sequencing to examine how ARID2 loss alters TFE3-dependent transcriptional regulation. Both in vitro and in vivo data demonstrated that ARID2 loss enhances tumor growth and migration. Transcriptomic analysis identified candidate therapeutic targets activated by TFE3 fusions and further enhanced by ARID2 deficiency, highlighting ARID2´s role in restraining TFE3-driven tumor progression.

[Disclosure of COI by Responsible researcher] Lead presenter is not the Responsible researcher.
[Lead presenter: Shoichiro Kuroda] We have no financial relationships to disclose.
[Principal Researcher: Masaya Baba] We have no financial relationships to disclose.

▲TOP

### [P-1065] I-P4-2
### Oncogenes & tumor-suppressor genes (1)

2025 / 9 / 25 (Thu) 16:45-17:30 Room P/(Motenashi Dome) Room P

---

### P-1065
### Modeling tumor-suppressive roles of ARID1A in early pancreatic tumorigenesis

[Speaker]Kazuya Koyama (Div of Gastroenterology, Dept of Med, Asahikawa Med Univ.) [Co-author]Kenji Takahashi (Div of Gastroenterology, Dept of Med, Asahikawa Med Univ.／Natl Inst of Biomed Innovation, Health and Nutrition)、Nobue Tamamura (Div of Gastroenterology, Dept of Med, Asahikawa Med Univ.)、Yu Otaki (Div of Gastroenterology, Dept of Med, Asahikawa Med Univ.)、Haruna Kawajiri (Div of Gastroenterology, Dept of Med, Asahikawa Med Univ.)、Tetsuhiro Okada (Div of Gastroenterology, Dept of Med, Asahikawa Med Univ.)、Hidemasa Kawabata (Div of Gastroenterology, Dept of Med, Asahikawa Med Univ.)、Hidetaka Iwamoto (Div of Gastroenterology, Dept of Med, Asahikawa Med Univ.)、Yohei Kitano (Div of Gastroenterology, Dept of Med, Asahikawa Med Univ.)、Yanpeng Sun (Div of Gastroenterology, Dept of Med, Asahikawa Med Univ.)、Hiroki Tanaka (Div of Gastroenterology, Dept of Med, Asahikawa Med Univ.／Natl Inst of Biomed Innovation, Health and Nutrition)、Taito Itoh (Div of Gastroenterology, Dept of Med, Asahikawa Med Univ.／Natl Inst of Biomed Innovation, Health and Nutrition)、Yusuke Ono (Div of Gastroenterology, Dept of Med, Asahikawa Med Univ.／Inst of Biomed Res, Sapporo Higashi Tokushukai Hosp.／Natl Inst of Biomed Innovation, Health and Nutrition)、Mikihiro Fujiya (Div of Gastroenterology, Dept of Med, Asahikawa Med Univ.)、Yusuke Mizukami (Div of Gastroenterology, Dept of Med, Asahikawa Med Univ.／Inst of Biomed Res, Sapporo Higashi Tokushukai Hosp.／Natl Inst of Biomed Innovation, Health and Nutrition)

ARID1A, a key component of the SWI/SNF chromatin remodeling complex, is recurrently mutated in ~8% of pancreatic ductal adenocarcinoma (PDA). Although murine models suggested a cooperative role with KRAS mutations in early tumorigenesis, its precise function in human pancreatic precancerous lesions remains unclear. To investigate this, we established ARID1A-knockout HPNE cells harboring KRAS G12D (HKG12DAK) using the LentiCRISPR v2-Blast system. Compared to parental HKG12D, HKG12DAK cells showed reduced proliferation and migration in vitro. RNA-sequencing identified 1029 upregulated and 411 downregulated genes upon ARID1A loss. Gene set enrichment analysis revealed activation of TGF-$\beta$ signaling, E2F targets, and G2/M checkpoint pathways. Notably, genes such as MYC, AURKA, E2F2, SMAD6/7, and IGFBP3 were upregulated, while NRK, TRHDE, and CCDN2 were downregulated. Contrary to previous reports suggesting oncogenic synergy, our data imply a tumor-suppressive phenotype associated with ARID1A deficiency in this early-stage model. This system provides a foundation for dissecting molecular interaction, including with TP53 mutations, that drive diversity of PDA progression.

[Disclosure of COI by Responsible researcher] Lead presenter is not the Responsible researcher.
[Lead presenter: Koyama Kazuya] We have no financial relationships to disclose.
[Principal Researcher: Kenji Takahashi] ■Research expenses: 日立ハイテク社, 合同会社HUグループ中央研究所

[P-1066] I-P4-2
Oncogenes & tumor-suppressor genes (1)

2025 / 9 / 25 (Thu) 16:45-17:30 Room P/(Motenashi Dome) Room P

### P-1066
### Combination of a folate antimetabolite and a topoisomerase I inhibitor has synergy on *ARID1A* -mutated ovarian cancer.

[Speaker]Mai Koizumi (Dept. of Obstetrics／Gynecology, The Univ. of Osaka) [Co-author]Yasuto Kinose (Dept. of Obstetrics／Gynecology, The Univ. of Osaka)、Yan Wang (Dept. of Obstetrics／Gynecology, The Univ. of Osaka)、Kanako Kasuya (Dept. of Obstetrics／Gynecology, The Univ. of Osaka)、Yuki Takemoto (Dept. of Obstetrics／Gynecology, The Univ. of Osaka)、Han (Univ. Osaka) Li (Dept. of Obstetrics／Gynecology, The Univ. of Osaka)、Aasa Shimizu (Dept. of Obstetrics／Gynecology, The Univ. of Osaka)、Erika Nakatsuka (Dept. of Obstetrics／Gynecology, The Univ. of Osaka)、Mahiru Kawano (Dept. of Obstetrics／Gynecology, The Univ. of Osaka)、Kenjiro Sawada (Dept. of Obstetrics／Gynecology, The Univ. of Osaka)、Tadashi Kimura (Dept. of Obstetrics／Gynecology, The Univ. of Osaka)、Michiko Kodama (Dept. of Obstetrics／Gynecology, The Univ. of Osaka)

[Objective] ***ARID1A*** mutations, known as tumor-suppressor genes, are found in a variety of cancers and detected in about 50% of ovarian clear cell carcinoma (OCCC), for which few effective treatments are available, especially in recurrent settings. Here we aim to explore the vulnerability of ***ARID1A***-mutated OCCC and develop novel therapies for OCCC. [Methods] We screened FDA-approved drug libraries by cell viability assay using ARID1A loss OCCC OV207 and the ARID1A-induced OV207 cells. We validated the anti-tumor effect of candidates using MTS, apoptosis, colony formation assay, and three-dimensional culture of human OCCC cells. [Results] High-throughput drug screening among 1134 compounds revealed that a folate antimetabolite, pemetrexed, and a topoisomerase I inhibitor, topotecan, were effective as a single agent in ARID1A loss OV207. The two drugs combination showed significant anti-tumor synergy and increased apoptotic cells in ***ARID1A***-mutant cells compared with ***ARID1A*** wild-type cells. [Conclusion] Based on the large-scale drug screening, we discovered a combination of pemetrexed and topotecan as a potential treatment for ***ARID1A***-mutant cancer.

[Disclosure of COI by Responsible researcher] Lead presenter is not the Responsible researcher.
[Lead presenter: Mai Koizumi] We have no financial relationships to disclose.
[Principal Researcher: Yasuto Kinose] We have no financial relationships to disclose.

[P-1076] I-P5-1
Extracellular/Intercellular signaling factors

2025 / 9 / 25 (Thu) 16:45-17:30 Room P/(Motenashi Dome) Room P

P-1076
### CD44 and EGFR may play a significant role in the cross talk between oral cancer stem cells and tumor microenvironment

[Speaker]Masakatsu Fukuda (Div. Biochem, Meikai Univ. Sch. Dent.) [Co-author]Kenjiro Bandow (Div. Biochem, Meikai Univ. Sch. Dent.)

CD44, a cell-surface glycoprotein, involves in cell-cell interactions, cell migration, and adhesion. For this reason, it plays a major role in tumorigenesis, invasion and metastasis. CD44 has been further described as a cancer stem cell marker in human oral squamous cell carcinoma (HOSCC) and can re-establish the tumor heterogeneity. This study aimed to analyze the relevance of CD44 in the invasion and proliferation of HOSCC; through an immunohistochemical study including correlation with the expression of FAS, SREBP1, E-FABP, EGFR, STAT3, JAK1 and with conventional clinicopathological variables in 50 cases of HOSCC. The positive immunoreactivity for CD44 were observed on the membrane of tumor cells in HOSCC tissues. The low CD44 expression was found in 44 %, moderate in 22 % and extensive in 34 %. CD44 expression in HOSCCs was positively correlated with SREBP1, E-FABP, FAS, EGFR, STAT3 and JAK1. These findings suggest that CD44 plays a significant role in the invasion and proliferation, probably with the cooperation of lipid metabolism in oral cancer. CD44 expression may be an indicator of poor prognosis, furthermore CD44 may serve as a potential therapeutic target in HOSCC.

[Disclosure of COI by Responsible researcher] Lead presenter is the Responsible researcher.
[Lead presenter: Masakatsu Fukuda] We have no financial relationships to disclose.

▲TOP

### [P-1077] I-P5-1
### Extracellular/Intercellular signaling factors

2025 / 9 / 25 (Thu) 16:45-17:30 Room P/(Motenashi Dome) Room P

### P-1077
### The role of ER-localized enzymes in the pathway of cancer stem cell formation

[Speaker]Shiori Yanai (Fac. Pharm. Sci., Hokkaido Univ.) [Co-author]Yusuke Ohta (Fac. Pharm. Sci., Hokkaido Univ.)、Takao Nomura (Fac. Pharm. Sci., Hokkaido Univ.)、Katsumi Maenaka (Fac. Pharm. Sci., Hokkaido Univ.／Fac. Pharm. Sci., Kyushu Univ.)

Endoplasmic reticulum oxidoreductin-1 alpha (ERO1α) is highly expressed in tumors and cancer cell lines, especially in cancer stem cells (CSCs), which cause relapses due to their stemness. ERO1α promotes disulfide bond formation in target proteins by regulating protein disulfide isomerase (PDI). Some ERO1&alpha-PDI target proteins have been reported in cancer, but not in CSCs, and the role of ERO1α in stemness remains unclear. In this study, we investigated the contribution of ERO1α to stemness using cell lines with different levels of ERO1α expression. Aldehyde dehydrogenase (ALDH) activity, an important CSC marker, correlated with ERO1α expression. Considering the Wnt/β-catenin pathway upstream of ALDH, we observed that nuclear localization of β-catenin and luciferase activity in reporter cells treated with supernatants decreased in correlation with ERO1α decline. In summary, our findings suggest that ERO1α contributes to stemness via Wnt/β-catenin pathway and downstream ALDH activity, possibly through its impact on Wnt-related molecules in the supernatant. We will conduct further analysis to elucidate the molecular mechanisms of the Wnt/β-catenin pathway mediated with ERO1α.

[Disclosure of COI by Responsible researcher] Lead presenter is not the Responsible researcher.
[Lead presenter: Shiori Yanai] We have no financial relationships to disclose.
[Principal Researcher: Katsumi Maenaka] We have no financial relationships to disclose.

### [P-1078] I-P5-1
### Extracellular/Intercellular signaling factors

2025 / 9 / 25 (Thu) 16:45-17:30 Room P/(Motenashi Dome) Room P

---

### P-1078
### The role of VEGFA and VEGFC in canine hemangiosarcoma

[Speaker]Keika Kishimoto (C.patho VetMed Hokkaido Univ.) [Co-author]Keisuke Aoshima (C.patho VetMed Hokkaido Univ.)、Tamami Suzuki (C.patho VetMed Hokkaido Univ.)、Takashi Kimura (C.patho VetMed Hokkaido Univ.)

Hemangiosarcoma (HSA) is a malignant endothelial tumor that frequently develops in the canine spleen with unknown molecular pathogenesis. VEGFA, a key angiogenic factor, has been considered a therapeutic target for HSA. However, our anti-VEGFA treatment in canine HSA patient-derived xenograft (PDX) models showed no significant effect. Similarly, other studies targeting VEGF-VEGFR axis have failed to demonstrate therapeutic benefit. To identify growth factors in HSA, we conducted single-cell RNA sequencing on canine HSA PDX tumors and revealed that HSA cells widely expressed VEGFC at higher levels than VEGFA. VEGFC was expressed in proliferating cells, whereas VEGFA was associated with hypoxia-associated gene expressions. Polyclonal knockout of VEGFC increased tumor cell proliferation in vitro but suppressed tumor growth in vivo. We will share mRNA-seq data on VEGFC knockout cells and tumor tissues with data from VEGFA knockout experiments at the conference.

[Disclosure of COI by Responsible researcher] Lead presenter is not the Responsible researcher.
[Lead presenter: Keika Kishimoto] We have no financial relationships to disclose.
[Principal Researcher: Keisuke Aoshima] We have no financial relationships to disclose.

### [P-1079] I-P5-1
### Extracellular/Intercellular signaling factors

2025 / 9 / 25 (Thu) 16:45-17:30 Room P/(Motenashi Dome) Room P

---

### P-1079
### Analysis of Transcriptomic Changes in Extracellular Vesicles Under In Vitro Coculture Conditions Using an EV Tracer

[Speaker]Yutaka Naito (Dept. Mol. Prevention., Inst. Adv. Med. Sci., Nippon Med. Sch.) [Co-author]Keisuke Yoshida (Dept. Mol. Prevention., Inst. Adv. Med. Sci., Nippon Med. Sch.)、Nami Miura (Dept. Mol. Prevention., Inst. Adv. Med. Sci., Nippon Med. Sch.)、Kashiro Ayumi (Dept. Mol. Prevention., Inst. Adv. Med. Sci., Nippon Med. Sch.)、Chiaki Hori (Dept. Mol. Prevention., Inst. Adv. Med. Sci., Nippon Med. Sch.／Dep. Oral Pathobiological Sci. Surg., Tokyo Dental College)、Kazufumi Honda (Dept. Mol. Prevention., Inst. Adv. Med. Sci., Nippon Med. Sch.／Dept. Mol. Prevention., Grad. Sch. Med., Nippon Med. Sch.)

Intercellular communication plays a crucial role in cancer initiation and progression through direct physical contact and indirect interactions. Recent studies have revealed that extracellular vesicles (EVs) are key regulatory mediators in such communication. However, few experimental systems are capable of monitoring the spatiotemporal dynamics of EV secretion by each cellular component in the presence of diverse cell types. In this study, we established cancer cell lines expressing CD63-Achilles GFP (CD63achGFP) and CD63-mScarlet (CD63exScarl), in which achGFP or mScarlet was fused to the extracellular domain of CD63. EVs derived from cancer cells expressing CD63achGFP or from fibroblasts expressing CD63exScarl were detectable by immunoprecipitation and the ExoCounter system using anti-GFP or anti-RFP antibodies. Furthermore, we found that EVs secreted by fibroblasts directly co-cultured with cancer cells exhibited a distinct gene expression compared to those in an indirect co-culture setting. Our system would contribute to a better understanding of the mechanisms underlying cancer progression.

[Disclosure of COI by Responsible researcher] Lead presenter is the Responsible researcher.
[Lead presenter: Yutaka Naito] We have no financial relationships to disclose.

▲TOP

### [P-1080] I-P5-1
### Extracellular/Intercellular signaling factors

2025 / 9 / 25 (Thu) 16:45-17:30 Room P/(Motenashi Dome) Room P

---

### P-1080
### Oleic acid-induced metastasis of KRAS/p53-mutant CRC relies on extracellular vesicles containing ICAM-1 and fibronectin

[Speaker]CHIHJIE SHEN (Research Center of Neuroscience, TMU, Taipei, Taiwan) [Co-author]Wen-Chang Chang (Graduate Institute of Medical Science, TMU, Taipei, Taiwan)、Ben-Kuen Chen (Department of Pharmacology, College of Medicine, NCKU, Tainan, Taiwan)

Hyperlipidemia caused chronic inflammation and involved in colorectal cancer metastasis. Extracellular vesicles (EVs) derived from cancer cells can bolster their metastatic and drug resistance capacities. Our research uncovers that EVs obtained from KRAS/P53 mutant cancer cells treated with oleic acid or oxaliplatin facilitate metastasis via ICAM-1 and fibronectin. Additionally, the CD36 receptor, a mediator of lipid uptake, specifically governs the presence of ICAM-1 and fibronectin on EVs, thereby influencing EV formation and the metastatic potential of cancer cells. These EVs originating from cancer cells within specific microenvironments can also influence the survival of neighboring cancer cells. Understanding the mechanism triggering EV formation through CD36 is pivotal for devising therapeutic strategies for malignant colorectal cancer (CRC). Targeting regulatory proteins like CD36, which are involved in the formation and release of EVs, shows promise in improving therapy for KRAS/P53 mutant CRC in the future.

[Disclosure of COI by Responsible researcher] Lead presenter is the Responsible researcher.
[Lead presenter: CHIHJIE SHEN] We have no financial relationships to disclose.

### [P-1081] I-P5-1
### Extracellular/Intercellular signaling factors

2025 / 9 / 25 (Thu) 16:45-17:30 Room P/(Motenashi Dome) Room P

---

### P-1081
### Development of a biotin proximity labeling method for cell surface proteins.

[Speaker]Masaya Yamazaki (Div. Carcinogenesis, Cancer Inst, JFCR) [Co-author]Takatsugu Ishimoto (Div. Carcinogenesis, Cancer Inst, JFCR)

Cellular functions are controlled not by individual proteins but by complex protein-protein interaction networks. In cancer, the dynamics of membrane microdomains promote the formation of receptor complexes, leading to aberrant and efficient signaling. However, conventional methods for comprehensive protein-protein interaction mapping, such as yeast two-hybrid and co-immunoprecipitation assays, are unsuitable for membrane proteins because of their methodological limitations. Here, we developed a biotin proximity labeling approach targeting receptor tyrosine kinases (RTKs) using biotin-phenol and HRP-conjugated antibody against ROR1, the cell surface RTK, for flow cytometry. This method revealed that ROR1 is close to several membrane proteins, including EGFR and other RTKs. Proximity ligation assays also showed the physical closeness of these candidates. This new method is a powerful tool for identifying membrane protein-protein interactions and contributes to the discovery of therapeutic targets that inhibit specific cell membrane complexes.

[Disclosure of COI by Responsible researcher] Lead presenter is the Responsible researcher.
[Lead presenter: Masaya Yamazaki] ■Contributions: 公益財団法人　花王芸術・科学財団、公益財団法人　ロッテ財団、公益財団法人　武田科学振興財団、公益財団法人　日本対がん協会、公益財団法人　興和生命科学振興財団、公益財団法人　鈴木謙三記念医科学応用研究財団、公益財団法人　新日本先進医療研究財団

Poster Session

### [P-1082] I-P5-1
### Extracellular/Intercellular signaling factors

2025 / 9 / 25 (Thu) 16:45-17:30 Room P/(Motenashi Dome) Room P

---

### P-1082
### Elucidation of the cause of malignant of endometrial adenocarcinoma caused by decreased progesterone receptor expression

[Speaker]Natsumi Hamasaki (Saga Univ.) [Co-author]Eisaburo Sueoka (Saga Univ.)、Hiroshi Miyamoto (Saga Univ.)、Takahiro Yaguchi (International Univ. of Health & Welfare)

It is well known that progesterone receptor (PGR) expression disappears with the malignancy of endometrial adenocarcinomas. The present study aimed to investigate the mechanism of loss of PGR expression, as well as the expression and localization of PGR-A and PGR-B, which differ in function. We constructed several 5' deletion mutants of PGR promoter and analyzed their response to 17-β-estradiol ($E_2$). In Ishikawa 3-H-12 (grade I) cells, deletion of the -498 bp to -246 bp promoter region attenuated $E_2$-induced PGR promoter activity. In HEC1 (grade II) cells, no PGR promoter activity was observed; in HEC50B (grade III) cells, promoter activity was observed by deleting the 5' upstream of PGR promoter (-742 bp to -498 bp). We examined the expression and localization of PGR-A and PGR-B, which differ in function. In Ishikawa 3-H-12 cells, $E_2$ attenuated the expression of PGR-B in nuclear. In HEC1 and HEC50B cells, PGR-A and PGR-B expression were not observed.The present study clearly demonstrated that the loss of PGR promoter activity and the altered PGR localization induced by $E_2$ led to malignant transformation of endometrial adenocarcinoma.

[Disclosure of COI by Responsible researcher] Lead presenter is not the Responsible researcher.
[Lead presenter: Natsumi Hamasaki] We have no financial relationships to disclose.
[Principal Researcher: Takahiro Yaguchi] We have no financial relationships to disclose.

### [P-1083] I-P5-1
### Extracellular/Intercellular signaling factors

2025 / 9 / 25 (Thu) 16:45-17:30 Room P/(Motenashi Dome) Room P

---

### P-1083
### Effect of ***Clostridium butyricum*** TO-A Culture Supernatant and n-Butyric Acid on Gene Expression in HeLa S3 cells

[Speaker]Fumiaki Uchiumi (Faculty of Pharm. Sci., Tokyo Univ. Sci.)

In this study, the ***Clostridium butyricum*** TO-A culture supernatant (CBCS) or butyric acid was added to a culture medium of human cervical carcinoma HeLa S3 cells, and changes in DNA-repair-related gene promoter activities were investigated. The HeLa S3 cells were transfected with a luciferase (Luc) expression vector containing the 5´-upstream regions of human genes and cultured with a medium containing CBCS (10%) or butyric acid (2.5 mM). A Luciferase assay showed that human ***ATM, PARP1***, and ***RB1*** gene promoter activities significantly increased. The amounts of ATM, PARP1 and RB1 proteins were shown to be increased. In order to analyze transcripts, total RNAs were extracted from cells that were treated with CBCS (10%) and were subjected to RNAseq analysis. The results showed that the expression of several inflammation- and DNA-replication/repair-related genes decreased markedly after 8 h. In contrast, that of the histone genes increased after 24 h. Elucidation of the mechanisms by which the CBCS and butyrate affect gene expression may contribute to the development of drugs to effectively prevent carcinogenesis, the risk of which rises in accordance with aging.

[Disclosure of COI by Responsible researcher] Lead presenter is the Responsible researcher.
[Lead presenter: Fumiaki Uchiumi] We have no financial relationships to disclose.

### [P-1084] I-P5-1
### Extracellular/Intercellular signaling factors

2025 / 9 / 25 (Thu) 16:45-17:30 Room P/(Motenashi Dome) Room P

### P-1084
### Withdrawn

No Abstract

### [P-1085] I-P5-1
### Extracellular/Intercellular signaling factors

2025 / 9 / 25 (Thu) 16:45-17:30 Room P/(Motenashi Dome) Room P

---

### P-1085
### Exploring the role of mechanical forces in chromosomal instability during metastasis

[Speaker]Satoru Shinriki (Department of Molecular Laboratory Medicine, Kumamoto University, Kumamoto, Japan／Cancer Genome Center, Kumamoto University Hospital, Kumamoto, Japan) [Co-author]Shiyang Deng (Department of Molecular Laboratory Medicine, Kumamoto University, Kumamoto, Japan／Department of Oral & Maxillofacial Surgery, Kumamoto Univ., Kumamoto, Japan)、Hideki Nakayama (Department of Oral & Maxillofacial Surgery, Kumamoto Univ., Kumamoto, Japan)、Yasuhito Tanaka (Department of Gastroenterology & Hepatology, Kumamoto Univ., Kumamoto, Japan)

Chromosomal instability (CIN) is known to be associated with metastasis. Recently, we have reported that only a subset of tumor cells efficiently formed circulating tumor cell (CTC) clusters stable in blood circulation and therefore dominantly colonize the distant organs. Then, we have proposed a new clonal selection mechanism called "mechanoselection". However, involvement of mechanostress during metastasis in genomic evolution remains unclear. Our analyses showed that suspension culture with fluid shear stress (FSS) rapidly induced cGAS-positive nuclear envelope (NE) rupture, which was associated with an altered expression of IFN-related genes. Furthermore, 2D culture after even a short-period suspension culture with FSS resulted in the increased tumor cells with DNA damage and micronucleus. Interestingly, these series of alterations were significant in single cells but not in clustered cells. Furthermore, RNA-seq analyses showed that cell clusters stable even under FSS conditions highly expressed ER- and NE-related genes compared to single cells. Collectively, transient mechanical loading during metastatic process may induce CIN and IFN responses, depending on the state of CTCs.

[Disclosure of COI by Responsible researcher] Lead presenter is the Responsible researcher.
[Lead presenter: Satoru Shinriki] We have no financial relationships to disclose.

### [P-1095] I-P9-1
### Metastasis & EMT (1)

2025 / 9 / 25 (Thu) 16:45-17:30 Room P/(Motenashi Dome) Room P

---

### P-1095
### TGF-&BetaR2 signaling regulates metastatic spreading by promoting invasive plasticity in nasopharyngeal carcinoma cells

[Speaker]Yuping Liu (Dept. of Med. Oncology, Cancer Hosp. of Guangxi Med. Univ.) [Co-author]Qianqing Fan (Dept. of Med. Oncology, Cancer Hosp. of Guangxi Med. Univ.)、Binli Cai (Dept. of Med. Oncology, Cancer Hosp. of Guangxi Med. Univ.)、Lihong Huang (Dept. of Otolaryngology & Head and Neck, People's Hosp. of Guangxi)、Xue Liu (Dept. of Clin. Nutrition, Cancer Hosp. of Guangxi Med. Univ.)、Yayan Deng (Dept. of Lab., First Affiliated Hosp. of Guangxi Med. Univ.)、Yujuan Huang (Dept. of Sci. Res., People's Hosp. of Guangxi)、Jiaxiang Ye (Dept. of Med. Oncology, Cancer Hosp. of Guangxi Med. Univ.)、Jiazhang Wei (Dept. of Otolaryngology & Head and Neck, People's Hosp. of Guangxi)、Jinyan Zhang (Dept. of Med. Oncology, Cancer Hosp. of Guangxi Med. Univ.)

The capacity for bi-directional epithelial-mesenchymal transition is a key invasive plasticity for tumor metastasis. Our previous work has indicated that distribution of the epithelial/mesenchymal (E/M)-hybrid circulating tumor cells (CTCs) correlates with clinical prognosis in patients with advanced nasopharyngeal carcinoma (NPC). However, the underlying pathway that regulates the phenotypic transition of NPC cells remains elusive. Here, using single-cell RNA ***in situ*** hybridization-based phenotypic classification, we show that TGF-&BetaR2 expression level is positively associated with the proportion of E/M-hybrid CTCs in NPC. Blockage of TGF-&Beta signaling by silencing TGF-&BetaR2 attenuates the TGF-&Beta-stimulated EMT in NPC cells. In addition, knockdown of TGF-&BetaR2 impairs clonogenic ability by reducing laminin-induced MET. Taking together, TGF-&BetaR2 signaling regulates invasive plasticity by maintaining the E/M-hybrid phenotype of the disseminated NPC cells.

[Disclosure of COI by Responsible researcher] Lead presenter is not the Responsible researcher.
[Lead presenter: Yuping Liu] We have no financial relationships to disclose.
[Principal Researcher: Yuping Liu] We have no financial relationships to disclose.

▲TOP

### [P-1096] I-P9-1
### Metastasis & EMT (1)

2025 / 9 / 25 (Thu) 16:45-17:30 Room P/(Motenashi Dome) Room P

---

### P-1096
### Palmitic acid inhibits migration and invasion in human oral squamous cell carcinoma

[Speaker]Jaehan Lee (Dept. of Oral Path., Sch. of Dent., SNU) [Co-author]Sung-Dae Cho (Dept. of Oral Path., Sch. of Dent., SNU)、Seong-Doo Hong (Dept. of Oral Path., Sch. of Dent., SNU)

Palmitic acid (PA) is a saturated fatty acid that is known to exert anti-inflammatory, metabolic regulation, and anti-tumor effects. In recent years, PA has been confirmed to be effective in colorectal cancer, liver cancer, and breast cancer via apoptosis and cell cycle arrest. Meanwhile, its efficacy on oral squamous cell carcinoma (OSCC) is unclear. Therefore, this study investigates the anti-cancer effects of PA in OSCC. Unexpectedly, treatment with fatty acids did not affect cell viability in HSC4 and HN22 cell lines. However, wound healing and transwell/matrigel assays showed that PA inhibited migration/invasion. Morphological analysis revealed a shift from a spindle-shaped mesenchymal phenotype to a round, epithelial-like form. Western blot analysis and immunocytochemistry demonstrated that PA significantly affected EMT markers. PA also downregulated EMT-associated transcription factors and inhibited their nuclear transport. As a result, these findings suggest that PA inhibits migration and invasion of OSCC by regulating the EMT-TFs and its potential as an anti-metastasis agent in OSCC. NRF grants (RS-2025-00553553 and RS-2024-00461097).

[Disclosure of COI by Responsible researcher] Lead presenter is the Responsible researcher.
[Lead presenter: Jaehan Lee] We have no financial relationships to disclose.

▲TOP

### [P-1097] I-P9-1
### Metastasis & EMT (1)

2025 / 9 / 25 (Thu) 16:45-17:30 Room P/(Motenashi Dome) Room P

### P-1097
### HMGA1 is a poor prognostic marker and confers the aggressiveness of cholangiocarcinoma

[Speaker]Kanlayanee Sawanyawisuth (Dept. of Biochem.,Ctr. for Translational Med.,Faculty of Med.,Khon Kaen Univ.／Cholangiocarcinoma Res. Inst.) [Co-author]Ratthaphong Phumphu (Dept. of Biochem.,Ctr. for Translational Med.,Faculty of Med.,Khon Kaen Univ.／Cholangiocarcinoma Res. Inst.)、Saowaluk Saisomboon (Dept. of Biochem.,Ctr. for Translational Med.,Faculty of Med.,Khon Kaen Univ.／Cholangiocarcinoma Res. Inst.)、Kulthida Vaeteewoottacharn (Dept. of Biochem.,Ctr. for Translational Med.,Faculty of Med.,Khon Kaen Univ.)、Sopit Wongkham (Dept. of Biochem.,Ctr. for Translational Med.,Faculty of Med.,Khon Kaen Univ.)、Seiji Okada (Div.of Hematopoiesis, Joint Res. Ctr.for Human Retrovirus Infection, Kumamoto Univ.)

High mobility group A1 (HMGA1) is a non-histone chromosomal protein that regulates the gene transcription. This study investigated the clinical impact and oncogenic roles of HMGA1 in cholangiocarcinoma (CCA) progression. Immunohistochemistry results revealed that high expression of HMGA1 was correlated with metastasis and shorter survival of 81 human CCA patients. Suppression of HMGA1 using specific small interfering RNA (siRNA) remarkably retarded cell growth via apoptosis. Moreover, knockdown of HMGA1 abated migration and invasion abilities via alteration of epithelial mesenchymal transition (EMT) in CCA cells. Recent study implied that HMGA1 was a poor prognostic marker and it promoted the aggressiveness of CCA. Inhibiting HMGA1 functions could control the progression and metastasis of CCA.

[Disclosure of COI by Responsible researcher] Lead presenter is the Responsible researcher.
[Lead presenter: Kanlayanee Sawanyawisuth] We have no financial relationships to disclose.

▲TOP

### [P-1098] I-P9-1
### Metastasis & EMT (1)

2025 / 9 / 25 (Thu) 16:45-17:30 Room P/(Motenashi Dome) Room P

### P-1098
### The organogermanium compound THGP suppresses norepinephrine-induced EMT in the gastric cancer cell line HGC-27 cells

[Speaker]Junya Azumi (Research Division, Asai Germanium Research Inst. Co.,Ltd.) [Co-author]Tomoya Takeda (Research Division, Asai Germanium Research Inst. Co.,Ltd.)、Yasuhiro Shimada (Research Division, Asai Germanium Research Inst. Co.,Ltd.)、Hisashi Aso (Research Division, Asai Germanium Research Inst. Co.,Ltd.／Animal Health Science Lab., Agricultural Science Grad., Tohoku Univ.)、Takashi Nakamura (Research Division, Asai Germanium Research Inst. Co.,Ltd.)

Background:3-(Trihydroxygermyl)propanoic acid (THGP) forms complexes with vicinal-diol moiety in the catechol, including norepinephrine (NE). NE, a stress hormone, induces epithelial-mesenchymal transition (EMT) in cancer cells. In this study, the effects of THGP on NE-induced EMT in human gastric cancer HGC-27 cells were evaluated.Methods:HGC-27 cells were treated with NE (30 μM) and THGP (500 μM). EMT markers, drug resistance, motility and tumorigenesis were analyzed by immunofluorescence, western blot, RT-PCR, cell viability, cell adhesion, migration, colony formation and spheroid assays.Results:THGP significantly reduced N-cadherin expression on NE-induced EMT by complexed with NE. NE increased resistance to 5-FU, cisplatin, and doxorubicin, but THGP restored drug sensitivity. Cell adhesion and migration assays showed NE promoted motility, while THGP suppressed these effects. Additionally, colony and spheroid assays demonstrated NE enhanced tumorigenic potential, whereas THGP reduced colony and spheroid sizes.Conclusion:THGP suppresses NE-induced EMT, potentially enhancing chemotherapy efficacy and preventing metastasis in cancer patients under stress.

[Disclosure of COI by Responsible researcher] Lead presenter is the Responsible researcher.
[Lead presenter: Junya Azumi] ■ employment by a company related to the presentation: 株式会社浅井ゲルマニウム研究所

▲TOP

### [P-1099] I-P9-1
### Metastasis & EMT (1)

2025 / 9 / 25 (Thu) 16:45-17:30 Room P/(Motenashi Dome) Room P

---

### P-1099
### EMT-promoted cell migration depends on the non-canonical activation of EphA2 via FGFR1

[Speaker]Yue Zhou (Dept. Cancer Cell Biol., Univ. Toyama) [Co-author]Leixin Song (Dept. Cancer Cell Biol., Univ. Toyama)、Tomohiro Yamamura (Dept. Cancer Cell Biol., Univ. Toyama)、Shoichiro Tange (Div. Med. Genome Sci., Sapporo Med. Univ.)、Satoru Yokoyama (Dept. Cancer Cell Biol., Univ. Toyama)、Takashi Tokino (Sapporo Univ. Health Sci.)、Hiroaki Sakurai (Dept. Cancer Cell Biol., Univ. Toyama)

EphA2 is a member of the receptor tyrosine kinase family that is overexpressed in malignant tumors. We previously reported that p90 ribosomal S6 kinase (RSK) phosphorylates EphA2 at Ser-897 (pS-EphA2) and induces cancer cell migration. The RSK-EphA2 signaling does not depend on its tyrosine kinase activity and has different functions from tyrosine kinase-dependent canonical activation; therefore, further study of non-canonically activated pS-EphA2 is essential for understanding the functions of EphA2 in cancer progression. Epithelial-to-mesenchymal transition (EMT) is also fundamental in cancer cell migration. The relationship between pS-EphA2 and EMT is unclear; therefore, we attempted to elucidate changes in the non-canonical activation of EphA2 during the process of EMT. We found that pS-EphA2 was induced during EMT in A549 lung cancer cells stimulated by TGF-$\beta$. This signaling was regulated by EMT-promoted activation of FGFR1. Interestingly, the FGFR1-ERK-RSK-EphA2 pathway promotes cell migration by forming the cell migration edge independent of EMT induction. These results revealed an important crosstalk between FGFR1 and pS-EphA2 in cancer cell migration.

[Disclosure of COI by Responsible researcher] Lead presenter is not the Responsible researcher.
[Lead presenter: Yue Zhou] We have no financial relationships to disclose.
[Principal Researcher: Hiroaki Sakurai] We have no financial relationships to disclose.

### [P-1100] I-P9-1
### Metastasis & EMT (1)

2025 / 9 / 25 (Thu) 16:45-17:30 Room P/(Motenashi Dome) Room P

---

### P-1100
### Efficacy and mechanism of action of Bergamottin for HB-EGF-induced EMT in esophageal Cancer

[Speaker]Moe Inoue (Grad. health & sports Sci. Toyo Univ.) [Co-author]Takumu Yamada (Grad. of Sci. and Eng. Dept. of BME Toyo Univ.)、Takumi Iwasawa (Inst. of Life Innovation Studies. Toyo Univ.)、Kazunori Kato (Grad. health & sports Sci. Toyo Univ.)

Esophageal squamous cell carcinoma (ESCC) is a highly aggressive malignancy with a strong tendency to metastasize and the lowest five-year survival rate among gastrointestinal cancers. Epithelial–mesenchymal transition (EMT) plays a key role in metastasis, and its inhibition is gaining attention as a therapeutic approach. EMT is regulated by various cytokines, including heparin–binding EGF–like growth factor (HB–EGF), which induces EMT in smooth muscle and epithelial cells. Bergamottin, a phytochemical found in bergamot orange peel, has been reported to reduce inflammation and inhibit cytochrome P450 3A4 (CYP3A4). In this study, we examined the effect of bergamottin on HB–EGF-induced EMT in ESCC cells. Results shown that bergamottin reduced the expression of EMT related transcription factors Snail1 and Snail2, and suppressed phosphorylation of STAT3. These findings suggest that bergamottin modulates EMT in ESCC cells, potentially through downregulation of the STAT3 pathway. This highlights the potential of phytochemicals as novel agents for targeting EMT in cancer treatment and prevention.

[Disclosure of COI by Responsible researcher] Lead presenter is not the Responsible researcher.
[Lead presenter: Moe Inoue] We have no financial relationships to disclose.
[Principal Researcher: Kazunori Kato] We have no financial relationships to disclose.

▲TOP

### [P-1101] I-P9-1
### Metastasis & EMT (1)

2025 / 9 / 25 (Thu) 16:45-17:30 Room P/(Motenashi Dome) Room P

### P-1101
### Oxaliplatin induces EMT via activation of KRAS/ERK pathway in KRAS-mutated colorectal cancer cells

[Speaker]Masanobu Tsubaki (Lab. Pharmacotherapy, Kagawa School of Pharmaceutical Sciences, Tokushima Bunri University) [Co-author]Shozo Nishida (Kindai University Faculty of Pharmacy)

<Objective> Oxaliplatin (L-OHP) is used for the treatment of colorectal cancer, but resistance is a factor contributing to poor prognosis. One of the factors contributing to this resistance is the induction of epithelial-mesenchymal transition (EMT) in tumor tissues after administration of anticancer drugs, which may be involved in drug resistance. In this study, we investigated the mechanism of EMT induction in colon cancer cells treated with L-OHP. <Methods> Cell viability was detected by trypan blue. The mRNA and protein activities were detected by real time PCR and western blotting. <Results> L-OHP at a clinically relevant steady-state concentration induced EMT in LoVo and DLD-1 cells (KRAS G13D mutant), but not in HT-29 and Caco-2 cells (wild-type KRAS). We also observed that L-OHP increased the expression of vimentin, N-cadherin, Twist, Slug, and Snail, and decreased the expression of E-cadherin. Furthermore, EMT cells showed decreased sensitivity to L-OHP. L-OHP also activated KRAS and ERK1/2 in KRAS-mutant colon cancer cells. <Discussion> These results suggest that activation of the KRAS/ERK pathway is important for EMT induction by L-OHP treatment.

[Disclosure of COI by Responsible researcher] Lead presenter is the Responsible researcher.
[Lead presenter: Masanobu Tsubaki] We have no financial relationships to disclose.

▲TOP

### [P-1109] I-P10-1
### Extracellular vesicles/Exosome (1)

2025 / 9 / 25 (Thu) 16:45-17:30 Room P/(Motenashi Dome) Room P

---

### P-1109
### Extracellular vesicles derived from mesenchymal stem cells inhibit tumor environment of triple negative breast cancer

[Speaker]Yunhsuan Chang (RIKEN IMS) [Co-author]Catkhanh Vuong (Inst. of Medicine, University of Tsukuba)、Nhathoang Ngo (Inst. of Medicine, University of Tsukuba)、Toshiharu Yamashita (Inst. of Medicine, University of Tsukuba)、Mizuho Fukushige (Inst. of Medicine, University of Tsukuba)、Xiucai Ye (Inst. of Systems and Information Engineering, University of Tsukuba)、Yasunori Futamura (Inst. of Systems and Information Engineering, University of Tsukuba)、Mana Obata (Inst. of Medicine, University of Tsukuba)、Hiromi Hamada (Inst. of Medicine, University of Tsukuba)、Motoo Osaka (Inst. of Medicine, University of Tsukuba)、Yuji Hiramatsu (Inst. of Medicine, University of Tsukuba)、Tetsuya Sakurai (Inst. of Systems and Information Engineering, University of Tsukuba)、Osamu Ohneda (Inst. of Medicine, University of Tsukuba)

Triple Negative Breast Cancer (TNBC) is the most malignant type of breast cancer with the high mortality. However, owing to the highly heterogeneous features, it requires much effort to develop the effective treatment for the TNBC patients. In the present study, we demonstrated an innovative strategy to remodeling the tumor microenvironment of TNBC by using mesenchymal stem cell-derived extracellular vesicles (EV). Our results suggest that the EV derived from Whartons Jelly MSC (WJ-EV) transformed the cellular behaviors of TNBC cells (BCC), attenuated the in vivo developmental and metastatic ability of BCC, and reduce the angiogenesis and the generation of cancer-associated fibroblasts. Besides, we identified the miRNA-125b, suggested as a key factor in WJ-EV, down-regulated the expression of HIF1$\alpha$, which is a crucial factor regulating various genes related to proliferation, epithelial-mesenchymal transition, and angiogenesis, in the BCC. In summary, our findings suggest that WJ-EV is a promising cell-free therapy that modifies cell behavior of cancer cells and remodels the tumor microenvironment, further attenuating tumor progression and metastasis in TNBC.

[Disclosure of COI by Responsible researcher] Lead presenter is the Responsible researcher.
[Lead presenter: Yunhsuan Chang] We have no financial relationships to disclose.

### [P-1110] I-P10-1
### Extracellular vesicles/Exosome (1)

2025 / 9 / 25 (Thu) 16:45-17:30 Room P/(Motenashi Dome) Room P

---

### P-1110
### Comparison of Urine exosome and Plasma exosome in colorectal cancer

[Speaker]Shintaro Kanaka (Department of Gastrointestinal and Hepato-Biliary-Pancreatic Surgery, Nippon Medical School) [Co-author]Takeshi Yamada (Department of Gastrointestinal and Hepato-Biliary-Pancreatic Surgery, Nippon Medical School)、Kay Uehara (Department of Gastrointestinal and Hepato-Biliary-Pancreatic Surgery, Nippon Medical School)、Seiichi Shinji (Department of Gastrointestinal and Hepato-Biliary-Pancreatic Surgery, Nippon Medical School)、Akihisa Matsuda (Department of Gastrointestinal and Hepato-Biliary-Pancreatic Surgery, Nippon Medical School)、Yasuyuki Yokoyama (Department of Gastrointestinal and Hepato-Biliary-Pancreatic Surgery, Nippon Medical School)、Goro Takahashi (Department of Gastrointestinal and Hepato-Biliary-Pancreatic Surgery, Nippon Medical School)、Takuma Iwai (Department of Gastrointestinal and Hepato-Biliary-Pancreatic Surgery, Nippon Medical School)、Toshimitsu Miyasaka (Department of Gastrointestinal and Hepato-Biliary-Pancreatic Surgery, Nippon Medical School)、Hiroshi(Nippon Med. Sch.) Yoshida (Department of Gastrointestinal and Hepato-Biliary-Pancreatic Surgery, Nippon Medical School)

Background: Regarding liquid biopsy, exosomes and CTCs are more stable than cell-free DNA or RNA in plasma, and they carry more information, enabling more complex analyses. This study aimed to compare exosomes isolated from urine and plasma in colorectal cancer patients and assess their utility for liquid biopsy. Methods: The study included colorectal cancer patients who underwent surgery between April 1, 2023, and December 31, 2024, with RAS/BRAF mutations identified in their tissue samples. Exosomes from urine (Ue) and plasma (Pe) were compared in terms of size, particle concentration, and protein content. Results: The study included 96 cases. The average particle size of Ue was significantly larger than that of Pe ( $p=0.04$), and Ue had a significantly lower particle concentration ($p=0.02$). DNA fragment length comparisons showed that Ue provided significantly longer DNA fragments ($p=0.01$). Conclusion: Urine exosomes included adequate concentration and length of DNA, and cancer-related proteins. Urine exosomes reflect the characteristics of colorectal cancer, suggesting that they can be effectively used for further analyses and as a promising non-invasive liquid biopsy.

[Disclosure of COI by Responsible researcher] Lead presenter is the Responsible researcher.
[Lead presenter: Shintaro Kanaka] We have no financial relationships to disclose.

### [P-1111] I-P10-1
### Extracellular vesicles/Exosome (1)

2025 / 9 / 25 (Thu) 16:45-17:30 Room P/(Motenashi Dome) Room P

---

### P-1111
### Development of breast cancer screening strategies by using extracellular vesicle in nipple discharge

[Speaker]Yuri Ozaki (Dept. of Breast & Endocrine Surg., Nagoya Univ.) [Co-author]Kosuke Yoshida (Dept. of Obstetrics & Gynecol., Nagoya Univ. Grad. Sch. of Med.)、Masami Kitagawa (Dept. of Obstetrics & Gynecol., Nagoya Univ. Grad. Sch. of Med.)、Masamichi Kato (KATO Surgery-Gynecology-Obstetrics-Breast Clinic)、Takao Yasui (Dept. of Life Sci. & Tech.,Inst. of Sci. Tokyo)、Hiroaki Kajiyama (Dept. of Obstetrics & Gynecol., Nagoya Univ. Grad. Sch. of Med.)、Norikazu Masuda (Dept. of Breast & Endocrine Surg., Nagoya Univ.／Dept. of Breast Surg., Kyoto Univ. Grad. Sch. of Med.)、Akira Yokoi (Dept. of Obstetrics & Gynecol., Nagoya Univ. Grad. Sch. of Med.)

Nipple discharge (ND) is one of major breast cancer symptoms, but screening methods are limited due to its difficulties to sample. We focused on extracellular vesicles (EVs) in ND to develop new biomarkers by using cellulose nanofiber (CNF) sheets which is the novel tool to capture EVs. Patients with ND or intracystic fluid (ICF) who visited two independent institutions were recruited, and EVs were captured by adsorbing ND or ICF into CNF sheets. miRNA profiles of ND-EVs, tissues and their surface EV were analyzed by small RNA sequencing. ND-EVs were collected from 23 samples (8 normal, 1 intraductal papilloma [IDP], 8 ductal carcinoma in situ [DCIS], 6 invasive ductal carcinoma), and we analyzed 23 ND-EV, 4 ICF-EV, and tissue/tissue surface EVs of 2 DCIS and 1 IDP. ND and ICF showed similar profiles, and DCIS-ND reflected tissue profile. Four miRNAs (miR-92b-3p, 375-3p, 182-5p, 96-5p) were elevated in DCIS tissue, tissue surface and ND. All 5 miRNAs were validated by qRT-PCR, and diagnostic performance of miRNA combination showed high performance (AUC : 0.902). In conclusion, ND-EV miRNAs using CNF sheet offer promising non-invasive biomarkers for early breast cancer screening.

[Disclosure of COI by Responsible researcher] Lead presenter is not the Responsible researcher.
[Lead presenter: Yuri Ozaki] We have no financial relationships to disclose.
[Principal Researcher: Akira Yokoi] We have no financial relationships to disclose.

▲TOP

### [P-1112] I-P10-1
### Extracellular vesicles/Exosome (1)

2025 / 9 / 25 (Thu) 16:45-17:30 Room P/(Motenashi Dome) Room P

---

### P-1112
### RNA-Seq of EVs-related genes analysis by t-SNE/PAM identifies molecular subtypes of bladder cancer

[Speaker]Eriko Nakano (Dept. Mol. Path., Tokyo Med. Univ.) [Co-author]Tomohiro Umezu (Dept. Mol. Path., Tokyo Med. Univ.)、Masahiko Kuroda (Dept. Mol. Path., Tokyo Med. Univ.)

Extracellular vesicles (EVs) mediate communication between cancer cells and the tumor microenvironment . We established molecular subtypes in Bladder Cancer based on EVs-related gene expression.Analyzing 412 TCGA data with 45 EVs-related genes from ExoBCD using t-SNE/PAM clustering, we identified two subtypes. Differential expression analysis identified 9 significant genes ($p<0.0001$). Multivariate Cox regression showed pathological staging as prognostic ($p=0.00014$), while subtypes alone were not. GSE31684 dataset validated these findings. Notably, subtype-staging interaction ($p=0.018$) enhanced prognostic prediction ($p=0.0019$). Early-stage patients showed survival differences between subtypes beyond about 1500 days, while advanced cases had similar outcomes.Heatmap analysis showed distinct biological processes, epithelial markers and growth/metabolic regulators in each subtype. This study provided novel insights into bladder cancer classification based on EVs-related genes. Future studies will integrate IMvigor trial data to analyze associations between our subtypes and immunotherapy response, aiming for more precise treatment selection.

[Disclosure of COI by Responsible researcher] Lead presenter is not the Responsible researcher.
[Lead presenter: Eriko Nakano] We have no financial relationships to disclose.
[Principal Researcher: Tomohiro Umezu, Masahiko Kuroda] We have no financial relationships to disclose.

### [P-1113] I-P10-1
### Extracellular vesicles/Exosome (1)

2025 / 9 / 25 (Thu) 16:45-17:30 Room P/(Motenashi Dome) Room P

### P-1113
### Cancer progression enhanced by sEVs released from gastric cancer cells during chemotherapy

[Speaker]Akane Sato (Waseda Univ. Dept. Biol.／BioPhenoMA Inc.) [Co-author]Kyo Okita (Waseda Univ. Dept. Biol.／BioPhenoMA Inc.)、Uno Oi (Waseda Univ. Dept. Biol.)、Etsuro Ito (Waseda Univ. Dept. Biol.／BioPhenoMA Inc.)

Although 5-FU and its prodrugs are commonly used as the first-line treatment for gastric cancer, drug tolerance hinders the treatment efficacy. GRP78 (glucose-regulated protein 78 kDa) expression is elevated in gastric cancer tumors and contributes to cancer progression via the release of sEVs. We hypothesized that GRP78 released through sEVs drives the malignancy of tumor cells by taking up sEVs to acquire stem cell-like properties, leading to 5-FU tolerance. First, using a thio-NAD cycling ELISA (TN-cyclon$^{TM}$), we detected GRP78 protein at a limit of 0.3 pg/mL. Our TN-cyclon$^{TM}$ showed that GRP78 levels in sEVs were approximately three times higher in 5-FU-treated cells compared to untreated cells. Moreover, tumor cells receiving sEVs acquired stem cell-like properties. Cell viability and wound healing assays revealed that tumor cells incorporating sEVs from 5-FU-treated cells exhibited enhanced migration and viability. The PI3K/Akt signaling pathway was implicated. These results suggest that 5-FU treatment promotes the release of GRP78-enriched sEVs, thereby promoting the development of cancer stem cell-like properties in recipient tumor cells.

[Disclosure of COI by Responsible researcher] Lead presenter is not the Responsible researcher.
[Lead presenter: Akane Sato] ■Research funds from company: 株式会社BioPhenoMA　■ employment by a company related to the presentation: 株式会社BioPhenoMA
[Principal Researcher: Etsuro Ito] ■Research funds from company: 株式会社BioPhenoMA　■employment by a company related to the presentation: 株式会社BioPhenoMA　■Profit of stock: 株式会社BioPhenoMA　■Patent fee: 株式会社BioPhenoMA

▲TOP

Poster Session

### [P-1120] I-P11-1
### Tumor antigens

2025 / 9 / 25 (Thu) 16:45-17:30 Room P/(Motenashi Dome) Room P

---

### P-1120
### Neoantigen identification with NESSIE for personalized cancer vaccine

[Speaker]Serina Tokita (Dept. Pathol., Sapporo Med. Univ.) [Co-author]Minami Fusagawa (Dept. Pathol., Sapporo Med. Univ.)、Toshihiko Torigoe (Dept. Pathol., Sapporo Med. Univ.)、Yoshihiko Hirohashi (Dept. Pathol., Sapporo Med. Univ.)、Takayuki Kanaseki (Dept. Pathol., Sapporo Med. Univ.)

Neoantigens arising from somatic mutations are tumor-specific and induce antitumor host T-cell responses. However, the frequency of immunogenic neoantigens that induce host responses is very low, and their sequences are individual-specific and therefore need to be identified for each patient for therapeutic applications. Here, we report a proteogenomic approach for neoantigen identification called Neoantigen Selection using a Surrogate Immunopeptidome (NESSIE) (Tokita S. et al. 2024 Science Advances). This approach uses an autologous wild-type immunopeptidome as a surrogate for the tumor immunopeptidome and allows HLA-agnostic identification of both HLA class I (HLA-I) and HLA class II (HLA-II) neoantigens. Fresh or frozen tumor samples are not required for analysis, making it applicable to many patients in clinical settings. We demonstrate efficient neoantigen identification in both clinical and preclinical samples. We show that neoantigen vaccination with NESSIE leads to tumor prevention in vivo in a preclinical mouse model. This approach may benefit personalized T cell-mediated immunotherapies.

[Disclosure of COI by Responsible researcher] Lead presenter is not the Responsible researcher.
[Lead presenter: Serina Tokita] ■Research funds from company: 日本学術振興会
[Principal Researcher: Takayuki Kanaseki] ■Research funds from company: 日本学術振興会、AMED

Poster Session

### [P-1121] I-P11-1
### Tumor antigens

2025 / 9 / 25 (Thu) 16:45-17:30 Room P/(Motenashi Dome) Room P

---

### P-1121
### Detection of neoepitopes from cancer cell lines using targeted mass spectrometry

[Speaker]Noriko Iwamoto (SHIMADZU Corporation) [Co-author]Eric Tran (Earle A. Chiles Research Inst. Providence Cancer Inst.)、Yoshinobu Koguchi (Pelotonia Inst. for Immuno-Oncology, Ohio State Univ.)、Takashi Shimada (SHIMADZU Corporation)

The immunopeptidome consists of all the peptides that are bound to major histocompatibility complexes (MHC) expressed at the surface of cells. Determining tumor-specific peptides such as mutated peptides (neoepitopes), that are expressed by cancer cells can facilitate the development of T-cell based immunotherapies to treat cancer. The peptide repertoire of the immunopeptidome has been studied using high-performance and high-resolution mass spectrometry (MS). However, it is often difficult to detect neoepitopes using non-targeted MS analysis due to the relatively low expression of neoepitopes compared to self-derived peptides.In this study, we developed a sensitive method to detect neoepitopes derived from hotspot mutations in proteins, such as KRAS, expressed in cancer cell lines using Evosep-multiple reaction monitoring MS analysis. The detection limit of KRAS-mutation derived synthetic peptides was 1 amol, which allows for the evaluation of these peptides in small sized clinical samples. Our technology could contribute to the establishment of a detection system for novel neoepitopes derived from oncogenic mutations and help prioritize the targets for immunotherapeutic.

[Disclosure of COI by Responsible researcher] Lead presenter is the Responsible researcher.
[Lead presenter: Noriko Iwamoto] We have no financial relationships to disclose.

[P-1122] I-P11-1
Tumor antigens

2025 / 9 / 25 (Thu) 16:45-17:30 Room P/(Motenashi Dome) Room P

### P-1122
### Bacterial infection induces transient melanoma dedifferentiation with attenuated antigenicity.

[Speaker]Sara Hatazawa (Dept. Microbiol., Fac. Med., Saitama Med. Univ.) [Co-author]Riko Kumatabara (Dept. Microbiol., Fac. Med., Saitama Med. Univ.)、Yukie Ando (Dept. Microbiol., Fac. Med., Saitama Med. Univ.)、Momo Mataki (Dept. Microbiol., Fac. Med., Saitama Med. Univ.)、Mio Nakajima (Dept. Microbiol., Fac. Med., Saitama Med. Univ.)、Rikuto Sone (Dept. Microbiol., Fac. Med., Saitama Med. Univ.)、Akihiro Nakamura (Dept. Microbiol., Fac. Med., Saitama Med. Univ.)、Mieko Tokano (Dept. Microbiol., Fac. Med., Saitama Med. Univ.)、Tomonaga Ichikawa (Dept. Microbiol., Fac. Med., Saitama Med. Univ.)、Takashi Murakami (Dept. Microbiol., Fac. Med., Saitama Med. Univ.)、Yutaka Horiuchi (Dept. Microbiol., Fac. Med., Saitama Med. Univ.)

Bacteria are attracting attention as living agents for the treatment of cancer. In particular, intracellular bacterial infection induces immunogenic cell death in tumor cells. In a previous study, we observed that most B16F10 melanoma cells infected with ***Salmonella typhimurium*** underwent cell death, but a few survived. Here, we investigated the phenotype and sensitivity to immunological elimination of B16F10 cells that survived intracellular infection with ***S. typhimurium*** (sisB16F10). sisB16F10 cells expressed higher levels of MHC class I and PD-L1 than uninfected control cells. We further investigated the immunological elimination of PD-L1 knockout sisB16F10 cells using melanocyte differentiation antigen (gp100)-specific cytotoxic T cells (MDAS-CTLs). The results showed that MDAS-CTLs failed to eliminate sisB16F10 cells. Protein expression analysis also revealed deletion of melanocyte differentiation antigens and high expression of neural crest cell (NCC) marker molecules. These results demonstrate that melanoma cells that escape infection-induced cell death can evade immunologic elimination by dedifferentiating into NCC-like phenotypes with their own attenuated antigenicity.

[Disclosure of COI by Responsible researcher] Lead presenter is not the Responsible researcher.
[Lead presenter: Sara Hatazawa] We have no financial relationships to disclose.
[Principal Researcher: Yutaka Horiuchi] ■Research funds from company: JSPS科研費 JP24K11482

▲TOP

### [P-1123] I-P11-1
### Tumor antigens

2025 / 9 / 25 (Thu) 16:45-17:30 Room P/(Motenashi Dome) Room P

### P-1123
### Frameshift Mutations as Sources of Neoantigens in Microsatellite-Stable Tumors

[Speaker]Peng Zhao (NIBN. Lab Immunogenomics CDIG／JFCR. Cancer Precision Med. Ctr.,／Science Tokyo. Grad.Sch.Med.Dent.Sci Genetics and Disease Diversity) [Co-author]Tosihiro Tanaka (Science Tokyo. Grad.Sch.Med.Dent.Sci Genetics and Disease Diversity)、Yusuke Nakamura (NIBN. Lab Immunogenomics CDIG)、Kazuma Kiyotani (NIBN. Lab Immunogenomics CDIG／JFCR. Cancer Precision Med. Ctr.,)

Frameshift mutations, caused by insertions or deletions disrupting the coding frame, are important sources of immunogenic neoantigens. Emerging evidence suggests that microsatellite-stable (MSS) cancers harbor lower-frequency frameshift events capable of generating immunogenic neoantigens. While immune checkpoint inhibitors show high efficacy in MSI-high cancers, but only limited benefit in unselected MSS tumors. Thus, identifying neoantigens in MSS tumors could offer new strategies to enhance immunotherapy. Using TCGA mutation data, we extracted frameshift insertions and deletions from MSS tumours and predicted their HLA class I neoantigens. Frameshift mutations frequently affected genes such as ***TP53*** and ***CDKN2A*** across pan-cancer samples, ***GATA3*** in BRCA, ***APC*** in COAD/READ, and ***VHL*** in KIRC. Certain neoantigen candidates were shared among different MSS patients, suggesting potential for broad application. These findings highlight frameshift mutations in MSS tumors as promising source of shared neoantigens, offering new opportunities for advancing immunotherapy.

[Disclosure of COI by Responsible researcher] Lead presenter is not the Responsible researcher.
[Lead presenter: Peng Zhao] We have no financial relationships to disclose.
[Principal Researcher: Kazuma Kiyotani] ■Advisor of the company and/or profit-making organization: Scientific advisor. Cancer Precision Medicine, Inc

### [P-1124] I-P11-1
### Tumor antigens

2025 / 9 / 25 (Thu) 16:45-17:30 Room P/(Motenashi Dome) Room P

---

### P-1124
### Withdrawn

No Abstract

### [P-1125] I-P11-1
### Tumor antigens

2025 / 9 / 25 (Thu) 16:45-17:30 Room P/(Motenashi Dome) Room P

---

### P-1125
### Neoantigen Prediction in Gliomas Enhanced by WHO CNS5 Reclassification and Multi-Omics Profiling

[Speaker]Anchih Wu (Lab. of Immunogenomics, CiDIG, NIBN, Ibaraki-shi, Osaka, Japan／International Master Program in Med. Neurosci., TMU, Taipei, Taiwan) [Co-author]Kazuma Kiyotani (Lab. of Immunogenomics, CiDIG, NIBN, Ibaraki-shi, Osaka, Japan)、Yusuke Nakamura (Lab. of Immunogenomics, CiDIG, NIBN, Ibaraki-shi, Osaka, Japan)

Glioblastoma (GBM), the most aggressive glioma, exhibits low tumor mutation burden (TMB) and an immunosuppressive microenvironment, limiting its response to immune checkpoint inhibitors. Identifying tumor-specific neoantigens - especially those shared across subtypes or from clonal driver mutations - holds promise for immunotherapy. However, datasets like TCGA were annotated using outdated histology-based criteria. With the 2021 WHO CNS5 classification incorporating molecular features, re-evaluation is needed. We reclassified TCGA-GBM and TCGA-LGG samples per CNS5 and integrated genomic (mutations, copy number changes) and epigenetic (promoter methylation) profiles. This allowed more accurate glioma subgrouping and revealed recurrent mutations, such as IDH1 R132H in IDH-mutant gliomas. While neoantigen prediction is ongoing, this reclassification provides a clearer basis for identifying immunologically relevant targets. We also analyzed TMB and immune cell composition across subtypes to assess the immunogenic landscape. These integrative efforts support neoantigen discovery aligned with CNS5-defined glioma subgroups.

[Disclosure of COI by Responsible researcher] Lead presenter is not the Responsible researcher.
[Lead presenter: Anchih Wu] We have no financial relationships to disclose.
[Principal Researcher: Yusuke Nakamura] We have no financial relationships to disclose.

### [P-1134] I-P11-3
### Antitumor immune response (2)

2025 / 9 / 25 (Thu) 16:45-17:30 Room P/(Motenashi Dome) Room P

---

### P-1134
### Identification of a novel immune-related factor associated with ICB resistance in EGFR-mutated lung adenocarcinoma

[Speaker]Wataru Nakajima (Dept. Mol. Oncol., Inst. Adv. Med. Sci., Nippon Med. Sch.) [Co-author]Kousuke Ishino (Dept. Path., Nippon Med. Sch)、Shingo Kitagawa (Dept. Pulmonol. & Oncol., Grad. Sch. Med., Nippon Med. Sch)、Ryuji Ohashi (Dept. Path., Nippon Med. Sch)、Hayashi Yamamoto (Dept. Mol. Oncol., Inst. Adv. Med. Sci., Nippon Med. Sch.)

Immune checkpoint blockade (ICB) therapy has shown clinical benefits in many cancers. However, its efficacy is limited in EGFR mutation-positive lung adenocarcinoma, and the mechanisms of resistance remain unclear. We previously identified a candidate gene X that may suppress antitumor immunity. In a 4T1 breast cancer mouse model, X knockdown suppressed tumor growth in immunocompetent mice, suggesting that X contributes to an immunosuppressive tumor microenvironment. In this study, we analyzed human EGFR-mutant lung adenocarcinoma samples to investigate the relevance of X in human disease. Using immunohistochemistry (IHC), we evaluated X expression in tumor tissues from EGFR mutation-positive patients. Preliminary results show variable expression of X among patients, suggesting a potential association with immune evasion. Further studies will examine correlations between X expression, immune cell infiltration, and clinical outcomes. These findings may clarify the immune landscape of EGFR-mutant lung cancer and highlight gene X as a novel therapeutic target to improve ICB response.

[Disclosure of COI by Responsible researcher] Lead presenter is the Responsible researcher.
[Lead presenter: Wataru Nakajima] ■Research funds from company: 科研費

### [P-1135] I-P11-3
### Antitumor immune response (2)

2025 / 9 / 25 (Thu) 16:45-17:30 Room P/(Motenashi Dome) Room P

### P-1135
### Effects of Anamorelin on Tumor Growth and Immune Modulation in Lung Cancer Cells

[Speaker]Kyohei Yano (Clinical Oncopharmacology, Faculty of Pham., Meijo Univ.) [Co-author]Yuya Ogimoto (Molecular & Cancer Meidicine, Faculty of Pham., Meijo Univ.)、Ayaka Mori (Dept. of Pham., Aichi Med. Univ. Hosp.)、Ryoko Kato (Dept. of Pham., Aichi Med. Univ. Hosp.)、Hideaki Ito (Dept. of Path., Aichi Med. Univ.)、Takashi Iwata (Oncology Ctr., Aichi Med. Univ. Hosp.)、Naoharu Mori (Dept. of Palliative & Supportive Med., Aichi Med. Univ.／Palliative Care Ctr., Aichi Med. Univ. Hosp.)、Hidesuke Yamamoto (Clinical Pharmacotherapeutics II, Faculty of Pham., Meijo Univ.)、Kiyoshi Yanagisawa (Molecular & Cancer Meidicine, Faculty of Pham., Meijo Univ.)、Ikuto Tsukiyama (Clinical Oncopharmacology, Faculty of Pham., Meijo Univ.／Dept. of Pham., Aichi Med. Univ. Hosp.)

**Introduction** Cancer cachexia has been reported to attenuate the efficacy of immune checkpoint inhibitors (ICIs). Anamorelin is a therapeutic agent for cancer cachexia, however its effects on hallmarks of tumor and the efficacy of ICIs remain unclear. In this study, we investigate the effects of anamorelin on tumor cell viability and the expression of immune checkpoint molecules.

**Methods** We employed MTT assay and western blotting for the analyses of tumor cell viability and expression levels of immune checkpoint molecules in A549 treated with anamorelin. Further, expressions of immune checkpoint molecules were examined in human lung, colon, pancreas and breast adenocarcinoma cell lines.

**Results** Anamorelin treatment at 1 μg/mL inhibited viability of A549, and 10 μg/mL of anamorelin induced cell death.In addition, anamorelin treatment increased the expression of LAG-3, while the expression levels of PD-L1 and VISTA remained unchanged.

**Discussion** The results shown here suggest that anamorelin treatment contributes the inhibition of A549 cell proliferation. Moreover, anamorelin treatment increased LAG-3 expression, which may influence the efficacy of related immunotherapies.

[Disclosure of COI by Responsible researcher] Lead presenter is not the Responsible researcher.
[Lead presenter: Kyohei Yano] We have no financial relationships to disclose.
[Principal Researcher: Ikuto Tsukiyama] We have no financial relationships to disclose.

### [P-1136] I-P11-3
### Antitumor immune response (2)

2025 / 9 / 25 (Thu) 16:45-17:30 Room P/(Motenashi Dome) Room P

### P-1136
### Research for immune response pathway related molecules following cancer radiation therapy

[Speaker]Junko Zenkoh (Grad. Sch. of Front. Sci., Univ. Tokyo) [Co-author]Yutaka Suzuki (Grad. Sch. of Front. Sci., Univ. Tokyo)、Ayako Suzuki (Grad. Sch. of Front. Sci., Univ. Tokyo)

To verify how local X-ray irradiation of tumor-bearing mice alters their systemic anti-tumor immune status, we performed single-cell transcriptome analysis of PBMCs from mice after irradiation. In mice with shrinking tumors, it was suggested that the systemic immune response was activated due to changes in the expression of genes associated with innate immune responses, heme metabolism, and oxygen uptake by erythrocytes. Among the genes with altered expression, we focused on Alas2, an enzyme that synthesizes 5-aminolevulinic acid (5-ALA). While Alas2 is known to be specifically expressed in erythrocytes, our analysis revealed that it was also expressed in a subset of other immune cells after tumor shrinkage. The up-regulation of Alas2 can activate the 5-ALA synthesis, which subsequently may lead to the synthesis of heme and the activation of cells. We examined the effect of 5-ALA-stimulated PBMCs on the proliferation of human lung cancer cells (A549). The results demonstrated that 5-ALA enhanced the anti-tumor activity of PBMCs. Furthermore, our findings suggested the presence of a sex-based difference in the effect of 5-ALA.

[Disclosure of COI by Responsible researcher] Lead presenter is the Responsible researcher.
[Lead presenter: Junko Zenkoh] We have no financial relationships to disclose.

### [P-1137] I-P11-3
### Antitumor immune response (2)

2025 / 9 / 25 (Thu) 16:45-17:30 Room P/(Motenashi Dome) Room P

### P-1137
### Targeting DHX9 as a therapeutic strategy in Human Gastric Cancer

[Speaker]You-zhen Wu (Dept. of Pharm., NTU) [Co-author]Bing-ze Yang (Dept. of Pharm., NTU)、Chingshyi P. Wu (Dept. of Pharm., NTU)

The RNA helicase DHX9 plays a critical role in RNA processing, RNA:DNA hybrid resolution, and genome stability. Recent studies show that DHX9 depletion induces replication stress, activates tumor-intrinsic immune responses, and reduces the viability of DHX9-dependent cancer cells, suggesting DHX9 as a potential therapeutic target. Gastric cancer (GC), a leading cause of cancer-related death worldwide, often resists treatment due to poor prognosis and molecular heterogeneity. Although chemotherapy, targeted therapy, and immunotherapy improve patient outcomes, their effectiveness remains limited. In this study, we analyze GC datasets from The Cancer Genome Atlas (TCGA) and find that DHX9 is significantly overexpressed across GC subtypes. Functional assays show that DHX9 loss in GC cells reduces viability, promotes apoptosis, and triggers interferon-stimulated gene expression, indicating a strong survival dependency on DHX9. These findings suggest that targeting DHX9 offers a promising strategy to selectively eliminate GC cells.

[Disclosure of COI by Responsible researcher] Lead presenter is the Responsible researcher.
[Lead presenter: You-zhen Wu] We have no financial relationships to disclose.

### [P-1138] I-P11-3
### Antitumor immune response (2)

2025 / 9 / 25 (Thu) 16:45-17:30 Room P/(Motenashi Dome) Room P

### P-1138
### CYLD regulates cGAS-STING pathway and metastasis in HNSCC

[Speaker]Shiyang Deng (Dept. of Mol. Lab. Med., Kumamoto Univ., Kumamoto, Japan) [Co-author]Satoru Shinriki (Dept. of Mol. Lab. Med., Kumamoto Univ., Kumamoto, Japan)、Yasuhito Tanaka (Dept. of Mol. Lab. Med., Kumamoto Univ., Kumamoto, Japan／Dept. of Gastroenterol. & Hepatol., Kumamoto Univ., Kumamoto, Japan)、Hideki Nakayama (Dept. of Mol. Lab. Med., Kumamoto Univ., Kumamoto, Japan／Dept. of Oral & Maxillofac. Surg., Kumamoto Univ., Kumamoto, Japan)

Metastasis is caused by disseminated tumor cells (DTCs) that initiate new tumors at distant organs. Mounting evidence suggests that after settling into distant organs, DTCs evade immune system and effectively remain dormant until as-yet-unknown trigger rouses them. We have previously reported that protein expression of Cylindromatosis (CYLD), a negative regulator of NF-kappaB pathway, was reduced in invasive HNSCC, which was significantly associated with poor prognosis. However, how CYLD expression is involved in metastatic process remains unknown. The aim of this study was to clarify the relationship between CYLD expression and the efficiency of distant metastasis. We found that suppression of CYLD expression by siRNA in HNSCC cell lines induced G0/G1 arrest. Accordingly, a human HNSCC cell line, SAS, with stable CYLD knockdown by shCYLD efficiently disseminated to distant organs in SCID mice while they remained dormant. Our preliminary studies showed that CYLD knockdown downregulated type-I IFN expression. These data suggest that downregulation of CYLD promotes tumor cell dissemination and persistence of the DTCs in HNSCC at least partly through the modulation of IFN responses.

[Disclosure of COI by Responsible researcher] Lead presenter is not the Responsible researcher.
[Lead presenter: Shiyang Deng] We have no financial relationships to disclose.
[Principal Researcher: Satoru Shinriki, Yasuhito Tanaka] We have no financial relationships to disclose.

### [P-1139] I-P11-3
### Antitumor immune response (2)

2025 / 9 / 25 (Thu) 16:45-17:30 Room P/(Motenashi Dome) Room P

---

### P-1139
### Cystatin A antitumor activity is mediated by Th1 lymphocytes and M1 macrophages in mouse models of pancreatic cancer

[Speaker]Shingo Inagaki (Graduate School of Advanced Preventive Medical Sciences, Kanazawa University) [Co-author]Alessandro Nasti (Information-Based Medicine Development, Graduate School of Medical Sciences, Kanazawa University)、Ho Yagi (Information-Based Medicine Development, Graduate School of Medical Sciences, Kanazawa University)、Akihiro Seki (Department of Gastroenterology, Kanazawa University Hospital)、Keiko Yoshida (Graduate School of Advanced Preventive Medical Sciences, Kanazawa University)、Kosuke Satomura (Graduate School of Advanced Preventive Medical Sciences, Kanazawa University)、Taro Yamashita (Graduate School of Advanced Preventive Medical Sciences, Kanazawa University／Department of Gastroenterology, Kanazawa University Hospital)、Yoshio Sakai (Graduate School of Advanced Preventive Medical Sciences, Kanazawa University)、Shuichi Kaneko (Graduate School of Advanced Preventive Medical Sciences, Kanazawa University／Information-Based Medicine Development, Graduate School of Medical Sciences, Kanazawa University／Department of Gastroenterology, Kanazawa University Hospital)

Pancreatic ductal adenocarcinoma (PDAC) is a disease with therapeutic limitations. Since cystatin A (CSTA) was identified as a PDAC biomarker, now we studied its antitumor properties. We established mouse intraperitoneal (IP) and subcutaneous (SC) PDAC models with PAN02 (NCI) tumor cell lines overexpressing CSTA. CSTA induced a longer survival in IP PDAC model ($P<0.001$), and its antitumor effect was mediated mainly by CD4+ cells ($P<0.0001$) and partly by CD8+ cells ($P<0.001$). We observed a decreased tumor growth in SC model treated with CSTA, coupled by an increased penetration of inflammatory CD4+ ($P<0.0001$) and CD8+ cells ($P<0.05$) in SC tumors. CSTA induced T helper type 1 (Th1) cell activity and frequency, as well as the activity of M1 macrophages, respectively, with higher Ifng and Tnf expression. DNA microarray analysis highlighted pathways related to Ifng induction and Th1 lymphocyte activation being induced by CSTA, as well as macrophages shift toward proinflammatory antitumor phenotypes. In conclusion, CSTA suppressed tumor growth; CSTA antitumor activity is mediated by Th1 lymphocytes and activated M1 macrophages. [Molecular Oncology, 2025. John Wiley & Sons Ltd]

[Disclosure of COI by Responsible researcher] Lead presenter is not the Responsible researcher.
[Lead presenter: Shingo Inagaki] We have no financial relationships to disclose.
[Principal Researcher: Alessandro Nasti] ■Research funds from company: 金沢大学大学院情報医学開発講座　■Endowed chair: 金沢大学大学院情報医学開発講座

[P-1140] I-P11-3
Antitumor immune response (2)

2025 / 9 / 25 (Thu) 16:45-17:30 Room P/(Motenashi Dome) Room P

### P-1140
### Comparing the efficacy of neoadjuvant immunotherapy for locally advanced gastric cancer

[Speaker]Yapeng Qi (Dept. of general surgery of Henan Cancer Hosp.) [Co-author]Chuang Shang (Dept. of general surgery of Henan Cancer Hosp.)、Zhimeng Li (Dept. of general surgery of Henan Cancer Hosp.)、Jian Yang (Dept. of general surgery of Henan Cancer Hosp.)、Meng Yu (Dept. of general surgery of Henan Cancer Hosp.)

This study aims to comparing the efficacy of neoadjuvant immunotherapy for locally advanced gastric cancer (LAGC) in the real-world.We conducted a retrospective analysis of clinical data from 323 patients diagnosed LAGC. Patients received either neoadjuvant chemotherapy or neoadjuvant immunotherapy followed by radical gastric surgery. We compared surgical outcomes and postoperative pathological responses between the two groups.Results: In the chemotherapy-only group, 24 patients achieved TRG0 (9.9%), 48 TRG1 (19.8%), 125 TRG2 (51.7%), and 45 TRG3 (18.6%). In contrast, the combined immunotherapy group had 21 patients with TRG0 (25.9%), 15 with TRG1 (18.5%), 36 with TRG2 (44.4%), and 9 with TRG3 (11.1%). The PCR rate was significantly higher in the immunotherapy group (25.9% vs. 9.9%, $p < 0.05$), as was the MPR rate (44.4% vs. 29.8%, $p < 0.05$).Stratified analysis revealed that neoadjuvant immunotherapy can NOT significantly improve the OS and RFS.This study provides that neoadjuvant immunotherapy significantly enhances the pathological response rate with LAGC,but can not to improve prognosis.

[Disclosure of COI by Responsible researcher] Lead presenter is the Responsible researcher.
[Lead presenter: Yapeng Qi] We have no financial relationships to disclose.

▲TOP

[P-1141] I-P11-3
Antitumor immune response (2)

2025 / 9 / 25 (Thu) 16:45-17:30 Room P/(Motenashi Dome) Room P

P-1141

### Combination therapy of cGAMP and agonistic anti-CD40 antibody suppresses tumor growth via activating CD8+ T-cells.

[Speaker]Nanami Ujiie (Dept. Pathol., Asahikawa Med. Univ.／Dept. Thoracic & Breast Surg., Asahikawa Med. Univ.) [Co-author]Takayuki Ohkuri (Dept. Pathol., Asahikawa Med. Univ.)、Ryusei Yoshino (Dept. Pathol., Asahikawa Med. Univ.／Dept. Thoracic & Breast Surg., Asahikawa Med. Univ.)、Takahiro Inoue (Dept. Pathol., Asahikawa Med. Univ.／Dept. Otolaryngology Head & Neck Surg., Asahikawa Med. Univ.)、Akemi Kosaka (Dept. Pathol., Asahikawa Med. Univ.)、Toshihiro Nagato (Dept. Pathol., Asahikawa Med. Univ.)、Masahiro Kitada (Dept. Thoracic & Breast Surg., Asahikawa Med. Univ.)、Hiroya Kobayashi (Dept. Pathol., Asahikawa Med. Univ.)

We previously demonstrated that combining an agonistic monoclonal antibody (mAb) against CD40 with the COX-2 inhibitor celecoxib, or the STING agonist cGAMP with an antagonistic mAb against CD47 effectively delayed tumor growth. In this study, we hypothesized that a combination with cGAMP and anti-CD40 mAb would show strong antitumor effects because we found that cGAMP-treated bone marrow-derived dendritic cells upregulated CD40 expression in a TNF$\alpha$-dependent manner in vitro. KM-plotter analysis revealed a positive correlation between high CD40 expression in breast cancer and patient prognosis, suggesting its potential as a hot tumor marker. In a mouse breast cancer model using E0771, the combination therapy significantly inhibited tumor growth in a STING or type I IFN-dependent manner. Moreover, the antitumor effect was canceled by depletion of $CD8^{+}$T-cells. We inoculated E0771 into both sides of mammary fat pads of WT mice and treated only the left-side tumor. The combination therapy significantly inhibited tumor growth on both sides and prolonged survival. These findings suggest that the therapy systemically activates T-cells and suppresses micrometastasis in breast cancer.

[Disclosure of COI by Responsible researcher] Lead presenter is the Responsible researcher.
[Lead presenter: Nanami Ujiie] We have no financial relationships to disclose.

▲TOP

### [P-1142] I-P11-3
### Antitumor immune response (2)

2025 / 9 / 25 (Thu) 16:45-17:30 Room P/(Motenashi Dome) Room P

---

### P-1142
### Enhancement of anti-tumor immune responses by anti-CD155 antibody

[Speaker]Yikun Lin (Grad. Sch Heal. & Sports Sci., Toyo Univ.) [Co-author]Takumu Yamada (Grad. Sch Heal. Sci. Eng., Toyo Univ.)、Yui Shimizu (Grad. Sch Heal. Sci. Eng., Toyo Univ.)、Takumi Iwasawa (Inst. of Life Innova. Stu., Toyo Univ.／Shizuoka Med. Res. Center for Disast., Juntendo Univ.)、Kazunori Kato (Grad. Sch Heal. & Sports Sci., Toyo Univ.／Inst. of Life Innova. Stu., Toyo Univ.)

CD155 (Poliovirus Receptor) is highly expressed in various cancer cells, and binds to immune checkpoint molecules TIGIT and CD96 on activated natural killer (NK) cells and T lymphocytes to suppress immune responses, while also interacting with the activating receptor DNAM-1. This study aimed to evaluate the immune checkpoint inhibitory effects of anti-CD155 antibodies with distinct recognition epitopes and enhance the anti-tumor ability of NK cells. We confirmed that TIGIT-Fc, CD96-Fc, and DNAM-1-Fc bind to CD155-positive cancer cells, and all three anti-CD155 antibodies completely inhibited TIGIT-Fc and CD96-Fc binding, whereas one of the clone 2H7 showed partial effects on DNAM-1-Fc binding. In co-cultures with IL-2-activated NK cells or KHYG-1 cells, 2H7-treated cancer cells exhibited significantly increased sensitivity to NK-mediated killing and activation compared to control antibodies. The immune activation effects were observed with anti-CD155 antibody 2H7 that most potently inhibited ligand chimera binding, suggesting a correlation between epitope specificity on CD155 and the therapeutic potential of these antibodies as immune checkpoint inhibitors.

[Disclosure of COI by Responsible researcher] Lead presenter is not the Responsible researcher.
[Lead presenter: Yikun Lin] We have no financial relationships to disclose.
[Principal Researcher: Kazunori Kato] We have no financial relationships to disclose.

▲TOP

### [P-1151] I-P11-5
### Tumor immune microenvironment/Tumor immune escape (1)

2025 / 9 / 25 (Thu) 16:45-17:30 Room P/(Motenashi Dome) Room P

---

### P-1151
### Elucidating immune-excluded microenvironment formation via integrative spatial and single-cell transcriptomics

[Speaker]Tomohiro Iwasawa (Keio Univ. Sch. of Med. Dept. of Urology ／Natl. Cancer Ctr. Div. of Cancer Immunol.) [Co-author]Nobuyuki Tanaka (Keio Univ. Sch. of Med. Dept. of Urology)、Kota Itahashi (Natl. Cancer Ctr. Div. of Cancer Immunol.)、Hiroyoshi Nishikawa (Natl. Cancer Ctr. Div. of Cancer Immunol.)、Mototsugu Oya (Keio Univ. Sch. of Med. Dept. of Urology)

Immune checkpoint blockades (ICBs) are widely used for advanced urothelial carcinoma (UC), but their efficacy remains unsatisfactory. UC often forms an immune-excluded tumor microenvironment (TME) with poor immune cell infiltration into the tumor core, yet the underlying mechanisms are unclear. We performed spatial transcriptomics (ST) and single-cell RNA sequencing (SC) on surgical specimens from advanced UC patients, including ICB-resistant cases. To assess spatial cell dynamics, we developed a novel method, "proximity scoring", which captures continuous phenotypic changes at single-cell resolution based on distance to tumor cells. This revealed that cancer-associated fibroblasts (CAFs) and tumor-associated macrophages (TAMs) show characteristic spatial distributions depending on their distance from tumor cells. Cell-cell interaction analysis indicated that specific TAMs and CAFs near tumor cells interact to form a stiff extracellular matrix, potentially limiting immune cell infiltration. This study has revealed part of the mechanism by which UC forms an immune excluded-type TME, and has indicated the possibility of new treatment targets.

[Disclosure of COI by Responsible researcher] Lead presenter is not the Responsible researcher.
[Lead presenter: Tomohiro Iwasawa] We have no financial relationships to disclose.
[Principal Researcher: Kota Itahashi] We have no financial relationships to disclose.

### [P-1152] I-P11-5
### Tumor immune microenvironment/Tumor immune escape (1)

2025 / 9 / 25 (Thu) 16:45-17:30 Room P/(Motenashi Dome) Room P

---

### P-1152
### EML4-ALK-rearrangement constructs a distinctive myeloid-cell-dominant immunosuppressive microenvironment in lung cancer

[Speaker]Kosuke Arai (Department of Immune Medicine, National Cancer Center Research Institute) [Co-author]Kazunori Aoki (Department of Immune Medicine, National Cancer Center Research Institute)、Hideaki Mizuno (Kamakura Research Laboratories, Chugai Pharmaceutical Co., Ltd)、Yukiko Sonobe (Kamakura Research Laboratories, Chugai Pharmaceutical Co., Ltd)、Yukari Nisito (Kamakura Research Laboratories, Chugai Pharmaceutical Co., Ltd)

Despite the initial efficacy of tyrosine kinase inhibitors (TKIs) in ALK fusion-gene-positive lung adenocarcinoma (ALK+ LUAD), acquired resistance inevitably occurs. Patients with ALK+ LUAD exhibit little or no response to immune checkpoint inhibitors and the underlying resistance mechanisms remain unknown. Here, we explored the immunological status of the tumor microenvironment (TME) in ALK+ LUAD tissues. Tumor-infiltrating leukocyte analysis revealed reduced numbers of effector T cells and increased myeloid-derived suppressor cells (MDSCs) relative to ALK- LUAD cases, constructing a myeloid cell-dominant immune-suppressive TME in ALK+ LUAD. A subset of macrophages expressed lower T cell-attractant chemokines (CXCL9, CXCL10, and CXCL11), and the macrophages were inactivated in ALK+ LUAD. ALK+ LUAD expressed MDSC-attractant chemokines (CXCL1 and CXCL8) and immunosuppressive cytokines (IL-6, VEGF, and HIF1A) than ALK- LUAD. The cytokine inhibitor transformed the TME in a murine ALK+ LUAD model, shifting it from an immunosuppressive to a T cell-dominant status. These results should help to guide the understanding of the immune TME and the direction of future research in ALK+ LUAD.

[Disclosure of COI by Responsible researcher] Lead presenter is not the Responsible researcher.
[Lead presenter: Kosuke Arai] We have no financial relationships to disclose.
[Principal Researcher: Hideaki Mizuno] We have no financial relationships to disclose.

### [P-1153] I-P11-5
### Tumor immune microenvironment/Tumor immune escape (1)

2025 / 9 / 25 (Thu) 16:45-17:30 Room P/(Motenashi Dome) Room P

---

### P-1153
### CD63 Recruits Tumor-Suppressive Macrophages in Tumor Microenvironment and Suppresses Metastasis in Breast Cancer

[Speaker]Masayuki Komura (Nagoya City Univ. Grad. Sch. Med. Sci.) [Co-author]Hiroyuki Kato (Nagoya City Univ. Grad. Sch. Med. Sci.)、Aya Naiki (Nagoya City Univ. Grad. Sch. Med. Sci.)、Shingo Inaguma (Nagoya City Univ. Grad. Sch. Med. Sci.／Nagoya City Univ. East Med. Ctr.)、Satoru(Nagoya City Univ.) Takahashi (Nagoya City Univ. Grad. Sch. Med. Sci.)

CD63, a member of the tetraspanin family, is widely expressed on cellular membranes; however, its function in the tumor microenvironment (TME) remains largely unknown. This study investigated the role of CD63 within the TME, particularly its association with tumor-associated macrophages (TAMs), using tissue microarrays from 547 patients with invasive breast cancer. High CD63 expression in tumor cells was associated with favorable prognosis and reduced distant metastasis. Immunohistochemical analyses revealed that both M1-like (typically tumor-suppressive) and M2-like (typically tumor-promoting) TAMs were elevated in the TME of CD63-high tumors. Furthermore, CD63-positive macrophage-like cells were abundant in the CD63-high group and were associated with better clinical outcomes and decreased metastasis. These CD63-positive cells were CD68-positive and CD163-negative, indicating that they represent a subset of M1-like TAMs. These findings suggest that CD63 expression in tumor cells may promote the recruitment of CD63-positive TAMs into the TME, and that these TAMs may play a role in suppressing tumor metastasis in breast cancer.

[Disclosure of COI by Responsible researcher] Lead presenter is the Responsible researcher.
[Lead presenter: Masayuki Komura] ■Research funds from company: 科学研究費助成事業

▲TOP

### [P-1154] I-P11-5
### Tumor immune microenvironment/Tumor immune escape (1)

2025 / 9 / 25 (Thu) 16:45-17:30 Room P/(Motenashi Dome) Room P

### P-1154
### Comprehensive NY-ESO and Immune Profiling across 150 Soft Tissue Sarcoma Cases

[Speaker]Hiroya Kondo (Dpt. of Musculoskeletal Oncol., NCCH) [Co-author]Koichi Ogura (Dpt. of Musculoskeletal Oncol., NCCH)、Akihiko Yoshida (Dpt. of Diagnostic Pathology, NCCH)、Tadashi Kondo (Div. of Rare Cancer Research, NCCRI)、Shingo Noguchi (Translational Science Department I, Daiichi Sankyo Co.)、Akemi Kita (Translational Research Lab., Daiichi Sankyo Co.)、Tomoki Tamura (Translational Research Lab., Daiichi Sankyo Co.)、Ryuichi Nakamura (Translational Research Lab., Daiichi Sankyo Co.)、Maki Kobayashi (Translational Research Lab., Daiichi Sankyo Co.)、Akira Kawai (Dpt. of Musculoskeletal Oncol., NCCH)

NY-ESO is a cancer-testis antigen predominantly expressed in germ cells,with high expression reported in malignancies.This study aimed to analyze NY-ESO expression and tumor microenvironment(TME)characteristics in soft tissue sarcomas to explore potential therapeutic strategies.We examined 50cases each of synovial sarcoma(SS),myxoid/round cell liposarcoma(MRCLS),and myxofibrosarcoma (MFS).Single and multiplex immunohistochemistry (mIHC)(CD4,CD8,PD-1,PD-L1,FoxP3,Pan-CK)were performed,with quantitative analysis using digital imaging systems.NY-ESO positivity rates were 86% in SS,98% in MRCLS,and 38% in MFS.CD3-positive cell densities varied:SS 73cells/mm2,MRCLS 136cells/mm2,and MFS 1320cells/mm2,revealing distinct Tcell infiltration patterns among subtypes.For SS,5cases were selected from the pre-and post-treatment(preoperative chemotherapy and/or radiotherapy)groups based on CD3 Tcell count,and mIHC was performed.The proportion of CD8-positive Tcells in total Tcells shows higher trend in post-treatment group than that in pre-treatment group.Our findings reveal that the NY-ESO expression and TME varies depending on the subtypes and the pre-or post-treatment in soft tissue sarcomas.

[Disclosure of COI by Responsible researcher] Lead presenter is not the Responsible researcher.
[Lead presenter: Hiroya Kondo] We have no financial relationships to disclose.
[Principal Researcher: Akira Kawai] We have no financial relationships to disclose.

### [P-1155] I-P11-5
### Tumor immune microenvironment/Tumor immune escape (1)

2025 / 9 / 25 (Thu) 16:45-17:30 Room P/(Motenashi Dome) Room P

### P-1155
### Single-cell profiling of neoantigen-reactive CD8+ T cells in sarcoma

[Speaker]Yukari Kobayashi (Dept. of Immunology, KINDAI Univ.) [Co-author]Koji Nagaoka (Dept. of Immunology, KINDAI Univ.)、Sachiko Okamoto (Takara Bio Inc.)、Kazuhiro Kakimi (Dept. of Immunology, KINDAI Univ.)

With the exception of certain subtypes, immune-based therapies for sarcoma remain largely undeveloped, and the mechanisms underlying neoantigen-specific T cell responses in the tumor microenvironment are not fully understood. We performed scRNA/TCR-seq of CD8+ T cells from sarcoma tissues. Neoantigen-specific TCRs were identified using computational prediction and validated through TCR-transduced reporter assays. This enabled detailed characterization of their spatial distribution and transcriptional states. Transcriptomic profiling revealed that these T cells exhibited an activated phenotype with upregulation of proliferative and glycolytic pathways. Concurrently, they expressed immunoregulatory molecules such as ENTPD1 and ADORA2A, suggesting functional adenosine-mediated immunosuppression via the ENTPD1 ADORA2A signaling pathway. This co-expression pattern indicates that T cells are subjected to both activating and inhibitory signals, potentially limiting their effector function. These findings emphasize how important it is to adjust the tumor immune microenvironment when using immunotherapy approaches, such as TCR-engineered T cells and immune checkpoint inhibitors.

[Disclosure of COI by Responsible researcher] Lead presenter is not the Responsible researcher.
[Lead presenter: Yukari Kobayashi] ■Research funds from company: 科学研究費助成事業
[Principal Researcher: Kazuhiro Kakimi] ■Research funds from company: 科学研究費助成事業　■Contributions: タカラバイオ株式会社、日油株式会社

▲TOP

### [P-1156] I-P11-5
### Tumor immune microenvironment/Tumor immune escape (1)

2025 / 9 / 25 (Thu) 16:45-17:30 Room P/(Motenashi Dome) Room P

---

### P-1156
### Investigation of the indicator related to fibrotic tumor microenvironment in gastric cancer

[Speaker]Yilin(Kumamoto Univ.) Tong (Kumamoto University Department of Gastroenterological Surgery) [Co-author]Takashi Semba (Cancer Institute, Japanese Foundation for Cancer Research)、Takatsugu Ishimoto (Cancer Institute, Japanese Foundation for Cancer Research)

Background: Cancer-associated fibroblasts (CAFs) in tumor microenvironment (TME) play important role in gastric cancer (GC). However, the mechanism of how gastric tumors foster CAF-rich microenvironment is still unclear. In this study, we sought the tumor-intrinsic markers that correlate with fibrotic TME.Methods: We analyzed public GC patient RNA-seq datasets by microenvironment cell populations-counter (MCP-counter). Multiplexed immunohistochemistry (mIHC) was used on tumor microarray (TMA) to examine the correlation between candidate gene and fibroblast-rich TME.Results: We found Frizzled class receptor 1 (FZD1) was highly expressed in fibroblast-enriched tumors, and was related to worse prognosis. The Fzd1 overexpressed tumors were significantly larger than ctrl tumors. mIHC on TMA showed FZD1 expression in tumor cells was positively associated with the number of fibroblasts. Conclusion: The expression of FZD1 on tumor cells was correlated with fibroblast-enriched TME and poorer prognosis of GC patients.

[Disclosure of COI by Responsible researcher] Lead presenter is not the Responsible researcher.
[Lead presenter: Yilin Tong] We have no financial relationships to disclose.
[Principal Researcher: Takatsugu Ishimoto] We have no financial relationships to disclose.

### [P-1157] I-P11-5
### Tumor immune microenvironment/Tumor immune escape (1)

2025 / 9 / 25 (Thu) 16:45-17:30 Room P/(Motenashi Dome) Room P

---

### P-1157
### DMC inhibits iCAF-mediated migration of esophageal squamous cell carcinoma cells via suppressing inflammatory chemokines

[Speaker]Kazuma Ito (Dept. Pharmacol, Sch. Med, UOEH) [Co-author]Masaki Arioka (Dept. Pharmacol, Sch. Med, UOEH)、Fumi Takahashi (Dept. Pharmacol, Sch. Med, UOEH)

Cancer-associated fibroblasts (CAFs) within the tumor stroma contribute to cancer progression. Inflammatory CAFs (iCAFs), a subset of CAFs, are characterized by the secretion of inflammatory cytokines. We have previously reported the anti-tumor effects of 2,5-dimethylcelecoxib (DMC), a celecoxib derivative; however, its impact on CAFs remains unclear. This study aimed to investigate the effects of DMC on CAF differentiation and function in the context of esophageal squamous cell carcinoma (ESCC). To achieve this, a co-culture model was established using the normal human dermal fibroblast cell line NB1RGB and the ESCC cell line TE-11. Co-culture did not alter the expression of myofibroblastic CAFs markers, but significantly increased the expression of iCAFs markers in NB1RGB. Notably, DMC did not influence the differentiation of NB1RGB into CAFs, but suppressed the mRNA and protein expression of inflammatory chemokines (CXCLs). Furthermore, DMC significantly reduced TE-11 cell migration by suppressing CXCL expression under co-culture conditions. These results suggest that DMC may inhibit the metastatic potential of ESCC by suppressing inflammatory chemokine secretion from iCAFs.

[Disclosure of COI by Responsible researcher] Lead presenter is not the Responsible researcher.
[Lead presenter: Kazuma Ito] We have no financial relationships to disclose.
[Principal Researcher: Masaki Arioka] We have no financial relationships to disclose.

### [P-1158] I-P11-5
### Tumor immune microenvironment/Tumor immune escape (1)

2025 / 9 / 25 (Thu) 16:45-17:30 Room P/(Motenashi Dome) Room P

---

### P-1158
### The associations of nutritional indicators and immune cells in microenvironment in colorectal cancer patients

[Speaker]Natsuko Sakaida (Dept. of Cell Path. Grad. Sch. of Med. Sci.Kumamoto Univ. ／Faculty of Environmental & Symbiotic Sci. Pref. Univ. of Kumamoto) [Co-author]Rin Yamada (Dept. of Cell Path. Grad. Sch. of Med. Sci.Kumamoto Univ.)、Seiya Shimoda (Faculty of Environmental & Symbiotic Sci. Pref. Univ. of Kumamoto)、Yoshihiro Komohara (Dept. of Cell Path. Grad. Sch. of Med. Sci.Kumamoto Univ.)

**Objectives**: To investigate the association between the nutritional status of colorectal cancer patients and the tumor immune microenvironment (TME), and to identify optimal nutritional indicators for predicting the TME.**Methods**: Tumor tissue paraffin sections from 116 colorectal cancer patients who underwent surgery were used to analyze tumor-infiltrating lymphocytes (TILs) and macrophages. Nutritional indicators (GNRI, mGPS, CONUT, NLR, PNI) were assessed for these cases, and their correlations with lymphocyte and macrophage counts, as well as other clinicopathological factors, were analyzed.**Results**: Patients with low GNRI, high mGPS, and low PNI (indicating malnutrition) had fewer FOXP3-positive regulatory T cells. Patients with high NLR (indicating malnutrition) had fewer CD3-positive lymphocytes, CD8-positive lymphocytes, and Iba1-positive macrophages. Overall, malnutrition was more prevalent among elderly people, and tumor markers and stage tended to be high.**Conclusion**: A favorable nutritional status is associated with tumor immune activation, suggesting that preoperative nutritional improvement is important not only for prognosis but also for tumor immunity.

[Disclosure of COI by Responsible researcher] Lead presenter is not the Responsible researcher.
[Lead presenter: Natsuko Sakaida] We have no financial relationships to disclose.
[Principal Researcher: Yoshihiro Komohara] We have no financial relationships to disclose.

▲TOP

### [P-1159] I-P11-5
### Tumor immune microenvironment/Tumor immune escape (1)

2025 / 9 / 25 (Thu) 16:45-17:30 Room P/(Motenashi Dome) Room P

---

### P-1159
### TNFR2 deficiency in M-MDSCs impairs immunosuppressive function in tumors

[Speaker]Yuta Tsuji (Grad. Sch. Pharm. Sci., Kobe Gakuin Univ) [Co-author]Masaki Inoue (Fac. Pharm. Sci., Kobe Gakuin Univ)、Shinichi Tsunoda (Grad. Sch. Pharm. Sci., Kobe Gakuin Univ／Fac. Pharm. Sci., Kobe Gakuin Univ)

Myeloid-derived suppressor cells (MDSCs) and regulatory T cells in the immunosuppressive tumor microenvironment play critical roles in tumor progression and therapeutic resistance. Developing strategies to modulate these cells could significantly enhance the efficacy of cancer immunotherapy. We previously demonstrated that tumor necrosis factor receptor type 2 (TNFR2) is highly expressed on monocytic MDSCs (M-MDSCs), a subset of MDSCs reported to be more suppressive than polymorphonuclear MDSCs. In this study, we investigated the role of TNFR2 in regulating the immunosuppressive activity of M-MDSCs. We observed significantly reduced tumor growth and lower numbers of tumor-infiltrating M-MDSCs in TNFR2-knockout mice than in wild-type mice. Furthermore, to evaluate whether TNFR2 regulates the T cell suppressive function of M-MDSCs, we performed a co-culture assay using M-MDSCs and splenocytes. TNFR2-knockout M-MDSCs exhibited diminished ability to suppress T-cell proliferation. Our results suggest that TNFR2 contributes to the immunosuppressive function of M-MDSCs and could be a potential target in cancer immunotherapy.

[Disclosure of COI by Responsible researcher] Lead presenter is not the Responsible researcher.
[Lead presenter: Yuta Tsuji] We have no financial relationships to disclose.
[Principal Researcher: Shinichi Tsunoda] We have no financial relationships to disclose.

▲TOP

### [P-1168] I-P11-7
### Tumor immune microenvironment/Tumor immune escape (3)

2025 / 9 / 25 (Thu) 16:45-17:30 Room P/(Motenashi Dome) Room P

---

### P-1168
### Distribution analysis of cancer immune-related PUFAs using MSI in a mouse subcutaneous tumor model

[Speaker]Kaoru Nakagawa (SCOE Analytical & Measuring Instruments Div. Shimadzu Corp.) [Co-author]Akiko Kubo (Div. of Dermatology, Kobe Univ. Grad. of Med.)、Yuki Katoh (Div. of Anatomical Sci., Nihon Univ. Sch. of Med.)、Manami Kobayashi (SCOE Analytical & Measuring Instruments Div. Shimadzu Corp.)

Ferroptosis is a form of regulated cell death characterized by iron-dependent lipid peroxidation of polyunsaturated fatty acids (PUFAs). While arachidonic acid has been reported to promote ferroptosis, its distribution in tumor microenvironments remains unclear. In this study, we visualized the spatial distribution of free fatty acids, including PUFAs, in a CT26 colon tumor-bearing mouse model using an iMScope QT atmospheric MALDI equipped with a light microscope and LCMS-9050. Our analysis revealed distinct distribution patterns of different fatty acids within the tumor microenvironment. Notably, palmitoleic acid was localized around neovascular vessels, while stearic acid accumulated in areas of immune cell infiltration and cancer cell necrosis. Most significantly, we observed accumulation of PUFAs, particularly arachidonic acid and docosahexaenoic acid, in regions where cancer cells and immune cells coexisted and where cancer cells were undergoing necrosis. These findings suggest a potential role for PUFAs in immune-mediated cancer cell death, demonstrating the utility of MALDI-MS imaging (MSI) for visualizing PUFAs in tumor microenvironments.

[Disclosure of COI by Responsible researcher] Lead presenter is the Responsible researcher.
[Lead presenter: Kaoru Nakagawa] ■ employment by a company related to the presentation: 株式会社島津製作所

### [P-1169] I-P11-7
### Tumor immune microenvironment/Tumor immune escape (3)

2025 / 9 / 25 (Thu) 16:45-17:30 Room P/(Motenashi Dome) Room P

---

### P-1169
### Interaction of Notch1 mutation and PD-L1 modulates tumour-infiltrating CD8+ T cells in oral squamous cell carcinoma

[Speaker]Kazuhiro Ogi (Dept. of Oral Surgery, Sapporo Medical University School of Medicine) [Co-author]Takahiro Iwamoto (Dept. of Oral Surgery, Sapporo Medical University School of Medicine)、Takashi Sasaya (Dept. of Oral Surgery, Sapporo Medical University School of Medicine)、Sho Miyamoto (Dept. of Oral Surgery, Sapporo Medical University School of Medicine)、Koyo Nishiyama (Dept. of Oral Surgery, Sapporo Medical University School of Medicine)、Mai Sugihara (Dept. of Oral Surgery, Sapporo Medical University School of Medicine／Division of Oral Surgery, Hakodate Municipal Hospital)、Akihiro Miyazaki (Dept. of Oral Surgery, Sapporo Medical University School of Medicine)

Objectives: To elucidate the interaction process between biological regulation of PD-L1, Notch1 mutation and tumour-infiltrating CD8+ T cells in oral squamous cell carcinoma (OSCC). Material and Methods: Localisation of PD-L1 was analysed by western blot (WB) and flow cytometry, and expression of PD-L1 was analysed through WB. In 47 OSCC patients, the number of between tumour infiltration CD8+ T cells and PD-L1 expression were analysed by immunohistochemical staining. Furthermore, TCGA databases were input into multiple online bioinformatic tools. Results: In Notch1 knockdown, expression of PD-L1 on tumour cell surface increased and the expression of intrinsic PD-L1 in cytoplasm decreased in mutated Notch1 phenotype. Moreover, in 47 OSCC patients, 7/47 exhibited Notch1 mutation, and these patients with both high tumour infiltration CD8+ T cells and Notch1 mutation were 5/7. It became a positive significant correlation both differentiated T cell CD8+ central memory cells and effector memory cells but a negative correlation among CD8+ naive T cells for wild type Notch1. Conclusion: The interaction between Notch1 mutation and PD-L1 modulates tumour-infiltrating CD8+ T cells.

[Disclosure of COI by Responsible researcher] Lead presenter is the Responsible researcher.
[Lead presenter: Kazuhiro Ogi] We have no financial relationships to disclose.

### [P-1170] I-P11-7
### Tumor immune microenvironment/Tumor immune escape (3)

2025 / 9 / 25 (Thu) 16:45-17:30 Room P/(Motenashi Dome) Room P

---

### P-1170
### Potentiation of PD-1 Blockade Efficacy via T Cell Activation Induced by Inhibition of Amino Acid Transporter LAT1

[Speaker]Minhui Xu (Grad. Sch. of Med., The Univ. of Osaka) [Co-author]Xinyu Zhou (Grad. Sch. of Med., The Univ. of Osaka)、Chunhuan Jin (Grad. Sch. of Med., The Univ. of Osaka)、Hiroki Okanishi (Grad. Sch. of Med., The Univ. of Osaka)、Ryuichi Ohgaki (Grad. Sch. of Med., The Univ. of Osaka／OTRI, The Univ. of Osaka)、Yoshikatsu Kanai (Grad. Sch. of Med., The Univ. of Osaka／OTRI, The Univ. of Osaka／WPI-PRIMe, The Univ. of Osaka／Ctr. for Society-Academia Collaboration, Fujita Health Univ.)

Although immune checkpoint inhibitors targeting PD-1 have achieved clinical success in various malignancies, their therapeutic efficacy is often hampered by the immunosuppressive tumor microenvironment. L-type amino acid transporter 1 (LAT1/SLC7A5), a key player in essential amino acid uptake, is upregulated in numerous cancers and has been implicated in tumor progression and immune modulation. In this study, we evaluated the immunomodulatory effects of selective LAT1 inhibitor nanvuranlat in combination with anti-PD-1 antibody therapy. Using syngeneic mouse tumor models, we observed that dual treatment with nanvuranlat and anti-PD-1 resulted in significantly enhanced tumor growth inhibition compared to monotherapies. Mechanistically, LAT1 inhibition promoted the infiltration and activation of CD8+ cytotoxic T lymphocytes and concurrently reduced the expression of immunosuppressive cytokines within the tumor microenvironment. These findings indicate that targeting LAT1 can reprogram the tumor immune landscape and potentiate the efficacy of PD-1 blockade. Our study highlights LAT1 as a promising combinatorial target for improving immune checkpoint therapy in cancer treatment.

[Disclosure of COI by Responsible researcher] Lead presenter is not the Responsible researcher.
[Lead presenter: Minhui Xu] We have no financial relationships to disclose.
[Principal Researcher: Yoshikatsu Kanai] We have no financial relationships to disclose.

▲TOP

### [P-1171] I-P11-7
### Tumor immune microenvironment/Tumor immune escape (3)

2025 / 9 / 25 (Thu) 16:45-17:30 Room P/(Motenashi Dome) Room P

---

### P-1171
### Effect of cancer cell-derived IL-6 on antitumor immune responses

[Speaker]Akemi Kosaka (Dept. Pathol., Asahikawa Med. Univ.,) [Co-author]Takayuki Ohkuri (Dept. Pathol., Asahikawa Med. Univ.,)、Nanami Ujiie (Dept. Pathol., Asahikawa Med. Univ.,／Dept. Surg., Asahikawa Med. Univ.)、Takahiro Inoue (Dept. Pathol., Asahikawa Med. Univ.,／Dept. Otolaryngology, Head and Neck Surg., Asahikawa Med. Univ.)、Ryusei Yoshino (Dept. Pathol., Asahikawa Med. Univ.,／Dept. Surg., Asahikawa Med. Univ.)、Hiroyoshi Nozaki (Dept. Pathol., Asahikawa Med. Univ.,／Dept. Dermatology, Asahikawa Med. Univ.)、Toshihiro Nagato (Dept. Pathol., Asahikawa Med. Univ.,)、Hiroya Kobayashi (Dept. Pathol., Asahikawa Med. Univ.,)

We previously reported that intratumoral administration of the STING agonist cGAMP exerts antitumor effects and have developed more effective combination therapies using mouse models. In light of these studies, we next investigated a combination therapy using cGAMP and an IL-6 receptor inhibitor since tumors expressing high levels of IL-6 have been reported to be associated with malignancy and poor prognosis. In TNBC mouse models, a combination therapy was not as effective as expected. Therefore, we generated IL-6 knockout (KO) cell lines to investigate how cancer cell-derived IL-6 affects antitumor immune responses. Compared with control tumors, IL-6 KO tumors showed enhanced growth and T-cell activation was reduced in the tumor-draining lymph nodes. Further studies are underway to elucidate its underlying mechanisms and to optimize the treatment regimen in order to maximize the efficacy of the combination.

[Disclosure of COI by Responsible researcher] Lead presenter is the Responsible researcher.
[Lead presenter: Akemi Kosaka] We have no financial relationships to disclose.

### [P-1172] I-P11-7
### Tumor immune microenvironment/Tumor immune escape (3)

2025 / 9 / 25 (Thu) 16:45-17:30 Room P/(Motenashi Dome) Room P

### P-1172
### The CCL17-CCR4 Axis Regulates TLS Formation and Anti-tumor Immunity with Prognostic Implications in PDAC

[Speaker]Tomoyuki Nakajima (Shinshu Univ. Hosp. Dept. of Lab. Med.) [Co-author]Shotaro Komamura (Shinshu Univ. Hosp. Dept. of Lab. Med.)、Mai Iwaya (Shinshu Univ. Hosp. Dept. of Lab. Med.)、Manaka Momose (Shinshu Univ. Hosp. Dept. of Lab. Med.)、Shinsuke Sugenoya (Shinshu Univ. Dept. of Surgery(GI, Transplanyt & Ped))、Hiroshi Sawaguchi (Shinshu Univ. Dept. of Gastroenterology)、Shunsuke Imamura (Shinshu Univ. Dept. of Gastroenterology)、Takeshi Uehara (Shinshu Univ. Hosp. Dept. of Lab. Med.)

**Introduction**

Pancreatic ductal adenocarcinoma (PDAC) is aggressive with poor prognosis. TME impacts outcome; TLSs and TILs improve survival. We investigated the CCL17-CCR4 axis's role in PDAC anti-tumor immunity via mregDCs, T cells, and TLSs.

**Materials and Methods**

Bulk RNA-seq (TCGA, n=130) assessed CCL17 prognosis. scRNA-seq (GSE205013, n=17) identified CCL17/CCR4 cells. Spatial CCL17 (RNA ISH) and CCR4/CD4/CD8 (IHC) determined on FFPE.

**Results**

High CCL17 expression significantly associated with improved overall survival ($p<0.001$, HR=1.91). Single-cell analysis identified CCL17 primarily in mregDCs. CCR4 found on T cell populations including Tfh, Tpex, CD4+ Tcm, and Treg. Spatial analysis localized CCL17 in/around TLSs. IHC revealed CCR4+, CD4+, and CD8+ T cells within TLSs.

**Discussion**

High CCL17 correlates with favorable PDAC prognosis. mregDCs, main CCL17 source, localize in/around TLS. We propose CCL17 recruits CCR4+ T cells, contributing to TLS formation and anti-tumor immunity enhancement. This analysis suggests the CCL17-CCR4 axis may represent an important regulatory loop governing TLS formation and anti-tumor immunity in PDACs.

[Disclosure of COI by Responsible researcher] Lead presenter is the Responsible researcher.
[Lead presenter: Tomoyuki Nakajima] We have no financial relationships to disclose.

### [P-1173] I-P11-7
### Tumor immune microenvironment/Tumor immune escape (3)

2025 / 9 / 25 (Thu) 16:45-17:30 Room P/(Motenashi Dome) Room P

---

### P-1173
### sTREM2-Bound Tumor Cell Debris Enhances Macrophage Phagocytosis in the TNBC Microenvironment

[Speaker]Zhaoliang SU (International Genome Center, Jiangsu University) [Co-author]Yan Wu (International Genome Center, Jiangsu University)、Jing Jin (International Genome Center, Jiangsu University)、Xiaoyun Ji (International Genome Center, Jiangsu University)

Triple-negative breast cancer (TNBC) is marked by an aggressive clinical course and a highly immunosuppressive tumor microenvironment. In our study, we identify a novel role for soluble TREM2 (sTREM2) in enhancing the phagocytic clearance of tumor cell debris by TAMs. We generated tumor cell debris from 4T1 TNBC cells and observed that, unlike intact or hypoxic tumor cells, these fragments do not induce TREM2 upregulation in primary macrophages. However, when recombinant sTREM2 was added to the system, a marked increase in the phagocytosis of tumor cell debris by TAMs was detected. Importantly, the phagocytic enhancement was significantly reduced upon the application of neutralizing anti-sTREM2 antibodies, underscoring the specificity of the sTREM2 effect. Our findings suggest that sTREM2 binds to tumor cell debris in a manner reminiscent of classical opsonins, thereby facilitating its recognition and clearance by TAMs. This dual function of sTREM2, enhancing TAM-mediated debris clearance provides new insights into its contribution to the TNBC microenvironment and reveals potential therapeutic targets to disrupt tumor-promoting immunosuppressive circuits.

[Disclosure of COI by Responsible researcher] Lead presenter is the Responsible researcher.
[Lead presenter: Zhaoliang SU] We have no financial relationships to disclose.

### [P-1174] I-P11-7
### Tumor immune microenvironment/Tumor immune escape (3)

2025 / 9 / 25 (Thu) 16:45-17:30 Room P/(Motenashi Dome) Room P

---

### P-1174
### Engineering a 3D Multilayered Tumor Model to Recapitulate Neutrophil Extracellular Trap-Coated Tumor Microenvironment

[Speaker]Chiu-Hua Kuo (School of Medicine, National Yang Ming Chiao Tung University, Taiwan) [Co-author]Chia-Yi Su (Institute of Biophotonics, National Yang Ming Chiao Tung University, Taiwan)

Neutrophils and their derivatives, neutrophil extracellular traps (NETs), play a critical role in tumor progression and frequently interact with tumor-associated extracellular matrix (ECM). However, the mechanisms underlying their interplay remain poorly understood, primarily due to the lack of physiologically relevant experimental models. In this study, we aim to develop a novel 3D tumor model with control over the layered deposition of cells and matrices to mimic a NETs-coated tumor microenvironment. The model features a concentric multilayered structure, with a tumor spheroid at the core, surrounded by a collagen I matrix serving as the ECM barrier, and an outer neutrophil-laden collagen layer to promote NETosis and neutrophil-tumor interactions. An additional alginate encapsulation layer forms the outermost shell, functioning as a containment barrier to maintain model integrity during long-term culture. Homogeneous coating and controlled layer thickness are achieved through serial liquid transfer and gentle mixing techniques. Through the innovative tumor model, we seek to unravel the complicated interplay between tumors, neutrophils, and the ECM.

[Disclosure of COI by Responsible researcher] Lead presenter is not the Responsible researcher.
[Lead presenter: Chiu-Hua Kuo] We have no financial relationships to disclose.
[Principal Researcher: Chiu-Hua Kuo] We have no financial relationships to disclose.

### [P-1175] I-P11-7
### Tumor immune microenvironment/Tumor immune escape (3)

2025 / 9 / 25 (Thu) 16:45-17:30 Room P/(Motenashi Dome) Room P

### P-1175
### Kasugamycin Derivatives: Modulating Chitinase Activity to Reprogram Immune Responses in the Tumor Microenvironment

[Speaker]An Ning Cheng (Genomics Res. Ctr. , Academia Sinica) [Co-author]Kuan-Hung Lin (Genomics Res. Ctr. , Academia Sinica)、Ning-Shian Hsu (Genomics Res. Ctr. , Academia Sinica)、Tsung-Lin Li (Genomics Res. Ctr. , Academia Sinica)、Alan Yueh-Luen Lee (Nat. Inst. of Cancer Res. , NHRI)

Cancer poses a significant global health challenge, highlighting the urgent need for innovative therapeutic strategies. We have identified kasugamycin, an amino cyclitol glycoside, as a promising candidate for cancer treatment. This study explores the therapeutic potential of kasugamycin and its analogs by targeting Glycoside Hydrolase Family 18 Chitinases, which play a pivotal role in cancer progression. We assessed the anticancer effects of kasugamycin and its analogs through in vitro assays focusing on angiogenesis, cell migration, and invasion in human cancer cell lines. Notably, kasugamycin suppresses angiogenesis via the FAK/ERK signaling pathway. Furthermore, kasugamycin significantly inhibits PD-L1 expression by targeting CHI3L1 in cancer cell lines and induces immune reprogramming to suppress tumor metastasis. Our findings provide compelling evidence for kasugamycin as a novel cancer therapeutic by inhibiting Glycoside Hydrolase Family 18 Chitinases, presenting a promising strategy for targeted cancer immunotherapy. Further investigations and clinical trials are essential to validate the efficacy and safety of kasugamycin in animal models.

[Disclosure of COI by Responsible researcher] Lead presenter is not the Responsible researcher.
[Lead presenter: An Ning Cheng] We have no financial relationships to disclose.
[Principal Researcher: An Ning Cheng] We have no financial relationships to disclose.

Poster Session

### [P-1184] I-P12-2
### Liquid biopsy (2)

2025 / 9 / 25 (Thu) 16:45-17:30 Room P/(Motenashi Dome) Room P

---

### P-1184
### Optimization of bile processing for liquid biopsy in cholangiocarcinoma focusing on ctDNA and protein stability

[Speaker]Jin Y. Han (Department of Surgery, Keimyung University Dongsan Medical Center.) [Co-author]Keun S Ahn (Department of Surgery, Keimyung University Dongsan Medical Center.)

Liquid biopsy using bile offers a promising non-invasive approach for molecular analysis in cholangiocarcinoma (CCA). However, the stability of key biomarkers such as proteins and circulating tumor DNA (ctDNA) at room temperature remains unclear. This study evaluated the temporal stability of proteins and ctDNA in bile under room temperature conditions to optimize pre-analytical handling. Bile samples from six CCA patients were analyzed at 1-, 3-, 5-, and 7-hour post-collection. Protein concentration, enzyme activity (E-Cadherin, N-Cadherin), and mutant KRAS ctDNA levels were measured using quantitative assays and droplet digital PCR. No significant degradation of proteins, enzyme activity, or KRAS ctDNA was observed over 7 hours. These findings suggest that bile samples retain molecular integrity for up to 7 hours at room temperature, supporting their utility in liquid biopsy and molecular diagnostics for CCA.

[Disclosure of COI by Responsible researcher] Lead presenter is not the Responsible researcher.
[Lead presenter: Jin Y. Han] We have no financial relationships to disclose.
[Principal Researcher: Keun S Ahn] We have no financial relationships to disclose.

### [P-1185] I-P12-2
### Liquid biopsy (2)

2025 / 9 / 25 (Thu) 16:45-17:30 Room P/(Motenashi Dome) Room P

### P-1185
### Withdrawn

No Abstract

### [P-1186] I-P12-2
### Liquid biopsy (2)

2025 / 9 / 25 (Thu) 16:45-17:30 Room P/(Motenashi Dome) Room P

---

### P-1186
### Exploring the Potential of Exosomal LAT1 as a Diagnostic Marker for Prostate Cancer Resistant to Standard Therapies

[Speaker]Takaaki Tamura (Chiba.Univ.Grad.Sch.Med.Dept.Urol.／Tokyo.Med.Univ.Inst.Med.Sci.Dept.Mol.Cel.Sci.) [Co-author]Yusuke Yoshioka (Tokyo.Med.Univ.Inst.Med.Sci.Dept.Mol.Cel.Sci.)、Takahiro Ochiya (Tokyo.Med.Univ.Inst.Med.Sci.Dept.Mol.Cel.Sci.)、Shinichi Sakamoto (Chiba.Univ.Grad.Sch.Med.Dept.Urol.)

Overexpression of nutrient transporters is well recognized in cancer cells. We have focused on L-type amino acid transporter 1 (LAT1, SLC7A5) and reported its association with tumor aggressiveness and prognosis in urological cancers. LAT1 facilitates amino acid uptake and is upregulated in many malignancies, with its high expression generally linked to poor outcomes. LAT1-specific PET probes and inhibitors are currently under clinical investigation.In prostate cancer, the acquisition of resistance to standard treatments, including hormonal therapy, represents a major clinical challenge. However, no reliable biomarkers are available to sensitively detect this transition. We hypothesize that LAT1 may serve as a promising biomarker for detecting resistance to standard therapies, particularly in prostate cancer.In this study, we focused on extracellular vesicles (EVs) in blood, a growing area in liquid biopsy research, and investigated whether LAT1 can be detected on EVs. We conducted preliminary experiments using prostate cancer cell lines and a small number of patient serum samples.

[Disclosure of COI by Responsible researcher] Lead presenter is not the Responsible researcher.
[Lead presenter: Tamura Takaaki] We have no financial relationships to disclose.
[Principal Researcher: Shinichi Sakamoto] We have no financial relationships to disclose.

▲TOP

### [P-1187] I-P12-2
### Liquid biopsy (2)

2025 / 9 / 25 (Thu) 16:45-17:30 Room P/(Motenashi Dome) Room P

---

### P-1187
### cfDNA-based mutational signature analysis reflects early carcinogenesis in esophageal squamous cell carcinoma

[Speaker]Tomohiro Umezu (Dept. Mol. Path., Tokyo Med. Univ.) [Co-author]Sakiko Naito (Dept. Gast. Hep., Tokyo Med. Univ.)、Takao Itoi (Dept. Gast. Hep., Tokyo Med. Univ.)、Masahiko Kuroda (Dept. Mol. Path., Tokyo Med. Univ.)

DNA damage induced by aging and environmental factors is implicated in the development of esophageal squamous cell carcinoma (ESCC). Such damage often leaves distinct mutational signatures across the genome. In this study, we performed exome sequencing of cfDNA from ESCC patients to evaluate somatic mutation profiles and their associated signatures. Principal component analysis revealed a distinct mutation pattern in Stage I tumors carrying both ALDH2-inactive and CYP2A6-reduced-function genotypes ($p = 0.036$). This pattern was characterized by high contributions of SBS5 (aging-related) and SBS92 (smoking-related). Moreover, SBS1, another aging-associated signature, was specifically enriched in Stage I cases, suggesting involvement in early carcinogenic processes. Somatic CYP2A6 mutations detected in cfDNA were significantly associated with clinical indicators of field cancerization, including alcohol history and multiple Lugol-voiding lesions. These findings suggest that mutational signatures reflect the interplay between genetic susceptibility and environmental exposure, and that cfDNA-based analysis can provide insights into early genomic alterations in ESCC.

[Disclosure of COI by Responsible researcher] Lead presenter is not the Responsible researcher.
[Lead presenter: Tomohiro Umezu] We have no financial relationships to disclose.
[Principal Researcher: Masahiko Kuroda] We have no financial relationships to disclose.

[P-1188] I-P12-2
Liquid biopsy (2)

2025 / 9 / 25 (Thu) 16:45-17:30 Room P/(Motenashi Dome) Room P

P-1188
### Utility of multi-body fluid integration analysis in liquid biopsy for pancreatic cancer.

[Speaker]Taisuke Imamura (Division of Digestive Surgery, Kyoto Prefectural University of Medicine) [Co-author]Shuhei Komatsu (Division of Digestive Surgery, Kyoto Prefectural University of Medicine)、Satoshi Hamada (Division of Digestive Surgery, Kyoto Prefectural University of Medicine)、Masateru Yamauchi (Division of Digestive Surgery, Kyoto Prefectural University of Medicine)、Rie Shibata (Division of Digestive Surgery, Kyoto Prefectural University of Medicine)、Ryo Ishida (Division of Digestive Surgery, Kyoto Prefectural University of Medicine)、Keiji Nishibeppu (Division of Digestive Surgery, Kyoto Prefectural University of Medicine)、Jun Kiuchi (Division of Digestive Surgery, Kyoto Prefectural University of Medicine)、Hidemasa Kubo (Division of Digestive Surgery, Kyoto Prefectural University of Medicine)、Yusuke Yamamoto (Division of Digestive Surgery, Kyoto Prefectural University of Medicine)、Ryo Morimura (Division of Digestive Surgery, Kyoto Prefectural University of Medicine)、Hirotaka Konishi (Division of Digestive Surgery, Kyoto Prefectural University of Medicine)、Hitoshi Fujiwara (Division of Digestive Surgery, Kyoto Prefectural University of Medicine)、Atsushi Shiozaki (Division of Digestive Surgery, Kyoto Prefectural University of Medicine)

Background: We evaluated the utility of integrating ctDNA analysis in plasma, urine, and ascitic fluid for pancreatic cancer (PC).Methods: We analyzed 30 patients who underwent surgery for PC. KRAS mutations in cfDNA from plasma, urine, and ascitic fluid were detected using droplet digital PCR with a KRAS multiplex kit (G12A, G12C, G12D, G12R, G12S, G12V, G13D). Samples with fractional abundance ≧ 0.1% were defined as ctDNA-positive.Results: KRAS mutations were detected in plasma, urine, and ascites in 8 (26%), 5 (17%), and 7 (23%) cases, respectively. Plasma ctDNA positivity was significantly associated with poor overall survival (OS) (P=0.004). Urine and ascitic ctDNA positivity showed no significant association with OS. In resected cases, plasma ctDNA also correlated with poor OS (P=0.030), high CA19-9 (P=0.005), and lymph node metastasis (P=0.022). No significant clinicopathological correlations were observed for urine or ascitic ctDNA.Conclusion: KRAS ctDNA was detectable in plasma, urine, and ascitic fluid, but only plasma ctDNA had prognostic relevance. Further studies are needed to validate ctDNA in urine and ascites.

[Disclosure of COI by Responsible researcher] Lead presenter is the Responsible researcher.
[Lead presenter: Taisuke Imamura] We have no financial relationships to disclose.

### [P-1189] I-P12-2
### Liquid biopsy (2)

2025 / 9 / 25 (Thu) 16:45-17:30 Room P/(Motenashi Dome) Room P

---

### P-1189
### Comparative Analysis of Extracellular Vesicle Surface Proteins Using Serum from Breast Cancer Patients

[Speaker]Sachiko Inubushi (Division of Breast Surgery, Graduate School of Medicine, Kobe University,) [Co-author]Tomonari Kunihisa (Division of Breast Surgery, Graduate School of Medicine, Kobe University,)、Shotaro Inoue (Division of Breast Surgery, Graduate School of Medicine, Kobe University,)、Mayuko Miki (Division of Breast Surgery, Graduate School of Medicine, Kobe University,)、Hirokazu Tanino (Department of Cardiovascular, Respiratory and Breast Surgery, Wakayama Medical University,)

Background: Extracellular vesicles (EVs, 30-150 nm), including exosomes, mediate intercellular communication and are involved in cancer metastasis. We focused on EV markers CD9, CD63, and HER2 to explore their potential as diagnostic/prognostic markers by quantifying EV surface proteins in serum from breast cancer patients before and after surgery.
Methods: Pre- and postoperative serum samples from eight breast cancer patients (>10 mm tumor) were analyzed using ExoCounter. Diluted serum (50 μL) was applied to antibody-coated discs to capture EVs, which were then detected using antibody-conjugated beads. Bead count represented EV levels.Results: CD63/CD9 double-positive EVs significantly decreased after surgery. In HER2 score 1+ to 3+ patients (n=5), CD9/HER2 double-positive EVs also significantly decreased postoperatively, unlike in score 0 patients (n=3).
Discussion: Tumor presence affects levels of CD63/CD9 and CD9/HER2 EVs. CD9/HER2-positive EVs may be useful for detecting minimal residual disease (MRD) in HER2-positive breast cancer. We also plan to investigate TROP2, a novel breast cancer target, using the same system.

[Disclosure of COI by Responsible researcher] Lead presenter is the Responsible researcher.
[Lead presenter: Sachiko Inubushi] We have no financial relationships to disclose.

▲TOP

### [P-1190] I-P12-2
### Liquid biopsy (2)

2025 / 9 / 25 (Thu) 16:45-17:30 Room P/(Motenashi Dome) Room P

### P-1190
### DNA extracted from peritoneal lavage fluid in Stage II/III colorectal cancer patients

[Speaker]Takanori Matsui (Department of Gastroenterological Surgery, Nippon Medical School) [Co-author]Takeshi Yamada (Department of Gastroenterological Surgery, Nippon Medical School)、Kay Uehara (Department of Gastroenterological Surgery, Nippon Medical School)、Koji Ueda (Department of Gastroenterological Surgery, Nippon Medical School)、Shintaro Kanaka (Department of Gastroenterological Surgery, Nippon Medical School)、Akihisa Matsuda (Department of Gastroenterological Surgery, Nippon Medical School)、Seiichi Shinji (Department of Gastroenterological Surgery, Nippon Medical School)、Yasuyuki Yokoyama (Department of Gastroenterological Surgery, Nippon Medical School)、Goro Takahashi (Department of Gastroenterological Surgery, Nippon Medical School)、Takuma Iwai (Department of Gastroenterological Surgery, Nippon Medical School)、Toshimitsu Miyasaka (Department of Gastroenterological Surgery, Nippon Medical School)、Sho Kuriyama (Department of Gastroenterological Surgery, Nippon Medical School)、Koki Hayashi (Department of Gastroenterological Surgery, Nippon Medical School)、Hiroshi(Nippon Med. Sch.) Yoshida (Department of Gastroenterological Surgery, Nippon Medical School)

Background: Cell-free tumor DNA (ctDNA) can be detected not only in blood but also in intraoperative peritoneal lavage fluid (PLF). This study aimed to investigate whether KRAS mutations detected using ctDNA in PLF can serve as prognostic markers in colorectal cancer. Method: A prospective study was conducted on colorectal cancer patients undergoing surgery between Nov 2018 and July 2020. PLF was collected prior to surgical intervention. DNA was extracted from surgical specimens and PLF, and the presence of KRAS mutations was examined by droplet digital PCR. Results: A total of 166 patients were enrolled, and KRAS mutations were identified in tissue samples from 74 patients and in PLF from 44 patients. KRAS mutations were identified in the PLF of 27 out of 74 patients (36.5%) with KRAS mutated in the tissue, and in the PLF of 17 out of 92 patients (18.5%) without KRAS mutations in the tissue. Among the 44 patients with KRAS mutations detected in PLF, 12 experienced recurrence (27.3%), whereas 21 out of 121 patients (17.4%) without KRAS mutations in PLF experienced recurrence (P=0.16). Conclusion: Patients in whom ctDNA was detected in PLF may have a poorer prognosis.

[Disclosure of COI by Responsible researcher] Lead presenter is not the Responsible researcher.
[Lead presenter: Takanori Matsui] We have no financial relationships to disclose.
[Principal Researcher: Takeshi Yamada] We have no financial relationships to disclose.

▲TOP

### [P-1199] I-P13-1
### Drug resistance (1)

2025 / 9 / 25 (Thu) 16:45-17:30 Room P/(Motenashi Dome) Room P

### P-1199
### Signaling Pathway Analysis of PD-L1 Regulation in Drug-Resistant Lung Cancer Cells

[Speaker]Tetsuya Fukui (Department of Thoracic Surgery, Graduate School of Medicine, Kyoto University) [Co-author]Shigeyuki Tamari (Department of Thoracic Surgery, Shizuoka city Shizuoka Hospital)、Yumeta Shimazu (Department of Thoracic Surgery, Graduate School of Medicine, Kyoto University)、Shigeto Nishikawa (Department of Thoracic Surgery, Graduate School of Medicine, Kyoto University)、Ryota Sumitomo (Department of Thoracic Surgery, Graduate School of Medicine, Kyoto University)、Toshi Menju (Department of Thoracic Surgery, Graduate School of Medicine, Kyoto University)

**Background:** In EGFR-mutated lung adenocarcinoma, the IFNγ/JAK/Stat1 and JAK/Stat3 pathways antagonize each other. PD-L1 expression is reported to be enhanced by IFNγ-induced Stat1 activation or Stat3 inhibition. However, how PD-L1 relates to Stat1/Stat3 in cells with acquired resistance to the EGFR-TKI osimertinib remains elusive. This study investigated whether PD-L1 expression in osimertinib-resistant lung cancer cells depends on Stat1. **Methods:** Osimertinib-resistant lines were developed from EGFR-mutant NSCLC H1975 (L858R, T790M) by gradual osimertinib exposure. Resistance was confirmed by IC50 assays. Protein levels of pStat1, pStat3, and PD-L1 were assessed by Western blot. Stattic, a Stat3 inhibitor, was also used. **Results:** Resistant H1975 cells showed elevated IC50 values. pStat3 was upregulated, while pStat1 and PD-L1 were downregulated. Stattic treatment suppressed pStat3 and increased both pStat1 and PD-L1. **Conclusion:** In osimertinib-resistant NSCLC cells, PD-L1 expression may depend on Stat1 and be suppressed by Stat3. This suggests a potential mechanism of immune checkpoint regulation and may help overcome immune evasion in EGFR-TKI resistance.

[Disclosure of COI by Responsible researcher] Lead presenter is not the Responsible researcher.
[Lead presenter: Tetsuya Fukui] We have no financial relationships to disclose.
[Principal Researcher: Toshi Menju] We have no financial relationships to disclose.

### [P-1200] I-P13-1
### Drug resistance (1)

2025 / 9 / 25 (Thu) 16:45-17:30 Room P/(Motenashi Dome) Room P

---

### P-1200
### Afatinib plus PEM and CBDCA overcome osimertinib resistance in EGFR-mutated NSCLC with high TSP-1 expression

[Speaker]Shinji Nakamichi (Department of Pulmonary Medicine and Oncology, Nippon Medical School) [Co-author]Naomi Onda (Department of Pulmonary Medicine and Oncology, Nippon Medical School)、Kakeru Hisakane (Department of Pulmonary Medicine and Oncology, Nippon Medical School)、Yasuhiro Kato (Department of Pulmonary Medicine and Oncology, Nippon Medical School)、Susumu Takeuchi (Department of Pulmonary Medicine and Oncology, Nippon Medical School)、Aya Fukuizumi (Department of Pulmonary Medicine and Oncology, Nippon Medical School)、Akihiko Miyanaga (Department of Pulmonary Medicine and Oncology, Nippon Medical School)、Kazuo Kasahara (Department of Pulmonary Medicine and Oncology, Nippon Medical School)、Masahiro Seike (Department of Pulmonary Medicine and Oncology, Nippon Medical School)

Acquired resistance to osimertinib remains an inevitable problem in epidermal growth factor receptor (EGFR)-mutated non-small-cell lung cancer (NSCLC) patients. In this study, we aimed to investigate osimertinib-resistant mechanisms and evaluate the combination therapy of afatinib and chemotherapy. We established osimertinib-resistant cell lines (PC-9-OR and H1975-OR) from EGFR-mutant lung adenocarcinoma cell lines. We found that thrombospondin-1 (TSP-1) expression was upregulated in resistant cells. We demonstrated that TSP-1 increases the expression of matrix metalloproteinases through integrin signaling and promotes tumor invasion in both resistant cells, and epithelial-to-mesenchymal transition (EMT) was involved in H1975-OR. Afatinib plus carboplatin (CBDCA) and pemetrexed (PEM) reversed TSP-1-induced invasion ability and EMT changes in resistant cells. In PC-9-OR xenograft mouse models, combination therapy of afatinib plus CBDCA and PEM strongly inhibited tumor growth. These results suggest that the combination of afatinib plus CBDCA and PEM, which effectively suppresses TSP-1 expression, may be a promising option in EGFR-mutated NSCLC patients after osimertinib resistance.

[Disclosure of COI by Responsible researcher] Lead presenter is the Responsible researcher.
[Lead presenter: Shinji Nakamichi] We have no financial relationships to disclose.

### [P-1201] I-P13-1
### Drug resistance (1)

2025 / 9 / 25 (Thu) 16:45-17:30 Room P/(Motenashi Dome) Room P

### P-1201
### Investigating the diversity of drug-tolerant persister cells in EGFR-Mutant NSCLC by single-cell clones

[Speaker]Mo Zhou (Grad. Sch. Pharm. Sci., Osaka Univ.) [Co-author]Yuya Haga (Grad. Sch. Pharm. Sci., Osaka Univ.／Sch. Pharm. Sci., Osaka Univ.)、Kazuma Higashisaka (Grad. Sch. Pharm. Sci., Osaka Univ.／Sch. Pharm. Sci., Osaka Univ.／IACS, Osaka Univ.)、Yasuo Tsutsumi (Grad. Sch. Pharm. Sci., Osaka Univ.／Sch. Pharm. Sci., Osaka Univ.／MEI Ctr., Osaka Univ.／OTRI., Osaka Univ.／INSD., Osaka Univ.)

Drug-sensitive cancer cells acquire drug resistance after prolonged treatment over months or years. During this process, drug-tolerant persister cells (DTPs) have been proposed as a drug-tolerant population with non-genetic mutational mechanisms following short-term drug exposure. However, the pre-existing diversity of DTPs before drug exposure remains unclear. In this study, we used single-cell clones to examine the differences in DTPs formation capacity, aiming to identify key factors involved in this process. We explored the gene expression differences between single-cell clones with varying DTP formation abilities before drug exposure. To continuously monitor cell number variation, we labeled the EGFR-mutant lung cancer cell line PC9 with Fucci fluorescent probes, allowing continuous observation of the cell cycle. After nine days of drug exposure, DTPs emerged, with some clones demonstrating a higher propensity for DTP formation, while others exhibited a lower capacity. Moving forward, we aim to identify key factors influencing DTPs formation to elucidate its underlying mechanisms and ultimately reduce DTPs emergence in clinical settings.

[Disclosure of COI by Responsible researcher] Lead presenter is not the Responsible researcher.
[Lead presenter: Mo Zhou] We have no financial relationships to disclose.
[Principal Researcher: Yuya Haga] We have no financial relationships to disclose.

### [P-1202] I-P13-1
### Drug resistance (1)

2025 / 9 / 25 (Thu) 16:45-17:30 Room P/(Motenashi Dome) Room P

---

### P-1202
### CD74 antibody-drug conjugate eliminates targeted therapy-tolerant persisters in oncogenic kinase-driven lung cancer

[Speaker]Motohiro Izumi (Beth Israel Deaconess Medical Center) [Co-author]Ikei Kobayashi (Beth Israel Deaconess Medical Center)、Vivian Ho (Beth Israel Deaconess Medical Center)、Meghan Lee (Beth Israel Deaconess Medical Center)、Daisuke Shibahara (Beth Israel Deaconess Medical Center)、Yusuke Otani (Beth Israel Deaconess Medical Center)、Masaki Maekawa (Beth Israel Deaconess Medical Center)、Atsushi Tanaka (Beth Israel Deaconess Medical Center)、Hibiki Udagawa (Exploratory Oncology Research and Clinical Trial Center, National Cancer Center／Department of Thoracic Oncology, National Cancer Center Hospital East)、Michael Roehrl (Beth Israel Deaconess Medical Center)、Ioannis Vlachos (Beth Israel Deaconess Medical Center)、Daniel Costa (Beth Israel Deaconess Medical Center)、Susumu Kobayashi (Beth Israel Deaconess Medical Center／Juntendo University Faculty of Medicine and Graduate School of Medicine)

The clinical efficacy of targeted therapies in oncogene-driven lung cancer is ultimately limited by development of acquired resistance. We hypothesize that targeting drug-tolerant persisters (DTPs), which are tumor cells tolerant to initial drug exposure, could eradicate tumor cells. We identify CD74 as a DTP marker in oncogenic kinase-driven lung cancer by analyzing scRNA-seq data from cell lines, a patient-derived xenograft model, and clinical samples, including untreated tumor cells and DTPs after tyrosine kinase inhibitor (TKI) treatment. Mechanistically, CD74 upregulation is due to increased BCL6 expression promoted by TKI-dependent ERK1/2 dephosphorylation. Spatial transcriptomics analysis in transgenic mice with EGFR-driven lung cancer enables evaluation of spatial heterogeneity and cell-cell interactions before and after TKI treatment and shows that TKI-treated DTP cells upregulate MIF-CD74 signaling to promote cell survival. Combining a TKI with an anti-CD74 antibody drug conjugate efficiently eliminates lung cancer cells with oncogenic kinase-drivers. CD74-based targeting approaches in combination with TKIs overcome drug resistance in oncogenic kinase-driven lung cancer.

[Disclosure of COI by Responsible researcher] Lead presenter is not the Responsible researcher.
[Lead presenter: Motohiro Izumi] We have no financial relationships to disclose.
[Principal Researcher: Susumu Kobayashi] ■Research funds from company: MiRXES ■Advisor of the company and/or profit-making organization: Taiho Therapeutics ■Patent fee: Signosis, Life Technologies ■Lecture fee: AstraZeneca, Boehringer Ingelheim, Bristol Meyers Squibb, Chugai Pharmaceutical, Takeda Pharmaceuticals

### [P-1203] I-P13-1
### Drug resistance (1)

2025 / 9 / 25 (Thu) 16:45-17:30 Room P/(Motenashi Dome) Room P

### P-1203
### PLK1-driven cell cycle dynamics contribute to osimertinib resistance in EGFR-mutated non-small cell lung cancer

[Speaker]Yuki Tsuneoka (Dept of Res Med, Grad Sch Med Sci, Kyushu Univ.) [Co-author]Daisuke Shibahara (Dept of Res Med, Grad Sch Med Sci, Kyushu Univ.)、Yasuto Yoneshima (Dept of Res Med, Grad Sch Med Sci, Kyushu Univ.)、Kohei Otsubo (Dept of Res Med, Grad Sch Med Sci, Kyushu Univ.)、Eiji Iwama (Dept of Res Med, Grad Sch Med Sci, Kyushu Univ.)、Isamu Okamoto (Dept of Res Med, Grad Sch Med Sci, Kyushu Univ.)

In EGFR-mutant non-small cell lung cancer (NSCLC), osimertinib, a third-generation EGFR tyrosine kinase inhibitor, exhibits significant therapeutic efficacy. However, resistance inevitably develops during treatment. To investigate the mechanisms underlying the acquisition of resistance to osimertinib, we performed single-cell RNA sequencing on PC-9 cells treated with osimertinib for 3 days, 7 days, or 3 days followed by a 3-day drug withdrawal. We found that Polo-like kinase 1 (PLK1), a key regulator of the cell cycle, was downregulated during the early phase of treatment, although some cell clusters restored PLK1 expression even under continued drug exposure. Osimertinib induced G1 phase cell cycle arrest, which was associated with reduced drug efficacy. Furthermore, PLK1 knockdown promoted resistance to osimertinib, while PLK1 overexpression enhanced its efficacy. These findings suggest that a slow-cycling state driven by PLK1 suppression may represent a critical feature of resistance acquisition. Understanding cell cycle dynamics during the initial response to targeted therapy may offer new strategies to overcome therapeutic resistance.

[Disclosure of COI by Responsible researcher] Lead presenter is not the Responsible researcher.
[Lead presenter: Yuki Tsuneoka] We have no financial relationships to disclose.
[Principal Researcher: Yasuto Yoneshima] We have no financial relationships to disclose.

### [P-1204] I-P13-1
### Drug resistance (1)

2025 / 9 / 25 (Thu) 16:45-17:30 Room P/(Motenashi Dome) Room P

### P-1204
### The role of METTL3 on osimertinib resistance in non-small cell lung cancer cells

[Speaker]Ryusuke Suzuki (Div. Func. Gen., Cancer Res. Inst., Kanazawa Univ.) [Co-author]Akihiko Ishimura (Div. Func. Gen., Cancer Res. Inst., Kanazawa Univ.)、Takeshi Suzuki (Div. Func. Gen., Cancer Res. Inst., Kanazawa Univ.)

Osimertinib, a third-generation EGFR tyrosine kinase inhibitor, has been used as first-line therapy for EGFR-mutated NSCLC. However, acquired drug resistance still remains as serious problem associated with poor clinical outcomes. METTL3, a key methyltransferase for N6-methyladenosine (m6A) modification of RNA, has been implicated in tumor development and cancer malignancy. In this study, we explored role of METTL3 in acquired resistance to osimertinib and investigated its therapeutic potential. We established METTL3 knockdown EGFR-mutated NSCLC cell lines and found that METTL3 downregulation suppressed development of osimertinib resistance. Microarray analysis and qRT-PCR identified ***CDC25A*** and ***AURKB*** as METTL3 downstream target genes, with METTL3 contributing to their mRNA stabilization. Downregulation of these METTL3 target gene also attenuated osimertinib resistance. Additionally, we examined effects of METTL3 inhibitor or CDC25A inhibitor on osimertinib treatment and both inhibitors increased drug efficacy by inducing apoptosis. Collectively, we demonstrated METTL3-mediated mechanism of acquired osimertinib resistance and therapeutic potential of targeting this machinery.

[Disclosure of COI by Responsible researcher] Lead presenter is not the Responsible researcher.
[Lead presenter: Ryusuke Suzuki] ■Research funds from company: 日本学術振興会科学研究費助成事業若手研究
[Principal Researcher: Takeshi Suzuki] ■Research funds from company: 日本学術振興会科学研究費助成事業基盤研究C

### [P-1205] I-P13-1
### Drug resistance (1)

2025 / 9 / 25 (Thu) 16:45-17:30 Room P/(Motenashi Dome) Room P

### P-1205
### Characterization of Drug-Tolerant Cells in EGFR-Mutant NSCLC Following Osimertinib Exposure

[Speaker]Hinata Chiba (Pharm. Sci., Chiba Univ.) [Co-author]Taichi Oshima (Grad. Sch. Pharm. Sci., Chiba Univ)、Shigeki Aoki (Grad. Sch. Pharm. Sci., Chiba Univ)

BackgroundIn EGFR-mutant non-small cell lung cancer (NSCLC), drug-tolerant cells (DTCs) that survive EGFR-TKI treatment without resistance mutations may contribute to later resistance. This study aimed to characterize the properties of DTCs emerging after Osimertinib exposure, with the goal of identifying therapeutic targets.MethodsEGFR-mutant NSCLC cell lines (PC9, H1975, H1650) were exposed to Osimertinib to induce drug tolerance. Cell viability was evaluated via MTT assay, mitochondrial membrane potential by JC-1 staining, and autophagy by LC3B expression in western blotting. Glutamine dependence was assessed using glutamine-free medium. Cellular senescence was examined using SA-b-gal staining.Results & DiscussionDTCs showed enhanced glutamine metabolism, elevated mitochondrial membrane potential, activated autophagy, and senescence-like features. Co-treatment with glutamine deprivation and autophagy inhibition induced cell death, suggesting both are essential for survival. These findings indicate that Osimertinib-induced DTCs survive via glutamine-dependent metabolism and autophagy in a senescence-like state, and these processes may represent therapeutic targets.

[Disclosure of COI by Responsible researcher] Lead presenter is the Responsible researcher.
[Lead presenter: Hinata Chiba] We have no financial relationships to disclose.

### [P-1206] I-P13-1
### Drug resistance (1)

2025 / 9 / 25 (Thu) 16:45-17:30 Room P/(Motenashi Dome) Room P

---

### P-1206
### Suppression of drug resistance development based on cystine metabolism alteration in drug-tolerant persister cells

[Speaker]Tomomiki Noda (Genome Res., Cancer Chemother. Ctr., JFCR／Dept. CBMS, Grad. Sch. Front. Sci., Uni. Tokyo) [Co-author]Hitomi Shirahama (Genome Res., Cancer Chemother. Ctr., JFCR)、Akihiro Tomida (Genome Res., Cancer Chemother. Ctr., JFCR／Dept. CBMS, Grad. Sch. Front. Sci., Uni. Tokyo)

Cancer cells produce excessive reactive oxygen species and rely on antioxidant systems. Cystine is an essential factor for the system through involving glutathione biosynthesis. Previously, we found that some cancer cells are sensitive to cystine depletion, while their drug-tolerant persister (DTP) state, developed after molecular targeted drug treatment, becomes resistant due to lower cystine demand. In this study, we focused on cystine depletion sensitivity of BRAF-mutant melanoma resistant cells, which emerge and proliferate from DTP under prolonged vemurafenib treatment. As a result, the resistant cells showed sensitivity to cystine depletion like parental cells, suggesting that cystine demand recovers as they acquire resistance. We hypothesized that cystine depletion might suppress the development of resistant cells from DTP. To confirm this, we conducted cotreatment experiments with vemurafenib and cystine depletion. DTP proliferated under vemurafenib treatment within a month, while growth of DTP was suppressed in combined treatment. These findings suggest that targeting cystine metabolism could provide new strategies to prevent the emergence of drug-resistant cancer cells.

[Disclosure of COI by Responsible researcher] Lead presenter is not the Responsible researcher.
[Lead presenter: Tomomiki Noda] We have no financial relationships to disclose.
[Principal Researcher: Akihiro Tomida] We have no financial relationships to disclose.

▲TOP

### [P-1207] I-P13-1
### Drug resistance (1)

2025 / 9 / 25 (Thu) 16:45-17:30 Room P/(Motenashi Dome) Room P

---

### P-1207
### AXL mediates intrinsic resistance to HER2 inhibitors via the SHC1BP-SHC1 pathway in HER2-aberrant cancers

[Speaker]Masaki Ishida (Department of Pulmonary Medicine, Kyoto Prefectural University of Medicine.) [Co-author]Tadaaki Yamada (Department of Pulmonary Medicine, Kyoto Prefectural University of Medicine.)、Yuki Katayama (Department of Pulmonary Medicine, Kyoto Prefectural University of Medicine.)、Kazuo Yasumoto (Department of Medical Oncology, Kanazawa Medical University, Uchinada, Ishikawa, Japan)、Mano Horinaka (Department of Drug Discovery Medicine, Kyoto Prefectural University of Medicine.)、Toshiyuki Sakai (Department of Drug Discovery Medicine, Kyoto Prefectural University of Medicine.)、Koichi Takayama (Department of Pulmonary Medicine, Kyoto Prefectural University of Medicine.)

IntroductionHER2 is an oncogenic driver in several cancers. Several HER2-TKIs have been developed for HER2-aberrant tumors, but complete remission remains difficult. Identifying optimal combination therapies is essential for better outcomes. This study examines adaptive resistance mechanisms to HER2-TKIs and strategies to overcome them.MethodsWe conducted in vitro and in vivo models using HER2-aberrant tumor cell lines to evaluate adaptive responses to HER2-TKIs. ResultsAXL signaling mediated adaptive resistance to HER2-TKIs in HER2-aberrant tumors, activated via GAS6 ligand production. AXL inhibition reduced AXL-overexpressing tumor cell viability by enhancing apoptosis. The Shc1BP-Shc1 axis contributed to this resistance. HER2-TKIs induced ShcBP1 dissociation from Shc1, promoted Shc1-AXL binding, and triggered ShcBP1 nuclear translocation. In xenograft models, AXL inhibition combined with HER2-TKIs significantly suppressed tumor growth and delayed recurrence compared to HER2-TKI monotherapy.ConclusionThese findings highlight the role of GAS6-AXL signaling in HER2-TKI resistance and suggest that targeting this pathway may improve HER2-targeted therapy efficacy.

[Disclosure of COI by Responsible researcher] Lead presenter is not the Responsible researcher.
[Lead presenter: Masaki Ishida] We have no financial relationships to disclose.
[Principal Researcher: Yuki Katayama] ■Research funds from company: 小野薬品、武田薬品、ヤンセンファーマ　■Lecture fee: 日本イーライリリー、中外製薬

### [P-1217] I-P13-3
### Drug resistance (3)

2025 / 9 / 25 (Thu) 16:45-17:30 Room P/(Motenashi Dome) Room P

### P-1217
### Analysis of lenvatinib resistance mechanisms to overcome its resistance in thyroid cancer

[Speaker]Masaki Kawamura (Division of Breast and Endocrine Surgery, Shinshu University) [Co-author]Masatsugu Amitani (Division of Breast and Endocrine Surgery, Shinshu University)、Tatsunori Chino (Division of Breast and Endocrine Surgery, Shinshu University)、Takaaki Oba (Division of Breast and Endocrine Surgery, Shinshu University)、Kenichi Ito (Division of Breast and Endocrine Surgery, Shinshu University)

Lenvatinib is used for advanced thyroid cancer (TC). However, little is known about its resistant mechanisms. To develop novel therapeutic strategies against lenvatinib-resistant TC, we analyzed the characteristics of lenvatinib-resistant TC. Drug sensitivity of TC cell lines was analyzed by WST assay. Expression and phosphorylation of signaling molecules were analyzed by western blotting. The effect of targeted drugs was analyzed by mouse model. We tested lenvatinib sensitivity in TC cell lines and found that ACT-1 and OCUT-1F showed a lower sensitivity to lenvatinib. When these cell lines were treated with lenvatinib, enhanced phosphorylation of EGFR, MEK, and ERK was observed. When a MEK inhibitor, trametinib, was administered simultaneously with lenvatinib, a synergistic growth inhibition was observed in these cells. In a mouse xenograft model, combination therapy with these drugs significantly inhibited the growth of lenvatinib-resistant tumors. These results suggest that combined use of MEK inhibitor with lenvatinib may enhance the growth inhibitory effect of lenvatinib in TC showing intrinsic resistance to lenvatinib and may represent a novel therapeutic strategy.

[Disclosure of COI by Responsible researcher] Lead presenter is not the Responsible researcher.
[Lead presenter: Masaki Kawamura] We have no financial relationships to disclose.
[Principal Researcher: Kenichi Ito] We have no financial relationships to disclose.

▲TOP

### [P-1218] I-P13-3
### Drug resistance (3)

2025 / 9 / 25 (Thu) 16:45-17:30 Room P/(Motenashi Dome) Room P

---

### P-1218
### CYP17A1-HDAC6 Inhibitor Overcomes Temozolomide Resistance in Glioblastoma

[Speaker]Siou-Lian Hu (Taipei Medical University) [Co-author]Tsung-I Hsu (Taipei Medical University)

Glioblastoma (GBM) is an aggressive brain tumor typically treated with surgery and temozolomide (TMZ) chemotherapy, though resistance to TMZ is common. Previous studies suggest that inhibiting cytochrome P450 17A1 (CYP17A1) and histone deacetylase 6 (HDAC6) individually can suppress GBM growth. Compound 12 is a dual inhibitor targeting both enzymes to overcome TMZ resistance. In vitro, Compound 12 inhibited GBM growth, assessed by the Cell Counting Kit-8 assay, and affected the cell cycle. In vivo, it was tested in xenograft and orthotopic GBM mouse models, where it showed strong anti-tumor effects in both TMZ-sensitive and resistant cells. Compound 12 was more cytotoxic than TMZ and selectively targeted cancer cells with minimal effects on normal astrocytes. At 10 $\mu$M, it induced apoptosis in TMZ-resistant cells. In animal models, Compound 12 extended survival and suppressed tumor growth. These findings suggest that Compound 12 is a promising strategy for overcoming TMZ resistance in GBM, with selective anti-tumor properties and low toxicity.

[Disclosure of COI by Responsible researcher] Lead presenter is the Responsible researcher.
[Lead presenter: Siou-Lian Hu] We have no financial relationships to disclose.

▲TOP

### [P-1219] I-P13-3
### Drug resistance (3)

2025 / 9 / 25 (Thu) 16:45-17:30 Room P/(Motenashi Dome) Room P

---

### P-1219
### Combinatorial drug strategies targeting histone deacetylases for the treatment of resistant glioblastoma

[Speaker]Hong-Yi Lin (Taipei Neuroscience Institute, New Taipei City 235, Taiwan／Taiwan Brain Disease Foundation, Taipei 100, Taiwan) [Co-author]Tsung-I Hsu (Taipei Medical University, Taipei 110, Taiwan)

Glioblastoma (GBM) is an aggressive brain tumor that remains resistant to standard therapies temozolomide (TMZ). Overexpression of histone deacetylases (HDACs) correlates with tumor recurrence and resistance to TMZ, making HDACs critical targets. This study investigates a combinatorial strategy targeting HDACs to overcome resistance to GBM. We developed two HDAC inhibitors, compound 1 and compound 6, designed to enhance efficacy and improve blood-brain barrier (BBB) penetration. Compound 1, modified with linezolid, efficiently targets HDACs, inducing acetylation and DNA damage. In orthotopic GBM mouse models, it significantly reduced tumor growth and extended survival. Compound 6, combined with abiraterone (Abi), showed potent anti-GBM activity, particularly against TMZ-resistant cell lines. This combination induced apoptosis, oxidative stress, and DNA damage, inhibiting tumor proliferation. In vivo, compound 6 significantly suppressed tumor growth and extended survival in both xenograft and orthotopic models. These findings highlight the potential of HDAC-targeting combinatorial therapies with Abi and linezolid to overcome GBM resistance, improving efficacy and survival outcomes.

[Disclosure of COI by Responsible researcher] Lead presenter is the Responsible researcher.
[Lead presenter: Hong-Yi Lin] We have no financial relationships to disclose.

### [P-1220] I-P13-3
### Drug resistance (3)

2025 / 9 / 25 (Thu) 16:45-17:30 Room P/(Motenashi Dome) Room P

---

### P-1220
### AKT inhibition enhances the efficacy of androgen receptor-targeted therapy in mouse hormone-sensitive prostate cancer

[Speaker]Marco Develasco (Dept. of Genome Biol. Kindai Univ. Faculty of Med.) [Co-author]Kazuko Sakai (Dept. of Genome Biol. Kindai Univ. Faculty of Med.)、Mamoru Hashimoto (Dept. of Urol.Kindai Univ. Faculty of Med.)、Shingo Toyoda (Dept. of Urol.Kindai Univ. Faculty of Med.)、Saizo Fujimoto (Dept. of Urol.Kindai Univ. Faculty of Med.)、Takafumi Minami (Dept. of Urol.Kindai Univ. Faculty of Med.)、Kazuhiro Yoshimura (Dept. of Urol.Kindai Univ. Faculty of Med.)、Hirotsugu Uemura (Dept. of Urol.Kindai Univ. Faculty of Med.)、Kazuto Nishio (Dept. of Genome Biol. Kindai Univ. Faculty of Med.)、Kazutoshi Fujita (Dept. of Genome Biol. Kindai Univ. Faculty of Med.)

Combining antiandrogen therapies with androgen deprivation therapy (ADT) has improved patient outcomes. However, the loss of the tumor suppressor PTEN, common in prostate cancer (PCa), increases AKT signaling and contributes to PCa progression. Capivasertib (Capiva, AZD5363) is being studied as an oral pan-AKT inhibitor combined with abiraterone and ADT (AA) to treat metastatic hormone-sensitive prostate cancer (HSPC) in patients with PTEN loss. We evaluated the antitumor and molecular responses to the combination of Abi and capivasertib using conditional knockout mouse models of PTEN-deficient PCa. Adding Capiva to AA enhanced tumor growth inhibition in both autochthonous and syngeneic models of Pten-null HSPC. Capiva reduced cancer cell proliferation and increased apoptosis. It also inhibited the phosphorylation of AKT effector molecules involved in cell survival and growth. Additionally, Capiva increased survival times and delayed tumor growth following progression to AA in a model of advanced HSPC. Our findings demonstrate that PTEN-deficient HSPCs are responsive to AKT inhibition. These models will be used to further identify the factors associated with treatment responses.

[Disclosure of COI by Responsible researcher] Lead presenter is the Responsible researcher.
[Lead presenter: Marco Develasco] ■Research funds from company: Astrazeneca

### [P-1221] I-P13-3
### Drug resistance (3)

2025 / 9 / 25 (Thu) 16:45-17:30 Room P/(Motenashi Dome) Room P

---

### P-1221
### Emergence of Progenitor Cell-Like Features in a CD20 Antibody-Resistant Subpopulation of Relapsed Mature B-Cell Lymphoma

[Speaker]Eiji Sugihara (Div. Gene Reg., Oncol. Innov. Center, Fujita Health Univ.／Open Facility Center, Fujita Health Univ.) [Co-author]Ryoko Kajiya (Hematology, Fujita Health Univ. School of Med.)、Yuko Hata (Open Facility Center, Fujita Health Univ.)、Naoe Goto (Hematology, Fujita Health Univ. School of Med.)、Chisako Iriyama (Hematology, Fujita Health Univ. School of Med.)、Kaori Ushida (Mol. Pathology, Fujita Health Univ. School of Med.)、Naoya Asai (Mol. Pathology, Fujita Health Univ. School of Med.)、Kazuyuki Shimada (Hematology, Nagoya Univ. School of Med.)、Hideyuki Saya (Div. Gene Reg., Oncol. Innov. Center, Fujita Health Univ.)、Akihiro Tomita (Hematology, Fujita Health Univ. School of Med.)

Relapse after molecular-targeted therapies remains a major challenge in cancer treatment. In this study, we focused on diffuse large B-cell lymphoma (DLBCL), a mature B-cell lymphoma with a high relapse rate of 30-40% after CD20 targeted therapy. Exome sequence revealed that relapsed DLBCL cell lines with low CD20 expression were classified as EZB subtype that carry mutations in epigenetic regulators. Compared to EZB-DLBCL lines with high CD20 expression, relapsed lines showed markedly reduced expression of mature B-cell markers such as CD20, BCL6 and CD40. In contrast, they exhibited high expression of progenitor B-cell markers, CD9, IGLL1, RAG1, and RAG2. Notably, their gene expression profiles resembled those of B-cell acute lymphoblastic leukemia. We confirmed that CD9 is highly expressed in lymphoma cells infiltrating the bone marrow of relapsed patients. To investigate the functional role of CD9, we generated CD9-knockout relapsed DLBCL cells. Although CD9 was not essential for proliferation, its loss significantly impaired infiltration ability. These findings suggest that subpopulation of DLBCL may evade CD20-targeted therapy by acquiring features of progenitor cells.

[Disclosure of COI by Responsible researcher] Lead presenter is the Responsible researcher.
[Lead presenter: Eiji Sugihara] ■Research funds from company: 科研費（20KK0364, 22K07179）

▲TOP

### [P-1222] I-P13-3
### Drug resistance (3)

2025 / 9 / 25 (Thu) 16:45-17:30 Room P/(Motenashi Dome) Room P

### P-1222
### NSC-3852 synergistically enhances the cytotoxicity of olaparib via ROS-induced DNA damage.

[Speaker]Yuka Sasaki (Dept. Pharmacology, Osaka Dental Univ.／Dept. Mol. Genomic Biomed. & CBMM, Grad.Sch. Biomed. Sci., Nagasaki Univ.) [Co-author]Takuma Inouchi (Dept. Pharmacology, Osaka Dental Univ.)、Chie Kise (Dept. Geriatric Dentistry, Osaka Dental Univ.)、Ryusuke Nakatsuka (Dept. Pharmacology, Osaka Dental Univ.)、Ryoji Taniguchi (Dept. Pharmacology, Osaka Dental Univ.)、Amane Inoue (Dept. Pharmacology, Osaka Dental Univ.)、Mitsuko Masutani (Dept. Mol. Genomic Biomed. & CBMM, Grad.Sch. Biomed. Sci., Nagasaki Univ.)、Tadashige Nozaki (Dept. Pharmacology, Osaka Dental Univ.)

PARP inhibitors are anti-cancer agents based on the concept of synthetic lethality and are used to treat patients with ***BRCAC1/2***-mutated breast, ovarian, pancreatic and prostate cancers, which exhibit homologous recombination repair deficiency. In this study, we screened enzyme inhibitors that enhance the efficacy of olaparib in oral squamous carcinoma cell lines, HSC-2 and SAS, with wild-type ***BRCAC1/2*** and identified the histone deacetylase inhibitor NSC-3852. The combination of NSC-3852 and olaparib synergistically reduced cell viability in HSC-2 and SAS cells. Treatment with N-acetyl-L-cysteine partially recovered cell survival after co-treatment with olaparib and NSC-3852. Moreover, the combination of NSC-3852 and olaparib rapidly upregulated γH2AX at 2 h, induced apoptosis at 6-24 h, and caused S-phase arrest at 24 h after treatment in HSC-2 cells. These results suggest that NSC-3852 enhances olaparib sensitivity by inducing apoptosis through accumulation of DNA damage via ROS generation. The combination of NSC-3852 and olaparib may be a useful therapeutic strategy for homologous recombination-proficient cancers.

[Disclosure of COI by Responsible researcher] Lead presenter is the Responsible researcher.
[Lead presenter: Yuka Sasaki] ■Research funds from company: 日本学術振興会 科学研究費助成事業 基盤研究(C) (22K10184, 25K13159)

### [P-1223] I-P13-3
### Drug resistance (3)

2025 / 9 / 25 (Thu) 16:45-17:30 Room P/(Motenashi Dome) Room P

---

### P-1223
### DNA damage enhances tumor immunogenicity via the Src-STAT1 pathway

[Speaker]Yuri Suzuki (Dept. Cancer Cell Biol., Univ. Toyama) [Co-author]Yue Zhou (Dept. Cancer Cell Biol., Univ. Toyama)、Atsushi Takahashi (Dept. Cancer Cell Biol., Univ. Toyama)、Soshi Nishibu (Dept. Cancer Cell Biol., Univ. Toyama)、Hiroaki Sakurai (Dept. Cancer Cell Biol., Univ. Toyama)、Satoru Yokoyama (Dept. Cancer Cell Biol., Univ. Toyama)

Immune checkpoint inhibitors have been approved for the treatment of various cancers; however, many patients do not experience sufficient clinical benefit due to therapeutic resistance. The most important factor contributing to this resistance is reduced tumor immunogenicity resulting from loss-of-function mutations in JAK1/2. Therefore, drugs that can enhance immunogenicity through JAK-independent mechanisms are being considered as potential combination therapies with immune checkpoint inhibitors. In this study, we focused on DNA-damaging chemotherapeutic agents and investigated their ability to enhance tumor immunogenicity independently of JAK signaling. We found that these agents induced the expression of IRF1, a key transcription factor involved in immunogenic responses, along with its downstream targets, PD-L1, PD-L2, and HLA-A/B/C. Notably, we confirmed that this induction of IRF1 was independent of JAK signaling but dependent on the Src-STAT1 pathway. These findings suggest that DNA-damaging chemotherapy enhances tumor immunogenicity via a Src-STAT1-dependent pathway across various cancer types, offering a promising strategy for combination with immune checkpoint inhibitors.

[Disclosure of COI by Responsible researcher] Lead presenter is not the Responsible researcher.
[Lead presenter: Yuri Suzuki] We have no financial relationships to disclose.
[Principal Researcher: Satoru Yokoyama] We have no financial relationships to disclose.

### [P-1224] I-P13-3
### Drug resistance (3)

2025 / 9 / 25 (Thu) 16:45-17:30 Room P/(Motenashi Dome) Room P

---

### P-1224
### Targeting HSF4 enhanced the Efficacy of Cabozantinib and Immune Checkpoint Inhibitors in Renal Cell Carcinoma

[Speaker]Saeki Saito (Department of Urology, Kagoshima University) [Co-author]Hirofumi Yoshino (Department of Urology, Kagoshima University)、Seiya Yokoyama (Department of Pathology, Kagoshima University)、Mitsuhiko Tominaga (Department of Urology, Kagoshima University)、Gang Li (Department of Urology, Kagoshima University)、Junya Arima (Department of Urology, Kagoshima University)、Ikumi Fukuda (Department of Urology, Kagoshima University)、Takashi Sakaguchi (Department of Urology, Kagoshima University)、Shuichi Tatarano (Department of Urology, Kagoshima University)、Hideki Enokida (Department of Urology, Kagoshima University)

This is an encore abstract presented at another conference. Recently, cabozantinib, a tyrosine kinase inhibitor (TKI) targeting MET, and immune checkpoint inhibitor (ICI) have been used to treat renal cell carcinoma (RCC). Previous reports showed that Heat shock factor 4 (HSF4) binds directly to the MET promoter to enhance MET expression in colon cancer cells, but its role in RCC is still unclear. This study aims to clarify the role of HSF4 and develop new therapeutic strategies for RCC. HSF4 expression was elevated in RCC tissue and its high expression was associated with poor overall survival significantly. Cell viabilities significantly decreased in HSF4-knockdown (KD) RCC cells. MET expression was decreased in the KD cells, whereas elevated in the sunitinib-resistant RCC cells. Cabozantinib in combination with HSF4-KD inhibited cell proliferation of the resistant cells more additively than each alone. In vivo, HSF4-KD combined with ICI showed additive suppression in tumor growth ($p<0.01$). These results indicated that HSF4 might play a crucial role in RCC. This strategy targeting HSF4 might enhance the efficacy of existing therapies such as cabozantinib and ICI.

[Disclosure of COI by Responsible researcher] Lead presenter is the Responsible researcher.
[Lead presenter: Saeki Saito] We have no financial relationships to disclose.

[P-1225] I-P13-3
Drug resistance (3)

2025 / 9 / 25 (Thu) 16:45-17:30 Room P/(Motenashi Dome) Room P

P-1225
### Extracellular domain mutations alter sensitivity to small molecule FLT3 kinase inhibitors

[Speaker]Kazuhiro Katayama (Lab. Mol. Target Therap., Sch. Pharm., Nihon Univ.)

Internal tandem duplication (ITD) mutations in the class III receptor tyrosine kinase FLT3 are major drivers of acute myeloid leukemia (AML). The development of FLT3 inhibitors (Quizartinib, Gilteritinib, Midostaurin) has improved outcomes in FLT3-ITD-positive cases. However, long-term treatment often leads to relapse and drug resistance. To investigate resistance mechanisms, we introduced random mutations into FLT3-ITD-positive cells using ethylnitrosourea, and established resistant cell lines by selection with Gilteritinib or Midostaurin. Sequencing revealed not only mutations in the kinase domain but also point mutations in the extracellular domain (ECD). Ba/F3 cells expressing the ECD-mutated FLT3-ITD showed resistance to Gilteritinib and Midostaurin but sensitivity to Quizartinib. Western blot analysis revealed that the ECD-mutated FLT3-ITD lacked glycosylation and was detected as an immature form of the protein. Quizartinib inhibits monomeric FLT3, while Gilteritinib and Midostaurin inhibit both monomeric and dimeric forms. These findings suggest that ECD mutations may affect dimerization, conferring resistance to Gilteritinib and Midostaurin.

[Disclosure of COI by Responsible researcher] Lead presenter is the Responsible researcher.
[Lead presenter: Kazuhiro Katayama] ■Research funds from company: 公的研究費

▲TOP

### [P-1235] I-P14-2
### Natural anticancer compounds (2)

2025 / 9 / 25 (Thu) 16:45-17:30 Room P/(Motenashi Dome) Room P

---

### P-1235
### Flower extract of ***Kalanchoe pinnata*** potentiates Bax-activated apoptosis of cancer cells and cell cycle arrest

[Speaker]Nanae Harashima (Div. Biometab. Chem., Univ. the Ryukyus Facult. Med.) [Co-author]Abdullah Aljwanieh (Div. Biometab. Chem., Univ. the Ryukyus Facult. Med.)

To overcome the drug resistance that many cancers acquire, there is a need to prove the anticancer effects of natural plant-derived ingredients and the drug efficacy of the ingredients. For this purpose, more elucidation of cell death mechanisms and complex signaling pathways will help solve challenges in prevention and treatment of cancer. In this study, we investigated the effects of different ***Kalanchoe pinnata*** flower extracts on human cancer cell death and its mechanism using several cancer cell lines. ***K. pinnata*** extracts significantly reduced cell viability with dose-dependent manner and reduced mitochondria membrane potential. Coculture with ***K. pinnata*** induced apoptotic cell death (decreased Bcl-2, and increased Bak, cleaved PARP, caspase-9 and 3), especially p18-Bax activation as well as cell cycle arrest. And then, apoptosis induced by ***K. pinnata*** was blocked with pan-caspase inhibitor. After treatment with ***K. pinnata*** extracts, cell cycle arrests were observed at G2/M or S phase, depending on the cell line. Our results indicate that ***K. pinnata*** extracts could have the potential to induce intrinsic apoptosis and cell cycle arrest in various cancers.

[Disclosure of COI by Responsible researcher] Lead presenter is the Responsible researcher.
[Lead presenter: Nanae Harashima] We have no financial relationships to disclose.

Poster Session

### [P-1236] I-P14-2
### Natural anticancer compounds (2)

2025 / 9 / 25 (Thu) 16:45-17:30 Room P/(Motenashi Dome) Room P

---

### P-1236
### Anti-tumor effect of the extracts from tea seed residue after oil extraction on gastrointestinal cancer.

[Speaker]Tetsushi Yamamoto (Patholo. and biomol. anal. lab., Faculty of Pharmacy, Kindai Univ.) [Co-author]Miyu Sakai (Patholo. and biomol. anal. lab., Faculty of Pharmacy, Kindai Univ.)、Mako Nakamura (Patholo. and biomol. anal. lab., Faculty of Pharmacy, Kindai Univ.)、Kuniko Mitamura (Patholo. and biomol. anal. lab., Faculty of Pharmacy, Kindai Univ.)、Atsushi Taga (Patholo. and biomol. anal. lab., Faculty of Pharmacy, Kindai Univ.／Antiaging center, Kindai Univ.)

Tea leaves are widely consumed as a daily beverage in worldwide. On the other hand, tea seed did not use to cultivate new tea leaves since grafting has become common for tea cultivation. Therfore, there is a problem about the environmental impact by disposing tea seed. Although the tea seed oil is used as material for cosmetics to resolve this problem, there is still problem about disposing of the tea seed residue after the oil extraction. In the present study, we investigate whether the extracts from tea seed residue after oil extraction is useful for gastrointestinal cancer treatment. The extracts from tea seed residue was prepared using 0.2% acetic acid contained 25% methanol. Then, we performed cell proliferation and scratch assays using the extracts from tea seed residue. Cell proliferaion and the migration ability of DLD-1 (colorectal cancer), OE33 (esophageal cancer) and PANC-1 (Pancreatic cancer) cells was significantly inhibited by the extracts from tea seed residue treatment. Therefore, the extracts from tea seed residue might be a useful source to develop novel pharmaceutical drugs for gastrointestinal cancer patients.

[Disclosure of COI by Responsible researcher] Lead presenter is the Responsible researcher.
[Lead presenter: Tetsushi Yamamoto] We have no financial relationships to disclose.

▲TOP

### [P-1237] I-P14-2
### Natural anticancer compounds (2)

2025 / 9 / 25 (Thu) 16:45-17:30 Room P/(Motenashi Dome) Room P

---

### P-1237
### Anticancer potentials of chaga and notoginseng against feline mammary tumor and canine hemangiosarcoma organoids

[Speaker]Yuta Shinohara (Pet Health & Food Div., Iskara Industry CO., LTD／Lab. of Vet. Pharmacology, Tokyo Univ. of Agri. and Tech.) [Co-author]Haru Yamamoto (Lab. of Vet. Pharmacology, Tokyo Univ. of Agri. and Tech.)、Yishian Liu (Lab. of Vet. Pharmacology, Tokyo Univ. of Agri. and Tech.)、Mohamed Elbadawy (Lab. of Vet. Pharmacology, Tokyo Univ. of Agri. and Tech.／Dept. of Pharmacology, Benha Univ.)、Kazuaki Sasaki (Lab. of Vet. Pharmacology, Tokyo Univ. of Agri. and Tech.)

Feline mammary tumors (FMT) are the third most common neoplasm in cats. Hemangiosarcoma (HAS) occurs at a higher frequency in dogs compared to humans. The prognosis of these tumors is poor due to their high malignancy, metastatic potential and therapy resistance.We previously revealed the anticancer effect of the traditional Chinese medical supplement Shibe-ria, which is a mixture of Chaga and notoginseng, against three-dimensional (3D) dog bladder cancer organoids.We also previously generated patient-derived FMT 2.5D organoids (FMTO) and canine HAS 2.5D organoids (CHASO). The 2.5D organoids exhibited similar characteristics to their original 3D organoids such as drug sensitivity and marker expression.In the present study, we examined the anticancer effect of Shibe-ria on FMTO and CHASO. Shibe-ria inhibited the cell viability of these organoids. Additionally, the invasion ability of FMTO and CHASO was significantly reduced with Shibe-ria treatment. While Shibe-ria significantly inhibited ***Cyclin D1*** expression in FMTO, it significantly suppressed ***Bax*** and ***Bcl2*** expression in CHASO.In conclusion, Shibe-ria may have potential to be a natural anticancer supplement in veterinary medicine.

[Disclosure of COI by Responsible researcher] Lead presenter is the Responsible researcher.
[Lead presenter: Yuta Shinohara] We have no financial relationships to disclose.

▲TOP

### [P-1238] I-P14-2
### Natural anticancer compounds (2)

2025 / 9 / 25 (Thu) 16:45-17:30 Room P/(Motenashi Dome) Room P

---

### P-1238
### Propolis reduces the colorectal cancer stemness

[Speaker]Nobuyuki Fujiwara (Lab. Drug Discov., Facult. Vet. Med., Okayama Univ. Sci.) [Co-author]Takashi Ito (Yamada Inst. Health Sci., Yamada Bee Company, Inc.)、Nobuaki Okumura (Yamada Inst. Health Sci., Yamada Bee Company, Inc.)、Noboru Fujitani (Okayama Univ. Sci.)

Background: Cancer stem cells (CSCs) represent a subset of cells within a tumor with stem cell characteristics,3 demonstrating resistance to anti-cancer drug. Therefore, it is necessary to develop therapies that target CSCs. Propolis is a resinous mixture obtained by honeybees from various plants. Among the various types of propolis, Brazilian green propolis has been reported to exhibit neuroprotective effects. Here, we investigated the effects of propolis on the CSC properties of colorectal cancer (CRC) cells.Results: To investigate whether propolis affect CSC properties, we performed colony formation assays. It was found that propolis treatment reduced the number of colonies in CRC cell lines. Additionally, the expression levels of ALDH1A1 and CD133, which are CSC markers in CRC, were also found to decrease following propolis treatment. Furthermore, we treated the CRC cells with propolis-containing compounds and examined their colony-forming ability. As a result, it was confirmed that propolis-containing compound X reduces colony-forming ability.Conclusion: It has been suggested that compound X, a component of propolis, reduces the stem cell properties of CRC cells.

[Disclosure of COI by Responsible researcher] Lead presenter is the Responsible researcher.
[Lead presenter: Nobuyuki Fujiwara] ■Research funds from company: 株式会社山田養蜂場

[P-1239] I-P14-2
Natural anticancer compounds (2)

2025 / 9 / 25 (Thu) 16:45-17:30 Room P/(Motenashi Dome) Room P

P-1239
### D-allose-induced glycolytic inhibition associates with an interaction of D-allose 6-phosphate and PGI

[Speaker]Asadur Rahman (Department of Pharmacology, Faculty of Medicine, Kagawa University) [Co-author]Nourin Jahan (Department of Pharmacology, Faculty of Medicine, Kagawa University)、Kento Kitada (Department of Pharmacology, Faculty of Medicine, Kagawa University)、Kazuya Akimitsu (Rare Sugar Research Center, Faculty of Agriculture, Kagawa University)、Akira Nishiyama (Department of Pharmacology, Faculty of Medicine, Kagawa University)

D-allose has demonstrated significant efficacy in reducing cell proliferation across various cancer cell lines. In recent investigations, we identified that D-allose induces a delayed cell cycle transition and inhibits glycolysis coinciding with alterations in phosphoglucose isomerase (PGI) activity in pancreatic ductal adenocarcinoma cells. Here, we aimed to elucidate the binding affinity between the active metabolite of D-allose, D-allose 6-phosphate (A6P) and PGI utilizing surface plasmon resonance (SPR) assay. PGI was immobilized on the sensor chip surface at a concentration of 50 $\mu$g/mL using sodium acetate buffers at pH 4.5 or higher. Upon injecting A6P over the sensor surface, we initially observed a weak binding response at a concentration of 6.67 mM. However, increasing the A6P concentration to 16.7 mM revealed a clear interaction with PGI. Consistent with our previous findings, these data suggest that A6P binds directly to PGI, leading to competitive inhibition of the enzyme and subsequent inhibition of glycolytic processes. This study underscores the potential of targeting the D-allose for developing therapeutic strategies against cancer.

[Disclosure of COI by Responsible researcher] Lead presenter is the Responsible researcher.
[Lead presenter: Asadur Rahman] We have no financial relationships to disclose.

### [P-1240] I-P14-2
### Natural anticancer compounds (2)

2025 / 9 / 25 (Thu) 16:45-17:30 Room P/(Motenashi Dome) Room P

### P-1240
### Anti-tumor effects of diosmetin in cholangiocarcinoma cells via inhibition of cancer cell growth and migration

[Speaker]Suchanya Boonruangtavorn (Cho-Kalaphruek Excellent Res. Projects, Faculty of Med., Khon Kaen Univ.) [Co-author]Waleeporn Kaewlert (Cho-Kalaphruek Excellent Res. Projects, Faculty of Med., Khon Kaen Univ.)、Supansa Buakaew (Cho-Kalaphruek Excellent Res. Projects, Faculty of Med., Khon Kaen Univ.)、Khin S. Win (Cho-Kalaphruek Excellent Res. Projects, Faculty of Med., Khon Kaen Univ.)、Panupong Mahalapbutr (Cho-Kalaphruek Excellent Res. Projects, Faculty of Med., Khon Kaen Univ.)、Chadamas Sakonsinsiri (Cho-Kalaphruek Excellent Res. Projects, Faculty of Med., Khon Kaen Univ.／Dept. of Biochem., Faculty of Med., Khon Kaen Univ.)、Pougrat Pakdeechote (Cholangiocarcinoma Res. Inst., Khon Kaen Univ.／Dept. of Physiol., Faculty of Med., Khon Kaen Univ.)、Raynoo Thanan (Cho-Kalaphruek Excellent Res. Projects, Faculty of Med., Khon Kaen Univ.)

Diosmetin, a natural bioflavonoid abundant in citrus fruits, demonstrates anti-tumor properties across multiple cancer types. Using SwissTargetPrediction, we identified CYP1B1 and ABCC1 as key molecular targets of diosmetin, leading to suppression of critical oncogenic pathways including PI3K and estrogen signaling. High expression of both genes correlated with poor prognosis in cholangiocarcinoma (CCA). We hypothesized that diosmetin might inhibit CCA aggressiveness. In vitro studies with CCA cell lines (KKU-100 and KKU-213A) confirmed that diosmetin treatment induced apoptosis, inhibited cell growth and migration, and increased cytotoxicity. Molecular docking studies supported these findings, demonstrating high binding affinity between diosmetin and both CYP1B1 and ABCC1 proteins. Our findings establish a novel link between diosmetin, CYP1B1, ABCC1, and CCA progression, identifying these proteins as potential therapeutic targets for CCA treatment.

[Disclosure of COI by Responsible researcher] Lead presenter is the Responsible researcher.
[Lead presenter: Suchanya Boonruangtavorn ] We have no financial relationships to disclose.

### [P-1241] I-P14-2
### Natural anticancer compounds (2)

2025 / 9 / 25 (Thu) 16:45-17:30 Room P/(Motenashi Dome) Room P

---

### P-1241
### New Pyrazoline Derivatives as a Potential of Antiproliferative Against Breast Cancer

[Speaker]Muchtaridi Muchtaridi (Faculty of Pharmacy, Universitas Padjadjaran, Indonesia.／Research Collaboration Centre for Radiopharmaceuticals Theranostic, BRIN, Indonesia.) [Co-author]Riska Prasetiawati (Faculty of Mathematics and Natural Science, Universitas Garut, Indonesia.)、Adel Zamri (Faculty of Mathematics and Natural Science, Universitas Riau, Indonesia.)、Mohamad N. Azmi (School of Chemical Sciences, Universiti Sains Malaysia, Malaysia.)、Shela Salsabila (Faculty of Pharmacy, Universitas Padjadjaran, Indonesia.)、Noval Herfindo (Faculty of Mathematics and Natural Science, Universitas Riau, Indonesia.)、Ritmaleni Ritmaleni (Faculty of Pharmacy, Universitas Gadjah Mada, Indonesia.)、Thierry Langer (Faculty of Life Sciences, University of Vienna, Austria.)

Tamoxifen is one of the oral anticancer medications used to treat breast cancer, however it has side effects that lower patients quality of life. To counteract this impact, some isolated natural compounds were altered. One substance extracted from the leaves of Eugenia aquea, chalcone, has been transformed into a pyrazoline-based substance. Following the in-silico analysis, the chalcone was chemically changed into pyrazoline derivatives. This pyrazoline derivatives were synthesized via a two-step reaction using chalcone as the starting material, followed by a cyclocondensation reaction with hydrazine. The pharmacophore fit score (%) for pyrazolines were 53.38, 53.36, and 53.68. Molecular docking study showed binding energy −10.09, −10.32, and −9.91, with GLU353 and ARG394 were recognized as the essential amino acid residues for pyrazolines 5a and 5b. ADMET predictions indicated favorable pharmacokinetic profiles. These pyrazolines were successfully synthesized and characterized by FTIR, HRMS, and NMR. The IC50 ($\mu$M) of pyrazolines 5a-c against MCF-7 cell lines were recorded as 13.17 ± 1.02, 16.42 ± 0.76, and 50.62 ± 6.84, which was in agreement with the in silico results.

[Disclosure of COI by Responsible researcher] Lead presenter is the Responsible researcher.
[Lead presenter: Muchtaridi Muchtaridi] We have no financial relationships to disclose.

### [P-1242] I-P14-2
### Natural anticancer compounds (2)

2025 / 9 / 25 (Thu) 16:45-17:30 Room P/(Motenashi Dome) Room P

---

### P-1242
### Regulation of Cancer Metabolism by Prodigiosin through Interaction with GLUT1

[Speaker]Tae-Hee Han (Korea Res. Inst. of Biosci. and Biotech. (KRIBB)／Korea Univ. of Sci. and Tech. (UST) [Co-author]Hyun-A Yang (Korea Res. Inst. of Biosci. and Biotech. (KRIBB)／Korea Univ. of Sci. and Tech. (UST))、Jinsu Kim (Korea Res. Inst. of Biosci. and Biotech. (KRIBB)／Korea Univ. of Sci. and Tech. (UST))、Joo-Young Im (Korea Res. Inst. of Biosci. and Biotech. (KRIBB))、Areum Seo (Korea Res. Inst. of Biosci. and Biotech. (KRIBB))、Tae-Su Han (Korea Res. Inst. of Biosci. and Biotech. (KRIBB)／Korea Univ. of Sci. and Tech. (UST))、Hyun Seung Ban (Korea Res. Inst. of Biosci. and Biotech. (KRIBB)／Korea Univ. of Sci. and Tech. (UST))

In contrast to normal cells, cancer cells predominantly utilize glycolysis for ATP generation under aerobic conditions, facilitating proliferation and metastasis. Targeting glycolysis is effective for cancer treatment. Prodigiosin (PDG) is a natural compound with various bioactivities, including anticancer effects. However, the precise action mechanisms and molecular targets of PDG, which has demonstrated efficacy in regulating glucose metabolism in cancer cells, remain elusive. Here, we aimed to investigate the anti-cancer activity of PDG and mechanism in cancer metabolism. PDG regulated cancer metabolism by suppressing intracellular ATP production rate and levels. It inhibited glycolysis and mitochondrial oxidative phosphorylation, impeding ATP production dependent on both glycolysis and mitochondrial respiration. Moreover, it inhibited cellular glucose uptake by directly interacting with glucose transporter 1 without affecting its mRNA or protein levels in HCT116 cells. We provide insights into the anti-cancer effects of PDG mediated via cancer metabolism regulation, suggesting its therapeutic potential for cancer. (Nat Prod Res. 2024 Jun 24:1-8.)

[Disclosure of COI by Responsible researcher] Lead presenter is not the Responsible researcher.
[Lead presenter: Tae-Hee Han] We have no financial relationships to disclose.
[Principal Researcher: Hyun Seung Ban] We have no financial relationships to disclose.

### [P-1249] I-P14-4
### Synthetic anticancer compounds (2)

2025 / 9 / 25 (Thu) 16:45-17:30 Room P/(Motenashi Dome) Room P

### P-1249
### Preclinical Efficacy Assessment of Brincidofovir against Glioblastoma

[Speaker]Shoji Nagatani (Translational Research Dept,, SymBio Pharmaceuticals, Ltd.) [Co-author]Seo Kyounghee (UCSF Brain Tumor Center Preclinical Therapeutic Testing Core)、Ozawa Tomoko (UCSF Brain Tumor Center Preclinical Therapeutic Testing Core)、Raleigh David (UCSF Brain Tumor Center Preclinical Therapeutic Testing Core)、Hazama Masatoshi (Translational Research Dept,, SymBio Pharmaceuticals, Ltd.)

The lipid-conjugated cidofovir (CDV), brincidofovir (BCV), is characterized by a lack of nephrotoxicity, as well as superior activity compared to CDV. The objectives of this study were to examine the preclinical efficacy of BCV against glioblastoma (GBM), and to identify biomarkers predicting the efficacy of BCV against GBM. In cytotoxicity assay, sub-micromolar BCV $IC_{50}$ values were obtained in three of 11 human GBM cell lines. In these highly sensitive cell lines, whole-transcriptome sequencing followed by differential gene expression analysis identified two genes, GPC6 and NYAP2, that predict high sensitivity to BCV. To examine the influence of MGMT expression on BCV sensitivity, monoclonal MGMT-overexpressing cell lines were established using lentiviral vector-based gene transfer followed by qPCR and western blot in U87MG cells. Compared to the parent MGMT-negative U87MG cells, MGMT-overexpressing cell lines exhibited lower $IC_{50}$ values. ***In vivo*** experiments demonstrated that peripherally administered BCV significantly decreased intracranial tumor growth and increased survival compared with the vehicle group. (Abstract #3043 in AACR 2025, presented with permission)

[Disclosure of COI by Responsible researcher] Lead presenter is not the Responsible researcher.
[Lead presenter: Shoji Nagatani] ■ employment by a company related to the presentation: シンバイオ製薬株式会社　■Profit of stock: シンバイオ製薬株式会社
[Principal Researcher: Hazama Masatoshi] ■employment by a company related to the presentation: シンバイオ製薬株式会社 ■Profit of stock: シンバイオ製薬株式会社

### [P-1250] I-P14-4
### Synthetic anticancer compounds (2)

2025 / 9 / 25 (Thu) 16:45-17:30 Room P/(Motenashi Dome) Room P

---

### P-1250
### Antitumor Effects and Mechanisms of Benzaldehyde, a Fig-Derived Compound, in Human Melanoma Cells

[Speaker]Yuri Nishimiya (Yokohama City Univ. Sch. of Med／Dept. Gastroenterology and Hepatology, Yokohama City Univ. Sch. of Med.) [Co-author]Shingo Kato (Dept. Gastroenterology and Hepatology, Yokohama City Univ. Sch. of Med.／Dept. Clin. Cancer genomics, Yokohama City Univ. Hosp.)

Nearly half a century ago, a researcher, inspired by a reference in the Bible, became interested in the anti-tumor properties of figs. His group extracted an anti-tumor compound from figs and identified it as benzaldehyde (BA). Although BA showed anti-tumor potential in various cell lines, its mechanism remained unclear. In this study, the anti-tumor effect of BA was evaluated both ***in vitro*** and ***in vivo*** using three human melanoma cell lines, and transcriptome analysis was conducted to investigate the underlying mechanisms. BA inhibited cell proliferation in all three melanoma cell lines ***in vitro***. ***In vivo***, intra-tumoral injection of BA suppressed tumor growth, whereas intraperitoneal administration showed no effect. RNA sequencing revealed that BA treatment led to the upregulation of several cytokines, including IL1$\alpha$ and IL1$\beta$. These findings suggest that BA exerts anti-tumor effects against melanoma cells, and that cytokine-mediated autocrine signaling may contribute to its mechanism of action. The lack of systemic efficacy might be due to insufficient intratumoral concentrations of BA. We will present these findings together with additional mechanistic analyses.

[Disclosure of COI by Responsible researcher] Lead presenter is not the Responsible researcher.
[Lead presenter: Yuri Nishimiya] We have no financial relationships to disclose.
[Principal Researcher: Shingo Kato] ■Lecture fee: コニカミノルタREALM

▲TOP

### [P-1251] I-P14-4
### Synthetic anticancer compounds (2)

2025 / 9 / 25 (Thu) 16:45-17:30 Room P/(Motenashi Dome) Room P

---

### P-1251
### Effects of siRNA on suppression of HPV16 E7 gene expression in cervical cancer

[Speaker]Mitsumasa Hasunuma (Waseda Univ., Dept. Biol.／Bio PhenoMA Inc) [Co-author]Etsuro Ito (Waseda Univ., Dept. Biol.／Bio PhenoMA Inc)

Cervical cancer is primarily caused by human papillomavirus (HPV) infection. If it is possible to suppress the expression of the E7 gene in HPV, it may lead to the suppression of cancer progression. Therefore, we transfected siRNA targeting HPV16 E7 into HPV16-positive cervical cancer cells and tested its efficacy. We also investigated whether there was an adjuvant effect when siRNA was used in combination with anti-cancer drugs. The results showed a significant decrease in E7 gene mRNA and E7 protein in cells treated with siRNA transfection. Furthermore, when siRNA was used with an anticancer drug (cisplatin), there was a significant decrease in E7 gene mRNA and a decreasing trend in E7 protein compared to the anticancer drug alone. These results suggest that siRNA may suppress cancer development in HPV-infected cells and play an adjuvant role in anticancer therapy.

[Disclosure of COI by Responsible researcher] Lead presenter is not the Responsible researcher.
[Lead presenter: Mitsumasa Hasunuma] ■ employment by a company related to the presentation: 株式会社BioPhenoMA
[Principal Researcher: Etsuro Ito] ■Research funds from company: 株式会社BioPhenoMA　■employment by a company related to the presentation: 株式会社BioPhenoMA　■Profit of stock: 株式会社BioPhenoMA　■Patent fee: 株式会社BioPhenoMA

### [P-1252] I-P14-4
### Synthetic anticancer compounds (2)

2025 / 9 / 25 (Thu) 16:45-17:30 Room P/(Motenashi Dome) Room P

---

### P-1252
### Effects of the number of ethylene glycol units on the efficacy of novel complex I inhibitor 9bw

[Speaker]Kyoko Fujiwara (Dept. Anatomy, Nihon Univ. Sch. Dent.) [Co-author]Motonori Tsuji (Inst. Molecular Function)、Kazuaki Sekimoto (Dept. Anatomy, Nihon Univ. Sch. Dent.)、Akiko Ohashi (Dept. Anatomy, Nihon Univ. Sch. Dent.)、Rei Fukui (Dept. Pathology, Nihon Univ. Sch. Dent.)、Hanaka Kinjo (Dept. Materials & Applied Chem., Coll. Sch. Tech., Nihon Univ.)、Mizuki Murakami (Dept. Materials & Applied Chem., Coll. Sch. Tech., Nihon Univ.)、Eri Nagasaki (Pediatr. Surg., Saitama Med. Cent., Jichi Med. Univ.)、Kazuhiro Ikeda (Div. System Med. & Gene Therap., Saitama Med. Univ.)、Kenichi Takayama (Systems Aging Sci. & Med., Tokyo Metropol. Inst. for Geriator. Gerontol.)、Satoshi Inoue (Div. System Med. & Gene Therap., Saitama Med. Univ.／Systems Aging Sci. & Med., Tokyo Metropol. Inst. for Geriator. Gerontol.)、Joe Otsuki (Dept. Materials & Applied Chem., Coll. Sch. Tech., Nihon Univ.)

4-Iodobiphenyl nonaethylene glycol ether (9bw) is a novel inhibitor for respiratory complex I (CI). Previously, we found that 9bw induces apoptosis in cancer cells but not in normal cells, indicating that 9bw is a potential antitumor agent with few adverse effects. However, the exact molecular mechanisms by which 9bw acts on CI are still elusive. 9bw is composed of a biphenyl unit and 9 ethylene glycol (EG) units, showing a little similarity with other known CI inhibitors. In this study, we tested the function of 9bw analogues, HIOP, I-BP-EG3, I-BP-EG6, and I-BP-EG12 containing 0, 3, 6, and 12 EG units, respectively. Our results showed that I-BP-EG6 and I-BP-EG12 inhibited CI to a similar extent as 9bw, whereas I-BP3 and HIOP showed no effect on them. However, HPLC analysis proved that both HIOP and I-BP-EG3 could be taken into mitochondria abundantly. Molecular docking analysis suggested that 4-iodo-biphenyl moiety of all compounds could bind at the entry portal of the Q-tunnel in CI, indicating that EG unit of HIOP and I-BP-EG3 may not be sufficiently long to reach the Q redox-active site. Thus, our results demonstrate that the number of EG units is crucial for 9bw activity.

[Disclosure of COI by Responsible researcher] Lead presenter is the Responsible researcher.
[Lead presenter: Kyoko Fujiwara] We have no financial relationships to disclose.

### [P-1253] I-P14-4
### Synthetic anticancer compounds (2)

2025 / 9 / 25 (Thu) 16:45-17:30 Room P/(Motenashi Dome) Room P

### P-1253
### Single-molecule observation system to identify NCYM inhibitors

[Speaker]Seigi Yamamoto (Lab. Evo. Oncol. Chiba Cancer Ctr. Res. Ins.) [Co-author]Ummi M. Zulfin (Lab. Evo. Oncol. Chiba Cancer Ctr. Res. Ins.／Grad. Sch. of Med. and Pharm. Sci., Chiba University)、Zafairo Shofa (Lab. Evo. Oncol. Chiba Cancer Ctr. Res. Ins.／Grad. Sch. of Med. and Pharm. Sci., Chiba University)、Daisuke Muto (Lab. Evo. Oncol. Chiba Cancer Ctr. Res. Ins.／Grad. Sch. of Med. and Pharm. Sci., Chiba University)、Kazuma Nakatani (Van Heesch group Princess Maxima Center for Pediatric Oncology)、Fumiaki Kono (Inst. for Quantum Life Sci., QST.)、Tatsuhito Matsuo (Faculty of Health Sci., Hiroshima International Univ.)、Koichi Matsuo (Res. Inst. for Synchrotron Radiation Science, Hiroshima University)、Taro Tamada (Inst. for Quantum Life Sci., QST.)、Yoshitaka Hippo (Lab. Evo. Oncol. Chiba Cancer Ctr. Res. Ins.)、Yusuke Suenaga (Lab. Evo. Oncol. Chiba Cancer Ctr. Res. Ins.)

NCYM is a human-specific ***de novo*** gene encoded in the antisense strand of MYCN. It encodes an oncoprotein that inhibits GSK3β, stabilizing MYCN in neuroblastoma cells. However, NCYM's flexible nature has hindered drug development. Our prior work developed a single-molecule observation system that visualizes NCYM's active conformation using a DNA aptamer, enhancing its interaction with GSK3β. This study aims to identify NCYM inhibitors from a library of 14 curcumin derivatives and 10 herbal compounds and examine their effects on NCYM structure and neuroblastoma cell survival. Purified NCYM proteins and DNA aptamers were fixed in DNA frames and analyzed by atomic force microscopy with each compound. Four compounds inhibited the binding of NCYM to DNA aptamers and significantly reduced neuroblastoma cell viability. Vacuum ultraviolet circular dichroism spectroscopy revealed that these compounds disrupt NCYM's secondary structure in the critical region for GSK3β inhibition. In conclusion, this system is a valuable tool for discovering NCYM inhibitors and possibly inhibitors for other flexible cancer-related proteins.

[Disclosure of COI by Responsible researcher] Lead presenter is the Responsible researcher.
[Lead presenter: Seigi Yamamoto] We have no financial relationships to disclose.

### [P-1264] I-P14-6
### Anticancer drug sensitivity/Drug resistance (2)

2025 / 9 / 25 (Thu) 16:45-17:30 Room P/(Motenashi Dome) Room P

---

### P-1264
### Chemotherapy-induced inflammation in oral cancer tissues alters the tumor microenvironment via E-selectin expression

[Speaker]Yukiko Kitahira (Osaka Univ.Dent.Oral&Maxillofacial Oncology and Surgery) [Co-author]Yoshihiro Morita (Osaka Univ.Dent.Oral&Maxillofacial Oncology and Surgery)、Emiko Ogasa (Osaka Univ.Dent.Oral&Maxillofacial Oncology and Surgery)、Ayano Nishimoto (Osaka Univ.Dent.Oral&Maxillofacial Oncology and Surgery)、Satoko Kishimoto (Osaka Univ.Dent.Oral&Maxillofacial Oncology and Surgery)、Hiroyasu Kameyama (Osaka Univ.Dent.Oral&Maxillofacial Oncology and Surgery)、Narikazu Uzawa (Osaka Univ.Dent.Oral&Maxillofacial Oncology and Surgery)

Inflammation in cancer tissues is induced also by biopsies, chemotherapy, and radiation therapy, and is closely related to the success of anticancer drug treatment. It has been reported that E-selectin, which is expressed in blood vessels in inflammation, is also expressed in cancer tissues and causes desmoplastic-like changes in the tumor microenvironment (TME). Desmoplastic-like changes have been reported as one of the factors of chemotherapy resistance. We investigated E-selectin expression in oral cancer and the relationship between inflammation induced by anticancer drugs and changes in the TME. First, immunohistological examination confirmed that E-selectin expression was increased before and after chemotherapy in human oral cancer tissues. When cisplatin was administered to tongue cancer-bearing mice, molecural analysis revealed that E-selectin expression was increased and the TME exhibited desmoplastic-like changes. Furthermore, when cisplatin was administered to tumor-bearing E-selectin knockout mice, desmoplastic-like changes were suppressed. These results suggest that E-selectin expression in oral cancer tissues may be a target that can suppress chemotherapy resistance.

[Disclosure of COI by Responsible researcher] Lead presenter is not the Responsible researcher.
[Lead presenter: Yukiko Kitahira] We have no financial relationships to disclose.
[Principal Researcher: Emiko Ogasa] We have no financial relationships to disclose.

### [P-1265] I-P14-6
### Anticancer drug sensitivity/Drug resistance (2)

2025 / 9 / 25 (Thu) 16:45-17:30 Room P/(Motenashi Dome) Room P

---

### P-1265
### Upregulation of ATF4 on LAT1 inhibition in pancreatic cancer cell lines

[Speaker]Yu Ma (Grad. Sch. of Med., The Univ. of Osaka) [Co-author]Suguru Okuda (Grad. Sch. of Med., The Univ. of Osaka)、Hiroki Okanishi (Grad. Sch. of Med., The Univ. of Osaka)、Minhui Xu (Grad. Sch. of Med., The Univ. of Osaka)、Chunhuan Jin (Grad. Sch. of Med., The Univ. of Osaka)、Ryuichi Ohgaki (Grad. Sch. of Med., The Univ. of Osaka／OTRI, The Univ. of Osaka)、Yoshikatsu Kanai (Grad. Sch. of Med., The Univ. of Osaka／OTRI, The Univ. of Osaka／WPI-PRIMe, The Univ. of Osaka／Ctr. for Society-Academia Collaboration, Fujita Health Univ.)

L-type amino acid transporter 1 (LAT1) has gained attention as a promising therapeutic target in cancer, yet how tumor cells adapt to its inhibition remains poorly understood. In this study, we examined how pancreatic ductal adenocarcinoma (PDAC) cells respond to LAT1 inhibition using nanvuranlat, a highly selective and potent LAT1 inhibitor. Through quantitative proteomic analysis, we observed a robust induction of the stress-related transcription factor ATF4, an indicator of amino acid deprivation, following LAT1 inhibition. Our results revealed that ATF4 activation is driven by the GCN2-eIF2$\alpha$ pathway, a central regulator of protein synthesis during nutrient stress. Functionally, ATF4 upregulation was found to attenuate the antiproliferative effects of nanvuranlat, enabling cells to adapt to the stress induced by LAT1 inhibition. Importantly, simultaneous inhibition of both LAT1 and ATF4 led to significantly greater suppression of PDAC cell growth than targeting LAT1 alone. These findings identify ATF4 as a key player in resistance to LAT1-targeted therapy and suggest that dual inhibition may offer a more effective approach in treating pancreatic cancer.

[Disclosure of COI by Responsible researcher] Lead presenter is not the Responsible researcher.
[Lead presenter: Yu Ma] We have no financial relationships to disclose.
[Principal Researcher: Yoshikatsu Kanai] We have no financial relationships to disclose.

### [P-1266] I-P14-6
### Anticancer drug sensitivity/Drug resistance (2)

2025 / 9 / 25 (Thu) 16:45-17:30 Room P/(Motenashi Dome) Room P

---

### P-1266
### Exploring a Novel Treatment for Drug-Resistant Prostate Cancer Based on the Susceptibility to Cell Death

[Speaker]Zhi Y. Zhang (Inst. of Biomed. Sci. and Eng., Tzu Chi Univ.) [Co-author]Hung J. Wang (Inst. of Biomed. Sci. and Eng., Tzu Chi Univ.／Inst. of Med. Sci., Tzu Chi Univ.)

Prostate cancer is the second leading cause of cancer–related death in men.With population aging, its incidence and mortality are increasing.Current treatments–mainly androgen deprivation therapy (ADT) and anti–androgen drugs–often result in castration–resistant prostate cancer (CRPC), a more aggressive and metastatic form.Although therapies delay progression, drug resistance remains a major issue, leading to relapse and death.Thus, novel strategies are urgently needed.
About 20–30% of CRPC cases have DNA damage repair (DDR) gene mutations, making PARP inhibitors a potential therapy.These drugs block DNA repair, causing cell death, but resistance limits their effect.This study explores ferroptosis, an iron- and ROS–dependent form of cell death, as an alternative.
Resistant cells showed reduced SLC7A11 and GPX4 expression, increasing sensitivity to ferroptosis.BODIPY 581/591 C11 staining and flow cytometry showed that arginine deprivation enhanced ferroptosis.Due to low ASS1 expression, cancer cells depend on external arginine.Arginine deprivation may thus be a promising approach against resistant CRPC.

[Disclosure of COI by Responsible researcher] Lead presenter is not the Responsible researcher.
[Lead presenter: Zhi Y. Zhang] We have no financial relationships to disclose.
[Principal Researcher: Hung J. Wang] We have no financial relationships to disclose.

▲TOP

### [P-1267] I-P14-6
### Anticancer drug sensitivity/Drug resistance (2)

2025 / 9 / 25 (Thu) 16:45-17:30 Room P/(Motenashi Dome) Room P

### P-1267
### Withdrawn

No Abstract

▲TOP

### [P-1268] I-P14-6
### Anticancer drug sensitivity/Drug resistance (2)

2025 / 9 / 25 (Thu) 16:45-17:30 Room P/(Motenashi Dome) Room P

### P-1268
### Decoding Spatiotemporal Complexity of the Tumor Microenvironment Driving Cancer Recurrence

[Speaker]Chia-Yi Su (Inst. of Biophotonics, National Yang Ming Chiao Tung University, Taiwan)

Tumor recurrence is a spatiotemporally complicated process modulated by the tumor microenvironment, yet it remains a major therapeutic gap. To understand how spatial and temporal cues drive drug tolerance, our lab aims to advance technologies that unravel the underlying mechanisms across various levels of complexity. At the human level, we have applied spatial transcriptomics to analyze post-neoadjuvant chemotherapy residual breast cancers, revealing how drug tolerance emerges during cell migration and treatment. At the tissue level, we have developed biomimetic 3D tumor models that recapitulate diverse microenvironmental contexts, such as tumor-vessel and tumor-immune interactions, enabling mechanistic studies of spatiotemporal evolutions of cell subpopulations. Together, we present a comprehensive approach to identify therapeutic strategies that prevent tumor recurrence and improve long-term outcomes.

[Disclosure of COI by Responsible researcher] Lead presenter is the Responsible researcher.
[Lead presenter: Chia-Yi Su] We have no financial relationships to disclose.

▲TOP

### [P-1269] I-P14-6
### Anticancer drug sensitivity/Drug resistance (2)

2025 / 9 / 25 (Thu) 16:45-17:30 Room P/(Motenashi Dome) Room P

---

### P-1269
### Modeling Cellular Plasticity During Tumor Invasion and Drug Treatment in a Biomimetic 3D Platform

[Speaker]Ya Hsin Hsu (Institute of Biophotonics, National Yang Ming Chiao Tung University, Taiwan) [Co-author]Chia Yi Su (Institute of Biophotonics, National Yang Ming Chiao Tung University, Taiwan)

Cellular plasticity that emerges during tumor progression is strongly influenced by microenvironmental cues, such as extracellular matrix (ECM) and drug treatment. A hallmark of invasive cancer is the directional migration of infiltrating cancer cells along aligned ECM fibers, frequently observed at drug therapy initiation and increasingly recognized as a contributor to therapeutic resistance. However, the interplay between ECM-guided invasion and drug treatment and their combined effect on cellular plasticity, which leads to drug tolerance, remains largely unexplored. In this study, we have developed an innovative 3D tumor model that integrates spatially aligned collagen fibers within a microchannel system coupled with a tumor spheroid formed in situ using the hanging drop technique. Our model enables the investigation of how the microenvironment modulates phenotypic switching and the emergence of drug-tolerant subpopulations during early phases of therapy.

[Disclosure of COI by Responsible researcher] Lead presenter is not the Responsible researcher.
[Lead presenter: Ya Hsin Hsu] We have no financial relationships to disclose.
[Principal Researcher: Ya Hsin Hsu] We have no financial relationships to disclose.

### [P-1270] I-P14-6
### Anticancer drug sensitivity/Drug resistance (2)

2025 / 9 / 25 (Thu) 16:45-17:30 Room P/(Motenashi Dome) Room P

---

### P-1270
### The Molecular Roles of Metformin: Potential to Reverse Docetaxel Resistance in Prostate Cancer Cells

[Speaker]Tzyh-Chyuan Hour (Depart. of Biochem., Sch. of Med., KMU, Kaohsiung, Taiwan) [Co-author]Yi-Ting Chen (Depart. of Biochem., Sch. of Med., KMU, Kaohsiung, Taiwan)、Ying-Fang Su (Depart. of Biochem., Sch. of Med., KMU, Kaohsiung, Taiwan)、His-Wen Chang (Depart. of Biochem., Sch. of Med., KMU, Kaohsiung, Taiwan)、Wen-Jeng Wu (Depart. of Urol., KMU Hosp., KMU, Kaohsiung, Taiwan)、Yeong-Shiau Pu (Depart. of Urol., NTU Hosp., Taipei, Taiwan)

Docetaxel was the first-line chemotherapeutic agent for castration resistant prostate cancer (CRPC) patients. Recent studies revealed metformin, a widely prescribed biguanide for type 2 diabetes was with a potential anti-cancer property in various cancers, including prostate cancer. In this study, we explored the chemoresistant mechanisms of metformin-induced cell death in prostate cancer (PCa). Our results showed PC/DX25 cells were more sensitive to metformin than PC3 cells, with the $IC_{50}$ value of PC3 being 3-fold higher than of PC/DX25. Metformin inhibited mitochondrial complex activity and activated the expression of phospho-AMPK (T172) in PCa cells. Metformin significantly induced S-phase arrest in PC/DX25 cells by downregulating CDK2 and cyclin A2. Metformin promoted the production of reactive oxygen species and specifically activated endoplasmic reticulum (ER) stress via the PERK pathway in PC/DX25 cells. Interestingly, we also found Metformin induced DNA damage by inhibition of DNA repair-related proteins in PC/DX25. These findings suggested metformin may serve as a promising therapeutic agent for treatment of docetaxel-resistant prostate cancer in future clinical.

[Disclosure of COI by Responsible researcher] Lead presenter is the Responsible researcher.
[Lead presenter: Tzyh-Chyuan Hour] We have no financial relationships to disclose.

▲TOP

### [P-1271] I-P14-6
### Anticancer drug sensitivity/Drug resistance (2)

2025 / 9 / 25 (Thu) 16:45-17:30 Room P/(Motenashi Dome) Room P

---

### P-1271
### Befotertinib restores chemosensitivity by inhibiting ABCB1-mediated drug efflux in resistant cancer cells

[Speaker]Yen Ching Li (Grad. Inst. of Biomed. Sci., Chang Gung Univ., Taiwan) [Co-author]Chung Pu Wu (Grad. Inst. of Biomed. Sci., Chang Gung Univ., Taiwan／Dept. of Physiol. & Pharmacology, Chang Gung Univ., Taiwan／Mol. Med. Res. Ctr., Chang Gung Univ., Taiwan／Dept. of Obstetrics & Gynecol., Taipei Chang Gung Memorial Hosp., Taiwan)

Chemotherapy remains a cornerstone of cancer treatment; however, its clinical effectiveness is frequently compromised by the development of multidrug resistance (MDR), a phenomenon predominantly driven by the overexpression of ATP-binding cassette (ABC) transporter ABCB1 (P-glycoprotein). Given the absence of clinically approved synthetic inhibitors capable of effectively reversing ABCB1-mediated MDR, drug repurposing represents a practical and promising alternative strategy. In this study, we identified befotertinib–a third-generation epidermal growth factor receptor (EGFR) tyrosine kinase inhibitor approved in China, as a direct inhibitor of ABCB1 efflux function. Befotertinib significantly enhanced cytotoxic drug-induced apoptosis and resensitized multidrug-resistant cancer cells in a concentration-dependent manner. Mechanistic investigations using vanadate-sensitive ATPase assays, complemented by molecular docking analysis, revealed key interactions between befotertinib and critical residues within the substrate-binding pocket of ABCB1. These findings support the potential of befotertinib as a candidate for combination therapies targeting tumors with elevated ABCB1 expression.

[Disclosure of COI by Responsible researcher] Lead presenter is not the Responsible researcher.
[Lead presenter: Yen Ching Li] We have no financial relationships to disclose.
[Principal Researcher: Chung Pu Wu] We have no financial relationships to disclose.

▲TOP

### [P-1272] I-P14-6
### Anticancer drug sensitivity/Drug resistance (2)

2025 / 9 / 25 (Thu) 16:45-17:30 Room P/(Motenashi Dome) Room P

---

### P-1272
### An epigenetic regulator drives regrowth of drug-tolerant persisters in colorectal cancer.

[Speaker]Roberto Coppo (Dept. Clin. Bio-Resource Res. Dev. Grad. Sch. Med. Kyoto University) [Co-author]Masahiro Inoue (Dept. Clin. Bio-Resource Res. Dev. Grad. Sch. Med. Kyoto University)

Drug-tolerant persisters (DTPs) are a subpopulation of tumor cells that survive therapy through reversible, non-genetic adaptive mechanisms. DTPs are in a slow-growing state after treatment and revert to a proliferative state upon therapy cessation, leading to tumor regrowth. Yet, the molecular mechanisms regulating their cellular plasticity remain unclear. A panel of patient-derived colorectal cancer (CRC) organoids exhibiting DTP phenotype under serial 5-FU treatments was selected. RNA-seq analyses were performed to profile both DTP and early re-growing cells. An epigenetic modulator (Gene X) was identified, which exhibited higher expression in early re-growing cells and low expression in untreated organoids and CRC patient samples. Gene X was unrelated to DNA damage response, druggable, and non-toxic in databases. GeneX inhibition alone had minimal effect on CRC organoid growth; however, its combination with chemotherapy attenuated DTP cell regrowth in both in vitro and in vivo models. These findings suggest that targeting the transition from the DTP state to the regrowth phase could improve therapeutic outcomes in CRC.

[Disclosure of COI by Responsible researcher] Lead presenter is not the Responsible researcher.
[Lead presenter: Roberto Coppo] We have no financial relationships to disclose.
[Principal Researcher: Masahiro Inoue] We have no financial relationships to disclose.

[P-1273] I-P14-6
Anticancer drug sensitivity/Drug resistance (2)

2025 / 9 / 25 (Thu) 16:45-17:30 Room P/(Motenashi Dome) Room P

P-1273
### Fatty Acid Signaling Promotes EGFR-Dependent Metastasis and Cisplatin Resistance in Hyperlipidemia-associated HNSCC

[Speaker]HSIN-MIN HUNG (Dept. of Pharm., NCKU) [Co-author]Shih-Hung Chan (Dept. of Pharm., NCKU)、Jing-Ting Chiou (Dept. of Pharm., NCKU)、Liang-Yi Chen (Dept. of Pharm., NCKU)、Wei-Ru Lin (Dept. of Pharm., NCKU)、Hsin-Fang Kao (Dept. of Pharm., NCKU)、Ting-Wei Chang (Dept. of Pharm., NCKU)、Bing-Han Chung (Dept. of Pharm., NCKU)、Wen-Chang Chang (Dept. of Pharm., NCKU)、Ben-Kuen Chen (Dept. of Pharm., NCKU)

Metabolic disorders have become increasingly prevalent in recent years, frequently associated with dysregulation of lipid metabolism. However, the molecular mechanisms in regulating the impact of hyperlipidemia on HNSCC remain unclear. This study investigated the effects of combined EGF and oleic acid (OA) treatment on metastasis and chemoresistance in HNSCC. Our results showed that EGF combined OA increased invasive capacity, accompanied by upregulation of PD-L1 and EMT markers. PD-L1 knockdown partially reversed these effects, while gefitinib fully restrained them. Co-treatment also reduced cisplatin-induced apoptosis; however, only gefitinib, not PD-L1 knockdown, could restore cisplatin sensitivity, indicating EGFR dependency in the chemoresistance mechanism. Interestingly, OA alone reduced apoptosis, suggesting that fatty acid metabolism may play an independent role in drug resistance. Mechanistically, OA treatment enhanced CPT1A expression, ROS levels and EGFR phosphorylation, which were inhibited by NAC. Taken together, our findings demonstrate the significance of PD-L1 and EGFR signaling in HNSCC metastasis and the chemoresistance in hyperlipidemia-associated HNSCC.

[Disclosure of COI by Responsible researcher] Lead presenter is the Responsible researcher.
[Lead presenter: HSIN-MIN HUNG] We have no financial relationships to disclose.

▲TOP

### [P-1279] I-P16-2
### Radiosensitivity/Efficacy enhancement

2025 / 9 / 25 (Thu) 16:45-17:30 Room P/(Motenashi Dome) Room P

### P-1279
### Changes in Radiosensitivity During Neuronal Differentiation of Human iPS Cell-Derived Neural Progenitor Cells

[Speaker]Takuto Nii (Kindai University Atomic Energy Research Institute) [Co-author]Toshiro Matsuda (Kindai University Atomic Energy Research Institute)

While the radiosensitivity of human tissues and organs has been studied, the radiosensitivity of differentiated cells remains poorly understood. Although differentiated cells are generally considered less radiosensitive than undifferentiated cells, how radiosensitivity changes during differentiation is still unclear.In this study, we aimed to evaluate changes in radiosensitivity during neuronal differentiation using human iPS cell-derived neural progenitor cells. Neurons were irradiated with X-rays at both early and post-differentiation stages. The survival rates were higher in the post-differentiation group compared to the early differentiation group. In the early differentiation irradiation group, axon and dendrite formation was suppressed, whereas the post-differentiation irradiation group exhibited a temporary loss of the neural network, followed by its restoration.Since radiosensitivity is believed to be linked to DNA repair, we focused on double-strand DNA breaks and compared the formation and resolution of $\gamma$-H2AX foci induced by X-irradiation between early and post-differentiated cells. The relationship between DNA repair capacity and radiosensitivity is also discussed.

[Disclosure of COI by Responsible researcher] Lead presenter is not the Responsible researcher.
[Lead presenter: Takuto Nii] We have no financial relationships to disclose.
[Principal Researcher: Toshiro Matsuda] We have no financial relationships to disclose.

▲TOP

[P-1280] I-P16-2
Radiosensitivity/Efficacy enhancement

2025 / 9 / 25 (Thu) 16:45-17:30 Room P/(Motenashi Dome) Room P

---

### P-1280
### SQAP, a Novel Radiation Sensitizer developed in Japan: From Veterinary Approval to Human Cancer Therapy Potential

[Speaker]Ayumi Hongo (M.T.3, Inc.) [Co-author]Daiki Kakinoki (Dept of Radiation Oncology, The Univ. Osaka Grad. Sch. Med.)、Kazumasa Minami (Dept of Radiation Oncology, The Univ. Osaka Grad. Sch. Med.)、Takaomi Fukuhara (M.T.3, Inc.)

Sulfoquinovosyl-acylpropanediol (SQAP) is a sulfur-containing glycolipid discovered in the intestines of sea urchins and red algae. SQAP, currently in development as a pharmaceutical for human cancer, was approved in 2023 as the world's first veterinary radiosensitizer. Derived from natural sources, SQAP is characterized by low toxicity and minimal side effects. It enhances the efficacy of To date, SQAP has been administered in over 400 companion animals, with significant tumor r radiotherapy by transiently increasing the partial pressure of oxygen within tumor tissues regression reported. Beyond its radiosensitizing effects, previous studies have shown that SQAP also inhibits angiogenesis and suppresses the expression of hypoxia-inducible factor 1-alpha (HIF-1 $\alpha$). More recently, SQAP has been found to inhibit the invasive and migratory capabilities in human osteosarcoma cells, suggesting its potential role in improving the tumor microenvironment and impacting cancer progression. In this presentation, we will report on both clinical experiences in dogs and cats and in vitro studies using human cancer cells to discuss the translational potential of SQAP for human application.

[Disclosure of COI by Responsible researcher] Lead presenter is not the Responsible researcher.
[Lead presenter: Ayumi Hongo] ■ employment by a company related to the presentation: 株式会社エム・ティー・スリー
[Principal Researcher: Takaomi Fukuhara] ■employment by a company related to the presentation: 株式会社エム・ティー・スリー

### [P-1281] I-P16-2
### Radiosensitivity/Efficacy enhancement

2025 / 9 / 25 (Thu) 16:45-17:30 Room P/(Motenashi Dome) Room P

---

### P-1281
### Effects of reoxygenation and fractionated X-ray irradiation on DNA damage and repair in osteosarcoma cells

[Speaker]Hiroko Ikeda (Rad Oncol., Dept. of Life Sci., Kindai Univ.) [Co-author]Yuka Amano (Mol Oncol., Dept. of Life Sci., Kindai Univ.)、Koki Hara (Mol Oncol., Dept. of Life Sci., Kindai Univ.)、Toshifumi Tsujiuchi (Mol Oncol., Dept. of Life Sci., Kindai Univ.)

Reoxygenation in radiation therapy is closely associated with the acquisition of radioresistance due to hypoxic stress in tumor core. This study aimed to elucidate DNA damage and repair in osteosarcoma MG-63 cells by altering the timing of reoxygenation and fractionated irradiation. MG-63 cells were cultured under hypoxic conditions (0.5% $O_2$ for 3 hours) using nBIONIX kits, and responses were compared to those under normoxia. After 2 Gy irradiation in hypoxia, γH2AX intensity at 6 hours post-irradiation was significantly reduced compared to normoxia. However, irradiation following reoxygenation resulted in DNA damage levels similar to those under normoxic conditions. At 24 hours post-irradiation, 53BP1 intensity decreased under both hypoxic and reoxygenated conditions, suggesting that distinct repair pathways may be activated. These results indicate that hypoxia impairs DNA repair, while reoxygenation enhances both DNA damage induction and repair activity, making cells more radiosensitive than in normoxia. Furthermore, hypoxia-induced radioresistance may be attenuated by reoxygenation, and further studies are needed to clarify its combined effect with fractionated irradiation.

[Disclosure of COI by Responsible researcher] Lead presenter is the Responsible researcher.
[Lead presenter: Hiroko Ikeda] We have no financial relationships to disclose.

[P-1282] I-P16-2
Radiosensitivity/Efficacy enhancement

2025 / 9 / 25 (Thu) 16:45-17:30 Room P/(Motenashi Dome) Room P

P-1282

### The early increase of long non-coding RNA SNHG12 expression after boron neutron capture therapy

[Speaker]Zhongming Gao (Dept. Mol. & Genomic Biomed. & CBMM, Grad. Sch. Biomed. Sci.Nagasaki Univ.) [Co-author]Ying Tong (Dept. Mol. & Genomic Biomed. & CBMM, Grad. Sch. Biomed. Sci.Nagasaki Univ.)、Shoji Imamichi (Dept. Mol. & Genomic Biomed. & CBMM, Grad. Sch. Biomed. Sci.Nagasaki Univ.／Central Radioisotope Div. Natl. Cancer Ctr. Res. Inst. ／Division of BNCT, EPOC, National Cancer Center)、Yu Sanada (Institute for Integrated Radiation and Nuclear Science, Kyoto University)、Minoru Suzuki (Institute for Integrated Radiation and Nuclear Science, Kyoto University)、Masamichi Ishiai (Central Radioisotope Div. Natl. Cancer Ctr. Res. Inst. ／Division of BNCT, EPOC, National Cancer Center)、Shinichiro Masunaga (Institute for Integrated Radiation and Nuclear Science, Kyoto University)、Mitsuko Masutani (Dept. Mol. & Genomic Biomed. & CBMM, Grad. Sch. Biomed. Sci.Nagasaki Univ.／Central Radioisotope Div. Natl. Cancer Ctr. Res. Inst. ／Division of BNCT, EPOC, National Cancer Center)

Boron neutron capture therapy (BNCT) is an innovative cancer treatment that selectively destroys tumor cells using thermal neutrons and the $^{10}$B-containing compound 4-boronophenylalanine (BPA). While BNCT is approved for head and neck cancers in Japan, early prediction of treatment efficacy remains a major challenge. This study focuses on the long non-coding RNA ***SNHG12*** as a potential biomarker for BNCT sensitivity. We previously found that ***SNHG12*** expression in SAS cells increased significantly after gamma-irradiation and treatment with methylmethanesulfonate within 24 hrs. Experiments using oral squamous carcinoma SAS cells revealed a rapid upregulation of ***SNHG12*** following BNCT. Functional assays with siRNA and plasmid vectors demonstrated that ***SNHG12*** expression levels modulate BNCT sensitivity, with similar trends observed in HSC3 and HeLa cells. Given its known role in microRNA (miRNA) regulation, we hypothesize that ***SNHG12*** acts as a competitive endogenous RNA, sequestering miRNAs and thereby influencing miRNA–mRNA networks involved in BNCT response. The function of ***SNHG12*** in responses after BNCT in comparison with other types of DNA damage will be discussed.

[Disclosure of COI by Responsible researcher] Lead presenter is not the Responsible researcher.
[Lead presenter: Zhongming Gao] We have no financial relationships to disclose.
[Principal Researcher: Mitsuko Masutani] We have no financial relationships to disclose.

### [P-1283] I-P16-2
### Radiosensitivity/Efficacy enhancement

2025 / 9 / 25 (Thu) 16:45-17:30 Room P/(Motenashi Dome) Room P

---

### P-1283
### Fusion between Macrophages and Cancer Cells Contributes to Radiation-Induced Malignant Progression by Activating SIRPb

[Speaker]Hui Ju Chang (National Institute of Cancer Research, Taiwan／Taipei Medical University, Taiwan／National Cheng Kung University, Taiwan) [Co-author]Yi-Chieh Tsai (National Institute of Cancer Research, Taiwan)、Ming-Chieh Hsin (National Institute of Cancer Research, Taiwan)、Ming Yao (National Taiwan University Hospital, Taiwan)、Shieh-Sheng Jiang (National Institute of Cancer Research, Taiwan)、Fang-Yu Tsai (National Institute of Cancer Research, Taiwan)、Yen-Lin Huang (National Taiwan University Hospital, Taiwan)、Shyh-Chi Lou (National Taiwan University Hospital, Taiwan)、I-Shiow Jan (National Taiwan University Hospital, Taiwan)、Yi-Lin Liu (National Institute of Cancer Research, Taiwan)、Ruei-Chin Liu (National Institute of Cancer Research, Taiwan)、Tze-Sing Huang (National Institute of Cancer Research, Taiwan)

Fusion between cancer cells and myeloid cells has been shown to enhance aggressive cancer phenotypes. The role of cancer-macrophage fusion hybrids to radiation-induced malignant progression remains unclear. We established dual fluorescence and Cre-loxP systems for in vitro and murine models to quantify fusion phenomenon. Transcriptomic analyses were conducted to identify differentially expressed signal pathways. Significant upregulation of the signal regulatory protein (SIRP) pathway was noted in fusion hybrids. Genetic or pharmacological inhibition of DAP12 and Pyk2 reduced fusion events and suppressed tumor growth. Radiation exposure was found to enhance the frequency of cancer-macrophage fusion hybrids mediated by TNFa, which physically interacts with SIRPb to activate the DAP12/Pyk2 pathway. Neutralizing TNFa or inhibiting SIRPb reduced cancer-macrophage fusion hybrids and mitigated tumor progression post-radiation.Cancer-macrophage fusion hybrids play a critical role in radiation-induced malignant progression. Targeting SIRPb/DAP12 provides a novel therapeutic approach to suppress radiation-enhanced cancer progression.

[Disclosure of COI by Responsible researcher] Lead presenter is the Responsible researcher.
[Lead presenter: Hui Ju Chang] We have no financial relationships to disclose.

### [P-1284] I-P16-2
### Radiosensitivity/Efficacy enhancement

2025 / 9 / 25 (Thu) 16:45-17:30 Room P/(Motenashi Dome) Room P

---

### P-1284
### Investigation of IL27RA as a potential target for radiosensitization

[Speaker]Zongxiang Zhang (Dept. Molecular Genomic Biomedicine, CBMM, GSBS, Nagasaki Univ.) [Co-author]Yongkang Shao (Dept. Molecular Genomic Biomedicine, CBMM, GSBS, Nagasaki Univ.)、Zhongming Gao (Dept. Molecular Genomic Biomedicine, CBMM, GSBS, Nagasaki Univ.)、Barkha Saraswat (Dept. Molecular Genomic Biomedicine, CBMM, GSBS, Nagasaki Univ.)、Abdullo Abduganiev (Dept. Molecular Genomic Biomedicine, CBMM, GSBS, Nagasaki Univ.)、Ying Tong (Dept. Molecular Genomic Biomedicine, CBMM, GSBS, Nagasaki Univ.)、Mitsuko Masutani (Dept. Molecular Genomic Biomedicine, CBMM, GSBS, Nagasaki Univ.)

The development of radiosensitization methods for cancer with low toxicity to normal cells is considered to contribute to improving cancer radiotherapy. Based on this concept, diverse molecular targets for radiosensitization have been investigated. From comprehensive screening of radiosensitization targets using shRNA library, we have been focusing ***IL27RA*** (interleukin 27 receptor subunit alpha) gene. IL27RA transduces signaling of cytokine IL27 and show both anti-tumor and protumor effects. IL6 and TGFβ expression was negatively regulated by IL27RA cascade. IL27RA was expressed at higher levels in lung cancer, glioblastoma, and breast cancer. By suppressing the expression of ***IL27RA*** using siRNA, it was confirmed that the radiosensitization was observed in lung cancer cell lines including adenocarcinoma A549 cells. The radiosensitization effect was also observed in oral squamous cell carcinoma SAS cells and cervical cancer HeLa cells. ***IL6***, ***IL8*** and ***TGFβ*** mRNA was induced after γ-irradiation in A549 ***IL27RA*** knockdown cells. Further mechanistic analysis was carried out using RNAseq. The functional inhibition of IL27RA may be useful for radiosensitization in particular cancer cells.

[Disclosure of COI by Responsible researcher] Lead presenter is not the Responsible researcher.
[Lead presenter: Zongxiang Zhang] We have no financial relationships to disclose.
[Principal Researcher: Mitsuko Masutani] We have no financial relationships to disclose.

### [P-1293] I-P20-1
### Immune Regulation & Tumor Microenvironment Dynamics in Cancer

2025 / 9 / 25 (Thu) 16:45-17:30 Room P/(Motenashi Dome) Room P

---

### P-1293
### A multi-task learning framework for TCR-antigen binding prediction guided by MHC-derived motif signature

[Speaker]Po-yuan Chen (Inst. of Bioinfo. and Sys. Biol., NYCU／Inst. of Biomed. Sci., Academia Sinica) [Co-author]Tai-Ming Ko (Inst. of Bioinfo. and Sys. Biol., NYCU／Inst. of Biomed. Sci., Academia Sinica／Dept. of Biol. Sci. and Tech., NYCU)

Accurate prediction of peptide-MHC-T cell receptor (pMHC-TCR) binding is critical for neoantigen identification in cancer immunotherapy. Existing models often overlook allele-specific binding preferences, typically representing MHCs solely by their amino acid sequences. To address this limitation, we propose a multi-task learning framework guided by MHC binding motif signatures. Rather than training on individual alleles, our method derives motif signatures from binding affinity assays and treats MHCs with similar motifs as tasks in the framework. This structure enables the model to learn conserved presentation patterns shared across MHCs with similar binding motifs, while adapting to allele-specific features influencing T cell recognition. The multi-task learning setup improves generalization in zero-shot settings. When evaluated on a neoantigen-specific TCR dataset, our method outperforms existing tools. By combining motif-guided signals with task-adaptive learning, it provides a biologically grounded and robust framework for TCR specificity prediction.

[Disclosure of COI by Responsible researcher] Lead presenter is the Responsible researcher.
[Lead presenter: Po-yuan Chen] We have no financial relationships to disclose.

▲TOP

### [P-1294] I-P20-1
### Immune Regulation & Tumor Microenvironment Dynamics in Cancer

2025 / 9 / 25 (Thu) 16:45-17:30 Room P/(Motenashi Dome) Room P

### P-1294
### Modeling Early Transcriptional Reprogramming of $CD8^+$ T Cells in Melanoma Following Anti-CTLA-4 Immunotherapy

[Speaker]Jing Rong Wang (Dept. of Biol. Sci. & Tech., Coll. of Eng. Biosci., NYCU) [Co-author]Tai Ming Ko (Dept. of Biol. Sci. & Tech., Coll. of Eng. Biosci., NYCU／Inst. of Bioinf. & Syst. Biol., Coll. of Eng. Biosci., NYCU／Intell. Drug Syst., NYCU／Inst. of Biomed. Sci., Academia Sinica／Sch. of Pharm., Coll. of Pharm., KMU)

Immune checkpoint inhibitors (ICIs) have advanced melanoma treatment, but predicting individual responses remains difficult. While many studies describe post-treatment T cell changes, few model transcriptional reprogramming from baseline. We developed a predictive framework to simulate early gene expression changes in $CD8^+$ T cells following anti-CTLA-4 therapy. Using public single-cell RNA-seq data, we focused on early activated $CD8^+$ T cells from patients receiving anti-CTLA-4 monotherapy. A variational autoencoder (VAE) model was trained on 2,310 cells from six patients (baseline and follow-up-1) and validated on 2,385 cells from three other patients (follow-up-2 to -4). The model predicts future transcriptomic states using only baseline features. To assess biological relevance, we identified differentially expressed genes (DEGs) across timepoints for each treatment (anti-CTLA-4, anti-PD-1, combination), extracted treatment- and stage-specific signatures, and compared them to predicted changes. The model recapitulates key activation and cytotoxic programs, supporting simulation of treatment-induced T cell reprogramming.

[Disclosure of COI by Responsible researcher] Lead presenter is not the Responsible researcher.
[Lead presenter: Jing Rong Wang] We have no financial relationships to disclose.
[Principal Researcher: Tai Ming Ko] We have no financial relationships to disclose.

▲TOP

### [P-1295] I-P20-1
### Immune Regulation & Tumor Microenvironment Dynamics in Cancer

2025 / 9 / 25 (Thu) 16:45-17:30 Room P/(Motenashi Dome) Room P

---

### P-1295
### Mapping TLS Architecture Across Cancer Types: Key Roles of Dendritic Cells and Chemokines in Their Formation

[Speaker]Ange Yan (Dept. Comp. Biol. Med. Sci., Grad. Frontier Sci., Univ. Tokyo) [Co-author]Tatsuhiko Tsunoda (Dept. Comp. Biol. Med. Sci., Grad. Frontier Sci., Univ. Tokyo／Dept.Biosci., Grad. Sch. Sci., Univ. Tokyo)、Artem Lysenko (Dept.Biosci., Grad. Sch. Sci., Univ. Tokyo)

This study characterizes the spatial structure of tertiary lymphoid structures across four cancer types using spatial transcriptomics. TLS regions were annotated by pathologists, and cell states were identified with EcoTyper. Spatial gene expression patterns were assessed using Morans I and Geographically Weighted Models.TLS regions were enriched in cell states belonging to CE10, both associated with high immune activity, and showed distinct profiles from nonTLS areas. Differences in cell state composition and gene expression patterns were observed between mature and immature TLS. Pathway analysis revealed immune activation in TLS, with mature TLS enriched for MHC class2 pathways and immature TLS associated with fibroblast related signaling. Notably, TLS with CE10 enrichment were observed across cancer types, except prostate cancer.

[Disclosure of COI by Responsible researcher] Lead presenter is the Responsible researcher.
[Lead presenter: Ange Yan] We have no financial relationships to disclose.

Poster Session

### [P-1296] I-P20-1
### Immune Regulation & Tumor Microenvironment Dynamics in Cancer

2025 / 9 / 25 (Thu) 16:45-17:30 Room P/(Motenashi Dome) Room P

---

### P-1296
### Mutation-Driven Heterogeneity of Tumor Microenvironment: Insights into Cancer Immune Evasion Mechanisms

[Speaker]Yuying Gao (Dept. Comp. Biol. Med. Sci., Grad. Frontier Sci., Univ. Tokyo) [Co-author]Artem Lysenko (Dept. Biosci., Grad. Sch. Sci., Univ. Tokyo／RIKEN Ctr. for Integrative Med. Sci.)、Tatsuhiko Tsunoda (Dept. Comp. Biol. Med. Sci., Grad. Frontier Sci., Univ. Tokyo／Dept. Biosci., Grad. Sch. Sci., Univ. Tokyo／RIKEN Ctr. for Integrative Med. Sci.)

While recent studies have characterized carcinoma ecotypes, the distinct tumor microenvironment (TME) states identified via the EcoTyper framework. Their association with clinical outcomes, such as immune checkpoint inhibitor (ICI) response, is increasingly recognized. However, the mechanisms driving inter-patient heterogeneity in ecotype distribution remain poorly understood. This study investigates the role of somatic mutational profiles in shaping ecotype-specific TME landscapes. By use machine learning-based predictive algorithms, I integrate multi-omics data, including tumor mutational profile and RNA data, to decode ecotype determinants. Preliminary analyses reveal distinct mutation hotspots and pathway enrichments correlated with specific ecotypes, suggesting cancer cell genomic drivers of reshaping TME. Furthermore, ecotype-phenotype associations are contextualized with clinical parameters, such as survival outcomes and therapy outcomes. This work advances our understanding of TME plasticity by bridging cancer cell genomic instability and TME heterogeneity, offering a systematic framework to stratify patients for personalised therapy strategies.

[Disclosure of COI by Responsible researcher] Lead presenter is not the Responsible researcher.
[Lead presenter: Yuying Gao] We have no financial relationships to disclose.
[Principal Researcher: Tatsuhiko Tsunoda] We have no financial relationships to disclose.

### [P-1297] I-P20-1
### Immune Regulation & Tumor Microenvironment Dynamics in Cancer

2025 / 9 / 25 (Thu) 16:45-17:30 Room P/(Motenashi Dome) Room P

---

### P-1297
### Elucidation of drug resistance mechanisms in multiple myeloma by single-cell meta-analysis

[Speaker]Hiroki Kataoka (Lab. of Integr. Oncol., Natl. Cancer Res. Inst.) [Co-author]Kazuya Nakamichi (Lab. of Integr. Oncol., Natl. Cancer Res. Inst.／Dept. Life Sci. & Med. Biosci., Waseda Univ.)、Yusuke Yamamoto (Lab. of Integr. Oncol., Natl. Cancer Res. Inst.)

Multiple myeloma is a hematopoietic tumor in which plasma cells become cancerous. Recently, proteasome inhibitors such as bortezomib and immunomodulatory agents such as lenalidomide have been approved, and the prognosis is improving. However, the disease is still refractory, with many relapses and a 5-year survival rate of 59.8%. Therefore, understanding the mechanisms of drug resistance acquisition and the development of drugs that target cells that have acquired resistance are essential for longer survival. The development of various subclones is thought to be a major obstacle to understanding the mechanisms of drug resistance acquisition in multiple myeloma. In this study, a total of more than 100 single-cell RNA-seq data samples were integrated to ensure sufficient sample size and successfully detected diverse subclones that arise during multiple myeloma progression. Experimental validation of the properties and functions of the subclones, many of which contain cells presumed to have acquired drug resistance, is currently underway. In this presentation, we will discuss new findings from single-cell meta-analysis of multiple myeloma patients.

[Disclosure of COI by Responsible researcher] Lead presenter is not the Responsible researcher.
[Lead presenter: Hiroki Kataoka] We have no financial relationships to disclose.
[Principal Researcher: Yusuke Yamamoto] ■Research expenses: 東芝、ニコン

### [P-1298] I-P20-1
### Immune Regulation & Tumor Microenvironment Dynamics in Cancer

2025 / 9 / 25 (Thu) 16:45-17:30 Room P/(Motenashi Dome) Room P

---

### P-1298
### Machine Learning-Based Identification of Metastasis-Associated Genes in Normal Adjacent Tissue (NAT)

[Speaker]Shinichiro Ono (Evolutionary Genomics, Grad. Sch. of Life Sci., Tohoku Univ.) [Co-author]Takashi Makino (Evolutionary Genomics, Grad. Sch. of Life Sci., Tohoku Univ.)

Understanding cancer biology requires comparison with normal tissue. Normal tissue Adjacent to Tumor (NAT), collected from the same organ and patient as the tumor, has long been used as a control. However, recent studies show that NAT exhibits gene expression distinct from healthy tissue and may be involved in disease processes. However, the relationship between NAT and metastasis remains unclear. In this study, we built a machine learning model to identify metastasis-associated genes in NAT. RNA-Seq data from 172 patients were obtained, and 32 patients developed metastasis during a 5-year follow-up. Differential gene expression analysis and Adaptive Elastic Net were used to identify biomarkers. To address class imbalance, we applied oversampling, and then constructed classification models using machine learning algorithms including Random Forest, XGBoost, and Neural Network. 18 genes were selected as biomarkers. The Random Forest model achieved highest performance with ROC-AUC = 0.796 and PR-AUC = 0.657, significantly outperforming the baseline (ROC-AUC = 0.5, PR-AUC = 0.186). These genes may serve as predictive markers and provide insights into metastasis mechanisms.

[Disclosure of COI by Responsible researcher] Lead presenter is not the Responsible researcher.
[Lead presenter: Shinichiro Ono] We have no financial relationships to disclose.
[Principal Researcher: Takashi Makino] We have no financial relationships to disclose.

### [P-1299] I-P20-1
### Immune Regulation & Tumor Microenvironment Dynamics in Cancer

2025 / 9 / 25 (Thu) 16:45-17:30 Room P/(Motenashi Dome) Room P

### P-1299
### Development of a Transformer-based Cell Type Deconvolution Model

[Speaker]Hanyong Ding (Dept. Comp. Biol. Med. Sci., Grad. Frontier Sci., Univ. Tokyo) [Co-author]Artem Lysenko (Dept. Biol. Sci., Sch. Sci., Univ. Tokyo)、Tatsuhiko Tsunoda (Dept. Comp. Biol. Med. Sci., Grad. Frontier Sci., Univ. Tokyo／Dept. Biol. Sci., Sch. Sci., Univ. Tokyo)

Accurate inference of cell type composition from bulk RNA–seq data is essential for studying tissue heterogeneity, disease mechanisms, and therapeutic response. However, existing deconvolution models are reference–based linear methods constrained by the limited availability of bulk reference datasets with known compositions. Generating realistic pseudo bulk profiles from scRNA–seq offers a viable training strategy, yet aligning synthetic and real data remains challenging. Here we frame deconvolution as a regression task using a Transformer that processes gene count embeddings via multi–head self–attention to capture complex gene–gene patterns and improve generalization. We improve pseudo bulk realism by integrating SMOTE and manifold oversampling and applying mRNA abundance corrections to reduce bias. On independent bulk RNA–seq benchmarks, our model achieves RMSE $< 0.1$ across diverse cell types, demonstrating superior accuracy and robustness. This scalable, reference–free framework enables precise tissue composition analysis and, by detecting more cell types with higher accuracy, will support critical research in cancer immunology and new therapy development.

[Disclosure of COI by Responsible researcher] Lead presenter is not the Responsible researcher.
[Lead presenter: Hanyong Ding] We have no financial relationships to disclose.
[Principal Researcher: Tatsuhiko Tsunoda] We have no financial relationships to disclose.

### [P-1300] I-P20-1
### Immune Regulation & Tumor Microenvironment Dynamics in Cancer

2025 / 9 / 25 (Thu) 16:45-17:30 Room P/(Motenashi Dome) Room P

---

### P-1300
### Evolutionary significance of endocytosis in CAF-TAM heterotypic nucleation: Insights from multi-agent simulation

[Speaker]Kenichi Inoue (Dokkyo Medical University Research Center for Advanced Medical Science) [Co-author]Masanori Hariyama (Tohoku University Graduate School of Information Sciences)、Satoko Kishimoto (Dokkyo Medical University Research Center for Advanced Medical Science)

Stromal cell dynamics in the tumor microenvironment play crucial roles in metastatic colonization and post-therapy recurrence. Previously, we used PhysiCell to simulate intercellular interactions between cancer-associated fibroblasts (CAFs) and tumor-associated macrophages (TAMs), confirming their coexistence in "heterotypic nucleation (Hetero-Nuc)." Further analysis revealed three essential conditions for Hetero-Nuc under hypoxic stress: (1) appropriate PDGF secretion by CAFs, (2) augmented PDGF secretion by macrophages in the absence of CSF (adaptive regulation), and (3) mutual negative feedback through endocytosis of PDGF and CSF molecules. Intriguingly, while "homotypic nucleation (Homo-Nuc)" of CAFs alone can be easily established merely by enhancing PDGF secretion, evolution has selected for the more complex Hetero-Nuc. Our findings suggest that Hetero-Nuc enhances robustness against gene expression fluctuations and optimizes survival strategies through functional specialization, potentially improving cellular survival during metastasis and recurrence processes.

[Disclosure of COI by Responsible researcher] Lead presenter is the Responsible researcher.
[Lead presenter: Kenichi Inoue] We have no financial relationships to disclose.

▲TOP

### [P-1310] I-P20-3
### AI & Computational Modeling for Cancer Prognosis & Personalization

2025 / 9 / 25 (Thu) 16:45-17:30 Room P/(Motenashi Dome) Room P

---

### P-1310
### A study on the treatment of continuous variables in statistical risk models predicting cancer treatment outcomes

[Speaker]Etsuro Bando (Shizuoka Cancer Center, Gastric Surgery Division) [Co-author]Michael Kattan (The Cleveland Clinic, Department of Quantitative Health Sciences,)、Xinge Ji (The Cleveland Clinic, Department of Quantitative Health Sciences,)、Yusuke Koseki (Shizuoka Cancer Center, Gastric Surgery Division)、Kenichiro Furukawa (Shizuoka Cancer Center, Gastric Surgery Division)、Keiichi Fujiya (Shizuoka Cancer Center, Gastric Surgery Division)、Yutaka Tanizawa (Shizuoka Cancer Center, Gastric Surgery Division)

[Background] With the development of multi-omics analysis, it has become possible to quantify various biomarkers. However, there is some debate about quantitative analysis. [Objective] The aim of this study is to explore optimal quantitative analysis by changing the handling of continuous variables in survival prediction models. [Methods] This study enrolled 5,231 patients with primary gastric cancer. Fifteen clinical variables including CEA/CA19-9 were used to create risk models. The handling of CEA and CA19-9 (continuous variables) was changed as follows, and each model was graphically evaluated. (A) natural logarithms transformation and restricted cubic spline (normal analysis) (B) non-logarithms and non-cubic spline (C) non-logarithms and cubic spline (D) categorization. [Results] (A) risk prediction was excellent and graphically correct. (B) tumor markers were not selected (C) Graphically incorrectly, and the model did not converge. (D) categorization; subjective and hard to standardize [Conclusion] Incorrect handling of continuous variables can lead to errors that are clearly visible intuitively.

[Disclosure of COI by Responsible researcher] Lead presenter is the Responsible researcher.
[Lead presenter: Etsuro Bando] We have no financial relationships to disclose.

### [P-1311] I-P20-3
### AI & Computational Modeling for Cancer Prognosis & Personalization

2025 / 9 / 25 (Thu) 16:45-17:30 Room P/(Motenashi Dome) Room P

### P-1311
### Virtual staining of fresh tissues with generative AI models

[Speaker]Shenglin Zhou (Dept. Comp. Biol. Med. Sci., Grad. Frontier Sci., Univ. Tokyo) [Co-author]Tatsuhiko Tsunoda (Dept. Comp. Biol. Med. Sci., Grad. Frontier Sci., Univ. Tokyo／Dept. Biol. Sci., Sch. Sci., Univ. Tokyo)、Artem Lysenko (Dept. Biol. Sci., Sch. Sci., Univ. Tokyo)

Histological staining is the gold benchmark for analyzing tissues in pathology and biomedical research. However, the traditional workflow requires time-consuming, laborious and costly procedures, leading to increased costs, wasted resources and delayed diagnoses. In recent years, with the development of deep learning techniques, generative AI models have been widely applied in pathological images. Virtual staining technology simplifies the staining process by using trained image generation models to digitally synthetize stained images, making it quicker and more cost-effective. Furthermore, staining procedures without chemical reagents can prevent damage to samples and reduce the errors caused by manual operation. In this study, we aim to design a staining transfer model that can understand the relationship between content and color in the images through deep neural networks to avoid the color mapping errors which often occur in current virtual staining technologies. This research is expected to provide pathologists with an efficient and accurate digital staining technique, further enhancing the efficiency and accuracy of diagnoses.

[Disclosure of COI by Responsible researcher] Lead presenter is not the Responsible researcher.
[Lead presenter: Shenglin Zhou] We have no financial relationships to disclose.
[Principal Researcher: Tatsuhiko Tsunoda] We have no financial relationships to disclose.

### [P-1312] I-P20-3
### AI & Computational Modeling for Cancer Prognosis & Personalization

2025 / 9 / 25 (Thu) 16:45-17:30 Room P/(Motenashi Dome) Room P

---

### P-1312
### Development of a CNN-Cox Integrated Model for Predicting Multiple Drug Responses in Multiple Myeloma Treatment

[Speaker]Shangru Jia (UTokyo,GSFS,CBMS,Tsunoda lab) [Co-author]Tatsuhiko Tsunoda (UTokyo,GSFS,CBMS,Tsunoda lab／UTokyo,School of Science,Biological Sciences,Tsunoda lab)、Artem Lysenko (UTokyo,School of Science,Biological Sciences,Tsunoda lab)

Multiple myeloma (MM) is a plasma cell tumor with significant genetic heterogeneity. In previous MM studies, drug response prediction models were mainly based on the expression of only 200 to 300 marker genes, which cannot fully capture patient differences. In this study, we will address this limitation by employing deep learning techniques to analyze comprehensive gene expression patterns. Based on the DeepInsight methodology developed in our laboratory (Sharma A., Tsunoda T. et al., Scientific Reports, 2019), which transforms non-image data into images, we will convert RNA-seq data from 995 patients in the MMRF Compass dataset into two-dimensional images. Paired samples from the same patients before and after drug administration are aslo incorporated. These transformed expression data will train a convolutional neural network (CNN) integrated with Cox proportional hazards modeling to create a comprehensive risk prediction model that correlates expression patterns with treatment outcomes. This study is expected to enable more precise patient stratification based on gene expression profiles and treatment history, ultimately promoting personalized medicine approaches in clinical.

[Disclosure of COI by Responsible researcher] Lead presenter is the Responsible researcher.
[Lead presenter: Shangru Jia] ■Research funds from company: JST SPRING-GXを先導する高度人材育成プロジェクト

[P-1313] I-P20-3

### AI & Computational Modeling for Cancer Prognosis & Personalization

2025 / 9 / 25 (Thu) 16:45-17:30 Room P/(Motenashi Dome) Room P

P-1313

### Pharmacokinetic Evaluation of a Lymphatic Drug Delivery System(LDDS) Targeting Clinically Occult N0 Lymph Nodes

[Speaker]Reito Miyazaki (Graduate School of Biomeidical Engineering, Tohoku University) [Co-author]Sukhbaatar Ariunbuyan (Graduate School of Biomeidical Engineering, Tohoku University／Graduate School of Dentistry, Tohoku University)、Takahiro Kusaka (Institute of Development, Aging and Cancer Tohoku University)、Katsunori Katagiri (Institute of Development, Aging and Cancer Tohoku University)、Ken Saijo (Department of Otorhinolaryngology, Iwate Medical University Hospital)、Hisato Kawakami (Department of Otorhinolaryngology, Iwate Medical University Hospital)、Chikashi Ishioka (Tohoku University Hospital)、Tsuyoshi Sugiura (Graduate School of Dentistry, Tohoku University)、Tetsuya Kodama (Graduate School of Biomeidical Engineering, Tohoku University)

Lymph node (LN) metastasis is a key determinant of cancer progression and patient prognosis. Among these, clinically occult non-metastatic (N0) LNs may serve as early reservoirs for metastatic spread. Early intervention targeting such nodes may offer significant therapeutic benefits. We developed a lymphatic drug delivery system (LDDS) designed to selectively and locally deliver drugs to N0 LNs, and quantitatively evaluated its pharmacokinetic behavior using non–compartmental analysis (NCA).A LN swelling mouse was used. Indocyanine green with varying viscosity and osmotic pressure were directly injected into the right subiliac LN. Time-lapse fluorescence imaging was performed, and converted to drug concentration profiles.Results showed that the peak concentration and residence time were dependent on both viscosity and osmotic pressure. Mathematical model provided a high degree of fit to the experimental data. These findings demonstrate that NCA is a valid and practical method for assessing drug kinetics within LNs when combined with mathematical modeling.This study provides quantitative support for LDDS as a proactive drug delivery strategy to clinically occult N0 lymph nodes.

[Disclosure of COI by Responsible researcher] Lead presenter is the Responsible researcher.
[Lead presenter: Reito Miyazaki] We have no financial relationships to disclose.

▲TOP

### [P-1314] I-P20-3
### AI & Computational Modeling for Cancer Prognosis & Personalization

2025 / 9 / 25 (Thu) 16:45-17:30 Room P/(Motenashi Dome) Room P

---

### P-1314
### Culturing personalized tumor cells in computer for predictive cancer genome medicine

[Speaker]Towa Fujii (Div. Bioinformatics, Res. Inst., NCC) [Co-author]Iurii Nagornov (Div. Bioinformatics, Res. Inst., NCC)、Eisaku Furukawa (Div. Bioinformatics, Res. Inst., NCC)、Shigehiro Yagishita (Div. Bioinformatics, Res. Inst., NCC)、Mamoru Kato (Div. Bioinformatics, Res. Inst., NCC)

Computer simulations for cancer-cell evolution have been developed so far. However, such models 1) do not directly aim at use in clinical practice, only aiming at the understanding of cancer, 2) and are abstract, not constructed with realistic settings such as real genes and genome structures with the exons and introns of the reference genome, and real time and size.
To overcome these difficulties, we are developing a simulation model called tugMediHall to predict whether or not, and how, molecularly-targeted drugs take effect on individual patients. The parameters for simulation are estimated via approximate Bayesian computation from VAF (variant allele frequency) data obtained in cancer genome medicine for an individual patient. The model has real exon and intron structures of the reference genome and the phenotypic effects emerge via the dominant/recessive mode depending on oncogene/suppressor of real genes. In addition to point mutations, CNAs with breakpoints occur on real genomic structures. We used data obtained from TCGA patients and from patient-derived xenografts (JPDX) to grow personalized virtual tumors and made predictions on computer.

[Disclosure of COI by Responsible researcher] Lead presenter is not the Responsible researcher.
[Lead presenter: Towa Fujii] We have no financial relationships to disclose.
[Principal Researcher: Shigehiro Yagishita] We have no financial relationships to disclose.

Poster Session

### [P-1315] I-P20-3
### AI & Computational Modeling for Cancer Prognosis & Personalization

2025 / 9 / 25 (Thu) 16:45-17:30 Room P/(Motenashi Dome) Room P

---

### P-1315
### Comparative modeling of HER2 signaling dynamics in breast cancer cells and iPS cell-derived cardiomyocytes

[Speaker]Shigeyuki Magi (Div. of Cell. Physiol., Sch. of Med., Toho Univ.) [Co-author]Atsuhiko Naito (Div. of Cell. Physiol., Sch. of Med., Toho Univ.)

Trastuzumab, a therapeutic antibody targeting HER2, is sometimes associated with adverse effects on non-tumor tissues, particularly the heart. To elucidate cell-type-specific responses to HER2 inhibition, we compared HER2 signaling dynamics and phenotypic outcomes between cancer cells and cardiomyocytes. We analyzed time-series responses to neuregulin-1 (NRG1) and trastuzumab in human breast cancer cell lines and human induced pluripotent stem cell-derived cardiomyocytes. Our findings revealed that trastuzumab strongly inhibited HER4 phosphorylation in cardiomyocytes, which exhibited a distinct signaling profile compared to cancer cells.To interpret these results, we constructed mathematical models that recapitulated HER2 signaling dynamics in cancer cells. However, applying the same parameter set to cardiomyocytes failed to replicate the signaling behavior, necessitating the development of a cell-type-specific model incorporating transcriptome data. Comparative analysis of the cardiomyocyte- and cancer cell-fitted models revealed distinct regulatory features, offering a basis for optimizing the benefit-risk balance of HER2-targeted therapies.

[Disclosure of COI by Responsible researcher] Lead presenter is not the Responsible researcher.
[Lead presenter: Shigeyuki Magi] We have no financial relationships to disclose.
[Principal Researcher: Atsuhiko Naito] We have no financial relationships to disclose.

### [P-1316] I-P20-3
### AI & Computational Modeling for Cancer Prognosis & Personalization

2025 / 9 / 25 (Thu) 16:45-17:30 Room P/(Motenashi Dome) Room P

---

### P-1316
### The integration of pathology images and multi-omics data to improve cancer prognosis prediction and explanation

[Speaker]Yifei Ma (Dept.Biosci., Grad. Sch. Sci., Univ. Tokyo) [Co-author]Tatsuhiko Tsunoda (Dept. Comp. Biol. Med. Sci., Grad. Frontier Sci., Univ. Tokyo／Dept.Biosci., Grad. Sch. Sci., Univ. Tokyo)、Artem Lysenko (Dept.Biosci., Grad. Sch. Sci., Univ. Tokyo／RIKEN Ctr. for Integrative Med.Sci.)

Although multi-omics analyses offer important molecular information, they lack spatial and morphological details. In comparison, pathology images can directly show tumor structures, including boundaries, necrosis, and invasion patterns, which are clinically meaningful and cost-effective. This study aims to combine pathology images and omics data to build a model for predicting cancer prognosis and recurrence. For image analysis, we extracted morphological features from the cell level up to the whole tissue level. Using these features together with omics data, we applied a Transformer model to predict disease-free survival and recurrence risk. Furthermore, by investigating the associations between morphological characteristics and molecular features at the gene and protein levels, we identified novel multimodal biomarkers and elucidated potential biological pathways involved in tumor progression and recurrence.

[Disclosure of COI by Responsible researcher] Lead presenter is the Responsible researcher.
[Lead presenter: Yifei Ma] We have no financial relationships to disclose.

▲TOP

### [P-1317] I-P20-3
### AI & Computational Modeling for Cancer Prognosis & Personalization

2025 / 9 / 25 (Thu) 16:45-17:30 Room P/(Motenashi Dome) Room P

### P-1317
### Generating Patient Documents from Electronic Health Records Using Generative Artificial Intelligence

[Speaker]Nobuaki Michihata (Cancer Prevention Ctr., Chiba Cancer Ctf. Res. Inst.) [Co-author]Hiroshi Ishii (Clinical Res. Ctr., Chiba Cancer Ctf.)、Hideki Tsujimura (Div. of Med. Oncology, Chiba Cancer Ctf.)、Aya Washio (Cancer Prevention Ctr., Chiba Cancer Ctf. Res. Inst.)、Yoshitaka Hippo (Cancer Prevention Ctr., Chiba Cancer Ctf. Res. Inst.)

Objective:Creating discharge summaries and referral letters is time-consuming and may detract from care. We assessed whether enterprise-grade generative artificial intelligence (AI) can automate this task using clinical records in a Japanese cancer hospital.Methods:We analyzed records of 61 adult patients at Chiba Cancer Center, excluding reports from other departments. GPT-4 Turbo generated 31 discharge summaries and 30 referral letters. Four evaluators (one physician, three administrative staff) scored them independently. A score of 80/100 was considered useful. One feedback cycle was conducted.Results:Documents scored about 60-70 points. After revision, 29% of discharge summaries and 17% of referral letters reached 80 points. Discharge scores showed no change (mean difference: -0.32, p=0.88), but referral scores improved (mean difference: 5.5, p=0.006).Conclusion:Generative AI may help reduce documentation workload, but performance depends on the quality of source records. Better structured clinical data are needed for real-world use.

[Disclosure of COI by Responsible researcher] Lead presenter is the Responsible researcher.
[Lead presenter: Nobuaki Michihata] We have no financial relationships to disclose.

### [P-1324] II-P2-1
### Colorectal cancer (1)

2025 / 9 / 25 (Thu) 16:45-17:30 Room P/(Motenashi Dome) Room P

### P-1324
### Withdrawn

No Abstract

### [P-1325] II-P2-1
### Colorectal cancer (1)

2025 / 9 / 25 (Thu) 16:45-17:30 Room P/(Motenashi Dome) Room P

---

### P-1325
### ARID5A promotes colorectal cancer tumorigenesis via STAT3 and YAP activation

[Speaker]Koji Taniguchi (Dep. Patho, Sch. Med., Hokkaido Univ.) [Co-author]Kazuya Hamada (Dep. Patho, Sch. Med., Hokkaido Univ.)、Kensuke Nakazono (Dep. Patho, Sch. Med., Hokkaido Univ.)、Shugo Tanaka (Dep. Patho, Sch. Med., Hokkaido Univ.)、Rino Saito (Dep. Patho, Sch. Med., Hokkaido Univ.)、Sari Iwasaki (Dep. Patho, Sch. Med., Hokkaido Univ.)、Satoshi Tanaka (Dep. Patho, Sch. Med., Hokkaido Univ.)

Chronic inflammation is important for cancer progression, and the inflammatory cytokine interleukin (IL)-6 contributes to this process. Revealing the regulatory mechanisms of inflammatory cytokine expression in cancer cells is crucial to develop novel therapies that target inflammation. In immune cells, AT-rich interactive domain-containing protein 5A (ARID5A) plays an important role in inflammation and regulation of IL6 and STAT3 expression. However, its role in colon cancer cells remains unclear. We explored the function of ARID5A in human colorectal cancer cell lines and revealed that ARID5A promotes cell proliferation, migration/invasion, and sphere formation as well as inhibits apoptosis via STAT3 and YAP activation. Our findings suggest that ARID5A may be a new therapeutic target in human colorectal cancer.

[Disclosure of COI by Responsible researcher] Lead presenter is the Responsible researcher.
[Lead presenter: Koji Taniguchi] We have no financial relationships to disclose.

[P-1326] II-P2-1
Colorectal cancer (1)

2025 / 9 / 25 (Thu) 16:45-17:30 Room P/(Motenashi Dome) Room P

---

### P-1326
### TRPV2 has an oncogenic role in colon cancer

[Speaker]Shingo Otsuji (Div. of Digestive Surg, Kyoto Pref. Univ. of Med.) [Co-author]Hiroki Shimizu (Div. of Digestive Surg, Kyoto Pref. Univ. of Med.)、Kento Kurashima (Div. of Digestive Surg, Kyoto Pref. Univ. of Med.)、Hiroyuki Inoue (Div. of Digestive Surg, Kyoto Pref. Univ. of Med.)、Naosuke Nakamichi (Div. of Digestive Surg, Kyoto Pref. Univ. of Med.)、Jun Kiuchi (Div. of Digestive Surg, Kyoto Pref. Univ. of Med.)、Taisuke Imamura (Div. of Digestive Surg, Kyoto Pref. Univ. of Med.)、Kenji Nanishi (Div. of Digestive Surg, Kyoto Pref. Univ. of Med.)、Tomohiro Arita (Div. of Digestive Surg, Kyoto Pref. Univ. of Med.)、Toshiyuki Kosuga (Div. of Digestive Surg, Kyoto Pref. Univ. of Med.)、Yusuke Yamamoto (Div. of Digestive Surg, Kyoto Pref. Univ. of Med.)、Hirotaka Konishi (Div. of Digestive Surg, Kyoto Pref. Univ. of Med.)、Ryo Morimura (Div. of Digestive Surg, Kyoto Pref. Univ. of Med.)、Hitoshi Fujiwara (Div. of Digestive Surg, Kyoto Pref. Univ. of Med.)、Atsushi Shiozaki (Div. of Digestive Surg, Kyoto Pref. Univ. of Med.)

IntroductionTRPV2 is a voltage-dependent calcium channel involved in cancer cell proliferation and apoptosis. While high TRPV2 expression is a poor prognostic factor in other cancers, its role in colorectal cancer remains unclear. This study evaluated the molecular biological and clinical significance of TRPV2 in colon cancer.MethodsColon cancer cell lines with high TRPV2 expression were selected. TRPV2 knockdown was performed using siRNA, followed by assays for proliferation, cell cycle, apoptosis, and wound healing. Gene expression profiles were analyzed using microarray. TRPV2 immunohistochemistry was performed on tumor samples from 200 patients who underwent curative surgery, and associations with clinical factors and prognosis were examined.ResultsTRPV2 knockdown inhibited proliferation, induced apoptosis, and reduced wound healing in colorectal cancer cells. It also blocked G1-to-S phase transition. Microarray analysis showed TRPV2 involvement in PI3K/AKT signaling. High TRPV2 expression was an independent poor prognostic factor for five-year recurrence-free survival ($p = 0.016$). These findings suggest TRPV2 may be a useful biomarker and therapeutic target in colon cancer.

[Disclosure of COI by Responsible researcher] Lead presenter is not the Responsible researcher.
[Lead presenter: Shingo Otsuji] We have no financial relationships to disclose.
[Principal Researcher: Hiroki Shimizu] We have no financial relationships to disclose.

▲TOP

### [P-1327] II-P2-1
### Colorectal cancer (1)

2025 / 9 / 25 (Thu) 16:45-17:30 Room P/(Motenashi Dome) Room P

---

### P-1327
### Clinicopathological analyses of colorectal cancer with neuroendocrine differentiation

[Speaker]Yuki Yasuda (Dept. of Surg., Tohoku Univ. Grad. Sch. of Med.) [Co-author]Keigo Murakami (Dept. of Investigative Path., Tohoku Univ. Grad. Sch. of Med.)、Hideaki Karasawa (Dept. of Surg., Tohoku Univ. Grad. Sch. of Med.)、Yukihiro Sato (Dept. of Surg., Tohoku Univ. Grad. Sch. of Med.)、Megumi Murakami (Dept. of Surg., Tohoku Univ. Grad. Sch. of Med.)、Tomoyuki Ono (Dept. of Surg., Tohoku Univ. Grad. Sch. of Med.)、Yoshihiro Sato (Dept. of Surg., Tohoku Univ. Grad. Sch. of Med.)、Hideyuki Suzuki (Dept. of Surg., Tohoku Univ. Grad. Sch. of Med.)、Kazuhiro Watanabe (Dept. of Surg., Tohoku Univ. Grad. Sch. of Med.)、Takashi Kamei (Dept. of Surg., Tohoku Univ. Grad. Sch. of Med.)、Shinobu Ohnuma (Dept. of Surg., Tohoku Univ. Grad. Sch. of Med.)、Toru Furukawa (Dept. of Investigative Path., Tohoku Univ. Grad. Sch. of Med.)、Michiaki Unno (Dept. of Surg., Tohoku Univ. Grad. Sch. of Med.)

Background: Some colorectal cancers (CRC) exhibit neuroendocrine differentiation (NED), which is thought to be associated with poorer prognosis than other CRCs. However, reports are limited and inconsistent. Objective: To investigate the clinicopathological characteristics of CRC with NED. Methods: Immunohistochemistry was performed on pStage II-IV CRCs resected between 2013 and 2017. Associations between neuroendocrine markers' expression and clinicopathological features including prognosis were retrospectively analyzed. Results: 162 patients underwent immunohistochemical evaluation. 17 were positive based on H-score≥4 for synaptophysin, including 9 males. Median age was 67 years. TNM staging: pStage II (n=4), III (n=7), IV (n=6). Immature desmoplastic reaction (n=10), lymphatic invasion (n=11), perineural invasion (n=11), and poorly differentiated clusters G2/G3 (n=8) were present and tended to be more frequent in the positive group. Median overall and relapse-free survival were 78 and 29 months, respectively. Relapse-free survival appeared poorer in CRC with NED than in those without NED. Conclusion: CRC with NED were tended to be associated with poor prognostic factors in CRC.

[Disclosure of COI by Responsible researcher] Lead presenter is not the Responsible researcher.
[Lead presenter: Yuki Yasuda] We have no financial relationships to disclose.
[Principal Researcher: Keigo Murakami] We have no financial relationships to disclose.

### [P-1328] II-P2-1
### Colorectal cancer (1)

2025 / 9 / 25 (Thu) 16:45-17:30 Room P/(Motenashi Dome) Room P

---

### P-1328
### Functional and clinical significance of Anoctamin 5 in colon cancer

[Speaker]Hiroki Shimizu (Department of Digestive Surgery, Kyoto Prefectural University of Medicine) [Co-author]Takayuki Miyoshi (Department of Digestive Surgery, Kyoto Prefectural University of Medicine)、Kento Kurashima (Department of Digestive Surgery, Kyoto Prefectural University of Medicine)、Hiroyuki Inoue (Department of Digestive Surgery, Kyoto Prefectural University of Medicine)、Jun Kiuchi (Department of Digestive Surgery, Kyoto Prefectural University of Medicine)、Taisuke Imamura (Department of Digestive Surgery, Kyoto Prefectural University of Medicine)、Kenji Nanishi (Department of Digestive Surgery, Kyoto Prefectural University of Medicine)、Tomohiro Arita (Department of Digestive Surgery, Kyoto Prefectural University of Medicine)、Toshiyuki Kosuga (Department of Digestive Surgery, Kyoto Prefectural University of Medicine)、Yusuke Yamamoto (Department of Digestive Surgery, Kyoto Prefectural University of Medicine)、Hirotaka Konishi (Department of Digestive Surgery, Kyoto Prefectural University of Medicine)、Ryo Morimura (Department of Digestive Surgery, Kyoto Prefectural University of Medicine)、Hitoshi Fujiwara (Department of Digestive Surgery, Kyoto Prefectural University of Medicine)、Atsushi Shiozaki (Department of Digestive Surgery, Kyoto Prefectural University of Medicine)

Background. Anoctamin 5 (ANO5) is a member of the calcium-activated chloride channel family and has been reported as an oncogene. However, its role in colon cancer remains poorly understood. Methods. We performed knockdown experiments on the human colon cancer cell lines HCT116 and LoVo using ANO5 siRNA, and performed cell function analysis. Gene expression changes were assessed using mRNA microarray analysis and validated. Additionally, we evaluated the expression of ANO5 by immunohistochemical staining (IHC) in 200 curatively resected patients with colon cancer, and analyzed the association between the expression of ANO5 and patients prognoses.Results. ANO5-depleted cells suppressed cell proliferation with G1 arrest, migration, and invasion, while inducing apoptosis. Microarray analysis showed altered expression of genes related to G1-S checkpoint control in ANO5-depleted HCT116 cells. Multivariate analysis identified ANO5 high expression by IHC as an independent prognostic factor for 5-year survival in colon cancer patients ($p=0.0002$).Conclusion. The present results reveal the function of ANO5 and indicate its potential as a prognostic biomarker for colon cancer.

[Disclosure of COI by Responsible researcher] Lead presenter is the Responsible researcher.
[Lead presenter: Hiroki Shimizu] We have no financial relationships to disclose.

▲TOP

### [P-1329] II-P2-1
### Colorectal cancer (1)

2025 / 9 / 25 (Thu) 16:45-17:30 Room P/(Motenashi Dome) Room P

### P-1329
### Regulation of colon cancer cell functions through LPA receptor signaling activation in cobalt chloride-induced hypoxia

[Speaker]Mao Yamamoto (Mol Oncol. Life Science, Kindai Univ.) [Co-author]Narumi Yashiro (Mol Oncol. Life Science, Kindai Univ.)、Yuka Kusumoto (Mol Oncol. Life Science, Kindai Univ.)、Moemi Tamura (Mol Oncol. Life Science, Kindai Univ.)、Shion Nagano (Mol Oncol. Life Science, Kindai Univ.)、Hiroko Ikeda (Mol Oncol. Life Science, Kindai Univ.)、Toshifumi Tsujiuchi (Mol Oncol. Life Science, Kindai Univ.)

In solid tumors, hypoxia and nutrient deprivation arise from abnormal blood vessels, promoting tumor progression. This study investigated the effects of lysophosphatidic acid (LPA) receptor signaling on malignant properties of colon cancer DLD-1 cells under cobalt chloride ($CoCl_2$)-induced hypoxia. $CoCl_2$ increased ***LPAR1***, ***LPAR2*** and ***LPAR3*** expression levels and enhanced DLD-1 cell motility, which was reduced by the HIF-1 inhibitor LW6. AM966 ($LPA_1$ antagonist) suppressed motility, while GRI-977143 ($LPA_2$ agonist) elevated it. Conversely, $CoCl_2$ decreased DLD-1 cell invasion. AM966 further inhibited cell invasion but GRI-977143 elevated it. $CoCl_2$-treated cells showed increased fluorouracil (5-FU) resistance, which AM966 and GRI-977143 enhanced. Under low-glucose conditions, ***LPAR1*** expression was upregulated compared to high-glucose media, while ***LPAR2*** expression was downregulated. Moreover, cell motility and invasion in $CoCl_2$-treated DLD-1 cells were further stimulated under low-glucose conditions. These findings suggest that LPA receptor signaling enhances the malignant potential of DLD-1 cells under hypoxic conditions mediated by $CoCl_2$-induced HIF-1 activation.

[Disclosure of COI by Responsible researcher] Lead presenter is not the Responsible researcher.
[Lead presenter: Mao Yamamoto] We have no financial relationships to disclose.
[Principal Researcher: Toshifumi Tsujiuchi] We have no financial relationships to disclose.

▲TOP

### [P-1330] II-P2-1
### Colorectal cancer (1)

2025 / 9 / 25 (Thu) 16:45-17:30 Room P/(Motenashi Dome) Room P

---

### P-1330
### Association Between Iron Accumulation by Colorectal Cancer Drugs and the Synergistic Effect of Pterostilbene

[Speaker]Yukiko Nishiguchi (Dep. of Mol. Pathol., Nara Med. Univ.) [Co-author]Rina Tani (Dep. of Mol. Pathol., Nara Med. Univ.)、Ruiko Ogata (Dep. of Mol. Pathol., Nara Med. Univ.)、Hitoshi Ohmori (Dep. of Mol. Pathol., Nara Med. Univ.)、Kiyomu Fujii (Dep. of Mol. Pathol., Nara Med. Univ.)、Takamitsu Sasaki (Dep. of Mol. Pathol., Nara Med. Univ.)、Yi Luo (Dep. of Mol. Pathol., Nara Med. Univ.)、Hiroki Kuniyasu (Dep. of Mol. Pathol., Nara Med. Univ.)

We previously showed that pterostilbene (PTE), a plant-derived stilbene, enhances the efficacy of iron-accumulating drugs like doxorubicin and lapatinib by inducing ER stress-mediated cell death. In this study, we examined whether the combination of PTE with 5-fluorouracil (5FU) or irinotecan (CPT11) -both used in colorectal cancer- correlates with iron accumulation. Using human colorectal cancer cell lines HCT116 and HT29, Ferrogreen assay revealed iron accumulation with CPT11 but not with 5FU. When combined with a non-cytotoxic dose of PTE, CPT11 showed enhanced growth inhibition, while no such additive effect was observed with 5FU. The CPT11+PTE-induced growth inhibition was not reversed by Z-VAD, NAC, or FER1, but was suppressed by the ER stress inhibitor 4PBA. Interestingly, DFO further enhanced the effect of CPT11+PTE, but had no effect with 5FU. These results suggest that CPT11, but not 5FU, promotes iron accumulation and shows increased anticancer efficacy when combined with PTE and DFO. CPT11+PTE also resulted in lower expression levels of stemness markers. Ongoing we aim to clarify the molecular mechanisms behind these differences.

[Disclosure of COI by Responsible researcher] Lead presenter is not the Responsible researcher.
[Lead presenter: Yukiko Nishiguchi] We have no financial relationships to disclose.
[Principal Researcher: Hiroki Kuniyasu] We have no financial relationships to disclose.

### [P-1338] II-P2-3
### Gastric cancer

2025 / 9 / 25 (Thu) 16:45-17:30 Room P/(Motenashi Dome) Room P

---

### P-1338
### Withdrawn

No Abstract

Poster Session

### [P-1339] II-P2-3
### Gastric cancer

2025 / 9 / 25 (Thu) 16:45-17:30 Room P/(Motenashi Dome) Room P

P-1339

### Neutrophil extracellular traps contributes to peritoneal and liver metastasis of gastric cancer in diabetic conditions

[Speaker]Rei Takahashi (Department of Gastrointestinal, General and Transplant Surgery, Jichi Medical University) [Co-author]Hideyo Miyato (Department of Clinical Oncology, Jichi Medical University)、Akira Saito (Department of Gastrointestinal, General and Transplant Surgery, Jichi Medical University)、Eri Suizu (Department of Obstetrics and Gynecology, Jichi Medical University)、Misaki Matsumiya (Department of Gastrointestinal, General and Transplant Surgery, Jichi Medical University)、Yuki Kaneko (Department of Gastrointestinal, General and Transplant Surgery, Jichi Medical University)、Yurie Futoh (Department of Gastrointestinal, General and Transplant Surgery, Jichi Medical University)、Kohei Tamura (Department of Obstetrics and Gynecology, Jichi Medical University)、Yuki Kimura (Department of Gastrointestinal, General and Transplant Surgery, Jichi Medical University)、Kazuya Takahashi (Department of Gastrointestinal, General and Transplant Surgery, Jichi Medical University)、Hirofumi Sonoda (Department of Gastrointestinal, General and Transplant Surgery, Jichi Medical University)、Hideyuki Ohzawa (Department of Clinical Oncology, Jichi Medical University)、Hiroharu Yamashita (Department of Gastrointestinal, General and Transplant Surgery, Jichi Medical University)、Hironori Yamaguchi (Department of Gastrointestinal, General and Transplant Surgery, Jichi Medical University)、Naohiro Sata (Department of Gastrointestinal, General and Transplant Surgery, Jichi Medical University)、Joji Kitayama (Department of Gastrointestinal, General and Transplant Surgery, Jichi Medical University)

Background: It is unknown how neutrophil extracellular traps (NETs) are involved in the process of liver and peritoneal metastasis of gastric cancer in diabetic conditions.
Methods: Neutrophils isolated from the bone marrow of C57BL/JHamSlc-ob/ob mice were stimulated with PMA. Peritoneal and liver metastasis of gastric cancer models were compared between ob/ob and lean mice. The prognosis of 1178 gastric cancer patients post curative gastrectomy (2010-2021) was compared between diabetic and non-diabetic patients.
Results: NETs formation was higher in ob/ob mice. Metastatic nodules of peritoneal metastases (23.5 (9-104) vs 15.5 (0-31), p=0.027, n=10) and liver metastases (123 (52-354) vs 1 (0-3), p=0.007, n=5) were higher in ob/ob mice than lean mice. Peritoneal metastasis was suppressed by DNAse1, but not in liver metastasis in this case. GSK484, a PAD4 inhibitor, suppressed liver metastases of ob/ob mice. NETs-forming neutrophils increased in metastases of ob/ob mice by immunohistochemistry. DFS (HR=1.66) and OS (HR=1.46) were worse in 203 diabetic vs 975 non-diabetic patients.
Conclusion: NETs formation in diabetes may promote gastric cancer metastasis to the liver and peritoneum.

[Disclosure of COI by Responsible researcher] Lead presenter is not the Responsible researcher.
[Lead presenter: Rei Takahashi] We have no financial relationships to disclose.
[Principal Researcher: Joji Kitayama] We have no financial relationships to disclose.

[P-1340] II-P2-3
Gastric cancer

2025 / 9 / 25 (Thu) 16:45-17:30 Room P/(Motenashi Dome) Room P

### P-1340
### Selective dual inhibition of MET and VEGFR2 as a therapeutic strategy for gastric cancer with malignant ascites

[Speaker]Akiko Inujima (Dept. of Medical Oncology, Kanazawa Med. Univ.) [Co-author]Naomi Kuwayama (Dept. of Medical Oncology, Kanazawa Med. Univ.)、Katsuya Sakai (Div. of Genome Biol., Cancer Res. Inst., Kanazawa Univ.)、Kunio Matsumoto (NanoLSI, Kanazawa Univ.)、Kazuo Yasumoto (Dept. of Medical Oncology, Kanazawa Med. Univ.)

Scirrhous gastric cancer (SGC) frequently accompanies malignant ascites and has an extremely poor prognosis. Malignant ascites is a fatal complication, yet its molecular pathogenesis remains unclear. We previously reported the involvement of the MET/HGF and VEGFR2/VEGF axes in this pathology using multi-kinase inhibitors (BBRC 2022). In this study, we aimed to clarify their specific roles by using novel selective MET inhibitors (capmatinib, tepotinib) and a specific VEGFR2 inhibitor (DC101) to evaluate their anti-tumor and ascites-suppressing effects. We employed two preclinical models: non-MET-amplified SGC cells (NUGC4) and MET-amplified gastric cancer cells (58As9). Selective MET inhibitors strongly suppressed the HGF-stimulated proliferation of NUGC4 and the high baseline proliferation of 58As9. In vivo, DC101 monotherapy significantly prolonged survival in both models, and combination therapy with MET inhibitors enhanced these effects. Our findings clarify the distinct and cooperative roles of MET and VEGFR2 in malignant ascites formation. Dual blockade of these pathways may serve as a promising therapeutic strategy for gastric cancer with malignant ascites.

[Disclosure of COI by Responsible researcher] Lead presenter is not the Responsible researcher.
[Lead presenter: Akiko Inujima] We have no financial relationships to disclose.
[Principal Researcher: Kazuo Yasumoto] We have no financial relationships to disclose.

▲TOP

[P-1341] II-P2-3
Gastric cancer

2025 / 9 / 25 (Thu) 16:45-17:30 Room P/(Motenashi Dome) Room P

P-1341
### Distinct modulation of gastric cancer cell function via LPA receptor signaling under $CoCl_2$-induced and 1% oxygen hypoxia

[Speaker]Narumi Yashiro (Mol Oncol. Life Science, Kindai Univ.) [Co-author]Mao Yamamoto (Mol Oncol. Life Science, Kindai Univ.)、Shion Nagano (Mol Oncol. Life Science, Kindai Univ.)、Yuka Kusumoto (Mol Oncol. Life Science, Kindai Univ.)、Moemi Tamura (Mol Oncol. Life Science, Kindai Univ.)、Hiroko Ikeda (Mol Oncol. Life Science, Kindai Univ.)、Toshifumi Tsujiuchi (Mol Oncol. Life Science, Kindai Univ.)

The tumor microenvironment enhances cancer progression through hypoxia. Lysophosphatidic acid (LPA) receptor signaling is involved in the pathogenesis of cancer cells. This study investigated the roles of LPA receptor-1 ($LPA_1$), $LPA_2$ and $LPA_3$ on cellular functions of gastric cancer AGS cells under cobalt chloride ($CoCl_2$)-induced and 1% $O_2$ hypoxia. $CoCl_2$ increased ***LPAR1*** and ***LPAR3*** expressions but reduced ***LPAR2*** expression, promoting cell motility. This effect was enhanced by AM966 ($LPA_1$ antagonist), GRI-977143 ($LPA_2$ agonist), and (2S)-OMPT ($LPA_3$ agonist). $CoCl_2$ also increased AGS cell viability in response to cisplatin (CDDP) with LPA, further enhanced by AM966, GRI-977143 and (2S)-OMPT. Under 1% $O_2$ conditions, ***LPAR1*** and ***LPAR3*** expression levels decreased, whereas ***LPAR2*** expression remained unchanged. AGS cells cultured under 1% $O_2$ showed higher motility than those maintained at 21% $O_2$. The viability of AGS cells treated with CDDP was inhibited at 1% $O_2$ in comparison to 21% $O_2$. These findings suggest that distinct modulation of LPA receptor signaling under $CoCl_2$-induced and 1% $O_2$ hypoxia influences AGS cell functions, resulting in differences in cell motility and drug response.

[Disclosure of COI by Responsible researcher] Lead presenter is not the Responsible researcher.
[Lead presenter: Narumi Yashiro] We have no financial relationships to disclose.
[Principal Researcher: Toshifumi Tsujiuchi] We have no financial relationships to disclose.

### [P-1342] II-P2-3
### Gastric cancer

2025 / 9 / 25 (Thu) 16:45-17:30 Room P/(Motenashi Dome) Room P

---

### P-1342
### Spatial Heterogeneity of CLDN18.2 Expression and Its Association with Clinicopathological Factors in Gastric Cancer

[Speaker]Takaomi Ozawa (First Department of Surgery, Faculty of Medicine, University of Yamanashi) [Co-author]Katsutoshi Shoda (First Department of Surgery, Faculty of Medicine, University of Yamanashi)、Akihito Mizukami (First Department of Surgery, Faculty of Medicine, University of Yamanashi)、Hiroto Tanaka (First Department of Surgery, Faculty of Medicine, University of Yamanashi)、Yudai Higuchi (First Department of Surgery, Faculty of Medicine, University of Yamanashi)、Koichi Matsuoka (First Department of Surgery, Faculty of Medicine, University of Yamanashi)、Suguru Maruyama (First Department of Surgery, Faculty of Medicine, University of Yamanashi)、Yoshihiko Kawaguchi (First Department of Surgery, Faculty of Medicine, University of Yamanashi)、Kazunori Takahashi (First Department of Surgery, Faculty of Medicine, University of Yamanashi)、Kensuke Shiraishi (First Department of Surgery, Faculty of Medicine, University of Yamanashi)、Shinji Furuya (First Department of Surgery, Faculty of Medicine, University of Yamanashi)、Ryo Saito (First Department of Surgery, Faculty of Medicine, University of Yamanashi)、Wataru Izumo (First Department of Surgery, Faculty of Medicine, University of Yamanashi)、Hidetake Amemiya (First Department of Surgery, Faculty of Medicine, University of Yamanashi)、Daisuke Ichikawa (First Department of Surgery, Faculty of Medicine, University of Yamanashi)

[Introduction]
Following the GLOW and SPOTLIGHT trials, zolbetuximab use for CLDN18.2–positive gastric cancer has expanded. Data on heterogeneity between primary and metastatic tumors are limited. This study evaluated CLDN18.2 expression by metastatic route and analyzed features of discordant cases.
[Methods]
We retrospectively analyzed 50 patients with unresectable gastric cancer (2005–2023) and paired primary/metastatic tissues. Immunohistochemistry with CLDN18.2 antibody (43–14A) was performed. Tumors with ≥75% expression were positive. Concordance was assessed, and features compared between concordant and discordant cases.
[Results]
Among 50 cases, 32 had peritoneal, 10 lymphatic, and 8 hematogenous metastases. CLDN18.2 positivity was 36% in primaries and 18% in metastases. Concordance was 76% (peritoneal: 72%, lymphatic: 80%, hematogenous: 100%). Discordant cases (n=11) had ≥N2 ($p=0.004$) and larger tumors (130 mm vs 70 mm, $p=0.02$). ROC cutoff was 90 mm; 81.8% of tumors ≥90 mm with ≥N2 were discordant.
[Conclusion]
CLDN18.2 expression varied by metastatic route. Tumor size and nodal status may influence discordance. Assessing metastases is key for zolbetuximab selection.

[Disclosure of COI by Responsible researcher] Lead presenter is not the Responsible researcher.
[Lead presenter: Takaomi Ozawa] ■Research funds from company: アステラス 研究者主導研究支援プログラム
[Principal Researcher: Katsutoshi Shoda] ■Research funds from company: アステラス 研究者主導研究支援プログラム

### [P-1343] II-P2-3
### Gastric cancer

2025 / 9 / 25 (Thu) 16:45-17:30 Room P/(Motenashi Dome) Room P

---

### P-1343
### Fascin1 as a potential therapeutic target for scirrhous gastric cancer

[Speaker]Canfeng Fan (Osaka Metro Univ, Grad Sch of Med, Mol Oncol & Ther) [Co-author]Qiang Wang (Osaka Metro Univ, Grad Sch of Med, Mol Oncol & Ther)、Kyoka Kawabata (Osaka Metro Univ, Grad Sch of Med, Mol Oncol & Ther)、Hinano Nishikubo (Osaka Metro Univ, Grad Sch of Med, Mol Oncol & Ther)、Tomoya Sano (Osaka Metro Univ, Grad Sch of Med, Mol Oncol & Ther)、Dongheng Ma (Osaka Metro Univ, Grad Sch of Med, Mol Oncol & Ther)、Daiki Imanishi (Osaka Metro Univ, Grad Sch of Med, Mol Oncol & Ther)、Takashi Sakuma (Osaka Metro Univ, Grad Sch of Med, Mol Oncol & Ther)、Koji Maruo (Osaka Metro Univ, Grad Sch of Med, Mol Oncol & Ther)、Gen Tsujio (Osaka Metro Univ, Grad Sch of Med, Mol Oncol & Ther)、Yurie Yamamoto (Osaka Metro Univ, Grad Sch of Med, Mol Oncol & Ther)、Tatsunari Fukuoka (Osaka Metro Univ, Grad Sch of Med, Mol Oncol & Ther)、Masakazu Yashiro (Osaka Metro Univ, Grad Sch of Med, Mol Oncol & Ther)

Fascin1 plays a critical role in the biological processes of tumor cell invasion and metastasis. Scirrhous gastric carcinoma (SGC) is an aggressive subtype of gastric cancer (GC) characterized by high invasiveness and a high mortality rate. However, no targeted therapies for SGC have been reported to date. In this study, we examined the clinico-pathologic significance of Fascin1 by immunohistochemical study using 689 gastric cancer tissue samples. Additionally, we evaluated the effects of two fascin1 inhibitors on reducing the number of filopodia and suppressing the invasion and metastasis capabilities in 4 gastric cancer cell lines. Fascin1 was highly expressed in cancer cells in 353 of 689 cases. The high expression of Fascin1 in cancer cells is associated with the scirrhous subtype, increased invasion depth, larger tumor size, lymph node metastasis, lymphatic invasion, venous invasion, advanced disease stage, higher rate of relapse, and poor prognosis. Fascin1 inhibitors NP-G2-044 and BDP-13176 significantly suppressed invasion, migration, and filopodia formation of SGC cells, but not non-SGC cells.Fascin1 may be one of the promising therapeutic targets for patients with SGC.

[Disclosure of COI by Responsible researcher] Lead presenter is not the Responsible researcher.
[Lead presenter: Canfeng Fan] We have no financial relationships to disclose.
[Principal Researcher: Masakazu Yashiro] ■Research funds from company: 日本学術振興会

[P-1344] II-P2-3
Gastric cancer

2025 / 9 / 25 (Thu) 16:45-17:30 Room P/(Motenashi Dome) Room P

### P-1344
### Spatial Transcriptomic Profiling Reveals Niche-Specific Interactions in Early Gastric Cancer Microenvironment

[Speaker]Keiko Nakamura (Dept. Gastroenterol. The Univ. Tokyo) [Co-author]Miwako Kakiuchi (Dept. Preventive Med. The Univ. Tokyo)、Ayumu Tsubosaka (Dept. Preventive Med. The Univ. Tokyo)、Kyohei Sano (Dept. Preventive Med. The Univ. Tokyo)、Daisuke Komura (Dept. Preventive Med. The Univ. Tokyo)、Keisuke Yamamoto (Dept. Gastroenterol. The Univ. Tokyo)、Chihiro Takeuchi (Dept. Gastroenterol. The Univ. Tokyo)、Yosuke Tsuji (Dept. Gastroenterol. The Univ. Tokyo)、Hiroto Katoh (Div. Path. Natl. Cancer Ctr.)、Shumpei Ishikawa (Dept. Preventive Med. The Univ. Tokyo／Div. Path. Natl. Cancer Ctr.)、Mitsuhiro Fujishiro (Dept. Gastroenterol. The Univ. Tokyo)

Gastric cancer remains a life-threatening disease, and understanding its early development is essential for improving diagnosis and treatment. The tumor microenvironment (TME) plays a crucial role in early-stage malignant transformation, yet its spatial organization and cellular interactions remain unclear. We performed single-cell spatial transcriptomic analysis on endoscopically resected early gastric cancer tissues. This allowed us to characterize distinct cellular niches based on histological subtypes and to identify context-specific interactions between epithelial and stromal cells. Alterations in epithelial-stromal crosstalk were observed compared to normal mucosa and across tumor progression. Our study highlights the utility of spatial transcriptomics in revealing the complex architecture and functional dynamics of the early gastric cancer microenvironment. These insights may help identify biomarkers and therapeutic targets specific to early-stage disease.

[Disclosure of COI by Responsible researcher] Lead presenter is not the Responsible researcher.
[Lead presenter: Keiko Nakamura] We have no financial relationships to disclose.
[Principal Researcher: Keisuke Yamamoto] We have no financial relationships to disclose.

Poster Session

### [P-1345] II-P2-3
### Gastric cancer

2025 / 9 / 25 (Thu) 16:45-17:30 Room P/(Motenashi Dome) Room P

### P-1345
### Identification of critical oncogenic genes and super enhancers by CRISPRi Screening

[Speaker]Qin Fan (Dept. Mol. Oncol., Grad. Sch. Med., Chib) [Co-author]Atsushi Okabe (Dept. Mol. Oncol., Grad. Sch. Med., Chib／Dept. Health and Disease Omics Center, Chiba Univ.)、Takayuki Hoshii (Dept. Mol. Oncol., Grad. Sch. Med., Chib)、Motoaki Seki (Dept. Mol. Oncol., Grad. Sch. Med., Chib)、Tianhui Zhu (Dept. Mol. Oncol., Grad. Sch. Med., Chib)、Masaki Fukuyo (Dept. Mol. Oncol., Grad. Sch. Med., Chib)、Bahityar Rahmutulla (Dept. Mol. Oncol., Grad. Sch. Med., Chib)、Atsushi Kaneda (Dept. Mol. Oncol., Grad. Sch. Med., Chib／Dept. Health and Disease Omics Center, Chiba Univ.)

Epstein-Barr Virus-positive (EBV(+)) gastric cancer (GC) shows extreme epigenetic changes, which are causally involved in EBV(+) GC development. Aberrant enhancers and super enhancers (SEs) activation contribute to the dysregulation of local and regional oncogene expression in various cancer. However, the roles of SEs and their interaction to active promoters in genesis of EBV(+) GC are yet to be fully clarified. Here, through analyzing ChIP-seq, H3K27ac HiChIP and RNA-seq, we determined 20 candidate genes with SE-promoter loop might contribute to tumorigenesis in EBV(+) GC. To assess the functional role of these genes, we constructed the dCas9 cell lines and pooled sgRNA library to perform CRISPRi screening. In addition, we included 15 sgRNAs targeting 5 known core cell essential genes as positive controls and 25 non-targeting controls. Following library transduction, cells were cultured for 28 days followed by DNA extraction and quantification of sgRNA abundance. CRISPRi screening revealed that suppression of several genes, including SOX9, KRT8, and BCL3, had a dramatic anti-proliferation effect. Our results indicated that SE interacting genes might contribute GC tumorigenesis.

[Disclosure of COI by Responsible researcher] Lead presenter is the Responsible researcher.
[Lead presenter: Qin Fan] ■Contributions: SRL Inc. Scrum Inc. Takeda Science Foundation. Uehara Memorial Foundation.

### [P-1346] II-P2-3
### Gastric cancer

2025 / 9 / 25 (Thu) 16:45-17:30 Room P/(Motenashi Dome) Room P

### P-1346
### Withdrawn

No Abstract

Poster Session

### [P-1349] II-P3-1
### Hepatocellular cancer

2025 / 9 / 25 (Thu) 16:45-17:30 Room P/(Motenashi Dome) Room P

### P-1349
### Investigation of Congestive Liver Microenvironment on Hepatocellular Carcinoma

[Speaker]Daisuke Taguchi (Osaka Univ., Dept. of Gastroenterological Surg.) [Co-author]Takehiro Noda (Osaka Univ., Dept. of Gastroenterological Surg.)、Hirofumi Akita (Osaka Univ., Dept. of Gastroenterological Surg.)、Yosuke Mukai (Osaka Univ., Dept. of Gastroenterological Surg.)、Kazuki Sasaki (Osaka Univ., Dept. of Gastroenterological Surg.)、Shinichiro Hasegawa (Osaka Univ., Dept. of Gastroenterological Surg.)、Daisaku Yamada (Osaka Univ., Dept. of Gastroenterological Surg.)、Yoshito Tomimaru (Osaka Univ., Dept. of Gastroenterological Surg.)、Shogo Kobayashi (Osaka Univ., Dept. of Gastroenterological Surg.)、Yuichiro Doki (Osaka Univ., Dept. of Gastroenterological Surg.)、Hidetoshi Eguchi (Osaka Univ., Dept. of Gastroenterological Surg.)

Background: While Fontan surgery has enabled the long-term survival of patients with congenital heart disease, the number of patients who develop hepatocellular carcinoma (HCC) due to congestive liver disease after Fontan surgery is increasing. The clarification of pathogenesis and improvement of patients' prognosis are urgently needed. This study aims to elucidate the tumor-promoting factors in congestive liver. Methods and Results: To establish HCC model with congestive liver, we performed partial inferior vena cava ligation (pIVCL) for mice and injected BNL-T (mouse HCC cell line) into the liver. The tumor diameter (8.9mm vs 20.4mm, p=0.014) and liver weight (1.99g vs 4.24g, p=0.004) were significantly larger in pIVCL group. Expecting the presence of tumor-promoting proteins derived from congestive liver, we performed the analysis of DIA proteomics of congestive liver and sham liver lysate, which showed significant changes in several proteins between the two groups. Conclusions: In pIVCL group, the tumor showed increased growth compared with sham group. Analyzing the data, we are trying to identify factors that can be targeted for the treatment of HCC with congestive liver.

[Disclosure of COI by Responsible researcher] Lead presenter is the Responsible researcher.
[Lead presenter: Daisuke Taguchi] We have no financial relationships to disclose.

### [P-1350] II-P3-1
### Hepatocellular cancer

2025 / 9 / 25 (Thu) 16:45-17:30 Room P/(Motenashi Dome) Room P

### P-1350
### Restoring Intestinal-LSEC Barriers Prevents Metabolic Dysfunction-Associated Steatohepatitis and Liver Cancer

[Speaker]Yi Cheng (Department of Pathophysiology, Graduate School of Medicine, Osaka Metropolitan University) [Co-author]Ryota Yamagishi (Department of Pathophysiology, Graduate School of Medicine, Osaka Metropolitan University)、Kanae Echizen (Department of Pathophysiology, Graduate School of Medicine, Osaka Metropolitan University)、Yoshiki Nonaka (Department of Pathophysiology, Graduate School of Medicine, Osaka Metropolitan University)、Naoko Ohtani (Department of Pathophysiology, Graduate School of Medicine, Osaka Metropolitan University)

Recent research highlights the role of the gut-liver axis in liver disease, where dysfunction of the intestinal and liver sinusoidal endothelial cell (LSEC) barriers contributes to pathogenesis. The LSEC acts as a hepatic barrier, and its impairment, alongside gut barrier disruption, may accelerate liver disease progression. These barrier repairments could offer novel preventive and therapeutic strategies for liver cancer. We explored the potential of junction-inducing peptides (JIPs), derived from alpha-1 antitrypsin protein, to restore gut and LSEC barriers in an obesity-associated hepatocellular carcinoma model. Our findings suggest that JIPs can repair LSEC barrier dysfunction by modulating fenestrae. Metabolome analysis of portal vein samples identified substances crossing the gut barrier, while spatial transcriptomics revealed enhanced actin filament organization following JIPs treatment, suggesting the fenestrae normalization. These results underscore the therapeutic potential of JIPs in barrier restoration. Elucidating their mechanism may lead to innovative strategies for preventing and treating liver disease. Further research is needed to understand barrier regulation.

[Disclosure of COI by Responsible researcher] Lead presenter is not the Responsible researcher.
[Lead presenter: Yi Cheng] ■Research funds from company: （科研）[基盤研究C]
[Principal Researcher: Naoko Ohtani] ■Research funds from company: 武田科学振興財団

[P-1351] II-P3-1
Hepatocellular cancer

2025 / 9 / 25 (Thu) 16:45-17:30 Room P/(Motenashi Dome) Room P

P-1351

### Transmembrane Protein 206 is a candidate driver gene for hepatocellular carcinoma

[Speaker]Kazuki Omachi (Kyusyu University Beppu Hospital, Depertment of Surgery) [Co-author]Hajime Otsu (Kyusyu University Beppu Hospital, Depertment of Surgery)、Shinsaku Itoyama (Kyusyu University Beppu Hospital, Depertment of Surgery)、Akinori Tsujimoto (Kyusyu University Beppu Hospital, Depertment of Surgery)、Takashi Ofuchi (Kyusyu University Beppu Hospital, Depertment of Surgery)、Koto Kawata (Kyusyu University Beppu Hospital, Depertment of Surgery)、Tomohiko Ikehara (Kyusyu University Beppu Hospital, Depertment of Surgery)、Shohei Shibuta (Kyusyu University Beppu Hospital, Depertment of Surgery)、Yuki Ando (Kyusyu University Beppu Hospital, Depertment of Surgery)、Kosuke Hirose (Kyusyu University Beppu Hospital, Depertment of Surgery)、Qingjiang Hu (Kyusyu University Beppu Hospital, Depertment of Surgery)、Yusuke Yonemura (Kyusyu University Beppu Hospital, Depertment of Surgery)、Koshi Mimori (Kyusyu University Beppu Hospital, Depertment of Surgery)

Background: We have focused on oncogenes localized on chromosomal genomes with copy number amplification in gastrointestinal cancers, and 1q and 8q copy number amplification is observed in hepatocellular carcinoma (HCC), and we aim to identify novel 1q localized driver gene candidate TMEM206 as a potential therapeutic target. Methods: Genes were selected from The Cancer Genome Atlas (TCGA) based on the following criteria. (1) mRNA levels were higher in tumor tissues than in normal tissues, (2) high mRNA expression group in tumor tissues had poorer prognosis than low mRNA expression group, and (3) DNA copy number and mRNA expression levels were positively correlated. TMEM206 was identified as a gene meeting the above criteria, and TMEM206 KO cell lines were generated and their proliferative ability was evaluated by colony formation assay. Results: TMEM206 expression was significantly upregulated in the tumor group in TCGA, and TMEM206-positive patients had a better prognosis. Discussion: TMEM206 has been reported to be involved in the Wnt/β-catenin pathway in other carcinomas, suggesting that a similar mechanism may be involved in HCC.

[Disclosure of COI by Responsible researcher] Lead presenter is not the Responsible researcher.
[Lead presenter: Kazuki Omachi] We have no financial relationships to disclose.
[Principal Researcher: Koshi Mimori] We have no financial relationships to disclose.

### [P-1352] II-P3-1
### Hepatocellular cancer

2025 / 9 / 25 (Thu) 16:45-17:30 Room P/(Motenashi Dome) Room P

---

### P-1352
### Identification of a Novel Therapeutic Target Gene TONSL in Hepatocellular Carcinoma

[Speaker]Akinori Tsujimoto (Dept. of Surgery, Kyushu Univ. Beppu Hosp.／Dept. of Surgery, Nara Med. Univ.) [Co-author]Hajime Otsu (Dept. of Surgery, Kyushu Univ. Beppu Hosp.)、Takashi Ofuchi (Dept. of Surgery, Kyushu Univ. Beppu Hosp.)、Tomohiko Ikehara (Dept. of Surgery, Kyushu Univ. Beppu Hosp.)、Takanari Tatsumi (Dept. of Surgery, Kyushu Univ. Beppu Hosp.／Dept. of Surgery, Nara Med. Univ.)、Koto Kawata (Dept. of Surgery, Kyushu Univ. Beppu Hosp.)、Shohei Shibuta (Dept. of Surgery, Kyushu Univ. Beppu Hosp.)、Yuki Ando (Dept. of Surgery, Kyushu Univ. Beppu Hosp.)、Kosuke Hirose (Dept. of Surgery, Kyushu Univ. Beppu Hosp.)、Takaaki Masuda (Dept. of Surgery, Kochi Univ.)、Qingjiang Hu (Dept. of Surgery, Kyushu Univ. Beppu Hosp.)、Yusuke Yonemura (Dept. of Surgery, Kyushu Univ. Beppu Hosp.)、Masayuki Sho (Dept. of Surgery, Nara Med. Univ.)、Koshi Mimori (Dept. of Surgery, Kyushu Univ. Beppu Hosp.)

Amplified genomic regions in cancer cells often harbor driver genes that promote tumorigenesis and represent therapeutic targets. We aimed to identify novel targetable oncogenes in hepatocellular carcinoma (HCC) using copy number analysis of the TCGA dataset (n = 372). Genes meeting all of the following were selected: (1) tumor mRNA overexpression (fold change > 2, $p < 0.05$); (2) association of high expression with poor prognosis ($p < 0.05$); (3) correlation between copy number and mRNA ($p < 0.05$, $r > 0.3$); (4) localization to amplified regions. Based on these, we identified TONSL. Immunohistochemistry showed high cytoplasmic expression in tumors. GSEA revealed positive correlations between TONSL expression and MYC targets, DNA repair, and cell cycle pathways (FDR q = 0.002, 0.009, < 0.001). Colony formation assays showed that cell proliferation was significantly suppressed in TONSL knockout cells. TONSL, a DNA repair factor involved in homologous recombination, may promote tumor growth via hyperactive DNA repair and impaired error surveillance. Further functional validation is underway.

[Disclosure of COI by Responsible researcher] Lead presenter is not the Responsible researcher.
[Lead presenter: Akinori Tsujimoto] We have no financial relationships to disclose.
[Principal Researcher: Koshi Mimori] We have no financial relationships to disclose.

### [P-1353] II-P3-1
### Hepatocellular cancer

2025 / 9 / 25 (Thu) 16:45-17:30 Room P/(Motenashi Dome) Room P

### P-1353
### Molecular biological significance of interleukin-11 in hepatocellular carcinoma

[Speaker]Yuto Yamahata (Dept. of Surg., The Jikei Univ. Sch. of Med.) [Co-author]Tomohiko Taniai (Dept. of Surg., The Jikei Univ. Sch. of Med.／Div. of Gene Therapy, The Jikei Univ. Sch. of Med.)、Mitsuru Yanagaki (Dept. of Surg., The Jikei Univ. Sch. of Med.／Div. of Gene Therapy, The Jikei Univ. Sch. of Med.)、Munetoshi Akaoka (Dept. of Surg., The Jikei Univ. Sch. of Med.／Div. of Gene Therapy, The Jikei Univ. Sch. of Med.)、Koichiro Haruki (Dept. of Surg., The Jikei Univ. Sch. of Med.)、Kenei Furukawa (Dept. of Surg., The Jikei Univ. Sch. of Med.)、Saki Matsushima (Div. of Gene Therapy, The Jikei Univ. Sch. of Med.)、Yohta Shimada (Div. of Gene Therapy, The Jikei Univ. Sch. of Med.)、Hiroshi Kobayashi (Div. of Gene Therapy, The Jikei Univ. Sch. of Med.)、Toru Ikegami (Dept. of Surg., The Jikei Univ. Sch. of Med.)

**Background**: Interleukin-11 (IL-11) has been reported to be a poor prognostic factor in hepatocellular carcinoma (HCC) due to the construction of abnormal vascular structures and tumor-infiltrating lymphocytes (Hepatology. 2025). However, the molecular biological significance of IL-11 in tumor cells has not been elucidated, we analyzed the molecular biological role of IL-11 in vitro using HCC cell lines.**Methods**: We measured the expression levels of IL-11 in six human HCC cell lines and selected those with high expression. We established cell lines with IL-11 knocked down (KD) using the siRNA method and evaluated their proliferation and migration abilities.**Results**: The expression levels of IL-11 in six types of human HCC cells were evaluated by Western blotting and qPCR. HepG2 and HLE showed high expression levels, and these cell lines were used in the following experiments. No significant differences were observed in migration and proliferation assay between parent cells and IL-11 KD cells.**Conclusion**: IL-11 did not affect the proliferation and migration of tumor cells in vitro. These results suggested that IL-11 may contribute to tumor growth by affecting the tumor microenvironment.

[Disclosure of COI by Responsible researcher] Lead presenter is the Responsible researcher.
[Lead presenter: Yuto Yamahata] We have no financial relationships to disclose.

▲TOP

### [P-1354] II-P3-1
### Hepatocellular cancer

2025 / 9 / 25 (Thu) 16:45-17:30 Room P/(Motenashi Dome) Room P

### P-1354
### Withdrawn

No Abstract

### [P-1355] II-P3-1
### Hepatocellular cancer

2025 / 9 / 25 (Thu) 16:45-17:30 Room P/(Motenashi Dome) Room P

---

### P-1355
### Withdrawn

No Abstract

### [P-1356] II-P3-1
### Hepatocellular cancer

2025 / 9 / 25 (Thu) 16:45-17:30 Room P/(Motenashi Dome) Room P

### P-1356
### Withdrawn

No Abstract

### [P-1364] II-P3-3
### Pancreatic cancer & endocrine tumor

2025 / 9 / 25 (Thu) 16:45-17:30 Room P/(Motenashi Dome) Room P

---

### P-1364
### p62 can be a useful for prognostic biomarker for the patients of pancreatic neuroendocrine tumors.

[Speaker]Takashi Matsumoto (Dept. of Anatomic Path., Kyushu Univ.) [Co-author]Takeo Yamamoto (Dept. of Anatomic Path., Kyushu Univ.)、Satoru Osajima (Dept. of Anatomic Path., Kyushu Univ.)、Yoshiyuki Nakanishi (Dept. of Anatomic Path., Kyushu Univ.)、Shoko Noguchi (Dept. of Anatomic Path., Kyushu Univ.)、Shinichi Aishima (Dept. of Scientific Path., Kyushu Univ.)、Masafumi Nakamura (Dept. of Surg. and Oncology, Kyushu Univ.)、Yoshinao Oda (Dept. of Anatomic Path., Kyushu Univ.)

Pancreatic neuroendocrine tumors (P-NETs) are relatively rare and exhibit marked heterogeneity. Only 30% of patients with progressive P-NET achieve stable disease, and few biomarkers are established. Notably, 15% of P-NETs harbor mutation in mammalian target of rapamycin (mTOR) pathway, associated with poor prognosis. Autophagy, a key homeostatic mechanism regulated by mTOR, can function as either a tumor suppressor or promoter, depending on the cancer type. However, its impact on P-NET clinical features remains unclear. Here, we evaluated autophagic activity in 193 P-NET cases resected at our hospital between 1994 and 2022. Immunohistochemical analysis revealed that high p62 expression, indicative of impaired autophagy, was significantly correlated with worse prognosis, lymph node metastasis, and lymphovascular invasion. Additionally, areas with elevated p62 showed reduced E-cadherin staining, suggesting a link between impaired autophagy, epithelial-mesenchymal transition, and metastasis. Collectively, these findings indicate that p62 may serve as an independent prognostic marker in P-NET.

[Disclosure of COI by Responsible researcher] Lead presenter is not the Responsible researcher.
[Lead presenter: Takashi Matsumoto] We have no financial relationships to disclose.
[Principal Researcher: Yoshinao Oda] We have no financial relationships to disclose.

[P-1365] II-P3-3
Pancreatic cancer & endocrine tumor

2025 / 9 / 25 (Thu) 16:45-17:30 Room P/(Motenashi Dome) Room P

### P-1365
### Hypoxic Primary Culture Identified Proliferating CAFs in Pancreatic Cancer: Distinct Growth and Spatial Features

[Speaker]Shuhei Komatsuzaki (Department of GI and HBP Surgery, University of Tsukuba) [Co-author]Yoshihiro Miyazaki (Department of GI and HBP Surgery, University of Tsukuba)、Hiromitsu Nakahashi (Department of GI and HBP Surgery, University of Tsukuba)、Osamu Shimomura (Department of GI and HBP Surgery, University of Tsukuba)、Tatsuya Oda (Department of GI and HBP Surgery, University of Tsukuba)

Pancreatic ductal adenocarcinoma (PDAC) is characterized by abundant stroma, with cancer-associated fibroblasts (CAFs) playing crucial roles in tumor progression and therapeutic resistance. CAFs are a heterogeneous population, complicating therapeutic strategies. While PDAC tumors are known to be highly hypoxic (0.7% $O_2$), CAFs are typically isolated under normoxia (21% $O_2$), possibly missing hypoxia-adapted subtypes. We isolated CAFs from surgical PDAC specimens under both normoxia (N-CAF) and hypoxia (1% $O_2$, H-CAF) and performed RNA-seq. H-CAFs showed upregulation of E2F targets, G2M checkpoint, and Mitotic spindle (MsigDB Human hallmarks), consistent with "Proliferating CAFs" reported in multi-cancer studies. Proliferation assays demonstrate its higher proliferation capacity. To determine the spatial localization of H-CAF-like populations within tumors, we employed spatial transcriptomics, which revealed that H-CAF signature-high CAFs were enriched in hypoxic, avascular regions.Our findings suggest that hypoxic isolation captures a distinct proliferative CAF subpopulation, potentially overlooked in conventional CAF research, and may offer novel therapeutic targets.

[Disclosure of COI by Responsible researcher] Lead presenter is not the Responsible researcher.
[Lead presenter: Shuhei Komatsuzaki] We have no financial relationships to disclose.
[Principal Researcher: Tatsuya Oda] We have no financial relationships to disclose.

▲TOP

### [P-1366] II-P3-3
### Pancreatic cancer & endocrine tumor

2025 / 9 / 25 (Thu) 16:45-17:30 Room P/(Motenashi Dome) Room P

---

### P-1366
### ECM1 and KRT6A Mediate Dexamethasone-Induced Tumor Progression and Chemoresistance in pancreatic cancer

[Speaker]Yoshiki Shinomiya (Ctr. for Development of Advanced Diagnostics, Hokkaido Univ. Hosp.) [Co-author]Yusuke Kouchi (Dept. of Mol. Pathol., Grad. Sch. of Med., Chiba Univ.)、Sakurako Kagitani (Dept. of Pathol., Chiba Univ. Hosp.)、Takayuki Ishige (Kazusa DNA Research Inst.／Div. of Lab. Med., Chiba Univ. Hosp.)、Kanako Hatanaka (Ctr. for Development of Advanced Diagnostics, Hokkaido Univ. Hosp.)、Yutaka Hatanaka (Ctr. for Development of Advanced Diagnostics, Hokkaido Univ. Hosp.)、Shigetsugu Takano (Dept. of General Surg., Grad. Sch. of Med., Chiba Univ.)、Masayuki Ohtsuka (Dept. of General Surg., Grad. Sch. of Med., Chiba Univ.)、Junichiro Ikeda (Dept. of Pathol., Chiba Univ. Hosp.／Dept. of Diagnostic Pathol., Grad. Sch. of Med., Chiba Univ.)、Takashi Kishimoto (Dept. of Mol. Pathol., Grad. Sch. of Med., Chiba Univ.)

Pancreatic ductal adenocarcinoma (PDAC) is a highly aggressive malignancy. Neoadjuvant chemotherapy is effective, but its adverse effects are managed with glucocorticoids such as dexamethasone (DEX), which also influence tumor biology. This study investigates the functional and gene expression changes induced by DEX in PDAC cells. PDAC cells were exposed to DEX, and their proliferation, migration, invasion, and gemcitabine (GEM) sensitivity were evaluated. RNA-Seq revealed upregulation of ECM1 and KRT6A. Their functional roles were assessed using overexpression models, and their clinicopathological significance analyzed in 130 PDAC cases.DEX enhanced migration and invasion while reducing GEM sensitivity, whereas proliferation decreased. ECM1 promoted migration, invasion, and GEM resistance, while KRT6A enhanced migration and invasion without affecting GEM sensitivity. Neither gene influenced proliferation. Immunohistochemistry showed KRT6A expression in poorly differentiated regions, and high ECM1 and KRT6A levels correlated with poor prognosis. These findings suggest that DEX alters PDAC cell function, with ECM1 and KRT6A potentially driving tumor progression and chemoresistance.

[Disclosure of COI by Responsible researcher] Lead presenter is not the Responsible researcher.
[Lead presenter: Yoshiki Shinomiya] We have no financial relationships to disclose.
[Principal Researcher: Takashi Kishimoto] We have no financial relationships to disclose.

▲TOP

### [P-1367] II-P3-3
### Pancreatic cancer & endocrine tumor

2025 / 9 / 25 (Thu) 16:45-17:30 Room P/(Motenashi Dome) Room P

---

### P-1367
### Elucidation of the mechanism of pancreatic cancer malignant transformation by stabilization of modified passenger miRNAs

[Speaker]Mayuka Ohkawa (YCU Grad Sch of NanoBiosci) [Co-author]Motomi Yamazaki (AIST Cell&Mol Bioltech Res inst)、Yuko Aihara (AIST Cell&Mol Bioltech Res inst)、Jun Koseki (AIST Cell&Mol Bioltech Res inst／Nagoya Univ Grad Sch of Med)、Shunsuke Kon (Tokyo Univ of Sci Res Inst for Biomed Sci)、Teppei Simamura (Nagoya Univ Grad Sch of Med／CLS Med Res Inst)、Masamitsu Konno (YCU Grad Sch of NanoBiosci／AIST Cell&Mol Bioltech Res inst)

Recently, RNA modifications have been reported to be involved in the malignant transformation of pancreatic cancer.In particular, among more than 170 types of RNA modifications, the functions of other RNA modifications other than m6A modification in cancer have been largely unknown.So in this study we aim to elucidate the mechanism of malignant transformation of pancreatic cancer by RNA-modifying enzymes.First, sequence analysis was performed to identify RNA-modifying enzymes whose expression is altered in pancreatic cancer.As a result, we identified a novel RNA modification enzyme whose expression is increased in pancreatic cancer.We found that RNA modified by the novel RNA-modifying enzyme avoids RNase degradation, thereby improving the stability of passenger RNA, which is degraded under normal conditions in cancer cells.Furthermore, when the proliferative ability was examined with and without the modification, it was found that the proliferation was enhanced in pancreatic cancer cells transfected with the modified RNA.

[Disclosure of COI by Responsible researcher] Lead presenter is not the Responsible researcher.
[Lead presenter: Mayuka Ohkawa] We have no financial relationships to disclose.
[Principal Researcher: Masamitu Konno] We have no financial relationships to disclose.

### [P-1368] II-P3-3
### Pancreatic cancer & endocrine tumor

2025 / 9 / 25 (Thu) 16:45-17:30 Room P/(Motenashi Dome) Room P

---

### P-1368
### RON kinase regulates HIF-1a-driven invasiveness in PDAC: Implications for targeted therapy

[Speaker]Akihisa Kato (Dept. Gastroenterol, Nagoya City Univ.) [Co-author]Kenji Urakabe (Dept. Gastroenterol, Nagoya City Univ.)、Yusuke Kito (Dept. Gastroenterol, Nagoya City Univ.)、Tadashi Toyohara (Dept. Gastroenterol, Nagoya City Univ.)、Michihiro Yoshida (Dept. Gastroenterol, Nagoya City Univ.)、Hiromi Kataoka (Dept. Gastroenterol, Nagoya City Univ.)

Pancreatic ductal adenocarcinoma (PDAC) is a highly lethal cancer with limited therapeutic options. This study identifies the RON tyrosine kinase receptor and hypoxia-inducible factor-1 alpha (HIF-1a) signaling axis as a key driver of PDAC invasiveness. RON and HIF-1a were co-expressed in PDAC tumors and cell lines, with RON levels positively correlating with HIF-1a expression. RON knockdown suppressed both HIF-1a expression and cell invasion, while ectopic RON expression in RON-null cells induced HIF-1a and enhanced invasiveness, which was blunted in the HIF-1a knockdown. Mechanistically, RON activated HIF-1a transcription via PI3K/AKT signaling and Sp1-mediated promoter activation. A small-molecule RON inhibitor blocked ligand (MSP)-induced HIF-1a upregulation and invasion, without affecting proliferation. In an orthotopic mouse model, RON knockdown significantly reduced tumor growth and metastatic spread (lymph node/liver metastasis), 80% in vector control vs. 26% in RON knockdown, with corresponding reduction of HIF-1a in tumors. These findings highlight the RON/HIF-1a axis as a novel and actionable therapeutic target in PDAC.

[Disclosure of COI by Responsible researcher] Lead presenter is the Responsible researcher.
[Lead presenter: Akihisa Kato] We have no financial relationships to disclose.

▲TOP

### [P-1369] II-P3-3
### Pancreatic cancer & endocrine tumor

2025 / 9 / 25 (Thu) 16:45-17:30 Room P/(Motenashi Dome) Room P

---

### P-1369
### Rap1b regulates cell cycle progression and proliferation in pancreatic cancer cells

[Speaker]Daigo Yoshimori (Dept. of Gastroenterological Surgery, Nippon medical school／Dept. of Integrated Diagnostic Pathology, Nippon medical school) [Co-author]Mitsuhiro Kudoh (Dept. of Integrated Diagnostic Pathology, Nippon medical school)、Kousuke Ishino (Dept. of Integrated Diagnostic Pathology, Nippon medical school)、Junji Ueda (Dept. of Gastroenterological Surgery, Nippon medical school)、Takahiro Haruna (Dept. of Gastroenterological Surgery, Nippon medical school)、Endo Endo (Dept. of Gastroenterological Surgery, Nippon medical school／Dept. of Integrated Diagnostic Pathology, Nippon medical school)、Yoko Kawamoto (Dept. of Integrated Diagnostic Pathology, Nippon medical school)、Taeko Kitamura (Dept. of Integrated Diagnostic Pathology, Nippon medical school)、Kiyoshi Teduka (Dept. of Integrated Diagnostic Pathology, Nippon medical school)、Takenori Fujii (Dept. of Integrated Diagnostic Pathology, Nippon medical school)、Yukako Domoto (Dept. of Integrated Diagnostic Pathology, Nippon medical school)、Ryuji Ohashi (Dept. of Integrated Diagnostic Pathology, Nippon medical school)

[Objectives] Rap1b is a small GTP-binding protein involved in adhesion, migration, and polarity. Its abnormal activation has been reported in various cancers and is linked to tumor progression. However, its role in proliferation and cell cycle regulation remains unclear. To address this, we modulated Rap1b expression in pancreatic cancer cells and analyzed molecular changes using proteomics.[Methods] Pancreatic cancer cell lines were transfected with siRap1b. Cell proliferation was assessed in vitro. Flow cytometry was used to analyze apoptosis and the cell cycle. Proteomics identified expression changes in response to Rap1b knockdown.[Results] Knockdown of Rap1b resulted in decreased cell proliferation. Flow cytometry analysis suggested cell cycle arrest at the G2/M phase, with no significant change in early apoptosis. Proteomic analysis showed a significant down-regulation of proteins involved in cytokinesis and mitosis following Rap1b suppression, suggesting that this may contribute to the observed M-phase arrest.[Conclusion] These findings suggest that Rap1b may influence the proliferative capacity of pancreatic cancer cells by regulating cell cycle progression.

[Disclosure of COI by Responsible researcher] Lead presenter is not the Responsible researcher.
[Lead presenter: Daigo Yoshimori] We have no financial relationships to disclose.
[Principal Researcher: Ryuji Ohashi] We have no financial relationships to disclose.

### [P-1370] II-P3-3
### Pancreatic cancer & endocrine tumor

2025 / 9 / 25 (Thu) 16:45-17:30 Room P/(Motenashi Dome) Room P

---

### P-1370
### Glucose-Induced Suppression of the STING Pathway Impairs Innate Immunity via Autophagy in Pancreatic Cancer

[Speaker]Yi-Ru Lin (Dept. of Biotech., China Med. Univ.) [Co-author]Wen-Chi Chou (Dept. of Biotech., China Med. Univ.)、Wei-Jan Wang (Dept. of Biotech., China Med. Univ.)

The link between diabetes and cancer is increasingly recognized. High glucose promotes tumor growth and alters the tumor microenvironment. However, its effect on immune evasion via innate immune signaling is unclear. STING is a key innate sensor that activates TBK1 and NF$\kappa$B, inducing type I interferons crucial for anti-tumor immunity. In this study, we show that high glucose boosts proliferation of glucose-sensitive cells but suppresses STING-TBK1-NF$\kappa$B signaling, reducing IFN$\beta$ mRNA expression. Treatment with STING agonist MIW815 or glucose analog 2-DG restored TBK1 activity under high glucose, partially reversing this effect. Autophagy also contributed to TBK1 regulation in high-glucose conditions, potentially aiding immune evasion in pancreatic cancer. In summary, high glucose suppresses innate immunity by inhibiting TBK1 and IFN$\beta$, promoting an immunosuppressive tumor environment. These findings highlight a mechanism linking diabetes to cancer progression and offer insights for future immunotherapy strategies.

[Disclosure of COI by Responsible researcher] Lead presenter is the Responsible researcher.
[Lead presenter: Yi-Ru Lin] We have no financial relationships to disclose.

### [P-1380] II-P4-2
### Head & neck tumors (2)

2025 / 9 / 25 (Thu) 16:45-17:30 Room P/(Motenashi Dome) Room P

### P-1380
### MTDH as a cancer fitness gene driving metastasis in oral squamous cell carcinoma

[Speaker]Sak Lee (Dept. of Oral Path., Sch. of Dent., SNU) [Co-author]Seong-Doo Hong (Dept. of Oral Path., Sch. of Dent., SNU)、Sung-Dae Cho (Dept. of Oral Path., Sch. of Dent., SNU)

Cancer fitness genes promote survival under the stresses encountered during metastasis but, unlike driver oncogenes, lack transforming ability and are largely dispensable in normal tissues, making them attractive therapeutic targets. Metadherin (MTDH), first identified as a metastasis mediator in breast cancer, is overexpressed in various cancers. Despite its known oncogenic potential, research elucidating the role of MTDH in OSCC remains insufficient. In the present study, in silico and in vitro analyses revealed significant overexpression of MTDH in oral squamous cell carcinoma (OSCC). Immunohistochemistry of 100 OSCC cases confirmed higher MTDH expression in tumors than in normal tissue, especially at the invasive front and in less differentiated tumors. Using HSC4 and Ca9.22 cell lines, MTDH knockout reduced invasion and migration but had no effect on proliferation or apoptosis. RNA sequencing and validation experiments suggested a potential association between MTDH and cytokeratin 14. These findings indicate that MTDH acts as a cancer fitness gene in OSCC and represents a promising therapeutic target. NRF grants (RS-2025-00553553, RS-2023-00247502).

[Disclosure of COI by Responsible researcher] Lead presenter is the Responsible researcher.
[Lead presenter: Sak Lee] We have no financial relationships to disclose.

### [P-1381] II-P4-2
### Head & neck tumors (2)

2025 / 9 / 25 (Thu) 16:45-17:30 Room P/(Motenashi Dome) Room P

---

### P-1381
### Improving risk stratification for oropharyngeal cancer by evaluating initial diagnosis risk of distant metastasis

[Speaker]Chuanhao Zhang (Grad. Sch. of Dalian Med. Univ.) [Co-author]Zhichao Cheng (Dept. of Med. Oncology, Affiliated Zhongshan Hosp. of Dalian Univ.)、Zhe Wang (Dept. of Med. Oncology, Affiliated Zhongshan Hosp. of Dalian Univ.)、Ruoyu Wang (Dept. of Med. Oncology, Affiliated Zhongshan Hosp. of Dalian Univ.)

Purpose:To identify factors linked to distant metastasis (DM) at diagnosis and survival outcomes in oropharyngeal cancer patients.Methods:This study analyzed oropharyngeal cancer cases from 2018 to 2021 using the SEER database, focusing on those with complete survival data. We used logistic and Cox regression to find clinical factors beyond HPV status and stage. Data analysis occurred from September 2024 to March 2025.Results:Out of 13,102 eligible patients, 440 (3.4%) were diagnosed with DM. Those with primary tumors in the tonsil (OR 0.58, $p<0.001$) or base of tongue (OR 0.56, $p<0.001$) had less DM risk compared to oropharynx. Widowed had higher DM risk (OR 1.70, $p<0.007$) compared to married. Furthermore, Tonsil (HR 0.74, $p<0.001$) or base of tongue (HR 0.80, $p<0.001$) had better overall survival (OS) than oropharynx. Single (HR 1.9, $p<0.001$), divorced (HR 1.62, $p<0.001$), widowed (HR 1.66, $p<0.001$) had worse OS compared to married. A similar trend was seen for cancer-specific survival.Conclusion:For those with oropharyngeal cancer, the tumor's location and marital status at diagnosis are key indicators of survival, potentially improving existing risk assessment methods.

[Disclosure of COI by Responsible researcher] Lead presenter is not the Responsible researcher.
[Lead presenter: Chuanhao Zhang] We have no financial relationships to disclose.
[Principal Researcher: Chuanhao Zhang] We have no financial relationships to disclose.

### [P-1382] II-P4-2
### Head & neck tumors (2)

2025 / 9 / 25 (Thu) 16:45-17:30 Room P/(Motenashi Dome) Room P

### P-1382
### Withdrawn

No Abstract

### [P-1383] II-P4-2
### Head & neck tumors (2)

2025 / 9 / 25 (Thu) 16:45-17:30 Room P/(Motenashi Dome) Room P

---

### P-1383
### Establishment of a Salivary Gland Tumorigenesis Model in salivary gland-specific EWSR1::ATF1 Transgenic Mice

[Speaker]Yuri Hirai (Dept. Head and Neck Surgery, Yokohama City University) [Co-author]Yoshitaka Utsumi (Department of Anatomical Pathology, Tokyo Medical University)、Daisuke Sano (Dept. Head and Neck Surgery, Yokohama City University)、Tatsu Kuwahara (Dept. Head and Neck Surgery, Yokohama City University)、Hisashi Hasumi (Department of Urology, Yokohama City University)、Satoshi Fujii (Dept. Molecular Pathology, Yokohama City University)、Toshitaka Nagao (Department of Anatomical Pathology, Tokyo Medical University)、Nobuhiko Oridate (Dept. Head and Neck Surgery, Yokohama City University)

Background: Salivary gland carcinoma is a rare malignancy with considerable pathologic, biological, and clinical diversity. A high frequency of fusion genes has been reported in certain histologic types of these malignancies, such as EWSR1::ATF1, which is found in 93% of hyalinizing clear cell carcinomas (HCCCs). We thus hypothesized that EWSR1::ATF1 is a driver mutation in HCCC. Methods: We generated EWSR1::ATF1 transgenic mice transfected with EWSR1::ATF1 through a breakpoint reported in HCCC. These mice were crossed with ***Lama parotid secretory protein-Cre*** transgenic mice expressing Cre recombinase in the salivary glands to generate salivary gland-specific EWSR1::ATF1 transgenic mice. Tumor specimens generated in the model mice were subjected to Western blotting, histopathologic evaluation, and RNA sequencing. Results: Three salivary gland-specific EWSR1::ATF1 transgenic mice showed more aggressive pathological characteristics in the major salivary glands compared to that of the HCCC do. Conclusions: This study suggests that salivary gland-specific expression of EWSR1::ATF1 plays an important role in malignant tumorigenesis in the major salivary glands.

[Disclosure of COI by Responsible researcher] Lead presenter is not the Responsible researcher.
[Lead presenter: Yuri Hirai] We have no financial relationships to disclose.
[Principal Researcher: Nobuhiko Oridate] We have no financial relationships to disclose.

▲TOP

### [P-1384] II-P4-2
### Head & neck tumors (2)

2025 / 9 / 25 (Thu) 16:45-17:30 Room P/(Motenashi Dome) Room P

---

### P-1384
### PLAU as a Prognostic Biomarker Based on Transcriptomic Analysis of P. gingivalis-Infected Head and Neck Cancer Cells

[Speaker]Masakazu Hamada (Dept of Oral Maxillofacial Oncology and Surgery UOsaka) [Co-author]Kyoko Nishiyama (Dept of Oral Maxillofacial Oncology and Surgery UOsaka)、Narikazu Uzawa (Dept of Oral Maxillofacial Oncology and Surgery UOsaka)

Periodontal disease is a risk factor for head and neck squamous cell carcinoma (HNSCC). Porphyromonas gingivalis, a major periodontal pathogen, is known as a microbial factor that increases the risk of cancer mortality. Gene expression in HNSCC due to P. g. infection affect the prognosis of HNSCC patients are not clarified. When P. g. was cultured with HNSCC cells, it enhanced their invasive ability. A transcriptome analysis of P. g. infected HNSCC cells showed that genes related to migration were highly regulated. The expression of up-regulated genes was examined using TCGA-HNSCC database, and the expression of 5 genes, including PLAU, was found to be higher in cancer tissue than in normal tissue. A Cox regression analysis showed that high PLAU expression levels were associated with a poor prognosis. Furthermore, the prognostic impact correlated with tumor size and lymph node metastasis. Collectively, these results suggest the potential of PLAU as a molecular prognostic marker in HNSCC patients. Further studies are needed to verify the findings of this study. Journal of Cellular and Molecular Medicine, 28, 0.1111/jcmm, 2024, Wiley. It is presented with permission of the publisher.

[Disclosure of COI by Responsible researcher] Lead presenter is the Responsible researcher.
[Lead presenter: Masakazu Hamada] We have no financial relationships to disclose.

### [P-1385] II-P4-2
### Head & neck tumors (2)

2025 / 9 / 25 (Thu) 16:45-17:30 Room P/(Motenashi Dome) Room P

---

### P-1385
### Elucidation of epigenetic mechanisms underlying carcinogenesis in maxillary sinus carcinoma

[Speaker]Ryo Arai (Dpt of Mol Oncology, Grad Sch of Med, Chiba Univ／Dept of Otorhinolaryngology ／ Head and Neck Surgery, Chiba Univ)
[Co-author]Takuya Nakagawa (Dpt of Mol Oncology, Grad Sch of Med, Chiba Univ／Dept of Otorhinolaryngology ／ Head and Neck Surgery, Chiba Univ／Dpt of Health and Disease Omics Center, Chiba Univ)、Masaki Fukuyo (Dpt of Mol Oncology, Grad Sch of Med, Chiba Univ)、Rahmutulla Bahityar (Dpt of Mol Oncology, Grad Sch of Med, Chiba Univ)、Toyoyuki Hanazawa (Dept of Otorhinolaryngology ／ Head and Neck Surgery, Chiba Univ)、Atsushi Kaneda (Dpt of Mol Oncology, Grad Sch of Med, Chiba Univ／Dpt of Health and Disease Omics Center, Chiba Univ)

Maxillary sinus carcinoma (MSC) exhibits higher recurrence and poorer prognosis compared to other head and neck cancers, with rare association to smoking, alcohol, or TP53 mutations. To elucidate underlying molecular mechanisms, we performed integrated analyses of transcriptome and epigenome in MSC cell line HSQ-89 and clinical MSC samples using RNA-seq and ChIP-seq. RNA-seq of HSQ-89 revealed 3324 upregulated genes compared to normal cell line NOKSI. ChIP-seq revealed 650 genes had activated promoter regions and 1923 genes had activated enhancer regions. Pathway analysis of 521 genes with upregulation and promoter activation revealed significant enrichment of the Wnt signaling pathway. Genes with activated enhancers showed comparable results. Motif analysis of both activated promoter/enhancer regions identified significant enrichment of LEF1, a downstream transcription factor of the Wnt signaling pathway. RNA-seq of clinical 11 MSC samples compared to 5 normal mucosa control samples shared 776 commonly upregulated genes with the HSQ-89/NOKSI comparison. These findings suggest the contribution of Wnt signaling pathway to genesis of MSC.

[Disclosure of COI by Responsible researcher] Lead presenter is not the Responsible researcher.
[Lead presenter: Ryo Arai] We have no financial relationships to disclose.
[Principal Researcher: Atsushi Kaneda] ■Research expenses: Takeda Science Foundation., Uehara Memorial Foundation.

▲TOP

### [P-1386] II-P4-2
### Head & neck tumors (2)

2025 / 9 / 25 (Thu) 16:45-17:30 Room P/(Motenashi Dome) Room P

---

### P-1386
### Integrated analysis of immune cells and fibroblasts in head and neck squamous cell carcinoma

[Speaker]Satoshi Owada (Dept. Oral Surg., Sapporo Med. Univ., Sch. Med.／Div. Mol. Biol., Dept. Biochem., Sapporo Med. Univ., Sch. Med.)
[Co-author]Akira Yorozu (Dept. Otolaryngol. Head Neck Surg., Sapporo Med. Univ., Sch. Med.)、Takeshi Niinuma (Div. Mol. Biol., Dept. Biochem., Sapporo Med. Univ., Sch. Med.)、Hiroshi Kitajima (Div. Mol. Biol., Dept. Biochem., Sapporo Med. Univ., Sch. Med.)、Kazuya Ishiguro (Div. Mol. Biol., Dept. Biochem., Sapporo Med. Univ., Sch. Med.)、Eiichiro Yamamoto (Div. Mol. Biol., Dept. Biochem., Sapporo Med. Univ., Sch. Med.)、Shohei Sekiguchi (Dept. Oral Surg., Sapporo Med. Univ., Sch. Med.)、Fumika Okazaki (Dept. Oral Surg., Sapporo Med. Univ., Sch. Med.)、Masahiro Kai (Div. Mol. Biol., Dept. Biochem., Sapporo Med. Univ., Sch. Med.)、Kenichi Takano (Dept. Otolaryngol. Head Neck Surg., Sapporo Med. Univ., Sch. Med.)、Akihiro Miyazaki (Dept. Oral Surg., Sapporo Med. Univ., Sch. Med.)、Hiromu Suzuki (Div. Mol. Biol., Dept. Biochem., Sapporo Med. Univ., Sch. Med.)

To improve the response to immune checkpoint inhibitors (ICIs) in head and neck squamous cell carcinoma (HNSCC), a deeper understanding of the tumor immune microenvironment (TIME) is essential. We analyzed the proportions of tumor-infiltrating immune cells and cancer-associated fibroblasts (CAFs) in HNSCC tumors surgically resected at our institution, including oral, oropharyngeal, hypopharyngeal, and laryngeal cancers. Immune cells and CAFs were quantified using flow cytometry and compared across tumor subsites. CAFs were classified into CAF-S1 to CAF-S4 based on FAP and CD29 (integrin-β1), following the method described by Costa et al. (Cancer Cell, 2018). As reported previously, immune cell infiltration was greater in tumor tissues than in adjacent normal tissues or lymph nodes. Notably, in oropharyngeal cancer, the abundance of CAF-S1 was inversely correlated with $CD4^+$ T-cell infiltration, while positive correlations were observed with $CD8^+$ T cells, granulocytes, and dendritic cells. Few studies have compared immune infiltration by tumor site in HNSCC. Our integrated analysis of immune and CAF subtypes may contribute to a more comprehensive understanding of the TIME in HNSCC.

[Disclosure of COI by Responsible researcher] Lead presenter is not the Responsible researcher.
[Lead presenter: Satoshi Owada] We have no financial relationships to disclose.
[Principal Researcher: Hiromu Suzuki] We have no financial relationships to disclose.

### [P-1387] II-P4-2
### Head & neck tumors (2)

2025 / 9 / 25 (Thu) 16:45-17:30 Room P/(Motenashi Dome) Room P

---

### P-1387
### Expression of CD74 and macrophage migration inhibitory factor in nasopharyngeal carcinoma

[Speaker]Toshihiro Nagato (Dept. Path., Asahikawa Med. Univ.) [Co-author]Takahiro Inoue (Dept. Path., Asahikawa Med. Univ.／Dept. Otolaryngology-Head and Neck Surg., Asahikawa Med. Univ.)、Nanami Ujiie (Dept. Path., Asahikawa Med. Univ.／Dept. Thoracic Surg. & Breast Surg., Asahikawa Med. Univ. Hosp.)、Ryusei Yoshino (Dept. Path., Asahikawa Med. Univ.／Dept. Thoracic Surg. & Breast Surg., Asahikawa Med. Univ. Hosp.)、Hiroyoshi Nozaki (Dept. Path., Asahikawa Med. Univ.／Dept. Dermatology, Asahikawa Med. Univ.)、Akemi Kosaka (Dept. Path., Asahikawa Med. Univ.)、Takayuki Ohkuri (Dept. Path., Asahikawa Med. Univ.)、Miki Takahara (Dept. Otolaryngology-Head and Neck Surg., Asahikawa Med. Univ.)、Hiroya Kobayashi (Dept. Path., Asahikawa Med. Univ.)

CD74, also known as major histocompatibility complex (MHC) class II-associated invariant chain, was initially identified to bind to MHC class II antigen-binding groove and regulate the trafficking of MHC class II in antigen presenting cells. Recent studies have revealed that CD74 also has functions as receptor for macrophage migration inhibitory factor (MIF) and that CD74-MIF pathway affects tumor proliferation and invasion. In the present study, we report that CD74 and MIF was expressed on the surface and in the culture supernatant of nasopharyngeal carcinoma (NPC) cell lines, respectively. In addition, CD74 was expressed by tumor cells in biopsy samples from NPC patients. Positive expression of CD74 by NPC cells was significantly correlated with Epstein-Barr virus infection. Furthermore, MIF was present in sera of NPC patients at higher levels as compared to healthy individuals. These results suggest that the CD74-MIF pathway may be an important factor in the development of NPC.

[Disclosure of COI by Responsible researcher] Lead presenter is the Responsible researcher.
[Lead presenter: Toshihiro Nagato] We have no financial relationships to disclose.

▲TOP

### [P-1388] II-P4-2
### Head & neck tumors (2)

2025 / 9 / 25 (Thu) 16:45-17:30 Room P/(Motenashi Dome) Room P

---

### P-1388
### In HNSCC, Trop2 is a marker of cancer stem cells and CD73 regulates metastasis.

[Speaker]Yohei Kawasaki (Otorhinolaryngology and Head and Neck Surgery, Akita University) [Co-author]Hitomi Suzuki (Otorhinolaryngology and Head and Neck Surgery, Akita University)、Yasufumi Omori (Molecular and Tumor Pathology, Akita University)

In head and neck squamous cell carcinoma (HNSCC), the presence of cancer stem cells (CSCs) and epithelial mesenchymal transition (EMT) as a cause of treatment resistance is a challenge to overcome in cancer therapy. A cell surface membrane protein called Trop2, which is overexpressed in malignant tumors, has attracted a great deal of attention. Furthermore, CD73 positive cells have been noted as inducing EMT. We examined Trop2 expression and its properties in HNSCC. The high expression group formed many spheroids and had increased invasion and migration ability. HNSCC cell lines were separated into CD73 positive and CD73 negative cells and cultured on Matrigel to observe the differences in morphology. The CD73 positive cells showed a very elongated cell morphology compared to CD73 negative cells, and appeared to have acquired mesenchymal cell characteristics. Trop2 high expressing CD73 positive cells showed enhanced spheroid formation, invasion, and migration, while Trop-2- high expressing CD73 negative cells maintained spheroid formation but suppressed invasion and migration, fully satisfying our hypothesis that Trop2 functions as a cancer stem cell and that CD73 induces EMT.

[Disclosure of COI by Responsible researcher] Lead presenter is the Responsible researcher.
[Lead presenter: Yohei Kawasaki] We have no financial relationships to disclose.

### [P-1008] I-P1-2
### Animal models for cancer (2)

2025 / 9 / 25 (Thu) 17:30-18:15 Room P/(Motenashi Dome) Room P

---

### P-1008
### LPO-caused cancer initiation to transform hepatocytes into GST-P(+) preneopastic single hepatocytes in rat livers

[Speaker]Kimihiko Satoh (Akita University of Nursing and Welfare, Medical Welfare)

LPO causes cancer initiation. Unforeeseeingly, new mechanism of cancer initiation that transforms hepatocytes into preneoplastic single hepatocytes heavily positive for glutathione S-transferase P-form (GST-P), precursors of foci and nodules, was identified, very recently[1,2]. GST-P was found to be rapidly excreted into bile of rat livers in response to strong carcinogenic stress caused by either promoters or initiators through 3D analysis using a vibratome. In this mechanism inititor carcinogens stimulate LPO, by which GST-P and other enzymes are induced to detoxicate the products. Carcinogens also act to decrease the excretion of GST-P into bile resulting in the accumulation of the marker enzyme in hepatocytes giving rise to GST-P(+) single hepatocytes. The initiation process was determined to be nongenetic because mutation is an invisible rare event. Details of the mechanism and the LPO action will be discussed. 1) Satoh, K. et al. Anal Biochem 2023, 672, 115168. 2) Satoh, K. New mechanism of cancer initiation to transform hepatocytes into preneoplastic single hepatocytes positive for GST-P in rat livers. Cancer Med 2024, 8, e70165.

[Disclosure of COI by Responsible researcher] Lead presenter is the Responsible researcher.
[Lead presenter: Kimihiko Satoh] We have no financial relationships to disclose.

▲TOP

### [P-1009] I-P1-2
### Animal models for cancer (2)

2025 / 9 / 25 (Thu) 17:30-18:15 Room P/(Motenashi Dome) Room P

### P-1009
### Early Gene Expression Alterations in DMH-Induced Rat Colon Carcinogenesis

[Speaker]Runjie Guo (Dept. Env. Risk Assess., Osaka Metropolitan Univ. Grad. Sch. Med) [Co-author]Arpamas Vachiraarunwong (Dept. Env. Risk Assess., Osaka Metropolitan Univ. Grad. Sch. Med)、Shugo Suzuki (Dept. Mol. Pathol., Osaka Metropolitan Univ. Grad. Sch. Med)、Masaki Fujioka (Dept. Mol. Pathol., Osaka Metropolitan Univ. Grad. Sch. Med)、Guiyu Qiu (Dept. Env. Risk Assess., Osaka Metropolitan Univ. Grad. Sch. Med)、Yurina Kawamura (Dept. Env. Risk Assess., Osaka Metropolitan Univ. Grad. Sch. Med)、Anna Kakehashi (Dept. Mol. Pathol., Osaka Metropolitan Univ. Grad. Sch. Med)、Hideki Wanibuchi (Dept. Env. Risk Assess., Osaka Metropolitan Univ. Grad. Sch. Med)、Min Gi (Dept. Env. Risk Assess., Osaka Metropolitan Univ. Grad. Sch. Med)

Early aberrant gene expression in the colonic mucosa is critical for understanding the initiation of colon carcinogenesis and identifying potential biomarkers for early detection. In this study, male F344 rats received four weekly subcutaneous injections of 1,2-dimethylhydrazine (DMH; 20 mg/kg body weight) and were sacrificed one week after the final treatment. Total RNA was extracted from the colonic mucosa and analyzed using microarray and Ingenuity Pathway Analysis. A total of 406 genes were significantly altered in the DMH-treated mucosa. The pathway activation profile revealed a complex response involving translational stress, immune dysregulation, oxidative and genotoxic stress, and oncogenic signalling that collectively contribute to tumour initiation. Additionally, Diseases and Functions analysis indicated upregulation of multiple genes associated with tumour cell proliferation. These findings characterize early-phase gene expression changes in DMH-induced colon carcinogenesis and may support the development of biomarkers and chemoprevention strategies.

[Disclosure of COI by Responsible researcher] Lead presenter is not the Responsible researcher.
[Lead presenter: Runjie Guo] We have no financial relationships to disclose.
[Principal Researcher: Min Gi] We have no financial relationships to disclose.

Poster Session

### [P-1010] I-P1-2
### Animal models for cancer (2)

2025 / 9 / 25 (Thu) 17:30-18:15 Room P/(Motenashi Dome) Room P

### P-1010
### Genomic mutation profiling of hepatocellular carcinoma induced by heavy-ion radiation in B6C3F1 mice

[Speaker]Yi Shang (Dept. Rad. Effects. Res., NIRS, QST) [Co-author]Atsuko Ishikawa (Dept. Rad. Effects. Res., NIRS, QST)、Takamitsu Morioka (Dept. Rad. Effects. Res., NIRS, QST)、Kazuhiro Daino (Dept. Rad. Effects. Res., NIRS, QST)、Shizuko Kakinuma (Dept. Rad. Effects. Res., NIRS, QST／IES)

Heavy-ion radiotherapy is an advanced strategy for cancer treatment. The most serious side effect of heavy-ion radiation is the increased risk of developing second cancer, especially in pediatric patients. Using mice, we found that childhood exposure to carbon-ion radiation increased the risk for hepatocellular carcinoma (HCC). To investigate the genomic mutation characteristics of carbon ion induced hepatocarcinogenesis, one-week-old mice were sham-irradiated or exposed to a single dose of 4 to 4.8 Gy of monoenergetic carbon ions (290 MeV/u) at nominal dose rates of 4.0 Gy/min. Liver tumors were collected for pathological and molecular analyses. We investigated genomic alterations in 10 spontaneous and 12 carbon ion radiation-induced HCCs. Whole-exome sequencing of the HCCs revealed frequent mutations in genes involved in the MAP kinase pathway. Several driver gene mutations observed in mice HCCs have also been well documented in human HCC. This study provides a landscape of genomic alterations in spontaneous and carbon ion radiation-induced HCCs, highlighting the potential of this mouse model using for human HCC studies.

[Disclosure of COI by Responsible researcher] Lead presenter is the Responsible researcher.
[Lead presenter: Yi Shang] We have no financial relationships to disclose.

### [P-1011] I-P1-2
### Animal models for cancer (2)

2025 / 9 / 25 (Thu) 17:30-18:15 Room P/(Motenashi Dome) Room P

---

### P-1011
### Mutational analysis of forestomach tumors induced by benzo[*a*]pyrene in wild-type and Pol κ-deficient mice

[Speaker]Junichi Akagi (Div. Path., Natl. Inst. Health Sci.) [Co-author]Youngman Cho (Div. Path., Natl. Inst. Health Sci.)、Takeshi Toyoda (Div. Path., Natl. Inst. Health Sci.)、Masayuki Yokoi (Biosignal Res. Ctr., Kobe Univ.)、Fumio Hanaoka (Biosignal Res. Ctr., Kobe Univ.／Natl. Inst. Genetics)、Kumiko Ogawa (Div. Path., Natl. Inst. Health Sci.／Dept. Toxicol., Hoshi Univ.)

Benzo[*a*]pyrene (BaP) is metabolized by CYP1A1 into a diol epoxide (BPDE), which forms DNA adducts such as $N^2$-BPDE-dG. Although DNA polymerase κ (Pol κ) efficiently bypasses $N^2$-BPDE-dG lesions and protects cells from cytotoxic and mutagenic effects of BPDE, our feeding study of BaP with or without α-naphthoflavone (ANF), a CYP1A1 antagonist, in male C57BL/6J ***Polk***$^{+/+}$ and ***Polk***$^{-/-}$ littermates showed that Pol κ deficiency had a limited impact on BaP-induced tumorigenesis. To further explore effects of BaP mutagenicity on tumorigenesis, we conducted whole genome sequencing on four BaP-induced tumors: papilloma in ***Polk***$^{+/+}$ with 0.01% BaP; squamous cell carcinoma (SCC) in ***Polk***$^{+/+}$ with 0.01% BaP+0.1% ANF; SCC in ***Polk***$^{-/-}$ with 0.01% BaP; SCC in ***Polk***$^{-/-}$ with 0.01% BaP+0.1% ANF. Despite differences in Pol κ status, treatment, or tumor malignancy, these tumors exhibited similar mutation spectra. In contrast to the mutation spectrum of BaP (C>A), these tumors mainly harbored C>T mutations, resembling COSMIC SBS5 (clock-like) signature. These findings suggest that tumors may acquire mutation spectra distinct from mutagenic profiles of initiating carcinogens during their proliferation and progression.

[Disclosure of COI by Responsible researcher] Lead presenter is the Responsible researcher.
[Lead presenter: Junichi Akagi] We have no financial relationships to disclose.

▲TOP

[P-1012] I-P1-2
Animal models for cancer (2)

2025 / 9 / 25 (Thu) 17:30-18:15 Room P/(Motenashi Dome) Room P

### P-1012
### Gene mutation analysis of amitrole-induced mouse thyroid benign tumor cell lines

[Speaker]Yotaro Shirai (Cancer Innovation Lab., NCI, NIH／Dept. Path., Sch. Med., Wakayama Med. Univ.) [Co-author]Nobuo Hoshi (Cancer Innovation Lab., NCI, NIH)、Jerrold M. Ward (Cancer Innovation Lab., NCI, NIH)、Huaitian Liu (Lab. Human Carcinogenesis, NCI, NIH)、Raul E. Cachau (Integrated Data Sciecnes Section, Resesarch Technology Branch, NIAID, NIH)、Maxwell P. Lee (Lab. Cancer Biology and Genetics, NCI, NIH)、Shogo Ehata (Dept. Path., Sch. Med., Wakayama Med. Univ.)、Shioko Kimura (Cancer Innovation Lab., NCI, NIH)

Gene mutation profiles of thyroid cancer cell lines have been of great value for the study of thyroid cancer. However, the availability of benign thyroid adenoma cell lines is limited and gene mutations involved in thyroid adenoma formation have not been well investigated. We established two cell lines with different NKX2-1 expression levels from thyroid adenomatous nodules developed in mice treated with goitrogen amitrole, named CAT458/458s (a subline of CAT458) and CAT459. Whole-genome sequencing analysis revealed the absence of prominent thyroid cancer-related genes such as ***Braf*** and ***Trp53***. On the other hand, missense mutations were identified in known possible tumor suppressor genes, ***Polk***, ***Thrb***, ***Tgfb1***, and ***Med12***. ***In silico*** mutation analysis suggested a possibility of association of the identified mutations with a disease, with prediction of deleterious mutations especially for ***Thrb*** and ***Tgfb1***. These findings suggest that the mutations found in CAT458s and 459 cells may contribute to thyroid adenoma formation. In addition, these cell lines would provide a tool to further clarify the process of thyroid multistep carcinogenesis.

[Disclosure of COI by Responsible researcher] Lead presenter is not the Responsible researcher.
[Lead presenter: Yotaro Shirai] We have no financial relationships to disclose.
[Principal Researcher: Shogo Ehata] We have no financial relationships to disclose.

[P-1013] I-P1-2
Animal models for cancer (2)

2025 / 9 / 25 (Thu) 17:30-18:15 Room P/(Motenashi Dome) Room P

P-1013
### The mechanism of leukemia development after radiation exposure in Pax5 heterozygous mice

[Speaker]Hirotaka Tachibana (CRIEPI／NIRS, QST) [Co-author]Kazuhiro Daino (NIRS, QST)、Yi Shang (NIRS, QST)、Chizuru Tsuruoka (NIRS, QST)、Masaaki Sunaoshi (NIRS, QST)、Takamitsu Morioka (NIRS, QST)、Daisuke Iizuka (NIRS, QST)、Tatsuhiko Imaoka (NIRS, QST)、Shizuko Kakinuma (NIRS, QST／IES)

Epidemiological studies have revealed an increased risk of developing acute lymphoblastic leukemia (ALL) by radiation exposure. However, the radiation carcinogenesis of ALL has not been elucidated. Recently, we identified hemizygous deletions of ***Pax5*** and mutations of ***Jak3*** as characteristics of radiation-induced mouse B-cell ALL (B-ALL). In this study, we irradiated ***Pax5*** heterozygous mice with 4 Gy of gamma-rays to reveal the effects of radiation on the development of ***Pax5*** driven B-ALL. The analysis of normal marrow and spleen showed no increase in the proportion of $B220^{+}$ B-cells due to ***Pax5*** heterozygosity and irradiation prior to the development of B-ALL. Nevertheless, irradiated ***Pax5*** heterozygous mice showed high incidence of B-ALL compared to mice without irradiation. Targeted RNA sequencing revealed that ***Jak3*** mutations occurred in about half of radiation-induced B-ALLs. These results suggest that radiation exposure may indirectly cause B-ALL by increasing the probability of ***Jak3*** mutations in ***Pax5*** heterozygous B cells. These works were supported by KAKENHI from JSPS (20K12176 and 24K20934) and a fund from the Radiation Disaster and Medical Science Research Center.

[Disclosure of COI by Responsible researcher] Lead presenter is the Responsible researcher.
[Lead presenter: Hirotaka Tachibana] We have no financial relationships to disclose.

### [P-1014] I-P1-2
### Animal models for cancer (2)

2025 / 9 / 25 (Thu) 17:30-18:15 Room P/(Motenashi Dome) Room P

---

### P-1014
### Metabolic signature of diethylnitrosamine-induced hepatocellular carcinoma development and sorafenib resistance in rats

[Speaker]Meng Chieh Yang (Ctr. of Biopharm. Sci. for Healthy Aging, Mahidol Univ.／Dept. of Anatomy, Faculty of Med., Srinakharinwirot Univ.) [Co-author]Nichawadee Sandech (Ctr. of Biopharm. Sci. for Healthy Aging, Mahidol Univ.)、Sucheewin Krobthong (Ctr. of Biopharm. Sci. for Healthy Aging, Mahidol Univ.)、Wisuit Pradidarcheep (Dept. of Anatomy, Faculty of Med., Srinakharinwirot Univ.)、Arnatchai Maiuthed (Ctr. of Biopharm. Sci. for Healthy Aging, Mahidol Univ.)

Chemically induced liver carcinogenesis in rats mimics characteristics of human liver tumors. Previous studies have linked hepatocellular carcinoma (HCC) to metabolic changes, making the investigation of these changes in HCC progression crucial for early risk management. Sorafenib resistance in HCC is hypothesized to arise through metabolic reprogramming. This study proposes that each step in HCC carcinogenesis and sorafenib-resistant HCC (SF-HCC) has distinct metabolic signatures that could aid in developing diagnostic tools and new therapeutic targets. Metabolomics analysis of rat liver tissue at various stages, including inflammation, fibrosis, cirrhosis, HCC, and SF-HCC, was conducted using LC-MS/MS. The results revealed unique metabolic shifts at each stage, with upregulation of distinct metabolites. Notably, HCC showed changes in galactose metabolism, while SF-HCC exhibited alterations in arachidonic acid metabolism. These findings suggest that metabolic patterns specific to each stage could contribute to novel diagnostic and therapeutic strategies, especially for early detection and monitoring of SF-HCC post-treatment.

[Disclosure of COI by Responsible researcher] Lead presenter is the Responsible researcher.
[Lead presenter: Meng Chieh Yang] We have no financial relationships to disclose.

▲TOP

[P-1023] I-P2-2
Animal model (2)

2025 / 9 / 25 (Thu) 17:30-18:15 Room P/(Motenashi Dome) Room P

P-1023
### Pak1 Deficiency in CD11c-positive Cells Suppresses Skin Carcinogenesis

[Speaker]Kazuhiro Okumura (Div. of Exp. Anim. Res., Chiba Cancer Center Res. Inst.,) [Co-author]Megumi Saito (Div. of Exp. Anim. Res., Chiba Cancer Center Res. Inst.,)、Sora Tanaka (Div. of Exp. Anim. Res., Chiba Cancer Center Res. Inst.,)、Keisuke Otoyama (Div. of Exp. Anim. Res., Chiba Cancer Center Res. Inst.,)、Kimi Araki (Div. of Dev. Genetics., Dev. and Analysis, Univ. Kumamoto)、Yuichi Wakabayashi (Div. of Exp. Anim. Res., Chiba Cancer Center Res. Inst.,)

Using a mouse genetic screening approach combined with DMBA/TPA-induced skin carcinogenesis, we identified p21-activated kinase 1 (***Pak1***), a downstream effector of ***Ras***, as a key cancer susceptibility gene. To investigate cell-type-specific functions of ***Pak1***, we generated ***Pak1***$^{fl/fl}$ mice using CRISPR/Cas9 and crossed them with ***Krt14Cre*** (epidermis-specific) and ***Cd11cCre*** (myeloid-specific) mice to generate conditional knockout (cKO) models. In the DMBA/TPA skin carcinogenesis model, both ***Krt14Cre*** and ***Cd11cCre Pak1***-cKO mice exhibited a significant reduction in tumor incidence and malignancy, indicating that Pak1 promotes tumor development in both epidermal and CD11c-positive cells. Notably, ***Cd11cCre Pak1***-cKO mice displayed reduced skin inflammation and immune cell infiltration during the early stages of tumor initiation. To further clarify the role of myeloid ***Pak1***, we generated ***LysMCre Pak1***-cKO mice, targeting monocytes/macrophages and granulocytes. These mice also showed markedly reduced tumor burden, similar to ***Cd11cCre*** cKOs. Ongoing genetic and immunological analyses aim to elucidate the molecular mechanisms underlying the tumor-suppressive effects of myeloid ***Pak1*** deficiency.

[Disclosure of COI by Responsible researcher] Lead presenter is not the Responsible researcher.
[Lead presenter: Kazuhiro Okumura] We have no financial relationships to disclose.
[Principal Researcher: Yuichi Wakabayashi] We have no financial relationships to disclose.

### [P-1024] I-P2-2
### Animal model (2)

2025 / 9 / 25 (Thu) 17:30-18:15 Room P/(Motenashi Dome) Room P

---

### P-1024
### Analysis of systemic exacerbation mechanisms linked to abnormal brain histamine pathways in cancer

[Speaker]Yukari Suda (Dept. Pharmacol., Hoshi Univ.／Lab. Cancer Pathophysiol., Natl. Cancer Ctr. Res. Inst.) [Co-author]Hina Miyata (Dept. Pharmacol., Hoshi Univ.)、Yutaro Takata (Dept. Pharmacol., Hoshi Univ.／Lab. Cancer Pathophysiol., Natl. Cancer Ctr. Res. Inst.)、Fukiko Matsuyama (Dept. Pharmacol., Hoshi Univ.／Japan Small Animal Medical Center)、Michiko Narita (Lab. Cancer Pathophysiol., Natl. Cancer Ctr. Res. Inst.)、Yusuke Hamada (Dept. Pharmacol., Hoshi Univ.／Lab. Cancer Pathophysiol., Natl. Cancer Ctr. Res. Inst.)、Hiroyuki Neyama (Center for Cancer Immunotherapy and Immunobiology, Kyoto Univ.)、Naoko Kuzumaki (Dept. Pharmacol., Hoshi Univ.／Lab. Cancer Pathophysiol., Natl. Cancer Ctr. Res. Inst.)、Yuki Sugiura (Center for Cancer Immunotherapy and Immunobiology, Kyoto Univ.)、Minoru Narita (Dept. Pharmacol., Hoshi Univ.／Lab. Cancer Pathophysiol., Natl. Cancer Ctr. Res. Inst.)

Cancer cachexia involves a debilitating inflammatory state closely linked to metabolic derangement and lacks effective therapy. As metabolic dysfunction is increasingly recognized as a defining feature of cachexia, deciphering brain-specific metabolic signatures may unlock new avenues for therapeutic breakthroughs. To investigate cachexia-associated metabolic alterations in the brain, we established a mouse model by transplanting pancreatic cancer cells. The cachexia group exhibited dynamic changes in the brain´s histidine and tryptophan metabolic pathways relative to controls. In contrast, plasma analysis revealed that while the tryptophan metabolic pathway was altered by cancer cachexia, the histidine metabolic pathway remained unaltered, indicating a brain-specific regulatory phenomenon. Subsequent imaging analysis demonstrated preferential localization of histamine in the brain, accompanied by region-specific changes in histamine concentration. Our data implicate dynamic remodeling of the hypothalamic histidine/tryptophan axis in the maladaptive neurophysiological changes that promote the onset and progression of cancer cachexia.

[Disclosure of COI by Responsible researcher] Lead presenter is the Responsible researcher.
[Lead presenter: Yukari Suda] We have no financial relationships to disclose.

### [P-1025] I-P2-2
### Animal model (2)

2025 / 9 / 25 (Thu) 17:30-18:15 Room P/(Motenashi Dome) Room P

---

### P-1025
### The proto-oncogene WWP1 promotes pancreatic tumorigenesis via acinar-to-ductal metaplasia

[Speaker]Genso Notoya (The Univ. of Tokyo Dept. of Gastroenterology) [Co-author]Takahiro Kishikawa (The Univ. of Tokyo Dept. of Gastroenterology)、Mitsuhiro Fujishiro (The Univ. of Tokyo Dept. of Gastroenterology)

The proto-oncogene WWP1 is overexpressed in various cancers and is linked to tumor progression and poor prognosis. We previously reported that WWP1 is upregulated in pancreatic cancer and that its inhibition suppresses tumor growth and cell proliferation. In this study, we explored the role of WWP1 in pancreatic tumorigenesis using genetically engineered mouse models (GEMMs) with pancreas-specific WWP1 overexpression. Analysis of human chronic pancreatitis tissues revealed that WWP1 expression is elevated in regions undergoing acinar-to-ductal metaplasia (ADM), a precursor lesion of pancreatic cancer. In GEMMs, WWP1 overexpression combined with Kras G12D mutation significantly accelerated mouse pancreatic intraepithelial neoplasia (mPanIN) development and reduced survival compared to KRAS G12D alone. Furthermore, WWP1 overexpression induced ADM in response to inflammatory stimuli or growth factors in vivo and ex vivo. Mechanistically, WWP1 induced SOX9 expression, a key ductal marker, in pancreatic acinar-derived 266-6 cells. These findings suggest that WWP1 promotes pancreatic tumorigenesis by facilitating ADM, playing an oncogenic role in the early stages of tumor development.

[Disclosure of COI by Responsible researcher] Lead presenter is not the Responsible researcher.
[Lead presenter: Genso Notoya] We have no financial relationships to disclose.
[Principal Researcher: Mitsuhiro Fujishiro] We have no financial relationships to disclose.

[P-1026] I-P2-2
Animal model (2)

2025 / 9 / 25 (Thu) 17:30-18:15 Room P/(Motenashi Dome) Room P

P-1026

### ***CblQ$^{367P}$*** cooperates with loss of ***Tet2*** to drive a CNL-like disease via the NF-κB pathway

[Speaker]Takuma Ohashi (Dept. Stem Cell & Immune Regulation, Yokohama City Univ.) [Co-author]Hiroyoshi Kunimoto (Dept. Stem Cell & Immune Regulation, Yokohama City Univ.)、Yoko Ino (Advanced Medical Research Center, Yokohama City Univ.)、Jotaro Harada (Dept. Molecular Pathology, Yokohama City Univ.)、Takayuki Sakuma (Dept. Stem Cell & Immune Regulation, Yokohama City Univ.)、Junji Ikeda (Dept. Stem Cell & Immune Regulation, Yokohama City Univ.)、Yuko Simosato (Div. Molecular Oncology, Graduate School of Frontier Sciences, Tokyo Univ.)、Keita Yamamoto (Div. Molecular Oncology, Graduate School of Frontier Sciences, Tokyo Univ.)、Yayoi Kimura (Advanced Medical Research Center, Yokohama City Univ.)、Susumu Goyama (Div. Molecular Oncology, Graduate School of Frontier Sciences, Tokyo Univ.)、Satoshi Fujii (Dept. Molecular Pathology, Yokohama City Univ.)、Tomohiko Tamura (Dept. Immunology, Yokohama City Univ.)、Hiroaki Honda (Inst. Laboratory Animals, Tokyo Women Med. Univ.)、Hideaki Nakajima (Dept. Stem Cell & Immune Regulation, Yokohama City Univ.)

In MDS, ***CBL*** and ***TET2*** mutations occur in 10% and 20% of patients respectively. To assess if ***Tet2*** loss and ***Cbl*** mutation functionally cooperate to drive myeloid transformation, we crossed ***Tet2*** conditional knockout mice (Mx1-Cre+ ***Tet2$^{flox/flox}$***) and ***Cbl*** mutant mice (Mx1-Cre+ ***Cbl$^{Q367P}$***) to generate mice with both disease alleles. At 16-20 weeks post-recombination, ***Tet2-/- Cbl$^{Q367P}$*** mice developed massive splenomegaly and chronic neutrophilic leukemia (CNL)-like disease in vivo which was serially transplantable. This CNL-like disease further transformed into AML and was lethal at six months after recombination. Proteomic analyses of BM CD11b+ leukemia cells demonstrated that ***Tet2*** loss and ***Cbl*** mutation cooperatively led to increases in activators of nuclear factor-kappa B (NFκB). IKK and IκB inhibitors abrogated leukemia cell growth in vitro and leukemia development in vivo using our murine model and patient samples. Taken together, our model represents a significant step toward building high fidelity and genetically tractable models of CNL and provides insights into how epigenetic and signaling factor mutations cooperate in myeloid transformation and a rationale for mechanism-based therapy.

[Disclosure of COI by Responsible researcher] Lead presenter is the Responsible researcher.
[Lead presenter: Takuma Ohashi] We have no financial relationships to disclose.

### [P-1027] I-P2-2
### Animal model (2)

2025 / 9 / 25 (Thu) 17:30-18:15 Room P/(Motenashi Dome) Room P

P-1027
### Antitumor effects and mechanisms of lenvatinib plus anti-PD-1 antibody in a novel endometrial cancer model.

[Speaker]Yoko Kashima (Kindai University,Obstetrics and Gynecology) [Co-author]Kosuke Murakami (Kindai University,Obstetrics and Gynecology)、Chiho Miyagawa (Kindai University,Obstetrics and Gynecology)、Yukari Kobayashi (Kindai University,Department of Immunology)、Koji Nagaoka (Kindai University,Department of Immunology)、Shiki Takamura (RIKEN Center for Integrative Medical Sciences)、Kazuhiro Kakimi (Kindai University,Department of Immunology)、Noriomi Matsumura (Kindai University,Obstetrics and Gynecology)

Combination therapy using immune checkpoint inhibitors (ICIs) with multikinase inhibitors or chemotherapy (chemo) has shown promise for advanced or recurrent endometrial cancer, but its antitumor mechanisms remain unclear. We established a syngeneic mouse endometrial cancer cell line harboring Arid1a and Pik3ca mutations. In a subcutaneous transplantation model, mice were divided into six groups: control, lenvatinib (Len), anti-PD-1 antibody (aPD-1ab), Len+aPD-1ab, chemo, and chemo+aPD-1ab. Tumor volume and survival were assessed, and the tumor immune microenvironment was analyzed using RNA sequencing and immunohistochemistry. Len and Len+aPD-1ab significantly reduced tumor size compared to control ($p<0.05$), and Len+aPD-1ab also prolonged survival ($p<0.0001$). Ido1 expression was significantly decreased in both Len and Len+aPD-1ab groups. In the Len+aPD-1ab group, infiltration of CD4+ and CD8+ T cells and PD-L1 expression were increased, while CD206+ tumor-associated macrophages were decreased. Chemo±aPD-1ab increased TILs but had no effect on PD-L1. These findings suggest that lenvatinib enhances ICI efficacy by reducing Ido1 and TAMs, thereby promoting antitumor immunity.

[Disclosure of COI by Responsible researcher] Lead presenter is not the Responsible researcher.
[Lead presenter: Yoko Kashima] ■Research expenses: エーザイ
[Principal Researcher: Kosuke Murakami] ■Research expenses: エーザイ、アストラゼネカ

### [P-1028] I-P2-2
### Animal model (2)

2025 / 9 / 25 (Thu) 17:30-18:15 Room P/(Motenashi Dome) Room P

---

### P-1028
### Pathological and molecular analyses of a newly established cancer cachexia mouse model

[Speaker]Masako Nakanishi (Dept. Path., Sch. Med., Wakayama Med. Univ.) [Co-author]Mizuki Nishikawa (Dept. Human Path., Sch. Med., Wakayama Med. Univ.)、Shinichi Murata (Dept. Human Path., Sch. Med., Wakayama Med. Univ.)、Hitomi Hamaoka (Dept. Anat. & Cell Biol., Osaka Med. & Pharm. Univ.)、Shogo Ehata (Dept. Path., Sch. Med., Wakayama Med. Univ.)

Cancer cachexia is an intractable complication characterized by progressive weight loss and systemic metabolic disorders. Cachexia reduced quality of life and accounts for over 30% of cancer mortalities. However, effective treatments have not yet been developed. We recently established a derivative line (EO771-LM) from mouse mammary carcinoma cell line EO771, by culturing lymph nodes with metastasis. EO771-LM-bearing mice exhibited gradual weight loss and severe fatty liver. Accumulation of lipid droplets was observed in hepatocytes, cardiomyocytes, and proximal renal tubule cells in EO771-LM-bearing mice. Biochemical analysis revealed abnormalities in hepatic and renal functions, as well as lipid metabolism in mice with EO771-LM cells, but not with parental EO771 cells. Muscle atrophy in EO771-LM-bearing mice was detected by microCT analysis and measurement of myofiber cross-section area. These findings suggest that EO771-LM may serve as a new model for studying cancer cachexia. To identify the cachexia-inducing factors, gene expression profiling and molecular analysis of EO771-LM cells are under conducting.

[Disclosure of COI by Responsible researcher] Lead presenter is not the Responsible researcher.
[Lead presenter: Masako Nakanishi] We have no financial relationships to disclose.
[Principal Researcher: Shogo Ehata] We have no financial relationships to disclose.

### [P-1029] I-P2-2
### Animal model (2)

2025 / 9 / 25 (Thu) 17:30-18:15 Room P/(Motenashi Dome) Room P

---

### P-1029
### Identification of genetic modifiers of Pak1 identified using a Japanese wild-derived inbred mouse strain, MSM/Ms

[Speaker]Yuichi Wakabayashi (Division of Experimental Animal Research, Chiba Cancer Center Research Institute) [Co-author]Kazuhiro Okumura (Division of Experimental Animal Research, Chiba Cancer Center Research Institute)、Megumi Saito (Division of Experimental Animal Research, Chiba Cancer Center Research Institute)、Keisuke Otoyama (Division of Experimental Animal Research, Chiba Cancer Center Research Institute)、Sora Tanaka (Division of Experimental Animal Research, Chiba Cancer Center Research Institute)

Previously, we generated backcross mice between FVB/N and MSM/Ms to search for tumor resistance loci of DMBA/TPA-induced skin tumors. Linkage analysis revealed strong peaks on chromosomes 7 (Stmm1, 2), 4 (Stmm3), and 6 (Stmm4). As a result, Pak1 (p21-activated kinase) was identified as a causative gene in the Stmm1 locus on chromosome 7. A polymorphism in Pak1 was detected only in the 3' untranslated region without any polymorphism in the coding region. The strong carcinogenic resistance of MSM/Ms was recapitulated in carcinogenesis experiments using mice in which the MSM/Ms sequence in the 3' untranslated region was knocked into the FVB sequence. As Pak1 is expressed not only in the skin cells but also in the immune cells such as dendritic cells and macrophages, skin carcinogenesis experiments were conducted after knocking out Pak1 in the respective cells. This resulted in the suppression of tumor formation in both cases. Furthermore, backcross mice in which Pak1 has been knocked out in the immune cells have been produced and skin carcinogenesis experiments have been conducted to identify potential genetic modifiers. We will discuss about progress of this experiment.

[Disclosure of COI by Responsible researcher] Lead presenter is the Responsible researcher.
[Lead presenter: Yuichi Wakabayashi] We have no financial relationships to disclose.

### [P-1030] I-P2-2
### Animal model (2)

2025 / 9 / 25 (Thu) 17:30-18:15 Room P/(Motenashi Dome) Room P

---

### P-1030
### Liver-specific DICER1 syndrome model mice develop cystic liver tumors with defective primary cilia

[Speaker]Shinichiro Ohno (Dept. Mol. Pathol., Tokyo Med. Univ., Sch. Med.) [Co-author]Yuichirou Harada (Dept. Mol. Pathol., Tokyo Med. Univ., Sch. Med.)、Masahiko Kuroda (Dept. Mol. Pathol., Tokyo Med. Univ., Sch. Med.)

Pathogenic variants in the DICER1 gene have been discovered in many rare cancers, including cystic liver tumors. The molecular mechanisms of liver lesions induced by DICER1 pathogenic variants remain unclear. In this study, we sought to elucidate their pathogenesis by generating a mouse model of liver-specific DICER1 syndrome. These model mice developed bile duct hyperplasia with fibrosis much like congenital hepatic fibrosis (CHF), and further developed intrahepatic cholangiocarcinoma (ICC), hepatocellular carcinoma (HCC), and Polycystic liver disease (PLD). Interestingly, the DICER1 syndrome model mice showed abnormal expression of genes involved in primary cilia and abnormal formation of primary cilia in the bile ducts. These results indicate that DICER1 gene mutation contributes to cystic liver tumors by inducing defective primary cilia. This finding contributes to our understanding of the potential mechanisms of tumorigenesis in DICER1 variants and will be useful for going a comprehensive understanding of DICER1 syndrome.

[Disclosure of COI by Responsible researcher] Lead presenter is not the Responsible researcher.
[Lead presenter: Shinichiro Ohno] We have no financial relationships to disclose.
[Principal Researcher: Masahiko Kuroda] We have no financial relationships to disclose.

▲TOP

### [P-1039] I-P3-1
### Viral Oncology: Basic

2025 / 9 / 25 (Thu) 17:30-18:15 Room P/(Motenashi Dome) Room P

---

### P-1039
### Characterization of high-risk HPVs through E6-associated chromosomal abnormalities

[Speaker]Kanako Hori (Div. Exp. Pathol. Cancer In／Tokyo Univ Sci. Faculty Sci. Tch.) [Co-author]Nanami Seshimo (Div. Exp. Pathol. Cancer In)、Ryusuke Nozawa (Div. Exp. Pathol. Cancer In)、Yasuhiro Hirano (Div. Exp. Pathol. Cancer In)、Toru Hirota (Div. Exp. Pathol. Cancer In)

Cervical cancer remains a significant global health concern, especially among young women, and is mostly related to high-risk HPV infection. There are two major groups of high-risk HPVs, namely HPV16 and 18, which show distinct clinical features of the disease. The HPV16 group typically causes dysplasia that precedes squamous cell carcinoma, while the HPV18 group de novo adenocarcinoma. However, what leads to these differences remains elusive. To investigate this, we focused on the cellular phenotype called ECAC (Ectopic Chromosome Around Centrosome), which is a characteristic chromosome congression defect observed in dysplasia. We found that oncogenic E6 of HPV16 group induces ECAC, and degrades CENP-E, a mitotic kinesin required for chromosome alignment at the metaphase plate, while no such effect was observed in HPV18 group. We could tell that CENP-E degradation is required for ECAC induction, as a non-degradable mutant rescued this phenotype. Furthermore, an HPV16 group E6 mutant having C-terminus of HPV18 group E6 lost the ability to degrade CENP-E. These findings suggest that specific structure of the C-terminus determines the substrates, and thereby different pathologies.

[Disclosure of COI by Responsible researcher] Lead presenter is not the Responsible researcher.
[Lead presenter: Kanako Hori] We have no financial relationships to disclose.
[Principal Researcher: Toru Hirota] We have no financial relationships to disclose.

### [P-1040] I-P3-1
### Viral Oncology: Basic

2025 / 9 / 25 (Thu) 17:30-18:15 Room P/(Motenashi Dome) Room P

---

### P-1040
### Elucidation of chromatin interaction between HPV-hybrid extrachromosomal DNA and host genome in head and neck cancer

[Speaker]Miki Sunagane (Dpt of Mol Oncology, Grad Sch of Med, Chiba Univ／Dept of Otorhinolaryngology ／ Head and Neck Surgery, Chiba Univ) [Co-author]Takuya Nakagawa (Dpt of Mol Oncology, Grad Sch of Med, Chiba Univ／Dept of Otorhinolaryngology ／ Head and Neck Surgery, Chiba Univ ／Dpt of Health and Disease Omics Center, Chiba Univ)、Atsushi Okabe (Dpt of Mol Oncology, Grad Sch of Med, Chiba Univ／Dpt of Health and Disease Omics Center, Chiba Univ)、Masato Mima (Dpt of Mol Oncology, Grad Sch of Med, Chiba Univ)、Kazuko Kita (Dpt of Mol Oncology, Grad Sch of Med, Chiba Univ)、Motoaki Seki (Dpt of Mol Oncology, Grad Sch of Med, Chiba Univ)、Masaki Fukuyo (Dpt of Mol Oncology, Grad Sch of Med, Chiba Univ)、Rahmutulla Bahityar (Dpt of Mol Oncology, Grad Sch of Med, Chiba Univ)、Toyoyuki Hanazawa (Dept of Otorhinolaryngology ／ Head and Neck Surgery, Chiba Univ)、Atsushi Kaneda (Dpt of Mol Oncology, Grad Sch of Med, Chiba Univ／Dpt of Health and Disease Omics Center, Chiba Univ)

Extrachromosomal DNA (ecDNA) formation is often observed and reportedly correlates with poor prognosis in cancer, including head and neck squamous cell carcinoma (HNSCC). Recently, ecDNA containing viral genomes was identified in HNSCC. While the importance of HPV hybrid ecDNA is recognized, its epigenetic contribution to HNSCC genesis remains unclear. This study investigates hybrid ecDNA-host genome interactions through integrative multi-omics analysis using the ecDNA (+) HNSCC cell line. We conducted integrated analyses of epigenome, 3D chromatin interaction, and transcriptome by ChIP-seq, ATAC-seq, Hi-C, 4C-seq, RNA-seq. ChIP-seq revealed novel enhancer regions on hybrid ecDNA absent in other cell lines. 4C-seq revealed interactions between these enhancers or HPV sequences on hybrid ecDNA and the host genome, identifying 30 interaction sites, 89% of which were transcriptionally active regions. Hi-C confirmed hybrid ecDNA-host genome interactions across multiple chromosomes. Genes on host chromosomes showing upregulated expression and interaction with ecDNA included oncogenes, e.g. CTHRC1, suggesting that hybrid ecDNA-host genome interactions may contribute to tumorigenesis.

[Disclosure of COI by Responsible researcher] Lead presenter is not the Responsible researcher.
[Lead presenter: Miki Sunagane] We have no financial relationships to disclose.
[Principal Researcher: Atsushi Kaneda] ■Research expenses: Takeda Science Foundation., Uehara Memorial Foundation.

[P-1041] I-P3-1
Viral Oncology: Basic

2025 / 9 / 25 (Thu) 17:30-18:15 Room P/(Motenashi Dome) Room P

### P-1041
### Sustained expression of intact ift81 mRNA despite intron-oriented HTLV-1 integration in an ATL cell line ED

[Speaker]Mayuko Yagi (OMPU, Pharm.) [Co-author]Yuka Hirosawa (OMPU, Pharm.)、Yusaku Sagisaka (OMPU, Pharm.)、Minami Hama (OMPU, Pharm.)、Satoshi Takeda (JIHS, NIID, Res Ctr Biosafety)、Jun Komano (OMPU, Pharm.)

Human T-cell leukemia virus type 1 (HTLV-1) establishes lifelong latency in T cells and is a known risk factor for adult T-cell leukemia/lymphoma (ATL). HTLV-1 frequently integrates into transcriptionally active regions of the host genome, often within introns due to their greater length compared to exons. In the ATL cell line ED, a single HTLV-1 provirus is inserted into intron 10 of the IFT81 gene, which has 21 exons.The viral hbz gene shares the same transcriptional orientation as ift81. Despite the integration, both ift81 and hbz mRNAs were actively transcribed. Notably, an ift81-hbz chimeric transcript was detected, but not hbz-ift81. This suggests that the splice acceptor and polyadenylation signal of hbz are inefficient when driven by the ift81 promoter, allowing full-length ift81 expression. Given the in cis enhancer-like activity of HTLV-1 and the biallelic expression of ift81, these results suggest a unique mode of host gene regulation by the integrated provirus. Such in cis regulation may increase the chance for the infected cells to survival, and thus, contribute to leukemogenesis, highlighting a less explored mechanism of HTLV-1 pathogenesis.

[Disclosure of COI by Responsible researcher] Lead presenter is not the Responsible researcher.
[Lead presenter: Mayuko Yagi] We have no financial relationships to disclose.
[Principal Researcher: Jun Komano] We have no financial relationships to disclose.

[P-1042] I-P3-1
Viral Oncology: Basic

2025 / 9 / 25 (Thu) 17:30-18:15 Room P/(Motenashi Dome) Room P

P-1042

### The identification of a novel viral factor that contributes to EBV-driven transformation

[Speaker]Takahiro Ukegawa (Dept. Virol., Nagoya Univ. Grad. Sch. Med.) [Co-author]Atsuko Sugimoto (Dept. Virol., Nagoya Univ. Grad. Sch. Med.)、Kamal M. Uddin (Dept. Virol., Nagoya Univ. Grad. Sch. Med.)、Takahiro Watanabe (Dept. Virol., Nagoya Univ. Grad. Sch. Med.)、Toi Daigo (Dept. Virol., Nagoya Univ. Sch. Med.)、Emi S. Mishiro (WPI-ITbM Mol. Structure Ctr., Nagoya Univ.)、Yuichi Abe (Immunoproteomics lab., iGCORE, Gifu Univ.)、Takayuki Murata (Dept. Virol., Fujita Health Univ. Sch. Med.)、Yoshitaka Sato (Dept. Virol., Nagoya Univ. Grad. Sch. Med.)、Hiroshi Kimura (Dept. Virol., Nagoya Univ. Grad. Sch. Med.)

Epstein-Barr virus (EBV) infection is widespread, affecting more than 90% of adults. EBV is also known as a human tumor virus, associated with various types of cancer. It efficiently infects primary B cells and induces cellular growth transformation, resulting in immortalization that allows for continuous proliferation. In this study, we focus on BBLF1, one of the EBV tegument proteins. EBV with the disruption of the BBLF1 gene in the EBV genome (ΔBBLF1) cannot induce EBV-driven transformation, suggesting that BBLF1 is an important factor in EBV-related oncogenesis. We aim to unveil the novel functions of BBLF1 in establishing EBV-driven transformation. First, we confirmed the role of BBLF1 in this process. ΔBBLF1 completely inhibited EBV-driven transformation, while ΔBBLF1 possessing BBLF1 protein in the particle enhanced transformation. Next, to identify the step in which BBLF1 is involved, we monitored the attachment of virions and nuclear transport. Our results indicated that the deletion of the BBLF1 gene impaired the nuclear transport of the viral genome. These findings suggest that BBLF1 plays important roles in EBV-driven transformation, particularly in nuclear transport.

[Disclosure of COI by Responsible researcher] Lead presenter is not the Responsible researcher.
[Lead presenter: Takahiro Ukegawa] We have no financial relationships to disclose.
[Principal Researcher: Hiroshi Kimura] We have no financial relationships to disclose.

### [P-1043] I-P3-1
### Viral Oncology: Basic

2025 / 9 / 25 (Thu) 17:30-18:15 Room P/(Motenashi Dome) Room P

---

### P-1043
### Establishment and analysis of a novel post-SVR HCC model by manipulating the expression of Core J1 in mouse liver

[Speaker]Maho Tsuda (Dept. of Pathophysiology, Grad. sch. of Med., Osaka Metropolitan Univ.) [Co-author]Yuki Hirai (Dept. of Pathophysiology, Grad. sch. of Med., Osaka Metropolitan Univ.)、Tomonori Kamiya (Dept. of Pathophysiology, Grad. sch. of Med., Osaka Metropolitan Univ.)、Yumi Ito (Dept. of Microbiology, Grad. sch. of Med., Juntendo Univ.)、Toru Okamoto (Dept. of Microbiology, Grad. sch. of Med., Juntendo Univ.)、Naoko Ohtani (Dept. of Pathophysiology, Grad. sch. of Med., Osaka Metropolitan Univ.)

Hepatitis C virus (HCV) selectively infects hepatocytes, causing chronic inflammation, steatosis, and cirrhosis, which often culminating in HCV-associated hepatocellular carcinoma (HCC). Direct-acting antivirals have achieved sustained virological response (SVR) in over 90% of patients, significantly reducing HCC risk. However, approximately 10% of SVR patients still develop HCC, yet mechanisms remain unclear. CoreJ1 is one of the major oncogenic proteins that constitutes HCV. Therefore, we first introduced the plasmids encoding floxed CoreJ1 into liver by hydrodynamic injection, and then the Cre recombinase was administered to remove it before visible tumor formation in mice, which we think as a "SVR model" system. As a result, we observed significant HCC formation in SVR model, suggesting that transient CoreJ1 expression enhanced the carcinogenic effect. A marked reduction in cytotoxic T cells was also observed in this SVR model, inversely correlating with CoreJ1 expression. Moreover, spatial transcriptomic analysis was performed to identify tumor-promoting factors in the SVR-HCC model, and the findings will be presented herein.

[Disclosure of COI by Responsible researcher] Lead presenter is not the Responsible researcher.
[Lead presenter: Maho Tsuda] ■Research funds from company: 武田科学振興財団
[Principal Researcher: Naoko Ohtani] We have no financial relationships to disclose.

### [P-1044] I-P3-1
### Viral Oncology: Basic

2025 / 9 / 25 (Thu) 17:30-18:15 Room P/(Motenashi Dome) Room P

---

### P-1044
### HPV18-related cancer model development and its application in the efficacy evaluation of a saRNA cancer vaccine

[Speaker]Zhongbao Song (in vivo pharmacology unit of WuXi AppTec) [Co-author]Guangxu Ding (in vivo pharmacology unit of WuXi AppTec)、Anran Xia (in vivo pharmacology unit of WuXi AppTec)、Wanqing Ma (in vivo pharmacology unit of WuXi AppTec)、Qiuhong Li (in vivo pharmacology unit of WuXi AppTec)、Xu Chen (in vivo pharmacology unit of WuXi AppTec)、Zhixiang Zhang (in vivo pharmacology unit of WuXi AppTec)、Qingyang Gu (in vivo pharmacology unit of WuXi AppTec)

Globally, about 10% of all cancers are caused by viral infection. After infection, these viruses manipulate the proliferation, metabolism, immunity and other life processes of the host cells through various mechanisms, resulting in malignant cell lesions. Therefore, targeting these viruses offers a novel therapeutic approach for virus-related cancers.In this study, codon optimization was performed on HPV18 E6 and E7 genes, which were then cloned into a lentivirus vector to establish an HPV18 E6/E7-overexpressing cell line. LLC1-HPV18 cells stably expressed HPV18 oncogenes E6 and E7 in vitro and in vivo, and induced tumor formation in mice. To further investigate the potential of this model, we designed a self-replicating RNA (saRNA) expressing HPV18 E6 and E7. Immunogenicity of the saRNA was validated after two rounds of immunizations, showing a marked increase in E6/E7-specific T cell response. Subsequently, the therapeutic effect was evaluated in the LLC1-HPV18 subcutaneous tumor model, where saRNA treatment remarkably inhibited tumor progression, improved infiltration of immune cells into the tumors post-saRNA treatment, especially E6/E7 specific IFN-&gamm + T cell subsets.

[Disclosure of COI by Responsible researcher] Lead presenter is the Responsible researcher.
[Lead presenter: Zhongbao Song] ■Research funds from company: WuXi AppTec ■ employment by a company related to the presentation: WuXi AppTec

▲TOP

### [P-1051] I-P4-1
### RAS-RB-E2F axis

2025 / 9 / 25 (Thu) 17:30-18:15 Room P/(Motenashi Dome) Room P

---

### P-1051
### Polo-like kinase 1 expression in colorectal cancer: association with RAS mutations

[Speaker]Yasushi Tanaka (Department of Surgery and Science, Kyushu University) [Co-author]Eiji Oki (Department of Surgery and Science, Kyushu University)、Ryota Nakanishi (Department of Surgery and Science, Kyushu University)、Tetsuro Kawazoe (Department of Surgery and Science, Kyushu University)、Kensuke Kudou (Department of Surgery and Science, Kyushu University)、Yoko Zaitsu (Department of Surgery and Science, Kyushu University)、Yuichi Hisamatsu (Department of Surgery and Science, Kyushu University)、Koji Ando (Department of Surgery and Science, Kyushu University)、Tomoharu Yoshizumi (Department of Surgery and Science, Kyushu University)

Polo-like kinase 1 (PLK1) controls mitotic spindle formation and cytokinesis. However, its role as a predictive biomarker in colorectal cancer (CRC) remains underexplored, particularly in the context of RAS mutations. We retrospectively analyzed PLK1 expression, clinicopathological factors, and survival in 225 CRC patients who underwent surgery. We also examined its association with survival after adjuvant chemotherapy and the impact of RAS mutations. PLK1 expression significantly correlated with histopathology ($p < 0.0001$) and perineural invasion ($p = 0.005$). High PLK1 expression was associated with worse relapse-free survival (RFS) in all patients ($p = 0.060$) and those with stage III disease ($p = 0.055$). Among stage III patients receiving adjuvant chemotherapy, high PLK1 was the only independent poor prognostic factor for RFS ($p = 0.01$). Patients with mutated RAS had significantly poorer outcomes than those with wild-type RAS ($p = 0.027$). High PLK1 expression was linked to poor survival after adjuvant therapy, with possible involvement of RAS mutations.

[Disclosure of COI by Responsible researcher] Lead presenter is not the Responsible researcher.
[Lead presenter: Yasushi Tanaka] We have no financial relationships to disclose.
[Principal Researcher: Eiji Oki] We have no financial relationships to disclose.

### [P-1052] I-P4-1
### RAS-RB-E2F axis

2025 / 9 / 25 (Thu) 17:30-18:15 Room P/(Motenashi Dome) Room P

---

### P-1052
### Enhancement of deregulated E2F1 activity enables induction of apoptosis in cancer cells via p53-independent pathways

[Speaker]Rinka Nakajima (Dept. of Biomed. Chem,, Kwansei Gaguin Univ) [Co-author]Kiyoshi Ohtani (Dept. of Biomed. Chem,, Kwansei Gaguin Univ)

pRB and p53 are two major tumor suppressors. pRB restricts cell proliferation by restraining the transcription factor E2F, which activates growth-related genes. Hence in almost all cancers, pRB function is disabled and E2F activity is enhanced (deregulated E2F), leading to aberrant cell proliferation. Upon such an event, deregulated E2F activates the tumor suppressor gene ***ARF***, upstream activator of p53, thereby inducing apoptosis to suppress tumorigenesis. Accordingly, p53 function is also disabled in almost all cancers. Of note, E2F can induce apoptosis independent of p53 by activating other tumor suppressor genes such as ***TAp73*** and ***Bim***. We thus searched for novel factors, which modulate deregulated E2F activity. We found that DDX5 enhanced E2F-induced tumor suppressor gene expression and apoptosis independent of p53. Conversely, cyclin D1 suppressed deregulated E2F activity, independent of CDK activity. These observations suggest possibility that enhancing deregulated E2F activity, either by enhancing function of facilitating factors such as DDX5 or by inhibiting function of suppressing factors such as cyclin D1, facilitates cell death even in p53-disabled cancer cells.

[Disclosure of COI by Responsible researcher] Lead presenter is not the Responsible researcher.
[Lead presenter: Rinka Nakajima] We have no financial relationships to disclose.
[Principal Researcher: Kiyoshi Ohtani] We have no financial relationships to disclose.

▲TOP

### [P-1053] I-P4-1
### RAS-RB-E2F axis

2025 / 9 / 25 (Thu) 17:30-18:15 Room P/(Motenashi Dome) Room P

---

### P-1053
### WDR1 functions as a coactivator of E2F1 in the nucleus and enhances tumor suppressive E2F1 activity

[Speaker]Mashiro Shirasawa (Dept. of Biomed. Chem., Kwansei Gakuin Univ.) [Co-author]Kiyoshi Ohtani (Dept. of Biomed. Chem., Kwansei Gakuin Univ.)

The transcription factor E2F1 is the principal target of the tumor suppressor pRB. E2F1 activated by dysfunction of pRB upon oncogenic changes (deregulated E2F1) activates pro-apoptotic tumor suppressor genes, which are not activated by physiological E2F1 induced by growth stimulation and suppresses tumorigenesis.
We identified WD repeat-containing protein 1 (WDR1), which has been known to facilitate actin depolymerization in the cytoplasm, as a novel E2F1-interacting factor using yeast two-hybrid system. Indeed, BiFC method showed that WDR1 and E2F1 interact in the nucleus. WDR1 enhanced deregulated E2F1 activation of target genes, indicating involvement of WDR1 in transcriptional regulation. Over-expression or knockdown of WDR1 enhanced or reduced E2F1-induced apoptosis, respectively, suggesting contribution of WDR1 in tumor suppressive function of E2F1.
We also found that over-expression of E2F1 increased WDR1 expression, suggesting that the ***WDR1*** gene may be an E2F1 target. Promoter analysis and ChIP assay showed that the ***WDR1*** gene is a target of deregulated E2F1 but not physiological E2F1, suggesting that WDR1 forms positive feedback loop in tumor suppressive E2F1 activity.

[Disclosure of COI by Responsible researcher] Lead presenter is not the Responsible researcher.
[Lead presenter: Mashiro Shirasawa] We have no financial relationships to disclose.
[Principal Researcher: Kiyoshi Ohtani] We have no financial relationships to disclose.

### [P-1054] I-P4-1
### RAS-RB-E2F axis

2025 / 9 / 25 (Thu) 17:30-18:15 Room P/(Motenashi Dome) Room P

---

### P-1054
### Deregulated E2F activity out of control by pRB discriminates cancer cells from normal growing cells

[Speaker]Yaxuan Zhou (Dept. of Biomed. Chem., Kwansei Gakuin Univ.) [Co-author]Kiyoshi Ohtani (Dept. of Biomed. Chem., Kwansei Gakuin Univ.)

The transcription factor E2F, principal target of the tumor suppressor pRB, plays central roles in cell proliferation and tumor suppression. E2F activated by growth stimulation, activates growth-associated genes to promote cell proliferation. E2F activated out of control by pRB (deregulated E2F) upon oncogenic changes, activates tumor suppressor genes such as ARF, to protect cells from tumorigenesis. Importantly, we found that E2F activated by growth stimulation does not activate the tumor suppressor genes using human normal fibroblasts. We show here that the tumor suppressor genes are regulated in the same manner in epithelial cells, from which 90% of cancer arises. Hence normal growing cells do not harbor deregulated E2F activity. In almost all cancers, in addition to the RB pathway, the ARF-p53 pathway is also disabled, enabling cancer cells to survive, raising the possibility that deregulated E2F activity specifically exist in cancer cells. Indeed, all of the cancer cell lines examined, but not normal growing cells, harbor deregulated E2F activity, showing that deregulated E2F activity serves as an important determinant to discriminate cancer cells from normal growing cells.

[Disclosure of COI by Responsible researcher] Lead presenter is not the Responsible researcher.
[Lead presenter: Yaxuan Zhou] We have no financial relationships to disclose.
[Principal Researcher: Kiyoshi Ohtani] We have no financial relationships to disclose.

### [P-1055] I-P4-1
### RAS-RB-E2F axis

2025 / 9 / 25 (Thu) 17:30-18:15 Room P/(Motenashi Dome) Room P

### P-1055
### Knockdown of Gene Y, Induced by Mutant KRAS, Inhibits Growth of Lung Cancer Cells

[Speaker]Ayana Iwabe (Dept. of Integrated Health Sciences, Nagoya University Graduate School Medicine) [Co-author]Nozomi Kawabe (Dept. of Integrated Health Sciences, Nagoya University Graduate School Medicine)、Masahito Katsuda (Dept. of Integrated Health Sciences, Nagoya University Graduate School Medicine)、Kazuki Komeda (Dept. of Respiratory Medicine, Nagoya University Graduate School of Medicine)、Ichidai Tanaka (Dept. of Respiratory Medicine, Nagoya University Graduate School of Medicine)、Miyoko Matsushima (Dept. of Integrated Health Sciences, Nagoya University Graduate School Medicine)、Tsutomu Kawabe (Dept. of Integrated Health Sciences, Nagoya University Graduate School Medicine)、Noriaki Sunaga (Dept. of Respiratory Medicine, Gunma University Graduate School of Medicine)、Mitsuo Sato (Dept. of Integrated Health Sciences, Nagoya University Graduate School Medicine)

Mutant KRAS is the most frequent driver oncogene in lung cancer, but only few molecular targeted drugs with limited efficacy have been applied clinically. Thus, this study aims to identify potential therapeutic targets, other than mutant KRAS itself, for mutant KRAS-driven lung cancer. We used the HBEC3-RIN2 cell line, which expresses mutant KRAS in a doxycycline dose-dependent manner, derived from immortalized normal human bronchial epithelial cells (HBECs). We performed next-generation sequencing to identify genes whose expression was upregulated by mutant KRAS expression. Furthermore, we assessed the growth-suppressive effects in a lung cancer cell line by individually silencing the expression of most of these genes. As a result, we identified gene Y as one of the most very promising targets. Knockdown of gene Y suppressed growth in three mutant KRAS lung cancer cell lines, as well as in a mutant EGFR lung cancer cell line. Additionally, gene Y expression was also increased in HBEC3-EIN, which expresses EGFR in a doxycycline-inducible manner. These results suggest that gene Y may be a potential therapeutic target for both mutant KRAS and mutant EGFR lung cancers.

[Disclosure of COI by Responsible researcher] Lead presenter is not the Responsible researcher.
[Lead presenter: Ayana Iwabe] We have no financial relationships to disclose.
[Principal Researcher: Mitsuo Sato] We have no financial relationships to disclose.

▲TOP

[P-1056] I-P4-1
RAS-RB-E2F axis

2025 / 9 / 25 (Thu) 17:30-18:15 Room P/(Motenashi Dome) Room P

### P-1056
### Functional analysis of BRAF V600E-induced protein kinase in colorectal cancer

[Speaker]Nagisa Furuno (Dept. of Med. Biochem., Grad. Sch. of Front. Biosci., Osaka) [Co-author]Hisakazu Kato (Dept. of Med. Biochem., Grad. Sch. of Med., Osaka Univ.)、Ryuta Ueda (Dept. of Med. Biochem., Grad. Sch. of Med., Osaka Univ.／Dept. of Gastroenterological Surg., Grad. Sch. of Med., Osaka Univ.)、Hidekazu Takahashi (Dept. of Gastroenterological Surg., Grad. Sch. of Med., Osaka Univ.)、Seiji Takashima (Dept. of Med. Biochem., Grad. Sch. of Front. Biosci., Osaka／Dept. of Med. Biochem., Grad. Sch. of Med., Osaka Univ.)

BRAFV600E-mutated colorectal cancer (CRC) has a poor prognosis due to its high malignancy and chemoresistance. In our previous study, we found that transduction of BRAFV600E led to growth arrest in the CRC cell line SW48. In these cells, BRAFV600E specifically induced the expression of a particular isoform of DCLK1, a known cancer stem cell marker. Based on this observation, we aim to elucidate the molecular functions of DCLK in BRAFV600E-mutated CRC. First, we established an in vitro kinase assay system using recombinant DCLK1 proteins to investigate the regulation of DCLK1 kinase activity. Phos-tag SDS-PAGE analysis revealed that the BRAFV600E increased the phosphorylation levels of DLCK1. Second, a sphere formation assay demonstrated that DCLK1 is partially essential for maintaining the stem-like properties of CRCs. Third, to identify novel substrates of DCLK1, we combined a radioisotope-based in vitro kinase assay with chromatographic protein purification and found several phosphoproteins as potential DCLK1 substrates. Our findings suggest that BRAFV600E contributes to the stem-like characteristics of CRCs by regulating both the expression and kinase activity of DCLK1.

[Disclosure of COI by Responsible researcher] Lead presenter is not the Responsible researcher.
[Lead presenter: Nagisa Furuno] We have no financial relationships to disclose.
[Principal Researcher: Seiji Takashima] We have no financial relationships to disclose.

### [P-1057] I-P4-1
### RAS-RB-E2F axis

2025 / 9 / 25 (Thu) 17:30-18:15 Room P/(Motenashi Dome) Room P

### P-1057
### Comprehensive and Rapid Identification of Driver Mutations via Single-Nucleotide Random Mutagenesis of Target Genes

[Speaker]Keisuke Yoshida (Dep. Mol. Prevention, Inst. Adv. Med. Sci., Nippon Med. Sch.) [Co-author]Kazufumi Honda (Dep. Mol. Prevention, Inst. Adv. Med. Sci., Nippon Med. Sch.／Dept. Mol. Prevention, Grad. Sch. Med., Nippon Med. Sch.)

Identification of driver mutations is critical for the diagnosis and development of effective treatment strategies in cancer. Currently, these are mainly explored through genomic analysis of clinical specimens, which is often time-consuming and costly. Here, we developed a novel method for comprehensive random mutagenesis of target genes using a genome editing technique. In this protocol, when a DNA amplicon of the target genomic region is prepared, cultured cells harboring random single-nucleotide variants can be established within 10 days. To evaluate the utility of this approach for driver mutation screening, we introduced random mutations into coding exons of endogenous oncogenes including ***KRAS***, ***EGFR***, and ***BRAF*** in HEK293. Spheroid formation and xenograft models revealed enhanced tumorigenicity, indicating acquisition of oncogenic potential by our method. Ampl-Seq analysis of spheroid DNA showed significant enrichment of pathogenic variants including KRAS G12/13X and Q61X. Moreover, 1.7% of variants of uncertain significance listed in ClinVar were also enriched. These findings demonstrate the utility of our mutagenesis platform for identifying functional driver mutations.

[Disclosure of COI by Responsible researcher] Lead presenter is the Responsible researcher.
[Lead presenter: Keisuke Yoshida] ■Research funds from company: 日本私立学校振興・共済事業団 「学術研究振興資金」
■Research expenses: 科研費 「学術変革(A)」・科研費 「基盤C」・受託研究 (株)アミノアップ

▲TOP

### [P-1067] I-P4-3
### Oncogenes & tumor-suppressor genes (2)

2025 / 9 / 25 (Thu) 17:30-18:15 Room P/(Motenashi Dome) Room P

---

### P-1067
### The role of TGF-β/Smad3/Smad4 in cancer immune evasion through suppression of STING

[Speaker]Takashi Yokoyama (Dept. Biochem., Grad. Sch. of Med., Univ. of Yamanashi) [Co-author]Masao Saitoh (Dept. Biochem., Grad. Sch. of Med., Univ. of Yamanashi／Ctr.for Med. Educ.&Sci., Grad. Sch. of Med., Univ. of Yamanashi)、Keiji Miyazawa (Dept. Biochem., Grad. Sch. of Med., Univ. of Yamanashi)

The TGF-β/Smad signaling pathway plays a critical role in the malignant progression of cancer through various mechanisms, including immune evasion. In this study, we focused on the downregulation of STING expression as a function of TGF-β/Smad in immune evasion of cancer cells. We demonstrated that stimulation of the human lung cancer cell line A549 with TGF-β decreased the STING mRNA and protein levels over 24-72 hours. Further investigation into the role of Smad family proteins revealed that knockout of ***SMAD3*** or ***SMAD4*** in A549 cells abrogated the TGF-β-induced suppression of STING, whereas A549-***SMAD2***-KO cells showed no such effect, indicating that TGF-β stimulation represses STING expression in a Smad3/Smad4-dependent manner. Notably, lung colonization of A549 cells in nude mice with tail vein injection was significantly suppressed by ***SMAD3*** KO. Therefore, A549-***SMAD3***-KO cells with STING knockdown were generated. We are further investigating the role of TGF-β/Smad3/Smad4-mediated suppression of STING on the induction of inflammatory cytokines, evasion of immune cells, and development of cancer cells in vivo.

[Disclosure of COI by Responsible researcher] Lead presenter is not the Responsible researcher.
[Lead presenter: Takashi Yokoyama] We have no financial relationships to disclose.
[Principal Researcher: Keiji Miyazawa] We have no financial relationships to disclose.

### [P-1068] I-P4-3
### Oncogenes & tumor-suppressor genes (2)

2025 / 9 / 25 (Thu) 17:30-18:15 Room P/(Motenashi Dome) Room P

---

### P-1068
### SPZ1-STING Axis Regulates Macrophage Polarization in the Lung Tumor Microenvironment

[Speaker]Chia W. Chou (Dept. of Life Sci., NTNU) [Co-author]Yu H. Huang (Dept. of Life Sci., NTNU)、Li T. Wang (Dept. of Life Sci., NTNU)、Shih H. Hsu (Grad. Inst. of Med., College of Med., KMU)

Lung cancer remains a leading cause of cancer mortality, with limited treatment efficacy despite therapeutic advances. Tumor–associated macrophages (TAMs), especially immunosuppressive M2–like macrophages, promote tumor progression and therapy resistance. Here, we identify SPZ1 as a key regulator of macrophage polarization. SPZ1 is highly expressed in M1–like macrophages, enhancing inflammatory responses, while its inhibition promotes M2–like polarization. Co–culturing cancer cells with SPZ1–overexpressing macrophages reduces tumor proliferation, whereas SPZ1 suppression increases tumor growth. Using macrophage–specific SPZ1 knockout mice, we observed impaired M1 responses and enhanced M2 functions. SPZ1 is regulated by the STING pathway, forming a positive feedback loop reinforcing M1 polarization. Single–cell RNA sequencing of SPZ1–deficient lung tumors revealed significant immune alterations, highlighting its role in shaping the tumor immune landscape. These findings establish the STING–SPZ1 axis as a key regulator of macrophage function and a potential target for enhancing anti–tumor immunity.

[Disclosure of COI by Responsible researcher] Lead presenter is the Responsible researcher.
[Lead presenter: Chia W. Chou] We have no financial relationships to disclose.

▲TOP

### [P-1069] I-P4-3
### Oncogenes & tumor-suppressor genes (2)

2025 / 9 / 25 (Thu) 17:30-18:15 Room P/(Motenashi Dome) Room P

---

### P-1069
### Targeting oxidative and immune signaling to suppress lymphovascular invasion in urothelial carcinoma

[Speaker]Jei-Ming Peng (Institute for Translational Research in Biomedicine, CGMH, Kaohsiung, Taiwan) [Co-author]Jia-Wun Luo (Institute for Translational Research in Biomedicine, CGMH, Kaohsiung, Taiwan)、Pei-Chia Wang (Institute for Translational Research in Biomedicine, CGMH, Kaohsiung, Taiwan)

Lymphovascular invasion (LVI), a strategy by tumors to enter lymphatic and blood vessels, is a key step in cancer metastasis, especially in upper tract urothelial carcinoma (UTUC). The role of tumor suppressor genes in this process remains underexplored. We report that PPP1R3C is tumor-suppressive and modulates cancer cell behavior under oxidative stress, metabolic changes, and LVI-associated progression. Loss of PPP1R3C alters expression of SOD3, SLC28A3, and NOD2 genes involved in oxidative defense, nucleotide transport, and immune signaling. While SOD3 upregulation may enhance oxidative stress response, downregulation of tumor suppressors like DIRAS1 and SHISA3 indicates impaired tumor suppression. These gene expression changes appear to promote tumor progression and LVI-related metastasis. Pathway analysis also revealed activation of NOD-like receptor and TNF signaling, potentially affecting clinical outcomes. Our findings reveal a novel mechanistic link between PPP1R3C loss and UTUC metastasis, and suggest these genes and downstream effectors as potential therapeutic targets.

[Disclosure of COI by Responsible researcher] Lead presenter is the Responsible researcher.
[Lead presenter: Jei-Ming Peng] We have no financial relationships to disclose.

### [P-1070] I-P4-3
### Oncogenes & tumor-suppressor genes (2)

2025 / 9 / 25 (Thu) 17:30-18:15 Room P/(Motenashi Dome) Room P

---

### P-1070
### CD47-ISX Axis Triggers Inflammasome Activation, Modulates Macrophage Polarization, and Tumor-Promoting Microenvironment

[Speaker]Li T. Wang (Department of Life Science, NTNU, Taipei, Taiwan) [Co-author]Shih H. Hsu (Graduate Institute of Medicine, College of Medicine, KMU, Kaohsiung, Taiwan)

Immunotherapies hold promise for cancer treatment but are limited by the evolving tumor microenvironment. This study explores the role of CD47 signaling, intestine-specific homeobox (ISX), and inflammasome-related genes (NLRPs) in hepatocellular carcinoma (HCC). We identified a strong correlation between these factors and key clinical parameters. Elevated CD47 and IL-18 drive M0 macrophage polarization into the M2-like phenotype, promoting disease progression. In liver-specific ISX knockout mice, M2-like macrophage depletion halted disease progression. Molecular analyses reveal that CD47 signaling enables ISX to interact with TWIST1's basic helix-loop-helix (bHLH) domain via its homeobox domain. This complex binds to base-degeneracy sequences (GGDWYR) in CD47 and inflammasome-related gene promoters, activating their expression and reinforcing M2-like polarization. These findings establish the CD47-ISX-NLRPs axis as a key driver of liver disease and tumor progression, offering new therapeutic targets to enhance immunotherapy.

[Disclosure of COI by Responsible researcher] Lead presenter is the Responsible researcher.
[Lead presenter: Li T. Wang] We have no financial relationships to disclose.

### [P-1071] I-P4-3
### Oncogenes & tumor-suppressor genes (2)

2025 / 9 / 25 (Thu) 17:30-18:15 Room P/(Motenashi Dome) Room P

### P-1071
### Formaldehyde Exposure Activates the RUNX1-ISX Signaling Axis to Promote Oncogenic Activity in B-ALL

[Speaker]Pei H. Huang (Dept. of Life Sci., NTNU) [Co-author]Li T. Wang (Dept. of Life Sci., NTNU)、Shih H. Hsu (Grad. Inst. of Med., College of Med., KMU)、Shyh S. Chiou (Department of Pediatrics, Faculty of Medicine, KMU)、Li H. Ye (Dept. of Life Sci., NTNU)

Formaldehyde, a colorless chemical used in industrial and household products, is linked to health issues, including respiratory problems, immune impairment, and cancer. Despite its widespread use, the molecular mechanisms underlying its oncogenic effects are poorly understood. This study shows that low–dose formaldehyde exposure (3 ppm) upregulates oncogenic markers such as RUNX1, ETV6, OCT4, and ISX in B–ALL cell lines. Transcriptomic analysis reveals that formaldehyde activates the RUNX1–ISX signaling axis, which enhances the expression of downstream oncogenic factors like YBX2, HES2, FOXH1, MYCL, FOS, and PBX4. This activation alters gene expression in cancer–associated pathways, promoting oncogenic activity and tumor formation in a xenograft model. Importantly, inhibiting the RUNX1–ISX axis effectively suppressed formaldehyde–induced oncogenic effects and tumor growth. Immunofluorescence staining and Pearson correlation analysis confirmed significant co–expression of RUNX1 and ISX in human samples. These findings highlight the RUNX1–ISX axis as a key mediator of formaldehyde–induced oncogenesis in B–ALL and suggest it as a potential therapeutic target.

[Disclosure of COI by Responsible researcher] Lead presenter is the Responsible researcher.
[Lead presenter: Pei H. Huang] We have no financial relationships to disclose.

▲TOP

### [P-1072] I-P4-3
### Oncogenes & tumor-suppressor genes (2)

2025 / 9 / 25 (Thu) 17:30-18:15 Room P/(Motenashi Dome) Room P

---

### P-1072
### ISX Axis Drives Bone Marrow Remodeling, Macrophage Polarization, and Cancer Stemness in BCP-ALL

[Speaker]Yu C. Lin (Dept. of Life Sci., NTNU) [Co-author]Chiung Y. Chiu (Dept. of Life Sci., NTNU)、Shih H. Hsu (Grad. Inst. of Med., College of Med., KMU)、Li T. Wang (Dept. of Life Sci., NTNU)

Acute lymphoblastic leukemia (ALL) is the most common pediatric malignancy, with BCP–ALL comprising 80% of cases. Despite therapeutic advances, drug resistance, relapse, and cancer stemness remain major challenges. ISX, a transcription factor overexpressed in multiple cancers, has an unclear role in BCP–ALL. Transcriptomic and functional analyses reveal that ISX activates Galectin–9 signaling, upregulating key stemness factors (Nanog, SOX2, OCT4) and promoting leukemia proliferation, transformation, and M2 macrophage polarization. Mechanistically, ISX directly binds to the LGALS9 promoter, enhancing Galectin–9 expression and reinforcing leukemic progression. An analysis of 64 BCP–ALL patient samples showed a significant correlation between ISX and LGALS9 expression, disease severity, and clinical outcomes. These findings establish the ISX–LGALS9 axis as a key regulator of leukemia progression and a potential therapeutic target.

[Disclosure of COI by Responsible researcher] Lead presenter is the Responsible researcher.
[Lead presenter: Yu C. Lin] We have no financial relationships to disclose.

### [P-1073] I-P4-3
### Oncogenes & tumor-suppressor genes (2)

2025 / 9 / 25 (Thu) 17:30-18:15 Room P/(Motenashi Dome) Room P

---

### P-1073
### Functional characterization of an endogenous retroviral gene expressed in melanoma

[Speaker]Takao Morinaga (Div. Cell Ther., Chiba Cancer Ctr. Res. Inst.) [Co-author]Yuki Nakamura (Div. Cell Ther., Chiba Cancer Ctr. Res. Inst.)、Takashi Inozume (Div. Cell Ther., Chiba Cancer Ctr. Res. Inst.／Dept. Derm., Grad. Med., Chiba univ.)、Masahito Kawazu (Div. Cell Ther., Chiba Cancer Ctr. Res. Inst.)

Endogenous retroviral genes make up about 8 % of the human genome, and while most of them are inactive in somatic cells, they are often active in germ cells. ERVFRD-1, one of these genes, is specifically activated in trophoblasts and plays a role in placenta formation. We previously analyzed the transcriptomes of cancer specimens and found that ERVFRD-1 expression was elevated in some cases. According to the TCGA data, the copy number of ERVFRD-1 varies especially in melanoma and correlates with poor prognosis. To examine the function of this gene, we transplanted an ERVFRD-1-transduced mouse cancer cells derived from MC38 cells into the dorsal back skin of C57BL/6 mice. As a result, tumors expressing ERVFRD-1 grew faster than the mock control, and the tumor infiltrated CD8+ T cells showed elevated expression of immunosuppressive markers. This study investigates the functional relevance of ERVFRD-1 expression in melanoma by utilizing ERVFRD-1 knockout cells and a custom-generated hybridoma producing an ERVFRD-1-specific antibody.

[Disclosure of COI by Responsible researcher] Lead presenter is not the Responsible researcher.
[Lead presenter: Takao Morinaga] ■Research funds from company: JSPS科研費 JP25K11596
[Principal Researcher: Masahito Kawazu] We have no financial relationships to disclose.

### [P-1074] I-P4-3
### Oncogenes & tumor-suppressor genes (2)

2025 / 9 / 25 (Thu) 17:30-18:15 Room P/(Motenashi Dome) Room P

---

### P-1074
### The FAM72A-ETV4 Complex Mediates HBx Signaling to Promote Oncogenic Activities and Accelerate HCC Progression

[Speaker]Li W. Tseng (Graduate Institute of Medicine, College of Medicine, KMU, Kaohsiung, Taiwan) [Co-author]Shih H. Hsu (Graduate Institute of Medicine, College of Medicine, KMU, Kaohsiung, Taiwan)、Kai T. Chang (Graduate Institute of Medicine, College of Medicine, KMU, Kaohsiung, Taiwan)

Despite the availability of hepatitis B virus (HBV) vaccines, HBV infection remains a major risk factor for chronic liver disease, including hepatocellular carcinoma (HCC). The HBV-encoded X protein (HBx) plays a crucial role in viral replication and disrupts cellular signaling pathways, contributing to HCC development. However, the mechanisms behind HCC initiation and progression are unclear. This study identifies the FAM72A-ETV4 complex as a key mediator of HBx signaling, driving oncogenic activity and worsening HCC malignancy. HBx upregulated FAM72A and ETV4 expression via the MAPK/ERK1/2 pathway. FAM72A interacted with the disordered domain of ETV4, facilitating its nuclear translocation and enhancing downstream oncogenic gene expression. Pearson correlation analysis and immunofluorescence staining confirmed the strong correlation between HBx, FAM72A, and ETV4 expression. Disrupting the FAM72A-ETV4 interaction significantly suppressed HBx-induced oncogenic activity and tumor formation in a xenograft model. These findings highlight the FAM72A-ETV4 complex as a crucial mediator of HBx-induced HCC progression, suggesting it as a potential therapeutic target.

[Disclosure of COI by Responsible researcher] Lead presenter is the Responsible researcher.
[Lead presenter: Li W. Tseng] We have no financial relationships to disclose.

### [P-1075] I-P4-3
### Oncogenes & tumor-suppressor genes (2)

2025 / 9 / 25 (Thu) 17:30-18:15 Room P/(Motenashi Dome) Room P

---

### P-1075
### The tumor suppressive function of MTSS1 and its potential mechanism

[Speaker]Nuo Li (Life Science Institute, Guangxi Medical University) [Co-author]Xiaoying Zhou (Life Science Institute, Guangxi Medical University)

The dual-gene characteristic of tumor metastasis suppressor factor 1 (MTSS1) in promoting cancer and inhibiting cancer in different tumors makes its regulatory mechanism a research hotspot. However, the expression and biological function of MTSS1 in renal clear cell carcinoma (ccRCC) have not been reported yet. We found that compared with normal tissues (n = 72), the expression level of MTSS1 gene was down-regulated in ccRCC (n = 532), and patients with low expression of MTSS1 in ccRCC had poorer survival status and prognosis. Moreover, we found that the CpG island in the DNA promoter region of MTSS1 was highly methylated in ccRCC, leading to transcriptional inhibition. Through bioinformatics analysis, we found that the gene most negatively correlated with MTSS1 was TMSB10. And the transcription level of MTSS1 was significantly positively correlated with epithelial cell markers EPCAM and CDH1, and negatively correlated with MMP7, suggesting that the loss of MTSS1 expression may promote tumor invasion through epithelial phenotype loss and matrix degradation. In summary, MTSS1 can be a new prognostic molecular marker and potential therapeutic target in ccRCC.

[Disclosure of COI by Responsible researcher] Lead presenter is the Responsible researcher.
[Lead presenter: Nuo Li] We have no financial relationships to disclose.

### [P-1086] I-P5-2
### Regulation of gene expression

2025 / 9 / 25 (Thu) 17:30-18:15 Room P/(Motenashi Dome) Room P

---

### P-1086
### Epigenomic Regulation of Enhancers Characteristic of ER-Positive Breast Cancers

[Speaker]Hiroaki Tachiwana (Hoshi Univ., Inst. for Advanced Life Sciences, Dept. of Epigenomics／The Cancer Institute of JFCR, Div. Cancer Biol.) [Co-author]Noriko Saitoh (The Cancer Institute of JFCR, Div. Cancer Biol.)

Breast cancer is classified into subtypes based on gene expression profiles, each linked to distinct therapeutic targets and clinical outcomes. Although these subtypes display unique transcriptional programs, the mechanisms maintaining these patterns are not fully understood. To explore this, we examined epigenomic regulation at enhancers-key elements in transcriptional control. Using ER-positive breast cancer cell lines, we identified subtype-specific enhancers affected by histone deacetylase (HDAC) inhibition. HDAC inhibitors repressed ER-positive defining genes. ChIP-seq of RNA Polymerase II showed reduced occupancy at promoters and adjacent intergenic regions of these genes. These regions co-localized with H3K27ac, H3K4me1, and eRNAs, indicating enhancer activity. Importantly, ATAC-seq of patient samples revealed that a subset of these enhancers exists in vivo and correlates with subtype-specific gene expression, highlighting clinical relevance. This presentation will discuss the formation of novel enhancers in breast cancer and mechanisms of their subtype-specific regulation.

[Disclosure of COI by Responsible researcher] Lead presenter is the Responsible researcher.
[Lead presenter: Hiroaki Tachiwana] We have no financial relationships to disclose.

### [P-1087] I-P5-2
### Regulation of gene expression

2025 / 9 / 25 (Thu) 17:30-18:15 Room P/(Motenashi Dome) Room P

---

### P-1087
### Inhibition of LRRC8A overcomes chemoresistance in the 3D spheroid model of breast cancer cells

[Speaker]Susumu Ohya (Dept. Pharmacol., Grad. Sch. Med. Sci., Nagoya City Univ.) [Co-author]Kajikuri Junko (Dept. Pharmacol., Grad. Sch. Med. Sci., Nagoya City Univ.)、Ryo Otsuka (Dept. Pharmacol., Grad. Sch. Med. Sci., Nagoya City Univ.)、Hinako Mitsui (Dept. Pharmacol., Grad. Sch. Med. Sci., Nagoya City Univ.)、Hiroaki Kito (Dept. Pharmacol., Grad. Sch. Med. Sci., Nagoya City Univ.)、Yohei Yamaguchi (Dept. Pharmacol., Grad. Sch. Med. Sci., Nagoya City Univ.)、Miki Matsui (Dept. Pharmacol., Grad. Sch. Med. Sci., Nagoya City Univ.)

The main component of volume-regulated chloride ($Cl^-$) channels, leucine-rich repeats containing 8A (LRRC8A) is amplified in several cancer cells and plays an important role in the acquisition of resistance to anti-cancer drugs and poor prognosis. The three-dimension (3D)-engineered cancer spheroid cells have been established as in vitro models for stemness and chemoresistance in solid cancers. In the present study, the resistance to doxorubicin (DOX) was acquired by the spheroid formation of human breast cancer cell line, YMB-1. The siRNA-mediated and pharmacological inhibition of LRRC8A overcame the resistance to DOX in the 3D spheroid model. The major drug-metabolizing enzyme, CYP3A4, and multidrug resistance-associated proteins, MRPs, were up-regulated following spheroid formation, and their expression levels were decreased by the inhibition of LRRC8A. Collectively, the present results suggest that the inhibition of LRRC8A may be a therapeutic strategy to overcome resistance to DOX through the transcriptional repression of CYP3A4 and MRPs in breast cancer stem cells.

[Disclosure of COI by Responsible researcher] Lead presenter is the Responsible researcher.
[Lead presenter: Susumu Ohya] We have no financial relationships to disclose.

▲TOP

### [P-1088] I-P5-2
### Regulation of gene expression

2025 / 9 / 25 (Thu) 17:30-18:15 Room P/(Motenashi Dome) Room P

---

### P-1088
### YAP1-2α isoform, fosters the formation of LLPS-mediated super-enhancers that increase cancer cell malignancy

[Speaker]Takuya Ooki (Lab of Microbial Carcinogenesis, Inst of Microbial Chemistry) [Co-author]Takeru Hayashi (Lab of Microbial Carcinogenesis, Inst of Microbial Chemistry)、Yui Yamamoto (Lab of Microbial Carcinogenesis, Inst of Microbial Chemistry)、Masanori Hatakeyama (Lab of Microbial Carcinogenesis, Inst of Microbial Chemistry／Ctr of Infection-associated Cancer, Inst for Genetic Med, Hokkaido Univ)

The acquisition of drug resistance is a key challenge for successful cancer treatment. Understanding the mechanisms by which cancer cells evade drug action is crucial for the development of effective therapies. PARP inhibitors (PARPi) induce synthetic lethality in BRCA-deficient cancer cells, eventually leading to PARPi resistance. We demonstrate that the splicing pattern of YAP1 pre-mRNA is altered in PARPi-resistant cancer cells, resulting in an elevated minor YAP1 isoform, YAP1-2α. Increased YAP1-2α specifically heterodimerizes with TAZ and triggers super-enhancers formation via liquid-liquid phase separation (LLPS). These super-enhancers are also activated in the CD44v$^{high}$ cancer stem-like population of parental cells. Disruption of YAP1 super-enhancers in both PARPi-resistant and CD44v$^{high}$ cancer cells abolishes cancer stem-like properties and drug resistance. Thus, activation of YAP1-2α super-enhancers reinforces the stemness of cancer cells, leading to drug resistance. Our study reveals that disrupting these super-enhancers represents a promising strategy to hinder neoplastic progression and prevent drug resistance in cancer cells.

[Disclosure of COI by Responsible researcher] Lead presenter is not the Responsible researcher.
[Lead presenter: Takuya Ooki] ■Research funds from company: 日本学術振興会
[Principal Researcher: Masanori Hatakeyama] ■Research funds from company: 日本学術振興会、AMED

### [P-1089] I-P5-2
### Regulation of gene expression

2025 / 9 / 25 (Thu) 17:30-18:15 Room P/(Motenashi Dome) Room P

---

### P-1089
### Analysis of the expression mechanism and molecular function of an oncogenic ERK-inducible tetraspanin protein in cancer

[Speaker]Junichiro Nashimoto (Div. Cell Signaling & Mol. Med., IMSUT) [Co-author]Yuji Kubota (Div. Cell Signaling & Mol. Med., IMSUT)、Mutsuhiro Takekawa (Div. Cell Signaling & Mol. Med., IMSUT)

Activating mutations in components of the ERK pathway induce tumorigenesis, but the underlying molecular mechanisms are not fully understood. In our previous study, we found that cancer-derived MEK1 mutants promote cell transformation by hyperactivation of ERK. Through transcriptome analysis, we identified several previously uncharacterized genes that are induced downstream of the MEK1 mutants. Among them, we focused on a protein X whose physiological and pathological function was largely unknown. First, we found that gene X is highly expressed in various cancer cell lines with oncogenic ERK activity. To elucidate the molecular function of protein X, we performed a comprehensive interactome analysis by mass spectrometry. This analysis identified several candidate interacting proteins that may contribute to apoptosis resistance. Consistent with this, ectopic expression of protein X in a non-small cell lung cancer cell line conferred resistance to apoptosis induced by UV irradiation and anticancer drugs. We are currently investigating the mechanism by which this ERK-inducible protein promotes apoptosis resistance in cancer and evaluating its potential as a novel therapeutic target.

[Disclosure of COI by Responsible researcher] Lead presenter is not the Responsible researcher.
[Lead presenter: Junichiro Nashimoto] We have no financial relationships to disclose.
[Principal Researcher: Mutsuhiro Takekawa] ■Research funds from company: アボット

▲TOP

### [P-1090] I-P5-2
### Regulation of gene expression

2025 / 9 / 25 (Thu) 17:30-18:15 Room P/(Motenashi Dome) Room P

---

### P-1090
### Liver cancer-specific repression of amino acid metabolism-related genes by the Wnt/β-catenin signaling

[Speaker]Saya Nakagawa (Div. Clin. Genome Res., Inst. Med. Sci.,) [Co-author]Kiyoshi Yamaguchi (Div. Clin. Genome Res., Inst. Med. Sci.,)、Yumiko Isobe (Div. Clin. Genome Res., Inst. Med. Sci.,)、Sho Tabata (Tsuruoka Metabolomics Lab., NCC)、Kiyoko Takane (Div. Clin. Genome Res., Inst. Med. Sci.,)、Yoichi Furukawa (Div. Clin. Genome Res., Inst. Med. Sci.,)

Aberrant activation of the Wnt/β-catenin signaling pathway plays a pivotal role in a wide range of human tumors including liver and colorectal cancer. Although a number of genes are transcriptionally upregulated by the β-catenin/TCF family complex, we previously found that histidine ammonia-lyase (***HAL***), an enzyme involved in histidine catabolism, was negatively regulated by the Wnt signaling in liver cancer cells. In this study, we demonstrated that ***HAL*** expression was transcriptionally regulated by CEBPA and FOXA1, and that these transcription factors were suppressed by Wnt/β-catenin signaling. Additionally, our integrated analysis of RNA-seq and ChIP-seq revealed that CEBPA and FOXA1 regulate the expression of arginase 1 (***ARG1***), suggesting that activated Wnt/β-catenin signaling suppresses the expression of both HAL and ARG1 in liver cancer cells. Our findings uncovered a novel transcriptional network downstream of Wnt/β-catenin signaling and highlighted its role in reprogramming cellular metabolism in liver cancer.

[Disclosure of COI by Responsible researcher] Lead presenter is not the Responsible researcher.
[Lead presenter: Saya Nakagawa] We have no financial relationships to disclose.
[Principal Researcher: Yoichi Furukawa] We have no financial relationships to disclose.

### [P-1091] I-P5-2
### Regulation of gene expression

2025 / 9 / 25 (Thu) 17:30-18:15 Room P/(Motenashi Dome) Room P

---

### P-1091
### The Role of a Hormone-Independent Novel GREB1 Transcript

[Speaker]Akikazu Harada (Ctr. Inf. Dis. Edu. Res., Univ. Osaka) [Co-author]Akira Kikuchi (Ctr. Inf. Dis. Edu. Res., Univ. Osaka)

GREB1 has been identified as a target gene of estrogen signaling and is thought to function as a transcriptional co-regulator of estrogen receptor. Beyond estrogen, GREB1 is also a target gene of other sex hormone signaling pathways, including androgen and progesterone, and is recognized as a tumor-promoting factor in hormone-dependent cancers. However, we previously observed that GREB1 is also expressed in hormone-independent tumors. Here, we discovered that the major transcript of GREB1 was not the one previously defined. We identified a transcription start site located approximately 60 kb upstream of the canonical GREB1 transcripts, which appears to be common across various tumor types. Therefore, to elucidate the function of this novel transcript, we conducted analyses using a neuroblastoma model that exclusively expresses the transcript. A comprehensive analysis of GREB1-interacting proteins revealed that GREB1 is a component of the NCOR2 complex. Moreover, our findings suggest that GREB1 switches the function of the NCOR2 complex–previously thought to repress transcription–toward transcriptional activation. We are also investigating the induction mechanism of this transcript.

[Disclosure of COI by Responsible researcher] Lead presenter is not the Responsible researcher.
[Lead presenter: Akikazu Harada] We have no financial relationships to disclose.
[Principal Researcher: Akira Kikuchi] We have no financial relationships to disclose.

### [P-1092] I-P5-2
### Regulation of gene expression

2025 / 9 / 25 (Thu) 17:30-18:15 Room P/(Motenashi Dome) Room P

### P-1092
### Analysis of Expressional Alterations of HER2 and Associated Phenotypes in Breast Cancer Cell Lines

[Speaker]Shota Katayama (Dept. of Life Sci. & Med. Biosci.,Waseda Univ.) [Co-author]Kentaro Semba (Dept. of Life Sci. & Med. Biosci.,Waseda Univ.／TR Ctr., Fukushima Med. Univ.)、Yusuke Yamamoto (Lab. Integr. Oncol., Natl. Cancer Ctr. Res. Inst.)、Jun(NCC) Nakayama (Dept. of Oncogenesis Growth Regulation, Osaka Intl. Cancer Inst.)

HER2 is a well-known as a negative prognostic factor and therapeutic target in breast cancer. However, dynamic alterations in HER2 expression during cancer progression make it to be difficult to complicate therapeutic decisions. Understanding the expressional plasticity of HER2 contributes to improve prognosis and clinical decisions in HER2-positive breast cancer.In this study, we performed the comparison of cancer phenotypes with HER2 expression, it was generated by sorting the top and bottom 10% of HER2-expressing cells from various breast cancer lines. While most cell lines reverted to their original HER2 profiles over time, MDA-MB-361 cells maintained stable expression in the both fractions. It suggested that the intrinsic stability of HER2 expression was maintained through epigenetic regulation. In vivo experiments, HER2-Low cells remained tumor-mas dormancy before rapid proliferation, whereas HER2-High cells proliferate immediately with enhanced sphere-forming ability. These findings may discover the mechanisms of HER2 dynamics and support the optimization of therapies with variable HER2 status.

[Disclosure of COI by Responsible researcher] Lead presenter is the Responsible researcher.
[Lead presenter: Shota Katayama] We have no financial relationships to disclose.

[P-1093] I-P5-2
Regulation of gene expression

2025 / 9 / 25 (Thu) 17:30-18:15 Room P/(Motenashi Dome) Room P

### P-1093
### Investigating the Impact of KIF12 Motor Protein on Extracellular Vesicle Phenomenon

[Speaker]Shan-Rong Wu (Dept. of Life Sci., NTNU) [Co-author]Li-Nian Chen (Dept. of Life Sci., NTNU)、Yi-Hsiang Hsieh (Dept. of Life Sci., NTNU)、Yun-Ju Lai (Dept. of Life Sci., NTNU)

Kinesin Family Member 12 (KIF12) is a microtubule-associated transport protein. It plays a crucial role in cell division and intracellular vesicle transport. Our previous studies have found that the expression level of KIF12 protein is associated with the proliferation, colony-formation ability, and autophagy capacity of colorectal cancer stem cells. To further investigate the impact of KIF12 on the exocytosis , we utilized SW480 cell with stably knockdown of the KIF12 gene (shKIF12) to explore its effects on extracellular vesicle secretion. In our preliminary results, we found that reducing the expression of KIF12 protein decreased the number of exosomes produced by SW480 cells and their tumospheroid cells, as well as the expression of exosome-related markers. Furthermore, we also found that a lower expression of KIF12 protein correlated with a lower levels of the stemness marker in SW480 tumorspheroid cells. In summary, KIF12 may have the potential as a therapeutic target for colorectal cancer.

[Disclosure of COI by Responsible researcher] Lead presenter is the Responsible researcher.
[Lead presenter: Shan-Rong Wu] We have no financial relationships to disclose.

▲TOP

### [P-1094] I-P5-2
### Regulation of gene expression

2025 / 9 / 25 (Thu) 17:30-18:15 Room P/(Motenashi Dome) Room P

---

### P-1094
### Suppression of EWS-FLI1 function by competitive inhibition of its binding to enhancer elements in Ewing sarcoma cells

[Speaker]Atsushi Takatori (Div. Innov. Cancer Therap., Chiba Cancer Ctr. Res. Inst.) [Co-author]Xiaoyi Lai (Div. Innov. Cancer Therap., Chiba Cancer Ctr. Res. Inst.／Grad. Sch. Med. & Pharm. Sci., Univ. Chiba)、Yuki Kida (Div. Innov. Cancer Therap., Chiba Cancer Ctr. Res. Inst.)

Genomic and epigenomic alterations are key events in tumor onset and progression. Among these, epigenomic reprogramming is an emerging research topic for the development of novel therapeutic approaches for malignant tumors. In Ewing sarcoma, the EWS-FLI1 oncogenic fusion protein activates the function of gene enhancer element containing GGAA repeats and upregulates its target genes. Previously, we developed a pyrrole-imidazole polyamide (PIP), CCC-040, which selectively binds to the enhancer element. In this study, we compared CCC-040 with a mismatch PIP, CCC-055, in Ewing sarcoma cells. The proliferation of Ewing sarcoma cell lines was significantly reduced by CCC-040 but not by CCC-055. A dual-luciferase reporter assay demonstrated that CCC-040 suppressed the activity of the enhancer element. Moreover, CCC-040 decreased the interaction of EWS-FLI1 protein with oligonucleotides containing the enhancer element and downregulated the expression of its target genes. These data suggest a novel targeted therapy that modulates oncogenic enhancer activity in malignant tumors.

[Disclosure of COI by Responsible researcher] Lead presenter is the Responsible researcher.
[Lead presenter: Atsushi Takatori] ■Research funds from company: 日本学術振興会

### [P-1102] I-P9-2
### Metastasis & EMT (2)

2025 / 9 / 25 (Thu) 17:30-18:15 Room P/(Motenashi Dome) Room P

---

### P-1102
### TGFβ1 induces Vasorin/slit-like2 protein and accelerates cellular migration in gastric cancer cells

[Speaker]Yoko Yasuda (Health Science Dept., Faculty of Medical Science, Kyushu Univ.) [Co-author]Tomoaki Koga (Med. Cell Biol., IMEG, Kumamoto Univ.)、Yuki Tateishi (Health Science Dept., Faculty of Medical Science, Kyushu Univ.)、Shinichi Mizuno (Health Science Dept., Faculty of Medical Science, Kyushu Univ.)、Mitsuyoshi Nakao (Med. Cell Biol., IMEG, Kumamoto Univ.)

Effective molecules that inhibit cellular migration for cancer therapy have not yet been identified. While current studies have reported that inflammatory cytokines, including TGFβ1, induce EMT, the molecular mechanisms underlying TGFβ1-induced EMT in gastric cancer remain largely unknown. In this study, we identified an EMT-related molecule, the type I transmembrane protein Vasorin/slit-like 2 (VASN/SLITL2), through ***in silico*** expression screening. To investigate the function of VASN in cellular migration, we performed a transwell migration assay using the gastric cancer cell line MKN-7. Interestingly, cells in which VASN expression was inhibited showed a significant decrease in migration, even in the presence of TGFβ1. Finally, we examined the relationship between VASN expression and clinical outcomes in gastric cancer patients. As a result, the expression levels of both VASN and TGFβ1 were found to be positively correlated with low-grade cancer stages. These findings suggest that an early shift in the cytokine environment toward TGFβ1 promotes EMT, and that VASN functions as an accelerator of this process.

[Disclosure of COI by Responsible researcher] Lead presenter is the Responsible researcher.
[Lead presenter: Yoko Yasuda] We have no financial relationships to disclose.

### [P-1103] I-P9-2
### Metastasis & EMT (2)

2025 / 9 / 25 (Thu) 17:30-18:15 Room P/(Motenashi Dome) Room P

---

### P-1103
### The role of INHBA in the EMT Induction of Oral squamous cell carcinoma

[Speaker]Yuriko Yawata (Dept. of Oral Biosci., Tokushima Univ. Grad. Sch. of BiomedSci.) [Co-author]Motoharu Sarubo (Dept. of Oral Biosci., Tokushima Univ. Grad. Sch. of BiomedSci.)、Yasusei Kudo (Dept. of Oral Biosci., Tokushima Univ. Grad. Sch. of BiomedSci.)

Recently, partial epithelial-to-mesenchymal transition (p-EMT), an intermediate and plastic state between epithelial and mesenchymal phenotypes, has been recognized as a key driver of cancer progression. However, the molecular mechanisms regulating p-EMT in oral squamous cell carcinoma (OSCC) remain poorly understood. Inhibin βA (INHBA), a member of the TGF-β superfamily, is upregulated in various cancers and has been proposed as a potential regulator of p-EMT. Nevertheless, its role in OSCC has not been fully elucidated. To investigate this, we assessed INHBA expression in 11 OSCC cell lines using qPCR. SKN-3 cells, which exhibited low endogenous INHBA expression, were selected for INHBA overexpression. Among the INHBA overexpressing clones, two distinct morphological phenotypes were observed: epithelial-like and mesenchymal-like. The mesenchymal-like clones showed decreased E-cadherin expression and increased levels of N-cadherin and SNAI2. Notably, these phenotypes correlated with the expression level of INHBA. These findings suggest that INHBA promotes p-EMT in OSCC cells through the SNAI2/E-cadherin axis, thereby contributing to tumor progression.

[Disclosure of COI by Responsible researcher] Lead presenter is not the Responsible researcher.
[Lead presenter: Yuriko Yawata] We have no financial relationships to disclose.
[Principal Researcher: Yasusei Kudo] We have no financial relationships to disclose.

### [P-1104] I-P9-2
### Metastasis & EMT (2)

2025 / 9 / 25 (Thu) 17:30-18:15 Room P/(Motenashi Dome) Room P

---

### P-1104
### RUNX3-MYC-p53 Axis: A Critical Regulatory Node of Cancer Metastasis and Therapeutic Targeting

[Speaker]Jungwon Lee (Cancer Science Inst. of Singapore, NUS) [Co-author]Junichi Matsuo (Cancer Science Inst. of Singapore, NUS)、Jangphattananont Nawaphat (Cancer Science Inst. of Singapore, NUS)、Manabu Takamatsu (The Cancer Institute of JFCR, Japan)、Tetsuo Noda (The Cancer Institute of JFCR, Japan)、Kazuto Suda (Juntendo University, Japan)、Atsushi Okabe (Dept. of Mol. Oncology, Chiba Cancer Center, Japan)、Atsushi Kaneda (Dept. of Mol. Oncology, Chiba Cancer Center, Japan)、Linda Shyue Huey Chuang (Cancer Science Inst. of Singapore, NUS)、Yoshiaki Ito (Cancer Science Inst. of Singapore, NUS)

Cancer metastasis is the leading cause of cancer deaths, yet underlying mechanisms remain unclear. Developmental transcription factor RUNX3 paradoxically acts as both tumor suppressor and metastasis driver. Early work showed that RUNX3 enhances TGF$\beta$-mediated cell cycle arrest and apoptosis. Lately, however, increasing evidence has linked high RUNX3 expression with metastatic burden. We found that p53 status dictates whether RUNX3 suppresses or promotes metastasis in gastric cancer. In a wildtype p53 background, RUNX3 cooperates with p53 to enhance CDKN1A transcription and disrupt MYC-mediated activation of pro-metastatic genes CD44 and WNT5A. Conversely, when p53 is deleted or mutated, RUNX3 directly interacts with MYC to induce CD44 and WNT5A expression, leading to epithelial-to-mesenchymal transition (EMT), invasion, and extracellular matrix remodeling. Moreover, point-mutant p53 forms a complex with RUNX3, thereby increasing MYC expression and fueling a more aggressive phenotype. Runx3 deletion in mouse models significantly reduces metastatic spread. These findings uncover the RUNX3-MYC-p53 axis as a critical regulator of metastasis, offering mechanistic insights for therapy.

[Disclosure of COI by Responsible researcher] Lead presenter is not the Responsible researcher.
[Lead presenter: Jungwon Lee] We have no financial relationships to disclose.
[Principal Researcher: ] We have no financial relationships to disclose.

▲TOP

### [P-1105] I-P9-2
### Metastasis & EMT (2)

2025 / 9 / 25 (Thu) 17:30-18:15 Room P/(Motenashi Dome) Room P

---

### P-1105
### MRC2 promotes gastric cancer malignancy through SCD-1-mediated modulation of epithelial-mesenchymal transition

[Speaker]Hsin-Ying Chiou (Genomics and Proteomics Core Laboratory, Kaohsiung Chang Gung Memorial Hospital) [Co-author]Pin-Chun Lu (Graduate Institute of Medicine, College of Medicine, Kaohsiung Medical University)、Ming-Hong Lin (Graduate Institute of Medicine, College of Medicine, Kaohsiung Medical University／Department of Microbiology and Immunology, Kaohsiung Medical University)

Gastric cancer (GC) is a significant contributor to cancer-related mortality worldwide, with metastatic GC exacerbating this issue. Evidence suggests that overexpression of mannose receptor C type 2 (MRC2) is linked to malignancy in various cancers. Our findings indicate that MRC2 protein levels are significantly elevated in cancerous tissues compared to adjacent normal tissues, correlating with poorer overall survival in patients. Ectopic expression of MRC2 enhances the migration and invasion of GC cells, while downregulation reduces these behaviors, consistent with changes in epithelial-mesenchymal transition (EMT) markers, including Vimentin, N-Cadherin, and Snail1. Additionally, MRC2 modulates the lipogenesis-related enzyme SCD-1 in conjunction with PPAR$\gamma$ and p38 signaling pathways, influencing cancer migration and invasion, as shown by the abrogation of these effects upon SCD-1 inhibition. Our in vivo experiments indicate that MRC2 impairment reduces tumor formation in mice, correlating with changes in lipogenesis, EMT, and proliferation markers. In conclusion, MRC2 plays a critical role in the growth and malignancy of gastric cancer both in vitro and in vivo.

[Disclosure of COI by Responsible researcher] Lead presenter is not the Responsible researcher.
[Lead presenter: Hsin-Ying Chiou] We have no financial relationships to disclose.
[Principal Researcher: Hsin-Ying Chiou] We have no financial relationships to disclose.

### [P-1106] I-P9-2
### Metastasis & EMT (2)

2025 / 9 / 25 (Thu) 17:30-18:15 Room P/(Motenashi Dome) Room P

---

### P-1106
### Mapping Tumor Metastasis and EMT-Associated Immune Changes in HNSCC Through Spatial Transcriptomic Analysis

[Speaker]Chih Hung Chung (CIRC,NYCU, Taipei, Taiwan) [Co-author]Muh Hwa Yang (CIRC,NYCU, Taipei, Taiwan／ICM, NYCU, Taipei, Taiwan／DMO, DO, TVGH, Taipei, Taiwan)

Head and neck squamous cell carcinoma (HNSCC) is often treated with surgery, radiation, or chemotherapy, but recurrent or metastatic cases respond poorly to immune checkpoint inhibitors (ICI). To improve therapeutic strategies, we investigated the immune microenvironment of HNSCC using spatial transcriptomic technologies. The GeoMx Digital Spatial Profiler (DSP) analyzed gene expression across tumor regions, including the invasive front, tumor core, and lymph node metastases. EMT feature clustering from Cancer Transcriptome Atlas (CTA) data revealed distinct EMT programs between primary and metastatic tumors. Notably, EMT markers such as ITGB1, CD44, and FN1 were elevated in metastases. We also applied 10x Visium spatial transcriptomics to validate EMT-associated gene expression. Tumor clusters with high EMT phenotypes exhibited enriched ITGB1 expression. NicheNet analysis revealed tumor-stroma network suggested that ITGB1-high EMT cells may modeling TME through suppress immune cell function through direct cell-cell contact. These findings highlight the immunosuppressive role of malignant EMT cells and offer insights for developing TME-targeted therapies.

[Disclosure of COI by Responsible researcher] Lead presenter is the Responsible researcher.
[Lead presenter: Chih Hung Chung] We have no financial relationships to disclose.

▲TOP

### [P-1107] I-P9-2
### Metastasis & EMT (2)

2025 / 9 / 25 (Thu) 17:30-18:15 Room P/(Motenashi Dome) Room P

---

### P-1107
### Elucidation of how the immunosuppressive microenvironment promotes peritoneal dissemination and its therapeutic targets

[Speaker]Atsuko Yonemura (Div. of Carcinogenesis, The cancer Inst., JFCR) [Co-author]Takashi Semba (Div. of Carcinogenesis, The cancer Inst., JFCR)、Takatsugu Ishimoto (Div. of Carcinogenesis, The cancer Inst., JFCR)

Malignant ascites associated with peritoneal dissemination contains a variety of factors and cell populations in addition to cancer cells; however, how the tumor microenvironment is shaped in ascites remains unclear. Single-cell proteomic profiling and comprehensive proteomic analysis were performed to comprehensively characterize malignant ascites. Here, we found defects in immune effectors along with an accumulation of immunosuppressive cells in ascites from gastric cancer (GC) patients and identified five distinct subpopulations of CD45(-)/EpCAM(-) cells. Mesothelial cells with a mesothelial-mesenchymal transition (MMT) phenotype in CD45(-)/EpCAM(-) cells are the predominant source of chemokines involved in immunosuppressive myeloid cell recruitment. Based on these findings, we further performed in vivo experiments treated with TGFBR1 inhibitor to block MMT induction in the peritoneal dissemination mouse model. As a result, TGFBR1 inhibitor significantly suppressed the peritoneal tumor formation. These findings highlight the existence of immunosuppressive microenvironment to promote peritoneal dissemination and the potential as a therapeutic target.

[Disclosure of COI by Responsible researcher] Lead presenter is not the Responsible researcher.
[Lead presenter: Atsuko Yonemura] We have no financial relationships to disclose.
[Principal Researcher: Takatsugu Ishimoto] We have no financial relationships to disclose.

▲TOP

Poster Session

### [P-1108] I-P9-2
### Metastasis & EMT (2)

2025 / 9 / 25 (Thu) 17:30-18:15 Room P/(Motenashi Dome) Room P

---

### P-1108
### MCE Suppresses Oral Cancer Metastasis by Inhibiting EMT through the β-Catenin/NOTCH1 pathways.

[Speaker]Tian Q. Zeng (Inst. of Pharmacology and Toxicology, Tzu Chi Univ.) [Co-author]Hsue Y. Hsu (Dept. of Post-Baccalaureate School of Chinese Medicine, Tzu Chi Univ.)

Metastasis is an important cause of high mortality and lethality of oral cancer. Epithelial-mesenchymal transition (EMT) is considered the initial step in the invasion-metastasis cascade. Based on the anticancer activity of ***Momordica charantia*** (MC) reported in cancer cells which oral cancer cells are included, we demonstrate the MC extract (MCE) suppresses epithelial-mesenchymal transition (EMT) in SAS oral squamous carcinoma cells by concurrently targeting the β-catenin and NOTCH1 signaling pathways. Wound-healing and transwell assays revealed a dose-dependent inhibition of SAS cell migration and invasion by MCE. Immunofluorescence analysis indicated that MCE effectively blocks the nuclear translocation of β-catenin, a key driver of EMT, while concurrently downregulating the NOTCH1 signaling cascade. Mechanistically, MCE treatment led to the decreased expression of β-catenin, NOTCH1, and EMT-associated transcription factors, including Snail and Twist. Collectively, these findings highlight MCE as a potent multi-target inhibitor of EMT, underscoring its therapeutic potential in limiting oral cancer metastasis.

[Disclosure of COI by Responsible researcher] Lead presenter is the Responsible researcher.
[Lead presenter: Tian Q. Zeng] We have no financial relationships to disclose.

### [P-1114] I-P10-2
### Extracellular vesicles/Exosome (2)

2025 / 9 / 25 (Thu) 17:30-18:15 Room P/(Motenashi Dome) Room P

---

### P-1114
### Assessment of Surface PD-L1 Expression in Small Extracellular Vesicles using High-Speed Atomic Force Microscopy

[Speaker]Reon Imakawa (Sch. of Biol. Sci. & Tech., Kanazawa Univ.) [Co-author]Keesiang Lim (WPI-Nano Life Sci. Inst., Kanazawa Univ.)、Koki Nagai (Sch. of Biol. Sci. & Tech., Kanazawa Univ.)、Noriko Goto (Div. of Cancer Cell Biol., Cancer Res. Inst., Kanazawa Univ.／Inst. for Frontier Sci. Initiative, Kanazawa Univ.)、Rikinari Hanayama (WPI-Nano Life Sci. Inst., Kanazawa Univ.／Dept. of Immunol., Kanazawa Univ. Grad. Sch. of Med. Sci.)、Richard Wong (WPI-Nano Life Sci. Inst., Kanazawa Univ.／Div. of Nano Life Sci., Kanazawa Univ.／Inst. for Frontier Sci. Initiative, Kanazawa Univ.／WISE Program for Nano-Precision Med., Sci. & Tech., Kanazawa Univ.／Cell-Bionomics Res. Unit, INFINITI, Kanazawa Univ.)

Immune checkpoint inhibitors (ICIs) enhance anti-tumor immunity, but cancer cells release PD-L1-expressing exosomes to suppress ICI efficacy and promote immune effector apoptosis. Thus, exosomal surface PD-L1 may serve as a predictive marker for ICI response. However, conventional methods, such as flow cytometry, have limited resolution for exosomal PD-L1 analysis. Here, we used high-speed atomic force microscopy (HS-AFM) to characterize exosomal PD-L1 from two breast cancer cell lines, MCF7 ($Exo^{MCF7}$) and MDA-MB-231 ($Exo^{MDA\text{-}MB\text{-}231}$), differing in endogenous PD-L1 levels. Western blotting showed higher PD-L1 expression in both cell and exosome lysates of MDA-MB-231 than MCF7. Moreover, real-time HS-AFM imaging revealed stable anti-PD-L1 antibody interaction with $Exo^{MDA\text{-}MB\text{-}231}$ but not $Exo^{MCF7}$. Our findings demonstrate that HS-AFM enables visualization of exosomal surface PD-L1 and supports its potential for monitoring dynamic PD-L1 changes in response to cytokine stimulation.

[Disclosure of COI by Responsible researcher] Lead presenter is not the Responsible researcher.
[Lead presenter: Reon Imakawa] We have no financial relationships to disclose.
[Principal Researcher: Richard Wong] ■Contributions: 武田科学振興財団

### [P-1115] I-P10-2
### Extracellular vesicles/Exosome (2)

2025 / 9 / 25 (Thu) 17:30-18:15 Room P/(Motenashi Dome) Room P

---

### P-1115
### FMR1-Mediated Selective Loading of Tumor-Promoting microRNAs into Extracellular Vesicles

[Speaker]Yue Dong (Div. of Cancer Cell regulation, Aichi Cancer Center Res. Inst.) [Co-author]Chitose Oneyama (Div. of Cancer Cell regulation, Aichi Cancer Center Res. Inst.／Target and Drug Discovery, Nagoya Univ., Grad. Sch. of Med.／ Oncology, Nagoya City Univ., Grad. Sch. of Pharm. Sci.)

Extracellular vesicles (EVs) are lipid bilayer-enclosed particles that facilitate intercellular communication by transferring various biomolecules, including proteins, RNAs, and lipids. EVs are increasingly recognized as active players in cancer development, metastasis, and therapeutic resistance. In this study, we discovered that Src-activated cancer cells release EVs enriched with specific tumor-promoting microRNAs (miRNAs). Through screening for RNA-binding proteins (RBPs) involved in this process, we identified Fragile X Messenger Ribonucleoprotein 1 (FMR1) as a key factor mediating the selective loading of these miRNAs into EVs. While FMR1 is known to regulate RNA metabolism and cell invasiveness, its role in EV-mediated miRNA sorting had not been previously elucidated. We show that FMR1 facilitates the packaging of oncogenic miRNAs into EVs and promotes their delivery to recipient cells, leading to enhanced invasiveness. These findings reveal a novel mechanism of EV-mediated communication and highlight FMR1 as a potential regulator of the tumor microenvironment and a target for future therapeutic strategies.

[Disclosure of COI by Responsible researcher] Lead presenter is not the Responsible researcher.
[Lead presenter: Yue Dong] We have no financial relationships to disclose.
[Principal Researcher: Chitose Oneyama] We have no financial relationships to disclose.

▲TOP

### [P-1116] I-P10-2
### Extracellular vesicles/Exosome (2)

2025 / 9 / 25 (Thu) 17:30-18:15 Room P/(Motenashi Dome) Room P

### P-1116
### Ouabain Selectively Inhibits Extracellular Vesicle Secretion in Cancer Cells and Suppresses Their growth

[Speaker]Haruki Adachi (Div. of Cancer Cell regulation, Aichi Cancer Center Res. Inst.／Dept. of Oncology, Nagoya City Univ.) [Co-author]Chitose Oneyama (Div. of Cancer Cell regulation, Aichi Cancer Center Res. Inst.／Dept. of Oncology, Nagoya City Univ.／Dept. of Target and Drug Discovery, Nagoya Univ.)

Extracellular vesicles (EVs) from cancer cells contribute to the tumor microenvironment, metastasis, and malignancy. As cancer cells often show elevated EV secretion, targeting this process is a potential therapeutic strategy. However, existing inhibitors like GW4869 lack cancer selectivity and also suppress EV release in normal cells, highlighting the need for more selective compounds. We screened ~400 signaling inhibitors using bioluminescent EV-reporter cancer cell lines and identified ouabain, a cardiac glycoside, as a novel EV secretion inhibitor. Ouabain, known to enhance myocardial contractility via $Na^+/K^+$-ATPase inhibition, markedly reduced EV release in HT-29 colon cancer cells, with autophagy induction and growth suppression. These effects were absent in normal colon myofibroblast CCD-18Co cells, suggesting cancer-selective action likely linked to $Na^+/K^+$-ATPase inhibition. Our results indicate that ouabain may serve as a cancer-selective EV secretion inhibitor and therapeutic candidate. In this presentation, we will discuss its mechanistic role and therapeutic implications.

[Disclosure of COI by Responsible researcher] Lead presenter is not the Responsible researcher.
[Lead presenter: Haruki Adachi] We have no financial relationships to disclose.
[Principal Researcher: Chitose Oneyama] We have no financial relationships to disclose.

### [P-1117] I-P10-2
### Extracellular vesicles/Exosome (2)

2025 / 9 / 25 (Thu) 17:30-18:15 Room P/(Motenashi Dome) Room P

---

### P-1117
### Extracellular vesicles-containing Regucalcin suppress tumor development by suppress M2-like macrophage.

[Speaker]Naoomi Tominaga (Yamaguchi Univ. Grad. Sch. of Med.) [Co-author]Saki Horie (Yamaguchi Univ. Grad. Sch. of Med.)、Risako Okada (Yamaguchi Univ. Grad. Sch. of Med.)、Kana Tominaga (Yamaguchi Univ. Grad. Sch. of Med.)、Tomiyasu Murata (Meijo Univ. Grad. Sch. of Pharm., Mol. Biol.)、Masayoshi Yamaguchi (Univ. of Hawaii Cancer Ctr.)

Regucalcin (RGN), essential for diverse cellular functions, exhibits elevated expression in prostate cancer, correlating with improved patient survival. Although established as a signaling suppressor, RGN's precise tumor suppressive mechanisms within the tumor microenvironment (TME) via intercellular communication remain elusive. We utilized PC3 cells stably expressing RGN or control. EVs isolated via ultracentrifugation were applied to J744α1 macrophages. M2 polarization was assessed by qRT-PCR/Western blot. PC3 xenografts received intratumoral EV injections in vivo. Tumor progression was monitored; excised tumors were analyzed for CD206 via immunofluorescence. RGN protein incorporation into EVs was confirmed. These RGN-containing EVs attenuated M2 macrophage polarization and inhibited tumorigenesis in vitro and in vivo. This suppression was recapitulated by recombinant RGN protein. Mechanistically, RGN-EVs impede M2 polarization, partly by reducing Akt/ERK1/2 phosphorylation/activation in vitro. This study elucidates a novel EV-mediated RGN mechanism governing macrophage polarization. These findings underscore the therapeutic potential of RGN-EVs in oncology.

[Disclosure of COI by Responsible researcher] Lead presenter is the Responsible researcher.
[Lead presenter: Naoomi Tominaga] ■ employment by a company related to the presentation: 株式会社ADDVEMO（取締役）
■Profit of stock: 株式会社ADDVEMO

### [P-1118] I-P10-2
### Extracellular vesicles/Exosome (2)

2025 / 9 / 25 (Thu) 17:30-18:15 Room P/(Motenashi Dome) Room P

### P-1118
### Withdrawn

No Abstract

### [P-1119] I-P10-2
### Extracellular vesicles/Exosome (2)

2025 / 9 / 25 (Thu) 17:30-18:15 Room P/(Motenashi Dome) Room P

### P-1119
### Withdrawn

No Abstract

### [P-1126] I-P11-2
### Antitumor immune response (1)

2025 / 9 / 25 (Thu) 17:30-18:15 Room P/(Motenashi Dome) Room P

### P-1126
### The difference in the affinity of antigenic peptides affects the antitumor effect of T-cell adoptive immunotherapy

[Speaker]Megumi Nakamura (Breast Center, Ehime Univ. Hosp.／Dept. of Immunol., Grad. Sch. of Med., Ehime Univ.) [Co-author]Yuko Matsuoka (Translational Res. Center, Ehime Univ. Hosp.／Dept. of Immunol., Grad. Sch. of Med., Ehime Univ.)、Haruna Noda (Breast Center, Ehime Univ. Hosp.)、Kana Taguchi (Breast Center, Ehime Univ. Hosp.)、Akari Murakami (Breast Center, Ehime Univ. Hosp.)、Yoshiaki Kamei (Breast Center, Ehime Univ. Hosp.)、Yuzo Umeda (Hepato-Biliary-Pancreatic Surg. and Breast Surg., Ehime Univ.)、Masakatsu Yamashita (Translational Res. Center, Ehime Univ. Hosp.／Dept. of Immunol., Grad. Sch. of Med., Ehime Univ.)

Adoptive T-cell immunotherapy is being developed based on the idea that increasing the affinity between TCR and tumor peptides induces strong intracellular signals, leading to a high anti-tumor effect, however, the optimal affinity is unknown. In this study, we fixed the TCR repertoire and investigated the impact of antigen peptide affinity on antitumor effects. Using a mouse tumor-bearing model with egg white albumin (OVA) as a pseudo-tumor antigen, we found that in the early stages, tumor growth was similarly suppressed in all groups of CD8 T cells activated with different affinities. However, from 12 days after transfer, the difference in tumor suppression became apparent, with tumor growth occurring in the order of low-, high-, and intermediate-affinity. In vitro analysis showed that, compared to low- and high-affinity CD8 T cells, intermediate-affinity CD8 T cells activated by medium-affinity peptides had a higher proportion of GzmA-, GzmB-, Tim3-positive, and CD62L-low cells, which are markers of terminal effector differentiation. These results indicate that high antigen peptide/TCR affinity do not always induce effector CD8 T cells with strong anti-tumor activity.

[Disclosure of COI by Responsible researcher] Lead presenter is not the Responsible researcher.
[Lead presenter: Megumi Nakamura] We have no financial relationships to disclose.
[Principal Researcher: Masakatsu Yamashita] We have no financial relationships to disclose.

### [P-1127] I-P11-2
### Antitumor immune response (1)

2025 / 9 / 25 (Thu) 17:30-18:15 Room P/(Motenashi Dome) Room P

---

### P-1127
### High expression of HNF1A in cancer cells promotes anti-tumor immunity

[Speaker]Tadayuki Nakashima (Dept. of Respiratory Med., Grad. Sch. of Med., Kyushu Univ.) [Co-author]Yasuto Yoneshima (Dept. of Respiratory Med., Grad. Sch. of Med., Kyushu Univ.)、Toshifumi Ninomiya (Dept. of Respiratory Med., Grad. Sch. of Med., Kyushu Univ.)、Daisuke Shibahara (Dept. of Respiratory Med., Grad. Sch. of Med., Kyushu Univ.)、Kohei Otsubo (Dept. of Respiratory Med., Grad. Sch. of Med., Kyushu Univ.)、Yoshimasa Shiraishi (Dept. of Respiratory Med., Grad. Sch. of Med., Kyushu Univ.)、Eiji Iwama (Dept. of Respiratory Med., Grad. Sch. of Med., Kyushu Univ.)、Isamu Okamoto (Dept. of Respiratory Med., Grad. Sch. of Med., Kyushu Univ.)

The efficacy of immune checkpoint inhibitors (ICIs) varies from patient to patient, and the biomarkers that can predict which patients will respond well to ICI treatment are not fully understood. HNF1A is a transcription factor belonging to the homeobox family that regulates the expression of several liver-specific genes. HNF1A expression is increased in some types of cancer, such as non-small cell lung cancer, compared to normal tissue. However, the effect of HNF1A on anti-tumor immunity is unknown. Here, we found that tumors with high HNF1A expression activate anti-tumor immunity. Lung cancer cells overexpressing HNF1A had increased cytotoxic activity compared to normal cells, when co-cultured with PBMC-derived T cells. The growth of mouse cancer cells overexpressing HNF1A was suppressed when injected subcutaneously into mice. Furthermore, FACS analysis of tumor infiltrating cells showed lymphocyte infiltration was activated in cancer cells overexpressing HNF1A. These results suggest the high expression of HNF1A in cancer cells promotes T lymphocyte-mediated anti-tumor immunity and indicate HNF1A may be a useful biomarker for predicting the efficacy of ICI therapy.

[Disclosure of COI by Responsible researcher] Lead presenter is not the Responsible researcher.
[Lead presenter: Tadayuki Nakashima] We have no financial relationships to disclose.
[Principal Researcher: Yasuto Yoneshima] We have no financial relationships to disclose.

### [P-1128] I-P11-2
### Antitumor immune response (1)

2025 / 9 / 25 (Thu) 17:30-18:15 Room P/(Motenashi Dome) Room P

### P-1128
### Anti-tumour effects of Naringin are due to the enhancement of the cross-presentation ability of antigen-presenting cells

[Speaker]Cheng Pan (Dept.Cell Path., Grad.Sch.Med.Sci., Fac.Life Sci., Kumamoto Univ.) [Co-author]Yukio Fujiwara (Dept.Cell Path., Grad.Sch.Med.Sci., Fac.Life Sci., Kumamoto Univ.)、Hiromu Yano (Dept.Tumor Pathol., Grad.Sch.Med.Sci., Fac.Life Sci., Kumamoto Univ.)、Yoshihiko Hirohashi (1st Dept. Path., Sapporo Med. Univ. Sch. Med.)、Yoshihiro Komohara (Dept.Cell Path., Grad.Sch.Med.Sci., Fac.Life Sci., Kumamoto Univ.)

Lymph node (LN) CD169+ sinus macrophage (M$\phi$) presents antigens to T cells to initiate tumour immunity. Naringin induces murine LN CD169 expression and tumour immunity. This study is aimed at investigating the effects of Naringin on the cross-presentation (XPT) of antigen-presenting cells (APCs). NY-ESO-1 specific T cells were co-cultured with A375-lysate-pulsed PBMC-derived M$\phi$, but IFN$\gamma$ secretion was not detected. By analysing the transcriptomes, the CD169+ sinus M$\phi$ were found similar to both PBMC-M$\phi$s and immature dendritic cells (iDCs). Naringin enhanced the IFN$\gamma$ secretion of NY-ESO-1-T cells co-cultured with A375 lysate-pulsed DCs. The co-culture of murine OT-I T cells and OVA-pulsed DCs or DC-skewed peritoneal M$\phi$s showed similar results. Naringin-treated iDCs down-regulated lysosomal structural genes, among which the decreases of LAMP1, LAMP2, and CD63 were confirmed at protein levels. Naringin also delayed the protein lysosomal degradation in iDCs. Taking together, Naringin may enhance the XPT of APCs by reducing lysosomal degradation of antigens. The anti-tumour effects of Naringin are probably due to the increased XPT of tumour antigens in DC-like LN CD169+ sinus M$\phi$s.

[Disclosure of COI by Responsible researcher] Lead presenter is the Responsible researcher.
[Lead presenter: Cheng Pan] We have no financial relationships to disclose.

▲TOP

### [P-1129] I-P11-2
### Antitumor immune response (1)

2025 / 9 / 25 (Thu) 17:30-18:15 Room P/(Motenashi Dome) Room P

### P-1129
### Identification of a Novel Immunoregulatory CD86high CD8 T Cell Subset in the Tumor Microenvironment

[Speaker]Hu Xin (NCCHD) [Co-author]Yifang Shui (NCCHD／The First Affiliated Hospital of Zhengzhou University)、Masayuki Fujino (NCCHD／National Institute of Infectious Diseases)、Xiaokang Li (NCCHD)

CD86 is a key costimulatory molecule typically expressed on antigen-presenting cells. In this study, we identified a subset of tumor-infiltrating CD8 T cells with high CD86 expression (CD86high CD8 T cells) within the tumor microenvironment (TME). These cells exhibited a regulatory phenotype, characterized by elevated IL-10 secretion and high expression of CD39 and CTLA-4. Conditional knockout of CD86 in CD8 T cells significantly suppressed tumor growth and enhanced the anti-tumor function of tumor-specific T cells. Mechanistically, CD86high CD8 T cells promoted the induction of regulatory dendritic cells (DCregs), and transcriptome analyses revealed increased expression of IRF4, IRF5, and IRF8. These findings identify CD86high CD8 T cells as a novel immunoregulatory subset within the TME and suggest that targeting these cells may offer a promising strategy to enhance anti-tumor immunity.

[Disclosure of COI by Responsible researcher] Lead presenter is not the Responsible researcher.
[Lead presenter: Hu Xin] We have no financial relationships to disclose.
[Principal Researcher: Xiaokang Li] We have no financial relationships to disclose.

▲TOP

### [P-1130] I-P11-2
### Antitumor immune response (1)

2025 / 9 / 25 (Thu) 17:30-18:15 Room P/(Motenashi Dome) Room P

### P-1130
### Differential Expression of Regulatory T cells Markers in lymphocytes of Oral Cancer Patients

[Speaker]Sanako Nakaya (Dept. Oral & Maxillofacial Surg. Aichi Gakuin Univ.) [Co-author]Shoya Ono (Dept. Oral & Maxillofacial Surg. Aichi Gakuin Univ.)、Susumu Suzuki (Res. Creation Support Ctr. Aichi Med. Univ.)、Yutaro Kondo (Dept. Oral & Maxillofacial Surg. Aichi Gakuin Univ.)、Ikuko Okubo (Res. Creation Support Ctr. Aichi Med. Univ.)、Hiroyuki Kinoshita (Dept. Oral & Maxillofacial Surg. JCHO Chukyo Hosp.)、Kou Kawahara (Dept. Oral & Maxillofacial Surg. Gifu Prefectural Tajimi Hosp.)、Terumi Saito (Dept. Oral & Maxillofacial Surg. Okazaki City Hosp.)、Hiroshi Watanabe (Dept. Oral & Maxillofacial Surg. Ichinomiya Municipal Hosp.)、Haruki Sato (Dept. Oral & Maxillofacial Surg. Chitahanto Med. Ctr.)、Tetsuya Ogawa (Dept. Otolaryngology Head and Neck Surg. Nippon Med. Sch.)、Toru Nagao (Dept. Oral & Maxillofacial Surg. Aichi Gakuin Univ.)、Ryuzo Ueda (Grad. Sch. Med. Sci. Nagoya Univ.)、Mitsuo Goto (Dept. Oral & Maxillofacial Surg. Aichi Gakuin Univ.)

Regulatory T cells (Tregs) are important targets in cancer immunotherapy. CCR4, CCR8, CTLA-4, and CD25 are known as representative molecules for Treg depleting. Therapeutic antibodies targeting these molecules are being investigated in clinical trials worldwide. However, direct comparable data on their expression in T cells is still limited. We analyzed the expression of these markers on Tregs from peripheral blood lymphocytes (PBLs) and tumor infiltrating lymphocytes (TILs) in 20 oral cancer patients using flow cytometry. In PBL, CCR4 was most highly expressed on effector Treg (eTreg) and also partially expressed other T-cell fraction, while the expression of CCR8 and CTLA-4 was low. CD25 expression was high in eTregs but low or not seen in the other fractions. In contrast, eTregs of TILs expressed significantly higher levels of CCR8 compared with PBLs, while the CCR4 expression was comparable. CTLA-4 and CD25 expressions were also significantly higher in TILs. These findings suggest that each molecule exhibits different expression patterns depending on the tissues obtained from each sample. Further assessment of TILs will be increasingly required in the future.

[Disclosure of COI by Responsible researcher] Lead presenter is not the Responsible researcher.
[Lead presenter: Sanako Nakaya] We have no financial relationships to disclose.
[Principal Researcher: Shoya Ono, Susumu Suzuki] We have no financial relationships to disclose.

▲TOP

### [P-1131] I-P11-2
### Antitumor immune response (1)

2025 / 9 / 25 (Thu) 17:30-18:15 Room P/(Motenashi Dome) Room P

### P-1131
### Antifolate pemetrexed activates innate immunity through STING regulation

[Speaker]Mizuki Homme (JFCR CPM Center Cancer Immunotherapy Development) [Co-author]Nobunari Sasaki (JFCR CPM Center Cancer Immunotherapy Development)、Shunsuke Kitajima (JFCR CPM Center Cancer Immunotherapy Development)

We previously reported that KRAS-LKB1 mutant (KL) non-small cell lung cancer (NSCLC), which is immune checkpoint inhibitor (ICI)-resistant, shows marked epigenetic silencing of STING, a central molecule in cytoplasmic dsDNA sensing. STING downstream cytokines such as IFN-β and CXCL10 are key molecules for T-cell recruitment into the tumor microenvironment (TME), and indeed, infiltration of T-cells into the TME is lower in KL-type NSCLC. Through an unbiased drug screening for their ability to re-activate STING, we identified antifolate pemetrexed as an optimal agent to prime the STING pathway in KL cells. Pemetrexed is well established as a first-line chemotherapy agent in combination with ICI for driver-negative lung adenocarcinoma. However, the mechanisms by which pemetrexed enhances ICI efficacy remain poorly understood. In this study, we focused on the immunomodulatory effects of pemetrexed, particularly activating STING signaling associated with remodeling of histone modification, including at STING gene loci. Our findings provide new insights into the epigenetic mechanisms underlying pemetrexed-induced immune activation.

[Disclosure of COI by Responsible researcher] Lead presenter is not the Responsible researcher.
[Lead presenter: Mizuki Homme] ■ employment by a company related to the presentation: 株式会社ワールドインテック
[Principal Researcher: Shunsuke Kitajima] ■Research funds from company: 日本ベーリンガーインゲルハイム

### [P-1132] I-P11-2
### Antitumor immune response (1)

2025 / 9 / 25 (Thu) 17:30-18:15 Room P/(Motenashi Dome) Room P

---

### P-1132
### Peptide modulation overrides glycan synergy in gold nanoparticle-based vaccines for cancer immunotherapy

[Speaker]Narumi(Kumamoto Univ.) Harada (Department of Urology Graduate School of Medical Sciences, Kumamoto University／Department of Molecular and Medical Pharmacology, Kumamoto University) [Co-author]Mayumi Niimura (Department of Molecular and Medical Pharmacology, Kumamoto University)、Yasuhisa Sakamoto (Department of Molecular and Medical Pharmacology, Kumamoto University)、Akihiro Nita (Division of Cellular Dynamics, Institute of Science Tokyo)、Mayuko Shimoda (Department of Molecular and Medical Pharmacology, Kumamoto University)、Shiho Wada (Department of Engineering, Chemistry and Biotechnology program, Kagoshima University)、Koki Murata (Department of Engineering, Chemistry and Biotechnology program, Kagoshima University)、Masahiro Wakao (Department of Engineering, Chemistry and Biotechnology program, Kagoshima University)、Tomomi Kamba (Department of Urology Graduate School of Medical Sciences, Kumamoto University)、Hiroyuki Shinchi (Department of Engineering, Chemistry and Biotechnology program, Kagoshima University)、Toshiro Moroishi (Division of Cellular Dynamics, Institute of Science Tokyo)

PurposeWe aimed to investigate how combinations of sugar chains and antigenic peptides affect the therapeutic efficacy and cellular uptake of integrated glyco-nanovaccines (iGNs), a gold nanoparticle (GNP)-based platform for cancer immunotherapy.MethodsiGNs were synthesized by conjugating GNPs with Toll-like receptor 7 ligands, the model antigenic peptide "SIINFEKL" and either α-mannose (miGN) or sialic acid (siGN). Cellular uptake was evaluated in bone marrow-derived dendritic cells (BMDCs), and type I interferon pathway activation and antigen-specific CD8+ T cell induction were assessed. In vivo antitumor efficacy was tested using an EG7 syngeneic tumor model.ResultsWithout SIINFEKL, α-mannose-modified GNPs showed efficient BMDC uptake, while sialic acid-modified GNPs showed limited uptake. Conjugation with SIINFEKL enhanced uptake for both miGN and siGN. In vivo, both iGN formulations induced similar CD8+ T cell responses and tumor suppression.ConclusionOur findings suggest antigenic peptides are more crucial than glycan modifications in determining immune uptake and efficacy, highlighting peptide modulation as a key strategy in nanovaccine design for cancer immunotherapy.

[Disclosure of COI by Responsible researcher] Lead presenter is not the Responsible researcher.
[Lead presenter: Narumi Harada] We have no financial relationships to disclose.
[Principal Researcher: Toshiro Moroishi] We have no financial relationships to disclose.

▲TOP

### [P-1133] I-P11-2
### Antitumor immune response (1)

2025 / 9 / 25 (Thu) 17:30-18:15 Room P/(Motenashi Dome) Room P

---

### P-1133
### Investigation of nivolumab-induced anti-tumor immune responses in a humanized NOG-Fcγ R-/- mouse model

[Speaker]Tatsuhito Ii (Immunol. Lab.,CIEM) [Co-author]Katsutoshi Sato (Immunol. Lab.,CIEM)、Ikumi Katano (Immunol. Lab.,CIEM)、Tingwei Yu (Immunol. Lab.,CIEM)、Iyo Ootsuka (Immunol. Lab.,CIEM)、Nao Suzuki (Immunol. Lab.,CIEM)、Motohito Goto (Anim. Resour. Cent.,CIEM)、Misa Mochizuki (Pathol. Anal. Cent.,CIEM)、Shumpei Ishikawa (Dept. Prev. Med.,UTokyo)、Masami Suzuki (TR Div.,CIEM)、Takeshi Takahashi (Immunol. Lab.,CIEM)

Improving the efficacy of nivolumab likely requires elucidating its immune mechanism, underscoring the need for a reliable animal model. Humanized NOG–Fcγ R-/- mice (NOGF mice) bearing HSC4 tumors exhibited tumor growth inhibition in response to nivolumab treatment. Using this model, we analyzed the immune cell populations, gene expression profiles, and associated biological pathways. Peripheral helper T (Tph) cells producing CXCL13 were increased in humanized NOGF mice by nivolumab treatment. GO analysis of Tph cells revealed significant enrichments in cytokine activity, cytokine receptor binding, and receptor-ligand activity. Cell-cell communication analysis suggested that Tph cells interact with other CXCR3-expressing T cells through CXCL10 and 13.The plasma concentrations of CXCL9,10,11 and 13 were significantly elevated in humanized NOGF mice. RNAscope revealed upregulation of CXCL9 and 10 in both inflammatory and tumor cells.Our results show that CXCL9, 10, and 13 are upregulated in humanized NOGF mice, contributing to anti-tumor immunity. These findings are consistent with previous observations in human cases, indicating that this model recapitulates human immune responses.

[Disclosure of COI by Responsible researcher] Lead presenter is not the Responsible researcher.
[Lead presenter: Tatsuhito Ii] We have no financial relationships to disclose.
[Principal Researcher: Takeshi Takahashi] We have no financial relationships to disclose.

[P-1143] I-P11-4
Antitumor immune response (3)

2025 / 9 / 25 (Thu) 17:30-18:15 Room P/(Motenashi Dome) Room P

### P-1143
### Expression analysis of representative regulatory T-cell (Treg) targeting candidate molecules in renal cell carcinoma

[Speaker]Susumu Suzuki (Res. Creation Support Ctr., Aichi Med.Univ.) [Co-author]Tomoaki Muramatsu (Dept.Urology, Aichi Med.Univ.)、Ikuko Okubo (Res. Creation Support Ctr., Aichi Med.Univ.)、Taishi Takahara (Dept.Surg.Pathol., Aichi Med.Univ.)、Toyonori Tsuzuki (Dept.Surg.Pathol., Aichi Med.Univ.)、Ryuzo Ueda (Grad.Sch.Med.Sci., Nagoya Univ.)、Naoto Sassa (Dept.Urology, Aichi Med.Univ.)

[Objective] CC chemokine receptor 4 (CCR4), CCR8, CD25 and CTLA-4 are Treg targeting candidates for cancer immunotherapy. We did the direct comparison analysis with flow cytometry to reveal discrepancy of expression pattern among these molecules using lymphocytes from peripheral blood (PBL), tumor (TIL) and normal tissue (NIL) in patients with renal cell carcinoma. [Results] CCR4 and CD25 were highly expressed in effector Treg(eTreg, CD4+CD45RA-FOXP3hi), whereas CCR8 expression was low and CTLA-4 expression was not almost seen. As for naive Treg (CD4+CD45RA+FOXP3lo), CCR4, CCR8 and CTLA-4 were not expressed except for CD25. In the other CD4+T-cells, CCR4 was partially expressed in CD45RA- cell, the expression of CCR8, CD25 and CTLA-4 were low or not seen. In TIL, CCR8 expression in eTreg was drastically higher than that of PBL. Although CCR4 and CD25 was also highly expressed in eTreg, CCR4 partially expressed in the other CD4+T-cells, CTLA-4 was also expressed in eTreg but the expression level was low. In NIL, the expression level was very low in all these molecules. [Conclusion] The expression pattern was variable among them. Application in situation is thought to be necessary.

[Disclosure of COI by Responsible researcher] Lead presenter is the Responsible researcher.
[Lead presenter: Susumu Suzuki] We have no financial relationships to disclose.

### [P-1144] I-P11-4
### Antitumor immune response (3)

2025 / 9 / 25 (Thu) 17:30-18:15 Room P/(Motenashi Dome) Room P

---

### P-1144
### Establishment of murine NK cell line having cytotoxicity and capability for genetic modification

[Speaker]Yuya Miyakura (Div. Mol. Cell. Oncol, Miyagi Cancer Ctr. Res. Inst.／Dept. of HNS., Miyagi Cancer Ctr. Hosp.／Dept. of ORL-HNS., Tohoku Univ. Hosp.) [Co-author]Kazunori Yamaguchi (Div. Mol. Cell. Oncol, Miyagi Cancer Ctr. Res. Inst.)、Shin Ito (Div. Mol. Cell. Oncol, Miyagi Cancer Ctr. Res. Inst.)、Satoshi Souma (Div. Mol. Cell. Oncol, Miyagi Cancer Ctr. Res. Inst.)、Takayuki Imai (Dept. of HNS., Miyagi Cancer Ctr. Hosp.)、Yukinori Asada (Dept. of HNS., Miyagi Cancer Ctr. Hosp.)、Yukio Katori (Dept. of ORL-HNS., Tohoku Univ. Hosp.)、Jun Yasuda (Div. Mol. Cell. Oncol, Miyagi Cancer Ctr. Res. Inst.)

The natural killer (NK) cell is an important member of the innate immune system and contributes to host homeostasis by eliminating infected or transformed cells. Recently, NK cells have gained attention as an alternative cell modality for cancer immunotherapy. The cells are expected to overcome the drawbacks of T-cell-based therapy such as high cost, long preparation time or immune-related adverse effects. Here, we established a mouse NK cell line that could be used to establish mouse models for NK cell-mediated cancer immunotherapy. Splenic NK cells were prepared from female Balb/c mice and cultured for more than one year in standard cell culture medium containing recombinant human IL-2. The cells were cloned by limiting dilution and evaluated by their cytotoxicity. The clone showing high killing activity against YAC-1 cells was further tested for growth characteristics, cell surface markers and karyotype. The cells could be cryopreserved and retained the capacity for genetic modification, suggesting that they may be useful for the development of mouse models for NK cell therapy as well as for the study of NK cell biology.

[Disclosure of COI by Responsible researcher] Lead presenter is not the Responsible researcher.
[Lead presenter: Yuya Miyakura] We have no financial relationships to disclose.
[Principal Researcher: Jun Yasuda] We have no financial relationships to disclose.

Poster Session

### [P-1145] I-P11-4
### Antitumor immune response (3)

2025 / 9 / 25 (Thu) 17:30-18:15 Room P/(Motenashi Dome) Room P

---

### P-1145
### Method to know patient-wise tumor-immune state making model in detail by sensitivity from added IL2, etc. for cure

[Speaker]Mitsuo Takase (LINFOPS Inc. development department)

A method to know patient-wise analytical model, from it tumor-immune state in detail and evidence for therapies through sensitivities to a solid tumor growth speed variation with IL2, TIL, etc. is shown. Added IL2 and TIL can, reportedly, work to cure tumor in papers of S. A. Rosenberg et al. To use analysis to know each tumor-immune state deepening it by sensitivities may be new. This model refers to equations from D. Kirschner paper modified. Sensitivities used for the model come from reading tumor growth speeds from therapy-survival curves, etc. From parameter values of a patient-wise model equations, followings can be known. 1. First of all, the distance to the explosive T cell increase (I call here local ignition.). 2. Then to know others. (1) activated T cell density from dendric cells. (2) hidden T cell density level as a tumor worsening speed. (3) effects of PD-1 and Treg. (4) chemokine effect from T cells dissipation. From these values, how to reach the explosive T cell state can be known. Here, the target is not to know the effect of a factor like PD-1 but to know patient-wise total state of tumor-immune system to reach the explosive T cell state. 3. Then to simulate.

[Disclosure of COI by Responsible researcher] Lead presenter is the Responsible researcher.
[Lead presenter: Mitsuo Takase] We have no financial relationships to disclose.

▲TOP

### [P-1146] I-P11-4
### Antitumor immune response (3)

2025 / 9 / 25 (Thu) 17:30-18:15 Room P/(Motenashi Dome) Room P

### P-1146
### Potential Novel Mesothelioma Therapy by Targeting VISTA

[Speaker]Lisa Ida (Aichi Cancer Center, Div. Cancer Biol) [Co-author]Tatsuhiro Sato (Aichi Cancer Center, Div. Cancer Biol)、Satomi Mukai (Aichi Cancer Center, Div. Cancer Biol)、Yoshitaka Sekido (Aichi Cancer Center, Div. Cancer Biol)

Mesothelioma is an extremely poor-prognosis tumor that is resistant to existing kinase inhibitors. In recent years, immune checkpoint inhibitors (ICIs) have been employed as chemotherapy for mesothelioma, but their response rates have not been as high as expected. One reason for this is the low expression of PD-L1 in mesothelioma cells. Using TCGA mesothelioma samples, we compared the expression of B7 family members to which PD-L1 belongs and found VISTA to be the most highly expressed. Furthermore, in epithelioid mesothelioma, the most common histological subtype, VISTA expression was high and negatively correlated with PD-L1 expression. When VISTA was knocked down by shRNA in mesothelioma cell lines, a marked growth inhibition was observed in cells expressing high levels of VISTA. In an in vivo experiments in which mesothelioma cells were transplanted subcutaneously into mice, VISTA shRNA significantly reduced tumor size. These results suggest that VISTA directly promotes mesothelioma cell growth. Future studies are needed to further investigate the molecular mechanisms by which VISTA regulates mesothelioma cell proliferation and its role in interactions with immune cells.

[Disclosure of COI by Responsible researcher] Lead presenter is not the Responsible researcher.
[Lead presenter: Lisa Ida] We have no financial relationships to disclose.
[Principal Researcher: Yoshitaka Sekido] We have no financial relationships to disclose.

### [P-1147] I-P11-4
### Antitumor immune response (3)

2025 / 9 / 25 (Thu) 17:30-18:15 Room P/(Motenashi Dome) Room P

---

### P-1147
### Relationship between HLA allelic polymorphism and prognosis in pan-cancer with immune-associated mutations

[Speaker]Akira Iizuka (Immunother. Div., Shizuoka Cancer Ctr. Res. Inst.) [Co-author]Tomoatsu Ikeya (Immunother. Div., Shizuoka Cancer Ctr. Res. Inst.)、Yasufumi Kikuchi (Immunother. Div., Shizuoka Cancer Ctr. Res. Inst.)、Takeshi Nagashima (Cancer Diagnostics Res. Div., Shizuoka Cancer Ctr. Res. Inst.)、Kenichi Urakami (Cancer Diagnostics Res. Div., Shizuoka Cancer Ctr. Res. Inst.)、Keiichi Ohshima (Medical Genetics Div., Shizuoka Cancer Ctr. Res. Inst.)、Ken(Shizuoka Cancer Ctr.) Yamaguchi (Shizuoka Cancer Ctr.)、Yasuto Akiyama (Immunother. Div., Shizuoka Cancer Ctr. Res. Inst.)

Pan-cancer immune-associated gene mutation analysis by whole exon sequencing (WES) was performed using tumor tissue and blood sample pairs from 6130 patients with survival data at the Shizuoka Cancer Centre. Comprehensive HLA typing by whole genome sequencing (WGS) was also performed in 353 primary cases to investigate the relationship between HLA type and prognosis. As previously reported, increased mutation rates in genes of both IFN signaling and antigen presentation pathway were observed in patients with TMB >20 mutation/Mb, and further significantly in genes of STING1 pathway and chemokines in patients with TMB >100 mutation/Mb. HLA-B*40:01 was detected to be a potentially poor prognostic factor in solid tumors, similar to previous report of ATL. Interestingly, HLA-C*15:02 a common polymorphism in Asians and minor in Europeans was found to be a potentially poor prognostic factor. Association between adverse events with EGFR inhibitors and HLA-C*15:02 was reported, which may affect disease outcomes.

[Disclosure of COI by Responsible researcher] Lead presenter is the Responsible researcher.
[Lead presenter: Akira Iizuka] We have no financial relationships to disclose.

[P-1148] I-P11-4
Antitumor immune response (3)

2025 / 9 / 25 (Thu) 17:30-18:15 Room P/(Motenashi Dome) Room P

### P-1148
### In vitro analysis of interaction between patient-derived organoids and tumor-specific T cells in renal cell carcinoma

[Speaker]Kayoko Tsuji (Dept. Cancer Immunotherapy, Kanagawa Cancer Ctr. Res. Inst.／Vaccin and Immunotherapy Ctr, Kanagawa Cancer Ctr.)
[Co-author]Taku Kouro (Dept. Cancer Immunotherapy, Kanagawa Cancer Ctr. Res. Inst.／Vaccin and Immunotherapy Ctr, Kanagawa Cancer Ctr.)、Shun Horaguchi (Dept. Cancer Immunotherapy, Kanagawa Cancer Ctr. Res. Inst.／Dep. of Pedeatric Surg., Sch. of Med., Nihon Univ.)、Mitsuru Komahashi (Dept. Cancer Immunotherapy, Kanagawa Cancer Ctr. Res. Inst.／Dep. of Pedeatric Surg., Sch. of Med., Nihon Univ.)、Feifei Wei (Dept. Cancer Immunotherapy, Kanagawa Cancer Ctr. Res. Inst.／Vaccin and Immunotherapy Ctr, Kanagawa Cancer Ctr.)、Hidetomo Himuro (Dept. Cancer Immunotherapy, Kanagawa Cancer Ctr. Res. Inst.／Vaccin and Immunotherapy Ctr, Kanagawa Cancer Ctr.)、Shinya Sato (Div. Mol. Pathol. & Genetics, Kanagawa Cancer Ctr. Res. Inst.／Morphological Information Lab., Kanagawa Cancer Ctr. Res. Inst)、Daisuke Hoshino (Dept. Cancer Biol., Kangawa Cancer Ctr. Res. Inst.)、Tetsuro Sasada (Dept. Cancer Immunotherapy, Kanagawa Cancer Ctr. Res. Inst.／Vaccin and Immunotherapy Ctr, Kanagawa Cancer Ctr.)

Renal cell carcinoma (RCC) is distinct from other cancers in that tumor-infiltrating lymphocytes (TILs) adversely affect patient prognosis. In this study, we developed a system to precisely analyze the interaction between T cells and tumor cells using organoids (PDOs) established from RCC patients in vitro. TILs were subjected to sequence analysis of TCRs, which were subsequently used to establish TCR-transduced T (TCR-T) cells. The TCR-T cells were co-cultured with PDOs derived from the same patients and used for screening a series of inhibitors for their effects on T cell responses. The assessment of T cell killing activity, as indicated by LDH release and interferon-γ secretion, revealed the presence of multiple inhibitors, which either augmented or diminished T cell responses to PDOs. As anticipated, a subset of the obtained results exhibited concordance with prior reports, thereby confirming that this co-culture system serves as a suitable experimental model for investigating immune responses between TILs and tumor cells. Further studies are currently underway to identify a novel regulatory mechanism of anti-tumor T cell responses.

[Disclosure of COI by Responsible researcher] Lead presenter is not the Responsible researcher.
[Lead presenter: Kayoko Tsuji] We have no financial relationships to disclose.
[Principal Researcher: Tetsuro Sasada] We have no financial relationships to disclose.

▲TOP

### [P-1149] I-P11-4
### Antitumor immune response (3)

2025 / 9 / 25 (Thu) 17:30-18:15 Room P/(Motenashi Dome) Room P

---

### P-1149
### Immunological characteristics of hyper-differentiated melanoma cells

[Speaker]Yukie Ando (Dept. Microbiol., Fac. Med., Saitama Med. Univ.) [Co-author]Sara Hatazawa (Dept. Microbiol., Fac. Med., Saitama Med. Univ.)、Momo Mataki (Dept. Microbiol., Fac. Med., Saitama Med. Univ.)、Riko Kumatabara (Dept. Microbiol., Fac. Med., Saitama Med. Univ.)、Mio Nakajima (Dept. Microbiol., Fac. Med., Saitama Med. Univ.)、Rikuto Sone (Dept. Microbiol., Fac. Med., Saitama Med. Univ.)、Akihiro Nakamura (Dept. Microbiol., Fac. Med., Saitama Med. Univ.)、Mieko Tokano (Dept. Microbiol., Fac. Med., Saitama Med. Univ.)、Tomonaga Ichikawa (Dept. Microbiol., Fac. Med., Saitama Med. Univ.)、Takashi Murakami (Dept. Microbiol., Fac. Med., Saitama Med. Univ.)、Yutaka Horiuchi (Dept. Microbiol., Fac. Med., Saitama Med. Univ.)

Malignant melanoma is a highly plastic tumor characterized by switching between different cell phenotypes. Recently, phenotype switching to a hyper-differentiated state has attracted attention as a critical factor in causing melanoma therapy resistance. However, the impact of phenotype switching on the immunological properties of melanoma cells has not yet been thoroughly investigated. Here, we show the immunological properties of hyper-differentiated melanoma cells (HDMCs) generated by exposing murine B16F10 melanoma cells to low-dose staurosporine. Staurosporine-induced hyper-differentiated B16F10 (sB16F10) cells were phagocytosed more efficiently than untreated cells in co-culture with phagocytic cells. In addition, activation of tumor antigen-specific lymphocytes was observed in mice inoculated with sB16F10 cells. However, sB16F10 cells expressed high levels of PD-L1 and were highly resistant to T lymphocyte cytotoxicity. To address this, anti-PD-1 antibody was administered to sB16F10-transplanted mice, resulting in a significant inhibition of tumor growth. These results demonstrate that HDMCs have opposing immunological properties: enhanced immunogenicity and immune evasion.

[Disclosure of COI by Responsible researcher] Lead presenter is not the Responsible researcher.
[Lead presenter: Yukie Ando] We have no financial relationships to disclose.
[Principal Researcher: Yutaka Horiuchi] ■Research funds from company: JSPS科研費 JP24K11482

▲TOP

### [P-1150] I-P11-4
### Antitumor immune response (3)

2025 / 9 / 25 (Thu) 17:30-18:15 Room P/(Motenashi Dome) Room P

### P-1150
### Expression of asialo-GM1 identifies the distinct subpopulation of NK cell lineage

[Speaker]Ka He (Inst. of Natural Med., Univ. of Toyama) [Co-author]Kazuyoshi Takeda (Lab. Cell Biol., Grad. Sch. of Med., Juntendo Univ.)、Soichiro Sasaki (Inst. of Natural Med., Univ. of Toyama)、Yoshihiro Hayakawa (Inst. of Natural Med., Univ. of Toyama)

The glycolipid asialo-GM1 (ASGM1) has been regarded as a pan marker of murine NK cells, and the ***in vivo*** depletion of NK cells using anti-ASGM1 polyclonal antibody (pAb) has been extensively utilized in the pre-clinical cancer research. However, due to the low specificity of the pAb cross-reacting with other structurally similar gangliosides, the exact expression and the role of ASGM1 on NK cell lineage have not been described. Recently, several clones of rabbit anti-ASGM1 monoclonal antibody (mAb) with distinct specificities have been established. In this study, we characterized those anti-ASGM1 monoclonal antibodies in their efficacy in depleting NK cells ***in vivo***, and identified a clone GA134 that has the highest specificity and efficacy. Using fluorescent-labeled GA134, we examined the expression of ASGM1 on NK cell lineage at various differentiation stages. Not only NK precursor but also the earliest immature NK cell subset exhibited both ASGM1$^{+}$ and ASGM1$^{-}$ populations. These results indicate that ASGM1 expression can discriminate the previously unknown subpopulation of NK cell lineage.

[Disclosure of COI by Responsible researcher] Lead presenter is not the Responsible researcher.
[Lead presenter: Ka He] We have no financial relationships to disclose.
[Principal Researcher: Yoshihiro Hayakawa] We have no financial relationships to disclose.

### [P-1160] I-P11-6
### Tumor immune microenvironment/Tumor immune escape (2)

2025 / 9 / 25 (Thu) 17:30-18:15 Room P/(Motenashi Dome) Room P

---

### P-1160
### The Innate Immune System Activated by Temozolomide Predicts Response to Immune Checkpoint Inhibitors in Glioblastoma

[Speaker]Natsuki Koide (Div. of Cancer Biol., Nagoya Univ. Sch. of Med.) [Co-author]Tatsunori Nishimura (Div. of Cancer Biol., Nagoya Univ. Grad. Sch. of Med.)、Miho Suzuki (Div. of Cancer Biol., Nagoya Univ. Grad. Sch. of Med.)、Keiko Shinjo (Div. of Cancer Biol., Nagoya Univ. Grad. Sch. of Med.)、Yutaka Kondo (Div. of Cancer Biol., Nagoya Univ. Grad. Sch. of Med.)

Immune checkpoint inhibitors (ICIs) have improved cancer treatment outcomes, but their effects in glioblastoma (GBM) are limited due to the low number of tumor-infiltrating lymphocytes (TILs). Temozolomide (TMZ), the first-line chemotherapeutic agent for GBM, has been reported to enhance ICI responses in some patients, but not all. Activation of type I interferon (IFN-I) signaling is known to recruit TILs, hence its strength can be reflected in the responsiveness. In this study, we aimed to identify biomarkers for effective ICI-TMZ combination therapy by elucidating the molecular mechanism of IFN-I activation under TMZ treatment. TMZ induced cytoplasmic double-stranded DNA (dsDNA) and RNA (dsRNA), which activate IFN-I signaling. In addition, the strength of IFN-I activation depended on the expression levels of their pattern recognition receptors (PRRs). In U251 GBM cells, which show low expression of the dsDNA sensor CGAS, the dsRNA sensor RIG-I was the major functioning PRR in TMZ-induced IFN-I activation. These results suggest that the expression levels of dsDNA and/or dsRNA PRRs could be useful biomarkers to predict the efficacy of ICI-TMZ combination therapy.

[Disclosure of COI by Responsible researcher] Lead presenter is not the Responsible researcher.
[Lead presenter: Natsuki Koide] We have no financial relationships to disclose.
[Principal Researcher: Yutaka Kondo] We have no financial relationships to disclose.

### [P-1161] I-P11-6
### Tumor immune microenvironment/Tumor immune escape (2)

2025 / 9 / 25 (Thu) 17:30-18:15 Room P/(Motenashi Dome) Room P

---

### P-1161
### Effects of Photodynamic Therapy Using Novel Light-Sensitive Substances on Tumor-Associated Macrophages

[Speaker]Sakiko Mizuno (Dept. Pathobiology, Nagoya City Univ., Sch. Phar.) [Co-author]Tatsuki Soyama (Dept. Pathobiology, Nagoya City Univ., Sch. Phar.)、Akira Sakuragi (Dept. Pathobiology, Nagoya City Univ., Sch. Phar.)、Oishi Daisuke (Dept. Pathobiology, Nagoya City Univ., Sch. Phar.)、Yuka Kimura (Dept. Pathobiology, Nagoya City Univ., Sch. Phar.)、Hiromasa Aoki (Dept. Pathobiology, Nagoya City Univ., Sch. Phar.)、Akihiro Nomoto (Dept. Applied Chemistry, Osaka Pref. Univ.)、Shigenobu Yano (KYOSEI Sci. Cent., Nara Women's Univ.)、Hiromi Kataoka (Dept. Gastroenterology and Metabolism, Nagoya City Univ. Med. Sci.)、Hirotada Nishie (Dept. Gastroenterology and Metabolism, Nagoya City Univ. Med. Sci.)、Mineyoshi Aoyama (Dept. Pathobiology, Nagoya City Univ., Sch. Phar.)

**Introduction:**Photodynamic therapy (PDT) is a minimally invasive cancer treatment that uses a photosensitizer with tumor-accumulating properties and laser irradiation. We developed mannose-conjugated chlorin e6 (M-chlorin e6) as a next-generation photosensitizer. Recently, tumor-associated macrophages (TAMs), which are M1-TAMs and M2-TAMs, are being noticed for a key role in the tumor microenvironment. This study investigated the effects of M-chlorin e6 PDT on tumor progression and TAM polarization.

**Methods:**A syngeneic tumor model was created by injecting CT26 colon cancer cells into BALB/c mice. M-chlorin e6 was administered intravenously, followed by LED irradiation of the tumor site. Tumors were excised and weighed 14 days after irradiation. TAM distribution was analyzed by flowcytometry.

**Results:**M-chlorin e6 PDT reduced tumor size and weight. Flow cytometric analysis showed a decrease in the proportion of M2-TAMs, accompanied by an increase in M1-TAMs.

**Discussion:**These findings suggest that M-chlorin e6 PDT not only has direct cytotoxic effects on tumor cells but also influences the tumor immune microenvironment by altering macrophage polarization.

[Disclosure of COI by Responsible researcher] Lead presenter is not the Responsible researcher.
[Lead presenter: Sakiko Mizuno] We have no financial relationships to disclose.
[Principal Researcher: Mineyoshi Aoyama] We have no financial relationships to disclose.

### [P-1162] I-P11-6
### Tumor immune microenvironment/Tumor immune escape (2)

2025 / 9 / 25 (Thu) 17:30-18:15 Room P/(Motenashi Dome) Room P

---

### P-1162
### Pan-cancer Immune Profiling Across Solid Cancers by Flow Cytometry

[Speaker]Azumi Ueyama (Pharm. Res. Div, Shionogi & Co., Ltd.) [Co-author]Hiroto Miwa (Pharm. Res. Div, Shionogi & Co., Ltd.／Dept. Clin. Res. Tumor Immunol, Grad. Sch. Med, Osaka Univ.)、Hitomi Morihara (Pharm. Res. Div, Shionogi & Co., Ltd.／Dept. Clin. Res. Tumor Immunol, Grad. Sch. Med, Osaka Univ.)、Yudai Sonoda (Pharm. Res. Div, Shionogi & Co., Ltd.／Dept. Clin. Res. Tumor Immunol, Grad. Sch. Med, Osaka Univ.)、Masaki Hagiwara (Pharm. Res. Div, Shionogi & Co., Ltd.／Dept. Res. Tumor Immunol, Grad. Sch. Med, Osaka Univ.)、Yoji Nagira (Pharm. Res. Div, Shionogi & Co., Ltd.)、Takuro Saito (Dept. Clin. Res. Tumor Immunol, Grad. Sch. Med, Osaka Univ.)、Hisashi Wada (Dept. Clin. Res. Tumor Immunol, Grad. Sch. Med, Osaka Univ.)

Recent advances in cancer immunotherapy have underscored the importance of understanding the tumor immune microenvironment. We analyzed tumor-infiltrating lymphocytes from 16 solid cancers using flow cytometry, focusing on granulocytes, macrophages, NK cells, and T cells. Immune profiles were heterogeneous even within the same cancer type, but distinct tendencies were observed. Ovarian cancer showed enhanced innate immunity with abundant macrophages and NK cells, while gastrointestinal cancers (colorectal, gastric, esophageal) had lower levels of these populations. Head and neck cancers showed strong immunosuppressive features with enriched Tregs and granulocytes. Liver cancers had high NK/NKT cells and low Tregs. The phenotypes of NK cells and macrophages resembled those in the surrounding normal tissues, suggesting local tissue environments influence tumor immunity. Unsupervised clustering identified six immune subtypes with variable distributions across cancer types. In some cancers, specific immune subtypes were associated with poor prognosis. Pan-cancer immune profiling may inform personalized immunotherapy and biomarker discovery.

[Disclosure of COI by Responsible researcher] Lead presenter is the Responsible researcher.
[Lead presenter: Azumi Ueyama] ■ employment by a company related to the presentation: 塩野義製薬株式会社　■Endowed chair: 大阪大学医学研究科 臨床腫瘍免疫学講座

### [P-1163] I-P11-6
### Tumor immune microenvironment/Tumor immune escape (2)

2025 / 9 / 25 (Thu) 17:30-18:15 Room P/(Motenashi Dome) Room P

---

### P-1163
### Restoring immunosuppressive tumor microenvironment by targeting HNF1beta in ovarian clear cellcarcinoma (OCCC)

[Speaker]Ayase Futatsugi (Div. of Clin. Physiol. & Therap. Keio Univ. Faculty of Pharm.) [Co-author]Hiroshi Nishio (Dept. of Obstet. & Gynecol. Keio Univ. Sch. of Med.)、Kaede Katagiri (Dept. of Obstet. & Gynecol. Keio Univ. Sch. of Med.)、Masaki Sugawara (Dept. of Obstet. & Gynecol. Keio Univ. Sch. of Med.)、Ryotaro Imagawa (Dept. of Immunol. and Genomic Med, Kyoto Univ.)、Risa Matsuda (Dept. of Obstet. & Gynecol. Keio Univ. Sch. of Med.)、Yuki Katoh (Dept. of Obstet. & Gynecol. Keio Univ. Sch. of Med.／Div. of Anat. Sci. Nihon Univ. Sch. of Med.)、Takashi Iwata (Dept. of Obstet. & Gynecol. Keio Univ. Sch. of Med.)、Wataru Yamagami (Dept. of Obstet. & Gynecol. Keio Univ. Sch. of Med.)、Maiko Matsushita (Div. of Clin. Physiol. & Therap. Keio Univ. Faculty of Pharm.)、Yutaka Kawakami (Dept. of Adv. Med. Res. Keio Univ. Sch. of Med.)、Tomonori Yaguchi (Dept. of Adv. Med. Res. Keio Univ. Sch. of Med.／Dept. of Immunol. and Genomic Med, Kyoto Univ.)

Immunological status correlates with responses to various cancer therapies including immune checkpoint blockade. We have been evaluating immunopathology of human ovarian clear cell carcinoma (OCCC), which produces high levels of immunosuppressive cytokines such as IL6 and IL8, and shows few T cell infiltration. We found that HNF-1β, a transcriptional factor preferentially expressed in OCCC and associated with malignant phenotypes, is the upstream for high production of IL6 and IL8 via NF-&kappaB; and STAT3 signal activation. From drug screening, we found that BET bromodomain inhibitor (BETi) is capable of inhibiting HNF1β expression and IL6 production by OCCC cell lines without affecting cell viability. Chip assay showed BETi suppressed HNF1β by binding a specific region (-1150bp to -850bp) indicating that HBPi epigenetically suppressed HNF-1β expression and inhibited IL-6 production. Finally, we validated anti-tumor effect of BETi with syngeneic OCCC mouse model. BETi oral administration showed anti-tumor effect trend with tumor weight (p=0.139). Inhibition of the HNF-1&beta pathway can improve immunosuppressive conditions of OCCC and is a useful target of immunotherapy for OCCC.

[Disclosure of COI by Responsible researcher] Lead presenter is not the Responsible researcher.
[Lead presenter: Ayase Futatsugi] We have no financial relationships to disclose.
[Principal Researcher: Kaede Katagiri] We have no financial relationships to disclose.

### [P-1164] I-P11-6
### Tumor immune microenvironment/Tumor immune escape (2)

2025 / 9 / 25 (Thu) 17:30-18:15 Room P/(Motenashi Dome) Room P

---

### P-1164
### Inhibition of TGFβ Type I Receptor Prevents M2 Polarization of TAMs and Suppresses Colorectal Cancer Progression

[Speaker]Tomoyoshi Watanabe (Dept. of Pathobiology, Nagoya City Univ. Grad. Sch) [Co-author]Akira Sakuragi (Dept. of Pathobiology, Nagoya City Univ. Grad. Sch)、Hiromasa Aoki (Dept. of Pathobiology, Nagoya City Univ. Grad. Sch)、Kohki Toriuchi (Dept. of Pathobiology, Nagoya City Univ. Grad. Sch)、Riri Kondo (Dept. of Pathobiology, Nagoya City Univ. Grad. Sch)、Yasumichi Inoue (Department of Cell Signaling, Nagoya City Univ. Grad. Sch)、Hidetoshi(Nagoya City Univ.) Hayashi (Department of Cell Signaling, Nagoya City Univ. Grad. Sch)、Satoru Takeshita (Dept. of Pathobiology, Nagoya City Univ. Grad. Sch／Department of Perinatal and Neonatal Medicine, Aichi Medical Univ)、Hiroki Kakita (Dept. of Pathobiology, Nagoya City Univ. Grad. Sch／Department of Perinatal and Neonatal Medicine, Aichi Medical Univ)、Yasumasa Yamada (Department of Perinatal and Neonatal Medicine, Aichi Medical Univ)、Mineyoshi Aoyama (Dept. of Pathobiology, Nagoya City Univ. Grad. Sch)

Macrophages are classified into two main phenotypes: pro-inflammatory M1 and anti-inflammatory M2. Tumor-associated macrophages (TAMs) are specific types of macrophage that infiltrates tumor tissues extensively, with the majority polarized to the M2 phenotype. M2-TAMs are known to promote tumor survival and progression through a variety of mechanisms. Transforming growth factor (TGF)β, which is abundant in tumor tissues and contributes to tumor progression, has been implicated in the M2 polarization of macrophages. In this study, we investigated how TGFβ regulates TAMs and evaluated its potential as a therapeutic target for colorectal cancer (CRC). The TGFβ type I receptor inhibitor SB431542 suppressed the M2 polarization of THP-1 derived macrophages induced by conditioned medium from CRC cells. In a syngeneic mouse model of CRC, SB431542 reduced both the volume and the weight of tumors and decreased the proportion of M2-TAMs. The antitumor effect of SB431542 was attenuated following TAM depletion, suggesting that its therapeutic effect on CRC is at least partly mediated by the reduction of M2-TAMs. These findings support targeting TAM polarization as a promising CRC therapy.

[Disclosure of COI by Responsible researcher] Lead presenter is not the Responsible researcher.
[Lead presenter: Tomoyoshi Watanabe] We have no financial relationships to disclose.
[Principal Researcher: Mineyoshi Aoyama] We have no financial relationships to disclose.

### [P-1165] I-P11-6
### Tumor immune microenvironment/Tumor immune escape (2)

2025 / 9 / 25 (Thu) 17:30-18:15 Room P/(Motenashi Dome) Room P

---

### P-1165
### Prognostic Significance of PDGFC and its role in myCAF-iCAF interactions in Pancreatic Ductal Adenocarcinoma

[Speaker]Shotaro Komamura (Shinshu Univ. Hosp. Dept. of Lab. Med.) [Co-author]Tomoyuki Nakajima (Shinshu Univ. Hosp. Dept. of Lab. Med.)、Mai Iwaya (Shinshu Univ. Hosp. Dept. of Lab. Med.)、Shinsuke Sugenoya (Shinshu Univ. Dept. of Gastroenterology)、Takeshi Uehara (Shinshu Univ. Hosp. Dept. of Lab. Med.)

**Introduction:** Pancreatic Ductal Adenocarcinoma (PDAC) has poor prognosis, influenced by the tumor microenvironment (TME). Cancer-associated fibroblasts (CAFs) with diverse subtypes are key TME players impacting progression. While Platelet-Derived Growth Factor C (PDGFC) has roles in cancer, its function regarding CAF subtypes in TME is unclear.
**Methods:** We analyzed the PDGFC expression/prognosis association using TCGA bulk RNA-seq. GEO scRNA-seq data were utilized to identify PDGFC producers and analyze CAF interactions via CellChat. Finally, PDGFC immunohistochemistry (IHC) was performed on PDAC surgical specimens.
**Results:** In TCGA data, high PDGFC expression correlated with poor prognosis (OS: p=0.002, DFS: p=0.019). scRNA-seq showed PDGFC mainly in myCAFs, receptors PDGFRA/B in fibroblast clusters. Strong PDGFC-PDGFRA interaction from myCAFs to iCAFs was identified via CellChat.
**Conclusions:** PDGFC is a poor prognostic marker in PDAC, likely reflecting pro-fibrotic CAF interactions that drive PDAC progression. myCAFs primarily produce PDGFC, mediating iCAF communication via PDGFRA. These findings enhance understanding of complex cellular interactions in the TME of PDAC.

[Disclosure of COI by Responsible researcher] Lead presenter is the Responsible researcher.
[Lead presenter: Shotaro Komamura] We have no financial relationships to disclose.

Poster Session

### [P-1166] I-P11-6
### Tumor immune microenvironment/Tumor immune escape (2)

2025 / 9 / 25 (Thu) 17:30-18:15 Room P/(Motenashi Dome) Room P

---

### P-1166
### Tumor microRNA induces regulatory T cells and affects their in vivo growth

[Speaker]Takashi Akazawa (Dept. Cancer Immunotherapy, ResCtr., Osaka International Cancer Institute／Dept. Cancer Drug Discovery Development, ResCtr., Osaka International Cancer Institute) [Co-author]Yu Mizote (Dept. Cancer Immunotherapy, ResCtr., Osaka International Cancer Institute／Dept. Cancer Drug Discovery Development, ResCtr., Osaka International Cancer Institute)、Tomoya Ekawa (Dept. Cancer Immunotherapy, ResCtr., Osaka International Cancer Institute／Dept. Cancer Drug Discovery Development, ResCtr., Osaka International Cancer Institute)、Hideaki Tahara (Dept. Cancer Drug Discovery Development, ResCtr., Osaka International Cancer Institute／Center for Clinical Research, Osaka International Cancer Institute)

We analyzed EG7-OVA subline that showed growth retardation in ***in vivo*** but not ***in vitro*** by comparing with the parental EG7-OVA cell and focused on one microRNA. Here, we investigated the effects and mechanisms of this microRNA-x (miR-x) expression in tumor on progression in ***in vivo***. The miR-x KO EG7 cells generated by CRISPR-Cas9 systems, showed growth retardation in ***in vivo***. The antibody depletion experiments and immunohistochemistry analysis indicated that the ability of the miR-x KO cells to induce regulatory T cells (Tregs) might be attenuated. When miR-x KO and WT cells were transplanted simultaneously into different sides of mice, respectively, the miR-x KO tumor showed no growth retardation. These results suggest that miR-x expressing tumors lead to systemic immune regulation by Treg induction in ***in v ivo***. Therefore, the regulation of tumor miR-x might be an effective strategy not only for treatment by enhancement of anti-tumor immunity but also for the preparation of tumor cell-based vaccine.

[Disclosure of COI by Responsible researcher] Lead presenter is the Responsible researcher.
[Lead presenter: Takashi Akazawa] We have no financial relationships to disclose.

### [P-1167] I-P11-6
### Tumor immune microenvironment/Tumor immune escape (2)

2025 / 9 / 25 (Thu) 17:30-18:15 Room P/(Motenashi Dome) Room P

---

### P-1167
### RIG-I-Driven Neutrophil Activation Induces Anti-Tumor Efficacy and Mitigates Immune Checkpoint Therapy Resistance

[Speaker]Chinyang Chang (The university of Osaka Gene and Stem Cell Regenerative Therapy) [Co-author]Jiayu Tai (The university of Osaka Impulse Science for Medicine)、Yudiao Kuan (The university of Osaka Impulse Science for Medicine)、0 Ju (The university of Osaka Gene and Stem Cell Regenerative Therapy)、Tomoyuki Nishika (The university of Osaka Impulse Science for Medicine)、Kunihiko Yamashita (The university of Osaka Impulse Science for Medicine)、Munehisa Shimamura (The university of Osaka Gene and Stem Cell Regenerative Therapy)

The neutrophils, classified as anti-tumor (N1) and tumor-associated immunosuppressive (IS-TANs), play distinct roles. IS-TANs, further divided into tumor-promoting (N2) and granulocyte-derived immunosuppressive cells (G-MDSC), suppress anti-tumor immunity, complicating tumor treatment and potentially neutralizing immune checkpoint inhibitors. If naive or N2 neutrophils could be stimulated to differentiate into N1 neutrophils, new treatments might emerge. Our research reveals that administering double-stranded RNA (poly I:C) to the 4T1 tumor model via intracellular delivery methods, induces neutrophil differentiation into N1 cells via the RIG-I pathway. This differentiation not only enhances anti-tumor effects but increases CTL numbers. Importantly, this RIG-I pathway mediated N1 differentiation can alleviate PD-1 antibody therapy resistance. Although poly I:C can stimulate both the RIG-I and TLR-3 pathways, only the RIG-I pathway increases N1 in the tumor. This study not only found that the RIG-I pathway can cause N1 cell differentiation and relieve the inhibitory effect of PD-1 therapy, but also highlighted the importance of the method of nucleic acid drugs administration.

[Disclosure of COI by Responsible researcher] Lead presenter is the Responsible researcher.
[Lead presenter: Chinyang Chang] We have no financial relationships to disclose.

### [P-1176] I-P12-1
### Liquid biopsy (1)

2025 / 9 / 25 (Thu) 17:30-18:15 Room P/(Motenashi Dome) Room P

---

### P-1176
### Circulating tumor DNA analysis based on CAPP-seq in patients with metastatic cutaneous angiosarcoma

[Speaker]Soichiro Sawamura (Dermatology and Plastic Surgery, Kumamoto University, Kumamoto, Japan) [Co-author]Kenichiro Tanaka (Dermatology and Plastic Surgery, Kumamoto University, Kumamoto, Japan)、Ikko Kajihara (Dermatology and Plastic Surgery, Kumamoto University, Kumamoto, Japan)、Toshihiro Kimura (Dermatology and Plastic Surgery, Kumamoto University, Kumamoto, Japan)、Azusa Miyashita (Dermatology and Plastic Surgery, Kumamoto University, Kumamoto, Japan)、Satoshi Fukushima (Dermatology and Plastic Surgery, Kumamoto University, Kumamoto, Japan)

Background: In dermatology, there are few reports of cell-free DNA (cfDNA)/ circulating tumor DNA (ctDNA) analyses using Cancer personalized profiling by deep sequencing (CAPP-seq).
Objective: To investigate the genomic mutational profiling of cfDNA using CAPP-seq and its clinical significance in patients with metastatic cutaneous angiosarcoma.
Methods: Plasma samples were obtained from five patients with metastatic cutaneous angiosarcoma. Variant allele frequencies (VAFs) of gene mutations were assessed using CAPP-seq and droplet digital polymerase chain reaction (ddPCR).
Results: Nine mutation sites in the genes ***TP53***, ***KDR***, ***DDR2***, ***NF2***, ***NTRK1***, ***SMO***, ***RNF43***, and ***FBXW7***, were detected by CAPP-seq in cfDNA. Four out of five patients had at least one nonsynonymous mutation. Although the same sites of gene alteration were not found in each case, two patients exhibited ***TP53*** mutation at different sites. The VAFs of some mutated genes varied with disease progression.
Conclusion: Mutational profiling of cfDNA using CAPP-seq and ddPCR may be useful for monitoring disease progression in patients with metastatic cutaneous angiosarcoma.

[Disclosure of COI by Responsible researcher] Lead presenter is the Responsible researcher.
[Lead presenter: Soichiro Sawamura] We have no financial relationships to disclose.

### [P-1177] I-P12-1
### Liquid biopsy (1)

2025 / 9 / 25 (Thu) 17:30-18:15 Room P/(Motenashi Dome) Room P

### P-1177
### Ultrasensitive measurement of HPV16 E6/E7 proteins in cervical cancer

[Speaker]Etsuro Ito (Waseda Univ., Dept. Biol.) [Co-author]Mitsumasa Hasunuma (Waseda Univ., Dept. Biol. ／BioPhenoMA Inc)、Takafumi Tasaki (Med. Res. Inst., Kanazawa Med. Univ.)、Toshiyuki Sasagawa (Sch. Med., Kanazawa Med. Univ.)

Cervical cancer is primarily caused by human papillomavirus (HPV) infection, with oncogenic progression largely attributed to the activity of HPV E6 and E7 proteins. Therefore, we propose a method to assess HPV activity by detecting and quantifying E6/E7 proteins using our thio-NAD cycling ELISA (TN-cyclon$^{TM}$). The thio-NAD cycling ELISA method combines the conventional sandwich ELISA and enzyme cycling method to amplify the signal, enabling ultrasensitive measurements. The measurement results showed that the limit of detection of HPV16 E6 protein was 5.23 pg/mL and that of HPV16 E7 protein was 0.905 pg/mL. The results are approximately 100 times more sensitive than conventional commercial ELISA kits. Now, trace amounts of E6/E7 proteins are found in urine. By measuring them, it may be possible to assess HPV activity without cytology.

[Disclosure of COI by Responsible researcher] Lead presenter is the Responsible researcher.
[Lead presenter: Etsuro Ito] ■Research funds from company: （株）BioPhenoMA ■ employment by a company related to the presentation: （株）BioPhenoMA ■Profit of stock: （株）BioPhenoMA ■Patent fee: （株）BioPhenoMA

### [P-1178] I-P12-1
### Liquid biopsy (1)

2025 / 9 / 25 (Thu) 17:30-18:15 Room P/(Motenashi Dome) Room P

---

### P-1178
### Development of a machine learning model for ovarian cancer using methylation liquid biopsy

[Speaker]Saki Aota (The University of Tokyo Hospital Obstetrics and Gynecology) [Co-author]Yusuke Toyohara (The University of Tokyo Hospital Obstetrics and Gynecology)、Kenbun Sone (The University of Tokyo Hospital Obstetrics and Gynecology)、Kengo Goto (FUJIFILM)、Naoko Yamaguchi (FUJIFILM)、Yusuke Kurimoto (FUJIFILM)、Maiko Wakita (FUJIFILM)、Ryo Shimura (FUJIFILM)、Kaito Takashima (FUJIFILM)、Yuna Kiyama (FUJIFILM)、Masahiro Sato (FUJIFILM)、Yuichiro Miyamoto (The University of Tokyo Hospital Obstetrics and Gynecology)、Mayuyo Mori (Tokyo Metropolitan Cancer and Infectious Diseases Center Komagome Hospital Gynecology)、Katsutoshi Oda (The University of Tokyo Hospital Department of Clinical Genomics)、Yutaka Osuga (Teikyo University Obstetrics and Gynecology)、Yasushi Hirota (The University of Tokyo Hospital Obstetrics and Gynecology)

Ovarian cancer lacks an effective screening method and is often diagnosed at advanced stages. We developed a methylation panel assay for detecting cancer-specific DNA methylation in cell-free DNA (cfDNA) and constructed a diagnostic model using machine learning. Plasma samples from 27 ovarian cancer patients (15 stage I, 2 stage II, 4 stage III, 6 stage IV), including 25 cases from our hospital (2021-2023), and 33 healthy controls were analyzed. A total of 673 CpG sites, identified as hypermethylated in cancer tissues compared to normal tissues and leukocytes, were used to train the model. The model achieved a specificity of 90.9%, sensitivity of 70.4%, and an AUC of 0.850. Sensitivity by subtype was 90.9% (serous), 25.0% (clear cell), and 83.3% (endometrioid). Stage-specific sensitivity was 67% (stage I), 75% (stage III), and 100% (stage IV). This cfDNA methylation assay enables sensitive detection of trace tumor DNA and shows promise for early ovarian cancer screening. Further validation in larger cohorts is needed.

[Disclosure of COI by Responsible researcher] Lead presenter is not the Responsible researcher.
[Lead presenter: Saki Aota] We have no financial relationships to disclose.
[Principal Researcher: Yusuke Toyohara] We have no financial relationships to disclose.

### [P-1179] I-P12-1
### Liquid biopsy (1)

2025 / 9 / 25 (Thu) 17:30-18:15 Room P/(Motenashi Dome) Room P

### P-1179
### Ongoing Efforts for Long-Term Expansion of Circulating Tumor Cells (CTCs)

[Speaker]Fumihiko Kakizaki (Dept. Personalized Cancer Med., Grad. Sch. Med. Kyoto Univ.) [Co-author]Kyoichi Oshiro (AFI corp.)、Yuya Enoki (Dept. Personalized Cancer Med., Grad. Sch. Med. Kyoto Univ.／Dept. of Surgery, Grad. Sch. of Med., Kyoto Univ.)、Kana Kawanishi (Dept. of Breast Surgery, Grad. Sch. of Med., Kyoto Univ.)、Norikazu Masuda (Dept. of Breast Surgery, Grad. Sch. of Med., Kyoto Univ.)、Hisatsugu Maekawa (Dept. Personalized Cancer Med., Grad. Sch. Med. Kyoto Univ.／Dept. of Surgery, Grad. Sch. of Med., Kyoto Univ.)、Jun Matsubayashi (Dept. Personalized Cancer Med., Grad. Sch. Med. Kyoto Univ.／Dept. of Surgery, Grad. Sch. of Med., Kyoto Univ.)、Masahiro Kawashima (Dept. of Breast Surgery, Grad. Sch. of Med., Kyoto Univ.)、Hiroyuki Miyoshi (Dept. Personalized Cancer Med., Grad. Sch. Med. Kyoto Univ.)、Yukitoshi Takemura (Dept. Personalized Cancer Med., Grad. Sch. Med. Kyoto Univ.)、Kazutaka Obama (Dept. Personalized Cancer Med., Grad. Sch. Med. Kyoto Univ.／Dept. of Surgery, Grad. Sch. of Med., Kyoto Univ.)

Malignant cancer cells invade surrounding tissues, reach nearby veins, and enter the bloodstream as circulating tumor cells (CTCs). Analyzing these CTCs offers several advantages in cancer diagnosis and treatment: (i) quantifying CTCs enables the prediction of metastatic progression and recurrence; (ii) monitoring longitudinal changes in CTC counts allows for the assessment of anticancer therapy efficacy; and (iii) genomic, transcriptomic, and phenotypic analyses of CTCs provide valuable insights into treatment strategies. Our aim is to enhance the clinical utility of CTCs by not only isolating them efficiently but also establishing long-term cultures and conducting drug sensitivity testing. In this study, we utilized the CROSSORTER (AFI Corporation). This device that isolates CTCs from blood based on their electrical properties using dielectrophoresis (DEP), while minimizing cellular stress during the separation process. Subsequently, we investigated long-term culture under various conditions, including different culture media, extracellular matrices, and supplements.

[Disclosure of COI by Responsible researcher] Lead presenter is not the Responsible researcher.
[Lead presenter: Fumihiko Kakizaki] ■Endowed chair: 株式会社ＳＣＲＥＥＮホールディングス、京ダイアグノスティクス株式会社、株式会社ＡＦＩテクノロジー
[Principal Researcher: Kazutaka Obama] ■Endowed chair: 株式会社SCREENホールディングス、京ダイアグノスティクス株式会社、株式会社AFIテクノロジー

### [P-1180] I-P12-1
### Liquid biopsy (1)

2025 / 9 / 25 (Thu) 17:30-18:15 Room P/(Motenashi Dome) Room P

---

### P-1180
### Potential role of circulating tumor cells and cell-free DNA as biomarkers for metastasis in oral squamous cell carcinoma

[Speaker]Shiori Wakita (Hiroshima University, Oral Oncology) [Co-author]Atsuko Hamada (Hiroshima University, Oral Oncology)、Natsuki Eboshida (Hiroshima University, Oral Oncology)、Suguru Hirota (Hiroshima University, Oral Oncology)、Fumitaka Obayashi (Hiroshima University, Oral Oncology)、Kota Morishita (Hiroshima University, Oral Oncology)、Sachiko Yamasaki (Hiroshima University, Oral Oncology)、Nanako Ito (Hiroshima University, Oral Oncology)、Koichi Koizumi (Hiroshima University, Oral Oncology)、Souichi Yanamoto (Hiroshima University, Oral Oncology)

Metastasis in patients with oral squamous cell carcinoma (OSCC) has been associated with a poor prognosis. However, sensitive tests for monitoring their occurrence are unavailable. Recent studies have highlighted the role of circulating tumor cells (CTCs) and cell-free DNA (cfDNA) in the distant metastasis of solid tumors, such as breast cancer, sparking growing interest in the potential of liquid biopsy as a diagnostic tool, but not yet in OSCC. To investigate the clinical significance of CTCs and cfDNA in OSCC, we analyzed them from 13 patients, who were diagnosed with OSCC in our hospital 2022-2023, at pre- or post-operation, and examined the relationship with the prognosis. We observed no correlation between the number of CTC and metastasis. However, the number of CTC clusters or the amount of preoperative cfDNA in metastatic cases was higher than that in non-metastatic cases. Moreover, as a result of next generation sequences of cfDNA, genetic mutations were identified in three out of the 13 cases, yet the two cases with metastasis shared several common genes. These results suggest that CTC clusters and cfDNA levels could provide valuable insights for managing OSCC metastasis.

[Disclosure of COI by Responsible researcher] Lead presenter is not the Responsible researcher.
[Lead presenter: Shiori Wakita] We have no financial relationships to disclose.
[Principal Researcher: Souichi Yanamoto] We have no financial relationships to disclose.

▲TOP

### [P-1181] I-P12-1
### Liquid biopsy (1)

2025 / 9 / 25 (Thu) 17:30-18:15 Room P/(Motenashi Dome) Room P

---

### P-1181
### Preliminary Use of a New Hybrid-Capture Panel for Liquid Biopsy of Bile cfDNA in Biliary Tract Cancer

[Speaker]Shin Ito (Div. Mol. & Cell. Oncol., Miyagi Cancer Ctr. Res. Inst.) [Co-author]Shuichi Aoki (Dept. Surg., Tohoku Univ.)、Jun Yasuda (Div. Mol. & Cell. Oncol., Miyagi Cancer Ctr. Res. Inst.)、Michiaki Unno (Dept. Surg., Tohoku Univ.)

Bile collected via endoscopic procedures contains substantially higher levels of cell-free DNA (cfDNA) than blood plasma. We previously extracted cfDNA from bile and plasma of patients with biliary tract cancer and analyzed them using the LiquidPlex multigene liquid biopsy panel. Our findings suggested that bile-derived cfDNA is compatible with plasma-oriented panels and may serve as a more reliable source than plasma for detecting tumor-derived driver mutations. Recently, several cfDNA-based multigene panels designed for liquid biopsy have become commercially available. One such system, employing hybrid-capture technology, offers rapid and specific target enrichment. In this study, we conducted a preliminary trial of this new panel using bile cfDNA samples from four patients in whom driver mutations had been previously detected with LiquidPlex. Libraries were prepared using the new platform, and the results were compared with those from the prior analysis. This pilot comparison aimed to examine the feasibility of applying an emerging liquid biopsy technology to bile-derived cfDNA and to evaluate its concordance with established platforms.

[Disclosure of COI by Responsible researcher] Lead presenter is not the Responsible researcher.
[Lead presenter: Shin Ito] We have no financial relationships to disclose.
[Principal Researcher: Jun Yasuda] We have no financial relationships to disclose.

### [P-1182] I-P12-1
### Liquid biopsy (1)

2025 / 9 / 25 (Thu) 17:30-18:15 Room P/(Motenashi Dome) Room P

---

### P-1182
### Proteomic exploration of new pharmacodynamic biomarkers of tankyrase inhibitors

[Speaker]Naoki Ueno (Div. Mol. Biother., Cancer Chemother. Ctr., JFCR.／Dept. Comp. Biol. Med. Sci., Grad. Frontier. Sci., Univ. Tokyo.) [Co-author]Tetsuo Mashima (Div. Mol. Biother., Cancer Chemother. Ctr., JFCR.)、Jin Lee (Div. Mol. Biother., Cancer Chemother. Ctr., JFCR.／Dept. Comp. Biol. Med. Sci., Grad. Frontier. Sci., Univ. Tokyo.)、Fumiyuki Shirai (Drug Disc. Chem. Platf. Unit, RIKEN Center Sustainable Res. Sci.)、Koji Ueda (Div. Anal. Biochem., Cancer Prec. Med. Ctr., JFCR)、Hiroyuki Seimiya (Div. Mol. Biother., Cancer Chemother. Ctr., JFCR.／Dept. Comp. Biol. Med. Sci., Grad. Frontier. Sci., Univ. Tokyo.)

Wnt signaling is activated in most colorectal cancers (CRCs). Tankyrase poly(ADP-ribose) (PAR) polymerase positively regulates this signaling through PARylation and subsequent degradation of AXIN, a β-catenin repressor. We developed a tankyrase inhibitor, RK-582, which blocks Wnt signaling and is under clinical trial for treatment of CRC patients. So far, however, its pharmacodynamics (PD) has been established only in cultured cells and xenograft tumors by monitoring the Wnt signaling factors. To identify more feasible PD biomarkers detectable by liquid biopsy, we performed proteome analyses of murine blood samples. First, mice with subcutaneously injected CRC cells were orally administered with RK-582. Proteome analysis of the plasma identified 132 proteins upregulated in the drug-treated groups. Metascape analysis revealed that RK-582 upregulated various cytoskeleton-related proteins with tankyrase-binding motifs, suggesting that PARylation-mediated degradation regulates the amounts of those proteins. These observations suggest that stabilization of tankyrase-binding proteins can be utilized as a less-invasive PD biomarker of tankyrase inhibitors.

[Disclosure of COI by Responsible researcher] Lead presenter is not the Responsible researcher.
[Lead presenter: Naoki Ueno] We have no financial relationships to disclose.
[Principal Researcher: Hiroyuki Seimiya] ■Research funds from company: JSPS科研費、AMED次世代がん、AMED革新がん、日本財団、武田科学振興財団、富士フイルムメディカル　■Research expenses: 日本財団　■Contributions: 日本財団、武田科学振興財団、富士フイルムメディカル

▲TOP

Poster Session

### [P-1183] I-P12-1
### Liquid biopsy (1)

2025 / 9 / 25 (Thu) 17:30-18:15 Room P/(Motenashi Dome) Room P

---

### P-1183
### Serum exosomal BCYRN1 as a potential biomarker and therapeutic target in renal cell cancer

[Speaker]Junya Arima (Dept. of Urology, Kagoshima univ.) [Co-author]Tomonao Naraki (Dept. of Urology, Kagoshima univ.)、Mitsuhiko Tominaga (Dept. of Urology, Kagoshima univ.)、Saeki Saito (Dept. of Urology, Kagoshima univ.)、Gang Li (Dept. of Urology, Kagoshima univ.)、Ikumi Fukuda (Dept. of Urology, Kagoshima univ.)、Takashi Sakaguchi (Dept. of Urology, Kagoshima univ.)、Hirofumi Yoshino (Dept. of Urology, Kagoshima univ.)、Shuichi Tatarano (Dept. of Urology, Kagoshima univ.)、Hideki Enokida (Dept. of Urology, Kagoshima univ.)

Renal cell cancer (RCC) is among the most prevalent cancers, yet lacks biomarkers for diagnosis or therapeutic evaluation. Recently, long non-coding RNAs (lncRNAs) have been proposed as biomarkers in various cancers. This study focuses on serum exosomal lncRNA BCYRN1 (seBCYRN1) in RCC patients and examines its impact on RCC cells. Serum samples collected pre-surgery from RCC patients revealed significantly higher seBCYRN1 levels compared to healthy donors, particularly in patients with advanced pT2+ stages. Loss-of-function assays utilizing siRNA demonstrated that BCYRN1 knockdown markedly suppressed proliferation, migration, invasion, and spheroid-forming abilities in vitro. Additionally, in vivo xenograft experiments confirmed reduced proliferation, with subcutaneous assays in mice showing suppression of tumor growth. RNA sequencing and GSEA analyses highlighted alterations in gene sets associated with cell cycle and apoptosis. BCYRN1 knockdown induced G2/M cell cycle arrest and apoptosis in RCC cells. As the first study to investigate seBCYRN1 in RCC, these findings suggest its potential as a novel diagnostic and therapeutic target.

[Disclosure of COI by Responsible researcher] Lead presenter is not the Responsible researcher.
[Lead presenter: Junya Arima] We have no financial relationships to disclose.
[Principal Researcher: Hirofumi Yoshino] We have no financial relationships to disclose.

Poster Session

### [P-1191] I-P12-3
### Genomic diagnostics / Panel sequencing

2025 / 9 / 25 (Thu) 17:30-18:15 Room P/(Motenashi Dome) Room P

---

### P-1191
### Li-Fraumeni syndrome caused by a 3.6 kb deletion in the TP53 gene suggested by additional data from the NCC Oncopanel

[Speaker]Satoshi Souma (Div. Mol. Cell. Oncol., Miyagi Cancer Ctr. Res. Inst.) [Co-author]Maki Ogawa (Div. Dev. Support, Miyagi Children's Hosp.／Dept. Breast Surg., Miyagi Cancer Ctr. Hosp.)、Shin Ito (Div. Mol. Cell. Oncol., Miyagi Cancer Ctr. Res. Inst.)、Kazunori Yamaguchi (Div. Mol. Cell. Oncol., Miyagi Cancer Ctr. Res. Inst.)、Haruna Fujimori (Div. Cancer Stem Cell, Miyagi Cancer Ctr. Res. Inst.)、Naoki Asano (Div. Cancer Stem Cell, Miyagi Cancer Ctr. Res. Inst.)、Koji Ohnuki (Dept. Breast Surg., Miyagi Cancer Ctr. Hosp.)、Mika Takeuchi (Div. Clin. Lab., Miyagi Cancer Ctr. Hosp.)、Kazunori Otsuka (Dept. Med. Oncol., Miyagi Cancer Ctr. Hosp.)、Hidekazu Shirota (Dept. Clin. Oncol., Tohoku Univ. Hosp.)、Jun Yasuda (Div. Mol. Cell. Oncol., Miyagi Cancer Ctr. Res. Inst.)

Li-Fraumeni syndrome (LFS) is a hereditary cancer predisposing disease caused by germline loss-of-functional mutations in the tumor suppressor gene TP53. In Japan, genetic testing for hereditary tumor syndromes is mostly not covered by insurance, making it costly. Cancer gene panel tests like the NCC Oncopanel, which include germline analyses, are clinically valuable but primarily focus on molecular targeted drug discovery, with limited insurance coverage for pathogenic germline variants. We report a novel 3.6 kb TP53 deletion identified through NCC Oncopanel data analysis. A woman with a history of breast cancer and a diagnosis of pancreatic cancer, had a family history suggesting LFS. While insurance-covered testing failed to detect pathogenic germline variants or actionable therapies, supplementary data (copy number distribution) indicated the germline TP53 deletion. Genetic testing confirmed a deletion spanning introns 1 to 6, and we characterized the molecular details of the deletion by the use of next generation sequencers. Our case suggests that NCC Oncopanel supplementary data supports decision-making for additional genetic testing, emphasizing its clinical significance.

[Disclosure of COI by Responsible researcher] Lead presenter is not the Responsible researcher.
[Lead presenter: Satoshi Souma] We have no financial relationships to disclose.
[Principal Researcher: Jun Yasuda] We have no financial relationships to disclose.

### [P-1192] I-P12-3
### Genomic diagnostics / Panel sequencing

2025 / 9 / 25 (Thu) 17:30-18:15 Room P/(Motenashi Dome) Room P

---

### P-1192
### Effects of a negative-pressure fixation method on nucleic acid preservation in FFPE tissue samples

[Speaker]Fumiaki Kinoshita (Grad. School of Health Sci., Kumiamoto Univ.) [Co-author]Hiromu Yano (Dept. Tumor Path., Faculty of Life Sci., Kumamoto Univ.)、Masahiro Yamamoto (Dept. Tumor Path., Faculty of Life Sci., Kumamoto Univ.)

Formalin-fixed paraffin-embedded (FFPE) tissue samples are essential resources for cancer genome analyses. However, the preservation of nucleic acid during the fixation process remains a major challenge. Therefore, the present study investigated the effects of a negative-pressure fixation method using a vacuum fixation device on nucleic acid. Forty-six resected lung cancer samples processed by the device-assisted or conventional method (n = 23 per group) were examined. The yield and quality of DNA and RNA in 5-μm-thick sections were assessed by the concentration of nucleic acid, DNA and RNA integrity numbers (DIN and RIN, respectively), and DV200. DNA and RNA yields were significantly higher in the device-assisted group than in the conventional group (DNA, 3.3 vs 1.2 $\mu g/mm^2$; RNA, 11.6 vs 6.8 $\mu g/mm^2$), whereas DIN values did not significantly differ. RIN and DV200 were significantly higher in the device-assisted group (RIN, 2.3 vs 1.7; DV200, 66.7% vs 51.2%). These results indicate that the negative-pressure fixation method increases nucleic acid yields and improves RNA quality in lung cancer specimens. Therefore, it is a valuable tissue fixation method for cancer genome analyses.

[Disclosure of COI by Responsible researcher] Lead presenter is not the Responsible researcher.
[Lead presenter: Kinoshita Fumiaki] We have no financial relationships to disclose.
[Principal Researcher: Masahiro Yamamoto] We have no financial relationships to disclose.

### [P-1193] I-P12-3
### Genomic diagnostics / Panel sequencing

2025 / 9 / 25 (Thu) 17:30-18:15 Room P/(Motenashi Dome) Room P

---

### P-1193
### Verification of the effectiveness of technology to improve the quality of library preparation for panel testing

[Speaker]Takahide Yokoi (Hitachi, Ltd. Research & Development Group) [Co-author]Ryota Kajihara (Hitachi, Ltd. Research & Development Group)、Mima Ogawa (Hitachi, Ltd. Research & Development Group)

In the success or failure of panel testing, (i) quality control of extracted DNA and (ii) quality control of the library created are important. (i) is difficult to control because it also depends on the condition of the sample. Therefore, in automation, it is thought that it is desirable to construct a technology that maintains high quality of (ii) while expanding the acceptable level of (i).We focused on the technology for fractionating DNA fragments as one of the factors that can cause problems in library preparation. The accurate DNA fractionation technology we developed, which is based on the principle of gel electrophoresis, was able to fractionate DNA molecules with correctly linked adapters. When we compared the libraries prepared using this technology with those prepared using the existing magnetic bead fractionation, we confirmed that the quality of the sequence data was improved using this technology. In this presentation, we will introduce the technology we developed and report on its effectiveness.

[Disclosure of COI by Responsible researcher] Lead presenter is the Responsible researcher.
[Lead presenter: Takahide Yokoi] ■ employment by a company related to the presentation: 株式会社日立製作所

▲TOP

### [P-1194] I-P12-3
### Genomic diagnostics / Panel sequencing

2025 / 9 / 25 (Thu) 17:30-18:15 Room P/(Motenashi Dome) Room P

### P-1194
### Detection of gene fusions in rare tumor cases

[Speaker]Yoji Kukita (Lab. Genomic Path., Osaka Int. Cancer Inst.) [Co-author]Harumi Nakamura (Div. Diagnostic Path., Shonan Kamakura General Hosp.)、Kenichi Yoshida (Dept. Diagnostic Path. Cyt., Osaka Int. Cancer Inst.)、Satoshi Takenaka (Dept. Orthop. Surg., Osaka Int. Cancer Inst.)、Toshinari Yagi (Dept. OP. Chemother., Osaka Int. Cancer Inst.)、Keiichiro Honma (Lab. Genomic Path., Osaka Int. Cancer Inst.／Dept. Diagnostic Path. Cyt., Osaka Int. Cancer Inst.)

Rare tumors are often challenging to diagnose, even for specialists, and research on them lags behind that of common cancers. To address this, it is essential to accumulate detailed clinical and molecular data. We are collecting rare tumor cases, mainly sarcomas, and conducting molecular pathological analyses using immunohistochemistry and targeted RNA sequencing. Among 134 cases analyzed, gene fusions were detected in 80 cases, of which 5 cases were reported as cases with novel fusions. We also reported 5 cases with known fusions as rare cases. Clustering analysis based on gene expression patterns showed that tumors with similar characteristics tended to group together, suggesting potential for molecular classification. In 29 cases, multiple fusions were identified; in some, these occurred on a single chromosome, indicating the involvement of complex genomic events such as chromothripsis or chromoplexy. Targeted RNA sequencing proved effective in detecting diagnostically relevant gene fusions in rare tumors. In addition to ongoing case collection, we are applying nanopore sequencing to identify gene fusions and structural variants in previously fusion-negative samples.

[Disclosure of COI by Responsible researcher] Lead presenter is the Responsible researcher.
[Lead presenter: Yoji Kukita] ■Research expenses: 合同会社ジーンメトリックス

[P-1195] I-P12-3
Genomic diagnostics / Panel sequencing

2025 / 9 / 25 (Thu) 17:30-18:15 Room P/(Motenashi Dome) Room P

---

### P-1195
### Comparison of genomic and clinical features of endometrial carcinoma between common and rare mutations detected in *POLE*

[Speaker]Takayuki Kawai (Div. Genome Biol., Natl. Cancer Ctr. Inst.／Dept. Obstet.Gynecol. Juntendo Univ.Faculty of Med.) [Co-author]Mayumi Kato (Div. Genome Biol., Natl. Cancer Ctr. Inst.／Dept. Gynecol. Natl.Cancer Ctr.Hosp.)、Maiko Yamaguchi (Div. Genome Biol., Natl. Cancer Ctr. Inst.／Dept. Obstet.Gynecol. Juntendo Univ.Faculty of Med.)、Hideki Okada (Med.& Biological Lab.Co,Ltd.(MBL))、Takuya Kondo (Med.& Biological Lab.Co,Ltd.(MBL))、Tetsuro Shiraishi (Div. Genome Biol., Natl. Cancer Ctr. Inst.／Dept. Obstet.Gynecol. Juntendo Univ.Faculty of Med.／Dept. Obstet.Gynecol. Keio Univ. Scl. Med.)、Daiki Higuchi (Div. Genome Biol., Natl. Cancer Ctr. Inst.／Dept. Obstet.Gynecol. Showa Univ. Scl. Med.)、Ai Ikki (Div. Genome Biol., Natl. Cancer Ctr. Inst.／Dept. Obstet.Gynecol. Showa Univ. Scl. Med.)、Taro Yamanaka (Dept. Medi. Oncol. Natl.Cancer Ctr.Hosp.)、Ryuji Hamamoto (Div. Med. AI Res. & Development, Natl.Cancer Ctr.Inst.／Cancer Translational Res. Team, RIKEN Ctr. For Advanced Intelligence Project)、Yasuhisa Terao (Dept. Obstet.Gynecol. Juntendo Univ.Faculty of Med.)、Emiko Yoshida (Dept. Obstet.Gynecol. Juntendo Univ.Faculty of Med.／Diagnostic Therap. Intractable Diseases,Intractable Disease Res. Ctr.Juntendo Univ)、Takashi Kohno (Div. Genome Biol., Natl. Cancer Ctr. Inst.)、Mitsuya Ishikawa (Dept. Gynecol. Natl.Cancer Ctr.Hosp.)、Hiroshi Yoshida (Dept. Diagnostic Pathol. Natl.Cancer Ctr.Hosp.)、Kouya Shiraishi (Div. Genome Biol., Natl. Cancer Ctr. Inst.／Dept.Clin. Genomics, Natl.Cancer Ctr.Inst.)

Endometrial carcinoma (EC) with hotspot mutations in *POLE* is characterized by distinct genomic features and favorable prognosis. However, their clinical significance in EC with rare mutations remains unclear. This study aimed to investigate the genomic and clinical features of *POLE*-mutated EC using whole-exome sequencing. Tumor samples were obtained between 1999 and 2023 at the National Cancer Center and Juntendo University Hospitals. 54 cases with *POLE* mutations, including 42 with hotspot mutations and 12 with rare mutations, were detected using the Luminex assay and whole-exome sequencing. Tumor mutation burden (TMB), mutational signatures, and clinical outcomes were compared between groups. The median TMB was 99.4 mutations/Mb [25.5-468.8] in the hotspot-mutated group and 205.6 mutations/Mb [20.2-435.4] in the rare-mutated group (P = 0.34). *POLE*-related mutational signatures were present in both groups. However, two recurrences occurred in the hotspot-mutated group. EC with rare *POLE* mutations demonstrated genomic and clinical features comparable to those of hotspot *POLE*-mutated EC. Patients with rare mutations in *POLE* may omit postoperative chemotherapy

[Disclosure of COI by Responsible researcher] Lead presenter is not the Responsible researcher.
[Lead presenter: Takayuki Kawai] We have no financial relationships to disclose.
[Principal Researcher: Kouya Shiraishi] We have no financial relationships to disclose.

### [P-1196] I-P12-3
### Genomic diagnostics / Panel sequencing

2025 / 9 / 25 (Thu) 17:30-18:15 Room P/(Motenashi Dome) Room P

---

### P-1196
### Automated NGS and dPCR for Malignant Lymphoma in a Community Setting: Use of the Liverpool Lymphoid Network Panel

[Speaker]Tomomi Yajima (Sapporo Dohto Hospital Medical Corporation) [Co-author]Fumitake Hata (Sapporo Dohto Hospital Medical Corporation)、Sei Kurokawa (Sapporo Dohto Hospital Medical Corporation)、Noriyuki Sato (Sapporo Dohto Hospital Medical Corporation)

The Liverpool Lymphoid Network Panel (LLNP) is a targeted NGS panel covering 60 genes for lymphoid hematologic malignancies. Digital PCR (dPCR) enables sensitive and cost-effective quantification of mutations for treatment monitoring. We report a 75-year-old male with relapsed mantle cell lymphoma who achieved remission after chemotherapy. Genomic analysis was performed on FFPE lymph node tissue using the Ion Torrent Genexus System, which automated extraction, sequencing, and analysis with the LLNP. Data were analyzed via the Ion Torrent Cancer Genome Report Service. Plasma for liquid biopsy was collected using PAXgene ccfDNA tubes, and mutation monitoring was conducted with the QuantStudio Absolute Q dPCR system.NGS identified 21 hotspot mutations within 2 days, including TP53 c.559+1G>A, which is associated with NCCN category 1 therapy and had been administered to the patient. This mutation was selected for dPCR-based follow-up. Serial liquid biopsies post-treatment showed no detectable TP53 mutation, consistent with clinical remission.This case demonstrates that automated NGS and dPCR enable rapid and effective molecular testing and disease monitoring in a community setting.

[Disclosure of COI by Responsible researcher] Lead presenter is the Responsible researcher.
[Lead presenter: Tomomi Yajima] We have no financial relationships to disclose.

### [P-1197] I-P12-3
### Genomic diagnostics / Panel sequencing

2025 / 9 / 25 (Thu) 17:30-18:15 Room P/(Motenashi Dome) Room P

---

### P-1197
### The benefit and limitation of Comprehensive Cancer Genome Panel Testing in Rural Areas

[Speaker]Yoshinaga Okugawa (Dept of Genomic Medicine, Mie University Hospital／Dept of Gastrointestinal and Pediatric Surgery) [Co-author]Takahito Kitajima (Dept of Genomic Medicine, Mie University Hospital／Dept of Gastrointestinal and Pediatric Surgery)、Tadanobu Shimura (Dept of Gastrointestinal and Pediatric Surgery)、Yuji Toiyama (Dept of Gastrointestinal and Pediatric Surgery)

Purpose:CGP should be regarded as a medical process, not merely a test. We evaluated its clinical utility, limitations, and quality management.Methods:We retrospectively analyzed 1555 patients who underwent insurance-covered CGP at our center.Results:CGP was performed using tissue-based(1185) or liquid-based(370) panels. The success rate was 95.3%, higher in blood-(98.1%) than tissue-CGP(94.4%, P=0.004). Drug accessibility was 13.8%, mostly to approved drugs. Patients receiving expert-recommended therapies had significantly better post-CGP survival(P<0.0001), with high PFS ratio(39.6%). Tissue CGP showed higher drug access than blood(14.7%, 11%, P=0.07). In pancreatic cancer, KRAS mutations were more often detected by tissue CGP(96.3%) than blood(54.2%, P<0.001), likely due to low ctDNA. Central review improved KRAS detection from 44.4% to 60%. Germline findings confirmed hereditary cancer syndromes in 2.6%. A satisfaction survey of 255 patients and referring doctors showed that drug inaccessibility tended to reduce satisfaction(P=0.07, 0.03), while secondary findings had no impact(P=0.91).Conclusion:CGP must be understood in context and delivered through expert-guided care.

[Disclosure of COI by Responsible researcher] Lead presenter is the Responsible researcher.
[Lead presenter: Yoshinga Okugawa] ■Lecture fee: 中外製薬、株式会社ツムラ

▲TOP

### [P-1198] I-P12-3
### Genomic diagnostics / Panel sequencing

2025 / 9 / 25 (Thu) 17:30-18:15 Room P/(Motenashi Dome) Room P

---

### P-1198
### Novel pathogenic variants of HBOC that were not reported by a genetic test, BRACAnalysis covered by National Insurance

[Speaker]Hidetaka Eguchi (Intractable Disease Res Ctr, Juntendo Univ Grad Sch Med) [Co-author]Sayaka Yoshioka (Dept Clin Genet, Juntendo Univ Grad Sch Med)、Ayumi Abe (Clin Genet Ctr, Toranomon Hospital)、Yoko Kobayashi (Clin Genet Ctr, Toranomon Hospital／Dept Breast Endocrine Surg, Toranomon Hospital)、Satoko Nakano (Dept Breast Surgery, Kawaguchi Municipal Med Ctr)、Takeshi Miyamoto (Dept Breast Oncol, Gunma Prefectural Cancer Ctr／Dept Clin Genet, Gunma Prefectural Cancer Ctr)、Mayu Aoki (Dept Breast Oncol, Gunma Prefectural Cancer Ctr／Div Breast Endocrine Surg, Grad Sch Med, Gunma Univ)、Hidetaka Kawabata (Clin Genet Ctr, Toranomon Hospital／Dept Breast Endocrine Surg, Toranomon Hospital)、Yasushi Okazaki (Intractable Disease Res Ctr, Juntendo Univ Grad Sch Med)、Masami Arai (Intractable Disease Res Ctr, Juntendo Univ Grad Sch Med／Dept Clin Genet, Juntendo Univ Grad Sch Med)

BRACAnalysis, a genetic test for hereditary breast and ovarian cancer covered by National Insurance, occasionally provides reports with "Inconclusive" or "Variant of Uncertain Significance (VUS)" results. We conducted additional genetic tests and enabled obtaining evidence to change the pathogenicity of such variants. The first female case suffered from left breast cancer at age 55. BRACAnalysis reported "inconclusive" with a possible gene rearrangement around exons 16-18 of ***BRCA1***. By conducting RNA sequencing using cultured T cells from her peripheral blood demonstrated unusual ***BRCA1*** transcripts retain sequence of unspliced intron 16 and fusion transcript of ***BRCA1*** and ***VAT1***, simultaneously. Further genetic analysis revealed integration of a transposon SVA in intron 16 of BRCA1. We also found 2 cases of altered splicing in patients with VUS that are outside of canonical ±1/2 positions, such as ***BRCA2***:c.7007+5G>C and ***BRCA2***:c.8487G>A. The former skipped exons 12 and 13 resulting in frameshift lacking C-terminus of BRCA2, while the latter skipped exon 19 giving inframe deletion of a domain important for DNA binding. These results highlighted importance of additional genetic tests.

[Disclosure of COI by Responsible researcher] Lead presenter is the Responsible researcher.
[Lead presenter: Hidetaka Eguchi] We have no financial relationships to disclose.

### [P-1208] I-P13-2
### Drug resistance (2)

2025 / 9 / 25 (Thu) 17:30-18:15 Room P/(Motenashi Dome) Room P

### P-1208
### Gene alteration of SKI gene affects ALK-TKI sensitivity in ALK-positive lung cancer

[Speaker]Hiroto Azuma (Div. Exp. Chemother., Cancer Chemother. Ctr., JFCR／Dept. CBMS, Grad. Sch. Front. Sci., The Univ. of Tokyo) [Co-author]Sumie Koike (Div. Exp. Chemother., Cancer Chemother. Ctr., JFCR)、Ken Uchibori (Div. Exp. Chemother., Cancer Chemother. Ctr., JFCR／Dept. Thoracic Med. Oncology, Cancer Inst. Hosp., JFCR)、Makoto Nishio (Dept. Thoracic Med. Oncology, Cancer Inst. Hosp., JFCR)、Ryohei Katayama (Div. Exp. Chemother., Cancer Chemother. Ctr., JFCR／Dept. CBMS, Grad. Sch. Front. Sci., The Univ. of Tokyo)

***ALK***-rearrangement (ALK+) is observed in 3-5% of non-small cell lung cancer (NSCLC), and ALK tyrosine kinase inhibitors (TKIs) largely contributed their prognosis. However, duration of response for ALK-TKIs varies due to multiple mechanisms such as the existence of co-mutation, or heterogeneity. In this study, we evaluated characteristics and genetic alterations of ALK+ NSCLC patients' samples by RNA sequencing. As a result, we found a gene rearrangement in ***SKI***, a protein homolog of avian sarcoma viral (***v-ski***) oncogene functioning as a TGF-β signaling modulator. In the SKI rearranged ALK+ cells, truncated SKI protein expressed, which is predicted to be loss of function mutation. To examine the importance of SKI protein, ALK-TKIs sensitivity was examined truncated type of SKI or SKI knocked-out (KO) cells or SKI overexpressed cells. SKI rearrangement-positive ALK+ cells showed greater sensitivity to ALK-TKIs. In contrast, full-length SKI expressed ALK+ cells induced partial resistance to ALK-TKIs. In addition, SKI KO cells markedly increased BIM expression, and showed increased apoptosis.
These findings suggest that loss of SKI protein may influence ALK-TKIs sensitivity in ALK+ NSCLC.

[Disclosure of COI by Responsible researcher] Lead presenter is not the Responsible researcher.
[Lead presenter: Hiroto Azuma] We have no financial relationships to disclose.
[Principal Researcher: Ryohei Katayama] ■employment by a company related to the presentation: 中外製薬、TOPPAN、UBE、BML　■Patent fee: 栄研化学

### [P-1209] I-P13-2
### Drug resistance (2)

2025 / 9 / 25 (Thu) 17:30-18:15 Room P/(Motenashi Dome) Room P

---

### P-1209
### Analyses of ROS1-rearranged lung adenocarcinoma cell lines with acquired resistance to entrectinib and repotrectinib

[Speaker]Yusuke Shima (Kyoto Univ. Grad. Sch. of Med.) [Co-author]Tomoko Funazo (Kyoto Univ. Grad. Sch. of Med.)、Hiroaki Ozasa (Kyoto Univ. Grad. Sch. of Med.)、Masahiro Oi (Kyoto Univ. Grad. Sch. of Med.)、Keiichiro Suminaga (Kyoto Univ. Grad. Sch. of Med.)、Kentaro Hashimoto (Kyoto Univ. Grad. Sch. of Med.)、Hiroshi(Kyoto Univ.) Yoshida (Kyoto Univ. Grad. Sch. of Med.)、Kazutaka Hosoya (Kyoto Univ. Grad. Sch. of Med.)、Hitomi Ajimizu (Kyoto Univ. Grad. Sch. of Med.)、Takashi Nomizo (Kyoto Univ. Grad. Sch. of Med.)、Hironori Yoshida (Kyoto Univ. Grad. Sch. of Med.)、Toyohiro Hirai (Kyoto Univ. Grad. Sch. of Med.)

Lung adenocarcinomas harboring rearrangements of the c-ros oncogene 1 (ROS1), a rare driver mutation, are typically treated with tyrosine kinase inhibitors (TKIs), such as entrectinib or repotrectinib. However, disease recurrence due to acquired resistance is common. The most well-characterized mechanism of resistance is the G2032R point mutation developed during TKI therapy. Nevertheless, alternative mechanisms of resistance remain unclarified. In this study, we established two paired cell lines, KTOR73 and KTOR74, derived from a patient with ROS1-rearranged lung adenocarcinoma. These were obtained at the time of disease progression following initial treatment with entrectinib and subsequent treatment with repotrectinib, respectively. KTOR73 and KTOR74 were further subdivived into a few sublines based on their morphological characters and differential drug sensitivities. Notably, none of these harbored the G2032R mutation. These results implicated the existence of intratumoral heterogeneities and the involvement of non-G2032R mutation or off-target resistance mechanism. In this presentation, we will discuss the potential alternative resistance mechanism based on our findings.

[Disclosure of COI by Responsible researcher] Lead presenter is not the Responsible researcher.
[Lead presenter: Yusuke Shima] We have no financial relationships to disclose.
[Principal Researcher: Ozasa Hiroaki] We have no financial relationships to disclose.

### [P-1210] I-P13-2
### Drug resistance (2)

2025 / 9 / 25 (Thu) 17:30-18:15 Room P/(Motenashi Dome) Room P

---

### P-1210
### STAT3 inhibition overcomes apoptotic resistance to KRAS-G12C inhibitors

[Speaker]Naohiro Yanagimura (Department of Respiratory Medicine and Infectious Diseases, Niigata University) [Co-author]Shinji Takeuchi (Department of Medical Oncology, Kanazawa University Hospital)、Seiji Yano (Department of Respiratory Medicine, Kanazawa University)、Kohei Kushiro (Department of Respiratory Medicine and Infectious Diseases, Niigata University)、Ryo Suzuki (Department of Respiratory Medicine and Infectious Diseases, Niigata University)、Miyuki Sato (Department of Respiratory Medicine and Infectious Diseases, Niigata University)、Tomohiro Tanaka (Department of Respiratory Medicine and Infectious Diseases, Niigata University)、Yu Saida (Department of Respiratory Medicine and Infectious Diseases, Niigata University)、Satoshi Watanabe (Department of Respiratory Medicine and Infectious Diseases, Niigata University)、Toshiaki Kikuchi (Department of Respiratory Medicine and Infectious Diseases, Niigata University)

Patients with KRASG12C-mutant lung cancer treated with KRAS-G12C inhibitors rarely achieve complete responses, as residual tumors often relapse with heterogeneous, resistant phenotypes. This highlights the need for novel therapeutic strategies to eliminate residual disease and prevent recurrence. In this study, we investigated the molecular mechanisms underlying treatment-induced adaptive cancer cell survival that contributes to residual tumor persistence following initial KRAS-G12C inhibitor therapy. Our findings demonstrate that the adaptive survival of KRASG12C-mutant lung cancer cells is predominantly dependent on STAT3 activity. Inhibition of STAT3 effectively suppressed this adaptive survival by enhancing KRAS-G12C inhibitor-induced apoptosis. This effect was mediated through transcriptional regulation of the anti-apoptotic factors MCL-1 and BCL-XL. In xenograft models, combination therapy significantly suppressed tumor growth. Collectively, these findings offer new insights into rational combination strategies for improving therapeutic outcomes in patients with KRASG12C-mutant lung cancer.

[Disclosure of COI by Responsible researcher] Lead presenter is the Responsible researcher.
[Lead presenter: Naohiro Yanagimura] We have no financial relationships to disclose.

### [P-1211] I-P13-2
### Drug resistance (2)

2025 / 9 / 25 (Thu) 17:30-18:15 Room P/(Motenashi Dome) Room P

### P-1211
### Approaches to Elucidate Mechanisms of Resistance to KRAS Inhibitors

[Speaker]Xin Lu (Department of Tumor Pathology, Nagoya University Graduate School of Medicine) [Co-author]Tadashi Iida (Department of Gastroenterology and Hepatology Nagoya University)、Hanwei Zhang (Department of Tumor Pathology, Nagoya University Graduate School of Medicine)、Takahiro Sugie (Department of Tumor Pathology, Nagoya University Graduate School of Medicine)、Takayuki Owaki (Department of Tumor Pathology, Nagoya University Graduate School of Medicine)、Atsushi Enomoto (Department of Tumor Pathology, Nagoya University Graduate School of Medicine)

Approaches to Elucidate Mechanisms of Resistance to KRAS InhibitorsBackgroundNext-generation inhibitors targeting non-G12C mutations (e.g., G12D, G12V) are also under investigation. However, resistance to KRAS inhibitor monotherapy remains a critical challenge. MethodsMouse tumor models harboring KRAS G12C or G12D mutations were used to evaluate changes in immune cell infiltration and fibrosis following KRAS inhibitor treatment.Cells were subcutaneously implanted into 6-8-week-old female C57BL/6J mice (n=5/group). Tumors were harvested on day 28 for analysis. Immune infiltration (CD3, CD8, CD163) was assessed via immunohistochemistry, and fibrosis via Sirius Red staining.ResultsCD8$^{+}$ T cells increased in G12C (p=0.008) and G12D (p=0.012) groups, as did CD3$^{+}$ T cells (G12C: p=0.005; G12D: p=0.018). CD163$^{+}$ macrophages showed no significant changes (p>0.05). Sirius Red staining revealed elevated fibrosis in both treatment groups (G12C: p=0.003; G12D: p=0.001).DiscussionIncreased CD8$^{+}$ T cells suggest KRAS inhibitors may enhance antitumor immunity. However, CD3$^{+}$ T cell expansion-potentially including immunosuppressive CD4$^{+}$ subsets-could contribute to resistance.

[Disclosure of COI by Responsible researcher] Lead presenter is not the Responsible researcher.
[Lead presenter: Xin Lu] We have no financial relationships to disclose.
[Principal Researcher: Atsushi Enomoto] We have no financial relationships to disclose.

### [P-1212] I-P13-2
### Drug resistance (2)

2025 / 9 / 25 (Thu) 17:30-18:15 Room P/(Motenashi Dome) Room P

### P-1212
### KRAS inhibitor tolerant cancer cells utilize glutamine metabolism for survival

[Speaker]Hiroki Furukawa (Grad. Sch. Pharm. Sci., Chiba Univ.) [Co-author]Taichi Oshima (Grad. Sch. Pharm. Sci., Chiba Univ.)、Shigeki Aoki (Grad. Sch. Pharm. Sci., Chiba Univ.)

(Purpose) Although KRAS-targeted therapies have advanced, resistance remains a major challenge. "Drug-tolerant cells," which initially survive drug exposure without resistance mutations, are increasingly recognized as a key obstacle. This study aims to elucidate the mechanisms of KRAS inhibitor tolerance and propose strategies to overcome it. (Methods) KRAS G12C-mutant pancreatic (MiaPaCa2) and lung (H358) cancer cells were treated with the KRAS G12C inhibitor Sotorasib. Cell viability was assessed by MTT assay. Mitochondrial membrane potential and glutamate dehydrogenase (GDH) expression were evaluated by JC-1 staining and western blotting, respectively. (Results and Discussion) After one week of Sotorasib exposure, most cells survived, showing enhanced mitochondrial activity and increased GDH expression. Combined treatment with Sotorasib and a GDH inhibitor induced cell death, which was rescued by $\alpha$-ketoglutarate supplementation. These findings suggest that drug-tolerant cells depend on glutamine metabolism-driven mitochondrial activity, and that co-targeting KRAS signaling and glutamine metabolism may overcome drug tolerance.

[Disclosure of COI by Responsible researcher] Lead presenter is the Responsible researcher.
[Lead presenter: Hiroki Furukawa] We have no financial relationships to disclose.

### [P-1213] I-P13-2
### Drug resistance (2)

2025 / 9 / 25 (Thu) 17:30-18:15 Room P/(Motenashi Dome) Room P

---

### P-1213
### Genome-wide CRISPR-Cas9 screen identifies genes involved in acquired KRAS G12C inhibitor resistance.

[Speaker]Ducanh Vu (Dept. Can. Biol., Inst. Biomed. Sci., Kansai Med. Univ.) [Co-author]Shiori Mori (Dept. Can. Biol., Inst. Biomed. Sci., Kansai Med. Univ.)、Noritaka Tanaka (Dept. Can. Biol., Inst. Biomed. Sci., Kansai Med. Univ.)、Yoshiki Yasukochi (Dept. Genome. Analysis., Inst. Biomed. Sci., Kansai Med. Univ.)、Kouichiro Higasa (Dept. Genome. Analysis., Inst. Biomed. Sci., Kansai Med. Univ.)、Takeharu Sakamoto (Dept. Can. Biol., Inst. Biomed. Sci., Kansai Med. Univ.)

Although KRAS G12C inhibitors demonstrate clinical efficacy in treating patients with KRAS G12C-mutated solid tumors, their effectiveness is often limited by multiple mechanisms of resistance. Therefore, there is a critical need to elucidate the underlying adaptive processes and develop novel therapeutic strategies. To identify potential resistance mechanisms, we established sotorasib-resistant cell lines and performed a genome-wide CRISPR-Cas9 loss-of-function screen. This analysis revealed the loss of multiple components of pathway X, including gene Y. Our initial in vitro results showed that depletion of gene Y using siRNAs and a specific pathway X inhibitor restored the sensitivity of resistant cells to sotorasib. These findings suggest that gene Y may serve as a promising target for combination therapies aimed at overcoming acquired resistance to sotorasib.

[Disclosure of COI by Responsible researcher] Lead presenter is not the Responsible researcher.
[Lead presenter: Ducanh Vu] We have no financial relationships to disclose.
[Principal Researcher: Takeharu Sakamoto] We have no financial relationships to disclose.

▲TOP

### [P-1214] I-P13-2
### Drug resistance (2)

2025 / 9 / 25 (Thu) 17:30-18:15 Room P/(Motenashi Dome) Room P

### P-1214
### TDO2 plays a role in mediating Lenvatinib resistance in hepatocellular carcinoma.

[Speaker]Taiki Hosoi (Faculty of Pharmaceutical Sciences Hokkaido Univ) [Co-author]Osamu Maehara (Faculty of Pharmaceutical Sciences Hokkaido Univ)、An Tsuji (Faculty of Pharmaceutical Sciences Hokkaido Univ)、Masatsugu Ohara (Graduate School of Medicine Hokkaido Univ)、Goki Suda (Graduate School of Medicine Hokkaido Univ)、Mitsuteru Natsuizaka (Graduate School of Medicine Hokkaido Univ)、Naoya Sakamoto (Graduate School of Medicine Hokkaido Univ)、Shunsuke Ohnishi (Faculty of Pharmaceutical Sciences Hokkaido Univ)

Lenvatinib, a multi-kinase inhibitor, is widely used as the first-line treatment for unresectable hepatocellular carcinoma (HCC). However, some patients develop resistance to Lenvatinib. The mechanism underlying this Lenvatinib resistance (LenR) remains unclear. This study aims to elucidate the mechanism of LenR and improve the therapeutic efficacy of Lenvatinib in HCC. We established Huh7-LenR cells through long-term exposure to Lenvatinib and identified genes with altered expression levels associated with the development of LenR using RNA sequencing. Additionally, we investigated the effects of combining Lenvatinib with inhibitors targeting potential candidate genes identified through RNA sequencing. RNA sequencing revealed that TDO2 (Tryptophan 2,3-dioxygenase) is upregulated in Huh7-LenR cells. Furthermore, the combined administration of Lenvatinib and a TDO2 inhibitor significantly reduced the viability of Huh7-LenR cells. These findings suggest that increased expression of TDO2 in HCC may contribute to Lenvatinib resistance, and targeting TDO2 could be a potential strategy to overcome LenR.

[Disclosure of COI by Responsible researcher] Lead presenter is not the Responsible researcher.
[Lead presenter: Taiki Hosoi] We have no financial relationships to disclose.
[Principal Researcher: Shunsuke Ohnishi] We have no financial relationships to disclose.

### [P-1215] I-P13-2
### Drug resistance (2)

2025 / 9 / 25 (Thu) 17:30-18:15 Room P/(Motenashi Dome) Room P

---

### P-1215
### Genome-wide CRISPR-Cas9 screen identifies genes conferring Lenvatinib resistance in hepatocellular carcinoma

[Speaker]Shuang Zhou (Molecular Oncology, Graduate School of Medical Sciences, NCU) [Co-author]Yuichi Abe (Immunoproteomics laboratory, iGCORE, Gifu University)、Hisanori Isomura (Molecular Oncology, Graduate School of Medical Sciences, NCU)、Miyako Tanaka (Research Institute of Environmental Medicine, Nagoya University)、Rui Yamaguchi (Division of Cancer Systems Biology, ACC)、Takayoshi Suganami (Research Institute of Environmental Medicine, Nagoya University)、Ayumu Taguchi (Molecular Oncology, Graduate School of Medical Sciences, NCU)

Resistance to tyrosine kinase inhibitors (TKIs) remains a major therapeutic obstacle in advanced hepatocellular carcinoma (HCC). To address this challenge, we established a novel murine HCC cell line derived from melanocortin-4 receptor-deficient mice fed a high-fat diet, which developed non-alcoholic steatohepatitis (NASH) and subsequent HCC, recapitulating key features of human NASH-driven hepatocarcinogenesis. The resulting HCC cells exhibited marked resistance to lenvatinib. To systematically identify genes driving this resistance, we conducted a genome-wide CRISPR-Cas9 knockout screen. The screen revealed that loss of PCIF1, an RNA methyltransferase, induced sensitivity to lenvatinib in HCC cells. Multi-omics analyses implicated PCIF1 in regulating nucleic acid metabolism enzymes, potentially through the transcription factor MYBL2. Strikingly, pharmacological inhibition of one such enzyme synergized with lenvatinib and other TKIs, suppressing cell proliferation across multiple cancer types. These findings provide novel insights into the molecular basis of lenvatinib resistance and highlight therapeutic vulnerabilities that may be exploited to overcome TKI resistance in HCC.

[Disclosure of COI by Responsible researcher] Lead presenter is not the Responsible researcher.
[Lead presenter: Shuang Zhou] ■Research funds from company: AMED　橋渡し研究プログラム
[Principal Researcher: Ayumu Taguchi] ■Research expenses: 小野薬品工業株式会社

### [P-1216] I-P13-2
### Drug resistance (2)

2025 / 9 / 25 (Thu) 17:30-18:15 Room P/(Motenashi Dome) Room P

---

### P-1216
### Exploring the role of YAP in BRAF inhibitors resistance in Melanoma

[Speaker]Min Han (Dep. Clinical Oncology, Juntendo University Graduate School of Medicine) [Co-author]Shigeaki Sunada (Dep. Clinical Oncology, Juntendo University Graduate School of Medicine)、Qianqian Guo (Dep. Clinical Oncology, Juntendo University Graduate School of Medicine／LC. Development and Research of Cancer Medicine, Juntendo University)、Jiwen Liu (Dep. Clinical Oncology, Juntendo University Graduate School of Medicine)、Toshiaki Ishikawa (Dep. Clinical Oncology, Juntendo University Graduate School of Medicine)、Shunsuke Kato (Dep. Clinical Oncology, Juntendo University Graduate School of Medicine)

YAP is an important component of the Hippo pathway, and its overexpression was reported to be associated with tumor progression, metastasis, and drug resistance. YAP acts as a transcriptional co-activator, promoting gene expression through interactions with DNA-binding transcription factors such as TEAD. However, the precise molecular mechanisms underlying drug resistance are not fully understood. We aimed to investigate the correlation between the YAP/TAZ pathway and resistance to BRAF inhibitors. We introduced a YAP/TAZ-TEAD reporter plasmid containing TEAD-binding sequences and mStrawberry into A375, a melanoma cell line, and subsequently established Dabrafenib-resistant cell lines. Compared to stable cells, the resistant cells exhibited increased YAP expression, however reporter activity was limited, it suggests that YAP may interact with transcription factors other than TEAD in resistant cells.Further studies are needed to identify these factors and to explore the potential of targeting these interactions as a strategy to overcome resistance. We are currently investigating the molecular mechanisms underlying this resistance.

[Disclosure of COI by Responsible researcher] Lead presenter is not the Responsible researcher.
[Lead presenter: Min Han] ■Research funds from company: 公的研究費(科学研究費: 24K10411)
[Principal Researcher: Shunsuke Kato] ■Research funds from company: 公的研究費(科学研究費: 24K10411)

▲TOP

Poster Session

### [P-1226] I-P14-1
### Natural anticancer compounds (1)

2025 / 9 / 25 (Thu) 17:30-18:15 Room P/(Motenashi Dome) Room P

---

### P-1226
### Harnessing Nature's Arsenal: Targeting Aggressive Cancers with Bioactive Natural Compounds

[Speaker]Wan-Chi Tsai (Dept. of Med. Lab. Sci. & Biotechnology, KMU／Drug Development & Value Creation Res. Ctr., KMU)

Natural products represent promising anticancer agents due to their diverse biological activities and unique mechanisms. Our recent studies highlight bioactive compounds from terrestrial plants and marine organisms effectively targeting aggressive cancers such as pancreatic ductal adenocarcinoma and non-small cell lung cancer. These compounds exhibit potent cytotoxicity, inducing apoptosis via oxidative stress, mitochondrial dysfunction, DNA damage, and disruption of critical signaling pathways (JAK2/STAT3, AKT, ERK). They effectively halt cancer cell proliferation by inducing cell-cycle arrest at key checkpoints, including G1, S, and G2/M phases, thereby interfering with tumor progression. Additionally, these natural agents significantly inhibit cancer cell migration/invasion, and display potent anti-angiogenic activity. Molecular targets, including tubulin polymerization and DNA topoisomerase I activity, have been identified, providing deeper mechanistic insight. Preclinical in vitro and in vivo models further validate their therapeutic potential, collectively underscoring natural products as promising candidates capable of addressing multiple cancer hallmarks simultaneously.

[Disclosure of COI by Responsible researcher] Lead presenter is the Responsible researcher.
[Lead presenter: Wan-Chi Tsai] We have no financial relationships to disclose.

▲TOP

### [P-1227] I-P14-1
### Natural anticancer compounds (1)

2025 / 9 / 25 (Thu) 17:30-18:15 Room P/(Motenashi Dome) Room P

### P-1227
### Withdrawn

No Abstract

### [P-1228] I-P14-1
### Natural anticancer compounds (1)

2025 / 9 / 25 (Thu) 17:30-18:15 Room P/(Motenashi Dome) Room P

---

### P-1228
### Withdrawn

No Abstract

### [P-1229] I-P14-1
### Natural anticancer compounds (1)

2025 / 9 / 25 (Thu) 17:30-18:15 Room P/(Motenashi Dome) Room P

### P-1229
### Withdrawn

No Abstract

### [P-1230] I-P14-1
### Natural anticancer compounds (1)

2025 / 9 / 25 (Thu) 17:30-18:15 Room P/(Motenashi Dome) Room P

---

### P-1230
### Senna garrettiana extract inhibits hepatocellular carcinoma through antiproliferation and apoptosis induction

[Speaker]Thitaporn Kompangjan (Dept. of Biochem. & Mol. Biol., Thammasat Univ.) [Co-author]Worawat Surarit (Dept. of Biotechnology, Thammasat Univ.)、Sumalee Panthong (Dept. of Applied Thai Traditional Med., Thammasat Univ.)、Unchalee Thonsri (Dept. of Preclinical. Sci. (Biochem), Thammasat Univ.)、Pintusorn Hansakul (Dept. of Preclinical. Sci. (Biochem), Thammasat Univ.)

Hepatocellular carcinoma is the 3rd deadliest cancer in the world. Plant-based phenolics have potential for cancer prevention and treatment with reduced adverse effects. Combined phenolics or whole phenolic extracts synergistically suppress cancer. Senna garrettiana extract (SGE) is traditionally employed to treat cancer and is reported to contain various phenolics. This study aimed to assess total contents of phenolics and flavonoids and anticancer activities of SGE. 95% ethanol SGE was prepared by maceration. Total contents of phenolics and flavonoids were quantified. Anti-proliferative, cytotoxic, anti-colony forming, and apoptosis induction effects on liver cancer HepG2 cells were determined. SGE contained high contents of phenolics and flavonoids and exhibited selective IC50 regarding anti-proliferation and cytotoxicity against HepG2 cells versus normal BNL CL.2 cells in a dose- and time-dependent manner. SGE inhibited colony formation of HepG2 in a dose- and time-dependent manner, except doses at IC25 and IC100 at 24 h. IC25, IC50 and IC75 of SGE induced apoptosis in the same manner. SGE showed anti-proliferative, anti-colony-forming, cytotoxic and apoptotic effects.

[Disclosure of COI by Responsible researcher] Lead presenter is the Responsible researcher.
[Lead presenter: Thitaporn Kompangjan] We have no financial relationships to disclose.

### [P-1231] I-P14-1
### Natural anticancer compounds (1)

2025 / 9 / 25 (Thu) 17:30-18:15 Room P/(Motenashi Dome) Room P

---

### P-1231
### E413-YJ, a Flavonoid Phytochemical, Exhibits Anti-Leukemic Effects in Human AML Cells and Zebrafish Xenograft Models

[Speaker]Trung Q. Phan (Inst. of Medical Sciences, Tzu Chi Univ.) [Co-author]Pei-Yi Chen (Lab. of Medical Genetics, Tzu Chi Hospital／Dept. of Molecular Biology and Human Genetics, Tzu Chi Univ.)、Jui-Hung Yen (Inst. of Medical Sciences, Tzu Chi Univ.／Dept. of Molecular Biology and Human Genetics, Tzu Chi Univ.)

Acute myeloid leukemia (AML) is a lethal hematologic malignancy, and resistance to cytarabine remains a major clinical challenge. E413-YJ (YJ), a flavonoid derived from Artemisia species, has demonstrated anticancer properties. This study aimed to investigate the antileukemic effects of YJ and its underlying mechanisms in human AML cells and zebrafish xenografts. YJ exhibited no cytotoxicity in normal PBMCs but significantly reduced the viability of human AML cells, with the MV4-11 cell line being the most sensitive. In both MV4-11 and cytarabine-resistant MV4-11 (MV4-11-CR) cells, YJ inhibited proliferation, induced cell cycle arrest, and promoted monocytic differentiation. In vivo, YJ markedly suppressed leukemia cell expansion in zebrafish xenograft models. Transcriptomic analysis revealed that YJ-regulated differentially expressed genes are associated with key biological processes and KEGG pathways implicated in cancer progression. Additionally, YJ synergized with standard chemotherapeutic agents to reduce the viability of MV4-11-CR cells. These findings highlight YJ as a promising therapeutic candidate for overcoming drug resistance and improving treatment outcomes in AML.

[Disclosure of COI by Responsible researcher] Lead presenter is not the Responsible researcher.
[Lead presenter: Trung Q. Phan] We have no financial relationships to disclose.
[Principal Researcher: Jui-Hung Yen] We have no financial relationships to disclose.

### [P-1232] I-P14-1
### Natural anticancer compounds (1)

2025 / 9 / 25 (Thu) 17:30-18:15 Room P/(Motenashi Dome) Room P

---

### P-1232
### Capsaicin: A Natural Therapeutic Agent Targeting Epstein-Barr Virus-Associated Epithelial Cancers

[Speaker]Chukkris Heawchaiyaphum (Dept. of Microbiol, Fac. of Med., Khon Kaen Univ.／HEC, Fac. of Med., Khon Kaen Univ.) [Co-author]Nutchanat Chatchawankanpanich (Dept. of Biotech, Fac. of Sci. & Tech., Thammasat Univ.)、Chamsai Pientong (Dept. of Microbiol, Fac. of Med., Khon Kaen Univ.／HEC, Fac. of Med., Khon Kaen Univ.)

Capsaicin, the primary bioactive compound in chili, exhibits diverse pharmacological activities, including anticancer potential. Epstein-Barr virus (EBV) infection is strongly implicated in the pathogenesis of epithelial malignancies such as gastric cancer (GC) and nasopharyngeal carcinoma (NPC), making EBV a relevant oncotherapeutic target. This study evaluated the anticancer effects of capsaicin on EBV-positive GC and NPC cells. Capsaicin significantly inhibited cell proliferation and migration, as demonstrated by CCK-8, wound healing, and transwell assays. Drug combination analysis showed that capsaicin alone exerted stronger cytotoxic effects than cisplatin or etoposide combinations. Furthermore, capsaicin suppressed EBV lytic and latent gene expression and reduced virion production. Proteomic analysis identified potential molecular mechanisms. Molecular docking predicted direct binding between capsaicin and viral proteins, LMP1, EBNA1, and Zta, suggesting functional inhibition. These findings highlight capsaicin's potential as a natural therapeutic agent for EBV-associated cancers by targeting viral persistence/reactivation and tumor progression.

[Disclosure of COI by Responsible researcher] Lead presenter is the Responsible researcher.
[Lead presenter: Chukkris Heawchaiyaphum] We have no financial relationships to disclose.

▲TOP

### [P-1233] I-P14-1
### Natural anticancer compounds (1)

2025 / 9 / 25 (Thu) 17:30-18:15 Room P/(Motenashi Dome) Room P

### P-1233
### Withdrawn

No Abstract

Poster Session

### [P-1234] I-P14-1
### Natural anticancer compounds (1)

2025 / 9 / 25 (Thu) 17:30-18:15 Room P/(Motenashi Dome) Room P

### P-1234
### Antitumor Effect of the Natural Compound Calebin A in Pancreatic Cance

[Speaker]Yuki Eguchi (Nagoya City Univ.) [Co-author]Yoichi Matsuo (Nagoya City Univ. East Med. Center)、Masaki Ishida (Nagoya City Univ.)、Yuriko Uehara (Nagoya City Univ.)、Saburo Sugita (Nagoya City Univ.)、Yuki Denda (Nagoya City Univ.)、Keisuke Nonoyama (Nagoya City Univ.)、Hiromichi Murase (Nagoya City Univ.)、Tomokatu Kato (Nagoya City Univ.)、Kenta Saito (Nagoya City Univ.)、Takafumi Sato (Nagoya City Univ.)、Yushi Yamakawa (Nagoya City Univ.)、Hiroyuki Sagawa (Nagoya City Univ.)、Ryo Ogawa (Nagoya City Univ.)、Shuji Takiguchi (Nagoya City Univ.)

Background:Pancreatic cancer (PaCa) is rapidly increasing, but treatment options remain limited. Gemcitabine (Gem) is a key drug, yet its efficacy declines over time. Gem resistance is a major hurdle, and its mechanism is unclear. Clarifying it may improve PaCa therapy.Methods: We established Gem-resistant PaCa cell lines and confirmed enhanced NF-$\kappa$B activity. Based on this, we focused on Calebin A, a turmeric-derived compound with anti-inflammatory properties. In collaboration with Sabinsa Japan, we examined its anti-tumor effect on PaCa.Results: Calebin A (10μM) significantly inhibited NF-$\kappa$B activity without affecting cell growth. Invasion assays showed Calebin A suppressed invasiveness. VEGF and IL-8 levels, elevated in resistant cells, were reduced by Calebin A. Tube formation in endothelial cells, enhanced by TNF-$\alpha$, was also suppressed by Calebin A.Conclusion: Calebin A may serve as a novel treatment for Gem-resistant PaCa. Further in vivo studies are planned. These findings suggest potential for a breakthrough in PaCa therapy.

[Disclosure of COI by Responsible researcher] Lead presenter is not the Responsible researcher.
[Lead presenter: Yuki Eguchi] We have no financial relationships to disclose.
[Principal Researcher: Masaki Ishida] We have no financial relationships to disclose.

▲TOP

### [P-1243] I-P14-3
### Synthetic anticancer compounds (1)

2025 / 9 / 25 (Thu) 17:30-18:15 Room P/(Motenashi Dome) Room P

---

### P-1243
### Inhibition of cell-intrinsic NF-κB activity and metastatic abilities of breast cancer by curcumin analogs

[Speaker]Sana Jabbar (Inst. of Natural Med., Univ. of Toyama) [Co-author]Sisca Ucche (Inst. of Natural Med., Univ. of Toyama／Faculty of Pharm., Univ. of Gadjah Mada)、Yue Zhou (Dept. Cancer Cell Biol., Univ. of Toyama)、Muhammad Irshad Farooq (Inst. of Natural Med., Univ. of Toyama)、Suthasinee Seephan (Inst. of Natural Med., Univ. of Toyama)、Yoshihiro Hayakawa (Inst. of Natural Med., Univ. of Toyama)

In breast cancer cells, the activity of inflammatory signal molecule NF-κB can be intrinsically elevated and regulates their growth, survival, and metastatic abilities. Curcumin, a natural compound derived from Curcuma Longa, has been extensively studied on its anti-cancer properties, particularly its inhibitory activity on NF-κB, although its systemic bioavailability is known to be extremely low. In this study, we investigated the effects of various curcumin analogs on the cell-intrinsic activity of NF-κB in breast cancer cells. Among 28 curcumin analogs, the 3,5-bis (2-ethoxy benzylidene) piperidine-4-one analog E145 exhibited potent NF-κB inhibitory activity. Subsequently, E145 exerted anti-proliferative and anti-metastatic effects in breast cancer cell lines in vitro. Mechanistically, E145 inhibited the expression of downstream molecules of NF-κB, such as VEGF-B, IL-6, MMP-9, and IL-1β in breast cancer cells. Collectively, this study demonstrates that E145 can be a potential lead compound to inhibit the cell-intrinsic NF-κB activity to suppress growth and metastasis in breast cancer cells.

[Disclosure of COI by Responsible researcher] Lead presenter is not the Responsible researcher.
[Lead presenter: Sana Jabbar] We have no financial relationships to disclose.
[Principal Researcher: Yoshihiro Hayakawa] We have no financial relationships to disclose.

### [P-1244] I-P14-3
### Synthetic anticancer compounds (1)

2025 / 9 / 25 (Thu) 17:30-18:15 Room P/(Motenashi Dome) Room P

---

### P-1244
### A potential indole compound for Glioblastoma therapy

[Speaker]Peter Lai (Dept. of Life Sci., NTNU) [Co-author]Ting-Ru Mu (Dept. of Life Sci., NTNU)、Ting-Wei Hsu (Dept. of Life Sci., NTNU)、Yun-Ju Lai (Dept. of Life Sci., NTNU)

Glioblastoma (GBM) is the most common and aggressive primary brain tumor, with current treatments offering limited effectiveness. Cancer stem cells (CSCs) contribute to this challenge due to their resistance to anti-cancer drugs and ability to regenerate tumors. In this study, we aim to identify a novel drug effective against glioblastoma stem cells (GSCs). Gavin 08 (G08), an indole-based compound, is hypothesized to penetrate the blood-brain barrier (BBB). Studies show many natural and synthetic indole derivatives have been discovered as promising anti-cancer agents. In vitro results demonstrate that G08 not only inhibits GBM cell proliferation but also suppresses GSC stemness. Further results reveals that G08 exerts its effects by arresting GBM and GSCs in the G1 phase. RNA sequencing indicates that this cell cycle disruption is closely linked to spliceosome activity. In vivo experiments further support its potential, showing that G08 significantly reduces tumor size and tumor cell density in xenografted GSC mouse models. These findings highlight G08 as a promising candidate for GBM co-treatment, offering a potential therapeutic strategy to both eliminate GBM and prevent relapse.

[Disclosure of COI by Responsible researcher] Lead presenter is the Responsible researcher.
[Lead presenter: Peter Lai] We have no financial relationships to disclose.

### [P-1245] I-P14-3
### Synthetic anticancer compounds (1)

2025 / 9 / 25 (Thu) 17:30-18:15 Room P/(Motenashi Dome) Room P

---

### P-1245
### ATF3 induces effects on Castration-resistant Prostate cancer and Olaparib-resistant cells

[Speaker]Linh T T. Nguyen (Inst. of Medical Sciences, Tzu Chi Univ.) [Co-author]Ru-Ping Lee (Inst. of Medical Sciences, Tzu Chi Univ.)、Hung-Jung Wang (Inst. of Medical Sciences, Tzu Chi Univ.／Inst. of Biomedical Sciences and Engineering, Tzu Chi Univ.)

Prostate cancer causes over 1.6 million cases and 366,000 deaths yearly. Androgen Deprivation Therapy is the primary treatment for advanced stages, but many progresses to castration-resistant prostate cancer (CRPC). Emerging resistance to current drugs like Enzalutamide and Olaparib highlights the urgent need for novel strategies to overcome therapeutic failure. Here, we investigate a synthetic Danshen-derived ATF3 inducer, ATF3a and its' cell killing mechanisms in the Olaparib-resistant and wild-type CRPC cells (CWR22Rv1, C4-2B). The WST-1 assays demonstrated reduced IC50 values in CRPC lines compared to normal RWPE1 cells, with heightened sensitivity in Olaparib-resistant variants. qPCR confirmed ATF3a-induced upregulation of ATF3 mRNA and ferroptosis-associated transcripts. Annexin V/BODIPY assays revealed apoptosis and lipid peroxidation in resistant C4-2B cells, while GSEA implicated heme metabolism modulation in CWR22Rv1. Transcriptomic profiling identified ferroptosis-related genes in treated CWR22Rv1 cells. These findings demonstrate ATF3 inducer's dual role in activating ATF3 and inducing ferroptosis, offering a promising therapeutic strategy for Olaparib-resistant CRPC.

[Disclosure of COI by Responsible researcher] Lead presenter is not the Responsible researcher.
[Lead presenter: Linh T T. Nguyen] We have no financial relationships to disclose.
[Principal Researcher: Hung-Jung Wang] We have no financial relationships to disclose.

### [P-1246] I-P14-3
### Synthetic anticancer compounds (1)

2025 / 9 / 25 (Thu) 17:30-18:15 Room P/(Motenashi Dome) Room P

### P-1246
### Withdrawn

No Abstract

### [P-1247] I-P14-3
### Synthetic anticancer compounds (1)

2025 / 9 / 25 (Thu) 17:30-18:15 Room P/(Motenashi Dome) Room P

### P-1247
### Withdrawn

No Abstract

### [P-1248] I-P14-3
### Synthetic anticancer compounds (1)

2025 / 9 / 25 (Thu) 17:30-18:15 Room P/(Motenashi Dome) Room P

P-1248
### Antitumor effects of p300-targeting curcumin derivatives on TNBC patient-derived organoids

[Speaker]Feng Cao (Chiba Cancer Center Research Institute Molecular Carcinogenesis／The Fourth Hospital of Hebei Medical University Radiation Oncology) [Co-author]Tatsuya Masuda (Chiba Cancer Center Research Institute Molecular Carcinogenesis)、Takayoshi Watanabe (Chiba Cancer Center Research Institute Molecular Carcinogenesis)、Ummi M. Zulfin (Cancer Chemoprevention Research Center ／Chiba Cancer Center Research Institute Evolutionary Oncology)、Rohmad Utomo (Cancer Chemoprevention Research Center ／Chiba Cancer Center Research Institute Evolutionary Oncology)、Edy Meiyanto (Cancer Chemoprevention Research Center)、Toshinori Ozaki (Chiba Cancer Center Research Institute Molecular Carcinogenesis)、Yusuke Suenaga (Chiba Cancer Center Research Institute Evolutionary Oncology)、Rikiya Nakamura (Chiba Cancer Center Hospital, Breast Surgery)、Yasuhiko Kamikubo (Chiba Cancer Center Research Institute Molecular Carcinogenesis)

Since triple-negative breast cancer (TNBC) has a poor response to the treatment, there is an urgent need to identify new therapeutic targets and develop an effective treatment strategy. In this study, we found that, among various subtypes of breast cancer, a high expression of p300 is restricted to TNBC, and its expression level is positively correlated to that of CBP. Consistently, a specific knockdown of p300 exhibited antitumor effects in vitro and xenograft tumor developed on CAM(chorioallantoic membrane) as well as mouse models in vivo. To ask how p300 could participate in the development of TNBC, we found p300-target genes based on in silico analysis. we have finally identified BIRC5 gene. A higher expression of BIRC5 was associated with a poor prognosis of TNBC patients. Notably, our novel curcumin derivatives (PGV-1 and CCA-1.1) significantly inhibited HAT activity of p300, down-regulated BIRC5 expression, and displayed a potent anti-proliferative effect on TNBC cells (nM level). Furthermore, these curcumin derivatives suppressed TNBC patient-derived organoids, suggesting further demonstrating the excellent efficacy of our p300 targeting treatment strategy on TNBC.

[Disclosure of COI by Responsible researcher] Lead presenter is not the Responsible researcher.
[Lead presenter: Feng Cao] We have no financial relationships to disclose.
[Principal Researcher: Yasuhiko Kamikubo] We have no financial relationships to disclose.

▲TOP

### [P-1254] I-P14-5
### Anticancer drug sensitivity/Drug resistance (1)

2025 / 9 / 25 (Thu) 17:30-18:15 Room P/(Motenashi Dome) Room P

---

### P-1254
### Investigation of survival mechanisms of doxorubicin tolerant persister cells in triple-negative breast cancer

[Speaker]Yuya Haga (Grad. Sch. Pharm. Sci., Osaka Univ.／Sch. Pharm. Sci., Osaka Univ.,) [Co-author]Suzuno Tanahashi (Sch. Pharm. Sci., Osaka Univ.,)、Kazuma Higashisaka (Grad. Sch. Pharm. Sci., Osaka Univ.／Sch. Pharm. Sci., Osaka Univ., ／IACS, Osaka Univ.,)、Yasuo Tsutsumi (Grad. Sch. Pharm. Sci., Osaka Univ.／Sch. Pharm. Sci., Osaka Univ., ／MEI Ctr., Osaka Univ., ／OTRI., Osaka Univ., ／INSD., Osaka Univ.)

Drug therapy has achieved significant outcomes in cancer treatment, but long-term exposure to chemotherapy or molecular targeted therapy leads to drug resistance. Recently, Drug Tolerant Persister cells (DTPs), which acquire reversible drug tolerance within days to weeks, have been identified. DTPs are considered the origin of long-term drug-resistant cells, making them a promising target for overcoming resistance. However, while studies on DTPs induced by molecular targeted drugs are advancing, research on chemotherapy-induced DTPs remains limited. We established DTPs by exposing 4T1 murine triple-negative breast cancer cells to doxorubicin for nine days. DTPs exhibited reduced drug sensitivity and increased senescence markers, including nuclear enlargement. Since PD-L1 is known to be upregulated in senescent cells, we evaluated its expression and found it elevated in DTPs. When STAT3 was inhibited during the formation of DTPs, the upregulation of PD-L1 was suppressed. These findings might suggest that immune evasion through PD-L1 upregulation and the acquisition of a senescence-like phenotype may contribute to chemotherapy resistance.

[Disclosure of COI by Responsible researcher] Lead presenter is the Responsible researcher.
[Lead presenter: Yuya Haga] We have no financial relationships to disclose.

### [P-1255] I-P14-5
### Anticancer drug sensitivity/Drug resistance (1)

2025 / 9 / 25 (Thu) 17:30-18:15 Room P/(Motenashi Dome) Room P

### P-1255
### Attenuation of SLC46A3 through methylation confers resistance to T-DM1 in gastric cancer N87 cells

[Speaker]Toshimitsu Yamaoka (Adv Cancer Trans Res Inst Showa Med Univ) [Co-author]Fumihiro Ishikawa (Ctr for Biotech Showa Med Univ)、Yuki Hasebe (Adv Cancer Trans Res Inst Showa Med Univ)、Rika Matsuo (Adv Cancer Trans Res Inst Showa Med Univ)、Hiroaki Ito (Adv Cancer Trans Res Inst Showa Med Univ)、Junji Tsurutani (Adv Cancer Trans Res Inst Showa Med Univ)

Anticancer agents targeting HER2 have been approved for certain cancers. T-DM1 is an antibody-drug conjugate consisting of anti-HER2 antibody, trastuzumab, and emtansine that is used to treat patients with HER2-positive advanced or metastatic breast cancer; however, acquired resistance inevitably develops. Using HER2-amplified N87 gastric cancer cells, we established cells that acquired resistance to T-DM1. T-DM1-resistant cells showed no change in HER2 expression or affinity for T-DM1 compared to parental N87 cells.In T-DM1-resistant N87 cells, elevated pH in lysosomes was observed after T-DM1 exposure. Moreover, mRNA level of the lysosomal membrane protein SLC46A3, which transports DM1- and linker-containing catabolites, was significantly reduced. Stable expression of SLC46A3 in T-DM1-resistant cells increased T-DM1-induced apoptosis and restored sensitivity to T-DM1. In the resistant cells, methylation of the CpG island of SLC46A3 suppressed SLC46A3 expression. Next, T-DXd was shown to inhibit proliferation of T-DM1-resistant cells and shrink xenograft tumors, suggesting that T-DXd is an effective treatment for T-DM1-resistant cases.

[Disclosure of COI by Responsible researcher] Lead presenter is the Responsible researcher.
[Lead presenter: Toshimitsu Yamaoka] ■Research funds from company: ベーリンガーインゲルハイム

▲TOP

### [P-1256] I-P14-5
### Anticancer drug sensitivity/Drug resistance (1)

2025 / 9 / 25 (Thu) 17:30-18:15 Room P/(Motenashi Dome) Room P

### P-1256
### Dissecting Chemotherapy Resistance in Gastric Cancer at Single-cell Resolution Using Orthotopic Syngeneic Mouse Model

[Speaker]Tsuyoshi Hirosue (Dept. Prev. Med., Grad. Sch., The Univ. of Tokyo／Dept. of Gastrointestinal Surg., Grad. Sch., Univ. of Tokyo) [Co-author]Miwako Kakiuchi (Dept. Prev. Med., Grad. Sch., The Univ. of Tokyo)、Kuniyo Sueyoshi (Dept. Prev. Med., Grad. Sch., The Univ. of Tokyo)、Ayumu Tsubosaka (Dept. Prev. Med., Grad. Sch., The Univ. of Tokyo)、Daisuke Komura (Dept. Prev. Med., Grad. Sch., The Univ. of Tokyo)、Hiroto Katoh (Div. Path., NCC Exploratory Oncol. Res. & Clin. Trial Ctr.)、Sachiyo Nomura (Dept. of Gastrointestinal Surg., Grad. Sch., Univ. of Tokyo)、Yoshifumi Baba (Dept. of Gastrointestinal Surg., Grad. Sch., Univ. of Tokyo)、Shumpei Ishikawa (Dept. Prev. Med., Grad. Sch., The Univ. of Tokyo)

Gastric cancer is the fourth leading cause of cancer-related death in Japan. Unresectable advanced and recurrent gastric cancer remains incurable, and systemic chemotherapy is the standard treatment. In recent years, adding molecular targeted agents and immune checkpoint inhibitors to chemotherapy has improved first-line therapy. However, the prognosis remains poor, largely because of acquired resistance to chemotherapy. Although mechanisms driving chemo-resistance, such as tumor cell-intrinsic changes and interactions with stromal cells, have been proposed, they are not fully understood. To investigate this, we developed an orthotopic, syngeneic mouse model using luciferase-labeled gastric cancer cells. This allowed visualization of tumor regression and regrowth during chemotherapy using in vivo imaging. Tumors were collected without treatment (No Therapy group), during regression (Chemo Sensitive group), and regrowth (Chemo Resistant group). Single-cell RNA sequencing was performed on cancer and stromal cells to examine gene expression and cell interactions. This analysis provides insights into chemoresistance mechanisms associated with distinct dynamism in gastric cancer TME.

[Disclosure of COI by Responsible researcher] Lead presenter is not the Responsible researcher.
[Lead presenter: Tsuyoshi Hirosue] We have no financial relationships to disclose.
[Principal Researcher: Shumpei Ishikawa] ■Research funds from company: 日本学術振興会　■Advisor of the company and/or profit-making organization: 新日本科学株式会社　■Research expenses: Chord is therapeutics株式会社・大鵬薬品工業株式会社・ロート製薬株式会社・アステラス製薬株式会社

### [P-1257] I-P14-5
### Anticancer drug sensitivity/Drug resistance (1)

2025 / 9 / 25 (Thu) 17:30-18:15 Room P/(Motenashi Dome) Room P

### P-1257
### Activated mitophagy endows hepatocellular carcinoma with resistance to lenvatinib

[Speaker]Munetoshi Akaoka (Div. of Hepato-Biliary-Pancreatic Surg., The Jikei Univ. Sch. of Med.) [Co-author]Mitsuru Yanagaki (Div. of Hepato-Biliary-Pancreatic Surg., The Jikei Univ. Sch. of Med.)、Tomohiko Taniai (Div. of Hepato-Biliary-Pancreatic Surg., The Jikei Univ. Sch. of Med.)、Yuto Yamahata (Div. of Hepato-Biliary-Pancreatic Surg., The Jikei Univ. Sch. of Med.)、Koichiro Haruki (Div. of Hepato-Biliary-Pancreatic Surg., The Jikei Univ. Sch. of Med.)、Kenei Furukawa (Div. of Hepato-Biliary-Pancreatic Surg., The Jikei Univ. Sch. of Med.)、Yosuke Igarashi (Div. of Hepato-Biliary-Pancreatic Surg., The Jikei Univ. Sch. of Med.)、Yoshihiro Shirai (Div. of Hepato-Biliary-Pancreatic Surg., The Jikei Univ. Sch. of Med.)、Michinori Matsumoto (Div. of Hepato-Biliary-Pancreatic Surg., The Jikei Univ. Sch. of Med.)、Takeshi Gocho (Div. of Hepato-Biliary-Pancreatic Surg., The Jikei Univ. Sch. of Med.)、Toru Ikegami (Div. of Hepato-Biliary-Pancreatic Surg., The Jikei Univ. Sch. of Med.)

Lenvatinib (LEN) has a high response rate in hepatocellular carcinoma (HCC). However, some HCCs develop LEN resistance. Several mechanisms of LEN resistance in HCC have been reported, but LEN resistance has not been overcome. The aim of this study is to reveal the mechanism of LEN resistance in HCC with a focus on mitophagy and to overcome LEN resistance. We increased the concentration of LEN in the culture fluids of human HCC cells, and created LEN-resistant (LEN-R) cells. LEN-R cells had a 10-fold higher $IC_{50}$ value of LEN than their parental cells. RNA sequencing and western blot showed that LEN-R cells had increased mitophagy. Transmission electron microscopy showed the accumulation of mitochondria in the cytoplasm of LEN-R cells. Fluorescence assays showed that the mitochondrial membrane potential was decreased and mitophagy and autophagosomes were increased in LEN-R cells. In in vitro and in vivo experiments using a LEN-R tumor xenograft mouse model, wortmannin, a PI3K inhibitor, inhibited mitophagy and improved sensitivity to LEN in LEN-R cells. In conclusion, LEN resistance in HCC might be associated with increased mitophagy and might be overcome by PI3K inhibitors.

[Disclosure of COI by Responsible researcher] Lead presenter is the Responsible researcher.
[Lead presenter: Munetoshi Akaoka] We have no financial relationships to disclose.

▲TOP

### [P-1258] I-P14-5
### Anticancer drug sensitivity/Drug resistance (1)

2025 / 9 / 25 (Thu) 17:30-18:15 Room P/(Motenashi Dome) Room P

---

P-1258

### Identification of HIF-$1^{+}$ M2-type macrophages as a chemoresistance niche component in ovarian clear cell carcinoma

[Speaker]Yusuke Kanda (Advanced Comprehensive Research Organization, Teikyo Univ.) [Co-author]Yutaro Mori (Dept. of Obstetrics & Gynecology, Niigata University)、Hiroaki Sakai (Advanced Comprehensive Research Organization, Teikyo Univ.)、Hirokazu Ohata (Advanced Comprehensive Research Organization, Teikyo Univ.)、Akinobu Hamada (Div. of Mo. Pharm., National Cancer Center Research Institute)、Kosuke Yoshihara (Dept. of Obstetrics & Gynecology, Niigata University)、Koji Okamoto (Advanced Comprehensive Research Organization, Teikyo Univ.)

Ovarian clear cell carcinoma (OCCC) is notoriously chemoresistant, yet its precise mechanisms remain unclear. We previously integrated single-cell and spatial transcriptomic analyses of OCCC samples and found that a cancer chemoresistance niche was composed of HIF-$1^{+}$ cancer cells and fibroblast activation protein $(FAP)^{+}$ cancer-associated fibroblasts (CAFs). In this study, we performed spatial analyses of the cellular neighborhood using CODEX (a multiplex antibody imaging platform) and confirmed the presence of chemoresistance-associated regions dominated by HIF-$1^{+}$ cancer cells or $FAP^{+}$ CAFs. Notably, HIF-$1^{+}$ M2-type macrophages also colocalized within the $FAP^{+}$ CAF-dominated region. Further examination of 86 OCCC FFPE samples using Vectra-based multiplex imaging demonstrated that patients whose tumors harbor higher proportions of both regions had significantly worse survival. Our results indicate that HIF-$1^{+}$ M2 macrophages may contribute to OCCC chemoresistance through interactions with subtypes of cancer cells and CAFs.

[Disclosure of COI by Responsible researcher] Lead presenter is not the Responsible researcher.
[Lead presenter: Yusuke Kanda] We have no financial relationships to disclose.
[Principal Researcher: Yutaro Mori] We have no financial relationships to disclose.

### [P-1259] I-P14-5
### Anticancer drug sensitivity/Drug resistance (1)

2025 / 9 / 25 (Thu) 17:30-18:15 Room P/(Motenashi Dome) Room P

---

### P-1259
### Reprogramming pancreatic stellate cells toward a quiescent-like state via hTCF21 overexpression in vitro.

[Speaker]Koki Kamo (Dept. Biosci., Tokyo Univ. Agric.) [Co-author]Tatsuya Kometani (Dept. Nutr. Sci. Food Safety, Tokyo Univ. Agric.)、Taku Chibazakura (Dept. Biosci., Tokyo Univ. Agric.)

Pancreatic cancer (PC) has an extremely poor prognosis, mainly due to its unique tumor microenvironment (TME). Cancer-associated fibroblasts, especially stellate cells (SCs), are key components of the TME. We have established an in vitro PC model using human iPSC-derived quiescent SCs (hQSCs) and activated SCs (hASCs), with normal pancreatic ductal cells (hNPDCs) and pancreatic cancer cells (hPCCs). This system has enabled us to show that both SCs contribute to chemoresistance in hNPDCs and hPCCs, while only hQSCs promote hPCC proliferation. In this study, we hypothesized that SC activation triggers PC chemoresistance. We focused on hTCF21, a transcription factor regulating SC activation, and established hTCF21-overexpressing SCs (hASC-Ts). qPCR analysis showed downregulation of activation markers (hACTA2, hCOL1A1), indicating SCs deactivation. Co-culture assays demonstrated that hASCs enhanced chemoresistance in hNPDCs, while hASC-Ts promoted not only chemoresistance but also proliferation in hPCCs. These findings suggest that hTCF21 overexpression induces hQSC-like features in SCs and that SCs may impede chemotherapeutic effects, providing new insights into PC TME and therapy.

[Disclosure of COI by Responsible researcher] Lead presenter is the Responsible researcher.
[Lead presenter: Koki Kamo] We have no financial relationships to disclose.

### [P-1260] I-P14-5
### Anticancer drug sensitivity/Drug resistance (1)

2025 / 9 / 25 (Thu) 17:30-18:15 Room P/(Motenashi Dome) Room P

### P-1260
### Overcoming Chemoresistance in Glioblastoma through Cell Death Induced by Lysosomal Membrane Permeabilization

[Speaker]Yuhang Yan (Cancer Research Institute of Kanazawa University) [Co-author]Atsushi Hirao (Cancer Research Institute of Kanazawa University)、Masahiko Kobayashi (Cancer Research Institute of Kanazawa University)、Yongwei Jing (Cancer Research Institute of Kanazawa University)

Conventional treatment for glioblastoma (GBM) includes surgery, temozolomide (TMZ), and radiation, forming the foundation of current therapeutic strategies. Although TMZ effectively induces GBM cell death, many patients exhibit resistance, often due to the activation of O6-methylguanine-DNA methyltransferase (MGMT), a DNA repair enzyme. To address this, we explored a lysosome-targeting strategy using the clinically available lysosomotropic agent ifenprodil in TMZ-resistant GBM cells. Ifenprodil induced multiple forms of cell death—including apoptosis, pyroptosis, and necroptosis—whereas TMZ primarily induced apoptosis. The cytotoxicity of ifenprodil, driven by lysosomal membrane permeabilization, was largely dependent on necroptosis and was enhanced by homoarginine, a lysosomal inhibitor. Combined treatment significantly suppressed tumor growth in TMZ-resistant GBM models. These findings suggest that activating diverse cell death pathways via lysosomal disruption may help overcome TMZ resistance in GBM.

[Disclosure of COI by Responsible researcher] Lead presenter is not the Responsible researcher.
[Lead presenter: Yuhang Yan] We have no financial relationships to disclose.
[Principal Researcher: Atsushi Hirao] ■Research funds from company: 科研費

### [P-1261] I-P14-5
### Anticancer drug sensitivity/Drug resistance (1)

2025 / 9 / 25 (Thu) 17:30-18:15 Room P/(Motenashi Dome) Room P

---

### P-1261
### Development of A3 Receptor Ligands as Modulators of the Multidrug Transporter ABCG2

[Speaker]Megumi Murakami (Dept. of Surg., Tohoku Univ. Grad. Sch. of Med.／Natl. Inst. of Health, USA) [Co-author]Dilip K. Tosh (Natl. Inst. of Health, USA)、Kenneth A. Jacobson (Natl. Inst. of Health, USA)、Shinobu Ohnuma (Dept. of Surg., Tohoku Univ. Grad. Sch. of Med.)、Michiaki Unno (Dept. of Surg., Tohoku Univ. Grad. Sch. of Med.)、Suresh V. Ambudkar (Natl. Inst. of Health, USA)

ABC transporters expressed on the cancer cell surface are one of the causes of cancer multidrug resistance. Understanding the interaction between drug substrates and ABC transporters is important to determine the mechanism of transport function. In this study, we focused on A3 adenosine receptor agonists, which have been developed for chronic disease treatment and have shown a possible interaction with ABC transporters. We synthesized derivatives of A3 adenosine ligands designed for ABCG2, one of the ABC transporters contributing to multidrug resistance. We analyzed the effect of 63 different A3 adenosine receptor ligands on the ATPase activity of ABCG2. Of these, compound 60, a 7-deaza-5 ´-ester derivative with low adenosine receptor affinity, was identified as a high-affinity ligand for ABCG2. This compound was also found to inhibit substrate transport by ABCG2 significantly. Adenine derivative 37c was also found to be a potent transport inhibitor. We were able to identify certain A3 adenosine receptor ligands as potential modulators of human ABCG2. This is an encore abstract that was presented at the Cancer Research and Drug Development in 2022.

[Disclosure of COI by Responsible researcher] Lead presenter is the Responsible researcher.
[Lead presenter: Megumi Murakami] We have no financial relationships to disclose.

▲TOP

### [P-1262] I-P14-5
### Anticancer drug sensitivity/Drug resistance (1)

2025 / 9 / 25 (Thu) 17:30-18:15 Room P/(Motenashi Dome) Room P

### P-1262
### Mechanism of therapy resistance mediated by the TFE3-lysosome axis in glioblastoma

[Speaker]Masahiko Kobayashi (Cancer Res. Inst., Kanazawa Univ.) [Co-author]Yongwei Jing (Cancer Res. Inst., Kanazawa Univ.)、Yuhang Yan (Cancer Res. Inst., Kanazawa Univ.)、Atsushi Hirao (Cancer Res. Inst., Kanazawa Univ.)

In glioblastoma (GBM), radiation and temozolomide (TMZ) are primary treatments, but recurrence often occurs due to the development of therapy resistance. To explore the mechanisms underlying this resistance, we focused on CD44, a known marker of therapy resistance. CD44-high subpopulation of a patient-derived GBM spheroid culture exhibited resistance to multiple treatments. RNA sequencing data showed that the lysosomal pathway was upregulated in the CD44-high subpopulation compared to the CD44-low subpopulation. Treatment with the lysosome inhibitor bafilomycin increased the sensitivity of the CD44-high subpopulation to therapy. We then focused on TFE3, a transcription factor involved in lysosomal biogenesis, which was found to be highly expressed in GBM cells. Analysis of TFE3 knockout and overexpression revealed its role in therapy resistance in GBM. Further investigation of upstream regulators of TFE3 revealed that AMPK activation increased TFE3 levels and enhanced resistance to TMZ, whereas inhibiting AMPK increased sensitivity to TMZ. In conclusion, our results suggest that AMPK may promote therapy resistance in GBM through the activation of TFE3.

[Disclosure of COI by Responsible researcher] Lead presenter is not the Responsible researcher.
[Lead presenter: Masahiko Kobayashi] ■Research funds from company: 科研費
[Principal Researcher: Atsushi Hirao] ■Research funds from company: 科研費

### [P-1263] I-P14-5
### Anticancer drug sensitivity/Drug resistance (1)

2025 / 9 / 25 (Thu) 17:30-18:15 Room P/(Motenashi Dome) Room P

---

### P-1263
### Identification of novel therapeutic targets for radioresistant OSCC focusing on tumor suppressor CYLD expression

[Speaker]Kou Yonemaru (Dept. of Clin. Pharm. Sci., Kumamoto Univ.) [Co-author]Naoki Suenaga (Dept. of Clin. Pharm. Sci., Kumamoto Univ.／Gofuku Pharm.)、Ayumi Kanemaru (Dept. of Clin. Pharm. Sci., Kumamoto Univ.／Clin. Res., Innov. & Educ. Ctr., Tohoku Univ. Hosp.)、Hirofumi Jono (Dept. of Clin. Pharm. Sci., Kumamoto Univ.／Dept. of Pharm., Kumamoto Univ. Hosp.)

Chemo/radio-resistance, frequently observed in oral squamous cell carcinoma (OSCC), is a major obstacle to effective treatment. In this study, we established clinically relevant radioresistant (CRR) cells exhibiting chemo/radio-resistance to identify critical factors in acquisition of resistance, and developed novel therapeutic strategies. Based on the results of comprehensive proteome analysis, we identified tumor suppressor gene CYLD downregulation, as a critical factor in chemo/radio-resistance, which has been also reported to be associated with poor prognosis in OSCC. Our results revealed that CYLD overexpression restored the chemo/radio-resistance in CRR cells, while its knockdown induced it in parental cells. Thus, CYLD downregulation contributes to the chemo/radio-resistance. Surprisingly, however, we found that it conversely enhances sensitivity to EGFR-TKIs in CRR-derived xenograft models. Moreover, low CYLD patient-derived xenograft (PDX) models showed resistance to standard therapies and sensitivity to EGFR-TKIs. These results suggested CYLD downregulation promotes chemo/radio-resistance and might act as a predictive biomarker for outcome of chemo/radiotherapy.

[Disclosure of COI by Responsible researcher] Lead presenter is not the Responsible researcher.
[Lead presenter: Kou Yonemaru] We have no financial relationships to disclose.
[Principal Researcher: Hirofumi Jono] ■Research funds from company: 株式会社日立ハイテク、株式会社ツムラ

### [P-1274] I-P16-1
### Radiation & particle therapy

2025 / 9 / 25 (Thu) 17:30-18:15 Room P/(Motenashi Dome) Room P

### P-1274
### GM-CSF mediates immune regulation after BNCT by activating M1 macrophages

[Speaker]Ying Tong (Dept. Molecular & Genomic Biomedicine, CBMM, Nagasaki University, Nagasaki, Japan／Radioisotope Division, National Cancer Center Research Institute, Tokyo, Japan) [Co-author]Zhongming Gao (Dept. Molecular & Genomic Biomedicine, CBMM, Nagasaki University, Nagasaki, Japan)、Barkha Saraswat (Dept. Molecular & Genomic Biomedicine, CBMM, Nagasaki University, Nagasaki, Japan)、Lichao Chen (Dept. Molecular & Genomic Biomedicine, CBMM, Nagasaki University, Nagasaki, Japan／Radioisotope Division, National Cancer Center Research Institute, Tokyo, Japan)、Shoji Imamichi (Dept. Molecular & Genomic Biomedicine, CBMM, Nagasaki University, Nagasaki, Japan／Radioisotope Division, National Cancer Center Research Institute, Tokyo, Japan)、Yu Sanada (Institute for Integrated Radiation and Nuclear Science, Kyoto University)、Yuka Sasaki (Dept. Molecular & Genomic Biomedicine, CBMM, Nagasaki University, Nagasaki, Japan／Department of Pharmacology, Osaka Dental University)、Tadashige Nozaki (Dept. Molecular & Genomic Biomedicine, CBMM, Nagasaki University, Nagasaki, Japan／Department of Pharmacology, Osaka Dental University)、Masamichi Ishiai (Radioisotope Division, National Cancer Center Research Institute, Tokyo, Japan／Division of BNCT, EPOC, National Cancer Center, Tokyo, Japan)、Minoru Suzuki (Institute for Integrated Radiation and Nuclear Science, Kyoto University)、Mitsuko Masutani (Dept. Molecular & Genomic Biomedicine, CBMM, Nagasaki University, Nagasaki, Japan／Radioisotope Division, National Cancer Center Research Institute, Tokyo, Japan／Division of BNCT, EPOC, National Cancer Center, Tokyo, Japan)

Boron-neutron capture therapy (BNCT) is a promising cancer treatment due to tumor-targeting capability. Previously, we found that ***CSF2*** gene expression, encoding granulocyte-macrophage colony-stimulating factor (GM-CSF), and GM-CSF concentration increased after BPA-based BNCT in SAS cells. GM-CSF promotes dendritic cell differentiation and enhances antigen presentation and immune responses. To study GM-CSF's role in tumor immunity after BNCT, mouse melanoma B16 cells were treated with BNCT and were cultured for 24 hours. Conditioned medium and bone marrow cells from untreated mice were cultured with or without recombinant mouse GM-CSF (rmGM-CSF) for 6 days. Analysis showed the cells differentiated into macrophages. Real-time PCR revealed increased M1 markers and decreased M2 markers, indicating pro-inflammatory, anti-tumor response with rmGM-CSF. When T cells from untreated mice were incubated with these macrophages, rmGM-CSF-treated macrophages enhanced T cell proliferation. These findings suggest M1-type macrophages may contribute to tumor immune responses when activated by GM-CSF. GM-CSF from cancer cells after BNCT may influence the immune response in the tumor environment.

[Disclosure of COI by Responsible researcher] Lead presenter is not the Responsible researcher.
[Lead presenter: Ying Tong] We have no financial relationships to disclose.
[Principal Researcher: Mitsuko Masutani] We have no financial relationships to disclose.

### [P-1275] I-P16-1
### Radiation & particle therapy

2025 / 9 / 25 (Thu) 17:30-18:15 Room P/(Motenashi Dome) Room P

### P-1275
### Evaluation of radiohalogenated anti-ADAM8 antibodies as theranostic agents for pancreatic cancer

[Speaker]Kengo Kaida (Grad. Sch. Pharm., Kanazawa Univ.) [Co-author]Takeshi Fuchigami (Grad. Sch. Pharm., Kanazawa Univ.)、Masayuki Munekane (Grad. Sch. Pharm., Kanazawa Univ.)、Kenji Mishiro (Grad. Sch. Pharm., Kanazawa Univ.)、Masashi Murakami (Institute for Rad. Sci., Osaka Univ.)、Kazuhiro Ooe (Institute for Rad. Sci., Osaka Univ.)、Atsushi Toyoshima (Institute for Rad. Sci., Osaka Univ.)、Kazuma Ogawa (Grad. Sch. Pharm., Kanazawa Univ.)

ADAM8 is highly expressed in early-stage pancreatic cancer but remains low in normal tissues. In this study, we prepared and evaluated $^{211}$At-labeled anti-ADAM8 antibody ([$^{211}$At]At-MAB1031) for targeted α-particle therapy (TAT) and $^{125}$I-labeled anti-ADAM8 antibody ([$^{125}$I]I-MAB1031) for companion diagnostic applications via SPECT (as an alternative radionuclide for $^{123}$I). Cell binding experiments demonstrated significantly higher accumulation of [$^{211}$At]At-MAB1031 and [$^{125}$I]I-MAB1031 in AsPC-1 (high ADAM8) and PANC-1_A8 (PANC-1 overexpressing ADAM8) compared to PANC-1 (low ADAM8). [$^{211}$At]At-MAB1031 also induced significant growth inhibition and DNA damage in AsPC-1 and PANC-1_A8 compared to PANC-1. In vivo biodistribution studies of [$^{211}$At]At-MAB1031 and [$^{125}$I]I-MAB1031 in tumor-bearing mice revealed significantly higher uptake in AsPC-1 tumor compared with PANC-1 tumor. [$^{211}$At]At-MAB1031 exhibited significantly higher anti-tumor efficacy in AsPC-1 tumor-bearing mice compared with nonradioactive MAB1031 and [$^{211}$At]At$^{-}$. In conclusion, radiohalogenated anti-ADAM8 antibodies have potential as theranostic agents for diagnostic imaging and TAT of pancreatic cancer.

[Disclosure of COI by Responsible researcher] Lead presenter is not the Responsible researcher.
[Lead presenter: Kengo Kaida] We have no financial relationships to disclose.
[Principal Researcher: Masayuki Munekane] ■Research funds from company: JST創発的研究支援事業 (2021-2028年度), 科研費 基盤研究(B) (2022-2024年度)

▲TOP

### [P-1276] I-P16-1
### Radiation & particle therapy

2025 / 9 / 25 (Thu) 17:30-18:15 Room P/(Motenashi Dome) Room P

---

### P-1276
### The radiosensitivity of tumors is predominantly determined by cancer cells, not stromal cells.

[Speaker]Yoshikazu Kuwahara (Radiat. Biol. & Med., Tohoku Med. & Pharm. Univ.) [Co-author]Kazuo Tomita (Applied Pharm. Kagoshima Univ. Grad. Sch. Med. & Dent. Sci.)、Tomoaki Sato (Applied Pharm. Kagoshima Univ. Grad. Sch. Med. & Dent. Sci.)、Akihiro Kurimasa (Radiat. Biol. & Med., Tohoku Med. & Pharm. Univ.)

A radioresistant SAS-R cell line, with the same genomic background as its parental SAS cell line, was successfully established. SAS-R cells continue to proliferate under exposure to 2 Gy/day of X-rays, a standard protocol for tumor radiotherapy. Whether tumor radiosensitivity is governed by stromal cells or tumor cells remains unresolved. To investigate this, we transplanted SAS and SAS-R cells into the same nude mouse and evaluated the effects of 2 Gy/day fractionated radiation (FR) on these tumors over 30 days. Starting from the 20th day of FR, the volume of SAS tumors began to decline. By the time the total radiation dose reached 60 Gy, the SAS tumor volume had reduced to less than half of its original size. In contrast, the volume of SAS-R tumors continued to increase and remained stable. Histological analysis after 30 days of FR revealed significant fibrosis in SAS tumors, whereas SAS-R tumors showed no notable fibrosis. These findings indicate that tumor radiosensitivity is predominantly determined by the cancer cells themselves, rather than the surrounding stromal cells.

[Disclosure of COI by Responsible researcher] Lead presenter is the Responsible researcher.
[Lead presenter: Yoshikazu Kuwahara] We have no financial relationships to disclose.

### [P-1277] I-P16-1
### Radiation & particle therapy

2025 / 9 / 25 (Thu) 17:30-18:15 Room P/(Motenashi Dome) Room P

### P-1277
### Evaluation of Early and Late Effects of BNCT in an Immunocompetent Mouse Model of Pelvic Recurrent Colorectal Cancer

[Speaker]Seita Hagihara (Dept of Surg, Osaka Med & Pharm Univ.) [Co-author]Jun Arima (Dept of Surg, Osaka Med & Pharm Univ.)、Kohei Taniguchi (Dept of Surg, Osaka Med & Pharm Univ.／Div of TR, Osaka Med & Pharm Univ.)、Yosuke Inomata (Dept of Surg, Osaka Med & Pharm Univ.)、Yasuhiko Ueda (Dept of Surg, Osaka Med & Pharm Univ.)、Mitsuhiro Asakuma (Dept of Surg, Osaka Med & Pharm Univ.)、Hideki Tomiyama (Dept of Surg, Osaka Med & Pharm Univ.)、Minoru Suzuki (Inst for Integrated Radiation & Nuclear Sci, Kyoto Univ.)、Sangwoong Lee (Dept of Surg, Osaka Med & Pharm Univ.)

Background: We previously demonstrated the anti-tumor effect of boron neutron capture therapy (BNCT) in a nude mouse model of pelvic recurrent colorectal cancer (CRC) (PMID: 36063646), but immune and late effects were not assessable due to limited observation and lack of immunity. This study evaluated pelvic BNCT in immunocompetent mice. Methods: A pelvic CRC recurrence model was created by injecting Colon-26-Luc cells into 7-week-old female BALB/c mice. Boronophenylalanine (BPA) was given intraperitoneally, achieving tumor levels of 37.3±5.6 ppm. Mice were grouped into Cold Control (no treatment), Hot Control (irradiation only), or BNCT (BPA+ irradiation). Non-tumor-bearing mice assessed long-term toxicity. Results: BNCT significantly prolonged survival (median: Cold 7 days, Hot 10, BNCT 16). All toxicity-study mice survived 189 days without severe adverse events except transient weight loss. Early mucosal shedding, degeneration, and apoptosis occurred in the cecum and iliopsoas but resolved over time. Conclusion: BNCT extended survival with minimal, reversible toxicity in an immunocompetent CRC model. Transcriptomic analysis is ongoing to clarify pelvic organ effects.

[Disclosure of COI by Responsible researcher] Lead presenter is not the Responsible researcher.
[Lead presenter: Seita Hagihara] We have no financial relationships to disclose.
[Principal Researcher: Kohei Taniguchi] ■Research funds from company: 国立研究開発法人日本医療研究開発機構（AMED）生命科学・創薬研究支援基盤事業創薬等先端技術支援基盤プラットフォーム（BINDS）JP25ama121052, JP25ama121054

▲TOP

### [P-1278] I-P16-1
### Radiation & particle therapy

2025 / 9 / 25 (Thu) 17:30-18:15 Room P/(Motenashi Dome) Room P

---

### P-1278
### Current BNCT & Precision BNCT based on Novel boron drug discovery strategies

[Speaker]Hiroyuki Michiue (Neutron Therapy Research Center, Okayama University, JAPAN)

Boron Neutron Capture Therapy (BNCT) emerged as a novel radiation treatment, gaining insurance approval in 2020 for inoperable or recurrent head and neck cancer. It is also being tested for malignant brain tumors and skin cancers, showing potential for broader use. BNCT employs a boron compound that targets cancer cells, combined with neutron irradiation at the tumor site. A significant advancement in BNCT is the shift from nuclear reactors to accelerator-based sources, enabling its integration into hospital settings. This transition emphasizes the importance of boron drugs, notably p-boronophenylalanine (BPA), approved in 2020, in enhancing BNCT's effectiveness. Despite its promise, the success of BPA-BNCT in cancers with low BPA uptake remains a concern. Our presentation will highlight the current state of BPA-BNCT and introduce a precision BNCT approach that targets specific genes and biomarkers. As a specific example, we will share our BNCT strategy using novel boron drug discovery based on precision BNCT targeting pancreatic cancer with high CA19-9 levels, which is an incurable cancer.

[Disclosure of COI by Responsible researcher] Lead presenter is the Responsible researcher.
[Lead presenter: Michiue Hiroyuki] We have no financial relationships to disclose.

### [P-1285] I-P16-3
### RI therapy/Others

2025 / 9 / 25 (Thu) 17:30-18:15 Room P/(Motenashi Dome) Room P

---

### P-1285
### CD154 change in peripheral blood after PRRT in NET patients

[Speaker]Eriko Katsuta (Dept. Hepatobiliary & Pancreatic Surgery, Inst. Science Tokyo) [Co-author]Masaru Takeuchi (Dept. Hepatobiliary & Pancreatic Surgery, Inst. Science Tokyo)、Satoshi Matsui (Dept. Hepatobiliary & Pancreatic Surgery, Inst. Science Tokyo)、Daisuke Asano (Dept. Hepatobiliary & Pancreatic Surgery, Inst. Science Tokyo)、Yoshiya Ishikawa (Dept. Hepatobiliary & Pancreatic Surgery, Inst. Science Tokyo)、Hiroki Ueda (Dept. Hepatobiliary & Pancreatic Surgery, Inst. Science Tokyo)、Keiichi Akahoshi (Dept. Hepatobiliary & Pancreatic Surgery, Inst. Science Tokyo)、Noritoshi Kobayashi (Dept. Oncology, Yokohama City University)、Yasushi Ichikawa (Dept. Oncology, Yokohama City University)、Daisuke Ban (Dept. Hepatobiliary & Pancreatic Surgery, Inst. Science Tokyo)

Background: Peptide receptor radionuclide therapy (PRRT) is a new treatment strategy for somatostatin receptor (SSTR) positive neuroendocrine tumor (NET), which is internal radiotherapy targeting SSTR on the tumor cell surface. External radiotherapy has shown to induce systematic immune reaction, such as abscopal effect. Therefore, we hypothesized that PRRT can induce immune reaction in NET patients. Methods: To test this hypothesis, we investigated serum cytokine change by cytokine array in NET patients who received PRRT. 12 pairs of serum from 9 patients were examined. Results: 9 pairs were from pre and post PRRT serum of 1st PRRT. 1 pair was from 2nd PRRT, remaining 2 were comparison pre and post whole course of PRRT. The results showed that, among 36 examined cytokines, only 12 were detectable by the cytokine array. Of those, CD154 showed significant changes in 7 pairs, in which 6 were downregulated and 1 was upregulated. IL-1ra was also upregulated in 3 pairs. Conclusion: We found that PRRT induce systemic cytokine change. Further study is needed to clarify the mechanism of the detected cytokine changes.

[Disclosure of COI by Responsible researcher] Lead presenter is the Responsible researcher.
[Lead presenter: Eriko Katsuta] We have no financial relationships to disclose.

### [P-1286] I-P16-3
### RI therapy/Others

2025 / 9 / 25 (Thu) 17:30-18:15 Room P/(Motenashi Dome) Room P

---

### P-1286
### Novel Carbonic Anhydrase IX targeting 64Cu- and 225Ac-PD-32766 are promising theranostic peptides for ccRCC and CRC

[Speaker]Hidetomo Kitamura (PeptiDream Inc.／PDRadiopharma Inc.) [Co-author]Shota Tsuchida (PeptiDream Inc.)、Yoshihide Mizukoshi (PeptiDream Inc.)、Hiroko Inaba (PeptiDream Inc.)、Tatsuro Kotake (PeptiDream Inc.)、Takanori Aoki (PeptiDream Inc.)、Yuichi Funase (PDRadiopharma Inc.)、Naoki Kanazawa (PDRadiopharma Inc.)、Hikaru Shimizu (PeptiDream Inc.)、Keisuke Matsuhara (PDRadiopharma Inc.)、Kentaro Suzuki (PDRadiopharma Inc.)、Hayato Yanagida (PeptiDream Inc.)、Takeru Ehara (PeptiDream Inc.)、Satoshi Matsushima (PDRadiopharma Inc.)、Masato Murakami (PeptiDream Inc.／PDRadiopharma Inc.)

Carbonic Anhydrase IX (CA9) is a zinc metalloenzyme involved in pH regulation to support tumor cell growth, and is overexpressed in colorectal cancer (CRC) and 95% of clear cell renal cell carcinoma (ccRCC) patients. PD-32766 is a novel macrocyclic peptide targeting CA9 and can be labeled with radionuclides ($^{64}$Cu and $^{225}$Ac) for PET imaging and targeted alpha therapy. Here we report the theranostic potential of PD-32766 in ccRCC.PD-32766 exhibits strong binding affinity with CA9 with a KD below 0.2 nM in SPR analysis. In biodistribution studies, $^{64}$Cu-PD-32766 and $^{225}$Ac-PD-32766 showed high and specific tumor accumulation in CA9 overexpressing HT-29 and VMRC-RCW xenograft models, with high retention at 48 hours after administration. PET imaging with $^{64}$Cu-PD-32766 clearly detected tumors, consistent with the biodistribution data. In therapeutic studies, $^{225}$Ac-PD-32766 was well tolerated and significantly prolonged overall survival in treated mice compared to controls, suggesting robust tumor growth inhibition with $^{225}$Ac-PD-32766 administration. In conclusion, PD-32766 is a promising novel radio-theranostic peptide for CA9 expressing tumors such as CRC and ccRCC.

[Disclosure of COI by Responsible researcher] Lead presenter is the Responsible researcher.
[Lead presenter: Hidetomo Kitamura] ■ employment by a company related to the presentation: ペプチドリーム株式会社

### [P-1287] I-P16-3
### RI therapy/Others

2025 / 9 / 25 (Thu) 17:30-18:15 Room P/(Motenashi Dome) Room P

---

### P-1287
### Development of targeted alpha therapeutics aimed at eliminating drug resistant cancers

[Speaker]Sifan Feng (Osaka Univ., Grad. Sch. Sci.) [Co-author]Kentaro Hisada (Osaka Univ., Grad. Sch. Sci.)、Haruna Yorifuji (Osaka Univ., Dept. Sci.)、Tadashi Watabe (Osaka Univ., Grad. Sch. Med.／Osaka Univ, IRS／Osaka Univ., Grad. Sch. Sci., FRC)、Yoshiyuki Manabe (Osaka Univ., Grad. Sch. Sci.／Osaka Univ, IRS／Osaka Univ., Grad. Sch. Sci., FRC)、Atsushi Shimoyama (Osaka Univ., Grad. Sch. Sci.／Osaka Univ, IRS／Osaka Univ., Grad. Sch. Sci., FRC)、Atsushi Toyoshima (Osaka Univ., Grad. Sch. Sci.／Osaka Univ, IRS／Osaka Univ., Grad. Sch. Sci., FRC)、Atsushi Shinohara (Osaka Univ., Grad. Sch. Sci., FRC)、Yoshikatsu Kanai (Osaka Univ, IRS／Osaka Univ, PRiMe／Osaka Univ., Grad. Sch. Sci., FRC)、Koichi Fukase (Osaka Univ, IRS／Osaka Univ., Grad. Sch. Sci., FRC)、Kazuko Kaneda (Osaka Univ., Grad. Sch. Sci.／Osaka Univ, IRS／Osaka Univ., Grad. Sch. Sci., FRC)

Nuclear medicine therapeutics is receiving much attention in the field of theranostics because of its high cytotoxic effect to the targeting cancer. Because of physiological uptakes in non-targeted organs might lead to the severe side-effects, we should consider about both maximizing the treatment efficacy of cancer and minimizing the side effects in the organs at risk. From their properties, nuclear medicine therapeutics is effective in the treatment of refractory cancers whose QOL is greatly reduced by surgery. We selected cancer type amino acid transporter, LAT1, as molecular target. It has highly specificity to several kinds of cancer tissues, certainly in rare cancer. We synthesized aromatic amino acid labeled with $At^{211}$ short-lived alpha emitting nuclide. To evaluate our compounds in anticancer drug-resistant cases, several examinations were conducted using drug-resistant strains. We confirmed the specificity and found the anti-cancer effects ***in vitro*** and ***in vivo*** study. As a result, we found the advantage which might be adoptive in acquired resistance cases.

[Disclosure of COI by Responsible researcher] Lead presenter is not the Responsible researcher.
[Lead presenter: Sifan Feng] We have no financial relationships to disclose.
[Principal Researcher: Kazuko Kaneda] We have no financial relationships to disclose.

### [P-1288] I-P16-3
### RI therapy/Others

2025 / 9 / 25 (Thu) 17:30-18:15 Room P/(Motenashi Dome) Room P

---

### P-1288
### PD-29875 is a promising radiotheranostic peptide targeting Claudin 18.2 for gastric and pancreatic cancers.

[Speaker]Shintaro Kato (PeptiDream Inc.) [Co-author]Yoshihide Mizukoshi (PeptiDream Inc.)、Shota Tsuchida (PeptiDream Inc.)、Hiroko Inaba (PeptiDream Inc.)、Rie Komura (PeptiDream Inc.)、Miho Satake (PDRadiopharma Inc.)、Yuki Sato (PDRadiopharma Inc.)、Naoki Kanazawa (PDRadiopharma Inc.)、Hayato Yanagida (PeptiDream Inc.)、Hidetomo Kitamura (PeptiDream Inc.)、Kentaro Suzuki (PDRadiopharma Inc.)、Satoshi Matsushima (PDRadiopharma Inc.)、Keisuke Matsuhara (PDRadiopharma Inc.)、Masato Murakami (PeptiDream Inc.／PDRadiopharma Inc.)

Claudin 18 (CLDN18, a tight junction protein) has two splicing variants (CLDN18.1 and 18.2). CLDN18.2 is overexpressed in gastric and pancreatic cancers and is exposed on the cell surface, indicating CLDN18.2 is a promising target of radio-theranostics. PD-29875 is a novel macrocyclic peptide binds to CLDN18.2 and can be labeled with 64Cu and 177Lu for PET imaging and radioligand therapy. Here we report the radio-theranostic potential of PD-29875 in gastric and pancreatic cancer models. Cell-based assay revealed that PD-29875 selectively bound to CLDN18.2 over CLDN18.1, and internalized. 64Cu- or 177Lu-PD-29875 was dosed to SNU-16 (gastric cancer) or BxPC3 (pancreatic cancer) overexpressing CLDN18.2 xenograft mice. 64Cu-PD-29875 clearly visualized tumors by PET imaging. In biodistribution studies, both 64Cu- and 177Lu-PD-29875 exhibited high tumor accumulation. These two compounds showed similar biodistribution, suggesting promising radio-theranostic profiles. In therapeutic studies, 177Lu-PD-29875 revealed remarkable anti-tumor effects in dose-dependent manners. In conclusion, PD-29875 is a promising radio-theranostic peptide for CLDN18.2 expressing gastric and pancreatic cancers.

[Disclosure of COI by Responsible researcher] Lead presenter is not the Responsible researcher.
[Lead presenter: Shintaro Kato] We have no financial relationships to disclose.
[Principal Researcher: Hidetomo Kitamura] We have no financial relationships to disclose.

[P-1289] I-P16-3
RI therapy/Others

2025 / 9 / 25 (Thu) 17:30-18:15 Room P/(Motenashi Dome) Room P

### P-1289
### Development of GPC3 antibody targeted delivery of isotope as a theranostic and diagnostic radionuclide platform for HCC

[Speaker]Yu-Chang Tyan (Dept. of Med. Imaging and Radiological Sci., Kaohsiung Med. Univ.,) [Co-author]Shih-Chang Chuang (Dept. of Surg., Kaohsiung Med. Univ. Hosp., Kaohsiung Med. Univ.)、Kuo-Chen Hung (Dept. of Surg., Kaohsiung Med. Univ. Hosp., Kaohsiung Med. Univ.)、Ming-Hui Yang (Dept. of Surg., Kaohsiung Med. Univ. Hosp., Kaohsiung Med. Univ.)

Hepatocellular carcinoma (HCC) is the second leading cause of cancer-related death. Glypican-3 (GPC3) antibody can be a promising treatment because of its high affinity for liver tumors. In this study, iodine-131 was labeled onto the GPC3 antibody and then its specificity and anti-tumor efficacy were evaluated in a spontaneous HCC mouse model. $^{131}$I-GPC3 antibody was specifically bound to the surface antigen of liver tumors in mice. The stability, biodistribution, and cytotoxicity of $^{131}$I-GPC3 antibodies were also determined. The stability of the isotope labeled GPC3 antibody was over 50% after five days. High levels of radioactivity accumulations in tumors were found. The cell viabilities diminished along with the increasing dosages of $^{131}$I-GPC3 antibody. An adjuvant treatment of $^{131}$I-GPC3 antibody will be a valuable diagnostic tool after liver tumor resection to reduce the recurrence rate and increase the overall survival. Our study not only demonstrates the usage of beta-ray as a radioimmunotherapy to achieve the purpose of treating cancer, but also develops the possibility of in vivo gamma-scintigraphic imaging.

[Disclosure of COI by Responsible researcher] Lead presenter is not the Responsible researcher.
[Lead presenter: Yu-Chang Tyan] We have no financial relationships to disclose.
[Principal Researcher: Ming-Hui Yang] We have no financial relationships to disclose.

▲TOP

### [P-1290] I-P16-3
### RI therapy/Others

2025 / 9 / 25 (Thu) 17:30-18:15 Room P/(Motenashi Dome) Room P

### P-1290
### Changes in Mesorectal CT Values and Histological Effect By Preoperative Treatments for Rectal Cancer

[Speaker]Hiroaki Nozawa (Dept. Surg. Oncol., Univ. Tokyo) [Co-author]So Kasuga (Dept. Surg. Oncol., Univ. Tokyo)、Soichiro Ishihara (Dept. Surg. Oncol., Univ. Tokyo)

The efficacy of preoperative treatment for lower rectal cancer is primarily determined by the effects in the primary tumor. We recently reported the change in mesorectal CT values (ΔCTVs) during chemoradiotherapy (CRT) was associated with histological tumor regression grade (TRG). In this study, we expanded the subjects to patients with rectal cancer receiving preoperative CRT (n=93) or systemic therapy (ST, n=16). The ΔCTV at 4 predetermined points within the mesorectum during preoperative treatment and clinicopathological factors were analyzed. The ΔCTV was higher in patients receiving CRT than those treated by ST (median: +8.7 vs -6.0 Hounsfield Unit, p=0.002). Multivariate analysis showed that CRT and positive value of ΔCTV were independent predictors of good TRG. In addition, a shorter operative time and smaller blood loss were observed in patients with positive ΔTV or good TRG. To conclude, rectal cancer was more likely to respond to CRT that generally increases ΔCTV, suggesting the induction of local edema. Despite of this unfavorable condition for dissecting surgical plane, the surgical outcomes were better in cases with good TRG presumably owing to good tumor shrinkage.

[Disclosure of COI by Responsible researcher] Lead presenter is the Responsible researcher.
[Lead presenter: Hiroaki Nozawa] We have no financial relationships to disclose.

### [P-1291] I-P16-3
### RI therapy/Others

2025 / 9 / 25 (Thu) 17:30-18:15 Room P/(Motenashi Dome) Room P

P-1291
### *P53*-independent bystander effects via secreted factors between carbon-ion irradiated tumor and unirradiated normal cells

[Speaker]Masao Suzuki (Dept. Charged Particle Therapy Res., QST Hospital, QST) [Co-author]Sei Sai (Dept. Charged Particle Therapy Res., QST Hospital, QST)

Communication from carbon-ion irradiated tumor to unirradiated normal cells is one of major concerns for heavy-ion radiotherapy. Human glioblastoma cells (mutated type *P53* )and human lung adenocarcinoma cells (wild-type *P53* ) were irradiated with carbon-ion microbeams (LET= 103keV/$\mu$m) generated with Takasaki Ion Accelerators for Advanced Radiation Application, National Institutes for Quantum Science and Technology (QST). The medium from the irradiated tumor cells was added to the flask inoculated on unirradiated normal human skin fibroblasts (wild-type *P53* ).The medium transfer resulted in increasing both cell killing and gene mutation in the normal cells. Furthermore, induced biological effects returned to control levels when using ascorbic acid (AsA), not dimethyl sulfoxide. There is clear evidence that the medium from the irradiated tumor cells enable to induce damage in the unirradiated normal cells via bystander effect mediated by secreted factor(s), which are scavenged by AsA. And *P53*-gene status of irradiated tumor cells is not participating in inducing secreted-factor-mediated bystander effects. (This is co-operative study with Drs. T. Funayama and M. Suzuki in QST.)

[Disclosure of COI by Responsible researcher] Lead presenter is the Responsible researcher.
[Lead presenter: Masao Suzuki] We have no financial relationships to disclose.

### [P-1292] I-P16-3
### RI therapy/Others

2025 / 9 / 25 (Thu) 17:30-18:15 Room P/(Motenashi Dome) Room P

---

### P-1292
### Assessing the potential of treating neuroblastoma by an isotopic compound

[Speaker]Ming-Hui Yang (Dept. of Surg., Kaohsiung Med. Univ. Hosp., Kaohsiung Med. Univ.) [Co-author]Shih-Chang Chuang (Dept. of Surg., Kaohsiung Med. Univ. Hosp., Kaohsiung Med. Univ.)、Kuo-Chen Hung (Dept. of Surg., Kaohsiung Med. Univ. Hosp., Kaohsiung Med. Univ.)、Yu-Chang Tyan (Dept. of Med. Imaging & Radiological Sci., Kaohsiung Med. Univ.)

Compounds with isotopic elements are applicable as diagnostic agents for oncological and neurological diseases or therapeutic agents as carriers to impair or eliminate tumors. Some radioactive compounds can play both roles depending on their decay mode. In this study, we synthesized a derivative of thioflavin-T and labeled it with iodine-125 using various labeling methods. Among them, the labeling efficiencies of isotope exchange and redox reactions were around 1 and 10 %. The labeling efficiency of Iodogen technique was over 95%. In addition, the radiolabeling using the Iodogen technique yielded over 90% radiolabeling at 2 days post-labeling. Then, neuroblastoma cells and normal cells were treated with the radioactive compound. The uptake rate of neuroblastoma cells was five-fold higher compared with that of normal cells. Compared with neuroblastoma cells treated with the non-radioactive compound, it was found that the cell viability decreased and the level of $\gamma$-H2AX increased along with increasing radiation doses. This study revealed that the Iodogen technique is effective for labeling $^{125}$I, and this radioactive compound may be utilized as a therapeutic agent.

[Disclosure of COI by Responsible researcher] Lead presenter is not the Responsible researcher.
[Lead presenter: Ming-Hui Yang] We have no financial relationships to disclose.
[Principal Researcher: Yu-Chang Tyan] We have no financial relationships to disclose.

### [P-1301] I-P20-2
### Frontiers in RNA Structure & Spatial Transcriptomics

2025 / 9 / 25 (Thu) 17:30-18:15 Room P/(Motenashi Dome) Room P

---

### P-1301
### Augmented Transcript-Level Quantification Enhances Tumor scRNA-seq Analysis

[Speaker]Yufei Zhang (Dept. Comp. Biol. Med. Sci., Grad. Frontier Sci., Univ. Tokyo) [Co-author]Tatsuhiko Tsunoda (Dept. Comp. Biol. Med. Sci., Grad. Frontier Sci., Univ. Tokyo／Dept. Biol. Sci., Sch. Sci., Univ. Tokyo)、Artem Lysenko (Dept. Biol. Sci., Sch. Sci., Univ. Tokyo)

Novel transcripts from alternative splicing events and transposon expression have been recognized for their role in shaping the tumor microenvironment and various biological processes. However, prior scRNA-seq studies focused on gene-level expression, overlooking transcript-level information. Recent full-length scRNA-seq tumor datasets enable investigation of transcript quantification. Furthermore, tools like Salmon, using the EM algorithm, demonstrate high accuracy in quantifying transcripts. Based on these insights, we developed an augmented transcript-level quantification pipeline for a reference transcriptome comprising 1 million well-annotated transcripts and 3 million transposon loci. To validate its reliability, we benchmarked with paired short- and long-read data and achieved a moderately strong correlation between their estimates. We also found the mapping rate increased across samples with our pipeline. Finally, our pipeline enhanced downstream analyses such as data imputation, leading to improved performance and higher resolution in recovering masked values. This pipeline offers a vital resource to dissect tumor heterogeneity and identify novel therapeutic targets.

[Disclosure of COI by Responsible researcher] Lead presenter is the Responsible researcher.
[Lead presenter: Yufei Zhang] We have no financial relationships to disclose.

▲TOP

### [P-1302] I-P20-2
### Frontiers in RNA Structure & Spatial Transcriptomics

2025 / 9 / 25 (Thu) 17:30-18:15 Room P/(Motenashi Dome) Room P

---

### P-1302
### Optimal detection of allele-specific copy number alterations and haplotype swapping from short-read sequences data

[Speaker]Yu Ito (Div. Genome Analysis Platform Development, Natl. Cancer Ctr. Res. Inst.／Dept. Respiratory Med., Grad. Sch. Med., Univ. Tokyo.) [Co-author]Yoshitaka Sakamoto (Div. Genome Analysis Platform Development, Natl. Cancer Ctr. Res. Inst.)、Hajime Suzuki (Div. Genome Analysis Platform Development, Natl. Cancer Ctr. Res. Inst.)、Yuichi Shiraishi (Div. Genome Analysis Platform Development, Natl. Cancer Ctr. Res. Inst.)

Cancer genes frequently exhibit copy number alterations (CNAs) at chromosomal or subchromosomal scales. For allele-specific CNA detection from short- or long-read data, haplotype phasing of each read and comparing haplotype-segregated read counts per segment is a common approach. However, regions having sparse variants or repeat sequences can disrupt continuous phasing of short-reads, allowing potential 'swapping' of inferred haplotypes between segments. Eliminating this swapping is essential for identifying true CNAs, yet no known algorithm can jointly search for both CNAs and swapping while guaranteeing a globally optimal solution. We extended the Pruned Exact Linear Time (PELT) algorithm, originally designed for one-dimensional time-series data, into a two-dimensional framework supporting multiple changepoint types with an optimal solution (named Scarpia). We also introduced an 'adaptive approximate pruning' method to speed computation. When applied to multiple cell lines, Scarpia efficiently detected these changepoints. This algorithm is expected to facilitate chromosome-scale haplotype phasing where haplotypes exhibit differing copy numbers.

[Disclosure of COI by Responsible researcher] Lead presenter is the Responsible researcher.
[Lead presenter: Yu Ito] We have no financial relationships to disclose.

### [P-1303] I-P20-2
### Frontiers in RNA Structure & Spatial Transcriptomics

2025 / 9 / 25 (Thu) 17:30-18:15 Room P/(Motenashi Dome) Room P

---

### P-1303
### Identifying copy number alterations from RNA-Seq data and evaluating their confidence

[Speaker]Eisuke Iwamoto (Natl. Hosp. Org. Nagoya Med. Ctr. Clin. Res. Ctr.) [Co-author]Masashi Sanada (Natl. Hosp. Org. Nagoya Med. Ctr. Clin. Res. Ctr.)、Takahiko Yasuda (Natl. Hosp. Org. Nagoya Med. Ctr. Clin. Res. Ctr.)

RNA-seq data is important for research and diagnosis of various lymphoid tumors because it can be used to identify fusion genes and disease subtypes. Copy number alterations (CNAs) are hallmarks of cancer. These alterations are usually detected using genomic DNA, while RNA-based approaches remain limited . It would be beneficial to determine copy number variations more accurately and more easily from RNA data. We developed a method to identify CNAs from RNA-Seq data, evaluating CNAs at the autosome arm level using gene read counts and SNP variant allele frequencies. Moreover, confidence evaluation is performed via the bootstrap method. Our method was validated using a DNA-based approach (CNACS) in 104 patients with ALL. Our method achieved 90% sensitivity (gain: 90%, loss: 91%) and 99% specificity using the CNACS results as a reference for comparison. Bootstrap-based confidence evaluation yielded a high AUC of 0.91 in the ROC curve. CNEX provides a method to identify copy number alterations even without DNA data, further enhancing the utility of RNA-seq in cancer diagnosis and research.

[Disclosure of COI by Responsible researcher] Lead presenter is not the Responsible researcher.
[Lead presenter: Eisuke Iwamoto] We have no financial relationships to disclose.
[Principal Researcher: Masashi Sanada] We have no financial relationships to disclose.

### [P-1304] I-P20-2
### Frontiers in RNA Structure & Spatial Transcriptomics

2025 / 9 / 25 (Thu) 17:30-18:15 Room P/(Motenashi Dome) Room P

### P-1304
### Unsupervised annotation of spatial transcriptomes based on vectorial information between transcripts

[Speaker]Ryosuke Nomura (National Cancer Center, Exploratory Oncology Research & Clinical Trial Center ／Graduate School of Frontier Sciences, The University of Tokyo) [Co-author]Shunsuke A. Sakai (National Cancer Center, Exploratory Oncology Research & Clinical Trial Center ／Graduate School of Frontier Sciences, The University of Tokyo)、Riu Yamashita (National Cancer Center, Exploratory Oncology Research & Clinical Trial Center ／Graduate School of Frontier Sciences, The University of Tokyo)、Shunichiro Kageyama (National Cancer Center, Exploratory Oncology Research & Clinical Trial Center)、Katsuya Tsuchihara (National Cancer Center, Exploratory Oncology Research & Clinical Trial Center ／Graduate School of Frontier Sciences, The University of Tokyo)

Spatial omics technologies have enabled to detect gene expressions with location information. However, existing algorithms remain limited in classifying spatial distribution patterns without biological knowledge. In this study, we introduced a method to cluster genes based on vectorial information between transcripts.We applied our method to Xenium data from lung and breast cancer samples. For each gene, we extracted the shortest vector between the tumor and an arbitrary gene. The vectors were transformed into polar coordinates. These values were converted into density maps, and the Jaccard index of the two was defined as the similarity of gene distribution. The scores were projected into two dimensions by PCA. As a result, expression patterns – within tumor cells, around them, and across tissue – were clustered. Immune-related genes such as CXCL9 or CXCL13 appeared in the peripheral group, indicating the activation of the immune response. We also observed distinct gene clusters in the PCA space, including those associated with lymphoid follicles and T cell markers. These results suggest our unsupervised method could extract functionally related genes without biological knowledge.

[Disclosure of COI by Responsible researcher] Lead presenter is not the Responsible researcher.
[Lead presenter: Ryosuke Nomura] We have no financial relationships to disclose.
[Principal Researcher: Shunsuke Sakai, Riu Yamashita] We have no financial relationships to disclose.

▲TOP

### [P-1305] I-P20-2
### Frontiers in RNA Structure & Spatial Transcriptomics

2025 / 9 / 25 (Thu) 17:30-18:15 Room P/(Motenashi Dome) Room P

---

### P-1305
### Resolving cancer-associated isoform landscapes with epitranscriptomic signatures

[Speaker]Boyi Yu (Advanced Data Science Div., RCAST, UTokyo) [Co-author]Genta Nagae (Genome Science & Medicine Div., RCAST, UTokyo)、Keisuke Yamada (Genome Science & Medicine Div., RCAST, UTokyo／Dept. of Neurosurgery, UTokyo)、Kenji Tatsuno (Genome Science & Medicine Div., RCAST, UTokyo)、Hiroyuki Aburatani (Genome Science & Medicine Div., RCAST, UTokyo)、Hiroki Ueda (Advanced Data Science Div., RCAST, UTokyo)

Aberrant RNA splicing in cancer generates transcript variants that contribute to tumor progression. While the mechanisms underlying splicing dysregulation remain incompletely understood, emerging evidence highlights the role of RNA modifications, such as m6A and m5C, in shaping alternative splicing. Understanding how these modifications interact with splicing machinery can provide new insights into cancer biology. Therefore, it is of great significance to develop a robust method for identification of modifications essential for splicing decisions. Here, we present a computational pipeline that jointly decodes modification and isoform profiles using Nanopore Direct RNA Sequencing. To improve accuracy, a novel filtering strategy is proposed to reduce false positive modification calls before splicing analysis. Applying this pipeline to U87 glioma cells, we profiled isoform-resolved epitranscriptomes under different m6A and splicing conditions. Our findings revealed that modulation of m6A levels alters the splicing landscape of cancer-related genes. In conclusion, the proposed pipeline shows great promise in advancing our understanding of modification-associated alternative splicing.

[Disclosure of COI by Responsible researcher] Lead presenter is not the Responsible researcher.
[Lead presenter: Boyi Yu] We have no financial relationships to disclose.
[Principal Researcher: Hiroki Ueda] We have no financial relationships to disclose.

### [P-1306] I-P20-2
### Frontiers in RNA Structure & Spatial Transcriptomics

2025 / 9 / 25 (Thu) 17:30-18:15 Room P/(Motenashi Dome) Room P

### P-1306
### Comprehensive Analysis of RNA Modifications in Coding and Non-Coding Transcripts Using Nanopore Sequencing

[Speaker]Hiroki Ueda (Advanced Data Science, RCAST, Univ. of Tokyo) [Co-author]Boyi Yu (Advanced Data Science, RCAST, Univ. of Tokyo)、Keisuke Yamada (Genome & Medicine, RCAST, Univ. of Tokyo)、Akihide Yoshimi (Div. Cancer RNA Research, NCC)、Genta Nagae (Genome & Medicine, RCAST, Univ. of Tokyo)、Hiroyuki Aburatani (Genome & Medicine, RCAST, Univ. of Tokyo)

RNA modifications regulate post-transcriptional processes and are increasingly linked to cancer. However, accurate and comprehensive detection remains difficult with short-read sequencing. Nanopore direct RNA sequencing (DRS) enables PCR-free, single-molecule detection of RNA modifications and is well suited for profiling cancer transcriptomes. We evaluated ONT's RNA004 flowcell and Dorado basecaller which detect m6A, m5C, Inosine, and Psude Uridine, but observed high false positive rates, especially in non-motif regions. To address this, we developed ModiScope, a filtering pipeline combining deep learning trained on IVT RNA with motif-based rules from known sites. For m6A, our method showed high concordance with GLORI data at DRACH motifs and reduced off-target calls elsewhere. Inosine detection improved by filtering ADAR targets like Alu elements. Additionally, we built a modification-agnostic tool to detect structural RNA modifications in non-coding RNAs such as rRNA and implemented a method to identify splicing events linked to RNA modifications. Our approach enables practical detection of cancer-related RNA modifications.

[Disclosure of COI by Responsible researcher] Lead presenter is the Responsible researcher.
[Lead presenter: Hiroki Ueda] ■Advisor of the company and/or profit-making organization: コニカミノルタREAKM株式会社

▲TOP

### [P-1307] I-P20-2
### Frontiers in RNA Structure & Spatial Transcriptomics

2025 / 9 / 25 (Thu) 17:30-18:15 Room P/(Motenashi Dome) Room P

---

### P-1307
### Mapping epigenomic states defining cancer cell states by integrating public single-cell datasets

[Speaker]Kohei Kumegawa (NEXT-Ganken Prog., JFCR) [Co-author]Reo Maruyama (NEXT-Ganken Prog., JFCR／Cancer Epigenomics, Cancer Inst., JFCR)

Understanding how cancer cell states are established by epigenomic variation is critical for interpreting tumor behavior and heterogeneity. Here, we integrated publicly available single-cell RNA-seq and ATAC-seq datasets across cancer types to identify epigenomic features associated with different cancer cell states. We first performed non-negative matrix factorization on single-cell transcriptomic profiles to define key cell states. We then examined chromatin accessibility profiles in corresponding cell populations using single-cell ATAC-seq datasets to determine whether specific epigenomic states are enriched in each cell state. Our analysis revealed that different cell states are often characterized by unique chromatin accessibility patterns, suggesting that epigenomic heterogeneity plays a key role in defining cancer cell states. This study provides a framework for linking transcriptomic and epigenomic landscapes and offers insights into the regulatory mechanisms underlying cancer cell plasticity.

[Disclosure of COI by Responsible researcher] Lead presenter is the Responsible researcher.
[Lead presenter: Kohei Kumegawa] We have no financial relationships to disclose.

### [P-1308] I-P20-2
### Frontiers in RNA Structure & Spatial Transcriptomics

2025 / 9 / 25 (Thu) 17:30-18:15 Room P/(Motenashi Dome) Room P

---

### P-1308
### Mining spatial transcriptomics datasets using DeepSpaceDB

[Speaker]Nupura M. Prabhune (Dept. of Life and Med. Biosci., Waseda Univ.／Dept. of Integrative Bioanalytics, IDAC, Tohoku Univ.) [Co-author]Yilin Du (Dept. of Integrative Bioanalytics, IDAC, Tohoku Univ.／Dept. of Rehabil. Med., Med. Sch., Tohoku Univ.)、Mugi R. Suzuki (Dept. of Integrative Bioanalytics, IDAC, Tohoku Univ.)、Yuichiro Nakajima (Dept. of Integrative Bioanalytics, IDAC, Tohoku Univ.)、Afeefa Zainab (LiMe., Kyoto Univ.)、Satoru Ebihara (Dept. of Rehabil. Med., Med. Sch., Tohoku Univ.)、Shinji Takeoka (Dept. of Life and Med. Biosci., Waseda Univ.)、Shinpei Kawaoka (Dept. of Integrative Bioanalytics, IDAC, Tohoku Univ.／Inter-Organ Comm. Res. Team, LiMe., Kyoto Univ.)、Alexis Vandenbon (LiMe., Kyoto Univ.／ILAS, Kyoto Univ.)

Spatial transcriptomics has wide-ranging applications in biological research, where gene expression variations under different experimental conditions can be analyzed across tissues while retaining positional information. Given its financial and logistical constraints, we introduce DeepSpaceDB, a comprehensive database for spatial transcriptomics data exploration. DeepSpaceDB provides access to manually annotated spatial transcriptomics data, with fine details of its origin, cell composition, spatially variable genes, and biological pathways. The platform enables intra- and inter-sample comparisons across organs, organisms, and conditions, allowing users to customize their analytical objectives. Here, we present a user-friendly protocol outlining the data mining process on DeepSpaceDB. We demonstrate the identification of immune activity signatures in regions of colorectal metastases in mouse livers. DeepSpaceDB serves as a critical resource for spatial transcriptomics research, facilitating deeper investigation into tissue organization and disease biology. We would be happy to discuss potential collaborations using this database. Access DeepSpaceDB at www.deepspacedb.com.

[Disclosure of COI by Responsible researcher] Lead presenter is the Responsible researcher.
[Lead presenter: Nupura M. Prabhune] We have no financial relationships to disclose.

Poster Session

### [P-1309] I-P20-2
### Frontiers in RNA Structure & Spatial Transcriptomics

2025 / 9 / 25 (Thu) 17:30-18:15 Room P/(Motenashi Dome) Room P

### P-1309
### Evaluation of copy number alteration detection tools using whole-genome sequence data

[Speaker]Hanako Ono (Dept. of Clin. Genomics, Natl. Cancer Ctr. Res. Inst.) [Co-author]Masahiro Gotoh (Dept. of Clin. Genomics, Natl. Cancer Ctr. Res. Inst.)、Mineko Ushiama (Dept. of Clin. Genomics, Natl. Cancer Ctr. Res. Inst.)、Kazuki Takahashi (Human Genome Ctr., Inst. Of Med. Sci., Univ. of Tokyo)、Takashi Kohno (Div. of Genome Biol., Natl. Cancer Ctr. Res. Inst.)、Kouya Shiraishi (Dept. of Clin. Genomics, Natl. Cancer Ctr. Res. Inst.／Div. of Genome Biol., Natl. Cancer Ctr. Res. Inst.)

DNA gains and losses are commonly detected in cancer, and emerge as a result of interrelated processes, particularly replication stress and mitotic errors, which can lead to chromosomal instability and aneuploidy. Copy number alterations (CNAs) contribute to cancer development, progression, and resistance to therapy and are important for accurate estimation. To evaluate the short-read-based CNA detection tools for whole genome sequence data, we selected several free software packages, which have been cited over 100 times, released from 2000 to 2021, and are suitable for whole genome sequencing analysis. We specifically focused on large copy number variants (over 1kb), and applied the selected programs to 20 tumor-normal paired clinical specimens with short-read sequencing data. Long-read data were obtained using both PacBio and Oxford Nanopore platforms, and CNAs were identified manually based on depth data. Concordance was examined not only between short-read and long-read, but also two systems for long-reads. Further details are provided in this presentation section.

[Disclosure of COI by Responsible researcher] Lead presenter is not the Responsible researcher.
[Lead presenter: Hanako Ono] ■ employment by a company related to the presentation: 第一三共株式会社（配偶者　従業員）
[Principal Researcher: Kouya Shiraishi] We have no financial relationships to disclose.

▲TOP

[P-1318] I-P20-4
Understanding Cancer Outcomes through Real-World & Multi-Factorial Analysis

2025 / 9 / 25 (Thu) 17:30-18:15 Room P/(Motenashi Dome) Room P

### P-1318
### Differences in the genome structure of ***Akkermansia muciniphila*** between elderly people in Ogimi Village and patients

[Speaker]Shinnosuke Agena (NIT Okinawa advanced course, Biores Engineer) [Co-author]Hidehiro Yokoda (Nagoya Univ, Grad Sch Med, Dept Biochem)、Yukitoshi Iha (Ryukyu Univ, Grad Sch Med, Dept Biochem)、Yuko Murayama (Munemasa Hosp.)、Kuniaki Aridome (Munemasa Hosp.)、Shinya Ikematsu (NIT Okinawa, Bioresources Engineering)

In this study, we focused on the intestinal bacterium ***Akkermansia muciniphila***, which has been reported as a beneficial bacterium that contributes to the improvement of intestinal environment, in the context of the rapid aging of society and the need to extend healthy life expectancy. A. muciniphila was detected by 16S amplicon analysis, we conducted shotgun metagenomic analysis using the NextSeq 1000 on fecal samples from 11 long-lived people (aged 80 or over) in Ogimi Village and four samples each from cancer patients (esophageal cancer and gastric cancer), and we obtained the genome of A. muciniphila. The intraspecific classification using the mycrobiome classification tools StrainPhlAn and PanPhlAn resulted in the creation of two major taxa (A and B).From these results, it is thought that the strains in Group A have functions that improve the health of the host more than those in Group B. In addition, the comparison of genes using PanPhlAn showed that Group A contained a large number of specific gene groups, while Group B contained a large number of different gene groups. This suggests that the gene group specific to taxon A may contribute particularly to healthy longevity.

[Disclosure of COI by Responsible researcher] Lead presenter is not the Responsible researcher.
[Lead presenter: Shinnosuke Agena] We have no financial relationships to disclose.
[Principal Researcher: Kuniaki Aridome] ■Research funds from company: 科学研究費　基盤研究B　■employment by a company related to the presentation: 株式会社iWellness　■Profit of stock: 株式会社iWellness

▲TOP

### [P-1319] I-P20-4
### Understanding Cancer Outcomes through Real-World & Multi-Factorial Analysis

2025 / 9 / 25 (Thu) 17:30-18:15 Room P/(Motenashi Dome) Room P

### P-1319
### Study on the Impact of Co-mutations on Survival in Patients with Biliary Tract Cancer using real-world data in Japan

[Speaker]Yuya Matsui (Division of Phamacotherapeutics, Keio Univ. Fac of Pharm.) [Co-author]Kazuki Ida (Division of Phamacotherapeutics, Keio Univ. Fac of Pharm.)、Yusei Urabe (Division of Phamacotherapeutics, Keio Univ. Fac of Pharm.)、Toshihide Muramatsu (Division of Phamacotherapeutics, Keio Univ. Fac of Pharm.)、Juntaro Matsuzaki (Res. Ctr. for Drug Discovery, Keio Univ. Fac. Pharm.)、Yoshimasa Saito (Division of Phamacotherapeutics, Keio Univ. Fac of Pharm.)

The CDKN2A tumor suppressor gene, frequently inactivated in malignancies such as glioblastoma, lung, and pancreatic cancers, is a crucial determinant of cancer progression and prognosis. While driver mutations in biliary tract cancer (BTC) are well-documented, the impact of CDKN2A loss in BTC remains poorly understood.To elucidate the genomic landscape of BTC in the Japanese population, we analyzed clinical and genomic data from 5,907 BTC patients registered in the Center for Cancer Genomics and Advanced Therapeutics (C-CAT) database between June 2019 and March 2025. We compared survival outcomes between CDKN2A loss and non-loss cohorts in intrahepatic cholangiocarcinoma (IHC), extrahepatic cholangiocarcinoma (EHC), and gallbladder cancer (GBC).Median overall survival (OS) with CDKN2A loss was 13.9months (IHC, n=508/2496), 20.1(EHC, n=303/1847), and 11.9(GBC, n=340/1564), compared to 14.4, 21.5, and 13.5 months in the non-loss group. Significant differences were seen in IHC(HR=0.84, P=0.022) and EHC(HR=0.78, P=0.0059), but not in GBC(HR=0.96, P=0.67).This genomic profiling highlights the subtype-specific impact of CDKN2A loss in BTC and informs the future of targeted therapies.

[Disclosure of COI by Responsible researcher] Lead presenter is not the Responsible researcher.
[Lead presenter: Yuya Matsui] We have no financial relationships to disclose.
[Principal Researcher: Yoshimasa Saito] We have no financial relationships to disclose.

### [P-1320] I-P20-4
### Understanding Cancer Outcomes through Real-World & Multi-Factorial Analysis

2025 / 9 / 25 (Thu) 17:30-18:15 Room P/(Motenashi Dome) Room P

### P-1320
### Data Visualizer for Proteome: Software for Interactive Analysis of Olink Proteomic Data

[Speaker]Daiki Shikata (RIKEN GENESIS CO.,LTD.) [Co-author]Takanori Washio (RIKEN GENESIS CO.,LTD.)

Proteomics plays a crucial role in understanding biological processes through large-scale protein analysis. In cancer research, changes in protein expression significantly affect tumor progression and treatment responses, driving the development of various tools. Recently, Olink's high-sensitivity protein quantification technology has gained attention. However, analyzing proteomic data requires advanced expertise, highlighting the need for user-friendly tools. Here, we developed Data Visualizer for Proteome, a software for interactive analysis of Olink proteomic data (available on GitHub). The software includes various features: It enables users to visually explore sample differences and identify outliers through PCA. Additionally, it provides GSEA results to help researchers identify gene sets strongly associated with expression variations and interpret their biological significance. This tool also offers results of two- or multi-group comparison, and allows users to re-run analyses.This software aims to simplify proteomic data analysis in various biological fields, including cancer research, and is expected to enhance the understanding of complex biological phenomena.

[Disclosure of COI by Responsible researcher] Lead presenter is the Responsible researcher.
[Lead presenter: Daiki Shikata] We have no financial relationships to disclose.

### [P-1321] I-P20-4
### Understanding Cancer Outcomes through Real-World & Multi-Factorial Analysis

2025 / 9 / 25 (Thu) 17:30-18:15 Room P/(Motenashi Dome) Room P

---

### P-1321
### Multi-omics analysis of cancers utilizing alternative lengthening of telomeres

[Speaker]Meng-ting Yeh (Inst. of Mol. & Cell. Biol., Natl. Taiwan Univ.) [Co-author]Hsueh-ping Chu (Inst. of Mol. & Cell. Biol., Natl. Taiwan Univ.)

Telomeres protect chromosome ends from being recognized as DNA damage. In normal somatic cells, telomere shortening limits replicative potential due to end replication problem. While most cancer cells overcome this by activating telomerase, a subset utilizes the alternative lengthening of telomeres (ALT) pathway, which is mechanistically distinct and may influence treatment response. In this study, we applied a multi-omic approach to investigate the molecular features of ALT+ cancer cells. Nanopore long-read sequencing of ALT+ cancer cells revealed frequent inter-telomeric recombination, suggesting genome instability. Whole-exome sequencing (WES) of sarcoma patients identified mutations in ALT-associated genes. Transcriptomic analysis further distinguished TERT+ and ALT+ cancer cells based on ALT-associated gene expression. Additionally, single-cell RNA-seq of glioblastoma revealed subpopulations with elevated ALT-associated gene expression, highlighting cellular heterogeneity. These findings offer structural and transcriptional insights into ALT and support the development of molecular markers for identifying ALT+ tumors in clinical samples.

[Disclosure of COI by Responsible researcher] Lead presenter is not the Responsible researcher.
[Lead presenter: Meng-ting Yeh] We have no financial relationships to disclose.
[Principal Researcher: Hsueh-ping Chu] We have no financial relationships to disclose.

▲TOP

### [P-1322] I-P20-4
### Understanding Cancer Outcomes through Real-World & Multi-Factorial Analysis

2025 / 9 / 25 (Thu) 17:30-18:15 Room P/(Motenashi Dome) Room P

### P-1322
### Interrupt time series analysis for psychiatric diseases on cancer survivors using real world data in Japan

[Speaker]Miki Nagata (Department of Hospital Pharmaceutics, School of Pharmacy, Showa Medical University／Department of Pharmacy, Showa Medical University East Hospital) [Co-author]Kenji Momo (Department of Hospital Pharmaceutics, School of Pharmacy, Showa Medical University／Department of Pharmacy, Showa Medical University Hospital)

Aim: A cancer diagnosis causes significant psychological distress. However, the temporal patterns of psychiatric comorbidities before and after diagnosis remain unclear. This study aimed to clarify their timing in cancer patients.
Method: We analyzed 224,184 cancer patients from DeSC Inc. using interrupted time series (ITS) analysis, with cancer diagnosis as the intervention. The sex- and age-standardized prevalence rate (PR) per 1,000 patients was calculated for major psychiatric comorbidities.
Results: The ITS analysis showed an increasing PR trend post-intervention (p=0.062). A significant increase was found in lung cancer (p=0.041), while breast (p=0.051) and gastrointestinal cancers (p=0.052) showed non-significant increasing trends. No significant trend change was observed over time, but cancer diagnosis caused a major level change in psychiatric comorbidities immediately after diagnosis.
Discussion: This study identified a structural change in psychiatric comorbidities right after cancer diagnosis, highlighting the need for timely mental health interventions.

[Disclosure of COI by Responsible researcher] Lead presenter is not the Responsible researcher.
[Lead presenter: Miki Nagata] We have no financial relationships to disclose.
[Principal Researcher: Kenji Momo] We have no financial relationships to disclose.

### [P-1323] I-P20-4
### Understanding Cancer Outcomes through Real-World & Multi-Factorial Analysis

2025 / 9 / 25 (Thu) 17:30-18:15 Room P/(Motenashi Dome) Room P

---

P-1323

### Prognostic value of perineural invasion in head and neck squamous cell carcinoma: A systematic review and meta-analysis

[Speaker]Luqing Zhang (Div. of Oral and Maxillofacial Surg., Niigata University, Niigata, Japan) [Co-author]Shu Zhu (College of Stomatology, North China University of Science and Technology)、Manabu Yamazaki (Div. of Oral Path., Niigata University, Niigata, Japan)、Hideaki Hirai (Div. of Oral and Maxillofacial Surg., Niigata University, Niigata, Japan)、Junichi Tanuma (Div. of Oral Path., Niigata University, Niigata, Japan)、Kei Tomihara (Div. of Oral and Maxillofacial Surg., Niigata University, Niigata, Japan)

Background: Head and neck squamous cell carcinoma (HNSCC) has been demonstrated to exhibit a high incidence of perineural invasion (PNI). Although several studies have reported an association between PNI and patient prognosis, the precise impact of PNI on clinical outcomes in HNSCC remains uncertain.Objective: This study reviews available literature to evaluate the prognostic value of PNI for the survival of patients with HNSCC.Methods: A systematic search of studies from 2015 to 2025 was conducted based on the Preferred Reporting Items for Systematic Reviews and Meta-analyses (PRISMA) and registered on PROSPERO (CRD 420250651830). Meta-analysis and subgroup analysis were performed to clarify the prognostic role of PNI. Results: Totally, 69 studies with 24,470 patients were included. Patients with PNI had a worse overall survival (HR: 1.85, 95%CI: 1.65-2.07), disease-free survival (HR: 1.77, 95%CI: 1.61-1.94), disease-specific survival (HR: 1.65, 95%CI: 1.49-1.84) and local recurrence (HR: 2.73, 95%CI: 1.82-4.10).Conclusions: The presence of PNI significantly affected overall survival, disease-free survival, disease-specific survival and local recurrence in patients with HNSCC.

[Disclosure of COI by Responsible researcher] Lead presenter is not the Responsible researcher.
[Lead presenter: Luqing Zhang] We have no financial relationships to disclose.
[Principal Researcher: ] We have no financial relationships to disclose.

### [P-1331] II-P2-2
### Colorectal cancer (2)

2025 / 9 / 25 (Thu) 17:30-18:15 Room P/(Motenashi Dome) Room P

### P-1331
### Functional analysis of Galectin-4 in colorectal cancer cells

[Speaker]Takashi Ogawa (Dept. Genome Med. Sci. Faculty of Med. Kagawa Univ.／Dept endocrin., Faculty of Med. Kagawa Univ.) [Co-author]Kensuke Kumamoto (Dept. Genome Med. Sci. Faculty of Med. Kagawa Univ.)、Takanori Nakamura (Dept endocrin., Faculty of Med. Kagawa Univ.)

Galectin-4 is highly expressed in normal intestinal tissues, but it is known that galectin-4 expression is rarely observed in invasive colorectal cancer . Therefore, we investigated the expression of galectin-4 at the protein lev el in colon cancer cell lines. As a result, we were able to classify the cel l lines into two groups: one expressing galectin-4 and the other not express ing galectin-4. To clarify the significance of the decreased expression of galectin-4 in colorectal cancer, we tried to induce the overexpression of galectin-4 in colon cancer cell lines that do not express galectin-4 and to in hibit its expression by siRNA in colon cancer cell lines that do express gal ectin-4. These results showed that galectin-4 inhibited the proliferation of colon cancer cells. In addition, when galectin-4 overexpressed cells were tra nsplanted into the abdominal cavity of mice, their tumorigenic potential was reduced compared to that of the control cells. Currently, we are searching for proteins whose expression is altered by galectin-4 knockdown or over-expression.

[Disclosure of COI by Responsible researcher] Lead presenter is the Responsible researcher.
[Lead presenter: Takashi Ogawa] We have no financial relationships to disclose.

### [P-1332] II-P2-2
### Colorectal cancer (2)

2025 / 9 / 25 (Thu) 17:30-18:15 Room P/(Motenashi Dome) Room P

---

### P-1332
### Dual inhibition of OXPHOS and glycolysis enhances antitumor effects by blocking metabolic shift in colorectal cancer

[Speaker]Akihiko Masuo (Dept. of Surgery, Kyoto Univ.) [Co-author]Nobu Oshima (Dept. of GI Surgery, Kobe City Medical Center General Hospital)、Shigeru Tsunoda (Dept. of Surgery, Kyoto Univ.)、Shigeo Hisamori (Dept. of Surgery, Kyoto Univ.)、Masayuki Matsuo (Dept. of Radiology, Gifu Univ.)、Kazutaka Obama (Dept. of Surgery, Kyoto Univ.)

[Introduction]Cancer cells depend on oxidative phosphorylation (OXPHOS) and glycolysis. Inhibiting one pathway may trigger compensatory adaptation and limit efficacy.[Objective]We tested if dual inhibition of OXPHOS and glycolysis enhances antitumor effects in colorectal cancer.[Methods]Colorectal cancer cells and xenografts were treated with IACS-010759 and NCI-006, alone or combined. Tumor growth, ATP, and pH were evaluated. Metabolism was analyzed by Seahorse XF96 and 13C-MRSI.[Results]IACS-010759 increased glycolysis and modestly suppressed tumor growth. NCI-006 showed limited or no effects in some models. Dual treatment caused ATP depletion, pH elevation, and synergistic tumor suppression. 13C-MRSI showed lactate elevation with IACS-010759, suggesting a metabolic shift, which was blocked by dual inhibition.[Discussion]Effects may result from energy loss and microenvironmental improvement. Blocking metabolic shift likely led to ATP depletion and pH changes. These findings suggest disrupting metabolic flexibility enhances therapeutic response in adaptive tumors.[Conclusion]Dual inhibition may effectively suppress tumor metabolism in colorectal cancer.

[Disclosure of COI by Responsible researcher] Lead presenter is not the Responsible researcher.
[Lead presenter: Akihiko Masuo] We have no financial relationships to disclose.
[Principal Researcher: Nobu Oshima] We have no financial relationships to disclose.

### [P-1333] II-P2-2
### Colorectal cancer (2)

2025 / 9 / 25 (Thu) 17:30-18:15 Room P/(Motenashi Dome) Room P

---

### P-1333
### Oncogenic role of EEPD1 via protein kinase B phosphorylation in colorectal cancer

[Speaker]Ruiya Ma (Dept of GIP Surg, Mie Univ) [Co-author]Tadanobu Shimura (Dept of GIP Surg, Mie Univ)、Yoshinaga Okugawa (Dept of GIP Surg, Mie Univ／Dept of Genomic medicine, Mie Univ Hosp)、Takahito Kitajima (Dept of GIP Surg, Mie Univ／Dept of Genomic medicine, Mie Univ Hosp)、Yuji Toiyama (Dept of GIP Surg, Mie Univ)

[Background and Methods] Endonuclease/exonuclease/phosphatase family domain-containing 1 (EEPD1) is involved in DNA repair and promotes the repair of oxidative-stressed replication forks. This study explored the oncological importance of EEPD1 in colorectal cancer (CRC). We evaluated the levels of EEPD1 mRNA by reverse transcription-quantitative PCR and of EEPD1 protein by immunohistochemistry in samples from 289 CRC patients. We investigated the cancer promoting function of EEPD1 by performing a series of in vitro assays in CRC cells (SW620 and WiDr). [Results] We revealed that the mRNA and protein levels of EEPD1 were remarkably higher in tumor tissues than in adjacent normal mucosa. Patients with high levels of EEPD1 showed significantly poorer overall survival and recurrence-free survival. Moreover, siRNA knockdown of EEPD1 significantly inhibited cell proliferation and migration activity, enhanced apoptotic activity, and reduced phosphorylation of protein kinase B (AKT) in CRC cells. [Conclusion] EEPD1 is a promising prognostic and recurrence-predictive biomarker in CRC patients, and is a potential therapeutic target in CRC.

[Disclosure of COI by Responsible researcher] Lead presenter is not the Responsible researcher.
[Lead presenter: Ruiya Ma] We have no financial relationships to disclose.
[Principal Researcher: Yuji Toiyama] ■Contributions: 日本イーライリリー株式会社

### [P-1334] II-P2-2
### Colorectal cancer (2)

2025 / 9 / 25 (Thu) 17:30-18:15 Room P/(Motenashi Dome) Room P

---

### P-1334
### Low-molecular-weight hyaluronan may cause vascular hypoplasia in the Immature-type stroma of colorectal cancer (CRC)

[Speaker]Kazuki Aihara (Department of Surgery, National Defense Medical College) [Co-author]Satsuki Mochizuki (Department of Surgery, National Defense Medical College)、Hiroyuki Yoshida (Biological Science Research, Kao Corporation)、Yoko Endo (Biological Science Research, Kao Corporation)、Takatsugu Ishimoto (Division of Carcinogenesis, The Cancer Institute, JFCR)、Koji Moriya (Department of Surgery, National Defense Medical College)、Masato Yamadera (Department of Surgery, National Defense Medical College)、Koichi Okamoto (Department of Surgery, National Defense Medical College)、Yoshiki Kajiwara (Department of Surgery, National Defense Medical College)、Yasunori Okada (Department of Pathophysiology, Juntendo University Graduate School of Medicine)、Hideki Ueno (Department of Surgery, National Defense Medical College)

Desmoplastic reaction (DR), morphologically classified as Mature, Intermediate and Immature, is correlated with prognosis of CRC patients. We previously reported that Immature stroma is characterized, albeit unknown mechanism, by decreased numbers of microvessels and immune cells. In this study, we investigated biochemical characteristics of Immature stroma, which affects prognosis most adversely. Using laser-capture microdissection and microarray panel, we found the high expression of hyaluronan (HA) synthase 1 in Immature stroma. Biochemical analyses indicated that low-molecular-weight HA is highly accumulated and HA-degrading enzyme HYBID is overexpressed in Immature stroma. RNA-seq analysis and barrier function assay showed that the expression levels of cell adhesion molecules such as cadherin and barrier function of endothelial cell sheet are significantly decreased by treating them with low-molecular-weight HA compared to high-molecular-weight HA. These results suggest that HYBID-mediated production of low-molecular-weight HA creates unfavorable stromal environment with vascular hypoplasia, which may cause chemoresistance and poor prognosis in CRC patients with Immature DR.

[Disclosure of COI by Responsible researcher] Lead presenter is the Responsible researcher.
[Lead presenter: Kazuki Aihara] We have no financial relationships to disclose.

▲TOP

### [P-1335] II-P2-2
### Colorectal cancer (2)

2025 / 9 / 25 (Thu) 17:30-18:15 Room P/(Motenashi Dome) Room P

---

### P-1335
### Genome-wide DNA methylation profiling of colitis-associated and sporadic colorectal cancer using FFPE tissues

[Speaker]Ayano Saito (Dept. Surgical Oncology, Univ. Tokyo) [Co-author]Genta Nagae (Genome Science Div., RCAST, Univ. Tokyo)、Yuichiro Yokoyama (Dept. Surgical Oncology, Univ. Tokyo)、Hiroyuki Aburatani (Genome Science Div., RCAST, Univ. Tokyo)、Soichiro Ishihara (Dept. Surgical Oncology, Univ. Tokyo)

Ulcerative colitis (UC) is a chronic inflammatory bowel disease of unknown etiology affecting the colon. The number of UC patients is increasing, and long–standing UC is associated with a higher risk of colorectal cancer (CRC). Although the adenoma–carcinoma sequence through the accumulation of genetic mutations in sporadic CRC (sCRC) has been well studied by multi–omics analyses, the molecular mechanism that colitis–associated cancer (CAC) develops through an inflammation–dysplasia–carcinoma sequence driven by chronic inflammation remains poorly understood. To uncover the difference of carcinogenic process between these CRCs, we conducted a comprehensive DNA methylation analysis using FFPE samples from CAC and sCRC patients who underwent surgery at the University of Tokyo Hospital between 2014 and 2024. We would like to report on the technical feasibility of FFPE samples for methylome analysis and discuss the comparative results of epigenetic dysregulation in CAC and sCRC.

[Disclosure of COI by Responsible researcher] Lead presenter is not the Responsible researcher.
[Lead presenter: Ayano Saito] We have no financial relationships to disclose.
[Principal Researcher: Soichiro Ishihara] We have no financial relationships to disclose.

### [P-1336] II-P2-2
### Colorectal cancer (2)

2025 / 9 / 25 (Thu) 17:30-18:15 Room P/(Motenashi Dome) Room P

### P-1336
### Synergistic Antitumor Effects of Aspirin and Metformin in Colorectal Cancer Organoids

[Speaker]Kojiro Inoue (Department of Gastroenterology and Hepatology, Yokohama City University) [Co-author]Naomi Morikubo (Department of Gastroenterology and Hepatology, Yokohama City University)、Tetsuya Matsuura (Department of Gastroenterology and Hepatology, Yokohama City University)、Takuma Higurashi (Department of Gastroenterology and Hepatology, Yokohama City University)

Chemoprevention is a strategy to reduce colorectal cancer mortality, with various agents studied for their preventive effects. Aspirin and metformin have shown individual efficacy via distinct mechanisms, but their synergistic potential remains unclear. We examined the antitumor effects of low-dose aspirin and metformin using patient-derived colorectal cancer organoids. Organoids were treated with aspirin (0.25-1.0 mM), metformin (0.25-1.0 mM), or both. Cell proliferation was assessed by cell counts; apoptosis and cell cycle markers were evaluated by immunohistochemistry, Western blotting, and quantitative real-time PCR. Combination treatment significantly inhibited growth even at 0.25 mM, where monotherapy showed little effect. Ki-67 expression was reduced and cleaved caspase-3 increased, indicating enhanced antiproliferative and pro-apoptotic activity. COX-2 expression was more strongly suppressed in the combination group. These results suggest a synergistic mechanism involving COX-2/PGE2 pathways. This study supports aspirin and metformin combination therapy as a potential chemopreventive strategy.

[Disclosure of COI by Responsible researcher] Lead presenter is not the Responsible researcher.
[Lead presenter: Kojiro Inoue] We have no financial relationships to disclose.
[Principal Researcher: Naomi Morikubo] We have no financial relationships to disclose.

[P-1337] II-P2-2
Colorectal cancer (2)

2025 / 9 / 25 (Thu) 17:30-18:15 Room P/(Motenashi Dome) Room P

### P-1337
### Sex-Dependent Prognostic Significance of CD99 in Tumor Cells and Cancer-Associated Fibroblasts in Colorectal Cancer

[Speaker]Chengbo Wang (Dept. Exp. Pathol. and Tumor Biol., Nagoya City Univ.) [Co-author]Masayuki Komura (Dept. Exp. Pathol. and Tumor Biol., Nagoya City Univ.／Dept. Pathol., Nagoya City Univ. East Med. Ctr.)、Hiroyuki Kato (Dept. Exp. Pathol. and Tumor Biol., Nagoya City Univ.)、Aya Nagano (Dept. Exp. Pathol. and Tumor Biol., Nagoya City Univ.)、Aya Naiki (Dept. Exp. Pathol. and Tumor Biol., Nagoya City Univ.)、Shingo Inaguma (Dept. Exp. Pathol. and Tumor Biol., Nagoya City Univ.／Dept. Pathol., Nagoya City Univ. East Med. Ctr.)、Satoru(Nagoya City Univ.) Takahashi (Dept. Exp. Pathol. and Tumor Biol., Nagoya City Univ.)

CD99 is a 32 kDa transmembrane glycoprotein involved in adhesion, migration, apoptosis and immune responses. In diagnostic pathology, CD99 immunohistochemistry is used to distinguish Ewing sarcoma from other small round cell tumors. This study analyzed CD99 expression in tumor cells (tCD99) and cancer-associated fibroblasts (fCD99) in 269 colorectal cancers (CRC). CD99 was detected in tumor cells, CAFs and normal mucosa. Loss of tCD99 occurred in 55% of cases and was linked to peritoneal ($p = 0.01$) and distant metastasis ($p = 0.014$) and proficient MMR ($p = 0.033$). High fCD99 (102 cases) was more common in males ($p < 0.001$) and associated with smaller size ($p = 0.019$), rectal location ($p = 0.014$), better differentiation ($p < 0.05$), less mucus ($p = 0.02$), lymph node metastasis ($p = 0.02$) and MMR deficiency ($p = 0.02$). Patients lacking tCD99 had poor 5-year survival ($p = 0.021$), more significantly in males ($p = 0.005$). High fCD99 showed no overall survival impact but predicted worse prognosis in females ($p = 0.036$). In vitro study, CD99 promoted proliferation but reduced migration of CRCs cells. CD99 may serve as a prognostic marker in CRC, with cell type- and sex-specific relevance.

[Disclosure of COI by Responsible researcher] Lead presenter is not the Responsible researcher.
[Lead presenter: Chengbo Wang] We have no financial relationships to disclose.
[Principal Researcher: Shingo Inaguma] We have no financial relationships to disclose.

### [P-1347] II-P2-4
### Eophageal cancer

2025 / 9 / 25 (Thu) 17:30-18:15 Room P/(Motenashi Dome) Room P

---

### P-1347
### Survival and recurrence characteristics of PCR in esophageal cancer after neoadjuvant immunochemotherapy

[Speaker]Feng Wang (The Dept of Thoracic Surgery, Beijing Chest Hospital) [Co-author]Yi Han (The Dept of Thoracic Surgery, Beijing Chest Hospital)

Background The study aims to investigates the survival and recurrence patterns of pCR in patients with esophageal cancer afterneoadjuvant immunochemotherapy.MethodsWe retrospectively collected 300 patients with esophageal cancer who underwent neoadjuvant immunochemotherapy between 2019 and 2024. The Cox proportional hazards model and the Kaplan Meier method after propensity score matching were used for survival analysis to evaluate the relationship between pCR and overall survival (OS) and recurrence free survival (RFS). ResultsIn the 300 patients assessed in the study, 80 individuals exhibited pCR. The Cox hazards model indicated that pCR was a statistically significant prognostic factor for overall survival and recurrence free survival. Following propensity score matching, survival analysis indicated that patients with pCR demonstrated superior RFS. The recurrence rate comprising a local and distant recurrence rate were significantly lower than those who did not achieve pCR.ConclusionFor patients with stage II-III esophageal cancer who underwent neoadjuvant immunochemotherapy, achieving pCR has a significant survival benefit and a lower local and distant recurrence rate.

[Disclosure of COI by Responsible researcher] Lead presenter is the Responsible researcher.
[Lead presenter: Feng Wang] We have no financial relationships to disclose.

### [P-1348] II-P2-4
### Eophageal cancer

2025 / 9 / 25 (Thu) 17:30-18:15 Room P/(Motenashi Dome) Room P

---

### P-1348
### Evaluation of Factors Influencing the Efficacy of Preoperative HIMAC Therapy in Esophageal Squamous Cell Carcinoma

[Speaker]Nobufumi Sekino (Dept. of Frontier Surg., Grad. Sch. of Med., Chiba Univ.) [Co-author]Takeshi Toyozumi (Dept. of Frontier Surg., Grad. Sch. of Med., Chiba Univ.)、Kentaro Murakami (Dept. of Frontier Surg., Grad. Sch. of Med., Chiba Univ.)、Masaya Uesato (Dept. of Frontier Surg., Grad. Sch. of Med., Chiba Univ.)、Akira Nakano (Dept. of Frontier Surg., Grad. Sch. of Med., Chiba Univ.)、Tadashi Shiraishi (Dept. of Frontier Surg., Grad. Sch. of Med., Chiba Univ.)、Koichi Hayano (Dept. of Frontier Surg., Grad. Sch. of Med., Chiba Univ.)、Yasunori Matsumoto (Dept. of Frontier Surg., Grad. Sch. of Med., Chiba Univ.)、Yoshihiro Kurata (Dept. of Frontier Surg., Grad. Sch. of Med., Chiba Univ.)、Tetsuro Isozaki (Dept of Radiology, Natl. Inst. QST Hospital)、Hitoshi Ishikawa (Dept of Radiology, Natl. Inst. QST Hospital)、Shigeru Yamada (Dept of Radiology, Natl. Inst. QST Hospital)、Hisahiro Matsubara (Dept. of Frontier Surg., Grad. Sch. of Med., Chiba Univ.)

[Background] Esophageal squamous cell carcinoma (ESCC) remains difficult to treat despite multimodal approaches. Our department uses preoperative heavy ion therapy at the Heavy Ion Medical Accelerator in Chiba (HIMAC), achieving favorable outcomes, though predictive factors for response remain unclear.[Aim] To investigate correlations between pre-HIMAC clinical parameters and pathological response in ESCC, aiming to identify factors influencing therapeutic efficacy.[Methods] We retrospectively analyzed 20 ESCC patients who underwent esophagectomy after preoperative HIMAC therapy from Sep 2004 to Oct 2022. Initial data including blood tests and PET SUV values were assessed. Parameters were dichotomized using the Youden Index, and their association with pathological response was examined.[Results] Pathological response was Grade 1b/2/3 in 1/9/10. Grade 3 response significantly correlated with low D-dimer (0.6) and low SUV (10.8). High BMI (24.0), low WBC (8200), and high total protein (7.3) showed trends.[Conclusion] Several pre-treatment factors were associated with better response to HIMAC therapy, potentially aiding in treatment planning and patient selection.

[Disclosure of COI by Responsible researcher] Lead presenter is the Responsible researcher.
[Lead presenter: Nobufumi Sekino] We have no financial relationships to disclose.

### [P-1357] II-P3-2
### Biliary tract cancer, pancreatic cancer

2025 / 9 / 25 (Thu) 17:30-18:15 Room P/(Motenashi Dome) Room P

### P-1357
### The Functional Role of the SWI/SNF Complex Subunit BRG1 in Biliary Carcinogenesis

[Speaker]Shinnosuke Nakayama (Kyoto University Hospital) [Co-author]Akihisa Fukuda (Kyoto University Hospital)、Sho Matsuyama (Kyoto University Hospital)、Kei Iimori (Kyoto University Hospital)、Naoki Aoyama (Kyoto University Hospital)、Kenta Mizukoshi (Kyoto University Hospital)、Munenori Kawai (Kyoto University Hospital)、Go Yamakawa (Kyoto University Hospital)、Kosuke Iwane (Kyoto University Hospital)、Mayuki Omatsu (Kyoto University Hospital)、Mio Namikawa (Kyoto University Hospital)、Makoto Sono (Kyoto University Hospital)、Munemasa Nagao (Kyoto University Hospital)、Takahisa Maruno (Kyoto University Hospital)、Yuki Nakanishi (Kyoto University Hospital)、Hiroshi Seno (Kyoto University Hospital)

Inactivating mutations of the SWI/SNF chromatin remodeling factors are observed in multiple human cancers, including cholangiocarcinoma (CCA). We investigated the role of BRG1, a subunit of the SWI/SNF complex, in biliary carcinogenesis. In human CCAs, BRG1 expression was lost in 12% of extrahepatic, 28% of intrahepatic (large duct type), and 14% of intrahepatic (small duct type) CCAs. To clarify the impact of BRG1 loss, we used Hnf1bCreER mice to generate biliary-specific knockout of Pten (HP), Brg1 (HB), or both (HPB). HP mice exhibited only biliary dysplasia and HB mice had no notable biliary lesions. In contrast, HPB mice developed invasive cholangiocarcinoma, accompanied by marked inflammation, increased ROS, and upregulated IL-1b/caspase-1 expression, indicating inflammasome activation. Moreover, pharmacological blockade of the inflammasome by disulfiram significantly suppressed biliary tumor formation in HPB mice, suggesting that inflammasome-driven inflammation contributes to biliary tumorigenesis in HPB mice. Our data indicate that concomitant PI3K/AKT activation and BRG1 inactivation cooperate to form cholangiocarcinoma partly through the inflammasome-driven inflammation.

[Disclosure of COI by Responsible researcher] Lead presenter is not the Responsible researcher.
[Lead presenter: Shinnosuke Nakayama] We have no financial relationships to disclose.
[Principal Researcher: Fukuda Akihisa] We have no financial relationships to disclose.

### [P-1358] II-P3-2
### Biliary tract cancer, pancreatic cancer

2025 / 9 / 25 (Thu) 17:30-18:15 Room P/(Motenashi Dome) Room P

---

### P-1358
### Expression and functional analysis of PODXL2 in intrahepatic cholangiocarcinoma cell lines

[Speaker]Minano Kayamori (Institute of Science Tokyo, Grad. Sch. Medical and Dental Sciences) [Co-author]Nozomi Takahashi (Institute of Science Tokyo, Grad. Sch. Medical and Dental Sciences)、Kaede Hatakeyama (Institute of Science Tokyo, Grad. Sch. Medical and Dental Sciences)、Kana Asanuma (Institute of Science Tokyo, Faculty of Med.)、Erina Iwabuchi (Institute of Science Tokyo, Grad. Sch. Medical and Dental Sciences)、Yurie Soejima (Institute of Science Tokyo, Grad. Sch. Medical and Dental Sciences)

Intrahepatic cholangiocarcinoma (iCCA) is a tumor arising above the second-order bile ducts and has a poor prognosis due to the lack of specific diagnostic markers. We have reported that integrin αvβ6 (β6), a cell adhesion molecule, may be a pathological diagnostic marker and therapeutic target molecule for iCCA. Furthermore, PODXL2 (podocalyxin-like 2) was identified as the most downregulated gene in ITGB6 knockout iCCA cell lines using RNA-seq analysis. PODXL2 is a member of the CD34 family, however, few reports have described its role in cancer. In this study, we aimed to analyze the expression and function of PODXL2 in iCCA cell lines and to clarify its association with integrins. First, PODXL2 was differentially expressed in several iCCA cell lines. Next, suppression of PODXL2 expression with siRNA reduced migration, invasion, and proliferation abilities, and expression of integrin β6 and its downstream signaling molecules. The proximity ligation assay (PLA) revealed protein-protein interactions between PODXL2 and integrin β6. These results suggest that PODXL2 may be a potential therapeutic target molecule for iCCA and may interact with integrin β6 to regulate its activation.

[Disclosure of COI by Responsible researcher] Lead presenter is not the Responsible researcher.
[Lead presenter: Minano Kayamori] We have no financial relationships to disclose.
[Principal Researcher: Yurie Soejima] We have no financial relationships to disclose.

[P-1359] II-P3-2
Biliary tract cancer, pancreatic cancer

2025 / 9 / 25 (Thu) 17:30-18:15 Room P/(Motenashi Dome) Room P

P-1359

### Development of a Novel Cholangioscopy System for 5-ALA based Photodynamic Diagnosis of Cholangiocarcinoma

[Speaker]Hiroaki Fujiwara (Inst. Med. Sci. Asahi Life Foundation Div. of Gastroenterology／The Univ. of Tokyo Dept. of Gastroenterology) [Co-author]Naminatsu Takahara (The Univ. of Tokyo Dept. of Gastroenterology)、Yousuke Nakai (The Univ. of Tokyo Dept. of Gastroenterology／Tokyo Women's Medical University Hospital Dept. of Gastroenterology)、Mitsuhiro Fujishiro (The Univ. of Tokyo Dept. of Gastroenterology)

Background: Improving the diagnostic accuracy of cholangiocarcinoma remains a challenge. 5-Aminolevulinic acid (5-ALA)-based photodynamic diagnosis (5-ALA-PDD), which detects red fluorescence from its metabolite protoporphyrin IX (Pp IX) under blue light, is clinically used in brain and bladder cancers. We previously demonstrated its potential in cholangiocarcinoma using patient-derived organoids (Surg Oncol. 2020), but no device is optimized for bile duct imaging. Methods: A prototype system was developed using a slim high-resolution camera and a light guide emitting white or blue light, compatible with a commercial cholangioscope. Fluorescence detection was evaluated in Pp IX-containing gels and 5-ALA-treated subcutaneous tumor models. Intensity was quantified via image analysis. Results: In gel models, Pp IX concentrations >5 μM produced fluorescence above the visible threshold (59 a.u.; observed >110.9 a.u.). In vivo, tumor fluorescence was significantly stronger than surrounding tissue (139.1 vs. 14.1 a.u., $P < 0.001$). Conclusion: Cholangioscopic 5-ALA-PDD is feasible. Further technical refinements are underway for clinical application.

[Disclosure of COI by Responsible researcher] Lead presenter is the Responsible researcher.
[Lead presenter: Fujiwara Hiroaki] ■Research funds from company: Japan Lifeline Co., Ltd

### [P-1360] II-P3-2
### Biliary tract cancer, pancreatic cancer

2025 / 9 / 25 (Thu) 17:30-18:15 Room P/(Motenashi Dome) Room P

---

### P-1360
### Hilar Cholangiocarcinoma Treated with Chemoimmunotherapy and Conversion Surgery

[Speaker]Norifumi Iseda (Department of Surgery, Japanese Red Cross Matsuyama Hospital) [Co-author]Ryosuke Minagawa (Department of Surgery, Japanese Red Cross Matsuyama Hospital)、Koichi Kimura (Department of Surgery, Japanese Red Cross Matsuyama Hospital)、Takashi Nishizaki (Department of Surgery, Japanese Red Cross Matsuyama Hospital)

(Background)Cholangiocarcinoma (CC) has a poor prognosis and few treatment options. Conversion surgery for unresectable CC has been frequently reported. In this study, we report the two cases of a patient with unresectable hilar CC who received durvalumab plus gemcitabine and cisplatin therapy and conversion surgery. (Case1) A 70-year-old man was diagnosed with hilar CC (cT3N1M1, Stage 4B). Metastasis was observed in the para-aortic lymph nodes. After durvalumab plus gemcitabine and cisplatin therapy, metastasis in para-aortic lymph nodes disappeared and right lobe hepatectomy and extrahepatic biliary reconstruction were performed as conversion surgeries. (Case2)A 74-year-old women was diagnosed with hilar CC (cT3N0M0, Stage 3A). CC centered on the left hepatic duct with hepatic S4 and right hepatic artery invasion. After durvalumab plus gemcitabine and cisplatin therapy, right lobe hepatectomy and extrahepatic biliary reconstruction were performed as conversion surgeries. (Conclusion) Immunotherapy with durvalumab, followed by conversion surgery, may improve the prognosis of patients with unresectable CC.

[Disclosure of COI by Responsible researcher] Lead presenter is the Responsible researcher.
[Lead presenter: Norifumi Iseda] We have no financial relationships to disclose.

[P-1361] II-P3-2

### Biliary tract cancer, pancreatic cancer

2025 / 9 / 25 (Thu) 17:30-18:15 Room P/(Motenashi Dome) Room P

---

P-1361

### Prognostic Value of Fibrosis in Metastatic Lymph Nodes After NAC and Surgery for Resectable Pancreatic Cancer

[Speaker]Hirofumi Shirakawa (Department of HepatoBiliary-Pancreatic Surgery, Tochigi Cancer Center) [Co-author]Hidenori Ojima (Department of Pathology, Tochigi Cancer Center)、Naoto Kubota (Department of Pathology, Tochigi Cancer Center)、Masatsugu Ishii (Department of HepatoBiliary-Pancreatic Surgery, Tochigi Cancer Center)

BackgroundFibrosis is common in pancreatic cancer, but its prognostic significance in metastatic lymph nodes after neoadjuvant chemotherapy (NAC) remains unclear. We investigated the association between nodal fibrosis and outcomes in patients treated with NAC and surgery.MethodsWe reviewed 39 patients with resectable pancreatic cancer and lymph node metastases who underwent NAC and surgery from 2019-2023. Fibrosis in metastatic nodes was evaluated histologically. Surgeries were PD/PPPD in 29 and distal pancreatectomy in 22, with portal vein resection in 11. About 78% of patients received adjuvant chemotherapy.ResultsFibrosis in metastatic nodes was observed in 65% of patients. Those with nodal fibrosis had significantly longer overall survival (28.7 vs 16.7 months) and recurrence-free survival (17.2 vs 8.3 months; p=0.001 for both), and this benefit was independent of nodal location.ConclusionFibrosis in metastatic lymph nodes may serve as a prognostic indicator in resectable pancreatic cancer after NAC and surgery.

[Disclosure of COI by Responsible researcher] Lead presenter is the Responsible researcher.
[Lead presenter: Shirakawa Hirofumi] We have no financial relationships to disclose.

### [P-1362] II-P3-2
### Biliary tract cancer, pancreatic cancer

2025 / 9 / 25 (Thu) 17:30-18:15 Room P/(Motenashi Dome) Room P

---

### P-1362
### The role of Actinin-4 and its phosphorylation in pancreatitis and pancreatic cancer

[Speaker]Hiroyuki Kato (Dep. Exp. Pathol. Tumor Biol., Nagoya City Univ.) [Co-author]Aya Naiki (Dep. Exp. Pathol. Tumor Biol., Nagoya City Univ.)、Masayuki Komura (Dep. Exp. Pathol. Tumor Biol., Nagoya City Univ.)、Shingo Inaguma (Dep. Exp. Pathol. Tumor Biol., Nagoya City Univ.)、Satoru(Nagoya City Univ.) Takahashi (Dep. Exp. Pathol. Tumor Biol., Nagoya City Univ.)

At the last JCA conference, we reported that upregulation of Actinin-4 (Actn4) and its phosphorylated form p-Actn4 (S160), identified via proteomic analysis in a mouse pancreatitis model, may contribute to acinar-ductal metaplasia (ADM) and pancreatic intraepithelial neoplasia (PanIN) development. In this study, we examined Actn4 expression in pancreatitis and pancreatic carcinoma (PDAC) tissue and investigated the effects of Actn4 and p-Actn4(S160) in vitro. In both mouse and human pancreas, Actn4 expression was elevated in ADM and PanIN compared to acinar cells, along with F-actin formation. In PDACs, high Actn4 expression was more frequent in well-differentiated tumors but was associated with poor prognosis. To study its function, we established AR42J cell lines expressing full-length Actn4, phospho-mimetic Actn4(S160D), and phosphor-mimetic Actn4(S160A). Increased Actn4 led to upregulation of $\beta$-actin, and Actn4(S160D)-expressing cells showed enhanced proliferation and migration. These findings suggest that S160 phosphorylation activates Actn4, enhancing actin remodeling and motility, and that high Actn4 expression may be useful as a poor prognostic marker in PDACs.

[Disclosure of COI by Responsible researcher] Lead presenter is the Responsible researcher.
[Lead presenter: Hiroyuki Kato] We have no financial relationships to disclose.

[P-1363] II-P3-2
Biliary tract cancer, pancreatic cancer

2025 / 9 / 25 (Thu) 17:30-18:15 Room P/(Motenashi Dome) Room P

P-1363
### Surfaceome-Based therapeutic Target Discovery in Pancreatic Ductal Adenocarcinoma.

[Speaker]Takayasu Azuma (Dept. Hepato-Biliary-Pancreatic. Transpl. Surg., Fukushima Med. Univ. Sch. Med.) [Co-author]Makoto Kobayashi (Dept. Basic. Pathol., Fukushima Med. Univ. Sch. Med.)、Kotaro Sugimoto (Dept. Basic. Pathol., Fukushima Med. Univ. Sch. Med.)、Minaka Ishibashi (Dept. Basic. Pathol., Fukushima Med. Univ. Sch. Med.)、Emi Mishiro (Inst. Transformative Biomolecules, Nagoya Univ.)、Aoto Ebe (Dept. Basic. Pathol., Fukushima Med. Univ. Sch. Med.)、Atsushi Ozeki (Dept. Hepato-Biliary-Pancreatic. Transpl. Surg., Fukushima Med. Univ. Sch. Med.)、Shigeyuki Tsukida (Dept. Hepato-Biliary-Pancreatic. Transpl. Surg., Fukushima Med. Univ. Sch. Med.)、Makoto Muto (Dept. Hepato-Biliary-Pancreatic. Transpl. Surg., Fukushima Med. Univ. Sch. Med.)、Yasuhide Kofunato (Dept. Hepato-Biliary-Pancreatic. Transpl. Surg., Fukushima Med. Univ. Sch. Med.)、Takashi Kimura (Dept. Hepato-Biliary-Pancreatic. Transpl. Surg., Fukushima Med. Univ. Sch. Med.)、Akira Kenjo (Dept. Hepato-Biliary-Pancreatic. Transpl. Surg., Fukushima Med. Univ. Sch. Med.)、Shigeru Marubashi (Dept. Hepato-Biliary-Pancreatic. Transpl. Surg., Fukushima Med. Univ. Sch. Med.)、Hideki Chiba (Dept. Basic. Pathol., Fukushima Med. Univ. Sch. Med.)

Pancreatic ductal adenocarcinoma (PDAC) remains one of the most fatal malignancies, underscoring the critical need for novel, targetable molecular interventions. Given their accessibility and pharmacologic tractability, cell surface proteins represent promising candidates for therapeutic exploitation. In this study, we performed a comprehensive proteomic interrogation of the PDAC surfaceome to uncover novel therapeutic targets. Utilizing a biotin-streptavidin enrichment strategy coupled with high-resolution mass spectrometry, we profiled surface proteins across six PDAC cell lines, identifying over 7,000 unique proteins. Among these, 92 proteins were consistently expressed across all six lines, highlighting their potential as broadly applicable therapeutic targets. Here, we present proof-of-concept findings focusing on one such candidate, elucidating its functional role in PDAC pathobiology and evaluating its viability as a novel molecular target.

[Disclosure of COI by Responsible researcher] Lead presenter is not the Responsible researcher.
[Lead presenter: Takayasu Azuma] We have no financial relationships to disclose.
[Principal Researcher: Kobayashi Makoto] We have no financial relationships to disclose.

▲TOP

### [P-1371] II-P4-1
### Head & neck tumors (1)

2025 / 9 / 25 (Thu) 17:30-18:15 Room P/(Motenashi Dome) Room P

P-1371

### Therapeutic targeting of STAT3 with BBI608 induces apoptosis in oral squamous cell carcinoma

[Speaker]Dongguk Park (Dept. of Oral Path., Sch. of Dent., SNU) [Co-author]Sung-Dae Cho (Dept. of Oral Path., Sch. of Dent., SNU)、Sujung Choi (Dept. of Oral Path., Sch. of Dent., SNU)

Signal transduction and activator of transcription 3 (STAT3) is abnormally activated in many cancers, with elevated nuclear levels of STAT3 strongly associated with poor prognosis in oral squamous cell carcinoma (OSCC). Despite ongoing progress in developing targeted therapies, no food and drug administration-approved drug currently targets STAT3 in OSCC. In this study, we observed that BBI608 inhibited cell proliferation in a concentration-dependent manner and triggered caspase 3-dependent apoptosis. Additionally, BBI608 reduced the nuclear translocation of p-STAT3. Mechanistically, it transcriptionally regulated survivin expression, while myeloid cell leukemia-1 expression was modulated post-translationally via proteasomal degradation. Consistent with results from 2D culture, BBI608 showed effective anti-cancer effects against OSCC spheroids. These results suggest that BBI608 effectively inhibits p-STAT3 expression in both 2D and 3D models, offering a promising therapeutic strategy and supporting its potential for repurposing in OSCC patients with elevated STAT3 activity. NRF grants (RS-2025-00553553 and RS-2024-00461097).

[Disclosure of COI by Responsible researcher] Lead presenter is the Responsible researcher.
[Lead presenter: Dongguk Park] We have no financial relationships to disclose.

### [P-1372] II-P4-1
### Head & neck tumors (1)

2025 / 9 / 25 (Thu) 17:30-18:15 Room P/(Motenashi Dome) Room P

### P-1372
### Withdrawn

No Abstract

### [P-1373] II-P4-1
### Head & neck tumors (1)

2025 / 9 / 25 (Thu) 17:30-18:15 Room P/(Motenashi Dome) Room P

---

### P-1373
### Transcriptome Analysis Identifies a Prognostic Gene Signature and Therapeutic Target in Oral Squamous Cell Carcinoma

[Speaker]Siwakorn Boonpok (Dept. of Microbiol, Fac. of Med., Khon Kaen Univ.／HEC, Fac. of Med., Khon Kaen Univ.) [Co-author]Sirinart Aromseree (Dept. of Microbiol, Fac. of Med., Khon Kaen Univ.／HEC, Fac. of Med., Khon Kaen Univ.)、Chukkris Heawchaiyaphum (Dept. of Microbiol, Fac. of Med., Khon Kaen Univ.／HEC, Fac. of Med., Khon Kaen Univ.)

Oral squamous cell carcinoma (OSCC) is the most common and aggressive form of head and neck cancer (HNC), with a high mortality rate. Understanding its molecular mechanisms is crucial for improving prognosis and therapeutic strategies. This study analyzed transcriptomic data to identify the potential candidate prognostic and therapeutic targets. Here, a total of 855 genes were upregulated, while 1943 were downregulated in OSCC. Upregulated genes were enriched in immune responses, particularly cytokine signaling, and cell cycle regulation, whereas downregulated genes were associated with muscle system processes and calcium signaling. Among the upregulated genes, CXCL10, IL6, AURKB, MELK, MMP1, and MMP9 were identified as hub genes. Notably, MMP1 was significantly upregulated and correlated with poor prognosis in HNC patients. Drug repurposing analysis identified chlordiazepoxide (-8.7 kcal/mol), triamterene (-8.7 kcal/mol), estazolam (-8.5 kcal/mol) as candidate drugs for HNC by targeting MMP1. These findings provide a molecular signature that could improve OSCC prognosis and guide targeted therapies.

[Disclosure of COI by Responsible researcher] Lead presenter is not the Responsible researcher.
[Lead presenter: Siwakorn Boonpok] We have no financial relationships to disclose.
[Principal Researcher: Chukkris Heawchaiyaphum] We have no financial relationships to disclose.

▲TOP

### [P-1374] II-P4-1
### Head & neck tumors (1)

2025 / 9 / 25 (Thu) 17:30-18:15 Room P/(Motenashi Dome) Room P

---

### P-1374
### Analysis for antitumor effect of Src inhibitors and their combination with cisplatin in oral squamous cell carcinoma

[Speaker]Yuri Ofusa (Div. of Genetic Therap., Jichi Med. Univ.) [Co-author]Tadahide Noguchi (Dept. of Dent, Oral & Maxillofacial Surg., Jichi Med. Univ.)、Hiroaki Mizukami (Div. of Genetic Therap., Jichi Med. Univ.)、Kenji Ohba (Div. of Genetic Therap., Jichi Med. Univ.)

Background: Cisplatin has been the first-line chemotherapy for oral squamous cell carcinoma (OSCC) for decades. However, serious side effects and drug resistance are problems. Approved drugs for OSCC are limited, suggesting that more effective drugs and treatment regimens with fewer side effects are needed to improve therapeutic effects and quality of life for patients. In our study, we focused on elevated Src activity, and investigated the efficacy of Src inhibitors in OSCC.Method: OSCC cell lines were treated with Src inhibitors (Dasatinib, Ponatinib, Vandetanib, Bosutinib) alone or in combination with cisplatin, and assessed by cell viability and cytotoxicity assays. Additionally, we evaluated the antitumor effects using a mouse model.Result: Antitumor effect differed in each Src inhibitor and cell line, but some inhibitors were commonly effective. Treatment of Src inhibitors reduced activation of p-Src and its downstream molecules, but these also had variables. The combination of Src inhibitors and cisplatin showed an additive antitumor effect.Conclusion: Our analyses suggest that Src inhibitors could be promising novel treatment for OSCC, including combination therapy.

[Disclosure of COI by Responsible researcher] Lead presenter is not the Responsible researcher.
[Lead presenter: Yuri Ofusa] We have no financial relationships to disclose.
[Principal Researcher: Kenji Ohba] ■Research funds from company: 日本医療研究開発機構(AMED), 日本学術振興会(科研費)
■Research expenses: タカラバイオ株式会社 ■Contributions: 武田科学振興財団

▲TOP

### [P-1375] II-P4-1
### Head & neck tumors (1)

2025 / 9 / 25 (Thu) 17:30-18:15 Room P/(Motenashi Dome) Room P

---

### P-1375
### Identification of bone marrow stem cell-derived oral squamous cell carcinoma and its verification in an animal model

[Speaker]Aya Koike (Dokkyo Medical Univ,Dept of Oral and Maxillofacial Surgery) [Co-author]Ryo Shiraishi (Dokkyo Medical Univ,Dept of Oral and Maxillofacial Surgery)、Toshiki Hyodo (Dokkyo Medical Univ,Dept of Oral and Maxillofacial Surgery)、Tomonori Hasegawa (Dokkyo Medical Univ,Dept of Oral and Maxillofacial Surgery)、Yuske Komiyama (Dokkyo Medical Univ,Dept of Oral and Maxillofacial Surgery)、Chonji Fukumoto (Dokkyo Medical Univ,Dept of Oral and Maxillofacial Surgery)、Takahiro Wakui (Dokkyo Medical Univ,Dept of Oral and Maxillofacial Surgery)、Hitoshi Kawamata (Dokkyo Medical Univ,Dept of Oral and Maxillofacial Surgery)

We hypothesized that Oral squamous cell carcinoma (OSCC) has three different origins: (1) epithelial stem cells, (2) oral mucosal stem cells in salivary gland, (3) bone marrow stem cells (BM). We attempted to identify OSCC originating from BM in clinical cases. We analyzed the sex chromosome of OSCC in patients who had undergone sex-mismatched BMtransplantation, and found that in 3 out of 6 OSCC cases (male), the proportion of X-only cells was high, suggesting that they were of donor origin. Additionally, in a transplanted female patient, 10.7% of the epithelial cells in the lichenoid lesion contained the Y chromosome, suggesting that they were of donor origin. The gene expression pattern of BM derived OSCC was clearly different from that of conventionally developed OSCC. BM derived OSCC showed poor prognosis. We are also investigating whether oral mucosal epithelium can regenerate from BM-derived cells by using the tongue injured wild-type mice (C57BL/6J) that BM transplantation has been done from GFP-expressing mice. Furthermore, by applying a low concentration of 4-NQO topically to these mice, we are examining whether GFP positive cells are present in the OSCC that develops.

[Disclosure of COI by Responsible researcher] Lead presenter is the Responsible researcher.
[Lead presenter: Aya Koike] We have no financial relationships to disclose.

[P-1376] II-P4-1
Head & neck tumors (1)

2025 / 9 / 25 (Thu) 17:30-18:15 Room P/(Motenashi Dome) Room P

### P-1376
### Cytotoxic T cell density and PD-L1 expression as predictors of anti-PD1 therapy in recurrent OSCC

[Speaker]Mayuko Yamashita (Grad.Sch.Med.Sci.,Kumamoto Univ.／Dept.Oral&Maxillofac.Surg.,Fac.Life Sci.,Kumamoto Univ.／Dept.Cell Path.Grad.Sch.Med.Sci.,Fac.Life Sci.,Kumamoto Univ.) [Co-author]Hiromu Yano (Dept.Cell Path.Grad.Sch.Med.Sci.,Fac.Life Sci.,Kumamoto Univ.／Dept.Tumor Path.,Grad. Sch.Health Sci.,Kumamoto Univ.)、Yoshihiro Komohara (Dept.Cell Path.Grad.Sch.Med.Sci.,Fac.Life Sci.,Kumamoto Univ.)、Rin Yamada (Dept.Cell Path.Grad.Sch.Med.Sci.,Fac.Life Sci.,Kumamoto Univ.)、Yukio Fujiwara (Dept.Cell Path.Grad.Sch.Med.Sci.,Fac.Life Sci.,Kumamoto Univ.)、Masatoshi Hirayama (Dept.Oral&Maxillofac.Surg.,Fac.Life Sci.,Kumamoto Univ.)、Yuki Seki (Dept.Oral&Maxillofac.Surg.,Fac.Life Sci.,Kumamoto Univ.)、Ryoji Yoshida (Dept.Oral&Maxillofac.Surg.,Fac.Life Sci.,Kumamoto Univ.)、Hideki Nakayama (Dept.Oral&Maxillofac.Surg.,Fac.Life Sci.,Kumamoto Univ.)

Background: The response to immune checkpoint inhibitors in recurrent oral cancer is low, and reliable biomarkers for immunotherapy efficacy are not identified. This study explores the relationship between treatment response and the expression of immune microenvironment molecules using pre-treatment biopsy specimens to identify useful biomarkers for recurrent oral cancer.Methods: From November 2017 to March 2023, 23 patients with recurrent OSCC treated with nivolumab or pembrolizumab were enrolled. Immunohistochemistry for CD3, CD8, FOXP3, CD163, $\beta$2m, HLA-class I, HLA-class II, and PD-L1 was performed.Results: No complete response (CR) was observed. Eight patients had partial response (PR), four had stable disease (SD), and 11 had progressive disease (PD). Responders showed higher CD8-positive T cell infiltration and PD-L1 expression than non-responders.Conclusion: CD8-positive T cell density and PD-L1 expression are useful for predicting favorable responses to anti-PD-1 therapy in recurrent OSCC, suggesting they could serve as potential biomarkers for treatment outcomes.

[Disclosure of COI by Responsible researcher] Lead presenter is not the Responsible researcher.
[Lead presenter: Mayuko Yamashita] We have no financial relationships to disclose.
[Principal Researcher: Yoshihiro Komohara] We have no financial relationships to disclose.

### [P-1377] II-P4-1
### Head & neck tumors (1)

2025 / 9 / 25 (Thu) 17:30-18:15 Room P/(Motenashi Dome) Room P

---

### P-1377
### Establish of FAT1 knockout cell lines for functional analyses in oral squamous cell carcinoma.

[Speaker]Yasutaka Hoshimoto (Dept. of Oral & Maxillofac. Surg, Tokai Univ. Sch. of Med.) [Co-author]Hisako Akatsuka (Dept. of Host Defense Mechanism, Tokai Univ. Sch. of Med.)、Kazuyoshi Hosomichi (Tokyo Univ. of Pharm. & Life Sci. Lab. of Computational Genomics.)、Masahiro Uchibori (Dept. of Oral & Maxillofac. Surg, Tokai Univ. Sch. of Med.)、Takayuki Aoki (Dept. of Oral & Maxillofac. Surg, Tokai Univ. Sch. of Med.)、Yoshihide Ota (Dept. of Oral & Maxillofac. Surg, Tokai Univ. Sch. of Med.)、Minoru Kimura (Tokai Univ.)

Background: Truncating mutations in the ***FAT1*** gene have been frequently reported in oral squamous cell carcinoma(OSCC). We established ***FAT1*** knockout cell lines to investigate the role of FAT1. Using these cell lines, we have examined the impact of ***FAT1*** loss through migration assays, xenograft experiments, and transcriptome analysis. Method: We used the CRISPR-Cas9 system to knock out the ***FAT1*** gene in HEK293 and HSC2 OSCC cell lines. Cas9 and ***FAT1***-targeting guide RNAs were delivered into these cells via plasmid transfection, resulting in partial deletions in the ***FAT1*** gene. Protein expression in ***FAT1*** mutant clones was assessed by Western blotting. Result: We successfully isolated 24 clones from HEK293 and 53 clones from HSC2. We identified three clones from HEK293 and one from HSC2 lacked FAT1 protein expression, fewer than we had expected. We will report the phenotypic analyses, and transcriptome analysis to elucidate differential gene expression in these ***FAT1*** mutant clones, with a particular focus on oncogenic pathways. Conclusion: We successfully generated FAT1 mutant clones lacking FAT1 expression using the CRISPR-Cas9 system.

[Disclosure of COI by Responsible researcher] Lead presenter is not the Responsible researcher.
[Lead presenter: Yasutaka Hoshimoto] We have no financial relationships to disclose.
[Principal Researcher: Yoshihide Ota] We have no financial relationships to disclose.

▲TOP

### [P-1378] II-P4-1
### Head & neck tumors (1)

2025 / 9 / 25 (Thu) 17:30-18:15 Room P/(Motenashi Dome) Room P

---

### P-1378
### Abnormal promoter methylation of smoking-related genes in oral squamous cell carcinoma and oral precancerous lesions

[Speaker]Masanobu Abe (Cancer Inst Hosp JFCR, Dept Dent & Oral Surg／Univ of Tokyo Hosp, Dept Oral & Maxillofac Surg) [Co-author]Ken Tomizuka (Cancer Inst Hosp JFCR, Dept Dent & Oral Surg)

Background: The development of oral cancer has many aspects in common with esophageal cancer, such as smoking and alcohol consumption. We analyzed DNA methylation abnormalities in oral squamous cell carcinoma (OSCC) and oral precancerous lesions (OPL) with respect to genes reported to accumulate DNA methylation abnormalities in esophageal cancer with a history of smoking and in normal tissues in non-cancerous areas. Materials and Methods: Quantitative methylation analysis of four smoking-related genes (HOXA9, MT1M, NEFH, and UCHL1) was performed by quantitative MSP using 24 oral leukoplakia (OL) tissues and 26 OSCC tissues. Results: All of the genes were significantly hypermethylated in OSCC and OL compared to normal tissues. On the other hand, there was no significant correlation between the degree of methylation of these genes and the patients' smoking history. Discussion: Although the four smoking-related genes were not correlated with the smoking history of oral cancer patients, they are likely to play an important role in the development of oral cancer, since they were found to be highly methylated in both OSCC and OPL.

[Disclosure of COI by Responsible researcher] Lead presenter is the Responsible researcher.
[Lead presenter: Masanobu Abe] We have no financial relationships to disclose.

▲TOP

### [P-1379] II-P4-1
### Head & neck tumors (1)

2025 / 9 / 25 (Thu) 17:30-18:15 Room P/(Motenashi Dome) Room P

### P-1379
### Effect of free fatty acids on the proliferation of oral squamous cell carcinoma: Mechanism of cell death induced by FFAs

[Speaker]Saeko Sakamoto (Dept. of Food and Nutritional Environment, Kinjo Gakuin Univ.) [Co-author]Sae Oyama (Dept. of Food and Nutritional Environment, Kinjo Gakuin Univ.)、Mina Ota (Dept. of Food and Nutritional Environment, Kinjo Gakuin Univ.)、Riko Ushie (Dept. of Food and Nutritional Environment, Kinjo Gakuin Univ.)、Miyu Sakai (Dept. of Food and Nutritional Environment, Kinjo Gakuin Univ.)、Sivasundaram Karnan (Dept. of Biochem., Aichi Med Univ.)、Yoshitaka Hosokawa (Dept. of Biochem., Aichi Med Univ.)、Akinobu Ota (Dept. of Food and Nutritional Environment, Kinjo Gakuin Univ.／Dept. of Biochem., Aichi Med Univ.)

Oral squamous cell carcinoma (OSCC) is a malignant oral cancer, which comprises nearly 90% of all oral cancers. Despite recent pharmacological development in molecular-targeted therapeutics, the overall survival of patients with OSCC remains approximately 50%. We previously showed that one of the free fatty acids (FFAs) palmitic acid (PA) preferentially suppresses the survival of malignant mesothelioma cell. However, little is known about the effect of PA on the survival of OSCC cells. Here, we examined the effect of saturated and unsaturated FFAs on the survival of human OSCC cells. In addition, the possible responsible FFAs receptors were examined by using reverse-transcribed polymerase chain reaction. MTT assay showed that FFAs dose-dependently decrease the survival of OSCC cell lines. In addition, the sensitivity to PA was different among the cell lines tested. Our findings indicate the possibility that treatment with specific FFAs suppresses the proliferation of OSCC cells, which might provide better treatment strategies to improve the clinical outcome of patients with OSCC. We would discuss the possible molecular mechanism underlying PA-induced cell death in OSCC cells.

[Disclosure of COI by Responsible researcher] Lead presenter is not the Responsible researcher.
[Lead presenter: Saeko Sakamoto] We have no financial relationships to disclose.
[Principal Researcher: Akinobu Ota] We have no financial relationships to disclose.

▲TOP

### [EL1] EL1
### Frontiers in the clinical and basic research of KRAS mutant cancer

2025 / 9 / 26 (Fri) 8:00-8:50 Room 3/(Hotel Nikko Kanazawa 4F Tsuru A) Room 3

[Chairpersons]Yoshihisa Kobayashi (National Cancer Center Research Institute)、Hiromichi Ebi (Aichi Cancer Center Research Institute)

---

### EL1
### Clinical and Basic Research of KRAS-Mutant Cancers: History and future

[Speaker]Tetsuya Mitsuidomi (Izumi City General Hospital)

KRAS, the first driver oncogene identified in human cancer in the early 1980s, encodes a 21kDa membrane-bound GTPase. Mutations in KRAS constitutively activate downstream pathways, driving malignant transformation. However, it has been deemed undruggable for decades until the discovery of KRAS G12C covalent inhibitors such as sotorasib and adagrasib. These agents are now clinically available in several countries. However, their efficacy is somewhat limited due to intrinsic resistance or early acquisition of resistance, leading to further optimization of this approach. While G12C predominates in lung cancer, G12D/V are more common in colorectal and pancreatic cancers, spurring the development of non-G12C KRAS inhibitors. Novel strategies, including molecular shielding, targeted protein degradation, and combination therapies with immune checkpoint inhibitors or the MAPK pathway, are under active investigation. Immunotherapeutic approaches, including TCR-T cell therapy, vaccines, and Hapimmune antibodies, are also emerging. This presentation will highlight recent advances in KRAS research, bridging basic science and clinical application in KRAS-mutated cancers.

[Disclosure of COI by Responsible researcher] Lead presenter is the Responsible researcher.
[Lead presenter: Tetsuya Mitsuidomi] ■Lecture fee: アストラゼネカ、アムジェン、ベーリンガーインゲルハイム、MSD. ノバルティス、中外製薬、小野薬品、

▲TOP

### [EL2] EL2
### Elucidation of immune escape mechanisms and development of novel therapeutic strategies

2025 / 9 / 26 (Fri) 8:00-8:50 Room 4/(Hotel Nikko Kanazawa 4F Tsuru B) Room 4

[Chairpersons]Yuka Maeda (Division of Cancer Immunology, National Cancer Center)、Shohei Koyama (National Cancer Center, Research Institute)

---

### EL2
### Elucidation of immune escape mechanisms and development of novel therapeutic strategies

[Speaker]Toshihiko Torigoe (1st Dept. Pathol., Sapporo Med. Univ. Sch. Med.)

All advanced cancers are believed to have impairments in the cancer immunity cycle, and cancer immunotherapy is a treatment that removes these inhibitory factors, thereby facilitating the smooth functioning of the cancer immunity cycle. Immune evasion mechanisms encompass both innate and adaptive immune evasion, which are triggered by mutations in the cancer genome and epigenome. In this educational lecture, I will introduce the principles and the latest findings regarding these immune evasion mechanisms and discuss prospective new treatment strategies to activate the cancer immunity cycle.

[Disclosure of COI by Responsible researcher] Lead presenter is the Responsible researcher.
[Lead presenter: Toshihiko Torigoe] ■Advisor of the company and/or profit-making organization: 札幌臨床検査センター（株） ■Lecture fee: 中外製薬（株）, メルクバイオファーマ（株） ■Research expenses: 札幌臨床検査センター（株） ■Contributions: 札幌臨床検査センター（株）

▲TOP

### [EL3] EL3
### Gut microbiota and cancer

2025 / 9 / 26 (Fri) 8:00-8:50 Room 5/(Hotel Nikko Kanazawa 4F Tsuru C) Room 5

[Chairperson]Shinichi Yachida (Graduate School of Medicine, The University of Osaka)

### EL3
### Gut microbiota and cancer

[Speaker]Naoko Ohtani (Dept. Pathophysiol.Grad Schl. Med. Osaka Metro. Univ)

The gut microbiota normally maintains a mutually beneficial relationship with the host, and its metabolic products and microbial components have significant effects on the host's homeostasis. These microbial metabolites not only exert their effects locally in the gut where the bacteria reside, but are also absorbed into the bloodstream and act on distant organs. In particular, the liver is thought to be continuously influenced by the gut microbiota-derived substances through the enterohepatic circulation. In recent years, it has become increasingly evident that certain metabolites, toxins, and components produced by gut bacteria can promote cancer development. Conversely, some gut bacterial species have been found to enhance the efficacy of immune checkpoint inhibitors, raising expectations for their therapeutic application. In this lecture, I will introduce recent findings on the relationship between the gut microbiota and cancer, a recent topical field in which the involvement of gut bacteria has been increasingly recognized.

[Disclosure of COI by Responsible researcher] Lead presenter is the Responsible researcher.
[Lead presenter: Naoko Ohtani] ■Research expenses: 武田科学振興財団

### [ML] ML
### Morning Lecture

2025 / 9 / 26 (Fri) 8:00-8:50 Room 8/(Hotel Nikko Kanazawa 3F Kujaku C) Room 8

[Chairperson]Reo Maruyama (Division of Cancer Epigenomics, Cancer Institute, Japanese Foundation for Cancer Research)

---

### ML
### How to write an engaging scientific paper in English

[Speaker]Takuro Nakamura (Tokyo Medical University)

No Abstract

### [EL4] EL4
### AI is opening up new frontiers in cancer research and medicine

2025 / 9 / 26 (Fri) 8:00-8:50 Room 11/(ANA Crowne Plaza Hotel Kanazawa 3F Ohtori (West)) Room 11

[Chairperson]Shumpei Ishikawa (The University of Tokyo/National Cancer Center)

---

### EL4
### Research trends in medical AI and efforts toward clinical application

[Speaker]Ryuji Hamamoto (Div. Medical AI Res. Dev., Natl. Cancer Ctr. Res. Inst.／Cancer Transl. Res. Team, RIKEN Ctr. for AIP project)

With the advancement of machine learning technologies centered on deep learning, expectations for artificial intelligence (AI) are rising. In particular, with the introduction of Transformer in 2017, generative AI technologies such as ChatGPT have made remarkable progress, and AI continues to evolve at a level that affects not only the scientific field but also the structure of society itself. The medical field is no exception, with over 1,000 AI-enabled medical devices approved by the US FDA, and many AI-enabled medical devices approved and used in clinical settings in Japan. In the field of cancer research, AI is widely used in a variety of applications, including medical image analysis of endoscopic images, radiological images, and pathological images; omics analysis of genomes and epigenomes; and extraction of important information from electronic medical records and medical papers using natural language processing. This lecture will introduce the current state of AI implementation in medical research, with a focus on cancer research, and discuss the challenges and future directions of medical AI.

[Disclosure of COI by Responsible researcher] Lead presenter is the Responsible researcher.
[Lead presenter: Ryuji Hamamoto] ■Research expenses: 富士フイルム株式会社、富士通株式会社

### [EL5] EL5
### Cancer Heterogeneity, Stem Cell Regulation, and Treatment Resistance

2025 / 9 / 26 (Fri) 8:00-8:50 Room 12/(ANA Crowne Plaza Hotel Kanazawa 3F Ohtori (Central)) Room 12

[Chairpersons]Noriko Gotoh (Cancer Research Institute, Kanazawa University)、Kohshi Mimori (Surgery, Kyushu University Beppu Hospital)

---

### EL5
### Cancer Heterogeneity, Stem Cell Regulation, and Treatment Resistance

[Speaker]Hideyuki Saya (Oncology Innovation Center, Fujita Health Univ.)

Normal tissues are organized in a hierarchical structure initiated by stem cells, generating diverse cell types that together form organs with complex functions. Cancer tissues similarly exhibit a hierarchy originating from cancer stem cells, and this structure is a fundamental basis for the heterogeneity observed in tumors.

Compared to normal tissues, cancer tissues accumulate a greater number of genetic mutations, which continue to increase over time. Consequently, tumor heterogeneity arises not only from diverse gene expression patterns but also from the expanding complexity of these genetic alterations. Moreover, the properties of cancer cells dynamically change in response to treatment and the tumor microenvironment, forming a central mechanism underlying treatment resistance.

In this educational lecture, I will explain the essential nature of cancer stem cells as the origin of tumor hierarchy, the actual landscape of heterogeneity among cancer cells derived from them, and the molecular mechanisms that govern treatment resistance. I will also introduce our ongoing efforts to develop novel therapeutic strategies targeting treatment-resistant cancer cell populations.

[Disclosure of COI by Responsible researcher] Lead presenter is the Responsible researcher.
[Lead presenter: Hideyuki Saya] ■Advisor of the company and/or profit-making organization: Dyamond Medino Capital株式会社、FerroptoCure株式会社、アルファフュージョン株式会社、StapleBio株式会社　■Research expenses: 株式会社医学生物学研究所、シスメックス株式会社、JSR株式会社

▲TOP

### [AACR2-1] AACR2
### Spatio-temporal regulation of cancer phenotypes by cancer epigenetics

2025 / 9 / 26 (Fri) 9:00-11:30 Room 1/(Ishikawa Ongakudo 2F Concert Hall) Room 1

[Chairpersons]Yutaka Kondo (Nagoya University Graduate School of Medicine)、Kristian Helin (The Institute of Cancer Research)

Genomic and epigenomic alterations play pivotal roles in driving cancer pathogenesis. Among them, the epigenome dynamically influences not only cancer cells but also the surrounding tumor microenvironment through multilayered regulatory mechanisms. These epigenetic processes contribute to critical phenotypic traits such as drug resistance and tissue heterogeneity-key challenges in cancer progression and treatment failure. Mechanistically, DNA methylation, histone modifications, chromatin remodeling, and more recently, non-coding RNAs have been found to shape the three-dimensional architecture of the genome, thereby orchestrating gene expression and other DNA-centered regulatory programs. With the advent of advanced technologies, our understanding of these mechanisms has evolved from static models to dynamic insights into the spatio-temporal regulation of epigenetic states. These breakthroughs are shedding light on how cancers evolve over time and in distinct tissue contexts, opening new avenues for precision diagnostics and targeted therapies. This symposium will explore the cutting edge of cancer epigenomics, with a particular focus on spatio-temporal regulation, and examine how this emerging perspective is transforming our understanding of cancer biology and informing the next generation of clinical strategies.

---

### AACR2-1
### Long Non-Coding RNAs: Illuminating Their Biological Functions and Potential as Novel Cancer Therapeutics

[Speaker]Yutaka Kondo (Div. Can. Biol., Nagoya Univ. Grad. Sch. Med.)

It is now widely recognized that a substantial portion of the human genome is transcribed into non-coding RNAs, which, despite lacking protein-coding potential, play critical regulatory roles in diverse cellular processes, including genetic and epigenetic regulation. Among them, long non-coding RNAs (lncRNAs), typically over 200 nucleotides in length, have emerged as important functional molecules. Our research has shown that lncRNAs significantly influence cancer cell phenotypes through various mechanisms. For example, we found that TUG1 maintains cancer cell homeostasis by regulating R-loops, which are frequently observed in cancer genomes. Clinical trials targeting TUG1 are currently underway. Recently, we identified a novel lncRNA that contributes to the regulation of WNT signaling in cancer cells, unveiling a previously unrecognized layer of control in this critical pathway. These results suggest that many previously overlooked lncRNAs play essential roles in tumorigenesis. In this symposium, we will share our latest findings on the roles of lncRNAs in cancer biology.

[Disclosure of COI by Responsible researcher] Lead presenter is the Responsible researcher.
[Lead presenter: Yutaka Kondo] ■Research funds from company: NANO MRNA株式会社

▲TOP

### [AACR2-2] AACR2
### Spatio-temporal regulation of cancer phenotypes by cancer epigenetics

2025 / 9 / 26 (Fri) 9:00-11:30 Room 1/(Ishikawa Ongakudo 2F Concert Hall) Room 1

[Chairpersons]Yutaka Kondo (Nagoya University Graduate School of Medicine)、Kristian Helin (The Institute of Cancer Research)

Genomic and epigenomic alterations play pivotal roles in driving cancer pathogenesis. Among them, the epigenome dynamically influences not only cancer cells but also the surrounding tumor microenvironment through multilayered regulatory mechanisms. These epigenetic processes contribute to critical phenotypic traits such as drug resistance and tissue heterogeneity-key challenges in cancer progression and treatment failure. Mechanistically, DNA methylation, histone modifications, chromatin remodeling, and more recently, non-coding RNAs have been found to shape the three-dimensional architecture of the genome, thereby orchestrating gene expression and other DNA-centered regulatory programs. With the advent of advanced technologies, our understanding of these mechanisms has evolved from static models to dynamic insights into the spatio-temporal regulation of epigenetic states. These breakthroughs are shedding light on how cancers evolve over time and in distinct tissue contexts, opening new avenues for precision diagnostics and targeted therapies. This symposium will explore the cutting edge of cancer epigenomics, with a particular focus on spatio-temporal regulation, and examine how this emerging perspective is transforming our understanding of cancer biology and informing the next generation of clinical strategies.

### AACR2-2
### Role of histone modifications in transcription, differentiation and disease

[Speaker]Kristian Helin (The Institute of Cancer Research)

Posttranslational modifications (PTMs) of histone proteins are widely believed to be essential for regulating chromatin structure and transcription. However, direct roles for various PTMs in these processes have mainly been inferred from correlative studies of disrupted enzymes that catalyze these modifications. Recent studies have demonstrated that chromatin-associated enzymes have many non-histone substrates as well as having functions decoupled from their enzymatic activity. Thus, more direct approaches are necessary to understand the roles of histone PTMs in regulating transcription.At the meeting, I will present our different strategies to address the functional roles of some of the most studied histone PTMs in regulating transcription. Specifically, I will talk about the methylation of lysines 4, 9, 27 and 36 on histone H3.

[Disclosure of COI by Responsible researcher] Lead presenter is the Responsible researcher.
[Lead presenter: Kristian Helin] ■Profit of stock: Dania Therapeutics ■Patent fee: University of Copenhagen

### [AACR2-3] AACR2
### Spatio-temporal regulation of cancer phenotypes by cancer epigenetics

2025 / 9 / 26 (Fri) 9:00-11:30 Room 1/(Ishikawa Ongakudo 2F Concert Hall) Room 1

[Chairpersons]Yutaka Kondo (Nagoya University Graduate School of Medicine)、Kristian Helin (The Institute of Cancer Research)

Genomic and epigenomic alterations play pivotal roles in driving cancer pathogenesis. Among them, the epigenome dynamically influences not only cancer cells but also the surrounding tumor microenvironment through multilayered regulatory mechanisms. These epigenetic processes contribute to critical phenotypic traits such as drug resistance and tissue heterogeneity-key challenges in cancer progression and treatment failure. Mechanistically, DNA methylation, histone modifications, chromatin remodeling, and more recently, non-coding RNAs have been found to shape the three-dimensional architecture of the genome, thereby orchestrating gene expression and other DNA-centered regulatory programs. With the advent of advanced technologies, our understanding of these mechanisms has evolved from static models to dynamic insights into the spatio-temporal regulation of epigenetic states. These breakthroughs are shedding light on how cancers evolve over time and in distinct tissue contexts, opening new avenues for precision diagnostics and targeted therapies. This symposium will explore the cutting edge of cancer epigenomics, with a particular focus on spatio-temporal regulation, and examine how this emerging perspective is transforming our understanding of cancer biology and informing the next generation of clinical strategies.

---

### AACR2-3
### Epimutation burden in stem cells precisely predicts gastric cancer risk

[Speaker]Toshikazu Ushijima (Dept. Epigenomics, Inst. Adv. Life Sci., Hoshi Univ.) [Co-author]Chihiro Takeuchi (Dept. Epigenomics, Inst. Adv. Life Sci., Hoshi Univ.／Dept. Gastroenterol., Grad. Sch. Med., Tokyo Univ.)、Harumi Yamada (Dept. Epigenomics, Inst. Adv. Life Sci., Hoshi Univ.)

Aberrant DNA methylation (epimutation) can be induced in a substantial fraction of cells in nonmalignant tissues. In gastric mucosa, DNA methylation is potently induced by Helicobacter pylori (HP) infection, and decreases after its eradication. This has been considered due to replacement of differentiated cells with DNA methylation by cells newly supplied from stem cells without. Using stem cell-enriched organoids and single cell ATAC-seq analysis of primary samples, stem cell-enriched population was shown to be resistant to DNA methylation induction and chromatin closure. Since the DNA methylation level in HP-eradicated people reflects that in stem cells, it can be used for precision cancer risk diagnosis. We used the DNA methylation level of a marker gene after HP eradication in a prospective study with 1,757 participants with open-type gastric atrophy. The highest quartile had a much higher gastric cancer incidence (hazard ratio of 7.7; 95%CI 1.8-33.7) compared with the lowest quartile [Yamada, Gut, online]. The measurement of epimutation burden in stem cells can be used for precision cancer risk diagnosis.

[Disclosure of COI by Responsible researcher] Lead presenter is the Responsible researcher.
[Lead presenter: Toshikazu Ushijima] ■Research expenses: シスメックス

### [AACR2-4] AACR2
### Spatio-temporal regulation of cancer phenotypes by cancer epigenetics

2025 / 9 / 26 (Fri) 9:00-11:30 Room 1/(Ishikawa Ongakudo 2F Concert Hall) Room 1

[Chairpersons]Yutaka Kondo (Nagoya University Graduate School of Medicine)、Kristian Helin (The Institute of Cancer Research)

Genomic and epigenomic alterations play pivotal roles in driving cancer pathogenesis. Among them, the epigenome dynamically influences not only cancer cells but also the surrounding tumor microenvironment through multilayered regulatory mechanisms. These epigenetic processes contribute to critical phenotypic traits such as drug resistance and tissue heterogeneity-key challenges in cancer progression and treatment failure. Mechanistically, DNA methylation, histone modifications, chromatin remodeling, and more recently, non-coding RNAs have been found to shape the three-dimensional architecture of the genome, thereby orchestrating gene expression and other DNA-centered regulatory programs. With the advent of advanced technologies, our understanding of these mechanisms has evolved from static models to dynamic insights into the spatio-temporal regulation of epigenetic states. These breakthroughs are shedding light on how cancers evolve over time and in distinct tissue contexts, opening new avenues for precision diagnostics and targeted therapies. This symposium will explore the cutting edge of cancer epigenomics, with a particular focus on spatio-temporal regulation, and examine how this emerging perspective is transforming our understanding of cancer biology and informing the next generation of clinical strategies.

---

### AACR2-4
### EPIGENETICS: A GATEKEEPER TO GENERATING EXTRACHROMOSOMAL DNA AMPLIFICATIONS & REARRANGEMENTS

[Speaker]Johnathan Whetstine (Fox Chase Cancer and Cancer Epigenetics Institute)

Aggressive and hard-to-treat malignancies contain critical DNA amplifications and genomic rearrangements, which promote heterogeneity and resistance. Recent advances uncovered a critical role for epigenetic states and their associated modulators in regulating ecDNA amplification. The question remained as to whether selective extrachromosomal copy gains could give rise to inherited genomic rearrangements. We have identified specific histone modifying enzymes that controls site-specific DNA copy gains, and in turn, genomic rearrangements. Our studies combined genetic and chemical tools coupled to cytological approaches and epigenome profiling to resolve this key question. While interrogating the histone modifying enzymes controlling the local epigenome, we have also begun to appreciate its impact on integrated, inherited genomic rearrangements. We have been able to establish relationships between these pathways and the cancer-associated amplifications and rearrangements. Collectively, our team established that key histone modifying enzymes generate and prevent these key genomic alterations. We will present our most recent studies addressing these observations at the meeting.

[Disclosure of COI by Responsible researcher] Lead presenter is the Responsible researcher.
[Lead presenter: Johnathan R. Whetstine] ■Advisor of the company and/or profit-making organization: Daiichi Sankyo, Inc; Qsonica ■Research expenses: Oryzon Genomics ■Endowed chair: Fox Chase Cancer Center Endowed Basic Science Chair ■Presents or other payment: Arima

### [YSA-1] YSA
### Canser Sicience　Young Scientists Awards Lectures

2025 / 9 / 26 (Fri) 9:00-10:40 Room 2/(Ishikawa Ongakudo 2F Hogaku Hall) Room 2

[Chairperson]Yutaka Kawakami (Department of Immunology, School of Medicine, International University of Health and Welfare)

### YSA-1
### Discovering cancer stem-like molecule, nuclear factor I X, using spatial transcriptome analysis in gastric cancer

[Speaker]Akira Ishikawa (Dept Mol Pathol, Hiroshima Univ) [Co-author]Takafumi Fukui (Dept Mol Pathol, Hiroshima Univ)、Kazuya Kuraoka (Dept Diag Pathol, Kure Med Ctr and Chugoku Cancer Ctr)、Narutaka Katsuya (Dept Mol Pathol, Hiroshima Univ)、Naohiro Uraoka (Dept Pathol, Kure Kyosai Hospital)、Takahisa Suzuki (Dept Surgery, Kure Med Ctr and Chugoku Cancer Ctr)、Shiro Oka (Dept Gastroenterol, Hiroshima Univ)、Hassan Ashktorab (Dept Med Cancer Ctr, Howard University College Med)、Duane Smoot (Dept Med, Meharry Medical College)、Wataru Yasui (Dept Mol Pathol, Hiroshima Univ／Div Pathology, Hiroshima City Med Association Clin Lab)、Shinji Mii (Dept Mol Pathol, Hiroshima Univ)

Gastric cancer (GC) ranks as the third leading cause of cancer-related deaths in Japan. Several subtype classifications have been proposed, such as The Cancer Genome Atlas scheme and mucin profile based classification. However, these approaches have had limited success due to the marked intratumoral heterogeneity characteristic of GC. To address this heterogeneity, single cell and spatial transcriptomic approaches are now being applied. We identified Nuclear Factor I X (NFIX) as a potential therapeutic target in GC through spatial transcriptomics (10x Visium) combined with morphological, CytoTRACE, and monocle3 trajectory analyses. NFIX was expressed in 51% of cases, correlated with pT and pM stages, and predicted poor prognosis. NFIX knockdown reduced proliferation and spheroid formation in GC cell lines, and RNA seq suggested it promotes progression via kinase activity. Spatial transcriptomics integrates genetic data with pathological morphology, enabling novel GC classification and identification of therapeutic targets such as NFIX.

[Disclosure of COI by Responsible researcher] Lead presenter is the Responsible researcher.
[Lead presenter: Akira Ishikawa] ■Research funds from company: 科学研究費

▲TOP

### [YSA-2] YSA
### Canser Sicience　Young Scientists Awards Lectures

2025 / 9 / 26 (Fri) 9:00-10:40 Room 2/(Ishikawa Ongakudo 2F Hogaku Hall) Room 2

[Chairperson]Yutaka Kawakami (Department of Immunology, School of Medicine, International University of Health and Welfare)

### YSA-2
### Circulating KRAS Mutations as Predictive Biomarkers for Targeted Therapy in Pancreatic Cancer

[Speaker]Mi Rim Lee (National Cancer Center Research Institute／National Cancer Center Graduate School of Cancer Science and Policy) [Co-author]Mi Rim Lee (National Cancer Center Research Institute／National Cancer Center Graduate School of Cancer Science and Policy)、Sang Myung Woo (National Cancer Center Research Institute／National Cancer Center Graduate School of Cancer Science and Policy／National Cancer Center Hospital)、Min Kyeong Kim (National Cancer Center Research Institute)、Sung-Sik Han (National Cancer Center Graduate School of Cancer Science and Policy／National Cancer Center Hospital)、Sang-Jae Park (National Cancer Center Hospital)、Woo Jin Lee (National Cancer Center Research Institute／National Cancer Center Hospital)、Dong-eun Lee (National Cancer Center Research Institute)、Sun Il Choi (National Cancer Center Research Institute／National Cancer Center Graduate School of Cancer Science and Policy)、Wonyoung Choi (National Cancer Center Research Institute／National Cancer Center Graduate School of Cancer Science and Policy／National Cancer Center Hospital)、Kyong-Ah Yoon (Konkuk University)、Jung Won Chun (National Cancer Center Research Institute／National Cancer Center Hospital)、Yun-Hee Kim (National Cancer Center Research Institute／National Cancer Center Graduate School of Cancer Science and Policy)、Sun-Young Kong (National Cancer Center Research Institute／National Cancer Center Graduate School of Cancer Science and Policy／National Cancer Center Hospital)

Kirsten rat sarcoma viral oncogene homolog (KRAS) mutations in circulating tumor DNA (ctDNA) are recognized as noninvasive prognostic markers for pancreatic ductal adenocarcinoma (PDAC). We evaluated single KRAS mutations as prognostic and predictive biomarkers, with a focus on their potential therapeutic implications. KRAS mutation status in tumor DNA and plasma ctDNA was assessed using droplet digital PCR (ddPCR), revealing higher concordance in advanced disease compared with early-stage PDAC. Prognostic prediction for both overall and progression-free survival was stronger using plasma ctDNA than tumor DNA. Furthermore, we tested the KRAS G12C inhibitor sotorasib in patient-derived organoid (PDO) and xenograft (PDX) models from a KRAS G12C-mutated patient, demonstrating selective antitumor effects and modulation of the tumor microenvironment. These findings support the use of plasma ctDNA KRAS mutation screening, combined with preclinical models, to guide precision therapy in PDAC.

[Disclosure of COI by Responsible researcher] Lead presenter is not the Responsible researcher.
[Lead presenter: Mi Rim Lee] We have no financial relationships to disclose.
[Principal Researcher: Mi Rim Lee] We have no financial relationships to disclose.

### [YSA-3] YSA
### Canser Sicience Young Scientists Awards Lectures

2025 / 9 / 26 (Fri) 9:00-10:40 Room 2/(Ishikawa Ongakudo 2F Hogaku Hall) Room 2

[Chairperson]Yutaka Kawakami (Department of Immunology, School of Medicine, International University of Health and Welfare)

---

### YSA-3
### Long noncoding RNA *TUG1* promotes cisplatin resistance in ovarian cancer via upregulation of DNA polymerase eta

[Speaker]Ryosuke Sonobe (Div. Can Biol., Nagoya Univ. Grad. Sch. Med.) [Co-author]Yang Peng (Div. Can Biol., Nagoya Univ. Grad. Sch. Med.／Dept. Gynecol., Nagoya Univ. Grad. Sch. Med.)、Miho Suzuki (Div. Can Biol., Nagoya Univ. Grad. Sch. Med.)、Keiko Shinjo (Div. Can Biol., Nagoya Univ. Grad. Sch. Med.)、Kenta Iijima (Div. Can Biol., Nagoya Univ. Grad. Sch. Med.)、Nobuhiro Nishiyama (Dept. Life Sci. and Tech., Inst. of Sci. Tokyo ／Innovation Ctr. of NanoMedicine, Kawasaki Inst. of Industrial Promotion)、Kanjiro Miyata (Dept. Mater. Eng., Sch. Eng. UTokyo)、Kazunori Kataoka (Innovation Ctr. of NanoMedicine, Kawasaki Inst. of Industrial Promotion)、Hiroaki Kajiyama (Dept. Gynecol., Nagoya Univ. Grad. Sch. Med.)、Yutaka Kondo (Div. Can Biol., Nagoya Univ. Grad. Sch. Med.)

The current standard of care for ovarian cancer (OVCA) remains suboptimal, particularly in recurrent cases driven by resistance to platinum-based chemotherapy. To address this therapeutic limitation, we focused on long non-coding RNAs (lncRNAs), which are increasingly recognized as key regulators of tumor progression and treatment resistance. Through transcriptomic profiling, we identified Taurine Upregulated Gene 1 (TUG1) as significantly upregulated in recurrent OVCA specimens. Functional studies revealed that antisense oligonucleotide (ASO)-mediated depletion of TUG1 enhanced cisplatin sensitivity in OVCA cells. TUG1 promotes the expression of the translesion synthesis enzyme DNA polymerase eta (POLH) by modulating a microRNA-dependent regulatory axis. Given that POLH facilitates DNA replication across cisplatin-induced DNA lesions, its upregulation contributes directly to chemoresistance. Notably, combination treatment with TUG1-targeting ASO and cisplatin produced a pronounced antitumor effect in OVCA xenograft models. Our findings establish TUG1 as a promising therapeutic target and provide a strong rationale for advancing ASO-based therapeutic strategies in ovarian cancer.

[Disclosure of COI by Responsible researcher] Lead presenter is not the Responsible researcher.
[Lead presenter: Ryosuke Sonobe] We have no financial relationships to disclose.
[Principal Researcher: Yutaka Kondo] We have no financial relationships to disclose.

▲TOP

### [YSA-4] YSA
### Canser Sicience　Young Scientists Awards Lectures

2025 / 9 / 26 (Fri) 9:00-10:40 Room 2/(Ishikawa Ongakudo 2F Hogaku Hall) Room 2

[Chairperson]Yutaka Kawakami (Department of Immunology, School of Medicine, International University of Health and Welfare)

### YSA-4
### Exploring the tumor microenvironment of colorectal cancer patients post renal transplantation by single-cell analysis

[Speaker]Jinghui Zhang (Department of General Surgery, Beijing Friendship Hospital／Department of Clinical Surgery and Oncology, Kyushu University) [Co-author]Yusuke Mizuuchi (Department of Clinical Surgery and Oncology, Kyushu University)、Kenoki Ohuchida (Department of Clinical Surgery and Oncology, Kyushu University)、Masafumi Nakamura (Department of Clinical Surgery and Oncology, Kyushu University)

Background and Aims: In renal transplant recipients, chronic allograft antigen exposure and immunosuppressants create a distinct immune milieu. Reports on CRC incidence in this population are inconsistent, yet suggest altered immune microenvironments. To better understand immune cell function and communication, we applied single cell RNA sequencing (scRNA seq) to clarify immune alterations in CRC post-transplantation. Methods: scRNA seq was performed on paired normal and tumor tissues from 4 post-transplant CRC patients, and compared to 23 sporadic CRC cases. We identified 22 cell clusters and analyzed functional pathways using GSEA. Results: CTLA4 was upregulated in post-transplant tumors. CD8+GZMH+ cytotoxic T cells showed reduced cytotoxicity and tumor reactivity. CD4+FOXP3+ Tregs maintained high suppressive signatures. Post-transplant B cells had elevated antigen presentation and enhanced T cell interactions. Conclusion: Our study reveals unique immune dysregulation in post-transplant CRC, offering novel insights into immune escape and informing future personalized immunotherapies.

[Disclosure of COI by Responsible researcher] Lead presenter is not the Responsible researcher.
[Lead presenter: Jinghui Zhang] We have no financial relationships to disclose.
[Principal Researcher: Yusuke Mizuuchi, Kenoki Ohuchida] We have no financial relationships to disclose.

### [S3-1] S3
### Frontiers in the clinical and basic research of KRAS mutant cancer

2025 / 9 / 26 (Fri) 9:00-11:30 Room 3/(Hotel Nikko Kanazawa 4F Tsuru A) Room 3

[Chairpersons]Yoshihisa Kobayashi (National Cancer Center Research Institute)、Hiromichi Ebi (Aichi Cancer Center Research Institute)

KRAS G12C inhibitors, sotorasib and adagrasib, have revolutionized treatment strategies for KRAS-mutant lung and colon cancers. Currently, novel drugs targeting other KRAS variants and pan-RAS inhibitors are under extensive development. This symposium brings together experts in lung, colon, and pancreatic cancers to present the latest clinical and basic research on KRAS. The session will provide a comprehensive overview of advancements in KRAS treatments, innovative drug discovery, resistance mechanisms, and challenges beyond the conventional targeting of KRAS GTP/GDP states. This diverse array of perspectives aims to foster interdisciplinary dialogue, bridging foundational research with the clinical translation of new KRAS-targeted therapies in terms of various cancer types and therapeutic approaches.

---

### S3-1
### Basic and clinical research on KRAS mutant lung cancer

[Speaker]Ferdinandos Skoulidis (University of Texas MD Anderson Cancer Center, Department of Thoracic and Head and Neck Medical Oncology)

No Abstract

### [S3-2] S3
### Frontiers in the clinical and basic research of KRAS mutant cancer

2025 / 9 / 26 (Fri) 9:00-11:30 Room 3/(Hotel Nikko Kanazawa 4F Tsuru A) Room 3

[Chairpersons]Yoshihisa Kobayashi (National Cancer Center Research Institute)、Hiromichi Ebi (Aichi Cancer Center Research Institute)

KRAS G12C inhibitors, sotorasib and adagrasib, have revolutionized treatment strategies for KRAS-mutant lung and colon cancers. Currently, novel drugs targeting other KRAS variants and pan-RAS inhibitors are under extensive development. This symposium brings together experts in lung, colon, and pancreatic cancers to present the latest clinical and basic research on KRAS. The session will provide a comprehensive overview of advancements in KRAS treatments, innovative drug discovery, resistance mechanisms, and challenges beyond the conventional targeting of KRAS GTP/GDP states. This diverse array of perspectives aims to foster interdisciplinary dialogue, bridging foundational research with the clinical translation of new KRAS-targeted therapies in terms of various cancer types and therapeutic approaches.

### S3-2
### Basic and clinical research on KRAS mutant pancreatic cancer

[Speaker]Andrew Aguirre (Dana-Farber Cancer Institute, Department of Medical Oncology)

No Abstract

### [S3-3] S3
### Frontiers in the clinical and basic research of KRAS mutant cancer

2025 / 9 / 26 (Fri) 9:00-11:30 Room 3/(Hotel Nikko Kanazawa 4F Tsuru A) Room 3

[Chairpersons]Yoshihisa Kobayashi (National Cancer Center Research Institute)、Hiromichi Ebi (Aichi Cancer Center Research Institute)

KRAS G12C inhibitors, sotorasib and adagrasib, have revolutionized treatment strategies for KRAS-mutant lung and colon cancers. Currently, novel drugs targeting other KRAS variants and pan-RAS inhibitors are under extensive development. This symposium brings together experts in lung, colon, and pancreatic cancers to present the latest clinical and basic research on KRAS. The session will provide a comprehensive overview of advancements in KRAS treatments, innovative drug discovery, resistance mechanisms, and challenges beyond the conventional targeting of KRAS GTP/GDP states. This diverse array of perspectives aims to foster interdisciplinary dialogue, bridging foundational research with the clinical translation of new KRAS-targeted therapies in terms of various cancer types and therapeutic approaches.

---

### S3-3
### ASP3082, a First-in-Class KRAS G12D Degrader: Nonclinical Efficacy and Early Clinical Evidence

[Speaker]Takeyuki Nagashima (Onc. Res., Astellas Pharma. Inc.) [Co-author]Yasutoshi Kuboki (Clin. Res. Coord. Div., Nat. Can. Cent. Hosp. East)

KRAS mutations are one of the most prevalent oncogenic drivers. While KRAS G12C inhibitors are available for clinical use, no targeted therapy has yet been approved for other KRAS mutations including G12D, which is common in pancreatic cancers.ASP3082 is a first-in-class KRAS G12D-targeted protein degrader. We have initiated the world's first clinical trial for patients with KRAS G12D-positive cancer, administering ASP3082 intravenously once a week.In nonclinical studies, ASP3082 selectively degraded KRAS G12D over wild-type KRAS, resulting in the inhibition of the downstream signaling pathway. ASP3082 inhibited the growth of human cancer cells. Furthermore, ASP3082 showed potent antitumor efficacy in various CDX and PDX models with sustained KRAS degradation. In a phase 1 study, early clinical data from patients with advanced solid tumors harboring KRAS G12D, including pancreatic and non-small cell lung cancers, demonstrated pharmacodynamic evidence of KRAS G12D degradation and preliminary antitumor activity with manageable safety.In this presentation, we describe our discovery and development process of ASP3082, along with nonclinical efficacy and early clinical evidence.

[Disclosure of COI by Responsible researcher] Lead presenter is the Responsible researcher.
[Lead presenter: Takeyuki Nagashima] ■ employment by a company related to the presentation: アステラス製薬

Symposia

### [S3-4] S3
### Frontiers in the clinical and basic research of KRAS mutant cancer

2025 / 9 / 26 (Fri) 9:00-11:30 Room 3/(Hotel Nikko Kanazawa 4F Tsuru A) Room 3

[Chairpersons]Yoshihisa Kobayashi (National Cancer Center Research Institute)、Hiromichi Ebi (Aichi Cancer Center Research Institute)

KRAS G12C inhibitors, sotorasib and adagrasib, have revolutionized treatment strategies for KRAS-mutant lung and colon cancers. Currently, novel drugs targeting other KRAS variants and pan-RAS inhibitors are under extensive development. This symposium brings together experts in lung, colon, and pancreatic cancers to present the latest clinical and basic research on KRAS. The session will provide a comprehensive overview of advancements in KRAS treatments, innovative drug discovery, resistance mechanisms, and challenges beyond the conventional targeting of KRAS GTP/GDP states. This diverse array of perspectives aims to foster interdisciplinary dialogue, bridging foundational research with the clinical translation of new KRAS-targeted therapies in terms of various cancer types and therapeutic approaches.

---

### S3-4
### ASP3082, a First-in-Class KRAS G12D Degrader: Nonclinical Efficacy and Early Clinical Evidence

[Speaker]Yasutoshi Kuboki (National Cancer Center Hospital East)

No Abstract

### [S3-5] S3
### Frontiers in the clinical and basic research of KRAS mutant cancer

2025 / 9 / 26 (Fri) 9:00-11:30 Room 3/(Hotel Nikko Kanazawa 4F Tsuru A) Room 3

[Chairpersons]Yoshihisa Kobayashi (National Cancer Center Research Institute)、Hiromichi Ebi (Aichi Cancer Center Research Institute)

KRAS G12C inhibitors, sotorasib and adagrasib, have revolutionized treatment strategies for KRAS-mutant lung and colon cancers. Currently, novel drugs targeting other KRAS variants and pan-RAS inhibitors are under extensive development. This symposium brings together experts in lung, colon, and pancreatic cancers to present the latest clinical and basic research on KRAS. The session will provide a comprehensive overview of advancements in KRAS treatments, innovative drug discovery, resistance mechanisms, and challenges beyond the conventional targeting of KRAS GTP/GDP states. This diverse array of perspectives aims to foster interdisciplinary dialogue, bridging foundational research with the clinical translation of new KRAS-targeted therapies in terms of various cancer types and therapeutic approaches.

---

### S3-5
### The mechanisms of reprogramming tumor immune microenvironment by KRAS inhibitor

[Speaker]Noritaka Tanaka (Div. of Mol. Ther, Aichi Cancer Ctr) [Co-author]Hiromichi Ebi (Div. of Mol. Ther, Aichi Cancer Ctr)

KRAS had long been undruggable until the first lead KRAS inhibiting compound had been reported in 2013. Recently, research aiming at the development of KRAS inhibitors has been surging in the world and variable achievements regarding the mechanisms of sensitivity/resistance to KRAS inhibitors have been reported. We have also identified several resistance mechanisms against KRAS inhibitors including EMT-mediated reactivation of PI3K pathways or MRAS-mediated reactivation of MAPK pathway. KRAS is also involved in functions besides signaling-mediated cell growth, which includes tumor microenvironments. Therefore, KRAS inhibitors could potentially modulate tumor microenvironments although not much is well elucidated, and our group focused on the point. We have identified that the treatment of KRAS G12C inhibitor sotorasib induces a molecule (molecule X) in lung cancer cells which could function as anti-immunogenic. The combination of inhibitory agents against molecule X and sotorasib improved the efficacy in vivo compared with sotorasib alone. These findings could potentially lead to the establishment of a novel strategy for lung cancer treatment utilizing KRAS inhibitors.

[Disclosure of COI by Responsible researcher] Lead presenter is not the Responsible researcher.
[Lead presenter: Noritaka Tanaka] We have no financial relationships to disclose.
[Principal Researcher: Hiromichi Ebi] ■Lecture fee: AMGEN　■Research expenses: アステラス、LOXO-Lilly、Servier

▲TOP

### [S3-6] S3
### Frontiers in the clinical and basic research of KRAS mutant cancer

2025 / 9 / 26 (Fri) 9:00-11:30 Room 3/(Hotel Nikko Kanazawa 4F Tsuru A) Room 3

[Chairpersons]Yoshihisa Kobayashi (National Cancer Center Research Institute)、Hiromichi Ebi (Aichi Cancer Center Research Institute)

KRAS G12C inhibitors, sotorasib and adagrasib, have revolutionized treatment strategies for KRAS-mutant lung and colon cancers. Currently, novel drugs targeting other KRAS variants and pan-RAS inhibitors are under extensive development. This symposium brings together experts in lung, colon, and pancreatic cancers to present the latest clinical and basic research on KRAS. The session will provide a comprehensive overview of advancements in KRAS treatments, innovative drug discovery, resistance mechanisms, and challenges beyond the conventional targeting of KRAS GTP/GDP states. This diverse array of perspectives aims to foster interdisciplinary dialogue, bridging foundational research with the clinical translation of new KRAS-targeted therapies in terms of various cancer types and therapeutic approaches.

---

### S3-6
### Novel CRISPR-based treatment strategy for KRAS mutant cancer

[Speaker]Kazuki Kato (Inst. of Integrated Research, Inst. of Sci. Tokyo)

CRISPR-Cas systems are prokaryotic adaptive immune systems that offer diverse molecular tools beyond genome editing. We discovered a novel CRISPR complex, Cas7-11-Csx29, possessing unique RNA-dependent protease activity (Kato et al, Science, 2022). Cas7-11-Csx29 recognizes a specific target RNA matching its guide RNA sequence, activating its protease activity.By harnessing this RNA-dependent protease activity alongside host cell death pathways, we are developing a novel "RNA-triggered programmed cell death" technology. The goal is to design systems that can precisely detect mutant KRAS mRNA within a cell and, upon detection, initiate host cell death. This strategy aims to selectively eliminate KRAS-mutant cancer cells while sparing healthy cells. This approach presents a promising new therapeutic avenue for KRAS-driven cancers, potentially overcoming challenges associated with directly inhibiting the KRAS protein and offering a way to target various KRAS mutations specifically at the RNA level.

[Disclosure of COI by Responsible researcher] Lead presenter is the Responsible researcher.
[Lead presenter: Kazuki Kato] We have no financial relationships to disclose.

### [S3-7] S3
### Frontiers in the clinical and basic research of KRAS mutant cancer

2025 / 9 / 26 (Fri) 9:00-11:30 Room 3/(Hotel Nikko Kanazawa 4F Tsuru A) Room 3

[Chairpersons]Yoshihisa Kobayashi (National Cancer Center Research Institute)、Hiromichi Ebi (Aichi Cancer Center Research Institute)

KRAS G12C inhibitors, sotorasib and adagrasib, have revolutionized treatment strategies for KRAS-mutant lung and colon cancers. Currently, novel drugs targeting other KRAS variants and pan-RAS inhibitors are under extensive development. This symposium brings together experts in lung, colon, and pancreatic cancers to present the latest clinical and basic research on KRAS. The session will provide a comprehensive overview of advancements in KRAS treatments, innovative drug discovery, resistance mechanisms, and challenges beyond the conventional targeting of KRAS GTP/GDP states. This diverse array of perspectives aims to foster interdisciplinary dialogue, bridging foundational research with the clinical translation of new KRAS-targeted therapies in terms of various cancer types and therapeutic approaches.

---

### S3-7
### KRAS-targeted therapy via splicing modulation using antisense oligonucleotides

[Speaker]Yoshihisa Kobayashi (Div. Mol. Path., Natl. Cancer Ctr. Res. Inst.)

While many KRAS mutant protein-targeted therapies are currently under active development, cancers often acquire resistance to these drugs. Therefore, it is critical to explore alternative therapeutic strategies to overcome KRAS-mutant cancers. In our investigation of KRAS mutations as resistance mechanisms to EGFR inhibitors, we serendipitously discovered that the KRAS Q61K mutation does not exhibit oncogenic activity unless accompanied by a silent mutation at codon G60. This finding led to the identification of a novel splicing vulnerability involving exonic splicing enhancer (ESE) motifs near the Q61 region of KRAS, as well as in NRAS and HRAS. In this presentation, we will discuss the interplay between KRAS mutations and alterations in splicing factors, and present preliminary data supporting the broader application of this splicing-targeted approach to other oncogenes, including additional RAS family mutations.

[Disclosure of COI by Responsible researcher] Lead presenter is the Responsible researcher.
[Lead presenter: Yoshihisa Kobayashi] ■Research funds from company: 公的研究費 (AMED、日本学術振興会、JST)　■Research expenses: 上原記念生命科学財団、SGH財団、鈴木謙三記念医科学応用研究財団、武田科学振興財団、がん研究振興財団、小林がん学術振興会、三重医学研究振興会、三菱財団、横山臨床薬理研究助成基金、中外創薬科学財団

### [S4-1] S4
### Elucidation of immune escape mechanisms and development of novel therapeutic strategies

2025 / 9 / 26 (Fri) 9:00-11:30 Room 4/(Hotel Nikko Kanazawa 4F Tsuru B) Room 4

[Chairpersons]Yuka Maeda (Division of Cancer Immunology, National Cancer Center)、Shohei Koyama (National Cancer Center, Research Institute)

It has been over a decade since immune checkpoint inhibitors became a standard treatment option in medical oncology. While these therapies offer the promise of durable responses, numerous challenges-such as therapeutic resistance and immune evasion-have become increasingly apparent.
This session aims to revisit the mechanisms of anti-tumor immune responses from the perspectives of both innate and adaptive immunity. We will explore recent insights into tumor immune evasion, focusing on factors within the tumor microenvironment and host-derived components. Furthermore, we will discuss emerging therapeutic strategies that target these mechanisms, emphasizing their translational potential and clinical relevance.

---

### S4-1
### Regulatory mechanisms of tumor-infiltrating innate immune cells

[Speaker]Osamu Takeuchi (Dept. Medical Chem., Grad Sch. Med, Kyoto Univ.)

Innate immune cells including macrophages play multiple roles in anti-microbial host defense and tissue repair via recognition of foreign substances by Toll-like receptors (TLRs). In the tumor microenvironment, tumor-associated macrophages (TAMs) show heterogeneity and have supportive and inhibitory influences on cancer. Tumor microenvironment is known to influence transcriptional and posttranscriptional profiles, and metabolic reprogramming in tumor-associated macrophages. We found that cyclin J, a TLR-inducible member of the cyclin family, reduced cytokine production in macrophages by coordinately controlling glycolysis and mitochondrial functions together with CDKs. In mice, cyclin J in macrophages limited the growth of transplanted tumors by inhibiting HIF-1a activation in TAMs. In addition, Thus, cyclin J-CDK signaling promotes antitumor immunity and the resolution of inflammation by opposing the metabolic changes that drive inflammatory responses in macrophages.

[Disclosure of COI by Responsible researcher] Lead presenter is the Responsible researcher.
[Lead presenter: Osamu Takeuchi] We have no financial relationships to disclose.

▲TOP

### [S4-2] S4
### Elucidation of immune escape mechanisms and development of novel therapeutic strategies

2025 / 9 / 26 (Fri) 9:00-11:30 Room 4/(Hotel Nikko Kanazawa 4F Tsuru B) Room 4

[Chairpersons]Yuka Maeda (Division of Cancer Immunology, National Cancer Center)、Shohei Koyama (National Cancer Center, Research Institute)

It has been over a decade since immune checkpoint inhibitors became a standard treatment option in medical oncology. While these therapies offer the promise of durable responses, numerous challenges-such as therapeutic resistance and immune evasion-have become increasingly apparent.
This session aims to revisit the mechanisms of anti-tumor immune responses from the perspectives of both innate and adaptive immunity. We will explore recent insights into tumor immune evasion, focusing on factors within the tumor microenvironment and host-derived components. Furthermore, we will discuss emerging therapeutic strategies that target these mechanisms, emphasizing their translational potential and clinical relevance.

---

### S4-2
### TBD

[Speaker]Andrea Ablasser (Swiss Federal Institute of Technology in Lausanne)

No Abstract

### [S4-3] S4
### Elucidation of immune escape mechanisms and development of novel therapeutic strategies

2025 / 9 / 26 (Fri) 9:00-11:30 Room 4/(Hotel Nikko Kanazawa 4F Tsuru B) Room 4

[Chairpersons]Yuka Maeda (Division of Cancer Immunology, National Cancer Center)、Shohei Koyama (National Cancer Center, Research Institute)

It has been over a decade since immune checkpoint inhibitors became a standard treatment option in medical oncology. While these therapies offer the promise of durable responses, numerous challenges-such as therapeutic resistance and immune evasion-have become increasingly apparent.
This session aims to revisit the mechanisms of anti-tumor immune responses from the perspectives of both innate and adaptive immunity. We will explore recent insights into tumor immune evasion, focusing on factors within the tumor microenvironment and host-derived components. Furthermore, we will discuss emerging therapeutic strategies that target these mechanisms, emphasizing their translational potential and clinical relevance.

---

### S4-3
### Identifying the mechanism of acquired resistance against cancer immunotherapy targeting innate immunity

[Speaker]Hitomi Nishinakamura (Division of Cancer Immunology, National Cancer Center Japan) [Co-author]Sayoko Shinya (Discovery and Research, Ono Pharmaceutical Company)、Yuka Maeda (Division of Cancer Immunology, National Cancer Center Japan)、Shohei Koyama (Department of Immuno genomic medicine, National Cancer Center Japan ／Department of Respiratory Medicine and Clinical Immunology, Osaka University)、Hiroyoshi Nishikawa (Division of Cancer Immunology, National Cancer Center Japan ／Department of Immunology, Nagoya University Graduate School of Medicine／Division of Cancer Immune Multicellular System Regulation, CCII, Kyoto University)

Immune checkpoint blockade therapy has been successfully applied in clinical settings as a standard therapy for many cancer types, but its clinical efficacy is restricted to patients with immunologically hot tumors. Various strategies to modify the tumor microenvironment (TME), such as Toll-like receptor (TLR) agonists that can stimulate innate immunity, have been explored but have not been successful. Here, we show a mechanism of acquired resistance to combination treatment consisting of an agonist for multiple TLRs, OK-432 (Picibanil), and PD-1 blockade. Adding the TLR agonist failed to convert the TME from immunogenically cold to hot and did not augment antitumor immunity, particularly $CD8^{+}$T cell responses, in multiple animal models. The failure was attributed to the coactivation of innate suppressive cells, such as polymorphonuclear myeloid-derived suppressor cells (PMN-MDSCs) expressing CXCR2, through high CXCL1 production by macrophages in the TME upon OK-432 treatment. We propose that successful combination cancer immunotherapy intended to stimulate innate antitumor immunity requires modulation of unwanted activation of innate immune suppressive cells, including PMN-MDSCs.

[Disclosure of COI by Responsible researcher] Lead presenter is not the Responsible researcher.
[Lead presenter: Hitomi Nishinakamura] We have no financial relationships to disclose.
[Principal Researcher: Hiroyoshi Nishikawa] ■Research funds from company: 小野薬品　■employment by a company related to the presentation: 富士フイルム、旭化成、日本BD、SRL、Meiji Seika ファルマ　■Profit of stock: ARC　Therapies、セルリアン・ビクロ　■Lecture fee: 小野薬品、BMS、MSD、アムジェン、中外製薬、エーザイ　■Research expenses: 日本BD、大日本住友製薬、富士フィルム、BMS、Rakuten Medical, Inc、日立製作所、小野薬品、大鵬薬品、ミラバイオロジクス、中外製薬、Janssen Research & Development、MSD、住友ファーマ、エーザイ、タイムラプス、ヘルスケア、ARCTherapies、リカケン　H.D.

▲TOP

### [S4-4] S4
### Elucidation of immune escape mechanisms and development of novel therapeutic strategies

2025 / 9 / 26 (Fri) 9:00-11:30 Room 4/(Hotel Nikko Kanazawa 4F Tsuru B) Room 4

[Chairpersons]Yuka Maeda (Division of Cancer Immunology, National Cancer Center)、Shohei Koyama (National Cancer Center, Research Institute)

It has been over a decade since immune checkpoint inhibitors became a standard treatment option in medical oncology. While these therapies offer the promise of durable responses, numerous challenges-such as therapeutic resistance and immune evasion-have become increasingly apparent.
This session aims to revisit the mechanisms of anti-tumor immune responses from the perspectives of both innate and adaptive immunity. We will explore recent insights into tumor immune evasion, focusing on factors within the tumor microenvironment and host-derived components. Furthermore, we will discuss emerging therapeutic strategies that target these mechanisms, emphasizing their translational potential and clinical relevance.

---

### S4-4
### Mitochondrial Transfer in the Tumor Microenvironment

[Speaker]Yosuke Togashi (Dept. TME., Okayama Univ.／Dept. Respir., Okayama Univ. Hosp.)

Mitochondria, through oxidative phosphorylation, play a pivotal role in T cell function, particularly in the formation of memory T cells. Impaired mitochondrial function has been reported in tumor-infiltrating lymphocytes (TILs). In this study, we identified mitochondrial DNA (mtDNA) mutations in TILs that were shared with tumor cells from the same patients. Experimental models confirmed mitochondrial transfer from cancer cells to TILs, with some TILs exhibiting complete replacement by mutant homoplasmic mitochondria. These mtDNA-mutant TILs showed accelerated senescence, functional impairment, and defective memory formation, suggesting a novel mechanism of immune evasion via mitochondrial transfer. Furthermore, we established an in vivo mouse model enabling the tracking of tumor-derived mitochondria. Using this model, we identified multiple immune cell types within the tumor microenvironment that acquired mitochondria from tumor cells. These recipient immune cells exhibited tumor-promoting properties.

[Disclosure of COI by Responsible researcher] Lead presenter is the Responsible researcher.
[Lead presenter: Yosuke Togashi] ■Research funds from company: AMED、JST、JSPS　■Lecture fee: 小野、BMS、中外、MSD、エーザイ、AZ　■Research expenses: 中外、AZ、武田、大鵬、ヤンセン

▲TOP

### [S4-5] S4
### Elucidation of immune escape mechanisms and development of novel therapeutic strategies

2025 / 9 / 26 (Fri) 9:00-11:30 Room 4/(Hotel Nikko Kanazawa 4F Tsuru B) Room 4

[Chairpersons]Yuka Maeda (Division of Cancer Immunology, National Cancer Center)、Shohei Koyama (National Cancer Center, Research Institute)

It has been over a decade since immune checkpoint inhibitors became a standard treatment option in medical oncology. While these therapies offer the promise of durable responses, numerous challenges-such as therapeutic resistance and immune evasion-have become increasingly apparent.
This session aims to revisit the mechanisms of anti-tumor immune responses from the perspectives of both innate and adaptive immunity. We will explore recent insights into tumor immune evasion, focusing on factors within the tumor microenvironment and host-derived components. Furthermore, we will discuss emerging therapeutic strategies that target these mechanisms, emphasizing their translational potential and clinical relevance.

---

### S4-5
### Single Cell Profiling of Tregs: Implications for Tumor Immunity and Cancer Therapy

[Speaker]Eliane Piaggio (Translational Immunotherapy Team. U932, Institut Curie, Paris; France)

$CD4^+$ T cells shape cancer immunity through opposing roles: conventional T cells (Tconvs) promote tumor rejection, whereas regulatory T cells (Tregs) enforce immunosuppression. To dissect this balance, we performed integrative single-cell transcriptomic, TCR, and chromatin accessibility profiling of Tconvs and Tregs from blood, lymph nodes (LNs), and tumors of treatment-naive NSCLC patients. This analysis revealed a BATF-driven subset of follicular and tissue-adapted (FTA) $CD4^+$ T cells, enriched for shared tumor specificities and localized to germinal centers and tertiary lymphoid structures, highlighting a role in anti-tumor responses. We also uncovered fundamental aspects of follicular Treg biology. Translationally, we identified a tumor-associated Treg subset characterized by clonal expansion, TCR activation, and a unique gene signature, enabling the development of novel therapeutic strategies currently being tested clinically. Together, these findings provide mechanistic insights and therapeutic avenues for targeting $CD4^+$ T cell subsets in cancer.

[Disclosure of COI by Responsible researcher] Lead presenter is the Responsible researcher.
[Lead presenter: Eliane Piaggio] ■Research funds from company: Egle Therapeutics ■Advisor of the company and/or profit-making organization: Co-founder and advisor of Egle Therapeutics ■Profit of stock: Egle Therapeutics

▲TOP

### [S4-6] S4
### Elucidation of immune escape mechanisms and development of novel therapeutic strategies

2025 / 9 / 26 (Fri) 9:00-11:30 Room 4/(Hotel Nikko Kanazawa 4F Tsuru B) Room 4

[Chairpersons]Yuka Maeda (Division of Cancer Immunology, National Cancer Center)、Shohei Koyama (National Cancer Center, Research Institute)

It has been over a decade since immune checkpoint inhibitors became a standard treatment option in medical oncology. While these therapies offer the promise of durable responses, numerous challenges-such as therapeutic resistance and immune evasion-have become increasingly apparent.
This session aims to revisit the mechanisms of anti-tumor immune responses from the perspectives of both innate and adaptive immunity. We will explore recent insights into tumor immune evasion, focusing on factors within the tumor microenvironment and host-derived components. Furthermore, we will discuss emerging therapeutic strategies that target these mechanisms, emphasizing their translational potential and clinical relevance.

---

### S4-6
### Interplay Between Gut Microbiota and the Tumor Immune Microenvironment

[Speaker]Shohei Koyama (Department of Immunogenomic Medicine, Research Institute, National Cancer Center／Osaka University Graduate School of Medicine)

Accumulating evidence across various cancer types has demonstrated that gut microbial diversity and the abundance of specific bacterial strains influence anti-tumor immune responses. Notably, fecal microbiota transplantation from immune checkpoint inhibitor (ICI)-responsive patients has been shown to overcome resistance. However, the mechanisms by which gut microbial profiles modulate the tumor immune microenvironment in distant organs remain poorly understood. We successfully isolated YB328, a member of the Ruminococcaceae family, from the feces of ICI-responsive patients. Using murine models, we found that YB328 promotes the differentiation, activation, and Toll-like receptor (TLR) expression of CD103 dendritic cells (DCs) in the gut. These activated DCs can migrate to distant tumor tissues and respond to a broad range of microbial TLR ligands, thereby enhancing anti-tumor immunity. Furthermore, YB328 administration was associated with increased diversity of the gut microbiota. Collectively, these findings suggest that YB328-stimulated CD103 DCs facilitate the induction of T cell responses against diverse antigens, contributing to enhance the efficacy of ICI treatment.

[Disclosure of COI by Responsible researcher] Lead presenter is the Responsible researcher.
[Lead presenter: Shohei Koyama] ■Research expenses: 中外製薬、大塚製薬、Meiji製菓ファルマ

### [S5-1] S5
### Gut microbiota and cancer

2025 / 9 / 26 (Fri) 9:00-11:30 Room 5/(Hotel Nikko Kanazawa 4F Tsuru C) Room 5

[Chairpersons]Naoko Ohtani (Graduate School of Medicine, Osaka Metropolitan University)、Shinichi Yachida (Graduate School of Medicine, The University of Osaka)

Owing to the next-generation sequencing technology, gut microbiota research has recently advanced, revealing that gut bacteria are associated with various human diseases. This session will focus on recent cutting-edge topics, particularly the relationship between cancer and gut microbiota. We will discuss the relationship between colorectal cancer and E. coli positive for the mutagenic bacterial toxin, colibactin, as well as the analysis of the hereditary colorectal tumors and the gut microbiota. Additionally, this session will cover the involvement of bacterial species present within tumors in cancer progression, which has been recently elucidated. Furthermore, we will discuss the bacterial species that enhance the efficacy of immune checkpoint inhibitors (ICIs) and the potential of phage therapy targeting specific causative bacteria. We are also accepting one presentation from the public.

---

### S5-1
### Colibactin, a toxin produced by intestinal bacteria, is involved in colorectal carcinogenesis

[Speaker]Tatsuhiro Shibata (Lab. of Molecular Med., IMSUT／ Div. Cancer Genomics, NCC)

The number of colorectal cancer (CRC) cases in Japan is one of the highest in the world, and the increase in younger cases (under 50 years old) is particularly important. Colibactin toxin secreted by intestinal bacteria directly induces mutations and is involved in colon carcinogenesis. In 2025, an international collaborative group including our group reported a geographical analysis of mutational signatures in CRC, and Japanese cases showed the highest frequency of mutational signatures caused by the colibactin toxin (Nature 2025). This signature also contributed more strongly to the development of younger CRC. In this session, we will present an analysis of combining whole-genome sequencing from a larger set of Japanese CRC with metagenomic sequencing of fecal samples. The mechanism of carcinogenesis by bacterial infection was known to be through chronic inflammation and DNA methylation, such as gastric carcinogenesis caused by H. pylori. However, DNA-damaging enteric bacteria are a new bacterial carcinogenesis mechanism that promotes cancer development by directly inducing DNA abnormalities. Furthermore, there is an ethical diversity in the effects of these factors.

[Disclosure of COI by Responsible researcher] Lead presenter is the Responsible researcher.
[Lead presenter: Tatsuhiro Shibata] We have no financial relationships to disclose.

### [S5-2] S5
### Gut microbiota and cancer

2025 / 9 / 26 (Fri) 9:00-11:30 Room 5/(Hotel Nikko Kanazawa 4F Tsuru C) Room 5

[Chairpersons]Naoko Ohtani (Graduate School of Medicine, Osaka Metropolitan University)、Shinichi Yachida (Graduate School of Medicine, The University of Osaka)

Owing to the next-generation sequencing technology, gut microbiota research has recently advanced, revealing that gut bacteria are associated with various human diseases. This session will focus on recent cutting-edge topics, particularly the relationship between cancer and gut microbiota. We will discuss the relationship between colorectal cancer and E. coli positive for the mutagenic bacterial toxin, colibactin, as well as the analysis of the hereditary colorectal tumors and the gut microbiota. Additionally, this session will cover the involvement of bacterial species present within tumors in cancer progression, which has been recently elucidated. Furthermore, we will discuss the bacterial species that enhance the efficacy of immune checkpoint inhibitors (ICIs) and the potential of phage therapy targeting specific causative bacteria. We are also accepting one presentation from the public.

---

### S5-2
### Colorectal cancer risk in familial adenomatous polyposis (FAP): The Impact of the Gut Microbiome

[Speaker]Sayaka Mizutani (Grad. Sch. Med., Juntendo Univ.／Sch. Life Sci. Tech., Inst. Sci. Tokyo)

Familial adenomatous polyposis (FAP) is a rare hereditary disease caused by germline mutations in the ***APC*** gene, leading to numerous gastrointestinal adenomas with a high risk of progressing to colorectal cancer (CRC). Despite shared genetics, phenotypic differences among patients suggest environmental factors, including the gut microbiota, contribute to CRC risk. To explore this, we conducted shotgun metagenomic and targeted metabolomic analyses on fecal samples from 130 FAP patients undergoing a colectomy-free endoscopic treatment allowing longitudinal study. While most patients had stable microbiota, those with high intra-individual variation showed changes in dysplasia grade. Cross-sectional comparisons with healthy controls revealed significant enrichment of ***Escherichia coli*** in FAP patients. Metabolomic profiling showed elevated amino acids and organic acids (malate, fumarate, succinate), potentially linked to excess mucus from polyps. Further analysis identified ***Solobacterium moorei*** enriched in advanced adenoma cases. This study in FAP highlights the role of gut microbes in early CRC development and suggests their potential use in risk assessment beyond genetic factors.

[Disclosure of COI by Responsible researcher] Lead presenter is the Responsible researcher.
[Lead presenter: Sayaka Mizutani] We have no financial relationships to disclose.

### [S5-3] S5
### Gut microbiota and cancer

2025 / 9 / 26 (Fri) 9:00-11:30 Room 5/(Hotel Nikko Kanazawa 4F Tsuru C) Room 5

[Chairpersons]Naoko Ohtani (Graduate School of Medicine, Osaka Metropolitan University)、Shinichi Yachida (Graduate School of Medicine, The University of Osaka)

Owing to the next-generation sequencing technology, gut microbiota research has recently advanced, revealing that gut bacteria are associated with various human diseases. This session will focus on recent cutting-edge topics, particularly the relationship between cancer and gut microbiota. We will discuss the relationship between colorectal cancer and E. coli positive for the mutagenic bacterial toxin, colibactin, as well as the analysis of the hereditary colorectal tumors and the gut microbiota. Additionally, this session will cover the involvement of bacterial species present within tumors in cancer progression, which has been recently elucidated. Furthermore, we will discuss the bacterial species that enhance the efficacy of immune checkpoint inhibitors (ICIs) and the potential of phage therapy targeting specific causative bacteria. We are also accepting one presentation from the public.

---

### S5-3
### Effect of Fusobacterium infection on ovarian cancer progression

[Speaker]Keiko Shinjo (Div. of Cancer Biol., Nagoya Univ. Grad. Sch. of Med.) [Co-author]Miho Suzuki (Div. of Cancer Biol., Nagoya Univ. Grad. Sch. of Med.)、Yutaka Kondo (Div. of Cancer Biol., Nagoya Univ. Grad. Sch. of Med.)

Recent studies have highlighted the impact of bacterial infection on carcinogenesis and treatment response. Fusobacterium, a gram-negative anaerobic bacterium, has been implicated in the pathogenesis of colorectal cancer and in modulating sensitivity to treatment. We previously reported that Fusobacterium infection contributes to the development of endometriosis. In our cohort, more than 60% of endometriosis cases were positive for Fusobacterium. The infection activates macrophages, and macrophage-derived TGF-$\beta$ induces fibroblasts into Transgelin (TAGLN)-positive myofibroblasts, thereby promoting the formation of endometriotic lesions. Since endometriosis is a known risk factor for ovarian cancer, we further investigated ovarian cancer tissues and found a high prevalence of Fusobacterium-positive cases. Notably, the average age at diagnosis was younger in Fusobacterium-positive than in negative cases, suggesting that Fusobacterium infection contributes to ovarian cancer development. Thus, targeting Fusobacterium infection may reduce the incidence of ovarian cancer and provide an innovative therapeutic approach for its treatment.

[Disclosure of COI by Responsible researcher] Lead presenter is not the Responsible researcher.
[Lead presenter: Keiko Shinjo] We have no financial relationships to disclose.
[Principal Researcher: Yutaka Kondo] We have no financial relationships to disclose.

### [S5-4] S5
### Gut microbiota and cancer

2025 / 9 / 26 (Fri) 9:00-11:30 Room 5/(Hotel Nikko Kanazawa 4F Tsuru C) Room 5

[Chairpersons]Naoko Ohtani (Graduate School of Medicine, Osaka Metropolitan University)、Shinichi Yachida (Graduate School of Medicine, The University of Osaka)

Owing to the next-generation sequencing technology, gut microbiota research has recently advanced, revealing that gut bacteria are associated with various human diseases. This session will focus on recent cutting-edge topics, particularly the relationship between cancer and gut microbiota. We will discuss the relationship between colorectal cancer and E. coli positive for the mutagenic bacterial toxin, colibactin, as well as the analysis of the hereditary colorectal tumors and the gut microbiota. Additionally, this session will cover the involvement of bacterial species present within tumors in cancer progression, which has been recently elucidated. Furthermore, we will discuss the bacterial species that enhance the efficacy of immune checkpoint inhibitors (ICIs) and the potential of phage therapy targeting specific causative bacteria. We are also accepting one presentation from the public.

---

### S5-4
### Innate lymphoid cells in tumor immunity and therapeutic potential

[Speaker]Naoko Satoh-Takayama (RIKEN ECL Unit, RIKEN IMS)

Innate lymphoid cells (ILCs) are a unique group of immune cells that have gained attention for their roles in immune regulation. ILCs are traditionally divided into groups 1, 2, and 3, based on their transcription factors and cytokine profiles. According to updated nomenclature, group 1 ILCs (ILC1s) no longer include natural killer (NK) cells. ILC1s are a distinct subset that functionally mirror Th1 cells, mainly producing interferon-&gamma. Unlike NK cells, ILC1s rely on the transcription factor T-bet, lack Eomes expression, and reside in tissues. NK cells are strongly cytotoxic, while ILC1s primarily have limited direct cytotoxicity. ILC1s are known as tissue-resident sentinels that protect the host from pathogens at the initial sites of infection. However, their interaction with the microbiota and the mechanisms regulating their activity remain largely unclear. The role of ILC1s in cancer has also been historically unclear, with limited and conflicting data. However, emerging evidence highlights their involvement in tumor immunity, and interest in their function is rapidly growing. In this session, I will present recent insights, including findings from our research.

[Disclosure of COI by Responsible researcher] Lead presenter is the Responsible researcher.
[Lead presenter: Naoko Satoh] We have no financial relationships to disclose.

### [S5-5] S5
### Gut microbiota and cancer

2025 / 9 / 26 (Fri) 9:00-11:30 Room 5/(Hotel Nikko Kanazawa 4F Tsuru C) Room 5

[Chairpersons]Naoko Ohtani (Graduate School of Medicine, Osaka Metropolitan University)、Shinichi Yachida (Graduate School of Medicine, The University of Osaka)

Owing to the next-generation sequencing technology, gut microbiota research has recently advanced, revealing that gut bacteria are associated with various human diseases. This session will focus on recent cutting-edge topics, particularly the relationship between cancer and gut microbiota. We will discuss the relationship between colorectal cancer and E. coli positive for the mutagenic bacterial toxin, colibactin, as well as the analysis of the hereditary colorectal tumors and the gut microbiota. Additionally, this session will cover the involvement of bacterial species present within tumors in cancer progression, which has been recently elucidated. Furthermore, we will discuss the bacterial species that enhance the efficacy of immune checkpoint inhibitors (ICIs) and the potential of phage therapy targeting specific causative bacteria. We are also accepting one presentation from the public.

---

### S5-5
### An Enterococcus phage-derived lytic enzyme suppresses graft-versus-host disease

[Speaker]Kosuke Fujimoto (RIMD, Osaka Univ.／Dept. Immunol. Genom., Sch. Med., Osaka Met. Univ.／Div. Metagenome Med., HGC, IMSUT, UTokyo)

Acute graft-versus-host disease (aGVHD) is a life-threatening complication of allogenic haematopoietic cell transplantation (allo-HCT) for haematological diseases. Disease-related alterations of the intestinal environment have been reported to be closely associated with aGVHD symptoms in clinical studies. We analysed the intestinal microbiome of allo-HCT patients and found that E. faecalis escaped elimination and proliferated in the intestine by forming biofilms. We isolated cytolysin-positive highly pathogenic E. faecalis from patient fecal samples and identified an anti-E. faecalis enzyme derived from E. faecalis-specific bacteriophages. The antibacterial enzyme had lytic activity against the biofilm of E. faecalis in vitro and in vivo. Furthermore, in aGVHD-induced gnotobiotic mice colonized with E. faecalis, or with patient fecal samples characterized by Enterococcus domination, the number of intestinal Enterococcus was decreased and survival was significantly increased in the E. faecalis-specific enzyme-treated group compared with the controls. Thus, administration of phage-derived antibacterial enzyme might be a novel approach for protection against aGVHD.

[Disclosure of COI by Responsible researcher] Lead presenter is the Responsible researcher.
[Lead presenter: Kosuke Fujimoto] We have no financial relationships to disclose.

### [S5-6] S5
### Gut microbiota and cancer

2025 / 9 / 26 (Fri) 9:00-11:30 Room 5/(Hotel Nikko Kanazawa 4F Tsuru C) Room 5

[Chairpersons]Naoko Ohtani (Graduate School of Medicine, Osaka Metropolitan University)、Shinichi Yachida (Graduate School of Medicine, The University of Osaka)

Owing to the next-generation sequencing technology, gut microbiota research has recently advanced, revealing that gut bacteria are associated with various human diseases. This session will focus on recent cutting-edge topics, particularly the relationship between cancer and gut microbiota. We will discuss the relationship between colorectal cancer and E. coli positive for the mutagenic bacterial toxin, colibactin, as well as the analysis of the hereditary colorectal tumors and the gut microbiota. Additionally, this session will cover the involvement of bacterial species present within tumors in cancer progression, which has been recently elucidated. Furthermore, we will discuss the bacterial species that enhance the efficacy of immune checkpoint inhibitors (ICIs) and the potential of phage therapy targeting specific causative bacteria. We are also accepting one presentation from the public.

---

### S5-6
### Development of a Catalog of Anticancer Drug Effects on the Human Gut Microbiome Across Cancer Types

[Speaker]Kyoko Kurihara (Integr. Biosci. Dept., Front. Sci., Univ. Tokyo／Transl. Inform. Div., EPOC, NCC) [Co-author]Shunsuke A. Sakai (Integr. Biosci. Dept., Front. Sci., Univ. Tokyo／Transl. Inform. Div., EPOC, NCC)、Naoko Iida (Transl. Res. Support Ofc., NCCE)、Kentaro Sawada (Med. Oncol. Dept., Kushiro Rosai Hosp.)、Satoshi Horasawa (Transl. Res. Support Ofc., NCCE)、Takao Fujisawa (Transl. Res. Support Ofc., NCCE／Head & Neck Med. Oncol. Dept., NCCE)、Yoshiaki Nakamura (Transl. Res. Support Ofc., NCCE／GI Oncol. Dept., NCCE)、Shunichiro Kageyama (Transl. Inform. Div., EPOC, NCC)、Katsuya Tsuchihara (Integr. Biosci. Dept., Front. Sci., Univ. Tokyo／Transl. Inform. Div., EPOC, NCC)、Riu Yamashita (Transl. Inform. Div., EPOC, NCC／Comput. Biol. & Med. Sci. Dept., Frontier Sci., Univ. Tokyo)

Evaluating the impact of anticancer drugs on the gut microbiome provides a basis for advancing microbiome-based therapeutics in cancer therapy. However, the effects of multiple anticancer drugs across cancer types remain poorly understood in previous studies. Here, we assessed changes in microbial composition induced by 22 anticancer drugs using 16S rRNA data of fecal samples collected at pre- and post-treatment (n=264) in the MONSTAR-SCREEN project. A multivariate regression model was employed to analyze the associations between each drug and gut bacteria. Principal component analysis on the coefficient matrix from the model showed subclusters of functionally similar drugs, such as immune checkpoint inhibitors (ICIs) (Nivolumab and Pembrolizumab) and anti-EGFR inhibitors (Panitumumab and Cetuximab). Specifically, Pembrolizumab decreased ***Faecalibaterium***, which has been reported to positively correlate with the response to ICIs. These results suggest that anticancer drugs could influence specific bacteria associated with treatment efficacy. Our study provides a catalog of anticancer drug effects on the gut microbiome, showing the potential targets of microbiome-based therapeutics.

[Disclosure of COI by Responsible researcher] Lead presenter is not the Responsible researcher.
[Lead presenter: Kyoko Kurihara] We have no financial relationships to disclose.
[Principal Researcher: Riu Yamashita] We have no financial relationships to disclose.

### [E-2001] I-E1
### DNA damage response & carcinogenesis

2025 / 9 / 26 (Fri) 9:00-10:15 Room 6/(Hotel Nikko Kanazawa 3F Kujaku A) Room 6

[Chairperson]Anna Kakehashi (Dept. Mol. Pathol., Osaka Metropol. Univ. Grad. Sch. Med.)

---

### E-2001
### Identification of novel regulator of ATR inhibitor sensitivity and splicing vulnerability in prostate cancer

[Speaker]Masahiko Ajiro (Div. of Cancer RNA Res., Natl. Cancer Ctr.) [Co-author]Kazuki Nishimura (Div. of Cancer RNA Res., Natl. Cancer Ctr.／Dept. of Urol. Brigham & Women's Hosp., Harvard Med. Univ.／Dept. of Urol. Osaka Med. & Pharm. Univ.)、Shoko Kinoshita (Dept. of Urol. Osaka Med. & Pharm. Univ.)、Takuya Tsujino (Dept. of Urol. Osaka Med. & Pharm. Univ.)、Van N. Trihn (Dept. of Exp. Rad. Oncol., MD Anderson Cancer Ctr.)、Jing Luo (Dept. of Urol. Brigham & Women's Hosp., Harvard Med. Univ.)、Muqing Li (Dept. of Urol. Brigham & Women's Hosp., Harvard Med. Univ.)、Haibo Yang (Dept. of Urol. Brigham & Women's Hosp., Harvard Med. Univ.)、Gui Fu (Dept. of Urol. Brigham & Women's Hosp., Harvard Med. Univ.)、Jhen H. Jhan (Dept. of Urol. Brigham & Women's Hosp., Harvard Med. Univ.)、Taraswi Mitra (Dept. of Urol. Brigham & Women's Hosp., Harvard Med. Univ.)、Marian Kalocsay (Dept. of Exp. Rad. Oncol., MD Anderson Cancer Ctr.)、Li Jia (Dept. of Urol. Brigham & Women's Hosp., Harvard Med. Univ.)、Akihide Yoshimi (Div. of Cancer RNA Res., Natl. Cancer Ctr.)

Castration-resistant prostate cancer has limited treatment options. Although Ataxia Telangiectasia and Rad3-related protein inhibitors (ATRi) have recently shown promising results in clinical trials, their biomarkers are not well understood. In this study, we performed CRISPR-Cas9 screening in two prostate cancer cell lines using ATRi VE-822 and identified loss of ***gene X*** as a potential therapeutic biomarker for ATRi sensitivity. Prostate cancer cell lines with ***gene X*** knockout (KO) indicated dysregulation of RNA-binding proteins and splicing factors, leading to a considerable increase in mis-splicing events, consistent with the observation of prostate cancers with ***gene X*** deletion in TCGA. Additionally, increased R-loop accumulation, associated with ATR activation, was confirmed using S9.6 immunofluorescence and Cut & Tag R-loop assays. We also assessed a set of splicing modulators, and found that splicing inhibitor H3B-8800 promoted ATRi sensitivity in prostate cancer cell lines with ***gene X*** KO.These findings highlight ***gene X*** loss as a novel biomarker for ATRi sensitivity and vulnerability to splicing inhibitor, potentially broadening its application in prostate cancer treatment.

[Disclosure of COI by Responsible researcher] Lead presenter is not the Responsible researcher.
[Lead presenter: Masahiko Ajiro] ■Research expenses: 武田科学振興財団
[Principal Researcher: Akihide Yoshimi] ■Research expenses: 第一三共株式会社、エーザイ株式会社、中外製薬株式会社

▲TOP

### [E-2002] I-E1
### DNA damage response & carcinogenesis

2025 / 9 / 26 (Fri) 9:00-10:15 Room 6/(Hotel Nikko Kanazawa 3F Kujaku A) Room 6

[Chairperson]Anna Kakehashi (Dept. Mol. Pathol., Osaka Metropol. Univ. Grad. Sch. Med.)

---

### E-2002
### Lung carcinogenicity of single-walled carbon nanotube and comparative analysis with multi-walled carbon nanotube in rats

[Speaker]Aya Naiki (Dept. Exp. Pathol. Tumor Biol., Nagoya City Univ.) [Co-author]Hiroyuki Kato (Dept. Exp. Pathol. Tumor Biol., Nagoya City Univ.)、Anna Kakehashi (Dept. Mol. Pathol., Osaka Metropolitan Univ. Grad. Sch. Med.)、Hiroyuki Tsuda (Dept. Exp. Pathol. Tumor Biol., Nagoya City Univ.／Nanotoxicology Lab Project, Nagoya City Univ.)、Satoru(Nagoya City Univ.) Takahashi (Dept. Exp. Pathol. Tumor Biol., Nagoya City Univ.)

Widespread industrial use of carbon nanotube (CNT) has raised toxicity concerns. While multi-walled CNT (MWCNT) are known to induce lung and mesothelial carcinogenicity, the single-walled CNT (SWCNT) carcinogenicity remains unclear. This study evaluated the long-term carcinogenic potential of SWCNT in comparison to MWCNTs (MWCNT-7, MWCNT-N). F344 rats were administered 0.1 or 0.5 mg of each CNT by intratracheal spraying. Pathological and molecular analyses were performed at weeks 4 and 104. All CNTs induced alveolar macrophage infiltration, upregulated cytokines and chemokines (***Ccl2***, ***Ccl3***, ***Ccl7***, Mmp12, Spp1, and Il-6), increased Ki-67 and γH2AX-positive alveolar epithelial cells in the lung at week 4. MWCNTs increased 8-OHdG-positive cells, while SWCNT induced neutrophil infiltration and 8-NG-positive cells. At week 104, alveolar adenocarcinomas were significantly increased by SWCNT and MWCNT-N. Malignant mesotheliomas were induced by MWCNTs but not by SWCNT. RNA-seq revealed cell cycle gene activation. This study demonstrates the lung carcinogenicity of SWCNT and identifies early toxicological markers that may predict CNT-related cancer risk.

[Disclosure of COI by Responsible researcher] Lead presenter is the Responsible researcher.
[Lead presenter: Aya Naiki] We have no financial relationships to disclose.

### [E-2003] I-E1
### DNA damage response & carcinogenesis

2025 / 9 / 26 (Fri) 9:00-10:15 Room 6/(Hotel Nikko Kanazawa 3F Kujaku A) Room 6

[Chairperson]Anna Kakehashi (Dept. Mol. Pathol., Osaka Metropol. Univ. Grad. Sch. Med.)

English Oral Session

### E-2003
### Identifying carcinogenic chemical agents based on genomic signatures in cancers of unknown primary

[Speaker]Sana Yokoi (div. Genet. Diagnostics, Chiba Cancer Center) [Co-author]Izumi Miyabe (div. Genet. Diagnostics, Chiba Cancer Center)

<Background> Cancers of unknown primary (CUP) often present with lymphadenopathy, mimicking lymphoma. When biopsy reveals epithelial origin, imaging and endoscopy are used to search for the primary. Genomic profiling is also used to estimate tissue of origin.
<Case & Results> A woman in her 70s with Fabry disease and kidney transplant was referred for suspected lymphoma. Biopsy showed poorly differentiated carcinoma without an obvious primary, leading to CUP diagnosis. NCC oncopanel test showed high tumor mutational burden (>60), mostly A>T transversions. Gnomic signature analysis indicated aristolochic acid exposure.
<Discussion> Aristolochic acid, found in Aristolochia herbs, causes T>A transversions and is linked to renal and liver cancers. The patient, a long-term Korean resident in Japan, had a habit of consuming imported herbal medicines. Her sibling also had renal failure.
<Conclusion> This CUP likely resulted from long-term aristolochic acid exposure. Genomic profiling should consider chemical carcinogenesis.

[Disclosure of COI by Responsible researcher] Lead presenter is the Responsible researcher.
[Lead presenter: Sana Yokoi] We have no financial relationships to disclose.

### [E-2004] I-E1
### DNA damage response & carcinogenesis

2025 / 9 / 26 (Fri) 9:00-10:15 Room 6/(Hotel Nikko Kanazawa 3F Kujaku A) Room 6

[Chairperson]Anna Kakehashi (Dept. Mol. Pathol., Osaka Metropol. Univ. Grad. Sch. Med.)

### E-2004
### OLA1 is phosphorylated by ATR and transported to centrosomes to promote DNA damage-induced centrosome amplification

[Speaker]Natsuko Chiba (Dept. Cancer Biol., IDAC, Tohoku Univ.) [Co-author]Tsechin Hsiao (Dept. Cancer Biol., IDAC, Tohoku Univ.)、Kazushi Sakaguchi (Dept. Cancer Biol., IDAC, Tohoku Univ.)、Yuki Yoshino (Dept. Cancer Biol., IDAC, Tohoku Univ.)、Zhenzhou Fang (Dept. Cancer Biol., IDAC, Tohoku Univ.)

Germline variants of ***BRCA1*** cause hereditary breast and ovarian cancer. Previously, we reported that BRCA1 is phosphorylated by ATM after DNA damage, transports the DNA damage response signal, and promotes DNA damage-induced centrosome amplification (DDICA) by activating polo-like kinase (PLK1) at the centrosome. We identified Obg-like ATPase 1 (OLA1) as a BRCA1-interacting protein and revealed that OLA1 functions in centrosome regulation together with BRCA1 and is also involved in DDICA after cisplatin (CDDP) treatment. In this study, we found that the nuclear export sequence (NES) of OLA1 is important for its centrosomal localization in physiological conditions and the increase in centrosomal OLA1 after CDDP treatment and DDICA. Furthermore, we found that phosphorylation of OLA1 by ATM and Rad3-related (ATR) is critical for OLA1 function in DDICA. Interestingly, OLA1 localization to centrosome after CDDP treatment is regulated independently of BRCA1. Therefore, these results indicate that ATR phosphorylates OLA1 after CDDP treatment in the nucleus, phosphorylated OLA1 moves to the cytoplasm, and locates centrosome independent of BRCA1 to induce centrosome amplification.

[Disclosure of COI by Responsible researcher] Lead presenter is the Responsible researcher.
[Lead presenter: Natsuko Chiba] ■Research funds from company: アストラゼネカ株式会社

▲TOP

### [E-2005] I-E1
### DNA damage response & carcinogenesis

2025 / 9 / 26 (Fri) 9:00-10:15 Room 6/(Hotel Nikko Kanazawa 3F Kujaku A) Room 6

[Chairperson]Anna Kakehashi (Dept. Mol. Pathol., Osaka Metropol. Univ. Grad. Sch. Med.)

---

### E-2005
### A DNA damage profiling of human blood cells using mass spectrometry with a chemical compound library of DNA adducts

[Speaker]Yuji Iwashita (Div. Cancer Epidemiol. Prev., Aichi Cancer Ctr. Res. Inst.) [Co-author]Yumiko Kasugai (Div. Cancer Epidemiol. Prev., Aichi Cancer Ctr. Res. Inst.)、Yuriko Koyanagi (Div. Cancer Epidemiol. Prev., Aichi Cancer Ctr. Res. Inst.)、Kazuya Shinmura (Dept. Tumor Pathol., Hamamatsu Univ. Sch. Med.)、Haruhiko Sugimura (Sasaki Inst.)、Keitaro Matsuo (Div. Cancer Epidemiol. Prev., Aichi Cancer Ctr. Res. Inst.)

Atypical chemical structure of DNAs altered by reactive chemicals and/or modification enzymes is a source of DNA damage, which causes DNA mutation in carcinogenesis. These atypical DNA molecules with similar chemical structures can be defined as DNA adducts. Several DNA adducts with individual specificity were identified by liquid chromatography coupled with tandem mass spectrometry using a chemical compound library of DNA adducts in our previous DNA adductome analysis. To profile damages of human blood DNAs with limited amount and large sample size, a method with high sensitivity and high throughput is required. We established an all-ion fragmentation method using an Orbitrap mass analyzer and simultaneously quantified 40 and more compounds of DNA adducts. We will report an application of this improved method of DNA adductomics with human blood DNAs collected under epidemiological design with lifestyle information like tobacco smoking and alcohol consumption and discuss potentials of DNA adducts as biomarkers reflecting premalignant step of carcinogenesis.

[Disclosure of COI by Responsible researcher] Lead presenter is not the Responsible researcher.
[Lead presenter: Yuji Iwashita] ■Research funds from company: JSPS
[Principal Researcher: Keitaro Matsuo] ■Research funds from company: JSPS

### [E-2006] I-E1
### DNA damage response & carcinogenesis

2025 / 9 / 26 (Fri) 9:00-10:15 Room 6/(Hotel Nikko Kanazawa 3F Kujaku A) Room 6

[Chairperson]Anna Kakehashi (Dept. Mol. Pathol., Osaka Metropol. Univ. Grad. Sch. Med.)

---

### E-2006
### Mutagenicity of 5-hydroxycytosine, an oxidized form of cytosine, in human cells

[Speaker]Hiroyuki Kamiya (Grad. Sch. Biomed. Hlth. Sci., Hiroshima Univ.) [Co-author]Tetsuya Suzuki (Grad. Sch. Biomed. Hlth. Sci., Hiroshima Univ.)

**Background:** Reactive oxygen species oxidize DNA bases. 5-Hydroxycytosine is a major oxidized cytosine base. The modified base is miscoding ***in vitro*** and mutagenic in ***Escherichia coli***. In this study, we examined the mutagenicity of 5-hydroxycytosine in human cells.
**Methods:** Shuttle plasmid DNA bearing 5-hydroxycytosine within the ***supF*** gene was constructed. The DNA was transfected into human U2OS cells by lipofection. The replicated plasmid DNA was recovered and mutations in the ***supF*** gene were analyzed.
**Results:** 5-Hydroxycytosine caused base substitution mutations, mainly the C to T transition, at the lesion site. This base seemed to induce untargeted base substitution mutations at positions distant from the 5-hydroxycytosine site.
**Conclusions:** These results suggested that 5-hydroxycytosine is mutagenic in human cells and contributes to reactive oxygen species-causing mutation signatures in human cancers.
**Acknowledgments:** We thank Ms. Ann Hirota and Prof. Shigenori Iwai for the mutagenicity experiments and the oligodeoxyribonucleotide synthesis, respectively.

[Disclosure of COI by Responsible researcher] Lead presenter is the Responsible researcher.
[Lead presenter: Hiroyuki Kamiya] We have no financial relationships to disclose.

### [E-2007] I-E12-3
### New cancer diagnostic technologies

2025 / 9 / 26 (Fri) 9:00-10:15 Room 7/(Hotel Nikko Kanazawa 3F Kujaku B) Room 7

[Chairperson]Yoshiki Murakumo (Dept. Pathol., Kitasato Univ. Sch. Med.)

---

### E-2007
### Development of AI model for 3D polygon data generation toward the XR navigation of gynecological cancer surgery

[Speaker]Takashi Natsume (Div. Med. AI Res. Dev., Natl. Cancer Ctr. Res. Inst.／Dept. Obstet. Gynecol, The Univ. of Tokyo) [Co-author]Masaaki Komatsu (Div. Med. AI Res. Dev., Natl. Cancer Ctr. Res. Inst.／Cancer Transl. Res. Team, RIKEN Ctr. for AIP project)、Katsuji Takeda (Cancer Transl. Res. Team, RIKEN Ctr. for AIP project)、Seiya Sato (Dept. Radiological Tech., Natl. Cancer Ctr. Hosp)、Nao Kikkawa (Dept. Diagnostic Radiology, Natl. Cancer Ctr. Hosp)、Mototaka Miyake (Dept. Diagnostic Radiology, Natl. Cancer Ctr. Hosp)、Mayumi Kato (Dept. Gynecol., Natl. Cancer Ctr. Hosp)、Kenbun Sone (Dept. Obstet. Gynecol, The Univ. of Tokyo)、Michihiro Tanikawa (Dept. Obstet. Gynecol, The Univ. of Tokyo)、Masaya Uno (Dept. Gynecol., Natl. Cancer Ctr. Hosp)、Tomoyasu Kato (Dept. Gynecol., Natl. Cancer Ctr. Hosp)、Mitsuya Ishikawa (Dept. Gynecol., Natl. Cancer Ctr. Hosp)、Yasushi Hirota (Dept. Obstet. Gynecol, The Univ. of Tokyo)、Ryuji Hamamoto (Div. Med. AI Res. Dev., Natl. Cancer Ctr. Res. Inst.／Cancer Transl. Res. Team, RIKEN Ctr. for AIP project)

Recently, the use of extended reality (XR) for surgical navigation and preoperative evaluation has been reported in several medical departments. We introduce artificial intelligence (AI) into 3D polygon data generation for surgical navigation models in gynecological cancer. The vessels from the renal artery and vein bifurcation to the pelvic vascular group were manually annotated in preoperative CT images (portal phase) of 200 gynecological cancer patients. We used CIS-UNet with shape-aware loss as the 3D segmentation model to generate 3D polygon data and the mean Dice coefficients (mDice) as a segmentation accuracy metric. As a result, the mDice for the arteries, veins, and renal vessels were 0.798, 0.775, and 0.682, respectively. The automatically generated 3D polygon data was projected into the real world as a hologram using the HoloLens 2(Microsoft, Redmond, WA, US) head-mounted display, and the XR platform was provided by the medical image display service, Holoeyes MD. Automatically generating 3D polygon data from portal phase images alone without multiphase imaging, we expect to reduce radiation exposure and significantly decrease the burden on radiographers.

[Disclosure of COI by Responsible researcher] Lead presenter is not the Responsible researcher.
[Lead presenter: Takashi Natsume] We have no financial relationships to disclose.
[Principal Researcher: Ryuji Hamamoto] We have no financial relationships to disclose.

### [E-2008] I-E12-3
### New cancer diagnostic technologies

2025 / 9 / 26 (Fri) 9:00-10:15 Room 7/(Hotel Nikko Kanazawa 3F Kujaku B) Room 7

[Chairperson]Yoshiki Murakumo (Dept. Pathol., Kitasato Univ. Sch. Med.)

---

### E-2008
### Clinical Insights from Pre-Biopsy Germline NGS Testing in Gynecologic Oncology

[Speaker]Min Kyu Kim (Department of Obstetrics and Gynecology, Chung-Ang University Hospital, Seoul, Korea) [Co-author]Dawn Chung (Department of Obstetrics and Gynecology, Chung-Ang University Hospital, Seoul, Korea)

Germline mutation testing is typically performed after cancer diagnosis, missing opportunities for early intervention. The clinical utility of pre-biopsy germline multigene panel testing was evaluated. From March 2018 to January 2024, gNGS was performed before surgery in 411 patients with suspected gynecologic cancers. Among them, 25 (6.1%) had pathogenic or likely pathogenic variants (P/LPV). The most frequently altered genes were BRCA1 and BRCA2 (n = 9, 36%), followed by SDHA, MSH6, MUTYH, RAD51C, MLH1, PALB2, ATM, PTCH1, and PMS2. 12 of 25 cases (48%) also carried variants of uncertain significance (VUS). Final pathology confirmed malignancy in 18 patients (72%), including breast, ovarian, endometrial, and cervical cancers. 7 patients (28%) had benign or premalignant conditions, with SDHA most frequently mutated. Although 56% of this subgroup had a family history of cancer - higher than the malignant group - the difference was not statistically significant (p = 0.200). Pre-diagnostic gNGS identified actionable variants, supporting its potential in early risk stratification and hereditary cancer management.

[Disclosure of COI by Responsible researcher] Lead presenter is the Responsible researcher.
[Lead presenter: Min Kyu Kim] We have no financial relationships to disclose.

### [E-2009] I-E12-3
### New cancer diagnostic technologies

2025 / 9 / 26 (Fri) 9:00-10:15 Room 7/(Hotel Nikko Kanazawa 3F Kujaku B) Room 7

[Chairperson]Yoshiki Murakumo (Dept. Pathol., Kitasato Univ. Sch. Med.)

### E-2009
### Constructing a Deep Learning-Based Proteomic Classifier for Unknown Primary Squamous Cell Carcinoma

[Speaker]Midie Xu (Department of Pathology, Fudan University Shanghai Cancer Center／Department of Oncology, Shanghai Medical College, Fudan University,／Institute of Pathology, Fudan University) [Co-author]Tianqi Gong (Department of Pathology, Fudan University Shanghai Cancer Center／Department of Oncology, Shanghai Medical College, Fudan University,／Institute of Pathology, Fudan University)、I. Weng Lao (Department of Pathology, Fudan University Shanghai Cancer Center／Department of Oncology, Shanghai Medical College, Fudan University,／Institute of Pathology, Fudan University)

Squamous cell carcinoma of unknown primary (SCCUP) constitutes 15% of CUP cases, posing substantial diagnostic and therapeutic challenges. Using formalin-fixed paraffin-embedded (FFPE) tissues from 387 patients across seven tertiary medical centers, we constructed a 39-protein biomarker classifier (39PBC) capable of accurately predicting the tissue of origin in primary/metastatic SCCs of cervical, esophageal, lung, nasopharyngeal, or head/neck origins. The 39PBC achieved an AUC of 0.924 with 87.8% accuracy in training datasets, and 0.961 AUC with 87.2% accuracy in internal validation. It demonstrated 87.3% classification accuracy (AUC=0.971) in an independent MCSCC cohort. Notably, the 39PBC retained high robustness in 12 challenging SCCUP cases. Additionally, an immunohistochemistry-based panel using P16, CCDC6, LGALS7, and LGALS9 markers showed comparable diagnostic performance. Our findings establish a novel proteomic classification framework, with both machine learning-derived 39PBC and IHC-based approaches providing accurate and clinically actionable solutions for SCCUP primary site prediction.

[Disclosure of COI by Responsible researcher] Lead presenter is the Responsible researcher.
[Lead presenter: Midie Xu] We have no financial relationships to disclose.

### [E-2010] I-E12-3
### New cancer diagnostic technologies

2025 / 9 / 26 (Fri) 9:00-10:15 Room 7/(Hotel Nikko Kanazawa 3F Kujaku B) Room 7

[Chairperson]Yoshiki Murakumo (Dept. Pathol., Kitasato Univ. Sch. Med.)

---

### E-2010
### Development of a treatment decision-making support system for intraductal papillary mucinous neoplasm

[Speaker]Masaaki Komatsu (Cancer Transl. Res. Team, RIKEN Ctr. for AIP project／Div. Med. AI Res. Dev., Natl. Cancer Ctr. Res. Inst.) [Co-author]Shota Harai (Dept. Hepatobiliary Pancreat. Oncol., Natl. Cancer Ctr. Hosp.)、Katsuji Takeda (Cancer Transl. Res. Team, RIKEN Ctr. for AIP project)、Naoki Teraya (Div. Med. AI Res. Dev., Natl. Cancer Ctr. Res. Inst.／Dept. Obstet. Gynecol, Showa Med. Univ. Sch. Med.)、Daiki Yamashige (Dept. Hepatobiliary Pancreat. Oncol., Natl. Cancer Ctr. Hosp.)、Naoaki Harada (Div. Med. AI Res. Dev., Natl. Cancer Ctr. Res. Inst.／NCC Cancer Sci., Grad. Sch., Inst. Sci. Tokyo)、Ken Asada (Cancer Transl. Res. Team, RIKEN Ctr. for AIP project／Div. Med. AI Res. Dev., Natl. Cancer Ctr. Res. Inst.)、Syuzo Kaneko (Cancer Transl. Res. Team, RIKEN Ctr. for AIP project／Div. Med. AI Res. Dev., Natl. Cancer Ctr. Res. Inst.)、Susumu Hijioka (Dept. Hepatobiliary Pancreat. Oncol., Natl. Cancer Ctr. Hosp.)、Ryuji Hamamoto (Cancer Transl. Res. Team, RIKEN Ctr. for AIP project／Div. Med. AI Res. Dev., Natl. Cancer Ctr. Res. Inst.／NCC Cancer Sci., Grad. Sch., Inst. Sci. Tokyo)

Intraductal papillary mucinous adenoma (IPMN) is one of the pancreatic cysts that have the potential for malignant transformation and therefore requires appropriate examination and follow-up. Although the guidelines provide surveillance methods, their detailed procedure makes it difficult for non-experts to follow the guidelines faithfully in daily clinical practice. The guidelines define high-risk stigmata as criteria for malignant transformation including the dilatation of the main pancreatic duct, and the size of mural nodules or solid components which are not often identified on CT or MRI but are only clearly detected on endoscopic ultrasonography (EUS). However, the quality of EUS depends on the skill levels of the examiners, so standardization of image assessment is crucial. Therefore, we aim to develop a multimodal AI model that combines EUS and MRI image information with clinical information from electronic medical records toward a simple, uniform, and highly accurate IPMN treatment decision-making support system. This system is expected to enable precision oncology, taking into account not only surgical resection but also the recommended follow-up period.

[Disclosure of COI by Responsible researcher] Lead presenter is not the Responsible researcher.
[Lead presenter: Masaaki Komatsu] We have no financial relationships to disclose.
[Principal Researcher: Ryuji Hamamoto] We have no financial relationships to disclose.

### [E-2011] I-E12-3
### New cancer diagnostic technologies

2025 / 9 / 26 (Fri) 9:00-10:15 Room 7/(Hotel Nikko Kanazawa 3F Kujaku B) Room 7

[Chairperson]Yoshiki Murakumo (Dept. Pathol., Kitasato Univ. Sch. Med.)

---

### E-2011
### A prospective observational study of oxygen saturation endoscopic imaging and radiosensitivity in head and neck cancer

[Speaker]Hidenari Hirata (Dept. Radiation Oncology Natl. Cancer Ctr. Hosp. East) [Co-author]Atsushi Motegi (Dept. Radiation Oncology Natl. Cancer Ctr. Hosp. East)、Kento Tomizawa (Dept. Radiation Oncology Natl. Cancer Ctr. Hosp. East)、Hiroki Yamashita (Dept. Gastroenterol. & Endoscopy Natl. Cancer Ctr. Hosp. East)、Hironori Sunakawa (Dept. Gastroenterol. & Endoscopy Natl. Cancer Ctr. Hosp. East)、Wataru Okano (Dept. Head & Neck Surg. Natl. Cancer Ctr. Hosp. East)、Tomohiro Enokida (Dept. Head & Neck Med. Oncology Natl. Cancer Ctr. Hosp. East)、Masaki Nakamura (Dept. Radiation Oncology Natl. Cancer Ctr. Hosp. East)、Seiichiro Abe (Endoscopy Div. Natl. Cancer Ctr. Hosp.)、Yutaka Saito (Endoscopy Div. Natl. Cancer Ctr. Hosp.)、Kazuto Matsuura (Dept. Head & Neck Surg. Natl. Cancer Ctr. Hosp. East)、Makoto Tahara (Dept. Head & Neck Med. Oncology Natl. Cancer Ctr. Hosp. East)、Tomonori Yano (Dept. Gastroenterol. & Endoscopy Natl. Cancer Ctr. Hosp. East)、Sadamoto Zenda (Dept. Radiation Oncology Natl. Cancer Ctr. Hosp. East)、Tetsuo Akimoto (Dept. Radiation Oncology The Jikei Univ. Sch. Med.)

Hypoxia is a cause of radioresistance in head and neck squamous cell carcinoma (HNSCC). We have developed an oxygen saturation endoscopic imaging (OXEI, FUJIFILM) method to assess real-time tissue oxygenation levels. To evaluate the effect of pretreatment tumor oxygen saturation ($StO_2$) quantified by OXEI on clinical outcomes of HNSCC patients receiving radiotherapy (RT), we performed a prospective study at 2 institutions. Of the 42 analyzed patients, the CR rate 8 weeks after RT (the primary endpoint) was not significantly different between the hypoxia ($StO_2 \leq 61\%$, n=22) and non-hypoxia ($StO_2 > 61\%$, n=20) groups. With a median follow-up of 36 (range, 9-53) months, the hypoxia group had significantly worse 1-year progression-free survival (77% vs. 95%) and overall survival (91% vs. 100%) and higher local failure rates (18% vs. 5%). RNA sequencing of 2 tumors showed $StO_2$ reflected expression-based hypoxia. The reactive oxygen species pathway was enriched in the samples of the hypoxic group, validated by 560 TCGA HNSCCs and 60 HNSCC cell lines. Our findings suggest pretreatment tumor $StO_2$ correlates with 1-year outcomes after RT, and OXEI could potentially predict RT response in HNSCC.

[Disclosure of COI by Responsible researcher] Lead presenter is the Responsible researcher.
[Lead presenter: Hidenari Hirata] We have no financial relationships to disclose.

▲TOP

### [E-2012] I-E12-3
### New cancer diagnostic technologies

2025 / 9 / 26 (Fri) 9:00-10:15 Room 7/(Hotel Nikko Kanazawa 3F Kujaku B) Room 7

[Chairperson]Yoshiki Murakumo (Dept. Pathol., Kitasato Univ. Sch. Med.)

### E-2012
### Withdrawn

No Abstract

### [E-2013] I-E10-2
### Metabolism/Metabolome (1)

2025 / 9 / 26 (Fri) 9:00-10:15 Room 8/(Hotel Nikko Kanazawa 3F Kujaku C) Room 8

[Chairperson]Hideki Makinoshima (Tsuruoka Metabolomics Lab., NCC)

---

### E-2013
### KEAP1 mutation in addition to KRAS-LKB1 mutation in NSCLC confers resistance to KRAS G12C inhibitor via anaplerosis

[Speaker]Renata Akhmetzianova (Div. of Oncol. Mol. Biol., CRI, Kanazawa Univ.) [Co-author]Chiaki Takahashi (Div. of Oncol. Mol. Biol., CRI, Kanazawa Univ.)、Shunsuke Kitajima (Cancer Immunotherapy Development, CPM Center of JFCR)、Susumu Kohno (Div. of Oncol. Mol. Biol., CRI, Kanazawa Univ.)

Despite the clinical promise of KRAS G12C inhibitors in treating KRAS G12C-type non-small cell lung cancer (NSCLC), a subset of patients harboring additional mutations in LKB1 and KEAP1 exhibits limited clinical response. The molecular mechanisms driving resistance in this subgroup remain poorly understood, necessitating further investigation to improve patient outcomes. In this study, we employed transcriptomic, metabolomic, and syngeneic murine models to elucidate the effects of mutations in KRAS G12C/LKB1 (KL) and KRAS G12C/LKB1/KEAP1 (KLK) NSCLC. Our findings confirmed resistance to KRAS G12C inhibitor sotorasib in KLK type in both in vitro and in vivo models. Metabolomic profiling, extra-flux analysis and 13C tracer assay revealed that KEAP1 mutation activates anaplerotic pathways due to upregulated expression of pyruvate carboxylase (PC). Notably, genetic inhibition of PC sensitized KLK-type NSCLC to sotorasib both in vitro and in vivo. These results suggest that targeting the anaplerotic pathway may represent a promising therapeutic strategy to overcome the therapy resistance in KLK-type NSCLC patients.

[Disclosure of COI by Responsible researcher] Lead presenter is not the Responsible researcher.
[Lead presenter: Renata Akhmetzianova] We have no financial relationships to disclose.
[Principal Researcher: Chiaki Takahashi, Shunsuke Kitajima, Susumu Kohno] We have no financial relationships to disclose.

### [E-2014] I-E10-2
### Metabolism/Metabolome (1)

2025 / 9 / 26 (Fri) 9:00-10:15 Room 8/(Hotel Nikko Kanazawa 3F Kujaku C) Room 8

[Chairperson]Hideki Makinoshima (Tsuruoka Metabolomics Lab., NCC)

---

### E-2014
### Suppressing glutaminase 1 expression synergizes the anti-tumor effects of metformin on cholangiocarcinoma

[Speaker]Charupong Saengboonmee (Dept. of Biochem., Faculty of Med., Khon Kaen Univ., Thailand／Cholangiocarcinoma Res. Inst., Khon Kaen Univ., Thailand) [Co-author]Kullanat Khawkhiaw (Dept. of Biochem., Faculty of Med., Khon Kaen Univ., Thailand／Cholangiocarcinoma Res. Inst., Khon Kaen Univ., Thailand)、Padcharee Yueangchantuek (Dept. of Biochem., Faculty of Med., Khon Kaen Univ., Thailand／Cholangiocarcinoma Res. Inst., Khon Kaen Univ., Thailand)、Kanyarat Thithuan (Dept. of Biochem., Faculty of Med., Khon Kaen Univ., Thailand／Cholangiocarcinoma Res. Inst., Khon Kaen Univ., Thailand)、Ronnakrit Trakoonsenathong (Dept. of Biochem., Faculty of Med., Khon Kaen Univ., Thailand／Cholangiocarcinoma Res. Inst., Khon Kaen Univ., Thailand)、Yu-wei Lu (Grad. Inst. of Metabolism&Obesity Sci. Taipei Med. Univ. Taiwan)、Ching-wen Chang (Grad. Inst. of Metabolism&Obesity Sci. Taipei Med. Univ. Taiwan)、Ching-feng Chiu (Grad. Inst. of Metabolism&Obesity Sci. Taipei Med. Univ. Taiwan)、Worachart Lert-itthiporn (Dept. of Biochem., Faculty of Med., Khon Kaen Univ., Thailand／Cholangiocarcinoma Res. Inst., Khon Kaen Univ., Thailand)、Seiji Okada (Joint Res. Ctr. for Human Retrovirus Infection, Kumamoto Univ., Japan)、Sopit Wongkham (Dept. of Biochem., Faculty of Med., Khon Kaen Univ., Thailand／Cholangiocarcinoma Res. Inst., Khon Kaen Univ., Thailand)

Metabolic reprogramming has been reported in cholangiocarcinoma (CCA) to support its malignancy. Glutaminase 1 (GLS1), a key enzyme supplying glutamine for cancer anaplerotic pathways, is upregulated in CCA. This study aimed to investigate the requirement of glutamine and GLS1 in CCA energy metabolism and the other metabolic networks. After knocking down GLS1, CCA cell proliferation and clonogenicity were significantly reduced. Western blots revealed that the phosphorylated AMPK was decreased, suggesting the disturbance of ATP production leading to retardation of CCA cell growth. Metformin treatment synergized the AMPK activation, resulting in additional effects on inhibiting CCA colony formation in vitro and significantly slower growth of CCA xenografts in a mouse model. Transcriptomic analyses using RNA sequencing showed that several pathways involving mitochondrial metabolisms were altered considerably. These suggested that glutamine contributed to substantial roles in CCA energy metabolism and is required for CCA cell growth. Targeting glutamine metabolism might be central to the anti-metabolic strategies for CCA treatment and might enhance the other anti-metabolic strategies.

[Disclosure of COI by Responsible researcher] Lead presenter is the Responsible researcher.
[Lead presenter: Charupong Saengboonmee] We have no financial relationships to disclose.

### [E-2015] I-E10-2
### Metabolism/Metabolome (1)

2025 / 9 / 26 (Fri) 9:00-10:15 Room 8/(Hotel Nikko Kanazawa 3F Kujaku C) Room 8

[Chairperson]Hideki Makinoshima (Tsuruoka Metabolomics Lab., NCC)

---

### E-2015
### Disruption of hepatocyte glutathione metabolism under breast cancer conditions: an integrated in vitro and in vivo study

[Speaker]Yilin Du (Dept. of Integrative Bioanalytics, IDAC, Tohoku Univ.／Dept. of Rehabil. Med., Med. Sch., Tohoku Univ.) [Co-author]Nupura Prabhune (Dept. of Integrative Bioanalytics, IDAC, Tohoku Univ.／Dept. of Life & Med. Biosci., Waseda Univ.)、Mayuko Yoda (Dept. of Integrative Bioanalytics, IDAC, Tohoku Univ.)、Shinji Takeoka (Dept. of Life & Med. Biosci., Waseda Univ.)、Satoru Ebihara (Dept. of Rehabil. Med., Med. Sch., Tohoku Univ.)、Shinpei Kawaoka (Dept. of Integrative Bioanalytics, IDAC, Tohoku Univ.／Inter-Organ Comm. Res. Team, LiMe., Kyoto Univ.)

Cancer progression significantly disrupts systemic metabolic homeostasis, with notable impacts on liver function. This study aimed to comprehensively characterize breast cancer-induced gene expression changes in hepatocytes, utilizing integrated transcriptomic datasets from ***in vivo*** and ***in vitro*** models. Transcriptome experiments identified DEGs (1,814 ***in vitro*** and 4,094 ***in vivo***), while WGCNA revealed modules of co-expressed genes associated with the breast cancer condition. Intersecting these data, we identified common DEGs in both ***in vitro*** and ***in vivo*** (37 upregulated, 100 downregulated). PPI network analysis revealed hub genes, including glutamate-cysteine ligase catalytic subunit (***Gclc***), suggesting increased vulnerability to oxidative stress and toxicity in hepatocytes under cancer conditions. Additionally, hepatocyte nuclear factor 1 beta (***Hnf1b***) emerged as a potential transcription factor regulating this anomaly, implicating a novel regulatory axis in hepatic dysfunction. These findings enhance understanding of how breast tumors influence liver metabolism and highlight key genes and pathways as potential diagnostic markers and therapeutic targets.

[Disclosure of COI by Responsible researcher] Lead presenter is the Responsible researcher.
[Lead presenter: Yilin Du] We have no financial relationships to disclose.

### [E-2016] I-E10-2
### Metabolism/Metabolome (1)

2025 / 9 / 26 (Fri) 9:00-10:15 Room 8/(Hotel Nikko Kanazawa 3F Kujaku C) Room 8

[Chairperson]Hideki Makinoshima (Tsuruoka Metabolomics Lab., NCC)

---

### E-2016
### Long chain fatty acid elongase 6 (ELOVL6) is a novel therapeutic target for breast cancer

[Speaker]Linxiang Gong (Div. Oncol. Mol. Biol., Cancer Res. Inst., Kanazawa Univ) [Co-author]Akio Hayashi (Center for Infectious Disease Education and Research, Osaka Univ)、Takashi Matauzaka (Department of Endocrinology and Metabolism, Institute of Medicine, Tsukuba Univ)、Hitoshi Shimano (Department of Endocrinology and Metabolism, Institute of Medicine, Tsukuba Univ)、Chiaki Takahashi (Div. Oncol. Mol. Biol., Cancer Res. Inst., Kanazawa Univ)、Susumu Kohno (Div. Oncol. Mol. Biol., Cancer Res. Inst., Kanazawa Univ)

Fatty acid elongation plays a pivotal role in generating the diversity in lipid species required for membrane synthesis and signaling. The ELOVL family enzymes catalyze the critical step by sequentially adding 2 carbon units to acyl-CoA substrates. Among these enzymes, ELOVL6 specifically converts C16 saturated and monounsaturated fatty acids into C18 species which is the rate-limiting step long-chain fatty acid synthesis. RB1 has previously been identified as a transcriptional regulator of ELOVL6, yet its impact on lipid metabolism remains unclear. Here, we demonstrate that BRCA cells with reduced RB1 expression exhibit increased ELOVL6 levels, which may link RB1 deficiency to lipid remodeling. Functional inhibition of ELOVL6 suppresses proliferation across all breast cancer subtypes in vitro and in vivo. Notably, RB1 null cells exhibited higher sensitivity to ELOVL6 depletion in inducing cell death. Lipidomic profiling reveals that ELOVL6 inhibition leads to a significant accumulation of intracellular ceramides. With respect to known cytotoxic functions of ceramide, ELOVL6 might be a promising therapeutic target in RB1-deficient breast cancer.

[Disclosure of COI by Responsible researcher] Lead presenter is not the Responsible researcher.
[Lead presenter: Linxiang Gong] We have no financial relationships to disclose.
[Principal Researcher: Chiaki Takahashi, Susumu Kohno] We have no financial relationships to disclose.

### [E-2017] I-E10-2
### Metabolism/Metabolome (1)

2025 / 9 / 26 (Fri) 9:00-10:15 Room 8/(Hotel Nikko Kanazawa 3F Kujaku C) Room 8

[Chairperson]Hideki Makinoshima (Tsuruoka Metabolomics Lab., NCC)

---

### E-2017
### Changes in glycosylation enzymes in mesothelial cells adjacent to adipose tissue and progression of ovarian cancer

[Speaker]Atsushi Kunishima (Dept. of Obstetrics & Gynecol, Nagoya Univ.) [Co-author]Shohei Iyoshi (Dept. of Obstetrics & Gynecol, Nagoya Univ.)、Marina Yoshikawa (Dept. of Obstetrics & Gynecol, Nagoya Univ.)、Emiri Miyamoto (Dept. of Obstetrics & Gynecol, Nagoya Univ.)、Hiroki Fujimoto (Dept. of Obstetrics & Gynecol, Nagoya Univ.／Dept. of Obstetrics & Gynecol, Aichi Medical Univ.)、Kosuke Yoshida (Dept. of Obstetrics & Gynecol, Nagoya Univ.)、Kazumasa Mogi (Dept. of Obstetrics & Gynecol, Nagoya Univ.)、Masato Yoshihara (Dept. of Obstetrics & Gynecol, Nagoya Univ.)、Satoshi Tamauchi (Dept. of Obstetrics & Gynecol, Nagoya Univ.)、Yukari Nagao (Dept. of Obstetrics & Gynecol, Nagoya Univ.)、Akira Yokoi (Dept. of Obstetrics & Gynecol, Nagoya Univ.)、Nobuhisa Yoshikawa (Dept. of Obstetrics & Gynecol, Nagoya Univ.)、Kaoru Niimi (Dept. of Obstetrics & Gynecol, Nagoya Univ.)、Hiroaki Kajiyama (Dept. of Obstetrics & Gynecol, Nagoya Univ.)

Ovarian cancer (OvCa) is often diagnosed with peritoneal dissemination, frequently involving fatty tissues like the omentum. Tumor- adipocyte interactions may promote OvCa dissemination, however, detailed mechanisms remain unelucidated. Here, we investigated how mesothelial cells (MCs) covering the surface of adipose tissue are altered by adipocytes and its effect on tumor progression. RNA-seq of mouse MCs co-cultured with 3T3-L1-induced adipocytes revealed that Hs3st3a1, a heparan sulfate-modifying enzyme, was upregulated upon indirect co-culturing with adipocytes. Glycomic analysis confirmed the increase of proteoglycan including heparan sulfate in co-cultured MCs. A highly metastatic subclone of ID8 mouse OvCa cell line (ID8-HM) was prepared and revealed that ID8-HM showed increased ECM production which strongly interact with proteoglycan-rich MCs. These results suggested that ECM production in OvCa cells and upregulation of Hs3st3a1 in MCs in the vicinity of adipose tissue contributed to the progression of OvCa at adipocyte-rich environment. The results of metabolomic/lipidomic analysis of adipocyte-conditioned media for exploring upstream regulators will be also presented.

[Disclosure of COI by Responsible researcher] Lead presenter is not the Responsible researcher.
[Lead presenter: Atsushi Kunishima] We have no financial relationships to disclose.
[Principal Researcher: Hiroaki Kajiyama] We have no financial relationships to disclose.

### [E-2018] I-E10-2
### Metabolism/Metabolome (1)

2025 / 9 / 26 (Fri) 9:00-10:15 Room 8/(Hotel Nikko Kanazawa 3F Kujaku C) Room 8

[Chairperson]Hideki Makinoshima (Tsuruoka Metabolomics Lab., NCC)

---

### E-2018
### High-Fat Diet inhibits ferroptosis and promotes cell proliferation of ovarian and endometrial cancer

[Speaker]Masashi Akada (Univ. of Osaka, Dept. of Obstet. & Gynecol.) [Co-author]Kosuke Hiramatsu (Univ. of Osaka, Dept. of Obstet. & Gynecol.)、Hitomi Mukaida (Univ. of Osaka, Dept. of Obstet. & Gynecol.)、Tatsuo Masuda (Univ. of Osaka, Dept. of Obstet. & Gynecol.)、Mamoru Kakuda (Univ. of Osaka, Dept. of Obstet. & Gynecol.)、Satoshi Nakagawa (Univ. of Osaka, Dept. of Obstet. & Gynecol.)、Tadashi Iwamiya (Univ. of Osaka, Dept. of Obstet. & Gynecol.)、Shinya Matsuzaki (Univ. of Osaka, Dept. of Obstet. & Gynecol.)、Yutaka Ueda (Univ. of Osaka, Dept. of Obstet. & Gynecol.)、Michiko Kodama (Univ. of Osaka, Dept. of Obstet. & Gynecol.)

Ovarian cancer (OC) and endometrial cancer (EC) are the common gynecologic malignancy. Recent decades, the relation of lipid metabolism and theses cancers has been reported. However, which lipid metabolic pathway regulates cell proliferation of theses cancers is still unclear. In this study, we focused on High-Fat Diet (HFD) and analyzed lipid molecules in HFD. In vivo, HFD significantly promoted tumor growth of OC ($p<0.05$), and HFD-fed mouse serum promoted cell proliferation of OC in vitro ($p<0.05$), suggesting that lipid molecules in HFD-fed mouse serum contribute to cell proliferation of OC. To identify the metabolites contributing to cell proliferation of OC, we performed metabolome analysis using HFD-fed mouse serum. By PLS discriminant analysis, alpha-tocopherol and oleic acid showed high score in HFD-fed mouse serum. Alpha-tocopherol and oleic acid significantly promoted cell proliferation of OC and EC cells ($p<0.05$, respectively). Moreover, alpha-tocopherol and oleic acid regulated the expression of proteins related to ferroptosis, respectively. These results showed that HFD inhibited ferroptosis and promoted subsequent cell proliferation in OC and EC.

[Disclosure of COI by Responsible researcher] Lead presenter is not the Responsible researcher.
[Lead presenter: Masashi Akada] We have no financial relationships to disclose.
[Principal Researcher: Hitomi Mukaida] We have no financial relationships to disclose.

▲TOP

### [E-2019] I-E19
### Cancer Prevention & Epidemiology (1)

2025 / 9 / 26 (Fri) 9:00-10:15 Room 9/(Hotel Nikko Kanazawa 3F Lumiere) Room 9

[Chairperson]Yuriko Koyanagi (Div. of Cancer Epidemiology and Prevention, Aichi Cancer Ctr. Res. Inst.)

---

### E-2019
### Prefectural Success in Smoking Target Projections Correlates with Minimum Wage & Density

[Speaker]Hasan Jamil (Div. of Population Data Science, NCC, Tokyo, Japan／GSPH, St. Luke's International University, Tokyo, Japan) [Co-author]Stuart Gilmour (GSPH, St. Luke's International University, Tokyo, Japan)、Kayo Togawa (Early Detection, Prevention, and Infections Branch, IARC／WHO, Lyon, France)、Kota Katanoda (Div. of Population Data Science, NCC, Tokyo, Japan)

Health Japan 21 targets 12% smoking prevalence by 2032. This study evaluates the probability of meeting this target nationally/prefecturally and the socioeconomic factors affecting the projected probability of meeting the target. We applied Bayesian linear regression to national and prefecture data (2001-2022) to calculate projected probabilities of target achievement, and examined the relationship between target achievement probability and prefecture minimum wage/population density quintiles. Japan nationally has a 64.3% probability of meeting the 2032 target, but 28/47 prefectures have less than 60% chance of target achievement, with an extreme range from Tokyo (99.3% of target achievement by 2026) to Fukushima (<1% chance of target achievement before 2053). Prefectures in the highest minimum wage/population density quintiles had higher achievement probabilities. While Japan's overall goal seems attainable, substantial regional disparities associated with socioeconomic indicators are evident, with faster progress in wealthier urban areas. These findings highlight the need for targeted tobacco control addressing regional socioeconomic contexts for equitable achievement.

[Disclosure of COI by Responsible researcher] Lead presenter is not the Responsible researcher.
[Lead presenter: Hasan Jamil] We have no financial relationships to disclose.
[Principal Researcher: Kota Katanoda] We have no financial relationships to disclose.

▲TOP

### [E-2020] I-E19
### Cancer Prevention & Epidemiology (1)

2025 / 9 / 26 (Fri) 9:00-10:15 Room 9/(Hotel Nikko Kanazawa 3F Lumiere) Room 9

[Chairperson]Yuriko Koyanagi (Div. of Cancer Epidemiology and Prevention, Aichi Cancer Ctr. Res. Inst.)

---

### E-2020
### Withdrawn

No Abstract

### [E-2021] I-E19
### Cancer Prevention & Epidemiology (1)

2025 / 9 / 26 (Fri) 9:00-10:15 Room 9/(Hotel Nikko Kanazawa 3F Lumiere) Room 9

[Chairperson]Yuriko Koyanagi (Div. of Cancer Epidemiology and Prevention, Aichi Cancer Ctr. Res. Inst.)

---

### E-2021
### Comparing Trends in Female Breast Cancer Incidence in Native Japanese and US Populations: An Age-Period-Cohort Analysis

[Speaker]Aminu K. Abubakar (Div. of Population Data Sci., Natl. Cancer Ctr.／Grad. Sch. of Public Health, St Luke's International Univ., Tokyo)
[Co-author]Kota Katanoda (Div. of Population Data Sci., Natl. Cancer Ctr.)、Hirokazu Tanaka (Div. of Population Data Sci., Natl. Cancer Ctr.)、Hasan Jamil (Div. of Population Data Sci., Natl. Cancer Ctr.／Grad. Sch. of Public Health, St Luke's International Univ., Tokyo)

Introduction: Breast cancer incidence has historically been higher in Western populations, but rates have recently risen among Asian women, including in Japan and the US, while declining among US White women. This study examines age, period, and cohort effects on breast cancer incidence among Japanese women, with comparisons to US populations.
Methods: Data for women aged 25–84 (1985–2019) were obtained from registries in Yamagata, Fukui, and Nagasaki. Comparative data came from the Hawaii registry (Japanese in Hawaii) and SEER 8 (US Black and White). The analysis was performed using the NCIs APC web tool.
Results: Incidence increased with age in all groups, with higher rates among US women. Period and cohort effects were most pronounced in native Japanese. Annual increases were greatest in native Japanese (3.77%) and Japanese–Americans (1.59%), with little change in US–Black (0.23%), and slight decline among US–White (–0.07%). The largest increases occurred among native Japanese aged 55–69.
Conclusion: The Rising breast cancer incidence in native Japanese highlights the need for targeted prevention and control strategies.

[Disclosure of COI by Responsible researcher] Lead presenter is the Responsible researcher.
[Lead presenter: Aminu K. Abubakar] We have no financial relationships to disclose.

### [E-2022] I-E19
### Cancer Prevention & Epidemiology (1)

2025 / 9 / 26 (Fri) 9:00-10:15 Room 9/(Hotel Nikko Kanazawa 3F Lumiere) Room 9

[Chairperson]Yuriko Koyanagi (Div. of Cancer Epidemiology and Prevention, Aichi Cancer Ctr. Res. Inst.)

### E-2022
### Exploring the Role of Helicobacter pylori in Biliary Tract and Pancreatic Cancers

[Speaker]Sayaka Yamamoto (Div. of Cancer Epidemiology and Prevention, Aichi Cancer Ctr.) [Co-author]Yuriko Koyanagi (Div. of Cancer Epidemiology and Prevention, Aichi Cancer Ctr.)、Yuji Iwashita (Div. of Cancer Epidemiology and Prevention, Aichi Cancer Ctr.)、Isao Oze (Div. of Cancer infromation and Control, Aichi Cancer Ctr.)、Yumiko Kasugai (Div. of Cancer Epidemiology and Prevention, Aichi Cancer Ctr.)、Yoshiaki Usui (Lab. for Genotyping Development, IMS, RIKEN)、Yukihide Momozawa (Lab. for Genotyping Development, IMS, RIKEN)、Issei Imoto (Aichi Cancer Ctr.)、Hidemi Ito (Div. of Cancer infromation and Control, Aichi Cancer Ctr.)、Keitaro Matsuo (Div. of Cancer Epidemiology and Prevention, Aichi Cancer Ctr.)

***Helicobacter pylori (H. pylori)*** infection, a well-established risk factor for gastric cancer, may also contribute to biliary tract cancer (BTC) and pancreatic cancer (PC). However, the evidence remains inconclusive. We conducted a case-control study of 116 BTC cases, 417 PC cases, and 1,788 controls. Odds ratios (ORs) and 95% confidence intervals (CIs) were estimated using logistic regression. ***H. pylori*** infection was serologically defined using antibody positivity, the pepsinogen test (a marker of atrophic gastritis, AG), and their combination.
Antibody seropositivity was significantly associated with BTC (OR 1.57, 95% CI: 1.02- 2.41) and non-significantly associated with PC (1.21, 0.92-1.58). AG was not associated with either cancer. Subsite analysis showed that antibody seropositivity was significantly associated with gallbladder (1.94, 1.01-3.70) and pancreatic body cancer (1.91, 1.11-3.27). No interaction was observed between ***H. pylori*** infection and pathogenic variants, smoking, or alcohol drinking for either cancer.
Our findings suggest that ***H. pylori*** is associated with both BTC and PC, however, the strength of the association may differ by anatomical subsite.

[Disclosure of COI by Responsible researcher] Lead presenter is not the Responsible researcher.
[Lead presenter: Sayaka Yamamoto] We have no financial relationships to disclose.
[Principal Researcher: Keitaro Matsuo] We have no financial relationships to disclose.

### [E-2023] I-E19
### Cancer Prevention & Epidemiology (1)

2025 / 9 / 26 (Fri) 9:00-10:15 Room 9/(Hotel Nikko Kanazawa 3F Lumiere) Room 9

[Chairperson]Yuriko Koyanagi (Div. of Cancer Epidemiology and Prevention, Aichi Cancer Ctr. Res. Inst.)

### E-2023
### Insulin-like growth factor-binding protein 3 and incidence of pancreatic cancer in a nested case-control study

[Speaker]Yasushi Adachi (Dept. of Gastroenterol., Sapporo Med. Univ., Sch. Med.／Div. Gastroenterol., Sapporo Shirakaba-dai Hosp.) [Co-author]Masanori Nojima (Inst. Med. Sci., Univ. of Tokyo)、Yingsong Lin (Aichi Med. Univ., Dept. Public Health)、Yoshiharu Masaki (Dept. of Gastroenterol., Sapporo Med. Univ., Sch. Med.)、Yasushi Sasaki (Div. Biology, Sapporo Med. Univ.)、Hiroshi Nakase (Dept. of Gastroenterol., Sapporo Med. Univ., Sch. Med.)、Yasuo Kato (Div. Gastroenterol., Sapporo Shirakaba-dai Hosp.)、Kenji Wakai (Dept. Preventive Med,, Nagoya Univ, Sch. Med.)、Mitsuru Mori (Hokkaido Chitose Coll. of Rehibilitation)、Akiko Tamakoshi (Dept. Public Helth, Hokkaido Univ. Sch. Med.)

Background: Insulin-like growth factors (IGF) are potent mitogens and IGF-binding protein 3 (IGFBP3) can regulate IGF activity. To elucidate relationships between IGF-related molecules and pancreatic cancer risk, we assessed these in a prospective, nested, case-control study within the JACC study.Methods: A baseline survey was conducted from 1988, during which period blood samples were obtained from 39,242 subjects. Subjects who had been diagnosed with pancreatic cancer by 1997 were regarded as cases. Conditional logistic regression was used to estimate odds ratios (OR) for the incidence of pancreatic cancer in relation to serum levels of IGF1 and IGFBP3.Results: This study included 72 cases and 216 controls. Free IGFBP3 (IGFBP3-IGF1) was associated with the risk of pancreatic cancer (P-trends=0.011), with the third tertile showing the highest risk (OR=3.42). None of total IGF1, free IGF1 (IGF1/IGFBP3), or total IGFBP3 were associated with risk of pancreatic cancer. This result remained unchanged in subanalyses of male, female, and older subjects (P-trends=0.017, 0.030, and 0.007, respectively). Conclusions: Free IGFBP3 is associated with the risk of pancreatic cancer.

[Disclosure of COI by Responsible researcher] Lead presenter is not the Responsible researcher.
[Lead presenter: Yasushi Adachi] We have no financial relationships to disclose.
[Principal Researcher: Akiko Tamakoshi] We have no financial relationships to disclose.

▲TOP

### [E-2024] I-E19
### Cancer Prevention & Epidemiology (1)

2025 / 9 / 26 (Fri) 9:00-10:15 Room 9/(Hotel Nikko Kanazawa 3F Lumiere) Room 9

[Chairperson]Yuriko Koyanagi (Div. of Cancer Epidemiology and Prevention, Aichi Cancer Ctr. Res. Inst.)

---

### E-2024
### Area-Level Socioeconomic Inequalities in Pancreatic Cancer Mortality in Japan: A Multilevel Analysis

[Speaker]Yasuhiro Sudo (Dept. of Med. Statistics, Osaka Med. & Pharm. Univ.) [Co-author]Masato Ota (Dept. of Med. Statistics, Osaka Med. & Pharm. Univ.)、Yuri Ito (Dept. of Med. Statistics, Osaka Med. & Pharm. Univ.)

**Background:** Pancreatic cancer deaths have risen globally, including in Japan, with marked area-level inequalities. To monitor these inequalities and pinpoint modifiable risk factors, we examined the link between pancreatic cancer mortality and regional characteristics.

**Methods:** We used Vital Statistics (2015-2022) and Population Census (2015-2020) for Japan. Employing a multilevel Poisson model with random intercepts at the municipality and secondary healthcare service area levels, we analyzed data sources such as the Areal Deprivation Index and Rurality Index for Japan.

**Results:** Our results showed that higher deprivation at the municipality level was associated with increased mortality in men under 75 (IRR = 1.060, 95% CI: 1.029-1.090), while greater rurality and higher smoking rates at the secondary healthcare service area level correlated with higher mortality in both men under 75 and women.

**Discussion:** These findings highlight the contribution of regional deprivation to pancreatic cancer death. Further research is needed based on cancer registry data linked to detailed data on local environment and medical records to inform prevention policy.

[Disclosure of COI by Responsible researcher] Lead presenter is not the Responsible researcher.
[Lead presenter: Yasuhiro Sudo] We have no financial relationships to disclose.
[Principal Researcher: Yuri Ito] We have no financial relationships to disclose.

▲TOP

### [SP1-1] SP1
### 10th Anniversary of the Survivor-Scientist Program (SSP)

2025 / 9 / 26 (Fri) 9:00-11:30 Room 10/(Art Hall) Room 10

[Chairpersons]Chiaki Takahashi (Division of Oncology and Molecular Biology, Kanazawa University, Cancer Research Institute)、Naomi Sakurai (CSR Project)

日本癌学会では、野田哲生 博士（公益財団法人がん研究会 顧問 兼 名誉研究所長）のご尽力により、がん研究を支援するサバイバー育成のためのプログラム「JCA-Survivor Scientist Program（略称JCA-SSP）」が2015年に創設され、同年の第74回日本癌学会学術総会時にトライアルがまず行われました。2016年以降、JCA-SSPプログラムは日本癌学会学術総会時に正式なプログラムとして毎年開催されています。⊠このJCA-SSPプログラムの創設にあたり参考とされたのが、米国癌学会（AACR）で1998年に創設され継続的に開催されている「米国癌学会－科学者・サバイバー プログラム（AACR-SSP）」です。このAACR-SSPは、がん研究者、がんサバイバー、そして患者アドボケート（擁護者・支援者）との協働が、がん研究の更なる発展には重要であるという認識のもと、アン・ベイカー博士とマーガレット・フォッティ博士によって創設され、1999年の第1回プログラムには25人のアドボケートが参加して開催されました。以降、AACR-SSPプログラムには日本を含む米国外からも数多くのアドボケートが参加しています。⊠10回目となる本年、通常のJCA-SSPプログラムに加え、10周年の節目を祝い、記念式典ならびに記念ワークショップを開催することとしました。マーガレット・フォッティ博士、アン・ベイカー博士、野田哲生博士ら、JCA-SSPの創設に関わった日米の関係者からのご祝辞、ご挨拶、功労者の表彰に続き、患者会代表者、がん研究者によって、活動の歴史と未来を考える、ワークショップを開催いたします。⊠本JCA-SSP10周年記念行事を通じて、がん研究者、がんサバイバー、そして患者アドボケートとの協働がより一層発展し、未来のがん治療に資するがん研究がより発展することを祈念しています。

---

### SP1-1
### TBD

[Speaker]Yoshiyuki Majima (Pancreatic Cancer Action Network Japan)

No Abstract

### [SP1-2] SP1
### 10th Anniversary of the Survivor-Scientist Program (SSP)

2025 / 9 / 26 (Fri) 9:00-11:30 Room 10/(Art Hall) Room 10

[Chairpersons]Chiaki Takahashi (Division of Oncology and Molecular Biology, Kanazawa University, Cancer Research Institute)、Naomi Sakurai (CSR Project)

日本癌学会では、野田哲生 博士（公益財団法人がん研究会 顧問 兼 名誉研究所長）のご尽力により、がん研究を支援するサバイバー育成のためのプログラム「JCA-Survivor Scientist Program（略称JCA-SSP）」が2015年に創設され、同年の第74回日本癌学会学術総会時にトライアルがまず行われました。2016年以降、JCA-SSPプログラムは日本癌学会学術総会時に正式なプログラムとして毎年開催されています。⊠このJCA-SSPプログラムの創設にあたり参考とされたのが、米国癌学会（AACR）で1998年に創設され継続的に開催されている「米国癌学会ー科学者・サバイバー プログラム（AACR-SSP）」です。このAACR-SSPは、がん研究者、がんサバイバー、そして患者アドボケート（擁護者・支援者）との協働が、がん研究の更なる発展には重要であるという認識のもと、アン・ベイカー博士とマーガレット・フォッティ博士によって創設され、1999年の第1回プログラムには25人のアドボケートが参加して開催されました。以降、AACR-SSPプログラムには日本を含む米国外からも数多くのアドボケートが参加しています。⊠10回目となる本年、通常のJCA-SSPプログラムに加え、10周年の節目を祝い、記念式典ならびに記念ワークショップを開催することとしました。マーガレット・フォッティ博士、アン・ベイカー博士、野田哲生博士ら、JCA-SSPの創設に関わった日米の関係者からのご祝辞、ご挨拶、功労者の表彰に続き、患者会代表者、がん研究者によって、活動の歴史と未来を考える、ワークショップを開催いたします。⊠本JCA-SSP10周年記念行事を通じて、がん研究者、がんサバイバー、そして患者アドボケートとの協働がより一層発展し、未来のがん治療に資するがん研究がより発展することを祈念しています。

---

### SP1-2
### TBD

[Speaker]Toshihiko Doi (National Cancer Center Hospital East)

No Abstract

▲TOP

### [SP1-3] SP1
### 10th Anniversary of the Survivor-Scientist Program (SSP)

2025 / 9 / 26 (Fri) 9:00-11:30 Room 10/(Art Hall) Room 10

[Chairpersons]Chiaki Takahashi (Division of Oncology and Molecular Biology, Kanazawa University, Cancer Research Institute)、Naomi Sakurai (CSR Project)

日本癌学会では、野田哲生 博士（公益財団法人がん研究会 顧問 兼 名誉研究所長）のご尽力により、がん研究を支援するサバイバー育成のためのプログラム「JCA-Survivor Scientist Program（略称JCA-SSP）」が2015年に創設され、同年の第74回日本癌学会学術総会時にトライアルがまず行われました。2016年以降、JCA-SSPプログラムは日本癌学会学術総会時に正式なプログラムとして毎年開催されています。☒このJCA-SSPプログラムの創設にあたり参考とされたのが、米国癌学会（AACR）で1998年に創設され継続的に開催されている「米国癌学会ー科学者・サバイバー プログラム（AACR-SSP）」です。このAACR-SSPは、がん研究者、がんサバイバー、そして患者アドボケート（擁護者・支援者）との協働が、がん研究の更なる発展には重要であるという認識のもと、アン・ベイカー博士とマーガレット・フォッティ博士によって創設され、1999年の第1回プログラムには25人のアドボケートが参加して開催されました。以降、AACR-SSPプログラムには日本を含む米国外からも数多くのアドボケートが参加しています。☒10回目となる本年、通常のJCA-SSPプログラムに加え、10周年の節目を祝い、記念式典ならびに記念ワークショップを開催することとしました。マーガレット・フォッティ博士、アン・ベイカー博士、野田哲生博士ら、JCA-SSPの創設に関わった日米の関係者からのご祝辞、ご挨拶、功労者の表彰に続き、患者会代表者、がん研究者によって、活動の歴史と未来を考える、ワークショップを開催いたします。☒本JCA-SSP10周年記念行事を通じて、がん研究者、がんサバイバー、そして患者アドボケートとの協働がより一層発展し、未来のがん治療に資するがん研究がより発展することを祈念しています。

---

### SP1-3
### TBD

[Speaker]Hideyuki Saya (Oncology Innovation Center, Fujita Health University)

No Abstract

▲TOP

### [SP1-4] SP1
### 10th Anniversary of the Survivor-Scientist Program (SSP)

2025 / 9 / 26 (Fri) 9:00-11:30 Room 10/(Art Hall) Room 10

[Chairpersons]Chiaki Takahashi (Division of Oncology and Molecular Biology, Kanazawa University, Cancer Research Institute)、Naomi Sakurai (CSR Project)

日本癌学会では、野田哲生 博士（公益財団法人がん研究会 顧問 兼 名誉研究所長）のご尽力により、がん研究を支援するサバイバー育成のためのプログラム「JCA-Survivor Scientist Program（略称JCA-SSP）」が2015年に創設され、同年の第74回日本癌学会学術総会時にトライアルがまず行われました。2016年以降、JCA-SSPプログラムは日本癌学会学術総会時に正式なプログラムとして毎年開催されています。⊠このJCA-SSPプログラムの創設にあたり参考とされたのが、米国癌学会（AACR）で1998年に創設され継続的に開催されている「米国癌学会ー科学者・サバイバー プログラム（AACR-SSP）」です。このAACR-SSPは、がん研究者、がんサバイバー、そして患者アドボケート（擁護者・支援者）との協働が、がん研究の更なる発展には重要であるという認識のもと、アン・ベイカー博士とマーガレット・フォッティ博士によって創設され、1999年の第1回プログラムには25人のアドボケートが参加して開催されました。以降、AACR-SSPプログラムには日本を含む米国外からも数多くのアドボケートが参加しています。⊠10回目となる本年、通常のJCA-SSPプログラムに加え、10周年の節目を祝い、記念式典ならびに記念ワークショップを開催することとしました。マーガレット・フォッティ博士、アン・ベイカー博士、野田哲生博士ら、JCA-SSPの創設に関わった日米の関係者からのご祝辞、ご挨拶、功労者の表彰に続き、患者会代表者、がん研究者によって、活動の歴史と未来を考える、ワークショップを開催いたします。⊠本JCA-SSP10周年記念行事を通じて、がん研究者、がんサバイバー、そして患者アドボケートとの協働がより一層発展し、未来のがん治療に資するがん研究がより発展することを祈念しています。

---

### SP1-4
### TBD

[Speaker]Hiroyuki Onishi (Rare Cancers Japan ／ NPO CureSarcoma)

No Abstract

▲TOP

### [SP1-5] SP1
### 10th Anniversary of the Survivor-Scientist Program (SSP)

2025 / 9 / 26 (Fri) 9:00-11:30 Room 10/(Art Hall) Room 10

[Chairpersons]Chiaki Takahashi (Division of Oncology and Molecular Biology, Kanazawa University, Cancer Research Institute)、Naomi Sakurai (CSR Project)

日本癌学会では、野田哲生 博士（公益財団法人がん研究会 顧問 兼 名誉研究所長）のご尽力により、がん研究を支援するサバイバー育成のためのプログラム「JCA-Survivor Scientist Program（略称JCA-SSP）」が2015年に創設され、同年の第74回日本癌学会学術総会時にトライアルがまず行われました。2016年以降、JCA-SSPプログラムは日本癌学会学術総会時に正式なプログラムとして毎年開催されています。⊠このJCA-SSPプログラムの創設にあたり参考とされたのが、米国癌学会（AACR）で1998年に創設され継続的に開催されている「米国癌学会ー科学者・サバイバー プログラム（AACR-SSP）」です。このAACR-SSPは、がん研究者、がんサバイバー、そして患者アドボケート（擁護者・支援者）との協働が、がん研究の更なる発展には重要であるという認識のもと、アン・ベイカー博士とマーガレット・フォッティ博士によって創設され、1999年の第1回プログラムには25人のアドボケートが参加して開催されました。以降、AACR-SSPプログラムには日本を含む米国外からも数多くのアドボケートが参加しています。⊠10回目となる本年、通常のJCA-SSPプログラムに加え、10周年の節目を祝い、記念式典ならびに記念ワークショップを開催することとしました。マーガレット・フォッティ博士、アン・ベイカー博士、野田哲生博士ら、JCA-SSPの創設に関わった日米の関係者からのご祝辞、ご挨拶、功労者の表彰に続き、患者会代表者、がん研究者によって、活動の歴史と未来を考える、ワークショップを開催いたします。⊠本JCA-SSP10周年記念行事を通じて、がん研究者、がんサバイバー、そして患者アドボケートとの協働がより一層発展し、未来のがん治療に資するがん研究がより発展することを祈念しています。

---

### SP1-5
### TBD

[Speaker]Kimie Sakurai (Nonprofit organization GISTERS)

No Abstract

▲TOP

### [SP1-6] SP1
### 10th Anniversary of the Survivor-Scientist Program (SSP)

2025 / 9 / 26 (Fri) 9:00-11:30 Room 10/(Art Hall) Room 10

[Chairpersons]Chiaki Takahashi (Division of Oncology and Molecular Biology, Kanazawa University, Cancer Research Institute)、Naomi Sakurai (CSR Project)

日本癌学会では、野田哲生 博士（公益財団法人がん研究会 顧問 兼 名誉研究所長）のご尽力により、がん研究を支援するサバイバー育成のためのプログラム「JCA-Survivor Scientist Program（略称JCA-SSP）」が2015年に創設され、同年の第74回日本癌学会学術総会時にトライアルがまず行われました。2016年以降、JCA-SSPプログラムは日本癌学会学術総会時に正式なプログラムとして毎年開催されています。⊠このJCA-SSPプログラムの創設にあたり参考とされたのが、米国癌学会（AACR）で1998年に創設され継続的に開催されている「米国癌学会－科学者・サバイバー プログラム（AACR-SSP）」です。このAACR-SSPは、がん研究者、がんサバイバー、そして患者アドボケート（擁護者・支援者）との協働が、がん研究の更なる発展には重要であるという認識のもと、アン・ベイカー博士とマーガレット・フォッティ博士によって創設され、1999年の第1回プログラムには25人のアドボケートが参加して開催されました。以降、AACR-SSPプログラムには日本を含む米国外からも数多くのアドボケートが参加しています。⊠10回目となる本年、通常のJCA-SSPプログラムに加え、10周年の節目を祝い、記念式典ならびに記念ワークショップを開催することとしました。マーガレット・フォッティ博士、アン・ベイカー博士、野田哲生博士ら、JCA-SSPの創設に関わった日米の関係者からのご祝辞、ご挨拶、功労者の表彰に続き、患者会代表者、がん研究者によって、活動の歴史と未来を考える、ワークショップを開催いたします。⊠本JCA-SSP10周年記念行事を通じて、がん研究者、がんサバイバー、そして患者アドボケートとの協働がより一層発展し、未来のがん治療に資するがん研究がより発展することを祈念しています。

---

### SP1-6
### TBD

[Speaker]Keiko KATSUI (Japan Agency for Medical Research and Development)

No Abstract

▲TOP

### [SP1-Panelist] SP1
### 10th Anniversary of the Survivor-Scientist Program (SSP)

2025 / 9 / 26 (Fri) 9:00-11:30 Room 10/(Art Hall) Room 10

[Chairpersons]Chiaki Takahashi (Division of Oncology and Molecular Biology, Kanazawa University, Cancer Research Institute)、Naomi Sakurai (CSR Project)

日本癌学会では、野田哲生 博士（公益財団法人がん研究会 顧問 兼 名誉研究所長）のご尽力により、がん研究を支援するサバイバー育成のためのプログラム「JCA-Survivor Scientist Program（略称JCA-SSP）」が2015年に創設され、同年の第74回日本癌学会学術総会時にトライアルがまず行われました。2016年以降、JCA-SSPプログラムは日本癌学会学術総会時に正式なプログラムとして毎年開催されています。⊠このJCA-SSPプログラムの創設にあたり参考とされたのが、米国癌学会（AACR）で1998年に創設され継続的に開催されている「米国癌学会－科学者・サバイバー プログラム（AACR-SSP）」です。このAACR-SSPは、がん研究者、がんサバイバー、そして患者アドボケート（擁護者・支援者）との協働が、がん研究の更なる発展には重要であるという認識のもと、アン・ベイカー博士とマーガレット・フォッティ博士によって創設され、1999年の第1回プログラムには25人のアドボケートが参加して開催されました。以降、AACR-SSPプログラムには日本を含む米国外からも数多くのアドボケートが参加しています。⊠10回目となる本年、通常のJCA-SSPプログラムに加え、10周年の節目を祝い、記念式典ならびに記念ワークショップを開催することとしました。マーガレット・フォッティ博士、アン・ベイカー博士、野田哲生博士ら、JCA-SSPの創設に関わった日米の関係者からのご祝辞、ご挨拶、功労者の表彰に続き、患者会代表者、がん研究者によって、活動の歴史と未来を考える、ワークショップを開催いたします。⊠本JCA-SSP10周年記念行事を通じて、がん研究者、がんサバイバー、そして患者アドボケートとの協働がより一層発展し、未来のがん治療に資するがん研究がより発展することを祈念しています。

---

### SP1-Panelist

[Panelist]Masanori Hatakeyama (Institute of Miocrobial Chemistry ／ Institute for Genetic Medicine, Hokkaido University)

No Abstract

▲TOP

### [SP1-Panelist] SP1
### 10th Anniversary of the Survivor-Scientist Program (SSP)

2025 / 9 / 26 (Fri) 9:00-11:30 Room 10/(Art Hall) Room 10

[Chairpersons]Chiaki Takahashi (Division of Oncology and Molecular Biology, Kanazawa University, Cancer Research Institute)、Naomi Sakurai (CSR Project)

日本癌学会では、野田哲生 博士（公益財団法人がん研究会 顧問 兼 名誉研究所長）のご尽力により、がん研究を支援するサバイバー育成のためのプログラム「JCA-Survivor Scientist Program（略称JCA-SSP）」が2015年に創設され、同年の第74回日本癌学会学術総会時にトライアルがまず行われました。2016年以降、JCA-SSPプログラムは日本癌学会学術総会時に正式なプログラムとして毎年開催されています。□このJCA-SSPプログラムの創設にあたり参考とされたのが、米国癌学会（AACR）で1998年に創設され継続的に開催されている「米国癌学会－科学者・サバイバー プログラム（AACR-SSP）」です。このAACR-SSPは、がん研究者、がんサバイバー、そして患者アドボケート（擁護者・支援者）との協働が、がん研究の更なる発展には重要であるという認識のもと、アン・ベイカー博士とマーガレット・フォッティ博士によって創設され、1999年の第1回プログラムには25人のアドボケートが参加して開催されました。以降、AACR-SSPプログラムには日本を含む米国外からも数多くのアドボケートが参加しています。□10回目となる本年、通常のJCA-SSPプログラムに加え、10周年の節目を祝い、記念式典ならびに記念ワークショップを開催することとしました。マーガレット・フォッティ博士、アン・ベイカー博士、野田哲生博士ら、JCA-SSPの創設に関わった日米の関係者からのご祝辞、ご挨拶、功労者の表彰に続き、患者会代表者、がん研究者によって、活動の歴史と未来を考える、ワークショップを開催いたします。□本JCA-SSP10周年記念行事を通じて、がん研究者、がんサバイバー、そして患者アドボケートとの協働がより一層発展し、未来のがん治療に資するがん研究がより発展することを祈念しています。

---

### SP1-Panelist

[Panelist]Shinsuke Amano ()

No Abstract

▲TOP

### [JS2-1] JS2
### AI is opening up new frontiers in cancer research and medicine.

2025 / 9 / 26 (Fri) 9:00-11:30 Room 11/(ANA Crowne Plaza Hotel Kanazawa 3F Ohtori (West)) Room 11

[Chairpersons]Ryuji Hamamoto (National Cancer Center Research Institute)、Shumpei Ishikawa (The University of Tokyo/National Cancer Center)

In recent years, expectations for artificial intelligence (AI) technology have been increasing due to the rapid progress of machine learning, centered around deep learning. Since the development of Transformer in 2017, there has been significant progress in the field of generative AI, including large language models (LLMs), and AI is now being used in various fields of social life, as well as being a research tool. The medical field is no exception, and AI is being introduced into a wide range of medical research, from basic research to clinical research. According to the latest data from the US FDA, there are over 950 AI-equipped medical devices that have been approved by the FDA and are actually being used in clinical practice. Under these circumstances, this symposium will be held in collaboration with the Japanese Association for Medical Artificial Intelligence to present the results of cancer research using cutting-edge AI, from basic research to clinical applications, and to discuss future directions and issues.

---

### JS2-1
### Data-Driven Analysis Using Cancer Histopathology

[Speaker]Shumpei Ishikawa (Dept. Preventive Medicine, The University of Tokyo／Div. Pathology, EPOC, NCC)

Recent advances in deep learning have greatly improved our ability to extract and quantify complex features from histopathological images, enabling large-scale comparisons across thousands of cases. This allows for data science-driven approaches similar to those in genomics and is transforming pathology into an evidence-based discipline that supports biomedical research and clinical practice. As a result, efforts are being made to address domain shift issues, such as inter-institutional variability, and to enhance the biological interpretability of image features. At the same time, large datasets are being leveraged worldwide to build foundational models in pathology. There is also growing interest in integrating histology data with genomic and clinical information, and analytical methods for such multimodal integration are actively being developed. In Europe and other regions, integrated databases combining histopathology and genomics are already underway. In Japan, similar efforts are progressing within the national Action Plan for Whole Genome Analysis, aiming to systematically collect pathology data, integrate it with genomic datasets, and apply these insights to patient care.

[Disclosure of COI by Responsible researcher] Lead presenter is the Responsible researcher.
[Lead presenter: Shumpei Ishikawa] ■Research funds from company: 日本医療研究開発機構（AMED）日本学術振興会　■Advisor of the company and/or profit-making organization: 新日本科学株式会社　■Research expenses: Chordia therapeutics株式会社 大鵬薬品工業株式会社 ロート製薬株式会社 アステラス製薬株式会社

### [JS2-2] JS2
### AI is opening up new frontiers in cancer research and medicine.

2025 / 9 / 26 (Fri) 9:00-11:30 Room 11/(ANA Crowne Plaza Hotel Kanazawa 3F Ohtori (West)) Room 11

[Chairpersons]Ryuji Hamamoto (National Cancer Center Research Institute)、Shumpei Ishikawa (The University of Tokyo/National Cancer Center)

In recent years, expectations for artificial intelligence (AI) technology have been increasing due to the rapid progress of machine learning, centered around deep learning. Since the development of Transformer in 2017, there has been significant progress in the field of generative AI, including large language models (LLMs), and AI is now being used in various fields of social life, as well as being a research tool. The medical field is no exception, and AI is being introduced into a wide range of medical research, from basic research to clinical research. According to the latest data from the US FDA, there are over 950 AI-equipped medical devices that have been approved by the FDA and are actually being used in clinical practice. Under these circumstances, this symposium will be held in collaboration with the Japanese Association for Medical Artificial Intelligence to present the results of cancer research using cutting-edge AI, from basic research to clinical applications, and to discuss future directions and issues.

---

### JS2-2
### Performance Comparison of Multiple Large Language Models for Cancer Gene Profiling Test Support System

[Speaker]Satoshi Takahashi (Cancer Translational Research Team, RIKEN Center for Advanced Intelligence Project／Division of Medical AI Research and Development, National Cancer Center) [Co-author]Takafumi Koyama (Department of Experimental Therapeutics, National Cancer Center Hospita)、Yuuji Uehara (Department of Experimental Therapeutics, National Cancer Center Hospita)、Ken Takasawa (Cancer Translational Research Team, RIKEN Center for Advanced Intelligence Project／Division of Medical AI Research and Development, National Cancer Center)、Nobuji Kouno (Cancer Translational Research Team, RIKEN Center for Advanced Intelligence Project／Division of Medical AI Research and Development, National Cancer Center ／Department of Gastrointestinal Surgery, Graduate School of Medicine, Kyoto University)、Yusuke Sakaguchi (Cancer Translational Research Team, RIKEN Center for Advanced Intelligence Project／Division of Medical AI Research and Development, National Cancer Center ／Department of Neurosurgery, The University of Tokyo Hospital)、Katsuji Takeda (Cancer Translational Research Team, RIKEN Center for Advanced Intelligence Project)、Masaaki Komatsu (Cancer Translational Research Team, RIKEN Center for Advanced Intelligence Project／Division of Medical AI Research and Development, National Cancer Center)、Ryuji Hamamoto (Cancer Translational Research Team, RIKEN Center for Advanced Intelligence Project／Division of Medical AI Research and Development, National Cancer Center)

Background: In cancer gene profiling tests, selecting appropriate clinical trials poses a significant burden on physicians. We developed ClinicalTrialV5, a clinical trial recommendation system utilizing large language models (LLMs), and compared performance among multiple models.Methods: The system comprises four modules: 1) Keyword generation: generates search keywords from patient descriptions, 2) Search and matching: hybrid search using BM25 and MedCPT encoder, 3) Eligibility evaluation: assesses inclusion/exclusion criteria, 4) Ranking: ranks trials by relevance and eligibility scores.We updated the data source to better capture Japanese clinical trials and compared GPT-4, Gemma3, Llama3, DeepSeek, and Phi4 models. A dedicated visualization application was also developed.Results: Several local LLMs demonstrated performance comparable to API-based GPT-4o. However, discrepancies with clinical physicians' recommendations were still observed.Discussion: Incorporating additional evaluation metrics may improve observed discrepancies. Future work will focus on prospective trial evaluation to validate system performance in real clinical settings.

[Disclosure of COI by Responsible researcher] Lead presenter is not the Responsible researcher.
[Lead presenter: Satoshi Takahashi] We have no financial relationships to disclose.
[Principal Researcher: Ryuuji Hamamoto] We have no financial relationships to disclose.

### [JS2-3] JS2
### AI is opening up new frontiers in cancer research and medicine.

2025 / 9 / 26 (Fri) 9:00-11:30 Room 11/(ANA Crowne Plaza Hotel Kanazawa 3F Ohtori (West)) Room 11

[Chairpersons]Ryuji Hamamoto (National Cancer Center Research Institute)、Shumpei Ishikawa (The University of Tokyo/National Cancer Center)

In recent years, expectations for artificial intelligence (AI) technology have been increasing due to the rapid progress of machine learning, centered around deep learning. Since the development of Transformer in 2017, there has been significant progress in the field of generative AI, including large language models (LLMs), and AI is now being used in various fields of social life, as well as being a research tool. The medical field is no exception, and AI is being introduced into a wide range of medical research, from basic research to clinical research. According to the latest data from the US FDA, there are over 950 AI-equipped medical devices that have been approved by the FDA and are actually being used in clinical practice. Under these circumstances, this symposium will be held in collaboration with the Japanese Association for Medical Artificial Intelligence to present the results of cancer research using cutting-edge AI, from basic research to clinical applications, and to discuss future directions and issues.

---

### JS2-3
### Cell State Analysis of Immune Cells in the Tumor Microenvironment with Deep Semi-NMF

[Speaker]Jiaxin Li (Dept. Comp. Biol. Med. Sci., Grad. Frontier. Sci., Univ. Tokyo) [Co-author]Lysenko Artem (Dept. Biol. Sci., Grad. Sch. Sci., Univ. Tokyo)、Tatsuhiko Tsunoda (Dept. Comp. Biol. Med. Sci., Grad. Frontier. Sci., Univ. Tokyo／Dept. Biol. Sci., Grad. Sch. Sci., Univ. Tokyo)

The tumor microenvironment (TME) influences cancer progression and immunotherapy response. Traditional bulk RNA–seq with deconvolution methods provides only averaged immune compositions, failing to capture distinct immune states. Ecotypes, representing functional immune ecosystems, remain poorly characterized in clinical significance.To address this, we introduce Deep Semi–NMF, a multi–layered non–negative matrix factorization approach that uncovers hierarchical immune patterns. Applying this to samples across 14 cancer types from TCGA, we identified diverse immune cell states with deeper layers capturing metabolic reprogramming and cytotoxic activity. These states were mapped to ecotypes, and some specific ecotypes correlated with improved survival, while others contributed to immune suppression and tumor immune evasion. Ecotype classification enables patient stratification based on immune landscape, offering applications in predicting immunotherapy response.By refining immune cell state resolution and linking ecotypes to clinical outcomes, this study advances precision tumor immunophenotyping and personalized cancer immunotherapy.

[Disclosure of COI by Responsible researcher] Lead presenter is the Responsible researcher.
[Lead presenter: Jiaxin Li] We have no financial relationships to disclose.

### [JS2-4] JS2
### AI is opening up new frontiers in cancer research and medicine.

2025 / 9 / 26 (Fri) 9:00-11:30 Room 11/(ANA Crowne Plaza Hotel Kanazawa 3F Ohtori (West)) Room 11

[Chairpersons]Ryuji Hamamoto (National Cancer Center Research Institute)、Shumpei Ishikawa (The University of Tokyo/National Cancer Center)

In recent years, expectations for artificial intelligence (AI) technology have been increasing due to the rapid progress of machine learning, centered around deep learning. Since the development of Transformer in 2017, there has been significant progress in the field of generative AI, including large language models (LLMs), and AI is now being used in various fields of social life, as well as being a research tool. The medical field is no exception, and AI is being introduced into a wide range of medical research, from basic research to clinical research. According to the latest data from the US FDA, there are over 950 AI-equipped medical devices that have been approved by the FDA and are actually being used in clinical practice. Under these circumstances, this symposium will be held in collaboration with the Japanese Association for Medical Artificial Intelligence to present the results of cancer research using cutting-edge AI, from basic research to clinical applications, and to discuss future directions and issues.

---

### JS2-4
### Integrating Transcriptomic Profiling and Machine Learning: A Prognostic Model for Infant Acute Myeloid Leukemia

[Speaker]YU TAO (Children's Hospital of Chongqing Medical Univ.) [Co-author]YALI SHEN (Children's Hospital of Chongqing Medical Univ.)、LI WEI (Women and Children's Hospital of Chongqing Medical Univ.)、HUI SHI (Children's Hospital of Chongqing Medical Univ.)、HUA YOU (Children's Hospital of Chongqing Medical Univ.)

Infant acute myeloid leukemia (AML) has poor prognostic outcomes and distinct biological characteristics requiring age-specific risk assessment. Expression data from 213 infant AML patients were analyzed using machine learning to develop the infant-prognostic-score (IPScore), or IPSgroup when categorized. Internal validation was done on 127 cases, and external validation on 32 patients from a different ethnic group. We compared its prognostic prediction with other AML models and explored its clinical decision-making value for infant AML patients. The IPSgroup independently predicted outcomes in infant AML, outperforming previous RNA expression-based models. High-risk patients in the IPSgroup may benefit from HSCT, while low-risk patients are not suitable for it. When combined with the pAML stratification system, IPSgroup re-stratified 43% of patients into more accurate risk groups. The model offers a valuable tool for clinical decision-making and treatment strategies, surpassing existing AML stratifications.

[Disclosure of COI by Responsible researcher] Lead presenter is the Responsible researcher.
[Lead presenter: YU TAO] We have no financial relationships to disclose.

### [JS2-5] JS2
### AI is opening up new frontiers in cancer research and medicine.

2025 / 9 / 26 (Fri) 9:00-11:30 Room 11/(ANA Crowne Plaza Hotel Kanazawa 3F Ohtori (West)) Room 11

[Chairpersons]Ryuji Hamamoto (National Cancer Center Research Institute)、Shumpei Ishikawa (The University of Tokyo/National Cancer Center)

In recent years, expectations for artificial intelligence (AI) technology have been increasing due to the rapid progress of machine learning, centered around deep learning. Since the development of Transformer in 2017, there has been significant progress in the field of generative AI, including large language models (LLMs), and AI is now being used in various fields of social life, as well as being a research tool. The medical field is no exception, and AI is being introduced into a wide range of medical research, from basic research to clinical research. According to the latest data from the US FDA, there are over 950 AI-equipped medical devices that have been approved by the FDA and are actually being used in clinical practice. Under these circumstances, this symposium will be held in collaboration with the Japanese Association for Medical Artificial Intelligence to present the results of cancer research using cutting-edge AI, from basic research to clinical applications, and to discuss future directions and issues.

### JS2-5
### Rational design of de novo proteins using large datasets and AI

[Speaker]Kotaro Tsuboyama (IIS UTokyo)

Proteins are essential for living organisms, playing diverse biological functions. They are polymers composed of 20 amino acids, and their amino acid sequences generally determine their structures and further define their functions. However, accurate prediction of protein structure/feature/function remains challenging.AlphaFold, developed by DeepMind, was revolutionary because it predicts the structures of diverse amino acid sequences with high accuracy. This model was developed using AI technology and training dataset (Protein Data Bank) including ~200,000 protein structures. Like in this successful case, with the advancement of AI technology, large and high-quality dataset has become more valuable in constructing highly accurate prediction models.However, in protein science, apart from structural databases and amino acid sequence databases, such databases are largely limited. In this talk, I would like to introduce method to quantify protein properties/functions at a scale and how we are now utilizing the large dataset.

[Disclosure of COI by Responsible researcher] Lead presenter is the Responsible researcher.
[Lead presenter: Kotaro Tsuboyama] ■Contributions: 財団など

▲TOP

### [JS3-1] JS3
### Cancer Heterogeneity, Stem Cell Regulation, and Treatment Resistance

2025 / 9 / 26 (Fri) 9:00-11:30 Room 12/(ANA Crowne Plaza Hotel Kanazawa 3F Ohtori (Central)) Room 12

[Chairpersons]Noriko Gotoh (Cancer Research Institute, Kanazawa University)、Kohshi Mimori (Surgery, Kyushu University Beppu Hospital)

This session is held as a joint session with the Japanese Society of Gastroenterological Carcinogenesis (JSGS). This society brings together a well-balanced mix of cross-disciplinary researchers, including those in clinical fields (surgery, internal medicine), pathology, and biochemistry, to engage in comprehensive discussions. Our key topics include, from a clinical research perspective: cancer genome medicine, large-scale clinical trials, immunology and immunotherapy, molecular targeted therapy, and liquid biopsy. In terms of basic research, we cover whole genome sequencing, epigenome and epitranscriptome analysis, (spatial) single-cell analysis, and omics analysis. Additionally, we focus on the development of humanized cancer models such as organoids and PDX, as well as microbiome analysis. From a mathematical statistics perspective, our research encompasses real-world evidence and real-world data, (generative) AI/information analysis, simulation, and bioimaging, among other areas. In terms of the theme provided by the Japanese Cancer Association, "Cancer Heterogeneity, Stem Cell Regulation, and Treatment Resistance," we look forward to presentations that explore foundational approaches, ultimately leading to diagnostic or therapeutic advancements.

---

### JS3-1
### AI-based analysis of pathologic images incorporating tumor heterogeneity and the microenvironment in pancreatic cancer

[Speaker]Miwako Kakiuchi (Dept. Prev. Med., Grad. Sch. Med., The Univ. of Tokyo) [Co-author]Maki Takao (Dept. Prev. Med., Grad. Sch. Med., The Univ. of Tokyo)、Daisuke Komura (Dept. Prev. Med., Grad. Sch. Med., The Univ. of Tokyo)、Yukihiko Hiroshima (Ctr. for Cancer Genome Med., Kanagawa Cancer Ctr.)、Yohei Miyagi (Mol. Pathol. Genetics Div., Kanagawa Cancer Ctr. Res. Inst.)、Koshi Mimori (Dept. Surg., Kyushu Univ. Beppu Hosp.)、Shumpei Ishikawa (Dept. Prev. Med., Grad. Sch. Med., The Univ. of Tokyo／Div. Path., NCC Exploratory Oncol. Res. & Clin. Trial Ctr.)

Histopathological diagnosis plays a key role in cancer diagnostics by providing cost-effective visualization of tissue structure, tumor heterogeneity, and immune cell distribution. This is especially valuable in pancreatic ductal adenocarcinoma (PDAC), where image-based analysis could accelerate treatment decisions for this aggressive cancer. In PDAC, AI models have shown promise in predicting molecular subtypes linked to chemotherapy response and identifying tumor regions from specimens. However, current methods struggle to capture morphological heterogeneity and the spatial complexity of the tumor microenvironment (TME), both crucial for prognosis and treatment outcomes. This highlights the need for more precise, quantitative approaches. We developed a method combining deep learning for tumor detection, TME cell segmentation, and texture analysis to quantify heterogeneity and TME components. Using PDAC specimens, we examined correlations between these features, genetic mutations, and systemic therapy response. Ultimately, we aim to improve prediction accuracy by integrating H&E and IHC images with genomic data in multimodal AI systems to forecast treatment resistance.

[Disclosure of COI by Responsible researcher] Lead presenter is not the Responsible researcher.
[Lead presenter: Miwako Kakiuchi] We have no financial relationships to disclose.
[Principal Researcher: Shumpei Ishikawa] ■Research funds from company: 日本医療研究開発機構（AMED） ■Advisor of the company and/or profit-making organization: 新日本科学株式会社 ■Research expenses: Chordia therapeutics株式会社、大鵬薬品工業株式会社、ロート製薬株式会社、アステラス製薬株式会社

Joint Symposia

### [JS3-2] JS3
### Cancer Heterogeneity, Stem Cell Regulation, and Treatment Resistance

2025 / 9 / 26 (Fri) 9:00-11:30 Room 12/(ANA Crowne Plaza Hotel Kanazawa 3F Ohtori (Central)) Room 12

[Chairpersons]Noriko Gotoh (Cancer Research Institute, Kanazawa University)、Kohshi Mimori (Surgery, Kyushu University Beppu Hospital)

This session is held as a joint session with the Japanese Society of Gastroenterological Carcinogenesis (JSGS). This society brings together a well-balanced mix of cross-disciplinary researchers, including those in clinical fields (surgery, internal medicine), pathology, and biochemistry, to engage in comprehensive discussions. Our key topics include, from a clinical research perspective: cancer genome medicine, large-scale clinical trials, immunology and immunotherapy, molecular targeted therapy, and liquid biopsy. In terms of basic research, we cover whole genome sequencing, epigenome and epitranscriptome analysis, (spatial) single-cell analysis, and omics analysis. Additionally, we focus on the development of humanized cancer models such as organoids and PDX, as well as microbiome analysis. From a mathematical statistics perspective, our research encompasses real-world evidence and real-world data, (generative) AI/information analysis, simulation, and bioimaging, among other areas. In terms of the theme provided by the Japanese Cancer Association, "Cancer Heterogeneity, Stem Cell Regulation, and Treatment Resistance," we look forward to presentations that explore foundational approaches, ultimately leading to diagnostic or therapeutic advancements.

---

### JS3-2
### Breast cancer cells leverage bone marrow cell plasticity at the single-cell level to drive metastasis initiation

[Speaker]Yasuto Takeuchi (Cancer Research Institute of Kanazawa University) [Co-author]Huazi Zhang (Cancer Research Institute of Kanazawa University)、Takahiko Murayama (Cancer Research Institute of Kanazawa University)、Kazuhiro Ikeda (Research Center for Genomic Medicine, Saitama Medical University)、Kuniko Horie (Research Center for Genomic Medicine, Saitama Medical University)、Satoshi Inoue (Research Center for Genomic Medicine, Saitama Medical University)、Masao Yano (Department of Breast Surgery, Minamimachida Hospital)、Tanabe Masahiko (Department of Breast & Endocrine Surgery, University of Tokyo)、Noriyuki Inaki (Department of Gastroenterological Surgery／Breast Surgery, Kanazawa University)、Keiichiro Tada (Nihon University School of Medicine)、Eishu Hirata (Cancer Research Institute of Kanazawa University)、Shuichi Tsukamoto (Department of Pathology, Kobe University)、Arinobu Tojo (Institute of Science Tokyo)、Koji Okamoto (Teikyo University Advanced Comprehensive Research Organization)、Noriko Gotoh (Cancer Research Institute of Kanazawa University)

Breast cancer remains a leading cause of cancer related deaths among women worldwide, largely due to the high risk of metastasis in HER2 negative and hormone receptor-negative triple negative breast cancer (TNBC) patients. Bone is a common metastatic site, but the mechanisms driving its initiation remain poorly understood. Given the stromal richness of TNBC, we explored interactions between cancer associated fibroblasts (CAFs) and breast cancer cells using a coculture system. RNA sequencing revealed strong upregulation of granulocyte colony stimulating factor (GCSF) in cocultured CAFs. We identified GCSF receptor positive cancer cells as a subpopulation of cancer stem cells (CSCs) with bone metastasis initiating potential. To investigate bone metastasis dynamics, we performed single cell RNA sequencing of metastatic bone marrow from patient derived xenograft models. These analyses revealed how CSCs exploit bone marrow plasticity to establish a metastatic niche. Our findings offer insights into recurrence and metastasis, suggesting therapeutic strategies to prevent bone metastasis.

[Disclosure of COI by Responsible researcher] Lead presenter is not the Responsible researcher.
[Lead presenter: Yasuto Takeuchi] We have no financial relationships to disclose.
[Principal Researcher: Noriko Gotoh] We have no financial relationships to disclose.

### [JS3-3] JS3
### Cancer Heterogeneity, Stem Cell Regulation, and Treatment Resistance

2025 / 9 / 26 (Fri) 9:00-11:30 Room 12/(ANA Crowne Plaza Hotel Kanazawa 3F Ohtori (Central)) Room 12

[Chairpersons]Noriko Gotoh (Cancer Research Institute, Kanazawa University)、Kohshi Mimori (Surgery, Kyushu University Beppu Hospital)

This session is held as a joint session with the Japanese Society of Gastroenterological Carcinogenesis (JSGS). This society brings together a well-balanced mix of cross-disciplinary researchers, including those in clinical fields (surgery, internal medicine), pathology, and biochemistry, to engage in comprehensive discussions. Our key topics include, from a clinical research perspective: cancer genome medicine, large-scale clinical trials, immunology and immunotherapy, molecular targeted therapy, and liquid biopsy. In terms of basic research, we cover whole genome sequencing, epigenome and epitranscriptome analysis, (spatial) single-cell analysis, and omics analysis. Additionally, we focus on the development of humanized cancer models such as organoids and PDX, as well as microbiome analysis. From a mathematical statistics perspective, our research encompasses real-world evidence and real-world data, (generative) AI/information analysis, simulation, and bioimaging, among other areas. In terms of the theme provided by the Japanese Cancer Association, "Cancer Heterogeneity, Stem Cell Regulation, and Treatment Resistance," we look forward to presentations that explore foundational approaches, ultimately leading to diagnostic or therapeutic advancements.

---

### JS3-3
### Clinical and Biological Implications of Fusobacterium nucleatum in Esophageal Cancer

[Speaker]Yoshifumi Baba (Dept. of Gastrointestinal surgery, The Univ. of Tokyo) [Co-author]Kazuki Omachi (Dept of Surgery, Beppu Hosp., Kyushu Univ)、Takashi Ofuchi (Dept of Surgery, Beppu Hosp., Kyushu Univ)、Koshi Mimori (Dept of Surgery, Beppu Hosp., Kyushu Univ)

Gut microbiome Fusobacterium nucleatum has emerged as a microbiome-derived factor linked to prognosis and immunity in human cancers. We quantified F. nucleatum DNA in 325 esophageal cancers and found its presence was associated with poor survival (Clin Cancer Res 2016, 2019). Mechanistic studies showed F. nucleatum promotes tumor aggressiveness via NF-kB activation (Cancer Lett 2022). Moreover, F. nucleatum-positive tumors had poor response to neoadjuvant chemotherapy, with in vitro data indicating bacterial invasion, autophagy induction, and drug resistance (Br J Cancer 2021). In cohorts from Kumamoto and MD Anderson, F. nucleatum positivity in gastric and gastroesophageal junction cancers was linked to worse outcomes and increased tumor cell growth (Br J Cancer 2024). In addition, F. nucleatum-positive tumors showed significantly reduced CD3+ lymphocyte infiltration, suggesting impaired local antitumor immunity (Br J Cancer 2023). TCGA-based metagenomic analysis revealed F. nucleatum correlates with CAF activation in ESCC. We are conducting spatial and single-cell transcriptomic analyses to explore its role in the tumor immune environment, and will present ongoing findings.

[Disclosure of COI by Responsible researcher] Lead presenter is the Responsible researcher.
[Lead presenter: Yoshifumi Baba] ■Lecture fee: 小野薬品工業　MSD株式会社　ミヤリサン製薬株式会社

### [JS3-4] JS3
### Cancer Heterogeneity, Stem Cell Regulation, and Treatment Resistance

2025 / 9 / 26 (Fri) 9:00-11:30 Room 12/(ANA Crowne Plaza Hotel Kanazawa 3F Ohtori (Central)) Room 12

[Chairpersons]Noriko Gotoh (Cancer Research Institute, Kanazawa University)、Kohshi Mimori (Surgery, Kyushu University Beppu Hospital)

This session is held as a joint session with the Japanese Society of Gastroenterological Carcinogenesis (JSGS). This society brings together a well-balanced mix of cross-disciplinary researchers, including those in clinical fields (surgery, internal medicine), pathology, and biochemistry, to engage in comprehensive discussions. Our key topics include, from a clinical research perspective: cancer genome medicine, large-scale clinical trials, immunology and immunotherapy, molecular targeted therapy, and liquid biopsy. In terms of basic research, we cover whole genome sequencing, epigenome and epitranscriptome analysis, (spatial) single-cell analysis, and omics analysis. Additionally, we focus on the development of humanized cancer models such as organoids and PDX, as well as microbiome analysis. From a mathematical statistics perspective, our research encompasses real-world evidence and real-world data, (generative) AI/information analysis, simulation, and bioimaging, among other areas. In terms of the theme provided by the Japanese Cancer Association, "Cancer Heterogeneity, Stem Cell Regulation, and Treatment Resistance," we look forward to presentations that explore foundational approaches, ultimately leading to diagnostic or therapeutic advancements.

---

### JS3-4
### Targeting tumor stromal components for therapeutic innovation in GI cancers with heterogeneous microenvironments

[Speaker]Takehiko Yokobori (Gunma Univ, Initiative for Advanc. Res.) [Co-author]Hiroshi Saeki (Dept. General Surg. Sci. Gunma Univ.)、Ken Shirabe (Dept. General Surg. Sci. Gunma Univ.)

Tumor heterogeneity is a major contributor to therapeutic resistance and poor clinical outcomes in gastrointestinal (GI) cancers. Within the heterogeneous tumor microenvironment (TME), cancer-associated fibroblasts (CAFs) consist of diverse subtypes. Among them, ECM-remodeling CAFs are a major source of latent transforming growth factor-beta binding protein 2 (LTBP2), whereas transforming growth factor-beta-induced (TGFBI) is secreted by multiple cell types, including inflammatory CAFs and tumor-associated macrophages (TAMs), contributing to TME heterogeneity. Based on our ongoing biomarker research, we have identified LTBP2 and TGFBI as key stromal regulators that promote intratumoral heterogeneity and therapeutic resistance. In murine GI cancer and patient-derived xenograft (PDX) models, neutralizing antibodies targeting TGFBI reduced cytokine levels and enhanced the efficacy of other therapeutic agents. Inhibition of LTBP2 suppressed tumor growth, further supporting its potential as a therapeutic target. These findings underscore the therapeutic promise of targeting stromal regulators TGFBI and LTBP2 to overcome resistance mechanisms in heterogeneous GI cancers.

[Disclosure of COI by Responsible researcher] Lead presenter is the Responsible researcher.
[Lead presenter: Takehiko Yokobori] ■Research expenses: 株式会社 The IT Lab、株式会社ペルセウスプロテオミクス

### [JS3-5] JS3
### Cancer Heterogeneity, Stem Cell Regulation, and Treatment Resistance

2025 / 9 / 26 (Fri) 9:00-11:30 Room 12/(ANA Crowne Plaza Hotel Kanazawa 3F Ohtori (Central)) Room 12

[Chairpersons]Noriko Gotoh (Cancer Research Institute, Kanazawa University)、Kohshi Mimori (Surgery, Kyushu University Beppu Hospital)

This session is held as a joint session with the Japanese Society of Gastroenterological Carcinogenesis (JSGS). This society brings together a well-balanced mix of cross-disciplinary researchers, including those in clinical fields (surgery, internal medicine), pathology, and biochemistry, to engage in comprehensive discussions. Our key topics include, from a clinical research perspective: cancer genome medicine, large-scale clinical trials, immunology and immunotherapy, molecular targeted therapy, and liquid biopsy. In terms of basic research, we cover whole genome sequencing, epigenome and epitranscriptome analysis, (spatial) single-cell analysis, and omics analysis. Additionally, we focus on the development of humanized cancer models such as organoids and PDX, as well as microbiome analysis. From a mathematical statistics perspective, our research encompasses real-world evidence and real-world data, (generative) AI/information analysis, simulation, and bioimaging, among other areas. In terms of the theme provided by the Japanese Cancer Association, "Cancer Heterogeneity, Stem Cell Regulation, and Treatment Resistance," we look forward to presentations that explore foundational approaches, ultimately leading to diagnostic or therapeutic advancements.

---

### JS3-5
### Application of Medium-Range Cell-Cell Interaction Analysis to Colorectal Cancer

[Speaker]Haruka Hirose (NCC) [Co-author]Yasuhiro Kojima (NCC)

The spatial diffusion range of ligands is a pivotal factor in cell-cell interactions, but remains largely unexplored in conventional analysis methods. This study aimed to develop a methodology for analyzing medium-range cell-cell interactions by estimating ligand diffusion distances from spatial transcriptome data. We developed an algorithm to model spatial ligand diffusion. Specifically, we constructed a method to learn the effective diffusion distance of each ligand through regression analysis of diffused ligand expression and downstream target gene expression. To evaluate performance, we applied it to Visium data of colorectal cancer tissue. The methodology successfully classified ligands into long-range and short-range types. In the tissue, the method detected medium-range interaction patterns between tumor and normal regions, revealing spatial intercellular network features that conventional methods cannot capture. We established a medium-range cell-cell interaction analysis method considering spatial ligand diffusion and demonstrated its effectiveness. This method is expected to enhance understanding of spatial cell-cell interactions in diverse diseases and tissues.

[Disclosure of COI by Responsible researcher] Lead presenter is the Responsible researcher.
[Lead presenter: Haruka Hirose] We have no financial relationships to disclose.

▲TOP

### [JS3-6] JS3
### Cancer Heterogeneity, Stem Cell Regulation, and Treatment Resistance

2025 / 9 / 26 (Fri) 9:00-11:30 Room 12/(ANA Crowne Plaza Hotel Kanazawa 3F Ohtori (Central)) Room 12

[Chairpersons]Noriko Gotoh (Cancer Research Institute, Kanazawa University)、Kohshi Mimori (Surgery, Kyushu University Beppu Hospital)

This session is held as a joint session with the Japanese Society of Gastroenterological Carcinogenesis (JSGS). This society brings together a well-balanced mix of cross-disciplinary researchers, including those in clinical fields (surgery, internal medicine), pathology, and biochemistry, to engage in comprehensive discussions. Our key topics include, from a clinical research perspective: cancer genome medicine, large-scale clinical trials, immunology and immunotherapy, molecular targeted therapy, and liquid biopsy. In terms of basic research, we cover whole genome sequencing, epigenome and epitranscriptome analysis, (spatial) single-cell analysis, and omics analysis. Additionally, we focus on the development of humanized cancer models such as organoids and PDX, as well as microbiome analysis. From a mathematical statistics perspective, our research encompasses real-world evidence and real-world data, (generative) AI/information analysis, simulation, and bioimaging, among other areas. In terms of the theme provided by the Japanese Cancer Association, "Cancer Heterogeneity, Stem Cell Regulation, and Treatment Resistance," we look forward to presentations that explore foundational approaches, ultimately leading to diagnostic or therapeutic advancements.

---

### JS3-6
### Targeting ECM-Secreted Matricellular Proteins to Overcome Immune Resistance in Aggressive Colorectal Cancer

[Speaker]Yuki Nakanishi (Kyoto Univ. Hosp., Dept. of Gastroenterology and Hepatology)

Accumulating evidence underscores the critical role of stroma in shaping the tumor microenvironment (TME) of colorectal cancer (CRC). A subset of CRC characterized by fibrotic stroma is typically microsatellite stable (MSS), highly desmoplastic, excludes CD8+ T cells, resists immunotherapy, and shows poor clinical outcomes. Identifying actionable targets within this stromal milieu is vital. Myeloid cells and cancer-associated fibroblasts (CAFs) dominate the immune and stromal landscape, respectively. Given the difficulty of directly targeting these cellular populations, we focused on matricellular proteins (MCP), non-structural ECM components secreted into the tumor stroma, as alternative therapeutic targets. Using aggressive mouse models of fibrotic CRC combined with transcriptomes at single cell level and spatial analyses, we identified specific tumor-associated MCP that mediate immune evasion and confer resistance to immune checkpoint blockade. Targeting these factors enhanced anti-tumor immunity and sensitized tumors to immunotherapy. Our findings reveal a promising strategy for overcoming immune resistance in fibrotic CRC through modulation of ECM-derived stromal signals.

[Disclosure of COI by Responsible researcher] Lead presenter is the Responsible researcher.
[Lead presenter: Yuki Nakanishi] We have no financial relationships to disclose.

### [AOSR3-1] AOSR3
### Urologic Cancer

2025 / 9 / 26 (Fri) 9:00-11:30 Room 13/(ANA Crowne Plaza Hotel Kanazawa 3F Ohtori (East)) Room 13

[Chairpersons]Atsushi Mizogami (Department of Integrative Cancer Therapy and Urology Kanazawa University, Graduate School of Medical Science)、Koji Ueda (Japanese Foundation for Cancer Research)

Treatment for genitourinary cancers (kidney, bladder, and prostate cancers) has been advancing rapidly, and there has been a clear improvement in life expectancy compared to 10 years ago. However, it is difficult to say that current treatments are working adequately. On the other hand, there have been remarkable developments in basic research technologies, such as genome analysis using next-generation sequencers, proteome analysis using mass spectrometers, and spatial multi-omics analysis, by which innovative new findings have been reported one after another in the field of genitourinary cancer. In this symposium, we will focus on renal, bladder, and prostate cancer, and hope that the latest results of basic research and future genitourinary cancer treatment based on these findings will be presented.

---

### AOSR3-1
### Development of precision medicine for kidney cancer

[Speaker]Hisashi Hasumi (Department of Urology, Yokohama City University) [Co-author]Ryosuke Jikuya (Department of Urology, Yokohama City University)、Go Noguchi (Department of Urology, Yokohama City University)、Sachi Kawaura (Department of Urology, Yokohama City University)、Kota Aomori (Department of Urology, Yokohama City University)、Yasuhiro Iribe (Department of Urology, Yokohama City University)、Kazuhide Makiyama (Department of Urology, Yokohama City University)、Wataru Obara (Department of Urology, Iwate Medical University)、Tomonori Habuchi (Department of Urology, Akita University)、Mitsuko Furuya (Department of Surgical Pathology, Hokkaido University)、Toyonori Tsuzuki (Department of Surgical Pathology, Aichi Medical University)、Yoji Nagashima (Department of Surgical Pathology, Tokyo Women's Medical University)、Satoshi Fujii (Department of Molecular Pathology, Yokohama City University)、Erika Muraoka (Department of Molecular Pathology, Yokohama City University)、Yuji Miura (Department of Medical Oncology, The Cancer Institute Hospital)、Hidewaki Nakagawa (RIKEN Center for Integrative Medical Science)

Kidney cancer is a complex disease with tumor heterogeneity, in which precision medicine is incompletely developed. To elucidate complex renal tumorigenesis, we identified several kidney cancer-associated genes, which led to the discovery of renal tumor suppression by FLCN/FNIP1/FNIP2 complex, a critical regulator for metabolic homeostasis (J Natl Cancer Inst.2012., PNAS.2015.). Recent technologies including WGS and single-cell or spatial RNA-seq furthered our understandings on complex renal tumorigenesis (EBioMedicine.2023., iScience.2022.). In this talk, we present our recent strategies for the development of precision medicine for kidney cancer, which include (a) the development of expression atlas of rare kidney cancers, (b) the development of imaging modalities visualizing hyperpolarized metabolites, and (c) the elucidation of renal tumorigenesis in the context of normal nephron developmental trajectory. Our aim is to provide foundations for precision surgical and pharmaceutical interventions.

[Disclosure of COI by Responsible researcher] Lead presenter is the Responsible researcher.
[Lead presenter: Hisashi Hasumi] We have no financial relationships to disclose.

▲TOP

### [AOSR3-2] AOSR3
### Urologic Cancer

2025 / 9 / 26 (Fri) 9:00-11:30 Room 13/(ANA Crowne Plaza Hotel Kanazawa 3F Ohtori (East)) Room 13

[Chairpersons]Atsushi Mizogami (Department of Integrative Cancer Therapy and Urology Kanazawa University, Graduate School of Medical Science)、Koji Ueda (Japanese Foundation for Cancer Research)

Treatment for genitourinary cancers (kidney, bladder, and prostate cancers) has been advancing rapidly, and there has been a clear improvement in life expectancy compared to 10 years ago. However, it is difficult to say that current treatments are working adequately. On the other hand, there have been remarkable developments in basic research technologies, such as genome analysis using next-generation sequencers, proteome analysis using mass spectrometers, and spatial multi-omics analysis, by which innovative new findings have been reported one after another in the field of genitourinary cancer. In this symposium, we will focus on renal, bladder, and prostate cancer, and hope that the latest results of basic research and future genitourinary cancer treatment based on these findings will be presented.

### AOSR3-2
### Identification of Predictive Biomarkers for the Efficacy of Immune Checkpoint Inhibitors through Multi-Omics Analysis

[Speaker]Taigo Kato (Dept of Urolgy, The University of Osaka, Faculty of Medicine) [Co-author]Yu Ishizuya (Dept of Urolgy, The University of Osaka, Faculty of Medicine)、Takuji Hayashi (Dept of Urolgy, The University of Osaka, Faculty of Medicine)、Yoshiyuki Yamamoto (Dept of Urolgy, The University of Osaka, Faculty of Medicine)、Koji Hatano (Dept of Urolgy, The University of Osaka, Faculty of Medicine)、Atsunari Kawashima (Dept of Urolgy, The University of Osaka, Faculty of Medicine)、Kazuma Kiyotani (National Institutes of Biomedical Innovation, Health and Nutrition)、Takao Fujisawa (Dept of Head and Neck Medical Oncology, NCC Hospital East／Dept of International Research Promotion Office, NCC Hospital East)、Hideaki Bando (Dept of International Research Promotion Office, NCC Hospital East／Dept of Gastroenterology and Gastrointestinal Oncology, NCC Hospital East)、Yoshiaki Nakamura (Dept of International Research Promotion Office, NCC Hospital East／Dept of Gastroenterology and Gastrointestinal Oncology, NCC Hospital East)、Takayuki Yoshino (Dept of Gastroenterology and Gastrointestinal Oncology, NCC Hospital East)、Norio Nonomura (Dept of Urolgy, The University of Osaka, Faculty of Medicine)

Immune checkpoint inhibitors (icis) have led to consistent response rates across various cancer types. However, objective response rates remain approximately 30% with monotherapy and 60% with combination therapy, highlighting the urgent need for the development of reliable predictive biomarkers for efficacy. Furthermore, icis are now being used not only for advanced cancers but also as adjuvant therapies after surgery in several tumors. Clinical trials guided by postoperative ctdna status (molecular residual disease) are currently underway. To achieve above, a deeper understanding of the tumor microenvironment is essential, incorporating not only cancer genomic and immune cell profiling (immunogenomics) but also advanced technologies such as spatial transcriptomics and single cell analysis to improve precision.In this lecture, we will provide an overview of future directions, focusing on urologic cancers, particularly kidney cancer, and introducing our findings from immunogenomic studies, as well as recent discoveries from the monstar-screen project, including novel biomarkers for predicting response.

[Disclosure of COI by Responsible researcher] Lead presenter is the Responsible researcher.
[Lead presenter: Taigo Kato] ■Lecture fee: MSD株式会社

### [AOSR3-3] AOSR3
### Urologic Cancer

2025 / 9 / 26 (Fri) 9:00-11:30 Room 13/(ANA Crowne Plaza Hotel Kanazawa 3F Ohtori (East)) Room 13

[Chairpersons]Atsushi Mizogami (Department of Integrative Cancer Therapy and Urology Kanazawa University, Graduate School of Medical Science)、Koji Ueda (Japanese Foundation for Cancer Research)

Treatment for genitourinary cancers (kidney, bladder, and prostate cancers) has been advancing rapidly, and there has been a clear improvement in life expectancy compared to 10 years ago. However, it is difficult to say that current treatments are working adequately. On the other hand, there have been remarkable developments in basic research technologies, such as genome analysis using next-generation sequencers, proteome analysis using mass spectrometers, and spatial multi-omics analysis, by which innovative new findings have been reported one after another in the field of genitourinary cancer. In this symposium, we will focus on renal, bladder, and prostate cancer, and hope that the latest results of basic research and future genitourinary cancer treatment based on these findings will be presented.

---

### AOSR3-3
### Development of bladder cancer urine liquid biopsy technique using mutant proteins in extracellular vesicles

[Speaker]Kazuma Sugimoto (Division of Analytical Biochemistry, CPM Center, JFCR／Urol., Surg. , Grad. school of med., The Uni. of Tokyo／Urol., The Univ. of Tokyo Hospital) [Co-author]Yuji Hakozaki (Urol., The Univ. of Tokyo Hospital)、Yuta Yamada (Urol., The Univ. of Tokyo Hospital)、Haruki Kume (Urol., Surg. , Grad. school of med., The Uni. of Tokyo／Urol., The Univ. of Tokyo Hospital)、Koji Ueda (Division of Analytical Biochemistry, CPM Center, JFCR)

Currently, urinary cytology is the only widely used biomarker for bladder cancer (BC), despite its limited sensitivity. To improve diagnostic accuracy and capture cancer-specific molecular signatures, we explored mutant proteins derived from somatic mutations present in urinary extracellular vesicles (u–EVs) as a novel biomarker source.In this study, whole exome sequencing was performed on genomic DNA from tumor tissues and matched blood samples of 15 BC patients to identify somatic mutations. Using liquid chromatography–mass spectrometry (LC–MS), we conducted a comprehensive proteomic analysis of cancer tissue, cultured tissue–derived EVs (CTD–EVs), and u-EVs. We identified 121 mutant proteins in cancer tissues, 106 in CTD–EVs, and 9 in u-EVs.Importantly, absolute quantification of these mutant proteins in pre- and postoperative u–EVs showed a marked decrease following surgical tumor removal, correlating with patient clinical outcomes. This study demonstrates a novel liquid biopsy approach that leverages mutant proteins in u–EVs, paving the way for highly personalized and non–invasive biomarkers for bladder cancer detection and monitoring.

[Disclosure of COI by Responsible researcher] Lead presenter is not the Responsible researcher.
[Lead presenter: Kazuma Sugimoto] We have no financial relationships to disclose.
[Principal Researcher: Koji Ueda] We have no financial relationships to disclose.

### [AOSR3-4] AOSR3
### Urologic Cancer

2025 / 9 / 26 (Fri) 9:00-11:30 Room 13/(ANA Crowne Plaza Hotel Kanazawa 3F Ohtori (East)) Room 13

[Chairpersons]Atsushi Mizogami (Department of Integrative Cancer Therapy and Urology Kanazawa University, Graduate School of Medical Science)、Koji Ueda (Japanese Foundation for Cancer Research)

Treatment for genitourinary cancers (kidney, bladder, and prostate cancers) has been advancing rapidly, and there has been a clear improvement in life expectancy compared to 10 years ago. However, it is difficult to say that current treatments are working adequately. On the other hand, there have been remarkable developments in basic research technologies, such as genome analysis using next-generation sequencers, proteome analysis using mass spectrometers, and spatial multi-omics analysis, by which innovative new findings have been reported one after another in the field of genitourinary cancer. In this symposium, we will focus on renal, bladder, and prostate cancer, and hope that the latest results of basic research and future genitourinary cancer treatment based on these findings will be presented.

---

### AOSR3-4
### A new era in the treatment of advanced urothelial cancer pioneered by basic cancer research

[Speaker]Kei Daizumoto (Dept.Urology, Tokushima Univ.Grad.Sch of Biomed.Sci.) [Co-author]Tomoya Fukawa (Dept.Urology, Tokushima Univ.Grad.Sch of Biomed.Sci.)、Jyunya Furukawa (Dept.Urology, Tokushima Univ.Grad.Sch of Biomed.Sci.)

The treatment paradigm for advanced urothelial carcinoma is undergoing a historic transformation. Over the past several decades, platinum-based chemotherapy has been the standard of care, but this era is now being replaced by the development of immunotherapy agents such as immune checkpoint inhibitors and antibody-drug conjugates. Amid this paradigm shift, there is an increasing need to focus on personalized medicine tailored to the biological characteristics of individual cases.FGFR inhibitors are emerging as treatment options, and antibody-drug conjugates targeting HER2 are being developed for HER2-high-expressing tumors. However, further understanding of the spatiotemporal heterogeneity of tumor tissues and genomic changes induced by treatment is essential to elucidate the underlying mechanisms of urothelial carcinoma.In this symposium, we will review our previous research findings and the latest literature reports on urothelial carcinoma research, and discuss the future prospects for cancer researchers to pioneer new treatment algorithms for advanced urothelial carcinoma.

[Disclosure of COI by Responsible researcher] Lead presenter is the Responsible researcher.
[Lead presenter: Kei Daizumoto] We have no financial relationships to disclose.

### [AOSR3-5] AOSR3
### Urologic Cancer

2025 / 9 / 26 (Fri) 9:00-11:30 Room 13/(ANA Crowne Plaza Hotel Kanazawa 3F Ohtori (East)) Room 13

[Chairpersons]Atsushi Mizogami (Department of Integrative Cancer Therapy and Urology Kanazawa University, Graduate School of Medical Science)、Koji Ueda (Japanese Foundation for Cancer Research)

Treatment for genitourinary cancers (kidney, bladder, and prostate cancers) has been advancing rapidly, and there has been a clear improvement in life expectancy compared to 10 years ago. However, it is difficult to say that current treatments are working adequately. On the other hand, there have been remarkable developments in basic research technologies, such as genome analysis using next-generation sequencers, proteome analysis using mass spectrometers, and spatial multi-omics analysis, by which innovative new findings have been reported one after another in the field of genitourinary cancer. In this symposium, we will focus on renal, bladder, and prostate cancer, and hope that the latest results of basic research and future genitourinary cancer treatment based on these findings will be presented.

---

### AOSR3-5
### The latest status and issues of prostate cancer: especially from the perspective of HBCO

[Speaker]Takeo Kosaka (Dept of Urology, Keio University School of medicine／HBOC center, Keio University Hospital／Oligo metastases center, Keio University Hospital) [Co-author]Mototsugu Oya (Dept of Urology, Keio University School of medicine)

Hereditary Breast and Ovarian Cancer syndrome (HBOC) is a familiar field for medical professionals involved in breast surgery, gynecology, and genetics, as its name includes breast cancer and ovarian cancer. However, in the field of urology in Japan, it was a relatively unknown field until its association with prostate cancer was revealed. Since the launch of the Japan Hereditary Breast and Ovarian Cancer Comprehensive Medical Care System in 2016, we have been actively engaged in awareness-raising activities regarding prostate cancer. In December 2020, PARP inhibitors became available for patients with castration-resistant prostate cancer with distant metastasis who are BRCA gene variant positive. Germline pathogenic variant in BRCA1 or BRCA2 have been linked to the risk of prostate cancer onset and progression, and have attracted particular attention, with gene panel testing and treatment being linked, and clinical implementation of HBOC treatment in the field of urology has begun. In this symposium, we would like to discuss the latest current situation and issues regarding prostate cancer as an HBOC-related tumor.

[Disclosure of COI by Responsible researcher] Lead presenter is the Responsible researcher.
[Lead presenter: Takeo Kosaka] ■Lecture fee: アステラス製薬　ヤンセンファーマ　武田薬品工業

▲TOP

### [AOSR3-6] AOSR3
### Urologic Cancer

2025 / 9 / 26 (Fri) 9:00-11:30 Room 13/(ANA Crowne Plaza Hotel Kanazawa 3F Ohtori (East)) Room 13

[Chairpersons]Atsushi Mizogami (Department of Integrative Cancer Therapy and Urology Kanazawa University, Graduate School of Medical Science)、Koji Ueda (Japanese Foundation for Cancer Research)

Treatment for genitourinary cancers (kidney, bladder, and prostate cancers) has been advancing rapidly, and there has been a clear improvement in life expectancy compared to 10 years ago. However, it is difficult to say that current treatments are working adequately. On the other hand, there have been remarkable developments in basic research technologies, such as genome analysis using next-generation sequencers, proteome analysis using mass spectrometers, and spatial multi-omics analysis, by which innovative new findings have been reported one after another in the field of genitourinary cancer. In this symposium, we will focus on renal, bladder, and prostate cancer, and hope that the latest results of basic research and future genitourinary cancer treatment based on these findings will be presented.

### AOSR3-6
### Mechanisms Underlying Double Negative CRPC: Interplay Between FGF and CCL2

[Speaker]Kouji Izumi (Department of Urology, Kanazawa Univ.)

Androgen deprivation therapy for advanced prostate cancer initially shows effectiveness but often leads to resistance, resulting in castration-resistant prostate cancer (CRPC). Traditionally, treatment focused on inhibiting the androgen receptor (AR) pathway; however, AR-independent prostate cancer cells are now recognized as key determinants of prognosis. Neuroendocrine differentiation (NED) is a well-known AR-independent phenotype, but recently, double negative CRPC (DNPC)-lacking both NED and AR activity-has garnered attention. Proposed in 2017, DNPC is increasing in prevalence. While platinum-based chemotherapy shows some efficacy against NED, DNPC lacks an identified target pathway, and no effective treatment exists. Understanding its progression mechanisms is crucial for overcoming prostate cancer. Though DNPC research remains limited, studies report the FGF signaling pathway sustains cell activity. Additionally, the CCL2 pathway contributes to DNPC metastasis and modulates the tumor microenvironment immune response. However, their relationship remains unclear. Our research focuses on this interplay, and we aim to present our findings alongside a comprehensive DNPC review.

[Disclosure of COI by Responsible researcher] Lead presenter is the Responsible researcher.
[Lead presenter: Kouji Izumi] ■Lecture fee: 小野薬品　BSM　■Research expenses: アストラゼネカ　ヤンセン　中外　ノバルティス

▲TOP

### [J-2025] I-J14-1
### Drug delivery system (1)

2025 / 9 / 26 (Fri) 9:00-10:15 Room 14/(ANA Crowne Plaza Hotel Kanazawa 3F Zuiun 1) Room 14

[Chairperson]Tetsuya Koama (Tohoku Univ. Grad School Biomed Eng)

Japanese Oral Session

---

### J-2025
### Enhancing anti-tumor effect by improving nanomedicine delivery with microbubbles and ultrasound

[Speaker]Ryo Suzuki (Teikyo Univ., Fac. Pharm. Sci.) [Co-author]Saori Kageyama (Teikyo Univ., Fac. Pharm. Sci.)、Lisa Munakata (Teikyo Univ., Fac. Pharm. Sci.)、Daiki Omata (Teikyo Univ., Fac. Pharm. Sci.)、Kazuo Maruyama (Teikyo Univ., Fac. Pharm. Sci.)

The passive targeting of nanomedicines, such as liposomal doxorubicin (DOXIL), to solid tumors via the Enhanced Permeability and Retention (EPR) effect remains inefficient due to inadequate capillary permeability in many solid tumor tissues. Thus, the development of strategies to enhance nanomedicine delivery to solid tumors is urgently needed. We have focused on improving vascular permeability through the oscillation and cavitation of microbubbles (MBs) under ultrasound exposure (US). In this study, we evaluated the feasibility of enhancing nanomedicine delivery to solid tumors using MBs and US. DOXIL was ***i.v.*** injected into Colon-26 cell-bearing mice, followed by MBs infusion and US. Tumor volume was measured to assess anti-tumor efficacy. The combination of MBs and US significantly enhanced anti-tumor efficacy compared to the DOXIL-alone. This improvement is likely attributable to the increased efficiency of DOXIL delivery to tumor tissues via the enhancement of vascular permeability. This finding suggests that the combination of MBs and US represents a promising strategy for nanomedicine delivery, improving the EPR effect by stimulating vascular permeability in tumor tissues.

[Disclosure of COI by Responsible researcher] Lead presenter is the Responsible researcher.
[Lead presenter: Ryo Suzuki] ■Research funds from company: 科研費　■Research expenses: 株式会社ウェルセラ

### [J-2026] I-J14-1
### Drug delivery system (1)

2025 / 9 / 26 (Fri) 9:00-10:15 Room 14/(ANA Crowne Plaza Hotel Kanazawa 3F Zuiun 1) Room 14

[Chairperson]Tetsuya Koama (Tohoku Univ. Grad School Biomed Eng)

---

### J-2026
### Therapy-oriented bioabsorbable nanofiber sheet toward location-specific targeted delivery of anti-cancer vesicles

[Speaker]Akira Yokoi (Nagoya univ. Med. Obst. & Gyne.) [Co-author]Kazuhiro Suzuki (Nagoya univ. Med. Obst. & Gyne.)、Masami Kitagawa (Nagoya univ. Med. Obst. & Gyne.)、Takao Yasui (Tokyo Inst. of Tech., Life Sci. and Tech.)、Hiroaki Kajiyama (Nagoya univ. Med. Obst. & Gyne.)

Extracellular vesicles (EVs) have emerged as promising therapeutic agents, particularly as biocompatible carriers for anti-cancer molecules, but efficient and targeted delivery remains challenging. We previously developed a nanofiber-based EV sheet capable of capturing EVs, and here, we aimed to invent a therapy-oriented version (tEV-sheet) designed for localized delivery of anti-tumor vesicles. We created tEV-sheets with bioabsorbable materials and confirmed sufficient EV loading capacity. tEV-sheets were placed in the peritoneal cavity and demonstrated full absorption within three months, indicating biocompatibility. Regarding biodistributions, tEV-sheet allowed for more targeted vesicle accumulation in specific peritoneal regions, compared to systemic delivery. tEV-sheets were loaded with lipid nanoparticles encapsulating carboplatin, and by using the mouse model mimicking site-specific peritoneal metastasis, the tEV-sheet adhered directly to metastatic tumors, resulting in localized tumor suppression without observable toxicity. These findings demonstrated tEV-sheet is a safe and effective platform for targeted delivery, with potential applications in a broad range of diseases.

[Disclosure of COI by Responsible researcher] Lead presenter is the Responsible researcher.
[Lead presenter: Akira Yokoi] We have no financial relationships to disclose.

### [J-2027] I-J14-1
### Drug delivery system (1)

2025 / 9 / 26 (Fri) 9:00-10:15 Room 14/(ANA Crowne Plaza Hotel Kanazawa 3F Zuiun 1) Room 14

[Chairperson]Tetsuya Koama (Tohoku Univ. Grad School Biomed Eng)

---

### J-2027
### Intraperitoneal administration of multilayer-structured anticancer nanoparticles prevents peritoneal dissemination

[Speaker]Susumu Hama (Res. Inst. Pharm.Sci., Musashino Univ.／Fac. Pharm., Musashino Univ.) [Co-author]Sae Shibatani (Fac. Pharm., Musashino Univ.)、Sumika Igarashi (Fac. Pharm., Musashino Univ.)

We previously demonstrated that tocopheryl succinate (TS) and egg phosphatidylcholine (EPC)-based nanoparticles (TS-EPC-NPs) exhibit anticancer effects following intraperitoneal (IP) administration in a murine model of peritoneal dissemination. However, limited IP retention remains a significant challenge. As larger nanoparticles tend to exhibit improved peritoneal retention, we aimed to develop larger TS-EPC-NPs using a layer-by-layer (LbL) assembly technique (LbL-TS-NPs). The formation of multilayers was confirmed by FRET analysis. In vivo, IP retention and antitumor efficacy were assessed in a mouse model of peritoneal dissemination through in vivo imaging system (IVIS) analysis and tumor weight measurements. Layer-by-layer assembly increased the particle size, and FRET analysis verified the interlayer interactions. Further multilayering (2LbL-TS-NPs) resulted in even larger nanoparticles with well-defined multilayered structures. Notably, 2LbL-TS-NPs demonstrated prolonged IP retention and enhanced antitumor effects compared to TS-EPC-NPs. These results suggest that IP administration of 2LbL-TS-NPs represents a promising strategy for the treatment of peritoneal dissemination.

[Disclosure of COI by Responsible researcher] Lead presenter is the Responsible researcher.
[Lead presenter: Susumu Hama] We have no financial relationships to disclose.

### [J-2028] I-J14-1
### Drug delivery system (1)

2025 / 9 / 26 (Fri) 9:00-10:15 Room 14/(ANA Crowne Plaza Hotel Kanazawa 3F Zuiun 1) Room 14

[Chairperson]Tetsuya Koama (Tohoku Univ. Grad School Biomed Eng)

---

### J-2028
### IL-7R-Targeted ADC Enables Non-Invasive Eradication of CNS-Infiltrated ALL via CSF-Mediated Pathway

[Speaker]Motochika Hamada (Division of Developmental Therapeutics, National Cancer Center／Graduate School of Frontier Sciences, the University of Tokyo) [Co-author]Hiroki Takashima (Division of Developmental Therapeutics, National Cancer Center)、Masahiro Yasunaga (Division of Developmental Therapeutics, National Cancer Center)

Central nervous system (CNS) infiltration is a major cause of relapse and poor prognosis in acute lymphoblastic leukemia (ALL). Intrathecal (IT) chemotherapy is the standard prophylaxis, but it is invasive and neurotoxic. Safer systemic strategies are urgently needed. We found that leukemic cells spread to the CNS via cerebrospinal fluid (CSF), first infiltrating the spinal cord, then the brain. This process is driven by high interleukin-7 receptor (IL-7R) expression, commonly seen in ALL. To exploit this, we developed an IL-7R-directed antibody-drug conjugate (ADC) carrying a pyrrolobenzodiazepine (PBD) payload. Without IT treatment, systemic intravenous delivery of the ADC eradicated CNS disease in ALL xenograft models, including leptomeningeal and parenchymal lesions. Pharmacokinetic analysis showed effective CNS penetration with minimal off-target toxicity due to selective IL-7R expression. The ADC also reduced leukemic burden in the CSF, supporting the CSF-mediated dissemination model. This study identifies a novel route of CNS invasion and offers a non-invasive, targeted therapy with strong translational potential for improving CNS control in high-risk ALL.

[Disclosure of COI by Responsible researcher] Lead presenter is not the Responsible researcher.
[Lead presenter: Motochika Hamada] ■Profit of stock: 株式会社凜研究所　■Research expenses: 第一三共株式会社、味の素株式会社、シスメックス株式会社、H.U.グループホールディングス、株式会社同仁グループ
[Principal Researcher: Masahiro Yasunaga] ■Profit of stock: 株式会社凜研究所　■Research expenses: 第一三共株式会社、味の素株式会社、シスメックス株式会社、H.U.グループホールディングス、株式会社同仁グループ

### [J-2029] I-J14-1
### Drug delivery system (1)

2025 / 9 / 26 (Fri) 9:00-10:15 Room 14/(ANA Crowne Plaza Hotel Kanazawa 3F Zuiun 1) Room 14

[Chairperson]Tetsuya Koama (Tohoku Univ. Grad School Biomed Eng)

---

### J-2029
### Mitochondria-targeted nanocapsules enhance photodynamic therapy efficacy by improving photosensitizer encapsulation

[Speaker]Yumi Kimura (Faculty of Pharm. Sci., Hokkaido Univ.) [Co-author]Yuta Takano (Res. Inst. for Electronic Sci., Hokkaido Univ.)、Yusuke Sato (Faculty of Pharm. Sci., Hokkaido Univ.)、Yuma Yamada (Faculty of Pharm. Sci., Hokkaido Univ.)

Photodynamic therapy (PDT) is a non-invasive therapeutic strategy used in the treatment of cancers. To enhance the efficacy of PDT and reduce its side effects, we have developed a drug delivery system targeting mitochondria as a major source of reactive oxygen species. We have succeeded in preparing MITO-Porter (MP), a mitochondria-targeted nanoparticles, encapsulating a photosensitizer rTPA. However, a key challenge remains that nanoparticles with higher rTPA loading exhibited reduced potency due to aggregation. In this study, we report the results of our efforts to prepare rTPA-encapsulated MP containing CAOM, a solubilizer we developed, aiming to inhibit rTPA aggregation and improve potency. Based on the evaluation of singlet oxygen generation and cancer-killing ability using HeLa cells, the inclusion of CAOM enabled higher potency with increasing rTPA content. Furthermore, CAOM was suggested to inhibit the aggregation of rTPA. These findings suggest that co-encapsulation of CAOM with photosensitizers in nanoparticles may represent a promising strategy to fully realize the therapeutic potential of conventional PDT agents.

[Disclosure of COI by Responsible researcher] Lead presenter is not the Responsible researcher.
[Lead presenter: Yumi Kimura] We have no financial relationships to disclose.
[Principal Researcher: Yuma Yamada] We have no financial relationships to disclose.

### [J-2030] I-J14-1
### Drug delivery system (1)

2025 / 9 / 26 (Fri) 9:00-10:15 Room 14/(ANA Crowne Plaza Hotel Kanazawa 3F Zuiun 1) Room 14

[Chairperson]Tetsuya Koama (Tohoku Univ. Grad School Biomed Eng)

---

### J-2030
### A multicenter phase II trial of CED of ACNU in children with DIPG: Comparison with other studies

[Speaker]Ryuta Saito (Department of Neurosurgery, Nagoya University Graduate School of Medicine) [Co-author]Fumiharu Ohka (Department of Neurosurgery, Nagoya University Graduate School of Medicine)、Masayuki Kanamori (Department of Neurosurgery, Tohoku University Graduate School of Medicine)、Yoshiki Arakawa (Department of Neurosurgery, Kyoto University Graduate School of Medicine)、Yohei Mineharu (Department of Neurosurgery, Kyoto University Graduate School of Medicine)、Yasuo Aihara (Department of Neurosurgery, Tokyo Women's Medical University)、Kentaro Chiba (Department of Neurosurgery, Tokyo Women's Medical University)、Toshihiro Kumabe (Department of Neurosurgery, Kitasato University School of Medicine)、Ichiyo Shibahara (Department of Neurosurgery, Kitasato University School of Medicine)、Yukihiko Sonoda (Department of Neurosurgery, Yamagata University)、Kenichiro Matsuda (Department of Neurosurgery, Yamagata University)、Manabu Kinoshita (Department of Neurosurgery, Asahikawa Medical University)、Aya Sato (Department of Neurosurgery, Nagoya University Graduate School of Medicine)、Fumiaki Takahashi (Department of Information Science, Iwate Medical University)、Teiji Tominaga (Department of Neurosurgery, Tohoku University Graduate School of Medicine)

Diffuse intrinsic pontine glioma (DIPG) is a challenging-to-treat pediatric malignant tumor, with a median survival time (MST) of less than 12 months. Convection-enhanced delivery (CED) allows for direct drug administration into the tumor site, showing potential as a novel therapy. Recently, we reported the result of our study that evaluated the efficacy of CED of nimustine hydrochloride (ACNU) in children with DIPG (Saito R, et al. Cancer Sci 2025). This phase 2, single-arm, multicenter study enrolled patients aged 3-21 years and diagnosed with DIPG. As a result, the 1-year survival rate from the start of radiotherapy was 60%, and the MST was 15 months. In this study, we compared the results with Japanese historical controls as well as international registries. Reported MST in Japanese historical control was 11 months, and in two international registries were 11.2 months and 11 months. In addition, we examined the previously reported prognostic factors for brainstem gliomas. No obvious differences in prognostic factors were found. Therefore, we concluded that the CED of ACNU in the brainstem of children with DIPG after radiotherapy appears to be an effective therapeutic strategy.

[Disclosure of COI by Responsible researcher] Lead presenter is the Responsible researcher.
[Lead presenter: Ryuta Saito] ■Lecture fee: 第一三共、エーザイ

### [E-2031] II-E3
### Hepatobiliary & pancreatic cancers

2025 / 9 / 26 (Fri) 9:00-10:15 Room 15/(ANA Crowne Plaza Hotel Kanazawa 3F Zuiun 2) Room 15

[Chairperson]Hideaki Ijichi (Clin. Nutr. Ctr., The Univ. of Tokyo Hosp.)

### E-2031
### Withdrawn

No Abstract

▲TOP

### [E-2032] II-E3
### Hepatobiliary & pancreatic cancers

2025 / 9 / 26 (Fri) 9:00-10:15 Room 15/(ANA Crowne Plaza Hotel Kanazawa 3F Zuiun 2) Room 15

[Chairperson]Hideaki Ijichi (Clin. Nutr. Ctr., The Univ. of Tokyo Hosp.)

### E-2032
### Withdrawn

No Abstract

### [E-2033] II-E3
### Hepatobiliary & pancreatic cancers

2025 / 9 / 26 (Fri) 9:00-10:15 Room 15/(ANA Crowne Plaza Hotel Kanazawa 3F Zuiun 2) Room 15

[Chairperson]Hideaki Ijichi (Clin. Nutr. Ctr., The Univ. of Tokyo Hosp.)

---

### E-2033
### Functional characterization of ATM variants of uncertain significance in patients with pancreatic cancer

[Speaker]Nicholas J. Roberts (Dept. of Pathology, The Johns Hopkins Univ. Sch. of Med.／Dept. of Oncology, The Johns Hopkins Univ. Sch. of Med.) [Co-author]Neha Nanda (Dept. of Pathology, The Johns Hopkins Univ. Sch. of Med.)、Valentyna Kryklyva (Dept. of Pathology, Radboud Univ. Medical Ctr.)、Hirokazu (Johns Hopkins Univ.) Kimura (Dept. of Pathology, The Johns Hopkins Univ. Sch. of Med.)、Tomoki Sugimoto (Dept. of Pathology, The Johns Hopkins Univ. Sch. of Med.)、Christian Gauthier (Dept. of Pathology, The Johns Hopkins Univ. Sch. of Med.)、Michael Skaro (Dept. of Pathology, The Johns Hopkins Univ. Sch. of Med.)、Michael Goggins (Dept. of Pathology, The Johns Hopkins Univ. Sch. of Med.／Dept. of Oncology, The Johns Hopkins Univ. Sch. of Med.／Dept. of Medicine, The Johns Hopkins Univ. Sch. of Med.)、James R. Eshleman (Dept. of Pathology, The Johns Hopkins Univ. Sch. of Med.／Dept. of Oncology, The Johns Hopkins Univ. Sch. of Med.)、Ralph H. Hruban (Dept. of Pathology, The Johns Hopkins Univ. Sch. of Med.／Dept. of Oncology, The Johns Hopkins Univ. Sch. of Med.)、Lodewijk A. A. Brosens (Dept. of Pathology, Radboud Univ. Medical Ctr.／Dept. of Pathology, Univ. Medical Ctr.)、Alison P. Klein (Dept. of Pathology, The Johns Hopkins Univ. Sch. of Med.／Dept. of Oncology, The Johns Hopkins Univ. Sch. of Med.)

Germline genetic testing of patients with pancreatic cancer is becoming more common. Genetic testing, however, identifies variants of uncertain significance (VUSs) that cannot be classified as either pathogenic or benign based on currently available data. VUSs are a significant cause of clinical uncertainty as individuals with a VUS are ineligible for cancer prevention and early detection initiatives. ***ATM*** VUSs are particularly prevalent, being identified in over 12% of patients with familial pancreatic cancer. To reclassify ***ATM*** VUSs, we developed and validated an in vitro functional assay to characterize ***ATM*** variants at the endogenous locus based on response to ionizing radiation. We characterized 35 ***ATM*** VUSs identified in patients with familial pancreatic cancer and found that 12 (34.3%) were functionally deleterious and reclassified as likely pathogenic using American College of Medical Genetics variant interpretation guidelines. We also used our functional classifications to assess performance of variant functional predictors. In conclusion, ***ATM*** VUSs are a frequent, underappreciated cause of inherited risk of pancreatic cancer.

[Disclosure of COI by Responsible researcher] Lead presenter is the Responsible researcher.
[Lead presenter: Nicholas J. Roberts] ■Research expenses: Research work supported by grants from US Deptartment of Defense.

▲TOP

### [E-2034] II-E3
### Hepatobiliary & pancreatic cancers

2025 / 9 / 26 (Fri) 9:00-10:15 Room 15/(ANA Crowne Plaza Hotel Kanazawa 3F Zuiun 2) Room 15

[Chairperson]Hideaki Ijichi (Clin. Nutr. Ctr., The Univ. of Tokyo Hosp.)

---

### E-2034
### Integrative multi-omics profiling of patient-derived cell line to define the network pharmacology for Precision Oncology

[Speaker]Sarinya Kongpetch (Dept. of Pharm., Khon Kaen Univ.／Cholangiocarcinoma Res.Inst.,Khon Kaen Univ.) [Co-author]Rattanaporn Jaidee (Dept. of Pharm., Khon Kaen Univ.／Cholangiocarcinoma Res.Inst.,Khon Kaen Univ.)、Piman Pocasap (Dept. of Pharm., Khon Kaen Univ.／Cholangiocarcinoma Res.Inst.,Khon Kaen Univ.)、Karnchanok Kaimuangpak (Dept. of Pharm., Khon Kaen Univ.／Cholangiocarcinoma Res.Inst.,Khon Kaen Univ.)、Apinya Jusakul (Cholangiocarcinoma Res.Inst.,Khon Kaen Univ.／Ctr.Res.Med.Diagnostic Lab., Khon Kaen Univ.)、Laddawan Senggunprai (Dept. of Pharm., Khon Kaen Univ.／Cholangiocarcinoma Res.Inst.,Khon Kaen Univ.)、Auemduan Prawan (Dept. of Pharm., Khon Kaen Univ.／Cholangiocarcinoma Res.Inst.,Khon Kaen Univ.)

Cancer cell lines serve as a powerful tool for defining the mechanism of carcinogenesis and developing therapeutic interventions to attack cancer vulnerabilities. Here, we present the model of cholangiocarcinoma (CCA), a biliary tract carcinoma with complex and highly heterogeneous malignancy. We established five patient-derived cell lines from CCA patients residing in Northeast Thailand, the endemic area of the liver fluke Opisthorchis viverrini. Those CCA cells were comprehensively genomics-profiling using whole-exome sequencing. The CCA cells exhibited numerous targetable mutations, including ARID1A, BRCA1, PIK3CA, KRAS, and ATR mutations. Then, we utilized synthetic lethality-based therapy to explore the sensitivity of poly (ADP-ribose) polymerase inhibitors (PARPi). CCA cells with BRCA1 deficiency and a high accumulation of epigenetic alterations (e.g., ARID1A) exhibited greater sensitivity to PARPi. Moreover, we performed the GSEA for CCA cells treated with PARPi and found that the enriched pathways were mTOR signaling and oxidative phosphorylation. These preclinical results could be a valuable tool for drug testing and the development of precision medicine in oncology.

[Disclosure of COI by Responsible researcher] Lead presenter is the Responsible researcher.
[Lead presenter: Sarinya Kongpetch] We have no financial relationships to disclose.

▲TOP

### [E-2035] II-E3
### Hepatobiliary & pancreatic cancers

2025 / 9 / 26 (Fri) 9:00-10:15 Room 15/(ANA Crowne Plaza Hotel Kanazawa 3F Zuiun 2) Room 15

[Chairperson]Hideaki Ijichi (Clin. Nutr. Ctr., The Univ. of Tokyo Hosp.)

---

### E-2035
### HER2 Amplification in PBM-Associated Gallbladder Cancer

[Speaker]Ming Zhu (Dept. of Surg., Tohoku Univ. Grad. Sch. of Med.) [Co-author]Daisuke Douchi (Dept. of Surg., Tohoku Univ. Grad. Sch. of Med.)、Mitsuhiro Shimura (Dept. of Surg., Tohoku Univ. Grad. Sch. of Med.)、Shuichi Aoki (Dept. of Surg., Tohoku Univ. Grad. Sch. of Med.)、Masamichi Mizuma (Dept. of Surg., Tohoku Univ. Grad. Sch. of Med.)、Michiaki Unno (Dept. of Surg., Tohoku Univ. Grad. Sch. of Med.)

Background: Pancreaticobiliary maljunction (PBM) contributes to gallbladder cancer (GBC) development, but its molecular basis remains unclear. This study investigated ERBB2-related oncogenesis and therapeutic potential in PBM-associated GBC. Methods: Among 107 patients, four groups were defined: A (normal), B (PBM without GBC), C (GBC without PBM), and D (GBC with PBM). Group D underwent whole-exome sequencing (WES). HER2 immunohistochemistry (IHC) was performed for all, with FISH used for equivocal IHC cases. Results: ERBB2 amplification was found in 43.8% (7/16) of Group D. HER2 expression (IHC>2+) was significantly higher in Group D (52.9%) than in other groups (0% in A/B, 17.0% in C; P<0.0001). HER2 positivity confirmed by IHC or FISH was also more frequent in Group D (35.3%) vs. Group C (13.0%) (P=0.0459). Conclusion: This study is the first to comprehensively evaluate HER2/ERBB2 expression in PBM-associated GBC through WES, IHC, and FISH. The significantly increased ERBB2 expression, driven by the synergistic interplay between GBC and PBM, underscores a critical molecular interaction to inform targeted therapeutic strategies.

[Disclosure of COI by Responsible researcher] Lead presenter is not the Responsible researcher.
[Lead presenter: Ming Zhu] We have no financial relationships to disclose.
[Principal Researcher: Daisuke Douchi] We have no financial relationships to disclose.

### [E-2036] II-E3
### Hepatobiliary & pancreatic cancers

2025 / 9 / 26 (Fri) 9:00-10:15 Room 15/(ANA Crowne Plaza Hotel Kanazawa 3F Zuiun 2) Room 15

[Chairperson]Hideaki Ijichi (Clin. Nutr. Ctr., The Univ. of Tokyo Hosp.)

### E-2036
### Diagnostic and Prognostic Utility of Bile-to-Serum exosomal miR-21-5p Ratio in Biliary Tract Cancer

[Speaker]Michihiro Yoshida (Nagoya City Univ., Sch. Med. Sci., Gastroenterology and Metabolism) [Co-author]Tadashi Toyohara (Nagoya City Univ., Sch. Med. Sci., Gastroenterology and Metabolism)、Akihisa Kato (Nagoya City Univ., Sch. Med. Sci., Gastroenterology and Metabolism)、Kenji Urakabe (Nagoya City Univ., Sch. Med. Sci., Gastroenterology and Metabolism)、Yasuhito Tanaka (Kumamoto Univ., Faculty of Life Sci., Gastroenterology and Hepatology)、Hiromi Kataoka (Nagoya City Univ., Sch. Med. Sci., Gastroenterology and Metabolism)

To address the challenge of standardization in miRNA analysis using bile-based liquid biopsy, a key step toward clinical application, this study examined the bile-to-serum exosomal expression ratio (B/S Ratio) of miR-21-5p as a potential biomarker for biliary tract cancer (BTC). Bile samples were obtained via initial ERCP from 55 BTC patients and 55 with benign biliary diseases (CTRL). miR-21-5p expression in bile and serum exosomes was quantified by qRT-PCR under standardized conditions, with normalization using Spike-In controls. The B/S Ratio was significantly higher in BTC than in CTRL (median: 12.26 vs 1.35; $p<0.0001$). ROC analysis showed high diagnostic performance (AUC: 0.870, cut-off: 2.040), exceeding CA19-9 (0.793) and serum miR-21-5p (0.628). Cox proportional hazards models adjusted for clinical variables identified B/S Ratio>13 as an independent predictor of poor prognosis (HR: 2.554, $p = 0.008$). These findings support B/S Ratio as a robust method for bile-derived miRNA analysis using serum as a reference, and demonstrate the potential of endoscopic bile sampling as a novel liquid biopsy for BTC diagnosis and prognosis.

[Disclosure of COI by Responsible researcher] Lead presenter is the Responsible researcher.
[Lead presenter: Michihiro Yoshida] We have no financial relationships to disclose.

### [J-2037] I-J2
### Patient-derived experimental models

2025 / 9 / 26 (Fri) 9:00-10:15 Room 16/(11F Visage) Room 16

[Chairperson]Kazuhiro Murakami (Div. of Epithelial Stem Cell Biol., Cancer Res. Inst, Kanazawa Univ.)

---

### J-2037
### Organoid library analysis of human esophageal squamous cell carcinoma reveals features of chemotherapy resistance.

[Speaker]Taku Sato (Nippon Medical School, Dept. Biochem. & Mol. Biol.) [Co-author]Shunsaku Nakagawa (Inst. Science Tokyo, Dept. Gastrointestinal Surg.)、Toshiaki Ohteki (Inst. Science Tokyo, Med. Res. Inst., Dept. Biodefence Res.)

Esophageal squamous cell carcinoma (ESCC) is a deadly cancer with a poor prognosis and a high recurrence rate after chemotherapy, posing a significant clinical challenge. To elucidate the molecular basis of chemotherapy (chemo)-resistance, we established an ESCC organoid (ESCCO) library from 24 ESCC patients of various stages, ages, and treatments. These ESCCOs faithfully recapitulate the oncogenic mutations observed in the original ESCC tissues and manifest tumorigenic properties when xenografted. The ESCCOs respond differently to cisplatin and 5-fluorouracil, chemotherapeutic agents commonly used to treat ESCC patients, with 7 ESCCOs exhibiting potent chemo-resistance. Notably, the chemo-resistant ESCCOs show higher genes involved in antioxidant stress response pathways and more accessible chromatin at their loci than the sensitive ESCCOs. These genes can serve as valuable biomarkers to stratify chemo-resistant ESCCs in histopathological specimens. Our human ESCCO model offers novel insights into the mechanism of chemo-resistance in ESCCs, which is critical for the development of effective therapeutic approaches to eradicate the recurrence of ESCCs. (Commun Biol. 2025,8:507.)

[Disclosure of COI by Responsible researcher] Lead presenter is not the Responsible researcher.
[Lead presenter: Taku Sato] We have no financial relationships to disclose.
[Principal Researcher: Toshiaki Ohteki] We have no financial relationships to disclose.

▲TOP

### [J-2038] I-J2
### Patient-derived experimental models

2025 / 9 / 26 (Fri) 9:00-10:15 Room 16/(11F Visage) Room 16

[Chairperson]Kazuhiro Murakami (Div. of Epithelial Stem Cell Biol., Cancer Res. Inst, Kanazawa Univ.)

---

### J-2038
### Establishment and characterization of novel patient-derived esophageal tumoroids with long-term cultivability

[Speaker]Takashi Urano (Department of General Surgery, Kawasaki Medical School) [Co-author]Etsuko Yokota (Department of General Surgery, Kawasaki Medical School)、Miki Iwai (General Medical Center Research Unit, , Kawasaki Medical School)、Takuro Yukawa (Department of General Surgery, Kawasaki Medical School)、Nagio Takigawa (Department of General Internal Medicine 4, Kawasaki Medical School)、Hideyo Fujiwara (Department of Pathology, Kawasaki Medical School)、Takashi Akiyama (Department of Pathology, Kawasaki Medical School)、Minoru Haisa (Kawasaki Medical School General Medical Center)、Yoshio Naomoto (Department of General Surgery, Kawasaki Medical School)、Takuya Fukazawa (Department of General Surgery, Kawasaki Medical School)、Tomoki Yamatsuji (Department of General Surgery, Kawasaki Medical School)

Esophageal cancer is an aggressive and fatal disease classified into two distinct histologic types, esophageal squamous cell carcinoma (ESCC) and esophageal adenocarcinoma (EAC). To develop novel therapeutic strategies, it is important to establish preclinical models of esophageal cancer. In this study, we successfully established three types of human esophageal cancer organoids (tumoroids) from surgical specimens for long-term culture. Two of the tumoroids were derived from ESCC and one from EAC, which arose from Barrett's esophagus. In addition to whole-exome analysis, copy number and immunohistochemical analyses demonstrated HER2 expression and amplification as well as HER3 expression and mutation in esophageal tumoroids derived from adenocarcinoma in Barrett's esophagus. HER2-targeting antibody-drug conjugates, trastuzumab deruxtecan and patritumab deruxtecan, effectively suppressed tumor viability. These findings indicate that the establishment of esophageal tumoroids with long-term cultivability makes it possible to obtain reproducible basic data, including drug sensitivity studies, which are important for the development of personalized therapies and treatment strategies.

[Disclosure of COI by Responsible researcher] Lead presenter is not the Responsible researcher.
[Lead presenter: Takashi Urano] We have no financial relationships to disclose.
[Principal Researcher: Takuya Fukazawa] We have no financial relationships to disclose.

### [J-2039] I-J2
### Patient-derived experimental models

2025 / 9 / 26 (Fri) 9:00-10:15 Room 16/(11F Visage) Room 16

[Chairperson]Kazuhiro Murakami (Div. of Epithelial Stem Cell Biol., Cancer Res. Inst, Kanazawa Univ.)

---

### J-2039
### Prediction of therapeutic Response Using ESCC Organoids and Recurrence Risk Assessment by MDR1 Expression Analysis

[Speaker]Yasushige Takeda (Dept. of Gastroenterological Surg., Okayama Univ.b) [Co-author]Kazuhiro Noma (Dept. of Gastroenterological Surg., Okayama Univ.b)、Hajime Kashima (Dept. of Gastroenterological Surg., Okayama Univ.b)、Akito Shimizu (Dept. of Gastroenterological Surg., Okayama Univ.b)、Tasuku Matsumoto (Dept. of Gastroenterological Surg., Okayama Univ.b)、Seitaro Nishimura (Dept. of Gastroenterological Surg., Okayama Univ.b)、Tatsuya Takahashi (Dept. of Gastroenterological Surg., Okayama Univ.b)、Hijiri Matsumoto (Dept. of Gastroenterological Surg., Okayama Univ.b)、Kento Kawasaki (Herbert Irving Comprehensive Cancer Research Center, New York, NY)、Tomoyoshi Kunitomo (Dept. of Gastroenterological Surg., Okayama Univ.b)、Satoru Kikuchi (Dept. of Gastroenterological Surg., Okayama Univ.b)、Toshiaki Ohara (Dept. of Gastroenterological Surg., Okayama Univ.b)、Hiroshi Tazawa (Dept. of Gastroenterological Surg., Okayama Univ.b)、Hiroshi Nakagawa (Herbert Irving Comprehensive Cancer Research Center, New York, NY)、Toshiyoshi Fujiwara (Dept. of Gastroenterological Surg., Okayama Univ.b)

Background: Esophageal squamous cell carcinoma (ESCC) often resists neoadjuvant therapy plus surgery, resulting in poor outcomes. Three-dimensional patient-derived organoids (3D-PDOs) are emerging as tumor models to assess tumor heterogeneity.Methods: We established 3D patient-derived organoids (3D-PDOs) from pre-treatment ESCC biopsies to assess morphology and drug sensitivity. We also performed immunohistochemistry for MDR1 on 78 ESCC biopsies from DCF-treated surgical patients, correlating expression with recurrence-free and overall survival.Results: ESCC 3D-PDOs formed irregular structures versus smooth spheroids in normal organoids. IHC on PDOs matched original tumor patterns. In two cases, PDO drug responses mirrored clinical outcomes. MDR1 expression varied among PDOs and was associated with chemoresistance. In biopsies, MDR1-positive patients (n=65) had shorter recurrence-free survival than MDR1-negatives (n=13); 5-year overall survival was similar.Conclusion: ESCC 3D-PDOs capture tumor heterogeneity and resistance. MDR1 analysis may predict recurrence risk and inform personalized therapy.

[Disclosure of COI by Responsible researcher] Lead presenter is not the Responsible researcher.
[Lead presenter: Yasushige Takeda] We have no financial relationships to disclose.
[Principal Researcher: Toshiyoshi Fujiwara] We have no financial relationships to disclose.

### [J-2040] I-J2
### Patient-derived experimental models

2025 / 9 / 26 (Fri) 9:00-10:15 Room 16/(11F Visage) Room 16

[Chairperson]Kazuhiro Murakami (Div. of Epithelial Stem Cell Biol., Cancer Res. Inst, Kanazawa Univ.)

---

### J-2040
### Organoid-Based Evaluation of Therapeutic Responses in Atypical Choroid Plexus Papilloma

[Speaker]Sachi Maeda (Dept. of Neurosurgery, Nagoya Univ. Grad. Sch. of Med.) [Co-author]Fumiharu Ohka (Dept. of Neurosurgery, Nagoya Univ. Grad. Sch. of Med.)、Kazuhito Takeuchi (Dept. of Neurosurgery, Nagoya Univ. Grad. Sch. of Med.)、Yuichi Nagata (Dept. of Neurosurgery, Nagoya Univ. Grad. Sch. of Med.)、Kosuke Aoki (Dept. of Neurosurgery, Nagoya Univ. Grad. Sch. of Med.)、Ryuta Saito (Dept. of Neurosurgery, Nagoya Univ. Grad. Sch. of Med.)

Atypical choroid plexus papilloma (aCPP) is a pediatric central nervous system tumor prone to recurrence and dissemination. Due to limited cases and models, its molecular basis remains poorly understood, although Notch and SHH pathways are dysregulated. We developed patient-derived organoids to assess their response to the SHH inhibitor for potential therapeutic use.Organoids were successfully generated from surgical specimens of two aCPP patients. Tissue fragments were embedded in Matrigel and cultured under defined conditions. Histological analysis revealed that the organoids retained key morphological features and expressed charachteristic markers of choroid plexus tumors. Molecular profiling by whole-exome and RNA sequencing demonstrated similar profilings in the organoids with those of their corresponding parental tumors. Upon administration of vismodegib, both organoid models exhibited a significant decrease in proliferation ($P<0.05$), accompanied by downregulation of SHH-associated genes.These organoids reproduce the molecular and histological characteristics of aCPP and offer a robust system for investigating pathogenesis and assessing therapeutic efficacy.

[Disclosure of COI by Responsible researcher] Lead presenter is the Responsible researcher.
[Lead presenter: Sachi Maeda] We have no financial relationships to disclose.

### [J-2041] I-J2
### Patient-derived experimental models

2025 / 9 / 26 (Fri) 9:00-10:15 Room 16/(11F Visage) Room 16

[Chairperson]Kazuhiro Murakami (Div. of Epithelial Stem Cell Biol., Cancer Res. Inst, Kanazawa Univ.)

---

### J-2041
### TFRC inhibitor antibody drugs have anti-tumor effects on metastatic liver tumor derived from colorectal cancer

[Speaker]Mizuki Endo (Dept. of Gene. Surg. Sci., Grad. Sch. Med., Gunma Univ.) [Co-author]Takehiko Yokobori (Initiative for advanced Res., Gunma Univ.)、Omar S. Ibrahim (Dept. of Gene. Surg. Sci., Grad. Sch. Med., Gunma Univ.)、Dorjkhorloo Gendensuren (Dept. of Gene. Surg. Sci., Grad. Sch. Med., Gunma Univ.)、Haruka Okami (Initiative for advanced Res., Gunma Univ.)、Gombodorj Navchaa (Initiative for advanced Res., Gunma Univ.)、Bilguun O. Erkhem (Initiative for advanced Res., Gunma Univ.)、Chika Katayama (Dept. of Gene. Surg. Sci., Grad. Sch. Med., Gunma Univ.)、Yuta Shibasaki (Dept. of Gene. Surg. Sci., Grad. Sch. Med., Gunma Univ.)、Chika Komine (Dept. of Gene. Surg. Sci., Grad. Sch. Med., Gunma Univ.)、Takuhisa Okada (Dept. of Gene. Surg. Sci., Grad. Sch. Med., Gunma Univ.)、Takuya Shiraishi (Dept. of Gene. Surg. Sci., Grad. Sch. Med., Gunma Univ.)、Akihiko Sano (Dept. of Gene. Surg. Sci., Grad. Sch. Med., Gunma Univ.)、Makoto Sakai (Dept. of Gene. Surg. Sci., Grad. Sch. Med., Gunma Univ.)、Ken Shirabe (Dept. of Gene. Surg. Sci., Grad. Sch. Med., Gunma Univ.)、Hiroshi Saeki (Dept. of Gene. Surg. Sci., Grad. Sch. Med., Gunma Univ.)

**Background:** The transferrin receptor (TFRC) is involved in iron uptake into cells, and its expression reportedly increases in cancerous tissues as cancer progresses.**Methods:** We analyzed the relationship between TFRC expression and clinicopathological factors in 77 pT4 colorectal cancer (CRC) patients who underwent curative resection. We examined the antitumor effects of TFRC-inhibiting antibody drugs in patient-derived xenograft (PDX) mouse models using metastatic liver tumors from rectal cancer patients and in liver metastasis mouse models using human CRC cell lines.**Results:** TFRC was expressed mainly on the cancer cell membrane in 76 cases. In the strongly positive group (n=61), there were significantly more distant metastases at the time of surgery (p=0.0341), especially liver metastases. In PDX model mice, the drug significantly reduced tumor size (p=0.0085) and Ki67 expression (p=0.0367). In liver metastasis model mice, pretreatment with the drug significantly suppressed the number of liver metastasis (p=0.0081).**Discussion:** TFRC may be a biomarker for distant metastasis, and inhibitory antibody drugs are expected to be a new treatment for CRC.

[Disclosure of COI by Responsible researcher] Lead presenter is not the Responsible researcher.
[Lead presenter: Mizuki Endo] We have no financial relationships to disclose.
[Principal Researcher: Takehiko Yokobori] ■Research funds from company: 株式会社ペルセウスプロテオミクス

### [J-2042] I-J2
### Patient-derived experimental models

2025 / 9 / 26 (Fri) 9:00-10:15 Room 16/(11F Visage) Room 16

[Chairperson]Kazuhiro Murakami (Div. of Epithelial Stem Cell Biol., Cancer Res. Inst, Kanazawa Univ.)

---

### J-2042
### Establishment of a high-risk AML cell line YCU-AML2 harboring ***KMT2A::MLLT3*** fusion and ***MECOM*** overexpression

[Speaker]Junji Ikeda (Dept. Pediatrics, Yokohama City Univ.) [Co-author]Norio Shiba (Dept. Pediatrics, Yokohama City Univ.)、Shota Kato (Dept. Pediatrics, The Univ. Tokyo)、Hiroyoshi Kunimoto (Dept. Hematol, Yokohama City Univ.)、Yusuke Saito (Div. Clin. Cancer Genomics, Hokkaido Univ.)、Maiko Sagisaka (Clin. Res. Inst., Kanagawa Children's Med. Ctr.)、Mieko Ito (Clin. Res. Inst., Kanagawa Children's Med. Ctr.)、Hiroaki Goto (Div. Hematol／Oncol, Kanagawa Children's Med. Ctr.)、Yusuke Okuno (Dept. Virol, Nagoya City Univ.)、Wataru Nakamura (Dept. Pediatrics, Yokohama City Univ.)、Masahiro Yoshitomi (Dept. Pediatrics, Yokohama City Univ.)、Masanobu Takeuchi (Dept. Pediatrics, Yokohama City Univ.)、Hideaki Nakajima (Dept. Hematol, Yokohama City Univ.)、Motohiro Kato (Dept. Pediatrics, The Univ. Tokyo)、Shinichi Tsujimoto (Dept. Pediatrics, Yokohama City Univ.)

The prognosis of acute myeloid leukemia (AML) with ***KMT2A::MLLT3*** rearrangement and ***MECOM*** overexpression is dismal, and the optimal treatment strategy remains unclear. However, a suitable model (such as a cell line or its xenograft model) for research on this subtype has not been established. We established a novel AML cell line, YCU-AML2, and its xenograft model harboring ***KMT2A::MLLT3*** rearrangement and ***MECOM*** overexpression. YCU-AML2 xenograft mice model developed AML and mimicked the clinical phenotype of the original patient. Intriguingly, YCU-AML2 exhibited high sensitivity to L-asparaginase (L-asp) with glutaminase activity. In line with a previous report (Saito et al. Haematologica, 2020), YCU-AML2 cells treated with L-asp exhibited much lower glutamine levels, basal and maximum oxygen consumption rates compared to non-treated cells, suggesting that YCU-AML2 is sensitive to L-asp due to the inhibition of oxidative phosphorylation activation via glutaminolysis. YCU-AML2 cell line and its xenograft model can serve as models to explore the molecular pathogenesis and develop new treatment strategies for high-risk AML with ***KMT2A::MLLT3*** rearrangement and ***MECOM*** overexpression.

[Disclosure of COI by Responsible researcher] Lead presenter is not the Responsible researcher.
[Lead presenter: Junji Ikeda] We have no financial relationships to disclose.
[Principal Researcher: Shinichi Tsujimoto] We have no financial relationships to disclose.

### [J-2001] I-J6
### DNA damage response & genomic instability

2025 / 9 / 26 (Fri) 10:15-11:30 Room 6/(Hotel Nikko Kanazawa 3F Kujaku A) Room 6

[Chairperson]Minji Jo (Div. Exp. Pathol., Cancer Inst., JFCR)

---

### J-2001
### Analysis of genomic instability and aging-inducing effects of glucocorticoids

[Speaker]Noriyuki Okudaira (Dept. Biochem. Med. Teikyo. Univ.) [Co-author]Miho Akimoto (Dept. Biochem. Med. Teikyo. Univ.)、Mimi Adachi (Dept. Biochem. Med. Teikyo. Univ.)

Glucocorticoid (GC), which regulates homeostasis, is a steroid hormone secreted by the zona fasciculata (zF) adrenal cortex. Negative feedback of the hypothalamic-pituitary-adrenal (HPA) axis allows for precise control of secretion and diurnal variation, including regulation of metabolism and immune response. We found that GC is a high secretion throughout the day in aged mice. The senescence-associated secretory phenotype (SASP) factor IL1β is secreted from senescence-zF-cells and acts on surrounding cells to promote GC secretion (Okudaira N et al. ***Aging Cell.*** 2024). We also showed that administration of Dexamethasone (Dex), which has similar effects to GC, in mice promotes accumulation of p16-positive cells in the liver and other organs, suggesting that GC has the effect of inducing senescence. Here we demonstrate that Dex treatment increases the frequency of retrotransposition (RTP) of Long Interspersed Element-1 (L1), in vitro. The 80-100 copies of L1, representing 17% of the human genome, are RTP sequences in normal cells. Thus, GCs have SASP-inducing and genomic instability effects associated with senescence and may be involved in the activating effects of cancer cells.

[Disclosure of COI by Responsible researcher] Lead presenter is not the Responsible researcher.
[Lead presenter: Noriyuki Okudaira] ■Research funds from company: 科研費、武田科学振興財団
[Principal Researcher: Mimi Adachi] ■Research funds from company: 科研費

### [J-2002] I-J6
### DNA damage response & genomic instability

2025 / 9 / 26 (Fri) 10:15-11:30 Room 6/(Hotel Nikko Kanazawa 3F Kujaku A) Room 6

[Chairperson]Minji Jo (Div. Exp. Pathol., Cancer Inst., JFCR)

---

### J-2002
### Inactivation of an $O_2/Fe^{2+}$-dependent enzyme causes cell cycle retardation at early S phase under hypoxia

[Speaker]Takakuni Dohkai (Cancer Cell Biol, Grad Sch of Biostudies, Kyoto Univ.) [Co-author]Minoru Kobayashi (Cancer Cell Biol, Grad Sch of Biostudies, Kyoto Univ.／Dept of Genomic Dynamics, Radiation Biology Center, GSB, Kyoto Uni)、Hiroshi Harada (Cancer Cell Biol, Grad Sch of Biostudies, Kyoto Univ.／Dept of Genomic Dynamics, Radiation Biology Center, GSB, Kyoto Uni)

Cancer cells in hypoxic regions of solid tumors delayed cell cycle progression and acquire resistance to chemoradiotherapies. Previous studies have reported that hypoxia induces G1 arrest at the G1 checkpoint. However, since many cancers lack this checkpoint, these studies cannot fully explain how the cell cycle is regulated under hypoxia. Here, through detailed cell cycle analysis, we show that hypoxia does not induce G1 arrest, but rather increases population of early S phase cells. Using a $Fe^{2+}$ chelator deferoxamine (DFO), we found that the accumulation of early S phase cells is due to reduced activity of an $O_2/Fe^{2+}$-dependent enzyme. DNA fiber assay revealed that both hypoxia and DFO limit DNA replication speed. Moreover, RPA2 foci were increased in S phase cells under hypoxia and in the presence of DFO even under normoxia. Our results demonstrate that the increase of early S phase cells under hypoxia is due to impaired DNA replication caused by the loss of activity of an $O_2/Fe^{2+}$-dependent enzyme. We are currently conducting a siRNA library screening based on the number of RPA2 foci in S phase cells to identify the responsible enzyme.

[Disclosure of COI by Responsible researcher] Lead presenter is not the Responsible researcher.
[Lead presenter: Takakuni Dohkai] We have no financial relationships to disclose.
[Principal Researcher: Hiroshi Harada] We have no financial relationships to disclose.

### [J-2003] I-J6
### DNA damage response & genomic instability

2025 / 9 / 26 (Fri) 10:15-11:30 Room 6/(Hotel Nikko Kanazawa 3F Kujaku A) Room 6

[Chairperson]Minji Jo (Div. Exp. Pathol., Cancer Inst., JFCR)

---

### J-2003
### Mechanism of the Selective Cytotoxic Antitumor Effect Induced by the Novel CDK Inhibitor Azalam4

[Speaker]Yoshimi Ohashi (Div. Mol. Pharmacology, Cancer Chemother. Ctr., JFCR) [Co-author]Tsutomu Fukuda (Div. Chemistry & Materials Sci., Grad. Sch. Engineering, Nagasaki Univ.)、Mutsumi Okamura (Div. Mol. Pharmacology, Cancer Chemother. Ctr., JFCR)、Naoyuki Nishiya (Dept. Clin. Pharm., Sch. Pharm., Iwate Med. Univ.)、Shingo Dan (Div. Mol. Pharmacology, Cancer Chemother. Ctr., JFCR)

Azalamellarin 4 (Azalam4) is a novel synthetic compound rationally designed based on the structure of the natural product Lamellarin N. We previously reported that Azalam4 acts as a pan-CDK inhibitor that induces selective cytotoxic effects in four of the JFCR39 cell lines tested, while exerting only cytostatic effects in the remaining cell lines. To investigate the molecular mechanisms underlying this differential response-between cell death and cell cycle arrest-we performed two experiments: RNA-seq-based gene expression profiling and a synthetic lethal screen using a genome-wide shRNA lentiviral library. Among the genes downregulated upon Azalam4 exposure, we identified Mcl-1 as a key mediator of selective cell death, as confirmed by siRNA-mediated knockdown in representative cell lines from both response types. Furthermore, the synthetic lethal screen revealed that knockdown of genes involved in maintaining the G2 phase sensitized cells to Azalam4-induced cell death. Together, these results suggest that G2-arrested cells exhibit a cytostatic response to Azalam4, and that release from G2 arrest can shift the response toward cytotoxicity, particularly when Mcl-1 is downregulated.

[Disclosure of COI by Responsible researcher] Lead presenter is not the Responsible researcher.
[Lead presenter: Yoshimi Ohashi] We have no financial relationships to disclose.
[Principal Researcher: Shingo Dan] We have no financial relationships to disclose.

▲TOP

### [J-2004] I-J6
### DNA damage response & genomic instability

2025 / 9 / 26 (Fri) 10:15-11:30 Room 6/(Hotel Nikko Kanazawa 3F Kujaku A) Room 6

[Chairperson]Minji Jo (Div. Exp. Pathol., Cancer Inst., JFCR)

---

### J-2004
### Cyclin E1 Overexpression Predicts Sensitivity to PLK1 Inhibition in Ovarian Cancer Cells

[Speaker]Akiko Kunita (Next- Generation Precision Medicine Development Laboratory, Grad.Sch.Med., Univ. ／Dept. Pathology., Grad.Sch.Med., Univ. Tokyo) [Co-author]Qian Xi (Integrative Genomics Div., Grad.Sch.Med., Univ. Tokyo)、Miho Ogawa (Next- Generation Precision Medicine Development Laboratory, Grad.Sch.Med., Univ.)、Mirei Ka (Integrative Genomics Div., Grad.Sch.Med., Univ. Tokyo／Dept. Respiratory Medicine, Grad.Sch.Med., Univ. Tokyo)、Saeko Nagai (Dept. Obstetrics and Gynecology., Grad.Sch.Med., Univ. Tokyo)、Kousuke Watanabe (Next- Generation Precision Medicine Development Laboratory, Grad.Sch.Med., Univ.)、Kenbun Sone (Dept. Obstetrics and Gynecology., Grad.Sch.Med., Univ. Tokyo)、Aya Ushiku (Dept. Pathology., Grad.Sch.Med., Univ. Tokyo)、Tetsuo Ushiku (Dept. Pathology., Grad.Sch.Med., Univ. Tokyo)、Yasushi Hirota (Dept. Obstetrics and Gynecology., Grad.Sch.Med., Univ. Tokyo)、Kazuhiro Katayama (Molecular Targeted Therapeutics, School of Pharmacy, Nihon University)、Hidenori Kage (Dept. Respiratory Medicine, Grad.Sch.Med., Univ. Tokyo)、Katsutoshi Oda (Integrative Genomics Div., Grad.Sch.Med., Univ. Tokyo)

Cyclin E1 overexpression is associated with poor prognosis and chemoresistance in ovarian cancer. Polo-like kinase 1 (PLK1), a key regulator of mitotic entry, spindle assembly, and chromosome segregation, has emerged as a potential therapeutic target. We investigated the effects of the PLK1 inhibitor volasertib on ovarian cancer cells with varying levels of Cyclin E1 expression. Volasertib significantly reduced cell viability in Cyclin E1-overexpressing cells compared with that in low-expressing cells. Cell cycle analysis revealed G1 phase reduction and G2/M phase accumulation with pronounced M phase arrest in high Cyclin E1 cells. Prolonged volasertib treatment increased the sub-G1 population, indicating enhanced apoptosis. Immunoblotting showed upregulation of cleaved caspase-3, cleaved PARP, and γH2AX in Cyclin E1-high cells following volasertib exposure. ***In vivo***, volasertib suppressed tumor growth in xenograft models derived from Cyclin E1-overexpressing cells. These findings suggest that volasertib selectively induces mitotic catastrophe in Cyclin E1-high ovarian cancer cells, highlighting PLK1 as a potential therapeutic target for this subset.

[Disclosure of COI by Responsible researcher] Lead presenter is not the Responsible researcher.
[Lead presenter: Akiko Kunita] ■Endowed chair: コニカミノルタ株式会社
[Principal Researcher: Katsutoshi Oda] We have no financial relationships to disclose.

### [J-2005] I-J6
### DNA damage response & genomic instability

2025 / 9 / 26 (Fri) 10:15-11:30 Room 6/(Hotel Nikko Kanazawa 3F Kujaku A) Room 6

[Chairperson]Minji Jo (Div. Exp. Pathol., Cancer Inst., JFCR)

---

### J-2005
### VCP contributes to DNA-PK-mediated repair of etoposide-induced DNA double-strand breaks

[Speaker]Ryo Sakasai (Biochem. I, Kanazawa Med. Univ.) [Co-author]Yumi Sunatani (Biochem. I, Kanazawa Med. Univ.)、Tadashi Matsui (Biochem. I, Kanazawa Med. Univ.)、Kuniyoshi Iwabuchi (Biochem. I, Kanazawa Med. Univ.)

DNA topoisomerase 2 (Top2) generates DNA double-strand breaks (DSBs) to relieve torsional stress during DNA replication and transcription. Etoposide (ETP), an anticancer drug, inhibits Top2 reactive intermediates, resulting in DSB accumulation. ETP-inhibited Top2 remains bound to DNA at the end of DSB to form Top2 DNA-protein crosslink (Top2-DPC), which is removed by the ubiquitin-proteasome pathway. DSBs are subsequently repaired using non-homologous end-joining (NHEJ). Therefore, we investigated the possibility that inhibition of Top2-DPC resolution affects DSB repair and the cell-killing effect of ETP.Inhibition of proteasome and VCP (p97), a selective chaperone of the proteasome, suppressed ETP-induced activation of DNA-PK, an NHEJ factor. Consistent with this finding, the repair of ETP-induced DSBs was reduced by VCP inhibition. These results suggest that the inhibition of Top2-DPC resolution suppresses DNA-PK-mediated NHEJ. Therefore, we investigated the effect of VCP inhibition on ETP cytotoxicity. Unexpectedly, ETP cytotoxicity was reduced by VCP inhibition, suggesting that VCP is also involved in other cellular pathways, such as the cell death signaling pathway.

[Disclosure of COI by Responsible researcher] Lead presenter is the Responsible researcher.
[Lead presenter: Ryo Sakasai] We have no financial relationships to disclose.

### [J-2006] I-J6
### DNA damage response & genomic instability

2025 / 9 / 26 (Fri) 10:15-11:30 Room 6/(Hotel Nikko Kanazawa 3F Kujaku A) Room 6

[Chairperson]Minji Jo (Div. Exp. Pathol., Cancer Inst., JFCR)

---

### J-2006
### CENP-E Inhibition Induces cGAS-STING Activation to Enhance Cancer Immunotherapy

[Speaker]Akihiro Ohashi (National Cancer Center, Exploratory Oncology Research & Clinical Trial Center) [Co-author]Ryo Kamata (National Cancer Center, Exploratory Oncology Research & Clinical Trial Center)、Hitoshi Saito (National Cancer Center, Exploratory Oncology Research & Clinical Trial Center)、Yumi Hakozaki (National Cancer Center, Exploratory Oncology Research & Clinical Trial Center)、Kumi Kinoshita (National Cancer Center, Exploratory Oncology Research & Clinical Trial Center)、Yukie Kashima (National Cancer Center, Exploratory Oncology Research & Clinical Trial Center)、Gaku Yamamoto (National Cancer Center, Exploratory Oncology Research & Clinical Trial Center)、Tomoko Morita (National Cancer Center, Exploratory Oncology Research & Clinical Trial Center)、Pinyi Lu (Frederick National Laboratory for Cancer Research)

Micronuclei generated by mitotic defects activate the cGAS-STING pathway, a key target for enhancing cancer immunotherapy. We investigated whether CENP-E inhibitors could enhance immune responses and sensitize tumors to immune checkpoint blockade (ICB). Treatment with CENP-E inhibitors significantly increased TBK1 and IRF3 phosphorylation, key markers of cGAS-STING activation, in parallel with micronucleus formation. RNA-seq analysis demonstrated enrichment of innate immune pathways, including cytosolic DNA sensing and cytokine signaling. Reporter assays confirmed robust activation of IRF and NF-kB pathways. Chemical screening identified mTOR inhibitors as potent suppressors of CENP-E inhibitor-induced IRF activation, suggesting potential combinatory strategies. Notably, combining CENP-E inhibition with ICB enhanced antitumor efficacy in mouse models. These findings highlight a novel approach to modulating tumor immunogenicity through mitotic perturbation, offering a promising strategy for cancer immunotherapy.

[Disclosure of COI by Responsible researcher] Lead presenter is the Responsible researcher.
[Lead presenter: Akihiro Ohashi] ■Research expenses: アステラス製薬、武田薬品工業、第一三共

### [J-2007] I-J12-2
### Artificial intelligence based diagnostics

2025 / 9 / 26 (Fri) 10:15-11:30 Room 7/(Hotel Nikko Kanazawa 3F Kujaku B) Room 7

[Chairperson]Shingo Sakashita (Natl.Cancer Ctr. EPOC, Pathology)

---

### J-2007
### Development of a deep learning model "DWI-ResViT" to accelerate acquisition time for Diffusion Kurtosis Imaging.

[Speaker]Yusuke Sakaguchi (Cancer Translational Research Team, RIKEN Center for Advanced Intelligence Project／Department of Neurosurgery, The University of Tokyo／Division of Medical AI Research and Development, National Cancer Center) [Co-author]Satoshi Takahashi (Cancer Translational Research Team, RIKEN Center for Advanced Intelligence Project／Division of Medical AI Research and Development, National Cancer Center)、Naoaki Ishiguro (Cancer Translational Research Team, RIKEN Center for Advanced Intelligence Project)、Yuichi Suzuki (Department of Radiology, The University of Tokyo Hospital)、Hirokazu Takami (Department of Neurosurgery, The University of Tokyo)、Masamichi Takahashi (Department of Neurosurgery, School of Medicine, Tokai University／Department of Neurosurgery and Neuro-Oncology, National Cancer Center Hospital)、Shunsaku Takayanagi (Department of Neuro-Oncology／Neurosurgery, Saitama Medical University International Medical Center)、Mototaka Miyake (Department of Diagnostic Radiology, National Cancer Center Hospital, Tokyo, Japan)、Masashi Nomura (Department of Neurosurgery, The University of Tokyo)、Yosuke Kitagawa (Department of Neurosurgery, The University of Tokyo)、Shota Tanaka (Department of Neurological Surgery, Okayama University Graduate School of Medicine)、Manabu Kinoshita (Department of Neurosurgery, Asahikawa Medical University)、Yoshitaka Narita (Department of Neurosurgery and Neuro-Oncology, National Cancer Center Hospital)、Nobuhito Saito (Department of Neurosurgery, The University of Tokyo)、Ryuji Hamamoto (Cancer Translational Research Team, RIKEN Center for Advanced Intelligence Project／Division of Medical AI Research and Development, National Cancer Center)

Diffusion Kurtosis Imaging (DKI)-derived Mean Kurtosis (MK) reflects tissue microstructure and is helpful for differential diagnosis and prognostication of malignant brain tumors, but requires multiple ***b***-values (>15 directions), prolonging scans and limiting clinical use. To address this, we developed "DWI-ResViT," a deep learning model that reconstructs DWI ***b***=2000 from ***b***=0 and 1000 (30 directions), aiming to reduce scan time while preserving image quality. We used 5-fold cross-validation on 157 DKI cases (2016-2024). Reconstruction accuracy was evaluated using structural similarity (SSIM) and peak signal-to-noise ratio (PSNR) between real and reconstructed ***b***=2000 images and MK maps. MK was compared between high-grade gliomas (HGG; n=67) and primary CNS lymphomas (PCNSL; n=15) via ROC analysis. Reconstructed ***b***=2000 images showed high similarity to actual images (PSNR 42.13±2.08 dB, SSIM 0.97±0.01). MK metrics were 37.80±4.11 dB and SSIM 0.96±0.02. MK was significantly lower in HGG than PCNSL (***p***<0.001, AUROC=0.79). DWI-ResViT accurately reconstructs ***b***=2000 images from ***b***=0 and ***b***=1000, suggesting its potential clinical value for differentiating HGG from PCNSL without additional scans.

[Disclosure of COI by Responsible researcher] Lead presenter is not the Responsible researcher.
[Lead presenter: Yusuke Sakaguchi] We have no financial relationships to disclose.
[Principal Researcher: Ryuji Hamamoto] We have no financial relationships to disclose.

### [J-2008] I-J12-2
### Artificial intelligence based diagnostics

2025 / 9 / 26 (Fri) 10:15-11:30 Room 7/(Hotel Nikko Kanazawa 3F Kujaku B) Room 7

[Chairperson]Shingo Sakashita (Natl.Cancer Ctr. EPOC, Pathology)

---

### J-2008
### Multimodal AI with CTC images and clinical information for postoperative prognosis prediction in colorectal cancer

[Speaker]Naoaki Harada (NCC Cancer Sci., Grad. Sch., Inst. Sci. Tokyo／Div. Med. AI Res. Dev., Natl. Cancer Ctr. Res. Inst.) [Co-author]Yudai Kumamoto (Dept. Colorectal Surg., Natl. Cancer Ctr. Hosp.)、Masaaki Komatsu (Div. Med. AI Res. Dev., Natl. Cancer Ctr. Res. Inst.／Cancer Transl. Res. Team, RIKEN Ctr. for AIP project)、Naoki Teraya (Div. Med. AI Res. Dev., Natl. Cancer Ctr. Res. Inst.／Dept. Obstet. Gynecol, Showa Med. Univ. Sch. Med.)、Katsuji Takeda (Cancer Transl. Res. Team, RIKEN Ctr. for AIP project)、Nobuji Kouno (Div. Med. AI Res. Dev., Natl. Cancer Ctr. Res. Inst.／Dept. GI Surg., Kyoto Univ., Sch. Med..)、Mototaka Miyake (Dept. Diagnostic Radiology, Natl. Cancer Ctr. Hosp.)、Yasuyuki Takamizawa (Dept. Colorectal Surg., Natl. Cancer Ctr. Hosp.)、Yukihide Kanemitsu (Dept. Colorectal Surg., Natl. Cancer Ctr. Hosp.)、Ryuji Hamamoto (NCC Cancer Sci., Grad. Sch., Inst. Sci. Tokyo／Div. Med. AI Res. Dev., Natl. Cancer Ctr. Res. Inst.／Cancer Transl. Res. Team, RIKEN Ctr. for AIP project)

Despite advance in colorectal cancer treatment, it remains difficult to predict early postoperative recurrence. CT colonography (CTC) is a medical imaging technique in which the intestine is inflated with carbon dioxide and CT images are taken. CTC demonstrates high accuracy in localizing colorectal cancer and is utilized in clinical practice as a tool for preoperative planning. Also, since the CT value of carbon dioxide is approximately -1000 HU (almost equivalent to air), it enhances tumor contrast, providing a significant advantage. In this study, we aim to develop a multimodal AI model that integrates preoperative CTC images with clinical data to accurately predict postoperative prognosis, including overall survival, recurrence-free survival, recurrence risk, and lymph node metastasis in colorectal cancer patients. Furthermore, to enhance the interpretability of the AI model, we will assess each input variable's impact on predictions using methods like SHAP. By accurately predicting postoperative prognosis and recurrence risk, we hope to support personalized treatment strategies and improve patient outcomes in colorectal cancer care.

[Disclosure of COI by Responsible researcher] Lead presenter is not the Responsible researcher.
[Lead presenter: Naoaki Harada] ■ employment by a company related to the presentation: 富士通株式会社
[Principal Researcher: Ryuji Hamamoto] We have no financial relationships to disclose.

### [J-2009] I-J12-2
### Artificial intelligence based diagnostics

2025 / 9 / 26 (Fri) 10:15-11:30 Room 7/(Hotel Nikko Kanazawa 3F Kujaku B) Room 7

[Chairperson]Shingo Sakashita (Natl.Cancer Ctr. EPOC, Pathology)

---

### J-2009
### Analysis of the Tumor Immune Microenvironment in Rectal Cancer Using Deep Learning-Based Imaging Cytometry

[Speaker]Yukari Adachi (Div. of Gastrointestinal Surg., Grad. Sch. of Med., Kobe Univ.) [Co-author]Kimihiro Yamashita (Dep. of Biophysics, Grad. Sch. of Health Sci., Kobe Univ.)、Toru Nagasaka (Association of Med. Artificial Intelligence Curation)、Tomoki Abe (Div. of Gastrointestinal Surg., Grad. Sch. of Med., Kobe Univ.)、Masayuki Ando (Div. of Gastrointestinal Surg., Grad. Sch. of Med., Kobe Univ.)、Yasunori Otowa (Div. of Gastrointestinal Surg., Grad. Sch. of Med., Kobe Univ.)、Hitoshi Harada (Div. of Gastrointestinal Surg., Grad. Sch. of Med., Kobe Univ.)、Naoki Urakawa (Div. of Gastrointestinal Surg., Grad. Sch. of Med., Kobe Univ.)、Hironobu Goto (Div. of Gastrointestinal Surg., Grad. Sch. of Med., Kobe Univ.)、Hiroshi Hasegawa (Div. of Gastrointestinal Surg., Grad. Sch. of Med., Kobe Univ.)、Shingo Kanaji (Div. of Gastrointestinal Surg., Grad. Sch. of Med., Kobe Univ.)、Takeru Matsuda (Div. of Gastrointestinal Surg., Grad. Sch. of Med., Kobe Univ.)、Yoshihiro Kakeji (Div. of Gastrointestinal Surg., Grad. Sch. of Med., Kobe Univ.)

BackgroundNeoadjuvant chemoradiotherapy (NACRT) is a key therapeutic strategy for rectal cancer. In the tumor immune microenvironment, increased infiltration CD8+ T cells after radiation therapy has been associated with favorable prognosis. This study investigates the prognostic significance of CD103+CD8+ T cells in rectal cancer patients treated with NACRT by using Cu-Cyto, our proprietary deep learning-based imaging cytometry platform.MethodWe retrospectively analyzed 40 patients with rectal cancer who underwent surgery after NACRT between 2005 and 2016. Cu-Cyto quantified the density of CD103+CD8+ T cells in the central tumor and stromal regions, and their correlation with recurrence-free survival (RFS) was assessed.ResultsHigh densities of CD103+CD8+ T cells in the stromal region showed significantly higher RFS than those with low densities (67.4% vs. 12.1%, P<0.001). The density of CD103+CD8+ T cell in the central tumor region showed no significant difference in RFS between the high- and low-density groups (37.0% vs. 55.2%, P=0.208).ConclusionCu-Cyto analysis showed that stromal CD103+CD8+ T cell density correlates with improved prognosis in rectal cancer patients after NACRT.

[Disclosure of COI by Responsible researcher] Lead presenter is not the Responsible researcher.
[Lead presenter: Yukari Adachi] We have no financial relationships to disclose.
[Principal Researcher: Toru Nagasaka] We have no financial relationships to disclose.

▲TOP

### [J-2010] I-J12-2
### Artificial intelligence based diagnostics

2025 / 9 / 26 (Fri) 10:15-11:30 Room 7/(Hotel Nikko Kanazawa 3F Kujaku B) Room 7

[Chairperson]Shingo Sakashita (Natl.Cancer Ctr. EPOC, Pathology)

---

### J-2010
### Radiomics-Based Difficulty-Aware Learning Algorithm for Robust Segmentation of Hard-to-Detect Lesions in PET/CT

[Speaker]Hiromi Matsumoto (Cancer Transl. Res. Team, RIKEN Ctr. for AIP Project) [Co-author]Ryota Shibaki (Div. Med. AI Res. Dev., Natl. Cancer Ctr. Res. Inst.)、Kazuma Kobayashi (Cancer Transl. Res. Team, RIKEN Ctr. for AIP Project／Div. Med. AI Res. Dev., Natl. Cancer Ctr. Res. Inst.／Digital Content Media Sci. Res. Div., Natl. Inst. of Informatics)、Ryuji Hamamoto (Cancer Transl. Res. Team, RIKEN Ctr. for AIP Project／Div. Med. AI Res. Dev., Natl. Cancer Ctr. Res. Inst.)

Background: Deep learning in medicine often uses disease-level labels, overlooking disease heterogeneity. This can hinder detection of important but atypical lesions. We propose a difficulty-aware learning algorithm based on Radiomics Features (RFs) to improve segmentation performance for such hard-to-detect lesions.Method: The AutoPET Dataset (1014 PET-CT scans) was split into training (N=710), validation (N=152), and test (N=152). First, based on a 3D U-Net that was trained in a standard manner, lesions with Dice < 0.7 were labeled as detection failures. Then, "hardness" for such a standard model was quantified as the probability of detection failure modeled by logistic regression using RFs of each lesion as predictors. Finally, a difficulty-aware Dice loss, which incorporates the lesion-wise hardness, was used for the model training of our approach.Results: On the test set, Dice improved from 0.677 (standard) to 0.695 (proposed). Average precision for failed lesions also improved from 0.513 to 0.621.Conclusion: The proposed RF-based difficulty-aware algorithm improved segmentation robustness for challenging lesions, showing promise for heterogeneous diseases such as cancer.

[Disclosure of COI by Responsible researcher] Lead presenter is not the Responsible researcher.
[Lead presenter: Hiromi Matsumoto] We have no financial relationships to disclose.
[Principal Researcher: Ryuji Hamamoto] ■Research expenses: 富士フイルム株式会社, 富士通株式会社

▲TOP

### [J-2011] I-J12-2
### Artificial intelligence based diagnostics

2025 / 9 / 26 (Fri) 10:15-11:30 Room 7/(Hotel Nikko Kanazawa 3F Kujaku B) Room 7

[Chairperson]Shingo Sakashita (Natl.Cancer Ctr. EPOC, Pathology)

---

### J-2011
### New method for tumor cytology: AI analysis of white light scattering spectrum

[Speaker]Fuka Takeuchi (Dept. of Path., Faculty of Med. Kindai Univ.) [Co-author]Yuka Tsuri (Div. of Materials Sci., Nara Inst. of Sci. & Tech.)、Man Hagiyama (Dept. of Path., Faculty of Med. Kindai Univ.)、Sachiyo Bando (Dept. of Path., Faculty of Med. Kindai Univ.)、Tomoko Wakasa (Dep. of Diagnostic Path., Kindai Univ. Nara Hosp.)、Yoichiroh Hosokawa (Div. of Materials Sci., Nara Inst. of Sci. & Tech.)、Akihiko Ito (Dept. of Path., Faculty of Med. Kindai Univ.)

At last year's meeting, we introduced a new tumor cytology method in which Papanicolaou-stained specimens are placed under dark-field illumination to measure the white light scattering spectrum of individual cells, which is then used for AI analysis, and reported that mesothelioma cells and reactive mesothelial cells could be distinguished with 95% accuracy in pleural fluid cytology. In the present study, we examined whether this method is also useful for cell clusters and pleural fluid cytology of other lung cancers. Light scattering spectra of 0.1-mm diameter circle of mesothelioma (206; n=3 ) and reactive mesothelial (58; n=1) cell clusters were measured and subjected to principal component analysis and support vector machine-learning algorithm, and both were discriminated with 93% accuracy. Lung adenocarcinoma (104; n=4) and small cell cancer (158; n=3 ) cells could be distinguished with 100% accuracy. These high accuracies may be attributed to the nature of the light scattering spectra, which provide information on intracellular microstructures with sizes ranging mainly from tens to hundreds of nanometers. This new method is expected to be a simple and powerful diagnostic aid.

[Disclosure of COI by Responsible researcher] Lead presenter is not the Responsible researcher.
[Lead presenter: Fuka Takeuchi] We have no financial relationships to disclose.
[Principal Researcher: Akihiko Ito] We have no financial relationships to disclose.

▲TOP

### [J-2012] I-J12-2
### Artificial intelligence based diagnostics

2025 / 9 / 26 (Fri) 10:15-11:30 Room 7/(Hotel Nikko Kanazawa 3F Kujaku B) Room 7

[Chairperson]Shingo Sakashita (Natl.Cancer Ctr. EPOC, Pathology)

---

### J-2012
### AI-Powered Cytological Diagnosis Support System Using Multi-Focus Image-Based 3D Recognition

[Speaker]Yukihiko Osawa (Faculty of Health Sci., Kyoto Tachibana Univ.／Res. Ctr. for Life & Health Sci., Kyoto Tachibana Univ.／Grad. Sch. of Health Sci., Kyoto Tachibana Univ.) [Co-author]Tomoyuki Miyamoto (Cancer Cell Inst., Kyushu Univ. of Med. Sci.／Sch. of Med. Life Sci., Kyushu Univ. of Med. Sci.／Grad. Sch. of HealthSci.Studies, Kyushu Univ. of Med. Sci.)、Takafumi Ohnishi (Faculty of Health Sci., Kyoto Tachibana Univ.／Res. Ctr. for Life & Health Sci., Kyoto Tachibana Univ.)、Manabu Hattori (Faculty of Health Sci., Kyoto Tachibana Univ.／Res. Ctr. for Life & Health Sci., Kyoto Tachibana Univ.／Grad. Sch. of Health Sci., Kyoto Tachibana Univ.)、Chikafumi Shoshi (Faculty of Health Sci., Kyoto Tachibana Univ.／Res. Ctr. for Life & Health Sci., Kyoto Tachibana Univ.／Grad. Sch. of Health Sci., Kyoto Tachibana Univ.)、Hiromasa Yakushiji (Cancer Cell Inst., Kyushu Univ. of Med. Sci.／Sch. of Med. Life Sci., Kyushu Univ. of Med. Sci.／Grad. Sch. of HealthSci.Studies, Kyushu Univ. of Med. Sci.)、Makoto Nishimori (Cancer Cell Inst., Kyushu Univ. of Med. Sci.／Sch. of Med. Life Sci., Kyushu Univ. of Med. Sci.／Grad. Sch. of HealthSci.Studies, Kyushu Univ. of Med. Sci.)、Kazuki Shibahara (Cancer Cell Inst., Kyushu Univ. of Med. Sci.／Sch. of Med. Life Sci., Kyushu Univ. of Med. Sci.／Grad. Sch. of HealthSci.Studies, Kyushu Univ. of Med. Sci.)、Yoshiya Shimao (Dept. of Path.,Miyazaki Pref. Nobeoka Hosp.)、Atsushi Hongo (Dept. of Obstetrics & Gynecology, Kawasaki Med. Sch.)、Hideki Hashimoto (Proassist, Ltd.)、Setsuyo Ohno (Res. Ctr. for Life & Health Sci., Kyoto Tachibana Univ.)、Eiji Ohno (Res. Ctr. for Life & Health Sci., Kyoto Tachibana Univ.)

Although image recognition technology using artificial intelligence (AI) has advanced rapidly, there are no reports of AI-based diagnostic systems being applied in actual cytological examinations. Recently, the development of virtual slide scanners capable of multi-focus imaging has enabled the acquisition of three-dimensional (3D) information from cytological preparations. In this study, we developed an AI-based cytological diagnostic support system using multi-focus image sequences obtained from a virtual slide scanner. The training data consisted of tens of thousands of multi-focus image sets extracted from cervical specimens (NILM, LSIL, HSIL and CANCER). The system can detect abnormal cells in scanned preparations and present them to the cytopathologist. Moreover, cytopathologist feedback on cell decision is reflected in the AI model, allowing continuous improvement of diagnostic accuracy. In this presentation, we will introduce the multi-focus imaging process, demonstrate the system. The AI training dataset developed for this study is publicly accessible via a dedicated website. This work was supported by JST AIP Acceleration Research JPMJCR23U4, Japan.

[Disclosure of COI by Responsible researcher] Lead presenter is not the Responsible researcher.
[Lead presenter: Yukihiko Osawa] We have no financial relationships to disclose.
[Principal Researcher: Eiji Ohno] We have no financial relationships to disclose.

### [J-2013] I-J10-2
### Metabolism/Metabolome (2)

2025 / 9 / 26 (Fri) 10:15-11:30 Room 8/(Hotel Nikko Kanazawa 3F Kujaku C) Room 8

[Chairperson]Hideaki Ogiwara (Division of Cancer Therapeutics, National Cancer Center Research Institute)

---

### J-2013
### Tumor Suppression Through Regular Exercise and PGC-1α-Driven Metabolic Changes in Hepatocellular Carcinoma

[Speaker]Ryota Yamagishi (Dept.PathoPhysiol., Osaka Metropolitan Unv.Sch,Med.) [Co-author]Kanae Echizen (Dept.PathoPhysiol., Osaka Metropolitan Unv.Sch,Med.)、Yoshiki Nonaka (Dept.PathoPhysiol., Osaka Metropolitan Unv.Sch,Med.)、Naoko Ohtani (Dept.PathoPhysiol., Osaka Metropolitan Unv.Sch,Med.)

Recently, the number of patients with steatosis-associated hepatocellular carcinoma (HCC) has been steadily increasing, highlighting the urgent need for preventive and therapeutic strategies. We previously developed a high-fat diet-induced HCC mouse model, showing that obesity-related metabolic and gut microbiota changes promote HCC. In this study, we explored the anti-tumor effects of exercise, finding that regular running significantly suppressed tumor formation in the HCC mouse model, without affecting body weight. Additionally, metabolome analysis revealed a reduction in the blood level of a tryptophan metabolite, known as an oncometabolite, suggesting that exercise may help degrade this oncometabolite. To further investigate, we prepared skeletal muscle-specific PGC-1α overexpressing mice and conducted similar hepatocarcinogenesis experiments. We confirmed that hepatocarcinogenesis was suppressed in these transgenic mice. Furthermore, we will present WGCNA results and identify the gene sets associated with tumor suppression. In this meeting, we will report our findings, focusing on exercise-induced metabolic changes.

[Disclosure of COI by Responsible researcher] Lead presenter is not the Responsible researcher.
[Lead presenter: Ryota Yamagishi] ■Research funds from company: 武田科学振興財団
[Principal Researcher: Naoko Ohtani] ■Research funds from company: 武田科学振興財団、AMED肝炎克服事業

▲TOP

### [J-2014] I-J10-2
### Metabolism/Metabolome (2)

2025 / 9 / 26 (Fri) 10:15-11:30 Room 8/(Hotel Nikko Kanazawa 3F Kujaku C) Room 8

[Chairperson]Hideaki Ogiwara (Division of Cancer Therapeutics, National Cancer Center Research Institute)

### J-2014
### Withdrawn

No Abstract

### [J-2015] I-J10-2
### Metabolism/Metabolome (2)

2025 / 9 / 26 (Fri) 10:15-11:30 Room 8/(Hotel Nikko Kanazawa 3F Kujaku C) Room 8

[Chairperson]Hideaki Ogiwara (Division of Cancer Therapeutics, National Cancer Center Research Institute)

---

### J-2015
### miR-216b-5p Functions as a Tumor Suppressor via the PTBP1/TYMS Axis in Pancreatic Cancer

[Speaker]Shigenori Suzuki (Osaka Med. & Pharm. Univ. Dept. of General Surg.) [Co-author]Kohei Taniguchi (Osaka Med. & Pharm. Univ. Div. of TR)、Yosuke Inomata (Osaka Med. & Pharm. Univ. Dept. of General Surg.)、Jun Arima (Osaka Med. & Pharm. Univ. Dept. of General Surg.)、Ryo Numoto (Osaka Med. & Pharm. Univ. Dept. of General Surg.)、Yuko Itoh (Osaka Med. & Pharm. Univ. Dept. of General Surg.)、Sangwoong Lee (Osaka Med. & Pharm. Univ. Dept. of General Surg.)

Previously we showed the Warburg effect, a feature of cancer metabolism, was induced by the suppression of some microRNAs (miRs) bind to PTBP1 (PMID: 33070451). We showed PTBP1-targeting miRs have extreme tissue specificity. But, more study is needed to elucidate the roles of PTBP1-targeting miRs in oncogenesis. The open data base suggested miR-216b-5p is mainly expressed in the pancreas. We tried to clarify the action and role of the miR-216b-5p/PTBP1 for pancreatic cancer (PC). We analyzed miR-216b-5p and PTBP1 expression in normal and PC, and suppressed miR-216b-5p in PC. We detected miR-216b-5p induced growth inhibition through the direct binding to PTBP1 in PC cell lines. Proteomic assay of cells with siR-PTBP1 identified thymidylate synthase (TYMS) as a downstream target of PTBP1. TYMS expression was elevated in surgical tissues and a TCGA-based analysis suggested reduced expression of PTBP1 and TYMS was associated with a longer prognosis in PC. Flow cytometry revealed the PC cell lines knockdowned PTBP1 or TYMS delayed S-phase progression. These suggest miR-216b-5p acts as a tumor suppressor by targeting PTBP1, leading to TYMS suppression and reduced PC cell proliferation.

[Disclosure of COI by Responsible researcher] Lead presenter is not the Responsible researcher.
[Lead presenter: Shigenori Suzuki] We have no financial relationships to disclose.
[Principal Researcher: Sangwoong Lee] ■Research funds from company: 科研費 21K08761, 25K02711, AMED JP25ama121052, JP25ama121054

### [J-2016] I-J10-2
### Metabolism/Metabolome (2)

2025 / 9 / 26 (Fri) 10:15-11:30 Room 8/(Hotel Nikko Kanazawa 3F Kujaku C) Room 8

[Chairperson]Hideaki Ogiwara (Division of Cancer Therapeutics, National Cancer Center Research Institute)

---

### J-2016
### Identification of anoikis resistant-related molecules in breast cancer stem cell models

[Speaker]Asahi Sami (Faculty Pharmaceutical Sciences Doshisha Women's College Liberal Arts) [Co-author]Kyoka Hashimoto (Faculty Pharmaceutical Sciences Doshisha Women's College Liberal Arts)、Aina Tsujihana (Faculty Pharmaceutical Sciences Doshisha Women's College Liberal Arts／Graduate School Pharmaceutical Sciences Doshisha Women's College Liberal Arts)、Rei Takahashi (Faculty Pharmaceutical Sciences Doshisha Women's College Liberal Arts)、Kiyotsugu Yoshikawa (Faculty Pharmaceutical Sciences Doshisha Women's College Liberal Arts)

**[Objective]**EMT promotes invasion, motility, and therapy resistance in cancer. EMT-induced breast cancer cells become resistant to anoikis. We aimed to identify metabolites and genes related to anoikis resistance using a breast cancer stem-like cell model.**[Methods]**RM cells (H-Ras G12V-transduced HMLE) exhibit $CD44^{hi}/CD24^{lo}$, high Vimentin promoter activity, and sphere-forming ability. Cells were cultured under 2D and ultra-low attachment 3D conditions. Metabolomic and RNA-seq analyses were performed to identify anoikis-related factors.**[Results]**In 3D vs 2D, RM cells showed increases in amino acid, nucleotide, carbohydrate, vitamin, lipid metabolism, and dipeptides. EMT-related oncometabolite 2-hydroxyglutarate was further elevated in 3D. 4-hydroxyproline was specifically increased. Hydroxylated-proline proteins implied HIF and ECM remodeling roles. PUFA levels also increased, suggesting greater membrane fluidity. CD26/DPP4 was upregulated by EMT and H-Ras, indicating a possible role in invasion.

[Disclosure of COI by Responsible researcher] Lead presenter is not the Responsible researcher.
[Lead presenter: Asahi Sami] We have no financial relationships to disclose.
[Principal Researcher: Kiyotsugu Yosikawa] We have no financial relationships to disclose.

▲TOP

### [J-2017] I-J10-2
### Metabolism/Metabolome (2)

2025 / 9 / 26 (Fri) 10:15-11:30 Room 8/(Hotel Nikko Kanazawa 3F Kujaku C) Room 8

[Chairperson]Hideaki Ogiwara (Division of Cancer Therapeutics, National Cancer Center Research Institute)

---

### J-2017
### HSD17B4-mediated lipid metabolism is involved in breast cancer drug response

[Speaker]Naoko Hattori (Inst. for Molecular&Cellular Regulation, Gunma Univ.／Inst. for Advanced Life Sci., Hoshi Univ.) [Co-author]Hideru Obinata (Education&Research Support Ctr., Grad. Sch. of Med., Gunma Univ.)、Nobuaki Arai (Dept. of Surgery, Kyorin Univ. Sch. of Med.)、Satoshi Yamashita (Faculty of Engineering, Maebashi Inst. of Tech.)、Kozue Kimura (Inst. for Molecular&Cellular Regulation, Gunma Univ.)、Genki Yamagishi (Inst. for Molecular&Cellular Regulation, Gunma Univ.)、Toshikazu Ushijima (Inst. for Advanced Life Sci., Hoshi Univ.)

HER2-positive breast cancer has a high chance of achieving a pathological complete response after HER2-directed chemotherapy when HSD17B4, responsible for peroxisomal β-oxidation of very long-chain fatty acids (VLCFA), is methylation-silenced [Yamashita et al., Sci Rep 2020]. We previously discovered the enhancement of glucose dependence as the major mechanism by which HSD17B4-silenced BT-474 cancer cells are sensitive to HER2 signal blockade [Arai et al., Breast Cancer Res Treatment 2023]. Here, we explored the role of HSD17B4-mediated lipid metabolism in HER2-targeted therapy. HSD17B4 depletion reduced polyunsaturated FAs, such as docosahexaenoic acid and arachidonic acid, along with decreased phospholipids containing these FAs in BT-474 and SK-BR-3 cells. This was accompanied by dihydroxyacetone phosphate accumulation and upregulation of phospholipase genes, including ***PLA2G2A*** and ***PLAAT3***. Decreased cellular elasticity in HSD17B4-depeleted cells was suggested by atomic force microscopy. These findings indicated that decreased PUFAs induced by HSD17B4 depletion lead to reduced ether-phospholipid contents in cellular membrane, potentially modulating HER2 oncogenic signaling.

[Disclosure of COI by Responsible researcher] Lead presenter is the Responsible researcher.
[Lead presenter: Naoko Hattori] ■Research funds from company: 公的研究費：JSPS、寄附：公益財団法人アステラス病態代謝研究会（主たる出捐者　アステラス製薬株式会社）、寄付： 公益財団法人日本応用酵素協会（主たる出捐者　田辺三菱製薬株式会社）
■Contributions: 公益財団法人アステラス病態代謝研究会

▲TOP

### [J-2018] I-J10-2
### Metabolism/Metabolome (2)

2025 / 9 / 26 (Fri) 10:15-11:30 Room 8/(Hotel Nikko Kanazawa 3F Kujaku C) Room 8

[Chairperson]Hideaki Ogiwara (Division of Cancer Therapeutics, National Cancer Center Research Institute)

### J-2018
### MUC1-C drives stemness by integrating metabolic reprogramming in triple-negative breast cancer

[Speaker]Nami Yamashita (Cancer Inst. Hosp. JFCR, Breast Oncology Ctr, Breast Surg. Oncology／Medical Oncology, Dana-Farber Cancer Institute, Harvard Medical School／JFCR, NEXT-Ganken Program) [Co-author]Yoshihiro Morimoto (Medical Oncology, Dana-Farber Cancer Institute, Harvard Medical School)、Uta Nakadaira (Cancer Inst. Hosp. JFCR, Breast Oncology Ctr, Breast Surg. Oncology)、Yuri Kimura (Cancer Inst. Hosp. JFCR, Breast Oncology Ctr, Breast Surg. Oncology)、Meiko Nishimura (Cancer Inst. Hosp. of JFCR, Breast Oncology Ctr, Breast Oncology)、Yukinori Ozaki (Cancer Inst. Hosp. of JFCR, Breast Oncology Ctr, Breast Oncology)、Asumi Iesato (JFCR, NEXT-Ganken Program)、Yuka Inoue (Cancer Inst. Hosp. JFCR, Breast Oncology Ctr, Breast Surg. Oncology)、Tetsuyo Maeda (Cancer Inst. Hosp. JFCR, Breast Oncology Ctr, Breast Surg. Oncology)、Natsue Uehiro (Cancer Inst. Hosp. JFCR, Breast Oncology Ctr, Breast Surg. Oncology)、Takayuki Kobayashi (Cancer Inst. Hosp. JFCR, Breast Oncology Ctr, Breast Surg. Oncology)、Takehiko Sakai (Cancer Inst. Hosp. JFCR, Breast Oncology Ctr, Breast Surg. Oncology)、Toshimi Takano (Cancer Inst. Hosp. JFCR, Breast Oncology Ctr, Breast Surg. Oncology)、Donald Kufe (Medical Oncology, Dana-Farber Cancer Institute, Harvard Medical School)、Takayuki Ueno (Cancer Inst. Hosp. JFCR, Breast Oncology Ctr, Breast Surg. Oncology／JFCR, NEXT-Ganken Program)

The Mucin1 (MUC1) is aberrantly expressed in triple-negative breast cancer (TNBC). MUC1 C-terminal (MUC1-C) subunit is activated by MUC1-N shedding (CA15-3) and promotes lineage plasticity, epigenetic reprogramming, and the cancer stem cell (CSC) state. However, little is known about the regulation of TNBC CSC metabolism. We enriched CSC populations by serial passage of TNBC mammospheres. This study demonstrated that MUC1-C is essential for integrating activation of glycolytic pathway genes (HK2, GLUT1 etc.) with self-renewal and tumorigenicity. Moreover, treatment with GO-203(MUC1-C inhibitor) and 2-deoxy-D-glucose (HK2 inhibitor), markedly decreased mammosphere formation. Futhermore, we found that by targeting MUC1-C with gene silencing and GO-203 treatment, MUC1-C suppressed mitochondrial DNA (mtDNA) genes. According to the clinical data from pre-operative TNBC patients, we found that serum CA-15-3 is positively correlated with (18)F-FDG PET/CT SUVmax ($r=0.44$, $p<0.001$).In summary, our findings in TNBC cells indicate that MUC1-C drives CSC self-renewal by integrating the activation of glycolysis in certain subpopulations with suppression of oxidative phospholylation.

[Disclosure of COI by Responsible researcher] Lead presenter is the Responsible researcher.
[Lead presenter: Nami Yamashita] We have no financial relationships to disclose.

### [J-2019] I-J19
### Cancer Prevention & Epidemiology (2)

2025 / 9 / 26 (Fri) 10:15-11:30 Room 9/(Hotel Nikko Kanazawa 3F Lumiere) Room 9

[Chairperson]Naoyuki Nishiya (Div.info., Dept. Clin. Pharm., Iwate Med. Univ. , Sch. Pham. )

---

### J-2019
### Cancer chemoprevention with DNA demethylating agents in oncovirus-associated leukemogenesis in mice.

[Speaker]Yuta Yamamoto (Division of Hematology, Respiratory Medicine and Oncology, Saga University／Department of Drug Discovery and Biomedical Sciences, Saga University) [Co-author]Tatsuro Watanabe (Division of Hematology, Respiratory Medicine and Oncology, Saga University)、Hiroshi Ureshino (Division of Hematology, Respiratory Medicine and Oncology, Saga University／Department of Drug Discovery and Biomedical Sciences, Saga University)、Keisuke Kidoguchi (Division of Hematology, Respiratory Medicine and Oncology, Saga University／Department of Drug Discovery and Biomedical Sciences, Saga University)、Ryo Yanagiya (Division of Hematology, Respiratory Medicine and Oncology, Saga University／Department of Drug Discovery and Biomedical Sciences, Saga University)、Shinya Kimura (Division of Hematology, Respiratory Medicine and Oncology, Saga University／Department of Drug Discovery and Biomedical Sciences, Saga University)

About 12-20% of human cancers are thought to be caused by viral infections. We have found that regional DNA hypermethylation accumulates in adult T-cell leukemia/lymphoma (ATL), which is initiated by the infection of human T-cell lymphotropic virus type 1 (HTLV-1), and that DNA demethylating agents inhibit ATL cell growth. Therefore, we hypothesized that oncovirus-associated carcinogenesis could be delayed by inhibiting DNA hypermethylation. In this study, we investigated the effects of the newly developed DNA demethylating agent OR2100 (OR) in AKR mice, which spontaneously develop retrovirus-associated lymphocytic leukemia, and found survival prolongation induced by OR treatment. Next, genome-wide DNA methylation and transcriptome analysis were performed using the cells isolated from the thymus of young (control) or aged (tumor) AKR mice. As expected, DNA hypermethylation of promoter CpG islands was observed in tumor cells. In addition, tumor cells aberrantly overexpressed MYC protein. Treatment of a leukemic cell line derived from AKR mice with OR decreased mRNA and protein expressions of MYC. Thus, we think that OR suppresses MYC and delayed leukemogenesis in AKR mice.

[Disclosure of COI by Responsible researcher] Lead presenter is not the Responsible researcher.
[Lead presenter: Yuta Yamamoto] We have no financial relationships to disclose.
[Principal Researcher: Shinya Kimura] ■Research expenses: 大原薬品工業株式会社

▲TOP

### [J-2020] I-J19
### Cancer Prevention & Epidemiology (2)

2025 / 9 / 26 (Fri) 10:15-11:30 Room 9/(Hotel Nikko Kanazawa 3F Lumiere) Room 9

[Chairperson]Naoyuki Nishiya (Div.info., Dept. Clin. Pharm., Iwate Med. Univ. , Sch. Pham. )

---

### J-2020
### Predicting colorectal cancer risk using genome-wide polygenic risk scores and lifestyle factors in a Japanese population

[Speaker]Shiori Nakano (Natl. Cancer Ctr. Inst. Cancer Control) [Co-author]Taiki Yamaji (Natl. Cancer Ctr. Inst. Cancer Control)、Tsuyoshi Hachiya (Div. Biomed. Information Analysis, Iwate Tohoku Medical Megabank Organization)、Aya Kuchiba (Biostatistics Div., Ctr. Res. Administration & Support, Natl. Cancer Ctr.／Teikyo Univ. Grad. Sch. Public Health)、Atsushi Shimizu (Div. Biomed. Information Analysis, Iwate Tohoku Medical Megabank Organization)、Norie Sawada (Natl. Cancer Ctr. Inst. Cancer Control)、Manami Inoue (Natl. Cancer Ctr. Inst. Cancer Control)、Shoichiro Tsugane (Natl. Cancer Ctr. Inst. Cancer Control／International Univ. Health & Welfare Grad. Sch. Public Health)、Motoki Iwasaki (Natl. Cancer Ctr. Inst. Cancer Control)

The predictive performance of colorectal cancer (CRC) risk prediction models that incorporate genome-wide polygenic risk scores (PRSs) and lifestyle factors remains unclear in Asian populations. This study aimed to develop and evaluate the models using a Japanese population-based prospective study.
We constructed 31 genome-wide PRSs and selected the best-performing PRS with the highest C-index in the development cohort. In the evaluation cohort, we assessed the discriminatory accuracy of models based on lifestyle factors, PRS, and their combination using five-fold cross-validation and estimated the 10-year absolute risk of CRC.
Among the 31 PRSs, the PRS incorporating 104,677 risk variants performed best in the development case-cohort. Integrating the PRS into the lifestyle-based model improved the C-index from 0.64 to 0.66 in men and from 0.61 to 0.63 in women. The estimated 10-year absolute risk of CRC was 3.3% for high-risk men and 1.6% for high-risk women, compared to 0.5% for both low-risk men and women.
This study suggests that the CRC risk prediction model utilizing genome-wide PRS for Asians is valuable; however, further improvement is needed before clinical implementation.

[Disclosure of COI by Responsible researcher] Lead presenter is not the Responsible researcher.
[Lead presenter: Shiori Nakano] We have no financial relationships to disclose.
[Principal Researcher: Motoki Iwasaki] We have no financial relationships to disclose.

[J-2021] I-J19

### Cancer Prevention & Epidemiology (2)

2025 / 9 / 26 (Fri) 10:15-11:30 Room 9/(Hotel Nikko Kanazawa 3F Lumiere) Room 9

[Chairperson]Naoyuki Nishiya (Div.info., Dept. Clin. Pharm., Iwate Med. Univ. , Sch. Pham. )

J-2021

### Association of plasma branched-chain amino acid levels with colorectal cancer risk in a nested case-control study

[Speaker]Taiki Yamaji (Natl. Cancer Ctr. Inst. Cancer Ctrl.) [Co-author]Izumi Hisada (Natl. Cancer Ctr. Inst. Cancer Ctrl.)、Norie Sawada (Natl. Cancer Ctr. Inst. Cancer Ctrl.)、Manami Inoue (Natl. Cancer Ctr. Inst. Cancer Ctrl.)、Shoichiro Tsugane (Natl. Cancer Ctr. Inst. Cancer Ctrl.／International Univ. Health & Welfare)、Motoki Iwasaki (Natl. Cancer Ctr. Inst. Cancer Ctrl.)

The association of plasma branched-chain amino acid (BCAA) levels with colorectal cancer (CRC) risk has not been fully evaluated. We conducted a prospective case-control study within a cohort of the JPHC Study, for which blood sample and questionnaire data were available. We identified 360 newly diagnosed CRC cases, and selected two matched controls for each case. We estimated odds ratio (OR) and 95% confidence interval (CI) for CRC using conditional logistic regression models adjusted for potential confounders. Increased plasma BCAA levels were not associated with CRC risk. Compared with the lowest quartile, OR (95% CI) for the highest quartile of plasma BCAA was 0.70 (0.47-1.05). After excluding cases diagnosed within the first 6 years of follow-up, plasma BCAA levels were significantly related to a decreased risk of CRC, with OR (95% CI) for the highest quartile being 0.58 (0.35-0.96). We found no statistically significant association between plasma BCAA levels and CRC risk in overall analyses, whereas on 6-year exclusion, plasma BCAA levels were associated with a decreased CRC risk. BCAAs in the plasma may play a protective role against colorectal carcinogenesis.

[Disclosure of COI by Responsible researcher] Lead presenter is not the Responsible researcher.
[Lead presenter: Taiki Yamaji] We have no financial relationships to disclose.
[Principal Researcher: Motoki Iwasaki] ■Research funds from company: Japan Society for the Promotion of Science

### [J-2022] I-J19
### Cancer Prevention & Epidemiology (2)

2025 / 9 / 26 (Fri) 10:15-11:30 Room 9/(Hotel Nikko Kanazawa 3F Lumiere) Room 9

[Chairperson]Naoyuki Nishiya (Div.info., Dept. Clin. Pharm., Iwate Med. Univ. , Sch. Pham. )

---

### J-2022
### Impact of Alcohol Consumption-Associated Loci on Alcohol-Related Cancer Risk: An Assessment by Mediation Analysis

[Speaker]Kengo Matsunaga (Div. of Cancer Epidemiology and Prevention, Aichi Cancer Ctr.) [Co-author]Yuriko Koyanagi (Div. of Cancer Epidemiology and Prevention, Aichi Cancer Ctr.)、Yumiko Kasugai (Div. of Cancer Epidemiology and Prevention, Aichi Cancer Ctr.)、Yuji Iwashita (Div. of Cancer Epidemiology and Prevention, Aichi Cancer Ctr.)、Isao Oze (Div. of Cancer Information and Control, Aichi Cancer Ctr.)、Hidemi Ito (Div. of Cancer Information and Control, Aichi Cancer Ctr.／Div. of Descriptive Cancer Epidemiology, Nagoya Univ. Grad. Sch. Med.)、Keitaro Matsuo (Div. of Cancer Epidemiology and Prevention, Aichi Cancer Ctr.／Div. of Cancer Epidemiology, Nagoya Univ. Grad. Sch. Med.)

Alcohol consumption is a well-known cancer risk factor. Our recent ***ALDH2*** (rs671, G>A) genotype-stratified genome-wide association study identified seven alcohol consumption-associated loci (***GCKR***, ***KLB***, ***ADH1B***, ***ALDH1B1***, ***ALDH1A1***, ***ALDH2***, and ***GOT2***) with genotype-specific effect (GG or GA). Here, we conducted case-control studies of alcohol-related cancers (4858 cases, 5770 controls) to evaluate whether these loci are associated with alcohol-related cancer risk directly or indirectly through changing drinking behavior. Using mediation analyses, we estimated the direct and indirect effect odds ratios (dOR and iOR) of each locus. Among the seven loci, ***GCKR***, ***KLB***, ***ADH1B***, and ***ALDH1B1*** showed significant iOR, and ***ADH1B*** showed a significant dOR, mainly for the upper digestive tract cancers (UDTC). In rs671 GG subjects, ***GCKR***, ***KLB***, and ***ADH1B*** were associated with UDTC in direct and/or indirect way, while all seven loci showed significant dOR and/or iOR for UDTC in GA subjects. Drinking-related loci demonstrated ***ALDH2*** genotype-specific association with alcohol-related cancers. Some loci may affect cancer risk through pathways other than drinking behavior, suggesting novel carcinogenic mechanisms.

[Disclosure of COI by Responsible researcher] Lead presenter is not the Responsible researcher.
[Lead presenter: Kengo Matsunaga] We have no financial relationships to disclose.
[Principal Researcher: Keitaro Matsuo] We have no financial relationships to disclose.

### [J-2023] I-J19
### Cancer Prevention & Epidemiology (2)

2025 / 9 / 26 (Fri) 10:15-11:30 Room 9/(Hotel Nikko Kanazawa 3F Lumiere) Room 9

[Chairperson]Naoyuki Nishiya (Div.info., Dept. Clin. Pharm., Iwate Med. Univ. , Sch. Pham. )

---

### J-2023
### Cardiovascular Disease Risk Among Childhood Leukemia Survivors: A retrospective matched cohort study

[Speaker]Naoto Kaneko (NEC Solution Innovators, Ltd.／Chiba Cancer Center Research Institute／Graduate School of Medical and Pharmaceutical Sciences, Chiba University) [Co-author]Shintaro Kato (NEC Solution Innovators, Ltd.)、Yohko Nakamura (Chiba Cancer Center Research Institute)、Yoshihide Terada (Chiba Cancer Center Research Institute／Faculty of Management and Governance, Shumei University)、Aya Washio (Chiba Cancer Center Research Institute)、Yoshitaka Hippo (Chiba Cancer Center Research Institute／Graduate School of Medical and Pharmaceutical Sciences, Chiba University)、Nobuaki Michihata (Chiba Cancer Center Research Institute)

**Background/Aim:** Treatment outcomes for childhood leukemia have improved, resulting in more survivors. However, long-term effects like cardiovascular disease (CVD) remain a concern. Epidemiological evidence in Japanese populations is limited. We aimed to evaluate the risk of CVD in childhood leukemia survivors compared to matched controls using hazard ratios (HRs).**Methods:** We conducted a retrospective matched cohort study using a large-scale medical claims database. The case group included individuals diagnosed with leukemia by age 16. Controls were matched 1:10 by birth year and sex from those without leukemia. The outcome was time to first diagnosis of CVD, including heart and cerebrovascular diseases. HRs were estimated using Cox proportional hazards models adjusted for age and sex.**Results:** We included 2,606 leukemia survivors and 20,737 matched controls. CVD incidence was significantly higher among leukemia survivors. The adjusted HR was 2.48 (95% CI: 2.03–3.03, p<0.001).**Conclusion:** Childhood leukemia survivors showed a significantly increased risk of CVD compared to matched controls. Further follow-up studies are needed to clarify late effects and inform prevention strategies.

[Disclosure of COI by Responsible researcher] Lead presenter is not the Responsible researcher.
[Lead presenter: Naoto Kaneko] ■ employment by a company related to the presentation: NECソリューションイノベータ株式会社
[Principal Researcher: Nobuaki Michihata] We have no financial relationships to disclose.

▲TOP

### [J-2024] I-J19
### Cancer Prevention & Epidemiology (2)

2025 / 9 / 26 (Fri) 10:15-11:30 Room 9/(Hotel Nikko Kanazawa 3F Lumiere) Room 9

[Chairperson]Naoyuki Nishiya (Div.info., Dept. Clin. Pharm., Iwate Med. Univ. , Sch. Pham. )

---

### J-2024
### Cancer survival varied by cancer type, regardless of rarity/commonality

[Speaker]Hiromi Sugiyama (Dept. of Epidemiology, Radiation Effects Res. Foundation) [Co-author]Kumiko Saika (Inst. for Cancer Control, National Cancer Ctr.)、Tomohiro Matsuda (Inst. for Cancer Control, National Cancer Ctr.)

We investigated the association between cancer rarity/commonality and survival. Using data from the National Cancer Registry, we analyzed 4,942,966 patients diagnosed with invasive cancer during 2016–2020. Cancer incidence and 4-year net survival were estimated for 216 Tier-2 cancers based on the RARECAREnet classification. Net survival varied among common cancers with crude incidence >6/100,000: e.g., 99.2% for adenocarcinoma of the prostate, and 15.2% for adenocarcinoma of the pancreas. Among rare cancers with crude incidence <6/100,000, survival also varied– e.g., 98.5% for seminomatous testicular cancer and 3.4% for undifferentiated carcinoma of the liver and intrahepatic bile duct. Regardless of rarity/commonality, cancers that are detectable early due to symptoms or are treatable with established therapies were associated with higher survival. In contrast, cancers that are asymptomatic, biologically aggressive, or diagnosed at advanced stages tended to have lower survival. Classifying cancers using the Rare Cancer Classification is crucial for identifying low-survival cancers that may be overlooked as common and helps to develop targeted strategies for rare cancer control.

[Disclosure of COI by Responsible researcher] Lead presenter is not the Responsible researcher.
[Lead presenter: Hiromi Sugiyama] ■Research funds from company: 厚生労働科学研究費補助金 がん対策推進総合研究事業（23EA1033）
[Principal Researcher: Tomohiro Matsuda] ■Research funds from company: 厚生労働科学研究費補助金 がん対策推進総合研究事業（23EA1033）

### [E-2025] I-E14-1
### Drug delivery system (2)

2025 / 9 / 26 (Fri) 10:15-11:30 Room 14/(ANA Crowne Plaza Hotel Kanazawa 3F Zuiun 1) Room 14

[Chairperson]Kanjiro Miyata (Dept. of Mater., Grad. Schl. Eng., UTokyo)

---

### E-2025
### Multifunctional Drug Delivery System to Inhibit the Proliferation of SW480 Cells

[Speaker]Kanpitcha Jiramitmongkon (Dept. of Chemistry, Thammasat Univ.) [Co-author]Pichayanoot Rotkrua (Dept. of Preclinical, Sci., Thammasat Univ.)、Paisan Khanchaitit (Natl. Nanotechnology Ctr., Natl. Sci. & Tech. Development Agency)、Jiraporn Arunpanichlert (Dept. of Chemistry, Thammasat Univ.)、Boonchoy Soontornworajit (Dept. of Chemistry, Thammasat Univ.)

Colorectal cancer is a global concern. Chemotherapy is commonly used but has limitations in efficiency. Targeted therapy offers improved specificity and reduced side effects. This study develops a multifunctional drug delivery system (MDS) by combining bioactive materials with targeting ligands for targeted therapy. The MDS includes the AS1411 aptamer, which targets nucleolin for selective cancer recognition; T9/U4 antisense oligonucleotide, which inhibits human telomerase RNA; and doxorubicin (Dox), a chemotherapeutic agent. AS1411 enhances specificity and uptake by binding to nucleolin on SW480 cells, while T9/U4 disrupts proliferation pathways. Dox induces apoptosis, ensuring a synergistic effect. The MDS was prepared via hybridization and characterized by gel electrophoresis. Binding to nucleolin was confirmed with fluorescence spectroscopy and flow cytometry. Anti-proliferation was assessed with MTS assay, and the protein expression (hTERT, vimentin, Bcl-2, and Bax) was analyzed by Western blot. The MDS showed improved tumor selectivity, reduced toxicity, and enhanced efficacy in treating SW480 cells, supporting its potential for targeted therapy.

[Disclosure of COI by Responsible researcher] Lead presenter is the Responsible researcher.
[Lead presenter: Kanpitcha Jiramitmongkon] We have no financial relationships to disclose.

### [E-2026] I-E14-1
### Drug delivery system (2)

2025 / 9 / 26 (Fri) 10:15-11:30 Room 14/(ANA Crowne Plaza Hotel Kanazawa 3F Zuiun 1) Room 14

[Chairperson]Kanjiro Miyata (Dept. of Mater., Grad. Schl. Eng., UTokyo)

### E-2026
### Withdrawn

No Abstract

### [E-2027] I-E14-1
### Drug delivery system (2)

2025 / 9 / 26 (Fri) 10:15-11:30 Room 14/(ANA Crowne Plaza Hotel Kanazawa 3F Zuiun 1) Room 14

[Chairperson]Kanjiro Miyata (Dept. of Mater., Grad. Schl. Eng., UTokyo)

---

### E-2027
### Inhalable ivermectin loaded lipid polymer hybrid nanoparticles against lung cancer with enhanced anticancer activity

[Speaker]Seyedehnegin Kassaee (Pharmacy discipline, School of Clinical Sciences, QUT, Brisbane, QLD, Australia／Center for Genomics and Personalized Health, QUT, Brisbane, Australia／Centre for Materials Science, QUT, Brisbane, QLD, Australia) [Co-author]Nazrul Islam (Pharmacy discipline, School of Clinical Sciences, QUT, Brisbane, QLD, Australia)、Derek Richard (Center for Genomics and Personalized Health, QUT, Brisbane, Australia)、Godwin Ayoko (Centre for Materials Science, QUT, Brisbane, QLD, Australia)

Lung cancer is a leading cause of cancer-related deaths globally. Its pervasive impact prompts exploration of novel drug delivery systems to overcome challenges such as multi-drug resistance, side effects, and high treatment costs associated with conventional chemotherapeutics. Ivermectin (IVM), originally used for treating parasitic infections, is being explored for its potential applications in cancer therapy. However, its low water solubility limits its oral bioavailability. To overcome this challenge, our research focused on developing IVM-loaded lipid polymer hybrid nanoparticles (LPHNPs) for pulmonary administration. Developed IVM-loaded LPHNPs demonstrated particles between 302 and 350 nm with drug entrapment efficiency between 68-80%. The in vitro aerosolization analysis revealed fine particle fractions (FPFs) ranging from 18.53% to 24.77%. In A549 lung cancer cells, IVM-loaded LPHNPs reduced IC50 values by about 20% compared to that of free IVM and significantly decreased cell migration. These findings suggest the potential of inhaled IVM-loaded LPHNPs for lung cancer therapy with improved efficacy.

[Disclosure of COI by Responsible researcher] Lead presenter is the Responsible researcher.
[Lead presenter: Seyedehnegin Kassaee] ■Research funds from company: Queensland University of Technology

### [E-2028] I-E14-1
### Drug delivery system (2)

2025 / 9 / 26 (Fri) 10:15-11:30 Room 14/(ANA Crowne Plaza Hotel Kanazawa 3F Zuiun 1) Room 14

[Chairperson]Kanjiro Miyata (Dept. of Mater., Grad. Schl. Eng., UTokyo)

---

### E-2028
### Nanomedicine-Enhanced PROTAC Delivery for Improved Glioblastoma Therapy

[Speaker]Thi Kim Dung Doan (Innovation Center of Nanomedicine, Kawasaki, Japan) [Co-author]Sabina Quader (Innovation Center of Nanomedicine, Kawasaki, Japan)、Xueying Liu (Innovation Center of Nanomedicine, Kawasaki, Japan)、Wangling Foo (Innovation Center of Nanomedicine, Kawasaki, Japan)、Hiroaki Kinoh (Innovation Center of Nanomedicine, Kawasaki, Japan)、Kazunori Kataoka (Innovation Center of Nanomedicine, Kawasaki, Japan)

Proteolysis-targeting chimeras (PROTACs) offer a promising approach for targeted protein degradation in cancer therapy. However, their clinical application is often limited by poor bioavailability, suboptimal tumor distribution, and potential off-target effects. In this study, we developed a nanomedicine formulation to enhance the delivery of ARV-825, a PROTAC targeting BRD4, in glioblastoma. The ARV-825-loaded micelles, with a size of 70 nm, demonstrated an approximately eight-fold decrease in IC50 compared to free ARV-825, suggesting improved cellular potency. Using an orthotopic GL-261 glioblastoma model, we demonstrated that the nanomedicine significantly inhibited tumor growth and prolonged survival compared to free ARV-825 treatment. Furthermore, combination therapy with the nanomedicine and temozolomide resulted in enhanced tumor suppression and further survival benefit. These findings highlight the potential of PROTAC-loaded nanocarriers to optimize drug delivery and advance precision oncology for glioblastoma.

[Disclosure of COI by Responsible researcher] Lead presenter is the Responsible researcher.
[Lead presenter: Thi Kim Dung Doan] We have no financial relationships to disclose.

### [E-2029] I-E14-1
### Drug delivery system (2)

2025 / 9 / 26 (Fri) 10:15-11:30 Room 14/(ANA Crowne Plaza Hotel Kanazawa 3F Zuiun 1) Room 14

[Chairperson]Kanjiro Miyata (Dept. of Mater., Grad. Schl. Eng., UTokyo)

### E-2029
### Broader therapeutic window via tumor-selective delivery of astatine-211 in radioimmunotherapy

[Speaker]Hiroki Takashima (Div. Developmental Therap., EPOC, Natl. Cancer Ctr.) [Co-author]Shino Manabe (Lab. Synthetic Biomol. Chemistry, Hoshi Univ.／Res. Ctr. for Pharm. Development, Tohoku Univ.／Glycometabolic Biochemistry Lab., RIKEN)、Ryo Tsumura (Div. Developmental Therap., EPOC, Natl. Cancer Ctr.)、Yoshikatsu Koga (Div. Developmental Therap., EPOC, Natl. Cancer Ctr.)、Takahiro Anzai (Div. Developmental Therap., EPOC, Natl. Cancer Ctr.)、Yousuke Kanayama (Nuclear Chemistry Group, Nishina Ctr. for Accelerator-Based Sci., RIKEN)、Nozomi Sato (Nuclear Chemistry Group, Nishina Ctr. for Accelerator-Based Sci., RIKEN)、Hiromichi Shimizu (Nuclear Chemistry Group, Nishina Ctr. for Accelerator-Based Sci., RIKEN)、Sayantani Mitra (Nuclear Chemistry Group, Nishina Ctr. for Accelerator-Based Sci., RIKEN)、Yudai Shigekawa (Nuclear Chemistry Group, Nishina Ctr. for Accelerator-Based Sci., RIKEN)、Akihiro Nambu (Nuclear Chemistry Group, Nishina Ctr. for Accelerator-Based Sci., RIKEN)、Keiko Watanabe (Nuclear Chemistry Group, Nishina Ctr. for Accelerator-Based Sci., RIKEN)、Hiromitsu Haba (Nuclear Chemistry Group, Nishina Ctr. for Accelerator-Based Sci., RIKEN)、Anri Inaki (Div. Functional Imaging, EPOC, Natl. Cancer Ctr.)、Masahiro Yasunaga (Div. Developmental Therap., EPOC, Natl. Cancer Ctr.)

Since alpha radiation is characterized by efficient deoxyribonucleic acid (DNA) double-strand breaks and a short path length in tissues, selective-tumor delivery of alpha emitter is pivotal in cancer therapy. In targeted alpha therapy, alpha emitters released from carriers for tumor targeting cause damage in normal organs.This study investigated the antitoxic effect of a sodium iodide symporter (NIS) inhibitor, which is expected to reduce free astatine-211 (At-211), an alpha emitter, accumulation in the stomach and thyroid, but whose antitoxic activity in radioimmunotherapy (RIT) remains unclear.In a xenograft model treated with At-211-RIT, pretreatment with the NIS inhibitor significantly reduced the uptake in stomach contents, stomach, and thyroid without attenuating tumor accumulation, which resulted in a significantly milder body weight loss with a more rapid recovery. The NIS blockade contributed to significantly fewer DNA double-strand breaks in these cells and minor changes in the histological structure of the thyroid.In astatine-211-RIT, tumor-selective delivery via NIS blockade significantly attenuates toxicity with less damage to stomach and thyroid cells.

[Disclosure of COI by Responsible researcher] Lead presenter is not the Responsible researcher.
[Lead presenter: Hiroki Takashima] We have no financial relationships to disclose.
[Principal Researcher: Masahiro Yasunaga] ■Profit of stock: 株式会社凜研究所

### [E-2030] I-E14-1
### Drug delivery system (2)

2025 / 9 / 26 (Fri) 10:15-11:30 Room 14/(ANA Crowne Plaza Hotel Kanazawa 3F Zuiun 1) Room 14

[Chairperson]Kanjiro Miyata (Dept. of Mater., Grad. Schl. Eng., UTokyo)

---

### E-2030
### Nano-designed carbon monoxide donor suppresses tumor via modulation of microenvironment and metabolism

[Speaker]Jun Fang (Fac. Pharm. Sci., Sojo Univ.) [Co-author]Shanghui Gao (Fac. Pharm. Sci., Sojo Univ.)、Jianrong Zhou (Fac. Pharm. Sci., Sojo Univ.)、Kazumi Yokomizo (Fac. Pharm. Sci., Sojo Univ.)

Tumors exhibit a unique metabolic phenotype known as the Warburg effect, relying heavily on glycolysis for energy. Therefore, altering the metabolic environment presents a rational strategy for tumor suppression. Recent studies have shown that activation of brown adipose tissue (BAT) can lower systemic glucose levels by increasing glucose consumption, thereby inhibiting tumor growth. In this study, we focused on carbon monoxide (CO), a gaseous signaling molecule known to activate BAT, and developed a nanoformulated CO donor (SMA@CORM2) to evaluate its therapeutic potential. In a murine S180 sarcoma model, SMA@CORM2 significantly suppressed tumor growth. Glucose measurement and tolerance tests revealed enhanced insulin secretion and reduced circulating glucose levels after SMA@CORM2 treatment. Histological analysis further indicated BAT activation. These findings suggest that CO donors may exert antitumor effects by modulating glucose metabolism and the tumor microenvironment. Further studies will investigate underlying molecular mechanisms.

[Disclosure of COI by Responsible researcher] Lead presenter is the Responsible researcher.
[Lead presenter: Jun Fang] We have no financial relationships to disclose.

▲TOP

### [J-2031] II-J3-1
### Hepatocellular cancer, biliary tract cancer

2025 / 9 / 26 (Fri) 10:15-11:30 Room 15/(ANA Crowne Plaza Hotel Kanazawa 3F Zuiun 2) Room 15

[Chairperson]Takahiro Kodama (Dept.Gastroenterol. Hepatol., Univ. Osaka., Grad. Sch. Med.)

---

### J-2031
### Evaluation of the Combined Effect of a Novel CompoundX and GC Therapy Using Patient-Derived Cholangiocarcinoma Organoids

[Speaker]Masahiro Umezu (Department of Gastroenterological Surgery Osaka Graduate School of Medicine) [Co-author]Shinichiro Hasegawa (Department of Gastroenterological Surgery Osaka Graduate School of Medicine)、Yosuke Mukai (Department of Gastroenterological Surgery Osaka Graduate School of Medicine)、Kazuki Sasaki (Department of Gastroenterological Surgery Osaka Graduate School of Medicine)、Daisaku Yamada (Department of Gastroenterological Surgery Osaka Graduate School of Medicine)、Yoshito Tomimaru (Department of Gastroenterological Surgery Osaka Graduate School of Medicine)、Takehiro Noda (Department of Gastroenterological Surgery Osaka Graduate School of Medicine)、Hirofumi Akita (Department of Gastroenterological Surgery Osaka Graduate School of Medicine)、Yuichiro Doki (Department of Gastroenterological Surgery Osaka Graduate School of Medicine)、Hidetoshi Eguchi (Department of Gastroenterological Surgery Osaka Graduate School of Medicine)

<Background>Biliary tract cancer (BTC) is a rare malignancy with diverse genetic backgrounds, limiting therapy development. We conducted drug screening using patient-derived BTC organoids retaining tumor traits and identified a novel compound X not used for BTC. The combined effect of compound X and standard gemcitabine-cisplatin (GC) therapy was evaluated using 3D organoid models.<Methods>BTC organoids were established from surgical specimens by the air-liquid interface method. After homogenization via 2D culture, drug screening of 1,143 FDA-approved compounds was performed. Organoids from 10 cases, including screening cases, were cultured in 3D and treated with compound X and GC. Cell viability was measured 72 hours after treatment using CellTiter-Glo 3D assay.<Results>Primary BTC organoids were established from 20 cases. Compound X showed strong antitumor effects. In 10 cases evaluated, compound X markedly inhibited tumor growth despite sensitivity differences. Combination of X and GC further reduced viability, suggesting enhanced effects. In vivo studies are ongoing.<Conclusion>The combination of novel compound X and GC therapy may offer a promising strategy for BTC.

[Disclosure of COI by Responsible researcher] Lead presenter is not the Responsible researcher.
[Lead presenter: Masahiro Umezu] We have no financial relationships to disclose.
[Principal Researcher: Yousuke Mukai] We have no financial relationships to disclose.

### [J-2032] II-J3-1
### Hepatocellular cancer, biliary tract cancer

2025 / 9 / 26 (Fri) 10:15-11:30 Room 15/(ANA Crowne Plaza Hotel Kanazawa 3F Zuiun 2) Room 15

[Chairperson]Takahiro Kodama (Dept.Gastroenterol. Hepatol., Univ. Osaka., Grad. Sch. Med.)

---

### J-2032
### Lipid profiling in metabolic dysfunction associated steatotic liver disease to elucidate hepatocarcinogenesis mechanisms

[Speaker]Junko Kuramoto (Dept. Pathol., Keio Univ., Sch. Med.) [Co-author]Eri Arai (Dept. Pathol., Keio Univ., Sch. Med.)、Hideo Shindo (Dept. Lipid Life Science, JIHS.／Medical Lipid Science, Graduate School of Med., Univ. of Tokyo.)、Yoshihiro Kita (LSCF, Graduate School of Med., Univ. of Tokyo)、Hiroki Muramoto (Dept. Pathol., Keio Univ., Sch. Med.)、Shunsuke Shichi (Dep. Gastroenterological Surg. I, Hokkaido Univ. Grad. Sch.)、Akinobu Taketomi (Dep. Gastroenterological Surg. I, Hokkaido Univ. Grad. Sch.)、Tatsuya Kanto (The Res. Cter. for Hepatitis and Immunology, JIHS.)、Yae Kanai (Dept. Pathol., Keio Univ., Sch. Med.)

This study aimed to investigate abnormalities in lipid metabolism involved in the mechanisms of metabolic dysfunction-associated steatotic liver disease (MASLD)-related hepatocarcinogenesis. We focused on differences between liver tissues showing MASLD from patients without hepatocellular carcinoma (HCC) (O) and those from patients with HCC (W). Multi-omics data from 160 liver tissue specimens were analyzed, with focus on lipid metabolomics. Partial least squares analysis revealed distinct lipid metabolic profiles between O and W. Notably, subclass distributions of triglyceride and phospholipid species differed between the two. These findings suggest not only alterations in the biosynthesis of triglycerides and phospholipids, but also possible differences in the synthesis and secretion of lipoproteins, including VLDL. RNA-seq analysis showed that lipid synthesis-related genes were suppressed, while gluconeogenesis-related genes were upregulated in W compared with O. These results indicate that hepatic lipid and glucose metabolism in MASLD changes in ways that may contribute to hepatocarcinogenesis and highlight the potential for identifying metabolic markers linked to cancer risk.

[Disclosure of COI by Responsible researcher] Lead presenter is the Responsible researcher.
[Lead presenter: Junko Kuramoto] We have no financial relationships to disclose.

### [J-2033] II-J3-1
### Hepatocellular cancer, biliary tract cancer

2025 / 9 / 26 (Fri) 10:15-11:30 Room 15/(ANA Crowne Plaza Hotel Kanazawa 3F Zuiun 2) Room 15

[Chairperson]Takahiro Kodama (Dept.Gastroenterol. Hepatol., Univ. Osaka., Grad. Sch. Med.)

---

### J-2033
### The impact of polyploidy on the pathogenesis of human intrahepatic cholangiocarcinoma

[Speaker]Kenji Nagata (Kobe University Department of Gastroenterology) [Co-author]Takanori Matsuura (Kobe University Department of Gastroenterology)、Taku Kitano (Kyoto University, Graduate School of Medicine, Department of Surgery)、Kazuki Hayashi (University of Osaka Department of Otorhinolaryngology & Head and Neck Surgery)、Miki Kawano (Kobe University Department of Gastroenterology)、Yuzo Kodama (Kobe University Department of Gastroenterology)、Eiji Hara (Department of Molecular Biology, RIMD, The University of Osaka)、Tomonori Matsumoto (Laboratory of Ploidy Pathology, FBS, The University of Osaka)

Intrahepatic cholangiocarcinoma (ICC) is the second most common type of primary liver cancer. Although polyploidization is thought to be closely associated with chromosomal instability, leading to poor prognosis, the impact of ploidy on the pathogenesis of human ICC remains unclear. We evaluated the ploidy of tumor cells and their intratumoral heterogeneity in human ICC tissues using fluorescence in situ hybridization targeting multiple chromosomes at the single-cell level. Among the 36 tumors examined, 19.4% (7/36) were predominantly composed of polyploid tumor cells. Notably, the median ploidy of tumor cells within a tumor is closely correlated with the degree of intratumoral heterogeneity of tumor cellular ploidy (r = 0.94). The presence of polyploid giant cancer cells was also positively correlated with the median and intratumoral heterogeneity of tumor cellular ploidy. Moreover, all polyploid ICCs were poorly differentiated and exhibited significantly higher Ki-67 positivity than diploid ICCs. These findings indicate that polyploidy is closely associated with intratumoral heterogeneity and determines a highly malignant subset of human ICC.

[Disclosure of COI by Responsible researcher] Lead presenter is not the Responsible researcher.
[Lead presenter: Kenji Nagata] We have no financial relationships to disclose.
[Principal Researcher: Tomonori Matsumoto] We have no financial relationships to disclose.

▲TOP

### [J-2034] II-J3-1
### Hepatocellular cancer, biliary tract cancer

2025 / 9 / 26 (Fri) 10:15-11:30 Room 15/(ANA Crowne Plaza Hotel Kanazawa 3F Zuiun 2) Room 15

[Chairperson]Takahiro Kodama (Dept.Gastroenterol. Hepatol., Univ. Osaka., Grad. Sch. Med.)

---

### J-2034
### Functional difference of MYC and MYCN in liver cancer: role of HNF1B in combined hepatocellular-cholangiocarcinoma

[Speaker]Masanori Goto (Div. of Tumor Pathol., Dept. of Pathol., Asahikawa Med. Univ.) [Co-author]Masahiro Yamamoto (Dept. of Tumor Pathol.,Grad. Schol. of Health Sci.,Kumamoto Univ.)、Hiroki Tanaka (Div. of Tumor Pathol., Dept. of Pathol., Asahikawa Med. Univ.)、Yumiko Fujii (Div. of Tumor Pathol., Dept. of Pathol., Asahikawa Med. Univ.)、Yuki Kamikokura (Div. of Tumor Pathol., Dept. of Pathol., Asahikawa Med. Univ.)、Masayo Kamikokura (Div. of Tumor Pathol., Dept. of Pathol., Asahikawa Med. Univ.)、Kumi Takasawa (Div. of Tumor Pathol., Dept. of Pathol., Asahikawa Med. Univ.)、Yuji Nishikawa (Div. of Tumor Pathol., Dept. of Pathol., Asahikawa Med. Univ.)、Akira Takasawa (Div. of Tumor Pathol., Dept. of Pathol., Asahikawa Med. Univ.)

MYC and MYCN oncogenes are upregulated in human liver cancers, yet their functional differences remain unclear. MYC is activated in human combined hepatocellular-cholangiocarcinoma (cHCC-CCA) and may be involved in its development. We previously reported that cHCC-CCAs were induced when introduced Myc or Mycn together with AKT and YAP genes using a transposon-mediated somatic gene integration system, and Mycn tumors exhibited a lower area of cholangiocarcinoma (CCA) components compared to Myc tumors. Here, to explore the underlying mechanisms, we analyzed the expression levels of genes involved in liver differentiation and found that Mycn tumors exhibited expression of HNF1B that is highly expressed in human CCA. When Hnf1B was co-introduced with AKT/YAP/Mycn, CCA area significantly increased. Moreover, these tumors exhibited increased expression of TEAD proteins and treatment with a TEAD inhibitor repressed the growth of Hnf1B-introduced tumors. Our findings indicate that MYC and MYCN play distinct roles in the phenotypic determination of liver tumors and suggest that their differential effect on Hnf1b expression and subsequent TEAD activation may be a key regulatory mechanism.

[Disclosure of COI by Responsible researcher] Lead presenter is not the Responsible researcher.
[Lead presenter: Masanori Goto] We have no financial relationships to disclose.
[Principal Researcher: Akira Takasawa] We have no financial relationships to disclose.

### [J-2035] II-J3-1
### Hepatocellular cancer, biliary tract cancer

2025 / 9 / 26 (Fri) 10:15-11:30 Room 15/(ANA Crowne Plaza Hotel Kanazawa 3F Zuiun 2) Room 15

[Chairperson]Takahiro Kodama (Dept.Gastroenterol. Hepatol., Univ. Osaka., Grad. Sch. Med.)

### J-2035
### Transcription factor JUNB promotes EMT of hepatocarcinoma stem cells and induces distant metastasis.

[Speaker]Yutaro Kawakami (Department of Gastroenterology, Kanazawa University Graduate School of Medical Science) [Co-author]Kouki Nio (Department of Gastroenterology, Kanazawa University Graduate School of Medical Science)、Taro Yamashita (Department of Gastroenterology, Kanazawa University Graduate School of Medical Science)

Background: We have previously reported the presence of AFP-producing epithelial (EpCAM-positive) stem cells and LG2m-producing, mesenchymal (CD90-positive) stem cells in HCC. In this study, we analyzed the interaction between EpCAM-positive cells and stromal cells. Methods: EpCAM-positive cells and fibroblasts were co-cultured, and changes in cell surface markers and gene expression were examined by Flow cytometry and RT-PCR. In addition, RNA-seq analysis was used to identify transcription factors that contribute to changes in cell phenotype. Results: A novel population of CD90-positive cells emerged from EpCAM-positive cells when co-cultured with fibroblasts. Furthermore, subcutaneous transplantation of EpCAM-positive cells co-cultured with fibroblasts into NOD SCID mice (N=5) showed significantly more lung metastases ($p=0.009$) compared to non-cultured cells. JUNB overexpression enhanced CD90, EMT-related markers and mice transplanted with JUNB-transfected hepatocarcinoma cells developed lung metastasis at a high rate. Conclusions: The transcription factor JUNB induces epithelial-mesenchymal transition and metastatic potential of EpCAM-positive cells.

[Disclosure of COI by Responsible researcher] Lead presenter is the Responsible researcher.
[Lead presenter: Yutaro Kawakami] We have no financial relationships to disclose.

▲TOP

### [J-2036] II-J3-1
### Hepatocellular cancer, biliary tract cancer

2025 / 9 / 26 (Fri) 10:15-11:30 Room 15/(ANA Crowne Plaza Hotel Kanazawa 3F Zuiun 2) Room 15

[Chairperson]Takahiro Kodama (Dept.Gastroenterol. Hepatol., Univ. Osaka., Grad. Sch. Med.)

### J-2036
### Withdrawn

No Abstract

### [E-2037] I-E2-2
### Organoid/Animal model (2)

2025 / 9 / 26 (Fri) 10:15-11:30 Room 16/(11F Visage) Room 16

[Chairperson]Daisuke Shiokawa (Ehime Univ. Hosp, Translational. Res. Ctr.)

---

### E-2037
### Elucidation of the mechanisms underlying drug resistance caused by heterogeneity of high-grade endometrial cancer

[Speaker]Yoshiaki Maru (Lab. Precis. Tumor Model. Syst., Chiba Cancer Ctr. Res. Inst.) [Co-author]Naotake Tanaka (Dept. Gynecol., Chiba Cancer Ctr.)、Yoshitaka Hippo (Lab. Precis. Tumor Model. Syst., Chiba Cancer Ctr. Res. Inst.)

Among endometrial cancer (EC), non-endometrioid (non-EM) type exhibits poorer clinical outcome. Although its underlying mechanism remains elusive, it can be caused by the drug-resistance due to heterogeneity, as often observed in many cancers. Since patient-derived organoids (PDOs) can recapitulate the original tumor's features, multiple PDOs from the same tumor might reflect its heterogeneity at high resolution. Therefore, we established 53 PDOs from 15 non-EM type ECs. These multiple PDOs indeed exhibited diversity in genomic profile, mitochondrial DNA mutations, and protein expression. More importantly, drug sensitivity also varied, which prompted us to conduct single cell RNA sequencing analysis to gain insights into the mechanisms underlying drug resistance. We identified several clusters of tumor cells in five PDOs obtained from a carcinosarcoma case, of which one cluster was enriched in drug resistant PDOs, suggesting that tumor heterogeneity may underlie drug resistance in this case and suggest that this might be also the case with other cases of non-EM type. Thus, using multiple PDOs per tumor may help link the heterogeneity to drug resistance in many high-grade cancers.

[Disclosure of COI by Responsible researcher] Lead presenter is not the Responsible researcher.
[Lead presenter: Yoshiaki Maru] ■Research funds from company: 日本学術振興会　■Research expenses: 第一三共株式会社
[Principal Researcher: Yoshitaka Hippo] We have no financial relationships to disclose.

▲TOP

### [E-2038] I-E2-2
### Organoid/Animal model (2)

2025 / 9 / 26 (Fri) 10:15-11:30 Room 16/(11F Visage) Room 16

[Chairperson]Daisuke Shiokawa (Ehime Univ. Hosp, Translational. Res. Ctr.)

---

### E-2038
### Transcription differences in hemangiosarcoma vs. nodular hyperplasia using organoids&xenograft models reveal new targets

[Speaker]Yishan Liu (TUAT Laboratory of Veterinary Pharmacology) [Co-author]Haru Yamamoto (TUAT Laboratory of Veterinary Pharmacology／Airdec mini Co.)、Mohamed Elbadawy (TUAT Laboratory of Veterinary Pharmacology／Department of Pharmacology, Faculty of Veterinary Medicine, Benha University)、Kazuaki Sasaki (TUAT Laboratory of Veterinary Pharmacology)

Angiosarcoma (AS) is an aggressive subtype of soft tissue sarcoma of endothelial cell origin with a propensity for local recurrence and metastasis. Preclinical models are necessary for AS new treatment discovery. Canine hemangiosarcoma (HSA) is considered a more pertinent preclinical model for AS than rodent models due to the similar pathology and molecular signatures. However, the HSA xenograft mouse model still needs standardization.In this study, we successfully generated 5 HSA 2.5D organoid lines from patient tumor tissues which were useful for drug screening. RNA sequencing compared HSA with benign tumor nodular hyperplasia (NH) showed pathways related to autophagy and lysosomal membranes were upregulated in HSA than NH. Also, we established an orthotopic xenograft model by spleen injection. Pathology results of xenograft tumors could maintain original patient tumor features. We also generated 3D organoids from xenograft tumors. Pathology and TEM micrograph results suggested that HSA 3D organoids maintain the microstructural feature of blood vessel sarcoma. In conclusion, this study offered a reference for HSA and AS therapeutic predictions in basic medical science.

[Disclosure of COI by Responsible researcher] Lead presenter is not the Responsible researcher.
[Lead presenter: Yishan Liu] We have no financial relationships to disclose.
[Principal Researcher: Kazuaki Sasaki] We have no financial relationships to disclose.

### [E-2039] I-E2-2
### Organoid/Animal model (2)

2025 / 9 / 26 (Fri) 10:15-11:30 Room 16/(11F Visage) Room 16

[Chairperson]Daisuke Shiokawa (Ehime Univ. Hosp, Translational. Res. Ctr.)

---

### E-2039
### The Iqgap3–Kras axis orchestrates early gastric tumorigenesis

[Speaker]Mitsuhiro Shimura (Dept. of Surg., Tohoku Univ. Grad. Sch. of Med.／Natl. Univ. of Singapore, Cancer Sci. Inst. of Singapore) [Co-author]Junichi Matsuo (Natl. Univ. of Singapore, Cancer Sci. Inst. of Singapore)、Daisuke Douchi (Dept. of Surg., Tohoku Univ. Grad. Sch. of Med.)、Michiaki Unno (Dept. of Surg., Tohoku Univ. Grad. Sch. of Med.)、Yoshiaki Ito (Natl. Univ. of Singapore, Cancer Sci. Inst. of Singapore)

IQGAP3 is a scaffold protein implicated in oncologic signalings. IQGAP3-positive cells are predominantly localized to the isthmus region, which harbors gastric stem/progenitor cells, and exhibit substantial overlap with Ki67-positive cells under both homeostatic and tissue injury status. Although aberrant upregulation of IQGAP3 has been documented in various advanced malignancies, its contribution to early carcinogenesis remains unclear. To elucidate the role of IQGAP3 in the initiation of gastric neoplasm, we employed transgenic mouse models, including ***eR1-cre;Kras***$^{G12D}$ and ***eR1-cre;Kras***$^{G12D}$***;Iqgap3***$^{flox/flox}$. After tamoxifen induction, ***Kras***$^{G12D}$ developed pre-cancerous metaplasia. Notably, Iqgap3 deletion significantly suppressed the frequency and the number of Ki67-positive cells per metaplasia. ***Kras***$^{G12D}$-induced p-ERK and Il-33 upregulation and metaplasia-related genes, such as Tff2, Cd44, and Sox9, were suppressed in Iqgap3-deficient mice. In parallel, the recruitment of Acta2-positive fibroblasts to the base of metaplasia was significantly attenuated. Collectively, these findings demonstrate that the Iqgap3-Kras axis plays a crucial role in early gastric tumorigenesis.

[Disclosure of COI by Responsible researcher] Lead presenter is the Responsible researcher.
[Lead presenter: Mitsuhiro Shimura] We have no financial relationships to disclose.

### [E-2040] I-E2-2
### Organoid/Animal model (2)

2025 / 9 / 26 (Fri) 10:15-11:30 Room 16/(11F Visage) Room 16

[Chairperson]Daisuke Shiokawa (Ehime Univ. Hosp, Translational. Res. Ctr.)

---

### E-2040
### NCG-MHC-dKO Mice - an Ideal Model for the Evaluation of Bispecific T Cell Engagers and in vivo CAR-T Therapies

[Speaker]Shiying Guo (GemPharmatech Co., Ltd.) [Co-author]Jun Xing (GemPharmatech Co., Ltd.)、Jianming Xu (GemPharmatech Co., Ltd.)、Hongyan Sun (GemPharmatech Co., Ltd.)、Xiang Gao (GemPharmatech Co., Ltd.)

To overcome graft-versus-host disease (GvHD) limitations in PBMC-reconstituted mice, we developed the NCG-MHC-dKO model by knocking out MHC class I/II genes, which enabling longer-term studies of immunotherapies without GvHD interference.
The NCG-MHC-dKO model shows strong translational value for evaluating bispecific T-cell engagers (TCEs). In proof-of-concept studies, NCG-MHC-dKO mice reconstituted with PBMCs and inoculated with NCI-H929 or SHP-77 cell lines exhibited potent tumor growth inhibition when treated with the TCEs Talquetamab or Tarlatamab, respectively. The treated mice maintained stable body weight and normal clinical parameters throughout the study without GvHD associated morbidity. Besides TCEs, the NCG-MHC-dKO model reconstituted with PBMCs is also appropriate for evaluating ***in vivo*** CAR-T therapy. In this model, we demonstrated the effectiveness of an anti-CD19 CAR lentiviral treatment for B-cell lymphoma. The results showed that the treated mice had strong tumor suppression, in contrast to the control group. Importantly, the absence of severe GvHD enabled continuous monitoring of CAR-T activity over a long period.

[Disclosure of COI by Responsible researcher] Lead presenter is the Responsible researcher.
[Lead presenter: Shiying Guo] We have no financial relationships to disclose.

### [E-2041] I-E2-2
### Organoid/Animal model (2)

2025 / 9 / 26 (Fri) 10:15-11:30 Room 16/(11F Visage) Room 16

[Chairperson]Daisuke Shiokawa (Ehime Univ. Hosp, Translational. Res. Ctr.)

---

### E-2041
### Preclinical evaluation of cancer immunotherapy targeting human macrophages in humanized mouse models

[Speaker]Yasuyuki Saito (Dept. Immunol. Faculty of Med. Shimane Univ.／Div. Biosignal Reg. Kobe Univ. Grad. Sch. Med.) [Co-author]Tania Afroj (Dept. Immunol. Faculty of Med. Shimane Univ.／Div. Biosignal Reg. Kobe Univ. Grad. Sch. Med.)、Tomoko Takai (Div. Biosignal Reg. Kobe Univ. Grad. Sch. Med.)、Takenori Kotani (Div. Mol. Cell. Signal. Kobe Univ. Grad. Sch. Med.)、Yoji Murata (Div. Mol. Cell. Signal. Kobe Univ. Grad. Sch. Med.)、Ikumi Katano (Immunol. Lab., Div. Multiverse Med. Sci., Central Inst. Exp. Med.)、Takeshi Takahashi (Immunol. Lab., Div. Multiverse Med. Sci., Central Inst. Exp. Med.)、Takashi Matozaki (Div. Biosignal Reg. Kobe Univ. Grad. Sch. Med.)

Macrophages (MPs) are the most abundant immune cells in the tumor microenvironment (TME). MPs highly express Ig-like receptor SIRP$\alpha$, which inhibits tumor cell phagocytosis upon interaction with CD47 on target tumor cells. We previously demonstrated that treatment with anti-mouse SIRP$\alpha$ antibody (Ab) enhances the anti-tumor effect by opsonizing tumor cells as well as blocking the CD47-SIRP$\alpha$ interaction against SIRP$\alpha$-expressing tumors such as renal cell carcinoma (RCC) and melanoma. However, the in vivo efficacy of anti-human (h) SIRP$\alpha$ Ab remains uncertain due to the limited specificity to human MPs in existing preclinical tumor models. Here, we generated a humanized mouse model by transplantation of human cord blood CD34+ hematopoietic stem cells into NOG-Fc$\gamma$ receptor knock-out (NOG-Fc$\gamma$RKO) mice, minimizing the influence of mouse-derived MPs in response to the Ab. We found increased infiltration of human MPs in the RCC tumor and significant inhibition of RCC growth following hSIRP$\alpha$ Ab treatment in humanized NOG-Fc$\gamma$RKO mice. Thus, our preclinical tumor model reveals the therapeutic potential of hSIRP$\alpha$ Ab in targeting human MPs within the TME in vivo.

[Disclosure of COI by Responsible researcher] Lead presenter is the Responsible researcher.
[Lead presenter: Saito Yasuyuki] We have no financial relationships to disclose.

▲TOP

### [E-2042] I-E2-2
### Organoid/Animal model (2)

2025 / 9 / 26 (Fri) 10:15-11:30 Room 16/(11F Visage) Room 16

[Chairperson]Daisuke Shiokawa (Ehime Univ. Hosp, Translational. Res. Ctr.)

---

### E-2042
### Meflin-positive cancer-associated fibroblasts exhibit anti-tumor effects in advanced ovarian cancer.

[Speaker]Emiri Miyamoto (Department of Obstetrics and Gynecology, Nagoya University) [Co-author]Masato Yoshihara (Department of Obstetrics and Gynecology, Nagoya University)、Shohei Iyoshi (Department of Obstetrics and Gynecology, Nagoya University／Institute for Advanced Research, Nagoya University)、Kazumasa Mogi (Department of Obstetrics and Gynecology, Nagoya University／Department of Medical Genomics Center, Nagoya University)、Kaname Uno (Department of Obstetrics and Gynecology, Nagoya University／Division of Oncology, Department of Clinical Sciences, Lund University)、Hiroki Fujimoto (Department of Obstetrics and Gynecology, Aichi Medical University)、Yoshihiro Koya (Department of Obstetrics and Gynecology, Nagoya University)、Kazuhisa Kitami (Department of Gynecologic Oncology, Aichi Cancer Center)、Atsushi Enomoto (Department of Pathology, Graduate School of Medicine, Nagoya University)、Hiroaki Kajiyama (Department of Obstetrics and Gynecology, Nagoya University)

Cancer-associated fibroblasts (CAFs) are known to play an important role in the acquisition of malignant hallmarks; however, the tumor-suppressive effect of meflin-positive CAFs has been noted in several carcinomas. This study aims to elucidate the function of meflin-positive CAFs in the peritoneal dissemination of advanced ovarian cancer (aOC/OC). We performed in situ hybridization (ISH) on aOC specimens (N=37) to examine the impact of Meflin-positive CAFs for prognosis. Next, we performed in vivo analysis with Meflin KO mice and AM80, a drug inducing meflin expression in CAFs. In ISH, we confirmed presence of Meflin-positive CAFs in the peritoneal dissemination of aOC (19/37 :51.3%). Higher proportion of Meflin-positive CAFs was significantly associated with longer overall survival (OS) {3-year OS rate: positive (N=12): 63.1%, negative (N=8): 44.4%, P=0.0497}. In Meflin KO mice, increased ascites volume and tumor weight were observed. Moreover, CBDCA+Am80 group showed decreased ascites volume and tumor weight compared with CBDCA group. The presence of meflin-positive CAFs may regulate peritoneal metastatic potential in aOC.

[Disclosure of COI by Responsible researcher] Lead presenter is not the Responsible researcher.
[Lead presenter: Emiri Miyamoto] We have no financial relationships to disclose.
[Principal Researcher: Hiroaki Kajiyama] We have no financial relationships to disclose.

### [IAL] IAL
### JCA International Award Lecture

2025 / 9 / 26 (Fri) 10:40-11:30 Room 2/(Ishikawa Ongakudo 2F Hogaku Hall) Room 2

[Chairperson]Hideyuki Yanai (Research Center for Advanced Science and Technology (RCAST), The University of Tokyo)

---

### IAL
### Updated perspectives on innate immune regulators in upper gastrointestinal cancers

[Speaker]J. Jenkins Brendan (South Australian immunoGENomics Cancer Institute (SAiGENCI), The University of Adelaide)

Overactivation of the innate immune system promotes chronic inflammation that is associated with one third of all cancers. In addition, it has emerged that innate immunity can modulate intrinsic tumor-promoting effects (e.g. proliferation) directly on cancer cells. Immunotherapy approaches in cancer are largely based on adaptive immune checkpoint inhibitors (e.g. anti-PD-1) and CAR-T cell therapy, however the clinical potential of targeting innate immune regulators in cancer is underexplored. Innate immune responses are triggered by families of cell surface, endosomal and cytosolic pattern recognition receptors (PRRs), such as Toll-like receptors (TLRs), Absent in melanoma 2 (AIM2)-like receptors (ALRs), and Nod-like receptors (NLRs). A subset of PRRs also form large multiprotein 'inflammasome' complexes which regulate the maturation and release of bioactive pro-inflammatory cytokines, IL-1$\beta$ and IL-18. Given the importance of inflammasome-associated PRRs to the net output of innate immune responses, recent advancements on the diverse role and potential therapeutic targeting of inflammasomes and their PRRs in inflammation-associated gastrointestinal cancers will be presented.

[Disclosure of COI by Responsible researcher] Lead presenter is the Responsible researcher.
[Lead presenter: Brendan J. Jenkins] We have no financial relationships to disclose.

▲TOP

### [LS13-1] LS13

### Team for advanced technical support and efficient research management in P-PROMOTE/ BINDS Activity Support Team for Collaboration Promotion Office / Innovative technologies or resources to support translational research in the field of drug development against cancer

2025 / 9 / 26 (Fri) 11:50-12:40 Room 1/(Ishikawa Ongakudo 2F Concert Hall) Room 1

[]Team for advanced technical support and efficient research management in P-PROMOTE/ BINDS Activity Support Team for Collaboration Promotion Office

[Chairperson]Kohei Miyazono (The University of Tokyo, Graduate School of Medicine, Applied Pathology)

---

### LS13-1

### 1) Advanced Technical Support by BINDS: New Initiatives to Accelerate Drug Discovery in Academia

[Speaker]Tsuyoshi Inoue (Osaka University, Graduate School of Pharmaceutical Sciences)

No Abstract

### [LS13-2] LS13

### Team for advanced technical support and efficient research management in P-PROMOTE/ BINDS Activity Support Team for Collaboration Promotion Office / Innovative technologies or resources to support translational research in the field of drug development against cancer

2025 / 9 / 26 (Fri) 11:50-12:40 Room 1/(Ishikawa Ongakudo 2F Concert Hall) Room 1
[]Team for advanced technical support and efficient research management in P-PROMOTE/ BINDS Activity Support Team for Collaboration Promotion Office

[Chairperson]Kohei Miyazono (The University of Tokyo, Graduate School of Medicine, Applied Pathology)

---

### LS13-2
### 2) An approach to preclinical R&D from seed identification to POC establishment for antitumor efficacy in academia (Drug discovery initiatives in P-PROMOTE)

[Speaker]Tetsuo Noda (Japanese Foundation for Cancer Research)

No Abstract

▲TOP

### [LS14] LS14

### CHUGAI PHARMACEUTICAL CO.,LTD. / Cell-free DNA Analysis in Malignant Lymphoma: Recent Findings and Clinical Applications

2025 / 9 / 26 (Fri) 11:50-12:40 Room 3/(Hotel Nikko Kanazawa 4F Tsuru A) Room 3
[]CHUGAI PHARMACEUTICAL CO.,LTD.

[Chairperson]Koichi Akashi (Director: Realization Hub for Biomedical Innovation, Senior Vice President, Kyushu University)

---

### LS14

### Cell-free DNA Analysis in Malignant Lymphoma: Recent Findings and Clinical Applications

[Speaker]Takeshi Sugio (Stanford University Division of Oncology)

No Abstract

▲TOP

### [LS15] LS15

### Amgen K.K. / Transforming Small Cell Lung Cancer Treatment ~Durable survival benefit from bispecific antibody~

2025 / 9 / 26 (Fri) 11:50-12:40 Room 4/(Hotel Nikko Kanazawa 4F Tsuru B) Room 4
[]Amgen K.K.

[Chairperson]Satoshi Oizumi (Department of Respiratory Medicine, NHO Hokkaido Cancer Center)

---

### LS15

### Transforming Small Cell Lung Cancer Treatment ~Durable survival benefit from bispecific antibody~

[Speaker]Hiroshi Kagamu (Department of Respiratory Medicine, Saitama Medical University International Medical Center)

No Abstract

### [LS16] LS16

### SHIBUYA CORPORATION / Future perspective of next-generation CAR-T cell therapy and automated cell manufacturing technologies

2025 / 9 / 26 (Fri) 11:50-12:40 Room 5/(Hotel Nikko Kanazawa 4F Tsuru C) Room 5
[]SHIBUYA CORPORATION

[Chairperson]Katsuya Tsuchihara (National Cancer Center Japan Exploratory Oncology Research & Clinical Trial Center)

---

### LS16

### Future perspective of next-generation CAR-T cell therapy and automated cell manufacturing technologies

[Speaker]Koji Tamada (Yamaguchi University Graduate School of medicine, Department of Immunology)

No Abstract

### [LS17] LS17

### Nippon Shinyaku Co., Ltd. / Current treatments for lymphoid neoplasms: The Impact of Pirtobrutinib on Treatment Strategies

2025 / 9 / 26 (Fri) 11:50-12:40 Room 6/(Hotel Nikko Kanazawa 3F Kujaku A) Room 6
[]Nippon Shinyaku Co., Ltd.

[Chairperson]Kazuhito Yamamoto (Aichi Cancer Center)

---

### LS17

### Current treatments for lymphoid neoplasms: The Impact of Pirtobrutinib on Treatment Strategies

[Speaker]Jun Takizawa (Department of Hematology, Endocrinology and Metabolism, Niigata University Faculty of Medicine)

No Abstract

▲TOP

### [LS18] LS18
### TAKARA BIO, Inc. / Technology Development for the expanding spatial analysis of cancers

2025 / 9 / 26 (Fri) 11:50-12:40 Room 7/(Hotel Nikko Kanazawa 3F Kujaku B) Room 7
[]TAKARA BIO, Inc.

[Chairperson]Akiyuki Satou (Genome Analysis Center, TAKARA BIO, Inc.)

---

### LS18
### Technology Development for the expanding spatial analysis of cancers

[Speaker]Yutaka Suzuki (Life Science Data Research Center, Graduate School of Frontier Sciences, The University of Tokyo)

No Abstract

### [LS19] LS19

### Kyowa Kirin Co., Ltd. / The Role of Gut Microbiota in the Era of Immune Checkpoint Inhibitor Chemotherapy Combination Therapy: Implications for Febrile Neutropenia Management with G-CSF

2025 / 9 / 26 (Fri) 11:50-12:40 Room 8/(Hotel Nikko Kanazawa 3F Kujaku C) Room 8
[]Kyowa Kirin Co., Ltd.

[Chairperson]Seiji Yano (Department of Respiratory Medicine, Faculty of Medicine, Institute of Medical, Pharmaceutical, and Health Sciences, Kanazawa University)

---

### LS19

### The Role of Gut Microbiota in the Era of Immune Checkpoint Inhibitor–Chemotherapy Combination Therapy: Implications for Febrile Neutropenia Management with G-CSF

[Speaker]Kiyoshi Yoshimura (Department of Clinical Immuno Oncology, Clinical Research Institute of Clinical Pharmacology and Therapeutics, Showa Medical University ／ Division of Medical Oncology, Department of Medicine, Showa Medical University School of Medicine)

No Abstract

### [LS20-1] LS20
### 10x Genomics / Pioneering cancer research: unlocking new frontiers with spatial multiomics

2025 / 9 / 26 (Fri) 11:50-12:40 Room 9/(Hotel Nikko Kanazawa 3F Lumiere) Room 9
[]10x Genomics

[Chairperson]Mio Tonouchi (10x Genomics, Regional Marketing, APAC)

---

### LS20-1
### 1) Spatial analysis reveals origin of cancer

[Speaker]Yosuke Tanaka (National Cancer Center Research Institute, Division of Cellular Signaling)

No Abstract

▲TOP

### [LS20-2] LS20
### 10x Genomics / Pioneering cancer research: unlocking new frontiers with spatial multiomics

2025 / 9 / 26 (Fri) 11:50-12:40 Room 9/(Hotel Nikko Kanazawa 3F Lumiere) Room 9
[]10x Genomics

[Chairperson]Mio Tonouchi (10x Genomics, Regional Marketing, APAC)

---

### LS20-2
### 2) Revolutionizing cancer research through spatial transcriptomics with Visium HD & Xenium

[Speaker]Yuji Sato (10x Genomics)

No Abstract

### [LS21] LS21
### AstraZeneca K.K. / BTK inhibition in the treatment of CLL: past and future

2025 / 9 / 26 (Fri) 11:50-12:40 Room 12/(ANA Crowne Plaza Hotel Kanazawa 3F Ohtori (Central)) Room 12
[]AstraZeneca K.K.

[Chairperson]Toshihiro Miyamoto (Department of Hematology, Faculty of Medicine, Institute of Medical Pharmaceutical and Health Sciences, Kanazawa University)

---

### LS21
### BTK inhibition in the treatment of CLL: past and future

[Speaker]Yoshikane Kikushige (Center for Cellular and Molecular Medicine, Kyushu University Hospital)

No Abstract

### [LS22] LS22
### Astellas Pharma Inc. / Treatment strategies for AML in the era of molecular targeted therapy

2025 / 9 / 26 (Fri) 11:50-12:40 Room 13/(ANA Crowne Plaza Hotel Kanazawa 3F Ohtori (East)) Room 13
[]Astellas Pharma Inc.

[Chairperson]Kato Motohiro (Professor, Department of Pediatrics, the University of Tokyo)

---

### LS22
### Treatment strategies for AML in the era of molecular targeted therapy

[Speaker]Yuichi Ishikawa (Lecture, Department of Hematology and Oncology, Nagoya University Graduate School of Medicine)

No Abstract

### [LS23] LS23

### Shimadzu Corporation / Visualization of Drug Distribution in cancer tissue by Mass Spectrometry Imaging— Exploring the Future of Cancer Research in Comparison with Raman Spectroscopic Analysis and Comprehensive Genetic Analysis —

2025 / 9 / 26 (Fri) 11:50-12:40 Room 14/(ANA Crowne Plaza Hotel Kanazawa 3F Zuiun 1) Room 14
[]Shimadzu Corporation

[Chairperson]Takushi Yamamoto (Shimadzu Corporation, Analytical and Measuring Instruments Division, Solutions COE, Healthcare Solutions Unit.)

---

### LS23

[Speaker]Yasuto Naoi (Kyoto Prefectural University of Medicine, Graduate School of Medical Science, Division of Endocrine and Breast Surgery)

No Abstract

▲TOP

### [LS24] LS24

### TOHO Holdings Co., Ltd.／REPROCELL Inc. / Perspectives for Adoptive Cellular Immunotherapy of Cancer -TIL, TCR-T, CAR-T-

2025 / 9 / 26 (Fri) 11:50-12:40 Room 15/(ANA Crowne Plaza Hotel Kanazawa 3F Zuiun 2) Room 15
[]TOHO Holdings Co., Ltd.／REPROCELL Inc.

[Chairperson]Hiroaki Ikeda (Professor, Department of Oncology, Nagasaki University Graduate School of Biomedical Sciences)

---

### LS24
### Perspectives for Adoptive Cellular Immunotherapy of Cancer -TIL, TCR-T, CAR-T-

[Speaker]Yutaka Kawakami (Professor,Department of Immunology,Graduate School of Medicine, International University of Health and Welfare(IUHW)

No Abstract

Special Symposia

### [SS4-1] SS4
### AMED Symposium "Aiming for prevention and cure of cancer-Challenges and prospects for cancer research in AMED's third term"

2025 / 9 / 26 (Fri) 12:50-15:20 Room 1/(Ishikawa Ongakudo 2F Concert Hall) Room 1

[Chairpersons]Toru Hirota (Japanese Foundation for Cancer Research, Cancer Instiutte)、Chizu Tanikawa (The University of Tokyo)

AMED's cancer research from the first to second term has achieved many results, with the goal of linking academic basic research to clinical practice and delivering it to patients as soon as possible, based on the "10-Year Cancer Research Strategy" formulated in 2014. The speed of new drug development over the past decade has been remarkable, and with medical advances, patient needs have become more diverse, calling for the enhancement of supportive and palliative care.⊠In the third term of AMED, based on the new "5th 10-Year Cancer Research Strategy", AMED aims to promote "cancer prevention, diagnosis, and treatment," actively support research projects that contribute to unmet medical needs such as intractable and rare cancers for which effective diagnostic and treatment methods have not yet been established, and continue to place emphasis on the practical application of drug discovery from academia, aiming to prevent and cure cancer.⊠This symposium will address two themes, "Realizing innovative cancer treatments" and "Cancer prevention and cure," and will discuss prospects for cancer prevention and cure. It is hoped that this symposium will lead to the realization of innovative cancer treatments for intractable and rare cancers, which still need adequate treatment, and to a cure for cancer.⊠⊠AMEDの第1期から第2期にかけてのがん研究は、2014年に策定された「がん研究10年戦略」に基づき、学術的基礎研究を臨床に繋げ、患者さんに一日も早く届けることを目標に、多くの成果を上げてきました。過去10年間の新薬開発のスピードは目覚ましく、医療の進歩に伴い患者さんのニーズは多様化し、支持療法や緩和ケアの充実が求められています。⊠AMED第3期では、新たな「第5期がん研究10年戦略」等に基づき、「がんの予防・診断・治療」を推進し、有効な診断・治療法が確立されていない難治がんや希少がんなど、アンメットメディカルニーズに資する研究プロジェクトを積極的に支援するとともに、がんの予防と治癒を目指し、アカデミア発の創薬の実用化に引き続き重点的に取り組んでいきます。⊠本シンポジムで取り上げる「革新的がん医療の実現」と「がんの予防と根治」の2つのテーマについて、がんの予防と根治を目指す将来展望について議論され、未だ充足されていない難治性がんや希少がんに対する革新的ながん医療の実現とがんの根治に繋がることを期待します。

---

### SS4-1
### Strategy for Controlling Metastatic Recurrence by Dormancy Induction

[Speaker]Jun(NCC) Nakayama (Dept. Oncogenesis Growth Regulation, Res. Inst., Osaka Intl. Cancer Inst.)

Distant metastasis and metastatic recurrence remain some of the leading causes of cancer mortality, and there is an urgent need to elucidate the mechanisms and establish therapeutic strategies. In breast cancer, most disseminated cancer cells in the distant organs stay in a dormant state. Cellular dormancy is generally considered as "staying in the reversible G0 phase" and "surviving for months, years, or decades in a hypometabolic state". However, the molecular definition of cellular dormancy has not yet been clearly defined.
We established a novel in vitro assay for elucidation of the dormant phenotype, named as in vitro Dormant assay. As a result, in vitro Dormant assay revealed 10 small molecules that regulated the cellular dormancy in breast cancer cell lines. In addition, omics analyses of the induced dormant cells revealed some of the molecular state in the dormancy. We consider a strategy that induces cellular dormancy could lead to an extension of the remission period and controlling metastasis. In this presentation, we will discuss the development of dormancy induction drugs and their current progress.

[Disclosure of COI by Responsible researcher] Lead presenter is the Responsible researcher.
[Lead presenter: Jun Nakayama] ■Research funds from company: AMED, 持田記念医学薬学振興財団, 安田記念医学財団, 千里ライフサイエンス振興財団

▲TOP

### [SS4-2] SS4
### AMED Symposium "Aiming for prevention and cure of cancer-Challenges and prospects for cancer research in AMED's third term"

2025 / 9 / 26 (Fri) 12:50-15:20 Room 1/(Ishikawa Ongakudo 2F Concert Hall) Room 1

[Chairpersons]Toru Hirota (Japanese Foundation for Cancer Research, Cancer Instiutte)、Chizu Tanikawa (The University of Tokyo)

AMED's cancer research from the first to second term has achieved many results, with the goal of linking academic basic research to clinical practice and delivering it to patients as soon as possible, based on the "10-Year Cancer Research Strategy" formulated in 2014. The speed of new drug development over the past decade has been remarkable, and with medical advances, patient needs have become more diverse, calling for the enhancement of supportive and palliative care.⊠In the third term of AMED, based on the new "5th 10-Year Cancer Research Strategy", AMED aims to promote "cancer prevention, diagnosis, and treatment," actively support research projects that contribute to unmet medical needs such as intractable and rare cancers for which effective diagnostic and treatment methods have not yet been established, and continue to place emphasis on the practical application of drug discovery from academia, aiming to prevent and cure cancer.⊠This symposium will address two themes, "Realizing innovative cancer treatments" and "Cancer prevention and cure," and will discuss prospects for cancer prevention and cure. It is hoped that this symposium will lead to the realization of innovative cancer treatments for intractable and rare cancers, which still need adequate treatment, and to a cure for cancer.⊠⊠AMEDの第1期から第2期にかけてのがん研究は、2014年に策定された「がん研究10年戦略」に基づき、学術的基礎研究を臨床に繋げ、患者さんに一日も早く届けることを目標に、多くの成果を上げてきました。過去10年間の新薬開発のスピードは目覚ましく、医療の進歩に伴い患者さんのニーズは多様化し、支持療法や緩和ケアの充実が求められています。⊠AMED第3期では、新たな「第5期がん研究10年戦略」等に基づき、「がんの予防・診断・治療」を推進し、有効な診断・治療法が確立されていない難治がんや希少がんなど、アンメットメディカルニーズに資する研究プロジェクトを積極的に支援するとともに、がんの予防と治癒を目指し、アカデミア発の創薬の実用化に引き続き重点的に取り組んでいきます。⊠本シンポジムで取り上げる「革新的がん医療の実現」と「がんの予防と根治」の2つのテーマについて、がんの予防と根治を目指す将来展望について議論され、未だ充足されていない難治性がんや希少がんに対する革新的ながん医療の実現とがんの根治に繋がることを期待します。

---

### SS4-2
### Controlling the origin of cancer clones for esophageal cancer prevention

[Speaker]Nobuyuki Kakiuchi (Dept. Gastroenterol., Hakubi Ctr., Kyoto Univ.) [Co-author]Akira Yokoyama (Dept. Clin. Oncology, Kyoto Univ.)

Recent studies have elucidated that normal tissues are remodeled by cells acquiring cancer driver mutations due to aging and exposure to environmental factors. This remodeling process, driven by clones harboring such mutations, is referred to as somatic mosaicism, and these driver-mutant clones are considered the origin of cancer. Esophageal squamous cell carcinoma (ESCC) is prevalent in Asia, including Japan. Endoscopic screening enables early detection and contributes to curative therapy. However, in high-risk patients, metachronous cancers frequently arise, posing a significant challenge to long-term outcomes. Our study aims to reduce the risk of developing ESCC by controlling precancerous clones. To this end, we investigated the mechanism underlying the expansion and malignant transformation of precancerous clones. In this presentation, we will share our recent findings and introduce a non-invasive method to assess ESCC risk by measuring the extent of somatic mosaicism in the buccal mucosa.

[Disclosure of COI by Responsible researcher] Lead presenter is the Responsible researcher.
[Lead presenter: Nobuyuki Kakiuchi] ■Research funds from company: 住友ファーマ

▲TOP

### [SS4-3] SS4
### AMED Symposium "Aiming for prevention and cure of cancer-Challenges and prospects for cancer research in AMED's third term"

2025 / 9 / 26 (Fri) 12:50-15:20 Room 1/(Ishikawa Ongakudo 2F Concert Hall) Room 1

[Chairpersons]Toru Hirota (Japanese Foundation for Cancer Research, Cancer Instiutte)、Chizu Tanikawa (The University of Tokyo)

AMED's cancer research from the first to second term has achieved many results, with the goal of linking academic basic research to clinical practice and delivering it to patients as soon as possible, based on the "10-Year Cancer Research Strategy" formulated in 2014. The speed of new drug development over the past decade has been remarkable, and with medical advances, patient needs have become more diverse, calling for the enhancement of supportive and palliative care.⌧In the third term of AMED, based on the new "5th 10-Year Cancer Research Strategy", AMED aims to promote "cancer prevention, diagnosis, and treatment," actively support research projects that contribute to unmet medical needs such as intractable and rare cancers for which effective diagnostic and treatment methods have not yet been established, and continue to place emphasis on the practical application of drug discovery from academia, aiming to prevent and cure cancer.⌧This symposium will address two themes, "Realizing innovative cancer treatments" and "Cancer prevention and cure," and will discuss prospects for cancer prevention and cure. It is hoped that this symposium will lead to the realization of innovative cancer treatments for intractable and rare cancers, which still need adequate treatment, and to a cure for cancer.⌧⌧AMEDの第1期から第2期にかけてのがん研究は、2014年に策定された「がん研究10年戦略」に基づき、学術的基礎研究を臨床に繋げ、患者さんに一日も早く届けることを目標に、多くの成果を上げてきました。過去10年間の新薬開発のスピードは目覚ましく、医療の進歩に伴い患者さんのニーズは多様化し、支持療法や緩和ケアの充実が求められています。⌧AMED第3期では、新たな「第5期がん研究10年戦略」等に基づき、「がんの予防・診断・治療」を推進し、有効な診断・治療法が確立されていない難治がんや希少がんなど、アンメットメディカルニーズに資する研究プロジェクトを積極的に支援するとともに、がんの予防と治癒を目指し、アカデミア発の創薬の実用化に引き続き重点的に取り組んでいきます。⌧本シンポジムで取り上げる「革新的がん医療の実現」と「がんの予防と根治」の2つのテーマについて、がんの予防と根治を目指す将来展望について議論され、未だ充足されていない難治性がんや希少がんに対する革新的ながん医療の実現とがんの根治に繋がることを期待します。

---

### SS4-3
### Unequal Segregation of Extrachromosomal DNA as a Driver of Intratumoral Heterogeneity

[Speaker]Reo Maruyama (Cancer Epigenomics, Cancer Inst., JFCR／NEXT-Ganken Prog., JFCR) [Co-author]Chikako Shibata (Cancer Epigenomics, Cancer Inst., JFCR)、Kohei Kumegawa (NEXT-Ganken Prog., JFCR)、Shoya Iwanami (iBLab, Div. Nat. Sci., Nagoya Univ.)、Kenichi Miyata (Cancer Epigenomics, Cancer Inst., JFCR)

Intratumoral heterogeneity (ITH) is a major driver of cancer progression and therapeutic resistance. Understanding its mechanisms and developing strategies for its control remain key challenges in cancer research. Recent studies have suggested that unequal segregation of extrachromosomal DNA (ecDNA) during cell division contributes to ITH and is associated with increased malignancy and poor prognosis. However, the underlying mechanisms remain largely unknown.To address this, we developed a multimodal experimental platform named IMPACH, which integrates cell barcoding, single-cell multi-omics analysis, and genetic perturbation. Using IMPACH, we established a high-throughput and high-precision system for quantifying ecDNA copy number at the single-cell level. In parallel, we are currently applying mathematical modeling approaches to investigate ecDNA segregation dynamics and to explore candidate regulatory factors.In this presentation, we will introduce our recent efforts to elucidate ecDNA-related heterogeneity and discuss the potential direction toward novel therapeutic strategies targeting ecDNA-mediated mechanisms.

[Disclosure of COI by Responsible researcher] Lead presenter is the Responsible researcher.
[Lead presenter: Reo Maruyama] We have no financial relationships to disclose.

### [SS4-4] SS4

### AMED Symposium "Aiming for prevention and cure of cancer-Challenges and prospects for cancer research in AMED's third term"

2025 / 9 / 26 (Fri) 12:50-15:20 Room 1/(Ishikawa Ongakudo 2F Concert Hall) Room 1

[Chairpersons]Toru Hirota (Japanese Foundation for Cancer Research, Cancer Instiutte)、Chizu Tanikawa (The University of Tokyo)

AMED's cancer research from the first to second term has achieved many results, with the goal of linking academic basic research to clinical practice and delivering it to patients as soon as possible, based on the "10-Year Cancer Research Strategy" formulated in 2014. The speed of new drug development over the past decade has been remarkable, and with medical advances, patient needs have become more diverse, calling for the enhancement of supportive and palliative care.☒In the third term of AMED, based on the new "5th 10-Year Cancer Research Strategy", AMED aims to promote "cancer prevention, diagnosis, and treatment," actively support research projects that contribute to unmet medical needs such as intractable and rare cancers for which effective diagnostic and treatment methods have not yet been established, and continue to place emphasis on the practical application of drug discovery from academia, aiming to prevent and cure cancer.☒This symposium will address two themes, "Realizing innovative cancer treatments" and "Cancer prevention and cure," and will discuss prospects for cancer prevention and cure. It is hoped that this symposium will lead to the realization of innovative cancer treatments for intractable and rare cancers, which still need adequate treatment, and to a cure for cancer.☒☒AMEDの第1期から第2期にかけてのがん研究は、2014年に策定された「がん研究10年戦略」に基づき、学術的基礎研究を臨床に繋げ、患者さんに一日も早く届けることを目標に、多くの成果を上げてきました。過去10年間の新薬開発のスピードは目覚ましく、医療の進歩に伴い患者さんのニーズは多様化し、支持療法や緩和ケアの充実が求められています。☒AMED第3期では、新たな「第5期がん研究10年戦略」等に基づき、「がんの予防・診断・治療」を推進し、有効な診断・治療法が確立されていない難治がんや希少がんなど、アンメットメディカルニーズに資する研究プロジェクトを積極的に支援するとともに、がんの予防と治癒を目指し、アカデミア発の創薬の実用化に引き続き重点的に取り組んでいきます。☒本シンポジムで取り上げる「革新的がん医療の実現」と「がんの予防と根治」の2つのテーマについて、がんの予防と根治を目指す将来展望について議論され、未だ充足されていない難治性がんや希少がんに対する革新的ながん医療の実現とがんの根治に繋がることを期待します。

---

### SS4-4

### Development of an AI Model for Early Diagnosis of Pancreatic and Bile Duct Cancer via Cachexia-Related Metabolic Changes

[Speaker]Taisuke Baba (Div Surg Oncol, Dept Surg, Nagoya Univ Grad Sch Med) [Co-author]Masaki Sunagawa (Div Surg Oncol, Dept Surg, Nagoya Univ Grad Sch Med)、Junpei Yamaguchi (Div Surg Oncol, Dept Surg, Nagoya Univ Grad Sch Med)、Toshio Kokuryo (Div Surg Oncol, Dept Surg, Nagoya Univ Grad Sch Med)、Hizuru Amano (Dept of Ped Surg, Nagoya Univ Grad Sch Med)、Akinari Hinoki (Dept of Ped Surg, Nagoya Univ Grad Sch Med)、Hiroo Uchida (Dept of Ped Surg, Nagoya Univ Grad Sch Med)、Tomoki Ebata (Div Surg Oncol, Dept Surg, Nagoya Univ Grad Sch Med)

Pancreatic and bile duct cancers have extremely poor prognoses, with 5-year survival rates of 10% and 28.7%. Early detection is critical, but tumor heterogeneity makes it difficult to identify universal biomarkers. Conventional single-biomarker approaches face limitations, highlighting the need for data-driven methods that account for this diversity.Mass spectrometry-based urinary metabolomics can analyze over 1800 metabolites from a single urine sample, but batch effects have hindered clinical application. To overcome this, we developed a single-sample classifier algorithm that enables robust cancer prediction across batches. Notably, our model appears to detect early cancer by recognizing cachexia-related metabolic changes. In this presentation, we will report the validation of our original AI model for predicting pancreatic and bile duct cancers, conducted with support from AMED and PRIMO. Additionally, using a pancreatic cancer mouse model, we demonstrated that cachexia induces metabolic changes at an early stage. Based on these findings, we will discuss how our AI model may enable the detection of early-stage cancers that are difficult to identify with conventional approaches.

[Disclosure of COI by Responsible researcher] Lead presenter is the Responsible researcher.
[Lead presenter: Taisuke Baba] We have no financial relationships to disclose.

### [SS4-5] SS4

### AMED Symposium "Aiming for prevention and cure of cancer-Challenges and prospects for cancer research in AMED's third term"

2025 / 9 / 26 (Fri) 12:50-15:20 Room 1/(Ishikawa Ongakudo 2F Concert Hall) Room 1

[Chairpersons]Junji Furuse (Kanagawa Cancer Center)、Koshi Mimori (Kyushu University Beppu Hospital)

---

### SS4-5
### Somatic Mosaicism and the Origins of Cancer

[Speaker]Seishi Ogawa (Department of Pathology and Tumor Biology, Kyoto University)

Over the past decade, technological advances in genome analysis have revealed that various histologically normal tissues and organs can become patchworks of clones carrying cancer-associated mutations, acquired through aging or chronic inflammation. This phenomenon, known as somatic mosaicism, is attracting increasing attention. Given the striking similarity between mutations observed in cancer and those found in somatic mosaic clones, it is now believed that these altered clones in normal tissues may represent the earliest cellular origins of cancer. As such, somatic mosaicism provides critical insights into the initial steps of tumorigenesis and holds significant implications for the early detection, intervention, and even prevention of cancer. In this session, I will introduce recent findings on somatic mosaicism across multiple human organs and discuss how these discoveries may reshape our understanding of cancer development and inform new strategies for its diagnosis, prevention, and treatment.

[Disclosure of COI by Responsible researcher] Lead presenter is the Responsible researcher.
[Lead presenter: Seishi Ogawa] ■Advisor of the company and/or profit-making organization: エーザイ株式会社、Chordia Therapeutics Inc.、Montage Bio, INC.　■Profit of stock: 朝日ゲノミクス株式会社　■Research expenses: エーザイ株式会社、Chordia Therapeutics Inc.　■Contributions: 日本新薬株式会社　■Endowed chair: Chordia Therapeutics Inc.、公益社団法人鹿児島共済会 南風病院

▲TOP

### [SS4-6] SS4
### AMED Symposium "Aiming for prevention and cure of cancer-Challenges and prospects for cancer research in AMED's third term"

2025 / 9 / 26 (Fri) 12:50-15:20 Room 1/(Ishikawa Ongakudo 2F Concert Hall) Room 1

[Chairpersons]Junji Furuse (Kanagawa Cancer Center)、Koshi Mimori (Kyushu University Beppu Hospital)

### SS4-6
### Development and Therapeutic Application of Next-Generation Nucleic Acid Drugs for Glioblastoma

[Speaker]Yutaka Kondo (Div. Can. Biol., Nagoya Univ. Grad. Sch. Med.)

Glioblastoma is a highly refractory tumor, and currently, effective treatments are extremely limited. It affects not only in middle-aged adults but also in children, imposing immense suffering on patients and their families, as well as considerable social and economic burdens. Therefore, the development of innovative therapeutic agents targeting novel mechanisms of action is an urgent challenge. In recent years, increasing attention has been paid to the role of long non-coding RNAs (lncRNAs) in tumorigenesis. These RNA molecules, transcribed from non-coding regions of the genome, have long remained functionally uncharacterized and thus were rarely considered viable therapeutic targets. Our research identified TUG1, a lncRNA highly expressed in glioblastoma, as essential for the maintenance and proliferation of glioblastoma cells. Moreover, we discovered that inhibition of TUG1 effectively induces glioma cell death. Based on these findings, we developed a nucleic acid-based therapeutic agent targeting TUG1, which has now progressed to clinical trials. In this symposium, I will present the path from basic discovery to clinical application of this novel lncRNA-targeted therapy.

[Disclosure of COI by Responsible researcher] Lead presenter is the Responsible researcher.
[Lead presenter: Yutaka Kondo] ■Research funds from company: NANO MRNA株式会社

### [SS4-7] SS4

### AMED Symposium "Aiming for prevention and cure of cancer-Challenges and prospects for cancer research in AMED's third term"

2025 / 9 / 26 (Fri) 12:50-15:20 Room 1/(Ishikawa Ongakudo 2F Concert Hall) Room 1

[Chairpersons]Junji Furuse (Kanagawa Cancer Center)、Koshi Mimori (Kyushu University Beppu Hospital)

---

### SS4-7

### Research and development aimed at eradicating refractory cancer

[Speaker]Keiichi I. Nakayama (Adv. Res. Initiative, Inst. Integrated Res., Inst. Sci. Tokyo)

We aim to improve outcomes for pancreatic and small-cell lung cancers, both with less than 10% five-year survival. Using our proprietary iMPAQT proteomics platform, we visualized cancer-associated metabolic shifts. Two major alterations were identified: (1) glucose-derived carbon flux (the Warburg effect), and (2) glutamine-derived nitrogen flux, which we term the "second Warburg effect." Among enzymes involved in the latter, PPAT showed the strongest correlation with malignancy in a meta-analysis of ~12,000 cancer cases. PPAT knockdown induced death in diverse cancer cell lines. We screened for PPAT inhibitors and optimized a hit compound into a potent lead. This compound suppressed tumor growth in xenograft models of pancreatic and small-cell lung cancer with minimal toxicity. In parallel, we are developing a photoimmunotherapy targeting ANTXR2, a receptor specifically expressed on pancreatic cancer stem cells. Both therapeutic approaches are currently being prepared for preclinical evaluation. These strategies aim to address metabolic and stemness-related vulnerabilities in hard-to-treat cancers.

[Disclosure of COI by Responsible researcher] Lead presenter is the Responsible researcher.
[Lead presenter: Keiichi I. Nakayama] ■Research funds from company: 株式会社アンチキャンサーテクノロジズ　■ employment by a company related to the presentation: 株式会社アンチキャンサーテクノロジズ　■Advisor of the company and/or profit-making organization: 株式会社アンチキャンサーテクノロジズ　■Profit of stock: 株式会社アンチキャンサーテクノロジズ/株式会社Qイノベーション　■Research expenses: 株式会社アンチキャンサーテクノロジズ

### [SS4-8] SS4
### AMED Symposium "Aiming for prevention and cure of cancer-Challenges and prospects for cancer research in AMED's third term"

2025 / 9 / 26 (Fri) 12:50-15:20 Room 1/(Ishikawa Ongakudo 2F Concert Hall) Room 1

[Chairpersons]Junji Furuse (Kanagawa Cancer Center)、Koshi Mimori (Kyushu University Beppu Hospital)

---

### SS4-8
### From discovery to clinical implementation of blood biomarker for early diagnosis of pancreatic cancer

[Speaker]Kazufumi Honda (Dept. Molecular Prevention, Nippon Med. Sch. Graduate Sch. Med.)

Pancreatic cancer is a rare and intractable disease. Developing blood-based biomarkers to identify high-risk individuals from the asymptomatic population remains an unmet medical need. Through proteomic analysis, we identified C-terminal truncation abnormalities in the apolipoprotein A2 dimer (apoA2 isoforms: apoA2-i) in the blood of pancreatic cancer patients. We previously reported their potential utility in diagnosing resectable pancreatic cancer. We developed ELISA reagents to measure apoA2-i for research use, and multiple studies have provided foundational evidence of their diagnostic value. In 2023, the ELISA was re-developed under Japanese Quality Management System regulations and received marketing authorization as an in vitro diagnostic (IVD) kit. This symposium presents our research trajectory from discovery using omics approaches to demonstrating clinical utility through multicenter and international studies. We also report on validating the IVD kit performance through rigorous trials under Japanese regulations. This includes MHLW approval, reimbursement listing, and recent U.S. activities.

[Disclosure of COI by Responsible researcher] Lead presenter is the Responsible researcher.
[Lead presenter: Kazufumi Honda] ■Manuscript fee: 東レ（株）

▲TOP

### [SS2-1] SS2
### Young Plenary Symposium

2025 / 9 / 26 (Fri) 12:50-15:20 Room 2/(Ishikawa Ongakudo 2F Hogaku Hall) Room 2

[Chairpersons]Eishu Hirata (Cancer Research Institute of Kanazawa University)、Yosuke Togashi (Okayama University/OKAYAMA UNIVERSITY HOSPITAL)

This symposium is designed to showcase outstanding recent achievements by early-career researchers across a broad spectrum of cancer research fields, including basic biology, translational science, and clinical oncology. The selected presenters have made significant and original contributions that not only advance our current understanding of cancer but also hold the potential to shape future directions in the field. The session aims to recognize scientific excellence at an early stage of career development and to provide a supportive platform for young investigators to present their work, exchange ideas, and receive feedback from peers and senior scientists alike. Through this exchange, the symposium seeks to foster interdisciplinary collaboration, stimulate critical discussion, and inspire the next generation of researchers. By emphasizing the creativity and potential of early-stage investigators, the session encourages broader engagement and supports the development of future leaders in the field of cancer research.

---

### SS2-1
### Immune evasion through mitochondrial transfer in the tumor microenvironment

[Speaker]Hideki Ikeda (Division of Cell Therapy, Chiba Cancer Center Research Institute,／Department of Respirology, Graduate School of Medicine, Chiba University) [Co-author]Masahito Kawazu (Division of Cell Therapy, Chiba Cancer Center Research Institute,)、Yosuke Togashi (Division of Cell Therapy, Chiba Cancer Center Research Institute,／Department of Tumor Microenvironment, Faculty of Medicine, Okayama University／Department of Allergy and Respiratory Medicine, Okayama University Hospital)

Cancer cells in the tumor microenvironment (TME) use various mechanisms to evade immune system. TME metabolic reprogramming and mitochondrial dysfunction in tumor-infiltrating lymphocytes (TILs) impair antitumor immunity, though mechanisms are unclear. We analyzed clinical specimens and identified shared mtDNA mutations in TILs and cancer cells. Further, cancer cell mitochondria transferred to TILs. Typically, mitochondria in TILs readily undergo mitophagy through reactive oxygen species. However, transferred cancer cell mitochondria resisted mitophagy, due to co-transferred mitophagy-inhibitory molecules. These molecules on mitochondria were co-transferred to TILs, causing homoplasmic replacement. T cells that acquired these cancer-derived mtDNA mutations showed metabolic abnormalities, senescence, and defective effector/memory functions. This impaired antitumor immunity ***in vitro*** and ***in vivo***. Accordingly, tumor mtDNA mutations predicted poor prognosis for immune checkpoint inhibitors in melanoma or non-small-cell lung cancer patients. These findings reveal a novel cancer immune evasion mechanism through mitochondrial transfer, aiding future cancer immunotherapy.

[Disclosure of COI by Responsible researcher] Lead presenter is not the Responsible researcher.
[Lead presenter: Hideki Ikeda] We have no financial relationships to disclose.
[Principal Researcher: Yosuke Togashi] ■Lecture fee: 中外製薬株式会社、エーザイ株式会社、アストラゼネカ株式会社、ブリストルマイヤーズスクイブ株式会社、MSD株式会社　■Research expenses: ヤンセンファーマ株式会社、アストラゼネカ、 WJOG、大鵬薬品工業株式会社、武田薬品工業ヤンセンファーマ株式会社、アストラゼネカ株式会社、西日本がん研究機構(WJOG)、大鵬薬品工業株式会社、武田薬品工業株式会社　■Contributions: （公財）小野薬品 がん・免疫・神経研究財団、（公財）小林がん学術振興会、（公財）武田科学振興財団、（公財）アステラス病態代謝研究会、（公財）上原記念生命科学財団

▲TOP

### [SS2-2] SS2
### Young Plenary Symposium

2025 / 9 / 26 (Fri) 12:50-15:20 Room 2/(Ishikawa Ongakudo 2F Hogaku Hall) Room 2

[Chairpersons]Eishu Hirata (Cancer Research Institute of Kanazawa University)、Yosuke Togashi (Okayama University/OKAYAMA UNIVERSITY HOSPITAL)

This symposium is designed to showcase outstanding recent achievements by early-career researchers across a broad spectrum of cancer research fields, including basic biology, translational science, and clinical oncology. The selected presenters have made significant and original contributions that not only advance our current understanding of cancer but also hold the potential to shape future directions in the field. The session aims to recognize scientific excellence at an early stage of career development and to provide a supportive platform for young investigators to present their work, exchange ideas, and receive feedback from peers and senior scientists alike. Through this exchange, the symposium seeks to foster interdisciplinary collaboration, stimulate critical discussion, and inspire the next generation of researchers. By emphasizing the creativity and potential of early-stage investigators, the session encourages broader engagement and supports the development of future leaders in the field of cancer research.

---

### SS2-2
### Maintenance of R-loop structures by phosphorylated hTERT preserves genome integrity

[Speaker]Mitsuhiro Machitani (Div. Cancer Stem Cell, Natl. Cancer Ctr. Res. Inst.) [Co-author]Kenkichi Masutomi (Div. Cancer Stem Cell, Natl. Cancer Ctr. Res. Inst.)

Since aberrant accumulation of RNA:DNA hybrids (R-loops) causes DNA damage and genome instability, cells express regulators of R-loop structures. Here we report that RNA dependent RNA polymerase (RdRP) activity of human telomerase reverse transcriptase (hTERT) regulates R-loop formation. We found that phosphorylated form of hTERT (p-hTERT) exhibits RdRP activity in nuclear speckles both in telomerase-positive cells and telomerase-negative cells with alternative lengthening of telomeres (ALT) activity. p-hTERT did not associate with telomerase RNA component in nuclear speckles but, instead, with TERRA RNAs to resolve R-loops. Targeting of the TERT gene in ALT cells ablated RdRP activity and impaired tumor growth. Using a genome-scale CRISPR loss-of-function screen, we identified Fanconi anemia/BRCA genes as synthetic lethal partners of hTERT RdRP. Inactivation of RdRP and Fanconi anemia/BRCA genes caused accumulation of R-loop structures and DNA damage. These findings indicate that RdRP activity of p-hTERT guards against genome instability by removing R-loop structures (Machitani et al. Nat. Cell Biol. 2024).

[Disclosure of COI by Responsible researcher] Lead presenter is not the Responsible researcher.
[Lead presenter: Mitsuhiro Machitani] ■Research funds from company: 公的研究費（日本医療研究開発機構）
[Principal Researcher: Kenkichi Masutomi] ■Research funds from company: 公的研究費（日本医療研究開発機構、日本学術振興会科研費）

### [SS2-3] SS2
### Young Plenary Symposium

2025 / 9 / 26 (Fri) 12:50-15:20 Room 2/(Ishikawa Ongakudo 2F Hogaku Hall) Room 2

[Chairpersons]Eishu Hirata (Cancer Research Institute of Kanazawa University)、Yosuke Togashi (Okayama University/OKAYAMA UNIVERSITY HOSPITAL)

This symposium is designed to showcase outstanding recent achievements by early-career researchers across a broad spectrum of cancer research fields, including basic biology, translational science, and clinical oncology. The selected presenters have made significant and original contributions that not only advance our current understanding of cancer but also hold the potential to shape future directions in the field. The session aims to recognize scientific excellence at an early stage of career development and to provide a supportive platform for young investigators to present their work, exchange ideas, and receive feedback from peers and senior scientists alike. Through this exchange, the symposium seeks to foster interdisciplinary collaboration, stimulate critical discussion, and inspire the next generation of researchers. By emphasizing the creativity and potential of early-stage investigators, the session encourages broader engagement and supports the development of future leaders in the field of cancer research.

### SS2-3
### Pan-cancer comparative and integrative analyses of driver alterations using Japanese and international genomic databases

[Speaker]Sara Horie (Div. Mol. Oncol., Natl. Cancer Ctr. Res. Inst.) [Co-author]Yuki Saito (Div. Mol. Oncol., Natl. Cancer Ctr. Res. Inst.／Div. Gastroenterol. Hepatol., Dept. Int. Med., Keio Univ. Sch. Med.)、Yasunori Kogure (Div. Mol. Oncol., Natl. Cancer Ctr. Res. Inst.)、Kota Mizuno (Div. Mol. Oncol., Natl. Cancer Ctr. Res. Inst.／Div. Hematol., Dept. Med., Keio Univ. Sch. Med.)、Yuta Ito (Div. Mol. Oncol., Natl. Cancer Ctr. Res. Inst.／Dept. Hematol., Natl. Cancer Ctr. Hosp.)、Mariko Tabata (Div. Cancer Evolution, Natl. Cancer Ctr. Res. Inst.)、Koichi Murakami (Div. Mol. Oncol., Natl. Cancer Ctr. Res. Inst.／Div. Hematol., Dept. Med., Keio Univ. Sch. Med.)、Junji Koya (Div. Mol. Oncol., Natl. Cancer Ctr. Res. Inst.／Div. Hematol., Dept. Med., Keio Univ. Sch. Med.)、Keisuke Kataoka (Div. Mol. Oncol., Natl. Cancer Ctr. Res. Inst.／Div. Hematol., Dept. Med., Keio Univ. Sch. Med.)

Using 48,627 samples from the Center for Cancer Genomics and Advanced Therapeutics (C-CAT), we present a pan-cancer landscape of driver alterations and their clinical actionability in Japanese patients. Comparison with White patients in Genomics Evidence Neoplasia Information Exchange (GENIE) demonstrates high TP53 mutation frequencies in Asian patients across multiple cancer types. Integration of C-CAT, GENIE, and The Cancer Genome Atlas data reveals many cooccurring and mutually exclusive relationships between driver mutations. At pathway level, mutations in epigenetic regulators frequently cooccur with PI3K pathway molecules. Furthermore, we found significant cooccurring mutations within the epigenetic pathway. Accumulation of mutations in epigenetic regulators causes increased proliferation-related transcriptomic signatures. Loss-of-function of many epigenetic drivers inhibits cell proliferation in their wild-type cell lines, but this effect is attenuated in those harboring mutations of not only the same but also different epigenetic drivers. Our analyses dissect various genetic properties and provide valuable resources for precision medicine in cancer.

[Disclosure of COI by Responsible researcher] Lead presenter is not the Responsible researcher.
[Lead presenter: Sara Horie] We have no financial relationships to disclose.
[Principal Researcher: Keisuke Kataoka] ■Profit of stock: 朝日ゲノミクス株式会社　■Lecture fee: 大塚製薬株式会社、Meiji Seika ファルマ株式会社、アッヴィ合同会社　■Research expenses: Meiji Seika ファルマ株式会社　■Contributions: エーザイ株式会社、協和キリン株式会社、中外製薬株式会社、大塚製薬株式会社、JCRファーマ株式会社、旭化成ファーマ株式会社

▲TOP

### [SS2-4] SS2
### Young Plenary Symposium

2025 / 9 / 26 (Fri) 12:50-15:20 Room 2/(Ishikawa Ongakudo 2F Hogaku Hall) Room 2

[Chairpersons]Eishu Hirata (Cancer Research Institute of Kanazawa University)、Yosuke Togashi (Okayama University/OKAYAMA UNIVERSITY HOSPITAL)

This symposium is designed to showcase outstanding recent achievements by early-career researchers across a broad spectrum of cancer research fields, including basic biology, translational science, and clinical oncology. The selected presenters have made significant and original contributions that not only advance our current understanding of cancer but also hold the potential to shape future directions in the field. The session aims to recognize scientific excellence at an early stage of career development and to provide a supportive platform for young investigators to present their work, exchange ideas, and receive feedback from peers and senior scientists alike. Through this exchange, the symposium seeks to foster interdisciplinary collaboration, stimulate critical discussion, and inspire the next generation of researchers. By emphasizing the creativity and potential of early-stage investigators, the session encourages broader engagement and supports the development of future leaders in the field of cancer research.

---

### SS2-4
### Differentiation of Tregs into Th1-Tregs induced by tumor-associated macrophages promotes tumor growth

[Speaker]Ayumi Kuratani (Dept. Immunoparasitology., RIMD., Osaka Univ.) [Co-author]Masahiro Yamamoto (Dept. Immunoparasitology., RIMD., Osaka Univ.／Lab. Immunoparasitology., iFReC., Osaka Univ.／CiDER., Osaka Univ.／CAMaD., Osaka Univ.)

The tumor microenvironment contains various immunosuppressive cells, including regulatory T cells (Tregs) and tumor-associated macrophages (TAMs). We have previously shown that one subset of Tregs, Th1-type Tregs (Th1-Tregs), significantly accumulates in tumors and suppresses antitumor immunity, though the mechanism was unclear. Recent studies suggested a link between TAMs and tumor-infiltrating Tregs. Using a novel mouse model with TAM-specific labeling and depletion, we found that TAM depletion reduced Th1-Tregs and suppressed tumor growth. TAMs promoted Treg differentiation into Th1-Tregs, and further analysis identified the chemokine PF4, highly expressed by TAMs, as a key factor. Administration of a PF4-neutralizing antibody decreased Th1-Tregs in tumors, activated antitumor immunity, and suppressed tumor growth without inducing systemic autoimmunity. These results indicate that TAM-derived PF4 promotes tumor growth by inducing Th1-Tregs and suppressing antitumor immunity, suggesting PF4 as a promising target for immunotherapy.

[Disclosure of COI by Responsible researcher] Lead presenter is not the Responsible researcher.
[Lead presenter: Ayumi Kuratani] We have no financial relationships to disclose.
[Principal Researcher: Masahiro Yamamoto] We have no financial relationships to disclose.

### [SS2-5] SS2
### Young Plenary Symposium

2025 / 9 / 26 (Fri) 12:50-15:20 Room 2/(Ishikawa Ongakudo 2F Hogaku Hall) Room 2

[Chairpersons]Eishu Hirata (Cancer Research Institute of Kanazawa University)、Yosuke Togashi (Okayama University/OKAYAMA UNIVERSITY HOSPITAL)

This symposium is designed to showcase outstanding recent achievements by early-career researchers across a broad spectrum of cancer research fields, including basic biology, translational science, and clinical oncology. The selected presenters have made significant and original contributions that not only advance our current understanding of cancer but also hold the potential to shape future directions in the field. The session aims to recognize scientific excellence at an early stage of career development and to provide a supportive platform for young investigators to present their work, exchange ideas, and receive feedback from peers and senior scientists alike. Through this exchange, the symposium seeks to foster interdisciplinary collaboration, stimulate critical discussion, and inspire the next generation of researchers. By emphasizing the creativity and potential of early-stage investigators, the session encourages broader engagement and supports the development of future leaders in the field of cancer research.

---

### SS2-5
### Genetic architecture of alcohol consumption identified by genotype-stratified GWAS and impact on esophageal cancer risk

[Speaker]Yuriko Koyanagi (Div. of Cancer Epidemiology and Prevention, Aichi Cancer Ctr.) [Co-author]Masahiro Nakatochi (Dept. of Integrated Sciences, Nagoya Univ. Grad. Sch. Med.)、Keitaro Matsuo (Div. of Cancer Epidemiology and Prevention, Aichi Cancer Ctr.／Div. of Cancer Epidemiology, Nagoya Univ. Grad. Sch. Med.)

An East Asian-specific variant in ***Aldehyde dehydrogenase 2*** (***ALDH2***), rs671 (G>A), is the major genetic determinant of alcohol consumption. Here, we performed an rs671 genotype-stratified genome-wide association study (GWAS) meta-analysis of alcohol consumption in 175,672 Japanese individuals to explore gene-gene interactions with rs671 behind drinking behavior. Three loci (***GCKR***, ***KLB***, and ***ADH1B***) reached genome-wide significance in wild-type homozygotes (GG), whereas six loci (***GCKR***, ***ADH1B***, ***ALDH1B1***, ***ALDH1A1***, ***ALDH2***, and ***GOT2***) did so in heterozygotes (GA). Of these, five loci showed genome-wide significant interactions with rs671. Many of the discovered loci showed significant associations with esophageal cancer risk, exhibiting the same direction of association as observed in the genotype-stratified GWAS, supporting their potential role in alcohol-related carcinogenesis. Our findings elucidate the genotype-specific genetic architecture underlying alcohol consumption in the Japanese population and highlight its impact on alcohol-related disease risk.

[Disclosure of COI by Responsible researcher] Lead presenter is the Responsible researcher.
[Lead presenter: Yuriko Koyanagi] We have no financial relationships to disclose.

▲TOP

### [SS2-6] SS2
### Young Plenary Symposium

2025 / 9 / 26 (Fri) 12:50-15:20 Room 2/(Ishikawa Ongakudo 2F Hogaku Hall) Room 2

[Chairpersons]Eishu Hirata (Cancer Research Institute of Kanazawa University)、Yosuke Togashi (Okayama University/OKAYAMA UNIVERSITY HOSPITAL)

This symposium is designed to showcase outstanding recent achievements by early-career researchers across a broad spectrum of cancer research fields, including basic biology, translational science, and clinical oncology. The selected presenters have made significant and original contributions that not only advance our current understanding of cancer but also hold the potential to shape future directions in the field. The session aims to recognize scientific excellence at an early stage of career development and to provide a supportive platform for young investigators to present their work, exchange ideas, and receive feedback from peers and senior scientists alike. Through this exchange, the symposium seeks to foster interdisciplinary collaboration, stimulate critical discussion, and inspire the next generation of researchers. By emphasizing the creativity and potential of early-stage investigators, the session encourages broader engagement and supports the development of future leaders in the field of cancer research.

---

### SS2-6
### IL33-ILC2s: Novel lymphoneogenic pathway

[Speaker]Masataka Amisaki (National Cancer Center Hospital East, Translational Research Support Office／Memorial Sloan Kettering Cancer Center, NY, USA) [Co-author]Abderezak Zebboudj (Memorial Sloan Kettering Cancer Center, NY, USA)、Rebecca Yu (Memorial Sloan Kettering Cancer Center, NY, USA)、Akihiro Ohmoto (Memorial Sloan Kettering Cancer Center, NY, USA)、Zachary Sethna (Memorial Sloan Kettering Cancer Center, NY, USA)、Luis Rojas (Memorial Sloan Kettering Cancer Center, NY, USA)、Pablo Guasp (Memorial Sloan Kettering Cancer Center, NY, USA)、Vinod Balachandran (Memorial Sloan Kettering Cancer Center, NY, USA)

Tertiary lymphoid structures (TLSs) are ectopic lymphoid aggregates that boost anti-tumor immunity, yet their induction machineries remain incompletely understood. Here, we identify IL33 as a key inflammatory signal that induces TLSs in pancreatic ductal adenocarcinoma (PDAC). Mechanistically, IL33 activates KLRG1$^{+}$ ILC2s expressing lymphotoxin (LT), which stimulates LT$\beta$R$^{+}$ myeloid cells which act as lymphoid tissue organizers that produce CXCL13 and IL33, forming a positive feedback loop. Using both loss- and gain-of-function mouse models, we show that IL33, ILC2s, and LT signaling are essential for TLS formation and tumor control. In human PDAC, TLSs enriched with IL33$^{+}$ cells and ILC2s associate with improved prognosis. A recombinant IL-33 protein expands ILC2s and TLSs in vivo, enhancing anti-tumor immunity. Our findings uncover a novel, druggable IL33-ILC2-LT axis driving TLS formation in cancer.

[Disclosure of COI by Responsible researcher] Lead presenter is not the Responsible researcher.
[Lead presenter: Masataka Amisaki] We have no financial relationships to disclose.
[Principal Researcher: Rebecca Yu] ■Advisor of the company and/or profit-making organization: Merck ■Lecture fee: Genentech, Abbvie ■Research expenses: Bristol Myers Squibb

### [SS2-7] SS2
### Young Plenary Symposium

2025 / 9 / 26 (Fri) 12:50-15:20 Room 2/(Ishikawa Ongakudo 2F Hogaku Hall) Room 2

[Chairpersons]Eishu Hirata (Cancer Research Institute of Kanazawa University)、Yosuke Togashi (Okayama University/OKAYAMA UNIVERSITY HOSPITAL)

This symposium is designed to showcase outstanding recent achievements by early-career researchers across a broad spectrum of cancer research fields, including basic biology, translational science, and clinical oncology. The selected presenters have made significant and original contributions that not only advance our current understanding of cancer but also hold the potential to shape future directions in the field. The session aims to recognize scientific excellence at an early stage of career development and to provide a supportive platform for young investigators to present their work, exchange ideas, and receive feedback from peers and senior scientists alike. Through this exchange, the symposium seeks to foster interdisciplinary collaboration, stimulate critical discussion, and inspire the next generation of researchers. By emphasizing the creativity and potential of early-stage investigators, the session encourages broader engagement and supports the development of future leaders in the field of cancer research.

---

### SS2-7
### Evolutionary histories of breast cancer and related clones

[Speaker]Tomomi Nishimura (Dep. of Surg., Japanese Red Cross Fukui Hosp.／Dep. of Breast Surg., Grad. Sch. of Med., Kyoto Univ.) [Co-author]Seishi Ogawa (Pathol. and Tumor Biol., Grad. Sch. of Med., Kyoto Univ.)

Recent studies have documented frequent evolution of clones carrying common cancer driver mutations in apparently normal tissues, which are implicated in cancer development. However, our knowledge is still missing regarding how these clones in normal tissues ultimately evolve to cancer. Here, using phylogenetic analyses of multiple microdissected samples from both cancer and non-cancer lesions located in the same breast, we show unique evolutionary histories of breast cancers harboring der(1;16), a common driver alteration found in estrogen-sensitive breast cancers. The approximate timing of early evolutionary events was estimated from the mutation rate measured in normal epithelial cells. In der(1;16)-positive cancers, the derivative chromosome was acquired from early puberty to late adolescence, followed by the emergence of a common ancestor by the patient's early 30s, from which both cancer and non-cancer clones evolved. Replacing the pre-existing mammary epithelium in the following years, these clones occupied a large area within the premenopausal breast tissues by the time of cancer diagnosis. Our findings provide new insight into how breast cancer evolves.

[Disclosure of COI by Responsible researcher] Lead presenter is not the Responsible researcher.
[Lead presenter: Tomomi Nishimura] ■Contributions: 住友財団、安田記念医学財団、SGH財団、高松宮妃癌研究基金　■Endowed chair: 南風病院
[Principal Researcher: Seishi Ogawa] ■Advisor of the company and/or profit-making organization: エーザイ株式会社、Chordia Therapeutics Inc.、Montage Bio, INC.　■Profit of stock: 朝日ゲノミクス株式会社　■Lecture fee: 三菱財団、中谷医工計測技術振興財団　■Research expenses: エーザイ株式会社、Chordia Therapeutics Inc.　■Contributions: 日本新薬株式会社　■Endowed chair: Chordia Therapeutics Inc.、鹿児島共済会 南風病院

▲TOP

### [AOSR4-1] AOSR4
### Recent advances in pediatic, AYA cancer research

2025 / 9 / 26 (Fri) 12:50-15:20 Room 3/(Hotel Nikko Kanazawa 4F Tsuru A) Room 3

[Chairpersons]Jyunko Takita (Department of Child Development and Pediatrics, Kyoto University)、kenichi Yoshida (National Cancer Center)

Recent advancement in treatment strategies has significantly improved the outcomes of pediatric cancers. However, there are still issues to be resolved, such as intractable cancers and late complications in pediatric cancer survivors. To understand the mechanisms of pediatric cancer development and to establish more specific and successful treatment strategies, many researchers have conducted extensive research on pediatric cancers using high-throughput sequencing technologies, functional assays and disease models. Through these studies, genetic and epigenetic basis of pediatric cancers have been elucidated and unique characteristics of pediatric cancers, such as the different underlying mutational processes and their origin in aberrantly developing tissue, were also highlighted. In this symposium, the researchers in the field of pediatric cancers will present the latest advances in basic research to understand the biology of pediatric cancers, including the genetics, the etiology and the origin.

---

### AOSR4-1
### The Coding and Non-Coding Mutational Landscapes of Pediatric Acute Myeloid Leukemia

[Speaker]Kaito Mimura (Division of Cancer Evolution, NCCRI) [Co-author]Norio Shiba (Department of Pediatrics, Yokohama City University)、Kentaro Watanabe (Department of Pediatrics, The University of Tokyo)、Takao Deguchi (Children's Cancer Center, NCCHD)、Kentaro Ohki (Department of Pediatric Hematology and Oncology Research, NCCHD)、Seiya Imoto (Laboratory of Sequence Analysis, IMSUT／Division of Health Medical Intelligence, IMSUT)、Akiko Saito (Clinical Research Center, NHO Nagoya Medical Center)、Yasuhiro Okamoto (Department of Pediatrics, Kagoshima University)、Souichi Adachi (School of Human Health Sciences, Kyoto University)、Daisuke Tomizawa (Children's Cancer Center, NCCHD)、Takashi Taga (Department of Pediatrics, Shiga university of Medical Science)、Yuichi Shiraishi (Division of Genome Analysis Platform Development, NCCRI)、Hiroshi Moritake (Division of Pediatrics, University of Miyazaki)、Motohiro Kato (Department of Pediatrics, The University of Tokyo)、Kenichi Yoshida (Division of Cancer Evolution, NCCRI)

In order to study the pathology and explore novel therapeutic targets of pediatric acute myeloid leukemia (pAML), we performed whole-genome sequencing (WGS) of Japanese primary pAML patients enrolled in Japan Children's Cancer Group (JCCG) AML-12/AML-D16 studies (n=95). WGS data from publicly available datasets were integrated, resulting in a total of 431 samples. Novel recurrent mutations were validated by targeted sequencing of an additional 472 patients. In coding regions, candidate driver genes such as ***DNM2, SI, TGM6, FBXL7, and FAM8A1*** were detected. In non-coding regions, somatic mutations were found to accumulate in intronic and intergenic hotspots, including the ***TERT*** promoter region (n=5), which is unusual for hematological malignancies. Structural variation analysis detected tandem duplications in the 'Blood enhancer cluster', located in the downstream of ***MYC***. Mutational signature analysis detected the reactive oxygen species (ROS)-related signature, SBS18, in AMLs with ***RUNX1::RUNX1T1*** and ***CBFB::MYH11***, with positive correlation with age (p=0.02177). In this study comprising 903 pAML patients, novel mutational events throughout the genome were discovered.

[Disclosure of COI by Responsible researcher] Lead presenter is not the Responsible researcher.
[Lead presenter: Kaito Mimura] We have no financial relationships to disclose.
[Principal Researcher: Kenichi Yoshida] ■Contributions: 公益財団法人持田記念医学薬学振興財団 公益財団法人内藤記念科学振興財団 公益財団法人第一三共生命科学研究振興財団 公益財団法人住友財団 公益財団法人安田記念医学財団 公益財団法人アステラス病態代謝研究会 公益財団法人MSD生命科学財団 一般社団法人日本血液学会 公益財団法人小児医学研究振興財団 公益財団法人上原記念生命科学財団 公益財団法人高松宮妃癌研究基金 公益信託日本白血病研究基金 公益財団法人小林がん学術振興会 公益財団法人武田科学振興財団 公益財団法人大和証券財団 公益財団法人ＳＧＨ財団 公益財団法人川野小児医学奨学財団 認定NPO法人ゴールドリボン・ネットワーク 公益財団法人先進医薬研究振興財団 公益財団法人テルモ生命科学振興財団 公益財団法人小野薬品がん・免疫・神経研究財団 公益財団法人細胞科学研究財団

### [AOSR4-2] AOSR4
### Recent advances in pediatic, AYA cancer research

2025 / 9 / 26 (Fri) 12:50-15:20 Room 3/(Hotel Nikko Kanazawa 4F Tsuru A) Room 3

[Chairpersons]Jyunko Takita (Department of Child Development and Pediatrics, Kyoto University)、kenichi Yoshida (National Cancer Center)

Recent advancement in treatment strategies has significantly improved the outcomes of pediatric cancers. However, there are still issues to be resolved, such as intractable cancers and late complications in pediatric cancer survivors. To understand the mechanisms of pediatric cancer development and to establish more specific and successful treatment strategies, many researchers have conducted extensive research on pediatric cancers using high-throughput sequencing technologies, functional assays and disease models. Through these studies, genetic and epigenetic basis of pediatric cancers have been elucidated and unique characteristics of pediatric cancers, such as the different underlying mutational processes and their origin in aberrantly developing tissue, were also highlighted. In this symposium, the researchers in the field of pediatric cancers will present the latest advances in basic research to understand the biology of pediatric cancers, including the genetics, the etiology and the origin.

---

### AOSR4-2
### Multi-omics analysis of pediatric mesenchymal liver tumors; UESL and MHL

[Speaker]Yoshinori Uchihara (Dept. Ped., Kyoto. Univ., Sch. Med.) [Co-author]Keiji Tasaka (Dept. Ped., Kyoto. Univ., Sch. Med.)、Katsutsugu Umeda (Dept. Ped., Kyoto. Univ., Sch. Med.)、Junko Takita (Dept. Ped., Kyoto. Univ., Sch. Med.)

Undifferentiated embryonal sarcoma of the liver (UESL) and mesenchymal hamartoma of the liver (MHL) are rare pediatric liver tumors. Although UESL occasionally arises from MHL, the relationship between these entities remains elusive. Through comprehensive multi-omics profiling of 18 UESL and 6 MHL cases, we identified structural variants involving C19MC miRNA cluster in all tumors, resulting in robust overexpression of C19MC miRNAs. While ***TP53*** mutations and/or copy number alterations were universally detected in all evaluable UESL, they were absent in MHL. Functional studies demonstrated that C19MC overexpression alone was insufficient for transformation, but synergized with TP53 loss to drive oncogenicity. Spatial transcriptomics, performed in the tumor sample including a composite MHL-UESL lesion, revealed pseudo-temporal gene expression trajectories and clonal diversification. Collectively, our findings delineate a molecular continuum from MHL to UESL and uncover the cooperative genetic events underlying malignant transformation in UESL.

[Disclosure of COI by Responsible researcher] Lead presenter is not the Responsible researcher.
[Lead presenter: Yoshinori Uchihara] We have no financial relationships to disclose.
[Principal Researcher: Junko Takita] We have no financial relationships to disclose.

### [AOSR4-3] AOSR4
### Recent advances in pediatic, AYA cancer research

2025 / 9 / 26 (Fri) 12:50-15:20 Room 3/(Hotel Nikko Kanazawa 4F Tsuru A) Room 3

[Chairpersons]Jyunko Takita (Department of Child Development and Pediatrics, Kyoto University)、kenichi Yoshida (National Cancer Center)

Recent advancement in treatment strategies has significantly improved the outcomes of pediatric cancers. However, there are still issues to be resolved, such as intractable cancers and late complications in pediatric cancer survivors. To understand the mechanisms of pediatric cancer development and to establish more specific and successful treatment strategies, many researchers have conducted extensive research on pediatric cancers using high-throughput sequencing technologies, functional assays and disease models. Through these studies, genetic and epigenetic basis of pediatric cancers have been elucidated and unique characteristics of pediatric cancers, such as the different underlying mutational processes and their origin in aberrantly developing tissue, were also highlighted. In this symposium, the researchers in the field of pediatric cancers will present the latest advances in basic research to understand the biology of pediatric cancers, including the genetics, the etiology and the origin.

---

### AOSR4-3
### Targeting MLL-rearranged leukemia by MENIN inhibitors

[Speaker]Akihiko Yokoyama (National Cancer Center. Tsuruoka Metabolomics Laboratory)

MLL gene rearrangements cause malignant leukemia especially in young patients. MLL is a transcriptional regulator. Gene rearrangements generate fusion of MLL and more than 80 different partner genes. MLL fusions often gain structures of various ENL/AF9-containg protein complexes such as AF4 and AF10, which confer constitutively active transcriptional activity. As a result, MLL fusions constitutively activate MLL-target genes such as HOXA9 and MEIS1, thereby transforming hematopoietic progenitors. Interactome analysis of MLL proteins identified a number of MLL cofactors, among which MENIN stood out as a common associated factor for both MLL fusions and wildtype MLL. The protein complex assembly of MENIN and MLL fusion is the critical event that must take place for leukemia induction, which provides opportunities for drug-mediated inhibition of the MLL fusion protein complex. Thus, small compounds that interfere MENIN-MLL interaction have been developed by several laboratories/pharmaceutical companies. These compounds are now in clinical trials and one of them has been approved by FDA. I will discuss the biological mechanism and therapeutic targeting of MLL-rearranged Leukemia.

[Disclosure of COI by Responsible researcher] Lead presenter is the Responsible researcher.
[Lead presenter: Akihiko Yokoyama] ■Research expenses: 住友ファーマ

### [AOSR4-4] AOSR4
### Recent advances in pediatic, AYA cancer research

2025 / 9 / 26 (Fri) 12:50-15:20 Room 3/(Hotel Nikko Kanazawa 4F Tsuru A) Room 3

[Chairpersons]Jyunko Takita (Department of Child Development and Pediatrics, Kyoto University)、kenichi Yoshida (National Cancer Center)

Recent advancement in treatment strategies has significantly improved the outcomes of pediatric cancers. However, there are still issues to be resolved, such as intractable cancers and late complications in pediatric cancer survivors. To understand the mechanisms of pediatric cancer development and to establish more specific and successful treatment strategies, many researchers have conducted extensive research on pediatric cancers using high-throughput sequencing technologies, functional assays and disease models. Through these studies, genetic and epigenetic basis of pediatric cancers have been elucidated and unique characteristics of pediatric cancers, such as the different underlying mutational processes and their origin in aberrantly developing tissue, were also highlighted. In this symposium, the researchers in the field of pediatric cancers will present the latest advances in basic research to understand the biology of pediatric cancers, including the genetics, the etiology and the origin.

---

### AOSR4-4
### The Landscape of Clonal Hematopoiesis and Structural Variation in Aplastic Anemia

[Speaker]Masanori Yoshida (St. Jude Children's Research Hospital, Department of Hematology／NCCHD, Department of Pediatric Hematology and Oncology Research) [Co-author]Marcin Wlodarski (St. Jude Children's Research Hospital, Department of Hematology)

Aplastic anemia (AA) features T-cell-mediated destruction of hematopoietic stem and progenitor cells. Persistent autoimmune attack drives clonal hematopoiesis via HLA loss, paroxysmal nocturnal hemoglobinuria, and clonal hematopoiesis of indeterminate potential (CHIP) mutations. Through comprehensive genomic analysis of 549 AA patients, comprising 189 pediatric and 360 adult cases, we identified somatic alterations in 70% of cases. While overall frequencies of somatic changes between children and adults were comparable, CHIP mutations displayed distinct patterns with BCOR/L1 and ASXL1 mutations more prevalent in pediatric patients. Meanwhile, adult patients showed a broader CHIP spectrum. Single-cell multi-omics profiling of 29 patients demonstrated highly diversified branching clonal landscape. We found up to 8 independent HLA loss events per patient converging to inactivate identical HLA risk alleles. Elevated CD34+ expression correlated with enhanced long-term clone fitness. Long-read and single-cell whole-genome sequencing precisely mapped UPD6p breakpoints, clone numbers. These findings reveal parallel clonal evolutionary pathways employed by hematopoietic cells in AA.

[Disclosure of COI by Responsible researcher] Lead presenter is not the Responsible researcher.
[Lead presenter: Masanori Yoshida] We have no financial relationships to disclose.
[Principal Researcher: Marcin Wlodarski] ■Research funds from company: Edward P. Evans MDS Foundation, The Vera and Joseph Dresner Foundation, The ALSAC foundation

▲TOP

### [AOSR4-5] AOSR4
### Recent advances in pediatic, AYA cancer research

2025 / 9 / 26 (Fri) 12:50-15:20 Room 3/(Hotel Nikko Kanazawa 4F Tsuru A) Room 3

[Chairpersons]Jyunko Takita (Department of Child Development and Pediatrics, Kyoto University)、kenichi Yoshida (National Cancer Center)

Recent advancement in treatment strategies has significantly improved the outcomes of pediatric cancers. However, there are still issues to be resolved, such as intractable cancers and late complications in pediatric cancer survivors. To understand the mechanisms of pediatric cancer development and to establish more specific and successful treatment strategies, many researchers have conducted extensive research on pediatric cancers using high-throughput sequencing technologies, functional assays and disease models. Through these studies, genetic and epigenetic basis of pediatric cancers have been elucidated and unique characteristics of pediatric cancers, such as the different underlying mutational processes and their origin in aberrantly developing tissue, were also highlighted. In this symposium, the researchers in the field of pediatric cancers will present the latest advances in basic research to understand the biology of pediatric cancers, including the genetics, the etiology and the origin.

---

### AOSR4-5
### Tracing the origin of pediatric cancer using single-cell whole genome sequencing

[Speaker]Ruben Vanboxtel (Princess Maxima Center for Pediatric Oncology)

Understanding how cancer arises is key to developing better prevention and treatment strategies. Our research focuses on uncovering the origins of cancer by analyzing DNA at the single-cell level. We aim to answer two fundamental questions: (1) why do children develop cancer despite minimal age-related DNA damage, and (2) what processes drive the DNA mutations that are required for carcinogenesis? To address these questions, we apply single-cell whole-genome sequencing to patient samples and use mutational patterns to reconstruct the earliest stages of cancer. This approach allows us to trace back its origins and identify critical events that lead to malignancy. In parallel, we use genome editing technologies to introduce cancer-driving mutations into in vitro stem cell models or expose them to potential carcinogens, modeling the mechanisms that shape cancer development. By combining retrospective lineage tracing with experimental validation, our work provides unique insights into the preventable causes of cancer and the molecular mechanisms that enable tumors to arise and evolve.

[Disclosure of COI by Responsible researcher] Lead presenter is the Responsible researcher.
[Lead presenter: Ruben Vanboxtel] ■Presents or other payment: Member of the Scientific Advisory Board of Bioskryb

### [AOSR4-6] AOSR4
### Recent advances in pediatic, AYA cancer research

2025 / 9 / 26 (Fri) 12:50-15:20 Room 3/(Hotel Nikko Kanazawa 4F Tsuru A) Room 3

[Chairpersons]Jyunko Takita (Department of Child Development and Pediatrics, Kyoto University)、kenichi Yoshida (National Cancer Center)

Recent advancement in treatment strategies has significantly improved the outcomes of pediatric cancers. However, there are still issues to be resolved, such as intractable cancers and late complications in pediatric cancer survivors. To understand the mechanisms of pediatric cancer development and to establish more specific and successful treatment strategies, many researchers have conducted extensive research on pediatric cancers using high-throughput sequencing technologies, functional assays and disease models. Through these studies, genetic and epigenetic basis of pediatric cancers have been elucidated and unique characteristics of pediatric cancers, such as the different underlying mutational processes and their origin in aberrantly developing tissue, were also highlighted. In this symposium, the researchers in the field of pediatric cancers will present the latest advances in basic research to understand the biology of pediatric cancers, including the genetics, the etiology and the origin.

---

### AOSR4-6
### Tracing the origins of childhood cancer through genome sequencing

[Speaker]Tim Coorens (European Bioinformatics Institute, European Molecular Biology Laboratory)

Somatic mutations continuously accumulate in cells and can therefore be used as natural barcodes to infer the developmental histories of cells. Using this principle, we can trace the origins of paediatric cancers to precursor clones in their underlying tissue bed by applying whole-genome sequencing to tumor samples and matched normal tissues. This approach can further delineate the ancestral history of multiple tumours from the same patient. In Wilms tumors, we find evidence of tissue-resident precursor clones driven by H19 hypermethylation that can seed multiple independent tumors. Furthermore, the trajectory of Wilms tumor evolution from its tissue context is dictated by the background predisposition mutations. We have applied these principles to study a variety of childhood cancers, including MRT, neuroblastoma and gliomas. Besides an overview of recent advances, I will speak to the next frontiers in dissecting the origins of pediatric cancer using genome sequencing.

[Disclosure of COI by Responsible researcher] Lead presenter is the Responsible researcher.
[Lead presenter: Tim Coorens] We have no financial relationships to disclose.

### [S6-1] S6
### Understanding the mechanism of cancer progression and metastasis orchestrated by stroma

2025 / 9 / 26 (Fri) 12:50-15:20 Room 4/(Hotel Nikko Kanazawa 4F Tsuru B) Room 4

[Chairpersons]Atsushi Enomoto (Nagoya University, Graduate School of Medicine)、Takatsugu Ishimoto (The Cancer Institute, Japanese Foundation for Cancer Research)

Tumor stroma is composed of various types of cells, including fibroblasts, vascular endothelial cells, and immune cells. Therefore, we need to examine the complexity caused by cross-talk between cancer cells and stromal cells to understand the whole picture of advanced cancer. Recent advances in single cell analysis and spatial transcriptomics techniques have elucidated the heterogeneity and complexity of the tumor stroma, making it a potential therapeutic target. In this session, 6 speakers from Asian countries (Japan, China and Korea) will present latest findings on the mechanisms underlying tumor stroma-induced cancer progression, resistance to therapy, and distant metastasis formation, as well as the impact of therapeutic strategies targeting the tumor stroma. We look forward to engaging significant presentation and fruitful discussion about the mechanism of cancer progression and metastasis orchestrated by stroma. This symposium would help us to make progress toward the novel cancer therapy targeting tumor stroma.

---

### S6-1
### CEP55 positive EVs Mediate Glucose-Driven Lung Metastasis via Fibroblast Reprogramming

[Speaker]Fu Lingfeng (Carcinogenesis, JFCR) [Co-author]Takatsugu Ishimoto (Carcinogenesis, JFCR)、Takashi Semba (Carcinogenesis, JFCR)、Masaya Yamazaki (Carcinogenesis, JFCR)、Atsuko Yonemura (Carcinogenesis, JFCR)

Tumor metastasis is a major cause of poor prognosis in cancer patients. Epidemiological data show that gastric cancer patients with diabetes have a significantly higher risk of lung metastasis. Although a high-glucose microenvironment is believed to promote metastasis by altering distant niches, the mechanisms remain unclear. In this study, we found that high glucose stimulates gastric cancer cells to secrete extracellular vesicles (EVs) that communicate with lung fibroblasts, inducing their differentiation into FAP-positive cancer-associated fibroblasts (CAFs). RNA-seq and proteomic analysis revealed that high glucose activates FoxM1, upregulating CEP55, which is packaged into EVs. These CEP55-positive EVs travel to the lungs, activate the CEP55-pFAK-FAP axis, and promote CAF formation. CEP55 also stimulates the pFAK-TNC pathway, leading to tenascin-C secretion, extracellular matrix remodeling, and pre-metastatic niche formation. Blocking pFAK signaling or TNC release significantly reduced lung metastasis. These findings identify a novel EV-mediated mechanism linking hyperglycemia and metastasis, suggesting new therapeutic strategies for diabetic gastric cancer patients.

[Disclosure of COI by Responsible researcher] Lead presenter is not the Responsible researcher.
[Lead presenter: Fu Lingfeng] We have no financial relationships to disclose.
[Principal Researcher: Takashi Semba] We have no financial relationships to disclose.

### [S6-2] S6
### Understanding the mechanism of cancer progression and metastasis orchestrated by stroma

2025 / 9 / 26 (Fri) 12:50-15:20 Room 4/(Hotel Nikko Kanazawa 4F Tsuru B) Room 4

[Chairpersons]Atsushi Enomoto (Nagoya University, Graduate School of Medicine)、Takatsugu Ishimoto (The Cancer Institute, Japanese Foundation for Cancer Research)

Tumor stroma is composed of various types of cells, including fibroblasts, vascular endothelial cells, and immune cells. Therefore, we need to examine the complexity caused by cross-talk between cancer cells and stromal cells to understand the whole picture of advanced cancer. Recent advances in single cell analysis and spatial transcriptomics techniques have elucidated the heterogeneity and complexity of the tumor stroma, making it a potential therapeutic target. In this session, 6 speakers from Asian countries (Japan, China and Korea) will present latest findings on the mechanisms underlying tumor stroma-induced cancer progression, resistance to therapy, and distant metastasis formation, as well as the impact of therapeutic strategies targeting the tumor stroma. We look forward to engaging significant presentation and fruitful discussion about the mechanism of cancer progression and metastasis orchestrated by stroma. This symposium would help us to make progress toward the novel cancer therapy targeting tumor stroma.

---

### S6-2
### Wnt-Induced hyaluronan expression promotes liver metastasis of gastric cancer

[Speaker]Hiroko Oshima (Div.Genetics., Cancer Res. Inst., Kanazawa Univ.) [Co-author]Yuichiro Furutani (Dept. Gastrointestinal Surg., Kanazawa Univ.)、Mizuho Nakayama (Div.Genetics., Cancer Res. Inst., Kanazawa Univ.)、Noriyuki Inaki (Dept. Gastrointestinal Surg., Kanazawa Univ.)、Masanobu Oshima (Div.Genetics., Cancer Res. Inst., Kanazawa Univ.)

Exogenous ligand-stimulated Wnt signaling has been implicated in the gastric cancer development; however, its role in metastasis remains poorly understood. To investigate this, we established gastric epithelial organoids from KTP mice carrying mutations in Kras, Tgfbr2, and p53, and from WKTP mice, which additionally express Wnt1. Remarkably, when transplanted into the spleen, WKTP cells developed liver metastases accompanied by a fibrotic microenvironment, whereas KTP organoids failed to metastasize. Spatial transcriptomic analysis revealed that Wnt signaling in the metastatic niche upregulates Has2, leading to hyaluronan accumulation in the stroma. Importantly, forced expression of hyaluronidase in WKTP cells abolished their metastatic potential, indicating a functional role of hyaluronan in liver metastasis. These findings suggest that Wnt-driven hyaluronan-rich stroma is critical for metastatic colonization in gastric cancer and may represent a potential therapeutic target.

[Disclosure of COI by Responsible researcher] Lead presenter is not the Responsible researcher.
[Lead presenter: Hiroko Oshima] ■Research funds from company: 科研費
[Principal Researcher: Masanobu Oshima] ■Research funds from company: 科研費

### [S6-3] S6
### Understanding the mechanism of cancer progression and metastasis orchestrated by stroma

2025 / 9 / 26 (Fri) 12:50-15:20 Room 4/(Hotel Nikko Kanazawa 4F Tsuru B) Room 4

[Chairpersons]Atsushi Enomoto (Nagoya University, Graduate School of Medicine)、Takatsugu Ishimoto (The Cancer Institute, Japanese Foundation for Cancer Research)

Tumor stroma is composed of various types of cells, including fibroblasts, vascular endothelial cells, and immune cells. Therefore, we need to examine the complexity caused by cross-talk between cancer cells and stromal cells to understand the whole picture of advanced cancer. Recent advances in single cell analysis and spatial transcriptomics techniques have elucidated the heterogeneity and complexity of the tumor stroma, making it a potential therapeutic target. In this session, 6 speakers from Asian countries (Japan, China and Korea) will present latest findings on the mechanisms underlying tumor stroma-induced cancer progression, resistance to therapy, and distant metastasis formation, as well as the impact of therapeutic strategies targeting the tumor stroma. We look forward to engaging significant presentation and fruitful discussion about the mechanism of cancer progression and metastasis orchestrated by stroma. This symposium would help us to make progress toward the novel cancer therapy targeting tumor stroma.

---

### S6-3
### The epigenetic dysregulation of SETD2 in pancreatic cancer: from tumor cells to tumor microenvironment

[Speaker]Jing Xue (Renji Hospital, Shanghai Jiaotong University School of Medicine, Shanghai , China) [Co-author]Ningning Niu (Renji Hospital, Shanghai Jiaotong University School of Medicine, Shanghai , China)

Epigenetic alterations are widespread and impact tumorigenesis. Emerging evidence underscores the influence of epigenetic dysregulation on the tumor microenvironment (TME), enabling adaptation to immune and metabolic stresses during tumor development. SETD2, a primary histone H3K36 trimethyltransferase.Our previous studies linked SETD2 deficiency to pancreatic tumorigenesis and immune evasion. However, how SETD2-deficient pancreatic tumor cells adapt to metabolic stress remains poorly understood.Through scRNA-seq, we identified a lipid-laden cancer-associated fibroblast (CAF) subpopulation marked by ABCA8 in Setd2-deficient pancreatic tumors. Our findings reveal that tumor-intrinsic SETD2 loss activates BMP2 signaling via ectopic gain of H3K27 acetylation (H3K27Ac), driving CAF differentiation toward a lipid-rich phenotype. These lipid-laden CAFs subsequently promote tumor progression by supplying lipids for mitochondrial oxidative phosphorylation through the ABCA8a transporter. Collectively, our study connects CAF heterogeneity to epigenetic dysregulation in tumor cells and uncovers a previously unrecognized metabolic crosstalk between CAFs and pancreatic tumor cells.

[Disclosure of COI by Responsible researcher] Lead presenter is the Responsible researcher.
[Lead presenter: Jing Xue] We have no financial relationships to disclose.

### [S6-4] S6
### Understanding the mechanism of cancer progression and metastasis orchestrated by stroma

2025 / 9 / 26 (Fri) 12:50-15:20 Room 4/(Hotel Nikko Kanazawa 4F Tsuru B) Room 4

[Chairpersons]Atsushi Enomoto (Nagoya University, Graduate School of Medicine)、Takatsugu Ishimoto (The Cancer Institute, Japanese Foundation for Cancer Research)

Tumor stroma is composed of various types of cells, including fibroblasts, vascular endothelial cells, and immune cells. Therefore, we need to examine the complexity caused by cross-talk between cancer cells and stromal cells to understand the whole picture of advanced cancer. Recent advances in single cell analysis and spatial transcriptomics techniques have elucidated the heterogeneity and complexity of the tumor stroma, making it a potential therapeutic target. In this session, 6 speakers from Asian countries (Japan, China and Korea) will present latest findings on the mechanisms underlying tumor stroma-induced cancer progression, resistance to therapy, and distant metastasis formation, as well as the impact of therapeutic strategies targeting the tumor stroma. We look forward to engaging significant presentation and fruitful discussion about the mechanism of cancer progression and metastasis orchestrated by stroma. This symposium would help us to make progress toward the novel cancer therapy targeting tumor stroma.

---

### S6-4
### Identification of CD44v6 as a marker for precancerous lesions in pancreatic cancer

[Speaker]Miho Sekai (Dept. Mol. Oncol., Kyoto Univ., Grad. Sch. Med.／Incubation Lab, Eisai Co., Ltd.) [Co-author]Yukihiro Shiraki (Dept. Patho., Nagoya Univ. Grad. Sch. Med.)、Mina Takahashi (Dept. Mol. Oncol., Kyoto Univ., Grad. Sch. Med.／Incubation Lab, Eisai Co., Ltd.)、Atsushi Enomoto (Dept. Patho., Nagoya Univ. Grad. Sch. Med.)、Yasuyuki Fujita (Dept. Mol. Oncol., Kyoto Univ., Grad. Sch. Med.)

Pancreatic cancer is characterized by early metastasis and poor therapeutic outcomes, making the development of early detection and intervention strategies an urgent issue. However, the absence of a specific marker for precancerous lesions has limited our understanding of early tumorigenesis. In a pancreatic cancer mouse model (KC; KrasG12D;Ptf1a-Cre), we have identified that CD44v6 is specifically expressed at precancerous acinar-to-ductal metaplasia (ADM) lesions. Notably, CD44v6 expression is also observed in cells retaining acinar characteristics, which are supposed to be the origin of ADM; we term this lesion as 'pre-ADM'. Furthermore, CD44v6 expression is upregulated in some of ADM lesions in human pancreatic tissues. We observed frequent accumulation of macrophages around pre-ADM/ADM, which promotes the ADM initiation/expansion. Single-cell RNA sequencing analysis of pancreatic macrophages has revealed a specific gene expression profile in the pre-ADM/ADM-associated macrophages. These findings indicate that CD44v6 represents a promising marker for identifying ADM lesions, and CD44v6 antibody may serve as a novel diagnostic approach for early detection of pancreatic cancer.

[Disclosure of COI by Responsible researcher] Lead presenter is the Responsible researcher.
[Lead presenter: Miho Sekai] We have no financial relationships to disclose.

### [S6-5] S6
### Understanding the mechanism of cancer progression and metastasis orchestrated by stroma

2025 / 9 / 26 (Fri) 12:50-15:20 Room 4/(Hotel Nikko Kanazawa 4F Tsuru B) Room 4

[Chairpersons]Atsushi Enomoto (Nagoya University, Graduate School of Medicine)、Takatsugu Ishimoto (The Cancer Institute, Japanese Foundation for Cancer Research)

Tumor stroma is composed of various types of cells, including fibroblasts, vascular endothelial cells, and immune cells. Therefore, we need to examine the complexity caused by cross-talk between cancer cells and stromal cells to understand the whole picture of advanced cancer. Recent advances in single cell analysis and spatial transcriptomics techniques have elucidated the heterogeneity and complexity of the tumor stroma, making it a potential therapeutic target. In this session, 6 speakers from Asian countries (Japan, China and Korea) will present latest findings on the mechanisms underlying tumor stroma-induced cancer progression, resistance to therapy, and distant metastasis formation, as well as the impact of therapeutic strategies targeting the tumor stroma. We look forward to engaging significant presentation and fruitful discussion about the mechanism of cancer progression and metastasis orchestrated by stroma. This symposium would help us to make progress toward the novel cancer therapy targeting tumor stroma.

### S6-5
### mRNA therapeutics for in situ cancer immunotherapy

[Speaker]Ji-Ho Park (Dept. of Bio & Brain Engineering, KAIST)

The global adoption of mRNA vaccines to combat the COVID-19 pandemic marked a major interdisciplinary milestone, highlighting the transformative potential of combining mRNA technology with nanomedicine. This innovative approach holds great promise as a next-generation therapeutic strategy for cancer immunotherapy. In this talk, I will present our strategies for engineering tumor-associated cells via mRNA delivery to enhance cancer immunotherapy. First, we developed an in situ tumor-infiltrating lymphocyte therapy, which simultaneously engineers tumor-associated macrophages (TAMs) and tumor cells within the tumor microenvironment (TME) using mRNA delivery. Second, we established an in situ CAR-macrophage therapy by selectively engineering TAMs using macrophage-targeted mRNA-LNPs. Lastly, we are currently developing an approach to engineer cancer-associated fibroblasts via mRNA delivery, aiming to recruit cytotoxic immune cells and diminish stromal barrier formation, thereby enhancing immune cell infiltration and improving therapeutic outcomes.

[Disclosure of COI by Responsible researcher] Lead presenter is the Responsible researcher.
[Lead presenter: Ji-Ho Park] We have no financial relationships to disclose.

### [S6-6] S6
### Understanding the mechanism of cancer progression and metastasis orchestrated by stroma

2025 / 9 / 26 (Fri) 12:50-15:20 Room 4/(Hotel Nikko Kanazawa 4F Tsuru B) Room 4

[Chairpersons]Atsushi Enomoto (Nagoya University, Graduate School of Medicine)、Takatsugu Ishimoto (The Cancer Institute, Japanese Foundation for Cancer Research)

Tumor stroma is composed of various types of cells, including fibroblasts, vascular endothelial cells, and immune cells. Therefore, we need to examine the complexity caused by cross-talk between cancer cells and stromal cells to understand the whole picture of advanced cancer. Recent advances in single cell analysis and spatial transcriptomics techniques have elucidated the heterogeneity and complexity of the tumor stroma, making it a potential therapeutic target. In this session, 6 speakers from Asian countries (Japan, China and Korea) will present latest findings on the mechanisms underlying tumor stroma-induced cancer progression, resistance to therapy, and distant metastasis formation, as well as the impact of therapeutic strategies targeting the tumor stroma. We look forward to engaging significant presentation and fruitful discussion about the mechanism of cancer progression and metastasis orchestrated by stroma. This symposium would help us to make progress toward the novel cancer therapy targeting tumor stroma.

---

### S6-6
### Retinoid-Driven CAF Reprogramming for Pancreatic Cancer: MIKE-1 and APPLE Trials

[Speaker]Yasuyuki Mizutani (Dept. Gastroenterol., Nagoya Univ. Hosp.) [Co-author]Atsushi Enomoto (Dept. Tumor Pathol., Nagoya Univ. Grad. Sch. Med.)

Pancreatic cancer has a stroma occupying ~90% of the tumor, mainly composed of cancer-associated fibroblasts (CAFs). This dense stroma elevates pressure, impedes drug delivery, and fosters an immunorestraining microenvironment. Converting CAFs into cancer-restraining CAFs is a new approach. We identified Meflin as a key marker of restraining CAFs. Meflin, a GPI-anchored fibroblast-specific protein, enhances BMP7 signaling to reduce fibrosis and inhibits lysyl oxidase to prevent tissue stiffening. We showed that Meflin-positive CAFs can switch to aSMA-positive, cancer-promoting CAFs. We discovered AM80 (tamibarotene) induces Meflin, reduces cancer-promoting CAF markers, and with gemcitabine boosts vascularization and softens tissues, yielding tumor shrinkage in murine pancreatic cancer. AM80 synergized with immune checkpoint inhibitors in pancreatic and gastric models. Nagoya University is conducting the MIKE-1 Phase I/II trial of AM80 plus gemcitabine/nab-paclitaxel for unresectable pancreatic cancer, and the APPLE trial with ICIs in refractory disease. We aim to proceed to Phase III and pursue global approval of this first-in-class stromal reprogramming therapy.

[Disclosure of COI by Responsible researcher] Lead presenter is the Responsible researcher.
[Lead presenter: Yasuyuki Mizutani] We have no financial relationships to disclose.

### [IS5-1] IS5
### Epigenetic modifications: from mechanisms to cancer

2025 / 9 / 26 (Fri) 12:50-15:20 Room 5/(Hotel Nikko Kanazawa 4F Tsuru C) Room 5

[Chairpersons]Noriko Saitoh (The Cancer Institute of JFCR)、Li-Jung Juan (Academia Sinica)

Disruption of epigenetic mechanisms, alongside genomic mutations, plays a crucial role in cancer initiation, progression, and recurrence. Post translational modifications of histones, non-histone proteins, DNA, and RNA are central to these processes. Enzymes that add or remove modifications, as well as factors that recognize these modifications and transmit signals, play a crucial role in cellular processes. The spatiotemporal regulation of these factors governs a wide range of molecular events, including chromatin remodeling, transcription, DNA replication, DNA damage repair, genomic imprinting, and so on. Additionally, they contribute to multiple levels of chromatin organization, from nucleosomes and chromatin loops to large-scale chromatin domains and nuclear architecture. Together, networks of the epigenetic modification factors regulate genome structure and function, ultimately influencing phenotypes, including oncogenesis. In this program, researchers from Asia-Pacific countries who are leaders in the field of epigenetic modification will be invited to share detailed mechanistic insights, novel in vivo, in vitro and in silico methods, as well as analyses of clinical samples to explore potential diagnostic and therapeutic targets. Young researchers will also be encouraged to present their work in this session.

---

### IS5-1
### Lysine hypoacetylation-defined enhancers in Luminar breast cancers

[Speaker]Noriko Saitoh (Div. Cancer Biol., The Cancer Institute of JFCR) [Co-author]Hiroaki Tachiwana (Div. Cancer Biol., The Cancer Institute of JFCR／Dept. of Epigenomics, Inst. for Advanced Life Sciences, Hoshi Univ.)

Enhancers shape transcriptome by activating cell type- and disease-specific gene expression, contributing to cell identification. At certain active genes, lysine deacetylases promote chromatin modification turnover by targeting histone and non-histone proteins in collaboration with histone acetyltransferases. Importantly, how a selected subset of enhancers is activated remains unclear. Here, we identified hypoacetylation-defined (HD) enhancers in MCF-7 cells, for genes associated with a cell identity as Luminal breast cancer. HD enhancers are defined as sites of bidirectional eRNA transcription and RNA polymerase II-dissociation by lysine deacetylation inhibition. HD enhancers are distinct from super-enhancers, and have extensive chromatin interactions. Further analysis of clinical datasets nailed down HD cluster 1 enhancers, whose formation in the patients correlates with transcriptome-defined breast cancer subtypes, and is associated with expression quantitative trait loci, eQTLs. Our study provides insight into cell identity enhancers and a strategy to identify key enhancer sets for potential therapeutic targets.

[Disclosure of COI by Responsible researcher] Lead presenter is the Responsible researcher.
[Lead presenter: Noriko Saitoh] We have no financial relationships to disclose.

▲TOP

### [IS5-2] IS5
### Epigenetic modifications: from mechanisms to cancer

2025 / 9 / 26 (Fri) 12:50-15:20 Room 5/(Hotel Nikko Kanazawa 4F Tsuru C) Room 5

[Chairpersons]Noriko Saitoh (The Cancer Institute of JFCR)、Li-Jung Juan (Academia Sinica)

Disruption of epigenetic mechanisms, alongside genomic mutations, plays a crucial role in cancer initiation, progression, and recurrence. Post translational modifications of histones, non-histone proteins, DNA, and RNA are central to these processes. Enzymes that add or remove modifications, as well as factors that recognize these modifications and transmit signals, play a crucial role in cellular processes. The spatiotemporal regulation of these factors governs a wide range of molecular events, including chromatin remodeling, transcription, DNA replication, DNA damage repair, genomic imprinting, and so on. Additionally, they contribute to multiple levels of chromatin organization, from nucleosomes and chromatin loops to large-scale chromatin domains and nuclear architecture. Together, networks of the epigenetic modification factors regulate genome structure and function, ultimately influencing phenotypes, including oncogenesis. In this program, researchers from Asia-Pacific countries who are leaders in the field of epigenetic modification will be invited to share detailed mechanistic insights, novel in vivo, in vitro and in silico methods, as well as analyses of clinical samples to explore potential diagnostic and therapeutic targets. Young researchers will also be encouraged to present their work in this session.

---

### IS5-2
### TERC interactome reveals its function beyond telomeres

[Speaker]Yin-Sian Li (Institute of Molecular and Cellular Biology, National Taiwan University) [Co-author]Liv W. Chen (Institute of Molecular and Cellular Biology, National Taiwan University)、Chia-Heng Fan (Institute of Molecular and Cellular Biology, National Taiwan University)、Po-Chen Yang (Institute of Molecular and Cellular Biology, National Taiwan University)、Yu-Syuan Tu (Institute of Molecular and Cellular Biology, National Taiwan University)、Yu-Hung Hsieh (Institute of Molecular and Cellular Biology, National Taiwan University)、HsuehPing C. Chu (Institute of Molecular and Cellular Biology, National Taiwan University)

The Telomerase RNA Component (TERC) serves as a template for telomere extension but may also function as a non-coding RNA in telomerase-independent processes. Here, we identify TERC interacting proteins using identification of direct interacting protein (iDRiP) mass spectrometry and find that TERC binds to proteins involved in rRNA synthesis, transcription regulation, cell cycle, and telomere maintenance. TERC depletion results in downregulation of a subset of genes involved in ribosomal biogenesis, transcription regulation, tumorigenesis, and cell cycle. We show that TERC binds to CCAR1 and Wdr82, and regulates their interactions with RNA polymerase II. TERC depletion using antisense oligos reduces histone H3.3 and H3K4me3 at transcription start sites (TSS), correlating with decreased Wdr82 occupancy. This suggests TERC facilitates Wdr82 recruitment to TSS and promotes H3K4 trimethylation. Notably, CCAR1 or Wdr82 depletion increases localization of TERC at telomeres, suggesting that CCAR1 and Wdr82 regulate TERC localization. These findings highlight TERC as an epigenetic regulator influencing transcription and chromatin status.

[Disclosure of COI by Responsible researcher] Lead presenter is not the Responsible researcher.
[Lead presenter: Yin-Sian Li] We have no financial relationships to disclose.
[Principal Researcher: HsuehPing C. Chu] We have no financial relationships to disclose.

### [IS5-3] IS5
### Epigenetic modifications: from mechanisms to cancer

2025 / 9 / 26 (Fri) 12:50-15:20 Room 5/(Hotel Nikko Kanazawa 4F Tsuru C) Room 5

[Chairpersons]Noriko Saitoh (The Cancer Institute of JFCR)、Li-Jung Juan (Academia Sinica)

Disruption of epigenetic mechanisms, alongside genomic mutations, plays a crucial role in cancer initiation, progression, and recurrence. Post translational modifications of histones, non-histone proteins, DNA, and RNA are central to these processes. Enzymes that add or remove modifications, as well as factors that recognize these modifications and transmit signals, play a crucial role in cellular processes. The spatiotemporal regulation of these factors governs a wide range of molecular events, including chromatin remodeling, transcription, DNA replication, DNA damage repair, genomic imprinting, and so on. Additionally, they contribute to multiple levels of chromatin organization, from nucleosomes and chromatin loops to large-scale chromatin domains and nuclear architecture. Together, networks of the epigenetic modification factors regulate genome structure and function, ultimately influencing phenotypes, including oncogenesis. In this program, researchers from Asia-Pacific countries who are leaders in the field of epigenetic modification will be invited to share detailed mechanistic insights, novel in vivo, in vitro and in silico methods, as well as analyses of clinical samples to explore potential diagnostic and therapeutic targets. Young researchers will also be encouraged to present their work in this session.

---

### IS5-3
### Epigenetic tumorigenesis by 3D interaction between external viral DNA and host genome

[Speaker]Atsushi Kaneda (Dept Mol Oncol, Grad Sch Med, Chiba Univ／Health and Disease Omics Center, Chiba Univ／Cancer Genome Center, Chiba Univ Hospital)

Cells alter epigenome due to various environments, e.g. viral infection. Epstein-Barr virus (EBV), for example, causes dense DNA hypermethylation in >3,000 gene promoters in gastric cells to silence tumor suppressor genes. But integrated interactome and epigenome analyses by Hi-C, 4C-seq, and ChIP-seq revealed that episomal EBV DNA bound to heterochromatin of the host genome, and induced B-to-A compartment shift in EBV-interacting regions with aberrant activation of enhancers and near-by proto-oncogenes in gastric, nasopharyngeal, and hematopoietic malignancies. Human papillomavirus (HPV) infection also causes epigenetic dysregulation in head and neck cancer. In HPV-interacting regions (HPVIRs) around integrated HPV genomes, 9% of HPVIRs were amplified and activated epigenetically forming super-enhancers while the remaining non-amplified HPVIRs showed increase of H3K27ac and upregulation of tumor-related genes. HPV genome also formed hybrid extrachromosomal DNA (ecDNA) with part of host genome; aberrantly activated enhancer regions on hybrid ecDNA not only activated E6/E7 viral oncogenes in cis interaction, but interacted with host chromosomes in trans to upregulate oncogenes.

[Disclosure of COI by Responsible researcher] Lead presenter is the Responsible researcher.
[Lead presenter: Atsushi Kaneda] ■Research expenses: 株式会社エスアールエル、株式会社スクラム　■Contributions: 武田科学振興財団、タムス岡本記念財団

### [IS5-4] IS5
### Epigenetic modifications: from mechanisms to cancer

2025 / 9 / 26 (Fri) 12:50-15:20 Room 5/(Hotel Nikko Kanazawa 4F Tsuru C) Room 5

[Chairpersons]Noriko Saitoh (The Cancer Institute of JFCR)、Li-Jung Juan (Academia Sinica)

Disruption of epigenetic mechanisms, alongside genomic mutations, plays a crucial role in cancer initiation, progression, and recurrence. Post translational modifications of histones, non-histone proteins, DNA, and RNA are central to these processes. Enzymes that add or remove modifications, as well as factors that recognize these modifications and transmit signals, play a crucial role in cellular processes. The spatiotemporal regulation of these factors governs a wide range of molecular events, including chromatin remodeling, transcription, DNA replication, DNA damage repair, genomic imprinting, and so on. Additionally, they contribute to multiple levels of chromatin organization, from nucleosomes and chromatin loops to large-scale chromatin domains and nuclear architecture. Together, networks of the epigenetic modification factors regulate genome structure and function, ultimately influencing phenotypes, including oncogenesis. In this program, researchers from Asia-Pacific countries who are leaders in the field of epigenetic modification will be invited to share detailed mechanistic insights, novel in vivo, in vitro and in silico methods, as well as analyses of clinical samples to explore potential diagnostic and therapeutic targets. Young researchers will also be encouraged to present their work in this session.

---

### IS5-4
### Toward Enhancing Precision Medicine Across Diverse Bile Duct Cancers

[Speaker]Jing Han Hong (Cancer and Stem Cell Biology Programme, Duke-NUS Medical School) [Co-author]Chern Han Yong (Department of Computer Science, National University of Singapore)、Hong Lee Heng (Laboratory of Cancer Epigenome, National Cancer Center Singapore)、Jason Chan (Cancer Discovery Hub, National Cancer Center Singapore)、Mai Chan Lau (Singapore Immunology Network, A*STAR)、Zhimei Li (Cancer Discovery Hub, National Cancer Center Singapore)、Peiyong Guan (Genome Institute of Singapore, A*STAR)、Pek Lim Chu (Cancer and Stem Cell Biology Programme, Duke-NUS Medical School)、Simona Dima (Center of Digestive Diseases and Liver Transplantation, Fundeni Clinical Institute)、Irinel Popescu (Center of Digestive Diseases and Liver Transplantation, Fundeni Clinical Institute)、Sen Yung Hsieh (Chang Gung Memorial Hospital and Chang Gung University)、Sarinya Kongpetch (Cholangiocarcinoma Research Institute, Khon Kaen University)、Apinya Jusakul (Cholangiocarcinoma Research Institute, Khon Kaen University)、Watcharin Loilome (Cholangiocarcinoma Research Institute, Khon Kaen University)、Jing Tan (Sun Yat-sen University Cancer Center)、Bin Tean Teh (Cancer and Stem Cell Biology Programme, Duke-NUS Medical School／Laboratory of Cancer Epigenome, National Cancer Center Singapore)

Cholangiocarcinoma (CCA) is an aggressive malignancy with extensive genomic and epigenomic heterogeneity. To advance precision medicine strategies, we characterized the genomic, transcriptomic, DNA methylation, and enhancer landscapes across CCA subtypes of diverse etiologies. Through integrated multi-omics analysis, we identified three distinct CCA clusters, each defined by specific enhancer dysregulation activities that impact steroid signaling, metabolism, or tumor immunogenicity. These alterations were leveraged to uncover subtype-specific therapeutic vulnerabilities. Using a panel of preclinical models, we performed functional assays and drug response studies to validate these pathways and to further investigate the associated vulnerabilities. To dissect immunogenicity within and across clusters, we utilized spatial transcriptomics, NanoString, and multiplex immunohistochemistry, revealing spatially resolved heterogeneity in mutational signatures and immune microenvironments. Our findings highlight enhancer dysregulation as a key driver of CCA subtype biology and provide a foundation for rational, subtype-guided therapeutic development.

[Disclosure of COI by Responsible researcher] Lead presenter is not the Responsible researcher.
[Lead presenter: Jing Han Hong] We have no financial relationships to disclose.
[Principal Researcher: Bin Tean Teh] We have no financial relationships to disclose.

### [IS5-5] IS5
### Epigenetic modifications: from mechanisms to cancer

2025 / 9 / 26 (Fri) 12:50-15:20 Room 5/(Hotel Nikko Kanazawa 4F Tsuru C) Room 5

[Chairpersons]Noriko Saitoh (The Cancer Institute of JFCR)、Li-Jung Juan (Academia Sinica)

Disruption of epigenetic mechanisms, alongside genomic mutations, plays a crucial role in cancer initiation, progression, and recurrence. Post translational modifications of histones, non-histone proteins, DNA, and RNA are central to these processes. Enzymes that add or remove modifications, as well as factors that recognize these modifications and transmit signals, play a crucial role in cellular processes. The spatiotemporal regulation of these factors governs a wide range of molecular events, including chromatin remodeling, transcription, DNA replication, DNA damage repair, genomic imprinting, and so on. Additionally, they contribute to multiple levels of chromatin organization, from nucleosomes and chromatin loops to large-scale chromatin domains and nuclear architecture. Together, networks of the epigenetic modification factors regulate genome structure and function, ultimately influencing phenotypes, including oncogenesis. In this program, researchers from Asia-Pacific countries who are leaders in the field of epigenetic modification will be invited to share detailed mechanistic insights, novel in vivo, in vitro and in silico methods, as well as analyses of clinical samples to explore potential diagnostic and therapeutic targets. Young researchers will also be encouraged to present their work in this session.

### IS5-5
### Molecular mechanisms of epigenetic regulation

[Speaker]Qi Zhang (South Australian immunoGENomics Cancer Institute (SAiGENCI), the University of Adelaide)

Chromatin modifiers play a crucial role in gene regulation by modulating chromatin structure, and their dysregulation is frequently implicated in cancer. Yet, the precise mechanisms underlying their recruitment and enzymatic regulation remain poorly understood. In this talk, I will present our published findings on the regulation of Polycomb Repressive Complex 2 (PRC2) and share unpublished insights from ongoing research on a distinct chromatin modifier.

[Disclosure of COI by Responsible researcher] Lead presenter is the Responsible researcher.
[Lead presenter: Qi Zhang] We have no financial relationships to disclose.

▲TOP

### [IS5-6] IS5
### Epigenetic modifications: from mechanisms to cancer

2025 / 9 / 26 (Fri) 12:50-15:20 Room 5/(Hotel Nikko Kanazawa 4F Tsuru C) Room 5

[Chairpersons]Noriko Saitoh (The Cancer Institute of JFCR)、Li-Jung Juan (Academia Sinica)

Disruption of epigenetic mechanisms, alongside genomic mutations, plays a crucial role in cancer initiation, progression, and recurrence. Post translational modifications of histones, non-histone proteins, DNA, and RNA are central to these processes. Enzymes that add or remove modifications, as well as factors that recognize these modifications and transmit signals, play a crucial role in cellular processes. The spatiotemporal regulation of these factors governs a wide range of molecular events, including chromatin remodeling, transcription, DNA replication, DNA damage repair, genomic imprinting, and so on. Additionally, they contribute to multiple levels of chromatin organization, from nucleosomes and chromatin loops to large-scale chromatin domains and nuclear architecture. Together, networks of the epigenetic modification factors regulate genome structure and function, ultimately influencing phenotypes, including oncogenesis. In this program, researchers from Asia-Pacific countries who are leaders in the field of epigenetic modification will be invited to share detailed mechanistic insights, novel in vivo, in vitro and in silico methods, as well as analyses of clinical samples to explore potential diagnostic and therapeutic targets. Young researchers will also be encouraged to present their work in this session.

---

### IS5-6
### Reactivation of embryonic programs in cancer

[Speaker]Wee Wei Tee (Institute of Molecular and Cell Biology (IMCB), A*STAR)

My lab has a long-standing interest in deciphering the role of chromatin regulators in shaping cell identity by governing gene expression programs. While chromatin stability preserves cellular identity, its intrinsic plasticity allows for dynamic cell state (re)programming, which is crucial for both normal development and disease progression. A key focus of our work is investigating the epigenetic mechanisms that regulate lineage plasticity during early mammalian development and understanding how these mechanisms are co-opted by cancer cells to drive tumorigenesis and phenotypic plasticity. Indeed, cancer cells frequently adopt embryonic-like traits by hijacking early developmental pathways to enhance phenotypic plasticity. By integrating developmental and cancer biology, we aim to uncover conserved regulatory principles and identify novel cancer vulnerabilities that may present potential therapeutic opportunities. In this talk, I will present our latest research on a group of largely unstudied embryonic factors that drive tumor development through previously unknown biochemical mechanisms.

[Disclosure of COI by Responsible researcher] Lead presenter is the Responsible researcher.
[Lead presenter: Wee Wei Tee] We have no financial relationships to disclose.

▲TOP

### [IS5-7] IS5
### Epigenetic modifications: from mechanisms to cancer

2025 / 9 / 26 (Fri) 12:50-15:20 Room 5/(Hotel Nikko Kanazawa 4F Tsuru C) Room 5

[Chairpersons]Noriko Saitoh (The Cancer Institute of JFCR)、Li-Jung Juan (Academia Sinica)

Disruption of epigenetic mechanisms, alongside genomic mutations, plays a crucial role in cancer initiation, progression, and recurrence. Post translational modifications of histones, non-histone proteins, DNA, and RNA are central to these processes. Enzymes that add or remove modifications, as well as factors that recognize these modifications and transmit signals, play a crucial role in cellular processes. The spatiotemporal regulation of these factors governs a wide range of molecular events, including chromatin remodeling, transcription, DNA replication, DNA damage repair, genomic imprinting, and so on. Additionally, they contribute to multiple levels of chromatin organization, from nucleosomes and chromatin loops to large-scale chromatin domains and nuclear architecture. Together, networks of the epigenetic modification factors regulate genome structure and function, ultimately influencing phenotypes, including oncogenesis. In this program, researchers from Asia-Pacific countries who are leaders in the field of epigenetic modification will be invited to share detailed mechanistic insights, novel in vivo, in vitro and in silico methods, as well as analyses of clinical samples to explore potential diagnostic and therapeutic targets. Young researchers will also be encouraged to present their work in this session.

---

### IS5-7
### Protein N-terminal Acetylase Naa10: From Development to Cancer and Metabolism

[Speaker]Li-Jung Juan (Academia Sinica)

Naa10 performs co-translational protein N-terminal acetylation and plays key roles in epigenetic and cellular regulation. We found that Naa10 promotes silencing of tumor suppressor genes through DNA methyltransferase 1 and is associated with poor prognosis in lung cancer. Naa10 also regulates energy metabolism by acetylating metabolic coactivators, and excessive Naa10 expression leads to reduced thermogenesis and increased fat accumulation, contributing to obesity. During development, Naa10 is essential for DNA methylation, genomic imprinting, and neural differentiation. Naa10 deficiency impairs neurite outgrowth, and its mutations are linked to neurodevelopmental disorders with growth and cardiac defects. These findings highlight Naa10 as a multifunctional regulator critical for epigenetic control, cancer, metabolism, development, and human disease.

### [ME3] ME3
### Nature Immunology

2025 / 9 / 26 (Fri) 12:50-13:30 Room 6/(Hotel Nikko Kanazawa 3F Kujaku A) Room 6

[Chairperson]Kazuo Okamoto (Division of Immune Environment Dynamics, Cancer Research Institute, Kanazawa University)

### ME3
### Nature Immunology

[Speaker]Laurie Dempsey (Senior Editor, Nature Immunology)

No Abstract

### [J-2049] I-J14-2
### Natural & Synthetic Anticancer Compounds

2025 / 9 / 26 (Fri) 12:50-14:05 Room 7/(Hotel Nikko Kanazawa 3F Kujaku B) Room 7

[Chairperson]Manabu Kawada (Lab. Oncology, IMC)

---

### J-2049
### Identification of plumbagin as a novel GGCT inhibitor by cell-based screening using a luminescence probe LISA-103

[Speaker]Keiko Taniguchi (Dept. Drug Discov. Med., Kyoto Pref. Univ. Med.) [Co-author]Yukie Nohara (Peptide Inst., Inc.)、Mano Horinaka (Dept. Drug Discov. Med., Kyoto Pref. Univ. Med.)、Kozue Nose (Dept. Clin. Oncol., Kyoto Pharm. Univ.)、Masaya Mori (Dept. Clin. Oncol., Kyoto Pharm. Univ.)、Hiromi Ii (Dept. Clin. Oncol., Kyoto Pharm. Univ.)、Susumu Nakata (Dept. Clin. Oncol., Kyoto Pharm. Univ.)、Taku Yoshiya (Peptide Inst., Inc.)、Toshiyuki Sakai (Dept. Drug Discov. Med., Kyoto Pref. Univ. Med.)

[Background] γ-Glutamylcyclotransferase (GGCT), an enzyme involved in glutathione metabolism, is highly expressed in various cancers, and its inhibition suppresses cancer growth. Thus, GGCT is a promising target for cancer therapy, but no GGCT-targeting drugs have progressed to clinical trials.
[Methods] We performed cell-based screening for GGCT inhibitors by its enzymatic chemiluminogenic probe LISA-103. The hit compound plumbagin was identified from 753 natural compounds. The direct binding of plumbagin to GGCT recombinant protein was tested using magnetic beads. The effects of plumbagin on the cell growth of MCF7 breast and HCT116 colon cancer cells were analyzed. The HCT116 xenograft mice were treated with plumbagin intraperitoneally.
[Results] Plumbagin bound directly to GGCT and inhibited the intracellular GGCT enzymatic activity. Plumbagin inhibited the cell cycle progression with p21 upregulation and dephosphorylating RB proteins. The tumor growth was significantly suppressed in plumbagin-treated xenograft mice. Here, we established a cell-based screening method for GGCT inhibitors and suggested that plumbagin is a potential novel GGCT inhibitor useful for cancer therapy.

[Disclosure of COI by Responsible researcher] Lead presenter is the Responsible researcher.
[Lead presenter: Keiko Taniguchi] We have no financial relationships to disclose.

### [J-2050] I-J14-2
### Natural & Synthetic Anticancer Compounds

2025 / 9 / 26 (Fri) 12:50-14:05 Room 7/(Hotel Nikko Kanazawa 3F Kujaku B) Room 7

[Chairperson]Manabu Kawada (Lab. Oncology, IMC)

---

### J-2050
### Anti-Cancer Effects of Coffee Diterpenes, Kahweol Acetate and Cafestol, on Taxane-Resistant Prostate Cancer

[Speaker]Tomohiro Hori (Department of Integrative Cancer Therapy and Urology, Kanazawa University) [Co-author]Hiroaki Iwamoto (Department of Integrative Cancer Therapy and Urology, Kanazawa University)、Taiki Kamijima (Department of Integrative Cancer Therapy and Urology, Kanazawa University)、Hiroshi Kano (Department of Integrative Cancer Therapy and Urology, Kanazawa University)、Tomoyuki Makino (Department of Integrative Cancer Therapy and Urology, Kanazawa University)、Renato Naito (Department of Integrative Cancer Therapy and Urology, Kanazawa University)、Hiroshi Yaegashi (Department of Integrative Cancer Therapy and Urology, Kanazawa University)、Kazuyoshi Shigehara (Department of Integrative Cancer Therapy and Urology, Kanazawa University)、Takahiro Nohara (Department of Integrative Cancer Therapy and Urology, Kanazawa University)、Kouji Izumi (Department of Integrative Cancer Therapy and Urology, Kanazawa University)、Atsushi Mizokami (Department of Integrative Cancer Therapy and Urology, Kanazawa University)

The coffee diterpenes kahweol acetate and cafestol have shown strong anti-proliferative effects and inhibited migration in human prostate cancer cells. Taxane-based chemotherapies like docetaxel and cabazitaxel are commonly used for advanced prostate cancer, but resistance eventually develops. Recently, increasing cases of prostate cancer progressing to neuroendocrine prostate cancer after becoming castration-resistant have been reported.Proliferation and migration assays were performed on docetaxel-resistant prostate cancer cells (DU145-TxR, PC3-TxR), cabazitaxel-resistant prostate cancer cells (DU145-TxRCxR, PC3-TxRCxR), and neuroendocrine prostate cancer cells (NCI-H660). Both kahweol acetate and cafestol demonstrated strong inhibition of cell proliferation, and their combination showed a synergistic effect in suppressing cell growth. Additionally, the combination of kahweol and cafestol also resulted in synergistic inhibition of cell migration.Kahweol acetate and cafestol may offer promising new treatments for castration-resistant prostate cancer resistant to taxane-based therapies.

[Disclosure of COI by Responsible researcher] Lead presenter is the Responsible researcher.
[Lead presenter: Tomohiro Hori] We have no financial relationships to disclose.

▲TOP

### [J-2051] I-J14-2
### Natural & Synthetic Anticancer Compounds

2025 / 9 / 26 (Fri) 12:50-14:05 Room 7/(Hotel Nikko Kanazawa 3F Kujaku B) Room 7

[Chairperson]Manabu Kawada (Lab. Oncology, IMC)

---

### J-2051
### Novel compounds inhibiting fibroblast-mediated pancreatic cancer stem-like cell growth

[Speaker]Daisuke Tatsuda (Inst. Microb. Chem., Lab. Onc.) [Co-author]Masahide Amemiya (Inst. Microb. Chem., Lab. Onc.)、Junjiro Yoshida (Inst. Microb. Chem., Lab. Onc.)、Tomokazu Ohishi (Inst. Microb. Chem., Lab. Onc.)、Manabu Kawada (Inst. Microb. Chem., Lab. Onc.)

Pancreatic cancer is one of the most difficult malignancies to treat due to poor early detection and high chemotherapy resistance, leading to an exceptionally low five-year survival rate. Pancreatic tumors contain many normal cells, including fibroblasts, that promote cancer progression and metastasis. To develop new therapeutic agents, we focused on cancer stem-like cells (CSCs), a subset of pancreatic cancer cells with self-renewal and differentiation abilities, believed to drive recurrence and treatment resistance. We aimed to identify compounds that inhibit CSC proliferation by disrupting interactions with fibroblasts. By screening microbial metabolites, we identified compounds that selectively inhibit anchorage-independent (3D) growth in fibroblast-conditioned medium (CM). We previously discovered adenopeptins B and C, and in this study, we identified two new oligomycin analogs. These compounds suppressed CM-dependent 3D growth of BxPC-3 and MIA PaCa-2 pancreatic cancer cells and induced ROS production via inhibition of mitochondrial complex V. We are currently investigating the mechanism underlying this selective 3D growth inhibition.

[Disclosure of COI by Responsible researcher] Lead presenter is not the Responsible researcher.
[Lead presenter: Daisuke Tatsuda] We have no financial relationships to disclose.
[Principal Researcher: Manabu Kawada] We have no financial relationships to disclose.

### [J-2052] I-J14-2
### Natural & Synthetic Anticancer Compounds

2025 / 9 / 26 (Fri) 12:50-14:05 Room 7/(Hotel Nikko Kanazawa 3F Kujaku B) Room 7

[Chairperson]Manabu Kawada (Lab. Oncology, IMC)

---

### J-2052
### 4-Octyl itaconate disrupts the antioxidant system in melanoma via glutathione metabolism disorders.

[Speaker]Ayaka Saeki (OBT Res Ctr, Fac Dent Sci, Kyushu Univ) [Co-author]Yoshikazu Hayashi (OBT Res Ctr, Fac Dent Sci, Kyushu Univ／Div Funct Struct, Fukuoka Dent Coll／Oral Med Res Ctr, Fukuoka Dent Coll)、Syohei Yoshimoto (Oral Med Res Ctr, Fukuoka Dent Coll／Div Pathol Struct, Fukuoka Dent Coll)、Masato Hirata (Oral Med Res Ctr, Fukuoka Dent Coll)、Eijiro Jimi (OBT Res Ctr, Fac Dent Sci, Kyushu Univ／Lab Mol Cell Biochem, Fac Dent Sci, Kyushu Univ)、Tomoyo Kawakuboyasukochi (OBT Res Ctr, Fac Dent Sci, Kyushu Univ)

Itaconate (IA) is a metabolite synthesized from cis-aconitate in the citric acid cycle via aconitate decarboxylase 1. Although most previous studies have used IA analogs such as 4-octyl itaconate (OI) to investigate the physiological roles of IA, the differences in biological activity between IA and OI have not been clarified. Therefore, in this study, we investigated the direct effects of IA and OI on cancer growth using human and mouse melanoma cells.OI, but not IA, significantly suppressed cell proliferation in vitro. RNA sequencing analysis revealed that OI treatment altered the gene expression pattern of glutathione metabolism-related genes in melanoma cells. Indeed, OI treatment decreased the intracellular glutathione concentration and induced the accumulation of reactive oxygen species, DNA damage, and cellular senescence. We also observed that OI treatment significantly reduced the mitochondrial respiratory capacity in melanoma cells. Furthermore, OI treatment significantly suppressed tumor growth via induction of DNA damage in vivo.This study demonstrated that OI disrupts the antioxidant system via the collapse of glutathione metabolism and inhibits melanoma proliferation.

[Disclosure of COI by Responsible researcher] Lead presenter is not the Responsible researcher.
[Lead presenter: Ayaka Saeki] We have no financial relationships to disclose.
[Principal Researcher: Tomoyo Kawakuboyasukochi] We have no financial relationships to disclose.

### [J-2053] I-J14-2
### Natural & Synthetic Anticancer Compounds

2025 / 9 / 26 (Fri) 12:50-14:05 Room 7/(Hotel Nikko Kanazawa 3F Kujaku B) Room 7

[Chairperson]Manabu Kawada (Lab. Oncology, IMC)

---

### J-2053
### Development of selective inhibitors of $Na^+/H^+$ exchanger 5 (NHE5) related in cancer metastasis

[Speaker]Yoshihiro Uto (Grad. Sch. Tech. Ind. Soc. Sci., Tokushima Univ.) [Co-author]Yusei Shinohara (Grad. Sch. Tech. Ind. Soc. Sci., Tokushima Univ.)、Yoshio Endo (Cancer Res. Inst., Kanazawa Univ.)、Takahisa Takino (Inst. of Liberal Arts and Sci., Kanazawa Univ.)

$Na^+/H^+$ exchanger (NHE) is an ion transport membrane protein that exchanges $Na^+$ and $H^+$ in a 1:1 ratio through biological membranes. Among NHE isoforms, NHE5 has been reported to be overexpressed in the cancer cell membrane and NHE5-positive cancer patients have a decreased survival rate. Therefore, we aimed to develop a NHE5 inhibitor by using the computational chemistry to design a novel UTX-143 derivative with high binding affinity to the sodium binding site of NHE5. The structure of NHE5 was predicted using AlphaFold, and the protein was preprocessed using Pymol. The structure of the UTX-143 derivative was then optimized using Avogadro, and the docking pose of NHE5 and the UTX-143 derivative was evaluated by docking score using AutoDock Vina. In addition, UTX-143 derivatives were organically synthesized and their NHE5 and NHE1 inhibitory activities were measured, respectively. In the result, the docking score of the UTX-143 derivatives for NHE5 showed a positive proportional relationship to the NHE5 inhibitory activity, and we succeeded in developing the potent inhibitor compound 6b ($IC_{50}$ = 77 nM).

[Disclosure of COI by Responsible researcher] Lead presenter is the Responsible researcher.
[Lead presenter: Yoshihiro Uto] We have no financial relationships to disclose.

### [J-2054] I-J14-2
### Natural & Synthetic Anticancer Compounds

2025 / 9 / 26 (Fri) 12:50-14:05 Room 7/(Hotel Nikko Kanazawa 3F Kujaku B) Room 7

[Chairperson]Manabu Kawada (Lab. Oncology, IMC)

### J-2054
### M4N as a Novel Therapeutic Approach for KDM6A-mutant Bladder Cancer

[Speaker]Nobuhito Muramoto (Inst. of Lab. Animals, Tokyo Women's Medical Univ.／Dept. of Urology, Juntendo Univ.) [Co-author]Yasuyuki Sera (Inst. of Lab. Animals, Tokyo Women's Medical Univ.)、Masayuki Iwasaki (Dept. of Urology, Hiroshima Univ.)、Kohei Kobatake (Dept. of Urology, Hiroshima Univ.)、Kotohiko Kimura (Dept. of Biol., Johns Hopkins Univ.)、Ruchih C. Huang (Dept. of Biol., Johns Hopkins Univ.)、Hisamitsu Ide (Dept. of Urology, Juntendo Univ.)、Shigeo Horie (Dept. of Urology, Juntendo Univ.)、Hiroaki Honda (Inst. of Lab. Animals, Tokyo Women's Medical Univ.)

Mutations in KDM6A (UTX) are common in bladder cancer (BCa) and contribute to carcinogenesis. Tetra-O-methyl-nordihydroguaiaretic acid (M4N), a methylated derivative of nordihydroguaiaretic acid, inhibits transcription factor Sp1 and exhibits anti-inflammatory activity through NF-κB pathway inhibition. Our previous research demonstrated that KDM6A deletion in BCa promotes inflammation-driven carcinogenesis. To investigate M4N's effect on KDM6A deletion, we established KDM6A knockout (KO) cells using CRISPR-Cas9 from mouse BCa cell line MB49. Syngeneic transplantation in mice showed significantly suppressed tumor growth in groups transplanted with M4N-treated KO cells compared to empty vector-transfected control cells. M4N administration to mice with KO cell transplants resulted in reduced IL-6 expression in subcutaneous tumors and decreased numbers of F4/80-positive macrophages. These findings suggest potential clinical application of M4N as a novel treatment approach for patients with KDM6A-mutant BCa.

[Disclosure of COI by Responsible researcher] Lead presenter is not the Responsible researcher.
[Lead presenter: Muramoto Nobuhito] We have no financial relationships to disclose.
[Principal Researcher: Ruchih C. Huang] We have no financial relationships to disclose.

### [SP2-1] SP2

### Achievement to cancer control no one left behind -cancer research focusing on the health inequalities-

2025 / 9 / 26 (Fri) 12:50-15:20 Room 8/(Hotel Nikko Kanazawa 3F Kujaku C) Room 8

[Chairpersons]Yuri Ito (Osaka Medical and Pharmaceutical University)、Shinsuke Amano ()

第4期がん対策推進基本計画の全体目標として「誰一人取り残さないがん対策を推進し、全ての国民とがんの克服を目指す」ことが掲げられた。がん対策における予防・検診・医療・共生のすべてのフェーズにおいて、個人の属性・社会的背景に関わらず、「誰一人取り残されることのない」がん対策を実現することを目指している。個人の要因だけでなくその人の属している社会地域ネットワークやこれまでに受けてきた教育、職業、収入、住居、保健医療サービスなど、社会・経済・文化・環境といった大きな枠組に起因するという健康の社会的決定要因（Social Determinants of Health: SDH）ががん対策の各フェーズに影響を与えており、健康格差につながっていることがわかってきた。本セッションでは、これまでの国内外のがん対策におけるSDHの影響に関するエビデンスを紹介し、健康格差の縮小に向けたディスカッションを行う場としたい。健康格差に取り組むためには、患者市民参画は不可欠である。学会参加者及びサバイバー・科学者プログラム（SSP）参加者とともに議論する機会としたい。

---

### SP2-1

### Approaches to Health Inequalities in Cancer Control Plans Abroad: A Review of Monitoring Indicators

[Speaker]Marisa Nishio (Dept. Med. Stat., Fac. Med., Osaka Med. Pharm. Univ.／Division of Prevention, Institute for Cancer Control, National Cancer Center)

健康格差の縮小は日本のがん対策における重要課題のひとつだが、格差のモニタリング指標や介入策に関する具体的な検討の参考となる資料は限られている。本研究では、日本を除くG7 Cancer参加国（米・英・豪・加・独・仏）のがん計画をレビューし、格差対策の位置付けとモニタリング指標を分析した。各国の計画では、主に、人種・民族、地域間の公平性の確保を重視していたが、実際のモニタリングは英国の地域レベル剥奪指標を除き、属性別のデータの収集に留まっていた。今後日本では、多様な集団を対象とした指標の設定、地域レベルの剥奪指標の活用、層別化データの整備等が必要であると考えられる。

[Disclosure of COI by Responsible researcher] Lead presenter is the Responsible researcher.
[Lead presenter: Marisa Nishio] We have no financial relationships to disclose.

### [SP2-2] SP2

### Achievement to cancer control no one left behind -cancer research focusing on the health inequalities-

2025 / 9 / 26 (Fri) 12:50-15:20 Room 8/(Hotel Nikko Kanazawa 3F Kujaku C) Room 8

[Chairpersons]Yuri Ito (Osaka Medical and Pharmaceutical University)、Shinsuke Amano ()

第4期がん対策推進基本計画の全体目標として「誰一人取り残さないがん対策を推進し、全ての国民とがんの克服を目指す」ことが掲げられた。がん対策における予防・検診・医療・共生のすべてのフェーズにおいて、個人の属性・社会的背景に関わらず、「誰一人取り残されることのない」がん対策を実現することを目指している。個人の要因だけでなくその人の属している社会地域ネットワークやこれまでに受けてきた教育、職業、収入、住居、保健医療サービスなど、社会・経済・文化・環境といった大きな枠組に起因するという健康の社会的決定要因（Social Determinants of Health: SDH）ががん対策の各フェーズに影響を与えており、健康格差につながっていることがわかってきた。本セッションでは、これまでの国内外のがん対策におけるSDHの影響に関するエビデンスを紹介し、健康格差の縮小に向けたディスカッションを行う場としたい。健康格差に取り組むためには、患者市民参画は不可欠である。学会参加者及びサバイバー・科学者プログラム（SSP）参加者とともに議論する機会としたい。

### SP2-2

### Monitoring for health inequalities and socioeconomic inequalities in cancer prevention (smoking)

[Speaker]Hirokazu Tanaka (NCCICC, Pop Data Sci)

健康格差は社会経済状況（教育歴・職業・所得など）によって集団間で健康状態に系統的な差があることを示す。健康格差の縮小に向けて健康政策の効果や進捗を検証するために、具体的な政策の実施と同時に健康格差の客観的なモニタリングが欠かせない。モニタリングの観点として、「社会経済状況を示す指標（曝露）」と「健康格差の大きさを測定する指標（結果）」の2つの側面が重要である。「社会経済状況を示す指標（曝露）」では、個人の社会経済的地位（教育歴・職業・所得など）や人口属性（婚姻状況・世帯人数など）と地域、地域指標（人口密度、地理的剥奪指標など）などが用いられる。また、「健康格差の大きさを測定する指標（結果）」では相対リスク、絶対リスクなどの疫学指標と格差勾配指数（SII: Slope index of inequalityおよびRII: Relative index of inequality）などが用いられる。一例として、厚生労働省の国民生活基礎調査のデータを二次解析した結果によると、2016年の教育歴別の喫煙率（25-64歳）は男性の中学卒業者で57.8%、高校卒業者で43.9%、大学以上卒業者で27.8%であり、女性では中学卒業者で34.7%、高校卒業者で15.9%、大学以上卒業者で5.6%であった。がん予防における健康格差について喫煙率の社会格差を中心にモニタリング事例を紹介するとともに、関連する統計や調査の課題や今後の活用方法について解説する。

[Disclosure of COI by Responsible researcher] Lead presenter is the Responsible researcher.
[Lead presenter: Hirokazu Tanaka] We have no financial relationships to disclose.

▲TOP

### [SP2-3] SP2

### Achievement to cancer control no one left behind -cancer research focusing on the health inequalities-

2025 / 9 / 26 (Fri) 12:50-15:20 Room 8/(Hotel Nikko Kanazawa 3F Kujaku C) Room 8

[Chairpersons]Yuri Ito (Osaka Medical and Pharmaceutical University)、Shinsuke Amano ()

第4期がん対策推進基本計画の全体目標として「誰一人取り残さないがん対策を推進し、全ての国民とがんの克服を目指す」ことが掲げられた。がん対策における予防・検診・医療・共生のすべてのフェーズにおいて、個人の属性・社会的背景に関わらず、「誰一人取り残されることのない」がん対策を実現することを目指している。個人の要因だけでなくその人の属している社会地域ネットワークやこれまでに受けてきた教育、職業、収入、住居、保健医療サービスなど、社会・経済・文化・環境といった大きな枠組に起因するという健康の社会的決定要因（Social Determinants of Health: SDH）ががん対策の各フェーズに影響を与えており、健康格差につながっていることがわかってきた。本セッションでは、これまでの国内外のがん対策におけるSDHの影響に関するエビデンスを紹介し、健康格差の縮小に向けたディスカッションを行う場としたい。健康格差に取り組むためには、患者市民参画は不可欠である。学会参加者及びサバイバー・科学者プログラム（SSP）参加者とともに議論する機会としたい。

### SP2-3

### Neighorhood-based inequalities in Human Papillomavirus vaccination

[Speaker]Emiko Oka (Department of Obstetrics and Gynecology, Osaka University／Department of Medical Statistics, Medical and Pharmaceutical University) [Co-author]Yuri Ito (Department of Medical Statistics, Medical and Pharmaceutical University)

子宮頸がんはHuman papillomavirus(HPV)ワクチン接種により予防可能ながんであるが、本邦のHPVワクチン接種割合は他の先進国よりも著しく低い。またワクチン接種割合上昇には接種割合が低い集団へ地域ベースの介入が重要であるが、本邦ではHPVワクチン接種割合の地域格差に着目した研究は見当たらない。そこで、HPVワクチン累積接種割合と地域の困窮度や都会度、HPVワクチン接種医療機関へのアクセスとの関連について評価した。e-Statより2020-2022年度 HPVワクチン接種者数データを取得し、市区町村別HPVワクチン累積接種割合と地域指標との関連を評価した結果、困窮度が低い地域や都会度が高い地域は、困窮度が高い地域や都会度が低い地域と比べ、累積接種割合が高かった。しかし、公開データは年度により対象者が異なり、定期・キャッチアップ接種を区別できない、など限界点が存在した。そこで大阪市より提供された2013～2022年度HPVワクチン接種個別データを用いて、町丁字単位、定期・キャッチアップ接種別の解析を行った。HPVワクチン累積接種割合は、地域の困窮度が低いほど高く、地域の代表点から500m圏域内のHPVワクチンを提供する医療機関の数が多いほど高かった。またこれらの格差は、定期接種で顕著であった。詳細な項目を含むデータを用いることで、格差の現状を適切に把握することが可能となった。本研究結果は、より強い介入を必要とする地域の選定に役立つと考えられる。

[Disclosure of COI by Responsible researcher] Lead presenter is not the Responsible researcher.
[Lead presenter: Emiko Oka] We have no financial relationships to disclose.
[Principal Researcher: Yuri Ito] We have no financial relationships to disclose.

### [SP2-4] SP2
### Achievement to cancer control no one left behind -cancer research focusing on the health inequalities-

2025 / 9 / 26 (Fri) 12:50-15:20 Room 8/(Hotel Nikko Kanazawa 3F Kujaku C) Room 8

[Chairpersons]Yuri Ito (Osaka Medical and Pharmaceutical University)、Shinsuke Amano ()

第4期がん対策推進基本計画の全体目標として「誰一人取り残さないがん対策を推進し、全ての国民とがんの克服を目指す」ことが掲げられた。がん対策における予防・検診・医療・共生のすべてのフェーズにおいて、個人の属性・社会的背景に関わらず、「誰一人取り残されることのない」がん対策を実現することを目指している。個人の要因だけでなくその人の属している社会地域ネットワークやこれまでに受けてきた教育、職業、収入、住居、保健医療サービスなど、社会・経済・文化・環境といった大きな枠組に起因するという健康の社会的決定要因（Social Determinants of Health: SDH）ががん対策の各フェーズに影響を与えており、健康格差につながっていることがわかってきた。本セッションでは、これまでの国内外のがん対策におけるSDHの影響に関するエビデンスを紹介し、健康格差の縮小に向けたディスカッションを行う場としたい。健康格差に取り組むためには、患者市民参画は不可欠である。学会参加者及びサバイバー・科学者プログラム（SSP）参加者とともに議論する機会としたい。

### SP2-4
### Health Disparities Revealed Through Cancer Screening and Their Social Context

[Speaker]Masayo Komatsu (The University of Osaka)

がん検診は、疾病の早期発見・早期治療を可能にする重要な手段であり、健康増進法（2002年施行）に基づき、市町村には住民に対するがん検診の実施が義務づけられている。一方で、がん検診の受診機会は市町村や勤務先をはじめ制度的に多様であり、全国民を一律に対象とする統一的なプログラムは存在しない。こうした構造のもと、がん検診の受診率には、個人の所属や社会的背景によって格差が生じている可能性がある。本研究では、2004年から2019年の国民生活基礎調査を用い、がん検診以外の健康診断（健康診断）の受診機会別に、がん検診受診の有無や受診者の属性を分析した。その結果、勤務先などを通じて健康診断を受けた群では、がん検診の受診率が高かったのに対し、市町村での健康診断受診群および未受診群では受診率が低く、特に健康診断未受診群では著しく低い受診率がみられた。ロジスティック回帰分析でも、健康診断を受けていない群は一貫して有意に低いオッズ比を示し、健康診断の受診機会ががん検診行動に大きな影響を与えていることが示唆された。制度上、がん検診の提供は法的に保障されていても、現実には健康診断の受診機会や個人の社会経済的条件により、検診アクセスに格差が生じている。今後は、家庭内の介護負担の有無や支援体制といった生活環境に根ざした要因を含め、健康格差の構造的理解と、公平ながん検診制度のあり方を再検討していく必要がある。

[Disclosure of COI by Responsible researcher] Lead presenter is the Responsible researcher.
[Lead presenter: Masayo Komatsu] We have no financial relationships to disclose.

### [SP2-5] SP2

### Achievement to cancer control no one left behind -cancer research focusing on the health inequalities-

2025 / 9 / 26 (Fri) 12:50-15:20 Room 8/(Hotel Nikko Kanazawa 3F Kujaku C) Room 8

[Chairpersons]Yuri Ito (Osaka Medical and Pharmaceutical University)、Shinsuke Amano ()

第4期がん対策推進基本計画の全体目標として「誰一人取り残さないがん対策を推進し、全ての国民とがんの克服を目指す」ことが掲げられた。がん対策における予防・検診・医療・共生のすべてのフェーズにおいて、個人の属性・社会的背景に関わらず、「誰一人取り残されることのない」がん対策を実現することを目指している。個人の要因だけでなくその人の属している社会地域ネットワークやこれまでに受けてきた教育、職業、収入、住居、保健医療サービスなど、社会・経済・文化・環境といった大きな枠組に起因するという健康の社会的決定要因（Social Determinants of Health: SDH）ががん対策の各フェーズに影響を与えており、健康格差につながっていることがわかってきた。本セッションでは、これまでの国内外のがん対策におけるSDHの影響に関するエビデンスを紹介し、健康格差の縮小に向けたディスカッションを行う場としたい。健康格差に取り組むためには、患者市民参画は不可欠である。学会参加者及びサバイバー・科学者プログラム（SSP）参加者とともに議論する機会としたい。

---

### SP2-5

### Inequalities in Cancer Medicine: Survival, Treatment Gaps, and Underlying Factors

[Speaker]Mariko Hanafusa (Div. of Cohort Research, NCC Institute for Cancer Control)

社会経済状況に起因するがん格差は、予防・検診だけでなく診断後の治療や予後にも及ぶ。日本ではがん登録・診療・社会情報が分断されており、全国がん登録と地域の困窮度指標を連結した解析の実現までに、多大な労力を要した。その結果、死亡率格差が特に顕著とされる肺がんでは、困窮地域の患者で手術機会と生存率も一貫して低く、これらの関連における腫瘍進展度の寄与は限定的であり、早期発見だけでは医療の格差は解消されないことが示された。海外では生存率格差の要因として、ガイドライン逸脱や併存疾患、緊急入院の多さが指摘されている。喫煙がCOPDや心血管疾患を介して治療選択を制限することから、発症前の体系的対策の重要性が強調される。また、スティグマは禁煙やガイドライン準拠治療を妨げる要因のひとつとして注目されており、それを低減するための医療者研修も実線されている。さらに、多職種ワンストップ外来に旅費補助・遠隔診療などを組み合わせた包括的プログラムの導入の報告もある。これらの取り組みは、直接的な格差是正効果に関するエビデンスはまだ限られるものの、社会的に不利な集団の情報的・心理的負担を軽減し得る点で、日本における格差縮小の実装モデルとして有望である。なお、対策の効果を適切に評価するためには、臨床情報と公的統計を安全に連結できる基盤の整備に加え、国民の理解と支持を得るための啓発活動を並行して進めることが不可欠である。

[Disclosure of COI by Responsible researcher] Lead presenter is the Responsible researcher.
[Lead presenter: Mariko Hanafusa] We have no financial relationships to disclose.

### [SP2-6] SP2
### Achievement to cancer control no one left behind -cancer research focusing on the health inequalities-

2025 / 9 / 26 (Fri) 12:50-15:20 Room 8/(Hotel Nikko Kanazawa 3F Kujaku C) Room 8

[Chairpersons]Yuri Ito (Osaka Medical and Pharmaceutical University)、Shinsuke Amano ()

第4期がん対策推進基本計画の全体目標として「誰一人取り残さないがん対策を推進し、全ての国民とがんの克服を目指す」ことが掲げられた。がん対策における予防・検診・医療・共生のすべてのフェーズにおいて、個人の属性・社会的背景に関わらず、「誰一人取り残されることのない」がん対策を実現することを目指している。個人の要因だけでなくその人の属している社会地域ネットワークやこれまでに受けてきた教育、職業、収入、住居、保健医療サービスなど、社会・経済・文化・環境といった大きな枠組に起因するという健康の社会的決定要因（Social Determinants of Health: SDH）ががん対策の各フェーズに影響を与えており、健康格差につながっていることがわかってきた。本セッションでは、これまでの国内外のがん対策におけるSDHの影響に関するエビデンスを紹介し、健康格差の縮小に向けたディスカッションを行う場としたい。健康格差に取り組むためには、患者市民参画は不可欠である。学会参加者及びサバイバー・科学者プログラム（SSP）参加者とともに議論する機会としたい。

---

### SP2-6
### The Current Landscape and Challenges of Financial Toxicity in Oncology

[Speaker]Kazunori Honda (Department of Clinical Oncology, Aichi Cancer Center)

近年、がん治療の進歩により高額な薬剤が増加し、治療期間も長期化している。その結果、患者・家族の経済的負担も増大し、「経済毒性（financial toxicity）」という概念が2013年に提唱された。経済毒性とは、がんに伴う経済的負担が患者や家族にもたらす精神的・社会的苦痛を指すもので、単なる医療費や経済学的課題にとどまらない。がんとの共生が求められるなか、経済毒性は生活の質や治療継続に影響を与える深刻な問題である。日本では高額療養費制度など公的保険制度が整備されているものの、経済毒性の影響は見過ごされてきた。米国で開発されたCOST質問紙（Comprehensive Score for financial Toxicity）の日本語版を用いた調査により、日本人がん患者では、若年層、貯蓄が少ない世帯、非正規雇用者、がんによる退職者などで経済毒性が特に強いことが明らかとなり、社会的・経済的な格差との関連も示唆された。この課題に対応するためには、薬剤費やLow-value careの見直しによる支出の適正化、就労支援による収入面の補完、医療費の事前提示や相談体制の整備など多面的な対策が必要である。今後、患者・医療者・社会が連携し、経済毒性の軽減と格差の是正に取り組むことで、がんとの共生を支える持続可能ながん医療の実現が求められている。

[Disclosure of COI by Responsible researcher] Lead presenter is the Responsible researcher.
[Lead presenter: Kazunori Honda] ■Contributions: ファイザー株式会社

### [SP2-7] SP2
### Achievement to cancer control no one left behind -cancer research focusing on the health inequalities-

2025 / 9 / 26 (Fri) 12:50-15:20 Room 8/(Hotel Nikko Kanazawa 3F Kujaku C) Room 8

[Chairpersons]Yuri Ito (Osaka Medical and Pharmaceutical University)、Shinsuke Amano ()

第4期がん対策推進基本計画の全体目標として「誰一人取り残さないがん対策を推進し、全ての国民とがんの克服を目指す」ことが掲げられた。がん対策における予防・検診・医療・共生のすべてのフェーズにおいて、個人の属性・社会的背景に関わらず、「誰一人取り残されることのない」がん対策を実現することを目指している。個人の要因だけでなくその人の属している社会地域ネットワークやこれまでに受けてきた教育、職業、収入、住居、保健医療サービスなど、社会・経済・文化・環境といった大きな枠組に起因するという健康の社会的決定要因（Social Determinants of Health: SDH）ががん対策の各フェーズに影響を与えており、健康格差につながっていることがわかってきた。本セッションでは、これまでの国内外のがん対策におけるSDHの影響に関するエビデンスを紹介し、健康格差の縮小に向けたディスカッションを行う場としたい。健康格差に取り組むためには、患者市民参画は不可欠である。学会参加者及びサバイバー・科学者プログラム（SSP）参加者とともに議論する機会としたい。

---

### SP2-7
### Expectations for Evidence-Based Policymaking

[Speaker]Naomi Sakurai (General Incorporated Association, CSR Project／Japan Federation of Cancer Patient Groups／Cancer Solutions, Inc.)

EBPM (エビデンス・ベースト・ポリシー・メイキング) とは、「政策立案に際して、経験や直感ではなく、客観的な証拠（エビデンス）に基づき、合理的根拠を重視する考え方です。具体的には、政策目的を明確化し、その達成に必要な政策手段とエビデンスを関連付けることで、より効果的な政策立案を目指します。」とされている。「パブリックヘルス」を考える上でＥＢＰＭを広めていくことは大変重要である。客観的なエビデンスに基づいた政策を立案していくことで、経験や直感、あるいは人間関係に依存した政策よりも、効果的に目標を達成、経過を検証、改善につなげることができる。こうした政策の透明性を高めることで、国民からの理解と信頼を得ることもでき、より政策、政治への興味も高まるものと考えられる。しかしながら、我が国をみると、そもそもとしてのデータがないこと、また、データがあっても活用にあたっての入り口規制が高いことなど、多くの課題が山積している。データを「利活用」することで、わたしたちの生活はどのように改善するのか、どのような課題がみえてくるのかについて、本セッションを通じて議論をしたい。

[Disclosure of COI by Responsible researcher] Lead presenter is the Responsible researcher.
[Lead presenter: Sakurai Naomi] ■ employment by a company related to the presentation: キャンサーソリューションズ株式会社
■Advisor of the company and/or profit-making organization: キャンサーソリューションズ株式会社

### [SP2-8] SP2

### Achievement to cancer control no one left behind -cancer research focusing on the health inequalities-

2025 / 9 / 26 (Fri) 12:50-15:20 Room 8/(Hotel Nikko Kanazawa 3F Kujaku C) Room 8

[Chairpersons]Yuri Ito (Osaka Medical and Pharmaceutical University)、Shinsuke Amano ()

第4期がん対策推進基本計画の全体目標として「誰一人取り残さないがん対策を推進し、全ての国民とがんの克服を目指す」ことが掲げられた。がん対策における予防・検診・医療・共生のすべてのフェーズにおいて、個人の属性・社会的背景に関わらず、「誰一人取り残されることのない」がん対策を実現することを目指している。個人の要因だけでなくその人の属している社会地域ネットワークやこれまでに受けてきた教育、職業、収入、住居、保健医療サービスなど、社会・経済・文化・環境といった大きな枠組に起因するという健康の社会的決定要因（Social Determinants of Health: SDH）ががん対策の各フェーズに影響を与えており、健康格差につながっていることがわかってきた。本セッションでは、これまでの国内外のがん対策におけるSDHの影響に関するエビデンスを紹介し、健康格差の縮小に向けたディスカッションを行う場としたい。健康格差に取り組むためには、患者市民参画は不可欠である。学会参加者及びサバイバー・科学者プログラム（SSP）参加者とともに議論する機会としたい。

---

### SP2-8

### Unheard Realities in Cancer Control: Insights from Frontline Patient Support Practice

[Speaker]Kazuho Hisamura (Dept. Epidemiology and Public Health, Kanazawa Med. Univ., Sch. Med.／Ishikawa Prefectural Cancer Support House)

がん対策における健康格差は、制度上の課題に留まらず、支援が届きにくい生活実態や社会的孤立に根ざしている。石川県がん安心生活サポートハウス（通称：はなうめ、済生会金沢病院運営）では、ピアサポート、就労・経済的困難の相談支援、進行がん患者や独居者のためのサポート・グループ等を通じて、患者・家族・遺族等の多様かつ長期的な支援ニーズに対応している。医療職に加え、ファイナンシャル・プランナー、司法書士、理美容師、哲学者など30を越える職種が協働している点も特徴である。　支援現場では、SDHが複雑に交錯している。特に独居、若年・中年期、日本語を母語としない患者・家族は、支援の手が届きにくく、制度の谷間に置かれている現実がある。近年はなうめで実施した独居進行がん患者へのインタビュー調査では、「全く孤独、世界で一人だけになった感じ」「どこでも”家族ありき”の話をされる」「孤独死するのではと不安」といった声が聞かれた。　本発言では、こうした支援実践と当事者の声を踏まえ、制度の枠外に置かれがちな声やデータに表れにくい課題を、研究や政策の中でいかに可視化し、反映できるかを考える。低頻度・高困難事例への対応には、地域に根ざした多様な“支え手・つなぎ手”が不可欠である。現場で蓄積された実践知やナラティブを定量データの補完資源と捉え、公平性の視点から“誰一人取り残さない”がん対策の実現に向けた共創を目指したい。

[Disclosure of COI by Responsible researcher] Lead presenter is the Responsible researcher.
[Lead presenter: Kazuho Hisamura] We have no financial relationships to disclose.

### [E-2055] I-E4-2
### Oncogenes & tumor-suppressor genes (3)

2025 / 9 / 26 (Fri) 12:50-14:05 Room 9/(Hotel Nikko Kanazawa 3F Lumiere) Room 9

[Chairperson]Ryota Kurimoto (Department of Molecular Diagnosis, Chiba University, Graduate School of Medicine／Department of Systems BioMedicine, Institute of Science Tokyo)

### E-2055
### Identification of a Novel Secretion Mechanism by Proximal-Dependent Interactomes for Endoplasmic Reticulum Therapy

[Speaker]Kohji Yamada (The Jikei univ Biochem)

Liver cancer is still a refractory cancer and has a lower response rate to drug therapy than other types of cancer. We have previously reported a novel secretion mechanism (Cancer-related unconventional protein secretion; CUPS) through analysis of proximal-dependent protein-protein interactions. CUPS is a mechanism in which nuclear proteins without signal sequences are secreted, and it is predominantly found in liver cancer, and in particular, serum PKC delta has higher diagnostic ability than existing markers for early-stage liver cancer. In addition, biological analysis has revealed that nuclear proteins are recruited to the endoplasmic reticulum (ER). Further analysis of the ER interactome by splitTurboID identified several ER proteins. Among them, CRP-2 (CUPS-related protein-2) deletion not only suppressed secretion of nuclear proteins, but also significantly inhibited the tumor growth/survival. In liver carcinogenesis models, CRP-2 suppression resulted in tumor reduction and disappearance. These findings suggest that a novel ER function called CUPS is involved in hepatocarcinogenesis and that CRP-2, a key factor in this interaction, is a potential therapeutic target.

[Disclosure of COI by Responsible researcher] Lead presenter is the Responsible researcher.
[Lead presenter: Kohji Yamada] ■Research funds from company: AMED次世代がん医療加速化研究事業、AMED革新的がん医療実用化研究事業、AMED創薬ブースター事業　■Contributions: アステラス製薬、安田財団、持田財団、武田財団、千里ライフサイエンス財団

▲TOP

### [E-2056] I-E4-2
### Oncogenes & tumor-suppressor genes (3)

2025 / 9 / 26 (Fri) 12:50-14:05 Room 9/(Hotel Nikko Kanazawa 3F Lumiere) Room 9

[Chairperson]Ryota Kurimoto (Department of Molecular Diagnosis, Chiba University, Graduate School of Medicine／Department of Systems BioMedicine, Institute of Science Tokyo)

### E-2056
### Pan-cancer analysis exploring the relationship between chromothripsis and global DNA hypomethylation

[Speaker]Keiichi Ohshima (Med. Genetics Div., Shizuoka Cancer Ctr. Res. Inst.) [Co-author]Takeshi Nagashima (Cancer Diagnostics Res. Div., Shizuoka Cancer Ctr. Res. Inst.／SRL Inc.)、Keiichi Hatakeyama (Cancer Multiomics Div., Shizuoka Cancer Ctr. Res. Inst.)、Fukumi Kamada (Cancer Diagnostics Res. Div., Shizuoka Cancer Ctr. Res. Inst.)、Kenichi Urakami (Cancer Diagnostics Res. Div., Shizuoka Cancer Ctr. Res. Inst.)、Yasuto Akiyama (Immunotherapy Div., Shizuoka Cancer Ctr. Res. Inst.)、Ken(Shizuoka Cancer Ctr.) Yamaguchi (Shizuoka Cancer Ctr.)

Whole genome sequencing (WGS) can identify structural chromosomal abnormalities. Among them, chromothripsis (CT) is a catastrophic event of genome instability where chromosomes break and assemble in a random order, resulting in complex chromosomal rearrangements. Global DNA hypomethylation (GDHO) is another event affecting genome instability and often occurs at repetitive elements. Both events play in a significant role in tumorigenesis. Here we aim to investigate the relationship between the two events using our own data of WGS and microarray-based DNA methylation analysis from 1355 patients across more than 100 tumor types. CT was detected in 534 cases (39%). GDHO level was elevated in CT-positive tumor samples from hepatocellular carcinoma, compared to CT-negative tumor tissue samples as well as non-cancerous tissue samples. However, the GDHO regions were not specific to regions where CT was detected, but were common to CT-positive tumors. We also examine these relationships in other tumor types, including from esophagus, colorectum, lung, and breast cancer, and the effects of CT and GDHO on gene expression.

[Disclosure of COI by Responsible researcher] Lead presenter is the Responsible researcher.
[Lead presenter: Keiichi Ohshima] We have no financial relationships to disclose.

### [E-2057] I-E4-2
### Oncogenes & tumor-suppressor genes (3)

2025 / 9 / 26 (Fri) 12:50-14:05 Room 9/(Hotel Nikko Kanazawa 3F Lumiere) Room 9

[Chairperson]Ryota Kurimoto (Department of Molecular Diagnosis, Chiba University, Graduate School of Medicine／Department of Systems BioMedicine, Institute of Science Tokyo)

### E-2057
### The role of FOXO3 in the mechanism of malignant progression of gastric cancer

[Speaker]Kazuyoshi Mitta (Department of Gastrointestinal Surgery, Kanazawa University Hospital) [Co-author]Toshikatsu Tsuji (Department of Gastrointestinal Surgery, Kanazawa University Hospital)、Haruka Kubo (Department of Gastrointestinal Surgery, Kanazawa University Hospital)、Hiroyuki Tanaka (Department of Gastrointestinal Surgery, Kanazawa University Hospital)、Shunsuke Takenaka (Department of Gastrointestinal Surgery, Kanazawa University Hospital)、Ryosuke Machi (Department of Gastrointestinal Surgery, Kanazawa University Hospital)、Hiroshi Saito (Department of Gastrointestinal Surgery, Kanazawa University Hospital)、Kenta Doden (Department of Gastrointestinal Surgery, Kanazawa University Hospital)、Yusuke Sakimura (Department of Gastrointestinal Surgery, Kanazawa University Hospital)、Hiroto Saito (Department of Gastrointestinal Surgery, Kanazawa University Hospital)、Daisuke Yamamoto (Department of Gastrointestinal Surgery, Kanazawa University Hospital)、Hideki Moriyama (Department of Gastrointestinal Surgery, Kanazawa University Hospital)、Jun Kinoshita (Department of Gastrointestinal Surgery, Kanazawa University Hospital)、Hiroko Oshima (Cancer Research Institute, Kanazawa University)、Noriyuki Inaki (Department of Gastrointestinal Surgery, Kanazawa University Hospital)、Masanobu Oshima (Cancer Research Institute, Kanazawa University)

**Background**: FOXO3 is considered a tumor suppressor, but some reports suggest it may promote cancer progression. We previously found that FOXO3 activation suppresses gastric cancer growth, but its role in progression remains unclear. **Objective**: To investigate the role of FOXO3 in the progression of gastric cancer. **Methods**: Gan-KTP mice, created by crossing Gan mice (spontaneous gastric cancer model) with KTP mice (mutations in KRAS, Tgfbr2, and P53), develop invasive gastric cancer. Gastric cancer cells were isolated from them, and a fusion gene encoding constitutively active FOXO3 and an estrogen receptor (ER) was transfected, creating cells capable of time-specific nuclear accumulation of active FOXO3 upon tamoxifen (Tam) treatment. They were cultured with Tam to assess the effect of FOXO3 on proliferation. Subcutaneous transplantation and liver metastasis models were used to compare tumor formation in FOXO3 nuclear accumulated versus non-accumulated groups.**Results**: FOXO3 nuclear accumulation significantly suppressed proliferation in vitro, and tumor formation and liver metastases in vivo. **Conclusion**: FOXO3 functions as a tumor suppressor in gastric cancer malignancy progression.

[Disclosure of COI by Responsible researcher] Lead presenter is not the Responsible researcher.
[Lead presenter: Kazuyoshi Mitta] We have no financial relationships to disclose.
[Principal Researcher: Toshikatsu Tsuji] We have no financial relationships to disclose.

### [E-2058] I-E4-2
### Oncogenes & tumor-suppressor genes (3)

2025 / 9 / 26 (Fri) 12:50-14:05 Room 9/(Hotel Nikko Kanazawa 3F Lumiere) Room 9

[Chairperson]Ryota Kurimoto (Department of Molecular Diagnosis, Chiba University, Graduate School of Medicine／Department of Systems BioMedicine, Institute of Science Tokyo)

---

### E-2058
### B4GALT3(Beta-1,4-Galactosyltransferase 3) Promotes Breast Cancer Cell Invasion via Integrin β1-Targeted Glycosylation.

[Speaker]Shohei Shibuta (Dept. of Surg. Kyushu Univ. Beppu Hosp.／Dept. of Surg. and Sci. Kyushu Univ.) [Co-author]Hajime Otsu (Dept. of Surg. Kyushu Univ. Beppu Hosp.／Dept. of Surg. and Sci. Kyushu Univ.)、Satoshi Higuchi (Dept. of Surg. Kyushu Univ. Beppu Hosp.／Dept of Gastroenterological Surg. Osaka Univ Hosp.)、Takashi Ofuchi (Dept. of Surg. Kyushu Univ. Beppu Hosp.)、Yuki Ando (Dept. of Surg. Kyushu Univ. Beppu Hosp.／Dept. of Surg. and Sci. Kyushu Univ.)、Tomohiko Ikehara (Dept. of Surg. Kyushu Univ. Beppu Hosp.)、Kosuke Hirose (Dept. of Surg. Kyushu Univ. Beppu Hosp.／Dept. of Surg. and Sci. Kyushu Univ.)、Akinori Tsujimoto (Dept. of Surg. Kyushu Univ. Beppu Hosp.)、Koto Kawata (Dept. of Surg. Kyushu Univ. Beppu Hosp.／Dept. of Surg. and Sci. Kyushu Univ.)、Kazuki Omachi (Dept. of Surg. Kyushu Univ. Beppu Hosp.)、Shinsaku Itoyama (Dept. of Surg. Kyushu Univ. Beppu Hosp.)、Yuya Ono (Dept. of Surg. Kyushu Univ. Beppu Hosp.／Dept. of Surg. and Sci. Kyushu Univ.)、Yu Takahashi (Dept. of Surg. Kyushu Univ. Beppu Hosp.／Dept. of Surg. and Sci. Kyushu Univ.)、Takaaki Masuda (Dept. of Breast and Endocrine Surg. Kochi Univ.／Dept. of Surg. and Sci. Kyushu Univ.)、Koshi Mimori (Dept. of Surg. Kyushu Univ. Beppu Hosp.／Dept. of Surg. and Sci. Kyushu Univ.)

Background: Chromosome arm-level amplifications correlate with driver gene density and are a hallmark of cancer evolution.
Objective: To identify novel breast cancer (BC) driver genes via copy number analysis.
Methods: Using TCGA data(n=1218), B4GALT3 was selected based on (1)correlation between DNA copy number and mRNA expression, (2)tumor-specific overexpression, and (3)poor prognosis in the high-expression group. Functional assays were conducted in B4GALT3-overexpressing(OE) MDAMB231 BC cell lines. A subcutaneous xenograft mouse model evaluate in vivo tumorigenesis. N-linked glycosylation inhibitor treatment assessed integrinβ1 regulation.
Results: B4GALT3, located on 1q, showed strong correlation between copy number and mRNA expression (r=0.69, p<0.01), higher tumor expression (p<0.01), and poor prognosis (p<0.05). B4GALT3 OE increased colony formation and activated integrin β1 and PI3K-Akt signaling. In vivo, B4GALT3 OE increased tumor growth. Glycosylation inhibitor reduced integrin β1 expression.
Conclusion: B4GALT3 may act as a novel breast cancer driver gene by promoting tumor progression via glycosylation-mediated integrin β1 activation.

[Disclosure of COI by Responsible researcher] Lead presenter is not the Responsible researcher.
[Lead presenter: Shohei Shibuta] We have no financial relationships to disclose.
[Principal Researcher: Takaaki Masuda] We have no financial relationships to disclose.

### [E-2059] I-E4-2
### Oncogenes & tumor-suppressor genes (3)

2025 / 9 / 26 (Fri) 12:50-14:05 Room 9/(Hotel Nikko Kanazawa 3F Lumiere) Room 9

[Chairperson]Ryota Kurimoto (Department of Molecular Diagnosis, Chiba University, Graduate School of Medicine／Department of Systems BioMedicine, Institute of Science Tokyo)

---

### E-2059
### Identification of URST1 as a novel biomarker and therapeutic target for lung cancer.

[Speaker]Atsushi Takano (Center Antibody Vaccine Therapy Institute Medical Science Univ. Tokyo) [Co-author]Yohei Miyagi (Molecular Pathology and Genetics Division, Kanagawa Cancer Center)、Yataro Daigo (Center Antibody Vaccine Therapy Institute Medical Science Univ. Tokyo／Depart. Medical Oncology and Cancer Center Shiga University Medical Science／Center for Advanced Medicine against Cancer Shiga University Medical Science)

We identified URST1 (Up-regulated in Solid Tumor 1) as a candidate biomarker and therapeutic target for lung cancer by gene expression database. URST1 mRNA and its gene product were highly expressed in most lung cancer cell lines and clinical tissues but barely detected in normal lung epithelial cells and tissues by real-time qPCR, western blotting, and immunocytochemistry. Immunohistochemical staining of URST1 showed that URST1 expression was observed in 231 (64.5%) of 358 non-small cell lung cancers (NSCLC). Strong URST1 expression was correlated with poor prognosis for NSCLC patients ($P = 0.0003$). Knockdown of URST1 expression by siRNAs against URST1 significantly suppressed the growth of lung cancer cells through induction of G2/M arrest and subsequent apoptosis of lung cancer cells. Several apoptosis-related proteins were activated after the suppression of URST1. Treatment of lung cancer cells with small molecule inhibitors against URST1 suppressed the cell growth. Microarray analysis and gene knock-down assays using lung cancer cells by siURST1 suggested various URST1-related oncogenic pathways. URST1 could be a potential biomarker and therapeutic target for lung cancer.

[Disclosure of COI by Responsible researcher] Lead presenter is not the Responsible researcher.
[Lead presenter: Atsushi Takano] We have no financial relationships to disclose.
[Principal Researcher: Yataro Daigo] We have no financial relationships to disclose.

▲TOP

### [E-2060] I-E4-2
### Oncogenes & tumor-suppressor genes (3)

2025 / 9 / 26 (Fri) 12:50-14:05 Room 9/(Hotel Nikko Kanazawa 3F Lumiere) Room 9

[Chairperson]Ryota Kurimoto (Department of Molecular Diagnosis, Chiba University, Graduate School of Medicine／Department of Systems BioMedicine, Institute of Science Tokyo)

### E-2060
### A functional assessment can identify therapeutic targets without either any expressional changes or genetic mutations

[Speaker]Hiroaki Konishi (Asahikawa Med Univ.) [Co-author]Takahiro Sasaki (Asahikawa Med Univ.)、Ryunosuke Hayashi (Asahikawa Med Univ.)、Katsuyoshi Ando (Asahikawa Med Univ.)、Kentaro Moriichi (Asahikawa Med Univ.)、Mikihiro Fujiya (Asahikawa Med Univ.)

RNA-binding proteins (RBPs) play crucial roles in organ development, differentiation, cell growth, and apoptosis in cancerous and normal cells. Bioinformatics analyses fail to detect functional abnormalities in RBPs that do not show changes in expression, leading to false negatives. To identify cancer-associated RBPs, we constructed an siRNA library based on the RBP database and evaluated their effects on the growth of colorectal, pancreatic, and esophageal cancer cells. A comprehensive functional screening using 1,198 siRNAs targeting 416 RBPs identified 41 RBPs that inhibited cancer cell growth by more than 50%. Among these, 12 RBPs showed no changes in mRNA expression and did not suppress the growth of non-cancerous cells when downregulated by specific siRNAs. Further analysis of these 12 RBPs revealed that SF3A1 promotes colorectal cancer progression by stabilizing syntaxin-12 (STX12) mRNA. This study demonstrated for the first time that functional assessment using an siRNA library is an effective strategy for identifying therapeutic targets from cancer-associated molecular groups, including RBPs that were not detected by expression or mutation analyses.

[Disclosure of COI by Responsible researcher] Lead presenter is not the Responsible researcher.
[Lead presenter: Hiroaki Konishi] ■Research funds from company: カムイファーマ株式会社　■Endowed chair: カムイファーマ株式会社
[Principal Researcher: Mikihiro Fujiya] ■Research funds from company: カムイファーマ株式会社　■Profit of stock: カムイファーマ株式会社　■Lecture fee: アッヴィ合同会社、武田薬品工業株式会社、EAファーマ株式会社　■Research expenses: 武田薬品工業株式会社、アッヴィ合同会社、あゆみ製薬株式会社、キッセイ薬品工業株式会社、株式会社ハート・オーガナイゼーション、セルトリオン、アルフレッサファーマ株式会社、ビオフェルミン株式会社、ヤンセンファーマ株式会社、キッセイ薬品工業株式会社、小野薬品工業株式会社 ■Contributions: ファイザー株式会社、日本イーライリリー株式会社　■Endowed chair: カムイファーマ株式会社、JA北海道厚生連農業協同組合連合会、医療法人社団　刀圭会　協立病院、社会医療法人 元生会　森山病院、深川市

▲TOP

### [S7-1] S7
### Cancer metabolism - Bench to Bedside

2025 / 9 / 26 (Fri) 12:50-15:20 Room 10/(Art Hall) Room 10

[Chairpersons]Atsushi Hirao (Kanazawa University, Cancer Research Institute)、Tomoyoshi Soga (Institute for Advanced Biosciences, Keio University)

Cancer-specific metabolic reprogramming is recognized as one of the hallmarks of cancer. Metabolic reprogramming provides cancer cells with most of the building blocks required for nucleotide, protein, and lipid synthesis through specific metabolic pathways such as glycolysis, glutamine metabolism, and one-carbon metabolism. In addition, several important metabolic pathways have recently been reported to be associated with cancer cell stemness and immune cell differentiation. On the other hand, onco-metabolites involved in carcinogenesis, such as 2-hydroxyglutaric acid, fumaric acid, succinic acid, and kynurenine have been found. Therefore, the development of anticancer drugs targeting cancer-specific metabolism has been vigorously pursued. In this symposium, we will discuss with leading researchers on metabolism involved in cell competition, cancer resistance, cachexia, acute myeloid leukemia, and prostate cancer, and demonstrate the latest cancer diagnostic and therapeutic methods using small molecule probes.

---

### S7-1
### TBD

[Speaker]Yasuyuki Fujita (Kyoto University Graduate School of Medicine)

No Abstract

### [S7-2] S7
### Cancer metabolism - Bench to Bedside

2025 / 9 / 26 (Fri) 12:50-15:20 Room 10/(Art Hall) Room 10

[Chairpersons]Atsushi Hirao (Kanazawa University, Cancer Research Institute)、Tomoyoshi Soga (Institute for Advanced Biosciences, Keio University)

Cancer-specific metabolic reprogramming is recognized as one of the hallmarks of cancer. Metabolic reprogramming provides cancer cells with most of the building blocks required for nucleotide, protein, and lipid synthesis through specific metabolic pathways such as glycolysis, glutamine metabolism, and one-carbon metabolism. In addition, several important metabolic pathways have recently been reported to be associated with cancer cell stemness and immune cell differentiation. On the other hand, onco-metabolites involved in carcinogenesis, such as 2-hydroxyglutaric acid, fumaric acid, succinic acid, and kynurenine have been found. Therefore, the development of anticancer drugs targeting cancer-specific metabolism has been vigorously pursued. In this symposium, we will discuss with leading researchers on metabolism involved in cell competition, cancer resistance, cachexia, acute myeloid leukemia, and prostate cancer, and demonstrate the latest cancer diagnostic and therapeutic methods using small molecule probes.

---

### S7-2
### One-carbon metabolism-polyamine axis controls tumor immune microenvironment and potential metastatic niche establishment

[Speaker]Noriko Gotoh (Cancer Res. Inst., Kanazawa Univ.)

One-carbon metabolism produces formic acid, which is essential for de novo nucleotide synthesis. We previously demonstrated that the mitochondrial enzyme MTHFD2 increases cancer cell growth and stemness. To investigate its role in the immune microenvironment, we generated ***Mthfd2***$^{fl/fl}$;MMTV-Cre mice, in which ***Mthfd2*** is specifically deleted in mammary tissues, and crossed them with the MMTV-ErbB2 tumor model. The mutant mice exhibited reduced tumorigenesis and lung metastasis. Immune profiling revealed a higher number of M2 macrophages in wild-type tumors. Metabolomic analysis showed increased polyamine levels in conditioned media from wild-type tumor cells. Furthermore, expression of Arginase 1, an upstream enzyme for polyamine production, was upregulated in macrophages cultured with polyamines, which also enhanced tumor sphere formation. Polyamine levels were elevated in blood and urine, indicating systemic circulation. These findings identify tumor-intrinsic MTHFD2 as a driver of a feed-forward loop of polyamine production, promoting M2 macrophage polarization, cancer stemness, and potential metastatic niche formation.

[Disclosure of COI by Responsible researcher] Lead presenter is the Responsible researcher.
[Lead presenter: Noriko Gotoh] ■Research expenses: セラバイオファーマ　■Contributions: 上原記念生命科学財団、高松宮妃癌研究基金、武田科学振興財団、中外創薬科学財団、安田記念医学財団

### [S7-3] S7
### Cancer metabolism - Bench to Bedside

2025 / 9 / 26 (Fri) 12:50-15:20 Room 10/(Art Hall) Room 10

[Chairpersons]Atsushi Hirao (Kanazawa University, Cancer Research Institute)、Tomoyoshi Soga (Institute for Advanced Biosciences, Keio University)

Cancer-specific metabolic reprogramming is recognized as one of the hallmarks of cancer. Metabolic reprogramming provides cancer cells with most of the building blocks required for nucleotide, protein, and lipid synthesis through specific metabolic pathways such as glycolysis, glutamine metabolism, and one-carbon metabolism. In addition, several important metabolic pathways have recently been reported to be associated with cancer cell stemness and immune cell differentiation. On the other hand, onco-metabolites involved in carcinogenesis, such as 2-hydroxyglutaric acid, fumaric acid, succinic acid, and kynurenine have been found. Therefore, the development of anticancer drugs targeting cancer-specific metabolism has been vigorously pursued. In this symposium, we will discuss with leading researchers on metabolism involved in cell competition, cancer resistance, cachexia, acute myeloid leukemia, and prostate cancer, and demonstrate the latest cancer diagnostic and therapeutic methods using small molecule probes.

---

### S7-3
### Systemic metabolic alterations in cancer cachexia revealed through multi-omics analysis

[Speaker]Masahiro Aoki (Aichi Cancer Center Research Institute／Nagoya University Graduate School of Medicine) [Co-author]Tomoyoshi Soga (Bio2Q, Keio University)、Yasushi Kojima (Aichi Cancer Center Research Institute)

Cancer cachexia, a complex metabolic syndrome affecting 50-80% of advanced cancer patients, is marked by body weight loss, muscle wasting, and anorexia. Despite its prevalence, the key metabolic features remain unclear, hampering early diagnosis and treatment development.Here, we explored systemic metabolic alterations associated with cachexia using multiple mouse cancer models with varying degrees of cachexia and a starvation model. Capillary electrophoresis-mass spectrometry (CE-MS)-based metabolomics revealed significant decrease of metabolites in key hepatic pathways in cachectic mice. Integrated metabolomic and proteomic analyses showed reduced levels of corresponding liver enzymes. These changes were accompanied by enhanced ribosome biogenesis and elevated acute-phase protein production in the liver. Notably, some of these metabolic and proteomic shifts were also detected in blood samples from cachectic mice and gastric cancer patients with cachexia. Our findings offer a comprehensive view of systemic metabolic disruption in cancer cachexia. Clarifying the mechanisms underlying these alterations could facilitate the development of new diagnostic and therapeutic approaches.

[Disclosure of COI by Responsible researcher] Lead presenter is the Responsible researcher.
[Lead presenter: Masahiro Aoki] We have no financial relationships to disclose.

### [S7-4] S7
### Cancer metabolism - Bench to Bedside

2025 / 9 / 26 (Fri) 12:50-15:20 Room 10/(Art Hall) Room 10

[Chairpersons]Atsushi Hirao (Kanazawa University, Cancer Research Institute)、Tomoyoshi Soga (Institute for Advanced Biosciences, Keio University)

Cancer-specific metabolic reprogramming is recognized as one of the hallmarks of cancer. Metabolic reprogramming provides cancer cells with most of the building blocks required for nucleotide, protein, and lipid synthesis through specific metabolic pathways such as glycolysis, glutamine metabolism, and one-carbon metabolism. In addition, several important metabolic pathways have recently been reported to be associated with cancer cell stemness and immune cell differentiation. On the other hand, onco-metabolites involved in carcinogenesis, such as 2-hydroxyglutaric acid, fumaric acid, succinic acid, and kynurenine have been found. Therefore, the development of anticancer drugs targeting cancer-specific metabolism has been vigorously pursued. In this symposium, we will discuss with leading researchers on metabolism involved in cell competition, cancer resistance, cachexia, acute myeloid leukemia, and prostate cancer, and demonstrate the latest cancer diagnostic and therapeutic methods using small molecule probes.

---

### S7-4
### Guanine nucleotides drive ribosome biogenesis and glycolytic reprogramming in acute myeloid leukemia stem cells

[Speaker]Yoshikane Kikushige (Center for Cellular and Molecular Medicine, Kyushu University Hospital)

Therapy resistance in acute myeloid leukemia (AML) remains a major clinical obstacle, particularly due to the persistence of leukemia stem cells (LSCs) capable of metabolic adaptation. While venetoclax (Ven) inhibits oxidative phosphorylation (OXPHOS), we found that Ven-resistant LSCs undergo glycolytic reprogramming to bypass OXPHOS inhibition. This metabolic shift is supported by enhanced ribosome biogenesis, sustained by upregulated de novo guanine nucleotide biosynthesis. Abundant guanine nucleotides suppress the impaired ribosome biogenesis checkpoint (IRBC), leading to TP53 destabilization and persistent MYC expression. Inhibition of inosine monophosphate dehydrogenases (IMPDH1/2) depletes guanine nucleotides, activates IRBC, stabilizes TP53, suppresses MYC, and impairs glycolysis. This metabolic rewiring disrupts LSC stemness and reduces leukemic reconstitution in vivo, even in Ven-resistant contexts. These findings reveal a mutation-independent mechanism of TP53 inactivation in AML and suggest that targeting guanine nucleotide biosynthesis may offer a clinically actionable strategy to eradicate therapy-resistant LSCs.

[Disclosure of COI by Responsible researcher] Lead presenter is the Responsible researcher.
[Lead presenter: Kikushige Yoshikane] ■Lecture fee: アステラス、ヤンセンファーマ　■Research expenses: アステラス、協和キリン

### [S7-5] S7
### Cancer metabolism - Bench to Bedside

2025 / 9 / 26 (Fri) 12:50-15:20 Room 10/(Art Hall) Room 10

[Chairpersons]Atsushi Hirao (Kanazawa University, Cancer Research Institute)、Tomoyoshi Soga (Institute for Advanced Biosciences, Keio University)

Cancer-specific metabolic reprogramming is recognized as one of the hallmarks of cancer. Metabolic reprogramming provides cancer cells with most of the building blocks required for nucleotide, protein, and lipid synthesis through specific metabolic pathways such as glycolysis, glutamine metabolism, and one-carbon metabolism. In addition, several important metabolic pathways have recently been reported to be associated with cancer cell stemness and immune cell differentiation. On the other hand, onco-metabolites involved in carcinogenesis, such as 2-hydroxyglutaric acid, fumaric acid, succinic acid, and kynurenine have been found. Therefore, the development of anticancer drugs targeting cancer-specific metabolism has been vigorously pursued. In this symposium, we will discuss with leading researchers on metabolism involved in cell competition, cancer resistance, cachexia, acute myeloid leukemia, and prostate cancer, and demonstrate the latest cancer diagnostic and therapeutic methods using small molecule probes.

---

### S7-5
### Targeting RB1-SUCLA2 Deletion in Advanced Prostate Cancer

[Speaker]Chiaki Takahashi (Div. Onc. Mol. Biol., Cancer Res. Inst., Kanazawa Univ.) [Co-author]Susumu Kohno (Div. Onc. Mol. Biol., Cancer Res. Inst., Kanazawa Univ.)、Ziheng Yao (Div. Onc. Mol. Biol., Cancer Res. Inst., Kanazawa Univ.)、Tomoyoshi Soga (Inst. Adv. Bio. and Grad Sch. Media. Govern., Keio Univ.)、Jun Koseki (Cell. Mol. Bio. Res. Inst., AIST)、Nobuyuki Okahashi (Grad. Sch. Info. Sci. Tech., Osaka Univ.)、Fumio Matsuda (Grad. Sch. Info. Sci. Tech., Osaka Univ.)、Kojiro Tashiro (Dep. Urol. The Jikei. Univ. Hosp.)、Takahiro Kimura (Dep. Urol. The Jikei. Univ. Hosp.)、Yoshihiro Watanabe (Kanazawa Univ. Hosp.)、Kenji Mishiro (INFINITI., Kanazawa Univ.)、Toshifumi Dohi (Dept. Pharma., Ritsumeikan Univ.)

The chromosomal loss at 13q14.2, associated with malignant progression in prostate cancer, encompasses genes including RB1, ITM2B, MED4, NUDT15 and SUCLA2. Among these, loss of SUCLA2 creates an irreversible metabolic vulnerability in cancer cells. We conducted a chemical library screen and identified 2-isopropyl-5-methylbenzo-1,4-quinone (thymoquinone, TQ) as a compound that selectively targets SUCLA2-deficient cancer cells. Further investigation revealed that TQ inhibits key enzymes involved in anaplerotic metabolism by promoting dissociation of enzyme complexes. The concurrent loss of SUCLA2 and TQ-targeted genes induces synthetic lethality driven by profound depletion of alfa-ketoglutarate. We subsequently synthesized a series of TQ derivatives that demonstrated enhanced bioactivity both in vitro and in vivo compared to the parent compound. To efficiently identify patients with SUCLA2 deletions, we are currently developing diagnostic methods including ctDNA digital PCR and biomarker assays. These efforts aim to establish a first-in-class therapeutic strategy targeting SUCLA2-deficient cancers including advanced prostate cancer.

[Disclosure of COI by Responsible researcher] Lead presenter is the Responsible researcher.
[Lead presenter: Chiaki Takahashi] We have no financial relationships to disclose.

### [S7-6] S7
### Cancer metabolism - Bench to Bedside

2025 / 9 / 26 (Fri) 12:50-15:20 Room 10/(Art Hall) Room 10

[Chairpersons]Atsushi Hirao (Kanazawa University, Cancer Research Institute)、Tomoyoshi Soga (Institute for Advanced Biosciences, Keio University)

Cancer-specific metabolic reprogramming is recognized as one of the hallmarks of cancer. Metabolic reprogramming provides cancer cells with most of the building blocks required for nucleotide, protein, and lipid synthesis through specific metabolic pathways such as glycolysis, glutamine metabolism, and one-carbon metabolism. In addition, several important metabolic pathways have recently been reported to be associated with cancer cell stemness and immune cell differentiation. On the other hand, onco-metabolites involved in carcinogenesis, such as 2-hydroxyglutaric acid, fumaric acid, succinic acid, and kynurenine have been found. Therefore, the development of anticancer drugs targeting cancer-specific metabolism has been vigorously pursued. In this symposium, we will discuss with leading researchers on metabolism involved in cell competition, cancer resistance, cachexia, acute myeloid leukemia, and prostate cancer, and demonstrate the latest cancer diagnostic and therapeutic methods using small molecule probes.

---

### S7-6
### Chemical Medicine: Development of novel chemistry-based and enzyme-driven theranostics technologies for cancer

[Speaker]Yasuteru Urano (Grad. Sch. Pharm. Sci., Univ. Tokyo／Grad. Sch. Med., Univ. Tokyo)

Toward overcoming clinical problems of cancer treatments during surgery and endoscopic procedure, our group has established intraoperative rapid fluorescence imaging methods for various kinds of tumors, and discovered biomarker enzymatic activities that are characteristic of the cancer site of each patient. More concretely, by applying our library of fluorogenic probes to real clinical cancerous and normal fresh specimens, biomarker enzymatic activities for various tumors including lung, ovarian, esophageal, hepatic, pancreatic, and brain cancers have been successfully found out and identified. For example, a fluorogenic probe for DPP-4 was found to be effective for esophageal cancer, and now human phase II trial is undergoing in Japan. Furthermore, we recently started to establish novel cancer theranostics technologies by developing prodrug-type and radiotherapeutic anticancer drugs which will realize personalized and precision medicine based on the biomarker enzymatic activity of each patient. In this talk, the latest chemistry (chemical biology) and its medical applications to realize new theranostics for various types of cancers (chemical medicine) will be showcased.

[Disclosure of COI by Responsible researcher] Lead presenter is the Responsible researcher.
[Lead presenter: Yasuteru Urano] We have no financial relationships to disclose.

### [E-2067] I-E20

### Advances in Cancer Stratification & Therapeutic Strategies: From Pathophysiology to Clinical Applications

2025 / 9 / 26 (Fri) 12:50-14:05 Room 11/(ANA Crowne Plaza Hotel Kanazawa 3F Ohtori (West)) Room 11

[Chairperson]Mamoru Kato (Div. Bioinformatics, Res. Inst., NCC)

---

### E-2067

### Adunctin E Suppresses Lung Cancer Cells by Targeting HSP90AA1

[Speaker]Iksen Iksen (Dept. of Pharm & Physiol., Chulalongkorn Univ.) [Co-author]Varisa Pongrakhananon (Dept. of Pharm & Physiol., Chulalongkorn Univ.)

Lung cancer ranks among the most lethal tumors globally, underscoring the pressing necessity for innovative and efficacious therapeutic approaches. Natural products provide a substantial reservoir of bioactive chemicals, and Aduntin E (AE), a flavonoid extracted from ***Conamomum rubidum***, has shown promising therapeutic properties. However, its anticancer activity remains predominantly unknown. This study aimed to examine the pro-apoptotic effects of AE and identify its molecular targets in lung cancer. A network pharmacology method identified 71 potential targets of AE, with enrichment analyses revealing their involvement in key cancer-related pathways. HSP90AA1, MAPK1, and PIK3CA were identified as regulators of apoptotic cell death. Structure–based computational docking and dynamic simulations demonstrated AE forms a stable complex with HSP90AA1. In vitro assays on A549 and H460 cells confirmed that AE induces apoptosis and downregulated HSP90AA1 protein levels. This fundings suggest that AE may exert its anticancer effects via targeting HSP90AA1, supporting its promise as a lead drug for lung cancer therapy.

[Disclosure of COI by Responsible researcher] Lead presenter is the Responsible researcher.
[Lead presenter: Iksen Iksen] We have no financial relationships to disclose.

### [E-2068] I-E20
### Advances in Cancer Stratification & Therapeutic Strategies: From Pathophysiology to Clinical Applications

2025 / 9 / 26 (Fri) 12:50-14:05 Room 11/(ANA Crowne Plaza Hotel Kanazawa 3F Ohtori (West)) Room 11

[Chairperson]Mamoru Kato (Div. Bioinformatics, Res. Inst., NCC)

---

### E-2068
### A Clinically Accessible Model for Predicting Induction Failure in pediatric acute myeloid leukemia

[Speaker]Rongqiu Liu (Children's Hospital of Chongqing Medical University)

Induction chemotherapy failure (IF) in pediatric acute myeloid leukemia (pAML) correlates with poor prognosis. To identify clinical/genetic risk factors, we analyzed 1,398 pAML cases (97 IF patients) from TARGET (AAML1031, AAML0531, AAML03P1) and CHCMU cohorts. AAML1031 patients were split into training/test cohorts to develop a logistic regression model; AAML0531/AAML03P1 (n=515) and CHCMU (n=515, 30 IF) served as external validation cohorts. IF incidence was less than 10%. The model incorporated age at diagnosis, t(6;9), monosomy7, and NUP98, outperforming existing risk stratification tools (Cyto Fusion Molecular Risk, LncScore) with AUCs of 91.24% (training), 86.69% (test), 89.77% (external cohort 1), and 92.82% (external cohort 2). Predicted non-IF groups consistently exhibited superior overall survival (OS) and event-free survival (EFS) vs. IF groups. This clinically applicable model enables early IF prediction to optimize risk-adapted therapies, validated by multi-cohort accuracy and prognostic stratification.

[Disclosure of COI by Responsible researcher] Lead presenter is the Responsible researcher.
[Lead presenter: Rongqiu Liu] We have no financial relationships to disclose.

### [E-2069] I-E20
### Advances in Cancer Stratification & Therapeutic Strategies: From Pathophysiology to Clinical Applications

2025 / 9 / 26 (Fri) 12:50-14:05 Room 11/(ANA Crowne Plaza Hotel Kanazawa 3F Ohtori (West)) Room 11

[Chairperson]Mamoru Kato (Div. Bioinformatics, Res. Inst., NCC)

---

### E-2069
### Stratifying Metastatic Breast Cancer by Systemic Symptoms: Biological Insights from Patient Clusters

[Speaker]Sumito Saeki (Cancer Epigenomics, Cancer Institute, JFCR／Breast Surgery, Cancer Institute Hospital, JFCR／Breast Oncology Center, Caner Institute Hospital, JFCR) [Co-author]Masahiro Kuno (Next-Ganken Program, Cancer Institute, JFCR／Breast Medical Oncology, Cancer Institute Hospital, JFCR／Department of Extended Intelligence for Medicine, Keio University)、Yukinori Ozaki (Breast Medical Oncology, Cancer Institute Hospital, JFCR)、Tomo Osako (Division of Pathology, Cancer Institute Hospital, JFCR)、Toshimi Takano (Breast Medical Oncology, Cancer Institute Hospital, JFCR)、Reo Maruyama (Cancer Epigenomics, Cancer Institute, JFCR／Next-Ganken Program, Cancer Institute, JFCR)、Takayuki Ueno (Breast Surgery, Cancer Institute Hospital, JFCR／Next-Ganken Program, Cancer Institute, JFCR)

We retrospectively analyzed 466 deceased metastatic breast cancer patients (2000-2023), using systemic symptoms and effusion status at the time of Best Supportive Care (BSC) decision. Latent class analysis and hierarchical clustering were performed to classify patient subgroups. Eight distinct clusters (C1-C8) were identified. Notably, C5 (carcinomatous lymphangiosis) and C7 (pleuritis) both showed high pleural effusion but exhibited distinct pathophysiological features, suggesting different mechanisms underlying fluid accumulation. These findings highlight the heterogeneity of disease manifestation in the end-of-life setting. To explore the biology of these subgroups, we established four organoid lines from pleural effusion samples and initiated single-cell RNA sequencing. These models are currently being used to validate the classification and may offer insights into effusion-related tumor behavior. Symptom-based stratification provides a clinically and biologically meaningful framework for understanding the systemic nature of metastatic breast cancer beyond metastatic site-specific effects.

[Disclosure of COI by Responsible researcher] Lead presenter is not the Responsible researcher.
[Lead presenter: Sumito Saeki] We have no financial relationships to disclose.
[Principal Researcher: Takayuki Ueno] We have no financial relationships to disclose.

### [E-2070] I-E20
### Advances in Cancer Stratification & Therapeutic Strategies: From Pathophysiology to Clinical Applications

2025 / 9 / 26 (Fri) 12:50-14:05 Room 11/(ANA Crowne Plaza Hotel Kanazawa 3F Ohtori (West)) Room 11

[Chairperson]Mamoru Kato (Div. Bioinformatics, Res. Inst., NCC)

---

### E-2070
### Computational Pathology with Topological Data Analysis

[Speaker]Haruhisa Oda (Dept. Preventive Med., Grad. Sch. Med., Univ. Tokyo) [Co-author]Ranny R. Herdiantoputri (Dept. Preventive Med., Grad. Sch. Med., Univ. Tokyo)、Daisuke Komura (Dept. Preventive Med., Grad. Sch. Med., Univ. Tokyo)、Shumpei Ishikawa (Dept. Preventive Med., Grad. Sch. Med., Univ. Tokyo)

Feature extraction from digital pathology images is a fundamental aspect of computational pathology and significantly influences cancer research from a data science perspective. Recent advancements in tools and frameworks based on deep neural networks have driven ongoing research in this field. Recently, topology, a branch of mathematics that studies spatial properties and patterns, has gained attention through Topological Data Analysis (TDA), which has been valuable in addressing complex scientific challenges.In this work, we present a topology-based framework for feature extraction from pathology images. Specifically, we calculate persistent homology--a key technique in TDA--at multiple scales for each input image patch. These outputs are then combined using vectorization methods to generate a feature vector. We will analyze the characteristics of the framework and compare its performance with existing approaches, aiming to enhance our understanding of their strengths and limitations in computational pathology. This study illustrates the potential of topology in computational pathology research while also highlighting challenges to be addressed in future work.

[Disclosure of COI by Responsible researcher] Lead presenter is not the Responsible researcher.
[Lead presenter: Haruhisa Oda] We have no financial relationships to disclose.
[Principal Researcher: Shumpei Ishikawa] ■Research funds from company: 日本医療研究開発機構　■Advisor of the company and/or profit-making organization: 新日本科学株式会社　■Research expenses: Chordia therapeutics株式会社　大鵬薬品工業株式会社　ロート製薬株式会社　アステラス製薬株式会社

### [E-2071] I-E20

### Advances in Cancer Stratification & Therapeutic Strategies: From Pathophysiology to Clinical Applications

2025 / 9 / 26 (Fri) 12:50-14:05 Room 11/(ANA Crowne Plaza Hotel Kanazawa 3F Ohtori (West)) Room 11

[Chairperson]Mamoru Kato (Div. Bioinformatics, Res. Inst., NCC)

---

### E-2071

### Spatial transcriptomic profiling to elucidate drug resistance via intercellular crosstalk in HGSOC

[Speaker]Hironori Suzuki (Dept. Obst. & Gynecol., Nagoya Univ. Grad. Sch. of Med.／Natl. Cancer Ctr. Res. Inst.) [Co-author]Kosuke Yoshida (Dept. Obst. & Gynecol., Nagoya Univ. Grad. Sch. of Med.／Inst. Adv. Res., Nagoya Univ.)、Akira Yokoi (Dept. Obst. & Gynecol., Nagoya Univ. Grad. Sch. of Med.／Inst. Adv. Res., Nagoya Univ.)、Kazuhiro Suzuki (Dept. Obst. & Gynecol., Nagoya Univ. Grad. Sch. of Med.)、Yuta Hirano (Natl. Cancer Ctr. Res. Inst.／Div. Respir. Dis., Jikei Univ. Grad. Sch. of Med.)、Hiroaki Kajiyama (Dept. Obst. & Gynecol., Nagoya Univ. Grad. Sch. of Med.)、Yusuke Yamamoto (Natl. Cancer Ctr. Res. Inst.)

High-grade serous ovarian carcinoma (HGSOC) accounts for nearly 80% of deaths from ovarian cancer and is usually detected at an advanced stage. The development of poly(ADP-ribose) polymerase inhibitors (PARPis) has improved the prognosis of patients with HGSOC. However, PARPis are not effective for all HGSOC patients. Spatial transcriptomics has attracted attention as a powerful tool for elucidating the functions of the tumor microenvironment (TME). In this study, we applied 10x Visium technology to formalin-fixed, paraffin-embedded (FFPE) samples from eight HGSOC patients receiving PARPi maintenance therapy. We captured 6,665 spots from the PARPi-sensitive cohort and 8,587 spots from the PARPi-resistant cohort. Through interspot interaction analysis, we identified upregulation of the midkine signaling pathway in interactions between CAF spots and malignant spots within resistant tumors. We then experimentally validated this finding in ovarian cancer cell lines. Furthermore, the analysis of intraspot interactions revealed the cell colocalization and interaction strength for each spot. These findings improve our understanding of how the TME contributes to PARPi resistance in HGSOC.

[Disclosure of COI by Responsible researcher] Lead presenter is not the Responsible researcher.
[Lead presenter: Hironori Suzuki] We have no financial relationships to disclose.
[Principal Researcher: Akira Yokoi, Hiroaki Kajiyama] We have no financial relationships to disclose.

### [E-2072] I-E20
### Advances in Cancer Stratification & Therapeutic Strategies: From Pathophysiology to Clinical Applications

2025 / 9 / 26 (Fri) 12:50-14:05 Room 11/(ANA Crowne Plaza Hotel Kanazawa 3F Ohtori (West)) Room 11

[Chairperson]Mamoru Kato (Div. Bioinformatics, Res. Inst., NCC)

### E-2072
### Design of T Cell Infusion Schedules Using a Mathematical Model of Adoptive Cell Therapy

[Speaker]Hiroki Kasai (Dept. Comp. Biol. Med. Sci., Grad. Frontier Sci., Univ. Tokyo) [Co-author]Koji Nagaoka (Dept. Immunol., Kindai Univ. Facult. Med.)、Artem Lysenko (Dept. Biol. Sci., Sch. Sci., Univ. Tokyo)、Kazuhiro Kakimi (Dept. Immunol., Kindai Univ. Facult. Med.)、Tatsuhiko Tsunoda (Dept. Comp. Biol. Med. Sci., Grad. Frontier Sci., Univ. Tokyo／Dept. Biol. Sci., Sch. Sci., Univ. Tokyo)

Adoptive cell therapy (ACT) for solid tumors exerts potent but transient antitumor effects. One major challenge is the accumulation of myeloid-derived suppressor cells (MDSCs) following ACT, which inhibits the function of transferred T cells. To improve therapeutic outcomes, optimizing treatment strategies is necessary. In this study, we developed a mathematical model based on experimental data from a B16F10 melanoma-bearing mouse model treated with tumor-specific cytotoxic T lymphocytes (CTLs). Using our mathematical model, we quantitatively evaluated the suppressive effects of MDSCs on transferred CTLs and simulated the efficacy of various therapeutic strategies. Our simulations showed that a single high-dose CTL injection was insufficient for tumor control due to strong MDSC-mediated suppression, whereas a fractionated dosing strategy improved antitumor efficacy. Moreover, we demonstrated that an intermittent dosing regimen could achieve sustained tumor suppression while reducing the total CTL dose. Our mathematical model provides a basis for optimizing ACT protocols.

[Disclosure of COI by Responsible researcher] Lead presenter is not the Responsible researcher.
[Lead presenter: Hiroki Kasai] We have no financial relationships to disclose.
[Principal Researcher: Tatsuhiko Tsunoda] We have no financial relationships to disclose.

### [S8-1] S8
### Foundational and innovative technologies to bridge basic cancer research to drug development

2025 / 9 / 26 (Fri) 12:50-15:20 Room 12/(ANA Crowne Plaza Hotel Kanazawa 3F Ohtori (Central)) Room 12

[Chairpersons]Hiroaki Taniguchi (Keio Cancer Center, Keio University School of Medicine)、Issay Kitabayashi (Fujita Health University)

In recent cancer research, increasing emphasis has been placed on how to deliver the outcomes of basic scientific studies to patients and realize their social implementation. To achieve this, it is essential to actively incorporate new technologies and conceptual advances, while fostering collaboration among researchers from diverse scientific and technological disciplines.
Drug discovery efforts have traditionally focused on molecules with enzymatic activity, which were considered tractable using conventional small-molecule approaches. However, the emergence and rapid development of various drug discovery modalities-such as nucleic acid-based drugs, targeted protein degraders, protein binding inhibitor and antibody-drug conjugates-have significantly diversified the therapeutic landscape. These advances are now enabling the targeting of molecules that were previously considered undruggable.
In this symposium, we will invite experts who are actively engaged in the development of foundational technologies and innovative therapeutic strategies aimed at bridging early-stage academic research to drug development and real-world clinical application. Through their presentations and ensuing discussions, we aim to explore new directions in drug discovery research that are appropriate for this evolving era, and to promote further innovation in the field.
We hope this session will stimulate meaningful dialogue and foster collaborations that advance translational cancer research and therapeutic implementation.

近年のがん研究においては、基礎研究の成果をいかに患者さんに届け、社会実装へと結びつけるかがこれまで以上に重要視されている。これを実現するためには、先進的な技術やコンセプトの積極的な導入と、多様な分野の研究者との協働が不可欠である。従来、創薬標的としては酵素活性を有する分子が重視されてきたが、近年のモダリティ技術の発展により、これまで標的化が困難とされてきた分子へのアプローチも可能となり、創薬の可能性は大きく広がっている。本シンポジウムでは、初期段階の医学的研究成果（シーズ）を社会実装に導くための基盤技術や革新的な創薬モダリティの開発に取り組む専門家をお招きし、新時代にふさわしい創薬研究の方向性を議論し、がん創薬における研究開発のさらなる活性化を図りたい。

---

### S8-1
### Utilization of the key technologies for translating Academia's Seeds to Industries through the iD3 Booster

[Speaker]Masuo Yamaoka (Department of Innovative Drug Discovery and Development, AMED)

The iD3 booster is a unique, unprecedented approach operated by the Department of Innovative Drug Discovery and Development (iD3) in AMED for accelerating the translation of promising basic research into innovative new medicines. This program is unique in that it is designed to provide academia scientists with the deep knowledge and industry experience of drug discovery experts, innovative technology and financial support necessary to proceed the drug discovery research as one-stop. The iD3 booster activity is promoted by the Drug Discovery Support Network consisting of RIKEN, NIBIOHN and AIST, and relationship with pharmaceutical companies and Bio-ventures through New Modality Consortium. Currently more than 70 approaches have been pursued for target validation, HTS and lead optimization as of May 2025. Examples of technologies offered to academia scientists will be shown in this presentation.

[Disclosure of COI by Responsible researcher] Lead presenter is the Responsible researcher.
[Lead presenter: Masuo Yamaoka] We have no financial relationships to disclose.

### [S8-2] S8
### Foundational and innovative technologies to bridge basic cancer research to drug development

2025 / 9 / 26 (Fri) 12:50-15:20 Room 12/(ANA Crowne Plaza Hotel Kanazawa 3F Ohtori (Central)) Room 12

[Chairpersons]Hiroaki Taniguchi (Keio Cancer Center, Keio University School of Medicine)、Issay Kitabayashi (Fujita Health University)

In recent cancer research, increasing emphasis has been placed on how to deliver the outcomes of basic scientific studies to patients and realize their social implementation. To achieve this, it is essential to actively incorporate new technologies and conceptual advances, while fostering collaboration among researchers from diverse scientific and technological disciplines.
Drug discovery efforts have traditionally focused on molecules with enzymatic activity, which were considered tractable using conventional small-molecule approaches. However, the emergence and rapid development of various drug discovery modalities-such as nucleic acid-based drugs, targeted protein degraders, protein binding inhibitor and antibody-drug conjugates-have significantly diversified the therapeutic landscape. These advances are now enabling the targeting of molecules that were previously considered undruggable.
In this symposium, we will invite experts who are actively engaged in the development of foundational technologies and innovative therapeutic strategies aimed at bridging early-stage academic research to drug development and real-world clinical application. Through their presentations and ensuing discussions, we aim to explore new directions in drug discovery research that are appropriate for this evolving era, and to promote further innovation in the field.
We hope this session will stimulate meaningful dialogue and foster collaborations that advance translational cancer research and therapeutic implementation.

近年のがん研究においては、基礎研究の成果をいかに患者さんに届け、社会実装へと結びつけるかがこれまで以上に重要視されている。これを実現するためには、先進的な技術やコンセプトの積極的な導入と、多様な分野の研究者との協働が不可欠である。従来、創薬標的としては酵素活性を有する分子が重視されてきたが、近年のモダリティ技術の発展により、これまで標的化が困難とされてきた分子へのアプローチも可能となり、創薬の可能性は大きく広がっている。本シンポジウムでは、初期段階の医学的研究成果（シーズ）を社会実装に導くための基盤技術や革新的な創薬モダリティの開発に取り組む専門家をお招きし、新時代にふさわしい創薬研究の方向性を議論し、がん創薬における研究開発のさらなる活性化を図りたい。

---

### S8-2
### Parallel epitope and affinity evaluation of antibodies via mammalian cell display and sequencing

[Speaker]Tetsuya Kadonosono (Science Tokyo)

Antibody drugs have become essential tools in treating diseases such as cancer. Their development requires optimization of various properties, including epitope, binding affinity, activity, and, solubility. Although high-throughput and automated systems for evaluating these characteristics have been developed, the number of antibodies that can be assessed simultaneously remains limited.To overcome this limitation, we are developing a machine learning-based prediction platform in combination with parallel evaluation technologies to identify high-performance antibodies more efficiently. Recently, we established technologies that allow simultaneous evaluation of epitope or binding affinity for a large number of antibodies. In our system, antibodies are displayed on the surface of mammalian cells via gene transduction, and their antigen-binding properties are analyzed by flow cytometry. Epitope and affinity profiles are then comprehensively analyzed using next-generation sequencing.This presentation introduces proof-of-concept studies using HER2 and anti-HER2 therapeutic antibodies, trastuzumab and pertuzumab, demonstrating the versatility and scalability of our platform.

[Disclosure of COI by Responsible researcher] Lead presenter is the Responsible researcher.
[Lead presenter: Tetsuya Kadonosono] ■ employment by a company related to the presentation: 協和キリン株式会社

### [S8-3] S8
### Foundational and innovative technologies to bridge basic cancer research to drug development

2025 / 9 / 26 (Fri) 12:50-15:20 Room 12/(ANA Crowne Plaza Hotel Kanazawa 3F Ohtori (Central)) Room 12

[Chairpersons]Hiroaki Taniguchi (Keio Cancer Center, Keio University School of Medicine)、Issay Kitabayashi (Fujita Health University)

In recent cancer research, increasing emphasis has been placed on how to deliver the outcomes of basic scientific studies to patients and realize their social implementation. To achieve this, it is essential to actively incorporate new technologies and conceptual advances, while fostering collaboration among researchers from diverse scientific and technological disciplines.
Drug discovery efforts have traditionally focused on molecules with enzymatic activity, which were considered tractable using conventional small-molecule approaches. However, the emergence and rapid development of various drug discovery modalities-such as nucleic acid-based drugs, targeted protein degraders, protein binding inhibitor and antibody-drug conjugates-have significantly diversified the therapeutic landscape. These advances are now enabling the targeting of molecules that were previously considered undruggable.
In this symposium, we will invite experts who are actively engaged in the development of foundational technologies and innovative therapeutic strategies aimed at bridging early-stage academic research to drug development and real-world clinical application. Through their presentations and ensuing discussions, we aim to explore new directions in drug discovery research that are appropriate for this evolving era, and to promote further innovation in the field.
We hope this session will stimulate meaningful dialogue and foster collaborations that advance translational cancer research and therapeutic implementation.

近年のがん研究においては、基礎研究の成果をいかに患者さんに届け、社会実装へと結びつけるかがこれまで以上に重要視されている。これを実現するためには、先進的な技術やコンセプトの積極的な導入と、多様な分野の研究者との協働が不可欠である。従来、創薬標的としては酵素活性を有する分子が重視されてきたが、近年のモダリティ技術の発展により、これまで標的化が困難とされてきた分子へのアプローチも可能となり、創薬の可能性は大きく広がっている。本シンポジウムでは、初期段階の医学的研究成果（シーズ）を社会実装に導くための基盤技術や革新的な創薬モダリティの開発に取り組む専門家をお招きし、新時代にふさわしい創薬研究の方向性を議論し、がん創薬における研究開発のさらなる活性化を図りたい。

---

### S8-3
### Next-Generation SNPD-siRNA Enables Allele-Specific Silencing of Mutant Genes

[Speaker]Kumiko Uitei (TIDE Center, Inst. Sci., Tokyo／Dept. Biol. Sci., Grad. Sch. Scil., Univ. Tokyo)

Recent advances in nucleic acid therapeutics have been remarkable, particularly in the development of small interfering RNA (siRNA) drugs. In recent years, seven siRNA-based drugs have been approved by the U.S. FDA, and four of them are also approved in Japan.siRNAs are short double-stranded RNAs that bind to complementary mRNA sequences and suppress gene expression via RNA interference (RNAi). However, none of the approved siRNA drugs currently target cancer. Cancer is often caused by point mutations in driver genes or by gene fusions, but suppressing wild-type genes can lead to harmful effects. Therefore, it is important to create siRNAs that specifically target and silence only the mutated gene, while leaving the normal gene untouched.Because siRNAs are short, distinguishing single-nucleotide differences is technically difficult, and cancer has been viewed as an "undruggable" target for siRNA therapeutics. Here, we present our development of next-generation SNPD-siRNAs that achieve allele-specific silencing at single-nucleotide resolution, with potential applications in cancer and genetic disorders.

[Disclosure of COI by Responsible researcher] Lead presenter is the Responsible researcher.
[Lead presenter: Uitei Kumiko] ■Research funds from company: ANRis Therapeutics ■Advisor of the company and/or profit-making organization: ANRis Therapeutics ■Profit of stock: ANRis Therapeutics ■Lecture fee: Alnylam Japan, Novartis ■Research expenses: ANRis Therapeutics ■Contributions: ANRis Therapeutics

### [S8-4] S8
### Foundational and innovative technologies to bridge basic cancer research to drug development

2025 / 9 / 26 (Fri) 12:50-15:20 Room 12/(ANA Crowne Plaza Hotel Kanazawa 3F Ohtori (Central)) Room 12

[Chairpersons]Hiroaki Taniguchi (Keio Cancer Center, Keio University School of Medicine)、Issay Kitabayashi (Fujita Health University)

In recent cancer research, increasing emphasis has been placed on how to deliver the outcomes of basic scientific studies to patients and realize their social implementation. To achieve this, it is essential to actively incorporate new technologies and conceptual advances, while fostering collaboration among researchers from diverse scientific and technological disciplines.
Drug discovery efforts have traditionally focused on molecules with enzymatic activity, which were considered tractable using conventional small-molecule approaches. However, the emergence and rapid development of various drug discovery modalities-such as nucleic acid-based drugs, targeted protein degraders, protein binding inhibitor and antibody-drug conjugates-have significantly diversified the therapeutic landscape. These advances are now enabling the targeting of molecules that were previously considered undruggable.
In this symposium, we will invite experts who are actively engaged in the development of foundational technologies and innovative therapeutic strategies aimed at bridging early-stage academic research to drug development and real-world clinical application. Through their presentations and ensuing discussions, we aim to explore new directions in drug discovery research that are appropriate for this evolving era, and to promote further innovation in the field.
We hope this session will stimulate meaningful dialogue and foster collaborations that advance translational cancer research and therapeutic implementation.

近年のがん研究においては、基礎研究の成果をいかに患者さんに届け、社会実装へと結びつけるかがこれまで以上に重要視されている。これを実現するためには、先進的な技術やコンセプトの積極的な導入と、多様な分野の研究者との協働が不可欠である。従来、創薬標的としては酵素活性を有する分子が重視されてきたが、近年のモダリティ技術の発展により、これまで標的化が困難とされてきた分子へのアプローチも可能となり、創薬の可能性は大きく広がっている。本シンポジウムでは、初期段階の医学的研究成果（シーズ）を社会実装に導くための基盤技術や革新的な創薬モダリティの開発に取り組む専門家をお招きし、新時代にふさわしい創薬研究の方向性を議論し、がん創薬における研究開発のさらなる活性化を図りたい。

---

### S8-4
### Drug discovery targeting KRAS mutants using targeted protein degradation

[Speaker]Chinatsu Sakata (Astellas Pharma Inc. Oncology Research)

Targeted protein degradation (TPD) is gaining attention as a novel approach in small molecule drug discovery. Unlike conventional small molecules that require strong binding to a target functional pocket, protein degraders induce degradation by bridging the target protein and a ubiquitin ligase, forming a ternary complex. This mechanism enables access to undruggable targets lacking functional pockets. A prominent example is Kirsten rat sarcoma viral oncogene homologue (KRAS), a key cancer driver long considered intractable. The breakthrough came in 2013 when Shokat et al. identified a compound covalently binding KRAS G12C, leading to two approved drugs. However, there are still no approved treatments for other KRAS mutants such as KRAS G12D and G12V, representing a major unmet need. We will highlight the development of ASP3082, the world's first selective KRAS G12D degrader, and discuss the future prospects of this groundbreaking technology.

[Disclosure of COI by Responsible researcher] Lead presenter is the Responsible researcher.
[Lead presenter: Chinatsu Sakata] ■ employment by a company related to the presentation: アステラス製薬株式会社　■Profit of stock: アステラス製薬株式会社　■Representative of organization for clinical study receiving research expenses from company: アステラス製薬株式会社

▲TOP

### [S8-5] S8
### Foundational and innovative technologies to bridge basic cancer research to drug development

2025 / 9 / 26 (Fri) 12:50-15:20 Room 12/(ANA Crowne Plaza Hotel Kanazawa 3F Ohtori (Central)) Room 12

[Chairpersons]Hiroaki Taniguchi (Keio Cancer Center, Keio University School of Medicine)、Issay Kitabayashi (Fujita Health University)

In recent cancer research, increasing emphasis has been placed on how to deliver the outcomes of basic scientific studies to patients and realize their social implementation. To achieve this, it is essential to actively incorporate new technologies and conceptual advances, while fostering collaboration among researchers from diverse scientific and technological disciplines.
Drug discovery efforts have traditionally focused on molecules with enzymatic activity, which were considered tractable using conventional small-molecule approaches. However, the emergence and rapid development of various drug discovery modalities-such as nucleic acid-based drugs, targeted protein degraders, protein binding inhibitor and antibody-drug conjugates-have significantly diversified the therapeutic landscape. These advances are now enabling the targeting of molecules that were previously considered undruggable.
In this symposium, we will invite experts who are actively engaged in the development of foundational technologies and innovative therapeutic strategies aimed at bridging early-stage academic research to drug development and real-world clinical application. Through their presentations and ensuing discussions, we aim to explore new directions in drug discovery research that are appropriate for this evolving era, and to promote further innovation in the field.
We hope this session will stimulate meaningful dialogue and foster collaborations that advance translational cancer research and therapeutic implementation.

近年のがん研究においては、基礎研究の成果をいかに患者さんに届け、社会実装へと結びつけるかがこれまで以上に重要視されている。これを実現するためには、先進的な技術やコンセプトの積極的な導入と、多様な分野の研究者との協働が不可欠である。従来、創薬標的としては酵素活性を有する分子が重視されてきたが、近年のモダリティ技術の発展により、これまで標的化が困難とされてきた分子へのアプローチも可能となり、創薬の可能性は大きく広がっている。本シンポジウムでは、初期段階の医学的研究成果（シーズ）を社会実装に導くための基盤技術や革新的な創薬モダリティの開発に取り組む専門家をお招きし、新時代にふさわしい創薬研究の方向性を議論し、がん創薬における研究開発のさらなる活性化を図りたい。

---

### S8-5
### Development of Enzomenib: A promising MENIN-MLL interaction inhibitor for precision medicine in acute myeloid leukemia.

[Speaker]Takafumi Shimizu (Cancer Drug Discovery Research Unit, Sumitomo Pharma Co Ltd) [Co-author]Ken Eguchi (Cancer Drug Discovery Research Unit, Sumitomo Pharma Co Ltd)、Daiki Kato (Cancer Drug Discovery Research Unit, Sumitomo Pharma Co Ltd)、Daiki Kusakabe (Cancer Drug Discovery Research Unit, Sumitomo Pharma Co Ltd)、Natsumi Okada (Cancer Drug Discovery Research Unit, Sumitomo Pharma Co Ltd)、Shah Jatin (Sumitomo Pharma America, Inc.)、Akihiko Yokoyama (National Cancer Center Tsuruoka Metabolomics Laboratory)、Issay Kitabayashi (Oncology Innovation Center, Center for Translational Research, Fujita Health University)、Hitoshi Ban (Cancer Drug Discovery Research Unit, Sumitomo Pharma Co Ltd)

The 11q23 abnormalities involving the mixed lineage leukemia (MLL) gene are frequently found in adult and pediatric patients with acute leukemia. MLL rearrangements (MLL-r) are associated with poor prognosis and these patients often show suboptimal responses to currently available therapies. Developing more effective therapies, particularly for this patient subpopulation, is urgently needed. The rearranged gene of MLL with various fusion partners, generates fusion proteins with leukemogenic activity that are critically dependent on direct interaction with MENIN. Therefore, we have generated a novel and orally bioavailable MENIN-MLL interaction inhibitor enzomenib (DSP-5336), which showed durable anti-leukemogenic activity in preclinical models of MLL-r and NPM1 mutant (NPM1m) acute leukemia. Enzomenib is being evaluated in an ongoing Phase I/II clinical trial that aims to fulfill unmet medical needs for acute leukemia. Preclinical studies and clinical data for enzomenib will be presented in the session.

[Disclosure of COI by Responsible researcher] Lead presenter is the Responsible researcher.
[Lead presenter: Takafumi Shimizu] ■ employment by a company related to the presentation: 住友ファーマ株式会社

### [S8-6] S8
### Foundational and innovative technologies to bridge basic cancer research to drug development

2025 / 9 / 26 (Fri) 12:50-15:20 Room 12/(ANA Crowne Plaza Hotel Kanazawa 3F Ohtori (Central)) Room 12

[Chairpersons]Hiroaki Taniguchi (Keio Cancer Center, Keio University School of Medicine)、Issay Kitabayashi (Fujita Health University)

In recent cancer research, increasing emphasis has been placed on how to deliver the outcomes of basic scientific studies to patients and realize their social implementation. To achieve this, it is essential to actively incorporate new technologies and conceptual advances, while fostering collaboration among researchers from diverse scientific and technological disciplines.
Drug discovery efforts have traditionally focused on molecules with enzymatic activity, which were considered tractable using conventional small-molecule approaches. However, the emergence and rapid development of various drug discovery modalities-such as nucleic acid-based drugs, targeted protein degraders, protein binding inhibitor and antibody-drug conjugates-have significantly diversified the therapeutic landscape. These advances are now enabling the targeting of molecules that were previously considered undruggable.
In this symposium, we will invite experts who are actively engaged in the development of foundational technologies and innovative therapeutic strategies aimed at bridging early-stage academic research to drug development and real-world clinical application. Through their presentations and ensuing discussions, we aim to explore new directions in drug discovery research that are appropriate for this evolving era, and to promote further innovation in the field.
We hope this session will stimulate meaningful dialogue and foster collaborations that advance translational cancer research and therapeutic implementation.

近年のがん研究においては、基礎研究の成果をいかに患者さんに届け、社会実装へと結びつけるかがこれまで以上に重要視されている。これを実現するためには、先進的な技術やコンセプトの積極的な導入と、多様な分野の研究者との協働が不可欠である。従来、創薬標的としては酵素活性を有する分子が重視されてきたが、近年のモダリティ技術の発展により、これまで標的化が困難とされてきた分子へのアプローチも可能となり、創薬の可能性は大きく広がっている。本シンポジウムでは、初期段階の医学的研究成果（シーズ）を社会実装に導くための基盤技術や革新的な創薬モダリティの開発に取り組む専門家をお招きし、新時代にふさわしい創薬研究の方向性を議論し、がん創薬における研究開発のさらなる活性化を図りたい。

---

### S8-6
### Development and Advancement of DXd ADC Platform

[Speaker]Yasuyuki Kaneta (Group II, Discovery Reseach Laboratories I, Daiichi Sankyo Co., Ltd)

The antibody-drug conjugate (ADC), which links an antibody that binds to cancer cells with a cytotoxic drug, is considered a next-generation antibody therapeutic that selectively and effectively eliminates cancer cells while reducing systemic toxicity. Although the concept of ADCs has been proposed for a long time, the path to success has been difficult, and the approval of drugs had not been achieved for some time. Daiichi Sankyo has successfully established the drug-linker technology (DXd ADC technology) using a novel derivative of the DNA topoisomerase I inhibitor exatecan (DXd) as the payload. This technology addresses issues faced by existing ADCs. It ensures high drug activity while maintaining safety through a short half-life. Moreover, even when the drug-to-antibody ratio is approximately 8, the linker is designed to maintain high stability in the blood. Additionally, the drug linker is selectively cleaved within tumors, allowing the released drug to exhibit activity not only against the antigen-expressing tumor cells but also against adjacent tumor cells due to its membrane permeability.I would like to discuss development of the DXd ADC pipeline and provide recent updates.

[Disclosure of COI by Responsible researcher] Lead presenter is the Responsible researcher.
[Lead presenter: Yasuyuki Kaneta] ■ employment by a company related to the presentation: 第一三共株式会社

### [IS6-1] IS6
### Host Factors in EBV-induced Carcinogenesis

2025 / 9 / 26 (Fri) 12:50-15:20 Room 13/(ANA Crowne Plaza Hotel Kanazawa 3F Ohtori (East)) Room 13

[Chairpersons]Ayako Arai (Department of Hematology and Oncology, St. Marianna University School of Medecine)、SeokJin Kim (Sungkyunkwan University)

Epstein-Barr virus (EBV) is a virus that infects nearly every human worldwide. While most individuals coexist with EBV without experiencing health issues, in some, it contributes to the development of life-threatening malignancies. What determines this difference? The answer lies not only in the virus but also in the host.
This symposium explores the critical role of host factors in EBV-induced carcinogenesis. Although EBV-related malignancies are found globally, they are particularly prevalent in Asia, underscoring the need for close collaboration and knowledge sharing among researchers in this region. To foster a multidisciplinary approach, we have invited leading experts from diverse fields, including hematology, pediatrics, otolaryngology, genomics, and regenerative medicine. Together, they will examine how genetic predisposition, immune response, and microenvironmental factors drive EBV-associated carcinogenesis.
How do EBV-infected cells evade host immunity and undergo malignant transformation? Can we predict, prevent, or even overcome EBV-related malignancies? Through cutting-edge research and dynamic discussions, we aim to uncover the answers. Tackling these challenges requires large-scale, longitudinal studies, and international collaboration will be key to accelerating progress.
We welcome both established experts and those new to the field to join this discussion, which may redefine our understanding of EBV and its role in malignancies. Together, let us explore the complex interplay between EBV and its host.

---

### IS6-1
### Host factors in carcinogenesis of nasopharyngeal carcinoma induced by Epstein-Barr virus

[Speaker]Satoru Kondo (Div. Otolaryngol. Head Neck Surg. Grad. Sch. Med,, Kanazawa Univ.)

Epstein-Barr virus (EBV) is strongly associated with the carcinogenesis and pathology of nasopharyngeal carcinoma (NPC). At the time EBV was discovered in 1970s, it was believed that the first trigger in the carcinogenesis of NPC began with EBV infection. However, recent studies have revealed that various cellular events, such as genomic abnormalities like p16 gene deletions and epigenomic abnormalities like DNA methylation-induced gene inactivation, which allow for persistent EBV infection, precede the establishment of EBV infection. Some reports showed that EBV infection occurs after these events. Furthermore, for the carcinogenesis of NPC, it is also crucial for EBV latent infection. In epithelial cells that transition to latent infection, the virus replicates along with the host DNA as the infected cells proliferate, ensuring the persistence of infection. Multiple EBV latent genes activation leads to downstream host gene cascades. Moreover, several studies have shown that lytic infection genes are associated carcinogenesis of NPC. In this presentation, I will discuss how lytic and latent EBV infections induce host gene cascades for carcinogenesis of NPC.

[Disclosure of COI by Responsible researcher] Lead presenter is the Responsible researcher.
[Lead presenter: Satoru Kondo] We have no financial relationships to disclose.

### [IS6-2] IS6
### Host Factors in EBV-induced Carcinogenesis

2025 / 9 / 26 (Fri) 12:50-15:20 Room 13/(ANA Crowne Plaza Hotel Kanazawa 3F Ohtori (East)) Room 13

[Chairpersons]Ayako Arai (Department of Hematology and Oncology, St. Marianna University School of Medecine)、SeokJin Kim (Sungkyunkwan University)

Epstein-Barr virus (EBV) is a virus that infects nearly every human worldwide. While most individuals coexist with EBV without experiencing health issues, in some, it contributes to the development of life-threatening malignancies. What determines this difference? The answer lies not only in the virus but also in the host.
This symposium explores the critical role of host factors in EBV-induced carcinogenesis. Although EBV-related malignancies are found globally, they are particularly prevalent in Asia, underscoring the need for close collaboration and knowledge sharing among researchers in this region. To foster a multidisciplinary approach, we have invited leading experts from diverse fields, including hematology, pediatrics, otolaryngology, genomics, and regenerative medicine. Together, they will examine how genetic predisposition, immune response, and microenvironmental factors drive EBV-associated carcinogenesis.
How do EBV-infected cells evade host immunity and undergo malignant transformation? Can we predict, prevent, or even overcome EBV-related malignancies? Through cutting-edge research and dynamic discussions, we aim to uncover the answers. Tackling these challenges requires large-scale, longitudinal studies, and international collaboration will be key to accelerating progress.
We welcome both established experts and those new to the field to join this discussion, which may redefine our understanding of EBV and its role in malignancies. Together, let us explore the complex interplay between EBV and its host.

---

### IS6-2
### Elucidating the mechanism of aberrant chromatin activation induced by the virus-host chromatin interactions

[Speaker]Atsushi Okabe (Health and Disease Omics Ctr., Chiba Univ.／Dept. of Mol. Oncol., Grad. Sch of Med., Chiba Univ.) [Co-author]Masaki Fukuyo (Dept. of Mol. Oncol., Grad. Sch of Med., Chiba Univ.)、Bahityar Rahmutulla (Dept. of Mol. Oncol., Grad. Sch of Med., Chiba Univ.)、Teru Kanda (Fac. of Med., Tohoku Med. and Pharm. Univ.)、Hironori Yoshiyama (Fac. of Med., Shimane Univ.)、Tan Patrick (Duke-NUS Med. Sch.)、Atsushi Kaneda (Health and Disease Omics Ctr., Chiba Univ.／Dept. of Mol. Oncol., Grad. Sch of Med., Chiba Univ.)

The Epstein-Barr virus (EBV) is a well-known cause of various tumors, including gastric cancer (GC) and lymphoma. We previously reported that direct interactions between the EBV genome and the host genome disrupt heterochromatin structures, leading to the aberrant activation of oncogenic pathways. However, the specific mechanisms of this heterochromatin disruption remain not fully understood.To investigate how interactions with the EBV genome disrupt the host's heterochromatin structure, we conducted further analyses of chromatin interactions and epigenetic state. We found that the interaction frequency with the EBV genome correlates with the transition from inactive to active chromatin shift. By focusing on the viral factor EBNA1, which mediates these interactions, we discovered that EBNA1 binds more frequently in EBV-interacting regions (EBVIRs). Besides, we analyzed various modifications related to heterochromatin and revealed that linker histone H1 is lost explicitly in the EBVIRs.This presentation will discuss our latest findings about the effect of the EBNA1-interaction on the host chromatin, highlighting the implications for understanding EBV-induced carcinogenesis.

[Disclosure of COI by Responsible researcher] Lead presenter is not the Responsible researcher.
[Lead presenter: Atsushi Okabe] ■Contributions: Nihonshinyaku. Takeda Science Foundation Mochida Memorial Foundation for Medical and Pharmaceutical Research
[Principal Researcher: Atsushi Kaneda] ■Contributions: SRL Inc. Scrum Inc. Takeda Science Foundation Uehara Memorial Foundation

### [IS6-3] IS6
### Host Factors in EBV-induced Carcinogenesis

2025 / 9 / 26 (Fri) 12:50-15:20 Room 13/(ANA Crowne Plaza Hotel Kanazawa 3F Ohtori (East)) Room 13

[Chairpersons]Ayako Arai (Department of Hematology and Oncology, St. Marianna University School of Medecine)、SeokJin Kim (Sungkyunkwan University)

Epstein-Barr virus (EBV) is a virus that infects nearly every human worldwide. While most individuals coexist with EBV without experiencing health issues, in some, it contributes to the development of life-threatening malignancies. What determines this difference? The answer lies not only in the virus but also in the host.
This symposium explores the critical role of host factors in EBV-induced carcinogenesis. Although EBV-related malignancies are found globally, they are particularly prevalent in Asia, underscoring the need for close collaboration and knowledge sharing among researchers in this region. To foster a multidisciplinary approach, we have invited leading experts from diverse fields, including hematology, pediatrics, otolaryngology, genomics, and regenerative medicine. Together, they will examine how genetic predisposition, immune response, and microenvironmental factors drive EBV-associated carcinogenesis.
How do EBV-infected cells evade host immunity and undergo malignant transformation? Can we predict, prevent, or even overcome EBV-related malignancies? Through cutting-edge research and dynamic discussions, we aim to uncover the answers. Tackling these challenges requires large-scale, longitudinal studies, and international collaboration will be key to accelerating progress.
We welcome both established experts and those new to the field to join this discussion, which may redefine our understanding of EBV and its role in malignancies. Together, let us explore the complex interplay between EBV and its host.

---

### IS6-3
### Intravesicular microRNA in EVs from ENKTL Patients: Prognostic and CAEBV-Associated Signatures

[Speaker]Seok Jin Kim (Sungkyunkwan University School of Medicine Samsung Medical Center) [Co-author]Kyung Ju Ryu (Samsung Advanced Institute of Health Sciences and Technology, Sungkyunkwan University)、Bon Park (Samsung Advanced Institute of Health Sciences and Technology, Sungkyunkwan University)、Sang Eung Yoon (Sungkyunkwan University School of Medicine Samsung Medical Center)、Won Seog Kim (Sungkyunkwan University School of Medicine Samsung Medical Center)

Epstein-Barr virus (EBV)-associated extranodal NK/T-cell lymphoma (ENKTL) is an aggressive malignancy with heterogeneous clinical outcomes. Emerging evidence suggests that extracellular vesicles (EVs) and their intravesicular microRNAs (miRNAs) may reflect the tumor microenvironment and host immune status. We performed comprehensive miRNA sequencing of intravesicular contents from plasma-derived EVs of ENKTL patients and analyzed their correlation with clinical outcomes and chronic active EBV infection (CAEBV)-related features. Specific miRNA signatures were enriched in EVs from patients with poor prognosis and overlapped with profiles observed in CAEBV, suggesting shared pathogenetic mechanisms. Notably, upregulation of EBV-related and immune-modulatory miRNAs, including miR-BART family members, was associated with disease progression and treatment resistance. Our findings highlight the prognostic and pathogenic relevance of EV-associated miRNAs in ENKTL and provide a potential link between ENKTL and CAEBV biology, offering novel biomarkers for risk stratification and therapeutic targeting.

[Disclosure of COI by Responsible researcher] Lead presenter is the Responsible researcher.
[Lead presenter: Seok Jin Kim] We have no financial relationships to disclose.

▲TOP

### [IS6-4] IS6
### Host Factors in EBV-induced Carcinogenesis

2025 / 9 / 26 (Fri) 12:50-15:20 Room 13/(ANA Crowne Plaza Hotel Kanazawa 3F Ohtori (East)) Room 13

[Chairpersons]Ayako Arai (Department of Hematology and Oncology, St. Marianna University School of Medecine)、SeokJin Kim (Sungkyunkwan University)

Epstein-Barr virus (EBV) is a virus that infects nearly every human worldwide. While most individuals coexist with EBV without experiencing health issues, in some, it contributes to the development of life-threatening malignancies. What determines this difference? The answer lies not only in the virus but also in the host.
This symposium explores the critical role of host factors in EBV-induced carcinogenesis. Although EBV-related malignancies are found globally, they are particularly prevalent in Asia, underscoring the need for close collaboration and knowledge sharing among researchers in this region. To foster a multidisciplinary approach, we have invited leading experts from diverse fields, including hematology, pediatrics, otolaryngology, genomics, and regenerative medicine. Together, they will examine how genetic predisposition, immune response, and microenvironmental factors drive EBV-associated carcinogenesis.
How do EBV-infected cells evade host immunity and undergo malignant transformation? Can we predict, prevent, or even overcome EBV-related malignancies? Through cutting-edge research and dynamic discussions, we aim to uncover the answers. Tackling these challenges requires large-scale, longitudinal studies, and international collaboration will be key to accelerating progress.
We welcome both established experts and those new to the field to join this discussion, which may redefine our understanding of EBV and its role in malignancies. Together, let us explore the complex interplay between EBV and its host.

---

### IS6-4
### Host Context and Early Clinical Features of Pediatric Chronic Active EBV Infection

[Speaker]Hyery Kim (University of Ulsan College of Medicine, Asan Medical Center)

Chronic active Epstein-Barr virus (CAEBV) infection in children and adolescents provides a unique clinical model to explore host-related susceptibility to EBV-induced carcinogenesis. Unlike typical self-limiting primary EBV infection, CAEBV is characterized by persistent symptoms, high EBV load, and progressive organ involvement, particularly in T or NK cells, which may evolve into overt lymphoid malignancies. In our institutional cohort, we observed heterogeneous clinical presentations at diagnosis, ranging from prolonged fever and hepatosplenomegaly to hemophagocytic lymphohistiocytosis (HLH). Although most patients had no relevant family history, occasional cases with hematologic or immune conditions among relatives raised the possibility of rare genetic predisposition. Longitudinal follow-up revealed variable clinical trajectories, with some cases progressing to lymphoma or severe organ dysfunction. These observations highlight the significance of early host immune context and clinical features in predicting disease course and carcinogenic potential. Further integration of immunogenetic profiling may refine risk stratification and management strategies in pediatric CAEBV.

[Disclosure of COI by Responsible researcher] Lead presenter is the Responsible researcher.
[Lead presenter: Hyery Kim] We have no financial relationships to disclose.

### [IS6-5] IS6
### Host Factors in EBV-induced Carcinogenesis

2025 / 9 / 26 (Fri) 12:50-15:20 Room 13/(ANA Crowne Plaza Hotel Kanazawa 3F Ohtori (East)) Room 13

[Chairpersons]Ayako Arai (Department of Hematology and Oncology, St. Marianna University School of Medecine)、SeokJin Kim (Sungkyunkwan University)

Epstein-Barr virus (EBV) is a virus that infects nearly every human worldwide. While most individuals coexist with EBV without experiencing health issues, in some, it contributes to the development of life-threatening malignancies. What determines this difference? The answer lies not only in the virus but also in the host.
This symposium explores the critical role of host factors in EBV-induced carcinogenesis. Although EBV-related malignancies are found globally, they are particularly prevalent in Asia, underscoring the need for close collaboration and knowledge sharing among researchers in this region. To foster a multidisciplinary approach, we have invited leading experts from diverse fields, including hematology, pediatrics, otolaryngology, genomics, and regenerative medicine. Together, they will examine how genetic predisposition, immune response, and microenvironmental factors drive EBV-associated carcinogenesis.
How do EBV-infected cells evade host immunity and undergo malignant transformation? Can we predict, prevent, or even overcome EBV-related malignancies? Through cutting-edge research and dynamic discussions, we aim to uncover the answers. Tackling these challenges requires large-scale, longitudinal studies, and international collaboration will be key to accelerating progress.
We welcome both established experts and those new to the field to join this discussion, which may redefine our understanding of EBV and its role in malignancies. Together, let us explore the complex interplay between EBV and its host.

---

### IS6-5
### Investigating the pathogenesis of CAEBV: our approaches today and future

[Speaker]Miwako Nishio (Hematology and Biophysical Systems, Institute of Science Tokyo) [Co-author]Mayumi Yoshimori (Hematology and Biophysical Systems, Institute of Science Tokyo／Blood Transfusion and Cell Therapy, Institute of Science Tokyo Hospital)、Saki Mizutani (Hematology and Biophysical Systems, Institute of Science Tokyo)、Yuri Maekawa (Hematology and Biophysical Systems, Institute of Science Tokyo)、Runa Shimomaki (Hematology and Biophysical Systems, Institute of Science Tokyo)、Kanoko Chihara (Hematology and Biophysical Systems, Institute of Science Tokyo)、Ayako Arai (Hematology and Biophysical Systems, Institute of Science Tokyo／Hematology and Oncology, St. Marianna University School of Medicine)

Systemic chronic active EBV disease (sCAEBV) is EBV-positive T- or NK-cell lymphoproliferative disorder characterized by persistent systemic inflammation driven by the activation of EBV-infected cells. sCAEBV is an Epstein-Barr virus (EBV)-infected T- or natural killer (NK) cell neoplasm, according to the World Health Organization (WHO) classification of tumors of hematopoietic and lymphoid tissues in 2022 (WHO 2022).
Why EBV persistently infects T or NK cells, leading to their immortalization and clonal expansion in sCAEBV, is unknown. Moreover, no effective drugs have been developed to eradicate EBV-infected T or NK cells in patients with sCAEBV. Hematopoietic stem cell transplantation (HSCT) is currently the only curative therapy for sCAEBV. Thus, there is an urgent need to elucidate the pathological mechanism and develop therapeutic agents.
The pathogenesis of sCAEBV starts with EBV infection of T or NK cells, followed by cellular immortalization, immune evasion by the host, and ultimately malignant transformation. We hypothesized that some host factors, as well as interactions between host and infected cells, are essential in this process. Here, we discuss our latest findings.

[Disclosure of COI by Responsible researcher] Lead presenter is the Responsible researcher.
[Lead presenter: Miwako Nishio] We have no financial relationships to disclose.

▲TOP

### [IS6-6] IS6
### Host Factors in EBV-induced Carcinogenesis

2025 / 9 / 26 (Fri) 12:50-15:20 Room 13/(ANA Crowne Plaza Hotel Kanazawa 3F Ohtori (East)) Room 13

[Chairpersons]Ayako Arai (Department of Hematology and Oncology, St. Marianna University School of Medecine)、SeokJin Kim (Sungkyunkwan University)

Epstein-Barr virus (EBV) is a virus that infects nearly every human worldwide. While most individuals coexist with EBV without experiencing health issues, in some, it contributes to the development of life-threatening malignancies. What determines this difference? The answer lies not only in the virus but also in the host.
This symposium explores the critical role of host factors in EBV-induced carcinogenesis. Although EBV-related malignancies are found globally, they are particularly prevalent in Asia, underscoring the need for close collaboration and knowledge sharing among researchers in this region. To foster a multidisciplinary approach, we have invited leading experts from diverse fields, including hematology, pediatrics, otolaryngology, genomics, and regenerative medicine. Together, they will examine how genetic predisposition, immune response, and microenvironmental factors drive EBV-associated carcinogenesis.
How do EBV-infected cells evade host immunity and undergo malignant transformation? Can we predict, prevent, or even overcome EBV-related malignancies? Through cutting-edge research and dynamic discussions, we aim to uncover the answers. Tackling these challenges requires large-scale, longitudinal studies, and international collaboration will be key to accelerating progress.
We welcome both established experts and those new to the field to join this discussion, which may redefine our understanding of EBV and its role in malignancies. Together, let us explore the complex interplay between EBV and its host.

### IS6-6
### Distinct Immune Landscapes in Endemic and Sporadic Burkitt Lymphoma Reflect EBV Association

[Speaker]Anand D. Jeyasekharan (Cancer Science Institute of Singapore, National University of Singapore, Singapore／Department of Haematology-Oncology, National University Cancer Institute, Singapore, Singapore ／NUS Centre for Cancer Research, National University of Singapore, Singapore) [Co-author]Zi Yan Charmaine Ong (Cancer Science Institute of Singapore, National University of Singapore, Singapore)、Liang Hong (Cancer Science Institute of Singapore, National University of Singapore, Singapore)、Min Liu (Cancer Science Institute of Singapore, National University of Singapore, Singapore／Department of Radiation Oncology, Chongqing University Cancer Hospital, Chongqing, China)、Chartsiam Tipgomut (Cancer Science Institute of Singapore, National University of Singapore, Singapore)、Patrick Jaynes (Cancer Science Institute of Singapore, National University of Singapore, Singapore)、Reiya Chua (Department of Haematology-Oncology, National University Cancer Institute, Singapore, Singapore)、Jaya Lakshmi (Department of Haematology-Oncology, National University Cancer Institute, Singapore, Singapore)、Yogeshini Batumalai (Department of Haematology-Oncology, National University Cancer Institute, Singapore, Singapore)、Sanjay De Mel (Department of Haematology-Oncology, National University Cancer Institute, Singapore, Singapore ／NUS Centre for Cancer Research, National University of Singapore, Singapore)、Limei Poon (Department of Haematology-Oncology, National University Cancer Institute, Singapore, Singapore ／NUS Centre for Cancer Research, National University of Singapore, Singapore)、Swee Shan Susan Hue (Department of Pathology, National University Hospital, Singapore)、Lam Ching Joyce Mei (Duke-NUS Medical School, Singapore／Department of Pathology and Laboratory Medicine, KK Hospital, Singapore／Haematology Laboratory and Blood Bank, KK Hospital, Singapore／Paediatric Haematology ／ Oncology Service, KK Hospital, Singapore)、Kenneth Chang (Duke-NUS Medical School, Singapore／Department of Pathology and Laboratory Medicine, KK Hospital, Singapore)、Alex Mremi (Kilimanjaro Christian Medical University College, Moshi, Tanzania)、Grace Moshi (Duke-NUS Medical School, Singapore／Department of Pathology and Laboratory Medicine, KK Hospital, Singapore／Haematology Laboratory and Blood Bank, KK Hospital, Singapore)

Burkitt lymphoma (BL) is an aggressive B-cell non-Hodgkin lymphoma with three forms: endemic (eBL), sporadic (sBL), and immunodeficiency-associated. eBL is linked to Epstein-Barr Virus (EBV) infection in >95% of cases, while sBL has an EBV association of ˜10-20%. Despite similar histological features, eBL responds well to low-intensity chemotherapy, whereas sBL requires more intensive treatment. This study compares the tumour immune microenvironment (TIME) of eBL and sBL using Digital Spatial Profiling across three cohorts: eBL from KCMUCo, Tanzania (N=15); sBL from NUH, Singapore (N=15) and KK Women's and Children's Hospital, Singapore (N=34). Our analysis revealed that sBL tumours had higher expression of CD66b (granulocyte), CD45 (pan-leukocyte), and CD68 (macrophage), indicating a myeloid-inflamed TIME that may reduce chemotherapy responsiveness. eBL samples showed increased levels of immune regulatory molecules, IDO1 and OX40L, as well as elevated GAPDH expression, which may correlate with favourable outcomes. These findings highlight differences in immune composition beyond viral aetiology among the subtypes, with implications for treatment response.

[Disclosure of COI by Responsible researcher] Lead presenter is the Responsible researcher.
[Lead presenter: Anand D. Jeyasekharan] We have no financial relationships to disclose.

▲TOP

### [J-2079] II-J4
### Head & neck tumors

2025 / 9 / 26 (Fri) 12:50-14:05 Room 14/(ANA Crowne Plaza Hotel Kanazawa 3F Zuiun 1) Room 14

[Chairperson]Nobuhiko Oridate (Dept Otolaryngology-Head & Neck Surgery Yokohama City Univ. Sch. Med.)

---

### J-2079
### Influence of the Oral Microenvironment on the Progression of Head and Neck Squamous Cell Carcinoma

[Speaker]Yurika Nakajima (Dept. Microbiol.& Immunol., Nihon Univ. Sch. Den.) [Co-author]Shogo Okazaki (Dept. Microbiol.& Immunol., Nihon Univ. Sch. Den.)、Shuichi Sato (Perio., Nihon Univ. Sch. Den.)、Kenichi Imai (Dept. Microbiol.& Immunol., Nihon Univ. Sch. Den.)

The progression of malignant tumors is influenced by the tumor microenvironment. However, many studies on head and neck squamous cell carcinoma (HNSCC) do not utilize models that reflect the unique conditions of the oral cavity. In this study, we investigated the role of the oral microenvironment in HNSCC progression. HSC-2 cells were transplanted into mice either orthotopically or subcutaneously, and tumor weights were measured two weeks later. Orthotopic tumors were significantly larger, suggesting that the oral environment promotes tumor growth. RNA-seq and immunofluorescence analyses revealed that orthotopic tumors exhibit a more undifferentiated and proliferative phenotype. TCGA analysis showed that the gene signature of orthotopic tumors closely correlates with gene expression patterns observed in clinical HNSCC samples, indicating that these tumors more accurately recapitulate the properties of clinical HNSCC.These findings underscore the critical role of the oral microenvironment in HNSCC progression and highlight the value of orthotopic models in HNSCC research for studying tumor biology under physiologically relevant conditions.

[Disclosure of COI by Responsible researcher] Lead presenter is not the Responsible researcher.
[Lead presenter: Yurika Nakajima] We have no financial relationships to disclose.
[Principal Researcher: Shogo Okazaki] We have no financial relationships to disclose.

▲TOP

### [J-2080] II-J4
### Head & neck tumors

2025 / 9 / 26 (Fri) 12:50-14:05 Room 14/(ANA Crowne Plaza Hotel Kanazawa 3F Zuiun 1) Room 14

[Chairperson]Nobuhiko Oridate (Dept Otolaryngology-Head & Neck Surgery Yokohama City Univ. Sch. Med.)

---

### J-2080
### HPV E6 and E7 synergize with CBP haploinsufficiency to drive head and neck tumorigenesis

[Speaker]Takuya Nakagawa (Health & Disease Omics Ctr., Chiba Univ. ／Dept Mol Oncol, Grad Sch Med, Chiba Univ. ／Dept Oto, Grad Sch Med, Chiba Univ.) [Co-author]Ryo Arai (Dept Mol Oncol, Grad Sch Med, Chiba Univ. ／Dept Oto, Grad Sch Med, Chiba Univ.)、Miki Sunagane (Dept Mol Oncol, Grad Sch Med, Chiba Univ. ／Dept Oto, Grad Sch Med, Chiba Univ.)、Kenta Saeda (Dept Mol Oncol, Grad Sch Med, Chiba Univ. ／Dept Oto, Grad Sch Med, Chiba Univ.)、Masaki Fukuyo (Dept Mol Oncol, Grad Sch Med, Chiba Univ.)、Toyoyuki Hanazawa (Dept Oto, Grad Sch Med, Chiba Univ.)、Atsushi Kaneda (Health & Disease Omics Ctr., Chiba Univ. ／Dept Mol Oncol, Grad Sch Med, Chiba Univ.)、Joseph Califano (Moores Cancer Ctr., UC San Diego)

While the oncogenic roles of HPV-derived E6 and E7 are well established, the molecular mechanisms of malignant transformation have not been well clarified. CBP, a histone acetyltransferase, is recurrently mutated in around 10% of HPV-associated head and neck cancer, but its role in HPV-mediated tumorigenesis is yet to be elucidated.We developed in vivo models of spontaneous tongue tumorigenesis by conditionally co-expressing E6 and E7 with CBP haploinsufficiency in tongue epithelium using a Krt14-CreERT system. Mice with E6 and E7 + CBP haploinsufficiency (n = 25) showed significantly earlier tumor onset than those with E6 and E7 alone (n = 36) (p = 0.0013), indicating cooperation between viral oncogenes and epigenetic disruption. CBP haploinsufficiency alone did not induce tumors, suggesting dependence on HPV signaling.ATAC-seq revealed distinct chromatin accessibility profiles between E6 and E7 and E6 and E7 + CBP haploinsufficiency groups. GO analysis of genes near by differentially accessible regions revealed enrichment of epithelial cell proliferation and mesenchyme development, implicating epithelial-mesenchymal pathways in HPV and CBP-mediated tumorigenesis.

[Disclosure of COI by Responsible researcher] Lead presenter is not the Responsible researcher.
[Lead presenter: Takuya Nakagawa] We have no financial relationships to disclose.
[Principal Researcher: Masaki Fukuyo] We have no financial relationships to disclose.

▲TOP

### [J-2081] II-J4
### Head & neck tumors

2025 / 9 / 26 (Fri) 12:50-14:05 Room 14/(ANA Crowne Plaza Hotel Kanazawa 3F Zuiun 1) Room 14

[Chairperson]Nobuhiko Oridate (Dept Otolaryngology-Head & Neck Surgery Yokohama City Univ. Sch. Med.)

---

### J-2081
### Depletion of nuclear protein X rescues the expression of malignancy-related genes in oral cancer cell lines

[Speaker]Shoichiro Tange (Div. Med. Genome Sci., Sapporo Med. Univ.) [Co-author]Shoko Goto (Div. Med. Genome Sci., Sapporo Med. Univ.)、Hideki Kawashima (Div. Med. Genome Sci., Sapporo Med. Univ.)、Masashi Idogawa (Div. Med. Genome Sci., Sapporo Med. Univ.)、Yasushi Sasaki (Biology, Medical Staff Dev. Center., Sappor Med. Univ.)、Takashi Tokino (Div. Med. Genome Sci., Sapporo Med. Univ.／Sapporo Univ. of Health Sci.)

Our analysis of clinical data using The Cancer Genome Atlas (TCGA), a public cancer database in the United States, showed a negative correlation between the expression level of the gene encoding nuclear protein coding gene ***X*** and recurrence-free survival (RFS) in oral cancer patients. We also found that the decreased expression of ***X*** is an independent prognosis indicator of shorter RFS in oral cancer using cox multivariate analysis. Of note, knockdown of ***X*** in oral cancer cell lines showed increased expression of genes that contribute to tumor malignancy and increased invasive potential. Gene-Set Enrichment Analysis (GSEA) showed upregulation of genes that are hallmarks of epithelial-mesenchymal transition (EMT), and ChIP assays revealed methylation of histone H3 Lys27 in the promoter regions of these genes, suggesting the accumulation of PRC2. In oral cancer, it has been reported that overexpression of ***HMGA 2*** and ***CLDN1*** correlate with poor prognosis and our analysis showed these genes are upregulated after knockdown of ***X***. These results suggest the existence of a novel function of ***X*** that suppresses tumor malignancy through suppression of the expression of the malignancy-related genes.

[Disclosure of COI by Responsible researcher] Lead presenter is the Responsible researcher.
[Lead presenter: Shoichiro Tange] We have no financial relationships to disclose.

▲TOP

### [J-2082] II-J4
### Head & neck tumors

2025 / 9 / 26 (Fri) 12:50-14:05 Room 14/(ANA Crowne Plaza Hotel Kanazawa 3F Zuiun 1) Room 14

[Chairperson]Nobuhiko Oridate (Dept Otolaryngology-Head & Neck Surgery Yokohama City Univ. Sch. Med.)

---

### J-2082
### Oral microenvironment-associated gene signature reflects high malignancy and tumor microenvironment changes in HNSCC

[Speaker]Shogo Okazaki (Dept. Microbiol. & Immunol., Nihon Univ. Sch. Dent.) [Co-author]Yurika Nakajima (Dept. Microbiol. & Immunol., Nihon Univ. Sch. Dent.／Dept. of Periodontol., Nihon Univ. Sch. Dent.)、Kenichi Imai (Dept. Microbiol. & Immunol., Nihon Univ. Sch. Dent.)

Head and neck squamous cell carcinoma (HNSCC) arises in the unique environment of the oral cavity, but the impact of this microenvironment remains unclear. We established a gene signature by RNA-seq analysis of tumors orthotopically or subcutaneously transplanted into mice, identifying genes upregulated in tongue tumors. Evaluation of signature scores in The Cancer Genome Atlas (TCGA) showed that the signature was more highly expressed in cancerous regions than in adjacent normal tissue, suggesting that tongue tumors better reflect clinical HNSCC than subcutaneous tumors. Moreover, cases with high signature scores had poorer prognosis, indicating higher tumor grade and malignancy. Single-cell RNA-seq data analysis of clinical HNSCC samples revealed that tumor cells with high signature scores exhibited features of high-grade malignancy. Tumors with high versus low signature scores also showed distinct microenvironmental profiles, particularly in fibroblast populations. These findings suggest that tongue transplantation models recapitulate the malignant properties of clinical HNSCC and provide a powerful tool for studying tumor biology under physiologically relevant conditions.

[Disclosure of COI by Responsible researcher] Lead presenter is the Responsible researcher.
[Lead presenter: Shogo Okazaki] We have no financial relationships to disclose.

### [J-2083] II-J4
### Head & neck tumors

2025 / 9 / 26 (Fri) 12:50-14:05 Room 14/(ANA Crowne Plaza Hotel Kanazawa 3F Zuiun 1) Room 14

[Chairperson]Nobuhiko Oridate (Dept Otolaryngology-Head & Neck Surgery Yokohama City Univ. Sch. Med.)

---

### J-2083
### Relationship between Self-care, Oral Environment, and Oral Mucositis during Chemoradiotherapy for Head and Neck Cancer

[Speaker]Shohei Okida (Kanazawa univ. Hosp. Dept. of Nursing) [Co-author]Kazuhiro Ogai (Grad. Sch. of Nursing, Ishikawa Pref. Nursing Univ.)、Mayumi Okuwa (Kanazawa Univ. Inst. of Med., Pharmaceutical and Health Sci.)

This study analyzed the associations between oral care methods, the oral environment, and the severity of mucositis during chemoradiotherapy in two cases of head and neck cancer.
These parameters were prospectively observed once a week during the treatment period. Oral bacterial samples were obtained from the hard palate, soft palate, and tip of the tongue. Bacterial DNA was extracted and analyzed using next-generation sequencing. Weekly variations in microbial composition were evaluated using the Bray-Curtis distance ($0 \leq$ dissimilarity $\leq 1$).
Intensity-modulated radiotherapy was administered in both cases, with a total dose of 70 Gy. The highest grade of oral mucositis observed was Grade 3 in Case A and Grade 1 in Case B. The maximum dissimilarity value, indicating changes in the oral microbiota from the previous week, was 0.8 from the hard palate in Case A and 0.66 from the tip of the tongue in Case B. Notably, Case A exhibited the highest microbial variation following the onset of fasting.
The findings suggest that fasting may contribute to changes in the oral microbiota, potentially increasing the severity of oral mucositis.

[Disclosure of COI by Responsible researcher] Lead presenter is not the Responsible researcher.
[Lead presenter: Shohei Okida] ■Research funds from company: 第6回SGHがん看護研究助成
[Principal Researcher: Mayumi Okuwa] We have no financial relationships to disclose.

### [J-2084] II-J4
### Head & neck tumors

2025 / 9 / 26 (Fri) 12:50-14:05 Room 14/(ANA Crowne Plaza Hotel Kanazawa 3F Zuiun 1) Room 14

[Chairperson]Nobuhiko Oridate (Dept Otolaryngology-Head & Neck Surgery Yokohama City Univ. Sch. Med.)

---

### J-2084
### Potential involvement of myoblasts as a cellular origin of cancer-associated fibroblasts in tongue cancer

[Speaker]Akira Yorozu (Dept. Otolaryngol. Head Neck Surg., Sapporo Med. Univ., Sch. Med.) [Co-author]Satoshi Owada (Div. Mol. Biol., Dept. Biochem., Sapporo Med. Univ., Sch. Med.／Dept. Oral Surg., Sapporo Med. Univ., Sch. Med)、Takeshi Niinuma (Dept. Oral Surg., Sapporo Med. Univ., Sch. Med)、Hiroshi Kitajima (Dept. Oral Surg., Sapporo Med. Univ., Sch. Med)、Kazuya Ishiguro (Dept. Oral Surg., Sapporo Med. Univ., Sch. Med)、Eiichiro Yamamoto (Dept. Oral Surg., Sapporo Med. Univ., Sch. Med)、Shohei Sekiguchi (Div. Mol. Biol., Dept. Biochem., Sapporo Med. Univ., Sch. Med.)、Fumika Okazaki (Div. Mol. Biol., Dept. Biochem., Sapporo Med. Univ., Sch. Med.)、Masahiro Kai (Dept. Oral Surg., Sapporo Med. Univ., Sch. Med)、Akihiro Miyazaki (Div. Mol. Biol., Dept. Biochem., Sapporo Med. Univ., Sch. Med.)、Kenichi Takano (Dept. Otolaryngol. Head Neck Surg., Sapporo Med. Univ., Sch. Med.)、Hiromu Suzuki (Dept. Oral Surg., Sapporo Med. Univ., Sch. Med)

Cancer-associated fibroblasts (CAFs) play key roles in the tumor microenvironment of tongue squamous cell carcinoma (TSCC), but their origin remains unclear. In this study, we investigated the potential of myoblasts as a cellular source of CAFs in TSCC. Using clinical specimens of head and neck cancers, we performed flow cytometric analysis of CAF markers FAP and CD29 following the method by Costa et al. TSCC samples exhibited a higher proportion of CAF-S1 cells ($FAP^{high}$, $CD29^{medium\text{-}high}$) compared to other head and neck cancers. Lingual muscle tissue and primary myoblasts derived from TSCC samples also showed a CAF-S1 marker pattern. We next co-cultured myoblasts, CAFs, and normal fibroblasts with TSCC cell lines in a 3D culture system and examined the expression of IL-6 and α-SMA, functional CAF markers. All stromal cell types, including myoblasts, showed early IL-6 upregulation, followed by α-SMA expression, and acquired CAF-S1 characteristics. Reanalysis of single-cell RNA-seq data from HNSCC also revealed a population of CAFs co-expressing IL-6 and α-SMA. These findings suggest that lingual myoblasts may transdifferentiate into CAF-like cells in the tumor microenvironment.

[Disclosure of COI by Responsible researcher] Lead presenter is not the Responsible researcher.
[Lead presenter: Akira Yorozu] We have no financial relationships to disclose.
[Principal Researcher: Hiromu Suzuki] We have no financial relationships to disclose.

▲TOP

### [J-2091] I-J11-4
### Tumor immune microenvironment/Tumor immune escape (3)

2025 / 9 / 26 (Fri) 12:50-14:05 Room 15/(ANA Crowne Plaza Hotel Kanazawa 3F Zuiun 2) Room 15

[Chairperson]Kazuhiro Kakimi (Dept. Immunology, Kindai Univ. Med)

---

### J-2091
### Opposing roles of Semaphorin 4A and Semaphorin 6D in regulating antitumor immunity

[Speaker]Yujiro Naito (Dept. of Respiratory Med. and Clin. Immunol., Univ. of Osaka) [Co-author]Shohei Koyama (Dept. of Respiratory Med. and Clin. Immunol., Univ. of Osaka／Dept. of Immuno Genomic Med, Natl. Cancer Ctr. Res. Inst.)、Takashi Hirai (Dept. of Otorhinolaryngology Head and Neck Surg., Univ. of Osaka)、Kentaro Masuhiro (Dept. of Respiratory Med. and Clin. Immunol., Univ. of Osaka)、Tomoki Kuge (Dept. of Respiratory Med. and Clin. Immunol., Univ. of Osaka)、Satoshi Miyamoto (Dept. of Respiratory Med. and Clin. Immunol., Univ. of Osaka)、Kazuo Yamashita (KOTAI Biotechnologies, Inc.)、Keita Aoi (Otsuka Pharmaceutical Co., Ltd.)、Hidenori Inohara (Dept. of Otorhinolaryngology Head and Neck Surg., Univ. of Osaka)、Yoshito Takeda (Dept. of Respiratory Med. and Clin. Immunol., Univ. of Osaka)、Atsushi Kumanogoh (Dept. of Respiratory Med. and Clin. Immunol., Univ. of Osaka)

Immune checkpoint inhibitors (ICIs) have caused revolutionary changes in cancer treatment, but low response rates remain a challenge. Semaphorins, initially identified as axon guidance molecules, have recently been recognized for their roles in immune regulation. Here, we demonstrate that two semaphorin family members, Sema4A and Sema6D, exert opposing effects on antitumor immunity and influence the efficacy of immune checkpoint inhibitors (ICIs). In non small cell lung cancer (NSCLC), ***SEMA4A*** expression in the tumor microenvironment (TME) correlated with improved response to ICIs. Mechanistically, Sema4A enhanced cytotoxicity and proliferation of tumor-specific $CD8^+$ T cells through mTORC1 activation and polyamine synthesis. In contrast, in a murine oral cancer model, nonhematopoietic cell derived Sema6D suppressed $CD8^+$ T cell infiltration and activation contributing to resistance to ICIs. Furthermore, analysis of human cancer datasets showed that ***SEMA6D*** expression was negatively correlated with T cell activation in the TME. These findings suggest that Sema4A may enhance ICI efficacy, while Sema6D targeting could help overcome resistance to cancer immunotherapy.

[Disclosure of COI by Responsible researcher] Lead presenter is not the Responsible researcher.
[Lead presenter: Yujiro Naito] ■Research funds from company: 大塚製薬株式会社　■Research expenses: 大塚製薬株式会社
[Principal Researcher: Atsushi Kumanogoh] ■Research funds from company: 大塚製薬株式会社　■Research expenses: 大塚製薬株式会社、中外製薬株式会社

### [J-2092] I-J11-4
### Tumor immune microenvironment/Tumor immune escape (3)

2025 / 9 / 26 (Fri) 12:50-14:05 Room 15/(ANA Crowne Plaza Hotel Kanazawa 3F Zuiun 2) Room 15

[Chairperson]Kazuhiro Kakimi (Dept. Immunology, Kindai Univ. Med)

---

### J-2092
### Immune Suppression by CAF-Cancer Cell Close Interaction Revealed Through 3D Bioprinting

[Speaker]Yuto Naoi (Okayama Univ. Dept. of Tumor microenvironment／Okayama Univ. Dept. of Otolaryngology-Head and Neck Surg.) [Co-author]Yumi Inukai (Okayama Univ. Dept. of Tumor microenvironment／Okayama Univ. Hosp. Dept. of Allergy and Respiratory Med.)、Tomoka Izumikawa (Okayama Univ. Dept. of Tumor microenvironment)、Joji Nagasaki (Okayama Univ. Dept. of Tumor microenvironment)、Youki Ueda (Okayama Univ. Dept. of Tumor microenvironment)、Ym Thu (Okayama Univ. Dept. of Tumor microenvironment)、Takamasa Ishino (Okayama Univ. Dept. of Tumor microenvironment)、Ken Suzawa (Okayama Univ. Dept. of Thoracic, Breast and Endocrinological Surg.)、Kenichi Yamamoto (Okayama Univ. Dept. of Cell Biol.)、Masakiyo Sakaguchi (Okayama Univ. Dept. of Cell Biol.)、Shuta Tomida (Okayama Univ. Hosp. Ctr. for Comprehensive Genomic Med.)、Yoshinobu Maeda (Okayama Univ. Dept. of Hematology, Oncology and Respiratory Med.)、Shinichi Toyooka (Okayama Univ. Dept. of Thoracic, Breast and Endocrinological Surg.)、Mizuo Ando (Okayama Univ. Dept. of Otolaryngology-Head and Neck Surg.)、Yosuke Togashi (Okayama Univ. Dept. of Tumor microenvironment／Okayama Univ. Hosp. Dept. of Allergy and Respiratory Med.／Kindai Univ. Faculty of Med.)

<Introduction>This study investigated how the spatial relationship between cancer-associated fibroblasts (CAFs) and cancer cells influences T cell-mediated antitumor immunity.<Methods>Using a 3D bioprinter, we generated two models: a "surrounding" model, where CAFs surrounded cancer cells as a physical barrier, and a "mixed" model, where CAFs and cancer cells were mixed. These models were co-cultured with antigen-specific T cells to evaluate infiltration and activation. Clinically, the association among CAF patterns, $CD8^+$ T cell infiltration, and outcomes of PD-1 blockade therapy was investigated.<Results>Compared to the surrounding model, the mixed model showed reduced T cell infiltration and activation, with increased crosstalk via TGF-$\beta$ and periostin. Postn (encoding periostin) knockdown in fibroblasts suppressed TGF-$\beta$ expression and restored T cell function in the mixed model. Clinically, tumors with mixed CAF distribution showed lower $CD8^+$ T cell infiltration and poorer prognosis.<Conclusion>These findings suggest that close CAF-cancer cell interactions promote immunosuppression and may serve as spatial biomarkers and therapeutic targets in cancer immunotherapy.

[Disclosure of COI by Responsible researcher] Lead presenter is not the Responsible researcher.
[Lead presenter: Yuto Naoi] We have no financial relationships to disclose.
[Principal Researcher: Yosuke Togashi] ■Research funds from company: オノ薬品工業、ブリストル・マイヤーズ スクイブ、中外製薬、アストラゼネカ、エーザイ、MSD

### [J-2093] I-J11-4
### Tumor immune microenvironment/Tumor immune escape (3)

2025 / 9 / 26 (Fri) 12:50-14:05 Room 15/(ANA Crowne Plaza Hotel Kanazawa 3F Zuiun 2) Room 15

[Chairperson]Kazuhiro Kakimi (Dept. Immunology, Kindai Univ. Med)

---

### J-2093
### Stromal Cell Diversity in Angioimmunoblastic T-cell Lymphoma through Spatial Transcriptomics at Single Cell Resolution

[Speaker]Yuki Shimizu (Grad. Sch. of Comprehensive Human Sci., Tsukuba Univ.) [Co-author]Yoshiaki Abe (Hematol. Depart., Inst. Med., Tsukuba Un／Hematol. Depart., Univ. of Tsukuba Hosp.)、Kenichi Makishima (Hematol. Depart., Univ. of Tsukuba Hosp.)、Sakurako Suma (Hematol. Depart., Univ. of Tsukuba Hosp.)、Yasuhito Suehara (Hematol. Depart., Inst. Med., Tsukuba Un／Hematol. Depart., Univ. of Tsukuba Hosp.)、Keiichiro Hattori (Hematol. Depart., Inst. Med., Tsukuba Un／Hematol. Depart., Univ. of Tsukuba Hosp.)、Tatsuhiro Sakamoto (Hematol. Depart., Inst. Med., Tsukuba Un／Hematol. Depart., Univ. of Tsukuba Hosp.)、Kyohei Sano (Preventive Med. Depart., Grad. Sch. of Med., Tokyo Univ.)、Ayumu Tsubosaka (Preventive Med. Depart., Grad. Sch. of Med., Tokyo Univ.)、Miwako Kakiuchi (Preventive Med. Depart., Grad. Sch. of Med., Tokyo Univ.)、Ayako Suzuki (Computational Biol. & Med. Sci. Dept., Tokyo Univ.)、Shumpei Ishikawa (Preventive Med. Depart., Grad. Sch. of Med., Tokyo Univ.)、Yutaka Suzuki (Computational Biol. & Med. Sci. Dept., Tokyo Univ.)、Mamiko Sakata-Yanagimoto (Grad. Sch. of Comprehensive Human Sci., Tsukuba Univ.／Hematol. Depart., Inst. Med., Tsukuba Un／Hematol. Depart., Univ. of Tsukuba Hosp.／Adv. Hemato-Oncology Div., Transborder Med. Res. Ctr., Tsukuba Univ.)

Background: Angioimmunoblastic T cell lymphoma (AITL) is a subtype of peripheral T-cell lymphoma, characterized by the proliferation of diverse stroma cells.Aim: We aimed to identify the heterogeneity of stromal cells in AITL microenvironments by spatial transcriptomics (ST) at single-cell resolution.Methods: We performed ST on 27 AITL by using Xenium in situ consisting of 289 genes.Results: We analyzed ST data of 2,020,192 cells and identified 15 stromal subclusters, 13 immune and tumor cell subclusters. Quantitative analysis demonstrated that C-aHEV1, in combination with follicular dendritic cells, constituted a tumor niche. On the other hand, C-aHEV2 was found to constitute an HEV niche in conjunction with other HEV subclusters. Of particular note, the number of C-aHEV2 cells per area was significantly higher in samples with G17V RHOA mutations than in those without.Conclusion/ Discussion: ST analysis suggested that C-aHEV1 is a stromal subcluster that constitutes the AITL microenvironments. Further investigation is needed to elucidate the mechanisms by which these two HEV niches interact with tumor cells and promote tumorigenesis.

[Disclosure of COI by Responsible researcher] Lead presenter is not the Responsible researcher.
[Lead presenter: Yuki Shimizu] We have no financial relationships to disclose.
[Principal Researcher: Mamiko Sakatayanagimoto] ■Research expenses: 大塚製薬（株）、エーザイ（株）、ブリストルマイヤーズスクイブ（株）、中外製薬（株）

▲TOP

### [J-2094] I-J11-4
### Tumor immune microenvironment/Tumor immune escape (3)

2025 / 9 / 26 (Fri) 12:50-14:05 Room 15/(ANA Crowne Plaza Hotel Kanazawa 3F Zuiun 2) Room 15

[Chairperson]Kazuhiro Kakimi (Dept. Immunology, Kindai Univ. Med)

### J-2094
### Cancer-associated fibroblast-targeted near-infrared photoimmunotherapy induces tumor-specific memory T cells from TDLNs

[Speaker]Akito Shimizu (Dept. of Gastroenterol. Surg., Okayama Univ. Grad. Sch. Med.) [Co-author]Kazuhiro Noma (Dept. of Gastroenterol. Surg., Okayama Univ. Grad. Sch. Med.)、Tatsuya Takahashi (Dept. of Gastroenterol. Surg., Okayama Univ. Grad. Sch. Med.)、Yohei Mizusawa (Dept. of Gastroenterol. Surg., Okayama Univ. Grad. Sch. Med.)、Tasuku Matsumoto (Dept. of Gastroenterol. Surg., Okayama Univ. Grad. Sch. Med.)、Seitaro Nishimura (Dept. of Gastroenterol. Surg., Okayama Univ. Grad. Sch. Med.)、Yasushige Takeda (Dept. of Gastroenterol. Surg., Okayama Univ. Grad. Sch. Med.)、Hijiri Matsumoto (Dept. of Gastroenterol. Surg., Okayama Univ. Grad. Sch. Med.)、Tomoyoshi Kunitomo (Dept. of Gastroenterol. Surg., Okayama Univ. Grad. Sch. Med.)、Hajime Kashima (Dept. of Gastroenterol. Surg., Okayama Univ. Grad. Sch. Med.)、Satoru Kikuchi (Dept. of Gastroenterol. Surg., Okayama Univ. Grad. Sch. Med.)、Toshiaki Ohara (Dept. of Gastroenterol. Surg., Okayama Univ. Grad. Sch. Med.)、Shunsuke Tanabe (Dept. of Gastroenterol. Surg., Okayama Univ. Grad. Sch. Med.)、Hiroshi Tazawa (Dept. of Gastroenterol. Surg., Okayama Univ. Grad. Sch. Med.)、Hisataka Kobayashi (Mol. Imaging Branch, CCR, NCI, NIH)、Toshiyoshi Fujiwara (Dept. of Gastroenterol. Surg., Okayama Univ. Grad. Sch. Med.)

Introduction:Cancer-associated fibroblasts (CAFs) suppress antitumor immunity in the tumor microenvironment (TME). We showed that CAF-targeted near-infrared photoimmunotherapy (PIT-CAF) remodels the TME, boosts immunity, and reduces tumor size. Dendritic cells (DCs) migrate to tumor-draining lymph nodes (TDLNs) to activate tumor-specific memory T cells. We hypothesized that CAFs block this process.Methods:We assessed memory T cells in esophageal squamous cell carcinoma by CD45RO immunostaining and analyzed their correlation with CAF area via A-SMA staining. In MC38 models, tumor-specific memory T cells and DCs were analyzed in the TME, TDLNs, and non-draining lymph nodes (NDLNs) using H-2Kb p15E tetramers. CAF-rich tumors were created by co-inoculating MEF.Results:High CD45RO+ density was linked to better prognosis and less CAF area. ($p<0.05$, $r=-0.315$) In mice, TDLNs had more tumor-specific T cells and DCs than NDLNs. ($p<0.05$) CAF-rich tumors showed fewer memory T cells, which were restored by PIT-CAF. TDLN resection before PIT-CAF reduced memory T cells and its effect. ($p<0.05$)
Conclusion:PIT-CAF promotes tumor-specific memory T cell induction from TDLNs via TME remodeling.

[Disclosure of COI by Responsible researcher] Lead presenter is not the Responsible researcher.
[Lead presenter: Akito Shimizu] We have no financial relationships to disclose.
[Principal Researcher: Kazuhiro Noma] We have no financial relationships to disclose.

▲TOP

### [J-2095] I-J11-4
### Tumor immune microenvironment/Tumor immune escape (3)

2025 / 9 / 26 (Fri) 12:50-14:05 Room 15/(ANA Crowne Plaza Hotel Kanazawa 3F Zuiun 2) Room 15

[Chairperson]Kazuhiro Kakimi (Dept. Immunology, Kindai Univ. Med)

---

### J-2095
### The effect of age-related accumulation of MDSCs on the progression of metastatic liver tumors

[Speaker]Takao Tsuneki (Division of Gastrointestinal Surgery, Department of Surgery, Kobe University) [Co-author]Kimihiro Yamashita (Division of Analytical Biomedical Sciences, Department of Biophysics, Kobe University)、Masafumi Saito (Department of Immunology and Microbiology, National Defense Medical College)、Yukari Adachi (Division of Gastrointestinal Surgery, Department of Surgery, Kobe University)、Yutaka Sugita (Division of Gastrointestinal Surgery, Department of Surgery, Kobe University)、Taro Ikea (Division of Gastrointestinal Surgery, Department of Surgery, Kobe University)、Yasuhumi Koterazawa (Division of Gastrointestinal Surgery, Department of Surgery, Kobe University)、Tomoaki Aoki (Division of Gastrointestinal Surgery, Department of Surgery, Kobe University)、Hitoshi Harada (Division of Gastrointestinal Surgery, Department of Surgery, Kobe University)、Yasunori Otowa (Division of Gastrointestinal Surgery, Department of Surgery, Kobe University)、Naoki Urakawa (Division of Gastrointestinal Surgery, Department of Surgery, Kobe University)、Hironobu Goto (Division of Gastrointestinal Surgery, Department of Surgery, Kobe University)、Hiroshi Hasegawa (Division of Gastrointestinal Surgery, Department of Surgery, Kobe University)、Shingo Kanaji (Division of Gastrointestinal Surgery, Department of Surgery, Kobe University)、Takeru Matsuda (Division of Gastrointestinal Surgery, Department of Surgery, Kobe University)、Yoshihiro Kakeji (Division of Gastrointestinal Surgery, Department of Surgery, Kobe University)

Aging reduces hematopoietic stem cells and their pluripotency, causing a shift toward myeloid over lymphoid lineages, leading to increased accumulation of myeloid-derived suppressor cells (MDSCs) with immunosuppressive functions in the tumor microenvironment of elderly individuals. This study investigated whether targeting MDSCs could suppress metastatic liver tumor progression in aged hosts. A liver metastasis model was established by injecting Luci-MC38 tumor cells ($3.0\times10^6$) into the spleens of young (8 weeks old) and old (100 weeks old) C57BL/6 mice. Tumor progression was monitored using an in vivo imaging system (IVIS), and tissues were analyzed three weeks post-injection. Results showed significantly more metastatic liver tumors in aged mice compared to young mice (2.167 vs. 19.63; 95% CI: 6.117-28.80; $P$= 0.0057). Immune profiling revealed a marked increase in PMN-MDSCs. PMN-MDSCs via anti-Ly6g antibody treatment significantly suppressed tumor progression and partially restored immune cell balance. These findings suggest that PMN-MDSC accumulation promotes immunosuppression and metastatic liver tumor progression in aging.

[Disclosure of COI by Responsible researcher] Lead presenter is the Responsible researcher.
[Lead presenter: Takao Tsuneki] We have no financial relationships to disclose.

### [J-2096] I-J11-4
### Tumor immune microenvironment/Tumor immune escape (3)

2025 / 9 / 26 (Fri) 12:50-14:05 Room 15/(ANA Crowne Plaza Hotel Kanazawa 3F Zuiun 2) Room 15

[Chairperson]Kazuhiro Kakimi (Dept. Immunology, Kindai Univ. Med)

---

### J-2096
### Type 17 immunity promotes cancer immune evasion through the induction of inflammation

[Speaker]Takumi Itoh (Graduate School of Medicine, Juntendo University) [Co-author]Yuta Hasegawa (Graduate School of Medicine, Juntendo University)、Ryo Hatano (Graduate School of Medicine, Juntendo University)、Yoshiya Horimoto (Graduate School of Medicine, Juntendo University／Tokyo Medical University)、Harumi Saeki (Graduate School of Medicine, Juntendo University)、Kazuyoshi Takeda (Graduate School of Medicine, Juntendo University)、Shogo Ehata (Wakayama Medical University)、Shuji Matsuoka (Graduate School of Medicine, Juntendo University)、Kei Ohnuma (Graduate School of Medicine, Juntendo University)、Chikao Morimoto (Graduate School of Medicine, Juntendo University)

Inflammation is a key driver of cancer progression. In particular, infiltration of neutrophils and myeloid-derived suppressor cells (MDSCs) impairs cytotoxic T lymphocyte (CTL) activity, leading to resistance to immune checkpoint blockade (ICB) therapy. However, the mechanisms promoting such inflammation remain unclear. We focused on type 17 cytokines (IL-17, IL-22, and IL-26) secreted by type 17 immune cells involved in neutrophil recruitment. Using transcriptomic analysis, we identified tumor-specific T cells highly expressing IL-26 in ICB-resistant colorectal cancer (CRC) patients. IL-26 was more abundantly expressed than other type 17 cytokines in CRC and enhanced chemokine expression and neutrophil infiltration. Moreover, IL-26 transgenic mice exhibited ICB resistance. Notably, IL-26 induced over 5000-fold chemokine production via epigenetic remodeling, a mechanism distinct from canonical cytokine signaling. As IL-26 is not expressed in mice and remains understudied, our findings highlight its potential as a therapeutic target in inflammation-associated cancers and chronic inflammatory diseases.

[Disclosure of COI by Responsible researcher] Lead presenter is the Responsible researcher.
[Lead presenter: Takumi Itoh] ■Endowed chair: 順天堂大学　免疫病・がん先端治療学講座

### [E-2091] I-E11-4
### Antibody therapy/Immune cell therapy (CAR-T, T cell therapy) (1)

2025 / 9 / 26 (Fri) 12:50-14:05 Room 16/(11F Visage) Room 16

[Chairperson]Shinichiro Motohashi (Dpt. Med. Immunol., Grad. Sch. Med., Chiba U.)

### E-2091
### An anti-CD44v10 antibody exerts antitumor activity in mouse xenograft models of oral squamous cell carcinomas

[Speaker]Ishikawa Kenichiro (Dept. Antibody Drug Development, Tohoku University Grad. Sch. of Med／Dept. Otolaryngology-Head-and-Neck Surgery, Tohoku University Grad. Sch. of Med) [Co-author]Hiroyuki Suzuki (Dept. Antibody Drug Development, Tohoku University Grad. Sch. of Med)、Tomokazu Ohishi (Inst. Microbial Chemistry (BIKAKEN), Laboratory of Oncology)、Manabu Kawada (Inst. Microbial Chemistry (BIKAKEN), Laboratory of Oncology)、Tomohiro Tanaka (Dept. Antibody Drug Development, Tohoku University Grad. Sch. of Med)、Mika K. Kaneko (Dept. Antibody Drug Development, Tohoku University Grad. Sch. of Med)、Akira Ohkoshi (Dept. Otolaryngology-Head-and-Neck Surgery, Tohoku University Grad. Sch. of Med)、Yukinari Kato (Dept. Antibody Drug Development, Tohoku University Grad. Sch. of Med)、Yukio Katori (Dept. Otolaryngology-Head-and-Neck Surgery, Tohoku University Grad. Sch. of Med)

CD44 regulates cell adhesion, proliferation, survival, and stemness, and it possesses the shortest CD44 standard (CD44s) and a variety of CD44 variant (CD44v) isoforms. It is known that the expression of CD44v is restricted in epithelial cells and carcinomas compared to CD44s. We previously developed an anti–CD44v10 monoclonal antibody (mAb), $C_{44}$Mab–18 (IgM, kappa), to recognize the variant exon 10–encoded region. This study generated a mouse $IgG_{2a}$ version of C44Mab–18 ($C_{44}$Mab–18–$mG_{2a}$) to evaluate the antitumor activities against CD44–positive cells. $C_{44}$Mab–18–mG $_{2a}$ exhibited higher reactivity compared with previously established anti–pan CD44 mAb, $C_{44}$Mab–46–$mG_{2a}$ to CD44v3–10–overexpressed CHO–K1 (CHO/CD44v3–10) and oral squamous cell carcinoma cell lines (HSC–2 and SAS) in flow cytometry. $C_{44}$Mab–18–$mG_{2a}$ exerted a high antibody–dependent cellular cytotoxicity against CHO/CD44v3–10, HSC–2, and SAS. Furthermore, administering $C_{44}$Mab–18–$mG_{2a}$ or $C_{44}$Mab–46–$mG_{2a}$ significantly suppressed CHO/CD44v3–10, HSC–2, and SAS xenograft tumor growth compared with the control mouse $IgG_{2a}$. These results indicate that $C_{44}$Mab–18–$mG_{2a}$ could be a promising therapeutic regimen for CD44v10–positive tumors.

[Disclosure of COI by Responsible researcher] Lead presenter is not the Responsible researcher.
[Lead presenter: Ishikawa Kenichiro] We have no financial relationships to disclose.
[Principal Researcher: Yukinari Kato] ■Patent fee: 小野薬品工業株式会社　■Endowed chair: 小野薬品工業株式会社、株式会社ファーマフーズ

▲TOP

### [E-2092] I-E11-4
### Antibody therapy/Immune cell therapy (CAR-T, T cell therapy) (1)

2025 / 9 / 26 (Fri) 12:50-14:05 Room 16/(11F Visage) Room 16

[Chairperson]Shinichiro Motohashi (Dpt. Med. Immunol., Grad. Sch. Med., Chiba U.)

---

### E-2092
### A cancer-specific anti-HER2 monoclonal antibody exhibits high reactivity in immunohistochemistry.

[Speaker]Hiroyuki Suzuki (Dept. Antibody Drug Development, Tohoku Univ. Grad. Sch. of Med.) [Co-author]Tomohiro Tanaka (Dept. Antibody Drug Development, Tohoku Univ. Grad. Sch. of Med.)、Tomokazu Ohishi (Inst. Microbial Chemistry (BIKAKEN), Lab. of Oncology)、Manabu Kawada (Inst. Microbial Chemistry (BIKAKEN), Lab. of Oncology)、Mika K. Kaneko (Dept. Antibody Drug Development, Tohoku Univ. Grad. Sch. of Med.)、Yukinari Kato (Dept. Antibody Drug Development, Tohoku Univ. Grad. Sch. of Med.)

Developing cancer-specific monoclonal antibodies (mAbs) with antitumor efficacy is critical for reducing the adverse effects. We established an anti-HER2 cancer-specific mAb (clone $H_2$CasMab-2, mouse $IgG_1$), which reacts with HER2-positive breast cancer cells, but not normal epithelial cells. In this study, we investigated the antibody-dependent cellular cytotoxicity (ADCC) and complement-dependent cellular cytotoxicity (CDC) using three versions of $H_2$CasMab-2: mouse $IgG_{2a}$ ($H_2$CasMab-2-$mG_{2a}$), mouse-human chimera ($H_2$CasMab-2-$hG_1$), and humanized $IgG_1$ (hum$H_2$CasMab-2). Every version of $H_2$CasMab-2 showed lower ADCC against the breast cancer cells than trastuzumab, whereas they exhibited significantly higher CDC in breast cancer cells than trastuzumab. Furthermore, they demonstrated compatible antitumor effects against tumor xenografts with trastuzumab. In immunohistochemistry, $H_2$CasMab-2 showed higher reactivity against several cancers than the clinically approved PATHWAY HER2 (4B5) test. These findings highlight the distinct roles of antitumor effects of $H_2$CasMab-2 and trastuzumab and suggest a potential direction for the clinical development of $H_2$CasMab-2 for HER2-positive tumors.

[Disclosure of COI by Responsible researcher] Lead presenter is not the Responsible researcher.
[Lead presenter: Hiroyuki Suzuki] ■Endowed chair: 小野薬品工業株式会社、株式会社ファーマフーズ
[Principal Researcher: Yukinari Kato] ■Research funds from company: 小野薬品工業株式会社 ■Patent fee: 小野薬品工業株式会社
■Endowed chair: 小野薬品工業株式会社、株式会社ファーマフーズ

▲TOP

### [E-2093] I-E11-4
### Antibody therapy/Immune cell therapy (CAR-T, T cell therapy) (1)

2025 / 9 / 26 (Fri) 12:50-14:05 Room 16/(11F Visage) Room 16

[Chairperson]Shinichiro Motohashi (Dpt. Med. Immunol., Grad. Sch. Med., Chiba U.)

---

### E-2093
### Development of a high-throughput platform for the rapid identification of neoantigens and their cognate TCRs

[Speaker]Koji Nagaoka (Department of Immunology, Kindai University Faculty of Medicine) [Co-author]Yukari Kobayashi (Department of Immunology, Kindai University Faculty of Medicine)、Kazuhiro Kakimi (Department of Immunology, Kindai University Faculty of Medicine)

Neoantigens (neoAgs) are key targets in cancer immunotherapy. While in silico prediction of their MHC class I binding has advanced, identifying those recognized by tumor-infiltrating lymphocytes (TILs) remains labor-intensive. We developed a high-throughput platform to simultaneously identify neoAgs and their cognate TCRs.
Jurkat reporter cells expressing CD8$\alpha\beta$, Cas9, and an NFAT-driven luciferase-P2A-EGFP cassette, and lacking CD4 and TRAC, were used for screening. Candidate TCRs from scRNA/TCR-Seq of $CD8^+$ TILs were synthesized as TCR$\alpha$-T2A-TCR$\beta$ cassettes tagged with one of four fluorescent proteins. A pool of these TCRs was knocked in simultaneously via HDR.
NeoAgs were predicted from WGS/WES/RNA-Seq data, synthesized as tandem minigenes (TMGs), introduced into EBV-transformed B cells, and co-cultured with reporter cells. Luciferase activity identified the reactive TCR pool and TMG. FACS analysis of $EGFP^+$ cells revealed the fluorescent tag, enabling identification of the TCR. Peptide synthesis from the TMG confirmed the neoAg.
Using this system, we identified neoAgs and cognate TCRs from a lung cancer patient. Our system provides a practical platform for advancing TCR-T cell therapy.

[Disclosure of COI by Responsible researcher] Lead presenter is not the Responsible researcher.
[Lead presenter: Koji Nagaoka] We have no financial relationships to disclose.
[Principal Researcher: Kazuhiro Kakimi] ■Research funds from company: タカラバイオ

English Oral Session

### [E-2094] I-E11-4
### Antibody therapy/Immune cell therapy (CAR-T, T cell therapy) (1)

2025 / 9 / 26 (Fri) 12:50-14:05 Room 16/(11F Visage) Room 16

[Chairperson]Shinichiro Motohashi (Dpt. Med. Immunol., Grad. Sch. Med., Chiba U.)

---

### E-2094
### Prevention of NK cell-mediated reaction against HLA-KO cells by using membrane bound mAbs for inhibitory NK receptors

[Speaker]Masao Itahara (Dept.of Immunology, Institute for Life and Medical Sciences, Kyoto Univ.) [Co-author]Kyoko Masuda (Dept.of Immunology, Institute for Life and Medical Sciences, Kyoto Univ.)、Koji Terada (Dept.of Biochemistry and Molecular Biology, Shiga Univ. of Medical Science)、Yuma Kato (Dept.of Immunology, Institute for Life and Medical Sciences, Kyoto Univ.)、Yasutoshi Agata (Dept.of Biochemistry and Molecular Biology, Shiga Univ. of Medical Science)、Hisashi Arase (Dept.of Immunochemistry, Research Institute for Microbial Diseases, Osaka Univ.)、Hiroshi Kawamoto (Dept.of Immunology, Institute for Life and Medical Sciences, Kyoto Univ.)

Recently, allogeneic CAR-T/TCR-T cell therapy has been well studied. Among them, regenerative cytotoxic T lymphocytes (CTLs) derived from ES/iPS cells are thought to be useful source for CAR-T/TCR-T therapy because of their stable quality. However, in the case of allogeneic transplantation, immune rejection takes place when HLA are mismatched. Although immune rejection mediated by T cells can be avoided if the regenerated grafts are derived from HLA knock-out (KO) ES/iPS cells, NK cells attack the grafts because NK cells sense the loss of HLA class1. Therefore, universal cells that can avoid the immune rejection mediated by both NK cells and CTLs has been expected. We developed four membrane bound monoclonal antibodies (mAbs) that can stimulate each of four inhibitory receptors of NK cells, NKG2A, KIR2DL1, KIR2DL3, and KIR3DL1. These 4 mAbs were able to significantly suppress NK cell activation when CTLs regenerated from HLA-KO ES cells transduced with 4 mAbs and NK cells from healthy donors were co-cultured. We expect that these four mAbs can be widely applied in the field of regenerative medicine and cell therapy.

[Disclosure of COI by Responsible researcher] Lead presenter is not the Responsible researcher.
[Lead presenter: Masao Itahara] We have no financial relationships to disclose.
[Principal Researcher: Hiroshi Kawamoto] ■Profit of stock: リバーセル株式会社　■Research expenses: リバーセル株式会社、大塚製薬株式会社、株式会社　日立製作所

### [E-2095] I-E11-4
### Antibody therapy/Immune cell therapy (CAR-T, T cell therapy) (1)

2025 / 9 / 26 (Fri) 12:50-14:05 Room 16/(11F Visage) Room 16

[Chairperson]Shinichiro Motohashi (Dpt. Med. Immunol., Grad. Sch. Med., Chiba U.)

---

### E-2095
### Development of an Antigen-Independent Dendritic Cell Therapy Using Tumor-Binding Antibodies Induced by Radiation Therapy

[Speaker]Tsuguhide Takeshima (Dept. of Charged Particle Therapy Research, QST) [Co-author]Sumitaka Hasegawa (Dept. of Charged Particle Therapy Research, QST)

Radiation therapy not only kills tumor cells but also modulates the immune microenvironment. In a B16F10-OVA tumor model, we found that tumor-binding antibodies (TBAs) increased in serum seven days after local X-ray irradiation at 8 Gy. Here, we developed an antigen-independent dendritic cell (DC) therapy using these TBAs. Serum collected on day 7 post-irradiation was used to isolate TBAs, which were incubated with bone marrow-derived DCs (BMDCs) via Fc receptor binding to generate TBA-loaded DCs (TBA-DCs). These showed enhanced antigen uptake and maturation. Intratumoral injection of TBA-DCs suppressed tumor growth and induced tumor-specific cytotoxic T lymphocytes (CTLs) more effectively than control DCs. As TBAs reflect each host's tumor antigens, this strategy enables DCs to present patient-specific antigens without prior profiling. This feature makes it suitable for tumors with unknown or variable antigens. By combining radiation-induced antibody responses with DC therapy, TBA-DC offers a promising approach to personalized cancer immunotherapy and may have future clinical potential.

[Disclosure of COI by Responsible researcher] Lead presenter is the Responsible researcher.
[Lead presenter: Takeshima Tsuguhide] We have no financial relationships to disclose.

[E-2096] I-E11-4
Antibody therapy/Immune cell therapy (CAR-T, T cell therapy) (1)

2025 / 9 / 26 (Fri) 12:50-14:05 Room 16/(11F Visage) Room 16

[Chairperson]Shinichiro Motohashi (Dpt. Med. Immunol., Grad. Sch. Med., Chiba U.)

E-2096

### The ***Runx3*** $^{R122C}$ Variant induces Effector $T_{EX}{}^{prog}$ Development and Improves CD8-T cell Anti-tumor Immunity.

[Speaker]Aneela Nomura (Lab. for Immunological Memory, RIKEN／Lab. for Transcriptional Regulation, RIKEN ／Dep of Immun., Tokyo Medical University／YCI lab. for Immunological Transcriptomics, RIKEN ／Lab. for Proteome Homeostasis Research Unit, RIKEN) [Co-author]Ei Wakamatsu (Dep of Immun., Tokyo Medical University)、Hideyuki Yoshida (YCI lab. for Immunological Transcriptomics, RIKEN)、Kazuki Okuyama (Lab. for Transcriptional Regulation, RIKEN)、Sawako Muroi (Lab. for Transcriptional Regulation, RIKEN)、Koshi Imami (Lab. for Proteome Homeostasis Research Unit, RIKEN)、Tadashi Yokosuka (Dep of Immun., Tokyo Medical University)、Ichiro Taniuchi (Lab. for Transcriptional Regulation, RIKEN)、Shiki Takamura (Lab. for Immunological Memory, RIKEN)

Immune checkpoint blockade (ICB) and CAR-T therapies have revolutionized cancer immunotherapy treatments, but patient response rates remain poor, due to CD8 $^{+}$ terminally exhausted T cells, causing clinical relapse. Critically, stem/progenitor-exhausted T cells ($T_{EX}{}^{prog}$) respond to ICB and provide favorable outcomes for patient survival, but the conditions required for their generation remain ill-defined. Here, we demonstrate that the ***Runx3*** $^{R122C}$ CD8$^{+}$ cells preferentially differentiated into tumoral $T_{EX}{}^{prog}$ cells. Interestingly, ***Runx3*** $^{R122C}$ $T_{EX}{}^{prog}$ cells significantly express the proinflammatory proteins IFN-$\gamma$ and Granzyme B, suggesting hybridization of both stemness and effector functions (herein effector $T_{EX}{}^{prog}$). Consequently, the ***Runx3*** $^{R122C}$ variant showed a stronger anti-tumor activity, which was further enhanced by ICB, demonstrating a strong clinical value for ICB treatments. Furthermore, generation of ***Runx3*** $^{R122C}$ CD19-CAR-T cells improved their anti-tumor function and ICB responses. Thus, we have identified a ***Runx3*** $^{R122C}$ variant that promotes the novel development of intra-tumoral effector $T_{EX}{}^{prog}$ cells and a potential route to improving CAR-T cell response to ICB.

[Disclosure of COI by Responsible researcher] Lead presenter is not the Responsible researcher.
[Lead presenter: Aneela Nomura] We have no financial relationships to disclose.
[Principal Researcher: Ei Wakamatsu, Hideyuki Yoshida] We have no financial relationships to disclose.

### [OS9-1] OS9
### Lipid metabolism in caner biology

2025 / 9 / 26 (Fri) 13:50-15:20 Room 6/(Hotel Nikko Kanazawa 3F Kujaku A) Room 6

[Chairpersons]Ai Kotani (Research Institute of Microbial Diseases, The University of Osaka)、Makoto Murakami (The University of Tokyo Graduate School of Medicine)

Alterations in lipid metabolism in cancer are understandable. The phospholipids that make up the cell membrane need to increase significantly due to the larger size of cancer cells. Increased cell proliferation also requires increased lipid synthesis. These may be regarded as requirements for oncogenesis.
On the other hand, in recent years, accumulated studies revealed that changes in lipid metabolism actively drive oncogenesis. For example, the phospholipid hydrolysing enzyme sPLA2, which is essential in lymphomagenesis, is produced by tumour-associated macrophages (TAMs), suggesting that it is involved in oncogenesis through active alterations in lipid metabolism. In hepatocellular carcinoma, lipid accumulation by palmitic acid upregulates PD-L1 expression, suggesting that it induces aggressive tumour cell immune deviation.
In this symposium, we hope to stimulate active discussion and deepen our understanding of oncogenesis actively driven by such changes in lipid metabolism.

---

### OS9-1
### Adrenal Lipoma Formation Induced by Aberrant PIP3 Metabolism

[Speaker]Junko Sasaki (Dept of Biochemical Pathophysiology MRL Science Tokyo) [Co-author]Shogo Yanai (Dept of Biochemical Pathophysiology MRL Science Tokyo)、Hiroaki Kajiho (Dept of Biochemical Pathophysiology MRL Science Tokyo)、Akira Suzuki (Dept of Biochemical Pathophysiology MRL Science Tokyo／Dept of Molecular and Cellular Biology Kobe Univ Sch Med)、Takehiko Sasaki (Dept of Biochemical Pathophysiology MRL Science Tokyo)

The adrenal gland is composed of the cortex and medulla and typically contains no adipose tissue. Intriguingly, lipomatous tumors can occasionally arise within the adrenal gland. Adrenal lipomas are benign lesions characterized by the presence of ectopic adipose tissue and may present clinical risks such as adrenal rupture or hemorrhage. However, the origin of this ectopic fat remains elusive, partly due to the lack of appropriate animal models. Phosphatidylinositol 3,4,5-trisphosphate (PIP3) is a critical mediator of cellular proliferation, differentiation, and metabolism. PIP3 levels are tightly regulated by the balance between synthesis by PI3-kinases and degradation by phosphatases. Dysregulation of PIP3 signaling is implicated in various pathologies, including cancer and metabolic disorders. However, its role in adrenal biology remains unclear. In this study, we report that genetic ablation of a PIP3 phosphatase in the adrenal cortex leads to the development of adrenal lipomas in mice. These findings suggest a novel link between PIP3 signaling and ectopic adipogenesis in the adrenal gland and provide a valuable animal model for further investigation.

[Disclosure of COI by Responsible researcher] Lead presenter is not the Responsible researcher.
[Lead presenter: Junko Sasaki] We have no financial relationships to disclose.
[Principal Researcher: Takehiko Sasaki] We have no financial relationships to disclose.

▲TOP

### [OS9-2] OS9
### Lipid metabolism in caner biology

2025 / 9 / 26 (Fri) 13:50-15:20 Room 6/(Hotel Nikko Kanazawa 3F Kujaku A) Room 6

[Chairpersons]Ai Kotani (Research Institute of Microbial Diseases, The University of Osaka)、Makoto Murakami (The University of Tokyo Graduate School of Medicine)

Alterations in lipid metabolism in cancer are understandable. The phospholipids that make up the cell membrane need to increase significantly due to the larger size of cancer cells. Increased cell proliferation also requires increased lipid synthesis. These may be regarded as requirements for oncogenesis.
On the other hand, in recent years, accumulated studies revealed that changes in lipid metabolism actively drive oncogenesis. For example, the phospholipid hydrolysing enzyme sPLA2, which is essential in lymphomagenesis, is produced by tumour-associated macrophages (TAMs), suggesting that it is involved in oncogenesis through active alterations in lipid metabolism. In hepatocellular carcinoma, lipid accumulation by palmitic acid upregulates PD-L1 expression, suggesting that it induces aggressive tumour cell immune deviation.
In this symposium, we hope to stimulate active discussion and deepen our understanding of oncogenesis actively driven by such changes in lipid metabolism.

---

### OS9-2
### Roles of the PLA2 family in cancer

[Speaker]Makoto Murakami (Grad. Sch. Med., Univ. Tokyo)

Among the phospholipase A2 (PLA2) superfamily, the secreted PLA2 (sPLA2) family consists of 11 isoforms in mammals. Individual sPLA2s have unique enzymatic specificity toward phospholipid substrates and display distinct tissue/cellular distributions, suggesting their distinct physiological roles. Recent studies using knockout mice for all sPLA2s have revealed their roles in various biological events. Mass spectrometric lipidomics has enabled us to identify target substrates and products of individual sPLA2s in given tissue microenvironments. sPLA2s hydrolyze not only phospholipids in the plasma membrane of neighboring cells, but also extracellular phospholipids such as those in extracellular vesicles (EVs). lipoproteins, food and microbiota, thereby exacerbating or ameliorating various diseases. In addition to facilitating the generation of fatty acid- or lysophospholipid-derived lipid mediators, sPLA2s can modify the secretion, uptake, and functions of EVs. In this talk, I will make an overview of our current understanding of the roles of sPLA2s in cancer in the context of sPLA2-driven modification of EVs.

[Disclosure of COI by Responsible researcher] Lead presenter is the Responsible researcher.
[Lead presenter: Makoto Murakami] ■Research funds from company: 小野薬品工業株式会社

### [OS9-3] OS9
### Lipid metabolism in caner biology

2025 / 9 / 26 (Fri) 13:50-15:20 Room 6/(Hotel Nikko Kanazawa 3F Kujaku A) Room 6

[Chairpersons]Ai Kotani (Research Institute of Microbial Diseases, The University of Osaka)、Makoto Murakami (The University of Tokyo Graduate School of Medicine)

Alterations in lipid metabolism in cancer are understandable. The phospholipids that make up the cell membrane need to increase significantly due to the larger size of cancer cells. Increased cell proliferation also requires increased lipid synthesis. These may be regarded as requirements for oncogenesis.
On the other hand, in recent years, accumulated studies revealed that changes in lipid metabolism actively drive oncogenesis. For example, the phospholipid hydrolysing enzyme sPLA2, which is essential in lymphomagenesis, is produced by tumour-associated macrophages (TAMs), suggesting that it is involved in oncogenesis through active alterations in lipid metabolism. In hepatocellular carcinoma, lipid accumulation by palmitic acid upregulates PD-L1 expression, suggesting that it induces aggressive tumour cell immune deviation.
In this symposium, we hope to stimulate active discussion and deepen our understanding of oncogenesis actively driven by such changes in lipid metabolism.

---

### OS9-3
### Lysophosphatidic acid-mediated cancer progression and targeting of its receptor LPAR1

[Speaker]Satoshi Takagi (Div. Exp. Chemother., Cancer Chemother. Ctr., JFCR) [Co-author]Sumie Koike (Div. Exp. Chemother., Cancer Chemother. Ctr., JFCR)、Ai Takemoto (Div. Exp. Chemother., Cancer Chemother. Ctr., JFCR)、Ryohei Katayama (Div. Exp. Chemother., Cancer Chemother. Ctr., JFCR／Dept. CBMS, Grad. Sch. Front. Sci., The Univ. of Tokyo)

Lysophosphatidic acid (LPA) is a bioactive phospholipid that is produced during the synthesis of cell membranes and is known as a robust extracellular signaling molecule present in all eukaryotic tissues and plasma. LPA exerts differential signaling through the interaction with its receptors, LPAR1-6, mediating cellular responses, such as cell proliferation, migration, inflammation, and wound healing. In particular, LPAR1 knockout mice are protected from bleomycin-induced lung fibrosis by reducing vascular leakage and fibroblast recruitment, suggesting the LPA-LPAR1 pathway as a promising therapeutic target for idiopathy pulmonary fibrosis. We have previously reported that LPAR1 is upregulated in osteosarcoma (OS), a primary malignant bone cancer with a high rate of pulmonary metastasis, and that pharmacological inhibition of LPAR1 prevents OS metastasis in xenograft models (Oncogene, 2021).In this symposium, we would like to introduce the important role of LPA-LPAR1 axis in OS and other cancer progression, and the utility of LPAR1 antagonists as a novel therapeutic intervention in cancer treatment. Collaborator: Keisuke Ae (Orthopedic Oncology, Cancer Inst Hosp, JFCR)

[Disclosure of COI by Responsible researcher] Lead presenter is not the Responsible researcher.
[Lead presenter: Satoshi Takagi] We have no financial relationships to disclose.
[Principal Researcher: Ryohei Katayama] ■Patent fee: 栄研化学　■Research expenses: 中外製薬、TOPPAN、UBE、日本化薬

### [OS9-4] OS9
### Lipid metabolism in caner biology

2025 / 9 / 26 (Fri) 13:50-15:20 Room 6/(Hotel Nikko Kanazawa 3F Kujaku A) Room 6

[Chairpersons]Ai Kotani (Research Institute of Microbial Diseases, The University of Osaka)、Makoto Murakami (The University of Tokyo Graduate School of Medicine)

Alterations in lipid metabolism in cancer are understandable. The phospholipids that make up the cell membrane need to increase significantly due to the larger size of cancer cells. Increased cell proliferation also requires increased lipid synthesis. These may be regarded as requirements for oncogenesis.
On the other hand, in recent years, accumulated studies revealed that changes in lipid metabolism actively drive oncogenesis. For example, the phospholipid hydrolysing enzyme sPLA2, which is essential in lymphomagenesis, is produced by tumour-associated macrophages (TAMs), suggesting that it is involved in oncogenesis through active alterations in lipid metabolism. In hepatocellular carcinoma, lipid accumulation by palmitic acid upregulates PD-L1 expression, suggesting that it induces aggressive tumour cell immune deviation.
In this symposium, we hope to stimulate active discussion and deepen our understanding of oncogenesis actively driven by such changes in lipid metabolism.

---

### OS9-4
### Lipid Metabolism Shapes the Onco-Immune Landscape and Determines Immunotherapy Response in Hepatocellular Carcinoma

[Speaker]Takahiro Kodama (Dept. Gastro Hepatol. Osaka Univ. Sch. Med)

Lipid metabolism is a key driver of hepatocellular carcinoma (HCC) pathogenesis. Using transposon mutagenesis in MASLD-HCC models, we identified candidate cancer genes, showing that aberrant lipid signaling promotes steatosis, oxidative stress, and progenitor activation. Loss of Hippo signaling via Sav1 deletion and Yap activation accelerated tumorigenesis. Multiomics analyses of human nonviral HCC revealed a steatotic subtype with an immune-enriched but immune-exhausted microenvironment, characterized by PD-L1 upregulation and infiltration of immunosuppressive macrophages and fibroblasts. Steatotic HCC was detectable by chemical-shift MRI and showed better responses to anti-PD-L1/VEGF therapy. RNA sequencing and spatial transcriptomics of pre-treatment tumors revealed that nonresponders showed TIL depletion and NRF2 activation. NRF2 promoted COX2-mediated immune exhaustion and resistance, reversible by NRF2 or COX2 inhibition. p62/NRF2 activation, exacerbated after anti-PD-1 therapy in NASH-HCC, underlies poor ICI efficacy. Targeting lipid-driven NRF2/COX2 signaling may improve outcomes in metabolic-associated HCC.

[Disclosure of COI by Responsible researcher] Lead presenter is the Responsible researcher.
[Lead presenter: Takahiro Kodama] ■Lecture fee: 中外製薬、アストラゼネカ　■Research expenses: アストラゼネカ

▲TOP

Open Symposia

### [OS9-5] OS9
### Lipid metabolism in caner biology

2025 / 9 / 26 (Fri) 13:50-15:20 Room 6/(Hotel Nikko Kanazawa 3F Kujaku A) Room 6

[Chairpersons]Ai Kotani (Research Institute of Microbial Diseases, The University of Osaka)、Makoto Murakami (The University of Tokyo Graduate School of Medicine)

Alterations in lipid metabolism in cancer are understandable. The phospholipids that make up the cell membrane need to increase significantly due to the larger size of cancer cells. Increased cell proliferation also requires increased lipid synthesis. These may be regarded as requirements for oncogenesis.
On the other hand, in recent years, accumulated studies revealed that changes in lipid metabolism actively drive oncogenesis. For example, the phospholipid hydrolysing enzyme sPLA2, which is essential in lymphomagenesis, is produced by tumour-associated macrophages (TAMs), suggesting that it is involved in oncogenesis through active alterations in lipid metabolism. In hepatocellular carcinoma, lipid accumulation by palmitic acid upregulates PD-L1 expression, suggesting that it induces aggressive tumour cell immune deviation.
In this symposium, we hope to stimulate active discussion and deepen our understanding of oncogenesis actively driven by such changes in lipid metabolism.

---

### OS9-5
### LGALS3BP Drives Tumorigenesis via sPLA2-X-Mediated EV Hydrolysis and TAM Uptake via Galectin-3

[Speaker]Akane Kanamori (Reg. Infect. Cancer, Res. Inst. for Microbial Diseases, Osaka Univ.／Dept. Mol. Life Sci., Tokai Univ. Sch. of Med.)
[Co-author]Kai Kudo (Dept. Mol. Life Sci., Tokai Univ. Sch. of Med.)、Ai Kotani (Reg. Infect. Cancer, Res. Inst. for Microbial Diseases, Osaka Univ.／Dept. Mol. Life Sci., Tokai Univ. Sch. of Med.)

Epstein-Barr virus (EBV) is linked to cancers with poor prognosis, including lymphoma. Recently, we found that tumor-associated macrophages (TAMs) in EBV-positive lymphoma express high levels of secretory phospholipase A2-X (sPLA2-X), which alters tumor-derived extracellular vesicles (EVs) by hydrolyzing their membrane phospholipids. Patient-derived EVs are more susceptible to hydrolysis by sPLA2-X compared to those from healthy individuals, despite similar phospholipid compositions. Proteomic analysis identified LGALS3BP as a patient-specific component. LGALS3BP, secreted by TAMs, forms a "protein corona" on EV membranes, enhancing sPLA2-X's hydrolytic activity via scaffolding interactions. While LGALS3BP alone increases EV uptake by target cells via Galectin-3, its synergy with sPLA2-X significantly amplifies this effect. Treatment with an sPLA2-X inhibitor in LGALS3BP knockout cells markedly reduced tumorigenesis. Thus, LGALS3BP promotes tumorigenesis by driving EV membrane hydrolysis through sPLA2-X activation and enhancing EV uptake via Galectin-3, which accelerates tumor microenvironment formation and malignant communication between tumor cells and TAMs.

[Disclosure of COI by Responsible researcher] Lead presenter is not the Responsible researcher.
[Lead presenter: Akane Kanamori] We have no financial relationships to disclose.
[Principal Researcher: Ai Kotani] We have no financial relationships to disclose.

### [E-2043] I-E14-2
### Cancer drug resistance & strategies to overcome it

2025 / 9 / 26 (Fri) 14:05-15:20 Room 7/(Hotel Nikko Kanazawa 3F Kujaku B) Room 7

[Chairperson]Akihiro Tomida (Genome Res., Cancer Chemother. Ctr., Jpn. Found. Cancer Res.)

---

### E-2043
### Role of miR-145-5p on cisplatin responsiveness in lung cancer cells

[Speaker]Natsaranyatron Singharajkomron (Dept. of Pharmacology & Physiol., Faculty of Pharm. Sci., Chulalongkorn Univ.) [Co-author]Varisa Pongrakhananon (Dept. of Pharmacology & Physiol., Faculty of Pharm. Sci., Chulalongkorn Univ.／Ctr. of Excellence of Preclinical Toxicity & Efficacy of Med. & Chemicals.)

In the last several years, cisplatin has served as a standard treatment for lung cancer. Nevertheless, the acquisition of cisplatin resistance continues, that limits its clinical efficacy. MicroRNAs (miRNAs), crucial epigenetic regulators, have been widely implicated in cancer progression and drug response. In this study, miRNAs that are potentially involved in the DNA damage response was identified, a key pathway associated with cisplatin sensitivity, through differential expression analysis using lung cancer data from TCGA database. Among the candidate miRNAs, miR-145-5p emerged as a notable suppressor, showing substantial downregulation in lung cancer tissues and a strong association with poor clinical outcomes. Functional analysis in lung cancer cell lines revealed that higher expression levels of miR-145-5p were associated with enhanced sensitivity to cisplatin. Importantly, the introduction of synthetic miR-145-5p mimics significantly increased the number of cell death, supporting its role in promoting cisplatin-induced apoptosis. These results support the potential of miR-145-5p as a novel target for future strategies aimed at sensitizing lung cancer cells to cisplatin.

[Disclosure of COI by Responsible researcher] Lead presenter is not the Responsible researcher.
[Lead presenter: Natsaranyatron Singharajkomron] We have no financial relationships to disclose.
[Principal Researcher: Varisa Pongrakhananon] We have no financial relationships to disclose.

### [E-2044] I-E14-2
### Cancer drug resistance & strategies to overcome it

2025 / 9 / 26 (Fri) 14:05-15:20 Room 7/(Hotel Nikko Kanazawa 3F Kujaku B) Room 7

[Chairperson]Akihiro Tomida (Genome Res., Cancer Chemother. Ctr., Jpn. Found. Cancer Res.)

---

### E-2044
### Anti-Cholangiocarcinoma Activity of the Repurposed Antiemetic Drug Thiethylperazine

[Speaker]Kyaw Z. Myint (Dept. of Biochem., Faculty of Sci., Mahidol Univ, Bangkok, Thailand) [Co-author]Sunhapas Soodvilai (Dept. of Physiol., Faculty of Sci., Mahidol Univ, Bangkok, Thailand)、Somchai Pinlaor (Dept. of Parasitolology, Khon Kaen Univ., Khon Kaen, Thailand)、Nattawadee Panyain (Dept. of Biochem., Faculty of Sci., Mahidol Univ, Bangkok, Thailand)、Sakonwan Kuhaudomlap (Dept. of Biochem., Faculty of Sci., Mahidol Univ, Bangkok, Thailand)、Porpimol Methinukul (Dept. of Veterinary Tech., Kasetsart Univ., Bangkok, Thailand)、Parichat Thammaratthi (Dept. of Clin. Chemistry, Chulalongkorn Univ., Bangkok, Thailand)、Surakit Nathisuwan (Dept. of Pharm., Faculty of Pharm., Mahidol Univ., Bangkok, Thailand)、Vorthon Sawaswong (Dept. of Biochem., Faculty of Sci., Mahidol Univ, Bangkok, Thailand)、Utid Suriya (Dept. of Biochem., Faculty of Sci., Mahidol Univ, Bangkok, Thailand)、David O. Bates (Div. of Cancer&Stem Cells, Sch. of Med., UON, Nottingham, UK)、Rutaiwan Tohtong (Dept. of Biochem., Faculty of Sci., Mahidol Univ, Bangkok, Thailand)

Cholangiocarcinoma (CCA) is an aggressive cancer with limited therapeutic options; hence identification of potent drugs is essential. Screening 2681 FDA-approved drugs identified the antiemetic Thiethylperazine (TEP) as a potent candidate. TEP selectively killed CCA cell lines while being less toxic to normal cholangiocytes cell line, confirmed by MTT, clonogenic, and spheroid assays. Target prediction analyses suggested interesting potential targets, which are involved in important cancer-related mechanisms. Among them, KIF11 (Kinesin Family Member 11) was found to be a potential TEP target. KIF11 is a molecular motor protein that plays a crucial role in mitosis, the process of cell division and involved in the formation and function of the mitotic spindle. KIF11 knockout proved lethal to CCA cells (Depmap database), and its mRNA is highly expressed in CCA tumors compared to normal tissue (TCGA database), indicating its importance in CCA progression. Further investigation into TEP's inhibition of KIF11 is warranted, as success could rapidly advance this repurposed drug towards clinical application, offering a new therapeutic avenue targeting CCA progression.

[Disclosure of COI by Responsible researcher] Lead presenter is not the Responsible researcher.
[Lead presenter: Kyaw Z. Myint] We have no financial relationships to disclose.
[Principal Researcher: Rutaiwan Tohtong] We have no financial relationships to disclose.

### [E-2045] I-E14-2
### Cancer drug resistance & strategies to overcome it

2025 / 9 / 26 (Fri) 14:05-15:20 Room 7/(Hotel Nikko Kanazawa 3F Kujaku B) Room 7

[Chairperson]Akihiro Tomida (Genome Res., Cancer Chemother. Ctr., Jpn. Found. Cancer Res.)

---

### E-2045
### Depletion of MLKL Augments Chemotherapy-Induced Apoptosis in Colorectal Cancer by Delaying TNFR-I Endosomal Trafficking

[Speaker]Sasiprapa Sonkaew (Faculty of Allied Health Sciences, Chulalongkorn University, Bangkok, Thailand) [Co-author]Nattaya Duangthim (Faculty of Allied Health Sciences, Chulalongkorn University, Bangkok, Thailand)、Choopet Nitsakulthong (Chulalongkorn University Laboratory Animal Center, Chulalongkorn University, Bangkok, Thailand)、Jeeraprapha Duangbupha (Chulalongkorn University Laboratory Animal Center, Chulalongkorn University, Bangkok, Thailand)、Swati Choksi (NCI, NIH, Bethesda, MD, United States)、Zheng-Gang Liu (NCI, NIH, Bethesda, MD, United States)、Siriporn Jitkaew (Faculty of Allied Health Sciences, Chulalongkorn University, Bangkok, Thailand)

Mixed Lineage Kinase domain-like protein (MLKL), a key regulator of necroptosis, has been reported to contribute to cancer progression and therapeutic response. Interestingly, our bioinformatics analysis found a significant association between high MLKL expression and poor therapeutic outcomes in cholangiocarcinoma (CCA) and colorectal cancer (CRC) patients treated with 5-Fluorouracil (5-FU), suggesting an important role of MLKL in 5-FU response. Although 5-FU is widely used for cancer treatment, its efficacy remains suboptimal. Therefore, understanding the mechanisms underlying 5-FU response is crucial for improving treatment outcomes. In this study, we identified MLKL as a crucial modulator that limits 5-FU efficacy. Depletion of MLKL promoted 5-FU-induced apoptosis in both in vitro and in vivo CRC models. Mechanistically, MLKL suppression promoted TNF-$\alpha$/TNFR-I-mediated apoptosis by prolonging TNFR-I localization in early endosomes and delaying its degradation after 5-FU treatment. Our findings provide insights into the role of MLKL in 5-FU response, suggesting MLKL as a potential therapeutic target to improve 5-FU efficacy in cancer treatment.

[Disclosure of COI by Responsible researcher] Lead presenter is not the Responsible researcher.
[Lead presenter: Sasiprapa Sonkaew] We have no financial relationships to disclose.
[Principal Researcher: Siriporn Jitkaew] We have no financial relationships to disclose.

### [E-2046] I-E14-2
### Cancer drug resistance & strategies to overcome it

2025 / 9 / 26 (Fri) 14:05-15:20 Room 7/(Hotel Nikko Kanazawa 3F Kujaku B) Room 7

[Chairperson]Akihiro Tomida (Genome Res., Cancer Chemother. Ctr., Jpn. Found. Cancer Res.)

---

### E-2046
### tRNA modifications drive glioma oncogenesis and are targets for therapy

[Speaker]Sherif Rashad (Graduate School of Biomedical Engineering, Tohoku University.) [Co-author]Atsushi Nakayashiki (Tohoku University Graduate School of Medicine.)、Arata Nagai (Tohoku University Graduate School of Medicine.)、Abdulrahman Mousa (Tohoku University Graduate School of Medicine.)、Kuniyasu Niizuma (Graduate School of Biomedical Engineering, Tohoku University.／Tohoku University Graduate School of Medicine.)

Glioblastoma (GB) has a median survival of 14 months despite modern advances in treatment. Thus, there is a pressing need to identify novel therapeutic targets and develop new modalities for GB therapy. tRNA modifications emerged as a potential therapeutic target for cancer treatment. We profiled tRNA modifications in various grades of glioma using high throughput mass spectrometry. We identified 20 significantly enriched modifications in GB and Grade IV astrocytoma compared to lower grade tumors. We identified a modification pathway modulated by ALKBH1 that synthesizes the modifications hm5C and f5C to be critical for glioma oncogenesis. Overexpressing ALKBH1 led to the induction of stem cell features in rodent and human glioma cell lines. ALKBH1 modulated mRNA translation in glioma cells via the impact of hm5C and f5C on codon decoding. ALKBH1 expression was associated with shorter survival in rodent glioma models. scRNA-seq analysis revealed that ALKBH1 enhanced glioma heterogeneity, immune-evasion, and induced a neuron-like phenotype in glioma cells, promoting their aggressiveness and oncogenic fitness. tRNA modifications appear to be promising targets for glioma therapy.

[Disclosure of COI by Responsible researcher] Lead presenter is the Responsible researcher.
[Lead presenter: Sherif Rashad] We have no financial relationships to disclose.

### [E-2047] I-E14-2
### Cancer drug resistance & strategies to overcome it

2025 / 9 / 26 (Fri) 14:05-15:20 Room 7/(Hotel Nikko Kanazawa 3F Kujaku B) Room 7

[Chairperson]Akihiro Tomida (Genome Res., Cancer Chemother. Ctr., Jpn. Found. Cancer Res.)

---

### E-2047
### Pre-clinical efficacy evaluation of combined AKT inhibition and chemotherapy in mouse prostate cancer

[Speaker]Hirotsugu Uemura (Dept. of Urol. Kindai Univ. Faculty of Med.) [Co-author]Marco Develasco (Dept. of Genome Biol. Kindai Univ. Faculty of Med.)、Kazuko Sakai (Dept. of Genome Biol. Kindai Univ. Faculty of Med.)、Takafumi Minami (Dept. of Urol. Kindai Univ. Faculty of Med.)、Saizo Fujimoto (Dept. of Urol. Kindai Univ. Faculty of Med.)、Mamoru Hashimoto (Dept. of Urol. Kindai Univ. Faculty of Med.)、Shingo Toyoda (Dept. of Urol. Kindai Univ. Faculty of Med.)、Kazuhiro Yoshimura (Dept. of Urol. Kindai Univ. Faculty of Med.)、Kazuto Nishio (Dept. of Genome Biol. Kindai Univ. Faculty of Med.)、Kazutoshi Fujita (Dept. of Urol. Kindai Univ. Faculty of Med.)

Taxane chemotherapy is the standard treatment for metastatic castration-resistant prostate cancer (mCRPC). However, hyperactivation of AKT due to PTEN loss may reduce the efficacy of taxane therapy. Capivasertib (Capiva, AZD5363) is a potent oral pan-AKT inhibitor that has shown promise against prostate cancer (PCa) in preclinical and early-phase clinical studies. Capiva is currently being evaluated along with docetaxel (Dtx) in phase 3 trials for treating patients with mCRPC. We assessed the efficacy of Dtx plus Capiva in transgenic mouse models of PTEN-deficient PCa. Adding Capiva to Dtx enhanced tumor growth inhibition (TGI) in PTEN-positive GDAs, with greater effects observed in PTEN-deficient GDAs. In autochthonous PCa models, adding Capiva to Dtx increased TGI in both the HSPC and CRPC settings. Capiva inhibited the phosphorylation of PRAS40 and S6 downstream AKT molecules in tumors. Adding capivasertib to Dtx resulted in longer survival and extended tumor doubling times compared with DTx alone after progression to ADT and apalutamide in a late-stage CRPC model. In conclusion, adding Capiva to DTx significantly enhances TGI and the therapeutic efficacy of PTEN-deficient PCa.

[Disclosure of COI by Responsible researcher] Lead presenter is the Responsible researcher.
[Lead presenter: Hirotsugu Uemura] ■Research funds from company: Astrazeneca, Ono Pharmaceutical, Astellas, and Takeda ■Lecture fee: Bayer, Bristol Myers Squibb, Eisai, Janssen, MSD, Pfizer, and Sanofi

### [E-2048] I-E14-2
### Cancer drug resistance & strategies to overcome it

2025 / 9 / 26 (Fri) 14:05-15:20 Room 7/(Hotel Nikko Kanazawa 3F Kujaku B) Room 7

[Chairperson]Akihiro Tomida (Genome Res., Cancer Chemother. Ctr., Jpn. Found. Cancer Res.)

### E-2048
### Targeting Telomeres by Lurbinectedin for ALT Cancer Therapy

[Speaker]HsuehPing C. Chu (Institute of Molecular and Cellular Biology, National Taiwan University) [Co-author]Liv W. Chen (Institute of Molecular and Cellular Biology, National Taiwan University)、ChenChia Lo (Institute of Molecular and Cellular Biology, National Taiwan University)、ChinHua Tai (Institute of Molecular and Cellular Biology, National Taiwan University)、KoJung Chen (Institute of Molecular and Cellular Biology, National Taiwan University)

Sustained telomere lengthening is a hallmark of cancer. While most cancers utilize telomerase to maintain telomeres, a subset relies on the Alternative Lengthening of Telomeres (ALT) pathway, a telomerase-independent, recombination-based mechanism. ALT is common in sarcomas, gliomas, and neuroendocrine tumors and is often linked to poor responses to conventional therapies.Our study shows that ALT-positive cancer cells are resistant to doxorubicin, cisplatin, and etoposide, but remain sensitive to lurbinectedin. Chem-seq analysis reveals that lurbinectedin preferentially binds to telomeres, GC-rich regions, and DNA G-quadruplexes in ALT cancer cells, enhancing telomeric R-loops, inducing DNA damage, and resulting in telomere dysfunction. The endonuclease XPF is recruited to R-loops and is essential for lurbinectedin-induced DNA breaks at telomeres.Notably, combining lurbinectedin with the ATR inhibitor AZD6738 synergistically reduces ALT cell viability by promoting telomere damage and blocking ATR-mediated DNA repair. These findings highlight a promising therapeutic strategy for targeting ALT-positive cancers through dual disruption of telomere maintenance and DNA repair pathways.

[Disclosure of COI by Responsible researcher] Lead presenter is the Responsible researcher.
[Lead presenter: HsuehPing C. Chu] We have no financial relationships to disclose.

### [J-2061] I-J4-2
### Oncogenes & tumor-suppressor genes (4)

2025 / 9 / 26 (Fri) 14:05-15:20 Room 9/(Hotel Nikko Kanazawa 3F Lumiere) Room 9

[Chairperson]Genta Nagae (Gen. Sci. Med., RCAST, Univ. Tokyo)

---

### J-2061
### Post-transcriptional regulation of the Hippo-YAP pathway

[Speaker]Junji Otani (Div. of MCB, Kobe Univ. Grad. Sch. of Med.) [Co-author]Riko Tokita (Div. of MCB, Kobe Univ. Grad. Sch. of Med.)、Miki Nishio (Div. of MCB, Kobe Univ. Grad. Sch. of Med.)、Tomohiko Maehama (Dept. of Biochem., Showa Univ. Sch. of Med.)、Akira Suzuki (Div. of MCB, Kobe Univ. Grad. Sch. of Med.)

Aberrant activation of the transcription coactivator YAP occurs in various cancers. In mice lacking Hippo pathway components that repress YAP, cancers develop early and frequently. These findings indicate that the Hippo-YAP pathway drives cancer. In this study, we identified drug target molecules affecting YAP transcriptional activity. We constructed a reporter cell line to monitor YAP activity and screened siRNA libraries targeting all human genes. Knockdown of SAMD4B, an RNA-binding protein involved in mRNA degradation and translational repression, most strongly suppressed YAP activity. Conversely, SAMD4B overexpression activated YAP. We found that VGLL4, a known YAP repressor, is suppressed by SAMD4B, which activates YAP via post-transcriptional regulation of VGLL4. In an intrahepatic cholangiocarcinoma (ICC) mouse model, liver-specific Samd4b overexpression caused liver enlargement and rapid tumor formation. ICC developed as early as two weeks after birth, showing the strong tumor-promoting effect of Samd4b in vivo.

[Disclosure of COI by Responsible researcher] Lead presenter is not the Responsible researcher.
[Lead presenter: Junji Otani] We have no financial relationships to disclose.
[Principal Researcher: Akira Suzuki] We have no financial relationships to disclose.

### [J-2062] I-J4-2
### Oncogenes & tumor-suppressor genes (4)

2025 / 9 / 26 (Fri) 14:05-15:20 Room 9/(Hotel Nikko Kanazawa 3F Lumiere) Room 9

[Chairperson]Genta Nagae (Gen. Sci. Med., RCAST, Univ. Tokyo)

---

### J-2062
### Secretoglobin superfamily protein SCGB3A1 suppresses rhabdomyosarcoma development.

[Speaker]Mitsuhiro Yoneda (Dept. of Oncology, Grad. Sch. of Biomed. Sci. Nagasaki Univ.) [Co-author]Kiyoshi Yasui (Dept. of Oncology, Grad. Sch. of Biomed. Sci. Nagasaki Univ.)、Hiroaki Kajiyama (Dept. of Obsterics & Gynecol., Grad. Sch. of med. Nagoya Univ.)、Ward M. Jerrold (Global VetPathology)、Shioko Kimura (Cancer Innovation Lab., Natl. Cancer Inst., Natl. Inst. of Health)、Hiroaki Ikeda (Dept. of Oncology, Grad. Sch. of Biomed. Sci. Nagasaki Univ.)

Secretoglobin (SCGB) superfamily consists of cytokine-like secreted proteins with small molecular weight. Among these, SCGB3A1 is highly expressed in the airway epithelial cells. SCGB3A1 expression is decreased in various cancer tissues due to promoter hypermethylation. However, the biological role of SCGB3A1 in cancer development remains largely unknown. We found that the spontaneous tumor incidence in ***Scgb3a1***$^{-/-}$ female mice was higher (12/27, 44.4 %) than that in wild-type mice (2/19, 10.5 %) at 18-month-old age. IHC staining with antibodies against myogenin, α-sarcomeric actin, and α-SMA was positive in tumors, demonstrating that all tumors developed in the mice were rhabdomyosarcomas. In addition, recombinant mouse SCGB3A1 protein suppressed the proliferation of rhabdomyosarcoma cells derived from wild-type mice ***in vitro***. Moreover, SCGB3A1 administration suppressed rhabdomyosarcoma development in ***Scgb3a1***$^{-/-}$ mice, whereas no ***in vivo*** growth inhibition was observed in wild-type mice.These results suggest that SCGB3A1 is a candidate tumor suppressor and a candidate target of novel anti-tumor therapy in rhabdomyosarcoma.

[Disclosure of COI by Responsible researcher] Lead presenter is not the Responsible researcher.
[Lead presenter: Mitsuhiro Yoneda] We have no financial relationships to disclose.
[Principal Researcher: Hiroaki Ikeda] We have no financial relationships to disclose.

### [J-2063] I-J4-2
### Oncogenes & tumor-suppressor genes (4)

2025 / 9 / 26 (Fri) 14:05-15:20 Room 9/(Hotel Nikko Kanazawa 3F Lumiere) Room 9

[Chairperson]Genta Nagae (Gen. Sci. Med., RCAST, Univ. Tokyo)

---

### J-2063
### GPR87 contributes to the malignant phenotype of esophageal squamous cell carcinoma by activating the Akt pathway.

[Speaker]Kosuke Hirose (Dept. of Surg., Kyushu Univ. Beppu Hosp.) [Co-author]Hajime Otsu (Dept. of Surg., Kyushu Univ. Beppu Hosp.)、Takashi Ofuchi (Dept. of Surg., Kyushu Univ. Beppu Hosp.)、Shohei Shibuta (Dept. of Surg., Kyushu Univ. Beppu Hosp.)、Yuki Ando (Dept. of Surg., Kyushu Univ. Beppu Hosp.)、Qingjiang Hu (Dept. of Surg., Kyushu Univ. Beppu Hosp.)、Yusuke Yonemura (Dept. of Surg., Kyushu Univ. Beppu Hosp.)、Koshi Mimori (Dept. of Surg., Kyushu Univ. Beppu Hosp.)

**Introduction** G-protein-coupled receptors (GPCRs) are key drug targets in cancer. The role of GPR87 in esophageal squamous cell carcinoma (ESCC) remains unclear. **Purpose** To investigate the significance of GPR87 expression in amplified chromosome in ESCC. **Methods** 1) TCGA analysis (n=71) identified amplified chromosomes in ESCC. We focused on GPR87 on chromosome 3, assessing its expression, copy number variation, prognosis, and pathways via Gene Set Enrichment Analysis (GSEA). 2)mRNA and protein expression levels were also analyzed in ESCC specimens. 3)GPR87-overexpressing cell lines were generated to assess phenotypic changes. **Results** Chromosome 3 amplification in ESCC was confirmed, consistent with a previous report. High GPR87 expression was a poor prognostic factor (HR=2.83 [1.01, 7.93], p=0.03).GPR87 mRNA was upregulated in tumors (p=0.067), and the protein was expressed heterogeneously in cytoplasm. GSEA revealed enrichment of P53 and PI3K-Akt-mTOR pathways. GPR87 overexpression enhanced proliferation and anti-apoptotic potential, increasing phosphorylated Akt levels. **Conclusion** GPR87 overexpression may promote ESCC malignancy via Akt activation.

[Disclosure of COI by Responsible researcher] Lead presenter is not the Responsible researcher.
[Lead presenter: Kosuke Hirose] We have no financial relationships to disclose.
[Principal Researcher: Koshi Mimori] We have no financial relationships to disclose.

▲TOP

### [J-2064] I-J4-2
### Oncogenes & tumor-suppressor genes (4)

2025 / 9 / 26 (Fri) 14:05-15:20 Room 9/(Hotel Nikko Kanazawa 3F Lumiere) Room 9

[Chairperson]Genta Nagae (Gen. Sci. Med., RCAST, Univ. Tokyo)

---

### J-2064
### Pathogenic annotation of variants of unknown significance (VUS) based on real-world data and machine learning tools

[Speaker]Teruki Tsunashima (Div. of Genome Biol., Natl. Cancer Ctr. Res. Inst.／Dept. of Clin Lab., Yokohama City Univ. Med. Ctr.) [Co-author]Kosuke Maruyama (Div. of Genome Biol., Natl. Cancer Ctr. Res. Inst.)、Shigenari Nukaga (Div. of Genome Biol., Natl. Cancer Ctr. Res. Inst.)、Yu Hamaguchi (Div. of Genome Biol., Natl. Cancer Ctr. Res. Inst.)、Takashi Kohno (Div. of Genome Biol., Natl. Cancer Ctr. Res. Inst.)、Takashi Nakaoku (Div. of Genome Biol., Natl. Cancer Ctr. Res. Inst.)

BACKGROUNDPathogenic annotation of VUS is essential for advancing cancer genomic medicine. Assuming structural activation mechanisms are shared among receptor-type tyrosine kinases (RTKs), we identified VUSs at conserved sites across RTKs as potential pathogenic mutations.METHODUsing the AACR GENIE v14.0 dataset, we analyzed missense and in-frame indel mutations in the kinase domains of 41 RTK genes in pan-cancers. We mapped mutations onto aligned sequences, detected significant mutations based on mutation frequency and cross-gene recurrence, and evaluated them using pathogenicity prediction tools.RESULTWe identified two conserved residues with recurrent mutations in multiple genes (e.g., ***PDGFRB***, ***FGFR2***) that remain poorly annotated. These VUSs were predicted as likely pathogenic by AlphaMissense and PrimateAI-3D. Analysis of kinase phosphorylation activity by transient expression in HEK293H cells showed that ***FGFR2***$^{D650G}$ exhibits kinase activity like driver mutations, which was suppressed by FGFR inhibitors.DISCUSSIONThis strategy enables the identification of oncogenic mutations from numerous VUSs. Further functional validation may support their clinical relevance.

[Disclosure of COI by Responsible researcher] Lead presenter is not the Responsible researcher.
[Lead presenter: Teruki Tsunashima] We have no financial relationships to disclose.
[Principal Researcher: Takashi Nakaoku] We have no financial relationships to disclose.

### [J-2065] I-J4-2
### Oncogenes & tumor-suppressor genes (4)

2025 / 9 / 26 (Fri) 14:05-15:20 Room 9/(Hotel Nikko Kanazawa 3F Lumiere) Room 9

[Chairperson]Genta Nagae (Gen. Sci. Med., RCAST, Univ. Tokyo)

---

### J-2065
### ARID1A loss promotes proliferation of normal hepatocytes but suppresses inflammation-driven hepatocarcinogenesis

[Speaker]Yosuke Fujii (Kyoto University, Graduate School of Medicine, Gastroenterology and Hepatology) [Co-author]Atsushi Takai (Kyoto University, Graduate School of Medicine, Gastroenterology and Hepatology)、Hiroshi Seno (Kyoto University, Graduate School of Medicine, Gastroenterology and Hepatology)

***ARID1A*** is one of the tumor suppressor genes frequently mutated in hepatocellular carcinoma. On the other hand, we have previously identified clonal ***ARID1A*** loss-of-function mutations in regenerative nodules of cirrhotic livers, raising a hypothesis that ***ARID1A*** loss in normal hepatocytes contributes to clonal cell expansion and inhibition of hepatocarcinogenesis. To investigate a role of ARID1A in hepatocytes for hepatocarcinogenesis, we generated liver-specific ***Arid1a*** knockout mice (***Alb-Cre;Arid1a***$^{floxed}$, ***Arid1a***-LKO) and analyzed the phenotype. Interestingly, ***Arid1a***-LKO mice showed enlarged livers with increased Ki67-positive cells compared to control mice. Transcriptome analysis of ***Arid1a***-LKO liver revealed upregulation of YAP pathway, and increase of nuclear YAP expression was verified by immunohistochemistry. In a chronic liver injury model using thioacetamide, ***Arid1a***-LKO mice exhibited reduced inflammatory cell infiltration and fibrosis. Furthermore, while 41% of control mice developed liver tumors, we could not find any tumors in ***Arid1a***-LKO mice. These results suggest ***ARID1A*** loss in hepatocytes could enhance proliferation but inhibit tumor formation under chronic inflammation.

[Disclosure of COI by Responsible researcher] Lead presenter is not the Responsible researcher.
[Lead presenter: Yosuke Fujii] We have no financial relationships to disclose.
[Principal Researcher: Hiroshi Seno] We have no financial relationships to disclose.

### [J-2066] I-J4-2
### Oncogenes & tumor-suppressor genes (4)

2025 / 9 / 26 (Fri) 14:05-15:20 Room 9/(Hotel Nikko Kanazawa 3F Lumiere) Room 9

[Chairperson]Genta Nagae (Gen. Sci. Med., RCAST, Univ. Tokyo)

---

### J-2066
### Novel candidate driver gene FIRRM (FIGNL1-interacting regulator of recombination and mitosis) in HCC

[Speaker]Yu Takahashi (Kyushu University Hospital Beppu Hospital Department of Surgery) [Co-author]Hajime Otsu (Kyushu University Hospital Beppu Hospital Department of Surgery／Department of Surgery and Science, Kyushu University)、Akinori Tsujimoto (Kyushu University Hospital Beppu Hospital Department of Surgery)、Kazuki Omachi (Kyushu University Hospital Beppu Hospital Department of Surgery)、Shinsaku Itoyama (Kyushu University Hospital Beppu Hospital Department of Surgery)、Koto Kawata (Kyushu University Hospital Beppu Hospital Department of Surgery／Department of Surgery and Science, Kyushu University)、Tomohiko Ikehara (Kyushu University Hospital Beppu Hospital Department of Surgery)、Takashi Ofuchi (Kyushu University Hospital Beppu Hospital Department of Surgery)、Shohei Shibuta (Kyushu University Hospital Beppu Hospital Department of Surgery／Department of Surgery and Science, Kyushu University)、Kosuke Hirose (Kyushu University Hospital Beppu Hospital Department of Surgery／Department of Surgery and Science, Kyushu University)、Qingjiang Hu (Kyushu University Hospital Beppu Hospital Department of Surgery／Department of Surgery and Science, Kyushu University)、Yusuke Yonemura (Kyushu University Hospital Beppu Hospital Department of Surgery／Department of Surgery and Science, Kyushu University)、Takaaki Masuda (Department of Breast and Endocrine Surgical Oncology, Kochi University)、Tomoharu Yoshizumi (Department of Surgery and Science, Kyushu University)、Koshi Mimori (Kyushu University Hospital Beppu Hospital Department of Surgery)

Aim: To identify candidate driver genes on amplified chromosomes in HCC.Methods: We analyzed copy number and mRNA data from 364 TCGA HCC cases using four criteria and identified FIRRM: 1. positive copy number/expression correlation; 2. higher tumor than normal expression; 3. worse overall survival in high-expression cases; 4. localization within amplified regions. We quantified FIRRM by qPCR in 55 HCC samples with multivariate analysis, localized protein by IHC and performed GSEA. FIRRM KO cell lines were evaluated for proliferation, mitotic figures (a-tubulin/pericentrin staining), cell cycle distribution, and pPLK1/other regulators by Western blot in vitro and in vivo.Results: FIRRM was upregulated in HCC and independently predicted poor prognosis. Immunohistochemistry showed strong cytoplasmic/membranous staining. GSEA linked FIRRM to G2/M checkpoint and mitotic spindle gene sets. FIRRM KO suppressed cell proliferation and tumor growth, decreased pPLK1 and other cell cycle proteins, and reduced metaphase numbers and G2/M phase cells.Conclusions: FIRRM enhances PLK1 kinase activity, promotes accurate chromosome segregation and G2 to M transition, and thus drives HCC progression.

[Disclosure of COI by Responsible researcher] Lead presenter is not the Responsible researcher.
[Lead presenter: Yu Takahashi] We have no financial relationships to disclose.
[Principal Researcher: Koshi Mimori] We have no financial relationships to disclose.

### [J-2067] I-J20
### Emerging Computational Approaches in Cancer Research: AI, Omics, & Precision Medicine

2025 / 9 / 26 (Fri) 14:05-15:20 Room 11/(ANA Crowne Plaza Hotel Kanazawa 3F Ohtori (West)) Room 11

[Chairperson]Atsushi Niida (IMSUT)

---

### J-2067
### Transcription Factor Binding Motif Analysis Method at the Single-Cell and Single-Gene Level Using Policy Gradient

[Speaker]Ken Murakami (Res. Inst. of Mol. Path.／Osaka Univ. Inst. for Protein Res.) [Co-author]Mariko Okada (Osaka Univ. Inst. for Protein Res.)

Cells respond to various external environments by adjusting the expression of different genes. This regulation is encoded by combinations of transcription factors, which activate the appropriate genes at the appropriate time. To understand these mechanisms, it is essential to evaluate transcription factor binding effects on gene expression at single-cell and single-gene levels. Recent advances in machine learning have enabled models to predict single-cell gene expression. By analyzing these models and identifying influential transcription factor motifs, researchers can indirectly assess transcription factor impact. Nevertheless, such indirect estimation methods based on regression models do not always provide accurate evaluations of transcription factor contributions. To address this, we propose a novel machine learning approach that more directly evaluates the effect of transcription factor binding motifs using a reinforcement learning technique known as policy gradient. This method enables the prediction of the effects of transcription factor binding motifs on gene expression at the single-cell and single-gene levels.

[Disclosure of COI by Responsible researcher] Lead presenter is not the Responsible researcher.
[Lead presenter: Ken Murakami] We have no financial relationships to disclose.
[Principal Researcher: Mariko Okada] We have no financial relationships to disclose.

▲TOP

### [J-2068] I-J20
### Emerging Computational Approaches in Cancer Research: AI, Omics, & Precision Medicine

2025 / 9 / 26 (Fri) 14:05-15:20 Room 11/(ANA Crowne Plaza Hotel Kanazawa 3F Ohtori (West)) Room 11

[Chairperson]Atsushi Niida (IMSUT)

---

### J-2068
### Computational Identification of lncRNAs Related to Hematological Malignancies via the ***ab initio*** GO method

[Speaker]Tsutomu Mori (Fukushima Med. Univ. Sch. Nur.) [Co-author]Susumu Goyama (Tokyo Univ. Grad. Sch. Front. Sci.)

The importance of long non-coding RNAs (lncRNAs) in hematological malignancies has received increasing attention. However, the identification of lncRNAs involved in hematological cancers has been insufficiently studied. Here, we applied the ***ab initio*** Genetic Orbital (GO) method (Pat. 6820621), an ***in silico*** approach for functional gene analysis without experiments. Using TCGA data, we identified 121 lncRNAs that showed statistically significant mutual information with other genes. Through GO-based calculations and Ingenuity Pathway Analysis (IPA), we identified three lncRNAs - LINC00635, LINC00636, and LINC01591 - that may play pivotal roles in leukemia progression. These molecules are involved in key biological processes, including cell cycle regulation, DNA repair, and immune modulation. Further investigation of these molecules may deepen our understanding of leukemia pathogenesis and suggest novel therapeutic targets. Our results highlight the potential of the ***ab initio*** GO method as a powerful tool in lncRNA research and cancer biology.

[Disclosure of COI by Responsible researcher] Lead presenter is the Responsible researcher.
[Lead presenter: Tsutomu Mori] We have no financial relationships to disclose.

### [J-2069] I-J20
### Emerging Computational Approaches in Cancer Research: AI, Omics, & Precision Medicine

2025 / 9 / 26 (Fri) 14:05-15:20 Room 11/(ANA Crowne Plaza Hotel Kanazawa 3F Ohtori (West)) Room 11

[Chairperson]Atsushi Niida (IMSUT)

---

### J-2069
### Anti-cancer drug response prediction with quantum-classical hybrid machine learning with proper normalization

[Speaker]Takafumi Ito (Dept. Comp. Biol. Med. Sci., Grad. Frontier Sci., Univ. Tokyo) [Co-author]Artem Lysenko (Dept. Biosci., Sch. Sci., Univ. Tokyo)、Tatsuhiko Tsunoda (Dept. Comp. Biol. Med. Sci., Grad. Frontier Sci., Univ. Tokyo／Dept. Biosci., Sch. Sci., Univ. Tokyo)

Quantum-classical Hybrid Machine Learning (QHML) is an emerging research field that combines deep learning and quantum computing and has received increasing attention in recent years. QHML is known to be able to make predictions with high generalization performance even for a small number of data and is expected to be used in predicting drug response, where it is difficult to increase the sample size. However, QHML studies using omics data used in drug response prediction are limited, and it is not clear how gene expression levels, a particularly important data set, can be effectively encoded in the quantum circuit. In this study, we propose a new normalization function that can efficiently pass gene expression data to the quantum circuit. By using gene expression data of cell lines and drug response values, we compared the prediction performance of classical deep learning models and QHML models that combine quantum circuits with those. The hybrid model with appropriate normalization performed better than the classical deep learning model without quantum circuits. This study suggests the possibility of omics data analysis using quantum computers.

[Disclosure of COI by Responsible researcher] Lead presenter is not the Responsible researcher.
[Lead presenter: Takafumi Ito] We have no financial relationships to disclose.
[Principal Researcher: Tatsuhiko Tsunoda] We have no financial relationships to disclose.

### [J-2070] I-J20
### Emerging Computational Approaches in Cancer Research: AI, Omics, & Precision Medicine

2025 / 9 / 26 (Fri) 14:05-15:20 Room 11/(ANA Crowne Plaza Hotel Kanazawa 3F Ohtori (West)) Room 11

[Chairperson]Atsushi Niida (IMSUT)

---

### J-2070
### Spatial dynamics of the tumor microenvironment linked to emerging resistance in EGFR-mutated non-small cell lung cancer

[Speaker]Satoshi Nakamura (Dept. of Respiratory Med., Kyushu Univ.) [Co-author]Daisuke Shibahara (Dept. of Respiratory Med., Kyushu Univ.)、Kentaro Tanaka (Dept. of Respiratory Med., Kyushu Univ.／Dept. of Pulmonary Med., Kagoshima Univ.)、Yasuyuki Kishikawa (Dept. of Respiratory Med., Kyushu Univ.)、Mikiko Hashisako (Dept. of Anatomic Path., Kyushu Univ.)、Keita Nakatomi (Dept. of Respiratory Med., Kyushu Central Hosp.)、Noriaki Nakagaki (Dept. of Respiratory Med., Fukuoka Red Cross Hosp.)、Mikihiro Kohno (Dept. of Surg. & Sci., Kyushu Univ.)、Koichi Azuma (Dept. of Internal Med., Kurume Univ. Sch. of Med.)、Ritsu Ibusuki (Dept. of Respiratory Med., Kyushu Univ.)、Kohei Otsubo (Dept. of Respiratory Med., Kyushu Univ.)、Yasuto Yoneshima (Dept. of Respiratory Med., Kyushu Univ.)、Eiji Iwama (Dept. of Respiratory Med., Kyushu Univ.)、Yoshinao Oda (Dept. of Anatomic Path., Kyushu Univ.)、Isamu Okamoto (Dept. of Respiratory Med., Kyushu Univ.)

Epidermal growth factor receptor (EGFR) tyrosine kinase inhibitors (TKIs) provide therapeutic benefit in EGFR mutation-positive non-small cell lung cancer, but some individuals develop early resistance. To investigate the mechanisms underlying such early resistance, we analyzed paired pre- and post-treatment tumor samples from the same patients using the Visium HD platform, with improved resolution at both single-cell and coordinate levels via StarDist-based image processing. Early resistance samples contained clusters with elevated TNF-$\alpha$/NF-$\kappa$B signaling, which is associated with osimertinib resistance, even before treatment. Maturity of tertiary lymphoid structures differed between early resistance and long-term response. Notably, the proportion of stromal cells decreased in long-term responders but increased in early resistance, which also showed active PDGF-mediated interactions between tumor cells and fibroblasts. These findings implicate tumor heterogeneity and an inflammatory tumor microenvironment in early EGFR-TKI resistance, providing insight into potential therapeutic strategies to improve treatment outcomes.

[Disclosure of COI by Responsible researcher] Lead presenter is not the Responsible researcher.
[Lead presenter: Satoshi Nakamura] We have no financial relationships to disclose.
[Principal Researcher: Kentaro Tanaka] We have no financial relationships to disclose.

### [J-2071] I-J20
### Emerging Computational Approaches in Cancer Research: AI, Omics, & Precision Medicine

2025 / 9 / 26 (Fri) 14:05-15:20 Room 11/(ANA Crowne Plaza Hotel Kanazawa 3F Ohtori (West)) Room 11

[Chairperson]Atsushi Niida (IMSUT)

---

### J-2071
### Application of SpatialKNifeY to Spatial Transcriptomics for Extracting Tissue Structure and Cataloging Microenvironment

[Speaker]Shunsuke A. Sakai (Univ. Tokyo, Front. Sci., Dept. Integr. Biosci.) [Co-author]Ryosuke Nomura (Univ. Tokyo, Front. Sci., Dept. Integr. Biosci.／Univ. Tokyo, Front. Sci., Dept. Comput. Biol. & Med. Sci.)、Motohiro Kojima (Kyoto Pref. Univ., Clin. & Diag. Pathol. Dept.)、Ayako Suzuki (Univ. Tokyo, Front. Sci., Dept. Comput. Biol. & Med. Sci.)、Yutaka Suzuki (Univ. Tokyo, Front. Sci., Dept. Comput. Biol. & Med. Sci.)、Katsuya Tsuchihara (Univ. Tokyo, Front. Sci., Dept. Integr. Biosci.／NCC, Div. Transl. Inform.)、Shunichiro Kageyama (NCC, Div. Transl. Inform.)、Riu Yamashita (NCC, Div. Transl. Inform.／Univ. Tokyo, Front. Sci., Dept. Comput. Biol. & Med. Sci.)

We have developed the SpatialKNifeY (SKNY) algorithm to analyze a local structure of a tissue, the "spatial domain," and their surrounding environment from spatial omics data (Sakai ***et al.***, ***PLOS CB***, 2025). In this study, we applied SKNY to Xenium data from lung and colorectal cancer samples. In lung cancer, we extracted tumor domains from surgical specimen and compared expression among the inside, peripheral, and outside domains. Immune exhaustion markers, such as ***PDCD1***, ***TIGIT***, and ***LAG3***, and cytotoxic molecule marker ***GZMB*** were enriched in the peripheral region, revealing a complex tumor immunity simultaneously accelerated and braked. For colorectal cancer, we focused on vascular domains extracted from radiation-treated specimen. The distance from these domains correlated with the expression of the hypoxia marker ***CA9***, supporting the presence of hypoxia in regions distant from the oxygen source. Given these circumstances, the microenvironment should be considered not only within the spatial domain, including vascular or tumor, but also in relation to the external region. We are now applying SKNY to other public datasets to construct a "Microenvironment Atlas."

[Disclosure of COI by Responsible researcher] Lead presenter is not the Responsible researcher.
[Lead presenter: Shunsuke A. Sakai] We have no financial relationships to disclose.
[Principal Researcher: Shunichiro Kageyama, Riu Yamashita] We have no financial relationships to disclose.

### [J-2072] I-J20

### Emerging Computational Approaches in Cancer Research: AI, Omics, & Precision Medicine

2025 / 9 / 26 (Fri) 14:05-15:20 Room 11/(ANA Crowne Plaza Hotel Kanazawa 3F Ohtori (West)) Room 11

[Chairperson]Atsushi Niida (IMSUT)

---

### J-2072

### Reconstruction of cancer genome structure by personalized reference genome-based cancer genome analysis pipeline

[Speaker]Yoshitaka Sakamoto (Div. Genome Analysis Platform Development, Natl. Cancer Ctr. Res. Inst.) [Co-author]Masahiro Sugawa (Div. Genome Analysis Platform Development, Natl. Cancer Ctr. Res. Inst.)、Ai Okada (Div. Genome Analysis Platform Development, Natl. Cancer Ctr. Res. Inst.)、Yotaro Ochi (Dept. Pathology and Tumor Biology, Grad. Sch. Med., Kyoto Univ.)、Yosuke Tanaka (Div. Cellular Signaling, Natl. Cancer Ctr. Res. Inst.)、Yasunori Kogure (Div. Molecular Oncology, Natl. Cancer Ctr. Res. Inst.)、Kenichi Chiba (Div. Genome Analysis Platform Development, Natl. Cancer Ctr. Res. Inst.)、Wataru Nakamura (Div. Genome Analysis Platform Development, Natl. Cancer Ctr. Res. Inst.)、Junji Koya (Div. Hematology, Dept. Med., Keio Univ. Sch. Med.／Div. Molecular Oncology, Natl. Cancer Ctr. Res. Inst.)、Hiroyuki Mano (Div. Cellular Signaling, Natl. Cancer Ctr. Res. Inst.)、Seishi Ogawa (Dept. Pathology and Tumor Biology, Grad. Sch. Med., Kyoto Univ.／Inst. for the Advanced Study of Human Biol., Kyoto Univ.／Kindai Univ. Faculty of Med.)、Keisuke Kataoka (Div. Hematology, Dept. Med., Keio Univ. Sch. Med.／Div. Molecular Oncology, Natl. Cancer Ctr. Res. Inst.)、Yuichi Shiraishi (Div. Genome Analysis Platform Development, Natl. Cancer Ctr. Res. Inst.)

Cancer genomes have point mutations and structural variations, which are curial for tumorigenesis and cancer progression. However, existing human reference genomes contain "dark regions", such as centromeres and segmental duplications, which make it difficult to comprehensively characterize cancer genomes.To overcome this issue, we propose Personalized Reference Genome-based Cancer Genome Analysis Pipeline (PRCGAP). In this pipeline, all cancer genome analyses are performed based on a personalized diploid reference genome, instead of the current human reference genomes, such as GRCh38.We applied PRCGAP to two cancer cell lines, H2009 and HCC1954. Personalized reference genomes for the cell lines were constructed by de novo assembly using their matched-normal cell line data. Point mutations and SVs identified using PRCGAP overlapped with more than 80% of those using GRCh38, while most of the remaining variants exhibited low variant frequencies. PRCGAP revealed centromere-involved complex SVs on chromosomes 4 and 19 in H2009 and those structures were reconstructed at the chromosome scale.In conclusion, PRCGAP enables a more comprehensive characterization of cancer genome structures.

[Disclosure of COI by Responsible researcher] Lead presenter is not the Responsible researcher.
[Lead presenter: Yoshitaka Sakamoto] We have no financial relationships to disclose.
[Principal Researcher: Yuichi Shiraishi] We have no financial relationships to disclose.

▲TOP

### [E-2079] II-E11
### Advances in pathogenesis & treatment of hematological malignancies

2025 / 9 / 26 (Fri) 14:05-15:20 Room 14/(ANA Crowne Plaza Hotel Kanazawa 3F Zuiun 1) Room 14

[Chairperson]Yasuhito Nannya (Dep. Hematol&Oncol Research Hospital IMSUT)

---

### E-2079
### Specific Impact of Non-DNMT3A/TET2 Clonal Hematopoiesis on the Risk of Severe COVID-19

[Speaker]Ryunosuke Saiki (Pathology and Tumor Biology, Kyoto University) [Co-author]Ryuya Edahiro (Statistical Genetics, Osaka University／Genome Informatics, The University of Tokyo／Respiratory Medicine and Clinical Immunology, Osaka University)、Kyuto Sonehara (Statistical Genetics, Osaka University／Genome Informatics, The University of Tokyo／iFremed, Osaka University)、Qingbo Wang (Statistical Genetics, Osaka University／WPI-IFReC, Osaka University)、Ho Namkoong (Department of Infectious Diseases, Keio University)、Takanori Hasegawa (M&D Data Science Center, IIR, Institute of Science Tokyo)、Shinichi Namba (Statistical Genetics, Osaka University／Genome Informatics, The University of Tokyo)、Yasuhito Nannya (Pathology and Tumor Biology, Kyoto University)、Nobuyuki Kakiuchi (Pathology and Tumor Biology, Kyoto University)、Akinori Kimura (Institute of Science Tokyo, Tokyo, Japan)、Seiya Imoto (HGC, the Institute of Medical Science, the University of Tokyo)、Satoru Miyano (M&D Data Science Center, IIR, Institute of Science Tokyo)、Takanori Kanai (Gastroenterology and Hepatology, Keio University School of Medicine)、Koichi Fukunaga (Division of Pulmonary Medicine, Keio University School of Medicine)、Yukinori Okada (Statistical Genetics, Osaka University／Genome Informatics, The University of Tokyo)、Seishi Ogawa (Pathology and Tumor Biology, Kyoto University／WPI-ASHBi, Kyoto University)

Age-related clonal hematopoiesis (CH) has been implicated not only in leukemia development, but also in non-malignant diseases, of which of particular interest is COVID-19. However, there are several conflicting reports regarding the association between CH and COVID-19, where generally small sample size and long interval between sample collection and COVID-19 onset might affected the results. We recruited a large cohort of COVID-19 patients (2,551 severe and 2,180 non-severe cases), in whom all samples were obtained at COVID-19 diagnosis. Overall, CH was significantly associated with severe COVID-19. However, the association was confined to non-DT (i.e., ***DNMT3A*** and ***TET2***) mutations (OR=1.76) with no significant impacts of DT mutations. To obtain mechanistic insight, we performed gene expression analysis on whole blood from 2,931 COVID-19 patients and 667 non-infected individuals. Enhanced inflammatory response, which was a marked feature of severe COVID-19 cases, was also observed in non-infected individuals with non-DT mutations, suggesting that non-DT mutations upregulated inflammatory response even before infection and predisposed individuals to severe disease upon infection.

[Disclosure of COI by Responsible researcher] Lead presenter is not the Responsible researcher.
[Lead presenter: Ryunosuke Saiki] ■Contributions: 日本新薬株式会社
[Principal Researcher: Seishi Ogawa] ■Advisor of the company and/or profit-making organization: エーザイ株式会社、Chordia Therapeutics Inc.、Montage Bio, INC.　■Profit of stock: 朝日ゲノミクス株式会社　■Lecture fee: (公財)三菱財団、(公財)中谷医工計測技術振興財団　■Research expenses: エーザイ株式会社、Chordia Therapeutics Inc.　■Contributions: 日本新薬株式会社　■Endowed chair: Chordia Therapeutics Inc.、(公財)鹿児島共済会 南風病院

▲TOP

### [E-2080] II-E11
### Advances in pathogenesis & treatment of hematological malignancies

2025 / 9 / 26 (Fri) 14:05-15:20 Room 14/(ANA Crowne Plaza Hotel Kanazawa 3F Zuiun 1) Room 14

[Chairperson]Yasuhito Nannya (Dep. Hematol&Oncol Research Hospital IMSUT)

---

### E-2080
### Genomic Landscape of Newly Diagnosed Adult Acute Lymphoblastic Leukemia

[Speaker]Hidehito Fukushima (Div. of Hem. Disease Control, IMSUT) [Co-author]Kaito Mimura (Div. of Cancer Evolution, NCCRI)、Koji Okazaki (Dept. Path. and Tumor Biol., Grad. Sch. Med.)、Hiroyuki Takamori (Dept. of HemOnc, Univ. of Osaka Grad. Sch. of Med.)、Ryunosuke Saiki (Dept. Path. and Tumor Biol., Grad. Sch. Med.)、Yotaro Ochi (Dept. Path. and Tumor Biol., Grad. Sch. Med.)、Kazuaki Yokoyama (Div. of Hem. Disease Control, IMSUT)、Kenichi Yoshida (Div. of Cancer Evolution, NCCRI)、Motohiro Kato (Dept. Pediatrics, Univ. of Tokyo, Tokyo, Japan)、Seishi Ogawa (Dept. Path. and Tumor Biol., Grad. Sch. Med.)、Yasuhito Nannya (Div. of Hem. Disease Control, IMSUT)

While pediatric acute lymphoblastic leukemia (ALL) has been extensively characterized through multi-omics sequencing, the genomic architecture of newly diagnosed adult ALL remains incompletely defined. We performed whole genome sequencing on 156 de-novo adult cases (126 B cell/MPAL, 30 T cell) using matched buccal swab germline controls. Compared to pediatric ALL, canonical subtypes such as ETV6::RUNX1, hyperdiploid, and hypodiploid are far less frequent in adult ALL. Notably, we identified recurrent structural variants in three relatively understudied genes, with recurrence rates of 45%, 18%, and 12% respectively, following well characterized alterations in IKZF1, CDKN2A, and PAX5.Alterations in these genes exhibit specific patterns of mutual exclusivity and coexistence with other drivers, were associated with distinct transcriptional states, and correlated with survival. These findings underscore heterogeneity in adult ALL and highlight the potential value of incorporating these genes' status into adult risk models and the design of future clinical trials.

[Disclosure of COI by Responsible researcher] Lead presenter is not the Responsible researcher.
[Lead presenter: Hidehito Fukushima] We have no financial relationships to disclose.
[Principal Researcher: Yasuhito Nannya] ■Lecture fee: ブリストルマイヤーズ、大塚製薬　■Contributions: 持田記念医学薬学振興財団 内藤記念科学振興財団 第一三共生命科学研究振興財団 住友財団 安田記念医学財団 アステラス病態代謝研究会 MSD生命科学財団 日本血液学会 小児医学研究振興財団 上原記念生命科学財団 高松宮妃癌研究基金 公益信託日本白血病研究基金 小林がん学術振興会 武田科学振興財団 大和証券財団 ＳＧＨ財団 川野小児医学奨学財団 ゴールドリボン・ネットワーク 先進医薬研究振興財団 テルモ生命科学振興財団 小野薬品がん・免疫・神経研究財団 細胞科学研究財団

▲TOP

### [E-2081] II-E11
### Advances in pathogenesis & treatment of hematological malignancies

2025 / 9 / 26 (Fri) 14:05-15:20 Room 14/(ANA Crowne Plaza Hotel Kanazawa 3F Zuiun 1) Room 14

[Chairperson]Yasuhito Nannya (Dep. Hematol&Oncol Research Hospital IMSUT)

---

### E-2081
### Cooperative Impact of Germline and Somatic ***DDX41*** Mutations on Myeloid Pathogenesis

[Speaker]Ayana Kon (Div. Hematology／Tumor Biology, Inst. Medical Science, Univ.Tokyo, Tokyo, Japan／Div.Stem Cell／Genome Biology, Inst. Medical Science, Univ.Tokyo, Tokyo, Japan／Dept. Pathology and Tumor Biology, Kyoto University, Kyoto, Japan) [Co-author]Masahiro M. Nakagawa (Dept. Pathology and Tumor Biology, Kyoto University, Kyoto, Japan)、Azumi Tomita (Div. Hematology／Tumor Biology, Inst. Medical Science, Univ.Tokyo, Tokyo, Japan)、Keisuke Kataoka (Div.Molecular Oncology, National Cancer Center Japan Research Inst., Tokyo,Japan／Dept. Hematology, Keio University School of Medicine, Tokyo, Japan)、Nobuyuki Kakiuchi (Dept. Pathology and Tumor Biology, Kyoto University, Kyoto, Japan)、Hideki Makishima (Dept. Hematology, Shinshu University School of Medicine, Shinshu, Japan)、Manabu Nakayama (Dept. Technology Development, Kazusa DNA Research Inst., Chiba, Japan)、Haruhiko Koseki (Lab. Developmental Genetics, RIKEN Center Integrative Medical Sciences, Yokohama, Japan)、Yasuhito Nannya (Dept. Hematology／Oncology, Inst. Medical Science, Univ.Tokyo, Tokyo, Japan)、Seishi Ogawa (Dept. Pathology and Tumor Biology, Kyoto University, Kyoto, Japan)

***DDX41*** mutations represent the most common germline predisposition to myeloid neoplasms. Typically, a germline loss-of-function allele is accompanied by a somatic R525H mutation in the helicase domain in the remaining allele. Using ***Ddx41*** mutant mouse models, we found that expression of compound loss-of-function and R525H alleles caused severe BM failure. In contrast, heterozygous ***Ddx41*** knock-out or R525H knock-in alleles supported hematopoiesis, albeit with impaired function and age-related MDS, potentially driven by attenuated inflammatory response and ribosome dysfunctions. As MDS clones harboring the ***DDX41*** somatic allele are often observed as minor subclones in patients, we co-transplanted ***Ddx41***$^{+/+}$- or ***Ddx41***$^{R525H/-}$-derived BM cells with ***Ddx41***$^{+/-}$ BM cells into recipient mice. Notably, recipients exhibited leukopenia when ***Ddx41***$^{R525H/-}$ BM cells were co-transplanted. To explore interactions between biallelic mutant hematopoietic cells and a monoallelic mutant microenvironment, we performed single cell RNA-seq of recipient BM, revealing molecular changes linked to ***Ddx41*** mutation. Our results provide insight into the non-cell-autonomous mechanisms of ***DDX41***-mutated myeloid neoplasms.

[Disclosure of COI by Responsible researcher] Lead presenter is not the Responsible researcher.
[Lead presenter: Ayana Kon] We have no financial relationships to disclose.
[Principal Researcher: Seishi Ogawa] ■Advisor of the company and/or profit-making organization: Eisai Co., Ltd., Chordia Therapeutics, Inc. ■Profit of stock: Asahi Genomics Co., Ltd. ■Contributions: Eisai Co., Ltd., Chordia Therapeutics, Inc., Otsuka Pharmaceutical Co., Ltd., Nippon Shinyaku Co., Ltd., Nanpuh Hospital

▲TOP

### [E-2082] II-E11
### Advances in pathogenesis & treatment of hematological malignancies

2025 / 9 / 26 (Fri) 14:05-15:20 Room 14/(ANA Crowne Plaza Hotel Kanazawa 3F Zuiun 1) Room 14

[Chairperson]Yasuhito Nannya (Dep. Hematol&Oncol Research Hospital IMSUT)

---

### E-2082
### Engineered metabolic induction of excessive mitochondrial ROS as a promising therapeutic approach in acute leukemia

[Speaker]Emi Soma (Lab. Cancer Pathobiology & Therapeutics, Ritsumeikan University, Shiga, Japan) [Co-author]Hikaru Shibata (Lab. Cancer Pathobiology & Therapeutics, Ritsumeikan University, Shiga, Japan)、Yusuke Nakanishi (Lab. Cancer Pathobiology & Therapeutics, Ritsumeikan University, Shiga, Japan)、Satoshi Takahashi (Lab. Cancer Pathobiology & Therapeutics, Ritsumeikan University, Shiga, Japan)、Yoshihiro Hayashi (Lab. Cancer Pathobiology & Therapeutics, Ritsumeikan University, Shiga, Japan)

Acute myeloid leukemia (AML) is a refractory hematological malignancy with a long-term survival rate of less than 50%. Growing evidence highlights that AML cell survival depends on mitochondria (but not glycolysis) and that mitochondria-derived reactive oxygen species (ROS) level influences the maintenance of leukemic stem cells. Here, we demonstrated that an engineered metabolic induction of ROS in mitochondria induces AML cell death in vitro and in vivo. To induce excessive ROS in mitochondria, we used a mitochondrial matrix-targeted D-amino acid oxidase (MTS-DAO) which catalyzes D-amino acids and produces hydrogen peroxide. We found that expression of the MTS-DAO in human AML cells causes mitochondrial mass reduction, resulting in cell cycle inhibition and apoptosis in a D-amino acid concentration-dependent manner. Furthermore, MTS-DAO-derived ROS completely eradicated leukemic clones in a mouse model of myeloid leukemia. These findings indicate that mitochondria are a vulnerability of AML cells and provide a clue for developing new treatments targeting mitochondria in AML.

[Disclosure of COI by Responsible researcher] Lead presenter is not the Responsible researcher.
[Lead presenter: Emi Soma] We have no financial relationships to disclose.
[Principal Researcher: Yoshihiro Hayashi] We have no financial relationships to disclose.

### [E-2083] II-E11
### Advances in pathogenesis & treatment of hematological malignancies

2025 / 9 / 26 (Fri) 14:05-15:20 Room 14/(ANA Crowne Plaza Hotel Kanazawa 3F Zuiun 1) Room 14

[Chairperson]Yasuhito Nannya (Dep. Hematol&Oncol Research Hospital IMSUT)

---

### E-2083
### Restoring serotonin signaling to alleviate cytopenia in MDS as a novel therapeutic approach.

[Speaker]Yasushige Kamimura (Lab. Oncology, Tokyo Univ. Pharm. & Life Sci., Tokyo, Japan／Dept. Stemcell & Immuneregulation, Yokohama City Univ. Yokohama, Japan) [Co-author]Kanako Yuki (Lab. Oncology, Tokyo Univ. Pharm. & Life Sci., Tokyo, Japan)、Yuka Harada (Lab. Oncology, Tokyo Univ. Pharm. & Life Sci., Tokyo, Japan／Clin. Lab., Tokyo Metropolitan Komagome Hosp., Tokyo, Japan)、Hiroki Kobayashi (Lab. Oncology, Tokyo Univ. Pharm. & Life Sci., Tokyo, Japan)、Yoshihiro Hayashi (Lab. Cancer Pathobiology & Therap. Ritsumeikan Univ., Shiga Japan)、Hideaki Nakajima (Dept. Stemcell & Immuneregulation, Yokohama City Univ. Yokohama, Japan)、Hironori Harada (Lab. Oncology, Tokyo Univ. Pharm. & Life Sci., Tokyo, Japan／Div. Hematology, Tokyo Metropolitan Komagome Hosp., Tokyo, Japan)

Myelodysplastic syndromes (MDS) are characterized by persistent cytopenia, genetic abnormalities, and dysplasia. Effective therapies for cytopenia are urgently needed, yet current therapeutic options remain limited. Cyclosporine A (CsA), an immunosuppressive agent, has shown clinical efficacy in some MDS patients, but its exact mechanism of action remains unclear. Using an MDS mouse model, we found that CsA improved anemia and thrombocytopenia independently of immunosuppression, suggesting CsA acts through undefined mechanisms. RNA-seq analysis revealed a distinct metabolic shift in MDS clones toward the kynurenine pathway, resulting in significantly reduced circulating serotonin (5HT). Notably, CsA corrected this imbalance by restoring 5HT, suggesting that increased 5HT levels may be critical for improving cytopenia. Treatment with a 5HT precursor markedly enhanced erythroid and megakaryocyte differentiation and effectively rescued cytopenia. Similar results were confirmed in MDS patient-derived cells. These findings indicate that CsA attenuates cytopenia in MDS by restoring 5HT through modulation of tryptophan metabolism, highlighting a promising new therapeutic strategy.

[Disclosure of COI by Responsible researcher] Lead presenter is not the Responsible researcher.
[Lead presenter: Yasushige Kamimura] We have no financial relationships to disclose.
[Principal Researcher: Hironori Harada] ■Lecture fee: ブリストル・マイヤーズスクイブ株式会社 , ノバルティス ファーマ株式会社

▲TOP

### [E-2084] II-E11
### Advances in pathogenesis & treatment of hematological malignancies

2025 / 9 / 26 (Fri) 14:05-15:20 Room 14/(ANA Crowne Plaza Hotel Kanazawa 3F Zuiun 1) Room 14

[Chairperson]Yasuhito Nannya (Dep. Hematol&Oncol Research Hospital IMSUT)

---

### E-2084
### Elucidating the unique pathogenesis of clonal hematopoiesis via single-cell multi-omics profiling.

[Speaker]Masahiro M. Nakagawa (Department of Pathology and Tumor Biology, Kyoto Univ.) [Co-author]Ryosaku Inagaki (Department of Pathology and Tumor Biology, Kyoto Univ.)、Yasuhito Nannya (Department of Pathology and Tumor Biology, Kyoto Univ.／Division of Hematopoietic Disease Control, IMS, Tokyo Univ.)、Masanori Motomura (Department of Pathology and Tumor Biology, Kyoto Univ.)、Ryunosuke Saiki (Department of Pathology and Tumor Biology, Kyoto Univ.)、Yotaro Ochi (Department of Pathology and Tumor Biology, Kyoto Univ.)、Ayana Kon (Department of Pathology and Tumor Biology, Kyoto Univ.／Division of Hematology and Tumor Biology, IMS, Tokyo Univ.)、Lanying Zhao (Department of Pathology and Tumor Biology, Kyoto Univ.／WPI-ASHBi, Kyoto University)、Koji Okazaki (Department of Pathology and Tumor Biology, Kyoto Univ.)、Xingxing Qi (Department of Pathology and Tumor Biology, Kyoto Univ.／WPI-ASHBi, Kyoto University)、Akinori Yoda (Department of Pathology and Tumor Biology, Kyoto Univ.)、Nobuyuki Kakiuchi (Department of Pathology and Tumor Biology, Kyoto Univ.／The Hakubi Center for Advanced Research, Kyoto Univ.)、Shuichi Matsuda (Department of Orthopedic Surgery in Kyoto Univ. Hosp.)、Seishi Ogawa (Department of Pathology and Tumor Biology, Kyoto Univ.／WPI-ASHBi, Kyoto University)

Although strongly associated with leukemogenesis, clonal hematopoiesis (CH) remains mechanistically elusive. We performed single cell sequencing of CD34+ cells from individuals with and without CH, using a platform that allowed for simultaneous detection of both somatic mutations and gene expression. In CH(+) samples, mutant cells showed upregulation of cell cycle-related genes compared to WT cells. By contrast, the response to proinflammatory cytokines was downregulated in mutant cells, which shows a sharp contrast to the observations in age-matched CH(-) bone marrow samples exhibiting marked upregulation of the latter pathways, suggesting that the differential responses to proinflammatory stimuli in aged bone marrow may contributes to evolution of CH cells. Notably, WT cells in CH(+) marrow showed upregulated cell cycle-related pathways and IFN-related gene expression compared to those from CH(-) individuals, which was recapitulated in competitive transplantation of ***Tet2*** mutant cells in mice. These findings provide insight in to the mechanism by which mutant cells obtain a selective advantage over the WT cells in aged BM.

[Disclosure of COI by Responsible researcher] Lead presenter is not the Responsible researcher.
[Lead presenter: Masahiro M. Nakagawa] We have no financial relationships to disclose.
[Principal Researcher: Seishi Ogawa] ■Advisor of the company and/or profit-making organization: エーザイ株式会社 Chordia Therapeutics Inc. Montage Bio, INC. ■Profit of stock: 朝日ゲノミクス株式会社 ■Lecture fee: 公益財団法人三菱財団 公益財団法人中谷医工計測技術振興財団 ■Research expenses: エーザイ株式会社 Chordia Therapeutics Inc. ■Contributions: 日本新薬株式会社 ■Endowed chair: Chordia Therapeutics Inc. 公益社団法人鹿児島共済会 南風病院

### [J-2097] I-J11-5
### Tumor immune microenvironment/Tumor immune escape (4)

2025 / 9 / 26 (Fri) 14:05-15:20 Room 15/(ANA Crowne Plaza Hotel Kanazawa 3F Zuiun 2) Room 15

[Chairperson]Hitomi Nishinakamura (NCC, RI, Cancer Immunol)

---

### J-2097
### Spatial Informatics and In Vivo Modeling Reveal Fibroblast-Mediated Immune Exclusion in Colorectal Cancer

[Speaker]Kosuke Iwane (Dept. Gastroenterology & Hepatology, Kyoto Univ. Grad. Sch. Med.) [Co-author]Yuki Nakanishi (Dept. Gastroenterology & Hepatology, Kyoto Univ. Grad. Sch. Med.)、Hiroshi Seno (Dept. Gastroenterology & Hepatology, Kyoto Univ. Grad. Sch. Med.)

Fibrotic colorectal cancers (CRCs) respond poorly to immune checkpoint inhibitors (ICIs), largely due to stromal barriers that exclude cytotoxic T cells. However, the molecular regulators of this immune-exclusionary environment remain unclear. Through single-cell and spatial transcriptomics with neighborhood analysis, we identified THBS2, a matricellular protein produced by cancer-associated fibroblasts (CAFs), as a key driver of T cell exclusion in fibrotic CRCs. THBS2 was enriched at the tumor front and associated with low $CD8^+$ T cell infiltration. In fibroblast-specific knockout models, THBS2 loss enhanced $CD8^+$ T cell entry and reshaped the tumor microenvironment, increasing cell–cell interactions between T cells and antigen-presenting cells. Neighborhood analyses further revealed expansion of immune-infiltrated regions and breakdown of exclusionary zones. Despite partial T cell exhaustion, THBS2-deficient tumors showed marked sensitivity to ICIs. These findings highlight THBS2 as a fibroblast-specific regulator of spatial immune heterogeneity and a promising therapeutic target for reprogramming the fibrotic tumor microenvironment.

[Disclosure of COI by Responsible researcher] Lead presenter is not the Responsible researcher.
[Lead presenter: Kosuke Iwane] We have no financial relationships to disclose.
[Principal Researcher: Yuki Nakanishi] We have no financial relationships to disclose.

▲TOP

### [J-2098] I-J11-5
### Tumor immune microenvironment/Tumor immune escape (4)

2025 / 9 / 26 (Fri) 14:05-15:20 Room 15/(ANA Crowne Plaza Hotel Kanazawa 3F Zuiun 2) Room 15

[Chairperson]Hitomi Nishinakamura (NCC, RI, Cancer Immunol)

---

### J-2098
### Mechanisms of hepatocellular carcinoma recurrence after liver transplantation in the immunosuppressed state

[Speaker]Tomohiko Ikehara (Department of Surgery, Beppu Hospital／Department of Surgery, Shinshu University School of Medicine) [Co-author]Masaya Onishi (Department of Computational Biology and Medical Sciences, Tokyo University)、Kiyotaka Hosoda (Department of Surgery, Shinshu University School of Medicine)、Kosuke Hirose (Department of Surgery, Beppu Hospital)、Shohei Shibuta (Department of Surgery, Beppu Hospital)、Takashi Ofuchi (Department of Surgery, Beppu Hospital)、Koto Kawata (Department of Surgery, Beppu Hospital)、Yuki Ando (Department of Surgery, Beppu Hospital)、Qingjiang Hu (Department of Surgery, Beppu Hospital)、Hajime Otsu (Department of Surgery, Beppu Hospital)、Yusuke Yonemura (Department of Surgery, Beppu Hospital)、Takeo Toshima (Department of Surgery, Kyushu University School of Medicine)、Takaaki Masuda (Department of Surgery, Kochi University School of Medicine)、Tomoharu Yoshizumi (Department of Surgery, Kyushu University School of Medicine)、Yuji Soejima (Department of Surgery, Shinshu University School of Medicine)、Koshi Mimori (Department of Surgery, Beppu Hospital)

Objective: To clarify the tumor immune microenvironment (TIME) of post-transplant hepatocellular carcinoma (HCC) recurrence under immunosuppressive conditions and to identify molecular mechanisms of cancer-stromal cell networks that may promote metastatic recurrence. Methods: Spatial analysis was performed on one of five FFPE sample pairs, each pair consisting of a specimen collected at liver transplantation (LT) for HCC and at rehepatectomy for recurrent HCC. The results were validated by immunohistochemistry (IHC) and in vitro studies.Results: HE staining revealed tumor progression into the sinusoids as a specific histopathological finding at the tumor margin in the recurrent tumor. Spatial analysis focused on this area extracted the CXCL12+liver sinusoidal endothelial cell (LSEC)/CXCR4+cancer-associated fibroblast (CAF) axis as a specific cell-cell interaction in recurrent foci. We also observed these signals by IHC in the local region where tumor progression into the sinusoids was detected, and demonstrated the aforementioned cell-cell communication by transwell migration assay.Conclusion: TIME remodelling via the CXCL12+LSEC/CXCR4+CAF axis may promote HCC recurrence after LT.

[Disclosure of COI by Responsible researcher] Lead presenter is not the Responsible researcher.
[Lead presenter: Tomohiko Ikehara] We have no financial relationships to disclose.
[Principal Researcher: Koshi Mimori] We have no financial relationships to disclose.

### [J-2099] I-J11-5
### Tumor immune microenvironment/Tumor immune escape (4)

2025 / 9 / 26 (Fri) 14:05-15:20 Room 15/(ANA Crowne Plaza Hotel Kanazawa 3F Zuiun 2) Room 15

[Chairperson]Hitomi Nishinakamura (NCC, RI, Cancer Immunol)

---

### J-2099
### Histamine H1 Receptor Antagonists Enhance Anti-PD-1 Therapy by Reducing Extracellular Matrix Production in TNBC

[Speaker]Aya Sasaki (Showa Med. Umiv. School of Med. Dept. of Pharm.／Showa Med. Umiv. Pharmacological Research Center／Showa Med. Umiv. Clinical Research Inst. ／Toho Univ. Ohashi Med.l Center Surg. Dept.) [Co-author]Masahiro Hosonuma (Showa Med. Umiv. School of Med. Dept. of Pharm.／Showa Med. Umiv. Pharmacological Research Center／Showa Med. Umiv. Clinical Research Inst.)、Atsuo Kuramasu (Showa Med. Umiv. Clinical Research Inst.)、Yuuki Maruyama (Showa Med. Umiv. School of Med. Dept. of Pharm.／Showa Med. Umiv. Pharmacological Research Center／Showa Med. Umiv. Clinical Research Inst.)、Eiji Funayama (Showa Med. Umiv. School of Med. Dept. of Pharm.／Showa Med. Umiv. Pharmacological Research Center／Showa Med. Umiv. Clinical Research Inst.)、Kouhei Tajima (Showa Med. Umiv. Clinical Research Inst.)、Hitoshi Toyoda (Showa Med. Umiv. School of Med. Dept. of Pharm.／Showa Med. Umiv. Pharmacological Research Center／Showa Med. Umiv. Clinical Research Inst.)、Toshiaki Tsurui (Showa Med. Umiv. School of Med. Dept. of Pharm.／Showa Med. Umiv. Pharmacological Research Center／Showa Med. Umiv. Clinical Research Inst.)、Yoshitaka Yamazaki (Showa Med. Umiv. Pharmacological Research Center)、Hikaru Isobe (Showa Med. Umiv. School of Med. Dept. of Pharm.／Showa Med. Umiv. Pharmacological Research Center)、Fumihiro Ishikawa (Showa Med, Univ. Recombinant DNA Lab.)、Kiyoshi Yoshimura (Showa Med. Umiv. Clinical Research Inst.)、Tatsunori Oguchi (Showa Med. Umiv. Pharmacological Research Center)

Triple-negative breast cancer (TNBC) has a poor prognosis due to frequent distant metastasis. Although anti-PD-1 antibody (αPD-1) therapy is approved for TNBC, its monotherapy effect is limited. Antihistamine use has been linked to better outcomes in breast cancer. We found that high HRH1 expression in TNBC, based on TCGA data, was associated with poor prognosis and extracellular matrix (ECM)-related pathway enrichment. Single-cell RNA-seq showed HRH1 and ECM-related genes were mainly expressed in cancer-associated fibroblasts (CAFs). In 4T1 TNBC mouse models, we examined the combination of cetirizine, a selective HRH1 antagonist, with αPD-1. The combination improved survival in the subcutaneous model but not in the lung metastasis model, suggesting effects on the tumor microenvironment. Bulk RNA-seq of tumors showed ECM pathway suppression in the combination group. In vitro, cetirizine reduced ECM gene expression in human CAFs. HRH1 blockade may enhance αPD-1 efficacy in TNBC by suppressing ECM production.

[Disclosure of COI by Responsible researcher] Lead presenter is not the Responsible researcher.
[Lead presenter: Aya Sasaki] We have no financial relationships to disclose.
[Principal Researcher: Kiyoshi Yoshimura] We have no financial relationships to disclose.

▲TOP

### [J-2100] I-J11-5
### Tumor immune microenvironment/Tumor immune escape (4)

2025 / 9 / 26 (Fri) 14:05-15:20 Room 15/(ANA Crowne Plaza Hotel Kanazawa 3F Zuiun 2) Room 15

[Chairperson]Hitomi Nishinakamura (NCC, RI, Cancer Immunol)

---

### J-2100
### Immunosuppressive mechanisms within the tumor microenvironment of Ewing's sarcoma mouse model

[Speaker]Sho Isoyama (Div. Mol. Pharmacol., Cancer Chemother. Ctr., JFCR) [Co-author]Miwa Tanaka (Proj. Cancer Epigenomics, Cancer Inst., JFCR)、Takuro Nakamura (Dept. Exp. Pathol., IMS, Tokyo Medical Univ.)、Shingo Dan (Div. Mol. Pharmacol., Cancer Chemother. Ctr., JFCR)

Immune checkpoint inhibitors are effective in various cancers. Recently, atezolizumab, an anti-PD-L1 antibody, showed significant efficacy in alveolar soft part sarcoma, leading to its approval. However, effective immunotherapies have not been developed for most other sarcoma subtypes. In this study, we focused on Ewing's sarcoma (ES) and investigated its immunosuppressive mechanisms using a mouse ES model. This model reproduces ES tumors by subcutaneously transplanting mouse ES cells, derived from osteochondrogenic progenitor cells transformed with the human EWSR1::FLI1 fusion gene, into syngeneic mice. Based on the hypothesis that the oncogenic fusion gene drives an immunosuppressive tumor microenvironment, we examined tumor-infiltrating immune cells following knockdown of fusion gene expression. Suppressing the fusion gene significantly decreased tumor-infiltrating regulatory T cells (Tregs) and increased NK cells. Depletion of Tregs with a specific antibody markedly suppressed tumor growth, whereas the anti-tumor effect was completely canceled by depletion of NK cells. These results suggest that ES escapes anti-tumor immunity by Tregs suppressing NK cells within the tumors.

[Disclosure of COI by Responsible researcher] Lead presenter is the Responsible researcher.
[Lead presenter: Sho Isoyama] We have no financial relationships to disclose.

### [J-2101] I-J11-5
### Tumor immune microenvironment/Tumor immune escape (4)

2025 / 9 / 26 (Fri) 14:05-15:20 Room 15/(ANA Crowne Plaza Hotel Kanazawa 3F Zuiun 2) Room 15

[Chairperson]Hitomi Nishinakamura (NCC, RI, Cancer Immunol)

---

### J-2101
### The Role of Fusobacterium nucleatum in the Tumor Microenvironment of Oral Squamous Cell Carcinoma

[Speaker]Tomonori Kamiya (Osaka Metro Univ, Grad Sch Med, Pathophysiol) [Co-author]Megumu Yano (Osaka Metro Univ, Grad Sch Med, Pathophysiol)、Yuki Yamamoto (Osaka Metro Univ, Grad Sch Med, Otolaryngol)、Miki Nishio (Kobe Univ, Grad Sch Med, Mol Biol)、Akira Suzuki (Kobe Univ, Grad Sch Med, Mol Biol)、Naoko Ohtani (Osaka Metro Univ, Grad Sch Med, Pathophysiol)

Oral squamous cell carcinoma (OSCC) cases are rising not only among smokers but also in young non-smokers and those with good oral hygiene, suggesting unidentified factors in OSCC pathogenesis. Microbiota dysbiosis, particularly oral bacteria, is implicated in tumor development. In colorectal cancer, tumor-residing bacteria often originate from the oral cavity, suggesting an oral microbiota-tumorigenesis link. This study investigates oral microbiota's role in OSCC, focusing on the tumor microenvironment (TME). Microbiome analysis reveals Fusobacterium nucleatum enrichment in OSCC tissues. An anaerobe, F. nucleatum may adapt to hypoxic TME, influencing host immunity. Immunohistochemistry of OSCC tissues from patients and mouse models shows notable regulatory T cell (Treg) accumulation. In vitro assays indicate metabolites from F. nucleatum culture supernatant promote Treg differentiation from naive CD4 T cells. In a Hippo pathway-activated OSCC model, F. nucleatum inoculation enhances nuclear YAP localization, coinciding with dysplastic cell proliferation.These findings suggest F. nucleatum fosters OSCC progression by inducing Tregs and modifying TME into a more tumorigenic state.

[Disclosure of COI by Responsible researcher] Lead presenter is not the Responsible researcher.
[Lead presenter: Tomonori Kamiya] ■Research funds from company: ヤクルトバイオサイエンス研究財団、ダノン健康栄養財団
[Principal Researcher: Naoko Ohtani] ■Research funds from company: 武田科学振興財団

### [J-2102] I-J11-5
### Tumor immune microenvironment/Tumor immune escape (4)

2025 / 9 / 26 (Fri) 14:05-15:20 Room 15/(ANA Crowne Plaza Hotel Kanazawa 3F Zuiun 2) Room 15

[Chairperson]Hitomi Nishinakamura (NCC, RI, Cancer Immunol)

---

### J-2102
### Immune and Molecular Profiling of NBNC HCC Based on Prior HBV Infection and Identification of Novel Therapeutic Targets

[Speaker]Himari Kurosu (Dept. Biomed. Eng., Sci. & Eng., Toyo Uinv.) [Co-author]Yuta Ouchi (Dept. Biomed. Eng., Sci. & Eng., Toyo Uinv.)、Wakana Nakaniwa (Dept. Biomed. Eng., Sci. & Eng., Toyo Uinv.)、Shunsuke Shichi (Dept. Gastroenterological Surg. I, Hokkaido Univ.)、Saori Kimura (Dept. Gastroenterological Surg. I, Hokkaido Univ.)、Hiroki Nakamoto (Dept. Gastroenterological Surg. I, Hokkaido Univ.)、Chisato Shirakawa (Dept. Gastroenterological Surg. I, Hokkaido Univ.)、Akinobu Taketomi (Dept. Gastroenterological Surg. I, Hokkaido Univ.)、Junya Ohtake (Res. Facility Ctr., Asaka, Toyo Univ.／Res. Ctr., Biomed. Eng., Toyo. Univ.)、Hidemitsu Kitamura (Dept. Biomed. Eng., Sci. & Eng., Toyo Uinv.／Res. Facility Ctr., Asaka, Toyo Univ.／Res. Ctr., Biomed. Eng., Toyo. Univ.／Dept. Biomed. Eng., Life Sci., Toyo Univ)

Recent advances in antiviral therapies have markedly improved the prognosis of hepatocellular carcinoma (HCC) patients with hepatitis virus infections. However, some HCC cases related to current or prior viral infection exhibit poor clinical outcomes. We investigated tumor tissues from non-B, non-C (NBNC) HCC patients with or without prior HBV exposure using IHC, transcriptome profiling, and gene signature analysis to address this issue. We found that HBc antibody-negative cases showed higher infiltration of $CD4^+$ T cells and lower infiltration of $CD8^+$ T cells compared to HBc antibody-positive cases. Furthermore, each group exhibited distinct gene expression profiles. Based on these findings, we developed a mouse HCC model and administered the inhibitor targeting a candidate factor identified from the transcriptomic data. This intervention increased infiltration of anti-tumor effector immune cells in the liver and significantly suppressed tumor formation. These results suggest that the candidate factor plays a crucial role in non-viral hepatocarcinogenesis and may serve as a promising therapeutic target for improving outcomes in patients with NBNC HCC.

[Disclosure of COI by Responsible researcher] Lead presenter is not the Responsible researcher.
[Lead presenter: Himari Kurosu] We have no financial relationships to disclose.
[Principal Researcher: Hidemitsu Kitamura] ■Contributions: テルモ生命科学振興財団

### [J-2103] I-J11-6
### Antibody therapy/Immune cell therapy (CAR-T, T cell therapy) (2)

2025 / 9 / 26 (Fri) 14:05-15:20 Room 16/(11F Visage) Room 16

[Chairperson]Yoshihiro Miyahara (Mie Univ. Grad. Sch. of Med.)

---

### J-2103
### Therapeutic potential of a monoclonal antibody targeting the receptor tyrosine kinase EphB4 in breast cancer

[Speaker]Tomokazu Ohishi (Institute of Microbial Chemistry (BIKAKEN), Laboratory of Oncology) [Co-author]Hiroyuki Suzuki (Dept. Antibody Drug Development, Tohoku University Grad. Sch. of Med.)、Mika K. Kaneko (Dept. Antibody Drug Development, Tohoku University Grad. Sch. of Med.)、Junjiro Yoshida (Institute of Microbial Chemistry (BIKAKEN), Laboratory of Oncology)、Daisuke Tatsuda (Institute of Microbial Chemistry (BIKAKEN), Laboratory of Oncology)、Shunichi Ohba (Institute of Microbial Chemistry (BIKAKEN), Numazu Branch)、Akiko Harakawa (Institute of Microbial Chemistry (BIKAKEN), Numazu Branch)、Tomohiro Tanaka (Dept. Antibody Drug Development, Tohoku University Grad. Sch. of Med.)、Yukinari Kato (Dept. Antibody Drug Development, Tohoku University Grad. Sch. of Med.)、Manabu Kawada (Institute of Microbial Chemistry (BIKAKEN), Laboratory of Oncology)

Breast cancer is the most common cancer among Japanese women. Ephrin type-B receptor 4 (EphB4), a receptor-type tyrosine kinase, promotes progression and is highly expressed in triple-negative, HER2-positive, and hormone receptor-positive subtypes. Ephrin-B2, a ligand of EphB4, activates downstream signaling including MAPK. Kaplan-Meier analysis showed that high EphB4 expression significantly correlates with poor prognosis. EphB4 overexpression was confirmed in several breast cancer cell lines. We developed a monoclonal antibody, Eb4Mab-7-mG2a, targeting EphB4, and tested its effect on Ephrin-B2-induced MAPK signaling in EphB4-positive MCF7 cells. Eb4Mab-7-mG2a inhibited MAPK activation via EphB4, while no effect was seen in EphB4-knockout (KO) MCF7 cells. In MCF10A, a normal mammary epithelial cell line, and in MCF7 and EphB4-KO MCF7 cells, the antibody did not affect proliferation. However, ADCC and CDC assays showed strong cytotoxicity against EphB4-positive cells. In a xenograft model, Eb4Mab-7-mG2a significantly suppressed tumor growth. These results suggest that EphB4-targeted antibody therapy may offer a promising strategy for treating EphB4-overexpressing breast cancer.

[Disclosure of COI by Responsible researcher] Lead presenter is the Responsible researcher.
[Lead presenter: Tomokazu Ohishi] We have no financial relationships to disclose.

### [J-2104] I-J11-6
### Antibody therapy/Immune cell therapy (CAR-T, T cell therapy) (2)

2025 / 9 / 26 (Fri) 14:05-15:20 Room 16/(11F Visage) Room 16

[Chairperson]Yoshihiro Miyahara (Mie Univ. Grad. Sch. of Med.)

---

### J-2104
### A cancer-specific anti-podoplanin monoclonal antibody exerts antitumor activities in human tumor xenograft models

[Speaker]Tomohiro Tanaka (Dept. Antibody Drug Development, Tohoku Univ. Grad. Sch. of Med.) [Co-author]Hiroyuki Suzuki (Dept. Antibody Drug Development, Tohoku Univ. Grad. Sch. of Med.)、Tomokazu Ohishi (Inst. Microbial Chemistry (BIKAKEN), Lab. of Oncology)、Manabu Kawada (Inst. Microbial Chemistry (BIKAKEN), Lab. of Oncology)、Mika K. Kaneko (Dept. Antibody Drug Development, Tohoku Univ. Grad. Sch. of Med.)、Yukinari Kato (Dept. Antibody Drug Development, Tohoku Univ. Grad. Sch. of Med.)

Overexpression of podoplanin (PDPN) is associated with poor clinical outcomes in various tumors. PDPN plays a crucial role in invasiveness, stemness, and metastasis of tumors. Therefore, PDPN is considered a promising target of antibody-based therapy. Because PDPN also plays an essential role in normal cells, cancer specificity is required to reduce adverse effects. We developed a cancer-specific anti-PDPN monoclonal antibody, PMab-117 (rat IgM, kappa), by immunizing rats with PDPN-overexpressed glioblastoma cells. The engineered recombinant mouse $IgG_{2a}$-type PMab-117 (PMab-117-$mG_{2a}$) and humanized PMab-117 (humPMab-117) reacted with the PDPN-positive PC-10 and LN319 cells, but not PDPN-knockout LN319 cells in flow cytometry. Importantly, PMab-117-$mG_{2a}$ and humPMab-117 did not react with normal epithelial cells derived from lung bronchus, mammary gland, corneal, and kidney podocytes. Moreover, PMab-117-$mG_{2a}$ and humPMab-117 exerted antibody-dependent cellular cytotoxicity and exhibited potent antitumor effects against human tumor xenografts. These results suggest the potential of PMab-117 as a therapeutic antibody for treating PDPN-positive tumors while reducing the adverse effects.

[Disclosure of COI by Responsible researcher] Lead presenter is not the Responsible researcher.
[Lead presenter: Tomohiro Tanaka] We have no financial relationships to disclose.
[Principal Researcher: Yukinari Kato] ■Patent fee: 小野薬品工業株式会社　■Endowed chair: 小野薬品工業株式会社、株式会社ファーマフーズ

### [J-2105] I-J11-6
### Antibody therapy/Immune cell therapy (CAR-T, T cell therapy) (2)

2025 / 9 / 26 (Fri) 14:05-15:20 Room 16/(11F Visage) Room 16

[Chairperson]Yoshihiro Miyahara (Mie Univ. Grad. Sch. of Med.)

---

### J-2105
### Development of SRG system for selective regulation of CAR-T cell kinetics ***in vivo***

[Speaker]Ryosuke Uchibori (Center for Gene Therapy Research, Jichi Med. Univ.) [Co-author]Ken Ohmine (Center for Gene Therapy Research, Jichi Med. Univ.／Div of Hematol., Jichi Med. Univ.)、Junichi Mineno (Takara Bio Inc.)、Keiya Ozawa (Center for Gene Therapy Research, Jichi Med. Univ.)

**[Background]** Despite the encouraging outcomes of CAR T-cell therapy, approximately half of patients relapse within a short period owing to the loss of proliferation and persistence of infused CAR-T cells. Therefore, we developed selective regulatory genes (SRGs) that allow selective control of CAR-T cell kinetics ***in vivo***. **[Methods]** SRGs encoding artificial chimeric erythropoietin (EPO)-responsive molecules consisting of the extracellular domain of the EPO receptor and the intracellular signaling domain of a receptor that controls T-cell proliferation and persistence were generated. Cellular characteristics were analyzed ***in vitro*** and ***in vivo***. **[Results]** T cells co-expressing SRG and CAR (SRG-CAR-T cells) proliferated selectively in an EPO-dependent manner both ***in vitro*** and ***in vivo***. When SRG-CAR-T cells were infused into tumor-bearing mice with the concomitant use of EPO, the number of SRG-CAR-T cells in the blood increased significantly, and the duration of response and mouse survival was significantly prolonged. **[Conclusions]** The SRG system is an innovative technology that enables the control of post-infusion cell kinetics, which is not possible with conventional CAR-T therapy.

[Disclosure of COI by Responsible researcher] Lead presenter is the Responsible researcher.
[Lead presenter: Ryosuke Uchibori] We have no financial relationships to disclose.

### [J-2106] I-J11-6
### Antibody therapy/Immune cell therapy (CAR-T, T cell therapy) (2)

2025 / 9 / 26 (Fri) 14:05-15:20 Room 16/(11F Visage) Room 16

[Chairperson]Yoshihiro Miyahara (Mie Univ. Grad. Sch. of Med.)

---

### J-2106
### Antibody-Dependent Cellular Cytotoxicity Mediated by Anti-GD2 mAb in iPSC-Derived NKT Cells for Neuroblastoma

[Speaker]Katsuhiro Nishimura (Dept of Medical Immunology, Chiba Univ Grad Sch of Medicine／Dept of Pediatric Surgery, Chiba Univ Grad Sch of Medicine) [Co-author]Takahiro Aoki (Dept of Medical Immunology, Chiba Univ Grad Sch of Medicine／Dept of Pediatrics, Chiba Univ Grad Sch of Medicine／Lab for Developmental Genetics, RIKEN IMS)、Midori Kobayashi (Dept of Medical Immunology, Chiba Univ Grad Sch of Medicine)、Mariko Takami (Dept of Medical Immunology, Chiba Univ Grad Sch of Medicine)、Daisuke Katsumi (Dept of Medical Immunology, Chiba Univ Grad Sch of Medicine／Dept of Pediatric Surgery, Chiba Univ Grad Sch of Medicine)、Ko Ozaki (Dept of Medical Immunology, Chiba Univ Grad Sch of Medicine)、Keita Ogawa (Dept of Medical Immunology, Chiba Univ Grad Sch of Medicine)、Hongxuan Wang (Dept of Medical Immunology, Chiba Univ Grad Sch of Medicine)、Haruhiko Koseki (Lab for Developmental Genetics, RIKEN IMS)、Tomoro Hishiki (Dept of Pediatric Surgery, Chiba Univ Grad Sch of Medicine)、Shinichiro Motohashi (Dept of Medical Immunology, Chiba Univ Grad Sch of Medicine)

Anti-GD2 monoclonal antibody (mAb) therapy improves survival in high-risk neuroblastoma (NB) by inducing antibody-dependent cellular cytotoxicity (ADCC), yet 40-50% of patients still die. We developed induced pluripotent stem cell-derived natural killer T (iPS-NKT) cells with NK-like cytotoxicity, but their ADCC potential remains unclear. Here, we evaluated the ADCC activity of iPS-NKT cells and their therapeutic effect in combination with anti-GD2 mAb. iPS-NKT cells expressed CD16 and mediated ADCC against NB cell lines in the presence of anti-GD2 mAb. CD16 stimulation induced cytotoxicity and secretion of interferon-γ, tumor necrosis factor, and granzyme B, whereas CD32 and CD64 had no effect. In a subcutaneous NB mouse model, combination therapy with iPS-NKT cells and anti-GD2 mAb significantly suppressed tumor growth compared to monotherapies or control. Additionally, iPS-NKT cells showed increased CD69 expression in the tumor microenvironment. These results demonstrate that iPS-NKT cells exert ADCC via CD16 and enhance the antitumor effect of anti-GD2 mAb, suggesting a promising immunotherapeutic strategy for NB.

[Disclosure of COI by Responsible researcher] Lead presenter is not the Responsible researcher.
[Lead presenter: Katsuhiro Nishimura] We have no financial relationships to disclose.
[Principal Researcher: Tomoro Hishiki] We have no financial relationships to disclose.

### [J-2107] I-J11-6
### Antibody therapy/Immune cell therapy (CAR-T, T cell therapy) (2)

2025 / 9 / 26 (Fri) 14:05-15:20 Room 16/(11F Visage) Room 16

[Chairperson]Yoshihiro Miyahara (Mie Univ. Grad. Sch. of Med.)

---

### J-2107
### Novel immunotherapy via induction of Tertiary Lymphoid Strcture

[Speaker]Taro Suzuki (Kyoto Univ. CiRA) [Co-author]Keitaro Kanie (Kyoto Univ. CiRA)、Tomoko Ishi (Kyoto Univ. CiRA)、Shin Kaneko (Kyoto Univ. CiRA)

Tertiary Lymphoid Structure (TLS) is a lymphoid tissue-like structure that occurs under chronic inflammation, such as a solid tumor. Some reports showed that TLS formation correlates with favorable prognosis of cancer patients. This study aims to develop novel cancer therapy via induction of TLS.Some important chemokines for TLS induction were injected into tumor-bearing mice.In the group treated with Chemokine combinations, flow cytometry analysis showed increased tumor-infiltrating lymphocytes (TILs) and the expression of TLS endothelial cell marker (PNAd). We have developed a novel evaluation method for TLS by integrating immunohistochemistry that showed co-localization of TILs and PNAd-expressing cells. and FACS. Then we also discovered that some T cell populations contribute to the anti-tumor effect of TLS, while dendritic cell population play a role in TLS formation. Furthermore, tumor growth suppression was also confirmed.It is suggested that the administration of specific chemokines induces TLS formation and anti-tumor effect and suppresses tumor growth through increased TIL infiltration. The future development of cancer therapy via TLS holds promise.

[Disclosure of COI by Responsible researcher] Lead presenter is not the Responsible researcher.
[Lead presenter: Taro Suzuki] We have no financial relationships to disclose.
[Principal Researcher: Shin Kaneko] We have no financial relationships to disclose.

▲TOP

### [J-2108] I-J11-6
### Antibody therapy/Immune cell therapy (CAR-T, T cell therapy) (2)

2025 / 9 / 26 (Fri) 14:05-15:20 Room 16/(11F Visage) Room 16

[Chairperson]Yoshihiro Miyahara (Mie Univ. Grad. Sch. of Med.)

### J-2108
### Development of novel adoptive T cell therapy that overcomes tumor heterogeneity with escape variant tumor clones

[Speaker]Kiyoshi Yasui (Nagasaki Univ.Grad. Sch. of Biomed. Sci., Dept. of Oncology) [Co-author]Daisuke Ehara (Nagasaki Univ.Grad. Sch. of Biomed. Sci., Dept. of Oncology／Nagasaki Univ.Grad. Sch. of Biomed. Sci., Dept. of Dermatology)、Mitsuhiro Yoneda (Nagasaki Univ.Grad. Sch. of Biomed. Sci., Dept. of Oncology)、Situo Deng (Nagasaki Univ.Grad. Sch. of Biomed. Sci., Dept. of Oncology)、Yasunori Amaishi (CDM Center 3, Takara Bio Inc.)、Sachiko Okamoto (CDM Center 3, Takara Bio Inc.)、Daisuke Muraoka (Aichi Cancer Ctr. Res. Inst., Div. of Translational Oncoimmunology)、Naohisa Ogo (Ctr. for Drug Discovery, Grad. Div. Pham., Univ. of Shizuoka)、Akira Asai (Ctr. for Drug Discovery, Grad. Div. Pham., Univ. of Shizuoka)、Hiroyuki Murota (Nagasaki Univ.Grad. Sch. of Biomed. Sci., Dept. of Dermatology)、Hiroaki Ikeda (Nagasaki Univ.Grad. Sch. of Biomed. Sci., Dept. of Oncology)

Tumor heterogeneity is a characteristic found in most tumors, showing the emergence of tumor cell clones with immune escape capacity by losing molecules dispensable for immunological eradication including antigens and/or antigen-presenting molecules. Tumor heterogeneity is one of the major problems of cancer immunotherapy. Our previous results suggest that ferroptosis inducer (FI) and compound 433 induce cell death in antigen-negative tumor cells, collaborating with antigen-specific immune responses. In this study, we established human cell lines without tumor antigens or MHC class I expression as antigen-negative tumor cells. Using MART-1 TCR-T or mesothelin CAR-T as antigen-specific T cells, combination of these T cells with FI or 433 showed effective eradication of antigen-negative tumor cells ***in vitro.*** In NOG mice, the combination therapy exhibited anti-tumor effects in a heterogeneity tumor model. Furthermore, the mechanism of antigen-negative tumor cell eradication by antigen-specific T cells with 433 appeared to involve an apoptotic pathway. These results suggest the potential benefit of combination therapy to overcome immunotherapy limitations due to tumor heterogeneity.

[Disclosure of COI by Responsible researcher] Lead presenter is the Responsible researcher.
[Lead presenter: Kiyoshi Yasui] ■Research expenses: Takara. Bio. Inc.

### [J-2043] I-J8
### Cancer epigenetics (3)

2025 / 9 / 26 (Fri) 15:20-16:35 Room 5/(Hotel Nikko Kanazawa 4F Tsuru C) Room 5

[Chairperson]Satoshi Fujii (Dept. Mol. Path. Yokohama City Univ. Grad. Sch. Med.)

---

### J-2043
### Proton pump inhibitors intake is associated with DNA methylation

[Speaker]Harumi Yamada (Department of Epigenomics, Hoshi University／Department of Surgery, Kyoto University) [Co-author]Toshikazu Ushijima (Department of Epigenomics, Hoshi University)

Cancer risk can be assessed by accumulation of genetic alterations, namely mutations, and epigenetic alterations such as DNA methylation in non-malignant tissue, and DNA methylation is more important for gastric cancer risk than mutation frequency [Yamashita, PNAS, 115:1328, 2018]. Proton pump inhibitors (PPIs), commonly used for gastric acid-related disorders, may increase gastric cancer risk after ***Helicobacter pylori*** (***HP***) eradication. Here, in our cohort with 1,624 healthy people after ***HP*** eradication, we measured DNA methylation levels of ***RIMS1***, which was established as a risk marker gene for gastric cancer [Maeda, Gastric Cancer, 21:745, 2017]. People taking PPIs (n=80) had higher DNA methylation levels of ***RIMS1*** compared to people without PPI (n=1,544) (PPI(+) vs (-);1.9-fold, ***P***<0.0001). On the other hand, people taking H2-blockers (H2B) (n=95) had similar methylation levels compared to people without H2B (n=1,529) (H2B(+) vs (-); ***P***=0.11). This result showed that PPIs are associated with high DNA methylation levels of a risk marker gene. Therefore, it is considered that PPIs increase gastric cancer risk via acceleration of aberrant DNA methylation.

[Disclosure of COI by Responsible researcher] Lead presenter is not the Responsible researcher.
[Lead presenter: Harumi Yamada] We have no financial relationships to disclose.
[Principal Researcher: Toshikazu Ushijima] We have no financial relationships to disclose.

### [J-2044] I-J8
### Cancer epigenetics (3)

2025 / 9 / 26 (Fri) 15:20-16:35 Room 5/(Hotel Nikko Kanazawa 4F Tsuru C) Room 5

[Chairperson]Satoshi Fujii (Dept. Mol. Path. Yokohama City Univ. Grad. Sch. Med.)

---

### J-2044
### SMARCC1 Loss Impairs Differentiation and Enhances Self-Renewal in ASXL1-Mutant Hematopoietic Cells

[Speaker]Taiki Ishida (Dept. of Hematol. & Oncol., the Univ. of Tokyo) [Co-author]Toshiya Hino (Dept. of Hematol. & Oncol., the Univ. of Tokyo)、Hiroki Hayashida (Dept. of Hematol. & Oncol., the Univ. of Tokyo)、Michiaki Sato (Dept. of Hematol. & Oncol., the Univ. of Tokyo)、Yosuke Masamoto (Dept. of Hematol. & Oncol., the Univ. of Tokyo／Dept. of Cell Therapy & Transplant., Univ. of Tokyo Hosp.)、Mineo Kurokawa (Dept. of Hematol. & Oncol., the Univ. of Tokyo／Dept. of Cell Therapy & Transplant., Univ. of Tokyo Hosp.)

ASXL1 is a histone modifier regulating hematopoiesis. Its mutations are common in secondary acute myeloid leukemia (AML), linked to poor prognosis, and found in clonal hematopoiesis, raising leukemia risk. However, the mechanisms driving leukemogenesis remain unclear. In this study, to identify genes whose loss contributes to leukemogenesis in ***ASXL1***-mutant cells, we screened ***Asxl1***-mutant mouse hematopoietic stem/progenitor cells using a CRISPR/Cas9 knockout library targeting putative tumor suppressors. Self-renewal was tested via serial colony-forming assay, and we found ***Smarcc1*** loss enhanced serial replating capacity, with greater effect in ***Asxl1***-mutant cells. Furthermore, its loss cooperates with ***ASXL1*** mutations to impair differentiation, revealed by the blunted expression of Gr-1. In conclusion, ***SMARCC1*** loss drives self-renewal and blocks differentiation in ***ASXL1***-mutant cells. As a subunit of SWI/SNF chromatin remodeling complex, ***SMARCC1*** may interact with ***ASXL1*** to alter epigenetic regulation, affecting self-renewal and differentiation genes. Their cooperative mechanisms may inform targeted therapies for ***ASXL1***-mutant leukemia, pending further validation.

[Disclosure of COI by Responsible researcher] Lead presenter is not the Responsible researcher.
[Lead presenter: Taiki Ishida] We have no financial relationships to disclose.
[Principal Researcher: Mineo Kurokawa] ■Lecture fee: アストラゼネカ株式会社、アッヴィ合同会社、ブリストルマイヤーズスクイブ株式会社　■Contributions: 協和キリン株式会社、アッヴィ合同会社

### [J-2045] I-J8
### Cancer epigenetics (3)

2025 / 9 / 26 (Fri) 15:20-16:35 Room 5/(Hotel Nikko Kanazawa 4F Tsuru C) Room 5

[Chairperson]Satoshi Fujii (Dept. Mol. Path. Yokohama City Univ. Grad. Sch. Med.)

---

### J-2045
### The pathophysiological role of Equilibrative Nucleoside Transporters (ENTs) in esophageal cancer

[Speaker]Chishou Mitsuura (Dept. of Gastroenterological Surg, Faculty of Life Sciences, Kumamoto Univ.／Dept. of Mol. Physiology Faculty of Life Sciences, Kumamoto Univ.) [Co-author]Yu Nagayoshi (Dept. of Mol. Physiology Faculty of Life Sciences, Kumamoto Univ.)、Hitomi Kaneko (Dept. of Mol. Physiology Faculty of Life Sciences, Kumamoto Univ.)、Ryosuke Yamamura (Dept. of Mol. Physiology Faculty of Life Sciences, Kumamoto Univ.)、Yuki Adachi (Dept. of Gastroenterological Surg, Faculty of Life Sciences, Kumamoto Univ.／Dept. of Mol. Physiology Faculty of Life Sciences, Kumamoto Univ.)、Akira Sasao (Dept. of Mol. Physiology Faculty of Life Sciences, Kumamoto Univ.)、Chihiro Matsumoto (Dept. of Gastroenterological Surg, Faculty of Life Sciences, Kumamoto Univ.)、Kohei Yamashita (Dept. of Gastroenterological Surg, Faculty of Life Sciences, Kumamoto Univ.)、Keisuke Kosumi (Dept. of Gastroenterological Surg, Faculty of Life Sciences, Kumamoto Univ.)、Kojiro Eto (Dept. of Gastroenterological Surg, Faculty of Life Sciences, Kumamoto Univ.)、Kazuto Harada (Dept. of Gastroenterological Surg, Faculty of Life Sciences, Kumamoto Univ.)、Satoshi Ida (Dept. of Gastroenterological Surg, Faculty of Life Sciences, Kumamoto Univ.)、Yuji Miyamoto (Dept. of Gastroenterological Surg, Faculty of Life Sciences, Kumamoto Univ.)、Kazuhito Tomizawa (Dept. of Mol. Physiology Faculty of Life Sciences, Kumamoto Univ.)、Masaaki Iwatsuki (Dept. of Gastroenterological Surg, Faculty of Life Sciences, Kumamoto Univ.)

RNAs have more than 100 chemical modifications. In a previous study, we found that modified nucleosides were finally secreted to extracellular spaces. We also found that ENTs are main transporters for the secretion of modified nucleosides. Although modified nucleosides have many physiological activities including induction of abnormal cell proliferation, the pathophysiological importance of ENTs in esophageal cancer cells were almost unclear. We found that ENT4 is significantly elevated in tumor area of clinical samples by RT-qPCR. In vitro experiments, we cultured human esophageal squamous cell carcinoma cell-lines under hypoxia conditions or acidic condition for 24 hours, and found that the expression levels of ENT4 were significantly elevated. Moreover, we found that the ENT4 expression under acidic condition was due to the histone modification by ChIP-qPCR. Whereas, we generated ENT4KO cell line by CRISPR-Cas9 and found decrease of cell proliferation, more apoptosis and cell cycle delay.From these results, the ENT4 expression in esophageal squamous cell cancer might be necessary in acidic condition by Warburg effect.

[Disclosure of COI by Responsible researcher] Lead presenter is not the Responsible researcher.
[Lead presenter: Chishou Mitsuura] We have no financial relationships to disclose.
[Principal Researcher: Masaaki Iwatsuki] We have no financial relationships to disclose.

▲TOP

### [J-2046] I-J8
### Cancer epigenetics (3)

2025 / 9 / 26 (Fri) 15:20-16:35 Room 5/(Hotel Nikko Kanazawa 4F Tsuru C) Room 5

[Chairperson]Satoshi Fujii (Dept. Mol. Path. Yokohama City Univ. Grad. Sch. Med.)

---

### J-2046
### Aberrant heterochromatin activation after oncovirus infection through H3K36me2 induction

[Speaker]Fumichika Sawada (Sch. of Med., Chiba Univ.／Dept. of Mol. Oncol., Grad. Sch of Med., Chiba Univ.) [Co-author]Atsushi Okabe (Dept. of Mol. Oncol., Grad. Sch of Med., Chiba Univ.／Health and Disease Omics Ctr., Chiba Univ.)、Masaki Fukuyo (Dept. of Mol. Oncol., Grad. Sch of Med., Chiba Univ.)、Bahityar Rahmutulla (Dept. of Mol. Oncol., Grad. Sch of Med., Chiba Univ.)、Keisuke Matsusaka (Dept. of Mol. Oncol., Grad. Sch of Med., Chiba Univ.)、Patrick Tan (Duke-NUS Med. Sch.)、Atsushi Kaneda (Dept. of Mol. Oncol., Grad. Sch of Med., Chiba Univ.／Health and Disease Omics Ctr., Chiba Univ.)

The Epstein-Barr virus (EBV) causes various cancers, including gastric cancer (GC), by inducing multiple epigenetic abnormalities. Previously, we reported that abnormal activation of heterochromatin after EBV infection contributes to EBV(+) GC carcinogenesis, although the precise mechanism remains unclear. In this study, we performed ChIP-seq and Hi-C to examine H3K36me2 and chromatin structure after EBV infection. We identified 327 regions with increased H3K36me2, and 77% of these regions also increased H3K27ac levels, suggesting that H3K36me2 increase may activate enhancers. Besides, the H3K36me2 increase correlated with inactive-to-active compartment shift. We then performed RNA-seq to assess the impact of these changes on transcription. Target genes of enhancer regions with higher H3K36me2 and H3K27ac showed significantly higher activation than those with only increased H3K27ac. These target genes were also cancer-related, particularly in epithelial-mesenchymal transition and KRAS signaling. We identified 14 cancer-related genes showed higher expression in both EBV(+) GC cell lines and tissues. H3K36me2 induction after EBV infection may contribute to oncogene activation.

[Disclosure of COI by Responsible researcher] Lead presenter is not the Responsible researcher.
[Lead presenter: Fumichika Sawada] We have no financial relationships to disclose.
[Principal Researcher: Atsushi Kaneda] ■Contributions: SRL Inc. Scrum Inc. Takeda Science Foundation. Uehara Memorial Foundation.

▲TOP

### [J-2047] I-J8
### Cancer epigenetics (3)

2025 / 9 / 26 (Fri) 15:20-16:35 Room 5/(Hotel Nikko Kanazawa 4F Tsuru C) Room 5

[Chairperson]Satoshi Fujii (Dept. Mol. Path. Yokohama City Univ. Grad. Sch. Med.)

---

### J-2047
### Dissecting the Mechanisms Underlying Cancer Cell Diversity Driven by Epigenomic Dysregulation

[Speaker]Kenichi Miyata (Div. of Cancer Epigenomics, Cancer Inst., JFCR／NEXT-Ganken Program, JFCR) [Co-author]Yuxiao Yang (Div. of Cancer Epigenomics, Cancer Inst., JFCR)、Chikako Shibata (Div. of Cancer Epigenomics, Cancer Inst., JFCR)、Takaaki Furukawa (Div. of Cancer Epigenomics, Cancer Inst., JFCR)、Reo Maruyama (Div. of Cancer Epigenomics, Cancer Inst., JFCR／NEXT-Ganken Program, JFCR)

In cancer, phenotypic diversity can arise even within clones sharing the same genetic background, which promotes the emergence of clones that acquire resistance to treatment and metastatic capabilities. This presents significant clinical challenges. Nevertheless, the mechanisms underlying such phenotypic diversity remain insufficiently elucidated. To address this, we have developed a novel platform that allows for the integrated analysis of clone dynamics and phenotypic diversity at the single-cell level.In this study, we focused on the frequently occurring loss-of-function mutations in the SWI/SNF complex across various cancers and analyzed them using our platform. Our findings reveal that this defectiveness induce chromatin destabilization, leading to increased transcriptional diversity. Furthermore, these alterations in transcriptional dynamics were found to induce a shift towards more malignant phenotypes in certain clones. This method not only serves as a powerful approach to elucidate the mechanisms generating intratumoral heterogeneity (ITH) but is also expected to serve as a platform technology for the development of novel therapeutic strategies targeting ITH.

[Disclosure of COI by Responsible researcher] Lead presenter is not the Responsible researcher.
[Lead presenter: Kenichi Miyata] We have no financial relationships to disclose.
[Principal Researcher: Reo Maruyama] We have no financial relationships to disclose.

▲TOP

### [J-2048] I-J8
### Cancer epigenetics (3)

2025 / 9 / 26 (Fri) 15:20-16:35 Room 5/(Hotel Nikko Kanazawa 4F Tsuru C) Room 5

[Chairperson]Satoshi Fujii (Dept. Mol. Path. Yokohama City Univ. Grad. Sch. Med.)

---

### J-2048
### Dynamics of mRNA modification in cancer microenvironment and EMT

[Speaker]Ryota Kurimoto (Department of Systems BioMedicine, Institute of Science Tokyo／Department of Medical Oncology, Chiba University)
[Co-author]Hiroshi Asahara (Department of Systems BioMedicine, Institute of Science Tokyo)

Epithelial-mesenchymal transition (EMT) and the tumor microenvironment (TME) are key drivers of cancer progression and therapy resistance, yet the role of RNA modifications (epitranscriptome) in regulating these processes remains unclear.In this study, we re-analyzed TCGA and public PDX transcriptomes to identify RNA-modifying enzymes associated with EMT. Several enzymes showed inverse correlations with EMT scores, implying potential EMT-suppressive roles.Using TGFb-induced EMT models in lung adenocarcinoma cells, mRNA LC/MS analysis revealed decreased levels of methylated GTP, mediated by a specific RNA-modifying enzyme. Site-specific incorporation of methylated GTP into synthetic mRNAs significantly reduced translation efficiency in vitro.Ribosome profiling further showed that knockdown of the enzyme enhanced translation of EMT- and TME-related genes, including chemokines.These findings suggest that this RNA modification enzyme suppresses EMT and TME remodeling by repressing translation in a modification-dependent manner, and may serve as a novel therapeutic target in cancer.

[Disclosure of COI by Responsible researcher] Lead presenter is the Responsible researcher.
[Lead presenter: Ryota Kurimoto] ■Research funds from company: 持田記念財団、がん研究振興財団、科研費

### [J-2055] I-J14-3
### Mechanisms of Cancer Drug Resistance & Strategies to Overcome It

2025 / 9 / 26 (Fri) 15:20-16:35 Room 7/(Hotel Nikko Kanazawa 3F Kujaku B) Room 7

[Chairperson]Shunsuke Kato (Dept. Clin. Oncol., Juntendo Univ., Grad. Sch. Med.)

---

### J-2055
### Effect of acquisition of gemcitabine resistance in pancreatic cancer cells on chromosomal instability

[Speaker]Keiko Yamakawa (Mol. Onco. Path., Dept. Path. Host-Defense, Fac. Med., Kagawa Univ.) [Co-author]Yuri Mukai (Mol. Onco. Path., Dept. Path. Host-Defense, Fac. Med., Kagawa Univ.)、Juanjuan Ye (Mol. Onco. Path., Dept. Path. Host-Defense, Fac. Med., Kagawa Univ.)、Saki Higuchi (Mol. Onco. Path., Dept. Path. Host-Defense, Fac. Med., Kagawa Univ.)、Kazuki Azuma (Mol. Onco. Path., Dept. Path. Host-Defense, Fac. Med., Kagawa Univ.)、Ken Nakagawa (Mol. Onco. Path., Dept. Path. Host-Defense, Fac. Med., Kagawa Univ.)、Sachiko Shokei (Mol. Onco. Path., Dept. Path. Host-Defense, Fac. Med., Kagawa Univ.)、Emi Ibuki (Mol. Onco. Path., Dept. Path. Host-Defense, Fac. Med., Kagawa Univ.)、Futoshi Suizu (Mol. Onco. Path., Dept. Path. Host-Defense, Fac. Med., Kagawa Univ.／Pathology, Dept. Med. Tech., Kagawa Pref. Univ. Health Sci.)、Hideyuki Tanabe (Res. Center Integ. Evol. Sci., Grad. Univ. Adv. Studies, SOKENDAI)、Kyuichi Kadota (Mol. Onco. Path., Dept. Path. Host-Defense, Fac. Med., Kagawa Univ.)

In chemotherapy for pancreatic ductal adenocarcinoma (PDAC), the acquisition of drug resistance in pancreatic cancer cells contributes to poor prognosis. In this study, we examined the effect on chromosomal instability related to drug resistance and cancer malignancy. Gemcitabine-resistant (GR) human pancreatic cancer cells were established by exposing PANC-1 and PK-1 to stepwise increasing concentrations of gemcitabine (PA-GR and PK-GR). Chromosomal analysis of parental and GR cells was performed using QFH-banding and multicolor FISH techniques. In parent cells, the proportion of translocated chromosomes was significantly higher in PK-1 than in PANC-1. In PK-GR, the proportion of chromosomes with two or more breakpoints was increased compared with that of the parental cells, but no change was observed in PA-GR. The chromosome modal number was significantly decreased in PK-GR compared with that of the parental cells. In addition, both GR cells were found to have more than 100 chromosomes (PA-GR: 2/17 cases, PK-GR: 1/18 cases). These data suggest that both numerical and structural chromosomal abnormalities are induced in GR cells during surviving cells.

[Disclosure of COI by Responsible researcher] Lead presenter is not the Responsible researcher.
[Lead presenter: Keiko Yamakawa] We have no financial relationships to disclose.
[Principal Researcher: Kyuichi Kadota] We have no financial relationships to disclose.

### [J-2056] I-J14-3
### Mechanisms of Cancer Drug Resistance & Strategies to Overcome It

2025 / 9 / 26 (Fri) 15:20-16:35 Room 7/(Hotel Nikko Kanazawa 3F Kujaku B) Room 7

[Chairperson]Shunsuke Kato (Dept. Clin. Oncol., Juntendo Univ., Grad. Sch. Med.)

---

### J-2056
### DCLK1 inhibition following gemcitabine decreases survival rate of pancreatic cancer cells escaped from senescence

[Speaker]Arata Nishimoto (Dept. of Pharm., Fac. of Pharm. Sci., Sanyo-Onoda City University) [Co-author]Yohei Miyaguni (Dept. of Pharm., Fac. of Pharm. Sci., Sanyo-Onoda City University)、Jinpei Hasegawa (Dept. of Pharm., Fac. of Pharm. Sci., Sanyo-Onoda City University)、Ami Tokushige (Dept. of Pharm., Fac. of Pharm. Sci., Sanyo-Onoda City University)、Satoka Yamanouchi (Dept. of Pharm., Fac. of Pharm. Sci., Sanyo-Onoda City University)

Cancer cells treated with gemcitabine cause DNA damage and growth arrest, leading to cellular senescence. Continuing cell culture following gemcitabine removal, cancer cells escaped from gemcitabine-induced senescence have been emerged. In this study, we examined the effect of gemcitabine and the inhibitor of DCLK1, a marker of pancreatic cancer stem-like cells, on the viability of PANC-1 cancer cells escaped from gemcitabine-induced senescence. DCLK1 inhibition following gemcitabine treatment significantly decreased the viability of PANC-1 cancer cells escaped from gemcitabine-induced senescence compared with individual treatment with gemcitabine, DCLK1 inhibitor or simultaneous treatment with gemcitabine and DCLK1 inhibitor. Furthermore, DCLK1 inhibition following gemcitabine treatment reduced sphere formation ability of PANC-1 cancer cells escaped from gemcitabine-induced senescence compared with individual treatment or simultaneous treatment with gemcitabine and DCLK1 inhibitor. Taken together, these results suggest that DCLK1 inhibition following gemcitabine treatment decreased survival rate of PANC-1 cancer cells escaped from gemcitabine-induced senescence.

[Disclosure of COI by Responsible researcher] Lead presenter is the Responsible researcher.
[Lead presenter: Arata Nishimoto] We have no financial relationships to disclose.

▲TOP

### [J-2057] I-J14-3
### Mechanisms of Cancer Drug Resistance & Strategies to Overcome It

2025 / 9 / 26 (Fri) 15:20-16:35 Room 7/(Hotel Nikko Kanazawa 3F Kujaku B) Room 7

[Chairperson]Shunsuke Kato (Dept. Clin. Oncol., Juntendo Univ., Grad. Sch. Med.)

---

### J-2057
### PDAC cell treated by gemcitabine acquire chemoresistance by transition of nucleotide metabolism via TME.

[Speaker]Kenta Takeuchi (IMSUT, Dept, of Rege. Med.) [Co-author]Yuya Yamamoto (Dept. of General surg. Chiba univ.)、Yohei Miyagi (Kanagawa Cancer Ctr. Res. Inst.)、Naoto Yamamoto (Kanagawa Cancer Ctr.)、Yoichi Furukawa (IMSUT, Div. of Clin. Genome Res.)、Kiyoshi Yamaguchi (IMSUT, Div. of Clin. Genome Res.)、Naoki Tanimizu (IMSUT, Dept, of Rege. Med.)、Hideki Taniguchi (IMSUT, Dept, of Rege. Med.／Dept. of Rege. Med. Yokohama City univ.)

We previously reported a pancreatic ductal adenocarcinoma (PDAC) organoid associated with TME that is named FPCO. Since FPCO recapitulates strong chemoresistance, we utilize the FPCO system to investigate how TME affects the drug resistance of PDAC cells. Gemcitabine(dFdC) widely used in a standard therapy for PDAC is incorporated to DNA as dFdCTP and shows therapeutic effect. However, even if the gemcitabine once shows therapeutic effect, it gradually loses the effect in most cases. Thus, PDAC cells probably become resistance against gemcitabine during the treatment. A recent study reported that PDAC cells upregulate ribonucleotide reductases (RRMs) that synthesize dCDP from CDP in response to gemcitabine treatment, which competes with dFdCTP. Consistently, we found that PDAC cells upregulate RRMs after gemcitabine treatment. However, RRMs inhibition alone did not affect chemoresistance. Since we found that gemcitabine treatment changed expression of several genes related to nucleotide metabolism, we hypothesize that PDAC cells may globally reconstruct nucleotide metabolism in response to gemcitabine. In this study, we report about additional transitions and discussed with them.

[Disclosure of COI by Responsible researcher] Lead presenter is not the Responsible researcher.
[Lead presenter: Kenta Takeuchi] We have no financial relationships to disclose.
[Principal Researcher: Yohei Miyagi] We have no financial relationships to disclose.

### [J-2058] I-J14-3
### Mechanisms of Cancer Drug Resistance & Strategies to Overcome It

2025 / 9 / 26 (Fri) 15:20-16:35 Room 7/(Hotel Nikko Kanazawa 3F Kujaku B) Room 7

[Chairperson]Shunsuke Kato (Dept. Clin. Oncol., Juntendo Univ., Grad. Sch. Med.)

### J-2058
### Exploring status of Drug-Resistant Cancer Cells with Teton system

[Speaker]Yasuhiko Haga (Grad. Sch. of Front. Sci., Univ. of Toky) [Co-author]Ayako Suzuki (Grad. Sch. of Front. Sci., Univ. of Toky)、Yutaka Suzuki (Grad. Sch. of Front. Sci., Univ. of Toky)

It is widely recognized that cancer recurrence frequently arises from drug-resistant persistent cancer cells. These cells evade treatment through diverse strategies, including genomic mutations and transcriptomic and epigenomic alterations. Understanding gene expression and epigenomic changes in drug resistance is vital for developing strategies against cancer recurrence. To elucidate drug resistance mechanisms in lung cancer, we subjected PC9 cells to a 3-day gefitinib treatment followed by a 3-day recovery period, repeating this cycle 20 times. The resulting drug-dosed PC9 cells were resistant to both gefitinib and osimertinib without secondary genomic mutations and displayed distinct morphology compared to parental PC9 cells. In this study, we investigated the relationship between molecular and morphological changes using the Teton system, which quantifies 350 RNA targets, 50 protein targets, and morphological features. We collected Teton data from parental, gefitinib-dosed, and resistant PC9 cells at six time points. The mean transcripts and proteins per cell were 1,218 and 171, respectively.

[Disclosure of COI by Responsible researcher] Lead presenter is the Responsible researcher.
[Lead presenter: Yasuhiko Haga] We have no financial relationships to disclose.

### [J-2059] I-J14-3
### Mechanisms of Cancer Drug Resistance & Strategies to Overcome It

2025 / 9 / 26 (Fri) 15:20-16:35 Room 7/(Hotel Nikko Kanazawa 3F Kujaku B) Room 7

[Chairperson]Shunsuke Kato (Dept. Clin. Oncol., Juntendo Univ., Grad. Sch. Med.)

---

### J-2059
### Auranofin resensitizes ferroptosis-resistant lung cancer cells to ferroptosis inducers

[Speaker]Hao Li (Department of Molecular and Medical Pharmacology, Kumamoto University／Biomedical Chemistry major, Kwansei Gakuin University) [Co-author]Toshiro Moroishi (Department of Molecular and Medical Pharmacology, Kumamoto University／Division of Cellular Dynamics, Institute of Science Tokyo)、Ikuko Yao (Biomedical Chemistry major, Kwansei Gakuin University)、Shuran Li (Department of Molecular and Medical Pharmacology, Kumamoto University)

SAS induced ferroptosis in vitro; however, its efficacy in vivo was limited, possibly because of factors, such as nutrient deprivation and high cell density, in the microenvironment that suppressed the activities of Yes-associated protein (YAP) and transcriptional coactivator with PDZ-binding motif (TAZ), key regulators of ferroptosis resistance. Screening of 2,483 drugs revealed AUR as a compound resensitizing the YAP/TAZ-deficient lung cancer cells to ferroptosis. Moreover, SAS and AUR combination significantly enhanced lipid peroxidation and reactive oxygen species accumulation, further driving ferroptosis in cells. This combination effectively inhibited tumor growth and enhanced survival in a murine lung cancer model.

[Disclosure of COI by Responsible researcher] Lead presenter is not the Responsible researcher.
[Lead presenter: Hao Li] We have no financial relationships to disclose.
[Principal Researcher: Toshiro Moroishi] We have no financial relationships to disclose.

▲TOP

### [J-2060] I-J14-3
### Mechanisms of Cancer Drug Resistance & Strategies to Overcome It

2025 / 9 / 26 (Fri) 15:20-16:35 Room 7/(Hotel Nikko Kanazawa 3F Kujaku B) Room 7

[Chairperson]Shunsuke Kato (Dept. Clin. Oncol., Juntendo Univ., Grad. Sch. Med.)

### J-2060
### Elucidating Exosome-Mediated Mechanisms of Tumor Progression in Drug-Resistant Ovarian Cancer

[Speaker]Mai Honjo (RCAST, Univ.Tokyo) [Co-author]Ayuko Hoshino (RCAST, Univ.Tokyo)

Ovarian cancer has the highest mortality among gynecological cancers. While serous carcinoma is globally common, clear cell carcinoma (CCC) predominates in Japan and is notable for its slow growth but high chemo-resistance and metastatic potential. We investigated the role of exosomes in tumor progression after chemo-resistance. Exosome (50~150 nm vesicles) carry selective proteins, lipids, and nucleic acids, influencing cancer behavior. Using CCC cells, we established a chemo-resistant line and compared IC50 values and exosome properties before and after resistance. While exosome size and total protein were similar, particle number was reduced in resistant cells. Biodistribution analysis revealed a shift: exosome from parental cells were taken up by the lung, while those from resistant cells showed greater liver uptake, though differences were not statistically significant. These findings suggest that as CCC acquires drug resistance, it may alter exosome-mediated organ targeting, potentially contributing to metastatic behavior.

[Disclosure of COI by Responsible researcher] Lead presenter is not the Responsible researcher.
[Lead presenter: Mai Honjo] We have no financial relationships to disclose.
[Principal Researcher: Ayuko Hoshino] We have no financial relationships to disclose.

### [E-2049] I-E15
### Evaluation & Prediction of Pharmacological Effects

2025 / 9 / 26 (Fri) 15:20-16:35 Room 8/(Hotel Nikko Kanazawa 3F Kujaku C) Room 8

[Chairperson]Riu Yamashita (EPOC, National Cancer Center)

---

### E-2049
### Pevonedistat response in Gastric Cancer: Proteomics for drug response prediction and biomarker discovery

[Speaker]Satoshi Muraoka (Natl. Inst. Of Biomed. Inov., Heal. & Nutl) [Co-author]Mayumi Ishida (Natl. Inst. Of Biomed. Inov., Heal. & Nutl)、Masayo Hirano (Natl. Inst. Of Biomed. Inov., Heal. & Nutl)、Narimi Takaai (Natl. Inst. Of Biomed. Inov., Heal. & Nutl)、Hidekazu Hirano (Dept. Gastrointestinal. Med. Oncology, Natl. Cancer. Ctr. Hosp.)、Hirokazu Shoji (Dept. Gastrointestinal. Med. Oncology, Natl. Cancer. Ctr. Hosp.)、Daisuke Takahari (Dept. Gastrointestinal. Cancer. Inst. Hosp. Of JFCR)、Akira Ooki (Dept. Gastrointestinal. Cancer. Inst. Hosp. Of JFCR)、Natsuko Okita (Dept. Gastrointestinal. Med. Oncology, Natl. Cancer. Ctr. Hosp.)、Kengo Nagashima (Biostatistics Unit, Clin. & Trans. Res. Ctr. Keio Univ. Hosp)、Kensei Yamaguchi (Dept. Gastrointestinal. Cancer. Inst. Hosp. Of JFCR)、Ken Kato (Dept. Gastrointestinal. Med. Oncology, Natl. Cancer. Ctr. Hosp.)、Narikazu Boku (Dept. Gastrointestinal. Med. Oncology, Natl. Cancer. Ctr. Hosp.／IMSUT Hosp., Inst. Of Med. Sci. Tokyo Univ)、Jun Adachi (Natl. Inst. Of Biomed. Inov., Heal. & Nutl／for Drug Dis., Grad. Sch. Of Pharm. Sci., Kyoto Univ.)

Based on synergistic antitumor effects of NEDD8 activating enzyme inhibitor and a platinum-based drug in preclinical studies, Phase I study was conducted to investigate the safety and tolerability of pevonedistat in combination with capecitabine plus oxaliplatin in patients with gastric cancer refractory or intolerant to fluoropyrimidines, platinum containing drugs and taxane. Here, we collected serial endoscopic biopsy specimens obtained before, during, after third-line treatment with drug and performed proteome analysis of the effect of pevonedistat. Furthermore, we tried to develop novel biomarkers in blood for drug response through proteomics analysis of serum EVs and serum collected at the same time as the biopsy specimens. The 413 tissue proteins were found to be a positive correlation with Progression Free Survival. Among them, we identified 4 proteins as biomarkers in serum EVs for the therapeutic effect of this combination chemotherapy. Our approach will provide substrate profiling of direct drug response in cancer tissue, and the serum or serum EVs proteome of the same patients could be shown as a potential monitoring biomarker for novel drug response molecules in blood.

[Disclosure of COI by Responsible researcher] Lead presenter is not the Responsible researcher.
[Lead presenter: Satoshi Muraoka] We have no financial relationships to disclose.
[Principal Researcher: Jun Adachi] ■Research expenses: タケダ薬品

### [E-2050] I-E15
### Evaluation & Prediction of Pharmacological Effects

2025 / 9 / 26 (Fri) 15:20-16:35 Room 8/(Hotel Nikko Kanazawa 3F Kujaku C) Room 8

[Chairperson]Riu Yamashita (EPOC, National Cancer Center)

### E-2050
### Risk Stratification and Therapeutic Suggestions for High-risk RUNX1::RUNX1T1-positive pAML patients

[Speaker]Hua You (Children's Hospital of Chongqing Medical University, China) [Co-author]Yang Xun (Children's Hospital of Chongqing Medical University, China)、Yali Shen (Children's Hospital of Chongqing Medical University, China)、Hua Yang (Children's Hospital of Chongqing Medical University, China)、Jinsong Yan (Children's Hospital of Chongqing Medical University, China)、Yu Tao (Children's Hospital of Chongqing Medical University, China)、Yance Feng (Children's Hospital of Chongqing Medical University, China)、Li Wei (Children's Hospital of Chongqing Medical University, China)、Huihan Wang (Children's Hospital of Chongqing Medical University, China)

Pediatric AML patients (pAML) with RUNX1::RUNX1T1 (RR) fusion gene are generally considered low-risk, yet a subset still faces poor survival outcome. We developed the RR pAML model, a prognostic tool incorporating two clinical factors at the first complete remission stage, validated in 284 RR+ pAML patients (training/internal validation at 7:3 ratio) and 214 external validation cases from Southwestern and Northeastern Chinese clinical centers. The model consistently identified over 30% RR+ high-risk patients with higher relapse and mortality rates with no benefit from stem cell transplantation. The c.kit exon 17 mutation adversely impacted the survival of high-risk patients, while dasatinib effectively improved the survival of these patients. For c.kit mutation-negative high-risk group, transcriptional profiling and drug sensitivity analysis suggested immune checkpoint inhibitors and small-molecular drugs as potential therapeutic options. This model enhanced risk stratification and supports more informed therapeutic decision-making for RR+ pAML patients.

[Disclosure of COI by Responsible researcher] Lead presenter is the Responsible researcher.
[Lead presenter: Hua You] We have no financial relationships to disclose.

▲TOP

### [E-2051] I-E15
### Evaluation & Prediction of Pharmacological Effects

2025 / 9 / 26 (Fri) 15:20-16:35 Room 8/(Hotel Nikko Kanazawa 3F Kujaku C) Room 8

[Chairperson]Riu Yamashita (EPOC, National Cancer Center)

---

### E-2051
### Association of ctDNA level with prognosis and efficacy in mCRC with anti-EGFR/anti-VEGF therapies: PARADIGM study

[Speaker]Katsuya Tsuchihara (National Cancer Center, EPOC) [Co-author]Riu Yamashita (National Cancer Center, EPOC)、Takayuki Yoshino (National Cancer Center Hospital East)、Kohei Shitara (National Cancer Center Hospital East)、Jun Watanabe (Kansai Medical University Hospital)、Kentaro Yamazaki (Shizuoka Cancer Center)、Eiji Oki (Kyushu University Hospital)、Takeo Sato (Kitasato University Hospital)、Takeshi Naitoh (Kitasato University Hospital)、Yoshito Komatsu (Hokkaido University Hospital)、Takeshi Kato (Osaka National Hospital)、Kouji Yamamoto (Yokohama City University)、Kazunori Yamanaka (Takeda Pharmaceutical Company Limited)、Ikuo Mori (Takeda Pharmaceutical Company Limited)、Junpei Soeda (Takeda Pharmaceutical Company Limited)、Kiwamu Akagi (International University of Health and Welfare)、Atsushi Ochiai (Tokyo University of Science)、Kei Muro (Aichi Cancer Center)、Hiroyuki Uetake (Disaster Medical Center)

In the phase 3 PARADIGM trial (NCT02394795), adding panitumumab (PAN) to FOLFOX6 improved median overall survival (mOS) compared with bevacizumab (BEV) in patients with RAS wild-type (WT) metastatic colorectal cancer (mCRC). This exploratory analysis investigates prognostic and therapeutic implications of circulating tumor (ct) DNA levels in patients receiving anti-EGFR vs anti-VEGF therapies.Patients were stratified into low, medium, and high baseline ctDNA level groups. With PAN treatment, mOS (95% CI) improved in a ctDNA-dependent manner (low: 55.1 months [36.3-NR], medium: 36.9 [26.5-51.3], high: 29.6 [25.7-34.8]). For BEV treatment, the longest mOS was observed in the low ctDNA group (49.6 months [42.9-66.6]), while the medium and high ctDNA groups had shorter and comparable mOS (medium: 30.9 (27.7-34.5), high: 27.0 [25.5-31.1]).Furthermore, longer post-treatment survival was observed with patients who had lower ctDNA levels at the post-treatment. More patients showed low ctDNA levels with PAN (36.7%) compared with BEV (30.6%).These results underscore differences in drug efficacy and ctDNA dynamics, which may inform personalized cancer treatment strategies.

[Disclosure of COI by Responsible researcher] Lead presenter is the Responsible researcher.
[Lead presenter: Katsuya Tsuchihara] ■Research funds from company: 武田薬品工業　■Research expenses: キヤノンメディカルシステムズ

▲TOP

### [E-2052] I-E15
### Evaluation & Prediction of Pharmacological Effects

2025 / 9 / 26 (Fri) 15:20-16:35 Room 8/(Hotel Nikko Kanazawa 3F Kujaku C) Room 8

[Chairperson]Riu Yamashita (EPOC, National Cancer Center)

---

### E-2052
### Multicohort Retrospective Validation of a Predictive Biomarker for Irinotecan

[Speaker]Koji Ando (Department of Surgery and Science, Kyushu University) [Co-author]Eiji Oki (Department of Surgery and Science, Kyushu University)、Tetsuro Kawazoe (Department of Surgery and Science, Kyushu University)、Sho Nambara (Department of Surgery and Science, Kyushu University)、Yasuo Tsuda (Department of Surgery and Science, Kyushu University)、Tomonori Nakanoko (Department of Surgery and Science, Kyushu University)、Tomoharu Yoshizumi (Department of Surgery and Science, Kyushu University)

Purpose: Irinotecan targets topoisomerase I (topoI) and is used for colorectal and gastric cancers. Response rates vary (10 to 30%) and no predictive biomarker exists. We developed P-topoI-Dx, an immunohistochemistry (IHC)-based test to stratify patients by response.Patients and Methods: We retrospectively studied a gastric cancer (GC) training set (n=79), a validation set (n=27), and eight colorectal cancer (CRC) cohorts (n=176). Progression-free survival (PFS) more than 6 months defined a positive response. Formalin-fixed, paraffin-embedded samples were stained with a phospho-specific topoI-Ser10 antibody (topoI-pS10) and analyzed.Results: In GC, a 35% threshold optimized performance. In the training set, sensitivity was 76.6, specificity 68.8%, positive predictive value (PPV) 92.5%, and negative predictive value (NPV) 42.3%. The validation set showed 82.4% sensitivity, 70.0% specificity, with PPV and NPV of 82.4% and 70.0%. In CRC, using a 40% threshold yielded 87.5% sensitivity, 70.0% specificity, 70.7% PPV, and 87.0% NPV.Conclusion: Retrospective analysis of 282 patients validated P-topoI-Dx as a predictive biomarker for irinotecan response.

[Disclosure of COI by Responsible researcher] Lead presenter is the Responsible researcher.
[Lead presenter: Koji Ando] We have no financial relationships to disclose.

### [E-2053] I-E15
### Evaluation & Prediction of Pharmacological Effects

2025 / 9 / 26 (Fri) 15:20-16:35 Room 8/(Hotel Nikko Kanazawa 3F Kujaku C) Room 8

[Chairperson]Riu Yamashita (EPOC, National Cancer Center)

---

### E-2053
### TLS predicts response and is associated with favorable outcomes in breast cancer patients receiving NAC

[Speaker]Masafumi Shimoda (Dept. Breast & Endocrine Surg., Grad. Sch. Med., Univ. Osaka) [Co-author]Sayumi Imamura (Dept. Breast & Endocrine Surg., Grad. Sch. Med., Univ. Osaka)、Yuichi Motoyama (Dept. Pathol., Grad. Sch. Med., Univ. Osaka)、Chieko Mishima (Dept. Breast & Endocrine Surg., Grad. Sch. Med., Univ. Osaka)、Kenzo Shimazu (Dept. Breast & Endocrine Surg., Grad. Sch. Med., Univ. Osaka)

Tertiary lymphoid structures (TLS) in tumor tissues regulate local immune responses, but their impact on therapeutic outcomes in breast cancer remains unclear. This retrospective cohort study investigated associations between TLS and pathological complete response in breast (bpCR) and recurrence following NAC. We analyzed 298 patients who underwent vacuum-assisted breast biopsy prior to NAC followed by surgery (2012-2022). Baseline characteristics: T2+ (84%), N+ (71%), HG3 (56%), ER+/HER2– (47%), HER2+ (34%), ER–/HER2– (19%). TLS density was dichotomized based on ROC analysis for bpCR, yielding TLS-high (41%) and TLS-low (59%) groups. Overall bpCR rate was 31%. Univariate analysis showed bpCR associated with premenopausal status, HG3, ER–, HER2+, TLS-high, and TIL-high. Multivariate analysis identified HG3, ER–, HER2+, and TLS-high as independent predictors of bpCR. Log-rank testing revealed significant associations between DRFS and T1, bpCR, and TLS-high. Cox regression identified TLS-high as the sole independent prognostic factor. Our findings suggest TLS density is both a predictive factor for bpCR and a potential prognostic factor in breast cancer patients receiving NAC.

[Disclosure of COI by Responsible researcher] Lead presenter is the Responsible researcher.
[Lead presenter: Masafumi Shimoda] ■Research funds from company: 科研費基盤C 24K11803

### [E-2054] I-E15
### Evaluation & Prediction of Pharmacological Effects

2025 / 9 / 26 (Fri) 15:20-16:35 Room 8/(Hotel Nikko Kanazawa 3F Kujaku C) Room 8

[Chairperson]Riu Yamashita (EPOC, National Cancer Center)

### E-2054
### 3D Bone Marrow Niche: Scalable and Predictive Drug Screening Platform for Acute Myeloid Leukemia and Multiple Myeloma

[Speaker]Talita Stessuk (Crown Bioscience Netherlands) [Co-author]Marrit Putker (Crown Bioscience Netherlands)、Dorrith Verstegen (Crown Bioscience Netherlands)、Sophia Ajala (Crown Bioscience Netherlands)、Hanna Vermeer (Crown Bioscience Netherlands)、Afsaneh Golestanil (Crown Bioscience Netherlands)、Jolie Flach (Crown Bioscience Netherlands)、Nataliia Beztsinna (Crown Bioscience Netherlands)、Ludovic Bourre (Crown Bioscience Netherlands)

Preclinical drug screening for Acute Myeloid Leukemia (AML) and Multiple Myeloma (MM) is limited by poor ex vivo viability of primary cells. Conventional short-term suspension cultures fail to mimic in vivo responses. Here, we developed a 3D bone marrow niche (BMN) platform using reproducible endothelial-mesenchymal networks to support malignant cell integration and drug response assessment.
Bone marrow and blood from newly diagnosed and relapsed/refractory AML and MM patients were processed to isolate CD34+ and CD138+ cells. Mesenchymal and endothelial cells were embedded in a 3D 384-well format to form networks. Cancer cells were added, followed by drug exposure for 6–7 days, and analyzed via high-content imaging. 3D BMNs were found homogeneous and to supported AML/MM cell outgrowth (confirmed via Ki67). IF staining showed BMN maturation (CD31, fibronectin), and niche phenotype and malignant cell proportion were confirmed by FACS. Dose-dependent Cytarabine and Daunorubicin responses were detected.
Our 3D BMN platform enables physiologically relevant, medium-throughput drug screening in primary hematologic malignancies and allows parallel assessment of effects on niche components.

[Disclosure of COI by Responsible researcher] Lead presenter is the Responsible researcher.
[Lead presenter: Talita Stessuk] ■ employment by a company related to the presentation: Crown Bioscience Netherlands

▲TOP

### [E-2061] I-E10-3
### Telomere/Aging/Senescence/Immortalization/Cell differentiation/Others

2025 / 9 / 26 (Fri) 15:20-16:35 Room 9/(Hotel Nikko Kanazawa 3F Lumiere) Room 9

[Chairperson]Akimitsu Konishi (Dept. Biochem., Dokkyo Med. Univ., Scho. Med.)

---

### E-2061
### HMMR Identifies a Transit-Amplifying Cell Population with TA-Specific Mitotic Behavior

[Speaker]Yuko M. Kiyosue (Dept. Mol. Genet., Inst. Biomed. Sci., Kansai Med. Univ.) [Co-author]Naoko Tokushige (Lab. Mol. Cell. Dyn., RIKEN BDR)、Shoko Sakai (Lab. Mol. Cell. Dyn., RIKEN BDR)、Akiko Hayashi (Lab. Mol. Cell. Dyn., RIKEN BDR)、Richi Nakatake (Dept. Pancreatobiliary Surg., Kansai Med. Univ.)、Toru Matsuura (Dept. Path., Kansai Med. Univ.)、Tomoatsu Hayashi (Lab. Mol. Genet. Inf., Inst. Quant. Biosci., Univ. Tokyo)、Tetsu Akiyama (Lab. Mol. Genet. Inf., Inst. Quant. Biosci., Univ. Tokyo)、Yoshihiro Kawasaki (Dept. Mol. Genet., Inst. Biomed. Sci., Kansai Med. Univ.／Div. Tumor Pathol., NIR-PIT Res. Inst., Kansai Med. Univ.)

In the intestinal epithelium, transit-amplifying (TA) cells arise from stem cells and undergo rapid proliferation before terminal differentiation. Despite their critical role in epithelial renewal, the lack of specific markers for TA cells has hindered their precise characterization. Here, we identify ***HMMR*** as a novel and specific marker of TA cells, initially discovered in mouse intestinal organoids and subsequently confirmed in human intestinal tissues. Unlike conventional proliferation-associated markers such as ***MKI67***, ***HMMR*** defines a distinct subset of epithelial cells with a unique spatial and transcriptional profile. We further identified a mitosis-related gene module co-expressed with ***HMMR*** but not with ***MKI67***, suggesting that TA cells may employ distinct regulatory mechanisms for mitotic control. Live imaging of mouse intestinal organoids revealed that mitotic TA cells predominantly divide with their spindle axis parallel to the basement membrane, a geometry indicative of symmetric division. Together, our findings establish ***HMMR*** as a robust TA cell marker and provide new insights into the proliferative dynamics and division orientation of these transient progenitors.

[Disclosure of COI by Responsible researcher] Lead presenter is the Responsible researcher.
[Lead presenter: Yuko M. Kiyosue] ■Research funds from company: 科学技術振興機構（JST）

▲TOP

### [E-2062] I-E10-3
### Telomere/Aging/Senescence/Immortalization/Cell differentiation/Others

2025 / 9 / 26 (Fri) 15:20-16:35 Room 9/(Hotel Nikko Kanazawa 3F Lumiere) Room 9

[Chairperson]Akimitsu Konishi (Dept. Biochem., Dokkyo Med. Univ., Scho. Med.)

---

### E-2062
### Osteocyte-like Differentiation of Osteosarcoma by Inorganic Phosphate treatment

[Speaker]Yuya Suzuki (Div. Experiment. Chemother., Cancer Chemother. Ctr., JFCR／Dept. Cancer Therapeutics, Grad. Sch. Pharm. Sci., Nagoya City Univ.) [Co-author]Makoto Takeutchi (Div. Experiment. Chemother., Cancer Chemother. Ctr., JFCR／Dept. Orthop. Surg., Fac. Med., Inst. Biomed. Sci., Tokushima Univ.)、Sumie Koike (Div. Experiment. Chemother., Cancer Chemother. Ctr., JFCR)、Ai Takemoto (Div. Experiment. Chemother., Cancer Chemother. Ctr., JFCR)、Ryohei Katayama (Div. Experiment. Chemother., Cancer Chemother. Ctr., JFCR／Dept. Cancer Therapeutics, Grad. Sch. Pharm. Sci., Nagoya City Univ.／CBMS, Grad. Sch. Front. Sci., Univ. of Tokyo)、Satoshi Takagi (Div. Experiment. Chemother., Cancer Chemother. Ctr., JFCR)

Osteosarcoma (OS) with pulmonary metastasis has a poor prognosis. OS cells deviate from the normal bone differentiation pathway and retain high invasive and metastatic potential. We previously showed that OS cells cultured in the osteogenic induction medium (OIM) differentiated into osteocyte-like cells. Here, we investigated the effect of inorganic phosphate (Pi), one of the OIM components and a key player in the mineralization of osteomalacia, on the differentiation of OS. To evaluate the effect of Pi on osteogenic differentiation and matrix calcification, we cultured OS cell lines in Pi-supplemented medium and found that several types of Pi increased mRNA levels of bone differentiation markers and calcium deposition by performing quantitative RT-PCR and Alizarin Red staining. Notably, supplementation of disodium phosphate (Na2HPO4) demonstrated potent differentiation inducing effects. Furthermore, a cell migration assay revealed that Pi-induced differentiation reduced OS cell motility. These results demonstrate that Pi is an important inducer of osteocyte-like differentiation in OS cells and might lead to the discovery of novel therapeutic strategies to suppress OS metastasis.

[Disclosure of COI by Responsible researcher] Lead presenter is not the Responsible researcher.
[Lead presenter: Yuya Suzuki] We have no financial relationships to disclose.
[Principal Researcher: Ryohei Katayama] ■Research funds from company: 中外製薬、TOPPAN、UBE、BML　■Patent fee: 栄研化学

▲TOP

### [E-2063] I-E10-3
### Telomere/Aging/Senescence/Immortalization/Cell differentiation/Others

2025 / 9 / 26 (Fri) 15:20-16:35 Room 9/(Hotel Nikko Kanazawa 3F Lumiere) Room 9

[Chairperson]Akimitsu Konishi (Dept. Biochem., Dokkyo Med. Univ., Scho. Med.)

---

### E-2063
### Selective targeting and disruption of the adaptive potential of tumor cells to the acidic pH microenvironment

[Speaker]Wunshaing W. Chang (Natl. Inst. Cancer Res., Natl. Health Res. Inst., Taiwan) [Co-author]Hualing Chen (Natl. Inst. Cancer Res., Natl. Health Res. Inst., Taiwan)、Peichu Tsai (Natl. Inst. Cancer Res., Natl. Health Res. Inst., Taiwan)、Sheng-Chieh Lin (Natl. Inst. Cancer Res., Natl. Health Res. Inst., Taiwan)

An acidic pH microenvironment is a common feature in most tumors. Neoplasms with an inverted pH gradient of extracellular pH lower than intracellular pH appears to impose selective pressures whereby tumor cells must adapt or perish. Unlike previous short-term analyses, our recent studies identified a novel mechanism of early rapid response and late reversible adaptation of tumor cells to the hostile stress of extracellular acidity. The presence of this distinctive yet slow mode of machinery fills an important knowledge gap in how neoplastic cells sense, respond, reprogram, and ultimately adapt to the persistent microenvironmental acidification. A more malignant state in chronically acid-acclimated tumor cells was detected with several key regulatory molecules found closely associated with disease staging and survival outcomes. Here, we further explore how tumor cells may coordinate various signaling responses and progressive adaptative strategies to cope with the long-lasting exterior acidic pH microenvironment, and to assess the potential interfering effects that may lead to the disruption of tumor cell adaptation to such extracellular acidotic stress.

[Disclosure of COI by Responsible researcher] Lead presenter is the Responsible researcher.
[Lead presenter: Wunshaing W. Chang] We have no financial relationships to disclose.

### [E-2064] I-E10-3
### Telomere/Aging/Senescence/Immortalization/Cell differentiation/Others

2025 / 9 / 26 (Fri) 15:20-16:35 Room 9/(Hotel Nikko Kanazawa 3F Lumiere) Room 9

[Chairperson]Akimitsu Konishi (Dept. Biochem., Dokkyo Med. Univ., Scho. Med.)

---

### E-2064
### Withdrawn

No Abstract

### [E-2065] I-E10-3
### Telomere/Aging/Senescence/Immortalization/Cell differentiation/Others

2025 / 9 / 26 (Fri) 15:20-16:35 Room 9/(Hotel Nikko Kanazawa 3F Lumiere) Room 9

[Chairperson]Akimitsu Konishi (Dept. Biochem., Dokkyo Med. Univ., Scho. Med.)

---

### E-2065
### Distinct Roles of Fanconi Anemia Pathway (FA) proteins on the Alternative Lengthening of Telomeres mechanism (ALT)

[Speaker]JESMIN AKTER (Res. Inst. for Clin. Oncology, Saitama Cancer Center, Saitama, Japan) [Co-author]Ritsuko Onuki (Res. Inst. for Clin. Oncology, Saitama Cancer Center, Saitama, Japan)、Hisanori Takenobu (Res. Inst. for Clin. Oncology, Saitama Cancer Center, Saitama, Japan)、Masayuki Haruta (Res. Inst. for Clin. Oncology, Saitama Cancer Center, Saitama, Japan)、Miki Ohira (Res. Inst. for Clin. Oncology, Saitama Cancer Center, Saitama, Japan)、Kiyohiro Ando (Res. Inst. for Clin. Oncology, Saitama Cancer Center, Saitama, Japan)、Takehiko Kamijo (Res. Inst. for Clin. Oncology, Saitama Cancer Center, Saitama, Japan)

ALT pathway is a telomerase-independent mechanism, often associated with poor prognosis and genomic instability in neuroblastoma. Emerging data highlight the important role of FA pathway proteins in the regulation of ALT. This study examines the expression patterns of key FA proteins in ALT (+) and (-) cell lines. Our study revealed that specific FA pathway components are upregulated in ALT (+) cells, potentially facilitating replication fork stability at telomeres. While FANCM is known to a suppressor of ALT, our findings indicate an opposing role for FANCD2. Publicly accessible data and experimental results consistently show elevated FANCD2 expression in ALT (+) cells and tumor samples, implying a potential regulatory role in ALT. Moreover, FANCD2 knocked-down in ALT (+) cells reduces C-circle production, a key biomarker of ALT activity. This contrasts with the previously reported function of FANCM, whose depletion enhances ALT-associated features. These results suggest that FANCD2 might help in telomere synthesis, contributing to the ALT process. Gaining insight into the differential regulation of ALT by FA pathway proteins could uncover novel targets for cancer therapies.

[Disclosure of COI by Responsible researcher] Lead presenter is the Responsible researcher.
[Lead presenter: JESMIN AKTER] We have no financial relationships to disclose.

### [E-2066] I-E10-3
### Telomere/Aging/Senescence/Immortalization/Cell differentiation/Others

2025 / 9 / 26 (Fri) 15:20-16:35 Room 9/(Hotel Nikko Kanazawa 3F Lumiere) Room 9

[Chairperson]Akimitsu Konishi (Dept. Biochem., Dokkyo Med. Univ., Scho. Med.)

---

### E-2066
### A polyamine improves anti-tumor T cell function by promoting BiP chaperone activity in aged mice

[Speaker]Yixin Zou (Dept. of Immunol. and Genomic Med., Kyoto Univ.) [Co-author]Tomonori Yaguchi (Dept. of Immunol. and Genomic Med., Kyoto Univ.／Department of Immuno-oncology PDT, Kyoto University Graduate School of Medicine)、Kazuhiro Sonomura (Center for Genomic Medicine, Kyoto University Graduate School of Medicine ／Life Science Research Center, Technology Research Laboratory, Shimadzu Corporation)、Ken (Kyoto Univ.) Matsumoto (Dept. of Immunol. and Genomic Med., Kyoto Univ.)、Kenji Morii (Dept. of Immunol. and Genomic Med., Kyoto Univ.)、Toshihiko Ogura (Dept. of Immunol. and Genomic Med., Kyoto Univ.)、Tasuku Honjo (Dept. of Immunol. and Genomic Med., Kyoto Univ.)、Kenji Chamoto (Dept. of Immunol. and Genomic Med., Kyoto Univ.／Department of Immuno-oncology PDT, Kyoto University Graduate School of Medicine)

T cells become dysfunctional with aging partly due to loss of proteostasis. However, the underlying mechanisms remain largely unknown. Here, we investigated the effect of the unfolded protein response of endoplasmic reticulum (UPRER) on the function of tumor-infiltrating lymphocytes (TILs). The expression of spliced X-box binding protein 1 (XBP1s), a classic marker of UPRER, was upregulated in dysfunctional aged CD8+ TILs, suggesting high ER stress in these T cells. Comprehensive metabolite analysis by mass spectrometry revealed a polyamine level was decreased in aged tumor-bearing mice. This polyamine supplementation could reduce Xbp1s expression and restore the dysfunctional status of aged CD8+ TILs. We then found that binding immunoglobulin protein (BiP), a key chaperone responsible for protein folding in the ER, could be a direct binding protein of this polyamine. Mechanistically, this polyamine could directly enhance the folding activity, substrate binding affinity and anti-aggregation capacity of BiP. When combined with this polyamine, PD-1 blockade therapy showed improved efficacy in aged mice. These findings provide a new strategy for the treatment of aged cancer patients.

[Disclosure of COI by Responsible researcher] Lead presenter is not the Responsible researcher.
[Lead presenter: Yixin Zou] We have no financial relationships to disclose.
[Principal Researcher: Tomonori Yaguchi, Kenji Chamoto] We have no financial relationships to disclose.

### [J-2073] I-J11-3
### Immune checkpoint inhibitors (2)

2025 / 9 / 26 (Fri) 15:20-16:35 Room 12/(ANA Crowne Plaza Hotel Kanazawa 3F Ohtori (Central)) Room 12

[Chairperson]Kiyoshi Yoshimura (Showa Medical University Clinical Immuno Oncology)

---

### J-2073
### ROSE12 , Fcγ Rs affinity-enhanced anti-CTLA-4 antibody, evokes tumor-selective immune response, activated by ATP in TME

[Speaker]Hiroki Hayashi (Chugai Pharmaceutical, co., ltd., Research Div) [Co-author]Kanako Tatsumi (Chugai Pharmaceutical, co., ltd., Research Div)、Hitoshi Katada (Chugai Pharmaceutical, co., ltd., Research Div)、Toshiaki Tsunenari (Chugai Pharmaceutical, co., ltd., Research Div)、Moe Yoshimoto (Chugai Pharmaceutical, co., ltd., Research Div)、Saki Michisaka (Chugai Pharmaceutical, co., ltd., Research Div)、Masaki Honda (Chugai Pharmaceutical, co., ltd., Translational Research Div.)、Hiroaki Susumu (Chugai Pharmaceutical, co., ltd., Research Div)、Kenji Nakagawa (Chugai Pharmaceutical, co., ltd., Research Div)、Hiroaki Nagano (Chugai Pharmaceutical, co., ltd., Research Div)、Chie Kato (Chugai Pharmaceutical, co., ltd., Translational Research Div.)、Junko Shinozuka (Chugai Pharmaceutical, co., ltd., Translational Research Div.)、Hiroshi Tanaka (Chugai Pharmaceutical, co., ltd., Research Div)、Takehisa Kitazawa (Chugai Pharmaceutical, co., ltd., Research Div)、Mika Sakurai (Chugai Pharmaceutical, co., ltd., Research Div)、Tomoyuki Igawa (Chugai Pharmaceutical, co., ltd., Research Div)

Tregs in tumors express high CTLA-4 levels. Fcγ Rs affinity-enhanced anti-CTLA-4 antibodies depleting Tregs via ADCC show promise for anti-tumor immunity. However, conventional approaches risk autoimmune side effects from systemic Treg depletion and have limited efficacy due to NK scarcity. Selective and potent intratumoral Treg depletion is required. We developed ROSE12, a Fcγ Rs affinity-enhanced anti-CTLA-4 antibody using our proprietary Switch-Ig™ technology. This enables ROSE12 to bind CTLA-4 only in the presence of ATP, which is elevated in tumor tissue but regulated in non-tumor tissue. ROSE12 also incorporates ART-Fc® technology for Fcγ Rs affinity. ROSE12 showed ATP-dependent CTLA-4 binding, stronger Fcγ RIIIa affinity, and enhanced ADCC activity with ATP. It showed anti-tumor efficacy and intratumoral Treg depletion without systemic T cell activation, unlike Fcγ Rs affinity-enhanced ipilimumab in mice. ROSE12 was well-tolerated in a cynomolgus monkey toxicity study. As a novel Fcγ Rs affinity-enhanced anti-CTLA-4 antibody, ROSE12 offers potent anti-tumor efficacy and a wide therapeutic window due to tumor-selective activity. ROSE12 is in phase 1 clinical study (NCT05907980).

[Disclosure of COI by Responsible researcher] Lead presenter is the Responsible researcher.
[Lead presenter: Hiroki Hayashi] ■ employment by a company related to the presentation: 中外製薬株式会社

▲TOP

### [J-2074] I-J11-3
### Immune checkpoint inhibitors (2)

2025 / 9 / 26 (Fri) 15:20-16:35 Room 12/(ANA Crowne Plaza Hotel Kanazawa 3F Ohtori (Central)) Room 12

[Chairperson]Kiyoshi Yoshimura (Showa Medical University Clinical Immuno Oncology)

---

### J-2074
### Activation of PD-1 antibody-bound progenitor-exhausted CD8+ T cells in lymph nodes after PD-1 blockade

[Speaker]Takuro Saito (Dept. Clin. Res. Tumor Immunol, Grad. Sch. Med, Osaka Univ.／Dept. Gastroenterol. Surg., Grad. Sch. Med, Osaka Univ.) [Co-author]Yohei Nose (Dept. Clin. Res. Tumor Immunol, Grad. Sch. Med, Osaka Univ.／Dept. Gastroenterol. Surg., Grad. Sch. Med, Osaka Univ.)、Yoshiaki Yasumizu (Dept. Basic Res. Tumor Immunol, Grad. Sch. Med, Osaka Univ.)、Azumi Ueyama (Dept. Clin. Res. Tumor Immunol, Grad. Sch. Med, Osaka Univ.／Pharm. Res. Div, Shionogi & Co., Ltd.)、Naganari Ohkura (Dept. Basic Res. Tumor Immunol, Grad. Sch. Med, Osaka Univ.)、Hisashi Wada (Dept. Clin. Res. Tumor Immunol, Grad. Sch. Med, Osaka Univ.)、Hidetoshi Eguchi (Dept. Gastroenterol. Surg., Grad. Sch. Med, Osaka Univ.)、Yuichiro Doki (Dept. Gastroenterol. Surg., Grad. Sch. Med, Osaka Univ.)

Introduction: Progenitor-exhausted T (Tpex) cells are essential for the efficacy of immune checkpoint inhibitors (ICIs). However, the dynamics of ICI-bound Tpex cells have not been fully elucidated. Here, we investigated the functional behavior of Tpex cells in patients with gastrointestinal cancer with ICIs.Methods: Tumor, LN, and blood samples were collected from patients with gastrointestinal cancer with or without ICIs. Purified lymphocytes were analyzed using flow cytometry and sequencing. In sequencing analysis, we employed an integrated sequencing strategy combining single-cell RNA sequencing, T cell receptor sequencing, and CITE-sequencing to identify cells bound by therapeutic anti-PD-1 antibodies.Results: Flow cytometric analysis revealed that CD8+ Tpex cells in LNs exhibited proliferation following ICI treatment. Sequencing analysis confirmed that ICI-bound Tpex cells in LNs underwent clonal expansion and shared TCR clonotypes with intratumoral exhausted T (Tex) cells.Conclusion: ICIs predominantly target PD-1+ Tpex cells in LNs, promoting their proliferation and migration to tumors, where they differentiate into Tex cells and mediate tumor clearance.

[Disclosure of COI by Responsible researcher] Lead presenter is the Responsible researcher.
[Lead presenter: Takuro Saito] ■Endowed chair: 塩野義製薬株式会社

### [J-2075] I-J11-3
### Immune checkpoint inhibitors (2)

2025 / 9 / 26 (Fri) 15:20-16:35 Room 12/(ANA Crowne Plaza Hotel Kanazawa 3F Ohtori (Central)) Room 12

[Chairperson]Kiyoshi Yoshimura (Showa Medical University Clinical Immuno Oncology)

---

### J-2075
### CLEC12A and CD200 mark tumor-reactive $CD8^+$ T-cell clones in tumor-draining lymph nodes

[Speaker]Satoshi Ueha (Tokyo University of Science, Research Institute for Biomedical Sciences) [Co-author]Masaki Kurosu (Tokyo University of Science, Research Institute for Biomedical Sciences)、Kouji Matsushima (Tokyo University of Science, Research Institute for Biomedical Sciences)

Tumor-draining lymph nodes (dLNs) play a key role in priming tumor-reactive $CD8^+$ T-cells during the anti-tumor immune response. However, dLNs also harbor many non-tumor-reactive T-cells, complicating analysis of tumor-specific clones. Here, we employed Fucci transgenic mice that label proliferating cells to trace $CD8^+$ T-cell clonal expansion in dLNs and tumors. Integration of bulk TCR-seq, single-cell RNA/TCR-seq, and CITE-seq revealed that dLN-proliferating clones expand further in tumors and contribute significantly to anti-tumor immunity. We identified CLEC12A and CD200 as novel markers that enrich for these tumor-reactive clones in dLNs more effectively than conventional markers such as $CD44^{hi}$ effector/memory or $PD\text{-}1^+$ $Ly108^+$ progenitor exhausted T cells. These markers enable improved isolation and evaluation of tumor-reactive T-cells in mice, offering a powerful tool for studying immune responses and enhancing immunotherapy strategies. Our study provides a foundation for refining the identification of tumor-reactive T-cells in secondary lymphoid organs, with broad implications for cancer immunotherapy development.

[Disclosure of COI by Responsible researcher] Lead presenter is the Responsible researcher.
[Lead presenter: Satoshi Ueha] ■Advisor of the company and/or profit-making organization: ImmunoGeneTeqs. Inc ■Profit of stock: ImmunoGeneTeqs. Inc

### [J-2076] I-J11-3
### Immune checkpoint inhibitors (2)

2025 / 9 / 26 (Fri) 15:20-16:35 Room 12/(ANA Crowne Plaza Hotel Kanazawa 3F Ohtori (Central)) Room 12

[Chairperson]Kiyoshi Yoshimura (Showa Medical University Clinical Immuno Oncology)

---

### J-2076
### Risk factors & prognosis of immune checkpoint inhibitor-induced pneumonia in lung cancer with interstitial lung disease

[Speaker]Tsukasa Ueda (Department of Respiratory Medicine, Kanazawa University Hospital) [Co-author]Shigeki Nanjo (Department of Respiratory Medicine, Kanazawa University Hospital)、Yuya Murase (Department of Respiratory Medicine, Kanazawa University Hospital)、Takahiro Shimizu (Department of Respiratory Medicine, Kanazawa University Hospital)、Shunichi Nomura (Department of Respiratory Medicine, Kanazawa University Hospital)、Yifeng Liu (Department of Respiratory Medicine, Kanazawa University Hospital)、Sachiko Arai (Department of Respiratory Medicine, Kanazawa University Hospital)、Kazumasa Kase (Department of Respiratory Medicine, Kanazawa University Hospital)、Hayato Koba (Department of Respiratory Medicine, Kanazawa University Hospital)、Satoshi Watanabe (Department of Respiratory Medicine, Kanazawa University Hospital)、Yuichi Tambo (Department of Respiratory Medicine, Kanazawa University Hospital)、Seiji Yano (Department of Respiratory Medicine, Kanazawa University Hospital)

Background: Lung cancer patients with interstitial lung disease (ILD) are known to be associated with a higher frequency of immune checkpoint inhibotor (ICI) induced pneumonitis. This study aimed to determine the risk factors and prognosis of ICI-induced pneumonitis in lung cancer patients with ILD treated with ICIs. Methods: We retrospectively analyzed 12 lung cancer patients with ILD out of 231 patients treated with ICIs at Kanazawa University Hospital from January 2019 to March 2023. Results: ICI-induced pneumonitis was observed in four patients (33%), but there was no Grade 3 or higher pneumonitis. Of the four cases with ICI-induced pneumonitis, 2 cases had honeycomb lung and 1 case had high antinuclear antibody levels, but %VC was better than 80% in all four cases. The proportion of patients with ICI-induced pneumonitis had high SP-D levels compared with patients without ICI-induced pneumonitis (75% and 0% respectively; p = 0.003 by Fisher's exact test). Conclusion: When ICIs are used for lung cancer patients with ILD, honeycomb lung, and a high SP-D level could be considered risk factors for ICI-induced pneumonitis.

[Disclosure of COI by Responsible researcher] Lead presenter is the Responsible researcher.
[Lead presenter: Tsukasa Ueda] We have no financial relationships to disclose.

[J-2077] I-J11-3

### Immune checkpoint inhibitors (2)

2025 / 9 / 26 (Fri) 15:20-16:35 Room 12/(ANA Crowne Plaza Hotel Kanazawa 3F Ohtori (Central)) Room 12

[Chairperson]Kiyoshi Yoshimura (Showa Medical University Clinical Immuno Oncology)

J-2077

### Investigation of the Activation Status and Significance of the cGAS-STING Pathway in Lung Squamous Cell Carcinoma

[Speaker]Hideki Terai (Keio Cancer Center, Keio University, School of Medicine) [Co-author]Junko Hamamoto (Division of Pulmonary Medicine, Keio University, School of Medicine)、Shunsuke Kitajima (Cancer Immunotherapy Development, Cancer Precision Medicine Center, JFCR)、Takashi Sato (Department of Respiratory Medicine, Kitasato University School of Medicine)、Hiroyuki Yasuda (Division of Pulmonary Medicine, Keio University, School of Medicine)、Kenzo Soejima (Department of Respiratory Medicine, University of Yamanashi)

The cGAS-STING pathway activates innate immunity by sensing cytosolic DNA. While its activation is linked to immune checkpoint inhibitor (ICI) sensitivity in lung adenocarcinoma, its role in lung squamous cell carcinoma (LUSC) remains unclear. We investigated the status and significance of this pathway in LUSC. Immunohistochemical analysis of thoracic malignancy tissue microarrays showed similar cGAS expression across subtypes, but STING expression was significantly lower in LUSC. Among ICI-treated LUSC patients, those with high STING expression tended to have better prognoses. In vitro, STING-high LUSC cell lines expressed high SOX2 and DNp63 and low BRN2, whereas STING-low lines showed low DNp63 and high BRN2. DNp63 overexpression restored STING expression, while BRN2 suppressed it. DNA methylation in the STING promoter contribute to its downregulation. In syngeneic mouse models, lung adenocarcinoma cells overexpressing Sox2 and Brn2 were more resistant to ICI therapy than those overexpressing Sox2 and DNp63. These findings suggest that STING downregulation contributes to ICI resistance in LUSC.

[Disclosure of COI by Responsible researcher] Lead presenter is the Responsible researcher.
[Lead presenter: Hideki Terai] We have no financial relationships to disclose.

▲TOP

### [J-2078] I-J11-3
### Immune checkpoint inhibitors (2)

2025 / 9 / 26 (Fri) 15:20-16:35 Room 12/(ANA Crowne Plaza Hotel Kanazawa 3F Ohtori (Central)) Room 12

[Chairperson]Kiyoshi Yoshimura (Showa Medical University Clinical Immuno Oncology)

---

### J-2078
### PD-1 blockade and hematological malignancy exacerbation in lung cancer

[Speaker]Toshifumi Ninomiya (Dept. of Resp. Med. Kyushu Univ.／Dept. of Tumor Microenvironment, Okayama Univ.) [Co-author]Caiyang Fang (Dept. of Tumor Microenvironment, Okayama Univ.)、Hirofumi Hamano (Dept. of Pharm. Okayama Univ. Hosp.)、Wenhao Zhou (Dept. of Tumor Microenvironment, Okayama Univ.／Dept. of Immunol. Nagoya Univ.／Dept. of Urol. Surg. Affiliated Hosp. of North-Sichuan Med. College)、Toshihiro Koyama (Dept. of Pharm. Data Sci. Okayama Univ.)、Sakura Miki (Dept. of Tumor Microenvironment, Okayama Univ.)、Tomoya Katsuta (Dept. of Resp. Med. Ehime Pref. Central Hosp.)、Kadoaki Ohashi (Dept. of Allerg. and Resp. Med. Okayama Univ. Hosp.)、Youki Ueda (Dept. of Tumor Microenvironment, Okayama Univ.)、Takamasa Ishino (Dept. of Tumor Microenvironment, Okayama Univ.)、Isamu Okamoto (Dept. of Resp. Med. Kyushu Univ.)、Yoshito Zamami (Dept. of Pharm. Okayama Univ. Hosp.)、Joji Nagasaki (Dept. of Tumor Microenvironment, Okayama Univ.／Dept. of Hem. Osaka Metropolitan Univ.)、Yosuke Togashi (Dept. of Tumor Microenvironment, Okayama Univ.／Dept. of Allerg. and Resp. Med. Okayama Univ. Hosp.)

[Background] Many patients with lung cancer have been treated with immune checkpoint inhibitors (ICIs). With long-term use, unexpected complications can emerge. We identified two cases of B cell lymphoma during PD-1 blockade therapy. However, the incidence of hematological malignancies and the mechanisms driving their progression remain unclear. [Methods] We performed immunohistochemical staining on samples from patients who developed B cell lymphoma during PD-1 blockade therapy. We also analyzed large-scale real-world datasets and investigated mechanisms through in vitro and in vivo experiments. [Results] Real-world data showed a higher incidence of hematological events, particularly lymphoma, in patients with lung cancer treated with PD-1 blockade. The identified lymphomas exhibited extensive CD4+ T follicular helper (TFH) cell infiltration. PD-1 blockade activated PD-1+ TFH cells, promoting lymphoma proliferation via IL-4/IL-4R, IL-21/IL-21R, and CD40L/CD40 pathways. These lymphomas highly expressed IL-4R, IL-21R, and CD40. [Conclusion] We propose "ICI-exacerbating B cell malignancy," emphasizing the need for vigilant monitoring in clinical settings where ICIs are used.

[Disclosure of COI by Responsible researcher] Lead presenter is not the Responsible researcher.
[Lead presenter: Toshifumi Ninomiya] We have no financial relationships to disclose.
[Principal Researcher: Yosuke Togashi] ■Research funds from company: Ono Pharmaceutical, Bristol-Myers Squibb, Daiichi-Sankyo, Janssen Pharmaceutical, AstraZeneca, KOTAI Biotechnologies Inc, and KORTUC ■Lecture fee: Ono Pharmaceutical, Bristol-Myers Squibb, Chugai Pharmaceutical, AstraZeneca, Eisai, MSD

▲TOP

### [E-2073] I-E7-1
### Advances in cancer research through genomic analysis

2025 / 9 / 26 (Fri) 15:20-16:35 Room 13/(ANA Crowne Plaza Hotel Kanazawa 3F Ohtori (East)) Room 13

[Chairperson]Yoshio Miki (Univ. of Tsukuba, PMC)

---

### E-2073
### Integrational analysis using somatic and germline alterations of lung cancer

[Speaker]Kouya Shiraishi (Div. Genome Biol., Natl. Cancer Ctr. Res. Inst.／Dept. Clin. Genomics., Natl. Cancer Ctr. Res. Inst.) [Co-author]Michiko Ueki (Div. Genome Biol., Natl. Cancer Ctr. Res. Inst.)、Jun Miyakoshi (Div. Genome Biol., Natl. Cancer Ctr. Res. Inst.／Dept. Thoracic Oncol., Natl. Cancer Ctr. Hosp.)、Masahiro Torasawa (Div. Genome Biol., Natl. Cancer Ctr. Res. Inst.／Dept. Thoracic Oncol., Natl. Cancer Ctr. Hosp.)、Akifumi Mochizuki (Div. Genome Biol., Natl. Cancer Ctr. Res. Inst.)、Kuniko Sunami (Div. Genome Biol., Natl. Cancer Ctr. Res. Inst.／Dept. Lab. Med., Natl. Cancer Ctr. Hosp.)、Hourin Cho (Div. Genome Biol., Natl. Cancer Ctr. Res. Inst.)、Yoko Ogawara (Dept. Clin. Genomics., Natl. Cancer Ctr. Res. Inst.)、Ryuji Hamamoto (Div. Med. AI Res. & Dept., Natl. Cancer Ctr. Res. Inst.)、Takashi Kohno (Div. Genome Biol., Natl. Cancer Ctr. Res. Inst.)

Lung adenocarcinoma (LUAD) is characterized by two distinctive characteristics in individuals of Asian ancestry. First, Asian patients with LADC are more frequently non-smokers than their European patients. Second, half of Asian patients with LADC have oncogenic mutations in the epidermal growth factor receptor (EGFR) gene, whereas this frequency is approximately 10% in European and American patients. Therefore, it is necessary to examine the somatic landscape considering germline variants. We conducted more than 1,500 WGS or WES analyses to elucidate the landscape of somatic mutations. Among these, a study focusing on young-onset LADC revealed that pathogenic variants of BRCA2 and TP53 were significantly associated with young-onset LADC and exhibited characteristic genetic mutation patterns. A germline ALKBH2 variant was associated with the risk of early-onset LADC, and patients with variant showed a correlation between SBS4-related somatic mutations and the Brinkman index. Differences in germline lineages may influence the characteristic somatic mutation patterns in tumor tissues and suggest potential therapeutic targets.

[Disclosure of COI by Responsible researcher] Lead presenter is not the Responsible researcher.
[Lead presenter: Kouya Shiraishi] We have no financial relationships to disclose.
[Principal Researcher: Takashi Kohno] ■Patent fee: 理研Genesis、Amoy、Foundation Medicine、サーモフィッシャーサイエンティフィック　■Lecture fee: 日本イーライリリー　■Contributions: 中外製薬、シスメックス、コニカミノルタ、ガーダントヘルス、イーライリリージャパン、ユーロフィンクリニカルジェネティクス

### [E-2074] I-E7-1
### Advances in cancer research through genomic analysis

2025 / 9 / 26 (Fri) 15:20-16:35 Room 13/(ANA Crowne Plaza Hotel Kanazawa 3F Ohtori (East)) Room 13

[Chairperson]Yoshio Miki (Univ. of Tsukuba, PMC)

---

### E-2074
### Differential immunogenic contributions of HLA-class I and -class II alleles to Lung Adenocarcinoma Development

[Speaker]Michiko Ueki (Division of Genome Biology, National Cancer Center Research Institute／Department of Respiratory Medicine, The University of Tokyo Hospital) [Co-author]Akiteru Goto (Department of Cellular and Organ Pathology Akita University)、Motonobu Saito (Dept. of Gastrointestinal Tract Surgery Fukushima Med. Univ.)、Takayuki Honda (Department of Respiratory Medicine, Instisute of Science Tokyo)、Kenji Wakai (Dept. of Preventive Med. Nagoya Univ. Grad. Sch. of Med.)、Masayuki Yamamoto (Tohoku Medical Megabank Organization, Tohoku University)、Motoki Iwasaki (Div. of Epidemiology Natl Cancer Ctr Inst. for Cancer Control)、Koichi Matsuda (Grad. School of Frontier Sciences The University of Tokyo)、Fumihiko Matsuda (Center for Genomic Medicine Graduate School of Medicine Kyoto University)、Keitaro Matsuo (Aichi Cancer Ctr. Div. of Cancer Epidemiology and Prevention)、Yasushi Yatabe (Department of Diagnostic Pathology, National Cancer Center Hospital)、Shunichi Watanabe (Department of Thoracic Surgery, National Cancer Center Hospital)、Ryuji Hamamoto (Div. Medical AI Res. Dev., Natl. Cancer Ctr. Res. Inst.)、Takashi Kohno (Division of Genome Biology, National Cancer Center Research Institute)、Kouya Shiraishi (Division of Genome Biology, National Cancer Center Research Institute)

Although there are racial differences in HLA diversity, HLA alleles have been reported to be associated with the risk of various types of cancer. However, their functional significance remains unclear. We conducted four genome-wide association studies (GWAS) involving 9,853 lung adenocarcinoma (LADC) cases and 151,070 controls, genotyped using Illumina SNP arrays. HLA alleles were imputed by SNP2HLA at 4-digit resolution for class I/II loci. A meta-analysis adjusted with Bonferroni correction ($p < 4.1 \times 10^{-4}$) identified five significant HLA risk alleles: HLA-C*12:02 (OR = 1.15), HLA-B*52:01 (OR = 1.15), HLA-DRB1*15:02 (OR = 1.18), HLA-DQB1*06:01 (OR = 1.13), and HLA-DPB1*09:01 (OR = 1.13). In addition, we obtained whole-exome sequencing data from 1,096 LADC fresh frozen tumor-normal pairs. We assessed the loss of heterozygosity (LOH) per tumor using these data. The LOH frequency in HLA-C*12:02, HLA-DRB1*15:02, HLA-DQA1*01:03, and HLA-DQA1*03:03 was significantly lower than that in the other HLA alleles ($p < 3.6 \times 10^{-3}$). These findings suggest that HLA risk alleles contribute to lung adenocarcinoma development through immune evasion.

[Disclosure of COI by Responsible researcher] Lead presenter is not the Responsible researcher.
[Lead presenter: Michiko Ueki] We have no financial relationships to disclose.
[Principal Researcher: Kouya Shiraishi] We have no financial relationships to disclose.

▲TOP

### [E-2075] I-E7-1
### Advances in cancer research through genomic analysis

2025 / 9 / 26 (Fri) 15:20-16:35 Room 13/(ANA Crowne Plaza Hotel Kanazawa 3F Ohtori (East)) Room 13

[Chairperson]Yoshio Miki (Univ. of Tsukuba, PMC)

### E-2075
### Intrapatient diversity of subclonal phenotypes in endometrial carcinomas

[Speaker]Masahito Kawazu (Chiba Cancer Ctr., Res. Inst., Div. of Cell Therap.) [Co-author]Suguru Miyata (Chiba Cancer Ctr., Res. Inst., Div. of Cell Therap.／Chiba Univ. Dept. Head and Neck Surg.)、Junsuke Muraoka (Chiba Cancer Ctr. Dept. of Gynecol.)、Yoshiaki Maru (Chiba Cancer Ctr., Res. Inst., Lab. of Precision Model)、Takao Morinaga (Chiba Cancer Ctr., Res. Inst., Div. of Cell Therap.)、Takahiro Sugiyama (Chiba Cancer Ctr. Dept. of Surg. Pathol.)、Akinobu Araki (Chiba Cancer Ctr. Dept. of Surg. Pathol.)、Toyoyuki Hanazawa (Chiba Univ. Dept. Head and Neck Surg.)、Makiko Itami (Chiba Cancer Ctr. Dept. of Surg. Pathol.)、Naotake Tanaka (Chiba Cancer Ctr. Dept. of Gynecol.)

The intratumoral heterogeneity of cancers and the presence of multiple subclones within tumors are widely recognized. However, the distinct characteristics of these subclones remain poorly understood. We performed whole-exome sequencing (WES) on multiple samples collected from the uterine cavity of patients with early-stage endometrioid endometrial carcinoma, along with single-cell transcriptome sequencing (scRNA-seq). Cancer cells exhibited epithelial phenotypes ranging from differentiated cells, such as ciliated and lumenal cells, to undifferentiated progenitor-like cells. The composition of these cell types varied not only between patients but also among different specimens from the same patient. Through integrated analysis, we successfully assigned individual cells identified by scRNA-seq to branches of phylogenetic trees constructed based on somatic mutation data obtained with WES. Our findings provide insights into the distinct characteristics of subclones shaped by both environmental factors and genetic backgrounds.

[Disclosure of COI by Responsible researcher] Lead presenter is the Responsible researcher.
[Lead presenter: Masahito Kawazu] ■Research funds from company: 次世代がん医療加速化研究事業、武田科学振興財団、高松宮妃癌研究基金、上原記念生命科学財団

### [E-2076] I-E7-1
### Advances in cancer research through genomic analysis

2025 / 9 / 26 (Fri) 15:20-16:35 Room 13/(ANA Crowne Plaza Hotel Kanazawa 3F Ohtori (East)) Room 13

[Chairperson]Yoshio Miki (Univ. of Tsukuba, PMC)

### E-2076
### Analysis of tumors with translocation breakpoints in the centromere using long read sequencing

[Speaker]Masahiro Sugawa (Div. of Genome Analysis Platform Dev., Natl. Cancer Ctr. Inst.／Dept. of Pediatr., The Univ. of Tokyo Hosp.) [Co-author]Rurika Okuda (Dept. of Path.／Tumor Biol., Grad. Sch. of Med., Kyoto Univ.／Inst. for the Advanced Study of Human Biol., Kyoto Univ.)、Keisuke Kimura (Dept. of Path.／Tumor Biol., Grad. Sch. of Med., Kyoto Univ.／Dept. of Neurosurg., Nagoya Univ., Grad. Sch. of Med.)、Yotaro Ochi (Dept. of Path.／Tumor Biol., Grad. Sch. of Med., Kyoto Univ.)、Yasuhito Nannya (Dept. of Hematology／Oncology, Inst. of Med. Sci., Univ. of Tokyo)、Yoshitaka Sakamoto (Div. of Genome Analysis Platform Dev., Natl. Cancer Ctr. Inst.)、Ai Okada (Div. of Genome Analysis Platform Dev., Natl. Cancer Ctr. Inst.)、Taro Tsujimura (Inst. for the Advanced Study of Human Biol., Kyoto Univ.)、Kazuhisa Chonabayashi (Ctr. for iPS Cell Res. and Application, Kyoto Univ.／Dept. of Hematology, Grad. Sch. of Med., Kyoto Univ.)、Takuya Yamamoto (Inst. for the Advanced Study of Human Biol., Kyoto Univ.／Ctr. for iPS Cell Res. and Application, Kyoto Univ.)、Yoshinori Yoshida (Ctr. for iPS Cell Res. and Application, Kyoto Univ.)、Motohiro Kato (Dept. of Pediatr., The Univ. of Tokyo Hosp.)、Ryuta Saito (Dept. of Neurosurg., Nagoya Univ., Grad. Sch. of Med.)、Akifumi Takaori (Dept. of Hematology, Grad. Sch. of Med., Kyoto Univ.)、Seishi Ogawa (Dept. of Path.／Tumor Biol., Grad. Sch. of Med., Kyoto Univ.／Inst. for the Advanced Study of Human Biol., Kyoto Univ.／Kindai Univ. Faculty of Med.)、Yuichi Shiraishi (Div. of Genome Analysis Platform Dev., Natl. Cancer Ctr. Inst.)

The centromere is crucial for chromosome inheritance, and its abnormalities are linked to genomic instability and disease. Its highly repetitive sequence composition has long hindered analysis, but recent advances in long-read sequencing technologies have enabled analysis of these previously intractable regions. In this study, we developed a novel analysis pipeline using long-read sequencing and identified the translocation breakpoints in two cancers with translocation breakpoints in the centromere: myelodysplastic syndrome (MDS) with der(1;7) and oligodendroglioma (ODG) with 1p19q codeletion. Analysis of the sequences around the breakpoints suggested that non-allelic homologous recombination between highly homologous sequences of the two chromosomes is involved in the generation of the translocation. In addition, we used publicly available data from the Human Pangenome Reference Consortium and found the race bias in centromere sequences. It appears to be associated with the cause of MDS with der(1;7) occurring frequently in East Asians and with the predisposition to ODG with 1p19q codeletion. This is the first report of the involvement of centromere polymorphisms in tumorigenesis.

[Disclosure of COI by Responsible researcher] Lead presenter is not the Responsible researcher.
[Lead presenter: Masahiro Sugawa] We have no financial relationships to disclose.
[Principal Researcher: Rurika Okuda] We have no financial relationships to disclose.

### [E-2077] I-E7-1
### Advances in cancer research through genomic analysis

2025 / 9 / 26 (Fri) 15:20-16:35 Room 13/(ANA Crowne Plaza Hotel Kanazawa 3F Ohtori (East)) Room 13

[Chairperson]Yoshio Miki (Univ. of Tsukuba, PMC)

---

### E-2077
### The genomic landscape of extrachromosomal DNA amplification in pediatric solid tumors

[Speaker]Owen S. Chapman (Dept. of Neuro-oncology, Inst. of Brain Sci., Nagoya City Univ.) [Co-author]Sunita Sridhar (Rady Children's Hosp. San Diego)、Eugene Chow (Sanford Burnham Prebys Med. Discovery Inst.)、Rishaan Kenkre (Sanford Burnham Prebys Med. Discovery Inst.)、Aditi Dutta (Dept. of Computer Sci., Univ. of California San Diego)、Shanqing Wang (Dept. of Computer Sci., Univ. of California San Diego)、Miguel Brown (Children's Hosp. of Philadelphia)、Jens Luebeck (Dept. of Computer Sci., Univ. of California San Diego)、Daisuke Kawauchi (Dept. of Neuro-oncology, Inst. of Brain Sci., Nagoya City Univ.)、Vineet Bafna (Dept. of Computer Sci., Univ. of California San Diego／Moores Cancer Ctr., Univ. of California San Diego)、Kevin Yip (Sanford Burnham Prebys Med. Discovery Inst.)、Megan Paul (Rady Children's Hosp. San Diego)、Jill P. Mesirov (Moores Cancer Ctr., Univ. of California San Diego／Dept. of Med., Univ. of California San Diego)、Lukas Chavez (Rady Children's Hosp. San Diego／Sanford Burnham Prebys Med. Discovery Inst.／Dept. of Med., Univ. of California San Diego)

Extrachromosomal DNA (ecDNA) has been linked to oncogene amplification, evolution of drug resistance, and poor outcomes in various adult cancers. However, the extent to which ecDNA contributes to oncogenesis of pediatric solid tumors has heretofore remained understudied. In addition, the relative importance of intra- versus extrachromosomal amplification remains an open question. We have therefore reanalyzed whole genome sequencing data to identify ecDNA sequences in a large retrospective cohort of 3,000 pediatric solid tumors. ecDNA was most frequent in aggressive tumor types and rare in low-grade or benign histologies. Recurrently ecDNA-amplified genomic regions included well-established oncogenes as well as genes with emerging evidence of possible oncogenic roles. Notably, multivariate Cox regression identifies additive effects of amplification and extrachromosomal amplification which contributed independently to poor patient outcomes. These results constitute a comprehensive map of the genetic and phenotypic diversity of extrachromosomal amplification in pediatric tumors, and underscore the utility of large genomic data resources to drive clinically relevant discoveries.

[Disclosure of COI by Responsible researcher] Lead presenter is not the Responsible researcher.
[Lead presenter: Owen S. Chapman] We have no financial relationships to disclose.
[Principal Researcher: Lukas Chavez] We have no financial relationships to disclose.

▲TOP

### [E-2078] I-E7-1
### Advances in cancer research through genomic analysis

2025 / 9 / 26 (Fri) 15:20-16:35 Room 13/(ANA Crowne Plaza Hotel Kanazawa 3F Ohtori (East)) Room 13

[Chairperson]Yoshio Miki (Univ. of Tsukuba, PMC)

### E-2078
### Repertoire of Non-coding Drivers Identified by Whole-genome Sequencing of 913 Myeloid Neoplasms

[Speaker]Koji Okazaki (Department of Pathology and Tumor Biology, Kyoto University／Department of Hematology, Kyoto University) [Co-author]Ryunosuke Saiki (Department of Pathology and Tumor Biology, Kyoto University)、Yotaro Ochi (Department of Pathology and Tumor Biology, Kyoto University／WPI-ASHBi, Kyoto University)、Masahiro M. Nakagawa (Department of Pathology and Tumor Biology, Kyoto University)、Masanori Motomura (Department of Pathology and Tumor Biology, Kyoto University)、Akinori Yoda (Department of Pathology and Tumor Biology, Kyoto University)、Rurika Okuda (Department of Pathology and Tumor Biology, Kyoto University／WPI-ASHBi, Kyoto University)、Lanying Zhao (Department of Pathology and Tumor Biology, Kyoto University／WPI-ASHBi, Kyoto University)、Hidehito Fukushima (Division of Hematopoietic Disease Control, IMSUT)、Kenichi Yoshida (Division of Cancer Evolution, National Cancer Center Research Institute)、Takayuki Ishikawa (Department of Hematology, Kobe City Medical Center General Hospital)、Senji Kasahara (Department of Hematology, Gifu Municipal Hospital)、Seiya Imoto (Division of Health Medical intelligence,IMSUT)、Akifumi Takaori (Department of Hematology, Kyoto University)、Yasuhito Nannya (Division of Hematopoietic Disease Control, IMSUT)、Seishi Ogawa (Department of Pathology and Tumor Biology, Kyoto University／WPI-ASHBi, Kyoto University)

During the past decades, our understanding of the molecular pathogenesis of myeloid neoplasms (MN) has dramatically been improved by comprehensive detection of a complete registry of driver mutations using next-generation sequencing. However, our knowledge about non-coding alterations and structural variations (SVs) has been less intensively studied using unbiased sequencing platforms such as whole genome sequencing (WGS). In this study, we performed WGS in a large cohort of patients with AML (n=494) and MDS (n=419) using paired tumor/normal DNA. Mutational burden was 0.53 /Mb and 0.44 /Mb in AML and MDS, respectively, which were dominated by SBS1 and SBS5 in both samples, while increased SBS18 suggestive of increased ROS activity were detected in CBF leukemia and ect... We detected most of the known driver mutations and recurrent translocations, as well as copy number alterations. In addition, we identified a total of 22 novel driver genes recurrently mutated albeit at low frequencies. Notably, we found a number of novel SVs causing enhancer hijacking inducing overexpression of ***GSX1*** and ***GSX2*** genes. Our results contribute to the improved understanding of the genetic basis of MN.

[Disclosure of COI by Responsible researcher] Lead presenter is not the Responsible researcher.
[Lead presenter: Koji Okazaki] We have no financial relationships to disclose.
[Principal Researcher: Seishi Ogawa] ■Advisor of the company and/or profit-making organization: エーザイ株式会社 Chordia Therapeutics Inc. Montage Bio, INC. ■Profit of stock: 朝日ゲノミクス株式会社 ■Lecture fee: 公益財団法人三菱財団 公益財団法人中谷医工計測技術振興財団 ■Research expenses: エーザイ株式会社 Chordia Therapeutics Inc. ■Contributions: 日本新薬株式会社 ■Endowed chair: Chordia Therapeutics Inc. 公益社団法人鹿児島共済会 南風病院

### [J-2085] II-J9
### Urological Cancer

2025 / 9 / 26 (Fri) 15:20-16:35 Room 14/(ANA Crowne Plaza Hotel Kanazawa 3F Zuiun 1) Room 14

[Chairperson]Shusuke Akamatsu (Dept. of Urology, Nagoya University)

---

### J-2085
### Genomic landscape identifies therapeutic vulnerabilities in urachal carcinoma

[Speaker]Toru Imai (Division of Cellular Signaling, National Cancer Center Research Institute／Department of Medical Oncology, National Cancer Center Hospital) [Co-author]Tatsunori Shimoi (Department of Medical Oncology, National Cancer Center Hospital)、Akiko Maeshima (Department of Diagnostic Pathology, National Cancer Center Hospital)、Hisashi Hashimoto (Division of Cellular Signaling, National Cancer Center Research Institute)、Jun Takahashi (Division of Cellular Signaling, National Cancer Center Research Institute)、Eijiro Nakamura (Department of Medical Oncology, National Cancer Center Hospital／Department of Urology and Retroperitoneal Surgery, National Cancer Center Hospital)、Yoshiyuki Matsui (Department of Urology and Retroperitoneal Surgery, National Cancer Center Hospital)、Hiroyuki Mano (Division of Cellular Signaling, National Cancer Center Research Institute)、Kan Yonemori (Department of Medical Oncology, National Cancer Center Hospital)、Yosuke Tanaka (Division of Cellular Signaling, National Cancer Center Research Institute)

Urachal carcinoma is an aggressive malignancy with a five-year survival rate of 8%. Although urachal carcinoma is thought to arise from the remnant of the embryonic urachus, the overview of the disease entity and its pathogenesis have not been elucidated due to its rarity. To investigate the molecular basis and potential therapeutic targets of urachal carcinoma, we performed comprehensive analyses using whole exome sequencing, RNA sequencing, and spatial transcriptome analyses of 48 cases. We identified gene mutations in the RTK/RAS/MAPK pathway in 61.3% of the cases, which could serve as potential therapeutic targets. The transcriptome-based classification revealed the subgroup-specific expression patterns which could further refine the histological classification. Interestingly, the expression profile of the claudin 18.2 isoform highlights its therapeutic potential in this disorder. Furthermore, spatial analysis and microdissection-based genomic analysis provided insights into the evolution from urachal remnant to urachal carcinoma. These findings elucidate the molecular characteristics of this rare cancer and propose a framework for novel therapeutic strategies.

[Disclosure of COI by Responsible researcher] Lead presenter is not the Responsible researcher.
[Lead presenter: Toru Imai] We have no financial relationships to disclose.
[Principal Researcher: Yosuke Tanaka] We have no financial relationships to disclose.

### [J-2086] II-J9
### Urological Cancer

2025 / 9 / 26 (Fri) 15:20-16:35 Room 14/(ANA Crowne Plaza Hotel Kanazawa 3F Zuiun 1) Room 14

[Chairperson]Shusuke Akamatsu (Dept. of Urology, Nagoya University)

---

### J-2086
### SLFN11 drives M1 macrophage activation and enhances response to immune checkpoint inhibitors in renal cell carcinoma

[Speaker]Yohei Okuda (Urology, The Univ. of Osaka Grad. Sch. of Med.) [Co-author]Taigo Kato (Urology, The Univ. of Osaka Grad. Sch. of Med.)、Tsuyoshi Takashima (Pathol., The Univ. of Osaka Grad. Sch. of Med.／Mol. Pathol., Hyogo Med. Univ.)、Atsuki Matsukawa (Urology, The Univ. of Osaka Grad. Sch. of Med.)、Masatoshi Konishi (Urology, The Univ. of Osaka Grad. Sch. of Med.)、Yutong Liu (Urology, The Univ. of Osaka Grad. Sch. of Med.)、Tomohiro Kanaki (Urology, The Univ. of Osaka Grad. Sch. of Med.)、Akihiro Yoshimura (Urology, The Univ. of Osaka Grad. Sch. of Med.)、Gaku Yamamichi (Urology, The Univ. of Osaka Grad. Sch. of Med.)、Yu Ishizuya (Urology, The Univ. of Osaka Grad. Sch. of Med.)、Takuji Hayashi (Urology, The Univ. of Osaka Grad. Sch. of Med.)、Yoshiyuki Yamamoto (Urology, The Univ. of Osaka Grad. Sch. of Med.)、Koji Hatano (Urology, The Univ. of Osaka Grad. Sch. of Med.)、Atsunari Kawashima (Urology, The Univ. of Osaka Grad. Sch. of Med.)、Junko Murai (Cell Growth and Tumor Regulation, Proteo-Sci. Ctr., Ehime Univ. ／Biochemistry and Mol. Genetics, Grad. Sch. of Med., Ehime Univ.)、Norio Nonomura (Urology, The Univ. of Osaka Grad. Sch. of Med.)

**Background:** ***SLFN11*** is reported to correlate with antitumor immunity in several cancers. However, its role in renal cell carcinoma (RCC) remains unclear. We aimed to explore its role in tumor microenvironment (TME) of RCC.
**Methods:** We analyzed transcriptome data and performed multiplex immunofluorescence using FFPE slides from human RCC samples to evaluate SLFN11 expression and immune contexture. ***In vitro***, we conducted co-culture assays with wild-type or ***SLFN11***-knockout (KO) RCC cell lines and macrophages.
**Results:** Transcriptome analysis revealed the enrichment of immune pathways and increased M1 macrophage infiltration in ***SLFN11***-high RCC tissues. ***SLFN11*** deletion in RCC cells significantly reduced GM-CSF expression. SLFN11-KO RCC cells decreased M1 markers in macrophages via GM-CSF after co-culture compared to wild-type. SLFN11-positive tumors had higher GM-CSF expression and more CD68+CD80+ M1 macrophages ($p < 0.01$) in TME. Moreover, high ***SLFN11*** expression was significantly associated with better progression-free survival in three-immune checkpoint inhibitor treated cohorts.
**Conclusion:** ***SLFN11*** promotes M1 macrophage activation via ***GM-CSF*** and may enhance antitumor response in RCC.

[Disclosure of COI by Responsible researcher] Lead presenter is the Responsible researcher.
[Lead presenter: Yohei Okuda] We have no financial relationships to disclose.

### [J-2087] II-J9
### Urological Cancer

2025 / 9 / 26 (Fri) 15:20-16:35 Room 14/(ANA Crowne Plaza Hotel Kanazawa 3F Zuiun 1) Room 14

[Chairperson]Shusuke Akamatsu (Dept. of Urology, Nagoya University)

---

### J-2087
### Genome-wide CRISPR screening identifies a novel therapeutic agent to enhance radiation sensitivity in prostate cancer

[Speaker]Koji Hatano (Urology, The University of Osaka Graduate School of Medicine) [Co-author]Toshiki Oka (Urology, The University of Osaka Graduate School of Medicine)、Shohei Katsuki (Radiation Oncology, The University of Osaka Graduate School of Medicine)、Masatoshi Konishi (Urology, The University of Osaka Graduate School of Medicine)、Masaru Tani (Urology, The University of Osaka Graduate School of Medicine)、Tomohiro Kanaki (Urology, The University of Osaka Graduate School of Medicine)、Yutong Liu (Urology, The University of Osaka Graduate School of Medicine)、Yu Ishizuya (Urology, The University of Osaka Graduate School of Medicine)、Takuji Hayashi (Urology, The University of Osaka Graduate School of Medicine)、Yoshiyuki Yamamoto (Urology, The University of Osaka Graduate School of Medicine)、Taigo Kato (Urology, The University of Osaka Graduate School of Medicine)、Atsunari Kawashima (Urology, The University of Osaka Graduate School of Medicine)、Keisuke Tamari (Radiation Oncology, The University of Osaka Graduate School of Medicine)、Keisuke Nimura (Gene Therapy Science, Gunma University Initiative for Advanced Research)、Norio Nonomura (Urology, The University of Osaka Graduate School of Medicine)

**Introduction:** Radiotherapy is widely used for prostate cancer (PCa), but the therapeutic efficacy varies widely among patients. Here, we used genome-wide CRISPR screens to identify genes that regulates radiosensitivity in PCa. We further identified novel agents that enhance PCa radiosensitivity. **Methods:** PCa cell lines, DU145, 22Rv1, and LNCaP, were used to establish CRISPR/Cas9 cell libraries. The cell libraries were irradiated (4 or 6 Gy) or untreated (control), and cultured for 11 days. Inhibitors for target genes were explored by in sillico analysis. **Results:** The most potent radiosensitizing target gene, DCLREX was identified by CRISPR screens of three PCa cell lines. Knockout of DCLREX significantly increased γH2AX positive cell rate 2.1-fold after irradiation in DU145 cells. Knockout of DCLREX reduced the radiation dose by 55% required to achieve 90% cell death. Notably, we identified a novel agent that enhances PCa radiosensitivity via the inhibition of DCLREX. **Conclusions:** The most potent radiosensitizing target gene, DCLREX, was identified by CRISPR screens. We identified a novel agent which enhances PCa radiosensitivity.

[Disclosure of COI by Responsible researcher] Lead presenter is the Responsible researcher.
[Lead presenter: Koji Hatano] ■Research funds from company: 科学研究費　基盤C　24K12482　■Lecture fee: アステラス製薬　ヤンセンファーマ　■Research expenses: アステラス製薬　バイエル薬品　ファイザー　アムジェン　MSD　アストラゼネカ

▲TOP

### [J-2088] II-J9
### Urological Cancer

2025 / 9 / 26 (Fri) 15:20-16:35 Room 14/(ANA Crowne Plaza Hotel Kanazawa 3F Zuiun 1) Room 14

[Chairperson]Shusuke Akamatsu (Dept. of Urology, Nagoya University)

---

### J-2088
### Phospholipid biosynthesis and oxidative stress interplay in the pathogenesis of renal cell carcinoma

[Speaker]Renpei Kato (Dept. of Urology, Iwate Med. Sch. of Med) [Co-author]Shigekatsu Maekawa (Dept. of Urology, Iwate Med. Sch. of Med)、Mitsugu Kanehira (Dept. of Urology, Iwate Med. Sch. of Med)、Yosuke Matsushita (Lab. of Bio. Med., Inst. Genome Res., Tokushima Univ.)、Tetsuro Yoshimaru (Lab. of Bio. Med., Inst. Genome Res., Tokushima Univ.)、Tomoya Fukawa (Dept. of Urology, Tokushima Univ. Grad. Sch.)、Toyomasa Katagiri (Lab. of Bio. Med., Inst. Genome Res., Tokushima Univ.)、Wataru Obara (Dept. of Urology, Iwate Med. Sch. of Med)

Renal cell carcinoma (RCC) is a major malignant tumor of the kidney. We identified a critical role for PRELI domain-containing 2 (PRELID2), a member of the PRELI protein family, which is specifically upregulated in clear cell RCC (ccRCC). RNA-seq analyses of the TCGA KIRC cohort and our own 178 ccRCC clinical samples revealed that mitochondrial respiratory chain subunit genes were downregulated in tumor tissues, while PRELID2 expression was negatively correlated with mitochondrially encoded gene expression. Gene set enrichment analysis further showed enrichment of mitochondrial-related molecular functions in PRELID2-high tumors.
In vitro experiments demonstrated that PRELID2 promotes cell proliferation and reduces ROS levels in ccRCC cells. Focusing on oxidative stress responses, we performed lipidomic profiling using 3D culture models and found a significant association between PRELID2 expression and phosphatidylglycerol levels. These findings suggest that PRELID2 contributes to mitochondrial homeostasis and oxidative stress regulation, highlighting its potential as a novel therapeutic target in ccRCC.

[Disclosure of COI by Responsible researcher] Lead presenter is the Responsible researcher.
[Lead presenter: Renpei Kato] We have no financial relationships to disclose.

### [J-2089] II-J9
### Urological Cancer

2025 / 9 / 26 (Fri) 15:20-16:35 Room 14/(ANA Crowne Plaza Hotel Kanazawa 3F Zuiun 1) Room 14

[Chairperson]Shusuke Akamatsu (Dept. of Urology, Nagoya University)

---

### J-2089
### Molecular and clinical relevance of mitochondrial respiratory supercomplex in prostate cancer tumorigenesis

[Speaker]Kazuhiro Ikeda (Div. Systems Med. & Gene Therapy, Saitama Med. Univ.) [Co-author]Takashi Suzuki (Dept. Anatomic Pathol., Tohoku Univ. Grad. Sch. Med.)、Kuniko Horie (Div. Systems Med. & Gene Therapy, Saitama Med. Univ.)、Satoshi Inoue (Div. Systems Med. & Gene Therapy, Saitama Med. Univ.／Dept. Systems Aging Sci. & Med., Tokyo Metropol. Inst. Geriatr. & Gerontol.)

Aerobic glycolysis was long considered as a major energy source in cancers. In contrast, we discovered that COX7RP (cytochrome ***c*** oxidase subunit 7a-related polypeptide), the unique mitochondrial respiratory supercomplex assembly factor, drives tumorigenesis of breast and endometrial cancers by enhancing the efficiency of mitochondrial oxidative phosphorylation (OXPHOS) [Nat Commun 2013, 2019]. Recently, we revealed that OXPHOS is a particular pathway upregulated in clinical castration-resistant prostate cancer tissues [Commun Biol 2020]. We here aimed to clarify the molecular and clinical relevance of COX7RP in prostate cancer. COX7RP silencing significantly suppressed prostate cancer cell proliferation and oxygen consumption rate, as well as tumor growth in vivo. Luciferase reporter assays demonstrated that ***COX7RP*** is transcriptionally regulated by estrogen-related receptor α (ERRα), a master regulator of OXPHOS. Clinically, high immunoreactivities of COX7RP and ERRα were associated with poor prognosis of prostate cancer patients. Overall, ERRα-COX7RP axis plays a pivotal role in regulating metabolic alterations and can be a potential therapeutic target for prostate cancer.

[Disclosure of COI by Responsible researcher] Lead presenter is not the Responsible researcher.
[Lead presenter: Kazuhiro Ikeda] We have no financial relationships to disclose.
[Principal Researcher: Satoshi Inoue] We have no financial relationships to disclose.

### [J-2090] II-J9
### Urological Cancer

2025 / 9 / 26 (Fri) 15:20-16:35 Room 14/(ANA Crowne Plaza Hotel Kanazawa 3F Zuiun 1) Room 14

[Chairperson]Shusuke Akamatsu (Dept. of Urology, Nagoya University)

---

### J-2090
### Whole-genome sequencing of 135 upper urinary tract urothelial carcinomas

[Speaker]Yoshiaki Kurokawa (Div. of Cancer Evolution, NCC／Dept. of Urology, The Univ. of Tokyo) [Co-author]Yoichi Fujii (Dept. of Urology, The Univ. of Tokyo)、Kaito Mimura (Div. of Cancer Evolution, NCC)、Ai Okada (Div. of Genome Analysis Platform Development, NCC)、Kenichi Chiba (Div. of Genome Analysis Platform Development, NCC)、Yusuke Sato (Dept. of Urology, The Univ. of Tokyo)、Kouya Shiraishi (Div. of Genome Biol., NCC)、Kotoe Katayama (Lab. of Sequence Analysis, The Univ. of Tokyo／Div. of Health Med. Intelligence, The Univ. of Tokyo)、Seiya Imoto (Lab. of Sequence Analysis, The Univ. of Tokyo)、Yuichi Shiraishi (Div. of Genome Analysis Platform Development, NCC)、Takashi Kohno (Div. of Genome Biol., NCC)、Haruki Kume (Dept. of Urology, The Univ. of Tokyo)、Seishi Ogawa (Dept. of Path. & Tumor Biol., Kyoto Univ.／Inst. for the Advanced Study of Human Biol., Kyoto Univ.)、Kenichi Yoshida (Div. of Cancer Evolution, NCC)

Upper urinary tract urothelial carcinoma (UTUC) is a poor-prognosis cancer of the renal pelvis and ureters. Recently our study identified five molecular subtypes of UTUC with different outcomes. To further understand the molecular pathogenesis of UTUC, we performed whole-genome analysis of 135 UTUCs from 135 patients. The median number of mutations was 11,000 mutations (range 2,700-780,000). 11 samples (8.5%) were hypermutated (>100,000 mutations), including 10 samples caused by DNA mismatch repair deficiency and one sample by APOBEC mutagenesis. Most frequently mutated driver genes were ***KMT2D*** (44.4%), ***TP53*** (35.6%) and ***FGFR3*** (31.1%), where some of the hot spot mutations were caused by APOBEC mutagenesis. Recurrent non-coding mutations were also identified, which include known driver mutations in the promoter and 5' UTR region of ***TERT*** as well as APOBEC-related hot spot mutations in ***TBC1D12*** and ***WDR74***. Extrachromosomal DNA (ecDNA) was frequently found in 58 samples (43.0%), including those containing oncogenes such as ***CCND1*** and ***MDM2***. Whole-genome sequencing highlights the significance of APOBEC mutagenesis and ecDNA in the pathogenesis of UTUC.

[Disclosure of COI by Responsible researcher] Lead presenter is not the Responsible researcher.
[Lead presenter: Yoshiaki Kurokawa] We have no financial relationships to disclose.
[Principal Researcher: Kenichi Yoshida] ■Contributions: 公益財団法人持田記念医学薬学振興財団、公益財団法人内藤記念科学振興財団、公益財団法人第一三共生命科学研究振興財団、公益財団法人住友財団、公益財団法人安田記念医学財団、公益財団法人アステラス病態代謝研究会、公益財団法人MSD生命科学財団、一般社団法人日本血液学会、公益財団法人小児医学研究振興財団、公益財団法人上原記念生命科学財団、公益財団法人高松宮妃癌研究基金、公益信託日本白血病研究基金、公益財団法人小林がん学術振興会、公益財団法人武田科学振興財団、公益財団法人大和証券財団、公益財団法人ＳＧＨ財団、公益財団法人川野小児医学奨学財団、認定NPO法人ゴールドリボン・ネットワーク、公益財団法人先進医薬研究振興財団、公益財団法人テルモ生命科学振興財団、公益財団法人小野薬品がん・免疫・神経研究財団、公益財団法人細胞科学研究財団

### [E-2085] I-E13-2
### New molecular targeted therapy (1)

2025 / 9 / 26 (Fri) 15:20-16:35 Room 15/(ANA Crowne Plaza Hotel Kanazawa 3F Zuiun 2) Room 15

[Chairperson]Hiroaki Sakurai (Dept. Cancer Cell Biol., Univ. Toyama)

---

### E-2085
### DOT1L inhibition reprograms innate immunity signaling to potentiate immunomodulatory drug responses in multiple myeloma

[Speaker]Kazuya Ishiguro (Div. Mol. Biol., Dept. Biochem., Sapporo Med. Univ., Sch. Med.／Div. Gastroenterol&Hepatol., Dept. Int. Med., Sapporo Med. Univ., Sch. Med.) [Co-author]Hiroshi Kitajima (Div. Mol. Biol., Dept. Biochem., Sapporo Med. Univ., Sch. Med.)、Takeshi Niinuma (Div. Mol. Biol., Dept. Biochem., Sapporo Med. Univ., Sch. Med.)、Reo Maruyama (Dept. Cancer Epigenome, Cancer Inst., Japanese Found. Cancer)、Tomohide Tsukahara (Div. 1st Pathol., Dept. Pathol., Sapporo Med. Univ., Sch. Med.)、Yoshihiko Hirohashi (Div. 1st Pathol., Dept. Pathol., Sapporo Med. Univ., Sch. Med.)、Kohei Kumegawa (Next-Ganken program, Cancer Inst., Japanese Found. Cancer)、Masahiro Kai (Div. Mol. Biol., Dept. Biochem., Sapporo Med. Univ., Sch. Med.)、Hiroshi Nakase (Div. Gastroenterol&Hepatol., Dept. Int. Med., Sapporo Med. Univ., Sch. Med.)、Hiromu Suzuki (Div. Mol. Biol., Dept. Biochem., Sapporo Med. Univ., Sch. Med.)

Immunomodulatory drugs (IMiDs) are the mainstay of multiple myeloma (MM) treatment, but their efficacy remains suboptimal. We previously reported that inhibition of DOT1L, a histone H3 lysine 79 methyltransferase, upregulates interferon (IFN)-regulated genes (IRGs) and exerts anti-myeloma effects in MM. In this study, we aimed to further elucidate the role of DOT1L in epigenetic dependency and the mechanism by which DOT1L inhibition induces IRGs. Analysis of DepMap portal data revealed that MM cells are selectively dependent on DOT1L among epigenetic genes for survival. DOT1L inhibition activated type I IFN responses and increased surface expression of human leukocyte antigen (HLA) class II molecules. Notably, DOT1L inhibition induced DNA damage and elevated cytoplasmic DNA levels in MM cells. CRISPR/Cas9-mediated knockout of STING1 attenuated IRG induction and reduced the anti-proliferative effect of DOT1L inhibition. Finally, DOT1L inhibition enhanced the anti-MM activity of IMiDs by further promoting IRG expression. These findings suggest that DOT1L is a promising epigenetic target in MM and that its inhibition can potentiate the therapeutic efficacy of IMiDs.

[Disclosure of COI by Responsible researcher] Lead presenter is not the Responsible researcher.
[Lead presenter: Kazuya Ishiguro] We have no financial relationships to disclose.
[Principal Researcher: Hiromu Suzuki] We have no financial relationships to disclose.

### [E-2086] I-E13-2
### New molecular targeted therapy (1)

2025 / 9 / 26 (Fri) 15:20-16:35 Room 15/(ANA Crowne Plaza Hotel Kanazawa 3F Zuiun 2) Room 15

[Chairperson]Hiroaki Sakurai (Dept. Cancer Cell Biol., Univ. Toyama)

---

### E-2086
### A novel abiraterone derivative suppresses glioblastoma through FLG-mediated glioma stem cell disruption

[Speaker]Hoang Yen Tran (School of Pharmacy, Taipei Medical University, Taipei, Taiwan／Pharmacology Department, Can Tho University of Medicine and Pharmacy, Vietnam) [Co-author]Jing-Ping Liou (School of Pharmacy, Taipei Medical University, Taipei, Taiwan)、Tsung-I Hsu (Ph.D. Program in Medical Neuroscience, Taipei Medical University, Taipei, Taiwan)

Filaggrin (FLG), a structural protein in the epidermis, is a key driver of skin cancer. Our gene database analysis showed reduced FLG level in glioblastoma (GBM), and our novel abiraterone derivative, Cp8, increased FLG expression in GBM cells. To investigate the effect of Cp8 on GBM, cell viability and colony formation assays, and RNA sequencing were performed. IHC staining and western blotting were conducted on patient tumor samples, mouse brains, and glioma cells. FLG knockdown and overexpression were performed to assess the functional role of FLG in GBM. The effects of Cp8 on glioma stem cell (GSC) enrichment, MGMT, and ROS metabolism were also evaluated. In orthotopic GBM mouse models, survival was recorded, and tumor growth was monitored using IVIS imaging and HE staining. Results showed that Cp8 inhibited GBM proliferation, induced apoptosis, and increased FLG levels. FLG deprivation elevated GBM viability and GSC levels, while FLG overexpression further enhanced cell viability. Cp8 also suppressed GSCs, MGMT expression, ROS metabolism, extended survival, and reduced tumor size with low toxicity. Our novel compound, Cp8, shows promise as an FLG-targeted GBM chemotherapeutic.

[Disclosure of COI by Responsible researcher] Lead presenter is the Responsible researcher.
[Lead presenter: Hoang Yen Tran] We have no financial relationships to disclose.

### [E-2087] I-E13-2
### New molecular targeted therapy (1)

2025 / 9 / 26 (Fri) 15:20-16:35 Room 15/(ANA Crowne Plaza Hotel Kanazawa 3F Zuiun 2) Room 15

[Chairperson]Hiroaki Sakurai (Dept. Cancer Cell Biol., Univ. Toyama)

---

### E-2087
### Dysadherin Inhibition Suppresses Hepatocellular Carcinoma and Enhances Combination Therapy

[Speaker]Da-Ye Lim (Department of Life Sciences, GIST, Gwangju, 61005, Republic of Korea) [Co-author]Hyeon-Ji Yun (Department of Life Sciences, GIST, Gwangju, 61005, Republic of Korea)、Eun-Ju Han (Department of Life Sciences, GIST, Gwangju, 61005, Republic of Korea)、Jang-Soo Chun (Inje University, Gimhae, 50834, Republic of Korea)、Jeong-Seok Nam (Department of Life Sciences, GIST, Gwangju, 61005, Republic of Korea)

Targeting protein-protein interactions is a promising strategy in cancer therapy due to its ability to disrupt essential oncogenic processes. Dysadherin, a cancer-associated glycoprotein, promotes tumor progression and therapeutic resistance by modulating integrin-mediated extracellular matrix adhesion. In this study, we explored the therapeutic potential of a dysadherin-inhibitory peptide (DIP) in hepatocellular carcinoma (HCC). Bioinformatics analyses of clinical genomic datasets revealed that dysadherin expression is significantly elevated in HCC and correlates with disease progression. Functional assays in HCC cell lines showed that DIP treatment suppresses proliferation, migration, metastatic potential, and tumor-initiating ability. In vivo xenograft models confirmed that DIP markedly inhibits tumor growth. Mechanistically, DIP disrupts the dysadherin-fibronectin-integrin axis. Moreover, DIP enhances the efficacy of combination therapies, including Sorafenib, both in vitro and in vivo. These findings identify dysadherin as a potential therapeutic target and support the clinical relevance of DIP as a novel strategy for improving treatment outcomes in HCC.

[Disclosure of COI by Responsible researcher] Lead presenter is not the Responsible researcher.
[Lead presenter: Da-Ye Lim] We have no financial relationships to disclose.
[Principal Researcher: Jang-Soo Chun] We have no financial relationships to disclose.

### [E-2088] I-E13-2
### New molecular targeted therapy (1)

2025 / 9 / 26 (Fri) 15:20-16:35 Room 15/(ANA Crowne Plaza Hotel Kanazawa 3F Zuiun 2) Room 15

[Chairperson]Hiroaki Sakurai (Dept. Cancer Cell Biol., Univ. Toyama)

### E-2088
### A bump & hole modification of IAP antagonist-IAP pair for a novel protein degradation system

[Speaker]Waka Sato (Graduate School of Pharmaceutical Sciences, The University of Tokyo) [Co-author]Yukihiro Furuuchi (National Institute of Health Sciences)、Yukari Oh (Graduate School of Pharmaceutical Sciences, The University of Tokyo)、Hidetomo Yokoo (National Institute of Health Sciences)、Yosuke Demizu (National Institute of Health Sciences)、Shigeo Murata (Graduate School of Pharmaceutical Sciences, The University of Tokyo)、Mikihiko Naito (Graduate School of Pharmaceutical Sciences, The University of Tokyo)

PROTACs are bifunctional molecules that induce ubiquitylation and proteasomal degradation of target proteins, and SNIPERs (IAP-based PROTACs) utilize the E3 ubiquitin ligase activity of IAPs. Since cells express several IAP family members to which LCL-161 module in the SNIPERs binds, the role and mechanism of each IAP family members in the SNIPER-mediated protein degradation are unclear. In this research, we employed the bump-and-hole strategy to engineer IAP binders that interact with mutant IAPs but not wild-type IAPs. Among the IAPs, cIAP1 is known to undergo autoubiquitination and degradation upon binding to IAP antagonists. Using this property as a readout, we successfully developed modified IAP antagonists that selectively induce degradation of cIAP1 mutants without affecting the wild-type protein. These customized ligands will be incorporated into SNIPERs to elucidate the role of each IAPs in targeted protein degradation.

[Disclosure of COI by Responsible researcher] Lead presenter is not the Responsible researcher.
[Lead presenter: Waka Sato] ■Endowed chair: エーザイ株式会社
[Principal Researcher: Mikihiko Naito] ■Endowed chair: エーザイ株式会社

### [E-2089] I-E13-2
### New molecular targeted therapy (1)

2025 / 9 / 26 (Fri) 15:20-16:35 Room 15/(ANA Crowne Plaza Hotel Kanazawa 3F Zuiun 2) Room 15

[Chairperson]Hiroaki Sakurai (Dept. Cancer Cell Biol., Univ. Toyama)

---

### E-2089
### Development of anti-metastatic drugs using synthetic short mRNA

[Speaker]Sachie Hiratsuka (Shinshu Univ. Sch. of Med.)

Most cancer deaths are caused by metastasis. In organs where cancer metastases occur, there are a small number of anti-metastatic immune cells. These cells express receptor proteins binding to mRNA on their surface, and when a special sequence of extracellular mRNA that is not wrapped in vesicles binds to them, the cells are activated regardless of translation (NatCommun,2021). However, natural mRNA has the disadvantage that it is quickly degraded in the body. Therefore, we identified the short sequence required for activation and newly synthesized mRNA with stabilizing modifications and administered it to individual mice, which suppressed lung metastasis of cancer cells. In this experiment, we found that synthetic mRNA has the advantage that it does not cause side effects such as cytokine storms or exhaustion of immune cells even when administered multiple times. Furthermore, when natural killer cells and cytotoxic T cells were isolated from cancer patients and activated with synthetic modified mRNA, these cells were confirmed to kill more human cancer cells than before activation (NatCommun,2025). We present a new strategy that can be used as an anti-metastatic drug.

[Disclosure of COI by Responsible researcher] Lead presenter is the Responsible researcher.
[Lead presenter: Sachie Hiratsuka] ■Research expenses: Bourbon Company, GF・Mille Co.,Ltd

### [E-2090] I-E13-2
### New molecular targeted therapy (1)

2025 / 9 / 26 (Fri) 15:20-16:35 Room 15/(ANA Crowne Plaza Hotel Kanazawa 3F Zuiun 2) Room 15

[Chairperson]Hiroaki Sakurai (Dept. Cancer Cell Biol., Univ. Toyama)

---

### E-2090
### Withdrawn

No Abstract

### [SL2] SL2
### Special Lecture2

2025 / 9 / 26 (Fri) 15:30-16:10 Room 1/(Ishikawa Ongakudo 2F Concert Hall) Room 1

[Chairperson]Noriko Gotoh (Cancer Research Institute, Kanazawa University)

---

### SL2
### TBD

[Speaker]Joseph Schlessinger (Yale Univerisity School of Medicine)

No Abstract

### [CS1] CS1

### CHUGAI PHARMACEUTICAL CO., LTD. / Tumor Microenvironment Remodeling by VEGF Inhibition and Immune Checkpoint Blockade : Unraveling the Mechanisms of Synergistic Effects

2025 / 9 / 26 (Fri) 15:30-16:20 Room 2/(Ishikawa Ongakudo 2F Hogaku Hall) Room 2
[]CHUGAI PHARMACEUTICAL CO., LTD.

[Chairperson]Makoto Maemondo (Jichi Medical University, Division of Pulmonary Medicine, Department of Medicine)

---

### CS1

### Tumor Microenvironment Remodeling by VEGF Inhibition and Immune Checkpoint Blockade : Unraveling the Mechanisms of Synergistic Effects

[Speaker]Ichidai Tanaka (Department of Respiratory Medicine, Nagoya University Hospital, Japan)

No Abstract

▲TOP

### [CS2] CS2

### Integrated DNA Technologies / Comprehensive and Customizable IDT NGS Portfolio to Accelerate Cancer Research

2025 / 9 / 26 (Fri) 15:30-16:20 Room 3/(Hotel Nikko Kanazawa 4F Tsuru A) Room 3
[]Integrated DNA Technologies

### CS2

[Speaker]Aarti Arun Gokhale (Integrated DNA Technologies)

No Abstract

### [ME4] ME4
### Cancer Science

2025 / 9 / 26 (Fri) 15:30-16:10 Room 6/(Hotel Nikko Kanazawa 3F Kujaku A) Room 6

[Chairperson]Daichi Inoue (Department of Cancer Pathology Graduate School of Medicine, The University of Osaka)

---

### ME4
### Cancer Science

[Speaker]Masanori Hatakeyama (Editor-in-Chief, Cancer Science)

No Abstract

### [CS3-1] CS3
### GeneBay, Inc. / Cancer Genome Analysis using Long Read Sequencing Technologies

2025 / 9 / 26 (Fri) 15:30-16:20 Room 11/(ANA Crowne Plaza Hotel Kanazawa 3F Ohtori (West)) Room 11
[]GeneBay, Inc.

[Chairperson]Seiichi Mori (Cancer Precision Medicine Center, Division of Cancer Genomics, Japanese Foundation for Cancer Research)

### CS3-1
### 1) Introduction of Nanopore Sequencing: From Sample Prep to Data Analysis

[Speaker]Yasuo Uemura (GeneBay, Inc.)

No Abstract

### [CS3-2] CS3
### GeneBay, Inc. / Cancer Genome Analysis using Long Read Sequencing Technologies

2025 / 9 / 26 (Fri) 15:30-16:20 Room 11/(ANA Crowne Plaza Hotel Kanazawa 3F Ohtori (West)) Room 11
[]GeneBay, Inc.

[Chairperson]Seiichi Mori (Cancer Precision Medicine Center, Division of Cancer Genomics, Japanese Foundation for Cancer Research)

---

### CS3-2
### 2) Cancer Genome Informatic Analysis Pipeline for Nanopore Long Read Genome Sequencing

[Speaker]Takanori Hasegawa (M&D Data Science Center, Institute of Integrated Research, Institute of Science Tokyo)

No Abstract

▲TOP

### [CS3-3] CS3
### GeneBay, Inc. / Cancer Genome Analysis using Long Read Sequencing Technologies

2025 / 9 / 26 (Fri) 15:30-16:20 Room 11/(ANA Crowne Plaza Hotel Kanazawa 3F Ohtori (West)) Room 11
[]GeneBay, Inc.

[Chairperson]Seiichi Mori (Cancer Precision Medicine Center, Division of Cancer Genomics, Japanese Foundation for Cancer Research)

---

### CS3-3
### 3) Landscape of Japanese Ovarian Cancer Genome

[Speaker]Seiichi Mori (Cancer Precision Medicine Center, Division of Cancer Genomics, Japanese Foundation for Cancer Research)

No Abstract

### [SSP-1] SSP
### Survivor Scientist Program

2025 / 9 / 26 (Fri) 16:45-18:15 Room P/(Motenashi Dome) Room P

[Chairperson]Masashi Sanada (Clin. Res. Ctr. NHO Nagoya Med.Ctr.)

Japanese Cancer Association (JCA) launched the JCA-Survivor Scientist Program (JCA-SSP) to train "research advocates" who will participate in cancer research from the perspective of cancer patients and their families by deepening the understanding of cancer research. In addition, the JCA-SSP program facilitates the collaboration between cancer scientists, cancer survivors, and patient-advocacy groups, which is essential to promote cancer research, thereby contributing to the improvement of cancer therapies.
At the annual meeting this year, we are planning to hold the 9th JCA-SSP program by inviting ~8 research advocates who will learn about cancer research and its current and future relevance to cancer therapeutics by inviting ~9research advocates who participated in the previous JCA-SSP program. Scientists and patient advocates who have attended the SSP program at American Association for Cancer Research (AACR) will also join this JCA-SSP program as scientific mentors and advocate mentors to assist participants throughout the 3-day program. Participants are requested to present their current activities and expectations for cancer research at poster presentation. Participants are divided into groups. Each group will present achievements of their group work on a given topic at the end of this program.

---

### SSP-1

[Speaker]Yasuko Azuma ()

No Abstract

▲TOP

### [SSP-2] SSP
### Survivor Scientist Program

2025 / 9 / 26 (Fri) 16:45-18:15 Room P/(Motenashi Dome) Room P

[Chairperson]Masashi Sanada (Clin. Res. Ctr. NHO Nagoya Med.Ctr.)

Japanese Cancer Association (JCA) launched the JCA-Survivor Scientist Program (JCA-SSP) to train "research advocates" who will participate in cancer research from the perspective of cancer patients and their families by deepening the understanding of cancer research. In addition, the JCA-SSP program facilitates the collaboration between cancer scientists, cancer survivors, and patient-advocacy groups, which is essential to promote cancer research, thereby contributing to the improvement of cancer therapies.
At the annual meeting this year, we are planning to hold the 9th JCA-SSP program by inviting ~8 research advocates who will learn about cancer research and its current and future relevance to cancer therapeutics by inviting ~9research advocates who participated in the previous JCA-SSP program. Scientists and patient advocates who have attended the SSP program at American Association for Cancer Research (AACR) will also join this JCA-SSP program as scientific mentors and advocate mentors to assist participants throughout the 3-day program. Participants are requested to present their current activities and expectations for cancer research at poster presentation. Participants are divided into groups. Each group will present achievements of their group work on a given topic at the end of this program.

---

### SSP-2

[Speaker]Akiko Igarashi ()

No Abstract

▲TOP

### [SSP-3] SSP
### Survivor Scientist Program

2025 / 9 / 26 (Fri) 16:45-18:15 Room P/(Motenashi Dome) Room P

[Chairperson]Masashi Sanada (Clin. Res. Ctr. NHO Nagoya Med.Ctr.)

Japanese Cancer Association (JCA) launched the JCA-Survivor Scientist Program (JCA-SSP) to train "research advocates" who will participate in cancer research from the perspective of cancer patients and their families by deepening the understanding of cancer research. In addition, the JCA-SSP program facilitates the collaboration between cancer scientists, cancer survivors, and patient-advocacy groups, which is essential to promote cancer research, thereby contributing to the improvement of cancer therapies.
At the annual meeting this year, we are planning to hold the 9th JCA-SSP program by inviting ~8 research advocates who will learn about cancer research and its current and future relevance to cancer therapeutics by inviting ~9research advocates who participated in the previous JCA-SSP program. Scientists and patient advocates who have attended the SSP program at American Association for Cancer Research (AACR) will also join this JCA-SSP program as scientific mentors and advocate mentors to assist participants throughout the 3-day program. Participants are requested to present their current activities and expectations for cancer research at poster presentation. Participants are divided into groups. Each group will present achievements of their group work on a given topic at the end of this program.

### SSP-3

[Speaker]Shinji Ishiwatari ()

No Abstract

▲TOP

### [SSP-4] SSP
### Survivor Scientist Program

2025 / 9 / 26 (Fri) 16:45-18:15 Room P/(Motenashi Dome) Room P

[Chairperson]Masashi Sanada (Clin. Res. Ctr. NHO Nagoya Med.Ctr.)

Japanese Cancer Association (JCA) launched the JCA-Survivor Scientist Program (JCA-SSP) to train "research advocates" who will participate in cancer research from the perspective of cancer patients and their families by deepening the understanding of cancer research. In addition, the JCA-SSP program facilitates the collaboration between cancer scientists, cancer survivors, and patient-advocacy groups, which is essential to promote cancer research, thereby contributing to the improvement of cancer therapies.
At the annual meeting this year, we are planning to hold the 9th JCA-SSP program by inviting ~8 research advocates who will learn about cancer research and its current and future relevance to cancer therapeutics by inviting ~9research advocates who participated in the previous JCA-SSP program. Scientists and patient advocates who have attended the SSP program at American Association for Cancer Research (AACR) will also join this JCA-SSP program as scientific mentors and advocate mentors to assist participants throughout the 3-day program. Participants are requested to present their current activities and expectations for cancer research at poster presentation. Participants are divided into groups. Each group will present achievements of their group work on a given topic at the end of this program.

### SSP-4

[Speaker]Satoshi Orimo ()

No Abstract

### [SSP-5] SSP
### Survivor Scientist Program

2025 / 9 / 26 (Fri) 16:45-18:15 Room P/(Motenashi Dome) Room P

[Chairperson]Masashi Sanada (Clin. Res. Ctr. NHO Nagoya Med.Ctr.)

Japanese Cancer Association (JCA) launched the JCA-Survivor Scientist Program (JCA-SSP) to train "research advocates" who will participate in cancer research from the perspective of cancer patients and their families by deepening the understanding of cancer research. In addition, the JCA-SSP program facilitates the collaboration between cancer scientists, cancer survivors, and patient-advocacy groups, which is essential to promote cancer research, thereby contributing to the improvement of cancer therapies.
At the annual meeting this year, we are planning to hold the 9th JCA-SSP program by inviting ~8 research advocates who will learn about cancer research and its current and future relevance to cancer therapeutics by inviting ~9research advocates who participated in the previous JCA-SSP program. Scientists and patient advocates who have attended the SSP program at American Association for Cancer Research (AACR) will also join this JCA-SSP program as scientific mentors and advocate mentors to assist participants throughout the 3-day program. Participants are requested to present their current activities and expectations for cancer research at poster presentation. Participants are divided into groups. Each group will present achievements of their group work on a given topic at the end of this program.

### SSP-5

[Speaker]Makiko Suzuki ()

No Abstract

▲TOP

### [SSP-6] SSP
### Survivor Scientist Program

2025 / 9 / 26 (Fri) 16:45-18:15 Room P/(Motenashi Dome) Room P

[Chairperson]Masashi Sanada (Clin. Res. Ctr. NHO Nagoya Med.Ctr.)

Japanese Cancer Association (JCA) launched the JCA-Survivor Scientist Program (JCA-SSP) to train "research advocates" who will participate in cancer research from the perspective of cancer patients and their families by deepening the understanding of cancer research. In addition, the JCA-SSP program facilitates the collaboration between cancer scientists, cancer survivors, and patient-advocacy groups, which is essential to promote cancer research, thereby contributing to the improvement of cancer therapies.
At the annual meeting this year, we are planning to hold the 9th JCA-SSP program by inviting ~8 research advocates who will learn about cancer research and its current and future relevance to cancer therapeutics by inviting ~9research advocates who participated in the previous JCA-SSP program. Scientists and patient advocates who have attended the SSP program at American Association for Cancer Research (AACR) will also join this JCA-SSP program as scientific mentors and advocate mentors to assist participants throughout the 3-day program. Participants are requested to present their current activities and expectations for cancer research at poster presentation. Participants are divided into groups. Each group will present achievements of their group work on a given topic at the end of this program.

---

### SSP-6

[Speaker]Tsutomu Takeuchi ()

No Abstract

### [SSP-7] SSP
### Survivor Scientist Program

2025 / 9 / 26 (Fri) 16:45-18:15 Room P/(Motenashi Dome) Room P

[Chairperson]Masashi Sanada (Clin. Res. Ctr. NHO Nagoya Med.Ctr.)

Japanese Cancer Association (JCA) launched the JCA-Survivor Scientist Program (JCA-SSP) to train "research advocates" who will participate in cancer research from the perspective of cancer patients and their families by deepening the understanding of cancer research. In addition, the JCA-SSP program facilitates the collaboration between cancer scientists, cancer survivors, and patient-advocacy groups, which is essential to promote cancer research, thereby contributing to the improvement of cancer therapies.
At the annual meeting this year, we are planning to hold the 9th JCA-SSP program by inviting ~8 research advocates who will learn about cancer research and its current and future relevance to cancer therapeutics by inviting ~9research advocates who participated in the previous JCA-SSP program. Scientists and patient advocates who have attended the SSP program at American Association for Cancer Research (AACR) will also join this JCA-SSP program as scientific mentors and advocate mentors to assist participants throughout the 3-day program. Participants are requested to present their current activities and expectations for cancer research at poster presentation. Participants are divided into groups. Each group will present achievements of their group work on a given topic at the end of this program.

---

### SSP-7

[Speaker]Masahiko Takeda ()

No Abstract

### [SSP-8] SSP
### Survivor Scientist Program

2025 / 9 / 26 (Fri) 16:45-18:15 Room P/(Motenashi Dome) Room P

[Chairperson]Masashi Sanada (Clin. Res. Ctr. NHO Nagoya Med.Ctr.)

Japanese Cancer Association (JCA) launched the JCA-Survivor Scientist Program (JCA-SSP) to train "research advocates" who will participate in cancer research from the perspective of cancer patients and their families by deepening the understanding of cancer research. In addition, the JCA-SSP program facilitates the collaboration between cancer scientists, cancer survivors, and patient-advocacy groups, which is essential to promote cancer research, thereby contributing to the improvement of cancer therapies.
At the annual meeting this year, we are planning to hold the 9th JCA-SSP program by inviting ~8 research advocates who will learn about cancer research and its current and future relevance to cancer therapeutics by inviting ~9research advocates who participated in the previous JCA-SSP program. Scientists and patient advocates who have attended the SSP program at American Association for Cancer Research (AACR) will also join this JCA-SSP program as scientific mentors and advocate mentors to assist participants throughout the 3-day program. Participants are requested to present their current activities and expectations for cancer research at poster presentation. Participants are divided into groups. Each group will present achievements of their group work on a given topic at the end of this program.

---

### SSP-8

[Speaker]Mitsuko Teraoka ()

No Abstract

### [SSP-9] SSP
### Survivor Scientist Program

2025 / 9 / 26 (Fri) 16:45-18:15 Room P/(Motenashi Dome) Room P

[Chairperson]Masashi Sanada (Clin. Res. Ctr. NHO Nagoya Med.Ctr.)

Japanese Cancer Association (JCA) launched the JCA-Survivor Scientist Program (JCA-SSP) to train "research advocates" who will participate in cancer research from the perspective of cancer patients and their families by deepening the understanding of cancer research. In addition, the JCA-SSP program facilitates the collaboration between cancer scientists, cancer survivors, and patient-advocacy groups, which is essential to promote cancer research, thereby contributing to the improvement of cancer therapies.
At the annual meeting this year, we are planning to hold the 9th JCA-SSP program by inviting ~8 research advocates who will learn about cancer research and its current and future relevance to cancer therapeutics by inviting ~9research advocates who participated in the previous JCA-SSP program. Scientists and patient advocates who have attended the SSP program at American Association for Cancer Research (AACR) will also join this JCA-SSP program as scientific mentors and advocate mentors to assist participants throughout the 3-day program. Participants are requested to present their current activities and expectations for cancer research at poster presentation. Participants are divided into groups. Each group will present achievements of their group work on a given topic at the end of this program.

---

### SSP-9

[Speaker]Takao Terasawa ()

No Abstract

▲TOP

### [SSP-10] SSP
### Survivor Scientist Program

2025 / 9 / 26 (Fri) 16:45-18:15 Room P/(Motenashi Dome) Room P

[Chairperson]Masashi Sanada (Clin. Res. Ctr. NHO Nagoya Med.Ctr.)

Japanese Cancer Association (JCA) launched the JCA-Survivor Scientist Program (JCA-SSP) to train "research advocates" who will participate in cancer research from the perspective of cancer patients and their families by deepening the understanding of cancer research. In addition, the JCA-SSP program facilitates the collaboration between cancer scientists, cancer survivors, and patient-advocacy groups, which is essential to promote cancer research, thereby contributing to the improvement of cancer therapies.
At the annual meeting this year, we are planning to hold the 9th JCA-SSP program by inviting ~8 research advocates who will learn about cancer research and its current and future relevance to cancer therapeutics by inviting ~9research advocates who participated in the previous JCA-SSP program. Scientists and patient advocates who have attended the SSP program at American Association for Cancer Research (AACR) will also join this JCA-SSP program as scientific mentors and advocate mentors to assist participants throughout the 3-day program. Participants are requested to present their current activities and expectations for cancer research at poster presentation. Participants are divided into groups. Each group will present achievements of their group work on a given topic at the end of this program.

---

### SSP-10

[Speaker]Mayumi Terada ()

No Abstract

### [SSP-11] SSP
### Survivor Scientist Program

2025 / 9 / 26 (Fri) 16:45-18:15 Room P/(Motenashi Dome) Room P

[Chairperson]Masashi Sanada (Clin. Res. Ctr. NHO Nagoya Med.Ctr.)

Japanese Cancer Association (JCA) launched the JCA-Survivor Scientist Program (JCA-SSP) to train "research advocates" who will participate in cancer research from the perspective of cancer patients and their families by deepening the understanding of cancer research. In addition, the JCA-SSP program facilitates the collaboration between cancer scientists, cancer survivors, and patient-advocacy groups, which is essential to promote cancer research, thereby contributing to the improvement of cancer therapies.
At the annual meeting this year, we are planning to hold the 9th JCA-SSP program by inviting ~8 research advocates who will learn about cancer research and its current and future relevance to cancer therapeutics by inviting ~9research advocates who participated in the previous JCA-SSP program. Scientists and patient advocates who have attended the SSP program at American Association for Cancer Research (AACR) will also join this JCA-SSP program as scientific mentors and advocate mentors to assist participants throughout the 3-day program. Participants are requested to present their current activities and expectations for cancer research at poster presentation. Participants are divided into groups. Each group will present achievements of their group work on a given topic at the end of this program.

### SSP-11

[Speaker]Hiromi Todoroki ()

No Abstract

▲TOP

### [SSP-12] SSP
### Survivor Scientist Program

2025 / 9 / 26 (Fri) 16:45-18:15 Room P/(Motenashi Dome) Room P

[Chairperson]Masashi Sanada (Clin. Res. Ctr. NHO Nagoya Med.Ctr.)

Japanese Cancer Association (JCA) launched the JCA-Survivor Scientist Program (JCA-SSP) to train "research advocates" who will participate in cancer research from the perspective of cancer patients and their families by deepening the understanding of cancer research. In addition, the JCA-SSP program facilitates the collaboration between cancer scientists, cancer survivors, and patient-advocacy groups, which is essential to promote cancer research, thereby contributing to the improvement of cancer therapies.
At the annual meeting this year, we are planning to hold the 9th JCA-SSP program by inviting ~8 research advocates who will learn about cancer research and its current and future relevance to cancer therapeutics by inviting ~9research advocates who participated in the previous JCA-SSP program. Scientists and patient advocates who have attended the SSP program at American Association for Cancer Research (AACR) will also join this JCA-SSP program as scientific mentors and advocate mentors to assist participants throughout the 3-day program. Participants are requested to present their current activities and expectations for cancer research at poster presentation. Participants are divided into groups. Each group will present achievements of their group work on a given topic at the end of this program.

---

### SSP-12

[Speaker]Kumiko Nishida ()

No Abstract

▲TOP

### [SSP-13] SSP
### Survivor Scientist Program

2025 / 9 / 26 (Fri) 16:45-18:15 Room P/(Motenashi Dome) Room P

[Chairperson]Masashi Sanada (Clin. Res. Ctr. NHO Nagoya Med.Ctr.)

Japanese Cancer Association (JCA) launched the JCA-Survivor Scientist Program (JCA-SSP) to train "research advocates" who will participate in cancer research from the perspective of cancer patients and their families by deepening the understanding of cancer research. In addition, the JCA-SSP program facilitates the collaboration between cancer scientists, cancer survivors, and patient-advocacy groups, which is essential to promote cancer research, thereby contributing to the improvement of cancer therapies.
At the annual meeting this year, we are planning to hold the 9th JCA-SSP program by inviting ~8 research advocates who will learn about cancer research and its current and future relevance to cancer therapeutics by inviting ~9research advocates who participated in the previous JCA-SSP program. Scientists and patient advocates who have attended the SSP program at American Association for Cancer Research (AACR) will also join this JCA-SSP program as scientific mentors and advocate mentors to assist participants throughout the 3-day program. Participants are requested to present their current activities and expectations for cancer research at poster presentation. Participants are divided into groups. Each group will present achievements of their group work on a given topic at the end of this program.

### SSP-13

[Speaker]Eiko Nishimura ()

No Abstract

### [SSP-14] SSP
### Survivor Scientist Program

2025 / 9 / 26 (Fri) 16:45-18:15 Room P/(Motenashi Dome) Room P

[Chairperson]Masashi Sanada (Clin. Res. Ctr. NHO Nagoya Med.Ctr.)

Japanese Cancer Association (JCA) launched the JCA-Survivor Scientist Program (JCA-SSP) to train "research advocates" who will participate in cancer research from the perspective of cancer patients and their families by deepening the understanding of cancer research. In addition, the JCA-SSP program facilitates the collaboration between cancer scientists, cancer survivors, and patient-advocacy groups, which is essential to promote cancer research, thereby contributing to the improvement of cancer therapies.
At the annual meeting this year, we are planning to hold the 9th JCA-SSP program by inviting ~8 research advocates who will learn about cancer research and its current and future relevance to cancer therapeutics by inviting ~9research advocates who participated in the previous JCA-SSP program. Scientists and patient advocates who have attended the SSP program at American Association for Cancer Research (AACR) will also join this JCA-SSP program as scientific mentors and advocate mentors to assist participants throughout the 3-day program. Participants are requested to present their current activities and expectations for cancer research at poster presentation. Participants are divided into groups. Each group will present achievements of their group work on a given topic at the end of this program.

### SSP-14

[Speaker]Mitsuko Hisada ()

No Abstract

### [SSP-15] SSP
### Survivor Scientist Program

2025 / 9 / 26 (Fri) 16:45-18:15 Room P/(Motenashi Dome) Room P

[Chairperson]Masashi Sanada (Clin. Res. Ctr. NHO Nagoya Med.Ctr.)

Japanese Cancer Association (JCA) launched the JCA-Survivor Scientist Program (JCA-SSP) to train "research advocates" who will participate in cancer research from the perspective of cancer patients and their families by deepening the understanding of cancer research. In addition, the JCA-SSP program facilitates the collaboration between cancer scientists, cancer survivors, and patient-advocacy groups, which is essential to promote cancer research, thereby contributing to the improvement of cancer therapies.
At the annual meeting this year, we are planning to hold the 9th JCA-SSP program by inviting ~8 research advocates who will learn about cancer research and its current and future relevance to cancer therapeutics by inviting ~9research advocates who participated in the previous JCA-SSP program. Scientists and patient advocates who have attended the SSP program at American Association for Cancer Research (AACR) will also join this JCA-SSP program as scientific mentors and advocate mentors to assist participants throughout the 3-day program. Participants are requested to present their current activities and expectations for cancer research at poster presentation. Participants are divided into groups. Each group will present achievements of their group work on a given topic at the end of this program.

---

### SSP-15

[Speaker]Suzue Yasui ()

No Abstract

▲TOP

### [SSP-16] SSP
### Survivor Scientist Program

2025 / 9 / 26 (Fri) 16:45-18:15 Room P/(Motenashi Dome) Room P

[Chairperson]Masashi Sanada (Clin. Res. Ctr. NHO Nagoya Med.Ctr.)

Japanese Cancer Association (JCA) launched the JCA-Survivor Scientist Program (JCA-SSP) to train "research advocates" who will participate in cancer research from the perspective of cancer patients and their families by deepening the understanding of cancer research. In addition, the JCA-SSP program facilitates the collaboration between cancer scientists, cancer survivors, and patient-advocacy groups, which is essential to promote cancer research, thereby contributing to the improvement of cancer therapies.
At the annual meeting this year, we are planning to hold the 9th JCA-SSP program by inviting ~8 research advocates who will learn about cancer research and its current and future relevance to cancer therapeutics by inviting ~9research advocates who participated in the previous JCA-SSP program. Scientists and patient advocates who have attended the SSP program at American Association for Cancer Research (AACR) will also join this JCA-SSP program as scientific mentors and advocate mentors to assist participants throughout the 3-day program. Participants are requested to present their current activities and expectations for cancer research at poster presentation. Participants are divided into groups. Each group will present achievements of their group work on a given topic at the end of this program.

---

### SSP-16

[Speaker]Seiji Yamazaki ()

No Abstract

▲TOP

### [SSP-17] SSP
### Survivor Scientist Program

2025 / 9 / 26 (Fri) 16:45-18:15 Room P/(Motenashi Dome) Room P

[Chairperson]Masashi Sanada (Clin. Res. Ctr. NHO Nagoya Med.Ctr.)

Japanese Cancer Association (JCA) launched the JCA-Survivor Scientist Program (JCA-SSP) to train "research advocates" who will participate in cancer research from the perspective of cancer patients and their families by deepening the understanding of cancer research. In addition, the JCA-SSP program facilitates the collaboration between cancer scientists, cancer survivors, and patient-advocacy groups, which is essential to promote cancer research, thereby contributing to the improvement of cancer therapies.
At the annual meeting this year, we are planning to hold the 9th JCA-SSP program by inviting ~8 research advocates who will learn about cancer research and its current and future relevance to cancer therapeutics by inviting ~9research advocates who participated in the previous JCA-SSP program. Scientists and patient advocates who have attended the SSP program at American Association for Cancer Research (AACR) will also join this JCA-SSP program as scientific mentors and advocate mentors to assist participants throughout the 3-day program. Participants are requested to present their current activities and expectations for cancer research at poster presentation. Participants are divided into groups. Each group will present achievements of their group work on a given topic at the end of this program.

---

### SSP-17

[Speaker]Naoko Yonemori ()

No Abstract

▲TOP

### [P-2001] I-P1-3
### Chemical carcinogens

2025 / 9 / 26 (Fri) 16:45-17:30 Room P/(Motenashi Dome) Room P

---

### P-2001
### Establishment of a chemical carcinogen-induced bladder cancer model using healthy dog bladder organoids

[Speaker]Yuko Nagashima (Laboratory of Veterinary Pharmacology, Tokyo University of Agriculture and Technology,) [Co-author]Haru Yamamoto (Laboratory of Veterinary Pharmacology, Tokyo University of Agriculture and Technology,)、Yoshiko Naito (Laboratory of Veterinary Pharmacology, Tokyo University of Agriculture and Technology,)、Ryouichi Tsunedomi (Department of Gastroenterological, Breast and Endocrine Surgery, Yamaguchi University／Research Institute for Cell Design Medical Science, Yamaguchi University)、Kazuaki Sasaki (Laboratory of Veterinary Pharmacology, Tokyo University of Agriculture and Technology,)

Dog bladder cancer (DBC) accounts for approximately 2% of all spontaneous dog cancers, and the majority of these are moderate to highly invasive bladder cancers. In terms of biological behavior, DBC is said to be similar to the cytological and molecular characteristics of human muscle-invasive BC, especially high-grade invasive transitional cell carcinoma. In recent years, cancer research in dogs has been used to define genetic and environmental risks and evaluate prevention and treatment approaches that will benefit not only dogs but also humans. An organoid is a miniaturized and simplified version of an organ produced ex vivo in three dimensions, mimicking the key functional, structural, and biological complexity of the actual organ. Organoids are attracting attention as complementary models to traditional animal models, and are often used in human and animal cancer research. In the current study, we developed a carcinogen-induced BC organoid model (DBCO) using organoids generated from healthy dog bladder tissue. DBCO showed elevated COL7A1 expression, which is upregulated in human bladder cancer.

[Disclosure of COI by Responsible researcher] Lead presenter is not the Responsible researcher.
[Lead presenter: Yuko Nagashima] We have no financial relationships to disclose.
[Principal Researcher: Kazuaki Sasaki] We have no financial relationships to disclose.

### [P-2002] I-P1-3
### Chemical carcinogens

2025 / 9 / 26 (Fri) 16:45-17:30 Room P/(Motenashi Dome) Room P

---

### P-2002
### Formation of the carcinogenic furan metabolite butenedial by hemin-induced degradation of alkadienals in fried foods

[Speaker]Hiroshi Kasai (Dept. Environ. Oncol., IIES, Univ. Occup. Environ. Health) [Co-author]Kazuaki Kawai (Dept. Environ. Oncol., IIES, Univ. Occup. Environ. Health)、Koichi Fujisawa (Dept. Environ. Oncol., IIES, Univ. Occup. Environ. Health)

High consumption of deep-fried foods is associated with various adverse health effects, such as cancer, atherosclerosis, and Alzheimer's disease. 2,4-Alkadienals (ADE) are significant toxic byproducts of lipid peroxidation commonly found in fried foods. In this study, we conclude that 1) 2-butene-1,4-dial (BDA) is efficiently generated from ADE in the presence of hemin under gastric conditions, and 2) BDA-derived Cys-pyrrole-Lys crosslink can also form under physiological conditions (pH 7.4) through the interaction of ADE, hemin, Cys, and Lys. BDA is recognized as the primary reactive metabolite of the suspected carcinogen furan (IARC, 2B). Given that human intake of ADE exceeds that of furan and acrylamide (IARC 2A) by several orders of magnitude, and the estimated hemin concentration in the stomach post-meal is comparable to the present study, a substantial amount of BDA may form in the stomach following consumption of fried foods and meat. The risk assessment of ADE warrants a thorough re-evaluation, based on the toxicity mechanism of BDA. (Genes and Environment, 2025, 47:8)

[Disclosure of COI by Responsible researcher] Lead presenter is the Responsible researcher.
[Lead presenter: Hiroshi Kasai] We have no financial relationships to disclose.

### [P-2003] I-P1-3
### Chemical carcinogens

2025 / 9 / 26 (Fri) 16:45-17:30 Room P/(Motenashi Dome) Room P

---

### P-2003
### Genotoxicity and mutational spectrum in lungs of *gpt* delta mice following inhalation exposure to heated tobacco products

[Speaker]Shinya Hasegawa (Environ. Health Sci., Hoshi Univ.) [Co-author]Kenichi Yoshida (Div. of Cancer Evolution, Natl. Cancer Ctr Res. Inst.)、Nobuyuki Kakiuchi (Dept. Path. & Tumor Biol., Kyoto Univ.)、Koichi Watanabe (Dept. Path. & Tumor Biol., Kyoto Univ.)、Shun Kawaguchi (Dept. Path. & Tumor Biol., Kyoto Univ.)、Seishi Ogawa (Dept. Path. & Tumor Biol., Kyoto Univ.／Inst., for the Advanced Study of Human Biol., Kyoto Univ.)、Yukari Totsuka (Environ. Health Sci., Hoshi Univ.)

The use of heated tobacco products (HTPs) is steadily increasing as a substitute for conventional cigarettes. Although HTPs are believed to produce fewer toxic substances than conventional cigarettes, their negative health effects are not yet fully understood. This study aimed to evaluate the in vivo genotoxicity of HTP exposure and provide scientific evidence regarding its potential impact on human health. In the study, male *gpt* delta mice were placed in a HTP inhalation exposure device developed by the National Institute of Public Health and exposed to HTP aerosols under two conditions: (1) five HTP sticks once per day, five days a week for four weeks, and (2) ten sticks twice per day, five days a week for four weeks. Twenty-four hours after the final exposure, lung tissue was collected for mutation analysis of the *gpt* gene. The mutation frequency was increased in the high dose group compared to the air-control group. Moreover, mutation spectrum analysis revealed an increase in G:C to A:T transitions and a trend toward increased deletions. Further investigation using error-corrected next-generation sequencing is currently underway.

[Disclosure of COI by Responsible researcher] Lead presenter is not the Responsible researcher.
[Lead presenter: Shinya Hasegawa] We have no financial relationships to disclose.
[Principal Researcher: Yukari Totsuka] We have no financial relationships to disclose.

### [P-2004] I-P1-3
### Chemical carcinogens

2025 / 9 / 26 (Fri) 16:45-17:30 Room P/(Motenashi Dome) Room P

---

### P-2004
### Comparative Gene Expression Analysis of Rat Lung Cancers Induced by Different Types of Carbon Nanotubes

[Speaker]Guiyu Qiu (Dept. Env. Risk Assess., Osaka Metropolitan Univ. Grad. Sch. Med.) [Co-author]Min Gi (Dept. Env. Risk Assess., Osaka Metropolitan Univ. Grad. Sch. Med.)、Shugo Suzuki (Dept. Mol. Path., Osaka Metropolitan Univ. Grad. Sch. Med.)、Masaki Fujioka (Dept. Mol. Path., Osaka Metropolitan Univ. Grad. Sch. Med.)、Arpamas Vachiraarunwong (Dept. Env. Risk Assess., Osaka Metropolitan Univ. Grad. Sch. Med.)、Runjie Guo (Dept. Env. Risk Assess., Osaka Metropolitan Univ. Grad. Sch. Med.)、Ikue Noura (Dept. Mol. Path., Osaka Metropolitan Univ. Grad. Sch. Med.)、Yurina Kawamura (Dept. Env. Risk Assess., Osaka Metropolitan Univ. Grad. Sch. Med.)、Hideki Wanibuchi (Dept. Env. Risk Assess., Osaka Metropolitan Univ. Grad. Sch. Med.／Dept. Mol. Path., Osaka Metropolitan Univ. Grad. Sch. Med.)

Carbon nanotubes (CNTs) have garnered significant attention due to concerns regarding their potential carcinogenicity. This study aimed to compare gene expression profiles of rat lung cancers induced by different types of CNTs. Multi-walled carbon nanotubes (MWCNT-7) were shown to induce both adenocarcinoma (ADC) and malignant mesothelioma (MM), while two types of double-walled carbon nanotubes (1 and 7 μm DWCNT) induced ADCs following intra-tracheal intra-pulmonary spraying administration. Pathway Analysis of differentially expressed genes revealed that the gene expression profiles and pathway alterations in ADCs induced by all CNTs were largely similar, while ADC induced by MWCNT-7 showed distinct activation of cholesterol biosynthesis pathways. In contrast, MM induced by MWCNT-7 showed unique activation of pathways such as actin cytoskeleton signaling and calcium signaling, clearly distinguishing it from the ADC profiles. These findings suggest shared carcinogenic mechanisms underlying lung ADCs induced by various types of CNTs. However, MWCNT-7 may induce lung ADC and MM through distinct molecular pathways.

[Disclosure of COI by Responsible researcher] Lead presenter is not the Responsible researcher.
[Lead presenter: Guiyu Qiu] We have no financial relationships to disclose.
[Principal Researcher: Min Gi] We have no financial relationships to disclose.

### [P-2005] I-P1-3
### Chemical carcinogens

2025 / 9 / 26 (Fri) 16:45-17:30 Room P/(Motenashi Dome) Room P

### P-2005
### Structure-Dependent Hepatotoxicity and Carcinogenic Potential of PFASs in Immortalized Human Liver Cells

[Speaker]Arpamas Vachiraarunwong (Dept. Environ. Risk Assess., Osaka Metropolitan Univ. Grad. Sch. Med.) [Co-author]Shugo Suzuki (Dept. Mol. Pathol., Osaka Metropolitan Univ. Grad. Sch. Med.)、Masaki Fujioka (Dept. Mol. Pathol., Osaka Metropolitan Univ. Grad. Sch. Med.)、Runjie Guo (Dept. Environ. Risk Assess., Osaka Metropolitan Univ. Grad. Sch. Med.)、Giuyu Qiu (Dept. Environ. Risk Assess., Osaka Metropolitan Univ. Grad. Sch. Med.)、Ikue Noura (Dept. Mol. Pathol., Osaka Metropolitan Univ. Grad. Sch. Med.)、Kawamura Yurina (Dept. Environ. Risk Assess., Osaka Metropolitan Univ. Grad. Sch. Med.)、Anna Kakehashi (Dept. Mol. Pathol., Osaka Metropolitan Univ. Grad. Sch. Med.)、Hideki Wanibuchi (Dept. Environ. Risk Assess., Osaka Metropolitan Univ. Grad. Sch. Med.)、Min Gi (Dept. Environ. Risk Assess., Osaka Metropolitan Univ. Grad. Sch. Med.)

Per- and polyfluoroalkyl substances (PFASs) are widely used in consumer products and are persistent in the environment. Perfluorooctanoic acid (PFOA) and perfluorooctane sulfonic acid (PFOS) are classified as human carcinogen (Group 1) and probably carcinogenic to human (Group 2A) by IARC, respectively, while the carcinogenic potential of other PFASs remains under evaluation. However, their toxicological mechanisms remain unclear. This study examined the toxic effects and gene expression alterations induced by carboxylate PFASs (PFOA, perfluorohexanoic acid (PFHxA), and undecafluoro-2-methyl-3-oxahexanoic acid (GenX)) and sulfonate PFASs (PFOS and perfluorohexasulfonic acid (PFHxS)) using immortalized human hepatocyte HepaMN cells. PFOS exhibited the strongest cytotoxicity, followed by PFHxS, PFOA, PFHxA, and GenX. Gene expression analysis revealed that all PFASs commonly activated integrated stress response, while only PFOS activated pathway related to epithelial cell proliferation. Our findings indicate that hepatotoxicity is structure-dependent, with sulfonate PFASs exhibiting greater toxicity than carboxylate PFASs. Moreover, PFOS may possess hepatocarcinogenic potential.

[Disclosure of COI by Responsible researcher] Lead presenter is not the Responsible researcher.
[Lead presenter: Arpamas Vachiraarunwong] We have no financial relationships to disclose.
[Principal Researcher: Min Gi] We have no financial relationships to disclose.

### [P-2006] I-P1-3
### Chemical carcinogens

2025 / 9 / 26 (Fri) 16:45-17:30 Room P/(Motenashi Dome) Room P

### P-2006
### Withdrawn

No Abstract

### [P-2007] I-P1-3
### Chemical carcinogens

2025 / 9 / 26 (Fri) 16:45-17:30 Room P/(Motenashi Dome) Room P

---

### P-2007
### Potential role of microplastics in oral cancer progression via oxidative stress

[Speaker]Yuuki Ohara (Nagoya Univ. Institute for Advanced Research／Nagoya Univ. Grad. Sch. of Med.) [Co-author]Syunsuke Togashi (Nagoya Univ. Sch. Med.)、Shinya Toyokuni (Nagoya Univ. Grad. Sch. of Med.)

Micro/nano-plastics (MPs/NPs) are global environmental and public health concerns. These particles, generated by plastic degradation, impact ecosystems and human health. MPs/NPs are also released from plastic bottles and food containers, leading to direct human exposure. They induce inflammation, oxidative stress, and metabolic changes in human cells. Given their ingestion via the oral and gastrointestinal tracts, we hypothesized that MPs may promote oral cancer progression through oxidative stress. Oral cancer cells were exposed to polystyrene MPs (5 μm, 3 μg/ml), and cell proliferation, oxidative stress, intracellular ferrous iron, migration, and transcriptome were analyzed. MPs did not affect proliferation but slightly increased oxidative stress and migration. Transcriptome analysis revealed MP-induced iron metabolism changes. Further studies using different particle types and sizes are needed to clarify MPs' impact on cancer progression.

[Disclosure of COI by Responsible researcher] Lead presenter is not the Responsible researcher.
[Lead presenter: Yuuki Ohara] We have no financial relationships to disclose.
[Principal Researcher: Shinya Toyokuni] We have no financial relationships to disclose.

### [P-2008] I-P1-3
### Chemical carcinogens

2025 / 9 / 26 (Fri) 16:45-17:30 Room P/(Motenashi Dome) Room P

---

### P-2008
### Tamoxifen metabolites-induced DNA damage and cell proliferation in relation to endometrial carcinogenesis

[Speaker]Yurie Mori (Dept. Environ. Mol. Med., Mie Univ. Grad. Sch. Med) [Co-author]Kaoru Midorikawa (Dept. Environ. Mol. Med., Mie Univ. Grad. Sch. Med)、Shinji Oikawa (Dept. Environ. Mol. Med., Mie Univ. Grad. Sch. Med)、Kiyoshi Fukuhara (Sch. Pherm. Showa Med Univ.)、Shosuke Kawanishi (Suzuka Univ. Med. Sci., Facl. Pharmaceutical Sci.)、Mariko Murata (Dept. Environ. Mol. Med., Mie Univ. Grad. Sch. Med／Suzuka Univ. Med. Sci., Grad. Sch. Health Sci.)

Tamoxifen (TAM) is a non-steroidal anti-estrogen used widely in the treatment and chemoprevention of breast cancer. However, long-term use of TAM increases the risk of developing endometrial cancer. To clarify the molecular mechanism of carcinogenesis, we examined DNA damage by TAM and its metabolites, 4-hydroxytamoxifen (4-OH-TAM), 3-hydroxytamoxifen (3-OH-TAM, also known as droloxifene) and 3,4-dihydroxytamoxifen (3,4-diOH-TAM). We also performed cell proliferation assay treated with TAM metabolites in human endometrial cancer cell line ECC-1 cells and breast cancer cell line MCF-7 cells. 3,4-Di-OH-TAM showed the ability to cause oxidative DNA damage in the presence of Cu(II) and NADH, while TAM, 4-OH-TAM and 3-OH-TAM did not. Cell proliferation assay revealed that 4-OH-TAM most efficiently inhibited 17$\beta$-estradiol-induced cell proliferation in MCF-7 cells, but showed no inhibitory effect on cell proliferation in ECC-1 cells. We conclude that oxidative DNA damage and cell proliferation by TAM metabolites, 3,4-diOH-TAM and 4-OH-TAM, respectively, may play roles in tumor initiation and promotion, leading to endometrial carcinogenesis.

[Disclosure of COI by Responsible researcher] Lead presenter is not the Responsible researcher.
[Lead presenter: Yurie Mori] We have no financial relationships to disclose.
[Principal Researcher: Mariko Murata] We have no financial relationships to disclose.

### [P-2009] I-P1-3
### Chemical carcinogens

2025 / 9 / 26 (Fri) 16:45-17:30 Room P/(Motenashi Dome) Room P

---

### P-2009
### Dissecting the Carcinogenesis Mechanisms of Aristolochic Acid in Cholangiocarcinoma

[Speaker]Pek Lim Chu (Duke-NUS Medical School, Singapore) [Co-author]Peiyong Guan (Genome Institute of Singapore, A*STAR)、Zihao Lin (Genome Institute of Singapore, A*STAR)、Zhimei Li (National Cancer Centre, Singapore)、Hong Lee Heng (National Cancer Centre, Singapore)、Jing Han Hong (Duke-NUS Medical School, Singapore)、Bin Tean Teh (Duke-NUS Medical School, Singapore／Genome Institute of Singapore, A*STAR／National Cancer Centre, Singapore)

Aristolochic acid (AA) is a potent mutagen found in commonly consumed herbal remedies, causing mutational signature (SBS22) that results in oncogenic transformation of tissues including that of hepatobiliary and genitourinary origins. However, the mechanistic role of AA in tumorigenesis remains unclear, limiting therapeutic strategy development for such cancers. Here, we established the world's first long-term AA exposed human cell models, which form xenograft tumors in animal models, and confirmed the recapitulation of SBS22a via genome sequencing. Multi-omics integrative analysis unveiled that AA exposure pathogenically reprogrammed the transcriptomic and epigenomic landscapes, to upregulate oncogenic metabolic pathways. Attenuating these pathways using gene silencing and drug inhibitors reduced tumor growth in preclinical models. Overall, our study reveals an actionable AA-specific pathway in CCA and potential therapeutic strategies, providing mechanistic insights and clinical relevance for tumors with AA signatures.

[Disclosure of COI by Responsible researcher] Lead presenter is not the Responsible researcher.
[Lead presenter: Pek Lim Chu] We have no financial relationships to disclose.
[Principal Researcher: Bin Tean Teh] We have no financial relationships to disclose.

### [P-2019] I-P2-4
### Organoid/Animal model (1)

2025 / 9 / 26 (Fri) 16:45-17:30 Room P/(Motenashi Dome) Room P

---

### P-2019
### Arid1a deficiency sensitizes pancreatic cancer to fatty acid synthase inhibition

[Speaker]Wen-Chun Hung (National Institute of Cancer Research, National Health Research Institutes) [Co-author]Tzu-Lei Kuo (National Institute of Cancer Research, National Health Research Institutes)、Ya-Chin Hou (Institute of Clinical Medicine, National Cheng Kung University)、Yan-Shen Shan (Institute of Clinical Medicine, National Cheng Kung University)、Li-Tzong Chen (Faculty of Medicine, College of Medicine, Kaohsiung Medical University)

Mutations in AT-rich interactive domain-containing protein 1A (ARID1A) gene are frequently found in pancreatic cancer. Previously, we generated genetically engineered mice with specific depletion of Arid1a gene in the pancreas and found that depletion of Arid1a at early developmental stage induced metabolic disturbance and diabetes mellitus. In this study, we established a mouse model with K-ras mutation and Arid1a depletion (KAR mice) in the pancreas and showed that the combination of these two genetic alterations induced pancreatic tumor formation. Compared to the tumors developed in mice with K-ras mutation and p53 deficiency (KPC mice), KAR tumors exhibited increased immune cell infiltration and reduced stromal activation. Our results demonstrated a significant upregulation of fatty acid metabolism and fatty acid synthase (FASN) in the KAR tumors, with ARID1A directly regulating FASN expression. Inhibition of FASN by chemical inhibitor reduced tumor cell viability and slowed tumor progression in mice. Collectively, FASN is a potential therapeutic target for ARID1A-deficient pancreatic cancer.

[Disclosure of COI by Responsible researcher] Lead presenter is the Responsible researcher.
[Lead presenter: Wen-Chun Hung] We have no financial relationships to disclose.

▲TOP

[P-2020] I-P2-4
Organoid/Animal model (1)

2025 / 9 / 26 (Fri) 16:45-17:30 Room P/(Motenashi Dome) Room P

### P-2020
### Establishment of Pancreatic Ductal Adenocarcinoma mouse model using phenotypically normal pancreatic ductal organoids

[Speaker]Mizuho Nakayama (Kanazawa UNIV, CRI, Div Gen／Kanazawa UNIV, NanoLSI) [Co-author]Kazuhiro Murakami (Kanazawa UNIV, CRI, Stem)、Hiroko Oshima (Kanazawa UNIV, CRI, Div Gen／Kanazawa UNIV, NanoLSI)、Masanobu Oshima (Kanazawa UNIV, CRI, Div Gen／Kanazawa UNIV, NanoLSI)

Pancreatic ductal adenocarcinoma (PDAC) is the lethal cancer because it evades detection until advanced stages. Genomic studies of PDAC identified KRAS and TP53 as frequently mutated driver genes. However, it has been not fully understood the process how pancreatic cancer develops after the driver gene mutations are expressed in pancreatic cells. To examine this, we generated ***Kras***$^{LSL\text{-}G12D}$ (K), ***Trp53***$^{LSL\text{-}R270H}$ (P), ***Rosa26-Cre***$^{ERT2}$ mice, then driver mutations were introduced by tamoxifen treatment. KP organoids were established from their pancreatic ducts. KP organoids were treated w/wo demethylating agent (5-aza-dC) ***in vitro***, and they were orthotopically transplanted into B6 mice pancreas. Both organoids survived in the pancreatic tissue, we collected these xenografted tissues and established 2$^{nd}$ organoid, each from w/wo 5-aza-dC pre-treatment. Interestingly, the 2$^{nd}$ organoids from pre-treated cells developed PDAC with liver metastasis after pancreatic intra-ductal transplantation, while the 2$^{nd}$ organoids from non-pre-treated cells did not develop tumors. These results suggest that epigenetic events with oncogenic mutations are required for promotion of pancreatic cancer development.

[Disclosure of COI by Responsible researcher] Lead presenter is not the Responsible researcher.
[Lead presenter: Mizuho Nakayama] We have no financial relationships to disclose.
[Principal Researcher: Masanobu Oshima] We have no financial relationships to disclose.

### [P-2021] I-P2-4
### Organoid/Animal model (1)

2025 / 9 / 26 (Fri) 16:45-17:30 Room P/(Motenashi Dome) Room P

---

### P-2021
### Dual Targeting of CXCR7 Signaling and HDAC-Mediated Epigenetic Reprogramming to Disrupt the Desmoplasia in PDAC

[Speaker]Shu-Yi Fang (Inst. of Biomed. Sci., National Chung Cheng University, Taiwan) [Co-author]Ching-Chieh Weng (Dept. of Biomed. Sci., National Chung Cheng University, Taiwan)、Michael W.Y. Chen (Dept. of Biomed. Sci., National Chung Cheng University, Taiwan)、Shaw-Jenq Tsai (Dept. of Biomed. Sci., National Chung Cheng University, Taiwan／Inst. of Basic Med. Sci., National Cheng Kung University, Taiwan)

PDAC remains one of the deadliest cancers, largely due to its late diagnosis, high metastatic potential, and strong resistance to therapy. These clinical challenges are strongly associated with its unique TME, characterized by desmoplastic stroma and the activation of pancreatic stellate cells (PSCs). Activated PSCs secrete SDF-1, which promote tumor progression and therapeutic resistance. Here, we propose to elucidate the function of CXCR7 in PSC-mediated tumor-stroma interactions and its contribution to PDAC progression. Moreover, recent evidence indicates that HDACs epigenetically regulate PSC activation, yet the mechanisms remain unclear. We aim to define the chromatin-modifying functions of HDACs in driving stromal activation and assess the therapeutic efficacy of selective HDACi in disrupting the desmoplastic TME. By targeting both tumor cells and the activated stroma, our study seeks to overcome therapeutic resistance and improve treatment outcomes in PDAC. This dual-targeted approach offers a promising strategy to reshape the tumor microenvironment and enhance the clinical efficacy of PDAC therapies.

[Disclosure of COI by Responsible researcher] Lead presenter is the Responsible researcher.
[Lead presenter: Shu-Yi Fang] We have no financial relationships to disclose.

### [P-2022] I-P2-4
### Organoid/Animal model (1)

2025 / 9 / 26 (Fri) 16:45-17:30 Room P/(Motenashi Dome) Room P

---

### P-2022
### In vivo reprogramming unveils the epigenetic trigger of anaplastic transformation in thyroid cancer

[Speaker]Hirofumi Shibata (Gifu University Department of Otolaryngology Head and Neck Surgery) [Co-author]Ryo Kawaura (Gifu University Department of Otolaryngology Head and Neck Surgery)、Hiroyuki Tomita (Gifu University Department of Tumor Pathology)、Takenori Ogawa (Gifu University Department of Otolaryngology Head and Neck Surgery)

Anaplastic thyroid carcinoma (ATC) is a rare but highly aggressive thyroid malignancy. To elucidate the mechanism underlying anaplastic transformation, we analyzed pluripotency-associated gene expression in human ATCs and applied in vivo reprogramming techniques. <Methods> Immunohistochemistry of SALL4 and LIN28 was conducted in 31 ATC cases. Public transcriptomic datasets were reanalyzed to identify pluripotency-related gene signatures. We developed a GEMM (TPO-Cre; LSL-Braf V600E; Rosa LSL-rtTA3; Col1a1 tetO-OSKM) to induce OSKM factor expression via a Tet-ON system. <Results> Human ATCs exhibited a hybrid pluripotent transcriptional state with upregulation of SOX2, MYC, LIN28B, and SALL4 alongside MAPK pathway activation. In mice, forced OSKM expression triggered anaplastic transformation of thyroid epithelium without Tp53 mutation, marked by Ttf-1 loss, Ki-67 elevation, ERK activation, and pluripotency marker expression. Notably, transient OSKM induction with Braf^V600E mutation was sufficient to induce ATC. <Conclusion> These findings suggest that epigenetic reprogramming drives anaplastic transformation in thyroid cancer and may be targeted for redifferentiation therapy.

[Disclosure of COI by Responsible researcher] Lead presenter is the Responsible researcher.
[Lead presenter: Hirofumi Shibata] We have no financial relationships to disclose.

[P-2023] I-P2-4

Organoid/Animal model (1)

2025 / 9 / 26 (Fri) 16:45-17:30 Room P/(Motenashi Dome) Room P

P-2023

### Orthotopic mouse model of distal bile duct cancer using tumor buds from genetically engineered bile duct organoids

[Speaker]Shingo Kato (Dept. Clin. Cancer Genomics, Yokohama City Univ. Hosp.) [Co-author]Junya Toyoda (Dept. Gastrological Surg, Yokohama City Univ. Grad. Sch. Med.)、Yoshitaka Hippo (Chiba Cancer Center, Res. Inst.)

Bile duct cancer (BTC) is an aggressive tumor from biliary epithelial cells. For advanced BTCs, effective treatments are urgently needed, yet lack of appropriate models hinders progress. This is especially true for extrahepatic BTCs due to technical challenges in selective gene manipulation and limitations of orthotopic xenografts in immunotherapy testing. To overcome this, we used a two-step approach previously applied to mouse gallbladder cancer. Normal common bile duct (CBD) organoids from ***Kras***$^{LSL\text{-}G12D/+}$ mice were engineered with Cre recombinase and CRISPR-Cas9-mediated p53 loss. These organoids organoids were inoculated in the subcutis of syngeneic immune-proficient mice. Developed tumors were subsequently fragmented as "tumor buds" and sutured to the CBD of recipient mice. Resulting distal BTCs mimicked key human disease features and enabled preclinical testing of anticancer drugs in a physiological setting. To mitigate complications associated with BTCs such as obstructive jaundice, we added a gallbladder-jejunal bypass, prolonging survival and allowing extended monitoring. This model offers scalability and genetic flexibility, providing a valuable platform for BTC research.

[Disclosure of COI by Responsible researcher] Lead presenter is the Responsible researcher.
[Lead presenter: Shingo Kato] ■Lecture fee: コニカミノルタREALM

### [P-2024] I-P2-4
### Organoid/Animal model (1)

2025 / 9 / 26 (Fri) 16:45-17:30 Room P/(Motenashi Dome) Room P

---

### P-2024
### The Effect of Mint3 on Bladder Cancer Carcinogenesis and Lung Metastasis

[Speaker]Tatsuki Ikoma (Department of Cancer Biology, Kansai Medical University／Department of Thoracic Oncology, Kansai Medical University／New Experimental Therapeutics, KMUH) [Co-author]Shiori Mori (Department of Cancer Biology, Kansai Medical University)、Takeharu Sakamoto (Department of Cancer Biology, Kansai Medical University)

It is known that Mint3 enhances cancer malignancy and metastatic ability by activating the hypoxia-responsive transcription factor HIF-1, but the influence of Mint3 on carcinogenesis and metastasis in bladder cancer remains unclear. We examined the effects of Mint3 on carcinogenesis to metastasis using a mouse model of bladder cancer induced by BBN. Eight-week-old Mint3-deficient male C57BL/6 mice and wild-type mice were given 0.05% BBN in drinking water for 12 weeks, and analysis was performed 8 weeks after the end of BBN administration. In Mint3-deficient mice, the carcinogenesis rate was lower compared to wild-type mice, and a significantly lower tendency for invasion was observed. Similarly, in the evaluation of lung fields in each individual, metastasis was observed in 6 mice (/9 mice) in the wild-type group and 2 mice (/3 mice) in the deficient group. In bulk RNA-seq of bladder tissue, Gene X expression was significantly increased in the wild-type group, and similar results were observed in immunostaining. The association between Mint3 and Gene X was suggested to not only increase bladder cancer malignancy but also promote invasion and metastasis.

[Disclosure of COI by Responsible researcher] Lead presenter is not the Responsible researcher.
[Lead presenter: Tatsuki Ikoma] We have no financial relationships to disclose.
[Principal Researcher: Takeharu Sakamoto] We have no financial relationships to disclose.

### [P-2025] I-P2-4
### Organoid/Animal model (1)

2025 / 9 / 26 (Fri) 16:45-17:30 Room P/(Motenashi Dome) Room P

---

### P-2025
### Withdrawn

No Abstract

### [P-2034] I-P2-6
### Organoid/animal model (3)

2025 / 9 / 26 (Fri) 16:45-17:30 Room P/(Motenashi Dome) Room P

---

### P-2034
### Lymphoproliferative Factors in the J-PDX Library: Role of Epstein-Barr Virus Infection and Rituximab Efficacy

[Speaker]Tomomi Yoshino (Department of pharmacology and therapeutics National Cancer Center Research Inst.) [Co-author]Shigehiro Yagishita (Department of pharmacology and therapeutics National Cancer Center Research Inst.／Division of Molecular Pharmacology, National Cancer Center Research Inst.)、Ako Takahashi (Division of Molecular Pharmacology, National Cancer Center Research Inst.)、Chiaki Ushie (Department of pharmacology and therapeutics National Cancer Center Research Inst.)、Misato Jinno (Division of Molecular Pharmacology, National Cancer Center Research Inst.)、Akinobu Hamada (Department of pharmacology and therapeutics National Cancer Center Research Inst.／Division of Molecular Pharmacology, National Cancer Center Research Inst.)

Patient-derived xenograft (PDX) models have only 30% establishment success rates. In failed cases, lymphoproliferation from patient-derived lymphocytes occurs, often due to Epstein-Barr virus (EBV)-infected B cells. Anti-CD20 antibody rituximab can suppress this phenomenon. We investigated rituximab's suppressive effect on lymphoproliferation and its association with EBV infection in PDX models. PDX models registered in the Japanese patient-derived xenograft (J-PDX) library were analyzed from August 2018 to March 2025. The rituximab group showed significantly suppressed lymphoproliferation compared to the non-treatment group ($p<0.001$). EBV DNA amplification was confirmed in 113/136 models (83%) with lymphoproliferation. Among these, 12 models received rituximab, with four models (33%) showing EBV DNA amplification and eight models (67%) showing none. These results suggest most lymphoproliferation in PDX models is attributed to EBV-infected B cells, while cases showing lymphoproliferation despite rituximab may involve non-B lymphocyte mechanisms. These findings enhance the understanding of lymphoproliferation in PDX models and may improve their establishment success rates.

[Disclosure of COI by Responsible researcher] Lead presenter is not the Responsible researcher.
[Lead presenter: Tomomi Yoshino] We have no financial relationships to disclose.
[Principal Researcher: Akinobu Hamada] ■Research funds from company: AMED、がん研究開発費、エーザイ株式会社、メディフォード株式会社

▲TOP

### [P-2035] I-P2-6
### Organoid/animal model (3)

2025 / 9 / 26 (Fri) 16:45-17:30 Room P/(Motenashi Dome) Room P

---

### P-2035
### Considerations for Designing Drug Efficacy Studies Based on Tumor Characteristics in PDX Models

[Speaker]Ako Takahashi (NCCRI Mol.) [Co-author]Shigehiro Yagishita (NCCRI Mol.／NCCRI FIOC)、Tomomi Yoshino (NCCRI FIOC)、Chiaki Ushie (NCCRI FIOC)、Koji Wada (NCCRI Mol.)、Nanami Fujita (NCCRI Mol.)、Minami Sekiguchi (NCCRI Mol.)、Naoko Matsura (NCCRI Mol.)、Akinobu Hamada (NCCRI Mol.／NCCRI FIOC)

Patient-derived xenograft (PDX) models are widely used preclinical models that retain tumor heterogeneity. However, differences in tumor growth rate and variability among models may affect drug efficacy study design.We analyzed 211 drug efficacy studies using 128 PDX models from 20 cancer types registered in the J-PDX library between May 2019 and November 2024. Time from tumor implantation to study start (time-to-testing, TTT) and the coefficient of variation (CV) in tumor volume at study initiation were evaluated by cancer type and model.Median TTT varied by cancer type, e.g. ovarian (202 days), breast (148), and rare cancers (141) required longer preparation, while sarcoma (81), gastric (94), and lung (95) were shorter. TTT variability was high in lung (CV 0.69) and biliary cancers (0.64), but lower in pancreatic (0.20) and ovarian (0.48). Tumor volume CV at study start ranged from 0.4 to 1.7 in ovarian and 0.4 to 1.9 in sarcoma.PDX models show substantial differences in tumor growth and variability. Considering these characteristics by cancer type and model is essential when designing drug efficacy studies to align with drug development timelines.

[Disclosure of COI by Responsible researcher] Lead presenter is not the Responsible researcher.
[Lead presenter: Ako Takahashi] ■ employment by a company related to the presentation: 国立がん研究センター
[Principal Researcher: Akinobu Hamada] ■employment by a company related to the presentation: 国立がん研究センター

### [P-2036] I-P2-6
### Organoid/animal model (3)

2025 / 9 / 26 (Fri) 16:45-17:30 Room P/(Motenashi Dome) Room P

---

### P-2036
### Evaluation of existing antibody drug efficacy based on survival time using PDX mouse models of hematopoietic tumors

[Speaker]Yuu Dobashi (Fukushima Translational Research Foundation, Fukushima, Japan) [Co-author]Junichi Imai (Fukushima Translational Research Foundation, Fukushima, Japan／Translational Research Center, Fukushima Medical University, Fukushima, Japan)、Shinya Watanabe (Translational Research Center, Fukushima Medical University, Fukushima, Japan)

The Fukushima PDX (F-PDX) Library was developed the Fukushima Project, a recovery initiative for the Great East Japan Earthquake. This study aims to establish an evaluation system for antibody drugs using the hematopoietic tumor F-PDX mouse models.The F-PDX mouse model was generated by transplanting two types of B-cell ALL ($2.5 \times 10^5$ cells/mouse) selected from the F-PDX library into the tail vein of immunodeficient NOG mice. Existing antibody drugs were selected based on comprehensive gene expression profile of F-PDX. Rituximab (3 or 10 mg/kg), daratumumab (3 or 8 mg/kg), and natalizumab (5 mg/kg) were administered intraperitoneally once a week for five weeks. To assess antibody-dependent cellular cytotoxicity, human NK cells ($1.0 \times 10^6$ or $1.0 \times 10^7$ cells/week for five weeks) were injected via the tail vein.As a result, administration of existing antibody drugs, either alone or in combination with NK cells, significantly prolonged survival compared to the control group, with variations depending on the drug type. Therefore, the hematopoietic tumor F-PDX mouse model is a valuable platform for evaluating differential efficacy of existing antibody drugs based on survival times.

[Disclosure of COI by Responsible researcher] Lead presenter is the Responsible researcher.
[Lead presenter: Yuu Dobashi] We have no financial relationships to disclose.

### [P-2037] I-P2-6
### Organoid/animal model (3)

2025 / 9 / 26 (Fri) 16:45-17:30 Room P/(Motenashi Dome) Room P

---

### P-2037
### Establishment of patient-derived xenograft and organoid models of pancreatic acinar cell carcinoma

[Speaker]Masashi Izumiya (Central Animal Div., Natl. Cancer Ctr. Res. Inst.／Grad. Sch. of Med., The Univ. of Tokyo) [Co-author]Mami Takahashi (Central Animal Div., Natl. Cancer Ctr. Res. Inst.／Dept. Pharmacol. Therapeutics, Natl. Cancer Ctr. Res. Inst.)、Yoshitaka Hippo (Chiba Cancer Ctr. Res. Inst.)、Nobuyoshi Hiraoka (Dep. Pathol., Natl. Cancer Ctr. Hosp.)、Susumu Hijioka (Dep. Hepatobiliary Pancreatic Oncol., Natl. Cancer Ctr. Hosp.)、Tomomi Yoshino (Dept. Pharmacol. Therapeutics, Natl. Cancer Ctr. Res. Inst.)、Shigehiro Yagishita (Dept. Pharmacol. Therapeutics, Natl. Cancer Ctr. Res. Inst.)、Akinobu Hamada (Dept. Pharmacol. Therapeutics, Natl. Cancer Ctr. Res. Inst.)、Hidetoshi Kassai (Central Animal Div., Natl. Cancer Ctr. Res. Inst.)

Pancreatic acinar cell carcinoma (ACC) is one of rare cancers of the pancreas, and there are no specific treatments for this disease. Human two-dimensional cultured cell lines of ACC are not available, and the establishment of an organoid model from a single case has been reported so far; the establishment of cultured cell lines in at least a few cases is desirable for the development of therapeutic treatment for ACC. The National Cancer Center has been constructing a library for rare cancers in the J-PDX Library and others. Three cases of ACC have been transplanted into immunodeficient mice, and patient-derived xenograft (PDX) models were successfully established in all three cases. Furthermore, as a result of attempts to culture organoids from xenograft tissues, we were able to establish passage-capable organoid strains in two cases in which xenograft growth was relatively rapid, and in one case in which xenograft growth was very slow, primary organoid culture was possible. In this report, we present the histological characteristics and STR analysis data of these models. We plan to conduct genomic analysis and drug sensitivity studies of these PDX/organoid strains in the future.

[Disclosure of COI by Responsible researcher] Lead presenter is the Responsible researcher.
[Lead presenter: Masashi Izumiya] ■Research funds from company: 科研費　基盤研究C

### [P-2038] I-P2-6
### Organoid/animal model (3)

2025 / 9 / 26 (Fri) 16:45-17:30 Room P/(Motenashi Dome) Room P

---

### P-2038
### Evaluation of Tumor Characteristic Retention in TNBC-PDX Models

[Speaker]Shotaro Inoue (Division of Breast Surgery, Kobe University Graduate School of Medicine) [Co-author]Sachiko Inubushi (Division of Breast Surgery, Kobe University Graduate School of Medicine)、Tomonari Kunihisa (Division of Breast Surgery, Kobe University Graduate School of Medicine)、Mayuko Miki (Division of Breast Surgery, Kobe University Graduate School of Medicine)、Hirokazu Tanino (Department of Cardiovascular, Respiratory and Breast Surgery, Wakayama Medical University)

Background: Patient-derived xenograft (PDX) models better reflect tumor biology than traditional cell lines and are useful for drug screening. However, they may not fully retain the traits of the original tumor, particularly in heterogeneous cancers like triple-negative breast cancer (TNBC). This study evaluated the consistency of tumor marker expression between TNBC-PDX models and patient tumors.Methods: Tumor tissues from TNBC patients were transplanted into nude mice to create PDX models. Immunohistochemistry for EGFR, AR, CK5/6, HER2, and Trop-2 was performed on both patient and PDX tumors.Results: EGFR and Trop-2 expression were preserved in all six cases. AR expression was not retained. CK5/6 expression varied among cases. HER2 expression was mostly maintained.Conclusion: TNBC-PDX models showed partial retention of tumor characteristics, reflecting the high heterogeneity of TNBC. It is important to verify whether key traits are preserved when using PDX models in drug research.

[Disclosure of COI by Responsible researcher] Lead presenter is not the Responsible researcher.
[Lead presenter: Shotaro Inoue] We have no financial relationships to disclose.
[Principal Researcher: Sachiko Inubushi] We have no financial relationships to disclose.

### [P-2039] I-P2-6
### Organoid/animal model (3)

2025 / 9 / 26 (Fri) 16:45-17:30 Room P/(Motenashi Dome) Room P

---

### P-2039
### Establishment of Two PDX Models from Organoids Derived from Circulating Tumor Cells of Prostate Cancer Patients

[Speaker]Kensuke Hikami (Kyoto University Graduate School of Medicine, Department of Urology) [Co-author]Takuro Sunada (Kurashiki Central Hospital, Department of Urology)、Noriyuki Makita (Kyoto University Graduate School of Medicine, Department of Urology)、Kodai Hattahara (Kyoto University Graduate School of Medicine, Department of Urology)、Maki Fujiwara (Kyoto University Graduate School of Medicine, Department of Urology)、Arinobu Fukunaga (Kyoto University Graduate School of Medicine, Department of Urology)、Tomohiro Fukui (Toyooka Public Hospital, Department of Urology)、Kei Mizuno (Kyoto University Graduate School of Medicine, Department of Urology)、Takayuki Sumiyoshi (Kyoto University Graduate School of Medicine, Department of Urology)、Takayuki Goto (Kyoto University Graduate School of Medicine, Department of Urology)、Shusuke Akamatsu (Nagoya University Graduate School of Medicine, Department of Urology)、Takashi Kobayashi (Kyoto University Graduate School of Medicine, Department of Urology)

The treatment of metastatic castration-sensitive prostate cancer (mCSPC) has improved with androgen receptor signaling inhibitors (ARSI), but progression to castration-resistant prostate cancer (CRPC) remains inevitable. Biopsies of metastatic tumors can help understand treatment resistance and identify new therapeutic targets, yet many patients lack accessible lesions. To address this, we aimed to establish patient-derived xenografts (PDXs) using circulating tumor cells (CTCs). CTCs were isolated from blood samples, embedded in Matrigel to form organoids, and then implanted into NOD/SCID mice. From 10 blood samples of 6 patients with mCSPC or mCRPC, two PDX models were successfully established from one patient before and after androgen deprivation therapy plus abiraterone. Histological analysis showed features of prostatic adenocarcinoma, and immunohistochemistry confirmed the expression of AR, PSA, and NKX3.1. These findings demonstrate the feasibility of generating CTC-derived PDXs, which may serve as valuable models for further genomic and transcriptomic analysis.

[Disclosure of COI by Responsible researcher] Lead presenter is not the Responsible researcher.
[Lead presenter: Kensuke Hikami] ■Research funds from company: 東ソー株式会社
[Principal Researcher: Takashi Kobayashi] ■Lecture fee: アステラス・ヤンセンファーマ・MSD・アストラゼネカ・メルクバイオファーマ

### [P-2040] I-P2-6
### Organoid/animal model (3)

2025 / 9 / 26 (Fri) 16:45-17:30 Room P/(Motenashi Dome) Room P

---

### P-2040
### Current status of human bone sarcoma cell line: publication and cell bank

[Speaker]Takashi Kondo (Mito Kyodo General Hosp.) [Co-author]Tadashi Kondo (Div. of Rare Cancer Res. , Natl. Cancer Ctr. Res. Inst.)

**Background** Bone sarcomas, such as osteosarcoma and Ewing sarcoma, are rare mesenchymal tumors that primarily affect adolescents and young adults. Their biological heterogeneity and low incidence pose significant challenges for clinical management and research. **Aim** To characterize existing bone sarcoma cell lines and identify gaps in available models. **Methods** A systematic search of the Cellosaurus database was performed to identify human-derived bone sarcoma cell lines. Searches were conducted using disease-specific terms, and results were categorized by publication status and availability in recognized cell banks. **Results** Only a limited number of cell lines have been established, and some of them were published. Of these, some are stored in cell banks, but distribution is often discontinued. As a result, few are available for research. **Conclusion** Bone sarcoma research is hindered by the limited and uneven availability of cell lines. Greater efforts in cell line development, preservation, and publication are essential to support future studies.

[Disclosure of COI by Responsible researcher] Lead presenter is not the Responsible researcher.
[Lead presenter: Takashi Kondo] We have no financial relationships to disclose.
[Principal Researcher: Tadashi Kondo] We have no financial relationships to disclose.

[P-2050] I-P4-5
Oncogenes & tumor-suppressor genes (4)

2025 / 9 / 26 (Fri) 16:45-17:30 Room P/(Motenashi Dome) Room P

---

### P-2050
### Loss of ANK3 promotes cell proliferation, migration, and structural disruption in renal cell carcinoma

[Speaker]Keerakarn Somsuan (Sch. of Med., Mae Fah Luang Univ.／Cancer & Immunol. Res. Unit, Mae Fah Luang Univ.) [Co-author]Artitaya Rongjumnong (Cancer & Immunol. Res. Unit, Mae Fah Luang Univ.)、Wararat Chiangjong (Dept. of Pediatrics, Faculty of Med., Mahidol Univ.,)、Atthapan Morchang (Sch. of Med., Mae Fah Luang Univ.／Cancer & Immunol. Res. Unit, Mae Fah Luang Univ.)、Arunothai Wanta (Sch. of Med., Mae Fah Luang Univ.／Cancer & Immunol. Res. Unit, Mae Fah Luang Univ.)、Ratirath Samol (Dept. of Anatomical Path., Sawanpracharak Hosp., Nakornsawan)、Natthiya Sakulsak (Dept. of Anatomical Path., Sawanpracharak Hosp., Nakornsawan)、Jatuporn Ngoenkam (Faculty of Med. Sci., Naresuan Univ.)、Sutatip Pongcharoen (Dept. of Med., Faculty of Med., Naresuan Univ.)、Siripat Aluksanasuwan (Sch. of Med., Mae Fah Luang Univ.／Cancer & Immunol. Res. Unit, Mae Fah Luang Univ.)

Renal cell carcinoma (RCC) is the most common form of kidney cancer, with limited effective treatments for advanced disease. Ankyrin 3 (ANK3), a multifunctional scaffold protein, has been linked to poor prognosis and immune infiltration in RCC, but its functional role remains unclear. In this study, ANK3-knockdown 786-O cells were generated using an shRNA approach to investigate its biological impact. Loss of ANK3 significantly enhanced cell proliferation, migration, and invasion compared to control cells. ANK3-knockdown cells exhibited increased nuclear size, G2/M phase arrest, and morphological changes, including a spindle-like shape and elevated cellular protrusions. Proteomic analysis identified 59 significantly altered proteins, largely involved in cell adhesion and structural integrity pathways. Western blot analysis confirmed disrupted expression of key junction proteins. Furthermore, reduced ANK3 expression was validated in RCC tissue samples. Collectively, these findings suggest that ANK3 acts as a tumor suppressor by maintaining cellular architecture and regulating proliferation and motility, highlighting its potential as a therapeutic target for RCC.

[Disclosure of COI by Responsible researcher] Lead presenter is the Responsible researcher.
[Lead presenter: Keerakarn Somsuan] ■Research funds from company: This project is funded by National Research Council of Thailand (NRCT) (Grant No. N42A660849).

▲TOP

### [P-2051] I-P4-5
### Oncogenes & tumor-suppressor genes (4)

2025 / 9 / 26 (Fri) 16:45-17:30 Room P/(Motenashi Dome) Room P

P-2051
### GSTM3 Suppresses Autophagy via PINK1/Parkin Pathway to Promote Nasopharyngeal Carcinoma Progression

[Speaker]CuiJuan Luo (Inst.Life Sci. Res., GXMU／Key Lab. Reg. High-Incidence Tumor., GXMU) [Co-author]Ying Xie (Key Lab. Reg. High-Incidence Tumor., GXMU／CLG.Intl. Edu., GXMU)

Nasopharyngeal carcinoma (NPC) exhibits distinct geographical prevalence in Southeast Asia. Our previous study identified glutathione S-transferase M3 (GSTM3) as a critical regulator in cisplatin-resistant NPC cells through epithelial-mesenchymal transition (EMT) modulation. Here, we mechanistically dissected GSTM3's oncogenic role through multi-modal analyses. Functional assays (CCK-8/EDU/colony formation) revealed GSTM3-driven proliferation ($p<0.01$) and cell cycle acceleration, complemented by apoptosis suppression via flow cytometry. Autophagy inhibition was confirmed by tfLC3 dynamics (reduced LC3-II) and TEM-validated autophagosome depletion. Mitochondrial dysfunction under GSTM3 overexpression manifested as MMP collapse (TMRE) and ROS surge (DCFH-DA/MitoSOX). Crucially, GSTM3 downregulated PINK1/Parkin expression($p<0.001$), impairing mitophagy. Rapamycin-induced autophagy reversed GSTM3's pro-tumor effects, normalizing proliferation/apoptosis metrics. This study uncovers a GSTM3-PINK1/Parkin axis wherein metabolic enzyme-driven mitophagy blockade promotes NPC progression, advancing targeted intervention strategies for this endemic malignancy.

[Disclosure of COI by Responsible researcher] Lead presenter is the Responsible researcher.
[Lead presenter: CuiJuan Luo] We have no financial relationships to disclose.

▲TOP

### [P-2052] I-P4-5
### Oncogenes & tumor-suppressor genes (4)

2025 / 9 / 26 (Fri) 16:45-17:30 Room P/(Motenashi Dome) Room P

---

### P-2052
### Neuromedin U Promotes Tumor Progression and Fibrotic Remodeling in Lung Adenocarcinoma

[Speaker]Shih-Miao Li (Natl. Inst. of Cancer Res., NHRI／Natl. Inst. of Infectious Diseases and Vaccinology, NHRI) [Co-author]Chi-Shuan Fan (Natl. Inst. of Cancer Res., NHRI)、Wen-Tsen Fang (Natl. Inst. of Cancer Res., NHRI)、Fang-Yu Tsai (Natl. Inst. of Cancer Res., NHRI)、Hui-Yu Jao (Natl. Inst. of Infectious Diseases and Vaccinology, NHRI)、Yi-Ju Wu (Inst. of Population Health Science, NHRI)、Zih-Syuan Cao (Natl. Inst. of Infectious Diseases and Vaccinology, NHRI)、Ko-Jiunn Liu (Natl. Inst. of Cancer Res., NHRI)、I-Shou Chang (Natl. Inst. of Cancer Res., NHRI)、Chao A. Hsiung (Inst. of Population Health Science, NHRI)、Shih Sheng Jiang (Natl. Inst. of Cancer Res., NHRI)

Neuromedin U (NMU) promotes lung adenocarcinoma (LUAD) progression through dual roles in tumor metabolism and fibrosis. Using patient datasets and survival analyses, we found that high NMU expression correlates with poor prognosis. Functional and transcriptomic analyses revealed that NMU enhances LUAD cell proliferation via upregulation of purine biosynthesis enzymes (***IMPDH1***, ***GMPS***, ***RRM1***). NMU also remodels the tumor microenvironment by activating fibroblasts and promoting TGF-β-driven fibrosis. NMU expression correlated with fibrosis markers (***COL1A1***, ***FN1***) and showed a biphasic relationship with ACTA2 and TGF-β activity. In xenograft models, NMU inhibition suppressed tumor growth and fibrosis. Notably, combining the NMU inhibitor SR48692 with osimertinib yielded synergistic anti-tumor and anti-fibrotic effects. These findings suggest that targeting NMU may enhance treatment efficacy and overcome EGFR-TKI resistance in LUAD.

[Disclosure of COI by Responsible researcher] Lead presenter is the Responsible researcher.
[Lead presenter: Shih-Miao Li] We have no financial relationships to disclose.

### [P-2053] I-P4-5
### Oncogenes & tumor-suppressor genes (4)

2025 / 9 / 26 (Fri) 16:45-17:30 Room P/(Motenashi Dome) Room P

---

### P-2053
### Allosteric SHP2 inhibitors inhibit non-canonical EphA2 activation in human lung adenocarcinoma cells

[Speaker]Khanh Nguyen (Dept. Cancer Cell Biology, Univ. Toyama／Dept. Medical Oncology and Palliative Care, Univ. Toyama) [Co-author]Yue Zhou (Dept. Cancer Cell Biology, Univ. Toyama)、Satoru Yokoyama (Dept. Cancer Cell Biology, Univ. Toyama)、Ryuji Hayashi (Dept. Medical Oncology and Palliative Care, Univ. Toyama)、Hiroaki Sakurai (Dept. Cancer Cell Biology, Univ. Toyama)

The Src homology phosphoribosyl phosphatase 2 (SHP2), which is encoded by the PTPN11 gene, is a non-receptor tyrosine phosphatase that positively regulates various receptor tyrosine kinase (RTK) signaling in cancer via regulating the RAS-MEK-ERK pathway. We have reported that non-canonical Ser-897 phosphorylation of EphA2 (pS-EphA2), a member of RTK family, plays a crucial role in cancer motility. However, the role of SHP2 in non-canonical activation of EphA2 remains unknown. We herein demonstrated that the allosteric SHP2 inhibitors suppressed both the TNF- and growth factor-induced non-canonical phosphorylation of EphA2 at Ser-897 via the ERK-RSK pathway. The significant impact of SHP2 inhibitors on non-canonical EphA2 activation in lung adenocarcinoma (LUAD) cells harboring the EGFR exon 19 deletion (PC-9, HCC827), EML4-ALK rearrangement (A925L), and KRAS mutation (A549) was also examined, and the results obtained showed a reduction in cell migration. Taken together, our study highlights the potential of allosteric SHP2 inhibitors as agents targeting the non-canonical activation of EphA2 in LUAD cells.

[Disclosure of COI by Responsible researcher] Lead presenter is not the Responsible researcher.
[Lead presenter: Khanh Nguyen] We have no financial relationships to disclose.
[Principal Researcher: Hiroaki Sakurai] We have no financial relationships to disclose.

### [P-2054] I-P4-5
### Oncogenes & tumor-suppressor genes (4)

2025 / 9 / 26 (Fri) 16:45-17:30 Room P/(Motenashi Dome) Room P

---

### P-2054
### Nuclear localization of insulin receptor substrate 1 (IRS1) correlates with estrogen signaling and CCA progression

[Speaker]Waleeporn Kaewlert (Dept. of Biochem., Fac. of Med., Khon Kaen Univ., Thailand.) [Co-author]Napat Armartmuntree (Dept. of Med. Sci., Fac. of Med., Mahidol Univ., Thailand.)、Panupong Mahalapbutr (Dept. of Biochem., Fac. of Med., Khon Kaen Univ., Thailand.)、Chadamas Sakonsinsiri (Dept. of Biochem., Fac. of Med., Khon Kaen Univ., Thailand.)、Mariko Murata (Dept. of Envi. and Mol. Med., Mie Univ., Japan.)、Raynoo Thanan (Dept. of Biochem., Fac. of Med., Khon Kaen Univ., Thailand.)

Cholangiocarcinoma (CCA), bile duct cancer, is progressed by estrogen signaling. Insulin receptor substrate 1 (IRS1), a cytosolic adaptor for the insulin receptor, has been observed to translocate into the nucleus in various cancers, including CCA. This study aimed to clarify the mechanism of IRS1 nuclear translocation and its role in CCA progression. Nuclear IRS1 was identified in the nuclei of CCA cells, in contrast to normal cells. Elevated levels of nuclear IRS1 in CCA tissues were associated with poor prognosis and the estrogen-producing enzyme aromatase expression. Nuclear IRS1 was also highly found in CCA cell lines compared to the cholangiocyte cell lines. The ***in silico*** and ***in vitro*** studies revealed the binding between IRS1 and estrogen receptors. Moreover, ChIP sequencing revealed IRS1-associated DNA regions on multiple oncogenes, and its mRNA levels positively correlated with IRS1 expression. In conclusion, the results suggest that activated estrogen receptors promote IRS1 nuclear translocation and lead to transcription control of oncogenes that contribute to CCA progression.

[Disclosure of COI by Responsible researcher] Lead presenter is the Responsible researcher.
[Lead presenter: Waleeporn Kaewlert] We have no financial relationships to disclose.

[P-2055] I-P4-5
Oncogenes & tumor-suppressor genes (4)

2025 / 9 / 26 (Fri) 16:45-17:30 Room P/(Motenashi Dome) Room P

P-2055
### DLL3 as a Common Molecular Marker in Neuroendocrine Carcinomas: Analysis in Esophageal NEC.

[Speaker]Ryo Numoto (Osaka Med. and Pharm. Univ. Dep of Gen. Surgery) [Co-author]Kentaro Matsuo (Osaka Med. and Pharm. Univ. Dep of Gen. Surgery)、Kohei Taniguchi (Osaka Med. and Pharm. Univ. Div. of TR)、Yosuke Inomata (Osaka Med. and Pharm. Univ. Dep of Gen. Surgery)、Mitsuhiro Asakuma (Osaka Med. and Pharm. Univ. Dep of Gen. Surgery)、Hideki Tomiyama (Osaka Med. and Pharm. Univ. Dep of Gen. Surgery)、Sangwoong Lee (Osaka Med. and Pharm. Univ. Dep of Gen. Surgery)

Background: Delta-like-3 (DLL3) is an inhibitory ligand of the Notch signaling pathway and is highly expressed in small cell lung cancer and pulmonary neuroendocrine carcinoma (NEC), making it a potential therapeutic target. We previously confirmed DLL3 overexpression in gastric and colorectal NEC (PMID: 31369178). However, its expression and function in esophageal NEC remains unclear. Methods: DLL3 expression was examined in esophageal NEC (TYUC-1) and squamous cell carcinoma (KYSE790) cell lines using Western blotting and RT-PCR. Immunohistochemical staining (IHC) was performed on esophageal NEC surgical specimens using chromogranin A, synaptophysin, CD56, and DLL3. Results: DLL3 expression was significantly higher in esophageal NEC cells compared to squamous cell carcinoma cells. IHC revealed DLL3 positively in a subset of esophageal NEC cases, while others showed DLL3 negatively despite expression of neuroendocrine marker. Conclusion: DLL3 is highly expressed in esophageal NEC, suggesting its ubiquitous expression across neuroendocrine carcinomas. We are currently conducting knockdown experiments to elucidate its function. In this presentation, we will share our latest finding.

[Disclosure of COI by Responsible researcher] Lead presenter is not the Responsible researcher.
[Lead presenter: Ryo Numoto] We have no financial relationships to disclose.
[Principal Researcher: Kentaro Matsuo] ■Research expenses: 科学研究費助成事業 25K18886

### [P-2056] I-P4-5
### Oncogenes & tumor-suppressor genes (4)

2025 / 9 / 26 (Fri) 16:45-17:30 Room P/(Motenashi Dome) Room P

---

### P-2056
### Identification of therapeutic targets against double-negative prostate cancer

[Speaker]Yasuhiko Kamikubo (Chiba Cancer Center Research Institute Div.ision of Molecular Carcinogenesis) [Co-author]Cao Feng (Chiba Cancer Center Research Institute Div.ision of Molecular Carcinogenesis／The Fourth Hospital of Hebei Medical University Department of Radiotherapy)、Takayoshi Watanabe (Chiba Cancer Center Research Institute Div.ision of Molecular Carcinogenesis)、Toshinori Ozaki (Chiba Cancer Center Research Institute Div.ision of Molecular Carcinogenesis)、Hiroshi Sugiyama (Kyoto University iCeMS)、Tatsuya Masuda (Chiba Cancer Center Research Institute Div.ision of Molecular Carcinogenesis)

Although FGFR signaling has been considered to be a biomarker for DNPC, its core oncogenic marker has remained unknown. Here, we found that Pan RUNX is highly expressed in DNPC patients. The inhibition of Pan RUNX strongly reduced DNPC cells, while the inhibition of FGFR downregulated Pan RUNX. To elucidate a possible significance of these observations, we found out gene responsible for the crosstalk between FGFR and Pan RUNX, and finally identified SOS1 gene. Based on the CRPC patient cohorts, the expression lof Pan RUNX and SOS1 was significantly higher in DNPC relative to the other subtypes. Pan RUNX transactivated SOS1 as examined by ChIP and reporter assays. Consistently, knockdown of Pan RUNX caused a reduction in SOS1 and dephosphorylation of FGFR, thereby attenuated the proliferation of DNPC cells. Thus, it is likely that Pan RUNX depletion-induced downregulation of FGFR signal and decrease in SOS1 play a vital role in the suppression of DNPC. Additionally, Our Pan RUNX inhibitor is effective on DNPC cell-derived mouse model. Taken together, we clarified a novel interaction between RUNX and FGFR signal in DNPC, which might contribute to the management of DNPC.

[Disclosure of COI by Responsible researcher] Lead presenter is the Responsible researcher.
[Lead presenter: Yasuhiko Kamikubo] We have no financial relationships to disclose.

▲TOP

### [P-2057] I-P4-5
### Oncogenes & tumor-suppressor genes (4)

2025 / 9 / 26 (Fri) 16:45-17:30 Room P/(Motenashi Dome) Room P

---

### P-2057
### The luciferase-based in vivo protein-protein interaction assay revealed that CHK1 promotes PP2A and PME-1 interaction.

[Speaker]Takashi Ohama (Joint Faculty of Vet. Medicine, Yamaguchi Univ.) [Co-author]Sana Ando (Joint Faculty of Vet. Medicine, Yamaguchi Univ.)、Koichi Sato (Joint Faculty of Vet. Medicine, Yamaguchi Univ.)

Protein phosphatase 2A (PP2A) is a crucial serine/threonine phosphatase, and its dysfunction is associated with cancer progression. PP2A functions as a trimeric holoenzyme, regulated by the methylation of its catalytic subunit (PP2Ac). PME-1, the sole PP2Ac methylesterase, is overexpressed in various cancer types and also serves as a PP2A inhibitor by binding to and suppressing PP2Ac. This dual role complicates drug development targeting the PME-1/PP2Ac axis. Using the NanoBiT system, we explored the molecular regulation of this interaction. Compound screening identified CHK1 inhibitors as disruptors of PME-1/PP2Ac binding without affecting PP2Ac methylation. CHK1 directly phosphorylates PP2Ac to enhance PME-1 association. Phospho-mass spectrometry and molecular dynamics simulation identified Thr219 as a critical site influencing this interaction. A phospho-Thr219 PP2Ac antibody confirmed CHK1-dependent phosphorylation in cells. Moreover, an in vitro phosphatase assay demonstrated that CHK1 is a PP2A substrate, with PME-1 inhibiting its dephosphorylation. These findings reveal a novel regulatory triad among PP2A, PME-1, and CHK1. (Ando S et al. JBC 2024, 300(5): 107277)

[Disclosure of COI by Responsible researcher] Lead presenter is the Responsible researcher.
[Lead presenter: Takashi Ohama] We have no financial relationships to disclose.

▲TOP

### [P-2058] I-P4-5
### Oncogenes & tumor-suppressor genes (4)

2025 / 9 / 26 (Fri) 16:45-17:30 Room P/(Motenashi Dome) Room P

---

### P-2058
### Functional analysis of TSC22 family proteins in malignant transformation of glioblastoma

[Speaker]Ling Zheng (Department of Neurosurgery, Faculty of Medicine, Saga University) [Co-author]Hiroki Namikawa (Department of Neurosurgery, Faculty of Medicine, Saga University)、Hiroshi Ito (Department of Neurosurgery, Faculty of Medicine, Saga University)、Atsushi Ogata (Department of Neurosurgery, Faculty of Medicine, Saga University)、Jun Masuoka (Department of Neurosurgery, Faculty of Medicine, Saga University)、Hiroyuki Suzuki (Department of Antibody DrugDevelopment, Tohoku University)、Tatsuya Abe (Department of Neurosurgery, Faculty of Medicine, Saga University)

Glioblastoma exhibits the most histopathologically undifferentiated cell morphology and is one of the tumors with the poorest prognosis among human malignancies. The main characteristics of glioblastoma include a high proliferative capacity, diffuse invasiveness, and strong angiogenic potential; however, its molecular mechanisms remain unclear. In this study, we focused on the molecular functions of TSC22 family proteins, which are expressed in many glioblastoma cells. We found that knockdown of TSC22 family proteins in brain tumors significantly reduced the proliferation, invasion, and sphere-forming ability of glioblastoma cells. Conversely, phosphorylation of TSC22 family proteins led to increased proliferation, motility, invasive potential, and morphological changes characteristic of cancer cells. These findings led us to hypothesize that overactivation of TSC22 family proteins in glioblastoma cells promotes tumorigenesis and malignant transformation. In this study, we further investigate the mechanisms by which these proteins enhance the proliferative and invasive potential of cancer cells, and evaluate their potential as molecular therapeutic targets.

[Disclosure of COI by Responsible researcher] Lead presenter is the Responsible researcher.
[Lead presenter: Ling Zheng] ■Research funds from company: 科研費　基盤(C)

### [P-2068] I-P5-3
### Non-coding RNAs (1)

2025 / 9 / 26 (Fri) 16:45-17:30 Room P/(Motenashi Dome) Room P

---

### P-2068
### Upregulation of LINC02154 contributes to esophageal cancer progression by enhancing cell cycle and EMT

[Speaker]Takeshi Niinuma (Div. Mol. Biol., Dept. Biochem., Sapporo Med. Univ.) [Co-author]Kotoha Shimote (Div. Mol. Biol., Dept. Biochem., Sapporo Med. Univ.)、Mitsunobu Saito (Div. Mol. Biol., Dept. Biochem., Sapporo Med. Univ.／Div. Gastroenterol. Hepatol., Dept. Intern. med., Sapporo Med. Univ.)、Kazuya Ishiguro (Div. Mol. Biol., Dept. Biochem., Sapporo Med. Univ.)、Hiroshi Kitajima (Div. Mol. Biol., Dept. Biochem., Sapporo Med. Univ.)、Masahiro Kai (Div. Mol. Biol., Dept. Biochem., Sapporo Med. Univ.)、Hiroshi Nakase (Div. Gastroenterol. Hepatol., Dept. Intern. med., Sapporo Med. Univ.)、Hiromu Suzuki (Div. Mol. Biol., Dept. Biochem., Sapporo Med. Univ.)

Long noncoding RNAs (lncRNAs) play crucial roles in the progression of human malignancies; however, their involvement in esophageal cancer (ESCA) remains incompletely understood. By using publicly available datasets including The Cancer Genome Atlas, we screened for lncRNAs upregulated in ESCA, and found that upregulation of LINC02154 is correlated with advanced T stages. Depletion of LINC02154 in ESCA cell lines suppressed cell proliferation and migration while ectopic expression of LINC02154 enhanced colony formation. Depletion of LINC02154 suppressed genes involved in various oncogenic properties including cell cycle, epithelial-mesenchyma transition (EMT), and metabolism. We also found that LINC02154 promotes EMT and enhances chemoresistance through, at least in part, suppressing miR-200b. Finally, RNA pulldown and mass spectrometry analysis revealed that LINC02154 interacts with proteins involved in the cornified envelope or desmosome. Our findings suggest that LINC02154 plays an oncogenic role through mediating multiple oncogenic signaling pathways in ESCA.

[Disclosure of COI by Responsible researcher] Lead presenter is not the Responsible researcher.
[Lead presenter: Takeshi Niinuma] We have no financial relationships to disclose.
[Principal Researcher: Hiroshi Nakase] We have no financial relationships to disclose.

### [P-2069] I-P5-3
### Non-coding RNAs (1)

2025 / 9 / 26 (Fri) 16:45-17:30 Room P/(Motenashi Dome) Room P

---

### P-2069
### Novel strategy to cure cancer through inhibition of long non-coding RNA

[Speaker]Tatsunori Nishimura (Div. of Cancer Biol. Nagoya Univ., Grad. Sch. of Med.) [Co-author]Keiko Shinjo (Div. of Cancer Biol. Nagoya Univ., Grad. Sch. of Med.)、Miho Suzuki (Div. of Cancer Biol. Nagoya Univ., Grad. Sch. of Med.)、Yutaka Kondo (Div. of Cancer Biol. Nagoya Univ., Grad. Sch. of Med.)

Immune checkpoint inhibitors have significantly enhanced the survival rates of cancer patients, through the inhibition of tumor proliferation by leukocytes. Previous research has indicated that glioblastoma multiform (GBM) exhibits a scarcity of tumor-infiltrating lymphocytes (TILs). In order to identify and target novel mode of action, we focused on long non-coding RNA (lncRNA) as a point of action instead of proteins, which have been targeted for cancer treatment to date. Employing antisense oligonucleotides (ASO), we have successfully knocked down lncRNAs and investigated whether this knockdown induces type I interferon and/or interferon stimulating genes (ISGs) including cytokines which recruit lymphocytes. As a result, ISGs were up-regulated under the treatment of ASO. We further examined the molecular mechanisms through which lncRNA modulates the type I interferon, and the synergy with temozolomide, which is the anticancer drug exploited for GBM patients.

[Disclosure of COI by Responsible researcher] Lead presenter is not the Responsible researcher.
[Lead presenter: Tatsunori Nishimura] We have no financial relationships to disclose.
[Principal Researcher: Yutaka Kondo] We have no financial relationships to disclose.

▲TOP

### [P-2070] I-P5-3
### Non-coding RNAs (1)

2025 / 9 / 26 (Fri) 16:45-17:30 Room P/(Motenashi Dome) Room P

---

### P-2070
### Androgen receptor-controlled microRNA-124-3p.2 suppresses prostate cancer progression via CCL2 inhibition

[Speaker]Shuhei Aoyama (Kanazawa Univ. Urology) [Co-author]Kouji Izumi (Kanazawa Univ. Urology)、Ryunosuke Nakagawa (Kanazawa Univ. Urology)、Taiki Kamijima (Kanazawa Univ. Urology)、Hiroshi Kano (Kanazawa Univ. Urology)、Tomoyuki Makino (Kanazawa Univ. Urology)、Renato Naito (Kanazawa Univ. Urology)、Hiroaki Iwamoto (Kanazawa Univ. Urology)、Hiroshi Yaegashi (Kanazawa Univ. Urology)、Atsushi Mizokami (Kanazawa Univ. Urology)

We reported that androgen receptor (AR) signaling blockade induces CCL2 secretion from prostate cancer cells and activates cancer cells through an autocrine manner. However, the mechanism on how AR negatively regulates CCL2 expression is still unclear. We hypothesized that there are AR-controlled miRs that regulate CCL2 production. Using LNCaP and C4-2B cells, miR-124-3p.2 was found as a potential miR from the miRNA PCR array and public database. The expression of miR-124-3p.2 was inhibited by knockdown AR, and the introduction of miR-124-3p.2 mimic decreased CCL2 expression and suppressed cell migration. To examine whether miR-124-3p.2 and CCL2 are involved in prostate cancer progression, their expression levels and clinical parameters were analyzed using prostate biopsy samples and whole blood RNAs obtained from the biopsied patients. Patients with low miR-124-3p.2 and high CCL2 levels showed a shorter time to castration-resistant prostate cancer development than those with high miR-124-3p.2 and low CCL2 levels. Our study, the miR-124-3p.2-CCL2 axis may be a novel therapeutic target and a useful blood prognostic biomarker for advanced prostate cancer.

[Disclosure of COI by Responsible researcher] Lead presenter is not the Responsible researcher.
[Lead presenter: Shuhei Aoyama] We have no financial relationships to disclose.
[Principal Researcher: Ryunosuke Nakagawa] We have no financial relationships to disclose.

### [P-2071] I-P5-3
### Non-coding RNAs (1)

2025 / 9 / 26 (Fri) 16:45-17:30 Room P/(Motenashi Dome) Room P

---

### P-2071
### Investigation into the feasibility of treating lymph node metastasis with microRNA using lymphatic drug delivery system

[Speaker]Akito Hirokawa (Division of Oral and Maxillofacial Oncology and Surgical Sciences) [Co-author]Yutaro Higashi (Division of Oral and Maxillofacial Oncology and Surgical Sciences)、Ariunbuyan Sukhbaatar (Division of Oral and Maxillofacial Oncology and Surgical Sciences／Laboratory of Biomedical Engineering for Cancer, Tohoku University)、Tetsuya Kodama (Laboratory of Biomedical Engineering for Cancer, Tohoku University)、Tsuyoshi Sugiura (Division of Oral and Maxillofacial Oncology and Surgical Sciences)

<Introduction>
We developed a lymphatic drug delivery system (LDDS) using a murine lymph node metastasis model with lymphadenopathy, and clinical trials for head and neck cancer are underway. In parallel, we identified lymph node metastasis-specific microRNAs (miRNAs) in oral squamous cell carcinoma patient serum, suggesting their use as biomarkers. This study aimed to identify such miRNAs in a murine model and evaluate their potential as therapeutic targets for LDDS.
<Methods>
A lymph node metastasis model was established by inoculating MXH10/Mo/lpr mice with FM3A-Luc tumor cells. Serum samples were collected from healthy and metastasis-bearing mice. miRNA profiling was performed using microarrays.
<Results>
Four miRNAs were upregulated over 8-fold, and 13 were downregulated in the metastasis group. miR-615, linked to epithelial-mesenchymal transition (EMT), was notably upregulated. Functions of other altered miRNAs remain unclear. Some mice also developed lung metastases, associated with co-upregulation of miR-615 and miR-1901.
<Conclusion>
Specific miRNAs were differentially expressed in lymph node metastasis and may serve as promising therapeutic targets for LDDS-based treatments.

[Disclosure of COI by Responsible researcher] Lead presenter is not the Responsible researcher.
[Lead presenter: Akito Hirokawa] We have no financial relationships to disclose.
[Principal Researcher: Tsuyoshi Sugiura] We have no financial relationships to disclose.

### [P-2072] I-P5-3
### Non-coding RNAs (1)

2025 / 9 / 26 (Fri) 16:45-17:30 Room P/(Motenashi Dome) Room P

---

### P-2072
### CircESRP1 is highly expressed in HGSOC and promotes cell proliferation via the miR-182-5p/CDK1 pathway.

[Speaker]Tadashi Oride (Osaka University) [Co-author]Kenjiro Sawada (Osaka University)、Yukako Oi (Osaka University)、Aasa Shimizu (Osaka University)、Mahiru Kawano (Osaka University)、Yasuto Kinose (Osaka University)、Michiko Kodama (Osaka University)

Objective: The functional relevance of circular RNAs (circRNAs), a subclass of non-coding RNAs, in high-grade serous ovarian carcinoma (HGSOC) remains largely unexplored. This study aimed to identify circRNAs aberrantly expressed in HGSOC and to elucidate their potential roles in tumorigenesis. Methods: A circRNA microarray identified candidates from HGSOC and matched normal tissue. The top circRNA was validated by RT-qPCR in cell lines and clinical samples. Its function was examined via siRNA knockdown, and the target miRNA/mRNA axis was investigated through in vitro analyses. Immunohistochemistry and mouse xenografts were used for further validation. Results: circESRP1 was highly upregulated in HGSOC. Its knockdown suppressed proliferation and induced apoptosis. circESRP1 functioned as a sponge for miR-182-5p, which targets CDK1 as documented by HGSOC clinical samples. Thus, its knockdown inhibited proliferation and promoted apoptosis of HGSOC cell lines through CDK1 inhibition. In vivo , knockdown of circESRP1 inhibited ovarian cancer progression. Conclusion: circESRP1 promotes HGSOC progression via the miR-182-5p/CDK1 axis and may serve as a potential therapeutic target.

[Disclosure of COI by Responsible researcher] Lead presenter is the Responsible researcher.
[Lead presenter: Tadashi Oride] We have no financial relationships to disclose.

### [P-2073] I-P5-3
### Non-coding RNAs (1)

2025 / 9 / 26 (Fri) 16:45-17:30 Room P/(Motenashi Dome) Room P

---

### P-2073
### Characterization of colorectal cancer-associated microproteins translated from small ORFs within non-coding RNAs

[Speaker]Hiroshi Kitajima (Div. Mol. Biol., Dept. Biochem., Sapporo Med. Univ.) [Co-author]Takeshi Niinuma (Div. Mol. Biol., Dept. Biochem., Sapporo Med. Univ.)、Kazuya Ishiguro (Div. Mol. Biol., Dept. Biochem., Sapporo Med. Univ.)、Akira Takasawa (Div. Tumor Pathol., Dept. Pathol., Asahikawa Med. Univ.)、Akira Yorozu (Dept. Otolaryngol., Sapporo. Med. Univ.)、Masahiro Kai (Div. Mol. Biol., Dept. Biochem., Sapporo Med. Univ.)、Hiroshi Nakase (Div. Gastroentrol., Hepatol., Dept. Int. Med., Sapporo. Med. Univ.)、Hiromu Suzuki (Div. Mol. Biol., Dept. Biochem., Sapporo Med. Univ.)

For a long time, small open reading frames (sORFs), which are less than 300 nucleotides, have generally been excluded from genome annotation due to the difficulty in accurately predicting functional sORFs. However, recent advances in ribosome profiling (Ribo-seq) and proteomics have begun to identify microproteins translated from unannotated sORFs. In this study, we aimed to identify functional microproteins translated from sORFs within noncoding RNAs (ncRNAs) in colorectal cancer (CRC). We obtained Ribo-seq and RNA-seq data of CRC cell lines from the NCBI Sequence Read Archive (SRA). By performing translatome analysis, we identified 557 sORFs potentially code for microproteins in 539 ncRNAs. We also obtained mass spectrometry data of CRC cells from the jPOST repository and identified a series of 1,202 candidate microproteins encoded in ncRNAs. Among them, we selected 47 sORFs derived from ncRNAs, including a long ncRNA which is upregulated in CRC and is associated with poor prognosis. Currently, we are experimentally investigating the translation potential of the sORFs and the molecular function of microproteins in CRC cells.

[Disclosure of COI by Responsible researcher] Lead presenter is not the Responsible researcher.
[Lead presenter: Hiroshi Kitajima] We have no financial relationships to disclose.
[Principal Researcher: Hiromu Suzuki] We have no financial relationships to disclose.

### [P-2074] I-P5-3
### Non-coding RNAs (1)

2025 / 9 / 26 (Fri) 16:45-17:30 Room P/(Motenashi Dome) Room P

---

### P-2074
### Malignant Alteration of Colorectal Cancer Cells Mediated by miR-4708 Expression

[Speaker]Ryoutaro Suzuki (Course Biomed. Eng., Grad. Sch. Life Sci, Toyo Univ.) [Co-author]Soma Todoriki (Course Biomed. Eng., Grad. Sch. Life Sci, Toyo Univ.)、Hikaru Sakamoto (Course Biomed. Eng., Grad. Sch. Life Sci, Toyo Univ.)、Junya Ohtake (Res. Facility Ctr., Asaka, Toyo Univ.／Res. Ctr., Biomed. Eng., Toyo. Univ.)、Hidemitsu Kitamura (Course Biomed. Eng., Grad. Sch. Life Sci, Toyo Univ.)

Elucidation of the regulatory mechanisms of malignant alteration of cancer cells is important for the development of more effective cancer therapies. We have identified miR-4708, a functional miRNA associated with IL-6 in the blood of colorectal cancer patients. In this study, we established human colon cancer cells with miR-4708 and verified the effect of miR-4708. First, the effect of miR-4708 on cell proliferation was examined in an in vitro culture system, and no significant difference was observed between miR-4708-transfected cells and Mock control cells in cell proliferation ability. The results of GSEA indicated that miR-4708-transfected cells were associated with a group of genes related to EMT. Furthermore, qPCR analysis verified that the expression levels of EMT-related genes, ZEB1, FN1, and SPARC were significantly increased in the miR-4708-transfected cells. These results indicate that miR-4708 expression is associated with the malignant alteration promoting metastasis of colorectal cancer cells by EMT-related gene regulations, suggesting that miR-4708 may be useful as a novel biomarker and therapeutic target in colorectal cancer patients.

[Disclosure of COI by Responsible researcher] Lead presenter is not the Responsible researcher.
[Lead presenter: Ryoutaro Suzuki] We have no financial relationships to disclose.
[Principal Researcher: Hidemitsu Kitamura] ■Contributions: テルモ生命科学振興財団

Poster Session

### [P-2075] I-P5-3
### Non-coding RNAs (1)

2025 / 9 / 26 (Fri) 16:45-17:30 Room P/(Motenashi Dome) Room P

---

### P-2075
### miR-100-5p and miR-203a-3p Suppress Esophageal Squamous Cell Carcinoma Progression by Targeting FKBP5

[Speaker]Hiroto Tanaka (Univ. of yamanashi, Dept. of digestive surgery) [Co-author]Suguru Maruyama (Univ. of yamanashi, Dept. of digestive surgery)、Katsutoshi Shoda (Univ. of yamanashi, Dept. of digestive surgery)、Takaomi Ozawa (Univ. of yamanashi, Dept. of digestive surgery)、Yudai Higuchi (Univ. of yamanashi, Dept. of digestive surgery)、Ryo Saito (Univ. of yamanashi, Dept. of digestive surgery)、Wataru Izumo (Univ. of yamanashi, Dept. of digestive surgery)、Koichi Takiguchi (Univ. of yamanashi, Dept. of digestive surgery)、Takashi Nakayama (Univ. of yamanashi, Dept. of digestive surgery)、Kensuke Shiraishi (Univ. of yamanashi, Dept. of digestive surgery)、Shinji Furuya (Univ. of yamanashi, Dept. of digestive surgery)、Yoshihiko Kawaguchi (Univ. of yamanashi, Dept. of digestive surgery)、Hidetake Amemiya (Univ. of yamanashi, Dept. of digestive surgery)、Hiromichi Kawaida (Univ. of yamanashi, Dept. of digestive surgery)、Daisuke Ichikawa (Univ. of yamanashi, Dept. of digestive surgery)

Background: In several poorly differentiated cancers (por), including esophageal squamous cell carcinoma (ESCC), the prognosis is worse than in well-differentiated types. In this study, we aimed to identify mRNAs targeted by specific microRNAs (miRNAs) involved in ESCC progression, focusing on their association with tumor differentiation.Methods: We selected miRNA candidates using a miRNA array and the GEO datasets to compare the expression levels between por and non-por ESCC. We examined the clinical significance and function of the selected miRNAs and their targeted mRNA in clinical samples and cell lines.Results: We identified miR-100-5p and miR-203a-3p as significantly downregulated in por ESCC patients, with strongly associated prognoses. Furthermore, FKBP5 was identified as a potential target of both miRNAs, and its expression level was reduced considerably by double-transfection with these miRNAs. Downregulation of FKBP5 significantly suppressed tumor aggressiveness in cell lines, and survival rates were significantly worse in the high-FKBP5 group in clinical samples.Conclusion: miR-100-5p and miR-203a-3p inhibited tumor progression by targeting FKBP5.

[Disclosure of COI by Responsible researcher] Lead presenter is not the Responsible researcher.
[Lead presenter: Hiroto Tanaka] We have no financial relationships to disclose.
[Principal Researcher: Daisuke Ichikawa] We have no financial relationships to disclose.

### [P-2076] I-P5-3
### Non-coding RNAs (1)

2025 / 9 / 26 (Fri) 16:45-17:30 Room P/(Motenashi Dome) Room P

---

### P-2076
### Elucidation of the Mechanism of miR-20b in Regulating the Malignant Alteration of Colorectal Cancer Cells

[Speaker]Soma Todoriki (Course Biomed. Eng., Grad. Sch. Life Sci, Toyo Univ) [Co-author]Ryoutaro Suzuki (Course Biomed. Eng., Grad. Sch. Life Sci, Toyo Univ)、Hikaru Sakamoto (Course Biomed. Eng., Grad. Sch. Life Sci, Toyo Univ)、Junya Ohtake (Res. Facility Ctr., Asaka, Toyo Univ.／Res. Ctr., Biomed. Eng., Toyo. Univ.)、Hidemitsu Kitamura (Course Biomed. Eng., Grad. Sch. Life Sci, Toyo Univ／Res. Facility Ctr., Asaka, Toyo Univ.／Res. Ctr., Biomed. Eng., Toyo. Univ.)

Suppressing the malignancy of colorectal cancer cells is crucial for the development of effective cancer therapies. In this study, we introduced miR-20b into human colorectal cancer cells to elucidate the mechanism of action of miR-20b in patients with colorectal cancer.We established HCT116 cells overexpressed with miR-20b (HCT116-20b) and mock control cells (Mock control), and conducted cell proliferation and migration assays. Furthermore, we performed GSEA based on the gene profiles. The candidate genes were validated using quantitative PCR.In this study, HCT116-20b cells exhibited suppressed cell proliferation and reduced migratory ability compared to Mock-control cells. Gene signature analysis suggested associations with EMT and KRAS in HCT116-20b cells. Furthermore, quantitative PCR confirmed a significant decrease in the expression level of the EMT-related gene ENO2 in HCT116-20b cells. The findings indicate that miR-20b expression in colorectal cancer cells suppresses malignancy, such as cancer cell proliferation and EMT, through the downregulation of ENO2 expression, suggesting that miR-20b may be a promising novel molecular target for suppressing cancer cell malignancy.

[Disclosure of COI by Responsible researcher] Lead presenter is not the Responsible researcher.
[Lead presenter: Soma Todoriki] We have no financial relationships to disclose.
[Principal Researcher: Hidemitsu Kitamura] ■Contributions: テルモ生命科学振興財団

### [P-2077] I-P5-3
### Non-coding RNAs (1)

2025 / 9 / 26 (Fri) 16:45-17:30 Room P/(Motenashi Dome) Room P

---

### P-2077
### Momordica charantia Extract Combined with Doxorubicin via lncRNA Regulation Overcomes Resistance in Breast Cancer Cells

[Speaker]Yu C. Kuo (Dept. of Biomedical Sciences and Engineering, Tzu-Chi Univ.) [Co-author]Hsue Y. Hsu (Dept. of Post-Baccalaureate School of Chinese Medicine, Tzu-Chi Univ.)

Doxorubicin (DOX) resistance remains a major challenge for breast cancer therapy despite advancements in drug development. Long non-coding RNAs (LncRNAs) are known to regulate breast cancer, but their role in triple-negative breast cancer cells (TNBC) is not well understood. We cultivated a specific strain of Momordica charantia (MC) and discovered that combining its extract (MCE) with DOX produced significant synergistic cytotoxicity in doxorubicin-resistant TNBC cells. This study investigated the role of LncRNAs in MCE-mediated cytotoxicity in DOX-resistant TNBC. Our findings showed that MCE induced apoptosis in both TNBC and DOX-resistant TNBC cells, as evidenced by PARP cleavage and caspase-3 activation. MCE downregulated drug-resistance-associated long non-coding RNAs, which correlated with enhanced doxorubicin sensitivity. This is the first report showing that MCE from this specific strain not only chemosensitizes cells to caspase-dependent apoptosis but also affects lncRNA regulatory networks, potentially overcoming TNBC chemoresistance. These findings provide novel mechanistic insights for combinatorial treatments against refractory TNBC.

[Disclosure of COI by Responsible researcher] Lead presenter is the Responsible researcher.
[Lead presenter: Yu C. Kuo] We have no financial relationships to disclose.

▲TOP

### [P-2087] I-P8-1
### DNA methylation

2025 / 9 / 26 (Fri) 16:45-17:30 Room P/(Motenashi Dome) Room P

---

### P-2087
### Epigenetic inactivation of DUOX1/DUOXA1 promotes tumor growth andmetastasis in nasopharyngeal carcinoma

[Speaker]QUANXIANG HAO (Department of Pathology, College of Stomatology／Key Lab. Regional. High-Incidence Tumor, GXMU) [Co-author]Haishan Zhang (Department of Pathology, College of Stomatology／Key Lab. Regional. High-Incidence Tumor, GXMU)、Xue Xiao (Key Lab. Regional. High-Incidence Tumor, GXMU)、Ping Li (Department of Pathology, College of Stomatology／Key Lab. Regional. High-Incidence Tumor, GXMU)

Dual oxidase 1 (DUOX1), a NADPH oxidase family member, generates airway reactive oxygen species (ROS) and shows tumor-suppressive properties. DUOX1 requires its maturation factor DUOXA1 to form a functional heterodimeric complex stabilized by disulfide bonds. In nasopharyngeal carcinoma (NPC), Our integrated bioinformatics and experimental analyses revealed consistent downregulation of DUOX1 and DUOXA1 at both mRNA and protein levels in NPC specimens. Mechanistically, promoter DNA hypermethylation was identified as a key driver of their transcriptional silencing. Critically, only co-expression of DUOX1/DUOXA1 (not individually) inhibited NPC cell proliferation, migration, and invasion while inducing apoptosis.This finding underscores DUOXA1's indispensable role in enabling DUOX1's tumor-suppressive activity and establishes the DUOX1-DUOXA1 complex as a functional unit in NPC pathogenesis. We further discovered that ketogenesis-mediated histone acetylation epigenetically activates DUOX1 expression. These results establish the DUOX1-DUOXA1 complex as an epigenetically regulated tumor suppressor in NPC, with therapeutic potential.

[Disclosure of COI by Responsible researcher] Lead presenter is the Responsible researcher.
[Lead presenter: QUANXIANG HAO] We have no financial relationships to disclose.

### [P-2088] I-P8-1
### DNA methylation

2025 / 9 / 26 (Fri) 16:45-17:30 Room P/(Motenashi Dome) Room P

---

### P-2088
### Collective Epigenetic Control in 14q32 miRNA Cluster of CRC: A Machine Learning Approach to Methylation and Expression

[Speaker]Masanori Nojima (Div. Advanced Med. Promotion, IMSUT)

The 14q32.2-32.31 region (Chr14) contains a large miRNA cluster with aberrant DNA methylation and expression in cancer. In colorectal cancer, early lesions show hypermethylation and reduced expression, while some advanced tumors may display hypomethylation and re-expression of certain miRNAs that suppress metastasis. Although prior studies often highlight single CpG sites, the complexity of this region suggests collective effects from multiple CpGs. We applied principal component analysis (PCA), partial least squares (PLS) regression, and network analysis to visualize how CpG methylation regulates miRNA expression using TCGA data. PCA indicated miR-127, miR-136, and miR-758 - all within 14q32 - as key features separating tumor from normal. Individual CpGs had weak expression correlations (R = 0.1-0.2), but PLS revealed that three CpG-derived composite variables explained nearly 50% of the variance in expression, highlighting the collective behavior of adjacent CpG sites in regulatory control. This supports a model where nearby CpGs act together to modulate miRNA levels. This presentation will introduce machine learning-based visualizations of such collective epigenetic regulation.

[Disclosure of COI by Responsible researcher] Lead presenter is the Responsible researcher.
[Lead presenter: Masanori Nojima] We have no financial relationships to disclose.

### [P-2089] I-P8-1
### DNA methylation

2025 / 9 / 26 (Fri) 16:45-17:30 Room P/(Motenashi Dome) Room P

### P-2089
### Characteristics of hypermethylated gastric intestinal metaplasia

[Speaker]Mari Teramura (Dept. of Gastroenterol. & Hepatol., Kyoto Univ. Grad. Sch. of Med.／Kyoto Katsura Hosp.) [Co-author]Takahiro Shimizu (Dept. of Gastroenterol. & Hepatol., Kyoto Univ. Grad. Sch. of Med.／Japan Baptist Hosp.)、Atsushi Takai (Dept. of Gastroenterol. & Hepatol., Kyoto Univ. Grad. Sch. of Med.)、Takahiko Ito (Dept. of Gastroenterol. & Hepatol., Kyoto Univ. Grad. Sch. of Med.／Japan Baptist Hosp.)、Haruka Amino (Dept. of Gastroenterol. & Hepatol., Kyoto Univ. Grad. Sch. of Med.)、Hiroshi Seno (Dept. of Gastroenterol. & Hepatol., Kyoto Univ. Grad. Sch. of Med.)

***H. pylori*** infection induces aberrant DNA methylation in the gastric mucosa, and methylation levels of several genes have been reported to correlate with the risk of gastric cancer (GC). However, the mechanism by which aberrant DNA methylation is involved in gastric carcinogenesis remains unclear. Here we isolated intestinal metaplasia (IM) and non-IM glands separately from inflamed mucosa and performed the comprehensive analysis for DNA methylation and gene expression on 64 samples. Genome-wide DNA methylation profiling revealed that gene promoter hypermethylation is more frequently detected in IM than non-IM. Interestingly, IM glands were classified into methylation-high (IM-high) and intermediate (IM-int) subgroups, and IM-high has numerous differential methylated genes (DMGs) shared with GC. We confirmed the IM classification by validating the methylation levels of eight representative DMGs using targeted bisulfite amplicon sequencing. IM-high was characterized by decreased expression of gastric differentiation marker genes and histologically severe atrophy. These results suggest that some types of IM glands could be at high risk of GC, especially the hypermethylated type.

[Disclosure of COI by Responsible researcher] Lead presenter is the Responsible researcher.
[Lead presenter: Mari Teramura] We have no financial relationships to disclose.

▲TOP

### [P-2090] I-P8-1
### DNA methylation

2025 / 9 / 26 (Fri) 16:45-17:30 Room P/(Motenashi Dome) Room P

### P-2090
### Analysis of somatic DNA variants and DNA methylation of tumor suppressor genes in colorectal cancer

[Speaker]Hisashi Nishiki (General and Digestive Surgery, Kanazawa Medical University) [Co-author]Takashi Miyata (General and Digestive Surgery, Kanazawa Medical University)、Hiroki Ura (Center for Clinical Genomics, Kanazawa Medical University Hospital)、Sumihito Toga (Center for Clinical Genomics, Kanazawa Medical University Hospital)、Hisayo Hatanaka (Center for Clinical Genomics, Kanazawa Medical University Hospital)、Akihumi Hashimoto (General and Digestive Surgery, Kanazawa Medical University)、Yasuto Tomita (General and Digestive Surgery, Kanazawa Medical University)、Koichi Okamoto (General and Digestive Surgery, Kanazawa Medical University)、Hideto Fujita (General and Digestive Surgery, Kanazawa Medical University)、Hiroyuki Takamura (General and Digestive Surgery, Kanazawa Medical University)、Yo Niida (Center for Clinical Genomics, Kanazawa Medical University Hospital)

In this study, we comprehensively analyzed DNA methylation and somatic variants of all gene regions across the genome of the major tumor suppressor genes, APC, TP53, SMAD4, and mismatch repair genes, MSH2, MSH6, MLH1, PMS2 in colorectal cancer using a novel next-generation sequencing-based analysis method. The Targeted Methyl Landscape shows that DNA hypermethylation patterns of these tumor suppressor genes in colorectal cancer are more complex and widespread than previously thought. Extremely high levels of DNA methylation were observed in relatively long regions around exon 1A of APC and exon 1 and surrounding region of MLH1. DNA hypermethylation occurred regardless of the somatic DNA variants were present in the tumor. Even in tumors where the loss of heterozygosity has been demonstrated by somatic variants alone, additional methylation of the same gene can occur. Our data demonstrate that somatic variants and hypermethylation of these tumor suppressor genes were considered independent, parallel events, not exclusive of each other or having one event affecting the other.

[Disclosure of COI by Responsible researcher] Lead presenter is not the Responsible researcher.
[Lead presenter: Hisashi Nishiki] We have no financial relationships to disclose.
[Principal Researcher: Hiroyuki Takamura, Yo Niida] We have no financial relationships to disclose.

### [P-2091] I-P8-1
### DNA methylation

2025 / 9 / 26 (Fri) 16:45-17:30 Room P/(Motenashi Dome) Room P

---

### P-2091
### Concurrent CGI hypermethylation and non-CGI hypomethylation drive gastric cancer risk after H. pylori eradication

[Speaker]Gota Sudo (Div. Mol. Biol., Dept. Biochem., Sapporo Med. Univ., Sch. Med.／Dept. Gastroenterol. Hepatol., Sapporo Med. Univ., Sch. Med.) [Co-author]Eiichiro Yamamoto (Div. Mol. Biol., Dept. Biochem., Sapporo Med. Univ., Sch. Med.)、Takeshi Niinuma (Div. Mol. Biol., Dept. Biochem., Sapporo Med. Univ., Sch. Med.)、Hiroshi Kitajima (Div. Mol. Biol., Dept. Biochem., Sapporo Med. Univ., Sch. Med.)、Kazuya Ishiguro (Div. Mol. Biol., Dept. Biochem., Sapporo Med. Univ., Sch. Med.)、Mitsunobu Saito (Div. Mol. Biol., Dept. Biochem., Sapporo Med. Univ., Sch. Med.／Dept. Gastroenterol. Hepatol., Sapporo Med. Univ., Sch. Med.)、Masahiro Kai (Div. Mol. Biol., Dept. Biochem., Sapporo Med. Univ., Sch. Med.)、Hironori Aoki (Div. Mol. Biol., Dept. Biochem., Sapporo Med. Univ., Sch. Med.)、Hiroo Yamano (Dept. Gastroenterol. Hepatol., Sapporo Med. Univ., Sch. Med.)、Hiroshi Nakase (Dept. Gastroenterol. Hepatol., Sapporo Med. Univ., Sch. Med.)、Hiromu Suzuki (Div. Mol. Biol., Dept. Biochem., Sapporo Med. Univ., Sch. Med.)

In this study, we performed comprehensive DNA methylation profiling of tumor and non-tumor gastric mucosa from patients who developed gastric cancer (GC) after Helicobacter pylori (H. pylori) eradication (post-eradication GC, PEGC). The normal mucosa of PEGC patients exhibited substantial CpG island (CGI) hypermethylation, comparable to that observed in H. pylori-infected tissues and intestinal metaplasia. We also identified 4081 CpG sites recurrently hypomethylated in GC using TCGA-STAD data, many of which were already hypomethylated in PEGC mucosa. Majority of the hypomethylated sites are located outside of CGIs, and several of them are located near oncogenes such as CDH17, HNF4A, and CD44, and their methylation levels inversely correlated with gene expression. Notably, we found a strong positive correlation between CGI hypermethylation and CpG hypomethylation, suggesting that both types of epigenetic alterations occur simultaneously from the early stages of carcinogenesis. These findings indicate that CGI hypermethylation and non-CGI hypomethylation represent a coordinated epigenetic landscape that may contribute to persistent GC risk after H. pylori eradication.

[Disclosure of COI by Responsible researcher] Lead presenter is not the Responsible researcher.
[Lead presenter: Gota Sudo] We have no financial relationships to disclose.
[Principal Researcher: Hiromu Suzuki] We have no financial relationships to disclose.

### [P-2092] I-P8-1
### DNA methylation

2025 / 9 / 26 (Fri) 16:45-17:30 Room P/(Motenashi Dome) Room P

---

### P-2092
### Search for therapeutic target genes of synthetic lethality in *CHFR*-methylated cancer cells

[Speaker]Yui Ohashi (Dept. of Epigenomics, Inst. for Adv. Life Sci., Hoshi Univ.) [Co-author]Hideyuki Takeshima (Dept. of Epigenomics, Inst. for Adv. Life Sci., Hoshi Univ.)、Yuyu Liu (Dept. of Epigenomics, Inst. for Adv. Life Sci., Hoshi Univ.)、Takahiro Ebata (Dept. of Epigenomics, Inst. for Adv. Life Sci., Hoshi Univ.)、Yumi Furuichi (Dept. of Epigenomics, Inst. for Adv. Life Sci., Hoshi Univ.)、Ryoma Mashiko (Dept. of Epigenomics, Inst. for Adv. Life Sci., Hoshi Univ.)、Teruaki Oku (Dept. of Microbiology, Hoshi Univ.)、Toshikazu Ushijima (Dept. of Epigenomics, Inst. for Adv. Life Sci., Hoshi Univ.)

Synthetic lethality enables cancer cell-specific therapy, and aberrant methylation, frequently present per one cancer cell, can be promising targets. Here, we identified therapeutic target genes by focusing on the methylation of ***CHFR***, a tumor-suppressor gene in gastrointestinal cancer. To mimic ***CHFR*** methylation, KO cells were established using gastric cancer cell lines, and infected with a lentivirus shRNA library targeting 2,161 genes (12,471 shRNAs). Therapeutic target genes were screened by comparing the number of shRNAs in cells before (P0) and after (P5) long-term culture. We extracted 132 shRNAs with decreases at P5 to 50% or less of that at P0 only in KO cells. Six genes, including ***BIRC5*** (Survivin), were targeted by multiple shRNAs of the 132 shRNAs. To validate the screening result, a growth inhibition assay using Survivin inhibitor, YM-155, was conducted. Cell growth was inhibited to 34±1.4% in KO cells, but 41±2.1% in WT cells. In addition, cell growth was inhibited to 33±1.4% in a ***CHFR***-methylated cell line, but to 58±4.5% in an unmethylated cell line. These results suggested that ***BIRC5*** is the potential therapeutic target gene in ***CHFR***-methylated cancer cells.

[Disclosure of COI by Responsible researcher] Lead presenter is not the Responsible researcher.
[Lead presenter: Yui Ohashi] We have no financial relationships to disclose.
[Principal Researcher: Toshikazu Ushijima] ■Research expenses: シスメックス株式会社

Poster Session

### [P-2093] I-P8-1
### DNA methylation

2025 / 9 / 26 (Fri) 16:45-17:30 Room P/(Motenashi Dome) Room P

### P-2093
### Defective chief cells in nodular gastritis in adults.

[Speaker]Yumi Furuichi (Department of Epigenomics, Institute for Advanced Life Sciences, Hoshi University／Department of Gastroenterological Surgery, Kagawa University) [Co-author]Yuyu Liu (Department of Epigenomics, Institute for Advanced Life Sciences, Hoshi University)、Akiko Sasaki (Department of Epigenomics, Institute for Advanced Life Sciences, Hoshi University／Gastroenterology Medicine Center, Shonan Kamakura General Hospital)、Hideyuki Takeshima (Department of Epigenomics, Institute for Advanced Life Sciences, Hoshi University)、Toshikazu Ushijima (Department of Epigenomics, Institute for Advanced Life Sciences, Hoshi University)

Nodular gastritis (NG) is characterized by severe lymphoid follicle in gastric antrum and induced by ***H. pylori*** (***HP***) infection. NG induces severe aberrant DNA methylation in gastric body and antral epithelial cells, and imposes a high risk of diffuse-type gastric cancer in gastric body. However, mechanisms of methylation induction and its consequences are still unclear. Two gastric body samples from NG patients (one with ***HP*** and one without), two from atrophic gastritis (AG) patients (one with ***HP*** and one without), and two normal gastric body tissues without ***HP*** were analyzed by snRNA-seq. Chief cells in NG had low expression of ***pepsinogen*** and other chief cell markers, and had high heterogeneity than those in AG. Methylation analysis showed that multiple genes related to gastric epithelial cell function were aberrantly methylated in NG. Decreased expression of ***TET2*** and ***TET3*** was observed in chief and neck cells of NG, which appeared to be caused by the lack of ***WNT5B*** and decreased ***BMP6*** in NG. Production of defective chief cells, along with inactivation of tumor-suppressor genes, due to severe methylation induction appear to underlie the high-risk status caused by NG.

[Disclosure of COI by Responsible researcher] Lead presenter is not the Responsible researcher.
[Lead presenter: Yumi Furuichi] We have no financial relationships to disclose.
[Principal Researcher: Toshikazu Ushijima] We have no financial relationships to disclose.

### [P-2102] I-P8-3
### Histone modification

2025 / 9 / 26 (Fri) 16:45-17:30 Room P/(Motenashi Dome) Room P

---

### P-2102
### β-hydroxybutyrate-induced HDAC2 β-hydroxybutyrylation suppresses nasopharyngeal carcinoma malignancy

[Speaker]WANQI WEI (Department of Otolaryngology-Head and Neck Surgery／Key Lab. Reg. High-Incidence Tumor, GXMU) [Co-author]Xiaoying Zhou (Key Lab. Reg. High-Incidence Tumor, GXMU)、Zhe Zhang (Department of Otolaryngology-Head and Neck Surgery／Key Lab. Reg. High-Incidence Tumor, GXMU)、Weilin Zhao (Department of Otolaryngology-Head and Neck Surgery)

Our study found significant β-hydroxybutyrate (β-HB) loss in nasopharyngeal carcinoma (NPC) versus normal tissues, linked to HMGCL/ACAT1 epigenetic silencing. β-HB supplementation inhibited NPC proliferation/invasion. Functionally, β-HB directly fuels lysine β-hydroxybutyrylation (Kbhb). Immunohistochemistry showed 62% pan-Kbhb reduction in NPC ($p=0.004$), reversed by β-HB dosing. Proteomic/Kbhb-modificomics identified histone acetylation pathway targets. HDAC2 harbored catalytic domain Kbhb sites (K439/457/458/468). β-HB elevated HDAC2 Kbhb 4.7-fold (Co-IP) and reduced deacetylation activity by 68% ($p=0.007$), sparing HDAC1. GEO data ($n=502$) tied HDAC2 overexpression to worse 5-year survival (HR=2.34, 95%CI 1.67-3.28). HDAC2 silencing cut 5-8F cell proliferation (71%) and migration (83%) ($p <0.001$). Starvation-induced ketosis boosted serum β-HB and slowed xenograft growth. This β-HB-Kbhb-HDAC2 axis disrupts HDAC2 deacetylation via catalytic site Kbhb modification, curbing tumorigenesis. HDAC2 Kbhb emerges as a therapeutic target, supporting ketogenic strategies for NPC epigenetic intervention.

[Disclosure of COI by Responsible researcher] Lead presenter is the Responsible researcher.
[Lead presenter: WANQI WEI] We have no financial relationships to disclose.

### [P-2103] I-P8-3
### Histone modification

2025 / 9 / 26 (Fri) 16:45-17:30 Room P/(Motenashi Dome) Room P

---

P-2103
### Histone lactylation drives metabolic reprogramming in response to glucose deprivation in canine hemangiosarcoma

[Speaker]Tamami Suzuki (Hokkaido Univ. Grad. Sch. of Vet. Med. Lab. of C.Path.) [Co-author]Jumpei Yamazaki (Hokkaido Univ. Grad. Sch. of Vet. Med. Vet. Teaching Hosp.)、Masaya Yamazaki (Div. of Carcinogenesis, The Cancer Inst, JFCR)、Kazuki Heishima (Gifu Univ. Grad. Sch. of Drug Discov. & Med. Info. Sci.)、Yuko Okamatsu (Hokkaido Univ. Grad. Sch. of Vet. Med. Lab. of Biochem.)、Takashi Kimura (Hokkaido Univ. Grad. Sch. of Vet. Med. Lab. of C.Path.)、Keisuke Aoshima (Hokkaido Univ. Grad. Sch. of Vet. Med. Lab. of C.Path.)

Hemangiosarcoma (HSA) is a malignant tumor that frequently develops in the canine spleens with unknown molecular pathogenesis. Given that HSA cells are derived from endothelial cells (ECs), which primarily rely on glycolysis for ATP production and produce abundant lactate, we hypothesized that lactate-mediated histone modification (histone lactylation: Hla) contribute to HSA pathogenesis. Using patient tissues and primary cells of HSA, we found that HSA cells exhibited higher global Hla levels than ECs. Glucose deprivation (Glc-dep) reduced global Hla levels and shifted ATP production toward mitochondrial respiration. Moreover, CUT&Tag revealed that Hla enrichment around transcription start sites in Glc-dep HSA cells, especially at genes involved in IFN responses and asparagine (Asn) metabolism. HSA cells promoted macrophage migration and induced M2-like polarization ***in vitro*** and in patient tissues. Furthermore, Asn supplementation partially rescued HSA cell growths in Glc-dep, which was not observed in ATF4-knockout cells. Overall, these findings suggest that HSA cells exploit Hla to reprogram tumor metabolisms in response to glucose shortage and reshape tumor microenvironment.

[Disclosure of COI by Responsible researcher] Lead presenter is not the Responsible researcher.
[Lead presenter: Suzuki Tamami] We have no financial relationships to disclose.
[Principal Researcher: Keisuke Aoshima] We have no financial relationships to disclose.

### [P-2104] I-P8-3
### Histone modification

2025 / 9 / 26 (Fri) 16:45-17:30 Room P/(Motenashi Dome) Room P

---

### P-2104
### Molecular mechanisms of ATAD2 degradation under hypoxic conditions

[Speaker]Takumi Yoshida (Cancer Cell Biol, Grad Sch of Biostudies, Kyoto Univ.) [Co-author]Minoru Kobayashi (Cancer Cell Biol, Grad Sch of Biostudies, Kyoto Univ.／Dept of Genomic Dynamics, Radiation Biology Center, GSB, Kyoto Univ.)、Takao Haitani (Cancer Cell Biol, Grad Sch of Biostudies, Kyoto Univ.)、Hiroshi Harada (Cancer Cell Biol, Grad Sch of Biostudies, Kyoto Univ.／Dept of Genomic Dynamics, Radiation Biology Center, GSB, Kyoto Univ.)

Hypoxia, a tumor microenvironment characterized by reduced oxygen availability, has been recognized as a therapeutic target due to its association with various malignant phenotypes. We previously reported that ATAD2 protein levels decrease dramatically under hypoxia, contributing to a retardation of cell cycle progression from early to late S phase and inducing chemoresistance in cancer cells. Moreover, we revealed that this reduction is mediated by the inactivation of $O_2$/$Fe^{2+}$/2-oxoglutarate dependent dioxygenases (2-OGDDs). However, the molecular mechanisms behind the decrease in ATAD2 expression under hypoxia have not yet been fully elucidated. Here, we show ATAD2 protein levels are regulated through the ubiquitin-proteasome system at the degradation step. Currently, we are trying to identify the E3 ligase responsible for ATAD2 proteolysis using siRNA library screening. In parallel, we are also trying to identify the 2-OGDD involved by employing a gRNA library to understand the molecular mechanisms driving cell cycle retardation under hypoxia. We will present the mechanism and biological significance of ATAD2 degradation, incorporating our most recent data.

[Disclosure of COI by Responsible researcher] Lead presenter is not the Responsible researcher.
[Lead presenter: Takumi Yoshida] We have no financial relationships to disclose.
[Principal Researcher: Hiroshi Harada] We have no financial relationships to disclose.

### [P-2105] I-P8-3
### Histone modification

2025 / 9 / 26 (Fri) 16:45-17:30 Room P/(Motenashi Dome) Room P

---

### P-2105
### Epigenetic regulation of CD8+ T cell-dependent antitumor immunity by histone H3K27 methyltransferase Ezh2

[Speaker]Kenji Takagi (Dept. Gastrointestinal Surg. Grad. Sch. Med. Ehime Univ.／Dept. Immunol. Grad. Sch. Med. Ehime Univ.) [Co-author]Junpei Suzuki (Dept. Immunol. Grad. Sch. Med. Ehime Univ.)、Makoto Kuwahara (Dept. Immunol. Grad. Sch. Med. Ehime Univ.)、Taro Oshikiri (Dept. Immunol. Grad. Sch. Med. Ehime Univ.)、Masakatsu Yamashita (Dept. Gastrointestinal Surg. Grad. Sch. Med. Ehime Univ.)

T-cell function is epigenetically regulated, however its precise role in tumor immunity remains unclear. Ezh2 promotes transcriptional silencing through trimethylation of histone H3K27. We previously reported that H3K27 methylation levels decreases in terminally differentiated and senescent T cells. In this study, we analyzed the role of Ezh2 in effector CD8+ T-cell differentiation and antitumor immunity. In a mouse tumor model, T-cell-specific Ezh2 KO mice exhibited reduced initial tumor growth compared to wild-type (WT) mice, but tumors grew rapidly after day 7. The number of CD8+ T cells in tumor-infiltrating lymphocytes (TILs) was significantly decreased in Ezh2 KO mice. We found that although the expression of effector molecules was rapidly increased in Ezh2 KO CD8+ T cells compared to WT cells, apoptotic cell death was also rapidly induced after activation. RNA-sequencing revealed that the expression of effector signature genes was upregulated in Ezh2 KO effector CD8+ T cells, along with the expression of apoptosis-related genes. These findings suggest that Ezh2 regulates antitumor immunity by controlling CD8+ T-cell differentiation, cell death and possibly trafficking.

[Disclosure of COI by Responsible researcher] Lead presenter is not the Responsible researcher.
[Lead presenter: Kenji Takagi] We have no financial relationships to disclose.
[Principal Researcher: Masakatsu Yamashita] We have no financial relationships to disclose.

### [P-2106] I-P8-3
### Histone modification

2025 / 9 / 26 (Fri) 16:45-17:30 Room P/(Motenashi Dome) Room P

---

### P-2106
### HDAC5, an early osimertinib-responsive gene, is a novel target for drug resistance in EGFR-mutant lung cancer cells.

[Speaker]Akihiko Ishimura (Kanazawa Univ. Cancer Res. Inst.) [Co-author]Hanbing Lyu (Kanazawa Univ. Cancer Res. Inst.)、Ryusuke Suzuki (Kanazawa Univ. Cancer Res. Inst.)、Takeshi Suzuki (Kanazawa Univ. Cancer Res. Inst.)

Aberrant epigenetic regulation contributes to early drug tolerance. We previously showed that an epigenetic initiator, rapidly induced by the third-generation EGFR tyrosine kinase inhibitor osimertinib, promotes tolerant cell formation. In this study, to identify novel epigenetic factors associated with osimertinib-tolerance, we performed a comprehensive screening of epigenetic factors whose expression is rapidly induced by osimertinib. Our results revealed that ***HDAC5***, a class IIa histone deacetylase (HDAC), is a prominently induced epigenetic regulator in several EGFR-mutant non-small cell lung cancer (NSCLC) cell lines during the early response to osimertinib. ***HDAC5*** knockdown significantly reduced the emergence of osimertinib-resistant cells. Furthermore, treatment with LMK235, a selective HDAC5 inhibitor, significantly increased global histone acetylation and enhanced osimertinib-induced apoptosis. These findings underscore the potential of HDAC5 as a novel therapeutic target to overcome osimertinib-resistance and propose LMK235 as a promising compound to provide significant therapeutic benefits for EGFR-mutant NSCLC patients undergoing osimertinib treatment.

[Disclosure of COI by Responsible researcher] Lead presenter is not the Responsible researcher.
[Lead presenter: Akihiko Ishimura] ■Research funds from company: 日本学術振興会　基盤研究(C)
[Principal Researcher: Takeshi Suzuki] ■Research funds from company: 日本学術振興会　基盤研究(C)

### [P-2107] I-P8-3
### Histone modification

2025 / 9 / 26 (Fri) 16:45-17:30 Room P/(Motenashi Dome) Room P

### P-2107
### Withdrawn

No Abstract

### [P-2108] I-P8-3
### Histone modification

2025 / 9 / 26 (Fri) 16:45-17:30 Room P/(Motenashi Dome) Room P

---

### P-2108
### Targeting EHMT2 Overcomes 5 Fluorouracil Resistance in Colorectal Cancer

[Speaker]IN HWAN TAE (Korea Research Institute of Bioscience and Biotechnology／Korea University of Science and Technology) [Co-author]Jinkwon Lee (Korea Research Institute of Bioscience and Biotechnology)、Yunsang Kang (Korea Research Institute of Bioscience and Biotechnology／Korea University of Science and Technology)、JeongMin Lee (Korea Research Institute of Bioscience and Biotechnology／Korea University of Science and Technology)、Jinsan Kim (Korea Research Institute of Bioscience and Biotechnology／Korea University of Science and Technology)、Dae-Soo Kim (Korea Research Institute of Bioscience and Biotechnology／Korea University of Science and Technology)、Mi-Young Son (Korea Research Institute of Bioscience and Biotechnology／Korea University of Science and Technology)、Hyun-Soo Cho (Korea Research Institute of Bioscience and Biotechnology／Korea University of Science and Technology)

5 Fluorouracil (5 FU) is a standard chemotherapeutic agent used in the treatment of colorectal cancer (CRC), but the emergence of drug resistance remains a major clinical obstacle. In our previous studies, we identified that the histone methyltransferase EHMT2 is upregulated in CRC cells. To elucidate its role in 5 FU resistance, we performed RNA sequencing, which revealed significantly elevated EHMT2 expression, and found that its inhibition induced apoptosis in resistant cells. Mechanistically, suppression of EHMT2 led to increased expression of protein phosphatase 1B (PPM1B), which disrupted cell cycle progression and triggered apoptotic pathways. These findings were corroborated in a xenograft mouse model, which showed consistent results with our 2D in vitro studies. Furthermore, we established 5 FU resistant colorectal cancer organoid (CCO) models derived from patient tissues and confirmed the involvement of the EHMT2, PPM1B, and p21 axis in mediating cell death. Collectively, our results suggest that EHMT2 plays a pivotal role in the development of 5 FU resistance in CRC and represents a promising therapeutic target for overcoming chemoresistance.

[Disclosure of COI by Responsible researcher] Lead presenter is the Responsible researcher.
[Lead presenter: IN HWAN TAE] We have no financial relationships to disclose.

### [P-2117] I-P9-4
### Cell adhesion & invasion (2)

2025 / 9 / 26 (Fri) 16:45-17:30 Room P/(Motenashi Dome) Room P

---

### P-2117
### Unravelling Cancer Evolution in a Gastric Cancer Mouse Model via SCNA Phylogeny from Single-Cell DNA Sequencing

[Speaker]Ichii Shirasuna (Dept. Prev. Med., Grad. Sch. Med., The Univ. of Tokyo) [Co-author]Daisuke Komura (Dept. Prev. Med., Grad. Sch. Med., The Univ. of Tokyo)、Miwako Kakiuchi (Dept. Prev. Med., Grad. Sch. Med., The Univ. of Tokyo)、Shumpei Ishikawa (Dept. Prev. Med., Grad. Sch. Med., The Univ. of Tokyo)

Somatic copy number alterations (SCNAs) are widespread in cancer. However, their functional roles remain elusive, largely because fitness effects of SCNAs are highly context-dependent. To address this, inferring the temporal order of SCNA events is essential. Here, we developed a Bayesian method to estimate SCNAs and their phylogenies from single-cell DNA sequencing data. Our model separately parameterizes focal and chromosome-level SCNA events, and estimates these parameters and phylogenetic tree using the Markov Chain Monte Carlo method. Our approach outperformed the existing method in accurately inferring ancestor-descendant and branching relationships between subclones in simulation data. Applying our method to data from a diffuse-type gastric cancer mouse model, we found that Nfib amplification occurs following a relative loss of Arid4b copy number with respect to ploidy. In vitro assays showed that ARID4B knockdown combined with NFIB overexpression enhances anoikis resistance. Our findings suggest that ARID4B downregulation heightens the oncogenic effect of NFIB amplification, underscoring the importance of reconstructing SCNA chronology to understand cancer evolution better.

[Disclosure of COI by Responsible researcher] Lead presenter is not the Responsible researcher.
[Lead presenter: Ichii Shirasuna] We have no financial relationships to disclose.
[Principal Researcher: Shumpei Ishikawa] ■Research funds from company: 日本学術振興会　■Advisor of the company and/or profit-making organization: 新日本科学株式会社　■Research expenses: Chordia therapeutics株式会社, 大鵬薬品工業株式会社, ロート製薬株式会社, アステラス製薬株式会社

### [P-2118] I-P9-4
### Cell adhesion & invasion (2)

2025 / 9 / 26 (Fri) 16:45-17:30 Room P/(Motenashi Dome) Room P

---

### P-2118
### CCL5 promotes collective invasion of human skin squamaus carcinoma cells

[Speaker]Toko Yamazaki (Grad. Sch. of Life Sci., Hokkaido Univ.) [Co-author]Yuji Kumagai (PHS & HB Div., Fred Hutchinson Cancer Ctr.)、Kosuke Maeda (Sch. of Sci., Hokkaido Univ.)、Tomoyasu Aizawa (Faculty of Advanced Life Sci., Hokkaido Univ.)、Seiichiro Ishihara (Faculty of Advanced Life Sci., Hokkaido Univ.)、Hisashi Haga (Faculty of Advanced Life Sci., Hokkaido Univ.)

Collective invasion, a process in which cancer cells form clusters and invade surrounding tissues, is associated with metastasis and poor prognosis of cancer patients. Therefore, inhibition of collective invasion is considered to improve cancer treatment. However, the mechanism of collective invasion in cancer cells is poorly understood. In this study, we investigated the differences of gene expression between a highly invasive subclone of A431 squamous carcinoma cells (A431-6 cells) and a low-invasive subclone of A431 cells (A431-7 cells). The three-dimensional collective invasive potential was quantified by analyzing sphericity of the cell clusters in collagen gels. We confirmed that A431-6 cells generated highly invasive cell clusters compared with A431-7 cells. CCL5, an inflammatory chemokine, was expressed higher in A431-6 cells than A431-7 cells in mRNA and protein levels. The collective invasive potential of A431-6 cells was decreased by CCL5 knockdown with siRNA transfection. In contrast, the collective invasive potential of A431-7 cells increased by treatment of recombinant CCL5. These results suggest that CCL5 promotes collective invasion of A431 cells.

[Disclosure of COI by Responsible researcher] Lead presenter is not the Responsible researcher.
[Lead presenter: Toko Yamazaki] We have no financial relationships to disclose.
[Principal Researcher: Hisashi Haga] We have no financial relationships to disclose.

### [P-2119] I-P9-4
### Cell adhesion & invasion (2)

2025 / 9 / 26 (Fri) 16:45-17:30 Room P/(Motenashi Dome) Room P

### P-2119
### Elucidating the Mechanism of COMT-Mediated Suppression of Invasion and Metastasis in Ovarian Cancer

[Speaker]Yuki Nishida (Keio University,IAB) [Co-author]Shojiro Kitajima (Keio University,IAB)、Tomoyoshi Soga (Keio University,Bio2Q)

Ovarian cancer is often linked to poor prognosis, especially in advanced stages. The 10-year relative survival rate drops by half from early (stage I/II) to late (stage III/IV) disease, highlighting the impact of invasion and metastasis. TCGA data show that high expression of catechol-O-methyltransferase (COMT), an estrogen-metabolizing enzyme, correlates with better outcomes. Yet, its molecular role remains unclear. We overexpressed COMT in ovarian cancer cell lines and found a marked reduction in invasiveness. Interestingly, this effect occurred regardless of estrogen presence, implying an estrogen-independent function. RNA-seq analysis revealed upregulation of desmoglein (DSG) genes and changes in metabolic pathways. CE-MS metabolomics further showed increased oxidative stress in COMT-overexpressing cells. These results suggest that COMT limits invasion and metastasis by promoting DSG-mediated cell adhesion and modulating redox homeostasis, which may underlie the favorable prognosis observed in ovarian cancer cases with high COMT expression.

[Disclosure of COI by Responsible researcher] Lead presenter is not the Responsible researcher.
[Lead presenter: Yuki Nishida] We have no financial relationships to disclose.
[Principal Researcher: Shojiro Kitajima] We have no financial relationships to disclose.

### [P-2120] I-P9-4
### Cell adhesion & invasion (2)

2025 / 9 / 26 (Fri) 16:45-17:30 Room P/(Motenashi Dome) Room P

---

### P-2120
### Isoform-specific localization of p120-catenin in colorectal cancer cells is regulated by adaptins and related proteins

[Speaker]Airi Tsuji (Appl. Biol. Chem., Kindai Univ. Grad. Sch. Agric.) [Co-author]Masahiro Aoki (Div. Pathophysiol., Aichi Cancer Ctr. Res. Inst.／Div. Cancer Physiol., Nagoya Univ. Grad. Sch. Med.)、Keiichiro Sakuma (Appl. Biol. Chem., Kindai Univ. Grad. Sch. Agric.／Dept. Food Sci. Nut., Fac. Agric., Kindai Univ.)

Skipping of exon B of the ***CTNND1*** gene, which encodes p120-catenin (p120ctn), is enhanced in colorectal cancer cells undergoing epithelial-mesenchymal transition, resulting in increased expression of p120ctn isoform 3A (3A) relative to isoform 3AB (3AB). 3A preferentially localizes at the leading edge of migrating DLD-1 cells and promotes matrigel invasion. The amino acid sequence encoded by exon B contains two potential adaptin-binding sites, and a mutant 3AB lacking the binding ability showed increased localization at the leading edge. In this study, we sought to identify the responsible adaptin among the five known members of the adaptin family (AP1-5). Based on their specific consensus binding motifs and our western blot analysis of colorectal cancer cell lines, AP1/2 emerged as a potential candidate. Knockout of AP1, as well as knockout of ARF1/6-essential GTPases for AP1/2 activation-resulted in translocation of 3AB to the leading edge of DLD-1 cells. We are currently addressing the mechanism by which 3A localizes to the leading edge, under the hypothesis that some unidentified factor(s) facilitate this translocation, while AP1/2 binds to 3AB and thereby interferes with it.

[Disclosure of COI by Responsible researcher] Lead presenter is not the Responsible researcher.
[Lead presenter: Airi Tsuji] We have no financial relationships to disclose.
[Principal Researcher: Keiichiro Sakuma] We have no financial relationships to disclose.

▲TOP

[P-2121] I-P9-4
Cell adhesion & invasion (2)

2025 / 9 / 26 (Fri) 16:45-17:30 Room P/(Motenashi Dome) Room P

P-2121
### *ANKRD17* Promotes Pro-Survival Signaling to Enhance Invasion and Migration in Hepatocellular Carcinoma

[Speaker]Elyse S.T. Chui (Dept. of Applied Biol. & Chemical Tech.) [Co-author]Xiao-Xiao Li (The Hong Kong Polytechnic Univ. Shenzhen Res. Inst.／Res. Inst. for Chinese Medicine Innovation, Hog Kong Polytechnic Univ.)、Vincent W Keng (Dept. of Applied Biol. & Chemical Tech.／The Hong Kong Polytechnic Univ. Shenzhen Res. Inst.)

Metastasis is the leading cause of high mortality in hepatocellular carcinoma (HCC). Our previous Sleeping Beauty insertional mutagenesis screen identified ankyrin repeat domain 17 (***ANKRD17***) as a key gene linked to HCC metastasis. In vivo and in vitro analyses showed that ***ANKRD17*** overexpression in mouse liver tumors increased tumor burden and EMT markers. This overexpression was associated with downregulation of Hippo and ***PTEN/PI3K/AKT*** pathways, indicated by increased ***YAP1*** and ***PIK3CA*** expression and decreased ***PTEN*** levels. In human liver cell lines, ***ANKRD17*** overexpression enhanced motility and invasion, while knockdown reversed these effects. Dysregulated pathways were linked to increased metastasis and cell survival. We also observed upregulation of the pro-metastatic ***DDR1*** gene, where ***DDR1*** inhibition reduced motility and invasion without affecting ***PTEN/PI3K/AKT*** signaling. Clinically, high ***ANKRD17*** expression correlates with aggressive HCC progression. Our findings suggest ***ANKRD17*** promotes HCC metastasis by activating multiple pro-metastatic and pro-survival pathways.

[Disclosure of COI by Responsible researcher] Lead presenter is not the Responsible researcher.
[Lead presenter: Elyse S.T. Chui ] We have no financial relationships to disclose.
[Principal Researcher: Vincent W Keng ] We have no financial relationships to disclose.

### [P-2122] I-P9-4
### Cell adhesion & invasion (2)

2025 / 9 / 26 (Fri) 16:45-17:30 Room P/(Motenashi Dome) Room P

---

### P-2122
### The Role of Extracellular Viscosity in Tumor Progression: From Single Cells to Spheroid Formation and Invasion

[Speaker]Yin-Quan Chen (CIRC, NYCU) [Co-author]Ming-Chung Wu (Biochem. Mol. Biol. , NYCU)、Helen Wenshin Yu (Biochem. Mol. Biol. , NYCU)、Jean-Cheng Kuo (Biochem. Mol. Biol. , NYCU)

Extracellular viscosity acts as a crucial mechanical cue within the tumor microenvironment. In this study, we systematically explore the impact of viscosity on tumor progression, ranging from single cells to 3D tumor spheroids and from cell adhesion to invasion. Our results reveal that high extracellular viscosity enhances membrane dynamics, particularly in terms of protrusion and retraction. Additionally, it promotes cell spreading, leading to the formation of actin stress fibers and focal adhesions. Notably, increased viscosity also stimulates 3D cancer spheroid formation by strengthening cell-cell junctions. Furthermore, elevated viscosity significantly boosts the invasive potential of cancer cells, facilitating their migration from tumor spheroids into the surrounding 3D collagen matrix. These findings underscore the pivotal role of extracellular viscosity in modulating critical aspects of cancer cell mechanics, including spheroid formation, spreading, and invasion, all of which are essential for tumor progression.

[Disclosure of COI by Responsible researcher] Lead presenter is the Responsible researcher.
[Lead presenter: Yin-Quan Chen] We have no financial relationships to disclose.

▲TOP

### [P-2123] I-P9-4
### Cell adhesion & invasion (2)

2025 / 9 / 26 (Fri) 16:45-17:30 Room P/(Motenashi Dome) Room P

### P-2123
### Isoeugenol inhibits the growth, migration and invasion in different stages of colorectal cancer cells

[Speaker]Yung-Hsi Kao (Dept. Life Sci., Natl Central Univ.) [Co-author]Zih-Shuan Liou (Dept. Life Sci., Natl Central Univ.)、Hui-Chen Ku (Dept Pediatrics, Taipei Tzu Chi Hospital)、Ching-Feng Cheng (Dept Pediatrics, Taipei Tzu Chi Hospital／Inst. Biomed Sci., Academia Sinica／Dept. Pediatrics, Tzu Chi Univ.)、Le Thi Bao Tran (Dept. Life Sci., Natl Central Univ.)、Yi-Qi Chen (Dept. Life Sci., Natl Central Univ.)、Li-Jane Shih (Natl. Defense Med. Center, Tri-Service General Hospital／Med. Lab., Taoyuan Armed Forces General Hospital)、Chien-Chih Yeh (Natl. Defense Med. Center, Tri-Service General Hospital／Div. Colon and Rectal Surgery, Dept. Surgery)、Chih-En Kao (Dept. Physiol., Natl. Cheng Kung Univ., Tainan, Taiwan)

Colorectal cancer (CRC) is the second common cause of cancer death in the world and can be regulated by herbal compounds. Using HCT-116, SW480 and SW620 CRC cells (CRCCs) for respective stages of Dukes A~C, we found that ***Piper betle*** isoeugenol inhibited cell growth, as evidenced by decreased cell viability and colony formation and, to a much lesser extent, affected normal FHC colon cell growth. Isoeugenol also inhibited the migration and invasion of all stages of CRCCs. Isoeugenol induced decreases in pAKT, CDK4, CDK6, cyclin D1, N-cadherin and Snail proteins and increased pAMPK, pJNK, pp38 MAPK and E-cadherin proteins. Isoeugenol stage-dependently affected isoeugenol on CDK2, cyclin D3, p21, p27, and pERK proteins. Isoeugenol inhibited MMP-2 and MMP-9 activities. Treatment with the inhibitor of either AMPK or p38 antagonized isoeugenol-altered cell growth, migration, invasion, pAMPK, pAKT, CDK4, CDK6, E-cadherin, pp38, and MMP2 and MMP9 activities. We conclude that isoeugenol may inhibit CRCC growth, migration and invasion through activations of AMPK and p38 pathways, as well as enabling CRCCs to suppress the epithelial features changing to mesenchymal ones.

[Disclosure of COI by Responsible researcher] Lead presenter is the Responsible researcher.
[Lead presenter: Yung-Hsi Kao] We have no financial relationships to disclose.

### [P-2124] I-P9-4
### Cell adhesion & invasion (2)

2025 / 9 / 26 (Fri) 16:45-17:30 Room P/(Motenashi Dome) Room P

---

### P-2124
### Intracellular and extracellular functions of Adipsin (CFD) promote adipocyte maturation and tumor invasion

[Speaker]Behnooush Khaledian (Dept. of Biochem., Sch. of Med., Fujita Health Univ.) [Co-author]Takanori Hayashi (Dept. of Biochem., Sch. of Med., Fujita Health Univ.)、Masahiro Mizuno (Dept. of Biochem., Sch. of Med., Fujita Health Univ.)、Masao Maeda (Dept. of Biochem., Sch. of Med., Fujita Health Univ.／Dept. Path., Sch. Med., Fujita Health Univ.)、Takashi(Fujita Health Univ.) Watanabe (Div. of Gene Regulation, Cancer Ctr., Fujita Health Univ.)、Kaori Ushida (Dept. Path., Sch. Med., Fujita Health Univ.)、Eiji Sugihara (Div. of Gene Regulation, Cancer Ctr., Fujita Health Univ.)、Kenji Kawada (Dept. of Med. Oncology, Sch. of Med., Fujita Health Univ.)、Naoya Asai (Dept. Path., Sch. Med., Fujita Health Univ.)、Yohei Shimono (Dept. of Biochem., Sch. of Med., Fujita Health Univ.)

Adipocytes are one of the major components of the breast cancer (BC) microenvironment. Previously, we demonstrated that adipsin (CFD) secreted from adipocytes promotes BC stemness and proliferation. Furthermore, adipocyte maturation induces tumor invasion in an adipsin-dependent manner. Here, we investigated the roles of adipsin in adipocyte maturation. When mammary adipose tissue-derived stem cells (mADSCs) were differentiated into adipocytes, Cfd-knockout (KO) adipocytes exhibited impaired lipid droplet maturation, with fewer large size lipid droplets, reducing adipocytes' ability to induce BC cell invasion. Restoration of wild-type CFD in Cfd KO mADSCs rescued the impaired lipid droplet morphology in mature adipocytes, whereas the secretion-deficient CFD variant lacking the signal sequence for extracellular secretion produced numerous small lipid droplets with less lipid accumulation. These findings suggest the presence of intracellular regulatory function of adipsin to promote adipocyte maturation.

[Disclosure of COI by Responsible researcher] Lead presenter is not the Responsible researcher.
[Lead presenter: Behnooush Khaledian] We have no financial relationships to disclose.
[Principal Researcher: Yohei Shimono] We have no financial relationships to disclose.

▲TOP

### [P-2136] I-P10-4
### Metabolism/Metabolome (1)

2025 / 9 / 26 (Fri) 16:45-17:30 Room P/(Motenashi Dome) Room P

### P-2136
### Plasma Lysophosphatidylcholine Levels Correlate with Prognosis and Immunotherapy Response in Squamous Cell Carcinoma

[Speaker]Tomoyuki Iwasaki (Department of Medical Oncology, Tohoku University Hospital) [Co-author]Eiji Hishunuma (Advanced Research Center for Innovations in Next-Generation Medicine, Tohoku University／Tohoku Medical Megabank Organization, Tohoku University)、Hidekazu Shirota (Department of Medical Oncology, Tohoku University Hospital)、Yuki Kasahara (Department of Medical Oncology, Tohoku University Hospital)、Ryo Saito (Department of Medical Oncology, Tohoku University Hospital)、Hisato Kawakami (Department of Medical Oncology, Tohoku University Hospital)

Cancer is a systemic disease rather than a localized pathology and is characterized by widespread effects, including whole-body exhaustion and chronic inflammation. To explore these dynamics, this study employed a comprehensive metabolomics of plasma samples from 149 patients with esophageal and head and neck squamous cell carcinoma (SCC). Among the metabolites identified, lysophosphatidylcholine (LPC) emerged as the sole biomarker strongly correlated with prognosis. Plasma LPC levels showed minimal correlation with patient-specific factors, such as tumor size and general condition, but showed significant association with the response to immunotherapy. Proteomic and cytokine analyses revealed that low plasma LPC levels reflected systemic chronic inflammation, characterized by high levels of inflammatory proteins, IL-6 and TNF-$\alpha$, and coagulation-related proteins. These findings indicate that plasma LPC levels may be used as a reliable biomarker for predicting prognosis and evaluating the efficacy of immunotherapy in patients with SCC. Integrating LPC metrics into clinical practice offers a novel approach to improving therapeutic outcomes and advancing personalized cancer treatment.

[Disclosure of COI by Responsible researcher] Lead presenter is not the Responsible researcher.
[Lead presenter: Tomoyuki Iwasaki] We have no financial relationships to disclose.
[Principal Researcher: Hisato Kawakami] ■Advisor of the company and/or profit-making organization: 第一三共、アステラス、アッヴィ　■Patent fee: 医学生物学研究所　■Lecture fee: ブリストル・マイヤーズ・スクイブ、イーライリリー、小野薬品、第一三共、武田薬品、帝人製薬、大塚製薬、グラクソ・スミスクライン、ノバルティス、バイエル、MSD、中外製薬、メルクバイオファーマ、ヤクルト、大鵬薬品、日本化薬、アムジェン、アステラス　■Research expenses: エーザイ、小林製薬、大鵬薬品、ブリストル・マイヤーズ・スクイブ、アステラス

### [P-2137] I-P10-4
### Metabolism/Metabolome (1)

2025 / 9 / 26 (Fri) 16:45-17:30 Room P/(Motenashi Dome) Room P

### P-2137
### Tumor heterogeneity of unique BCAA metabolism regulates the aggressive nature in human triple-negative breast cancer

[Speaker]Kenkyo Matsuura (Inst. Life Med. Sci., Kyoto Univ.) [Co-author]Ririko Shinonaga (Inst. Life Med. Sci., Kyoto Univ.／Grad. Sch. Pharm. Sci., Kyoto Univ.)、Mizuki Yamamoto (Inst. Med. Sci., Univ. Tokyo)、Yukako Watanabe (Dept. Breast Surg., Sch. Med., Intl. Univ. Health and Welfare)、Mamoru Takada (Dept. Gen. Surg., Grad. Sch. Med., Chiva Univ.)、Junichiro Inoue (UTOPIA, Univ. Tokyo)、Ayuna Hattori (Inst. Life Med. Sci., Kyoto Univ.)、Hiromi Imamura (Grad. Sch. Biostudies, Kyoto Univ.／Org. Res. Init., Yamaguchi Univ.)、Takahiro Ito (Inst. Life Med. Sci., Kyoto Univ.)

Triple-negative breast cancer (TNBC) remains a clinically challenging type of cancer, making it essential to identify molecular targets for effective treatment. We examined whether branched-chain amino acid (BCAA) metabolism controls TNBC. We found that TNBC cells are more dependent on BCAA for their growth than other subtype breast cancer cells. By using a BCAA biosensor, cellular BCAA concentration was found to be highly heterogenous within tumor in vivo. Cells with elevated BCAAs exhibit increased proliferation activity in vitro, and greater tumor-forming potential in a mouse xenograft model. Altered expressions of metabolic enzymes in the BCAA pathway contributes to the enhanced BCAA levels in TNBC cells. Patient dataset analysis supported the expression alteration in vivo. Suppression of metabolic enzymes to modulate BCAA metabolic flux decreased cancer cell proliferation. Our results indicate that BCAA metabolism and aggressive features of the cancer is controlled by both enhanced BCAA production and suppressed branched-chain ketoacid breakdown that collectively affect the flux to elevate BCAA levels. This mechanism can be a novel targetable vulnerability in TNBC.

[Disclosure of COI by Responsible researcher] Lead presenter is not the Responsible researcher.
[Lead presenter: Kenkyo Matsuura] We have no financial relationships to disclose.
[Principal Researcher: Takahiro Ito] We have no financial relationships to disclose.

▲TOP

### [P-2138] I-P10-4
### Metabolism/Metabolome (1)

2025 / 9 / 26 (Fri) 16:45-17:30 Room P/(Motenashi Dome) Room P

### P-2138
### Lysine acylation of plasma proteins as a potential biomarker for cancer cachexia

[Speaker]Yasushi Kojima (Div. Pathophysiol., Aichi Cancer Ctr Res. Inst.) [Co-author]Emi Mishiro (WPI-ITbM, Nagoya Univ.)、Teruaki Fujishita (Div. Pathophysiol., Aichi Cancer Ctr Res. Inst.)、Rie Kajino (Div. Pathophysiol., Aichi Cancer Ctr Res. Inst.)、Makoto M. Taketo (Pers. Canc. Therap., MIC, Kyoto Univ.)、Tomoyoshi Soga (Inst. Adv. Biosci. Keio Univ.)、Masahiro Aoki (Div. Pathophysiol., Aichi Cancer Ctr Res. Inst.)

Cancer cachexia is a multifactorial syndrome characterized by involuntary weight and muscle loss, significantly affecting prognosis and quality of life in patients with advanced cancer. Despite extensive research, the metabolic characteristics of cachexia remain unclear, impeding the development of effective biomarkers. To address this knowledge gap, we analyzed metabolic and proteomic changes in mouse models of cancer cachexia and gastric cancer patients.Our study revealed a marked reduction in enzymes associated with pantothenate (vitamin B5)-dependent acyl-CoA metabolism in cachectic liver tissue, along with altered protein acetylation and malonylation profiles. Notably, liver-derived plasma proteins, especially apolipoproteins, exhibited consistently decreased acetylation and malonylation levels in both mouse cachexia models and gastric cancer patients.These findings highlight altered lysine acylation of plasma proteins as a potential metabolic hallmark of cancer cachexia. Future research should focus on developing blood-based biomarkers derived from protein modification profiles to facilitate early diagnosis and improve clinical management of cancer cachexia.

[Disclosure of COI by Responsible researcher] Lead presenter is the Responsible researcher.
[Lead presenter: Yasushi Kojima] We have no financial relationships to disclose.

### [P-2139] I-P10-4
### Metabolism/Metabolome (1)

2025 / 9 / 26 (Fri) 16:45-17:30 Room P/(Motenashi Dome) Room P

---

### P-2139
### Cancer-derived HMGB1 impairs cardiac mitochondria via mtDNA damage and CKMT2 suppression

[Speaker]Rina Tani (Nara Med. Univ., Mol. Path.) [Co-author]Shota Nukaga (Nara Med. Univ., Mol. Path.)、Isao Kawahara (Nara Med. Univ., Mol. Path.)、Ryoichi Nishida (Nara Med. Univ., Mol. Path.)、Ruiko Ogata (Nara Med. Univ., Mol. Path.)、Hiroki Kuniyasu (Nara Med. Univ., Mol. Path.)

Cancer-induced myocardial damage has been attributed not only to chemotherapy but also to cancer itself. We previously established both in vivo and in vitro cancer cachexia-induced myocardial damage models using the colorectal cancer cell lines RCN-9 and ACL-15. Using these models, we demonstrated mitochondrial dysfunction associated with elevated serum HMGB1 levels, a feature also observed in patients with advanced cancer. This study aimed to elucidate the mechanism by which HMGB1 contributes to mitochondrial damage. Treatment of H9c2 cardiomyoblasts with HMGB1 (10 ng/mL) led to decreased protein levels of mitochondrial complexes I, II, IV, and V. In contrast, mitochondrial OGG1, a base excision repair gene, was upregulated, potentially reflecting a compensatory response to mtDNA point mutations. Furthermore, in cardiomyotubes differentiated from H9c2 cells, HMGB1 reduced the expression of CKMT2 mRNA. These findings suggest that HMGB1 may induce mitochondrial dysfunction through mtDNA damage and suppression of CKMT2.

[Disclosure of COI by Responsible researcher] Lead presenter is the Responsible researcher.
[Lead presenter: Rina Tani] We have no financial relationships to disclose.

▲TOP

### [P-2140] I-P10-4
### Metabolism/Metabolome (1)

2025 / 9 / 26 (Fri) 16:45-17:30 Room P/(Motenashi Dome) Room P

---

### P-2140
### Cancer-derived Wnt ligand possibly inhibits adipogenesis via Wnt/β-catenin signaling pathway in preadipocytes.

[Speaker]Shota Ikeda (Dept of Surg, Saga Univ Fac of Med) [Co-author]Yoshihiko Kitazima (NHO Saga Hosp, Res & Tes Dept)、Tomokazu Tanaka (Dept of Surg, Saga Univ Fac of Med)、Hirokazu Noshiro (Dept of Surg, Saga Univ Fac of Med)

Cancer-associated cachexia (CAC) accounts for more than 20% of mortality in cancer patients worldwide. CAC is well known for the loss of skeletal muscle mass and/or fat mass. Adipose tissue in CAC is characterized by adipocyte atrophy, mainly due to impairment of adipogenesis and increased lipolysis. However, the precise mechanism of adipocyte atrophy in patients with CAC is largely unknown. The objective of this study is to evaluate the biological effect of cancer-derived factor on adipogenesis in mouse pre-adipocyte 3T3-L1 cells. We first explored whether culture medium (CM) from human cancer cell lines inhibits adipogenesis in 3T3-L1. Among digestive cancer cell lines, HT29 (colon cancer), HepG2 (HCC) and Hep3B (HCC) strongly inhibited adipogenic differentiation of 3T3L1, by which accumulation of lipid droplets and the expression of adipogenesis-related genes was impaired. Furthermore, drug treatment with Wnt/β-catenin signaling inhibitor IWP-2 restored the adipogenic differentiation in 3T3L1. This study strongly suggested that some Wnt ligand was secreted from HT29, HepG2 and Hep3B cells, and inhibited adipogenesis in 3T3-L1 cells via Wnt/β-catenin signaling pathway.

[Disclosure of COI by Responsible researcher] Lead presenter is not the Responsible researcher.
[Lead presenter: Shota Ikeda] We have no financial relationships to disclose.
[Principal Researcher: Hirokazu Noshiro] We have no financial relationships to disclose.

▲TOP

### [P-2141] I-P10-4
### Metabolism/Metabolome (1)

2025 / 9 / 26 (Fri) 16:45-17:30 Room P/(Motenashi Dome) Room P

---

### P-2141
### Metabolic reprogramming through enhanced cholesterol synthesis in CAFs of CMS4 colorectal cancer.

[Speaker]Iguru Omori (Dept. of Gastroenterological Surg, Osaka Metropolitan Univ.) [Co-author]Hiroaki Kasashima (Dept. of Gastroenterological Surg, Osaka Metropolitan Univ.)、Yukina Kusunoki (Dept. of Pathophysiology, Osaka Metropolitan Univ.)、Yasuhiro Fukui (Dept. of Gastroenterological Surg, Osaka Metropolitan Univ.)、Zizhou Wang (Dept. of Gastroenterological Surg, Osaka Metropolitan Univ.)、Nobuhiro Naito (Dept. of Gastroenterological Surg, Osaka Metropolitan Univ.)、Hideki Tanda (Dept. of Gastroenterological Surg, Osaka Metropolitan Univ.)、Tomoya Tsukida (Dept. of Gastroenterological Surg, Osaka Metropolitan Univ.)、Yuki Seki (Dept. of Gastroenterological Surg, Osaka Metropolitan Univ.)、Kenji Kuroda (Dept. of Gastroenterological Surg, Osaka Metropolitan Univ.)、Yuichiro Miki (Dept. of Gastroenterological Surg, Osaka Metropolitan Univ.)、Mami Yoshii (Dept. of Gastroenterological Surg, Osaka Metropolitan Univ.)、Tatsuro Tamura (Dept. of Gastroenterological Surg, Osaka Metropolitan Univ.)、Masatsune Shibutani (Dept. of Gastroenterological Surg, Osaka Metropolitan Univ.)、Takahiro Toyokawa (Dept. of Gastroenterological Surg, Osaka Metropolitan Univ.)、Kiyoshi Maeda (Dept. of Gastroenterological Surg, Osaka Metropolitan Univ.)

Cholesterol is essential for cancer, particularly through its roles in membrane integrity and intracellular signaling. Dysregulation of cholesterol homeostasis has been implicated in cancer progression. Recent studies have shown that activation of SREBP2, a key transcription factor for cholesterol biosynthesis, contributes to the development of aggressive serrated colorectal tumors characterized by desmoplasia. However, the metabolic state of cancer-associated fibroblasts (CAFs), particularly regarding cholesterol metabolism, remains unclear.We analyzed bulk RNA sequencing data from 10 colorectal cancer samples. Gene set enrichment analysis (GSEA) was performed to compare CAFs from CMS4 tumors with those from other CMS subtypes.Our results revealed that CMS4-CAFs exhibited significant enrichment of gene sets related to cholesterol homeostasis. Furthermore, expression levels of SREBP2 and its downstream targets were markedly upregulated in CMS4-CAFs, suggesting a cholesterol biosynthesis-driven metabolic reprogramming specific to this subtype.These findings highlight a potential metabolic feature of CAFs in CMS4 colorectal cancer that may contribute to tumor aggressiveness.

[Disclosure of COI by Responsible researcher] Lead presenter is not the Responsible researcher.
[Lead presenter: Iguru Omori] We have no financial relationships to disclose.
[Principal Researcher: Kiyoshi Maeda] We have no financial relationships to disclose.

### [P-2142] I-P10-4
### Metabolism/Metabolome (1)

2025 / 9 / 26 (Fri) 16:45-17:30 Room P/(Motenashi Dome) Room P

P-2142
### A novel organelle based nutrient sensing system in cancer cells

[Speaker]Tomoya Matsushita (CHEMBIO) [Co-author]Sumire Nakagawa (CHEMBIO)、Mayu Asao (RCAST)、Sho Aki (CHEMBIO／RCAST)、Tsuyoshi Osawa (CHEMBIO／RCAST)

In solid tumors, the formation of abnormal vasculature leads to the development of a tumor microenvironment characterized by nutrient deprivation, hypoxia, and low pH. Adaptation to these harsh conditions is deeply involved in cancer malignancy. One well-known cellular adaptation mechanism to nutrient deficiency is the nutrient-sensing pathway mediated by the mammalian target of rapamycin (mTOR). We propose that integrating the analysis of nutrient deprivation and organelle remodeling using our unique nutrient-deficient culture system can provide novel insights into nutrient sensing mechanisms.This study aims to elucidate cancer cell-specific nutrient sensing mechanisms driven by organelle remodeling in the tumor microenvironment. The applicant has already identified nutrient-dependent Golgi apparatus remodeling and its associated stress response. Building upon these findings, the following three objectives will be pursued

[Disclosure of COI by Responsible researcher] Lead presenter is not the Responsible researcher.
[Lead presenter: Tomoya Matsushita] We have no financial relationships to disclose.
[Principal Researcher: Tsuyoshi Osawa] We have no financial relationships to disclose.

▲TOP

### [P-2151] I-P10-6
### Metabolism/Metabolome (3)

2025 / 9 / 26 (Fri) 16:45-17:30 Room P/(Motenashi Dome) Room P

---

### P-2151
### Inhibition of PSMD14 by 7MeERT Suppresses Cancer Energy Metabolism via Regulation of PKM2 Ubiquitination

[Speaker]Sonoko Atsumi (Institute of Microbial Chemistry Laboratory of Oncology) [Co-author]Takefumi Onodera (Institute of Microbial Chemistry Numazu Branch)、Masabumi Shibuya (Jobu university)、Manabu Kawada (Institute of Microbial Chemistry Laboratory of Oncology)

PSMD14 (Proteasome 26S Subunit, Non-ATPase 14) is highly expressed in pancreatic and breast cancers, contributing to tumor progression and correlating with poor prognosis. It promotes PKM2 (pyruvate kinase M2) nuclear translocation and glycolytic shift via deubiquitination, playing a central role in cancer-specific metabolism.We analyzed the effect of 7MeERT, a compound initially found to inhibit 3D sphere formation of murine EGFRvIII -transformed cells, on PSMD14 and metabolic regulation in cancer cells.A DARTS assay using pronase resistance suggested binding of 7MeERT to PSMD14. 7MeERT suppressed glycolysis in and growth of pancreatic and, breast cancer, and glioblastoma cells. Similar effects were observed with PSMD14 inhibitors, capzimin and O-phenanthroline. Both 7MeERT and capzimin increased PKM2 ubiquitination and inhibited its nuclear translocation.These findings suggest that 7MeERT targets PSMD14 and suppresses cancer metabolism. This study indicates the therapeutic potential of 7MeERT as a novel PSMD14-targeting agent. (Coauthors) Ho Jeong Kwon, Se In Park

[Disclosure of COI by Responsible researcher] Lead presenter is the Responsible researcher.
[Lead presenter: Sonoko Atsumi] We have no financial relationships to disclose.

### [P-2152] I-P10-6
### Metabolism/Metabolome (3)

2025 / 9 / 26 (Fri) 16:45-17:30 Room P/(Motenashi Dome) Room P

---

### P-2152
### Targeting Carboxylation Pathway in RB1- SUCLA2 -Deficient Advanced Prostate Cancer

[Speaker]Ziheng Yao (Div. Oncol. Mol. Biol., Cancer Res. Inst., Kanazawa Univ) [Co-author]Susumu Kohno (Div. Oncol. Mol. Biol., Cancer Res. Inst., Kanazawa Univ)、Chiaki Takahashi (Div. Oncol. Mol. Biol., Cancer Res. Inst., Kanazawa Univ)

The RB1 tumor suppressor gene is frequently deleted in advanced prostate cancer, causing castration resistance and/or metastasis. We previously demonstrated that the SUCLA2 gene loci are collaterally deleted with RB1, particularly in advanced prostate cancer SUCLA2 deletion in prostate cancer cells aberrates the central carbon metabolism, creating a metabolic vulnerability that could be pharmacologically targeted. In this study, we demonstrated that Thymoquinone (TQ), a compound containing a quinone, selectively kills SUCLA2-deficient prostate cancer cells. Through biochemical approaches, we identified two mitochondrial enzymes that are inhibited by TQ, providing a strategy to exploit the metabolic weaknesses associated with SUCLA2 deletion. Additionally, we redesigned and optimized the chemical structure of TQ to develop novel compounds with improved efficacy and selectivity. Here we provide a new strategy to treat prostate cancer patients in advanced stages using small molecules targeting metabolic vulnerability associated with genetic aberration specific in cancers.

[Disclosure of COI by Responsible researcher] Lead presenter is not the Responsible researcher.
[Lead presenter: Ziheng Yao] We have no financial relationships to disclose.
[Principal Researcher: Susumu Kohno, Chiaki Takahashi] We have no financial relationships to disclose.

### [P-2153] I-P10-6
### Metabolism/Metabolome (3)

2025 / 9 / 26 (Fri) 16:45-17:30 Room P/(Motenashi Dome) Room P

---

### P-2153
### Involvement of ER stress in the suppression of pancreatic cancer cell proliferation via FADS2 inhibition

[Speaker]Emi Nishijima (Grad. Sch. of Humanities & Sci., Ochanomizu Univ.) [Co-author]Daisuke Yamane (Tokyo Metropolitan Inst. of Med. Sci.)、Rieko Ohki (Lab. of Fundamental Oncology, Natl. Cancer Ctr. Res. Inst.)、Makoto Shimizu (Natl. Sci. Div., Faculty of Core Res., Ochanomizu Univ.)、Ikuyo Ichi (Natl. Sci. Div., Faculty of Core Res., Ochanomizu Univ.)

FADS2 is highly expressed in various cancer cells, and the unsaturated fatty acids produced via FADS2 are thought to contribute to cancer cell proliferation by serving as components of biological membranes and bioactive substances. We previously demonstrated that inhibition of FADS2, either through siRNA or a chemical inhibitor, reduced cell viability in the pancreatic cancer cell line Panc1 and the phosphorylation of Akt, a key regulator of cell proliferation. In this study, it was confirmed that phosphorylation of ERK, which is activated in cancer cells, was not affected by FADS2 inhibition. Since lipid unsaturation plays a crucial role in maintaining endoplasmic reticulum (ER) homeostasis, we then examined the CHOP expression, a marker of ER stress. CHOP expression was increased upon FADS2 inhibition. Additionally, the expression of TRB3, a CHOP target gene known to inhibit Akt activation, was also increased. Furthermore, the expression of LC3-II, an autophagy marker downstream of Akt signaling, was also increased. These finding suggest that FADS2 inhibition suppresses Akt signaling via the CHOP-TRB3 pathway, and may promote cell death by inducing autophagy.

[Disclosure of COI by Responsible researcher] Lead presenter is not the Responsible researcher.
[Lead presenter: Emi Nishijima] We have no financial relationships to disclose.
[Principal Researcher: Ikuyo Ichi] We have no financial relationships to disclose.

▲TOP

### [P-2154] I-P10-6
### Metabolism/Metabolome (3)

2025 / 9 / 26 (Fri) 16:45-17:30 Room P/(Motenashi Dome) Room P

---

### P-2154
### Mitochondrial one-carbon metabolic enzyme MTHFD1L is a novel molecular target for breast cancer

[Speaker]Hirokazu Kusunoki (Div. of Cancer Cell Biol. Cancer Res. Inst, Kanazawa Univ.／School of Natural Science and Technology, Kanazawa Univ.) [Co-author]Tsunaki Hongu (Div. of Cancer Cell Biol. Cancer Res. Inst, Kanazawa Univ.)、Tatsunori Nishimura (Div. of Cancer Biol., Nagoya Univ. Grad. Sch. of Med.)、Yasuto Takeuchi (Div. of Cancer Cell Biol. Cancer Res. Inst, Kanazawa Univ.)、Koji Okamoto (Advanced Comprehensive Res. Org. Teikyo Univ.)、Noriko Gotoh (Div. of Cancer Cell Biol. Cancer Res. Inst, Kanazawa Univ.)

The one-carbon metabolism, which is carried out in both the cytoplasm and mitochondria, is an important intracellular metabolic pathway for the synthesis of purines and pyrimidines used in DNA replication. It has been found that mitochondrial one-carbon metabolic enzymes are higher activated in cancer cells than in normal cells. However, the role of these enzymes in the cancer stemness and metastasis remains unclear. Therefore, we analyzed the function of MTHFD1L, one of the mitochondrial one-carbon metabolic enzymes, in breast cancer metastasis and stemness. By suppressing the expression of ***MTHFD1L*** using shRNA, it was suggested that MTHFD1L plays an important role in cancer cell growth and stemness in vitro. Moreover, we generated triple-negative patient-derived breast cancer cells in which ***MTHFD1L*** could be knocked out in a Doxycyclin-inducible manner using the CRISPR/Cas9 system. Knocking out ***MTHFD1L*** after tumor formation in mice significantly suppressed tumor growth in vivo. In a lung metastasis model, lung metastasis was suppressed in ***MTHFD1L*** KO cells. Therefore, MTHFD1L is a promising target in breast cancer for treating primary tumor formation and preventing metastasis.

[Disclosure of COI by Responsible researcher] Lead presenter is not the Responsible researcher.
[Lead presenter: Hirokazu Kusunoki] We have no financial relationships to disclose.
[Principal Researcher: Noriko Gotoh] We have no financial relationships to disclose.

### [P-2155] I-P10-6
### Metabolism/Metabolome (3)

2025 / 9 / 26 (Fri) 16:45-17:30 Room P/(Motenashi Dome) Room P

---

### P-2155
### IDH1 upregulation contributes hepatoma cell survival via glutamine metabolism under the glucose-deprived condition.

[Speaker]Keita Kanki (Dep. Biosci., Okayama Univ. Sci.)

To understand possible role of isocitrate dehydrogenase (IDH1) on cancer cell survival, we investigate hepatoma cell survival and gene expression profile under glucose deprivation (GD). Rapid cell death occurred in HLF cells cultured under GD, while sub-population which survived GD condition were observed. These cells showed upregulation of glutamine metabolism-related genes including IDH1 and SLC1A5. We established IDH1-overexpressing (OE) cells in HLF cells and showed that IDH1-OE cells showed increased viability compared to control cells under GD condition. In spheroid culture using ultra-low attachment plate, IDH1-OE cells yielded greater number of spheroids with larger size than control cells. IDH1-OE cells inoculated into nude mice formed tumor earlier than control cells. Interestingly, HLF cells cultured under glutamine-deprived condition showed enhanced motility and invasive property accompanied with MMP upregulation. Our results suggested that HLF cells basically rely on glucose as metabolic source while IDH1 upregulation confers an ability to survive under the glucose-depleted condition.

[Disclosure of COI by Responsible researcher] Lead presenter is the Responsible researcher.
[Lead presenter: Keita Kanki] We have no financial relationships to disclose.

### [P-2156] I-P10-6
### Metabolism/Metabolome (3)

2025 / 9 / 26 (Fri) 16:45-17:30 Room P/(Motenashi Dome) Room P

P-2156

### Preclinical evaluation of a NAD-targeting therapy for SCLC: potent antitumor activity and tolerable toxicities in mice.

[Speaker]Kyoji Tsurumi (Div. of Cancer Chemother., Miyagi Cancer Center Res. Inst.／Dept. of Biochemical Oncology, Tohoku Univ. Grad. Sch. of Med.／Dept. of Respiratory Medicine., Miyagi Cancer Center Hops.) [Co-author]Miyuki Nomura (Div. of Cancer Chemother., Miyagi Cancer Center Res. Inst.)、Yoji Yamashita (Dept. of Neurosurgery, Miyagi Cancer Center Hosp.)、Mami Morita (Div. of Cancer Chemother., Miyagi Cancer Center Res. Inst.)、Nobuhiro Tanuma (Div. of Cancer Chemother., Miyagi Cancer Center Res. Inst.／Dept. of Biochemical Oncology, Tohoku Univ. Grad. Sch. of Med.)

NAMPT plays a major role in NAD biosynthesis in many cancers, including small cell lung cancer (SCLC). We previously reported that SCLC is significantly vulnerable to NAMPT inhibitor (NAMPTi), particularly when combined with the restriction of dietary niacin in CDX models in mice. This combination, termed NAD-targeting therapy, offers a promising strategy to combat SCLC. Yet, further validation using animal models is warranted to facilitate clinical translation. Here we report that the NAD-targeting therapy is also effective against SCLC PDX models with minimal toxicity in mice. We tested the efficacy of the NAD-targeting therapy against three PDX lines, including two that were chemo-refractory, and found that the therapy promoted tumor regression in all cases. Most of the tumors receiving the therapy exhibited sustained regression even after the final dosing of NAMPTi, suggesting cytotoxic effects. Acute toxicity evaluations identified transient neutropenia as the most significant adverse effect; however, the therapy was well tolerated. These results represent an important stepping stone toward translating NAD-targeting therapy into the clinic.

[Disclosure of COI by Responsible researcher] Lead presenter is not the Responsible researcher.
[Lead presenter: Kyoji Tsurumi] We have no financial relationships to disclose.
[Principal Researcher: Nobuhiro Tanuma] We have no financial relationships to disclose.

### [P-2157] I-P10-6
### Metabolism/Metabolome (3)

2025 / 9 / 26 (Fri) 16:45-17:30 Room P/(Motenashi Dome) Room P

---

### P-2157
### Analysis of G-body formation in quiescent cancer cells using a reversible cell model

[Speaker]Gele Teri (Sch. of Life Sci. and Tech., Inst. of Sci. Tokyo) [Co-author]Kotaro Miyamoto (Sch. of Life Sci. and Tech., Inst. of Sci. Tokyo)、Natsuko Miura (Grad. Sch. of Agriculture, Osaka Metropolitan Univ.)、Tetsuya Kadonosono (Sch. of Life Sci. and Tech., Inst. of Sci. Tokyo)

Quiescent cancer cells (QCCs) are cancer cells that enter a non-dividing state while remaining metabolically active at a minimal level under hypoxic and low-nutrient tumor microenvironments. Since QCCs resume proliferation when surrounding conditions improve, they are considered one of the factors contributing to cancer relapse. In previous study, we identified the non-small cell lung cancer cell line H2228, which can be induced into a reversible quiescent state under hypoxia and serum-starved (HSS) conditions, suppressing glycolysis.In this study, we aimed to analyze the formation of glycolytic granules (G-bodies) in H2228 cells, because G-bodies are known to enhance glycolytic efficiency in cancer cells. By immunofluorescence staining, we found G-bodies were formed in H2228 cells under both hypoxia-only and HSS conditions. However, the number of G-bodies was lower, and their size smaller under HSS compared to hypoxia-only conditions. These results highlight the potential influence of G-body formation on metabolic activity in QCCs. Further investigation will reveal the G-body functions in QCCs and establish their potential as a therapeutic target.

[Disclosure of COI by Responsible researcher] Lead presenter is not the Responsible researcher.
[Lead presenter: Gele Teri] We have no financial relationships to disclose.
[Principal Researcher: Tetsuya Kadonosono] We have no financial relationships to disclose.

### [P-2158] I-P10-6
### Metabolism/Metabolome (3)

2025 / 9 / 26 (Fri) 16:45-17:30 Room P/(Motenashi Dome) Room P

---

### P-2158
### The Role of NNMT in Liver Metabolic Diseases and Its Therapeutic Applications

[Speaker]Xi Chen (Division of Molecular Genetics, Cancer Research Institute, Kanazawa University) [Co-author]Hiroki Sugiyama (Department of Pharmacy Yamagata University Hospital)、Yuhang Yan (Division of Molecular Genetics, Cancer Research Institute, Kanazawa University)、Masahiko Kobayashi (Division of Molecular Genetics, Cancer Research Institute, Kanazawa University)、Hiroshi Arakawa (Pharmaceutical Sciences, Medical Pharmaceutical and Health Sciences, Kanazawa University)、Tomoyoshi Soga (Institute for Advanced Biosciences, Keio University)、Keisuke Yaku (Molecular and Medical Pharmacology, Faculty of Medicine, University of Toyama)、Takashi Nakagawa (Molecular and Medical Pharmacology, Faculty of Medicine, University of Toyama)、Shinpei Kawaoka (Bioinformatics, Institute of Development Aging and Cancer, Tohoku University)、Atsushi Hirao (Division of Molecular Genetics, Cancer Research Institute, Kanazawa University／WPI Nano Life Science Institute, Kanazawa University)

Nicotinamide adenine dinucleotide (NAD+) is a key coenzyme involved in cellular metabolism, energy homeostasis, and redox balance. Its depletion has been linked to various pathological conditions, particularly liver-related diseases. In this study, we examined NAD+ levels across different disease models, including cancer cachexia, liver fibrosis, and alcoholic fatty liver disease. We observed consistent abnormalities in nicotinamide metabolism, particularly a persistent reduction in NAD+ levels, suggesting its involvement in disease progression. To counteract NAD+ depletion, we supplemented with nicotinamide (NAM), which significantly improved disease phenotypes in multiple models, indicating its therapeutic potential. Additionally, we identified a critical role for nicotinamide N-methyltransferase (NNMT) in these disease models. We are currently investigating how NNMT influences NAD+ levels in these diseases, which may lead to new therapeutic strategies.

[Disclosure of COI by Responsible researcher] Lead presenter is not the Responsible researcher.
[Lead presenter: Xi Chen] We have no financial relationships to disclose.
[Principal Researcher: Atsushi Hirao] We have no financial relationships to disclose.

### [P-2159] I-P10-6
### Metabolism/Metabolome (3)

2025 / 9 / 26 (Fri) 16:45-17:30 Room P/(Motenashi Dome) Room P

---

### P-2159
### Single-cell multiomics technology for the comprehensive analysis of transcriptomics and cancer driver mutation

[Speaker]Tadashi Imafuku (Dept. Mol. Pathophysiol, Wakayama Med. Univ.) [Co-author]Kyohei Matsumoto (Dept. Mol. Pathophysiol, Wakayama Med. Univ.／2nd. Dept. Surg, Wakayama Med. Univ.)、Shigeyuki Shichino (Div. Regulation of Inflammation, Inst. Biomed. Sci, Tokyo Univ. Sci.)、Manabu Kawai (2nd. Dept. Surg, Wakayama Med. Univ.)、Shinichi Hashimoto (Dept. Mol. Pathophysiol, Wakayama Med. Univ.)

Somatic mutations are a primary cause of cancer and have been investigated using bulk DNA-seq. Recent advancements in single-cell genome mutation analysis have revealed the presence of cancer cells with diverse mutation patterns in the tumor microenvironment. However, the relationship between these diverse mutation patterns and their contributions to cancer pathogenesis remains unclear. In this study, we have developed a micro-well device capable of single-cell genomic mutation analysis by the microbeads incorporating oligonucleotide sequences from a cancer panel targeting driver mutations. These microbeads also enable concurrent analysis of gene expression, thus facilitating comprehensive single-cell multiomics analysis. We aimed to establish a protocol for single-cell multiomics analysis using pancreatic cancer cell lines. As a result, we succeeded in classifying cancer cells by different gene mutation patterns and analyzing their unique gene expression. In addition, this protocol was also available for clinical cancer tissue samples. Our technology contributes to the understanding of the role of cancer mutations and the identification of the novel therapeutic target.

[Disclosure of COI by Responsible researcher] Lead presenter is not the Responsible researcher.
[Lead presenter: Tadashi Imafuku] We have no financial relationships to disclose.
[Principal Researcher: Shinichi Hashimoto] We have no financial relationships to disclose.

### [P-2160] I-P10-6
### Metabolism/Metabolome (3)

2025 / 9 / 26 (Fri) 16:45-17:30 Room P/(Motenashi Dome) Room P

### P-2160
### Unveiling a Novel Role of Mesothelial Glycocalyx in Peritoneal Metastasis of Ovarian Cancer

[Speaker]Kazumasa Mogi (Nagoya University, Department of Obstetrics and Gynecology／Nagoya University Hospital, Department of Medical Genomics Center) [Co-author]Marina Yoshikawa (Nagoya University, Department of Obstetrics and Gynecology)、Atsushi Kunishima (Nagoya University, Department of Obstetrics and Gynecology)、Emiri Miyamoto (Nagoya University, Department of Obstetrics and Gynecology)、Yoshihiro Koya (Nagoya University, Department of Obstetrics and Gynecology)、Yoshihiko Yamakita (Nagoya University, Department of Obstetrics and Gynecology)、Hiroki Fujimoto (Aichi Medical University, Department of Obstetrics and Gynecology)、Shohei Iyoshi (Nagoya University, Department of Obstetrics and Gynecology)、Kosuke Yoshida (Nagoya University, Department of Obstetrics and Gynecology)、Masato Yoshihara (Nagoya University, Department of Obstetrics and Gynecology)、Yukari Nagao (Nagoya University, Department of Obstetrics and Gynecology)、Satoshi Tamauchi (Nagoya University, Department of Obstetrics and Gynecology)、Akira Yokoi (Nagoya University, Department of Obstetrics and Gynecology)、Nobuhisa Yoshikawa (Nagoya University, Department of Obstetrics and Gynecology)、Kaoru Niimi (Nagoya University, Department of Obstetrics and Gynecology)、Hiroaki Kajiyama (Nagoya University, Department of Obstetrics and Gynecology)

Objective: The peritoneal surface is covered with a glycocalyx of mesothelial cells. The glycocalyx plays a critical role in cell adhesion and signaling, but its involvement in ovarian cancer metastasis remains unclear. This study aims to investigate the function of the mesothelial glycocalyx in peritoneal dissemination. Methods: We used electron microscopy and lectin staining to analyze the structure of the mesothelial glycocalyx. Transcriptome analysis was performed in normal and TGF-$\beta$ induced cancer-associated mesothelial cells (CAM). Results: Electron microscopy and lectin staining revealed slight differences in the mesothelial glycocalyx from different organs, in particular, surface sialylation, the key element in reducing peritoneal dissemination, was much higher in the parietal peritoneum than in the omentum (intensity 1.62 vs. 0.55). Transcriptome analysis showed that CAM was downregulated in syndecan1-4, a core protein of the glycocalyx, compared to normal mesothelial cells. Conclusion: In the ovarian cancer microenvironment, the mesothelial glycocalyx may play a critical role and be a potential therapeutic target.

[Disclosure of COI by Responsible researcher] Lead presenter is the Responsible researcher.
[Lead presenter: Kazumasa Mogi] We have no financial relationships to disclose.

[P-2169] I-P11-9
Tumor immune microenvironment/Tumor immune escape (5)

2025 / 9 / 26 (Fri) 16:45-17:30 Room P/(Motenashi Dome) Room P

### P-2169
### Immunophenotypes of tumor microenvironment (TME) as prognostic factor for head and neck squamous cell carcinoma (HNSCC)

[Speaker]Rui Sano (Aichi Med. Univ. Dept. of Otorhinolaryngology, Head and Neck Surg.／Chubu Rosai Hosp. Dept. of Otorhinolaryngology)
[Co-author]Susumu Suzuki (Aichi Med. Univ. Research Creation Support Ctr.)、Daisuke Inukai (Aichi Med. Univ. Dept. of Otorhinolaryngology, Head and Neck Surg.)、Hiroki Okamoto (Aichi Med. Univ. Dept. of Otorhinolaryngology, Head and Neck Surg.)、Shunpei Yamanaka (Aichi Med. Univ. Dept. of Otorhinolaryngology, Head and Neck Surg.)、Taishi Takahara (Aichi Med. Univ. Dept. of Surgical Path.)、Akira Satou (Aichi Med. Univ. Dept. of Surgical Path.)、Kazuhiro Yoshikawa (Aichi Med. Univ. Research Creation Support Ctr.)、Yasushi Fujimoto (Aichi Med. Univ. Dept. of Otorhinolaryngology, Head and Neck Surg.)、Toyonori Tsuzuki (Aichi Med. Univ. Dept. of Surgical Path.)、Ryuzo Ueda (Nagoya Univ. Graduate Sch. of Med. Dept. of Immunol.)、Tetsuya Ogawa (Aichi Med. Univ. Dept. of Otorhinolaryngology, Head and Neck Surg.)

TME immunophenotype is known to relate with prognosis for malignancies. Our purpose is to clarify relationship of clinical factors and immunophenotypes in HNSCC.We examined 82 cases of HNSCC with primary resection. Immunophenotypes was evaluated by tumor immune microenvironment classification with Hematoxylin-Eosin staining (HE) and CD8 staining (CD8S); "Immune-Desert (I-D)" in which very few lymphocytes infiltrated into cancer tissues, "Immune-Excluded (I-E)" in which lymphocytes infiltrated into stromal areas but not into the nest, and "Immune-Inflamed (I-I)" in which lymphocytes infiltrated into the nest and stroma.18(22%), 57(69.5%), and 7(8.5%) in total and 10 (52.6%), 8(42.1%), and 1(5.3%) in cases previously treated with radiotherapy were classified for I-D, I-E, and I-I respectively with HE. Previous radiotherapy was associated to I-D ($p<0.05$) in HE, but not in CD8S. The group of I-I and I-E showed better 5-year disease free survivals (HE, 43.4%, $p<0.05$; CD8S, 42.7%, $p<0.05$) than I-D (16.7%, 24.3%).Radiotherapy might affect reducing lymphocytes other than CD8+ T cells in cancer lesions. Lymphocyte infiltration in tumor site predicts preferable survival in HNSCC.

[Disclosure of COI by Responsible researcher] Lead presenter is the Responsible researcher.
[Lead presenter: Rui Sano] We have no financial relationships to disclose.

▲TOP

### [P-2170] I-P11-9
### Tumor immune microenvironment/Tumor immune escape (5)

2025 / 9 / 26 (Fri) 16:45-17:30 Room P/(Motenashi Dome) Room P

---

### P-2170
### Epigenetic modifications of low blood miR-5193 as an immunotherapy target to PD-L1 in gastric and esophageal cancer

[Speaker]Ryo Ishida (Department of Digestive Surgery, Kyoto Prefectural University of Medicine) [Co-author]Shuhei Komatsu (Department of Digestive Surgery, Kyoto Prefectural University of Medicine)、Hajime Kamiya (Department of Digestive Surgery, Kyoto Prefectural University of Medicine)、Hiroshi Arakawa (Department of Digestive Surgery, Kyoto Prefectural University of Medicine)、Masateru Yamauchi (Department of Digestive Surgery, Kyoto Prefectural University of Medicine)、Satoshi Hamada (Department of Digestive Surgery, Kyoto Prefectural University of Medicine)、Keiji Nishibeppu (Department of Digestive Surgery, Kyoto Prefectural University of Medicine)、Jun Kiuchi (Department of Digestive Surgery, Kyoto Prefectural University of Medicine)、Taisuke Imamura (Department of Digestive Surgery, Kyoto Prefectural University of Medicine)、Hirotaka Konishi (Department of Digestive Surgery, Kyoto Prefectural University of Medicine)、Hitoshi Fujiwara (Department of Digestive Surgery, Kyoto Prefectural University of Medicine)、Atsushi Shiozaki (Department of Digestive Surgery, Kyoto Prefectural University of Medicine)

BACKGROUND: Epigenetic regulation of microRNAs (miRNAs) is involved in cancer immune escape. Our studies showed that low levels of tumor-suppressive miRNAs in blood are linked to tumor progression and poor prognosis. We identified a PD-L1-targeting miRNA significantly downregulated in gastric carcinoma (GC) and esophageal squamous cell carcinoma (ESCC), and evaluated its clinical relevance. METHODS: Candidate miRNAs putatively targeting PD-L1 were screened via NCBI and miRNA databases. RESULTS: Plasma miR-5193 levels were significantly lower in GC/ESCC patients than in healthy controls. Low levels were associated with advanced tumor depth, pathological stage, and poor prognosis. miR-5193 overexpression suppressed PD-L1 on tumor cells even after IFN-$\gamma$ stimulation. In co-culture with anti-CD3/CD28-activated T cells, miR-5193 enhanced T-cell anti-tumor activity by downregulating PD-L1. CONCLUSIONS: Low circulating miR-5193 is linked to GC/ESCC progression and immune evasion, and may be a target for nucleic acid-based immunotherapy.

[Disclosure of COI by Responsible researcher] Lead presenter is not the Responsible researcher.
[Lead presenter: Ryo Ishida] We have no financial relationships to disclose.
[Principal Researcher: Shuhei Komatsu] We have no financial relationships to disclose.

### [P-2171] I-P11-9
### Tumor immune microenvironment/Tumor immune escape (5)

2025 / 9 / 26 (Fri) 16:45-17:30 Room P/(Motenashi Dome) Room P

---

### P-2171
### LncRNA ***NEAT1v1*** confers resistance to immune checkpoint inhibitor therapy via CD24

[Speaker]Hiroyuki Tsuchiya (Div. Regen. Med. & Ther., Fac. Med., Tottori Univ.)

**Aims:** LincRNA ***NEAT1v1*** has been shown to upregulate the expression of CD24, the immune checkpoint molecule against macrophages, via the miR-320a-SP1 axis. In the present study, the effect of ***NEAT1v1*** on tumor immunity, especially on tumor-associated macrophages (TAMs), was investigated.
**Methods:** Cellular interaction was studied using heterospheroids of hepatocellular carcinoma (HCC) cells and human primary macrophages. Tumor immunity was assessed in a syngeneic HCC model.
**Results:** ***NEAT1v1*** expression was significantly upregulated in tumor tissue and plasma of patients with HCC. ***NEAT1v1*** in HCC cells suppressed the expression of M1 markers (CD80 and HLA-DR) in macrophages in heterospheroids while upregulating M2 markers (CD163 and CD206). ***NEAT1v1*** overexpression promoted tumor growth and increased M2-like TAMs ***in vivo***. In addition, ***NEAT1v1*** conferred resistance to anti-Pd-1 and anti-Cd24 antibodies; however, their combination treatment significantly suppressed tumor growth.
**Conclusions:** These findings suggest that ***NEAT1v1*** is responsible for immune checkpoint inhibitor resistance in HCC and underscore the efficacy of CD24 blockade in combination with current immune checkpoint inhibitors.

[Disclosure of COI by Responsible researcher] Lead presenter is the Responsible researcher.
[Lead presenter: Hiroyuki Tsuchiya] We have no financial relationships to disclose.

▲TOP

### [P-2172] I-P11-9
### Tumor immune microenvironment/Tumor immune escape (5)

2025 / 9 / 26 (Fri) 16:45-17:30 Room P/(Motenashi Dome) Room P

### P-2172
### T cell Repertoire of CML Patients Who Attempt Discontinuation of TKIs; the ISAC-TFR study

[Speaker]Mai Fujita (Saga Universiry Hematology and Oncology) [Co-author]Hiroshi Ureshino (Saga Universiry Hematology and Oncology／Saga Universiry Drug Discovery and Biomedical Sciences)、Shinya Kimura (Saga Universiry Hematology and Oncology／Saga University Drug Discovery and Biomedical Sciences)

While tyrosine kinase inhibitor (TKI) discontinuation is an established therapeutic goal. Immune surveillance, particularly T cell and natural killer (NK) cell responses, may influence treatment-free remission (TFR), although no definitive biomarkers for predicting sustained TFR have been identified. This retrospective study included 57 chronic myeloid leukemia (CML) patients who attempted TKI discontinuation. Clinical outcomes after TFR attempts were analyzed, and T cell receptor (TCR) and B cell receptor (BCR) repertoire analyses were conducted on peripheral blood samples from 14 patients to investigate the immune landscape associated with TFR. TFR1 at 1 year was 67.9% (95% confidence interval [CI], 53.9 - 78.4%). Sixteen patients attempted a second discontinuation, achieving a 1-year TFR2 rate of 31.2% (95% CI, 11.4 - 53.6%). Patients maintaining BCR::ABL1 mRNA levels below MR4.5 at 3 months post-TKI discontinuation had a significantly lower risk of relapse (HR, 0.099; 95% CI, 0.012 - 0.829; p = 0.033). TCR repertoire analysis did not identify CML-specific T cell clones, but a significant age-related decline in T cell diversity was observed.

[Disclosure of COI by Responsible researcher] Lead presenter is not the Responsible researcher.
[Lead presenter: Mai Fujita] We have no financial relationships to disclose.
[Principal Researcher: Hiroshi Ureshino] ■Lecture fee: ファイザー、大塚製薬、ノバルティス、ブリストルマイヤーズ　■Research expenses: ファイザー、ブリストルマイヤーズ、大原薬品工業

### [P-2173] I-P11-9
### Tumor immune microenvironment/Tumor immune escape (5)

2025 / 9 / 26 (Fri) 16:45-17:30 Room P/(Motenashi Dome) Room P

---

### P-2173
### The immunosuppressive role of $CCR8^+$ Tregs in gastric cancer

[Speaker]Koichi Jinushi (Department of Gastroenterological Surgery, Graduate School of Medicine, Osaka University) [Co-author]Takuro Saito (Department of Gastroenterological Surgery, Graduate School of Medicine, Osaka University／Department of Clinical Research in Tumor Immunology, Osaka University)、Azumi Ueyama (Pharmaceutical Research Division, Shionogi & Co., Ltd.)、Koichi Morishita (Pharmaceutical Research Division, Shionogi & Co., Ltd.)、Yukinori Kurokawa (Department of Gastroenterological Surgery, Graduate School of Medicine, Osaka University)、Tsuyoshi Takahashi (Department of Gastroenterological Surgery, Graduate School of Medicine, Osaka University)、Takaomi Hagi (Department of Gastroenterological Surgery, Graduate School of Medicine, Osaka University)、Kota Momose (Department of Gastroenterological Surgery, Graduate School of Medicine, Osaka University)、Kotaro Yamashita (Department of Gastroenterological Surgery, Graduate School of Medicine, Osaka University)、Koji Tanaka (Department of Gastroenterological Surgery, Graduate School of Medicine, Osaka University)、Tomoki Makino (Department of Gastroenterological Surgery, Graduate School of Medicine, Osaka University)、Hidetoshi Eguchi (Department of Gastroenterological Surgery, Graduate School of Medicine, Osaka University)、Yuichiro Doki (Department of Gastroenterological Surgery, Graduate School of Medicine, Osaka University)

Background: Regulatory T cells (Tregs) suppress antitumor immunity, and CCR8 has been identified as a marker for activated tumor-infiltrating Tregs. However, the role of $CCR8^+$ Tregs in gastric cancer remains unclear. Methods: Eighty patients with gastric cancer who underwent curative surgical resection at our hospital in 2016 were included. Double immunohistochemistry was performed for CCR8 and Foxp3, as well as granzyme B and CD8. Positive cell counts and spatial distribution were assessed using the HALO system. We examined the correlation between the infiltration of $CCR8^+$ Tregs and $CD8^+$ T cells and patient prognosis. Results: The density of $CCR8^+$ Treg correlated with that of $CD8^+$ T cells, but a low $CD8^+$ T cell to $CCR8^+$ Treg ratio was associated with shorter recurrence-free survival. In $CCR8^+$ Treg hotspots, $CD8^+$ T cells exhibited reduced granzyme B expression. Tregs were enriched at the tumor invasion front, and fewer $CD8^+$ T cells were less frequently located near $CCR8^+$ Tregs than near $CCR8^-$ Tregs. Conclusions: $CCR8^+$ Tregs may attenuate local antitumor immunity by excluding $CD8^+$ T cells. Targeting $CCR8^+$ Tregs could improve the efficacy of immunotherapy in gastric cancer.

[Disclosure of COI by Responsible researcher] Lead presenter is not the Responsible researcher.
[Lead presenter: Koichi Jinushi] ■Endowed chair: 臨床腫瘍免疫学教室の所属
[Principal Researcher: Azumi Ueyama] ■employment by a company related to the presentation: 塩野義製薬株式会社　■Endowed chair: 臨床腫瘍免疫学教室の所属

▲TOP

[P-2174] I-P11-9
Tumor immune microenvironment/Tumor immune escape (5)

2025 / 9 / 26 (Fri) 16:45-17:30 Room P/(Motenashi Dome) Room P

### P-2174
### Analysis of tumor-infiltrating lymphocytes and their clinical significance in ovarian clear cell carcinoma

[Speaker]Tomomi Taguchi (Dept. of Clin. Res. in Tumor Immunol, Osaka Univ.／Dept. of OB-GYN, Osaka Univ.) [Co-author]Azumi Ueyama (Dept. of Clin. Res. in Tumor Immunol, Osaka Univ.／Biopharmaceutical Res. Div, Shionogi & Co. Ltd.)、Tatsuo Masuda (Dept. of OB-GYN, Osaka Univ.)、Mamoru Kakuda (Dept. of OB-GYN, Osaka Univ.)、Satoshi Nakagawa (Dept. of OB-GYN, Osaka Univ.)、Kosuke Hiramatsu (Dept. of OB-GYN, Osaka Univ.)、Tadashi Iwamiya (Dept. of OB-GYN, Osaka Univ.)、Shinya Matsuzaki (Dept. of OB-GYN, Osaka Univ.)、Takuro Saito (Dept. of Clin. Res. in Tumor Immunol, Osaka Univ.)、Hisashi Wada (Dept. of Clin. Res. in Tumor Immunol, Osaka Univ.)、Yutaka Ueda (Dept. of OB-GYN, Osaka Univ.)、Michiko Kodama (Dept. of OB-GYN, Osaka Univ.)

Background: Ovarian clear cell carcinoma (OCCC) is prevalent in Asian women and has poorer prognosis than High grade serous carcinoma (HGSC) in advanced cases. This study aimed to clarify the immune phenotype and clinical relevance of tumor-infiltrating lymphocytes (TILs) in OCCC. Methods: TILs from 64 patients (HGSC: 27, OCCC: 37) undergoing primary surgery at Osaka University Hospital (2016–2024) were analyzed by flow cytometry. RNA-seq data from 119 OCCC cases (Clin Cancer Res, 2022) were evaluated for associations between T cell exhaustion markers and clinical outcomes. Results: Two immune clusters were identified: "Activated" (high Treg and T cell exhaustion markers) and "Inactivated". HGSC was enriched in the Activated cluster, and OCCC in the Inactivated cluster ($p<0.01$). In OCCC, advanced or recurrent cases showed higher Tim-3 expression on CD8+ T cell and reduced IL-2 production ($p<0.05$). RNA-seq analysis confirmed that high Tim-3/CD8 expression correlated with worse progression free survival ($p<0.05$). Conclusion: OCCC shows lower immune activation than HGSC. In advanced OCCC, T cell exhaustion may contribute to disease progression via immune evasion.

[Disclosure of COI by Responsible researcher] Lead presenter is not the Responsible researcher.
[Lead presenter: Tomomi Taguchi] ■Endowed chair: 大阪大学医学系研究科　臨床腫瘍免疫学
[Principal Researcher: Yutaka Ueda] We have no financial relationships to disclose.

▲TOP

Poster Session

### [P-2175] I-P11-9
### Tumor immune microenvironment/Tumor immune escape (5)

2025 / 9 / 26 (Fri) 16:45-17:30 Room P/(Motenashi Dome) Room P

---

### P-2175
### Role of ANGPTL2 in multicellular network-mediated regulation of anti-tumor immunity in the tumor microenvironment

[Speaker]Haruki Horiguchi (Grad. Sch. of Med. Sci., Kumamoto Univ.) [Co-author]Yuichi Oike (Grad. Sch. of Med. Sci., Kumamoto Univ.)

The tumor microenvironment is composed not only of cancer cells but also of various stromal cell types, including cancer-associated fibroblasts, immune cells, and blood vessels. Notably, recent updates to the cancer immunity cycle have incorporated intercellular interactions within tertiary lymphoid structures (TLS). Previously, we identified a family of secreted proteins structurally similar to angiopoietins and designated them angiopoietin-like proteins (ANGPTLs). Among the eight ANGPTL family members, ANGPTL2 plays a role in maintaining tissue homeostasis (EMBO J [2017]). In cancer pathology, ANGPTL2 exhibits a dual role in tumor promotion (Cancer Sci [2014, 2024]; Oncogene [2022], etc.) and tumor suppression (Genes Dev [2019]; Cancer Gene Ther [2024], etc.), depending on the expressing and target cells (Reviewed in Cancer Sci [2025]). In this presentation, we will discuss these findings along with our latest insights into the tumor immune regulatory mechanisms controlled by multicellular networks in the tumor microenvironment, with a particular focus on the potential involvement of ANGPTL2 in TLS formation and function.

[Disclosure of COI by Responsible researcher] Lead presenter is the Responsible researcher.
[Lead presenter: Haruki Horiguchi] We have no financial relationships to disclose.

### [P-2176] I-P11-9
### Tumor immune microenvironment/Tumor immune escape (5)

2025 / 9 / 26 (Fri) 16:45-17:30 Room P/(Motenashi Dome) Room P

---

### P-2176
### Blockade of SIRPα enhances macrophage-dependent antitumor activity against head and neck squamous cell carcinoma

[Speaker]Takashi Ueda (Div. Mol. Cell. Signaling, Kobe Univ. Grad. Schol. Med.／Dept. Otolaryngol. Head Neck Surg., Kobe Univ. Grad. Schol. Med.／Div. Biosignal. Reg., Kobe Univ. Grad. Schol. Med.) [Co-author]Yoji Murata (Div. Mol. Cell. Signaling, Kobe Univ. Grad. Schol. Med.)、Daisuke Tanaka (Div. Biosignal. Reg., Kobe Univ. Grad. Schol. Med.)、Yasuyuki Saito (Dept. Immunology, Shimane Univ. Grad. Schol. Med.)、Takenori Kotani (Div. Mol. Cell. Signaling, Kobe Univ. Grad. Schol. Med.)、Kenichi Nibu (Dept. Otolaryngol. Head Neck Surg., Kobe Univ. Grad. Schol. Med.)、Takashi Matozaki (Div. Biosignal. Reg., Kobe Univ. Grad. Schol. Med.)

The transmembrane protein SIRPα and its ligand CD47 acts as an innate immune checkpoint that prevents macrophage (MΦ)-mediated tumor elimination. The interaction between SIRPα on MΦs and CD47 on cancer cells inhibits the phagocytic activity of MΦs. We have demonstrated that blockade of the CD47-SIRPα interaction using an anti-SIRPα antibody (Ab) enhances MΦ-mediated phagocytosis of cancer cells opsonized with tumor-targeting Abs including rituximab. This antibody also potentiated the anti-tumor effects of tumor-targeting antibodies in murine cancer models. Here we show that SIRPα blockade with an anti-SIRPα Ab inhibits tumor growth in murine models of head and neck squamous cell carcinoma (HNSCC). Tumor growth was also attenuated in Sirpα-deficient mice compared with wild-type controls. Moreover, the anti-SIRPα Ab enhanced both the phagocytic and cytotoxic activities of MΦs against HNSCC cells. Similarly, genetic ablation of SIRPα in MΦs or CD47 in tumor cells also augmented these activities of MΦs. Collectively, these findings suggest that SIRPα blockade promotes MΦ-mediated killing of HNSCC cells and may represent a promising immunotherapeutic approach for HNSCC.

[Disclosure of COI by Responsible researcher] Lead presenter is not the Responsible researcher.
[Lead presenter: Takashi Ueda] We have no financial relationships to disclose.
[Principal Researcher: Takashi Matozaki] ■Research expenses: JCRファーマ株式会社

▲TOP

### [P-2186] I-P11-11
### Immune checkpoint inhibitors (1)

2025 / 9 / 26 (Fri) 16:45-17:30 Room P/(Motenashi Dome) Room P

---

### P-2186
### Identification of predictive peripheral blood biomarkers for NSCLC patient treated with anti-PD-1 antibody using PEA

[Speaker]Shigeki Ohta (Dept. Immunology, IUHW Sch Med) [Co-author]Atsushi Ikemoto (JSR Corp, JKiC)、Seiki Wakui (JSR Corp, JKiC)、Tetsuya(JSR) Takimoto (JSR Corp, JKiC)、Ryosuke Satomi (Dept. Respiratory Med, Tokyo Med Ctr, Natl Hosp Org)、Shinnosuke Ikemura (Dept. Respiratory Med, Yamanashi Univ Sch Med)、Kenzo Soejima (Dept. Respiratory Med, Yamanashi Univ Sch Med)、Yutaka Kawakami (Dept. Immunology, IUHW Sch Med)

The efficacy of immune checkpoint inhibitors (ICIs) in NSCLC (Non-small cell lung cancer) patients is still limited. Therefore, it is important to develop diagnostic methods to predict ICI responders at baseline or early in ICI treatment. We analysed 2925 proteins in plasma from 10 healthy donors and NSCLC patients (PR10, PD10) at baseline and after the first administration of anti-PD-1 antibody (5 paired samples) using PEA (Proximity Extension Assay). Significantly higher plasma CXCL10 which is a chemokine produced by various immune cells that attracts activated T cells to tumor tissue, was observed in PR patients than in PD patients at baseline (***P***=0.02), and some proteins were upregulated after anti-PD-1 antibody administration in PR patients. We also performed bulk RNA sequencing of PBMC from ICI-treated NSCLC patients (n=31), and found a similar trend of higher CXCL10 expression in PBMC from PR patients with favourable PFS at baseline (***P***=0.01). These results suggest that PEA is a useful method for comprehensive analysis to identify plasma protein biomarkers, and that plasma CXCL10 may be an attractive biomarker for ICI-treated NSCLC patients.

[Disclosure of COI by Responsible researcher] Lead presenter is not the Responsible researcher.
[Lead presenter: Shigeki Ohta] We have no financial relationships to disclose.
[Principal Researcher: Yutaka Kawakami] ■Contributions: カルナバイオサイエンス

### [P-2187] I-P11-11
### Immune checkpoint inhibitors (1)

2025 / 9 / 26 (Fri) 16:45-17:30 Room P/(Motenashi Dome) Room P

---

### P-2187
### Exploratory study for the immunomodulatory effects of Juzentaihoto in preclinical cancer models

[Speaker]Kanata Yamaguchi (Inst. of Nat. Med., Univ. of Toyama) [Co-author]Takeshi Susukida (Inst. of Nat. Med., Univ. of Toyama)、Soichiro Sasaki (Inst. of Nat. Med., Univ. of Toyama)、Yoshihiro Hayakawa (Inst. of Nat. Med., Univ. of Toyama)

Juzentaihoto (JTT) is a Kampo medicine widely used in clinics as part of supportive care for cancer patients. We have previously reported that JTT potentiates the anti-tumor effect of anti-PD-1 (aPD-1) therapy and modulates the gut microbiota composition in the B16-OVA model. However, the pharmacological mechanism of action by which Juzentaihoto modulates anti-tumor immune responses remains unexplored. In the 4T1 breast cancer model, the combination of aPD-1 and JTT significantly inhibited tumor growth compared to aPD-1 monotherapy and showed a trend to inhibit the expansion of Tregs in tumor-draining lymph nodes. Considering JTT is formulated as a combination of Shimotsuto and Shikunshito, we have been testing which component of JTT is responsible for its immunomodulatory effect. Additionally, we further analyzed the involvement of gut microbiome in the immunomodulatory effect of JTT using mice treated with the antibiotics. By updating these results, we will discuss the pharmacological mechanisms of JTT to exert its immunomodulatory effects.

[Disclosure of COI by Responsible researcher] Lead presenter is not the Responsible researcher.
[Lead presenter: Kanata Yamaguchi] We have no financial relationships to disclose.
[Principal Researcher: Yoshihiro Hayakawa] ■Research expenses: 株式会社ツムラ

### [P-2188] I-P11-11
### Immune checkpoint inhibitors (1)

2025 / 9 / 26 (Fri) 16:45-17:30 Room P/(Motenashi Dome) Room P

---

### P-2188
### Investigation of predictive biomarkers for immune checkpoint inhibitor efficacy in esophageal squamous cell carcinoma

[Speaker]Kosuke Kanemitsu (Department of Cell Pathology, Kumamoto University) [Co-author]Yoshiyuki Tagayasu (Department of Cell Pathology, Kumamoto University／Department of Gastroenterological Surgery, Kumamoto University)、Cheng Pan (Department of Cell Pathology, Kumamoto University)、Hirotake Tsukamoto (Division of Clinical Immunology and Cancer Immunotherapy, Kyoto University)、Hiromu Yano (Department of Cell Pathology, Kumamoto University)、Daiki Yoshii (Department of Cell Pathology, Kumamoto University)、Takuya Shiota (Department of Diagnostic Pathology, Kumamoto University Hospital)、Yukio Fujiwara (Department of Cell Pathology, Kumamoto University)、Masaaki Iwatsuki (Department of Gastroenterological Surgery, Kumamoto University)、Yoshihiro Komohara (Department of Cell Pathology, Kumamoto University／ Center for Metabolic Regulation of Healthy Aging, Kumamoto University)

As immunotherapy has become the first-line treatment for unresectable advanced or recurrent esophageal squamous cell carcinoma (ESCC), identifying predictive markers of therapeutic response is of great clinical significance. We performed immunohistochemical analysis using resected specimens from patients who received immunotherapy for recurrent ESCC. In the responder group, higher expression of HLA class II in tumor cells and PD-L1 in macrophages was observed, suggesting their potential as predictive markers of treatment efficacy. Furthermore, while aging of lymph nodes has been reported to reduce immune competence due to a decrease in CD169-positive macrophages, a higher proportion of senescent lymph nodes was found in progressive disease (PD) cases. These findings suggest that HLA class II expression in tumor cells, PD-L1 expression in macrophages, and the proportion of senescent lymph nodes may serve as potential predictive markers for the efficacy of immunotherapy.

[Disclosure of COI by Responsible researcher] Lead presenter is not the Responsible researcher.
[Lead presenter: Kanemitsu Kosuke] We have no financial relationships to disclose.
[Principal Researcher: Yoshihiro Komohara] We have no financial relationships to disclose.

▲TOP

### [P-2189] I-P11-11
### Immune checkpoint inhibitors (1)

2025 / 9 / 26 (Fri) 16:45-17:30 Room P/(Motenashi Dome) Room P

---

### P-2189
### Tissue-resident memory T cell infiltration predicts response to anti-PD-L1 therapy in extrahepatic cholangiocarcinoma

[Speaker]Yoshiyuki Tagayasu (Department of Cell Pathology, Graduate School of Medicine, Kumamoto University／Department of Gastroenterological Surgery, Graduate School of Medicine, Kumamoto University) [Co-author]Rin Yamada (Department of Cell Pathology, Graduate School of Medicine, Kumamoto University)、Kosuke Kanemitsu (Department of Cell Pathology, Graduate School of Medicine, Kumamoto University／Department of Gastroenterological Surgery, Graduate School of Medicine, Kumamoto University)、Yoshihiko Kondo (Department of Cell Pathology, Graduate School of Medicine, Kumamoto University)、Yukio Fujiwara (Department of Cell Pathology, Graduate School of Medicine, Kumamoto University)、Takumi Tanizaki (Department of Gastroenterological Surgery, Graduate School of Medicine, Kumamoto University)、Rumi Itoyama (Department of Gastroenterological Surgery, Graduate School of Medicine, Kumamoto University)、Yuki Kitano (Department of Gastroenterological Surgery, Graduate School of Medicine, Kumamoto University)、Masaaki Iwatsuki (Department of Gastroenterological Surgery, Graduate School of Medicine, Kumamoto University)、Yoshihiro Komohara (Department of Cell Pathology, Graduate School of Medicine, Kumamoto University)

[Background]Durvalumab plus gemcitabine/cisplatin (GC) was approved for unresectable extrahepatic cholangiocarcinoma (eCCA) in 2022 based on the TOPAZ-1 trial. ICIs are more effective in "hot tumors" with high T cell infiltration. In resectable eCCA, $CD3^+/CD8^+$ T cell density is a prognostic factor. This study aimed to identify predictive immune biomarkers for ICI response. [Methods]We analyzed 15 eCCA patients treated with ICIs for postoperative recurrence from Jan 2023 to Apr 2024. Exclusions: intrahepatic CCA (n=4), mixed-type (n=2), and lost to follow-up (n=3). Immunohistochemistry assessed PD-L1, T cell markers, and HLA in surgical specimens. Response was evaluated at 6 months using RECIST 1.1.[Results]Among 15 cases: PR=5, SD=5, PD=5. No significant differences in background, tumor markers, or stage. $CD8^+$T cells were higher in PR/SD vs PD (PR:184, SD:289, PD:38/mm$^2$; p<0.05). $CD103^+$T cells were also higher in SD vs PD (88 vs 18; P=0.03), and tended to be higher in PR (92; P=0.14). Responders (PR+SD) had more $CD8^+$(236 vs 38; P=0.002) and $CD103^+$(90 vs 18; P=0.007) than non-responders. [Conclusion]Intratumoral $CD103^+CD8^+$ T cell infiltration may predict ICI efficacy in eCCA.

[Disclosure of COI by Responsible researcher] Lead presenter is the Responsible researcher.
[Lead presenter: Yoshiyuki Tagayasu] We have no financial relationships to disclose.

▲TOP

### [P-2190] I-P11-11
### Immune checkpoint inhibitors (1)

2025 / 9 / 26 (Fri) 16:45-17:30 Room P/(Motenashi Dome) Room P

---

### P-2190
### CD80 is positively correlated with the efficacy of neoadjuvant PD-1 Ab combined with chemotherapy for esophagus cancer

[Speaker]Tianyi Liu (Chinese PLA General Hosp.) [Co-author]Liangliang Wu (Chinese PLA General Hosp.)、Guoqing Zhang (Chinese PLA General Hosp.)

The aim of this study was to investigate peripheral blood biomarkers and explore the underlying mechanism. Baseline serum samples were collected from esophagus cancer patients receiving neoadjuvant PD-1 blockade plus chemotherapy (discovery cohort n = 46, validation cohort n = 28). Transcriptome sequencing and PD-L1 immunohistochemistry were analyzed pre- and post-treatment. Five serum circulating cells and 50 proteins were quantified. In the discovery cohort, patients with high CD80 levels showed a higher response rate (AUC = 0.686). This predictive value of serum CD80 was confirmed in the validation cohort (AUC = 0.778). Furthermore, CD80-high patients had higher CD8 T cell and lower neutrophil levels in the tumor microenvironment. During treatment, immunosuppressive factors such as iTreg and PD-L1 expression increased significantly, while MHC class I signature decreased in CD80-low patients. Circulating soluble CD80 protein in peripheral blood may serve as a potential non-invasive biomarker to predict clinical benefit in esophagus cancer patients receiving neoadjuvant PD-1 blockade with chemotherapy.

[Disclosure of COI by Responsible researcher] Lead presenter is not the Responsible researcher.
[Lead presenter: Tianyi Liu] We have no financial relationships to disclose.
[Principal Researcher: Guoqing Zhang] We have no financial relationships to disclose.

### [P-2191] I-P11-11
### Immune checkpoint inhibitors (1)

2025 / 9 / 26 (Fri) 16:45-17:30 Room P/(Motenashi Dome) Room P

---

### P-2191
### Elucidation of the function of proline isomerase Pin1 on esophageal squamous cell carcinoma

[Speaker]Sho Hirano (Dept. of Frontier Surg., Grad. Sch. of Med., Chiba Univ.／Dept. of Lab. Med., Chiba Univ. Hosp.) [Co-author]Nobufumi Sekino (Dept. of Frontier Surg., Grad. Sch. of Med., Chiba Univ.)、Takeshi Toyozumi (Dept. of Frontier Surg., Grad. Sch. of Med., Chiba Univ.)、Yasunori Matsumoto (Dept. of Frontier Surg., Grad. Sch. of Med., Chiba Univ.)、Koichiro Okada (Dept. of Frontier Surg., Grad. Sch. of Med., Chiba Univ.)、Tadashi Shiraishi (Dept. of Frontier Surg., Grad. Sch. of Med., Chiba Univ.)、Kazuya Kinoshita (Dept. of Frontier Surg., Grad. Sch. of Med., Chiba Univ.)、Tenshi Makiyama (Dept. of Frontier Surg., Grad. Sch. of Med., Chiba Univ.)、Yuri Nishioka (Dept. of Frontier Surg., Grad. Sch. of Med., Chiba Univ.)、Masanari Yamada (Dept. of Frontier Surg., Grad. Sch. of Med., Chiba Univ.)、Akane Morimoto (Dept. of Frontier Surg., Grad. Sch. of Med., Chiba Univ.)、Kazuyuki Matsushita (Dept. of Lab. Med., Chiba Univ. Hosp.)、Hisahiro Matsubara (Dept. of Frontier Surg., Grad. Sch. of Med., Chiba Univ.)

Background: Since anti-PD-1 (αPD-1) therapy alone shows inadequate response in esophageal squamous cell carcinoma (ESCC), combination therapies are worth exploring. The proline isomerase Pin1, highly expressed in various cancers, including ESCC, plays a role in malignant traits. Studies in other cancers have shown that Pin1 inhibition enhances PD-L1 expression and boosts the antitumor efficacy of αPD-1 antibodies. This study investigates the impact of Pin1 inhibition on ESCC.
Methods: Pin1 inhibitors (Pin1i) were added to ESCC cell lines to evaluate protein expression and proliferation rates. Additionally, pre-treatment biopsy specimens from patients who underwent chemoradiotherapy followed by αPD-1 therapy were analyzed to assess Pin1 immunostaining patterns and the response to αPD-1 therapy.
Results & Discussion: Pin1i reduced Pin1 expression while increasing E-cadherin and PD-L1 expression, alongside a decreased proliferation rate. Immunostaining revealed that high Pin1 expression correlated with poor αPD-1 therapy responses. Thus, Pin1 inhibition reduces malignancy and enhances PD-L1 expression in ESCC, suggesting that combining αPD-1 therapy with Pin1i could be effective.

[Disclosure of COI by Responsible researcher] Lead presenter is not the Responsible researcher.
[Lead presenter: Sho Hirano] We have no financial relationships to disclose.
[Principal Researcher: Nobufumi Sekino] We have no financial relationships to disclose.

### [P-2192] I-P11-11
### Immune checkpoint inhibitors (1)

2025 / 9 / 26 (Fri) 16:45-17:30 Room P/(Motenashi Dome) Room P

### P-2192
### Development of personalized immunotherapy for metastatic renal cell carcinoma using integrative genomic analyses

[Speaker]Go Noguchi (Dept. of Urology, Yokohama City University School of Medicine) [Co-author]Ryosuke Jikuya (Dept. of Urology, Yokohama City University School of Medicine)、Erika Muraoka (Dept. of Molecular pathology, Yokohama City University School of Medicine)、Mitsuru Komeya (Dept. of Urology, Yokohama City University School of Medicine)、Kota Aomori (Dept. of Urology, Yokohama City University School of Medicine)、Hirotaka Nagasaka (Dept. of Urology, Yokohama City University School of Medicine)、Sachi Kawaura (Dept. of Urology, Yokohama City University School of Medicine)、Yasuhiro Iribe (Dept. of Urology, Yokohama City University School of Medicine)、Hiroki Ito (Dept. of Urology, Yokohama City University School of Medicine)、Yusuke Ito (Dept. of Urology, Yokohama City University School of Medicine)、Kentaro Muraoka (Dept. of Urology, Yokohama City University School of Medicine)、Tomoyuki Tatenuma (Dept. of Urology, Yokohama City University School of Medicine)、Kota Kobayashi (Dept. of Urology, Yokohama City University School of Medicine)、Satoshi Fujii (Dept. of Molecular pathology, Yokohama City University School of Medicine)、Kazuhide Makiyama (Dept. of Urology, Yokohama City University School of Medicine)、Hisashi Hasumi (Dept. of Urology, Yokohama City University School of Medicine)

A personalized ICI therapy for RCC is incompletely developed, because most of studies including biomarker analyses from CheckMate214 had analyzed FFPE specimens with low DNA or RNA quality. Here, we report a novel companion diagnostic algorithm for ICI therapy. We conducted WES and RNA-seq analyses using frozen specimens from 38 metastatic clear cell RCC patients, who were treated with CTLA-4 inhibitor and/or PD1 inhibitor. Using GSEA analysis comparing CR/PR cases and PD cases, we found that the integrative status of immune response and Wnt signaling pathways might be associated with an efficacy of CTLA-4 inhibitor. Indeed, cases with high immune response and low Wnt signaling pathways exhibited prolonged PFS by administrating CTLA-4 inhibitor in addition to PD1 inhibitor, harbored ***PBRM1*** alteration with low frequency and exhibited downregulated PPAR signaling pathway. We identified several human endogenous retroviruses, which may be possible neoantigens for immune cells. These data provide a foundation for the development of precision immunotherapy for metastatic RCC patients.

[Disclosure of COI by Responsible researcher] Lead presenter is not the Responsible researcher.
[Lead presenter: Go Noguchi] We have no financial relationships to disclose.
[Principal Researcher: Hisashi Hasumi] ■Research funds from company: 第一三共

### [P-2193] I-P11-11
### Immune checkpoint inhibitors (1)

2025 / 9 / 26 (Fri) 16:45-17:30 Room P/(Motenashi Dome) Room P

P-2193

### Immunotherapy outcomes and immune microenvironment profiles of patients with non-clear cell renal cell carcinoma

[Speaker]Hiroki Ishihara (Dept. of Urology, Saiseikai Kawaguchi General Hosp.／Dept. of Urology, Tokyo Women's Med. Univ.) [Co-author]Hironori Fukuda (Dept. of Urology, Tokyo Women's Med. Univ.)、Riu Yamashita (Div. of Translational Informatics, Natl. Cancer Ctr.)、Kazunori Aoki (Div. of Immune Med., Natl. Cancer Ctr.)、Koichi Nishimura (Dept. of Urology, Tokyo Women's Med. Univ. Adachi Med. Ctr.)、Shinsuke Mizoguchi (Dept. of Urology, Saiseikai Kazo Hosp.)、Yuki Nemoto (Dept. of Urology, Jyoban Hosp.)、Hiroaki Shimmura (Dept. of Urology, Jyoban Hosp.)、Yasunobu Hashimoto (Dept. of Urology, Saiseikai Kawaguchi General Hosp.)、Takayuki Nakayama (Dept. of Urology, Tokyo Women's Med. Univ.)、Kazuhiko Yoshida (Dept. of Urology, Tokyo Women's Med. Univ.)、Toshihito Hirai (Dept. of Urology, Tokyo Women's Med. Univ.)、Junpei Iizuka (Dept. of Urology, Tokyo Women's Med. Univ.)、Daisuke Tokita (Dept. of Urology, Tokyo Women's Med. Univ.)、Tsunenori Kondo (Dept. of Urology, Tokyo Women's Med. Univ. Adachi Med. Ctr.)、Toshio Takagi (Dept. of Urology, Tokyo Women's Med. Univ.)

**Backgrounds**: Data regarding the outcomes of immune checkpoint inhibitor (ICI) treatment and tumor immune microenvironment (TIME) profiles of patients with non-clear-cell renal cell carcinoma (nccRCC) are limited.**Methods**: Using clinical data from 234 patients treated with ICIs, we compared outcome profiles between patients with clear-cell renal cell carcinoma (ccRCC) and those with nccRCC. Next, using 116 RCC samples, gene expression and tumor-infiltrating immune cell (TIIC) profiles were also compared using RNA-seq and flow cytometry.**Results**: Fifty-four patients (23%) were diagnosed with nccRCC. Progression-free and overall survival rates were lower in patients with nccRCC than in those with ccRCC. Flow cytometry and RNA-seq showed a decrease in TIICs, including CD8+ T cells, and downregulation of immune-related pathways in patients with nccRCC compared with in those with ccRCC.**Conclusions**: Clinical and molecular analyses indicated that patients with nccRCC harbored an immune-cold TIME phenotype, characterized by a decrease in TIICs and downregulation of immune-related pathways, potentially resulting in inferior clinical outcomes following ICI treatment.

[Disclosure of COI by Responsible researcher] Lead presenter is the Responsible researcher.
[Lead presenter: Hiroki Ishihara] ■Research funds from company: 日本学術振興会 科学研究費助成事業; 日本泌尿器科学会; 一般財団法人 土谷記念医学振興基金 土谷記念医学振興基金; 公益社団法人　日本透析医会

### [P-2203] I-P11-13
### Immune checkpoint inhibitors (3)

2025 / 9 / 26 (Fri) 16:45-17:30 Room P/(Motenashi Dome) Room P

---

### P-2203
### Does LDDS inhibit lymph node metastasis and the development of distant tumors? Importance of LDDS delivered Docetaxel

[Speaker]Ariunbuyan Sukhbaatar (Oral and Maxillofacial Oncology and Surg. Sci., Tohoku Univ.／Lab. of Biomed. Engineering for Cancer, Tohoku Univ. Div.／Biomed. Engineering Cancer Res. Ctr, Tohoku Univ.) [Co-author]Shiro Mori (Oral and Maxillofacial Oncology and Surg. Sci., Tohoku Univ.／Lab. of Biomed. Engineering for Cancer, Tohoku Univ. Div.／Biomed. Engineering Cancer Res. Ctr, Tohoku Univ.)、Katsunori Katagiri (Dep. ORL-H&N Surg., Iwate Med. Univ. Hosp)、Tsuyoshi Sugiura (Biomed. Engineering Cancer Res. Ctr, Tohoku Univ.)、Tetsuya Kodama (Lab. of Biomed. Engineering for Cancer, Tohoku Univ. Div.／Biomed. Engineering Cancer Res. Ctr, Tohoku Univ.)

Lymphatic Drug Delivery System (LDDS) is undergoing its first clinical trial in Japan. Briefly, anticancer drugs are directly injected into a lymph node (LN) to inhibit tumor growth at the injection site and in its downstream LNs. Previous experiments using LN metastatic mouse models described that tumor inhibition in the lymphatic system improves with increased osmotic pressure and viscosity of the drug. LDDS is independent of increased intranodal pressure and a focal defect in the marginal sinus that occurs in the early stage of LN metastasis. Here, we show the antitumor activity of docetaxel in combination with immune checkpoint inhibitors (ICIs) in a mouse model of LN resection-induced distant metastasis, along with the occurrence of immune-related Adverse Events (irAEs). The results showed a significant improvement in tumor inhibition within the combination therapy groups compared to the monotherapy groups. Notably, the combination therapies not only led to more substantial tumor inhibition but also exhibited a reduced frequency of irAEs. These findings confirm that integrating docetaxel with ICIs can lead to superior clinical outcomes in the management of metastatic cancer.

[Disclosure of COI by Responsible researcher] Lead presenter is the Responsible researcher.
[Lead presenter: Ariunbuyan Sukhbaatar] We have no financial relationships to disclose.

### [P-2204] I-P11-13
### Immune checkpoint inhibitors (3)

2025 / 9 / 26 (Fri) 16:45-17:30 Room P/(Motenashi Dome) Room P

---

### P-2204
### Impact of immediate inflammatory cytokines elevation after chemotherapy and pembrolizumab in advanced non-squamous NSCLC

[Speaker]Mitsuo Osuga (Ctr. for Biomed. Sci., Wakayama Med. Univ.) [Co-author]Yuichi Ozawa (Internal Med. III, Wakayama Med. Univ.／Dept. of Respiratory Med., Hamamatsu Med. Ctr.／Med. Oncology, Hamamatsu Med. Ctr.)、Hiroaki Akamatsu (Internal Med. III, Wakayama Med. Univ.)、Ryota Shibaki (Internal Med. III, Wakayama Med. Univ.)、Atsushi Hayata (Internal Med. III, Wakayama Med. Univ.)、Masanori Nakanishi (Internal Med. III, Wakayama Med. Univ.)、Nobuyuki Yamamoto (Ctr. for Biomed. Sci., Wakayama Med. Univ.／Internal Med. III, Wakayama Med. Univ.)、Yasuhiro Koh (Ctr. for Biomed. Sci., Wakayama Med. Univ.／Internal Med. III, Wakayama Med. Univ.)

Cytokine fluctuations immediately after PD-1/L1 inhibitors with chemotherapy and its correlation with efficacy remain elusive.
A prospective observational study at Wakayama Medical University enrolled 110 patients with thoracic malignancies receiving PD-1/L1 inhibitors as first-line therapy from Oct 2019 to Nov 2023 and 41 with advanced non-squamous non-small cell lung cancer treated with platinum, pemetrexed, and pembrolizumab were analyzed. Serum samples were collected at baseline, Day 3, 7, and 42, and analyzed for 40 cytokines with Milliplex MAP system.
Seven inflammation-associated cytokines (CXCL10, CXCL13, IL-6, MCP-1, etc.) showed ≥50% elevation at Day 3. A univariate Cox analysis revealed an increase in IL-6 or MCP-1 at Day 3 (Day 3/0 ratio >1) was associated with longer PFS [IL-6: HR 0.41 (95%CI 0.17-0.97), p=0.049; MCP-1: HR 0.43 (95% CI 0.19-0.97), p=0.042]. In the multivariate analysis adjusted for age, PS, and PD-L1 TPS, MCP-1 remained significant (HR 0.36, 95% CI 0.13-0.97, p=0.043), showing median PFS 463 vs. 201 days in MCP-1 increased and decreased cases, respectively (p=0.036).
The immediate increase in MCP-1 after treatment may serve as a predictive biomarker.

[Disclosure of COI by Responsible researcher] Lead presenter is not the Responsible researcher.
[Lead presenter: Mitsuo Osuga] We have no financial relationships to disclose.
[Principal Researcher: Yasuhiro Koh] ■Research funds from company: 第一三共株式会社、日本ゼオン株式会社、武田薬品工業株式会社、アストラゼネカ株式会社、中外製薬株式会社、日本ベーリンガーインゲルハイム株式会社　■Lecture fee: Amgen Inc.

### [P-2205] I-P11-13
### Immune checkpoint inhibitors (3)

2025 / 9 / 26 (Fri) 16:45-17:30 Room P/(Motenashi Dome) Room P

---

### P-2205
### Evaluation of combination therapy with anti-PD-1 and anti-CD47 antibody in non-small cell lung cancer liver metastasis

[Speaker]Hiroshi(Kyoto Univ.) Yoshida (Department of Respiratory Medicine, Kyoto University Graduate School of Medicine) [Co-author]Hiroaki Ozasa (Department of Respiratory Medicine, Kyoto University Graduate School of Medicine)、Hitomi Ajimizu (Department of Respiratory Medicine, Kyoto University Graduate School of Medicine)、Kazutaka Hosoya (Department of Respiratory Medicine, Kyoto University Graduate School of Medicine)、Yusuke Shima (Department of Respiratory Medicine, Kyoto University Graduate School of Medicine)、Keiichiro Suminaga (Department of Respiratory Medicine, Kyoto University Graduate School of Medicine)、Kentaro Hashimoto (Department of Respiratory Medicine, Kyoto University Graduate School of Medicine)、Tomoko Funazo (Department of Respiratory Medicine, Kyoto University Graduate School of Medicine)、Takashi Nomizo (Department of Respiratory Medicine, Kyoto University Graduate School of Medicine)、Hironori Yoshida (Department of Respiratory Medicine, Kyoto University Graduate School of Medicine)、Toyohiro Hirai (Department of Respiratory Medicine, Kyoto University Graduate School of Medicine)

Liver metastasis is a significant poor prognostic factor in non-small cell lung cancer (NSCLC) and contributes to resistance against immune checkpoint inhibitors. The aim of this study was to identify novel therapeutic target factors for liver metastasis of lung cancer from an immunological mechanism perspective. We established a mouse liver metastasis model by intrasplenic injection of CMT167 tumor cells into CX3CR1-GFP C57BL/6 mice. Using two-photon microscopy and bioluminescence imaging, we observed immune cell dynamics during metastatic progression. CX3CR1-expressing macrophages were found to engage and phagocytose tumor cells. Since CD47 suppresses macrophage-mediated phagocytosis, we investigated the impact of PD-1 antibody treatment in mice bearing CMT167-CD47 knockout (CD47KO) tumors. The combination of CD47 deletion and PD-1 blockade significantly inhibited liver metastasis progression.The study is testing whether combination therapy with CD47 and PD-1 antibodies is useful in lung cancer liver metastases. Furthermore, we report that immunostaining and flowcytometry were performed to evaluate the tumor immune environment in lung cancer liver metastases in mice.

[Disclosure of COI by Responsible researcher] Lead presenter is not the Responsible researcher.
[Lead presenter: Hiroshi Yoshida] We have no financial relationships to disclose.
[Principal Researcher: Hiroaki Ozasa] ■Research funds from company: 日本ベーリンガーインゲルハイム株式会社

### [P-2206] I-P11-13
### Immune checkpoint inhibitors (3)

2025 / 9 / 26 (Fri) 16:45-17:30 Room P/(Motenashi Dome) Room P

---

### P-2206
### Clinical Data and Mouse Model of NSCLC Brain Metastasis Reveal Efficacy of Anti-PD-1 + anti-CTLA-4 Antibody Therapy.

[Speaker]Kazutaka Hosoya (Kyoto Univ. Dept. of Respiratory Med.) [Co-author]Hiroaki Ozasa (Kyoto Univ. Dept. of Respiratory Med.)、Takahiro Tsuji (Kyoto Univ. Dept. of Respiratory Med.)、Hitomi Ajimizu (Kyoto Univ. Dept. of Respiratory Med.)、Hiroshi(Kyoto Univ.) Yoshida (Kyoto Univ. Dept. of Respiratory Med.)、Yusuke Shima (Kyoto Univ. Dept. of Respiratory Med.)、Takashi Nomizo (Kyoto Univ. Dept. of Respiratory Med.)、Hironori Yoshida (Kyoto Univ. Dept. of Respiratory Med.)、Hiroyuki Katsuragawa (Kyoto Univ. Dept. of Diagnostic Path.)、Kentaro Tsuji (Kyoto Univ. Dept. of Diagnostic Path.)、Shigeto Nishikawa (Kyoto Univ. Dept. of Thoracic Surg.)、Toshi Menju (Kyoto Univ. Dept. of Thoracic Surg.)、Akihiko Yoshizawa (Nara Med. Univ. Dept. of Diagnostic Path.)、Yoshiki Arakawa (Kyoto Univ. Dept. of Neurosurgery)、Toyohiro Hirai (Kyoto Univ. Dept. of Respiratory Med.)

Brain metastasis (BrM) significantly impacts NSCLC patients' outcomes. The distinct immunosuppressive microenvironment in BrM requires further investigation using clinically relevant models to assess treatment efficacy and mechanisms.Using clinical data, we showed that NSCLC BrM showed lower infiltration of cytotoxic T lymphocytes (CTLs) and lower formation of tertiary lymphoid structures, and anti-PD-1 plus anti-CTLA-4 combination therapy was associated with better central nervous system outcomes.Moreover, we developed a mouse BrM model by introducing syngeneic mouse lung adenocarcinoma cells into immunocompetent mice. Combined anti-PD-1 and anti-CTLA-4 therapy showed superior efficacy compared to monotherapy, as evaluated by bioluminescence imaging and mouse survival. In the functional analysis, the efficacy of combined therapy could be primarily attributed to changes in CTL numbers and cytotoxicity.Our results suggested the efficacy and importance of anti-PD-1 + anti-CTLA-4 combination therapy against NSCLC BrM. This combination therapy could overcome the immunosuppressive microenvironment of NSCLC BrM.

[Disclosure of COI by Responsible researcher] Lead presenter is not the Responsible researcher.
[Lead presenter: Kazutaka Hosoya] ■Research funds from company: 日本ベーリンガーインゲルハイム　■Research expenses: 日本学術振興会
[Principal Researcher: Takahiro Tsuji] ■Research funds from company: 日本ベーリンガーインゲルハイム

### [P-2207] I-P11-13
### Immune checkpoint inhibitors (3)

2025 / 9 / 26 (Fri) 16:45-17:30 Room P/(Motenashi Dome) Room P

---

### P-2207
### CALLY Index as a Predictive Biomarker for ICI Plus Chemotherapy in Advanced HER2(-) Gastric Cancer: A First Report

[Speaker]Masahiro Shimokawa (Department of Medical Oncology, Showa Medical University) [Co-author]Atsushi Horiike (Department of Medical Oncology, Showa Medical University)、Emiko Mura (Department of Medical Oncology, Showa Medical University)、Risako Suzuki (Department of Medical Oncology, Showa Medical University)、Toshiaki Tsurui (Department of Medical Oncology, Showa Medical University)、Nana Iriguchi (Department of Medical Oncology, Showa Medical University)、Tomoyuki Ishiguro (Department of Medical Oncology, Showa Medical University)、Yuya Hirasawa (Department of Medical Oncology, Showa Medical University)、Ryotaro Ohkuma (Department of Medical Oncology, Showa Medical University)、Goh Ikeda (Department of Medical Oncology, Showa Medical University)、Hirotsugu Ariizumi (Department of Medical Oncology, Showa Medical University)、Yutaro Kubota (Department of Medical Oncology, Showa Medical University)、Yasuyuki Kojima (Department of Medical Oncology, Showa Medical University／Institute for Clinical Genetics and Genomics, Showa Medical University)、Satoshi Wada (Department of Medical Oncology, Showa Medical University／CRI for Clinical Pharmacology and Therapeutics, Showa Medical University)、Kiyoshi Yoshimura (Department of Medical Oncology, Showa Medical University／CRI for Clinical Pharmacology and Therapeutics, Showa Medical University)、Takuya Tsunoda (Department of Medical Oncology, Showa Medical University)

Immune checkpoint inhibitor plus platinum-based chemotherapy is now standard 1L treatment for UR/recurrent HER2-negative gastric cancer (GC), but responders remain limited. Established biomarkers such as PD-L1 CPS, NLR, PNI, omit at least one critical host factor (inflammation, immunity or nutrition) and reliable stratification is lacking. We therefore propose the CALLY index (Albumin x Lymphocyte count / CRP) on GC patients received nivolumab + platinum therapy (n=45). Baseline CALLY values correlated with OS (p=0.0106) and PFS (p=0.0492). Using an ROC-defined cut-off of 44, high CALLY predicted markedly longer OS (16.4 vs 6.1mo, p=0.0006) and PFS (10.2 vs 2.1mo, p=0.0009) in patients younger than 72 years; no significant differences were seen in those elder than 72 years. In both subgroups, high CALLY was also associated with higher objective response rate (81.8 vs 30.0%, p=0.0001) and disease control rate (100 vs 70.0%, p=0.0252). These results demonstrate, for the first time, that a pre-treatment host-status index can predict ICI response in GC. Prospective multi-center validation against established benchmarks is warranted.

[Disclosure of COI by Responsible researcher] Lead presenter is the Responsible researcher.
[Lead presenter: Masahiro Shimokawa] We have no financial relationships to disclose.

▲TOP

### [P-2208] I-P11-13
### Immune checkpoint inhibitors (3)

2025 / 9 / 26 (Fri) 16:45-17:30 Room P/(Motenashi Dome) Room P

---

### P-2208
### Evaluation of Chemoimmunotherapy for Metastatic Lymph Nodes via Treatment of Normal Lymph Nodes

[Speaker]Ryuma Suzuki (Grad. sch. of Biomed. Engineering, Tohoku Univ.) [Co-author]Ariunbuyan Sukhbaatar (Grad. sch, of Dent., Tohoku Univ.)、Shiro Mori (Grad. sch, of Dent., Tohoku Univ.)、Tetsuya Kodama (Grad. sch. of Biomed. Engineering, Tohoku Univ.)

The treatment of metastatic lymph node (MLNs) are crucial for reducing cancer-associated mortality. However, intravenous (i.v.) injection, which is commonly used in clinical settings, has several limitations in targeting MLNs due to their unique characteristics.Our laboratory has developed a novel treatment method called lymphatic drug delivery system (LDDS) to improve MLN treatment efficacy. LDDS involves injecting drugs into the sentinel lymph node (SLN) to target both the SLN and its downstream lymph nodes. In our previous study, we demonstrated that chemotherapy and immunotherapy using LDDS achieved superior therapeutic effects compared to i.v. injection.Although clinical trials of LDDS are currently being conducted, a major challenge in clinical application is the difficulty of identifying the SLN. To address this issue, we investigated the efficacy of injecting Docetaxel and anti-CTLA-4 monoclonal antibody into normal lymph nodes.Our results showed a therapeutic effect in these treatment groups, suggesting that LDDS could be implemented in clinical practice without identifying the SLN. This advancement could facilitate broader clinical adoption of LDDS.

[Disclosure of COI by Responsible researcher] Lead presenter is the Responsible researcher.
[Lead presenter: Ryuma Suzuki] We have no financial relationships to disclose.

### [P-2209] I-P11-13
### Immune checkpoint inhibitors (3)

2025 / 9 / 26 (Fri) 16:45-17:30 Room P/(Motenashi Dome) Room P

---

### P-2209
### Immune checkpoint inhibitor response and clinicopathological features of SMARCA4-deficient tumors

[Speaker]Norito Suzuki (Dept. Med. Oncology, Sapporo Med. Univ.) [Co-author]Tomohiro Kubo (Dept. Med. Oncology, Sapporo Med. Univ.)、Joji Muramatsu (Dept. Med. Oncology, Sapporo Med. Univ.)、Ryota Yokoyama (Dept. Med. Oncology, Sapporo Med. Univ.)、Yohei Arihara (Dept. Med. Oncology, Sapporo Med. Univ.)、Shintaro Sugita (Dept. Surg. Path. Sapporo Med. Univ.)、Ayako Murota (Dept. Gastroenterology and Hepatology, Sapporo Med. Univ.)、Kazuyuki Murase (Dept. Med. Oncology, Sapporo Med. Univ.)、Masashi Idogawa (Dept. Med. Research Institute for Frontier Med, Sapporo Med. Univ.)、Kohichi Takada (Dept. Med. Oncology, Sapporo Med. Univ.)

[Background]The SWI/SNF complex regulates chromatin structure. ***SMARCA4***-deficient tumors, due to BRG-1 loss, have a poor prognosis and often show an undifferentiated, rhabdoid morphology but details are unclear.
[Methods]We retrospectively analyzed 7 ***SMARCA4***-deficient tumors identified via cancer genomic profiling (CGP) (Mar 2020-Jun 2024).
[Results]Primary tumor sites included lung (n=3), biliary tract, pancreas, esophagus, and unknown origin (n=1 each). Histology showed well-differentiated adenocarcinomas in 4 cases, and undifferentiated carcinomas in 3. Rhabdoid morphology were observed in 2 cases. Combined Positive Score (CPS) was high in 4 cases. CGP detected high tumor mutational burden (TMB) in 3 cases. Six cases underwent immune checkpoint inhibitors (ICIs), with objective response rate of 83% and median progression-free survival of 329 days.
[Conclusion]***SMARCA4***-deficient tumors tend to have a high CPS/TMB and respond well to ICIs. While ***SMARCA4***-deficient tumors often showed an undifferentiated with rhabdoid features, some lack these traits. CGP is useful for detecting SMARCA4 deficiency in such cases.

[Disclosure of COI by Responsible researcher] Lead presenter is not the Responsible researcher.
[Lead presenter: Norito Suzuki] We have no financial relationships to disclose.
[Principal Researcher: Kohichi Takada] We have no financial relationships to disclose.

### [P-2210] I-P11-13
### Immune checkpoint inhibitors (3)

2025 / 9 / 26 (Fri) 16:45-17:30 Room P/(Motenashi Dome) Room P

---

### P-2210
### The Impact of $PGD_2$-CRTH2 Signaling on the Anti-Tumor Effects of Immune Checkpoint Inhibitors

[Speaker]Takahisa Murata (Tokyo University, Animal Radiology／Tokyo University, Food and Animal Systemics／Tokyo University, Veterinary Pharmacology) [Co-author]Kanan Yasui (Tokyo University, Animal Radiology)、Akane Hayashi (Tokyo University, Animal Radiology)、Yui Kobayashi (Tokyo University, Animal Radiology)、Koji Kobayashi (Tokyo University, Food and Animal Systemics)

Although immune checkpoint inhibitors show promise in cancer therapy, many patients fail to respond. We previously showed that $PGD_2$ modulates T cell polarity via its receptor CRTH2. This study investigated how $PGD_2$-CRTH2 signaling affects the anti-tumor effects of anti-PD-1 therapy. B16F1 melanoma cells were implanted in wild-type (WT) and CRTH2-deficient ($CRTH2^{-/-}$) mice, followed by anti-PD-1 antibody (5 mg/kg) administration. Tumor growth was unchanged in WT mice but was significantly suppressed in $CRTH2^{-/-}$ mice. Histological analysis revealed larger necrotic areas in tumors from $CRTH2^{-/-}$ mice treated with anti-PD-1. Flow cytometry showed fewer $CD11c^+$ tumor-infiltrating cells in $CRTH2^{-/-}$ mice regardless of treatment. RT-qPCR showed increased IFN-$\gamma$ expression in anti-PD-1-treated WT mice, with even higher levels in $CRTH2^{-/-}$ mice. Furthermore, combining ramatroban (30 mg/kg), a CRTH2 inhibitor, with anti-PD-1 in WT mice led to significant tumor regression, while anti-PD-1 alone had no effect. These findings suggest that blocking $PGD_2$-CRTH2 signaling enhances anti-PD-1 efficacy by promoting a Th1-polarized tumor microenvironment and may expand its benefit to unresponsive patients.

[Disclosure of COI by Responsible researcher] Lead presenter is the Responsible researcher.
[Lead presenter: Takahisa Murata] ■Endowed chair: 株式会社リバンプ

▲TOP

### [P-2211] I-P11-13
### Immune checkpoint inhibitors (3)

2025 / 9 / 26 (Fri) 16:45-17:30 Room P/(Motenashi Dome) Room P

---

### P-2211
### Enhanced efficacy of immune checkpoint inhibitors in combination with Japanese herbal medicine "TSUDOSAN"

[Speaker]Hidetomo Himuro (Div Cancer Immunotherapy, Kanagawa Cancer Ctr Res Inst／Cancer Vaccine and Immunotherapy Ctr, Kanagawa Cancer Ctr) [Co-author]Taku Kouro (Div Cancer Immunotherapy, Kanagawa Cancer Ctr Res Inst／Cancer Vaccine and Immunotherapy Ctr, Kanagawa Cancer Ctr)、Feifei Wei (Div Cancer Immunotherapy, Kanagawa Cancer Ctr Res Inst／Cancer Vaccine and Immunotherapy Ctr, Kanagawa Cancer Ctr)、Kayoko Tsuji (Div Cancer Immunotherapy, Kanagawa Cancer Ctr Res Inst／Cancer Vaccine and Immunotherapy Ctr, Kanagawa Cancer Ctr)、Mitsuru Komahashi (Div Cancer Immunotherapy, Kanagawa Cancer Ctr Res Inst／Cancer Vaccine and Immunotherapy Ctr, Kanagawa Cancer Ctr／ Dept. of Pediatric Surgery, Nihon Univ. School of Medicine)、Tomoya Matsui (Div Cancer Immunotherapy, Kanagawa Cancer Ctr Res Inst／Cancer Vaccine and Immunotherapy Ctr, Kanagawa Cancer Ctr／Dept. of Obstetrics & Gynecology, Keio University School of Medicine)、Tetsuro Sasada (Div Cancer Immunotherapy, Kanagawa Cancer Ctr Res Inst／Cancer Vaccine and Immunotherapy Ctr, Kanagawa Cancer Ctr)

BackgroundThe development of strategies to enhance the efficacy of immune checkpoint inhibitors (ICIs) is critical, and the gut microbiota has been identified as one of the key targets. Traditional herbal therapies have been shown to interact with the gut microbiota. In traditional medicine, "OKETSU" refers to a pathological condition caused by impaired microcirculation, with tumors being considered a manifestation of this condition. Tsudosan is a herbal remedy used to improve OKETSU.ObjectiveThis study aimed to evaluate the effect of combining PD-1 antibody (Ab) with Tsudosan on tumor growth and to explore its relationship with gut microbiota and immune responses.Methods and ResultsIn a Lewis lung carcinoma transplantation mouse model, the combination of PD-1 Ab with Tsudosan resulted in enhanced suppression of tumor growth. Metagenomic analysis revealed significant changes in the gut microbiota, while RNA sequencing suggested activation of immune pathways.DiscussionTsudosan may enhance the antitumor effects of PD-1 Ab by modulating the gut microbiota and activating immune responses. Further research is needed to understand these relationships and to improve the efficacy of ICIs.

[Disclosure of COI by Responsible researcher] Lead presenter is the Responsible researcher.
[Lead presenter: Himuro Hidetomo] We have no financial relationships to disclose.

### [P-2221] I-P12-5
### Pathological analysis & Biomarkers

2025 / 9 / 26 (Fri) 16:45-17:30 Room P/(Motenashi Dome) Room P

---

### P-2221
### Transgelin in Cancer Activated Fibroblasts Regulates Stromal Contractility and Correlates with Colon Cancer Progression

[Speaker]Ryuji Okamoto (Department of Pathology and Clinical Laboratories, NCCHE／Department of Gastroenterology and Endoscopy, NCCHE／Course of Advanced Clinical Research, Cancer Juntendo University) [Co-author]Tetsuro Hiroi (Department of Pathology and Clinical Laboratories, NCCHE)、Yuka Nakamura (Division of Innovative Pathology and Laboratory Medicine, EPOC)、Hiroko Hashimoto (Division of Innovative Pathology and Laboratory Medicine, EPOC)、Shingo Sakashita (Division of Innovative Pathology and Laboratory Medicine, EPOC)、Naoya Sakamoto (Division of Innovative Pathology and Laboratory Medicine, EPOC)、Shumpei Ishikawa (Division of Innovative Pathology and Laboratory Medicine, EPOC／Department of Preventive Medicine, Graduate School of Medicine, Tokyo University)、Horacio Cabral (Department of Bioengineering, Graduate School of Engineering, Tokyo University)、Triantafyllos Stylianopoulos (Department of Mechanical and Manufacturing Engineering, Cyprus University)、Yuichiro Tsukada (Department of Colorectal Surgery, NCCHE)、Atsushi Ochiai (Research Institute for Biomedical Sciences, Tokyo University of Science)、Tomonori Yano (Department of Gastroenterology and Endoscopy, NCCHE／Course of Advanced Clinical Research, Cancer Juntendo University)、Masaaki Ito (Department of Colorectal Surgery, NCCHE)、Genichiro Ishii (Department of Pathology and Clinical Laboratories, NCCHE／Course of Advanced Clinical Research, Cancer Juntendo University／Division of Innovative Pathology and Laboratory Medicine, EPOC)、Motohiro Kojima (Department of Pathology and Clinical Laboratories, NCCHE／Division of Pathology, EPOC／Department of Surgical Pathology, Kyoto Prefectural University of Medicine)

Previously, we reported that transgelin mRNA expression is a prognostic marker in colon cancer. However, the utility of its protein expression and distribution in tumor tissues is controversial. The biological function of transgelin in cancer is unknown. In this study, we identified that transgelin protein expression is identical to other solid tumors, including pancreas, liver, and lung cancers. We performed a clinicopathological analysis in 359 colon cancer cases and found that transgelin expression in cancer stroma was associated with poor prognosis. Human colonic fibroblasts were obtained and cultivated, and transgelin short-hairpin knockdown (KD) was performed. Transgelin KD did not influence the upregulation of ACTA2, COL1A1, and TNC after a cancer cell-conditioned medium was stimulated. On the other hand, transgelin KD reduced collagen gel contractility. In vivo studies by co-transplanting cancer cells and fibroblasts into immunodeficient mice showed that transgelin KD inhibited tumor growth significantly. These findings highlight transgelin as a key regulator of the tumor microenvironment and a promising therapeutic target for the treatment of colorectal cancer.

[Disclosure of COI by Responsible researcher] Lead presenter is not the Responsible researcher.
[Lead presenter: Ryuji Okamoto] We have no financial relationships to disclose.
[Principal Researcher: Motohiro Kojima] We have no financial relationships to disclose.

### [P-2222] I-P12-5
### Pathological analysis & Biomarkers

2025 / 9 / 26 (Fri) 16:45-17:30 Room P/(Motenashi Dome) Room P

---

### P-2222
### Clinical significance of tertiary lymphoid structures in oral squamous cell carcinoma

[Speaker]Takaharu Doi (Oral and Maxillofacial Surg, Ehime Univ Grad Sch of Med) [Co-author]Kohichi Nakashiro (Oral and Maxillofacial Surg, Ehime Univ Grad Sch of Med)、Nobuyuki Kuribayashi (Oral and Maxillofacial Surg, Ehime Univ Grad Sch of Med)、Mie Kurata (Analytical Path , Ehime Univ Grad Sch of Med)、Maya Nakagawa (Oral and Maxillofacial Surg, Ehime Univ Grad Sch of Med)、Daisuke Uchida (Oral and Maxillofacial Surg, Ehime Univ Grad Sch of Med)

In previous study, we showed that C-X-C motif chemokine 13 (CXCL13) was commonly overexpressed in oral squamous cell carcinoma (OSCC). It is well known that CXCL13 is an essential factor for tertiary lymphoid structures (TLS) formation. TLS are ectopic lymphoid formations that arise in non-lymphoid tissues under chronic inflammation or cancer. In this study, we evaluated the maturity of TLS in primary tumor tissues of 51 OSCC cases using H&E and CD20 immunohistochemical staining, and classified each TLS into three maturation: early TLS (E-TLS), primary follicle-like TLS (PFL-TLS), and secondary follicle-like TLS (SFL-TLS). Peritumoral TLS was found in 90.2% (46/51) of all cases, including E-TLS in 39.2% (20/51), PFL-TLS in 35.3% (18/51), and SFL-TLS in 15.7% (8/51). Subsequently, we analyzed the association between TLS and prognosis using the Kaplan-Meier method with log-rank test. In disease-free survival, OSCC cases with no TLS and E-TLS showed significantly worse prognosis compared to PFL- and SFL-TLS positive cases ($P < 0.05$). These findings suggest that the maturity of TLS is a useful parameter for predicting the recurrence of OSCC.

[Disclosure of COI by Responsible researcher] Lead presenter is not the Responsible researcher.
[Lead presenter: Takaharu Doi] We have no financial relationships to disclose.
[Principal Researcher: Kohichi Nakashiro] We have no financial relationships to disclose.

Poster Session

### [P-2223] I-P12-5
### Pathological analysis & Biomarkers

2025 / 9 / 26 (Fri) 16:45-17:30 Room P/(Motenashi Dome) Room P

---

### P-2223
### Role of zinc transporters in the immune microenvironment of the breast cancer

[Speaker]Erina Iwabuchi (Dept. Pathol.&Anat. Sci., Grad. Sch. Med. & Dent. Sci., Science Tokyo) [Co-author]Yasuhiro Miki (Dept. Anat. Pathol., Tohoku Univ. Grad. Sch. Med.)、Katsuhiko Ono (Dept. Anat. Pathol., Tohoku Univ. Grad. Sch. Med.)、Asumi Yamazaki (Dept. Breast & Endocrine Surg. Oncology, Tohoku Univ. Grad. Sch. Med.)、Takanori Ishida (Dept. Breast & Endocrine Surg. Oncology, Tohoku Univ. Grad. Sch. Med.／Tohoku Kosai Hosp., Breast Surg.)、Takashi Suzuki (Dept. Anat. Pathol., Tohoku Univ. Grad. Sch. Med.／Dept. Pathol. & Histotech., Tohoku Univ. Grad. Sch. Med.／Dept. Pathol., Tohoku Univ. Hosp.)、Yurie Soejima (Dept. Pathol.&Anat. Sci., Grad. Sch. Med. & Dent. Sci., Science Tokyo)

Zinc levels in breast cancer tissues are higher than those in normal breast tissues, and the expression of multiple zinc transporters is involved in cancer cell progression in breast cancer. At the 83rd Annual Meeting of the Japanese Cancer Association, we reported the association between zinc transporters (ZnT5, ZnT6, Zip1) and breast cancer progression. In this study, we investigated the relationship between zinc transporter ZnT1 and the tumor immune microenvironment in breast cancer. We performed immunohistochemistry on 157 breast cancer surgical specimens at Tohoku University Hospital. Immunohistochemical analysis revealed that there was no significance association between ZnT1 expression and T-cells (CD4-positive, CD8-positive) in all 157 cases; however, ZnT1-positive cases were significantly lower in patients with CD4-positive T cells in estrogen receptor (ER) positive breast cancer. Furthermore, in MCF-7 ER-positive breast cancer cells, CXCL12 expression was upregulated by treatment with 100 $\mu$M $ZnCl_2$. These results indicate that zinc transporters or intratumoral zinc concentrations could be involved in immune cell migration in ER-positive breast cancer.

[Disclosure of COI by Responsible researcher] Lead presenter is the Responsible researcher.
[Lead presenter: Erina Iwabuchi] We have no financial relationships to disclose.

### [P-2224] I-P12-5
### Pathological analysis & Biomarkers

2025 / 9 / 26 (Fri) 16:45-17:30 Room P/(Motenashi Dome) Room P

---

### P-2224
### Identification of urinary free *N* -glycan tumor marker having difucosylated type-1 structure

[Speaker]Miki Okamoto (Dept. Glyco-Oncol., Osaka Intl. Cancer Inst.) [Co-author]Ken Hanzawa (Glycoanal. Chem. Lab., iGCORE, Gifu Univ.)、Takashi Yamamoto (Dept. Clin. Lab., Osaka Intl. Cancer Inst.)、Kazuyoshi Ohkawa (Dept. Hepatobiliary and Pancreatic Oncol., Osaka Intl. Cancer Inst.)、Kenichi Yoshida (Dept. Diag. Path. & Cytol., Osaka Intl. Cancer Inst.)、Satoshi Takenaka (Dept. Orthopedic Surg., Osaka Intl. Cancer Inst.)、Masayuki Ohue (Dept. Gastroenterol. Surg., Osaka Intl. Cancer Inst.)、Tomoyuki Yamasaki (Dept. Clin. Lab., Osaka Intl. Cancer Inst.)、Yasuhide Miyamoto (Dept. Clin. Lab., Osaka Intl. Cancer Inst.)

In our previous studies, many free-glycan tumor marker candidates were identified based on structural analysis of human cancer tissues. In this study, to identify novel glycan tumor markers, we performed precise glycan structural analysis focusing on neutral glycans in colon cancer tissues. One free-glycan that is significantly increased in colorectal cancer cells compared to the corresponding normal epithelial cells was detected. The structural analysis of this glycan revealed that it was free-***N***-glycan having difucosylated type-1 structure (Lewis B antigen), but not conventional type-2 structure. The presence of ***N***-glycans with type-1 structure has not been reported in humans. To examine the clinical usefulness, the urinary level of this free-glycan in the urine of 121 healthy subjects, 141 gastric cancer patients, 87 colorectal cancer patients, 55 pancreatic cancer patients, 13 bile duct cancer patients and 79 rare cancer patients were measured using LC/MS/MS (selected reaction monitoring). This marker candidate was found to be elevated in rare types of cancer, such as AFP-producing gastric cancer.

[Disclosure of COI by Responsible researcher] Lead presenter is not the Responsible researcher.
[Lead presenter: Miki Okamoto] We have no financial relationships to disclose.
[Principal Researcher: Yasuhide Miyamoto] We have no financial relationships to disclose.

### [P-2225] I-P12-5
### Pathological analysis & Biomarkers

2025 / 9 / 26 (Fri) 16:45-17:30 Room P/(Motenashi Dome) Room P

---

### P-2225
### Enhanced Specificity of 6-Color digital PCR with 3D Analysis for Multiplex Detection of Pancreatic Cancer Mutations

[Speaker]Yusuke Ono (Inst. Biomed. Res., Sapporo Higashi Tokushukai Hosp.／Div. Gast., Dept. Int. Med. Asahikawa Med. Univ.／Natl. Inst. Biomed. Innov., Hlth & Nutr) [Co-author]Chiho Maeda (Inst. Biomed. Res., Sapporo Higashi Tokushukai Hosp.)、Kenji Takahashi (Div. Gast., Dept. Int. Med. Asahikawa Med. Univ.／Natl. Inst. Biomed. Innov., Hlth & Nutr)、Hidemasa Kawabata (Div. Gast., Dept. Int. Med. Asahikawa Med. Univ.)、Kazuya Koyama (Div. Gast., Dept. Int. Med. Asahikawa Med. Univ.)、Takuya Yamamoto (Inst. Biomed. Res., Sapporo Higashi Tokushukai Hosp.／Natl. Inst. Biomed. Innov., Hlth & Nutr)、Yusuke Mizukami (Inst. Biomed. Res., Sapporo Higashi Tokushukai Hosp.／Div. Gast., Dept. Int. Med. Asahikawa Med. Univ.／Natl. Inst. Biomed. Innov., Hlth & Nutr)

In our previous research, we developed a six-color multiplex digital PCR (dPCR) assay to detect 14 hotspot mutations in driver genes (***KRAS*** G12D/V/R/C/A/S, Q61H/K/L/R, and ***GNAS*** R201C/H) associated with pancreatic adenocarcinoma on the Bio-Rad QX600 system. This assay method achieved high accuracy in identifying mutations with minimal sample quantities. To overcomelimitations in cluster identification in 2D projections, we extended the multiplex analysis in the 3D matrix. We constructed a pipeline that processes droplet intensity data from each fluorophore in 3D space, enabling separation and counting of clusters individually. By measuring the fluorescence amplitudes of three different dyes for each variant, we distinguished clusters based on their spatial positions in the 3D scatter plot, significantly improving separation specificity and reducing overlap with background noise. The 3D-multiplex-dPCR method is expected to provide high-sensitivity detection of trace driver mutations in a single-well PCR setup, holding promise for clinical applications like liquid biopsies and high-sensitivity mutation research, supporting early cancer detection and personalized medicine.

[Disclosure of COI by Responsible researcher] Lead presenter is not the Responsible researcher.
[Lead presenter: Yusuke Ono] ■Research expenses: 株式会社日立ハイテク
[Principal Researcher: Yusuke Mizukami] ■Research expenses: 株式会社日立ハイテク

▲TOP

Poster Session

### [P-2226] I-P12-5
### Pathological analysis & Biomarkers

2025 / 9 / 26 (Fri) 16:45-17:30 Room P/(Motenashi Dome) Room P

### P-2226
### Withdrawn

No Abstract

### [P-2227] I-P12-5
### Pathological analysis & Biomarkers

2025 / 9 / 26 (Fri) 16:45-17:30 Room P/(Motenashi Dome) Room P

### P-2227
### Withdrawn

No Abstract

### [P-2228] I-P12-5
### Pathological analysis & Biomarkers

2025 / 9 / 26 (Fri) 16:45-17:30 Room P/(Motenashi Dome) Room P

### P-2228
### Withdrawn

No Abstract

### [P-2229] I-P12-5
### Pathological analysis & Biomarkers

2025 / 9 / 26 (Fri) 16:45-17:30 Room P/(Motenashi Dome) Room P

### P-2229
### Withdrawn

No Abstract

### [P-2239] I-P13-5
### Signal transduction inhibitor

2025 / 9 / 26 (Fri) 16:45-17:30 Room P/(Motenashi Dome) Room P

---

### P-2239
### USP8 Inhibition Induces Synthetic Lethality via FGFR2 Degradation in ARID1A-Deficient Ovarian Clear Cell Carcinoma

[Speaker]Hideaki Ogiwara (Cancer Therapeutics, NCCRI) [Co-author]Ryosuke Saito (Cancer Therapeutics, NCCRI)、Makoto Fukushima (Cancer Therapeutics, NCCRI)、Mariko Sasaki (Cancer Therapeutics, NCCRI)

Over 50% of ovarian clear cell carcinoma (OCCC) cases harbor ARID1A deficiency, contributing to chemotherapy resistance and underscoring the need for novel therapies. We analyzed the DepMap database to identify synthetic lethal targets for ARID1A-deficient ovarian cancer. Among several candidates, USP8, a deubiquitinating enzyme, emerged as a key vulnerability in ARID1A-deficient OCCC cell lines and mouse xenografts. Notably, USP8 inhibitors showed higher selectivity than other candidate drugs under clinical evaluation. Mechanistically, USP8 inhibition led to proteasomal degradation of FGFR2, a receptor upstream of the STAT3 pathway. While ARID1A loss disrupts STAT3 signaling, USP8 suppression further reduced STAT3 pT705 phosphorylation and triggered apoptosis. These findings highlight USP8 as a promising therapeutic target for ARID1A-deficient OCCC via modulation of FGFR2-STAT3 signaling.

[Disclosure of COI by Responsible researcher] Lead presenter is the Responsible researcher.
[Lead presenter: Hideaki Ogiwara] ■Research funds from company: 共同研究費：住友ファーマ　■Research expenses: 共同研究費：住友ファーマ

### [P-2240] I-P13-5
### Signal transduction inhibitor

2025 / 9 / 26 (Fri) 16:45-17:30 Room P/(Motenashi Dome) Room P

### P-2240
### Development of a novel covalent drug that allosterically inhibits the pro-oncogenic phosphatase SHP2

[Speaker]Takeru Hayashi (Lab. of Microbial Carcinogenesis, Inst. of Microbial Chem.) [Co-author]Wataru Ikeda (Grad. Sch. of Chem. Sci. & Eng., Hokkaido Univ.)、Miki Senda (Struct. Biol. Res. Ctr., IMSS, KEK)、Takuya Ooki (Lab. of Microbial Carcinogenesis, Inst. of Microbial Chem.)、Toshiya Senda (Struct. Biol. Res. Ctr., IMSS, KEK)、Keiji Tanino (Dept. of Chem., Faculty of Sci., Hokkaido Univ.)、Masanori Hatakeyama (Lab. of Microbial Carcinogenesis, Inst. of Microbial Chem.)

The pro-oncogenic phosphatase SHP2 promotes Ras-MAP kinase signaling and serves as a downstream mediator of PD-1. In this study, we aimed to develop a novel allosteric inhibitor of SHP2 for anticancer therapy. High-throughput screening identified HA001, which showed SHP2-specific inhibition with little effect on its paralog SHP1. Given this, we systematically conducted structure-activity relationship analysis through organic chemical synthesis and obtained compound HA079, which showed greater inhibition of SHP2 ***in vitro*** compared to the first-in-class allosteric inhibitor SHP099. Treatment with HA079 significantly reduced the active form of ERK1/2 in multiple cancer cell lines and inhibited the ***in vivo*** growth of xenograft tumors in nude mice. Crystallographic and biochemical analyses revealed that, unlike other SHP2 inhibitors, HA079 covalently binds to SHP2 through amide bond formation between the side chain of R260 in SHP2 and the cyclic ester in HA079 to exhibit allosteric inhibition. In conclusion, we have developed a novel allosteric compound that might be a potential SHP2 inhibitor with an unprecedented mode of action.

[Disclosure of COI by Responsible researcher] Lead presenter is not the Responsible researcher.
[Lead presenter: Takeru Hayashi] ■Research funds from company: 公的研究費（日本学術振興会）
[Principal Researcher: Masanori Hatakeyama] ■Research funds from company: 公的研究費（AMED、日本学術振興会）

▲TOP

### [P-2241] I-P13-5
### Signal transduction inhibitor

2025 / 9 / 26 (Fri) 16:45-17:30 Room P/(Motenashi Dome) Room P

---

### P-2241
### Lymphatic Drug Delivery System for Metastatic Lymph Nodes Using an HDAC/PI3K Dual Inhibitor

[Speaker]Erika Tada (Tohoku Univ. Grad. Sch. of Dent.) [Co-author]Ariunbuyan Sukhbaatar (Tohoku Univ. Grad. Sch. of Dent.)、Shiro Mori (Tohoku Univ. Grad. Sch. of Dent.／Tohoku Univ. Grad. Sch. of Biomed. Eng.)、Ken Saijo (Tohoku Univ. Grad. Sch. of Med.)、Chikashi Ishioka (Tohoku Univ. Grad. Sch. of Med.)、Hisato Kawakami (Tohoku Univ. Grad. Sch. of Med.)、Tsuyoshi Sugiura (Tohoku Univ. Grad. Sch. of Dent.)、Tetsuya Kodama (Tohoku Univ. Grad. Sch. of Biomed. Eng.)

Histone deacetylase (HDAC) and phosphoinositide 3-kinase (PI3K) inhibitors are promising molecular targeted cancer drugs. The combination of these agents has shown superior antitumor activity, with FK-A11 being the most potent dual inhibitor identified to date. Our laboratory has developed a new treatment method for metastatic lymph node (LN) known as the lymphatic drug delivery system (LDDS), in which drugs are directly injected into the LN. In this study, we applied FK-A11 to LDDS to evaluate its therapeutic effect.
Tumor cells were inoculated into the sentinel LN of MXH54/Mo/lpr mice, inducing metastasis to the downstream LN. FK-A11 was then administered via LDDS. Tumor growth was assessed by measuring luciferase activity and LN volume.
The luciferase activity in both the sentinel and downstream LNs was significantly lower in the treated group. Additionally, LN volume increase was suppressed in the treated group, indicating the antitumor efficacy of FK-A11. These findings suggest that FK-A11 treatment via LDDS exhibits potent antitumor activity in metastatic LNs. In clinical applications, FK-A11 holds promise as a therapeutic agent for LN-targeted cancer treatment using LDDS.

[Disclosure of COI by Responsible researcher] Lead presenter is not the Responsible researcher.
[Lead presenter: Erika Tada] We have no financial relationships to disclose.
[Principal Researcher: Tetsuya Kodama] We have no financial relationships to disclose.

### [P-2242] I-P13-5
### Signal transduction inhibitor

2025 / 9 / 26 (Fri) 16:45-17:30 Room P/(Motenashi Dome) Room P

---

### P-2242
### DOT1L inhibition exerts anti-tumor effects through activating interferon signaling in breast cancer cells

[Speaker]Hiromu Suzuki (Div. Mol. Biol., Dept. Biochem., Sapporo Med. Univ., Sch. Med.) [Co-author]Kazuya Ishiguro (Div. Mol. Biol., Dept. Biochem., Sapporo Med. Univ., Sch. Med.)、Takeshi Niinuma (Div. Mol. Biol., Dept. Biochem., Sapporo Med. Univ., Sch. Med.)、Hiroshi Kitajima (Div. Mol. Biol., Dept. Biochem., Sapporo Med. Univ., Sch. Med.)、Reo Maruyama (Project Cancer Epigenome, The Cancer Inst., Japanese Found. Cancer Res.)、Kohei Kumegawa (NEXT-Ganken, Japanese Found. Cancer Res.)、Tomohide Tsukahara (Div. Pathol. 1, Dept. Pathol., Sapporo Med. Univ., Sch. Med.)、Mitsunobu Saito (Div. Mol. Biol., Dept. Biochem., Sapporo Med. Univ., Sch. Med.／Dept. Gastroenterol. Hepatol., Sapporo Med. Univ., Sch. Med.)、Eiichiro Yamamoto (Div. Mol. Biol., Dept. Biochem., Sapporo Med. Univ., Sch. Med.)、Masahiro Kai (Div. Mol. Biol., Dept. Biochem., Sapporo Med. Univ., Sch. Med.)、Hiroshi Nakase (Dept. Gastroenterol. Hepatol., Sapporo Med. Univ., Sch. Med.)

DOT1L, a histone H3 lysine 79 (H3K79) methyltransferase, is a potential therapeutic target in various cancer. In this study, we aimed to clarify the effects of DOT1L inhibition in breast cancer. Treatment with a DOT1L inhibitor suppressed proliferation and induced cell cycle arrest and apoptosis in estrogen receptor (ER)-positive/HER2-negative cancer cells (MCF7) as well as in ER-negative/HER2-positive cancer cells (SKBR3). Transcriptome and epigenome analysis revealed that DOT1L inhibition activated transcription of a number of interferon (IFN)-related genes (IRGs) in both cell lines. Notably, DOT1L inhibition induced DNA damage and upregulated levels of cytoplasmic DNA in breast cancer cells. CRISPR/Cas9-mediated knockout of STING in breast cancer cells significantly suppressed the IFN signaling activated by DOT1L inhibition and attenuated the anti-tumor effects. Moreover, single-cell ATAC-seq analysis revealed that DOT1L inhibition suppressed expression of ERBB2 in HER2-positive cells. These findings suggest that the anti-breast cancer cell effects of DOT1L inhibition are mediated by multiple mechanisms, including activation of innate immune signaling.

[Disclosure of COI by Responsible researcher] Lead presenter is the Responsible researcher.
[Lead presenter: Hiromu Suzuki] We have no financial relationships to disclose.

### [P-2243] I-P13-5
### Signal transduction inhibitor

2025 / 9 / 26 (Fri) 16:45-17:30 Room P/(Motenashi Dome) Room P

---

### P-2243
### JAK inhibitors paradoxically activate an active JAK mutant, leading to discontinuation syndrome

[Speaker]Makio Higuchi (Dept. of Fundamental Biosci. Shiga University of Med. Sci.)

ATP-competitive kinase inhibitors have achieved significant success as anticancer agents and have become one of the mainstays of cancer therapy. However, unexpected side effects remain, and the underlying mechanisms are not fully understood. In myelofibrosis, active mutation JAK2-V617F is frequently observed and JAK inhibitors are commonly used for treatment. Several reports showed that discontinuation of JAK inhibitors occasionally lead to a severe adverse effects, however the molecular mechanisms of the adverse effect remain poorly understood.I investigated the molecular-level effects of JAK inhibitors on JAK family, a member of the JAK family, using JAK1 mutants and a newly developed FRET probe. These findings revealed that JAK inhibitors have an allosteric effect on JAK1, resulting in excessive phosphorylation of JAK. Furthermore, this allosteric effect paradoxically activates the JAK1-V658F mutant which is a variant homologous to JAK2-V617F and a reduction in inhibitor concentration leads to excessive activation of STATs.These findings showed the molecular mechanisms of discontinuation adverse effect, providing potentially important clues for the safe JAK inhibitors treatment.

[Disclosure of COI by Responsible researcher] Lead presenter is the Responsible researcher.
[Lead presenter: Makio Higuchi] ■Research funds from company: 武田科学振興財団　研究助成金、科研費（23K19480）

Poster Session

### [P-2244] I-P13-5
### Signal transduction inhibitor

2025 / 9 / 26 (Fri) 16:45-17:30 Room P/(Motenashi Dome) Room P

---

### P-2244
### Development of novel anticancer agent targeting nucleolar stress pathway

[Speaker]Yoshimi Shimizu (Dept Biochem, Showa Med Univ Sch Med／Res Ctr Biochem, Showa Med Univ) [Co-author]Miki Nishio (Div Mol Cell Biol, Kobe Univ Grad Sch Med)、Junji Otani (Div Mol Cell Biol, Kobe Univ Grad Sch Med)、Akira Suzuki (Dept Biochem, Showa Med Univ Sch Med／Div Mol Cell Biol, Kobe Univ Grad Sch Med)、Tomohiko Maehama (Dept Biochem, Showa Med Univ Sch Med／Res Ctr Biochem, Showa Med Univ)

Nucleolar stress is the cellular response toward impaired ribosome biogenesis by the inhibition of ribosomal RNA synthesis and/or the imbalance of ribosomal protein levels. The nucleolar stress eventually activates p53, leading to cell cycle arrest and/or cell death. The nucleolar stress pathway has been a promising anticancer drug target since p53 activation by this response proceeds without DNA damage. A nucleolar protein PICT1 plays a key role in the nucleolar stress response. The nucleolar stress induces PICT1 degradation that leads to RPL11 protein translocation from the nucleolus to the nucleoplasm, followed by MDM2 inhibition and p53 activation. We recently found a ribosomal protein RPx bound to and stabilized PICT1 protein under non-stressed conditions, suggesting PICT1-RPx disruption triggers the nucleolar stress to activate p53. Chemical library screening extracted 8 compounds as the PICT1-RPx disruptors, and R-11 compound induced PICT1 decrease and p53 activation without DNA damage response in U2OS cells. R-11 repressed U2OS cell proliferation with IC50 of 0.78 uM. In this report, we will discuss the in vivo effect of R-11 and hit compounds other than R-11.

[Disclosure of COI by Responsible researcher] Lead presenter is not the Responsible researcher.
[Lead presenter: Yoshimi Shimizu] We have no financial relationships to disclose.
[Principal Researcher: Tomohiko Maehama] ■Research expenses: 小野医学研究財団研究助成金

### [P-2254] I-P14-8
### Combinational therapy/Adjuvant therapy

2025 / 9 / 26 (Fri) 16:45-17:30 Room P/(Motenashi Dome) Room P

---

### P-2254
### Amoxicillin, a β-lactam antibiotic, enhances cisplatin sensitivity in cancer cells affecting mitochondria.

[Speaker]Kazuo Tomita (Applied Pharm. Kagoshima Univ. Grad. Sch. Med. & Dent. Sci.) [Co-author]Yoshikazu Kuwahara (Applied Pharm. Kagoshima Univ. Grad. Sch. Med. & Dent. Sci.／Radiat. Biol. & Med. Tohoku Med. & Pharm. Univ.)、Kento Igarashi (Applied Pharm. Kagoshima Univ. Grad. Sch. Med. & Dent. Sci.)、Akihiro Kurimasa (Radiat. Biol. & Med. Tohoku Med. & Pharm. Univ.)、Tomoaki Sato (Applied Pharm. Kagoshima Univ. Grad. Sch. Med. & Dent. Sci.)

We investigated whether amoxicillin (AMPC) enhances anticancer effects. Compared with controls, treatment of cervical cancer (HeLa) and oral squamous cell carcinoma (SAS) cells with 10 μg/mL AMPC significantly increased mitochondrial reactive oxygen species levels, decreased mitochondrial membrane potential, and reduced mitochondrial $Fe^{2+}$ levels. The viability of HeLa and SAS cells was significantly reduced when cisplatin was administrated after AMPC; however, this effect was not observed in normal human fibroblast-like lung cells or human periodontal ligament fibroblast cells. AMPC did not enhance the anticancer effect of docetaxel. The AMPC-enhanced anticancer effect of cisplatin was blocked by the iron-chelating agent phenanthroline, indicating that the effect was primarily driven by ferroptosis, an iron-dependent form of cell death.AMPC transporters PEPT1 and PEPT2 expressions were higher in HeLa and SAS cells than in the normal cells examined in this study. This phenomenon facilitates greater AMPC uptake and mitochondrial dysfunction in cancer cells. We conclude that AMPC enhances the sensitivity of cancer cells to chemotherapy by impairing mitochondrial function.

[Disclosure of COI by Responsible researcher] Lead presenter is the Responsible researcher.
[Lead presenter: Kazuo Tomia] We have no financial relationships to disclose.

### [P-2255] I-P14-8
### Combinational therapy/Adjuvant therapy

2025 / 9 / 26 (Fri) 16:45-17:30 Room P/(Motenashi Dome) Room P

### P-2255
### Effect of Synbiotics on Cancer Sarcopenia

[Speaker]Isao Kawahara (Dept. Mol. Pathol., Nara. Med. Univ.／Div. Rehab., Hanna Central Hosp.) [Co-author]Rina Tani (Dept. Mol. Pathol., Nara. Med. Univ.)、Shota Nukaga (Dept. Mol. Pathol., Nara. Med. Univ.／Div. Rehab., Hanna Central Hosp.)、Ryoichi Nishida (Dept. Mol. Pathol., Nara. Med. Univ.)、Chie Nakashima (Dept. Mol. Pathol., Nara. Med. Univ.)、Kei Goto (Dept. Mol. Pathol., Nara. Med. Univ.)、Yi Luo (Dept. Mol. Pathol., Nara. Med. Univ.)、Bhawal Ujjal (Rios, Nihon Univ., Dental Sci. at Matsudo)、Kaoru Kubo (Dept. Mol. Pathol., Nara. Med. Univ.)、Hiroki Kuniyasu (Dept. Mol. Pathol., Nara. Med. Univ.)

Cancer sarcopenia contributes to poor prognosis in cancer patients, making the development of effective treatments a critical priority. Dysbiosis associated with cancer has been suggested to contribute to the development of cancer cachexia and sarcopenia. In this study, we employed a mouse model of cancer cachexia using the CT26 mouse colon cancer cell line, and conducted a nutritional intervention focusing on synbiotics. As a synbiotics, we used GFOB diet; a mixture of Glutamine, dietary Fiber, and Oligosaccharides and Bifidobacterium longum. Compared to mice fed a standard diet, the GFOB fed group did not show improvement in body weight loss or skeletal muscle atrophy, but adipose tissue mass was preserved. Fecal short-chain fatty acids increased 2.3-fold, and blood ketone body levels increased 1.8-fold. In skeletal muscle, the expression of MYH7 and MYH4, markers of muscle maturation, was increased in the gastrocnemius muscle, a representative fast-twitch muscle. These findings suggest that modulation of the gut microbiota via synbiotics may exert a protective effect on slow-twitch muscles, which are particularly vulnerable to cancer sarcopenia.

[Disclosure of COI by Responsible researcher] Lead presenter is not the Responsible researcher.
[Lead presenter: Kawahara Isao] We have no financial relationships to disclose.
[Principal Researcher: Kuniyasu Hiroki] We have no financial relationships to disclose.

### [P-2256] I-P14-8
### Combinational therapy/Adjuvant therapy

2025 / 9 / 26 (Fri) 16:45-17:30 Room P/(Motenashi Dome) Room P

---

### P-2256
### Targeting LIG1 and PARP for synthetic lethality-based treatment in prostate cancer

[Speaker]Masaru Tani (Department of Urology, The University of Osaka) [Co-author]Koji Hatano (Department of Urology, The University of Osaka)、Yu Ishizuya (Department of Urology, The University of Osaka)、Keisuke Nimura (Gunma University Initiative for Advanced Research)、Atsuki Matsukawa (Department of Urology, The University of Osaka)、Tomohiro Kanaki (Department of Urology, The University of Osaka)、Liu Yutong (Department of Urology, The University of Osaka)、Akihiro Yoshimura (Department of Urology, The University of Osaka)、Toshiki Oka (Department of Urology, The University of Osaka)、Takuji Hayashi (Department of Urology, The University of Osaka)、Yoshiyuki Yamamoto (Department of Urology, The University of Osaka)、Taigo Kato (Department of Urology, The University of Osaka)、Atsunari Kawashima (Department of Urology, The University of Osaka)、Norio Nonomura (Department of Urology, The University of Osaka)

[Background]In prostate cancer (PCa), PARP inhibitors (PARPis) are approved only for patients with BRCA mutations. Identifying genes exhibiting synthetic lethality with PARP may broaden therapeutic options. We aimed to discover such genes and establish novel combination therapies.[Methods]A genome-wide CRISPR-Cas9 screen was conducted in PCa cell lines (DU145, 22Rv1, LNCaP) treated with olaparib. Candidate genes were knocked out to assess effects on proliferation and DNA damage. Combination therapy was tested in vitro and in vivo.[Results]Seven genes, including LIG1, were identified (FDR<0.01). LIG1 knockout enhanced sensitivity to PARPis and increased DNA damage (p<0.001). Combined LIG1 and PARP inhibition reduced cell growth with synergistic effects. In vivo, combination therapy suppressed tumor growth more than either monotherapy (p<0.001). LIG1 expression was higher in castration resistant PCa tissues than in normal or localized hormone-sensitive PCa (p<0.001).[Conclusions]LIG1 is a novel synthetic lethality gene with PARP. Its inhibition enhances olaparib efficacy in vitro and in vivo, supporting its potential as a therapeutic target in PCa, regardless of BRCA.

[Disclosure of COI by Responsible researcher] Lead presenter is not the Responsible researcher.
[Lead presenter: Masaru Tani] We have no financial relationships to disclose.
[Principal Researcher: Yu Ishizuya] We have no financial relationships to disclose.

### [P-2257] I-P14-8
### Combinational therapy/Adjuvant therapy

2025 / 9 / 26 (Fri) 16:45-17:30 Room P/(Motenashi Dome) Room P

---

### P-2257
### Targeting DNA Repair Pathways in TNBC via Combined FAK and PARP Inhibition Independent of BRCA1 Status

[Speaker]Fumiaki Sato (Juntendo Univ. Faculty of Pharm.) [Co-author]Hideyuki Takeshima (Hoshi Univ. Sch. of Pharm. Sci.)、Yoko Tabe (Juntendo Univ. Faculty of Pharm.)

Triple-negative breast cancer (TNBC) is an aggressive subtype of breast cancer that lacks hormone receptors and HER2 expression, and thus has limited treatment options. While PARP inhibitors have shown efficacy in patients with BRCA1/2 mutations, their benefit in BRCA1/2 wild-type TNBC remains limited. In this study, we aimed to evaluate the therapeutic potential of combined FAK and PARP inhibition in TNBC with either wild-type or mutant BRCA1. FAK inhibitor induced apoptosis and cell cycle arrest, thereby reducing TNBC cell proliferation. Transcriptomic analysis by RNA-seq revealed that FAK inhibition downregulated the expression of genes associated with DNA repair, as well as cell cycle-regulated genes. In BRCA1 wild-type TNBC cells, FAK inhibition decreased BRCA1 expression, possibly due to mitochondrial dysfunction, and increased γH2AX levels, indicating enhanced DNA damage. Notably, the combination of FAK inhibitor and PARP inhibitor significantly suppressed cell proliferation in both BRCA1 wild-type and mutant TNBC cells. These results suggest that dual inhibition of FAK and PARP may represent a promising treatment strategy for TNBC, irrespective of BRCA1 mutation status.

[Disclosure of COI by Responsible researcher] Lead presenter is the Responsible researcher.
[Lead presenter: Fumiaki Sato] We have no financial relationships to disclose.

### [P-2258] I-P14-8
### Combinational therapy/Adjuvant therapy

2025 / 9 / 26 (Fri) 16:45-17:30 Room P/(Motenashi Dome) Room P

---

### P-2258
### Targeting EGFR with Two DNA Intercalators to Inhibit Breast Cancer Metastasis

[Speaker]Chih-Chun Chang (Graduate Institute of Biotechnology, NCHU)

Chemotherapeutic drugs are commonly used to treat cancers lacking targeted therapy options. However, their low specificity limits their treatment effectiveness. We report here that the cooperative binding of Doxorubicin (Dox) with Actinomycin D (ActD) enhances the specificity for consecutive GCCG sites in DNA. Using X-ray crystallography, we determined the crystal structure of ActD and Dox bound to d(AGCCGT)2 DNA. ActD intercalation at the GpC site induces a novel Dox binding mode at the adjacent CpG step. This ensures a snug fit, avoids steric clashes, and enhances the specificity. Transcriptome analysis revealed that combining Dox with ActD synergistically down-regulates EGFR in TNBC cells. Additionally, it reduces EGFR promoter activity. In vivo, the combination significantly suppresses tumor growth and outperforms the standard Dox and cyclophosphamide regimen in inhibiting metastasis. This study highlights targeting the activated EGFR pathway with sequence-specific DNA-targeting drug combinations as a potential TNBC treatment.

[Disclosure of COI by Responsible researcher] Lead presenter is not the Responsible researcher.
[Lead presenter: Chih-Chun Chang] We have no financial relationships to disclose.
[Principal Researcher: ] We have no financial relationships to disclose.

### [P-2259] I-P14-8
### Combinational therapy/Adjuvant therapy

2025 / 9 / 26 (Fri) 16:45-17:30 Room P/(Motenashi Dome) Room P

---

### P-2259
### Withdrawn

No Abstract

### [P-2260] I-P14-8
### Combinational therapy/Adjuvant therapy

2025 / 9 / 26 (Fri) 16:45-17:30 Room P/(Motenashi Dome) Room P

---

### P-2260
### Dimethoxyflavones potentiate the antitumor effects of microtubule polymerization inhibitors

[Speaker]Tomoya Masuoka (Grad. Sch. Health. Sport. Sci., Toyo Univ.,) [Co-author]Kenshin Oba (Grad. Sch. Health. Sport. Sci., Toyo Univ.,)、Takumu Yamada (Grad. Sch. Sci. Eng., Toyo Univ.,)、Takumi Iwasawa (Inst. of Life Inovation Studies., Toyo Univ.,)、Kazunori Kato (Grad. Sch. Health. Sport. Sci., Toyo Univ.,／Inst. of Life Inovation Studies., Toyo Univ.,)

We previously demonstrated that nobiletin (5,6,7,8,3´,4´-Hexamethoxyflavone), a polymethoxyflavone (PMF) in citrus fruit peels, enhances the antitumor efficacy of the microtubule polymerization inhibitor DM1 (Mertansine). Screening 73 species of PMFs led to the identification of novel PMFs: 2´,4´-Dimethoxyflavone (2´,4´-DiMF) and 2´,5´-DiMF with antitumor enhancing effect. We also showed that PMFs enhanced the tumor-specific effect of DM1-conjugated antibody-drug conjugates (ADCs), suggesting that nobiletin and DiMFs act via distinct mechanisms. In this study, we aimed to determine whether the antitumor effects of chemotherapeutic agents could be enhanced by DiMFs. As expected, DiMFs significantly enhanced the effect of microtubule polymerization inhibitors but had no effect when combined with antimetabolites or topoisomerase I inhibitors. We found that DiMFs increased the G2/M phase cell population and induced M-phase arrest. Our results show that DiMFs enhance microtubule polymerization inhibition, suggesting that combining microtubule polymerization inhibitors with PMFs could be a promising chemotherapeutic adjuvant strategy.

[Disclosure of COI by Responsible researcher] Lead presenter is not the Responsible researcher.
[Lead presenter: Tomoya Masuoka] We have no financial relationships to disclose.
[Principal Researcher: Kazunori Kato] We have no financial relationships to disclose.

### [P-2261] I-P14-8
### Combinational therapy/Adjuvant therapy

2025 / 9 / 26 (Fri) 16:45-17:30 Room P/(Motenashi Dome) Room P

---

### P-2261
### Shortening of the G2 phase avoids senescence and provides synergistic cytotoxicity with DNA replication stress inducers

[Speaker]Makoto Iimori (Oral Med. Res.Ctr., Fukuoka Dent. Col.) [Co-author]Kentaro Nonaka (Dept. Surg.Sci., Grad. Sch. Med. Sci., Kyushu Univ.)、Hiroyuki Kitao (Oral Med. Res.Ctr., Fukuoka Dent. Col.)

The cellular response to DNA replication stress (DRS) provoked by anticancer drugs involves activation of the G2/M checkpoint, which promotes transient cell cycle arrest at G2 phase and DNA repair, and consequent induction of apoptosis or senescence. Here, we found that the G2-phase cells undergo senescence transition (indicated by mitosis skip) via prolonged G2 duration due to activation of p53-p21 and ATR. The senescence transition requires sufficient G2 duration and the temporal regulation by G2/M checkpoint inhibitors causes a shortening of the prolonged G2 duration in cells treated with DRS-inducing drugs. The shortening of G2 duration by G2/M checkpoint inhibitors leads not only to a switch in cell fate from senescence to mitotic entry, but also to effective cell death through the carry-over of chromosomal aberrations generated by DRS-inducing drugs into mitosis and subsequent mitotic progression. Such enhanced cell death is also observed in p53 deficient cells which do not originally undergo senescence. We propose that the temporal regulation of G2 phase is an approach for synergistic effects of DRS-inducing drugs in a manner that is independent of the p53 status.

[Disclosure of COI by Responsible researcher] Lead presenter is the Responsible researcher.
[Lead presenter: Makoto Iimori] ■Research funds from company: 大鵬薬品工業株式会社

▲TOP

### [P-2262] I-P14-8
### Combinational therapy/Adjuvant therapy

2025 / 9 / 26 (Fri) 16:45-17:30 Room P/(Motenashi Dome) Room P

---

### P-2262
### Development of novel combination cancer therapy with a catalytic inhibitor of PARP1

[Speaker]Qianqian Guo (Juntendo Univ. Grad. Sch. of Med. Dept. Clin. Oncology ／Juntendo Univ. LC. Development and Res. of cancer Med.)
[Co-author]Shigeaki Sunada (Juntendo Univ. Grad. Sch. of Med. Dept. Clin. Oncology ／Juntendo Univ. JARIHES)、Min Han (Juntendo Univ. Grad. Sch. of Med. Dept. Clin. Oncology)、Shunsuke Kato (Juntendo Univ. Grad. Sch. of Med. Dept. Clin. Oncology)

Combination therapy offers a promising strategy to overcome the limitations of monotherapy in cancer treatment. We performed phenotypic screening to identify novel DNA repair regulators that enhance the synergistic effect when combined with DNA damaging agents. Among the hit compounds, C#6 showed resistance when combined with the PARP inhibitor talazoparib. Further analysis revealed that C#6 reduced PARP1-mediated Poly(ADP-ribosyl)ation and directly interacted with PARP1, suggesting it functions as a catalytic PARP1 inhibitor rather than a PARP trapper. As such, C#6 is likely to suppress single-strand break (SSB) repair. We then assessed the combination of C#6 with the DNA-alkylating agent methyl methanesulfonate (MMS), which generates SSB intermediates during base excision repair. The combination synergistically increased DNA double-strand breaks (DSBs), indicating enhanced cytotoxicity. These findings support the potential of C#6 as part of a novel combination therapy targeting DNA repair pathways.

[Disclosure of COI by Responsible researcher] Lead presenter is not the Responsible researcher.
[Lead presenter: Qianqian Guo] We have no financial relationships to disclose.
[Principal Researcher: Shigeaki Sunada] ■Research funds from company: 文部科学省卓越研究員事業（ JPMXS0320210041）

Poster Session

### [P-2272] I-P17-2
### Multimodal treatment in surgery

2025 / 9 / 26 (Fri) 16:45-17:30 Room P/(Motenashi Dome) Room P

---

### P-2272
### Outcomes of Conversion Surgery After Combined Immunotherapy for cM1 and Recurrent Esophageal Cancer

[Speaker]Tomoki Makino (Department of Gastroenterological Surgery, The University of Osaka) [Co-author]Shigeto Nakai (Department of Gastroenterological Surgery, The University of Osaka)、Takaomi Hagi (Department of Gastroenterological Surgery, The University of Osaka)、Kota Momose (Department of Gastroenterological Surgery, The University of Osaka)、Kotaro Yamashita (Department of Gastroenterological Surgery, The University of Osaka)、Takuro Saito (Department of Gastroenterological Surgery, The University of Osaka)、Koji Tanaka (Department of Gastroenterological Surgery, The University of Osaka)、Tsuyoshi Takahashi (Department of Gastroenterological Surgery, The University of Osaka)、Yukinori Kurokawa (Department of Gastroenterological Surgery, The University of Osaka)、Hidetoshi Eguchi (Department of Gastroenterological Surgery, The University of Osaka)、Yuichiro Doki (Department of Gastroenterological Surgery, The University of Osaka)

Background) ICIs are standard first-line therapy for cM1 or recurrent esophageal cancer, but global data on conversion surgery post-ICI are limited.Methods) We retrospectively analyzed 26 patients who underwent conversion surgery after ICI therapy from Dec 2021 to Dec 2024.Results) Median age was 67.5 (range: 42-82), 17 males and 9 females. PS was 0 in 23, 1 in 3. Twenty had unresectable disease (cM1a: 5, cM1b: 17, cT3br-T4: 3; overlaps included), 6 had recurrence (regional LN: 5, distant: 1). Median FP+ICI courses: 3.5 (1-7). Best responses: CR/PR/SD/PD = 1/22/2/1. Surgery reasons: cM1 disappearance (13), solitary recurrence (6), others (7). R0 resection in all but one (R1, tracheal invasion). Pathologic grades: 0/1a/1b/2/3/NA = 0/7/3/4/8/4. Postop treatment: surgery alone (13), continued ICI (12), non-curative surgery (1). Recurrence in 7 (27%): local (3), distant LN (2), metastases (4), peritoneal (1). Median OS not reached; 1-year OS: 85.3%. Unresectable/recurrent groups: OS not reached/86.8%, 20.8 mo/83.3%.Conclusion) Conversion surgery after ICI may be effective in selected cM1/recurrent esophageal cancer cases. We will present surgical strategies, videos, and future outlook.

[Disclosure of COI by Responsible researcher] Lead presenter is the Responsible researcher.
[Lead presenter: Tomoki Makino] ■Lecture fee: 小野薬品工業･ﾌﾞﾘｽﾄﾙﾏｲﾔｰｽﾞ スクイブ、MSD株式会社

### [P-2273] I-P17-2
### Multimodal treatment in surgery

2025 / 9 / 26 (Fri) 16:45-17:30 Room P/(Motenashi Dome) Room P

### P-2273
### Outcome of conversion surgery for HER2-negative Stage IV gastric cancer

[Speaker]Hiroshi Nakade (Nara Medical University Department of Surgery) [Co-author]Sohei Matumoto (Nara Medical University Department of Surgery)、Tomohiro Kunishige (Nara Medical University Department of Surgery)、Shintaro Miyao (Nara Medical University Department of Surgery)、Satoko Aoki (Nara Medical University Department of Surgery)、Takanari Tatsumi (Nara Medical University Department of Surgery)、Masahiro Soga (Nara Medical University Department of Surgery)、Masayuki Sho (Nara Medical University Department of Surgery)

[Background] Conversion surgery (CS) for Stage IV gastric cancer is recommended when chemotherapy is effective and R0 resection is possible. For HER2-negative gastric cancer, the outcomes of Nivolumab+chemotherapy followed by surgery remain unclear.[Objective] To assess CS outcomes in Stage IV gastric cancer.[Methods] Retrospective analysis of 31 CS patients among 86 HER2-negative Stage IV gastric cancer cases (2010-2023).[Results] Of 86 patients, 64 received chemotherapy alone, and 22 received Nivolumab+chemotherapy. CS was performed in 31 cases. R0:27, R1/2:4. Preoperative regimens: chemotherapy:25, Nivo+SOX:6. CS group showed better OS/PFS than non-resection group (P=0.005/P<0.001). The Nivo+Chemo group had a higher Grade 2+ histological response.[Conclusion] CS may provide long-term survival benefit. Nivo+Chemo showed better histological response, but further studies needed.

[Disclosure of COI by Responsible researcher] Lead presenter is the Responsible researcher.
[Lead presenter: Hiroshi Nakade] We have no financial relationships to disclose.

### [P-2274] I-P17-2
### Multimodal treatment in surgery

2025 / 9 / 26 (Fri) 16:45-17:30 Room P/(Motenashi Dome) Room P

---

### P-2274
### Simultaneous colorectal and liver resection for colorectal cancer with synchronous liver metastasis

[Speaker]Yukiharu Hiyoshi (Department of Gastroenterological Surgery, Kumamoto University) [Co-author]Yuji Miyamoto (Department of Gastroenterological Surgery, Kumamoto University)、Kohei Yamashita (Department of Gastroenterological Surgery, Kumamoto University)、Kota Arima (Department of Gastroenterological Surgery, Kumamoto University)、Keisuke Kosumi (Department of Gastroenterological Surgery, Kumamoto University)、Kazuto Harada (Department of Gastroenterological Surgery, Kumamoto University)、Kojiro Eto (Department of Gastroenterological Surgery, Kumamoto University)、Satoshi Ida (Department of Gastroenterological Surgery, Kumamoto University)、Hiromitsu Hayashi (Department of Gastroenterological Surgery, Kumamoto University)、Masaaki Iwatsuki (Department of Gastroenterological Surgery, Kumamoto University)

AimsTo elucidate the safety and utility of simultaneous colorectal and liver resection for patients with colorectal cancer with synchronous liver metastasis.MethodsShort- and long-term outcomes of 121 patients who underwent simultaneous resection were analyzed.ResultsThe primary site was right colon/left colon/rectum: 31/54/36. The number of liver metastasis (median): 2, tumor diameter (median): 24 mm. The resection approach (colon/liver) was Lap/Lap: 48 (including Ro/Ro: 2 cases), Lap/Open: 33, Open/Open: 28, Open/Lap: 3, and other: 9. Short-term results were operative time (median): 584 minutes, blood loss (median): 395 g. Complications of CD grade 3 or higher were observed in 28 patients (23%). In 48 complete laparoscopic resection cases, median operative time: 544 minutes, median blood loss 135 g, 4 cases (8.3%) with CD grade 3 or higher complications. The 3-year PFS and 3-year OS were 32% and 75%, respectively.ConclusionsSimultaneous colorectal and liver resection for colorectal cancer with synchronous liver metastasis was safely performed. Complete laparoscopic resection has been shown to reduce blood loss and postoperative complication rates and may be a useful option.

[Disclosure of COI by Responsible researcher] Lead presenter is the Responsible researcher.
[Lead presenter: Yukiharu Hiyoshi] We have no financial relationships to disclose.

### [P-2275] I-P17-2
### Multimodal treatment in surgery

2025 / 9 / 26 (Fri) 16:45-17:30 Room P/(Motenashi Dome) Room P

---

### P-2275
### Repeated R0 resection in cases of synchronous liver metastasis in colorectal cancer

[Speaker]Tetsuro Kawazoe (Dept. of Surgery and Science, Kyushu Univ.) [Co-author]Koji Ando (Dept. of Surgery and Science, Kyushu Univ.)、Yuichi Hisamatsu (Dept. of Surgery and Science, Kyushu Univ.)、Eiji Oki (Dept. of Surgery and Science, Kyushu Univ.)、Tomoharu Yoshizumi (Dept. of Surgery and Science, Kyushu Univ.)

Background: Guidelines recommend resection of primary and liver metastatic lesions for CRC with resectable liver metastases (LM). However, the significance of repeated liver resection has not been clearly defined. Objective: To assess outcomes of CRC with synchronous LM, focusing on the impact of R0 and repeat R0 resections.Methods: We reviewed 135 patients with synchronous LM who began treatment between Jan 2013 and Mar 2023. Median age: 65 ; 56% male. Liver-limited H1 cases (n=33) were judged resectable. Among 102 initially unresectable cases, 31 (30%) received R0 after chemotherapy.Results: R0 resection was an independent favorable prognostic factor for OS (HR=0.14, p<0.01). Among 62 patients with R0, 50 (81%) relapsed, and 28 (56%) underwent repeat R0. Repeat R0 resection led to significantly better OS (HR=0.32, p=0.02) and was more common in patients with initial liver-limited disease (p=0.04).Conclusion: R0 resection improves prognosis in CRC with synchronous LM. Repeat R0 surgery also contributes to favorable survival, especially in patients with liver-limited disease.

[Disclosure of COI by Responsible researcher] Lead presenter is the Responsible researcher.
[Lead presenter: Tetsuro Kawazoe] We have no financial relationships to disclose.

### [P-2276] I-P17-2
### Multimodal treatment in surgery

2025 / 9 / 26 (Fri) 16:45-17:30 Room P/(Motenashi Dome) Room P

---

### P-2276
### Conversion surgery for unresectable pancreatic ductal adenocarcinoma

[Speaker]Koji Kubota (Department of surgery, Shinshu University School of Medicine) [Co-author]Akira Shimizu (Department of surgery, Shinshu University School of Medicine)、Tuyoshi Notake (Department of surgery, Shinshu University School of Medicine)、Kiyotaka Hosoda (Department of surgery, Shinshu University School of Medicine)、Hiroki Sakai (Department of surgery, Shinshu University School of Medicine)、Hikaru Hayashi (Department of surgery, Shinshu University School of Medicine)、Koya Yasukawa (Department of surgery, Shinshu University School of Medicine)、Shinsuke Sugenoya (Department of surgery, Shinshu University School of Medicine)、Shiori Yamazaki (Department of surgery, Shinshu University School of Medicine)、Hidenori Tomida (Department of surgery, Shinshu University School of Medicine)、Reina Miyazawa (Department of surgery, Shinshu University School of Medicine)、Yuji Soejima (Department of surgery, Shinshu University School of Medicine)

Backgrounds: The long-term outcomes of conversion surgery (CS) for unresectable pancreatic ductal adenocarcinoma (UR-PDAC) remain unclear.Methods: A retrospectively analysis was conducted on 78 patients with UR-PDAC treated from 2012 to 2024. Patients were divided into a CS group (n=9) and a non-CS (NCS) group (n=71) to compare treatment outcomes.Results: There were no significant differences between the two groups in tumor location, the proportion of UR-LA/UR-M, or CEA levels, whereas CA19-9 levels were significantly lower in the CS group (19.8 vs 173.9 U/ml, p<0.001). In the CS group, GnP was more frequently selected as first-line therapy (78% vs. 15%, p<0.001). PD was performed in 7 (78%), whereas DP in 2 (22%). Portal vein resection was necessary in 7 (78%). Pathological staging was as follows: ypStage: 0 (n=1), IIA (n=5), IIB (n=2), IV (n=1). OS was significantly better in the CS than the NCS group (MST: not reached vs 489 days , p<0.001). Multivariate analysis identified CS (HR 0.161, 95% CI 0.049-0.527, p = 0.003) and CEA<4 ng/ml (HR 0.440, 95% CI 0.260-0.744, p = 0.002) were the independent prognostic factor for OS.Conclusion: CS may improve long-term prognosis in UR-PDAC.

[Disclosure of COI by Responsible researcher] Lead presenter is not the Responsible researcher.
[Lead presenter: Koji Kubota] We have no financial relationships to disclose.
[Principal Researcher: Yuji Soejima] We have no financial relationships to disclose.

Poster Session

### [P-2277] I-P17-2
### Multimodal treatment in surgery

2025 / 9 / 26 (Fri) 16:45-17:30 Room P/(Motenashi Dome) Room P

---

### P-2277
### Conversion Surgery for unresectable pancreatic cancer

[Speaker]Yoshihiro Shirai (Division of Hepatobiliary and Pancreas Surgery, Jikei University) [Co-author]Takeshi Gocho (Division of Hepatobiliary and Pancreas Surgery, Jikei University)、Michinori Matsumoto (Division of Hepatobiliary and Pancreas Surgery, Jikei University)、Yoshiaki Tanji (Division of Hepatobiliary and Pancreas Surgery, Jikei University)、Norimitsu Okui (Division of Hepatobiliary and Pancreas Surgery, Jikei University)、Ryoga Hamura (Division of Hepatobiliary and Pancreas Surgery, Jikei University)、Masashi Tsunematsu (Division of Hepatobiliary and Pancreas Surgery, Jikei University)、Yosuke Igarashi (Division of Hepatobiliary and Pancreas Surgery, Jikei University)、Taro Sakamoto (Division of Hepatobiliary and Pancreas Surgery, Jikei University)、Tadashi Uwagawa (Division of Hepatobiliary and Pancreas Surgery, Jikei University)、Tomohiko Taniai (Division of Hepatobiliary and Pancreas Surgery, Jikei University)、Mitsuru Yanagaki (Division of Hepatobiliary and Pancreas Surgery, Jikei University)、Koichiro Haruki (Division of Hepatobiliary and Pancreas Surgery, Jikei University)、Kenei Furukawa (Division of Hepatobiliary and Pancreas Surgery, Jikei University)、Toru Ikegami (Division of Hepatobiliary and Pancreas Surgery, Jikei University)

The standard treatment for unresectable pancreatic cancer is multimodal therapy including chemotherapy. Even if the disease is controlled by multimodal therapy, the probability of complete response is extremely low. Surgical resection is a potentially curative treatment, but the indications for surgery have been limited. Recent advances in chemotherapy have increased the number of cases that can be converted from unresectable to resectable. We describe a case in which conversion surgery was indicated due to the response to preoperative treatment, and indicate the importance of multidisciplinary treatment. The patient is a 76-year-old woman. Serum CA19-9 level was 60 IU/ml. The pancreatic body tumor invaded the celiac artery and portal vein, and a diagnosis of URLA pancreatic cancer was made. Seven courses of gemcitabine and nab-paclitaxel therapy were administered. Additional radiation therapy (54 Gy) with gemcitabine was also induced. Distal pancreatectomy with celiac axis resection and partial gastrectomy were performed. And the patient achieved R0 resection. Postoperative adjuvant chemotherapy was introduced, and the patient has been recurrence-free for 8 months.

[Disclosure of COI by Responsible researcher] Lead presenter is the Responsible researcher.
[Lead presenter: Yoshihiro Shirai] We have no financial relationships to disclose.

### [P-2278] I-P17-2
### Multimodal treatment in surgery

2025 / 9 / 26 (Fri) 16:45-17:30 Room P/(Motenashi Dome) Room P

---

### P-2278
### The significance of neoadjuvant therapy for pancreatic cancer

[Speaker]Hirofumi Akita (Department of Gastroenterological Surgery,The Univarsity of Osaka) [Co-author]Yosuke Mukai (Department of Gastroenterological Surgery,The Univarsity of Osaka)、Kazuki Sasaki (Department of Gastroenterological Surgery,The Univarsity of Osaka)、Shinichiro Hasegawa (Department of Gastroenterological Surgery,The Univarsity of Osaka)、Daisaku Yamada (Department of Gastroenterological Surgery,The Univarsity of Osaka)、Yoshito Tomimaru (Department of Gastroenterological Surgery,The Univarsity of Osaka)、Takehiro Noda (Department of Gastroenterological Surgery,The Univarsity of Osaka)、Hidenori Takahashi (Department of Gastroenterological Surgery,The Univarsity of Osaka)、Shogo Kobayashi (Department of Gastroenterological Surgery,The Univarsity of Osaka)、Yuichiro Doki (Department of Gastroenterological Surgery,The Univarsity of Osaka)、Hidetoshi Eguchi (Department of Gastroenterological Surgery,The Univarsity of Osaka)

(Background)Prognosis of pancreatic cancer (PC) is still poor and neoadjuvant therapy (NAT) is commonly performed for better prognosis. (Patients) We reviewed the outcome of 442 PC patients receiving radical surgery from 2007 to 2022 in our institute. (Result)This study included 313 resectable (R), 95 boderline resectable (BR) and 34 unresectable (UR) PC patients. NAT was performed in 63.3%, 88.4%, and 97.1% of each status respectively. The NAT period was 2.9 months, 4.2 months and 8.3 months respectively, which was significantly longer according to resectability status ($p<0.001$). In R-PC patients, 5-year overall survival rate (5y-OS) of patients receiving NAT was 42.0%, significantly better than patients without NAT (28.7%, $p=0.018$). In 315 PC patients receiving NAT, 5y-OS of R-PC was significantly better than BR-PC (42.0% vs 28.0%, $p=0.048$), partially because distant recurrence rate of BR-PC was marginally higher than R-PC ($p=0.058$). 5y-OS of UR-PC was 29.8%, which was similar to BR-PC, showing the indication of conversion surgery could be admissible. (Conclusion)NAT for PC could improve the prognosis, but further modification based on resectability status should be necessary.

[Disclosure of COI by Responsible researcher] Lead presenter is not the Responsible researcher.
[Lead presenter: Akita Hirofumi] We have no financial relationships to disclose.
[Principal Researcher: Hidetoshi Eguchi] We have no financial relationships to disclose.

### [P-2279] I-P17-2
### Multimodal treatment in surgery

2025 / 9 / 26 (Fri) 16:45-17:30 Room P/(Motenashi Dome) Room P

---

### P-2279
### Indications and Outcomes of Conversion Surgery after Chemotherapy for Biliary Tract Cancer

[Speaker]Keishi Sugimachi (Hepatobiliary and Pancreatic Surgery, Kyushu Cancer Center) [Co-author]Takahiro Tomino (Hepatobiliary and Pancreatic Surgery, Kyushu Cancer Center)、Takeshi Kurihara (Hepatobiliary and Pancreatic Surgery, Kyushu Cancer Center)、Emi Onishi (Hepatobiliary and Pancreatic Surgery, Kyushu Cancer Center)、Masaru Morita (Kyushu Cancer Center)

Background: With the improved response rates of chemotherapy, reports of conversion surgery (CS) for initially unresectable biliary tract cancer (BTC) are increasing. We present outcomes of CS cases for BTC. Methods: We reviewed 142 patients who underwent resection for BTC. 14 cases (10%) were unresectable at initial diagnosis and underwent CS after chemotherapy. Results: These cases included 2 perihilar, 5 distal, 3 intrahepatic, 1 ampullary, and 3 gallbladder carcinomas. Reasons for unresectability included distant metastases (n=8) and locally advanced disease (n=6). Preoperative chemotherapy regimens included GEM+CDDP (n=3), GC+S-1 (n=9), and GC+Durvalumab (n=2). Compared with non-CS cases, CS cases had significantly longer operative times, but no differences in blood loss or complications. One- and three-year overall survival rates for CS cases were 70% and 60%, and recurrence-free survival rates were 70% and 56%, respectively, which revealed no significant differences compared with non-CS cases. Conclusion: In patients with favorable response to chemotherapy, CS may offer comparable prognostic outcomes to those of initially resectable BTC.

[Disclosure of COI by Responsible researcher] Lead presenter is the Responsible researcher.
[Lead presenter: Keishi Sugimachi] We have no financial relationships to disclose.

### [P-2283] I-P19-1
### Cancer Prevention & Epidemiology (1)

2025 / 9 / 26 (Fri) 16:45-17:30 Room P/(Motenashi Dome) Room P

---

### P-2283
### Efficacy of Caffeic acid in Coffee on Colon and Liver cancer cells

[Speaker]Kanpa Rodted (Dept. of Emergency Med. Operation, Sch. of Med.,Univ. of Phayao) [Co-author]Thatsani Sri-pho (Dept. of Emergency Med. Operation, Sch. of Med.,Univ. of Phayao)、Chatchadaporn Changkham (Dept. of Emergency Med. Operation, Sch. of Med.,Univ. of Phayao)、Bussakorn Phimsalee (Dept. of Emergency Med. Operation, Sch. of Med.,Univ. of Phayao)、Nantaporn Duangsungnoen (Dept. of Emergency Med. Operation, Sch. of Med.,Univ. of Phayao)、Anchalee Rawangkan (Dept. of Microbiology, Sch. of Med. Sci.,Univ. of Phayao)、Acharaphon Duangjai (Dept. of Physiol., Sch. of Med. Sci.,Univ. of Phayao)、Ratsada Praphasawat (Dept. of Path., Sch. of Med.,Univ. of Phayao)

**Background:**Caffeic acid has received much attention owing to their diverse biological properties. However, the anti-cancer effect of caffeic acid needs more study.
**Objectives:**We performed ***in vitro*** study to investigate whether caffeic acid shows anti-cancer activity and potential its cellular mechanisms.
**Methods:**Cells were exposed to caffeic acid at concentrations 125-1000 μM for 24-48 h and their cytotoxicity were evaluated using MTT test. Then, cell proliferation, cell migration and invasion abilities were analyzed using Colony formation, Wound healing and Transwell chamber assay. The cellular mechanism was confirmed using Cell death detection ELISA.
**Results:**Our results showed that caffeic acid significantly inhibits cell viability and clonogenic property and displays $IC_{50}$values as 851.66 and 307.82 μM for HCT116 and HepG2, respectively (at 48 h). Caffeic acid abrogates the motility and invasive property of those cancerous cells. Interestingly, cells death through apoptosis induction was found in HCT116 upon caffeic acid treatment.
**Conclusion:**This study provides a positive trend that caffeic acid are potential as anti-cancer benefits of colon and liver cancers.

[Disclosure of COI by Responsible researcher] Lead presenter is not the Responsible researcher.
[Lead presenter: Kanpa Rodted] We have no financial relationships to disclose.
[Principal Researcher: Ratsada Praphasawat] We have no financial relationships to disclose.

### [P-2284] I-P19-1
### Cancer Prevention & Epidemiology (1)

2025 / 9 / 26 (Fri) 16:45-17:30 Room P/(Motenashi Dome) Room P

---

### P-2284
### Dinactin Exerts Anti-cancer Activity and Its Cellular Mechanism of Action

[Speaker]Ratsada Praphasawat (Dept. of Pathology, Sch. of Med.,Univ. of Phayao) [Co-author]Anchalee Rawangkan (Dept. of Microbial., School of Med. Sci. Univ.of Phayao)、Nantaporn Duangsungnoen (Dept. of Pathology, Sch. of Med.,Univ. of Phayao)

**Purpose:**Dinactin, a secondary metabolite antibiotic, strongly exhibited bactericidal potential and anti-fungal activities. However, the anti-cancer activity and the potential anti-cancer mechanisms are not fully explored yet. We aim to investigate the anti-cancer effect of dinactin and its cellular mechanisms in colon cancer cells.
**Methods:**Colon cancer cells (HCT116 and HT29) were treated with increasing concentrations of dinactin at different times. Cell-viability and self-renewal abilities were assessed by MTT and cell proliferation colony-assay. The cellular pathways that are crucial for carcinogenesis including apoptosis and cell cycle progression were measured using AnnexinV/PI staining and cell cycle analysis.
**Results:** Dinactin reduced cell-viability in HCT116 and HT29 and their ability to form colonies at concentrations from 0.5-1 nM. Mechanically, we observed that dinactin treatments to those cancer cell lines induced apoptosis but had no effect on cell cycle arrest.
**Conclusion:**Dinactin exerts anti-cancer effects on both human colon cancer cells through the induction of apoptosis and could be promising a targeted therapy for treatment of colon cancer.

[Disclosure of COI by Responsible researcher] Lead presenter is the Responsible researcher.
[Lead presenter: Ratsada Praphasawat] We have no financial relationships to disclose.

[P-2285] I-P19-1
Cancer Prevention & Epidemiology (1)

2025 / 9 / 26 (Fri) 16:45-17:30 Room P/(Motenashi Dome) Room P

P-2285
### Protective Effects of Lignosus rhinocerotis Extract Against Lipid Accumulation in Palmitic Acid-Induced HepG2 Cells

[Speaker]Wanwisa Tachakunpranee (Dept. of Biochem.&Mol.Biol.,Thammasat Uni.) [Co-author]Boonchoy Soontornworajit (Dept. of Chemistry, Thammasat Univ.)、Pichayanoot Rotkrua (Dept. of Preclinical, Sci.,Thammasat Univ.)

Non-alcoholic fatty liver disease (NAFLD) is a major chronic liver disease marked by excessive hepatic lipid accumulation, which may progress to more severe conditions. A key factor in NAFLD is lipid overload from elevated free fatty acids, leading to fat deposition and inflammation in liver cells. Lignosus rhinocerotis, used in traditional Asian medicine, has shown antioxidant and lipid-lowering properties. However, its exact mechanism in reducing hepatic lipid buildup remains unclear. This study investigates the antioxidant activity of L. rhinocerotis extract (LR-C) and its effects on lipid accumulation in palmitic acid (PA)-induced HepG2 cells. Cells were treated with 0.2 mM PA and co-treated with LR-C. LR-C showed strong DPPH scavenging activity (IC50 = 302.453 micrograms/millilitre), was non-toxic, and significantly reduced lipid buildup. These results highlight LR-C's potential as a natural agent for NAFLD management and prevention of disease progression.

[Disclosure of COI by Responsible researcher] Lead presenter is the Responsible researcher.
[Lead presenter: Wanwisa Tachakunpranee] We have no financial relationships to disclose.

[P-2286] I-P19-1
Cancer Prevention & Epidemiology (1)

2025 / 9 / 26 (Fri) 16:45-17:30 Room P/(Motenashi Dome) Room P

P-2286
### Evaluation of the impact of health screening on mortality: A study using the Japanese Cancer Registry Database

[Speaker]Yoshihide Terada (Chiba Cancer Ctr. Res. Inst., Cancer Prev. Ctr.／Fac. of Mgmt. & Governance, Shumei Univ.) [Co-author]Aya Washio (Chiba Cancer Ctr. Res. Inst., Cancer Prev. Ctr.)、Naoto Kaneko (Chiba Cancer Ctr. Res. Inst., Cancer Prev. Ctr.／NEC Solution Innovators)、Yohko Nakamura (Chiba Cancer Ctr. Res. Inst., Cancer Prev. Ctr.)、Yoshitaka Hippo (Chiba Cancer Ctr. Res. Inst., Cancer Prev. Ctr.)、Nobuaki Michihata (Chiba Cancer Ctr. Res. Inst., Cancer Prev. Ctr.)

Background: Cancer screening and health checkups help reduce cancer mortality through early detection, but their effectiveness by cancer type, sex, and age is unclear.Objective: This study examined whether detection through health checkups is associated with mortality and how this association varies by sex, age, and cancer type.Methods: We conducted a population-based cohort study using Chiba Cancer Registry data. The study included 285,025 patients diagnosed between 2013 and 2020. Patients were grouped into screening-targeted, non-screening-targeted, and pancreatic cancers. Cox proportional hazards models estimated hazard ratios for all-cause mortality associated with detection through health checkups, including interaction terms for sex and age.Results: Detection through health checkups reduced mortality in all groups. The reduction was greater in females (screening-targeted) and males (non-screening-targeted). The interaction between detection and age was significant in screening-targeted and non-screening-targeted cancers but not in pancreatic cancer.Conclusion: Detection through health checkups reduced mortality, with differences by cancer type, sex, and age.

[Disclosure of COI by Responsible researcher] Lead presenter is not the Responsible researcher.
[Lead presenter: Yoshihide Terada] We have no financial relationships to disclose.
[Principal Researcher: Nobuaki Michihata] We have no financial relationships to disclose.

### [P-2287] I-P19-1
### Cancer Prevention & Epidemiology (1)

2025 / 9 / 26 (Fri) 16:45-17:30 Room P/(Motenashi Dome) Room P

P-2287

### Joinpoint analysis of age-standardized cancer mortality and comparison of annual percent change among prefectures

[Speaker]Kosuke Kubota (Shizuoka Cancer Center Department of Nursing) [Co-author]Megumi Hori (Institute for Cancer Control, National Cancer Center)、Ayami Suzuki (University of Shizuoka School of Nursing)

This study aimed to reveal the trend of cancer mortality in Japan and compare national and prefectural rates.Mortality data from 1995-2023 was extracted from age - standardized mortality rate (National Cancer Center Japan). Seven cancer sites (stomach, liver, pancreas, lung, colon, breast, uterus) were analyzed. The annual percent change (APC) was derived using joinpoint regression analysis. We subtracted the national APCs from the prefectural one and examined whether there were significant differences at the 95 percent confidence interval.Nationwide mortality showed a decreasing trend for stomach, liver, lung, and colon cancer, but an increasing trend for pancreas, breast, and uterus cancer. Five prefectures for the liver cancer and two prefectures for colon showed a greater trend toward a decline in APC than the national average and then followed a similar course to the national rate. In contrast, five prefectures saw a slower decrease in liver cancer mortality than the national rate and then followed a similar course to the national rate.Further investigation is needed to determine the cause of these results, including a closer look at prefectural cancer prevention measures.

[Disclosure of COI by Responsible researcher] Lead presenter is the Responsible researcher.
[Lead presenter: Kosuke Kubota] We have no financial relationships to disclose.

### [P-2294] I-P19-3
### Cancer Prevention & Epidemiology (3)

2025 / 9 / 26 (Fri) 16:45-17:30 Room P/(Motenashi Dome) Room P

### P-2294
### Withdrawn

No Abstract

[P-2295] I-P19-3
Cancer Prevention & Epidemiology (3)

2025 / 9 / 26 (Fri) 16:45-17:30 Room P/(Motenashi Dome) Room P

P-2295
### Right versus left lung cancer: Findings from Chiba Prefecture Cancer Registry Database

[Speaker]Yohko Nakamura (Chiba Cancer Ctr. Res. Inst., Cancer Prevention Center) [Co-author]Aya Washio (Chiba Cancer Ctr. Res. Inst., Cancer Prevention Center)、Yoshihide Terada (Chiba Cancer Ctr. Res. Inst., Cancer Prevention Center)、Naoko Takahashi (Chiba Cancer Ctr. Res. Inst., Cancer Prevention Center)、Yoshitaka Hippo (Chiba Cancer Ctr. Res. Inst., Cancer Prevention Center)、Nobuaki Michihata (Chiba Cancer Ctr. Res. Inst., Cancer Prevention Center)

**Purpose**
Biological left-right differences are key in cancer development, with asymmetric incidence in symmetrical organs. In this study, we aimed to analyze the laterality of lung cancer, its incidence rate, and its association with prognosis using Chiba Prefecture cancer registry database.
**Methods**
Using the cancer registry database from 2013 to 2020, we extracted all lung cancer cases. We selected cases with clear laterality and excluded those with simultaneous bilateral occurrence. Kaplan-Meier survival curves were performed with all-cause mortality as the outcome. In addition, the Cox proportional hazards model, adjusted for age, sex, cancer stage, histological type, and other covariates, was used to evaluate the difference in mortality between the left and right sides.
**Results**
Finally, 36,359 patients were analyzed. Of these, the incidence rate of right lung cancer was 60%. The Cox proportional hazards model showed that the mortality rate of right lung cancer was significantly higher (hazard ratio 1.1, 95% confidence interval 1.0–1.1, p = 0.003).
**Conclusions**
Cancer incidence and mortality were higher in the right lung, possibly due to anatomical, genetic, and environmental factors.

[Disclosure of COI by Responsible researcher] Lead presenter is not the Responsible researcher.
[Lead presenter: Yohko Nakamura] We have no financial relationships to disclose.
[Principal Researcher: Nobuaki Michihata] We have no financial relationships to disclose.

### [P-2296] I-P19-3
### Cancer Prevention & Epidemiology (3)

2025 / 9 / 26 (Fri) 16:45-17:30 Room P/(Motenashi Dome) Room P

---

P-2296

### Null effects of diabetes mellitus on the prognosis of meningioma: A retrospective cohort study from a single center

[Speaker]Chirabhat Tantraporn (Faculty of Med., Khon kaen Univ., Thailand) [Co-author]Mingkhun Visetsang (Faculty of Med., Khon kaen Univ., Thailand)、Teetawat Ratanaanagechai (Faculty of Med., Khon kaen Univ., Thailand)、Nontaphon Piyawattanametha (Dept. of Surg., Faculty of Med., Khon Kaen Univ., Thailand／Ctr. for Translational Med., Faculty of Med.Khon Kaen Univ.)、Charupong Saengboonmee (Dept. of Biochem., Faculty of Med., Khon Kaen Univ., Thailand／Ctr. for Translational Med., Faculty of Med.Khon Kaen Univ.)

Meningioma is the most common type of primary intracranial tumor, with favorable outcomes but a high recurrent rate. However, the roles of systemic co-morbidities such as diabetes mellitus (DM) on meningioma prognosis remain under-explored and controversial among studies. This study preliminarily investigated whether DM and levels of glycemic control affect survival and were associated with clinico-pathological characteristics of patients with meningioma. A Retrospective cohort study of 44 patients with histologically confirmed meningioma at Srinagarind Hospital, Khon Kaen University, was conducted (EC Approval No. HE671646). The prevalence of DM was significantly associated with age >60 years (P<0.05). However, DM was neither associated with recurrent free survival of patients nor the other clinico-pathological data, i.e., sex, tumor location, recurrence, WHO classification, and the surgical extent, in the present cohort. Further investigation in a larger cohort with longer follow-up is needed to clarify the effects of DM on meningioma progression and patients' prognoses.

[Disclosure of COI by Responsible researcher] Lead presenter is not the Responsible researcher.
[Lead presenter: Chirabhat Tantraporn] We have no financial relationships to disclose.
[Principal Researcher: Charupong Saengboonmee] We have no financial relationships to disclose.

▲TOP

### [P-2297] I-P19-3
### Cancer Prevention & Epidemiology (3)

2025 / 9 / 26 (Fri) 16:45-17:30 Room P/(Motenashi Dome) Room P

---

### P-2297
### A cross-sectional study on post-marketing requirements for anticancer drugs approved in Japan from 2001 to 2024.

[Speaker]Aina Tsuno (Regulatory Sci., Meiji Pharm. Univ.) [Co-author]Hiroe Kitagaki (Regulatory Sci., Meiji Pharm. Univ.／Astellas Pharma Inc.)、Hideki Maeda (Regulatory Sci., Meiji Pharm. Univ.)

In 2023, the U.S. FDA has announced "Project Optimus" to optimize the dosage of anticancer drugs and issued draft guidance on dosage optimization. The FDA anticipates an increase in post-marketing requirement (PMRs) regarding dosage. In light of these developments, we conducted a survey on the conditions for approval proposed by the PMDA for anticancer drugs approved in Japan. The survey included drugs with new drugs and new indications approved in Japan between January 2001 and June 2024. The results showed that 328 of the 471 approved drugs (69.6%) had conditions for approval, the most common being the development of a drug risk management plan (RMP). Post-marketing clinical trials were rarely included as a condition of approval, particularly post-marketing clinical trials on dosage for only two drugs (0.4%). In contrast, 12% of anticancer drugs in the U.S. have PMR or PMC related to dosage between 2010 and 2023, suggesting a difference in approach between Japan and the U.S. In the future, there may be an increase in approval conditions related to Project Optimus, and consideration should be given to the differences between the two countries.

[Disclosure of COI by Responsible researcher] Lead presenter is not the Responsible researcher.
[Lead presenter: Aina Tsuno] We have no financial relationships to disclose.
[Principal Researcher: Hideki Maeda] We have no financial relationships to disclose.

### [P-2298] I-P19-3
### Cancer Prevention & Epidemiology (3)

2025 / 9 / 26 (Fri) 16:45-17:30 Room P/(Motenashi Dome) Room P

---

### P-2298
### A Novel Strategy to Bridge the Gap Between Cancer Screening and Treatment Research : Evaluation of a Behavioral Model

[Speaker]Masaki Okazaki (Juntendo University Graduate School of Medicine, Department of Immunodiagnostics／Keio Research Institute at SFC, Keio) [Co-author]Shuji Matsuoka (Juntendo University Graduate School of Medicine, Department of Immunodiagnostics)、Yuki Sawai (Juntendo University Graduate School of Medicine, Department of Immunodiagnostics)

Despite significant progress in cancer treatment research, screening rates in Japan remain low, highlighting a gap between prevention and clinical implementation. This study evaluated a behavioral intervention strategy to address this issue. We conducted a school-based program for sixth-grade students, including lectures from cancer researchers on therapeutic antibody development. Students learned about cancer prevention, early detection, and treatment options. They then created outreach materials (posters, slogans, handouts) and shared them with their families.Pre- and post-intervention surveys in four elementary schools showed a significant increase in parental awareness of screening importance (45.5% to 52.1%, $p<0.05$). Free-text responses revealed increased motivation toward screening. This model goes beyond health education, functioning as a translational strategy to promote societal uptake of cancer research. Collaboration between medical and educational sectors may help establish community-based prevention strategies aligned with national cancer control policies.

[Disclosure of COI by Responsible researcher] Lead presenter is not the Responsible researcher.
[Lead presenter: Masaki Okazaki] ■Research funds from company: 科研費（研究活動スタート支援）
[Principal Researcher: Shuji Matsuoka] We have no financial relationships to disclose.

▲TOP

### [P-2299] I-P19-3
### Cancer Prevention & Epidemiology (3)

2025 / 9 / 26 (Fri) 16:45-17:30 Room P/(Motenashi Dome) Room P

### P-2299
### Withdrawn

No Abstract

### [P-2302] II-P1
### Brain tumor

2025 / 9 / 26 (Fri) 16:45-17:30 Room P/(Motenashi Dome) Room P

### P-2302
### Spatial and genomic analyses for glioblastoma microenvironment with tertiary lymphoid structures

[Speaker]Ryo Mizuta (Department of Neurological Surgery, Okayama University Graduate School of Medicine／Division of Translational Oncoimmunology, Aichi Cancer Center Research Institute) [Co-author]Daisuke Muraoka (Division of Translational Oncoimmunology, Aichi Cancer Center Research Institute)、Ayako Okamura (Division of Translational Oncoimmunology, Aichi Cancer Center Research Institute)、Takanari Okamoto (Division of Translational Oncoimmunology, Aichi Cancer Center Research Institute)、Yoshihiro Otani (Department of Neurological Surgery, Okayama University Graduate School of Medicine)、Joji Ishida (Department of Neurological Surgery, Okayama University Graduate School of Medicine)、Shota Tanaka (Department of Neurological Surgery, Okayama University Graduate School of Medicine)、Hirokazu Matsushita (Division of Translational Oncoimmunology, Aichi Cancer Center Research Institute)

Background: Tertiary lymphoid structure (TLS), composed mainly of intratumoral B cells, has been reported as a favorable prognostic factor in various types of cancer, however, TLS has not been well studied in glioblastoma. Therefore, we performed spatial and genomic analyses focusing on TLS.
Methods: We evaluated lymphocytic infiltration by immunostaining in 50 cases. RNA-sequencing (RNA-seq) was performed in 39 cases to compare those with and without TLS by differential expression genes (DEGs) and Gene Set Enrichment Analysis (GSEA). Spatial transcriptomic analysis by Visium was performed.
Results: TLS was detected in 4 of 50 cases (8.0%). RNA-seq analysis showed increased gene expression related to the T cell activation marker (***TNFRSF9***), chemokine (***CCL5***) etc. in patients with TLS. GSEA also confirmed the activation of immune responses such as adaptive immune response and B cell receptor signaling pathway. Visium showed that elevated expression of T cell activation markers and chemokines etc. seen in RNA-seq analysis was concentrated in the TLS portion.
Conclusion: We analyzed a total of 50 cases of glioblastoma. Glioblastoma with TLS were also immune activated.

[Disclosure of COI by Responsible researcher] Lead presenter is not the Responsible researcher.
[Lead presenter: Ryo Mizuta] We have no financial relationships to disclose.
[Principal Researcher: Hirokazu Matsushita] ■Research expenses: 日本電気株式会社

▲TOP

### [P-2303] II-P1
### Brain tumor

2025 / 9 / 26 (Fri) 16:45-17:30 Room P/(Motenashi Dome) Room P

---

### P-2303
### Effects of MTAP deletion on tumor survival and growth mechanisms in glioblastoma.

[Speaker]Ryoji Imoto (Department of Neurological Surgery, Okayama University Graduate School of Medicine) [Co-author]Yoshihiro Otani (Department of Neurological Surgery, Okayama University Graduate School of Medicine)、Yasuki Suruga (Department of Neurological Surgery, Okayama University Graduate School of Medicine)、Shuichiro Hirano (Department of Neurological Surgery, Okayama University Graduate School of Medicine)、Madoka Hokama (Department of Neurological Surgery, Okayama University Graduate School of Medicine)、Tsuyoshi Umeda (Department of Neurological Surgery, Okayama University Graduate School of Medicine)、Ryosuke Ikemachi (Department of Neurological Surgery, Okayama University Graduate School of Medicine)、Shohei Nishigaki (Department of Neurological Surgery, Okayama University Graduate School of Medicine)、Naoya Kemmotsu (Department of Neurological Surgery, Okayama University Graduate School of Medicine)、Ryo Mizuta (Department of Neurological Surgery, Okayama University Graduate School of Medicine)、Ryo Omae (Department of Neurological Surgery, Okayama University Graduate School of Medicine)、Joji Ishida (Department of Neurological Surgery, Okayama University Graduate School of Medicine)、Oltea Sampetrean (Keio University Human Biology-Microbiome-Quantum Research Center)、Hideyuki Saya (Oncology Innovation Center, Fujita Health University)、Shota Tanaka (Department of Neurological Surgery, Okayama University Graduate School of Medicine)

Introduction: CDKN2A is a tumor suppressor gene involved in the cell cycle, and homozygous deletion of CDKN2A/B is associated with poor prognosis in gliomas. Methylthioadenosine phosphorylase (MTAP), a gene located near CDKN2A, is deleted in approximately 45% of gliomas. MTAP is involved in methionine metabolism, and its loss leads to reduced methionine synthesis and potential alterations in methylation, which may impact tumor cells and the tumor microenvironment. However, the detailed mechanisms are unclear. This study aimed to investigate how MTAP deletion affects the biology of glioma.Methods: MTAP-deficient murine glioma cell lines were established using shRNA and CRISPR/Cas9. Functional analyses were performed in vitro and in vivo. Bulk RNA sequencing was conducted.Results: MTAP-deficient tumor cells showed increased proliferation in vitro. In vivo, MTAP-deficient tumors exhibited shorter survival time. RNA-sequencing revealed downregulation of programmed cell death-related genes in MTAP-deficient cells. These cells were resistant to ferroptosis induction by elastin.Conclusion: MTAP deletion in glioblastoma promotes tumor proliferation and is linked to ferroptosis resistance.

[Disclosure of COI by Responsible researcher] Lead presenter is not the Responsible researcher.
[Lead presenter: Imoto Ryoji] We have no financial relationships to disclose.
[Principal Researcher: Suruga Yasuki] We have no financial relationships to disclose.

### [P-2304] II-P1
### Brain tumor

2025 / 9 / 26 (Fri) 16:45-17:30 Room P/(Motenashi Dome) Room P

### P-2304
### Withdrawn

No Abstract

### [P-2305] II-P1
### Brain tumor

2025 / 9 / 26 (Fri) 16:45-17:30 Room P/(Motenashi Dome) Room P

---

### P-2305
### Chromatin Modification Abnormalities by CHD7 and KMT2C Loss Promote Medulloblastoma Progression

[Speaker]Wanchen Wang (Dept. of Neuro-oncology, NCU) [Co-author]Kohei Kumegawa (Project for Cancer Epigenomics, Cancer Inst., JFCR)、Owen Chapman (Dept. of Neuro-oncology, NCU)、Zhize Xiao (Dept. of Neuro-oncology, NCU)、Konstantin Okonechnikov (German Cancer Research Center)、Stefan Pfister (German Cancer Research Center)、Naofumi Uesaka (Inst. of Science Tokyo)、Reo Maruyama (Project for Cancer Epigenomics, Cancer Inst., JFCR)、Daisuke Kawauchi (Dept. of Neuro-oncology, NCU)

Medulloblastoma, a common malignant pediatric brain tumor arising in the cerebellum, is characterized by mutations in chromatin modifiers, highlighting the significance of epigenomic abnormalities in its progression. While animal models have effectively demonstrated this, a comprehensive evaluation of the oncogenic potential of these mutations remains incomplete. In this study, we use CRISPR-mediated gene editing to knockout chromatin modifier genes mutated in human SHH medulloblastoma, along with the ***Ptch1*** gene, in cerebellar granule neuron progenitors of neonatal mice. This reveals that depletion of ***Chd7*** and ***Kmt2c*** accelerates tumor growth. Multi-layered omics analysis uncovers that epigenetic inhibition of the neuronal differentiation program is a key signaling pathway in tumor progression. Additionally, forced expression of ***Neurod1***, a common target of these chromatin modifiers inhibits proliferation and promotes differentiation. These findings highlight converging epigenomic abnormalities in SHH medulloblastoma and suggest that epigenetic drugs activating neuronal genes have significant potential as novel treatments.

[Disclosure of COI by Responsible researcher] Lead presenter is the Responsible researcher.
[Lead presenter: Wanchen Wang] We have no financial relationships to disclose.

▲TOP

### [P-2306] II-P1
### Brain tumor

2025 / 9 / 26 (Fri) 16:45-17:30 Room P/(Motenashi Dome) Room P

---

### P-2306
### Genetic analysis of H3K27-altered glioma utilizing the C-CAT data

[Speaker]Yasuhito Kegoya (Department of Neurological Surgery, Okayama University Graduate School of Medicine) [Co-author]Yoshihiro Otani (Department of Neurological Surgery, Okayama University Graduate School of Medicine)、Ryosuke Ikemachi (Department of Neurological Surgery, Okayama University Graduate School of Medicine)、Ryo Omae (Department of Neurological Surgery, Okayama University Graduate School of Medicine)、Shohei Nishigaki (Department of Neurological Surgery, Okayama University Graduate School of Medicine)、Yohei Inoue (Department of Neurological Surgery, Okayama University Graduate School of Medicine)、Tsuyoshi Umeda (Department of Neurological Surgery, Okayama University Graduate School of Medicine)、Madoka Hokama (Department of Neurological Surgery, Okayama University Graduate School of Medicine)、Ryo Mizuta (Department of Neurological Surgery, Okayama University Graduate School of Medicine)、Ryoji Imoto (Department of Neurological Surgery, Okayama University Graduate School of Medicine)、Naoya Kemmotsu (Department of Neurological Surgery, Okayama University Graduate School of Medicine)、Shuichiro Hirano (Department of Neurological Surgery, Okayama University Graduate School of Medicine)、Joji Ishida (Department of Neurological Surgery, Okayama University Graduate School of Medicine)、Shuta Tomida (Center for Comprehensive Genomic Medicine, Okayama University Hospital)、Daisuke Ennishi (Center for Comprehensive Genomic Medicine, Okayama University Hospital)、Shota Tanaka (Department of Neurological Surgery, Okayama University Graduate School of Medicine)

**[Introduction]** Diffuse midline glioma, H3K27-altered (DMG), is a rare disease of pediatric and adolescents and young adults (AYA) with poor prognosis. Its genetic features beyond driver genes and their prognostic impact remain unclear. We analyzed the genetic characteristics of H3K27-altered gliomas using Cancer Genome Information Management Center (C-CAT) data.
**[Methods]** We analyzed 139 cases with ***H3F3A K28M*** mutations among 2,687 brain tumor cases registered with C-CAT between June 2019 and November 2024.
**[Results]** Among the 139 cases, 20 cases (14.4%) had ***FGFR1*** mutations, and 8 cases (5.8%) had ***BRAF V600E*** mutations. Patients with ***FGFR1*** mutations were significantly older than those with ***FGFR1*** wild-type (median: 29.5 vs 22 years, P = 0.023), and they had a longer overall survival (median: 25.4 vs 15 months, P = 0.02). Additionally, the ***FGFR1*** mutation positive group had significantly higher frequencies of ***CDK4*** amplification (4/20), ***MDM2*** amplification (4/20), and ***ATRX*** mutation (13/20), and significantly fewer ***TP53*** mutation (3/20).
**[Conclusion]** We investigated the genetic characteristics of H3K27-altered gliomas. Cases with ***FGFR1*** co-mutations were older and had a better prognosis.

[Disclosure of COI by Responsible researcher] Lead presenter is not the Responsible researcher.
[Lead presenter: Yasuhito Kegoya] We have no financial relationships to disclose.
[Principal Researcher: Yoshihiro Otani] We have no financial relationships to disclose.

### [P-2307] II-P1
### Brain tumor

2025 / 9 / 26 (Fri) 16:45-17:30 Room P/(Motenashi Dome) Room P

### P-2307
### Molecular backgrounds of diffuse pediatric–type high-grade glioma, H3–wildtype and IDH–wildtype

[Speaker]Yohei Inoue (Department of Neurological Surgery, Okayama University Graduate School of Medicine／Department of Pathology, Kyorin University Faculty of Medicine) [Co-author]Yoshihiro Otani (Department of Neurological Surgery, Okayama University Graduate School of Medicine)、Joji Ishida (Department of Neurological Surgery, Okayama University Graduate School of Medicine)、Akira Miyahira (Department of Pathology, Kyorin University Faculty of Medicine／Department of Pediatrics, Juntendo University Faculty of Medicine)、Asuka Fujii (Department of Pathology, Kyorin University Faculty of Medicine／Department of Pediatrics, Juntendo University Faculty of Medicine)、Yuko Hibiya (Department of Pathology, Kyorin University Faculty of Medicine)、Yuko Matsushita (Department of Pathology, Kyorin University Faculty of Medicine)、Kaishi Satomi (Department of Pathology, Kyorin University Faculty of Medicine)、Kohei Fukuoka (Department of Hematology／Oncology, Saitama Children's Medical Center)、Kai Yamasaki (Department of Pediatric Hematology and Oncology, Osaka City General Hospital)、Yoshiko Nakano (Department of Pediatrics, The University of Tokyo Hospital)、Takako Yoshioka (Department of Pathology, National Center for Child Health and Development)、Junko Hirato (Department of Pathology, Public Tomioka General Hospital)、Shota Tanaka (Department of Neurological Surgery, Okayama University Graduate School of Medicine)、Koichi Ichimura (Department of Pathology, Kyorin University Faculty of Medicine)

Introduction: Diffuse pediatric–type high–grade glioma, H3–wildtype and IDH–wildtype (DPHGH3/IDHWT), was previously classified as glioblastoma (GBM) or anaplastic astrocytoma based on histology. It was defined as a new entity in the WHO Classification, 5th edition. However, its molecular profile remains uncovered.
Methods: We analyzed cases classified as DPHGH3/IDHWT by genome–wide DNA methylation profiling conducted in the central pathology review by the Japan Children's Cancer Group.
Results: Twenty–four cases (M:F = 12:12; median age: 10 years, range: 0–29) were included. Tumor locations were cerebral hemispheres (n=16), cerebellum (n=3), and others (n=5). Methylation classification revealed 1 case as DPHGH3/IDHWT subtype A, 11 as RTK1 subtype, 6 as RTK2 subtype, and 6 as MYCN subtype. Calibrated score > 0.84 was observed in 13 cases. Chromosome 7 gain and 10 loss were detected in 2 cases. ***MGMT*** promoter methylation was present in 5 cases. ***TERT*** mutations were identified in 6 cases, 5 of which were RTK2 subtype.
Conclusion: DPHGH3/IDHWT shows a unique molecular profile, distinct from GBM. Further integrated molecular and clinical studies are needed.

[Disclosure of COI by Responsible researcher] Lead presenter is not the Responsible researcher.
[Lead presenter: Yohei Inoue] We have no financial relationships to disclose.
[Principal Researcher: Koichi Ichimura] ■Research expenses: 第一三共、理研ジェネシス　■Endowed chair: イドルシアファーマシューティカル

▲TOP

### [P-2314] II-P9-1
### Prostate cancer (1)

2025 / 9 / 26 (Fri) 16:45-17:30 Room P/(Motenashi Dome) Room P

---

### P-2314
### ASCT2/xCT Axis Promotes Castration-Resistant Prostate Cancer Progression viaMetabolic and Stress-Response Modulation

[Speaker]Jiaxing Wei (Department of Urology, Chiba University Graduate School of Medicine) [Co-author]Yasutaka Yamada (Department of Urology, Chiba University Graduate School of Medicine)、Masahiro Sugiura (Department of Urology, Chiba University Graduate School of Medicine／Department of Molecular Oncology, Chiba University Graduate School of Medicine)、Tomokazu Sazuka (Department of Urology, Chiba University Graduate School of Medicine)、Yusuke Imamura (Department of Urology, Chiba University Graduate School of Medicine)、Shinichi Sakamoto (Department of Urology, Chiba University Graduate School of Medicine)

ASCT2, a glutamine transporter, is highly expressed in prostate cancer. Silencing ASCT2inhibits cell proliferation, migration, invasion, and amino acid uptake. ASCT2 is regulated bythe androgen receptor (AR), and its knockdown activates apoptosis, p53, MAPK, and cell cyclepathways, leading to G2/M arrest. Importantly, ASCT2 suppression upregulates xCT(SLC7A11), while xCT silencing does not alter ASCT2, suggesting xCT acts as downstream ofASCT2. Combined inhibition of ASCT2 and xCT enhances p53 and cleaved caspase activation.Pharmacologic targeting of ASCT2 using V-9302 recapitulates these effects, and combinationtherapy with the xCT inhibitor Erastin exerts synergistic antitumor activity. In vivo, V-9302suppresses xenograft tumor growth and increases xCT expression. Clinically, ASCT2 and xCTare overexpressed in prostate cancer tissues, and high ASCT2 is a predictor of shorterrecurrence-free survival. These findings highlight the ASCT2/xCT axis as a critical modulatorof metabolic adaptation and stress signaling in CRPC, and a potential therapeutic target.

[Disclosure of COI by Responsible researcher] Lead presenter is the Responsible researcher.
[Lead presenter: Jiaxing Wei] We have no financial relationships to disclose.

### [P-2315] II-P9-1
### Prostate cancer (1)

2025 / 9 / 26 (Fri) 16:45-17:30 Room P/(Motenashi Dome) Room P

---

### P-2315
### Adipokines are involved in the proliferation, cell death, and biochemical recurrence of prostate cancer

[Speaker]Atsuto Suzuki (Kanagawa Cancer Center, Department of Urology) [Co-author]Shinya Sato (Kanagawa Cancer Centre, Clinical Research Institute.)

Biochemical recurrence (BCR) is a precursor of castration-resistant prostate cancer (CRPC). We analyzed the correlation between concentrations of Adiponectin and Leptin with BCR in 99 patients with adipose invasive prostate cancer (pT3pN0) at our institution. Also, to explore the significance of Adipokine receptor-mediated stimulation in prostate cancer, we administered Adiponectin receptor stimulants to the CRPC cell line PC3 and the castration-sensitive prostate cancer (CSPC) cell line LNCaP.There were significantly more BCR-positive cases in patients with Adiponectin6<μg/ml and Leptin>4 ng/ml (p<0.05). Adipokine stratification was a significant risk factor for BCR on multivariate analysis, along with Initial PSA>15, Gleason Score>8, and positive surgical margins (HR 4.03, p<0.05). In both the PC3 and LNCaP, inhibition of cell proliferation and increased apoptosis were observed in the adiponectin receptor stimulator groups (p<0.05, p<0.01).Our results suggest that the interaction between cancer and adipocyte-secreted factors may regulate prostate cancer progression. To elucidate these interactions, we plan to report the results of histological and genetic analysis.

[Disclosure of COI by Responsible researcher] Lead presenter is not the Responsible researcher.
[Lead presenter: Atsuto Suzuki] ■Research funds from company: 木原財団
[Principal Researcher: Shinya Sato] We have no financial relationships to disclose.

▲TOP

### [P-2316] II-P9-1
### Prostate cancer (1)

2025 / 9 / 26 (Fri) 16:45-17:30 Room P/(Motenashi Dome) Room P

### P-2316
### Tumor-associated macrophages promote bladder cancer metastasis through the CCL20-CCR6 axis

[Speaker]Ryunosuke Nakagawa (Kanazawa university) [Co-author]Kouji Izumi (Kanazawa university)、Shuhei Aoyama (Kanazawa university)、Taiki Kamijima (Kanazawa university)、Hiroshi Kano (Kanazawa university)、Tomoyuki Makino (Kanazawa university)、Renato Naito (Kanazawa university)、Hiroaki Iwamoto (Kanazawa university)、Hiroshi Yaegashi (Kanazawa university)、Atsushi Mizokami (Kanazawa university)

We investigated the interaction between bladder cancer cells and tumor associated macrophages. Coculture of UMUC3 and T24 cells with M2 like macrophages derived from THP1 cells reduced CD68 and increased CD206 expression, promoting cancer cell migration and invasion. These macrophages secreted more CCL20, which enhanced cancer cell motility via the MEK and ERK pathway through paracrine effects. Coculture also increased CCR6 expression in cancer cells, indicating greater sensitivity to CCL20. In human bladder cancer tissues, high CCR6 expression correlated with poor prognosis. Inhibiting CCR6 suppressed cancer metastasis in vitro and in vivo. Additionally, CXCL1 from cancer cells promoted M2 like polarization of macrophages and further enhanced cancer cell migration and invasion via autocrine and indirect actions. These findings suggest that CCL20 and CXCL1 are key mediators in the interaction between bladder cancer cells and macrophages and may serve as targets for therapy.

[Disclosure of COI by Responsible researcher] Lead presenter is not the Responsible researcher.
[Lead presenter: Ryunosuke Nakagawa] We have no financial relationships to disclose.
[Principal Researcher: Shuhei Aoyama] We have no financial relationships to disclose.

### [P-2317] II-P9-1
### Prostate cancer (1)

2025 / 9 / 26 (Fri) 16:45-17:30 Room P/(Motenashi Dome) Room P

---

### P-2317
### CD169-Positive Macrophages and the Tumor Microenvironment in Regional Lymph Nodes of Prostate Cancer

[Speaker]Yojiro Ozaki (Dept. of Urology, Kumamoto Univ. Hosp.／Dept. of Cell Pathology, Kumamoto Univ Hosp.) [Co-author]Toshiki Anami (Dept. of Urology, Kumamoto Univ. Hosp.)、Yuki Ibe (Dept. of Urology, Kumamoto Univ. Hosp.／Dept. of Cell Pathology, Kumamoto Univ Hosp.)、Hiromu Yano (Dept. of Cell Pathology, Kumamoto Univ Hosp.)、Yukio Fujiwara (Dept. of Cell Pathology, Kumamoto Univ Hosp.)、Takanobu Motoshima (Dept. of Urology, Kumamoto Univ. Hosp.)、Yoshihiro Komohara (Dept. of Cell Pathology, Kumamoto Univ Hosp.)、Tomomi Kamba (Dept. of Urology, Kumamoto Univ. Hosp.)

The global incidence of prostate cancer is increasing. While immune checkpoint inhibitors are effective in many cancers, they show limited efficacy in prostate cancer. Lymph node sinus macrophages (LSMs), as antigen-presenting cells, play a vital role in antitumor immunity. CD169 expression in LSMs has been linked to immune responses in several malignancies. We examined the relationship between the tumor immune microenvironment and LSM activation in 42 patients with high-risk localized prostate cancer who underwent robot-assisted radical prostatectomy and lymph node dissection (2017-2021). CD169 expression was assessed by immunohistochemistry. Expression was significantly lower in older patients, but showed no correlation with other clinicopathological factors. CD3- and CD8-positive lymphocyte infiltration in tumor tissues was also analyzed. Although CD169^high cases tended to show greater T cell infiltration, the difference was not statistically significant. These findings suggest that reduced CD169 expression in LSMs may be associated with impaired antitumor immunity, particularly in elderly prostate cancer patients.

[Disclosure of COI by Responsible researcher] Lead presenter is the Responsible researcher.
[Lead presenter: Yojiro Ozaki] We have no financial relationships to disclose.

### [P-2318] II-P9-1
### Prostate cancer (1)

2025 / 9 / 26 (Fri) 16:45-17:30 Room P/(Motenashi Dome) Room P

---

### P-2318
### FABP4 promotes HFD-induced prostate cancer progression via inflammatory and metabolic pathways in TRAMP mice

[Speaker]Hiromi Sato (Dept. of Urology Akita Univ. Grad. Sch. of Med.) [Co-author]Mingguo Huang (Dept. of Urology Akita Univ. Grad. Sch. of Med.／Dept. of Organ Anatomy and Nanomedicine, Grad. Sch. of Med.)、Shintaro Narita (Dept. of Urology Akita Univ. Grad. Sch. of Med.)、Yuya Sekine (Dept. of Urology Akita Univ. Grad. Sch. of Med.)、Mizuki Kobayashi (Dept. of Urology Akita Univ. Grad. Sch. of Med.)、Soki Kashima (Dept. of Urology Akita Univ. Grad. Sch. of Med.)、Ryohei Yamamoto (Dept. of Urology Akita Univ. Grad. Sch. of Med.)、Atsushi Koizumi (Omagari Kousei Med. Ctr.)、Taketoshi Nara (Akita Red Cross Hosp.)、Kazuyuki Numakura (Dept. of Urology Akita Univ. Grad. Sch. of Med.)、Mitsuru Saito (Dept. of Urology Akita Univ. Grad. Sch. of Med.)、Hiroshi Nanjyo (Dept. of Clin. Pathology, Akita Univ. Hosp.)、Tomonori Habuchi (Dept. of Urology Akita Univ. Grad. Sch. of Med.)

Fatty acid binding protein 4 (FABP4) is an important adipokine involved in inflammatory responses and metabolic regulation. Although our previous study suggests that FABP4 plays a key role in high-fat diet (HFD)- and/or obesity-induced prostate cancer (PCa) progression, the underlying mechanism remains unclear. In this study, we generated transgenic adenocarcinoma of the mouse prostate (TRAMP) mice with FABP4 knockout (FABP4-/-). FABP4 knockout inhibited HFD-induced PCa progression through the suppression of body weight gain, systemic cytokine secretions (including CXCL13, CXCL5, and MMP9), and periprostatic adipocyte infiltration. Comprehensive metabolomic analysis revealed that FABP4 knockout decreased nine essential and ten non-essential amino acids in the tumor. Furthermore, similar antitumor effects were observed with pharmacological inhibition of FABP4. These findings indicate that FABP4 promotes HFD-induced PCa progression by modulating systemic cytokine production and tumor metabolic reprogramming, and may represent a promising therapeutic and preventive target for HFD-related PCa.

[Disclosure of COI by Responsible researcher] Lead presenter is not the Responsible researcher.
[Lead presenter: Hiromi Sato] We have no financial relationships to disclose.
[Principal Researcher: Shintaro Narita] We have no financial relationships to disclose.

### [P-2319] II-P9-1
### Prostate cancer (1)

2025 / 9 / 26 (Fri) 16:45-17:30 Room P/(Motenashi Dome) Room P

---

### P-2319
### EndoMT in the tumor microenvironment promotes neuroendocrine differentiation of prostate cancer via GM-CSF signaling

[Speaker]Takumi Kageyama (Nepro-Urologic Surg. & Andrology, Mie Univ. Hosp.) [Co-author]Manabu Kato (Urology, Aichi Cancer Ctr.)、Ryota Ikadai (Nepro-Urologic Surg. & Andrology, Mie Univ. Grad. Sch. Med.)、Shiori Miyachi (Nepro-Urologic Surg. & Andrology, Mie Univ. Grad. Sch. Med.)、Xin Bao (Nepro-Urologic Surg. & Andrology, Mie Univ. Grad. Sch. Med.)、Sho Sekito (Nepro-Urologic Surg. & Andrology, Mie Univ. Grad. Sch. Med.／ASHBi, Kyoto Univ.)、Yusuke Sugino (Nepro-Urologic Surg. & Andrology, Mie Univ. Grad. Sch. Med.)、Takeshi Sasaki (Nepro-Urologic Surg. & Andrology, Mie Univ. Hosp.)、Kouhei Nishikawa (Nepro-Urologic Surg. & Andrology, Mie Univ. Grad. Sch. Med.)、Yasuhiro Murakawa (ASHBi, Kyoto Univ.)、Takahiro Inoue (Nepro-Urologic Surg. & Andrology, Mie Univ. Grad. Sch. Med.)

**Introduction:** We observed the proximity of endothelial cells (ECs) and neuroendocrine prostate cancer (NEPC) cells in human tissues. We hypothesized that ECs contribute to NEPC through endothelial-mesenchymal transition (EndoMT).**Methods:** EndoMT was induced in human umbilical vein endothelial cells (HUVECs) and co-cultured with LNCaP cells. NE marker expression, proliferation, and migration in LNCaP were assessed. RNA-seq of EndoMT-induced HUVECs was conducted to identify NED-inducing factors.**Results:** LNCaP co-cultured with EndoMT-HUVECs exhibited increased NE markers and enhanced migration and proliferation. GM-CSF was upregulated in EndoMT-HUVECs. GM-CSF stimulation activated NE markers, p-STAT3, and MYC in LNCaP. Under androgen deprivation, LNCaP cells promoted EndoMT and GM-CSF expression in ECs, suggesting a feedback loop toward treatment-emergent NEPC (t-NEPC).**Conclusion:** EC-derived GM-CSF induced via EndoMT may facilitate treatment-emergent NEPC (t-NEPC) development in androgen-deprived PC via the CSF2RA/STAT3/MYC axis. Targeting this pathway could offer therapeutic strategies against resistance to hormone therapy.

[Disclosure of COI by Responsible researcher] Lead presenter is the Responsible researcher.
[Lead presenter: Takumi Kageyama] We have no financial relationships to disclose.

### [P-2327] II-P9-3
### Urothelial cancer

2025 / 9 / 26 (Fri) 16:45-17:30 Room P/(Motenashi Dome) Room P

---

### P-2327
### Elucidation of RNA modification function in upper tract urothelial carcinoma through multi-omics analysis

[Speaker]Yoshiyuki Yamamoto (Dept. Urology, UOsaka. Grad. Sch. Med.) [Co-author]Atsunari Kawashima (Dept. Urology, UOsaka. Grad. Sch. Med.)、Hiroaki Hase (Stem Cell Reg. And Adap. UOsaka. Grad. Sch. Pharma.)、Yuki Horibe (Dept. Urology, UOsaka. Grad. Sch. Med.)、Yohei Miyazaki (Mol Cell Physiol. UOsaka. Grad. Sch. Pharma.)、Hiromu Horitani (Dept. Urology, UOsaka. Grad. Sch. Med.)、Shunsuke Inoguchi (Dept. Urology, UOsaka. Grad. Sch. Med.)、Nesrine Sassi (Dept. Urology, UOsaka. Grad. Sch. Med.)、Akinaru Yamamoto (Dept. Urology, UOsaka. Grad. Sch. Med.)、Toshiki Oka (Dept. Urology, UOsaka. Grad. Sch. Med.)、Yu Ishizuya (Dept. Urology, UOsaka. Grad. Sch. Med.)、Takuji Hayashi (Dept. Urology, UOsaka. Grad. Sch. Med.)、Taigo Kato (Dept. Urology, UOsaka. Grad. Sch. Med.)、Koji Hatano (Dept. Urology, UOsaka. Grad. Sch. Med.)、Kazutake Tsujikawa (Dept. Urology, UOsaka. Grad. Sch. Med.)、Norio Nonomura (Dept. Urology, UOsaka. Grad. Sch. Med.)

Objective: RNA modifications (RNAMs) have gained increasing attention in cancer biology. This study aimed to explore the biological significance of RNAMs in upper tract urothelial carcinoma (UTUC).Methods: RNAMs were quantified in UTUC and adjacent normal uroepithelium (NU) tissues from 61 patients using mass spectrometry. RNA sequencing (RNA-seq) was performed to assess gene expression, and gene set enrichment analysis (GSEA) was used for pathway analysis. Results: In the discovery cohort, 2-O-methylcytidine (Cm) showed the highest discriminatory ability between cancer and NU (AUC=0.79) and was significantly reduced in cancer ($p<0.001$). The validation cohort confirmed these findings (AUC=0.74, $p<0.001$). Cm levels were not significantly associated with pT stage, lymphovascular invasion, or the presence of FGFR3 or TP53 mutations. GSEA revealed that Cm-low tumors were enriched in pathways related to the cell cycle and DNA replication.Conclusion: Cm levels are reduced in UTUC and are associated with activation of proliferation-related pathways. RNAM profiles may reflect distinct molecular features independent of conventional pathological or genomic factors.

[Disclosure of COI by Responsible researcher] Lead presenter is the Responsible researcher.
[Lead presenter: Yoshiyuki Yamamoto] We have no financial relationships to disclose.

### [P-2328] II-P9-3
### Urothelial cancer

2025 / 9 / 26 (Fri) 16:45-17:30 Room P/(Motenashi Dome) Room P

---

### P-2328
### PDGFR beta-STAT3 axis is involved in sphere-forming ability through regulating CD44 expression in bladder cancer

[Speaker]Kiyohiro Ando (Res. Inst. Clin. Oncol., Saitama Cancer Ctr.) [Co-author]Hirotaka Fuchizawa (Dept. Urol. Saitama Cancer Ctr.)、Noriko Motoi (Dept. Path., Saitama Cancer Ctr.)、Toshihiko Iizuka (Dept. Path., Saitama Cancer Ctr.)、Yuta Sano (Dept. Urol. Saitama Cancer Ctr.)、Hisanori Takenobu (Res. Inst. Clin. Oncol., Saitama Cancer Ctr.)、Masayuki Haruta (Res. Inst. Clin. Oncol., Saitama Cancer Ctr.)、Ritsuko Onuki (Res. Inst. Clin. Oncol., Saitama Cancer Ctr.)、Yoh Matsuoka (Dept. Urol. Saitama Cancer Ctr.)、Hiroaki Kanda (Dept. Path., Saitama Cancer Ctr.)、Yukio Kageyama (Res. Inst. Clin. Oncol., Saitama Cancer Ctr.／Dept. Urol. Saitama Cancer Ctr.)、Takehiko Kamijo (Res. Inst. Clin. Oncol., Saitama Cancer Ctr.)

Combination treatment with an immune checkpoint inhibitor and conventional chemotherapies can improve survival in patients with advanced bladder cancer (BLDC), but most patients experience relapse. Platelet-derived growth factor receptor beta (PDGFRβ) is closely associated with various tumor progression, yet their connection with BLDC remains unclear. This study aims to address a potential role of PDGFRβ, along with the impact of stemness and epithelial-mesenchymal plasticity in BLDC. Increased expression of PDGFRβ was associated with poor prognosis in patients with BLDC. JMSU1 BLDC cell line exhibited an in vitro sphere-forming ability, while T24 BLDC cell line did not. Remarkably, either PDGFRβ knockout or treatment with sunitinib in JMSU1 cells showed decrease in sphere formation capability. Furthermore, either PDGFRβ or STAT3 was necessary for maintaining expression of the cell adhesion molecule CD44, which was a widely accepted marker for cancer stem cells, thus identifying a role of PDGFRβ-STAT3 axis may provide insights into mechanisms aiding escape from current standard therapies in advanced BLDC.

[Disclosure of COI by Responsible researcher] Lead presenter is the Responsible researcher.
[Lead presenter: Kiyohiro Ando] We have no financial relationships to disclose.

▲TOP

### [P-2329] II-P9-3
### Urothelial cancer

2025 / 9 / 26 (Fri) 16:45-17:30 Room P/(Motenashi Dome) Room P

P-2329

### PRAME as a Prognostic Marker and Predictor of BCG Therapy Response in Bladder Cancer

[Speaker]Moritoshi Sakamoto (Div. Cancer RNA Reseach, NCCRI／Dept. of Urology, Osaka Medical and Pharmaceutical University) [Co-author]Maiko Seki (Div. Cancer RNA Reseach, NCCRI)、Ryoichi Maenosono (Div. Cancer RNA Reseach, NCCRI／Dept. of Urology, Osaka Medical and Pharmaceutical University)、Masahiko Ajiro (Div. Cancer RNA Reseach, NCCRI)、Kazuki Nishimura (Div. Cancer RNA Reseach, NCCRI／Dept. of Urology, Osaka Medical and Pharmaceutical University)、Asuka Kawachi (Div. Cancer RNA Reseach, NCCRI)、Junko Mukohyama (Div. Cancer RNA Reseach, NCCRI／Department of Surgery, IMS, The University of Tokyo)、Mitsuaki Ishida (Department of Pathology, Osaka Medical and Pharmaceutical University)、Kazumasa Komura (Department of Urology, Kawasaki Medical School)、Haruhito Azuma (Dept. of Urology, Osaka Medical and Pharmaceutical University)、Akihide Yoshimi (Div. Cancer RNA Reseach, NCCRI)

PRAME, a tumor-associated antigen, plays a role in tumor progression and immune-mediated therapy resistance. Transcriptomic, genetic, and clinical data from 459 bladder cancer (BC) patients were analyzed to evaluate the potential biological and clinical impact of PRAME. High PRAME expression was linked to significantly worse RFS, PFS, and CSS in non-muscle-invasive bladder cancer (NMIBC), supported by in vitro findings showing enhanced proliferation and migratory potential of PRAME-high cells. Interestingly, PRAME-high cases had worse RFS among BCG-treated NMIBC patients, suggesting its role in BCG resistance. In MIBC, PRAME-high cases sex- and subtype-specific clinical implications. In female MIBC patients, high PRAME expression was significantly associated with worse OS, CSS, and RPF. Additionally, in LumP MIBC, high PRAME expression predicted a better response to ICI, with transcriptomic deconvolution suggesting the involvement of B-cell infiltration. These findings highlight PRAME as a potential biomarker for prognosis and guiding treatment selection in BC, especially for BCG and ICI treatment strategies, supporting its role in future precision medicine approaches.

[Disclosure of COI by Responsible researcher] Lead presenter is not the Responsible researcher.
[Lead presenter: Sakamoto Moritoshi] We have no financial relationships to disclose.
[Principal Researcher: Akihide Yoshimi] ■Research funds from company: 第一三共株式会社、エーザイ株式会社、中外製薬株式会社

▲TOP

[P-2330] II-P9-3
Urothelial cancer

2025 / 9 / 26 (Fri) 16:45-17:30 Room P/(Motenashi Dome) Room P

P-2330

### Therapeutic strategies targeting miR-424-5p-dependent CCNE1 amplification in cisplatin-resistant bladder cancer

[Speaker]Takashi Sakaguchi (Dept. of Urol., Grad. Sch. of Med., Kagoshima Univ.) [Co-author]Wataru Fukumoto (Dept. of Urol., Grad. Sch. of Med., Kagoshima Univ.)、Hirofumi Yoshino (Dept. of Urol., Grad. Sch. of Med., Kagoshima Univ.)、Junya Arima (Dept. of Urol., Grad. Sch. of Med., Kagoshima Univ.)、Saeki Saito (Dept. of Urol., Grad. Sch. of Med., Kagoshima Univ.)、Mitsuhiko Tominaga (Dept. of Urol., Grad. Sch. of Med., Kagoshima Univ.)、Ikumi Fukuda (Dept. of Urol., Grad. Sch. of Med., Kagoshima Univ.)、Gang Li (Dept. of Urol., Grad. Sch. of Med., Kagoshima Univ.)、Shuichi Tatarano (Dept. of Urol., Grad. Sch. of Med., Kagoshima Univ.)、Hideki Enokida (Dept. of Urol., Grad. Sch. of Med., Kagoshima Univ.)

Cisplatin-based chemotherapy is recommended for the treatment of advanced bladder cancer, but its effectiveness is limited due to acquiring resistance. The aim of this study is to identify and characterize microRNAs involved in cisplatin resistance. In Small RNA sequencing analysis, we identified ***miR-424-5p*** as a candidate microRNA that was downregulated in cisplatin-resistant (CDDP-R) strains compared with parental strains. In gain-of-function studies, ***miR-424-5p*** suppressed the proliferative ability of CDDP-R bladder cancer (CDDP-R BC) and restored sensitivity to cisplatin. Among several candidate target genes identified by RNA sequencing analysis, cyclin E1 (***CCNE1***) was chosen for subsequent analyses because its expression was upregulated in CDDP-R BC cells compared with parental cells and because recent studies have shown that ***CCNE1*** amplification is synthetic lethal with PKMYT1 kinase inhibition. We performed functional analysis using the PKMYT1 inhibitor RP-6306 and demonstrated that RP-6306 inhibited cell growth and restored cisplatin sensitivity in CDDP-R BC. In conclusion, our findings provided insights into the development of novel therapeutic strategies for CDDP-R BC.

[Disclosure of COI by Responsible researcher] Lead presenter is the Responsible researcher.
[Lead presenter: Takashi Sakaguchi] We have no financial relationships to disclose.

### [P-2331] II-P9-3
### Urothelial cancer

2025 / 9 / 26 (Fri) 16:45-17:30 Room P/(Motenashi Dome) Room P

---

### P-2331
### Tumor Microenvironment in Photodynamic Therapy for Upper Tract Urothelial Carcinoma

[Speaker]Yuki Ibe (Department of Cell Pathology, Faculty of Life Sciences, Kumamoto University) [Co-author]Yukio Fujiwara (Department of Cell Pathology, Faculty of Life Sciences, Kumamoto University)、Toshiki Anami (Department of Cell Pathology, Faculty of Life Sciences, Kumamoto University／Department of Urology, Faculty of Life Sciences, Kumamoto University)、Hiromu Yano (Department of Cell Pathology, Faculty of Life Sciences, Kumamoto University)、Takanobu Motoshima (Department of Urology, Faculty of Life Sciences, Kumamoto University)、Tomomi Kamba (Department of Urology, Faculty of Life Sciences, Kumamoto University)、Yoshihiro Komohara (Department of Cell Pathology, Faculty of Life Sciences, Kumamoto University)

In Japan, nephroureterectomy is the standard treatment for non-metastatic upper urothelial carcinoma. However, patients with a solitary kidney or chronic renal insufficiency may require dialysis after surgery, especially those with low recurrence risk. Renal-sparing options like laser ablation or partial ureteral resection exist but have high recurrence rates. Therefore, we investigated photodynamic therapy (PDT), used in other cancers, as a local treatment alternative.MB49 murine urothelial carcinoma cells were injected subcutaneously into mice. Four days later, talaporfin sodium was administered intraperitoneally, followed by red laser irradiation of the tumor two hours later. Compared to controls, tumor volume was significantly reduced. Immunohistochemistry showed increased infiltration of CD8-positive cytotoxic T cells and CD4-positive helper T cells in the tumor microenvironment, while macrophage levels remained unchanged. Flow cytometry confirmed elevated CD8-positive T cells in lymph nodes and tumors.Since the immune response elicited by photodynamic therapy (PDT) in urothelial carcinoma remains insufficiently characterized, we present our findings.

[Disclosure of COI by Responsible researcher] Lead presenter is not the Responsible researcher.
[Lead presenter: Yuki Ibe] We have no financial relationships to disclose.
[Principal Researcher: Yoshihiro Komohara] We have no financial relationships to disclose.

### [P-2332] II-P9-3
### Urothelial cancer

2025 / 9 / 26 (Fri) 16:45-17:30 Room P/(Motenashi Dome) Room P

---

P-2332

### Loss of wild-type p53 drives immune evasion in a novel urothelial organoid model harboring p53 missense mutation

[Speaker]Akihiro Hamada (Dept. of Urology, Kyoto Univ. Grad. Sch. of Med.) [Co-author]Yuki Kita (Dept. of Urology, Kyoto Univ. Grad. Sch. of Med.)、Toru Sakatani (Dept. of Urology, Kyoto Univ. Grad. Sch. of Med.)、Kenji Nakamura (Dept. of Urology, Kyoto Univ. Grad. Sch. of Med.)、Hideaki Takada (Dept. of Urology, Kyoto Univ. Grad. Sch. of Med.)、Ryosuke Ikeuchi (Dept. of Urology, Kyoto Univ. Grad. Sch. of Med.)、Norihiko Masuda (Dept. of Urology, Kyoto Univ. Grad. Sch. of Med.)、Kaoru Murakami (Dept. of Urology, Kyoto Univ. Grad. Sch. of Med.)、Takeshi Sano (Dept. of Urology, Kyoto Univ. Grad. Sch. of Med.／Dept. of Urology and Andrology, Kansai Med. Univ.)、Takayuki Goto (Dept. of Urology, Kyoto Univ. Grad. Sch. of Med.)、Ryoichi Saito (Dept. of Urology, Kyoto Univ. Grad. Sch. of Med.)、Yuki Teramoto (Dept. of Diagnostic Pathology, Kyoto Univ. Grad. Sch. of Med.)、Masakazu Fujimoto (Dept. of Diagnostic Pathology, Kyoto Univ. Grad. Sch. of Med.)、Mayumi Kamada (Dept. of Biomedical Data Intelligence, Kyoto Univ.)、Osamu Ogawa (Dept. of Urology, Kyoto Univ. Grad. Sch. of Med.)、Takashi Kobayashi (Dept. of Urology, Kyoto Univ. Grad. Sch. of Med.)

Missense mutations in p53 account for over 60% of alterations in bladder cancer, while homozygous deletions (HOM) are seen in less than 5% of cases. However, most existing mouse models are deficient of p53, limiting research on the role of missense mutations over HOM. Organoids from Krt5-expressing mouse urothelium revealed loss of Pten drives loss of wild-type Trp53 allele (Trp53R172H/LOH), promoting tumorigenicity in athymic mice. These tumors mimicked histological features of the basal-squamous subtype of human bladder cancer. Both Trp53R172H/-; Pten-/- and Trp53-/-; Pten-/- organoids formed tumors in athymic mice, but only the former formed tumors in immunocompetent mice. The absence of wild-type p53 led to increased proliferative signaling, while the presence of a mutant p53 allele resulted in an immune-excluded microenvironment. This study underscores the functional role of p53 mutant LOH in bladder cancer, contributing to key cancer hallmarks like sustaining proliferation and evading immune destruction. It also introduces a novel immunocompetent mouse model of urothelial carcinoma harboring p53 mutations as a novel tool for cancer immunology research.

[Disclosure of COI by Responsible researcher] Lead presenter is not the Responsible researcher.
[Lead presenter: Akihiro Hamada] We have no financial relationships to disclose.
[Principal Researcher: Takashi Kobayashi] We have no financial relationships to disclose.

### [P-2333] II-P9-3
### Urothelial cancer

2025 / 9 / 26 (Fri) 16:45-17:30 Room P/(Motenashi Dome) Room P

---

### P-2333
### Improved diagnostic yield for non-muscle invasive bladder cancer using urinary cell-free DNA

[Speaker]Shohei Nagakawa (Dep.of Urology,Grad.Sch.of Med.Sci.,Kyushu Univ.) [Co-author]Masaki Shiota (Dep.of Urology,Grad.Sch.of Med.Sci.,Kyushu Univ.)、Shigehiro Tsukahara (Dep.of Urology,Grad.Sch.of Med.Sci.,Kyushu Univ.)、Jun Mutaguchi (Dep.of Urology,Grad.Sch.of Med.Sci.,Kyushu Univ.)、Tokiyoshi Tanegashima (Dep.of Urology,Grad.Sch.of Med.Sci.,Kyushu Univ.)、Shunsuke Goto (Dep.of Urology,Grad.Sch.of Med.Sci.,Kyushu Univ.)、Satoshi Kobayashi (Dep.of Urology,Grad.Sch.of Med.Sci.,Kyushu Univ.)、Takashi Matsumoto (Dep.of Urology,Grad.Sch.of Med.Sci.,Kyushu Univ.)、Masatoshi Eto (Dep.of Urology,Grad.Sch.of Med.Sci.,Kyushu Univ.)

Non-muscle invasive bladder cancer (NMIBC) accounts for 70% of bladder cancers. Urine cytology has a high specificity, but sensitivity varies from 35% to 70%, especially for low atypicality and recurrent tumors. Digital PCR is suitable for early detection of cell-free DNA (cfDNA) mutations, but an efficient method using urinary cfDNA has not been established. In this study, we detected several hot-spot mutations in non-coding regions and efficiently analyzed the mutant allele frequency (VAF) of cfDNA to predict the disease status of NMIBC.43 patients diagnosed with NMIBC at Kyushu University Hospital after 2020 were included in the study. NGS was performed to confirm somatic mutations using tumor and leukocyte DNA. Urine was collected and cfDNA was extracted prior to transurethral resection of bladder tumors.We established a method to detect PLEKHS1 promoter mutations by digital PCR and analyzed VAFs for several hotspot mutations, including that mutation.Seven cases of recurrence were identified during the maximum 3-year observation period. Particular attention was paid to VAF at diagnosis, which, in combination with urine cytology, was shown to increase diagnostic efficiency.

[Disclosure of COI by Responsible researcher] Lead presenter is not the Responsible researcher.
[Lead presenter: Shohei Nagakawa] We have no financial relationships to disclose.
[Principal Researcher: Masaki Shiota] We have no financial relationships to disclose.

### [P-2342] II-P10
### Skin tumors

2025 / 9 / 26 (Fri) 16:45-17:30 Room P/(Motenashi Dome) Room P

---

### P-2342
### Analysis of the impact of a germline POLE mutation on voriconazole-associated cutaneous squamous cell carcinoma

[Speaker]Kohei Yamakawa (Department of Environmental Immuno-Dermatology Yokohama City Univ) [Co-author]Shingo Kato (Department of Clinical Cancer Genomics, Yokohama City University Hospital)、Yukie Yamakguchi (Department of Environmental Immuno-Dermatology Yokohama City Univ)

Long-term voriconazole (VRCZ) use increases photosensitivity and cutaneous squamous cell carcinoma (cSCC) risk. We encountered a case of aggressive cSCC with rapid postoperative recurrences in a patient who had undergone long-term VRCZ administration. Cancer genomic profiling test revealed that this patient had a germline POLE mutation (c1503_1504del: p.C501*). Although there have been multiple reports of missense germline POLE mutations, truncating mutations are rare and of unknown significance. Given POLE's DNA repair role, we investigated its potential synergy with VRCZ phototoxicity. Using CRISPR-Cas9, we generated heterozygous POLE mutant keratinocytes that precisely matched the patient's mutation. POLE protein expression and in vitro cell proliferation assays showed no significant differences between the POLE wild-type parental cells and mutant cells. Planned studies will evaluate UVB interactions: comparing DNA damage response, and tumorigenicity in UVB-exposed xenografts. This functional analysis clarifies whether this POLE mutation exacerbates genomic instability in photodamaged cells, potentially explaining accelerated carcinogenesis in VRCZ users.

[Disclosure of COI by Responsible researcher] Lead presenter is not the Responsible researcher.
[Lead presenter: Kohei Yamakawa] We have no financial relationships to disclose.
[Principal Researcher: Yukie Yamakguchi] We have no financial relationships to disclose.

### [P-2343] II-P10
### Skin tumors

2025 / 9 / 26 (Fri) 16:45-17:30 Room P/(Motenashi Dome) Room P

---

### P-2343
### NF-κB signaling is suppressed by diacylglycerol kinase γ in melanoma cells.

[Speaker]Masahiro Kai (Dept. Mol. Biol., Sapporo Med. Univ., Sch. Med.) [Co-author]Akiko Sato (Dept. Mol. Biol., Sapporo Med. Univ., Sch. Med.)、Eiichiro Yamamoto (Dept. Mol. Biol., Sapporo Med. Univ., Sch. Med.)、Takeshi Niinuma (Dept. Mol. Biol., Sapporo Med. Univ., Sch. Med.)、Hiroshi Kitajima (Dept. Mol. Biol., Sapporo Med. Univ., Sch. Med.)、Kazuya Ishiguro (Dept. Mol. Biol., Sapporo Med. Univ., Sch. Med.)、Akira Yorozu (Dept. Mol. Biol., Sapporo Med. Univ., Sch. Med.)、Hiromu Suzuki (Dept. Mol. Biol., Sapporo Med. Univ., Sch. Med.)

In the last annual JCA meeting, we reported that diacylglycerol kinase γ (DGKG) was downregulated in both melanoma cell lines and primary melanoma tissues and that ectopic expression of DGKG in melanoma cells significantly repressed cell migration and invasion, suggesting that DGKG is a putative tumor suppressor in melanoma. To clarify its biological function, microarray assay was performed and we found that DGKG may modulate expression of NF-κB signaling-related genes in melanoma cells. In particular, ectopic expression of a constitutively active form of DGKG significantly downregulated ***NFKB1*** and ***RELB***, suggesting that DGKG may attenuate NF-κB signaling. In addition, luciferase reporter assays confirmed that DGKG significantly suppressed tumor necrosis factor (TNF)-α-induced NF-κB signaling in melanoma cells. These results suggest that DGKG may play a tumor suppressor role via repressing NF-κB signaling in melanoma cells. We will discuss the mechanism that DGKG regulate NF-κB signaling.

[Disclosure of COI by Responsible researcher] Lead presenter is the Responsible researcher.
[Lead presenter: Masahiro Kai] We have no financial relationships to disclose.

### [P-2344] II-P10
### Skin tumors

2025 / 9 / 26 (Fri) 16:45-17:30 Room P/(Motenashi Dome) Room P

---

### P-2344
### Enhanced tumor endothelial marker 8 protein predicts poor prognosis in angiosarcoma.

[Speaker]Ikko Kajihara (Department of Dermatology, Kumamoto University) [Co-author]Soichiro Sawamura (Department of Dermatology, Kumamoto University)、Satoshi Fukushima (Department of Dermatology, Kumamoto University)

Tumor endothelial cells (TECs) have the ability to proliferate/migrate, and resistance to anticancer drugs. TECs express several tumor endothelium-specific markers, including tumor endothelial marker 8 (TEM8). The purpose of this study was to investigate the expression level of TEM8 and its clinical significance in angiosarcoma. We determined whether angiosarcoma cells have upregulated TEM8 protein levels when compared with senile angioma tissue and normal vessel cells by immunohistochemical staining. TEM8 protein was strongly expressed in angiosarcoma tissues when compared with senile angioma tissue and normal vessel cells. Most angiosarcoma cells from all 30 tissue samples were positive for TEM8. A high TEM8 protein level in angiosarcoma tissue was a statistically significant poor prognosis factor for overall survival time. Our study revealed that TEM8, which is a marker of TECs, is detected prominently in angiosarcoma, and overexpression of TEM8 indicates a poor clinical course in patients with angiosarcoma.

[Disclosure of COI by Responsible researcher] Lead presenter is the Responsible researcher.
[Lead presenter: Ikko Kajihara] We have no financial relationships to disclose.

### [P-2345] II-P10
### Skin tumors

2025 / 9 / 26 (Fri) 16:45-17:30 Room P/(Motenashi Dome) Room P

---

### P-2345
### Treatment of advanced melanoma using IPS cell-derived myeloid cells overexpressing OX40 ligand

[Speaker]Toshihiro Kimura (Department of Dermatology and Plastic Surgery, Kumamoto University) [Co-author]Soichiro Sawamura (Department of Dermatology and Plastic Surgery, Kumamoto University)、Ikko Kajihara (Department of Dermatology and Plastic Surgery, Kumamoto University)、Azusa Miyashita (Department of Dermatology and Plastic Surgery, Kumamoto University)、Rong Zhang (Division of Cancer Immunotherapy, National Cancer Center (NCC))、Yasushi Uemura (Division of Cancer Immunotherapy, National Cancer Center (NCC))、Satoshi Fukushima (Department of Dermatology and Plastic Surgery, Kumamoto University)

We previously reported the efficacy of immune cell therapy using immortalized myeloid cells derived from induced pluripotent stem cells (iPSC-pMC). In this study, we generated OX40L-overexpressing iPSC-pMC (iPSC-pMC-OX40L) and investigated their characteristics and in vivo efficacy against murine melanoma. We found that iPSC-pMC-OX40L suppressed the progression of B16-BL6 melanoma, and prolonged the survival of mice with ovalbumin (OVA)-expressing B16 melanoma (MO4). The number of antigen-specific CD8+ T cells was higher in spleen cells treated with OVA-peptide-pulsed iPSC-pMC-OX40L than in those without OX40L. Furthermore, the OVA-peptide-pulsed iPSC-pMC-OX40L significantly increased the number of tumor-infiltrating lymphocytes (TILs) in MO4 tumor. Flow-cytometry analysis showed decreased regulatory T cells but increased effector T cells among the TILs. Collectively, these results indicate that iPSC-pMC-OX40L therapy might be a new method for antigen-specific cancer immunotherapy.

[Disclosure of COI by Responsible researcher] Lead presenter is the Responsible researcher.
[Lead presenter: Toshihiro Kimura] We have no financial relationships to disclose.

### [P-2352] II-P11-2
### Hematological malignancies (2)

2025 / 9 / 26 (Fri) 16:45-17:30 Room P/(Motenashi Dome) Room P

---

### P-2352
### Response to valemetostat is linked to the induction of anti-HTLV-1 immune responses in adult T-cell leukemia (ATL)

[Speaker]Yuki Okamoto (Department of Hematology, Rheumatology and Infectious Diseases, Kumamoto University) [Co-author]Takafumi Shichijo (Department of Hematology, Rheumatology and Infectious Diseases, Kumamoto University)、Shinya Endo (Department of Hematology, Rheumatology and Infectious Diseases, Kumamoto University)、Takahisa Nakamura (Department of Hematology, Rheumatology and Infectious Diseases, Kumamoto University)、Nobukazu Watanabe (Department of Hematology, Rheumatology and Infectious Diseases, Kumamoto University)、Toshiro Kawakita (Department of Hematology, National Hospital Organization Kumamoto Medical Center)、Masao Matsuoka (Department of Hematology, Rheumatology and Infectious Diseases, Kumamoto University)、Junichiro Yasunaga (Department of Hematology, Rheumatology and Infectious Diseases, Kumamoto University)

ATL is a hematologic malignancy caused by HTLV-1 infection with a poor prognosis. Valemetostat, a recently approved EZH1/2 inhibitor, suppresses H3K27 trimethylation and may exert efficacy through epigenetic regulation. However, its involvement in host immunity remains unclear. This study explored the impact of valemetostat on immune responses in ATL patients. Peripheral blood mononuclear cells were obtained pre- and on-treatment from ATL patients receiving valemetostat. ELISpot assays and flow cytometry were performed to evaluate anti-HTLV-1 cellular immunity and HLA class I expression, respectively. Nine ATL patients received valemetostat. Among six responders, three showed increased ELISpot counts (9.3 to 75.7) , indicating restored anti-HTLV-1 immunity. Flow cytometry was then performed on samples from these patients. Two of the three responders showed increased HLA class I expression (MFI: 44,064 to 67,849) in HTLV-1-infected cells, including ATL cells, while the two non-responders showed decreased expression. In conclusion, valemetostat enhanced host immunity by increasing HLA class I expression in responders, suggesting immune-mediated mechanisms of valemetostat in ATL.

[Disclosure of COI by Responsible researcher] Lead presenter is not the Responsible researcher.
[Lead presenter: Yuki Okamoto] We have no financial relationships to disclose.
[Principal Researcher: Yasunaga Yasunaga] ■Research expenses: エーザイ

Poster Session

### [P-2353] II-P11-2
### Hematological malignancies (2)

2025 / 9 / 26 (Fri) 16:45-17:30 Room P/(Motenashi Dome) Room P

---

### P-2353
### Analysis of the mechanism by which vitamin K2 induces an integrated stress response in AML cell lines.

[Speaker]Naoharu Takano (Dept. of Biochem., Tokyo Med. Univ.) [Co-author]Rina Saikawa (Sch. of Med., Tokyo Med. Univ.)、Shota Moriya (Dept. of Biochem., Tokyo Med. Univ.)、Shiori Yoshimura (Sch. of Med., Tokyo Med. Univ.)、Hiromi Kazama (Dept. of Biochem., Tokyo Med. Univ.)、Michiko Itoh (Dept. of Biochem., Tokyo Med. Univ.)、Keisuke Miyazawa (Dept. of Biochem., Tokyo Med. Univ.)

We have reported that vitamin K2 (VK2) sensitizes acute myeloid leukemia (AML) cells and multiple myeloma (MM) cells against the BCL-2 inhibitor, Venetoclax (VEN). We also demonstrated that VK2 produces ROS and following NOXA induction which are critical to sensitize the AML and MM cells against VEN. However, the mechanisms of how VK2 produces ROS and induces NOXA expression have not been elucidated. Initially, we searched for the source of ROS production by VK2-administration and identified mitochondrial complex II. Although the transcription of NOXA was activated in a ROS-dependent manner, mitochondrial ROS from complex II did not upregulate NOXA mRNA but stabilized NOXA protein. Next, we tried to identify the VK2-responsible elements on the NOXA gene by luciferase reporter assay. The assay identified a novel ATF4 binding site on the NOXA gene. Additionally, we found that VK2 treatment increased p-eIF2$\alpha$ and ATF4 expression, indicating that VK2 induced integrated stress response (ISR). Our findings demonstrated that VK2 activates NOXA expression at the protein level by mitochondrial ROS and also at the transcription level by the ISR activation in AML cell lines.

[Disclosure of COI by Responsible researcher] Lead presenter is the Responsible researcher.
[Lead presenter: Naoharu Takano] ■Research funds from company: 科研費、学内研究費

▲TOP

### [P-2354] II-P11-2
### Hematological malignancies (2)

2025 / 9 / 26 (Fri) 16:45-17:30 Room P/(Motenashi Dome) Room P

---

### P-2354
### Impact of Clinico-genetic Factors on Prognosis After allogeneic HSCT in Acute Lymphoblastic Leukemia

[Speaker]Sadato Daichi (Clinical Res. and Trials Center, Tokyo Metropolitan Komagome Hospital) [Co-author]Kana Kato (Div. Hematology, Tokyo Metropolitan Komagome Hospital)、Takashi Toya (Div. Hematology, Tokyo Metropolitan Komagome Hospital)、Chizuko Hirama (Clinical Res. and Trials Center, Tokyo Metropolitan Komagome Hospital)、Yuho Najima (Div. Hematology, Tokyo Metropolitan Komagome Hospital)、Hiroaki Shimizu (Div. Hematology, Tokyo Metropolitan Komagome Hospital)、Noriko Doki (Div. Hematology, Tokyo Metropolitan Komagome Hospital)、Tomomi Toubai (Clinical Res. and Trials Center, Tokyo Metropolitan Komagome Hospital／Div. Hematology, Tokyo Metropolitan Komagome Hospital)、Hironori Harada (Div. Hematology, Tokyo Metropolitan Komagome Hospital／Lab. Oncology, Tokyo University of Pharmacy & Life Sciences)、Yuka Harada (Clinical Res. and Trials Center, Tokyo Metropolitan Komagome Hospital)

The clinico-genetic factors associated with allogeneic hematopoietic stem cell transplantation (HSCT) outcomes in acute lymphoblastic leukemia (ALL) remain unclear. In this study, we investigate the prognostic impact of clinical factors and pre-transplant genomic alterations on outcomes in patients with ALL receiving allogeneic HSCT. Genomic analysis was performed using amplicon sequencing, targeting 85 gene mutations and 687 chimeras. A total of 73 pre–HSCT tumor samples were analyzed. Consequently, non-complete remission at transplantation, ***CDKN2A/2B*** deletions and non-Philadelphia chromosome status were identified as independent factors for relapse. A risk stratification system based on these factors revealed distinct prognostic differentiation, with 1–year overall survival rates of 88.1%, 60.2%, and 0.0% for scores of 0–1, 2, and 3, respectively, and 1–year cumulative incidence of relapse of 4.8%, 33.3%, and 100%, respectively. These results suggest that our model is useful for post-transplantation prognostic stratification. These pre–transplant factors could be adopted to determine transplant eligibility and to guide peri-transplant treatment strategies.

[Disclosure of COI by Responsible researcher] Lead presenter is not the Responsible researcher.
[Lead presenter: Daichi Sadato] We have no financial relationships to disclose.
[Principal Researcher: Chizuko Hirama] We have no financial relationships to disclose.

### [P-2355] II-P11-2
### Hematological malignancies (2)

2025 / 9 / 26 (Fri) 16:45-17:30 Room P/(Motenashi Dome) Room P

---

P-2355
### Differential regulatory T cell-like activity of novel paired HTLV-1 infected cell lines with and without CD13 expression

[Speaker]Tomonori Higuchi (Dept. Microbiol. Infect., Kochi med. Sch., Kochi Univ.) [Co-author]Yumiko Hashida (Dept. Microbiol. Infect., Kochi med. Sch., Kochi Univ.)、Masanori Daibata (Dept. Microbiol. Infect., Kochi med. Sch., Kochi Univ.)

Adult T-cell leukemia/lymphoma (ATL) occurs after human T-cell leukemia virus type-1 (HTLV-1) infection with a long latency period, and this implies the presence of immune evasion mechanisms for HTLV-1-infected T cells. Although ATL cells have an immune phenotype similar to that of regulatory T cells (Tregs), they do not always possess the immunosuppressive functions of Tregs. Previous studies have demonstrated the presence of a $CD13^{+}$ Treg subpopulation with enhanced immunosuppressive activity. We established the paired $CD13^{-}$ (designated as MT-50.1) and $CD13^{+}$ (MT-50.4) HTLV-1-infected non-leukemic T-cell lines derived from a single ATL patient, and evaluated the immunosuppressive activity of these cell lines. MT-50.4 cells expressed higher levels of Foxp3, secreted higher levels of IL-10 and TGF-$\beta$, and strongly suppressed $CD4^{+}CD25^{-}$ T cell proliferation than MT-50.1 cells. Furthermore, the CD13 inhibitor bestatin significantly suppressed MT-50.4 cell growth, but not MT-50.1 cells. These findings suggest that CD13 expression may be involved in the increased Treg-like activity of MT-50.4 cells, and MT-50.4 cells will be useful for in-depth studies of $CD13^{+}Foxp3^{+}$ HTLV-1-infected cells.

[Disclosure of COI by Responsible researcher] Lead presenter is not the Responsible researcher.
[Lead presenter: Tomonori Higuchi] ■Research funds from company: 日本学術振興会　科研費（課題番号：23K07786）
[Principal Researcher: Masanori Daibata] ■Research funds from company: 日本学術振興会　科研費（課題番号：23K07786）

▲TOP

Poster Session

### [P-2356] II-P11-2
### Hematological malignancies (2)

2025 / 9 / 26 (Fri) 16:45-17:30 Room P/(Motenashi Dome) Room P

---

### P-2356
### CRISPR-based inducible knockout system in human blood cancer xenograft models

[Speaker]Yuki Itatani (Life & Med. Sci. Inst., Kyoto Univ.) [Co-author]Kazunari Aoki (Life & Med. Sci. Inst., Kyoto Univ.)、Kosuke Yusa (Life & Med. Sci. Inst., Kyoto Univ.)

CRISPR screening is one of the most powerful tools to comprehensively search for genes that cause a phenotype of interest. It has been used for exploring genes essential for survival in various types of human cancer cells, and novel therapeutic targets have been identified. However, since most of these screens were performed ***in vitro***, the effects of interactions between cancer cells and surrounding cells, including mesenchymal and endothelial cells, were not considered. To explore genes essential for cancer cell growth ***in vivo***, we constructed a new system, in which target genes can be inducibly knocked out in human blood cancer cells. We employed the FlpERT2-FRT system to induce Cas9 expression via site-specific recombination in a tamoxifen-dependent manner. Cells carrying this system were transduced with gRNA-expressing lentivirus and then transplanted into immunodeficient mice. We confirmed that target genes were efficiently knocked out following intraperitoneal injection of tamoxifen. This new system enables us to perform ***in vivo*** CRISPR screening, by which novel therapeutic target genes for human cancer cells would be identified.

[Disclosure of COI by Responsible researcher] Lead presenter is not the Responsible researcher.
[Lead presenter: Yuki Itatani] We have no financial relationships to disclose.
[Principal Researcher: Kosuke Yusa] We have no financial relationships to disclose.

### [P-2357] II-P11-2
### Hematological malignancies (2)

2025 / 9 / 26 (Fri) 16:45-17:30 Room P/(Motenashi Dome) Room P

---

### P-2357
### Ambroxol induces myeloma cell death via inhibiting late stage of autophagy.

[Speaker]Yamato Miyashita (Div. of Clin. Physiol. & Therap., Keio Univ. Faculty of Pharm.) [Co-author]Taiga Okaue (Div. of Clin. Physiol. & Therap., Keio Univ. Faculty of Pharm.)、Shinsuke Shibata (Grad. Sch. of Med. and Dent. Sci., Niigata Univ.)、Taketo Yamada (Dept. of Path., Saitama Med. Univ.)、Tomohumi Yamamoto (Div. of Clin. Physiol. & Therap., Keio Univ. Faculty of Pharm.)、Kouhei Yamazaki (Dept. of Hematology, Tokyo Saiseikai Central Hosp.)、Hisako Kunieda (Dept. of Hematology, Tokyo Saiseikai Central Hosp.)、Hideyuki Saya (Oncology Innovation Ctr, Fujita Health Univ.)、Yutaka Hattori (Div. of Clin. Physiol. & Therap., Keio Univ. Faculty of Pharm.／Dept. of Hematology, Tokyo Saiseikai Central Hosp.／Keikokai Med. Corp., P-one Clinic.／Dept. of Hematology, International Univ. of Health and Welfare Hosp.)、Maiko Matsushita (Div. of Clin. Physiol. & Therap., Keio Univ. Faculty of Pharm.)

Since multiple myeloma(MM) remains an incurable hematologic tumor, exploitation of new drugs that potentiate anti-MM activities of existing drugs is mandatory. We found through drug repurposing that ambroxol(Abx), a cough medicine, induces MM cell death. Abx increased expression of LC3-II and p62/SQSTM1 in MM cells in turnover assay and reporter assay. Transmission electron microscopy observation also showed that Abx significantly increased accumulation of autophagic vacuoles in the cytoplasm of MM cells, indicating that Abx inhibits the late stage of autophagy. Among existing anti-MM drugs, Panobinostat(Pan), a histone deacetylase inhibitor, induces autophagy, whereas bortezomib and lenalidomide do not. Abx synergistically delayed MM cells growth with Pan in SCID-xenograft model, likely due to Abx blocking the activation of Pan-induced autophagy. Interestingly, Abx also ameliorated Pan-induced diarrhea. GSEAs showed that Abx increased the gene expression for autophagy inhibition and unfolded protein response. In conclusion, autophagy is a promising therapeutic target for MM. Autophagy inhibitor may overcome the drug-resistance and toxicities of existing anti-neoplastic drugs.

[Disclosure of COI by Responsible researcher] Lead presenter is not the Responsible researcher.
[Lead presenter: Yamato Miyashita] We have no financial relationships to disclose.
[Principal Researcher: Maiko Matsushita] We have no financial relationships to disclose.

▲TOP

### [P-2364] II-P13
### Challenges in elucidating the nature of rare cancers & exploring new treatment options

2025 / 9 / 26 (Fri) 16:45-17:30 Room P/(Motenashi Dome) Room P

---

### P-2364
### Development of hypoxically activated Magic Bullet having ultrahigh tumor-specificity

[Speaker]Yutaka Ikeda (Kanazawa University Cancer research institute) [Co-author]Taisuke Furuta (Hiroshima University Graduate School of Biomedical and Health Sciences(Medical))

Hypoxia is a feature of solid tumors and tumor hypoxia is well known to promote mutagenesis, invasiveness and to be associated with a worse prognosis. A strategy for targeting the hypoxic region of tumors is highly required; however, impaired drug penetration in hypoxic regions has hampered drug development. We have developed a novel prodrug system in which antitumor agent is delivered to hypoxic region and released under hypoxic condition (Ikeda et. al. Mol. Pharm (2016), WO2013122112). A variety of drugs including molecularly targeted drugs have been modified and evaluated in vitro and in vivo. Newly designed hypoxically activated prodrugs (HAPs) drastically reduced the toxicity of original anticancer drugs and improved therapeutic effects in sarcoma tumor-bearing mouse model. Interestingly, combination of HAPs with radiation further improved therapeutic efficiency and reduced toxicity which is often observed in the chemoradiotherapy (PCT/JP2022/046076). Biodistribution study showed HAPs reduced the accumulation of anticancer drugs at the organs other than tumor. These results showed the potency of newly designed HAPs as novel antitumor reagents targeting tumor hypoxia

[Disclosure of COI by Responsible researcher] Lead presenter is the Responsible researcher.
[Lead presenter: Yutaka Ikeda] We have no financial relationships to disclose.

### [P-2365] II-P13
### Challenges in elucidating the nature of rare cancers & exploring new treatment options

2025 / 9 / 26 (Fri) 16:45-17:30 Room P/(Motenashi Dome) Room P

---

### P-2365
### Epigenetic suppression of GPX7 in pseudomyxoma peritonei may promote mucin production via enhanced PKA signaling

[Speaker]Kiyoko Takane (Div. Clin. Genome Res., Inst. Med. Sci., Univ. Tokyo) [Co-author]Tingwei Cai (Div. Clin. Genome Res., Inst. Med. Sci., Univ. Tokyo)、Kiyoshi Yamaguchi (Div. Clin. Genome Res., Inst. Med. Sci., Univ. Tokyo)、Takahiro Oshina (Div. Clin. Genome Res., Inst. Med. Sci., Univ. Tokyo)、Yasunori Ohta (Dept. of Path., IMSUT Hosp.)、Yoshimasa Gohda (Dept. of Surg. Natl. Ctr. for Global health & Med.)、Tomomichi Kiyomatsu (Dept. of Surg. Natl. Ctr. for Global health & Med.)、Hideaki Yano (Dept. of Surg. Natl. Ctr. for Global health & Med.)、Toru Igari (Path. Div., Natl. Ctr. for Global health & Med.)、Masaki Fukuyo (Dept. of Mol. Oncology Grad. Sch. of Med. Chiba Univ.)、Motoaki Seki (Dept. of Mol. Oncology Grad. Sch. of Med. Chiba Univ.)、Atsushi Kaneda (Dept. of Mol. Oncology Grad. Sch. of Med. Chiba Univ.／Health and Disease Omics Ctr., Chiba Univ.)、Yoichi Furukawa (Dept. of Mol. Oncology Grad. Sch. of Med. Chiba Univ.／Health and Disease Omics Ctr., Chiba Univ.)

Pseudomyxoma peritonei (PMP) is a rare malignancy characterized by mucinous ascites, most commonly originating from appendiceal tumors. While KRAS and GNAS mutations have been implicated in PMP tumorigenesis, the contribution of epigenetically silenced genes remains unclear. To identify such genes, we integrated genome-wide DNA methylation and RNA-seq data, revealing six genes downregulated in PMP due to promoter hypermethylation. Among them, GPX7 was significantly suppressed in PMP tissues compared to normal colonic mucosa and colorectal cancer. Treatment of patient-derived PMP organoids with the DNMT inhibitor 5-Azacytidine restored GPX7 expression, suggesting epigenetic silencing. Furthermore, overexpression of GPX7 in colon cancer cells reduced phosphorylation of PKA substrates and MUC2 expression. These findings suggest that reduced GPX7 may enhance mucin production via PKA pathway activation and highlight its potential as a therapeutic target to suppress mucin overproduction in PMP.

[Disclosure of COI by Responsible researcher] Lead presenter is not the Responsible researcher.
[Lead presenter: Kiyoko Takane] ■Research funds from company: JSPS KAKENHI Grant Numbers JP:20K17641, 23K08108
[Principal Researcher: Tingwei Cai] We have no financial relationships to disclose.

[P-2366] II-P13

Challenges in elucidating the nature of rare cancers & exploring new treatment options

2025 / 9 / 26 (Fri) 16:45-17:30 Room P/(Motenashi Dome) Room P

P-2366

### Development of a Comprehensive Specimen Collection Timepoint Management System for Spatiotemporal Multi-omics Analysis

[Speaker]Mari Takahashi (TR Sample Management Office,NCC Hosp.East.) [Co-author]Fubuki Omoya (TR Sample Management Office,NCC Hosp.East.／Department of Clinical Laboratory,NCC Hosp.East.)、Yuki Iino (TR Sample Management Office,NCC Hosp.East.)、Yasuko Tada (TR Sample Management Office,NCC Hosp.East.)、Hitomi Nakai (TR Sample Management Office,NCC Hosp.East.)、Yumie Takeshima (TR Sample Management Office,NCC Hosp.East.)、Mana Shimamura (TR Sample Management Office,NCC Hosp.East.／Department of Clinical Laboratory,NCC Hosp.East.)、Asako Takahata (TR Sample Management Office,NCC Hosp.East.)、Tadayoshi Hashimoto (TR Support Office,NCC Hosp.East.／Department of Gastrointestinal Oncology,NCC Hosp.East.)、Shin Kobayashi (Department of Hepatobiliary and Pancreatic Surgery,NCC Hosp.East.)、Hideaki Bando (TR Support Office,NCC Hosp.East.／Department of Gastrointestinal Oncology,NCC Hosp.East.)、Tomonori Yano (Endoscopy Center.,NCC Hosp.East.)、Takao Fujisawa (TR Sample Management Office,NCC Hosp.East.／TR Support Office,NCC Hosp.East.／Department of Head and Neck Medical Oncology,Ncc Hosp.East.)、Takayuki Yoshino (Department of Gastrointestinal Oncology,NCC Hosp.East.)

Spatiotemporal multi-omics research, which involves longitudinal collection of diverse biological specimens, has advanced our understanding of comprehensive molecular mechanism dynamics. However, managing multiple specimen types across multiple timepoints presents substantial logistical challenges. The MONSTAR-SCREEN-3 study is a spatiotemporal multi-omics investigation comprising 3 independent cohorts with 4 specimen types at up to 10 timepoints. To address complex specimen management requirements, we developed a comprehensive management system for coordinating collection timepoints and established a multidisciplinary framework for specimen handling in our hospital. This system accommodates various cohort patterns while enabling real-time tracking of collection status for each specimen type. The system also generates alerts as collection dates approach to prevent missed collections. To benefit other participating institutions, we regularly share our operational experience through scheduled meetings. This comprehensive specimen collection management system provides a valuable model for specimen management in future spatiotemporal multi-omics research studies.

[Disclosure of COI by Responsible researcher] Lead presenter is not the Responsible researcher.
[Lead presenter: Mari Takahashi] We have no financial relationships to disclose.
[Principal Researcher: Takayuki Yoshino] We have no financial relationships to disclose.

### [P-2367] II-P13
### Challenges in elucidating the nature of rare cancers & exploring new treatment options

2025 / 9 / 26 (Fri) 16:45-17:30 Room P/(Motenashi Dome) Room P

---

### P-2367
### Uncovering Prognostic Biomarkers for Enhanced Risk Stratification in Extraskeletal Myxoid Chondrosarcoma

[Speaker]Amphun Chaiboonchoe (SiCorePM&SP,Dept of Pharm, Faculty of Medicine Siriraj Hosp, Mahidol Univ.) [Co-author]Jantappapa Chanthercrob (SiCorePM&SP,Dept of Pharm, Faculty of Medicine Siriraj Hosp, Mahidol Univ.)、Romgase Sakamula (SiCorePM&SP,Dept of Pharm, Faculty of Medicine Siriraj Hosp, Mahidol Univ.)、Thanaphon Likhityungyuen (SiCorePM&SP,Dept of Pharm, Faculty of Medicine Siriraj Hosp, Mahidol Univ.)、Sorranart Maungsomboon (Dept. of Pathology, Faculty of Medicine Siriraj Hosp., Mahidol Univ.)、Jomjit Chantharasamee (Div of Med.Onco, Faculty of Med Siriraj Hosp., Mahidol Univ.)、Frabizio Remotti (Dept of Path.&Cell Biology, College of Physician&Surg., Columbia Univ.)、Francis Young-in Lee (Dept of Orthopaedics and Rehabilitation, Yale University, Connecticut, USA)、Pannin Thanapipatsiri (Dept of Orthopaedic Surg, Faculty of Med.Siriraj Hosp, Mahidol Univ.)、Rapin Phimolsarnti (Dept of Orthopaedic Surg, Faculty of Med.Siriraj Hosp, Mahidol Univ.)、Apichat Asavamongkolkul (Dept of Orthopaedic Surg, Faculty of Med.Siriraj Hosp, Mahidol Univ.)、Somponnat Sampattavanich (SiCorePM&SP,Dept of Pharm, Faculty of Medicine Siriraj Hosp, Mahidol Univ.)、Chandhanarat Chandhanayingyong (Dept of Orthopaedic Surg, Faculty of Med.Siriraj Hosp, Mahidol Univ.)

Extraskeletal Myxoid Chondrosarcoma (EMC) is a rare soft tissue sarcoma with unique clinical behavior and molecular heterogeneity. Developing prognosis biomarkers is challenging, and this study aimed to identify potential biomarkers to predict prognosis more accurately in EMC patients.
We sequenced the transcriptome (RNA) of 12 EMC patients. Differential gene expression analysis was performed, followed by gene set enrichment analysis. Gene set enrichment analysis showed that allograft rejection, mitotic spindle, and TNF$\alpha$ signaling via NF$\kappa$B underlie the prognosis prediction. We identified H1FX, PXN, and TYMS as potential prognostic biomarkers using LASSO-Cox regression, with higher expression indicating lower survival rates. The study identifies 550 genes associated with varying prognoses. The study also developed a risk score to differentiate prognosis between high-risk and low-risk subgroups. Immune cell prediction revealed T cell enrichment in high-risk groups, while low-risk groups showed higher B cell presence.
This research advances prognostic stratification in EMC patients and offers a foundation for personalized treatment strategies.

[Disclosure of COI by Responsible researcher] Lead presenter is the Responsible researcher.
[Lead presenter: Amphun Chaiboonchoe] We have no financial relationships to disclose.

### [P-2368] II-P13
### Challenges in elucidating the nature of rare cancers & exploring new treatment options

2025 / 9 / 26 (Fri) 16:45-17:30 Room P/(Motenashi Dome) Room P

---

### P-2368
### The effect of free fatty acids on the survival of malignant mesothelioma cells: Mechanism of cell death induced by FFAs

[Speaker]Hinako Matsumuro (Dept. of Food and Nutritional Environment, Kinjo Gakuin Univ.) [Co-author]Rin Minami (Dept. of Food and Nutritional Environment, Kinjo Gakuin Univ.)、Nozomi Yoshizawa (Dept. of Food and Nutritional Environment, Kinjo Gakuin Univ.)、Sivasundaram Karnan (Dept. of Biochem., Aichi Med Univ.)、Yoshitaka Hosokawa (Dept. of Biochem., Aichi Med Univ.)、Akinobu Ota (Dept. of Food and Nutritional Environment, Kinjo Gakuin Univ.／Dept. of Biochem., Aichi Med Univ.)

Malignant mesothelioma (MMe) is a rare and fatal cancer type associated with asbestos exposure. Despite recent clinical advances in molecular-targeted therapeutics, MMe remains an incurable disease. We previously showed that one of the free FAs (FFAs) palmitic acid (PA) preferentially suppresses the proliferation of human MMe cells compared with normal mesothelial cells. However, the precise molecular mechanism by which PA suppresses the growth of MMe cells is obscure. Here, we examined the effect of saturated and unsaturated FFAs on the survival of human MMe cells. In addition, the expression of possible genes related to PA metabolism were examined by using reverse-transcribed polymerase chain reaction. Furthermore, the effect of PA on the ferroptosis of MMe cells were examined by using flowcytometry analysis. MTT assay showed that PA readily decrease the survival, whereas a monounsaturated FA oleic acid slightly suppresses the proliferation in MMe cell lines. Our findings suggest that treatment with saturated FAs suppresses the proliferation of MMe cells stronger than other FFAs. We would discuss the possible molecular mechanism by which PA decreases the survival of MMe cells.

[Disclosure of COI by Responsible researcher] Lead presenter is not the Responsible researcher.
[Lead presenter: Hinako Matsumuro] We have no financial relationships to disclose.
[Principal Researcher: Akinobu Ota] We have no financial relationships to disclose.

### [P-2369] II-P13
### Challenges in elucidating the nature of rare cancers & exploring new treatment options

2025 / 9 / 26 (Fri) 16:45-17:30 Room P/(Motenashi Dome) Room P

---

### P-2369
### Pre-existing autoimmune disease as a risk factor for irAEs in cancer patients receiving immune checkpoint inhibitors

[Speaker]Hidetoshi Sumimoto (Dep. Med. Oncol., Shiga Univ. Med. Sci.／Cancer Centr., Shiga Univ. Med. Sci.／Centr. Adv. Med. Cancer, Shiga Univ. Med. Sci.) [Co-author]Atsushi Takano (Dep. Med. Oncol., Shiga Univ. Med. Sci.／Cancer Centr., Shiga Univ. Med. Sci.／Centr. Adv. Med. Cancer, Shiga Univ. Med. Sci.／Centr. Antibody Vaccine, Inst. Med. Sci. Univ. Tokyo)、Koji Teramoto (Dep. Med. Oncol., Shiga Univ. Med. Sci.／Cancer Centr., Shiga Univ. Med. Sci.／Centr. Adv. Med. Cancer, Shiga Univ. Med. Sci.)、Yataro Daigo (Dep. Med. Oncol., Shiga Univ. Med. Sci.／Cancer Centr., Shiga Univ. Med. Sci.／Centr. Adv. Med. Cancer, Shiga Univ. Med. Sci.／Centr. Antibody Vaccine, Inst. Med. Sci. Univ. Tokyo)

Immune checkpoint inhibitors (ICIs) are applied to various cancers but can cause irAEs leading to discontinuation. Due to the increased irAE risk, patients with pre-existing autoimmune diseases (AI) are often advised against ICIs, but objective risk assessment for AI is insufficient. We conducted a logistic regression analysis of 478 cases treated with ICIs at our hospital. Among them, 28 (5.9%) had pre-existing AI. Among several independent factors, the adjusted odds ratio (OR) of AI for irAE was 2.52 [95% CI: 1.08–5.86] (p=0.033). Patients with pre-existing AI had a significantly shorter irAE-free survival time than those without AI (median: 5.7 months [3.5–7.8] vs. 10.4 months [7.9–12.9], p=0.035). Frequent irAEs in the full ICI cohort, such as dermatitis (7.5%), pneumonitis (7.1%), hepatitis (4.6%), and hypothyroidism (4.2%), were often accompanied by pre-existing AI. AI flared up in 6 cases (37.5% of AI-positive irAE-positive cases), but AI activity was not related to irAE occurrence. Grade 3 or higher irAEs were observed in 6 of 20 (30.0%) AI-accompanied irAE cases. Although AI increases irAE risk, it may not be a contraindication for ICI treatment if closely monitored.

[Disclosure of COI by Responsible researcher] Lead presenter is not the Responsible researcher.
[Lead presenter: Hidetoshi Sumimoto] We have no financial relationships to disclose.
[Principal Researcher: Yataro Daigo] We have no financial relationships to disclose.

### [P-2370] II-P13
### Challenges in elucidating the nature of rare cancers & exploring new treatment options

2025 / 9 / 26 (Fri) 16:45-17:30 Room P/(Motenashi Dome) Room P

---

### P-2370
### Cross-Tumor Single-cell RNA-Seq Analysis Reveals Cancer-Specific Cellular and Molecular Features

[Speaker]Riku Okamoto (Dept. of Adv. Surg. Oncol., Kitasato Univ. Sch. of Med.) [Co-author]Shunpei Shibaki (Dept. of Adv. Surg. Oncol., Kitasato Univ. Sch. of Med.)、Kanako Naito (Dept. of Adv. Surg. Oncol., Kitasato Univ. Sch. of Med.)、Hiroyuki Minoura (Dept. of Adv. Surg. Oncol., Kitasato Univ. Sch. of Med.)、Kyonosuke Ikemura (Dept. of Adv. Surg. Oncol., Kitasato Univ. Sch. of Med.)、Kota Okuno (Dept. of Adv. Surg. Oncol., Kitasato Univ. Sch. of Med.)、Yu Kuroda (Dept. of Adv. Surg. Oncol., Kitasato Univ. Sch. of Med.)、Akiko Watanabe (Dept. of Adv. Surg. Oncol., Kitasato Univ. Sch. of Med.)、Keiko Oki (Dept. of Adv. Surg. Oncol., Kitasato Univ. Sch. of Med.)、Shiori Fujino (Dept. of Adv. Surg. Oncol., Kitasato Univ. Sch. of Med.)、Yusuke Nie (Dept. of Adv. Surg. Oncol., Kitasato Univ. Sch. of Med.)、Yousuke Oizumi (Dept. of Surgery, Sagamihara Hosp.)、Naoki Hiki (Dept. of Upper GI Surg., Kitasato Univ. Sch. of Med.)、Keishi Yamashita (Dept. of Adv. Surg. Oncol., Kitasato Univ. Sch. of Med.)

[Background]Single-cell RNA sequencing (scRNA-seq) reveals cellular heterogeneity in cancers, but cross-tumor studies remain limited. We aimed to identify tumor-specific cellular and gene expression features using public scRNA-seq data.[Methods]We analyzed 10x Genomics-based scRNA-seq data from colorectal (n=16), gastric (n=26), esophageal (n=60), pancreatic (n=15), liver (n=25), thyroid (n=7), and breast (n=26) cancers. Cells with <2500 nFeature_RNA and <10% mitochondrial reads were included. Doublet removal and batch correction were applied as needed.[Results]Thyroid cancer showed the highest tumor cell ratio; plasma cells were enriched in gastric cancer. Mast cells were absent in breast and thyroid cancers. Pancreatic cancer lacked endothelial/pericyte cells. Stellate cells in liver cancer were annotated as pericytes; CAFs were not found. ***CXCR1***/***CXCR2***+ TANs appeared only in pancreatic cancer. The tumor suppressor ***HOPX*** was highly expressed in thyroid cancer and enhanced tumor suppression in vitro.[Conclusion]Cross-tumor scRNA-seq revealed unique cellular and molecular traits. ***HOPX*** may be a therapeutic target.

[Disclosure of COI by Responsible researcher] Lead presenter is not the Responsible researcher.
[Lead presenter: Riku Okamoto] We have no financial relationships to disclose.
[Principal Researcher: Keishi Yamasita] We have no financial relationships to disclose.

### [P-2371] II-P13
### Challenges in elucidating the nature of rare cancers & exploring new treatment options

2025 / 9 / 26 (Fri) 16:45-17:30 Room P/(Motenashi Dome) Room P

---

### P-2371
### Two neuroendocrine tumors of kidney with ghrelin expression

[Speaker]Akane Naruoka (Drug Discovery and Development Div. Shizuoka Cancer Ctr. Res. Inst.) [Co-author]Keiichi Ohshima (Drug Discovery and Development Div. Shizuoka Cancer Ctr. Res. Inst.／SRL Inc.)、Takashi Sugino (Div. of Pathology, Shizuoka Cancer Ctr Hosp.)、Koji Muramatsu (Div. of Pathology, Shizuoka Cancer Ctr Hosp.)、Takeshi Nagashima (Cancer Diagnostics Res. Div. Shizuoka Cancer Ctr. Res. Inst.／SRL Inc.)、Sumiko Ohnami (Cancer Diagnostics Res. Div. Shizuoka Cancer Ctr. Res. Inst.)、Shumpei Ohnami (Cancer Diagnostics Res. Div. Shizuoka Cancer Ctr. Res. Inst.)、Masakuni Serizawa (Drug Discovery and Development Div. Shizuoka Cancer Ctr. Res. Inst.)、Keiichi Hatakeyama (Exp. Animal Facility. Shizuoka Cancer Ctr. Res. Inst.)、Kouji Maruyama (Cancer Multiomics Div. Shizuoka Cancer Ctr. Res. Inst.)、Yasue Horiuchi (Cancer Diagnostics Res. Div. Shizuoka Cancer Ctr. Res. Inst.)、Yuji Shimoda (Cancer Diagnostics Res. Div. Shizuoka Cancer Ctr. Res. Inst.)、Akira Iizuka (Immunotherapy Div. Shizuoka Cancer Ctr. Res. Inst.)、Kenichi Urakami (Cancer Diagnostics Res. Div. Shizuoka Cancer Ctr. Res. Inst.)、Yasuto Akiyama (Immunotherapy Div. Shizuoka Cancer Ctr. Res. Inst.)、Ken(Shizuoka Cancer Ctr.) Yamaguchi (Shizuoka Cancer Center)

We have been conducting a multi-omics analysis of cancer patients undergoing surgery at a single institution since January 2014 and have now enrolled and analyzed over 10,000 cases. In this cohort, we observed two ghrelin-expressing neuroendocrine tumors of the kidney (kideny-NET). Both cases were women with horseshoe kidney, aged 73 and 69 years. These two cases of kideny-NET were found to have remarkable expression of the ghrelin gene in the gene expression analysis of 10,000 cases. Immunohistochemical staining for ghrelin in these cases confirmed ghrelin staining in the tumor tissue. Kideny-NET is a rare cancer and the number of reported cases is very small. Therefore, the results of whole genome and gene expression analysis are limited, and there are no previous reports on the ability to produce ghrelin. However, in this study, microarray-based gene expression analysis and immunohistochemical staining clearly confirmed ghrelin expression and identified ghrelin-producing Kideny-NET. Furthermore, we believe that further analysis and case accumulation will be required to elucidate the role of ghrelin in these tumors.

[Disclosure of COI by Responsible researcher] Lead presenter is the Responsible researcher.
[Lead presenter: Akane Naruoka] We have no financial relationships to disclose.

### [P-2010] I-P1-4
### Evaluation of Chemical & Radiation Carcinogenesis

2025 / 9 / 26 (Fri) 17:30-18:15 Room P/(Motenashi Dome) Room P

---

### P-2010
### Leveraging mathematical models for extrapolation from laboratory animal data to human cancer risk

[Speaker]Tatsuhiko Imaoka (QST, Inst. for Radiol. Sci., Dept. of Radiat. Effects Res.／QST, Inst. for Quantum Life Sci., Quantum Cancer Sci. Team) [Co-author]Kento Nagata (QST, Inst. for Radiol. Sci., Dept. of Radiat. Effects Res.／QST, Inst. for Quantum Life Sci., Quantum Cancer Sci. Team)、Yuzuki Nakamura (QST, Inst. for Radiol. Sci., Dept. of Radiat. Effects Res.／Tokyo Metropolitan Univ., Grad. Sch. Human Health Sci., Radiol. Sci.／JSPS Research Fellow DC)、Yukiko Nishimura-Yano (QST, Inst. for Radiol. Sci., Dept. of Radiat. Effects Res.)、Keito Asano (QST, Inst. for Radiol. Sci., Dept. of Radiat. Effects Res.／Tokyo Metropolitan Univ., Grad. Sch. Human Health Sci., Radiol. Sci.)、Masaru Takabatake (QST, Inst. for Radiol. Sci., Dept. of Radiat. Effects Res.／Tokyo Metropolitan Univ., Grad. Sch. Human Health Sci., Radiol. Sci.)

Animal models are essential for assessing the risk of carcinogens and benefits of preventive measures, despite limitations in extrapolating to human risk. We used a mathematical model to formulate the relationship between exposure-related cancer acceleration and mutation rates. Analysis of cancer mortality data from atomic bomb survivors and radiation-exposed laboratory mice revealed ~100-fold greater cancer acceleration in mice at the same radiation dose; available data on radiation mutagenesis and age-related somatic mutations showed that this 100-fold difference is inversely related to the somatic mutation rate between species (Int J Cancer 2024;155:1101). A similar mathematical approach identified the same trend in breast cancer incidence in humans and rats exposed to radiation. Analysis of breast cancer incidence in humans and rats carrying BRCA1 pathogenic variants predicted that the variants increased spontaneous and radiation-induced mutations by ~3-fold, allowing estimation of radiation risk in variant carriers. Our finding of an association between cancer risk and somatic mutation rate across species provides a basis for extrapolating animal data to human cancer risk.

[Disclosure of COI by Responsible researcher] Lead presenter is the Responsible researcher.
[Lead presenter: Tatsuhiko Imaoka] ■Research funds from company: 環境省、文部科学省

▲TOP

Poster Session

### [P-2011] I-P1-4
### Evaluation of Chemical & Radiation Carcinogenesis

2025 / 9 / 26 (Fri) 17:30-18:15 Room P/(Motenashi Dome) Room P

---

### P-2011
### Robustness of the four maker genes (Bax, Btg2, Ccng1, and Cdkn1a) for predicting genotoxicity of hepatocarcinogens

[Speaker]Takayoshi Suzuki (Natl Inst Helth Sci, Genome Safety Sci) [Co-author]Keiichi Sugiyama (Natl Inst Helth Sci, Genome Safety Sci)、Chie Furihata (Aoyama Gakuin Univ)

Previously, we proposed 12 genotoxic marker genes to discriminate genotoxichepatocarcinogens (GTHCs) from non-genotoxic hepatocarcinogens (NGTHCs) and non-genotoxic non-hepatocarcinogens (NGTNHCs) in mice. Four of the 12 genes overlappedwith seven marker genes proposed by the U.S. Environmental Protection Agency (USEPA). Then, we validated these four common marker genes (Bax, Btg2, Ccng1, andCdkn1a) with the rat toxicogenomic database (Open TG-GATEs). These four genes provedto be highly effective in distinguishing five typical GTHCs from seven typical NGTHCs and11 NGTNHCs by the principal component analysis (PCA) at 24 h after a singleadministration with 100% accuracy. Now, we also perform similar validation with the DrugMatrix database. Despite the large SD in the raw data of the experimental group andthe vehicle control group in the DrugMatrix data, PCA distinguished four typical GTHCsfrom six typical NGTHCs and optional nine NGTNHCs with the four genes. These resultsdemonstrated the four marker genes' effectiveness and robustness in predicting hepatocarcinogen genotoxicity.

[Disclosure of COI by Responsible researcher] Lead presenter is the Responsible researcher.
[Lead presenter: Takayoshi Suzuki] We have no financial relationships to disclose.

### [P-2012] I-P1-4
### Evaluation of Chemical & Radiation Carcinogenesis

2025 / 9 / 26 (Fri) 17:30-18:15 Room P/(Motenashi Dome) Room P

---

### P-2012
### Direct visualization of clustered DNA damage using atomic force microscope in TK6 cells exposed on irradiation.

[Speaker]Toshiaki Nakano (QST) [Co-author]Ken Akamatsu (QST)、Naoya Shikazono (QST)

Radiation-induced clustered DNA damage is closely linked to the biological effects of radiation. In this study, atomic force microscopy was used to evaluate the complexity of clustered DNA damage in genomic DNA from TK6 cells exposed to low-dose ionizing radiation. By isolating and concentrating damaged DNA molecules, we estimated the extent of damage clustering in vivo. Our results showed that X-rays and Fe-ion beams induced clustered DNA damage, whereas Fentons reagent did not, reinforcing its significance as a unique signature of ionizing radiation. Notably, Fe-ion beams generated more complex clustered damage, often consisting of more than two lesions, which was rare in X-ray exposure. Repair analysis revealed that non-DSB clustered damage was efficiently repaired in TK6 cells after both X-ray and Fe-ion irradiation. However, complex DSBs, which were frequently induced by Fe-ion beams, exhibited poor repair efficiency. These findings emphasize the distinct nature and repair challenges of clustered DNA damage caused by high-LET radiation.

[Disclosure of COI by Responsible researcher] Lead presenter is the Responsible researcher.
[Lead presenter: Toshiaki Nakano] We have no financial relationships to disclose.

Poster Session

### [P-2013] I-P1-4
### Evaluation of Chemical & Radiation Carcinogenesis

2025 / 9 / 26 (Fri) 17:30-18:15 Room P/(Motenashi Dome) Room P

---

### P-2013
### Effect of free fatty acid receptor signaling on X-ray-induced DNA damages in osteosarcoma cells

[Speaker]Yuka Amano (Mol Oncol., Dept. of Life Sci., Kindai Univ.) [Co-author]Koki Hara (Mol Oncol., Dept. of Life Sci., Kindai Univ.)、Toshifumi Tsujiuchi (Mol Oncol., Dept. of Life Sci., Kindai Univ.)、Hiroko Ikeda (Rad Oncol., Dept. of Life Sci., Kindai Univ.)

Osteosarcoma is a highly malignant and treatment-resistant cancer, often characterized by its acquisition of resistance to anticancer drugs and radiation. G-protein-coupled receptors (GPCRs) have emerged as key regulators of cellular functions in cancers. In this study, we focused on free fatty acid receptors (FFARs), known as one of GPCRs, to explore intracellular signaling involved in the progression of osteosarcoma MG-63 cells after X-ray irradiation. Treatment with TUG-770 (FFAR1 agonist) or TUG-891 (FFAR4 agonist) suppressed the proliferation of MG-63 cells. In micronucleus (MN) assay which is used to assess chromosomal instability, the MN frequency in binucleated cells irradiated with X-ray (1-2 Gy) was reduced by TUG-770 treatment. In contrast, FFAR1 knockdown cells irradiated with 2 Gy showed a significant increase in MN formation compared to control cells. In addition, intensities of $\gamma$H2AX foci known as a marker of DNA damages were enhanced by TUG-770 treatment following X-ray exposure, whereas TUG-891 had no impact. These findings suggest that FFAR signaling contributes to the regulation of osteosarcoma MG-63 cell responses to X-ray irradiation.

[Disclosure of COI by Responsible researcher] Lead presenter is not the Responsible researcher.
[Lead presenter: Yuka Amano] We have no financial relationships to disclose.
[Principal Researcher: Hiroko Ikeda] We have no financial relationships to disclose.

▲TOP

[P-2014] I-P1-4
### Evaluation of Chemical & Radiation Carcinogenesis

2025 / 9 / 26 (Fri) 17:30-18:15 Room P/(Motenashi Dome) Room P

P-2014
### Effect of radiation quality on Ptch1 allele deletion in radiation-induced mouse medulloblastoma

[Speaker]Chizuru Tsuruoka (Dept. Radiat. Effects Res., QST) [Co-author]Yi Shang (Dept. Radiat. Effects Res., QST)、Kazuhiro Daino (Dept. Radiat. Effects Res., QST)、Tatsuhiko Imaoka (Dept. Radiat. Effects Res., QST)、Shizuko Kakinuma (Dept. Radiat. Effects Res., QST／IES)

Cancer is an important late effect of radiation exposure. However, the effects of radiation quality on radiation carcinogenesis are not fully elucidated. We have shown that gamma-ray-induced medulloblastoma (G-MB) develops via loss of heterozygosity (LOH) affecting the normal ***Ptch1*** allele, and that the genome copy number of the LOH region reduced to one. Therefore, the G-MBs were likely caused by interstitial deletion affecting ***Ptch1***. We also reported that neutron (N) and carbon-ion (C)-induced MBs show the same LOH pattern, although chromosomal alterations involved in the LOH remain to be determined. This study aims to elucidate the feature of LOH in G-, N-, and C-MBs. The copy number of the LOH region was reduced in N- and C-MBs as in G-MBs. The size of the regions of copy number loss depended on the age at autopsy rather than the radiation quality. The analysis of the breakpoint junction sequences showed evidence of interstitial deletion generated by non-homologous end joining in G-MBs. The N- and C-MBs exhibited different sequences compared to the G-MBs. These results suggest that the feature of LOH in the region of the tumor suppressor gene is radiation quality dependent.

[Disclosure of COI by Responsible researcher] Lead presenter is the Responsible researcher.
[Lead presenter: Chizuru Tsuruoka] ■Research funds from company: 科学研究費助成事業（21H04932、24H00764）

### [P-2015] I-P1-4
### Evaluation of Chemical & Radiation Carcinogenesis

2025 / 9 / 26 (Fri) 17:30-18:15 Room P/(Motenashi Dome) Room P

### P-2015
### Potential roles of chronic inflammation in radiation-induced mammary carcinogenesis

[Speaker]Daisuke Iizuka (Dept. Rad. Effects Res., NIRS, QST) [Co-author]Haruka Izawa (Dept. Rad. Effects Res., NIRS, QST／Grad. Sch. Human Health Sci., Tokyo Metropolitan Univ.)、Mari Ogawa (Dept. Rad. Effects Res., NIRS, QST)、Chizuru Tsuruoka (Dept. Rad. Effects Res., NIRS, QST)、Yi Shang (Dept. Rad. Effects Res., NIRS, QST)、Masaaki Sunaoshi (Dept. Rad. Effects Res., NIRS, QST)、Hirotaka Tachibana (Dept. Rad. Effects Res., NIRS, QST／CRIEPI)、Takamitsu Morioka (Dept. Rad. Effects Res., NIRS, QST)、Shizuko Kakinuma (Dept. Rad. Effects Res., NIRS, QST／IES)、Masaru Takabatake (Dept. Rad. Effects Res., NIRS, QST／Grad. Sch. Human Health Sci., Tokyo Metropolitan Univ.)、Tatsuhiko Imaoka (Dept. Rad. Effects Res., NIRS, QST／Grad. Sch. Human Health Sci., Tokyo Metropolitan Univ.)

The mechanism of radiation-induced mammary carcinogenesis is still unclear. This study aimed to elucidate the role of the microenvironment in the mechanism of radiation-induced mammary carcinogenesis in female BALB/c-***Trp53***$^{+/-}$ mice. The study found that radiation exposure led to an increase in mammary cancer incidence and a reduction in the latency period. Loss of heterozygosity (LOH) and array comparative genomic hybridization analysis showed that LOH events caused by deletion of the wild-type ***Trp53*** allele occurred in many mammary cancers regardless of irradiation. In the comprehensive gene expression analysis, several changes related to immunity were noted in the radiation-induced mammary cancers and showed a tendency toward inflammation. In addition, significant upregulation of inflammation-related genes was observed in the mammary glands of mice 23 weeks after irradiation. This upregulation was accompanied by an enrichment of gene sets related to adipogenesis and adipocyte differentiation. This study has demonstrated that chronic inflammation, facilitated by aberrant lipid metabolism, plays a potential role in the development of radiation-induced mammary cancer.

[Disclosure of COI by Responsible researcher] Lead presenter is the Responsible researcher.
[Lead presenter: Daisuke Iizuka] We have no financial relationships to disclose.

### [P-2016] I-P1-4
### Evaluation of Chemical & Radiation Carcinogenesis

2025 / 9 / 26 (Fri) 17:30-18:15 Room P/(Motenashi Dome) Room P

---

### P-2016
### A Tumor-Suppressive miRNA Induces Senescence via Targeting DNA Replication and Repair genes in Oral Cancer

[Speaker]Ting Yu Lai (Inst. of Bioinform. & Struct. Biol., Natl. Tsing Hua Univ., Taiwan／Natl. Inst. of Cancer Res., Natl. Health Res. Inst., Taiwan) [Co-author]Cih Yuang Fang (Natl. Inst. of Cancer Res., Natl. Health Res. Inst., Taiwan／Inst. of Biotech., Natl. Tsing Hua Univ., Taiwan)、Guan Hsun Wu (Natl. Inst. of Cancer Res., Natl. Health Res. Inst., Taiwan)、Shine Gwo Shiah (Natl. Inst. of Cancer Res., Natl. Health Res. Inst., Taiwan)

In recent years, several studies have highlighted the critical roles of microRNAs (miRNAs) in regulating cancer progression. One key downregulated miRNA was identified by analysis of 40 oral squamous cell carcinoma (OSCC) microarrays and The Cancer Genome Atlas data. This miRNA was not only low expressing in tumor but also negatively correlated with patient survival and cancer stage. Overexpress this miRNA reduced OSCC cells proliferation, migration, invasion and in vivo tumor growth. To understand its function, mRNA sequencing was performed and revealed significant alterations in cell cycle and DNA repair-related pathways in cells with high miRNA expression. Follow-up assays based on bioinformatic analyses demonstrated that this miRNA induced cellular senescence through the accumulation of DNA damage. Because of their short nucleotide sequences to identified mRNA, miRNAs typically had multiple targets. However, a set of DNA replication and repair proteins were identified as direct targets of this miRNA, confirmed at both mRNA and protein levels. Combining this miRNA, targeted genes and recent chemical drugs, we provided a novel miRNA-guided therapeutic strategy for OSCC.

[Disclosure of COI by Responsible researcher] Lead presenter is not the Responsible researcher.
[Lead presenter: Ting Yu Lai] We have no financial relationships to disclose.
[Principal Researcher: ] We have no financial relationships to disclose.

### [P-2017] I-P1-4
### Evaluation of Chemical & Radiation Carcinogenesis

2025 / 9 / 26 (Fri) 17:30-18:15 Room P/(Motenashi Dome) Room P

---

### P-2017
### WDR82-Mediated Regulation of LncRNA Expression and R-loop Formation at Bidirectional Promoters in Response to UV Damage

[Speaker]Chien-Ping Yen (Genome and Systems Biology Degree Program, National Taiwan University) [Co-author]HsuehPing C. Chu (Institute of Molecular and Cellular Biology, National Taiwan University)

DNA damage can stall RNA polymerase II (RNAPII), creating transcription-blocking lesions and inducing R-loops-structures formed by a DNA:RNA hybrid and displaced single-stranded DNA. Persistent R-loops threaten genome stability and are processed into double-strand breaks by transcription-coupled nucleotide excision repair (TC-NER) factors. After UV exposure, while most transcripts are downregulated, a subset of long non-coding RNAs (lncRNAs), particularly antisense lncRNAs, is upregulated. These lncRNAs often originate from bidirectional promoters of repressed protein-coding genes. The mechanisms behind this selective induction remain unclear. We show that UV-induced dissociation of WDR82 from RNAPII promotes lncRNA transcription and causes R-loop accumulation at their promoters. RNAPII occupancy shifts from protein-coding gene promoters to those of lncRNAs. Furthermore, depletion of the endonuclease XPF, which resolves R-loops, reduces lncRNA induction. These findings suggest that UV stress redirects RNAPII toward lncRNA loci via WDR82 and XPF, revealing a regulatory axis for non-coding RNA expression under genotoxic stress.

[Disclosure of COI by Responsible researcher] Lead presenter is not the Responsible researcher.
[Lead presenter: Chien-Ping Yen] We have no financial relationships to disclose.
[Principal Researcher: HsuehPing C. Chu] We have no financial relationships to disclose.

▲TOP

### [P-2018] I-P1-4
### Evaluation of Chemical & Radiation Carcinogenesis

2025 / 9 / 26 (Fri) 17:30-18:15 Room P/(Motenashi Dome) Room P

### P-2018
### The vulnerability of NRF2-activated cancer cells

[Speaker]Hiroshi Kitamura (Div. of Microbiol., Sch. of Med., TMPU) [Co-author]Teru Kanda (Div. of Microbiol., Sch. of Med., TMPU)

The KEAP1-NRF2 system is a crucial biological defense mechanism that governs redox balance, in which KEAP1 negatively regulates the activation of the transcription factor NRF2. This persistent activation confers resistance to radiotherapy and chemotherapy on cancer cells, and contributes to tumor malignancy. We use mouse fibroblasts and lung epithelial cells to clarify why NRF2-activated cancer cells are refractory and identify therapeutic targets for NRF2-activated cancers. While it has been reported that RAS signaling can activate NRF2 by upregulating NRF2 transcription, we found that in our NRF2-activated model cancer cells, overexpression of RAS signaling suppresses activation of NRF2 target genes. This tendency is particularly pronounced when the NRF2-activated model cancer cells are transplanted into mice. These results suggest that under specific in vivo conditions, RAS signaling could downregulate NRF2 transcriptional activation independently of KEAP1. Currently, we are investigating how RAS signaling could suppress NRF2 transcriptional activation.

[Disclosure of COI by Responsible researcher] Lead presenter is the Responsible researcher.
[Lead presenter: Hiroshi Kitamura] We have no financial relationships to disclose.

### [P-2026] I-P2-5
### Organoid/Animal model (2)

2025 / 9 / 26 (Fri) 17:30-18:15 Room P/(Motenashi Dome) Room P

---

P-2026

### A lung cancer patient-derived xenograft with the ***KRAS*** allelic imbalance showed a cancer stem cell property

[Speaker]Yasuyoshi Mizutani (Dept. Mol. Oncol., Fujita Health Univ. Sch. Med.) [Co-author]Toshiyuki Takeuchi (Dept. Mol. Oncol., Fujita Health Univ. Sch. Med.)、Atsuko Niimi (Dept. Mol. Oncol., Fujita Health Univ. Sch. Med.)、Siripan Limsirichaikul (Dept. Mol. Oncol., Fujita Health Univ. Sch. Med.)、Dat Q. Tran (Dept. Mol. Oncol., Fujita Health Univ. Sch. Med.)、Kazuya Shiogama (Dept. Morpho. Path. Diag., Fujita Health Univ. Sch. Med. Sci.)、Eiji Sugihara (Div. Gene Reg., Oncol. Innovation Ctr., Fujita Health Univ.)、Yusuke Okuno (Dept. Virol., Nagoya City Univ. Grad. Sch. Med. Sci.)、Noriko Hara (Supp. Ofc. Bioresc. Res., Fujita Health Univ.)、Yukika Hasegawa (Supp. Ofc. Bioresc. Res., Fujita Health Univ.)、Naoki Yamamoto (Ctr. Soc. Acad. Collab., Fujita Health Univ.)、Atsushi Enomoto (Dept. Path., Nagoya Univ. Grad. Sch. Med.)、Tetsunari Hase (Dept. Respir Med., Nagoya Univ. Grad. Sch. Med.)、Hisanori Isomura (Div. Mol. Diag. Aichi Cancer Ctr. Res inst.)、Motoshi Suzuki (Dept. Mol. Oncol., Fujita Health Univ. Sch. Med.)

Patient-derived tumor xenografts (PDXs) provide a major tool for evaluation of ***in vivo*** -cancer properties, although majority of human solid PDXs do not metastasize when they are grown subcutaneously in immunocompromised mice. Here we report a lung cancer LC309 PDX that was frequently associated with metastatic lesions in lymph nodes, vessels and lungs. We further established a patient-derived tumor cell (PDC) line and found that the PDC showed similar pathological-phenotypes. They were associated with KRAS p.G12C and TP53 p.P278L mutations. It also was found that the PDX and PDC, but not the clinical tissue, lost the wild type ***KRAS*** allele, while all were associated with the allelic imbalance. Interestingly, LC309 PDC formed spheroids under a three-dimensional culture condition, where ***SOX2*** expression was significantly increased. In accord with this finding, LC309 PDC formed tumors in mice by a transplantation with a limited number of cells. Additionally, RNA-seq analysis showed that in spheroids multiple genes and pathways are highly expressed and activated, respectively, suggesting the underlying mechanisms of the cancer stemness and cancer metastasis-prone properties.

[Disclosure of COI by Responsible researcher] Lead presenter is not the Responsible researcher.
[Lead presenter: Yasuyoshi Mizutani] We have no financial relationships to disclose.
[Principal Researcher: Motoshi Suzuki] We have no financial relationships to disclose.

### [P-2027] I-P2-5
### Organoid/Animal model (2)

2025 / 9 / 26 (Fri) 17:30-18:15 Room P/(Motenashi Dome) Room P

---

### P-2027
### Development and application of a 3D organotypic in vitro oral cancer model composed of patient-derived cells

[Speaker]Yuka Aizawa (Div. Oral Maxillofacial Surg., Niigata Univ.／Div. Biomimetics, Niigata Univ.) [Co-author]Kenta Haga (Div. Reconstructive Surg. Oral Maxillofacial Reg., Niigata Univ.)、Tatsuya Abe (Div. Oral Pathol., Niigata Univ.)、Satoshi Maruyama (Oral Pathol. Section, Div. Oral Surg. Pathology, Niigata Univ. Hospital)、Manabu Yamazaki (Oral Pathol. Section, Div. Oral Surg. Pathology, Niigata Univ. Hospital)、Junichi Tanuma (Oral Pathol. Section, Div. Oral Surg. Pathology, Niigata Univ. Hospital)、Putri P. Larasati (Div. Biomimetics, Niigata Univ.)、Kenji Izumi (Div. Biomimetics, Niigata Univ.)、Kei Tomihara (Div. Oral Maxillofacial Surg., Niigata Univ.)

Objective: In oral squamous cell carcinoma(OSCC), tumor-stromal interactions are pivotal in shaping cancer behavior. To investigate this interface, we developed a 3D organotypic culture model incorporating four distinct cell types, including patient-derived cancer-associated fibroblasts(PD-CAFs), to recreate the tumor microenvironment(TME) in vitro. Methods: We established a novel protocol using a stainless-steel ring to spatially compartmentalize cancerous stroma and normal oral connective tissue within the stromal layer, and OSCC cell layer and normal oral mucosa within the overlying epithelial layer. This design mimicked the histologic organization of OSCC in the oral mucosa. Results: Within this model, PD-CAFs expressing $\alpha$-smooth muscle actin localized adjacent to the OSCC cell layer, indicating active tumor-stromal crosstalk. Moreover, the loss of linear laminin-$\gamma$2 expression between the OSCC cell layer and the underlying cancer stromal layer suggested basement membrane disruption, a key feature of malignant invasion. Conclusions: This 3D model offers a platform technique to study OSCC pathophysiology and provides a useful tool to evaluate novel therapies targeting the TME.

[Disclosure of COI by Responsible researcher] Lead presenter is the Responsible researcher.
[Lead presenter: Yuka Aizawa] We have no financial relationships to disclose.

▲TOP

### [P-2028] I-P2-5
### Organoid/Animal model (2)

2025 / 9 / 26 (Fri) 17:30-18:15 Room P/(Motenashi Dome) Room P

P-2028

### Development of Predictive Biomarkers Using Multiple In Vitro Models: Pseudo-organoid and Monolayer Cultured Cells

[Speaker]Yomogi Shiota (NCCRI, Div. Rare Cancer) [Co-author]Sumio Otsuki (Kumamoto Univ., Pharmaceutical Microbiology Lab.)、Julia Osaki (NCCRI, Div. Rare Cancer)、Anna Yoshida (Knowledge Palette, Inc.)、Sakura Hayashi (Knowledge Palette, Inc.)、Keiko Kobayashi (Knowledge Palette, Inc.)、Ikumi Fujita (Knowledge Palette, Inc.)、Masakazu Fukuda (Knowledge Palette, Inc.)、Tadashi Kondo (NCCRI, Div. Rare Cancer)

Identification of genes associated with differential therapeutic responses is a crucial step in developing predictive biomarkers.This study explored gene/protein expression profiles and drug responses in different culture systems using genetically identical sarcoma cells.We established a pseudo-organoid model with specialized medium and low-attachment plates. In this 3D model, sarcoma cells formed structures resembling native tumor tissue through intercellular interactions.Despite identical genetic backgrounds, cells in the pseudo-organoid model exhibited distinct gene/protein expression and drug response profiles compared to conventional 2D monolayer cultures.By integrating gene expression and drug response data, we identified specific genes and proteins whose expression levels correlated with treatment sensitivity or resistance.These molecular signatures represent promising candidates for predictive biomarkers that may complement genome-based diagnostics and support precision oncology approaches for sarcoma therapy.

[Disclosure of COI by Responsible researcher] Lead presenter is not the Responsible researcher.
[Lead presenter: Yomogi Shiota] We have no financial relationships to disclose.
[Principal Researcher: Tadashi Kondo] ■Research expenses: 日本ゼオン株式会社、第一三共株式会社、ノバセラム株式会社

### [P-2029] I-P2-5
### Organoid/Animal model (2)

2025 / 9 / 26 (Fri) 17:30-18:15 Room P/(Motenashi Dome) Room P

---

### P-2029
### Establishment and Characterization of a Cancer Organoid from Esophageal SCC with Large Cytoplasmic Vacuoles

[Speaker]Shunsuke Fujita (Dept. Gastroenterological and Pediatric Surgery, Faculty of Med., Oita Univ.) [Co-author]Yoshiyuki Tsukamoto (Dept. Molecular Pathology, Faculty of Med., Oita Univ.)、Tomotaka Shibata (Dept. Gastroenterological and Pediatric Surgery, Faculty of Med., Oita Univ.)、Haruto Nishida (Dept. Diagnostic Pathology, Faculty of Med., Oita Univ.)、Shusaku Kurogi (Dept. Molecular Pathology, Faculty of Med., Oita Univ.)、Shigeo Ninomiya (Dept. Gastroenterological and Pediatric Surgery, Faculty of Med., Oita Univ.)、Tsuyoshi Etoh (Research Center for GLOBAL and LOCAL Infectious Diseases, Oita Univ.)、Tsutomu Daa (Dept. Diagnostic Pathology, Faculty of Med., Oita Univ.)、Naoki Hijiya (Dept. Molecular Pathology, Faculty of Med., Oita Univ.)、Masafumi Inomata (Dept. Gastroenterological and Pediatric Surgery, Faculty of Med., Oita Univ.)

Esophageal squamous cell carcinoma (ESCC) accounts for over 90% of esophageal cancers in Japan. Although vacuole formation is rarely seen in squamous cell carcinoma, rare cases with large vacuoles have been reported in various organs. These tumors, lacking mucin, have been referred to as signet ring cell squamous carcinoma, though not classified as a distinct subtype by the WHO. Due to their rarity and the absence of appropriate models, their biological features remain unclear. Organoids are 3D cultures derived from patient tumors, with ESCC organoids showing a relatively low success rate (~25%) but preserving genetic and phenotypic traits of the original tumor. We successfully established an ESCC organoid with large vacuoles resembling the primary tumor. It showed mucin-negative vacuole formation, active proliferation, and transplantability into immunodeficient mice, where vacuoles were also observed. We report its morphological features, live-cell staining, and drug sensitivity as a novel patient-derived ESCC model.

[Disclosure of COI by Responsible researcher] Lead presenter is the Responsible researcher.
[Lead presenter: Shunsuke Fujita] We have no financial relationships to disclose.

### [P-2030] I-P2-5
### Organoid/Animal model (2)

2025 / 9 / 26 (Fri) 17:30-18:15 Room P/(Motenashi Dome) Room P

---

### P-2030
### A model of peritoneal dissemination using co-culture of gastric cancer organoids and mesothelial cells

[Speaker]Hiroyuki Uematsu (Dept. Clin. Bio-Resource Res. Dev. Grad. Sch. Med. Kyoto Univ.／Dept. Organoid Research. KBBM Inc.) [Co-author]Kunishige Onuma (Dept. Clin. Bio-Resource Res. Dev. Grad. Sch. Med. Kyoto Univ.)、Shota Shimizu (Dept. Gastrointestinal and Pediatric Surg. Tottori Univ. Hosp.)、Yoshiyuki Fuziwara (Dept. Gastrointestinal and Pediatric Surg. Tottori Univ. Hosp.)、Masahiro Inoue (Dept. Clin. Bio-Resource Res. Dev. Grad. Sch. Med. Kyoto Univ.)

Prognosis of gastric cancer patients with peritoneal dissemination is poor due to lack of effective treatment against peritoneal dissemination. Until now, models of adhesion and invasion into peritoneal mesothelium using established cancer cell lines have been widely used, but it has become clear that cancer cell clusters in ascites are more important for the establishment of metastasis than single cells. In this study, we established a model of peritoneal metastasis by co-culturing organoids derived from ascites of gastric cancer patients with human mesothelial cells. The model for the stage before peritoneal dissemination is a layered culture of gastric cancer organoids on cultured mesothelial cells, and the adhesion of the organoids and mesothelial cells was evaluated. Antibodies against integrin β1 suppressed organoid adhesion. The model for the stage after peritoneal dissemination is a layered culture of mesothelial cells on collagen embedded with organoids. Organoid growth was suppressed by co-culture and culture with mesothelial cell culture supernatant. The co-culture model of gastric cancer organoids and mesothelial cells can be a useful research tool.

[Disclosure of COI by Responsible researcher] Lead presenter is not the Responsible researcher.
[Lead presenter: Hiroyuki Uematsu] ■ employment by a company related to the presentation: 株式会社KBBM
[Principal Researcher: Masahiro Inoue] We have no financial relationships to disclose.

### [P-2031] I-P2-5
### Organoid/Animal model (2)

2025 / 9 / 26 (Fri) 17:30-18:15 Room P/(Motenashi Dome) Room P

---

### P-2031
### Orthotopic Lung Cancer Model with Endoscopic Cell Delivery for Evaluating Tumor Environment and Drug Effects

[Speaker]Chiyoko Nishime (Dept. of Development Research) [Co-author]Toshio Imai (Dept. of Development Research)、Masayuki Komatsu (Dept. of Development Research)、Taichi Yamamoto (Dept. of Development Research)、Masami Suzuki (Dept. of Development Research)

This study evaluated the efficacy of anticancer agents and tumor microenvironment features using both orthotopic (ORT) and subcutaneous (SC) lung cancer models. To promote tumor engraftment, 3% Lauryl 10 solution was administered intratracheally to the left lung lobe on day -3. On day 0, $1 \times 10^5$ luciferase-expressing lung cancer cells (A549) were transplanted into the left lung intrathecally. Two weeks later, the cytotoxic agents carboplatin and paclitaxel were administered intraperitoneally, and antitumor effects were evaluated by IVIS imaging (ORT) and tumor volume measurement (SC). Tumor microenvironment features were analyzed histologically and molecularly.Carboplatin showed no efficacy in the SC or ORT models, while paclitaxel demonstrated antitumor activity in both. Tumor area measurements on digital pathology slides corroborated these results. Histological features such as alveolar growth and focal necrosis reflected a more physiological environment in the ORT model, which was further supported by molecular findings. These results highlight the ORT model as a relevant platform for evaluating cancer-chemo therapies targeting the tumor microenvironment.

[Disclosure of COI by Responsible researcher] Lead presenter is the Responsible researcher.
[Lead presenter: Chiyoko Nishime] We have no financial relationships to disclose.

▲TOP

### [P-2032] I-P2-5
### Organoid/Animal model (2)

2025 / 9 / 26 (Fri) 17:30-18:15 Room P/(Motenashi Dome) Room P

---

### P-2032
### Bladder Cancer Xenografts in Mice and Zebrafish: Comparative Human Gene Expression In Vitro and In Vivo

[Speaker]Yusuke Sugino (Dept. of Nephro-Urologic Surgery & Andrology, Mie Univ.) [Co-author]Sho Sekito (Dept. of Nephro-Urologic Surgery & Andrology, Mie Univ.／Inst. for the Advanced Study of Human Biology, Kyoto Univ.)、Xin Bao (Dept. of Nephro-Urologic Surgery & Andrology, Mie Univ.)、Raku Son (RIKEN-IFOM Joint Lab. for Cancer Genomics)、Shiori Miyachi (Dept. of Nephro-Urologic Surgery & Andrology, Mie Univ.)、Takumi Kageyama (Dept. of Nephro-Urologic Surgery & Andrology, Mie Univ.)、Takeshi Sasaki (Dept. of Nephro-Urologic Surgery & Andrology, Mie Univ.)、Toshio Tanaka (Dept. of Systems Pharmacology, Mie Univ.)、Yasuhiro Murakawa (Inst. for the Advanced Study of Human Biology, Kyoto Univ.／RIKEN-IFOM Joint Lab. for Cancer Genomics)、Takahiro Inoue (Dept. of Nephro-Urologic Surgery & Andrology, Mie Univ.)

Patient-derived xenograft (PDX) models replicate human cancer and support personalized medicine. Mice are standard PDX hosts, but their high cost and slow engraftment limit clinical use. Zebrafish enable real-time tumor observation and rapid experiments. We previously showed zebrafish PDX predicts cisplatin sensitivity in bladder cancer. This study compares differentially expressed genes (DEGs) in vitro and in vivo and evaluates zebrafish and mice as PDX hosts.
We analyzed DEGs in UMUC3 bladder cancer cells in vitro and in zebrafish and mouse xenografts. RNA was extracted from cultured cells, zebrafish xenografts, and mouse xenografts. Cap Analysis of Gene Expression (CAGE) was performed, and gene expression was analyzed with GENCODE version 43.
Human RNA was detected in both in vivo models. In total, 1,277 DEGs were identified in zebrafish and mouse models vs. in vitro, with 113 linked to tumor microenvironmental stress, including hypoxia, nutrient deprivation, oxidative/mechanical stress, immune response, and pH stress.
Zebrafish PDX shows promise as an avatar model replicating the human tumor microenvironment.

[Disclosure of COI by Responsible researcher] Lead presenter is not the Responsible researcher.
[Lead presenter: Yusuke Sugino] ■Research funds from company: 科研費 25K12543, 横山臨床薬理研究助成基金 YRY-2323, 武田科学振興財団 2024042769
[Principal Researcher: Takahiro Inoue] ■Research funds from company: 科研費 21H03066, 24K02576　■Endowed chair: 株式会社MZT：産学連携講座費

### [P-2033] I-P2-5
### Organoid/Animal model (2)

2025 / 9 / 26 (Fri) 17:30-18:15 Room P/(Motenashi Dome) Room P

---

P-2033
### Proteogenomics of patient-derived cancer cachexia models: toward organ-targeted therapeutic strategies

[Speaker]Julia Osaki (Div. Rare Cancer Res., Natl. Cancer Ctr. Res. Inst.) [Co-author]Kazuyoshi Yanagihara (Div. Rare Cancer Res., Natl. Cancer Ctr. Res. Inst.)、Yomogi Shiota (Div. Rare Cancer Res., Natl. Cancer Ctr. Res. Inst.)、Ikumi Fujita (Knowledge Palette)、Anna Yoshida (Knowledge Palette)、Yuki Suyama (Knowledge Palette)、Masakazu Fukuda (Knowledge Palette)、Hiroki Danno (Knowledge Palette)、Masaki Matsumoto (Dept. Omics & Syst. Biol., Niigata Univ.)、Tadashi Kondo (Div. Rare Cancer Res., Natl. Cancer Ctr. Res. Inst.)

Cancer cachexia is a debilitating syndrome characterized by progressive weight loss and muscle wasting, for which no fundamental treatment has been established. Recent studies have revealed that malignant tumors impair not only skeletal muscle but also various organs, such as the gastrointestinal tract, endocrine organs, and metabolic organs. Current therapies primarily address symptoms like appetite loss and weight reduction, and no strategies exist to restore the function of affected organs. To better understand the systemic effects of cachexia and to explore therapeutic directions, we established three cachexia mouse models using patient-derived cancer cell lines. Proteomic and transcriptomic analyses were conducted on four organs from each model to identify cachexia-associated molecular changes. We further performed Connectivity Map analysis based on transcriptomic profiles and identified candidate compounds that may reverse the cachexia-induced gene expression patterns. This study demonstrates the utility of multi-organ omics approaches in investigating cancer-induced systemic organ damage and provides a foundation for developing future organ-targeted therapeutic strategies.

[Disclosure of COI by Responsible researcher] Lead presenter is not the Responsible researcher.
[Lead presenter: Julia Osaki] We have no financial relationships to disclose.
[Principal Researcher: Tadashi Kondo] ■Research expenses: 日本ゼオン株式会社、ノバセラム株式会社、第一三共株式会社

### [P-2041] I-P4-4
### Oncogenes & tumor-suppressor genes (3)

2025 / 9 / 26 (Fri) 17:30-18:15 Room P/(Motenashi Dome) Room P

---

### P-2041
### Novel regulatory mechanisms for c-Myc oncoprotein degradation by the PP2A-B55α complex

[Speaker]Sana Ando (Grad. Sch., Vet. Sci., Yamaguchi Univ.) [Co-author]Shunta Ikeda (Vet. Med., Yamaguchi Univ.)、Koichi Sato (Vet. Med., Yamaguchi Univ.)、Takashi Ohama (Vet. Med., Yamaguchi Univ.)

The transcription factor c-Myc is an oncoprotein that regulates at least 15% of all transcription. Its stability and activation are enhanced by Ser62 phosphorylation, while subsequent Thr58 phosphorylation promotes ubiquitination by the E3 ligase FBXW7. In the classical model, PP2A, a key tumor suppressor, was thought to dephosphorylate Ser62, facilitating FBXW7-mediated c-Myc degradation. This model was supported by the fact that PP2A activation promotes c-Myc degradation; however, recent studies indicate that FBXW7 preferentially ubiquitinates c-Myc when both Thr58 and Ser62 are phosphorylated. Therefore, the regulatory mechanism of c-Myc degradation by PP2A remains unclear. In this study, we demonstrated that reduced PP2A-B55α induces FBXW7-mediated c-Myc degradation via Thr58 phosphorylation, whereas increased PP2A-B55α triggers UBR5-mediated degradation independent of phosphorylation. PP2A-B55α associates with UBR5 and dephosphorylates its N-terminal region, while B55δ, despite sharing core structure, neither binds UBR5 nor promotes c-Myc degradation. These findings clarify the link between PP2A activation and c-Myc degradation.

[Disclosure of COI by Responsible researcher] Lead presenter is not the Responsible researcher.
[Lead presenter: Sana Ando] We have no financial relationships to disclose.
[Principal Researcher: Takashi Ohama] We have no financial relationships to disclose.

### [P-2042] I-P4-4
### Oncogenes & tumor-suppressor genes (3)

2025 / 9 / 26 (Fri) 17:30-18:15 Room P/(Motenashi Dome) Room P

---

### P-2042
### Identification of an NCYM-targeting compound that induces mitotic and apoptotic cell death

[Speaker]Ummi M. Zulfin (Lab. of Evolutionary Oncology, Chiba Cancer Ctr. Res. Inst., Japan／Grad. Sch. of Med. and Pharm. Sci., Chiba University, Japan) [Co-author]Kazuma Nakatani (Lab. of Evolutionary Oncology, Chiba Cancer Ctr. Res. Inst., Japan／Grad. Sch. of Med. and Pharm. Sci., Chiba University, Japan)、Daisuke Muto (Lab. of Evolutionary Oncology, Chiba Cancer Ctr. Res. Inst., Japan／Grad. Sch. of Med. and Pharm. Sci., Chiba University, Japan)、Seigi Yamamoto (Lab. of Evolutionary Oncology, Chiba Cancer Ctr. Res. Inst., Japan)、Hiroyuki Kogashi (Lab. of Evolutionary Oncology, Chiba Cancer Ctr. Res. Inst., Japan)、Kazuto Kugou (Dept. of Applied Genomics, Kazusa DNA Res. Inst., Japan)、Osamu Shimozato (Lab. of Oncogenomics, Chiba Cancer Ctr. Res. Inst., Japan)、Rohmad Y. Utomo (Cancer Chemoprevention Res. Ctr., Universitas Gadjah Mada, Indonesia)、Edy Meiyanto (Cancer Chemoprevention Res. Ctr., Universitas Gadjah Mada, Indonesia)、Yoshitaka Hippo (Lab. of Evolutionary Oncology, Chiba Cancer Ctr. Res. Inst., Japan／Grad. Sch. of Med. and Pharm. Sci., Chiba University, Japan／Lab. Precision Tumor Model Systems, Chiba Cancer Ctr. Res. Inst.)、Yusuke Suenaga (Lab. of Evolutionary Oncology, Chiba Cancer Ctr. Res. Inst., Japan)

***NCYM***, a ***cis***-antisense gene of ***MYCN***, produces an oncoprotein that stabilizes MYCN by inhibiting GSK3β, promoting neuroblastoma progression. Currently, there are no available drugs that directly inhibit NCYM. In this study, we identified CCA-1.1, a curcumin analog, as a potential NCYM inhibitor and evaluate its anticancer potential in ***MYCN***-amplified neuroblastoma cells. Using a holographic microscope, we observed that CCA-1.1 caused both apoptotic and mitotic cell death. Moreover, CCA-1.1 reduced NCYM and MYCN expression and induced apoptosis via activation of GSK3β-JNK signaling cascade. This compound was also found to disrupt anaphase entry by GSK3β-dependent KIF2B induction, ultimately inducing mitotic cell death. In xenograft models, CCA-1.1 (10 mg/kg BW, twice weekly) markedly suppressed tumor growth without weight loss. These results propose CCA-1.1 as the first NCYM inhibitor with therapeutic potential in ***MYCN***-amplified neuroblastoma cells.

[Disclosure of COI by Responsible researcher] Lead presenter is not the Responsible researcher.
[Lead presenter: Ummi M. Zulfin] We have no financial relationships to disclose.
[Principal Researcher: Yusuke Suenaga] We have no financial relationships to disclose.

### [P-2043] I-P4-4
### Oncogenes & tumor-suppressor genes (3)

2025 / 9 / 26 (Fri) 17:30-18:15 Room P/(Motenashi Dome) Room P

---

### P-2043
### Photochemical method inhibiting expression of c-Myc gene by using photo-crosslinkable antisense oligonucleotides

[Speaker]Zumila Hailili (Biofunctional Medical Engineering Research Area, JAIST) [Co-author]Qingyuan Huang (Biofunctional Medical Engineering Research Area, JAIST)、Kenzo Fujimoto (Biofunctional Medical Engineering Research Area, JAIST)

Overexpression of c-Myc is observed in many human cancer cells, and its inhibition is considered one of the therapeutic strategies for treating cancer. FIR is a transcriptional repressor that suppresses c-Myc transcription. However, its splicing variant FIRΔexon2, skips exon 2 which is considered to contain critical functional domains, leading to the loss of its repression ability. Previously, we have confirmed that antisense oligonucleotides containing our original ultra-fast photo-cross-linker $^{CNV}$K can effectively suppress GFP protein expression in GFP-HeLa cells. In this study, we introduce an antisense method to repress c-Myc expression using photochemical antisense oligonucleotides (pcASOs) containing $^{CNV}$K. We designed 2 types of pcASO probes targeting FIRΔexon2 and c-Myc separately, and confirmed that both probes successfully reduced c-Myc expression, FIRΔexon2-targeting probe more effectively than c-Myc -targeting one. Additionally, while the suppression effect of c-Myc -targeting pcASO is concentration-dependent, FIRΔexon2-targeting pcASO maintained its suppressing ability even at remarkably low concentration, down to the nanomolar level.

[Disclosure of COI by Responsible researcher] Lead presenter is not the Responsible researcher.
[Lead presenter: Zumila Hailili] We have no financial relationships to disclose.
[Principal Researcher: ] We have no financial relationships to disclose.

### [P-2044] I-P4-4
### Oncogenes & tumor-suppressor genes (3)

2025 / 9 / 26 (Fri) 17:30-18:15 Room P/(Motenashi Dome) Room P

### P-2044
### Functional analysis implicates oncogenic potential of SNIP1 in 1p34 risk locus in ovarian cancer.

[Speaker]Saki Ishii (Osaka Univ. Obstetrics & Gynecology) [Co-author]Koji Nakamura (Osaka Univ. Obstetrics & Gynecology)、Aasa Shimizu (Osaka Univ. Obstetrics & Gynecology)、Mahiru Kawano (Osaka Univ. Obstetrics & Gynecology)、Yasuto Kinose (Osaka Univ. Obstetrics & Gynecology)、Kenjiro Sawada (Osaka Univ. Obstetrics & Gynecology)、Michiko Kodama (Osaka Univ. Obstetrics & Gynecology)

Previously we identified chromosome 1p34.3 as an ovarian cancer risk locus in which several putative oncogenes including SNIP1. Here we performed functional validation of SNIP1 as a novel oncogene in ovarian cancer development.
SNIP1 expression of ovarian cancer cell lines was compared with that of benign epithelial cell lines. SNIP1 was transiently silenced to assess its effects on proliferation and colony formation in ovarian cancer cell lines. Since SNIP1 had been reported as a co-activator of c-Myc, c-Myc expression after SNIP1 silencing was tested.
Kaplan-Meier plotter revealed increased SNIP1 expression was associated with shorter survival in ovarian cancer patients. SNIP1 was upregulated at both mRNA and protein levels in ovarian cancer cell lines versus benign cell lines. SNIP1 silencing impaired proliferation and colony formation in ovarian cancer cell lines. c-Myc protein expression was downregulated by SNIP1 silencing. Moreover, silencing of SNIP1 in cancer cells resulted in marked inhibition of tumor growth in a xenograft model.
Our findings suggest SNIP1 is a potential oncogene at the 1p34 locus, with future studies focusing on its link to c-Myc.

[Disclosure of COI by Responsible researcher] Lead presenter is not the Responsible researcher.
[Lead presenter: Saki Ishii] We have no financial relationships to disclose.
[Principal Researcher: Michiko Kodama] We have no financial relationships to disclose.

### [P-2045] I-P4-4
### Oncogenes & tumor-suppressor genes (3)

2025 / 9 / 26 (Fri) 17:30-18:15 Room P/(Motenashi Dome) Room P

---

### P-2045
### HSPD1 acts as an oncogenic driver in lung adenocarcinoma: Insights from proteomic analysis and functional validation

[Speaker]Siripat Aluksanasuwan (Sch. of Med., Mae Fah Luang Univ., Thailand／Cancer & Immunol. Res. Unit, Mae Fah Luang Univ., Thailand)
[Co-author]Keerakarn Somsuan (Sch. of Med., Mae Fah Luang Univ., Thailand／Cancer & Immunol. Res. Unit, Mae Fah Luang Univ., Thailand)、Artitaya Rongjumnong (Cancer & Immunol. Res. Unit, Mae Fah Luang Univ., Thailand)、Wararat Chiangjong (Faculty of Med., Mahidol Univ., Thailand)、Jatuporn Ngoenkam (Faculty of Med. Sci., Naresuan Univ., Thailand)、Atthapan Morchang (Sch. of Med., Mae Fah Luang Univ., Thailand／Cancer & Immunol. Res. Unit, Mae Fah Luang Univ., Thailand)、Papawarin Karamart (Cancer & Immunol. Res. Unit, Mae Fah Luang Univ., Thailand)、Somchai Chutipongtanate (Univ. of Cincinnati College of Med., USA)、Sutatip Pongcharoen (Faculty of Med., Naresuan Univ., Thailand)

Lung adenocarcinoma (LUAD) is the most common histological subtype of lung cancer. Previous studies have demonstrated that the expression of heat shock protein family D member 1 (HSPD1 or HSP60) is associated with poor survival outcomes and immune cell infiltration in LUAD. Knockdown of HSPD1 in A549 cells using a shRNA approach altered their secretome and reduced their ability to promote cancer-associated fibroblast activation and angiogenesis. In this study, we conducted proteomic analysis and functional assays on HSPD1-knockdown and control A549 cells. The results showed that HSPD1 knockdown suppressed cell proliferation, colony formation, migration, and invasion in A549 cells. Proteomic analysis identified 32 altered proteins in HSPD1-knockdown cells, primarily involved in translation and the ribosome pathway. Reduced levels of ribosomal proteins and diminished protein synthesis were confirmed by Western blot and protein synthesis assays. Moreover, HSPD1 knockdown sensitized the cells to the ribosome inhibitor homoharringtonine. These findings suggest that HSPD1 functions as an oncogenic driver in LUAD by modulating the ribosome pathway and protein synthesis.

[Disclosure of COI by Responsible researcher] Lead presenter is the Responsible researcher.
[Lead presenter: Siripat Aluksanasuwan] ■Research funds from company: Thailand Science Research and Innovation (TSRI) (grant no. 682A07015)

▲TOP

### [P-2046] I-P4-4
### Oncogenes & tumor-suppressor genes (3)

2025 / 9 / 26 (Fri) 17:30-18:15 Room P/(Motenashi Dome) Room P

### P-2046
### SPZ1 as an Oncogene: Its Role in Tumorigenesis and Stemness Regulation in NSCLC

[Speaker]Wei T. Hsu (Dept. of Life Sci., NTNU) [Co-author]Li T. Wang (Dept. of Life Sci., NTNU)、Shih H. Hsu (Grad. Inst. of Med., College of Med., KMU)

Lung cancer has been the leading cause of mortality worldwide until 2023. Of all cases of lung cancer, 80% are diagnosed as non–small cell lung cancer (NSCLC). The mutation of oncogenes has been identified as the primary cause of NSCLC. Consequently, there is a necessity for research to reveal the mechanism of oncogenes. In this study, a novel oncogene, Spermatogenic Leucine Zipper 1 (SPZ1), was found to be highly expressed in NSCLC. In previous studies, SPZ1 has been indicated to play an important role in tumourigenesis. Furthermore, our NGS data demonstrated that SPZ1 can bind to the promoter of NANOG, a stem factor that regulates stem cell phenotype. Cancer stem cells (CSCs) are a group of cancer cells which exhibit higher levels of pluripotency and self–renewal ability, and are associated with a longer duration of disease progression and a worse prognosis. The present study further indicates that SPZ1 increases stem-like property through promoting the expression of NANOG. This study not only underscores the role of SPZ1 in tumorigenesis, but also highlights its significance in stem–like property.

[Disclosure of COI by Responsible researcher] Lead presenter is the Responsible researcher.
[Lead presenter: Wei T. Hsu] We have no financial relationships to disclose.

▲TOP

### [P-2047] I-P4-4
### Oncogenes & tumor-suppressor genes (3)

2025 / 9 / 26 (Fri) 17:30-18:15 Room P/(Motenashi Dome) Room P

---

### P-2047
### Downregulation of KRTAP2-3 suppresses proliferation and tumor formation in oral cancer cells

[Speaker]Qianqian Miao (Science Tokyo, Grad. Sch. Med. Dent. Sci., Biochemistry) [Co-author]Zhijun Huang (Science Tokyo, Grad. Sch. Med. Dent. Sci., Biochemistry)、Katarzyna A. Inoue (Science Tokyo, Grad. Sch. Med. Dent. Sci., Biochemistry)、Kazuki Takahashi (Science Tokyo, Grad. Sch. Med. Dent. Sci., Biochemistry)、Kohei Miyazono (The Univ. of Tokyo, Grad. Sch. Med., Dept Applied Pathol.)、Tetsuro Watabe (Science Tokyo, Grad. Sch. Med. Dent. Sci., Biochemistry)

The progression of oral squamous cell carcinoma (OSCC), the most common type of oral cancer, is driven by complex molecular mechanisms, including epithelial-mesenchymal transition (EMT) and various proliferative signaling pathways. We recently reported that keratin-associated protein 2-3 (KRTAP2-3) is involved in regulation of OSCC progression. However, its molecular mechanisms have not yet been fully elucidated. In the present study, we aimed to determine molecular mechanisms by which KRTAP2-3 regulates OSCC progression, focusing on its effect on cell proliferation, EMT and tumor formation. Using gene knockout and knockdown approaches in multiple OSCC cell lines, we showed that downregulation of KRTAP2-3 induces mesenchymal-epithelial transition and reduces cell proliferation. Using RNA sequencing analysis, we identified a factor playing an important role in KRTAP2-3-mediated tumor progression. Furthermore, we found that decreased expression of KRTAP2-3 significantly suppressed tumor formation of oral cancer cells. Our results suggested that the inhibition of KRTAP2-3 expression is a promising therapeutic strategy for OSCC to suppress both cancer cell proliferation and metastasis.

[Disclosure of COI by Responsible researcher] Lead presenter is not the Responsible researcher.
[Lead presenter: Qianqian Miao] We have no financial relationships to disclose.
[Principal Researcher: Tetsuro Watabe] We have no financial relationships to disclose.

[P-2048] I-P4-4
Oncogenes & tumor-suppressor genes (3)

2025 / 9 / 26 (Fri) 17:30-18:15 Room P/(Motenashi Dome) Room P

P-2048

### Regulatory mechanism of BRCA1 ubiquitination and centrosomal localization by E3 activity of Aurora A

[Speaker]Yuuki Shibutani (Dept. Cancer Biol., IDAC, Tohoku Univ.) [Co-author]Zhenzhou Fang (Dept. Cancer Biol., IDAC, Tohoku Univ.)、Xingming Li (Dept. Cancer Biol., IDAC, Tohoku Univ.)、Shun Iwasaki (Dept. Cancer Biol., IDAC, Tohoku Univ.)、Yuki Yoshino (Dept. Cancer Biol., IDAC, Tohoku Univ.)、Natsuko Chiba (Dept. Cancer Biol., IDAC, Tohoku Univ.)

***Breast cancer-associated gene 1*** (***BRCA1***) is a gene responsible for hereditary breast and ovarian cancer syndrome, and its gene product functions in centrosome regulation. BRCA1 localizes to the centrosome throughout the cell cycle, whereas its localization decreases in G2 phase. Previously, we reported that the E3 activity of Aurora A promotes centrosome maturation by ubiquitinating BRCA1-interacting protein, Obg-like ATPase 1 (OLA1), in G2 phase. Adaptor protein X (APX) is involved in signaling pathways such as cell survival and proliferation and has been shown to be localized to the centrosome. However, its function at the centrosome remains unclear. In this study, we found that the E3 activity of Aurora A decreases centrosomal BRCA1 through Aurora A-mediated polyubiquitination during S and G2 phases. APX interacts with Aurora A, and these overexpressions cause a cumulative effect, further reducing the expression and centrosomal localization of BRCA1 compared to Aurora A and APX individually. Furthermore, centrosomal localization of APX peaks in G2 phase. These results suggest that Aurora A polyubiquitinates BRCA1 and reduces centrosomal BRCA1 together with APX.

[Disclosure of COI by Responsible researcher] Lead presenter is not the Responsible researcher.
[Lead presenter: Yuuki Shibutani] We have no financial relationships to disclose.
[Principal Researcher: Natsuko Chiba] We have no financial relationships to disclose.

▲TOP

### [P-2049] I-P4-4
### Oncogenes & tumor-suppressor genes (3)

2025 / 9 / 26 (Fri) 17:30-18:15 Room P/(Motenashi Dome) Room P

---

### P-2049
### BARD1 $\beta$ increases the OLA1 polyubiquitination by Aurora A to promote centrosome maturation

[Speaker]Xingming Li (Dept. Cancer Biol., IDAC, Tohoku Univ.) [Co-author]Zhenzhou Fang (Dept. Cancer Biol., IDAC, Tohoku Univ.)、Rin Oya (Dept. Cancer Biol., IDAC, Tohoku Univ.)、Minori Watanabe (Dept. Cancer Biol., IDAC, Tohoku Univ.)、Mai Morooka (Dept. Cancer Biol., IDAC, Tohoku Univ.)、Mahiru Kusaka (Dept. Cancer Biol., IDAC, Tohoku Univ.)、Yuki Yoshino (Dept. Cancer Biol., IDAC, Tohoku Univ.)、Natsuko Chiba (Dept. Cancer Biol., IDAC, Tohoku Univ.)

BRCA1-interacting protein BARD1 functions in centrosome regulation. We identified Obg-like ATPase 1 (OLA1) as a BARD1-interacting protein. OLA1 localizes to the centrosome throughout the cell cycle, whereas its localization decreases in G2 phase. We revealed that OLA1 is polyubiquitinated by mitotic kinase Aurora A, followed by proteasomal degradation in G2 phase. The reduction of centrosomal OLA1 in G2 phase promotes the recruitment of pericentriolar material (PCM) proteins. In addition, we revealed that BARD1 suppresses OLA1 polyubiquitination by Aurora A. In this study, we found that a BARD1 isoform, BARD1 $\beta$ promoted OLA1 polyubiquitination by Aurora A. Co-expression of BARD1 $\beta$ decreased OLA1, which was abolished by treatment with the proteasome inhibitor MG132. BARD1 $\beta$ localizes to centrosome throughout the cell cycle, whereas its localization increased during G2 and M phases. Overexpression of BARD1 $\beta$ suppresses the interaction between BARD1 and OLA1, reduced the centrosomal OLA1, and increased the recruitment of PCM proteins in G2 phase. These suggest that BARD1 $\beta$ enhances the OLA1 polyubiquitination by Aurora A and promotes centrosome maturation.

[Disclosure of COI by Responsible researcher] Lead presenter is not the Responsible researcher.
[Lead presenter: Xingming Li] We have no financial relationships to disclose.
[Principal Researcher: Natsuko Chiba] We have no financial relationships to disclose.

### [P-2059] I-P4-6
### Oncogenes & tumor-suppressor genes (5)

2025 / 9 / 26 (Fri) 17:30-18:15 Room P/(Motenashi Dome) Room P

---

### P-2059
### Functional Characterization and Clinical Relevance of KCNB1 in Esophageal Cancer

[Speaker]Naofumi Takami (Division of Digestive Surgery, Kyoto Prefectural University of Medicine) [Co-author]Hiroki Shimizu (Division of Digestive Surgery, Kyoto Prefectural University of Medicine)、Atsuki Ota (Division of Digestive Surgery, Kyoto Prefectural University of Medicine)、Hiroyuki Inoue (Division of Digestive Surgery, Kyoto Prefectural University of Medicine)、Kento Kurashima (Division of Digestive Surgery, Kyoto Prefectural University of Medicine)、Hidemasa Kubo (Division of Digestive Surgery, Kyoto Prefectural University of Medicine)、Jun Kiuchi (Division of Digestive Surgery, Kyoto Prefectural University of Medicine)、Taisuke Imamura (Division of Digestive Surgery, Kyoto Prefectural University of Medicine)、Kenji Nanishi (Division of Digestive Surgery, Kyoto Prefectural University of Medicine)、Tomohiro Arita (Division of Digestive Surgery, Kyoto Prefectural University of Medicine)、Toshiyuki Kosuga (Division of Digestive Surgery, Kyoto Prefectural University of Medicine)、Yusuke Yamamoto (Division of Digestive Surgery, Kyoto Prefectural University of Medicine)、Hirotaka Konishi (Division of Digestive Surgery, Kyoto Prefectural University of Medicine)、Ryo Morimura (Division of Digestive Surgery, Kyoto Prefectural University of Medicine)、Hitoshi Fujiwara (Division of Digestive Surgery, Kyoto Prefectural University of Medicine)、Atsushi Shiozaki (Division of Digestive Surgery, Kyoto Prefectural University of Medicine)

Background:Potassium Voltage-Gated Channel Subfamily B Member 1 (KCNB1) is a voltage-gated potassium channel that regulates ion transport. Its role in esophageal cancer is unclear. This study investigated the link between KCNB1 expression and tumor progression in esophageal cancer.Methods:KCNB1 expression was knocked down using siRNA in esophageal cancer cell lines. Cell proliferation, cell cycle, apoptosis, migration, and invasion were analyzed. Immunohistochemistry (IHC) was performed on 129 resected cases to evaluate associations between KCNB1 expression, clinicopathological features, and prognosis.Results:Among eight cell lines, TE5 and KYSE70 showed high KCNB1 expression. Knockdown led to reduced proliferation, increased apoptosis, and suppressed migration and invasion. Cell cycle analysis revealed G2-M phase arrest. High KCNB1 expression was associated with worse 5-year recurrence-free survival (28.5% vs. 58.1%, $p = 0.0016$). Multivariate analysis of IHC results identified KCNB1 as an independent poor prognostic factor ($p = 0.0197$).Conclusion:KCNB1 promotes malignancy in esophageal cancer and may serve as a prognostic biomarker and therapeutic target.

[Disclosure of COI by Responsible researcher] Lead presenter is not the Responsible researcher.
[Lead presenter: Naofumi Takami] We have no financial relationships to disclose.
[Principal Researcher: Hiroki Shimizu, Atsushi Shiozaki] We have no financial relationships to disclose.

### [P-2060] I-P4-6
### Oncogenes & tumor-suppressor genes (5)

2025 / 9 / 26 (Fri) 17:30-18:15 Room P/(Motenashi Dome) Room P

---

### P-2060
### Characteristics of Gastric Cancer Revealed by Single-Cell RNA Sequencing

[Speaker]Hiroyuki Minoura (Division of Advanced Surgical Oncology, Kitasato University School of Medicine) [Co-author]Kota Okuno (Division of Advanced Surgical Oncology, Kitasato University School of Medicine)、Shunpei Shibaki (Division of Advanced Surgical Oncology, Kitasato University School of Medicine)、Kanako Naito (Division of Advanced Surgical Oncology, Kitasato University School of Medicine)、Kyosuke Ikemura (Division of Advanced Surgical Oncology, Kitasato University School of Medicine)、Riku Okamoto (Division of Advanced Surgical Oncology, Kitasato University School of Medicine)、Akiko Watanabe (Division of Advanced Surgical Oncology, Kitasato University School of Medicine)、Keiko Oki (Division of Advanced Surgical Oncology, Kitasato University School of Medicine)、Yusuke Nie (Division of Advanced Surgical Oncology, Kitasato University School of Medicine)、Shiori Fujino (Division of Advanced Surgical Oncology, Kitasato University School of Medicine)、Keishi Yamashita (Division of Advanced Surgical Oncology, Kitasato University School of Medicine)

Background:We performed single-cell RNA sequencing to explore molecular features of gastric cancer (GC).Methods:Single-cell data from 26 primary tumors (Stage I-IV: 3/6/14/3 cases, GSE183904) were analyzed. Cells with 500-2000 genes and <10% mitochondrial genes were included (Seurat of R language).Results:UMAP and marker-based annotation (2664 cells/case) identified canonical tumor components (7.7% cancer cells). Plasma cells (17%) and mast cells (2.9%) were abundant, whereas Ki67-positive cells were few. CAFs and pericytes were clearly separate in GC, differently from colorectal cancer (CRC), although expression of 115 CAF-associated genes was similar. CellChat analysis showed strongest interactions between CAFs and FCN1 TAMs, and TREM2 TAMs and FCN1 TAMs. FCN1 TAMs received ANXA1, C3, MDK, and PTN from CAFs, and SPP1, LGALS9, and ANXA1 from TREM2 TAMs, and interacted with endothelia via CXCL genes, NAMPT, SPP1, and ADM. Plasma cells uniquely interacted with endothelia via IGF1. Cancer cells were activated by PTN, MDK, and ANGPTL family genes.Conclusion:Single-cell analysis revealed molecular characteristics of GC, warranting further validation.

[Disclosure of COI by Responsible researcher] Lead presenter is not the Responsible researcher.
[Lead presenter: Hiroyuki Minoura] We have no financial relationships to disclose.
[Principal Researcher: Keishi Yamashita] We have no financial relationships to disclose.

▲TOP

### [P-2061] I-P4-6
### Oncogenes & tumor-suppressor genes (5)

2025 / 9 / 26 (Fri) 17:30-18:15 Room P/(Motenashi Dome) Room P

---

### P-2061
### Collaboration between Wnt Signaling and E-Cadherin Loss in the Development of Diffuse-Type Gastric Cancer

[Speaker]Ardyan S. Putra (Kanazawa Univ. CRI) [Co-author]Dong(Kanazawa Univ.) Wang (Kanazawa Univ. CRI／Kanazawa Univ. NanoLSI)、Hiroko Oshima (Kanazawa Univ. CRI／Kanazawa Univ. NanoLSI)、Masanobu Oshima (Kanazawa Univ. CRI／Kanazawa Univ. NanoLSI)

Diffuse-type gastric cancer (DGC) is a highly aggressive subtype of gastric cancer (GC). While Wnt signaling drives gastric tumorigenesis, the mechanistic interaction between Wnt pathway activation and mutations in ***CDH1*** and ***PIGR***-both frequently altered in DGC-remains unclear. To investigate this, we established mouse GC-derived organoids carrying Kras, Tgfbr2, and Trp53 (KTP) mutations and subsequently disrupted ***Cdh1*** or both ***Cdh1*** and ***Pigr*** to model DGC. Loss of ***Cdh1*** alone impaired glandular morphology, promoted invasiveness and proliferation compared to parental KTP organoids. In contrast, ***Pigr*** loss had minimal effect on these ***Cdh1***-loss-induced tumorigenic phenotypes. As expected, ***Cdh1***-deficient organoids exhibited marked activation of Wnt signaling, characterized by increased nuclear β-catenin accumulation. However, they remained dependent on GSK3β inhibition or exogenous Wnt ligand stimulation for survival and growth, indicating that ligand-dependent Wnt activation is essential for DGC progression with ***CDH1*** mutations. Notably, we observed no distinct tumorigenic role of ***Pigr*** disruption, suggesting its mutation may be relevant to early, preneoplastic phase of DGC development.

[Disclosure of COI by Responsible researcher] Lead presenter is not the Responsible researcher.
[Lead presenter: Ardyan S. Putra] We have no financial relationships to disclose.
[Principal Researcher: Masanobu Oshima] We have no financial relationships to disclose.

### [P-2062] I-P4-6
### Oncogenes & tumor-suppressor genes (5)

2025 / 9 / 26 (Fri) 17:30-18:15 Room P/(Motenashi Dome) Room P

---

### P-2062
### Significance of ***CCNE1*** Amplification in Lymph Node Metastasis of Gastric Cancer

[Speaker]Hinano Nishikubo (Molecular Oncology and Therapeutics, Osaka Metropolitan University Graduate School) [Co-author]Kyoka Kawabata (Molecular Oncology and Therapeutics, Osaka Metropolitan University Graduate School)、Saki Kanei (Molecular Oncology and Therapeutics, Osaka Metropolitan University Graduate School)、Rika Aoyama (Molecular Oncology and Therapeutics, Osaka Metropolitan University Graduate School)、Tomoya Sano (Molecular Oncology and Therapeutics, Osaka Metropolitan University Graduate School)、Daiki Imanishi (Molecular Oncology and Therapeutics, Osaka Metropolitan University Graduate School)、Takashi Sakuma (Molecular Oncology and Therapeutics, Osaka Metropolitan University Graduate School)、Koji Maruo (Molecular Oncology and Therapeutics, Osaka Metropolitan University Graduate School)、Yurie Yamamoto (Molecular Oncology and Therapeutics, Osaka Metropolitan University Graduate School)、Dongheng Ma (Molecular Oncology and Therapeutics, Osaka Metropolitan University Graduate School)、Canfeng Fan (Molecular Oncology and Therapeutics, Osaka Metropolitan University Graduate School)、Masakazu Yashiro (Molecular Oncology and Therapeutics, Osaka Metropolitan University Graduate School)

[Background] Lymph node (LN) metastasis is one of most frequent metastatic patterns in patients with gastric cancer (GC), but few genes predictive of the LN status in GC have been identified. We aimed to identify candidate genes for association with LN metastasis by analyzing data from a multi-cancer genome profiling database. [Methods] Genetic abnormalities were identified in GC patients with LN metastasis. And immunohistochemical staining was performed on surgical specimens from 482 GC patients who underwent gastrectomy at our hospital.[Results] Gene amplification in ***ERBB2***, ***CCNE1*** and ***MYC*** were frequent in GC with LN metastasis. CCNE1 expression was found in 43.1% of the 482 GC samples. LN metastasis and lymphatic invasion were significantly more frequent in CCNE1-positive compared to CCNE1-negative patients. In addition, the CCNE1-positive group had a poorer prognosis than the CCNE1-negative group. This was especially true for patients with clinical stage I GC, in whom CCNE1-positivity was significantly correlated with postoperative recurrence. [Conclusion] These findings suggest that CCNE1 expression might be a promising therapeutic target for GC patients with LN metastasis.

[Disclosure of COI by Responsible researcher] Lead presenter is not the Responsible researcher.
[Lead presenter: Hinano Nishikubo] We have no financial relationships to disclose.
[Principal Researcher: Masakazu Yashiro] We have no financial relationships to disclose.

▲TOP

### [P-2063] I-P4-6
### Oncogenes & tumor-suppressor genes (5)

2025 / 9 / 26 (Fri) 17:30-18:15 Room P/(Motenashi Dome) Room P

---

### P-2063
### Investigating the role of ADAMTS6 in gastric cancer developing

[Speaker]Chunxiao Liu (Hengqin Hosp., Gastrointestinal Surg.)

Gastric carcinoma is a common malignancy with a high incidence, particularly in the Chinese population, where its insidious onset often leads to diagnosis at intermediate to advanced stages and consequently a high mortality rate, with surgical resection remaining the primary treatment supplemented by adjuvant chemotherapy and radiotherapy. Previous studies have identified integrin and metalloproteinase (ADAMTS) family thrombospondin motifs in gastric carcinoma, revealing that ADAMTS6 expression is significantly correlated with patient prognosis such that elevated levels are associated with the aggressive and malignant characteristics of the disease. The tumor microenvironment (TME), a critical factor in gastric cancer metastasis involving cancer cell adaptation, proliferation, and new nodule formation in distant sites with fibroblasts as a major component, is influenced by ADAMTS6

[Disclosure of COI by Responsible researcher] Lead presenter is the Responsible researcher.
[Lead presenter: Chunxiao Liu] We have no financial relationships to disclose.

### [P-2064] I-P4-6
### Oncogenes & tumor-suppressor genes (5)

2025 / 9 / 26 (Fri) 17:30-18:15 Room P/(Motenashi Dome) Room P

---

### P-2064
### CKAP4 is highly expressed in malignant pulmonary mesothelioma and regulates AKT and tumor growth

[Speaker]Mariko Kawachi (Lab. Mol. Can. Med., Meijo Univ. Fac. Pharm.) [Co-author]Kiyoshi Yanagisawa (Lab. Mol. Can. Med., Meijo Univ. Fac. Pharm.)

Malignant pulmonary mesothelioma (MPM) is a form of tumor that develops from the protective lining that covers the lung and usually caused by exposure to asbestos. Asbestos has been used in industry for many years in Europe and Asia, and so persistent increase in deaths from MPM has been observed even today. However, no effective standard treatment has been established for MPM. Previously, we employed shotgun proteomics together with peptide labeling technology to analyze comprehensive protein expression profiles of MPM and identified an MPM related molecule, CKAP4 (cytoskeleton associated preotein 4). We found that CKAP4 is aberrantly up-regulated in MPM and is useful for discrimination of MPM from lung adenocarcinoma.In this study, we conducted molecular functional analyses of CKAP4 and revealed that it regulated activities of AKT and related molecules, which plays a central role in tumor proliferative and anti-apoptotic response. Thus our findings shed light on the underlying mechanisms how the MPM regulates tumor growth. Further research holds promise for the development of novel therapeutic agents for MPM.

[Disclosure of COI by Responsible researcher] Lead presenter is not the Responsible researcher.
[Lead presenter: Mariko Kawachi] We have no financial relationships to disclose.
[Principal Researcher: Yanagisawa Kiyoshi] We have no financial relationships to disclose.

### [P-2065] I-P4-6
### Oncogenes & tumor-suppressor genes (5)

2025 / 9 / 26 (Fri) 17:30-18:15 Room P/(Motenashi Dome) Room P

---

### P-2065
### AHNAK2 promotes proliferation and survival of pancreatic cancer cells through enhanced p21 expression

[Speaker]Daisuke Katsuchi (St. Mary's Res. Ctr.) [Co-author]Taichi Matsumoto (St. Mary's Res. Ctr.)、Yuichi Murakami (St. Mary's Res. Ctr.)、Kuon Kanazawa (St. Mary's Res. Ctr.)、Michihiko Kuwano (St. Mary's Res. Ctr.)

Pancreatic ductal adenocarcinoma (PDAC) remains one of the most lethal cancers, with limited effective treatment options. Transcriptome analysis of patient-derived PDAC samples identified AHNAK2 as consistently upregulated genes in tumor tissues relative to adjacent non-tumor tissues, with higher expression correlating with poor survival. Functional studies using eight PDAC cell lines demonstrated that siRNA-mediated knockdown of AHNAK2 significantly suppressed cell proliferation, altered cell cycle progression, and induced apoptosis. Mechanistically, AHNAK2 silencing elevated p21 protein levels without a corresponding increase in mRNA expression, suggesting post-transcriptional regulation. Cycloheximide chase assays further confirmed that AHNAK2 promotes p21 protein degradation, suggesting a role of AHNAK2 in p21 protein stability. In addition, AHNAK2 knockdown induced apoptotic cell death in a subset of PDAC cell lines. Collectively, these findings may indicate AHNAK2 as a key regulator of PDAC cell growth and survival, and highlight its potential as a novel therapeutic target in pancreatic cancer.
Collaborators: Kazuhiro Koikawa (Kyushu Univ.), Mayumi Ono (St. Mary's Res. Ctr.)

[Disclosure of COI by Responsible researcher] Lead presenter is not the Responsible researcher.
[Lead presenter: Daisuke Katsuchi] We have no financial relationships to disclose.
[Principal Researcher: Michihiko Kuwano] We have no financial relationships to disclose.

### [P-2066] I-P4-6
### Oncogenes & tumor-suppressor genes (5)

2025 / 9 / 26 (Fri) 17:30-18:15 Room P/(Motenashi Dome) Room P

---

### P-2066
### Elucidation of the function of mRNA nuclease Regnase-1 in renal cell carcinoma

[Speaker]Akihiro Yoshimura (The Univ. Osaka Grad. Sch. of Med. Dept. of Urol.) [Co-author]Taigo Kato (The Univ. Osaka Grad. Sch. of Med. Dept. of Urol.)、Takuya Uehata (Univ. Kyoto Grad. Sch. of Med. Lab. of Chem.)、Atsuki Matsukawa (The Univ. Osaka Grad. Sch. of Med. Dept. of Urol.)、Masaru Tani (The Univ. Osaka Grad. Sch. of Med. Dept. of Urol.)、Yohei Okuda (The Univ. Osaka Grad. Sch. of Med. Dept. of Urol.)、Toshiki Oka (The Univ. Osaka Grad. Sch. of Med. Dept. of Urol.)、Gaku Yamamichi (The Univ. Osaka Grad. Sch. of Med. Dept. of Urol.)、Yu Ishizuya (The Univ. Osaka Grad. Sch. of Med. Dept. of Urol.)、Takuji Hayashi (The Univ. Osaka Grad. Sch. of Med. Dept. of Urol.)、Yoshiyuki Yamamoto (The Univ. Osaka Grad. Sch. of Med. Dept. of Urol.)、Koji Hatano (The Univ. Osaka Grad. Sch. of Med. Dept. of Urol.)、Atsunari Kawashima (The Univ. Osaka Grad. Sch. of Med. Dept. of Urol.)、Osamu Takeuchi (Univ. Kyoto Grad. Sch. of Med. Lab. of Chem.)、Norio Nonomura (The Univ. Osaka Grad. Sch. of Med. Dept. of Urol.)

**Background** Regnase-1, encoded by ***ZC3H12A***, is an endonuclease that regulates gene expression by degrading mRNAs in various cell types. In the immune system, it plays a critical role in controlling inflammatory responses by degrading target genes such as ***IL6***. However, the role of Regnase-1 in cancer cells is unknown. **Material and method** We analyzed public and in house data of patients with renal cell carcinoma (RCC) to determine whether Regnase-1 expression is associated with clinical prognosis. By generating ***Regnase-1*** knockout cells from two RCC cell lines (Caki-1, 769-P), we investigated its function by evaluating cell proliferation, wound healing, and invasion. **Results** In both public and in house data, the high Regnase-1 expression was associated with worse prognosis ($p$ = 0.004 and $p$ = 0.009). ***Regnase-1*** deficiency exhibited a significant reduction in proliferative capacity (Caki-1; $p$ = 0.020, 769-P; $p$ = 0.020). In ***Regnase-1***-deficient cells, migratory capacity and invasive capacity were impaired compared to control cells (Caki-1; $p < 0.001$, 769-P; $p$ = 0.002 for migration and 769-P; $p$ = 0.004 for invasion). **Conclusion** Deletion of Regnase-1 suppresses the function of RCC cells.

[Disclosure of COI by Responsible researcher] Lead presenter is not the Responsible researcher.
[Lead presenter: Akihiro Yoshimura] We have no financial relationships to disclose.
[Principal Researcher: Taigo Kato] We have no financial relationships to disclose.

▲TOP

### [P-2067] I-P4-6
### Oncogenes & tumor-suppressor genes (5)

2025 / 9 / 26 (Fri) 17:30-18:15 Room P/(Motenashi Dome) Room P

---

### P-2067
### Development of Rapid Variant Assessment Method Using Zebrafish

[Speaker]Masashi Miyamoto (Okayama Univ Grad Sch of Med, Dept of Clin Pharm.) [Co-author]Yoshito Zamami (Okayama Univ Hosp, Dept of Clin Pharm.)、Akira Hirasawa (Okayama Univ Grad Sch of Med, Clin Genomic Med.)、Yasuyuki Hosono (Okayama Univ Grad Sch of Med, Dept of Pharmacology.)

Recent advances in next-generation sequencing (NGS) have enabled practical use of multigene panel testing (MGPT), which allows simultaneous analysis of many genes. However, frequent detection of variants of uncertain significance (VUS) remains a challenge. While various functional assays exist, most rely on specific outputs from cell lines. For multifunctional genes like ***BRCA2*** , determining true pathogenicity is difficult. Reliable and rapid functional evaluation systems are needed, but conventional animal models are not suited for short-term assessment.Zebrafish have long been used as a model organism in various fields of research due to the advantage that, being externally fertilized, their organ development and formation processes, which occur at a remarkably rapid pace, can be visually observed from the single-cell stage.In this study, we established a zebrafish model in which hyperpigmentation/melanoma is observed within an exceptionally short time frame. We then applied it to newly evaluate ***BRCA2*** variants previously classified as variants of uncertain significance (VUS).

[Disclosure of COI by Responsible researcher] Lead presenter is not the Responsible researcher.
[Lead presenter: Masashi Miyamoto] ■Research funds from company: 次世代研究者挑戦的研究プログラム（SPRING）
[Principal Researcher: Yasuyuki Hosono] ■Research funds from company: AMED　令和4年度ゲノム創薬基盤推進研究事業

▲TOP

[P-2078] I-P5-4
Non-coding RNAs (2)

2025 / 9 / 26 (Fri) 17:30-18:15 Room P/(Motenashi Dome) Room P

P-2078

### Induction of APOBEC3B expression via the TGFβ-Smad pathway is regulated by ***lincNMR***

[Speaker]Satoshi Sakai (Department of Molecular Biology, Hamamatsu University School of Medicine) [Co-author]Masatoshi Kitagawa (Department of Molecular Biology, Hamamatsu University School of Medicine)

Long non-coding RNAs (lncRNAs) are involved in various cellular processes in human cells, whereas their functions remain to be elucidated. We are focusing on lncRNAs regulated by TGFβ and analyzing their functions.We performed RNA-sequence analysis of Huh7 cells treated with TGFβ, and picked up 19 lncRNAs induced by TGFβ. Among them, we found that ***lincNMR***, which contributes to nucleotide metabolism and is highly expressed in various cancer cell lines, was induced by TGFβ. The TGFβ-mediated induction of ***lincNMR*** was decreased by depletion of Smad2/3, suggesting that the TGFβ-Smad pathway is involved in ***lincNMR*** expression. Moreover, we found that APOBEC3B (A3B), which promotes C to U mutation and highly expressed in various human cancers, was one of target genes of ***lincNMR***. ***LincNMR*** bound to endogenous Smad2. The increase in A3B promoter activity induced by Smad2/3 overexpression was suppressed by ***lincNMR*** depletion. These data suggest that ***lincNMR*** is involved in A3B expression by collaborating with TGFβ-Smad pathway and may contribute to tumor malignancy via enhanced gene mutation through A3B expression.

[Disclosure of COI by Responsible researcher] Lead presenter is not the Responsible researcher.
[Lead presenter: Satoshi Sakai] ■Research funds from company: 科研費
[Principal Researcher: Masatoshi Kitagawa] ■Research funds from company: 科研費

▲TOP

### [P-2079] I-P5-4
### Non-coding RNAs (2)

2025 / 9 / 26 (Fri) 17:30-18:15 Room P/(Motenashi Dome) Room P

---

### P-2079
### A high-throughput 3'UTR reporter screening identifies microRNAs targeting ASF1B in pancreatic adenocarcinoma

[Speaker]Lisa Akiyama (Cell. & Mol. Biol., Grad. Sch. Biomed. & Health Sci., Hiroshima Univ.) [Co-author]Ryouu Takahashi (Cell. & Mol. Biol., Grad. Sch. Biomed. & Health Sci., Hiroshima Univ.)、Kimiyoshi Yano (Cell. & Mol. Biol., Grad. Sch. Biomed. & Health Sci., Hiroshima Univ.)、Yuki Yamamoto (Cell. & Mol. Biol., Grad. Sch. Biomed. & Health Sci., Hiroshima Univ.)、Hidetoshi Tahara (Cell. & Mol. Biol., Grad. Sch. Biomed. & Health Sci., Hiroshima Univ.)

Anti-silencing function 1B (ASF1B) which is one of the histone chaperones, promotes tumor cell proliferation. While elevated expression of ASF1B correlates with poor prognosis in pancreatic adenocarcinoma (PAAD), little is known about the factors controlling ASF1B expression. MicroRNAs (miRNAs) regulate gene expression mainly by binding to the 3' untranslated region (3'UTR) of their targets. While in silico-based algorithms enable us to predict miRNA targets, it is still difficult to elucidate the true regulatory network between miRNAs and their targets. In this study, we developed a high-throughput screening system based on a luciferase reporter assay and established MIA PaCa-2 cells expressing firefly luciferase with ASF1B 3'UTR (MIA Paca-2 ASF1B-3'UTR). After transfection of an miRNA library (2028 mimics), several miRNAs significantly reduced the luciferase activity in MIA Paca-2 ASF1B-3'UTR. Candidate miRNAs also reduced ASF1B expression at protein level. These results suggest that our system well worked for understanding the interaction between miRNA and their targets and miRNAs targeting ASF1B have therapeutic potential for pancreatic cancer treatment.

[Disclosure of COI by Responsible researcher] Lead presenter is not the Responsible researcher.
[Lead presenter: Lisa Akiyama] We have no financial relationships to disclose.
[Principal Researcher: Hidetoshi Tahara] ■Research funds from company: PURMX Therapeutics ■employment by a company related to the presentation: PURMX Therapeutics ■Profit of stock: PURMX Therapeutics

### [P-2080] I-P5-4
### Non-coding RNAs (2)

2025 / 9 / 26 (Fri) 17:30-18:15 Room P/(Motenashi Dome) Room P

---

### P-2080
### The Role of miR-224 in Hepatocellular Carcinoma: An Integrated In Vitro and In Vivo Analysis

[Speaker]Liang Teng (Dept. of Surg. I, Yamagata Univ. Fac. of Med.) [Co-author]Xuhong Zhang (Dept. of Biochem. Mol Biol., Yamagata Univ. Fac. of Med.)、Hisayoshi Watanabe (Dept. of Gastro., Yamagata Univ. Fac. of Med.)、Akiyoshi Hirayama (Inst. for Adv.. Biosci., Keio Univ.)、Naoki Fukuda (Lab. Animal Cent., Yamagata Univ. Fac. of Med.)、Tsunekata Ito (Lab. Animal Cent., Yamagata Univ. Fac. of Med.)、Naing Yeaung (Dept, of Path., Yamagata Univ. Fac. of Med.)、Koji Tezuka (Dept. of Surg. I, Yamagata Univ. Fac. of Med.)、Wataru Kimura (Dept. of Surg. I, Yamagata Univ. Fac. of Med.)、Yoshiyuki Ueno (Dept. of Gastro., Yamagata Univ. Fac. of Med.)、Tsuneo Konta (Dept. of Pub. Health & Hygiene, Yamagata Univ. Fac. of Med.)、Ri Sho (Dept. of Pub. Health & Hygiene, Yamagata Univ. Fac. of Med.)

MicroRNAs are short non-coding RNAs that regulate gene expression, including cancer pathways. Although miR-224 upregulation has been shown to acts as an oncogene in hepatocellular carcinoma (HCC), its precise role in the hallmarks of HCC and the underlying mechanisms remain unclear. We conducted integrated in vitro and in vivo analyses of miR-224 in HCC. Gain- and loss-of-function assays using transfection of miR-224 mimics or inhibitors into various HCC cell lines and human umbilical vein endothelial cells (HUVECs) demonstrated that miR-224 promotes tumor cell migration and angiogenesis, with minimal impact on proliferation and apoptosis. CE-TOF MS metabolic profiling of miR-224-transfected HuH7 cells indicated modulation of the TCA cycle as well as amino acid and nucleotide metabolism, rather than the Warburg effect, to enhance bioenergetics. In HLF xenograft mouse models, intratumoral delivery of a miR-224 inhibitor reduced tumor growth and vascular formation. Furthermore, molecular analyses revealed that these effects are mechanistically linked to suppression of HOXD10 and CIN85. Taken together, miR-224 represents a promising therapeutic target in HCC.

[Disclosure of COI by Responsible researcher] Lead presenter is not the Responsible researcher.
[Lead presenter: Liang Teng] We have no financial relationships to disclose.
[Principal Researcher: Ri Sho] We have no financial relationships to disclose.

### [P-2081] I-P5-4
### Non-coding RNAs (2)

2025 / 9 / 26 (Fri) 17:30-18:15 Room P/(Motenashi Dome) Room P

### P-2081
### Withdrawn

No Abstract

### [P-2082] I-P5-4
### Non-coding RNAs (2)

2025 / 9 / 26 (Fri) 17:30-18:15 Room P/(Motenashi Dome) Room P

---

### P-2082
### Withdrawn

No Abstract

### [P-2083] I-P5-4
### Non-coding RNAs (2)

2025 / 9 / 26 (Fri) 17:30-18:15 Room P/(Motenashi Dome) Room P

---

### P-2083
### Tumor suppressive effect and potential as a molecular target of miR-224 in colorectal cancer

[Speaker]Junya Ohtake (Res. Ctr., Biomed. Eng., Toyo. Univ.／Res. Facility Ctr., Asaka, Toyo Univ.) [Co-author]Isana Oguri (Dept. Biomed. Eng., Sci. & Eng., Toyo Univ.)、Soma Todoriki (Course Biomed. Eng., Grad. Sch. Life Sci, Toyo Univ.)、Ryoutaro Suzuki (Course Biomed. Eng., Grad. Sch. Life Sci, Toyo Univ.)、Hikaru Sakamoto (Course Biomed. Eng., Grad. Sch. Life Sci, Toyo Univ.)、Hidemitsu Kitamura (Res. Ctr., Biomed. Eng., Toyo. Univ.／Res. Facility Ctr., Asaka, Toyo Univ.／Dept. Biomed. Eng., Sci. & Eng., Toyo Univ.／Course Biomed. Eng., Grad. Sch. Life Sci, Toyo Univ.)

Colorectal cancer is the leading cause of death among Japanese men and women. Although various therapeutic strategies have advanced in recent years, they have yet to be effective for many advanced patients. We previously identified miR-224 as a functional miRNA involved in the production of IFN-γ, a cytokine that suppresses tumor growth and metastasis by regulating immune cell functions. However, it remains unclear how miR-224 plays its role in cancer cells.In this study, human colorectal cancer cells (HCT116) introducing miR-224 were used to evaluate cell proliferation and perform gene expression profiling of malignancy-related genes. The results showed that the introduction of miR-224 significantly inhibited cancer cell proliferation. Furthermore, it appeared to downregulate EMT-related factors such as IL-6, TWIST1, TWIST2, and fibronectin 1, thereby regulating epithelial-mesenchymal transition (EMT) and suppressing angiogenesis through reduced VEGF expression.These findings suggest that miR-224 not only enhances anti-tumor immunity but also inhibits tumor malignancy and improves the tumor immune microenvironment.

[Disclosure of COI by Responsible researcher] Lead presenter is not the Responsible researcher.
[Lead presenter: Junya Ohtake] We have no financial relationships to disclose.
[Principal Researcher: Hidemitsu Kitamura] ■Contributions: テルモ生命科学振興財団

### [P-2084] I-P5-4
### Non-coding RNAs (2)

2025 / 9 / 26 (Fri) 17:30-18:15 Room P/(Motenashi Dome) Room P

P-2084

### circPPFIA1 induces cell growth in squamous cell carcinoma by modulating cancer-associated gene expression.

[Speaker]Kiyoshi Masuda (Kawasaki Med. Sch)

Covalently closed circular RNAs (circRNAs) are a large class of non-coding RNAs generated by a process called back-splicing. Recent evidence has shown that circRNAs participate in many pathological processes such as diabetes mellitus, therosclerosis, chronic inflammatory diseases, and cancer. Here, we report that a novel circRNA, circ, induces the cell growth of esophageal squamous cell carcinoma (ESCC) through regulating the expression of cell cycle- and apoptosis-regulating genes. We initially screened RNA-seq data of tumors and non-tumorous tissues (23 ESCCs and 23 normal esophageal tissues) obtained from GEO and found that circPPFIA1 was overexpressed in ESCC. By silencing endogenous circPPFIA1 using siRNA, cell proliferation was significantly suppressed in KYSE140 and KYSE180 cells. RNA-seq of circPPFIA1-knockdown KYSE180 cells showed that 471 and 577 genes were up- and -down-regulated, respectively, compared with control cells. Enrichment analysis using Metascape ranked "cell division" and "regulation of mitotic cell cycle" as significantly suppressed biological processes. Our results indicate that circPPFIA1 might be a crucial molecular target of ESCC.

[Disclosure of COI by Responsible researcher] Lead presenter is the Responsible researcher.
[Lead presenter: Kiyoshi Masuda] We have no financial relationships to disclose.

### [P-2085] I-P5-4
### Non-coding RNAs (2)

2025 / 9 / 26 (Fri) 17:30-18:15 Room P/(Motenashi Dome) Room P

---

### P-2085
### The utility of plasma miR-1254 as chemosensitivity biomarker in esophageal squamous cell carcinoma

[Speaker]Satoshi Hamada (Div. Digestive Surg., Kyoto Prefectural Univ. Med., Kyoto) [Co-author]Shuhei Komatsu (Div. Digestive Surg., Kyoto Prefectural Univ. Med., Kyoto)、Yusuke Takashima (Div. Digestive Surg., Kyoto Prefectural Univ. Med., Kyoto)、Taisuke Imamura (Div. Digestive Surg., Kyoto Prefectural Univ. Med., Kyoto)、Masateru Yamauchi (Div. Digestive Surg., Kyoto Prefectural Univ. Med., Kyoto)、Ryo Ishida (Div. Digestive Surg., Kyoto Prefectural Univ. Med., Kyoto)、Hiroshi Arakawa (Div. Digestive Surg., Kyoto Prefectural Univ. Med., Kyoto)、Keiji Nishibeppu (Div. Digestive Surg., Kyoto Prefectural Univ. Med., Kyoto)、Jun Kiuchi (Div. Digestive Surg., Kyoto Prefectural Univ. Med., Kyoto)、Hiroki Shimizu (Div. Digestive Surg., Kyoto Prefectural Univ. Med., Kyoto)、Tomohiro Arita (Div. Digestive Surg., Kyoto Prefectural Univ. Med., Kyoto)、Yusuke Yamamoto (Div. Digestive Surg., Kyoto Prefectural Univ. Med., Kyoto)、Hirotaka Konishi (Div. Digestive Surg., Kyoto Prefectural Univ. Med., Kyoto)、Ryo Morimura (Div. Digestive Surg., Kyoto Prefectural Univ. Med., Kyoto)、Hitoshi Fujiwara (Div. Digestive Surg., Kyoto Prefectural Univ. Med., Kyoto)、Atsushi Shiozaki (Div. Digestive Surg., Kyoto Prefectural Univ. Med., Kyoto)

Background: This study investigated novel tumor suppressor microRNAs (miRNAs) that decrease in plasma and predict chemosensitivity to neoadjuvant chemotherapy (NAC) for esophageal squamous cell carcinoma (ESCC) and revealed their usefulness as novel therapeutic agents. Methods and results: We identified miR-1254, which was more highly elevated in pre-treatment plasma of ESCC patients with a high histopathological response than in those with a low histopathological response (P = 0.0021) through the microRNA microarray approach. High plasma miR-1254 levels were an independent factor predicting a higher response to chemotherapy (P = 0.0022) and better prognosis (P = 0.0235). Overexpressing miR-1254 in ESCC cells significantly enhanced chemosensitivity to cisplatin through the transcriptional regulation of ABCC1 in vitro. Moreover, increased plasma miR-1254 levels by subcutaneous injection significantly improved responses to cisplatin in mice.Conclusion: Plasma miR-1254 might be a useful biomarker for predicting responses to NAC, and the restoration of plasma miR-1254 levels might improve chemosensitivity in ESCC.

[Disclosure of COI by Responsible researcher] Lead presenter is not the Responsible researcher.
[Lead presenter: Satoshi Hamada] We have no financial relationships to disclose.
[Principal Researcher: Shuhei Komatsu] We have no financial relationships to disclose.

[P-2086] I-P5-4
Non-coding RNAs (2)

2025 / 9 / 26 (Fri) 17:30-18:15 Room P/(Motenashi Dome) Room P

### P-2086
### Circular RNA circWDPCP_0026 and Its Regulatory Network in Cervical Cancer

[Speaker]Sirinart Aromseree (Dept. of Microbiol, Fac. of Med., Khon Kaen Univ.／HEC Res. Group, Fac. of Med., Khon Kaen Univ.) [Co-author]Thananon Srihabua (Dept. of Microbiol, Fac. of Med., Khon Kaen Univ.／HEC Res. Group, Fac. of Med., Khon Kaen Univ.)、Chukkris Heawchaiyaphum (Dept. of Microbiol, Fac. of Med., Khon Kaen Univ.／HEC Res. Group, Fac. of Med., Khon Kaen Univ.)、Juthamas Subin (Dept. of Microbiol, Fac. of Med., Khon Kaen Univ.／HEC Res. Group, Fac. of Med., Khon Kaen Univ.)、Chamsai Pientong (Dept. of Microbiol, Fac. of Med., Khon Kaen Univ.／HEC Res. Group, Fac. of Med., Khon Kaen Univ.)

Cervical cancer (CC) is a leading cause of cancer-related deaths among women worldwide. Circular RNAs (circRNAs) have emerged as key regulators in cancer hallmarks, yet their roles in CC remain largely elusive. Here, we investigated the expression profiles of circRNAs and their regulatory network in CC. Raw RNA sequencing data from 3 of the Gene Expression Omnibus (GEO) datasets were analyzed. We identified 9,115 differentially expressed circRNAs, with 201 upregulated and 88 downregulated, in CC tissues. Upregulated circRNAs were prioritized as potential biomarkers and therapeutic targets. CircWDPCP_0026 showed the highest expression in CC tissues and targeted 5 miRNAs, with hsa-miR-3916 exhibiting the highest binding affinity. To construct a circRNA-miRNA-mRNA regulatory network, 33 upregulated genes were identified as targets of hsa-miR-3916. Among these genes, 6 hub genes were spotted. Notably, ***NCAPH***, one of the hub genes, was correlated with tumor progression, cell proliferation, apoptosis inhibition, and epithelial-mesenchymal transition. Our findings implied that circWDPCP_0026 plays a crucial role in CC and might serve as a potential biomarker and therapeutic target.

[Disclosure of COI by Responsible researcher] Lead presenter is the Responsible researcher.
[Lead presenter: Sirinart Aromseree] We have no financial relationships to disclose.

▲TOP

### [P-2094] I-P8-2
### DNA methylation & others

2025 / 9 / 26 (Fri) 17:30-18:15 Room P/(Motenashi Dome) Room P

### P-2094
### Cell-fate reprogramming of gastric cells with tumorigenic aberrant epigenome by transient Yamanaka factor expression

[Speaker]Tomoka Okada (Dept. Mol Oncol, Grad. Sch. Med., Chiba Univ.) [Co-author]Takayuki Hoshii (Dept. Mol Oncol, Grad. Sch. Med., Chiba Univ.)、Atsushi Okabe (Dept. Mol Oncol, Grad. Sch. Med., Chiba Univ. ／Health and Disease Omics Ctr., Chiba Univ.)、Motoaki Seki (Dept. Mol Oncol, Grad. Sch. Med., Chiba Univ.)、Daichi Komiyama (Dept. Mol Oncol, Grad. Sch. Med., Chiba Univ.)、Masaki Fukuyo (Dept. Mol Oncol, Grad. Sch. Med., Chiba Univ.)、Yasuhiro Yamada (Dept. Mol Path., Grad. Sch. Med., The Univ. Tokyo.)、Atsushi Kaneda (Dept. Mol Oncol, Grad. Sch. Med., Chiba Univ. ／Health and Disease Omics Ctr., Chiba Univ.)

Environmental factors such as viral infections play a crucial role in cancer development. Epstein-Barr virus (EBV)-associated gastric cancer exhibits promoter DNA hypermethylation on tumor suppressor genes and dynamic alteration of enhancer-related H3K27 acetylation. Although EBV is a key driver to alter epigenome and cell fate, it remains unclear whether these changes are reversible. While Yamanaka 4 factors (Oct4, Sox2, Klf4, c-Myc; OSKM) are known to induce cell-fate reprogramming, we infected gastric cells (GES1) with EBV (GES1_EB) and examined the perturbation with transient OSKM expression, repeating a cycle with 3 days on and 4 days off by a Doxycycline inducible system. Apoptosis-related genes were epigenetically repressed in GES1_EB but re-expressed and retained after transient OSKM expression. Re-expression of densely hypermethylated genes were rarely observed, though. Contrary, genes e.g. interferon-inflammatory signaling genes were epigenetically activated in GES1_EB, but re-suppressed and maintained after transient OSKM expression. These suggest that transient cell-fate reprogramming may serve as a therapeutic approach to restore cellular state, at least partially.

[Disclosure of COI by Responsible researcher] Lead presenter is not the Responsible researcher.
[Lead presenter: Tomoka Okada] We have no financial relationships to disclose.
[Principal Researcher: Atsushi Kaneda] ■Research expenses: SRL Inc., Scrum Inc., Tsubakimoto Chain Co. ■Contributions: Takeda Science Foundation., Uehara Memorial Foundation.

### [P-2095] I-P8-2
### DNA methylation & others

2025 / 9 / 26 (Fri) 17:30-18:15 Room P/(Motenashi Dome) Room P

---

### P-2095
### Causal roles of DNA hypermethylation in better treatment response of the head and neck carcinoma

[Speaker]Kenta Saeda (Dpt of Mol Oncology, Grad Sch of Med, Chiba Univ／Dpt of otorhinolaryngology, Head and Neck Surgery, chiba University Hospital) [Co-author]Atsushi Okabe (Dpt of Mol Oncology, Grad Sch of Med, Chiba Univ／Dpt of Health and Disease Omics Center)、Takuya Nakagawa (Dpt of Mol Oncology, Grad Sch of Med, Chiba Univ／Dpt of otorhinolaryngology, Head and Neck Surgery, chiba University Hospital／Dpt of Health and Disease Omics Center)、Tomoya Kurokawa (Dpt of Mol Oncology, Grad Sch of Med, Chiba Univ／Dpt of otorhinolaryngology, Head and Neck Surgery, chiba University Hospital)、Kazuko Kita (Dpt of Mol Oncology, Grad Sch of Med, Chiba Univ)、Motoaki Seki (Dpt of Mol Oncology, Grad Sch of Med, Chiba Univ)、Masaki Fukuyo (Dpt of Mol Oncology, Grad Sch of Med, Chiba Univ)、Rahmutulla Bahityar (Dpt of Mol Oncology, Grad Sch of Med, Chiba Univ)、Toyoyuki Hanazawa (Dpt of otorhinolaryngology, Head and Neck Surgery, chiba University Hospital)、Atsushi Kaneda (Dpt of Mol Oncology, Grad Sch of Med, Chiba Univ／Dpt of Health and Disease Omics Center)

While favorable prognosis is generally observed in HPV+ head and neck squamous cell carcinoma (HNSCC), particularly oropharyngeal carcinoma (OPC), a subset of HPV+OPC exhibits resistance to chemotherapy. We previously conducted stratification of clinical OPC cases through DNA methylome analysis, and found correlation between high DNA methylation levels and favorable treatment response. We here elucidate the mechanisms of this correlation. We performed integrated methylome analyses of 77 HPV+ HNSCC cases in TCGA and 30 HPV+ OPC in our cohort. We extracted 115 and 73 genes showing high DNA methylation in good responders in TCGA and our cohort, respectively. Among these genes, ROBO1 commonly showed high DNA methylation levels in good responders. ROBO1 knockdown in HNSCC cells lead to reduction of cell proliferation. Combination of cisplatin treatment with ROBO1 knockdown resulted in synergistic reduction of proliferation ($P<0.01$). Overexpression of ROBO1 in HNSCC cells lead to resistance to cisplatin. These results suggest that DNA hypermethylation is causally important in chemosensitivity of HNSCC, and silencing of ROBO1 might help enhancing chemotherapy efficacy.

[Disclosure of COI by Responsible researcher] Lead presenter is not the Responsible researcher.
[Lead presenter: Kenta Saeda] We have no financial relationships to disclose.
[Principal Researcher: Atushi Kaneda] ■Research funds from company: SRL Inc., Scrum Inc., Tsubakimoto Chain Co.　■Research expenses: SRL Inc., Scrum Inc.　■Contributions: Takeda Science Foundation., Uehara Memorial Foundation.

Poster Session

### [P-2096] I-P8-2
### DNA methylation & others

2025 / 9 / 26 (Fri) 17:30-18:15 Room P/(Motenashi Dome) Room P

---

### P-2096
### Methylated HOXA1 with conventional tumor markers shows high diagnostic sensitivity for early hepatocellular carcinoma

[Speaker]Yuki Kunimune (Dept. of Oncology & Lab.Med., Yamaguchi Univ.Grad. Sch. of Med／Div. of Lab., Yamaguchi Univ. Hosp) [Co-author]Yutaka Suehiro (Dept. of Oncology & Lab.Med., Yamaguchi Univ.Grad. Sch. of Med／Div. of Lab., Yamaguchi Univ. Hosp)、Issei Saeki (Dept. of Gastroenterology & Hepatology, Yamaguchi Univ.Grad. Sch. of Med)、Yurika Yamauchi (Dept. of Gastroenterology & Hepatology, Yamaguchi Univ.Grad. Sch. of Med)、Norikazu Tanabe (Div. of Lab., Yamaguchi Univ. Hosp／Dept. of Gastroenterology & Hepatology, Yamaguchi Univ.Grad. Sch. of Med)、Toshihiko Matsumoto (Dept. of Gastroenterology & Hepatology, Yamaguchi Univ.Grad. Sch. of Med)、Mitsuaki Nishioka (Div. of Lab., Yamaguchi Univ. Hosp)、Kiyoshi Ichihara (Dept. of Clin. Lab. Sciences, Yamaguchi Univ.Grad. Sch. of Med)、Hiroaki Nagano (Dept. of Gastroenterological, Breast,&Endocrine Surg., Yamaguchi Univ.Grad. Sch. of Med)、Taro Takami (Dept. of Gastroenterology & Hepatology, Yamaguchi Univ.Grad. Sch. of Med)、Takahiro Yamasaki (Dept. of Oncology & Lab.Med., Yamaguchi Univ.Grad. Sch. of Med／Div. of Lab., Yamaguchi Univ. Hosp)

[Background] Non-viral hepatocellular carcinoma (HCC) is increasing in Japan, but there is no surveillance system for it. Therefore, we performed the present study.[Methods] We enrolled 516 participants (healthy subjects, n=154; chronic liver disease [CLD], n=93; HCC, n=269). Serum methylated HOXA1 (m-HOXA1) copy numbers were quantified using our proprietary methylation-sensitive restriction enzyme assay. Alpha-fetoprotein (AFP) and des-gamma-carboxy prothrombin (DCP) were measured. In this study, to develop a prediction model for HCC, we performed multivariate analysis with cross-validation.[Results] The multivariate analysis identified age, sex, DCP, AFP and m-HOXA1 as independent factors for HCC. Based on the result, we developed a prediction formula named ASDAm-H1 index, which attained an average AUC of 0.96 by cross-validation. The sensitivity for early-stage overall, non-viral, and viral HCC was 76.4%, 76.6%, and 76.2%, respectively, with 93.9% specificity.[Conclusions] We showed clinical utility of the ASDAm-H1 index to detect early-stage non-viral HCC. This index may be applicable to a non-viral HCC surveillance system.

[Disclosure of COI by Responsible researcher] Lead presenter is not the Responsible researcher.
[Lead presenter: Yuki Kunimune] We have no financial relationships to disclose.
[Principal Researcher: Takahiro Yamasaki] We have no financial relationships to disclose.

### [P-2097] I-P8-2
### DNA methylation & others

2025 / 9 / 26 (Fri) 17:30-18:15 Room P/(Motenashi Dome) Room P

### P-2097
### Identification of the structural alterations causing epigenetic aberrations by long-read sequencing in mesothelioma

[Speaker]Yuan Chen (Genome Science, RCAST, Univ. of Tokyo) [Co-author]Genta Nagae (Genome Science, RCAST, Univ. of Tokyo)、Hiroki Ueda (Biological Data Science, RCAST, Univ. of Tokyo)、Kenji Tatsuno (Genome Science, RCAST, Univ. of Tokyo)、Shinichi Morishita (Dept. CBMS., Grad. Sch. of Frontier Sci., Univ. of Tokyo)、Yoshitaka Sekido (Div. Cancer Biology, Aichi Cancer Ctr. Res. Ins.)、Hiroyuki Aburatani (Genome Science, RCAST, Univ. of Tokyo)

Although cancer genome analysis using short-read sequencing (SRS) has revealed a comprehensive landscape of single-nucleotide variants (SNVs) and structural variants (SVs), the variants in repetitive regions remain poorly characterized. In this study, we analyzed nine pairs of malignant pleural mesothelioma (MPM) samples with Oxford Nanopore Technology (ONT) to identify the somatic SVs and aberrant allele-specific methylation (ASM) that influence chromatin state regulation.We detected the somatic SVs by ONT data using NanomonSV and validated these SVs with PacBio HiFi data. The cancer-specific ASM (somatic ASM, sASM) was defined by phasing the methylation frequency into two alleles with the germline SNVs. In total, we detected 1,259 somatic SVs and 1,958 genes having sASM. Next, screening with the aberrant ASM with overexpression in a larger sample size of the SRS data (81 MPMs and 7 normal samples), we narrowed down to tens of candidate genes with sASM, allele-specific expression (ASE), abnormal expression in the tumor, and SV nearby. We would like to introduce the integrative approach to identify the mechanism of cancer gene dysregulation in MPMs.

[Disclosure of COI by Responsible researcher] Lead presenter is not the Responsible researcher.
[Lead presenter: Yuan Chen] We have no financial relationships to disclose.
[Principal Researcher: Hiroyuki Aburatani] We have no financial relationships to disclose.

### [P-2098] I-P8-2
### DNA methylation & others

2025 / 9 / 26 (Fri) 17:30-18:15 Room P/(Motenashi Dome) Room P

P-2098

### The Impact of SLC25A22-Regulated Metabolic Pathways on the epigenetic regulation of NRXN1 in Gastric Cancer Progression

[Speaker]Fu-Ting Jiang (Dept. of Biomedical Sci.) [Co-author]Kit-San Yu (Dept. of Biomedical Sci.)、Michael Chan (Dept. of Biomedical Sci.)

The mitochondrial glutamate transporter SLC25A22 plays a key role in glutaminolysis, contributing to cancer progression and metabolic reprogramming. Although SLC25A22 is frequently overexpressed in various cancers, its role in gastric cancer (GC) remains unclear. TCGA RNA-Seq analysis reveals that NRXN1, a neuronal-immune network gene, is significantly downregulated in GC tissues compared to normal tissues, with further suppression observed in tumors with high SLC25A22 expression. Knockdown of SLC25A22 in GC cells significantly reduces proliferation in colony formation assays, supporting its oncogenic potential. Given reports that SLC25A22 may inhibit TET-mediated DNA demethylation, it is hypothesized that SLC25A22 contributes to NRXN1 silencing via epigenetic mechanisms. Current studies focus on assessing NRXN1 promoter methylation and the impact of NRXN1 loss on the tumor microenvironment. These findings suggest that targeting the SLC25A22-NRXN1 axis could offer a novel therapeutic strategy for gastric cancer.

[Disclosure of COI by Responsible researcher] Lead presenter is the Responsible researcher.
[Lead presenter: Fu-Ting Jiang] We have no financial relationships to disclose.

### [P-2099] I-P8-2
### DNA methylation & others

2025 / 9 / 26 (Fri) 17:30-18:15 Room P/(Motenashi Dome) Room P

---

### P-2099
### Heterogeneity and biological implications of critical transcription factor in hepatoblastoma development

[Speaker]Wataru Kudo (Health and Disease Omics Center, Chiba Univ.／Dept. Mol. Oncol., Grad. Sch. Med., Chiba Univ.／Dept. Ped. Surg., Grad. Sch. Med., Chiba Univ.) [Co-author]Mizuki Minami (Health and Disease Omics Center, Chiba Univ.)、Ryoya Furugane (Dept. Mol. Oncol., Grad. Sch. Med., Chiba Univ.／Dept. Ped. Surg., Grad. Sch. Med., Chiba Univ.)、Takuya Nakagawa (Health and Disease Omics Center, Chiba Univ.／Dept. Ped. Surg., Grad. Sch. Med., Chiba Univ.)、Genki Usui (Dept. Mol. Oncol., Grad. Sch. Med., Chiba Univ.)、Takayuki Hoshii (Dept. Mol. Oncol., Grad. Sch. Med., Chiba Univ.)、Motoaki Seki (Dept. Mol. Oncol., Grad. Sch. Med., Chiba Univ.)、Atsushi Okabe (Health and Disease Omics Center, Chiba Univ.／Dept. Mol. Oncol., Grad. Sch. Med., Chiba Univ.)、Masaki Fukuyo (Dept. Mol. Oncol., Grad. Sch. Med., Chiba Univ.)、Rahmutulla Bahityar (Dept. Mol. Oncol., Grad. Sch. Med., Chiba Univ.)、Tomoro Hishiki (Dept. Ped. Surg., Grad. Sch. Med., Chiba Univ.)、Atsushi Kaneda (Health and Disease Omics Center, Chiba Univ.／Dept. Mol. Oncol., Grad. Sch. Med., Chiba Univ.)

Hepatoblastoma (HB) is the most common malignant liver tumor in children, and development of novel therapies for poor-prognosis cases is the urgent issue. Here we conducted integrated analyses of transcriptome and epigenome combined with database analysis and identified a transcription factor (TFX) as a potential therapeutic target regulating metabolic and cell cycle-related genes. We further assessed the association between TFX expression in clinical HB samples and pathological features using immunostaining with whole slide imaging. TFX expression was frequently elevated in tumor regions, with a significant negative correlation with serum alpha-fetoprotein levels during chemotherapy ($r = -0.79$, $P = 0.01$). Spatial transcriptome analysis revealed five tumor clusters, with high TFX-expressing clusters showing activated metabolism-related pathways and cell cycle profiles. Knockdown of TFX or its downstream metabolism-related gene lead to decrease of cellular growth synergistically with cisplatin treatment. These findings suggest that elevation of TFX may contribute to HB development, e.g. via chemoresistance, and potentially be a novel druggable target.

[Disclosure of COI by Responsible researcher] Lead presenter is not the Responsible researcher.
[Lead presenter: Wataru Kudo] ■Research funds from company: 公的研究費：科研費（基盤C）
[Principal Researcher: Atsushi Kaneda] ■Research expenses: SRL Inc., Scrum Inc. ■Contributions: Takeda Science Foundation., Uehara Memorial Foundation.

### [P-2100] I-P8-2
### DNA methylation & others

2025 / 9 / 26 (Fri) 17:30-18:15 Room P/(Motenashi Dome) Room P

### P-2100
### Improvement of prognostic marker assessment and application to patient stratification

[Speaker]Miwako K. Homma (Fukushima Medical University, Biomolecular Sciences／Southern Tohoku Research Institute) [Co-author]Hinata Kishino (Fukushima Medical University, Biomolecular Sciences)、Yuko Hashimot (Fukushima Medical University, Diagnostic Pathology)、Tadashi Nomizu (Hoshi General Hospital, Surgery)

We previously reported that IHC evaluation of nucleolar CK2$\alpha$, a protein kinase, was a reliable predictor for poor prognosis for breast cancer and lung adenocarcinoma: an independent variable for the length of RFS also for patients in early p Stage. We demonstrated epigenetic function of CK2$\alpha$ on active gene loci, and a group of proteins in the nucleolar CK2 complexes. Based on these physiological interactions which were delineated in reconstituted systems, this study explored the way to evaluate multiple stains to validate the prognostic marker for greater accuracy. The histochemical staining of CK2$\alpha$ along with several cooperating molecules were performed by using biopsy specimens from TN breast cancer patients who had undergone preoperative chemotherapy. CK2 nucleolus-positive cases showed a significantly lower percentage of RFS with 58% in the 5-year, and a much larger difference by combining the staining evaluation of CK2-linked molecules. These results suggest highly reliable evaluation could be utilized in terms of stratification of treatment targets, especially in the case of immune checkpoint inhibitors (ICIs) in the perioperative treatment of patients with TNBC.

[Disclosure of COI by Responsible researcher] Lead presenter is the Responsible researcher.
[Lead presenter: Miwako K. Homma] We have no financial relationships to disclose.

### [P-2101] I-P8-2
### DNA methylation & others

2025 / 9 / 26 (Fri) 17:30-18:15 Room P/(Motenashi Dome) Room P

---

### P-2101
### SEgene: A platform for analyzing cancer-related super-enhancers based on epigenetic correlation data and public database

[Speaker]Norio Shinkai (Cancer Transl. Res. Team, RIKEN Ctr. for AIP project／Div. Med. AI Res. Dev., Natl. Cancer Ctr. Res. Inst.) [Co-author]Syuzo Kaneko (Cancer Transl. Res. Team, RIKEN Ctr. for AIP project／Div. Med. AI Res. Dev., Natl. Cancer Ctr. Res. Inst.)、Ken Asada (Cancer Transl. Res. Team, RIKEN Ctr. for AIP project／Div. Med. AI Res. Dev., Natl. Cancer Ctr. Res. Inst.)、Ken Takasawa (Cancer Transl. Res. Team, RIKEN Ctr. for AIP project／Div. Med. AI Res. Dev., Natl. Cancer Ctr. Res. Inst.)、Masaaki Komatsu (Cancer Transl. Res. Team, RIKEN Ctr. for AIP project／Div. Med. AI Res. Dev., Natl. Cancer Ctr. Res. Inst.)、Hidenori Machino (Cancer Transl. Res. Team, RIKEN Ctr. for AIP project／Div. Med. AI Res. Dev., Natl. Cancer Ctr. Res. Inst.)、Satoshi Takahashi (Cancer Transl. Res. Team, RIKEN Ctr. for AIP project／Div. Med. AI Res. Dev., Natl. Cancer Ctr. Res. Inst.)、Nobuji Kouno (Cancer Transl. Res. Team, RIKEN Ctr. for AIP project／Div. Med. AI Res. Dev., Natl. Cancer Ctr. Res. Inst.)、Ryuji Hamamoto (Cancer Transl. Res. Team, RIKEN Ctr. for AIP project／Div. Med. AI Res. Dev., Natl. Cancer Ctr. Res. Inst.／NCC Cancer ., Grad. Sci. Med. Dent. Sci., Sci. Tokyo. Univ.)

Super-enhancers (SEs) are genomic structures that are believed to strongly drive gene expression. We have developed SEgene (https://github.com/hamamoto-lab/SEgene), an SE analysis platform designed to evaluate SE regions based on sample group information and integration with public database. This system evaluates SE regions (initially identified by ROSE method) that functionally contribute to gene expression based on correlation information between enhancers and genes across sample groups and visualizes network structures based on correlation patterns. Additionally, SEgene incorporates public databases to characterize and assess the properties of identified SE regions. Using SEgene, we successfully extracted SE accumulation region across multiple samples (merge-SE) with strong cancer associations from both public cancer patient datasets and the NCC pan-negative lung cancer cohort, revealing potential cancer-specific SE activity patterns. In this presentation, we report these findings and our approach to enhance analysis accuracy through integration of omics data and machine learning. We also discuss the benefits of this system for cancer-related SE analysis, with demonstrations.

[Disclosure of COI by Responsible researcher] Lead presenter is not the Responsible researcher.
[Lead presenter: Norio Shinkai] We have no financial relationships to disclose.
[Principal Researcher: Ryuji Hamamoto] We have no financial relationships to disclose.

### [P-2109] I-P9-3
### Cell adhesion & invasion (1)

2025 / 9 / 26 (Fri) 17:30-18:15 Room P/(Motenashi Dome) Room P

---

### P-2109
### Avasimibe's pan-cancer action: Blocking lipid accumulation and STAT1/3 signaling

[Speaker]Lixian Deng (Life Sci. Res. Inst., GXMU／Key Lab. Regional. High-Incidence Tumor., GXMU) [Co-author]Tingting Huang (Key Lab. Regional. High-Incidence Tumor., GXMU)、Zhe Zhang (Key Lab. Regional. High-Incidence Tumor., GXMU)、Xiaoying Zhou (Life Sci. Res. Inst., GXMU／Key Lab. Regional. High-Incidence Tumor., GXMU)

Lipid metabolic reprogramming has been proved to play a critical role in tumorigenesis and cancer progression. In this study, we aim to screen out and identifiy lipid-modulating molecules as promising therapuetic agents for tumor treatment.We screened 410 compounds (Selleckchem) across four tumor models - renal (786-O), hepatic (SNU449), nasopharyngeal (TW03), and oral squamous (SAS) carcinomas - identifying Avasimibe as a pan-cancer inhibitor. Validation in Caki-1, 5-8F, and CAL27 cells confirmed broad activity (IC50:40-65 μM). Avasimibe, a SOAT1 inhibitor, reduces cholesteryl esters. TCGA showed SOAT1 upregulation in cancers. Network pharmacology and docking identified STAT1/STAT3 as targets. Functional assays linked Avasimibe to lipid modulation, cell cycle arrest (G1-phase in RCC/HCC/OSCC; S-phase in NPC, $p<0.05$), and apoptosis. In vivo, Avasimibe reduced NPC xenograft volume ($p<0.05$) and systemic cholesterol ($p<0.001$). These results advance our understanding of lipid metabolism in oncogenesis while offering concrete translational potential, though further mechanistic exploration remains warranted.

[Disclosure of COI by Responsible researcher] Lead presenter is the Responsible researcher.
[Lead presenter: Lixian Deng] We have no financial relationships to disclose.

▲TOP

### [P-2110] I-P9-3
### Cell adhesion & invasion (1)

2025 / 9 / 26 (Fri) 17:30-18:15 Room P/(Motenashi Dome) Room P

---

### P-2110
### Angulin-1/LSR suppresses vasculogenic mimicry in human breast cancer isoform-specific manner

[Speaker]Yuma Yoshioka (Faculty of Science and Technology, Keio University) [Co-author]Tomokazu Ohishi (Inst. Microbial Chemistry (BIKAKEN), Laboratory of Oncology)、Daisuke Tatsuda (Inst. Microbial Chemistry (BIKAKEN), Laboratory of Oncology)、Minami Nakajima (Faculty of Science and Technology, Keio University)、Naoto Kubota (Division of Molecular Pathology,Tochigi Cancer Center)、Jiro Ando (Department of Breast Surgery, Tochigi Cancer Center)、Manabu Kawada (Inst. Microbial Chemistry (BIKAKEN), Laboratory of Oncology)、Hidenori Ojima (Division of Molecular Pathology,Tochigi Cancer Center)、Siro Simizu (Faculty of Science and Technology, Keio University)

Vasculogenic mimicry (VM) is a process in which cancer cells form blood vessel-like structures to obtain nutrition and oxygen. VM is associated with patient poor prognosis, but the mechanism has not elucidated in detail. Cell-cell junction such as tight junctions might be important for VM formation. Thus, we focus on angulin-1/LSR (Ang-1), which is a tricellular tight junction protein, and examined its role for VM. ***In vitro***, when we performed VM formation assay using Ang-1 knockout (KO) breast cancer cells established, their VM formations were significantly promoted. The Uniprot database posts that Ang-1 has 6 isoforms, and we determined to identify endogenous isoform of Ang-1 in T47D breast cancer cells. Notably, we detected new isoform of Ang-1, and sought to re-express all isoforms, including the new isoform, in Ang-1 KO cells. As a result, not all isoforms but some specific isoforms inhibited VM formation in the cells. Moreover, Ang-1 KO xenograft tumors were significantly heavier than control via increasing VM vessels, not angiogenesis tubes. Therefore, we proposed that angulin-1/LSR might be a novel VM regulator and an interesting marker for breast cancer diagnosis.

[Disclosure of COI by Responsible researcher] Lead presenter is not the Responsible researcher.
[Lead presenter: Yuma Yoshioka] We have no financial relationships to disclose.
[Principal Researcher: Siro Simizu] We have no financial relationships to disclose.

### [P-2111] I-P9-3
### Cell adhesion & invasion (1)

2025 / 9 / 26 (Fri) 17:30-18:15 Room P/(Motenashi Dome) Room P

---

### P-2111
### Zyxin is mainly involved in persistent cell migration during migration and invasion.

[Speaker]Hiroto Yuasa (Dept. Oncologic Path. Mie Univ. Grad. Sch. Med.／Path. Div. Mie Univ. Hosp.) [Co-author]Hitoshi Nakano (Biobank Ctr. Mie Univ. Hosp.)、Hiroshi Imai (Path. Div. Mie Univ. Hosp.／Biobank Ctr. Mie Univ. Hosp.)、Yoshifumi Hirokawa (Dept. Oncologic Path. Mie Univ. Grad. Sch. Med.)、Masatoshi Watanabe (Dept. Oncologic Path. Mie Univ. Grad. Sch. Med.／Dept. Diag. Pathol., Saiseikai Matsusaka General Hosp.)

The incidence of metastatic prostate cancer appears to have increased in all races and age groups over the past decade. There is a need to improve treatment outcomes for metastatic prostate cancer. During metastasis, cancer cell migration is one of important processes. Migrating cancer cells depend on the surrounding matrix, genetic mutations, cell polarity and others. The role of zyxin in prostate cancer behavior is poorly understood. In this study, we investigated the effect of zyxin on behavior of prostate cancer cell line PC3 using siRNA-zyxin. Zyxin knockdown cells were analyzed in various migration and invasion assays including Timelapse microscopy. Expression level of zyxin mRNA and protein was significantly decreased via siRNA transfection. The migration, invasion, and wound healing assay revealed that ZYX knockdown significantly reduced motility, invasion, and wound closure of PC3 cells respectively. In addition, timelapse microscopy showed that persistent cell migration was affected. Zyxin may affect migration and invasion, especially persistent cell migration. These findings indicate that zyxin has the potential to be a target for metastatic prostate cancer treatment.

[Disclosure of COI by Responsible researcher] Lead presenter is not the Responsible researcher.
[Lead presenter: Hiroto Yuasa] We have no financial relationships to disclose.
[Principal Researcher: Masatoshi Watanabe] We have no financial relationships to disclose.

▲TOP

### [P-2112] I-P9-3
### Cell adhesion & invasion (1)

2025 / 9 / 26 (Fri) 17:30-18:15 Room P/(Motenashi Dome) Room P

---

### P-2112
### EVs of cancer cells induce FOXP3+ fibroblasts and facilitate tumor invasion via Wnt3-b-catenin pathway

[Speaker]Masamitsu Tanaka (Dept. Mol Med & Biochem., Akita Univ., Sch. Med.) [Co-author]Tomoaki Kimura (Dept. Mol Med & Biochem., Akita Univ., Sch. Med.)、Kurara Takagane (Dept. Mol Med & Biochem., Akita Univ., Sch. Med.)、Go Itoh (Dept. Mol Med & Biochem., Akita Univ., Sch. Med.)、Sei Kuriyama (Dept. Mol Med & Biochem., Akita Univ., Sch. Med.)、Sachiyo Nomura (Clinic Pharma Sci Hoshi Univ)、Motonobu Saito (Gastrointest Tract Surg Fukushima Med Univ)、Akiteru Goto (Dept. Cell Organ Pathol, Akita Univ Sch Med)

FOXP3 is a key transcription factor in Tregs. However, its expression and significance in non-immune stromal cells in the tumor microenvironment remain unclear. Here, we demonstrated FOXP3 expression in stromal fibroblasts of mouse and human gastrointestinal tumors. Immunohistological examination revealed FOXP3 expression in aSMA+ myofibroblasts. In the mouse omentum inoculated with gastric cancer cells, cytokeratin(-)/CD45(-)/FoxP3(+) stromal cells were identified, and high FOXP3 expression was noted in fibroblasts surrounding the tumor glands, where CD8+ T cells were exclusively infiltrated. Extracellular vesicles (EVs) from mouse gastric cancer cells upregulated Foxp3 transcription in fibroblasts. FOXP3 overexpression in NIH/3T3 fibroblasts enhanced Wnt3a-induced b-catenin responses, accompanied by cell growth and tumor invasion in mice stomach. FOXP3 induced CDH11 expression in fibroblasts, which augmented the Wnt3/b-catenin pathway, and blocking of CDH11 suppressed tumor invasion mediated by FOXP3(+) fibroblasts. Our results suggest that cancer cell-derived EVs regulate FOXP3 expression in stromal fibroblasts, attenuating antitumor immunity, and facilitating tumor invasion.

[Disclosure of COI by Responsible researcher] Lead presenter is the Responsible researcher.
[Lead presenter: Masamitsu Tanaka] We have no financial relationships to disclose.

### [P-2113] I-P9-3
### Cell adhesion & invasion (1)

2025 / 9 / 26 (Fri) 17:30-18:15 Room P/(Motenashi Dome) Room P

---

### P-2113
### PTEN regulates MT1-MMP-mediated proMMP-2 activation in cancer cells.

[Speaker]Takahisa Takino (Inst. Liberal Arts & Science, Kanazawa Univ.) [Co-author]Takeshi Suzuki (Cancer Res. Inst., Kanazawa Univ.)

Phosphatase and tensin homolog deleted on chromosome 10 (PTEN) is one of the most frequently mutated tumor suppressor genes in human cancer. PTEN inhibits the PI3K/Akt pathways by acting as both a lipid phosphatase and a protein phosphatase. The loss of PTEN causes aberrant activation of the PI3K/Akt pathways, which promotes tumorigenesis and malignant conversion. In this study, we examined the role of PTEN in MT1-MMP-mediated proMMP-2 activation in cancer cells. Knockdown of PTEN in cancer cells facilitated proMMP-2 activation, which was blocked by the treatment with MMP inhibitor or MT1-MMP knockdown. Treatment of PTEN-silenced cells with ferric ammonium citrate resulted in the promotion of proMMP-2 activation, which was abrogated by either treatment with MMP inhibitor or antioxidant N-acetyl-L-cysteine, suggesting the involvement of reactive oxygen species in MT1-MMP-mediated proMMP-2 activation in PTEN-silenced cancer cells. PTEN knockdown stimulated the invasion of cancer cells, which was blocked by the treatment with MMP inhibitor. Our study suggests that PTEN may regulate the invasiveness of cancer cells through MT1-MMP-MMP-2 axis.

[Disclosure of COI by Responsible researcher] Lead presenter is the Responsible researcher.
[Lead presenter: Takahisa Takino] We have no financial relationships to disclose.

▲TOP

### [P-2114] I-P9-3
### Cell adhesion & invasion (1)

2025 / 9 / 26 (Fri) 17:30-18:15 Room P/(Motenashi Dome) Room P

---

### P-2114
### TFEC inhibits the motility of renal cell carcinoma through enhanced cell-cell adhesion by upregulation of Ksp-cadherin

[Speaker]Keito Fujiwara (Lab. Mol. Oncol., Biores. Sci., Akita Pref. Univ.) [Co-author]Jun Murata (Lab. Mol. Oncol., Biores. Sci., Akita Pref. Univ.)

We previously reported that a neuropeptide, arginine vasopressin (AVP) suppressed the motility of mouse Renca renal cell carcinoma by enhancing intercellular adhesion. AVP also induced the expression of both kidney specific (Ksp)-cadherin and transcription factor EC (TFEC), which binds to E-box (CANNTG) in the promoters of target genes. TFEC overexpression suppressed Renca cell motility, whereas TFEC knockdown promoted it. In this study, we investigated the effects of TFEC on intercellular adhesion and expression of Ksp-cadherin in Renca cells. TFEC overexpression promoted intercellular adhesion of Renca cells, while TFEC knockdown inhibited it. The enhanced cell-cell adhesion induced by TFEC overexpression was attenuated by anti-Ksp-cadherin antibody. TFEC overexpression stimulated the expression of Ksp-cadherin in Renca cells at the mRNA and protein levels, whereas TFEC knockdown suppressed it. Chromatin immunoprecipitation-qPCR revealed that TFEC specifically bound to the promoter region of Ksp-cadherin gene, which contains E-box. These results suggest that TFEC may inhibit Renca cell motility by enhancing intercellular adhesion through upregulated expression of Ksp-cadherin.

[Disclosure of COI by Responsible researcher] Lead presenter is not the Responsible researcher.
[Lead presenter: Keito Fujiwara] We have no financial relationships to disclose.
[Principal Researcher: Jun Murata] We have no financial relationships to disclose.

### [P-2115] I-P9-3
### Cell adhesion & invasion (1)

2025 / 9 / 26 (Fri) 17:30-18:15 Room P/(Motenashi Dome) Room P

### P-2115
### Contribution of FAK to pS-EphA2-mediated cell migration

[Speaker]Yuuki Oguchi (Dept. Cancer Cell Biol., Univ. Toyama) [Co-author]Yue Zhou (Dept. Cancer Cell Biol., Univ. Toyama)、Ryoma Taniguchi (Dept. Cancer Cell Biol., Univ. Toyama)、Satoru Yokoyama (Dept. Cancer Cell Biol., Univ. Toyama)、Hiroaki Sakurai (Dept. Cancer Cell Biol., Univ. Toyama)

The receptor tyrosine kinase EphA2 is overexpressed in various cancer cells. We have reported that EphA2 phosphorylation at Ser-897 (pS-EphA2) via p90 ribosomal S6 kinase (RSK) promotes cell migration by lamellipodia formation. The focal adhesion kinas FAK also regulates lamellipodia formation. However, the relationship between pS-EphA2 and FAK is unclear. Here, we investigated their interaction. Cell migration was suppressed by RSK inhibitor or EphA2-knockdown in human breast cancer cells MDA-MB-231. An FAK inhibitor defactinib also blocked cell migration, suggesting that pS-EphA2 and FAK collaboratively regulate cell migration. By using immunofluorescence staining of pS-EphA2 and FAK, we found that pS-EphA2 was partially co-localized with FAK in lamellipodia. In addition, the aggregation of FAK in focal adhesion was detected; however, it was blocked in EphA2 Ser-897-to Ala substituted mutant cells. These results suggested that pS-EphA2 regulated focal adhesion to induce cell migration.

[Disclosure of COI by Responsible researcher] Lead presenter is not the Responsible researcher.
[Lead presenter: Yuuki Oguchi] We have no financial relationships to disclose.
[Principal Researcher: Hiroaki Sakurai] We have no financial relationships to disclose.

### [P-2116] I-P9-3
### Cell adhesion & invasion (1)

2025 / 9 / 26 (Fri) 17:30-18:15 Room P/(Motenashi Dome) Room P

### P-2116
### SOX10 regulates melanoma metastasis through ITGA3 and EphA2.

[Speaker]Mayu Nishizawa (Dept. Cancer Cell Biol., Univ. Toyama) [Co-author]Soichiro Sasaki (Insti. of Nat. Med., Univ. Toyama)、Yue Zhou (Dept. Cancer Cell Biol., Univ. Toyama)、Yoshihiro Hayakawa (Insti. of Nat. Med., Univ. Toyama)、Hiroaki Sakurai (Dept. Cancer Cell Biol., Univ. Toyama)、Satoru Yokoyama (Dept. Cancer Cell Biol., Univ. Toyama)

The transcription factor SOX10 is expressed in cutaneous melanoma and regulates a melanoma-specific oncogene MITF. Recent studies have shown that SOX10 modulates tumor immunogenicity independently of MITF; but, MITF-independent functions of SOX10 remain unclear.Melanoma heterogeneity presents a major challenge for treatment. While $MITF^{hi}/AXL^{lo}$ cells exhibit proliferative phenotypes, $MITF^{lo}/AXL^{hi}$ cells display invasive characteristics. However, the mechanisms driving the transition to an invasive phenotype are not well understood.Here, we identify SOX10 as a key regulator of melanoma metastasis. Reduced SOX10 expression enhances both adhesion and motility through integrin-$\alpha$3 (ITGA3) and the receptor tyrosine kinase EphA2, respectively. This regulation is mediated by a transcription factor, IRF1. Furthermore, we demonstrate that ITGA3-mediated FAK signaling is activated in the melanoma cells after SOX10 knockdown, and that pharmacological inhibition of FAK with defactinib effectively suppresses metastasis ***in vivo***. Our findings establish a novel SOX10-IRF1-ITGA3/EphA2 pathway that drives melanoma invasiveness and highlight FAK as a potential therapeutic target for metastatic melanoma.

[Disclosure of COI by Responsible researcher] Lead presenter is not the Responsible researcher.
[Lead presenter: Mayu Nishizawa] We have no financial relationships to disclose.
[Principal Researcher: Satoru Yokoyama] We have no financial relationships to disclose.

▲TOP

### [P-2125] I-P10-3
### Telomere/Aging/Senescence/Immortalization

2025 / 9 / 26 (Fri) 17:30-18:15 Room P/(Motenashi Dome) Room P

### P-2125
### The crosstalk between NK cells and senescent fibroblasts

[Speaker]Xintong Zheng (Dept. of Molecular Cell Biology, Graduate school of Medicine, UTokyo／Division of Cancer Cell Biology, IMSUT) [Co-author]David Wang (Division of Cancer Cell Biology, IMSUT)、Makoto Nakanishi (Division of Cancer Cell Biology, IMSUT)

Cellular senescence is a biological process describing a permanent cell-cycle arrest triggered by various cellular stresses. The accumulation of senescent cells has been widely proven to be involved in age-related dysfunction. NK cells are a component of the innate immune system that rapidly responds to virally infected cells. In a previous report, human senescent cells can stimulate NKG2D of NK cells by expressing NKG2D ligand, thereby inducing cytotoxicity. On the other hand, NKG2D blockade treatment partially impairs NK surveillance on senescent cells in the mouse liver fibrosis model. However, the mechanism of up-regulating NKG2D ligands during the cellular senescence process is still largely unknown. In this study, we conducted an in vitro co-culture platform of mouse primary pulmonary fibroblasts and splenic NK cells to study the NK surveillance mechanism. Compared with quiescent cells, senescent cells expressed a higher level of RAE1 and were significantly attacked by activated NK cells. Combining transcriptome analysis and transcription factor binding motif bioinformatic prediction, we hypothesized that NF-$\kappa$ B and/or AP-1 pathway may highly contribute to RAE1 expressing.

[Disclosure of COI by Responsible researcher] Lead presenter is not the Responsible researcher.
[Lead presenter: Xintong Zheng] We have no financial relationships to disclose.
[Principal Researcher: Makoto Nakanishi] ■Profit of stock: 株式会社エアウィーヴ社　reverSASP Therapeutics株式会社

### [P-2126] I-P10-3
### Telomere/Aging/Senescence/Immortalization

2025 / 9 / 26 (Fri) 17:30-18:15 Room P/(Motenashi Dome) Room P

### P-2126
### Human Artificial Chromosome-based identification of an mTert suppression gene in pancreatic cancer cells.

[Speaker]Takahito Ohira (Div. Chromosome Biomed Eng. Fac. of Med., Tottori Univ.／Chromosome Eng Res Center., Tottori Univ.) [Co-author]Haruka Ooi (Div. Chromosome Biomed Eng. Fac. of Med., Tottori Univ.)、Hiroyuki Kugoh (Div. Chromosome Biomed Eng. Fac. of Med., Tottori Univ.／Chromosome Eng Res Center., Tottori Univ.)

The catalytic component of telomerase, telomerase reverse transcriptase (TERT), is reactivated in immortalized cells and plays a crucial role in cancer development. Pancreatic cancer (PC) is frequently observed with loss of heterozygosity (LOH) on the short arm of human chromosome 3 (3p). Previously, we identified putative TERT suppressor genes (TSGs) within the 3p21.3 region in PC using chromosome engineering. In this study, we performed functional analysis using a human artificial chromosome (HAC) carrying only the 3p21.3 region (3p21.3-HAC) to directly determine whether this region harbors TSGs. To identify the specific TSG within 3p21.3, we performed RNA sequencing analysis using two cell lines: (1) murine LTPA PC cells expressing mouse Tert (mTert) and containing an empty HAC vector, and (2) microcell hybrid cells in which mTert expression is suppressed by the introduction of 3p21.3-HAC. Here, we report the identification of TMEM115 as a novel TSG. Functional analysis showed that TMEM115 suppresses both human and mouse TERT. Thus, TMEM115, a novel regulator of telomerase activity, may contribute to PC development through the suppression of TERT.

[Disclosure of COI by Responsible researcher] Lead presenter is the Responsible researcher.
[Lead presenter: Takahito Ohira] We have no financial relationships to disclose.

### [P-2127] I-P10-3
### Telomere/Aging/Senescence/Immortalization

2025 / 9 / 26 (Fri) 17:30-18:15 Room P/(Motenashi Dome) Room P

---

### P-2127
### The Role of Cellular Senescence in Fibrogenesis Phenotype

[Speaker]Kexin He (Department of Biological Sciences Graduate School of Science, UTokyo／The Institute of Medical Science, UTokyo) [Co-author]David Wang (The Institute of Medical Science, UTokyo)、Makoto Nakanishi (The Institute of Medical Science, UTokyo)

Cellular senescence is a state of permanent cell cycle arrest of the cell, induced by responding to endogenous and exogenous stresses, including oxidative stress, telomere attrition, and DNA damage. It serves as an important tumor suppression mechanism. Notably, cellular senescence has been widely implicated in fibrogenesis, which may trigger organ dysfunction, since growing evidence suggests that cellular senescence markers are detectable in fibrotic tissue, and the elimination of senescent cells improves the pathological signs in aged mice. Paradoxically, senescent fibroblasts in dermal tissue exhibit downregulated expression of collagen and other ECM components, whose excessive deposition is a driver of organ fibrosis. Thus, the molecular insight of how senescent cells involve in fibrogenesis should be explored to further understand the role of aging in various disorders, which may also provide the anti-fibrosis therapeutic potential of senolytic agents.

[Disclosure of COI by Responsible researcher] Lead presenter is not the Responsible researcher.
[Lead presenter: Kexin He] We have no financial relationships to disclose.
[Principal Researcher: Makoto Nakanishi] ■Profit of stock: 株式会社エアウィーヴ社、reverSASP Therapeutics株式会社

### [P-2128] I-P10-3
### Telomere/Aging/Senescence/Immortalization

2025 / 9 / 26 (Fri) 17:30-18:15 Room P/(Motenashi Dome) Room P

---

### P-2128
### SIN3A/SAP3-mediated hTERT upregulation in human oral squamous cell carcinoma cells.

[Speaker]Haruka Ooi (Chr. Biomed Eng., Grad. Sch. of Med. Sci., Tottori Univ.) [Co-author]Takahito Ohira (Div. Chromosome Biomed Eng., Fac. of Med., Tottori Univ.／Chromosome Eng Res Center., Tottori Univ.)、Hiroyuki Kugoh (Div. Chromosome Biomed Eng., Fac. of Med., Tottori Univ.／Chromosome Eng Res Center., Tottori Univ.)

Telomeres at the ends of chromosomes shorten with each cell division in somatic cells. Telomerase, regulated by the catalytic subunit hTERT, maintains telomeres and plays a crucial role in cell immortality in cancer. Elucidating the regulatory mechanisms of hTERT is crucial for advancing cancer research and development of effective therapy.Previous studies have shown that SIN3A suppresses hTERT expression by binding to its promoter region. While the SIN3A/SAP30 complex is known to regulate multiple genes, its specific role in controlling hTERT expression in oral squamous cell carcinoma (OSCC) remained unclear.In this study, we investigated the roles of SIN3A and SAP30 in hTERT regulation in OSCC. Knockdown of SIN3A and SAP30 significantly reduced hTERT expression. Chromatin immunoprecipitation (ChIP) assays revealed the localization of SIN3A and SAP30 at the hTERT core promoter. Furthermore, reporter assays identified the region from -15 to +15 base pairs relative to the transcription start site as critical for hTERT transcription activation. These findings suggest that the SIN3A/SAP30 complex directly interacts with the hTERT core promoter to regulate transcription in OSCC.

[Disclosure of COI by Responsible researcher] Lead presenter is not the Responsible researcher.
[Lead presenter: Haruka Ooi] We have no financial relationships to disclose.
[Principal Researcher: Takahito Ohira, Hiroyuki Kugoh] We have no financial relationships to disclose.

### [P-2129] I-P10-3
### Telomere/Aging/Senescence/Immortalization

2025 / 9 / 26 (Fri) 17:30-18:15 Room P/(Motenashi Dome) Room P

---

### P-2129
### TERRA RNA regulates cellular senescence

[Speaker]Hong-Jhih Shen (Inst. of Mol. and Cell. Biol. , NTU) [Co-author]Jen-Lung Yang (Inst. of Mol. and Cell. Biol. , NTU)、Hsueh-Ping Chu (Inst. of Mol. and Cell. Biol. , NTU)

Cellular senescence is an irreversible growth arrest regulated by p53/p21 and p16/Rb. Telomere shortening causes intermediate-state telomeres, activating the DNA damage response and leading to senescence. Oxidative stress accelerates telomere shortening, triggering stress-induced premature senescence (SIPS). While telomere shortening is known to drive senescence, other factors that cause telomere damage are less discussed. Our data showed higher TERRA levels in elderly human blood cells, senescent mouse embryonic fibroblasts (MEFs), and oxidative stress-induced HeLa cells, with elevated telomeric R-loops. To investigate TERRA's role in senescence, we induced senescence in human primary fibroblasts (BJ cells) via continuous culture or oxidative stress. Both methods showed increased TERRA levels and TERRA RNA-DNA hybrids in the nucleus and cytoplasm, along with type I interferon activation. Mimicking senescence by CTC1 depletion also increased TERRA expression and interferon activation. Depleting TERRA with locked nucleic acid antisense oligos abolished the oxidative stress-induced senescent phenotype. These results suggest TERRA may be a potential target for anti-aging.

[Disclosure of COI by Responsible researcher] Lead presenter is not the Responsible researcher.
[Lead presenter: Hong-Jhih Shen] We have no financial relationships to disclose.
[Principal Researcher: Hsueh-Ping Chu] We have no financial relationships to disclose.

### [P-2130] I-P10-3
### Telomere/Aging/Senescence/Immortalization

2025 / 9 / 26 (Fri) 17:30-18:15 Room P/(Motenashi Dome) Room P

---

### P-2130
### RNA acetylation enzyme NAT10 regulates telomerase activity

[Speaker]Yu-Sheng Yang (Institute of Molecular and Cellular Biology, National Taiwan University) [Co-author]Chia-Heng Fan (Institute of Molecular and Cellular Biology, National Taiwan University)、HsuehPing C. Chu (Institute of Molecular and Cellular Biology, National Taiwan University)

Telomeres are repetitive DNA sequences (TTAGGG)n at chromosome ends, protected by the shelterin complex to prevent recognition as DNA damage. Telomerase, a ribonucleoprotein enzyme, counteracts telomere shortening. It includes TERT (the catalytic subunit), TERC (the RNA template), and accessory proteins. Using the identification of direct RNA-interacting proteins (iDRiP) method, we identified TERC interacting proteins, including the RNA acetylation protein NAT10. To investigate the role of NAT10 in regulating telomerase activity, we used NAT10 knockout cells and found that TERC levels were decreased in NAT10 knockout cells. Telomere FISH staining and telomere qPCR showed a reduction in telomere length in NAT10 knockout cells. Additionally, the TRAP-qPCR assay revealed a decrease in telomerase activity. These results suggest that NAT10 acts as a positive regulator of telomerase, supporting the hypothesis that NAT10 acetylates TERC to promote telomere lengthening.

[Disclosure of COI by Responsible researcher] Lead presenter is not the Responsible researcher.
[Lead presenter: Yu-Sheng Yang] We have no financial relationships to disclose.
[Principal Researcher: Chia-Heng Fan, HsuehPing C. Chu] We have no financial relationships to disclose.

▲TOP

### [P-2131] I-P10-3
### Telomere/Aging/Senescence/Immortalization

2025 / 9 / 26 (Fri) 17:30-18:15 Room P/(Motenashi Dome) Room P

### P-2131
### The role of CREB1 in keratinocyte differentiation: Insights from a calcium-induced differentiation model

[Speaker]Nozomi Mihara (Dept. Biochem., Nippon Dent. Univ. Sch. Life Dent. at Tokyo) [Co-author]Kazushi Imai (Dept. Biochem., Nippon Dent. Univ. Sch. Life Dent. at Tokyo)

The imbalanced expression of transcription factors that regulate epithelial proliferation and differentiation contributes to oncogenesis. CREB1, a widely distributed transcription factor, is highly expressed in carcinomas and is associated with tumor dynamics, but its role in epithelial differentiation remains unclear.We analyzed endogenous CREB1 expression using a calcium-induced keratinocyte differentiation model. CREB1 expression decreased after switching to high-calcium medium, occurring before the upregulation of differentiation markers. In low-calcium medium, the expression of differentiation markers showed little increase, and CREB1 levels gradually declined. Keratinocytes exposed to calcium-depleted medium prior to proliferation arrest showed no significant changes in CREB1 expression or in the expression of differentiation markers.These results indicate that CREB1 downregulation occurs before calcium-induced differentiation begins. Calcium removal did not decrease CREB1 expression, and only minimal differentiation was observed, suggesting that early CREB1 downregulation may trigger differentiation.

[Disclosure of COI by Responsible researcher] Lead presenter is the Responsible researcher.
[Lead presenter: Nozomi Mihara] ■Research funds from company: 日本学術振興会（JSPS）科研費

### [P-2132] I-P10-3
### Telomere/Aging/Senescence/Immortalization

2025 / 9 / 26 (Fri) 17:30-18:15 Room P/(Motenashi Dome) Room P

---

### P-2132
### Exploring the mechanisms regulating malignant micropapillary patterns of lung adenocarcinoma via epigenomic profiling

[Speaker]Ryosuke Inoue (Department of Respiratory Medicine, Kitasato University School of Medicine) [Co-author]Takashi Sato (Department of Respiratory Medicine, Kitasato University School of Medicine)、Hiromi Matsuo (Department of Respiratory Medicine, Kitasato University School of Medicine)、Yukiko Matsuo (Department of Thoracic Surgery, Kitasato University School of Medicine)、Masashi Mikubo (Department of Thoracic Surgery, Kitasato University School of Medicine)、Yuki Akazawa (Department of Respiratory Medicine, Kitasato University School of Medicine)、Hiroki Yamamoto (Department of Respiratory Medicine, Kitasato University School of Medicine)、Yuri Yagami (Department of Respiratory Medicine, Kitasato University School of Medicine)、Katsuhiko Naoki (Department of Respiratory Medicine, Kitasato University School of Medicine)

Lung adenocarcinoma (LUAD) exhibits distinct histologic patterns. Among these, micropapillary pattern (MIP) is characterized by tuft cells growing without fibrovascular cores and floating in alveolar space and/or stroma. The presence of this pattern has been reported to be a poor prognostic factor. Despite its clinical importance, the mechanisms of MIP development have not been elucidated. In this study, we adopted KU-Lu-MPPt3 which is a unique cell line established from MIP LUAD as a model to explore the mechanisms. This cell line grows into adhesive monolayer, adhesive tufts and suspended tufts under the same culture condition. We hypothesized that the morphologic heterogeneity occurs due to transcriptional regulation of differentiation factors and to test this, firstly we compared the transcriptional programs between adhesive and suspended populations by CUT&Tag-seq targeting H3K27ac. Differential peaks were identified at genetic loci of several transcription factors. Subsequent DNA motif enrichment analysis suggested differential occupancies of the same transcription factors. Our findings implied the involvement of the candidate transcription factors in the regulation of MIP.

[Disclosure of COI by Responsible researcher] Lead presenter is not the Responsible researcher.
[Lead presenter: Ryosuke Inoue] ■Research funds from company: 公的研究費　日本学術振興会
[Principal Researcher: Katsuhiko Naoki] ■Lecture fee: honoraria for lectures(AstraZeneca K.K.),　honoraria for lectures(Chugai Pharmaceutical Co. Ltd.)　■Research expenses: research funding(Chugai Pharmaceutical Co. Ltd.),　research funding(Taiho Pharmaceutical Co. Ltd.),　research funding(Boehringer-Ingelheim Japan Inc.),　research funding(Ono Pharmaceutical Co. Ltd.),　research funding(Abbvie Inc.),　research funding(Syneos Health)　■Contributions: research funding(Chugai Pharmaceutical Co. Ltd.),　research funding(Taiho Pharmaceutical Co. Ltd.)

### [P-2133] I-P10-3
### Telomere/Aging/Senescence/Immortalization

2025 / 9 / 26 (Fri) 17:30-18:15 Room P/(Motenashi Dome) Room P

---

### P-2133
### Role of PDZD8 and MFN2 in MAM Remodeling During Neuroblastoma Differentiation

[Speaker]Ayaka Sasaki (Dept. Mol. Pathol., Nara Med. Univ.) [Co-author]Rina Tani (Dept. Mol. Pathol., Nara Med. Univ.)、Ruiko Ogata (Dept. Mol. Pathol., Nara Med. Univ.)、Rika Sasaki (Dept. Mol. Pathol., Nara Med. Univ.)、Hiroki Kuniyasu (Dept. Mol. Pathol., Nara Med. Univ.)

In high-risk neuroblastoma (per INRG classification), differentiation therapy is applied. We focused on MAM (mitochondria-associated membrane) proteins at the mitochondria-ER interface, particularly PDZD8 and MFN2, as potential factors in differentiation. Our previous study showed low PDZD8 expression in normal gastric and colonic mucosa and adenomas, but high expression in adenocarcinomas, suggesting a transition from PDZD8 to MFN2 during differentiation. In this study, neuroblastoma cell lines (IMR-32, NB1, Neuro-2a) were subjected to differentiation induction, and MAM-related gene/protein expression was analyzed by RT-PCR and Western blotting. Differentiation promoted neurite outgrowth, increased neuronal markers, and reduced stemness in IMR-32 and NB1, but not in Neuro-2a. The PDZD8/MFN2 protein ratio shifted to 0.80 (IMR-32), 0.85 (NB1), and 2.76 (Neuro-2a). Expression of ZIP8, ABCB8, and ISCU - transporters for Zn, Fe, and Fe-S clusters, respectively - was also upregulated in IMR-32 and NB1. These findings suggest a link between MAM dynamics and neuroblastoma cell differentiation.

[Disclosure of COI by Responsible researcher] Lead presenter is not the Responsible researcher.
[Lead presenter: Ayaka Sasaki] We have no financial relationships to disclose.
[Principal Researcher: Hiroki Kuniyasu] We have no financial relationships to disclose.

▲TOP

[P-2134] I-P10-3
Telomere/Aging/Senescence/Immortalization

2025 / 9 / 26 (Fri) 17:30-18:15 Room P/(Motenashi Dome) Room P

P-2134
### Relation between MAM-associated proteins and differentiation in colorectal cancer cells

[Speaker]Ayaka Ikemoto (Nara Med Univ, Dept Mol Pathol) [Co-author]Rina Tani (Nara Med Univ, Dept Mol Pathol)、Ruiko Ogata (Nara Med Univ, Dept Mol Pathol)、Hiroki Kuniyasu (Nara Med Univ, Dept Mol Pathol)

Mitochondria-associated membrane (MAM) connect mitochondria and endoplasmic reticulum (ER), enabling inter-organelle material exchange. In human colorectal cancer (CRC), the MAM protein PDZD8 was more highly expressed than in normal mucosa or adenomas. Here, we examined MAM dynamics, stemness, and mitochondrial differentiation using two human CRC cell lines, HT29 and HCT116, under differentiation induced by sodium butyrate (NaBu) or nocodazole (Noco). In HT29 cells treated with NaBu, PGC1α increased relative to c-Myc, and stem cell markers were reduced. Both PDZD8 and MFN2 increased, but the PDZD8/MFN2 ratio declined. pDRP1 and the pDRP1/MFN2 ratio also decreased. Noco synchronized the cell cycle, increased p21Waf1 expression, and induced differentiation. Again, PGC1α increased relative to c-Myc, and stem markers declined. At the mRNA level, PDZD8 and MFN2 changed little, but their protein levels decreased. The PDZD8/MFN2 ratio rose in HT29 but fell in HCT116. These results suggest that both treatments promote stemness loss and mitochondrial maturation, though MAM protein changes differed by method. This may reflect divergent differentiation pathways, warranting further study.

[Disclosure of COI by Responsible researcher] Lead presenter is not the Responsible researcher.
[Lead presenter: Ayaka Ikemoto] We have no financial relationships to disclose.
[Principal Researcher: Hiroki Kuniyasu] We have no financial relationships to disclose.

### [P-2135] I-P10-3
### Telomere/Aging/Senescence/Immortalization

2025 / 9 / 26 (Fri) 17:30-18:15 Room P/(Motenashi Dome) Room P

---

### P-2135
### Spatial Multi-omics Analysis Reveals Distinctive Molecular Events During Colorectal Adenoma-Carcinoma Transition

[Speaker]Mamoru Arai (Grad. Sch. of Front. Sci., Univ. Tokyo) [Co-author]Junko Zenkoh (Grad. Sch. of Front. Sci., Univ. Tokyo)、Tomoko Dai (Diag. Pathol., Ins. Med., Univ. Tsukuba)、Masayuki Noguchi (Shonan Kamakura Gen. Hosp.)、Yutaka Suzuki (Grad. Sch. of Front. Sci., Univ. Tokyo)、Ayako Suzuki (Grad. Sch. of Front. Sci., Univ. Tokyo)

Colorectal cancer (CRC) is a leading cause of cancer-related deaths worldwide with poor prognosis, especially in advanced stages. Many CRCs develop through the adenoma-carcinoma sequence, a well-established progression model from benign adenomas. Understanding this transition at molecular and cellular levels is essential for early detection, risk prediction, and prevention of CRCs. However, the role of the tumor microenvironment (TME) in this transition remains unclear.
In this study, we focused a special type CRC called as "carcinoma in adenoma" that includes both adenoma and carcinoma in one lesion and conducted integrative analyses of whole-exome sequencing, spatial transcriptomics (Xenium), and multiplex immunostaining (PhenoCycler) on eight cases of colorectal adenoma intra-adenocarcinoma. Clustering and pseudotime analyses of Xenium data revealed tumor cells exhibiting diverse lineage differentiation trajectories, such as cancer stem and transient amplifying-like. We are now focusing on TME cells, including fibroblasts, that shows specific molecular features changed along tumor cell trajectories from adenomas to carcinomas.

[Disclosure of COI by Responsible researcher] Lead presenter is not the Responsible researcher.
[Lead presenter: Mamoru Arai] We have no financial relationships to disclose.
[Principal Researcher: Junko Zenkoh] We have no financial relationships to disclose.

### [P-2143] I-P10-5
### Metabolism/Metabolome (2)

2025 / 9 / 26 (Fri) 17:30-18:15 Room P/(Motenashi Dome) Room P

---

### P-2143
### Combination therapeutic strategies targeting a glutamine transporter in colorectal cancer

[Speaker]Kazuha Kimura (Department of Molecular Pathology, Faculty of Medicine, Hyogo Medical University) [Co-author]Tsuyoshi Takashima (Department of Molecular Pathology, Faculty of Medicine, Hyogo Medical University)、Kenji Ohshima (Department of Molecular Pathology, Faculty of Medicine, Hyogo Medical University)

Advancements in technologies such as metabolome analysis have facilitated the development of agents that target cancer cell metabolism. However, these new agents face challenges in showing efficacy as monotherapies. To address this, we developed combination therapeutic strategies targeting the glutamine transporter SLC1A5 by elucidating compensatory signaling or metabolic adaptations in response to SLC1A5 inhibition. The SLC1A5 inhibitor reduced colorectal cancer (CRC) cell proliferation in vitro as a single agent. Notably, concomitant RNA sequencing and metabolome analyses revealed that cells cultured with the inhibitor for extended periods exhibited increased serine levels and decreased glutathione levels, accompanied by upregulation of their synthetic enzymes and increased phosphorylation of Akt. Combination therapies pairing the SLC1A5 inhibitor with inhibitors of glutathione or serine synthetic enzymes, or with an Akt inhibitor, synergistically suppressed CRC cell proliferation in vitro. These findings suggest that inhibiting glutamine metabolism, in combination with targeting compensatory signaling or metabolic adaptations, may offer an effective therapeutic approach for CRC.

[Disclosure of COI by Responsible researcher] Lead presenter is not the Responsible researcher.
[Lead presenter: Kazuha Kimura] We have no financial relationships to disclose.
[Principal Researcher: Kenji Ohshima] We have no financial relationships to disclose.

### [P-2144] I-P10-5
### Metabolism/Metabolome (2)

2025 / 9 / 26 (Fri) 17:30-18:15 Room P/(Motenashi Dome) Room P

---

### P-2144
### Functional linkage in mitochondrial electron transport, glycolysis, and AMPK signaling underlying cancer cell survival

[Speaker]Masato Higurashi (Dept. CancerCellBiol., SHOWA Med. Univ. Grad. Sch. Pharm.) [Co-author]Momoko Uchida (Dept. CancerCellBiol., SHOWA Med. Univ. Grad. Sch. Pharm.)、Kazunori Mori (Dept. CancerCellBiol., SHOWA Med. Univ. Grad. Sch. Pharm.)、Hidetsugu Nakagawa (Dept. CancerCellBiol., SHOWA Med. Univ. Grad. Sch. Pharm.)、Fumihiro Ishikawa (Ctr. for Biotechnol., SHOWA Med. Univ.)、Motoko Shibanuma (Dept. CancerCellBiol., SHOWA Med. Univ. Grad. Sch. Pharm.)

Many tumors arising from various tissue types exhibit decreased mitochondrial DNA (mtDNA) copy number relative to their normal counterparts. In this study, we compared hepatocellular carcinoma cell lines with low mtDNA content (mt-Low) with those exhibiting normal levels (mt-Normal) to determine the effect of decreased mtDNA on the biochemical properties of the cells. Although the two groups were equally competent at generating ATP, mt-Low cells with low electron transport chain (ETC) levels were deficient in glycolytic activity and vulnerable to ETC inhibition. In particular, these cells died following treatment with ETC inhibitors at lower doses. In contrast, mt-Normal cells upregulated glycolysis, activated AMPK, and survived under the same conditions. Moreover, the inhibition of glycolysis and AMPK activation resulted in cell death in mt-Normal cells. In conclusion, the results suggest that there is a functional linkage between glycolysis, AMPK, and ETC, in which glycolysis and AMPK are upregulated in response to changes in ETC levels, and this is important for cell survival. Interference with this linkage causes cell death and provides a potential target for cancer therapy.

[Disclosure of COI by Responsible researcher] Lead presenter is not the Responsible researcher.
[Lead presenter: Masato Higurashi] We have no financial relationships to disclose.
[Principal Researcher: Motoko Shibanuma] We have no financial relationships to disclose.

### [P-2145] I-P10-5
### Metabolism/Metabolome (2)

2025 / 9 / 26 (Fri) 17:30-18:15 Room P/(Motenashi Dome) Room P

---

P-2145

### Ferroptosis Overcomes AMG510 Resistance in ***KRAS***$^{G12C}$ /***KEAP1***-Mutant Lung Cancer via NRF2-Driven Glutathione Metabolism

[Speaker]Hironari Matsuda (Dept. of Respiratory Med., Juntendo Univ. Grad. Sch. of Med.／Res. Insti. for Diseases of Old Age, Juntendo Univ.)
[Co-author]Yoichiro Mituishi (Dept. of Respiratory Med., Juntendo Univ. Grad. Sch. of Med.／Res. Insti. for Diseases of Old Age, Juntendo Univ.)、Isana Katayama (Dept. of Respiratory Med., Juntendo Univ. Grad. Sch. of Med.／Res. Insti. for Diseases of Old Age, Juntendo Univ.)、Masahiro Fujioka (Dept. of Respiratory Med., Juntendo Univ. Grad. Sch. of Med.／Res. Insti. for Diseases of Old Age, Juntendo Univ.)、Yousuke Miyashita (Dept. of Respiratory Med., Juntendo Univ. Grad. Sch. of Med.／Res. Insti. for Diseases of Old Age, Juntendo Univ.)、Arisa Komatsu (Dept. of Respiratory Med., Juntendo Univ. Grad. Sch. of Med.／Res. Insti. for Diseases of Old Age, Juntendo Univ.)、Adityo Wibowo (Dept. of Respiratory Med., Juntendo Univ. Grad. Sch. of Med.／Res. Insti. for Diseases of Old Age, Juntendo Univ.)、Bagus Amien (Dept. of Respiratory Med., Juntendo Univ. Grad. Sch. of Med.／Res. Insti. for Diseases of Old Age, Juntendo Univ.)、Yoshiki Miura (Lab. of Proteomics and Biomolecular Sci, Juntendo Univ.)、Takehito Syukuya (Dept. of Respiratory Med., Juntendo Univ. Grad. Sch. of Med.／Res. Insti. for Diseases of Old Age, Juntendo Univ.)、Ken Tajima (Dept. of Respiratory Med., Juntendo Univ. Grad. Sch. of Med.／Res. Insti. for Diseases of Old Age, Juntendo Univ.)、Shunsuke Kitajima (Cancer Immunotherapy Development, Cancer Precision Medicine Center, JFCR)、Kazuhisa Tkahashi (Dept. of Respiratory Med., Juntendo Univ. Grad. Sch. of Med.／Res. Insti. for Diseases of Old Age, Juntendo Univ.)

***KRAS/KEAP1*** co-mutations in non-small cell lung cancer (NSCLC) are associated with a poor prognosis. However, the underlying mechanisms remain unclear. In this study, we investigated the mechanisms of primary resistance to KRAS G12C inihibitor,AMG510, using ***KRAS***$^{G12C}$/***KEAP1*** co-mutant cell lines. Our results indicate that these cells exhibit enhanced glutathione metabolism. Transcriptome analysis revealed an increase upregulation of genes involved in the glutathione synthesis pathway, driven by NRF2 stabilaization. Metabolomic profiling further confirmed the elevated levels of reduced glutathione in ***KRAS/KEAP1***-knockout cells. Inhibition of xCT, a cystine transporter involved in glutathione metabolism, rendered ***KRAS/KEAP1*** co-mutant cells susceptible to ferroptosis. The combination of AMG510 with ferroptosis inducers enhanced the therapeutic sensitivity, supporting the notion that inducing ferroptosis could improve treatment efficacy in ***KRAS/KEAP1*** co-mutant NSCLC. These findings highlight the role of glutathione metabolism in early resistance to KRAS G12C inhibitors and present a promising strategy to enhance treatment response in patients with ***KRAS/KEAP1*** co-mutations.

[Disclosure of COI by Responsible researcher] Lead presenter is not the Responsible researcher.
[Lead presenter: Hironari Matsuda] We have no financial relationships to disclose.
[Principal Researcher: Yoichiro Mituishi] We have no financial relationships to disclose.

▲TOP

### [P-2146] I-P10-5
### Metabolism/Metabolome (2)

2025 / 9 / 26 (Fri) 17:30-18:15 Room P/(Motenashi Dome) Room P

---

### P-2146
### Analysis of cancer cachexia mechanisms using severely immune deficient mice

[Speaker]Mayuko Yoda (Tohoku Univ. IDAC, Dept.of integrative Bioanalytics) [Co-author]Kosuke Kawaguchi (Breast Center, Mie University Hospital)、Shinpei Kawaoka (Tohoku Univ. IDAC, Dept.of integrative Bioanalytics)

Cancer cachexia is a cancer-induced systemic metabolic disorder that causes abnormalities in the liver, fat, muscle, weight loss, and decreased sensitivity to therapy. However, the molecular mechanisms by which cancer affects the host organism remain unclear. In this study, we analyzed how human cancer affects the host organism using an immunodeficient mouse model. The NOG mice (NOD.Cg-PrkdcscidIl2rgtm1Sug/ShiJic) used in this study lack T cells, B cells, and natural killer cells but retain certain innate immune cells, such as neutrophils. After transplanting human breast cancer cell lines into these NOG mice, we observed that the expression of genes involved in iron homeostasis was suppressed in the liver of cancer-bearing mice, leading to significant iron accumulation. Furthermore, RNA-seq analysis revealed that the gene expression profile in the liver of cancer-bearing NOG mice closely resembled that of NOG-dKO mice, which mimic hereditary hemochromatosis (mice with a deletion of β2m, an important cofactor of the HFE gene, in the NOG background). In this presentation, I will discuss the molecular mechanisms underlying human breast cancer-dependent iron overload in the liver.

[Disclosure of COI by Responsible researcher] Lead presenter is not the Responsible researcher.
[Lead presenter: Mayuko Yoda] We have no financial relationships to disclose.
[Principal Researcher: Shinpei Kawaoka] We have no financial relationships to disclose.

### [P-2147] I-P10-5
### Metabolism/Metabolome (2)

2025 / 9 / 26 (Fri) 17:30-18:15 Room P/(Motenashi Dome) Room P

---

### P-2147
### Glutamine-polyamine metabolic axis promotes cell growth in cancer cells.

[Speaker]Mayu Asao (Dept. Eng., Univ. of Tokyo／Dept. of Integrative Nutriomics and Oncology RCAST, Univ. of Tokyo) [Co-author]Tomoya Matsushita (Dept. Eng., Univ. of Tokyo／Dept. of Integrative Nutriomics and Oncology RCAST, Univ. of Tokyo)、Sho Aki (Dept. Eng., Univ. of Tokyo／Dept. of Integrative Nutriomics and Oncology RCAST, Univ. of Tokyo／Osaka University, Premium Research Institute for Human Metaverse Medicine)、Tsuyoshi Osawa (Dept. Eng., Univ. of Tokyo／Dept. of Integrative Nutriomics and Oncology RCAST, Univ. of Tokyo)

The tumor microenvironment, such as hypoxia and nutrient starvation (NS), stimulates cancer malignancy. In cancer cells, glutamine(Gln) metabolism promotes cancer malignancy, but the mechanism is still unclear. We found that Gln contributes to cancer cell growth by using an NS medium supplemented with each amino acids. We aimed to elucidate the mechanism of Gln-enhanced cancer cell growth. Capillary electrophoresis-mass spectrometry (CE-MS) analysis revealed that Gln restored metabolites of the polyamine pathway. Gln upregulated the expression of ODC1, the rate-limiting enzyme in polyamine metabolism. ODC1 was suggested to be regulated by Gln through both mechanisms of promoting its translation and inhibiting its degradation. In ODC1 knockdown cells, cell growth and MAPK signaling were reduced, which were restored by the addition of putrescine, synthesized by ODC1. These results suggest that Gln may promote cell growth by activating polyamine metabolism via the upregulation of ODC1 expression. We will further investigate the role of the glutamine-polyamine metabolic axis in cancer malignancy, with the goal of uncovering its potential as a therapeutic target for cancer treatment.

[Disclosure of COI by Responsible researcher] Lead presenter is not the Responsible researcher.
[Lead presenter: Mayu Asao] We have no financial relationships to disclose.
[Principal Researcher: Tsuyoshi Osawa] We have no financial relationships to disclose.

### [P-2148] I-P10-5
### Metabolism/Metabolome (2)

2025 / 9 / 26 (Fri) 17:30-18:15 Room P/(Motenashi Dome) Room P

---

### P-2148
### Involvement of FUT8 in EMT and cancer stemness via behavior of HDAC in pancreatic cancer

[Speaker]Taiki Kuribara (Research Center, Osaka International Cancer Institute) [Co-author]Yuki Ohkawa (Research Center, Osaka International Cancer Institute)、Naoyuki Taniguchi (Research Center, Osaka International Cancer Institute)

Cancer cells have altered glycan patterns on the cell surface compared to normal cells, and an increase in fucosylated glycans have been observed in various cancers. More than ten enzymes have been reported to transfer fucose to glycans, and core fucosylation is generated by the action of $\alpha$-1,6 fructosyltransferase FUT8, which transfers fucose to the innermost GlcNAc redisue of N-glycans. FUT8 has been reported to be involved in the cancer stemness and EMT (endothelial mesenchymal transition) in cancer cells. In addition, core fucosylation level is increased in pancreatic cancer patients. However, the underlined mechanism of FUT8 in cancer has not been well understood. In this study we used a pancreatic cancer cell BxPC-3 and generated FUT8-KO cells. The results showed even though FUT8-KO had no effect on the cell growth. Single cell migration ability was increased and sphere formation ability was decreased. We found that the underlying mechanism by which FUT8 is implicated in stemness and EMT is due to the upregulation of HDAC (histone deacetylase) genes and downregulation of DNA repair- and telomere-related genes from RNA-seq analysis.

[Disclosure of COI by Responsible researcher] Lead presenter is the Responsible researcher.
[Lead presenter: Taiki Kuribara] We have no financial relationships to disclose.

### [P-2149] I-P10-5
### Metabolism/Metabolome (2)

2025 / 9 / 26 (Fri) 17:30-18:15 Room P/(Motenashi Dome) Room P

### P-2149
### Withdrawn

No Abstract

### [P-2150] I-P10-5
### Metabolism/Metabolome (2)

2025 / 9 / 26 (Fri) 17:30-18:15 Room P/(Motenashi Dome) Room P

### P-2150
### Effects of Synbiotics on Cancer Cachexia-Induced Tongue Atrophy

[Speaker]Chie Nakashima (Dept. Mol. Pathol., Nara Med. Univ.) [Co-author]Rina Tani (Dept. Mol. Pathol., Nara Med. Univ.)、Shota Nukaga (Dept. Mol. Pathol., Nara Med. Univ.)、Ryoichi Nishida (Dept. Mol. Pathol., Nara Med. Univ.)、Yoshihiro Miyagawa (Dept. Mol. Pathol., Nara Med. Univ.)、Kei Goto (Dept. Mol. Pathol., Nara Med. Univ.)、Isao Kawahara (Dept. Mol. Pathol., Nara Med. Univ.)、Hitoshi Ohmori (Dept. Mol. Pathol., Nara Med. Univ.)、Kaoru Kubo (Dept. Mol. Pathol., Nara Med. Univ.)、Hiroki Kuniyasu (Dept. Mol. Pathol., Nara Med. Univ.)

Cancer sarcopenia worsens prognosis in cancer patients, highlighting the need for effective treatments. The gut-muscle axis has recently been proposed, indicating a link between gut microbiota and skeletal muscle health. Cancer-related dysbiosis is believed to contribute to cachexia and sarcopenia. In this study, we used a CT26 colon cancer mouse model of cachexia and evaluated a synbiotics-based nutritional intervention. The GFOB diet contained glutamine, fiber, and oligosaccharides (5%; prebiotics) along with Bifidobacterium longum (1%; probiotic). Compared to the control diet, GFOB did not prevent body weight loss or muscle atrophy but preserved adipose tissue. Fecal short-chain fatty acids increased 5.6-fold, and blood ketone bodies rose 1.8-fold. In the CT26 group, muscle fiber atrophy and fibrosis were observed, while these were improved in the GFOB group. MYH7-positive slow-twitch fibers, low in the control, increased in CT26 but decreased in GFOB, suggesting GFOB inhibited cachexia-induced slow-twitch transition in tongue muscle.

[Disclosure of COI by Responsible researcher] Lead presenter is not the Responsible researcher.
[Lead presenter: Chie Nakashima] We have no financial relationships to disclose.
[Principal Researcher: Hiroki Kuniyasu] We have no financial relationships to disclose.

### [P-2161] I-P11-8
### Tumor immune microenvironment/Tumor immune escape (4)

2025 / 9 / 26 (Fri) 17:30-18:15 Room P/(Motenashi Dome) Room P

---

### P-2161
### Evaluation of a Novel Dipeptide PD1 Inhibitor Across Three Syngeneic Models Reveals Broad Immune Modulatory Activity

[Speaker]Rebecca J. Boohaker (Pre-clinical Operations, Southern Research) [Co-author]Fusataka Koide (Pre-clinical Operations, Southern Research)、Jessica Keith (Pre-clinical Operations, Southern Research)、Kayton Cherry (Pre-clinical Operations, Southern Research)、Ayodeji Ipinmorati (Pre-clinical Operations, Southern Research)、Zaina Tomasino (Pre-clinical Operations, Southern Research)、Heather Dorman (Pre-clinical Operations, Southern Research)、Synthia Mayfield (Pre-clinical Operations, Southern Research)

We evaluated a novel dipeptide small molecule PD-1 inhibitor across three syngeneic mouse tumor models to assess its immunomodulatory activity. In the MC38 colon carcinoma model, treatment enhanced CD8 T cell infiltration and induced robust memory responses upon tumor rechallenge. In the 4T1 triple-negative breast cancer model, spatial transcriptomics revealed reprogramming of the tumor microenvironment, with increased immune cell engagement and altered expression of immunoregulatory pathways. In the Lewis lung carcinoma model, we employed a CD206-targeted radiotracer for longitudinal PET imaging to noninvasively monitor tumor-associated macrophage (TAM) polarization. As PD-1 signaling is increasingly implicated in regulating macrophage plasticity, reduced CD206 signal suggests a functional shift toward a pro-inflammatory, anti-tumor phenotype. Collectively, these results demonstrate the compound's capacity to modulate both adaptive and innate immunity, supporting its potential as a next-generation immune checkpoint inhibitor.

[Disclosure of COI by Responsible researcher] Lead presenter is the Responsible researcher.
[Lead presenter: Rebecca J. Boohaker] We have no financial relationships to disclose.

### [P-2162] I-P11-8
### Tumor immune microenvironment/Tumor immune escape (4)

2025 / 9 / 26 (Fri) 17:30-18:15 Room P/(Motenashi Dome) Room P

---

### P-2162
### Etoposide-Induced tumor derived DAMPs upregulate Ccl2 and Ccl7 expression in macrophages

[Speaker]JIAN X. ZHAO (Dept. of inflammology, RCAST, The Univ. of Tokyo) [Co-author]H. Yanai (Dept. of inflammology, RCAST, The Univ. of Tokyo)

Tumor cells undergoing cellular damage or stress can release tumor–derived molecules termed damage-associated molecular patterns (DAMPs). It has been known that DAMPs are involved in the modulation of tumor immune microenvironment (TIME). Unraveling the mechanisms of DAMP–driven immune modulation may contribute to improved tumor control and treatment. In this study, nutrient deprivation, hypoxia, chemotherapeutic drug treatment, and irradiation were employed to induce tumor cell death. Conditional media (C.M.) from these dying cells were collected and used to stimulate mouse bone marrow-derived macrophages (BMDMs) to investigate how DAMPs released regulate macrophage gene expression. RNA–seq analysis revealed that BMDMs stimulated with C.M. derived from etoposide–treated B16F10 melanoma cells specifically exhibited upregulated expression of ***Ccl2*** and ***Ccl7***. We are currently attempting to identify the DAMP molecule and elucidate its underlying mechanism. In this poster presentation, I would like to present our recent data, and discuss the chemotherapeutic drug–induced specific DAMPs and how they contribute to TIME remodeling.

[Disclosure of COI by Responsible researcher] Lead presenter is not the Responsible researcher.
[Lead presenter: JIAN X. ZHAO] We have no financial relationships to disclose.
[Principal Researcher: H. Yanai] We have no financial relationships to disclose.

### [P-2163] I-P11-8
### Tumor immune microenvironment/Tumor immune escape (4)

2025 / 9 / 26 (Fri) 17:30-18:15 Room P/(Motenashi Dome) Room P

### P-2163
### Withdrawn

No Abstract

### [P-2164] I-P11-8
### Tumor immune microenvironment/Tumor immune escape (4)

2025 / 9 / 26 (Fri) 17:30-18:15 Room P/(Motenashi Dome) Room P

---

### P-2164
### Glutamine Deprivation Suppresses cGAS/TBK1 Signaling and Impairs Antitumor Immunity in KRAS-Mutant Lung Cancer

[Speaker]Wei-Jan Wang (Dept. of Biol. Sci. & Tech., CMU) [Co-author]Yu-Hsuan Chen (Dept. of Biol. Sci. & Tech., CMU)、Wan-Jou Shen (Grad. Inst. of Biomed. Sci., CMU)

KRAS mutations are common in non-small cell lung cancer (NSCLC), a disease often diagnosed at advanced stages with poor prognosis. Although immune checkpoint blockade (ICB) has improved treatment outcomes, nearly 70% of patients with advanced NSCLC fail to respond, often due to impaired T cell activation and a suppressive tumor microenvironment (TME). The cGAS/TBK1 pathway, a key innate immune sensor of cytosolic DNA, promotes type I interferon signaling and T cell-mediated antitumor immunity. However, nutrient availability in the TME, particularly glutamine, plays a crucial role in modulating both tumor and immune cell function. In this study, we demonstrate that glutamine deprivation inhibits cGAS/p-TBK1 activation in lung cancer cells. Low glutamine levels reduce TBK1 phosphorylation and suppress interferon-gamma (IFN-gamma) expression, impairing T cell activation. These findings reveal a novel mechanism by which nutrient limitation in the TME suppresses innate immune signaling and contributes to immune evasion. Targeting metabolic stress pathways may improve immunotherapy efficacy in KRAS-mutant NSCLC.

[Disclosure of COI by Responsible researcher] Lead presenter is the Responsible researcher.
[Lead presenter: Wei-Jan Wang] We have no financial relationships to disclose.

### [P-2165] I-P11-8
### Tumor immune microenvironment/Tumor immune escape (4)

2025 / 9 / 26 (Fri) 17:30-18:15 Room P/(Motenashi Dome) Room P

---

### P-2165
### SERBP1 Drives Immune Evasion via STING-Mediated Galectin-9 Upregulation in Pancreatic Cancer

[Speaker]Wan-Jou Shen (Grad. Inst. of Biomed. Sci., CMU) [Co-author]Shu-He Wang (Grad. Inst. of Biomed. Sci., CMU)、Wei-Jan Wang (Dept. of Biol. Sci. & Tech., CMU)

SERPINE1 mRNA Binding Protein 1 (SERBP1) is an RNA-binding protein that regulates gene expression by blocking the mRNA entry site of the 80S ribosome. It has been implicated in cancer cell proliferation, migration, and invasion. GEPIA2 database analysis shows high SERBP1 expression in pancreatic cancer, correlating with poor patient survival. However, its role in tumor progression remains unclear. KEGG analysis revealed that SERBP1 co-expressed genes are enriched in viral infection pathways, including the cGAS/STING axis, a key innate immune pathway that induces type I interferons (IFNs) and regulates antitumor immunity. Our study found that SERBP1 upregulates STING, leading to increased Galectin-9 expression and secretion in pancreatic cancer cells. Secreted Galectin-9 can bind to the Tim-3 receptor on T cells, suppressing their activation and promoting immune evasion. These results suggest that SERBP1 contributes to an immunosuppressive microenvironment via the STING/Galectin-9/Tim-3 axis and may serve as a potential biomarker and therapeutic target in pancreatic cancer.

[Disclosure of COI by Responsible researcher] Lead presenter is the Responsible researcher.
[Lead presenter: Wan-Jou Shen] We have no financial relationships to disclose.

### [P-2166] I-P11-8
### Tumor immune microenvironment/Tumor immune escape (4)

2025 / 9 / 26 (Fri) 17:30-18:15 Room P/(Motenashi Dome) Room P

---

### P-2166
### Complement c5: A Possible Linkage Molecule among diabetes and inflammation-mediated Progression of cholangiocarcinoma

[Speaker]Charat Kusoltech (Dept. of Biochem., Faculty of Med., Khon Kaen Univ., Thailand／Cholangiocarcinoma Res. Inst., Khon Kaen Univ., Thailand) [Co-author]Kullanat Khawkhiaw (Dept. of Biochem., Faculty of Med., Khon Kaen Univ., Thailand／Cholangiocarcinoma Res. Inst., Khon Kaen Univ., Thailand)、Kanyarat Thithuan (Dept. of Biochem., Faculty of Med., Khon Kaen Univ., Thailand／Cholangiocarcinoma Res. Inst., Khon Kaen Univ., Thailand)、Charupong Saengboonmee (Dept. of Biochem., Faculty of Med., Khon Kaen Univ., Thailand／Cholangiocarcinoma Res. Inst., Khon Kaen Univ., Thailand)

Diabetes is an established risk factor for cholangiocarcinoma (CCA) development and progression, but the underlying mechanisms are not fully understood. Multiple inflammatory mediators are also involved in promoting CCA. Previous studies indicated that high glucose (HG) upregulated inflammatory cytokines in CCA cells, leading to a cytokine array screening in KKU-213A under HG and normal glucose (NG) conditions in the present study. Levels of complement component 5 (c5) were found to be highest in the conditioned medium of KKU-213A in HG compared with the NG condition. Western blotting also verified the upregulation of c5 in CCA cells cultured in HG. Immunohistochemistry revealed that high c5 expression was significantly associated with the metastasis and advanced stages of CCA, which are the independent prognostic factors for CCA patients. Altogether, c5 might be a linking molecule among diabetes, inflammation, carcinogenesis, and progression of CCA. A better understanding of the functional roles of c5 in the aggressive phenotypes of CCA might suggest the development of CCA prevention and treatment both in diabetes- and inflammation-mediated carcinogenesis and advancement.

[Disclosure of COI by Responsible researcher] Lead presenter is not the Responsible researcher.
[Lead presenter: Charat Kusoltech] We have no financial relationships to disclose.
[Principal Researcher: Charupong Saengboonmee] We have no financial relationships to disclose.

### [P-2167] I-P11-8
### Tumor immune microenvironment/Tumor immune escape (4)

2025 / 9 / 26 (Fri) 17:30-18:15 Room P/(Motenashi Dome) Room P

### P-2167
### Withdrawn

No Abstract

▲TOP

### [P-2168] I-P11-8
### Tumor immune microenvironment/Tumor immune escape (4)

2025 / 9 / 26 (Fri) 17:30-18:15 Room P/(Motenashi Dome) Room P

---

### P-2168
### Single-cell RNA sequencing identifies immune evasion mechanisms in endometriosis resembling cancer

[Speaker]Ya-Ching Chou (School of Medicine, National Tsing Hua University／Institute of Molecular Medicine, National Tsing Hua University／Department of Medical Science, National Tsing Hua University)

Endometriosis shares pathological features with cancer, including immune evasion and chronic inflammation. We conducted a meta-analysis of single-cell RNA sequencing data from over 250,000 cells (ectopic, eutopic, and normal endometrial tissues). We found increased infiltration of NK cells with impaired cytotoxicity due to reduced killer inhibitory receptor expression, resembling tumor immune evasion strategies. Hormone treatments further suppressed NK cell function. Lesions were enriched with immunosuppressive M2 macrophages, while untreated lesions showed elevated pro-inflammatory M1 macrophages. Notably, IGFBP2, frequently implicated in cancer progression, was upregulated, indicating potential therapeutic targets. This study highlights immune dysfunction parallels between endometriosis and cancer, guiding future therapeutic strategies.

[Disclosure of COI by Responsible researcher] Lead presenter is the Responsible researcher.
[Lead presenter: Ya-Ching Chou] We have no financial relationships to disclose.

▲TOP

### [P-2177] I-P11-10
### Tumor immune microenvironment/Tumor immune escape (6)

2025 / 9 / 26 (Fri) 17:30-18:15 Room P/(Motenashi Dome) Room P

---

### P-2177
### Carcinogenic mechanism of IFN-STAT1-dependent hepatocellular carcinoma in past HBV infection

[Speaker]Shouta Kanari (Dept. Biomed. Eng., Sci. & Eng., Toyo Univ.) [Co-author]Yuta Ouchi (Dept. Biomed. Eng., Sci. & Eng., Toyo Univ.)、Wakana Nakaniwa (Dept. Biomed. Eng., Sci. & Eng., Toyo Univ.)、Shunsuke Shichi (Dept. Gastroenterological Surg. I, Hokkaido Univ. Grad. Sch. Med.)、Saori Kimura (Dept. Gastroenterological Surg. I, Hokkaido Univ. Grad. Sch. Med.)、Hiroki Nakamoto (Dept. Gastroenterological Surg. I, Hokkaido Univ. Grad. Sch. Med.)、Chisato Shirakawa (Dept. Gastroenterological Surg. I, Hokkaido Univ. Grad. Sch. Med.)、Akinobu Taketomi (Dept. Gastroenterological Surg. I, Hokkaido Univ. Grad. Sch. Med.)、Junya Ohtake (Res. Ctr., Biomed. Eng., Toyo. Univ.／Res. Ctr., Biomed. Eng., Toyo. Univ.)、Hidemitsu Kitamura (Dept. Biomed. Eng., Sci. & Eng., Toyo Univ.／Res. Ctr., Biomed. Eng., Toyo. Univ.／Res. Ctr., Biomed. Eng., Toyo. Univ.／Dept. Biomed. Eng., Life Sci., Toyo Univ.)

Understanding the carcinogenic mechanisms of hepatocellular carcinoma (HCC) is essential for the development of effective therapies. In this study, we analyzed HCC cases with metabolic syndrome, classified into three groups: HBc Ab+ NBNC, HBc Ab- NBNC, and HBV-associated cases. Comprehensive gene expression profiling and immune signature analysis using gene set enrichment analysis (GSEA) were performed. Additionally, the expression of immune-related factors was validated by immunohistochemical staining. The resolved HBV infection group showed activation of cytokine signaling pathways including IFN-$\alpha$, IFN-$\gamma$, and TNF-$\alpha$, as well as enhanced adaptive immune responses. Persistent activation of the IFN-STAT1 pathway was observed, which transiently promoted CD8+ T cell recruitment but subsequently led to chronic inflammation and immune evasion, potentially contributing to tumor progression. These findings suggest that targeting aberrant IFN-STAT1 signaling could be a promising therapeutic strategy for HCC. This study indicates that distinct hepatocarcinogenic mechanisms may exist in NBNC-HCC with metabolic syndrome, depending on the presence or absence of prior HBV infection.

[Disclosure of COI by Responsible researcher] Lead presenter is not the Responsible researcher.
[Lead presenter: Shouta Kanari] We have no financial relationships to disclose.
[Principal Researcher: Hidemitsu Kitamura] ■Contributions: テルモ生命科学振興財団

### [P-2178] I-P11-10
### Tumor immune microenvironment/Tumor immune escape (6)

2025 / 9 / 26 (Fri) 17:30-18:15 Room P/(Motenashi Dome) Room P

---

### P-2178
### Tertiary Lymphoid Structures in CRC: Clinical Significance and Interaction with CAFs and the Immune Microenvironment

[Speaker]Zizhou Wang (Dept. of Gastroenterological Surg.,Grad. Sch. of Med.,Osaka Metropolitan Univ.) [Co-author]Hiroaki Kasashima (Dept. of Gastroenterological Surg.,Grad. Sch. of Med.,Osaka Metropolitan Univ.)、Yukina Kusunoki (Dept. of Pathophysiol., Grad. Sch. of Med., Osaka Metropolitan. Univ.)、Yasuhiro Fukui (Dept. of Gastroenterological Surg.,Grad. Sch. of Med.,Osaka Metropolitan Univ.)、Iguru Omori (Dept. of Gastroenterological Surg.,Grad. Sch. of Med.,Osaka Metropolitan Univ.)、Hideki Tanda (Dept. of Gastroenterological Surg.,Grad. Sch. of Med.,Osaka Metropolitan Univ.)、Yuki Seki (Dept. of Gastroenterological Surg.,Grad. Sch. of Med.,Osaka Metropolitan Univ.)、Kenji Kuroda (Dept. of Gastroenterological Surg.,Grad. Sch. of Med.,Osaka Metropolitan Univ.)、Yuichiro Miki (Dept. of Gastroenterological Surg.,Grad. Sch. of Med.,Osaka Metropolitan Univ.)、Mami Yoshii (Dept. of Gastroenterological Surg.,Grad. Sch. of Med.,Osaka Metropolitan Univ.)、Tatsuro Tamura (Dept. of Gastroenterological Surg.,Grad. Sch. of Med.,Osaka Metropolitan Univ.)、Masatsune Shibutani (Dept. of Gastroenterological Surg.,Grad. Sch. of Med.,Osaka Metropolitan Univ.)、Takahiro Toyokawa (Dept. of Gastroenterological Surg.,Grad. Sch. of Med.,Osaka Metropolitan Univ.)、Naoko Ohtani (Dept. of Pathophysiol., Grad. Sch. of Med., Osaka Metropolitan. Univ.)、Kiyoshi Maeda (Dept. of Gastroenterological Surg.,Grad. Sch. of Med.,Osaka Metropolitan Univ.)

Colorectal cancer (CRC) is a major malignancy with high global mortality. The tumor immune microenvironment, especially tertiary lymphoid structures (TLS), is increasingly recognized for its role in antitumor responses. However, their specific functions in CRC and the prognostic relevance of cells within TLS remain unclear. In this study, we analyzed 166 CRC patients who underwent curative resection, evaluating TLS using HE staining and immunohistochemistry for CD20, A-SMA, CD8, and CD103. Patients were categorized by TLS density and immune cell infiltration levels. TLS were mostly located at invasive margins, and higher TLS density correlated with better prognosis ($p<0.05$). High CAF density in TLS was linked to poorer outcomes($p<0.01$), while high $CD8^{+}$ T cell presence predicted improved survival. These results suggest that TLS and their cellular composition influence CRC prognosis. In particular, CAF accumulation within TLS may modulate the immune microenvironment, possibly impairing antitumor T cell activity.

[Disclosure of COI by Responsible researcher] Lead presenter is not the Responsible researcher.
[Lead presenter: Zizhou Wang] We have no financial relationships to disclose.
[Principal Researcher: Hiroaki Kasashima] We have no financial relationships to disclose.

▲TOP

### [P-2179] I-P11-10
### Tumor immune microenvironment/Tumor immune escape (6)

2025 / 9 / 26 (Fri) 17:30-18:15 Room P/(Motenashi Dome) Room P

---

### P-2179
### Regulation of CD73 by Pyra-Metho-Carnil in Tumor Microenvironment and Its Link to Tumor-Associated Macrophage Maturation

[Speaker]Takanori Kitaguchi (Dept.Cell Biol., Fac. Med., Fukuoka Univ.) [Co-author]Toshiyuki Tsunoda (Dept.Cell Biol., Fac. Med., Fukuoka Univ.)、Kazumasa Yoshida (Dept.Cell Biol., Fac. Med., Fukuoka Univ.)、Senji Shirasawa (Dept.Cell Biol., Fac. Med., Fukuoka Univ.)

Pyra-Metho-Carnil (PMC) is a small molecule that inhibits tumor growth even in nude mice lacking T and B cells, suggesting its antitumor effect is independent of adaptive immunity. We examined PMC's impact on macrophage differentiation using a co-culture model of THP-1 monocytes with normal or tumor-mimicking spheroids. PMC suppressed THP-1 differentiation and maturation. RNA sequencing revealed downregulation of genes related to cell motility, cytokine signaling, and the NF κ B pathway. Among these, NT5E, encoding CD73 - an ecto-5'-nucleotidase producing extracellular adenosine - was downregulated by PMC. CD73 promotes immune evasion by converting ATP into immunosuppressive adenosine in the tumor microenvironment (TME). CD73 expression was elevated in KRAS-mutant colorectal cancer (CRC) cell lines in vitro, and CD73-positive macrophages were detected in KRAS-mutant human CRC tissues. Although CD73 inhibitors (CD73i) did not affect THP-1 or tumor cell proliferation, CD73i suppressed THP-1 differentiation and maturation similarly to PMC. These findings suggest that PMC exerts its antitumor effects by modulating the immunosuppressive TME via the CD73 pathway.

[Disclosure of COI by Responsible researcher] Lead presenter is not the Responsible researcher.
[Lead presenter: Takanori Kitaguchi] We have no financial relationships to disclose.
[Principal Researcher: Toshiyuki Tsunoda] We have no financial relationships to disclose.

### [P-2180] I-P11-10
### Tumor immune microenvironment/Tumor immune escape (6)

2025 / 9 / 26 (Fri) 17:30-18:15 Room P/(Motenashi Dome) Room P

### P-2180
### ICOS expression in the tumor immune microenvironment of lung adenocarcinoma

[Speaker]Hiroyuki Yamada (Cell path., Grad. Sch. of Med. Sci., Kumamoto／Thorac. & Breast Surg., Grad. Sch. of Med. Sci., Kumamoto) [Co-author]Yoshihiro Komohara (Cell path., Grad. Sch. of Med. Sci., Kumamoto)、Hirotake Tsukamoto (Clin. Immunol. & Cancer Immunother., Grad. Sch. of Med., Kyoto)、Hiromu Yano (Tumor path., Grad. Sch. of Med. Sci., Kumamoto)、Yukio Fujiwara (Cell path., Grad. Sch. of Med. Sci., Kumamoto)、Koei Ikeda (Thorac. & Breast Surg., Grad. Sch. of Med. Sci., Kumamoto)、Makoto Suzuki (Thorac. & Breast Surg., Grad. Sch. of Med. Sci., Kumamoto)

Lung cancer is the leading cause of cancer-related death worldwide. Inducible T-cell costimulator (ICOS), upregulated upon T-cell activation, has dual roles in promoting both antitumor responses and immunosuppression via regulatory T cells (Tregs). However, its role in lung adenocarcinoma (LUAD) remains unclear. HLA-DR, a class II human leukocyte antigen, is typically expressed on antigen-presenting cells but is also found on tumor cells, including LUAD. Its expression has been linked to with $CD4^+$ and $CD8^+$ tumor-infiltrating lymphocytes (TILs), suggesting possible $CD4^+$ T cell recognition. We analyzed paraffin-embedded tumor tissues from 228 LUAD patients who underwent resection at Kumamoto University Hospital between 2010 and 2013. ICOS expression and $CD4^+$ TIL density were positively correlated with HLA-DR expression. Moreover, high ICOS expression was associated with favorable postoperative survival. These findings suggest that ICOS may serve as a prognostic marker in LUAD and could interact with tumor-expressed HLA class II. Further investigations focusing on ICOS expression in Tregs and the association between ICOS expression and responsiveness to immunotherapy are ongoing.

[Disclosure of COI by Responsible researcher] Lead presenter is not the Responsible researcher.
[Lead presenter: Hiroyuki Yamada] We have no financial relationships to disclose.
[Principal Researcher: Yoshihiro Komohara] We have no financial relationships to disclose.

▲TOP

### [P-2181] I-P11-10

### Tumor immune microenvironment/Tumor immune escape (6)

2025 / 9 / 26 (Fri) 17:30-18:15 Room P/(Motenashi Dome) Room P

---

### P-2181

### Inhibition of TGF-β signals suppresses tumor formation by regulating multiple components of tumor microenvironments

[Speaker]Haruka Ibi (Inst.Science Tokyo, Grad. Sch. Med. Dent. Sci., Oral Surg. Oncology／Inst.Science Tokyo, Grad. Sch. Med. Dent. Sci., Biochemistry) [Co-author]Kai Ishihara (Inst.Science Tokyo, Grad. Sch. Med. Dent. Sci., Biochemistry／Inst.Science Tokyo, Grad. Sch. Med. Dent. Sci., Maxillofacial Surgery)、Katarzyna A. Inoue (Inst.Science Tokyo, Grad. Sch. Med. Dent. Sci., Biochemistry)、Kazuki Takahashi (Inst.Science Tokyo, Grad. Sch. Med. Dent. Sci., Biochemistry)、Takehisa Matsumoto (RIKEN Ctr. for Biosystems Dynamics Res.)、Mikako Shirouzu (RIKEN Ctr. for Biosystems Dynamics Res.)、Hiroyuki Harada (Inst.Science Tokyo, Grad. Sch. Med. Dent. Sci., Oral Surg. Oncology)、Kohei Miyazono (The Univ. of Tokyo, Grad. Sch. Med., Dept Applied Pathol.)、Tetsuro Watabe (Inst.Science Tokyo, Grad. Sch. Med. Dent. Sci., Biochemistry)

TGF-β signaling in the tumor microenvironment promotes cancer progression through various mechanisms, by inducing epithelial-mesenchymal transition (EMT) of epithelial cancer cells, promoting angiogenesis and inhibiting tumor immunity. However, the precise mechanisms how TGF-β elicits its effects in the tumor microenvironment of squamous cell carcinoma (SCC) have not been fully elucidated. In this study, we examined the effects of TGF-β inhibition on tumor progression using in vitro and in vivo immunocompetent model of mouse SCC cells. Treatment of SCC cells with a kinase inhibitor of TGF-β receptor resulted in the suppression of EMT and reduced cell migration. Furthermore, administration of the recombinant Fc chimera receptor targeting all isoforms of TGF-β significantly suppressed the tumor formation of SCC cells in vivo. We found that inhibition of TGF-β signals suppressed tumor angiogenesis, a critical factor in tumor formation, and increased the infiltration of CD8-positive T cells in the tumor microenvironment. Future studies will allow an identification of factors regulated by TGF-β signals and will lead to the development of effective treatments for SCC.

[Disclosure of COI by Responsible researcher] Lead presenter is not the Responsible researcher.
[Lead presenter: Haruka Ibi] We have no financial relationships to disclose.
[Principal Researcher: Tetsuro Watabe] We have no financial relationships to disclose.

### [P-2182] I-P11-10
### Tumor immune microenvironment/Tumor immune escape (6)

2025 / 9 / 26 (Fri) 17:30-18:15 Room P/(Motenashi Dome) Room P

### P-2182
### The impact of novel NLRC5-binding protein on anti-cancer immune response.

[Speaker]Tsutomu Tanaka (Dept. of Immunol., Grad. Sch. of Med., Hokkaido Univ.／The Inst. for Vaccine Res. & Dept (IVReD), Hokkaido Univ.)
[Co-author]Ryota Ouda (Dept. of Immunol., Grad. Sch. of Med., Hokkaido Univ.)、Noyuri Zama (Dept. of Immunol., Grad. Sch. of Med., Hokkaido Univ.)、Xin Sun (Dept. of Immunol., Grad. Sch. of Med., Hokkaido Univ.)、Koichi S. Kobayashi (Dept. of Immunol., Grad. Sch. of Med., Hokkaido Univ.／The Inst. for Vaccine Res. & Dept (IVReD), Hokkaido Univ.／Dept. of Microbial pathogenesis and Immunol., Texas A&M Univ.)

MHC-I is crucial molecule to initiate immune response against tumors. Its expression is regulated by NLRC5. We investigated a role of a novel NLRC5-binding protein, "X" in anti-cancer immune responses. [Methods] HEK293T cells were transfected with reporter gene of HLA-B promoter together with NLRC5 and gene "***X***" expression vectors, and the promoter activity was measured by luciferase assay. MHC-I expressions in control and gene "***X***" knockout (KO) HeLa cells were compared by RT-qPCR and flow cytometry. In vivo tumor growth was compared in control and gene "***X***" KO mouse breast cancer cell line, E0771. The Number and activation status of tumor infiltrating CD8 T cells were determined by flow cytometry. [Result] Protein X overexpression induced NLRC5-dependent HLA-B promoter activity. Gene "***X***" deficient HeLa and E0771 cells exhibited impaired expression of MHC-I. Increased tumor growth accompanied with poor infiltration of CD8 T cells was observed in the mice transplanted with Gene "***X***" deficient E0771 cells. [Discussion] Novel NLRC5-binding protein, "X" induces anti-tumor immune response through the regulation of MHC-I expression in cooperation with NLRC5.

[Disclosure of COI by Responsible researcher] Lead presenter is not the Responsible researcher.
[Lead presenter: Tsutomu Tanaka] We have no financial relationships to disclose.
[Principal Researcher: Koichi S. Kobayashi] We have no financial relationships to disclose.

Poster Session

### [P-2183] I-P11-10
### Tumor immune microenvironment/Tumor immune escape (6)

2025 / 9 / 26 (Fri) 17:30-18:15 Room P/(Motenashi Dome) Room P

---

### P-2183
### Immunological assessment for right-sided colon cancer

[Speaker]Naoyuki Sakamoto (Asahi University Hospital, Department of Gastroenterology) [Co-author]Mikiko Matsuo (Asahi University Hospital, Department of Pathology)、Shigeyuki Sugie (Asahi University Hospital, Department of Pathology)、Nobuaki Yagi (Asahi University Hospital, Department of Gastroenterology)

Background; Recent studies have reported that right-sided and left-sided colon differ in their characteristics due to its embryological differences. In this study, we focus on right-sided mismatch repair-deficient(R-dMMR) colon cancer, and then investigate its expression of immune checkpoint molecules of the tumor microenvironment. Methods; This study examined 152 samples of colon cancers obtained between November 2019 and July 2024. Immunohistochemical staining for DNA mismatch repair proteins were performed on them. Additionally, we investigated expressions of immune checkpoint molecules of the tumor microenvironment by immunohistochemical staining of 13 samples that were diagnosed R-dMMR colon cancer. Results; The frequency of R-dMMR colon cancer among RCC was 29.0%. We found some high PD-L1 Combined Positive Score (CPS) cases in patients with R-dMMR colon cancer. In some of these cases, immune checkpoint molecules such as TIM-3, LAG-3, and Galectin-9 were expressed in the tumor microenvironment. Conclusion; Combining PD-1/PD-L1 inhibitors with other immune checkpoint inhibitors may improve outcome of patients with R-dMMR colon cancer.

[Disclosure of COI by Responsible researcher] Lead presenter is the Responsible researcher.
[Lead presenter: Naoyuki Sakamoto] ■Research funds from company: 科研費

### [P-2184] I-P11-10
### Tumor immune microenvironment/Tumor immune escape (6)

2025 / 9 / 26 (Fri) 17:30-18:15 Room P/(Motenashi Dome) Room P

---

### P-2184
### GSEA of FGF pathway signature in TME-evaluable specimens of ccRCC

[Speaker]Takafumi Narisawa (Department of Urology, Faculty of Medicine, Yamagata University) [Co-author]Suguru Ito (Department of Urology, Faculty of Medicine, Yamagata University)、Shinta Suenaga (Department of Urology, Faculty of Medicine, Yamagata University)、Yuki Takai (Department of Urology, Faculty of Medicine, Yamagata University)、Satoshi Takai (Department of Urology, Faculty of Medicine, Yamagata University)、Mayu Yagi (Department of Urology, Faculty of Medicine, Yamagata University)、Hiromi Ito (Department of Urology, Faculty of Medicine, Yamagata University)、Sei Naito (Department of Urology, Faculty of Medicine, Yamagata University)、Norihiko Tsuchiya (Department of Urology, Faculty of Medicine, Yamagata University)

Background: The role of the FGF/FGFR pathway in ccRCC and its relationship with oncobiological function and TME remain poorly understood. Objective: To explore differences in gene expression profiles according to FGF/FGFR pathway activity and trends. Methods: RNA-seq data from 78 FFPE ccRCC specimens were analyzed. Signature scores based on the PID_FGF_pathway (MSigDB) were calculated using ssGSEA, and cases were stratified into high and low groups. GSEA was performed using CellMarker, KEGG, Reactome and results were compared with immune cell infiltration quantified by IHC. Results: The high FGF signature group showed significant enrichment of macrophage-related signatures. Although transcript levels correlated with CD68/CD163-positive cell counts, infiltration levels did not differ between groups. MSR1, IL10, and VSIG4 were significantly upregulated in the high group. IL- and chemokine-related pathways were enriched in GSEA, with increased expression of CXCL1, CXCL8, CCL2, CCL7, CCL8, CCL18, JAK2, LYN, and CDC42. Conclusion: FGF/FGFR pathway activation may promote local immunosuppressive function through inflammatory signaling, suggesting a new direction for future research.

[Disclosure of COI by Responsible researcher] Lead presenter is the Responsible researcher.
[Lead presenter: Takafumi Narisawa] ■Research funds from company: エーザイ株式会社（RNA抽出およびNGSについて、他研究で知財共有したもの

### [P-2185] I-P11-10
### Tumor immune microenvironment/Tumor immune escape (6)

2025 / 9 / 26 (Fri) 17:30-18:15 Room P/(Motenashi Dome) Room P

---

### P-2185
### CD155 expression in pathological stage I lung adenocarcinoma

[Speaker]Kazuki Takada (Dept. of Thorac. Surg., Kyushu Univ. Hosp.) [Co-author]Kyoto Matsudo (Dept. of Thorac. Surg., Kitakyushu Municipal Med. Ctr.)、Fumihiko Kinoshita (Dept. of Thorac. Surg., Kyushu Univ. Hosp.)、Naoya Iwamoto (Dept. of Thorac. Surg., Kyushu Univ. Hosp.)、Taichi Matsubara (Dept. of Thorac. Surg., Kyushu Univ. Hosp.)、Tomoyoshi Takenaka (Dept. of Thorac. Surg., Kyushu Univ. Hosp.)、Tomoharu Yoshizumi (Dept. of Surg. and Sci., Kyushu Univ.)

Background: CD155, a nectin-like transmembrane protein, is often overexpressed in cancers, but its role in stage I lung adenocarcinoma remains unclear.Methods: We retrospectively analyzed 320 patients with pathological stage I lung adenocarcinoma who underwent surgery at Kyushu University Hospital (from 2006 to 2015). CD155 expression was assessed by immunohistochemistry and classified as high or low. Clinical features and outcomes were compared between groups.Results: EGFR mutation status was available for 237 patients: 106 (33.1%) were wild-type and 131 (40.9%) mutant-type. CD155 expression was high in 77 (24.1%) and low in 243 (75.9%). High CD155 expression was independently associated with pleural invasion and EGFR wild-type status. Patients with high CD155 expression had significantly worse recurrence-free and overall survival. Multivariate analysis confirmed CD155 as an independent poor prognostic factor. In subgroup analyses, prognostic impact of CD155 expression was evident only in EGFR wild-type cases.Conclusions: High CD155 expression is linked to EGFR wild-type and may indicate poor prognosis in stage I lung adenocarcinoma, especially in EGFR wild-type patients.

[Disclosure of COI by Responsible researcher] Lead presenter is the Responsible researcher.
[Lead presenter: Kazuki Takada] We have no financial relationships to disclose.

▲TOP

### [P-2194] I-P11-12
### Immune checkpoint inhibitors (2)

2025 / 9 / 26 (Fri) 17:30-18:15 Room P/(Motenashi Dome) Room P

### P-2194
### Immune status and the efficacy of immune checkpoint inhibitors in non-small cell lung cancer patients with sarcopenia

[Speaker]Ryo Yamazaki (Dept. of Respiratory Med. & Infectious Dis., Niigata Univ.) [Co-author]Satoshi Watanabe (Dept. of Respiratory Med. & Infectious Dis., Niigata Univ.)、Takaaki Masuda (Dept. of Respiratory Med. & Infectious Dis., Niigata Univ.)、Kensuke Yanai (Dept. of Respiratory Med. & Infectious Dis., Niigata Univ.)、Yumi Ando (Dept. of Respiratory Med. & Infectious Dis., Niigata Univ.)、Tomoya Wakabayashi (Dept. of Respiratory Med. & Infectious Dis., Niigata Univ.)、Kunihiro Shono (Sado General Hosp.)、Kohei Kushiro (Dept. of Respiratory Med. & Infectious Dis., Niigata Univ.)、Ryo Suzuki (Dept. of Respiratory Med. & Infectious Dis., Niigata Univ.)、Naohiro Yanagimura (Dept. of Respiratory Med. & Infectious Dis., Niigata Univ.)、Miyuki Sato (Dept. of Respiratory Med. & Infectious Dis., Niigata Univ.)、Tomohiro Tanaka (Dept. of Respiratory Med. & Infectious Dis., Niigata Univ.)、Koichiro Nozaki (Dept. of Respiratory Med. & Infectious Dis., Niigata Univ.)、Yu Saida (Dept. of Respiratory Med. & Infectious Dis., Niigata Univ.)、Toshiaki Kikuchi (Dept. of Respiratory Med. & Infectious Dis., Niigata Univ.)

**Background:** Our previous study showed that non-small cell lung cancer (NSCLC) patients (pts) with sarcopenia had poor responses to immune checkpoint inhibitors (ICIs). Herein, we evaluated the association between immune status and ICI efficacy in NSCLC pts with sarcopenia. **Method:** Pts with advanced-NSCLC receiving ICI as first-line treatment at Niigata University Medical and Dental Hospital were eligible. Before treatment, peripheral blood mononuclear cells (PBMCs) were collected, and immune data was obtained by flow cytometry. **Result:** PBMCs were obtained from 44 pts from 2019 through 2024. Eight and nine pts were diagnosed with pre-sarcopenia and sarcopenia, respectively. The pts with pre- or sarcopenia had significantly higher % of monocytic myeloid-derived suppressor cells (MDSCs) and polymorphonuclear-MDSCs, and higher % of these cells were significantly associated with shorter OS (median, 14.4 months (95% CI, 6.67-31.7) vs 44.5 months (95% CI, 12-NR); HR 2.6; P=0.0451). **Conclusion:** MDSCs may contribute to immune suppression in NSCLC pts with sarcopenia, leading to poor OS. Strategies to overcome MDSC-mediated suppression are warranted to improve survival outcomes.

[Disclosure of COI by Responsible researcher] Lead presenter is not the Responsible researcher.
[Lead presenter: Ryo Yamazaki] We have no financial relationships to disclose.
[Principal Researcher: Satoshi Watanabe] We have no financial relationships to disclose.

▲TOP

### [P-2195] I-P11-12
### Immune checkpoint inhibitors (2)

2025 / 9 / 26 (Fri) 17:30-18:15 Room P/(Motenashi Dome) Room P

---

### P-2195
### Development of an in vivo ICI evaluation system using humanized NOG-ΔMHC mice with colorectal cancer-derived PDX

[Speaker]Asami Hanazawa (Central Inst. for Exp. Med. and Life Sci. (CIEM) [Co-author]Seinosuke Sakai (Central Inst. for Exp. Med. and Life Sci. (CIEM))、Chiyoko Nishime (Central Inst. for Exp. Med. and Life Sci. (CIEM))、Naohisa Ogo (Grad. Sch. of Pharm. Societies, Univ. of Shizuoka)、Masami Suzuki (Central Inst. for Exp. Med. and Life Sci. (CIEM))、Junichi Hata (Central Inst. for Exp. Med. and Life Sci. (CIEM))、Akira Asai (Grad. Sch. of Pharm. Societies, Univ. of Shizuoka)、Taichi Yamamoto (Central Inst. for Exp. Med. and Life Sci. (CIEM))

We previously reported an in vivo immune checkpoint inhibitor (ICI) evaluation system using an immune humanized mouse model engrafted with a lung adenocarcinoma-derived PDXs. However, when evaluating the efficacy of ICIs, it is crucial to select appropriate tumor strains based on the cancer type. To expand the range of cancer types within this system, we have established a colorectal cancer-derived PDX-engrafted animal model. Using human PBMC-transplanted MHC class I and II-deficient NOG (NOG-ΔMHC) mice engrafted with PDX-derived cell lines COL-05C or COL-08C, we evaluated the efficacy of the anti-PD-1 antibody and a novel compound, the IDO1/TDO2 dual inhibitor. Pathological analysis revealed enhanced infiltration of human $CD8^{+}$ T cells into tumor nests in both the monotherapy and combination therapy groups. In contrast, in the untreated control group, these cells remained localized in the stroma. Furthermore, the combination therapy group demonstrated significant destruction of tumor structure and showed a reduction in tumor volume. In this in vivo evaluation system, we successfully demonstrated the efficacy of ICIs against colorectal cancer.

[Disclosure of COI by Responsible researcher] Lead presenter is not the Responsible researcher.
[Lead presenter: Asami Hanazawa] We have no financial relationships to disclose.
[Principal Researcher: Taichi Yamamoto] ■employment by a company related to the presentation: インビボサイエンス株式会社
■Advisor of the company and/or profit-making organization: インビボサイエンス株式会社

[P-2196] I-P11-12
Immune checkpoint inhibitors (2)

2025 / 9 / 26 (Fri) 17:30-18:15 Room P/(Motenashi Dome) Room P

---

### P-2196
### Galectin-1 expression predicts the efficacy of anti-PD-1 therapy for non-small cell lung cancer patients

[Speaker]Kota Iwahori (Osaka International Cancer Inst. Dept. Cancer Immunotherapy) [Co-author]Mari Tone (Osaka International Cancer Inst. Dept. Cancer Immunotherapy／Osaka Univ. Dept. Respiratory Med. & Clin. Immunol.)、Yukihiro Yano (Osaka Toneyama Med. Ctr. Dept. Thoracic Oncology)、Junji Uchida (Osaka Toneyama Med. Ctr. Dept. Thoracic Oncology)、Masahide Mori (Osaka Toneyama Med. Ctr. Dept. Thoracic Oncology)、Yoshito Takeda (Osaka Univ. Dept. Respiratory Med. & Clin. Immunol.)、Atsushi Kumanogoh (Osaka Univ. Dept. Respiratory Med. & Clin. Immunol.)

Objectives: We examined the potential of galectin-1 expression to predict the efficacy of anti-PD-1 therapy for patients with non-small cell lung cancer (NSCLC).
Methods: This retrospective cohort study included NSCLC patients treated with anti-PD-1 monotherapy between November 2017 and August 2019. Galectin-1 expression in the plasma and tumor tissues of NSCLC patients was analyzed using ELISA and immunohistochemistry. The cytotoxicity of peripheral human T cells against tumor cells was assessed using bispecific T-cell engager (BiTE) technology (BiTE assay).
Results: Median progression-free survival with anti-PD-1 therapy was slightly longer in the group with low galectin-1 expression in both plasma and tumor tissues than in the group with high galectin-1 expression. T-cell cytotoxicity in the BiTE assay was stronger against galectin-1 knockout tumor cells than wild-type tumor cells.
Conclusions: Galectin-1 expression correlated with the efficacy of anti-PD-1 therapy in NSCLC patients.

[Disclosure of COI by Responsible researcher] Lead presenter is the Responsible researcher.
[Lead presenter: Kota Iwahori] ■Research funds from company: 国立研究開発法人日本医療研究開発機構

### [P-2197] I-P11-12
### Immune checkpoint inhibitors (2)

2025 / 9 / 26 (Fri) 17:30-18:15 Room P/(Motenashi Dome) Room P

---

### P-2197
### Identification and functional analysis of immune suppressor in liver cancer

[Speaker]Chihiro Nakatsutsumi (Grad. Sch. of Med. Sci., Kanazawa Univ.) [Co-author]Masao Honda (Grad. Sch. of Med. Sci., Kanazawa Univ.)、Kazuhisa Murai (Grad. Sch. of Med. Sci., Kanazawa Univ.)、Kureha Takara (Grad. Sch. of Med. Sci., Kanazawa Univ.)、Moe Kitamura (Grad. Sch. of Med. Sci., Kanazawa Univ.)

[Background]
The response rate of combination therapies such as atezolizumab plus bevacizumab or durvalumab plus tremelimumab, approved as first–line treatments for advanced liver cancer, remains around 20–30%. This study aimed to identify tumor cell–intrinsic factors causing resistance to immune checkpoint inhibitors (ICIs).
[Methods]
Inflamed "hot" tumors with high lymphocyte infiltration respond well to ICIs. GeneX was identified as inversely correlated with CD3, a T–cell marker, by DNA chip analysis of liver cancer tissues. WT and GeneX knockout (KO) MHCF5 cells (a house–established mouse liver cancer cell line) were transplanted into syngeneic C57BL/6 mice.
[Results]
GeneX KO tumors showed marked regression and increased infiltration of CD4+ T cells, CD8+ T cells, B cells, and dendritic cells compared to WT tumors. Metabolomic analysis revealed elevated intratumoral glutamine in KO tumors, suggesting enhanced immune activation.
[Conclusions]
We identified GeneX as a tumor immunosuppressor in liver cancer. Combining ICIs with GeneX–targeted therapy may offer a promising immunotherapeutic strategy.

[Disclosure of COI by Responsible researcher] Lead presenter is not the Responsible researcher.
[Lead presenter: Chihiro Nakatsutsumi] We have no financial relationships to disclose.
[Principal Researcher: Masao Honda] We have no financial relationships to disclose.

### [P-2198] I-P11-12
### Immune checkpoint inhibitors (2)

2025 / 9 / 26 (Fri) 17:30-18:15 Room P/(Motenashi Dome) Room P

---

### P-2198
### scRNA and scTCR sequencing of liver-infiltrating T cells and antigen search in fulminant hepatitis after ICI therapy

[Speaker]Hiroki Kobayashi (Dept. Pathol., Sapporo Med. Univ., Sch. Med.) [Co-author]Naoki Shijubou (Dept. Pathol., Sapporo Med. Univ., Sch. Med.)、Terufumi Kubo (Dept. Pathol., Sapporo Med. Univ., Sch. Med.)、Kenji Murata (Dept. Pathol., Sapporo Med. Univ., Sch. Med.)、Kenta Sasaki (Dept. Pathol., Sapporo Med. Univ., Sch. Med.)、Toshihiko Torigoe (Dept. Pathol., Sapporo Med. Univ., Sch. Med.)、Yoshihiko Hirohashi (Dept. Pathol., Sapporo Med. Univ., Sch. Med.)

Immune checkpoint inhibitors (ICIs) can elicit remarkable clinical responses, but are frequently associated with immune-related adverse events (irAEs). While antigen-specific T cell responses have been implicated in irAE pathogenesis, the characteristics of T cells infiltrating affected organs and the antigens they recognize remain largely unknown.We analyzed a case of renal cell carcinoma that showed a favorable response to first-line combination therapy with ipilimumab and nivolumab but resulted in death due to fulminant hepatitis as an irAE. Using liver tissue obtained from autopsy, we performed single-cell RNA sequencing (scRNA-seq) and single-cell T cell receptor sequencing (scTCR-seq) on infiltrating T cells to investigate their transcriptional profiles and clonal expansion. scTCR-seq revealed prominent clonal expansion of CD8 positive T cells. The second most abundant TCR clone exhibited a memory T cell phenotype, and TCR-transduced T cells derived from this clone demonstrated a specific immune response against the hepatoblastoma cell line HepG2. Further investigations are underway to identify the cognate antigen recognized by these T cells.

[Disclosure of COI by Responsible researcher] Lead presenter is not the Responsible researcher.
[Lead presenter: Hiroki Kobayashi] We have no financial relationships to disclose.
[Principal Researcher: Terufumi Kubo] We have no financial relationships to disclose.

▲TOP

### [P-2199] I-P11-12
### Immune checkpoint inhibitors (2)

2025 / 9 / 26 (Fri) 17:30-18:15 Room P/(Motenashi Dome) Room P

---

### P-2199
### Development of NK humanized mouse models for the in vivo evaluation of NK cell-based therapeutics

[Speaker]Dong(WuXi AppTec) Wang (In Vivo Pharmacology Unit, WuXi Biology, WuXi AppTec, China) [Co-author]Zhi X. Zhang (In Vivo Pharmacology Unit, WuXi Biology, WuXi AppTec, China)、Zhong B. Song (In Vivo Pharmacology Unit, WuXi Biology, WuXi AppTec, China)、Xiang N. Qiang (In Vivo Pharmacology Unit, WuXi Biology, WuXi AppTec, China)、Qing Y. Gu (In Vivo Pharmacology Unit, WuXi Biology, WuXi AppTec, China)

In this study, we developed a humanized mouse model for the specific reconstitution of functional and durable human NK cells within a murine system. In this model, human NK cells were purified from peripheral blood mononuclear cells (PBMCs) using magnetic bead separation, expanded in vitro, and subsequently engrafted into human IL-15 transgenic NOG mice. We collected blood samples from these mice to monitor human NK cell kinetics and profile using flow cytometry. The therapeutic efficacy of an anti-CD20 antibody was then evaluated in NK-humanized model using the SU-DHL-2 subcutaneous xenograft tumor model. Results indicated that anti-CD20 treatment was ineffective in non-NK-humanized mice. In contrast, in NK-humanized mice, human NK cells significantly inhibited tumor growth via antibody-dependent cellular cytotoxicity (ADCC). Additionally, we assessed NK cell intratumoral infiltration and the expression of activation markers at the end of the in vivo efficacy study, providing further insights into the immune response in the tumor microenvironment.

[Disclosure of COI by Responsible researcher] Lead presenter is the Responsible researcher.
[Lead presenter: Dong Wang] We have no financial relationships to disclose.

### [P-2200] I-P11-12
### Immune checkpoint inhibitors (2)

2025 / 9 / 26 (Fri) 17:30-18:15 Room P/(Motenashi Dome) Room P

### P-2200
### Withdrawn

No Abstract

### [P-2201] I-P11-12
### Immune checkpoint inhibitors (2)

2025 / 9 / 26 (Fri) 17:30-18:15 Room P/(Motenashi Dome) Room P

### P-2201
### Withdrawn

No Abstract

### [P-2202] I-P11-12
### Immune checkpoint inhibitors (2)

2025 / 9 / 26 (Fri) 17:30-18:15 Room P/(Motenashi Dome) Room P

---

### P-2202
### Immune Checkpoint Inhibitors for Advanced Triple Negative Breast Cancer Patients: A Bayesian Network Meta Analysis

[Speaker]I Gede Wikania Wira Wiguna (Graduated Student, Faculty of Medicine, Udayana University, Indonesia) [Co-author]I Gede Krisna Arim Sadeva (Graduated Student, Faculty of Medicine, Udayana University, Indonesia)、Christo Timothy Mamangdean (Graduated Student, Faculty of Medicine, Udayana University, Indonesia)、Ngakan Putu Krishna Mahayana (Graduated Student, Faculty of Medicine, Udayana University, Indonesia)、Suparada Khanaruksombat (Department of Immunology, Faculty of Medicine, Siriraj Hospital, Mahidol University／Siriraj Center of Research Excellence for Cancer Immunotherapy, Research Department)、Nattiya Hirankarn (Department of Microbiology, Faculty of Medicine, Chulalongkorn University, Bangkok, Thailand)、May Soe Thu (Department of Microbiology, Faculty of Medicine, Chulalongkorn University, Bangkok, Thailand)、Putu Mirah Wahyu Subagia Putri (Undergraduated Student, Faculty of Medicine, Udayana University, Indonesia)、Kadek Meryndha Kumala Tungga (Undergraduated Student, Faculty of Medicine, Udayana University, Indonesia)、Agung Brahmanthya Nadine Kepakisan (Undergraduated Student, Faculty of Medicine, Udayana University, Indonesia)、Komang Indra Parama Arta (Undergraduated Student, Faculty of Medicine, Udayana University, Indonesia)、I Komang Raditya Putra Pratama (Undergraduated Student, Faculty of Medicine, Udayana University, Indonesia)

Introduction: Limited evidence compares the relative efficacy of different immune checkpoint inhibitor (ICI) regimens, and the FDA is currently reconsidering the approval of ICIs for advanced TNBC, highlighting the need for further studies. Methods: A systematic review via Bayesian network meta analysis was conducted following the PRISMA guidelines. Randomized controlled trial (RCT) studies reporting survival outcomes in patients with advanced TNBC were included. Progression free survival (PFS) was analyzed using hazard ratio (HR) and synthesized via indirect comparison using R Software v.4.4.0. Results: Four RCTs were included (n=1144). Indirect comparison for both treatments compared to placebo showed a better 1-year PFS in PD-L1$^{+}$ (atezolizumab; intention to treat (ITT) HR=0.73; CrI 0.56-0.95 vs PD-L1$^{+}$ HR=0.66; CrI 0.46-0.93; pembrolizumab; ITT HR=0.66; CrI 0.46-0.94 vs PD-L1$^{+}$ HR=0.59; CrI 0.39-0.89). Forest plot comparing both treatments showed no significant difference in ITT and PD-L1$^{+}$ population. Conclusion: Both atezolizumab and pembrolizumab demonstrated comparable efficacy in improving PFS in patients with advanced TNBC, particularly among those with PD-L1 positive tumors.

[Disclosure of COI by Responsible researcher] Lead presenter is the Responsible researcher.
[Lead presenter: I Gede Wikania Wira Wiguna] We have no financial relationships to disclose.

### [P-2212] I-P12-4
### Diagnosis by tumor markers & biomarkers

2025 / 9 / 26 (Fri) 17:30-18:15 Room P/(Motenashi Dome) Room P

---

### P-2212
### Prognostic Significance of EMT Markers in ARID1A-Deficient Colorectal Cancer: A Clinicopathological Study

[Speaker]Phattarapon Sonthi (Bioinform. & Genom., Adv. Prev. Med. Sci., Kanazawa Univ.) [Co-author]Keerakarn Somsuan (Sch. of Med., Mae Fah Luang Univ.,Thailand／Cancer & Immunol. Res. Unit, Mae Fah Luang Univ.,Thailand)、Ratirath Samol (Unit of Path., Sawan Pracharak Hosp.,Thailand)、Natthiya Sakulsak (Dept. of Anat., Faculty of Med. Sci., Naresuan Univ., Thailand／Faculty of Med., Praboromarajchanok Inst. Ministry of Public Health, Thailand)

Metastasis, driven by epithelial-mesenchymal transition (EMT), has been suggested as one of the major causes of mortality in colorectal cancer (CRC) and regulated by ARID1A, a tumour suppressor. However, its prognostic significance in CRC remains unclear. The immunoreactivity of EMT markers; ZO-1, fibronectin (FN), and TWIST1 in CRC tissues with low ARID1A expression and their clinicopathological relevance were evaluated in 30 CRC samples. It showed significantly decreased ZO-1 whereas increased FN and TWIST1 expressions in tumour compared to those in adjacent non-cancerous areas and significantly correlated with ARID1A expression. Low ZO-1 and high TWIST1 levels were associated with advanced clinicopathological features in Thai CRC patients. Kaplan-Meier analysis revealed that patients with low ZO-1 and high FN and TWIST1 had significantly shorter progression-free survival. Multivariate Cox regression identified ZO-1, histological type, and AJCC stage IV as independent prognostic factors. These findings clarify the association between ARID1A and EMT markers expressions, suggesting their potential utility as prognostic biomarkers to enhance clinical outcomes in CRC.

[Disclosure of COI by Responsible researcher] Lead presenter is not the Responsible researcher.
[Lead presenter: Phattarapon Sonthi] We have no financial relationships to disclose.
[Principal Researcher: Natthiya Sakulsak] ■Research expenses: This study was supported by Thailand Science Research and Innovation (TSRI); grant number R2564B002 and Faculty of Medical Science, Naresuan University, Phitsanulok, Thailand.

### [P-2213] I-P12-4
### Diagnosis by tumor markers & biomarkers

2025 / 9 / 26 (Fri) 17:30-18:15 Room P/(Motenashi Dome) Room P

---

### P-2213
### Withdrawn

No Abstract

▲TOP

### [P-2214] I-P12-4
### Diagnosis by tumor markers & biomarkers

2025 / 9 / 26 (Fri) 17:30-18:15 Room P/(Motenashi Dome) Room P

---

### P-2214
### Combination of urinary free-glycan markers for the diagnosis of various malignant tumors

[Speaker]Yasuhide Miyamoto (Dept. Clin. Lab., Osaka Intl. Cancer Inst.) [Co-author]Miki Okamoto (Dept. Glyco-Oncol., Osaka Intl. Cancer Inst.)、Ken Hanzawa (Glycoanal. Chem. Lab., iGCORE, Gifu Univ.)、Takashi Yamamoto (Dept. Clin. Lab., Osaka Intl. Cancer Inst.)、Tomoki Michida (Dept. Gastrointestinal Oncol., Osaka Intl. Cancer Inst.)、Kazuyoshi Ohkawa (Dept. Hepatobiliary and Pancreatic Oncol., Osaka Intl. Cancer Inst.)、Kazumi Nishino (Dept. Thoracic Oncol., Osaka Intl. Cancer Inst.)、Takafumi Yokota (Dept. Hematol., Osaka Intl. Cancer Inst.)、Michihide Maeda (Dept. Gynecol., Osaka Intl. Cancer Inst.)、Kenichi Yoshida (Dept. Diag. Path. and Cytol., Osaka Intl. Cancer Inst.)、Satoshi Takenaka (Dept. Orthopedic Surg., Osaka Intl. Cancer Inst.)、Masayuki Ohue (Dept. Gastroenterol. Surg., Osaka Intl. Cancer Inst.)、Tomoyuki Yamasaki (Dept. Clin. Lab., Osaka Intl. Cancer Inst.)

Many urinary free-glycan markers were identified in our previous studies. The clinical utility of these markers was examined. Urine samples taken from 120 healthy subjects and 503 patients with various malignant tumors were analyzed. Four lactose-core glycans containing N-glycolylglucosamine (GlcNGc) were synthesized and used as internal standards (ISs). Free-glycans were isolated, pyridylaminated, and subjected to LC-selected reaction monitoring. Twelve markers composed of three sialyl lactose-core glycans, three sulfated glycans, four Gn1-core free-N-glycans, and two glycans with unusual structures were selected to investigate their effectiveness for cancer diagnosis. Markers were simultaneously measured and the relative area ratio (marker/IS) quantified. ROC curve analyses revealed that individual markers were not sufficient for highly accurate diagnosis. However, a combination of four markers resulted in higher AUC of 0.910, 0.867, and 0.914 for gastric, colorectal, and pancreatic cancers, respectively. Moreover, levels of these markers were elevated in various other malignancies. These analyses demonstrated high clinical utility of the free-glycan markers.

[Disclosure of COI by Responsible researcher] Lead presenter is the Responsible researcher.
[Lead presenter: Yasuhide Miyamoto] We have no financial relationships to disclose.

[P-2215] I-P12-4

### Diagnosis by tumor markers & biomarkers

2025 / 9 / 26 (Fri) 17:30-18:15 Room P/(Motenashi Dome) Room P

---

P-2215

### Serum D-asparagine concentration adjusted for eGFR is a promising blood-based screening marker for urothelial carcinoma

[Speaker]Shunsuke Inoguchi (Department of Urology, The University of Osaka) [Co-author]Akinaru Yamamoto (Department of Urology, The University of Osaka)、Atsunari Kawashima (Department of Urology, The University of Osaka)、Hiromu Horitani (Department of Urology, The University of Osaka)、Sassi Nesrine (Department of Urology, The University of Osaka)、Yuki Horibe (Department of Urology, The University of Osaka)、Toshihiro Uemura (Department of Urology, The University of Osaka)、Yu Ishizuya (Department of Urology, The University of Osaka)、Takuji Hayashi (Department of Urology, The University of Osaka)、Yoshiyuki Yamamoto (Department of Urology, The University of Osaka)、Taigo Kato (Department of Urology, The University of Osaka)、Koji Hatano (Department of Urology, The University of Osaka)、Yoichi Kakuta (Department of Urology, The University of Osaka)、Tomonori Kimura (Department of Nephrology, The University of Osaka)、Norio Nonomura (Department of Urology, The University of Osaka)

Introduction & objective: While urinary-based tests such as occult blood or cytology are used for urothelial carcinoma (UC) screening, their sensitivity is limited, especially in early-stage disease. This study aimed to establish a novel blood-based screening method for UC.Method: We measured 22 D-amino acids (DAAs) level in 108 patients with UC and 192 patients without UC in derivation cohort, and 15 patients with UC and 25 patients without UC in validation cohort. Serum DAAs were quantified using a 2D-HPLC platform. We conducted five-fold cross-validation to assess the diagnostic ability of DAAs for UC.Results: Only D-alanine, D-asparagine, D-proline, and D-serine could be detected in human serum. These DAAs level were significantly higher in patients with UC than those in patients without UC. Serum D-asparagine level had the best diagnostic ability (sensitivity: 79.6%, specificity: 79.7%). Serum D-asparagine level adjusted for log10(eGFR) showed high performance to diagnose UC (AUC-ROC: 0.869, sensitivity: 80.6%, specificity: 82.7%).Conclusions: Serum D-asparagine level adjusted for log10(eGFR) could be a novel useful screening tool for UC.

[Disclosure of COI by Responsible researcher] Lead presenter is not the Responsible researcher.
[Lead presenter: Shunsuke Inoguchi] We have no financial relationships to disclose.
[Principal Researcher: Atsunari Kawashima] We have no financial relationships to disclose.

### [P-2216] I-P12-4
### Diagnosis by tumor markers & biomarkers

2025 / 9 / 26 (Fri) 17:30-18:15 Room P/(Motenashi Dome) Room P

---

### P-2216
### Proteogenomics-based comprehensive profiling of autoantibody-bound novel cancer antigens

[Speaker]Hisanori Isomura (Nagoya City Univ. Grad. Sch. of Med. Sci. Molecular Oncology) [Co-author]Yasuhide Okumoto (Nagoya City Univ. Grad. Sch. of Med. Sci. Molecular Oncology)、Yongwoon Han (Nagoya City Univ. Grad. Sch. of Med. Sci. Molecular Oncology)、Ayumu Taguchi (Nagoya City Univ. Grad. Sch. of Med. Sci. Molecular Oncology)

Colorectal cancer (CRC) shows a steep drop in 5 year survival, from over 95% in Stage I to ~20% in Stage IV. Most CRCs develop from adenomas, and early-stage lesions are treatable by endoscopic resection. Early detection is thus crucial for minimally invasive, cost-effective intervention. Circulating autoantibodies against tumor antigens emerge at early stages of tumorigenesis, making them attractive biomarkers for early detection. Recent studies have shown that functional microproteins can arise from circRNAs and lncRNAs, previously considered non-coding, and that non AUG translation can yield novel proteins. These noncanonical proteins are often tumor-specific and immunogenic, and likely targets of humoral immune response.We developed a mass spectrometry based platform that profiles over 1,000 autoantibody-bound antigens per sample, along with a proteogenomic pipeline to detect noncanonical proteins. In CRC patient plasma, we identified 398 peptides from circRNA and lncRNA derived proteins. Selected candidates were validated by qPCR and targeted proteomics. This strategy is being applied to other cancers to build a pancancer autoantibody antigen atlas for early detection.

[Disclosure of COI by Responsible researcher] Lead presenter is not the Responsible researcher.
[Lead presenter: Hisanori Isomura] ■Research funds from company: 科研費　若手研究
[Principal Researcher: Ayumu Taguchi] ■Research expenses: 小野薬品工業株式会社

[P-2217] I-P12-4
Diagnosis by tumor markers & biomarkers

2025 / 9 / 26 (Fri) 17:30-18:15 Room P/(Motenashi Dome) Room P

---

### P-2217
### Identification of candidate molecules for disease glycobiomarkers by data-driven analysis

[Speaker]Hidemi Arakawa (Graduate School of Science and Engineering, Soka University) [Co-author]Kiyohiko Angata (Glycan & Life Systems Integration Center ／ GalSIC)、Kiyoko F. Kinoshita (Glycan & Life Systems Integration Center ／ GalSIC)、Akiko Tozawa (Department of Obstertrics and Gynecology, St.Marianna University School of Medicine)、Akira Togayachi (Graduate School of Science and Engineering, Soka University／Glycan & Life Systems Integration Center ／ GalSIC)

Cervical cancer (CC) has various examination methods including cytological and colposcopic diagnosis using magnification. However, these methods impose a physical burden on patients and sometimes fail to confirm the diagnosis. In this study, we focused on CC as the target disease, the relationship between pathological conditions and changes in glycan structures and identify for potential tumor glycobiomarkers.Using public database information, we analyzed the expression levels of glycan-related genes and their alterations in cultured cell lines and cancer patient samples.Subsequently, we further analyzed the changes in glycan biosynthesis pathways for each histological subtype of CC (e.g. squamous cell carcinoma: SCC, adenocarcinoma: AC) separately for ***N***-glycan and ***O***-glycan synthesis pathways.Our finding revealed that the respective gene expression levels of ***N***-glycan and ***O***-glycan in SCC and AC tended to be higher in specific synthetic pathways, such as blood group glycoprotein antigens.Furthermore, bioinformatics analyses were utilized to identify candidate molecules for tumor markers (specific glycan structures) by visualizing differences in tumorigenesis and each tissue subtype.

[Disclosure of COI by Responsible researcher] Lead presenter is not the Responsible researcher.
[Lead presenter: Hidemi Arakawa] We have no financial relationships to disclose.
[Principal Researcher: Akira Togayachi] We have no financial relationships to disclose.

### [P-2218] I-P12-4
### Diagnosis by tumor markers & biomarkers

2025 / 9 / 26 (Fri) 17:30-18:15 Room P/(Motenashi Dome) Room P

---

### P-2218
### MicroRNA-Based Identification of Novel Basal-like Subtypes in Post-Treatment Pancreatic Cancer

[Speaker]Kota Nakamura (Department of Surger, Nara Medical University) [Co-author]Minako Nagai (Department of Surger, Nara Medical University)、Satoshi Yasuda (Department of Surger, Nara Medical University)、Yasuko Matsuo (Department of Surger, Nara Medical University)、Yuichiro Kohara (Department of Surger, Nara Medical University)、Shunsuke Doi (Department of Surger, Nara Medical University)、Takeshi Sakata (Department of Surger, Nara Medical University)、Masayuki Sho (Department of Surger, Nara Medical University)

Background: Neoadjuvant therapy alters pancreatic cancer genetics and post-radiotherapy nucleic acid degradation challenges traditional subtyping. We used microRNAs, resistant to degradation, to develop a novel clustering method for pancreatic cancer subtypes.Methods: Key miRNAs were identified using TCGA (N=150) and CPTAC (N=123) datasets. We analyzed RNA from 167 post-chemoradiotherapy surgical samples with molecular and clinical analyses.Results: Five miRNAs were highly expressed in the basal-like subtype. Four prognostic miRNAs revealed two subtypes: Neo-Basal-like (NBL, N=81) and Neo-Classical (NC, N=86). NBL showed higher CA19-9 levels and worse survival (43 vs 66 months, P=0.004). Post-recurrence survival differed significantly (13 vs 19 months, P=0.016), suggesting NBL chemotherapy resistance. Multivariate analysis confirmed NBL as an independent poor prognostic factor.Conclusion: miRNA-based clustering reproduced basal-like characteristics in post-chemoradiation tissues, identifying a prognostic factor with implications for future pancreatic cancer therapeutic strategies.

[Disclosure of COI by Responsible researcher] Lead presenter is the Responsible researcher.
[Lead presenter: Kota Nakamura] We have no financial relationships to disclose.

[P-2219] I-P12-4

Diagnosis by tumor markers & biomarkers

2025 / 9 / 26 (Fri) 17:30-18:15 Room P/(Motenashi Dome) Room P

P-2219

### ADAR1 can be a effective biomarker for colorectal cancer patients with synchronous liver metastasis

[Speaker]Kazuya Moriwake (Dept. of Gastroenterological Surg. Okayama Univ.) [Co-author]Kunitoshi Shigeyasu (Dept. of Gastroenterological Surg. Okayama Univ.)、Kaori Nitta (Dept. of Gastroenterological Surg. Okayama Univ.)、Yuhei Kondo (Dept. of Gastroenterological Surg. Okayama Univ.)、Eiki Miyake (Dept. of Gastroenterological Surg. Okayama Univ.)、Yoshitaka Kondo (Dept. of Gastroenterological Surg. Okayama Univ.)、Yuki Matsumi (Dept. of Gastroenterological Surg. Okayama Univ.)、Tomokazu Fuji (Dept. of Gastroenterological Surg. Okayama Univ.)、Kazuya Yasui (Dept. of Gastroenterological Surg. Okayama Univ.)、Kosei Takagi (Dept. of Gastroenterological Surg. Okayama Univ.)、Masashi Kayano (Dept. of Gastroenterological Surg. Okayama Univ.)、Shunsuke Nakamura (Dept. of Gastroenterological Surg. Okayama Univ.)、Nobuhiko Kanaya (Dept. of Gastroenterological Surg. Okayama Univ.)、Hiroshi Tazawa (Dept. of Gastroenterological Surg. Okayama Univ.)、Shunsuke Kagawa (Dept. of Gastroenterological Surg. Okayama Univ.)、Toshiyoshi Fujiwara (Dept. of Gastroenterological Surg. Okayama Univ.)

Background: Colorectal cancer (CRC) with synchronous liver metastasis often has aggressive clinical course, and effective biomarkers to predict prognosis and treatment response remain limited. RNA editing by adenosine deaminases acting on RNA (ADAR) enzymes plays a role in cancer progression; however, its clinical significance in metastatic CRC remains unclear.Methods: We evaluated ADAR1 protein expression in tumor tissues from 40 patients with Stage IV CRC and synchronous liver metastases and analyzed clinicopathological outcomes, overall survival (OS), and responsibility to chemotherapy.Results: High ADAR1 expression was significantly associated with multiple liver metastases and reduced responsibility to chemotherapy. Patients with high nucleus ADAR1 expression had significantly shorter OS. In multivariate analysis, ADAR1 expression was an independent prognostic factor. Additionally, low ADAR1 expression correlated with better responsibility to chemotherapy.Conclusion: ADAR1 has potential utility as a dual-purpose biomarker for predicting prognosis and optimizing treatment intensity.

[Disclosure of COI by Responsible researcher] Lead presenter is not the Responsible researcher.
[Lead presenter: Kazuya Moriwake] We have no financial relationships to disclose.
[Principal Researcher: Kunitoshi Shigeyasu] We have no financial relationships to disclose.

[P-2220] I-P12-4
Diagnosis by tumor markers & biomarkers

2025 / 9 / 26 (Fri) 17:30-18:15 Room P/(Motenashi Dome) Room P

P-2220
### A novel index including age, sex, hTERT, and methylated RUNX3 is useful for predicting early gastric cancer

[Speaker]Yutaka Suehiro (Department of Oncology and Laboratory Medicine, Yamaguchi University／Division of Laboratory, Yamaguchi University Hospital／Division of Medical Genetics, Yamaguchi University Hospital) [Co-author]Katsuhiko Nakamura (Department of Gastroenterology and Hepatology, Yamaguchi University)、Yuki Kunimune (Division of Laboratory, Yamaguchi University Hospital)、Koichi Hamabe (Department of Gastroenterology and Hepatology, Yamaguchi University)、Shunsuke Ito (Department of Gastroenterology and Hepatology, Yamaguchi University)、Yuko Yamaoka (Department of Gastroenterology and Hepatology, Yamaguchi University)、Atsushi Goto (Department of Gastroenterology and Hepatology, Yamaguchi University)、Shinichi Hashimoto (Division of Endoscopy, Yamaguchi University Hospital)、Mitsuaki Nishioka (Division of Laboratory, Yamaguchi University Hospital)、Jun Nishikawa (Faculty of Laboratory Science, Yamaguchi University)、Taro Takami (Department of Gastroenterology and Hepatology, Yamaguchi University)、Takahiro Yamasaki (Department of Oncology and Laboratory Medicine, Yamaguchi University／Division of Laboratory, Yamaguchi University Hospital)

**Purpose:** We conducted this study to develop a simple blood test for early gastric cancer (GC).
**Methods:** We collected serum samples from 319 participants, including 225 healthy subjects without GC and 94 with early GC. After evaluating the copy number of serum hTERT and methylated RUNX3 (m-RUNX3) using our proprietary methylation-sensitive restriction enzyme assay, the combined restriction digital PCR (CORD) assay, we evaluated the diagnostic performance of hTERT and m-RUNX3 for early GC.
**Results:** Serum levels of hTERT and m-RUNX3 were significantly higher in the early GC group than in the control group. Multivariate logistic regression analysis revealed that age, sex, hTERT copy number, and m-RUNX3 copy number were independent factors for early GC. We then developed a prediction formula, named the ASTEm-R3 index. The area under the receiver operating characteristic curve of the ASTEm-R3 index was 0.93 with 79.7% sensitivity and 91.1% specificity.
**Conclusions:** We demonstrated the excellent performance of the ASTEm-R3 index using the CORD assay for the detection of early GC. This index may be a promising alternative to upper gastrointestinal endoscopy.

[Disclosure of COI by Responsible researcher] Lead presenter is the Responsible researcher.
[Lead presenter: Suehiro Yutaka] ■Research expenses: 栄研化学(株)　■Contributions: 栄研化学(株)

[P-2230] I-P13-4
### Molecular targeted therapy

2025 / 9 / 26 (Fri) 17:30-18:15 Room P/(Motenashi Dome) Room P

---

P-2230
### Evaluation of the Effects of the NAMPT Inhibitor FK866 on Cell Proliferation in Colorectal Cancer Cells

[Speaker]Dongyu Luo (Natl. Cancer Ctr. Res. Inst.) [Co-author]Haruna Takeda (Natl. Cancer Ctr. Res. Inst.)

Colorectal cancer (CRC) is the second most common cancer in the world, and APC mutations are observed in nearly 80% of CRC. Recently, the NAD+ synthesis pathway has attracted attention as a therapeutic target, and FK866, which targets the NAMPT enzyme, has been investigated as a candidate anticancer drug. However, the effects of FK866 on CRC have not been fully elucidated. In this study, we used APC wild-type CRC cell lines, APC knockout cell lines, and cell lines carrying mutations in APC for further analysis. FK866 was added, and the effect on cell proliferation was evaluated. We also analyzed the expression level of NAMPT protein. We found that HCT116 cells were more sensitive to FK866, but RKO and SW480 cells were less sensitive, indicating that the effect of FK866 differed among cell lines. Furthermore, APC knockout cells were more sensitive to FK866 than the wild-type counterpart, possibly due to decreased NAMPT expression. This suggests that Wnt signaling overactivation may be involved in the decreased NAMPT expression. We will analyze the regulatory mechanisms of APC mutations and NAMPT expression to stratify colorectal cancers predicted to benefit from FK866 therapy.

[Disclosure of COI by Responsible researcher] Lead presenter is not the Responsible researcher.
[Lead presenter: Dongyu Luo] We have no financial relationships to disclose.
[Principal Researcher: Haruna Takeda] We have no financial relationships to disclose.

### [P-2231] I-P13-4
### Molecular targeted therapy

2025 / 9 / 26 (Fri) 17:30-18:15 Room P/(Motenashi Dome) Room P

---

### P-2231
### Targeting EGFR and STING Synergistically Enhances Pancreatic Cancer Therapy

[Speaker]Ze-Syuan Chen (Dept. of Biol. Sci. & Tech., CMU) [Co-author]Yu-Hsin Hsu (Dept. of Biol. Sci. & Tech., CMU)、Wei-Jan Wang (Dept. of Biol. Sci. & Tech., CMU)

Pancreatic cancer is often diagnosed at advanced stages, leading to poor prognosis and limited treatment efficacy. While surgical resection offers the best outcome, few patients qualify, leaving most reliant on systemic therapies with modest survival benefits. EGFR is overexpressed in 30%-95% of pancreatic cancers and is linked to disease progression and metastasis, making it a key therapeutic target. However, current EGFR inhibitors only extend survival by 2-4 weeks. In this study, we show that EGFR inhibition upregulates STING, triggering immune and metabolic activation in tumor cells. Increased STING expression enhances EGFR inhibitor sensitivity and cytotoxicity. Co-inhibition of EGFR and STING further increases cancer cell death, suggesting STING as a modulator of EGFR-driven tumorigenesis. EGFR inhibition also reduces $\alpha$-ketoglutarate ($\alpha$-KG), indicating its role in glutamine metabolism. Notably, combined EGFR/STING inhibition significantly suppresses tumor growth in wild-type p53 (wtp53) cells, but not in mutant p53 (mtp53) cells. These9 findings support STING-based therapies to improve EGFR-targeted treatment in pancreatic cancer.

[Disclosure of COI by Responsible researcher] Lead presenter is the Responsible researcher.
[Lead presenter: Ze-Syuan Chen ] We have no financial relationships to disclose.

### [P-2232] I-P13-4
### Molecular targeted therapy

2025 / 9 / 26 (Fri) 17:30-18:15 Room P/(Motenashi Dome) Room P

P-2232

### PVZB1077 Induces Mitochondrial Fragmentation and Cell Death in Nutrient-Stressed Pancreatic Cancer Cells

[Speaker]Yuka Unno (Div. Mol. & Cell. Oncol., Miyagi Cancer Ctr. Res. Inst.／Ctr. Drug Discovery, Grad. Sch. Pharm. Sci., Univ. Shizuoka) [Co-author]Haruna Fujimori (Div. Cancer Stem Cell., Miyagi Cancer Ctr. Res. Inst.)、Yoko Yashiroda (RIKEN CSRS)、Naohisa Ogo (Ctr. Drug Discovery, Grad. Sch. Pharm. Sci., Univ. Shizuoka)、Nao Miyoshi (Ctr. Drug Discovery, Grad. Sch. Pharm. Sci., Univ. Shizuoka)、Mami Yoshimura (RIKEN CSRS)、Kaori Honda (RIKEN CSRS)、Junichi Sawada (Ctr. Drug Discovery, Grad. Sch. Pharm. Sci., Univ. Shizuoka)、Hiroyuki Osada (RIKEN CSRS)、Naoshi Domae (RIKEN CSRS)、Naoki Asano (Div. Cancer Stem Cell., Miyagi Cancer Ctr. Res. Inst.)、Minoru Yoshida (RIKEN CSRS)、Jun Yasuda (Div. Mol. & Cell. Oncol., Miyagi Cancer Ctr. Res. Inst.)、Akira Asai (Ctr. Drug Discovery, Grad. Sch. Pharm. Sci., Univ. Shizuoka)

PVZB1077 is a novel small-molecule compound that selectively induces cytotoxicity in PANC-1 pancreatic cancer cells under hypoxic and glucose-deprived conditions. This compound was identified through a screening of approximately 100,000 chemicals. PVZB1077 was found to interact with DRP1, a key regulator of mitochondrial fission, as indicated by LC-MS/MS analysis of proteins pulled down with PVZB1077-conjugated beads. In contrast, a structurally related compound, PVZB1078, showed no such interaction. PVZB1077 promoted mitochondrial fragmentation, which was followed by ATP depletion, loss of mitochondrial membrane potential, and increased mitochondrial ROS production, ultimately leading to cell death. These effects were not observed with PVZB1078. The data suggest that PVZB1077 may exert its selective cytotoxicity by perturbing mitochondrial dynamics through a potential interaction with DRP1. This mechanism offers a promising strategy for targeting therapy-resistant pancreatic cancer cells under nutrient stress.

[Disclosure of COI by Responsible researcher] Lead presenter is the Responsible researcher.
[Lead presenter: Yuka Unno] We have no financial relationships to disclose.

### [P-2233] I-P13-4
### Molecular targeted therapy

2025 / 9 / 26 (Fri) 17:30-18:15 Room P/(Motenashi Dome) Room P

---

### P-2233
### Potential of Autotaxin-Targeted Therapy for Biliary Tract Cancer

[Speaker]Kenji Urakabe (Department of Gastroenterology and Metabolism, NCU Graduate School) [Co-author]Hidenori Sahashi (Department of Gastroenterology and Metabolism, NCU Graduate School)、Michihiro Yoshida (Department of Gastroenterology and Metabolism, NCU Graduate School)、Yusuke Kito (Department of Gastroenterology and Metabolism, NCU Graduate School)、Tadashi Toyohara (Department of Gastroenterology and Metabolism, NCU Graduate School)、Akihisa Kato (Department of Gastroenterology and Metabolism, NCU Graduate School)、Hiromi Kataoka (Department of Gastroenterology and Metabolism, NCU Graduate School)

Autotaxin (ATX) is an enzyme that hydrolyzes lysophosphatidylcholine (LPC) to generate lysophosphatidic acid (LPA), a growth factor. Overexpression of ATX has been reported to contribute to cancer cell growth. This study aimed to explore the potential of targeting ATX as a novel molecular therapy for biliary tract cancer (BTC). In patients with BTC, serum ATX levels were significantly higher compared to those in the control group. In vitro, treatment with LPA significantly enhanced the proliferation and migration of BTC cell lines (HuCCT-1 and RBE). In contrast, treatment with ATX inhibitors (PF-8380 and GLPG1690) significantly suppressed cell proliferation and migration. LPA stimulation enhanced ERK phosphorylation, while ATX inhibitors suppressed this phosphorylation. In an in vivo model using immunodeficient mice subcutaneously implanted with HuCCT-1 cells, intraperitoneal administration of ATX inhibitors suppressed tumor growth compared to the control group. These findings suggest that ATX could serve as a novel therapeutic target for biliary tract cancer.

[Disclosure of COI by Responsible researcher] Lead presenter is not the Responsible researcher.
[Lead presenter: Kenji Urakabe] We have no financial relationships to disclose.
[Principal Researcher: Michihiro Yoshida] We have no financial relationships to disclose.

### [P-2234] I-P13-4
### Molecular targeted therapy

2025 / 9 / 26 (Fri) 17:30-18:15 Room P/(Motenashi Dome) Room P

P-2234

### Targeting STAMBPL1 with a Novel Mushroom-Derived Peptide Sensitizes Cholangiocarcinoma to Apoptosis-Inducing Therapy

[Speaker]Supattra Khansong (Department of Biochemistry, Faculty of Medical Science, NU, Thailand) [Co-author]Sucheewin Krobthong (Faculty of Pharmacy, MU, Thailand)、Nopawit Khamto (Department of Biochemistry, Faculty of Medical Science, NU, Thailand)、Worasak Kaewkong (Department of Biochemistry, Faculty of Medical Science, NU, Thailand)

As drug resistance continues to limit the success of chemotherapy in cholangiocarcinoma (CCA), identifying novel apoptotic regulators is critical. This study is the first to identify STAMBPL1, a deubiquitinating enzyme, as a key mediator of chemoresistance in CCA, with high expression levels associated with poor overall survival based on GEPIA and TCGA analyses. STAMBPL1 knockdown moderately induced apoptosis, but subsequent treatment with an apoptosis-inducing drug synergistically enhanced cell death, as demonstrated by MTT assay, Annexin V/PI staining, and apoptotic markers activation. To therapeutically inhibit STAMBPL1, we screened natural peptides from Pleurotus djamor and identified a candidate that downregulated STAMBPL1 without cytotoxic effects on normal cells. Molecular docking suggested that the peptide and STAMBPL1's catalytic domain would interact in a stable manner. Peptide treatment mimicked siRNA effects, inducing apoptosis and altering the secretome profile, including reduction of survival-associated proteins. These results highlight STAMBPL1 as a potential therapeutic target and propose a nature-derived peptide as a promising chemo-sensitizing agent in CCA.

[Disclosure of COI by Responsible researcher] Lead presenter is the Responsible researcher.
[Lead presenter: Supattra Khansong] We have no financial relationships to disclose.

### [P-2235] I-P13-4
### Molecular targeted therapy

2025 / 9 / 26 (Fri) 17:30-18:15 Room P/(Motenashi Dome) Room P

---

### P-2235
### Exploring Anti-Angiogenic Drug Repurposing as a Synergistic Approach with CDK4/6 Inhibitors in Cholangiocarcinoma

[Speaker]Orawan Suppramote (Princess Srisavangavadhana Faculty of Med., Chulabhorn Royal Academy, Bangkok, Thailand) [Co-author]Nontaphat Leerach (Princess Srisavangavadhana Faculty of Med., Chulabhorn Royal Academy, Bangkok, Thailand)、Hattapark Dejakaisaya (Princess Srisavangavadhana Faculty of Med., Chulabhorn Royal Academy, Bangkok, Thailand)、Sunisa Prasopporn (Dept. of Pharmacology, Siriraj Hosp., Mahidol Univ., Bangkok, Thailand)、Pongsakorn Buraphat (Faculty of Med., Siriraj Hosp., Mahidol Univ., Bangkok, Thailand)、Jiradej Makjaroen (Dept. of Transfusion Med. and Clin. Microbiology, Chulalongkorn Univ.)、Siwanon Jirawatnotai (Dept. of Pharmacology, Siriraj Hosp., Mahidol Univ., Bangkok, Thailand)

Cholangiocarcinoma (CCA) is an aggressive cancer with high incidence in Asia. Surgery is the only treatment to cure the disease, but only a limited number of patients qualify for this option. Effective pharmacological therapies are crucial in the fight against CCA. CDK4/6 inhibitors are important drugs being evaluated across many cancer types. Our previous study identified an acquired vulnerability in CDK4/6 inhibitor-resistant cells to ribosome biogenesis stress inducers. These resistant clones displayed an upregulation of RPL29, a ribosomal protein. In this study, we explored the potential effects of RPL29 in CDK4/6 inhibitor-resistant CCA cells, utilizing unbiased transcriptomics and proteomics approaches. Our analysis indicated that drug resistance also resulted in the upregulation of angiogenesis-related pathways, driven by the activation of the VEGF, PI3K/AKT/mTOR, and MAPK pathways. Consequently, co-inhibition of these pathways using repurposed VEGF inhibitors or drugs targeting the PI3K/AKT/mTOR and MAPK pathways, already approved for other indications, may represent a scientifically rational and economical strategy to overcome resistance to CDK4/6 inhibitors in CCA.

[Disclosure of COI by Responsible researcher] Lead presenter is the Responsible researcher.
[Lead presenter: Orawan Suppramote] We have no financial relationships to disclose.

### [P-2236] I-P13-4
### Molecular targeted therapy

2025 / 9 / 26 (Fri) 17:30-18:15 Room P/(Motenashi Dome) Room P

---

### P-2236
### TELO2 targeting therapy with ivermectin inhibits proliferation of malignant rhabdoid tumor cells

[Speaker]Honami Yonezawa (Div. Health Chem., Iwate Med. Univ., Sch. Pharm.) [Co-author]Haruki Ujiie (Div. Info., Iwate Med. Univ., Sch. Pharm.)、Yoshimasa Uehara (Div. Info., Iwate Med. Univ., Sch. Pharm.)、Naoyuki Nishiya (Div. Info., Iwate Med. Univ., Sch. Pharm.)

SMARCB1-deficient malignant rhabdoid tumor (MRT) is an aggressive childhood tumor without a standard effective treatment. MRTs are genetically defined as tumors inactivated SMARCB1, a subunit of the SWI/SNF chromatin remodeling complex. We report that telomere length regulation protein TEL2 homolog (TELO2), a stabilizer of the ATR or ATM protein kinases regulating the DNA damage response signaling pathway, is a candidate of therapeutic target for MRTs. In our previous studies, we found that ivermectin (IVM) specifically bound to and reduced TELO2 protein in HEK293 cells. Database analysis revealed that survival of MRT cell lines has a tendency to depend on TELO2. In fact, TELO2 knockdown and knockout suppressed survival of a MRT cell line, G401. IVM also reduced TELO2, ATM and ATR protein levels and inhibited MRT cell proliferation. MRT cells expressing TELO2 K749T, a mutant lost affinity for IVM, resisted IVM-induced MRT cell death. Similarly, exogenous SMARCB1 expression conferred IVM-resistance to MRT cells. These data suggest that TELO2 targeting therapy causes synthetic lethality to SMARCB1-deficient MRTs.

[Disclosure of COI by Responsible researcher] Lead presenter is not the Responsible researcher.
[Lead presenter: Honami Yonezawa] We have no financial relationships to disclose.
[Principal Researcher: Naoyuki Nishiya] We have no financial relationships to disclose.

### [P-2237] I-P13-4
### Molecular targeted therapy

2025 / 9 / 26 (Fri) 17:30-18:15 Room P/(Motenashi Dome) Room P

---

### P-2237
### The role of cetuximab in modulating calcium dynamics in oral cancer cells

[Speaker]Kazumasa Ohyumi (Grad. Sch. Med. Sci., Kumamoto Univ.／Dept. Oral & Maxillofac. Surg., Fac. Life Sci., Kumamoto Univ.) [Co-author]Kenta Kawahara (Dept. Oral & Maxillofac. Surg., Fac. Life Sci., Kumamoto Univ.)、Yusei Todoroki (Grad. Sch. Med. Sci., Kumamoto Univ.／Dept. Oral & Maxillofac. Surg., Fac. Life Sci., Kumamoto Univ.)、Kosuke Shinohara (Dept. Oral & Maxillofac. Surg., Fac. Life Sci., Kumamoto Univ.)、Yuki Seki (Dept. Oral & Maxillofac. Surg., Fac. Life Sci., Kumamoto Univ.)、Masatoshi Hirayama (Dept. Oral & Maxillofac. Surg., Fac. Life Sci., Kumamoto Univ.)、Akiyuki Hirosue (Dept. Oral & Maxillofac. Surg., Fac. Life Sci., Kumamoto Univ.)、Ryoji Yoshida (Dept. Oral & Maxillofac. Surg., Fac. Life Sci., Kumamoto Univ.)、Hideki Nakayama (Dept. Oral & Maxillofac. Surg., Fac. Life Sci., Kumamoto Univ.)

Background: EGFR overexpression is associated with poor prognosis in oral cancer. Although the anti-EGFR monoclonal antibody cetuximab (CTX) is a therapeutic option, resistance often occurs, even in cases with high EGFR levels. Emerging evidence suggests calcium signaling abnormalities may play a role in this resistance. This study investigated how CTX influences calcium dynamics in oral cancer cells.Materials and Methods: TCGA RNA-seq data were analyzed to assess the link between EGFR expression and patient outcomes. Several human oral cancer cell lines with varying EGFR levels were treated with CTX, and intracellular calcium kinetics were measured.Results: High EGFR expression was associated with worse prognosis. Cell lines showed differing EGFR levels and responded to CTX with distinct changes in calcium signaling, depending on EGFR expression.Conclusion: These findings suggest a functional link between EGFR signaling and calcium homeostasis. Modulating calcium pathways may enhance CTX efficacy in oral cancer treatment.

[Disclosure of COI by Responsible researcher] Lead presenter is not the Responsible researcher.
[Lead presenter: Kazumasa Ohyumi] We have no financial relationships to disclose.
[Principal Researcher: Hideki Nakayama] We have no financial relationships to disclose.

### [P-2238] I-P13-4
### Molecular targeted therapy

2025 / 9 / 26 (Fri) 17:30-18:15 Room P/(Motenashi Dome) Room P

---

### P-2238
### Rapid TKIs sensitivity profiling in CML cells using the opto-diagnostic FRET biosensor Pickles

[Speaker]Tatsuaki Mizutani (Dept. Cell Physiol. Faculty of Med. Hokkaido Univ.) [Co-author]Aya O. Satoh (Dept. Cell Physiol. Faculty of Med. Hokkaido Univ.)、Mari Fujioka (HILO, Co. Ltd)、Yoichiro Fujioka (Dept. Cell Physiol. Faculty of Med. Hokkaido Univ.)、Maho Amano (HILO, Co. Ltd)、Yusuke Ohba (Dept. Cell Physiol. Faculty of Med. Hokkaido Univ.)

Chronic myeloid leukemia (CML) is driven by constitutive tyrosine kinase activity of the BCR::ABL1 fusion protein. The introduction of tyrosine kinase inhibitors (TKIs) has significantly advanced the clinical management of CML; however, the emergence of drug-resistant BCR::ABL1 mutations remains a major clinical issue. Rapid and precise evaluation of TKI sensitivity is therefore crucial for optimizing therapeutic strategies. We have developed an opto-diagnostic biosensor based on the principle of Forster resonance energy transfer (FRET), which allows direct measurement of BCR::ABL1 kinase activity in living cells. In this study, we established CML cells harboring representative drug-resistant BCR::ABL1 mutations using the CRISPR/Cas9 system, and applied Pickles to evaluate their responses to different TKIs in mixed cell populations. Our opto-diagnostic system could detect resistant cells comprising as little as 1% of the total population. These findings demonstrate that Pickles provides high sensitivity and specificity, supporting its potential as a powerful tool for personalized treatment decision-making in CML.

[Disclosure of COI by Responsible researcher] Lead presenter is not the Responsible researcher.
[Lead presenter: Tatsuaki Mizutani] ■Research funds from company: ノバルティスファーマ株式会社　■ employment by a company related to the presentation: HILO株式会社
[Principal Researcher: Yusuke Ohba] ■Research funds from company: ノバルティスファーマ株式会社　■Profit of stock: HILO株式会社　■Contributions: オプトライン株式会社

### [P-2245] I-P14-7
### Anticancer drug & cell death

2025 / 9 / 26 (Fri) 17:30-18:15 Room P/(Motenashi Dome) Room P

---

### P-2245
### Withdrawn

No Abstract

### [P-2246] I-P14-7
### Anticancer drug & cell death

2025 / 9 / 26 (Fri) 17:30-18:15 Room P/(Motenashi Dome) Room P

### P-2246
### Lysosome-dependent cell death mediates the cytotoxic effects of paroxetine in A549 lung adenocarcinoma cells

[Speaker]Yi-Ching Huang (Inst. of Biomed. Sci., NCHU, Taichung, Taiwan) [Co-author]Show-Mei Chuang (Inst. of Biomed. Sci., NCHU, Taichung, Taiwan)

Non-small cell lung cancer (NSCLC) is often diagnosed at advanced stages. The anticancer mechanisms of paroxetine remain unclear in NSCLC. This study demonstrates that paroxetine reduced cell viability, but apoptosis was not involved in A549. RNA sequencing revealed that several lysosomal-related genes were upregulated following paroxetine treatment, suggesting lysosome-dependent cell death (LDCD). RT-qPCR confirmed an increase in mRNA levels of lysosome-specific enzymes ***CTSB*** and ***CTSD.*** Further, paroxetine treatment caused lysosome enlargement, accumulation of immature CTSB and CTSD, decreased CTSB-LAMP1 colocalization, increased Galectin-3 puncta, and reduced phospho-TFEB levels, indicating lysosomal damage and enhanced lysosomal membrane permeabilization (LMP). We also observed that paroxetine led to the accumulation of ROS, resulting in lipid peroxidation. In contrast, NAC alleviated ROS accumulation, lipid peroxidation, and LMP, while restoring cell viability. In conclusion, paroxetine induces LDCD in A549 through ROS accumulation and lysosomal damage, highlighting its potential as a therapeutic agent for lung cancer.

[Disclosure of COI by Responsible researcher] Lead presenter is the Responsible researcher.
[Lead presenter: Yi-Ching Huang] We have no financial relationships to disclose.

▲TOP

[P-2247] I-P14-7
Anticancer drug & cell death

2025 / 9 / 26 (Fri) 17:30-18:15 Room P/(Motenashi Dome) Room P

P-2247
### FUT8 Promotes TBK1 Stability via Autophagy to Enhance Lung Cancer Cell Survival

[Speaker]Pin-Chieh Liu (Dept. of Biol. Sci. & Tech., CMU) [Co-author]Wan-Jou Shen (Grad. Inst. of Biomed. Sci., CMU)、Zi-Xuan Kuo (Dept. of Biol. Sci. & Tech., CMU)、Wei-Jan Wang (Dept. of Biol. Sci. & Tech., CMU)

Fucosyltransferase 8 (FUT8), the sole enzyme mediating core fucosylation of N-glycoproteins, is upregulated in several cancers, including lung cancer. This post-translational modification is crucial for cell development and differentiation, and aberrant fucosylation is linked to increased malignancy and reduced immunotherapy efficacy. However, the role of FUTs in immune modulation, especially in lung cancer, remains unclear. Immune checkpoint inhibitors (ICIs) are central to treating non-small cell lung cancer (NSCLC), yet over half of patients respond poorly, highlighting the need for better therapies. Innate immune signaling, particularly via TANK-binding kinase 1 (TBK1), regulates tumor immune evasion. TBK1 also promotes cancer cell survival, but its regulation remains unknown. Multi-omics analyses (Oncomine, TCGA, Kaplan-Meier) show FUT8 is highly expressed in NSCLC and linked to poor survival. Silencing FUT8 reduced phosphorylated TBK1 (p-TBK1), while proteasome and autolysosome inhibition restored it. Thus, FUT8 regulates TBK1 via non-classical, autophagy-related pathways, suggesting FUT8 as a target to enhance immunotherapy in lung cancer.

[Disclosure of COI by Responsible researcher] Lead presenter is the Responsible researcher.
[Lead presenter: Pin-Chieh Liu] We have no financial relationships to disclose.

### [P-2248] I-P14-7
### Anticancer drug & cell death

2025 / 9 / 26 (Fri) 17:30-18:15 Room P/(Motenashi Dome) Room P

P-2248

### Loperamide Enhances AMG-510 Efficacy via Autophagy-Mediated GPX4 Degradation in KRAS G12C-Mutant Lung Cancer

[Speaker]Yu Fang Ho (Dept. of Biol. Sci. & Tech., CMU) [Co-author]Wei-Jan Wang (Dept. of Biol. Sci. & Tech., CMU)

Lung cancer is a leading cause of cancer deaths worldwide. In non-small cell lung cancer (NSCLC), KRAS mutations occur in ~25% of cases, with KRAS G12C accounting for 13%. AMG-510 (Sotorasib), a KRAS G12C inhibitor, shows clinical promise but faces limitations due to resistance and modest efficacy in ~50% of patients. Ferroptosis, an iron-dependent cell death, contributes to lung cancer progression and is driven by reactive oxygen species (ROS), lipid peroxidation, and mitochondrial dysfunction. However, how ferroptosis affects AMG-510 efficacy remains unclear. Loperamide, an opioid receptor agonist used for cancer-associated diarrhea, has shown antitumor effects in solid tumors. Its ability to enhance AMG-510 efficacy is unexplored. Our data show Loperamide boosts AMG-510's cytotoxicity and induces ferroptosis in H23 cells. It promotes GPX4 degradation via autophagy and copper ion regulation. Inhibiting autophagy or lysosomes reverses this effect. These findings offer insight into optimizing KRAS-targeted therapies and overcoming resistance.

[Disclosure of COI by Responsible researcher] Lead presenter is the Responsible researcher.
[Lead presenter: Yu Fang Ho] We have no financial relationships to disclose.

### [P-2249] I-P14-7
### Anticancer drug & cell death

2025 / 9 / 26 (Fri) 17:30-18:15 Room P/(Motenashi Dome) Room P

---

### P-2249
### A Novel Eupatilin-Derived Multi-Kinase Inhibitor Enhances Therapeutic Efficacy in Triple-Negative Breast Cancer

[Speaker]Choong-Jae Lee (Targeted Therapy Branch, NCC／Cancer Outcome & Quality Improvement Branch, NCC) [Co-author]Jinyoung Park (Targeted Therapy Branch, NCC)、Sun-Young Kong (Targeted Therapy Branch, NCC／Dept. of Cancer Biomedical Science, NCC GCSP／Dept. of Laboratory Medicine, NCC)

Triple-negative breast cancer (TNBC) is characterized by an aggressive phenotype and poor clinical outcomes due to the lack of effective therapeutic strategies. In this study, we developed a new compound targeting TNBC, ONG41008 and ONG41003 which were designed with distinct structural modifications at specific glucuronidation sites of multi-kinase inhibitor Eupatilin to enhance its metabolic stability. Treatment with these drugs in TNBC cells reduced cell viability and proliferation, accompanied by G2/M phase arrest and increased apoptosis. Furthermore, ONG41008 and ONG41003 treatment inhibited cell migration and invasion. Notably, the dose-effects of the drug combination were assessed, and concurrent treatment with these drugs and either doxorubicin or paclitaxel may induce synergistic anticancer effects in TNBC cells. Collectively, our findings suggest a promising therapeutic candidate for TNBC, with the potential to enhance treatment efficacy when used in combination with conventional chemotherapeutic agents. Based on these findings, we are currently conducting an in vivo study as further study.This study was supported by grant from Osteoneurogen (Grant No. NCC-202300190002).

[Disclosure of COI by Responsible researcher] Lead presenter is not the Responsible researcher.
[Lead presenter: Choong-Jae Lee] We have no financial relationships to disclose.
[Principal Researcher: ] We have no financial relationships to disclose.

[P-2250] I-P14-7
Anticancer drug & cell death

2025 / 9 / 26 (Fri) 17:30-18:15 Room P/(Motenashi Dome) Room P

### P-2250
### Targeting of PTX3 impairs tumor metastasis by disruption of the tumor microenvironment in drug-tolerant persister cells

[Speaker]Jing-Ting Chiou (Grad. Inst. Of Med. Sci.,Taipei Med. Univ.) [Co-author]Hsiao-Yu Chang (Dept. of Pharm.,Natl. Cheng Kung Univ.)、Ming-Yao Chung (Dept. of Pharm.,Natl. Cheng Kung Univ.)、An-Jhen Yan (Dept. of Pharm.,Natl. Cheng Kung Univ.)、Wen-Chang Chang (Grad. Inst. Of Med. Sci.,Taipei Med. Univ.)、Ben-Kuen Chen (Dept. of Pharm.,Natl. Cheng Kung Univ.)

Human cancer cells elicit a tolerant response when exposed to commonly used chemotherapy regimens, and some tumor cells can transform into drug-tolerant persisters (DTPs). However, the molecular mechanism involved in metastasis and chemoresistance of DTP cells derived from head and neck squamous cell carcinoma (HNSCC) remains unclear. Here, we show that surviving HNSCC cells after cisplatin treatment acquired DTP (DTP-cis) characteristics, including dormancy, senescence, stem-like phenotype, and drug resistance. We also found that the upregulation and secretion of cytokines, such as pentraxin-3 (PTX3) from DTP-cis cells, promoted tumor metastasis. The conditioned medium (CM) harvested from DTP-cis or endothelial cells significantly triggered spheroid formation and invasion of tumor cells and upregulated angiogenesis. Notably, the DTP-cis CM-enhanced effects were inhibited by the depletion of PTX3. These results demonstrate the role of PTX3 in regulating DTP-cis metastasis and its impact on angiogenesis. Targeting PTX3 may prevent or eliminate the metastasis of chemo-surviving cells, thereby improving therapy for advanced HNSCC.

[Disclosure of COI by Responsible researcher] Lead presenter is the Responsible researcher.
[Lead presenter: Jing-Ting Chiou] We have no financial relationships to disclose.

### [P-2251] I-P14-7
### Anticancer drug & cell death

2025 / 9 / 26 (Fri) 17:30-18:15 Room P/(Motenashi Dome) Room P

### P-2251
### Withdrawn

No Abstract

### [P-2252] I-P14-7
### Anticancer drug & cell death

2025 / 9 / 26 (Fri) 17:30-18:15 Room P/(Motenashi Dome) Room P

---

### P-2252
### Tri–Synergy Metabo–Epi–Immune Therapy:Fe–Cu DAzymes & β–Alanine for tumors

[Speaker]Xi Tao (Provincial-Ministry Collaborative Centre for Regenerative Medicine／Institute of Life Sciences／College of International Education)

The aberrant accumulation of lactate in the tumor microenvironment drives immune evasion, bone metastasis, and therapeutic resistance through dual mechanisms: microenvironment acidification and lactate-dependent lysine lactylation, a novel post–translational modification. Current metabolic interventions, such as monocarboxylate transporter inhibitors, face limitations due to compensatory lactylation–mediated pro–tumor signaling. To address this challenge, we propose a groundbreaking "metabolic–epigenetic–immune" tri–axis therapeutic strategy by integrating Fe–Cu diatomic nanozymes with β–alanine. This innovative approach aims to simultaneously disrupt lactate metabolism, suppress lactylation–driven oncogenesis, and reprogram the immunosuppressive TME, with a specific focus on overcoming bone metastasis.
This project combines nanotechnology, epigenetics, and immunometabolism to develop a multifunctional therapeutic platform. By targeting the dynamic interplay between lactate metabolism and lactylation modifications, we seek to establish a new paradigm for precision oncology and metastatic disease treatment.

[Disclosure of COI by Responsible researcher] Lead presenter is the Responsible researcher.
[Lead presenter: Xi Tao] We have no financial relationships to disclose.

### [P-2253] I-P14-7
### Anticancer drug & cell death

2025 / 9 / 26 (Fri) 17:30-18:15 Room P/(Motenashi Dome) Room P

---

### P-2253
### Expression and roles of KIF18A in progression and survival of gemcitabine-resistance cholangiocarcinoma cells

[Speaker]Kitti Intuyod (Dept. of Path., Faculty of Med., Khon Kaen Univ.／Cholangiocarcinoma Res. Inst., Khon Kaen Univ.) [Co-author]Pakornkiat Tanasuka (Dept. of Path., Faculty of Med., Khon Kaen Univ.／Cholangiocarcinoma Res. Inst., Khon Kaen Univ.)、Phonpilas Thongpon (Dept. of Parasitology., Faculty of Med., Khon Kaen Univ.／Cholangiocarcinoma Res. Inst., Khon Kaen Univ.)、Sasitorn Chomwong (Dept. of Path., Faculty of Med., Khon Kaen Univ.／Cholangiocarcinoma Res. Inst., Khon Kaen Univ.)、Thatsanapong Pongking (Biomed. Sci. Program, Grad. Sch., Khon Kaen Univ.／Cholangiocarcinoma Res. Inst., Khon Kaen Univ.)、Chawalit Pairojkul (Dept. of Path., Faculty of Med., Khon Kaen Univ.／Cholangiocarcinoma Res. Inst., Khon Kaen Univ.)、Somchai Pinlaor (Dept. of Parasitology., Faculty of Med., Khon Kaen Univ.／Cholangiocarcinoma Res. Inst., Khon Kaen Univ.)

Cholangiocarcinoma (CCA) is a malignant cancer of bile duct epithelium characterized by poor prognosis due to late diagnosis and irresponsive to gemcitabine-based chemotherapy and thus, requiring novel and effective therapeutic targets especially for those who do not respond to gemcitabine treatment. This study revealed that Kinesin Family Member 18A (KIF18A) is highly expressed in CCA tissues and significantly associated with anatomical and histological types. KIF18A expression levels were also significantly increased in CCA cells, particularly gemcitabine-resistant CCA cells (KKU-213B$^{GemR}$). Suppression of KIF18A significantly inhibited colony formation, migration and invasion and induced early apoptotic cell death of KKU-213B$^{GemR}$ cells. Mechanistically, suppression of KIF18A expression significantly downregulated the expression of key oncogenic and survival signaling proteins, including phosphorylated PI3K (p-PI3K), AKT (p-AKT), mTOR (total and p-mTOR), NF-κB (total and p-NF-κB), and BCL-2 in KKU-213B$^{GemR}$ cells. Taken together, our findings suggest that KIF18A plays a crucial role in promoting CCA progression and survival partly through modulating PI3K/AKT/mTOR pathway.

[Disclosure of COI by Responsible researcher] Lead presenter is the Responsible researcher.
[Lead presenter: Kitti Intuyod] We have no financial relationships to disclose.

▲TOP

### [P-2263] I-P17-1
### Surgical intervention

2025 / 9 / 26 (Fri) 17:30-18:15 Room P/(Motenashi Dome) Room P

---

### P-2263
### The emerging message from animal experiments on lymph node resection suggests what may happen in the real-world

[Speaker]Tetsuya Kodama (Lab. of Biomed. Engineering for Cancer, Tohoku Univ.／Biomed. Engineering Cancer Res. Ctr, Tohoku Univ.) [Co-author]Ariunbuyan Sukhbaatar (Lab. of Biomed. Engineering for Cancer, Tohoku Univ.／Biomed. Engineering Cancer Res. Ctr, Tohoku Univ.／Div. Oral and Maxillofacial Oncology and Surg. Sci., Tohoku Univ.)、Shiro Mori (Lab. of Biomed. Engineering for Cancer, Tohoku Univ.／Biomed. Engineering Cancer Res. Ctr, Tohoku Univ.／Div. Oral and Maxillofacial Oncology and Surg. Sci., Tohoku Univ.)、Tsuyoshi Sugiura (Biomed. Engineering Cancer Res. Ctr, Tohoku Univ.／Div. Oral and Maxillofacial Oncology and Surg. Sci., Tohoku Univ.)

Sentinel lymph node (SLN) resection/biopsy is the primary diagnostic tool for oncologists, and the status of the SLN at the tumor site is a crucial factor in determining prognosis, metastatic status, and treatment choices for cancer patients. Yet, the effect of SLNB on cancer metastasis progression was unclear, such as how it may alter the tumor-immune microenvironment and interventions with other treatments. Here, we attempted to answer these questions using a mouse model with a swollen LN by inoculating luciferase-expressing tumor cells directly into the LN to induce metastasis into its downstream LN, referred to as the LN metastasis mouse model. Tumor-free/tumor-positive ipsilateral LN were resected 7 days after tumor inoculation to assess the impact of LN resection on metastasis progression in the lymphatic system and in distant organs based on changes in luciferase expression over time. We found that activation of distant metastases occurred in both tumor-free/positive LN resection groups, regardless of the tumor type. This highlights the complexity of the tumor-immune microenvironment and raises important considerations for oncologists when making surgical decisions.

[Disclosure of COI by Responsible researcher] Lead presenter is the Responsible researcher.
[Lead presenter: Tetsuya Kodama] We have no financial relationships to disclose.

### [P-2264] I-P17-1
### Surgical intervention

2025 / 9 / 26 (Fri) 17:30-18:15 Room P/(Motenashi Dome) Room P

---

### P-2264
### Efforts to Prevent Postoperative Complications in Esophageal Cancer Surgery to Improve Oncologic Outcomes

[Speaker]Kenoki Ohuchida (Dept of Surgery and Oncology, Kyushu Univ) [Co-author]Kohei Horioka (Dept of Surgery and Oncology, Kyushu Univ)、Sokichi Matsumoto (Dept of Surgery and Oncology, Kyushu Univ)、Tomonori Nakanoko (Dept of Surgery and Science, Kyushu Univ)、Kinuko Nagayoshi (Dept of Surgery and Oncology, Kyushu Univ)、Yusuke Mizuuchi (Dept of Surgery and Oncology, Kyushu Univ)、Eiji Oki (Dept of Surgery and Science, Kyushu Univ)、Tomoharu Yoshizumi (Dept of Surgery and Science, Kyushu Univ)、Masafumi Nakamura (Dept of Surgery and Oncology, Kyushu Univ)

It has become clear that postoperative complications impact long-term oncologic outcomes. Major complications following esophagectomy include pneumonia, chylothorax, anastomotic leakage, and so on. To reduce these risks, we routinely choose robotic-assisted surgery as our primary approach. This not only facilitates meticulous dissection but also enhances recognition of fine anatomical structures, thereby helping to prevent complications such as recurrent laryngeal nerve palsy (which may lead to pneumonia), chylothorax, and pneumothorax. Recurrent laryngeal nerve palsy is often caused by traction. Now, we employ NIM, allowing real-time adjustment of traction based on neural response. The incidence of anastomotic leakage and stricture has also improved with the introduction of advanced surgical devices. Furthermore, we routinely conduct preoperative 3D simulations to understand patient-specific surgical anatomy. This information aids in optimizing the surgical approach, selecting the reconstruction route, evaluating aspiration risk, and planning postoperative positioning. Here, we will share our strategies for preventing postoperative complications in robotic-assisted esophagectomy.

[Disclosure of COI by Responsible researcher] Lead presenter is the Responsible researcher.
[Lead presenter: Kenoki Ohuchida] We have no financial relationships to disclose.

### [P-2265] I-P17-1
### Surgical intervention

2025 / 9 / 26 (Fri) 17:30-18:15 Room P/(Motenashi Dome) Room P

---

### P-2265
### Innovations and practice of laparoscopic gastric-tube reconstruction.

[Speaker]Yasuo Tsuda (Dept of Surgery and Science, Kyushu University) [Co-author]Tomonori Nakanoko (Dept of Surgery and Science, Kyushu University)、Tetsuro Kawazoe (Dept of Surgery and Science, Kyushu University)、Sho Nambara (Dept of Surgery and Science, Kyushu University)、Koji Ando (Dept of Surgery and Science, Kyushu University)、Eiji Oki (Dept of Surgery and Science, Kyushu University)、Tomoharu Yoshizumi (Dept of Surgery and Science, Kyushu University)

[Background] Since 2011, our department has been performing thoracoscopic subtotal esophagectomy and laparoscopic gastric tube reconstruction. [Method] In our department, the stomach wall on the lesser curvature side that is grasped is cut off when the gastric tube is created. The trick is to dissect the left edge of the esophagus (left side of the crus of the diaphragm) thoroughly. If the left esophagus and crus of the diaphragm are dissected thoroughly first, the time required to pull out the esophagus can be shortened. In the posterior mediastinal route, a 4 cm incision is made at the umbilicus, and the esophagus and stomach are pulled out of the body from the umbilicus. A thin gastric tube with a horizontal diameter of 3.5 cm is used. [Results] 308 cases of laparoscopic gastric tube reconstruction were performed. The operative time required for gastric tube reconstruction was 97 (50-125) minutes. Only in one case we needed reoperation for gastric tube trouble. [Conclusion] Laparoscopic gastric tube reconstruction can be performed safely, and is expected to result in high cosmetic results and early postoperative recovery due to its minimal invasiveness.

[Disclosure of COI by Responsible researcher] Lead presenter is not the Responsible researcher.
[Lead presenter: Yasuo Tsuda] We have no financial relationships to disclose.
[Principal Researcher: Tomoharu Yoshizumi] We have no financial relationships to disclose.

Poster Session

### [P-2266] I-P17-1
### Surgical intervention

2025 / 9 / 26 (Fri) 17:30-18:15 Room P/(Motenashi Dome) Room P

---

### P-2266
### Prognostic Significance of PLR and PNI in Remnant Gastric Cancer: A Multicenter Study

[Speaker]Kensuke Kudou (Dept. of Gastroenterological Surg, The Cancer Institute Hospital of JFCR／Dept. of Surgery and Science, Kyushu Univ.)
[Co-author]Mitsuhiko Ota (Dept. of Gastroenterological Surg, Kyushu Medical Center)、Kippei Ogaki (Department of Surgery, Kyushu Central Hospital)、Yasue Kimura (Dept. of Gastroenterological Surg, Kyushu Cancer Center)、Yuta Kasagi (Dept. of Gastroenterological Surg, Kyushu Cancer Center)、Naomichi Koga (Dept. of Gastroenterological Surg, Kyushu Cancer Center)、Hirofumi Hasuda (Dept. of Gastroenterological Surg, Kyushu Medical Center)、Hirotada Tajiri (Dept. of Gastroenterological Surg, Kyushu Medical Center)、Tetsuro Kawazoe (Dept. of Surgery and Science, Kyushu Univ.)、Tomonori Nakanoko (Dept. of Surgery and Science, Kyushu Univ.)、Koji Ando (Dept. of Surgery and Science, Kyushu Univ.)、Eiji Oki (Dept. of Surgery and Science, Kyushu Univ.)、Tomoharu Yoshizumi (Dept. of Surgery and Science, Kyushu Univ.)

[Background]Inflammation-based prognostic scores (IBPSs), which reflect nutritional status and chronic inflammation, have been reported to be prognostic indicators in various malignancies. However, few studies have addressed their significance in remnant gastric cancer (RGC). [Methods]We retrospectively analyzed 126 patients who underwent curative resection for RGC between 2013 and 2024 at five affiliated institutions. We investigated the associations between various IBPSs and long-term outcomes. [Results]Kaplan-Meier survival analyses were performed based on each IBPS. Patients with a platelet–lymphocyte ratio (PLR) ≥195 and a prognostic nutritional index (PNI) <43.5 had significantly poor overall survival (OS) ($P = 0.008$ and $P < 0.001$, respectively). Multivariate analysis further identified PLR ≥195 and PNI <43.5 as independent prognostic factors for poor OS ($P = 0.0050$ and $P = 0.0076$). Patients with both high PLR and low PNI had significantly lower 5–year OS ($P < 0.001$).[Conclusion]In patients who underwent curative resection for RGC, both PLR and PNI showed significant associations with OS and RFS. The subgroup with both high PLR and low PNI had a particularly poor prognosis.

[Disclosure of COI by Responsible researcher] Lead presenter is the Responsible researcher.
[Lead presenter: Kensuke Kudou] We have no financial relationships to disclose.

### [P-2267] I-P17-1
### Surgical intervention

2025 / 9 / 26 (Fri) 17:30-18:15 Room P/(Motenashi Dome) Room P

### P-2267
### Short-term Outcomes of Robot-assisted Left Hemicolectomy in Our Institution

[Speaker]Yoshiaki Fujimoto (Department of Surgery, Saiseikai Fukuoka General Hospital) [Co-author]Taichi Nagano (Department of Surgery, Saiseikai Fukuoka General Hospital)、Kanrin Oh (Department of Surgery, Saiseikai Fukuoka General Hospital)、Tomoko Shigechi (Department of Surgery, Saiseikai Fukuoka General Hospital)、Fumihiko Hirai (Department of Surgery, Saiseikai Fukuoka General Hospital)、Takuya Honbo (Department of Surgery, Saiseikai Fukuoka General Hospital)、Noboru Harada (Department of Surgery, Saiseikai Fukuoka General Hospital)、Noriaki Sadanaga (Department of Surgery, Saiseikai Fukuoka General Hospital)

Background: Left hemicolectomy is a standard procedure for transverse colon cancer near the splenic flexure and descending colon cancer. Robot-assisted surgery enables precise maneuvers in minimally invasive procedures. This study evaluates short-term outcomes of robot-assisted left hemicolectomy at our institution.Methods: We retrospectively analyzed six patients who underwent robot-assisted left hemicolectomy from April 2023 to October 2024. Parameters included operative time, blood loss, complications, harvested lymph nodes, and hospital stay. All surgeries used the da Vinci Xi system.Results: One had transverse colon cancer, five had descending colon cancer. Anastomoses included five functional end-to-end and one overlap method; four were intracorporeal. Mean operative time was 226 min (191 to 284), blood loss 10 mL (5 to 38). No complications occurred. Mean lymph nodes: 12 (2 to 21). Average hospital stay: 8.5 days (7 to 10).Conclusion: Robot-assisted surgery enabled safe, precise left hemicolectomy despite anatomical complexity. Minimal blood loss and no complications suggest benefits in recovery and shorter hospital stay. It appears safe and feasible, meriting further study.

[Disclosure of COI by Responsible researcher] Lead presenter is the Responsible researcher.
[Lead presenter: Yoshiaki Fujimoto] We have no financial relationships to disclose.

### [P-2268] I-P17-1
### Surgical intervention

2025 / 9 / 26 (Fri) 17:30-18:15 Room P/(Motenashi Dome) Room P

---

### P-2268
### Surgical treatment using ICG fluorescence imaging for extrahepatic metastasis of hepatocellular carcinoma

[Speaker]Shoichi Inokuchi (Dept. of Surg. , Oita prefectural Hosp.) [Co-author]Satoshi Tsutsumi (Dept. of Surg. , Oita prefectural Hosp.)、Kenji Umeda (Dept. of Surg. , Oita prefectural Hosp.)、Takahiro Terashi (Dept. of Surg. , Oita prefectural Hosp.)、Masahiko Ikebe (Dept. of Surg. , Oita prefectural Hosp.)、Toshio Bandoh (Dept. of Surg. , Oita prefectural Hosp.)、Tohru Utsunomiya (Dept. of Surg. , Oita prefectural Hosp.)

Method: Four cases of HCC with single extrahepatic metastasis who underwent tumor resection in our department between Jan.2018 and Apr.2025 were included. In all cases, ICG 0.5mg/kg was administered intravenously 4 to 8 days before surgery. Result: The details of surgical procedures was as follows: 2 case of diaphragmatic tumor resection, 1 case of right adrenal tumor resection, and 1 case of right retroperitoneal tumor resection. The median time from previous hepatectomy to recurrence was 15 months (range, 3-26). In all cases, the tumor and margins were difficult to identify due to adhesion of previous surgery. Three cases were positive for ICG fluorescence during the current resection, and in 1 case the tumor fluorescence was unclear due to the strong fluorescence of the adjacent lesion. Surgical margins were negative in all patients, and the patients were discharged without postoperative complications. One patient developed liver recurrence 1 year after surgery, and died of another disease 3 years after surgery. The other three patients are alive and free of recurrence. Conclusion: Surgery for extrahepatic metastases of HCC using ICG is useful in some cases.

[Disclosure of COI by Responsible researcher] Lead presenter is the Responsible researcher.
[Lead presenter: Shoichi Inokuchi] We have no financial relationships to disclose.

### [P-2269] I-P17-1
### Surgical intervention

2025 / 9 / 26 (Fri) 17:30-18:15 Room P/(Motenashi Dome) Room P

---

### P-2269
### A study of short-term outcomes of laparoscopic hepatectomy for four or more metastatic lesions

[Speaker]Ryosuke Minagawa (Department of surgery Matsuyama Red Cross Hospital) [Co-author]Koichi Kimura (Department of surgery Matsuyama Red Cross Hospital)、Norifumi Iseda (Department of surgery Matsuyama Red Cross Hospital)、Yoshinari Nobuto (Department of surgery Matsuyama Red Cross Hospital)、Hiroko Yano (Department of surgery Matsuyama Red Cross Hospital)、Yuichiro Kajiwara (Department of surgery Matsuyama Red Cross Hospital)、Kazuhito Minami (Department of surgery Matsuyama Red Cross Hospital)、Takashi Nishizaki (Department of surgery Matsuyama Red Cross Hospital)

Although the number of LHs has been increasing in recent years and the indications for LH seem to be expanding, there are few studies on the upper limit of the number of resections.The purpose of this study was to evaluate the short-term postoperative outcomes of patients who underwent LH of four or more sites for metastatic liver cancers.We compared the background factors and postoperative outcomes of 3 patients who underwent >4 LHs(Group1) with those of 92 patients who underwent <3 LHs(Group2) or with those of 8 patients who underwent >4 OHs(Group3) at our hospital during the same period.Results: (1) The mean number of resections in G1 was 6.3, and the operation time, pringle time , hospital stay were significantly longer, and the incidence of complications(>CD2) was significantly higher. (2) The mean number of resections in G3 was 5.4, and Pringle time was significantly longer in G1, but the length of hospital stay was similar. Conclusion: LH may be selected more frequently as part of multidisciplinary treatment with the advancement of drug therapy.

[Disclosure of COI by Responsible researcher] Lead presenter is the Responsible researcher.
[Lead presenter: Ryosuke Minagawa] We have no financial relationships to disclose.

Poster Session

### [P-2270] I-P17-1
### Surgical intervention

2025 / 9 / 26 (Fri) 17:30-18:15 Room P/(Motenashi Dome) Room P

---

### P-2270
### Analysis of Perigallbladder Lymphatic Flow as a Pathway of Gallbladder Cancer Progression

[Speaker]Atsushi Yamamoto (Dept. of Surg. Tsuru Municipal General Hosp.) [Co-author]Hirotaka Okamoto (Dept. of Surg. Tsuru Municipal General Hosp.)

Introduction: Direct invasion, hematogenous metastasis, and lymphatic metastasis have been reported as pathways of gallbladder cancer progression. In this study, lymphatic flow around the gallbladder was identified during cholecystectomy and analyzed for the pathway of gallbladder cancer progression. Methods: This study included patients who underwent cholecystectomy for cholecystitis, cholecystolithiasis, and gallbladder cancer. Intraoperatively, indocyanine green (ICG) was injected into the serosa of the gallbladder fundus and neck, and the lymphatic flow pathways were compared by observing fluorescence in the hepatoduodenal ligament and the S4/5 of the liver. Results: When inflammation or cancer spread to the neck of the gallbladder, lymphatic vessels in the hepatoduodenal ligament showed fluorescence in fewer cases, while the S4/5 of the liver showed fluorescence in more cases. This confirmed that lymphatic flow pathways are altered when lymphatic vessels become obstructed. Conclusion: It is suggested that lymphatic flow pathways may be altered by lymphatic obstruction around the gallbladder, which may contribute to the formation of another pathway of cancer progression.

[Disclosure of COI by Responsible researcher] Lead presenter is the Responsible researcher.
[Lead presenter: Atsushi Yamamoto] We have no financial relationships to disclose.

### [P-2271] I-P17-1
### Surgical intervention

2025 / 9 / 26 (Fri) 17:30-18:15 Room P/(Motenashi Dome) Room P

---

### P-2271
### Severe complications in gynecological oncosurgery.

[Speaker]Yoshitomo Ashitate (Urakawa Red Cross Hospital)

A 68-year-old woman, with a history of hysterectomy and pelvic lymphadenectomy for uterine cancer 3 years ago, presented with abdominal pain. Enhanced CT showed a strangulated loop of the small bowel below the left external iliac artery, for which emergency open surgery was performed based on the diagnosis of strangulated bowel obstruction. Intraoperative findings revealed that the small bowel had herniated into the space between the left external iliac artery and vein. The strangulation was released. And the strangulated small bowel and the left external iliac artery was preserved. The gap was covered with a sigmoid colon to prevent recurrence. Here, we describe our experience with a case of small bowel strangulation caused by migration of the intestine into the space after pelvic lymphadenectomy, with a review of previous literature describing similar cases.

[Disclosure of COI by Responsible researcher] Lead presenter is the Responsible researcher.
[Lead presenter: Yoshitomo Ashitate] We have no financial relationships to disclose.

### [P-2280] I-P18
### Advances in Cell & Gene Therapy Technologies

2025 / 9 / 26 (Fri) 17:30-18:15 Room P/(Motenashi Dome) Room P

---

### P-2280
### Development of Nucleic Acid Therapeutics Targeting the Warburg Effect

[Speaker]Keita Matsumoto (Department of Gastrointestinal Surgery, Gifu University Hospital) [Co-author]Hirokatsu Hayashi (Department of Gastrointestinal Surgery, Gifu University Hospital)、Masashi Kuno (Department of Gastrointestinal Surgery, Gifu University Hospital)、Seito Fujibayashi (Department of Gastrointestinal Surgery, Gifu University Hospital)、Noriki Mitsui (Department of Gastrointestinal Surgery, Gifu University Hospital)、Takeshi Horaguchi (Department of Gastrointestinal Surgery, Gifu University Hospital)、Yuji Hatanaka (Department of Gastrointestinal Surgery, Gifu University Hospital)、Ryoma Yokoi (Department of Gastrointestinal Surgery, Gifu University Hospital)、Masahiro Fukada (Department of Gastrointestinal Surgery, Gifu University Hospital)、Ryuichi Asai (Department of Gastrointestinal Surgery, Gifu University Hospital)、Itaru Yasufuku (Department of Gastrointestinal Surgery, Gifu University Hospital)、Yuta Sato (Department of Gastrointestinal Surgery, Gifu University Hospital)、Jesse Y. Tajima (Department of Gastrointestinal Surgery, Gifu University Hospital)、Yoshihiro Tanaka (Department of Gastrointestinal Surgery, Gifu University Hospital)、Katsutoshi Murase (Department of Gastrointestinal Surgery, Gifu University Hospital)、Nobuhisa Matsuhashi (Department of Gastrointestinal Surgery, Gifu University Hospital)

[Background]Cancer cells rely on glycolysis for energy, known as the Warburg effect, which supports survival by promoting nucleotide synthesis, reducing oxidative stress, and inhibiting apoptosis. Polypyrimidine tract-binding protein 1 (PTBP1) regulates pyruvate kinase muscle (PKM1/PKM2) splicing, maintaining a PKM2-dominant state. Suppressing PTBP1 shifts metabolism to a PKM1-dominant state, inhibiting glycolysis and promoting apoptosis. We developed optimized siR-PTBP1 derivatives to evaluate their therapeutic potential.[Methods]Chemically modified siR-PTBP1 derivatives were tested in DLD-1 colorectal cancer cells. Cell viability was assessed by CVS assay, and PTBP1, PKM1/PKM2, and cleaved PARP protein levels were analyzed by Western blotting and immunostaining.[Results]Derivative X significantly suppressed PTBP1, increased PKM1 expression, and raised the PKM1/PKM2 ratio. Increased cleaved PARP expression indicated apoptosis induction and Warburg effect suppression.[Conclusion]Optimized siR-PTBP1 derivatives suppressed glycolysis and induced apoptosis, highlighting their potential as nucleic acid therapeutics. Future research will refine derivatives and assess in vivo efficacy.

[Disclosure of COI by Responsible researcher] Lead presenter is the Responsible researcher.
[Lead presenter: Keita Matsumoto] We have no financial relationships to disclose.

### [P-2281] I-P18
### Advances in Cell & Gene Therapy Technologies

2025 / 9 / 26 (Fri) 17:30-18:15 Room P/(Motenashi Dome) Room P

---

### P-2281
### Withdrawn

No Abstract

### [P-2282] I-P18
### Advances in Cell & Gene Therapy Technologies

2025 / 9 / 26 (Fri) 17:30-18:15 Room P/(Motenashi Dome) Room P

---

### P-2282
### Development of epigenome editing therapy for acute myeloid leukemia

[Speaker]Yoshitaka Sunami (IMS, Tokyo Med. Univ.) [Co-author]Miwa Tanaka (IMS, Tokyo Med. Univ.／Dept. Cancer Epigenomics, Cancer Inst, JFCR.)、Takuro Nakamura (IMS, Tokyo Med. Univ.)

Aberrant expression of oncogenes via activated enhancers contributes to myeloid leukemogenesis. We previously demonstrated that the pseudokinase Trib1 promotes Hoxa9-induced acute myeloid leukemia (AML) through modification of the super-enhancers (SEs) associated with oncogenes such as Erg. These SEs could be therapeutic targets of JQ1, a BRD4 inhibitor. However, there are limitations in current epigenetic therapies targeting trans-acting factors due to their effects for SEs important for normal hematopoiesis. To overcome the non-specific effects of epigenetic drugs, we aimed to develop a novel therapeutic strategy for AML in which epigenetic aberrations play an essential role by targeting SEs and active enhancers through epigenome editing using CRISPR/dCas9. We introduced a gRNA mini-library targeting enhancer regions surrounding MLL-AF9 breakpoints into THP-1 cells expressing dCas9-KRAB-MeCP2 and identified MLL-specific enhancers. Suppression of MLL-AF9 expression was effectively achieved, resulting in growth arrest and granulocytic differentiation of THP-1 cells. These results underscore the power of epigenome editing as a promising therapeutic approach for AML.

[Disclosure of COI by Responsible researcher] Lead presenter is not the Responsible researcher.
[Lead presenter: Yoshitaka Sunami] We have no financial relationships to disclose.
[Principal Researcher: Takuro Nakamura] We have no financial relationships to disclose.

[P-2288] I-P19-2
Cancer Prevention & Epidemiology (2)

2025 / 9 / 26 (Fri) 17:30-18:15 Room P/(Motenashi Dome) Room P

P-2288

### Cancer chemopreventive effect of ***Piper sarmentosum*** leaf extract on the early stage of liver and colon carcinogenesis

[Speaker]Beilin Li (Dept. of Biochem,CMU) [Co-author]Sirinya Taya (MDRI, CMU)、Jetsada Ruangsuriya (Dept. of Biochem,CMU)、Teera Chewonarin (Dept. of Biochem,CMU)

***Piper sarmentosum*** Roxb. is a fragrant herbaceous species with a rich culinary heritage in Thai gastronomy. The leaves of ***P. sarmentosum*** possess several phytochemicals exhibiting a range of pharmacological effects. The cancer chemopreventive efficacy of ***P. sarmentosum*** leaf extract (PSE) remains unexplored. The objective of this study is to examine the cancer chemopreventive effects of PSE on liver and colon carcinogenesis and its inhibitory mechanisms. Male Wistar rats received three injections of DEN to induce hepatocarcinogenesis and two injections of DMH to induce colon carcinogenesis. PSE was administered orally to rats for a duration of 10 weeks. Preneoplastic lesions, such as $GSTP^{+}$ foci in the liver and ACF in the colon, were assessed as endpoint markers. PSE significantly diminished $GSTP^{+}$ foci in comparison to carcinogen-treated rats. Nonetheless, PSE exhibited no notable changes in the suppression of ACF in the colon. In conclusion, PSE demonstrates potential as a chemopreventive agent against liver carcinogenesis by diminishing preneoplastic lesions in the liver. The mechanisms of inhibition, including cell proliferation and death, are now under investigation.

[Disclosure of COI by Responsible researcher] Lead presenter is not the Responsible researcher.
[Lead presenter: Beilin Li] We have no financial relationships to disclose.
[Principal Researcher: Sirinya Taya, Teera Chewonarin] We have no financial relationships to disclose.

[P-2289] I-P19-2
Cancer Prevention & Epidemiology (2)

2025 / 9 / 26 (Fri) 17:30-18:15 Room P/(Motenashi Dome) Room P

P-2289
### miR-101 mediates the inhibitory effects of active components from ***Antrodia cinnamomea*** mycelia on skin cancer progression

[Speaker]Hui-En Hsu (Institute of Medicine, Chung Shan Medical University) [Co-author]Yun-Ching Chang (Institute of Medicine, Chung Shan Medical University／Department of Health Industry Technology, Chung Shan Medical University)

Skin cancer, among the ten most common cancers according to the WHO, includes basal cell carcinoma, squamous cell carcinoma, and melanoma. Melanoma, the deadliest form, has limited treatment options due to resistance and side effects. Antrodin B, a bioactive compound derived from ***Antrodia cinnamomea***, has demonstrated anti-inflammatory and anti-cancer effects. This study investigates its potential in melanoma, focusing on its interaction with miR-101, a key regulator of the cell cycle, apoptosis, and differentiation. In vitro, 80 μM antrodin B reduced B16-F10 cell viability by 30 %, activated Caspase-3, downregulated Bcl-2, and upregulated miR-101. In vivo, antrodin B (15 and 30 mg/kg) treatment for four weeks resulted in a 60 % reduction in tumor volume and a 22 % decrease in tumor weight. Histological analysis showed increased tumor cell death, reduced neovascularization, and confirmed miR-101 upregulation. Immunohistochemistry confirmed Caspase-3 activation and Bcl-2 downregulation. These results suggest that antrodin B could be a promising melanoma treatment by modulating apoptosis and regulating miR-101, potentially overcoming resistance and improving therapeutic outcomes.

[Disclosure of COI by Responsible researcher] Lead presenter is the Responsible researcher.
[Lead presenter: Hui-En Hsu] We have no financial relationships to disclose.

▲TOP

### [P-2290] I-P19-2
### Cancer Prevention & Epidemiology (2)

2025 / 9 / 26 (Fri) 17:30-18:15 Room P/(Motenashi Dome) Room P

Poster Session

---

### P-2290
### Growth Inhibitory Effect of Oxylipin on Human Lung Adenocarcinoma Cell Line PC-9

[Speaker]Hinako Suga (Sch. Health Sci. Odawara, Intl. Univ. Health Welfare) [Co-author]Kyoichi Takao (Nihon Univ. Sch. Med.／Juntendo Univ. Grad. Sch. Med.)、Masaharu Nomura (Shingo Central Clinic)、Noriko Gotoh (Kanazawa Univ. Cancer Res. Inst.)

[Introduction] Oxylipins are bioactive molecules derived from the unsaturated fatty acids, involved in inflammation, immune responses, regulation of cell cycle and apoptosis. They contribute to various diseases including cancer. One such oxylipin, 9-oxo-octadecadienoic acid (9-oxo-ODA) has been reported to suppress ovarian and cervical cancer cell proliferation (Mogi, K. et al., 2023). We investigated whether 9-oxo-ODA inhibits proliferation of human lung adenocarcinoma cell line PC-9, which harbor EGFR mutation.
[Methods] PC-9 cells were cultured with 9-oxo-ODA (Cayman), and cell numbers were measured using an automated cell counter (Bio-Rad).
[Results & Discussion] 9-oxo-ODA inhibited proliferation of PC-9 cells in a concentration-dependent manner, causing growth suppression and morphological changes at 40 μg/mL, where cell viability remained at 93 ± 2.5%. These findings suggest that 9-oxo-ODA suppresses the growth of lung adenocarcinoma cells with EGFR mutation and acts mainly as a cytostatic agent.
[Acknowledgments] This work was partly supported by Extramural Collaborative Research Grant of Cancer Research Institute, Kanazawa University.

[Disclosure of COI by Responsible researcher] Lead presenter is the Responsible researcher.
[Lead presenter: Hinako Suga] We have no financial relationships to disclose.

### [P-2291] I-P19-2
### Cancer Prevention & Epidemiology (2)

2025 / 9 / 26 (Fri) 17:30-18:15 Room P/(Motenashi Dome) Room P

### P-2291
### Withdrawn

No Abstract

### [P-2292] I-P19-2
### Cancer Prevention & Epidemiology (2)

2025 / 9 / 26 (Fri) 17:30-18:15 Room P/(Motenashi Dome) Room P

### P-2292
### Withdrawn

No Abstract

### [P-2293] I-P19-2
### Cancer Prevention & Epidemiology (2)

2025 / 9 / 26 (Fri) 17:30-18:15 Room P/(Motenashi Dome) Room P

---

### P-2293
### Prognostic value of HER2 discordance between primary and metastatic/recurrent breast cancer: a SR and MA

[Speaker]Shunsuke Nakatani (Regulatory Sci., Meiji Pharm. Univ.／Daiichi Sankyo Co., Ltd.) [Co-author]Takuya Hayashi (Daiichi Sankyo Co., Ltd.)、Aoi Hosaka (Regulatory Sci., Meiji Pharm. Univ.)、Hideki Maeda (Regulatory Sci., Meiji Pharm. Univ.)

Approximately 10% of metastatic/recurrent (m/r) breast cancer (BC) shows HER2 discordance (loss/gain) between primary and m/r tumors. The impact of it on prognosis remains unclear. Therefore, we aimed to investigate the prognostic value of it through a systematic review and meta-analysis.
Studies were searched from PubMed and Cochrane Library. The key eligibility criteria were as follows: m/r BC; available HER2 status of both primary and m/r tumors; and reported OS, PRS, or PFS.
Of 1,374 studies that were initially identified, after conducting 2nd screening on 109 out of them, 7 met the eligibility criteria. HER2 discordance was associated with shorter OS than HER2 concordance; HR: 1.72 (95% CI: 0.82, 3.58; p=0.1491). Additionally, subgroup analysis focusing on HER2 loss revealed that it significantly worsens OS compared to HER2 retained; HR: 3.53 (95% CI: 1.91, 6.54; p< .0001).
These findings suggest that HER2 discordance may be a potential risk factor for prognosis. Moreover, HER2 loss is indicated as a prognostic factor in patients with HER2-positive m/r BC. One possible factor contributing to the poorer prognosis in HER2 loss may be the ineffectiveness of anti-HER2 therapies.

[Disclosure of COI by Responsible researcher] Lead presenter is not the Responsible researcher.
[Lead presenter: Shunsuke Nakatani] ■Profit of stock: 第一三共株式会社
[Principal Researcher: Hideki Maeda] We have no financial relationships to disclose.

### [P-2300] I-P21
### Palliative medicine

2025 / 9 / 26 (Fri) 17:30-18:15 Room P/(Motenashi Dome) Room P

---

### P-2300
### Relationship Between Opioid Dose and Opioid-Induced Constipation in Cancer Patients: A Post Hoc Analysis of OIC-J Study

[Speaker]Hisao Imai (Dept. of Respiratory Med., Saitama Med. Univ. International Med. Ctr.) [Co-author]Soichi Fumita (Dept. of Med. Oncology, Kindai Univ. Nara Hosp.)、Toshiyuki Harada (Ctr. for Respiratory Diseases, JCHO Hokkaido Hosp.)、Yusaku Akashi (Dept. of Med. Oncology, Kindai Univ. Nara Hosp.)、Yuichi Koretaka (Data Sci. Dept., Shionogi & Co., Ltd.)、Yasuhide Morioka (Med. Affairs Dept., Shionogi & Co., Ltd.)、Yoshiyuki Kizawa (Inst. of Med., Univ. of Tsukuba)、Akihiro Tokoro (Dept. of Psychosomatic Internal Med. Kinki-Chuo Chest Med. Ctr.)

This post-hoc analysis of the OIC-J observational study (UMIN000025864) investigated the incidence of opioid-induced constipation (OIC) in cancer patients. The study included patients without pre-existing constipation who initiated strong opioid analgesics without prophylactic laxatives (n=100). The relationship between daily opioid dosage (oral morphine milligram equivalent [MME]) and OIC incidence was assessed. Within two weeks of opioid initiation, 66% developed OIC. The mean (±SD) dosage was 22.5±15.7 MME/day in the OIC group and 17.8±13.0 MME/day in the non-OIC group, with no significant difference (***p***=0.1380). The OIC incidence increased numerically from 54.2% (95% CI: 32.8-74.5) at ≤10 mg/day to 80.0% (44.4-97.5) at >40-80 mg/day. In logistic regression analysis, the adjusted odds ratio and p-value for daily opioid dosage (per 10 mg) were 1.339 (95% CI: 0.949-1.890) and 0.096, suggesting that daily opioid dosage was not a statistically significant factor in the OIC incidence. OIC should be managed in all patients initiating opioid analgesia, regardless of dosage, given that approximately half of the lowest-dose group patients without pre-existing constipation developed OIC.

[Disclosure of COI by Responsible researcher] Lead presenter is the Responsible researcher.
[Lead presenter: Hisao Imai] We have no financial relationships to disclose.

### [P-2301] I-P21
### Palliative medicine

2025 / 9 / 26 (Fri) 17:30-18:15 Room P/(Motenashi Dome) Room P

---

### P-2301
### Investigation of the hypothalamic dysfunction in cancer cachexia aiming to discover novel druggable targets

[Speaker]Takumi Kobayashi (Grad. Sch. of Pharm. Sci. Chiba Univ.) [Co-author]Miaki Uzu (Grad. Sch. of Pharm. Sci. Chiba Univ.)

Cancer cachexia is a systemic metabolic syndrome characterized by unintentional anorexia, and loss of skeletal muscle mass with or without the loss of fat mass. This syndrome results in poor quality of life in cancer patients, thus, new effective treatments are required for better palliative care. We established a novel animal model of cachexia that meets clinical criteria by s.c injecting the murine Renal Cancer cell line RenCa into mice. This study aims to explore novel druggable targets for cancer cachexia using the RenCa-induced cachexia model, focusing on the hypothalamus, which regulates the appetite and the secretion of hormones involved in muscle loss. Despite a time-dependent reduction of food intake, an increase of orexigenic peptide Npy was detected in the hypothalamus in concurrence with decrease of anorexigenic peptide Pomc. Furthermore, the neural activity marker c-fos was decreased in the hypothalamus. These results indicate that abnormalities in neuronal activity in the hypothalamus occur with the progression of cachexia. Therefore, we are now investigating the mechanisms that peripherally located RenCa affects the neuronal activity in the hypothalamus.

[Disclosure of COI by Responsible researcher] Lead presenter is not the Responsible researcher.
[Lead presenter: Takumi Kobayashi] We have no financial relationships to disclose.
[Principal Researcher: Miaki Uzu] We have no financial relationships to disclose.

[P-2308] II-P8
Bone & soft tissue tumors

2025 / 9 / 26 (Fri) 17:30-18:15 Room P/(Motenashi Dome) Room P

P-2308
### Identification of sufficient conditions for osteosarcoma development in juvenile mice

[Speaker]Masato Saito (Dept. of Mol. Pathol., The Univ. of Tokyo／Dept. of Orthop. Surg., Keio Univ.) [Co-author]Nao Sankoda (Dept. of Mol. Pathol., The Univ. of Tokyo)、Fumie Nakasuka (Dept. of Mol. Pathol., The Univ. of Tokyo)、Robert Nakayama (Dept. of Orthop. Surg., Keio Univ.)、Masaya Nakamura (Dept. of Orthop. Surg., Keio Univ.)、Yasuhiro Yamada (Dept. of Mol. Pathol., The Univ. of Tokyo)

Using a ***p21*** reporter mouse model, we unexpectedly observed frequent ***p21***-expressing cells in the metaphysis (MP) of long bones in normal juvenile mice. This study aims to elucidate ***p21*** expression patterns in the MP and to determine whether ***p21***-expressing cells can give rise to osteosarcoma (OS), as human OSs most commonly arise in the MP during juvenile to adolescence. We show that ***p21*** is expressed in proliferating osteoblasts (OBs) of juvenile mice through replication stress and that OS arises from ***p21***-expressing OBs following ***Trp53*** inactivation, a common mutation in human OSs. However, OS develops only in aged mice and appears as a solitary tumor, indicating that ***Trp53*** mutation alone is insufficient for OS development from immature ***p21***-expressing OBs. To uncover additional factors driving OS, we focused on c-Myc, which is frequently amplified in human OSs. ***c-Myc*** induction transiently boosts the proliferation of ***p21***-expressing OBs in juvenile mice, but this effect is not sustained after the growth plate maturation. Finally, ***Trp53*** inactivation confers sustained proliferative ability in the ***c-Myc***-induced ***p21***-expressing OBs in juvenile mice, leading to rapid metastasis to the lungs.

[Disclosure of COI by Responsible researcher] Lead presenter is not the Responsible researcher.
[Lead presenter: Masato Saito] We have no financial relationships to disclose.
[Principal Researcher: Yasuhiro Yamada] ■Patent fee: 住友ファーマ

▲TOP

[P-2309] II-P8
Bone & soft tissue tumors

2025 / 9 / 26 (Fri) 17:30-18:15 Room P/(Motenashi Dome) Room P

P-2309
### Identification of an antigen recognized by tumor-infiltrating lymphocyte in a patient with osteosarcoma

[Speaker]Takahide Itabashi (Department of Pathology, Sapporo Medical University, School of Medicine) [Co-author]Kenji Murata (Department of Pathology, Sapporo Medical University, School of Medicine)、Tomohide Tsukahara (Department of Pathology, Sapporo Medical University, School of Medicine)、Terufumi Kubo (Department of Pathology, Sapporo Medical University, School of Medicine)、Takayuki Kanaseki (Department of Pathology, Sapporo Medical University, School of Medicine)、Yoshihiko Hirohashi (Department of Pathology, Sapporo Medical University, School of Medicine)、Toshihiko Torigoe (Department of Pathology, Sapporo Medical University, School of Medicine)

Antigenicity of osteosarcoma (OS) in tumor microenvironment is still remain uncertain. In this study, we tried to identify antigens recognized by T cell receptor (TCR) derived from tumor-infiltrating lymphocyte (TIL) in OS.
TCR genes were obtained by scRNAseq of the biopsy specimen of an OS patient, followed by transduction into T cells to establish TCR-T cells. B2M-knocked out OS cell lines were established using CRISPER-Cas9 and transfected with each HLA class I allele matched with the patient. cDNA library was constructed from the OS cell line and the reactivity of TCR-T cells against 293T cells transfected with HLA and cDNA library was screened based on 41BB expression.
Five TCR-T cells were established. Among them, one TCR-T cells was reacted with OS cell line expressing HLA-B*40:02. After screening of 54,000 cDNA library clones, we identified a cDNA encoding an antigen recognized by the TCR.
We identified the antigen recognized by TIL-derived TCR. It is useful for elucidation of immune surveillance in patients with OS.

[Disclosure of COI by Responsible researcher] Lead presenter is not the Responsible researcher.
[Lead presenter: Takahide Itabashi] We have no financial relationships to disclose.
[Principal Researcher: Toshihiko Torigoe] We have no financial relationships to disclose.

### [P-2310] II-P8
### Bone & soft tissue tumors

2025 / 9 / 26 (Fri) 17:30-18:15 Room P/(Motenashi Dome) Room P

---

### P-2310
### Megakaryoblastic leukemia 1 (MKL1) contributes to metastatic progression in osteosarcoma

[Speaker]Nobusue Hiroyuki (Div. Gene. Reg, Oncology Innovation Center., Fujita Health Univ.) [Co-author]Takatsune Shimizu (Dep. Pathophysiology, Hoshi Univ.)、Tetsuya(Fujita Health Univ.) Takimoto (Div. Gene. Reg, Oncology Innovation Center., Fujita Health Univ.)、Megumi Uetaki (Div. Gene. Reg, Oncology Innovation Center., Fujita Health Univ.)、Reiko Hagiwara (Div. Gene. Reg, Oncology Innovation Center., Fujita Health Univ.)、Seiji Yamada (Div. Gene. Reg, Oncology Innovation Center., Fujita Health Univ.)、Hideyuki Saya (Div. Gene. Reg, Oncology Innovation Center., Fujita Health Univ.)

Metastasis involves sequential processes-local invasion, systemic dissemination, and colonization at distant sites. While individual molecules contributing to each step have been identified and described, mechanisms that integrate and orchestrate these processes remain unclear. Here, we show that megakaryoblastic leukemia 1 (MKL1), a transcriptional coactivator involved in actin cytoskeleton regulation, acts as an integrative driver of metastatic progression in osteosarcoma (OS). MKL1 activity was significantly elevated in OS cells derived from metastatic lung lesions compared to those from primary tumors. Forced activation of MKL1 in mouse OS cells led to the upregulation of gene signatures associated with invasion and resulted in a marked increase in Matrigel invasion capacity. Furthermore, MKL1-induced OS cells also exhibited increased sphere-forming capacity, suggesting a role in metastatic colonization. Together, our findings demonstrate that MKL1 coordinates the invasion, dissemination, and colonization steps of osteosarcoma metastasis through its regulation of actin cytoskeletal dynamics, and may represent a promising therapeutic target for the treatment of metastatic OS.

[Disclosure of COI by Responsible researcher] Lead presenter is not the Responsible researcher.
[Lead presenter: Hiroyuki Nobusue] We have no financial relationships to disclose.
[Principal Researcher: Hideyuki Saya] ■Advisor of the company and/or profit-making organization: Diamont Medino Capital株式会社　■Research expenses: 株式会社医学生物学研究所、シスメックス株式会社、三洋化成株式会社

### [P-2311] II-P8
### Bone & soft tissue tumors

2025 / 9 / 26 (Fri) 17:30-18:15 Room P/(Motenashi Dome) Room P

---

### P-2311
### Molecular Pathology of the Tumor Microenvironment of Giant Cell Tumor of Bone by Spatial Transcriptomic Analysis

[Speaker]Yo Kimura (Division of Cellular Signaling, National Cancer Center Research Institute) [Co-author]Masachika Ikegami (Division of Cellular Signaling, National Cancer Center Research Institute)、Akihiko Yoshida (Department of Diagnostic Pathology, National Cancer Center Hospital)、Satoshi Kamio (Division of Cellular Signaling, National Cancer Center Research Institute／Department of Musculoskeletal Oncology and Rehabilitation, National Cancer Center Hospital)、Eisuke Kobayashi (Department of Musculoskeletal Oncology and Rehabilitation, National Cancer Center Hospital)、Akira Kawai (Department of Musculoskeletal Oncology and Rehabilitation, National Cancer Center Hospital)、Yoshiyuki Suehara (Division of Cellular Signaling, National Cancer Center Research Institute／Department of Musculoskeletal Oncology and Orthopedic Surgery, Tochigi Cancer Center／Department of Orthopedic Surgery, Juntendo University, Graduate School of Medicine)、Shinji Kohsaka (Division of Cellular Signaling, National Cancer Center Research Institute)

**Introduction:** Giant cell tumor of bone (GCTB) is a locally aggressive osteolytic tumor of intermediate malignancy treated with surgery and/or Denosumab, a RANKL inhibitor. However, its molecular characteristics and tumor heterogeneity remain poorly understood. This study aimed to analyze GCTB using spatial transcriptomics and single-cell RNA sequencing to uncover tumor heterogeneity and drug-related molecular changes.

**Methods:** 10 Frozen and 11 FFPE samples from GCTB patients, both pre- and post-Denosumab treatment, were analyzed. Spatial transcriptomics was performed using 10x Genomics Visium HD, and single-cell analysis used the Chromium platform. Sequencing was conducted on the Illumina NovaSeq 6000.

**Results:** UMAP analysis revealed spatially distinct and heterogeneous gene expression across tumor regions. We identified transcription factors involved in monocyte-to- osteoclast differentiation and analyzed intercellular communication between osteoclasts and tumor cells.

**Conclusions:** This study revealed spatial and cellular heterogeneity in GCTB and provided new insights into the molecular mechanisms underlying Denosumab's therapeutic effects.

[Disclosure of COI by Responsible researcher] Lead presenter is not the Responsible researcher.
[Lead presenter: Yo Kimura] We have no financial relationships to disclose.
[Principal Researcher: Shinji Kohsaka] We have no financial relationships to disclose.

▲TOP

### [P-2312] II-P8
### Bone & soft tissue tumors

2025 / 9 / 26 (Fri) 17:30-18:15 Room P/(Motenashi Dome) Room P

---

### P-2312
### Analysis of Tumor Microenvironment and Angiogenesis in Fibrosarcomatous Dermatofibrosarcoma Protuberans

[Speaker]Sakura Shiraishi (Department of Anatomic Pathology, Kyushu University) [Co-author]Takeshi Iwasaki (Department of Anatomic Pathology, Kyushu University)、Ken Takigawa (Department of Medicine and biosystem Science, Kyushu University)、Hiroki Sonoda (Department of Anatomic Pathology, Kyushu University)、Taro Mori (Department of Anatomic Pathology, Kyushu University)、Kohta Miyawaki (Department of Medicine and biosystem Science, Kyushu University)、Masatoshi Shimo (Department of Medicine and biosystem Science, Kyushu University)、Makoto Endo (Department of Orthopedic Surgery, Kyushu University)、Yasuharu Nakashima (Department of Orthopedic Surgery, Kyushu University)、Yoshinao Oda (Department of Anatomic Pathology, Kyushu University)

Fibrosarcomatous DFSP (FS-DFSP) is a more aggressive variant of DFSP characterized by the presence of fibrosarcomatous components. The tumor immune microenvironment and metabolism influence tumor behavior, but their roles in DFSP remain unclear. We analyzed 84 DFSP and 38 FS-DFSP cases. Immune markers (CD8, CD20, PD-L1, CTLA4, TIM3, CD163, SIRP-α), metabolic marker IDO-1, and CAF markers (FAP, periostin) were evaluated by immunohistochemistry. FS-DFSP was assessed by separating conventional and FS components. Angiogenesis was assessed with αSMA and CD31 to identify vessel maturity. Gene expression profiling was performed with nCounter®.CD163-positive macrophages were significantly increased in FS areas. T and B cell infiltration did not differ significantly. PD-L1 was positive in 2/38 FS-DFSP (5%). IDO-1 was more frequently expressed in FS-DFSP ($p<0.05$). CTLA4, TIM3 and SIRP-α were not detected. CAFs markers were more abundant at the invasive front of FS-DFSP. FS areas also showed more immature vessels.These findings suggest that the TME differs between FS and DFSP components, potentially contributing to tumor progression. Gene expression data will also be discussed.

[Disclosure of COI by Responsible researcher] Lead presenter is not the Responsible researcher.
[Lead presenter: Sakura Shiraishi] We have no financial relationships to disclose.
[Principal Researcher: Yoshinao Oda] We have no financial relationships to disclose.

▲TOP

### [P-2313] II-P8
### Bone & soft tissue tumors

2025 / 9 / 26 (Fri) 17:30-18:15 Room P/(Motenashi Dome) Room P

---

### P-2313
### Immunohistochemical expression of SETD5 as a pathological factor for dedifferentiation and prognosis in liposarcoma

[Speaker]Makoto Abe (Tochigi cancer center, research institute, division of molecular pathology) [Co-author]Naoto Kubota (Tochigi cancer center, research institute, division of molecular pathology)、Ken Yamazaki (Tochigi cancer center, research institute, division of molecular pathology)、Eisuke Miura (Tochigi cancer center, research institute, division of molecular pathology)、Masatsugu Ishii (Tochigi cancer center, research institute, division of molecular pathology)、Hirofumi Shirakawa (Tochigi cancer center, research institute, division of molecular pathology)、Hidenori Ojima (Tochigi cancer center, research institute, division of molecular pathology)

SETD5 (SET Domain Containing 5), a chromatin regulator involved in adipocytic differentiation, has been identified in various cancers, but its immunohistochemical expression and prognostic significance in liposarcoma remain unclear.
This study examined SETD5 immunohistochemical expression in liposarcomas and its prognostic significance. SETD5 expression was analyzed in 100 adipocytic tumors using immunohistochemistry, including 24 dedifferentiated liposarcoma (DDLPS), 26 well differentiated liposarcoma (WDLPS), 10 myxoid liposarcomas, 5 pleomorphic liposarcomas, and 35 benign adipocytic tumors.
SETD5 expression was significantly higher in WDLPS than in lipomas ($p = 0.01$) and markedly elevated in the dedifferentiated component of DDLPS compared to the well-differentiated component ($p<0.001$). In DDLPS, high SETD5 expression in the dedifferentiated component correlated with worse overall survival ($p<0.001$) but not disease-free survival ($p = 0.086$).
SETD5 immunohistochemical expression significantly correlates with prognosis in DDLPS, and may serve as a biomarker of dedifferentiation and prognosis to guide treatment strategy.

[Disclosure of COI by Responsible researcher] Lead presenter is not the Responsible researcher.
[Lead presenter: Makoto Abe] We have no financial relationships to disclose.
[Principal Researcher: Hidenori Ojima] We have no financial relationships to disclose.

### [P-2320] II-P9-2
### Prostate cancer (2)

2025 / 9 / 26 (Fri) 17:30-18:15 Room P/(Motenashi Dome) Room P

---

### P-2320
### Exercise alters arginine metabolism in the tumor microenvironment and inhibits prostate cancer growth

[Speaker]Toshiki Oka (The Department of Urology, The University of Osaka) [Co-author]Koji Hatano (The Department of Urology, The University of Osaka)、Hiroaki Hase (Laboratory of Cell Biology and Physiology, Osaka University)、Masatoshi Konishi (The Department of Urology, The University of Osaka)、Tomohiro Kanaki (The Department of Urology, The University of Osaka)、Yutong Liu (The Department of Urology, The University of Osaka)、Masaru Tani (The Department of Urology, The University of Osaka)、Yohei Okuda (The Department of Urology, The University of Osaka)、Gaku Yamamichi (The Department of Urology, The University of Osaka)、Yu Ishizuya (The Department of Urology, The University of Osaka)、Takuji Hayashi (The Department of Urology, The University of Osaka)、Yoshiyuki Yamamoto (The Department of Urology, The University of Osaka)、Taigo Kato (The Department of Urology, The University of Osaka)、Atsunari Kawashima (The Department of Urology, The University of Osaka)、Kazutake Tsujikawa (Laboratory of Cell Biology and Physiology, Osaka University)、Norio Nonomura (The Department of Urology, The University of Osaka)

[Objective]
Exercise improves the prognosis of prostate cancer (PCa) patients, but the mechanism is unclear. Therefore, we aimed to elucidate the mechanism by which exercise inhibits PCa growth using a mouse model.
[Methods]
We used prostate-specific ***Pten***-knockout mice [***Pb***-Cre+; ***Pten***(fl/fl)], spontaneously developing PCa. We divided the 12 mice into a voluntary exercise group and a control group. Exercise-induced changes in PCa tissues were analyzed using transcriptomic and metabolomic analysis.
[Results]
All mice in both groups showed carcinogenesis of PCa. Prostate weight (0.127 g vs 0.170 g, p=0.008) and Ki-67 positive cell rate (6.9% vs 16.5%, p=0.026) were significantly reduced in the exercise group compared to the control group. Exercise most significantly altered arginine-related pathways in transcriptome as well as metabolomic analysis. Specifically, the enzyme that converts arginine to ornithine, arginase1 (Arg1), was suppressed by exercise, leading to higher arginine in PCa tissue.
[Conclusions]
We have established a novel mouse model in which exercise inhibits the growth of PCa. Exercise suppressed Arg1 expression and altered arginine metabolism in PCa tissues.

[Disclosure of COI by Responsible researcher] Lead presenter is not the Responsible researcher.
[Lead presenter: Toshiki Oka] ■Research funds from company: 公的研究費（科研費）
[Principal Researcher: Koji Hatano] ■Research funds from company: 公的研究費（科研費）

▲TOP

### [P-2321] II-P9-2
### Prostate cancer (2)

2025 / 9 / 26 (Fri) 17:30-18:15 Room P/(Motenashi Dome) Room P

---

### P-2321
### Association of PRC1.1 components in tumorigenesis of prostate cancer

[Speaker]Masahiro Sugiura (Chiba University Graduate School of Medicine department of Molecular Oncology／Chiba University Graduate School of Medicine department of Urology) [Co-author]Sanji Kanaoka (Chiba University Graduate School of Medicine department of Urology)、Manato Kanesaka (Chiba University Graduate School of Medicine department of Urology)、Wataru Kudo (Chiba University Graduate School of Medicine department of Molecular Oncology)、Takayuki Hoshii (Chiba University Graduate School of Medicine department of Molecular Oncology)、Masaki Fukuyo (Chiba University Graduate School of Medicine department of Molecular Oncology)、Bahityar R. Nawai (Chiba University Graduate School of Medicine department of Molecular Oncology)、Atsushi Kaneda (Chiba University Graduate School of Medicine department of Molecular Oncology)

Prostate cancer is expected to be the most common cancer among men in Japan in near future. While PRC2 member, EZH2 can activate the prostate cancer oncogenic signal independent from epigenetic inactivation mechanism via PRC2, contribution of PRC1 or PRC1 component remains unknown. BCOR is a component protein of PRC1.1 and is known to be a tumor suppressor gene in various tumors, especially hematologic tumors. However, BCOR gene was reported as a possible oncogene in prostate cancer in 2020. Therefore, we started research to elucidate the function of BCOR and other PRC1.1 proteins in prostate cancer. Here we analyzed the several databases. Among the PRC1.1 components, BCOR and PCGF1 were upregulated in prostate cancer, especially CRPC, and their upregulation were significantly associated with poor survival. In prostate cancer cell lines, these components were also upregulated in CRPC, and knockdown of these components reduced proliferative capacity. These downstream target genes are now analyzed by ChIP-seq and RNA-seq. It is suggested that PRC1.1 may critically contribute to prostate cancer and might be potentially druggable targets.

[Disclosure of COI by Responsible researcher] Lead presenter is not the Responsible researcher.
[Lead presenter: Masahiro Sugiura] We have no financial relationships to disclose.
[Principal Researcher: Sanji Kanaoka] ■Research expenses: Takeda Science Foundation, Uehara Memorial Foundation.

▲TOP

### [P-2322] II-P9-2
### Prostate cancer (2)

2025 / 9 / 26 (Fri) 17:30-18:15 Room P/(Motenashi Dome) Room P

---

### P-2322
### Elucidation of the tumor microenvironment that determines the chemotherapy sensitivity of bladder cancer

[Speaker]Ryosuke Ikeuchi (Department of Urology, Graduate School of Medicine, Kyoto University) [Co-author]Yuki Kita (Department of Urology, Graduate School of Medicine, Kyoto University)、Hajime Takamori (Department of Urology, Graduate School of Medicine, Kyoto University)、Hideaki Takada (Department of Urology, Graduate School of Medicine, Kyoto University)、Kenji Nakamura (Department of Urology, Graduate School of Medicine, Kyoto University)、Toshihiro Magaribuchi (Department of Urology, Graduate School of Medicine, Kyoto University)、Toru Sakatani (Department of Urology, Graduate School of Medicine, Kyoto University)、Akihiro Hamada (Department of Urology, Graduate School of Medicine, Kyoto University)、Kei Mizuno (Department of Urology, Graduate School of Medicine, Kyoto University)、Takayuki Sumiyoshi (Department of Urology, Graduate School of Medicine, Kyoto University)、Kimihiko Masui (Department of Urology, Graduate School of Medicine, Kyoto University)、Takayuki Goto (Department of Urology, Graduate School of Medicine, Kyoto University)、Ryoichi Saito (Department of Urology, Hyogo Medical University)、Takashi Kobayashi (Department of Urology, Graduate School of Medicine, Kyoto University)

**Introduction**

In recent years, it has been reported that the tumor microenvironment, including immune cells and cancer-associated fibroblasts(CAFs), plays an important role in chemosensitivity. This study aims to clarify the influence of CAFs on the efficacy of chemotherapy for bladder cancer.

**Methods**

We used 21 cell lines derived from 2 murine bladder cancer models (UPPL; ***Upk3a-CreERT2; Trp53***$^{L/L}$***; Pten***$^{L/L}$, and BBN carcinogenesis model). Using these cell lines, we identified the cell lines with and without resistance to cisplatin, specifically ***in vivo***. We also examined the differences in CAF populations between these two groups by single cell RNA-seq.

**Results**

We identified 6 cell lines that could be subcutaneously transplanted at high rates into B6 mice. Although the IC50 of cisplatin ***in vitro*** was comparable among the cell lines, the growth inhibitory effect of cisplatin differed among these subcutaneous tumors. We selected two lines each of responder and non-responder. Single cell RNA-seq revealed the differences in CAF polpulations between the two groups.

**Conclusion**

Single cell RNA-seq data suggested the possibility that the specific CAF population was related to chemoresistance.

[Disclosure of COI by Responsible researcher] Lead presenter is not the Responsible researcher.
[Lead presenter: Ryosuke Ikeuchi] We have no financial relationships to disclose.
[Principal Researcher: Takashi Kobayashi] ■Lecture fee: アステラス製薬・ヤンセンファーマ・MSD・アストラゼネカ・メルクバイオファーマ

▲TOP

### [P-2323] II-P9-2
### Prostate cancer (2)

2025 / 9 / 26 (Fri) 17:30-18:15 Room P/(Motenashi Dome) Room P

---

### P-2323
### The critical role of the BIG3-PHB2 complex as a novel therapeutic target in prostate cancer

[Speaker]Fengyu He (Natl. Inst. of Biomed. Innovation, Health & Nutrition／Dept. of Med. Nutrition, Tokushima Univ.) [Co-author]Keiji Uchiyama (Natl. Inst. of Biomed. Innovation, Health & Nutrition)、Tetsuro Yoshimaru (Natl. Inst. of Biomed. Innovation, Health & Nutrition／Inst. of Advanced Med. Sci, Tokushima Univ.)、Yosuke Matsushita (Natl. Inst. of Biomed. Innovation, Health & Nutrition)、Yasuhiko Nishioka (Dept. of Respiratory Med. & Rheumatology, Tokushima Univ.)、Toyomasa Katagiri (Natl. Inst. of Biomed. Innovation, Health & Nutrition／Inst. of Advanced Med. Sci, Tokushima Univ.)

Prostate cancer (PC) initially responds well to androgen deprivation therapy (ADT). However, resistance inevitably develops, underscoring the urgent need for new therapies. Our previous studies demonstrated that, in ER-positive breast cancer (BC) cells, the cancer specific scaffold protein BIG3(brefeldin A-inhibited guanine nucleotide-exchange protein 3) binds to the tumor suppressor PHB2 (prohibitin2), causing estrogen-signaling in BC cells. TCGA database analysis revealed that BIG3 is frequently upregulated in PC, similar to BC. Here we report the critical role of BIG3-PHB2 complex as a novel therapeutic target for AR-positive PC. We confirmed high BIG3 expression in AR-positive PC cell lines, LNCap and 22Rv1, and observed that BIG3 knockdown markedly suppressed cell growth. Importantly, we demonstrated that BIG3 forms a complex with PHB2 in AR-positive PC, and stERAP, a specific peptide inhibitor of BIG3-PHB2 interaction, effectively inhibited cancer cell proliferation. These findings suggest that the BIG3-PHB2 complex plays a pivotal role in the proliferation of PC cells, and that targeting this interaction represents a promising therapeutic strategy for AR-positive PC.

[Disclosure of COI by Responsible researcher] Lead presenter is not the Responsible researcher.
[Lead presenter: Fengyu He] We have no financial relationships to disclose.
[Principal Researcher: Fengyu He, Keiji Uchiyama, Tetsuro Yoshimaru] ■Research funds from company: Oncotherapy Science Co., Ltd. ■Advisor of the company and/or profit-making organization: Oncotherapy Science Co., Ltd. ■Contributions: Tokushima Breast Care Clinic

▲TOP

### [P-2324] II-P9-2
### Prostate cancer (2)

2025 / 9 / 26 (Fri) 17:30-18:15 Room P/(Motenashi Dome) Room P

---

### P-2324
### Impact of androgen receptor signaling suppression on prostate cancer progression through KRAS activation

[Speaker]Taiki Kamijima (Dept. of Integrative Cancer Therapy & Urology, Kanazawa Univ.) [Co-author]Kouji Izumi (Dept. of Integrative Cancer Therapy & Urology, Kanazawa Univ.)、Ryunosuke Nakagawa (Dept. of Integrative Cancer Therapy & Urology, Kanazawa Univ.)、Hiroshi Kano (Dept. of Integrative Cancer Therapy & Urology, Kanazawa Univ.)、Tomoyuki Makino (Dept. of Integrative Cancer Therapy & Urology, Kanazawa Univ.)、Renato Naito (Dept. of Integrative Cancer Therapy & Urology, Kanazawa Univ.)、Hiroaki Iwamoto (Dept. of Integrative Cancer Therapy & Urology, Kanazawa Univ.)、Hiroshi Yaegashi (Dept. of Integrative Cancer Therapy & Urology, Kanazawa Univ.)、Atsushi Mizokami (Dept. of Integrative Cancer Therapy & Urology, Kanazawa Univ.)

[Background]Prostate cancer (PCa) cells are highly dependent on androgens and are treated by suppressing androgen receptor (AR) signaling. But many patients relapse and develop castration-resistant prostate cancer (CRPC). Double negative CRPC(DNPC) is AR-independent and without neuroendocrine differentiation, and no effective treatment is available now. [Methods] We used the castration-sensitive PCa cell line LNCaP and CRPC cell lines LNCaP-SF and DU145 to evaluate the effects of KRAS inhibition and activation. [Results] The clinical database revealed KRAS signaling activation in both AR-independent and metastatic prostate cancer. In LNCaP-SF and DU145, KRAS inhibition suppressed proliferation and migration, but in LNCaP did not. The inhibition of AR in LNCaP increased the expression of FGFR3, which is easily bound to FGF8b, and activated KRAS signaling. The prostate stromal cells secrete FGF8b, and that cells derived from DNPC patients secrete more FGF8b. [Conclusion] As prostate cancer cells become AR-independent, stimulation of FGF8b from stromal cells in the prostate microenvironment activates KRAS in prostate cancer cells, leading to cancer progression.

[Disclosure of COI by Responsible researcher] Lead presenter is not the Responsible researcher.
[Lead presenter: Taiki Kamijima] We have no financial relationships to disclose.
[Principal Researcher: Ryunosuke Nakagawa] ■Research funds from company: 科研費 22K09495

[P-2325] II-P9-2
Prostate cancer (2)

2025 / 9 / 26 (Fri) 17:30-18:15 Room P/(Motenashi Dome) Room P

P-2325
### The effects of microRNAs induced by luteolin on prostate cancer.

[Speaker]Akihiro Murakami (Dept. Exp. Pathol. Tumor Biol., Nagoya City Univ.) [Co-author]Aya Naiki (Dept. Exp. Pathol. Tumor Biol., Nagoya City Univ.)、Taku Naiki (Dept. Exp. Pathol. Tumor Biol., Nagoya City Univ.／Dept. Nephro-urol., Nagoya City Univ.)、Hiroyuki Kato (Dept. Exp. Pathol. Tumor Biol., Nagoya City Univ.)、Satoru(Nagoya City Univ.) Takahashi (Dept. Exp. Pathol. Tumor Biol., Nagoya City Univ.)

[Background] We have previously reported that luteolin (lut) suppress prostate carcinogenesis and castration-resistant prostate cancer (CRPC) in animal models. In this study, we examined the effects of miR-29, which is upregulated by lut and targets androgen receptor (AR) and IGF-1R, on prostate cancer.[Methods] [1] MiR-29 was introduced into a hormone-sensitive human prostate cancer cell line (LNCaP) and a human CRPC cell line (22Rv1) and their effects on cell growth and gene expression were examined. [2] Five patients with low-intermediate risk prostate cancer and under active surveillance were administered with 50 mg of lut orally. All patients underwent a protocol biopsy, and the expression of miRNAs in serum and prostate tissue were analyzed. [Results] [1] MiR-29 suppressed cell proliferation and decreased the expression of AR and IGF-1R. [2] Two out of five patients showed a favorable response. In these patients, there was a trend toward increased miR-29 expression in serum and prostate and decreased AR, NKX3.1 expression in normal prostate glands.[Conclusion] MiR-29 suppressed prostate cancer by down-regulation of AR and IGF-1R.

[Disclosure of COI by Responsible researcher] Lead presenter is not the Responsible researcher.
[Lead presenter: Akihiro Murakami] We have no financial relationships to disclose.
[Principal Researcher: Aya Naiki] We have no financial relationships to disclose.

### [P-2326] II-P9-2
### Prostate cancer (2)

2025 / 9 / 26 (Fri) 17:30-18:15 Room P/(Motenashi Dome) Room P

---

### P-2326
### GDF15 propeptide as a novel theranostic target in PSMA-negative and neuroendocrine prostate cancer

[Speaker]Atsuki Matsukawa (Dept. Urology., Osaka Univ. Grad. Sch. Med.) [Co-author]Gaku Yamamichi (Dept. Urology., Osaka Univ. Grad. Sch. Med.)、Taigo Kato (Dept. Urology., Osaka Univ. Grad. Sch. Med.)、Masatoshi Konishi (Dept. Urology., Osaka Univ. Grad. Sch. Med.)、Tomohiro Kanaki (Dept. Urology., Osaka Univ. Grad. Sch. Med.)、Akihiro Yoshimura (Dept. Urology., Osaka Univ. Grad. Sch. Med.)、Toshiki Oka (Dept. Urology., Osaka Univ. Grad. Sch. Med.)、Yohei Okuda (Dept. Urology., Osaka Univ. Grad. Sch. Med.)、Yu Ishizuya (Dept. Urology., Osaka Univ. Grad. Sch. Med.)、Takuji Hayashi (Dept. Urology., Osaka Univ. Grad. Sch. Med.)、Yoshiyuki Yamamoto (Dept. Urology., Osaka Univ. Grad. Sch. Med.)、Koji Hatano (Dept. Urology., Osaka Univ. Grad. Sch. Med.)、Atsunari Kawashima (Dept. Urology., Osaka Univ. Grad. Sch. Med.)、Tadashi Watabe (Dept. Nuclear Med., Osaka Univ. Grad. Sch. Med.)、Norio Nonomura (Dept. Urology., Osaka Univ. Grad. Sch. Med.)

Objective: PSMA-targeted radioligand therapy (RLT) is effective for metastatic castration-resistant prostate cancer (mCRPC). However, 20% of these cases are PSMA-negative, and RLT is not recommended for neuroendocrine prostate cancer (NEPC). Thus, we evaluated GDF15 propeptide (GDPP), a novel marker, as a potential theranostic target for PSMA-negative mCRPC and NEPC.Methods: We examined the expression of PSMA and GDPP in CRPC and NEPC tissues by immunohistochemistry. An anti-GDPP antibody was labeled with Zirconium-89, and then intravenously administered to a prostate cancer bone metastases mouse models (n=3). The model mice were established using the PSMA-negative, GDPP-positive cell line PC3. Accumulation in bone metastases was assessed via PET-CT for 7 weeks.Results: In CRPC tissues, 16.2% of the sites were PSMA-positive, whereas 45.3% were GDPP-positive. In the mouse at 7weeks, the mean SUV max in bone metastases was 2.64 one day post-antibody administration, decreasing to 1.17 four days later.Conclusion: GDPP expression was confirmed in PSMA-negative CRPC/NEPC. GDPP represents a theranostic target for these refractory subtypes, potentially enabling future GDPP-targeted RLT.

[Disclosure of COI by Responsible researcher] Lead presenter is the Responsible researcher.
[Lead presenter: Atsuki Matsukawa] We have no financial relationships to disclose.

### [P-2334] II-P9-4
### Kidney/adrenal/testis cancer

2025 / 9 / 26 (Fri) 17:30-18:15 Room P/(Motenashi Dome) Room P

---

### P-2334
### PSMA-positive Tumo Vasculature in Renal Cell Carcinoma: An Angiogenic Phenotype Driven by the Microenvironment

[Speaker]Ryuta Watanabe (Department of Urology, Ehime University Hospital) [Co-author]Keito Kagimoto (Department of Urology, Ehime University Hospital)、Mami Chousei (Department of Biochemistry and Molecular Genetics, Ehime University)、Tomohisa Sakaue (Department of Cardiovascular and Thoracic Surgery, Ehime University／Division of Cell Growth and Tumor Regulation, Proteo-Science Center)、Mie Kurata (Department of Analytical Pathology, Ehime University／Department of Pathology, Ehime University Proteo-Science Center)、Noriyoshi Miura (Department of Urology, Ehime University Hospital)、Riko Kitazawa (Division of Diagnostic Pathology, Ehime University Hospital)、Tadahiko Kikugawa (Department of Urology, Ehime University Hospital)、Shigeki Higashiyama (Department of Biochemistry and Molecular Genetics, Ehime University／Department of Oncogenesis and Growth Regulation, Osaka International Cancer Institute)、Takashi Saika (Department of Urology, Ehime University Hospital)

PSMA is a marker of tumor vasculature, but its regulation in non-prostatic cancers is unclear. We examined PSMA expression in renal cell carcinoma (RCC) and its clinical and biological significance. In 45 RCC specimens, PSMA expression was assessed by immunohistochemistry and correlated with recurrence. Conditioned medium (CM) and 10,000 g pellet fractions from RCC cell lines were added to HUVECs. PSMA expression and angiogenic activity were evaluated by Western blotting, immunofluorescence, and tube formation. RNA-seq was used to assess transcriptomic changes. Spatial transcriptomics compared gene expression between tumor and normal vessels. Mass spectrometry analyzed proteins in the pellet. PSMA was localized to peritumoral vessels and associated with recurrence. CM induced PSMA and tube formation in HUVECs. The 10,000 g pellet had strong effects, suggesting microvesicle involvement. GDF15 and other growth factors were detected by mass spectrometry. RNA-seq showed activation of angiogenesis pathways. Spatial transcriptomics identified vascular clusters with high FOLH1 and angiogenic signals. PSMA-positive vessels may serve as biomarkers and targets.

[Disclosure of COI by Responsible researcher] Lead presenter is the Responsible researcher.
[Lead presenter: Ryuta Watanabe] ■Contributions: 日本新薬　研究助成300万円

### [P-2335] II-P9-4
### Kidney/adrenal/testis cancer

2025 / 9 / 26 (Fri) 17:30-18:15 Room P/(Motenashi Dome) Room P

---

### P-2335
### Alternative Lengthening of Telomeres in Chromophobe RCC: Prognostic Significance and Therapeutic Implications

[Speaker]Masayuki Haruta (Res. Inst. for Clin. Oncol., Saitama Cancer Ctr.) [Co-author]Riko Ikeda (Dept. Urol., Saitama Cancer Ctr.／Dept. Urol., Science Tokyo)、Yuta Sano (Dept. Urol., Saitama Cancer Ctr.)、Yuki Nakamura (Dept. Urol., Science Tokyo)、Hirotaka Fuchizawa (Dept. Urol., Saitama Cancer Ctr.)、Naoki Inoue (Dept. Urol., Saitama Cancer Ctr.)、Ayataka Ishikawa (Dept. Path., Saitama Cancer Ctr.)、Toshihiko Iizuka (Dept. Path., Saitama Cancer Ctr.)、Soichiro Yoshida (Dept. Urol., Science Tokyo)、Ritsuko Onuki (Res. Inst. for Clin. Oncol., Saitama Cancer Ctr.)、Miki Ohira (Res. Inst. for Clin. Oncol., Saitama Cancer Ctr.)、Kiyohiro Ando (Res. Inst. for Clin. Oncol., Saitama Cancer Ctr.)、Yoh Matsuoka (Dept. Urol., Saitama Cancer Ctr.)、Yasuhisa Fujii (Dept. Urol., Science Tokyo)、Yukio Kageyama (Dept. Urol., Saitama Cancer Ctr.)、Takehiko Kamijo (Res. Inst. for Clin. Oncol., Saitama Cancer Ctr.)

Chromophobe renal cell carcinoma (chRCC) is a rare histological subtype usually associated with a good prognosis; however, about 10% of patients relapse or die of the disease. To improve patient outcomes, there is a need to elucidate molecular mechanisms, identify prognostic factors to identify malignant chRCC, and develop novel therapeutic strategies. Alternative lengthening of telomeres (ALT) has been associated with a poor prognosis in some cancers. Our previous studies suggested that ALT(+) chRCCs are more aggressive and linked to worse outcomes (progression-free survival ***p***=0.0002). To investigate the molecular characteristics of ALT(+) chRCC, we performed exome sequencing and a comparative genomic hybridization analysis in 3 ALT(+) and 3 ALT(-) chRCCs.Our results suggested the potential therapeutic approaches for ALT(+) chRCC: case 1, ***SMARCAL1*** homozygous deletion (etoposide and radiation therapy); case 2, ***PTEN*** and ***TBC1D7*** homozygous deletion (mTOR inhibitors); and case 3, TMB high and ***PTEN*** mutation (immune checkpoint inhibitors and mTOR inhibitors). An ALT analysis, exome sequencing, and chromosomal aberration analysis may assist in selecting treatment options for ALT(+) chRCC.

[Disclosure of COI by Responsible researcher] Lead presenter is not the Responsible researcher.
[Lead presenter: Masayuki Haruta] We have no financial relationships to disclose.
[Principal Researcher: Takehiko Kamijo] We have no financial relationships to disclose.

### [P-2336] II-P9-4
### Kidney/adrenal/testis cancer

2025 / 9 / 26 (Fri) 17:30-18:15 Room P/(Motenashi Dome) Room P

---

### P-2336
### FAXC regulates tumor progression via c-MET pathway in renal cell carcinoma

[Speaker]Masato Konno (Div. Cancer Stem Cell, Miyagi Cancer Ctr. Res. Inst.／Div. Urology, Miyagi Cancer Ctr.／Div. Urology, Tohoku Univ. Grad. Sch. Med.) [Co-author]Haruna Fujimori (Div. Cancer Stem Cell, Miyagi Cancer Ctr. Res. Inst.)、Rie Takahashi (Div. Cancer Stem Cell, Miyagi Cancer Ctr. Res. Inst.)、Mai Mochizuki (Div. Cancer Stem Cell, Miyagi Cancer Ctr. Res. Inst.)、Shigekazu Murakami (Div. Cancer Stem Cell, Miyagi Cancer Ctr. Res. Inst.)、Kazunori Yamaguchi (Div. Mol. & Cell. Oncol., Miyagi Cancer Ctr. Res. Inst.)、Jun Yasuda (Div. Mol. & Cell. Oncol., Miyagi Cancer Ctr. Res. Inst.)、Hisanobu Adachi (Div. Urology, Miyagi Cancer Ctr.)、Akihiro Ito (Div. Urology, Tohoku Univ. Grad. Sch. Med.)、Keiichi Tamai (Div. Cancer Stem Cell, Miyagi Cancer Ctr. Res. Inst.)、Naoki Asano (Div. Cancer Stem Cell, Miyagi Cancer Ctr. Res. Inst.)

Renal cell carcinoma (RCC) is a common cancer worldwide and there is an urgent need for novel therapeutic targets. We investigated the role of Failed Axon Connections Homolog (FAXC), a novel tumor factor that we recently identified, in RCC. In RCC cells, FAXC knockdown enhanced proliferation through activation of ERK. Knockdown of FAXC in RCC cells augmented c-MET expression and phosphorylation, and the increased proliferation in FAXC-knockdown cells was abolished by the inhibition of c-Met phosphorylation. On the other hand, FAXC-overexpressing RCC cells exhibited suppressed cell proliferation and c-MET expression. We further analyzed the intracellular distribution of FAXC and found that FAXC localized to the mitochondria in RCC cells. Finally, we investigated FAXC expression in RCC specimens and found that FAXC expression negatively correlated with c-MET phosphorylation, and that the expression of FAXC was lower in tumor tissues compared with that in adjacent normal tissues. In conclusion, our data revealed that FAXC depletion leads to the promotion of cell proliferation via activation of c-MET in RCC cells. This study provides a novel mechanism of RCC progression.

[Disclosure of COI by Responsible researcher] Lead presenter is not the Responsible researcher.
[Lead presenter: Masato Konno] We have no financial relationships to disclose.
[Principal Researcher: Naoki Asano] We have no financial relationships to disclose.

### [P-2337] II-P9-4
### Kidney/adrenal/testis cancer

2025 / 9 / 26 (Fri) 17:30-18:15 Room P/(Motenashi Dome) Room P

---

### P-2337
### YAP inhibits angiogenesis via suppression of HIF expression in clear cell renal cell carcinoma

[Speaker]Yuki Takai (Dept. of Urology, Yamagata Univ. Fac. of Med.) [Co-author]Hiromi Ito (Dept. of Urology, Yamagata Univ. Fac. of Med.)、Takafumi Narisawa (Dept. of Urology, Yamagata Univ. Fac. of Med.)、Sei Naito (Dept. of Urology, Yamagata Univ. Fac. of Med.)、Norihiko Tsuchiya (Dept. of Urology, Yamagata Univ. Fac. of Med.)

(1) Background; YAP, that is an effector of Hippo pathway, is known as a major oncogenic molecule. It is also reported that YAP is degraded VHL-proteasome in lung cancer. We investigated the expression and functions of YAP in clear cell renal cell carcinoma (ccRCC). (2) Method; On in vitro studies with RCC cell lines, we used immunoblotting, RT-qPCR, MTS assay, cell cycle analysis using FACS, transwell assay and HUVEC tube formation assay. (3) Results; The expression levels of YAP were decreased by prolyl hydroxylase 2 (PHD2) inhibitor only in VHL wild-type RCC cell lines. This result revealed that YAP is degraded via PHD2/VHL-proteasome and accumulated in VHL-mutant ccRCC. In ccRCC cell lines, YAP knockdown induced accumulation of HIF-2$\alpha$ and VEGF, a transcriptional target of HIF-2$\alpha$. HIF2 inhibition by PT2385 reduced VEGF elevation by YAP knockdown. HUVEC tube formation was decreased by coculture with 786-O with YAP knockdown. Whereas, YAP knockdown suppressed cell viability, cell cycle and motility of RCC cells. (4) Conclusion; YAP functions as an oncogenic molecule in RCC cell viability and motility, whereas YAP inhibits angiogenesis via suppression of HIF expression.

[Disclosure of COI by Responsible researcher] Lead presenter is the Responsible researcher.
[Lead presenter: Yuki Takai] We have no financial relationships to disclose.

### [P-2338] II-P9-4
### Kidney/adrenal/testis cancer

2025 / 9 / 26 (Fri) 17:30-18:15 Room P/(Motenashi Dome) Room P

---

### P-2338
### Investigation of the relationship between αE-catenin, migration ability, and intercellular adhesion in ccRCC

[Speaker]Shinta Suenaga (Yamagata University Faculty of Medicine, Department of Urology) [Co-author]Takafumi Narisawa (Yamagata University Faculty of Medicine, Department of Urology)、Suguru Ito (Yamagata University Faculty of Medicine, Department of Urology)、Yuki Takai (Yamagata University Faculty of Medicine, Department of Urology)、Mayu Yagi (Yamagata University Faculty of Medicine, Department of Urology)、Hiromi Ito (Yamagata University Faculty of Medicine, Department of Urology)、Sei Naito (Yamagata University Faculty of Medicine, Department of Urology)、Norihiko Tsuchiya (Yamagata University Faculty of Medicine, Department of Urology)

In clear cell renal cell carcinoma (ccRCC), a copy number gain in the long arm of chromosome 5 is observed at a frequency of approximately 60%, but its biological significance is not yet understood. We focused on αE-catenin, which has high mRNA expression on the same gene region and has not been previously reported in ccRCC. We studied 57 metastatic ccRCC samples from TCGA and our institution. Human renal cancer cell lines A-498, 789-O, and 769-P were used for the study. The siRNA method knocked down αE-catenin, and in the invasion assay, invasive capacity was significantly decreased; forced expression with A-498 tended to increase invasive capacity. Fluorescence staining and confocal microscopy revealed that αE-catenin co-localized with N-cadherin in intercellular spaces. β-catenin and N-cadherin, which had been localized intercellularly, were scattered throughout the cytoplasm. It has been reported that during cell migration, αE-catenin activate RHoA and myosin IIB, and participate in cell migration and invasion. Our results may support that in ccRCCs, αE-catenin is involved in cell adhesion together with N-cadherin, but also contributes to cell invasion and migration capacity.

[Disclosure of COI by Responsible researcher] Lead presenter is not the Responsible researcher.
[Lead presenter: Shinta Suenaga] We have no financial relationships to disclose.
[Principal Researcher: Norihiko Tsuchiya] We have no financial relationships to disclose.

### [P-2339] II-P9-4
### Kidney/adrenal/testis cancer

2025 / 9 / 26 (Fri) 17:30-18:15 Room P/(Motenashi Dome) Room P

---

### P-2339
### Transcriptomic Features of Metabolically Classified Clear Cell Renal Cell Carcinoma Reveal Distinct Biological Pathways

[Speaker]Sei Naito (Department of Urology, Yamagata University Faculty of Medicine) [Co-author]Takafumi Narisawa (Department of Urology, Yamagata University Faculty of Medicine)、Hiromi Ito (Department of Urology, Yamagata University Faculty of Medicine)、Yuki Takai (Department of Urology, Yamagata University Faculty of Medicine)、Shinta Suenaga (Department of Urology, Yamagata University Faculty of Medicine)、Mayu Yagi (Department of Urology, Yamagata University Faculty of Medicine)、Norihiko Tsuchiya (Department of Urology, Yamagata University Faculty of Medicine)

Objectives: This study aimed to characterize transcriptomic features of clear cell renal cell carcinoma (ccRCC) metabolic clusters using RNA expression analysis, building on previously established metabolic classifications. Methods: RNA sequencing was conducted on 31 ccRCC surgical specimens with existing metabolomic data. Gene set enrichment analysis identified pathways associated with each cluster.Results: Cluster A, defined by high levels of sugars, lactate, amino acids, and glutamine, showed elevated expression of hypoxia-related gene sets. Clusters B, C, and D were enriched in gene sets linked to apical junctions, mitotic spindle, TGF-b signaling, and K-RAS signaling. Cluster B, characterized by high fatty acid and pyruvate levels, had low expression of gene sets for IFN-g/a responses, complement activation, EMT, inflammation, allograft rejection, and myogenesis. Cluster D, distinct from Cluster C by higher glutamine and sugar levels, showed enrichment in oxidative phosphorylation, mTORC1 signaling, glycolysis, and ROS pathways. Conclusions: Transcriptomic profiles reveal molecular differences among ccRCC clusters, highlighting heterogeneity and therapeutic implications.

[Disclosure of COI by Responsible researcher] Lead presenter is the Responsible researcher.
[Lead presenter: Sei Naito] ■Lecture fee: BMS株式会社

### [P-2340] II-P9-4
### Kidney/adrenal/testis cancer

2025 / 9 / 26 (Fri) 17:30-18:15 Room P/(Motenashi Dome) Room P

---

### P-2340
### Elucidating the pathology of adrenocortical carcinoma using RNA-sequencing and exome sequencing.

[Speaker]Kentaro Yoshihara (Lab of Integrative Oncology Natl. Cancer Ctr. Res. Inst.) [Co-author]Fumihiko Urabe (Lab of Integrative Oncology Natl. Cancer Ctr. Res. Inst.／Dept. Urology, Jikei Univ., Sch, Med.)、Kagenori Ito (Lab of Integrative Oncology Natl. Cancer Ctr. Res. Inst.／Dept. Urology, Jikei Univ., Sch, Med.)、Hironori Suzuki (Lab of Integrative Oncology Natl. Cancer Ctr. Res. Inst.)、Yuki Kojima (Dept. of Med. Oncology, Natl. Cancer Ctr. Hosp.)、Ryouu Takahashi (Grad. Sch. of Biomed. & Health Sci., Hiroshima Univ.)、Takahiro Kimura (Dept. Urology, Jikei Univ., Sch, Med.)、Yusuke Yamamoto (Lab of Integrative Oncology Natl. Cancer Ctr. Res. Inst.)

Adrenocortical carcinoma (ACC) is an extremely rare cancer, occurring in about two people per million. It often develops in childhood and adulthood and is known for its high malignancy. Genetic factors, including TP53 mutations, are considered to be involved. Mitotane is available as a pharmacological treatment, but it isn't easy to continue due to its side effects in many cases. In this study, our goal was to identify prognostic biomarkers and novel therapeutic targets for ACC. We performed RNA-sequencing and exome-sequencing on both ACC and adrenocortical adenoma specimens. We characterized DNA mutation in ACC. Additionally, we analyzed comprehensively RNA expression and focused on the differences in gene expression between adenoma and cancer. Furthermore, using Curio Trekker (Curio Bioscience), we conducted single-cell spatial transcriptomic analysis of frozen ACC specimens. This approach revealed a distinct gene expression profile specific to cancer regions. In this presentation, we will report our findings on ACC-related gene expression and cell-cell interactions, highlighting the identified genes of interest.

[Disclosure of COI by Responsible researcher] Lead presenter is not the Responsible researcher.
[Lead presenter: Kentaro Yoshihara] We have no financial relationships to disclose.
[Principal Researcher: Yusuke Yamamoto] ■Research expenses: 東芝, NIKON

[P-2341] II-P9-4
Kidney/adrenal/testis cancer

2025 / 9 / 26 (Fri) 17:30-18:15 Room P/(Motenashi Dome) Room P

P-2341

### Automated spatial analysis reveals proximity of HLA class-I positive tumor cells and CD8 positive cells in seminoma.

[Speaker]Takuto Ogasawara (Dept. of Patology, Sapporo Medical University, Sapporo, Japan) [Co-author]Terufumi Kubo (Dept. of Patology, Sapporo Medical University, Sapporo, Japan)、Kenta Sasaki (Dept. of Patology, Sapporo Medical University, Sapporo, Japan)、Kenji Murata (Dept. of Patology, Sapporo Medical University, Sapporo, Japan)、Hashimoto Kohei (Dept. of Urology, Sapporo Medical University, Sapporo, Japan)、Takayuki Kanaseki (Dept. of Patology, Sapporo Medical University, Sapporo, Japan)、Tomohide Tsukahara (Dept. of Patology, Sapporo Medical University, Sapporo, Japan)、Yoshihiko Hirohashi (Dept. of Patology, Sapporo Medical University, Sapporo, Japan)、Naoya Masumori (Dept. of Urology, Sapporo Medical University, Sapporo, Japan)、Toshihiko Torigoe (Dept. of Patology, Sapporo Medical University, Sapporo, Japan)

[Background] The testis is known to be an immune-privileged organ due to its lack of HLA class I (HLA I) expression. However, the expression status of HLA I in seminoma, which is histologically characterized by lymphocytic infiltration, remains unclear. [Material and methods] We performed immunohistochemistry to quantify HLA I expression and its spatial relationship with CD8-positive cells in 20 cases of stage I seminoma. Each specimen was initially scored manually, followed by analysis using the HALO platform (Indica Labs, USA). [Results] Manual assessment and HALO detection of HLA I in seminoma showed 80% concordance, with 13 cases (65%) identified as HLA I positive. Based on the level of HLA I expression, we classified tumor lesions into three categories. The number of CD8-positive cells infiltrating the tumor correlated significantly with the level of HLA I expression. Furthermore, measuring the distance between tumor cells expressing HLA I and CD8-positive cells showed that smaller distances were associated with higher levels of HLA I expression.[Conclusion] Spatial analysis revealed that seminoma cells expressing HLA I were observed in close proximity to CD8-positive cells.

[Disclosure of COI by Responsible researcher] Lead presenter is not the Responsible researcher.
[Lead presenter: Takuto Ogasawara] We have no financial relationships to disclose.
[Principal Researcher: Yoshihiko Hirohashi] We have no financial relationships to disclose.

### [P-2346] II-P11-1
### Hematological malignancies (1)

2025 / 9 / 26 (Fri) 17:30-18:15 Room P/(Motenashi Dome) Room P

### P-2346
### Withdrawn

No Abstract

[P-2347] II-P11-1
Hematological malignancies (1)

2025 / 9 / 26 (Fri) 17:30-18:15 Room P/(Motenashi Dome) Room P

P-2347
### Investigation of the anti-cancer effects of Vitamin D3 (Rocaltrol) against primary effusion lymphoma (PEL)

[Speaker]Maako Ifuku (School of Medicine, Kumamoto Univ.／Joint Research Center for Human Retrovirus Infection) [Co-author]Prin Sungwan (Joint Research Center for Human Retrovirus Infection／graduate school of medical sciences, Kumamoto Univ.)、Seiji Okada (Joint Research Center for Human Retrovirus Infection／graduate school of medical sciences, Kumamoto Univ.)

Primary effusion lymphoma (PEL) is a rare B-cell lymphoma caused by human herpes virus8 (HHV-8) infection with a poor prognosis. Since PEL is resistant for standard chemotherapy, alternative treatment is needed. Active vitamin D3 (Rocaltrol) offers chemoprevention and potential anti-cancer effects against several malignancies. This study aims to examine the effect of Rocaltrol against PEL. The effect of Rocaltrol against PEL cell lines (BCBL-1, TY-1, GTO) was examined using MTT. Then, cell cycle status and apoptosis (subG1) were analyzed using propidium Iodide (PI) staining using Flowcytometry. Apoptosis was also investigated using Annexin V/PI staining. Rocaltrol strongly promoted G1 cell cycle arrest and induced apoptosis as shown by subG1 analysis and Annexin V/PI staining at the nanomolar level. IC50s of each cells are as follows: BCBL-1 0.56nM, TY-1 80.5nM, GTO 0.78nM. The underlying mechanisms of PEL response, including production of IL-6, IL-8, and PEL differentiation status, are being explored. Rocaltol exerted potent anti-cancer effects against PEL at the nanomolar level. VitaminD3 and its derivative might be the alternative potential treatment/chemoprevention for PEL.

[Disclosure of COI by Responsible researcher] Lead presenter is not the Responsible researcher.
[Lead presenter: Maako Ifuku] We have no financial relationships to disclose.
[Principal Researcher: Seiji Okada] We have no financial relationships to disclose.

### [P-2348] II-P11-1
### Hematological malignancies (1)

2025 / 9 / 26 (Fri) 17:30-18:15 Room P/(Motenashi Dome) Room P

---

### P-2348
### SREBF1 promotes the development of AML with poor prognosis by accelerating cholesterol metabolism.

[Speaker]Kazutsune Yamagata (Dept. Biochem. Fujita Health Univ. Sch. Med.) [Co-author]Yoko Kuroki (Ctr. Translational Res. Fujita Health Univ.)、Issay Kitabayashi (Ctr. Translational Res. Fujita Health Univ.)

Acute myeloid leukemia (AML) is among the most aggressive types of hematological malignancies, the subgroup with poor prognosis has a lesss than 25 % of 5-year survival rate, highlighting the need for the extensive studies to explore the mechanism of development of AML with poor prognosis. We observed that overexpression of SREBF1 correlates with a poor prognosis in AML patients. Indeed, SREBF1 was demonstrated to be crucial for the development of AML, and its overexpression was confirmed to accelerate AML development. While SREBF1 is primarily recognized for its role in regulating fatty acid metabolism, within AML cells, it contributes to AML pathogenesis by promoting cholesterol metabolism, specifically through enhanced cholesterol synthesis and LDL uptake. Our findings have established that SREBF1 overexpression increases intracellular cholesterol levels in AML cells, leading to an increase in lipid rafts in the cell membrane. This, in turn, results in the activation of the AKT pathway and inactivation of the TGF$\beta$ pathway. Our studies strongly suggest that SREBF1 may contribute to the development and unfavorable prognosis of AML by modulating these two signaling cascades.

[Disclosure of COI by Responsible researcher] Lead presenter is not the Responsible researcher.
[Lead presenter: Kazutsune Yamagata] We have no financial relationships to disclose.
[Principal Researcher: Issay Kitabayashi] We have no financial relationships to disclose.

▲TOP

### [P-2349] II-P11-1
### Hematological malignancies (1)

2025 / 9 / 26 (Fri) 17:30-18:15 Room P/(Motenashi Dome) Room P

### P-2349
### Clinico-genomic characteristics of chromosome 7 aberrations in AML and MDS treated with stem cell transplantation

[Speaker]Kentaro Gosho (Hematology Div., Tokyo Metro. Komagome Hosp.) [Co-author]Sadato Daichi (Clin. Res. & Trials Ctr., Tokyo Metro. Komagome Hosp.)、Takashi Toya (Hematology Div., Tokyo Metro. Komagome Hosp.)、Chizuko Hirama (Clin. Res. & Trials Ctr., Tokyo Metro. Komagome Hosp.)、Kaoru Morita (Hematology Div., Tokyo Metro. Komagome Hosp.)、Hiroaki Shimizu (Hematology Div., Tokyo Metro. Komagome Hosp.)、Yuho Najima (Hematology Div., Tokyo Metro. Komagome Hosp.)、Tomomi Toubai (Hematology Div., Tokyo Metro. Komagome Hosp.／Clin. Res. & Trials Ctr., Tokyo Metro. Komagome Hosp.)、Hironori Harada (Hematology Div., Tokyo Metro. Komagome Hosp.／Lab. Oncology, Tokyo Univ. of Pharm. & Life Sci.)、Yuka Harada (Clin. Res. & Trials Ctr., Tokyo Metro. Komagome Hosp.)、Noriko Doki (Hematology Div., Tokyo Metro. Komagome Hosp.)

Chromosome 7 aberrations (ch7ab) are known to be adverse prognostic factors in acute myeloid leukemia (AML) and myelodysplastic syndromes (MDS), and are indications for hematopoietic stem cell transplantation (HSCT), but their clinical and genomic features in the context of HSCT remain unclear. We performed targeted sequencing on 593 patients with AML and MDS. Of the 593 patients, 58 (9.8%) had ch7ab. Within this group, mutations in ***TP53***, ***ASXL1***, and ***DNMT3A*** were detected in 50%, 19%, and 14% of cases, respectively. Compared to patients without ch7ab, those with ch7ab had a significantly higher frequency of ***TP53***, ***ASXL1*** and ***NF1*** mutations, and a significantly lower frequency of ***NPM1***, ***FLT3*** and ***SF3B1*** mutations. Furthermore, in patients with ***TP53*** mutations, the variant allele frequency of ***TP53*** was significantly higher in patients with ch7ab than that in those without. Among 274 patients who underwent HSCT, ch7ab were identified in 34 cases (12%). In multivariable analysis of overall survival, ch7ab was not a significant factor (HR, 1.31; P = 0.35). These findings suggest that the adverse prognostic impact of ch7ab may be overcome by HSCT.

[Disclosure of COI by Responsible researcher] Lead presenter is not the Responsible researcher.
[Lead presenter: Kentaro Gosho] We have no financial relationships to disclose.
[Principal Researcher: Takashi Toya] We have no financial relationships to disclose.

Poster Session

### [P-2350] II-P11-1
### Hematological malignancies (1)

2025 / 9 / 26 (Fri) 17:30-18:15 Room P/(Motenashi Dome) Room P

---

### P-2350
### Serial gene panel testing is useful in the clinical management of hematopoietic neoplasms: insights from 1,500 analyses

[Speaker]Yuka Harada (Clin. Lab., Tokyo Metropolitan Komagome Hosp., Tokyo, Japan／Clin. Res. Trials Ctr., Tokyo Metropolitan Komagome Hosp., Tokyo, Japan) [Co-author]Sadato Daichi (Clin. Res. Trials Ctr., Tokyo Metropolitan Komagome Hosp., Tokyo, Japan)、Chizuko Hirama (Clin. Res. Trials Ctr., Tokyo Metropolitan Komagome Hosp., Tokyo, Japan)、Takashi Toya (Div. Hematology, Tokyo Metropolitan Komagome Hosp., Tokyo, Japan)、Hironori Harada (Div. Hematology, Tokyo Metropolitan Komagome Hosp., Tokyo, Japan／Lab. Oncology, Tokyo Univ. Pharm. & Life Sci., Tokyo, Japan)

The implementation of gene panel testing for hematopoietic neoplasms has enabled the detection of genetic abnormalities essential for molecular diagnosis and prognostic prediction. We performed clinical sequencing in approximately 1,500 samples across various diseases and stages. Our findings are as follows: 1) At disease onset, gene panel testing identified alterations corresponding to the genetic classification of hematopoietic neoplasms, which aided disease classification, treatment selection, and prognosis estimation. 2) Germline pathogenic variants such as those in DDX41 were more frequent than expected, even without family history, indicating the need for genetic counseling and family screening. 3) Serial analyses during progression or relapse/refractory phases revealed dynamics of mutant clones driven by disease course or treatment, including emergence of mutations associated with resistance. 4) In post-transplant samples, SNP patterns enabled clear identification of tumor origin. This real-world dataset highlights the clinical value of serial gene panel testing in understanding refractory diseases and informing treatment decisions.

[Disclosure of COI by Responsible researcher] Lead presenter is the Responsible researcher.
[Lead presenter: Yuka Harada] ■Research funds from company: 科研費、AMED研究費

▲TOP

### [P-2351] II-P11-1
### Hematological malignancies (1)

2025 / 9 / 26 (Fri) 17:30-18:15 Room P/(Motenashi Dome) Room P

---

### P-2351
### Distinctive Molecular Landscape of *NUTM1*-rearranged Infant Leukemias: Insights from Clinical and Animal Models

[Speaker]Koutarou Nishimura (Dept Cancer Path, Grad Sch Med, Univ Osaka／Lab Cancer Path, Grad Sch Frontier Biosciences, Univ Osaka)
[Co-author]Tomoya Isobe (Dept Pediatrics, Grad Sch Med, Univ Tokyo／Dept Hematology, Wellcome-MRC Cambridge Stem Cell Inst, Univ Cambridge)、Takako Miyamura (Dept Pediatrics, Grad Sch Med, Univ Osaka／Infant Leukemia Sub-committee, Japan Children's Cancer Group)、Masatoshi Takagi (Dept of Pediatrics, Neonatal and Maternal Med, Inst Science Tokyo)、Daichi Inoue (Dept Cancer Path, Grad Sch Med, Univ Osaka／Lab Cancer Path, Grad Sch Frontier Biosciences, Univ Osaka)

NUTM1-rearrangement (NUTM1-r) represents a significant subset of infant B-ALL lacking KMT2A-r. However, the underlying molecular mechanisms for NUTM1-r leukemia remain poorly elucidated. Our integrated analysis reveals that infant leukemias harboring NUTM1-r, particularly the BRD9-NUTM1 (BN1) fusion, exhibit a unique transcriptional and epigenetic landscape associated with B-lineage commitment with stem cell properties, thereby defining a unique leukemia subtype within infant ALL. Expression of BN1 is sufficient to induce serially-transplantable prepro-B-like leukemia, recapitulating key features of human NUTM1-r B-ALL, such as skewed B cell transcriptional programs, and low CD34 expression. Mechanistically, BN1 expression creates distinctive open chromatin regions with enhanced H3K27ac, particularly at NF-kB and posterior HoxA gene loci. Notably, NUTM1-r leukemic cells display stemness characteristics yet lose these properties during chemotherapy due to their reliance on active transcription, contributing to the favorable outcome seen in NUTM1-r leukemic patients. These findings highlight the unique molecular and clinical profile and enhance our understanding of NUTM1-r leukemias.

[Disclosure of COI by Responsible researcher] Lead presenter is not the Responsible researcher.
[Lead presenter: Koutarou Nishimura] We have no financial relationships to disclose.
[Principal Researcher: Daichi Inoue] ■Research expenses: 住友ファーマ株式会社, Abbvie Inc.　■Contributions: 田辺三菱製薬株式会社

### [P-2358] II-P12
### Advances in Pediatric & AYA Cancers

2025 / 9 / 26 (Fri) 17:30-18:15 Room P/(Motenashi Dome) Room P

---

### P-2358
### Potential novel classification of pediatric osteosarcoma based on DNA methylation profiles and copy number aberrations

[Speaker]Kyohei Inoue (Department of Pathology, Keio University School of Medicine／Department of Pediatrics, Keio University School of Medicine／Department of Pediatrics, Tokyo Dental College Ichikawa General Hospital) [Co-author]Eri Arai (Department of Pathology, Keio University School of Medicine)、Mao Fujimoto (Department of Pathology, Keio University School of Medicine)、Hiroyuki Shimada (Department of Pediatrics, Keio University School of Medicine)、Satoshi Narumi (Department of Pediatrics, Keio University School of Medicine)、Yae Kanai (Department of Pathology, Keio University School of Medicine)

Osteosarcoma is a rare cancer that occurs most frequently in children and young adults. Therapeutic innovations, which have not been achieved in more than 30 years, require a detailed understanding of the molecular aberrations.We evaluated genome-wide methylation profiles and copy number aberrations in 16 pediatric osteosarcoma samples. Hierarchical clustering analysis using methylome profiles clarified an osteosarcoma group characterized by DNA hypomethylation in open sea. The number of probes showing DNA hypomethylation was correlated with that of copy number aberrations. The osteosarcoma group characterized by accumulation of DNA hypomethylation and copy number aberrations included all patients with tumor recurrence and many of whom had a poor response to neoadjuvant chemotherapy. Genome-wide DNA hypomethylation may induce copy number abnormalities due to chromosomal instability, which may determine the clinicopathological features of osteosarcoma.We discuss the possible molecular mechanisms of DNA hypomethylation in osteosarcoma based on the expression of DNA methylation-related molecules, e.g. DNMTs and TETs.

[Disclosure of COI by Responsible researcher] Lead presenter is not the Responsible researcher.
[Lead presenter: Kyohei Inoue] ■Research funds from company: 研究助成金（NPO法人ゴールドリボン・ネットワーク）、博士課程学生研究支援プログラム（慶應義塾大学医学部）
[Principal Researcher: Eri Arai] We have no financial relationships to disclose.

### [P-2359] II-P12
### Advances in Pediatric & AYA Cancers

2025 / 9 / 26 (Fri) 17:30-18:15 Room P/(Motenashi Dome) Room P

---

### P-2359
### Forkhead box O (FOXO) signal pathway activation in pulmonary metastasis-positive pediatric osteosarcoma

[Speaker]Yasutoshi Tatsumi (Lab. Ped. Ref. Cancer, Chiba Cancer Ctr. Res. Inst.) [Co-author]Miki Ohira (Res. Inst. Clin. Oncol., Saitama Cancer Ctr.)、Naohiro Makise (Div. Surg. Pathol., Chiba Cancer Ctr.)、Makiko Itami (Div. Surg. Pathol., Chiba Cancer Ctr.)、Hideyuki Kinoshita (Div. Orthopedic Surg., Chiba Cancer Ctr.)、Tsukasa Yonemoto (Div. Orthopedic Surg., Chiba Cancer Ctr.)、Shintaro Iwata (Dept. Musculoskelet. Oncol. & Rehabil., Natl. Cancer Ctr. Hosp.)

Pulmonary metastasis (PM) is a major cause of cancer-related deaths in patients with pediatric osteosarcoma (OS), which is the most prevalent malignant bone tumor in children and adolescents. However, reliable biomarkers for predicting PM remain elusive.To elucidate the molecular profiles of PM-positive primary OS, we conducted whole-exome sequencing (WES) and comprehensive gene expression analysis of the primary tumors obtained through pretherapeutic biopsies from 27 patients with high-grade OS (PM-positive = 17, PM-negative = 10). WES results revealed a significant enrichment of nonsynonymous somatic mutations in the genes with bone and skeletal muscle homeostasis in patients with PM-positive OS. Moreover, PM-positive OS had a higher frequency of T>C mutations, caused by adenine deamination induced by nitrogen dioxide radicals derived from neutrophils and macrophages. Hence, we confirmed a relationship between inflammation and PM via gene expression profiling, identifying significant upregulation of the FOXO signal pathway mediated by IL-6 in PM-positive OS.These results provide a potential basis for identifying biomarkers and treatment targets for patients with PM-positive OS.

[Disclosure of COI by Responsible researcher] Lead presenter is the Responsible researcher.
[Lead presenter: Yasutoshi Tatsumi] We have no financial relationships to disclose.

### [P-2360] II-P12
### Advances in Pediatric & AYA Cancers

2025 / 9 / 26 (Fri) 17:30-18:15 Room P/(Motenashi Dome) Room P

---

### P-2360
### The cellular and temporal landscape of neuroblastoma development

[Speaker]Shoma Tsubota (Dept of Mol Biol, Nagoya Univ Grad of Med) [Co-author]Kenji Kadomatsu (Institute for Glyco-core Research)

Neuroblastoma is a pediatric solid tumor that originates from genomic or epigenomic abnormalities during the development of neural crest-derived tissues. However, its precise cellular origin and timing of initiation remain unclear. Using an in vitro spheroid culture system, we identified a MYCN-responsive population in the adrenal glands of postnatal day 1 (P1) mice that exhibited strong tumorigenic potential. This population markedly decreased with age. To investigate the temporal and cellular specificity of neuroblastoma initiation, we performed single-cell RNA sequencing on cells from the adrenal gland at different postnatal stages. Transcriptomic analysis revealed a distinct Th (tyrosine hydroxylase)-positive population enriched at P1. Further clustering and marker gene expression analyses suggested that chromaffin cells, a subset of Th-positive adrenal medullary cells, represent the major cell type susceptible to MYCN-induced transformation. These findings indicate that chromaffin cells in the early postnatal adrenal medulla may serve as the cells of origin for neuroblastoma, providing novel insights into the developmental mechanisms of tumor initiation.

[Disclosure of COI by Responsible researcher] Lead presenter is the Responsible researcher.
[Lead presenter: Shoma Tsubota] We have no financial relationships to disclose.

### [P-2361] II-P12
### Advances in Pediatric & AYA Cancers

2025 / 9 / 26 (Fri) 17:30-18:15 Room P/(Motenashi Dome) Room P

---

### P-2361
### Telomere maintenance abnormalities in high-risk neuroblastoma and their molecular subtypes

[Speaker]Miki Ohira (Res. Inst. Clin. Oncol., Saitama Cancer Ctr.) [Co-author]Masayuki Haruta (Res. Inst. Clin. Oncol., Saitama Cancer Ctr.)、Ritsuko Onuki (Res. Inst. Clin. Oncol., Saitama Cancer Ctr.)、Jesmin Akter (Res. Inst. Clin. Oncol., Saitama Cancer Ctr.)、Yasutoshi Tatsumi (Lab. Pediatr. & Refract. Cancer, Chiba Cancer Ctr. Res. Inst.)、Yohko Nakamura (Cancer Prev. Ctr., Chiba Cancer Ctr. Res. Inst.)、Atsushi Takatori (Div. Innov. Cancer Therap., Chiba Cancer Ctr. Res. Inst.)、Atsuko Nakazawa (Div. Clin. Res., Saitama Child. Med. Ctr.)、Tetsuya Takimoto (Dept. Child. Cancer Data Manag., Natl. Ctr. Child Health & Dev.)、Tatsuro Tajiri (Dept. Pediatr. Surg., Grad. Sch. Med. Sci., Kyushu Univ.)、Tomoko Iehara (Dept. Pediatr., Kyoto Pref. Univ. Med., Grad. Sch. Med. Sci.)、Akira Nakagawara (SAGA HIMAT)、Takehiko Kamijo (Res. Inst. Clin. Oncol., Saitama Cancer Ctr.)

Telomere maintenance abnormalities are a hallmark of refractory high-risk neuroblastoma (NB) , mainly classified into ***TERT*** reactivation and alternative lengthening of telomeres (ALT). While ALT-positive NBs frequently harbor mutations in ***ATRX*** or ***DAXX***, other factors are less known. In the present study, we classified 138 NB cases into 5 groups by multi-omics analyses and searched for molecular characteristics of each group: A(n=47): ***TERT***-high or ***MYCN*** amplification; B(n=37): ALT(+), ***ATRX*** abnormal; C(n=10): ALT(+), ***ATRX*** wild-type; D(n=24): ALT(-), stage 4; E(n=16): ALT(-), non-stage 4. All groups except group E showed comparably poor 10-year overall survival rates (A: 44%, B: 33%, C: 38%, D: 53% vs E: 100%). In group C, mutations in DNA repair and histone modifiers were found, suggesting an association between these pathways and ALT. Comparison of gene expression revealed high expression of ***TERC*** in group A, high expression of ***H3F3B*** and ***RAD51C*** in group B, and low expression of ***TP53*** in group C. Given the increasing interest in identifying therapeutic vulnerabilities in ALT-positive NBs, further subclassification of ALT subtypes may facilitate the development of more effective therapies.

[Disclosure of COI by Responsible researcher] Lead presenter is the Responsible researcher.
[Lead presenter: Miki Ohira] We have no financial relationships to disclose.

[P-2362] II-P12

Advances in Pediatric & AYA Cancers

2025 / 9 / 26 (Fri) 17:30-18:15 Room P/(Motenashi Dome) Room P

P-2362

### Pharmacological blockade of BMI1 induces the p53 pathway, suppressing MYCN-amplified neuroblastoma.

[Speaker]Masahiro Hirayama (Dept. of Pathobiology, Nagoya City Univ. Grad. Sch.) [Co-author]Eri Yamada (Dept. of Pathobiology, Nagoya City Univ. Grad. Sch.)、Hiromasa Aoki (Dept. of Pathobiology, Nagoya City Univ. Grad. Sch.)、Kazuya Izumi (Dept. of Pathobiology, Nagoya City Univ. Grad. Sch.)、Ayumi Amano (Dept. of Pathobiology, Nagoya City Univ. Grad. Sch.)、Kohki Toriuchi (Dept. of Pathobiology, Nagoya City Univ. Grad. Sch.)、Koichi Ogami (Mol. Oncol., Neuro Dis. & Cancer Ctr., Nagoya Univ.)、Mai Nagasaka (Dept. of Cell Signaling, Nagoya City Univ. Pharm. Grad. Sch.)、Yasumichi Inoue (Dept. of Cell Signaling, Nagoya City Univ. Pharm. Grad. Sch.)、Hidetoshi(Nagoya City Univ.) Hayashi (Dept. of Cell Signaling, Nagoya City Univ. Pharm. Grad. Sch.)、Satoru Takeshita (Dept. of Pathobiology, Nagoya City Univ. Grad. Sch.／Dept. of Perinatal & Neonatal Med., Aichi Med. Univ.)、Hiroki Kakita (Dept. of Pathobiology, Nagoya City Univ. Grad. Sch.／Dept. of Perinatal & Neonatal Med., Aichi Med. Univ.)、Yasumasa Yamada (Dept. of Perinatal & Neonatal Med., Aichi Med. Univ.)、Mineyoshi Aoyama (Dept. of Pathobiology, Nagoya City Univ. Grad. Sch.)

Neuroblastoma, a common childhood cancer originating from neural crest cells, often forms in the sympathetic ganglia and adrenal medulla. Neuroblastomas with MYCN gene amplification are classified as high-risk tumors, associated with poor therapy response rates. BMI1, a component of PRC1, regulates transcription via H2A Lys119 monoubiquitination and is upregulated by MYCN binding to its promoter. Currently, BMI1 inhibitors like PTC-028 and PTC-209 are expected. Therefore, we investigated the efficacy of BMI1 inhibitors as a potential therapy for high-risk MYCN-amplified neuroblastoma.We tested the effect of BMI1 inhibitors on MYCN-amplified and non-amplified neuroblastoma cell lines, finding reduced viability specifically in MYCN-amplified cells. PTC-028 induced cell cycle arrest and apoptosis. RNA-seq analysis revealed that PTC-028 induces the p53 pathway, and proteins involved in the p53 pathway, such as p21, were upregulated. Significantly, in a xenograft model, PTC-028 treatment suppressed tumor growth. These results suggest that BMI1 inhibitors, especially PTC-028, may be effective in the treatment of high-risk MYCN-amplified neuroblastoma.

[Disclosure of COI by Responsible researcher] Lead presenter is not the Responsible researcher.
[Lead presenter: Masahiro Hirayama] We have no financial relationships to disclose.
[Principal Researcher: Mineyoshi Aoyama] We have no financial relationships to disclose.

### [P-2363] II-P12
### Advances in Pediatric & AYA Cancers

2025 / 9 / 26 (Fri) 17:30-18:15 Room P/(Motenashi Dome) Room P

---

### P-2363
### Identification of a Synthetic Lethal Target in SMARCB1-Deficient Cancers

[Speaker]Mariko Sasaki (Cancer Therapeutics, NCCRI) [Co-author]Daiki Kato (Cancer Research Unit, Sumitomo Pharma Co., Ltd,)、Karin Murakami (Cancer Research Unit, Sumitomo Pharma Co., Ltd,)、Tsuguteru Otsubo (Cancer Research Unit, Sumitomo Pharma Co., Ltd,)、Hideaki Ogiwara (Cancer Therapeutics, NCCRI)

SMARCB1, a subunit of the SWI/SNF chromatin remodeling complex, is the causative gene of pediatric and juvenile cancers. Here, we used a method based on the "simultaneous inhibition of a paralog pair" and identified a synthetic lethal target comprising acetyltransferases CBP and p300 in SMARCB1-deficient cancers. CBP/p300 dual inhibitors treatment suppressed growth of cell lines and tumor xenografts derived from SMARCB1-deficient cells but not proficient cells. SMARCB1-containing SWI/SNF complexes localized with H3K27me3 (a marker of transcriptional repression) and its methyltransferase, EZH2, at the promotor region of the KREMEN2 locus. By contrast, SMARCB1 deficiency led to localization of H3K27ac (a marker of transcriptional promotion) and recruitment of CBP and p300 there, resulting in transcriptional upregulation of KREMEN2, which cooperates with the SMARCA1 chromatin remodeling complex instead of SMARCB1-deficient residual SWI/SNF complexes. Inhibition of CBP/p300 led to transcriptional downregulation of KREMEN2 and then induced apoptosis. Our findings indicate that simultaneous inhibitors of CBP/p300 could be promising therapeutic agents for SMARCB1-deficient cancers.

[Disclosure of COI by Responsible researcher] Lead presenter is not the Responsible researcher.
[Lead presenter: Mariko Sasaki] We have no financial relationships to disclose.
[Principal Researcher: Hideaki Ogiwara] ■Research funds from company: 共同研究費　住友ファーマ株式会社　■Research expenses: 共同研究費　住友ファーマ株式会社

### [EL6] EL6
### Cellular senescence: From Guardians to Enablers of Cancer

2025 / 9 / 27 (Sat) 8:00-8:50 Room 3/(Hotel Nikko Kanazawa 4F Tsuru A) Room 3

[Chairperson]Akiko Takahashi (Division of Cellular Senescence, Cancer Institute, Japanese Foundation for Cancer Research)

### EL6
### Cellular Senescence at a Crossroads: Toward More Rigorous and Reproducible Research

[Speaker]Eiji Hara (Dept. Mol. Biol., Res. Inst. Microb. Dis., Univ. Osaka)

While cellular senescence was initially defined as a tumor-suppressive mechanism due to its ability to irreversibly arrest the cell cycle, subsequent studies have revealed that senescent cells actively influence their microenvironment by secreting pro-inflammatory and tissue-remodeling factors. This phenotype, known as the senescence-associated secretory phenotype (SASP), has dramatically reshaped our understanding of cellular senescence from a purely tumor-suppressive process to one that can also drive chronic inflammation, tissue dysfunction, and even tumor progression. However, this conceptual expansion has made it increasingly difficult to define the precise roles of senescence in vivo. Indeed, contradictory findings and issues with reproducibility have emerged, particularly concerning the identification and elimination of senescent cells and the biological consequences of their clearance. These inconsistencies have contributed to ongoing uncertainty in the field. In this educational lecture, I will explore the underlying causes of these discrepancies and propose strategies to address them, aiming to promote more rigorous and meaningful progress in cellular senescence research.

[Disclosure of COI by Responsible researcher] Lead presenter is the Responsible researcher.
[Lead presenter: Eiji Hara] ■Research expenses: 江崎グリコ株式会社　■Contributions: 江崎グリコ株式会社

### [EL7] EL7
### Antigen-specific immunotherapy: cancer vaccine and cell therapy

2025 / 9 / 27 (Sat) 8:00-8:50 Room 4/(Hotel Nikko Kanazawa 4F Tsuru B) Room 4

[Chairpersons]Koji Tamada (Research Institute for Cell Design Medical Science, Yamaguchi University)、Yuki Kagoya (Keio University School of Medicine)

---

### EL7
### TBD

[Speaker]Hiroaki Ikeda (Department of Tumor Biology, Graduate School of Biomedical Sciences, Nagasaki University)

No Abstract

### [EL8] EL8
### Chemical Biology for Cancer Research

2025 / 9 / 27 (Sat) 8:00-8:50 Room 5/(Hotel Nikko Kanazawa 4F Tsuru C) Room 5

[Chairpersons]Hozumi Motohashi (Tohoku University Graduate School of Medicine)、Yasuteru Urano (The University of Tokyo)

---

### EL8
### Life sciences and drug discovery based on chemical biology

[Speaker]Minoru Yoshida (RIKEN CSRS／Univ. Tokyo, Office Univ. Prof.)

Chemical biology is a field of study that has developed to elucidate various phenomena in living organisms using chemistry-driven approaches. Chemical probes for chemical biology can be roughly divided into two types. The first type enables the observation and measurement of normally invisible phenomena occurring inside living organisms. The second type intervenes in the regulatory mechanisms through bioactive compounds, thereby deepening our understanding of life sciences. In this lecture, I will mainly focus on the usefulness of the latter. Bioactive compounds always have specific targets, and their identification not only leads to biologically important discoveries but also provides valuable information for drug discovery. Chemical genetics, a chemical approach that replaces genetic mutations with compounds, enables the elucidation of mechanisms of action at the molecular and genetic level. I will present the birth and progress of chemical genetics and its application to cancer research, as well as the latest chemical genomics techniques that analyze comprehensive chemical-genetic interactions with compounds in yeast and animal cells, and their application to drug screening.

[Disclosure of COI by Responsible researcher] Lead presenter is the Responsible researcher.
[Lead presenter: Minoru Yoshida] We have no financial relationships to disclose.

### [EL9] EL9
### The role of the vascular microenvironment in cancer development and progression

2025 / 9 / 27 (Sat) 8:00-8:50 Room 11/(ANA Crowne Plaza Hotel Kanazawa 3F Ohtori (West)) Room 11

[Chairpersons]Yukiko Matsunaga (The University of Tokyo)、Miwa Tanaka (The Cancer Institute, Japanese Foundation for Cancer Research)

---

### EL9
### Tumor Blood Vessels Driving Cancer Progression and Metastasis: Cutting-Edge Research and Novel Therapeutic Strategies

[Speaker]Tetsuro Watabe (Dept. Biochem., Grad. Sch. Med. Dent. Sci., Inst. Sci. Tokyo)

Blood vessels in the tumor microenvironment play a key role in cancer progression and metastasis. Tumors form many new vessels distinct from normal ones, supplying oxygen and nutrients to cancer cells and serving as routes for metastasis. However, abnormalities in these vessels cause hypoxia and limit immune cell infiltration. Endothelial cells in tumor vessels are influenced by factors secreted by cancer cells, changing their properties and functions. This promotes angiogenesis and increases vascular permeability, creating conditions that support tumor growth and spread. Some endothelial cells undergo morphological and functional changes that affect surrounding tissues and promote tumor progression. While anti-angiogenic therapies like Avastin have gained attention, newer strategies target unique features of tumor vessels. The tumor blood vessel environment is a complex network formed by interactions between cancer cells and stromal components. Understanding its molecular mechanisms is crucial for clarifying metastasis and immune evasion and developing novel therapies. This lecture reviews recent advances in tumor blood vessel pathology and emerging therapeutic approaches.

[Disclosure of COI by Responsible researcher] Lead presenter is the Responsible researcher.
[Lead presenter: Tetsuro Watabe] We have no financial relationships to disclose.

### [EL10] EL10
### Opening Up New Avenues for p53 Research

2025 / 9 / 27 (Sat) 8:00-8:50 Room 12/(ANA Crowne Plaza Hotel Kanazawa 3F Ohtori (Central)) Room 12

[Chairpersons]Rieko Ohki (National Cancer Center Research Institute)、Koji Itahana (Duke-NUS Medical School, Singapore)

---

### EL10
### New approach to targeting mutant p53 cancers

[Speaker]Christine M. Eischen (Department of Medical Oncology, Thomas Jefferson University)

All cells are protected from an array of stresses and insults, including cellular alterations that lead to cancer development. Activation of the p53 tumor suppressor protein protects cells and safeguards their genomes. However, half of human cancers mutate p53, allowing cells with alterations that should die to survive and grow, and increasing cancer aggressiveness and resistance to treatments. Cancers also can suppress p53 activity by overexpressing Mdm2, a negative regulator of p53. Due to the complexity of p53, specifically targeting mutant p53 cancers has been very challenging. However, recent research advances by my laboratory and others have opened new avenues for mutant p53 cancer treatments. We discovered Mdm2 was an unexpected vulnerability in p53 mutant cancer cells. Deletion, knockdown, or targeted degradation of Mdm2 in p53 mutant cancers induced apoptotic cell death, revealing Mdm2 is required for their continued survival. We are developing novel Mdm2-targeted degraders for therapeutic use for p53 mutant cancers. Other scientists are targeting specific p53 mutations with novel compounds as well as testing drugs to cause synthetic lethality in mutant p53 cancers.

[Disclosure of COI by Responsible researcher] Lead presenter is the Responsible researcher.
[Lead presenter: Christine M. Eischen] ■Endowed chair: Thomas Jefferson University

▲TOP

### [SS1-1] SS1
### JCA-KCA Joint Symposium

2025 / 9 / 27 (Sat) 8:30-11:30 Room 1/(Ishikawa Ongakudo 2F Concert Hall) Room 1

[Chairpersons]Masanobu Oshima (Division of Genetics, Cancer Research Institute, Kanazawa University)、SunYoung Rha (Yonsei Cancer Center, Yonsei Universtiy College of Medicine)

The Japanese Cancer Association (JCA) maintains a close and collaborative relationship with the Korean Cancer Association (KCA) and is committed to advancing cancer research through bilateral scientific exchange. At this year's JCA Annual Meeting, we are pleased to host the JCA-KCA Joint Symposium.
This symposium aims to expand the collaboration between Japanese and Korean cancer researchers to include the wider Asia-Pacific region. Leading scientists at the forefront of both basic and translational cancer research from five countries - Japan, Korea, China, Singapore, and USA - will come together to present under the theme: "Cancer Research in Asia, from Bench to Bedside." The JCA Annual Meeting attracts a large number of international participants, particularly from across Asia. We warmly encourage overseas attendees to actively participate in this symposium and take advantage of this opportunity to learn how cancer research in these countries is being translated into clinical applications. We also hope the event will serve as a new opportunity for future international collaboration. This symposium is co-organized with MEXT Promotion of Development of a Joint Usage/Research System Project: Coalition of Universities for Research Excellence (CURE) Program "Extending Healthy Lifespan" and Kanazawa International Tumor Biology.

### SS1-1
### Molecular Changes Underlying Cholangiocarcinoma Heterogeneity and their Clinical Implications

[Speaker]Bin T. Teh (National Cancer Centre Singapore)

Cholangiocarcinoma (CCA) is a highly lethal adenocarcinoma of the hepatobiliary system, which can be classified as intrahepatic, perihilar and distal. It is a heterogeneous malignancy with high mortality and dismal prognosis, and an urgent clinical need for effective new therapies. It is therefore important to understand the genomic, epigenomic, proteomic and metabolomic changes underlying the heterogeneity of CCA and identify potential therapeutic targets based on these changes. We previously characterized the genomic alterations by profiling CCA of different etiological groups. Approved therapies based on some of these genomic changes will be reviewed in this talk. More recently, we studied the CCA epigenome by looking at both aberrant DNA methylation and dysregulation of enhancer activities which are known to affect carcinogenesis and potentially targetable therapeutically. Our study elucidates the mechanisms underlying enhancer dysregulation and deepens understanding of different tumorigenesis processes in distinct CCA subtypes, with potential significant therapeutics and clinical implications. Finally, latest advances in CCA research will also be reviewed.

[Disclosure of COI by Responsible researcher] Lead presenter is the Responsible researcher.
[Lead presenter: Bin T. Teh] We have no financial relationships to disclose.

### [SS1-2] SS1
### JCA-KCA Joint Symposium

2025 / 9 / 27 (Sat) 8:30-11:30 Room 1/(Ishikawa Ongakudo 2F Concert Hall) Room 1

[Chairpersons]Masanobu Oshima (Division of Genetics, Cancer Research Institute, Kanazawa University)、SunYoung Rha (Yonsei Cancer Center, Yonsei Universtiy College of Medicine)

The Japanese Cancer Association (JCA) maintains a close and collaborative relationship with the Korean Cancer Association (KCA) and is committed to advancing cancer research through bilateral scientific exchange. At this year's JCA Annual Meeting, we are pleased to host the JCA-KCA Joint Symposium.
This symposium aims to expand the collaboration between Japanese and Korean cancer researchers to include the wider Asia-Pacific region. Leading scientists at the forefront of both basic and translational cancer research from five countries - Japan, Korea, China, Singapore, and USA - will come together to present under the theme: "Cancer Research in Asia, from Bench to Bedside." The JCA Annual Meeting attracts a large number of international participants, particularly from across Asia. We warmly encourage overseas attendees to actively participate in this symposium and take advantage of this opportunity to learn how cancer research in these countries is being translated into clinical applications. We also hope the event will serve as a new opportunity for future international collaboration. This symposium is co-organized with MEXT Promotion of Development of a Joint Usage/Research System Project: Coalition of Universities for Research Excellence (CURE) Program "Extending Healthy Lifespan" and Kanazawa International Tumor Biology.

---

### SS1-2
### To Be or Not to Be: The Existentialism of PIK3CA Mutation in Lung Cancer

[Speaker]Hongbin Ji (School of Medicine, Westlake University, Hangzhou, Zhejiang, China)

PIK3CA mutation is frequently concurrent with known oncogenic drivers such as EGFR mutation in lung cancer, raising an interesting question about its real function. Cachexia is a systemic disease involving complex interaction between primary tumors and distant organs, significantly contributing to cancer-related mortality. Through integrative study of genetically engineered mouse models (GEMMs) and clinical data, we find concurrent PIK3CA mutant preferentially drives cachexia in EGFR-mutant lung cancer, promoting malignant progression instead of cancer initiation. PIK3CA mutant-mediated cachexia could be overcome by osimertinib (Osi) treatment in Osi-sensitive GEMM. In contrast, chemotherapy, routinely used in clinic for those relapsed from Osi therapy, fails to ameliorate cachexia in Osi-resistant GEMM despite notable tumor suppression. PIK3CA mutant-driven cachexia is mediated through NF-kB activation and could be dampened by combined aspirin treatment. This work uncovers the biological function of PIK3CA mutant and mechanism behinds its clinical impacts, and proposes a potentially effective strategy for clinical management.

[Disclosure of COI by Responsible researcher] Lead presenter is the Responsible researcher.
[Lead presenter: Hongbin Ji] We have no financial relationships to disclose.

▲TOP

### [SS1-3] SS1
### JCA-KCA Joint Symposium

2025 / 9 / 27 (Sat) 8:30-11:30 Room 1/(Ishikawa Ongakudo 2F Concert Hall) Room 1

[Chairpersons]Masanobu Oshima (Division of Genetics, Cancer Research Institute, Kanazawa University)、SunYoung Rha (Yonsei Cancer Center, Yonsei Universtiy College of Medicine)

The Japanese Cancer Association (JCA) maintains a close and collaborative relationship with the Korean Cancer Association (KCA) and is committed to advancing cancer research through bilateral scientific exchange. At this year's JCA Annual Meeting, we are pleased to host the JCA-KCA Joint Symposium.
This symposium aims to expand the collaboration between Japanese and Korean cancer researchers to include the wider Asia-Pacific region. Leading scientists at the forefront of both basic and translational cancer research from five countries - Japan, Korea, China, Singapore, and USA - will come together to present under the theme: "Cancer Research in Asia, from Bench to Bedside." The JCA Annual Meeting attracts a large number of international participants, particularly from across Asia. We warmly encourage overseas attendees to actively participate in this symposium and take advantage of this opportunity to learn how cancer research in these countries is being translated into clinical applications. We also hope the event will serve as a new opportunity for future international collaboration. This symposium is co-organized with MEXT Promotion of Development of a Joint Usage/Research System Project: Coalition of Universities for Research Excellence (CURE) Program "Extending Healthy Lifespan" and Kanazawa International Tumor Biology.

### SS1-3
### Precision Immunotherapy for Gastric Cancer: Insights from the Tumor Microenvironment

[Speaker]SunYoung Rha (Yonsei Cancer Center, Yonsei Universtiy College of Medicine)

Precision medicine is becoming feasible in gastric cancer(GC) with accumulation of immune-genomic information. Based on TCGA molecular classification, current NGS technology makes it feasible for proper subgrouping toward precision treatment of advanced GC. However, the progress of precision immunotherapy in metastatic GC has been slow and somewhat disappointing. The potential reasons for these limitations are tumor heterogeneity and difficulties in proper sample availability for reliable translational researches, and complex clinical behavior with unproven immune-genomic biology behind. Recently, we have increasing data with new technologies such as RNA transcriptomics, single cell genomics and spatial transcriptomics. Even though there is still lack of success in genome stable type GC, now we have more opportunities for precision medicine in GC with novel targets such as claudin 18.2 and various combination treatment with immunotherapy. Also, recent clinical trials data supports changing strategy of systemic treatment for metastatic GC. Here I will share the advances of immunotherapy of GC with more precise stratification of the patients based on tumor microenvironment.

[Disclosure of COI by Responsible researcher] Lead presenter is the Responsible researcher.
[Lead presenter: Sun Young Rha] ■Research funds from company: Astallas, MSD, Daichii-Sankyo, Astra-Zeneca ■Representative of organization for clinical study receiving research expenses from company: Amgen, Astallas, MSD, Ono, Diachii-Sankyo, BeOne, Astra-Zeneca, Taiho, Roche, Jazz,

### [SS1-4] SS1
### JCA-KCA Joint Symposium

2025 / 9 / 27 (Sat) 8:30-11:30 Room 1/(Ishikawa Ongakudo 2F Concert Hall) Room 1

[Chairpersons]Masanobu Oshima (Division of Genetics, Cancer Research Institute, Kanazawa University)、SunYoung Rha (Yonsei Cancer Center, Yonsei Universtiy College of Medicine)

The Japanese Cancer Association (JCA) maintains a close and collaborative relationship with the Korean Cancer Association (KCA) and is committed to advancing cancer research through bilateral scientific exchange. At this year's JCA Annual Meeting, we are pleased to host the JCA-KCA Joint Symposium.
This symposium aims to expand the collaboration between Japanese and Korean cancer researchers to include the wider Asia-Pacific region. Leading scientists at the forefront of both basic and translational cancer research from five countries - Japan, Korea, China, Singapore, and USA - will come together to present under the theme: "Cancer Research in Asia, from Bench to Bedside." The JCA Annual Meeting attracts a large number of international participants, particularly from across Asia. We warmly encourage overseas attendees to actively participate in this symposium and take advantage of this opportunity to learn how cancer research in these countries is being translated into clinical applications. We also hope the event will serve as a new opportunity for future international collaboration. This symposium is co-organized with MEXT Promotion of Development of a Joint Usage/Research System Project: Coalition of Universities for Research Excellence (CURE) Program "Extending Healthy Lifespan" and Kanazawa International Tumor Biology.

---

### SS1-4
### AACR Initiatives in Asia and Asia Pacific Regions

[Speaker]Lillian Siu (Princess Margaret Cancer Centre, Toronto, Canada)

In the first part of the presentation, ongoing and new initiatives from the American Association for Cancer Research (AACR) in Asia and Asia Pacific regions will be highlighted. In addition, objectives and activities of the AACR's Asian/Asian American, Native Hawaiian and Pacific Islander (AANHPI) task force will be discussed. In the second part of the presentation, an example of bench to bedside research with a disease focus will be showcased. Specifically, a translational research project encompassing spatial transcriptomic sequencing, circulating tumor DNA kinetics and T cell receptor repertoire profiling in patients with recurrent or metastatic head and neck squamous cell cancer treated with immune checkpoint blockade will be described.

[Disclosure of COI by Responsible researcher] Lead presenter is the Responsible researcher.
[Lead presenter: Lillian Siu] ■Advisor of the company and/or profit-making organization: Voronoi ■Endowed chair: BMO Chair in Precision Genomics ■Representative of organization for clinical study receiving research expenses from company: Clinical trials support for institution: Merck, Novartis, Bristol-Myers Squibb, Pfizer/SeaGen, Boerhinger-Ingelheim, GlaxoSmithKline, Roche/Genentech, AstraZeneca/Medimmune, Bayer, Abbvie, Amgen, EMD Serono, Daiichi Sankyo, Gilead, Marengo, Incyte, LegoChem, Loxo/Eli Lilly, Medicenna, Takara

### [SS1-5] SS1
### JCA-KCA Joint Symposium

2025 / 9 / 27 (Sat) 8:30-11:30 Room 1/(Ishikawa Ongakudo 2F Concert Hall) Room 1

[Chairpersons]Masanobu Oshima (Division of Genetics, Cancer Research Institute, Kanazawa University)、SunYoung Rha (Yonsei Cancer Center, Yonsei Universtiy College of Medicine)

The Japanese Cancer Association (JCA) maintains a close and collaborative relationship with the Korean Cancer Association (KCA) and is committed to advancing cancer research through bilateral scientific exchange. At this year's JCA Annual Meeting, we are pleased to host the JCA-KCA Joint Symposium.
This symposium aims to expand the collaboration between Japanese and Korean cancer researchers to include the wider Asia-Pacific region. Leading scientists at the forefront of both basic and translational cancer research from five countries - Japan, Korea, China, Singapore, and USA - will come together to present under the theme: "Cancer Research in Asia, from Bench to Bedside." The JCA Annual Meeting attracts a large number of international participants, particularly from across Asia. We warmly encourage overseas attendees to actively participate in this symposium and take advantage of this opportunity to learn how cancer research in these countries is being translated into clinical applications. We also hope the event will serve as a new opportunity for future international collaboration. This symposium is co-organized with MEXT Promotion of Development of a Joint Usage/Research System Project: Coalition of Universities for Research Excellence (CURE) Program "Extending Healthy Lifespan" and Kanazawa International Tumor Biology.

---

### SS1-5
### Trials for early detection of pancreatic cancer

[Speaker]Hiroshi Seno (Dept. Gastroenterol & Hepatol, Kyoto Univ. Grad. Sch. Med.) [Co-author]Munenori Kawai (Dept. Gastroenterol & Hepatol, Kyoto Univ. Grad. Sch. Med.)、Akihisa Fukuda (Dept. Gastroenterol & Hepatol, Kyoto Univ. Grad. Sch. Med.)

Pancreatic cancer is the third or fourth leading cause of cancer-related death in Japan, with a five-year survival rate around 12%, making early detection crucial for improving prognosis. While patients with tumors smaller than 10 mm confined to the pancreas can have 90% five-year survival, early-stage pancreatic cancer is often asymptomatic and lacks reliable biomarkers. To address this problem, we conducted a multi-center study, analyzing blood miRNAs from pancreatic cancer patients and healthy individuals by next-generation sequencing. We developed a machine learning model using highly expressed 100 miRNAs. The model achieved higher diagnostic accuracy than the conventional biomarkers, particularly for early and asymptomatic cases, with an AUC of 0.99 when combined with CA19-9. The model demonstrated robustness across different sequencing platforms. This minimally invasive approach shows promise not only for pancreatic cancer but also potentially for detecting other gastrointestinal cancers, given the role of miRNAs in various situations. Future studies may explore its application to high-risk groups and broader cancer screening.

[Disclosure of COI by Responsible researcher] Lead presenter is the Responsible researcher.
[Lead presenter: Hiroshi Seno] We have no financial relationships to disclose.

▲TOP

### [SS1-6] SS1
### JCA-KCA Joint Symposium

2025 / 9 / 27 (Sat) 8:30-11:30 Room 1/(Ishikawa Ongakudo 2F Concert Hall) Room 1

[Chairpersons]Masanobu Oshima (Division of Genetics, Cancer Research Institute, Kanazawa University)、SunYoung Rha (Yonsei Cancer Center, Yonsei Universtiy College of Medicine)

The Japanese Cancer Association (JCA) maintains a close and collaborative relationship with the Korean Cancer Association (KCA) and is committed to advancing cancer research through bilateral scientific exchange. At this year's JCA Annual Meeting, we are pleased to host the JCA-KCA Joint Symposium.
This symposium aims to expand the collaboration between Japanese and Korean cancer researchers to include the wider Asia-Pacific region. Leading scientists at the forefront of both basic and translational cancer research from five countries - Japan, Korea, China, Singapore, and USA - will come together to present under the theme: "Cancer Research in Asia, from Bench to Bedside." The JCA Annual Meeting attracts a large number of international participants, particularly from across Asia. We warmly encourage overseas attendees to actively participate in this symposium and take advantage of this opportunity to learn how cancer research in these countries is being translated into clinical applications. We also hope the event will serve as a new opportunity for future international collaboration. This symposium is co-organized with MEXT Promotion of Development of a Joint Usage/Research System Project: Coalition of Universities for Research Excellence (CURE) Program "Extending Healthy Lifespan" and Kanazawa International Tumor Biology.

### SS1-6
### Strategic drug development using in vitro and in vivo screening to overcome cancer heterogeneity

[Speaker]Shinji Kohsaka (Division of Cellular Signaling., National Cancer Center Research Institute)

The identification of predictive biomarker is crucial step for the new drug development. We utilized in vivo patient-derived xenograft (PDX) mouse models established from 42 patients with solid tumors to identify biomarkers of the antitumor efficacy of E7820, a splicing modulator. Exome sequencing revealed that loss-of-function mutations in genes of the HRR system were significantly enriched in tumors that responded to E7820. Interestingly, E7820-mediated double-strand breaks in DNA were increased in tumors with BRCA2 dysfunction, and knockdown or knockout of HRR genes sensitized cancer cells to E7820. Transcriptomic analyses revealed that E7820 treatment resulted in the intron retention of mRNAs and decreased transcription, especially for HRR genes. This induced HRR malfunction probably leads to the synthetic lethality of tumor cells with homologous recombination deficiency (HRD). Furthermore, E7820 in combination with olaparib exerted a synergistic effect, and E7820 was even effective in an olaparib-resistant cell line. In conclusion, HRD is a promising predictive biomarker of E7820 efficacy and has high potential to improve the prognosis of patients with HRD-positive cancers.

[Disclosure of COI by Responsible researcher] Lead presenter is the Responsible researcher.
[Lead presenter: Shinji Kohsaka] ■Research expenses: Boehringer Ingelheim, Chordia Therapeutics, Eisai, Konica Minolta, CIMIC, H.U. Group Research Institute

▲TOP

### [SS1-7] SS1
### JCA-KCA Joint Symposium

2025 / 9 / 27 (Sat) 8:30-11:30 Room 1/(Ishikawa Ongakudo 2F Concert Hall) Room 1

[Chairpersons]Masanobu Oshima (Division of Genetics, Cancer Research Institute, Kanazawa University)、SunYoung Rha (Yonsei Cancer Center, Yonsei Universtiy College of Medicine)

The Japanese Cancer Association (JCA) maintains a close and collaborative relationship with the Korean Cancer Association (KCA) and is committed to advancing cancer research through bilateral scientific exchange. At this year's JCA Annual Meeting, we are pleased to host the JCA-KCA Joint Symposium.
This symposium aims to expand the collaboration between Japanese and Korean cancer researchers to include the wider Asia-Pacific region. Leading scientists at the forefront of both basic and translational cancer research from five countries - Japan, Korea, China, Singapore, and USA - will come together to present under the theme: "Cancer Research in Asia, from Bench to Bedside." The JCA Annual Meeting attracts a large number of international participants, particularly from across Asia. We warmly encourage overseas attendees to actively participate in this symposium and take advantage of this opportunity to learn how cancer research in these countries is being translated into clinical applications. We also hope the event will serve as a new opportunity for future international collaboration. This symposium is co-organized with MEXT Promotion of Development of a Joint Usage/Research System Project: Coalition of Universities for Research Excellence (CURE) Program "Extending Healthy Lifespan" and Kanazawa International Tumor Biology.

---

### SS1-7
### Never-smoker Lung Cancer in Asians: Epidemiology, Molecular Features, and Clinical Implications

[Speaker]Se-Hoon Lee (Dept. of Medicine, Samsung Medical Center)

A significant proportion of lung cancer cases occur in never-smoker, particularly among Asian populations. The incidence of lung cancer in never-smokers is notably higher in Asia compared to Western countries, highlighting the need for tailored research and clinical management strategies. Never-smoker lung cancers in Asians are predominantly adenocarcinomas and exhibit distinct molecular profiles compared to those in smokers. EGFR mutations are found in up to 50 to 60% of Asian never-smoker patients and other actionable genetic alterations, such as ALK, ROS1 and RET rearrangements, and ERBB2 mutations are also more common in this group. Early detection remains a challenge due to the lack of specific screening guidelines for never-smokers. Taiwan has a national lung cancer screening program with low-dose CT scans that uniquely includes never-smokers at high risk. Understanding its unique characteristics is crucial for optimizing care and advancing research. Multidisciplinary and multinational efforts are required to address the rising burden of this disease in Asia and to ensure equitable access to precision oncology.

[Disclosure of COI by Responsible researcher] Lead presenter is the Responsible researcher.
[Lead presenter: Se-Hoon Lee] ■Research funds from company: AstraZeneca, Daiichi Sankyo, Lunit, and MSD

### [SP3-1] SP3
### JCA Work-Life-Balance Grand Conference - Achieving a well-being researcher life with JCA -

2025 / 9 / 27 (Sat) 9:00-11:30 Room 2/(Ishikawa Ongakudo 2F Hogaku Hall) Room 2

[Chairpersons]Yushi Otsuki (Oncology Innovation Center, Fujita Health University)、Naoko Otani (Graduate School of Medicine, Osaka Metropolitan University)

研究に打ち込む毎日。やりがいはあるけれど、「時間の使い方」「生活とのバランス」「これからの研究者としての生き方」について、不安や悩みを感じたことはありませんか？☒日本癌学会では、これまでに3回、総会にてワークライフバランスをテーマとしたセッションを開催してきました。多くの方から貴重なご意見をいただき、研究と生活の両立に関する課題が多くの研究者に共通するものであることを再確認しています。☒今回開催するオープンミーティングでは、これまでの議論や声を踏まえつつ、さらに多くの立場の方々と一緒に、研究と生活のより良いバランスについて考える場としたいと考えています。☒また、この企画を通じて、日本癌学会としてワークライフバランスに関する方針を明確にし、研究環境の持続可能性を高めるための具体的な施策づくりをしていきたいと思います。☒このテーマは、次世代の研究者が安心してキャリアを築いていけるようにするためだけでなく、日本全体の研究者コミュニティの健全な発展にもつながるものです。☒学生、若手研究者、子育て中の方、ベテランの方など、さまざまな立場の声をお聞きしたいと思っています。どうぞお気軽にご参加ください！

---

### SP3-1
### Back to the Heart of Cancer Research: Integrating Mission and Researcher Well-being

[Speaker]Hideko Yamauchi (University of Hawai'i Cancer Center)

本セッションではまず、私たちは「なぜ研究を行うのか」「誰のために研究を行っているのか」という根源的な問いから出発したいと考えています。がん研究に携わる多くの皆様と共有すべき最も本質的なミッションは、がんによって命を落とす人を一人でも減らすことであり、その実現のために、私たちはそれぞれの立場で日々取り組んでいます。この目的意識の共有こそが、研究活動における原動力であり、また研究者のウェルビーイングを考える際にも重要な出発点となります。研究費の確保、キャリア形成、臨床との両立、時間的・精神的負担など、研究者を取り巻く課題は多岐にわたりますが、それらを乗り越えて研究を継続する意味や価値を再確認する機会としたいと考えています。私は日本で乳がん診療と臨床研究に従事した後、現在はアメリカ・ハワイにて医療と研究に携わっており、日米における研究環境や制度の違いを実感しています。本セッションでは、国や制度の違いを超えて、研究者が心身ともに健全な状態で使命感を持ち、持続可能な形で研究を続けるには何が必要か、皆さまと率直に意見を交わしたいと思います。研究者一人ひとりのウェルビーイングが、最終的には患者と社会の利益につながるという視点から、建設的なディスカッションができれば幸いです。

[Disclosure of COI by Responsible researcher] Lead presenter is the Responsible researcher.
[Lead presenter: Hideko Yamauchi] We have no financial relationships to disclose.

▲TOP

### [SP3-2] SP3
### JCA Work-Life-Balance Grand Conference - Achieving a well-being researcher life with JCA -

2025 / 9 / 27 (Sat) 9:00-11:30 Room 2/(Ishikawa Ongakudo 2F Hogaku Hall) Room 2

[Chairpersons]Yushi Otsuki (Oncology Innovation Center, Fujita Health University)、Naoko Otani (Graduate School of Medicine, Osaka Metropolitan University)

研究に打ち込む毎日。やりがいはあるけれど、「時間の使い方」「生活とのバランス」「これからの研究者としての生き方」について、不安や悩みを感じたことはありませんか？日本癌学会では、これまでに3回、総会にてワークライフバランスをテーマとしたセッションを開催してきました。多くの方から貴重なご意見をいただき、研究と生活の両立に関する課題が多くの研究者に共通するものであることを再確認しています。今回開催するオープンミーティングでは、これまでの議論や声を踏まえつつ、さらに多くの立場の方々と一緒に、研究と生活のより良いバランスについて考える場としたいと考えています。また、この企画を通じて、日本癌学会としてワークライフバランスに関する方針を明確にし、研究環境の持続可能性を高めるための具体的な施策づくりをしていきたいと思います。このテーマは、次世代の研究者が安心してキャリアを築いていけるようにするためだけでなく、日本全体の研究者コミュニティの健全な発展にもつながるものです。学生、若手研究者、子育て中の方、ベテランの方など、さまざまな立場の声をお聞きしたいと思っています。どうぞお気軽にご参加ください！

---

### SP3-2
### Mindset for Leading a Research Team: Balancing Personal and Team Well-being

[Speaker]Hideyuki Saya (ONIC, Fujita Health Univ.)

これまで私は医学部の講義のなかで、学生たちに「mission（社会的使命）」と「vision（将来像）」の両方を常に意識し、それらを並行して進めていくことの大切さを伝えてきた。医師や研究者として歩むうえで、これらはキャリア形成の軸となる。さらに、それらを正しい方向へ導いてくれる「value（価値観・行動規範）」も欠かすことのできないバックボーンである。研究チームを率いる立場にある者には、チーム全体とメンバー一人ひとりが、こうした要素を自らの拠りどころとしながら、安心して取り組める環境を整えることが求められる。そのためには、各メンバーの「well-being（幸福感・満足感）」を理解し、それを支援する姿勢が求められる。研究者としての人生を悔いなく歩むために、そして共に働く仲間の力を引き出すために、どのようなマインドセットが必要なのか。本講演では、その一端を考えてみたい。

[Disclosure of COI by Responsible researcher] Lead presenter is the Responsible researcher.
[Lead presenter: Hideyuki Saya] ■Advisor of the company and/or profit-making organization: Dyamond Medino Capital株式会社、FerroptoCure株式会社、アルファフュージョン株 式会社、StapleBio株式会社 ■Research expenses: 株式会社医学生物学研究所、シスメックス株式会社、JSR株式会社

### [SP3-3] SP3
### JCA Work-Life-Balance Grand Conference - Achieving a well-being researcher life with JCA -

2025 / 9 / 27 (Sat) 9:00-11:30 Room 2/(Ishikawa Ongakudo 2F Hogaku Hall) Room 2

[Chairpersons]Yushi Otsuki (Oncology Innovation Center, Fujita Health University)、Naoko Otani (Graduate School of Medicine, Osaka Metropolitan University)

研究に打ち込む毎日。やりがいはあるけれど、「時間の使い方」「生活とのバランス」「これからの研究者としての生き方」について、不安や悩みを感じたことはありませんか？⊠日本癌学会では、これまでに3回、総会にてワークライフバランスをテーマとしたセッションを開催してきました。多くの方から貴重なご意見をいただき、研究と生活の両立に関する課題が多くの研究者に共通するものであることを再確認しています。⊠今回開催するオープンミーティングでは、これまでの議論や声を踏まえつつ、さらに多くの立場の方々と一緒に、研究と生活のより良いバランスについて考える場としたいと考えています。⊠また、この企画を通じて、日本癌学会としてワークライフバランスに関する方針を明確にし、研究環境の持続可能性を高めるための具体的な施策づくりをしていきたいと思います。⊠このテーマは、次世代の研究者が安心してキャリアを築いていけるようにするためだけでなく、日本全体の研究者コミュニティの健全な発展にもつながるものです。⊠学生、若手研究者、子育て中の方、ベテランの方など、さまざまな立場の声をお聞きしたいと思っています。どうぞお気軽にご参加ください！

---

### SP3-3
### Where is utopia for us scientists?

[Speaker]Toru Hirota (Cancer Institute, Japanese Foundation for Cancer Research)

大学卒業後の３年間を消化器外科医として臨床に携わったのちに、熊本大学（当時）の佐谷秀行先生の大学院生にしていただき、私は、研究者としての道を歩みはじめました。私たち学生は乏しい知識と浅い経験しかないくせに、明るく、バイタリティにあふれ、サイエンスに興じていたように思います。「ワーク・ライフ・バランス」は完全に破綻していましたが、がむしゃらに頑張れば、何か大きな発見ができるとみんな信じ、何かに取り憑かれたように、日夜、実験に明け暮れていました。学生は、日本癌学会学術総会で発表することを共通の目標に掲げ、晴れ舞台に登壇することに心を躍らせていました。こうして「研究者とはなんて素晴らしい仕事だろう」と、覚醒しながらにして夢をみて、充実した日々を過ごしていたことを思うと、ウェルビーングに最も必要なことは何か、自ずと答えが得られそうです。年と経験を重ねるとともに、夢は大きく責任は少ない佳い時期はいつしか過ぎ去り、さらに今日では、進歩した科学技術も得られる情報量も一昔前とは隔世の感がありますが、私たちを駆り立てる最も根源には、いつの時代も、キャリアにかかわらず、普遍的なものがあるのではないでしょうか。本ワークショップでは、全世代の研究者にとって、日本癌学会が、「真に魅力的な学会」であり続け、研究者としての「ウェルビーングを肌で感じられる学会」であるために肝心なことは何か、その探求を試みます。

[Disclosure of COI by Responsible researcher] Lead presenter is the Responsible researcher.
[Lead presenter: Toru Hirota] We have no financial relationships to disclose.

### [SP3-4] SP3
### JCA Work-Life-Balance Grand Conference - Achieving a well-being researcher life with JCA -

2025 / 9 / 27 (Sat) 9:00-11:30 Room 2/(Ishikawa Ongakudo 2F Hogaku Hall) Room 2

[Chairpersons]Yushi Otsuki (Oncology Innovation Center, Fujita Health University)、Naoko Otani (Graduate School of Medicine, Osaka Metropolitan University)

研究に打ち込む毎日。やりがいはあるけれど、「時間の使い方」「生活とのバランス」「これからの研究者としての生き方」について、不安や悩みを感じたことはありませんか？⊠日本癌学会では、これまでに3回、総会にてワークライフバランスをテーマとしたセッションを開催してきました。多くの方から貴重なご意見をいただき、研究と生活の両立に関する課題が多くの研究者に共通するものであることを再確認しています。⊠今回開催するオープンミーティングでは、これまでの議論や声を踏まえつつ、さらに多くの立場の方々と一緒に、研究と生活のより良いバランスについて考える場としたいと考えています。⊠また、この企画を通じて、日本癌学会としてワークライフバランスに関する方針を明確にし、研究環境の持続可能性を高めるための具体的な施策づくりをしていきたいと思います。⊠このテーマは、次世代の研究者が安心してキャリアを築いていけるようにするためだけでなく、日本全体の研究者コミュニティの健全な発展にもつながるものです。⊠学生、若手研究者、子育て中の方、ベテランの方など、さまざまな立場の声をお聞きしたいと思っています。どうぞお気軽にご参加ください！

---

### SP3-4
### Considering work-life balance in basic medical research

[Speaker]Noriko Saitoh (The Cancer Institute of JFCR)

ウェルビーイングな研究者はどうしたら生まれるか。研究者個人としてできることと、社会としての取り組みのふたつの観点から考える。まず個人としては、目の前の研究に邁進することに加え、学会などに参加して、なるべく積極的に研究・プライベートなどの話ができる知り合いをつくることは重要で、それを自分の財産・宝物であると感じられること、その結果人的ネットワークができることは素晴らしいことと思う。ワーク・ライフ・バランスの根幹が多様性であることを理解することも重要である。そもそもウェルビーイングな研究者とは主観的なものであり、楽観的になる努力もまた必要かもしれない。一方、社会として、この十数年で労働に対する意識は随分変化した。働き方改革、コンプライアンス遵守、有給取得義務化などを頻繁に耳にする。今や「子どもが生まれたので育休を取ります」という男性がいても違和感はない。内閣府の政策として「仕事と生活の調和（ワーク・ライフ・バランス）憲章」が決定され、問題提起や取り組みの提案がされている。足りないことはまだ多いが、社会が変われることは確かなようである。研究者の労働環境に焦点を絞ると、研究費の増加、任期付きポジションの改定やPIの研究室運営のトレーニングなど課題は多い。これらについて自由に語りあう場があることがさらなる前進の一歩と考えて本セッションに臨みたい。

[Disclosure of COI by Responsible researcher] Lead presenter is the Responsible researcher.
[Lead presenter: Noriko Saitoh] We have no financial relationships to disclose.

### [S9-1] S9
### Cellular senescence: From Guardians to Enablers of Cancer

2025 / 9 / 27 (Sat) 9:00-11:30 Room 3/(Hotel Nikko Kanazawa 4F Tsuru A) Room 3

[Chairpersons]Akiko Takahashi (Division of Cellular Senescence, Cancer Institute, Japanese Foundation for Cancer Research)、Eiji Hara (Research Institute for Microbial Diseases, Osaka University)

Cellular senescence is a state of stable cell-cycle arrest triggered by various potentially oncogenic stimuli, serving as a key defense mechanism against cancer. However, because senescent cells do not immediately undergo cell death, any genetic or epigenetic inactivation of genes responsible for inducing senescence can allow these cells to escape senescence and become cancerous. Recent studies have further revealed that in addition to secreting pro-tumorigenic proteins-such as inflammatory cytokines, chemokines, growth factors, and extracellular matrix-degrading enzymes-through a phenomenon known as the senescence-associated secretory phenotype (SASP), senescent cells also release small extracellular vesicles (sEVs) and infectious endogenous retroviruses. These secretions can affect not only the senescent cells themselves but also neighboring or distant cells. Thus, understanding the diverse functions of senescent cells and developing strategies to control them holds great promise for advancing cancer diagnosis, prevention, and treatment. This symposium brings together five leading scientists to explore the multifaceted roles of cellular senescence and their potential in cancer control. We hope this symposium will contribute to the advancement of cancer research by shedding light on its underlying mechanisms and identifying new strategies for intervention.

---

### S9-1
### Induction of Cellular Senescence and Ploidy Alterations in Cancer Evolution

[Speaker]Tomonori Matsumoto (Grad. Sch. of Frontier Biosciences, Univ. Osaka) [Co-author]Eiji Hara (Res. Inst. for Microbial Dis., Univ. Osaka)

Cellular senescence and ploidy alterations play critical roles in the clonal evolution of cancer. Senescence induction serves as a tumor-suppressive mechanism by irreversibly arresting the proliferation of cells with genomic damage. However, when senescence-associated genes are dysregulated, precancerous cells may evade senescence and eventually undergo malignant transformation. A similar pattern of senescence induction followed by escape has also been observed in the development of resistance to cancer therapies, where ploidy alterations have recently been implicated. Moreover, senescence induction in stromal cells, such as cancer-associated fibroblasts, contributes to cancer pathogenesis via the senescence-associated secretory phenotype. In this study, we investigated the dynamics of senescence induction and ploidy alterations in both cancer cells and cancer-associated fibroblasts using human clinical specimens. We also used cultured cells to examine the impact of ploidy alterations on senescence. We will discuss the involvement of cellular senescence and ploidy alterations in the evolution of cancer.

[Disclosure of COI by Responsible researcher] Lead presenter is the Responsible researcher.
[Lead presenter: Tomonori Matsumoto] We have no financial relationships to disclose.

### [S9-2] S9
### Cellular senescence: From Guardians to Enablers of Cancer

2025 / 9 / 27 (Sat) 9:00-11:30 Room 3/(Hotel Nikko Kanazawa 4F Tsuru A) Room 3

[Chairpersons]Akiko Takahashi (Division of Cellular Senescence, Cancer Institute, Japanese Foundation for Cancer Research)、Eiji Hara (Research Institute for Microbial Diseases, Osaka University)

Cellular senescence is a state of stable cell-cycle arrest triggered by various potentially oncogenic stimuli, serving as a key defense mechanism against cancer. However, because senescent cells do not immediately undergo cell death, any genetic or epigenetic inactivation of genes responsible for inducing senescence can allow these cells to escape senescence and become cancerous. Recent studies have further revealed that in addition to secreting pro-tumorigenic proteins-such as inflammatory cytokines, chemokines, growth factors, and extracellular matrix-degrading enzymes-through a phenomenon known as the senescence-associated secretory phenotype (SASP), senescent cells also release small extracellular vesicles (sEVs) and infectious endogenous retroviruses. These secretions can affect not only the senescent cells themselves but also neighboring or distant cells. Thus, understanding the diverse functions of senescent cells and developing strategies to control them holds great promise for advancing cancer diagnosis, prevention, and treatment. This symposium brings together five leading scientists to explore the multifaceted roles of cellular senescence and their potential in cancer control. We hope this symposium will contribute to the advancement of cancer research by shedding light on its underlying mechanisms and identifying new strategies for intervention.

---

### S9-2
### Plasma membrane damage-dependent senescence and its implications for cancer biology

[Speaker]Keiko Kono (Okinawa Inst. Sci. Tech. Grad. Univ.)

Plasma membrane damage (PMD) is common in all cell types and is caused by environmental perturbations and cell-autonomous activities. However, the long-term cellular consequences of PMD remain largely unknown, except for recovery or cell death. Recently, we identified cellular senescence, a stable cell cycle arrest that contributes to organismal aging, as a long-term cellular consequence of PMD. In normal human fibroblasts, PMD induces premature senescence via the $Ca^{2+}$-p53 signaling axis. Consistently, upregulation of plasma membrane repair suppresses PMD-dependent senescence. Transcriptomic profiling revealed that PMD-dependent senescent cells share a molecular signature with senescent cells near tissue injury sites. These results support our hypothesis that PMD may explain the origin of certain senescent cell populations in vivo. Furthermore, we recently found the potential significance of PMD-dependent senescent cells in the context of cancer biology. PMD-dependent senescence is a previously unrecognized cellular consequence with potential implications for tissue regeneration, aging, and cancer.

[Disclosure of COI by Responsible researcher] Lead presenter is the Responsible researcher.
[Lead presenter: Keiko Kono] ■Research funds from company: SUNTORY Holdings

### [S9-3] S9
### Cellular senescence: From Guardians to Enablers of Cancer

2025 / 9 / 27 (Sat) 9:00-11:30 Room 3/(Hotel Nikko Kanazawa 4F Tsuru A) Room 3

[Chairpersons]Akiko Takahashi (Division of Cellular Senescence, Cancer Institute, Japanese Foundation for Cancer Research)、Eiji Hara (Research Institute for Microbial Diseases, Osaka University)

Cellular senescence is a state of stable cell-cycle arrest triggered by various potentially oncogenic stimuli, serving as a key defense mechanism against cancer. However, because senescent cells do not immediately undergo cell death, any genetic or epigenetic inactivation of genes responsible for inducing senescence can allow these cells to escape senescence and become cancerous. Recent studies have further revealed that in addition to secreting pro-tumorigenic proteins-such as inflammatory cytokines, chemokines, growth factors, and extracellular matrix-degrading enzymes-through a phenomenon known as the senescence-associated secretory phenotype (SASP), senescent cells also release small extracellular vesicles (sEVs) and infectious endogenous retroviruses. These secretions can affect not only the senescent cells themselves but also neighboring or distant cells. Thus, understanding the diverse functions of senescent cells and developing strategies to control them holds great promise for advancing cancer diagnosis, prevention, and treatment. This symposium brings together five leading scientists to explore the multifaceted roles of cellular senescence and their potential in cancer control. We hope this symposium will contribute to the advancement of cancer research by shedding light on its underlying mechanisms and identifying new strategies for intervention.

---

### S9-3
### p53 loss enables breast cancer escape from CDK4/6 inhibitor-induced senescence

[Speaker]Rei Kudo (Proj. Cancer Cell Commun., NEXT-Ganken, JFCR)

Inhibition of CDK4/6 kinases has led to improved outcomes in breast cancer. Nevertheless, only a minority of patients achieve long-term disease control. Using a large, clinically annotated cohort of patients with metastatic hormone receptor-positive (HR+) breast cancer, we identified TP53 loss as genetic alterations associated with poor long-term response. p53 loss did not affect CDK4/6i-induced G1 arrest but instead promotes drug-insensitive phosphorylation of p130 by CDK2. Persistent phospho-p130 inhibits DREAM complex assembly, thereby enabling cell-cycle re-entry and tumor progression. CDK2 inhibitors can restore growth arrest in p53-deficient cells, inducing geroconversion and senescence phenotypes. Our findings suggest that complete inhibition of both CDK4/6 and CDK2 may be required to achieve durable responses across genomically diverse HR+ breast cancers. Furthermore, we observed that even among cases in the prolonged response group, disease progression eventually occurred after long-term administration of CDK4/6 inhibitors. We are currently investigating the long-term impact of CDK4/6 inhibition on the senescence-associated secretory phenotype (SASP).

[Disclosure of COI by Responsible researcher] Lead presenter is the Responsible researcher.
[Lead presenter: Rei Kudo] We have no financial relationships to disclose.

### [S9-4] S9
### Cellular senescence: From Guardians to Enablers of Cancer

2025 / 9 / 27 (Sat) 9:00-11:30 Room 3/(Hotel Nikko Kanazawa 4F Tsuru A) Room 3

[Chairpersons]Akiko Takahashi (Division of Cellular Senescence, Cancer Institute, Japanese Foundation for Cancer Research)、Eiji Hara (Research Institute for Microbial Diseases, Osaka University)

Cellular senescence is a state of stable cell-cycle arrest triggered by various potentially oncogenic stimuli, serving as a key defense mechanism against cancer. However, because senescent cells do not immediately undergo cell death, any genetic or epigenetic inactivation of genes responsible for inducing senescence can allow these cells to escape senescence and become cancerous. Recent studies have further revealed that in addition to secreting pro-tumorigenic proteins-such as inflammatory cytokines, chemokines, growth factors, and extracellular matrix-degrading enzymes-through a phenomenon known as the senescence-associated secretory phenotype (SASP), senescent cells also release small extracellular vesicles (sEVs) and infectious endogenous retroviruses. These secretions can affect not only the senescent cells themselves but also neighboring or distant cells. Thus, understanding the diverse functions of senescent cells and developing strategies to control them holds great promise for advancing cancer diagnosis, prevention, and treatment. This symposium brings together five leading scientists to explore the multifaceted roles of cellular senescence and their potential in cancer control. We hope this symposium will contribute to the advancement of cancer research by shedding light on its underlying mechanisms and identifying new strategies for intervention.

---

### S9-4
### A double-edged sword: cellular senescence in mammary gland development and breast cancer.

[Speaker]Han (Inst. Pasteur) Li (Developmental and Stem Cell Biology Department, Institut Pasteur, Paris, France)

Cellular senescence is a conserved stress response implicated in both tissue repair and aging-related pathologies. While its role in wound healing is established, it remains unclear whether physiological senescence can be exploited to promote tumorigenesis. Postpartum mammary gland involution, a major tissue remodeling event resembling wound healing and associated with postpartum breast cancer (PPBC), offers a relevant model to address this question. We show that senescence is induced in alveolar luminal cells during involution in a p16-dependent manner. Ablation of these senescent cells delays involution, indicating their role in normal tissue remodeling. However, in a PPBC mouse model where oncogenic activation coincides with involution, eliminating senescent cells extends tumor latency. Mechanistically, we find that senescent cells enhance tumor-initiating cell plasticity via paracrine signaling, promoting invasion and metastasis. These results reveal that senescence during involution is essential for physiological remodeling but can be co-opted to fuel cancer progression, highlighting senescence as a potential mechanistic link between tissue repair and tumorigenesis.

[Disclosure of COI by Responsible researcher] Lead presenter is the Responsible researcher.
[Lead presenter: Han Li] We have no financial relationships to disclose.

### [S9-5] S9
### Cellular senescence: From Guardians to Enablers of Cancer

2025 / 9 / 27 (Sat) 9:00-11:30 Room 3/(Hotel Nikko Kanazawa 4F Tsuru A) Room 3

[Chairpersons]Akiko Takahashi (Division of Cellular Senescence, Cancer Institute, Japanese Foundation for Cancer Research)、Eiji Hara (Research Institute for Microbial Diseases, Osaka University)

Cellular senescence is a state of stable cell-cycle arrest triggered by various potentially oncogenic stimuli, serving as a key defense mechanism against cancer. However, because senescent cells do not immediately undergo cell death, any genetic or epigenetic inactivation of genes responsible for inducing senescence can allow these cells to escape senescence and become cancerous. Recent studies have further revealed that in addition to secreting pro-tumorigenic proteins-such as inflammatory cytokines, chemokines, growth factors, and extracellular matrix-degrading enzymes-through a phenomenon known as the senescence-associated secretory phenotype (SASP), senescent cells also release small extracellular vesicles (sEVs) and infectious endogenous retroviruses. These secretions can affect not only the senescent cells themselves but also neighboring or distant cells. Thus, understanding the diverse functions of senescent cells and developing strategies to control them holds great promise for advancing cancer diagnosis, prevention, and treatment. This symposium brings together five leading scientists to explore the multifaceted roles of cellular senescence and their potential in cancer control. We hope this symposium will contribute to the advancement of cancer research by shedding light on its underlying mechanisms and identifying new strategies for intervention.

---

### S9-5
### Elucidation of ferroptosis resistance mechanisms in pancreatic cancer and senescent stromal cells

[Speaker]Akiko Takahashi (Div. Cellular Senescence, Cancer Inst., JFCR／Proj. Cancer Cell Commun., NEXT-Ganken, JFCR) [Co-author]Tzemun Loo (Div. Cellular Senescence, Cancer Inst., JFCR)、Xiangyu Zhou (Div. Cellular Senescence, Cancer Inst., JFCR)、Yoko Tanaka (Div. Cellular Senescence, Cancer Inst., JFCR)、Sho Sugawara (Div. Cellular Senescence, Cancer Inst., JFCR)、Shota Yamauchi (Div. Cellular Senescence, Cancer Inst., JFCR)、Takashi Kamatani (Dept. AI Tech., M&D Data Science Center, ISCT)

Senescent cells, characterized by irreversible cell cycle arrest and inflammatory factor secretion, promote various age-related pathologies. Senescent cells exhibit resistance to ferroptosis, a form of iron-dependent cell death; however, the underlying mechanisms remain unclear. Here, we discovered that lysosomal acidity was crucial for lipid peroxidation and ferroptosis induction by cystine deprivation. In senescent cells, lysosomal alkalinization causes the aberrant retention of ferrous iron in lysosomes, resulting in resistance to ferroptosis. Treatment with the V-ATPase activator restored lysosomal acidity and ferroptosis sensitivity in senescent cells. A similar ferroptosis resistance mechanism involving lysosomal alkalinization was observed in pancreatic cancer cell lines. The V-ATPase activator treatment prevented pancreatic cancer development in xenograft and Kras mutant mouse models. Our findings reveal a link between lysosomal dysfunction and the regulation of ferroptosis, suggesting a novel therapeutic strategy for the treatment of cancers.

[Disclosure of COI by Responsible researcher] Lead presenter is the Responsible researcher.
[Lead presenter: Akiko Takahashi] We have no financial relationships to disclose.

▲TOP

### [S10-1] S10
### Antigen-specific immunotherapy: cancer vaccine and cell therapy

2025 / 9 / 27 (Sat) 9:00-11:30 Room 4/(Hotel Nikko Kanazawa 4F Tsuru B) Room 4

[Chairpersons]Koji Tamada (Research Institute for Cell Design Medical Science, Yamaguchi University)、Yuki Kagoya (Keio University School of Medicine)

Cancer immunotherapy is an established treatment for a growing number of cancers. In addition to immune checkpoint blockade therapy, adoptive immunotherapy and cancer vaccines are promising modalities to induce robust antigen-specific immune response against tumor cells.
Based on the remarkable efficacy of chimeric antigen receptor (CAR)-T cell therapy for hematologic malignancies, numerous strategies have been developed to maximize immune cell activity using the synthetic biology-based approaches. In addition to effector T cells, other immune cells such as NK, NKT, and myeloid cells play essential roles in the regulation of antitumor immunity, which can be exploited as therapeutic modalities. On the other hand, high-throughput analysis provides us with a better understanding of the overall architecture of tumor antigens, which has enabled us to induce potent endogenous antitumor immune responses in a logical manner, including the development of novel cancer vaccines. In this symposium, we will discuss how these emerging modalities have the potential to cure refractory cancers. We will also dissect the detailed molecular and cellular mechanisms of these therapeutics, based on an understanding of the interactions between tumor cells and immune cells at the single cell resolution.

---

### S10-1
### First in human study of allogeneic iPS-NKT cell infusion in patients with recurrent or advanced head and neck cancer

[Speaker]Shinichiro Motohashi (Dept. Med. Immunol., Grad. Sch. Med., Chiba Univ.) [Co-author]Tomohisa Iinuma (Dept. Otorhinolaryngol., Grad. Sch.Med., Chiba Univ.)、Takahiro Aoki (Dept. Med. Immunol., Grad. Sch. Med., Chiba Univ.)、Haruhiko Koseki (RIKEN, IMS)

Invariant natural killer T (iNKT) cells demonstrate anti-tumor activity through their intrinsic cytotoxicity and adjuvant properties. We conducted a phase 1 investigator-initiated clinical trial to evaluate the safety and efficacy of clinical-grade allogeneic invariant natural killer T (iNKT) cells generated from induced pluripotent stem cells (iPSC-iNKT cells) in patients with recurrent head and neck cancer (jRCT2033200116). The primary endpoint was the incidence of dose-limiting toxicities (DLT) at each dose level. The secondary endpoints were to assess safety and efficacy, as well as to evaluate immunological dynamics. iPSC-iNKT cells were administered intra-arterially to 10 patients. One subject developed grade 3 skin rash at the second dose, identified as DLT. Circulating mononuclear cells exhibited IFN-$\gamma$ pathway activation in CD4+ and CD8+ T cells after iPSC-iNKT cell administration. Clonal expansion of memory- and effector-phenotype CD8+ T cells was associated with tumor regression. These findings support the safety and therapeutic potential of iPSC-iNKT cells as an immunotherapy for solid tumors.

[Disclosure of COI by Responsible researcher] Lead presenter is the Responsible researcher.
[Lead presenter: Shinichiro Motohashi] ■Research expenses: ブライトパス・バイオ株式会社

### [S10-2] S10
### Antigen-specific immunotherapy: cancer vaccine and cell therapy

2025 / 9 / 27 (Sat) 9:00-11:30 Room 4/(Hotel Nikko Kanazawa 4F Tsuru B) Room 4

[Chairpersons]Koji Tamada (Research Institute for Cell Design Medical Science, Yamaguchi University)、Yuki Kagoya (Keio University School of Medicine)

Cancer immunotherapy is an established treatment for a growing number of cancers. In addition to immune checkpoint blockade therapy, adoptive immunotherapy and cancer vaccines are promising modalities to induce robust antigen-specific immune response against tumor cells.
Based on the remarkable efficacy of chimeric antigen receptor (CAR)-T cell therapy for hematologic malignancies, numerous strategies have been developed to maximize immune cell activity using the synthetic biology-based approaches. In addition to effector T cells, other immune cells such as NK, NKT, and myeloid cells play essential roles in the regulation of antitumor immunity, which can be exploited as therapeutic modalities. On the other hand, high-throughput analysis provides us with a better understanding of the overall architecture of tumor antigens, which has enabled us to induce potent endogenous antitumor immune responses in a logical manner, including the development of novel cancer vaccines. In this symposium, we will discuss how these emerging modalities have the potential to cure refractory cancers. We will also dissect the detailed molecular and cellular mechanisms of these therapeutics, based on an understanding of the interactions between tumor cells and immune cells at the single cell resolution.

### S10-2
### Cytokine signaling to boost CAR-T cell therapy

[Speaker]Yuki Kagoya (Keio University School of Medicine)

Adoptive immunotherapy using chimeric antigen receptor (CAR)-engineered T cells has demonstrated remarkable efficacy against B-cell malignancies and multiple myeloma. However, durable responses remain limited to a narrow range of tumor types. Various strategies have been explored to enhance the proliferation, persistence, resistance to exhaustion of CAR-T cells. Beyond efficacy, safety concerns such as cytokine release syndrome (CRS) continue to pose significant challenges. To address these issues, we and others have developed approaches to artificially modulate cytokine signaling, aiming to improve both the efficacy and safety of CAR-T cells. Given that cytokines act through distinct signaling pathways across diverse immune cell types, a detailed understanding of these molecular mechanisms is critical for the rational design of effective interventions.In this presentation, I will introduce our synthetic immunology approach that provides optimal cytokine signaling. This approach enhances antitumor activity while minimizing the risk of CRS, and offers broad applicability across different CAR-T cell therapies.

[Disclosure of COI by Responsible researcher] Lead presenter is the Responsible researcher.
[Lead presenter: Yuki Kagoya] ■Research funds from company: タカラバイオ株式会社

▲TOP

### [S10-3] S10
### Antigen-specific immunotherapy: cancer vaccine and cell therapy

2025 / 9 / 27 (Sat) 9:00-11:30 Room 4/(Hotel Nikko Kanazawa 4F Tsuru B) Room 4

[Chairpersons]Koji Tamada (Research Institute for Cell Design Medical Science, Yamaguchi University)、Yuki Kagoya (Keio University School of Medicine)

Cancer immunotherapy is an established treatment for a growing number of cancers. In addition to immune checkpoint blockade therapy, adoptive immunotherapy and cancer vaccines are promising modalities to induce robust antigen-specific immune response against tumor cells.
Based on the remarkable efficacy of chimeric antigen receptor (CAR)-T cell therapy for hematologic malignancies, numerous strategies have been developed to maximize immune cell activity using the synthetic biology-based approaches. In addition to effector T cells, other immune cells such as NK, NKT, and myeloid cells play essential roles in the regulation of antitumor immunity, which can be exploited as therapeutic modalities. On the other hand, high-throughput analysis provides us with a better understanding of the overall architecture of tumor antigens, which has enabled us to induce potent endogenous antitumor immune responses in a logical manner, including the development of novel cancer vaccines. In this symposium, we will discuss how these emerging modalities have the potential to cure refractory cancers. We will also dissect the detailed molecular and cellular mechanisms of these therapeutics, based on an understanding of the interactions between tumor cells and immune cells at the single cell resolution.

---

### S10-3
### Development of novel cancer mRNA vaccine therapy using shared cancer antigens

[Speaker]Tetsuya Nakatsura (Div. Cancer Immunother., EPOC, National Cancer Center)

We conducted a clinical trial of a peptide vaccine derived from glypican-3 (GPC3). There have been cases of advanced cancer patients whose cancers shrank after just a few doses. Seven refractory childhood cancer patients have survived for 10 years without recurrence after receiving the vaccine. On the other hand, there have been several cases of GPC3-negative cancer recurrence. In other words, there are limitations to using a peptide vaccine with a single antigen. We have collected 10 shared cancer antigens, such as GPC3. These antigens are frequently expressed in various solid cancers but are not expressed in almost non-cancerous normal organs adjacent to the cancer. We are currently developing a vaccine that combines full-length mRNA of these antigens. It has already been demonstrated that administering these mRNA vaccines to mice expressing human HLA induces antigen-specific T cell responses. In this human HLA-expressing mouse model system, we are currently conducting experiments in which we transplant cancer cells expressing each antigen and verify whether these cancer cells can be eliminated by administering these cancer mRNA vaccines.

[Disclosure of COI by Responsible researcher] Lead presenter is the Responsible researcher.
[Lead presenter: Tetsuya Nakatsura] ■Profit of stock: ノイルイミューン・ バイオテック株式会社　■Research expenses: サイアス（シノビ）、ブライトパス・バイオ、NA Vaccine Institute、小野薬品工業、メディネット、タカラバイオ、MaxCyte、オプティアム・バイオテクノロジーズ、Logomix、ダイセル、Heartseed、NapaJen Pharma、東芝、テルモ

### [S10-4] S10
### Antigen-specific immunotherapy: cancer vaccine and cell therapy

2025 / 9 / 27 (Sat) 9:00-11:30 Room 4/(Hotel Nikko Kanazawa 4F Tsuru B) Room 4

[Chairpersons]Koji Tamada (Research Institute for Cell Design Medical Science, Yamaguchi University)、Yuki Kagoya (Keio University School of Medicine)

Cancer immunotherapy is an established treatment for a growing number of cancers. In addition to immune checkpoint blockade therapy, adoptive immunotherapy and cancer vaccines are promising modalities to induce robust antigen-specific immune response against tumor cells.
Based on the remarkable efficacy of chimeric antigen receptor (CAR)-T cell therapy for hematologic malignancies, numerous strategies have been developed to maximize immune cell activity using the synthetic biology-based approaches. In addition to effector T cells, other immune cells such as NK, NKT, and myeloid cells play essential roles in the regulation of antitumor immunity, which can be exploited as therapeutic modalities. On the other hand, high-throughput analysis provides us with a better understanding of the overall architecture of tumor antigens, which has enabled us to induce potent endogenous antitumor immune responses in a logical manner, including the development of novel cancer vaccines. In this symposium, we will discuss how these emerging modalities have the potential to cure refractory cancers. We will also dissect the detailed molecular and cellular mechanisms of these therapeutics, based on an understanding of the interactions between tumor cells and immune cells at the single cell resolution.

---

### S10-4
### Engineering TCR-controlled fuzzy logic into CAR T cells enhances therapeutic specificity

[Speaker]Taisuke Kondo (Keio University School of Medicine)

Chimeric antigen receptor (CAR) T cell immunotherapy represents a breakthrough in the treatment of hematological malignancies, but poor specificity has limited its applicability to solid tumors. By contrast, natural T cells harboring T cell receptors (TCRs) can discriminate between neoantigen-expressing cancer cells and self-antigen-expressing healthy tissues but have limited potency against tumors. We used a high-throughput platform to systematically evaluate the impact of co-expressing a TCR and CAR on the same CAR T cell. While strong TCR-antigen interactions enhanced CAR activation, weak TCR-antigen interactions actively antagonized their activation. Mathematical modeling captured this TCR-CAR crosstalk in CAR T cells, allowing us to engineer dual TCR/CAR T cells targeting neoantigens and human epithelial growth factor receptor 2 (HER2) ligands, respectively. These T cells exhibited superior anti-cancer activity and minimal toxicity against healthy tissue compared with conventional CAR T cells in a humanized solid tumor mouse model. Harnessing pre-existing inhibitory crosstalk between receptors, therefore, paves the way for the design of more precise cancer immunotherapies.

[Disclosure of COI by Responsible researcher] Lead presenter is the Responsible researcher.
[Lead presenter: Taisuke Kondo] We have no financial relationships to disclose.

### [S10-5] S10
### Antigen-specific immunotherapy: cancer vaccine and cell therapy

2025 / 9 / 27 (Sat) 9:00-11:30 Room 4/(Hotel Nikko Kanazawa 4F Tsuru B) Room 4

[Chairpersons]Koji Tamada (Research Institute for Cell Design Medical Science, Yamaguchi University)、Yuki Kagoya (Keio University School of Medicine)

Cancer immunotherapy is an established treatment for a growing number of cancers. In addition to immune checkpoint blockade therapy, adoptive immunotherapy and cancer vaccines are promising modalities to induce robust antigen-specific immune response against tumor cells.
Based on the remarkable efficacy of chimeric antigen receptor (CAR)-T cell therapy for hematologic malignancies, numerous strategies have been developed to maximize immune cell activity using the synthetic biology-based approaches. In addition to effector T cells, other immune cells such as NK, NKT, and myeloid cells play essential roles in the regulation of antitumor immunity, which can be exploited as therapeutic modalities. On the other hand, high-throughput analysis provides us with a better understanding of the overall architecture of tumor antigens, which has enabled us to induce potent endogenous antitumor immune responses in a logical manner, including the development of novel cancer vaccines. In this symposium, we will discuss how these emerging modalities have the potential to cure refractory cancers. We will also dissect the detailed molecular and cellular mechanisms of these therapeutics, based on an understanding of the interactions between tumor cells and immune cells at the single cell resolution.

---

### S10-5
### Targeting the cytosolic nucleic acid sensing pathway to drive anti-tumor immunity

[Speaker]Shunsuke Kitajima (Cancer Immunotherapy Development, CPM Center, JFCR)

The clinical success of anti-PD-(L)1 antibodies demonstrates that, if we can accurately identify and appropriately disrupt the specific immune evasion strategies that each cancer type relies on, it would be possible to achieve effective immune-mediated tumor control, even in cancers currently considered resistant to immunotherapy. We particularly focus on the inactivation of the cytosolic DNA sensing pathway, cGAS/STING, as a key mechanism of immune evasion. Since cancer cells typically exhibit rapid proliferation and dysregulated DNA replication and repair mechanisms, leading to the accumulation of DNA damage, inactivation of the cGAS/STING pathway attenuates DNA-damage-dependent innate immune responses, resulting in lower immunogenicity. To address this issue, we tried to induce the accumulation of highly immunogenic nucleic acids, such as cytosolic DNA and double-stranded RNA, within cancer cells. Moreover, we evaluate clinically approved anticancer drugs in terms of their ability to accumulate immunogenic nucleic acids. Through these works, we aim to identify predictive biomarkers and to develop rational combination therapies to enhance the efficacy of cancer immunotherapy.

[Disclosure of COI by Responsible researcher] Lead presenter is the Responsible researcher.
[Lead presenter: Shunsuke Kitajima] We have no financial relationships to disclose.

### [JS4-1] JS4
### Chemical Biology for Cancer Research

2025 / 9 / 27 (Sat) 9:00-11:30 Room 5/(Hotel Nikko Kanazawa 4F Tsuru C) Room 5

[Chairpersons]Hozumi Motohashi (Tohoku University Graduate School of Medicine)、Yasuteru Urano (The University of Tokyo)

Recent advancements in analytical and synthetic technologies have driven significant innovations in chemical biology, with notable applications in cancer research. These advancements include the development of probes that target cancer cell-specific molecular markers and metabolic pathways, progress in chemical proteomics for examining protein functions and interactions through small molecules, and the creation of theranostics that combine diagnosis with treatment. These innovations are broadening the possibilities for novel diagnostic and therapeutic methods. By leveraging chemical biology approaches, we can deepen our understanding of cancer from perspectives that are challenging to achieve with traditional molecular biology techniques, and further progress is anticipated. In this session, five pioneering researchers in the field of chemical biology will introduce their latest research findings on innovative approaches and applications in cancer research. We will explore the new perspectives and technological advancements that chemical biology offers to the field of cancer research.

---

### JS4-1
### Synergistic Use of Functional Small Molecules and Proteins for Cancer Antigen Imaging

[Speaker]Ryosuke Kojima (Grad. Sci. Med. Univ. Tokyo.) [Co-author]Mamiko Nakadate (Grad. Sci. Med. Univ. Tokyo.)、Naoki Seike (Grad. Sci. Pharm. Sci. Univ. Tokyo.)、Ziyi Wang (Grad. Sci. Med. Univ. Tokyo.)、Rikuki Kiji (Grad. Sci. Pharm. Sci. Univ. Tokyo.)、Yasuteru Urano (Grad. Sci. Med. Univ. Tokyo.／Grad. Sci. Pharm. Sci. Univ. Tokyo.)

Complete surgical removal of cancer can lead to a cure, but identifying and eliminating microscopic cancers is difficult, often leading to recurrence. If cancerous areas can be clearly visualized using activatable fluorescent probes that emit light only upon recognizing cancer, complete resection becomes feasible. Our lab has developed probes that emit signals only after activation by cancer-specific hydrolases, enabling rapid, high-contrast imaging in clinical samples. However, due to cancer diversity, suitable enzymatic targets are not always found. As an alternative, activatable detection of surface antigens has been explored using antibodies labeled with small-molecule fluorophores. Yet, current methods face issues such as slow activation, weak signal, and high background. To address these, we are developing a new strategy for cancer antigen detection combining functional proteins with fluorogenic small molecules. This talk introduces probes that show strong fluorescence increase through new molecular design, along with a bio-orthogonal probe-enzyme pair for low-background cancer imaging.

[Disclosure of COI by Responsible researcher] Lead presenter is the Responsible researcher.
[Lead presenter: Ryosuke Kojima] We have no financial relationships to disclose.

▲TOP

### [JS4-2] JS4
### Chemical Biology for Cancer Research

2025 / 9 / 27 (Sat) 9:00-11:30 Room 5/(Hotel Nikko Kanazawa 4F Tsuru C) Room 5

[Chairpersons]Hozumi Motohashi (Tohoku University Graduate School of Medicine)、Yasuteru Urano (The University of Tokyo)

Recent advancements in analytical and synthetic technologies have driven significant innovations in chemical biology, with notable applications in cancer research. These advancements include the development of probes that target cancer cell-specific molecular markers and metabolic pathways, progress in chemical proteomics for examining protein functions and interactions through small molecules, and the creation of theranostics that combine diagnosis with treatment. These innovations are broadening the possibilities for novel diagnostic and therapeutic methods. By leveraging chemical biology approaches, we can deepen our understanding of cancer from perspectives that are challenging to achieve with traditional molecular biology techniques, and further progress is anticipated. In this session, five pioneering researchers in the field of chemical biology will introduce their latest research findings on innovative approaches and applications in cancer research. We will explore the new perspectives and technological advancements that chemical biology offers to the field of cancer research.

---

### JS4-2
### Therapeutic In Vivo Synthetic Chemistry

[Speaker]Katsunori Tanaka (Dept. Chem. Sci. & Eng., Sch. Mat. & Chem. Technol., Science Tokyo／RIKEN PRI)

The long-term goal of our research is to develop the working tools and methodologies that will form the foundation of "Therapeutic In Vivo Synthetic Chemistry". The main benefit of this approach is that synthetic transformations can be directly performed at target regions within the body to generate molecules that elicit localized biological effects. This method should largely circumvent off-target binding and instability issues associated with current drug administration techniques. The first approach is through the usage of glycosylated artificial metalloenzymes, where the primary aim is to exploit the chemoselectivity of embedded, non-natural transition metal catalysts for the synthesis/release of bioactive molecules. The second approach is rather centered on discovering chemical probes with novel and selective reactivity to biological metabolites naturally overexpressed in cancer cells. Once developed, the objective is then to adapt them for synthesizing diagnostic probes or anticancer drugs. A part of our strategies has already met with successful outcome in clinical trials and could be applied to pharmaceutical fields and hospitals.

[Disclosure of COI by Responsible researcher] Lead presenter is the Responsible researcher.
[Lead presenter: Katsunori Tanaka] ■Research funds from company: 科研費、AMED、三菱財団自然科学研究助成、公益財団法人アステラス病態代謝研究会研究助成、（株）糖鎖工学研究所共同研究費

### [JS4-3] JS4
### Chemical Biology for Cancer Research

2025 / 9 / 27 (Sat) 9:00-11:30 Room 5/(Hotel Nikko Kanazawa 4F Tsuru C) Room 5

[Chairpersons]Hozumi Motohashi (Tohoku University Graduate School of Medicine)、Yasuteru Urano (The University of Tokyo)

Recent advancements in analytical and synthetic technologies have driven significant innovations in chemical biology, with notable applications in cancer research. These advancements include the development of probes that target cancer cell-specific molecular markers and metabolic pathways, progress in chemical proteomics for examining protein functions and interactions through small molecules, and the creation of theranostics that combine diagnosis with treatment. These innovations are broadening the possibilities for novel diagnostic and therapeutic methods. By leveraging chemical biology approaches, we can deepen our understanding of cancer from perspectives that are challenging to achieve with traditional molecular biology techniques, and further progress is anticipated. In this session, five pioneering researchers in the field of chemical biology will introduce their latest research findings on innovative approaches and applications in cancer research. We will explore the new perspectives and technological advancements that chemical biology offers to the field of cancer research.

---

### JS4-3
### Identification of Redox and Druggable Vulnerabilities in Cancer

[Speaker]Liron Barpeled (MGH Cancer Center)

Molecular glues that engage protein complexes have transformed the study of cell biology and have had a direct impact on clinical oncology. However, the identification of new glue classes and their corresponding protein complexes has remained largely serendipitous. To overcome this challenge, we report the development of molecular COUPLrs, elaborated small molecules flanked by two cysteine-reactive warheads, as well as CONNECT, an integrated chemical proteomic platform for target deconvolution. By profiling a library of molecular COUPLrs across 13 cancer cell lines, we uncovered hundreds of proteins that can be coupled together, including in some cases in mutant selective fashions. We develop an advanced COUPLr for the oncogene EML4-ALK, which engages the fusion outside of its kinase domain, restricts protein dynamics, and disrupts EML4-ALK signaling. Collectively, molecular COUPLrs substantially expand the scope of proteins that can be chemically connected, providing an unbiased approach to identify small molecules that target protein complexes.

[Disclosure of COI by Responsible researcher] Lead presenter is the Responsible researcher.
[Lead presenter: Liron Barpeled] We have no financial relationships to disclose.

### [JS4-4] JS4
### Chemical Biology for Cancer Research

2025 / 9 / 27 (Sat) 9:00-11:30 Room 5/(Hotel Nikko Kanazawa 4F Tsuru C) Room 5

[Chairpersons]Hozumi Motohashi (Tohoku University Graduate School of Medicine)、Yasuteru Urano (The University of Tokyo)

Recent advancements in analytical and synthetic technologies have driven significant innovations in chemical biology, with notable applications in cancer research. These advancements include the development of probes that target cancer cell-specific molecular markers and metabolic pathways, progress in chemical proteomics for examining protein functions and interactions through small molecules, and the creation of theranostics that combine diagnosis with treatment. These innovations are broadening the possibilities for novel diagnostic and therapeutic methods. By leveraging chemical biology approaches, we can deepen our understanding of cancer from perspectives that are challenging to achieve with traditional molecular biology techniques, and further progress is anticipated. In this session, five pioneering researchers in the field of chemical biology will introduce their latest research findings on innovative approaches and applications in cancer research. We will explore the new perspectives and technological advancements that chemical biology offers to the field of cancer research.

---

### JS4-4
### Design and evaluation of target-selective covalent inhibitors for cancer treatment

[Speaker]Ojida Akio (Grad. Scl. Pharm. Sci., Kyushu Univ.)

Irreversible inhibition of proteins is a powerful approach to increase the potency and persistence of small-molecule drugs. In this presentation, I will present the new design of covalent inhibitors for the selective modification of proteins. We have developed several cysteine-reactive warheads, such as alpha-chlorofluoroacetamide (CFA), bicyclobutanamide (BCB-amide), and beta-fluorovinylsulfone (FVS), and applied them to the covalent inhibition of various protein kinases. We have also developed cyanoarylsulfonamide (CNS) as a new lysine-reactive warhead. By exploiting the lysine-selective reactivity of CNS, we have successfully developed the CNS-based covalent ligands towards the cancer-related proteins. We evaluated the protein reactivity profiles of the covalent inhibitors by chemical biology approaches, such as ABPP (activity-based protein profiling) and chemical proteomics analysis. I would like to discuss these data in the presentation.

[Disclosure of COI by Responsible researcher] Lead presenter is the Responsible researcher.
[Lead presenter: Ojida Akio] We have no financial relationships to disclose.

### [JS4-5] JS4
### Chemical Biology for Cancer Research

2025 / 9 / 27 (Sat) 9:00-11:30 Room 5/(Hotel Nikko Kanazawa 4F Tsuru C) Room 5

[Chairpersons]Hozumi Motohashi (Tohoku University Graduate School of Medicine)、Yasuteru Urano (The University of Tokyo)

Recent advancements in analytical and synthetic technologies have driven significant innovations in chemical biology, with notable applications in cancer research. These advancements include the development of probes that target cancer cell-specific molecular markers and metabolic pathways, progress in chemical proteomics for examining protein functions and interactions through small molecules, and the creation of theranostics that combine diagnosis with treatment. These innovations are broadening the possibilities for novel diagnostic and therapeutic methods. By leveraging chemical biology approaches, we can deepen our understanding of cancer from perspectives that are challenging to achieve with traditional molecular biology techniques, and further progress is anticipated. In this session, five pioneering researchers in the field of chemical biology will introduce their latest research findings on innovative approaches and applications in cancer research. We will explore the new perspectives and technological advancements that chemical biology offers to the field of cancer research.

---

### JS4-5
### Precision Control of Cellular Functions via Photothermal Nanoheating Platforms

[Speaker]Satoshi Arai (Kanazawa University, Nano Life Science Institute)

Our research aims to elucidate and manipulate cellular functions through thermodynamic principles. To achieve this, we have developed microscopic tools for precise intracellular thermometry and localized heating. For example, we created a palette of fluorescent thermometers capable of detecting organelle-specific temperature changes using fluorescence lifetime imaging microscopy (FLIM). In parallel, we established a localized heating method using near-infrared (NIR)-absorbing dyes that efficiently convert light into heat. Recently, we found that photothermal heating of lipid bilayers embedded with unique photothermal dyes increases membrane permeability. Based on this finding, we engineered photothermal dye-embedded liposomes encapsulating neurotransmitters or anticancer drugs, enabling NIR-triggered release. In this talk, I will present our latest progress in these thermodynamic tools, along with other quantitative biosensors for signaling molecules. While not directly focused on cancer, I hope that the tools we present will provide useful perspectives that may contribute to future developments in cancer research.

[Disclosure of COI by Responsible researcher] Lead presenter is the Responsible researcher.
[Lead presenter: Satoshi Arai] ■Research funds from company: JST-FOREST　旭硝子財団　科研費基盤研究B

### [E-3001] I-E8-1
### Cancer epigenetics (1)

2025 / 9 / 27 (Sat) 9:00-10:15 Room 6/(Hotel Nikko Kanazawa 3F Kujaku A) Room 6

[Chairperson]Atsushi Kaneda (Dept Mol Oncol, Grad Sch Med, Chiba Univ)

### E-3001
### Synthetic lethality using DNA methylation silencing

[Speaker]Takahiro Ebata (Dep of Epi, Hoshi Univ.) [Co-author]Yumi Furuichi (Dep of Epi, Hoshi Univ.)、Hideyuki Takeshima (Dep of Epi, Hoshi Univ.)、Yui Ohashi (Dep of Epi, Hoshi Univ.)、Ryoma Mashiko (Dep of Epi, Hoshi Univ.)、Qichun Wang (Dep of Epi, Hoshi Univ.)、Toshikazu Ushijima (Dep of Epi, Hoshi Univ.)

Synthetic lethality with methylation-silenced gene can provide new combination with a target gene. Using methylation data of 11,737 genes and dependency data assessed by knockdown of 16,836 genes from database, we identified a paralog combination that methylation silencing of FERMT2 was synthetic lethality with FERMT1 suppression. We confirmed that sensitivity to FERMT1 knockdown was dependent upon the FERMT2 methylation status and overexpression of FERMT2 rescued its sensitivity. Mechanistically, suppression of FERMT family caused adhesion deficiency which inactivates YAP by the change of its localization from nucleus to cytosol. In a xenograft model, FERMT1 suppression resulted in tumor repression in FERMT2 methylated cancer cells and the repression was rescued by FERMT2 expression. Our research provided new therapeutic strategy to cause synthetic lethality with FERMT1 suppression in FERMT2 methylated cancers.

[Disclosure of COI by Responsible researcher] Lead presenter is not the Responsible researcher.
[Lead presenter: Takahiro Ebata] We have no financial relationships to disclose.
[Principal Researcher: Toshikazu Ushijima] We have no financial relationships to disclose.

### [E-3002] I-E8-1
### Cancer epigenetics (1)

2025 / 9 / 27 (Sat) 9:00-10:15 Room 6/(Hotel Nikko Kanazawa 3F Kujaku A) Room 6

[Chairperson]Atsushi Kaneda (Dept Mol Oncol, Grad Sch Med, Chiba Univ)

---

### E-3002
### Establishment of a single-cell epigenomic profiling pipeline

[Speaker]Syuzo Kaneko (Div. of Med. AI Res. and Dev., Natl. Cancer Ctr.／Cancer Transl. Res. Team, RIKEN Ctr. for AIP project) [Co-author]Ryuji Hamamoto (Div. of Med. AI Res. and Dev., Natl. Cancer Ctr.／Cancer Transl. Res. Team, RIKEN Ctr. for AIP project)

Tumor tissues contain not only malignant cells but also immune and stromal cells, whose interactions critically influence cancer progression and therapy resistance. To unravel these complex intercellular dynamics, single-cell omics analyses are essential. We developed a single-cell epigenomic analysis pipeline targeting genomic structural abnormalities, utilizing a split-and-pool barcoding strategy. By combining concanavalin A (ConA)-coated magnetic beads with barcoding, we efficiently isolated nuclei and assigned unique barcodes to over 100,000 single cells with >50% labeling efficiency. This framework was extended to single-cell Hi-C for detecting chromatin contacts and structural variants. Furthermore, we implemented a generative adversarial network (GAN)-based approach to predict high-resolution contact maps from sparse single-cell Hi-C data. Comparisons with bulk Hi-C confirmed the quality of the predictions. These results advance the development of single-cell epigenomics tools and provide a foundation for identifying novel therapeutic targets through chromatin architecture analysis.

[Disclosure of COI by Responsible researcher] Lead presenter is the Responsible researcher.
[Lead presenter: Syuzo Kaneko] ■Research funds from company: CREST多細胞C3、科研費基盤（B）

▲TOP

### [E-3003] I-E8-1
### Cancer epigenetics (1)

2025 / 9 / 27 (Sat) 9:00-10:15 Room 6/(Hotel Nikko Kanazawa 3F Kujaku A) Room 6

[Chairperson]Atsushi Kaneda (Dept Mol Oncol, Grad Sch Med, Chiba Univ)

---

### E-3003
### Early-life epigenetic plasticity is shaped by dynamic chromatin accessibility changes and stem cell diversity

[Speaker]Yuyu Liu (Dept. of Epigenomics, Inst. for Advanced Life Sci., Hoshi Univ.) [Co-author]Chihiro Takeuchi (Dept. of Epigenomics, Inst. for Advanced Life Sci., Hoshi Univ.)、Toshikazu Ushijima (Dept. of Epigenomics, Inst. for Advanced Life Sci., Hoshi Univ.)

High epigenetic plasticity in youth is thought to contribute to the aggressive nature of cancers in adolescent and young adults. However, whether this plasticity arises from intrinsic chromatin accessibility changes or greater stem cell diversity remains unclear. To investigate this, we examined organoids derived from murine antral tissues across different life stages. Infant-derived organoids uniquely resisted IFN-γ-induced apoptosis and remained unaffected by MEK inhibitor, confirming their high epigenetic plasticity. Single-cell ATAC-seq revealed that IFN-γ treatment induced the highest number of differentially accessible chromatin regions in infant-derived organoids, suggesting extensive chromatin accessibility changes. Further single-cell ATAC-seq analysis of murine antrum-pyloric tissues identified that infant mice had the greatest stem cell diversity. These findings indicate that the epigenetic plasticity observed in early-life gastric stem cells is shaped by both their ability to undergo dynamic accessibility changes and the inherent diversity within the stem cells population.

[Disclosure of COI by Responsible researcher] Lead presenter is not the Responsible researcher.
[Lead presenter: Yuyu Liu] We have no financial relationships to disclose.
[Principal Researcher: Toshikazu Ushijima] We have no financial relationships to disclose.

▲TOP

### [E-3004] I-E8-1
### Cancer epigenetics (1)

2025 / 9 / 27 (Sat) 9:00-10:15 Room 6/(Hotel Nikko Kanazawa 3F Kujaku A) Room 6

[Chairperson]Atsushi Kaneda (Dept Mol Oncol, Grad Sch Med, Chiba Univ)

---

### E-3004
### Simultaneous single cell gene mutation and chromatin accessibility analysis of myeloid precursor lesions

[Speaker]Masanori Motomura (Dept. of Path. & Tumor Biol., Kyoto Univ.) [Co-author]Yotaro Ochi (Dept. of Path. & Tumor Biol., Kyoto Univ.)、Yasuhito Nannya (Dept. of Path. & Tumor Biol., Kyoto Univ.／Div. of Hematopoietic Disease Control, The Univ. of Tokyo)、Nobuyuki Kakiuchi (Dept. of Path. & Tumor Biol., Kyoto Univ.／The Hakubi Ctr. for Advanced Res., Kyoto Univ.)、Nobuo Sezaki (Dept. of Hematology, Chugoku Central Hosp.)、Senji Kasahara (Dept. of Hematology, Gifu Municipal Hosp.)、Tomoe Nakagawa (Dept. of Path. & Tumor Biol., Kyoto Univ.)、Lanying Zhao (Dept. of Path. & Tumor Biol., Kyoto Univ.／Inst. for the Advanced Study of Human Biol., Kyoto Univ.)、Shuichi Matsuda (Dept. of Orthopedic Surg., Kyoto Univ.)、Masahiro M. Nakagawa (Dept. of Path. & Tumor Biol., Kyoto Univ.)、Seishi Ogawa (Dept. of Path. & Tumor Biol., Kyoto Univ.／Inst. for the Advanced Study of Human Biol., Kyoto Univ.)

Age-related clonal hematopoiesis (CH) and clonal cytopenia of undetermined significance (CCUS) frequently harbor mutations in epigenetic regulators. However, how these mutations alter epigenetic profiles remains unclear. To investigate this, we analyzed ***TET2***-mutated (***TET2***-MUT) CH/CCUS samples using our novel Mission Bio Tapestri-based single-cell multi-omics platform enabling simultaneous detection of gene mutations and chromatin accessibility. A total of 33,000 cells from ***TET2***-MUT CH (n=4) and CCUS (n=5) cases were analyzed. ***TET2***-MUT hematopoietic stem and progenitor cells (HSPCs) showed increased accessibility to the motifs of myeloid differentiation-related transcription factors (TFs), compared to ***TET2*** wild-type (***TET2***-WT) HSPCs, consistent with myeloid skewing of ***TET2*** mutants. ***TET2***-MUT B cells exhibited decreased accessibility to the motifs for TFs related to B cell differentiation, compared to ***TET2***-WT B cells. In terminally differentiated erythroblasts, ***TET2***-MUT cells had increased accessibility to cell proliferation-related genes, compared to ***TET2***-WT cells. Our results provide new insights into how ***TET2*** mutation disrupts chromatin landscapes at single-cell level in CH/CCUS.

[Disclosure of COI by Responsible researcher] Lead presenter is not the Responsible researcher.
[Lead presenter: Masanori Motomura] We have no financial relationships to disclose.
[Principal Researcher: Yotaro Ochi, Tomoe Nakagawa] ■Advisor of the company and/or profit-making organization: エーザイ株式会社　Chordia Therapeutics Inc.　Montage Bio, INC.　■Profit of stock: 朝日ゲノミクス株式会社　■Lecture fee: 公益財団法人三菱財団　公益財団法人中谷計測技術振興財団　■Research expenses: エーザイ株式会社　Chordia Therapeutics Inc.　■Contributions: 日本新薬株式会社　■Endowed chair: Chordia Therapeutics Inc.　公益社団法人鹿児島共済会 南風病院

### [E-3005] I-E8-1
### Cancer epigenetics (1)

2025 / 9 / 27 (Sat) 9:00-10:15 Room 6/(Hotel Nikko Kanazawa 3F Kujaku A) Room 6

[Chairperson]Atsushi Kaneda (Dept Mol Oncol, Grad Sch Med, Chiba Univ)

---

### E-3005
### Non-coding RNA ELEANOR2-driven super-enhancer regulation of ***ESR1*** with genomic amplification

[Speaker]Maierdan Palihati (Division of Cancer Biology, The Cancer Institute of JFCR) [Co-author]Yuichi Ichikawa (Division of Cancer Biology, The Cancer Institute of JFCR)、Noriko Saitoh (Division of Cancer Biology, The Cancer Institute of JFCR)

In estrogen receptor (ER)-positive breast cancer, which constitutes ~70% of cases, the ESR1 gene encoding ER is activated by a cluster of non-coding RNAs (ncRNAs) termed ELEANORs, forming an RNA cloud. The role of ELEANOR2, a highly expressed ncRNA from an intronic sequence of CCDC170, is poorly understood. Using CHIRP-seq and RADICL-seq, we demonstrate that ELEANOR2 binds a super-enhancer (SE) located 15 kb downstream of its transcription site and 100 kb upstream of ***ESR1***. This SE, enriched with H3K27ac and BRD4, interacts with ELEANOR2 to form a biomolecular condensate, driving ***ESR1*** transcription via a positive feedback loop. Notably, in ER-positive HCC1428 cells and patient tissues, the ELEANOR2-***ESR1***-SE region is focally amplified, enhancing RNA cloud formation and ESR1 activation. This amplification may contribute to genome rearrangements that promote cancer progression and resistance to endocrine therapies, which often fail due to acquired estrogen independence. These findings elucidate a novel mechanism where chromatin-associated ncRNAs, via interactions with epigenetic factors like BRD4, regulate gene expression and drive oncogenic genomic alterations in breast cancer.

[Disclosure of COI by Responsible researcher] Lead presenter is not the Responsible researcher.
[Lead presenter: Maierdan Palihati] We have no financial relationships to disclose.
[Principal Researcher: Noriko Saitoh] We have no financial relationships to disclose.

### [E-3006] I-E8-1
### Cancer epigenetics (1)

2025 / 9 / 27 (Sat) 9:00-10:15 Room 6/(Hotel Nikko Kanazawa 3F Kujaku A) Room 6

[Chairperson]Atsushi Kaneda (Dept Mol Oncol, Grad Sch Med, Chiba Univ)

---

### E-3006
### Oncogenic RAS activation drives transcriptional alterations through regulating chromatin dynamics

[Speaker]Ryusuke Nozawa (Div. Exp. Path. Cancer Inst., JFCR) [Co-author]Saho Matsui (Div. Exp. Path. Cancer Inst., JFCR)、Ren Hanake (Dept. of Life Sci. and Tech., Science Tokyo)、Takehiko Itoh (Dept. of Life Sci. and Tech., Science Tokyo)、Toru Hirota (Div. Exp. Path. Cancer Inst., JFCR)

Global transcriptional alterations characterize cancer cells and drive the acquisition and progression of malignant phenotypes. However, how oncogenic growth signals relate to aberrations in transcriptional regulation remains poorly understood. Here, to elucidate the pathological mechanisms underlying transcriptional changes in cancer cells, we examined chromatin dynamics induced by active RAS. First, we identified genes exhibiting acute transcriptional changes upon short-term HRAS-G12V expression in non-transformed human RPE1 cells. Notably, upregulated genes involved increased accessibility at transcription start sites as expected, while the level of accessibility of downregulated genes remained unchanged. To go beyond the chromatin states underlying these distinct responses, we conducted super-resolution imaging using STORM and found that accessible chromatin forms clusters associated with transcriptional activation, and RAS stimuli suppress this cluster formation. These observations suggest that RAS activation induces transcriptional alterations in at least two layers; not only by modulating local chromatin accessibility but also by regulating chromatin conformation.

[Disclosure of COI by Responsible researcher] Lead presenter is not the Responsible researcher.
[Lead presenter: Ryusuke Nozawa] We have no financial relationships to disclose.
[Principal Researcher: Toru Hirota] We have no financial relationships to disclose.

### [J-3001] I-J12-3
### Liquid biopsy in cancer diagnosis

2025 / 9 / 27 (Sat) 9:00-10:15 Room 7/(Hotel Nikko Kanazawa 3F Kujaku B) Room 7

[Chairperson]Satoshi Nishizuka (Iwate Med. Univ. Inst. Biomed. Sci.)

---

### J-3001
### GeMSort: Algorithm to Identify Germline Pathogenic Variants in Liquid Genomic Profiling - Insights from C-CAT Database

[Speaker]Satoyo Oda (Dept. Lab. Med., Natl. Cancer Ctr. Hosp.／Dept. Genet. Med. & Services, Natl. Cancer Ctr. Hosp.) [Co-author]Manami Matsukawa (Dept. Genet. Med. & Services, Natl. Cancer Ctr. Hosp.)、Chikako Tomozawa (Dept. Genet. Med. & Services, Natl. Cancer Ctr. Hosp.)、Noriko Tanabe (Dept. Genet. Med. & Services, Natl. Cancer Ctr. Hosp.／Dept. Clin. Genet., Saitama Med. Ctr.)、Tomoko Watanabe (Dept. Genet. Med. & Services, Natl. Cancer Ctr. Hosp.)、Takafumi Koyama (Dept. Exp. Therap., Natl. Cancer Ctr. Hosp)、Teruhiko Yoshida (Dept. Genet. Med. & Services, Natl. Cancer Ctr. Hosp.)、Makoto Hirata (Dept. Genet. Med. & Services, Natl. Cancer Ctr. Hosp.)

**Background**Liquid biopsy comprehensive genomic profiling (LB-CGP) tests are performed to identify somatic mutations and guide treatment strategy. The CGP tests can also identify germline findings associated with hereditary tumor syndromes (HTS). Differentiating presumed germline pathogenic variants (PGPVs) from somatic mutations remains difficult in LB-CGP tests.**Materials and Methods**Using the C-CAT database, we analyzed 169,370 variants detected in 11,399 patients registered with FoundationOne Liquid CDx to develop an algorithm to identify PGPVs in LB-CGP tests.**Results**By extracting variants that are presumed predominantly somatic (e.g., hotspot driver mutations) or germline (e.g., common polymorphisms), we established a highly accurate criterion for VAF assessment. Further investigation of other variants led to the development of an algorithm for differentiating somatic/germline variants of HTS-associated genes. Based on this algorithm, 726 variants from 710 patients were extracted as PGPVs among 26 HTS-associated genes.**Conclusion**This algorithm should help to differentiate the PGPVs from somatic mutations, although further analyses are required to validate the algorithm.

[Disclosure of COI by Responsible researcher] Lead presenter is not the Responsible researcher.
[Lead presenter: Satoyo Oda] We have no financial relationships to disclose.
[Principal Researcher: Makoto Hirata] We have no financial relationships to disclose.

### [J-3002] I-J12-3
### Liquid biopsy in cancer diagnosis

2025 / 9 / 27 (Sat) 9:00-10:15 Room 7/(Hotel Nikko Kanazawa 3F Kujaku B) Room 7

[Chairperson]Satoshi Nishizuka (Iwate Med. Univ. Inst. Biomed. Sci.)

---

### J-3002
### cfDNA-Based Monitoring and MRD Prediction in ***BRAF***$^{V600E}$ Mutant Colorectal Cancer: A Precision Medicine Biomarker Study

[Speaker]Takuma Iwai (Department of Gastroenterological Surgery, Nippon Medical School) [Co-author]Takeshi Yamada (Department of Gastroenterological Surgery, Nippon Medical School)、Kay Uehara (Department of Gastroenterological Surgery, Nippon Medical School)、Seiichi Shinji (Department of Gastroenterological Surgery, Nippon Medical School)、Akihisa Matsuda (Department of Gastroenterological Surgery, Nippon Medical School)、Yasuyuki Yokoyama (Department of Gastroenterological Surgery, Nippon Medical School)、Goro Takahashi (Department of Gastroenterological Surgery, Nippon Medical School)、Toshimitsu Miyasaka (Department of Gastroenterological Surgery, Nippon Medical School)、Hiroshi(Nippon Med. Sch.) Yoshida (Department of Gastroenterological Surgery, Nippon Medical School)

**Background/Objectives:*****BRAF***$^{V600E}$-mutant colorectal cancer (CRC) has a poor prognosis, and despite BEACON therapy, challenges remain in real-time monitoring and minimal residual disease (MRD) evaluation. This study evaluates the clinical utility of cfDNA ***BRAF***$^{V600E}$ as a biomarker for treatment response and recurrence prediction.

**Methods:**This study included 37 patients with ***BRAF***$^{V600E}$-mutant and MSS CRC, divided into: Cohort 1 (Stage IV, n=14): Treatment monitoringCohort 2 (Stage I–III, n=23): Recurrence risk prediction. cfDNA ***BRAF*** were analyzed using droplet digital PCR to quantify variant allele frequency (VAF).

**Results:**Cohort 1: Pre-treatment cfDNA ***BRAF***$^{V600E}$ was detected in 93%. A VAF decrease post-treatment correlated with longer survival (511 vs. 189 days, p=0.03).Cohort 2: cfDNA ***BRAF***$^{V600E}$ was detected in 4/23 (17.4%) at 1 month post-surgery, with superior recurrence prediction vs. CEA (100% vs. 18.8%, p=0.004). cfDNA ***BRAF***$^{V600E}$ positivity predicted shorter recurrence-free survival (179 vs. 840 days, p=0.04).

**Conclusions:**cfDNA ***BRAF***$^{V600E}$ is a promising biomarker for treatment monitoring and MRD detection, supporting precision oncology and personalized treatment strategies.

[Disclosure of COI by Responsible researcher] Lead presenter is the Responsible researcher.
[Lead presenter: Takuma Iwai] We have no financial relationships to disclose.

▲TOP

### [J-3003] I-J12-3
### Liquid biopsy in cancer diagnosis

2025 / 9 / 27 (Sat) 9:00-10:15 Room 7/(Hotel Nikko Kanazawa 3F Kujaku B) Room 7

[Chairperson]Satoshi Nishizuka (Iwate Med. Univ. Inst. Biomed. Sci.)

---

### J-3003
### Circulating tumor DNA detects minimal residual disease and predicts outcomes in esophageal cancer after esophagectomy

[Speaker]Qingjiang Hu (Department of Surgery, Kyushu University Beppu Hospital／Department of Surgery and Science, Kyushu University) [Co-author]Yasue Kimura (Department of Surgery and Science, Kyushu University)、Tomonori Nakanoko (Department of Surgery and Science, Kyushu University)、Mitsuhiko Ota (Department of Surgery and Science, Kyushu University)、Tomoharu Yoshizumi (Department of Surgery and Science, Kyushu University)、Koshi Mimori (Department of Surgery, Kyushu University Beppu Hospital)、Eiji Oki (Department of Surgery and Science, Kyushu University)

Background: Circulating tumor DNA (ctDNA) monitoring shows promise for detecting minimal residual disease (MRD) and predicting prognosis in various cancers. This study evaluated ctDNA for detecting MRD and predicting outcomes in esophageal cancer (EC) post-esophagectomy. Methods: A observational study included 40 EC patients undergoing upfront surgery or neoadjuvant chemotherapy (NAC) followed by esophagectomy. Plasma samples (n = 124) were collected at six time points, and ctDNA was assessed by next-generation sequencing using an in-house cancer panel. Associations with clinical outcomes were analyzed. Results: Pre-therapy ctDNA levels correlated with tumor stage (P = 0.01). Changes in ctDNA levels predicted tumor progression with an area under the curve of 0.77. Postsurgical ctDNA positivity predicted recurrence earlier than radiographic evidence (median: 90 days) and was associated with shorter recurrence-free survival (RFS) and progression-free survival (PFS) across all patients (RFS hazard ratio [HR]: 11.1, P = 0.006; PFS HR: 12.6, P = 0.002). Conclusions: ctDNA assessment is a reliable tool for detecting MRD and predicting outcomes in EC patients after esophagectomy.

[Disclosure of COI by Responsible researcher] Lead presenter is not the Responsible researcher.
[Lead presenter: Qingjiang Hu] We have no financial relationships to disclose.
[Principal Researcher: Eiji Oki] We have no financial relationships to disclose.

### [J-3004] I-J12-3
### Liquid biopsy in cancer diagnosis

2025 / 9 / 27 (Sat) 9:00-10:15 Room 7/(Hotel Nikko Kanazawa 3F Kujaku B) Room 7

[Chairperson]Satoshi Nishizuka (Iwate Med. Univ. Inst. Biomed. Sci.)

---

### J-3004
### Utility of Plasma and Urine ctDNA as Minimally Invasive Biomarkers in Advanced Esophageal Cancer

[Speaker]Rie Shibata (Department of Surgery, Kyoto Prefectural University of Medicine) [Co-author]Hirotaka Konishi (Department of Surgery, Kyoto Prefectural University of Medicine)、Kenji Nanishi (Department of Surgery, Kyoto Prefectural University of Medicine)、Tomohiro Arita (Department of Surgery, Kyoto Prefectural University of Medicine)、Hiroyuki Inoue (Department of Surgery, Kyoto Prefectural University of Medicine)、Keiji Nishibeppu (Department of Surgery, Kyoto Prefectural University of Medicine)、Jun Kiuchi (Department of Surgery, Kyoto Prefectural University of Medicine)、Hidemasa Kubo (Department of Surgery, Kyoto Prefectural University of Medicine)、Taisuke Imamura (Department of Surgery, Kyoto Prefectural University of Medicine)、Hiroki Shimizu (Department of Surgery, Kyoto Prefectural University of Medicine)、Ryo Morimura (Department of Surgery, Kyoto Prefectural University of Medicine)、Shuhei Komatsu (Department of Surgery, Kyoto Prefectural University of Medicine)、Hitoshi Fujiwara (Department of Surgery, Kyoto Prefectural University of Medicine)、Atsushi Shiozaki (Department of Surgery, Kyoto Prefectural University of Medicine)

Objective: Circulating tumor DNA(ctDNA) is a promising biomarker in various cancers. Detection of ctDNA in plasma and urine was evaluated in esophageal squamous cell carcinoma(ESCC).
Methods: We studied 94 patients with advanced ESCC undergoing esophagectomy from 2019 to 2023. Two PIK3CA mutations(E545K, H1047R) were examined using droplet digital PCR. First, in 47 cases, we assessed mutation rates in tumor tissue and sensitivity and specificity of ctDNA detection in plasma and urine. Next, we analyzed associations between plasma ctDNA detection and clinicopathological factors. In 16 preoperative ctDNA-positive cases, postoperative ctDNA changes and prognoses were evaluated.
Results: Mutation rates were 14.9%(E545K) and 10.6%(H1047R). Sensitivity/specificity of plasma ctDNA detection was 42.9%/90.0% (E545K) and 80.0%/97.6%(H1047R); in urine, 0.0%/92.5% and 60.0%/83.3%. Preoperative ctDNA positivity was associated with pT>3, R1/2 resection, and tumor size >45mm. After surgery, 13 of 16 ctDNA-positive cases turned negative; persistent positivity was linked to poor prognosis.
Conclusion: Plasma and urine ctDNA detection is feasible and may be a useful minimally invasive biomarker in ESCC.

[Disclosure of COI by Responsible researcher] Lead presenter is not the Responsible researcher.
[Lead presenter: Rie Shibata] We have no financial relationships to disclose.
[Principal Researcher: Kenji Nanishi] We have no financial relationships to disclose.

▲TOP

### [J-3005] I-J12-3
### Liquid biopsy in cancer diagnosis

2025 / 9 / 27 (Sat) 9:00-10:15 Room 7/(Hotel Nikko Kanazawa 3F Kujaku B) Room 7

[Chairperson]Satoshi Nishizuka (Iwate Med. Univ. Inst. Biomed. Sci.)

---

### J-3005
### Serial circulating tumor DNA analysis reveals clonal dynamics and prognostic alterations in high-risk neuroblastoma.

[Speaker]Ryunosuke Tojo (Dept of Ped, Grad Sch of Med, Kyoto Univ.) [Co-author]Hirohito Kubota (Dept of Ped, Grad Sch of Med, Kyoto Univ.)、Shuro Nishio (Dept of Ped, Grad Sch of Med, Kyoto Univ.)、Kazuki Mitani (Dept of Ped, Grad Sch of Med, Kyoto Univ.)、Shohei Azumi (Dept of Ped, Grad Sch of Med, Kyoto Univ.)、Hirokazu Kobushi (Dept of Ped, Grad Sch of Med, Kyoto Univ.)、Yoshinori Uchihara (Dept of Ped, Grad Sch of Med, Kyoto Univ.)、Ryo Akazawa (Dept of Ped, Grad Sch of Med, Kyoto Univ.)、Tatsuya Kamitori (Dept of Ped, Grad Sch of Med, Kyoto Univ.)、Takashi Mikami (Dept of Ped, Grad Sch of Med, Kyoto Univ.)、Satoshi Saida (Dept of Ped, Grad Sch of Med, Kyoto Univ.)、Itaru Kato (Dept of Ped, Grad Sch of Med, Kyoto Univ.)、Katsutsugu Umeda (Dept of Ped, Grad Sch of Med, Kyoto Univ.)、Junko Takita (Dept of Ped, Grad Sch of Med, Kyoto Univ.)

Introduction: High-risk neuroblastoma (HR-NB) remains a therapeutic challenge due to acquired treatment resistance, often driven by tumor clonal evolution. Non-invasive monitoring using circulating tumor DNA (ctDNA) offers a promising approach to track these genetic changes. Experimental design: We conducted serial ctDNA analysis using next-generation sequencing in 10 HR-NB patients (57 plasma samples) to detect emerging alterations and assess their clinical significance.Results: Pathogenic clonal and subclonal mutations were detected in ctDNA from all patients. Eight relapsed or refractory patients had newly detected driver genetic alterations at progression, which were absent in matched primary or relapsed tissue samples. The emergence of clonal mutations affecting the RAS/MAPK pathway (n=3, MAPK1, NF1, FGFR1) was associated with an aggressive clinical course and shorter subsequent survival (median 154 days [range 83-154]) compared to other pathway mutations (n=5, median 394 days [124-527]). Conclusions: Serial ctDNA analysis effectively captures HR-NB clonal evolution dynamics. Emerging RAS/MAPK pathway mutations may reflect a shift towards aggressive disease phenotype.

[Disclosure of COI by Responsible researcher] Lead presenter is not the Responsible researcher.
[Lead presenter: Ryunosuke Tojo] We have no financial relationships to disclose.
[Principal Researcher: Junko Takita] We have no financial relationships to disclose.

### [J-3006] I-J12-3
### Liquid biopsy in cancer diagnosis

2025 / 9 / 27 (Sat) 9:00-10:15 Room 7/(Hotel Nikko Kanazawa 3F Kujaku B) Room 7

[Chairperson]Satoshi Nishizuka (Iwate Med. Univ. Inst. Biomed. Sci.)

---

### J-3006
### TALnc1 (LINC01869) as a potential biomarker for liquid biopsy in colorectal cancer

[Speaker]Mitsuaki Nishioka (Div. of Lab., Yamaguchi Univ. Hosp.) [Co-author]Yutaka Suehiro (Div. of Lab., Yamaguchi Univ. Hosp.／Dept. of Oncology & Lab.Med., Yamaguchi Univ.Grad. Sch. of Med)、Yuki Kunimune (Div. of Lab., Yamaguchi Univ. Hosp.)、Masaki Kodama (Div. of Lab., Yamaguchi Univ. Hosp.)、Koichi Hamabe (Dept. of Gastroenterology & Hepatology, Yamaguchi Univ.Grad. Sch. of Med)、Shinichi Hashimoto (Dept. of Gastroenterology & Hepatology, Yamaguchi Univ.Grad. Sch. of Med)、Taro Takami (Dept. of Gastroenterology & Hepatology, Yamaguchi Univ.Grad. Sch. of Med)、Hiroaki Nagano (Dept. of Gastroenterological, Breast,&Endocrine Surg., Yamaguchi Univ.Grad. Sch. of Med)、Takahiro Yamasaki (Div. of Lab., Yamaguchi Univ. Hosp.／Dept. of Oncology & Lab.Med., Yamaguchi Univ.Grad. Sch. of Med)

Background: We previously reported that LINC01869, tentatively named TALnc1 (TROY and LGR5-associated long non-coding RNA 1), was significantly downregulated in colorectal cancer (CRC) tissues. The aim of this study was to identify the tumor-specific methylated regions of TALnc1 and apply it to a liquid biopsy. Methods: To identify the tumor-specific methylated sites, bisulfite sequencing was performed on paired tumor and non-tumor tissues. TALnc1 methylation levels in cell-free DNA (cfDNA) were quantified using our proprietary combined digital restriction PCR (CORD) assay and compared between the early CRC and control groups in the pilot cohort (X1 controls and Y1 CRC patients) and the validation cohort (X2 and Y2, respectively). Results: CpG #8 in the TALnc1 promoter region had significantly higher methylation levels in tumor tissues than in normal tissues ($p = 0.034$). Regarding cfDNA, the methylation level of CpG #8 was also higher in the CRC group than in the control group (pilot study: $p = 0.0002$ and AUC = 0.837; validation study: $p < 0.0001$ and AUC = 0.823). Conclusions: Our findings suggest that TALnc1 may be a promising marker for liquid biopsy in CRC.

[Disclosure of COI by Responsible researcher] Lead presenter is the Responsible researcher.
[Lead presenter: Mitsuaki Nishioka] ■Research funds from company: 科研費 基盤研究(C) 研究課題番号: 23K06885、令和3年度公益信託臨床検査医学研究振興基金

### [IS7-2] IS7
### UICC: Inequality and Cancer Outcome

2025 / 9 / 27 (Sat) 9:00-11:30 Room 8/(Hotel Nikko Kanazawa 3F Kujaku C) Room 8

[Chairpersons]Keitaro Matsuo (Aichi Cancer Center)、Salvatore Vaccarella (International Agency for Research on Cancer)

---

### IS7-2
### Socioeconomic Inequalities and Cancer Outcomes at the Community Level: A Spatial Epidemiological Approach

[Speaker]Hidemi Ito (Div of Cancer Information and Control)

Socioeconomic disparities at the community level play an important role in cancer incidence and mortality. Factors such as income, education, and social support shape daily life, living environments, and access to health care. These differences create unequal opportunities for prevention, early detection, treatment, and follow-up, leading to disparities in cancer outcomes. Such inequalities are not limited to rural or underserved areas. They may also vary by the degree of urbanization. Urban areas often appear to provide good access because of the high density of medical facilities, yet substantial social and economic divides within the same municipality can create "hidden inequalities" that affect health status and cancer outcomes.In this presentation, I will introduce our spatial epidemiological research examining the association between community-level socioeconomic deprivation and cancer outcomes, with attention to differences by urbanicity. By clarifying the pathways through which deprivation is associated with cancer incidence and mortality, our work provides evidence to support strategies aimed at reducing regional inequalities in cancer outcomes.

[Disclosure of COI by Responsible researcher] Lead presenter is the Responsible researcher.
[Lead presenter: Hidemi Ito] We have no financial relationships to disclose.

### [IS7-3] IS7
### UICC: Inequality and Cancer Outcome

2025 / 9 / 27 (Sat) 9:00-11:30 Room 8/(Hotel Nikko Kanazawa 3F Kujaku C) Room 8

[Chairpersons]Keitaro Matsuo (Aichi Cancer Center)、Salvatore Vaccarella (International Agency for Research on Cancer)

---

### IS7-3
### Social disparities in cancer through the dual lens of place

[Speaker]Tomoki Nakaya (Grad. Sch. Environ. Stud., Tohoku Univ.／Grad. Sch. Sci., Tohoku Univ.)

Geographic disparities in cancer outcomes are increasingly recognised as manifestations of broader social inequalities. Drawing on population-level data from Japan, this presentation examines how place-based deprivation shapes disparities across the cancer spectrum, from prevention and early detection to treatment and survivorship. Despite nationwide declines in age-adjusted cancer mortality, socioeconomic inequalities by residential area have persisted or even widened. Area deprivation reflects the socioeconomic composition of residents as well as contextual factors such as the physical environment, access to services and local social conditions, all of which influence health behaviours including smoking, physical activity and mental health. In sum, place acts as a dual lens: one for observing cancer disparities, and another for uncovering the structural and environmental factors that generate them, making its design a critical concern for advancing equitable cancer control.

[Disclosure of COI by Responsible researcher] Lead presenter is the Responsible researcher.
[Lead presenter: Tomoki Nakaya] ■Contributions: 東日本旅客鉄道株式会社

▲TOP

### [IS7-4] IS7
### UICC: Inequality and Cancer Outcome

2025 / 9 / 27 (Sat) 9:00-11:30 Room 8/(Hotel Nikko Kanazawa 3F Kujaku C) Room 8

[Chairpersons]Keitaro Matsuo (Aichi Cancer Center)、Salvatore Vaccarella (International Agency for Research on Cancer)

---

### IS7-4
### Current situations of socioeconomic inequalities in cancer mortality in Japan

[Speaker]Hirokazu Tanaka (NCCICC, Pop Data Sci)

Regional inequalities in cancer mortality and incidence are often cited in Japan. However, Japan lacks a framework for collecting and evaluating outcomes by individual socioeconomic indicators (e.g., educational level, occupation, and income). Therefore, we developed the 2020 Japanese census-linked mortality database, which linked the 2020 Census and death records (2020-2022) based on mortality data linked to resident registration information in European countries. This mortality database revealed the educational inequalities in all-cancer were 1.58 times (95% confidence interval: 1.56-1.60) for men and 1.43 times (1.40-1.46) for women. When examining the contribution of each cancer-site to inequalities in all-cancer mortality, the most significant contributors were lung (34.6%), stomach (17.5%), and liver (10.0%) in men, and lung (23.0%), stomach (12.0%), and colon (11.7%) in women. Socioeconomic inequalities in cancer mortality were small for pancreas, breast, and brain/central nervous system. The National Cancer Registry has no system for linking data with the Census. A framework for comprehensively monitoring socioeconomic inequalities in cancer outcomes is required.

[Disclosure of COI by Responsible researcher] Lead presenter is the Responsible researcher.
[Lead presenter: Hirokazu Tanaka] We have no financial relationships to disclose.

### [IS7-1] IS7
### UICC: Inequality and Cancer Outcome

2025 / 9 / 27 (Sat) 9:00-11:30 Room 8/(Hotel Nikko Kanazawa 3F Kujaku C) Room 8

[Chairpersons]Keitaro Matsuo (Aichi Cancer Center)、Salvatore Vaccarella (International Agency for Research on Cancer)

---

### IS7-1
### Underuse and overuse of medical cancer in cancer

[Speaker]Salvatore Vaccarella (Cancer Surveillance Branch, International Agency for Research on Cancer)

Cancer is not equally distributed across countries and individuals. The scale and profile of cancer evolve over time, in parallel with the socioeconomic development of societies. While transitions towards better economic/living conditions provides many advantages, they also result in greater exposure to various cancer risk factors. However, it is always the most disadvantaged who are disproportionately hit harder. The role of the context and of the inefficiencies in delivering health-care services are crucial. Whereas disadvantaged individuals, who are most in need, cannot fully benefit from health-care services, citizens with higher access to the health system sometimes receive too much medical care. Overdiagnosis and overtreatment harm many individuals and represent a waste of resources, thereby preventing the reduction of the cancer burden and compromising the sustainability of health systems.

### [IS7-5] IS7
### UICC: Inequality and Cancer Outcome

2025 / 9 / 27 (Sat) 9:00-11:30 Room 8/(Hotel Nikko Kanazawa 3F Kujaku C) Room 8

[Chairpersons]Keitaro Matsuo (Aichi Cancer Center)、Salvatore Vaccarella (International Agency for Research on Cancer)

---

### IS7-5
### Socioeconomic Disparities in Six Common Cancer Survival Rates in South Korea

[Speaker]Chang Kyun Choi (Natl. Cancer Ctr. Inst, Natl. Cancer Ctr. Korea) [Co-author]JinWook Lee (Natl. Cancer Ctr. Inst, Natl. Cancer Ctr. Korea／Dept. Public Health, Grad. Sch., Yonsei Univ.)、JuWon Park (Natl. Cancer Ctr. Inst, Natl. Cancer Ctr. Korea)、Nayeon Kim (Natl. Cancer Ctr. Inst, Natl. Cancer Ctr. Korea)、Fatima Nari (Natl. Cancer Ctr. Inst, Natl. Cancer Ctr. Korea)、Seowoo Bae (Natl. Cancer Ctr. Inst, Natl. Cancer Ctr. Korea)、Hyeon Ji Lee (Natl. Cancer Ctr. Inst, Natl. Cancer Ctr. Korea)、Mingyu Lee (Natl. Cancer Ctr. Inst, Natl. Cancer Ctr. Korea)、Jae Kwan Jun (Natl. Cancer Ctr. Inst, Natl. Cancer Ctr. Korea)、Kui Son Choi (Natl. Cancer Ctr. Inst, Natl. Cancer Ctr. Korea)、Mina Suh (Natl. Cancer Ctr. Inst, Natl. Cancer Ctr. Korea)

Background: Despite overall survival gains in South Korea, socioeconomic disparities remain. We examined survival differences across six major cancers by socioeconomic status (SES).Methods: This nationwide retrospective cohort study used the Korea Central Cancer Registry (KCCR) and National Health Information Database (NHID), core resources of Korea's cancer control system. Patients with stomach, colorectal, liver, lung, breast, or cervical cancers diagnosed between 2014-2018 were followed until 2021. Survival was analyzed using Kaplan-Meier curves and Cox models.Results: Lower income and higher regional deprivation significantly increased mortality risk across all six cancers, most notably in breast cancer (HR 2.19 for medical aid vs. highest income). Advanced diagnostic stage was also strongly linked to higher mortality.Conclusions: SES substantially influences cancer survival disparities in South Korea. Targeted strategies, including improved early detection and treatment access for vulnerable groups, are needed to reduce these inequities.

[Disclosure of COI by Responsible researcher] Lead presenter is not the Responsible researcher.
[Lead presenter: Chang Kyun Choi] We have no financial relationships to disclose.
[Principal Researcher: Mina Suh] We have no financial relationships to disclose.

▲TOP

### [E-3013] I-E10-4
### Cancer stem cells (1)

2025 / 9 / 27 (Sat) 9:00-10:15 Room 9/(Hotel Nikko Kanazawa 3F Lumiere) Room 9

[Chairperson]Mahito Sadaie (Dept. Appl. Biol. Sci., Tokyo Univ. Sci.)

---

### E-3013
### Metabolic vulnerability of ancestor-like cancer stem cells in radio-resistance

[Speaker]Masahiro Yamazaki (Div. of Cancer Cell Biol., CRI, Kanazawa Univ.／Dept. Radiology, Kanazawa Univ.) [Co-author]Mengjiao Li (Div. of Cancer Cell Biol., CRI, Kanazawa Univ.)、Tsunaki Hongu (Div. of Cancer Cell Biol., CRI, Kanazawa Univ.)、Tatsunori Nishimura (Div. of Cancer Cell Biol., CRI, Kanazawa Univ.)、Shigeyuki Takamatsu (Dept. Radiology, Kanazawa Univ.)、Toshifumi Gabata (Dept. Radiology, Kanazawa Univ.)、Masaya Ueno (Div. of Mol. Genetics, CRI, Kanazawa Univ.)、Atsushi Hirao (Div. of Mol. Genetics, CRI, Kanazawa Univ.)、Susumu Kohno (Div. of Oncology and Mol. Biol., CRI, Kanazawa Univ.)、Chiaki Takahashi (Div. of Oncology and Mol. Biol., CRI, Kanazawa Univ.)、Kuniko Horie (Division of Systems Med. and Gene Therapy, Saitama Med. Univ.)、Kazuhiro Ikeda (Division of Systems Med. and Gene Therapy, Saitama Med. Univ.)、Satoshi Inoue (Division of Systems Med. and Gene Therapy, Saitama Med. Univ.)、Noriko Gotoh (Div. of Cancer Cell Biol., CRI, Kanazawa Univ.／Inst. for Frontier Sci. Initiative, Kanazawa Univ.)

Patients with triple-negative breast cancer (TNBC) face poor prognoses, often experiencing local or distant recurrence despite receiving standard surgery and radiation. The impact of cancer stem cells (CSCs) on radiotherapy efficacy remains unclear. Recently, we identified a subset of CSCs, termed ancestor-like CSCs, characterized by high expression of the plasma membrane protein FXYD3 (Li M, Gotoh N et al., J Clin Invest, 2023). These cells exhibit resistance to chemotherapy and persist after neoadjuvant chemotherapy (NAC). Following radiation treatment of TNBC patient-derived cancer cells, we observed an enrichment of ancestor-like CSCs compared to other cell populations, suggesting potential radiotherapy resistance. RNA sequencing and gene set enrichment analysis (GSEA) using irradiated patient-derived cancer cells revealed upregulated pathways in ancestor-like CSCs that confer radio-resistance. Inhibiting these pathways in combination with radiotherapy could decrease radioresistant cancer cells in vitro and in vivo. The findings can lead to novel clinical strategies for TNBC.

[Disclosure of COI by Responsible researcher] Lead presenter is not the Responsible researcher.
[Lead presenter: Masahiro Yamazaki] We have no financial relationships to disclose.
[Principal Researcher: Noriko Gotoh] We have no financial relationships to disclose.

### [E-3014] I-E10-4
### Cancer stem cells (1)

2025 / 9 / 27 (Sat) 9:00-10:15 Room 9/(Hotel Nikko Kanazawa 3F Lumiere) Room 9

[Chairperson]Mahito Sadaie (Dept. Appl. Biol. Sci., Tokyo Univ. Sci.)

---

### E-3014
### G-CSF promotes tumorigenesis, metastasis, and macrophage recruitment via G-CSFR in CAF-driven breast cancer.

[Speaker]Huazi Zhang (Cancer cell biology, Cancer Research Institute, Kanazawa University) [Co-author]Yasuto Takeuchi (Cancer cell biology, Cancer Research Institute, Kanazawa University)、Takahiko Murayama (Cancer cell biology, Cancer Research Institute, Kanazawa University)、Satoshi Inoue (Division of Systems Medicine and Gene Therapy, Saitama Medical University)、Masao Yano (Department of Breast Surgery, Minamimachida Hospital)、Tanabe Masahiko (Department of Breast & Endocrine Surgery, University of Tokyo)、Terakawa Hirofumi (Department of Gastroenterological Surgery／Breast Surgery, Kanazawa University)、Noriyuki Inaki (Department of Gastroenterological Surgery／Breast Surgery, Kanazawa University)、Susaki Etsuo (Department of Biochemistry and Systems Biomedicine, Juntendo university／Nakatani Biomedical Spatialomics Hub, Juntendo University Graduate School of Medicine)、Eishu Hirata (Division of Tumor Cell Biology and Bioimaging, Kanazawa University)、Hiroshi Watarai (Department of Immunology and Stem Cell Biology, Kanazawa University)、Arai Fumio (Department of Stem Cell Biology, Kyushu University)、Toshio Suda (National University of Singapore Cancer Science Institute)、Kuniko Horie (Division of Systems Medicine and Gene Therapy, Saitama Medical University)、Koji Okamoto (Teikyo University Advanced Comprehensive Research Organization)、Noriko Gotoh (Cancer cell biology, Cancer Research Institute, Kanazawa University)

Breast cancer tissues are rich in cancer-associated fibroblasts (CAFs). To explore their role in regulating breast cancer stem cells (BCSCs), we conducted transcriptome analysis of CAFs cultured alone and with patient-derived breast cancer cells (BCCs). Co-culture induced upregulation of granulocyte colony-stimulating factor (G-CSF) in CAFs. We then found that G-CSF receptor (G-CSFR) expression was induced in BCCs upon co-culture with CAFs. Notably, G-CSFR expression was further enhanced under sphere-forming conditions, suggesting a link to BCSC tumor-initiating capacity. Tail artery injection of high or low G-CSFR-expressing cells into mice showed significantly increased bone metastasis in the high-G-CSFR group, indicating that G-CSFR high cells possess metastatic BCSC traits. Immunohistochemical analysis of patient-derived breast tumors also revealed that the G-CSF&G-CSFR axis involves not only CAFs but also immune components, particularly macrophages, within the tumor microenvironment.

[Disclosure of COI by Responsible researcher] Lead presenter is not the Responsible researcher.
[Lead presenter: Huazi Zhang] We have no financial relationships to disclose.
[Principal Researcher: Gotoh Noriko] We have no financial relationships to disclose.

### [E-3015] I-E10-4
### Cancer stem cells (1)

2025 / 9 / 27 (Sat) 9:00-10:15 Room 9/(Hotel Nikko Kanazawa 3F Lumiere) Room 9

[Chairperson]Mahito Sadaie (Dept. Appl. Biol. Sci., Tokyo Univ. Sci.)

---

### E-3015
### Formation of chemo-resistant gastric cancer microenvironment by ALDH1A3 through regulation of extracellular matrix (ECM)

[Speaker]Tetsuo Mashima (Div. Mol. Biother., Cancer Chemother. Ctr., JFCR) [Co-author]Naomi Kawata (Div. Mol. Biother., Cancer Chemother. Ctr., JFCR／Dept. Gastroenterol. Chemother., Cancer Inst. Hosp., JFCR)、Saori Inaba (Div. Mol. Biother., Cancer Chemother. Ctr., JFCR)、Jin Lee (Div. Mol. Biother., Cancer Chemother. Ctr., JFCR／Dept. Comp. Biol. Med. Sci., Grad. Frontier Sci., Univ. Tokyo)、Noriko Yamamoto (Div. Pathol., Cancer Inst., JFCR)、Takeru Wakatsuki (Dept. Gastroenterol. Chemother., Cancer Inst. Hosp., JFCR)、Koshi Kumagai (Dept. Upper Gastrointest. Surg., Kitasato Univ. Sch. Med)、Kensei Yamaguchi (Dept. Gastroenterol. Chemother., Cancer Inst. Hosp., JFCR)、Hiroyuki Seimiya (Div. Mol. Biother., Cancer Chemother. Ctr., JFCR／Dept. Comp. Biol. Med. Sci., Grad. Frontier Sci., Univ. Tokyo)

ALDH1A3 is a cancer stemness-related aldehyde dehydrogenase, involved in retinoic acid (RA) biosynthesis. We recently reported that this enzyme is frequently upregulated in gastric cancer (GC) persister cells after chemotherapy through histone H3K27 acetylation and BRD4 recruitment to the gene promoter, causing drug resistance. However, it is elusive how ALDH1A3 mediates such cell tolerance. In this study, we tried to identify molecular mechanisms of ALDH1A3-dependent drug tolerance. We found that ALDH1A3-RA pathway was essential for GC patient-derived cell (PDC) growth in a three-dimensional cell culture model. Transcriptome analysis revealed that the ALDH1A3-RA axis regulates the expression of extracellular matrix (ECM)-related genes in GC PDCs. In GC tissues, ALDH1A3 expression correlated with expression of a collagen-crosslinking enzyme. Stable knockdown of the enzyme in a GC PDC xenograft model caused a marked degradation of intratumor collagen structure and repressed residual tumor growth in 5-fluorouracil-treated mice. Collectively, these results indicate that ALDH1A3 is involved in GC persister cell survival after chemotherapy by regulating the intratumor ECM status.

[Disclosure of COI by Responsible researcher] Lead presenter is the Responsible researcher.
[Lead presenter: Tetsuo Mashima] We have no financial relationships to disclose.

### [E-3016] I-E10-4
### Cancer stem cells (1)

2025 / 9 / 27 (Sat) 9:00-10:15 Room 9/(Hotel Nikko Kanazawa 3F Lumiere) Room 9

[Chairperson]Mahito Sadaie (Dept. Appl. Biol. Sci., Tokyo Univ. Sci.)

---

### E-3016
### Identification of MUC1-C as a Novel Oncogenic Regulator in Merkel Cell Carcinoma

[Speaker]Yoshihiro Morimoto (Osaka General Med. Ctr.／Dana Farber Cancer Ctr.) [Co-author]Nami Yamashita (Dana Farber Cancer Ctr.)、Tatsuaki Daimon (Dana Farber Cancer Ctr.)、Naoki Haratake (Dana Farber Cancer Ctr.)、Donald Kufe (Dana Farber Cancer Ctr.)

Merkel cell carcinoma (MCC) is a rare and aggressive neuroendocrine skin cancer with poor prognosis and limited therapeutic options. The oncogenic protein MUC1-C has not been implicated in MCC progression before. Here, we demonstrate that MUC1-C is widely expressed in both Merkel cell polyomavirus-positive (MCCP) and -negative (MCCN) tumors, with higher levels in MCCP. Functional studies revealed that MUC1-C regulates core transcriptional programs associated with RNA processing, pluripotency, and neuroendocrine lineage commitment. MUC1-C interacts with MYCL, a key regulator of MCC progression, and is essential for maintaining expression of pluripotency factors such as OCT4, SOX2, KLF4, MYC, and NANOG, and neuroendocrine lineage markers including NEUROD1, BRN2, and ATOH1. Moreover, MUC1-C suppresses replication stress and inhibits the p53-mediated apoptotic pathway, promoting MCC cell survival. Genetic and pharmacologic inhibition of MUC1-C impairs MCC cell self-renewal and tumorigenicity. These findings uncover a novel role for MUC1-C in MCC pathogenesis and suggest it may serve as a therapeutic target for both MCCP and MCCN subtypes.

[Disclosure of COI by Responsible researcher] Lead presenter is the Responsible researcher.
[Lead presenter: Yoshihiro Morimoto] We have no financial relationships to disclose.

### [E-3017] I-E10-4
### Cancer stem cells (1)

2025 / 9 / 27 (Sat) 9:00-10:15 Room 9/(Hotel Nikko Kanazawa 3F Lumiere) Room 9

[Chairperson]Mahito Sadaie (Dept. Appl. Biol. Sci., Tokyo Univ. Sci.)

---

### E-3017
### Perivascular niche generates glioblastoma stem cells *via* calcium ion-NFAT-c-Myc driven reprogramming

[Speaker]Masumi Tsuda (Dept. of Path., Fac. of Med. Hokkaido Univ.／WPI-ICReDD) [Co-author]Yuheng Nie (Dept. of Path., Fac. of Med. Hokkaido Univ.)、Lei Wang (Dept. of Path., Fac. of Med. Hokkaido Univ.／WPI-ICReDD)、Atsuko Takamura (Dept. of Path., Fac. of Med. Hokkaido Univ.)、Yoshitaka Oda (Dept. of Path., Fac. of Med. Hokkaido Univ.)、Zenichi Tanei (Dept. of Path., Fac. of Med. Hokkaido Univ.／Dept. of Surg. Path., Hokkaido Univ. Hosp.)、Tasuku Nakajima (WPI-ICReDD／Fac. of Adv. Life Sci., Hokakido Univ.)、Jian P. Gong (WPI-ICReDD／Fac. of Adv. Life Sci., Hokakido Univ.)、Shinya Tanaka (Dept. of Path., Fac. of Med. Hokkaido Univ.／WPI-ICReDD／Dept. of Surg. Path., Hokkaido Univ. Hosp.)

Glioblastoma (GBM) has a poor prognosis and a markedly low five-year survival rate. Glioma stem cells (GSCs) are regarded as a critical factor in recurrence, but the molecular mechanisms of GSCs generation in tumor microenvironment remain unclear. Recently, we identified several genes, including ***Col6A2***, as novel GSC markers by integrating scRNAseq of GSCs generated by hydrogel-activated reprogramming (HARP) phenomena (Nature Index: Cancer, 2025) and spatial transcriptome analysis of GBM patient tissues using Xenium. Col6A2-expressing cells were specifically localized in the perivascular region of GBM tissues. Furthermore, we demonstrated that cationic hydrogel mimicking positively charged GBM tissues induced GSC characteristics such as stemness marker induction, glial-to-mesenchymal transition, and temozolomide resistance, through calcium ion-NFAT-c-Myc-driven glioma reprogramming. c-Myc-positive cells were localized in the perivascular region surrounding palisading necrosis in GBM tissues. Thus, we have revealed the tissue localization and characteristics of GSCs using HARP phenomena, and inhibition of the GSC generation process may provide a potent strategy for GBM treatment.

[Disclosure of COI by Responsible researcher] Lead presenter is not the Responsible researcher.
[Lead presenter: Masumi Tsuda] We have no financial relationships to disclose.
[Principal Researcher: Shinya Tanaka] We have no financial relationships to disclose.

### [E-3018] I-E10-4
### Cancer stem cells (1)

2025 / 9 / 27 (Sat) 9:00-10:15 Room 9/(Hotel Nikko Kanazawa 3F Lumiere) Room 9

[Chairperson]Mahito Sadaie (Dept. Appl. Biol. Sci., Tokyo Univ. Sci.)

### E-3018
### Lipid Droplet Profiling and Visualization of Lipid Metabolism in Ovarian Cancer by Fluorescence Lifetime Imaging

[Speaker]Shohei Iyoshi (Dept. Obstet. Gynecol. Nagoya Univ. Sch. Med.／Inst. Adv. Res. Nagoya Univ.) [Co-author]Masayasu Taki (iGCORE, Gifu Univ.)、Atsushi Kunishima (Dept. Obstet. Gynecol. Nagoya Univ. Sch. Med.)、Marina Yoshikawa (Dept. Obstet. Gynecol. Nagoya Univ. Sch. Med.)、Emiri Miyamoto (Dept. Obstet. Gynecol. Nagoya Univ. Sch. Med.)、Hiroki Fujimoto (Dept. Obstet. Gynecol. Aichi Medical Univ.)、Kazumasa Mogi (Dept. Obstet. Gynecol. Nagoya Univ. Sch. Med.)、Masato Yoshihara (Dept. Obstet. Gynecol. Nagoya Univ. Sch. Med.)、Hiroaki Kajiyama (Dept. Obstet. Gynecol. Nagoya Univ. Sch. Med.)

Ovarian cancer (OvCa) is among the deadliest gynecologic malignancies, often diagnosed at advanced stages with dissemination to adipose tissues such as omentum, however, the dynamics of lipid metabolism within disseminating tumor cells remain unclear. We employed a fluorescence lifetime imaging microscopy (FLIM)-based approach using the polarity-sensitive lipid droplet fluorescent probe to evaluate dynamics in lipid metabolism in OvCa. When comparing 2D and 3D spheroid culture conditions, OvCa cells in spheroid form exhibited shorter and more heterogeneous LD fluorescence lifetimes, indicating enhanced lipid metabolism in physiologically relevant spheroid forms. Upon addition of malignant ascites, both 2D and 3D models showed rapid reductions in LD lifetime, suggesting accelerated lipid remodeling. Also, when comparing highly metastatic subclone of mouse OvCa cells (ID8-HM) to its original clone, prolonged lifetimes in ID8-HM were observed after oleic acid treatment, suggesting enhanced energy storage or reduced lipolytic activity in highly metastatic cells. These findings demonstrated that FLIM-based LD profiling can reveal metabolic adaptations associated with tumor progression.

[Disclosure of COI by Responsible researcher] Lead presenter is the Responsible researcher.
[Lead presenter: Shohei Iyoshi] We have no financial relationships to disclose.

### [SP4-reviewer1] SP4
### Special Program4

2025 / 9 / 27 (Sat) 9:00-11:30 Room 10/(Art Hall) Room 10

[Chairpersons]Yasuhiko Yamamoto (Kanazawa University)、Tetsushi Mizuno (Faculty of Medicine, Institute of Medical, Pharmaceutical and Health Sciences, Kanazawa University)

本セッションは、医学生や高校生といった次世代の研究を担う若手が主役の発表の場として、大島会長に特別にセッティングしていただきました。「たまご・ひよこ」は、研究者として歩き始めたばかりの学生（ひよこ）と、研究者としての孵化を待つ高校生（たまご）をイメージしています。
今回は全国の医学生から、がんについては勿論、領域を超えて疫学調査や自閉症の研究などを含め計７演題が集まりました。発表後にはレジェンドの先生方にも講評を頂く予定にしており、高校生も交えた活発な議論を期待しています。本セッション終了後には、医学生と高校生、そして、研究者・教員の交流を目的とした「たまご・ひよこふれあいタイム」を設けています。高校生からのさまざまな疑問や相談を自由に受け付ける時間となっています。たまご・ひよこの皆さんが頑張っている姿を「成鳥」である研究者・教員の先生方にも是非ご覧いただきエールをお願いしたいと思います。

---

### SP4-reviewer1

[SP4-reviewer1]Atsushi Hirao (Cancer Res. Inst., Kanazawa Univ.)

No Abstract

▲TOP

### [SP4-reviewer2] SP4
### Special Program4

2025 / 9 / 27 (Sat) 9:00-11:30 Room 10/(Art Hall) Room 10

[Chairpersons]Yasuhiko Yamamoto (Kanazawa University)、Tetsushi Mizuno (Faculty of Medicine, Institute of Medical, Pharmaceutical and Health Sciences, Kanazawa University)

本セッションは、医学生や高校生といった次世代の研究を担う若手が主役の発表の場として、大島会長に特別にセッティングしていただきました。「たまご・ひよこ」は、研究者として歩き始めたばかりの学生（ひよこ）と、研究者としての孵化を待つ高校生（たまご）をイメージしています。
今回は全国の医学生から、がんについては勿論、領域を超えて疫学調査や自閉症の研究などを含め計７演題が集まりました。発表後にはレジェンドの先生方にも講評を頂く予定にしており、高校生も交えた活発な議論を期待しています。本セッション終了後には、医学生と高校生、そして、研究者・教員の交流を目的とした「たまご・ひよこふれあいタイム」を設けています。高校生からのさまざまな疑問や相談を自由に受け付ける時間となっています。たまご・ひよこの皆さんが頑張っている姿を「成鳥」である研究者・教員の先生方にも是非ご覧いただきエールをお願いしたいと思います。

### SP4-reviewer2

[SP4-reviewer2]Etsuko Kiyokawa (Dept. Oncol. Pathol., Kanazawa Med. Univ.)

No Abstract

### [SP4-1] SP4
### Special Program4

2025 / 9 / 27 (Sat) 9:00-11:30 Room 10/(Art Hall) Room 10

[Chairpersons]Yasuhiko Yamamoto (Kanazawa University)、Tetsushi Mizuno (Faculty of Medicine, Institute of Medical, Pharmaceutical and Health Sciences, Kanazawa University)

本セッションは、医学生や高校生といった次世代の研究を担う若手が主役の発表の場として、大島会長に特別にセッティングしていただきました。「たまご・ひよこ」は、研究者として歩き始めたばかりの学生（ひよこ）と、研究者としての孵化を待つ高校生（たまご）をイメージしています。
今回は全国の医学生から、がんについては勿論、領域を超えて疫学調査や自閉症の研究などを含め計７演題が集まりました。発表後にはレジェンドの先生方にも講評を頂く予定にしており、高校生も交えた活発な議論を期待しています。本セッション終了後には、医学生と高校生、そして、研究者・教員の交流を目的とした「たまご・ひよこふれあいタイム」を設けています。高校生からのさまざまな疑問や相談を自由に受け付ける時間となっています。たまご・ひよこの皆さんが頑張っている姿を「成鳥」である研究者・教員の先生方にも是非ご覧いただきエールをお願いしたいと思います。

### SP4-1
### Comprehensive molecular profiling of 84 cases of endometrial cancer.

[Speaker]Ayumi Imai (Department of Molecular and Cellular Pathology, Graduate School of Medical Sciences, Kanazawa University) [Co-author]Masafumi Horie (Department of Molecular and Cellular Pathology, Graduate School of Medical Sciences, Kanazawa University／Kobe University Graduate School of Medicine, Department of Pathology, Division of Molecular and Genomic Pathology)、Yukinobu Ito (Department of Molecular and Cellular Pathology, Graduate School of Medical Sciences, Kanazawa University)、Maki Sakaguchi (Department of Molecular and Cellular Pathology, Graduate School of Medical Sciences, Kanazawa University／Department of Diagnostic Pathology, Nara Medical University)、Daichi Maeda (Department of Molecular and Cellular Pathology, Graduate School of Medical Sciences, Kanazawa University／Kobe University Graduate School of Medicine, Department of Pathology, Division of Molecular and Genomic Pathology／Garvan Institute of Medical Research)

子宮内膜癌の分子病理学的分類は、TCGA研究をふまえてWHO分類第5版に採用され、2023年にFIGO分類に導入された。具体的には***POLEmut***群、MMR-d群、p53abn群、NSMP群の4群に分けることで予後を予測するというものである。現状、本邦において大規模研究は限られており、***POLE***変異内膜癌の症例蓄積も不十分である。今回、我々は子宮内膜癌の手術例84例を対象に、サンガー法での***POLE***、***KRAS***、***CTNNB1***の変異検出、MMR蛋白とp53の免疫組織化学を施行した。その結果、***POLE***変異は6例（7.1%）において検出され、そのうち2例はMMR-dでもあった。MMR-d群は10例、p53abn群は16例、NSMP群は52例であった。本研究における***POLE***変異内膜癌の病理組織学的特徴、および新FIGO分類を適用した際にStageがどのように改訂されるかについて具体例を提示し、考察したい。

[Disclosure of COI by Responsible researcher] Lead presenter is not the Responsible researcher.
[Lead presenter: Ayumi Imai] We have no financial relationships to disclose.
[Principal Researcher: Daichi Maeda] We have no financial relationships to disclose.

▲TOP

### [SP4-2] SP4
### Special Program4

2025 / 9 / 27 (Sat) 9:00-11:30 Room 10/(Art Hall) Room 10

[Chairpersons]Yasuhiko Yamamoto (Kanazawa University)、Tetsushi Mizuno (Faculty of Medicine, Institute of Medical, Pharmaceutical and Health Sciences, Kanazawa University)

本セッションは、医学生や高校生といった次世代の研究を担う若手が主役の発表の場として、大島会長に特別にセッティングしていただきました。「たまご・ひよこ」は、研究者として歩き始めたばかりの学生（ひよこ）と、研究者としての孵化を待つ高校生（たまご）をイメージしています。
今回は全国の医学生から、がんについては勿論、領域を超えて疫学調査や自閉症の研究などを含め計７演題が集まりました。発表後にはレジェンドの先生方にも講評を頂く予定にしており、高校生も交えた活発な議論を期待しています。本セッション終了後には、医学生と高校生、そして、研究者・教員の交流を目的とした「たまご・ひよこふれあいタイム」を設けています。高校生からのさまざまな疑問や相談を自由に受け付ける時間となっています。たまご・ひよこの皆さんが頑張っている姿を「成鳥」である研究者・教員の先生方にも是非ご覧いただきエールをお願いしたいと思います。

---

### SP4-2
### DNA Damage Tolerance and Therapy Resistance in Polyploid Cancer Cells

[Speaker]Yuta Ogawa (Laboratory of Ploidy Pathology, Graduate School of Frontier Biosciences, The University of Osaka) [Co-author]Kazuki Hayashi (Laboratory of Ploidy Pathology, Graduate School of Frontier Biosciences, The University of Osaka)、Taku Kitano (Laboratory of Ploidy Pathology, Graduate School of Frontier Biosciences, The University of Osaka)、Kenji Nagata (Laboratory of Ploidy Pathology, Graduate School of Frontier Biosciences, The University of Osaka)、Miki Kobayashi (Laboratory of Ploidy Pathology, Graduate School of Frontier Biosciences, The University of Osaka)、Eiji Hara (Department of Molecular Microbiology, Research Institute for Microbial Diseases,The University of Osaka)、Tomonori Matsumoto (Laboratory of Ploidy Pathology, Graduate School of Frontier Biosciences, The University of Osaka)

多倍体化(全ゲノム倍化)は多くのがんで見られるゲノム異常である。このような多倍体がん細胞は種々のストレスに対して耐性を持ち、がんの治療抵抗性獲得にも寄与していると考えられているが、その詳しい機序については未だ明らかとなっていないところが多い。そこで本研究では、多倍体化と抗がん剤治療、ゲノム損傷ストレスとの関係について検討した。ヒト肝がん細胞株を用いて樹立した二倍体および多倍体がん細胞株に対して、薬剤処理による多倍体化を惹起し、比較したところ、多倍体細胞はより多くのDNA損傷を受けやすいことが明らかとなった。しかし一方で、多倍体細胞はその影響に対して高い耐性を示していた。以上により、DNA損傷を蓄積しつつもその影響を軽減するという多倍体細胞の二面性が明らかとなり、この特性は多倍体がんが抗がん剤に高い耐性を持つ一因となっていると考えられる。

[Disclosure of COI by Responsible researcher] Lead presenter is not the Responsible researcher.
[Lead presenter: Yuta Ogawa] We have no financial relationships to disclose.
[Principal Researcher: Tomonori Matsumoto] We have no financial relationships to disclose.

▲TOP

### [SP4-3] SP4
### Special Program4

2025 / 9 / 27 (Sat) 9:00-11:30 Room 10/(Art Hall) Room 10

[Chairpersons]Yasuhiko Yamamoto (Kanazawa University)、Tetsushi Mizuno (Faculty of Medicine, Institute of Medical, Pharmaceutical and Health Sciences, Kanazawa University)

本セッションは、医学生や高校生といった次世代の研究を担う若手が主役の発表の場として、大島会長に特別にセッティングしていただきました。「たまご・ひよこ」は、研究者として歩き始めたばかりの学生（ひよこ）と、研究者としての孵化を待つ高校生（たまご）をイメージしています。
今回は全国の医学生から、がんについては勿論、領域を超えて疫学調査や自閉症の研究などを含め計７演題が集まりました。発表後にはレジェンドの先生方にも講評を頂く予定にしており、高校生も交えた活発な議論を期待しています。本セッション終了後には、医学生と高校生、そして、研究者・教員の交流を目的とした「たまご・ひよこふれあいタイム」を設けています。高校生からのさまざまな疑問や相談を自由に受け付ける時間となっています。たまご・ひよこの皆さんが頑張っている姿を「成鳥」である研究者・教員の先生方にも是非ご覧いただきエールをお願いしたいと思います。

---

### SP4-3
### Association between Green Tea Intake and bone strength and its sex differences -one example of Shika study-

[Speaker]Chigusa Suma (Kanazawa University) [Co-author]Hiromasa Tsujiguchi (Kanazawa University)、Akinari Hara (Kanazawa University)、Yasuhiro Kambayashi (Okayama University of Science)、Fumihiko Suzuki (Ohu University)、Koichi Hayashi (Mukogawa Women's University)、Hirohito Tsuboi (Kanazawa University)、Atsushi Tajima (Kanazawa University)、Toshinari Takamura (Kanazawa University)、Hiroyuki Nakamura (Kanazawa University)

本研究は緑茶摂取と骨強度との関係とその性差を明らかにすることを目的とした。
対象者は志賀町在住の40歳以上の住民で2013～2019年度までに実施された検診を受診した990人(男性467人, 女性523人、平均年齢61.6±11.3歳) である。
緑茶摂取（150ml以下/日、150ml超/日）を独立変数、音響的骨評価（正常/異常）を従属変数、BMI、エネルギー摂取、年齢、喫煙、飲酒を共変量として、ロジスティック回帰分析を行なった。
男性では150ml/日超の緑茶摂取は骨強度異常リスクを有意に低下させた（オッズ比=0.619, p=0.047）が、女性では有意差がなかった。
本研究は、特に男性に対する緑茶の骨強度に及ぼす有益な作用について新たな見解を提供する。今後はさらに喫煙との関係も視野に入れて解析を進めたい。

[Disclosure of COI by Responsible researcher] Lead presenter is not the Responsible researcher.
[Lead presenter: Chigusa Suma] We have no financial relationships to disclose.
[Principal Researcher: Hiromasa Tsujiguchi] We have no financial relationships to disclose.

### [SP4-4] SP4
### Special Program4

2025 / 9 / 27 (Sat) 9:00-11:30 Room 10/(Art Hall) Room 10

[Chairpersons]Yasuhiko Yamamoto (Kanazawa University)、Tetsushi Mizuno (Faculty of Medicine, Institute of Medical, Pharmaceutical and Health Sciences, Kanazawa University)

本セッションは、医学生や高校生といった次世代の研究を担う若手が主役の発表の場として、大島会長に特別にセッティングしていただきました。「たまご・ひよこ」は、研究者として歩き始めたばかりの学生（ひよこ）と、研究者としての孵化を待つ高校生（たまご）をイメージしています。
今回は全国の医学生から、がんについては勿論、領域を超えて疫学調査や自閉症の研究などを含め計７演題が集まりました。発表後にはレジェンドの先生方にも講評を頂く予定にしており、高校生も交えた活発な議論を期待しています。本セッション終了後には、医学生と高校生、そして、研究者・教員の交流を目的とした「たまご・ひよこふれあいタイム」を設けています。高校生からのさまざまな疑問や相談を自由に受け付ける時間となっています。たまご・ひよこの皆さんが頑張っている姿を「成鳥」である研究者・教員の先生方にも是非ご覧いただきエールをお願いしたいと思います。

---

### SP4-4
### The Innate Immune System Activated by Temozolomide Predicts Response to Immune Checkpoint Inhibitors in Glioblastoma

[Speaker]Natsuki Koide (Div. of Cancer Biol., Nagoya Univ. Sch. of Med.) [Co-author]Tatsunori Nishimura (Div. of Cancer Biol., Nagoya Univ. Grad. Sch. of Med.)、Miho Suzuki (Div. of Cancer Biol., Nagoya Univ. Grad. Sch. of Med.)、Keiko Shinjo (Div. of Cancer Biol., Nagoya Univ. Grad. Sch. of Med.)、Yutaka Kondo (Div. of Cancer Biol., Nagoya Univ. Grad. Sch. of Med.)

免疫チェックポイント阻害剤（ICI）はがん治療成績を向上させてきたが、膠芽腫（GBM）では腫瘍浸潤リンパ球（TILs）の少なさなどの要因により効果が限定的である。GBM治療の第一選択薬であるテモゾロミド（TMZ）は、一部でICIの反応性を高めるが、詳細な機序は不明である。本研究では、TMZにより二本鎖DNA（dsDNA）および二本鎖RNA（dsRNA）が細胞質内に増加し、I型インターフェロン（IFN-I）シグナルを活性化することとその強さはパターン認識受容体（PRR）の発現量に依存することを明らかにした。特にdsDNAの受容体であるCGASの発現が低いU251細胞では、dsRNAの受容体であるRIG-Iが活性化され、INF-Iシグナルを活性化していた。これらの結果は、dsDNAまたはdsRNAのPRR発現量がICI-TMZ併用療法の有効性を予測するバイオマーカーとなり得ることを示唆する。

[Disclosure of COI by Responsible researcher] Lead presenter is not the Responsible researcher.
[Lead presenter: Natsuki Koide] We have no financial relationships to disclose.
[Principal Researcher: Yutaka Kondo] We have no financial relationships to disclose.

### [SP4-5] SP4
### Special Program4

2025 / 9 / 27 (Sat) 9:00-11:30 Room 10/(Art Hall) Room 10

[Chairpersons]Yasuhiko Yamamoto (Kanazawa University)、Tetsushi Mizuno (Faculty of Medicine, Institute of Medical, Pharmaceutical and Health Sciences, Kanazawa University)

本セッションは、医学生や高校生といった次世代の研究を担う若手が主役の発表の場として、大島会長に特別にセッティングしていただきました。「たまご・ひよこ」は、研究者として歩き始めたばかりの学生（ひよこ）と、研究者としての孵化を待つ高校生（たまご）をイメージしています。
今回は全国の医学生から、がんについては勿論、領域を超えて疫学調査や自閉症の研究などを含め計７演題が集まりました。発表後にはレジェンドの先生方にも講評を頂く予定にしており、高校生も交えた活発な議論を期待しています。本セッション終了後には、医学生と高校生、そして、研究者・教員の交流を目的とした「たまご・ひよこふれあいタイム」を設けています。高校生からのさまざまな疑問や相談を自由に受け付ける時間となっています。たまご・ひよこの皆さんが頑張っている姿を「成鳥」である研究者・教員の先生方にも是非ご覧いただきエールをお願いしたいと思います。

---

### SP4-5
### Identification of neural circuits responsible for abnormal social behavior in a mouse model of autism

[Speaker]Taisei Nagadomi (Department of Histology and Cell Biology, Graduate School of Medical Sciences, Kanazawa University) [Co-author]Reika Kuribayashi (Department of Histology and Cell Biology, Graduate School of Medical Sciences, Kanazawa University)、Mayu Harada (Department of Histology and Cell Biology, Graduate School of Medical Sciences, Kanazawa University)、Yu Yoneda (Department of Histology and Cell Biology, Graduate School of Medical Sciences, Kanazawa University)、Ayumu Tashiro (Department of Histology and Cell Biology, Graduate School of Medical Sciences, Kanazawa University)、Masaaki Nishiyama (Department of Histology and Cell Biology, Graduate School of Medical Sciences, Kanazawa University／Social Brain Development Research Unit, Institute for Frontier Science Initiative, Kanazawa University)

自閉スペクトラム症は、社会性行動異常を中核症状とする非常に発症頻度の高い発達障害であり（全人口の約2.8%）、発症メカニズムの解明と治療法の開発が強く求められている。自閉症患者は他者の顔や名前を覚えにくいという特性があり、自閉症モデルマウスは馴染みのある（既知）マウスと馴染みのない（未知）マウスを正確に区別できないのではないかという仮説を立てた。正常マウスは既知マウスよりも未知マウスとの接触時間が長いのに対し、自閉症モデルマウスでは接触時間に差が見られなかった。また行動中の内側前頭前野の興奮性ニューロンの活動を測定すると、正常マウスと比較して自閉症モデルマウスでは相手マウスが既知か未知かに関わらず、接触する際に過度な神経活動が観察された。これらの結果から、自閉症モデルマウスでは内側前頭前野の興奮性ニューロンが過剰に活動することで、社会性行動異常を引き起こしている可能性が示唆された。

[Disclosure of COI by Responsible researcher] Lead presenter is not the Responsible researcher.
[Lead presenter: Taisei Nagadomi] We have no financial relationships to disclose.
[Principal Researcher: Masaaki Nishiyama] We have no financial relationships to disclose.

### [SP4-6] SP4
### Special Program4

2025 / 9 / 27 (Sat) 9:00-11:30 Room 10/(Art Hall) Room 10

[Chairpersons]Yasuhiko Yamamoto (Kanazawa University)、Tetsushi Mizuno (Faculty of Medicine, Institute of Medical, Pharmaceutical and Health Sciences, Kanazawa University)

本セッションは、医学生や高校生といった次世代の研究を担う若手が主役の発表の場として、大島会長に特別にセッティングしていただきました。「たまご・ひよこ」は、研究者として歩き始めたばかりの学生（ひよこ）と、研究者としての孵化を待つ高校生（たまご）をイメージしています。
今回は全国の医学生から、がんについては勿論、領域を超えて疫学調査や自閉症の研究などを含め計７演題が集まりました。発表後にはレジェンドの先生方にも講評を頂く予定にしており、高校生も交えた活発な議論を期待しています。本セッション終了後には、医学生と高校生、そして、研究者・教員の交流を目的とした「たまご・ひよこふれあいタイム」を設けています。高校生からのさまざまな疑問や相談を自由に受け付ける時間となっています。たまご・ひよこの皆さんが頑張っている姿を「成鳥」である研究者・教員の先生方にも是非ご覧いただきエールをお願いしたいと思います。

---

### SP4-6
### Investigation of the Mechanism of Radiosensitization by Bafilomycin

[Speaker]Ayaha Mizoguchi (The University of Osaka Faculty of Medicine) [Co-author]Shohei Katsuki (Department of Medical Physics and Engineering, The University of Osaka Graduate School of Medicine)、Keisuke Tamari (Department of Radiation Oncology, The University of Osaka Graduate School of Medicine)、Kazuhiko Ogawa (Department of Radiation Oncology, The University of Osaka Graduate School of Medicine)

液胞型$H^+$-ATPaseはリソソームなどに存在し、プロトン輸送を通じて細胞内pH制御に関わっている。液胞型$H^+$-ATPase阻害剤であるBafilomycin（Baf）は放射線増感作用が示唆されているが、メカニズムは明らかとなっていない。本研究はBafによる増感作用メカニズムの解明を目的とした。A549（ヒト肺腺癌細胞）にBafを24時間暴露後、放射線照射を行い、コロニーアッセイで生存率を評価した。また、放射線照射後、複数タイムポイントにおけるDNA二重鎖切断マーカー（γH2AX）の発現を解析した。結果、Baf添加により4Gyで生存率が15%、8Gyで35%低下し、照射後0.5, 1時間でγH2AXの発現量が約5倍に増加した。Bafは放射線照射直後のDNAダメージを増加させることによって、細胞の放射線感受性を高めることが示唆された。

[Disclosure of COI by Responsible researcher] Lead presenter is not the Responsible researcher.
[Lead presenter: Ayaha Mizoguchi] We have no financial relationships to disclose.
[Principal Researcher: Keisuke Tamari] We have no financial relationships to disclose.

▲TOP

### [SP4-7] SP4
### Special Program4

2025 / 9 / 27 (Sat) 9:00-11:30 Room 10/(Art Hall) Room 10

[Chairpersons]Yasuhiko Yamamoto (Kanazawa University)、Tetsushi Mizuno (Faculty of Medicine, Institute of Medical, Pharmaceutical and Health Sciences, Kanazawa University)

本セッションは、医学生や高校生といった次世代の研究を担う若手が主役の発表の場として、大島会長に特別にセッティングしていただきました。「たまご・ひよこ」は、研究者として歩き始めたばかりの学生（ひよこ）と、研究者としての孵化を待つ高校生（たまご）をイメージしています。
今回は全国の医学生から、がんについては勿論、領域を超えて疫学調査や自閉症の研究などを含め計７演題が集まりました。発表後にはレジェンドの先生方にも講評を頂く予定にしており、高校生も交えた活発な議論を期待しています。本セッション終了後には、医学生と高校生、そして、研究者・教員の交流を目的とした「たまご・ひよこふれあいタイム」を設けています。高校生からのさまざまな疑問や相談を自由に受け付ける時間となっています。たまご・ひよこの皆さんが頑張っている姿を「成鳥」である研究者・教員の先生方にも是非ご覧いただきエールをお願いしたいと思います。

---

### SP4-7
### A study on changes in mindset and learning among medical students participating in a cancer genomic medicine practicum

[Speaker]Sho Asano (Faculty of Medicine, The University of Tokyo) [Co-author]Manazumi Okubo (Faculty of Medicine, The University of Tokyo)、Masashi Izumiya (International Research Center for Medical Education, Graduate School of Medicine, The University of Tokyo)、Tetsuo Ushiku (The Department of Clinical Genomics, The University of Tokyo Hospital／Department of Pathology, Pathology, Immunology and Microbiology, Graduate School of Medicine, The University of Tokyo)、Katsutoshi Oda (The Department of Clinical Genomics, The University of Tokyo Hospital／Department of Biomedical Engineering, Radiology and Biomedical Engineering, Graduate School of Medicine, The University of Tokyo)、Masato Eto (International Research Center for Medical Education, Graduate School of Medicine, The University of Tokyo)

高度ながん医療・研究を担う人材の育成は喫緊の課題であり、がんゲノム医療の教育はその中核をなす。本研究では、東京大学医学部において、今年より導入されたがんゲノム医療実習に参加した医学部5年生を対象に、教育効果の評価を目的としたアンケート調査を実施した。実習は5日間で構成され、クルズスや症例演習を通じて、がん遺伝子パネル検査を中心としたゲノム医療の概要を学ぶ内容であった。実習前後の意識や理解の変化を把握するため、Likert尺度等による定量評価に加え、自由記述を用いた定性評価を行った。また、同意が得られた学生にはインタビューも実施する。
上記の調査結果は、医学生に対するがんゲノム医療教育の有効性や課題を検証するための先行的資料となることが期待される。さらに、学生からの実際の感想を教育側へフィードバックすることで、今後のカリキュラム設計や人材育成戦略の立案に資する知見の提供を目指す。

[Disclosure of COI by Responsible researcher] Lead presenter is not the Responsible researcher.
[Lead presenter: Sho Asano] We have no financial relationships to disclose.
[Principal Researcher: Masashi Izumiya] We have no financial relationships to disclose.

### [JS5-1] JS5
### The role of the vascular microenvironment in cancer development and progression

2025 / 9 / 27 (Sat) 9:00-11:30 Room 11/(ANA Crowne Plaza Hotel Kanazawa 3F Ohtori (West)) Room 11

[Chairpersons]Yukiko Matsunaga (The University of Tokyo)、Miwa Tanaka (The Cancer Institute, Japanese Foundation for Cancer Research)

Recent studies have demonstrated that the tumor angiogenesis is a highly complex and heterogenous process. Over 20 angiogenic factors are known to be secreted by cancer cells of different origins. Moreover, additional cytokines/growth factors produced by immune cells and cancer-associated fibroblasts induce structural alterations of tumor blood vessels. As a result, tumor vasculatures are quite different among many cancer types, and phenotypic alterations of endothelial cells affect the biological behavior of cancer cells. Nevertheless, there is no doubt that tumor angiogenesis is a key process for metastasis and an important target of the therapy. To get better insights into tumor angiogenesis and to develop innovative anti-angiogenic therapies, we would like to present recent advances in tumor angiogenesis research. Invited speakers will introduce unique model systems that cover both in vitro and in vivo studies using novel imaging, tissue engineering and molecular dissections. We will expect constructive and fruitful discussions to defeat cancer angiogenesis and to suppress cancer progression.

---

### JS5-1
### How tumor cells cross endothelial barrier

[Speaker]Yukiko T. Matsunaga (IIS, UTokyo)

Metastasis involves local invasion, intravasation into vessels, and colonization of distant sites. Among these, intravasation is a critical early step, but the spatiotemporal dynamics by which tumor cells, especially circulating tumor cell (CTC) clusters, penetrate the endothelial barrier remain unclear due to resolution limits in conventional in vivo models. To address this, we developed a microfluidic 3D coculture platform for live imaging of tumor/vessel interactions. In this system, intestinal tumor organoids with defined mutations are arranged around microvessels. We observed collective invasion toward vessels, vascular co-option, and release of CTC clusters. Mechanistic analysis showed that TGF$\beta$ and activin from endothelial cells induce endothelial to mesenchymal transition (EndoMT), compromising vascular integrity and facilitating tumor cell entry into the circulation. These findings suggest that dynamic tumor/vessel interactions and endothelial plasticity are central to the intravasation process.

[Disclosure of COI by Responsible researcher] Lead presenter is the Responsible researcher.
[Lead presenter: Yukiko Matsunaga] We have no financial relationships to disclose.

### [JS5-2] JS5
### The role of the vascular microenvironment in cancer development and progression

2025 / 9 / 27 (Sat) 9:00-11:30 Room 11/(ANA Crowne Plaza Hotel Kanazawa 3F Ohtori (West)) Room 11

[Chairpersons]Yukiko Matsunaga (The University of Tokyo)、Miwa Tanaka (The Cancer Institute, Japanese Foundation for Cancer Research)

Recent studies have demonstrated that the tumor angiogenesis is a highly complex and heterogenous process. Over 20 angiogenic factors are known to be secreted by cancer cells of different origins. Moreover, additional cytokines/growth factors produced by immune cells and cancer-associated fibroblasts induce structural alterations of tumor blood vessels. As a result, tumor vasculatures are quite different among many cancer types, and phenotypic alterations of endothelial cells affect the biological behavior of cancer cells. Nevertheless, there is no doubt that tumor angiogenesis is a key process for metastasis and an important target of the therapy. To get better insights into tumor angiogenesis and to develop innovative anti-angiogenic therapies, we would like to present recent advances in tumor angiogenesis research. Invited speakers will introduce unique model systems that cover both in vitro and in vivo studies using novel imaging, tissue engineering and molecular dissections. We will expect constructive and fruitful discussions to defeat cancer angiogenesis and to suppress cancer progression.

---

### JS5-2
### Inflammaging of endothelial cells and cancer progression

[Speaker]Kyoko Hida (Vasc. Biol. Mol. Pathol., Dent. Med., Hokkaido Univ.)

It is well known that vascular endothelial cells exhibit phenotypic diversity, including morphological and genetic changes. We have previously studied the characteristics of tumor endothelial cells (TECs) and reported that they acquire various properties depending on the microenvironment. In particular, they exhibit oxidative stress effects such as reactive oxygen species accumulation, increased expression of inflammatory cytokines including SASP factors, and other features indicative of cellular aging. These properties are induced by microenvironmental factors such as hypoxic environments, tumor-derived extracellular vesicles containing miRNAs, etc. Additionally, TECs activate inflammatory pathways accompanied by NF-kB activation, thrombosis pathways, neutrophil activation, and promotion of NETs formation. Such vascular characteristics are not limited to vascular endothelial cells within the primary tumor but also exhibit inflammatory changes in blood vessels of metastatic organs due to drugs, oral microbiota, tumor-associated factors, and stress. These vascular changes contribute to cancer metastasis and the induction of thrombotic complications.

[Disclosure of COI by Responsible researcher] Lead presenter is the Responsible researcher.
[Lead presenter: Kyoko Hida] We have no financial relationships to disclose.

### [JS5-3] JS5
### The role of the vascular microenvironment in cancer development and progression

2025 / 9 / 27 (Sat) 9:00-11:30 Room 11/(ANA Crowne Plaza Hotel Kanazawa 3F Ohtori (West)) Room 11

[Chairpersons]Yukiko Matsunaga (The University of Tokyo)、Miwa Tanaka (The Cancer Institute, Japanese Foundation for Cancer Research)

Recent studies have demonstrated that the tumor angiogenesis is a highly complex and heterogenous process. Over 20 angiogenic factors are known to be secreted by cancer cells of different origins. Moreover, additional cytokines/growth factors produced by immune cells and cancer-associated fibroblasts induce structural alterations of tumor blood vessels. As a result, tumor vasculatures are quite different among many cancer types, and phenotypic alterations of endothelial cells affect the biological behavior of cancer cells. Nevertheless, there is no doubt that tumor angiogenesis is a key process for metastasis and an important target of the therapy. To get better insights into tumor angiogenesis and to develop innovative anti-angiogenic therapies, we would like to present recent advances in tumor angiogenesis research. Invited speakers will introduce unique model systems that cover both in vitro and in vivo studies using novel imaging, tissue engineering and molecular dissections. We will expect constructive and fruitful discussions to defeat cancer angiogenesis and to suppress cancer progression.

---

### JS5-3
### The roles of endothelial-mesenchymal transition (EndoMT) in tumor progression and metastasis

[Speaker]Kazuki Takahashi (Dept. Biochem, Grad. Sc Med. & Dent., Science Tokyo.) [Co-author]Mizuki Tanaka (Dept. Biochem, Grad. Sc Med. & Dent., Science Tokyo.)、Miho Kobayashi (Dept. Biochem, Grad. Sc Med. & Dent., Science Tokyo.)、Yusuke Yoshioka (Div. Mol. Cell. Med., Inst. Med. Sci., Tokyo Med. Univ.)、Katarzyna A. Inoue (Dept. Biochem, Grad. Sc Med. & Dent., Science Tokyo.)、Takahiro Ochiya (Div. Mol. Cell. Med., Inst. Med. Sci., Tokyo Med. Univ.)、Yukiko T. Matsunaga (Inst. Ind. Sci., The Univ. of Tokyo.)、Tetsuro Watabe (Dept. Biochem, Grad. Sc Med. & Dent., Science Tokyo.)

During cancer metastasis to distant organs, cancer cells destabilize integrity of blood vessels for efficient intravasation at primary tumor and extravasation at metastatic sites. TGF-$\beta$ is abundant in many types of cancer and induces epithelial-mesenchymal transition (EMT) of epithelial cancer cells and endothelial-mesenchymal transition (EndoMT) of endothelial cells (ECs). During EndoMT, ECs lose their properties and differentiate into mesenchymal cells through a partial EndoMT state, which reduces endothelial barrier function. While previous studies have reported that the cancer cells in primary tumor remodel distant vascular environments, the lack of appropriate experimental system has hampered to elucidate its mechanisms. In this study, we established a novel EndoMT reporter cell system, the EndoMT reporter endothelial cells (EMRECs). EMRECs allowed visualization of sequential changes during EndoMT. Furthermore, we show that extracellular vesicles secreted from cancer cells undergoing TGF-$\beta$-induced EMT reduced intercellular adhesion between ECs and induced localized EndoMT. These findings show the importance of vascular microenvironments in cancer progression and metastasis.

[Disclosure of COI by Responsible researcher] Lead presenter is not the Responsible researcher.
[Lead presenter: Kazuki Takahashi] We have no financial relationships to disclose.
[Principal Researcher: Tetsuro Watabe] We have no financial relationships to disclose.

### [JS5-4] JS5
### The role of the vascular microenvironment in cancer development and progression

2025 / 9 / 27 (Sat) 9:00-11:30 Room 11/(ANA Crowne Plaza Hotel Kanazawa 3F Ohtori (West)) Room 11

[Chairpersons]Yukiko Matsunaga (The University of Tokyo)、Miwa Tanaka (The Cancer Institute, Japanese Foundation for Cancer Research)

Recent studies have demonstrated that the tumor angiogenesis is a highly complex and heterogenous process. Over 20 angiogenic factors are known to be secreted by cancer cells of different origins. Moreover, additional cytokines/growth factors produced by immune cells and cancer-associated fibroblasts induce structural alterations of tumor blood vessels. As a result, tumor vasculatures are quite different among many cancer types, and phenotypic alterations of endothelial cells affect the biological behavior of cancer cells. Nevertheless, there is no doubt that tumor angiogenesis is a key process for metastasis and an important target of the therapy. To get better insights into tumor angiogenesis and to develop innovative anti-angiogenic therapies, we would like to present recent advances in tumor angiogenesis research. Invited speakers will introduce unique model systems that cover both in vitro and in vivo studies using novel imaging, tissue engineering and molecular dissections. We will expect constructive and fruitful discussions to defeat cancer angiogenesis and to suppress cancer progression.

---

### JS5-4
### Vascular encapsulation shields tumor clusters from immune cells in macrotrabecular-massive hepatocellular carcinoma

[Speaker]Shu Shimada (Dept. Mol. Oncol., Inst. Sci. Tokyo) [Co-author]Tomohiko Taniai (Dept. Mol. Oncol., Inst. Sci. Tokyo／Dept. Surg., The Jikei Univ. Sch. Med.)、Yoshimitsu Akiyama (Dept. Mol. Oncol., Inst. Sci. Tokyo)、Keiichi Akahoshi (Dept. Hepato-Biliary-Pancreatic Surg., Inst. Sci. Tokyo)、Daisuke Ban (Dept. Hepato-Biliary-Pancreatic Surg., Inst. Sci. Tokyo)、Toru Ikegami (Dept. Surg., The Jikei Univ. Sch. Med.)、Shinji Tanaka (Dept. Mol. Oncol., Inst. Sci. Tokyo／Dept. Hepato-Biliary-Pancreatic Surg., Inst. Sci. Tokyo)

Abnormal tumor vasculature facilitates carcinogenesis through various biological processes. Macrotrabecular-massive hepatocellular carcinoma (MTM-HCC) accounts for 20-30% of cases and is characterized by aggressive metastasis and poor prognosis. Comprehensive bulk and single-cell analyses of human HCC tissues along with additional clinical validation revealed enrichment of ***TP53*** mutations, activation of MYC signaling, formation of vessels encapsulating tumor clusters (VETC), and depletion of cytotoxic T lymphocytes in MTM-HCC. Mouse HCC cells with ***Trp53*** knockout and ***MYC*** overexpression demonstrated highly tumorigenic and metastatic phenotypes with the MTM/VETC structure and an immunosuppressive microenvironment. Genetic and pharmacological inhibition of angiogenesis disrupted the MTM/VETC structure and recruited T cells to the tumor tissues, and its combination with anti-PD-1 antibody achieved remission. These findings suggest that cancer cells attract vascular endothelial cells to surround and shield themselves from immune cells in MTM-HCC, and that angiogenesis inhibition and immune checkpoint blockade synergistically enhanced anti-tumor effects in this subtype (***Hepatology*** 2025).

[Disclosure of COI by Responsible researcher] Lead presenter is not the Responsible researcher.
[Lead presenter: Shu Shimada] ■Research funds from company: 武田科学振興財団
[Principal Researcher: Shinji Tanaka] We have no financial relationships to disclose.

### [JS5-5] JS5
### The role of the vascular microenvironment in cancer development and progression

2025 / 9 / 27 (Sat) 9:00-11:30 Room 11/(ANA Crowne Plaza Hotel Kanazawa 3F Ohtori (West)) Room 11

[Chairpersons]Yukiko Matsunaga (The University of Tokyo)、Miwa Tanaka (The Cancer Institute, Japanese Foundation for Cancer Research)

Recent studies have demonstrated that the tumor angiogenesis is a highly complex and heterogenous process. Over 20 angiogenic factors are known to be secreted by cancer cells of different origins. Moreover, additional cytokines/growth factors produced by immune cells and cancer-associated fibroblasts induce structural alterations of tumor blood vessels. As a result, tumor vasculatures are quite different among many cancer types, and phenotypic alterations of endothelial cells affect the biological behavior of cancer cells. Nevertheless, there is no doubt that tumor angiogenesis is a key process for metastasis and an important target of the therapy. To get better insights into tumor angiogenesis and to develop innovative anti-angiogenic therapies, we would like to present recent advances in tumor angiogenesis research. Invited speakers will introduce unique model systems that cover both in vitro and in vivo studies using novel imaging, tissue engineering and molecular dissections. We will expect constructive and fruitful discussions to defeat cancer angiogenesis and to suppress cancer progression.

---

### JS5-5
### Endothelial heterogeneity in organogenesis and tissue repair

[Speaker]Yoshiaki Kubota (Keio University School of Medicine)

In vertebrates, the vascular network develops throughout the body to meet tissue demands for oxygen and nutrients, and to secrete organotypic paracrine molecules, known as angiocrine factors, which drive cell differentiation and tissue morphogenesis. The skeletal system consists of bones and teeth, both of which are hardened via mineralization to support daily physical activity and mastication. Recent advances in histological technology have significantly enhanced our understanding of the spatio-temporal association between angiogenesis and osteogenesis during embryonic and postnatal bone development. In this presentation, I will discuss our recent findings on cell-to-cell communication regulating angiogenesis of the skeletal system and tumor angiogenesis.

[Disclosure of COI by Responsible researcher] Lead presenter is the Responsible researcher.
[Lead presenter: Yoshiaki Kubota] We have no financial relationships to disclose.

### [JS5-6] JS5
### The role of the vascular microenvironment in cancer development and progression

2025 / 9 / 27 (Sat) 9:00-11:30 Room 11/(ANA Crowne Plaza Hotel Kanazawa 3F Ohtori (West)) Room 11

[Chairpersons]Yukiko Matsunaga (The University of Tokyo)、Miwa Tanaka (The Cancer Institute, Japanese Foundation for Cancer Research)

Recent studies have demonstrated that the tumor angiogenesis is a highly complex and heterogenous process. Over 20 angiogenic factors are known to be secreted by cancer cells of different origins. Moreover, additional cytokines/growth factors produced by immune cells and cancer-associated fibroblasts induce structural alterations of tumor blood vessels. As a result, tumor vasculatures are quite different among many cancer types, and phenotypic alterations of endothelial cells affect the biological behavior of cancer cells. Nevertheless, there is no doubt that tumor angiogenesis is a key process for metastasis and an important target of the therapy. To get better insights into tumor angiogenesis and to develop innovative anti-angiogenic therapies, we would like to present recent advances in tumor angiogenesis research. Invited speakers will introduce unique model systems that cover both in vitro and in vivo studies using novel imaging, tissue engineering and molecular dissections. We will expect constructive and fruitful discussions to defeat cancer angiogenesis and to suppress cancer progression.

---

### JS5-6
### Trafficking inhibition to improve the vascular microenvironment in sarcoma

[Speaker]Miwa Tanaka (Div. Cancer Epigenomics, Cancer Inst., JFCR／Dept. Exp. Pathol., IMS, Tokyo Medical Univ.)

Intra-cytoplasmic trafficking in cancer cells plays an important role in remodeling tumor microenvironment. Alveolar soft part sarcoma (ASPS) and translocation renal cell carcinoma are characterized by abundant vasculature and high metastatic potential, for which TFE3 fusion proteins are responsible as oncogenic drivers. Using epigenomic CRISPR screening, we identified RAB27A and SYTL2 as transcriptional targets of the ASPSCR1::TFE3 in ASPS tumorigenesis and angiogenesis. RAB27, a small GTPase, and its binding effector proteins of the SYTL family mediate intracellular trafficking of vesicles and membrane receptors to the cell surface. Although this pathway is frequently activated in cancer, its role in angiogenesis is largely unknown. Inducible silencing of RAB27A or SYTL2 significantly inhibited interaction between sarcoma cells and capillary blood vessels, resulting in drastic degeneration of tumors in vivo. We are currently developing novel therapeutic agents targeting the RAB27A-SYTL2 interaction. These studies will provide novel insights into cell-cell interaction mediated by the RAB27A-regulated intracellular trafficking.

[Disclosure of COI by Responsible researcher] Lead presenter is the Responsible researcher.
[Lead presenter: Tanaka Miwa] We have no financial relationships to disclose.

### [S11-1] S11
### Opening Up New Avenues for p53 Research

2025 / 9 / 27 (Sat) 9:00-11:30 Room 12/(ANA Crowne Plaza Hotel Kanazawa 3F Ohtori (Central)) Room 12

[Chairpersons]Rieko Ohki (National Cancer Center Research Institute)、Koji Itahana (Duke-NUS Medical School, Singapore)

This symposium highlights recent advances in basic p53 research while also showcasing the development of clinical studies targeting p53, including therapies focused on the MDM2-p53 pathway.

---

### S11-1
### Cancer therapy based on co-targeting CKI&alpha and transcriptional kinases

[Speaker]Yinon Ben-Neriah (Lautenberg Center for Immunology and Cancer Research, Hebrew University, Jerusalem／Department of Medical Oncology, Dana-Farber Cancer Institute／Sarcoma Center, Dana-Farber Cancer Institute ／Ludwig Center at Harvard, Harvard Medical School, Boston, Massachusetts／David Liposarcoma Research Initiative, Dana-Farber Cancer Institute／Harvard Medical School, Boston, Massachusetts／Deptartment of Molecular Cell Biology, Weizmann Institute of Science, Rehovot) [Co-author]Irit Snir-Alkalay (Lautenberg Center for Immunology and Cancer Research, Hebrew University, Jerusalem)、Avanthika Venkatachalam (Lautenberg Center for Immunology and Cancer Research, Hebrew University, Jerusalem)、Luriano Peters (Lautenberg Center for Immunology and Cancer Research, Hebrew University, Jerusalem)、Eric Hung (Lautenberg Center for Immunology and Cancer Research, Hebrew University, Jerusalem)、Chava Brim (Lautenberg Center for Immunology and Cancer Research, Hebrew University, Jerusalem)、Anyi Chen (Lautenberg Center for Immunology and Cancer Research, Hebrew University, Jerusalem)、Nicole Solimini (Department of Medical Oncology, Dana-Farber Cancer Institute／Sarcoma Center, Dana-Farber Cancer Institute ／Ludwig Center at Harvard, Harvard Medical School, Boston, Massachusetts／David Liposarcoma Research Initiative, Dana-Farber Cancer Institute)、Renyan Liu (Department of Medical Oncology, Dana-Farber Cancer Institute／Ludwig Center at Harvard, Harvard Medical School, Boston, Massachusetts／David Liposarcoma Research Initiative, Dana-Farber Cancer Institute／Harvard Medical School, Boston, Massachusetts)、Geoffrey Shapiro (Department of Medical Oncology, Dana-Farber Cancer Institute／Ludwig Center at Harvard, Harvard Medical School, Boston, Massachusetts／David Liposarcoma Research Initiative, Dana-Farber Cancer Institute／Harvard Medical School, Boston, Massachusetts)、George Demetri (Department of Medical Oncology, Dana-Farber Cancer Institute／Sarcoma Center, Dana-Farber Cancer Institute ／Ludwig Center at Harvard, Harvard Medical School, Boston, Massachusetts／David Liposarcoma Research Initiative, Dana-Farber Cancer Institute／Harvard Medical School, Boston, Massachusetts)、Moshe Oren (Deptartment of Molecular Cell Biology, Weizmann Institute of Science, Rehovot)、Eli Pikarsky (Lautenberg Center for Immunology and Cancer Research, Hebrew University, Jerusalem)

We identified CKI&alpha as a key component in the destruction complex of &beta-catenin, controlling the WNT signaling pathway and found that its deletion in the mouse epithelium results in stabilization of both &beta-catenin and the tumor suppressor protein p53. This finding was a basis for developing small molecule inhibitors of CKI&alpha. Among the many CKI&alpha inhibitors developed, those that most strongly stabilized p53 were found to co-target two key transcriptional kinases, CDK7 and CDK9, with similar potency. Their inhibition disrupts the transcription of many genes, particularly those driven by super-enhancers (SEs). SEs emerge de novo in many cancer types and control the transcription of multiple oncogenes, including MDM2, and our inhibitors disrupt their structure and activity. This additional inhibitors' facet with co-silencing MDM2 transcription explains the robust stabilization and activation of p53, compared to CKI&alpha deletion. One of the inhibitors is being evaluated in phase I/II clinical trials in certain cancer types, based on the target availability of the drug. I will elaborate on the relevant preclinical data and the rationale for these clinical trials.

[Disclosure of COI by Responsible researcher] Lead presenter is the Responsible researcher.
[Lead presenter: Yinon Ben-Neriah] We have no financial relationships to disclose.

▲TOP

### [S11-2] S11
### Opening Up New Avenues for p53 Research

2025 / 9 / 27 (Sat) 9:00-11:30 Room 12/(ANA Crowne Plaza Hotel Kanazawa 3F Ohtori (Central)) Room 12

[Chairpersons]Rieko Ohki (National Cancer Center Research Institute)、Koji Itahana (Duke-NUS Medical School, Singapore)

This symposium highlights recent advances in basic p53 research while also showcasing the development of clinical studies targeting p53, including therapies focused on the MDM2-p53 pathway.

---

### S11-2
### RUNX3-MYC-p53 Axis Governs Metastatic Switch in Gastric Cancer

[Speaker]Yoshiaki Ito (Cancer Science Institute (CSI), Natl Univ of Singapore (NUS)

Our multistage mouse model for gastric cancer revealed that following deletion of p53 in KrasG12G/+;Apc-/- background, the mice manifested aggressive metastatic tumors with high Runx3 expression. Human gastric cancer with liver metastases (collaboration with T.Noda, Tokyo) revealed high RUNX3 expression in 60% of metastatic tissues. RUNX3, classically a tumor suppressor, exhibits pro-metastatic behavior in a mutant p53 background. Splenic inoculation of RUNX3-expressing gastric cancer cells into immunodeficient mice resulted in robust liver metastasis, whereas RUNX3 knockout led to markedly reduced metastatic capacity. Mechanistically, RUNX3 cooperates with wildtype p53 to induce CDKN1A, suppressing proliferation. In contrast, with mutant or null p53, RUNX3 interacts with MYC to activate CD44 and WNT5A, promoting EMT, invasion, and metastatic niche formation. Notably, mutant p53 forms a complex with RUNX3 that enhances MYC expression, further reinforcing this axis. These findings define a p53-dependent switch in RUNX3 function and identify the RUNX3-MYC-p53 axis as a key regulator of metastatic progression. RUNX3 inhibition may benefit patients with p53-mutant gastric cancer.

[Disclosure of COI by Responsible researcher] Lead presenter is the Responsible researcher.
[Lead presenter: Yoshiaki Ito] We have no financial relationships to disclose.

▲TOP

### [S11-3] S11
### Opening Up New Avenues for p53 Research

2025 / 9 / 27 (Sat) 9:00-11:30 Room 12/(ANA Crowne Plaza Hotel Kanazawa 3F Ohtori (Central)) Room 12

[Chairpersons]Rieko Ohki (National Cancer Center Research Institute)、Koji Itahana (Duke-NUS Medical School, Singapore)

This symposium highlights recent advances in basic p53 research while also showcasing the development of clinical studies targeting p53, including therapies focused on the MDM2-p53 pathway.

---

### S11-3
### A tailored combination of a ROS inducer and a DDR inhibitor complements p53 loss, resulting in p53 synthetic lethality

[Speaker]Tomoo Iwakuma (Department of Pediatrics, Children's Mercy Research Institute, Kansas City／Department of Cancer Biology, University of Kansas Medical Center) [Co-author]Alejandro Parrales (Department of Pediatrics, Children's Mercy Research Institute, Kansas City／Department of Cancer Biology, University of Kansas Medical Center)、Whitney Nolte (Department of Pediatrics, Children's Mercy Research Institute, Kansas City)、Shigeto Nishikawa (Department of Pediatrics, Children's Mercy Research Institute, Kansas City／Department of Cancer Biology, University of Kansas Medical Center)、Atul Ranjan (Department of Pediatrics, Children's Mercy Research Institute, Kansas City／Department of Cancer Biology, University of Kansas Medical Center)、Anuradha Roy (High Throughput Screening Laboratory, University of Kansas, Lawrence, KS, USA)、Frank Schoenen (Higuchi Biosciences Center, University of Kansas, Lawrence, KS, USA)、Melinda Broward (Institute for Advancing Medical Innovation, University of Kansas Medical Center)、Michael Baltezor (Institute for Advancing Medical Innovation, University of Kansas Medical Center)、Stefan Bossmann (Department of Cancer Biology, University of Kansas Medical Center)、Thais Sielekie (Institute for Advancing Medical Innovation, University of Kansas Medical Center)、Scott Weir (Institute for Advancing Medical Innovation, University of Kansas Medical Center)、Joy Fulbright (Department of Pediatrics, Children's Mercy Research Institute, Kansas City)

Mutations or deletions in the tumor suppressor p53 (p53 deficiency) are the most common cancer-specific events. Developing compounds and strategies to induce selective cytotoxicity in p53-deficient cancers (p53 synthetic lethality) could lead to safer and more efficient anti-cancer therapies. To this end, a high-throughput screening of a chemical library using p53-deficient osteosarcoma cell lines, alongside a p53-proficient osteoblast cell line as a negative control, identified a novel compound, KU. Knockdown of wild-type p53 significantly enhanced KU-induced mitotic arrest and cell death. KU demonstrated selective cytotoxicity in p53-deficient cells by inducing reactive oxygen species (ROS)-dependent DNA double-strand breaks (DSBs) and inhibiting DNA damage response (DDR), including ATM phosphorylation. Furthermore, specific cytotoxicity to p53-deficient cancer cells was observed when ATM inhibition was combined with treatment using a ROS inducer, but not other chemotherapy agents. This study underscores the potential of combining ROS inducers and DDR inhibitors as a tailored approach to exploit p53 deficiency and achieve p53 synthetic lethality.

[Disclosure of COI by Responsible researcher] Lead presenter is the Responsible researcher.
[Lead presenter: Tomoo Iwakuma] ■Research funds from company: NIH/NCI ■Contributions: Braden's Hope for Childhood Cancer

### [S11-4] S11
### Opening Up New Avenues for p53 Research

2025 / 9 / 27 (Sat) 9:00-11:30 Room 12/(ANA Crowne Plaza Hotel Kanazawa 3F Ohtori (Central)) Room 12

[Chairpersons]Rieko Ohki (National Cancer Center Research Institute)、Koji Itahana (Duke-NUS Medical School, Singapore)

This symposium highlights recent advances in basic p53 research while also showcasing the development of clinical studies targeting p53, including therapies focused on the MDM2-p53 pathway.

---

### S11-4
### The novel role of p53 in regulating macrophage iron metabolism for host defense

[Speaker]Koji Itahana (Cancer & Stem Cell Biology Program, Duke-NUS Medical School, Singapore)

Macrophages are key innate immune cells involved in tumor suppression and pathogen defense. While p53 activation in macrophages is known to enhance anti-tumor immunity, its role in anti-pathogenic responses remains unclear, despite evidence that p53 is activated by viruses and bacteria. Our unpublished data show that activating p53 lowers intracellular iron in macrophages, critical regulators in maintaining iron balance in the body, whereas p53 knockout increases iron levels. We further demonstrate that p53 promotes phagocytosis and IL-1$\beta$ production, an essential cytokine in host defense, by modulating iron levels. Supplementing iron or feeding iron-rich reticulocytes to macrophages suppresses both phagocytosis and IL-1$\beta$ expression. These findings suggest that p53 prevents iron overload in macrophages during infection, thereby promoting their immune functions. This mechanism may also contribute to anti-tumor immunity. Additionally, I will discuss a potential novel mechanism by which p53 modulates macrophage iron homeostasis to support immune responses.

[Disclosure of COI by Responsible researcher] Lead presenter is the Responsible researcher.
[Lead presenter: Koji Itahana] We have no financial relationships to disclose.

▲TOP

### [S11-5] S11
### Opening Up New Avenues for p53 Research

2025 / 9 / 27 (Sat) 9:00-11:30 Room 12/(ANA Crowne Plaza Hotel Kanazawa 3F Ohtori (Central)) Room 12

[Chairpersons]Rieko Ohki (National Cancer Center Research Institute)、Koji Itahana (Duke-NUS Medical School, Singapore)

This symposium highlights recent advances in basic p53 research while also showcasing the development of clinical studies targeting p53, including therapies focused on the MDM2-p53 pathway.

---

### S11-5
### Extrinsic induction of apoptosis and tumor suppression via the p53- Reprimo-Hippo-YAP-p73 pathway

[Speaker]Rieko Ohki (Laboratory of Fundamental Oncology, NCCRI)

The most commonly mutated gene in human cancer is the p53 tumor suppressor gene, which plays a pivotal role in tumor suppression. We have characterized and reported the molecular function of several novel p53 target genes, including Noxa, AEN, FUCA1, IER5, and PHLDA3 (Science, 2000; Cell, 2009; PNAS, 2014, etc). Reprimo is a p53 target gene known to be involved in tumor suppression, but its molecular function has remained elusive. Here we report that Reprimo protein is secreted and extrinsically induces apoptosis in recipient cells. We further identified FAT1, FAT4, CELSR1, CELSR2, and CELSR3, members of the protocadherin family, as receptors for Reprimo. Subsequent analyses revealed that Reprimo induces apoptosis through the Hippo-YAP-p73 axis by transactivating pro-apoptotic p73 target genes. We also demonstrated that Reprimo can shift YAP-mediated signaling from tumor-promoting to tumor-suppressing by inhibiting expression of tumor-promoting genes regulated by YAP. In vivo analyses further support the tumor-suppressive effects of secreted Reprimo. These findings identify p53-Reprimo-Hippo-YAP-p73 as a novel, extrinsic apoptosis pathway crucial for tumor suppression.

[Disclosure of COI by Responsible researcher] Lead presenter is the Responsible researcher.
[Lead presenter: Rieko Ohki] We have no financial relationships to disclose.

### [S11-6] S11
### Opening Up New Avenues for p53 Research

2025 / 9 / 27 (Sat) 9:00-11:30 Room 12/(ANA Crowne Plaza Hotel Kanazawa 3F Ohtori (Central)) Room 12

[Chairpersons]Rieko Ohki (National Cancer Center Research Institute)、Koji Itahana (Duke-NUS Medical School, Singapore)

This symposium highlights recent advances in basic p53 research while also showcasing the development of clinical studies targeting p53, including therapies focused on the MDM2-p53 pathway.

---

### S11-6
### Constitutive activation of hepatocyte p53 promotes liver carcinogenesis via connective tissue growth factor (CTGF).

[Speaker]Yuki Makino (Department of Gastroenterology and Hepatology, University of Osaka) [Co-author]Yasuharu Kawamoto (Department of Gastroenterology and Hepatology, University of Osaka)、Jihyun Sung (Department of Gastroenterology and Hepatology, University of Osaka)、Seiya Kato (Department of Gastroenterology and Hepatology, University of Osaka)、Yoshinobu Saito (Department of Gastroenterology and Hepatology, University of Osaka)、Yoshito Hayashi (Department of Gastroenterology and Hepatology, University of Osaka)、Takahiro Kodama (Department of Gastroenterology and Hepatology, University of Osaka)、Hayato Hikita (Department of Gastroenterology and Hepatology, University of Osaka)

Aim: In chronic liver diseases (CLDs), hepatocytes are constantly exposed to cellular stress, which activates p53 signaling. This study aimed to clarify the significance of p53 activation on liver carcinogenesis.

Methods: Mdm2, a negative regulator of p53, p53, or CTGF were deleted in hepatocytes (Alb-Cre Mdm2$^{fl/fl}$, p53$^{fl/fl}$, or CTGF$^{fl/fl}$, respectively). Liver biopsy samples were obtained from 182 CLD patients.
Results: Loss of Mdm2 activated p53 in hepatocytes and accelerated diethylnitrosamine (DEN)-induced liver carcinogenesis, together with the enrichment of inflammation-related gene signatures. These phenotypes were abrogated by further deletion of p53 in hepatocytes. Mdm2 deletion upregulated hepatic CTGF, which was a downstream target of p53 in vitro. Further deletion of hepatocyte CTGF suppressed DEN-induced liver carcinogenesis in Mdm2-knockout mice, along with the downregulation of inflammation-related gene signatures. Human CLD liver samples revealed that CTGF was positively correlated with p21, as well as several inflammation-related genes.
Conclusion: Activation of hepatocyte p53 promoted liver carcinogenesis via CTGF, a potential mediator of inflammatory response.

[Disclosure of COI by Responsible researcher] Lead presenter is not the Responsible researcher.
[Lead presenter: Yuki Makino] We have no financial relationships to disclose.
[Principal Researcher: Hayato Hikita] We have no financial relationships to disclose.

▲TOP

### [AOSR5-1] AOSR5
### The World of Hematologic Malignancy Research Unveiled by Emerging Technologies

2025 / 9 / 27 (Sat) 9:00-11:30 Room 13/(ANA Crowne Plaza Hotel Kanazawa 3F Ohtori (East)) Room 13

[Chairpersons]Toshihiro Miyamoto (Department of Hematology, Faculty of Medicine, Institute of Medical Pharmaceutical and Health Sciences, Kanazawa University)、Mamiko Sakata (Department of Hematology, Institute of Medicine, University of Tsukuba)

Huge advance in multi-omics approaches including genomics, epigenomics, transcriptomics, proteomics, and metabolomics has unveiled new perspectives for disease diagnosis and treatment in hematologic malignancies. Recent development of spatially resolved multi-omics profiling in single-cell reveal considerable heterogeneity in tissue environments, which accelerate systematically understanding of the precise mechanisms of multi-step development in hematologic carcinogenesis. Integrative multi-layer omics-based information would construct platform for precision medicine.

---

### AOSR5-1
### Liquid Biopsy-Based Monitoring of Disease Dynamics and Immune Landscape in Lymphoma

[Speaker]Takeshi Sugio (Stanford University Division of Oncology)

Circulating cell-free DNA (cfDNA) originates from dead cells throughout the body and reflects the dynamics of cellular turnover. Similarly, circulating cell-free RNA (cfRNA) derives from a variety of tissues. By removing background signals from platelets and erythroid cells, it is possible to reconstruct tissue-specific transcriptomic profiles from plasma cfRNA enabling noninvasive monitoring of tumor-specific gene expression and dynamic changes in the immune landscape. Leveraging these characteristics, cfDNA and cfRNA allow comprehensive and timely profiling not only of tumor cells but also of systemic immune status, including non-circulating subsets in the tumor tissues. In this presentation, we highlight the distinct features of cfDNA and cfRNA for disease and immune monitoring, respectively. We further discuss how these complementary modalities can be integrated to enhance noninvasive characterization of lymphoma biology, with a focus on applications in disease burden assessment and immune profiling.

[Disclosure of COI by Responsible researcher] Lead presenter is the Responsible researcher.
[Lead presenter: Takeshi Sugio] We have no financial relationships to disclose.

▲TOP

### [AOSR5-2] AOSR5
### The World of Hematologic Malignancy Research Unveiled by Emerging Technologies

2025 / 9 / 27 (Sat) 9:00-11:30 Room 13/(ANA Crowne Plaza Hotel Kanazawa 3F Ohtori (East)) Room 13

[Chairpersons]Toshihiro Miyamoto (Department of Hematology, Faculty of Medicine, Institute of Medical Pharmaceutical and Health Sciences, Kanazawa University)、Mamiko Sakata (Department of Hematology, Institute of Medicine, University of Tsukuba)

Huge advance in multi-omics approaches including genomics, epigenomics, transcriptomics, proteomics, and metabolomics has unveiled new perspectives for disease diagnosis and treatment in hematologic malignancies. Recent development of spatially resolved multi-omics profiling in single-cell reveal considerable heterogeneity in tissue environments, which accelerate systematically understanding of the precise mechanisms of multi-step development in hematologic carcinogenesis. Integrative multi-layer omics-based information would construct platform for precision medicine.

### AOSR5-2
### Uncovering Epigenetic Heterogeneity in Leukemia: Insights from Integrative Profiling

[Speaker]Yotaro Ochi (Department of Pathology and Tumor Biology, Kyoto University／WPI-ASHBi, Kyoto University)

Acute myeloid leukemia (AML) is a heterogeneous hematological malignancy. Although genomic studies have advanced our understanding of its pathogenesis, genetic alterations alone do not fully explain the clinical and molecular heterogeneity of AML. To address this, we performed ATAC-seq on >1,500 AML samples, integrated with targeted-seq, RNA-seq, ChIP-seq, drug screening, and single-cell multi-omics. ATAC-seq analysis identified 16 epigenetic subgroups of AML, each with distinct epigenetic and transcriptomic profiles, co-mutation patterns, clinical outcomes, and drug sensitivities. Integrated bulk RNA- and ATAC-seq analysis revealed that each subgroup was characterized by a unique gene regulatory network (GRN) driven by distinct combinations of activated transcription factors (TFs), many of which were regulated by super-enhancers and formed core autoregulatory circuits. Single-cell GRN and pseudotime trajectory analysis further demonstrated that TF activity peaked at distinct stages of differentiation in a subgroup-specific manner. In summary, integrative epigenomic profiling refines AML classification and provides a framework for mechanistic insight and therapeutic stratification.

[Disclosure of COI by Responsible researcher] Lead presenter is the Responsible researcher.
[Lead presenter: Yotaro Ochi] We have no financial relationships to disclose.

### [AOSR5-3] AOSR5
### The World of Hematologic Malignancy Research Unveiled by Emerging Technologies

2025 / 9 / 27 (Sat) 9:00-11:30 Room 13/(ANA Crowne Plaza Hotel Kanazawa 3F Ohtori (East)) Room 13

[Chairpersons]Toshihiro Miyamoto (Department of Hematology, Faculty of Medicine, Institute of Medical Pharmaceutical and Health Sciences, Kanazawa University)、Mamiko Sakata (Department of Hematology, Institute of Medicine, University of Tsukuba)

Huge advance in multi-omics approaches including genomics, epigenomics, transcriptomics, proteomics, and metabolomics has unveiled new perspectives for disease diagnosis and treatment in hematologic malignancies. Recent development of spatially resolved multi-omics profiling in single-cell reveal considerable heterogeneity in tissue environments, which accelerate systematically understanding of the precise mechanisms of multi-step development in hematologic carcinogenesis. Integrative multi-layer omics-based information would construct platform for precision medicine.

---

### AOSR5-3
### Novel technology to understand and target aberrant RNA processing in cancer

[Speaker]Akihide Yoshimi (Div. Cancer RNA Research, National Cancer Center Research Institute)

Aberrant RNA splicing caused by cis- or trans-regulatory mutations is a novel cancer hallmark. While trans-regulatory mutations in splicing factors have enabled the development of spliceosome inhibitors, cis-acting splice site mutations directly disrupt the splicing of the mutated gene. Screening 230,000 RNA-seq datasets identified frequent intron retention-associated variants (IRAVs), especially in TP53 (3.0% of cancers). Although antisense oligonucleotides (ASOs) are promising tools to correct such mis-splicing, rational design is hindered by limited knowledge of splicing regulation. To overcome this, we developed hareCLIP-seq, a highly reproducible CLIP method applicable to splicing factors. Profiling SRSF and hnRNP families, we discovered that SR proteins bind to shared motifs cooperatively or competitively, and that m6A modulates SR binding affinity. hareCLIP-seq identified a TP53 intronic splicing regulatory element. ASO#2 targeting this site corrected splicing and restored p53/p21. In TP53-IRAV CDX models, ASO#2 with cisplatin caused tumor regression and cures. LNP-encapsulated ASO#2 also showed systemic efficacy, offering a strategy to target undruggable mutations.

[Disclosure of COI by Responsible researcher] Lead presenter is the Responsible researcher.
[Lead presenter: Akihide Yoshimi] ■Research expenses: 第一三共株式会社、中外製薬株式会社、エーザイ株式会社

### [AOSR5-4] AOSR5
### The World of Hematologic Malignancy Research Unveiled by Emerging Technologies

2025 / 9 / 27 (Sat) 9:00-11:30 Room 13/(ANA Crowne Plaza Hotel Kanazawa 3F Ohtori (East)) Room 13

[Chairpersons]Toshihiro Miyamoto (Department of Hematology, Faculty of Medicine, Institute of Medical Pharmaceutical and Health Sciences, Kanazawa University)、Mamiko Sakata (Department of Hematology, Institute of Medicine, University of Tsukuba)

Huge advance in multi-omics approaches including genomics, epigenomics, transcriptomics, proteomics, and metabolomics has unveiled new perspectives for disease diagnosis and treatment in hematologic malignancies. Recent development of spatially resolved multi-omics profiling in single-cell reveal considerable heterogeneity in tissue environments, which accelerate systematically understanding of the precise mechanisms of multi-step development in hematologic carcinogenesis. Integrative multi-layer omics-based information would construct platform for precision medicine.

---

### AOSR5-4
### Advances in SETBP1-Mutated Leukemia: Genomic, Epigenetic, and Therapeutic Insights

[Speaker]Daichi Inoue (Dept. Cancer Pathology, Grad. Sch. Med., Univ. Osaka)

SETBP1 mutations, frequently found in myeloid malignancies, are associated with adverse clinical outcomes, yet the pathogenic mechanisms remain poorly understood. Here, we generated a blood-specific Setbp1 mutant locus knock-in (KI) mouse. Although the single KI did not alter stem cell properties in vivo, the double mutant model with Asxl1 KI showed pronounced monocytosis, with a distinct transcriptional program featuring posterior HoxA and Setbp1 expression. This model spontaneously acquired RAS pathway activating hotspot mutations in Nras, Flt3, and Ptpn11, resulting in monocytic AML with serial transplantability, faithfully recapitulating the clinically observed co-occurrence of ASXL1, SETBP1, and RAS mutations. Through integrated CRISPR/drug screening, we identified an unexpected vulnerability in a specific gene. Pharmacologic and genetic inhibition induced monocytic differentiation followed by apoptosis, with an FDA-approved drug achieving sustained remission in the preclinical model by downregulating Setbp1 target genes through the unrecognized chromatin regulation. These findings reveal novel pathogenic insights and therapeutic opportunities in SETBP1-mutated leukemia.

[Disclosure of COI by Responsible researcher] Lead presenter is the Responsible researcher.
[Lead presenter: Daichi Inoue] ■Research funds from company: The Sumitomo Foundation, The Mitsubishi Foundation, KAKETSUKEN, Bristol Myers Squibb Foundation, Astellas Foundation for Research on Metabolic Disorders, Mochida Memorial Foundation for Medical and Pharmaceutical Research ■Contributions: AbbVie, Sumitomo Pharma, Mitsubishi Tanabe Pharma

### [AOSR5-5] AOSR5
### The World of Hematologic Malignancy Research Unveiled by Emerging Technologies

2025 / 9 / 27 (Sat) 9:00-11:30 Room 13/(ANA Crowne Plaza Hotel Kanazawa 3F Ohtori (East)) Room 13

[Chairpersons]Toshihiro Miyamoto (Department of Hematology, Faculty of Medicine, Institute of Medical Pharmaceutical and Health Sciences, Kanazawa University)、Mamiko Sakata (Department of Hematology, Institute of Medicine, University of Tsukuba)

Huge advance in multi-omics approaches including genomics, epigenomics, transcriptomics, proteomics, and metabolomics has unveiled new perspectives for disease diagnosis and treatment in hematologic malignancies. Recent development of spatially resolved multi-omics profiling in single-cell reveal considerable heterogeneity in tissue environments, which accelerate systematically understanding of the precise mechanisms of multi-step development in hematologic carcinogenesis. Integrative multi-layer omics-based information would construct platform for precision medicine.

---

### AOSR5-5
### Single-cell and spatial multi-omics reveals tumor heterogeneity and microenvironment diversity in T-cell lymphoma

[Speaker]Sakurako Suma (Dept. of Hematol., Univ. of Tsukuba Hosp.,Tsukuba, Japan.) [Co-author]Yuki Shimizu (Graduate School of Comprehensive Human Sciences, Tsukuba Univ., Tsukuba, Japan.)、Mamiko Sakata-Yanagimoto (Dept. of Hematol., Univ. of Tsukuba Hosp.,Tsukuba, Japan.／Dept. of Hematol., Inst. of Med., Univ. of Tsukuba, Japan.／Advanced Hemato-Oncol., Transborder Med. Res. Ctr., Univ. of Tsukuba, Japan.)

T follicular helper cell lymphomas (TFHLs) are intractable hematological malignancies marked by immune-cell infiltration and stromal-cell proliferation in tumor tissues, with tumor cells exhibiting TFH-like characteristics. We conducted single-cell RNA sequencing and spatial multi-omics analysis and found that tumor cells showed significant heterogeneity, with copy number and genetic alterations potentially driving clonal evolution towards TFH-like and proliferation phenotypes. Further analysis of immune cell clustering and spatial networks showed interactions between tumor cells and an immunosuppressive microenvironment with clonally expanded exhausted CD8 T cells and increased Tregs in relapsed/refractory cases.
Moreover, single-cell spatial transcriptomics identified 15 stromal subclusters, including two distinctive BEC subsets transitioning between capillary BECs and activated high endothelial venules ("C-aHEVs"): one increased in TFHL tumor tissues correlated with TFHL-specific mutations and the other located near tumor cells, contributing to the TFHL niche.
Our data highlight the diverse microenvironment of T-cell lymphomas and the importance of dissecting this spatially.

[Disclosure of COI by Responsible researcher] Lead presenter is not the Responsible researcher.
[Lead presenter: Sakurako Suma] We have no financial relationships to disclose.
[Principal Researcher: Mamiko Sakata] ■Research funds from company: 大塚製薬（株）、エーザイ（株）、ブリストルマイヤーズスクイブ（株）、中外製薬（株）

### [J-3019] I-J10-4
### Cell culture system/Organoid

2025 / 9 / 27 (Sat) 9:00-10:15 Room 14/(ANA Crowne Plaza Hotel Kanazawa 3F Zuiun 1) Room 14

[Chairperson]Hideki Terai (Keio Cancer Center, School of Medicine, Keio University)

---

### J-3019
### Creation of a new pancreatic cancer organoid model using tissue resident macrophages derived from human iPS cell

[Speaker]Yuya Yamamoto (IMSUT, Dept, of Rege. Med.／Dept. of General Surgery, Chiba Univ.) [Co-author]Kenta Takeuchi (IMSUT, Dept, of Rege. Med.)、Shunsuke Tabe (IMSUT, Dept, of Rege. Med.／Dept. of General Surgery, Chiba Univ.)、Yohei Miyagi (Molecular Pathology and Genetics Div. Kanagawa Cancer Center)、Naoto Yamamoto (Dept. of Gastrointestinal Surgery, Kanagawa Cancer Center)、Naoki Tanimizu (IMSUT, Dept, of Rege. Med.)、Masayuki Ohtsuka (Dept. of General Surgery, Chiba Univ.)、Hideki Taniguchi (IMSUT, Dept, of Rege. Med.／Dept. of Rege. Med. Yokohama City univ.)

Pancreatic ductal adenocarcinoma (PDAC) is one of the most aggressive cancer, characterized by an immunosuppressive tumor microenvironment (TME). We previously reported a PDAC organoid associated with TME that is named FPCO and further generated FPCO with tumor associated macrophages (TAMs) derived from bone marrow-derived macrophages, which have been suggested to be the major source of TAMs. However, recent scRNA–seq have identified a distinct subset of TAMs that are derived from tissue resident macrophages (TRMs) originating from yolk sac hematopoietic and erythromyeloid progenitors and implicated their roles in PDAC malignancy. Indeed, we found that the frequency of TRMs is significantly associated with poor prognosis by performing immunostaining on PDAC patients´ tissue. To improve our understanding about roles of TRM–TAMs, we induced TRM–like cells from hiPSC (hiPSC–TRMs) and incorporated them into the FPCO system. We confirmed that, within FPCO, hiPSC–TRMs acquire transcriptional features of TAMs derived from TRMs identified in human PDAC tissues by scRNA–seq. Currently, we are investigating how TRM–TAMs affect PDAC characteristics using TRM–FPCO system.

[Disclosure of COI by Responsible researcher] Lead presenter is not the Responsible researcher.
[Lead presenter: Yuya Yamamoto] We have no financial relationships to disclose.
[Principal Researcher: Naoki Tanimizu] We have no financial relationships to disclose.

### [J-3020] I-J10-4
### Cell culture system/Organoid

2025 / 9 / 27 (Sat) 9:00-10:15 Room 14/(ANA Crowne Plaza Hotel Kanazawa 3F Zuiun 1) Room 14

[Chairperson]Hideki Terai (Keio Cancer Center, School of Medicine, Keio University)

---

### J-3020
### The effect of oxygen in pancreatic organoid growth and anti-cancer drug responses revealed by live imaging

[Speaker]Toru Hiratsuka (Dept. of Oncogen and Growth Reg, Osaka Int Cancer Inst／Dept of Mol Oncol, Grad Sch of Med, Osaka Univ) [Co-author]Huaze Ye (Dept. of Oncogen and Growth Reg, Osaka Int Cancer Inst／Dept of Mol Oncol, Grad Sch of Med, Osaka Univ)、Akihito Machinaga (Oncol Tsukuba Res Dept, Discov, Med Creation, OBG, Eisai Co.)、Nobuyuki Kakiuchi (Dept of Gastroent and Hepatol, Kyoto Univ／Inst for the Adv Study of Human Biol, Kyoto Univ)、Seishi Ogawa (Pathol and Tumor Biol, Grad Sch of Med, Kyoto Univ)、Hiroshi Seno (Cell Cycle Reg, Grad Sch of Biostudies, Kyoto Univ)、Shigeki Higashiyama (Dept. of Oncogen and Growth Reg, Osaka Int Cancer Inst)、Michiyuki Matsuda (Cell Cycle Reg, Grad Sch of Biostudies, Kyoto Univ)

Organoid culture of patient-derived cancer cells often suffers from slow growth rate or even complete growth suspension, which is a major drawback for biological assays such as drug efficacy evaluation.Here we used an organoid culture system where pancreatic ductal adenocarcinoma (PDAC) patient-derived organoids were cultured on a gas-permeable culture membrane plate. We found the organoid growth is significantly enhanced in the gas-permeable plate. Our wide-area live imaging revealed increased total cell number, organoid formation efficiency, and size of the largest organoid under the organoid culture system. We live imaged AMP-activated protein kinase (AMPK) activity and found the enhanced organoid growth was can be attributed to increased AMPK activity. Organoids culture on the gas-permeable plate or a regular plastic plate revealed different responses to PD0325901 and hydroxychloroquine, revealing the effect of oxygen in the drug responses.Our findings of the effect of oxygen in the efficient organoid culture and drug assessment will expand the application of organoid culture for biochemical assays, which is key for the development of personalized medicine for cancer patients.

[Disclosure of COI by Responsible researcher] Lead presenter is the Responsible researcher.
[Lead presenter: Toru Hiratsuka] We have no financial relationships to disclose.

▲TOP

### [J-3021] I-J10-4
### Cell culture system/Organoid

2025 / 9 / 27 (Sat) 9:00-10:15 Room 14/(ANA Crowne Plaza Hotel Kanazawa 3F Zuiun 1) Room 14

[Chairperson]Hideki Terai (Keio Cancer Center, School of Medicine, Keio University)

---

### J-3021
### Integrating Spatial Transcriptomics with Hypoxia-Based Organoid to Unveil Heterogeneity of Pancreatic Cancer

[Speaker]Hiromitsu Nakahashi (Gastroenterological and Hepatobiliary Surgery, University of Tsukuba) [Co-author]Koichirou Kumano (Gastroenterological and Hepatobiliary Surgery, University of Tsukuba)、Yoshihiro Miyazaki (Gastroenterological and Hepatobiliary Surgery, University of Tsukuba)、Osamu Shimomura (Gastroenterological and Hepatobiliary Surgery, University of Tsukuba)、Osamu Shimomura (Gastroenterological and Hepatobiliary Surgery, University of Tsukuba)、Kazuto Kamohara (Gastroenterological and Hepatobiliary Surgery, University of Tsukuba)、Shuhei Komatsuzaki (Gastroenterological and Hepatobiliary Surgery, University of Tsukuba)、Yoshimasa Akashi (Gastroenterological and Hepatobiliary Surgery, University of Tsukuba)、Tomoaki Furuta (Gastroenterological and Hepatobiliary Surgery, University of Tsukuba)、Yukihito Kuroda (Gastroenterological and Hepatobiliary Surgery, University of Tsukuba)、Tomohiro Kurokawa (Department of Medical Epigenomics Reseach, Fukushima Medical University)、Tatsuya Oda (Gastroenterological and Hepatobiliary Surgery, University of Tsukuba)

Basal-like molecular subtypes are key to therapeutic strategies for the highly aggressive pancreatic ductal adenocarcinoma (PDAC). In this study, we analyzed intratumoral heterogeneity in 12 PDAC specimens using spatial transcriptomics with the Xenium10X platform. All tumors exhibited mixed classical and basal-like subtype signatures, with five classical-dominant and four basal-like-dominant. Paired pancreatic cancer organoids (PCOs) were derived under normoxic (NORM-PCO) and hypoxic (HYPO-PCO) conditions. Transcriptomic analysis showed that HYPO-PCOs exhibited basal-like expression, whereas NORM-PCOs tended toward classical patterns. Comparative analysis of primary tumors, NORM-PCOs, and HYPO-PCOs on the same Xenium platform revealed that HYPO-PCOs reproduced the basal-like subtype distribution and gene expression of the original tumors. Notably, these expression profiles remained irreversible after oxygen conditions were switched, indicating selection of distinct clonal populations. The HYPO-PCO platform enables modeling of tumor heterogeneity under more physiological conditions and may serve as a system for discovering patient-specific biomarkers and therapeutic targets in PDAC.

[Disclosure of COI by Responsible researcher] Lead presenter is the Responsible researcher.
[Lead presenter: Hiromitsu Nakahashi] We have no financial relationships to disclose.

### [J-3022] I-J10-4
### Cell culture system/Organoid

2025 / 9 / 27 (Sat) 9:00-10:15 Room 14/(ANA Crowne Plaza Hotel Kanazawa 3F Zuiun 1) Room 14

[Chairperson]Hideki Terai (Keio Cancer Center, School of Medicine, Keio University)

---

### J-3022
### Investigation of interactions between cancer cells and CAFs in desmoplastic reaction subtypes of colorectal cancer

[Speaker]Yota Akamori (Dept. of Surg.,Tohoku Univ. Grad. Sch. of Med.) [Co-author]Keigo Murakami (Investigative Path.,Tohoku Univ. Grad. Sch. of Med.)、Hideaki Karasawa (Dept. of Surg.,Tohoku Univ. Grad. Sch. of Med.)、Yuki Yasuda (Dept. of Surg.,Tohoku Univ. Grad. Sch. of Med.)、Yukihiro Sato (Dept. of Surg.,Tohoku Univ. Grad. Sch. of Med.)、Megumi Murakami (Dept. of Surg.,Tohoku Univ. Grad. Sch. of Med.)、Tomoyuki Ono (Dept. of Surg.,Tohoku Univ. Grad. Sch. of Med.)、Yoshihiro Sato (Dept. of Surg.,Tohoku Univ. Grad. Sch. of Med.)、Hideyuki Suzuki (Dept. of Surg.,Tohoku Univ. Grad. Sch. of Med.)、Kazuhiro Watanabe (Dept. of Surg.,Tohoku Univ. Grad. Sch. of Med.)、Takashi Kamei (Dept. of Surg.,Tohoku Univ. Grad. Sch. of Med.)、Shinobu Ohnuma (Dept. of Surg.,Tohoku Univ. Grad. Sch. of Med.)、Toru Furukawa (Investigative Path.,Tohoku Univ. Grad. Sch. of Med.)、Michiaki Unno (Dept. of Surg.,Tohoku Univ. Grad. Sch. of Med.)

[Background]The desmoplastic reaction (DR) of colorectal cancer (CRC) is recognized as a prognostic marker. DR is classified into mature, immature, and intermediate types based on morphology. Cancer-associated fibroblasts (CAFs), the main DR component, are linked to poor prognosis, especially in immature DR, but the mechanisms remain unclear. We investigated CRC cell-CAF interactions using a 3D co-culture model of CRC organoids and CAFs.[Methods]Tissue samples from advanced CRC patients were used to establish CRC organoids and CAFs. DR type was assessed by H&E staining. Organoid proliferation was evaluated by total area measurement. Protein characteristic of each DR type was investigated using mass spectrometry.[Results]Co-cultures included organoids with normal fibroblasts, mature CAFs, or immature CAFs in addition to organoid alone. Fibroblast co-culture increased organoid proliferation, most notably with immature CAFs. OPLS-DA analysis revealed distinct protein profiles by DR type. [Conclusion]Immature CAFs markedly enhance CRC cell proliferation. Further RNA-seq and spatial transcriptomic analyses are planned.

[Disclosure of COI by Responsible researcher] Lead presenter is not the Responsible researcher.
[Lead presenter: Yota Akamori] We have no financial relationships to disclose.
[Principal Researcher: Keigo Murakami] We have no financial relationships to disclose.

### [J-3023] I-J10-4
### Cell culture system/Organoid

2025 / 9 / 27 (Sat) 9:00-10:15 Room 14/(ANA Crowne Plaza Hotel Kanazawa 3F Zuiun 1) Room 14

[Chairperson]Hideki Terai (Keio Cancer Center, School of Medicine, Keio University)

### J-3023
### An Ex Vivo Lung Cancer Model Using Bioengineered Rat Lungs

[Speaker]Satoshi Mizoguchi (Nagasaki University, Department of Surgical Oncology) [Co-author]Ryoichiro Doi (Nagasaki University, Department of Surgical Oncology)、Tomohiro Obata (Nagasaki University, Department of Surgical Oncology)、Ryosuke Ogata (Nagasaki University, Department of Surgical Oncology)、Soichiro Kiya (Nagasaki University, Department of Surgical Oncology)、Daisuke Taniguchi (Nagasaki University, Department of Surgical Oncology)、Koichiro Shimoyama (Nagasaki University, Department of Surgical Oncology)、Takuro Miyazaki (Nagasaki University, Department of Surgical Oncology)、Tomoshi Tsuchiya (University of Toyama, Department of Thoracic Surgery)、Keitaro Matsumoto (Nagasaki University, Department of Surgical Oncology)

[Introduction] Cancer tissues exist on complex tissue structures and coexist with native cells, interacting with each other via cell-to-cell signaling. Such cancer microenvironment is a critical factor that greatly influences the characteristics and behavior of tumors.[Methods] Harvested rat lungs were decellularized using the SDS method, and recellularized with rat lung cells. Cultured human lung cancer cells were then seeded by local injection and cultured in a designed bioreactor for 5 days. The samples were assessed histopathologically, and transcriptomic analysis was performed by bulk RNA sequencing to reveal gene profiles in the model.[Results] The cancer model revealed various morphological features according to cell type such as glandular structure formation, mucus production, and high MUC1 expression. RNA-seq analysis of A549 cancer model revealed that various kinds of drug resistance related genes were upregulated/downregulated compared with cells in 2D culture.[Conclusion] We developed a novel ex vivo lung cancer model with a complex cancer microenvironment similar to in vivo cancer tissue using bioengineered lungs.

[Disclosure of COI by Responsible researcher] Lead presenter is not the Responsible researcher.
[Lead presenter: Satoshi Mizoguchi] We have no financial relationships to disclose.
[Principal Researcher: Tomohiro Obata] We have no financial relationships to disclose.

### [J-3024] I-J10-4
### Cell culture system/Organoid

2025 / 9 / 27 (Sat) 9:00-10:15 Room 14/(ANA Crowne Plaza Hotel Kanazawa 3F Zuiun 1) Room 14

[Chairperson]Hideki Terai (Keio Cancer Center, School of Medicine, Keio University)

---

### J-3024
### Development of a long-term primary culture model using prostate cancer needle biopsies: a new tool for target discovery

[Speaker]Akihito Inoko (Dept. Path., Aichi Med. Univ., Sch. Med.) [Co-author]Minako Suzuki (Dept. Path., Aichi Med. Univ., Sch. Med.)、Takahiro Kojima (Aichi Cancer Center)、Goushi Kitano (Aichi Cancer Center)、Jun Tsuchimoto (Inst. Mol. Sci. Med., Aichi Med. Univ.)、Koji Ohnishi (Dept. Path., Aichi Med. Univ., Sch. Med.)、Kenji Kasai (Dept. Path., Aichi Med. Univ., Sch. Med.)、Waki Hosoda (Aichi Cancer Center)、Chitose Oneyama (Aichi Cancer Center)、Naomi Kuroiwa (Sinshu Univ.)、Shinya Nishio (Sinshu Univ.)、Tsutomu Miyamoto (Sinshu Univ.)、Tomoki Kosho (Sinshu Univ.)

Prostate cancer in Japan has rapidly increased with aging populations, yet therapeutic optimization remains limited, partly due to a lack of predictive biological models. Recently, we developed a primary culture method enabling long-term expansion of basal stem/progenitor cells from bilayered epithelial tissues, using a compound cocktail named "YDAC" (Inoko et al., Sci. Rep., In press). This method supports (1) inducible epithelial differentiation upon YDAC removal, (2) identification of differentiation-specific markers through comprehensive analysis, and (3) organoid formation in 3D culture.
We further optimized this culture system for various cancer specimens, including all grades of prostate cancer and clear cell ovarian carcinoma (AMED et al., unpublished). Tumor origin was validated by karyotyping, AMACR expression, Rb/p53 loss, tumorigenicity in mice, and NGS. Ongoing proteomic analyses are identifying stratification and patient-specific biomarkers. These "patient-derived cells" recapitulate patient-specific traits, enable PDX, and support primary tumor analysis, suggesting high fidelity, scalability, and translational relevance, thereby facilitating robust clinical research.

[Disclosure of COI by Responsible researcher] Lead presenter is the Responsible researcher.
[Lead presenter: Akihito Inoko] ■Research funds from company: 科研費、AMED

▲TOP

### [J-3031] II-J3-2
### Pancreatic cancer

2025 / 9 / 27 (Sat) 9:00-10:15 Room 15/(ANA Crowne Plaza Hotel Kanazawa 3F Zuiun 2) Room 15

[Chairperson]Yusuke Mizukami (Natl. Inst. Biomed. Innovation, Health & Nutrition)

---

### J-3031
### NUAK2 inhibition improves macromolecular delivery in a 3D model of the fibrotic pancreatic tumor microenvironment

[Speaker]Hiroyoshi Y. Tanaka (Faculty Med. Dent. Pharm. Sci., Okayama Univ.) [Co-author]Misaki Nakamura (Sch. Pharm. Sci., Okayama Univ.)、Mayu Ohira (Sch. Pharm. Sci., Okayama Univ.)、Atsushi Masamune (Div. Gastroenterol., Grad. Sch. Med., Tohoku Univ.)、Mitsunobu R. Kano (Faculty Interdisciplinary Sci. Engin. Health Syst., Okayama Univ.)

Pancreatic ductal adenocarcinoma (PDAC) is refractory to therapy, due partly to its dense, fibrotic stroma impeding drug delivery. Pancreatic stellate cells (PSCs) drive fibrosis via excessively secreting extracellular matrix proteins such as collagen I. Targeting fibrotic signaling in PSCs may be useful in overcoming this fibrotic barrier. The AMP-activated protein kinase-related kinases NUAK1/2 have recently gained interest as promoters of fibrosis, but whether they are pro-fibrotic in PSCs remains unknown. Here, we utilized immortalized PSCs to assess NUAK1/2 involvement. Using a 3D culture model of PDAC fibrotic tissue, we found that NUAK1/2 inhibition diminished collagen I expression in PSCs and enhanced macromolecular permeability, via a mechanism largely independent of established pathways involving transforming growth factor-$\beta$ and yes-associated protein (YAP). Isoform-specific knockdown revealed that NUAK2 more strongly contributes to fibrotic signaling than NUAK1. Mechanistically, actin stress fiber regulation by NUAK2 was crucial. Altogether, we show ***in vitro*** that pro-fibrotic signaling mediated by NUAK2 in PSCs may be targeted to enhance macromolecular delivery in PDAC.

[Disclosure of COI by Responsible researcher] Lead presenter is the Responsible researcher.
[Lead presenter: Hiroyoshi Y. Tanaka] ■Research funds from company: 科研費、ハーモニック伊藤財団、ウエスコ学術振興財団、薬学研究奨励財団、稲盛財団

### [J-3032] II-J3-2
### Pancreatic cancer

2025 / 9 / 27 (Sat) 9:00-10:15 Room 15/(ANA Crowne Plaza Hotel Kanazawa 3F Zuiun 2) Room 15

[Chairperson]Yusuke Mizukami (Natl. Inst. Biomed. Innovation, Health & Nutrition)

---

### J-3032
### Establishment of Precancerous Lesions of Pancreatic Cancer Organoids and Their Genomic and Transcriptomic Analysis

[Speaker]Masahiro Tsujimae (Div. of Gastroenterology, Dept. of Internal Medicine, Kobe Univ.) [Co-author]Atsuhiro Masuda (Div. of Gastroenterology, Dept. of Internal Medicine, Kobe Univ.)、Yosuke Irie (Div. of Gastroenterology, Dept. of Internal Medicine, Kobe Univ.)、Noriko Juri (Div. of Gastroenterology, Dept. of Internal Medicine, Kobe Univ.)、Yoshiyuki Harada (Div. of Gastroenterology, Dept. of Internal Medicine, Kobe Univ.)、Mika Miki (Div. of Gastroenterology, Dept. of Internal Medicine, Kobe Univ.)、Noriko Inomata (Div. of Gastroenterology, Dept. of Internal Medicine, Kobe Univ.)、Masanori Gonda (Div. of Gastroenterology, Dept. of Internal Medicine, Kobe Univ.)、Arata Sakai (Div. of Gastroenterology, Dept. of Internal Medicine, Kobe Univ.)、Takashi Kobayashi (Div. of Gastroenterology, Dept. of Internal Medicine, Kobe Univ.)、Norimitsu Uza (Div. of Gastroenterology, Dept. of Internal Medicine, Kobe Univ.)、Yuzo Kodama (Div. of Gastroenterology, Dept. of Internal Medicine, Kobe Univ.)

Background: Early diagnosis of pancreatic cancer is challenging, and the limited availability of samples hinders molecular analysis. This study aimed to establish organoids from precancerous lesions of pancreatic cancer and perform molecular biological analyses.Methods: Between October 2021 and August 2023, cases with pancreatic duct brushing or juice cytology under ERCP were included. Organoids were established from cell suspensions, and genomic analyses, along with bulk and single-cell transcriptomic analyses, were conducted.Results: Out of 32 sessions, 14 cases were diagnosed with adenocarcinoma by cytology. The organoid establishment rate was 75% (24/32), with 31% (10/32) successfully cultured long-term, revealing genomic abnormalities consistent with the diagnosis. Single-cell analysis revealed high heterogeneity, with basal-like cells present even at early stages.Conclusion: Establishing organoids from precancerous lesions of pancreatic cancer provides a novel approach for research. The unexpectedly high heterogeneity of pancreatic cancer was revealed, including the presence of basal-like cells at early stages.

[Disclosure of COI by Responsible researcher] Lead presenter is the Responsible researcher.
[Lead presenter: Masahiro Tsujimae] ■Research funds from company: KAKENHI

### [J-3033] II-J3-2
### Pancreatic cancer

2025 / 9 / 27 (Sat) 9:00-10:15 Room 15/(ANA Crowne Plaza Hotel Kanazawa 3F Zuiun 2) Room 15

[Chairperson]Yusuke Mizukami (Natl. Inst. Biomed. Innovation, Health & Nutrition)

---

### J-3033
### Decoding the Mechanisms of Pancreatic Tumorigenesis through Spatial Analysis of Ultra-Early Resected Pancreatic Cancer

[Speaker]Kazuto Kamohara (Gastrointestinal Surgery, University of Tsukuba) [Co-author]Shuhei Komatsuzaki (Gastrointestinal Surgery, University of Tsukuba)、Koichiro Kumano (Gastrointestinal Surgery, University of Tsukuba)、Hiromitsu Nakahashi (Gastrointestinal Surgery, University of Tsukuba)、Tomoaki Furuta (Gastrointestinal Surgery, University of Tsukuba)、Kinji Furuya (Gastrointestinal Surgery, University of Tsukuba)、Yoshihiro Miyazaki (Gastrointestinal Surgery, University of Tsukuba)、Osamu Shimomura (Gastrointestinal Surgery, University of Tsukuba)、Yoshimasa Akashi (Gastrointestinal Surgery, University of Tsukuba)、Tatsuya Oda (Gastrointestinal Surgery, University of Tsukuba)

Early detection is crucial for improving survival in pancreatic cancer, yet reliable early diagnostic markers are lacking. In Japan, specialists detect ultra-early pancreatic cancer based on subtle imaging findings like localized atrophy and ductal stenosis. Surgical specimens without prior therapy provide rare opportunities to study cancer initiation. We collected untreated Stage 0 or I pancreatic cancer specimens from multiple Japanese institutions and performed spatial transcriptomic analyses to profile gene expression in early lesions and surrounding microenvironments. Regions of interest, including normal ducts, PanIN, PDAC, and stroma, were analyzed using a panel of 5000 genes plus 100 pancreatic cancer-related genes. Integrating spatial data enabled finer subclassification and identification of molecular events linked to progression. Spatial interactions between epithelial cells and microenvironmental components like vessels, T cells, and macrophages were also assessed. Our findings reveal spatial dynamics of ultra-early pancreatic tumorigenesis and may support discovery of early diagnostic biomarkers.

[Disclosure of COI by Responsible researcher] Lead presenter is not the Responsible researcher.
[Lead presenter: Kazuto Kamohara] We have no financial relationships to disclose.
[Principal Researcher: Tatsuya Oda] We have no financial relationships to disclose.

### [J-3034] II-J3-2
### Pancreatic cancer

2025 / 9 / 27 (Sat) 9:00-10:15 Room 15/(ANA Crowne Plaza Hotel Kanazawa 3F Zuiun 2) Room 15

[Chairperson]Yusuke Mizukami (Natl. Inst. Biomed. Innovation, Health & Nutrition)

---

### J-3034
### Investigation of tumor mutation burden in de novo malignancies after liver/pancreas transplantation in our institution

[Speaker]Yoshito Tomimaru (Dept. of Gastroenterological Surgery, Grad. Sch. of Med., Osaka Univ.) [Co-author]Hirofumi Akita (Dept. of Gastroenterological Surgery, Grad. Sch. of Med., Osaka Univ.)、Hiroki Imamura (Dept. of Gastroenterological Surgery, Grad. Sch. of Med., Osaka Univ.)、Shogo Kobayashi (Dept. of Gastroenterological Surgery, Grad. Sch. of Med., Osaka Univ.)、Kazuki Sasaki (Dept. of Gastroenterological Surgery, Grad. Sch. of Med., Osaka Univ.)、Shinichiro Hasegawa (Dept. of Gastroenterological Surgery, Grad. Sch. of Med., Osaka Univ.)、Daisaku Yamada (Dept. of Gastroenterological Surgery, Grad. Sch. of Med., Osaka Univ.)、Takehiro Noda (Dept. of Gastroenterological Surgery, Grad. Sch. of Med., Osaka Univ.)、Hidenori Takahashi (Dept. of Gastroenterological Surgery, Grad. Sch. of Med., Osaka Univ.)、Yuichiro Doki (Dept. of Gastroenterological Surgery, Grad. Sch. of Med., Osaka Univ.)、Hidetoshi Eguchi (Dept. of Gastroenterological Surgery, Grad. Sch. of Med., Osaka Univ.)

Background: We evaluated the incidence of de novo malignancies after liver and pancreas transplantation in our center, and investigated tumor mutation burden (TMB), a marker potentially linked to tumor immunogenicity under post-transplant immunosuppression. Methods: We analyzed cases with de novo malignancies in transplant recipients treated until 2024. TMB was measured in the specimens from selected cases using capture sequencing of 783 cancer-related genes. Variants were mapped to the reference genome, filtered (AF>0.05, Depth>10), and TMB was calculated. Results: De novo cancers occurred in 21 of 189 liver (11.1%) and 8 of 73 pancreas (11.0%) transplant cases. Multiple cancers developed in 3 patients. Malignancies included: colorectal (n=8), renal (5), breast (3), lung (2), gastric (1), pancreatic (1), prostate (1), skin (1), leukemia (1), and PTLD (9). Excluding PTLD, mean age at diagnosis was 57±13. TMB was analyzed in 14 cases, yielding 242±263 mut/Mb (median 106, range 27-768); all were TMB-High (≥10 mut/Mb). Conclusion: A high TMB rate was observed in post-transplant de novo malignancies, suggesting a link to the unique immune environment in these patients.

[Disclosure of COI by Responsible researcher] Lead presenter is the Responsible researcher.
[Lead presenter: Yoshito Tomimaru] We have no financial relationships to disclose.

▲TOP

### [J-3035] II-J3-2
### Pancreatic cancer

2025 / 9 / 27 (Sat) 9:00-10:15 Room 15/(ANA Crowne Plaza Hotel Kanazawa 3F Zuiun 2) Room 15

[Chairperson]Yusuke Mizukami (Natl. Inst. Biomed. Innovation, Health & Nutrition)

---

### J-3035
### Autophagy inhibition enhances oncolytic virus-mediated antitumor immunity in pancreatic cancer via MHC-I upregulation

[Speaker]Yosuke Takahashi (Dept. Gastroenterological Surg., Okayama Univ. Grad. Sch. Med. Dent. Pharm.) [Co-author]Hiroshi Tazawa (Dept. Gastroenterological Surg., Okayama Univ. Grad. Sch. Med. Dent. Pharm.／Ctr Innovative Clinical Med., Okayama Univ. Hosp.)、Shuto Fujita (Dept. Gastroenterological Surg., Okayama Univ. Grad. Sch. Med. Dent. Pharm.)、Naohiro Okada (Dept. Gastroenterological Surg., Okayama Univ. Grad. Sch. Med. Dent. Pharm.)、Nobuhiko Kanaya (Dept. Gastroenterological Surg., Okayama Univ. Grad. Sch. Med. Dent. Pharm.)、Yoshihiko Kakiuchi (Dept. Gastroenterological Surg., Okayama Univ. Grad. Sch. Med. Dent. Pharm.)、Satoru Kikuchi (Dept. Gastroenterological Surg., Okayama Univ. Grad. Sch. Med. Dent. Pharm.)、Shinji Kuroda (Dept. Gastroenterological Surg., Okayama Univ. Grad. Sch. Med. Dent. Pharm.)、Yasuo Urata (Oncolys BioPharma, Inc.)、Shunsuke Kagawa (Dept. Gastroenterological Surg., Okayama Univ. Grad. Sch. Med. Dent. Pharm.)、Toshiyoshi Fujiwara (Dept. Gastroenterological Surg., Okayama Univ. Grad. Sch. Med. Dent. Pharm.)

Pancreatic ductal adenocarcinoma (PDAC) shows high basal rate of autophagy, which supports tumor cell survival and promotes immune evasion via lysosomal degradation of MHC-I molecule. Therefore, autophagy inhibition appears to be a promising strategy for treating PDAC. We developed an oncolytic adenovirus expressing tumor suppressor p53 (OBP-702) to induce apoptosis and antitumor immunity in PDAC. Here we show the therapeutic potential of an autophagy inhibitor chloroquine (CQ) to enhance the MHC-I expression and antitumor effect of OBP-702 in PDAC. CQ increased the MHC-I expression on the surface of murine PDAC cells (PAN02) and human PDAC cells (MIAPaCa-2). Combination therapy of CQ and OBP-702 showed a synergistic antitumor effect via CQ-mediated enhancement of OBP-702-induced apoptosis. In vivo experiment further revealed that combination therapy of CQ and OBP-702 significantly enhanced the suppression of PAN02 tumor growth compared with monotherapy via enhancement of tumor-infiltrating cytotoxic CD8+ T cells and CD11c+ dendritic cells. Our results suggest that autophagy inhibition enhances oncolytic virus-mediated antitumor immune response in PDAC via MHC-I upregulation.

[Disclosure of COI by Responsible researcher] Lead presenter is not the Responsible researcher.
[Lead presenter: Yosuke Takahashi] We have no financial relationships to disclose.
[Principal Researcher: Hiroshi Tazawa] We have no financial relationships to disclose.

### [J-3036] II-J3-2
### Pancreatic cancer

2025 / 9 / 27 (Sat) 9:00-10:15 Room 15/(ANA Crowne Plaza Hotel Kanazawa 3F Zuiun 2) Room 15

[Chairperson]Yusuke Mizukami (Natl. Inst. Biomed. Innovation, Health & Nutrition)

---

### J-3036
### Targeting Collagen Signaling via DDR1 Enhances Macromolecular Drug Delivery in a 3D Pancreatic Cancer Fibrosis Model

[Speaker]Mayu Ohira (Sch. Pharm. Sci., Okayama Univ.) [Co-author]Moe Kitamura (Sch. Pharm. Sci., Okayama Univ.)、Atsushi Masamune (Div. Gastroenterol., Grad. Sch. Med., Tohoku Univ.)、Mitsunobu R. Kano (Faculty Interdisciplinary Sci. Engin. Health Syst., Okayama Univ.)、Hiroyoshi Y. Tanaka (Faculty Med. Dent. Pharm. Sci, Okayama Univ.)

Fibrosis poses a barrier to drug delivery in pancreatic ductal adenocarcinoma (PDAC) and contributes to its dismal prognosis. Pancreatic stellate cells (PSCs) drive fibrosis by excessively secreting extracellular matrix proteins such as collagen I. Collagen I is thought to physically obstruct the delivery of macromolecules, such as albumin, antibodies, and nanomedicines. Apart from its structural role, collagen signals through dedicated receptors such as the discoidin domain receptors (DDR) 1/2. However, whether and how collagen signaling contributes to the fibrotic barrier remains uncharacterized. Here, we utilized a 3D culture model of PDAC fibrosis constructed from immortalized PSCs to assess the contribution of DDR1/2-mediated collagen signaling. DDR1/2 inhibition diminished collagen I expression in PSCs to enhance macromolecular delivery. Through isoform-specific targeting, inhibiting DDR1, but not DDR2, was found to be effective. Downstream of DDR, we discovered the involvement of the PI3K/AKT/mTOR pathway. Altogether, we show ***in vitro*** that DDR1-mediated collagen signaling exacerbates the fibrotic barrier and may be targeted to enhance macromolecular drug delivery in PDAC.

[Disclosure of COI by Responsible researcher] Lead presenter is not the Responsible researcher.
[Lead presenter: Mayu Ohira] We have no financial relationships to disclose.
[Principal Researcher: Hiroyoshi Y. Tanaka] ■Research funds from company: 科研費、ウエスコ学術振興財団、ハーモニック伊藤財団、日本応用酵素協会、稲盛財団、ホクト生物化学振興財団、薬学研究奨励財団

▲TOP

### [E-3025] I-E7-2
### Insights into cancer progression & pathogenesis based on genomic analysis

2025 / 9 / 27 (Sat) 9:00-10:15 Room 16/(11F Visage) Room 16

[Chairperson]Nobuyuki Kakiuchi (Gastroenterol., Hakubi Ctr., Kyoto Univ.)

### E-3025
### Novel molecular classification of colorectal cancer based on Wnt pathway alterations

[Speaker]Yoshikage Inoue (Dept. Tumor Biology, Kyoto Univ., Sch. Med.／Dept.GI Surg., Kyoto Univ., Sch. Med.) [Co-author]Nobuyuki Kakiuchi (Dept. Tumor Biology, Kyoto Univ., Sch. Med.／Hakubi Center, Kyoto Univ.／Dept. of Gastroenterology, Kyoto Univ., Grad. Sch. of Med.)、Kenichi Yoshida (Div. of Cancer Evolution, Natl. Cancer Center Res. Inst.)、Koichi Watanabe (Dept. of Ob／Gyn, Kyoto Univ., Grad. Sch. of Med.)、Ryunosuke Saiki (Dept. Tumor Biology, Kyoto Univ., Sch. Med.)、Yuichi Shiraishi (Div. of Genome Informatics Development, Natl. Cancer Center Res. Inst.)、Kenichi Chiba (Div. of Genome Informatics Development, Natl. Cancer Center Res. Inst.)、Yasuhito Nanya (Dept. of Hematol. & Oncol., Univ. of Tokyo, Fac. of Med.)、Tetuichi Yoshizato (Dept. of Medicine, Karolinska Inst.)、Satoshi Nagayama (Dept. of Gastroenterol. Surg., Uji Tokushukai Hosp.)、Kazutaka Obama (Dept.GI Surg., Kyoto Univ., Sch. Med.)、Seishi Ogawa (Dept. Tumor Biology, Kyoto Univ., Sch. Med.)

Colorectal cancer (CRC) is broadly classified into hypermutated and non-hypermutated (NHM) types, but subclassifying NHM-CRC is challenging due to co-occurring driver mutations. We analyzed mutations and copy number alterations in 4,437 CRCs, and gene expression and fusion transcripts in 2,833 tumors. Among 3,986 NHM-CRCs, we stratified tumors into APC-mutated (APCmut) and APC-unmutated (APCwt) groups, based on mutual exclusivity between APC and other Wnt pathway alterations. APCwt tumors were further classified into CTNNB1 (2.4%), RNF43 (1.8%), RSPO fusion (1.7%), and Wnt-negative (~1.5%) subtypes, each with poor prognosis and distinct molecular profiles. Based on the selection strength of binned bases in APC, we segmented the gene into five regions, enabling classification of 99% of APCmut into four subtypes. These differed in Wnt activation, co-mutations (TP53, RAS, PI3K, TGFβ, non-APC Wnt genes), and clinical characters. Applying this classification to precancerous lesions revealed subtype-specific patterns in APCmut. These findings suggest that CRC evolution is influenced by initial WNT pathway alterations, resulting in heterogeneity in molecular and clinical characteristics.

[Disclosure of COI by Responsible researcher] Lead presenter is not the Responsible researcher.
[Lead presenter: Yoshikage Inoue] We have no financial relationships to disclose.
[Principal Researcher: Seishi Ogawa] ■Advisor of the company and/or profit-making organization: エーザイ株式会社、Chordia Therapeutics ■Profit of stock: 朝日ゲノミクス株式会社 ■Research expenses: エーザイ株式会社、Chordia Therapeutics Inc.、大塚製薬株式会社 ■Contributions: 日本新薬株式会社 ■Endowed chair: Chordia Therapeutics Inc. 公益社団法人鹿児島共済会 南風病院

▲TOP

### [E-3026] I-E7-2
### Insights into cancer progression & pathogenesis based on genomic analysis

2025 / 9 / 27 (Sat) 9:00-10:15 Room 16/(11F Visage) Room 16

[Chairperson]Nobuyuki Kakiuchi (Gastroenterol., Hakubi Ctr., Kyoto Univ.)

---

### E-3026
### Multi-omics analyses of *ELOC*-mutated renal cell carcinoma identify the distinctive molecular features

[Speaker]Akihiko Fukagawa (Dept. of Pathology, The Univ. of Tokyo) [Co-author]Yasuhito Arai (Div. of Cancer Genomics, Natl. Cancer Ctr. Res. Inst.)、Akiko Maeshima (Dept. of Diagnostic Pathology, Natl. Cancer Ctr. Hosp.)、Natsuko Hama (Div. of Cancer Genomics, Natl. Cancer Ctr. Res. Inst.)、Yukio Hokazono (Dept. of Pathology, The Univ. of Tokyo／Div. of Cancer Genomics, Natl. Cancer Ctr. Res. Inst.)、Eijiro Nakamura (Dept. of Urology, Natl. Cancer Ctr. Hosp.)、Yoshiyuki Matsui (Dept. of Urology, Natl. Cancer Ctr. Hosp.)、Shungo Adachi (Dept. of Proteomics, Natl. Cancer Ctr. Res. Inst.)、Tetsuo Ushiku (Dept. of Pathology, The Univ. of Tokyo)、Shinichi Yachida (Dept. of Cancer Genome Informatics, Osaka Univ.)、Tatsuhiro Shibata (Dept. of Pathology, The Univ. of Tokyo／Lab. of Mol. Med., The Univ. of Tokyo)

***ELOC***-mutated renal cell carcinoma (RCC) is a rare tumor with only approximately 30 cases reported and has a molecular background similar to that of clear cell RCC (ccRCC) in terms of HIF-$\alpha$ protein accumulation. ***ELOC***-mutated RCC exhibits prominent leiomyomatous stromal growth and indolent growth compared to ccRCC. Gene and protein expression studies of ***ELOC***-mutated RCC have been limited, and the molecular mechanisms underlying the unique histological features of this tumor remain largely unclear. We identified five ***ELOC***-mutated RCC cases using whole genome sequencing in our previous study for Japanese RCCs (Fukagawa ***et al.***, ***Nat Commun.*** 2023). We performed spatial transcriptomic and proteomic analyses on these five cases to elucidate the molecular mechanisms underlying the distinctive histopathological features. Here, we represent the genomic and transcriptomic characteristics and the distinctive metabolic system of ***ELOC***-mutated RCC.

[Disclosure of COI by Responsible researcher] Lead presenter is not the Responsible researcher.
[Lead presenter: Fukagawa Akihiko] We have no financial relationships to disclose.
[Principal Researcher: Tstsuhiro Shibata] We have no financial relationships to disclose.

### [E-3027] I-E7-2
### Insights into cancer progression & pathogenesis based on genomic analysis

2025 / 9 / 27 (Sat) 9:00-10:15 Room 16/(11F Visage) Room 16

[Chairperson]Nobuyuki Kakiuchi (Gastroenterol., Hakubi Ctr., Kyoto Univ.)

---

### E-3027
### DDX3Y is a synthetic dependency for therapy in high-grade B-cell lymphoma harbouring damaging DDX3X mutations.

[Speaker]Sam A. Greenall (Dept. of Med., Monash Univ., Clayton., Australia) [Co-author]Matteo Costacurta (Monash Health, Clayton, Australia)、Natalie Borg (Sch. of Health and Biomed. Sci., RMIT Univ., Bundoora., Australia)、Jake Shortt (Dept. of Med., Monash Univ., Clayton., Australia／Monash Health, Clayton, Australia)

DDX3X is an essential RNA helicase that is often mutated in high-grade B-cell lymphoma (HGBL), resulting in loss of function. In females, viability is maintained via unsilencing of the inactivated second X-chromosome allele; in male HGBL, the normally testis restricted Y-chromosome paralogue, DDX3Y, is re-expressed. The crosstalk between DDX3X and DDX3Y in male cancers and potential interdependencies are incompletely understood. Accordingly, we generated a suite of genetic tools such as DDX3X-Y targeting CRISPR Cas9 sgRNA and endogenous knock in of mAID and dTAG degron technologies onto DDX3X and DDX3Y loci in a range of HGBL lines. We show that male HGBLs harbouring loss-of-function DDX3X mutations are highly dependent on DDX3Y for survival. Eliminating DDX3Y protein with dTAG-13 degron targeting induced rapid and widespread DNA damage and apoptosis, resulting in the complete death of DDX3X mutated HGBL cells. DDX3Y degradation also led to universal transcriptome perturbation, especially involving DNA damage, RNA splicing and translational pathways by GSEA. Thus, DDX3Y as a synthetic lethal dependency in DDX3X mutated cancer could be selectively targeted for therapeutic gain.

[Disclosure of COI by Responsible researcher] Lead presenter is the Responsible researcher.
[Lead presenter: Sam A. Greenall] We have no financial relationships to disclose.

▲TOP

### [E-3028] I-E7-2
### Insights into cancer progression & pathogenesis based on genomic analysis

2025 / 9 / 27 (Sat) 9:00-10:15 Room 16/(11F Visage) Room 16

[Chairperson]Nobuyuki Kakiuchi (Gastroenterol., Hakubi Ctr., Kyoto Univ.)

---

### E-3028
### Whole genome landscape of asymptomatic HTLV-1 carriers with potential risk of ATL progression

[Speaker]Kako Suzuki (Dept. CBMS, Grad. Sch. Front. Sci., Univ. Tokyo) [Co-author]Yuta Kuze (Dept. CBMS, Grad. Sch. Front. Sci., Univ. Tokyo)、Koji Jimbo (Dept. Hematology & Oncology, IMSUT, Univ. Tokyo)、Kazu Okuma (Dept. Microbiology, Fac. Med., Kansai Medical Univ.)、Rei Gibu (Dept. CBMS, Grad. Sch. Front. Sci., Univ. Tokyo)、Koju Kamoi (Dept. Ophthalmology, Inst. Science Tokyo)、Yasuhito Nannya (Dept. Hematology & Oncology, IMSUT, Univ. Tokyo)、Yutaka Suzuki (Dept. CBMS, Grad. Sch. Front. Sci., Univ. Tokyo)、Kaoru Uchimaru (Dept. CBMS, Grad. Sch. Front. Sci., Univ. Tokyo)、Makoto Yamagishi (Dept. CBMS, Grad. Sch. Front. Sci., Univ. Tokyo)

Persistent HTLV-1 infection and multiple genetic abnormalities are involved in multistep tumorigenesis in adult T-cell leukemia (ATL). However, comprehensive genomic analysis of asymptomatic HTLV-1 carriers has been limited. Here we comprehensively performed WGS (n=43) and ultra-deep sequencing (n=148) on enriched infected cells from HTLV-1-infected carriers and patients. WGS uncovered 0.3-4.4 mutations per megabase in carriers, indicative of genomic instability in a preleukemic state. Tumor mutation burden positively correlated with age, proviral load, and clonality. The mutational signature was characterized by clock-like mutational processes. Genomic analyses ***in vitro*** and ***in vivo*** HTLV-1 infection models demonstrated that genomic instability arises immediately after infection. Furthermore, ultra-deep sequencing detected the accumulation of abnormalities in specific genes even before disease onset, thereby identifying predictive risk factors in the pre-onset state. The comprehensive genome study supports that genomic instability is definitely not a late occurrence in tumorigenesis and underscores its crucial role in developing evidence-based risk assessments for ATL progression.

[Disclosure of COI by Responsible researcher] Lead presenter is not the Responsible researcher.
[Lead presenter: Kako Suzuki] We have no financial relationships to disclose.
[Principal Researcher: Makoto Yamagishi] ■Patent fee: 第一三共株式会社、田辺三菱製薬　■Lecture fee: 第一三共株式会社
■Research expenses: 第一三共株式会社

### [E-3029] I-E7-2
### Insights into cancer progression & pathogenesis based on genomic analysis

2025 / 9 / 27 (Sat) 9:00-10:15 Room 16/(11F Visage) Room 16

[Chairperson]Nobuyuki Kakiuchi (Gastroenterol., Hakubi Ctr., Kyoto Univ.)

---

### E-3029
### Genomic landscape of Japanese ovarian cancer: genomic features and driver mutations per histotype and molecular subtype

[Speaker]Osamu Gotoh (JFCR. CPM Ctr.) [Co-author]Akiko Tonooka (JFCR. Cancer Inst. Dept. Path.)、Norio Tanaka (JFCR. CPM Ctr.)、Tetsuo Noda (JFCR. CPM Ctr.)、Seiichi Mori (JFCR. CPM Ctr.)

Ovarian cancer (OV) is an intractable disease with multiple histotypes and exhibits heterogeneity even within a same histotype. While genomic features can support patient stratification, prior studies have mainly focused on high-grade serous, a major histotype in Caucasian. The other histotypes such as clear cell, more common in East Asian, have not been well studied. To address this, we conducted whole-genome sequencing of 1,412 Japanese OVs across 6 major histotypes: clear cell, high-grade serous, endometrioid, mucinous, carcinosarcoma, and sex-cord stromal tumors. We identified 5 molecular subtypes: POLE, MSI, CNH-A, CNH-B, and CNL. We present genomic features including germline mutations, somatic driver events and mutational signatures in association with histotypes and molecular subtypes. CNH-B showed distinct characteristics such as frequent ***ATM*** mutations and copy number alterations with heterozygosity. Pathogenic variants in hereditary genes showed histotype-specific patterns, with high HRD scores in tumors with biallelic HR gene alterations and distinct somatic CNVs/SVs. These results highlight the genomic landscape of OV and offer insights for future personalized therapy.

[Disclosure of COI by Responsible researcher] Lead presenter is not the Responsible researcher.
[Lead presenter: Osamu Gotoh] We have no financial relationships to disclose.
[Principal Researcher: Akiko Tonooka] ■Research funds from company: 公的資金(AMED): G-CARD

### [E-3030] I-E7-2
### Insights into cancer progression & pathogenesis based on genomic analysis

2025 / 9 / 27 (Sat) 9:00-10:15 Room 16/(11F Visage) Room 16

[Chairperson]Nobuyuki Kakiuchi (Gastroenterol., Hakubi Ctr., Kyoto Univ.)

---

### E-3030
### Evolution of Aneuploid Karyotypes during Malignant Transformation

[Speaker]Shengyue Lei (Div. Exp. Pathol., Cancer Inst., JFCR／Dept. JFCR., Institute of Science Tokyo) [Co-author]Minji Jo (Div. Exp. Pathol., Cancer Inst., JFCR)、Utako Kato (Div. Exp. Pathol., Cancer Inst., JFCR)、Toru Hirota (Div. Exp. Pathol., Cancer Inst., JFCR／Dept. JFCR., Institute of Science Tokyo)

Cancer cells exhibit progressive karyotype changes, resulting in tumors with a plethora of aneuploid populations. However, what karyotypic patterns associate with malignant phenotypes and how these patterns affect transcriptional shifts, remain unclear. To address this, we generated p53-depleted aneuploid RPE1 cells and monitored their aneuploid populations. Copy number variation (CNV) analysis, based on scRNA-seq data, revealed cellular karyotypes were dynamically changed: initially varied karyotypic patterns converged to near-euploid states over several passages. However, in later, distinct aneuploid CNV patterns emerged and became dominant. Intriguingly, these cells showed enhanced proliferation and adaptability to the 3D culture condition, suggesting altered CNV associates with the acquisition of malignant features. These cells also transcriptomically upregulated MAPK signaling-related genes that possibly contribute to malignant transformation. Moreover, we have adopted cell barcodes for lineage tracing to elucidate the clonal dynamics of karyotypic evolution. These findings provide insights into how aneuploidy promotes malignancy, transcriptional shifts and clonal evolution.

[Disclosure of COI by Responsible researcher] Lead presenter is not the Responsible researcher.
[Lead presenter: Shengyue Lei] We have no financial relationships to disclose.
[Principal Researcher: Toru Hirota] We have no financial relationships to disclose.

### [E-3007] I-E8-2
### Cancer epigenetics (2)

2025 / 9 / 27 (Sat) 10:15-11:30 Room 6/(Hotel Nikko Kanazawa 3F Kujaku A) Room 6

[Chairperson]Yasuhiro Yamada (Dept. Mol. Pathol. Univ. Tokyo)

---

### E-3007
### The Role of Ezh2 of PCR2 in Driving Tumor-associated Macrophages toward Tumor Promoting Phenotypes in Liver Cancer Model

[Speaker]Tanapat Palaga (Dept. Microbiology, Faculty of Science, Chulalongkorn University ／Center of Excellence in Immunology and Immune-mediated Diseases, Chulalongkorn University) [Co-author]Kittin Weerasopon (Dept. Microbiology, Faculty of Science, Chulalongkorn University ／Graduate Program in Biotechnology, Faculty of Science, Chulalongkorn University)、Benjawan Saechue (Faculty of Veterinary Science, Mahasarakham University)、Atsadang Boonmee (Dept. Microbiology, Faculty of Medicine Siriraj Hospital, Mahidol University)、Thitiporn Pattarakankul (Dept. Microbiology, Faculty of Science, Chulalongkorn University ／Center of Excellence in Materials and Biointerfaces, Chulalongkorn University)、Benjawan Wongprom (Dept. Microbiology, Faculty of Science, Chulalongkorn University ／Center of Excellence in Immunology and Immune-mediated Diseases, Chulalongkorn University)

Ezh2, a subunit of the Polycomb Repressive Complex 2 (PRC2), is essential for depositing a repressive epigenetic mark of H3K27me3. The specific role of Ezh2 in myeloid cells and its contribution to tumorigenesis remains largely unexplored. This study investigated the effects of myeloid-specific ***Ezh2*** KO on liver tumorigenesis using an ***in vitro*** and mouse models. An ***in vitro*** study revealed that conditioned medium (CM) from Hepa1-6 cell line programed ***Ezh2*** KO macrophages to become more pro-tumorigenic such as increasing expression of Arginase 1 and CD206. RNA seq and ATAC-seq of ***Ezh2*** KO macrophages cocultured with Hepa1-6 CM identified glycolysis pathway as enriched pathway. In an ***in vivo*** model using using Dt81Hepa1-6 cell line showed more liver tumor burden in ***Ezh2*** KO mice than wild-type controls, accompanying with elevated TNF-&alpha in the liver tissues of ***Ezh2*** KO mice, indicating a heightened inflammatory response. The results from both ***in vitro*** and ***in vivo*** models collaborated that Ezh2 in TAM plays important role in controlling tumor growth of liver cancer. Targeting PRC2/Ezh2 may provide alternative approaches to treat liver cancer.

[Disclosure of COI by Responsible researcher] Lead presenter is the Responsible researcher.
[Lead presenter: Tanapat Palaga] We have no financial relationships to disclose.

### [E-3008] I-E8-2
### Cancer epigenetics (2)

2025 / 9 / 27 (Sat) 10:15-11:30 Room 6/(Hotel Nikko Kanazawa 3F Kujaku A) Room 6

[Chairperson]Yasuhiro Yamada (Dept. Mol. Pathol. Univ. Tokyo)

---

### E-3008
### Epigenetic modulation elicits an NK cell-mediated Immune Response in Urothelial Carcinoma

[Speaker]Michael Chan (Dept. of Biomedical Sci., Natl. Chung Cheng Univ., Taiwan／Epigenomics & Human Diseases Ctr., Natl. Chung Cheng Univ., Taiwan) [Co-author]Himani Kumari (Dept. of Biomedical Sci., Natl. Chung Cheng Univ., Taiwan／Epigenomics & Human Diseases Ctr., Natl. Chung Cheng Univ., Taiwan)、Ciao-Ni Chen (Dept. of Biomedical Sci., Natl. Chung Cheng Univ., Taiwan／Epigenomics & Human Diseases Ctr., Natl. Chung Cheng Univ., Taiwan)、Cheng-Huang Shen (Dept. of Urology, Chiayi Christian Hosp., Taiwan)

Urothelial carcinoma (UC) evades NK cell-mediated killing through various pathways, necessitating innovative therapies. We investigated cyproheptadine (CPH) and entinostat (ENT) as epigenetic modifiers enhancing NK cell cytotoxicity against UC. Pre-treatment with CPH or ENT significantly increased NK cell-mediated killing of UC cells, demonstrated through co-culture with NK-92 and human primary NK cells. This enhancement is linked to epigenetic activation of NKG2D ligands, notably through increased H3K27ac and decreased H2K27me3 at the ULBP2 promoter region. Ectopic ULBP2 expression further enhanced susceptibility to NK cell killing. In a syngeneic mouse model using MB49 cells, CPH or ENT treatments significantly reduced tumor growth by promoting NK cell infiltration, likely due to elevated CCL3 levels. These findings highlight the potential of CPH and ENT to enhance NK cell activity by modulating epigenetic pathways, providing promising strategies for UC treatment.

[Disclosure of COI by Responsible researcher] Lead presenter is the Responsible researcher.
[Lead presenter: Michael Chan] We have no financial relationships to disclose.

### [E-3009] I-E8-2
### Cancer epigenetics (2)

2025 / 9 / 27 (Sat) 10:15-11:30 Room 6/(Hotel Nikko Kanazawa 3F Kujaku A) Room 6

[Chairperson]Yasuhiro Yamada (Dept. Mol. Pathol. Univ. Tokyo)

---

### E-3009
### Loss of SIRT1 drives a positive feedback loop pathway of H19-glycolysis-histone lactylation in gastric cancer

[Speaker]Yoshimitsu Akiyama (Dept. Mol. Oncol., Institute of Science Tokyo) [Co-author]Shu Tsukihara (Dept. Mol. Oncol., Institute of Science Tokyo／Dept. Surg., Jikei Univ. Sch. of Med.)、Shu Shimada (Dept. Mol. Oncol., Institute of Science Tokyo)、Megumi Hatano (Dept. Mol. Oncol., Institute of Science Tokyo)、Kinugasa Yusuke (Dept. Gastrointestinal Surg., Institute of Science Tokyo)、Ken Eto (Dept. Surg., Jikei Univ. Sch. of Med.)、Shinji Tanaka (Dept. Mol. Oncol., Institute of Science Tokyo)

Histone lactylation, a novel epigenetic modification, is regulated by lactate produced during glycolysis that is activated in various cancers, including gastric cancer (GC). Here, we examined the molecular mechanism and clinical significance of histone lactylation in GC. Histone H3K18 lactylation (H3K18la) was elevated in GC, correlating with worse prognosis. H3K18la levels were decreased in SIRT1-overexpressed GC cells but increased in SIRT1 knockdown-cells. RNA-seq analysis showed that lncRNA H19 was markedly downregulated in GC cells with SIRT1 overexpression and in those cultured under glucose-free conditions, confirming decreased H3K18la levels at its promoter region. Knockdown of H19 reduced the expression of LDHA and H3K18la, while LDHA knockdown impaired the expression of H19 and H3K18la, suggesting an H19/glycolysis/H3K18la positive feedback loop. The SIRT1-low/H3K18la-high signature was found to be an independent prognostic factor in GC patients. Therefore, SIRT1 acts as a histone delactylase for H3K18, and the loss of SIRT1 triggers a positive feedback loop of H19/glycolysis/H3K18la. Targeting this pathway represents a novel therapeutic strategy for GC treatment.

[Disclosure of COI by Responsible researcher] Lead presenter is the Responsible researcher.
[Lead presenter: Yoshimitsu Akiyama] We have no financial relationships to disclose.

### [E-3010] I-E8-2
### Cancer epigenetics (2)

2025 / 9 / 27 (Sat) 10:15-11:30 Room 6/(Hotel Nikko Kanazawa 3F Kujaku A) Room 6

[Chairperson]Yasuhiro Yamada (Dept. Mol. Pathol. Univ. Tokyo)

---

### E-3010
### Non canonical Polycomb group proteins epigenetically regulate tumorigenesis during lymphocyte development

[Speaker]Mayumi Hirakawa (Tokyo University of Science Research Institute for Biomedical Sciences) [Co-author]Lisa Hirano (Tokyo University of Science Research Institute for Biomedical Sciences)、Tomokatsu Ikawa (Tokyo University of Science Research Institute for Biomedical Sciences)

Polycomb group (PcG) proteins are crucial epigenetic factors in development. The PcG protein is classified into polycomb repressor complex 1 (PRC1) and PRC2, and the PRC1 mediates monoubiquitination of histone H2A lysine 119 to negatively regulate transcription. PRC1 is divided into 6 complexes according to the containing PCGF proteins (PCGF1-6). Canonical PRC1 is required for the maintenance of hematopoietic stem cells and normal lymphocyte development. However, the function of non-canonical (nc)PRC1, such as PCGF1, 3 and 5 in the lymphocyte development is unknown. Therefore, the purpose of this study is to clarify the function of PCGF1, 3, and 5 during lymphocyte differentiation. To study this, T-cell or B-cell specific PCGF 1-, 3-, and 5- single and triple knockout (Lck-Cre or Mb1-Cre Pcgf1fl/fl, Pcgf3fl/fl, Pcgf5fl/fl TKO) mice were generated and analyzed for bone marrow, thymus and spleen. B-cell specific TKO mice had abnormal pro B cell development in bone marrow and spleen. Immature single CD8 positive T cells were proliferated in the thymus and spleen of T-cell specific TKO mice. This is first report that ncPRC1 controls cell lineage differentiation and tumorigenesis.

[Disclosure of COI by Responsible researcher] Lead presenter is the Responsible researcher.
[Lead presenter: Mayumi Hirakawa] ■Research funds from company: 科研費・若手研究

### [E-3011] I-E8-2
### Cancer epigenetics (2)

2025 / 9 / 27 (Sat) 10:15-11:30 Room 6/(Hotel Nikko Kanazawa 3F Kujaku A) Room 6

[Chairperson]Yasuhiro Yamada (Dept. Mol. Pathol. Univ. Tokyo)

---

### E-3011
### The DNMT inhibitor enhance the effects of EZH2 inhibitors through MYCN instability

[Speaker]Hisanori Takenobu (Research Institute for Clinical Oncology, Saitama Cancer Center) [Co-author]Yuki Endo (Research Institute for Clinical Oncology, Saitama Cancer Center／Department of Pediatric Surgery, Tohoku University)、Ryuichi Sugino (Research Institute for Clinical Oncology, Saitama Cancer Center)、Miki Ohira (Research Institute for Clinical Oncology, Saitama Cancer Center)、Masayuki Haruta (Research Institute for Clinical Oncology, Saitama Cancer Center)、Shunpei Satoh (Research Institute for Clinical Oncology, Saitama Cancer Center)、Kyosuke Mukae (Research Institute for Clinical Oncology, Saitama Cancer Center)、Jesmin Akter (Research Institute for Clinical Oncology, Saitama Cancer Center)、Tomoko Wada (Research Institute for Clinical Oncology, Saitama Cancer Center)、Ritsuko Onuki (Research Institute for Clinical Oncology, Saitama Cancer Center)、Atsuko Nakazawa (Department of Clinical Research, Saitama Children's Medical Center)、Naoko Hattori (Institute for Molecular and Cellular Regulation, Gunma University)、Toshikazu Ushijima (Department of Epigenomics, Institute for Advanced Life Sciences, Hoshi University)、Takehiko Kamijo (Research Institute for Clinical Oncology, Saitama Cancer Center／Department of Graduate School of Science and Engineering, Saitama University)

The polycomb repressor complex (PRC) 2 molecule EZH2 and EED inhibitors have recently been developed; however, the effectiveness and underlying molecular mechanisms in many malignancies have not been elucidated precisely. We examined the antitumor effect of EZH2 inhibitor (EZH2i) using a WST assay. However, several cell lines, including neuroblastoma (NB) cells, were resistant to EZH2i. Transcriptome analysis revealed the de-repression of gene sets related to differentiation and cell cycle arrest. Genes such as ***TRIM63, VSTM2L, GPNMB***, and ***TIMP3***, which are de-repressed by EZH2is in sensitive cells, may serve as EZH2i sensitivity biomarkers. Additionally, the combined treatment with EZH2i and 5-aza-dC significantly inhibited cell proliferation with MYCN destabilization at protein level and induced a robust differentiation phenotype. These results confirm the importance of epigenetic regulation in NB biology and provide information for developing epigenetic therapies for patients with advanced NB.

[Disclosure of COI by Responsible researcher] Lead presenter is the Responsible researcher.
[Lead presenter: Hisanori Takenobu] We have no financial relationships to disclose.

### [E-3012] I-E8-2
### Cancer epigenetics (2)

2025 / 9 / 27 (Sat) 10:15-11:30 Room 6/(Hotel Nikko Kanazawa 3F Kujaku A) Room 6

[Chairperson]Yasuhiro Yamada (Dept. Mol. Pathol. Univ. Tokyo)

---

### E-3012
### Regulation of H3K4me3 breadth and MYC expression by the SETD1B catalytic domain in MLL-rearranged leukemia

[Speaker]Takayuki Hoshii (Mol. Oncol., Grad. Sch. Med., Chiba Univ., Chiba, Japan) [Co-author]Shintaro Izumi (Mol. Oncol., Grad. Sch. Med., Chiba Univ., Chiba, Japan／Dept. End. Hem. and Ger., Chiba Univ, Chiba, Japan)、Ko Ohtani (Mol. Oncol., Grad. Sch. Med., Chiba Univ., Chiba, Japan／Dept. End. Hem. and Ger., Chiba Univ, Chiba, Japan)、Makoto Matsumoto (Mol. Oncol., Grad. Sch. Med., Chiba Univ., Chiba, Japan)、Seito Shibata (Mol. Oncol., Grad. Sch. Med., Chiba Univ., Chiba, Japan)、Bahityar Rahmutulla (Mol. Oncol., Grad. Sch. Med., Chiba Univ., Chiba, Japan)、Masaki Fukuyo (Mol. Oncol., Grad. Sch. Med., Chiba Univ., Chiba, Japan)、Mitsutaka Nishimoto (Dep. Hem., Osaka Met. Univ., Osaka, Japan)、Hideo Miyagawa (Prev. Med. and Env. Health, Osaka Met. Univ., Osaka, Japan)、Emiko Sakaida (Dept. End. Hem. and Ger., Chiba Univ, Chiba, Japan)、Koutaro Yokote (Dept. End. Hem. and Ger., Chiba Univ, Chiba, Japan)、Issay Kitabayashi (Oncol. Innov. Center, Fujita Health Univ., Aichi, Japan)、Kimi Araki (Div. Dev. Genet., IRDA, Kumamoto Univ., Kumamoto, Japan／Center for Metab. Reg. Healthy Aging, Kumamoto Univ,Kumamoto, Japan)、Atsushi Kaneda (Mol. Oncol., Grad. Sch. Med., Chiba Univ., Chiba, Japan／Health and Disease Omics Center, Chiba University, Chiba,Japan)

Histone H3 lysine 4 trimethylation (H3K4me3) is abundant in mixed-lineage leukemia-rearranged (MLL-r) acute myeloid leukemia (AML) cells; however, the responsible enzymes and their roles remain unclear. This study aimed to identify the modifier responsible for high H3K4me3 modification in MLL-r leukemia and its downstream targets essential for the cell proliferation. Here, we performed a CRISPR-tiling screen against known H3K4 methylation modifiers in an MLL-r AML model. Disrupting the SETD1B catalytic SET domain caused depletion of FLT3-ITD or NrasG12D-expressing AML cells, and gene expression downregulation, particularly in the MYC pathway. SETD1B SET domain loss results in a significant decrease in H3K4me3 breadth. Exogenous MYC expression or disrupting H3K4 demethylase KDM5C significantly restored growth defects in SETD1B SET domain-mutant cells. These data indicated that SETD1B was required for H3K4me3 breadth and MYC expression. Thus, a thorough understanding of SETD1B-mediated H3K4me3 breadth is critical for developing markers and therapies for MYC-dependent leukemia subtypes.

[Disclosure of COI by Responsible researcher] Lead presenter is the Responsible researcher.
[Lead presenter: Hoshii Takayuki] We have no financial relationships to disclose.

▲TOP

### [J-3007] I-J12-4
### Diagnosis by expression analysis

2025 / 9 / 27 (Sat) 10:15-11:30 Room 7/(Hotel Nikko Kanazawa 3F Kujaku B) Room 7

[Chairperson]Mitsuru Futakuchi (Dept. of Pathol. Faculty of Med. Yamagata Univ. )

---

### J-3007
### Heterogeneity of lung adenosquamous carcinoma visualized by histopathological images and spatial analysis using Xenium

[Speaker]Keiichi Hatakeyama (Cancer. Multiomics Div., Shizuoka Cancer Ctr. Res. Inst.) [Co-author]Takuya Kawata (Div. of Path., Shizuoka Cancer Ctr.)、Takashi Sugino (Div. of Path., Shizuoka Cancer Ctr.)、Takeshi Nagashima (Cancer Diagnostics Res. Div., Shizuoka Cancer Ctr. Res. Inst.／SRL Inc.)、Keiichi Ohshima (Med. Genetics Div., Shizuoka Cancer Ctr. Res. Inst.)、Sumiko Ohnami (Cancer Diagnostics Res. Div., Shizuoka Cancer Ctr. Res. Inst.)、Shumpei Ohnami (Cancer Diagnostics Res. Div., Shizuoka Cancer Ctr. Res. Inst.)、Kouji Maruyama (Experimental Animal Facility, Shizuoka Cancer Ctr. Res. Inst.)、Akane Naruoka (Drug Discovery and Development Div., Shizuoka Cancer Ctr. Res. Inst.)、Masakuni Serizawa (Drug Discovery and Development Div., Shizuoka Cancer Ctr. Res. Inst.)、Akira Iizuka (Immunother. Div., Shizuoka Cancer Ctr. Res. Inst.)、Tadashi Ashizawa (Immunother. Div., Shizuoka Cancer Ctr. Res. Inst.)、Hirotsugu Kenmotsu (Div. of Thoracic Oncol., Shizuoka Cancer Ctr.)、Ken(Shizuoka Cancer Ctr.) Yamaguchi (Shizuoka Cancer Ctr.)

Single-cell level gene expression analysis, which preserves cell position information, is called spatial analysis and allows visualization of tumor heterogeneity and the tumor microtubule environment. In this study, we integrated the histopathological image and spatial transcriptome in lung adenosquamous carcinoma. First, we stained AE1/AE3 pan-keratin, TTF-1, and P40 based on immunohistochemistry (IHC). Next, we performed spatial transcriptome analysis using Xenium (10xGenomics) on the serial section. We used IHC to distinguish between adeno- and squamous-dominant regions in the tumor area and evaluated gene expression in these regions at the single cell level. Our analysis identified specific gene expressions in each region that were not detected by previous bulk analysis, and was also able to visualize the heterogeneity within the tumor that could not be isolated by IHC. The integration of histopathological images and spatial transcriptome will not only clarify the diversity of tumor cells, but will also become a technology that can expand the possibilities of pathological diagnosis.

[Disclosure of COI by Responsible researcher] Lead presenter is the Responsible researcher.
[Lead presenter: Keiichi Hatakeyama] We have no financial relationships to disclose.

### [J-3008] I-J12-4
### Diagnosis by expression analysis

2025 / 9 / 27 (Sat) 10:15-11:30 Room 7/(Hotel Nikko Kanazawa 3F Kujaku B) Room 7

[Chairperson]Mitsuru Futakuchi (Dept. of Pathol. Faculty of Med. Yamagata Univ. )

---

### J-3008
### Unlocking the Past: A Comparative Study of Three Extraction Protocols for Proteomics of 30-Year Archival FFPE Samples

[Speaker]Rei Noguchi (Dep. Pharmacology, TWMU／Div. Rare Cancer Res, NCC RI) [Co-author]Yuki Adachi (Div. Rare Cancer Res, NCC RI)、Sumio Otsuki (Lab of Phama Micro, Kumamoto Univ)、Yuki Yoshimatsu (Tochigi Cancer Center RI)、Tadashi Kondo (Div. Rare Cancer Res, NCC RI)

Formalin-fixed paraffin-embedded (FFPE) tissues are essential for retrospective translational research. However, extracting high-quality proteins from aged FFPE tissues remains challenging due to cross-linking. In this study, we compared three protein extraction methods using FFPE liver cancer samples stored for over 30 years. We analyzed eight hepatocellular carcinoma (HCC) cases, isolating tumor and adjacent regions by macro-dissection. Protein extraction was performed using adaptive focused acoustics (AFA), pressure cycling technology (PCT), and SP3 paramagnetic bead-based clean-up. Proteomic analysis was conducted via data-independent acquisition mass spectrometry on a ZenoTOF 7600. All methods enabled LC-MS/MS-based analysis. Among them, AFA consistently yielded more peptides and proteins with better reproducibility. These findings suggest that even decades-old FFPE samples can be suitable for proteomic profiling with optimized methods, supporting broader applications in translational research, particularly in rare cancer studies where fresh tissues are scarce.

[Disclosure of COI by Responsible researcher] Lead presenter is the Responsible researcher.
[Lead presenter: Rei Noguchi] We have no financial relationships to disclose.

### [J-3009] I-J12-4
### Diagnosis by expression analysis

2025 / 9 / 27 (Sat) 10:15-11:30 Room 7/(Hotel Nikko Kanazawa 3F Kujaku B) Room 7

[Chairperson]Mitsuru Futakuchi (Dept. of Pathol. Faculty of Med. Yamagata Univ. )

---

### J-3009
### Utilization of an Extracellular Vesicle Encapsulated RNA Panel as a Liquid Biopsy for the Diagnosis of Pancreatic Cancer

[Speaker]Kenji Takahashi (Div. of Gastroenterology, Dept. of Internal Medicine, Asahikawa Medical University) [Co-author]Fumi Asai (H.U. Group Research Inst. G.K.)、Yuta Shimizu (H.U. Group Research Inst. G.K.)、Kazuya Koyama (Div. of Gastroenterology, Dept. of Internal Medicine, Asahikawa Medical University)、Yu Otaki (Div. of Gastroenterology, Dept. of Internal Medicine, Asahikawa Medical University)、Ayari Kozuka (H.U. Group Research Inst. G.K.)、Haruna Kawajiri (Div. of Gastroenterology, Dept. of Internal Medicine, Asahikawa Medical University)、Tetsuhiro Okada (Div. of Gastroenterology, Dept. of Internal Medicine, Asahikawa Medical University)、Hidemasa Kawabata (Div. of Gastroenterology, Dept. of Internal Medicine, Asahikawa Medical University)、Hidetaka Iwamoto (Div. of Gastroenterology, Dept. of Internal Medicine, Asahikawa Medical University)、Yohei Kitano (Div. of Gastroenterology, Dept. of Internal Medicine, Asahikawa Medical University)、Yusuke Ono (Inst. of Biomedical Research, Sapporo Higashi Tokushukai Hospital)、Tatsutoshi Inuzuka (H.U. Group Research Inst. G.K.)、Mikihiro Fujiya (Div. of Gastroenterology, Dept. of Internal Medicine, Asahikawa Medical University)、Yusuke Mizukami (Div. of Gastroenterology, Dept. of Internal Medicine, Asahikawa Medical University)

Circulating extracellular vesicle (EV) encapsulated RNAs show potential as pancreatic ductal adenocarcinoma (PDAC) biomarkers. We have recently developed a novel immunoprecipitation method called EViSTEP$^{R}$ to isolate EVs from body fluids. We aimed to identify candidate genes using our system to establish an EV-RNA panel for PDAC diagnosis. Serum EVs from healthy controls (HC, n=29), IPMN (n=28), and PDAC patients (n=25) were isolated via EViSTEP. EV-RNA sequencing was performed using Illumina Nextseq 550, followed by bioinformatics analysis. Twelve RNAs, including CXCL8 and G0S2, were significantly upregulated in PDAC compared to HC/IPMN. Four RNAs were elevated in stage I PDAC. Individual RNA diagnostic accuracy (AUC in ROC curve) ranged 0.55-0.74; the combination of 12 RNAs achieved AUC 0.83 and when combined with CA19-9, AUC increased to 0.98. Moreover, we examined the gene expression signatures of IPMN samples with and without worrisome features (WF). Five RNAs showed increased levels in IPMN with WF. Identified PDAC-specific EV encapsulated RNAs would contribute to a clinical diagnostic panel for early PDAC detection.

[Disclosure of COI by Responsible researcher] Lead presenter is the Responsible researcher.
[Lead presenter: Kenji Takahashi] ■Research expenses: HUグループ中央研究所、日立ハイテク社

### [J-3010] I-J12-4
### Diagnosis by expression analysis

2025 / 9 / 27 (Sat) 10:15-11:30 Room 7/(Hotel Nikko Kanazawa 3F Kujaku B) Room 7

[Chairperson]Mitsuru Futakuchi (Dept. of Pathol. Faculty of Med. Yamagata Univ. )

---

### J-3010
### Mass spectrometry-based exploration of glycan biomarkers for predicting cancer immunotherapy response

[Speaker]Yoshimi Haga (Div. Analytical Biochemistry, CPM Ctr., JFCR) [Co-author]Kengo Takeuchi (Div. Pathology, Cancer Inst., JFCR／Pathology Project for Molecular Targets, Cancer Inst., JFCR／Dept. Pathology, Cancer Institute Hospital, JFCR)、Koji Ueda (Div. Analytical Biochemistry, CPM Ctr., JFCR)

Cancer immunotherapy targeting immune checkpoint molecules is a promising approach for cancer treatment. However, the difficulty in predicting therapeutic efficacy and the occurrence of severe irAEs in some patients remain significant challenges. To improve patient outcomes, accurate companion diagnostics capable of predicting treatment response before administration are essential.We aim to develop a reliable biomarker for predicting the efficacy of anti-PD-L1 antibodies, based on the hypothesis that variations in PD-L1 N-glycan structures influence antibody binding. Using ultra-sensitive mass spectrometry, we analyzed glycan heterogeneity of PD-L1 and identified glycan structures in lung cancer cell lines and patient tissues that may affect drug response. Furthermore, we found that the frequency of specific glycan groups on PD-L1 significantly correlates with OS or PFS in patients treated with anti-PD-L1 therapies.These findings suggest that site-specific glycan analysis of PD-L1 may provide an important basis for development of companion diagnostics, enabling better prediction of efficacy and patient stratification before treatment.

[Disclosure of COI by Responsible researcher] Lead presenter is not the Responsible researcher.
[Lead presenter: Yoshimi Haga] ■Research funds from company: AMED 次世代がん医療加速化研究事業
[Principal Researcher: Kengo Takeuchi] We have no financial relationships to disclose.

### [J-3011] I-J12-4
### Diagnosis by expression analysis

2025 / 9 / 27 (Sat) 10:15-11:30 Room 7/(Hotel Nikko Kanazawa 3F Kujaku B) Room 7

[Chairperson]Mitsuru Futakuchi (Dept. of Pathol. Faculty of Med. Yamagata Univ. )

---

### J-3011
### Identification of Disease-Relevant Tumor Subpopulations in Pancreatic Cancer Microenvironment Using AbSeq Profiling

[Speaker]Takahiro Tomiyama (Natl Inst of Biomed Innovation, Health & Nutrition (NIBN) [Co-author]Michihiro Aomatsu (Natl Inst of Biomed Innovation, Health & Nutrition (NIBN))、Shokichi Takahama (Natl Inst of Biomed Innovation, Health & Nutrition (NIBN))、Yuta Ngatsuka (Natl Inst of Biomed Innovation, Health & Nutrition (NIBN))、Hirotomo Murakami (Natl Inst of Biomed Innovation, Health & Nutrition (NIBN))、Masahiko Kubo (Dept of Gastroenterological Surgery, Osaka International Cancer Institute)、Kunihito Gotoh (Dept of Gastroenterological Surgery, Osaka International Cancer Institute)、Hirofumi Akita (Dept of Gastroenterological Surgery, Osaka International Cancer Institute／Dept of Gastroenterological Surgery, Osaka Univ Grad Med Sch)、Yuichiro Doki (Dept of Gastroenterological Surgery, Osaka Univ Grad Med Sch)、Hidetoshi Eguchi (Dept of Gastroenterological Surgery, Osaka Univ Grad Med Sch)、Takuya Yamamoto (Natl Inst of Biomed Innovation, Health & Nutrition (NIBN)／Lab of Aging & Immune Regulation, Osaka Univ Pharm Sci／Dept of Virology & Immunology, Osaka Univ Grad Med Sch)

Background: Pancreatic cancer is highly lethal, and new therapies are urgently needed. mRNA vaccines show promise, but only half of patients benefit. Identifying tumor antigens and understanding subpopulations are key. Conventional scRNA-seq lacks surface protein data, limiting utility.Methods: Tumor specimens from patients receiving neoadjuvant chemo(radio)therapy were processed into single-cell suspensions. A cocktail of 142 AbSeq antibodies was applied, and BD Rhapsody was used to obtain transcriptomic and surface protein data.Results: Of 142 antibodies, 141 were detected. Cell proportions were consistent between AbSeq and flow cytometry. AbSeq clustering revealed six CD45- and three CD45+ clusters. CD45- clusters expressed EpCAM and were enriched in cell cycle and EMT pathways; CD45+ clusters showed immune-related enrichment. Reclustering identified seven tumor subgroups with distinct surface profiles: epithelial-like, stem-like, immune-evasive, and proliferative EMT-type. Each subgroup had unique tumor antigens and functional diversity. Many matched fluorescent antibodies, enabling validation by high-parameter flow cytometry and sorting to identify therapeutic targets.

[Disclosure of COI by Responsible researcher] Lead presenter is not the Responsible researcher.
[Lead presenter: Takahiro Tomiyama] We have no financial relationships to disclose.
[Principal Researcher: Takuya Yamamoto] ■Research expenses: BD

▲TOP

### [J-3012] I-J12-4
### Diagnosis by expression analysis

2025 / 9 / 27 (Sat) 10:15-11:30 Room 7/(Hotel Nikko Kanazawa 3F Kujaku B) Room 7

[Chairperson]Mitsuru Futakuchi (Dept. of Pathol. Faculty of Med. Yamagata Univ. )

---

### J-3012
### Gene expression profiling of metaplastic breast carcinoma using spatial transcriptomics

[Speaker]Masashi Akiya (Div. Path., Cancer Inst. JFCR／Dept. Path., Cancer Inst. Hosp., JFCR) [Co-author]Asumi Iesato (Cancer Cell Diversity Prj., NEXT-Ganken Program, Cancer Inst., JFCR)、Kazutaka Otsuji (Cancer Cell Diversity Prj., NEXT-Ganken Program, Cancer Inst., JFCR)、Sumito Saeki (Div. Cancer Epigenomics, Cancer Inst., JFCR)、Takayuki Ueno (Dept. Breast Surg., Cancer Inst. Hosp., JFCR)、Tomo Osako (Div. Path., Cancer Inst. JFCR／Dept. Path., Cancer Inst. Hosp., JFCR)、Reo Maruyama (Cancer Cell Diversity Prj., NEXT-Ganken Program, Cancer Inst., JFCR／Div. Cancer Epigenomics, Cancer Inst., JFCR)

Background: Metaplastic breast carcinoma (MpBC) is a rare and aggressive breast cancer subtype that is further classified into histologic subtypes such as spindle cell carcinoma (SpCC) and matrix-producing carcinoma (MTXC) based on the type of metaplastic differentiation. Due to the complexity of its histologic architecture, limited progress has been made in elucidating its molecular basis. We employed spatial transcriptomics to explore the gene expression profiles of MpBC.Methods: This study focused on SpCC and MTXC. Two cases of each subtype were selected, each containing areas of metaplastic and non-metaplastic components. Spatial transcriptomic analysis was performed on all 4 areas.Results: In the comparison between metaplastic and non-metaplastic components, 352 and 220 genes were upregulated in MTXC cases, and 2015 and 530 genes were upregulated in SpCC cases, respectively. Twelve genes were commonly upregulated across all 4 cases, and these genes were enriched in pathways associated with extracellular matrix composition and degradation.Conclusion: We identified genes that were highly expressed in MpBC. These genes may provide new insights into the molecular basis of MpBC.

[Disclosure of COI by Responsible researcher] Lead presenter is the Responsible researcher.
[Lead presenter: Masashi Akiya] We have no financial relationships to disclose.

### [J-3013] I-J10-3
### Cancer stem cells (2)

2025 / 9 / 27 (Sat) 10:15-11:30 Room 9/(Hotel Nikko Kanazawa 3F Lumiere) Room 9

[Chairperson]Chitose Oneyama (Div. of Cancer Cell Reg., Aichi Cancer Ctr. Res. Inst.)

---

### J-3013
### Asymmetric cell division of ALDH1-positive cancer stem cells generates glycolytic metabolically diverse cell populations

[Speaker]Shoma Tamori (Pharm., Tokyo Univ. of Sci) [Co-author]Takahiro Kasai (Pharm., Tokyo Univ. of Sci)、Shigeo Ohno (Inst. Disease of Old Age, Juntendo Univ. Sch. of Med)、Kazunori Sasaki (Inst. Disease of Old Age, Juntendo Univ. Sch. of Med)、Kazunori Akimoto (Pharm., Tokyo Univ. of Sci)

Metabolic heterogeneity in cancer cells within a tumor promotes resistance to therapy and enhances recurrence and metastasis. However, the mechanisms underlying this heterogeneity remain unclear. Here, we show that PKCλ-dependent asymmetric division of ALDH1-positive cancer stem cells (CSCs) results in uneven glycolytic capacity, providing insight into metabolic heterogeneity. The rate-limiting enzyme PFKP and glycolytic probe CDG codistributed with ALDH1A3 after cell division, suggesting a mechanism for generating metabolic diversity. We identified 28 distinct distribution patterns of PFKP and CDG, reflecting the complexity of this heterogeneity. PKCλ deficiency reduced the asymmetric distribution of these proteins in $ALDH1^{high}$ cells, indicating a pivotal role of PKCλ in glycolytic heterogeneity. Moreover, a probabilistic model validated that PKCλ deficiency decreased glycolytic diversity in cancer cell colonies derived from ALDH1-positive CSCs. These findings suggest that PKCλ-dependent asymmetric division is essential for glycolytic heterogeneity in tumor cells, offering potential therapeutic targets to overcome tumor resistance and metastasis.

[Disclosure of COI by Responsible researcher] Lead presenter is not the Responsible researcher.
[Lead presenter: Tamori Shoma] We have no financial relationships to disclose.
[Principal Researcher: Kazunori Akimoto] We have no financial relationships to disclose.

### [J-3014] I-J10-3
### Cancer stem cells (2)

2025 / 9 / 27 (Sat) 10:15-11:30 Room 9/(Hotel Nikko Kanazawa 3F Lumiere) Room 9

[Chairperson]Chitose Oneyama (Div. of Cancer Cell Reg., Aichi Cancer Ctr. Res. Inst.)

---

### J-3014
### Expression of ALDH1 during serial transplantation of PDX for small bowel adenocarcinoma

[Speaker]Yuri Nishioka (Dept. of Frontier Surg., Grad. Sch. of Med., Chiba Univ.) [Co-author]Yasunori Matsumoto (Dept. of Frontier Surg., Grad. Sch. of Med., Chiba Univ.)、Takeshi Toyozumi (Dept. of Frontier Surg., Grad. Sch. of Med., Chiba Univ.)、Koichiro Okada (Dept. of Frontier Surg., Grad. Sch. of Med., Chiba Univ.)、Nobufumi Sekino (Dept. of Frontier Surg., Grad. Sch. of Med., Chiba Univ.)、Tadashi Shiraishi (Dept. of Frontier Surg., Grad. Sch. of Med., Chiba Univ.)、Kazuya Kinoshita (Dept. of Frontier Surg., Grad. Sch. of Med., Chiba Univ.)、Tenshi Makiyama (Dept. of Frontier Surg., Grad. Sch. of Med., Chiba Univ.)、Masanari Yamada (Dept. of Frontier Surg., Grad. Sch. of Med., Chiba Univ.)、Akane Morimoto (Dept. of Frontier Surg., Grad. Sch. of Med., Chiba Univ.)、Hisahiro Matsubara (Dept. of Frontier Surg., Grad. Sch. of Med., Chiba Univ.)

Background: Small bowel adenocarcinoma (SBA) is a rare malignancy with poor prognosis and no standard treatment. Cancer stemness is associated with recurrence and resistance, and is essential for establishing patient-derived xenograft (PDX). We established PDXs of SBA and analyzed expression of ALDH1, a known cancer stem cell marker, during serial transplantation.
Materials and Methods: Female BALB/c Slc-nu/nu mice (6-8 weeks old) were used for PDX establishment. The first passage was defined as F0. ALDH1 expression in PDX tissues at the early passage (F1) and late passage (F5) was analyzed by qRT-PCR.
Results and Discussion: The PDX establishment rate for SBA was 67% (4/6), higher than that for gastric cancer (18%, 2/11). Four PDXs (PDX5, 14, 15, 16) retained similar histological features to the original tumors on HE staining. STR analysis confirmed genetic identity with the original tumors. Relative ALDH1 expression (F5/F1) were as follows: PDX5: 6.13, P=0.0006; PDX14: 0.90, P=0.58; PDX15: 4.46, P=0.0006; PDX16: 2.16, P=0.046. ALDH1 was elevated in the F5 PDX tissues in 3/4 cases, suggesting that stemness may be enriched during serial transplantation.

[Disclosure of COI by Responsible researcher] Lead presenter is not the Responsible researcher.
[Lead presenter: Yuri Nishioka] We have no financial relationships to disclose.
[Principal Researcher: Hisahiro Matsubara] We have no financial relationships to disclose.

▲TOP

### [J-3015] I-J10-3
### Cancer stem cells (2)

2025 / 9 / 27 (Sat) 10:15-11:30 Room 9/(Hotel Nikko Kanazawa 3F Lumiere) Room 9

[Chairperson]Chitose Oneyama (Div. of Cancer Cell Reg., Aichi Cancer Ctr. Res. Inst.)

---

### J-3015
### Regulation of γ-glutamylcyclotransferase (GGCT) expression and anti-proliferative effect by its knockdown

[Speaker]Kozue Nose (Lab. of Clinical Oncology, Kyoto Pharm. Univ.) [Co-author]Keiko Taniguchi (Kyoto Prefectural Univ of Med.)、Mitsugu Fujita (Faculty of Med, Kindai Univ.)、Chiami Moyama (Lab. of Clinical Oncology, Kyoto Pharm. Univ.)、Masaya Mori (Lab. of Clinical Oncology, Kyoto Pharm. Univ.)、Mizuki Ishita (Lab. of Clinical Oncology, Kyoto Pharm. Univ.)、Tatsushi Yoshida (Kyoto Prefectural Univ of Med.)、Hiromi Ii (Lab. of Clinical Oncology, Kyoto Pharm. Univ.)、Toshiyuki Sakai (Kyoto Prefectural Univ of Med.)、Susumu Nakata (Lab. of Clinical Oncology, Kyoto Pharm. Univ.)

Glioblastoma stem cells (GSCs) have been reported to cause the glioblastoma aggressiveness. γ-Glutamylcyclotransferase (GGCT) is highly expressed in various cancer cells including glioblastoma, and knockdown of GGCT inhibits proliferation of cancer cells. However, its function in GSCs is unknown. In this study, we used GSCs established from a murine glioblastoma model and human glioblastoma cells (U251, A172). We performed phenotypic analyses by target knockdown using RNAi. Knockdown of c-Jun reduced expression levels of GGCT and the activity of the GGCT promoter via the AP-1 consensus sequence. GGCT knockdown significantly inhibited proliferation and tumor growth of the GSCs. Microarray analyses identified the Delta-Notch signaling pathway as a pathway significantly suppressed by GGCT knockdown. Knockdown of Notch1 also significantly inhibited proliferation of GSCs. Overexpression of N1ICD significantly restored the antiproliferative effects by GGCT knockdown. Moreover, event-free survival of the transplanted glioblastoma mouse model was significantly prolonged by GGCT knockdown. These results suggest that GGCT may represent a novel therapeutic target for attacking GSCs.

[Disclosure of COI by Responsible researcher] Lead presenter is not the Responsible researcher.
[Lead presenter: Kozue Nose] We have no financial relationships to disclose.
[Principal Researcher: Susumu Nakata] We have no financial relationships to disclose.

### [J-3016] I-J10-3
### Cancer stem cells (2)

2025 / 9 / 27 (Sat) 10:15-11:30 Room 9/(Hotel Nikko Kanazawa 3F Lumiere) Room 9

[Chairperson]Chitose Oneyama (Div. of Cancer Cell Reg., Aichi Cancer Ctr. Res. Inst.)

---

### J-3016
### Ferroptosis sensitivity in adipose-like differentiated cells derived from mesenchymal breast cancer cells.

[Speaker]Aina Tsujihana (Faculty of Pharmaceutical Sciences Doshisha Woman's College of Liberal Arts／Graduate School of Pharmaceutical Sciences DWCLA) [Co-author]Ukina Isomoto (Faculty of Pharmaceutical Sciences Doshisha Woman's College of Liberal Arts)、Momoko Kashiwagi (Faculty of Pharmaceutical Sciences Doshisha Woman's College of Liberal Arts)、Asahi Sami (Faculty of Pharmaceutical Sciences Doshisha Woman's College of Liberal Arts)、Tamami Fujiwara (Faculty of Pharmaceutical Sciences Doshisha Woman's College of Liberal Arts)、Karin Matsumoto (Faculty of Pharmaceutical Sciences Doshisha Woman's College of Liberal Arts)、Matsuri Matsuoka (Faculty of Pharmaceutical Sciences Doshisha Woman's College of Liberal Arts)、Sayaka Aoki (Faculty of Pharmaceutical Sciences Doshisha Woman's College of Liberal Arts)、Rei Takahashi (Faculty of Pharmaceutical Sciences Doshisha Woman's College of Liberal Arts)、Kiyotsugu Yoshikawa (Faculty of Pharmaceutical Sciences Doshisha Woman's College of Liberal Arts)

[Objective]EMT-derived mesenchymal breast cancer cells possess stem-like features and are resistant to therapy. Our prior study showed that epithelial induction alone does not suppress growth, and PPAR$\gamma$ mRNA is highly expressed in mesenchymal cells. We investigated the link between adipocyte-like differentiation, epithelial features, and ferroptosis sensitivity.[Methods]SUM159 and H-Ras-transduced HMLE (HMLER) cells were treated with rosiglitazone to activate PPAR$\gamma$. Epithelialization was induced via shRNA. EMT status was assessed by CD44/CD24 and E-cad/Vim promoter reporters. Adipocyte-like differentiation was measured via CD36 and BODIPY uptake. RNA-seq analyzed gene expression changes.[Results]Rosiglitazone increased CD36 and BODIPY uptake. Epithelialization and adipocyte-like differentiation were inversely correlated. Epithelial status did not clearly correlate with ferroptosis sensitivity. Adipocyte-like differentiation did not affect SUM159 cells but reduced ferroptosis sensitivity in mesenchymal HMLER cells. RNA-seq suggested altered PUFA and MUFA enzyme expression may underlie this phenotype.

[Disclosure of COI by Responsible researcher] Lead presenter is not the Responsible researcher.
[Lead presenter: Aina Tsujihana] We have no financial relationships to disclose.
[Principal Researcher: Kiyotsugu Yoshikawa] We have no financial relationships to disclose.

### [J-3017] I-J10-3
### Cancer stem cells (2)

2025 / 9 / 27 (Sat) 10:15-11:30 Room 9/(Hotel Nikko Kanazawa 3F Lumiere) Room 9

[Chairperson]Chitose Oneyama (Div. of Cancer Cell Reg., Aichi Cancer Ctr. Res. Inst.)

---

### J-3017
### Contribution between breast cancer stem cells and cancer-associated fibroblasts in breast cancer formation.

[Speaker]Risako Okada (Yamaguchi Univ. Grad. Sch. of Med.) [Co-author]Kana Tominaga (Yamaguchi Univ. Grad. Sch. of Med.)、Naoomi Tominaga (Yamaguchi Univ. Grad. Sch. of Med.)

Growth factors secreted by cancer stem cells help maintain their undifferentiated state by activating the tumor microenvironment, including fibroblasts and endothelial cells. The Neuropilin 1 (Nrp1) receptor, specifically expressed in breast cancer stem-like cells (BCSCs), is involved in their symmetric division and tumor progression. Cancer-associated fibroblasts (CAFs) are also related to promote cancer invasion and metastasis. Here, to solve the relationship between BCSCs and CAFs during the tumor progression, we tried to investigate whether CAFs interacted with Nrp1-positive BCSCs. First, we analyzed three breast cancer cell lines to confirm the BCSCs population, Nrp1/Numb double positive cells. Next, cultured cells were sorted into Nrp1-positive and Nrp1-negative populations using flow cytometry, and then transplanted into immunodeficient mice. Nrp1-positive BCSCs had high tumor formation compared to Nrp1-negative cells and CAFs interacted with Nrp1-positive BCSCs were high proliferation rates. Finally, RNA-seq revealed five genes were upregulated in CAFs from tumors transplanted with Nrp1-positive BCSCs. Those were possible to be key factors contributing to cancer malignancy.

[Disclosure of COI by Responsible researcher] Lead presenter is not the Responsible researcher.
[Lead presenter: Risako Okada] We have no financial relationships to disclose.
[Principal Researcher: Kana Tominaga, Naoomi Tominaga] ■employment by a company related to the presentation: 株式会社ADDVEMO（取締役） ■Profit of stock: 株式会社ADDVEMO

▲TOP

### [J-3018] I-J10-3
### Cancer stem cells (2)

2025 / 9 / 27 (Sat) 10:15-11:30 Room 9/(Hotel Nikko Kanazawa 3F Lumiere) Room 9

[Chairperson]Chitose Oneyama (Div. of Cancer Cell Reg., Aichi Cancer Ctr. Res. Inst.)

---

### J-3018
### Development of Neuroblastoma Tumorigenesis Model from MYCN/ALK-Overexpressing Cranial Neural Crest Cells

[Speaker]Kyosuke Mukae (Res. Inst. for Clin. Oncol., Saitama Cancer Ctr.) [Co-author]Tianyuan Shi (Res. Inst. for Clin. Oncol., Saitama Cancer Ctr.／Dept. Graduate School of Science and Engineering., Saitama Univ.)、Maya Shindo (Res. Inst. for Clin. Oncol., Saitama Cancer Ctr.／Dept. Graduate School of Science and Engineering., Saitama Univ.)、Ritsuko Onuki (Res. Inst. for Clin. Oncol., Saitama Cancer Ctr.)、Atsuko Nakazawa (Dep. of Clin. Res., Saitama Children's Medical Ctr.)、Hisanori Takenobu (Res. Inst. for Clin. Oncol., Saitama Cancer Ctr.)、Miki Ohira (Res. Inst. for Clin. Oncol., Saitama Cancer Ctr.)、Takehiko Kamijo (Res. Inst. for Clin. Oncol., Saitama Cancer Ctr.)

Neuroblastoma (NB) is a pediatric solid tumor arising from neural crest cells (NCCs), precursors of the sympathetic nervous system. MYCN amplification is a key factor in poor prognosis and is strongly linked to aggressive progression. ALK mutations and amplifications drive oncogenic pathways, such as MAPK signaling, further increasing malignancy under MYCN expression. NCCs are mainly classified into cranial NCCs (cNCCs) and trunk NCCs (tNCCs), and recent studies have reported NB development from tNCCs. However, the potential for NB development from cNCCs remains unexplored. In this study, we established a NB model using human iPSC-derived cNCCs overexpressing MYCN and ALK. Upon subcutaneous injection into immunodeficient mice, these cells formed NB-like tumors, demonstrating their utility as an in vitro model. Gene expression profiling and whole-exome sequencing revealed key NB features, including inhibition of cell death, activation of proliferative pathways, mutations in NB-related genes, and 17q gain. This model provides a valuable platform for studying ALK/MYCN-driven tumorigenesis in cNCC-derived NB and exploring therapeutic approaches to improve patient outcomes.

[Disclosure of COI by Responsible researcher] Lead presenter is not the Responsible researcher.
[Lead presenter: Kyosuke Mukae] We have no financial relationships to disclose.
[Principal Researcher: Takehiko Kamijo] We have no financial relationships to disclose.

### [J-3025] I-J16
### Radiation therapy

2025 / 9 / 27 (Sat) 10:15-11:30 Room 14/(ANA Crowne Plaza Hotel Kanazawa 3F Zuiun 1) Room 14

[Chairperson]Kiyoshi Miyagawa (Lab. Mol. Radiol., Grad. Sch. Med., Univ. Tokyo)

---

### J-3025
### Spatiotemporal metabolomic mapping of the brains of mouse glioma model treated with Boron Neutron Capture Therapy

[Speaker]Natsuko Kondo (Institute for Integrated Radiation and Nuclear Science, Kyoto University) [Co-author]Nao Kobayashi (Faculty of Life and Medical Sciences, Doshisha University)、Yoshinori Sakurai (Institute for Integrated Radiation and Nuclear Science, Kyoto University)、Minoru Suzuki (Institute for Integrated Radiation and Nuclear Science, Kyoto University)、Shinici Yamaguchi (SHIMADZU Corporation)、Masaya Ikegawa (Faculty of Life and Medical Sciences, Doshisha University)

Glioblastoma is the most lethal primary brain tumor. Boron Neutron Capture Therapy (BNCT) is a tumor-selective particle radiation therapy in which high-LET $\alpha$-particles and recoil $^{7}Li$ are produced by the capture reaction between $^{10}B$ and thermal neutrons, and it has extended the prognosis of glioblastomas. Purine and pyrimidine are fundamental energy source of tumor cells and metabolites of them function as critical signaling. However, the effects of BNCT on their metabolism in glioma and surrounding normal brain remain unknown. We performed BNCT on C57BL/6 mice orthotopically implanted with GL261 glioma cells and conducted a spatial investigation of the metabolites in the brains 2, 7, and 14 days after BNCT, using mass spectrometry imaging (MSI) and immunohistochemistry. Histopathological analysis revealed a tumor mass extinction after BNCT. We visualized tumor-specific metabolites such as UDP-GlucNac, ADP, etc. Brain adenylate metabolites elevated in the normal brain after BNCT which decreased with time, while they decreased inside the tumor after BNCT. MSI mapped the specific metabolites reflecting tumor dynamics, BNCT efficacy and response of normal brain after BNCT.

[Disclosure of COI by Responsible researcher] Lead presenter is the Responsible researcher.
[Lead presenter: Natsuko Kondo] We have no financial relationships to disclose.

### [J-3026] I-J16
### Radiation therapy

2025 / 9 / 27 (Sat) 10:15-11:30 Room 14/(ANA Crowne Plaza Hotel Kanazawa 3F Zuiun 1) Room 14

[Chairperson]Kiyoshi Miyagawa (Lab. Mol. Radiol., Grad. Sch. Med., Univ. Tokyo)

---

### J-3026
### STING pathway activation enhances anti-tumor effect of photodynamic therapy through reactive oxygen species production

[Speaker]Makiko Sasaki (Nagoya City Univ. Gastroenterology & Metabolism Dept.) [Co-author]Mamoru Tanaka (Nagoya City Univ. Gastroenterology & Metabolism Dept.)、Yasunari Sasaki (Nagoya City Univ. Gastroenterology & Metabolism Dept.)、Yuki Kojima (Nagoya City Univ. Gastroenterology & Metabolism Dept.)、Taketo Suzuki (Nagoya City Univ. Midorimunicipal Hosp. Gastroenterology Dept.)、Hirotada Nishie (Nagoya City Univ. Midorimunicipal Hosp. Gastroenterology Dept.)、Takaya Shimura (Nagoya City Univ. Gastroenterology & Metabolism Dept.)、Eiji Kubota (Nagoya City Univ. Gastroenterology & Metabolism Dept.)、Hiromi Kataoka (Nagoya City Univ. Gastroenterology & Metabolism Dept.)

**BACKGROUND&AIM** Photodynamic therapy (PDT) is an anti-cancer treatment to generate ROS and induce tumor cell death. STING activation is a key immunotherapeutic strategy, but its role in ROS regulation remains unclear. We hypothesized that STING controls ROS generation in PDT, and its loss alters ROS homeostasis leading to therapeutic resistance. **METHODS** STING KO HCT116 cells were established to compare talaporfin sodium (TS)-PDT efficacy with parental cells. Cell death (MTT assay), ROS generation (DCFDA assay), DNA damage (comet assay) and STING signaling activation (WB) were analyzed. The effect of combining STING agonists (cGAMP, ADU-S100) with TS-PDT was investigated ***in vivo***. **RESULTS** STING KO reduced TS-PDT-induced cell death (IC50: 12.5 vs. 14.5 $\mu$mol/L), ROS levels (fluorescence intensity: 4491 vs. 1706), and DNA damage (Total Comet Score; 86.0 vs. 41.3). TS-PDT activated STING signaling, but disappeared in KO cells. The combination of STING agonist and TS-PDT significantly inhibited tumor growth compared to monotherapy. **CONCLUSION** STING regulates ROS homeostasis and tumor susceptibility to PDT. STING agonists enhance PDT efficacy, suggesting potential for clinical applications.

[Disclosure of COI by Responsible researcher] Lead presenter is not the Responsible researcher.
[Lead presenter: Makiko Sasaki] We have no financial relationships to disclose.
[Principal Researcher: Mamoru Tanaka] We have no financial relationships to disclose.

▲TOP

### [J-3027] I-J16
### Radiation therapy

2025 / 9 / 27 (Sat) 10:15-11:30 Room 14/(ANA Crowne Plaza Hotel Kanazawa 3F Zuiun 1) Room 14

[Chairperson]Kiyoshi Miyagawa (Lab. Mol. Radiol., Grad. Sch. Med., Univ. Tokyo)

---

### J-3027
### Nonclinical study of the Combined Therapeutic Approach of Bortezomib and Carbon-Ion Irradiation

[Speaker]Nakako Nakajima (QST QST Hospital Department of Charged Particle Therapy Research) [Co-author]Sumitaka Hasegawa (QST QST Hospital Department of Charged Particle Therapy Research)、Takashi Shimokawa (QST Institute for Quantum Medical Science)

The local control rate of osteosarcoma treated with carbon-ion beam therapy ranges from 50% to 80%, indicating favorable therapeutic outcomes. Carbon-ion irradiation (CIR) induces DNA damage that is not repaired by classical non-homologous end-joining (cNHEJ), effectively leading proliferating tumor cells to cell death. However, in quiescent cells, the DNA damage induced by CIR is repaired slowly through the ATM-RNF8-53BP1-dependent NHEJ pathway, resulting in cancer cell regrowth. This study investigated the effect of the proteasome inhibitor bortezomib, which inhibits 53BP1 accumulation at sites of DNA damage, on the sensitization of osteosarcoma cells to CIR. Our findings indicate that bortezomib impedes DNA damage repair, reduces post-CIR cell repopulation rates in human osteosarcoma U2OS cells, and enhances the sensitivity of U2OS cells to CIR. In vivo experiments with C3H/He mice transplanted with LM8 cells demonstrated significant tumor growth suppression following combined treatment with bortezomib and CIR. These findings suggest that the integration of carbon-ion beam therapy with proteasome inhibitors may improve therapeutic efficacy in the treatment of osteosarcoma.

[Disclosure of COI by Responsible researcher] Lead presenter is the Responsible researcher.
[Lead presenter: Nakako Nakajima] We have no financial relationships to disclose.

### [J-3028] I-J16
### Radiation therapy

2025 / 9 / 27 (Sat) 10:15-11:30 Room 14/(ANA Crowne Plaza Hotel Kanazawa 3F Zuiun 1) Room 14

[Chairperson]Kiyoshi Miyagawa (Lab. Mol. Radiol., Grad. Sch. Med., Univ. Tokyo)

---

### J-3028
### Radiation Therapy Enhancement Using Magnetosome@RGD via Lymphatic Drug Delivery System for Metastatic Lymph Nodes

[Speaker]Taiki Shimano (Grad. Sch. of Biomed. Eng., Tohoku Univ.) [Co-author]Reito Miyazaki (Grad. Sch. of Biomed. Eng., Tohoku Univ.)、Ariunbuyan Sukhbaatar (Grad. Sch. of Biomed. Eng., Tohoku Univ.／Grad. Sch of Dent., Tohoku Univ.)、Sandra Preveral (CEA, SBVME)、Shiro Mori (Grad. Sch. of Biomed. Eng., Tohoku Univ.／Grad. Sch of Dent., Tohoku Univ.)、Georges Vassaux (IPMC (UMR6097, CNRS Univ. Cote d Azur))、Beatrice Cambien (TIRO (UMR E 4320 Univ. Cote d Azur)／Federation Claude Lalanne (Univ. Cote d Azur))、Tetsuya Kodama (Grad. Sch. of Biomed. Eng., Tohoku Univ.)

Radiotherapy is a standard cancer treatment, with 50% of patients receiving ionizing radiation. However, collateral damage to healthy tissue remains a significant drawback. Radioenhancers such as iron oxide nanoparticles can improve therapeutic effects at lower doses. Yet, for the treatment of metastatic lymph nodes (LNs), systemic delivery often fails clinically due to the tumor growth mediated loss of blood vessels and lymphatic sinuses, increased intranodal pressure, and the lack of tumor neovasculature.In this study, we applied a lymphatic drug delivery system (LDDS) to directly administer a bacteria-derived iron oxide nanoparticle, magnetosome@RGD, into the sentinel LN of a mouse LN metastasis model. This was followed by 2 Gy of localized X-ray irradiation.Treatment with magnetosome@RGD significantly enhanced the oncocidal effect of radiation on metastatic LNs. Moreover, it prevented the spread of metastasis to the lungs and liver.These findings suggest that combining LDDS with magnetosome@RGD-based radioenhancement offers a promising strategy to improve the efficacy and safety of radiotherapy in metastatic cancer.

[Disclosure of COI by Responsible researcher] Lead presenter is the Responsible researcher.
[Lead presenter: Taiki Shimano] We have no financial relationships to disclose.

▲TOP

Japanese Oral Session

### [J-3029] I-J16
### Radiation therapy

2025 / 9 / 27 (Sat) 10:15-11:30 Room 14/(ANA Crowne Plaza Hotel Kanazawa 3F Zuiun 1) Room 14

[Chairperson]Kiyoshi Miyagawa (Lab. Mol. Radiol., Grad. Sch. Med., Univ. Tokyo)

---

### J-3029
### Radiotheranostics of pentadecanoic acid conjugated $^{111}In/^{225}Ac$-RGD peptide

[Speaker]Mitsuyoshi Yoshimoto (NCC EPOC FID) [Co-author]Shunichi Wada (OUPS)、Yukie Yoshii (LinqMed Inc.)、Anri Inaki (NCC EPOC FID)、Hirofumi Fujii (NCC EPOC FID)

Too rapid clearance of peptides is unsuitable for peptide receptor radionuclide therapy. To overcome this, we developed RGD peptides with pentadecanoic acid (PD) as an albumin binding moiety. In this study, we investigated the pharmacokinetics and therapeutic effects of radiolabeled PD-conjugated RGD peptides. The conjugation of PD to RGD peptides significantly increased the binding ability to HSA. The HSA binding of PD-K($^{111}$In-DOTA)-RGD was higher than that of PD-K($^{111}$In-DOTA)-$RGD_2$. The biodistribution study indicated that the PD conjugation significantly increased the radioactivity in the blood and tumor and slowed the clearance. In the therapeutic experiment using BxPC-3 xenograft mice, PD-K($^{225}$n-DOTA)-RGD successfully suppressed tumor growth. The hepatic toxicity dose-dependently appeared though the renal toxicity was not obvious. In this study, the conjugation of PD to DOTA-c(RGDfK) successfully improved the pharmacokinetics of DOTA-c(RGDfK). In addition, PD-K($^{225}$Ac-DOTA)-RGD inhibited the tumor growth. These results suggested that PD-K($^{111}$In/$^{225}$Ac-DOTA)-RGD has great potential for radiotheranostics of pancreatic cancers.

[Disclosure of COI by Responsible researcher] Lead presenter is the Responsible researcher.
[Lead presenter: Mitsuysohi Yoshimoto] ■Research expenses: 日立製作所

▲TOP

### [J-3030] I-J16
### Radiation therapy

2025 / 9 / 27 (Sat) 10:15-11:30 Room 14/(ANA Crowne Plaza Hotel Kanazawa 3F Zuiun 1) Room 14

[Chairperson]Kiyoshi Miyagawa (Lab. Mol. Radiol., Grad. Sch. Med., Univ. Tokyo)

---

### J-3030
### Efficacy of the combination of ICG/Au/Resovist conjugated cell-permeable peptide and mild hyperthermia for oral cancer.

[Speaker]Shigeru Amano (Meikai Univ. Res. Inst. of Odontology) [Co-author]Kanya Ogura (Div. Oral and Maxillofacial Surg. Meikai Uni. Sch. of Dent.)、Hiroshi Sakagami (Meikai Univ. Res. Inst. of Odontology)、Shinji Yamamoto (Div. Oral and Maxillofacial Surg. Meikai Uni. Sch. of Dent.)、Kenji Mitsudo (Dept. of Oral and Maxillofacial Surg. Yokohama City Univ.)、Iwai Tohnai (Meikai Univ. Sch. of Dent.)

The cell-penetrating peptide (CPP) (JP6932340) binds to CD13 on the cell membrane and enters the cell cytoplasm, causing nuclear translocation and cell death in CD13-positive cancer cells but not in CD13-positive normal cells. Shotgun analysis of FITC-CPP interacting proteins revealed histones, ribosomal proteins, and B23 on FITC-CPP stimulated HT-1080 cells. FITC-CPP-induced apoptosis was inhibited by siB23 electroporation but not by siGFP, suggesting a role for B23 as a cancer cell-specific nuclear migration factor. Surgery is the first-line therapy for oral cancer, followed by combination therapy with radiotherapy or chemotherapy. However, patients' quality of life (QOL) often declines after surgery. Therefore, a new therapeutic strategy is required for oral cancer patients due to impaired QOL. The therapeutic efficacy of ICG and Au/Resovist-conjugated CPP was evaluated against Ca9-22 cells ***in vitro*** and ***in vivo***. The combination of CPP and mild hyperthermia may be a new cancer treatment method because of the appearance of a DNA ladder and cleaved caspase 3 and an increase in sub-G1 phase cells, thereby reducing tumor volume in mice bearing Ca9-22 cells.

[Disclosure of COI by Responsible researcher] Lead presenter is not the Responsible researcher.
[Lead presenter: Shigeru Amano] We have no financial relationships to disclose.
[Principal Researcher: Iwai Tohnai] We have no financial relationships to disclose.

▲TOP

### [E-3019] II-E1
### Brain tumor

2025 / 9 / 27 (Sat) 10:15-11:30 Room 15/(ANA Crowne Plaza Hotel Kanazawa 3F Zuiun 2) Room 15

[Chairperson]Akitake Mukasa (Dept. of Neurosurgery, Grad. Sch. of Med. Sci., Kumamoto Univ.)

---

### E-3019
### Dissecting p53 Regulation by Nuclear Pores in Glioblastoma: From Genomics to Nanoscale Imaging

[Speaker]Richard Wong (WPI-NanoLSI Kanazawa University)

Nuclear pore complexes (NPCs) are essential hubs for nucleocytoplasmic transport and emerging regulators of cancer progression. While NPC dysregulation is implicated in various cancers, its role in glioblastoma (GBM) remains unclear. We recently identified genomic amplification and overexpression of NUP107, a scaffold nucleoporin critical for NPC integrity, in human GBM. This amplification co-occurs with increased MDM2, an E3 ligase promoting p53 degradation. NUP107 depletion in GBM cells stabilizes p53 and suppresses tumor growth. Mechanistically, NPCs act as export-coupled platforms tethering the 26S proteasome to mediate p53 degradation. Loss of NUP107 disrupts NPC architecture and reduces perinuclear proteasome localization, impairing this degradation pathway. To explore NPC alterations at nanometer resolution, we are collaborating with neurosurgeons in our university hospital to apply high-speed atomic force microscopy (HS-AFM) to GBM cell lines and patient-derived samples. This ongoing work seeks to reveal GBM-specific changes in nuclear pore structure and dynamics, offering new nanobiological insights into p53 regulation and potential diagnostic strategies.

[Disclosure of COI by Responsible researcher] Lead presenter is the Responsible researcher.
[Lead presenter: Richard Wong] We have no financial relationships to disclose.

### [E-3020] II-E1
### Brain tumor

2025 / 9 / 27 (Sat) 10:15-11:30 Room 15/(ANA Crowne Plaza Hotel Kanazawa 3F Zuiun 2) Room 15

[Chairperson]Akitake Mukasa (Dept. of Neurosurgery, Grad. Sch. of Med. Sci., Kumamoto Univ.)

### E-3020
### Integrated Genomic and Transcriptomic Analysis Reveals Molecular Diversity and Novel Drivers in Japanese Medulloblastoma

[Speaker]Tsubasa Miyauchi (Div. of Brain Tumor Translational Res., Natl. Cancer Ctr. Inst.／Dept. of Neurosurgery, Keio Univ. Sch. of Med.)
[Co-author]Takuma Nakashima (Div. of Brain Tumor Translational Res., Natl. Cancer Ctr. Inst.)、Miho Kato (Natl. Ctr. for Child Health and Development)、Hajime Okita (Dept. of Diagnostic Path., Natl Cancer Ctr. Inst.)、Yonehiro Kanemura (Inst. for Clin. Res., Natl. Hosp. Organization Osaka Natl. Hosp.)、Yuko Watanabe (Dept. of Pediatric Oncology, Natl. Cancer Center Hosp.)、Masahiro Toda (Dept. of Neurosurgery, Keio Univ. Sch. of Med.)、Hiromichi Suzuki (Div. of Brain Tumor Translational Res., Natl. Cancer Ctr. Inst.)

Background: Medulloblastoma (MB) is the most common malignant pediatric brain tumor. While most genetic studies have primarily focused on Western populations, data on Japanese patients remain limited. To elucidate inter-patient and -ethnic heterogeneity, we analyzed 200 Japanese MB cases.
Methods: We conducted targeted DNA and RNA sequencing on Japanese Children's Cancer Group (JCCG) cases. Publicly available whole-genome and RNA sequencing from 462 Western MB patients were used for comparison.
Results: Subgroup-specific driver mutations were detected with no significant ethnic differences. A novel coding mutation was detected, albeit at a low frequency. It has previously been identified as a cancer driver in other tumor types, suggesting its oncogenic role. Prognostic analysis confirmed the significance of chromosomal aberrations, supporting genetic risk classification. Additionally, intronic mutations causing aberrant splicing were identified, demonstrating the benefit of integrating DNA and RNA analyses.
Discussion: Our findings reinforce MB's molecular heterogeneity and the need for genomic analysis to refine risk stratification.

[Disclosure of COI by Responsible researcher] Lead presenter is not the Responsible researcher.
[Lead presenter: Tsubasa Miyauchi] We have no financial relationships to disclose.
[Principal Researcher: Hiromichi Suzuki] We have no financial relationships to disclose.

### [E-3021] II-E1
### Brain tumor

2025 / 9 / 27 (Sat) 10:15-11:30 Room 15/(ANA Crowne Plaza Hotel Kanazawa 3F Zuiun 2) Room 15

[Chairperson]Akitake Mukasa (Dept. of Neurosurgery, Grad. Sch. of Med. Sci., Kumamoto Univ.)

---

### E-3021
### Identification of potentially novel therapeutic targets in supratentorial ependymoma

[Speaker]Zhize Xiao (Dept. of Neuro-oncology, Inst. of Brain Sci., Nagoya City Univ.／Grad. Sch. of Med. & Dent., Sci., Inst. of Sci.) [Co-author]Yukako Handa (Inst. for Clin. Res., NHO Osaka Natl. Hosp.)、Konstantin Okonechnikov (German Cancer Res. Ctr. (DKFZ) and Heidelberg Univ. Hosp.)、Owen Chapman (Dept. of Neuro-oncology, Inst. of Brain Sci., Nagoya City Univ.)、Naofumi Uesaka (Grad. Sch. of Med. & Dent., Sci., Inst. of Sci.)、Stefan M. Pfister (German Cancer Res. Ctr. (DKFZ) and Heidelberg Univ. Hosp.)、Kristian Pajtler (German Cancer Res. Ctr. (DKFZ) and Heidelberg Univ. Hosp.)、Yonehiro Kanemura (Inst. for Clin. Res., NHO Osaka Natl. Hosp.)、Daisuke Kawauchi (Dept. of Neuro-oncology, Inst. of Brain Sci., Nagoya City Univ.)

ZFTA fusion subtype is the most common and aggressive form of supratentorial ependymomas. Current treatments are limited to surgery and radiotherapy, underlining the urgent need for novel therapeutic targets. L1CAM is known as a surrogate marker for this tumor. To assess its functional role, we performed EdU and Annexin V assays after shRNA-mediated knockdown in the ZFTA fusion-positive EP1NS cell line. The results showed that L1CAM promotes tumor cell proliferation and survival. In line with a previous report linking L1CAM to Hippo signaling, our high throughput drug screening using an FDA-approved drug library identified a YAP1 inhibitor as a promising candidate. This compound significantly suppressed tumor growth both in vitro and in vivo. Also, L1CAM knockdown reduces nuclear translocation of YAP1. Together, our findings reveal a novel L1CAM YAP1 signaling axis in ZFTA fusion positive ST-EPNs and suggest that targeting YAP1 could be an effective strategy to inhibit tumor progression.

[Disclosure of COI by Responsible researcher] Lead presenter is not the Responsible researcher.
[Lead presenter: Zhize Xiao] We have no financial relationships to disclose.
[Principal Researcher: Zhize Xiao] We have no financial relationships to disclose.

▲TOP

### [E-3022] II-E1
### Brain tumor

2025 / 9 / 27 (Sat) 10:15-11:30 Room 15/(ANA Crowne Plaza Hotel Kanazawa 3F Zuiun 2) Room 15

[Chairperson]Akitake Mukasa (Dept. of Neurosurgery, Grad. Sch. of Med. Sci., Kumamoto Univ.)

### E-3022
### Inhibition of Anti-apoptotic Bcl-2 Family Members Induces Synergistic Cell death under ER-Stress in Glioblastoma

[Speaker]Tianyi Huang (Div. of Exp. Chemother. Cancer Chemother. Ctr. JFCR／GSFS, Tokyo Univ.) [Co-author]Satoshi Takagi (Div. of Exp. Chemother. Cancer Chemother. Ctr. JFCR)、Sumie Koike (Div. of Exp. Chemother. Cancer Chemother. Ctr. JFCR)、Ryohei Katayama (Div. of Exp. Chemother. Cancer Chemother. Ctr. JFCR／GSFS, Tokyo Univ.)

Glioblastoma (GBM) is the most aggressive type of brain tumor with remaining therapeutic difficulty, highlighting the urgent need for novel treatment.In this study, we used an in-house inhibitor library and a chemical library with over 350 compounds to identify promising targets for GBM. Obatoclax, a pan-Bcl-2 inhibitor, exclusively suppressed GBM cell growth, emphasizing the essential roles of Mcl-1 and Bcl-xL in GBM survival. Double knockdown of Mcl-1 and Bcl-xL further enhanced growth inhibition and apoptosis, with detectable mitochondrial depolarization. The anti-tumor activity of obatoclax was further synergistically enhanced when combined with several ER-stress inducers such as tunicamycin. We demonstrated that when combined with tunicamycin, obatoclax disrupted protective autophagy to enhance ER-stress, and thus upregulated ATF-4, a transcription factor promoting apoptosis under severe stress conditions. Upregulation of CHOP and Bim were also observed which potentially contributed to the synergistic cell death. Our findings reveal a unique cellular mechanism mediated via the ER-stress level and mitochondrial apoptosis, offering a potential therapeutic strategy for GBM.

[Disclosure of COI by Responsible researcher] Lead presenter is not the Responsible researcher.
[Lead presenter: Tianyi Huang] We have no financial relationships to disclose.
[Principal Researcher: Satoshi Takagi] We have no financial relationships to disclose.

▲TOP

### [E-3023] II-E1
### Brain tumor

2025 / 9 / 27 (Sat) 10:15-11:30 Room 15/(ANA Crowne Plaza Hotel Kanazawa 3F Zuiun 2) Room 15

[Chairperson]Akitake Mukasa (Dept. of Neurosurgery, Grad. Sch. of Med. Sci., Kumamoto Univ.)

---

### E-3023
### Prediction of Malignant Transformation in IDH-mutant Glioma Using Pre- and Postoperative Tumor Volumes

[Speaker]Kosuke Aoki (Department of Neurosurgery, Nagoya University Graduate School of Medicine／Department of Neurosurgery, Kasugai Municipal Hospital) [Co-author]Tomohide Nishikawa (Department of Neurosurgery, Nagoya University Graduate School of Medicine)、Fumiharu Ohka (Department of Neurosurgery, Nagoya University Graduate School of Medicine)、Shoichi Deguchi (Department of Neurosurgery, Nagoya University Graduate School of Medicine／Division of Neurosurgery, Shizuoka Cancer Center Hospital)、Sachi Maeda (Department of Neurosurgery, Nagoya University Graduate School of Medicine)、Yoshiki Shiba (Department of Neurosurgery, Nagoya University Graduate School of Medicine)、Hideo Nakamura (Department of Neurosurgery, Kumamoto University／Department of Neurosurgery, Kurume University)、Masamichi Takahashi (Department of Neurosurgery and Neuro-oncology, National Cancer Center Hospital／Department of Neurosurgery, Tokai University)、Yoshitaka Narita (Department of Neurosurgery and Neuro-oncology, National Cancer Center Hospital)、Mitsutoshi Nakada (Department of Neurosurgery, Kanazawa University, Kanazawa)、Masahiro Mizoguchi (Department of Neurosurgery, Graduate School of Medical Sciences Kyushu University／Department of Neurosurgery, National Hospital Organization Kyushu Medical Center)、Yosutomo Momii (Department of Neurosurgery, Oita University)、Yoshihiro Muragaki (Department of Neurosurgery, Tokyo Women's Medical University／Department of Medical Device Engineering, Kobe University)、Tatsuya Abe (Department of Neurosurgery, Faculty of Medicine, Saga University)、Hiroshi Haeno (Research Institute for Biomedical Sciences, Tokyo University of Science)、Ryuta Saito (Department of Neurosurgery, Nagoya University Graduate School of Medicine)

Low-grade gliomas with IDH mutations (IDHmut-LGGs) exhibit slow progression, but frequently undergo malignant transformation (MT) during the disease course. Previously, we demonstrated that IDHmut-LGGs follow an exponential growth pattern, and the risk of MT varies with treatment modalities, using a tumor volume-based model. In this study, we simplified this model by predicting tumor progression and MT risk using only pre- and postoperative tumor volumes as inputs. We applied this model to 269 cases of IDHmut-LGG (118 oligodendrogliomas and 151 astrocytomas). We compared the simplified model with a model that used all tumor volume data, and with a model that only incorporated the number of days since diagnosis. The results showed that the simplified model accurately predicted the MT timing, with performance comparable to the full volume-based model and superior to the days-based model. Our findings suggest that the timing of MT in IDHmut-LGGs can be predicted with high precision using this simplified approach. We are currently developing a software tool to predict MT timing using only pre- and postoperative tumor volumes, which can aid in predicting MT timing for each case.

[Disclosure of COI by Responsible researcher] Lead presenter is the Responsible researcher.
[Lead presenter: Kosuke Aoki] We have no financial relationships to disclose.

### [E-3024] II-E1
### Brain tumor

2025 / 9 / 27 (Sat) 10:15-11:30 Room 15/(ANA Crowne Plaza Hotel Kanazawa 3F Zuiun 2) Room 15

[Chairperson]Akitake Mukasa (Dept. of Neurosurgery, Grad. Sch. of Med. Sci., Kumamoto Univ.)

---

### E-3024
### Basic research and investigator-initiated trial with NS-REIC treatment for glioblastoma

[Speaker]Yoshihiro Otani (Department of Neurological Surgery, Okayama University) [Co-author]Kazuhiko Kurozumi (Department of Neurosurgery, Hamamatsu University School of Medicine)、Joji Ishida (Department of Neurological Surgery, Okayama University)、Ryo Mizuta (Department of Neurological Surgery, Okayama University／Division of Translational Oncoimmunology, Aichi Cancer Center Research Institute)、Yasuki Suruga (Department of Neurological Surgery, Okayama University)、Ryoji Imoto (Department of Neurological Surgery, Okayama University)、Ryosuke Ikemachi (Department of Neurological Surgery, Okayama University)、Shohei Nishigaki (Department of Neurological Surgery, Okayama University)、Naoya Kemmotsu (Department of Neurological Surgery, Okayama University)、Tsuyoshi Umeda (Department of Neurological Surgery, Okayama University)、Atsushi Fujimura (Molecular Physiology & Biophysics, Faculty of Medicine, Kagawa University,)、Hirokazu Matsushita (Division of Translational Oncoimmunology, Aichi Cancer Center Research Institute)、Daisuke Ennishi (Center for Genomic Medicine, Okayama University Hospital)、Hironobu Tan (Center for Innovative Clinical Medicine, Okayama University)、Hiromi Kumon (Innovation Center Okayama for Nanobio-targeted Therapy, Okayama University)、Shota Tanaka (Department of Neurological Surgery, Okayama University)

A viral gene therapy incorporating the tumor suppressor gene REIC/Dkk-3 discovered at Okayama University was conducted from basic research to investigator-initiated clinical trials. Here, we present the results from basic research to clinical application.In 2014, we confirmed the antitumor effects of the first-generation Ad-REIC product, Ad-CAG-REIC, in both in vitro and in vivo experiments. Subsequently, we developed the second-generation formulation, Ad-SGE-REIC, which enhances the expression of the REIC/Dkk-3 gene. Ad-SGE-REIC induced stronger endoplasmic reticulum stress and higher levels of cell death. Significant infiltration of immune cells were observed, suggesting immunogenic cell death. Furthermore, the combination of Ad-SGE-REIC with Bevacizumab enhanced the therapeutic effect.Based on these results, we initiated a Phase I/IIa investigator-initiated clinical trial (jRCT2063190013) for recurrent malignant glioma in 2019, and completed patient enrollment in 2022. We evaluated the safety and efficacy of this trial. Additionally, we have explored biomarkers for Ad-REIC. Ad-SGE-REIC has the potential to be a novel treatment for recurrent glioblastoma.

[Disclosure of COI by Responsible researcher] Lead presenter is the Responsible researcher.
[Lead presenter: Yoshihiro Otani] ■Research funds from company: AMED次世代がん医療加速化研究事業、AMED再生・細胞医療・遺伝子治療実現加速化プログラム

### [J-3037] I-J13-2
### New molecular targeted therapy (2)

2025 / 9 / 27 (Sat) 10:15-11:30 Room 16/(11F Visage) Room 16

[Chairperson]Ryohei Katayama (Div of Experimental Chemotherapy, Cancer Chemotherapy Center, Japanese Foundation for Cancer Research)

### J-3037
### Proteogenomic analysis to explore therapeutic target signaling pathways for advanced myxoid liposarcoma

[Speaker]Kiminori Hori (Div. of Analytical Biochem., Cancer Precision Med Ctr., JFCR) [Co-author]Yuki Funauchi (Dept. of Orthopedic Surg., Inst. of Sci. Tokyo)、Takashi Kamatani (M&D Data Sci. Ctr., Inst. of Sci. Tokyo)、Makoto Konishi (Div. of Analytical Biochem., Cancer Precision Med Ctr., JFCR)、Kyoko Yamashita (Div. of Pathology, Cancer Inst., JFCR／Dept. of Pathology, Cancer Inst. Hosp., JFCR)、Makiko Yamashita (Advanced Med. Development Ctr., Cancer Inst. Hosp., JFCR)、Shigehisa Kitano (Advanced Med. Development Ctr., Cancer Inst. Hosp., JFCR／Clin. Chemother., Cancer Chemother. Ctr., JFCR)、Kengo Takeuchi (Div. of Pathology, Cancer Inst., JFCR／Dept. of Pathology, Cancer Inst. Hosp., JFCR／Pathology Project for Mol. Targets, Cancer Inst., JFCR)、Tatsuhiko Tsunoda (Dept. of Biol. Sci., Sch. Sci., Univ. of Tokyo／Dept. of CBMS, Grad. Sch. Frontier Sci., Univ. of Tokyo)、Koichi Matsuda (Dept. of CBMS, Grad. Sch. Frontier Sci., Univ. of Tokyo／Human Genome Ctr., Inst. of Med. Sci., Univ. of Tokyo)、Keisuke Ae (Dept. of Orthopedic Oncology, Cancer Inst. Hosp., JFCR)、Koji Ueda (Div. of Analytical Biochem., Cancer Precision Med Ctr., JFCR)

Myxoid liposarcoma (MLS) with metastases has a poor prognosis, and these advanced cases are treated with drug therapy. However, the response rate of existing drugs is low (8-27%), so the development of novel drugs is desirable. In this study, to comprehensively explore therapeutic targets for advanced MLS, we performed proteogenomic analysis, including whole exome sequence (n = 43), RNA-seq (n = 72), total/phospho- proteome analysis (n = 38, 70), using surgically resected tissue samples. Overall, we detected 317 somatic mutations and identified 41,719 transcripts, 5,739 proteins, and 4,944 phosphorylated sites. We then performed unsupervised clustering to characterize the features of advanced MLS. As a result, the phosphorylation profile suggested that a specific pathway critical for cell survival is significantly activated in advanced MLS. Since some drugs targeting this pathway has been clinically available, we evaluated the efficacy of them. We found that the drugs showed antitumor activity against MLS cell lines at the same concentration of Cmax at the clinical dose. These results indicate that the drug has potential for use in advanced MLS with a minimal risk of toxicity.

[Disclosure of COI by Responsible researcher] Lead presenter is not the Responsible researcher.
[Lead presenter: Kiminori Hori] We have no financial relationships to disclose.
[Principal Researcher: Koji Ueda] We have no financial relationships to disclose.

### [J-3038] I-J13-2
### New molecular targeted therapy (2)

2025 / 9 / 27 (Sat) 10:15-11:30 Room 16/(11F Visage) Room 16

[Chairperson]Ryohei Katayama (Div of Experimental Chemotherapy, Cancer Chemotherapy Center, Japanese Foundation for Cancer Research)

---

### J-3038
### Antibody-drug conjugate targeting glypican-1 shows an antitumor effect on gemcitabine-resistant pancreatic cancer

[Speaker]Kazuki Shimizu (Sch. of Med., Iwate Med. Univ.) [Co-author]Satoshi Serada (Inst. for Biomed. Sci. Mol. Pathophysiol. Iwate Med. Univ.)、Yuji Suzuki (Inst. for Biomed. Sci. Mol. Pathophysiol. Iwate Med. Univ.／Division of Allergy & Rheumatology, Sch. of Med., Iwate Med. Univ.)、Tetsuji Naka (Inst. for Biomed. Sci. Mol. Pathophysiol. Iwate Med. Univ.／Division of Allergy & Rheumatology, Sch. of Med., Iwate Med. Univ.)

Pancreatic cancer (PC) is one of the most difficult malignancies to treat due to de novo and acquired resistance to chemotherapy such as gemcitabine (GEM). Thus, new therapies for GEM-resistant PC are needed. We previously reported that glypican-1 (GPC1) is a promising cancer antigen targeted by an antibody-drug conjugate (ADC) in preclinical GPC1-positive solid tumor models, including PC. Here, we evaluated the efficacy of GPC1-ADC in GEM-resistant PC. We established two GEM-resistant GPC1-positive PC cell lines with an $IC_{50}$ value from 105.19 to 210.80-fold higher than parental cell lines by stepwise exposure to GEM. Flow cytometric analysis confirmed the retention of GPC1 expression in GEM-resistant pancreatic cancer cell lines. GPC1-ADC significantly reduced cell viability compared to a control-ADC ***in vitro***. GPC1-ADC significantly increased caspase-3/7 activity, suggesting induction of apoptosis. GPC1-ADC showed significant antitumor activity in a subcutaneous xenograft model of GEM-resistant PC ***in vivo***. These findings suggest that GPC1-ADC may be a promising therapeutic strategy for overcoming GEM-resistant PC, warranting further clinical investigation.

[Disclosure of COI by Responsible researcher] Lead presenter is not the Responsible researcher.
[Lead presenter: Kazuki Shimizu] We have no financial relationships to disclose.
[Principal Researcher: Tetsuji Naka] ■Research funds from company: 日本医療研究開発機構

### [J-3039] I-J13-2
### New molecular targeted therapy (2)

2025 / 9 / 27 (Sat) 10:15-11:30 Room 16/(11F Visage) Room 16

[Chairperson]Ryohei Katayama (Div of Experimental Chemotherapy, Cancer Chemotherapy Center, Japanese Foundation for Cancer Research)

---

### J-3039
### Small molecule inhibitors targeting PSF modulate p53 signaling and suppress hormone therapy-resistant cancer growth

[Speaker]Kenichi Takayama (Dept. Syst. Aging Sci. Med. Tokyo Metro. Inst. Geron. Geriat.) [Co-author]Satoshi Inoue (Dept. Syst. Aging Sci. Med. Tokyo Metro. Inst. Geron. Geriat.／Div. Syst. Med. Gene Ther, Saitama Med. Univ.)

Proline- and glutamine-rich protein, PTB-associated splicing factor (PSF), has been identified as a key driver of aggressive cancers. PSF promotes oncogene expression by modulating epigenetic and splicing mechanisms. Through a small molecule library screen, we discovered a compound, No.10-3, and its optimized analog, C-30, as potent PSF inhibitors. C-30 treatment suppressed the growth of hormone therapy-resistant breast and prostate cancer cells harboring mutant p53. Moreover, C-30 activated a distinct set of p53-regulated genes in these cancer cells. A comprehensive analysis of PSF and p53-binding regions revealed enhanced PSF binding at genomic regions corresponding to p53-binding peaks in p53 wild-type cancer cells treated with C-30, even in cancer cells expressing mutant p53.Treatment with C-30 reduced PSF binding, leading to enhanced histone acetylation and transcriptional activation. We demonstrated that C-30 impaired tumor growth and upregulated the expression of p53-target genes in vivo. Our findings highlight the tumor-suppressive effects of these inhibitors, providing strong rationale for targeting PSF as a promising therapeutic strategy for treatment-resistant cancers.

[Disclosure of COI by Responsible researcher] Lead presenter is the Responsible researcher.
[Lead presenter: Kenichi Takayama] We have no financial relationships to disclose.

### [J-3040] I-J13-2
### New molecular targeted therapy (2)

2025 / 9 / 27 (Sat) 10:15-11:30 Room 16/(11F Visage) Room 16

[Chairperson]Ryohei Katayama (Div of Experimental Chemotherapy, Cancer Chemotherapy Center, Japanese Foundation for Cancer Research)

### J-3040
### Anti-CSC Drug Discovery targeted for ER-stress Responsive Enzyme

[Speaker]Takao Nomura (Grad. Sch. Pharm. Sci. Hokkaido Univ.) [Co-author]Shiori Yanai (Grad. Sch. Life Sci. Hokkaido Univ.)、Shoki Fujita (Grad. Sch. Life Sci. Hokkaido Univ.)、Yusuke Ohta (Grad. Sch. Pharm. Sci. Hokkaido Univ.)、Takanori Matsumaru (Fuc. Sci. Tech. Keio Univ.)、Katsumi Maenaka (Grad. Sch. Pharm. Sci. Hokkaido Univ.／Grad. Sch. Pharm. Sci. Kyushu Univ.)

Endoplasma reticulum oxidoreductin 1 α (ERO1α) consumes oxygen and acts as a regulatory enzyme of protein disulfide isomerase that catalyzes disulfide bond formation of various proteins. ERO1α was found to be highly expressed in cancer cell lines, tissues, and also cancer stem cells. ERO1α is involved in tumorigenesis and plays an important role in regulating growth factor production. In other words, it has been suggested that suppressing the expression of ERO1α can control tumor growth, leading to the eradication of cancer. In this study, we aimed to develop ERO1α inhibitors that could be novel anticancer agents. We carried out the in vitro screening for ERO1α binder by physicochemical assays. We eliminated some binders and optimized the structure of the strongest inhibitor for ERO1α enzyme activity. After characterizations with analog synthesis ERO1α inhibitor leads was developed, furthermore, this compound exhibited the anti-tumor effect. One of these candidates suppressed the cell growth of CSC-like cells in vitro and xenograft model of CSC-like cells. These novel drug developments would be expected to eradicate cancer tissue, including cancer stem cells.

[Disclosure of COI by Responsible researcher] Lead presenter is the Responsible researcher.
[Lead presenter: Takao Nomura] We have no financial relationships to disclose.

### [J-3041] I-J13-2
### New molecular targeted therapy (2)

2025 / 9 / 27 (Sat) 10:15-11:30 Room 16/(11F Visage) Room 16

[Chairperson]Ryohei Katayama (Div of Experimental Chemotherapy, Cancer Chemotherapy Center, Japanese Foundation for Cancer Research)

---

### J-3041
### Development of therapeutic sustainable PPI inhibitor peptides targeting the BIG3-PHB2 complex in breast cancer

[Speaker]Tetsuro Yoshimaru (Natl. Inst. Biomed. Innovation, Natl. Inst. Biomed. Innovation. Health. Nutrition／Div. Genome Med., Inst. Adv. Med. Sci., Tokushima Univ.) [Co-author]Yosuke Matsushita (Natl. Inst. Biomed. Innovation, Natl. Inst. Biomed. Innovation. Health. Nutrition／Div. Genome Med., Inst. Adv. Med. Sci., Tokushima Univ.)、Keiji Uchiyama (Natl. Inst. Biomed. Innovation, Natl. Inst. Biomed. Innovation. Health. Nutrition)、Yasuo Miyoshi (Dept. Surg., Hyogo College Med.)、Mitsunori Sasa (Dept. Surg., Tokushima Breast Care Clin.)、Toyomasa Katagiri (Natl. Inst. Biomed. Innovation, Natl. Inst. Biomed. Innovation. Health. Nutrition)

We have identified cancer-specific scaffold complex BIG3-PHB2 as a key contributor to breast cancer malignancy and developed a stapled peptide inhibitor, stERAP, targeting this complex. This stERAP offers the advantage of prolonged antitumor activity. Here, we report the novel pathophysiological roles of BIG3-PHB2 and the molecular mechanism of long-term inhibition of stERAP in HER2-positive breast cancer with BIG3 overexpression. We found that BIG3-PHB2 regulates the extracellular secretion of cancer-related proteins in trans-Golgi network, whereas stERAP inhibits this secretion. Furthermore, stERAP disrupts BIG3-PHB2 interaction, leading to the nuclear-translocation of PHB2, where it binds to SUMOylated proteins. This SUMOylation-dependent process contributes to prolonged antitumor effect. In contrast, in trastuzumab-resistant HER2-positive breast cancer, BIG3-PHB2 undergoes a drastic shift in intracellular localization to plasma membrane. Notably, stERAP effectively disrupts HER2-EGFR formation, thereby suppressing tumor proliferation. These results suggest that stERAP exerts a sustained antitumor effect by modulating BIG3-PHB2 complex in HER2-positive breast cancer progression.

[Disclosure of COI by Responsible researcher] Lead presenter is not the Responsible researcher.
[Lead presenter: Tetsuro Yoshimaru] We have no financial relationships to disclose.
[Principal Researcher: Toyomasa Katagiri] ■Research funds from company: オンコセラピー・サイエンス株式会社　■Advisor of the company and/or profit-making organization: オンコセラピー・サイエンス株式会社、株式会社Cancer Precision Medicine ■Contributions: とくしまブレストケアクリニック

### [J-3042] I-J13-2
### New molecular targeted therapy (2)

2025 / 9 / 27 (Sat) 10:15-11:30 Room 16/(11F Visage) Room 16

[Chairperson]Ryohei Katayama (Div of Experimental Chemotherapy, Cancer Chemotherapy Center, Japanese Foundation for Cancer Research)

---

### J-3042
### Withdrawn

No Abstract

### [LS25] LS25

### Otsuka Pharmaceutical Co., Ltd. - Medical Affairs / Mechanisms of T Cell Activation and Inhibition in the Tumor Microenvironment

2025 / 9 / 27 (Sat) 11:50-12:40 Room 4/(Hotel Nikko Kanazawa 4F Tsuru B) Room 4
[]Otsuka Pharmaceutical Co., Ltd. - Medical Affairs

[Chairperson]Takahiro Maeda (Division of Precision Medicine, Graduate School of Medical Sciences, Kyushu University)

---

### LS25
### Mechanisms of T Cell Activation and Inhibition in the Tumor Microenvironment

[Speaker]Hiroyoshi Nishikawa (Division of Cancer Immune Multicellular System Regulation, Center for Cancer Immunotherapy and Immunobiology (CCII) Graduate School of Medicine, Kyoto University ／ Division of Cancer Immunology, Research Institute／ Exploratory Oncology Research and Clinical Trial Center (EPOC), National Cancer Center ／ Department of Immunology, Nagoya University Graduate School of Medicine)

No Abstract

### [LS26] LS26

### Janssen Pharmaceutical K.K. / Mastering Bispecific Antibody ~Novel therapeutic options for EGFR-Mutant NSCLC~

2025 / 9 / 27 (Sat) 11:50-12:40 Room 6/(Hotel Nikko Kanazawa 3F Kujaku A) Room 6
[]Janssen Pharmaceutical K.K.

[Chairperson]Seiji Yano (Department of Respiratory Medicine, Faculty of Medicine, Institute of Medical, Pharmaceutical, and Health Sciences, Kanazawa University)

---

### LS26

### Mastering Bispecific Antibody ~Novel therapeutic options for EGFR-Mutant NSCLC~

[Speaker]Hidehito Horinouchi (Department of Thoracic Oncology, National Cancer Center Hospital)

No Abstract

### [LS27] LS27

### AbbVie GK / Real-world data of VEN+AZA therapy in Japanese patients with acute myeloid leukemia useful for clinical practice

2025 / 9 / 27 (Sat) 11:50-12:40 Room 7/(Hotel Nikko Kanazawa 3F Kujaku B) Room 7
[]AbbVie GK

[Chairperson]Seishi Ogawa (Department of Pathology and Tumor Biology, Kyoto University)

---

### LS27

### Real-world data of VEN+AZA therapy in Japanese patients with acute myeloid leukemia useful for clinical practice

[Speaker]Hiroki Yamaguchi (Department of Hematology, Nippon Medical School)

No Abstract

▲TOP

### [LS28-1] LS28
### Bio-Rad Laboratories K.K. / The Forefront of Multiplex Digital PCR Analysis in Cancer Research

2025 / 9 / 27 (Sat) 11:50-12:40 Room 8/(Hotel Nikko Kanazawa 3F Kujaku C) Room 8
[]Bio-Rad Laboratories K.K.

[Chairperson]Masatoshi Soejima (Bio-Rad Laboratories K.K.)

---

### LS28-1
### 1) Basics of droplet digital PCR

[Speaker]Ayumi Miyakawa (Bio-Rad Laboratories K.K.)

No Abstract

### [LS28-2] LS28
### Bio-Rad Laboratories K.K. / The Forefront of Multiplex Digital PCR Analysis in Cancer Research

2025 / 9 / 27 (Sat) 11:50-12:40 Room 8/(Hotel Nikko Kanazawa 3F Kujaku C) Room 8
[]Bio-Rad Laboratories K.K.

[Chairperson]Masatoshi Soejima (Bio-Rad Laboratories K.K.)

---

### LS28-2
### 2) Multiplex ddPCR analysis toward cancer genome diagnosis at a private hospital research institute

[Speaker]Yusuke Ono (Institute of Biomedical Research, Sapporo Higashi Tokushukai Hospital)

No Abstract

### [LS29] LS29

### KONICA MINOLTA REALM, INC. / Comprehensive analysis using DNA/RNA twin panels and its development to cancer genomic medicine

2025 / 9 / 27 (Sat) 11:50-12:40 Room 9/(Hotel Nikko Kanazawa 3F Lumiere) Room 9
[]KONICA MINOLTA REALM, INC.

[Chairperson]Katsutoshi Oda (Division of Integrative, Genomics, Graduate School of Medicine, The University of Tokyo)

---

### LS29

### Comprehensive analysis using DNA/RNA twin panels and its development to cancer genomic medicine

[Speaker]Kousuke Watanabe (Next-Generation Precision Medicine Development Laboratory, Graduate School of Medicine, The University of Tokyo)

No Abstract

▲TOP

### [LS30] LS30

### DAIICHI SANKYO COMPANY, LIMITED / Pathogenesis of Malignant Lymphoma Explored by Single-Cell and Spatial Omics Analyses

2025 / 9 / 27 (Sat) 11:50-12:40 Room 11/(ANA Crowne Plaza Hotel Kanazawa 3F Ohtori (West)) Room 11
[]DAIICHI SANKYO COMPANY, LIMITED

[Chairperson]Motoko Yamaguchi (Department of Hematological Malignancies Mie University Graduate School of Medicine)

---

### LS30

### Pathogenesis of Malignant Lymphoma Explored by Single-Cell and Spatial Omics Analyses

[Speaker]Mamiko Sakata-Yanagimoto (Department of Hematology, Institute of Medicine, University of Tsukuba)

No Abstract

▲TOP

### [LS31] LS31

### CyberomiX Inc. / The truth about the tumor microenvironment revealed by high-resolution spatial transcriptomics analysis

2025 / 9 / 27 (Sat) 11:50-12:40 Room 12/(ANA Crowne Plaza Hotel Kanazawa 3F Ohtori (Central)) Room 12
[]CyberomiX Inc.

[Chairperson]Akira Watanabe (CyberomiX Inc.)

---

### LS31

### The truth about the tumor microenvironment revealed by high-resolution spatial transcriptomics analysis

[Speaker]Shinji Kohsaka (National Cancer Center Research Institute)

No Abstract

### [LS32] LS32

### GlaxoSmithKline.K.K. / Fundamentals and Potential of Antibody-Drug Conjugates (ADCs) Learned through Belantamab mafodotin

2025 / 9 / 27 (Sat) 11:50-12:40 Room 13/(ANA Crowne Plaza Hotel Kanazawa 3F Ohtori (East)) Room 13
[]GlaxoSmithKline.K.K.

[Chairperson]Tadao Ishida (Japanese Red Cross Medical Center)

---

### LS32

### Fundamentals and Potential of Antibody-Drug Conjugates (ADCs) Learned through Belantamab mafodotin

[Speaker]Yusuke Furukawa (Teikyo University of Science Medical Education Center)

No Abstract

▲TOP

### [LS33-1] LS33
### Cosmo Bio Co., Ltd.／Proteintech Japan, Co., Ltd. /

2025 / 9 / 27 (Sat) 11:50-12:40 Room 14/(ANA Crowne Plaza Hotel Kanazawa 3F Zuiun 1) Room 14
[]Cosmo Bio Co., Ltd.／Proteintech Japan, Co., Ltd.

---

### LS33-1
### 1) Advancing Single-Cell Multi-Omics Analysis with Intracellular CITE-seq

[Speaker]Saki Imura (Proteintech Japan, Co., Ltd.)

No Abstract

▲TOP

### [LS33-2] LS33
### Cosmo Bio Co., Ltd.／Proteintech Japan, Co., Ltd. /

2025 / 9 / 27 (Sat) 11:50-12:40 Room 14/(ANA Crowne Plaza Hotel Kanazawa 3F Zuiun 1) Room 14
[]Cosmo Bio Co., Ltd.／Proteintech Japan, Co., Ltd.

---

### LS33-2
### 2) Genotype-integrated single-cell transcriptomics reveals the pathogenesis of myeloid neoplasms

[Speaker]Hirotaka Matsui (Department of Laboratory Medicine, National Cancer Center Hospital)

No Abstract

### [LS34] LS34

### Mediford Corporation / Translational Research contributing to cancer research by using PDX models PDX Models as a Platform for Drug Development and Translational Research

2025 / 9 / 27 (Sat) 11:50-12:40 Room 15/(ANA Crowne Plaza Hotel Kanazawa 3F Zuiun 2) Room 15
[]Mediford Corporation

[Chairperson]Kosei Hasegawa (Department of Gynecologic Oncology, Saitama Medical University International Medical Center)

---

### LS34

### Translational Research contributing to cancer research by using PDX models PDX Models as a Platform for Drug Development and Translational Research

[Speaker]Akinobu Hamada (Division of Molecular Pharmacology, National Cancer Center Research Institute)

No Abstract

▲TOP

### [TYPL] TYPL
### The Tomizo Yoshida Prize Lecture

2025 / 9 / 27 (Sat) 12:50-14:40 Room 1/(Ishikawa Ongakudo 2F Concert Hall) Room 1

[Chairperson]Masanobu Oshima (Division of Genetics, Cancer Research Institute, Kanazawa University)

---

### TYPL
### Fall down seven times, stand up eight with cancer epigenetics

[Speaker]Toshikazu Ushijima (Hoshi Univ.)

It was, and even is, common to use nonmalignant tissues of cancer patients as normal controls. In 2006, we showed that a substantial amount of DNA methylation was present even in nonmalignant tissue of gastric cancer patients in association with ***Helicobacter pylori*** (***HP***) infection, and that the DNA methylation level in people without current ***HP*** infection was correlated with cancer risk. It was not ***HP*** itself but the resultant inflammation that induced aberrant DNA methylation. We further revealed molecular mechanisms of methylation induction, namely TET down-regulation due to NF-kB activation and increased DNMT activity due to NO production, which appeared to be applicable to other inflammation-associated cancers. The decrease in the DNA methylation level after ***HP*** eradication is now explained by the resistance of stem cells to aberrant DNA methylation induction. All these mechanisms support the use of DNA methylation levels after ***HP*** eradication for cancer risk diagnosis, and we demonstrated that it can be done by two large-scale prospective studies. Now, it is becoming common sense that the nonmalignant tissue of cancer patients harbors a lot of epigenetic and genetic alterations.

[Disclosure of COI by Responsible researcher] Lead presenter is the Responsible researcher.
[Lead presenter: Toshikazu Ushijima] ■Research funds from company: AMED　■Research expenses: シスメックス

### [MNPL] MNPL
### The Matayo Nagayo Prize Lecture

2025 / 9 / 27 (Sat) 12:50-14:40 Room 1/(Ishikawa Ongakudo 2F Concert Hall) Room 1

[Chairperson]Seishi Ogawa (Department of Pathology and Tumor Biology, Kyoto University)

---

### MNPL
### Development and implementation of oncolytic virus therapy using genetically engineered herpes viruses

[Speaker]Tomoki Todo (Div. Innovative Cancer Therapy, Inst. Med. Sci., Univ. Tokyo)

Oncolytic virus therapy is a new therapeutic modality using genetically engineered viruses that selectively replicate in and kill cancer cells. The virus not only exerts direct cytocidal effects but also triggers a specific antitumor immune response as virally destroyed tumor cells are cleared by the host immune system. A triple-mutated oncolytic herpes simplex virus type 1 (HSV-1), G47Δ, was developed with enhanced replication capability in all solid cancer, efficient induction of antitumor immunity, and high safety. An investigator-initiated phase II trial for recurrent or residual glioblastoma showed survival benefit and potential cures, leading to 2021 drug approval in Japan. A groundbreaking recombinant HSV-1 generation system was further developed, enabling insertion of desired transgenes into the G47Δ backbone. Among many armed triple-mutated HSV-1 created is human interleukin-12-expressing oncolytic HSV-1, which recently demonstrated significant efficacy in an investigator-initiated phase II trial for progressive malignant melanoma. I believe oncolytic virus therapy may soon revolutionize cancer medicine and become a standard treatment option for all cancer patients.

[Disclosure of COI by Responsible researcher] Lead presenter is the Responsible researcher.
[Lead presenter: Tomoki Todo] ■Contributions: デンカ株式会社

▲TOP

### [JWAS] JWAS
### JCA Woman Scientists Awards

2025 / 9 / 27 (Sat) 12:50-13:20 Room 2/(Ishikawa Ongakudo 2F Hogaku Hall) Room 2

[Chairperson]Noriko Gotoh (Cancer Research Institute, Kanazawa University)

---

### JWAS
### Molecular Pathogenesis of Malignant Lymphoma and Its Application to Precision Medicine

[Speaker]Mamiko Sakata-Yanagimoto (Dept. of Hematol., Inst. of Med., Univ. of Tsukuba)

悪性リンパ腫の微小環境に着目し、最先端解析技術と臨床医学を統合したトランスレーショナルリサーチを展開してきた。特に、クローン性造血（clonal hematopoiesis, CH）に由来する体細胞変異のある免疫細胞が腫瘍発症を制御する多段階発がんモデルを示してきた。T/NK細胞リンパ腫のうち世界的に最も頻度の高いT濾胞ヘルパー細胞リンパ腫（TFHL）において、p.Gly17Val（G17V）RHOA変異を発見し（Nat Genet 2014）、G17V RHOA VAV1シグナル経路が腫瘍発症の駆動因子かつ治療標的となりうることを示した（Leukemia 2018, Blood 2020, Cancer Res 2020）。さらに、CHに由来するTET2変異を持つ炎症性細胞が腫瘍ニッチを形成し、進展を促すことを報告した（Blood 2022, Leukemia 2024, 2025）。一方、成熟B細胞リンパ腫で2番目に多い濾胞性リンパ腫（FL）では、一細胞RNAおよび空間トランスクリプトーム解析により間質細胞アトラスを構築（Nat Cell Biol 2022）、予後良好と関連する新規濾胞性T細胞サブセットを同定した（Cancer Cell 2025）。これらの成果は、腫瘍進化と微小環境の相互作用の重要性を示し、臨床応用が期待される。

[Disclosure of COI by Responsible researcher] Lead presenter is the Responsible researcher.
[Lead presenter: Mamiko Sakatayanagimoto] ■Research funds from company: 科研費、AMED、エーザイ、中外製薬、ブリストルマイヤーズ　■Research expenses: 大塚製薬

### [OS10-1] OS10
### Cutting edge research on chronic inflammation and tumor microenvironment

2025 / 9 / 27 (Sat) 12:50-14:20 Room 3/(Hotel Nikko Kanazawa 4F Tsuru A) Room 3

[Chairpersons]Takeharu Sakamoto (Institute of Biomedical Science, Kansai Medical University)、Yoshikazu Johmura (Cancer Research Institute, Kanazawa University)

Although the importance of chronic inflammation and the cancer microenvironment in cancer progression has long been understood, we are still in the process of understanding and applying to therapy the complex systems in which multiple cells and different extracellular environments interact in space and time. This symposium will feature up-and-coming researchers from Japan and abroad, who will share their unique perspectives and techniques with the conference participants, and share their cutting-edge findings in chronic inflammation and cancer microenvironment research, including novel mechanisms of fibroblasts-mediated inflammation and carcinogenesis; overcoming chronic inflammation and carcinogenesis by targeting cellular senescence; development of novel therapies for liver inflammation and carcinogenesis targeting the tumor microenvironment; development of novel technologies to visualize cell-cell interactions in vivo; and development of brain tumor therapies by engineering cell-cell interactions.

---

### OS10-1
### Chronic Inflammation and Tumor Microenvironment Research: Past and Future.

[Speaker]Takeharu Sakamoto (Dept. Cancer Biol., Inst. Biomed. Sci., Kansai Med. Univ.)

The relationship between chronic inflammation and cancer was first proposed by Rudolf Virchow in 1863, when he observed immune cells in tumor tissues, suggesting that inflammation might promote cancer. However, this idea remained marginal until the late 20th century. By the 1990s and early 2000s, epidemiological studies revealed clear links between chronic inflammatory diseases such as ulcerative colitis and hepatitis and cancers like colorectal and liver cancer. Around the same time, the concept of the tumor microenvironment (TME) emerged. Research showed that cancer progression is not driven by tumor cells alone, but also by surrounding immune cells, stromal cells, cytokines, and extracellular matrix components. Key inflammatory mediators such as NF-κB, STAT3, and IL-6 were found to promote tumor growth, immune evasion, and metastasis. Today, modulating inflammation within the TME has become a vital strategy in cancer treatment, especially in enhancing immunotherapy efficacy. Here, I review the history of research on chronic inflammation and the TME to date, as well as the possibilities for future research, along with the work of the speakers in this symposium.

[Disclosure of COI by Responsible researcher] Lead presenter is the Responsible researcher.
[Lead presenter: Takeharu Sakamoto] We have no financial relationships to disclose.

### [OS10-2] OS10
### Cutting edge research on chronic inflammation and tumor microenvironment

2025 / 9 / 27 (Sat) 12:50-14:20 Room 3/(Hotel Nikko Kanazawa 4F Tsuru A) Room 3

[Chairpersons]Takeharu Sakamoto (Institute of Biomedical Science, Kansai Medical University)、Yoshikazu Johmura (Cancer Research Institute, Kanazawa University)

Although the importance of chronic inflammation and the cancer microenvironment in cancer progression has long been understood, we are still in the process of understanding and applying to therapy the complex systems in which multiple cells and different extracellular environments interact in space and time. This symposium will feature up-and-coming researchers from Japan and abroad, who will share their unique perspectives and techniques with the conference participants, and share their cutting-edge findings in chronic inflammation and cancer microenvironment research, including novel mechanisms of fibroblasts-mediated inflammation and carcinogenesis; overcoming chronic inflammation and carcinogenesis by targeting cellular senescence; development of novel therapies for liver inflammation and carcinogenesis targeting the tumor microenvironment; development of novel technologies to visualize cell-cell interactions in vivo; and development of brain tumor therapies by engineering cell-cell interactions.

---

### OS10-2
### Dual Functions of IL-11+ Fibroblasts: Cancer Promotion versus Colitis Protection in Colorectal Disease

[Speaker]Takashi Nishina (Dept. Biochem., Toho Univ., Fucult. Med) [Co-author]Masaki Ohmuraya (Dept. Genet., Hyogo Med. Coll.)、Yutaro Kumagai (Cell. Mol. Biotechnol. Res. Inst., AIST)、Yutaka Suzuki (Grad. Sch. Front. Sci., Univ. Tokyo)、Soh Yamazaki (Dept. Biochem., Toho Univ., Fucult. Med)、Tetuo Mikami (Dept. Pathol., Toho Univ., Facult. Med.)、Katsuhide Okunishi (Dept. Biochem., Toho Univ., Fucult. Med)、Hiroyasu Nakano (Unit Host Defense, Toho Univ., Facult. Med.)

Fibroblasts are crucial for extracellular matrix remodeling and secreting soluble factors that influence tissue repair and cancer progression. Interleukin-11 (IL-11), an IL-6 family cytokine, drives key cellular responses in colon cancer pathogenesis. We previously identified IL-11+ fibroblasts in murine acute colitis, colitis-associated colorectal cancer, and human tumors, but their origin and function remained unclear. Using novel lineage tracing and single-cell multi-omics analysis, we demonstrate that tissue-resident fibroblasts acquire IL-11 production capacity and transdifferentiate into cancer-associated fibroblasts. Notably, while IL-11+ fibroblasts promote colorectal cancer, disrupting IL-11/IL-11R signaling worsens dextran sulfate sodium-induced colitis. These findings reveal the paradoxical dual roles of IL-11+ fibroblasts as cancer promoters yet colitis protectors, providing new insights into colorectal disease mechanisms.

[Disclosure of COI by Responsible researcher] Lead presenter is the Responsible researcher.
[Lead presenter: Takashi Nishina] ■Research funds from company: 公益財団法人細胞科学研究財団、公益財団法人第一三共生命科学研究振興財団、公益財団法人武田科学振興財団

### [OS10-3] OS10
### Cutting edge research on chronic inflammation and tumor microenvironment

2025 / 9 / 27 (Sat) 12:50-14:20 Room 3/(Hotel Nikko Kanazawa 4F Tsuru A) Room 3

[Chairpersons]Takeharu Sakamoto (Institute of Biomedical Science, Kansai Medical University)、Yoshikazu Johmura (Cancer Research Institute, Kanazawa University)

Although the importance of chronic inflammation and the cancer microenvironment in cancer progression has long been understood, we are still in the process of understanding and applying to therapy the complex systems in which multiple cells and different extracellular environments interact in space and time. This symposium will feature up-and-coming researchers from Japan and abroad, who will share their unique perspectives and techniques with the conference participants, and share their cutting-edge findings in chronic inflammation and cancer microenvironment research, including novel mechanisms of fibroblasts-mediated inflammation and carcinogenesis; overcoming chronic inflammation and carcinogenesis by targeting cellular senescence; development of novel therapies for liver inflammation and carcinogenesis targeting the tumor microenvironment; development of novel technologies to visualize cell-cell interactions in vivo; and development of brain tumor therapies by engineering cell-cell interactions.

---

### OS10-3
### Senescent cells regulate the progression of metabolic dysfunction-related steatohepatitis and hepatocellular carcinoma

[Speaker]Yasuhiro Nakano (Dev. Cancer Senescence Biol., Cancer Res. Inst., Kanazawa Univ.／Integrated Systems Aging Res., Inst. Front. Sci Initiative, Kanazawa Univ.) [Co-author]Zixue Zhang (Dev. Cancer Senescence Biol., Cancer Res. Inst., Kanazawa Univ.)、Soichiro Kumamoto (Dev. Cancer Senescence Biol., Cancer Res. Inst., Kanazawa Univ.)、Yoshikazu Johmura (Dev. Cancer Senescence Biol., Cancer Res. Inst., Kanazawa Univ.／Integrated Systems Aging Res., Inst. Front. Sci Initiative, Kanazawa Univ.)

Senescent cells are characterized by irreversible growth arrest and acquisition of the senescence-associated secretory phenotype (SASP). Their accumulation in tissues is thought to promote cancer and age-related diseases via SASP-mediated mechanisms, though the details remain unclear. In this study, we aimed to characterize p16$^{Ink4a}$-expressing cells in metabolic dysfunction-associated steatohepatitis (MASH) and hepatocellular carcinoma (HCC), and to investigate their molecular contribution to disease progression.

Using p16-CreER-tdTomato mice, which enable lineage tracing of p16$^{Ink4a}$-expressing cells, we induced MASH and HCC via a choline-deficient high-fat diet. Histological analysis revealed accumulation of p16$^{Ink4a}$-lineage-positive (p16-Lin(+)) cells in the liver during MASH, primarily hepatocytes, which localized near tumor margins in HCC. We isolated p16-Lin(+) and p16-Lin(-) cells by FACS and performed scRNA-seq. At the tumor stage, p16-Lin(+) hepatocytes showed upregulation of multiple SASP factors compared to p16-Lin(-) hepatocytes. In this meeting, we will discuss the potential roles of these SASP factors secreted by senescent hepatocytes in HCC progression.

[Disclosure of COI by Responsible researcher] Lead presenter is the Responsible researcher.
[Lead presenter: Yasuhiro Nakano] We have no financial relationships to disclose.

### [OS10-4] OS10
### Cutting edge research on chronic inflammation and tumor microenvironment

2025 / 9 / 27 (Sat) 12:50-14:20 Room 3/(Hotel Nikko Kanazawa 4F Tsuru A) Room 3

[Chairpersons]Takeharu Sakamoto (Institute of Biomedical Science, Kansai Medical University)、Yoshikazu Johmura (Cancer Research Institute, Kanazawa University)

Although the importance of chronic inflammation and the cancer microenvironment in cancer progression has long been understood, we are still in the process of understanding and applying to therapy the complex systems in which multiple cells and different extracellular environments interact in space and time. This symposium will feature up-and-coming researchers from Japan and abroad, who will share their unique perspectives and techniques with the conference participants, and share their cutting-edge findings in chronic inflammation and cancer microenvironment research, including novel mechanisms of fibroblasts-mediated inflammation and carcinogenesis; overcoming chronic inflammation and carcinogenesis by targeting cellular senescence; development of novel therapies for liver inflammation and carcinogenesis targeting the tumor microenvironment; development of novel technologies to visualize cell-cell interactions in vivo; and development of brain tumor therapies by engineering cell-cell interactions.

---

### OS10-4
### TEAD Inhibition as a Therapeutic Strategy Targeting Hepatocellular Carcinoma and Its Fibrotic Microenvironment

[Speaker]Yoshinobu Saito (Dept. Gastro and Hep, Grad Med., The Univ. Osaka／iFremed, OTRI, The University of Osaka)

Hepatocellular carcinoma (HCC) frequently arises from chronic liver diseases such as viral hepatitis and MASLD, typically in the context of advanced liver fibrosis. The fibrotic microenvironment, characterized by increased extracellular matrix stiffness, plays a central role in tumor initiation and progression through activation of the TAZ-TEAD transcriptional pathway, as we previously demonstrated. Emerging evidence also suggests that HCC can develop in MASH even without significant fibrosis, implicating metabolic reprogramming in hepatocarcinogenesis. We identified the mevalonate pathway as a metabolic trigger for TAZ-TEAD activation, underscoring its importance beyond mechanical cues. Given that most HCC patients have cirrhosis and reduced hepatic reserve, and that current therapies do not improve the fibrotic background, dual-targeted strategies addressing both the tumor and its microenvironment are urgently needed. We show that TEAD inhibition prolongs survival in HCC models, attenuates steatosis and fibrosis in MASH, and restores Rspo3 expression-a key regulator of liver regeneration and homeostasis. TEAD inhibition may represent a transformative approach for HCC treatment.

[Disclosure of COI by Responsible researcher] Lead presenter is the Responsible researcher.
[Lead presenter: Yoshinobu Saito] ■Research funds from company: Grant-in-Aid, AMED, 高松宮妃癌研究基金

▲TOP

### [OS10-5] OS10
### Cutting edge research on chronic inflammation and tumor microenvironment

2025 / 9 / 27 (Sat) 12:50-14:20 Room 3/(Hotel Nikko Kanazawa 4F Tsuru A) Room 3

[Chairpersons]Takeharu Sakamoto (Institute of Biomedical Science, Kansai Medical University)、Yoshikazu Johmura (Cancer Research Institute, Kanazawa University)

Although the importance of chronic inflammation and the cancer microenvironment in cancer progression has long been understood, we are still in the process of understanding and applying to therapy the complex systems in which multiple cells and different extracellular environments interact in space and time. This symposium will feature up-and-coming researchers from Japan and abroad, who will share their unique perspectives and techniques with the conference participants, and share their cutting-edge findings in chronic inflammation and cancer microenvironment research, including novel mechanisms of fibroblasts-mediated inflammation and carcinogenesis; overcoming chronic inflammation and carcinogenesis by targeting cellular senescence; development of novel therapies for liver inflammation and carcinogenesis targeting the tumor microenvironment; development of novel technologies to visualize cell-cell interactions in vivo; and development of brain tumor therapies by engineering cell-cell interactions.

---

### OS10-5
### Interaction-activated transcriptomics dissects molecular crosstalk in cancer cell - microenvironment

[Speaker]Misa Minegishi (LiMe, Kyoto Univ.／Fac. Eng., Kyoto Univ.) [Co-author]Keiji Nozaki (LiMe, Kyoto Univ.／Fac. Eng., Kyoto Univ.)、Keisuke Kondo (Fac. Eng., Kyoto Univ.)、Kaori Nishikawa (LiMe, Kyoto Univ.)、Takahiro Kuchimaru (Ctr. Mol. Med., Jichi Med. Univ.)、Hirofumi Shintaku (LiMe, Kyoto Univ.／Fac. Eng., Kyoto Univ.)

Metastasis involves complex interactions between cancer cells and their surrounding microenvironment. Here, we present two tools developed to investigate these interactions.
First, ***s***GRAPHIC labels tissue-resident cells that neighbor on cancer cells using split fluorescent protein reconstitution, enabling their isolation and characterization. Applying to a liver metastasis mouse model, ***s***GRAPHIC revealed inflammation-related gene expression in neighboring hepatocytes and identified ***Lgals3*** as a potential pro-metastatic factor.
Second, we developed a high-throughput system for longitudinal analysis of single cancer cells under controlled mechanical confinement by encapsulating in micro-hydrogel beads. Encapsulated breast cancer cells showed cell-cycle arrest in G1 phase. Upon confinement release, arrested cells resumed proliferation, suggesting that confinement induces dormancy. RNA-seq analysis revealed that mechanical confinement induces cytoskeletal remodeling, leading to cell-cycle arrest and metabolic regression.
Together, these two tools offer powerful platforms to dissect cancer cells - microenvironment interactions and advance understanding of metastasis.

[Disclosure of COI by Responsible researcher] Lead presenter is not the Responsible researcher.
[Lead presenter: Misa Minegishi] We have no financial relationships to disclose.
[Principal Researcher: Hirofumi Shintaku] ■Research funds from company: 中島記念国際交流財団、公益財団法人JKA
■Contributions: 株式会社アナナスジャパン　■Presents or other payment: 中谷財団（中谷賞貢献賞）

### [OS10-6] OS10
### Cutting edge research on chronic inflammation and tumor microenvironment

2025 / 9 / 27 (Sat) 12:50-14:20 Room 3/(Hotel Nikko Kanazawa 4F Tsuru A) Room 3

[Chairpersons]Takeharu Sakamoto (Institute of Biomedical Science, Kansai Medical University)、Yoshikazu Johmura (Cancer Research Institute, Kanazawa University)

Although the importance of chronic inflammation and the cancer microenvironment in cancer progression has long been understood, we are still in the process of understanding and applying to therapy the complex systems in which multiple cells and different extracellular environments interact in space and time. This symposium will feature up-and-coming researchers from Japan and abroad, who will share their unique perspectives and techniques with the conference participants, and share their cutting-edge findings in chronic inflammation and cancer microenvironment research, including novel mechanisms of fibroblasts-mediated inflammation and carcinogenesis; overcoming chronic inflammation and carcinogenesis by targeting cellular senescence; development of novel therapies for liver inflammation and carcinogenesis targeting the tumor microenvironment; development of novel technologies to visualize cell-cell interactions in vivo; and development of brain tumor therapies by engineering cell-cell interactions.

### OS10-6
### Mesenchymal transition in glioblastoma driven by inflammatory cells

[Speaker]Nako Maishi (Dept Neurosurg. Med. Univ. Michigan. USA／BI. Univ. Michigan. USA／Rogel Cancer Center Univ. Michigan. USA) [Co-author]Toshiro Hara (Dept Neurosurg. Med. Univ. Michigan. USA／BI. Univ. Michigan. USA／Rogel Cancer Center Univ. Michigan. USA)

The mesenchymal subtype of glioblastoma is associated with increased aggressiveness, therapeutic resistance, and poor clinical outcomes. This phenotype is believed to arise from a combination of intrinsic alterations in cancer cells and extrinsic cues from the tumor microenvironment; however, the mechanisms governing the transition to this state remain incompletely understood. In this study, we investigate glioblastoma – microenvironment interactions by integrating single-cell RNA sequencing of human tumors and model systems with functional assays. Our findings reveal that multiple inflammatory cell types within the tumor microenvironment promote mesenchymal states in glioblastoma cells through the secretion of soluble factors. We will discuss how these microenvironmental signals shape glioblastoma cell plasticity and invasive potential, offering new insights into the mechanisms driving disease progression.

[Disclosure of COI by Responsible researcher] Lead presenter is not the Responsible researcher.
[Lead presenter: Nako Maishi] We have no financial relationships to disclose.
[Principal Researcher: Toshiro Hara] We have no financial relationships to disclose.

### [IS8-1] IS8

### Triangular dynamics among iron, sulfur and oxygen in cancer: novel redox concepts toward cancer prevention and therapeutics

2025 / 9 / 27 (Sat) 12:50-15:20 Room 4/(Hotel Nikko Kanazawa 4F Tsuru B) Room 4

[Chairpersons]Shinya Toyokuni (Department of Pathology and Biological Responses, Nagoya University Graduate School of Medicine)、Young-Joon SURH (Seoul National University)

The intricate interplay among iron, sulfur, and oxygen represents a fundamental yet often overlooked aspect of cancer biology. This **triangular redox dynamics** provides a fresh perspective on how oxidative and reductive forces shape tumor development and progression. **Iron**, a double-edged sword in cellular metabolism, fuels oxidative stress through the **Fenton reaction**, generating reactive oxygen species (ROS) that induce DNA damage and genomic instability. **Oxygen**, while essential for cellular respiration, also amplifies oxidative stress under dysregulated conditions, exacerbating cancer risk. In contrast, **sulfur-containing compounds**, such as glutathione and persulfides, counteract this oxidative burden by maintaining a reductive environment and supporting antioxidant defense mechanisms.
Understanding the balance among these three elements is critical, as it dictates cancer cell survival, proliferation, and therapeutic resistance. Emerging concepts such as **ferroptosis**, a form of iron-dependent cell death, and the role of **sulfur metabolism in redox regulation**, offer promising avenues for intervention. By targeting iron metabolism, modulating sulfur pathways, and controlling oxidative stress, novel strategies for cancer prevention and therapy may be developed, opening new frontiers in redox-based oncology.

---

### IS8-1

### Pseudohypoxic Stabilization of HIF-1α by NRF2: Implications for Metabolic Reprogramming of Hepatoma Cells

[Speaker]Young-Joon Surh (College of Pharmacy, Seoul National University, Seoul, South Korea)

Hypoxia-inducible factor-1α (HIF-1α) and nuclear factor erythroid 2-related factor 2 (NRF2) are constitutively overactivated in hepatocellular carcinoma (HCC). In the present study, we investigated their possible complementary roles in growth and progression of HCC cells and underlying molecular mechanisms. While silencing HIF-1α in HepG2 human hepatoma cells did not alter the protein expression of NRF2, NRF2 knockdown markedly reduced the nuclear accumulation of HIF-1α without influencing its mRNA expression. In diethylnitrosamine-induced murine hepatocarcinogenesis, there was elevated NRF2 expression with concomitant upregulation of HIF-1α. NRF2 and HIF-1α co-localize and physically interact with each other. In addition, the interaction between NRF2 and HIF-1α was found in specimens obtained from HCC patients. In normoxia, HIF-1α undergoes hydroxylation by a specific HIF-prolyl hydroxylase domain protein (PHD), which facilitates its ubiquitination and proteasomal degradation. These findings suggest that NRF2 directly binds to HIF-1α, which hampers PHD2-mediated hydroxylation of HIF-1α, leading to prolonged stabilization of the latter transcription factor.

[Disclosure of COI by Responsible researcher] Lead presenter is the Responsible researcher.
[Lead presenter: Young-Joon Surh] We have no financial relationships to disclose.

▲TOP

### [IS8-2] IS8

### Triangular dynamics among iron, sulfur and oxygen in cancer: novel redox concepts toward cancer prevention and therapeutics

2025 / 9 / 27 (Sat) 12:50-15:20 Room 4/(Hotel Nikko Kanazawa 4F Tsuru B) Room 4

[Chairpersons]Shinya Toyokuni (Department of Pathology and Biological Responses, Nagoya University Graduate School of Medicine)、Young-Joon SURH (Seoul National University)

The intricate interplay among iron, sulfur, and oxygen represents a fundamental yet often overlooked aspect of cancer biology. This **triangular redox dynamics** provides a fresh perspective on how oxidative and reductive forces shape tumor development and progression. **Iron**, a double-edged sword in cellular metabolism, fuels oxidative stress through the **Fenton reaction**, generating reactive oxygen species (ROS) that induce DNA damage and genomic instability. **Oxygen**, while essential for cellular respiration, also amplifies oxidative stress under dysregulated conditions, exacerbating cancer risk. In contrast, **sulfur-containing compounds**, such as glutathione and persulfides, counteract this oxidative burden by maintaining a reductive environment and supporting antioxidant defense mechanisms.
Understanding the balance among these three elements is critical, as it dictates cancer cell survival, proliferation, and therapeutic resistance. Emerging concepts such as **ferroptosis**, a form of iron-dependent cell death, and the role of **sulfur metabolism in redox regulation**, offer promising avenues for intervention. By targeting iron metabolism, modulating sulfur pathways, and controlling oxidative stress, novel strategies for cancer prevention and therapy may be developed, opening new frontiers in redox-based oncology.

---

### IS8-2

### NRF2-Driven Reductive Stress Impairs Anti-Tumor Immunity in the Tumor Microenvironment

[Speaker]Madoka Kawaguchi (Dept. Med. Biochem., Tohoku Univ., Grad. Sch. Med.) [Co-author]Shohei Murakami (Dept. Med. Biochem., Tohoku Univ., Grad. Sch. Med.)、Keito Okazaki (Dept. Med. Biochem., Tohoku Univ., Grad. Sch. Med.／Dept. Gene Expr. Regul., IDAC, Tohoku Univ.)、Shaoting Pan (Dept. Med. Biochem., Tohoku Univ., Grad. Sch. Med.)、Haruna Takeda (Dept. Med. Biochem., Tohoku Univ., Grad. Sch. Med.)、Shigeyuki Shichino (Div. Mol. Regul. Inflamm. & Immune Dis., RIBS, TUS)、Kazuki Hayasaka (Dept. Med. Biochem., Tohoku Univ., Grad. Sch. Med.／Dept. Thorac. Surg., IDAC, Tohoku Univ.)、Chikara Sakai (Dept. Med. Biochem., Tohoku Univ., Grad. Sch. Med.／Dept. Thorac. Surg., IDAC, Tohoku Univ.)、Tomoaki Ida (Dept. Redox Mol. Med., Tohoku Univ., Grad. Sch. Med.)、Tsuyoshi Takata (Dept. Redox Mol. Med., Tohoku Univ., Grad. Sch. Med.)、Yuki Mochida (Dept. Adv. Nanomed. Eng., Inst. Integr. Res., Inst. Sci. Tokyo)、Yoshinori Okada (Dept. Thorac. Surg., IDAC, Tohoku Univ.)、Takashi Suzuki (Dept. Anat. Pathol., Tohoku Univ., Grad. Sch. Med.)、Takaaki Akaike (Dept. Redox Mol. Med., Tohoku Univ., Grad. Sch. Med.)、Hozumi Motohashi (Dept. Med. Biochem., Tohoku Univ., Grad. Sch. Med.／Dept. Gene Expr. Regul., IDAC, Tohoku Univ.)

Recent advances in analytical chemistry have identified supersulfides as conserved, endogenously produced biomolecules with strong antioxidant and anti-inflammatory properties. Emerging evidence suggests their abundance is associated with cancer malignancy. However, underlying mechanisms for the detrimental effects of supersulfides in tumor microenvironment (TME) remain unclear. Here, we investigated NRF2-activated cancer cells, characterized by enhanced sulfur metabolism. We found that NRF2 promotes supersulfide synthesis and the excretion of glutathione (GSH) and glutathione persulfide (GSSH). These supersulfides suppressed immune cell migration, inhibited $CD8^+$ T cell activation, and reduced the survival of $CD8^+$ T cells and dendritic cells, indicating strong immunosuppressive effects.Additionally, GSH polarized macrophages toward a tumor-promoting phenotype, further inhibiting $CD8^+$ T cell function. Together, our findings suggest that a reductive, supersulfide-rich TME impairs anti-tumor immunity. Importantly, targeting this disrupted redox balance may represent a promising therapeutic strategy for NRF2-driven cancers.

[Disclosure of COI by Responsible researcher] Lead presenter is not the Responsible researcher.
[Lead presenter: Madoka Kawaguchi] We have no financial relationships to disclose.
[Principal Researcher: Hozumi Motohashi] We have no financial relationships to disclose.

### [IS8-3] IS8

### Triangular dynamics among iron, sulfur and oxygen in cancer: novel redox concepts toward cancer prevention and therapeutics

2025 / 9 / 27 (Sat) 12:50-15:20 Room 4/(Hotel Nikko Kanazawa 4F Tsuru B) Room 4

[Chairpersons]Shinya Toyokuni (Department of Pathology and Biological Responses, Nagoya University Graduate School of Medicine)、Young-Joon SURH (Seoul National University)

The intricate interplay among iron, sulfur, and oxygen represents a fundamental yet often overlooked aspect of cancer biology. This **triangular redox dynamics** provides a fresh perspective on how oxidative and reductive forces shape tumor development and progression. **Iron**, a double-edged sword in cellular metabolism, fuels oxidative stress through the **Fenton reaction**, generating reactive oxygen species (ROS) that induce DNA damage and genomic instability. **Oxygen**, while essential for cellular respiration, also amplifies oxidative stress under dysregulated conditions, exacerbating cancer risk. In contrast, **sulfur-containing compounds**, such as glutathione and persulfides, counteract this oxidative burden by maintaining a reductive environment and supporting antioxidant defense mechanisms.
Understanding the balance among these three elements is critical, as it dictates cancer cell survival, proliferation, and therapeutic resistance. Emerging concepts such as **ferroptosis**, a form of iron-dependent cell death, and the role of **sulfur metabolism in redox regulation**, offer promising avenues for intervention. By targeting iron metabolism, modulating sulfur pathways, and controlling oxidative stress, novel strategies for cancer prevention and therapy may be developed, opening new frontiers in redox-based oncology.

### IS8-3

### Spatial transcriptomic morphometrics unveil renal carcinogenic shift via oxidative stress and BRCA1 haploinsufficiency

[Speaker]Yingyi Kong (Dept. Pathol. & Biol., Nagoya.Univ., Grad. Sch. Med.) [Co-author]Yukihiro Shiraki (Dept. Tumor Pathol., Nagoya.Univ., Grad. Sch. Med.)、Atsushi Enomoto (Dept. Tumor Pathol., Nagoya.Univ., Grad. Sch. Med.)、Tatsuhiko Imaoka (Dept. Radiation Effects Research, NIRS, QST)、Shinya Toyokuni (Dept. Pathol. & Biol., Nagoya.Univ., Grad. Sch. Med.)

BRCA1 haploinsufficiency accelerates iron-driven renal tumorigenesis, but the initiating spatial events remain undefined. Using Xenium in-situ spatial transcriptomics on $Brca1^{+/-}$ rat kidneys, we mapped single cells with matched nuclear morphometrics and biological signatures.
In mutants, we observed an early, selective expansion of injury-linked proximal-tubule subsets, up-regulating pro-oncogenic and ferroptosis-resistant programs, while homeostatic transport genes collapsed. Concurrently, a karyomegalic epithelial population (cells with enlarged, dysmorphic nuclei) emerged with unique patterns in mutants. Graph-based spatial analysis revealed preferential stromal cell recruitment around these altered tubules in mutant cortex, forming permissive niches that may drive malignant progression. Under identical iron stress, wild-type kidneys exhibited a markedly lower incidence of stromal-epithelial remodeling.
Together, these data define an early, BRCA1-sensitive route to renal carcinogenesis: integrating epithelial reprogramming, nuclear dysmorphology, and microenvironmental remodeling, showcase the power of spatial transcriptomics to pinpoint actionable disease microdomains.

[Disclosure of COI by Responsible researcher] Lead presenter is not the Responsible researcher.
[Lead presenter: Yingyi Kong] We have no financial relationships to disclose.
[Principal Researcher: Shinya Toyokuni] We have no financial relationships to disclose.

▲TOP

### [IS8-4] IS8

### Triangular dynamics among iron, sulfur and oxygen in cancer: novel redox concepts toward cancer prevention and therapeutics

2025 / 9 / 27 (Sat) 12:50-15:20 Room 4/(Hotel Nikko Kanazawa 4F Tsuru B) Room 4

[Chairpersons]Shinya Toyokuni (Department of Pathology and Biological Responses, Nagoya University Graduate School of Medicine)、Young-Joon SURH (Seoul National University)

The intricate interplay among iron, sulfur, and oxygen represents a fundamental yet often overlooked aspect of cancer biology. This **triangular redox dynamics** provides a fresh perspective on how oxidative and reductive forces shape tumor development and progression. **Iron**, a double-edged sword in cellular metabolism, fuels oxidative stress through the **Fenton reaction**, generating reactive oxygen species (ROS) that induce DNA damage and genomic instability. **Oxygen**, while essential for cellular respiration, also amplifies oxidative stress under dysregulated conditions, exacerbating cancer risk. In contrast, **sulfur-containing compounds**, such as glutathione and persulfides, counteract this oxidative burden by maintaining a reductive environment and supporting antioxidant defense mechanisms.
Understanding the balance among these three elements is critical, as it dictates cancer cell survival, proliferation, and therapeutic resistance. Emerging concepts such as **ferroptosis**, a form of iron-dependent cell death, and the role of **sulfur metabolism in redox regulation**, offer promising avenues for intervention. By targeting iron metabolism, modulating sulfur pathways, and controlling oxidative stress, novel strategies for cancer prevention and therapy may be developed, opening new frontiers in redox-based oncology.

---

### IS8-4

### Hijacking Ferroptosis with Selenium: A Redox Therapeutic Avenue

[Speaker]Namgyu Lee (Department of Biomedical Science & Systems Biology, Dankook University, Korea) [Co-author]Ihyeon Ahn co first (Department of Biomedical Science & Systems Biology, Dankook University, Korea)、Sung jin Park co first (PMM, University of Massachusetts Chan Medical School, USA)、Christopher Switzer (Department of Molecular and Cell Biology, University of Leicester, England)、Sena Atici (Rudolf Virchow Zentrum, University of Wuerzburg, Wuerzburg, Germany)、Inhwan Yoo (Department of Biomedical Science & Systems Biology, Dankook University, Korea／Department of Microbiology and Biotechnology, Dankook University, Cheonan, South Korea)、Jumi Lee (Department of Biomedical Science & Systems Biology, Dankook University, Korea)、Lenny H.E. Winkel (Institute of Biogeochemistry and Pollutant Dynamics, ETH, Zurich, Switzerland)、Paul L. Greer (PMM, University of Massachusetts Chan Medical School, USA)、Jose pedro Friedmann angeli (Rudolf Virchow Zentrum, University of Wuerzburg, Wuerzburg, Germany)、Heather R. Keys (Whitehead Institute for Biomedical Research, Cambridge, MA, USA)、Sunggyu Lee (Department of Biomedical Laboratory Science, Dankook University, Cheonan, Korea)、Seung-soon Im (Department of Physiology, Keimyung University School of Medicine, Korea)、Jessica B. Spinelli (PMM, University of Massachusetts Chan Medical School, USA)、Dohoon Kim (MCCB, University of Massachusetts Chan Medical School, USA)

Selenium is an essential micronutrient with redox activity similar to that of sulfur, yet it exhibits a paradoxical role in ferroptosis, protecting cells at physiological levels while inducing oxidative cell death at higher concentrations. Our recent work has uncovered a noncanonical mechanism in which selenium, in its reduced form as hydrogen selenide produced by sulfide quinone oxidoreductase (SQOR), modulates mitochondrial redox dynamics independently of GPX4. Interestingly, our ongoing research indicates that this same pathway also mediates selenium-induced cytotoxicity, revealing a concentration-dependent dual role for selenium in the regulation of ferroptosis. In this presentation, I will highlight how hijacking ferroptosis through the modulation of selenium metabolism offers a novel and selective therapeutic strategy for exploiting redox vulnerabilities in cancer cells.

[Disclosure of COI by Responsible researcher] Lead presenter is the Responsible researcher.
[Lead presenter: Namgyu Lee] We have no financial relationships to disclose.

▲TOP

### [IS8-5] IS8

### Triangular dynamics among iron, sulfur and oxygen in cancer: novel redox concepts toward cancer prevention and therapeutics

2025 / 9 / 27 (Sat) 12:50-15:20 Room 4/(Hotel Nikko Kanazawa 4F Tsuru B) Room 4

[Chairpersons]Shinya Toyokuni (Department of Pathology and Biological Responses, Nagoya University Graduate School of Medicine)、Young-Joon SURH (Seoul National University)

The intricate interplay among iron, sulfur, and oxygen represents a fundamental yet often overlooked aspect of cancer biology. This **triangular redox dynamics** provides a fresh perspective on how oxidative and reductive forces shape tumor development and progression. **Iron**, a double-edged sword in cellular metabolism, fuels oxidative stress through the **Fenton reaction**, generating reactive oxygen species (ROS) that induce DNA damage and genomic instability. **Oxygen**, while essential for cellular respiration, also amplifies oxidative stress under dysregulated conditions, exacerbating cancer risk. In contrast, **sulfur-containing compounds**, such as glutathione and persulfides, counteract this oxidative burden by maintaining a reductive environment and supporting antioxidant defense mechanisms.
Understanding the balance among these three elements is critical, as it dictates cancer cell survival, proliferation, and therapeutic resistance. Emerging concepts such as **ferroptosis**, a form of iron-dependent cell death, and the role of **sulfur metabolism in redox regulation**, offer promising avenues for intervention. By targeting iron metabolism, modulating sulfur pathways, and controlling oxidative stress, novel strategies for cancer prevention and therapy may be developed, opening new frontiers in redox-based oncology.

---

### IS8-5

### A lactate-lipid peroxidation-acetate metabolic axis between TAMs and cancer cells fuels HCC metastasis

[Speaker]Ming Lu (Shanghai Institute of Nutrition and Health, CAS, Shanghai, China) [Co-author]Li Shen (Shanghai Institute of Nutrition and Health, CAS, Shanghai, China)

Increased acetyl-CoA generation facilitates cancer metastasis and represents a critical metabolic characteristic in metastatic cancers. To maintain high abundance of acetyl-CoA, cancer cells exhibit an enhanced uptake of acetate for the biosynthesis of acetyl-CoA in various cancers. However, the source of acetate picked up by cancer cells remains largely unknown. Here, using hepatocellular carcinoma (HCC) models, we demonstrate that tumor-associated macrophages (TAMs) promote acetate accumulation in HCC cells by secreting acetate into the cancer microenvironment. Mechanistically, lipid peroxidation-ALDH2 pathway is responsible for the acetate production in TAMs. Silencing ALDH2 in TAMs abrogates acetate-induced migration of HCC cells in vitro. In mice HCC model, genetic ablation of ALDH2 in TAMs reduces acetate levels in HCC cells of primary tumors, and significantly diminishes lung metastasis in vivo. Finally, we identify lactate as the inducer of lipid peroxidation-ALDH2 pathway in TAMs. Collectively, our findings reveal a lactate-lipid peroxidation-acetate metabolic interaction between HCC cells and TAMs, which positions TAMs as an acetate reservoir to fuel HCC metastasis.

[Disclosure of COI by Responsible researcher] Lead presenter is the Responsible researcher.
[Lead presenter: Ming Lu] We have no financial relationships to disclose.

▲TOP

### [IS8-6] IS8

### Triangular dynamics among iron, sulfur and oxygen in cancer: novel redox concepts toward cancer prevention and therapeutics

2025 / 9 / 27 (Sat) 12:50-15:20 Room 4/(Hotel Nikko Kanazawa 4F Tsuru B) Room 4

[Chairpersons]Shinya Toyokuni (Department of Pathology and Biological Responses, Nagoya University Graduate School of Medicine)、Young-Joon SURH (Seoul National University)

The intricate interplay among iron, sulfur, and oxygen represents a fundamental yet often overlooked aspect of cancer biology. This **triangular redox dynamics** provides a fresh perspective on how oxidative and reductive forces shape tumor development and progression. **Iron**, a double-edged sword in cellular metabolism, fuels oxidative stress through the **Fenton reaction**, generating reactive oxygen species (ROS) that induce DNA damage and genomic instability. **Oxygen**, while essential for cellular respiration, also amplifies oxidative stress under dysregulated conditions, exacerbating cancer risk. In contrast, **sulfur-containing compounds**, such as glutathione and persulfides, counteract this oxidative burden by maintaining a reductive environment and supporting antioxidant defense mechanisms.
Understanding the balance among these three elements is critical, as it dictates cancer cell survival, proliferation, and therapeutic resistance. Emerging concepts such as **ferroptosis**, a form of iron-dependent cell death, and the role of **sulfur metabolism in redox regulation**, offer promising avenues for intervention. By targeting iron metabolism, modulating sulfur pathways, and controlling oxidative stress, novel strategies for cancer prevention and therapy may be developed, opening new frontiers in redox-based oncology.

---

### IS8-6

### Oxidative stress adaptation and its role in cholangiocarcinogenesis: the EBF1-IRS1 axis in focus

[Speaker]Raynoo Thanan (Dept. of Biochem., Fac. of Med., Khon Kaen Univ., Thailand.) [Co-author]Waleeporn Kaewlert (Dept. of Biochem., Fac. of Med., Khon Kaen Univ., Thailand.)、Napat Armartmuntree (Dept. of Med. Sci., Amnatcharoen Camp., Mahidol Univ.,Thailand.)、Chadamas Sakonsilsiri (Dept. of Biochem., Fac. of Med., Khon Kaen Univ., Thailand.)、Worachart Lert-itthiporn (Dept. of Biochem., Fac. of Med., Khon Kaen Univ., Thailand.)、Panupong Mahalapbutr (Dept. of Biochem., Fac. of Med., Khon Kaen Univ., Thailand.)、Somchai Pinlaor (Dept. of Parasit., Fac. of Med., Khon Kaen Univ., Thailand.)、Mariko Murata (Dept. of Envi&Mol. Med., Mie Univ., Japan.)

Oxidative stress, caused by an imbalance between oxidants and antioxidants, contributes to cholangiocarcinoma (CCA) development. We hypothesized that some cells adapt to chronic oxidative stress, acquiring survival and tumor-promoting traits. A hydrogen peroxide-resistant cholangiocyte cell line was developed, and it showed enhanced proliferation, migration, and stem-like properties, with downregulated early B cell factor 1 (EBF1) and upregulated insulin receptor substrate 1 (IRS1). These changes, also seen in CCA tissues, correlated with poor prognosis. EBF1 down regulation increased estrogen response and IRS1 expression, promoting oxidative stress resistance and stemness. Transcriptomic analysis revealed activation of PI3K and MEKK3 pathways. Targeting these pathways reduced CCA aggressiveness in vitro and in vivo. Our findings reveal a novel link between oxidative stress adaptation, estrogen signaling, and cancer stemness in liver fluke-associated CCA, identifying EBF1 and IRS1 as potential therapeutic targets.

[Disclosure of COI by Responsible researcher] Lead presenter is the Responsible researcher.
[Lead presenter: Raynoo Thanan] We have no financial relationships to disclose.

### [E-3031] I-E11-5
### Cancer vaccines/Other immunotherapies (1)

2025 / 9 / 27 (Sat) 12:50-14:05 Room 5/(Hotel Nikko Kanazawa 4F Tsuru C) Room 5

[Chairperson]Hideshi Ishii (CoMIT. Univ. Osaka, Sch. Med.)

---

### E-3031
### Neo-DCVac in Combination with Immune Checkpoint Inhibitors for the Treatment of ICI-Resistant Advanced Lung Cancer

[Speaker]Juanyan Liao (Dept. of Biotherapy, West China Hspt., Sichuan Univ.) [Co-author]Qing Li (Dept. of Biotherapy, West China Hspt., Sichuan Univ.)、Yinyin Xue (Lung Cancer Ctr., West China Hspt., Sichuan Univ.)、Heng Xu (State Key Lab. of Biotherapy, West China Hspt., Sichuan Univ.)、Li Yang (State Key Lab. of Biotherapy, West China Hspt., Sichuan Univ.)、Zhenyu Ding (Dept. of Biotherapy, West China Hspt., Sichuan Univ.)

Background: Immune checkpoint inhibitors (ICIs) improve outcomes in advanced lung cancer, but resistance persists. This prompted a clinical trial evaluating the efficacy and safety of Neo-DCVac(a neoantigen dendritic cell vaccine) plus ICIs in ICI-resistant advanced lung cancer.Methods: Eligible patients received Neo-DCVac while continuing ICIs during vaccine preparation and administration. A full immunization course comprised five doses. Efficacy was assessed post-cycle: responders continued treatment, while those with confirmed progression discontinued therapy.Results: Of 15 screened patients, 9 received Neo-DCVac. One patient died of pericardial effusion, 1 died of COVID and 3 experienced progression post-treatment. Four patients continued therapy. By March 2025, median PFS was 10.5 months, and OS was 14.37 months. Among those receiving 10-12 doses, 44.4% (n = 4) had > 20% tumor regression, with two showing > 50% reduction. Post-vaccination analysis indicated antigen-specific immune activation and elevated IFN$\gamma$ levels. Conclusion: Neo-DCVac plus ICIs demonstrates clinical benefit in ICI-resistant lung cancer and warrants further study.Note: This is an encore submission.

[Disclosure of COI by Responsible researcher] Lead presenter is not the Responsible researcher.
[Lead presenter: Juanyan Liao] We have no financial relationships to disclose.
[Principal Researcher: Juanyan Liao] We have no financial relationships to disclose.

### [E-3032] I-E11-5
### Cancer vaccines/Other immunotherapies (1)

2025 / 9 / 27 (Sat) 12:50-14:05 Room 5/(Hotel Nikko Kanazawa 4F Tsuru C) Room 5

[Chairperson]Hideshi Ishii (CoMIT. Univ. Osaka, Sch. Med.)

---

### E-3032
### EZH2 inhibitor combined with radiotherapy induces immune response by activating cGAS-STING pathway in prostate cancer

[Speaker]Tian-Qi Du (Cancer Center, People's Hospital of Yangjiang) [Co-author]Gang Chen (Cancer Center, People's Hospital of Yangjiang)

The enhancer of zeste homolog 2 (EZH2) is overexpressed in prostate cancer (PCa) and correlates with a poor prognosis. In this study, we investigated the immune response induced by EZH2 inhibitors (EZH2i) in combination with radiotherapy in PCa and its underlying mechanisms. We observed cell surface localization of MHC-1, PD-L1 and calreticulin, as well as accumulation of dsDNA in the cytoplasm with a radiation dose-dependent increase by combination. Further we confirmed that dsDNA activates the cGAS-STING pathway. The same expression trend was also observed in TNF-$\alpha$, IFN-$\alpha$, IFN-$\beta$, IL-2, IL-6, CCL-5, and CXCL-10. ***In vivo*** experiments showed that the combination group increased the proportion of $CD4^+$ and $CD8^+$ T cells and decreased the proportion of MDSCs and Tregs in peripheral blood, spleen, and tumors, and enhanced the response to anti-PD-1 with a survival benefit. These results were not observed in ***Cgas***$^{-/-}$ mice. EZH2i combined with radiotherapy mediates the reshaping of the immune microenvironment in PCa through the cGAS-STING pathway, which has a positive modulatory effect on tumor immunity and contributes to the enhancement of response to immune checkpoint blockade.

[Disclosure of COI by Responsible researcher] Lead presenter is the Responsible researcher.
[Lead presenter: Tian-Qi Du] We have no financial relationships to disclose.

English Oral Session

### [E-3033] I-E11-5
### Cancer vaccines/Other immunotherapies (1)

2025 / 9 / 27 (Sat) 12:50-14:05 Room 5/(Hotel Nikko Kanazawa 4F Tsuru C) Room 5

[Chairperson]Hideshi Ishii (CoMIT. Univ. Osaka, Sch. Med.)

---

### E-3033
### Novel combination immunotherapy to enhance the efficacy of immune checkpoint inhibitors against hepatocellular carcinoma

[Speaker]Masao Nakajima (Dept. of Gastroenterological Surgery, Yamaguchi Univ..) [Co-author]Ryouichi Tsunedomi (Dept. of Gastroenterological Surgery, Yamaguchi Univ..)、Takahiro Ozasa (Dept. of Gastroenterological Surgery, Yamaguchi Univ..)、Yuta Kimura (Dept. of Gastroenterological Surgery, Yamaguchi Univ..)、Hiroto Matsui (Dept. of Gastroenterological Surgery, Yamaguchi Univ..)、Yukio Tokumitsu (Dept. of Gastroenterological Surgery, Yamaguchi Univ..)、Yoshitaro Shindo (Dept. of Gastroenterological Surgery, Yamaguchi Univ..)、Yusaku Watanabe (Dept. of Gastroenterological Surgery, Yamaguchi Univ..)、Shinobu Tomochika (Dept. of Gastroenterological Surgery, Yamaguchi Univ..)、Michihisa Iida (Dept. of Gastroenterological Surgery, Yamaguchi Univ..)、Hidenori Takahashi (Dept. of Gastroenterological Surgery, Yamaguchi Univ..)、Tatsuya Ioka (Dept. of Immunology, Yamaguchi Univ.)、Koji Tamada (Oncology center, Yamaguchi university hospital)、Hiroaki Nagano (Dept. of Gastroenterological Surgery, Yamaguchi Univ..)

Background: HCC frequently recurs after curative resection. ICIs are effective in immunologically "hot" tumors with substantial lymphocyte infiltration. We developed a novel immunotherapy combining two adjuvants (hLAG-3Ig, Poly-ICLC) with peptides of Heat Shock Protein 70 and Glypican-3 to induce a "hot" tumor state. A phase I trial confirmed perioperative safety for resectable HCC.
Methods: Twenty resectable HCC pts. received 6 preoperative and 10 postoperative doses of this therapy. Tumor-infiltrating lymphocytes of resected tumors were analyzed via IHC and mass cytometry (CyTOF). Twenty surgical patients without preoperative treatment served as controls.
Results: IHC revealed significantly higher CD8+PD-1+ cell infiltration in 60% of treated pts. versus 20% controls. CyTOF detected activated antigen-specific T cells exclusively in treated pts. Overall survival trended positively in treated pts. (p=0.056). Among those receiving ICIs for recurrence, four of six treated pts. responded (including 3 pts. with CR), compared to one of seven controls.
Conclusion: This therapy may convert HCC tumors to a "hot" state, enhancing responsiveness to ICIs against recurrence.

[Disclosure of COI by Responsible researcher] Lead presenter is not the Responsible researcher.
[Lead presenter: Masao Nakajima] We have no financial relationships to disclose.
[Principal Researcher: Hiroaki Nagano] ■Research funds from company: Toyo Kohan Co., Ltd. ■Contributions: TAIHO PHARMACEUTICAL CO., LTD., Shionogi & Co., Ltd., MSD K.K, Otsuka Pharmaceutical Factory, Inc., Otsuka Pharmaceutical Co., Ltd., TSUMURA & CO., Kyowa Hakko Kirin Co.,Ltd., Kaken Pharmaceutical Co., Ltd., NIPPON KAYAKU Co., Ltd., DAIICHI SANKYO COMPANY, LIMITED., Japan Blood Products Organization, Chugai Pharmaceutical Co., Ltd.,

### [E-3034] I-E11-5
### Cancer vaccines/Other immunotherapies (1)

2025 / 9 / 27 (Sat) 12:50-14:05 Room 5/(Hotel Nikko Kanazawa 4F Tsuru C) Room 5

[Chairperson]Hideshi Ishii (CoMIT. Univ. Osaka, Sch. Med.)

---

### E-3034
### Development of a novel immunomodulation therapy for pancreatic ductal adenocarcinoma targeting complement factor B

[Speaker]Daren Zhou (Dept. General Surg., Sch., Med., Chiba Univ.) [Co-author]Shigetsugu Takano (Dept. General Surg., Sch., Med., Chiba Univ.)、Kensuke Suzuki (Dept. General Surg., Sch., Med., Chiba Univ.)、Takashi Mishima (Dept. General Surg., Sch., Med., Chiba Univ.)、Takanori Konishi (Dept. General Surg., Sch., Med., Chiba Univ.)、Masayuki Ohtsuka (Dept. General Surg., Sch., Med., Chiba Univ.)

We have discovered that complement factor B (CFB) is secreted by pancreatic ductal adenocarcinoma (PDAC) cells. We performed immunohistochemistry (IHC) for 113 resected PDAC tissues and divided into high/low stromal CFB expression groups. Median survival time was shorter (p=0.014), and the frequency of hematogenous recurrence was more (p=0.0083) in high CFB group. High CFB expression was one of the independent prognostic factors in multivariate analysis. We also found that immunosuppressive cells such as $CD8^{+}$/$Foxp3^{+}$ regulatory T-cells (Tregs) are more expressed in high CFB tissues. We next investigated the impact of selective CFB inhibitor, Iptacopan, by administration to orthotopic transplantation mice. Tumor volume in Iptacopan group was decreased compared to that in control group (p<0.0001). The combination of Iptacopan with gemcitabine and nab-paclitaxel (GnP) group showed less tumor volume than that in GnP group (p=0.019). We next performed IHC and revealed that Tregs are less observed in Iptacopan groups (p<0.001). Also, $CD8^{+}$ T-cells are much observed in Iptacopan groups (p<0.001). As a result, Iptacopan might be a future complement-based immunomodulation therapy for PDAC.

[Disclosure of COI by Responsible researcher] Lead presenter is not the Responsible researcher.
[Lead presenter: Daren Zhou] We have no financial relationships to disclose.
[Principal Researcher: Shigetsugu Takano] ■Research funds from company: デンカ株式会社

▲TOP

### [E-3035] I-E11-5
### Cancer vaccines/Other immunotherapies (1)

2025 / 9 / 27 (Sat) 12:50-14:05 Room 5/(Hotel Nikko Kanazawa 4F Tsuru C) Room 5

[Chairperson]Hideshi Ishii (CoMIT. Univ. Osaka, Sch. Med.)

---

### E-3035
### Near-infrared photoimmunotherapy using an affibody for EGFR-positive malignant brain tumor

[Speaker]Haruka Yamaguchi (Dept. of Biochem. Niigata, Nippon Dental Univ.) [Co-author]Masayasu Okada (Dept. of Neurosurgery, Brain Res. Inst. Niigata Univ.／Dept. of Brain Tumor Biol. Brain Res. Inst. Niigata Univ.)、Takuya Otani (Near InfraRed Photo-ImmunoTherapy Res. Inst. Kansai Med. Univ.)、Takamasa Suzuki (Faculty of Engineering, Niigata Univ.)、Akihiro Ishikawa (Shimadzu, Startup incubation center)、Hideyuki Sakata (Shimadzu, Startup incubation center)、Takao Morita (Dept. of Biochem. Niigata, Nippon Dental Univ.)、Manabu Natsumeda (Dept. of Neurosurgery, Brain Res. Inst. Niigata Univ.／Advanced Treatment of Neurological Diseases, Brain Res. Inst. Niigata Univ.)

**Introduction**: Near-infrared photoimmunotherapy (NIR-PIT) is a promising cancer treatment that uses a monoclonal antibody (mAb) conjugated to a photosensitizer (IR700). In this study, we investigated NIR-PIT for EGFR-positive malignant brain tumors using an affibody (6 kDa). **Materials and Methods**: RSV-M (EGFR-positive mouse brain tumor cells) was injected into the brains of C3H/HeSlc mice. Two weeks after cell injection, the EGFR Affibody-IR700 conjugate was administered via the tail vein, and real-time fluorescence imaging was performed. Subsequently, the brain tumors were irradiated with near-infrared light. After fixation, the brain tissues were analyzed using hematoxylin and eosin (HE) staining and immunohistochemistry (IHC).**Results**: Fluorescence signals appeared in the cranial region within 10 seconds of conjugate injection. HE staining confirmed the presence of cancer cells in areas with significant IR700 accumulation. IHC identified microglial cells within the tumor mass.**Conclusion**: The EGFR Affibody-IR700 conjugate crossed the blood-brain barrier and accumulated within the brain tumor. This approach can improve the efficiency of NIR-PIT in the treatment of brain tumors.

[Disclosure of COI by Responsible researcher] Lead presenter is the Responsible researcher.
[Lead presenter: Haruka Yamaguchi] We have no financial relationships to disclose.

### [E-3036] I-E11-5
### Cancer vaccines/Other immunotherapies (1)

2025 / 9 / 27 (Sat) 12:50-14:05 Room 5/(Hotel Nikko Kanazawa 4F Tsuru C) Room 5

[Chairperson]Hideshi Ishii (CoMIT. Univ. Osaka, Sch. Med.)

---

### E-3036
### Anti-cancer cell therapy using CCL19-expressing mesenchymal stem cells exerts robust anti-tumor effect

[Speaker]Yuichi Iida (Department of Immunology, Faculty of Medicine, Shimane University) [Co-author]Mamoru Harada (Department of Immunology, Faculty of Medicine, Shimane University)、Yasuyuki Saito (Department of Immunology, Faculty of Medicine, Shimane University)

Immune checkpoint blockade (ICB) is a promising anti-cancer therapy, whereas the presence of T cells in tumor sites is indispensable for its therapeutic efficacy. To promote the infiltration of T cells and dendritic cells (DCs) into the tumor, we previously proposed a cell therapy using chemokine (c-c motif) ligand 19 (CCL19)-expressing immortalized mesenchymal stem cells (iMSC/CCL19). In this study, we developed iMSC/CCL19 cell therapy using several tumor mice models. iMSC/CCL19 therapy exerted drastic anti-tumor effects via promoting the infiltration of $CD103^{+}$ interleukin (IL) -12-producing DCs and priming of $CD8^{+}$ T cells at tumor sites. The anti-tumor effect of iMSC/CCL19 therapy was not influenced by fingolimod, a sphingosine 1-phosphate receptor modulator, implying no involvement of draining lymph nodes in the priming of tumor-specific T cells. iMSC/CCL19 therapy could be a promising approach to anticancer therapy, particularly for overcoming dysfunction in the cancer-immunity cycle.

[Disclosure of COI by Responsible researcher] Lead presenter is the Responsible researcher.
[Lead presenter: Iida Yuichi] We have no financial relationships to disclose.

▲TOP

### [ME5] ME5
### Cancer Cell

2025 / 9 / 27 (Sat) 12:50-13:30 Room 6/(Hotel Nikko Kanazawa 3F Kujaku A) Room 6

[Chairperson]Hideko Isozaki (Kanazawa University Cancer Research Institute)

---

### ME5
### Cancer Cell

[Speaker]Steve Mao (Cancer Cell, Cell Place)

No Abstract

▲TOP

### [E-3043] I-E6-1
### Cell cycle & DNA damage response

2025 / 9 / 27 (Sat) 12:50-14:05 Room 7/(Hotel Nikko Kanazawa 3F Kujaku B) Room 7

[Chairperson]Junko Murai (Proteo-Science Center, Ehime Univ.)

---

### E-3043
### Identification of Flavonols as PARP1 Inhibitors in Cholangiocarcinoma: Insights from Molecular Dynamics and Multi-Omics

[Speaker]Rattanaporn Jaidee (Dept. of Pharm., Faculty of Med., Khon Kaen Univ.／Cholangiocarcinoma Res. Inst., Khon Kaen Univ.) [Co-author]Piman Pocasap (Dept. of Pharm., Faculty of Med., Khon Kaen Univ.／Cholangiocarcinoma Res. Inst., Khon Kaen Univ.)、Karnchanok Kaimuangpak (Dept. of Pharm., Faculty of Med., Khon Kaen Univ.／Cholangiocarcinoma Res. Inst., Khon Kaen Univ.)、Apinya Jusakul (Cholangiocarcinoma Res. Inst., Khon Kaen Univ.／Centre for Res. and Dept. of Med. Diagnostic Lab.)、Laddawan Senggunprai (Dept. of Pharm., Faculty of Med., Khon Kaen Univ.／Cholangiocarcinoma Res. Inst., Khon Kaen Univ.)、Auemduan Prawan (Dept. of Pharm., Faculty of Med., Khon Kaen Univ.／Cholangiocarcinoma Res. Inst., Khon Kaen Univ.)、Sarinya Kongpetch (Dept. of Pharm., Faculty of Med., Khon Kaen Univ.／Cholangiocarcinoma Res. Inst., Khon Kaen Univ.)

Genetic alterations in DNA damage repair (DDR) genes contribute to cholangiocarcinoma (CCA) carcinogenesis. Poly(ADP-ribose) polymerase (PARP) inhibitors (PARPi) are effective in cancers with DDR deficiencies, such as ***BRCA*** mutations. Flavonoids have been demonstrated to inhibit PARP1, providing a strategy to target DDR defects in CCA. This study investigates the antitumor effects of flavonoids as PARP1 inhibitors. Molecular docking identified flavonols as potential PARPi. Sensitivity testing revealed that quercetin was more effective than fisetin and myricetin, with KKU-023 (***BRCA***-defective) cells exhibiting higher sensitivity to quercetin than KKU-100 (***BRCA***-intact) cells. Quercetin decreased phosphorylation of DDR proteins, including ATM, ATR, and NBS1. Fourier-transform infrared spectroscopy confirmed molecular changes in flavonol-treated cells. GSEA showed enrichment of G2M checkpoint, E2F target genes, and KRAS signaling, consistent with immunoblot results showing higher G2M cell cycle arrest in quercetin-treated cells compared to controls. These results suggest that flavonols could be a potential synthetic lethal therapy for CCA patients harboring DDR mutations.

[Disclosure of COI by Responsible researcher] Lead presenter is the Responsible researcher.
[Lead presenter: Rattanaporn Jaidee] We have no financial relationships to disclose.

### [E-3044] I-E6-1
### Cell cycle & DNA damage response

2025 / 9 / 27 (Sat) 12:50-14:05 Room 7/(Hotel Nikko Kanazawa 3F Kujaku B) Room 7

[Chairperson]Junko Murai (Proteo-Science Center, Ehime Univ.)

---

### E-3044
### Targeting Motor Protein X to Disrupt Centrosome Clustering: A Therapeutic Strategy for Small Cell Lung Cancer (SCLC)

[Speaker]Natsuki Nakagawa (Departments of Respiratory Medicine, The University of Tokyo) [Co-author]Masakatsu Tokunaga (Departments of Respiratory Medicine, The University of Tokyo)、Mirei Ka (Division of Integrative Genomics, The University of Tokyo)、Takahiro Iida (Thoracic Surgery, Graduate School of Medicine, The University of Tokyo)、Takahiro Ando (Departments of Respiratory Medicine, The University of Tokyo)、Akiko Kunita (Next-Generation Precision Medicine Development Laboratory, The University of Tokyo)、Kousuke Watanabe (Next-Generation Precision Medicine Development Laboratory, The University of Tokyo)、Yasunori Ohta (Department of Pathology, Research Hospital, The University of Tokyo)、Xi Liu (Molecular Pharmacology Program, Frederick National Laboratory for Cancer Research)、Ethan Dmitrovsky (Molecular Pharmacology Program, Frederick National Laboratory for Cancer Research)、Hidenori Kage (Departments of Respiratory Medicine, The University of Tokyo)、Masanori Kawakami (Departments of Respiratory Medicine, The University of Tokyo)

Centrosome amplification is a hallmark of cancer. Cancer cells cluster supernumerary centrosomes to ensure bipolar mitosis. We previously reported that CDK2 inhibition disrupts this clustering, inducing multipolar cell division and apoptotic death, termed anaphase catastrophe. Motor protein X facilitates centrosome clustering but is dispensable in normal cells. SCLC, characterized by p53 inactivation and frequent centrosome amplification, may be particularly vulnerable to mitotic disruption. We investigated protein X as a therapeutic target in SCLC.SCLC cells exhibited higher centrosome amplification and overexpressed protein X compared to normal bronchial epithelial (NHBE) cells. Genetic and pharmacological inhibition of protein X reduced proliferation and induced apoptosis in SCLC cells, with minimal effects on NHBE cells. Immunofluorescence analysis revealed decreased centrosome clustering and increased multipolar mitosis upon protein X inhibition, indicating anaphase catastrophe. The in vivo effects of protein X inhibition were confirmed using a xenograft model.Prevention of supernumerary centrosome clustering by targeting protein X is a promising therapeutic strategy for SCLC.

[Disclosure of COI by Responsible researcher] Lead presenter is not the Responsible researcher.
[Lead presenter: Natsuki Nakagawa] ■Research expenses: JSPS KAKENHI Grant Number 25K23836
[Principal Researcher: Masanori Kawakami] ■Research expenses: JSPS KAKENHI Grant Number 21H02776 and 24K02316

### [E-3045] I-E6-1
### Cell cycle & DNA damage response

2025 / 9 / 27 (Sat) 12:50-14:05 Room 7/(Hotel Nikko Kanazawa 3F Kujaku B) Room 7

[Chairperson]Junko Murai (Proteo-Science Center, Ehime Univ.)

---

### E-3045
### Tissue-specificity in centriole duplication regulation by BRCA1-associated protein RACK1

[Speaker]Yuki Yoshino (Dept. Cancer Biology, IDAC, Tohoku Univ.) [Co-author]Yukako Yoshizaki (Dept. Cancer Biology, IDAC, Tohoku Univ.)、Rena Fujisawa (Dept. Cancer Biology, IDAC, Tohoku Univ.)、Shin Kikuta (Dept. Cancer Biology, IDAC, Tohoku Univ.)、Natsuko Chiba (Dept. Cancer Biology, IDAC, Tohoku Univ.)

Proper centriole duplication is essential for maintaining centrosome numbers in the cell cycle. BRCA1, of which defect is responsible for hereditary breast cancer, contributes to the regulation of centriole duplication in mammary epithelial cells; however, its molecular mechanisms are not well understood yet. We previously reported that BRCA1-associated protein RACK1 also regulates centriole duplication in mammary epithelial cells. In this study, we found that RACK1 stabilized polo-like kinase 4 (PLK4) to promote centriole duplication. RACK1 regulated the expression level of PLK4 in various cells, regardless of their tissue of origin. Interestingly, centriole duplication was suppressed by RACK1 knockdown only in mammary epithelium-derived cells. Similarly, centriole duplication in mammary epithelium-derived cells was more efficiently suppressed by PLK4 knockdown. These data suggested that centriole duplication in mammary epithelium cells is highly sensitive to changes in PLK4 expression level. This difference in sensitivity may cause tissue-specific centrosome aberration in BRCA1-associated cancers.

[Disclosure of COI by Responsible researcher] Lead presenter is the Responsible researcher.
[Lead presenter: Yuki Yoshino] ■Research funds from company: ハーモニック伊藤財団、小林財団

### [E-3046] I-E6-1
### Cell cycle & DNA damage response

2025 / 9 / 27 (Sat) 12:50-14:05 Room 7/(Hotel Nikko Kanazawa 3F Kujaku B) Room 7

[Chairperson]Junko Murai (Proteo-Science Center, Ehime Univ.)

---

### E-3046
### A novel strategy for targeting chromosomal instability in cancer

[Speaker]Motoko Takahashi (Division of Exp. Pathol. The Cancer Inst. JFCR) [Co-author]Minji Jo (Division of Exp. Pathol. The Cancer Inst. JFCR)、Nana Kamakura (Division of Exp. Pathol. The Cancer Inst. JFCR)、Chang Liu (Division of Exp. Pathol. The Cancer Inst. JFCR)、Utako Kato (Division of Exp. Pathol. The Cancer Inst. JFCR)、Toru Hirota (Division of Exp. Pathol. The Cancer Inst. JFCR)

Targeting cancer-specific vulnerabilities is crucial to the development of effective anti-cancer drugs, and one promising candidate is mitotic chromosomal instability (CIN). CIN is a common feature of cancer caused by mitotic defects such as abnormal kinetochore-microtubule attachments and subsequent chromosome missegregation. While CIN accelerates intra-tumor heterogeneity, leading to metastasis, drug resistance and poor prognosis, aneuploidy itself is known to suppress cell growth, leading to the idea that extreme ploidy alteration by targeting CIN must be detrimental to cancer cells.Plk1 and Aurora B kinases ensure the proper kinetochore-microtubule attachment in mitosis by stabilizing or destabilizing normal or abnormal attachment. We have previously reported that AurB activity is compromised, leading to chromosome missegregation in a variety of cancer cells. In this study, we aimed to manipulate the Plk1 activity and found that tipping the balance towards Plk1 can induce lethal levels of chromosome segregation errors predominantly in cancer cells. We will discuss the efficacy of the drug candidates targeting CIN.

[Disclosure of COI by Responsible researcher] Lead presenter is not the Responsible researcher.
[Lead presenter: Motoko Takahashi] We have no financial relationships to disclose.
[Principal Researcher: Toru Hirota] We have no financial relationships to disclose.

### [E-3047] I-E6-1
### Cell cycle & DNA damage response

2025 / 9 / 27 (Sat) 12:50-14:05 Room 7/(Hotel Nikko Kanazawa 3F Kujaku B) Room 7

[Chairperson]Junko Murai (Proteo-Science Center, Ehime Univ.)

---

### E-3047
### Dysregulation of supercoil dynamics associated with replication stress in cancer

[Speaker]Yoshiharu Kusano (Cancer Institute, Japanese Foundation For Cancer Research) [Co-author]Ryusuke Nozawa (Cancer Institute, Japanese Foundation For Cancer Research)、Toru Hirota (Cancer Institute, Japanese Foundation For Cancer Research)

Many human cancer cells are often subjected to replication stress (RS), which disrupts progression of the replication fork. During fork progression, the twisting properties of DNA duplexes inevitably result in the formation of twisted DNA structures, or supercoils around the fork. Superhelical tension stored in supercoils impedes fork progression, unless released by topoisomerase (Top). How these supercoils, which dynamically diffuse along the DNA, are regulated and released remains unknown. Here, we report that the SMC5/6 is required to stabilize supercoil dynamics during replication. This stabilization of supercoils promotes Top2A recruitment to them, ensuring efficient supercoil release and rapid fork progression. Remarkably, we also find that cancer cell lines with high RS tend to exhibit greater supercoil accumulation. Among them, HeLa cells show dysregulated supercoil dynamics and impaired Top2A recruitment to supercoils due to insufficient SMC5/6 supply to newly generated supercoils. This is likely due to the extensive use of SMC5/6 for over-accumulated supercoils. These findings highlight the relevance of dysregulated supercoil dynamics in response to RS in cancer cells.

[Disclosure of COI by Responsible researcher] Lead presenter is not the Responsible researcher.
[Lead presenter: Yoshiharu Kusano] We have no financial relationships to disclose.
[Principal Researcher: Toru Hirota] We have no financial relationships to disclose.

### [E-3048] I-E6-1
### Cell cycle & DNA damage response

2025 / 9 / 27 (Sat) 12:50-14:05 Room 7/(Hotel Nikko Kanazawa 3F Kujaku B) Room 7

[Chairperson]Junko Murai (Proteo-Science Center, Ehime Univ.)

---

### E-3048
### Synergism of Glutamine and the Calcium responsive NFATc1 transcription factor in pancreatic cancer progression

[Speaker]Volker Ellenrieder (Deptartment of Gastroenterology, University Medical Center, Goettingen, Germany) [Co-author]Laura Huhnold (Deptartment of Gastroenterology, University Medical Center, Goettingen, Germany)、Ivan Bogeski (Deptartment of Physiology, University Medical Center, Goettingen, Germany)、Umair Latif (Deptartment of Gastroenterology, University Medical Center, Goettingen, Germany)

Background: The mechanisms of metabolic reprogramming in pancreatic cancer progression and resistance remain elusive. Here, we assessed the role of Nuclear Factor of Activated T-Cells 1 (NFATc1) in metabolic reprogramming and regulation of PDAC survival.Methods: We employed human PDAC cell lines, patient-derived-xenografts and organoids to de-termine gene regulation, stress responses and metabolism in resistance. RNA-seq, HPLC/MS, seahorse and Ca2+ trafficking experiments were carried out to define glutamine/NFATc1 driven transcription and metabolic reprogramming. Result: NFATc1 regulates expression and activity of key enzymes in glutamine metabolism and controls oxidative phosphorylation and redox. Moreover, NFATc1 inactivation synergizes with glutamine pathway impairment in regulation of metabolic responses and pancreatic cancer growth and resistance. Discussion: This study shows that NFATc1 mediates addiction to glutamine in pancreatic cancer and controls cellular stress responses, Krebs cycle activity and OXPHOS, and offers therapeutic vulnerability in a particularly aggressive subtype in pancreatic cancer, characterized by nuclear accumulation of NFATc1.

[Disclosure of COI by Responsible researcher] Lead presenter is the Responsible researcher.
[Lead presenter: Volker Ellenrieder] ■Research expenses: DFG, German Cancer Aid

### [IS9-1] IS9
### Elucidating the regulatory mechanisms involved in tumor tissue heterogeneity through advanced informatics technologies

2025 / 9 / 27 (Sat) 12:50-15:20 Room 8/(Hotel Nikko Kanazawa 3F Kujaku C) Room 8

[Chairpersons]Yasuhiro Kojima (Laboratory of Computational Life Science, National Cancer Center Research Institute)、Mo Lotfollahi (Wellcome Sanger institute, University of Cambridge)

In recent years, rapid advances in high-resolution omics technologies-such as single-cell and spatial transcriptomics-have ushered in an era where comprehensive molecular profiling can illuminate the heterogeneity of cancer tissues both within and between tumors. At the same time, the growing complexity of these datasets has driven the development of diverse analytical methods-including deep learning, probabilistic modeling, and mathematical modeling-to extract meaningful insights. These approaches enable the identification of gene programs associated with clinical features, tracing the differentiation trajectories of cancer cells, and characterizing the population structures within tumor microenvironments. As a result, these computational strategies have become essential for elucidating and interpreting tumor heterogeneity in cancer biology.
This symposium will bring together researchers who develop and apply a variety of computational methods to explore cutting-edge techniques and findings that deepen our understanding of tumor heterogeneity through the analysis of high-resolution omics data. Through this exchange, we aim to envision the future of computational analyses in high-resolution omics and to investigate the transformative impact these sophisticated approaches can have on cancer biology.

---

### IS9-1
### Deriving microenvironment associated with cell state distribution by transformer-based self supervised learning

[Speaker]Yasuhiro Kojima (Comp. Lif. Lab., Nat. Canc. Ctr. Res. Inst,)

Recent advances in single cell resolution spatial omics are poised to elucidate the diversity of the tumor microenvironment (TME) by directly linking it to the heterogeneity of its constituent cells. Representation-learning approaches for capturing TME states, however, typically learn embeddings of averaged expression profiles within user defined neighborhoods, thereby failing to exploit the full resolution of single-cell data.To address this limitation, we introduce Mievformer, a deep learning framework that leverages the masked self-supervised learning paradigm to infer microenvironmental states. The model predicts the cellular state observed at the masked central location, thus learning microenvironment embeddings that are intrinsically coupled to the probability distribution of cellular states at the center.We benchmarked Mievformer against existing tools for TME state estimation using both simulated and real datasets, demonstrating its superior performance. Moreover, we showcase several downstream analyses enabled by these distribution-aware microenvironment embeddings, including single-cell co-localization analysis and clustering based on spatial distribution within tissues.

[Disclosure of COI by Responsible researcher] Lead presenter is the Responsible researcher.
[Lead presenter: Yasuhiro Kojima] We have no financial relationships to disclose.

### [IS9-2] IS9

### Elucidating the regulatory mechanisms involved in tumor tissue heterogeneity through advanced informatics technologies

2025 / 9 / 27 (Sat) 12:50-15:20 Room 8/(Hotel Nikko Kanazawa 3F Kujaku C) Room 8

[Chairpersons]Yasuhiro Kojima (Laboratory of Computational Life Science, National Cancer Center Research Institute)、Mo Lotfollahi (Wellcome Sanger institute, University of Cambridge)

In recent years, rapid advances in high-resolution omics technologies-such as single-cell and spatial transcriptomics-have ushered in an era where comprehensive molecular profiling can illuminate the heterogeneity of cancer tissues both within and between tumors. At the same time, the growing complexity of these datasets has driven the development of diverse analytical methods-including deep learning, probabilistic modeling, and mathematical modeling-to extract meaningful insights. These approaches enable the identification of gene programs associated with clinical features, tracing the differentiation trajectories of cancer cells, and characterizing the population structures within tumor microenvironments. As a result, these computational strategies have become essential for elucidating and interpreting tumor heterogeneity in cancer biology.
This symposium will bring together researchers who develop and apply a variety of computational methods to explore cutting-edge techniques and findings that deepen our understanding of tumor heterogeneity through the analysis of high-resolution omics data. Through this exchange, we aim to envision the future of computational analyses in high-resolution omics and to investigate the transformative impact these sophisticated approaches can have on cancer biology.

---

### IS9-2
### Withdrawn

No Abstract

### [IS9-3] IS9
### Elucidating the regulatory mechanisms involved in tumor tissue heterogeneity through advanced informatics technologies

2025 / 9 / 27 (Sat) 12:50-15:20 Room 8/(Hotel Nikko Kanazawa 3F Kujaku C) Room 8

[Chairpersons]Yasuhiro Kojima (Laboratory of Computational Life Science, National Cancer Center Research Institute)、Mo Lotfollahi (Wellcome Sanger institute, University of Cambridge)

In recent years, rapid advances in high-resolution omics technologies-such as single-cell and spatial transcriptomics-have ushered in an era where comprehensive molecular profiling can illuminate the heterogeneity of cancer tissues both within and between tumors. At the same time, the growing complexity of these datasets has driven the development of diverse analytical methods-including deep learning, probabilistic modeling, and mathematical modeling-to extract meaningful insights. These approaches enable the identification of gene programs associated with clinical features, tracing the differentiation trajectories of cancer cells, and characterizing the population structures within tumor microenvironments. As a result, these computational strategies have become essential for elucidating and interpreting tumor heterogeneity in cancer biology.
This symposium will bring together researchers who develop and apply a variety of computational methods to explore cutting-edge techniques and findings that deepen our understanding of tumor heterogeneity through the analysis of high-resolution omics data. Through this exchange, we aim to envision the future of computational analyses in high-resolution omics and to investigate the transformative impact these sophisticated approaches can have on cancer biology.

---

### IS9-3
### Geospatial Analysis of Liver Cancer Microenvironment for Comprehensive Insight and Drug Discovery

[Speaker]Kanae Echizen (Dept.of Pathophysiology, Osaka Metropolitan Univ. Grad. Sch. Medicine) [Co-author]Yoshiki Nonaka (Dept.of Pathophysiology, Osaka Metropolitan Univ. Grad. Sch. Medicine)、Tomonori Kamiya (Dept.of Pathophysiology, Osaka Metropolitan Univ. Grad. Sch. Medicine)、Takahiro Kodama (Gastroenterology and Hepatology, Grad. Sch.)、Yoshimi Muto (Dept. of Hepatology, Grad. Sch. Medicine, Osaka Metropolitan Univ.)、Hideki Fujii (Dept. of Hepatology, Grad. Sch. Medicine, Osaka Metropolitan Univ.)、Kenichi Kohashi (Dept. of Pathology, Grad. Sch. Medicine, Osaka Metropolitan Univ.)、Naoko Ohtani (Dept.of Pathophysiology, Osaka Metropolitan Univ. Grad. Sch. Medicine)

Hepatocellular carcinoma (HCC) associated with metabolic dysfunction-associated steatotic liver disease (MASLD) has been increasing in incidence in recent years, highlighting the urgent need to better understand its pathogenesis for therapeutic development. Our group has conducted single–cell and spatial transcriptomic analyses to uncover cellular features and heterogeneity. In particular, intertumoral heterogeneity is reported to contribute to therapeutic resistance, and spatial transcriptomics is a powerful approach to dissect such complexity.In this study, we developed a spatial analytical framework that combines pathway activity scores with geostatistical methods and distance metrics from histopathological structures, calculated using a digital unroll method, to identify region–specific cellular populations within tumor tissues. Furthermore, we analyzed cell–cell communication patterns to elucidate localized network architectures within the tumor microenvironment. Additionally, we identified secreted factors from specific cell tyles and evaluated their potential as non–invasive biomarkers.Our findings offer new spatial insights for therapy and diagnostics.

[Disclosure of COI by Responsible researcher] Lead presenter is not the Responsible researcher.
[Lead presenter: Kanae Echizen] We have no financial relationships to disclose.
[Principal Researcher: Naoko Ohtani] ■Research funds from company: 武田科学振興財団

### [IS9-4] IS9
### Elucidating the regulatory mechanisms involved in tumor tissue heterogeneity through advanced informatics technologies

2025 / 9 / 27 (Sat) 12:50-15:20 Room 8/(Hotel Nikko Kanazawa 3F Kujaku C) Room 8

[Chairpersons]Yasuhiro Kojima (Laboratory of Computational Life Science, National Cancer Center Research Institute)、Mo Lotfollahi (Wellcome Sanger institute, University of Cambridge)

In recent years, rapid advances in high-resolution omics technologies-such as single-cell and spatial transcriptomics-have ushered in an era where comprehensive molecular profiling can illuminate the heterogeneity of cancer tissues both within and between tumors. At the same time, the growing complexity of these datasets has driven the development of diverse analytical methods-including deep learning, probabilistic modeling, and mathematical modeling-to extract meaningful insights. These approaches enable the identification of gene programs associated with clinical features, tracing the differentiation trajectories of cancer cells, and characterizing the population structures within tumor microenvironments. As a result, these computational strategies have become essential for elucidating and interpreting tumor heterogeneity in cancer biology.
This symposium will bring together researchers who develop and apply a variety of computational methods to explore cutting-edge techniques and findings that deepen our understanding of tumor heterogeneity through the analysis of high-resolution omics data. Through this exchange, we aim to envision the future of computational analyses in high-resolution omics and to investigate the transformative impact these sophisticated approaches can have on cancer biology.

---

### IS9-4
### Intelligent Decoding of Spatial Biology

[Speaker]Shihua Zhang (Academy of Mathematics and Systems Science, Chinese Academy of Sciences)

Technological advances in spatial transcriptomics are critical for better understanding the structures and functions of tissues in biological research. The combination of intelligent or statistical algorithms and spatial transcriptomics has emerged to pave the way for deciphering tissue architecture. We have made great efforts to advance intelligent spatial transcriptomics and developed a group of STA- tools such as STAGATE, STAligner, STAMarker, STAGE, STASCAN, STALocaor, and STAMapper. In this talk, I will take these tools as examples to introduce how to utilize deep learning to model spatial transcriptomics data effectively. Moreover, I will shed light on some recent progress in spatial multi-omics integration with STAMO, directionally or temporally variable gene detection with STAVAG, and high-fidelity simulation and full-view 3D modeling of spatial transcriptomics with STADiffuser.

[Disclosure of COI by Responsible researcher] Lead presenter is the Responsible researcher.
[Lead presenter: Shihua Zhang] We have no financial relationships to disclose.

### [IS9-5] IS9

### Elucidating the regulatory mechanisms involved in tumor tissue heterogeneity through advanced informatics technologies

2025 / 9 / 27 (Sat) 12:50-15:20 Room 8/(Hotel Nikko Kanazawa 3F Kujaku C) Room 8

[Chairpersons]Yasuhiro Kojima (Laboratory of Computational Life Science, National Cancer Center Research Institute)、Mo Lotfollahi (Wellcome Sanger institute, University of Cambridge)

In recent years, rapid advances in high-resolution omics technologies-such as single-cell and spatial transcriptomics-have ushered in an era where comprehensive molecular profiling can illuminate the heterogeneity of cancer tissues both within and between tumors. At the same time, the growing complexity of these datasets has driven the development of diverse analytical methods-including deep learning, probabilistic modeling, and mathematical modeling-to extract meaningful insights. These approaches enable the identification of gene programs associated with clinical features, tracing the differentiation trajectories of cancer cells, and characterizing the population structures within tumor microenvironments. As a result, these computational strategies have become essential for elucidating and interpreting tumor heterogeneity in cancer biology.
This symposium will bring together researchers who develop and apply a variety of computational methods to explore cutting-edge techniques and findings that deepen our understanding of tumor heterogeneity through the analysis of high-resolution omics data. Through this exchange, we aim to envision the future of computational analyses in high-resolution omics and to investigate the transformative impact these sophisticated approaches can have on cancer biology.

---

### IS9-5

### Spatial omics approaches to understand the evolution and therapeutic response of gastrointestinal cancers

[Speaker]Jong-Eun Park (Graduate School of Medical Science and Engineering, KAIST)

No Abstract

### [IS9-6] IS9

### Elucidating the regulatory mechanisms involved in tumor tissue heterogeneity through advanced informatics technologies

2025 / 9 / 27 (Sat) 12:50-15:20 Room 8/(Hotel Nikko Kanazawa 3F Kujaku C) Room 8

[Chairpersons]Yasuhiro Kojima (Laboratory of Computational Life Science, National Cancer Center Research Institute)、Mo Lotfollahi (Wellcome Sanger institute, University of Cambridge)

In recent years, rapid advances in high-resolution omics technologies-such as single-cell and spatial transcriptomics-have ushered in an era where comprehensive molecular profiling can illuminate the heterogeneity of cancer tissues both within and between tumors. At the same time, the growing complexity of these datasets has driven the development of diverse analytical methods-including deep learning, probabilistic modeling, and mathematical modeling-to extract meaningful insights. These approaches enable the identification of gene programs associated with clinical features, tracing the differentiation trajectories of cancer cells, and characterizing the population structures within tumor microenvironments. As a result, these computational strategies have become essential for elucidating and interpreting tumor heterogeneity in cancer biology.
This symposium will bring together researchers who develop and apply a variety of computational methods to explore cutting-edge techniques and findings that deepen our understanding of tumor heterogeneity through the analysis of high-resolution omics data. Through this exchange, we aim to envision the future of computational analyses in high-resolution omics and to investigate the transformative impact these sophisticated approaches can have on cancer biology.

---

### IS9-6

### The Multi-Layered Transcriptional Architecture of Glioblastoma Ecosystems

[Speaker]Masashi Nomura (The University of Tokyo, Department of Neurosurgery／Massachusetts General Hospital and Harvard Medical School, Molecular Pathology／Broad Institute of Harvard and MIT) [Co-author]Avishay Spitzer (Weizmann Institute of Science, Department of Molecular Cell Biology)、Kevin C. Johnson (Yale School of Medicine, Department of Neurosurgery)、Luciano Garbanzo (University of Miami, Sylvester Comprehensive Cancer Center)、Anna Lasorella (University of Miami, Sylvester Comprehensive Cancer Center)、Roel Verhaak (Yale School of Medicine, Department of Neurosurgery)、Antonio Iavarone (University of Miami, Sylvester Comprehensive Cancer Center)、Itay Tirosh (Weizmann Institute of Science, Department of Molecular Cell Biology)、Mario L. Suva (Massachusetts General Hospital and Harvard Medical School, Molecular Pathology／Broad Institute of Harvard and MIT)

In glioblastoma (GBM), cellular heterogeneity exists across and within tumors and may drive therapeutic resistance. Here, under Cellular Analysis of Resistance and Evolution (CARE) consortium, we analyzed 121 GBM samples by single-nucleus RNA-sequencing and bulk DNA sequencing, characterizing transcriptional heterogeneity across three levels. First, GBMs can be classified by their broad cellular composition, encompassing malignant, immune, vascular, neuronal, and glial cell types. Second, within each cell type we describe the diversity of cellular states. In particular, we describe an expanded set of malignant cell states, including glial progenitor cell-like, neuronal-like, and cilia-like states. Third, after controlling for the frequencies of cellular states, we find that the remaining variation between GBMs highlights three baseline gene expression programs which we labeled Neuronal, Glial, and ECM. These three layers of heterogeneity are inter-related and partially associated with specific genetic aberrations, thereby defining three stereotypic ecosystems in GBM. This work provides an unparalleled view of the multi-layered transcriptional architecture of GBM.

[Disclosure of COI by Responsible researcher] Lead presenter is not the Responsible researcher.
[Lead presenter: Masashi Nomura] We have no financial relationships to disclose.
[Principal Researcher: Mario L. Suva] ■employment by a company related to the presentation: Immunitas Therapeutics ■Advisor of the company and/or profit-making organization: Immunitas Therapeutics

### [E-3055] I-E10-5
### Cell-to-cell interaction (1)

2025 / 9 / 27 (Sat) 12:50-14:05 Room 9/(Hotel Nikko Kanazawa 3F Lumiere) Room 9

[Chairperson]Shinichi Hashimoto (Inst. Adv. Med, Wakayama Med. Univ., Sch. Med.)

---

### E-3055
### Integrating spatial dissection of differentiation heterogeneity in immature-type gastric carcinoma

[Speaker]Genta Nagae (Gen Sci & Med., RCAST, Univ. Tokyo) [Co-author]Kazuhiro Osawa (Gen Sci & Med., RCAST, Univ. Tokyo／Dept. Pathol., Univ. Tokyo)、Aya Nonaka (Gen Sci & Med., RCAST, Univ. Tokyo)、Tetsuo Ushiku (Dept. Pathol., Univ. Tokyo)、Hiroyuki Aburatani (Gen Sci & Med., RCAST, Univ. Tokyo)

Gastric cancer (GC) is characterized by diverse histological phenotypes, sometimes with marked plasticity exhibiting fetal gut/liver-like morphology or with coexisting fibrous stroma. To molecularly define the entity in an inclusive concept and understand the pathogenesis of the immature type of this carcinoma, we performed spatial transcriptomics of FFPE tissue sections using a 10X Visium system. Data integration along with the core pathway genes could reduce inter-tumoral bias and highlight the lineage trajectory involving enterocytic differentiation or retained stemness. We also deconvoluted these spatial data using scRNA-seq profiles to infer the cell composition and validate the specific interactions and cell states using Xenium in a single cell resolution. Multiplex protein profiling using phenocycler fusion could reveal the subcellular localization of transcription factors and secreting factors that are involved in cellular differentiation. We would like to introduce an integrative analytical approach to explain the complex and heterogeneous phenotype of gastric cancer tissues and to provide a bridge to quantitative digital pathology.

[Disclosure of COI by Responsible researcher] Lead presenter is the Responsible researcher.
[Lead presenter: Genta Nagae] ■Endowed chair: 中外製薬株式会社

### [E-3056] I-E10-5
### Cell-to-cell interaction (1)

2025 / 9 / 27 (Sat) 12:50-14:05 Room 9/(Hotel Nikko Kanazawa 3F Lumiere) Room 9

[Chairperson]Shinichi Hashimoto (Inst. Adv. Med, Wakayama Med. Univ., Sch. Med.)

---

### E-3056
### A prospective study on the effect of preoperative rehabilitation on blood levels of a tumor-suppressor miRNA

[Speaker]Jun Kiuchi (Division of Digestive Surgery, Kyoto Prefectural University of Medicine) [Co-author]Shuhei Komatsu (Division of Digestive Surgery, Kyoto Prefectural University of Medicine)、Takeshi Kobota (Division of Digestive Surgery, Kyoto Prefectural University of Medicine)、Yudai Nakabayashi (Division of Digestive Surgery, Kyoto Prefectural University of Medicine)、Naosuke Nakamichi (Division of Digestive Surgery, Kyoto Prefectural University of Medicine)、Taisuke Imamura (Division of Digestive Surgery, Kyoto Prefectural University of Medicine)、Kenji Nanishi (Division of Digestive Surgery, Kyoto Prefectural University of Medicine)、Hiroki Shimizu (Division of Digestive Surgery, Kyoto Prefectural University of Medicine)、Tomohiro Arita (Division of Digestive Surgery, Kyoto Prefectural University of Medicine)、Toshiyuki Kosuga (Division of Digestive Surgery, Kyoto Prefectural University of Medicine)、Yusuke Yamamoto (Division of Digestive Surgery, Kyoto Prefectural University of Medicine)、Hirotaka Konishi (Division of Digestive Surgery, Kyoto Prefectural University of Medicine)、Ryo Morimura (Division of Digestive Surgery, Kyoto Prefectural University of Medicine)、Hitoshi Fujiwara (Division of Digestive Surgery, Kyoto Prefectural University of Medicine)、Eigo Otsuji (Division of Digestive Surgery, Kyoto Prefectural University of Medicine)、Atsushi Shiozaki (Division of Digestive Surgery, Kyoto Prefectural University of Medicine)

As a mechanism by which sarcopenia is associated with poor oncological prognosis in cancers, we have focused on miR-133b, a tumor-suppressor microRNA derived from skeletal muscle, and reported that a decrease in its blood levels is associated with tumor progression. In addition, we found that an increase in blood miR-133b levels induced by exercise suppressed tumor progression in vivo. Therefore, we are now prospectively analyzing the effects of preoperative rehabilitation on blood miR-133b levels in gastric and colorectal cancer (GC and CRC) patients.
We performed a 2-week rehabilitation prior to radical surgery for GC and CRC, and observed the changes in blood miR-133b levels.
Rehabilitation have been performed for 10 GC and 5 CRC cases. Of the 10 GC cases, 6 showed an increase in blood miR-133b levels. In CRC cases, increased blood miR-133b levels were observed in 2 of the 5 cases. The group with high miR-133b levels tended to include patients with high pre-albumin levels.
Preoperative rehabilitation for GC and CRC patients may increase blood tumor-suppressor miR-133b levels. Currently, we are accumulating cases in a prospective study.
This is an encore presentation.

[Disclosure of COI by Responsible researcher] Lead presenter is the Responsible researcher.
[Lead presenter: Jun Kiuchi] We have no financial relationships to disclose.

### [E-3057] I-E10-5
### Cell-to-cell interaction (1)

2025 / 9 / 27 (Sat) 12:50-14:05 Room 9/(Hotel Nikko Kanazawa 3F Lumiere) Room 9

[Chairperson]Shinichi Hashimoto (Inst. Adv. Med, Wakayama Med. Univ., Sch. Med.)

---

### E-3057
### Withdrawn

No Abstract

▲TOP

### [E-3058] I-E10-5
### Cell-to-cell interaction (1)

2025 / 9 / 27 (Sat) 12:50-14:05 Room 9/(Hotel Nikko Kanazawa 3F Lumiere) Room 9

[Chairperson]Shinichi Hashimoto (Inst. Adv. Med, Wakayama Med. Univ., Sch. Med.)

---

### E-3058
### Functional Characterization of Tumor-Educated Platelets in Hepatocellular Carcinoma Progression

[Speaker]Hiroki Tanaka (Div. Tumor Pathology., Asahikawa Med. Univ.) [Co-author]Kie Horioka (Dept. Legal Med., Univ. of Oulu)、Masanori Goto (Div. Tumor Pathology., Asahikawa Med. Univ.)、Kumi Takasawa (Div. Tumor Pathology., Asahikawa Med. Univ.)、Akira Takasawa (Div. Tumor Pathology., Asahikawa Med. Univ.)、Kyohei Oyama (Dept. Cardiac Surgery., Asahikawa Med. Univ.)、Aina Hirofuji (Dept. Cardiac Surgery., Asahikawa Med. Univ.)、Taito Itoh (Lab. Mol. Diag.Thera., Nat. Inst. Biomed. Inno.)、Yusuke Mizukami (Lab. Mol. Diag.Thera., Nat. Inst. Biomed. Inno.)

We previously reported that platelets promote hepatocellular carcinoma (HCC). In cancer patients, platelets are influenced by tumor-derived humoral factors and are termed tumor-educated platelets (TEPs), though their role in carcinogenesis remains unclear. In this study, we investigated the functional differences between TEPs and normal platelets. When rat HCC cell lines were co-cultured with platelets from normal rats (normal-PLTs) or HCC model rats (HCC-PLTs), HCC-PLTs significantly promoted HCC cell growth. RNA sequencing revealed that HCC-PLTs were enriched in transcripts for ribosomal proteins. Flow cytometry showed that HCC-PLTs had enhanced protein synthesis capacity compared to normal-PLTs. Furthermore, megakaryocytes in HCC model rats were significantly larger than in controls. These findings suggest that tumor-derived factors alter megakaryocyte biology and platelet function, enhancing de novo protein synthesis in TEPs, which may contribute to cancer progression.

[Disclosure of COI by Responsible researcher] Lead presenter is the Responsible researcher.
[Lead presenter: Hiroki Tanaka] We have no financial relationships to disclose.

### [E-3059] I-E10-5
### Cell-to-cell interaction (1)

2025 / 9 / 27 (Sat) 12:50-14:05 Room 9/(Hotel Nikko Kanazawa 3F Lumiere) Room 9

[Chairperson]Shinichi Hashimoto (Inst. Adv. Med, Wakayama Med. Univ., Sch. Med.)

### E-3059
### Cancer-associated lymphatic endothelial cells facilitate lymphatic metastasis: interactions with cancer organoids

[Speaker]Asumi Iesato (Cancer Cell Diversity Project, NEXT-Ganken Program, JFCR) [Co-author]Kazutaka Otsuji (Cancer Cell Diversity Project, NEXT-Ganken Program, JFCR)、Masashi Akiya (Division of Pathology, Cancer Institute, JFCR)、Tomo Osako (Division of Pathology, Cancer Institute, JFCR)、Tetsuyo Maeda (Breast Oncology Center, Cancer Institute Hospital, JFCR)、Nami Yamashita (Breast Oncology Center, Cancer Institute Hospital, JFCR)、Yuka Inoue (Breast Oncology Center, Cancer Institute Hospital, JFCR)、Kohei Kumegawa (Department of Cancer Epigenomics, The Cancer Institute, JFCR)、Sumito Saeki (Department of Cancer Epigenomics, The Cancer Institute, JFCR)、Miwa Tanaka (Department of Cancer Epigenomics, The Cancer Institute, JFCR)、Takehiko Sakai (Breast Oncology Center, Cancer Institute Hospital, JFCR)、Tetsuo Noda (Cancer Cell Diversity Project, NEXT-Ganken Program, JFCR)、Takayuki Ueno (Cancer Cell Diversity Project, NEXT-Ganken Program, JFCR／Breast Oncology Center, Cancer Institute Hospital, JFCR)、Reo Maruyama (Cancer Cell Diversity Project, NEXT-Ganken Program, JFCR／Department of Cancer Epigenomics, The Cancer Institute, JFCR)

Lymphatic invasion (Ly) is a poor prognostic factor in cancer. Based on pathological observations of breast cancer (BC) spheroids within Ly and migration assay results, we hypothesized that lymphatic endothelial cells (LECs) facilitate lymphatic invasion. We established LEC cell lines from four BC patients (two Ly+ and two Ly-). LECs from Ly- cases exhibited enhanced tubule formation, further augmented by cancer-conditioned medium. A mathematical analysis of time-lapse co-cultures of BC organoids and LECs revealed that organoids moved more substantially when aligned with the cumulative vector of surrounding LECs, weighted by proximity. Conversely, co-cultures with HUVEC did not alter organoid motility. Additionally, through scRNA-seq analysis of normal-derived and cancer-derived LECs, we identified gene expression patterns common across various cancer types, suggesting the existence of cancer-associated LECs. Moreover, we identified interactions between LECs and BC that could serve as potential therapeutic targets. This study suggests that understanding these interactions could enable the development of novel therapeutic approaches.

[Disclosure of COI by Responsible researcher] Lead presenter is the Responsible researcher.
[Lead presenter: Asumi Iesato] ■Research expenses: 興和生命科学振興財団 ・中外創薬科学財団

▲TOP

### [E-3060] I-E10-5
### Cell-to-cell interaction (1)

2025 / 9 / 27 (Sat) 12:50-14:05 Room 9/(Hotel Nikko Kanazawa 3F Lumiere) Room 9

[Chairperson]Shinichi Hashimoto (Inst. Adv. Med, Wakayama Med. Univ., Sch. Med.)

---

### E-3060
### Exploring Mechanisms Driving Glioblastoma Invasion

[Speaker]Hirotaka Inoue (Dept. Prev. Med., Grad. Sch. Med., The Univ. of Tokyo／Dept. Neurosurg., Kumamoto Univ.) [Co-author]Miwako Kakiuchi (Dept. Prev. Med., Grad. Sch. Med., The Univ. of Tokyo)、Masashi Nomura (Dept. Neurosurg., Tokyo Univ.)、Kazuhito Tanaka (Dept. Diagnostic Path., Kumamoto Univ.)、Mieko Ochi (Dept. Prev. Med., Grad. Sch. Med., The Univ. of Tokyo)、Ayumu Tsubosaka (Dept. Prev. Med., Grad. Sch. Med., The Univ. of Tokyo)、Kyohei Sano (Dept. Prev. Med., Grad. Sch. Med., The Univ. of Tokyo)、Hiroyuki Sueyoshi (Dept. Neurosurg., Kumamoto Univ.)、Kenji Fujimoto (Dept. Neurosurg., Kumamoto Univ.)、Takahiro Yamamoto (Dept. Neurosurg., Kumamoto Univ.)、Junichiro Kuroda (Dept. Neurosurg., Kumamoto Univ.)、Hiroyuki Uetani (Dept. Diagnostic Radiology, Kumamoto Univ.)、Daisuke Komura (Dept. Prev. Med., Grad. Sch. Med., The Univ. of Tokyo)、Hiroto Katoh (Div. Path., NCC Exploratory Oncol. Res. & Clin. Trial Ctr.)、Akitake Mukasa (Dept. Neurosurg., Kumamoto Univ.)、Shumpei Ishikawa (Dept. Prev. Med., Grad. Sch. Med., The Univ. of Tokyo／Div. Path., NCC Exploratory Oncol. Res. & Clin. Trial Ctr.)

Glioblastoma cells change their cellular state to adapt to the tumor microenvironment (TME) and invade healthy brain. To investigate the interaction between invasive tumor cells and normal cells in the TME, we focused on tumor edge heterogeneity in imaging. Each region exhibiting varied invasiveness was precisely and individually harvested by navigation-guided resection and evaluated by three neuropathologists. The differences in invasiveness were further analyzed using single-cell spatial transcriptomics, a robust tool that allows the integration of histological morphology and gene expression. We identified a high-invasive TME with gene expression related to lipid-laden macrophages, which are known to promote tumor proliferation and immunosuppression, as they not only supply tumor cells with myelin-derived lipids but also shelter them from lipotoxicity. Also, the high-invasive tumor cells increased gene expression related to cell migration and PI3K-Akt signaling pathway, which promotes cholesterol uptake. Our study provides insight into immune and metabolic mechanisms driving glioblastoma invasion, suggesting a potential strategy to target metabolic vulnerabilities.

[Disclosure of COI by Responsible researcher] Lead presenter is not the Responsible researcher.
[Lead presenter: Hirotaka Inoue] We have no financial relationships to disclose.
[Principal Researcher: Shumpei Ishikawa] ■Research funds from company: 日本学術振興会　■Advisor of the company and/or profit-making organization: 新日本科学株式会社　■Research expenses: Chordia therapeutics株式会社、大鵬薬品工業株式会社、ロート製薬株式会社、アステラス製薬株式会社

### [OS11-1] OS11
### Cancer Biology and Therapeutic Strategies in Cross-Organ Neuroendocrine Neoplasm

2025 / 9 / 27 (Sat) 12:50-15:20 Room 11/(ANA Crowne Plaza Hotel Kanazawa 3F Ohtori (West)) Room 11

[Chairpersons]Kenta Kawasaki (Keio University School of Medicine (Memorial Sloan Kettering Cancer Center)、Yasuo Hamamoto (Institution of Science Tokyo)

Neuroendocrine neoplasms (NENs) are a heterogeneous group of diseases characterized by the expression of neuroendocrine markers. NENs encompass a spectrum ranging from indolent neuroendocrine tumors (NETs) to highly aggressive neuroendocrine carcinomas (NECs). NENs can arise in a wide range of organs including gastrointestinal tract, pancreas, lung (notably through EGFR-treatment induced transformation), prostate (associated with transformation due to resistance to hormonal therapy), as well as the head and neck, ovaries, and other sites. Recent advancements in clinical diagnostic methodologies coupled with increased opportunities for tumor re-biopsy, have contributed to a rising incidence of NEN diagnoses. Despite their shared characteristic phenotype across different organs, the biological underpinnings of NENs are insufficiently understood, largely due to the scarcity of clinical materials and the limited availability of suitable animal models which also hinders the novel therapeutic developments. The recent application of organoid technologies and the development of patient-derived xenografts (PDXs) in NEN research have provided valuable tools for investigating the biological dynamics underlying these tumors, such as lineage plasticity (neuroendocrine transformation from adenocarcinoma) and the exceptional metastatic potential of these malignancies. This symposium aims to advance the biological understanding of neuroendocrine malignancies and explore their therapeutic applications through cross-organ and interdisciplinary discussions by bridging the gap between basic and clinical frameworks.

---

### OS11-1
### Elucidation of the factors involved in the development of neuroendocrine neoplasm and its metastasis

[Speaker]Kenta Kawasaki (Keio University School of Medicine, Keio Cancer Center)

Gastroenteropancreatic Neuroendocrine Neoplasms (GEP-NENs) consist of GEP-NETs (Gastroenteropancreatic Neuroendocrine Tumors) and GEP-NECs (Gastroenteropancreatic Neuroendocrine Carcinomas). Both tumors are metastatic, and GEP-NECs present more drastic metastatic potential which results in poor prognosis. Opportunities for tissue collection are limited due to their rapid progression, and animal models which facilitates our biological understanding of this tumors remains inadequate. Given this background, we employed organoid technology, an epithelial culture system with high establishment efficiency, and successfully established a library of 25 GEP-NEN organoids. Applying the result from comprehensive analysis, we succeeded in replicating GEP-NEC from normal colonic epithelial cells. While the unique morphology was reproduced, the metastatic potential could not be replicated. The factor which might be contributing during the metastatic process is identified through analysis using clinical specimens of small cell lung carcinoma. In this study, we focus on the gastrointestinal and pulmonary organs to discuss the mechanisms of tumorigenesis and metastasis in neuroendocrine cancer.

[Disclosure of COI by Responsible researcher] Lead presenter is the Responsible researcher.
[Lead presenter: Kenta Kawasaki] We have no financial relationships to disclose.

▲TOP

### [OS11-2] OS11
### Cancer Biology and Therapeutic Strategies in Cross-Organ Neuroendocrine Neoplasm

2025 / 9 / 27 (Sat) 12:50-15:20 Room 11/(ANA Crowne Plaza Hotel Kanazawa 3F Ohtori (West)) Room 11

[Chairpersons]Kenta Kawasaki (Keio University School of Medicine (Memorial Sloan Kettering Cancer Center)、Yasuo Hamamoto (Institution of Science Tokyo)

Neuroendocrine neoplasms (NENs) are a heterogeneous group of diseases characterized by the expression of neuroendocrine markers. NENs encompass a spectrum ranging from indolent neuroendocrine tumors (NETs) to highly aggressive neuroendocrine carcinomas (NECs). NENs can arise in a wide range of organs including gastrointestinal tract, pancreas, lung (notably through EGFR-treatment induced transformation), prostate (associated with transformation due to resistance to hormonal therapy), as well as the head and neck, ovaries, and other sites. Recent advancements in clinical diagnostic methodologies coupled with increased opportunities for tumor re-biopsy, have contributed to a rising incidence of NEN diagnoses. Despite their shared characteristic phenotype across different organs, the biological underpinnings of NENs are insufficiently understood, largely due to the scarcity of clinical materials and the limited availability of suitable animal models which also hinders the novel therapeutic developments. The recent application of organoid technologies and the development of patient-derived xenografts (PDXs) in NEN research have provided valuable tools for investigating the biological dynamics underlying these tumors, such as lineage plasticity (neuroendocrine transformation from adenocarcinoma) and the exceptional metastatic potential of these malignancies. This symposium aims to advance the biological understanding of neuroendocrine malignancies and explore their therapeutic applications through cross-organ and interdisciplinary discussions by bridging the gap between basic and clinical frameworks.

---

### OS11-2
### Patient-derived small cell lung cancer organoid library and elucidation of its biological dynamics

[Speaker]Fumimaro Ito (Division of Pulmonary Medicine, Keio University School of Medicine) [Co-author]Junko Hamamoto (Division of Pulmonary Medicine, Keio University School of Medicine)、Takahiro Fukushima (Division of Pulmonary Medicine, Keio University School of Medicine)、Kazuhito Horie (Division of Pulmonary Medicine, Keio University School of Medicine)、Lisa Shigematsu (Division of Pulmonary Medicine, Keio University School of Medicine)、Hatsuyo Takaoka (Division of Pulmonary Medicine, Keio University School of Medicine)、Shigenari Nukaga (Division of Pulmonary Medicine, Keio University School of Medicine)、Tetsuo Tani (Division of Pulmonary Medicine, Keio University School of Medicine)、Hideki Terai (Division of Pulmonary Medicine, Keio University School of Medicine)、Koichi Fukunaga (Division of Pulmonary Medicine, Keio University School of Medicine)、Hiroyuki Yasuda (Division of Pulmonary Medicine, Keio University School of Medicine)、Toshiro Sato (Integrated Medicine and Biochemistry, Keio University School of Medicine)

Small cell lung cancer (SCLC) is a devastating disease with limited therapeutic advancements. Recent studies have classified SCLC into four molecular subtypes, namely ASCL1, NEUROD1, POU2F3 and YAP1. However, a lack of clinically relevant and tractable SCLC models has hampered molecular understanding and treatment development of SCLC. To understand the biological dynamics of SCLC, we established patient-derived SCLC organoids. To establish SCLC organoids efficiently, we developed a method of deriving SCLC organoids from non-invasively obtained samples including sputum or circulating tumor cells. We developed an organoid library consisting of 102 SCLC organoid lines (56 patients) with predominant TP53 and RB1 alteration using a variety of clinical samples. We confirmed that the library faithfully recapitulated pathological and molecular characteristics of original tumors. Interestingly, our molecular profiling and phenotype analysis revealed previously unrecognized therapeutic vulnerabilities in SCLC. We would like to share our recent data to provide a clinically relevant and tractable research model for SCLC.

[Disclosure of COI by Responsible researcher] Lead presenter is not the Responsible researcher.
[Lead presenter: Fumimaro Ito] ■Research funds from company: 日本学術振興会　科研費
[Principal Researcher: Hiroyuki Yasuda] ■Research funds from company: 日本学術振興会　科研費

### [OS11-3] OS11
### Cancer Biology and Therapeutic Strategies in Cross-Organ Neuroendocrine Neoplasm

2025 / 9 / 27 (Sat) 12:50-15:20 Room 11/(ANA Crowne Plaza Hotel Kanazawa 3F Ohtori (West)) Room 11

[Chairpersons]Kenta Kawasaki (Keio University School of Medicine (Memorial Sloan Kettering Cancer Center)、Yasuo Hamamoto (Institution of Science Tokyo)

Neuroendocrine neoplasms (NENs) are a heterogeneous group of diseases characterized by the expression of neuroendocrine markers. NENs encompass a spectrum ranging from indolent neuroendocrine tumors (NETs) to highly aggressive neuroendocrine carcinomas (NECs). NENs can arise in a wide range of organs including gastrointestinal tract, pancreas, lung (notably through EGFR-treatment induced transformation), prostate (associated with transformation due to resistance to hormonal therapy), as well as the head and neck, ovaries, and other sites. Recent advancements in clinical diagnostic methodologies coupled with increased opportunities for tumor re-biopsy, have contributed to a rising incidence of NEN diagnoses. Despite their shared characteristic phenotype across different organs, the biological underpinnings of NENs are insufficiently understood, largely due to the scarcity of clinical materials and the limited availability of suitable animal models which also hinders the novel therapeutic developments. The recent application of organoid technologies and the development of patient-derived xenografts (PDXs) in NEN research have provided valuable tools for investigating the biological dynamics underlying these tumors, such as lineage plasticity (neuroendocrine transformation from adenocarcinoma) and the exceptional metastatic potential of these malignancies. This symposium aims to advance the biological understanding of neuroendocrine malignancies and explore their therapeutic applications through cross-organ and interdisciplinary discussions by bridging the gap between basic and clinical frameworks.

---

### OS11-3
### Treatment strategies for neuroendocrine carcinoma arising from head and neck, and gastrointestinal area.

[Speaker]Yoshitaka Honma (National Cancer Center Hospital)

Neuroendocrine carcinoma (NEC) is a heterogeneous and highly malignant neoplasm. Due to its rarity, no standard approach according to primary sites has been established for extrapulmonary NEC (epNEC). While treatment guidelines recommend a multidisciplinary approach for localized epNEC, supporting evidence remains limited, and appropriate local therapy varies depending on the primary site. For metastatic epNEC, palliative chemotherapy, modeled after small-cell lung cancer, is used as the 1st-line therapy. However, no established regimen exists for salvage-line settings, particularly for cases outside the gastro-entero-pancreatic area. Most NEC in the head and neck area originates in the nasal/paranasal cavity and is diagnosed at a locally advanced stage. Similar malignancies, such as olfactory neuroblastoma, often arise in the nasal/paranasal cavity, making differentiation using biopsy specimens particularly challenging. Given these complexities, numerous clinical questions surrounding epNEC remain unresolved. In this session, I hope to gain insights into treatment strategies for head and neck and gastrointestinal NEC by engaging in discussions with experts from different fields.

[Disclosure of COI by Responsible researcher] Lead presenter is the Responsible researcher.
[Lead presenter: Yoshitaka Honma] ■Lecture fee: 帝人ファーマ、メルクバイオファーマ　■Research expenses: アステラス製薬

▲TOP

### [OS11-4] OS11
### Cancer Biology and Therapeutic Strategies in Cross-Organ Neuroendocrine Neoplasm

2025 / 9 / 27 (Sat) 12:50-15:20 Room 11/(ANA Crowne Plaza Hotel Kanazawa 3F Ohtori (West)) Room 11

[Chairpersons]Kenta Kawasaki (Keio University School of Medicine (Memorial Sloan Kettering Cancer Center)、Yasuo Hamamoto (Institution of Science Tokyo)

Neuroendocrine neoplasms (NENs) are a heterogeneous group of diseases characterized by the expression of neuroendocrine markers. NENs encompass a spectrum ranging from indolent neuroendocrine tumors (NETs) to highly aggressive neuroendocrine carcinomas (NECs). NENs can arise in a wide range of organs including gastrointestinal tract, pancreas, lung (notably through EGFR-treatment induced transformation), prostate (associated with transformation due to resistance to hormonal therapy), as well as the head and neck, ovaries, and other sites. Recent advancements in clinical diagnostic methodologies coupled with increased opportunities for tumor re-biopsy, have contributed to a rising incidence of NEN diagnoses. Despite their shared characteristic phenotype across different organs, the biological underpinnings of NENs are insufficiently understood, largely due to the scarcity of clinical materials and the limited availability of suitable animal models which also hinders the novel therapeutic developments. The recent application of organoid technologies and the development of patient-derived xenografts (PDXs) in NEN research have provided valuable tools for investigating the biological dynamics underlying these tumors, such as lineage plasticity (neuroendocrine transformation from adenocarcinoma) and the exceptional metastatic potential of these malignancies. This symposium aims to advance the biological understanding of neuroendocrine malignancies and explore their therapeutic applications through cross-organ and interdisciplinary discussions by bridging the gap between basic and clinical frameworks.

---

### OS11-4
### New treatment strategies for pancreatic neuroendocrine neoplasms (NEN)

[Speaker]Chigusa Morizane (Department of Hepatobiliary and Pancreatic Oncology, National Cancer Center Hospital)

Malignant potential of neuroendocrine tumors (NET) range widely from grade 1 to 3. Neuroendocrine carcinoma (NEC) is characterized by morphologically poor differentiation and extremely high malignancy. Treatment regimens for NETs have been involved cytotoxic drugs such as streptozocin and temozolomide, molecularly targeted drugs such as everolimus and sunitinib, and hormone therapy using somatostatin analogs. And more recently, peptide receptor radionuclide therapy has shown to be effective. NEC has been treated with regimens for small cell lung cancer, such as etoposide (or irinotecan) plus platinum. In recent years, combination therapy with immune checkpoint inhibitors and targeted agents for DLL3 and 41BB are under development. It is clinically well known that the malignant transformation from NETG1 to G2 or G3 is often observed, but the transformation from NET to NEC is seldom known. However, recent reports have indicated that gene mutations in RB1 or TP53 can lead to NEC-like transformation from NET G3. There is insufficient information on appropriate treatment strategies for cases exhibiting NEC-like transformation.

[Disclosure of COI by Responsible researcher] Lead presenter is the Responsible researcher.
[Lead presenter: Chigusa Morizane] ■Lecture fee: AstraZeneca、MSD K.K.、Taiho Pharmaceutical ■Research expenses: Eisai Yakult Honsha ONO Pharmaceutical Taiho Pharmaceutical AstraZeneca Daiichi Sankyo HITACHI Boehringer Ingelheim Chugai Labcorp

### [OS11-5] OS11
### Cancer Biology and Therapeutic Strategies in Cross-Organ Neuroendocrine Neoplasm

2025 / 9 / 27 (Sat) 12:50-15:20 Room 11/(ANA Crowne Plaza Hotel Kanazawa 3F Ohtori (West)) Room 11

[Chairpersons]Kenta Kawasaki (Keio University School of Medicine (Memorial Sloan Kettering Cancer Center)、Yasuo Hamamoto (Institution of Science Tokyo)

Neuroendocrine neoplasms (NENs) are a heterogeneous group of diseases characterized by the expression of neuroendocrine markers. NENs encompass a spectrum ranging from indolent neuroendocrine tumors (NETs) to highly aggressive neuroendocrine carcinomas (NECs). NENs can arise in a wide range of organs including gastrointestinal tract, pancreas, lung (notably through EGFR-treatment induced transformation), prostate (associated with transformation due to resistance to hormonal therapy), as well as the head and neck, ovaries, and other sites. Recent advancements in clinical diagnostic methodologies coupled with increased opportunities for tumor re-biopsy, have contributed to a rising incidence of NEN diagnoses. Despite their shared characteristic phenotype across different organs, the biological underpinnings of NENs are insufficiently understood, largely due to the scarcity of clinical materials and the limited availability of suitable animal models which also hinders the novel therapeutic developments. The recent application of organoid technologies and the development of patient-derived xenografts (PDXs) in NEN research have provided valuable tools for investigating the biological dynamics underlying these tumors, such as lineage plasticity (neuroendocrine transformation from adenocarcinoma) and the exceptional metastatic potential of these malignancies. This symposium aims to advance the biological understanding of neuroendocrine malignancies and explore their therapeutic applications through cross-organ and interdisciplinary discussions by bridging the gap between basic and clinical frameworks.

---

### OS11-5
### Elucidation of Neuroendocrine Differentiation Mechanisms and Therapeutic Strategies for Prostate Cancer

[Speaker]Yasutaka Yamada (Department of Urology, Chiba University Graduate School of Medicine)

Most neuroendocrine prostate cancer (NEPC) undergo morphologic transformation from adenocarcinoma to small cell carcinoma during hormonal therapy, a disease status known as treatment-induced NEPC (t-NEPC). The frequency of de novo NEPCs with small cell carcinoma from diagnosis is very rare, about 1%. t-NEPC can be induced by potent androgen receptor pathway inhibitors (ARPI), and its frequency is increasing due to recent upfront ARPI treatment. Mutations in TP53, RB1, and PTEN, epigenomic aberrations such as histone modifications and DNA methylation, and activation of neuroendocrine-related transcription factors (e.g., NeuroD1, INSM1) have been identified as factors facilitating neuroendocrine differentiation from castration-resistant prostate cancer (CRPC), and therapeutic strategies targeting these factors are being examined. Recently, antibody-drug conjugates (ADCs) targeting B7-H3, DLL3, and CEACAM5, which are cell surface proteins highly expressed in NEPC, have been tested in clinical trials and have shown some efficacy. Based on the insight and evidence accumulated to date, I will discuss molecular mechanism and therapeutic strategies for NEPC.

[Disclosure of COI by Responsible researcher] Lead presenter is the Responsible researcher.
[Lead presenter: Yasutaka Yamada] We have no financial relationships to disclose.

▲TOP

### [OS11-6] OS11
### Cancer Biology and Therapeutic Strategies in Cross-Organ Neuroendocrine Neoplasm

2025 / 9 / 27 (Sat) 12:50-15:20 Room 11/(ANA Crowne Plaza Hotel Kanazawa 3F Ohtori (West)) Room 11

[Chairpersons]Kenta Kawasaki (Keio University School of Medicine (Memorial Sloan Kettering Cancer Center)、Yasuo Hamamoto (Institution of Science Tokyo)

Neuroendocrine neoplasms (NENs) are a heterogeneous group of diseases characterized by the expression of neuroendocrine markers. NENs encompass a spectrum ranging from indolent neuroendocrine tumors (NETs) to highly aggressive neuroendocrine carcinomas (NECs). NENs can arise in a wide range of organs including gastrointestinal tract, pancreas, lung (notably through EGFR-treatment induced transformation), prostate (associated with transformation due to resistance to hormonal therapy), as well as the head and neck, ovaries, and other sites. Recent advancements in clinical diagnostic methodologies coupled with increased opportunities for tumor re-biopsy, have contributed to a rising incidence of NEN diagnoses. Despite their shared characteristic phenotype across different organs, the biological underpinnings of NENs are insufficiently understood, largely due to the scarcity of clinical materials and the limited availability of suitable animal models which also hinders the novel therapeutic developments. The recent application of organoid technologies and the development of patient-derived xenografts (PDXs) in NEN research have provided valuable tools for investigating the biological dynamics underlying these tumors, such as lineage plasticity (neuroendocrine transformation from adenocarcinoma) and the exceptional metastatic potential of these malignancies. This symposium aims to advance the biological understanding of neuroendocrine malignancies and explore their therapeutic applications through cross-organ and interdisciplinary discussions by bridging the gap between basic and clinical frameworks.

---

### OS11-6
### Organoid analysis revealed differentiation plasticity in cervical neuroendocrine cancer

[Speaker]Masahiro Inoue (Kyoto Univ. Grad. Sch. Med., Clinical Bioresource Research and Development)

Small-cell neuroendocrine carcinoma (SCNEC) of the cervix is a rare disease characterized by a high incidence of mixed tumors with other types of cancer. We established a panel of organoid lines from patients with SCNEC of the cervix. The organoids and xenograft tumors exhibited substantial intra-tumor and intra-organoid variation in the expression levels of SCNEC markers. Under hypoxic conditions, the marker expression was suppressed by HIF1-$\alpha$ and Notch-dependent mechanisms. In one line, a clear distinction between SCNEC and adenocarcinoma was recognized in some areas but was unclear in other areas. Tracking single cells in culture indicated the existence of cells with bipotential differentiation toward SCNEC and adenocarcinomas. Single-cell transcriptional analysis revealed that human papillomavirus 18 E6 was enriched in the SCNEC-like cluster, which showed higher proliferation and lower levels of the p53 pathway. After treatment with anticancer drugs, the expression of adenocarcinoma markers increased, whereas that of SCNEC decreased. These data suggest that mixed SCNEC/cervical tumors have a clonal origin and are characterized by an ambiguous and flexible differentiation state.

[Disclosure of COI by Responsible researcher] Lead presenter is the Responsible researcher.
[Lead presenter: Masahiro Inoue] ■Endowed chair: KBBM

### [OS12-1] OS12
### Frontier research on regulation of cancer progression deciphered by organelle dynamics

2025 / 9 / 27 (Sat) 12:50-15:20 Room 12/(ANA Crowne Plaza Hotel Kanazawa 3F Ohtori (Central)) Room 12

[Chairpersons]Atsushi Saito (Department of Frontier Science and Interdisciplinary Research, Faculty of Medicine, Kanazawa University)、Toyomasa Katagiri (National Institutes of Biomedical Innovation, Health and Nutrition)

The tumor microenvironment is intermingled with a variety of cells, including immune cells and cancer-associated fibroblasts, which induce abnormal biological responses, resulting in promoting malignant transformation, invasion, and metastasis of cancer. Thus, understanding the subsets of cells, and the mechanisms underlying the formation of specific tissue environments in and around cancer tissues is essential for establishing fundamental therapeutic strategies against cancer. On the other hand, there are organelles that perform specific functions in micro area inside a cell. Recently, it has become clear that organelle dynamics and the functional crosstalk among organelles, or their disruption cause the changes not only in individual cells but also in intercellular communication and the tumor microenvironment, leading to malignant transformation of cancer. We focus on organelles and cellular structures such as endoplasmic reticulum, Golgi apparatus, mitochondria, nuclear membrane, exosomes, and plasma membrane as a platform for cancer progression, and introduce the latest findings involved in the malignant evolution of cancer. Additionally, the discussion regarding the potential for drug discovery is included in the aim of this symposium.

---

### OS12-1
### Tumor-driven mitochondrial "hijacking" suppresses antitumor immunity

[Speaker]Takamasa Ishino (Dept. Tumor microenvironment, Okayama Univ.) [Co-author]Hideki Ikeda (Dept. Respiratory Med., Chiba Univ.)、Yosuke Togashi (Dept. Tumor microenvironment, Okayama Univ.／Dept. Allergy and Respiratory Med., Okayama Univ. Hosp.)

Cancer immunotherapy, including immune checkpoint inhibitors (ICIs), has significantly improved patient survival. However, over half of patients do not respond, highlighting the need to elucidate immune evasion mechanisms. Mitochondria are essential organelles playing multifaceted roles, including energy metabolism. Tumor microenvironment is typically characterized by hypoxia and nutrient deprivation, leading to mitochondrial dysfunction in tumor-infiltrating lymphocytes (TILs). However, the detail mechanisms behind the metabolic reprogramming remain unclear. Accumulating evidence shows that mitochondria can transfer between cells, modulating the recipient cell metabolism. Although such transfer can occur in TILs, direct evidence has been lacking. Here, we analyzed clinical samples and identified mtDNA mutations in TILs that were shared with cancer cells. We demonstrated that these mutations were acquired via mitochondria transfer from cancer cells. The mutated T cells exhibited mitochondrial dysfunction leading to senescence and terminal exhaustion, reducing ICI efficacy. We identified mitochondria transfer as a novel strategy which cancer cells use to evade antitumor immunity.

[Disclosure of COI by Responsible researcher] Lead presenter is not the Responsible researcher.
[Lead presenter: Takamasa Ishino] ■Contributions: 公益財団法人　上原記念生命科学財団
[Principal Researcher: Yosuke Togashi] ■Lecture fee: 中外製薬株式会社、エーザイ株式会社、アストラゼネカ株式会社、ブリストルマイヤーズスクイブ株式会社、MSD株式会社　■Research expenses: ヤンセンファーマ株式会社、アストラゼネカ株式会社、WJOG、大鵬薬品工業株式会社、武田薬品工業株式会社　■Contributions: 公益財団法人　小野薬品 がん・免疫・神経研究財団、公益財団法人　小林がん学術振興会、公益財団法人　武田科学振興財団、公益財団法人　アステラス病態代謝研究会、公益財団法人　上原記念生命科学財団

### [OS12-2] OS12
### Frontier research on regulation of cancer progression deciphered by organelle dynamics

2025 / 9 / 27 (Sat) 12:50-15:20 Room 12/(ANA Crowne Plaza Hotel Kanazawa 3F Ohtori (Central)) Room 12

[Chairpersons]Atsushi Saito (Department of Frontier Science and Interdisciplinary Research, Faculty of Medicine, Kanazawa University)、Toyomasa Katagiri (National Institutes of Biomedical Innovation, Health and Nutrition)

The tumor microenvironment is intermingled with a variety of cells, including immune cells and cancer-associated fibroblasts, which induce abnormal biological responses, resulting in promoting malignant transformation, invasion, and metastasis of cancer. Thus, understanding the subsets of cells, and the mechanisms underlying the formation of specific tissue environments in and around cancer tissues is essential for establishing fundamental therapeutic strategies against cancer. On the other hand, there are organelles that perform specific functions in micro area inside a cell. Recently, it has become clear that organelle dynamics and the functional crosstalk among organelles, or their disruption cause the changes not only in individual cells but also in intercellular communication and the tumor microenvironment, leading to malignant transformation of cancer. We focus on organelles and cellular structures such as endoplasmic reticulum, Golgi apparatus, mitochondria, nuclear membrane, exosomes, and plasma membrane as a platform for cancer progression, and introduce the latest findings involved in the malignant evolution of cancer. Additionally, the discussion regarding the potential for drug discovery is included in the aim of this symposium.

---

### OS12-2
### Unraveling Cancer Progression Through Exosome-Mediated Intercellular Communication

[Speaker]Ayuko Hoshino (RCAST the University of Tokyo)

Exosomes play pivotal roles in cancer progression and diagnosis. We demonstrated that cancer cells secrete exosomes to alter the microenvironment, creating favorable conditions for metastasis. Through plasma-derived exosomal proteomics, we established robust methods to distinguish cancer patients from healthy individuals and further classify cancer types. Intriguingly, while analyzing tumor tissue-derived exosomes, we discovered that plasma exosomal proteins distinguishing cancer from healthy states originate not only from tumor tissues but also from distant, tumor-free organs, highlighting systemic intercellular communication in cancer. Additionally, we revealed that aging significantly impacts plasma exosome composition, with organ-specific exosomal changes occurring throughout the aging process. These findings underscore exosomes as critical mediators of cancer progression, powerful diagnostic biomarkers, and key players in age-related physiological changes, opening new avenues for cancer detection, monitoring, and therapeutic intervention.

[Disclosure of COI by Responsible researcher] Lead presenter is the Responsible researcher.
[Lead presenter: Ayuko Hoshino] ■Contributions: 稲盛財団　■Fees of testimony judgement comment etc.: 株式会社トヨタコンポン研究所

### [OS12-3] OS12

### Frontier research on regulation of cancer progression deciphered by organelle dynamics

2025 / 9 / 27 (Sat) 12:50-15:20 Room 12/(ANA Crowne Plaza Hotel Kanazawa 3F Ohtori (Central)) Room 12

[Chairpersons]Atsushi Saito (Department of Frontier Science and Interdisciplinary Research, Faculty of Medicine, Kanazawa University)、Toyomasa Katagiri (National Institutes of Biomedical Innovation, Health and Nutrition)

The tumor microenvironment is intermingled with a variety of cells, including immune cells and cancer-associated fibroblasts, which induce abnormal biological responses, resulting in promoting malignant transformation, invasion, and metastasis of cancer. Thus, understanding the subsets of cells, and the mechanisms underlying the formation of specific tissue environments in and around cancer tissues is essential for establishing fundamental therapeutic strategies against cancer. On the other hand, there are organelles that perform specific functions in micro area inside a cell. Recently, it has become clear that organelle dynamics and the functional crosstalk among organelles, or their disruption cause the changes not only in individual cells but also in intercellular communication and the tumor microenvironment, leading to malignant transformation of cancer. We focus on organelles and cellular structures such as endoplasmic reticulum, Golgi apparatus, mitochondria, nuclear membrane, exosomes, and plasma membrane as a platform for cancer progression, and introduce the latest findings involved in the malignant evolution of cancer. Additionally, the discussion regarding the potential for drug discovery is included in the aim of this symposium.

---

### OS12-3

### Onset of glioblastoma by failure of alignment between ER and nucleus, and potential as a therapeutic target.

[Speaker]Atsushi Saito (Dept. Frontier Sci. Interdisciplinary Res., Fac. Med., Kanazawa Univ.) [Co-author]Tetsuro Yoshimaru (Natl. Inst. Biomed. Innov., Health Nut., Natl. Inst. Biomed. Innov.)、Yosuke Matsushita (Natl. Inst. Biomed. Innov., Health Nut., Natl. Inst. Biomed. Innov.)、Toyomasa Katagiri (Natl. Inst. Biomed. Innov., Health Nut., Natl. Inst. Biomed. Innov.)、Kazunori Imaizumi (The Osaka Med. Res. Foundation Intractable Dis.／Dept. Child Dev. Mol. Sci., Grad. Child Dev., Osaka Univ.)

Old Astrocyte Specifically Induced Substance (OASIS) is an endoplasmic reticulum (ER)-resident transmembrane transcription factor. OASIS is cleaved in response to ER stress, subsequently generating the N-terminal fragments containing a bZIP-type DNA binding domain, which act as a transcription factor. We found that OASIS is recruited to the impaired structure of nuclear envelope (NE) to repair the disrupted NE. Simultaneously, the induction of DNA damage by the rupture of NE activates OASIS, eventually, arresting cell cycle at G2/M phase via direct induction of p21. Intriguingly, a definite number of glioma and glioblastoma patients was included in the category featuring quite low expression level of OASIS due to DNA methylation in its promoter. The several number of glioblastoma cell lines also showed the low expression level of OASIS accompanied by hypermethylation of its promoter. Specific removal of this hypermethylation in glioblastoma transplanted to nude mice by epigenomic engineering suppressed its tumorigenesis. Taken together, OASIS is novel cell cycle inhibitor, and has potential to act as tumor suppressor for glioblastoma.

[Disclosure of COI by Responsible researcher] Lead presenter is the Responsible researcher.
[Lead presenter: Atsushi Saito] We have no financial relationships to disclose.

### [OS12-4] OS12
### Frontier research on regulation of cancer progression deciphered by organelle dynamics

2025 / 9 / 27 (Sat) 12:50-15:20 Room 12/(ANA Crowne Plaza Hotel Kanazawa 3F Ohtori (Central)) Room 12

[Chairpersons]Atsushi Saito (Department of Frontier Science and Interdisciplinary Research, Faculty of Medicine, Kanazawa University)、Toyomasa Katagiri (National Institutes of Biomedical Innovation, Health and Nutrition)

The tumor microenvironment is intermingled with a variety of cells, including immune cells and cancer-associated fibroblasts, which induce abnormal biological responses, resulting in promoting malignant transformation, invasion, and metastasis of cancer. Thus, understanding the subsets of cells, and the mechanisms underlying the formation of specific tissue environments in and around cancer tissues is essential for establishing fundamental therapeutic strategies against cancer. On the other hand, there are organelles that perform specific functions in micro area inside a cell. Recently, it has become clear that organelle dynamics and the functional crosstalk among organelles, or their disruption cause the changes not only in individual cells but also in intercellular communication and the tumor microenvironment, leading to malignant transformation of cancer. We focus on organelles and cellular structures such as endoplasmic reticulum, Golgi apparatus, mitochondria, nuclear membrane, exosomes, and plasma membrane as a platform for cancer progression, and introduce the latest findings involved in the malignant evolution of cancer. Additionally, the discussion regarding the potential for drug discovery is included in the aim of this symposium.

---

### OS12-4
### Adaptation to the tumor microenvironment through ER-Golgi collaboration: A new therapeutic strategy

[Speaker]Keiji Uchiyama (National Institutes of Biomedical Innovation, Health and Nutrition) [Co-author]Tetsuro Yoshimaru (National Institutes of Biomedical Innovation, Health and Nutrition)、Yosuke Matsushita (National Institutes of Biomedical Innovation, Health and Nutrition)、Koji Ueda (Japanese foundation for cancer research, Cancer precision medical center)、Masayuki Nagahashi (Hyogo College of Medicine, Dept. Breast and Endocrine Surgery)、Yasuo Miyoshi (Hyogo College of Medicine, Dept. Breast and Endocrine Surgery)、Mitsunori Sasa (Tokushima Breast Care Clinic)、Toyomasa Katagiri (National Institutes of Biomedical Innovation, Health and Nutrition)

In solid tumors, including breast cancer (BC), the tumor microenvironment (TME) is crucial for cell proliferation and resistance to therapies. TME stresses (e.g., hypoxia, glucose starvation) continuously induce endoplasmic reticulum (ER) stress. Cancer cells counteract potential death by constitutively activating the unfolded protein response (UPR). In this study, we uncovered that functional ER-Golgi collaboration is essential for BC cell adaptation to the TME. Notably, we focused on O-linked glycosyltransferases (GALNTx and GALNTy), which are frequently overexpressed in the Golgi apparatus of BC cells, to investigate sustained UPR in response to TME-induced stress. Our comprehensive analysis revealed that GALNTx, and GALNTy contribute to sustained UPR activation by modifying IRE1, a key ER stress sensor located in the ER. Furthermore, suppressing this ER-Golgi collaboration significantly reduced BC cell proliferation under persistent ER stress. These findings suggest that O-glycosylation of IRE1, facilitated by ER-Golgi collaboration, is crucial for BC cell adaptation to chronic ER stress in the TME. This molecular mechanism presents a potential novel therapeutic target for BC.

[Disclosure of COI by Responsible researcher] Lead presenter is not the Responsible researcher.
[Lead presenter: Keiji Uchiyama] We have no financial relationships to disclose.
[Principal Researcher: Mitsunori Sasa] ■Research funds from company: オンコセラピー・サイエンス株式会社　■Advisor of the company and/or profit-making organization: オンコセラピー・サイエンス株式会社、株式会社Cancer Precision Medicine
■Contributions: とくしまブレストケアクリニック

▲TOP

### [OS12-5] OS12
### Frontier research on regulation of cancer progression deciphered by organelle dynamics

2025 / 9 / 27 (Sat) 12:50-15:20 Room 12/(ANA Crowne Plaza Hotel Kanazawa 3F Ohtori (Central)) Room 12

[Chairpersons]Atsushi Saito (Department of Frontier Science and Interdisciplinary Research, Faculty of Medicine, Kanazawa University)、Toyomasa Katagiri (National Institutes of Biomedical Innovation, Health and Nutrition)

The tumor microenvironment is intermingled with a variety of cells, including immune cells and cancer-associated fibroblasts, which induce abnormal biological responses, resulting in promoting malignant transformation, invasion, and metastasis of cancer. Thus, understanding the subsets of cells, and the mechanisms underlying the formation of specific tissue environments in and around cancer tissues is essential for establishing fundamental therapeutic strategies against cancer. On the other hand, there are organelles that perform specific functions in micro area inside a cell. Recently, it has become clear that organelle dynamics and the functional crosstalk among organelles, or their disruption cause the changes not only in individual cells but also in intercellular communication and the tumor microenvironment, leading to malignant transformation of cancer. We focus on organelles and cellular structures such as endoplasmic reticulum, Golgi apparatus, mitochondria, nuclear membrane, exosomes, and plasma membrane as a platform for cancer progression, and introduce the latest findings involved in the malignant evolution of cancer. Additionally, the discussion regarding the potential for drug discovery is included in the aim of this symposium.

---

### OS12-5
### Elucidation of novel nutrient sensing mechanisms through organelle dynamics

[Speaker]Tsuyoshi Osawa (RCAST, The University of Tokyo)

Understanding cancer and disease pathogenesis requires integrating complex organic compounds including nucleic acids, carbohydrates, lipids, and proteins, termed multi-omics. Next-generation sequencers and mass spectrometers now enable comprehensive analysis of cancer cells at single-cell and organellar levels, examining genomic sequences, transcription, translation, metabolism, and protein complexes. This mega-data era demands sophisticated analytical approaches for cancer pathogenesis elucidation. Using nutriomics, we capture cellular changes leading to pathogenesis through integrated omics analysis of nutrition and metabolism, identifying metabolic pathways for novel cancer and lifestyle disease therapies. Recently, intercellular and inter-organellar interactions, beyond cancer cell omics variations, critically influence malignancy. Organellar regulation emerges as a promising therapeutic target. This symposium introduces nutriomics analysis of cancer malignancy focusing on metabolites, and discusses metabolic connections and therapeutic strategies spanning cellular to multicellular to inter-organellar levels, presenting cutting-edge integrated omics research.

[Disclosure of COI by Responsible researcher] Lead presenter is the Responsible researcher.
[Lead presenter: Tsuyoshi Osawa] We have no financial relationships to disclose.

Open Symposia

### [OS12-6] OS12
### Frontier research on regulation of cancer progression deciphered by organelle dynamics

2025 / 9 / 27 (Sat) 12:50-15:20 Room 12/(ANA Crowne Plaza Hotel Kanazawa 3F Ohtori (Central)) Room 12

[Chairpersons]Atsushi Saito (Department of Frontier Science and Interdisciplinary Research, Faculty of Medicine, Kanazawa University)、Toyomasa Katagiri (National Institutes of Biomedical Innovation, Health and Nutrition)

The tumor microenvironment is intermingled with a variety of cells, including immune cells and cancer-associated fibroblasts, which induce abnormal biological responses, resulting in promoting malignant transformation, invasion, and metastasis of cancer. Thus, understanding the subsets of cells, and the mechanisms underlying the formation of specific tissue environments in and around cancer tissues is essential for establishing fundamental therapeutic strategies against cancer. On the other hand, there are organelles that perform specific functions in micro area inside a cell. Recently, it has become clear that organelle dynamics and the functional crosstalk among organelles, or their disruption cause the changes not only in individual cells but also in intercellular communication and the tumor microenvironment, leading to malignant transformation of cancer. We focus on organelles and cellular structures such as endoplasmic reticulum, Golgi apparatus, mitochondria, nuclear membrane, exosomes, and plasma membrane as a platform for cancer progression, and introduce the latest findings involved in the malignant evolution of cancer. Additionally, the discussion regarding the potential for drug discovery is included in the aim of this symposium.

---

### OS12-6
### Targeting metabolic vulnerabilities to treat pancreatic ductal adenocarcinoma

[Speaker]Masahiro Sonoshita (Div. Biomedical Oncology, Inst. for Genetic Medicine, Hokkaido Univ.)

Recent studies have greatly deepened our understanding of the pathogenesis of pancreatic ductal adenocarcinoma (PDAC). To facilitate systemic identification of novel therapeutic targets and accelerate drug discovery, we established a ***Drosophila*** model that recapitulates the key genetic alterations that are associated with the most dismal clinical outcomes. Leveraging this "***4-hit***" model in a genetic screening approach, we identified candidate strategies that exploit metabolic vulnerabilities as potential therapeutic interventions for PDAC. This ***Drosophila***-based platform provides a powerful, whole-organism assay that complements existing systems and enables the discovery of new therapeutic targets in PDAC.

[Disclosure of COI by Responsible researcher] Lead presenter is the Responsible researcher.
[Lead presenter: Masahiro Sonoshita] ■ employment by a company related to the presentation: 株式会社FlyWorks、FlyWorks America Inc. ■Profit of stock: 株式会社FlyWorks

### [AOSR7-1] AOSR7
### Advances in Cancer Biology and Translational Research for Primary and Metastatic Brain Tumors

2025 / 9 / 27 (Sat) 12:50-15:20 Room 13/(ANA Crowne Plaza Hotel Kanazawa 3F Ohtori (East)) Room 13

[Chairpersons]Mitsutoshi Nakata (Kanazawa University)、Hiromichi Suzuki (National Cancer Center Research Institute)

Brain tumors are notoriously refractory to treatment and are associated with dismal prognoses, underscoring the critical need for the development of novel therapies. In recent years, intensive research efforts have led to a rapid expansion of molecular understanding in brain tumors, leading to personalized medicine based on molecular abnormality and induction of molecularly targeted therapies. As well as primary brain tumors, the advancement of understanding of systemic cancers has enabled us to induce personalized therapies for metastatic brain tumors based on the molecular characteristics of the primary organ. However, these therapies contribute to the limited number of patients, with the remaining patients with dismal prognoses yet. This session will focus on recent advances in basic and translational research on malignant and metastatic brain tumors, highlighting the latest insights to understand brain tumor pathogenesis and advance novel therapeutic development. The program welcomes early-career investigators and graduate students and calls for abstracts from younger researchers and clinicians.

---

### AOSR7-1
### Unraveling the complexity of glioblastoma invasion

[Speaker]Toshiro Hara (Dept. Neurosurg., U Michigan, School. Med.)

The limited efficacy of surgical resection and high rate of tumor recurrence in diffuse gliomas underscores the urgency of better understanding tumor cell invasion in the brain. We present a method to investigate glioblastoma (GBM) invasion using human GBM spheroid lines in mouse intracranial xenografts. To validate this approach with 20 models, we performed single-cell RNA sequencing (scRNA-seq) to identify models with varying abilities to form tumors and infiltrate tissue. We discovered that the OPC-like cell state is a key feature associated with tumor cells that invade the opposite side of the brain. We then examined invading cells near the original tumor site and those reaching distant brain areas using scRNA-seq. This analysis revealed two distinct gene expression programs related to invasion, both enriched in the OPC-like state. Finally, we used spatial transcriptomics to confirm these invasion-related programs and uncovered a unique relationship between the tumor microenvironment and invading cells. Our findings illuminate the complex relationship between cell development states and GBM invasion, providing insights for targeted therapies against this severe disease.

[Disclosure of COI by Responsible researcher] Lead presenter is the Responsible researcher.
[Lead presenter: Toshiro Hara] We have no financial relationships to disclose.

### [AOSR7-2] AOSR7
### Advances in Cancer Biology and Translational Research for Primary and Metastatic Brain Tumors

2025 / 9 / 27 (Sat) 12:50-15:20 Room 13/(ANA Crowne Plaza Hotel Kanazawa 3F Ohtori (East)) Room 13

[Chairpersons]Mitsutoshi Nakata (Kanazawa University)、Hiromichi Suzuki (National Cancer Center Research Institute)

Brain tumors are notoriously refractory to treatment and are associated with dismal prognoses, underscoring the critical need for the development of novel therapies. In recent years, intensive research efforts have led to a rapid expansion of molecular understanding in brain tumors, leading to personalized medicine based on molecular abnormality and induction of molecularly targeted therapies. As well as primary brain tumors, the advancement of understanding of systemic cancers has enabled us to induce personalized therapies for metastatic brain tumors based on the molecular characteristics of the primary organ. However, these therapies contribute to the limited number of patients, with the remaining patients with dismal prognoses yet. This session will focus on recent advances in basic and translational research on malignant and metastatic brain tumors, highlighting the latest insights to understand brain tumor pathogenesis and advance novel therapeutic development. The program welcomes early-career investigators and graduate students and calls for abstracts from younger researchers and clinicians.

---

### AOSR7-2
### Genomic heterogeneity within WNT medulloblastoma

[Speaker]Evan Y. Wang (University of Toronto, Canada／The Hospital for Sick Children, Canada／National Cancer Center Research Institute, Japan) [Co-author]Jessica Taylor (Cancer Reseach UK Cambridge Insititute, United Kingdom)、Hiromichi Suzuki (National Cancer Center Research Institute, Japan)、Richard Gilbertson (Cancer Reseach UK Cambridge Insititute, United Kingdom)、Michael D. Taylor (University of Toronto, Canada／The Hospital for Sick Children, Canada／Baylor College of Medicine, USA／Texas Children's Hospital, USA)

Medulloblastoma is the most common malignant pediatric brain tumor. Recent molecular analyses have revealed further heterogeneity within medulloblastoma subgroups, yet our understanding of the smallest subgroup (WNT MB) has been limited by lack of power due to small cohort sizes. To examine if such heterogeneity also exists within WNT MB, we performed RNA-sequencing on 74 WNT MB, the largest cohort assembled to date. We confirm CTNNB1 mutation and loss of chromosome 6 (monosomy 6) as the most frequent genetic alterations in this subgroup. Clustering of transcriptomes revealed distinct patient subsets associated with transcriptional signatures, mutation patterns, and clinical features. Our analysis reveals recurrent mutations converging on the SHH signaling pathway at a much greater prevalence than previously appreciated. We also characterize recurrent mutations in chromosome 6 genes, which represent potential targets of monosomy 6. Our analyses uncover remarkable genetic heterogeneity within WNT-MB and potential therapeutic avenues.

[Disclosure of COI by Responsible researcher] Lead presenter is the Responsible researcher.
[Lead presenter: Evan Y. Wang] We have no financial relationships to disclose.

### [AOSR7-3] AOSR7
### Advances in Cancer Biology and Translational Research for Primary and Metastatic Brain Tumors

2025 / 9 / 27 (Sat) 12:50-15:20 Room 13/(ANA Crowne Plaza Hotel Kanazawa 3F Ohtori (East)) Room 13

[Chairpersons]Mitsutoshi Nakata (Kanazawa University)、Hiromichi Suzuki (National Cancer Center Research Institute)

Brain tumors are notoriously refractory to treatment and are associated with dismal prognoses, underscoring the critical need for the development of novel therapies. In recent years, intensive research efforts have led to a rapid expansion of molecular understanding in brain tumors, leading to personalized medicine based on molecular abnormality and induction of molecularly targeted therapies. As well as primary brain tumors, the advancement of understanding of systemic cancers has enabled us to induce personalized therapies for metastatic brain tumors based on the molecular characteristics of the primary organ. However, these therapies contribute to the limited number of patients, with the remaining patients with dismal prognoses yet. This session will focus on recent advances in basic and translational research on malignant and metastatic brain tumors, highlighting the latest insights to understand brain tumor pathogenesis and advance novel therapeutic development. The program welcomes early-career investigators and graduate students and calls for abstracts from younger researchers and clinicians.

### AOSR7-3
### Translational Research for Central Nervous System Lymphomas with paired Patient Tumors and PDX models

[Speaker]Kensuke Tateishi (Dept. Neurosurg., Yokohama City Univ.／Grad. Sch. Med. Sci., Yokohama City Univ.)

Central nervous system lymphoma (CNSL) has a poor prognosis despite intensive chemotherapy, highlighting the urgent need for novel therapeutic strategies and reliable preclinical models. However, the scarcity of suitable CNSL models has hindered progress in translational research.To address this challenge, we have established the largest panel of CNSL patient-derived xenograft (PDX) models, comprising 51 models derived from individual patients. These models faithfully recapitulate the phenotypic and genetic characteristics of the original tumors, providing a valuable resource for preclinical studies.In this presentation, I will introduce the histopathological, genomic, and transcriptomic features of these 51 CNSL-PDX models and discuss their potential applications in future translational research. I will also present our current projects, including metabolic imaging of CNSL, the in vitro propagation of PDX-derived cells, and the exploration of novel therapeutic strategies.

[Disclosure of COI by Responsible researcher] Lead presenter is the Responsible researcher.
[Lead presenter: Kensuke Tateishi] ■Research funds from company: 小野薬品工業株式会社

### [AOSR7-4] AOSR7
### Advances in Cancer Biology and Translational Research for Primary and Metastatic Brain Tumors

2025 / 9 / 27 (Sat) 12:50-15:20 Room 13/(ANA Crowne Plaza Hotel Kanazawa 3F Ohtori (East)) Room 13

[Chairpersons]Mitsutoshi Nakata (Kanazawa University)、Hiromichi Suzuki (National Cancer Center Research Institute)

Brain tumors are notoriously refractory to treatment and are associated with dismal prognoses, underscoring the critical need for the development of novel therapies. In recent years, intensive research efforts have led to a rapid expansion of molecular understanding in brain tumors, leading to personalized medicine based on molecular abnormality and induction of molecularly targeted therapies. As well as primary brain tumors, the advancement of understanding of systemic cancers has enabled us to induce personalized therapies for metastatic brain tumors based on the molecular characteristics of the primary organ. However, these therapies contribute to the limited number of patients, with the remaining patients with dismal prognoses yet. This session will focus on recent advances in basic and translational research on malignant and metastatic brain tumors, highlighting the latest insights to understand brain tumor pathogenesis and advance novel therapeutic development. The program welcomes early-career investigators and graduate students and calls for abstracts from younger researchers and clinicians.

---

### AOSR7-4
### Induction of cancer cell death by microglia and its regulatory mechanisms

[Speaker]Eishu Hirata (Tumor Cell Biology and Bioimaging, CRI of Kanazawa Univ.)

Cancer cells that metastasize to the brain are immediately recognized by astrocytes and microglia. However, much remains unknown about the role of these glial cells and their functional regulation. Here, we found that primary microglia co-cultured with cancer cells in a soft substrate exhibit robust tumoricidal activity. Mechanistically, these microglia are able to recognize and enwrap cancer cells, deliver specific DNA into the cancer cells, and induce active cell death. In addition, our results indicate that cell death is induced by IFI16-mediated inflammasome formation, resulting in caspase-1-dependent cell death. We also found that cancer cells engineered to enhance their ability to metastasize to the brain acquired the ability to evade microglial induction of cell death. Surprisingly, this ability was not solely intrinsic, but was mediated by astrocytes. These findings suggest that glial networks possess a mechanism for eliminating cancer cells, and that brain metastatic cancer cells have acquired specific strategies to evade this mechanism.

[Disclosure of COI by Responsible researcher] Lead presenter is the Responsible researcher.
[Lead presenter: Eishu Hirata] We have no financial relationships to disclose.

### [AOSR7-5] AOSR7
### Advances in Cancer Biology and Translational Research for Primary and Metastatic Brain Tumors

2025 / 9 / 27 (Sat) 12:50-15:20 Room 13/(ANA Crowne Plaza Hotel Kanazawa 3F Ohtori (East)) Room 13

[Chairpersons]Mitsutoshi Nakata (Kanazawa University)、Hiromichi Suzuki (National Cancer Center Research Institute)

Brain tumors are notoriously refractory to treatment and are associated with dismal prognoses, underscoring the critical need for the development of novel therapies. In recent years, intensive research efforts have led to a rapid expansion of molecular understanding in brain tumors, leading to personalized medicine based on molecular abnormality and induction of molecularly targeted therapies. As well as primary brain tumors, the advancement of understanding of systemic cancers has enabled us to induce personalized therapies for metastatic brain tumors based on the molecular characteristics of the primary organ. However, these therapies contribute to the limited number of patients, with the remaining patients with dismal prognoses yet. This session will focus on recent advances in basic and translational research on malignant and metastatic brain tumors, highlighting the latest insights to understand brain tumor pathogenesis and advance novel therapeutic development. The program welcomes early-career investigators and graduate students and calls for abstracts from younger researchers and clinicians.

---

### AOSR7-5
### Exploring the Therapeutic Potential of CAR-Engineered Immune Cells Using a Syngeneic GBM Model

[Speaker]Ryusuke Hatae (Department of Neurological Surgery, University of California, San Francisco／Department of Neurosurgery, Graduate School of Medical Sciences, Kyushu University) [Co-author]Payal Watchmaker (Department of Neurological Surgery, University of California, San Francisco)、Akane Yamamichi (Department of Neurological Surgery, University of California, San Francisco)、Keith Kyewalabye (Department of Neurological Surgery, University of California, San Francisco)、Masaki Terabe (Neuro-Oncology Branch, National Cancer Institute, NIH)、Hideho Okada (Department of Neurological Surgery, University of California, San Francisco／The Parker Institute for Cancer Immunotherapy)

CAR-based therapies have yet to achieve meaningful success in solid tumors such as glioblastoma (GBM). To address this, we developed a transgenic mouse model suitable for syngeneic GBM studies, expressing an EGFRvIII-targeting CAR in all immune cell types. This platform enables comparative evaluation of CAR-immune subsets and offers insights into their therapeutic potential. CAR-T cells infiltrating the hypoxic tumor microenvironment showed mitochondrial dysfunction and impaired effector function, both of which were restored by enhancing cellular metabolism. CAR-macrophages carrying a CAR with CD3 zeta as the sole signaling domain increased TNF production and phagocytosis in vitro but failed to suppress tumors in vivo. CAR-NK cells were similarly active in vitro but ineffective in vivo. In contrast, CAR-NKT cells exhibited strong antitumor activity and high cytokine secretion both in vitro and in vivo. Strikingly, combined treatment with CAR-T and CAR-NKT cells enhanced IFN-$\gamma$ secretion in vitro and improved tumor control in vivo.
This model supports functional dissection of CAR immune cells and the development of synergistic immunotherapies for solid tumors.

[Disclosure of COI by Responsible researcher] Lead presenter is the Responsible researcher.
[Lead presenter: Ryusuke Hatae] We have no financial relationships to disclose.

▲TOP

### [AOSR7-6] AOSR7
### Advances in Cancer Biology and Translational Research for Primary and Metastatic Brain Tumors

2025 / 9 / 27 (Sat) 12:50-15:20 Room 13/(ANA Crowne Plaza Hotel Kanazawa 3F Ohtori (East)) Room 13

[Chairpersons]Mitsutoshi Nakata (Kanazawa University)、Hiromichi Suzuki (National Cancer Center Research Institute)

Brain tumors are notoriously refractory to treatment and are associated with dismal prognoses, underscoring the critical need for the development of novel therapies. In recent years, intensive research efforts have led to a rapid expansion of molecular understanding in brain tumors, leading to personalized medicine based on molecular abnormality and induction of molecularly targeted therapies. As well as primary brain tumors, the advancement of understanding of systemic cancers has enabled us to induce personalized therapies for metastatic brain tumors based on the molecular characteristics of the primary organ. However, these therapies contribute to the limited number of patients, with the remaining patients with dismal prognoses yet. This session will focus on recent advances in basic and translational research on malignant and metastatic brain tumors, highlighting the latest insights to understand brain tumor pathogenesis and advance novel therapeutic development. The program welcomes early-career investigators and graduate students and calls for abstracts from younger researchers and clinicians.

---

### AOSR7-6
### Extended Follow-Up of Phase 2 Trial on Survival Outcomes of Boron Neutron Capture Therapy in Recurrent Glioblastoma

[Speaker]Shinji Kawabata (Dept. of Neurosurgery, Osaka Med. & Pharm. Univ.) [Co-author]Shinichi Myatake (Kansai BNCT Med. Ctr., Osaka Med. & Pharm. Univ.)、Hdeki Kashiwagi (Dept. of Neurosurgery, Osaka Med. & Pharm. Univ.)、Naosuke Nonoguchi (Dept. of Neurosurgery, Osaka Med. & Pharm. Univ.)、Motomasa Furuse (Dept. of Neurosurgery, Osaka Med. & Pharm. Univ.)、Kohei Tsujino (Dept. of Neurosurgery, Osaka Med. & Pharm. Univ.)、Natsuko Kondo (Kyoto Univ. Inst. for Integrated Radiation and Nuclear Sci.)、Minoru Suzuki (Kyoto Univ. Inst. for Integrated Radiation and Nuclear Sci.)、Koji Ono (Kansai BNCT Med. Ctr., Osaka Med. & Pharm. Univ.／Kyoto Univ. Inst. for Integrated Radiation and Nuclear Sci.)、Masahiko Wanibuchi (Dept. of Neurosurgery, Osaka Med. & Pharm. Univ.)

**Purpose:** The Phase II JG002 trial of **accelerator-based boron neutron capture therapy (AB-BNCT)** in recurrent glioblastoma demonstrated a 1-year survival rate of 79.2% (95% CI: 57.0 - 90.8). This study extends follow-up to assess long-term outcomes.
**Materials and Methods:** Long-term survival data from patients in the JG002 trial were analyzed, including those with recurrent glioblastoma treated with standard therapies but no prior bevacizumab. Kaplan-Meier survival analysis and survival models (exponential, Weibull) were used, considering both overall distribution and individual variations.
**Results:** Kaplan-Meier analysis showed a 1-year survival rate of 79.2(57.0 - 90.8) % and median overall survival of 19.2 (13.1 - 24.8) months. Survival rates at 2 and 3 years were 33.3% and 20.8%, respectively. Weibull modeling better reflected the progressive nature of glioblastoma.
**Conclusion:** AB-BNCT showed long-term survival benefits and an acceptable safety profile in recurrent glioblastoma. The extended follow-up confirms the efficacy of BNCT in prolonging survival. We are planning further trials to enhance our understanding and add valuable insights to this evolving treatment approach.

[Disclosure of COI by Responsible researcher] Lead presenter is the Responsible researcher.
[Lead presenter: Shinji Kawabata] We have no financial relationships to disclose.

### [E-3061] I-E13-3
### Molecular targeted therapy (2)

2025 / 9 / 27 (Sat) 12:50-14:05 Room 14/(ANA Crowne Plaza Hotel Kanazawa 3F Zuiun 1) Room 14

[Chairperson]Noritaka Tanaka (Div. of Cancer Target TR, Aichi Cancer Ctr)

### E-3061
### The Oncogenic Impact of KRAS in Cholangiocarcinoma

[Speaker]Siwanon Jirawatnotai (Department of Pharmacology, Faculty of Medicine, Siriraj, Mahidol University, Thailand) [Co-author]Sunisa Prasopporn (Department of Pharmacology, Faculty of Medicine, Siriraj, Mahidol University, Thailand／SiCORE for PM&SP Faculty of Medicine, Siriraj, Mahidol University, Thailand)、Makamas Chanda (Department of Pharmacology, Faculty of Medicine, Siriraj, Mahidol University, Thailand／SiCORE for PM&SP Faculty of Medicine, Siriraj, Mahidol University, Thailand)、Amphun Chaiboonchoe (Department of Pharmacology, Faculty of Medicine, Siriraj, Mahidol University, Thailand／SiCORE for PM&SP Faculty of Medicine, Siriraj, Mahidol University, Thailand)、Yuh-Pyng Sher (Graduate Institute of Biomedical Sciences, China Medical University, Taiwan／Center for Molecular Medicine, China Medical University Hospital, Taiwan／Institute of Biochemistry and Molecular Biology, China Medical University, Taiwan)

Cholangiocarcinoma (CCA) is a rare cancer originating in the bile duct, affecting up to 6 per 100,000 people in countries such as Thailand, Korea, and China. Several mutation targets have been identified in CCA, including FGFR2, IDH1, and HER2. However, these targets are rare and found in a small population of CCA patients. To identify a better target for CCA therapy, we investigated the potential of the KRAS-targeting approach in CCA. We found in clinical databases that CCA with high expression of KRAS, both mutant and wild-type, correlated with a significantly shorter patient survival. Inducible expression of both wild-type and mutant KRAS protein in CCA cell lines promoted cell proliferation in low serum conditions and cell migration. Conversely, KRAS depletion by siRNA or a KRAS degrader, ADAM9 inhibitor, resulted in suppressed CCA proliferation. Suppression of KRAS expression by the ADAM9 inhibitor also effectively blocked tumor growth in CCA xenograft and patient-derived xenograft models. Therefore, we propose that CCA depends on KRAS for its tumorigenesis and survival, and inhibition of KRAS expression may be an effective therapeutic strategy for CCA.

[Disclosure of COI by Responsible researcher] Lead presenter is the Responsible researcher.
[Lead presenter: Siwanon Jirawatnotai] We have no financial relationships to disclose.

▲TOP

### [E-3062] I-E13-3
### Molecular targeted therapy (2)

2025 / 9 / 27 (Sat) 12:50-14:05 Room 14/(ANA Crowne Plaza Hotel Kanazawa 3F Zuiun 1) Room 14

[Chairperson]Noritaka Tanaka (Div. of Cancer Target TR, Aichi Cancer Ctr)

### E-3062
### Resistance mechanism to KRAS Inhibitor in patient-derived xenograft with KRAS G12C mutation

[Speaker]Yifeng Liu (Dept. of Respiratory Medicine, Kanazawa Univ. Hosp.) [Co-author]Shigeki Nanjo (Dept. of Respiratory Medicine, Kanazawa Univ. Hosp.)、Yuya Murase (Dept. of Respiratory Medicine, Kanazawa Univ. Hosp.)、Sachiko Arai (Dept. of Respiratory Medicine, Kanazawa Univ. Hosp.)、Koji Fukuda (Dept. of Medical Oncology, Kanazawa Univ. Cancer Research Institute)、Tsukasa Ueda (Dept. of Respiratory Medicine, Kanazawa Univ. Hosp.)、Shunichi Nomura (Dept. of Respiratory Medicine, Kanazawa Univ. Hosp.)、Hayato Koba (Dept. of Respiratory Medicine, Kanazawa Univ. Hosp.)、Yuichi Tambo (Dept. of Respiratory Medicine, Kanazawa Univ. Hosp.)、Seiji Yano (Dept. of Respiratory Medicine, Kanazawa Univ. Hosp.)

**Background**: KRAS G12C inhibitors, such as Sotorasib and Adagrasib, represent a breakthrough in treating KRAS G12C-mutant non-small cell lung cancer (NSCLC). However, intrinsic and acquired resistance remain major challenges, demanding new strategies.**Methods**: We established an intrinsically resistant model, KU-001, a patient-derived xenograft (PDX) that exhibited clinical resistance to Sotorasib, enabling mechanistic investigation and therapeutic evaluation.**Results**: Our findings identify EGFR activation as a key resistance driver. Elevated phosphorylated EGFR (p-EGFR) in KU-001 sustained oncogenic signaling despite KRAS inhibition. Silencing EGFR restored sensitivity to KRAS inhibitors. Notably, combining an EGFR TKI with KRAS inhibitor led to marked tumor suppression both in vitro and in vivo, indicating a promising approach to improve treatment efficacy.**Conclusion**: These results underscore the potential of co-targeting EGFR to overcome KRAS G12C inhibitor resistance in NSCLC. Clinical validation could enhance patient response and expand therapeutic options for this difficult-to-treat cancer subset.

[Disclosure of COI by Responsible researcher] Lead presenter is not the Responsible researcher.
[Lead presenter: Yifeng Liu] ■Research funds from company: 金沢大学Hakase+プロジェクト
[Principal Researcher: Shigeki Nanjo] ■Lecture fee: AstraZeneca

### [E-3063] I-E13-3
### Molecular targeted therapy (2)

2025 / 9 / 27 (Sat) 12:50-14:05 Room 14/(ANA Crowne Plaza Hotel Kanazawa 3F Zuiun 1) Room 14

[Chairperson]Noritaka Tanaka (Div. of Cancer Target TR, Aichi Cancer Ctr)

### E-3063
### Perspectives on novel combination strategies for KRAS mutant lung cancer

[Speaker]Kosuke Tanaka (NCC, Research Institute, Division of Cancer Immunology) [Co-author]Yasuki Adachi (NCC, Research Institute, Division of Cancer Immunology)、Gaku Yamamoto (NCC, Hospital East, Department of Thoracic Oncology)、Shohei Koyama (NCC, Research Institute, Division of Cancer Immunology)、Akihiro Ohashi (NCC, EPOC, Division of Collaborative Research and Developments)、Hiroyoshi Nishikawa (NCC, Research Institute, Division of Cancer Immunology)

Despite the initial success of novel KRAS(G12C) inhibitors (KRASi) in NSCLC, the rapid emergence of drug resistance underscores the urgent need to develop combination therapeutic strategies with KRASi to improve clinical outcomes. Here, we present novel combination approaches in preclinical models. 1) Targeting tumor-intrinsic mechanisms: Through high-throughput screening, we identified WEE1i as a top candidate for combination with sotorasib. Dual inhibition of WEE1 and KRAS demonstrated synergistic antiproliferative activities both in vitro and in vivo. These findings are supported by previous studies. 2) Enhancing antitumor immunity: We investigated the potential for KRAS inhibition to augment the efficacy of immunotherapy. KRAS/MEKi promoted innate immunity, particularly when combined with the mitophagy inhibitor CQ. This effect was dependent on the cGAS-STING pathway. The triple combination with ICI further enhanced antitumor immunity by remodeling the TME in mouse models. These results highlight a novel therapeutic paradigm of KRASi that induces innate immunity to augment the ICI efficacy. Together, we will discuss the perspectives on novel combinations for KRAS mutant NSCLC.

[Disclosure of COI by Responsible researcher] Lead presenter is the Responsible researcher.
[Lead presenter: Kosuke Tanaka] ■Research funds from company: AMED（次世代がん・革新がん）、MSD、武田、アステラス、SGH

▲TOP

### [E-3064] I-E13-3
### Molecular targeted therapy (2)

2025 / 9 / 27 (Sat) 12:50-14:05 Room 14/(ANA Crowne Plaza Hotel Kanazawa 3F Zuiun 1) Room 14

[Chairperson]Noritaka Tanaka (Div. of Cancer Target TR, Aichi Cancer Ctr)

---

### E-3064
### The inhibition of abnormal protein processing mechanisms suppresses the proliferation of drug-resistant cancer

[Speaker]Toshiyuki Tsunoda (Dept.Cell Biol., Fac. Med., Fukuoka Univ.／Cent. Res. Inst. for Adv. Mol. Med., Fukuoka Univ.) [Co-author]Kazumasa Yoshida (Dept.Cell Biol., Fac. Med., Fukuoka Univ.／Cent. Res. Inst. for Adv. Mol. Med., Fukuoka Univ.)、Takanori Kitaguchi (Dept.Cell Biol., Fac. Med., Fukuoka Univ.)、Senji Shirasawa (Dept.Cell Biol., Fac. Med., Fukuoka Univ.／Cent. Res. Inst. for Adv. Mol. Med., Fukuoka Univ.)

Drug resistance has been observed in refractory cancers. For instance, mutant (mt) KRAS inhibitors develop drug resistance after six months. In a screen of compounds that preferentially suppress the growth of cancer spheroids containing mtKRAS (G13D) over those of a normal model (wild-type KRAS), we identified pyra-metho-carnil (PMC), which inhibits cancer spheroid growth with various driver gene mutations in vitro and HCT116 xenograft growth in vivo. Furthermore, pull-down assays identified KDELRs as PMC targets. KDELRs, downstream effectors of MAPK signaling, act as trigger molecules for abnormal protein processing. Aggresome accumulation has been observed after KRAS inhibitor (KRASi) treatment but disappears upon drug resistance acquisition, suggesting its role in overcoming resistance.Notably, KDELR inhibitors (KDELRi) induce ER stress, promoting aggresome accumulation and subsequent cell death. Additionally, KDELRi and KRASi synergistically suppress KRASi-resistant cell growth and enhance aggresome formation. These findings indicate KDELRs as promising targets for drug-resistant cancers.

[Disclosure of COI by Responsible researcher] Lead presenter is the Responsible researcher.
[Lead presenter: Toshiyuki Tsunoda] We have no financial relationships to disclose.

▲TOP

### [E-3065] I-E13-3
### Molecular targeted therapy (2)

2025 / 9 / 27 (Sat) 12:50-14:05 Room 14/(ANA Crowne Plaza Hotel Kanazawa 3F Zuiun 1) Room 14

[Chairperson]Noritaka Tanaka (Div. of Cancer Target TR, Aichi Cancer Ctr)

---

### E-3065
### Anti-tumor activity of orally available cyclic peptide LUNA18 through direct RAS inhibition

[Speaker]Hitoshi Sase (Chugai Pharm Co., LTD. Translational Res Div.) [Co-author]Saki Michisaka (Chugai Pharm Co., LTD. Res Div.)、Yukako Tachibana (Chugai Pharm Co., LTD. Res Div.)、Masami Hasegawa (Chugai Pharm Co., LTD. Res Div.)、Tomoko Kanei (Chugai Pharm Co., LTD. Res Div.)、Naoaki Murao (Chugai Pharm Co., LTD. Res Div.)、Shino Kuramoto (Chugai Pharm Co., LTD. Res Div.)、Norihito Shibahara (Chugai Pharm Co., LTD. Translational Res Div.)、Atsushi Ohta (Chugai Pharm Co., LTD. Res Div.)、Mikimasa Tanada (Chugai Pharm Co., LTD. Res Div.)、Takuya Shiraishi (Chugai Pharm Co., LTD. Res Div.)、Hitoshi Iikura (Chugai Pharm Co., LTD. Translational Res Div.)、Takehisa Kitazawa (Chugai Pharm Co., LTD. Res Div.)、Hiroshi Tanaka (Chugai Pharm Co., LTD. Res Div.)

RAS is the frequently altered gene in cancers, representing unmet medical needs in RAS-mutated cancers. LUNA18 is a cyclic peptide binding to RAS mutants and wildtype (WT), thereby inhibiting protein-protein interaction between the inactive form of RAS and GEFs. LUNA18 decreased the active form of KRAS, ERK and AKT, resulting in anti-proliferation of RAS-mutated cells. LUNA18 exhibited favorable oral bioavailability in several species. Oral administration of LUNA18 led to RAS signal inhibition and tumor regression in RAS-mutated xenograft models. Moreover, LUNA18 showed robust efficacy in KRAS-amplified tumors. These findings suggested LUNA18 is applicable to a wide range of RAS-altered tumors. Identifying effective combination partners is crucial for enhancing efficacy. Combining cetuximab augmented efficacy of LUNA18 through profound inhibition of the RAS/MAPK pathway in a colorectal cancer model. We also discovered addition of LUNA18 to KRAS G12C inhibitors (G12C-i) delayed emergence of resistance to G12C-i. These findings offer a promising therapeutic strategy for overcoming RAS-mutated cancers by utilizing LUNA18. Copyright permission confirmed for AACR abstract #1654, 2024.

[Disclosure of COI by Responsible researcher] Lead presenter is not the Responsible researcher.
[Lead presenter: Hitoshi Sase] ■ employment by a company related to the presentation: 中外製薬株式会社
[Principal Researcher: Saki Michisaka] ■employment by a company related to the presentation: 中外製薬株式会社

### [E-3066] I-E13-3
### Molecular targeted therapy (2)

2025 / 9 / 27 (Sat) 12:50-14:05 Room 14/(ANA Crowne Plaza Hotel Kanazawa 3F Zuiun 1) Room 14

[Chairperson]Noritaka Tanaka (Div. of Cancer Target TR, Aichi Cancer Ctr)

---

### E-3066
### Combination of RAS inhibitor LUNA18 and KRAS G12C inhibitors enhances anti-tumor effects by blocking MAPK reactivation.

[Speaker]Saki Michisaka (Chugai Pharmaceutical Co., LTD. Res Div.) [Co-author]Hitoshi Sase (Chugai Pharmaceutical Co., LTD. Translational Res Div.)、Yukako Tachibana (Chugai Pharmaceutical Co., LTD. Res Div.)、Masami Hasegawa (Chugai Pharmaceutical Co., LTD. Res Div.)、Shino Kuramoto (Chugai Pharmaceutical Co., LTD. Res Div.)、Norihito Shibahara (Chugai Pharmaceutical Co., LTD. Translational Res Div.)、Atsushi Ohta (Chugai Pharmaceutical Co., LTD. Res Div.)、Mikihisa Tanada (Chugai Pharmaceutical Co., LTD. Res Div.)、Takuya Shiraishi (Chugai Pharmaceutical Co., LTD. Res Div.)、Hitoshi Iikura (Chugai Pharmaceutical Co., LTD. Translational Res Div.)、Takehisa Kitazawa (Chugai Pharmaceutical Co., LTD. Res Div.)、Hiroshi Tanaka (Chugai Pharmaceutical Co., LTD. Res Div.)

KRAS G12C inhibitors (G12C-i) are FDA-approved RAS-targeting agents but show limited monotherapy efficacy likely due to MAPK pathway reactivation. Here we report LUNA18, a first-in-class orally available cyclic peptide that acts as a pan-RAS GDP inhibitor binding to both wildtype (WT) RAS and various RAS mutants. Notably, under long-term treatment and growth factor stimulation, LUNA18 induced more potent and sustained signal inhibition than G12C-i. This enhanced inhibition is attributed to LUNA18's ability to persistently suppress MAPK signaling by inactivating WT RAS, which plays a key role in MAPK activation in KRAS-mutated cancers. Furthermore, in vitro studies demonstrated stronger growth inhibition with combination of LUNA18 and G12C-i than single agents. In vivo models, LUNA18 and G12C-i combination suppressed regrowth that observed G12C-i monotherapy. Our data demonstrate that MAPK pathway reactivation via WT RAS activation drives resistance to G12C-i. LUNA18's ability to inhibit WT RAS contributes to its efficacy, making it a promising candidate for combination therapy with G12C-i to overcome resistance mechanisms. Copyright permission confirmed for AACR2024 abstract #1664.

[Disclosure of COI by Responsible researcher] Lead presenter is the Responsible researcher.
[Lead presenter: Saki Michisaka] ■ employment by a company related to the presentation: 中外製薬株式会社

### [J-3055] I-J7-2
### Clinical cancer genomics

2025 / 9 / 27 (Sat) 12:50-14:05 Room 15/(ANA Crowne Plaza Hotel Kanazawa 3F Zuiun 2) Room 15

[Chairperson]Kuniko Sunami (Dept. Lab. Med., Natl. Cancer. Ctr.)

---

### J-3055
### Genetic architecture and clinical significance of clonal hematopoiesis among older Japanese individuals

[Speaker]Yoshiaki Usui (IMS, RIKEN) [Co-author]Kazuo Miyazawa (IMS, RIKEN)、Hanae Iijima (IMS, RIKEN)、Mikiko Endo (IMS, RIKEN)、Yusuke Iwasaki (IMS, RIKEN)、Tomomi Aoi (IMS, RIKEN)、Kaoru Ito (IMS, RIKEN)、Hidewaki Nakagawa (IMS, RIKEN)、Koichi Matsuda (Grad. Sch. of Frontier Sci., The Univ. of Tokyo)、Yukihide Momozawa (IMS, RIKEN)

Clonal hematopoiesis of indeterminate potential (CHIP) is a unique phenomenon related to somatic mosaicism in which cells can freely mix and expand. In older individuals, CHIP may represent a natural experiment in saturation mutagenesis in humans. Moreover, it may enhance the statistical power of genome-wide association studies (GWAS) by increasing the number of identifiable carriers. In this study, we investigated CHIP in approximately 25,000 individuals aged 80 years and older without hematological malignancies from BioBank Japan, using high-coverage sequencing data. GWAS revealed the genetic architecture of CHIP in the Japanese population. These CHIP-related genetic factors showed locus-specific associations with different types of cancer in phenome-wide association studies. Regarding the positive selection of somatic mutations, ***PPM1D*** was enriched in males, regardless of cancer status or environmental factors. Furthermore, CHIP was associated with poor prognosis even in older individuals, with the number of mutations correlating with adverse outcomes. These findings provide valuable insights into somatic evolution during aging and improve our understanding of CHIP dynamics.

[Disclosure of COI by Responsible researcher] Lead presenter is the Responsible researcher.
[Lead presenter: Yoshiaki Usui] ■Research funds from company: 公的研究費：科学研究費助成事業（文部科学省）、民間学術助成金：安田記念医学財団、上原記念生命科学財団（大正製薬ホールディングス株式会社）、武田科学振興財団（武田薬品工業株式会社）、MSD生命科学財団（MSD株式会社）、その他：基礎科学特別研究員制度（理化学研究所）　■Research expenses: 安田記念医学財団、上原記念生命科学財団、武田科学振興財団、MSD生命科学財団

### [J-3056] I-J7-2
### Clinical cancer genomics

2025 / 9 / 27 (Sat) 12:50-14:05 Room 15/(ANA Crowne Plaza Hotel Kanazawa 3F Zuiun 2) Room 15

[Chairperson]Kuniko Sunami (Dept. Lab. Med., Natl. Cancer. Ctr.)

---

### J-3056
### Clinical utility of detecting hereditary tumor-related pathogenic variants in ovarian cancer using adaptive sampling

[Speaker]Ai Ikki (Dept. Clin. Genomics., Natl. Cancer Ctr. Res. Inst.／Dept. Reproductive Medicine, Perinatology and Gynecologic Oncology., Nippon Med. Sch.) [Co-author]Daiki Higuchi (Div. Genome Biol., Natl. Cancer Ctr. Res. Inst.)、Ayako Suzuki (CBMS, Grad. Sch. of Fr. Sci., Univ. of Tokyo.)、Maiko Yamaguchi (Div. Genome Biol., Natl. Cancer Ctr. Res. Inst.)、Takayuki Kawai (Div. Genome Biol., Natl. Cancer Ctr. Res. Inst.)、Tetsuro Shiraishi (Dept. Gynecology., Natl. Cancer Ctr. Hosp.)、Mayumi Kato (Dept. Gynecology., Natl. Cancer Ctr. Hosp.)、Shunji Suzuki (Dept. Reproductive Medicine, Perinatology and Gynecologic Oncology., Nippon Med. Sch.)、Masahiro Gotoh (Dept. Clin. Genomics., Natl. Cancer Ctr. Res. Inst.)、Ryuji Hamamoto (Div. Medical AI Res. Dev., Natl. Cancer Ctr. Res. Inst.)、Hiroshi Yoshida (Dept. Diagnostic Path., Natl. Cancer Ctr. Hosp.)、Takashi Kohno (Div. Genome Biol., Natl. Cancer Ctr. Res. Inst.)、Mitsuya Ishikawa (Dept. Gynecology., Natl. Cancer Ctr. Hosp.)、Yutaka Suzuki (CBMS, Grad. Sch. of Fr. Sci., Univ. of Tokyo.)、Kouya Shiraishi (Dept. Clin. Genomics., Natl. Cancer Ctr. Res. Inst.／Div. Genome Biol., Natl. Cancer Ctr. Res. Inst.)

Ovarian cancer is frequently diagnosed at an advanced stage and often has a poor prognosis. Therefore, it is crucial to identify ovarian cancer before its onset accurately. In this study, we focused on 202 genes associated with hereditary cancer risk and performed adaptive sampling using PromethION for the +10 kb region at both termini of each gene-coding region. Nanopore sequencing technology can be used to detect various genomic alterations. We assessed the prevalence of pathogenic variants in 266 patients diagnosed with ovarian cancer at the National Cancer Center Hospital between 2016 and 2021 (IRB number 2015-278). We utilized blood-derived DNA to prepare libraries according to the manufacturer´s protocol and sequenced them. The average depth of the target region exceeded 15. A total of 55 cases (20.7%) detected pathogenic variants, including BRCA1 (26 cases), BRCA2 (11 cases), and MSH6 (six cases). The frequency of pathogenic variants observed in Japanese patients with ovarian cancer was consistent with the findings of other studies. We also examined structural variants and DNA methylation alterations, and their frequencies will be announced in this presentation.

[Disclosure of COI by Responsible researcher] Lead presenter is not the Responsible researcher.
[Lead presenter: Ai Ikki] We have no financial relationships to disclose.
[Principal Researcher: Mitsuya Ishikawa] We have no financial relationships to disclose.

### [J-3057] I-J7-2
### Clinical cancer genomics

2025 / 9 / 27 (Sat) 12:50-14:05 Room 15/(ANA Crowne Plaza Hotel Kanazawa 3F Zuiun 2) Room 15

[Chairperson]Kuniko Sunami (Dept. Lab. Med., Natl. Cancer. Ctr.)

---

### J-3057
### Comprehensive HER2/ERBB2 landscape and its association with clinicopathology in bladder cancer

[Speaker]Kensuke Hirosuna (Department of Urology, Osaka Medical and Pharmaceutical University) [Co-author]Kazuki Nishimura (Department of Urology, Osaka Medical and Pharmaceutical University／Division of Cancer RNA Research, National Cancer Center Research Institute)、Mitsuaki Ishida (Department of Pathology, Osaka Medical and Pharmaceutical University)、Ryoichi Maenosono (Department of Urology, Osaka Medical and Pharmaceutical University／Division of Cancer RNA Research, National Cancer Center Research Institute)、Moritoshi Sakamoto (Department of Urology, Osaka Medical and Pharmaceutical University／Division of Cancer RNA Research, National Cancer Center Research Institute)、Kazumasa Komura (Department of Urology, Kawasaki Medical School)、Haruhito Azuma (Department of Urology, Osaka Medical and Pharmaceutical University)、Akihide Yoshimi (Division of Cancer RNA Research, National Cancer Center Research Institute)

Trastuzumab deruxtecan (T-DXd), a HER2-targeting antibody-drug conjugate, showed a clinically meaningful response in bladder carcinoma (BC). However, the relationships between HER2 expression, ***ERBB2*** alterations, and prognosis in BC remain underexplored.
We comprehensively analyzed clinicopathological data, HER2 IHC, whole-exome sequencing (WES), and RNA sequencing data in 459 BC patients. HER2 IHC revealed that 30.0% of patients exhibited IHC scores of 1+/2+/3+, whereas 8.6% displayed IHC 0 with membranous staining. WES identified ***ERBB2*** mutations in 15.3% of patients. No significant associations were observed between HER2 expression, ***ERBB2*** mutations, and patient prognosis. In contrast, ***ERBB2*** amplification was detected in 7.6% of patients. Among muscle-invasive BC (MIBC) cases, ***ERBB2*** amplification was significantly enriched in the consensus luminal unstable (LumU) subtype and associated with poor prognosis regardless of HER2 expression.
These findings suggest the potential utility of T-DXd in BC and highlight ***ERBB2*** amplification as a critical prognostic marker in MIBC patients.

[Disclosure of COI by Responsible researcher] Lead presenter is not the Responsible researcher.
[Lead presenter: Kensuke Hirosuna] We have no financial relationships to disclose.
[Principal Researcher: Akihide Yoshimi] ■Research funds from company: 第一三共株式会社、中外製薬株式会社、エーザイ株式会社

▲TOP

### [J-3058] I-J7-2
### Clinical cancer genomics

2025 / 9 / 27 (Sat) 12:50-14:05 Room 15/(ANA Crowne Plaza Hotel Kanazawa 3F Zuiun 2) Room 15

[Chairperson]Kuniko Sunami (Dept. Lab. Med., Natl. Cancer. Ctr.)

---

### J-3058
### An exploration of the advantages of whole-genome and transcriptome sequencing in cancer genomic medicine

[Speaker]Masakuni Serizawa (Shizuoka Cancer Ctr. Res. Inst.) [Co-author]Takeshi Nagashima (Shizuoka Cancer Ctr. Res. Inst.／SRL Inc.)、Maki Mizuguchi (Shizuoka Cancer Ctr. Res. Inst.)、Ryo Ko (Div. of Genomic Medicine Promotion, Shizuoka Cancer Ctr.)、Hirotsugu Kenmotsu (Div. of Genomic Medicine Promotion, Shizuoka Cancer Ctr.)、Takuma Oishi (Div. of Pathology, Shizuoka Cancer Ctr.)、Takashi Sugino (Div. of Pathology, Shizuoka Cancer Ctr.)、Yoshimi Kiyozumi (Div. of Genomic Medicine Promotion, Shizuoka Cancer Ctr.)、Hiroyuki Matsubayashi (Div. of Genomic Medicine Promotion, Shizuoka Cancer Ctr.)、Keiichi Ohshima (Shizuoka Cancer Ctr. Res. Inst.)、Masanori Terashima (Div. of Gastric Surgery, Shizuoka Cancer Ctr.)、Ken(Shizuoka Cancer Ctr.) Yamaguchi (Shizuoka Cancer Ctr.)、Yasuto Akiyama (Shizuoka Cancer Ctr. Res. Inst.)、Kenichi Urakami (Shizuoka Cancer Ctr. Res. Inst.)

**Background:** This study evaluated the potential advantages of whole-genome and transcriptome sequencing (WGTS) in the context of cancer genomic medicine.

**Methods:** An informatic assessment was performed to investigate the benefits of WGTS. The study analyzed genetic alterations identified in 587 patients, as assessed by an expert panel based on WGTS findings, and compared them to the coverage provided by cancer genome profiling tests (CGPs).

**Results:** Most driver genetic alterations, including variants and fusions detected through WGTS, were predicted to be identifiable by CGP. However, CGP might miss approximately 20% of copy number gains (amplifications) and about 26% of copy number losses (deletions). Significant discrepancies were observed in the detection of chromosomal rearrangements of tumor suppressor genes, suggesting that CGPs may overlook more than 57% of the alterations identified by WGTS.

**Conclusions:** WGTS is crucial for detecting clinically significant genetic alterations and comprehensive identification of cancer-related genetic changes. Additionally, it can provide patients with insights into the biological mechanisms driving carcinogenesis and tumor progression.

[Disclosure of COI by Responsible researcher] Lead presenter is the Responsible researcher.
[Lead presenter: Masakuni Serizawa] We have no financial relationships to disclose.

▲TOP

### [J-3059] I-J7-2
### Clinical cancer genomics

2025 / 9 / 27 (Sat) 12:50-14:05 Room 15/(ANA Crowne Plaza Hotel Kanazawa 3F Zuiun 2) Room 15

[Chairperson]Kuniko Sunami (Dept. Lab. Med., Natl. Cancer. Ctr.)

---

### J-3059
### Therapeutic and diagnostic utility of clinical whole genome sequencing in cancer genome medicine.

[Speaker]Norio Tanaka (JFCR. CPM Ctr.) [Co-author]Osamu Gotoh (JFCR. CPM Ctr.)、Ippei Fukada (JFCR Cancer Inst. Hosp. Dept. Genomic Med.)、Arisa Ueki (JFCR Cancer Inst. Hosp. Dept. Clinical Genetic Oncology)、Takayuki Ueno (JFCR Cancer Inst. Hosp. Dept. Cancer Genomic Med. Development.)、Shunji Takahashi (JFCR Cancer Inst. Hosp. Dept. Genomic Med.)、Seiichi Mori (JFCR. CPM Ctr.)、Tetsuo Noda (JFCR. CPM Ctr.)

We performed WGS and RNA-Seq on 1,050 surgical specimens from 1,049 patients treated at the Cancer Institute Hospital of JFCR. SNVs, Indels, CNVs, SVs, and virus-derived sequences were identified and analyzed with clinical data.Sarcoma was the most common cancer (n=155), followed by rectal (153), oral (106), and pancreatic (64). A total of 127 samples, including 26 pancreatic, had <25% tumor content and were excluded from somatic analysis. Pathogenic variants in hereditary cancer genes were found in 93 cases, with 26 showing LOH or somatic truncating mutations likely to be drivers. Among somatic variants, TP53 was most frequent (n=610), followed by CDKN2A (267), APC (196), and TERT (193), including 122 promoter mutations.Viral reads from HPV, EBV, and HBV were detected in 10, 10, and 3 cases, respectively; EBV in a salivary gland tumor aided subtype classification. In 136 recurrent/metastatic cases, actionable mutations were found in 122 (89.7%), therapy was recommended in 63 (46.3%), and given in 12 (8.8%), similar to panel-based profiling.These findings support the implementation of WGS in cancer genomic medicine.

[Disclosure of COI by Responsible researcher] Lead presenter is not the Responsible researcher.
[Lead presenter: Norio Tanaka] We have no financial relationships to disclose.
[Principal Researcher: Seiichi Mori] We have no financial relationships to disclose.

▲TOP

### [J-3060] I-J7-2
### Clinical cancer genomics

2025 / 9 / 27 (Sat) 12:50-14:05 Room 15/(ANA Crowne Plaza Hotel Kanazawa 3F Zuiun 2) Room 15

[Chairperson]Kuniko Sunami (Dept. Lab. Med., Natl. Cancer. Ctr.)

---

### J-3060
### Whole-Genome Sequencing vs. Comprehensive Genomic Profiling in Solid Tumors: High Concordance and Expanded Detection

[Speaker]Hiroyuki Fujii (Department of Laboratory Medicine, National Cancer Center Hospital／Department of Pulmonary Medicine, Kyoto Prefectural University of Medicine) [Co-author]Takashi Kubo (Department of Laboratory Medicine, National Cancer Center Hospital)、Makoto Hirata (Department of Genetic Medicine and Services, National Cancer Center Hospital)、Satoyo Oda (Department of Laboratory Medicine, National Cancer Center Hospital／Department of Genetic Medicine and Services, National Cancer Center Hospital)、Kayoko Tao (Department of Genetic Medicine and Services, National Cancer Center Hospital)、Takafumi Koyama (Department of Experimental Therapeutics, National Cancer Center Hospital)、Kenichi Chiba (Division of Genome Analysis Platform Development, National Cancer Center Research-Institute)、Yasushi Yatabe (Department of Pathology, National Cancer Center Hospital)、Yuichi Shiraishi (Division of Genome Analysis Platform Development, National Cancer Center Research-Institute)、Takashi Kohno (Division of Genome Biology, National Cancer Center Research-Institute)、Noboru Yamamoto (Department of Experimental Therapeutics, National Cancer Center Hospital)、Kuniko Sunami (Department of Laboratory Medicine, National Cancer Center Hospital／Division of Genome Biology, National Cancer Center Research-Institute)

**Background:** Whole-genome sequencing (WGS) is being considered for implementation in cancer care under the ***Action Plan for Whole Genome Analysis 2022.*** We evaluated its variant detection performance compared to comprehensive genomic profiling (CGP).
**Methods:** WGS and CGP (NCC Oncopanel) data generated from the same DNA samples derived from 62 solid tumors were compared. Variants were called from WGS data using our in-house G-CAT workflow (https://github.com/ncc-gap/GCATWorkflow), focusing on specific 1147 cancer-related genes.
**Results:** Of the 843 SNVs/INDELs identified by CGP, 738 (88%) were also detected by WGS, with a concordance rate of 90% for 303 actionable variants. WGS uniquely detected 334 pathogenic and 18 actionable variants. Variants detected by CGP alone tended to have low VAFs. All 25 CNVs identified by CGP were confirmed by WGS, which detected an additional 31 CNVs. Of the 22 FDA-approved drug-associated SVs involving CGP-listed genes detected by WGS, only 2 were reported by CGP.
**Conclusions:** WGS demonstrates high concordance with CGP while revealing additional pathogenic and druggable alterations beyond the CGP boundaries, supporting its utility in practice.

[Disclosure of COI by Responsible researcher] Lead presenter is not the Responsible researcher.
[Lead presenter: Hiroyuki Fujii] We have no financial relationships to disclose.
[Principal Researcher: Kuniko Sunami] We have no financial relationships to disclose.

### [J-3067] I-J9-3
### Metastasis & cell adhesion

2025 / 9 / 27 (Sat) 12:50-14:05 Room 16/(11F Visage) Room 16

[Chairperson]Hideki Yamaguchi (Dept. Cancer Cell Res., Sasaki Inst.)

---

### J-3067
### Role of Claudin-11/MT1-MMP Axis in Small Cell Lung Cancer

[Speaker]Shuichi Sakamoto (Numazu branch, IMC, MCRF) [Co-author]Hiroyuki Inoue (Numazu branch, IMC, MCRF)、Takahisa Takino (Inst. Liberal Arts & Sci, Kanazawa Univ.)、Yasuko Kohda (Numazu branch, IMC, MCRF)、Junjiro Yoshida (Lab. of Oncology, IMC, MCRF)、Shunichi Ohba (Numazu branch, IMC, MCRF)、Ihomi Usami (Numazu branch, IMC, MCRF)、Takeshi Suzuki (Cancer Res. Inst., Kanazawa Univ.)、Manabu Kawada (Lab. of Oncology, IMC, MCRF)、Masanori Hatakeyama (Lab. of Microbial Carcinogenesis, IMC, MCRF)

Small cell lung cancer (SCLC) accounts for 15% of lung cancer cases and is highly refractory with frequent distant metastases. While initial chemotherapy is effective, surgical treatment is rarely an option, and patient-derived samples are difficult to obtain. Therefore, developing a clinically relevant mouse model is crucial.We established a spontaneous metastasis model using the human SCLC cell line DMS273 and identified a highly metastatic subclone. Analysis revealed that Claudin-11 promotes invasion and metastasis by interacting with membrane-type matrix metalloproteinase MT1-MMP. Immunoprecipitation confirmed their co-precipitation, and gelatin zymography showed increased MT1-MMP activity. MT1-MMP-knockdown suppressed invasion and metastasis, and Claudin-11 overexpression did not enhance these abilities in MT1-MMP-deficient cells, suggesting a Claudin-11/MT1-MMP dependent mechanism. Immunohistochemical analysis of a human tumor tissue array detected Claudin-11 expression in some SCLC patients, and gene expression data showed that high CLDN11 expression correlated with poor prognosis.These findings suggest that the Claudin-11/MT1-MMP axis plays a key role in SCLC metastasis.

[Disclosure of COI by Responsible researcher] Lead presenter is the Responsible researcher.
[Lead presenter: Shuichi Sakamoto] We have no financial relationships to disclose.

### [J-3068] I-J9-3
### Metastasis & cell adhesion

2025 / 9 / 27 (Sat) 12:50-14:05 Room 16/(11F Visage) Room 16

[Chairperson]Hideki Yamaguchi (Dept. Cancer Cell Res., Sasaki Inst.)

---

### J-3068
### Laminin-γ2 fusion protein promotes metastatic potential in EGFR-wildtype non-small cell lung carcinoma

[Speaker]Nobuaki Funahashi (Sch. of Life sci. & Tech., Inst. of Sci. Tokyo) [Co-author]Ryo Kaneko (Sch. of Life sci. & Tech., Inst. of Sci. Tokyo)、Naohiko Koshikawa (Sch. of Life sci. & Tech., Inst. of Sci. Tokyo)

Laminin-γ2 fusion gene (LAMC2-NR6A1, termed Lm-γ2F) arises from a chromosomal translocation between chromosomes 1 and 9, and functions as a novel ligand for EGFR. In this study, we investigated the role of Lm-γ2F in promoting malignant progression of non-small cell lung carcinoma (NSCLC) lacking EGFR mutations. Using EKVX cells stably expressing Lm-γ2F, we found that Lm-γ2F enhances phosphorylation of EGFR and activates downstream signaling pathways including AKT and ERK, compared to control cells. To evaluate the metastatic potential conferred by Lm-γ2F, we conducted Matrigel invasion assays and in vivo lung metastasis experiments using BALB/c nude mice. Forced expression of Lm-γ2F significantly increased invasive activity in vitro and the number of metastatic foci in the lungs in vivo. Importantly, these effects were attenuated by treatment with the EGFR inhibitor gefitinib, suggesting EGFR-dependence. Our findings indicate that Lm-γ2F acts as a potent driver of metastasis in EGFR-wildtype NSCLC, and may represent a novel therapeutic target.

[Disclosure of COI by Responsible researcher] Lead presenter is not the Responsible researcher.
[Lead presenter: Nobuaki Funahashi] We have no financial relationships to disclose.
[Principal Researcher: Naohiko Koshikawa] ■Research expenses: Abbott laboratories

### [J-3069] I-J9-3
### Metastasis & cell adhesion

2025 / 9 / 27 (Sat) 12:50-14:05 Room 16/(11F Visage) Room 16

[Chairperson]Hideki Yamaguchi (Dept. Cancer Cell Res., Sasaki Inst.)

---

### J-3069
### Glycosylation of tetraspanin CD151 defines invasive phenotype of human breast cancer cells

[Speaker]Yuki Ohkawa (OICI Dept Glyco-Oncology) [Co-author]Yuanqin Yin (OICI Dept Glyco-Oncology／First Affili Hosp China Dept Tumor Bio)、Miyako Nakano (Hiroshima Univ Grad Sch of Integ Sci)、Noriko Kanto (OICI Dept Glyco-Oncology)、Shiho Ohno (Tohoku Med and Pharma Univ Div of Struc Glyco)、Yoshiki Yamaguchi (Tohoku Med and Pharma Univ Div of Struc Glyco)、Naoyuki Taniguchi (OICI Dept Glyco-Oncology)

Tetraspanin CD151 and is a transmembrane glycoprotein that forms a complex with integrins. Previous studies have shown that CD151 plays very different roles in cancers. For this, we attempted to elucidate the role of ***N***-glycosylation of CD151 in relation to cell behavior. The expression of CD151 varied in different types of cancer cells and was very low in a human breast cancer cell line MCF-7. To clarify the role of ***N***-glycosylation, we established wild-type CD151 (CD151/WT)-expressing cells and CD151-non-glycosylation mutant (CD151/NQ)-expressing cells in MCF-7 cells, and we found that the invasion activity of CD151/NQ cells was dramatically enhanced. The ***N***-glycan structure of CD151/WT was rich in oligomannose-type, which may inhibit the interaction with integrin α3 according to 3D structural models. In addition, gene expression analyses using public genomics data sets showed that invasive breast cancers exhibit low expression levels of the ***STT3B*** gene: a key player in the production of ***N***-glycans. These results suggest that expression of the non-gylcosylation form of CD151 confers an invasive phenotype to breast cancer cells.

[Disclosure of COI by Responsible researcher] Lead presenter is the Responsible researcher.
[Lead presenter: Yuki Ohkawa] We have no financial relationships to disclose.

Japanese Oral Session

### [J-3070] I-J9-3
### Metastasis & cell adhesion

2025 / 9 / 27 (Sat) 12:50-14:05 Room 16/(11F Visage) Room 16

[Chairperson]Hideki Yamaguchi (Dept. Cancer Cell Res., Sasaki Inst.)

---

### J-3070
### Gemcitabine-induced $\alpha 6\beta 4$ integrin confers gemcitabine resistance to PDAC cells and promotes PDAC progression

[Speaker]Yoshinobu Kariya (Dept. Biochem, Fukushima Med. Univ., Sch. Med.) [Co-author]Haruto Suzuki (Dept. Biochem, Fukushima Med. Univ., Sch. Med.)、Yukiko Kariya (Dept. Biochem, Fukushima Med. Univ., Sch. Med.)、Michiru Nishita (Dept. Biochem, Fukushima Med. Univ., Sch. Med.)

While some integrin expressions are known to be induced by anticancer drug stimulation in cancer cells, the molecular mechanism and the impact of this on cancer progression are poorly understood. We found that gemcitabine (GEM)-resistant pancreatic ductal adenocarcinoma (PDAC) cells have enhanced malignant properties, including invasion, metastasis, tumorigenesis, and stemness compared with parental cells, and that GEM treatment induces $\alpha 6\beta 4$ integrin expression in PDAC cells. ShRNA knockdown of $\beta 4$ integrin decreased malignancy in PDAC cells, whereas $\alpha 6\beta 4$ integrin overexpression increased malignancy and GEM resistance in PDAC cells. Consistent with these findings, PDAC patients with high $\beta 4$ integrin expression had a poor prognosis. Furthermore, the $\alpha 6\beta 4$ integrin/laminin-332 axis enhanced cell motility through Src activation. Finally, GEM-induced $\alpha 6\beta 4$ integrin expression was found to be epigenetically regulated through increased $\beta$-catenin expression, nuclear translocation, and subsequent histone acetylation at $\beta 4$ integrin promoter site. These data suggest that GEM-induced $\alpha 6\beta 4$ integrin confers GEM resistance to PDAC cells and also contributes to PDAC progression.

[Disclosure of COI by Responsible researcher] Lead presenter is the Responsible researcher.
[Lead presenter: Yoshinobu Kariya] We have no financial relationships to disclose.

### [J-3071] I-J9-3
### Metastasis & cell adhesion

2025 / 9 / 27 (Sat) 12:50-14:05 Room 16/(11F Visage) Room 16

[Chairperson]Hideki Yamaguchi (Dept. Cancer Cell Res., Sasaki Inst.)

---

### J-3071
### RSK/GSK3 mediated phosphorylation of FilGAP regulates chemotactic cancer invasion

[Speaker]Koji Tsutsumi (Kitasato Univ. Sch. Sci.) [Co-author]Yasutaka Ohta (Kitasato Univ. Sch. Sci.)

Cancer cell migration is a fundamental process in tumor invasion and metastasis. In response to chemoattractants like growth factors, cells form protrusions and move toward the signal. FilGAP, a Rac-specific GTPase-activating protein (GAP), suppresses lamellipodia formation and regulates tumor cell migration. We found that FilGAP is phosphorylated downstream of epidermal growth factor (EGF) signaling. Upon EGF stimulation, FilGAP is phosphorylated at Ser625 by p90 ribosomal S6 kinase (RSK), then at Ser621 by glycogen synthase kinase 3 (GSK3). These phosphorylations induce FilGAP dissociation from actin filaments. We identified a novel actin-localization domain in FilGAP that stabilizes cell adhesion. Moreover, phosphorylation inhibits the ability of FilGAP to suppress lamellipodia. Finally, expression of a non-phosphorylatable FilGAP mutant, but not wild-type, reduced migration speed and persistence toward the EGF gradient. These results suggest that EGF-dependent FilGAP phosphorylation regulates chemotactic tumor cell migration by modulating adhesion and membrane protrusion.

[Disclosure of COI by Responsible researcher] Lead presenter is the Responsible researcher.
[Lead presenter: Koji Tsutsumi] We have no financial relationships to disclose.

### [J-3072] I-J9-3
### Metastasis & cell adhesion

2025 / 9 / 27 (Sat) 12:50-14:05 Room 16/(11F Visage) Room 16

[Chairperson]Hideki Yamaguchi (Dept. Cancer Cell Res., Sasaki Inst.)

---

### J-3072
### SUSD2 enhances tumor growth and metastasis by upregulating integrin-FAK signaling in pancreatic cancer cells

[Speaker]Junjiro Yoshida (Laboratory of Oncology, Institute of Microbial Chemistry (BIKAKEN) [Co-author]Tomokazu Ohishi (Laboratory of Oncology, Institute of Microbial Chemistry (BIKAKEN))、Daisuke Tatsuda (Laboratory of Oncology, Institute of Microbial Chemistry (BIKAKEN))、Shunichi Ohba (Numazu Branch, Institute of Microbial Chemistry (BIKAKEN))、Kyohei Kurosawa (Numazu Branch, Institute of Microbial Chemistry (BIKAKEN))、Akiko Harakawa (Numazu Branch, Institute of Microbial Chemistry (BIKAKEN))、Hiroyuki Inoue (Numazu Branch, Institute of Microbial Chemistry (BIKAKEN))、Isao Momose (Numazu Branch, Institute of Microbial Chemistry (BIKAKEN))、Manabu Kawada (Laboratory of Oncology, Institute of Microbial Chemistry (BIKAKEN))

Tumor tissues of pancreatic cancer develop with abundant cancer-associated fibroblasts (CAFs), which promote tumor progression. We have previously found that conditioned medium of CAFs induced the expression of sushi domain containing 2 (SUSD2), a single transmembrane protein and enhanced the invasive potential of pancreatic cancer cells. In this study we report that SUSD2 binds to integrin β1 and enhances the motility of pancreatic cancer cells through the phosphorylation of FAK promoting the formation of focal adhesion complexes in cells adhered to collagen I. Orthotopic transplantation of SUSD2-overexpressing pancreatic cancer cells into mouse pancreas enhances liver metastasis of Panc-1 cells, but it increases primary tumor growth of KP2 cells without effect on metastasis. In spheroid cultures of KP2 cells, forced expression of SUSD2 increases phosphorylation of FAK independent of cell adhesion, suggesting that SUSD2 promotes the cell proliferation even in non-metastatic cells. These findings indicate that high expression of SUSD2 in cancer cells contributes to the tumor growth and metastasis suggesting SUSD2 as a therapeutic target in pancreatic cancer.

[Disclosure of COI by Responsible researcher] Lead presenter is not the Responsible researcher.
[Lead presenter: Junjiro Yoshida] ■Research funds from company: JSPS、科研費、JP 23K07407
[Principal Researcher: Manabu Kawada] We have no financial relationships to disclose.

### [AOSR6-1] AOSR6
### Ongoing development of targeted radionuclide therapy in Japan

2025 / 9 / 27 (Sat) 13:20-15:50 Room 2/(Ishikawa Ongakudo 2F Hogaku Hall) Room 2

[Chairpersons]Seigo Kinuya (Department of Nuclear Medicine, Kanazawa University)、Anri Inaki (National Cancer Center)

Targeted radionuclide therapy (TRT) is currently called "theranostics". This is a relatively new terminology which is a combination of therapeutics and diagnostics. Diagnosis with PET or SPECT preceding TRT enables patient selection by positive delineation of lesions, quantification of radiation dose and elimination of adverse reaction by unfavorable distribution. New radiopharmaceuticals are under development in the world. We can see this trend in Japanese institutions as well. NaAt(211At) is tested in a clinical trial for thyroid cancer refractory to radioiodine in Osaka University. The trial of 211At-PSMA-5 has been initiated for prostate cancer. 211At-MABG therapy is in a trial for pheochromocytoma/paraganglioma refractory to 131I-MIBG at Fukushima Medical College. 64Cu-ATSM which targets hypoxic area in tumors moved into phase 3 trial for brain tumors led by LinqMed, Inc. Needless to say, several industrial developments are also on their way. Currently, Japanese government is supporting these activities especially in domestic production of radionuclides. 225Ac is planned to be produced by the fast experimental reactor, Joyo, of the Japan Atomic Energy Agency. 211At is delivered by several domestic production sites including Riken. In this session, these on-going achievements will be demonstrated for the further domestic development of TRTs.

---

### AOSR6-1
### Development and Clinical Translation of Targeted Alpha Therapy using Astatine in Osaka

[Speaker]Tadashi Watabe (Graduate School of Medicine, The University of Osaka／Institute for Radiation Sciences, The University of Osaka)

Astatine ($^{211}$At) is an alpha-particle-emitting radionuclide with uptake capability via the sodium iodide symporter (NIS), similar to iodine. Alpha particles exert stronger antitumor effects than conventional beta particles and have the advantage of not requiring inpatient isolation in specialized rooms. We have evaluated the safety and preliminary efficacy of [$^{211}$At]NaAt in patients with differentiated thyroid cancer refractory to conventional [$^{131}$I]NaI therapy (jRCT2051210144). A single intravenous administration of [$^{211}$At]NaAt was well tolerated, and some patients demonstrated more than 50% reduction in serum thyroglobulin levels along with decreased uptake on [$^{131}$I] imaging, indicating therapeutic potential. Currently, we are conducting a clinical trial of [$^{211}$At]PSMA-5 in patients with metastatic castration-resistant prostate cancer, which has shown promising early results (EJNMMI 2024). In addition, we are investigating radiolabeled agents targeting pan-tumor markers such as LAT1, FAP, and EphA2. Astatine, alongside actinium, is expected to serve as a cyclotron-produced alpha emitter in theranostics applications targeting a wide range of cancers.

[Disclosure of COI by Responsible researcher] Lead presenter is the Responsible researcher.
[Lead presenter: Tadashi Watabe] ■Research expenses: アルファフュージョン社

### [AOSR6-2] AOSR6
### Ongoing development of targeted radionuclide therapy in Japan

2025 / 9 / 27 (Sat) 13:20-15:50 Room 2/(Ishikawa Ongakudo 2F Hogaku Hall) Room 2

[Chairpersons]Seigo Kinuya (Department of Nuclear Medicine, Kanazawa University)、Anri Inaki (National Cancer Center)

Targeted radionuclide therapy (TRT) is currently called "theranostics". This is a relatively new terminology which is a combination of therapeutics and diagnostics. Diagnosis with PET or SPECT preceding TRT enables patient selection by positive delineation of lesions, quantification of radiation dose and elimination of adverse reaction by unfavorable distribution. New radiopharmaceuticals are under development in the world. We can see this trend in Japanese institutions as well. NaAt(211At) is tested in a clinical trial for thyroid cancer refractory to radioiodine in Osaka University. The trial of 211At-PSMA-5 has been initiated for prostate cancer. 211At-MABG therapy is in a trial for pheochromocytoma/paraganglioma refractory to 131I-MIBG at Fukushima Medical College. 64Cu-ATSM which targets hypoxic area in tumors moved into phase 3 trial for brain tumors led by LinqMed, Inc. Needless to say, several industrial developments are also on their way. Currently, Japanese government is supporting these activities especially in domestic production of radionuclides. 225Ac is planned to be produced by the fast experimental reactor, Joyo, of the Japan Atomic Energy Agency. 211At is delivered by several domestic production sites including Riken. In this session, these on-going achievements will be demonstrated for the further domestic development of TRTs.

---

### AOSR6-2
### Development of Novel Alpha-Emitting Therapeutic Agents at Fukushima Medical University.

[Speaker]Tohru Shiga (Avvanced clinical Reserch center , Fukushima medical University)

At Fukushima Medical University, we are actively advancing the development of novel alpha-emitting radiopharmaceuticals for targeted cancer therapy. Our first-in-human Phase I clinical trial of At-211 meta-astatobenzylguanidine (At-211 MABG)-an alpha-emitting analogue of I-131 MIBG targeting the norepinephrine transporter-successfully completed patient enrollment in April 2025. This study focused on patients with malignant pheochromocytoma and paraganglioma.In parallel, we are preparing to initiate a clinical trial of At-211 labeled PSMA-targeted compounds (At-211 NpG-PSMA) for the treatment of metastatic castration-resistant prostate cancer. Additionally, At-211 labeled microspheres are under development for intra-arterial therapy in hepatic tumors.Through these initiatives, Fukushima Medical University is pioneering the clinical application of At-211 based therapeutics, aiming to establish innovative treatment options for refractory cancers. This presentation will offer a comprehensive overview of our translational research, ongoing clinical development, and future directions in alpha-emitter-based radiotherapy.

[Disclosure of COI by Responsible researcher] Lead presenter is the Responsible researcher.
[Lead presenter: Tohru Shiga] ■Endowed chair: 株式会社沖縄臨床検査センター、一般財団法人温知会 会津中央病院

### [AOSR6-3] AOSR6
### Ongoing development of targeted radionuclide therapy in Japan

2025 / 9 / 27 (Sat) 13:20-15:50 Room 2/(Ishikawa Ongakudo 2F Hogaku Hall) Room 2

[Chairpersons]Seigo Kinuya (Department of Nuclear Medicine, Kanazawa University)、Anri Inaki (National Cancer Center)

Targeted radionuclide therapy (TRT) is currently called "theranostics". This is a relatively new terminology which is a combination of therapeutics and diagnostics. Diagnosis with PET or SPECT preceding TRT enables patient selection by positive delineation of lesions, quantification of radiation dose and elimination of adverse reaction by unfavorable distribution. New radiopharmaceuticals are under development in the world. We can see this trend in Japanese institutions as well. NaAt(211At) is tested in a clinical trial for thyroid cancer refractory to radioiodine in Osaka University. The trial of 211At-PSMA-5 has been initiated for prostate cancer. 211At-MABG therapy is in a trial for pheochromocytoma/paraganglioma refractory to 131I-MIBG at Fukushima Medical College. 64Cu-ATSM which targets hypoxic area in tumors moved into phase 3 trial for brain tumors led by LinqMed, Inc. Needless to say, several industrial developments are also on their way. Currently, Japanese government is supporting these activities especially in domestic production of radionuclides. 225Ac is planned to be produced by the fast experimental reactor, Joyo, of the Japan Atomic Energy Agency. 211At is delivered by several domestic production sites including Riken. In this session, these on-going achievements will be demonstrated for the further domestic development of TRTs.

### AOSR6-3
### Development of $^{64}$Cu Radiotherapeutics Ongoing in Japan

[Speaker]Yukie Yoshii (LinqMed Inc.)

Cancer remains the leading cause of death worldwide. Radiotheranostics, an approach that integrates diagnosis and therapy, has gained significant attention. We focuses on $^{64}$Cu, a radionuclide that can be used for both positron emission tomography (PET) imaging and radionuclide therapy with a single drug. $^{64}$Cu emits positrons for imaging purposes and $\beta^-$ particles and Auger electrons for therapeutic purposes, making it an ideal candidate for theranostic applications. One of our lead compounds, $^{64}$Cu-ATSM, is currently being investigated in clinical trials in Japan for the treatment of malignant brain tumors. $^{64}$Cu–ATSM selectively accumulates in hypoxic tumors, which are often resistant to conventional therapies, exerting cytotoxic effects. Preclinical studies have demonstrated its strong therapeutic efficacy in glioblastoma models. A Phase I clinical trial of $^{64}$Cu–ATSM (STAR–64) has been conducted in Japan to evaluate its safety and potential efficacy, and a Phase III clinical trial (STEP–64) is currently ongoing. In this presentation, we will highlight recent progress in the preclinical and clinical development of $^{64}$Cu-ATSM, and discuss our efforts to produce the drug.

[Disclosure of COI by Responsible researcher] Lead presenter is the Responsible researcher.
[Lead presenter: Yukie Yoshii] ■ employment by a company related to the presentation: リンクメッド株式会社　■Profit of stock: リンクメッド株式会社

▲TOP

### [AOSR6-4] AOSR6
### Ongoing development of targeted radionuclide therapy in Japan

2025 / 9 / 27 (Sat) 13:20-15:50 Room 2/(Ishikawa Ongakudo 2F Hogaku Hall) Room 2

[Chairpersons]Seigo Kinuya (Department of Nuclear Medicine, Kanazawa University)、Anri Inaki (National Cancer Center)

Targeted radionuclide therapy (TRT) is currently called "theranostics". This is a relatively new terminology which is a combination of therapeutics and diagnostics. Diagnosis with PET or SPECT preceding TRT enables patient selection by positive delineation of lesions, quantification of radiation dose and elimination of adverse reaction by unfavorable distribution. New radiopharmaceuticals are under development in the world. We can see this trend in Japanese institutions as well. NaAt(211At) is tested in a clinical trial for thyroid cancer refractory to radioiodine in Osaka University. The trial of 211At-PSMA-5 has been initiated for prostate cancer. 211At-MABG therapy is in a trial for pheochromocytoma/paraganglioma refractory to 131I-MIBG at Fukushima Medical College. 64Cu-ATSM which targets hypoxic area in tumors moved into phase 3 trial for brain tumors led by LinqMed, Inc. Needless to say, several industrial developments are also on their way. Currently, Japanese government is supporting these activities especially in domestic production of radionuclides. 225Ac is planned to be produced by the fast experimental reactor, Joyo, of the Japan Atomic Energy Agency. 211At is delivered by several domestic production sites including Riken. In this session, these on-going achievements will be demonstrated for the further domestic development of TRTs.

---

### AOSR6-4
### Domestic production of Ac-225 using the experimental fast reactor Joyo

[Speaker]Hiroyuki Ohshima (JAEA, Nucl. Energy R&D Domain) [Co-author]Shigetaka Maeda (JAEA, Oarai Nucl. Eng. Institute)

Targeted alpha particle therapy is gaining attention in the field of nuclear medicine. This treatment combines short-lived alpha-emitting radionuclides with antibodies or peptides that selectively accumulate in cancer cells. In 2016, the therapeutic efficacy of Actinium-225 (Ac-225) as an alpha-emitting radionuclide for prostate cancer was reported, and its potential application to other cancers is also currently under investigation. As a result, the demand for Ac-225 is rapidly increasing in the world. Internationally, production methods such as generator-based and accelerator-based approaches are being developed. In Japan, domestic production using nuclear reactors and accelerators is being promoted under the Medical and Other Radioisotope Production and Utilization Promotion Action Plan of the Atomic Energy Commission. The fast experimental reactor Joyo, owned by JAEA, holds promise for large-scale production, with plans to demonstrate production by the JFY2026. This presentation will report on the production principles, production demonstration plan, and prospects for producing enough Ac-225 to meet domestic demand in the future.

[Disclosure of COI by Responsible researcher] Lead presenter is the Responsible researcher.
[Lead presenter: Hiroyuki Ohshima] We have no financial relationships to disclose.

### [AOSR6-5] AOSR6
### Ongoing development of targeted radionuclide therapy in Japan

2025 / 9 / 27 (Sat) 13:20-15:50 Room 2/(Ishikawa Ongakudo 2F Hogaku Hall) Room 2

[Chairpersons]Seigo Kinuya (Department of Nuclear Medicine, Kanazawa University)、Anri Inaki (National Cancer Center)

Targeted radionuclide therapy (TRT) is currently called "theranostics". This is a relatively new terminology which is a combination of therapeutics and diagnostics. Diagnosis with PET or SPECT preceding TRT enables patient selection by positive delineation of lesions, quantification of radiation dose and elimination of adverse reaction by unfavorable distribution. New radiopharmaceuticals are under development in the world. We can see this trend in Japanese institutions as well. NaAt(211At) is tested in a clinical trial for thyroid cancer refractory to radioiodine in Osaka University. The trial of 211At-PSMA-5 has been initiated for prostate cancer. 211At-MABG therapy is in a trial for pheochromocytoma/paraganglioma refractory to 131I-MIBG at Fukushima Medical College. 64Cu-ATSM which targets hypoxic area in tumors moved into phase 3 trial for brain tumors led by LinqMed, Inc. Needless to say, several industrial developments are also on their way. Currently, Japanese government is supporting these activities especially in domestic production of radionuclides. 225Ac is planned to be produced by the fast experimental reactor, Joyo, of the Japan Atomic Energy Agency. 211At is delivered by several domestic production sites including Riken. In this session, these on-going achievements will be demonstrated for the further domestic development of TRTs.

### AOSR6-5
### Production and supply of astatin-211 for targeted alpha-particle therapy

[Speaker]Hiromitsu Haba (RIKEN Nishina Center)

Astatine-211, with the half-life of 7.214 h, is one of the most promising radionuclides for targeted alpha-particle therapy. The 5.87- and 7.45-MeV $\alpha$-particle emissions occur in intensities of 41.8% and 58.2%, respectively, associated with the $^{211}$At decay. Due to the appropriate ranges of these $\alpha$-particles in tissue, the $^{211}$At-labeled radiopharmaceuticals are effective in destroying cancer cells. $^{211}$At is produced via the $^{209}$Bi($^{4}$He,2n) $^{211}$At reaction at accelerator facilities. We developed an $^{211}$At production system on the beamline of the RIKEN AVF cyclotron (AVF). The metallic $^{209}$Bi target is irradiated by the 28-MeV $^{4}He^{2+}$ beam from AVF. With a maximum beam current of 50 $\mu$A, up to 1.2 GBq of $^{211}$At can be produced during a one-hour irradiation. After irradiation, $^{211}$At is purified from the $^{209}$Bi target and byproducts using a dry distillation method. We currently distribute $^{211}$At to approximately 20 universities, research institutes, and companies throughout Japan. A large-scale production technology of $^{211}$At is under development to enable future production of clinically relevant quantities of $^{211}$At using a >200 $\mu$A $^{4}$He beam.

[Disclosure of COI by Responsible researcher] Lead presenter is the Responsible researcher.
[Lead presenter: Hiromitsu Haba] We have no financial relationships to disclose.

### [J-3043] I-J11-7
### Cancer vaccines/Other immunotherapies (2)

2025 / 9 / 27 (Sat) 14:05-15:20 Room 5/(Hotel Nikko Kanazawa 4F Tsuru C) Room 5

[Chairperson]Shin-ichiro Fujii (Laboratory for Immunotherapy, RIKEN Center for Integrative Medical Sciences )

---

### J-3043
### Novel Immune Drug Combination Induces Tumour Microenvironment Remodelling and Reduces the Dosage of Anti-PD-1 Antibody

[Speaker]Takahiro Ozasa (Dept. of Gastroenterological and Breast and Endocrine Surg. Yamaguchi Univ.) [Co-author]Masao Nakajima (Dept. of Gastroenterological and Breast and Endocrine Surg. Yamaguchi Univ.)、Ryouichi Tsunedomi (Dept. of Gastroenterological and Breast and Endocrine Surg. Yamaguchi Univ.／Research Institute for Cell Design Medical Science)、Shunsuke Goto (Department of Urology, Graduate School of Medical Sciences, Kyushu University,)、Keishi Adachi (Yamaguchi Univ. Grad. Sch. of Med. Dept. of Immunol.／Research Institute for Cell Design Medical Science)、Hidenori Takahashi (Dept. of Gastroenterological and Breast and Endocrine Surg. Yamaguchi Univ.)、Koji Tamada (Yamaguchi Univ. Grad. Sch. of Med. Dept. of Immunol.／Research Institute for Cell Design Medical Science)、Hiroaki Nagano (Dept. of Gastroenterological and Breast and Endocrine Surg. Yamaguchi Univ.／Research Institute for Cell Design Medical Science)

Background: Dose reduction of ICIs may mitigate immune-related adverse events and a financial burden; however, it could compromise the therapeutic efficacy. Using two adjuvants (poly(I:C) and LAG-3-Ig) combined with three neoantigen peptides (Comb), we examined whether Comb could enhance the efficacy of a reduced dose of αPD-1 mAb (RD-αPD-1 mAb).Methods: We performed this assessment in an MC38 murine CRC model. We conducted flow cytometry and integrated single-cell RNA sequencing (scRNA-seq) with single-cell T-cell receptor (scTCR) to evaluate immune cell dynamics in the tumor. Results: Comb addition to RD-αPD-1 mAb enhanced the treatment efficacy. Flow cytometry and scRNA-seq revealed decreased immuno-suppressive macrophages and increased antigen-presenting plasmacytoid DCs in the tumor treated with Comb. A potent infiltration of CD8+ tumor-infiltrating lymphocytes (TILs) with an effector profile was observed in RD-αPD-1 mAb with Comb. ScTCR repertoire analysis underscored an oligoclonal expansion of CD8+ TILs following treatment with RD-αPD-1 mAb with Comb. Conclusion: Comb may be a promising strategy to reduce αPD-1 mAb dosage while preserving antitumor efficacy.

[Disclosure of COI by Responsible researcher] Lead presenter is not the Responsible researcher.
[Lead presenter: Takahiro Ozasa] We have no financial relationships to disclose.
[Principal Researcher: Hiroaki Nagano] ■Research funds from company: TAIHO PHARMACEUTICAL CO., LTD., Shionogi & Co., Ltd., MSD K.K, Otsuka Pharmaceutical Factory, Inc., Otsuka Pharmaceutical Co., Ltd., TSUMURA & CO., Kyowa Hakko Kirin Co.,Ltd., Kaken Pharmaceutical Co., Ltd., NIPPON KAYAKU Co., Ltd., DAIICHI SANKYO COMPANY, LIMITED., Japan Blood Products Organization,Chugai Pharmaceutical Co., Ltd., ■Research expenses: CompanyCYTLIMIC Inc., Toyo Kohan Co., Ltd., NEC Corporation, Eisai, Incyte, Astellas Pharma Inc.

### [J-3044] I-J11-7
### Cancer vaccines/Other immunotherapies (2)

2025 / 9 / 27 (Sat) 14:05-15:20 Room 5/(Hotel Nikko Kanazawa 4F Tsuru C) Room 5

[Chairperson]Shin-ichiro Fujii (Laboratory for Immunotherapy, RIKEN Center for Integrative Medical Sciences )

---

### J-3044
### Dynamic analysis of long-term T cell immunity of TCR-T Vax, resulting in cure and recurrence suppression of solid tumors

[Speaker]Fumiyasu Momose (Dept. Personalized Cancer Immunotherapy, Mie Univ. Grad. Sch. Med.) [Co-author]Makiko Yamane (Dept. Personalized Cancer Immunotherapy, Mie Univ. Grad. Sch. Med.)、Junko Nakamura (Dept. Personalized Cancer Immunotherapy, Mie Univ. Grad. Sch. Med.)、Linan Wang (Dept. Personalized Cancer Immunotherapy, Mie Univ. Grad. Sch. Med.)、Keiki Nagaharu (Lund Stem Cell Center, Lund University)、Kohei Yabuuchi (New Product Development Office, Healthcare Materials Div., Asahi Kasei Corporation)、Shogo Aso (New Product Development Office, Healthcare Materials Div., Asahi Kasei Corporation)、Takero Kurosawa (New Product Development Office, Healthcare Materials Div., Asahi Kasei Corporation／DiveRadGel Co., Ltd.)、Toru Katsumata (New Product Development Office, Healthcare Materials Div., Asahi Kasei Corporation)、Tsuyoshi Shimoboji (New Product Development Office, Healthcare Materials Div., Asahi Kasei Corporation)、Takashi Nakai (New Product Development Office, Healthcare Materials Div., Asahi Kasei Corporation／DiveRadGel Co., Ltd.)、Yoshihiro Miyahara (Dept. Personalized Cancer Immunotherapy, Mie Univ. Grad. Sch. Med.)

Recently, we have developed a novel cancer vaccine based on hyaluronic acid nanogel (HANG-Vax). Our previous studies showed the treatment of infused TCR-T cells with HANG-Vax induced complete eradication of B16F10 and CMS5a tumors in all mice, and mice that achieved complete remission (CR) survived for long periods. In this study, scRNA/TCR seq analysis revealed that HANG-Vax enhanced the proliferation, activation, differentiation, and metabolic status of infused TCR-T cells. In addition, spatial gene expression analysis revealed that various chemokines, including***Ccl5*, *Cxcl9***, were expressed at high levels in B16F10 tumor tissue, and that $CD8^+$ T cells expressing ***Gzmb*** infiltrated into the tumor along with APCs. CR mice rejected rechallenged CMS5a, even in mice that survived for approximately 2 years after induction of CR. Surprisingly, even long-term survived CR mice rejected rechallenge of not only parental CMS5a but also mERK2-KO CMS5a, indicating that TCR-T Vax induced the "Antigen-spreading". These results indicate that HANG-Vax can potently maximize the efficacy of TCR-T therapy, leading to the cure of immunotherapy-resistant solid tumors and long-term prevention of recurrence.

[Disclosure of COI by Responsible researcher] Lead presenter is not the Responsible researcher.
[Lead presenter: Fumiyasu Momose] ■Research funds from company: 旭化成株式会社、DiveRadGel株式会社、科研費　■Research expenses: 旭化成株式会社、DiveRadGel株式会社　■Endowed chair: ティーセルヌーヴォー株式会社
[Principal Researcher: Yoshihiro Miyahara] ■Research funds from company: 旭化成株式会社、DiveRadGel株式会社　■Research expenses: 旭化成株式会社、DiveRadGel株式会社、コージンバイオ株式会社　■Endowed chair: ティーセルヌーヴォー株式会社

### [J-3045] I-J11-7
### Cancer vaccines/Other immunotherapies (2)

2025 / 9 / 27 (Sat) 14:05-15:20 Room 5/(Hotel Nikko Kanazawa 4F Tsuru C) Room 5

[Chairperson]Shin-ichiro Fujii (Laboratory for Immunotherapy, RIKEN Center for Integrative Medical Sciences )

---

### J-3045
### Anti-tumor effects of nucleic acid assembly triggered by miRNAs

[Speaker]Akimitsu Okamoto (Grad. Sch. Eng., Univ. Tokyo) [Co-author]Kunihiko Morihiro (Grad. Sch. Eng., Univ. Tokyo)

Double-stranded nucleic acids have attracted attention as cancer immunotherapeutic materials because they can be recognized by various nucleic acid sensors and stimulate innate immune responses. However, it has been difficult to use nucleic acid molecules for cancer immunotherapy because of their low selectivity for cancer cells and the systemic immunotoxicity they produce. We have developed a DNA assembly technology that enables induction of cytotoxicity through cancer-selective immune activation. The DNA hairpin pairs we designed formed an assembly structure triggered by intracellular microRNA-21 (miR-21), which is overexpressed in various types of cancer cells, resulting in long double-stranded DNA. This intracellular nucleic acid assembly triggered miR-21-dependent innate immune activation (cGAS-STING pathway), resulting in suppression of tumor growth in mice.

[Disclosure of COI by Responsible researcher] Lead presenter is the Responsible researcher.
[Lead presenter: Akimitsu Okamoto] ■Research funds from company: 科研費基盤研究(A)23H00317、AMED創薬基盤推進研究事業JP22ym0126805j0001 ■ employment by a company related to the presentation: 東京核酸合成株式会社 ■Advisor of the company and/or profit-making organization: 東京核酸合成株式会社 ■Profit of stock: 東京核酸合成株式会社

### [J-3046] I-J11-7
### Cancer vaccines/Other immunotherapies (2)

2025 / 9 / 27 (Sat) 14:05-15:20 Room 5/(Hotel Nikko Kanazawa 4F Tsuru C) Room 5

[Chairperson]Shin-ichiro Fujii (Laboratory for Immunotherapy, RIKEN Center for Integrative Medical Sciences )

### J-3046
### Intestinal bacterial metabolites enhance the efficacy of immune checkpoint inhibitor therapy for gastric cancer

[Speaker]Takumi Iwasawa (Inst. of Life Innova. Stu., Toyo Univ.／Grad. Sch Heal. & Sports Sci., Toyo Univ.／Shizuoka Med. Res. Center for Disast., Juntendo Univ.) [Co-author]Suguru Yamauchi (Dept. Surg., Johns Hopkins Univ.)、Tomoaki Ito (Shizuoka Hospital, Juntendo Univ.)、Kazunori Kato (Inst. of Life Innova. Stu., Toyo Univ.／Grad. Sch Heal. & Sports Sci., Toyo Univ.)

Recently, immune checkpoint inhibitors (ICIs) have been attracting attention as a new molecular targeted drug for cancer, but not all cancer cases respond to ICIs. In this context, it has been reported that certain intestinal bacteria enhance antitumor immunity and aid the function of ICIs. FMT has been a challenge as there are few cases in which the donor's intestinal microbiota can be stably transplanted, and it is also known that the above action requires live bacteria to form colonies and metabolize. Therefore, in this study, we aim to investigate in vivo and in vitro whether the effect of ICIs in gastric cancer, where ICIs are less effective, can be enhanced by ingesting metabolites (LM) made by co-cultivating 16 types of 31 strains of human-derived intestinal bacteria.As a result, antitumor immunity against primary tumors and peritoneal dissemination was confirmed only when LM was combined with ICI. Furthermore, LM assisted the activity of improving T cell exhaustion. Metabolomic analysis revealed that it contained 465 types of metabolites. AI analysis was able to extract several candidates for active ingredients.

[Disclosure of COI by Responsible researcher] Lead presenter is the Responsible researcher.
[Lead presenter: Takumi Iwasawa] We have no financial relationships to disclose.

▲TOP

### [J-3047] I-J11-7
### Cancer vaccines/Other immunotherapies (2)

2025 / 9 / 27 (Sat) 14:05-15:20 Room 5/(Hotel Nikko Kanazawa 4F Tsuru C) Room 5

[Chairperson]Shin-ichiro Fujii (Laboratory for Immunotherapy, RIKEN Center for Integrative Medical Sciences )

---

### J-3047
### Enhancement of STING-targeting immune responses through co-treatment with the low molecular weight compound KIN1148

[Speaker]Takayuki Ohkuri (Dept. Pathol., Asahikawa Med. Univ.) [Co-author]Akemi Kosaka (Dept. Pathol., Asahikawa Med. Univ.)、Nanami Ujiie (Dept. Pathol., Asahikawa Med. Univ.)、Takahiro Inoue (Dept. Pathol., Asahikawa Med. Univ.)、Ryusei Yoshino (Dept. Pathol., Asahikawa Med. Univ.)、Hiroyoshi Nozaki (Dept. Pathol., Asahikawa Med. Univ.)、Toshihiro Nagato (Dept. Pathol., Asahikawa Med. Univ.)、Hiroya Kobayashi (Dept. Pathol., Asahikawa Med. Univ.)

KIN1148 is known to act as an agonist of RIG-I, leading to activation of IRF3 signaling. Nevertheless, whether it directly modulates STING stimulation has not yet been determined. Therefore, we aimed to evaluate whether co-treatment with KIN1148 enhance immune responses by stimulated with cGAMP, an agonist of STING. We found that when stimulated with KIN1148 and cGAMP, the human monocytic leukemia cell line THP-1 synergistically enhanced production levels of IFN-$\beta$, CXCL10, and CCL5. In the analyses using the reporter cell lines, NF-kB signaling was synergistically activated by co-treatment with KIN1148 and cGAMP. In addition, the production of type I IFNs was also enhanced in mouse bone marrow-derived dendritic cells by co-treatment with KIN1148 and cGAMP. In the breast cancer mouse model using E0771, the combination therapy demonstrated the greatest inhibition of tumor growth compared to each monotherapy. Furthermore, the antitumor effect was abrogated in STING-deficient mice as well as in IFNAR1-KO mice. These results suggest that co-treatment with KIN1148 represents a rational strategy for STING-targeted cancer immunotherapy.

[Disclosure of COI by Responsible researcher] Lead presenter is the Responsible researcher.
[Lead presenter: Takayuki Ohkuri] We have no financial relationships to disclose.

### [J-3048] I-J11-7
### Cancer vaccines/Other immunotherapies (2)

2025 / 9 / 27 (Sat) 14:05-15:20 Room 5/(Hotel Nikko Kanazawa 4F Tsuru C) Room 5

[Chairperson]Shin-ichiro Fujii (Laboratory for Immunotherapy, RIKEN Center for Integrative Medical Sciences )

---

### J-3048
### Cell fusion and immunostimulatory cytokines extremely impact on immunotherapeutic potential of oncolytic vaccinia virus

[Speaker]Motomu Nakatake (Div. Genomic Medicine, Faculty of Med. Tottori University) [Co-author]Ryou Ishiguro (Div. Genomic Medicine, Faculty of Med. Tottori University)、Mami Takahashi (Dept. Pharmacology and Therap., Natl. Cancer Center Res. Inst.)、Hajime Kurosaki (Div. Genomic Medicine, Faculty of Med. Tottori University)、Takafumi Nakamura (Div. Genomic Medicine, Faculty of Med. Tottori University)

Oncolytic virus induces systemic antitumor immune response following local oncolysis. We have developed fusogenic oncolytic vaccinia virus (FUVAC) exerted stronger antitumor functions depending on its cell-cell fusion induction. However, the treatment of distant untreated tumors relying on antitumor immune response is insufficient owing to the lack of direct oncolysis. To improve the systemic antitumor immune response, we engineered FUVAC armed with two immunostimulatory cytokines, interleukin 12 (IL-12) and C-C motif chemokine ligand 21 (CCL21).
Unilateral injection of FUVAC-IL-12/CCL21 increased distant tumor regression (achieved 72% CR) compared to non-armed FUVAC or cytokine-armed non-fusogenic virus in mice bearing bilateral CT26 tumors. Single cell RNA-seq revealed that FUVAC-IL-12/CCL21 increased infiltration of diverse tumor-reactive effector memory $CD8^+$ T cells into distant tumors. In orthotopic pancreatic KMPC tumor model, intraperitoneal injection of FUVAC-IL12/CCL21 also prolonged the survival of mice by increasing $CD8^+$ T cells while decreasing tumors and stromal infiltration.
Thus, FUVAC armed with IL-12 and CCL21 remarkably improved its immunotherapeutic potential.

[Disclosure of COI by Responsible researcher] Lead presenter is not the Responsible researcher.
[Lead presenter: Motomu Nakatake] We have no financial relationships to disclose.
[Principal Researcher: Takafumi Nakamura] We have no financial relationships to disclose.

▲TOP

### [E-3037] I-E11-6
### Tumor immune microenvironment/Tumor immune escape (5)

2025 / 9 / 27 (Sat) 14:05-15:20 Room 6/(Hotel Nikko Kanazawa 3F Kujaku A) Room 6

[Chairperson]Heiichiro Udono (Dept.Metabolic Immune Regulation, Okayama Univ.)

---

### E-3037
### Functional analysis of apCAF and mesothelial cells in colorectal cancer tumor microimmunity

[Speaker]Yasuhiro Fukui (Dept. of Gastroenterological Surg., Osaka Metropolitan Univ.) [Co-author]Hiroaki Kasashima (Dept. of Gastroenterological Surg., Osaka Metropolitan Univ.)、Yukina Kusunoki (Department of Pathophysiology, Osaka Metropolitan Univ.)、Zizhou Wang (Dept. of Gastroenterological Surg., Osaka Metropolitan Univ.)、Iguru Omori (Dept. of Gastroenterological Surg., Osaka Metropolitan Univ.)、Yuki Seki (Dept. of Gastroenterological Surg., Osaka Metropolitan Univ.)、Kenji Kuroda (Dept. of Gastroenterological Surg., Osaka Metropolitan Univ.)、Yuichiro Miki (Dept. of Gastroenterological Surg., Osaka Metropolitan Univ.)、Mami Yoshii (Dept. of Gastroenterological Surg., Osaka Metropolitan Univ.)、Tatsuro Tamura (Dept. of Gastroenterological Surg., Osaka Metropolitan Univ.)、Masatsune Shibutani (Dept. of Gastroenterological Surg., Osaka Metropolitan Univ.)、Takahiro Toyokawa (Dept. of Gastroenterological Surg., Osaka Metropolitan Univ.)、Masakazu Yashiro (Molecular Oncology and Therapeutics, Osaka Metropolitan Univ.)、Yuki Nakanishi (Dept. of Gastroenterology and Hepatology, Kyoto Univ.)、Naoko Ohtani (Department of Pathophysiology, Osaka Metropolitan Univ.)、Kiyoshi Maeda (Dept. of Gastroenterological Surg., Osaka Metropolitan Univ.)

Cancer-associated fibroblasts (CAFs) contribute to tumor progression. Among them, antigen-presenting CAFs (apCAFs), likely originate from mesothelial cells, exhibit immunosuppressive functions. We investigated the roles of apCAFs and mesothelial cells in colorectal cancer (CRC) using a mouse orthotopic model and clinical specimens. Flow cytometry revealed that apCAFs peaked at week 6 after transplantation, preceding the expantion of myofibroblastic CAFs. We established cancer-associated mesothelial cells (CAmeso) from ascites of CRC patients with peritoneal metastasis and normal mesothelial cells (Nmeso) from those without peritoneal metastasis. CAmeso exhibited higher expression of apCAF markers. Co-transplantation with colon cancer cells increased tumor growth ***in vivo***. In mixed lymphocyte reactions, CAmeso co-cultured with dendritic cells suppressed $CD4^+$ T cell activation. scRNA-seq identified CAF subsets with high SLPI expression, confirmed by αSMA/SLPI immunofluorescence. SLPI was also highly expressed in CAmeso. Importantly, $SLPI^+$ $\alpha SMA^+$ stromal cells were associated with poor prognosis. $SLPI^+$ CAFs may represent apCAFs and serve as biomarkers and therapeutic targets.

[Disclosure of COI by Responsible researcher] Lead presenter is not the Responsible researcher.
[Lead presenter: Yasuhiro Fukui] We have no financial relationships to disclose.
[Principal Researcher: Kiyoshi Maeda] We have no financial relationships to disclose.

▲TOP

### [E-3038] I-E11-6
### Tumor immune microenvironment/Tumor immune escape (5)

2025 / 9 / 27 (Sat) 14:05-15:20 Room 6/(Hotel Nikko Kanazawa 3F Kujaku A) Room 6

[Chairperson]Heiichiro Udono (Dept.Metabolic Immune Regulation, Okayama Univ.)

---

### E-3038
### Immune cells derived from Tet2-mutant clonal haematopoiesis suppress liver metastasis of colorectal carcinoma

[Speaker]AI NHAN THI LE (Graduate School of Comprehensive Human Sciences, University of Tsukuba) [Co-author]Tran Bich Nguyen (Department of Hematology, Institute of Medicine, University of Tsukuba)、Robert J. Vanner (Princess Margaret Cancer Centre, University Health Network, Toronto, Canada／Institute of Medical Science, Faculty of Medicine, University of Toronto)、Manabu Fujisawa (Department of Hematology, Institute of Medicine, University of Tsukuba／Centre for Lymphoid Cancer, BC Cancer, Vancouver, BC, Canada)、Yoshiaki Abe (Department of Hematology, Institute of Medicine, University of Tsukuba／Department of Hematology, University of Tsukuba Hospital, Tsukuba, Japan)、Yen T. M. Nguyen (Department of Hematology, Institute of Medicine, University of Tsukuba／Department of Hematology, University of Tsukuba Hospital, Tsukuba, Japan)、Sakurako Suma (Graduate School of Comprehensive Human Sciences, University of Tsukuba／Department of Hematology, Institute of Medicine, University of Tsukuba)、Yasuhito Suehara (Department of Hematology, Institute of Medicine, University of Tsukuba／Department of Hematology, University of Tsukuba Hospital, Tsukuba, Japan)、Fumihito Miura (Graduate School of Frontier Sciences, the University of Tokyo, Kashiwa)、Hiromitsu Araki (Department of Biochemistry, Kyushu University Graduate School of Medical Sciences)、Kenichi Makishima (Graduate School of Comprehensive Human Sciences, University of Tsukuba／Department of Hematology, Institute of Medicine, University of Tsukuba)、Tatsuhiro Sakamoto (Department of Hematology, Institute of Medicine, University of Tsukuba／Department of Hematology, University of Tsukuba Hospital, Tsukuba, Japan)、Shigeru Chiba (Department of Hematology, Institute of Medicine, University of Tsukuba／Department of Hematology, University of Tsukuba Hospital, Tsukuba, Japan)、Masanobu Oshima (Division of Genetics, Cancer Research Institute, Kanazawa University, Kanazawa, Japan／WPI Nano Life Science Institute, Kanazawa University, Kanazawa, Japan)、Shumpei Ishikawa (Department of Preventive Medicine, The University of Tokyo)、Mamiko Sakata-Yanagimoto (Department of Hematology, Institute of Medicine, University of Tsukuba／Department of Hematology, University of Tsukuba Hospital, Tsukuba, Japan／Transborder Medical Research Center, University of Tsukuba)

Backgrounds: In the 82nd JCA Annual Meeting, we reported that ***Tet2***-mutant clonal hematopoiesis of indeterminate potential (CHIP)-derived immune cells suppressed colorectal cancer liver metastasis (CRCLM) in mice via CD8 tumor-infiltrating T lymphocytes (TILs). Here, we further explore how ***Tet2*** deficiency shapes CD8 TIL differentiation in CRCLM. Methods: $\boldsymbol{AKTP^{M/LOH}}$ CRC cells were injected into spleens of $\boldsymbol{Vav1^{Cre}Tet2KO}$ and $\boldsymbol{Cd4^{Cre}Tet2KO}$ mice. Anti-CD8α antibody was given to $\boldsymbol{Cd4^{Cre}Tet2KO}$ mice. Liver metastatic tumor burden (MTB) was the mean tumor-to-section area ratio. TILs were analyzed by scRNAseq. Anti-PD1 treatment was tested in $\boldsymbol{Vav1^{Cre}Tet2KO}$ mice. $\boldsymbol{Tet2^{flox/flox}}$ mice served as controls (ctrl). Results: $\boldsymbol{CD4^{Cre}Tet2KO}$ mice had lower MTB than ctrl under vehicle, but this difference was diminished by anti-CD8α antibody treatment. scRNAseq showed downregulation of exhaustion genes, upregulation of effector genes, fewer terminally exhausted T cells, and broader TCR clone distribution across effector states in ***Tet2KO*** CD8 TILs compared to ctrl CD8 TILs. Anti-PD1 eradicated metastasis in both $\boldsymbol{Vav1^{Cre}Tet2KO}$ and ctrl mice. Conclusion: ***Tet2*** deficiency limits CD8 TIL exhaustion in CRCLM.

[Disclosure of COI by Responsible researcher] Lead presenter is not the Responsible researcher.
[Lead presenter: AI NHAN THI LE] We have no financial relationships to disclose.
[Principal Researcher: Mamiko Sakata-Yanagimoto] ■Research funds from company: Eisai Co., Ltd.; Muldipharma K.K.; Otsuka Pharmaceutical Co., Ltd. ■Lecture fee: Kyowa Kirin Co., Ltd.

### [E-3039] I-E11-6
### Tumor immune microenvironment/Tumor immune escape (5)

2025 / 9 / 27 (Sat) 14:05-15:20 Room 6/(Hotel Nikko Kanazawa 3F Kujaku A) Room 6

[Chairperson]Heiichiro Udono (Dept.Metabolic Immune Regulation, Okayama Univ.)

---

### E-3039
### PCSK9 from Mixed-Polarized Macrophages Induces CD8+ T Cell Exhaustion and Immune Resistance in DGC

[Speaker]Jun Zhang (Dept. of Gastric Cancer)

We have previously reported that M1/M2 mixed-polarized tumor-associated macrophages (M1/2), which are widely present in Diffuse-type gastric cancer (DGC), promote tumor invasive growth. Spatial transcriptomic analysis revealed that M1/2 macrophages specifically express high levels of PCSK9, and clinical samples demonstrated a positive correlation between M1/2, PCSK9, and terminally exhausted CD8+ T cells. Furthermore, knocking out PCSK9 in macrophages significantly enhanced the therapeutic efficacy of PD-1 blockade. Further mechanistic investigations revealed that M1/2-derived PCSK9 degrades LDLR in CD8+ T cells, and the loss of LDLR disrupts mitochondrial-endoplasmic reticulum structural coupling (MAMs), leading to mitochondrial dysfunction. This process drives CD8+ T cells into terminal exhaustion, ultimately resulting in immunotherapy resistance. Preclinical experiments demonstrated that combining PCSK9 monoclonal antibodies with PD-1 blockade effectively enhances the efficacy of immunotherapy. We propose a novel combination therapy strategy, providing new insights for precision immunotherapy in diffuse-type gastric cancer.

[Disclosure of COI by Responsible researcher] Lead presenter is the Responsible researcher.
[Lead presenter: Jun Zhang] We have no financial relationships to disclose.

English Oral Session

### [E-3040] I-E11-6
### Tumor immune microenvironment/Tumor immune escape (5)

2025 / 9 / 27 (Sat) 14:05-15:20 Room 6/(Hotel Nikko Kanazawa 3F Kujaku A) Room 6

[Chairperson]Heiichiro Udono (Dept.Metabolic Immune Regulation, Okayama Univ.)

---

### E-3040
### Secreted Peroxidasin Regulates Cancer Cell Immune Evasion by Modulating NK Cell-Mediated Cytotoxicity

[Speaker]Ivan Bogeski (University Medical Center, Goettingen, Germany) [Co-author]Hsu-Ming Sung (University Medical Center, Goettingen, Germany)、David Bickel (Vrije Universiteit Brussel, Brussels, Belgium)、Lena Krause (University Medical Center, Goettingen, Germany)、Christian Ickes (University Medical Center, Goettingen, Germany)、Joris Messens (Vrije Universiteit Brussel, Brussels, Belgium)、Volker Ellenrieder (University Medical Center, Goettingen, Germany)

Peroxidasin (PXDN), a peroxidase involved in extracellular matrix formation, has been implicated in cancer progression. However, the underlying molecular mechanisms remain insufficiently understood. We demonstrate that PXDN upregulation correlates with poor prognosis and decreased survival in melanoma and PDAC patients. Depleting PXDN impaired cancer cell growth, disrupted cell cycle, and decreased migration and invasion. From a therapeutic perspective, we identified PXDN as a key regulator of cancer cell susceptibility to natural killer (NK) cell-mediated cytotoxicity. Structural modeling revealed a trimeric PXDN architecture, with peroxidase domains interconnected by disulfide bonds. Moreover, molecular dynamics simulations indicated a suppressing interaction between PXDN's N-terminal leucine-rich repeat domain and the NK cell receptor NKG2D. Collectively, this study uncovers a novel redox-independent function of PXDN and establishes the peroxidase as a critical regulator of melanoma and PDAC progression and immune evasion. These findings position PXDN as a promising therapeutic target for NK cell-based immunotherapy in solid tumors.

[Disclosure of COI by Responsible researcher] Lead presenter is the Responsible researcher.
[Lead presenter: Ivan Bogeski] We have no financial relationships to disclose.

### [E-3041] I-E11-6
### Tumor immune microenvironment/Tumor immune escape (5)

2025 / 9 / 27 (Sat) 14:05-15:20 Room 6/(Hotel Nikko Kanazawa 3F Kujaku A) Room 6

[Chairperson]Heiichiro Udono (Dept.Metabolic Immune Regulation, Okayama Univ.)

---

### E-3041
### Molecular mechanisms of IL-23 overproduction by tumor-derived lactic acid

[Speaker]Takashi Baba (Dept. of Mol. Genet., Wakayama Med. Univ.) [Co-author]Hongwu Ying (Dept. of Mol. Genet., Wakayama Med. Univ.)、Kosuke Yusa (Stem Cell Genet., Kyoto Univ.)、Norimitsu Inoue (Dept. of Mol. Genet., Wakayama Med. Univ.)

Cancer cells generate environments with high lactic acid (LA) levels by enhancing glycolysis. LA promotes the expression of the ***Il23a*** gene, which encodes the p19 subunit of the protumorigenic cytokine IL-23, in macrophages stimulated by TLR2 or TLR4 ligands. IL-23 levels are indeed elevated in malignant tissues, including colorectal carcinoma. However, mechanisms underlying the LA-mediated ***Il23a*** transcription are poorly understood. We performed a genome-wide CRISPR screening to identify the regulatory genes of ***Il23a*** expression under high LA conditions. We found that ***Nfkb1***, ***Nfkbiz***, ***Ube2m*** or ***Ube2f*** disruption impaired the ***Il23a*** induction in response to LA. In macrophage-like J774.1 cell lines and bone-marrow-derived macrophages, costimulation with LA and TLR2 or TLR4 ligands enhanced the nuclear translocation of p50 and IκBζ, encoded by ***Nfkb1*** and ***Nfkbiz***. The neddylation E2 proteins UBE2M and UBE2F were found to regulate the elevated nuclear translocation of IκBζ in J774.1 cells. These results suggest that high lactate levels are involved in the regulation of IL-23 expression in tumors by controlling the nuclear localization of p50 and IκBζ in macrophages via UBE2M or UBE2F activity.

[Disclosure of COI by Responsible researcher] Lead presenter is not the Responsible researcher.
[Lead presenter: Takashi Baba] ■Research funds from company: 科学研究費助成事業、武田科学振興財団、持田記念医学薬学振興財団
[Principal Researcher: Norimitsu Inoue] ■Research funds from company: 科学研究費助成事業

### [E-3042] I-E11-6
### Tumor immune microenvironment/Tumor immune escape (5)

2025 / 9 / 27 (Sat) 14:05-15:20 Room 6/(Hotel Nikko Kanazawa 3F Kujaku A) Room 6

[Chairperson]Heiichiro Udono (Dept.Metabolic Immune Regulation, Okayama Univ.)

### E-3042
### The transcription factor MAFB defines the immunosuppressive nature of tumor-associated macrophages in pancreatic cancer

[Speaker]Kuniyuki Kawano (Dept. of Immunol, Yokohama City Univ.／Dept. of Gastroenterology, Yokohama City Univ.) [Co-author]Hiroshi Okuda (Dept. of Immunol, Yokohama City Univ.)、Juri Ichikawa (Dept. of Immunol, Yokohama City Univ.)、Shingo Kato (Dept. Clin. Cancer genomics, Yokohama City Univ. Hosp.)、Daisuke Kurotaki (Dept. of Immunol, Yokohama City Univ.／Lab. of Chromatin Organization in Immune Cell Development, Kumamoto Univ.)、Waka Mochizuki (Dept. of Immunol, Yokohama City Univ.)、Wataru Kawase (Dept. of Immunol, Yokohama City Univ.／Advanced Cancer Therapy Div., Kanagawa Cancer Ctr. Res. Inst.)、Yukihiko Hiroshima (Advanced Cancer Therapy Div., Kanagawa Cancer Ctr. Res. Inst.)、Michito Hamada (Ctr. for Medical Sci., Ibaraki Pref. Univ. of Health Sci.)、Satoru(Univ. of Tsukuba) Takahashi (Dept. of Anatomy and Embryology, Univ. of Tsukuba)、Itaru Endo (Dept. of Gastrological Surg., Yokohama City Univ.)、Shin Maeda (Dept. of Gastroenterology, Yokohama City Univ.)、Tomohiko Tamura (Dept. of Immunol, Yokohama City Univ.)

Tumor-associated macrophages (TAMs) are predominant immune cells infiltrating pancreatic ductal adenocarcinoma (PDAC) lesions, with a high abundance of M2 subsets being associated with poor prognosis. Therefore, reprogramming TAM phenotypes represents a potential strategy for improving PDAC treatment. Here, we identified the transcription factor MAFB as a key regulator of pancreatic TAM phenotype. Single-cell RNA sequencing of human PDAC revealed that ***MAFB*** was highly expressed in the M2 subset of TAMs. Myeloid-specific deletion of ***Mafb*** in the murine PDAC model significantly suppressed tumor progression and prolonged survival. ***Mafb*** deficiency induced a shift in TAM phenotypes from M2 to M1 and enhanced cytotoxicity of tumor-infiltrating $CD8^+$ T cells while attenuating their exhaustion. Additionally, $CD8^+$ T cells co-cultured with ***Mafb***-deficient TAMs exhibited increased Interferon-$\gamma$ production. In human PDAC, ***MAFB*** gene expression in TAMs positively correlated with exhaustion maker gene expression in $CD8^+$ T cells. These findings indicate that MAFB plays a crucial role in defining the tumor-promoting nature of TAMs and may be a promising therapeutic target for PDAC.

[Disclosure of COI by Responsible researcher] Lead presenter is not the Responsible researcher.
[Lead presenter: Kuniyuki Kawano] We have no financial relationships to disclose.
[Principal Researcher: Tomohiko Tamura] ■Research funds from company: Grant/A joint research fund from Eisai Co., Ltd. to T. Tamura

### [E-3049] I-E6-2
### Genomic instability

2025 / 9 / 27 (Sat) 14:05-15:20 Room 7/(Hotel Nikko Kanazawa 3F Kujaku B) Room 7

[Chairperson]Yasukazu Daigaku (Cancer Genome Dynamics, Cancer Institute, Japanese Foundation for Cancer Research)

---

### E-3049
### UV-Associated Genome Instability is Increased by DNA Replication in Cells with CPD UV Photoproducts in Active Genes

[Speaker]Kenichi Yoshioka (Lab. Genome Stability Maintenance, NCCRI) [Co-author]Yusuke Matsuno (Lab. Genome Stability Maintenance, NCCRI)

UV light primarily induces UV photoproducts CPDs and 6-4PPs, which increases the risk of melanoma developing with genome instability. However, it is unclear how these photoproducts increase genome instability. Here, we show that replication stress-associated double-strand breaks (DSBs) that increase genome instability are induced by CPDs in transcriptionally active genes, in which clonal evolution of cells with genome instability could be induced. 6-4PPs, which increased gammaH2AX expression and delayed S-phase entry, were repaired before the onset of DNA replication, while CPDs were not. Replication stress-associated DSBs are generated in association with transcription-coupled repair, increasing the risk of genome instability. DSBs decreased after CPDs were partially repaired in active chromatin regions, but not in heterochromatin regions where unrepaired CPDs largely remained. The UV-associated single-base substitution signature SBS7 was induced in a structural variant (SV)-dependent manner in UV-irradiated cells. Importantly, those genomic alterations are also seen in human melanoma. These findings reveal the importance of CPD repair to suppress UV-associated genome instability.

[Disclosure of COI by Responsible researcher] Lead presenter is the Responsible researcher.
[Lead presenter: Kenichi Yoshioka] We have no financial relationships to disclose.

### [E-3050] I-E6-2
### Genomic instability

2025 / 9 / 27 (Sat) 14:05-15:20 Room 7/(Hotel Nikko Kanazawa 3F Kujaku B) Room 7

[Chairperson]Yasukazu Daigaku (Cancer Genome Dynamics, Cancer Institute, Japanese Foundation for Cancer Research)

---

### E-3050
### Molecular mechanism of centromeric HP1 enrichment ensuring mitotic fidelity

[Speaker]Saho Matsui (Div. Exp. Pathol. Cancer Inst. JFCR／Dept. JFCR., Tokyo Med. & Dent. Univ.) [Co-author]Ryusuke Nozawa (Div. Exp. Pathol. Cancer Inst. JFCR)、Anna Suzuki (Div. Exp. Pathol. Cancer Inst. JFCR／Dept. JFCR., Tokyo Med. & Dent. Univ.)、Toru Hirota (Div. Exp. Pathol. Cancer Inst. JFCR／Dept. JFCR., Tokyo Med. & Dent. Univ.)

Chromosomal instability (CIN) is a hallmark of cancer cells that is acquired through chromosome missegregation in mitosis. Faithful segregation depends on the concentrated activity of Aurora B at centromeres and its complex formation with heterochromatin protein HP1. Our previous study has shown that centromeric HP1 is widely reduced in cancer cells, leading to chromosome missegregation; yet little is known about the underlying mechanism. To elucidate what causes its dispersion in cancer cells, here we addressed how mitotic HP1 is concentrated at centromeres. We found that the HP1 enrichment requires centromeric RNA, and its electrostatic interaction with positively charged amino acids in the HP1 hinge region. Notably, HP1 dispersion in cancer cells is associated with altered transcripts which seem to disrupt this interaction. We further examined if the oncogenic RAS signaling affects the centromeric transcription and thus Aurora B regulation. Interestingly, the activation of growth signals altered centromeric structure and transcription, resulting in reduced centromeric HP1. These observations provide new insight into how cell growth signaling is linked to CIN in cancer cells.

[Disclosure of COI by Responsible researcher] Lead presenter is not the Responsible researcher.
[Lead presenter: Saho Matsui] We have no financial relationships to disclose.
[Principal Researcher: Toru Hirota] We have no financial relationships to disclose.

### [E-3051] I-E6-2
### Genomic instability

2025 / 9 / 27 (Sat) 14:05-15:20 Room 7/(Hotel Nikko Kanazawa 3F Kujaku B) Room 7

[Chairperson]Yasukazu Daigaku (Cancer Genome Dynamics, Cancer Institute, Japanese Foundation for Cancer Research)

---

### E-3051
### Investigation of protein network involving ARID1A in SWI/SNF chromatin remodeler, and its function in genome stability

[Speaker]Ayako Ui (Institute of Development, Aging and Cancer, Tohoku University) [Co-author]Shinichiro Kanno (Institute of Development, Aging and Cancer, Tohoku University)、Takayasu Kobayashi (Institute of Development, Aging and Cancer, Tohoku University)、Reiko Watanabe (Institute for Protein Research, Osaka University)、Akihiro Kurimasa (Tohoku Medical and Pharmaceutical University)、Kozo Tanaka (Institute of Development, Aging and Cancer, Tohoku University)、Akira Yasui (Institute of Development, Aging and Cancer, Tohoku University)

The SWI/SNF chromatin-remodeling complex that comprises multiple subunits orchestrates diverse cellular processes, including gene expression, DNA repair, and DNA replication, by sliding and releasing nucleosomes. ARID1A and ARID1B (ARID1A/B), a pivotal subunit, have significant relevance in cancer management because they are frequently mutated in a broad range of cancer types. To delineate the protein network involving ARID1A/B, we investigated the interactions of this with other proteins. The ARID domain of ARID1A/B interacts with proteins involved in transcription and DNA/RNA metabolism. Several proteins are responsible for genome integrity maintenance, including DNA-dependent protein kinase catalytic subunit (DNA-PKcs), bound to the armadillo (ARM) domain of ARID1A/B. Introducing a knock-in mutation at the binding amino acid of DNA-PKcs in HCT116 cells reduced the autophosphorylation of DNA-PKcs and the recruitment of LIG4 in response to ionizing radiation. Our findings suggest that within the SWI/SNF complex, ARID1A couples DNA double-strand break repair processes with chromatin remodeling via the ARM domains to directly engage with DNA-PKcs to maintain genome stability.

[Disclosure of COI by Responsible researcher] Lead presenter is the Responsible researcher.
[Lead presenter: Ayako Ui] We have no financial relationships to disclose.

### [E-3052] I-E6-2
### Genomic instability

2025 / 9 / 27 (Sat) 14:05-15:20 Room 7/(Hotel Nikko Kanazawa 3F Kujaku B) Room 7

[Chairperson]Yasukazu Daigaku (Cancer Genome Dynamics, Cancer Institute, Japanese Foundation for Cancer Research)

### E-3052
### Chromosomal Instability Is Associated with cGAS-STING Activation in EGFR-TKI Refractory Non-Small-Cell Lung Cancer

[Speaker]Kimio Yonesaka (Dept. of Med. Oncology, Kindai Univ. Faculty of Med.) [Co-author]Takashi Kurosaki (Dept. of Med. Oncology, Kindai Univ. Faculty of Med.)、Junko Tanizaki (Dept. of Med. Oncology, Kindai Univ. Faculty of Med.／Dept. of Med. Oncology, Kishiwada City Hosp.)、Hisato Kawakami (Dept. of Med. Oncology, Kindai Univ. Faculty of Med.)、Kaoru Tanaka (Dept. of Med. Oncology, Kindai Univ. Faculty of Med.)、Osamu Maenishi (Dept. of Path., Kindai Univ. Faculty of Med.)、Kazuko Sakai (Dept of Genome Biol., Kindai Univ. Faculty of Med.)、Yasutaka Chiba (Clin. Res. Ctr, Kindai Univ. Hosp.)、Takeshi Teramura (Inst. of Advanced Clin. Med., Kindai Univ. Faculty of Med.)、Masanori Funabashi (R&D Div., Daiichi Sankyo Co., Ltd.)、Yasuki Kamai (R&D Div., Daiichi Sankyo Co., Ltd.)、Takashi Kagari (R&D Div., Daiichi Sankyo Co., Ltd.)、Kazuto Nishio (Dept of Genome Biol., Kindai Univ. Faculty of Med.)、Kazuhiro Kakimi (Dept. of Immunol., Kindai Univ. Faculty of Med.)、Hidetoshi(Kindai Univ.) Hayashi (Dept. of Med. Oncology, Kindai Univ. Faculty of Med.)

Epidermal growth factor receptor tyrosine kinase inhibitors (EGFR-TKIs) are standard therapies for EGFR-mutated non-small-cell lung cancer (NSCLC). However, their efficacy is inconsistent and resistance mechanisms have not been fully identified. Chromosomal instability (CIN) is a hallmark of cancer and results in genetic diversity. In this study, we demonstrated by transcriptomic analysis that CIN activates the cGAS-STING signaling, which leads to EGFR-TKI refractoriness in some patients with EGFR-mutation. Furthermore, EGFR-mutated H1975dnMCAK cells, which frequently underwent chromosomal mis-segregation, showed refractoriness to the EGFR-TKI compared to control. Second, H1975dnMCAK cells exhibited activation of cGAS-STING signaling and its downstream signaling, including tumor-promoting IL-6. Finally, chromosomally unstable EGFR-mutated NSCLC exhibited enhanced epithelial-mesenchymal transition (EMT). Blockade of cGAS-STING-TBK1 signaling reversed EMT, resulting in restored susceptibility to EGFR-TKIs in vitro and in vivo. These results suggest that CIN may lead to the activation of cGAS-STING signaling in some EGFR-mutated NSCLC, resulting in EMT-associated EGFR-TKI resistance.

[Disclosure of COI by Responsible researcher] Lead presenter is the Responsible researcher.
[Lead presenter: Kimio Yonesaka] ■Research funds from company: 第一三共株式会社

### [E-3053] I-E6-2
### Genomic instability

2025 / 9 / 27 (Sat) 14:05-15:20 Room 7/(Hotel Nikko Kanazawa 3F Kujaku B) Room 7

[Chairperson]Yasukazu Daigaku (Cancer Genome Dynamics, Cancer Institute, Japanese Foundation for Cancer Research)

---

### E-3053
### How Plk1 activity is regulated by conformational transition

[Speaker]Chang Liu (Div. Exp. Pathol., Cancer Inst., JFCR／Path. Immunol. & Microbiol., Grad. Sch. of Med., Univ. Tokyo) [Co-author]Motoko Takahashi (Div. Exp. Pathol., Cancer Inst., JFCR)、Yasuhiro Hirano (Div. Exp. Pathol., Cancer Inst., JFCR／Grad. Sch. of Frontier Biosci., Univ. Osaka)、Toru Hirota (Div. Exp. Pathol., Cancer Inst., JFCR／Path. Immunol. & Microbiol., Grad. Sch. of Med., Univ. Tokyo)

Polo-like kinase 1 (Plk1) is a mitotic kinase that plays a pivotal role in the stable transmission of chromosomes. Perturbation of its activity therefore contributes to chromosomal instability, a hallmark of many cancers, and is also a promising target for intervention of the disease. Plk1 comprises a kinase domain (KD) and two Polo-box domains (PBD), and the regulation of its kinase activity is thought to involve the KD-PBD interaction and the phosphorylation within the molecule; but the relevance of this mechanism remains unaddressed. Here we investigate this conformational activation of Plk1 using structural and cell biological approaches. Unexpectedly, we found that crosslinking of purified recombinant Plk1 formed dimer, in addition to the monomer species. The phosphorylation of Plk1 at Thr210, which triggers its enzymatic activation, was found exclusively in monomeric Plk1. Besides the interaction of KD and PBD within the molecule, our results importantly suggest that inter-molecular dimer/monomer transition is also involved in Plk1 activation. These understandings of Plk1 activation should pave the way for the design of drug targets of Plk1 activity for therapeutic purposes.

[Disclosure of COI by Responsible researcher] Lead presenter is not the Responsible researcher.
[Lead presenter: Chang Liu] We have no financial relationships to disclose.
[Principal Researcher: Toru Hirota] We have no financial relationships to disclose.

### [E-3054] I-E6-2
### Genomic instability

2025 / 9 / 27 (Sat) 14:05-15:20 Room 7/(Hotel Nikko Kanazawa 3F Kujaku B) Room 7

[Chairperson]Yasukazu Daigaku (Cancer Genome Dynamics, Cancer Institute, Japanese Foundation for Cancer Research)

---

### E-3054
### Sensitizing effects of inhibition of mitochondrial RNA polymerase on DNA damage-inducing radiotherapy

[Speaker]Noriko Hosoya (Lab. Mol. Radiol., Grad. Sch. of Med.,The Univ. of Tokyo) [Co-author]Yukina Osawa (Lab. Mol. Radiol., Grad. Sch. of Med.,The Univ. of Tokyo)

Radioresistance is a serious limitation of current DNA-damaging cancer radiotherapy. Therefore, new therapeutic strategies are needed to improve the efficacy of radiotherapy. Mitochondria are major cellular sites crucial for energy production depending on oxidative phosphorylation and have emerged as promising targets for cancer therapies. Mitochondria have their own DNA known as mitochondrial DNA, encoding 13 proteins essential for oxidative phosphorylation. Mitochondrial RNA polymerase (POLRMT) is a polymerase specifically required for mitochondrial DNA transcription. We found that the expression levels of POLRMT are increased in various cancer cells compared to those in non-cancerous cells. Furthermore, its expression levels were found to have significant correlations with poor overall survival, presence of metastasis, and advanced cancer stage. Then, we inhibited POLRMT by using a recently-developed inhibitor IMT1 in combination with ionizing radiation in both cancer cells and normal cells. We found that IMT1 induces cancer-specific increased apoptosis when combined with ionizing radiation. The effects of IMT1 addition on mitochondrial respiration will be presented.

[Disclosure of COI by Responsible researcher] Lead presenter is the Responsible researcher.
[Lead presenter: Noriko Hosoya] ■Research funds from company: 日本学術振興会　科研費

### [J-3049] I-J10-5
### Cell-to-cell interaction (2)

2025 / 9 / 27 (Sat) 14:05-15:20 Room 9/(Hotel Nikko Kanazawa 3F Lumiere) Room 9

[Chairperson]Tatsunori Nishimura (Div. Cancer Biol., Nagoya Univ., Grad. Sch. Med.)

---

### J-3049
### Analysis of miRNA Expression Changes Induced by Kras$^{G12D}$ Mutant Cells in Pancreatic Ductal Progenitor Cells

[Speaker]Yuto Ohshima (Res. Ctr. for Drug Discovery, Keio Univ. Fac. Pharm.) [Co-author]Juntaro Matsuzaki (Res. Ctr. for Drug Discovery, Keio Univ. Fac. Pharm.)、Chihiro Oikawa (Div. Pharmacotherap., Keio Univ. Fac. Pharm.)、Rina Shibagaki (Div. Pharmacotherap., Keio Univ. Fac. Pharm.)、Taisuke Koreeda (Div. Pharmacotherap., Keio Univ. Fac. Pharm.)、Takahiro Ochiya (Dept. Mol. & Cell. Med., Inst. Med. Sci. Tokyo Med. Univ.)、Takeshi Katsuda (Dept. Chem. Sys. Eng. Univ. of Tokyo.)、Yoshimasa Saito (Div. Pharmacotherap., Keio Univ. Fac. Pharm.)

Cell competition, where fitter cells eliminate less fit neighbors, contributes to both cancer progression and suppression. In pancreatic cancer, however, it remains unclear which mutations affect cell fitness or how such differences are sensed and acted upon. In this study, we established mouse pancreatic ductal progenitor (mPP) cells using a chemical reprogramming method originally developed to convert mature hepatocytes into hepatic progenitor cells (Cell Stem Cell, 2017). We also introduced the Kras$^{G12D}$ mutation, a driver mutation in pancreatic cancer, into these mPP cells using CRISPR/Cas9. Wild-type and Kras$^{G12D}$ mutant cells, each labeled with different fluorescent proteins, were co-cultured in a 6-well plate. Since microRNAs (miRNAs) can transfer between cells via extracellular vesicles, we hypothesized that co-culture might alter in miRNA expression. We isolated and collected cells by flow cytometry, extracted total RNA, and performed miRNA sequencing. Several miRNAs exhibited differential expression compared to monoculture, suggesting that these miRNAs may respond to fitness information recognized between neighboring cells and regulate gene expression accordingly.

[Disclosure of COI by Responsible researcher] Lead presenter is not the Responsible researcher.
[Lead presenter: Yuto Ohshima] We have no financial relationships to disclose.
[Principal Researcher: Juntaro Matsuzaki] ■Research funds from company: ISM株式会社、レグリア株式会社、フェムトディプロイメンツ株式会社

### [J-3050] I-J10-5
### Cell-to-cell interaction (2)

2025 / 9 / 27 (Sat) 14:05-15:20 Room 9/(Hotel Nikko Kanazawa 3F Lumiere) Room 9

[Chairperson]Tatsunori Nishimura (Div. Cancer Biol., Nagoya Univ., Grad. Sch. Med.)

---

### J-3050
### The role of tumor endothelial cells in tumor microenvironment in liver cancer

[Speaker]Takehiro Noda (Dept of Gastroenterological Surg, The Univ of Osaka) [Co-author]Hirofumi Akita (Dept of Gastroenterological Surg, The Univ of Osaka)、Yoshihiro Sakano (Dept of Gastroenterological Surg, The Univ of Osaka)、Daijiro Matoba (Dept of Gastroenterological Surg, The Univ of Osaka)、Yosuke Mukai (Dept of Gastroenterological Surg, The Univ of Osaka)、Kazuki Sasaki (Dept of Gastroenterological Surg, The Univ of Osaka)、Shinichiro Hasegawa (Dept of Gastroenterological Surg, The Univ of Osaka)、Daisaku Yamada (Dept of Gastroenterological Surg, The Univ of Osaka)、Yoshito Tomimaru (Dept of Gastroenterological Surg, The Univ of Osaka)、Shogo Kobayashi (Dept of Gastroenterological Surg, The Univ of Osaka)、Hisashi Wada (Dept of Gastroenterological Surg, The Univ of Osaka／Dept of Clin Res Tumor Immunol, The Univ of Osaka)、Yuichiro Doki (Dept of Gastroenterological Surg, The Univ of Osaka)、Hidetoshi Eguchi (Dept of Gastroenterological Surg, The Univ of Osaka)

Hepatocellular carcinoma (HCC) is the primary liver neoplasm with hyper vascular feature. Tumor endothelial cells (TECs) in tumor tissue have different characteristics and functions compared with normal endothelial cells. Our recent studies showed that TECs showed the interaction for microenvironmental immune cells, such as tumor infiltrating T cells and tumor-associated macrophages (TAM). TECs induced tumor-infiltrating T cell exhaustion via glycoprotein nonmetastatic melanoma protein B (GPNMB) expression and the silencing of GPNMB induced the suppression of invasiveness and tumor development and restored the T cells exhaustion. Moreover, TECs and TAMs were closely correlated and the patients with high TECs and high TAM showed poor prognosis. The supernatant of TEC included large amount of IL-4 and TECs could induce M2 macrophage polarization through IL-4 secretion, leading to immune suppression and cancer progression. TECs play the major roles of HCC tumor progression by influencing various types of immune cells in tumor microenvironment.

[Disclosure of COI by Responsible researcher] Lead presenter is the Responsible researcher.
[Lead presenter: Takehiro Noda] We have no financial relationships to disclose.

### [J-3051] I-J10-5
### Cell-to-cell interaction (2)

2025 / 9 / 27 (Sat) 14:05-15:20 Room 9/(Hotel Nikko Kanazawa 3F Lumiere) Room 9

[Chairperson]Tatsunori Nishimura (Div. Cancer Biol., Nagoya Univ., Grad. Sch. Med.)

---

### J-3051
### Regulation of ***transforming growth factor-beta*** in cancer-associated fibroblasts in the hypoxic tumor microenvironment

[Speaker]Koji Teramoto (Shiga Univ. Med. Sci., Dept. Med. Oncol.) [Co-author]Hidetoshi Sumimoto (Shiga Univ. Med. Sci., Dept. Med. Oncol.)、Yataro Daigo (Shiga Univ. Med. Sci., Dept. Med. Oncol.)

**Background:** Tumor stroma is composed of multiple cell types, and both cancer-associated fibroblasts (CAFs) and tumor-associated macrophages (TAMs) are major cell types there. In this study, we focused on transforming growth factor-beta (TGF-$\beta$) that contributes to tumor growth and elucidated the interaction of CAFs and TAMs in the hypoxic tumor microenvironment. **Methods:** M2 macrophages were exposed to deferoxamine mesylate, an inhibitor of hypoxia-inducible factor 1$\alpha$ degradation. ***Transforming growth factor-alpha*** (***TGF-α***) mRNAs levels were measured by real time qPCR. In addition, CAFs were cultured with TGF-$\alpha$ under mimic hypoxic conditions, and the level of ***TGF-β*** mRNA in CAFs was measured. **Results:** The level of ***TGF-α*** mRNA in M2 macrophages was elevated after HIF1$\alpha$ accumulation. The level of ***TGF-β*** mRNA in CAFs was additively upregulated by TGF-$\alpha$ supplementation in a dose-dependent manner. **Discussion:** The data suggest that ***TGF-β*** expression in CAFs may be upregulated by TGF-$\alpha$ from TAMs in the hypoxic tumor microenvironment. Although further validation is needed, an interaction between TAMs and CAFs through the cytokines might contribute to the generation of a pro-tumor microenvironment.

[Disclosure of COI by Responsible researcher] Lead presenter is the Responsible researcher.
[Lead presenter: Koji Teramoto] We have no financial relationships to disclose.

▲TOP

### [J-3052] I-J10-5
### Cell-to-cell interaction (2)

2025 / 9 / 27 (Sat) 14:05-15:20 Room 9/(Hotel Nikko Kanazawa 3F Lumiere) Room 9

[Chairperson]Tatsunori Nishimura (Div. Cancer Biol., Nagoya Univ., Grad. Sch. Med.)

---

### J-3052
### Functional distinctions between primary and metastatic cancer-associated fibroblasts in head and neck cancer

[Speaker]Kazuaki Chikamatsu (Dept. of Otolaryngology-Head & Neck Surgery, Faculty of Med. Gunma Univ.) [Co-author]Hiroyuki Hagiwara (Dept. of Otolaryngology-Head & Neck Surgery, Faculty of Med. Gunma Univ.)、Hideyuki Takahashi (Dept. of Otolaryngology-Head & Neck Surgery, Faculty of Med. Gunma Univ.)、Hiroe Tada (Dept. of Otolaryngology-Head & Neck Surgery, Faculty of Med. Gunma Univ.)、Miho Uchida (Dept. of Otolaryngology-Head & Neck Surgery, Faculty of Med. Gunma Univ.)

Head and neck squamous cell carcinoma (HNSCC) shows limited prognostic improvement despite intensive multidisciplinary treatments. Regional lymph node metastases are generally more treatment-resistant than primary tumors and are key prognostic factors. We investigated biological differences in cancer-associated fibroblasts (CAFs) between primary tumors and lymph node metastases.
Eight paired CAFs were established from primary tumors and matched metastases. Metastasis-derived CAFs (mCAFs) expressed significantly higher $\alpha$-SMA than primary tumor-derived CAFs (pCAFs). mCAFs showed lower proliferation and migration. HNSCC cells co-cultured with mCAFs exhibited increased expression of stemness-related genes and tumor sphere formation, suggesting a role for mCAFs in treatment resistance. RNA sequencing of five CAF pairs revealed that mCAFs predominantly expressed myofibroblast-related genes. IPA indicated activation of fibrosis-related pathways in mCAFs.
These findings suggest that CAF differences between primary tumors and metastases may influence malignancy and resistance. Further investigation of mCAF characteristics and therapeutic targeting may improve outcomes in HNSCC.

[Disclosure of COI by Responsible researcher] Lead presenter is the Responsible researcher.
[Lead presenter: Kazuaki Chikamatsu] We have no financial relationships to disclose.

### [J-3053] I-J10-5
### Cell-to-cell interaction (2)

2025 / 9 / 27 (Sat) 14:05-15:20 Room 9/(Hotel Nikko Kanazawa 3F Lumiere) Room 9

[Chairperson]Tatsunori Nishimura (Div. Cancer Biol., Nagoya Univ., Grad. Sch. Med.)

---

### J-3053
### Adipose-derived adipokines regulate cancer proliferation and metabolism, thereby determining cancer malignancy.

[Speaker]Shinya Sato (Kanagawa Cancer Center Resaerch Institute Morphological Information Analysis Lab) [Co-author]Kentaro Odani (Kanagawa Cancer Center Resaerch Institute Morphological Information Analysis Lab／Kanagawa Cancer Center Resaerch Institute Molecular Pathology And Genetics Division)、Yohei Miyagi (Kanagawa Cancer Center Resaerch Institute Molecular Pathology And Genetics Division／Kanagawa Cancer Center Pathology Division)

Adipokines secreted from adipocytes, widespread in the cancer microenvironment, have diverse physiological activities. We performed a cross-organ gene expression analysis of approximately 40 types of adipokines and their receptors and lipid metabolism regulators in cancer cells. As a result, significant changes in the expression of some adipokine receptors, including adiponectin receptors, were observed in cancer. Adiponectin receptors are highly expressed in breast cancer, and receptor stimulation significantly controls cancer cells' and tissues' proliferation. In addition, adiponectin receptor stimulation activated cell death and lipid metabolism signals in breast cancer cells. Next, the effect of blood levels of adipokines on cancer phenotype was analyzed. Blood adiponectin levels were inversely correlated with the frequency of lymph node metastasis. In addition, cancer patients with low blood adiponectin levels and high leptin levels had a significantly increased recurrence after treatment. From the above, we believe that adipokines determine the malignancy of cancer through cell proliferation and metabolic control and are promising therapeutic targets.

[Disclosure of COI by Responsible researcher] Lead presenter is the Responsible researcher.
[Lead presenter: Shinya Sato] We have no financial relationships to disclose.

### [J-3054] I-J10-5
### Cell-to-cell interaction (2)

2025 / 9 / 27 (Sat) 14:05-15:20 Room 9/(Hotel Nikko Kanazawa 3F Lumiere) Room 9

[Chairperson]Tatsunori Nishimura (Div. Cancer Biol., Nagoya Univ., Grad. Sch. Med.)

---

### J-3054
### Elucidation of the mechanism of tumor fibrosis through interorgan crosstalk

[Speaker]Akiho Nishimura (Div. of Carcinogenesis, The Cancer Inst., JFCR／Dept. of Obstetrics and Gynecol., Kumamoto Univ.) [Co-author]Atsuko Yonemura (Div. of Carcinogenesis, The Cancer Inst., JFCR)、Takashi Semba (Div. of Carcinogenesis, The Cancer Inst., JFCR)、Takeshi Motohara (Dept. of Obstetrics and Gynecol., Kumamoto Univ.)、Goro Sashida (IRCMS, Kumamoto Univ.)、Takatsugu Ishimoto (Div. of Carcinogenesis, The Cancer Inst., JFCR／IRCMS, Kumamoto Univ.)

Diffuse–type gastric cancer (DGC) shows strong interactions between cancer-associated fibroblasts (CAFs) and cancer cells through PDGF/PDGFR signaling. While PDGFR is expressed in CAFs, PDGF ligands were believed to be mainly secreted by cancer cells. However, DGC cohort analysis revealed a correlation between stromal fibrosis and platelet aggregation, suggesting that platelets may serve as an alternative PDGF source. This study aims to elucidate the mechanisms of interorgan crosstalk through GC fibrosis–bone marrow (BM) hematopoiesis–platelet axis. IL6 was particularly upregulated in DGC and correlated with fibrosis. We established an orthotopic model using IL6–overexpressing murine GC cells, which developed fibrotic tumors. Notably, platelets in peripheral blood of these mice showed increased count, size, and PDGFD concentration. Cell population analysis in BM revealed increased megakaryocyte progenitors and decreased erythroid progenitors, indicating IL6–induced hematopoietic imbalance. These findings suggest that IL6 promotes PDGFD–rich platelet production, contributing to tumor fibrosis. This study highlights a key role of BM–derived platelets to enhance tumor fibrosis in DGC.

[Disclosure of COI by Responsible researcher] Lead presenter is not the Responsible researcher.
[Lead presenter: Akiho Nishimura] We have no financial relationships to disclose.
[Principal Researcher: Takatsugu Ishimoto] We have no financial relationships to disclose.

▲TOP

### [E-3067] I-E13-4
### Molecular targeted therapy (3)

2025 / 9 / 27 (Sat) 14:05-15:20 Room 14/(ANA Crowne Plaza Hotel Kanazawa 3F Zuiun 1) Room 14

[Chairperson]Hidenori Kage (Dept. Respir. Med., Univ. Tokyo)

---

### E-3067
### Osimertinib resistance via MET activation without amplification identified in a EGFR-mutant lung cancer synegeneic model

[Speaker]Masato Fujitani (Div. Exp. Chemother., Cancer Chemother. Ctr., JFCR／Dept. CBMS, Grad. Sch. Front. Sci., Univ. of Tokyo) [Co-author]Ai Takemoto (Div. Exp. Chemother., Cancer Chemother. Ctr., JFCR)、Kadoaki Ohashi (Dept. Allergy and Respiratory Med, Okayama Univ.)、Ryohei Katayama (Div. Exp. Chemother., Cancer Chemother. Ctr., JFCR／Dept. CBMS, Grad. Sch. Front. Sci., Univ. of Tokyo)

Osimertinib, an EGFR tyrosine kinase inhibitor, is effective in NSCLC harboring EGFR-activating mutations such as exon-19 deletion (Del19), but resistance is inevitable. In recent years, the tumor microenvironment (TME) has been reported to play a critical role in resistance. We previously established a syngeneic mEGFR-mutant lung cancer model harboring Del19 (mDEL), and also established an Osimertinib-resistant (OR) tumor by treating Trp53/Rb1$^{-/-}$ mDEL tumor with Osimertinib for 6 months in vivo. Unexpectedly, OR tumor did not show SCLC transformation, but showed MET signaling dependent resistance, as demonstrated by the combination of Osimertinib with MET inhibitor Capmatinib overcame the resistance. Increased MET phosphorylation was detected in this OR tumor, but no increase in MET gene copy number or total MET protein expression was observed. In addition, OR cells were found to lose their resistance by long-term in vitro culture, although Osimertinib resistance was maintained in vivo. Interestingly, Osimertinib-sensitive mDEL tumors also showed resistance to Osimertinib when transplanted adjacent to OR tumors, suggesting that alteration of the TME is the cause of resistance.

[Disclosure of COI by Responsible researcher] Lead presenter is not the Responsible researcher.
[Lead presenter: Masato Fujitani] We have no financial relationships to disclose.
[Principal Researcher: Ryohei Katayama] ■Research funds from company: 中外製薬、TOPPAN、UBE、BML ■Patent fee: 栄研科学

### [E-3068] I-E13-4
### Molecular targeted therapy (3)

2025 / 9 / 27 (Sat) 14:05-15:20 Room 14/(ANA Crowne Plaza Hotel Kanazawa 3F Zuiun 1) Room 14

[Chairperson]Hidenori Kage (Dept. Respir. Med., Univ. Tokyo)

---

### E-3068
### YAP-mediated regulation of HER3-driven adaptive resistance to RET inhibitors in RET-altered cancer

[Speaker]Tadaaki Yamada (Department of Pulmonary Medicine, Kyoto Prefectural University of Medicine) [Co-author]Yuki Katayama (Department of Pulmonary Medicine, Kyoto Prefectural University of Medicine)、Seiji Yano (Kanazawa University Graduate School of Medical Sciences)、Mano Horinaka (Department of Drug Discovery Medicine, Kyoto Prefectural University of Medicine)、Toshiyuki Sakai (Department of Drug Discovery Medicine, Kyoto Prefectural University of Medicine)、Koichi Takayama (Department of Pulmonary Medicine, Kyoto Prefectural University of Medicine)

Background: Rearranged during transfection (RET) gene aberrations are key oncogenic drivers in cancers, targeted by RET tyrosine kinase inhibitors (TKIs). However, resistance mechanisms limit efficacy. This study investigates the emergence of drug-tolerant cells in RET-aberrant cancers treated with RET-TKIs.Methods: Four RET-aberrant cancer cell lines were tested for sensitivity to selpercatinib and pralsetinib. Resistance mechanisms were explored using RNA sequencing, phospho-RTK arrays, ChIP, and luciferase assays. Clinical specimens from RET fusion-positive lung cancer patients were analyzed for YAP expression and its correlation with treatment outcomes.Results: RET-aberrant cancer cells with high YAP expression showed activated YAP-HER3 signaling, promoting resistance to RET-TKIs. Co-treatment with afatinib and YAP/TEAD inhibitors verteporfin and K-975 sensitized these cells to RET-TKIs. High pretreatment YAP expression in clinical specimens was linked to poor outcomes in RET-TKI-treated patients.Conclusion: The YAP-HER3 axis contributes to resistance in RET-aberrant cancer cells treated with RET-TKIs. Targeting YAP and HER3 with RET-TKIs may help overcome resistance.

[Disclosure of COI by Responsible researcher] Lead presenter is the Responsible researcher.
[Lead presenter: Tadaaki Yamada] ■Lecture fee: 日本イーライリリー、中外製薬　■Research expenses: アストラゼネカ、小野薬品

### [E-3069] I-E13-4
### Molecular targeted therapy (3)

2025 / 9 / 27 (Sat) 14:05-15:20 Room 14/(ANA Crowne Plaza Hotel Kanazawa 3F Zuiun 1) Room 14

[Chairperson]Hidenori Kage (Dept. Respir. Med., Univ. Tokyo)

---

### E-3069
### Dynamic Insights from MD Simulations Enable Prediction and Mechanistic Exploration of RET Inhibitor Resistance

[Speaker]Kosuke Maruyama (Div. of Genome Biol., NCC／Grad. Sch. of Med., The Univ. of Tokyo) [Co-author]Mitsugu Araki (Grad. Sch. of Med., Kyoto Univ.)、Biao Ma (RIKEN CCS)、Yasushi Okuno (Grad. Sch. of Med., Kyoto Univ.／RIKEN CCS)、Takashi Kohno (Div. of Genome Biol., NCC)、Takashi Nakaoku (Div. of Genome Biol., NCC)

Targeted therapies play a central role in cancer treatment, but resistance limits long-term survival. To understand and predict resistance, we integrated in vitro (GI50, thermal shift assay) and in silico (molecular dynamics simulation) analyses of 31 resistance mutations across 15 residues in the RET kinase domain against five RET inhibitors (2 selective, 3 multi-kinase). FP-II bridging loop mutations specifically conferred resistance to selective inhibitors, while solvent front mutations caused broad resistance. Resistance levels were accurately predicted by relative binding free energy changes (ddG) calculated using the MutationFEP method, revealing mechanisms based on drug-protein interactions. Resistance driven by steric clashes from mutations proximal to the inhibitors was quantitatively assessed. Notably, FP-II loop mutations conferred resistance to selective inhibitors through a novel mechanism: increased kinase flexibility destabilizing the drug-protein complex, as confirmed by thermal shift assays and RMSF analysis. These findings suggest selective inhibitors can drive the emergence of novel resistance mutations through previously unrecognized, dynamics-based mechanisms.

[Disclosure of COI by Responsible researcher] Lead presenter is not the Responsible researcher.
[Lead presenter: Kosuke Maruyama] We have no financial relationships to disclose.
[Principal Researcher: Takashi Kohno] ■Patent fee: 理研Genesis、Amoy、Foundation Medicine、サーモフィッシャーサイエンティフィック　■Lecture fee: 日本イーライリリー　■Research expenses: 中外製薬、シスメックス、コニカミノルタ、ガーダントヘルス、イーライリリージャパン、ユーロフィンクリニカルジェネシス

### [E-3070] I-E13-4
### Molecular targeted therapy (3)

2025 / 9 / 27 (Sat) 14:05-15:20 Room 14/(ANA Crowne Plaza Hotel Kanazawa 3F Zuiun 1) Room 14

[Chairperson]Hidenori Kage (Dept. Respir. Med., Univ. Tokyo)

---

### E-3070
### Exploration of drug tolerance mechanisms in ALK-rearranged lung cancer using ***in vivo*** CRISPR screening

[Speaker]Marie Kawahara (Div. Exp. Chemother., Cancer Chemother. Ctr., JFCR／Dept. CBMS. Grad. Sch. Front. Sci., The Univ. of Tokyo) [Co-author]Mai Nagasaka (Div. Exp. Chemother., Cancer Chemother. Ctr., JFCR)、Ken Uchibori (Dept. Thoracic Med. Oncology, Cancer Inst. Hosp., JFCR)、Makoto Nishio (Dept. Thoracic Med. Oncology, Cancer Inst. Hosp., JFCR)、Ryohei Katayama (Div. Exp. Chemother., Cancer Chemother. Ctr., JFCR／Dept. CBMS. Grad. Sch. Front. Sci., The Univ. of Tokyo)

ALK-rearranged lung cancer, accounting for 3-5% of non-small cell lung cancer, initially responds to ALK tyrosine kinase inhibitors (ALK-TKIs), but resistance inevitably emerges. Drug-tolerant persister (DTP) cells, which exhibit lower drug sensitivity and evade apoptosis, can arise during treatment and eventually lead to emergence of resistant cells. To identify regulators of DTP cells in the context of the tumor microenvironment, we performed ***in vivo*** CRISPR activation screening. Patient-derived ALK-rearranged lung cancer cells expressing dCas9 fused to VP64 transcriptional activation domain and a genome-wide sgRNA library were transplanted into mice, followed by ALK-TKI treatment. Analysis of sgRNA abundance in residual tumors revealed some of the enriched genes encode tumor microenvironment-dependent factors, distinct from those identified through ***in vitro*** screening. Notably, ***KIT***, previously reported as crizotinib resistance mechanisms in patients, was identified only through ***in vivo*** screening, suggesting the requirement of its ligand, stem cell factor. Thus, ***in vivo*** CRISPR screening enables to identify factors contributing to DTP cell´s survival in tumor microenvironment.

[Disclosure of COI by Responsible researcher] Lead presenter is not the Responsible researcher.
[Lead presenter: Marie Kawahara] We have no financial relationships to disclose.
[Principal Researcher: Ryohei Katayama] ■Research funds from company: 中外製薬、TOPPAN、UBE、BML　■Patent fee: 栄研化学

▲TOP

### [E-3071] I-E13-4
### Molecular targeted therapy (3)

2025 / 9 / 27 (Sat) 14:05-15:20 Room 14/(ANA Crowne Plaza Hotel Kanazawa 3F Zuiun 1) Room 14

[Chairperson]Hidenori Kage (Dept. Respir. Med., Univ. Tokyo)

---

### E-3071
### Patient-derived organoid-aided development of invasive cancer cells and angiogenesis dual targeting therapeutic antibody

[Speaker]Kelvin K. Tsai (Dept. of Medical Sciences, College of Medicine, Taipei Medical University／TMU Research Center of Cancer Translational Medicine, Taipei Medical University)

A proteomic screening of the invadosomes of stem-like cancer cells in gastric cancer (GC) patient-derived organoids (PDOs) led to the identification of an invadosome membrane glycoprotein (imGP), which is also expressed on the podosome membrane of endothelial cells. Molecular, biochemical, and imaging analyses revealed that imGP+ cancer cells are mesenchymal-like, invasive, extravasating, and pro-metastatic. Mechanistically, imGP promotes invadosome formation by initiating invadosome-localized kinase cascades. We developed a first-in-class imGP-targeted functional inhibiting humanized antibody, efficiently inhibiting cancer cell invasion and neovascularization. Systemic administrations of the antibody abolished the metastasis and spreading of GC and non-small cell lung cancer. This antibody also markedly inhibited tumor growth and synergized with anti-PD1 therapy in orthotopic, patient-derived xenografts, and transgenic tumor models. Our findings illuminate a novel molecular underpinning of cancer invasiveness and angiogenesis, the targeting of which holds great promise for developing a breakthrough strategy to treat advanced-stage and aggressive human cancers.

[Disclosure of COI by Responsible researcher] Lead presenter is the Responsible researcher.
[Lead presenter: Kelvin K. Tsai] We have no financial relationships to disclose.

### [E-3072] I-E13-4
### Molecular targeted therapy (3)

2025 / 9 / 27 (Sat) 14:05-15:20 Room 14/(ANA Crowne Plaza Hotel Kanazawa 3F Zuiun 1) Room 14

[Chairperson]Hidenori Kage (Dept. Respir. Med., Univ. Tokyo)

---

### E-3072
### A novel heavy-chain antibody-drug conjugate targeting CEACAM6 for cancer therapy

[Speaker]Ming H. Wu (Taipei Medical University, Taiwan) [Co-author]Yao T. Tsai (Taipei Medical University, Taiwan)、Chee V. Yap (Taipei Medical University, Taiwan)、Chang Y. Liu (Taipei Medical University, Taiwan)

Current antibody-drug conjugates (ADCs) are often too specific to certain cancer subtypes, yet insufficiently selective for cancer-specific antigens. To address the challenges, we developed a heavy-chain antibody (HCAb) targeting cancer glycosylated CEACAM6 from a llama immune library. Compared to conventional antibodies, the HCAb exhibits superior binding affinity, enhanced thermal stability, and improved tissue penetration and epitope recognition. We engineered an HCAb-drug conjugate (HCAb-ADC) carrying tubulin or topoisomerase 1 inhibitors. This HCAb-ADC selectively killed CEACAM6-expressing cells of colorectal, lung, pancreatic, and bile duct carcinomas and demonstrated greater anti-tumor efficacy than currently approved ADCs. In addition, HCAb-ADC robustly inhibited the growth of colorectal, lung and pancreatic tumors in CDX and PDX models, even at a low dose (0.04 mg/kg) and outperformed existing therapeutic regimens. Importantly, it showed minimal toxicity toward normal cells. In summary, the potent anti-tumor activity across multiple epithelial cancer types highlight the clinical potential of HCAb-ADC as a next-generation therapeutic for epithelial-origin cancers.

[Disclosure of COI by Responsible researcher] Lead presenter is the Responsible researcher.
[Lead presenter: Ming H. Wu] ■Profit of stock: Ji Yan Biomedical Co.　■Research expenses: Ji Yan Biomedical Co.

### [J-3061] I-J18
### Advances in Cell & Gene Therapy Technologies

2025 / 9 / 27 (Sat) 14:05-15:20 Room 15/(ANA Crowne Plaza Hotel Kanazawa 3F Zuiun 2) Room 15

[Chairperson]Fumitaka Takeshita (Dept. Transl. Oncol., Natl. Cancer Ctr. Res. Inst.)

---

### J-3061
### A transcriptomic model accurately predicting stem cell transplantation survival in pediatric acute myeloid leukemia

[Speaker]Hua You (Children's Hospital of Chongqing Medical University) [Co-author]Yance Feng (Children's Hospital of Chongqing Medical University)

Hematopoietic stem cell transplantation (HSCT) is a critical treatment for pediatric acute myeloid leukemia (pAML), particularly for high-risk patients. However, the heterogeneity of pAML leads to variable responses to HSCT, rendering some patients deriving limited benefit from this costly therapy. To address this clinical challenge, we developed a 64-gene transcriptomic model, HSCT-64, based on RNA-seq data from bone marrow samples collected at diagnosis from 249 pAML patients who received HSCT and 1,398 who did not. The HSCT-64 model can predict survival at diagnosis, without the need for minimal residual disease (MRD) testing. Performance evaluation using internal repeatedly split test data shows a median AUC of 0.84 for predicting 5-year overall survival, marking a significant improvement over several existing models. The generalization performance of HSCT-64 is successfully validated with external test data. Furthermore, functional enrichment analysis of the 64 prognostic genes reveals a notable association between HSCT survival and lipid metabolism. Whether targeting lipid metabolism-related genes can enhance the treatment efficacy of HSCT is under intensive investigation.

[Disclosure of COI by Responsible researcher] Lead presenter is the Responsible researcher.
[Lead presenter: Hua You] We have no financial relationships to disclose.

### [J-3062] I-J18
### Advances in Cell & Gene Therapy Technologies

2025 / 9 / 27 (Sat) 14:05-15:20 Room 15/(ANA Crowne Plaza Hotel Kanazawa 3F Zuiun 2) Room 15

[Chairperson]Fumitaka Takeshita (Dept. Transl. Oncol., Natl. Cancer Ctr. Res. Inst.)

### J-3062
### Exploring potential benefits of exploiting TCR machinery to CAR-T cells

[Speaker]Meiou Liu (Dept. of Personalized Cancer Immunotherapy, Grad. Sch. Med., Mie Univ.) [Co-author]Yasushi Akahori (Dept. of Personalized Cancer Immunotherapy, Grad. Sch. Med., Mie Univ.)、Yoshihiro Miyahara (Dept. of Personalized Cancer Immunotherapy, Grad. Sch. Med., Mie Univ.)

The lack of suitable target antigens is one of the primary limitation of CAR-T cell therapy for solid tumors. Targeting intracellular antigens through peptide MHC complex can be a promising strategy to overcome this challenge in CAR-T cell therapy. In the previous study, we developed the peptide/MHC-specific CAR-T cells that enable CAR-T cells to recognize intracellular tumor-specific antigens. In addition, we designed a novel form of CAR (Hybrid-TCR) by fused TCR alpha and beta constant regions with VL and VH domains of the scFv antibody. The results revealed that Hybrid-TCR-T cells significantly improved both in vivo and in vitro functionalities compared to the CAR-T cells. However, the mechanisms behind this improvement remain unclear. Since Hybrid-TCR shares the same target specificity and recognition domain as CAR-T cells, it offers an ideal model to explore the differences and potential benefits of employing TCR machinery to CAR-T cells. In this study, through using CAR and Hybrid-TCR targeting the same pMHC as a model, we tried to explore the potential functional advantages of Hybrid-TCR over CAR. Our results might lead to development of more effective cell therapies.

[Disclosure of COI by Responsible researcher] Lead presenter is not the Responsible researcher.
[Lead presenter: Meiou Liu] ■Endowed chair: ティーセルヌーヴォー株式会社
[Principal Researcher: Yoshihiro Miyahara] ■Research funds from company: 科学研究費　■Research expenses: ティーセルヌーヴォー株式会社、旭化成株式会社、コージンバイオ株式会社　■Endowed chair: ティーセルヌーヴォー株式会社

### [J-3063] I-J18
### Advances in Cell & Gene Therapy Technologies

2025 / 9 / 27 (Sat) 14:05-15:20 Room 15/(ANA Crowne Plaza Hotel Kanazawa 3F Zuiun 2) Room 15

[Chairperson]Fumitaka Takeshita (Dept. Transl. Oncol., Natl. Cancer Ctr. Res. Inst.)

---

### J-3063
### Disruption of the IGF2BP1–EWS::FLI1 Loop via ASO Therapy with Machine Learning-Optimized LNP Delivery in Ewing Sarcoma

[Speaker]Asuka Kawachi (NCCRI Div. of Cancer RNA Research) [Co-author]Masahiko Ajiro (NCCRI Div. of Cancer RNA Research)、Shigehiro Yagishita (NCCRI Div. of Molecular Pharmacology)、Nanami Yamano (NCCRI Div. of Cancer RNA Research)、Tatsunori Shimoi (NCCH Dept. of Med. Oncol.)、Mina Yoshida (NCCRI Div. of Cancer RNA Research)、Kazuki Nishimura (NCCRI Div. of Cancer RNA Research)、Ryoichi Maenosono (NCCRI Div. of Cancer RNA Research)、Hirotaka Matsui (NCCRI Div. of Cancer RNA Research／NCCH Dept. of Laboratory Medicine)、Junko Mukohyama (NCCRI Div. of Cancer RNA Research／IMSUT Dept. of Surgery)、Eisuke Kobayashi (NCCH Musculoskeletal Oncology)、Akihiko Yoshida (NCCH Dept. of Diagnostic Pathology)、Mitsuko Sugano (Toshiba Corporation)、Akinobu Hamada (NCCRI Div. of Molecular Pharmacology)、Kan Yonemori (NCCH Dept. of Med. Oncol.)、Akihide Yoshimi (NCCRI Div. of Cancer RNA Research)

**Background:** Ewing sarcoma is defined by the EWS::FLI1 fusion, a transcription factor that reprograms cellular identity. We aimed to identify post-transcriptional regulators stabilizing EWS::FLI1 mRNA and develop RNA-targeted therapeutic strategies. **Results:** A CRISPR–Cas9 screen identified IGF2BP1, an m6A reader protein, as essential for maintaining EWS::FLI1 transcript stability. Knockout of IGF2BP1 reduced EWS::FLI1 expression and triggered tumor cell death. In turn, chromatin profiling showed EWS::FLI1 binding to an IGF2BP1 enhancer, revealing a positive feedforward loop. We developed an antisense oligonucleotide (ASO) that disrupts this interaction, resulting in reduced EWS::FLI1 levels and apoptosis in Ewing sarcoma cells. To enhance systemic delivery, we applied machine learning to optimize lipid nanoparticle (LNP) formulations and successfully identified a tumor–targeting LNP composition. Intravenous administration of ASO-LNP complexes achieved potent anti–tumor effects in vivo. **Conclusion:** Our findings highlight IGF2BP1 as a therapeutic vulnerability and support RNA–based ASO therapy with optimized LNP delivery as a next–generation strategy for fusion-driven solid tumors.

[Disclosure of COI by Responsible researcher] Lead presenter is not the Responsible researcher.
[Lead presenter: Asuka Kawachi] We have no financial relationships to disclose.
[Principal Researcher: Akihide Yoshimi] ■Research funds from company: 第一三共株式会社、Eisai株式会社、中外製薬株式会社

### [J-3064] I-J18
### Advances in Cell & Gene Therapy Technologies

2025 / 9 / 27 (Sat) 14:05-15:20 Room 15/(ANA Crowne Plaza Hotel Kanazawa 3F Zuiun 2) Room 15

[Chairperson]Fumitaka Takeshita (Dept. Transl. Oncol., Natl. Cancer Ctr. Res. Inst.)

---

### J-3064
### Mesenchymal stem cell (MSC)-based virotherapy for peritoneal metastasis in gastrointestinal cancers

[Speaker]Yu Mikane (Dept. Gastroenterological Surg., Okayama Univ. Grad. Sch. Med. Dent. Pharm.) [Co-author]Nobuhiko Kanaya (Dept. Gastroenterological Surg., Okayama Univ. Grad. Sch. Med. Dent. Pharm.)、Shinji Kuroda (Dept. Gastroenterological Surg., Okayama Univ. Grad. Sch. Med. Dent. Pharm.)、Hitoshi Minagi (Dept. Gastroenterological Surg., Okayama Univ. Grad. Sch. Med. Dent. Pharm.)、Makoto Matsumoto (Dept. Gastroenterological Surg., Okayama Univ. Grad. Sch. Med. Dent. Pharm.)、Tetsuya Katayama (Dept. Gastroenterological Surg., Okayama Univ. Grad. Sch. Med. Dent. Pharm.)、Daisuke Kadowaki (Dept. Gastroenterological Surg., Okayama Univ. Grad. Sch. Med. Dent. Pharm.)、Shunsuke Hanzawa (Dept. Gastroenterological Surg., Okayama Univ. Grad. Sch. Med. Dent. Pharm.)、Atsuki Taniguchi (Dept. Gastroenterological Surg., Okayama Univ. Grad. Sch. Med. Dent. Pharm.)、Yoshihiko Kakiuchi (Dept. Gastroenterological Surg., Okayama Univ. Grad. Sch. Med. Dent. Pharm.)、Masashi Hashimoto (Dept. Gastroenterological Surg., Okayama Univ. Grad. Sch. Med. Dent. Pharm.)、Satoru Kikuchi (Dept. Gastroenterological Surg., Okayama Univ. Grad. Sch. Med. Dent. Pharm.)、Shunsuke Kagawa (Dept. Gastroenterological Surg., Okayama Univ. Grad. Sch. Med. Dent. Pharm.)、Hiroshi Tazawa (Dept. Gastroenterological Surg., Okayama Univ. Grad. Sch. Med. Dent. Pharm.)、Yasuo Urata (Oncolys BioPharma, Inc.)、Toshiyoshi Fujiwara (Dept. Gastroenterological Surg., Okayama Univ. Grad. Sch. Med. Dent. Pharm.)

Peritoneal metastasis has a poor prognosis due to the limited efficacy of systemic therapies. Although intraperitoneal chemotherapy has been explored, its benefits remain controversial.We previously developed OBP-702, a telomerase-specific oncolytic adenovirus armed with the p53 gene, which demonstrated potent antitumor effects and induced abscopal responses when combined with immune checkpoint inhibitors. However, intraperitoneal administration of OBP-702 alone showed limited efficacy.Recently, our preclinical studies identified that mesenchymal stem cells (MSCs) loaded with oncolytic herpes simplex virus home to brain metastasis from melanoma and non-small cell lung cancer, resulting in significant therapeutic efficacy (Kanaya et al, Sci Transl Med, 2023).We created MSCs loaded with OBP-702 (MSC-OBP-702) as an effective therapeutic agent targeting peritoneal metastasis. Intraperitoneal administration of MSC-OBP-702 effectively suppressed tumor growth, and significantly prolonged overall survival via the activation of T cells and M1 macrophages as well as oncolytic cell death. Our results highlight MSC-OBP-702 as a novel and effective approach for treating peritoneal metastasis.

[Disclosure of COI by Responsible researcher] Lead presenter is not the Responsible researcher.
[Lead presenter: Yu Mikane] We have no financial relationships to disclose.
[Principal Researcher: Nobuhiko Kanaya] We have no financial relationships to disclose.

▲TOP

### [J-3065] I-J18
### Advances in Cell & Gene Therapy Technologies

2025 / 9 / 27 (Sat) 14:05-15:20 Room 15/(ANA Crowne Plaza Hotel Kanazawa 3F Zuiun 2) Room 15

[Chairperson]Fumitaka Takeshita (Dept. Transl. Oncol., Natl. Cancer Ctr. Res. Inst.)

---

### J-3065
### Development of innovative Triple-regulated oncolytic virus of effective and safer therapy of peritoneal carcinomatosis.

[Speaker]Hiroaki Ijuin (Gene Ther. & Regen. Med., Kagoshima Univ. Grad. Sch. Med.) [Co-author]Yuya Nishikawaji (Gene Ther. & Regen. Med., Kagoshima Univ. Grad. Sch. Med.)、Satoshi Nagano (Orthopaedic Surg., Kagoshima Univ. Grad. Sch. Med. ／Ctr. Innov. Ther. Res. App., Kagoshima Univ. Grad. Sch. Med.／South Kyushu Ctr. Innov. Med. Res. App., Kagoshima Univ.／Fac. Med. Sch. Health Sci., Kagoshima Univ.／Cog. & Mol. Res. Inst. of Brain Diseases, Kurume Univ.)、Junichi Kamizono (Gene Ther. & Regen. Med., Kagoshima Univ. Grad. Sch. Med.／Orthopaedic Surg., Kagoshima Univ. Grad. Sch. Med. ／Cog. & Mol. Res. Inst. of Brain Diseases, Kurume Univ.)、Yoshiharu Horikawa (Gene Ther. & Regen. Med., Kagoshima Univ. Grad. Sch. Med.／Orthopaedic Surg., Kagoshima Univ. Grad. Sch. Med.)、Kaoru Mitsui (Gene Ther. & Regen. Med., Kagoshima Univ. Grad. Sch. Med.／Ctr. Innov. Ther. Res. App., Kagoshima Univ. Grad. Sch. Med.／South Kyushu Ctr. Innov. Med. Res. App., Kagoshima Univ.)、Eriko Matsuda (Gene Ther. & Regen. Med., Kagoshima Univ. Grad. Sch. Med.)、Kenichiro Kosai (Gene Ther. & Regen. Med., Kagoshima Univ. Grad. Sch. Med.／Ctr. Innov. Ther. Res. App., Kagoshima Univ. Grad. Sch. Med.／South Kyushu Ctr. Innov. Med. Res. App., Kagoshima Univ.／Ctr. Clin. Transl. Res., Kagoshima Univ. Hosp.／Cog. & Mol. Res. Inst. of Brain Diseases, Kurume Univ.)

An ideal conditionally replicating adenovirus (CRA) that selectively replicates in and kills cancer cells has not been developed for gastric cancer-derived peritoneal carcinomatosis. Using our platform technology of CRA regulated and treating tumors with multiple factors (m-CRA), we generated new survivin-responsive m-CRAs, Surv.m-CRA/CEAp, consisting of E1A downstream of the survivin promoter, and the mutated E1B gene downstream of the carcinoembryonic antigen promoter. While both Surv.m-CRA/CEAp and conventional Surv.m-CRA/CMVp exhibited potent cytotoxic effects on gastric cancer in vitro, Surv.m-CRA/CEAp significantly reduced cytotoxicity to normal cells compared to Surv.m-CRA/CMVp. In addition, all mice receiving Surv.m-CRA/CEAp or Surv.m-CRA/CMVp-infectedgastric cancer increased their body weight and survived 120 days. Thus, the triple-regulated Surv.m-CRA-CEAp enhances cancer specificity (i.e., safety) without reducing the potent therapeutic effect for gastric cancer-derived peritoneal carcinomatosis. The altered E1B promoter strategy of CRA facilitates the development of novel CRAs for the effective and safe treatment of a variety of intractable cancers.

[Disclosure of COI by Responsible researcher] Lead presenter is not the Responsible researcher.
[Lead presenter: Hiroaki Ijuin] We have no financial relationships to disclose.
[Principal Researcher: Kenichiro Kosai] ■Research funds from company: サーブ・バイオファーマ株式会社　■Advisor of the company and/or profit-making organization: サーブ・バイオファーマ株式会社　■Profit of stock: サーブ・バイオファーマ株式会社　■Research expenses: サーブ・バイオファーマ株式会社

### [J-3066] I-J18
### Advances in Cell & Gene Therapy Technologies

2025 / 9 / 27 (Sat) 14:05-15:20 Room 15/(ANA Crowne Plaza Hotel Kanazawa 3F Zuiun 2) Room 15

[Chairperson]Fumitaka Takeshita (Dept. Transl. Oncol., Natl. Cancer Ctr. Res. Inst.)

---

### J-3066
### Development of a sensor system to selectively induce cell death in chromosome 19 microRNA cluster-positive tumors

[Speaker]Masato Sakamoto (Dept. Pharmacotherapeutics, Keio Univ. Fac. Pharm.) [Co-author]Juntaro Matsuzaki (Div. Gen. Nano., Res. Ctr. Drug Discov., Keio Univ. Pharm.)、Zixiang Ye (Dept. Pharmacotherapeutics, Keio Univ. Fac. Pharm.)、Toshihide Muramatsu (Dept. Pharmacotherapeutics, Keio Univ. Fac. Pharm.)、Yoshimasa Saito (Dept. Pharmacotherapeutics, Keio Univ. Fac. Pharm.)

The chromosome 19 microRNA cluster (C19MC) is a primate-specific region that contains 46 miRNA genes, all of which are located within an area of less than 100 kbp. It is specifically expressed in some malignant tumors, including cholangiocarcinoma (CCA). We considered C19MC to be an ideal target for cancer therapy and created a sensor system that selectively induces cell death in C19MC-expressing tumors. MiRNAs usually negatively regulate the expression of target genes. However, this sensor amplifies a slight increase in miRNA expression through positive feedback, and when a specific threshold is exceeded, the expression of the cytotoxin increases dramatically. This allows the expression of cytotoxins in positive correlation with the miRNA expression levels. To demonstrate the C19MC expression-dependent function of this sensor, we introduced an inducible CRISPR-dCas9 activation system (iCRISPRa) that enables on/off control of C19MC expression in CCA cells. The expected functionality of iCRISPRa was confirmed by miRNA-seq. Finally, we successfully validated the sensor system's ability to induce cell death in CCA cells with the iCRISPRa system in response to C19MC expression levels.

[Disclosure of COI by Responsible researcher] Lead presenter is not the Responsible researcher.
[Lead presenter: Masato Sakamoto] We have no financial relationships to disclose.
[Principal Researcher: Juntaro Matsuzaki] ■Research funds from company: ISM株式会社、レグリア株式会社、フェムトディプロイメンツ株式会社

▲TOP

### [J-3073] I-J9-4
### Metastasis models & EMT

2025 / 9 / 27 (Sat) 14:05-15:20 Room 16/(11F Visage) Room 16

[Chairperson]Satoshi Takagi (Div. Exp. Chemother., Cancer Chemother. Ctr., JFCR)

---

### J-3073
### Colorectal cancer co-opts the wound healing program to form disseminated tumor cells during metastasis

[Speaker]Ryoji Yao (Dept. Cell Biol., Cancer Institute, JFCR)

During metastasis, tumor cells detach from the primary lesion and disseminate to distant tissues, where they stay dormant before initiating metastatic outgrowth. However, little is known about the disseminated tumor cells (DTCs) owing to difficulties in their clinical detection. We have developed a mouse model of metastasis by orthotopic transplantation of PDOs from CRC patients, and established organoids from DTCs in distant organs. We found they had lost the expression of cancer stem cell markers, and instead expressed wound-inducible genes. Mechanistically, they are induced by the combination of epigenomic modification and YAP signaling. In the clinical setting, tumor cells expressing wound-inducible genes are found exclusively in tumors and are localized at the leading edge of primary tumors. These observations demonstrate that CRC co-opts the wound-healing program to produce DTCs during metastasis. Our findings provide new insights into the plasticity of cancer cells during metastasis and are expected to lay the groundwork for developing effective prevention and treatment strategies for metastatic CRC.

[Disclosure of COI by Responsible researcher] Lead presenter is the Responsible researcher.
[Lead presenter: Ryoji Yao] We have no financial relationships to disclose.

### [J-3074] I-J9-4
### Metastasis models & EMT

2025 / 9 / 27 (Sat) 14:05-15:20 Room 16/(11F Visage) Room 16

[Chairperson]Satoshi Takagi (Div. Exp. Chemother., Cancer Chemother. Ctr., JFCR)

---

### J-3074
### Rho signaling is a critical driver of cancer stemness in metastatic colorectal cancer

[Speaker]Teruaki Fujishita (Div. Pathophysiol., Aichi Cancer Ctr. Res. Inst.) [Co-author]Emi Mishiro (Inst. Trans.Bio-Mol., Nagoya Univ.)、Yanqing Niu (Div. Pathophysiol., Aichi Cancer Ctr. Res. Inst.／Div. Cancer Physiol., Nagoya Univ. Grad. Sch. Med.)、Rie Kajino (Div. Pathophysiol., Aichi Cancer Ctr. Res. Inst.)、Yasushi Kojima (Div. Pathophysiol., Aichi Cancer Ctr. Res. Inst.)、Makoto M. Taketo (Pers. Canc. Ther., MIC, Kyoto Univ. Grad. Sch. Med.)、Masahiro Aoki (Div. Pathophysiol., Aichi Cancer Ctr. Res. Inst.／Div. Cancer Physiol., Nagoya Univ. Grad. Sch. Med.)

Cancer metastasis is the leading cause of cancer-related deaths, highlighting the urgent need to understand its molecular mechanisms and identify novel therapeutic targets. Given that colorectal cancer (CRC) is one of the most commonly diagnosed cancers, we previously developed an autochthonous mouse model of metastatic CRC by introducing sporadic mutations in four key CRC-associated genes: ***Ctnnb1***, ***Kras***, ***Trp53*** and ***Smad4*** (CKPS mice). Analysis of this model revealed that cancer stem cell (CSC) traits are crucial for CRC metastasis. To explore the molecular mechanisms underlying CSC-associated metastasis, we performed proteomic analysis using CRC cell lines derived from CKPS mice (CKPS cells) cultured under 2D and 3D conditions. We found that RhoC was highly expressed in CSC-like CKPS cells and was linked to enhanced liver metastatic potential. Further analysis revealed that other Rho family members and certain protein kinases also significantly contributed to maintaining CSC traits in CKPS cells. These findings highlight Rho signaling as a critical regulator of cancer stemness driving CRC metastasis, suggesting its potential as a promising therapeutic target against metastatic CRC.

[Disclosure of COI by Responsible researcher] Lead presenter is the Responsible researcher.
[Lead presenter: Teruaki Fujishita] ■Research funds from company: 科研費・基盤研究C

▲TOP

### [J-3075] I-J9-4
### Metastasis models & EMT

2025 / 9 / 27 (Sat) 14:05-15:20 Room 16/(11F Visage) Room 16

[Chairperson]Satoshi Takagi (Div. Exp. Chemother., Cancer Chemother. Ctr., JFCR)

---

### J-3075
### Epithelial and Mesenchymal Malignant Cells in Pleural Mesothelioma and Their Role in Intralesional Heterogeneity

[Speaker]Kuniyo Sueyoshi (Dept. Prev. Med., Grad. Sch. Med., Univ. of Tokyo／Dept. Thorac. Surg., Inst. Sci. Tokyo) [Co-author]Miwako Kakiuchi (Dept. Prev. Med., Grad. Sch. Med., Univ. of Tokyo)、Daisuke Komura (Dept. Prev. Med., Grad. Sch. Med., Univ. of Tokyo)、Hiroto Katoh (Dev. Patholo., Exp. Oncol. Res. Clin. Trial Ctr., NCC)、Hironori Ishibashi (Dept. Thorac. Surg., Inst. Sci. Tokyo)、Kenichi Okubo (Dept. Thorac. Surg., Inst. Sci. Tokyo)、Shumpei Ishikawa (Dept. Prev. Med., Grad. Sch. Med., Univ. of Tokyo／Dev. Patholo., Exp. Oncol. Res. Clin. Trial Ctr., NCC)

Pleural mesothelioma (PM) is an aggressive tumor featured by marked intra- and intertumor heterogeneity, spanning epithelioid to sarcomatoid subtype, which is indicative of EMT. Classical analytical approaches have struggled to identify transitional tumor cells due to their similarity with reactive stromal components. To address this, we performed whole genome and single-cell RNA sequencing (scRNA-seq) on tumors from 11 patients, diagnosed as non-sarcomatoid PMs. scRNA-seq revealed a distinct fibroblast-like malignant subpopulation (PM-F) with altered copy number variations (CNVs) patterns and elevated vimentin expression, suggesting an intratumor transition toward the mesenchymal phenotype. Still, PM-F exhibited uncommitted transcriptional profiles, lacking clear correspondence to either subtype. In silico analysis revealed marked stromal interaction with PM-F, whose ECM remodeling-enriched gene signature correlates with poor prognosis in the TCGA cohort. Together, we propose that PM-F represents a transitional malignant population contributing to fibrous intratumor heterogeneity. Further studies are needed to clarify its role in therapy resistance and tumor evolution.

[Disclosure of COI by Responsible researcher] Lead presenter is not the Responsible researcher.
[Lead presenter: Kuniyo Sueyoshi] ■Research funds from company: 日本学術振興会
[Principal Researcher: Shumpei Ishikawa] ■Research funds from company: 日本学術振興会　■Advisor of the company and/or profit-making organization: 新日本科学株式会社　■Research expenses: Chordia therapeutics株式会社, 大鵬薬品工業株式会社, ロート製薬株式会社, アステラス製薬株式会社

### [J-3076] I-J9-4
### Metastasis models & EMT

2025 / 9 / 27 (Sat) 14:05-15:20 Room 16/(11F Visage) Room 16

[Chairperson]Satoshi Takagi (Div. Exp. Chemother., Cancer Chemother. Ctr., JFCR)

---

### J-3076
### Impact of Increased Interstitial Pressure on Tumor Progression in Malignant Pleural Mesothelioma

[Speaker]Aosa Sasada (Dept. of Pulmonary Med., Kyoto Prefectural University of Medicine) [Co-author]Shinsaku Tokuda (Dept. of Pulmonary Med., Kyoto Prefectural University of Medicine)、Mototaka Fukui (Dept. of Pulmonary Med., Kyoto Prefectural University of Medicine)、Keisuke Onoi (Dept. of Pulmonary Med., Kyoto Prefectural University of Medicine)、Yoshitaka Sekido (Div. of Cancer Biol., Aichi Cancer Center Research Institute)、Tadaaki Yamada (Dept. of Pulmonary Med., Kyoto Prefectural University of Medicine)、Koichi Takayama (Dept. of Pulmonary Med., Kyoto Prefectural University of Medicine)

Background: Malignant pleural mesothelioma (MPM) is a refractory tumor with limited response to chemotherapy. Increased interstitial pressure has been reported in most cancers and is considered to promote tumor progression. A similar association is expected in MPM, but the mechanism remains unclear. Methods: We used several human mesothelioma cell lines and simulated increased interstitial pressure by applying hydrostatic pressure to the basal side of cultured cell sheets on Transwell filters. The effects on tumor behavior were then evaluated. Results: Pressure from the basal side caused epithelial stratification and disrupted cell polarity. Both cell motility and proliferation were enhanced. To explore the relationship between motility and stratification, we inhibited vimentin, actin, and myosin. Myosin inhibition led to partial changes, but stratification remained. In contrast, vimentin knockout completely suppressed stratification. These findings suggest that vimentin plays a key role not only in motility but also in proliferation. Conclusion: Increased interstitial pressure may promote mesothelioma progression, with vimentin potentially playing a critical role.

[Disclosure of COI by Responsible researcher] Lead presenter is not the Responsible researcher.
[Lead presenter: Aosa Sasada] We have no financial relationships to disclose.
[Principal Researcher: Shinsaku Tokuda] We have no financial relationships to disclose.

### [J-3077] I-J9-4
### Metastasis models & EMT

2025 / 9 / 27 (Sat) 14:05-15:20 Room 16/(11F Visage) Room 16

[Chairperson]Satoshi Takagi (Div. Exp. Chemother., Cancer Chemother. Ctr., JFCR)

### J-3077
### Withdrawn

No Abstract

### [J-3078] I-J9-4
### Metastasis models & EMT

2025 / 9 / 27 (Sat) 14:05-15:20 Room 16/(11F Visage) Room 16

[Chairperson]Satoshi Takagi (Div. Exp. Chemother., Cancer Chemother. Ctr., JFCR)

---

### J-3078
### Metastatic mechanism of pancreatic cancer based on the analysis of circulating tumor cells in portal vein blood

[Speaker]Naoko Sekiguchi (Department of Gastroenterological Surgery, Osaka University Graduate School of Medicine) [Co-author]Hidenori Takahashi (Department of Gastroenterological Surgery, Osaka University Graduate School of Medicine)、Kazuki Sasaki (Department of Gastroenterological Surgery, Osaka University Graduate School of Medicine)、Shinichiro Hasegawa (Department of Gastroenterological Surgery, Osaka University Graduate School of Medicine)、Daisaku Yamada (Department of Gastroenterological Surgery, Osaka University Graduate School of Medicine)、Yoshito Tomimaru (Department of Gastroenterological Surgery, Osaka University Graduate School of Medicine)、Hirofumi Akita (Department of Gastroenterological Surgery, Osaka University Graduate School of Medicine)、Takehiro Noda (Department of Gastroenterological Surgery, Osaka University Graduate School of Medicine)、Shogo Kobayashi (Department of Gastroenterological Surgery, Osaka University Graduate School of Medicine)、Yuichiro Doki (Department of Gastroenterological Surgery, Osaka University Graduate School of Medicine)、Hidetoshi Eguchi (Department of Gastroenterological Surgery, Osaka University Graduate School of Medicine)、Hirofumi Yamamoto (Department of Gastroenterological Surgery, Osaka University Graduate School of Medicine)

Pancreatic ductal adenocarcinoma cancer (PDC) has a poor prognosis, and about 70% patients develop metastatic recurrence after surgery. Circulating tumor cells (CTCs) in peripheral blood are extremely rare in PDC, but reported to be abundant in portal vein blood (PVB). In this study, we aimed to reveal the genetic characteristics of CTCs in PVB of PDC.We performed intraoperative PVB sampling in 18 PDC patients and measured the expression level of epithelial markers (KRT-19), and investigated its relationship with pathological findings and recurrence rate. Compared to peripheral blood in healthy volunteers, KRT19 level was significantly higher in 10 patients (55%). The KRT19 - high group had higher incidence of venous invasion (p=0.006), lymph node metastasis (p=0.005), and distant metastatic recurrence after surgery (p=0.033). Finally, we compared gene expression profile between CTCs and primary cancer cells in 4 out of 18 patients, in whom single cell analysis for CTCs was performed using C1 System (Fluidlike, Inc). We here present the preliminary results.

[Disclosure of COI by Responsible researcher] Lead presenter is not the Responsible researcher.
[Lead presenter: Naoko Sekiguchi] ■Research funds from company: 住友ゴム工業株式会社
[Principal Researcher: Hirofumi Yamamoto] ■Research funds from company: 住友ゴム工業株式会社

### [OS13-1] OS13
### New horizons of tumor microenvironment research via spatial single-cell analyses

2025 / 9 / 27 (Sat) 14:20-15:50 Room 3/(Hotel Nikko Kanazawa 4F Tsuru A) Room 3

[Chairpersons]Koji Okamoto (Teikyo University, Advanced Comprehensive Research Organization)、Yutaka Suzuki (Life Science Data Research Center, Graduate School of Frontier Sciences, The University of Tokyo)

空間1細胞解析は、組織標本上で1細胞レベルの発現解析を行う革新的技術であるが、解析可能な遺伝子数の増加に伴いその重要性が飛躍的に高まりつつある。加えてたんぱくレベルでの多重抗体解析なども可能になってきており、これらの解析技術と組織病理学との融合による空間マルチオミクス解析も構築されつつある。これらの新たなモダリティは、組織標本上での細胞ネットワークの可視化を通じて、これまで難しかったがん微小環境の包括的理解を可能にし、ひいては様々ながん腫における診断治療に貢献すると期待される。本シンポジウムではがん組織の空間発現解析を精力的に行っている研究者に講演いただき、がん微小環境研究の理解を深めるとともに、当分野の発展に寄与することをめざす。

---

### OS13-1
### Towards Multi-Omics Spatial Analysis to Reveal Cancer Heterogeneity

[Speaker]Yutaka Suzuki (LiSDaC, Grad. Sch. of Fr. Sci., Univ. of Tokyo.)

The interaction between tumor cells and their microenvironment is believed to be a decisive factor for tumor development. Herein, we characterize spatial RNA profiles obtained from 30 lung adenocarcinoma patients at various stages, including non-invasive and later invasive stages. These samples were subjected to the spatial transcriptome sequencing analysis in conjunction with higher resolution analysis using in situ RNA profiling. The detailed inspection on each case and the following computational modeling based on the observed diverse profiles revealed that the drastic changes in phenotypic appearances of tumor cells are frequently triggered by their interaction with immune cells. Rapid progress in the spatial analytic platforms have further allowed us to expand the spatial analysis to the multi-omics layers. The multiple-immunohistochemistry analysis using PhenoCycler is revealing how the observed gene expressions are associated with protein profiles. Very recently, even spatial open chromatin analysis is enabled using Trekker of Curio Bio. I'd like to discuss how the multi-omics data generated from the respective platforms should be integrated.

[Disclosure of COI by Responsible researcher] Lead presenter is the Responsible researcher.
[Lead presenter: Yutaka Suzuki] We have no financial relationships to disclose.

▲TOP

### [OS13-2] OS13
### New horizons of tumor microenvironment research via spatial single-cell analyses

2025 / 9 / 27 (Sat) 14:20-15:50 Room 3/(Hotel Nikko Kanazawa 4F Tsuru A) Room 3

[Chairpersons]Koji Okamoto (Teikyo University, Advanced Comprehensive Research Organization)、Yutaka Suzuki (Life Science Data Research Center, Graduate School of Frontier Sciences, The University of Tokyo)

空間1細胞解析は、組織標本上で1細胞レベルの発現解析を行う革新的技術であるが、解析可能な遺伝子数の増加に伴いその重要性が飛躍的に高まりつつある。加えてたんぱくレベルでの多重抗体解析なども可能になってきており、これらの解析技術と組織病理学との融合による空間マルチオミクス解析も構築されつつある。これらの新たなモダリティは、組織標本上での細胞ネットワークの可視化を通じて、これまで難しかったがん微小環境の包括的理解を可能にし、ひいては様々ながん腫における診断治療に貢献すると期待される。本シンポジウムではがん組織の空間発現解析を精力的に行っている研究者に講演いただき、がん微小環境研究の理解を深めるとともに、当分野の発展に寄与することをめざす。

---

### OS13-2
### Unraveling the ecosystem of follicular lymphoma at Single-Cell and Spatial Resolution

[Speaker]Mamiko Sakata-Yanagimoto (Dept. of Hematol. Institute of Medicine, Univ. Tsukuba)

Follicular lymphoma (FL) is an indolent lymphoma. When tumor burden is low, a "watch and wait" strategy is often adopted, during which spontaneous regression may occur, This phenomenon suggests the importance of continuous stimuli from the tumor microenvironment (TME). To unravel the complex FL TME, we employed multi-omics approaches, integrating single-cell transcriptomics, spatial transcriptomics, and multiplexed tissue imaging.In particular, we focus on the heterogeneity of T cells within the neoplastic follicles, which may mediate immune pressure, affecting tumor dynamics. We identified transcriptionally and spatially distinct novel T-cell subsets expanded in FL. These subsets exhibited enhanced anti-tumorigenic properties, and formed unique immune niches, suggesting the existence of discrete self-regulatory immune ecosystems. Moreover, these subsets robustly stratified patient prognoses independently of existing clinical indicators in multiple FL cohorts. Our integrative analysis highlights the complexity of the FL TME and provides new insights into immune regulation and potential therapeutic strategies.

[Disclosure of COI by Responsible researcher] Lead presenter is the Responsible researcher.
[Lead presenter: Mamiko Sakata] We have no financial relationships to disclose.

### [OS13-3] OS13
### New horizons of tumor microenvironment research via spatial single-cell analyses

2025 / 9 / 27 (Sat) 14:20-15:50 Room 3/(Hotel Nikko Kanazawa 4F Tsuru A) Room 3

[Chairpersons]Koji Okamoto (Teikyo University, Advanced Comprehensive Research Organization)、Yutaka Suzuki (Life Science Data Research Center, Graduate School of Frontier Sciences, The University of Tokyo)

空間1細胞解析は、組織標本上で1細胞レベルの発現解析を行う革新的技術であるが、解析可能な遺伝子数の増加に伴いその重要性が飛躍的に高まりつつある。加えてたんぱくレベルでの多重抗体解析なども可能になってきており、これらの解析技術と組織病理学との融合による空間マルチオミクス解析も構築されつつある。これらの新たなモダリティは、組織標本上での細胞ネットワークの可視化を通じて、これまで難しかったがん微小環境の包括的理解を可能にし、ひいては様々ながん腫における診断治療に貢献すると期待される。本シンポジウムではがん組織の空間発現解析を精力的に行っている研究者に講演いただき、がん微小環境研究の理解を深めるとともに、当分野の発展に寄与することをめざす。

---

### OS13-3
### Comprehensive analysis of the cancer microenvironment in MSI-H colorectal cancer

[Speaker]Takanari Tatsumi (Dept. of Surg., Kyushu Univ. Beppu Hosp.／Dept. of Surg., Nara Medical Univ.) [Co-author]Masayuki Sho (Dept. of Surg., Nara Medical Univ.)、Koshi Mimori (Dept. of Surg., Kyushu Univ. Beppu Hosp.)

Background: While immune checkpoint inhibitors (ICIs) are effective in microsatellite instability-high (MSI-H) colorectal cancer (CRC), a subset of patients exhibits treatment resistance, and the underlying mechanisms remain unclear. This study used spatial transcriptomics (ST) to investigate the tumor microenvironment (TME) of MSI-H CRC and identify ICI resistance factors.Methods: We analyzed newly acquired ST (n=12) from MSI-H CRC and performed an integrated analysis with TCGA-COAD and single-cell RNA-seq.Results: MSI-H CRC showed a significant enrichment of CD8-positive T cells (TCD8), which were localized to specific regions within the tumor tissue. The abundance of TCD8 was associated with ICI sensitivity. We identified a gene X-positive tumor epithelial cell subcluster that contributes to the immune evasion of TCD8, leading to ICI resistance. In vitro/vivo studies confirmed that gene X expression is involved in TCD8 evasion and tumor growth, and that a gene X inhibitor showed additive effects with ICI.Conclusion: Gene X likely drives immune regulatory mechanisms in the MSI-H CRC TME, contributing to ICI resistance and representing a potential novel therapeutic target.

[Disclosure of COI by Responsible researcher] Lead presenter is not the Responsible researcher.
[Lead presenter: Takanari Tatsumi] We have no financial relationships to disclose.
[Principal Researcher: Koshi Mimori] We have no financial relationships to disclose.

### [OS13-4] OS13
### New horizons of tumor microenvironment research via spatial single-cell analyses

2025 / 9 / 27 (Sat) 14:20-15:50 Room 3/(Hotel Nikko Kanazawa 4F Tsuru A) Room 3

[Chairpersons]Koji Okamoto (Teikyo University, Advanced Comprehensive Research Organization)、Yutaka Suzuki (Life Science Data Research Center, Graduate School of Frontier Sciences, The University of Tokyo)

空間1細胞解析は、組織標本上で1細胞レベルの発現解析を行う革新的技術であるが、解析可能な遺伝子数の増加に伴いその重要性が飛躍的に高まりつつある。加えてたんぱくレベルでの多重抗体解析なども可能になってきており、これらの解析技術と組織病理学との融合による空間マルチオミクス解析も構築されつつある。これらの新たなモダリティは、組織標本上での細胞ネットワークの可視化を通じて、これまで難しかったがん微小環境の包括的理解を可能にし、ひいては様々ながん腫における診断治療に貢献すると期待される。本シンポジウムではがん組織の空間発現解析を精力的に行っている研究者に講演いただき、がん微小環境研究の理解を深めるとともに、当分野の発展に寄与することをめざす。

---

### OS13-4
### Understanding cellular networks in refractory cancers through spatial omics.

[Speaker]Koji Okamoto (Teikyo Univ., Adv. Comp. Res. Org.)

Malignant behaviors of cancer, such as chemoresistance and metastasis, are closely associated with intratumoral cellular networks. Within the tumor microenvironment, various non-cancerous cells, such as cancer-associated fibroblasts (CAFs) and macrophages, are thought to interact with cancer cells and establish distinct niches that promote their own survival and proliferation. To investigate such intercellular interactions, we are conducting integrated analyses combining single-cell sequencing, spatial transcriptomics, and CODEX multiplexed antibody imaging, focusing on clinical specimens of ovarian cancer and colorectal cancer xenografts. Our findings aimed at elucidating the intercellular networks that contribute to cancer refractoriness will be presented.

[Disclosure of COI by Responsible researcher] Lead presenter is the Responsible researcher.
[Lead presenter: Koji Okamoto] We have no financial relationships to disclose.

▲TOP

### [Public Open Seminar] OPS
### Public Open Seminar

2025 / 9 / 27 (Sat) 15:00-18:00 Room 10/(Art Hall) Room 10

[Chairperson]Seiji Yano (Department of Respiratory Medicine, Faculty of Medicine, Institute of Medical, Pharmaceutical, and Health Sciences, Kanazawa University)

### Public Open Seminar

[Speaker]Seiji Yano (Department of Respiratory Medicine, Kanazawa University.／WPI Nano Life Science Institute (WPI-NanoLSI), Kanazawa University)

No Abstract

▲TOP

### [Public Open Seminar] OPS
### Public Open Seminar

2025 / 9 / 27 (Sat) 15:00-18:00 Room 10/(Art Hall) Room 10

[Chairperson]Seiji Yano (Department of Respiratory Medicine, Faculty of Medicine, Institute of Medical, Pharmaceutical, and Health Sciences, Kanazawa University)

### Public Open Seminar

[Speaker]Noriyuki Inaki ()

No Abstract

### [Public Open Seminar] OPS
### Public Open Seminar

2025 / 9 / 27 (Sat) 15:00-18:00 Room 10/(Art Hall) Room 10

[Chairperson]Seiji Yano (Department of Respiratory Medicine, Faculty of Medicine, Institute of Medical, Pharmaceutical, and Health Sciences, Kanazawa University)

### Public Open Seminar

[Speaker]Masafumi Iguchi ()

No Abstract

### [Public Open Seminar] OPS
### Public Open Seminar

2025 / 9 / 27 (Sat) 15:00-18:00 Room 10/(Art Hall) Room 10

[Chairperson]Seiji Yano (Department of Respiratory Medicine, Faculty of Medicine, Institute of Medical, Pharmaceutical, and Health Sciences, Kanazawa University)

### Public Open Seminar

[Speaker]Hiroaki Taniguchi ()

No Abstract

### [Public Open Seminar] OPS
### Public Open Seminar

2025 / 9 / 27 (Sat) 15:00-18:00 Room 10/(Art Hall) Room 10

[Chairperson]Seiji Yano (Department of Respiratory Medicine, Faculty of Medicine, Institute of Medical, Pharmaceutical, and Health Sciences, Kanazawa University)

---

### Public Open Seminar

[Speaker]Atsushi Hirao (Cancer Res. Inst., Kanazawa Univ.)

No Abstract

### [P-3001] I-P3-3
### Bacteria & Cancer

2025 / 9 / 27 (Sat) 15:30-16:15 Room P/(Motenashi Dome) Room P

---

### P-3001
### The association between tumoral pks+ Escherichia coli expression and oncological outcomes in colorectal cancer patients.

[Speaker]Takahito Kitajima (The Department of Genomic Medicine, Mie University Hospital／The Department of Gastrointestinal and Pediatric Surgery, Mie University) [Co-author]Yoshinaga Okugawa (The Department of Genomic Medicine, Mie University Hospital／The Department of Gastrointestinal and Pediatric Surgery, Mie University)、Ruiya Ma (The Department of Gastrointestinal and Pediatric Surgery, Mie University)、Tadanobu Shimura (The Department of Gastrointestinal and Pediatric Surgery, Mie University)、Yuji Toiyama (The Department of Gastrointestinal and Pediatric Surgery, Mie University)

[Background] Gut bacteria may promote colorectal cancer (CRC) development by producing genotoxins. Escherichia coli harboring the pks genomic island (pks+) produce colibactin. But, the clinical relevance of tumoral pks+ expression in CRC remains unclear. We investigated the association between pks+ expression and oncological outcomes in CRC patients.[Methods] We collected clinicopathological data from 84 CRC patients, and analyzed pks+ mRNA expression in both cancerous and normal tissues. We analyzed the relationships between pks+ expression, clinicopathological characteristics, and oncological outcomes.[Results] Among the 84 CRC patients, tumoral pks+ expression was detected in 56 patients. pks+ expression was significantly associated with distant metastasis ($p=0.04$). pks+ positive patients had lower lymphocyte counts ($p=0.04$) and higher CRP levels ($p=0.04$). Kaplan-Meier curves showed no significant association between pks+ expression and OS or DFS. [Conclusions] While pks+ expression in CRC was associated with distant metastasis and inflammation, it was not associated with poor prognosis. Further larger patients are needed to clarify its clinical impact of pks+ expression in CRC.

[Disclosure of COI by Responsible researcher] Lead presenter is not the Responsible researcher.
[Lead presenter: Takahito Kitajima] We have no financial relationships to disclose.
[Principal Researcher: Yuji Toiyama] We have no financial relationships to disclose.

▲TOP

[P-3002] I-P3-3
Bacteria & Cancer

2025 / 9 / 27 (Sat) 15:30-16:15 Room P/(Motenashi Dome) Room P

### P-3002
### Prognostic significance of Fusobacterium nucleatum abundance differs between right- and left-sided colon cancers.

[Speaker]Toshimitsu Miyasaka (NMS Dept. Gastroenterological Surg.) [Co-author]Takeshi Yamada (NMS Dept. Gastroenterological Surg.)、Seiichi Shinji (NMS Dept. Gastroenterological Surg.)、Akihisa Matsuda (NMS Dept. Gastroenterological Surg.)、Yasuyuki Yokoyama (NMS Dept. Gastroenterological Surg.)、Goro Takahashi (NMS Dept. Gastroenterological Surg.)、Takuma Iwai (NMS Dept. Gastroenterological Surg.)、Shintaro Kanaka (NMS Dept. Gastroenterological Surg.)、Hiroshi(Nippon Med. Sch.) Yoshida (NMS Dept. Gastroenterological Surg.)

**Background:*****Fusobacterium nucleatum*** (***F. nucleatum***) is a well-known bacterium associated with colon cancer. We measured the abundance of ***F. nucleatum*** in tumor tissues to investigate its prognostic significance.**Methods:**Patients with colon cancer who underwent surgical resection were included. The absolute copy number of ***F. nucleatum*** DNA extracted from tumor tissues was quantified by droplet digital PCR.**Results:**A total of 352 patients were included, and ***F. nucleatum*** was detected in 224 (63.6%). The copy number was significantly higher in right-sided than in left-sided colon cancer (p = 0.01). The abundance was significantly lower in stage I compared to stage II, III, and IV tumors. Patients were divided into high and low ***F. nucleatum*** groups. High abundance was associated with worse cancer-specific survival overall (p = 0.023) and in right-sided cases (p = 0.048), but not in left-sided cases (p = 0.28).**Conclusion:*****F. nucleatum*** may have a greater impact on the prognosis of right-sided than left-sided colon cancer.

[Disclosure of COI by Responsible researcher] Lead presenter is the Responsible researcher.
[Lead presenter: Toshimitsu Miyasaka] We have no financial relationships to disclose.

[P-3003] I-P3-3
Bacteria & Cancer

2025 / 9 / 27 (Sat) 15:30-16:15 Room P/(Motenashi Dome) Room P

### P-3003
### The effect of secretory DNA degrading enzymes of ***Prevotella intermedia*** on squamous cell carcinoma

[Speaker]Fumi Tetsuo (Dept. Microbiol & Oral Infection. Nagasaki Univ. Grad. Sch.) [Co-author]Naoki Katase (Dept. Oral Pathol. Nagasaki Univ. Grad. Sch.)、Mariko Naito (Dept. Microbiol & Oral Infection. Nagasaki Univ. Grad. Sch.)

***Prevotella intermedia (P. int)***, a gram-negative obligate anaerobic bacterium, has strong DNase activity compared to other periodontal pathogens. We recently established a method for creating a mutant strain of ***P. int*** and succeeded in creating a DNase-deficient strain. It has been reported that ***P. int*** is involved not only in periodontal disease but also in the progression of cancer. Neutrophil extracellular traps (NETs), mesh-like structures of DNA and proteins from neutrophils, can also promote the progression and metastasis of cancer. We investigated whether DNase activity of ***P. int*** affect cancer and NETs formation. In this study, we used tongue squamous cell carcinoma SAS cells to examine the involvement of DNase secreted by ***P. int***. The results are as follows: 1) The culture supernatant of ***P. int*** promoted cell migration, and that the culture supernatant of DNase mutant strains even more strongly promoted cell migration. 2) The DNase mutant strain did not inhibit NETs formation. In the future, we would like to investigate the detail mechanism of promoting migration and how ***P. int*** culture supernatant affects the interaction between cancer and NETs.

[Disclosure of COI by Responsible researcher] Lead presenter is the Responsible researcher.
[Lead presenter: Fumi Tetsuo] ■Research funds from company: 日本学術振興会 科学研究費助成事業 若手研究

[P-3004] I-P3-3
Bacteria & Cancer

2025 / 9 / 27 (Sat) 15:30-16:15 Room P/(Motenashi Dome) Room P

P-3004

### Glycosylation changes in macrophages induced by ***Fusobacterium*** within the endometriosis microenvironment

[Speaker]Ryosuke Sonobe (Div. Can. Biol., Nagoya Univ., Sch. Med.) [Co-author]Miho Suzuki (Div. Can. Biol., Nagoya Univ., Sch. Med.)、Keiko Shinjo (Div. Can. Biol., Nagoya Univ., Sch. Med.)、Hirokazu Yagi (Grad. Sch. Pharm. Sci., Nagoya City Univ.)、Yutaka Kondo (Div. Can. Biol., Nagoya Univ., Sch. Med.)

Glycosylation has emerged as an important mechanism regulating cell–cell interactions. In particular, glycosylation in immune cells is increasingly recognized as a key determinant of immune cell behavior and the establishment of specific immune states in the tumor microenvironment. Endometriosis is a precursor lesion of ovarian cancer. We previously reported that intrauterine infection by the anaerobic bacterium ***Fusobacterium*** (***Fuso***) triggers cytokine release from macrophages, which promotes fibroblast transformation and endometriosis development. In this study, we investigated whether ***Fuso*** alters glycosylation patterns in macrophages. RAW264.7 macrophages were exposed to ***Fuso*** for three days, followed by RNA sequencing, which revealed significant upregulation of a specific glycosyltransferase. Glycomic analysis of ***Fuso***-treated cells showed a marked increase in N-glycans branched by this enzyme, with a characteristic terminal modification. These findings suggest that ***Fuso***-exposed macrophages may contribute to chronic inflammation by modifying glycosylation profiles, thereby remodeling the immune microenvironment and promoting malignant transformation of endometriotic lesions.

[Disclosure of COI by Responsible researcher] Lead presenter is not the Responsible researcher.
[Lead presenter: Ryosuke Sonobe] We have no financial relationships to disclose.
[Principal Researcher: Yutaka Kondo] We have no financial relationships to disclose.

### [P-3005] I-P3-3
### Bacteria & Cancer

2025 / 9 / 27 (Sat) 15:30-16:15 Room P/(Motenashi Dome) Room P

### P-3005
### Bacteria residing in the ovarian cancer microenvironment

[Speaker]Miho Suzuki (Div. Cancer Biol., Nagoya Univ. Grad. Sch. Med.) [Co-author]Keiko Shinjo (Div. Cancer Biol., Nagoya Univ. Grad. Sch. Med.)、Ryosuke Sonobe (Div. Cancer Biol., Nagoya Univ. Grad. Sch. Med.)、Tatsunori Nishimura (Div. Cancer Biol., Nagoya Univ. Grad. Sch. Med.)、Yutaka Kondo (Div. Cancer Biol., Nagoya Univ. Grad. Sch. Med.)

Bacterial infections contribute to various human diseases. We previously reported bacteria in the uterus-long considered sterile-and their link to gynecologic disorders. Given that bacteria in menstrual blood may reach the ovaries via retrograde menstruation, we investigated their involvement in ovarian cancer. Using DNA FISH with 16S rRNA probes, we detected bacteria in ~60% of ovarian cancer tissues. In serous and mucinous carcinoma, bacteria were mainly localized in tumor areas (72% and 75%, respectively), while in clear cell carcinoma, they were enriched in the stroma (68%). In endometrioid carcinoma, both patterns were observed, with tumor localization being slightly more frequent (61%). To assess their role, bacteria from human ovarian cancer tissues were injected intraperitoneally into mice, followed by transplantation of mouse ovarian cancer cells. Tumor growth was significantly enhanced in the bacteria-exposed group, suggesting a tumor-promoting effect. These findings suggest that bacterial colonization may affect cancer cells directly or modulate the tumor microenvironment via stromal interactions such as inflammation, immune alteration, or fibroblast reprogramming.

[Disclosure of COI by Responsible researcher] Lead presenter is not the Responsible researcher.
[Lead presenter: Miho Suzuki] We have no financial relationships to disclose.
[Principal Researcher: Yutaka Kondo] We have no financial relationships to disclose.

### [P-3006] I-P3-3
### Bacteria & Cancer

2025 / 9 / 27 (Sat) 15:30-16:15 Room P/(Motenashi Dome) Room P

### P-3006
### Withdrawn

No Abstract

### [P-3007] I-P3-3
### Bacteria & Cancer

2025 / 9 / 27 (Sat) 15:30-16:15 Room P/(Motenashi Dome) Room P

### P-3007
### Withdrawn

No Abstract

### [P-3008] I-P3-3
### Bacteria & Cancer

2025 / 9 / 27 (Sat) 15:30-16:15 Room P/(Motenashi Dome) Room P

---

### P-3008
### Inflammatory Signaling and RAGE in Pancreatic Cancer: A Potential Therapeutic Target

[Speaker]Takahiro Araki (Department of Hepato-Biliary-Pancreatic Surgery and Transplantation, Kanazawa University) [Co-author]Satoshi Takada (Department of Hepato-Biliary-Pancreatic Surgery and Transplantation, Kanazawa University)、Seiichi Munesue (Department of Biochemistry and Molecular Vascular Biology, Kanazawa University)、Kazuki Kato (Department of Hepato-Biliary-Pancreatic Surgery and Transplantation, Kanazawa University)、Hironori Minami (Department of Hepato-Biliary-Pancreatic Surgery and Transplantation, Kanazawa University)、Ryohei Takei (Department of Hepato-Biliary-Pancreatic Surgery and Transplantation, Kanazawa University)、Kaichiro Kato (Department of Hepato-Biliary-Pancreatic Surgery and Transplantation, Kanazawa University)、Mitsuyoshi Okazaki (Department of Hepato-Biliary-Pancreatic Surgery and Transplantation, Kanazawa University)、Shinichi Nakanuma (Department of Hepato-Biliary-Pancreatic Surgery and Transplantation, Kanazawa University)、Isamu Makino (Department of Hepato-Biliary-Pancreatic Surgery and Transplantation, Kanazawa University)、Yasuhiko Yamamoto (Department of Biochemistry and Molecular Vascular Biology, Kanazawa University)、Shintaro Yagi (Department of Hepato-Biliary-Pancreatic Surgery and Transplantation, Kanazawa University)

Background and Aim: Inflammatory signaling is implicated in pancreatic cancer progression. RAGE, a receptor for AGEs, also binds HMGB1, S100, and LPS, activating NF-$\kappa$B, MAPK, and other tumor-promoting pathways. We investigated the role of RAGE in vivo and the therapeutic potential of its inhibition. Methods: Subcutaneous and orthotopic xenograft models using Panc-1 cells were created in nude mice. Mice were assigned to control (C), LPS-treated (L), and LPS + RAGE inhibitor Compound 11-treated (R) groups. Tumor volume and distant metastasis were assessed. Flow cytometry was used to evaluate RAGE expression. Results: At week 14, tumor volumes were 1208±739 $mm^3$ (C) and 2623±1627 $mm^3$ (L). At week 9 in the orthotopic model, 1959±1189 $mm^3$ (C) and 4714±1645 $mm^3$ (L). Distant metastasis occurred in 10% (C) vs. 82% (L). Group R showed reduced volume (2043±467 $mm^3$) and RAGE expression (3.8±4.2%) compared to Group L (9.3±4.6%). Conclusion: Repeated LPS exposure promoted RAGE expression and tumor progression, while RAGE inhibition suppressed both, highlighting its therapeutic potential.

[Disclosure of COI by Responsible researcher] Lead presenter is not the Responsible researcher.
[Lead presenter: Takahiro Araki] We have no financial relationships to disclose.
[Principal Researcher: Shintaro Yagi] We have no financial relationships to disclose.

▲TOP

### [P-3009] I-P3-3
### Bacteria & Cancer

2025 / 9 / 27 (Sat) 15:30-16:15 Room P/(Motenashi Dome) Room P

---

### P-3009
### The impacts of appendectomy for cancer development and disease control in patients with ulcerative colitis

[Speaker]Takayuki Ogino (Department of Gastroenterological Surgery, The University of Osaka) [Co-author]Yusuke Izutani (Department of Gastroenterological Surgery, The University of Osaka)、Yuki Sekido (Department of Gastroenterological Surgery, The University of Osaka)、Mitsunobu Takeda (Department of Gastroenterological Surgery, The University of Osaka)、Tsuyoshi Hata (Department of Gastroenterological Surgery, The University of Osaka)、Atsushi Hamabe (Department of Gastroenterological Surgery, The University of Osaka)、Norikatsu Miyoshi (Department of Gastroenterological Surgery, The University of Osaka)、Mamoru Uemura (Department of Gastroenterological Surgery, The University of Osaka)、Hirofumi Yamamoto (Department of Gastroenterological Surgery, The University of Osaka)、Yuichiro Doki (Department of Gastroenterological Surgery, The University of Osaka)、Hidetoshi Eguchi (Department of Gastroenterological Surgery, The University of Osaka)

Background: Ulcerative colitis (UC) is an idiopathic inflammatory disease involving genetic, environmental, and immunological factors. While appendectomy may influence UC onset and activity, the timing effect remains unclear. This study examined the impact of appendectomy on UC disease activity and cancer development.Methods: A retrospective analysis of 368 UC patients at Osaka University Hospital (2008-2023) compared appendectomy and non-appendectomy groups regarding clinical features, disease activity, medication use, and colorectal cancer development.Results: 18 patients (4.9%) had undergone appendectomy. They developed UC at an older age (42.0 vs. 34.3 years, $p=0.03$). Appendectomy was associated with lower partial Mayo scores (4.5 vs. 8.0, $p<0.001$), fewer relapses ($p=0.016$), and reduced steroid ($p=0.032$) and biologic use ($p=0.006$). Although colorectal cancer incidence was similar, cancer developed later in the appendectomy group (29.3 vs. 16.0 years, $p=0.03$). Appendectomy before UC onset delayed UC development ($p=0.004$).Conclusion: Appendectomy was linked to milder UC activity and delayed cancer development, suggesting a suppressive effect on disease progression.

[Disclosure of COI by Responsible researcher] Lead presenter is the Responsible researcher.
[Lead presenter: Takayuki Ogino] ■Lecture fee: 武田薬品工業　■Endowed chair: 医療法人錦秀会

▲TOP

### [P-3018] I-P4-7
### Cancer biology driven by TP53 abnormality

2025 / 9 / 27 (Sat) 15:30-16:15 Room P/(Motenashi Dome) Room P

---

### P-3018
### Cell Cycle-Related Gene Mutations and the Prognosis of Endometrial Cancer Patient: A Bayesian Network Meta-Analysis

[Speaker]I Gede Krisna Arim Sadeva (Graduated Student, Faculty of Medicine, Udayana University, Indonesia) [Co-author]Pande Kadek Aditya Prayudi (Obstetric and Gynaecology Department, Faculty of Medicine, Udayana University, Indonesia)、I Gede Putu Supadmanaba (Biochemistry Department, Faculty of Medicine, Udayana University, Indonesia)

Introduction: Mutations in genes related to the cell cycle in cancer can increase progression or serve as markers in determining appropriate therapy. This study aims to evaluate cell cycle-related gene mutations and the prognosis of endometrial cancer (EC) patients.Methods: The Preferred Reporting Items for Systematic Reviews and Meta-Analysis (PRISMA) protocol was used. Cohort studies reporting the survival of EC patients were included. The primary outcome, overall survival (OS), was calculated in the hazard ratio (HR) using R Statistical Software v.4.4.0.Results: This study included six prospective cohort studies (n=2714). All patients in the included studies were EC patients who had received adjuvant therapy. Forest plot comparing TP53 and CTNNB1 mutation showed that CTNNB1 mutations had worse 5-years OS (HR=2.67; 95%CrI 1.03-16.43) than TP53 (HR=1.22; 95%CrI 0.93-1.64). In the 5-years OS analysis, PTEN gene mutations showed better prognosis (HR=0.83; 95%CrI 0.54-1.25) than POLE (HR=0.90; 95%CrI 0.65-1.17).Conclusion: CTNNB1 gene mutations in EC patients have worse prognosis, while PTEN mutations are associated with better prognosis.

[Disclosure of COI by Responsible researcher] Lead presenter is the Responsible researcher.
[Lead presenter: I Gede Krisna Arim Sadeva] We have no financial relationships to disclose.

### [P-3019] I-P4-7
### Cancer biology driven by TP53 abnormality

2025 / 9 / 27 (Sat) 15:30-16:15 Room P/(Motenashi Dome) Room P

### P-3019
### Crosstalk from Wnt signaling to p53 in multistep tumorigenesis

[Speaker]Tadasuke Tsukiyama (Hokkaido Univ Grad Schol Med)

In the multi-step carcinogenesis model, it is believed that cancer progresses in the following order: activation of Wnt signaling by APC mutations, then activation of the RAS pathway by KRAS mutations, and finally inactivation of the p53 pathway by TP53 mutations. Each of these events has been studied in detail for a long time and understood deeply. However, there is still insufficient information to make a clear judgment as to whether the events necessary for this carcinogenesis are independent of each other, or whether their mutual interaction is essential for carcinogenesis progression. In the evidence of the interaction, it has been reported that Wnt signaling stabilizes the Ras pathway, and the Ras-MAPK pathway enhances Wnt signaling. We have also previously reported that the stem cell-specific ubiquitin ligase RNF43, a direct target gene of Wnt signaling, not only functions to terminate Wnt signaling but also suppresses the p53 pathway. Here, we explore a little deeper into the molecular mechanism of p53 suppression by RNF43, which has been unknown, and discuss the possibility that this event functions not only in carcinogenesis but also in stem cell maintenance.

[Disclosure of COI by Responsible researcher] Lead presenter is the Responsible researcher.
[Lead presenter: Tadasuke Tsukiyama] We have no financial relationships to disclose.

### [P-3020] I-P4-7
### Cancer biology driven by TP53 abnormality

2025 / 9 / 27 (Sat) 15:30-16:15 Room P/(Motenashi Dome) Room P

---

### P-3020
### *TP53* Status as a Determinant of Response to Gemcitabine-Based Regimens in Pancreato-Biliary cancers

[Speaker]Makoto Sugimori (Div. of Cancer Genome Medicine, Yokohama City University Medical Center) [Co-author]Akane Hirotani (Div. of Cancer Genome Medicine, Yokohama City University Medical Center／Dept. of Gastroenterology, Yokohama City University Medical Center)、Hiromi Tsuchiya (Dept. of Gastroenterology, Yokohama City University Medical Center)、Akihiro Funaoka (Dept. of Gastroenterology, Yokohama City University Medical Center)、Kazuki Endo (Dept. of Gastroenterology, Yokohama City University Medical Center)、Ritsuko Oishi (Dept. of Gastroenterology, Yokohama City University Medical Center)、Yuichi Suzuki (Dept. of Gastroenterology, Yokohama City University Medical Center)、Yushi Kanemaru (Dept. of Gastroenterology, Yokohama City University Medical Center)、Yoshimasa Suzuki (Dept. of Gastroenterology, Yokohama City University Medical Center)、Sho Onodera (Dept. of Gastroenterology, Yokohama City University Medical Center)、Haruo Miwa (Dept. of Gastroenterology, Yokohama City University Medical Center)、Akito Nozaki (Dept. of Gastroenterology, Yokohama City University Medical Center)、Manabu Morimoto (Dept. of Gastroenterology, Yokohama City University Medical Center)、Makoto Kudo (Div. of Cancer Genome Medicine, Yokohama City University Medical Center／Dept. of Respiratory Disease Center, Yokohama City University Medical Center)、Shin Maeda (Dept. of Gastroenterology, Yokohama City University Graduate School of Medicine)

Aim: Although *TP53* is a tumor suppressor, pathogenic missense mutations are more common than loss-of-function variants. This study examined the association between *TP53* status and chemotherapy outcomes in pancreato-biliary cancers using C-CAT real-world data.
Methods: We analyzed 4,379 biliary tract and 5,025 pancreatic cancer cases with genomic profiling. PFS after first-line therapy was compared by *TP53* status. Pathogenic missense mutations were classified as SNVs, and frameshifts, or nonsense mutations as truncating (Trun.). Drug sensitivity data were obtained from the Sanger Institute.
Results: *TP53* alterations were seen in 55.3% of biliary and 75.1% of pancreatic cancers. In both, PFS with gemcitabine-based therapy was shorter in patients with *TP53* alterations (biliary: 184.0 vs. 148.0 days; pancreatic: 213.5 vs. 176.0 days; both $p < 0.0001$). In pancreatic cancer, Trun. mutations showed worse PFS than SNVs (161.5 vs. 181.0 days; $p = 0.0206$). In pancreatic cancer cell-lines, gemcitabine IC50 was significantly higher in Trun. vs. SNV and wild-type (81.1 vs. 2.38 vs. 14.0 μM; $p < 0.01$).
Conclusion: *TP53* status may predict gemcitabine response in pancreato-biliary cancers.

[Disclosure of COI by Responsible researcher] Lead presenter is the Responsible researcher.
[Lead presenter: Makoto Sugimori] We have no financial relationships to disclose.

### [P-3021] I-P4-7
### Cancer biology driven by TP53 abnormality

2025 / 9 / 27 (Sat) 15:30-16:15 Room P/(Motenashi Dome) Room P

---

### P-3021
### Combined p53 Activation and Wnt Signaling Inhibition Synergistically Induce Cell Death in Colorectal Cancer Cells

[Speaker]Hisakazu Kato (Univ. Osaka Grad. Sch. Med. Dept. Med. Biochem.) [Co-author]Hiroto Seto (Univ. Osaka Grad. Sch. Med. Dept. Gastroent.Surg.)、Hidekazu Takahashi (Univ. Osaka Grad. Sch. Med. Dept. Gastroent.Surg.)、Seiji Takashima (Univ. Osaka Grad. Sch. Med. Dept. Med. Biochem.)

The ***TP53*** gene, encoding the tumor suppressor p53, is frequently mutated in colorectal cancers (CRCs). However, about half of CRCs retain wild–type ***TP53***. While p53–activating therapies are promising, severe side effects have hindered clinical application. We previously showed that combining the MDM2 inhibitor HDM201 with a MEK inhibitor synergistically suppressed growth of CRC cells with wild–type ***TP53***. However, MDM2 inhibitors induce little cell death in these cells, despite their anti–proliferative effect.To identify compounds that enhance cytotoxicity, we screened 1,200 molecules and found compound PP, which synergized with HDM201 at 1 µM. PP is a known Wnt pathway inhibitor. Cell viability assay using CRC cell lines with Wnt pathway mutations showed that the combination´s effect increased in a βcatenin–dependent manner. Notably, a specific βcatenin inhibitor recapitulated the cytotoxic effects of PP when combined with HDM201.These findings suggest that in CRC cells with active Wnt signaling, dual targeting of p53 activation and Wnt inhibition can cooperatively trigger cell death, offering a potential strategy for treating ***TP53*** wild–type tumors.

[Disclosure of COI by Responsible researcher] Lead presenter is not the Responsible researcher.
[Lead presenter: Hisakazu Kato] We have no financial relationships to disclose.
[Principal Researcher: Seiji Takashima] We have no financial relationships to disclose.

Poster Session

### [P-3022] I-P4-7
### Cancer biology driven by TP53 abnormality

2025 / 9 / 27 (Sat) 15:30-16:15 Room P/(Motenashi Dome) Room P

---

### P-3022
### Identification of HSP40 members that stabilize conformational mutant p53

[Speaker]Atsushi Kaida (Dept. of Dental Radiology & Oncology, Institute of Science Tokyo) [Co-author]Hitomi Nojima (Dept. of Dental Radiology & Oncology, Institute of Science Tokyo)、Masahiko Miura (Dept. of Dental Radiology & Oncology, Institute of Science Tokyo)、Tomoo Iwakuma (Children's Mercy Research Institute)

Mutations in tumor suppressor p53 impair its transcriptional and tumor-suppressive function. However, p53 mutants acquire cancer-promoting properties, known as gain of function (GOF). Accumulation of mutant p53 (mutp53) is crucial for GOF activity. We previously showed that DNAJA1, an HSP40 member, specifically interacts with and stabilizes conformational mutp53, but not the DNA contact type, promoting migration and metastasis of cancer cells. Given the heterogeneity of mutp53 GOF and the diversity of HSP40s, we hypothesized that different HSP40s selectively bind distinct mutp53 variants to alter GOF. To test this, we performed an siRNA screen to assess their ability to deplete mutp53 variants. This screen identified HSP40 member X as a potential stabilizer of mutp53 variant $p53^{C176F}$. Proximity ligation and co-immunoprecipitation assays confirmed interaction between factor X and $p53^{C176F}$. Knockdown of factor X significantly reduced migration of $p53^{C176F}$-expressing head and neck cancer cell line HN31, suggesting that factor X regulates mutp53-driven malignancy. These findings suggest that factor X may be a therapeutic target in $p53^{C176F}$-expressing cancers via mutp53 GOF depletion.

[Disclosure of COI by Responsible researcher] Lead presenter is the Responsible researcher.
[Lead presenter: Atsushi Kaida] We have no financial relationships to disclose.

### [P-3023] I-P4-7
### Cancer biology driven by TP53 abnormality

2025 / 9 / 27 (Sat) 15:30-16:15 Room P/(Motenashi Dome) Room P

### P-3023
### Secreted PAD7 Shapes a Tumor-Suppressive Microenvironment by Inhibiting Lipid Metabolism in MASH-Driven Liver Cancer

[Speaker]Airi Nakano (Lab. of Fundamental Oncology, Natl. Cancer Ctr. Res. Inst.／Grad. Sch. of Biomed. Sci, Nagasaki Univ.) [Co-author]Yuko Tabata (Lab. of Fundamental Oncology, Natl. Cancer Ctr. Res. Inst.)、Masaki Okudaira (Lab. of Fundamental Oncology, Natl. Cancer Ctr. Res. Inst.／Tokyo College of Biotech.)、Yuri Mitsui (Lab. of Fundamental Oncology, Natl. Cancer Ctr. Res. Inst.／Tokyo College of Biotech.)、Masahiro Takikawa (Lab. of Fundamental Oncology, Natl. Cancer Ctr. Res. Inst.／Dept. of Urology, Grad. Sch. of Medicine, Nagoya Univ.)、Atsushi Okabe (Dept. of Mol. Oncol., Grad. Sch. of Medicine, Chiba Univ.／Health & Disease Omics Ctr., Chiba Univ.)、Yuichi Wakabayashi (Div. of Exp. Animal Res., Chiba Cancer Ctr. Res. Inst.)、Naoko Ohtani (Dept. of Pathophysiol., Grad. Sch. of Medicine, Osaka Metro. Univ.)、Ikuyo Ichi (Fac. of Core Res., Ochanomizu Univ.)、Emi Nishijima (Grad. Sch. of Humanities & Sci., Ochanomizu Univ.)、Atsushi Kaneda (Dept. of Mol. Oncol., Grad. Sch. of Medicine, Chiba Univ.／Health & Disease Omics Ctr., Chiba Univ.)、Kimi Araki (Inst. of Res. Dev. & Analysis, Kumamoto Univ.)、Masamichi Ishiai (Grad. Sch. of Biomed. Sci, Nagasaki Univ.／Central Radioisotope Div., Natl. Cancer Ctr. Res. Inst.)、Rieko Ohki (Lab. of Fundamental Oncology, Natl. Cancer Ctr. Res. Inst.)

PAD7 is downregulated in many cancers and has tumor-suppressive function. Since PAD7 protein is secreted extracellularly, we hypothesized that PAD7 suppresses cancer by modulating the tumor microenvironment. In liver cancer derived from metabolic dysfunction-associated steatohepatitis (MASH), chronic inflammation caused by obesity and lipid metabolism abnormalities promotes tumor progression. RNA-seq analysis showed that PAD7 treatment suppresses genes involved in adipogenesis and fatty acid synthesis. To explore the role of PAD7 in MASH-derived liver cancer, we established MEFs from wild-type and PAD7-KO mouse embryos and induced adipocyte differentiation. In PAD7 KO MEFs, adipogenesis and lipid synthesis were significantly enhanced. We then induced MASH-derived hepatocellular carcinoma (HCC) in mice by DMBA treatment, followed by a high-fat diet and 30% sucrose water. At 40 weeks, PAD7 deficiency led to an increased number of liver tumors. In non-tumor liver regions, PAD7 suppressed expression of expression of lipid metabolism genes. These findings suggest PAD7 inhibits lipid synthesis and inflammation, supporting a tumor-suppressive microenvironment in MASH-derived HCC.

[Disclosure of COI by Responsible researcher] Lead presenter is not the Responsible researcher.
[Lead presenter: Airi Nakano] We have no financial relationships to disclose.
[Principal Researcher: Rieko Ohki] We have no financial relationships to disclose.

### [P-3024] I-P4-7
### Cancer biology driven by TP53 abnormality

2025 / 9 / 27 (Sat) 15:30-16:15 Room P/(Motenashi Dome) Room P

---

### P-3024
### The p53-PHLDA3 pathway promotes Liver cancer.

[Speaker]Naoya Kobayashi (Laboratory of Fundamental Oncology, National Cancer Center Research Institute, Tokyo.／Department of NCC Cancer Science, Institute of Science Tokyo.) [Co-author]Kiyono Aoki (Laboratory of Fundamental Oncology, National Cancer Center Research Institute, Tokyo.)、Musashi Kagaya (Laboratory of Fundamental Oncology, National Cancer Center Research Institute, Tokyo.／Department of Bioscience, School of Science, Kitasato University.)、Yu Chen (Laboratory of Fundamental Oncology, National Cancer Center Research Institute, Tokyo.)、Daisuke Shiokawa (Translational Research Center, Ehime University Hospital.)、Masahiro Yamamoto (Department of Tumor Pathology, Kumamoto University.)、Rieko Ohki (Laboratory of Fundamental Oncology, National Cancer Center Research Institute, Tokyo.)

The tumor suppressor gene p53, frequently mutated in human cancer, functions as a transcription factor regulating genes involved in tumor suppression. While known for tumor suppression, p53 may also promote tumorigenesis in cancers with low mutation rates, such as liver cancer. However, p53 target gene responsible for this tumor promoting activity in liver cancer is unknown.We identified PHLDA3 as a p53 target gene that encodes an Akt repressor, previously shown to act as a tumor suppressor gene in lung and pancreatic cancers. However, its role in liver cancer remains unclear. Using hydrodynamic tail vein injection and the Sleeping Beauty transposon system, we investigated the role of PHLDA3 in mouse hepatocarcinogenesis. We found that PHLDA3 expression is elevated in liver cancer, and its overexpression accelerates tumorigenesis. Notably, PHLDA3 overexpression suppressed Akt activation while activating the YAP pathway, suggesting that PHLDA3 may promote hepatocarcinogenesis through these mechanisms. To further explore the p53 – PHLDA3 – Akt – YAP axis in liver cancer, we are also analyzing PHLDA3-deficient mice.

[Disclosure of COI by Responsible researcher] Lead presenter is not the Responsible researcher.
[Lead presenter: Naoya Kobayashi] We have no financial relationships to disclose.
[Principal Researcher: Rieko Ohki] ■Research expenses: 科研費

### [P-3025] I-P4-7
### Cancer biology driven by TP53 abnormality

2025 / 9 / 27 (Sat) 15:30-16:15 Room P/(Motenashi Dome) Room P

---

### P-3025
### Mutant p53 Promotes Breast Cancer Malignancy through Transcriptional Regulation of the Mevalonate Pathway

[Speaker]Akitoshi Nakayama (Dept. Mol. Diagnosis, Grad. Sch. of Med., Chiba Univ.) [Co-author]Masataka Yokoyama (Dept. Mol. Diagnosis, Grad. Sch. of Med., Chiba Univ.)、Tomoaki Tanaka (Dept. Mol. Diagnosis, Grad. Sch. of Med., Chiba Univ.／Res. Inst. of Disaster Med. Chiba Univ.)

Gain-of-function mutations in ***TP53***, a key tumor suppressor gene, are linked to cancer progression, but the underlying molecular mechanisms remain unclear. Here, we investigated how mutant p53 promotes malignancy in breast cancer using gene-modified MDA-MB-231 cells, on-top 3D culture, single-cell RNA (scRNA)-seq, and ChIP-seq. Mutant p53 expression induced malignant morphologies in 3D culture. scRNA-seq revealed upregulation of lipid metabolism genes, particularly the mevalonate (MVA) pathway. Modulating extracellular cholesterol levels altered the frequency of malignant morphologies, indicating a functional link to cholesterol metabolism. We identified SREBP2, a central transcription factor of the MVA pathway, as a downstream effector of mutant p53. ChIP-seq analysis revealed SCAP, the ER-to-Golgi transporter of SREBP2, as a direct transcriptional target of mutant p53. This mutant p53-SCAP-SREBP2 axis activated MVA pathway and increased geranylgeranyl pyrophosphate levels, promoting filopodia formation via small GTPase activation. These findings demonstrate that mutant p53 enhances breast cancer malignancy through metabolic reprogramming and cytoskeletal remodeling.

[Disclosure of COI by Responsible researcher] Lead presenter is not the Responsible researcher.
[Lead presenter: Akitoshi Nakayama] We have no financial relationships to disclose.
[Principal Researcher: Tomoaki Tanaka] We have no financial relationships to disclose.

▲TOP

### [P-3026] I-P4-7
### Cancer biology driven by TP53 abnormality

2025 / 9 / 27 (Sat) 15:30-16:15 Room P/(Motenashi Dome) Room P

### P-3026
### p53 and Cellular Senescence in Cancer Cell Lines

[Speaker]Hiroaki Kuribayashi (Univ.Tokyo) [Co-author]Satoshi Kawakami (IMSUT)、Tehwei Wang (IMSUT)、Makoto Nakanishi (IMSUT)

When stressed by various stimuli such as DNA damage, proliferative cells induce a process called cellular senescence, which leads to a stable cell cycle arrest. Our laboratory has previously reported that the activation of the p53-p21 pathway at the G2/M phase is sufficient to induce cellular senescence in human fibroblast cell line, and that senescent cells are tetraploid cells resulting from mitotic skip. Although p53 mutations occur frequently in tumor cells, p53 mutant cancer cell lines still exhibit senescent-like phenotypes and increased ploidy. Thus, contrary to the importance of p53 in canonical cellular senescence, senescence-like processes in cancer cells can be initiated in a p53-independent manner. Unexpectedly, we found that some cancer cell lines without functional p53 were unable to survive stably for long periods of time after a chemotherapy treatment. Does partial retention of p53 function favor drug resistance in cancer cells? Now we would like to discuss the potential importance of p53 in therapy-induced senescence.

[Disclosure of COI by Responsible researcher] Lead presenter is not the Responsible researcher.
[Lead presenter: Hiroaki Kuribayashi] We have no financial relationships to disclose.
[Principal Researcher: Makoto Nakanishi] ■Profit of stock: 株式会社エアウィーヴ社、reverSASP Therapeutics株式会社

### [P-3036] I-P5-6
### Intracellular signal transduction (2)

2025 / 9 / 27 (Sat) 15:30-16:15 Room P/(Motenashi Dome) Room P

### P-3036
### RTK endocytois-dependent glycolysis regulation in ALK-expressing neuroblastoma

[Speaker]Ryouhei Tsutsumi (Dept. Biochem., Kitasato Univ. Sch. Med.) [Co-author]Ryuichi Sakai (Dept. Biochem., Kitasato Univ. Sch. Med.)

Overexpression or activating mutations of receptor tyrosine kinases (RTKs) are often associated with tumorigenesis, tumor malignancy, and acquired drug resistance. While activation-dependent endocytosis of RTKs typically leads to receptor degradation, some intracellular signaling is thought to originate from RTK-containing endosomes formed through endocytosis. In addition to their roles in intracellular growth and survival signaling, we recently discovered that the transport of RTK-containing endosomes to the vicinity of mitochondria upon ligand-induced receptor endocytosis mediates cellular glycolysis. In this study, we investigated whether RTK endocytosis-mediated glycolysis contributes to cancer cell metabolism, focusing on anaplastic lymphoma kinase (ALK)-overexpressing or mutated neuroblastoma. Our results suggest a novel therapeutic strategy targeting endosome-dependent metabolic regulation of RTK-overexpressing or mutated cancers.

[Disclosure of COI by Responsible researcher] Lead presenter is the Responsible researcher.
[Lead presenter: Ryouhei Tsutsumi] ■Research funds from company: 上原記念生命科学財団、住友財団

▲TOP

### [P-3037] I-P5-6
### Intracellular signal transduction (2)

2025 / 9 / 27 (Sat) 15:30-16:15 Room P/(Motenashi Dome) Room P

---

### P-3037
### Midkine promotes tumor growth and Cisplatin resistance through AKT pathway activation in small cell lung cancer

[Speaker]Shotaro Ito (Dept. of Respiratory Med., Faculty of Med., Hokkaido Univ.) [Co-author]Jun Sakakibara (Dept. of Respiratory Med., Faculty of Med., Hokkaido Univ.)、Mineyoshi Sato (Dept. of Respiratory Med., Faculty of Med., Hokkaido Univ.)、Tetsuaki Shoji (Dept. of Respiratory Med., Faculty of Med., Hokkaido Univ.)、Megumi Furuta (Dept. of Respiratory Med., Faculty of Med., Hokkaido Univ.)、Hirofumi Takahashi (Dept. of Respiratory Med., Faculty of Med., Hokkaido Univ.)、Kosuke Tsuji (Dept. of Respiratory Med., Faculty of Med., Hokkaido Univ.)、Daisuke Morinaga (Dept. of Respiratory Med., Faculty of Med., Hokkaido Univ.)、Masahiro Kashima (Dept. of Respiratory Med., Faculty of Med., Hokkaido Univ.)、Hidenori Kitai (Dept. of Respiratory Med., Faculty of Med., Hokkaido Univ.)、Kanako Hatanaka (Ctr. Development of Advanced Diagnostics, Hokkaido Univ. Hosp.)、Yutaka Hatanaka (Ctr. Development of Advanced Diagnostics, Hokkaido Univ. Hosp.)、Kyoko Hida (Vascular Biol. Mol. Pathol., Faculty of. Dent. Med., Hokkaido Univ.)、Takuro Noguchi (Dept. of Med. Oncology, Hokkaido Univ. Grad. Sch. of Med.)、Satoshi Konno (Dept. of Respiratory Med., Faculty of Med., Hokkaido Univ.)

Background: Although target therapies for DLL3 or several molecules have been developed in small cell lung cancer (SCLC), those remain scarce. Midkine (MDK) is upregulated in various cancers and involved in cell proliferation and chemotherapy resistance. Cisplatin (CDDP) is a key drug in SCLC, and it has been reported that suppression of MDK enhances the efficacy of CDDP in other tumors, which suggest that combination therapy with MDK inhibitor might be effective in SCLC. Objective: We analyzed the expression and function of MDK in SCLC and evaluated its potential as a therapeutic and diagnostic target. Results: Serum MDK level of SCLC patients tended to reflect tumor volume. Proliferative capacities of SCLC cell lines were increased by overexpression and decreased by inhibition of MDK. Overexpression or knockdown of MDK modulated AKT pathways. Overexpression of MDK attenuated the antitumor effect of CDDP and suppression of MDK enhanced CDDP antitumor effect. Additionally, the combination of CDDP and MDK inhibitor showed synergistic effects in vitro and in vivo. Conclusion: In SCLC, MDK is involved in tumor growth and CDDP resistance and may be a therapeutic target.

[Disclosure of COI by Responsible researcher] Lead presenter is not the Responsible researcher.
[Lead presenter: Shotaro Ito] We have no financial relationships to disclose.
[Principal Researcher: Jun Sakakibara] We have no financial relationships to disclose.

### [P-3038] I-P5-6
### Intracellular signal transduction (2)

2025 / 9 / 27 (Sat) 15:30-16:15 Room P/(Motenashi Dome) Room P

---

### P-3038
### The regulation and function of SAPK signaling pathways in the anticancer drug-induced genotoxic stress responses

[Speaker]Yuji Kubota (Div. Cell Signaling & Mol. Med., Inst. Med. Sci., Univ. Tokyo) [Co-author]Shuri Komai (Div. Cell Signaling & Mol. Med., Inst. Med. Sci., Univ. Tokyo)、Yukari Shiozaki (Div. Cell Signaling & Mol. Med., Inst. Med. Sci., Univ. Tokyo)、Mutsuhiro Takekawa (Div. Cell Signaling & Mol. Med., Inst. Med. Sci., Univ. Tokyo)

The stress-activated p38 and JNK signaling pathways, collectively known as SAPK pathways, consists of three types of protein kinases (SAPKKK, SAPKK, and SAPK) and are highly conserved across eukaryotes from yeast to mammals. In response to distinct sets of environmental stresses, different SAPKKKs are activated and phosphorylate their downstream SAPKKs, which in turn activate their cognate SAPKs. The activated SAPKs eventually induce a variety of cellular responses, such as cell cycle arrest, inflammation, and apoptosis. The chemotherapeutic drug etoposide induces DNA double-strand breaks (DSBs), leading to apoptosis via SAPK activation, but the detailed molecular mechanism has not been fully elucidated. Analysis of SAPKKK-knockout (KO) cell lines generated by the CRISPR-Cas9 system revealed that DSBs induce sustained activation of SAPK via coordinated activation of multiple SAPKKK molecules, thereby promoting DSB responses. Moreove, RNA-seq analysis suggested that this SAPK signal is involved in regulating the expression of DSB-responsive genes. Our results provide insights into a molecular mechanism by which chemotherapeutic agents efficiently induce apoptosis via SAPK signaling.

[Disclosure of COI by Responsible researcher] Lead presenter is not the Responsible researcher.
[Lead presenter: Yuji Kubota] We have no financial relationships to disclose.
[Principal Researcher: Mutsuhiro Takekawa] ■Research funds from company: アボット

▲TOP

### [P-3039] I-P5-6
### Intracellular signal transduction (2)

2025 / 9 / 27 (Sat) 15:30-16:15 Room P/(Motenashi Dome) Room P

---

### P-3039
### Significance of ULK1 in tolerance to EGFR TKI

[Speaker]Ayako Morita (Department of Allergy and Respiratory Medicine, Okayama University Hospital) [Co-author]Tatsuya Nishi (Department of Respiratory Medicine, NHO Iwakuni Clinical Center)、Naofumi Hara (Center for Graduate Medical Education, Okayama University Hospital)、Go Makimoto (Department of Allergy and Respiratory Medicine, Okayama University Hospital)、Kiichiro Ninomiya (Center for Comprehensive Genomic Medicine, Okayama University Hospital)、Kanmei Rai (Center of Innovative Clinical Medicine , Okayama University Hospital)、Kadoaki Ohashi (Department of Allergy and Respiratory Medicine, Okayama University Hospital)、Katsuyuki Hotta (Center of Innovative Clinical Medicine , Okayama University Hospital)、Yosuke Togashi (Department of Allergy and Respiratory Medicine, Okayama University Hospital)、Yoshinobu Maeda (Okayama University Graduate School of Medicine, Dentistry and Pharmaceutical Sciences)、Eiki Ichihara (Center for Clinical Oncology, Okayama University Hospital)

Background: Drug-tolerant persister (DTP) cells limit the efficacy of EGFR tyrosine kinase inhibitors (TKIs) in EGFR-mutant non-small cell lung cancer (NSCLC).Because autophagy is a potential target for eliminating DTP cells, we assessed whether inhibition of ULK1, a specific autophagy target, could overcome resistance to EGFR TKIs in EGFR-mutated NSCLC cells.Methods: DTP cells were generated by treating EGFR-mutant NSCLC cell lines with osimertinib. The impact of pharmacological and genetic ULK1 inhibition was assessed in vitro using proliferation and crystal violet assays, and in vivo using xenograft mouse models.Results: Pharmacological or genetic inhibition of ULK1 suppressed autophagy, reduced the emergence of DTP cells, and enhanced the antitumor effect of osimertinib both in vitro and in vivo. ULK1 inhibition was effective even after resistance had developed. Serum starvation-induced autophagy through ULK1 also conferred tolerance to EGFR TKI, further supporting its role.Conclusion: ULK1-mediated autophagy contributes to drug tolerance in EGFR-mutant NSCLC. Targeting ULK1 is a promising strategy to enhance the efficacy of EGFR TKIs and delay or overcome resistance.

[Disclosure of COI by Responsible researcher] Lead presenter is not the Responsible researcher.
[Lead presenter: Ayako Morita] We have no financial relationships to disclose.
[Principal Researcher: Eiki Ichihara] ■Research expenses: ヤンセンファーマ

### [P-3040] I-P5-6
### Intracellular signal transduction (2)

2025 / 9 / 27 (Sat) 15:30-16:15 Room P/(Motenashi Dome) Room P

---

### P-3040
### Reciprocal regulation of ERK and MKP activities promotes mitogenic signaling and tumorigenesis

[Speaker]Ryoko Ando (Div. Cell Signaling & Mol. Med., IMSUT) [Co-author]Yuji Kubota (Div. Cell Signaling & Mol. Med., IMSUT)、Mutsuhiro Takekawa (Div. Cell Signaling & Mol. Med., IMSUT)

The ERK signaling pathway mediates mitogenic signals and is critical for cell proliferation, differentiation and carcinogenesis. ERK-specific MAPK phosphatases (MKPs) inactivate ERK signaling by dephosphorylating the activating-phosphorylation sites on ERK, thereby acting as tumor suppressors. However, the detailed regulatory mechanisms of MKPs remain unclear. In this study, we identified a constitutively expressed MKP, MKP-A, as a novel substrate of ERK. MKP-A was phosphorylated at a specific Ser residue in an ERK dependent manner, and this phosphorylation reduced its phosphatase activity. Using a non-phosphorylatable MKP-A mutant, we found that ERK-mediated phosphorylation and subsequent inactivation of MKP-A are crucial for robust ERK activation and modulation of downstream gene expression in response to growth factor stimulation. Furthermore, MKP-A was constitutively phosphorylated in cancer cells with ERK-activating oncogenes, and the expression of non-phosphorylatable MKP-A in these cancer cells suppressed tumor growth in mice. Our data indicate that ERK and MKP-A form a hitherto unrecognized regulatory system mediated by reciprocal phosphorylation and dephosphorylation.

[Disclosure of COI by Responsible researcher] Lead presenter is not the Responsible researcher.
[Lead presenter: Ryoko Ando] We have no financial relationships to disclose.
[Principal Researcher: Mutsuhiro Takekawa] ■Research funds from company: アボット

### [P-3041] I-P5-6
### Intracellular signal transduction (2)

2025 / 9 / 27 (Sat) 15:30-16:15 Room P/(Motenashi Dome) Room P

---

### P-3041
### Regulation of CTGF Protein Levels via AKT Signaling Activated by PDGF Receptor in Malignant Mesothelioma

[Speaker]Tomoya Suehiro (Grad. Sch. of Biomed. & Health Sci., Hiroshima Univ.) [Co-author]Bingwen Xu (Grad. Sch. of Biomed. & Health Sci., Hiroshima Univ.)、Yuichi Mine (Grad. Sch. of Biomed. & Health Sci., Hiroshima Univ.)、Makiko Fujii (Grad. Sch. of Biomed. & Health Sci., Hiroshima Univ.)

Malignant mesothelioma (MM) is a rare, aggressive cancer caused by asbestos exposure. Although asbestos use was banned in Japan in 2006, MM incidence is expected to rise due to its long latency of 20 to 50 years. Surgical resection is the only potentially curative treatment, while chemotherapy remains ineffective for remission.Previous studies have shown a correlation between connective tissue growth factor (CTGF) protein expression and MM malignancy. In this study, we examined CTGF regulation in MM cells. We found that PDGF-BB, a ligand for platelet-derived growth factor receptor (PDGFR), significantly increased CTGF protein levels without affecting CTGF mRNA levels. Inhibiting PDGFR reduced CTGF expression, indicating its key role in this pathway. PDGF-BB also activated AKT signaling, and suppressing AKT phosphorylation blocked PDGFR-induced CTGF expression. Furthermore, PDGFR activation promoted mTOR and 4E-BP1 phosphorylation, crucial regulators of mRNA translation downstream of AKT.These findings suggest that PDGFR regulates CTGF expression post-transcriptionally via the AKT–mTOR pathway, supporting CTGF as a potential therapeutic target in MM.

[Disclosure of COI by Responsible researcher] Lead presenter is not the Responsible researcher.
[Lead presenter: Tomoya Suehiro] We have no financial relationships to disclose.
[Principal Researcher: Makiko Fujii] We have no financial relationships to disclose.

Poster Session

### [P-3042] I-P5-6
### Intracellular signal transduction (2)

2025 / 9 / 27 (Sat) 15:30-16:15 Room P/(Motenashi Dome) Room P

---

### P-3042
### Taste 2 receptor-mediated signaling suppresses the malignancy of colorectalcancer cells

[Speaker]Kotoha Nozaki (Dept. Biomed. Eng., Sci. & Eng., Toyo Univ.) [Co-author]Taiga Yunoue (Course Biomed. Eng., Grad. Sch. Life Sci, Toyo Univ.)、Junya Ohtake (Res. Facility Ctr., Asaka, Toyo Univ.／Res. Ctr., Biomed. Eng., Toyo. Univ.)、Hidemitsu Kitamura (Dept. Biomed. Eng., Sci. & Eng., Toyo Univ.／Course Biomed. Eng., Grad. Sch. Life Sci, Toyo Univ.／Res. Facility Ctr., Asaka, Toyo Univ.／Res. Ctr., Biomed. Eng., Toyo. Univ.)

Advanced cases of colorectal cancer (CRC), such as those with recurrence ormetastasis, still present poor outcomes. Therefore, elucidating the detailed mechanisms underlying CRC malignancy is crucial for developing more effective treatments. Our recent study found that taste 2 receptors (TAS2Rs) are expressed in CRC cells. Accordingly, we investigated the role of TAS2R-mediated signaling in the malignant alteration of CRC cells. Using an in vitro culture system with human colorectal cancer HCT116 cells, we treated the cells with amygdalin or arbutin as ligands. This treatment led to a significant, dose-dependent suppression of cell proliferation. Furthermore, gene expression profiling of the amygdalin- or arbutin-treated cells revealed a marked downregulation of gene clusters associated with angiogenesis compared to control cells. These findings suggest that activation of the TAS2R-mediated signaling pathway suppresses the malignant alteration of CRC cells. Our study indicates that regulation of TAS2R-mediated signaling may represent a novel and promising therapeutic strategy for advanced and recurrent colorectal cancer.

[Disclosure of COI by Responsible researcher] Lead presenter is not the Responsible researcher.
[Lead presenter: Kotoha Nozaki] We have no financial relationships to disclose.
[Principal Researcher: Hidemitsu Kitamura] ■Contributions: テルモ生命科学振興財団

Poster Session

### [P-3043] I-P5-6
### Intracellular signal transduction (2)

2025 / 9 / 27 (Sat) 15:30-16:15 Room P/(Motenashi Dome) Room P

---

### P-3043
### ERK mediated up-regulation of ErbB2/ErbB3 heterodimer in the LNCaP human prostate cancer cells

[Speaker]Yuki Kawasaki (Lab. Pub. Health, Fac. Pharm., Takasaki Univ. Health & Welfare) [Co-author]Aoi Nagashima (Lab. Pub. Health, Fac. Pharm., Takasaki Univ. Health & Welfare)

Antiandrogens were developed for prostate cancer (PCa) therapy. However, the acquired resistances obstruct such therapies. The receptor tyrosine kinase ErbB family expression and its involvement in PCa have been reported. The detailed ErbB regulatory mechanisms and its relationship to the acquired resistances in PCa are still unclear. In this study, we examined ErbB regulation in androgen receptor (AR)-expressing human PCa cells. The protein expression of ErbB family (EGFR, ErbB2, ErbB3, ErbB4) was analyzed by WB. The ErbB downstream pathway ERK was activated in a ligand-independent manner with TPA treatment, and the influence on ErbB tyrosine autophosphorylation was analyzed. Expression of EGFR, ErbB2, and ErbB3 was confirmed in the LNCaP human PCa cells, ErbB2 and ErbB3 were highly expressed. Lapatinib, ErbB2 inhibitor treatment decreased ErbB2/ErbB3 tyrosine autophosphorylation. ErbB2/ErbB3 tyrosine phosphorylation was increased with TPA treatment, suggesting the increased ErbB2/ErbB3 heterodimer activity. These results suggested ERK pathway-mediated EbrB2/ErbB3 heterodimer activation may affect PCa cell growth.

[Disclosure of COI by Responsible researcher] Lead presenter is the Responsible researcher.
[Lead presenter: Yuki Kawasaki] We have no financial relationships to disclose.

### [P-3052] I-P5-8
### Intracellular signal transduction (4)

2025 / 9 / 27 (Sat) 15:30-16:15 Room P/(Motenashi Dome) Room P

---

### P-3052
### Functional Analysis of the Stress-Responsive MAP Kinase Pathway in Epithelial-Mesenchymal Transition (EMT)

[Speaker]Noriko Tokai (IMS, The Univ of Tokyo) [Co-author]Hisashi Moriizumi (IMS, The Univ of Tokyo)、Mutsuhiro Takekawa (IMS, The Univ of Tokyo)

Epithelial-mesenchymal transition (EMT) is a fundamental biological process where epithelial cells acquire mesenchymal traits, contributing to tissue repair, regeneration, and cancer metastasis. EMT enhances cellular resistance to apoptosis, but the molecular mechanisms remain unclear. The stress-activated MAP kinase (SAPK) pathway, known for regulating stress responses and cell fate, may be involved. In this study, we investigated the role of the SAPK pathway in EMT. In epithelial cells, the intracellular localization of a specific component of the SAPK pathway markedly changed during EMT, with increased nuclear accumulation observed after EMT. Furthermore, post-EMT cells exhibited reduced activation of the SAPK pathway in response to stress stimuli, suggesting that EMT alters cellular stress responses by modulating SAPK signaling. To assess the physiological relevance, we created a mouse model in which EMT-dependent SAPK regulation is impaired. In a wound healing assay, these mice exhibited delayed tissue repair. These findings indicate that EMT-associated regulation of SAPK signaling may contribute to tissue regeneration and cancer metastasis.

[Disclosure of COI by Responsible researcher] Lead presenter is not the Responsible researcher.
[Lead presenter: Noriko Tokai] We have no financial relationships to disclose.
[Principal Researcher: Mutsuhiro Takekawa] ■Research funds from company: アボット

### [P-3053] I-P5-8
### Intracellular signal transduction (4)

2025 / 9 / 27 (Sat) 15:30-16:15 Room P/(Motenashi Dome) Room P

---

### P-3053
### Spatiotemporal regulation of KSR1 in RAS/MAPK signal propagation revealed by super-resolution microscopy

[Speaker]Ryo Yoshizawa (Natl. Cancer Ctr. Res. Inst.) [Co-author]Kenichi Suzuki (Natl. Cancer Ctr. Res. Inst. ／iGCORE, Gifu Univ.)

The growth factor-induced receptor tyrosine kinase (RTK)/RAS/MAPK signaling pathway plays critical roles in tumor progression. While numerous mutations in this pathway have been identified across various cancers, recent studies suggest that non-genetic factors such as the spatial localization and dynamic recruitment of signaling proteins are also critical for oncogenic signaling. For instance, knockdown of the scaffold protein KSR1 notably suppresses oncogenic signaling, implicating KSR1 as a key organizer of spatially coordinated signaling events. In this study, we investigated the role of KSR1 in spatiotemporal regulation of the RAS/MAPK pathway using super-resolution microscopy (dSTORM). Upon EGF stimulation, KSR1 molecules translocate to the plasma membrane, where they accumulate and form distinct cluster-like structures. We are currently investigating how these membrane-localized KSR1 clusters modulate the recruitment and dynamic behavior of downstream effectors, including RAF and RAS proteins. In future work, we aim to elucidate how the spatiotemporal regulation of KSR1 contributes to cancer progression and to evaluate its potential as a target for therapeutic intervention.

[Disclosure of COI by Responsible researcher] Lead presenter is not the Responsible researcher.
[Lead presenter: Ryo Yoshizawa] We have no financial relationships to disclose.
[Principal Researcher: Kenichi Suzuki] We have no financial relationships to disclose.

### [P-3054] I-P5-8
### Intracellular signal transduction (4)

2025 / 9 / 27 (Sat) 15:30-16:15 Room P/(Motenashi Dome) Room P

---

### P-3054
### HER2/HER3 signaling enhances activation of the integrated stress response in breast cancer cell lines

[Speaker]Miku Otsuka (Div. Genome Res., Cancer Chemother. Ctr. JFCR／Grad. Sch. Front. Sci., Univ. Tokyo) [Co-author]Yuka Okamoto (Div. Genome Res., Cancer Chemother. Ctr. JFCR／Lab. Genome Tch. , Human Genome. Ctr., IMSUT)、Akihiro Tomida (Div. Genome Res., Cancer Chemother. Ctr. JFCR／Grad. Sch. Front. Sci., Univ. Tokyo)

Under conditions of malnutrition or hypoxia due to inadequate vascularization, tumor cells activate adaptive responses including the integrated stress response (ISR) to survive and proliferate. We have previously shown that oncogenic signals such as activating mutations in EGFR enhance ISR programs. Another EGFR family member, HER3, is considered to lack kinase activity and forms heterodimers with other member like HER2 to promote tumor development or therapeutic resistance. In this study, we assessed whether HER2/3 signal is involved in ISR regulation in breast cancer cell lines; HER2-amplified SKBR3 cells and HER2-non-amplified MCF7 cells. We examined induction of ATF4, a marker and transcription factor of ISR, in cells treated with chemical stressors (tunicamycin; TM or L-histidinol; His) alone or in combination with a HER3 ligand, heregulin (HRG) for 4 hours. ATF4 induction under TM or His treatment was enhanced by HRG co-treatment in both cell lines. This enhancement was inhibited by knockdown of HER3 as well as by HER2 kinase inhibition. These results indicate that HER2/3 signal can enhance the ISR.

[Disclosure of COI by Responsible researcher] Lead presenter is not the Responsible researcher.
[Lead presenter: Miku Otsuka] We have no financial relationships to disclose.
[Principal Researcher: Akihiro Tomida] We have no financial relationships to disclose.

▲TOP

### [P-3055] I-P5-8
### Intracellular signal transduction (4)

2025 / 9 / 27 (Sat) 15:30-16:15 Room P/(Motenashi Dome) Room P

---

### P-3055
### ALPK2 Drives Cell Proliferation in Oral Squamous Cell Carcinoma

[Speaker]Yume Ueda (Dept. of Oral Oncology, Grad. Sch. of Medicine, Hiroshima Univ.) [Co-author]Nanako Kataoka (Ctr. for Oral Clinical Examination, Hiroshima Univ. Hosp.)、Kento Okamoto (Dept. of Oral Oncology, Grad. Sch. of Medicine, Hiroshima Univ.)、Kai Omachi (Dept. of Oral Oncology, Grad. Sch. of Medicine, Hiroshima Univ.)、Yuga Maeda (Dept. of Oral Oncology, Grad. Sch. of Medicine, Hiroshima Univ.)、Yukina Kobayashi (Ctr. for Oral Clinical Examination, Hiroshima Univ. Hosp.)、Mikihito Kajiya (Ctr. for Oral Clinical Examination, Hiroshima Univ. Hosp.)、Souichi Yanamoto (Dept. of Oral Oncology, Grad. Sch. of Medicine, Hiroshima Univ.)、Toshinori Ando (Ctr. for Oral Clinical Examination, Hiroshima Univ. Hosp.)

Aberrantly activated YAP/TAZ co-transcription factors drive the growth of oral squamous cell carcinoma (OSCC). However, the key genes driving proliferation in YAP/TAZ-activated OSCC cells remains unidentified. This study aims to identify and elucidate the role of the principal growth-promoting gene in YAP/TAZ-activated OSCC cells. We identified ALPK2 as one of the most upregulated kinases upon YAP/TAZ activation through RNA-seq analysis. Gene set enrichment analysis using cancer cell lines and tissues revealed that ALPK2 expression was enriched in YAP/TAZ-target gene sets. Knockdown of ALPK2 in YAP/TAZ-activated OSCC cells significantly inhibited cell proliferation. Mechanistically, RNA-seq analysis of ALPK2 knockdown cells showed enrichment of MAPK pathway-related gene sets. Furthermore, FOSL1, a downstream effector of the MAPK pathway, was reduced by ALPK2 knockdown, whereas FOSL1 was increased by YAP/TAZ activation. We demonstrated that YAP/TAZ-induced ALPK2 promotes cell proliferation by enhancing the transcription of FOSL1. Further investigations are needed to elucidate the detailed mechanisms underlying ALPK2-mediated proliferation and to develop therapeutic agents.

[Disclosure of COI by Responsible researcher] Lead presenter is not the Responsible researcher.
[Lead presenter: Yume Ueda] We have no financial relationships to disclose.
[Principal Researcher: Toshinori Ando] We have no financial relationships to disclose.

### [P-3056] I-P5-8
### Intracellular signal transduction (4)

2025 / 9 / 27 (Sat) 15:30-16:15 Room P/(Motenashi Dome) Room P

---

### P-3056
### YAP reactivation drives ER stress-mediated dormancy under EGFR inhibition in oral squamous cell carcinoma

[Speaker]Nanako Kataoka (Ctr. for Oral Clinical Examination, Hiroshima Univ. Hosp.) [Co-author]Yume Ueda (Dept. of Oral Oncology, Grad. Sch. of Medicine, Hiroshima Univ.)、Kento Okamoto (Dept. of Oral Oncology, Grad. Sch. of Medicine, Hiroshima Univ.)、Yukina Kobayashi (Ctr. for Oral Clinical Examination, Hiroshima Univ. Hosp.)、Kai Omachi (Dept. of Oral Oncology, Grad. Sch. of Medicine, Hiroshima Univ.)、Yuga Maeda (Dept. of Oral Oncology, Grad. Sch. of Medicine, Hiroshima Univ.)、Souichi Yanamoto (Dept. of Oral Oncology, Grad. Sch. of Medicine, Hiroshima Univ.)、Mikihito Kajiya (Ctr. for Oral Clinical Examination, Hiroshima Univ. Hosp.)、Toshinori Ando (Ctr. for Oral Clinical Examination, Hiroshima Univ. Hosp.)

Aberrantly activated YAP is critical for cancer cell proliferation. We previously reported that EGFR aberrantly activates YAP to promote cancer growth. Accordingly, EGFR inhibitors transiently inactivate YAP, suppressing proliferation. However, the response rate to EGFR inhibitors remains low in oral squamous cell carcinoma (OSCC). Dormant cancer cells, which are non-proliferative yet survive during EGFR inhibitor treatment, may lead to relapse. We hypothesized that YAP is reactivated during dormancy, contributing to recurrence. This study aims to clarify the role of YAP during EGFR inhibitor-induced dormancy. We found that YAP was initially inactivated by EGFR inhibition but strongly reactivated during dormancy following prolonged treatment. This reactivation occurred alongside an endoplasmic reticulum (ER) stress response. RNA-seq of YAP-activated OSCC cells revealed enrichment of ER lumen-related gene sets. These cells also showed ER stress markers. We demonstrated that YAP reactivation induces ER stress, which is essential for dormancy under EGFR inhibition. These findings suggest a novel therapeutic strategy targeting YAP-driven dormant cells during EGFR inhibitor treatment.

[Disclosure of COI by Responsible researcher] Lead presenter is not the Responsible researcher.
[Lead presenter: Nanako Kataoka] We have no financial relationships to disclose.
[Principal Researcher: Toshinori Ando] We have no financial relationships to disclose.

### [P-3057] I-P5-8
### Intracellular signal transduction (4)

2025 / 9 / 27 (Sat) 15:30-16:15 Room P/(Motenashi Dome) Room P

---

### P-3057
### Withdrawn

No Abstract

### [P-3058] I-P5-8
### Intracellular signal transduction (4)

2025 / 9 / 27 (Sat) 15:30-16:15 Room P/(Motenashi Dome) Room P

---

### P-3058
### Transcription-Dependent Autophagy Triggered by Nutrient Deprivation: Role of the IGF1R/PKC$\beta$/CREB1 Axis

[Speaker]Jo-Ying Chang (Department of biological science and environmental biology, KMU) [Co-author]Yung-Ding Bow (Ph.D program in life science, KMU)、Ruei-Nian Li (Department of biological science and environmental biology, KMU／Ph.D program in life science, KMU)

Autophagy regulates cell growth, death, and survival in cancer by recycling damaged organelles and proteins during nutrient deprivation. This process is linked to cancer malignancy, including metastasis. Protein kinase C beta (PKCbeta), a canonical member of the protein kinase C family, plays a vital role in metastasis, autophagy, and oncogenesis. In this study, Insulin-like Growth Factor 1 Receptor (IGF1R), a key regulator of cancer growth, influences autophagic flux via PKCbeta. CREB1, a transcriptional activator, is predicted to be activated by PKCbeta, promoting autophagy-related gene expression. Nutrient starvation triggers autophagy through the IGF1R/PKCbeta/CREB1 axis in OSCC. IGF1R inhibition reduced cancer migration and PKC substrate phosphorylation during starvation, suggesting the role of IGF1R in PKCbeta activity. Linsitinib increased LC3B conversion and p62 degradation, indicating autophagy activation, though impaired lysosomal function was noted. PKCbeta inhibitor Enzastaurin hindered lysosomal function and reduced CREB1 expression, emphasizing the role of PKCbeta in autophagy regulation. Targeting IGF1R and PKCbeta offers novel strategies for cancer treatment.

[Disclosure of COI by Responsible researcher] Lead presenter is not the Responsible researcher.
[Lead presenter: Jo-Ying Chang] We have no financial relationships to disclose.
[Principal Researcher: ] We have no financial relationships to disclose.

### [P-3065] I-P6-2
### DNA damage response

2025 / 9 / 27 (Sat) 15:30-16:15 Room P/(Motenashi Dome) Room P

---

### P-3065
### What is Lynch-like syndrome? Molecular pathology of MSI-H tumors.

[Speaker]Kokichi Sugano (Dpt. Genetic Medicine, Sasaki Foundation Kyoundo Hospital) [Co-author]Miho Ando (Dpt. Genetic Medicine, Sasaki Foundation Kyoundo Hospital)、Keiichi Iwaya (Dpt. Pathology Sasaki Foundation Kyoundo Hospital,)、Haruhiko Sugimura (Director, Sasaki Foundation Kyoundo Hospital)、Masaru Sakamoto (Full-time advisor, Sasaki Foundation Kyoundo Hospital)、Teruhiko Yoshida (Dept. Genetic Medicine and Services, National Cancer Center Hospital)、Makoto Hirata (Dept. Genetic Medicine and Services, National Cancer Center Hospital)

[Purpose] Lynch syndrome is a hereditary cancer syndrome caused by pathogenic germline variants (PGV) of mismatch repair (MMR) genes. Lynch syndrome-related tumor show microsatellite instability-high (MSI-H) phenotype, while MSI is also caused by somatic variants of MMR genes or promoter hypermethylation of ***MLH1*** . We performed matched-pair analysis of peripheral blood lymphocytes (PBL) and tumor samples. [Materials and Methods] Twenty-one MSI-H tumor samples (colon and rectum (n=16) and endometrium(n=5)) were examined, along with germline DNA testing of the MMR genes. [Results and Conclusion] PGVs of MMR genes were detected in 4/21 cases (19.0%), i.e., ***MLH1*** (n=2), ***MSH6*** (n=1) and ***PMS2***(n=1). Methylation of ***MLH1*** promoter region was detected in 11 cases (52.4%) of which one case showed constitutive methylation. Pathogenic variants of ***MLH1*** gene were detected in 7 cases, of which 2 cases as germline and 5 cases as somatic. Four cases had allelic loss of the wild type allele. Three cases harboring In/Del or frameshift SVs in ***MLH1*** gene due to MSI without allelic loss were considered as Lynch-like syndrome where the tumor showing MSI-H phenotype, but no recognizable MMR gene abnormality.

[Disclosure of COI by Responsible researcher] Lead presenter is not the Responsible researcher.
[Lead presenter: Kokichi Sugano] We have no financial relationships to disclose.
[Principal Researcher: Masaru Sakamoto] We have no financial relationships to disclose.

[P-3066] I-P6-2
DNA damage response

2025 / 9 / 27 (Sat) 15:30-16:15 Room P/(Motenashi Dome) Room P

### P-3066
### The mechanisms of the recruitment of SLX4-XPF in the response to replication stress induced by lacO-LacI interaction

[Speaker]Yoko Katsuki (Grad. Sch. Pharm. Sci., Kyushu Univ.) [Co-author]Takuma Okano (Grad. Sch. Pharm. Sci., Kyushu Univ.)、Soki Haruta (Grad. Sch. Pharm. Sci., Kyushu Univ.)、Junpei Fujii (Grad. Sch. Pharm. Sci., Kyushu Univ.)、Tomoki Matsumura (Grad. Sch. Pharm. Sci., Kyushu Univ.)、Yasunori Noguchi (Grad. Sch. Pharm. Sci., Kyushu Univ.)、Miyako Shiraishi (Grad. Sch. Pharm. Sci., Kyushu Univ.)、Kazumasa Yoshida (Grad. Sch. Pharm. Sci., Kyushu Univ.)、Masatoshi Fujita (Grad. Sch. Pharm. Sci., Kyushu Univ.)

In human cells, few model systems are available to analyze repair pathways for endogenous replication impediments, and the molecular mechanisms underlying these processes remain poorly understood. We have established a system where DNA-protein complexes are formed via lacO-LacI interaction, inducing replication stress. The SLX4-XPF nuclease complex has been identified as an early response factor in replication stress response.The N-terminal UBZ4 ubiquitin-binding domains or C-terminal SUMO-interacting motifs (SIMs) are required for SLX4 localization to stalled forks in mammals; however, the detailed mechanisms remain unclear. Following LacI binding, the N-terminal half of SLX4 localizes to the lacO region in a UBZ4-dependent manner but fails to recruit XPF despite the presence of the XPF-binding domain. We found that SUMOylation is critical for the localization of endogenous SLX4 and XPF and that the C-terminal half of SLX4 is also recruited to the stalled forks via SIMs. Furthermore, we discuss the possible implications of this mechanism in the recruitment of SLX4 to the centromere, one of the difficult-to-replicate regions in the genome.

[Disclosure of COI by Responsible researcher] Lead presenter is not the Responsible researcher.
[Lead presenter: Yoko Katsuki] We have no financial relationships to disclose.
[Principal Researcher: Masatoshi Fujita] We have no financial relationships to disclose.

### [P-3067] I-P6-2
DNA damage response

2025 / 9 / 27 (Sat) 15:30-16:15 Room P/(Motenashi Dome) Room P

### P-3067
### Regulatory mechanism of BRCA1/BARD1 stability mediated by calcineurin.

[Speaker]Yosei Miki (Lab. of Veterinary Physiol. & Biochem., Yamaguchi Univ.) [Co-author]Makoto Habara (Lab. of Veterinary Physiol. & Biochem., Yamaguchi Univ.)、Shoma Tsubota (Dept. of Mol. Biol., Grad. Sch. of Med., Nagoya Univ.)、Shigeaki Sugiyama (Dept. of Mol. Biol., Grad. Sch. of Med., Nagoya Univ.)、Yanjun Chen (Dept. of Mol. Biol., Grad. Sch. of Med., Nagoya Univ.)、Akari Sugiura (Dept. of Mol. Biol., Grad. Sch. of Med., Nagoya Univ.)、Harukatsu Kitajima (Dept. of Mol. Biol., Grad. Sch. of Med., Nagoya Univ.)、Midori Shimada (Dept. of Mol. Biol., Grad. Sch. of Med., Nagoya Univ.)

Dysregulation of intracellular $Ca^{2+}$ levels has been implicated in tumor initiation, progression and metastasis. Calcineurin is a $Ca^{2+}$-dependent Ser/Thr phosphatase involved in immune activation and cell division. We previously demonstrated that high calcineurin expression was associated with recurrence in breast cancer patients receiving endocrine therapy. We also found that calcineurin inhibitors enhanced the anti-proliferative effects of PARP inhibitors in breast cancer cells.
In this study, we focused on the BRCA1/BARD1 complex, a key factor in DNA double-strand break repair, and analyzed the mechanism regulating its degradation. Calcineurin inhibition promoted the degradation of BRCA1 and BARD1, reducing their protein levels. We identified a ubiquitin ligase responsible for BARD1 polyubiquitination. We are further investigating how BRCA1/BARD1 phosphorylation relates to DNA damage. Although BRCA1/BARD1 act as tumor suppressors, their expression can promote resistance to PARP inhibitors and platinum-based agents. Elucidating how calcineurin regulates their stability may help to develop strategies to overcome therapy resistance.

[Disclosure of COI by Responsible researcher] Lead presenter is not the Responsible researcher.
[Lead presenter: Yosei Miki] We have no financial relationships to disclose.
[Principal Researcher: Harukatsu Kitajima] We have no financial relationships to disclose.

### [P-3068] I-P6-2
### DNA damage response

2025 / 9 / 27 (Sat) 15:30-16:15 Room P/(Motenashi Dome) Room P

---

### P-3068
### Targeting the POLD4-PRIMPOL axis in non-small cell lung cancer

[Speaker]Atsuko Niimi (Dept. Mol. Oncol., Fujita Health Univ., Sch. Med) [Co-author]Siripan Limsirichaikul (Dept. Mol. Oncol., Fujita Health Univ., Sch. Med)、Dat Q. Tran (Dept. Mol. Oncol., Fujita Health Univ., Sch. Med)、Yasuyoshi Mizutani (Dept. Mol. Oncol., Fujita Health Univ., Sch. Med)、Toshiyuki Takeuchi (Dept. Mol. Oncol., Fujita Health Univ., Sch. Med)、Motoshi Suzuki (Dept. Mol. Oncol., Fujita Health Univ., Sch. Med)

Lung cancer has become the leading cause of cancer death in Japan, with non-small cell lung cancer (NSCLC) accounting for about 80-85% of all cases. Research to identify new pathological pathways and to develop molecular-targeted therapies is desired to be urgently conducted.
Previously, we analyzed gene expression profiles of 149 lung cancer specimens and found DNA polymerase delta subunit POLD4-low patients were associated with genomic instability and poor prognosis. In POLD4-high cells, RPA and CHK1 were highly phosphorylated under replication stress, resulting in the activation of DNA damage response (DDR) to suppress genomic instability.
In this study, we analyzed the mechanism of the POLD4-dependent DDR pathway for therapeutic strategies against POLD4-high cancer, the majority of NSCLC. DNA fiber assay showed single-stranded DNA gaps were accumulated in POLD4-high cells in a PRIMPOL-dependent manner, suggesting POLD4 promoted the PRIMPOL-mediated repriming pathway. We also found a lethal phenotype was triggered by targeting this particular pathway in both in vitro- and in mice- analyses. These results suggest the POLD4-PRIMPOL axis may be a novel druggable pathway in NSCLC.

[Disclosure of COI by Responsible researcher] Lead presenter is not the Responsible researcher.
[Lead presenter: Atsuko Niimi] We have no financial relationships to disclose.
[Principal Researcher: Motoshi Suzuki] We have no financial relationships to disclose.

▲TOP

### [P-3069] I-P6-2
### DNA damage response

2025 / 9 / 27 (Sat) 15:30-16:15 Room P/(Motenashi Dome) Room P

---

P-3069

### An ATR-PrimPol axis maintains chronic heterochromatin-associated replication stress tolerance in KRAS-driven cancer cell

[Speaker]Taichi Igarashi (Lab. of Genome Stress Response, Kyoto Univ.) [Co-author]Kimiyoshi Yano (Grad. Sch. of Biomed. Health Sci., Hiroshima Univ.)、Lee Zou (Dep. of Pharm. and Cancer Biol., Duke Univ.)、Takaaki Yasuhara (Lab. of Genome Stress Response, Kyoto Univ.)、Bunsyo Shiotani (Dep. of Genome Stress Signaling, Tokyo Med. Univ.)

DNA replication stress (RS), which is induced by chemicals, irradiation, and oncogenes activation, confers dysregulation of accurate DNA duplication. Regardless of their origins, cells have developed replication stress tolerance (RST) to avoid deleterious consequences. Recently, we have reported that the ATR-PrimPol pathway plays a critical role in the tolerance of heterochromatin-associated RS induced by oncogenic KRAS during the initial step of cancerous clonal expansion (PMID: 37591859). However, it remains elucidated whether oncogenic KRAS induces RS chronically in KRAS-driven transformed cells. Moreover, despite the availability of other RST, such as fork reversal and trans-lesion DNA synthesis (TLS), the mechanisms by which ATR priorities PrimPol-mediated repriming remain unclear. Here, we will discuss a model in which ATR orchestrates RST in lung adenocarcinoma cells by regulating PrimPol in its kinase activity dependent manner. These findings would contribute to the understanding of the molecular mechanisms of cancer development and progression.

[Disclosure of COI by Responsible researcher] Lead presenter is not the Responsible researcher.
[Lead presenter: Taichi Igarashi] We have no financial relationships to disclose.
[Principal Researcher: Bunsyo Shiotani] We have no financial relationships to disclose.

### [P-3070] I-P6-2
### DNA damage response

2025 / 9 / 27 (Sat) 15:30-16:15 Room P/(Motenashi Dome) Room P

---

### P-3070
### Functional analysis of ubiquitin ligase RFWD3 in DNA damage tolerance in human cells

[Speaker]Rie Kanao (Res. Inst. Environ. Med., Nagoya Univ. ／Nagoya Univ. Grad. Sch. Med.) [Co-author]Chikahide Masutani (Res. Inst. Environ. Med., Nagoya Univ. ／Nagoya Univ. Grad. Sch. Med.)

Genomic instability is a hallmark of most cancers. DNA damage is one of the key contributors to genomic instability, as it impairs DNA replication. Cells possess multiple DNA damage tolerance pathways, including translesion synthesis (TLS), to overcome replication blockage. PCNA ubiquitination is crucial for DNA damage tolerance. DNA damage tolerance also contributes to resistance to cancer chemotherapy, such as cisplatin treatment. We identified that ubiquitin ligase RFWD3 is involved in DNA damage tolerance outside of TLS. Although RFWD3 does not directly ubiquitinate PCNA, PCNA ubiquitination is necessary for the RFWD3-dependent DNA damage tolerance pathway. RFWD3 mutant defective in chromatin translocation and interaction with RPA exhibited impaired DNA damage tolerance. We found a novel RFWD3 mutant with chromatin translocation activity comparable to wild-type RFWD3 but with disrupted interaction with RPA. HeLa cells expressing the mutant RFWD3 exhibited increased sensitivity to cisplatin treatment. Our results suggest that the interaction between RFWD3 and RPA is a potential target for enhancing cancer chemotherapy with DNA-damaging agents.

[Disclosure of COI by Responsible researcher] Lead presenter is not the Responsible researcher.
[Lead presenter: Rie Kanao] ■Research funds from company: 公的研究費（科研費） ■Contributions: 公益財団法人武田科学振興財団、公益財団法人大幸財団
[Principal Researcher: Chikahide Masutani] We have no financial relationships to disclose.

### [P-3071] I-P6-2
### DNA damage response

2025 / 9 / 27 (Sat) 15:30-16:15 Room P/(Motenashi Dome) Room P

---

### P-3071
### Functional analysis of ARID1A in DNA repair

[Speaker]Usaki Arai (Grad. Sch. Nanobiosci., Yokohama City Univ.) [Co-author]Shinta Saito (Grad. Sch. Nanobiosci., Yokohama City Univ.)、Noritaka Adachi (Grad. Sch. Nanobiosci., Yokohama City Univ.)

ARID1A (AT-rich interactive domain-containing protein 1A) is a component of the SWI/SNF complex, which is one of the chromatin remodeling factors. The SWI/SNF complex plays an important role in regulating the development and differentiation by facilitating DNA binding of transcription factors through ARID1A-mediated structural alterations. Recent reports suggest that ARID1A may be involved in homologous recombination (HR) and mismatch repair (MMR), but details remain unclear. In this study, we generated human ARID1A gene knockout cell lines to analyze the impact of ARID1A deficiency on DNA repair. Loss of ARID1A resulted in a significant reduction in HR activity, although this did not lead to increased sensitivity to PARP inhibitors. Intriguingly, we also found that ARID1A deficiency resulted in an ~95% reduction in MMR activity. Consistent with this finding, temozolomide sensitivity was reduced in ARID1A knockout cells. Our findings reveal that the loss of ARID1A function results in a reduction in HR and, more pronouncedly, MMR. Our findings suggest that reduced MMR activity could be used as a new biomarker for ARID1A-deficient tumors.

[Disclosure of COI by Responsible researcher] Lead presenter is not the Responsible researcher.
[Lead presenter: Usaki Arai] We have no financial relationships to disclose.
[Principal Researcher: Noritaka Adachi] We have no financial relationships to disclose.

Poster Session

### [P-3072] I-P6-2
### DNA damage response

2025 / 9 / 27 (Sat) 15:30-16:15 Room P/(Motenashi Dome) Room P

---

### P-3072
### RNA helicase DHX8 promotes DNA damage response

[Speaker]Kuanlin Chiu (Dept. & Grad. Inst. of Pharm., Natl. Taiwan Univ., Taipei, Taiwan) [Co-author]Yun Chen (Inst. of Mole. & Cell. Biol., Natl. Taiwan Univ., Taipei, Taiwan)、Peichi Liao (Inst. of Mole. & Cell. Biol., Natl. Taiwan Univ., Taipei, Taiwan)、Tingchia Chang (Inst. of Mole. & Cell. Biol., Natl. Taiwan Univ., Taipei, Taiwan)、Yiting Wu (Dept. & Grad. Inst. of Pharm., Natl. Taiwan Univ., Taipei, Taiwan／Inst. of Mole. & Cell. Biol., Natl. Taiwan Univ., Taipei, Taiwan)、Ihsin Liao (Inst. of Atomic & Mole. Sciences, Academia Sinica, Taipei, Taiwan)、Hsiangyin Hsueh (Dept. & Grad. Inst. of Pharm., Natl. Taiwan Univ., Taipei, Taiwan)、Chien-Ping Yen (Inst. of Mole. & Cell. Biol., Natl. Taiwan Univ., Taipei, Taiwan)、Chialung Hsieh (Inst. of Atomic & Mole. Sciences, Academia Sinica, Taipei, Taiwan)、Hsuehping Catherine Chu (Inst. of Mole. & Cell. Biol., Natl. Taiwan Univ., Taipei, Taiwan)、Chingshyi Peter Wu (Dept. & Grad. Inst. of Pharm., Natl. Taiwan Univ., Taipei, Taiwan)

DHX8/Prp22 is a DEAH-box RNA helicase known for its role in splicing, particularly in the release of mature mRNA from the spliceosome. Here, we report an unexpected and essential function of DHX8 in the DNA damage response. DHX8 depletion severely impairs RPA foci formation and ATR-Chk1 pathway activation following camptothecin (CPT) treatment, while yH2AX foci formation remains unaffected. Using time-lapse live-cell imaging and immunofluorescence microscopy, we demonstrate that DHX8 is recruited to sites of DNA damage. Further analyses reveal that DHX8 ablation leads to the accumulation of RNA:DNA hybrids, defects in DNA end resection, and increased sensitivity to genotoxic stress. Notably, cells expressing a helicase-dead DHX8 mutant fail to rescue ATR-Chk1 activation, unlike those expressing wild-type DHX8, highlighting the critical requirement for DHX8's helicase activity. Together, these findings uncover a previously unrecognized role for DHX8 in promoting DNA end resection and ATR pathway activation during the DNA damage response.

[Disclosure of COI by Responsible researcher] Lead presenter is not the Responsible researcher.
[Lead presenter: Kuanlin Chiu] We have no financial relationships to disclose.
[Principal Researcher: Chingshyi Peter Wu] We have no financial relationships to disclose.

▲TOP

[P-3073] I-P6-2

DNA damage response

2025 / 9 / 27 (Sat) 15:30-16:15 Room P/(Motenashi Dome) Room P

### P-3073

### Deletion of the deubiquitinating enzyme USP10 leads to prolonged DNA repair and chromosomal instability.

[Speaker]Kohichi Utani (Kanazawa.Med.Univ. Sch.Med. Microbio.) [Co-author]Masaya Higuchi (Kanazawa.Med.Univ. Sch.Med. Microbio.)、Ryo Sakasai (Kanazawa.Med.Univ. Sch.Med. Biochem.)、Kuniyoshi Iwabuchi (Kanazawa.Med.Univ. Sch.Med. Biochem.)

USP10 is known as a various stress responsible deubiquitinase. The most well-studied intracellular function of USP10 is dissolvement of stress granules, which is inducible to a small proteasome in the cytoplasm by oxidative stress. On the other hand, it has been reported that USP10 is also involved in DNA damage repair. Our previous report showed that USP10 knockout mice exhibit severe anemia because of reduction of hematopoietic stem cells. In this study, we found depletion of USP10 retard Homologous Recombination (HR) dependent DNA repair and exhibit Fanconi Anemia (FA) like chromosome abnormality in USP10 depleted mouse embryonic fibroblast and human cancer cell lines. Furthermore, we showed that those HR repair abnormalities in USP10 depleted cells could be eliminated by either depletion or inhibition of DNA-PK, PP6 and SAPS2. It is a novel finding that USP10 regulates the switch DNA repair pathway choice and could be an important piece of evidence for understanding USP10-mediated tumorigenesis, FA and hematopoietic cell development.

[Disclosure of COI by Responsible researcher] Lead presenter is not the Responsible researcher.
[Lead presenter: Kohichi Utani] We have no financial relationships to disclose.
[Principal Researcher: Higuchi Masaya] We have no financial relationships to disclose.

### [P-3081] I-P7-1
### Cancer genomics

2025 / 9 / 27 (Sat) 15:30-16:15 Room P/(Motenashi Dome) Room P

---

### P-3081
### Variation in Gene Expression and Methylation Profiling in Gingival and Tongue Cancers

[Speaker]Takafumi Kashiwagi (Dept. of Cancer Genome Informatics, Osaka Univ.) [Co-author]Yasushi Totoki (Dept. of Cancer Genome Informatics, Osaka Univ.)、Hidenori Inohara (Dept. of Otorhinolaryngology-Head & Neck Surg., Osaka Univ.)、Narikazu Uzawa (Dept. of Oral & Maxillofacial Oncology and Surg., Osaka Univ.)、Shinichi Yachida (Dept. of Cancer Genome Informatics, Osaka Univ.)

Encore; Introduction:In oral squamous cell carcinoma (OSCC), mortality remains high and prognostic and molecular differences are observed based on tumor location. This study aimed to characterize gene expression changes, underlying mechanisms, and tumor immune profiles in gingival squamous cell carcinoma (GSCC) and tongue squamous cell carcinoma (TSCC). Methods:Whole transcriptome and tumor immune profiling were conducted on 31 GSCC and 27 TSCC cases, and DNA methylation analysis was performed on 30 GSCC and 27 TSCC cases. Results:Gene expression analysis revealed that cell cycle and antibacterial humoral immunity pathways were upregulated in GSCC, while immune response pathways were upregulated in TSCC. High HOXC13 expression could be associated with prognosis in GSCC. DNA methylation analysis identified hypermethylation of the HOXD11 gene's 3'UTR region, leading to increased expression in both GSCC and TSCC. Tumor immune profiling showed altered proportions of inflammatory cells between normal and tumoral areas of GSCC. Conclusion:Tailored therapeutic strategies and standardized treatment protocols based on site-specific characteristics are crucial to improving patient outcomes.

[Disclosure of COI by Responsible researcher] Lead presenter is not the Responsible researcher.
[Lead presenter: Takafumi Kashiwagi] We have no financial relationships to disclose.
[Principal Researcher: Narikazu Uzawa, Shinichi Yachida] We have no financial relationships to disclose.

[P-3082] I-P7-1
Cancer genomics

2025 / 9 / 27 (Sat) 15:30-16:15 Room P/(Motenashi Dome) Room P

### P-3082
### Immunohistochemical and Molecular Evolutionary Characteristics of Jejunoileal Adenocarcinoma

[Speaker]Rei Ishikawa (Tumor Pathol., Hamamatsu Univ. Sch. Med.) [Co-author]Satoshi Baba (Pathol., Hamamatsu Univ. Hosp.)、Yoshifumi Arai (Pathol., Toyohashi Mun. Hosp.)、Yoshiro Otsuki (Pathol., Seirei Hamamatsu Hosp.)、Hiroshi Ogawa (Pathol., Seirei Mikatahara Hosp.)、Hiroki Mori (Pathol., Hamamatsu Med. Center)、Shioto Suzuki (Pathol., Iwata City Hosp.)、Makoto Suzuki (Pathol., Shizuoka Gen. Hosp.)、Haruhiko Sugimura (Sasaki Inst.)、Kazuya Shinmura (Tumor Pathol., Hamamatsu Univ. Sch. Med.)

Jejunoileal adenocarcinoma (JIAC) is an extremely rare malignancy, accounting for less than 1% of all cancers. In this study, we analyzed 52 cases of JIAC (55 tumors) and compared them with 182 cases of CRAC (192 tumors) using immunohistochemistry. Additionally, for 8 cases, we conducted whole exome sequencing (WES) on 40 tumor samples to investigate key features of JIAC. (1) JIAC frequently expressed oncofetal proteins at higher rates than CRAC (28.8% vs. 1.6%, $P < 0.001$). Furthermore, SALL4 expression was strongly correlated with Chromogranin A expression ($\delta > 0.60$). (2) Mutations in TP53 and ARID2 were frequently observed in JIAC, and these mutations were identified as significant driver gene mutations ($P < 0.001$). Moreover, ARID2 mutations correlated with the expression of oncofetal proteins ($P = 0.043$). (3) The evolutionary tree of JIAC resembled advanced CRAC (long trunk - short branches type), however, the variant allele frequency (VAF) distribution indicated features of early-stage CRAC (less neutral tails: VAF < 0.15; $P < 0.001$). Given that JIAC's evolutionary process is a critical finding for future research and treatment strategies.

[Disclosure of COI by Responsible researcher] Lead presenter is not the Responsible researcher.
[Lead presenter: Ishikawa Rei] We have no financial relationships to disclose.
[Principal Researcher: Kazuya Shinmura] We have no financial relationships to disclose.

▲TOP

### [P-3083] I-P7-1
### Cancer genomics

2025 / 9 / 27 (Sat) 15:30-16:15 Room P/(Motenashi Dome) Room P

---

P-3083

### Distinctive Immune Microenvironment in Pancreatic Cancer: Comparative Single-Cell Analysis with Gastrointestinal Cancers

[Speaker]Kyonosuke Ikemura (Div. Adv. Surg. Oncol., R&D Ctr., Kitasato Univ. Sch. Med) [Co-author]Riku Okamoto (Div. Adv. Surg. Oncol., R&D Ctr., Kitasato Univ. Sch. Med)、Shunpei Shibaki (Div. Adv. Surg. Oncol., R&D Ctr., Kitasato Univ. Sch. Med)、Hiroyuki Minoura (Div. Adv. Surg. Oncol., R&D Ctr., Kitasato Univ. Sch. Med)、Kanako Naito (Div. Adv. Surg. Oncol., R&D Ctr., Kitasato Univ. Sch. Med)、Akiko Watanabe (Div. Adv. Surg. Oncol., R&D Ctr., Kitasato Univ. Sch. Med)、Keiko Oki (Div. Adv. Surg. Oncol., R&D Ctr., Kitasato Univ. Sch. Med)、Kota Okuno (Div. Adv. Surg. Oncol., R&D Ctr., Kitasato Univ. Sch. Med)、Shiori Fujino (Div. Adv. Surg. Oncol., R&D Ctr., Kitasato Univ. Sch. Med)、Takafumi Sangai (Dept. of Breast & Thyroid Surg., Kitasato Univ. Sch. of Med)、Yusuke Kumamoto (Dept. Gen. Hepatobiliary, Pancreatic & Pediatr. Surg., Kitasato Univ)、Naoki Hiki (Dept. of Upper Gastrointest. Surg., Kitasato Univ. Sch. of Med)、Takeshi Naito (Dept. of Lower Gastrointest. Surg., Kitasato Univ. Sch. of Med.)、Keishi Yamashita (Div. Adv. Surg. Oncol., R&D Ctr., Kitasato Univ. Sch. Med)

Background: Pancreatic cancer has a dismal prognosis, partly due to its complex tumor microenvironment (TME). Single-cell RNA sequencing (scRNA-seq) enables detailed analysis of TME cell populations and interactions.Objective: To characterize immune and stromal cells in the pancreatic cancer TME and reveal tumor-specific immune features.Methods: We analyzed scRNA-seq data from 15 pancreatic cancer cases (GSE155698). Seurat was used for clustering and annotation, and CellChat was applied to infer cell-cell communication.Results: UMAP identified major TME cell types. Endothelial cells were few and overlapped with CAFs but expressed VWF and CD34. Myeloid cells comprised 42%, including CXCR2+ tumor-associated neutrophils (TANs), absent in other gastrointestinal cancers. CellChat revealed TREM2+ TAMs strongly signaled to Tregs, which communicated with cancer cells via TWEAK-TNFRSF12A and SPP1-CD44 axes. TANs interacted with infiltrating cells but were less abundant.Conclusion: The pancreatic cancer TME forms an immunosuppressive network involving CAFs, TREM2+ TAMs, Tregs, and TANs, highlighting potential therapeutic targets.

[Disclosure of COI by Responsible researcher] Lead presenter is not the Responsible researcher.
[Lead presenter: Kyonosuke Ikemura] We have no financial relationships to disclose.
[Principal Researcher: Keishi Yamashita] We have no financial relationships to disclose.

[P-3084] I-P7-1
Cancer genomics

2025 / 9 / 27 (Sat) 15:30-16:15 Room P/(Motenashi Dome) Room P

### P-3084
### Chemotherapy resistance mechanisms of CF-based NAC in esophageal cancer

[Speaker]Masataka Hirasaki (Dept. of Clinical Cancer Genomics, SMUIMC.) [Co-author]Yutaka Miyawaki (Dept. of Gastroenterol. Surg., SMUIMC.)、Yasuo Kamakura (Dept. of Clinical Cancer Genomics, SMUIMC.)、Tomonori Kawasaki (Dept. of Diagnostic Pathology, SMUIMC.)、Hiroshi Sato (Dept. of Gastroenterol. Surg., SMUIMC.)、Tetsuya Hamaguchi (Dept. of Clinical Cancer Genomics, SMUIMC.／Dept. of Gastroenterol. Oncol., SMUIMC.)

Cisplatin plus 5-FU (CF) remains a key backbone in the treatment of locally advanced and unresectable esophageal cancer (EC). Understanding the mechanisms of chemotherapy resistance to CF therapy is critical for improving clinical outcomes. We performed RNA sequencing on paired tumor specimens (pre-neoadjuvant chemotherapy [NAC] biopsy and post-NAC surgical) from patients with EC receiving CF-based NAC. Differential gene expression analysis was conducted across the four groups defined by sample timing (pre vs. post) and recurrence status.Genes involved in glutathione metabolism were highly expressed in the recurrent PreNAC and PostNAC groups. Notably, GSTP1 and GSTM3 were significantly upregulated in the PostNAC recurrent group. In the recurrent group, 53 genes were upregulated and 54 downregulated post-NAC compared to pre-NAC. Although we hypothesized that NAC would induce new resistance mechanisms, the number of differentially expressed genes was limited, suggesting that resistance-related molecular features may already be present at the PreNAC stage. These findings highlight the potential importance of pretreatment molecular profiling for optimizing CF-based chemotherapy in EC.

[Disclosure of COI by Responsible researcher] Lead presenter is the Responsible researcher.
[Lead presenter: Masataka Hirasaki] We have no financial relationships to disclose.

### [P-3085] I-P7-1
### Cancer genomics

2025 / 9 / 27 (Sat) 15:30-16:15 Room P/(Motenashi Dome) Room P

---

### P-3085
### Genomic and epigenomic analysis of lung large cell neuroendocrine carcinoma using a long read sequencing technology

[Speaker]Yoshiki Otsuka (Grad. Sch. of Front. Sci., Univ. Tokyo) [Co-author]Megumi Tateishi (Grad. Sch. of Front. Sci., Univ. Tokyo)、Junko Zenkoh (Grad. Sch. of Front. Sci., Univ. Tokyo)、Ryota Matsuoka (Dept. Diag. Pathol., Inst. Med., Univ. Tsukuba)、Daisuke Matsubara (Dept. Diag. Pathol., Inst. Med., Univ. Tsukuba)、Yutaka Suzuki (Grad. Sch. of Front. Sci., Univ. Tokyo)、Ayako Suzuki (Grad. Sch. of Front. Sci., Univ. Tokyo)

Large cell neuroendocrine carcinoma (LCNEC) is a high-grade tumor with poor prognosis, and their molecular characteristics remain incompletely understood. We analyzed genomic and epigenomic aberrations of 21 LCNEC cases using long-read sequencing (tumor N50 read length: 14.5 kb, average depth: 58.5). These cases were classified by transcription factor expressions into four subtypes (ASCL1, NEUROD1, POU2F3, YAP1). From the obtained long-read data, we detected an average of 326 structural variants (SVs). Two YAP1 cases harbored a large number of insertion SVs, and the one of them had 635 LINE-1 insertions. Extrachromosomal DNAs (ecDNAs) were identified in 5 cases specifically in ASCL1/POU2F3 subtypes. The ecDNAs mainly contained MYC family genes. Methylation analysis revealed genome-wide hypomethylation in tumor tissues although two POU2F3 cases exhibited hypermethylation. We also found that methylation statuses especially in CpG island shores classified POU2F3 cases into a distinct group, suggesting subtype-specific epigenetic aberrations in this subtype. These findings reveal previously unidentified differences of complex genomic and epigenomic aberrations among the LCNEC subtypes.

[Disclosure of COI by Responsible researcher] Lead presenter is the Responsible researcher.
[Lead presenter: Yoshiki Otsuka] We have no financial relationships to disclose.

▲TOP

[P-3086] I-P7-1
Cancer genomics

2025 / 9 / 27 (Sat) 15:30-16:15 Room P/(Motenashi Dome) Room P

P-3086
### Exploration of telomere maintenance mechanisms associated genetic abnormalities in 321 neuroblastomas (JCCG-JNBSG)

[Speaker]Ritsuko Onuki (Research Institute for Clinical Oncology, Saitama Cancer Center) [Co-author]Masayuki Haruta (Research Institute for Clinical Oncology, Saitama Cancer Center)、Miki Ohira (Research Institute for Clinical Oncology, Saitama Cancer Center)、Motoki Sugawara (Research Institute for Clinical Oncology, Saitama Cancer Center)、Jesmin Akter (Research Institute for Clinical Oncology, Saitama Cancer Center)、Atsuko Nakazawa (Division of Clinical Research, Saitama Children's Medical Center)、Takako Yoshioka (Department of Pathology, National Center for Child Health and Development)、Hajime Okita (Division of Diagnostic Pathology, Keio University School of Medicine)、Miho Kato (Childhood Cancer Center, National Center for Child Health and Development)、Tetsuya Takimoto (Childhood Cancer Center, National Center for Child Health and Development)、Kentaro Watanabe (Department of Pediatrics, The University of Tokyo)、Seiya Imoto (Human Genome Center, The Institute of Medical Science)、Junko Takita (Department of Pediatrics, Graduate School of Medicine, Kyoto University)、Motohiro Kato (Department of Pediatrics, The University of Tokyo)、Tomoko Iehara (Department of Pediatrics, Kyoto Prefectural University of Medicine)、Takehiko Kamijo (Research Institute for Clinical Oncology, Saitama Cancer Center)

Telomere maintenance mechanisms (TMM) abnormalities are frequent in high-risk neuroblastoma, characterized by high expression of TERT (TERT-high) or alternative lengthening of telomeres (ALT). In this study, we analyzed 321 neuroblastic tumors registered in the JCCG-JNBSG for the presence of TMM and genetic abnormalities in ALT-positive tumors. RT-qPCR identified TERT-high in 64 NB and 7 GNB cases and c-circle assay found 38 ALT-positive NB and 6 GNB cases. ATRX and DAXX mutations are known in ALT-positive NB; however, other ALT-related genes are less understood. Therefore, we performed custom CGH+SNP array and Sanger sequencing on ALT-positive tumors, covering the ATRX genomic region, and found ATRX deletions and/or mutations in 34 cases. For the remaining 9 of 10 cases without ATRX mutations, further analysis using AMED whole genome sequencing (WGS) data was conducted. After filtering SNVs/indels detected by Mutect2 and adding clinical annotations, 277 non-silent SNVs and 48 SNVs in promoter/enhancer regions were extracted as candidate mutations that may be involved in ALT. Verification of the candidate mutations will be performed using gene expression from RNA-seq data.

[Disclosure of COI by Responsible researcher] Lead presenter is not the Responsible researcher.
[Lead presenter: Ritsuko Onuki] We have no financial relationships to disclose.
[Principal Researcher: Takehiko Kamijo] We have no financial relationships to disclose.

### [P-3087] I-P7-1
### Cancer genomics

2025 / 9 / 27 (Sat) 15:30-16:15 Room P/(Motenashi Dome) Room P

---

### P-3087
### Search for the conditions of library preparation aimed at obtaining optimized data using nanopore sequence

[Speaker]Mineko Ushiama (Dept. Clin. Genomics, Natl. Cancer Ctr. Res. Inst.) [Co-author]Masahiro Gotoh (Dept. Clin. Genomics, Natl. Cancer Ctr. Res. Inst.)、Hanako Ono (Dept. Clin. Genomics, Natl. Cancer Ctr. Res. Inst.)、Ai Ikki (Dept. Clin. Genomics, Natl. Cancer Ctr. Res. Inst.)、Daiki Higuchi (Div. Genome Biol., Natl. Cancer Ctr. Res. Inst.)、Ryuji Hamamoto (Med. AI Res. and Dev., Natl. Cancer Ctr. Res. Inst.)、Takashi Kohno (Div. Genome Biol., Natl. Cancer Ctr. Res. Inst.)、Kouya Shiraishi (Dept. Clin. Genomics, Natl. Cancer Ctr. Res. Inst.／Div. Genome Biol., Natl. Cancer Ctr. Res. Inst.)

Nanopore sequencing technology, which is simple, fast, and requires minimal pre-treatment, has become widely available, making long-read sequencing analysis easier. However, the amount of data obtained is influenced by factors, such as DNA extraction and fragmentation conditions during library preparation, and it may not be possible to obtain the expected amount of data. This study identified the factors influencing data volume and investigated the conditions for improving the data acquisition. Sixty-nine clinical specimens of DNA derived from peripheral blood, tumors, and normal tissues were analyzed. DNA fragmentation was performed using the needle method and g-TUBE (Covaris). Library preparation was performed according to the standard protocol of Oxford Nanopore Technologies, and sequencing was performed using PromethION48 and Flow Cell R10.4.1. The Estimated bases (Gb) calculated using MinKNOW software indicated the amount of data obtained. In this study, DNA fragmentation using g-TUBE was shown to be important for optimal data acquisition (needle: 58 Gb vs g-TUBE: 80 Gb). In this presentation, we report more detailed experimental conditions and results.

[Disclosure of COI by Responsible researcher] Lead presenter is not the Responsible researcher.
[Lead presenter: Mineko Ushiama] We have no financial relationships to disclose.
[Principal Researcher: Kouya Shiraishi] We have no financial relationships to disclose.

▲TOP

### [P-3088] I-P7-1
### Cancer genomics

2025 / 9 / 27 (Sat) 15:30-16:15 Room P/(Motenashi Dome) Room P

---

### P-3088
### Integrated analysis of Whole exome sequencing(WES) and Target capture sequencing

[Speaker]Sou Nakatani (Cancer Diagnostics Res. Div. Shizuoka Cancer Ctr.Res. inst.) [Co-author]Yuji Shimoda (Cancer Diagnostics Res. Div. Shizuoka Cancer Ctr.Res. inst.)、Fukumi Kamada (Cancer Diagnostics Res. Div. Shizuoka Cancer Ctr.Res. inst.)、Takeshi Nagashima (Cancer Diagnostics Res. Div. Shizuoka Cancer Ctr.Res. inst.／SRL Inc.)、Kenichi Urakami (Cancer Diagnostics Res. Div. Shizuoka Cancer Ctr.Res. inst.)、Shumpei Ohnami (Cancer Diagnostics Res. Div. Shizuoka Cancer Ctr.Res. inst.)、Sumiko Ohnami (Cancer Diagnostics Res. Div. Shizuoka Cancer Ctr.Res. inst.)、Maki Mizuguchi (Cancer Diagnostics Res. Div. Shizuoka Cancer Ctr.Res. inst.)、Ken(Shizuoka Cancer Ctr.) Yamaguchi (Cancer Diagnostics Res. Div. Shizuoka Cancer Ctr.Res. inst.)

Project HOPE is a research project aimed at investigating the genetic changes that cause cancer, and using these changes to aid in diagnosis and treatment.In DNA sequencing, tumor content and intratumor heterogeneity are relevant to mutation detection, and samples with low tumor content and high intratumor heterogeneity are more likely to have undetected mutations that are originally present.To improve the mutation detection rate, we added a probe targeting pathogenic mutations in 410 cancer-related genes (Custom Probe) to the WES target capture process.In a comparison of 11 cases with and without custom probe, the detection of pathogenic and possibly pathogenic mutations increased by 40% in the presence of custom probe, with new or stable mutations detected in the presence of custom probe.Using this method, WES was performed in 2400 cases, and the depth enrichment rate in the custom probe region was 8-fold, and 106 mutations that could not be detected by conventional WES were newly detected.The improved detection rate of important mutations in this study is expected to contribute to improved accuracy of diagnosis and treatment.

[Disclosure of COI by Responsible researcher] Lead presenter is the Responsible researcher.
[Lead presenter: Sou Nakatani] We have no financial relationships to disclose.

### [P-3089] I-P7-1
### Cancer genomics

2025 / 9 / 27 (Sat) 15:30-16:15 Room P/(Motenashi Dome) Room P

---

### P-3089
### Withdrawn

No Abstract

### [P-3096] I-P7-3
### Cancer genomic medicine

2025 / 9 / 27 (Sat) 15:30-16:15 Room P/(Motenashi Dome) Room P

### P-3096
### Identification of Actionable Mutations and Prognostic factors using Cancer Genome Profiling in Biliary Tract Cancer

[Speaker]Tomohiro Kubo (Dept. Med. Oncology, Sapporo Med. Univ.) [Co-author]Norito Suzuki (Dept. Med. Oncology, Sapporo Med. Univ.)、Ryota Yokoyama (Dept. Med. Oncology, Sapporo Med. Univ.)、Joji Muramatsu (Dept. Med. Oncology, Sapporo Med. Univ.)、Kazuma Ishikawa (Dept. Med. Oncology, Sapporo Med. Univ.)、Ayako Murota (Dept. Gastroenterology and Hepatology, Sapporo Med. Univ.)、Kohichi Takada (Dept. Med. Oncology, Sapporo Med. Univ.)

<Background> Recent studies have demonstrated the efficacy of genotype-matched therapy (GMT) for biliary tract cancer (BTC). Cancer genome profiling (CGP) has gained attention as a powerful tool for identifying potential molecular targets in BTC. However, the utility of CGP has not been fully elucidated.
<Methods> We prospectively analyzed data of 108 patients with advanced BTC who underwent CGP from August 2019 to March 2025.
<Results> Actionable mutations were detected in 24% of cases, included TMB-high (7 cases), ***ERBB2*** amplification (7 cases), ***BRCA1/2*** mutation (6 cases), ***IDH1*** mutation (4 cases), ***FGFR2*** fusion (1 case), and ***BRAF V600E*** mutation (1 case). 7 cases (6.4%) received GMT. In analysis of genetic alterations and overall survival, ***CDKN2A/2B*** loss was identified as a poor prognostic factor in intrahepatic cholangiocarcinoma.
<Conclusion> While access to GMT remains limited, the availability of targeted therapies, such as ***ERBB2*** amplification, is expected to increase the rate of access to GMT. Furthermore, this study suggests that ***CDKN2A2/B*** loss is a poor prognostic factor in BTC. CGP may be beneficial for treatment decisions and the prediction of clinical outcomes in BTC.

[Disclosure of COI by Responsible researcher] Lead presenter is not the Responsible researcher.
[Lead presenter: Tomohiro Kubo] We have no financial relationships to disclose.
[Principal Researcher: Kohichi Takada] We have no financial relationships to disclose.

### [P-3097] I-P7-3
### Cancer genomic medicine

2025 / 9 / 27 (Sat) 15:30-16:15 Room P/(Motenashi Dome) Room P

---

### P-3097
### Genomic analysis of meningiomas in Japan using C-CAT data

[Speaker]Ryosuke Ikemachi (Dept. of Neurological Surgery, Okayama Univ. Grad. Sch. of Med.) [Co-author]Yoshihiro Otani (Dept. of Neurological Surgery, Okayama Univ. Grad. Sch. of Med.)、Yasuhito Kegoya (Dept. of Neurological Surgery, Okayama Univ. Grad. Sch. of Med.)、Shuichiro Hirano (Dept. of Neurological Surgery, Okayama Univ. Grad. Sch. of Med.)、Joji Ishida (Dept. of Neurological Surgery, Okayama Univ. Grad. Sch. of Med.)、Shohei Nishigaki (Dept. of Neurological Surgery, Okayama Univ. Grad. Sch. of Med.)、Ryo Omae (Dept. of Neurological Surgery, Okayama Univ. Grad. Sch. of Med.)、Madoka Hokama (Dept. of Neurological Surgery, Okayama Univ. Grad. Sch. of Med.)、Tsuyoshi Umeda (Dept. of Neurological Surgery, Okayama Univ. Grad. Sch. of Med.)、Ryo Mizuta (Dept. of Neurological Surgery, Okayama Univ. Grad. Sch. of Med.)、Ryoji Imoto (Dept. of Neurological Surgery, Okayama Univ. Grad. Sch. of Med.)、Naoya Kemmotsu (Dept. of Neurological Surgery, Okayama Univ. Grad. Sch. of Med.)、Shuta Tomida (Ctr. for Comprehensive Genomic Med., Okayama Univ. Hosp.)、Daisuke Ennishi (Ctr. for Comprehensive Genomic Med., Okayama Univ. Hosp.)、Shota Tanaka (Dept. of Neurological Surgery, Okayama Univ. Grad. Sch. of Med.)

Introduction: The 2021 WHO Classification of CNS Tumors (WHO CNS 5) introduced molecular-based grading for meningiomas, emphasizing molecular diagnostics. We analyzed the genetic features and prognosis of meningiomas using the Center for Cancer Genomics and Advanced Therapeutics (C-CAT) data.
Methods: We reviewed 48 grade 2 or 3 meningiomas registered in C-CAT from June 2019 to December 2024. In this study, genomic alterations with an evidence level of D or higher, according to the C-CAT classification, were defined as actionable alterations.
Results: Of the 48 cases, 26 were grade 2 (24 atypical, 2 clear cell) and 22 were grade 3 (anaplastic). TERT promoter mutations were found in 1 grade 3 and 2 grade 2 cases. CDKN2A/B deletions were found in 10 grade 3 and 7 grade 2 cases. Actionable alterations were seen in 15 cases (17 mutations). In the WHO CNS5 classification, considering TERT mutations or CDKN2A/B deletions, grade 3 had shorter survival than grade 2 (median: 25 months vs not reached, p = 0.005).
Conclusion: This study utilizing C-CAT data identified genetic features of meningiomas in Japan, suggesting that molecular diagnostics may provide a more accurate prognosis.

[Disclosure of COI by Responsible researcher] Lead presenter is not the Responsible researcher.
[Lead presenter: Ryosuke Ikemachi] We have no financial relationships to disclose.
[Principal Researcher: Yoshihiro Otani] We have no financial relationships to disclose.

### [P-3098] I-P7-3
### Cancer genomic medicine

2025 / 9 / 27 (Sat) 15:30-16:15 Room P/(Motenashi Dome) Room P

---

### P-3098
### Varporter software streamlines the evaluation of cancer gene panel test results

[Speaker]Masashi Idogawa (Med. Genome Sci., Dept. Genomic Preventive Med., Sapporo Med. Univ.) [Co-author]Shoichiro Tange (Med. Genome Sci., Dept. Genomic Preventive Med., Sapporo Med. Univ.)、Kohichi Takada (Div. Med. Oncol., Sapporo Med. Univ.)、Takashi Tokino (Med. Genome Sci., Dept. Genomic Preventive Med., Sapporo Med. Univ.)、Akihiro Sakurai (Genome Med. Ctr., Caress Memorial Hosp.／Clin. Genomics, Dept. Genomic Preventive Med., Sapporo Med. Univ.)

The evaluation of cancer gene panel test results requires a large amount of manual work. To improve the efficiency of this process, we developed Varporter, a software support tool for cancer gene panel test analysis. The software automatically incorporates mutation information and enables filtering by secondary findings disclosure recommendations for germline variants. The Varporter can also link to drug therapy databases such as CIViC and OncoKB, and import C-CAT reports to display the level of evidence, in addition to the pathogenicity of ClinVar. The first effect of Varporter is a significant reduction in the time required for evaluation. Secondly, the elimination of simple tasks such as copy-pasting and searching has reduced the mental burden on the evaluator by eliminating the risk of making errors. Furthermore, the ability to refer to the results of various related databases by simply clicking on the links has an educational effect on the evaluators. The Varporter has made it possible to automate and streamline complicated manual tasks related to cancer gene panel evaluation, and to redirect human resources to more essential tasks.

[Disclosure of COI by Responsible researcher] Lead presenter is the Responsible researcher.
[Lead presenter: Masashi Idogawa] We have no financial relationships to disclose.

### [P-3099] I-P7-3
### Cancer genomic medicine

2025 / 9 / 27 (Sat) 15:30-16:15 Room P/(Motenashi Dome) Room P

### P-3099
### Molecular and Cellular Profiling of Patient-Derived Hypopharyngeal Carcinoma Cell Lines Toward Clinical Application

[Speaker]Kazue Ito (Miygai Cancer Ctr. Dept. Head & Neck Caner Oncology) [Co-author]Satoshi Souma (Miyagi Caner Ctr. Int. Div. of Mol. & Cell. Oncology)、Shin Ito (Miyagi Caner Ctr. Int. Div. of Mol. & Cell. Oncology)、Norihisa Shindo (Miyagi Caner Ctr. Int. Div. of Mol. & Cell. Oncology)、Takayuki Imai (Miygai Cancer Ctr. Head & Neck Surg.)、Tomoko Yamazaki (Saitama Med. Univ. International Med. Ctr. Dept. Head & Neck)、Kazunori Yamaguchi (Miyagi Caner Ctr. Int. Div. of Mol. & Cell. Oncology)、Jun Yasuda (Miyagi Caner Ctr. Int. Div. of Mol. & Cell. Oncology)

Hypopharyngeal carcinoma (HPC) is a major cancer in head and neck cancer, with limited availability of effective molecularly targeted therapies. The underlying oncogenic mechanisms remain poorly understood. We established patient-derived cell (PDC) lines from surgically resected HPC samples to explore their utility in identifying patient-specific molecular targets. Comprehensive analyses including driver gene mutation profiling via a multi-gene panel, transcriptomic profiling, and karyotyping were conducted. Findings were compared to established HPC cell lines (BICR6, FaDu). The PDCs displayed cancer-associated genomic alterations, primarily copy number gains. Amplifications of EGFR and CCND1 were associated with overexpression, suggesting potential driver roles. A PDC with a PIK3CA activating mutation showed sensitivity to the PI3K inhibitor alpelisib. Both PDCs exhibited aneuploidy and chromosomal instability (CIN). As CIN-positive cells are known to depend on KIF18A, we tested KIF18A inhibition and observed differing responses between the strains. These results support the value of HPC-derived PDCs as platforms for precision oncology research.

[Disclosure of COI by Responsible researcher] Lead presenter is the Responsible researcher.
[Lead presenter: Kazue Ito] We have no financial relationships to disclose.

[P-3100] I-P7-3
Cancer genomic medicine

2025 / 9 / 27 (Sat) 15:30-16:15 Room P/(Motenashi Dome) Room P

P-3100

### Clinical relevance of non-synonymous variants in ***BRCA2***-related 6 cancer types across ***in vitro*** and ***in silico*** analysis

[Speaker]Tatsuki Yamazoe (Lab. for Genotyping Development, IMS, RIKEN) [Co-author]Kentaro Arai (Lab. for Genotyping Development, IMS, RIKEN)、Toshiki Tanigawa (Precision Immune Regulation RIKEN ECL Research Unit, IMS, RIKEN)、Mikiko Endo (Lab. for Genotyping Development, IMS, RIKEN)、Yusuke Iwasaki (Lab. for Genotyping Development, IMS, RIKEN)、Shigeaki Sunada (JARIHES, Juntendo University)、Yoshio Miki (Research and Development Center for Precision Medicine, University of Tsukuba)、Yukihide Momozawa (Lab. for Genotyping Development, IMS, RIKEN)

Clinical genetic testing has identified numerous variants of uncertain significance (VUSs) in ***BRCA2***. Despite the development of various ***in vitro*** and ***in silico*** methods to assess the pathogenicity of VUSs, their application in clinical settings remains challenging. In this study we conducted two distinct ***in vitro*** analysis: Homologous recombination assay and Minigene splicing assay. Furthermore, we evaluated the association between functionally abnormal variants, spliceognic variants and 6 ***BRCA2***-related cancer risks in case-control study consisting of 36,088 cases and 38,842 controls. Similarly, we performed the same case-control study using previous functional data obtained by other ***in vitro*** or ***in silico*** methods. For almost all functional analysis, abnormal non-synonymous variants within the DNA-binding domain (DBD) were associated with a higher breast cancer risk. Likewise, spliceognic variants were associated with breast cancer risk with lower odds ratio than functionally abnormal DBD variants. We predicted that our assessment of clinical relevance for non-synonymous variants in ***BRCA2*** can provide useful information for appropriate clinical management of individuals with VUSs.

[Disclosure of COI by Responsible researcher] Lead presenter is not the Responsible researcher.
[Lead presenter: Tatsuki Yamazoe] We have no financial relationships to disclose.
[Principal Researcher: Yukihide Momozawa] We have no financial relationships to disclose.

### [P-3101] I-P7-3
### Cancer genomic medicine

2025 / 9 / 27 (Sat) 15:30-16:15 Room P/(Motenashi Dome) Room P

---

### P-3101
### Integrative Analysis of GATA3 Tissue Array and Genetic Variants Reveals Prognostic Implications in Urothelial Carcinoma

[Speaker]Chia-Min Chung (Grad. Inst. of Biomed. Sci., China Med. Univ., Taichung, Taiwan／Ctr. for Addiction, China Med.Univ. Hosp. , Taichung, Taiwan) [Co-author]Han C. (Dept.of Pathology, CMUH)、ChaoHsiang C. (Dept.of Urology, CMUH)、YiHuei C. (Dept.of Urology, CMUH)、ChiJung C. (Dept. of Public Health,CMU／Dept. of Med Res. ,CMUH)

Background:GATA3 is a key marker of urothelial differentiation, but its prognostic value in urothelial carcinoma(UC) remains uncertain despite associations with tumor grade and invasiveness. To elucidate GATA3's role in UC, we integrated immunohistochemistry (IHC) and SNP genotyping to explore links between genetic variants, protein expression, and patient survival.Methods: We conducted a case-control study involving 486 UC patients and 610 controls, analyzing five GATA3 SNPs and tumor GATA3 expression to evaluate the prognostic impact of SNP and IHC status using logistic regression and survival analysis.Results:The GATA3 rs1244159 G allele was associated with reduced UC risk (adjusted OR = 0.48, p = 0.0231) and higher GATA3 expression (p = 0.0173). High GATA3 expression predicted better overall survival (p = 0.0092), particularly in G allele carriers (p = 0.0071), with no significant effect in A allele carriers (p = 0.1824).Conclusion:GATA3 polymorphism affects expression and outcomes in UC, with elevated GATA3, particularly in G allele carriers, serving as a potential protective prognostic biomarker.

[Disclosure of COI by Responsible researcher] Lead presenter is the Responsible researcher.
[Lead presenter: Chia-Min Chung] We have no financial relationships to disclose.

### [P-3102] I-P7-3
### Cancer genomic medicine

2025 / 9 / 27 (Sat) 15:30-16:15 Room P/(Motenashi Dome) Room P

---

### P-3102
### Withdrawn

No Abstract

### [P-3103] I-P7-3
### Cancer genomic medicine

2025 / 9 / 27 (Sat) 15:30-16:15 Room P/(Motenashi Dome) Room P

---

### P-3103
### Significance of Pancreas Surveillance for HBOC Patients

[Speaker]Tomoya Sano (Molecular Oncology and Therapeutics, Osaka Metropolitan University Graduate School ／Gastroenterological Surgery, Osaka Metropolitan University Graduate School) [Co-author]Masakazu Yashiro (Molecular Oncology and Therapeutics, Osaka Metropolitan University Graduate School ／Gastroenterological Surgery, Osaka Metropolitan University Graduate School)、Kyoka Kawabata (Molecular Oncology and Therapeutics, Osaka Metropolitan University Graduate School)、Hinano Nishikubo (Molecular Oncology and Therapeutics, Osaka Metropolitan University Graduate School)、Dongheng Ma (Molecular Oncology and Therapeutics, Osaka Metropolitan University Graduate School)、Canfeng Fan (Molecular Oncology and Therapeutics, Osaka Metropolitan University Graduate School)、Yurie Yamamoto (Molecular Oncology and Therapeutics, Osaka Metropolitan University Graduate School)、Daiki Imanishi (Molecular Oncology and Therapeutics, Osaka Metropolitan University Graduate School ／Gastroenterological Surgery, Osaka Metropolitan University Graduate School)、Takashi Sakuma (Molecular Oncology and Therapeutics, Osaka Metropolitan University Graduate School ／Gastroenterological Surgery, Osaka Metropolitan University Graduate School)、Koji Maruo (Molecular Oncology and Therapeutics, Osaka Metropolitan University Graduate School ／Gastroenterological Surgery, Osaka Metropolitan University Graduate School)、Tatsunari Fukuoka (Molecular Oncology and Therapeutics, Osaka Metropolitan University Graduate School ／Gastroenterological Surgery, Osaka Metropolitan University Graduate School)、Kiyoshi Maeda (Gastroenterological Surgery, Osaka Metropolitan University Graduate School)

Hereditary breast and ovarian cancer (HBOC) syndrome, which is caused by the germline pathogenic mutation of BRCA1/BRCA2 genes, shows frequent development of breast, ovarian, prostate, or pancreatic cancer. The aim of this study is to clarify the significance of pancreas surveillance for HBOC patients without pancreatic cancer. A total of 75 HBOC patients was enrolled from 2018 to 2025. Of the 75 cases, 2 cases were excluded in this study because pancreatic cancer was found at the time of registration in our hospital. In 73 HBOC cases, 40 cases were breast cancer, 11 cases were ovarian cancer, 4 cases were prostate cancer, 4 cases were other types of cancer, and 14 cases were asymptomatic. MRCP (magnetic resonance cholangiopancreatography) was performed for 41 cases, and abdominal CT (computed tomography) was performed for 56 cases. IPMN (intraductal papillary mucinous neoplasm) was diagnosed in 4 cases of the 41 cases underwent MRCP. On the other hand, IPMN was detected by CT in only one case of the 4 IPMN cases. These findings suggested that IPMN might be frequently detected in HBOC patients, and MRCP is useful for pancreas surveillance for HBOC patients.

[Disclosure of COI by Responsible researcher] Lead presenter is not the Responsible researcher.
[Lead presenter: Tomoya Sano] We have no financial relationships to disclose.
[Principal Researcher: Kiyoshi Maeda] We have no financial relationships to disclose.

### [P-3104] I-P7-3
### Cancer genomic medicine

2025 / 9 / 27 (Sat) 15:30-16:15 Room P/(Motenashi Dome) Room P

---

### P-3104
### Sample Management System at TR Sample Management Office, National Cancer Center Hospital East.

[Speaker]Yasuko Tada (TR Sample Management Office, NCC Hosp. East.) [Co-author]Mari Takahashi (TR Sample Management Office, NCC Hosp. East.／Endoscopy Ctr., NCC Hosp. East.)、Hitomi Nakai (TR Sample Management Office, NCC Hosp. East.)、Yumie Takeshima (TR Sample Management Office, NCC Hosp. East.)、Mana Shimamura (TR Sample Management Office, NCC Hosp. East.／Department of Clin. Lab., NCC Hosp. East.)、Fubuki Omoya (TR Sample Management Office, NCC Hosp. East.／Department of Clin. Lab., NCC Hosp. East.)、Yuki Iino (TR Sample Management Office, NCC Hosp. East.)、Asako Takahata (TR Sample Management Office, NCC Hosp. East.)、Kana Sonoki (TR Sample Management Office, NCC Hosp. East.)、Yukari Nakagawa (TR Sample Management Office, NCC Hosp. East.)、Michiteru Yamagishi (TR Sample Management Office, NCC Hosp. East.／Department of Clin. Lab., NCC Hosp. East.)、Takao Fujisawa (TR Sample Management Office, NCC Hosp. East.／TR Support Office, NCC Hosp. East.／Department of Head and Neck Medical Oncology, NCC Hosp.East.)

Many clinical studies using human-derived samples are conducted in the National Cancer Center Hospital East and the Exploratory Oncology Research & Clinical Trial Center (Kashiwa Campus). We classified the clinical studies into two management levels: level A, central management due to our TR Sample Management Section, and level B, local management due to researchers themselves. To standardize Level A sample management, we have developed SOPs and operating manuals. Sample collection, processing, and storage are recorded based on these SOPs to maintain quality and traceability.In FY2022, we acquired ISO 9001 certification and have since worked toward continuous improvement. In addition, we prepare a manual for each study to meet its specific requirements. As advances in cancer genome research have increased the need for high-quality biospecimens, proper sample management has become even more critical to ensure reliable analysis results.In this presentation, we will introduce our sample management approach, share our experience in operating under a quality management system, and discuss future challenges.

[Disclosure of COI by Responsible researcher] Lead presenter is not the Responsible researcher.
[Lead presenter: Yasuko Tada] We have no financial relationships to disclose.
[Principal Researcher: Takao Fujisawa] We have no financial relationships to disclose.

▲TOP

### [P-3110] I-P8-4
### Histone modification & others

2025 / 9 / 27 (Sat) 15:30-16:15 Room P/(Motenashi Dome) Room P

### P-3110
### MOA study of gut microbiome-derived propionate for colon cancer treatment

[Speaker]Jinsan Kim (Korea Research Institude of Bioscience and Biotechnology／Korea University of Science and Technology) [Co-author]Jinkwon Lee (Korea Research Institude of Bioscience and Biotechnology)、Yunsang Kang (Korea Research Institude of Bioscience and Biotechnology／Korea University of Science and Technology)、Jeongmin Lee (Korea Research Institude of Bioscience and Biotechnology／Korea University of Science and Technology)、In Hwan Tae (Korea Research Institude of Bioscience and Biotechnology)、Dae-Soo Kim (Korea Research Institude of Bioscience and Biotechnology／Korea University of Science and Technology)、Mi-Young Son (Korea Research Institude of Bioscience and Biotechnology／Korea University of Science and Technology)、Hyun-Soo Cho (Korea Research Institude of Bioscience and Biotechnology／Korea University of Science and Technology)

Colorectal cancer (CRC) is one of the most common malignancies worldwide, ranking among the top causes of cancer-related mortality. Recently studies suggests that the gut microbiome plays a significant role in CRC development and progression. Despite increasing interest in these microbiome, the role of metabolites such as propionate and mode of action (MOA) in CRC remain poorly understood. In this study, we performed RNA sequencing analysis following propionate treatment and observed a significant downregulation of several histone methyltransferase (HMT) genes. Interestingly, these HMT genes were found to be upregulated in colorectal cancer patient samples from the cancer genome atlas (TCGA) portal. siRNA-mediated knockdown of selected HMT genes resulted in the regulation of genes associated with cell proliferation and apoptosis. These findings suggest that propionate may suppress CRC proliferation by downregulating HMT genes involved in proliferation and apoptosis. Thus, we suggested that propionate, a gut microbiome-derived metabolite, may exert a synergistic effect with HMT inhibition, offering a potential novel therapeutic strategy for colorectal cancer.

[Disclosure of COI by Responsible researcher] Lead presenter is the Responsible researcher.
[Lead presenter: Jinsan Kim] We have no financial relationships to disclose.

### [P-3111] I-P8-4
### Histone modification & others

2025 / 9 / 27 (Sat) 15:30-16:15 Room P/(Motenashi Dome) Room P

### P-3111
### Lacticaseibacillus rhamnosus is involved apoptosis in Renal cell carcinoma by targeting EHMT2

[Speaker]Jeongmin Lee (Korea Research Institute of Bioscience and Biotechnology／Korea University of Science and Technology) [Co-author]Jinkwon Lee (Korea Research Institute of Bioscience and Biotechnology)、Yunsang Kang (Korea Research Institute of Bioscience and Biotechnology／Korea University of Science and Technology)、In Hwan Tae (Korea Research Institute of Bioscience and Biotechnology)、Jinsan Kim (Korea Research Institute of Bioscience and Biotechnology／Korea University of Science and Technology)、Dae-Soo Kim (Korea Research Institute of Bioscience and Biotechnology／Korea University of Science and Technology)、Mi-Young Son (Korea Research Institute of Bioscience and Biotechnology／Korea University of Science and Technology)、Hyun-Soo Cho (Korea Research Institute of Bioscience and Biotechnology／Korea University of Science and Technology)

Renal cell carcinoma (RCC) is the most common type of kidney cancer and a typical malignant tumor in the urinary system. Although surgery is key, new therapeutic targets and drugs are needed due to high relapse and low five-year survival rates. Microbiota therapy is emerging in RCC. In this study, Lacticaseibacillus rhamnosus supernatant (J01) was applied to RCC cell lines A498 and Caki1, inducing apoptosis. GO term analysis indicated involvement in apoptotic processes and cell cycle regulation. Cell growth assays and FACS analysis confirmed apoptosis. RNA-seq revealed EHMT2, a histone methyltransferase, was downregulated by J01. Knockdown of EHMT2 also induced apoptosis. Furthermore, DDIT3, an apoptosis-related gene, was upregulated after J01 treatment, and RNA-seq confirmed that EHMT2 directly regulates DDIT3. In 3D spheroid models and xenografts, suppression of EHMT2 reduced tumor size. Thus, J01 induces apoptosis in RCC by increasing DDIT3 via EHMT2 suppression. These findings suggest Lacticaseibacillus rhamnosus as a potential therapeutic strategy for RCC.

[Disclosure of COI by Responsible researcher] Lead presenter is the Responsible researcher.
[Lead presenter: Jeongmin Lee] We have no financial relationships to disclose.

### [P-3112] I-P8-4
### Histone modification & others

2025 / 9 / 27 (Sat) 15:30-16:15 Room P/(Motenashi Dome) Room P

---

### P-3112
### Lactobacillus reuteri Induces Apoptosis in HCC via SUV39H2 Modulation

[Speaker]Yunsang Kang (Korea Research Institute of Bioscience and Biotechnology, Daejeon, Korea.／Korea University of Science and Technology, Daejeon, Korea.) [Co-author]In Hwan Tae (Korea Research Institute of Bioscience and Biotechnology, Daejeon, Korea.)、Jinkwon Lee (Korea Research Institute of Bioscience and Biotechnology, Daejeon, Korea.)、Jeongmin Lee (Korea Research Institute of Bioscience and Biotechnology, Daejeon, Korea.／Korea University of Science and Technology, Daejeon, Korea.)、Jinsan Kim (Korea Research Institute of Bioscience and Biotechnology, Daejeon, Korea.／Korea University of Science and Technology, Daejeon, Korea.)、Dae-Soo Kim (Korea Research Institute of Bioscience and Biotechnology, Daejeon, Korea.／Korea University of Science and Technology, Daejeon, Korea.)、Mi-Young Son (Korea Research Institute of Bioscience and Biotechnology, Daejeon, Korea.／Korea University of Science and Technology, Daejeon, Korea.)、Hyun-Soo Cho (Korea Research Institute of Bioscience and Biotechnology, Daejeon, Korea.／Korea University of Science and Technology, Daejeon, Korea.)

Hepatocellular carcinoma (HCC) is one of the most lethal cancers worldwide. Recent studies suggest that the gut microbiota affects liver cancer development via the gut liver axis. Here, we evaluated the therapeutic potential of Lactobacillus reuteri, a gut-resident bacterium, in HCC. RNA sequencing after treatment with L. reuteri supernatant revealed downregulation of SUV39H2, a histone methyltransferase highly overexpressed in HCC and linked to poor prognosis. Hence, we investigated the function of SUV39H2 in HCC cell lines. SUV39H2 inhibition by siRNA transfection and a specific inhibitor significantly reduced cell growth. RNA sequencing analysis showed that this effect was associated with activation of the apoptosis pathway. Furthermore, the induction of apoptosis following SUV39H2 inhibition was validated using a 3D spheroid culture system. Our findings highlight the therapeutic potential of L. reuteri in HCC through downregulation of SUV39H2, resulting in apoptosis induction. These results may provide new insights for liver cancer treatment strategies

[Disclosure of COI by Responsible researcher] Lead presenter is not the Responsible researcher.
[Lead presenter: Yunsang Kang] We have no financial relationships to disclose.
[Principal Researcher: Yunsang Kang] We have no financial relationships to disclose.

▲TOP

### [P-3113] I-P8-4
### Histone modification & others

2025 / 9 / 27 (Sat) 15:30-16:15 Room P/(Motenashi Dome) Room P

---

P-3113
### DUB-mediated KDM6 stabilization drives mitochondrial metabolism in lung adenocarcinoma

[Speaker]Soo Yeon Oh (College of Pharmacy, Yonsei Univ.) [Co-author]Jung Yoo (College of Pharmacy, Yonsei Univ.)、Yu Hyun Jeon (College of Pharmacy, Yonsei Univ.)、Sang Wu Lee (College of Pharmacy, Yonsei Univ.)、So Hee Kwon (College of Pharmacy, Yonsei Univ.)

Globally, lung cancer is the second most commonly diagnosed cancer, with an estimated 2.2 million new cases and 1.8 million deaths in 2020. Non-small cell lung cancer (NSCLC) accounts for about 85% of cases, yet the five-year survival rate remains only 27%. Thus, novel and effective therapeutic strategies are urgently needed. KDM6, H3K27 demethylases, are overexpressed in NSCLC and promote oncogenesis by activating metabolic programs. However, the mechanisms that control their protein stability remain poorly understood. Given the crucial role of deubiquitinases (DUBs) in protein turnover, we established a DUB screening platform by overexpressing 59 individual DUBs. Candidate DUBs were further evaluated via transcriptomic correlation and docking-based interaction modeling. To better understand the role of KDM6 in tumorigenesis, we analyzed GSE118319 RNA-seq data in NSCLC cells treated with GSK-J4, a KDM6-specific inhibitor. Surprisingly, KDM6 inhibition downregulated mitochondrial genes in lung cancer. These findings reveal a novel DUB-KDM6 regulatory axis linked to mitochondrial function in NSCLC and provide new insights into the epigenetic control of cancer metabolism.

[Disclosure of COI by Responsible researcher] Lead presenter is not the Responsible researcher.
[Lead presenter: Soo Yeon Oh] We have no financial relationships to disclose.
[Principal Researcher: Jung Yoo] We have no financial relationships to disclose.

### [P-3114] I-P8-4
### Histone modification & others

2025 / 9 / 27 (Sat) 15:30-16:15 Room P/(Motenashi Dome) Room P

### P-3114
### The role of SYTL3 in restoring anti-tumor immunity in bladder cancer

[Speaker]Yu-Chi Yang (Dept. of Biomed. Sci., Natl. Chung Cheng Univ., Chiayi, Taiwan) [Co-author]Jie-Ting Low (Dept. of Biomed. Sci., Natl. Chung Cheng Univ., Chiayi, Taiwan)、Michael W.Y. Chan (Dept. of Biomed. Sci., Natl. Chung Cheng Univ., Chiayi, Taiwan)

Bladder cancer is the ninth most common malignancy worldwide, with urothelial carcinoma (UC) accounting for ~90% of cases. UC is divided into non-muscle invasive (NMIBC) and muscle-invasive (MIBC) types, with MIBC posing higher risk of progression. While immune checkpoint blockade (ICB) benefits some patients, its efficacy remains limited by the immunosuppressive tumor microenvironment, especially in tumors with low immune infiltration. Analysis of TCGA bladder cancer RNA-seq data identified SYTL3, a gene involved in vesicle trafficking and chemokine secretion. SYTL3 expression correlated with CXCL9, CXCL10, and CD80, suggesting a role in recruiting $CD8^+$ T cells and M1 macrophages. SYTL3 overexpression suppressed tumor growth in a syngeneic mouse model. Epigenetic analysis showed that treatment with 5azaDC, and cyproheptadine restored SYTL3 expression, implicating that promoter methylation and histone acetylation may regulate its transcription. In conclusion, SYTL3 may contribute to immune cell infiltration in the TME. Further investigation into the epigenetic reprogramming of SYTL3 could provide insight into enhancing the efficacy of ICB-based immunotherapy in bladder cancer.

[Disclosure of COI by Responsible researcher] Lead presenter is not the Responsible researcher.
[Lead presenter: Yu-Chi Yang] We have no financial relationships to disclose.
[Principal Researcher: ] We have no financial relationships to disclose.

▲TOP

### [P-3115] I-P8-4
### Histone modification & others

2025 / 9 / 27 (Sat) 15:30-16:15 Room P/(Motenashi Dome) Room P

---

### P-3115
### Characteristics of chromatin accessibility in gastric cancer cells with fetal phenotype

[Speaker]Daizo Koinuma (Dept. Pathol. Grad. Sch. Med., Univ. Tokyo.) [Co-author]Amane Yamamoto (Dept. Pathol. Grad. Sch. Med., Univ. Tokyo.)、Tetsuo Ushiku (Dept. Pathol. Grad. Sch. Med., Univ. Tokyo.)

Chromosomal instability (CIN) is the most frequent molecular subtype of gastric caner. CIN cases show relatively better prognosis, however, tumors with enteroblastic differentiation, often categorized as CIN, have poor prognosis. Either genomic or epigenomic characteristics that distinguish gastric cancer with the fetal gut phenotype from the other CIS have not been fully elucidated. In this study, we evaluated chromatin accessibility of three AFP-positive gastric cancer cell lines by ATAC-seq and acquired RNA-seq data to reveal their epigenomic and transcriptional characteristics. We identified 5,335 accessible chromatin regions common to three gastric cancers with fetal phenotype, Fu97, GCIY, and Takigawa. Of those, 3,236 regions are not shared by other type of gastric cancer cells, MKN7, MKN45, and MKN74. We identified several transcription factor binding motifs, e.g. E2F, enriched in the euchromatin regions of tumors with fetal phenotype. Specific regulatory process through the motifs and downstream target genes were identified, which may determine the charachteristics of this type of gastric cancer.

[Disclosure of COI by Responsible researcher] Lead presenter is not the Responsible researcher.
[Lead presenter: Daizo Koinuma] We have no financial relationships to disclose.
[Principal Researcher: Tetsuo Usiku] We have no financial relationships to disclose.

### [P-3116] I-P8-4
### Histone modification & others

2025 / 9 / 27 (Sat) 15:30-16:15 Room P/(Motenashi Dome) Room P

### P-3116
### Analysis of epitranscriptomic regulation of RNA demethylase ALKBH3 in pancreatic cancer

[Speaker]Manami Yamada (Lab. Stem Cell Regen. Adapt., Grad. Sch. Pharm. Sci., UOsaka) [Co-author]Shuntaro Aoi (Lab. Stem Cell Regen. Adapt., Sch. Pharm. Sci., UOsaka)、Honoka Kitamura (Lisc, Sch. Pharm. Sci., UOsaka)、Kaori Kitae (Lisc, Sch. Pharm. Sci., UOsaka)、Hiroaki Hase (Lab. Stem Cell Regen. Adapt., Grad. Sch. Pharm. Sci., UOsaka)、Yuko Ueda (Lisc, Sch. Pharm. Sci., UOsaka)、Kentaro Jingushi (Lab. Stem Cell Regen. Adapt., Grad. Sch. Pharm. Sci., UOsaka)、Soichiro Fukada (Lab. Stem Cell Regen. Adapt., Grad. Sch. Pharm. Sci., UOsaka)、Kazutake Tsujikawa (Lisc, Sch. Pharm. Sci., UOsaka)

Pancreatic cancer is an intractable cancer, and the development of new therapeutic agents is desired. AlkB homolog 3 (ALKBH3) cloned in our laboratory is known to be highly expressed in pancreatic cancer. Our previous studies using clinical samples showed a correlation between high ALKBH3 expression and poor prognosis. In addition, analysis using pancreatic cancer cell lines showed that ALKBH3 siRNAs exhibited cell growth inhibition and anti-tumor effects. These results suggest ALKBH3 is a promising molecular target for pancreatic cancer. In this study, we aim to analyze the role of ALKBH3 in pancreatic cancer using the developed compound that inhibits ALKBH3 demethylase activity as a tool compound.The tool compound inhibited proliferation and induced apoptosis in pancreatic cancer cells. To elucidate the molecular mechanism, we performed proteomic analysis and identified 378 proteins whose expression was decreased upon compound treatment. The differentially expressed proteins were mainly enriched in terms related to mitochondria.ALKBH3 may contribute to the promotion of pancreatic cancer cell proliferation through the regulation of mitochondrial function by RNA m1A demethylation.

[Disclosure of COI by Responsible researcher] Lead presenter is not the Responsible researcher.
[Lead presenter: Manami Yamada] We have no financial relationships to disclose.
[Principal Researcher: Kazutake Tsujikawa] ■Research funds from company: 株式会社HOIST　■Profit of stock: 株式会社HOIST　■Patent fee: 株式会社HOIST　■Research expenses: 株式会社HOIST、クニミネ工業株式会社、東ソー株式会社、株式会社Dot Aqua、日産化学株式会社　■Contributions: クニミネ工業株式会社

▲TOP

### [P-3117] I-P8-4
### Histone modification & others

2025 / 9 / 27 (Sat) 15:30-16:15 Room P/(Motenashi Dome) Room P

### P-3117
### Functional significance and epigenetic regulation of RXRG in head and neck squamous cell carcinoma

[Speaker]Masaaki Higashihara (Dpt of Mol Oncology, Grad Sch of Med, Chiba Univ) [Co-author]Takuya Nakagawa (Dpt of Mol Oncology, Grad Sch of Med, Chiba Univ／Dpt of Health and Disease Omics Center／Dpt Otorhinolaryngology Dead and Neck Surgery)、Atsushi Okabe (Dpt of Mol Oncology, Grad Sch of Med, Chiba Univ／Dpt of Health and Disease Omics Center)、Motoaki Seki (Dpt of Mol Oncology, Grad Sch of Med, Chiba Univ)、Masaki Fukuyo (Dpt of Mol Oncology, Grad Sch of Med, Chiba Univ)、Rahmutulla Bahityar (Dpt of Mol Oncology, Grad Sch of Med, Chiba Univ)、Takayuki Hoshii (Dpt of Mol Oncology, Grad Sch of Med, Chiba Univ)、Atsushi Kaneda (Dpt of Mol Oncology, Grad Sch of Med, Chiba Univ／Dpt of Health and Disease Omics Center)

RXRG is a member of the retinoid X receptor (RXR) family. Our previous studies revealed frequent hypermethylation of the RXRG promoter in head and neck squamous cell carcinoma (HNSCC). Although retinoic acid has been traditionally considered ineffective in HNSCC, this may be due to frequent hypermethylation of the RXRG promoter, leading to its reduced expression and impaired retinoic acid signaling.In this study, we investigated the correlation between the methylation level of the RXRG promoter and its mRNA expression, and evaluated the effects of retinoic acid treatment in HNSCC with RXRG expression.RXRG promoter hypermethylation occurred in 44% of our cohort and 25% of TCGA HNSCC cases, with significantly lower mRNA expression in hypermethylated tumors ($P = 4.4\times10^{-4}$), while 10% cases showed high level of RXRG expression with unmethylated promoter.Because RXRG expression was low in all the HNSCC cell lines available, we overexpressed RXRG and tested their response to retinoic acid. Treatment with bexarotene, an RXR-selective retinoid, significantly inhibited cell proliferation (P =0.027).These findings suggest RXRG might be a novel therapeutic target in HNSCC with RXRG expression.

[Disclosure of COI by Responsible researcher] Lead presenter is not the Responsible researcher.
[Lead presenter: Higashihara Masaaki] We have no financial relationships to disclose.
[Principal Researcher: Atsushi Kaneda] We have no financial relationships to disclose.

### [P-3126] I-P9-5
### Metastasis & vascular biology

2025 / 9 / 27 (Sat) 15:30-16:15 Room P/(Motenashi Dome) Room P

---

### P-3126
### Proteinase-3 impairs anti-VEGF antibody efficacy via tumor angiogenesis in colorectal cancer

[Speaker]Yuki Nishida (Dept. of Gastroenterological, Breast and Endocrine Surg. Yamaguchi Univ.) [Co-author]Masao Nakajima (Dept. of Gastroenterological, Breast and Endocrine Surg. Yamaguchi Univ.)、Ryouichi Tsunedomi (Dept. of Gastroenterological, Breast and Endocrine Surg. Yamaguchi Univ.／Yamaguchi Univ. Res. Inst. for Cell Design Med. Sci.)、Mitsuo Nishiyama (Dept. of Gastroenterological, Breast and Endocrine Surg. Yamaguchi Univ.)、Yoshitaro Shindo (Dept. of Gastroenterological, Breast and Endocrine Surg. Yamaguchi Univ.)、Yukio Tokumitsu (Dept. of Gastroenterological, Breast and Endocrine Surg. Yamaguchi Univ.)、Hiroto Matsui (Dept. of Gastroenterological, Breast and Endocrine Surg. Yamaguchi Univ.)、Yusaku Watanabe (Dept. of Gastroenterological, Breast and Endocrine Surg. Yamaguchi Univ.)、Shinobu Tomochika (Dept. of Gastroenterological, Breast and Endocrine Surg. Yamaguchi Univ.)、Michihisa Iida (Dept. of Gastroenterological, Breast and Endocrine Surg. Yamaguchi Univ.)、Hidenori Takahashi (Dept. of Gastroenterological, Breast and Endocrine Surg. Yamaguchi Univ.)、Tatsuya Ioka (Yamaguchi Univ. Hosp. Cancer Ctr.)、Hiroaki Nagano (Dept. of Gastroenterological, Breast and Endocrine Surg. Yamaguchi Univ.／Yamaguchi Univ. Res. Inst. for Cell Design Med. Sci.)

Background: Proteinase-3 (PRTN3) is a serine protease secreted by neutrophils. Previous studies have suggested that elevated serum PRTN3 levels may be associated with the therapeutic response to anti-VEGF antibody (anti-VEGFab) in metastatic colorectal cancer (CRC). This study aimed to investigate the effects of PRTN3 on tumor growth, angiogenesis, and antitumor efficacy of anti-VEGFab in murine CRC models.
Methods: Mice were subcutaneously transplanted with SW480 (human; xenogenic) or MC38 (mouse; syngenic) CRC cell lines, followed by the administration of PRTN3 (human or mouse) and/or anti-VEGFab, and assessed tumor growth, angiogenesis, and comprehensive expressional change using tumor, CD31-based microvessel density (MVD), and RNA sequencing, respectively.
Results: In the xenograft model, only murine PRTN3 significantly promoted tumor growth, increased CD31-positive MVD, and upregulated angiogenesis-related pathways, including the MAPK cascade. These effects were not observed with human PRTN3. In the syngeneic model, murine PRTN3 attenuated the antitumor efficacy of anti-VEGFab.
Conclusion: PRTN3 may promote resistance to anti-VEGFab therapy by enhancing tumor angiogenesis.

[Disclosure of COI by Responsible researcher] Lead presenter is not the Responsible researcher.
[Lead presenter: Yuki Nishida] ■Research funds from company: CYTLIMIC Inc., Toyo Kohan Co., Ltd., NEC Corporation, Eisai, Incyte, Astellas Pharma Inc.,TAIHO PHARMACEUTICAL CO., LTD., Shionogi & Co., Ltd., MSD K.K, Otsuka Pharmaceutical Factory, Inc., Otsuka Pharmaceutical Co.,Ltd., TSUMURA & CO.,Kyowa Hakko Kirin Co.,Ltd., Kaken Pharmaceutical Co., Ltd., NIPPON KAYAKU Co., Ltd., DAIICHI SANKYO COMPANY, LIMITED., Japan Blood Products Organization,Chugai Pharmaceutical Co., Ltd.
[Principal Researcher: Masao Nakajima] ■Research funds from company: CYTLIMIC Inc., Toyo Kohan Co., Ltd., NEC Corporation, Eisai, Incyte, Astellas Pharma Inc.,TAIHO PHARMACEUTICAL CO., LTD., Shionogi & Co., Ltd., MSD K.K, Otsuka Pharmaceutical Factory, Inc., Otsuka Pharmaceutical Co.,Ltd., TSUMURA & CO.,Kyowa Hakko Kirin Co.,Ltd., Kaken Pharmaceutical Co., Ltd., NIPPON KAYAKU Co., Ltd., DAIICHI SANKYO COMPANY, LIMITED., Japan Blood Products Organization,Chugai Pharmaceutical Co., Ltd.

### [P-3127] I-P9-5
### Metastasis & vascular biology

2025 / 9 / 27 (Sat) 15:30-16:15 Room P/(Motenashi Dome) Room P

---

### P-3127
### The study of the inhibitory effects of IL-1R antagonists on pancreatic cancer invasion and angiogenesis.

[Speaker]Yuki Denda (Nagoya City University Graduate School of Medical Sciences) [Co-author]Yoichi Matsuo (Nagoya City University Graduate School of Medical Sciences)、Yuki Eguchi (Nagoya City University Graduate School of Medical Sciences)

[Background] IL-1 is one of the inflammatory cytokines and has been reported to play an important role in tumor metastasis and angiogenesis. The IL-1/IL-1RI pathway is the main signal that activates the NF-kB, which is strongly involved in cancer progression. In this study, we focused on IL-1R antagonist (IL-1Ra), which suppresses this IL-1a/IL-1RI signaling, and analyzed it. [Methods] (1) Western blotting was used to confirm the expression of IL-1RI in each pancreatic cancer cell line. (2) IL-1a and IL-1Ra were used to examine the changes in invasive and angiogenic activity due to IL-1a/IL-1RI signaling. (3) ELISA was used to evaluate changes in NF-kB activity due to IL-1a/IL-1RI signaling. (4) Liver metastasis was evaluated using a intrasplenic injection model in BALB/c nu/nu mice. [Results] Stimulation with IL-1a enhanced invasive and angiogenic activity, which were suppressed by IL-1Ra treatment. Similar changes were observed in NF-kB ELISA. In the transsplenic liver metastasis model, administration of IL-1Ra significantly suppressed liver metastasis. [Conclusion] IL-1Ra is expected to be a new therapeutic agent for pancreatic cancer.

[Disclosure of COI by Responsible researcher] Lead presenter is not the Responsible researcher.
[Lead presenter: Yuki Denda] We have no financial relationships to disclose.
[Principal Researcher: Yoichi Matsuo] ■Research funds from company: 日本学術振興会

### [P-3128] I-P9-5
### Metastasis & vascular biology

2025 / 9 / 27 (Sat) 15:30-16:15 Room P/(Motenashi Dome) Room P

---

### P-3128
### The effects of beta-blockers on metastasis via their effects on vascular endothelial cells

[Speaker]Takeshi Munekata (Vascular Biol. & Mol. Pathol., Hokkaido Univ.／Oral Diagnosis & Med., Hokkaido Univ.) [Co-author]Aya Matsuda (Vascular Biol. & Mol. Pathol., Hokkaido Univ.)、Nako Maishi (Vascular Biol. & Mol. Pathol., Hokkaido Univ.)、Ikuya Miyamoto (Oral Diagnosis & Med., Hokkaido Univ.)、Yasuhiro Hida (Advanced Robotic & Endoscopic Surg., Fujita Health Univ.)、Kyoko Hida (Vascular Biol. & Mol. Pathol., Hokkaido Univ.)

It has been reported that surgical operations in cancer treatment can increase the blood concentration of norepinephrine (NE), a stress hormone, and the number of CTCs, due to surgical invasion. It has been shown that NE enhances the metastatic potential of tumor cells via beta adrenergic receptors, whereas beta-blockers (BBLs), which are used as antihypertensive agents, suppress it. However, the effects of BBLs on metastasis, particularly the effects on host vascular endothelial cells (ECs) that make up the metastatic pathway, are still unclear. In this study, we aimed to analyze the effects of BBLs on ECs and investigate their effects on metastasis. Mouse ECs were treated with NE and BBLs, and changes in gene expression were analyzed by RT-qPCR. The expression of adhesion molecules was upregulated by NE treatment, whereas BBLs suppressed these expressions. When NE was administered before tumor cell administration in a mouse lung metastasis model, metastasis was enhanced, but when BBLs were administered beforehand, metastasis was suppressed. These results suggest that BBLs may act on host ECs and contribute to the suppression of metastasis by suppressing adhesion of tumor cells.

[Disclosure of COI by Responsible researcher] Lead presenter is not the Responsible researcher.
[Lead presenter: Takeshi Munekata] We have no financial relationships to disclose.
[Principal Researcher: Kyoko Hida] We have no financial relationships to disclose.

### [P-3129] I-P9-5
### Metastasis & vascular biology

2025 / 9 / 27 (Sat) 15:30-16:15 Room P/(Motenashi Dome) Room P

---

### P-3129
### EphA2-mediated signaling is involved in the formation of vasculogenic mimicry structures constructed by cancer cells

[Speaker]Tetsuya Nomura (Lab. Pharm., Showa Pharm Univ) [Co-author]Naoki Utoguchi (Showa Pharm Univ)

Vasculogenic Mimicry (VMs) are periodate-Schiff (PAS)-stain positive and CD31 negative blood vessel-like structures constructed by cancer cells. We have previously developed an ***in vitro*** model of melanoma cell-derived VMs and demonstrated that the malignancy of cancer cells correlates with their ability to form VMs. However, the mechanism of VM formation remains unclear. In this study, we examined the possible involvement of signaling through EphA2, a receptor associated with cell proliferation, in VM formation by culturing seven cancer cell lines (B16F10, B16BL6, CT26, 203G, GL261, Colon26, 4T1) in the presence of Matrigel. B16F10, B16BL6, CT26, and 203G formed reticulate luminal structures. The luminal structures were positive for PAS staining and negative for CD31, and were characterized as VMs. In addition, the formation of the luminal structure revealed increased expression of EphA2. Furthermore, inhibitors of PI3K and Akt, which are downstream of EphA2, suppressed lumen formation. In addition, siRNA downregulation of EphA2 expression similarly inhibited the formation of lumenal structures. Thus, it is clear that EphA2-mediated signaling is involved in the construction of VMs.

[Disclosure of COI by Responsible researcher] Lead presenter is the Responsible researcher.
[Lead presenter: Tetsuya Nomura] ■Research funds from company: 若手研究者研究助成

▲TOP

### [P-3130] I-P9-5
### Metastasis & vascular biology

2025 / 9 / 27 (Sat) 15:30-16:15 Room P/(Motenashi Dome) Room P

---

### P-3130
### The angiostatic effects of clock gene inhibitors in pleural mesothelioma

[Speaker]Bayarmaa Agarzandan (Department of Respiratory Medicine and Rheumatology, Tokushima University) [Co-author]Atsushi Mitsuhashi (Department of Respiratory Medicine and Rheumatology, Tokushima University)、Hirokazu Ogino (Department of Respiratory Medicine and Rheumatology, Tokushima University)、Aito Yoshida (Department of Respiratory Medicine and Rheumatology, Tokushima University)、Yuki Tsukazaki (Department of Respiratory Medicine and Rheumatology, Tokushima University)、Ryohiko Ozaki (Department of Respiratory Medicine and Rheumatology, Tokushima University)、Yutaka Morita (Department of Respiratory Medicine and Rheumatology, Tokushima University)、Masaki Hanibuchi (Departments of Community Medicine for Respirology, Tokushima University)、Yasuhiko Nishioka (Department of Respiratory Medicine and Rheumatology, Tokushima University)

Pleural mesothelioma (PM) is known as an aggressive tumor due to its resistance to chemotherapy. The efficacy of anti-angiogenic reagents against PM has been reported in preclinical models. Thus, angiogenesis is a key target to overcome PM. Circadian rhythms are controlled by clock genes such as CLOCK/BMAL1 and their negative feedback regulators PER/CRY. While several clock gene inhibitors have been developed for metabolic diseases, their antitumor effects remain unclear. To evaluate the efficacy of clock gene inhibitors in PM, human PM cells were inoculated into the thoracic cavity of immune-deficient mice and treated with CRY stabilizer KL001. KL001 suppressed tumor progression and angiogenesis in PM-bearing mice. However, the clock gene inhibitor did not show direct cytotoxic effects on PM cells and vessel endothelial cells in vitro. The tumor tissue treated with KL001 showed significant upregulation of CXCL9, known as an angiostatic chemokine. Treatments with KL001 or the CLOCK/BMAL1 inhibitor CLK8 upregulated CXCL9 gene expression in PM cell lines in vitro. These results suggest that clock genes are promising therapeutic targets in PM.

[Disclosure of COI by Responsible researcher] Lead presenter is not the Responsible researcher.
[Lead presenter: Bayarmaa Agarzandan] We have no financial relationships to disclose.
[Principal Researcher: Yasuhiko Nishioka] ■Lecture fee: 日本ベーリンガーインゲルハイム株式会社　■Research expenses: SANSHO株式会社、株式会社ティムス　■Contributions: 中外製薬株式会社、大鵬薬品工業株式会社、旭化成ファーマ株式会社、日本イーライリリー株式会社、日本ベーリンガーインゲルハイム株式会社

### [P-3131] I-P9-5
### Metastasis & vascular biology

2025 / 9 / 27 (Sat) 15:30-16:15 Room P/(Motenashi Dome) Room P

---

### P-3131
### Cancer-associated mesothelial cells exhibit vascular differentiation potential in the ovarian tumor microenvironment

[Speaker]Marina Yoshikawa (Nagoya University Graduate School of Medicine) [Co-author]Masato Yoshihara (Nagoya University Graduate School of Medicine)、Kazumasa Mogi (Nagoya University Graduate School of Medicine)、Yoshihiko Yamakita (Nagoya University Graduate School of Medicine)、Emiri Miyamoto (Nagoya University Graduate School of Medicine)、Kaname Uno (Nagoya University Graduate School of Medicine)、Kazuhisa Kitami (Nagoya University Graduate School of Medicine)、Shohei Iyoshi (Nagoya University Graduate School of Medicine)、Hiroki Fujimoto (Nagoya University Graduate School of Medicine)、Hiroaki Kajiyama (Nagoya University Graduate School of Medicine)

[Introduction]Cancer-associated mesothelial cells (CAMs) are characterized by their enhanced pro-tumorigenic properties. Although morphological changes have previously been reported, their functional roles remain unclear. Here we traced CAMs using a conditional knock-in mouse model and analyzed their phenotype.[Methods]Wt1CreERT2/ROSA26-LSL-tdTomato mice in which mesothelial cells are selectively labeled with red fluorescence were observed following intraperitoneal injection of ID8 ovarian cancer cells. Immunofluorescence analysis of tumor sections was performed. In vitro experiments to induce differentiation of mesothelial cells into vascular endothelial cells by treatment with cAMP and VEGF were also conducted.[Results]Labeled CAMs accounted for 20.6% of the disseminated tumor area and formed multilayered structures. Notably, red fluorescence was also observed in intratumoral blood vessels, suggesting a potential mesothelial origin. Furthermore, inducted vascular endothelial cells showed increased expression of Desmin, vascular smooth muscle marker and CD31, vascular endothelial marker.[Conclusion] The diversity and broad differentiation potential of CAMs were suggested.

[Disclosure of COI by Responsible researcher] Lead presenter is the Responsible researcher.
[Lead presenter: Marina Yoshikawa] We have no financial relationships to disclose.

### [P-3132] I-P9-5
### Metastasis & vascular biology

2025 / 9 / 27 (Sat) 15:30-16:15 Room P/(Motenashi Dome) Room P

---

### P-3132
### Withdrawn

No Abstract

### [P-3133] I-P9-5
### Metastasis & vascular biology

2025 / 9 / 27 (Sat) 15:30-16:15 Room P/(Motenashi Dome) Room P

---

### P-3133
### FJX1 secreted by metastasis associated endothelial cells promote breast cancer cells stemness at the lung

[Speaker]Dan Shan (Div. of Cancer Cell Biol., Cancer Res. Inst., Kanazawa Univ.) [Co-author]Tsunaki Hongu (Div. of Cancer Cell Biol., Cancer Res. Inst., Kanazawa Univ.)、Noriko Gotoh (Div. of Cancer Cell Biol., Cancer Res. Inst., Kanazawa Univ.)

Inhibiting breast cancer(BC) metastasis has always been a thorny issue in clinical. It is known that BC cells have metastasized to distant tissues by utilizing support from surrounding niche cells at the metastatic site. Recent studies have revealed that metastasis associated endothelial cells (ECs) secret several factors in metastasis environment for cancer cells disseminated to distant organs. In this study, we aim to identify vascular niche-associated functional factors involved in lung metastasis of BC. we found that Four jointed box kinase 1 (Fjx1) is significantly upregulated in lung ECs at metastasis site. Furthermore, recombinant FJX1 protein induces sphere formation ability of BC cells in vitro, indicating that FJX1 can promote stemness of BC cells. These results suggest that metastasis associated lung ECs provide stem cell niche to BC cells at the metastatic site by producing FJX1. Discovering the molecular mechanism of FJX1 function and its regulation in metastasis site of BC may provide an opportunity of novel target therapy for metastatic BC at the lung.

[Disclosure of COI by Responsible researcher] Lead presenter is not the Responsible researcher.
[Lead presenter: Dan Shan] We have no financial relationships to disclose.
[Principal Researcher: Noriko Gotoh] We have no financial relationships to disclose.

### [P-3134] I-P9-5
### Metastasis & vascular biology

2025 / 9 / 27 (Sat) 15:30-16:15 Room P/(Motenashi Dome) Room P

---

### P-3134
### Squalene Epoxidase Drives Vascular Co-option and Astrocyte Reprogramming in Breast Cancer Brain Metastasis

[Speaker]Jia-Yun Yeh (Institute of Biosci., Academia Sinica) [Co-author]Shu-Ping Wang (Institute of Biosci., Academia Sinica)、Yung-Lung Yu (Institute of Biosci, China Medical University)

Breast cancer brain metastases (BCBM) account for 30% of breast cancer mortality, especially in HER2+ and triple negative subtypes, and represent an unmet clinical need. The mechanisms that mediate metastatic cancer growth in brain parenchyma are largely unknown. Our unbiased transcriptomic analysis of patient-derived samples revealed that the cholesterol synthesis gene Squalene Epoxidase (SQLE) is differentially upregulated in the BCBM group. Functionally, SQLE high cells that cross the ***blood-brain*** barrier form perivascular sheaths, while SQLE low cells fail to ***co-opt*** vessels and exhibit impaired brain colonization. Our data show that (a) high SQLE expression correlates with poor prognosis and increased brain metastasis; (b) SQLE inhibition reduces BBB transmigration and vascular co-option; (c) SQLE activates astrocytes into a prometastatic, ***anti-inflammatory*** phenotype and suppresses microglial phagocytosis; and (d) SQLE knockdown reduces viability in brain metastatic cells. By protecting cancer cells from phagocytosis and fostering vascular ***co-option***, target SQLE may provides a potential mechanism for the initiation of brain metastasis in breast cancers.

[Disclosure of COI by Responsible researcher] Lead presenter is the Responsible researcher.
[Lead presenter: Jia-Yun Yeh] We have no financial relationships to disclose.

### [P-3142] I-P9-7
### Metastasis models & circulating tumor cells

2025 / 9 / 27 (Sat) 15:30-16:15 Room P/(Motenashi Dome) Room P

---

### P-3142
### Identification of RAS Mutations Using Circulating Tumor Cells, Circulating Tumor DNA, and Exosomes

[Speaker]Koki Hayashi (Department of Gastroenterological Surgery, Nippon Medical School) [Co-author]Takeshi Yamada (Department of Gastroenterological Surgery, Nippon Medical School)、Kay Uehara (Department of Gastroenterological Surgery, Nippon Medical School)、Seiichi Shinji (Department of Gastroenterological Surgery, Nippon Medical School)、Akihisa Matsuda (Department of Gastroenterological Surgery, Nippon Medical School)、Yasuyuki Yokoyama (Department of Gastroenterological Surgery, Nippon Medical School)、Goro Takahashi (Department of Gastroenterological Surgery, Nippon Medical School)、Takuma Iwai (Department of Gastroenterological Surgery, Nippon Medical School)、Toshimitsu Miyasaka (Department of Gastroenterological Surgery, Nippon Medical School)、Shintaro Kanaka (Department of Gastroenterological Surgery, Nippon Medical School)、Takanori Matsui (Department of Gastroenterological Surgery, Nippon Medical School)、Hiroshi(Nippon Med. Sch.) Yoshida (Department of Gastroenterological Surgery, Nippon Medical School)

[Introduction]
Circulating tumor cells (CTCs) are expected to provide various types of information about tumors. However, their low abundance in the bloodstream poses a challenge to obtaining a sufficient quantity for analysis. Here, we report the results of a comparative analysis of mutation identification sensitivity using CTCs, ctDNA, and exosomes.
[Methods]
This study targeted patients with RAS-mutant colorectal cancer. CTCs were isolated using the ELESTA CROSSORTER (AFI Technology). This device isolates CTCs using size-based separation and electrophoretic separation. RAS mutations in DNA extracted from CTCs, exosomes, and ctDNA were identified using droplet digital PCR.
[Results]
The study included 30 patients. RAS mutations were detected in 8 cases using CTCs, in 15 cases using ctDNA, and in 11 cases using exosomes. Patients with identified mutations were comprehensively covered by ctDNA and CTCs.
[Discussion]
Improvements in blood collection methods, microchannel design, and sheath fluid composition enabled enhanced CTC isolation accuracy. The isolated CTCs contain numerous white blood cells, and their efficient removal remains a challenge for future work.

[Disclosure of COI by Responsible researcher] Lead presenter is not the Responsible researcher.
[Lead presenter: Koki Hayashi] We have no financial relationships to disclose.
[Principal Researcher: Takeshi Yamada] ■Research funds from company: 株式会社AFIテクノロジー

### [P-3143] I-P9-7
### Metastasis models & circulating tumor cells

2025 / 9 / 27 (Sat) 15:30-16:15 Room P/(Motenashi Dome) Room P

---

### P-3143
### Establishment of the detection and collection method of circulating tumor cells in sarcoma using a microfluidic device.

[Speaker]Mami Iwasaki (Department of Mechanical Engineering, Faculty of Engineering, University of Toyama) [Co-author]Takashi Ohnaga (Second Department of Surgery, University of Occupational and Environmental Health／Cytona Corporation)、Kayo Suzuki (Department of Orthopaedic Surgery, Faculty of Medicine, University of Toyama)、Atsushi Kase (Department of Mechanical Engineering, Faculty of Engineering, University of Toyama)

Purpose: Circulating tumor cells (CTCs) have gathered attention as a biomarker for carcinomas. However, very few studies have been conducted on CTCs in sarcomas. This study aims to establish high-precision detection and collection method for CTCs in sarcomas. Methods: The microfluidic device, called polymer CTC-chip enables to capture specific cells with an antibody attached to microposts. In this study, the antibody against Cell Surface Vimentin (CSV) was conjugated onto the chip, and capture efficiency was examined using sarcoma cell lines (MG63 and Saos2). And the captured cells were released and collected physically by flowing air foam.Results: The average capture efficiencies of MG63 and Saos2 were 82 % and 70 % (flow rate: 0.5 mL/h), and 63 % and 70 % (flow rate: 1.0 mL/h). And the collected cells were undamaged and sufficient for culture and any analysis. Discussion: The success of highly efficient capture of cells with CSV using polymer CTC-chip suggests the possibility of detection of CTCs in sarcoma patients. And the continuous process of CTCs detection and collection is expected to lead to both early cancer detection and molecular analyses for oncogene identification.

[Disclosure of COI by Responsible researcher] Lead presenter is the Responsible researcher.
[Lead presenter: Mami Iwasaki] ■Research funds from company: 公益財団法人富山県ひとづくり財団

### [P-3144] I-P9-7
### Metastasis models & circulating tumor cells

2025 / 9 / 27 (Sat) 15:30-16:15 Room P/(Motenashi Dome) Room P

---

### P-3144
### A novel 9-gene classifier for predicting distant metastasis of soft-tissue sarcoma and multiple malignancies

[Speaker]Atsushi Tanabe (Lab. Highly-Adv. Vet. Med. Tech., Vet. Tech. Hosp. Aazabu Univ.) [Co-author]Jerry Ndzinu (Lab. Biol., Aazabu Univ. Sch. Vet. Med.)、Hiroeki Sahara (Lab. Biol., Aazabu Univ. Sch. Vet. Med.)

Soft-tissue sarcoma (STS) is a malignant tumor that arises in mesenchymal tissues. Distant metastasis of STS occurs within 5 years in approximately 50% of high-risk cases. Chemotherapy with doxorubicin in combination with ifosfamide is the standard treatment for metastatic STS, but once distant metastases occur, the prognosis is very poor. In this study, we performed a comprehensive analysis of gene expression profiles of STS samples available from public databases in conjunction with patient prognostic information. By combining the expression profiles of metastasis-related genes with simple machine learning algorithms, we developed a new classifier, designated the 9-gene classifier. The accuracy of the 9-gene classifier was higher than that of existing gene signatures in predicting distant metastasis of STS. In addition to STS, the 9-gene classifier was also used to predict the prognostic risk of breast cancer, kidney clear cell carcinoma, and uveal melanoma. In summary, the 9-gene classifier provides prognostic information relevant to a variety of cancer types and thus could be useful clinically in making treatment decisions.

[Disclosure of COI by Responsible researcher] Lead presenter is the Responsible researcher.
[Lead presenter: Atsushi Tanabe] ■Endowed chair: Malignant Tumor Treatment Technologies, Inc.

### [P-3145] I-P9-7
### Metastasis models & circulating tumor cells

2025 / 9 / 27 (Sat) 15:30-16:15 Room P/(Motenashi Dome) Room P

---

### P-3145
### A mitochondrial one-carbon metabolism promotes breast cancer tumorigenesis and lung metastasis

[Speaker]Tsunaki Hongu (Cancer Res Inst, Kanazawa Univ) [Co-author]Yuming Wang (Cancer Res Inst, Kanazawa Univ)、Tatsunori Nishimura (Cancer Res Inst, Kanazawa Univ)、Takiko Daikoku (Res Ctr Exp Model of Human Dis, Kanazawa Univ)、Ryoji Yao (Cancer Inst, Japanese Found for Cancer Res)、Satoshi Kojo (Inst of Med, Pharm & Health Sci, Kanazawa Univ)、Hiroshi Watarai (Inst of Med, Pharm & Health Sci, Kanazawa Univ)、Tomoyoshi Soga (Institute for Advanced Biosciences, Keio University)、Noriko Gotoh (Cancer Res Inst, Kanazawa Univ／InFiniti, Kanazawa Univ)

Mitochondrial one-carbon metabolism is pivotal for embryonic development and de novo nucleotide synthesis. However, its contribution to tumorigenesis and metastasis is largely rudimentary. Here, we unveil that mitochondrial one-carbon metabolic enzyme MTHFD2 is essential for breast cancer tumorigenesis and lung metastasis. We developed ***Mthfd2****flox/flox* mice and deleted ***Mthfd2*** gene specifically in mammary tissues. Although specific deletion of ***Mthfd2*** in mouse mammary epithelial cells led to a modest delay in mammary gland development, they displayed significant delays in tumor formation, reduced growth, and notably fewer lung metastasis when these mice were bred with MMTV-neu mammary tumor model mice. We found impaired spheroid forming ability in the mutant MTHFD2 tumor cells in vitro, indicating that MTHFD2 is important for maintenance of cancer stem cell-like properties. Furthermore, MTHFD2 deletion significantly attenuates polarization of tumor-associated macrophage that supports tumor progression and metastasis. Thus, one-carbon metabolism profoundly impacts breast cancer progression via both cell-autonomous and non-cell autonomous mechanisms.

[Disclosure of COI by Responsible researcher] Lead presenter is not the Responsible researcher.
[Lead presenter: Tsunaki Hongu] We have no financial relationships to disclose.
[Principal Researcher: Noriko Gotoh] We have no financial relationships to disclose.

▲TOP

### [P-3146] I-P9-7
### Metastasis models & circulating tumor cells

2025 / 9 / 27 (Sat) 15:30-16:15 Room P/(Motenashi Dome) Room P

---

### P-3146
### Analysis of the biological property of peritoneal cancer cell clusters in gastric carcinoma

[Speaker]Makoto Miyazaki (Lab. Pediatric and Refractory Cancer, Chiba Cancer Ctr. Res. Inst.／Dept. Cancer Cell Res., Sasaki Inst., Sasaki Foundation) [Co-author]Hideki Yamaguchi (Dept. Cancer Cell Res., Sasaki Inst., Sasaki Foundation)

Multiclonal metastasis contributes intra-tumor heterogeneity. Cancer cell clusters may serve as a source of multiclonal metastasized tumors; however, the mechanisms underlying cluster formation and the acquisition of metastatic potential remain poorly understood. We previously reported that cancer cells induce fibrin formation via tissue factor to form clusters in the peritoneal cavity, which are the source of peritoneal metastasized tumors. Thus, cancer cell clusters represent a potential therapeutic target for peritoneal metastasis. However, the molecular mechanisms underlying cell clustering-mediated peritoneal metastasis remain largely unknown.
In this study, we performed transcriptome analysis of gastric cancer cell clusters to elucidate their biological properties and targetable vulnerabilities. Gene ontology analysis revealed that the oxidative phosphorylation pathway was altered in cancer cell clusters compared to single-cell cancer cells. We also found that cancer cell clusters rely more on glycolysis for ATP production than single-cell cancer cells. These results suggest that metabolic alterations in cancer cell clusters contributes to peritoneal metastasis.

[Disclosure of COI by Responsible researcher] Lead presenter is not the Responsible researcher.
[Lead presenter: Makoto Miyazaki] We have no financial relationships to disclose.
[Principal Researcher: Hideki Yamaguchi] ■Research funds from company: 車両競技公益資金記念財団

### [P-3147] I-P9-7
### Metastasis models & circulating tumor cells

2025 / 9 / 27 (Sat) 15:30-16:15 Room P/(Motenashi Dome) Room P

---

### P-3147
### Non-Target Organ Perturbation Enhances Pre-Metastatic Niche Conditioning in Experimental Metastasis Models

[Speaker]Hiroki Sato (Grad. Sch. Nanobiosci., Yokohama City Univ.) [Co-author]Shoichi Higashi (Grad. Sch. Nanobiosci., Yokohama City Univ.)

Cancer metastasis is a major cause of cancer-related mortality, with organ-specific dissemination (organotropism) often associated with the location of the primary tumor. The seed and soil hypothesis posits that tumor-secreted factors promote pre-metastatic microenvironment formation in specific organs prior to metastatic colonization. However, given that cancer is a systemic disease accompanied by paraneoplastic syndromes, this model may not fully capture the complexity of metastatic progression. To investigate systemic tumor-organ communication, we developed an artificial tumor-bearing mouse model by delivering biotinylated tumor-secreted factors via osmotic pumps. These factors accumulated in multiple organs, including the kidney, liver, and brain. In a B16F10 hematogenous metastasis model, we observed recruitment of F4/80+ macrophages and αSMA+ myofibroblasts in the kidney, indicative of an inflammatory microenvironment. Notably, mice subjected to unilateral ureteral obstruction exhibited a trend toward increased metastasis. These findings suggest that systemic physiological disturbances in non-target organs may facilitate the formation of pre-metastatic microenvironments.

[Disclosure of COI by Responsible researcher] Lead presenter is the Responsible researcher.
[Lead presenter: Hiroki Sato] We have no financial relationships to disclose.

▲TOP

[P-3148] I-P9-7
Metastasis models & circulating tumor cells

2025 / 9 / 27 (Sat) 15:30-16:15 Room P/(Motenashi Dome) Room P

P-3148
### The autophagy-activated PTX3 glycosylation enhances HNSCC anoikis resistance and metastasis

[Speaker]Ting-Wei Chang (Inst. of Basic Medical Science, National Cheng Kung University) [Co-author]Shih-Hung Chan (Division of Cardiology, National Cheng Kung University)、Jhih-Peng Tsai (Division of Cardiology, National Cheng Kung University／Inst. of Bioinformatics and Biosignal Transduction, National Cheng Kung University)、Ying-Zhu Meow (Dept. of Pharmacology, National Cheng Kung University)、Wen-Chang Chang (Graduate Inst. of Medical Sciences, Taipei Medical University)、Ben-Kuen Chen (Inst. of Basic Medical Science, National Cheng Kung University／Dept. of Pharmacology, National Cheng Kung University)

Tumor metastasis is the major cause of tumor-related death. However, the molecular mechanisms in regulating suspended head and neck squamous cell carcinoma (HNSCC) cell survival-associated metastasis remain unclear. This study established anoikis-resistant (AR) cells by a repeated switch from suspension to attachment. We found that AR cells acquired enhanced spheroid formation, migration, invasion, and extravasation. In addition, the next-generation sequencing of AR cells indicates the enhancement of EMT signature and upregulation of PTX3. Intriguingly, the depletion of PTX3 by RNAi in AR cells significantly reduced the spheroid formation, survival signal, and metastasis both in vitro and in vivo studies. Moreover, PTX3 was heavily glycosylated in suspended HNSCC cells, which was correlated with spheroid formation. In addition, the interference of autophagy by knockdown of ATG5 and 3-MA treatment significantly reduced the expression of PTX3. Autophagy inhibitors also blocked PTX3 expression and spheroid formation, triggering apoptosis in AR cells. These results suggest that targeting PTX3 may be a potential therapeutic approach to treating anoikis-resistant HNSCC metastasis.

[Disclosure of COI by Responsible researcher] Lead presenter is the Responsible researcher.
[Lead presenter: Ting-Wei Chang] We have no financial relationships to disclose.

### [P-3149] I-P9-7
### Metastasis models & circulating tumor cells

2025 / 9 / 27 (Sat) 15:30-16:15 Room P/(Motenashi Dome) Room P

### P-3149
### Analysis of Gene Regulatory Networks in Breast Cancer Dormancy

[Speaker]Eilma Yasuoka (Dept. of Oncogenesis Growth Regulation, Res. Inst., OICI) [Co-author]Ryoichi Nishida (Dept. of Oncogenesis Growth Regulation, Res. Inst., OICI)、Yusuke Yamamoto (Lab. On Integr. Oncol., Natl. Cancer Ctr. Res. Inst.)、Shigeki Higashiyama (Dept. of Oncogenesis Growth Regulation, Res. Inst., OICI)、Jun(NCC) Nakayama (Dept. of Oncogenesis Growth Regulation, Res. Inst., OICI／Lab. On Integr. Oncol., Natl. Cancer Ctr. Res. Inst.)

Metastatic recurrence is major causes of cancer mortality, emphasizing the need to understand its mechanisms and develop effective treatments. Metastasis involves multiple steps, including invasion, dormancy, and metastatic outgrowth. In breast cancer, many disseminated tumor cells in distant organs enter a dormant state, which plays a critical role in recurrence. Thus, uncovering the mechanisms of cellular dormancy is vital for cancer prevention and therapy. Cellular dormancy is considered as "staying in the reversible G0 phase" and "surviving for months, years, or decades in a hypometabolic state". However, its molecular definition remains unclear.This study focuses on the orphan nuclear receptor NR2F1, known to regulate dormancy in breast cancer and head and neck cancer. To clarify the gene regulatory network of NR2F1, we conducted a comprehensive interactome analysis using a human protein array combined with cell-free protein synthesis. The screening identified the novel candidates that interact with NR2F1. In this presentation, we would like to discuss the results of the interactome analysis of nuclear receptor that regulates cellular dormancy and their regulatory mechanisms.

[Disclosure of COI by Responsible researcher] Lead presenter is not the Responsible researcher.
[Lead presenter: Eilma Yasuoka] We have no financial relationships to disclose.
[Principal Researcher: Jun Nakayama] ■Research funds from company: 公益財団法人持田記念医学薬学振興財団,国立研究開発法人日本医療研究開発機構

### [P-3150] I-P9-7
### Metastasis models & circulating tumor cells

2025 / 9 / 27 (Sat) 15:30-16:15 Room P/(Motenashi Dome) Room P

---

### P-3150
### Exploration of novel metastasis mechanisms using metastatic lung adenocarcinoma cell lines derived from CIEA-PDX

[Speaker]Seinosuke Sakai (Central Inst. for Exp. Med. and Life Sci.) [Co-author]Masayuki Komatsu (Central Inst. for Exp. Med. and Life Sci.)、Eiko Nishinaka (Central Inst. for Exp. Med. and Life Sci.)、Chiyoko Nishime (Central Inst. for Exp. Med. and Life Sci.)、Asami Hanazawa (Central Inst. for Exp. Med. and Life Sci.)、Misa Mochizuki (Central Inst. for Exp. Med. and Life Sci.)、Daisuke Komura (Grad. Sch. of Med, The Univ. of Tokyo)、Miwako Kakiuchi (Grad. Sch. of Med, The Univ. of Tokyo)、Shumpei Ishikawa (Grad. Sch. of Med, The Univ. of Tokyo／Exploratory Oncology Res. & Clin. Trial Ctr., Natl. Cancer Ctr.)、Masami Suzuki (Central Inst. for Exp. Med. and Life Sci.)、Junichi Hata (Central Inst. for Exp. Med. and Life Sci.)、Taichi Yamamoto (Central Inst. for Exp. Med. and Life Sci.)

To elucidate the metastasis mechanisms of lung adenocarcinoma (LUAD), we newly established three PDX-derived cell lines (PDXC) LC-06C, LC-49C, LC-58C from CIEA-PDX models of LUAD. Among them, only LC-58C exhibited remarkable pulmonary metastasis when implanted either subcutaneously or orthotopically, indicating its potential as a valuable model for metastasis research. To further investigate novel metastasis mechanisms, we performed RNA-seq analysis on five LUAD PDXs, including the metastatic strains LC-07 and LC-58. From the analysis of gene expression data and prognostic information from a public database, four genes including ***SQLE*** were identified as potential drivers of metastatic property. Subsequent pathway analysis revealed significant activation of the cholesterol synthesis pathway, in which SQLE functions as a rate-limiting enzyme. Currently, we are generating stable knockdowned PDXCs for the identified candidate genes to further evaluate their roles in metastasis using the ***in vivo*** model.

[Disclosure of COI by Responsible researcher] Lead presenter is not the Responsible researcher.
[Lead presenter: Seinosuke Sakai] We have no financial relationships to disclose.
[Principal Researcher: Junichi Hata] We have no financial relationships to disclose.

▲TOP

### [P-3160] I-P10-8
### Cell culture system/Organoid (2)

2025 / 9 / 27 (Sat) 15:30-16:15 Room P/(Motenashi Dome) Room P

### P-3160
### NCYM promotes cholangiocarcinogenesis *via* activating of autophagy

[Speaker]Daisuke Muto (Grad. Sch. of Med. & Pharm. Sci., Chiba Univ.／Evolutionary Oncology, Chiba Cancer Ctr. Res. Ins.) [Co-author]Kazuma Nakatani (Grad. Sch. of Med. & Pharm. Sci., Chiba Univ.／Evolutionary Oncology, Chiba Cancer Ctr. Res. Ins.)、Hiroyuki Kogashi (Grad. Sch. of Med. & Pharm. Sci., Chiba Univ.／Evolutionary Oncology, Chiba Cancer Ctr. Res. Ins.)、Ummi M. Zulfin (Grad. Sch. of Med. & Pharm. Sci., Chiba Univ.／Evolutionary Oncology, Chiba Cancer Ctr. Res. Ins.)、Akiko Endo (Evolutionary Oncology, Chiba Cancer Ctr. Res. Ins.)、Harumi Saida (Evolutionary Oncology, Chiba Cancer Ctr. Res. Ins.)、Akane Sada (Evolutionary Oncology, Chiba Cancer Ctr. Res. Ins.)、Kazuto Kugo (Dept. of Applied Genomics, Kazusa DNA Res. Inst.)、Yoshinori Hasegawa (Dept. of Applied Genomics, Kazusa DNA Res. Inst.)、Yusuke Suenaga (Evolutionary Oncology, Chiba Cancer Ctr. Res. Ins.)、Yoshitaka Hippo (Grad. Sch. of Med. & Pharm. Sci., Chiba Univ.／Evolutionary Oncology, Chiba Cancer Ctr. Res. Ins.／Lab. of Precision Tumor Model Systems, CCCRI)

***NCYM*** is encoded by the antisense strand of the ***MYCN*** oncogene, and its high expression is associated with poor prognosis in cholangiocarcinoma. In this study, we show that NCYM promotes cholangiocarcinogenesis through activation of autophagy. Based on the result that NCYM expression levels correlate with Ras signaling in cholangiocarcinoma, we developed a mouse bile duct organoid carcinogenesis model expressing KrasG12D. Organoids with NCYM overexpression formed subcutaneous tumors in nude mice at a higher frequency (79%) compared to those expressing only KrasG12D (52%). In contrast, overexpression of a Japanese-specific NCYM SNP variant (V71D) led to a lower tumor formation rate (36%). Long-read RNA sequencing revealed elevated expression of autophagy-related genes in organoids expressing wild-type NCYM. Additionally, LC3 was stabilized in wild-type NCYM-expressing organoids but not in those expressing the SNP variant. Instead, NCYM SNP variants caused accumulation of p62 and autolysosomes. These findings suggest that wild-type NCYM enhances cholangiocarcinogenesis by promoting autophagy, whereas the SNP variant suppresses it by inhibiting autophagy.

[Disclosure of COI by Responsible researcher] Lead presenter is not the Responsible researcher.
[Lead presenter: Daisuke Muto] We have no financial relationships to disclose.
[Principal Researcher: Yusuke Suenaga, Yoshitaka Hippo] We have no financial relationships to disclose.

### [P-3161] I-P10-8
### Cell culture system/Organoid (2)

2025 / 9 / 27 (Sat) 15:30-16:15 Room P/(Motenashi Dome) Room P

### P-3161
### Investigation of an anti-tumor drug efficacy evaluation system using PDC

[Speaker]Amane Tateishi (Mediford Corporation) [Co-author]Misato Kakita (Mediford Corporation)、Shigenori Enoki (Mediford Corporation)、Kousuke Morizumi (Mediford Corporation)

Patient-Derived Cells (PDC) are cells collected from the ascites of cancer patients and stocked after low-passage cultures (Tanaka Y, Chiwaki F, et al., Nature Cancer 2021). PDCs have properties closer to clinical cancer than conventional cell lines and show high predictive value for clinical trials; therefore, they may be useful for developing anti-tumor drugs. We obtained gastric PDC from the National Cancer Center Research Institute and evaluated the Antibody-Dependent Cellular Cytotoxicity (ADCC) using PDC as target cells. As a result, the ADCC activity of anti-tumor drugs could be assessed similarly to conventional cell lines. The evaluation system we established may become an efficient tool for the development of anti-tumor drugs.

[Disclosure of COI by Responsible researcher] Lead presenter is not the Responsible researcher.
[Lead presenter: Amane Tateishi] ■ employment by a company related to the presentation: メディフォード株式会社
[Principal Researcher: Kousuke Morizumi] ■employment by a company related to the presentation: メディフォード株式会社

### [P-3162] I-P10-8
### Cell culture system/Organoid (2)

2025 / 9 / 27 (Sat) 15:30-16:15 Room P/(Motenashi Dome) Room P

### P-3162
### NOTCH2NL is a context-dependent mediator of intestinal homeostasis in human

[Speaker]Tsukasa Oda (Gunma Univ., IMCR, Lab of MED) [Co-author]Karin Ninomiya (Gunma Univ., IMCR, Lab of MED)、Moe Kondo (Gunma Univ., IMCR, Lab of MED)、Chihiro Mogi (Gunma Univ., IMCR, Lab of MED)、Ikuo Suzuki (The Univ. Tokyo, Grad. Sch. of Sci., Dep. Biol. Sci.)、Nobuo Sasaki (Gunma Univ., IMCR, Lab of MED)

A human-specific gene N2NL(NOTCH2NL) has two key features: 1) activating Notch signaling in neural progenitor cells, and 2) mutated in various cancers. These findings suggest N2NL involved in carcinogenesis by regulating Notch signaling, but its precise function is unclear. Using human and mouse colon organoids, we studied its effects on cell proliferation and differentiation in the colon epithelium. N2NL knockdown or overexpression didn't significantly alter organoids' size or growth rate. However, N2NL overexpression increased Goblet cell marker expression, while knockdown diminished it. These results suggest N2NL acts as a negative regulator of the Notch signaling in the colonic epithelium because Notch signaling inhibition promotes Goblet cell differentiation. FACS analysis indicated that N2NL reduces NOTCH1 surface levels, suggesting its role in suppressing Notch signaling. Together, our findings indicate N2NL negatively modulates Notch signaling in colonic stem cells and is crucial for sustaining goblet cell homeostasis, contrasting with neurogenic stem cells in the brain. We continue investigating N2NL's role in colorectal cancer using our tumor organoid biobank.

[Disclosure of COI by Responsible researcher] Lead presenter is the Responsible researcher.
[Lead presenter: Tsukasa Oda] We have no financial relationships to disclose.

▲TOP

### [P-3163] I-P10-8
### Cell culture system/Organoid (2)

2025 / 9 / 27 (Sat) 15:30-16:15 Room P/(Motenashi Dome) Room P

---

### P-3163
### Comparative Proteomic and Immunocytochemical Study of Squamous Components in 2D- and 3D-Cultured PDAC Cell Lines

[Speaker]Yuuki Shichi (Res. Team for Geriatr. Pathol., Tokyo Met. Inst. Geriatr. Gerontol.) [Co-author]Hiroki Tsumoto (Res. Team for Mech. Aging, Tokyo Met. Inst. Geriatr. Gerontol.)、Masakazu Fujiwara (Res. Team for Geriatr. Pathol., Tokyo Met. Inst. Geriatr. Gerontol.)、Yasuko Hasegawa (Res. Team for Geriatr. Pathol., Tokyo Met. Inst. Geriatr. Gerontol.)、Seiichi Shinji (Res. Team for Geriatr. Pathol., Tokyo Met. Inst. Geriatr. Gerontol.／Dept. of Gastroenterological Surg., Nippon Med. Sch. Hosp.)、Kimimasa Takahashi (Dept. Vet. Pathol., Nippon Vet. and Life Sci. Univ.)、Yuri Miura (Res. Team for Mech. Aging, Tokyo Met. Inst. Geriatr. Gerontol.)、Toshiyuki Ishiwata (Res. Team for Geriatr. Pathol., Tokyo Met. Inst. Geriatr. Gerontol.)

Pancreatic ductal adenocarcinoma (PDAC) exhibits epithelial-mesenchymal heterogeneity linked to malignancy. We examined the expression of ductal and squamous markers in eight PDAC cell lines classified as epithelial or mesenchymal, using LC-MS/MS-based proteomics and immunocytochemistry under 2D and 3D cultures. CK7 was expressed in epithelial but not mesenchymal lines, while CK20 was absent in all lines. CK5/6 was strongly expressed in T3M-4 and PK-1, with distinct localization between 2D and 3D. p63 was highly expressed in PK-8, PK-1, and T3M-4, but decreased in 3D cultures of PK-8 and T3M-4. Its isoform p40 was detected under 2D in the same lines, but was undetectable or peripherally localized in 3D. Mesenchymal lines showed low or no expression of squamous markers. These results suggest that epithelial PDAC cells maintain adenocarcinoma features and exhibit squamous traits depending on the culture microenvironment, providing insight into mechanisms of squamous differentiation in PDAC.

[Disclosure of COI by Responsible researcher] Lead presenter is not the Responsible researcher.
[Lead presenter: Yuuki Shichi] We have no financial relationships to disclose.
[Principal Researcher: Toshiyuki Ishiwata] We have no financial relationships to disclose.

▲TOP

### [P-3164] I-P10-8
### Cell culture system/Organoid (2)

2025 / 9 / 27 (Sat) 15:30-16:15 Room P/(Motenashi Dome) Room P

---

### P-3164
### Interaction between breast cancer cells and tumor microenvironment in breast cancer organoids

[Speaker]Yuto Oshima (Kanazawa Univ. Cancer Research Inst.) [Co-author]Yasuto Takeuchi (Kanazawa Univ. Cancer Research Inst.)、Noriko Gotoh (Kanazawa Univ. Cancer Research Inst.)

Cancer cells in solid tumors, including breast cancer, grow in a complex tumor microenvironment with cancer-associated fibroblasts (CAFs), endothelial cells, and immune cells. Organoid culture provides a physiologically relevant in vitro model by preserving spatial architecture. Recent studies have demonstrated the utility of organoids for analyzing molecular mechanisms in the tumor microenvironment and for drug screening. We established patient-derived breast cancer organoids using the hanging drop method. To better model the tumor microenvironment, we co-cultured patient-derived breast cancer cells with CAFs, endothelial cells, and macrophages in organoids. Immunohistochemical analyses were performed to investigate the spatial interactions among these cells using anti-SMA for CAFs, anti-CK4 for breast cancer cells, anti-CD31 for endothelial cells, and anti-F4/80 antibodies for macrophages. Our results show diverse interactions among these cell types within organoids.

[Disclosure of COI by Responsible researcher] Lead presenter is not the Responsible researcher.
[Lead presenter: Yuto Oshima] We have no financial relationships to disclose.
[Principal Researcher: Noriko Gotoh] We have no financial relationships to disclose.

[P-3165] I-P10-8

Cell culture system/Organoid (2)

2025 / 9 / 27 (Sat) 15:30-16:15 Room P/(Motenashi Dome) Room P

P-3165

### Targeting rare cancers: drug discovery using patient-derived organoids of small bowel adenocarcinoma.

[Speaker]Koya Ichikawa (Lab. of Cancer Cell Systems, National Cancer Center Research Institute) [Co-author]Haruto Sugasawa (Lab. of Cancer Cell Systems, National Cancer Center Research Institute)、Hiroyuki Fujii (Dept. of Gastrointestinal Medical Oncology, National Cancer Center Hospital／Dept. of Laboratory Medicine, National Cancer Center Hospital)、Shungo Adachi (Dept. of Proteomics, National Cancer Research Institute)、Yosuke Tanaka (Div. of Cellular Signaling, National Cancer Center Research Institute)、Kyoko Inoue (Lab. of Cancer Cell Systems, National Cancer Center Research Institute)、Obata Yuuki (Lab. of Intracellular Traffic Oncology, National Cancer Center Research Institute)、Hirokazu Shoji (Dept. of Gastrointestinal Medical Oncology, National Cancer Center Hospital／Dept. of Experimental Therapeutics, National Cancer Center Hospital)、Jun Sato (Dept. of Experimental Therapeutics, National Cancer Center Hospital)、Keisuke Sekine (Lab. of Cancer Cell Systems, National Cancer Center Research Institute)

Rare cancers affect fewer than 6 per 100,000 people but comprise about 22% of all cases, making clinical trials difficult. This study aimed to establish small bowel adenocarcinoma (SBA) organoids and conduct drug screening.
SBA is a rare cancer treated with FOLFOX or CapeOX, but response rates are under 50%, and no established second-line therapies exist, highlighting the need for new treatments. Using biopsy samples, we established three SBA organoids that formed xenograft tumors resembling patient tissues, with preserved genetic features confirmed by exome sequencing. We initially tested five standard chemotherapeutic agents; however, their ability to inhibit cell proliferation was limited.We then screened 360 compounds and identified 12 with significant growth-inhibitory effects. Among them, an HSP90 inhibitor showed promising, concentration-dependent suppression of organoid growth. Further in vivo experiments confirmed its tumor growth inhibition. Proteomic and RNA sequencing suggested synthetic lethality mechanisms, and synergistic effects were observed when combined with other agents. Further investigation into the detailed mechanisms is ongoing.

[Disclosure of COI by Responsible researcher] Lead presenter is not the Responsible researcher.
[Lead presenter: Koya Ichikawa] We have no financial relationships to disclose.
[Principal Researcher: Keisuke Sekine] We have no financial relationships to disclose.

### [P-3166] I-P10-8
### Cell culture system/Organoid (2)

2025 / 9 / 27 (Sat) 15:30-16:15 Room P/(Motenashi Dome) Room P

### P-3166
### Activin Signaling Promotes Colon Cancer Cell Motility

[Speaker]Soya Arai (Natl. Cancer Ctr. Res. Inst. Lab. Mol. Genetics／Hoshi Univ. Dept. Pharmacol) [Co-author]Haruna Takeda (Natl. Cancer Ctr. Res. Inst. Lab. Mol. Genetics)、Minoru Narita (Hoshi Univ. Dept. Pharmacol)

Activin belongs to the TGF$\beta$ superfamily and is a factor involved in regulating cell differentiation. Although Activin expression is highly induced in malignant colorectal cancers (CRC), its function has not been well characterized compared to TGF$\beta$, which induces EMT. Therefore, we investigated the function of activin using colon tumor-derived organoids. First, we investigated the genes regulated by activin by RNA-seq and identified 267 genes. Among these, genes involved in interaction with the ECM, and EMT have been enriched, as well as genes encoding membrane proteins of unknown function. Next, we found that organoids stimulated by Activin showed increased cell motility, but adding an Activin receptor inhibitor inhibited cell motility. We use this culture system to identify novel Activin-induced membrane proteins involved in cell motility for further detailed functional analysis. Such molecules may be involved in the malignant progression of CRC and thus may be potential therapeutic targets.

[Disclosure of COI by Responsible researcher] Lead presenter is not the Responsible researcher.
[Lead presenter: Soya Arai] We have no financial relationships to disclose.
[Principal Researcher: Haruna Takeda] We have no financial relationships to disclose.

### [P-3167] I-P10-8
### Cell culture system/Organoid (2)

2025 / 9 / 27 (Sat) 15:30-16:15 Room P/(Motenashi Dome) Room P

---

### P-3167
### Clinical significance *in vitro* drug sensitivity profiling using pancreatic cancer organoids from EUS-FNA saline flushes

[Speaker]Tomoya Ekawa (Dept. Cancer Drug Discovery & Development, Osaka Intl. Cancer Inst.／Dept. Cancer Immunotherapy, Res. Ctr., Osaka Intl. Cancer Inst.) [Co-author]Kenji Ikezawa (Dept. Hepatobiliary & Pancreatic Oncol., Osaka Intl. Cancer Inst.)、Yoji Kukita (Lab. Genomic Pathology, Res. Ctr., Osaka Intl. Cancer Inst.)、Makiko Urabe (Dept. Hepatobiliary & Pancreatic Oncol., Osaka Intl. Cancer Inst.)、Yugo Kai (Dept. Hepatobiliary & Pancreatic Oncol., Osaka Intl. Cancer Inst.)、Ryoji Takada (Dept. Hepatobiliary & Pancreatic Oncol., Osaka Intl. Cancer Inst.)、Takashi Akazawa (Dept. Cancer Drug Discovery & Development, Osaka Intl. Cancer Inst.／Dept. Cancer Immunotherapy, Res. Ctr., Osaka Intl. Cancer Inst.)、Yu Mizote (Dept. Cancer Drug Discovery & Development, Osaka Intl. Cancer Inst.／Dept. Cancer Immunotherapy, Res. Ctr., Osaka Intl. Cancer Inst.)、Kumiko Tatsumi (Dept. Cancer Drug Discovery & Development, Osaka Intl. Cancer Inst.／Dept. Surgery, Grad. Sch. of Med., Kyoto Univ.／Dept. Med. Dev., iACT, Kyoto Univ. Hosp.)、Shigenori Nagata (Dept. Diagnostic Pathology & Cytology, Osaka Intl. Cancer Inst.)、Hisataka Ogawa (Nitto Dept. Nucleic Acid Med., Osaka Intl. Cancer Inst.)、Shinichiro Hasegawa (Dept. Gastroenterol. Surgery, Osaka Intl. Cancer Inst.)、Hidenori Takahashi (Dept. Gastroenterol. Surgery, Osaka Intl. Cancer Inst.)、Kazuyoshi Ohkawa (Dept. Hepatobiliary & Pancreatic Oncol., Osaka Intl. Cancer Inst.)、Hideaki Tahara (Dept. Cancer Drug Discovery & Development, Osaka Intl. Cancer Inst.／Clin. Res. Ctr., Osaka Intl. Cancer Inst.)

We previously reported a method for establishing pancreatic cancer organoids using residual samples from saline flushes (RSSF) collected from needle rinses during diagnostic endoscopic ultrasound-guided fine-needle aspiration (EUS-FNA). In this study, we employed this non-invasive approach on newly collected clinical samples and conducted *in vitro* drug sensitivity testing on the established patient-derived cancer organoids (PDCOs). In addition, we assessed whether their drug responses correlated with the observed clinical outcomes of the corresponding patients. Organoid establishment was successful in most RSSF cases. Established organoids harbored oncogenic mutations, and mutational profiles remained stable in organoids re-established from xenograft tumors in immunodeficient mice. Drug responses varied among PDCOs, suggesting patient-specific differences, and showed general consistency with clinical outcomes.
These results suggest that this model may contribute to the development of personalized therapeutic strategies in pancreatic cancer.

[Disclosure of COI by Responsible researcher] Lead presenter is the Responsible researcher.
[Lead presenter: Tomoya Ekawa] We have no financial relationships to disclose.

### [P-3176] I-P10-10
### Cancer stem cells (2)

2025 / 9 / 27 (Sat) 15:30-16:15 Room P/(Motenashi Dome) Room P

---

### P-3176
### Identification of pancreatic cancer stem cells and development of specific ablation therapy

[Speaker]Osamu Sugahara (Anticancer Strategies Lab., Advanced Res. Inst., Inst. Sci. Tokyo) [Co-author]Tsunaki Higa (Anticancer Strategies Lab., Advanced Res. Inst., Inst. Sci. Tokyo)、Keiichi I. Nakayama (Anticancer Strategies Lab., Advanced Res. Inst., Inst. Sci. Tokyo)

Pancreatic ductal adenocarcinoma (PDAC) is a clinically challenging disease that acquires resistance to conventional anticancer treatments and relapses frequently. Given that cancer stem cells (CSCs) give rise to heterogeneous cancer cell populations and can cause recurrence after initial treatments, therapies targeting CSCs would be of great clinical benefit for the curative treatment of PDAC. However, the lack of a full understanding of their identity in PDAC limits the development of such therapies. Here, we analyzed single-cell RNA sequencing data of pancreatic tumors using the stem cell inference algorithm CytoTRACE to identify chemoresistant CSCs (CytoTRACE-high cells). We discovered a plasma membrane protein specifically expressed in CSCs and modified its gene in pancreatic cancer organoids to allow visualization and ablation of CSCs. CytoTRACE-high cells showed high stemness and chemoresistance, and their ablation eradicated tumors, which is proof of concept for future therapeutic applications. To translate these findings into clinical applications, we are developing near-infrared photoimmunotherapy (NIR-PIT) targeting CSCs.

[Disclosure of COI by Responsible researcher] Lead presenter is not the Responsible researcher.
[Lead presenter: Osamu Sugahara] We have no financial relationships to disclose.
[Principal Researcher: Keiichi I. Nakayama] We have no financial relationships to disclose.

### [P-3177] I-P10-10
### Cancer stem cells (2)

2025 / 9 / 27 (Sat) 15:30-16:15 Room P/(Motenashi Dome) Room P

### P-3177
### SLC14A1 regulates stemness and tumorigenicity of osteosarcoma stem cells

[Speaker]Soji Hayashida (Gifu Pharm. Univ. Lab. of Pharmacology) [Co-author]Kazuya Tokumura (Gifu Pharm. Univ. Lab. of Pharmacology)、Makoto Yoshimoto (Gifu Pharm. Univ. Lab. of Pharmacology)、Shohei Tsuji (Gifu Pharm. Univ. Lab. of Pharmacology)、Eiichi Hinoi (Gifu Pharm. Univ. Lab. of Pharmacology)

**Objective:** Osteosarcoma is a primary malignant bone tumor. Osteosarcoma stem cells (OSCs), subpopulations of osteosarcoma cells, have the ability to self-renew and differentiate. OSCs play an important role in tumor recurrence and metastasis. Therefore, targeting OSCs is necessary to cure osteosarcoma completely. In this study, we aimed to identify and explore the function of a novel stemness regulator of OSCs. **Methods:** We used 143B cells, a human-derived osteosarcoma cell line. 143B cells were cultured under floating condition (for OSCs) or adherent condition (for differentiated osteosarcoma cells), followed by ***in silico***, ***in vitro*** and ***in vivo*** analyses. **Results:** The expression of ***Solute Carrier Family 14 Member 1*** (***SLC14A1***) encoding a B-type urea transporter was significantly upregulated in OSCs rather than in differentiated osteosarcoma cells. Moreover, the genetic inactivation of ***SLC14A1*** by RNA interference abrogated self-renewal potential of OSCs ***in vitro***, but also tumorigenicity in a heterotopic xenograft model. This study suggests that SLC14A1 could play a pivotal role in the regulation of OSC properties and osteosarcoma malignancy.

[Disclosure of COI by Responsible researcher] Lead presenter is not the Responsible researcher.
[Lead presenter: Soji Hayashida] We have no financial relationships to disclose.
[Principal Researcher: Eiichi Hinoi] We have no financial relationships to disclose.

### [P-3178] I-P10-10
### Cancer stem cells (2)

2025 / 9 / 27 (Sat) 15:30-16:15 Room P/(Motenashi Dome) Room P

---

### P-3178
### Role of PLOD2 in regulating osteosarcoma stem cell properties

[Speaker]Yuki Yamawaki (Gifu Pharm. Univ. Lab. of Pharmacology) [Co-author]Ryoma Osumi (Gifu Pharm. Univ. Lab. of Pharmacology)、Kazuya Tokumura (Gifu Pharm. Univ. Lab. of Pharmacology)、Ryotaro Yamakawa (Gifu Pharm. Univ. Lab. of Pharmacology)、Makoto Yoshimoto (Gifu Pharm. Univ. Lab. of Pharmacology)、Shohei Tsuji (Gifu Pharm. Univ. Lab. of Pharmacology)、Eiichi Hinoi (Gifu Pharm. Univ. Lab. of Pharmacology)

Osteosarcoma stem cells (OSCs) are characterized by their self-renewal and multilineage differentiation abilities, contributing to the pathogenesis of osteosarcoma, which is the most common malignant primary bone tumor. The significance and pivotal role collagen, a major extracellular matrix component, in OSC phenotypes are largely unknown. This study aimed to investigate the role of collagen modification in OSCs through ***in silico***, ***in vitro***, ***in vivo*** analyses. The expression of ***Procollagen-Lysine, 2-Oxoglutarate 5-Dioxygenase 2***, which is an enzyme that hydroxylates the lysine residues of collagen precursor molecules, was significantly upregulated in OSC populations and was associated with poor survival in osteosarcoma patients. Moreover, the genetic inactivation of ***PLOD2*** by RNA interference abrogated self-renewal potential and migration of OSCs ***in vitro***, but also tumorigenicity in a heterotopic xenograft model. Collectively, these findings suggest a pivotal role for ***PLOD2*** in the regulation of OSC phenotypes and osteosarcoma malignancy, thereby providing a potential target for OSC-directed therapy.

[Disclosure of COI by Responsible researcher] Lead presenter is not the Responsible researcher.
[Lead presenter: Yuki Yamawaki] We have no financial relationships to disclose.
[Principal Researcher: Eiichi Hinoi] We have no financial relationships to disclose.

### [P-3179] I-P10-10
### Cancer stem cells (2)

2025 / 9 / 27 (Sat) 15:30-16:15 Room P/(Motenashi Dome) Room P

---

### P-3179
### Identification of a novel molecular regulator of chemoresistance in colorectal cancer

[Speaker]Hirokazu Ohata (Teikyo Univ., ACRO, Div. Health Sci.) [Co-author]Daisuke Shiokawa (Ehime Univ. Hosp, Translational. Res. Ctr.)、Yusuke Kanda (Teikyo Univ., ACRO, Div. Health Sci.)、Koji Okamoto (Teikyo Univ., ACRO, Div. Health Sci.)

Chemoresistance is often attributed to the presence of intratumoral heterogeneity. In our previous study, we identified the heterogeneity within colorectal cancer patient-derived organoids and xenografted tumors. We demonstrated that the NOX1-mTORC1 signaling axis promotes proliferation in a fast-cycling subpopulation of cancer cells, whereas the transcription factor PROX1 is essential for maintaining a slow-cycling, persister-like population of cancer stem cells (CSCs).
Importantly, PROX1 induction attenuates NOX1-mediated mTORC1 activation, thereby promoting a quiescent state via feedback regulation. This persister-like state is associated with elevated autophagic activity and enhanced resistance to chemotherapy, highlighting the critical importance of targeting slow-cycling CSCs to overcome chemoresistance.
To elucidate key regulators of the persister-like state, we recently performed a functional screening and identified novel molecular regulators implicating in mediating chemoresistance in the autophagy-high, slow-cycling CSC population. The potential role of newly identified candidate genes in conferring chemoresistance will be discussed.

[Disclosure of COI by Responsible researcher] Lead presenter is not the Responsible researcher.
[Lead presenter: Hirokazu Ohata] ■Research funds from company: 科研費（基盤C）
[Principal Researcher: Koji Okamoto] We have no financial relationships to disclose.

### [P-3180] I-P10-10
### Cancer stem cells (2)

2025 / 9 / 27 (Sat) 15:30-16:15 Room P/(Motenashi Dome) Room P

---

### P-3180
### Compound Screening Reveals a Key Signaling Pathway Involved in the Regulation of Colorectal Cancer Stemness

[Speaker]Yanqing Niu (Div. Pathophysiol., Aichi Cancer Ctr. Res. Inst.／Div. Cancer Physiol., Nagoya Univ. Grad. Sch. Med.) [Co-author]Teruaki Fujishita (Div. Pathophysiol., Aichi Cancer Ctr. Res. Inst.)、Yasushi Kojima (Div. Pathophysiol., Aichi Cancer Ctr. Res. Inst.)、Rie Kajino (Div. Pathophysiol., Aichi Cancer Ctr. Res. Inst.)、Emi Mishiro (Inst. Trans.Bio-Mol., Nagoya Univ.)、Makoto M. Taketo (Pers. Canc. Ther., MIC, Kyoto Univ. Grad. Sch. Med.)、Masahiro Aoki (Div. Pathophysiol., Aichi Cancer Ctr. Res. Inst.／Div. Cancer Physiol., Nagoya Univ. Grad. Sch. Med.)

Despite therapeutic advances, colorectal cancer (CRC) remains a major cause of cancer-related mortality, largely due to distant metastasis. To investigate the mechanisms underlying CRC metastasis, we established a genetically engineered mouse model named CKPS mice, harboring mutations in four driver genes-***Ctnnb1***, ***Kras***, ***Trp53*** and ***Smad4***-that develop CRC with spontaneous liver metastasis. Our previous analyses using CKPS mice and a derived CRC cell line (CKPS cells) showed that the cAMP/PKA/CREB and TGF$\beta$/SMAD4 pathways regulate their stemness and metastatic ability. To further explore mechanisms maintaining CRC stemness, we screened ~500 compounds using 3D spheroid cultures of CKPS cells, monitoring the expression of CRC stemness markers ALCAM (CD166) and PROM1 (CD133). QNZ, an inhibitor known to target NF-kB, markedly reduced ALCAM and PROM1 expression and suppressed CKPS cell metastasis. However, mechanistic analysis showed that this effect was independent of NF-kB signaling. Further investigation revealed an alternative pathway mediating the action of QNZ. We will discuss its possible role in the regulating stemness and metastasis in CKPS cells.

[Disclosure of COI by Responsible researcher] Lead presenter is not the Responsible researcher.
[Lead presenter: Yanqing Niu] We have no financial relationships to disclose.
[Principal Researcher: Masahiro Aoki] ■Research funds from company: 科研費　基盤研究B

▲TOP

### [P-3181] I-P10-10
### Cancer stem cells (2)

2025 / 9 / 27 (Sat) 15:30-16:15 Room P/(Motenashi Dome) Room P

---

### P-3181
### Development of novel targeted therapy for ovarian cancer stem cells using hydrogel

[Speaker]Lei Wang (Department of Cancer Pathology, Hokkaido University, Sapporo, Japan／WPI-ICReDD, Hokkaido University, Sapporo, Japan) [Co-author]Tianyue Zhai (Department of Obstetrics and Gynecology, Hokkaido University, Sapporo, Japan.)、Masumi Tsuda (Department of Cancer Pathology, Hokkaido University, Sapporo, Japan／WPI-ICReDD, Hokkaido University, Sapporo, Japan)、Hidemichi Watari (Department of Obstetrics and Gynecology, Hokkaido University, Sapporo, Japan.)、Shinya Tanaka (Department of Cancer Pathology, Hokkaido University, Sapporo, Japan／WPI-ICReDD, Hokkaido University, Sapporo, Japan)

Background: Despite indisputable progress in the treatment of ovarian cancer, the problems of chemo-resistance and recurrent disease are the main obstacles for successful therapy. Frequent recurrence and chemoresistance are driven by cancer stem cells (CSCs), thus it has become one of the most important targets for cancer treatments. In our previous studies, it has successfully reprogrammed the cancer cells into CSCs on hydrogels originally developed by Prof. Jian Ping Gong in Hokkaido University. So far, therapeutic targets Ovarian cancer CSCs have not been able to be detected for improlved treatment and prognosis of Ovarian cancer.Results: Hydrogel-cultured cells exhibited spheroid formation and significantly higher expression of CSC markers compared to standard culture. In vivo, hydrogel-induced CSCs formed tumors within 49 days, whereas control cells did not. Metabolic analysis showed increased oxidative respiration and reserve capacity, indicating higher adaptability to microenvironmental changes. Chemoresistance assays demonstrated that hydrogel-induced CSCs were more resistant to cisplatin compared to control cells.

[Disclosure of COI by Responsible researcher] Lead presenter is the Responsible researcher.
[Lead presenter: Lei Wang] We have no financial relationships to disclose.

### [P-3182] I-P10-10
### Cancer stem cells (2)

2025 / 9 / 27 (Sat) 15:30-16:15 Room P/(Motenashi Dome) Room P

---

### P-3182
### CD80 Enhances Stemness and Environmental Adaptability in Rapidly Growing Oral Squamous Cell Carcinoma

[Speaker]Yu-Lin Chen (Natl. Inst. of Cancer Res., NHRI, Miaoli, Taiwan) [Co-author]Shih-Han Huang (Natl. Inst. of Cancer Res., NHRI, Miaoli, Taiwan)、Shih-Sheng Jiang (Natl. Inst. of Cancer Res., NHRI, Miaoli, Taiwan)、Fang-Yu Tsai (Natl. Inst. of Cancer Res., NHRI, Miaoli, Taiwan)、Ssu-Han Wang (Natl. Inst. of Cancer Res., NHRI, Miaoli, Taiwan)、Yen-Chung Chiu (Natl. Inst. of Cancer Res., NHRI, Miaoli, Taiwan)、Daw-Yang Huang (Natl. Inst. of Cancer Res., NHRI, Tainan, Taiwan)、Ko-Jiunn Liu (Natl. Inst. of Cancer Res., NHRI, Tainan, Taiwan)、Tsung-Hsien Chuang (Immunol. Res. Ctr., NHRI, Miaoli, Taiwan)、Yu-Wen Su (Immunol. Res. Ctr., NHRI, Miaoli, Taiwan)、Ya-Wen Chen (Natl. Inst. of Cancer Res., NHRI, Miaoli, Taiwan)

Tumor growth rates in oral squamous cell carcinoma (OSCC) vary across patients, affecting prognosis and treatment response. To investigate contributing factors, we used the NHRI-HN1 mouse OSCC cell line and derived a faster-growing variant, M1-2sph-T, via homologous orthotopic injection. RNA sequencing (RNAseq) and single-cell RNA sequencing (scRNAseq) revealed that M1-2sph-T cells express higher levels of CD80. Tumor cells with elevated CD80 showed enhanced stress responses and metabolic activity, suggesting an intracellular regulatory role beyond immunological function. CD80 high-expressing cells displayed increased proliferation, migration, sphere formation, and stemness-associated transcription factor expression. CD80 knockdown reduced these aggressive traits, and CD80-neutralizing antibody inhibited sphere formation, indicating CD80's role in pluripotency and adaptability. These findings identify CD80 as a driver of OSCC aggressiveness and a potential therapeutic target.

[Disclosure of COI by Responsible researcher] Lead presenter is the Responsible researcher.
[Lead presenter: Yu-Lin Chen] We have no financial relationships to disclose.

### [P-3183] I-P10-10
### Cancer stem cells (2)

2025 / 9 / 27 (Sat) 15:30-16:15 Room P/(Motenashi Dome) Room P

---

### P-3183
### Depleting Anillin in 3D Culture Reveals Abnormal Cell Division of Enriched Triple-Negative Breast Cancer Stem-Like Cells

[Speaker]Nuriza Ulul Azmi (Lab. Exp. Path, University of Tsukuba, Japan) [Co-author]Yukari Okita (Lab. Exp. Path, University of Tsukuba, Japan)、Mitsuyasu Kato (Lab. Exp. Path, University of Tsukuba, Japan)

Triple-negative breast cancer (TNBC) shows poor prognosis with a high population of cancer stem cells (CSC). CSCs are hypothesized to exhibit unique cell division patterns, which contribute to tumor heterogeneity; yet, their regulation remains unclear. Anillin, an actin-binding protein, is known to be essential for the process of cytokinesis. However, the involvement of anillin in regulating CSC remains poorly understood. This study aimed to investigate the role of anillin in regulating cell division of enriched TNBC stem-like cells. We depleted anillin in TNBC cells to observe subsequent phenotypic outcomes and cell dynamics during division in a 3D spheroid culture model. We found that silencing anillin significantly suppressed sphere growth and reduced the frequency of stem cells. Moreover, we observed abnormal cell morphology during cell division in 3D spheroid culture conditions, which is distinct from that in 2D monolayer culture. We successfully visualized the dynamics of anillin localization during cell division in 3D spheroid culture. The results suggest that anillin plays an important role in regulating enriched-TNBC stem-like cells during cell division.

[Disclosure of COI by Responsible researcher] Lead presenter is not the Responsible researcher.
[Lead presenter: Nuriza Ulul Azmi] We have no financial relationships to disclose.
[Principal Researcher: Mitsuyasu Kato] We have no financial relationships to disclose.

### [P-3184] I-P10-10
### Cancer stem cells (2)

2025 / 9 / 27 (Sat) 15:30-16:15 Room P/(Motenashi Dome) Room P

### P-3184
### Withdrawn

No Abstract

▲TOP

### [P-3196] I-P10-12
### Cell death (1)

2025 / 9 / 27 (Sat) 15:30-16:15 Room P/(Motenashi Dome) Room P

---

### P-3196
### Induction of Ferroptosis by Dual Inhibition of GGCT and xCT Targeting Cysteine Metabolism in Glioblastoma

[Speaker]Masaya Mori (Lab. of Clinical Oncology, Kyoto Pharma. Univ) [Co-author]Hiromi Ii (Lab. of Clinical Oncology, Kyoto Pharma. Univ)、Yuhi Sone (Lab. of Clinical Oncology, Kyoto Pharma. Univ)、Kozue Nose (Lab. of Clinical Oncology, Kyoto Pharma. Univ)、Takahiro Matsumoto (Lab. of Public Health, Kyoto Pharma. Univ)、Mitsugu Fujita (Faculty of Med, Kindai Univ)、Susumu Nakata (Lab. of Clinical Oncology, Kyoto Pharma. Univ)

Aim: Glioblastoma (GBM) is an aggressive brain cancer resistant to standard therapies. This study investigated the ferroptosis-inducing effects of the combined treatment with the GGCT inhibitor pro-GA and the xCT inhibitor erastin and their anti-GBM efficacy in vivo.Results: GGCT was highly expressed in GBM cells. The combined treatment showed a synergistic antiproliferative effects, which were restored by ferrostatin-1 and deferoxamine, confirming induction of ferroptosis. Accumulation of membrane lipid peroxidation was observed by the co-treatment with a decrease in cellular cysteine levels, and N-acetylcysteine rescued their effects. Co-treatment with pro-GA and IKE inhibited tumor growth in vivo with increased expression of 4-HNE, a lipid peroxidation marker. Histological analysis confirmed suppression of tumor growth without significant body weight loss or evident toxicity to liver and kidney.Conclusion: Dual inhibition of GGCT and xCT efficiently inhibits cysteine availability and induces ferroptosis by disrupting redox homeostasis. In vivo results confirmed tumor suppression and increased lipid peroxidation, supporting the therapeutic potential of this novel strategy.

[Disclosure of COI by Responsible researcher] Lead presenter is not the Responsible researcher.
[Lead presenter: Masaya Mori] We have no financial relationships to disclose.
[Principal Researcher: Susumu Nakata] We have no financial relationships to disclose.

### [P-3197] I-P10-12
### Cell death (1)

2025 / 9 / 27 (Sat) 15:30-16:15 Room P/(Motenashi Dome) Room P

---

### P-3197
### Regulation of cell death by tRNA modification enzyme, CDKAL1, in hepatocellular carcinoma

[Speaker]Yuki Adachi (Department of Molecular Physiology, Kumamoto University) [Co-author]Yu Nagayoshi (Department of Molecular Physiology, Kumamoto University)、Hitomi Kaneko (Department of Molecular Physiology, Kumamoto University)、Chishou Mitsuura (Department of Molecular Physiology, Kumamoto University)、Takeshi Chujo (Department of Molecular Physiology, Kumamoto University)、Masaaki Iwatsuki (Gastroenterological surgery, Kumamoto university hospital)、Kazuhito Tomizawa (Department of Molecular Physiology, Kumamoto University)

RNA has various post-transcriptional chemical modifications and these modifications regulates protein translation efficiency and RNA stability. CDKAL1 thiomethylates tRNALys and the dysfunction of this enzyme cause type 2 diabetes through Lysine mistranslation. Hepatocellular carcinoma (HCC) is one of the most malignant neoplasms. Recently, the background disease of HCC is shifting from viral hepatitis to non-alcoholic hepatitis caused by obesity and diabetes. However, the importance of CDKAL1 and RNA thiomethylation in HCC remains largely unknown. Firstly, CDKAL1 expression was significantly correlated with prognosis in HCC from the open data base analysis. Next, we performed CDKAL1 knockdown (KD) experiments using human HCC cell lines and we found that CDKAL1 KD induced significantly reduced cell proliferation. Moreover, we found CDKAL1 KD also induced cell death by LDH assay. To investigate the molecular mechanisim, we conducted proteomic assay and we found CDKAL1 related to ferroptosis. In this study, CDKAL1 affects the long-term prognosis of HCC patients and inhibits the progression of ferroptosis and may serve as a prognostic marker and potential therapeutic target in HCC.

[Disclosure of COI by Responsible researcher] Lead presenter is the Responsible researcher.
[Lead presenter: Yuki Adachi] We have no financial relationships to disclose.

[P-3198] I-P10-12
Cell death (1)

2025 / 9 / 27 (Sat) 15:30-16:15 Room P/(Motenashi Dome) Room P

### P-3198
### The mechanism through which BACH1 inhibition induces iron-deficient cell death in head and neck cancer cells

[Speaker]Mitsuyo Matsumoto (Dept. of Biochem., Tohoku Univ. Grad. Sch. Med.／IMRAM, Tohoku Univ.) [Co-author]Kazuki Nakamura (Dept. of Biochem., Tohoku Univ. Grad. Sch. Med.／Dept. of Otolaryngology-HNS, Tohoku Univ. Grad. Sch. Med.)、Masahiro Rokugo (Dept. of Biochem., Tohoku Univ. Grad. Sch. Med.／Dept. of Otolaryngology-HNS, Tohoku Univ. Grad. Sch. Med.)、Hironari Nishizawa (Dept. of Biochem., Tohoku Univ. Grad. Sch. Med.)、Hiroki Shima (Dept. of Biochem., Tohoku Univ. Grad. Sch. Med.)、Takumi Akiyama (IMRAM, Tohoku Univ.)、Akihiko Muto (Dept. of Biochem., Tohoku Univ. Grad. Sch. Med.)、Yukio Katori (Dept. of Otolaryngology-HNS, Tohoku Univ. Grad. Sch. Med.)、Kazuhiko Igarashi (Dept. of Biochem., Tohoku Univ. Grad. Sch. Med.／Ctr. for Reg. Epi. & Dis., Tohoku Univ. Grad. Sch. Med.)

We reported at last year's annual meeting that the BACH1 knockdown (KD) in HSC-2 cells derived from head and neck squamous cell carcinoma (HNSCC) cells, induce mitochondrial dysfunction and subsequent cell death by decreasing the mitochondrial iron ($Fe^{2+}$), accompanied by impaired autophagy. However, the mechanism by which BACH1 regulates intracellular iron in HNSCC cells remains unclear. To investigate this, we examined intracellular distribution of iron in the HSC-2 cells, and found that the BACH1 KD hindered iron trafficking within cells. While $Fe^{2+}$ in mitochondria became lower, it was accumulated within lysosome. Furthermore, BACH1 KD was found to cause autophagy dysfunction by preventing proper lysosomal acidification. Importantly, we discovered that the total cellular iron content was rather increased by 20-30% upon BACH1 KD. These findings suggest that cell death in BACH1KD HSC-2 cells is not due to a decrease in the overall iron levels, but rather due to functional $Fe^{2+}$ deficiency caused by its sequestration within autophagosomes. These results highlight the potential of BACH1 as a novel therapeutic target for HNSCC.

[Disclosure of COI by Responsible researcher] Lead presenter is the Responsible researcher.
[Lead presenter: Mitsuyo Matsumoto] We have no financial relationships to disclose.

▲TOP

### [P-3199] I-P10-12
### Cell death (1)

2025 / 9 / 27 (Sat) 15:30-16:15 Room P/(Motenashi Dome) Room P

---

### P-3199
### Analysis of JTC-801-induced cell death mechanisms in Osteosarcoma

[Speaker]Yusuke Imagawa (Osaka Intl. Cancer Inst., Res. Ctr., Dept. Oncog. Growth Regul.／Osaka Univ., Grad. Sch. of Pharm. Sci., Clin. Exp. Pathophysiol.) [Co-author]Keiko Takano (Osaka Intl. Cancer Inst., Res. Ctr., Dept. Oncog. Growth Regul.)、Yuki Nakashima (Osaka Intl. Cancer Inst., Res. Ctr., Dept. Oncog. Growth Regul.／Osaka Univ., Grad. Sch. of Pharm. Sci., Clin. Exp. Pathophysiol.)、Hironari Tamiya (Osaka Intl. Cancer Inst., Dept. of Orthopedic Surg.／Osaka Intl. Cancer Inst., Dept. of Rehub.)、Yoshihide Tsujimoto (Osaka Intl. Cancer Inst., Res. Ctr.)

Many anti-cancer therapies induce apoptosis in cancer cells. However, many types of cancer remain resistant to current treatment strategies. Osteosarcoma, a rare and intractable cancer, is one such example. Recently, various forms of non-apoptotic (necrotic) regulated cell death have been reported, and our group has also identified and reported several novel types of cell death.In this study, we assessed the antitumor effects of several necrotic cell death inducers, including inducers of novel types of cell death we identified in various osteosarcoma cell lines. As a result, we found that JTC-801, an inducer of alkaliptosis–a non-apoptotic type of cell death–demonstrated a strong ability to induce cell death. Notably, JTC-801 was more effective at inducing cell death in osteosarcoma cells compared to various other cancer types. Additionally, anti-osteosarcoma activity was confirmed in vivo analysis using a xenograft model. We investigated the molecular mechanisms underlying JTC-801-induced cell death in osteosarcoma cells. Our findings suggest that lysosomal dysfunction, resulting from excessive lysosomal acidification, may trigger cell death.

[Disclosure of COI by Responsible researcher] Lead presenter is the Responsible researcher.
[Lead presenter: Yusuke Imagawa] ■Research funds from company: 科研費・基盤C　■Research expenses: 一般社団法人Advalife Science

### [P-3200] I-P10-12
### Cell death (1)

2025 / 9 / 27 (Sat) 15:30-16:15 Room P/(Motenashi Dome) Room P

### P-3200
### Tolerance to Extracellular Acidic pH Facilitates Tumor Plasticity

[Speaker]Manami Hasegawa (Dept. of Chemistry & Biotechnology, Eng., Univ. of Tokyo／Div. of Integrative Nutriomics & Oncology, RCAST, Univ. of Tokyo) [Co-author]Bo Xu (Div. of Integrative Nutriomics & Oncology, RCAST, Univ. of Tokyo)、Feifei Cai (Div. of Integrative Nutriomics & Oncology, RCAST, Univ. of Tokyo)、Keisuke Maede (Div. of Integrative Nutriomics & Oncology, RCAST, Univ. of Tokyo)、Rika Tsuchida (Div. of Integrative Nutriomics & Oncology, RCAST, Univ. of Tokyo)、Kazuyuki Yamagata (Div. of Integrative Nutriomics & Oncology, RCAST, Univ. of Tokyo)、Shinsuke Sando (Dept. of Chemistry & Biotechnology, Eng., Univ. of Tokyo)、Tsuyoshi Osawa (Dept. of Chemistry & Biotechnology, Eng., Univ. of Tokyo／Div. of Integrative Nutriomics & Oncology, RCAST, Univ. of Tokyo／Dept. of Biology, Grad. Sch. of Science, Univ. of Tokyo)

Cancer cells maintain glycolytic metabolism despite oxygen availability, resulting in an acidic microenvironment mediated by enhanced proton and lactate secretion. However, the mechanisms governing cell survival under acidic conditions are poorly understood. Here, we demonstrated that severe acidification (pH 5.6) triggered necroptotic cell death, which was prevented under acidic conditions (pH 6.8) for anchorage-independent cell survival and tumor initiation. Through RNA sequencing analysis, we identified the activation of the respiratory chain complex pathway in suspended cells at pH 6.8, suggesting a metabolic adaptation mechanism. Tumorigenicity was assessed by subcutaneous injection of pH 6.8- and pH 7.4-floating MIA-PaCa2 cells into SCID/SCID mice, and only pH 6.8-floating cells initiated tumors. Furthermore, a genome-wide CRISPR-Cas9 knockout screening in PANC1 cells under chronic acidic conditions identified genes essential for pH tolerance and survival. Notably, these gene set was correlated with poor prognosis in TCGA data. Our findings reveal a previously uncharacterized pathway that enables necroptotic avoidance and cellular plasticity under acidic stress.

[Disclosure of COI by Responsible researcher] Lead presenter is not the Responsible researcher.
[Lead presenter: Manami Hasegawa] We have no financial relationships to disclose.
[Principal Researcher: Tsuyoshi Osawa] We have no financial relationships to disclose.

### [P-3201] I-P10-12
### Cell death (1)

2025 / 9 / 27 (Sat) 15:30-16:15 Room P/(Motenashi Dome) Room P

---

### P-3201
### Effect of caprylic acid on colon cancer cells

[Speaker]Ruiko Ogata (Nara Med. Univ. Mol. Path.) [Co-author]Rina Tani (Nara Med. Univ. Mol. Path.)、Shingo Kishi (Kansai Med. Univ. Path.)、Ryoichi Nishida (Nara Med. Univ. Mol. Path.)、Shota Nukaga (Nara Med. Univ. Mol. Path.)、Hiroki Kuniyasu (Nara Med. Univ. Mol. Path.)

We previously demonstrated that caprylic acid (C8), a medium-chain fatty acid, exerts various physiological effects, including enhanced mitochondrial function and promotion of muscle maturation. In this study, we investigated the effects of C8 on HT29 human colorectal cancer cells. C8 induced cell death in a dose-dependent manner. Notably, C8 treatment decreased mitochondrial membrane potential and ROS levels, increased lipid peroxidation, and reduced cardiolipin content. C8-induced cell death was not prevented by inhibitors of apoptosis, ferroptosis, or necroptosis. Interestingly, the iron chelator deferoxamine further accelerated cell death, suggesting an iron-dependent mechanism. Iron transfer from the ER to mitochondria was also implied. Moreover, the expression of mitochondrial iron metabolism-related genes (***HO-1***, ***Bach1***, ***FECH***) was upregulated. Meanwhile, C8 treatment enhanced lysosomal activity and modestly increased autophagy. These findings suggest that C8 may induce a unique form of lipid peroxide -and mitochondrial iron- associated cell death distinct from ferroptosis. Further studies are ongoing to clarify this mechanism.

[Disclosure of COI by Responsible researcher] Lead presenter is not the Responsible researcher.
[Lead presenter: Ruiko Ogata] We have no financial relationships to disclose.
[Principal Researcher: Hiroki Kuniyasu] We have no financial relationships to disclose.

### [P-3202] I-P10-12
### Cell death (1)

2025 / 9 / 27 (Sat) 15:30-16:15 Room P/(Motenashi Dome) Room P

---

### P-3202
### Elucidating the role of MyD88 and its related factors in colorectal cancer progression

[Speaker]Rie Kajino (Div. Pathophysiology, Aichi Cancer Ctr. Res. Inst.) [Co-author]Teruaki Fujishita (Div. Pathophysiology, Aichi Cancer Ctr. Res. Inst.)、Yasushi Kojima (Div. Pathophysiology, Aichi Cancer Ctr. Res. Inst.)、Makoto M. Taketo (Pers. Canc. Ther., MIC, Kyoto Univ. Grad. Sch. Med.)、Masahiro Aoki (Div. Pathophysiology, Aichi Cancer Ctr. Res. Inst.／Div. Cancer Physiol., Nagoya Univ. Grad. Sch. Med.)

Despite advances in chemotherapy for colorectal cancer (CRC), therapeutic efficacy remains suboptimal, highlighting the need for new therapeutic strategies and target molecules. Our research aims to identify potential therapeutic targets for CRC using ***Apc***$^{+/\Delta716}$ (***Apc***) mice, a mouse model of early-stage CRC.
We previously reported that MyD88, a critical adaptor protein in the TLR and IL-1 pathways, is essential for proliferation and survival of ***Apc***-mutated intestinal epithelial cells. Additionally, inhibition of MyD88 function suppressed the growth of colon tumor organoids with CRC driver mutations. In our exploration of MyD88-related factors in CRC progression, we identified several candidate molecules, including HIF. MyD88 knockout reduced HIF protein accumulation in ***Apc*** polyps, resulting in the down-regulation of HIF target genes. Analysis of ***Hif1α***$^{F/F}$;***Apc***;***Villin-Cre***$^{ERT2}$ and ***Hif2α***$^{F/F}$;***Apc***;***Villin-Cre***$^{ERT2}$ compound mutant mice suggested that Hif2α plays a more significant role in tumor formation than Hif1α. Characterization of the MyD88-related candidate molecules, including HIF, may provide potential therapeutic targets for CRCs.

[Disclosure of COI by Responsible researcher] Lead presenter is the Responsible researcher.
[Lead presenter: Rie Kajino] We have no financial relationships to disclose.

▲TOP

### [P-3203] I-P10-12
### Cell death (1)

2025 / 9 / 27 (Sat) 15:30-16:15 Room P/(Motenashi Dome) Room P

---

### P-3203
### Elucidation of Cell Death Mechanisms Induced by NUDT5 Inhibition

[Speaker]Rika Sasaki (Dept. of Mol. Pathol., Nara Med.Univ.) [Co-author]Rina Tani (Dept. of Mol. Pathol., Nara Med.Univ.)、Shingo Kishi (Dept. of Mol. Pathol., Nara Med.Univ.／Department of Pathology, Kansai Medical University)、Ruiko Ogata (Dept. of Mol. Pathol., Nara Med.Univ.)、Hiroki Kuniyasu (Dept. of Mol. Pathol., Nara Med.Univ.)

Nudix hydrolase 5 (NUDT5) hydrolyzes ADP-ribose to produce ATP, contributing to cellular energy metabolism. We previously reported that NUDT5 inhibition exerts anti-tumor effects in colorectal cancer (CRC) cells, although the underlying mechanism remains unclear. In this study, we focused on mitochondria to clarify how NUDT5 inhibition suppresses tumor growth. NUDT5 inhibition led to increased mitochondrial ROS and decreased mitochondrial membrane potential. It also reduced mRNA expression of mitochondrial glutathione peroxidase 4, which protects against lipid peroxidation, and promoted mitochondrial iron accumulation. These findings suggest ferroptosis involvement; however, ferroptosis inhibition did not rescue cell viability. In addition, NUDT5 inhibition activated mitophagy with enhanced lysosomal function at early stages. Interestingly, long-term NUDT5 inhibition reduced both mitophagy and lysosomal activity. These results suggest that NUDT5 inhibition was suggested to induce cell death in CRC cells through different mechanisms at early and late stages. Further studies are needed to elucidate the detailed mitochondrial mechanisms involved in NUDT5 inhibition-induced cell death.

[Disclosure of COI by Responsible researcher] Lead presenter is not the Responsible researcher.
[Lead presenter: Rika Sasaki] We have no financial relationships to disclose.
[Principal Researcher: Hiroki Kuniyasu] We have no financial relationships to disclose.

### [P-3204] I-P10-12
### Cell death (1)

2025 / 9 / 27 (Sat) 15:30-16:15 Room P/(Motenashi Dome) Room P

---

### P-3204
### Hybrid liposomes exhibit remarkable growth-inhibitory effects on esophageal cancer cells through apoptosis

[Speaker]Junna Takai (Div. Appl. Life Sci., Grad. Sch. Eng., Sojo Univ.) [Co-author]Masaki Okumura (Div. Appl. Life Sci., Grad. Sch. Eng., Sojo Univ.)、Koichi Goto (Div. Appl. Life Sci., Grad. Sch. Eng., Sojo Univ.)、Yoko Matsumoto (Div. Appl. Life Sci., Grad. Sch. Eng., Sojo Univ.)、Hideaki Ichihara (Div. Appl. Life Sci., Grad. Sch. Eng., Sojo Univ.)

Hybrid liposomes (HL) are nanoparticles formed by sonication of vesicles of L-$\alpha$-dimyristoylphosphatidylcholine (DMPC) and micellar molecules of polyoxyethylene (25) dodecyl ether ($C_{12}(EO)_{25}$). HL have been effective for inhibiting tumor growth ***in vitro***, ***in vivo***, and in clinical applications without observed side effects. In this study, we investigated the inhibitory effects of HL on esophageal cancer cells, focusing on apoptosis. HL were prepared by ultrasonic irradiation in a 5% glucose solution and remained stable for over one month, maintaining a hydrodynamic diameter of less than 100 nm. The growth inhibitory effect of HL on esophageal cancer cells was assessed using the WST-8 assay. Flow cytometric analysis revealed an increase in apoptotic cells following HL treatment. Fluorescence intensity measurements of caspase-3, -8, and -9 substrates indicated that HL induce apoptosis through caspase activation. Additionally, time-dependent accumulation of HL containing a fluorescent probe was observed in esophageal cancer cells using fluorescence microscopy. These findings indicate that HL exert a significant growth-inhibitory effect on esophageal cancer cells through apoptosis.

[Disclosure of COI by Responsible researcher] Lead presenter is not the Responsible researcher.
[Lead presenter: Junna Takai] We have no financial relationships to disclose.
[Principal Researcher: Hideaki Ichihara] We have no financial relationships to disclose.

### [P-3214] I-P11-14
### Antibody therapy/Cancer vaccines (1)

2025 / 9 / 27 (Sat) 15:30-16:15 Room P/(Motenashi Dome) Room P

---

### P-3214
### Enhancing T Cell-Mediated Tumor Killing via 4-1BB Agonism & CD3/Tumor Antigen Targeting to Improve Cancer Immunotherapy

[Speaker]Chenwei Wang (Division of Developmental Therapeutics, National Cancer Center／Grad. Sch. of Frontier Sci., The Univ. of Tokyo) [Co-author]Tsubasa Aoki (Ajinomoto Co., Inc.)、Tomohiro Fujii (Ajinomoto Co., Inc.)、Ryutaro Asano (Department of Biomolecular Engineering, Graduate School of Engineering, Tohoku University)、Masahiro Yasunaga (Division of Developmental Therapeutics, National Cancer Center／Grad. Sch. of Frontier Sci., The Univ. of Tokyo)

T cell-based immunotherapies show promise for cancer treatment but are limited by insufficient activation and persistence, which can be improved by a key costimulatory receptor 4-1BB, making it an attractive target. Meanwhile, we identified a novel tumor antigen(TA) with high cancer cell expression and low normal tissue expression. CD3/TA targeting facilitates tumor-specific T cell activation. We aim to investigate the combined effects of 4-1BB agonism and CD3/TA targeting on T cell-mediated tumor killing to optimize immunotherapies.
Among tested 4-1BB agonism conditions, the optimal combination was 4-1BBL trimer and Urelumab, as it upregulated 4-1BB expression and significantly enhanced cytotoxicity when combined with BsAb hEx3 and Solitomab. To assess the potential of TA targeting, we evaluated the cytotoxicity of a novel CD3×TA BsAb, observing potent efficacy, further enhanced by 4-1BB agonism, which also boosted T cell migration and immune synapse formation.
This study identified synergistic effects of 4-1BB agonism and CD3/TA targeting in enhancing T cell tumor killing, supporting the development of a 4-1BB×CD3×TA trispecific antibody to further improve cancer immunotherapy.

[Disclosure of COI by Responsible researcher] Lead presenter is not the Responsible researcher.
[Lead presenter: Chenwei Wang] We have no financial relationships to disclose.
[Principal Researcher: Masahiro Yasunaga] ■Profit of stock: Rin Institute Inc. ■Research expenses: Daiichi Sankyo Co., Ltd., Ajinomoto Co., Inc., Sysmex Corporation, H.U. Group Holdings, Inc., and Dojin Group Co., Ltd.

### [P-3215] I-P11-14
### Antibody therapy/Cancer vaccines (1)

2025 / 9 / 27 (Sat) 15:30-16:15 Room P/(Motenashi Dome) Room P

---

### P-3215
### Effects of EMT-inducing condition on the expression of cancer-associated membrane proteins

[Speaker]Kouki Okita (Advanced Design for Pharmaceuticals, Fac Pharm, Kindai Univ／Carna Biosciences, Inc) [Co-author]Takashi Nakano (Grad Sch Health & Sports Sci, Toyo Univ)、Eri Kitadai (Linkgenomics, Inc.)、Yoko Sasaki (Linkgenomics, Inc.)、Hidemi Hayashi (Linkgenomics, Inc.)、Kazunori Kato (Grad Sch Health & Sports Sci, Toyo Univ)、Yoshihisa Tomioka (Lab Oncol Pharm Prac & Sci, Grad Sch, Tohoku Univ)、Noriaki Nagai (Advanced Design for Pharmaceuticals, Fac Pharm, Kindai Univ)、Hideyuki Saya (Oncology Innovation Center, Fujita Medical University)、Shinichiro Niwa (Linkgenomics, Inc.)、Takashi Masuko (Advanced Design for Pharmaceuticals, Fac Pharm, Kindai Univ／Oncology Innovation Center, Fujita Medical University)

Epithelial to mesenchymal transition (EMT) is involved in cell differentiation, wound healing and cancer metastasis, and can be induced by in vitro TGF-Β stimulation. EMT is known to promote cancer cell invasion during the early stages of metastasis. In this study, we analyzed the effects of EMT-inducing condition on the expression of various cancer-associated membrane proteins (transporters, adhesion molecules, growth factor receptors) in ARPE-19 retinal pigment epithelial cells. TGF-Β2 and TNF-Α stimulation of ARPE-19 cells resulted in increased mRNA expression of amino acid transporters (CD98, xCT, LAT1, ASCT2, CAT1), TfR, CD44 and HER1, but decreased mRNA expression of HER2, HER4 and MET, although effects of monoclonal antibodies against these membrane proteins on EMT status were not observed.

[Disclosure of COI by Responsible researcher] Lead presenter is not the Responsible researcher.
[Lead presenter: Kouki Okita] We have no financial relationships to disclose.
[Principal Researcher: Takashi Masuko] We have no financial relationships to disclose.

### [P-3216] I-P11-14
### Antibody therapy/Cancer vaccines (1)

2025 / 9 / 27 (Sat) 15:30-16:15 Room P/(Motenashi Dome) Room P

---

### P-3216
### The Role of Periostin Splicing Variants in Solid Tumors and Their Potential as Targets for Therapy

[Speaker]Kana Shibata (Osaka Univ., Sch. of Med.,Advanced Molecular Therapy ／PerioTherapia Co.,Ltd.) [Co-author]Yuko Kanemoto (Osaka Univ.,Sch.of Med.,Breast and Endocrine Surgery)、Kenzo Shimazu (Osaka Univ.,Sch.of Med.,Breast and Endocrine Surgery)、Yoshiaki Taniyama (Osaka Univ., Sch. of Med.,Advanced Molecular Therapy)

Alternative splicing allows a single gene to generate multiple mRNA variants with distinct sequences. Our research has shown that periostin (PN) splicing plays both physiological and pathological roles, influencing disease progression either positively or negatively. Specifically, we investigated the C-terminal splicing variant of PN, an extracellular matrix protein, and found that its alterations contribute to cancer metastasis, radiation resistance, and drug resistance.In this study, we aim to confirm that the expression of PN exon17 and 21 variants is elevated in therapy-resistant cancers, such as triple-negative breast cancer and malignant melanoma. We will also evaluate the antitumor efficacy of neutralizing antibodies targeting these variants. Pathological PN promotes cancer proliferation, enhances the tumor microenvironment, recruits bone marrow-derived cells, drives epithelial-mesenchymal transition, and facilitates metastasis. Selective inhibition of PN variants could serve as a promising, tumor-specific therapeutic strategy with a favorable safety profile.

[Disclosure of COI by Responsible researcher] Lead presenter is not the Responsible researcher.
[Lead presenter: Kana Shibata] ■Research funds from company: ペリオセラピア株式会社　科研費　AMED
[Principal Researcher: Yoshiaki Taniyama] ■Research funds from company: ペリオセラピア株式会社　科研費　AMED　■Profit of stock: ペリオセラピア株式会社

### [P-3217] I-P11-14
### Antibody therapy/Cancer vaccines (1)

2025 / 9 / 27 (Sat) 15:30-16:15 Room P/(Motenashi Dome) Room P

### P-3217
### Investigation of the mechanism of cytotoxicity of the anti-pan HLA class 2 mAb 4713 against ATL cells.

[Speaker]Takeshi Hirano (Dept. of Immunological Diagnosis, Grad.Sch. of Med., Juntendo／Dept. of Latic acid bacteria biological function res., Juntendo) [Co-author]Shuji Matsuoka (Dept. of Latic acid bacteria biological function res., Juntendo／Div. of Cancer Immnotherapy, Natl. Cancer Ctr.)、Natsuko Mizutani (Dept. of Latic acid bacteria biological function res., Juntendo)、Yasuhiko Ito (Dept. of Latic acid bacteria biological function res., Juntendo)

The anti-pan-HLA class II mAb, mAb 4713, showed direct cytotoxic activity against advanced adult T-cell leukemia (IL-2 independent ATL) cells, but did not show similar cytotoxic activity against not advanced (IL-2 dependent) ATL cells. Therefore, we investigated how mAb 4713 could treat patients with IL-2 dependent ATL. We hypothesized that ATL cell lines with high levels of HLA class II molecules would be selectively induced to anapocosis by mAb4713 alone. However, several IL-2 dependent ATL cell lines were resistant to mAb4713 treatment despite high expression of HLA class II. We investigated the possibility that IL-2 receptor signaling and amphotericin B (AMB) may contribute to the activation of mAb 4713 cytotoxicity against IL-2-dependent ATL cell lines. Incubation of ATL cell lines with anti-IL-2 antibody or AMB for a certain time and treatment with mAb4713 activated cytotoxicity of mAb 4713 in a concentration-dependent manner. We found that mAb4713 treatment with blockade of IL-2 receptor signaling or in combination with AMB and mAb 4713 has potential for patients with early stage ATL.

[Disclosure of COI by Responsible researcher] Lead presenter is not the Responsible researcher.
[Lead presenter: Takeshi Hirano] We have no financial relationships to disclose.
[Principal Researcher: Shuuji Matsuoka] ■Profit of stock: 日本抗体医薬株式会社

### [P-3218] I-P11-14
### Antibody therapy/Cancer vaccines (1)

2025 / 9 / 27 (Sat) 15:30-16:15 Room P/(Motenashi Dome) Room P

---

### P-3218
### Trastuzumab deruxtecan resistance caused by increased expression of drug transporter inHER2-positive gastric cancer

[Speaker]Yuya Murase (Department of Respiratory Medicine, Kanazawa University.) [Co-author]Shigeki Nanjo (Department of Respiratory Medicine, Kanazawa University.)、Hayato Koba (Department of Respiratory Medicine, Kanazawa University.)、Sachiko Arai (WPI Nano Life Science Institute (WPI-NanoLSI), Kanazawa University)、Tsukasa Ueda (Department of Respiratory Medicine, Kanazawa University.)、Yuichi Tambo (Department of Respiratory Medicine, Kanazawa University.)、Yifeng Liu (Department of Respiratory Medicine, Kanazawa University.)、Seiji Yano (Department of Respiratory Medicine, Kanazawa University.／WPI Nano Life Science Institute (WPI-NanoLSI), Kanazawa University)

Trastuzumab deruxtecan (T-DXd), a HER2 targeting antibody drug conjugate (ADC), has demonstrated remarkable clinical efficacy in HER2-positive gastric cancer. However, the development of acquired resistance remains a major therapeutic challenge. In this study, we established a T-DXd resistant HER2 amplified gastric cancer cell line (N87 AR) through repeated in vivo drug exposure and investigated the underlying resistance mechanisms. N87 AR cells exhibited cross-resistance to T-DXd, its cytotoxic payload DXd, despite preserved HER2 expression and unchanged drug internalization. Notably, the drug transporter expression was significantly upregulated in resistant cells. Genetic knockdown or pharmacologic inhibition of this restored sensitivity to T-DXd. Furthermore, Disitamab vedotin, a HER2 ADC with an MMAE payload not effluxed by this transporter, overcame resistance in vitro. These findings demonstrate that drug transporter-mediated efflux contributes to T-DXd resistance and suggest that combining T-DXd with its inhibitors or using alternative ADCs with distinct payloads may represent effective therapeutic strategies for refractory HER2-positive gastric cancer.

[Disclosure of COI by Responsible researcher] Lead presenter is not the Responsible researcher.
[Lead presenter: Yuya Murase] We have no financial relationships to disclose.
[Principal Researcher: Seiji Yano] ■Lecture fee: Chugai Pharmaceutical Co., Ltd., ONO PHARMACEUTICAL CO., LTD., Pfizer Inc., Eli Lilly Japan K.K., Daiichi Sankyo Co., Ltd., MSD, and Eisai Co., Ltd., AstraZeneca K.K., TAIHO PHARMACEUTICAL CO., LTD.. ■Research expenses: Takeda Pharmaceutical Company Limited, Janssen Pharmaceutical K.K., Bliss Bio Co., Ltd., Eisai Co., Ltd.

### [P-3219] I-P11-14
### Antibody therapy/Cancer vaccines (1)

2025 / 9 / 27 (Sat) 15:30-16:15 Room P/(Motenashi Dome) Room P

---

### P-3219
### The cancer-specific anti-human podoplanin antibody chLpMab-2f exerts antitumor effects on pleural mesothelioma.

[Speaker]Aito Yoshida (Department of Respiratory Medicine & Rheumatology, Tokushima University) [Co-author]Shinji Abe (Department of Clinical Pharmacy Practice Pedagogy, Tokushima University)、Atsushi Mitsuhashi (Department of Respiratory Medicine & Rheumatology, Tokushima University)、Takaaki Yamamoto (Department of Clinical Pharmacy Practice Pedagogy, Tokushima University)、Kei Kawada (Department of Clinical Pharmacy Practice Pedagogy, Tokushima University)、Chiemi Sato (Department of Clinical Pharmacy Practice Pedagogy, Tokushima University)、Hirokazu Ogino (Department of Respiratory Medicine & Rheumatology, Tokushima University)、Mika K. Kaneko (Department of Antibody Drug Development, Tohoku University)、Yukinari Kato (Department of Antibody Drug Development, Tohoku University)、Yasuhiko Nishioka (Department of Respiratory Medicine & Rheumatology, Tokushima University)

The prognosis of pleural mesothelioma (PM) is poor because of the resistance to various cytotoxic chemotherapies. To develop a novel treatment for PM, we focused on podoplanin (PDPN), a transmembrane glycoprotein. Anti-PDPN antibodies exhibited antitumor effects on PDPN-expressing PM in preclinical studies. However, these antibodies have the risk to cause adverse events because PDPN is also expressed in normal tissues. To solve this problem, the cancer-specific monoclonal antibody (CasMab)-producing technology was developed. We established an anti-human PDPN CasMab (chLpMab-2f) that reacted to cancers but did not react to normal human organs. Here, we evaluated the antitumor effects of chLpMab-2f on PM. We confirmed that chLpMab-2f recognized the cell surface PDPN on PM cell lines and human PM tissues. This antibody exerted antibody-dependent cellular cytotoxicity and complement-dependent cytotoxicity to PDPN-expressing PM cells. In addition, chLpMab-2f inhibited tumor progression in human PM cells-bearing immune deficient mice. These data suggest that PDPN-targeting immunotherapy using chLpMab-2f might provide an effective therapeutic strategy against PM.

[Disclosure of COI by Responsible researcher] Lead presenter is not the Responsible researcher.
[Lead presenter: Aito Yoshida] We have no financial relationships to disclose.
[Principal Researcher: Yasuhiko Nishioka] ■Lecture fee: 日本ベーリンガーインゲルハイム株式会社　■Research expenses: SANSHO株式会社、株式会社ティムス　■Contributions: 中外製薬株式会社、大鵬薬品工業株式会社、旭化成ファーマ株式会社、日本イーライリリー株式会社、日本ベーリンガーインゲルハイム株式会社

### [P-3220] I-P11-14
### Antibody therapy/Cancer vaccines (1)

2025 / 9 / 27 (Sat) 15:30-16:15 Room P/(Motenashi Dome) Room P

---

### P-3220
### Enhancing The Efficacy of T Cell-Dependent Bispecific Antibodies for Solid Tumors by Overcoming T Cell Exhaustion

[Speaker]Shoko Hamamoto (Grad. Sch. Frontier Sci., Tokyo Univ.／Div. Developmental Therap., Natl Cancer Ctr.) [Co-author]Ryo Tsumura (Div. Developmental Therap., Natl Cancer Ctr.)、Ryutaro Asano (Inst. Engineering, Tokyo Univ. of Agriculture & Tech.)、Masahiro Yasunaga (Grad. Sch. Frontier Sci., Tokyo Univ.／Div. Developmental Therap., Natl Cancer Ctr.)

T cell-dependent bispecific antibodies (TDBs) are transforming cancer immunotherapy, yet their success remains largely confined to hematologic malignancies. A key barrier in solid tumors is T cell exhaustion, a dysfunctional state driven by chronic antigen stimulation in the tumor microenvironment. To address this, we developed a reproducible in vitro model using PBMCs and repeated CD3/CD28 stimulation, reliably inducing exhausted T cells expressing PD-1, TIM-3, and LAG-3 across donors. This method overcomes donor variability seen in conventional assays and enables consistent exhaustion induction. It mimics chronic activation and allows robust evaluation of exhaustion-targeted interventions. RNA-seq analysis identified anti-exhaustion candidate genes, including cytokine receptors and TCR regulators. Lentiviral transduction of selected genes significantly reduced exhaustion phenotypes, with synergistic effects observed upon combination. This model provides a platform to dissect exhaustion mechanisms and identify therapeutic targets. Our findings offer a strategy to overcome immune resistance in solid tumors and advance next-generation TDB therapies.

[Disclosure of COI by Responsible researcher] Lead presenter is not the Responsible researcher.
[Lead presenter: Shoko Hamamoto] We have no financial relationships to disclose.
[Principal Researcher: Ryo Tsumura, Ryutaro Asano] ■Profit of stock: 株式会社凜研究所　■Research expenses: 第一三共株式会社、味の素株式会社、シスメックス株式会社、H.U.グループホールディングス、株式会社同仁グループ

[P-3221] I-P11-14
Antibody therapy/Cancer vaccines (1)

2025 / 9 / 27 (Sat) 15:30-16:15 Room P/(Motenashi Dome) Room P

---

P-3221
### Development of a Bispecific T-cell engaging antibody (BiTE) HLA-A2/Claspin Peptide Complex for Platinum-Resistant Cancer

[Speaker]Yuka Mizue (Dept. of Path., Sapporo Med. Univ. Sch. of Med.) [Co-author]Yoshihiko Hirohashi (Dept. of Path., Sapporo Med. Univ. Sch. of Med.)、Tomohide Tsukahara (Dept. of Path., Sapporo Med. Univ. Sch. of Med.)、Takayuki Kanaseki (Dept. of Path., Sapporo Med. Univ. Sch. of Med.)、Terufumi Kubo (Dept. of Path., Sapporo Med. Univ. Sch. of Med.)、Kenji Murata (Dept. of Path., Sapporo Med. Univ. Sch. of Med.)、Toshihiko Torigoe (Dept. of Path., Sapporo Med. Univ. Sch. of Med.)

We identified Claspin (CLSPN) as specific to bladder cancer stem-like cells and observed its elevated expression in cisplatin-resistant cell lines (CDDP-R). HLA-A2/CLSPN peptide complex-specific cytotoxic T lymphocytes recognized CLSPN-positive cells. We thus developed a single-chain variable fragment (scFv) targeting the HLA-A2/CLSPN peptide complex. The scFv was then used to engineer into a human IgG1 format (HLA-A2/CLSPN-hIgG), a Bispecific T-cell engaging antibody (BiTE), and a Chimeric Antigen Receptor-T cells (CAR-T). In this study, we investigated the reactivities of each antibody and CAR-T ***in vitro*** . FACS analysis confirmed its specific binding to Claspin-expressing HLA-A2-positive tumor cell lines. Furthermore, co-culturing these cancer cells with PBMCs and adding the BiTE induced IFN-γ production as measured by T-cell responses in an ELISPOT assay. And also, BiTE-mediated cytotoxicity against these tumor cell lines was facilitated by cytotoxic T cells from PBMCs. These results suggested that targeting the HLA-A2/CLSPN peptide complex, including BiTE, holds potential as an effective therapeutic strategy for platinum-resistant cancer.

[Disclosure of COI by Responsible researcher] Lead presenter is not the Responsible researcher.
[Lead presenter: Yuka Mizue] We have no financial relationships to disclose.
[Principal Researcher: Yoshihiko Hirohashi] We have no financial relationships to disclose.

### [P-3222] I-P11-14
### Antibody therapy/Cancer vaccines (1)

2025 / 9 / 27 (Sat) 15:30-16:15 Room P/(Motenashi Dome) Room P

---

### P-3222
### Analysis of cetuximab-induced EGFR interactome using proximity biotinylation AirID

[Speaker]Hinako Fujisaki (Proteo-Science Center, Ehime University) [Co-author]Kohdai Yamada (Proteo-Science Center, Ehime University)、Tatsuya Sawasaki (Proteo-Science Center, Ehime University)

Cetuximab is a monoclonal antibody that targets EGFR for anticancer activity. Cetuximab, which binds to the extracellular domain of EGFR, prevents cell proliferation by inhibiting ligand-induced dimerization and downstream signaling. However, the detailed mechanism of how cetuximab works to inhibit the formation of EGFR complex remain unclear. In this study, we analyzed changes in EGFR-related proteins in response to cetuximab administration by AirID. Cetuximab was applied to EGFR-AirID-expressing lung cancer-derived NCI-H226 cells and colorectal cancer-derived HT-29 cells. Phosphorylation and biotinylation of membrane-bound EGFR were analyzed by immunoblotting, and interacting proteins were identified by mass spectrometry. Cetuximab treatment decreased tyrosine phosphorylation and biotinylation, indicating inhibition of EGFR activation. Mass spectrometry showed that cetuximab treatment inhibited biotinylation of 15 proteins in NCI-H226 cells and 169 proteins in HT-29 cells. GO analysis showed that EGF signalling was suppressed by the addition of cetuximab in HT-29 cells. This may suggest why cetuximab is effective in the treatment of colorectal cancer.

[Disclosure of COI by Responsible researcher] Lead presenter is not the Responsible researcher.
[Lead presenter: Hinako Fujisaki] We have no financial relationships to disclose.
[Principal Researcher: Tatsuya Sawasaki] ■Advisor of the company and/or profit-making organization: 株式会社セルフリーサイエンス

### [P-3232] I-P11-16
### Immune cell therapy (CAR-T, T cell therapy) (1)

2025 / 9 / 27 (Sat) 15:30-16:15 Room P/(Motenashi Dome) Room P

---

### P-3232
### Vγ9Vδ2 T cell-mediated lung cancer cytotoxicity and NK cell-mediated mesothelioma cytotoxicity

[Speaker]Hiromi Tomono (Dept. Resp. Med. Nagasaki Univ. Hosp.／Nagasaki Univ. Ctr. for Med. Innovation) [Co-author]Kazumasa Akagi (Dept. Resp. Med. Nagasaki Univ. Hosp.／Clin. Oncol. Ctr, Nagasaki Univ. Hosp.)、Noritaka Honda (Dept. Resp. Med. Nagasaki Univ. Hosp.)、Yasuhiro Umeyama (Sasebo City General Hosp.)、Yosuke Dotsu (Dept. Resp. Med. Nagasaki Univ. Hosp.)、Hirokazu Taniguchi (Dept. Resp. Med. Nagasaki Univ. Hosp.／Clin. Oncol. Ctr, Nagasaki Univ. Hosp.)、Midori Matsuo (Dept. Resp. Med. Nagasaki Univ. Hosp.／Cli. Res. Ctr. Nagasaki Univ. Hosp.)、Shinnosuke Takemoto (Dept. Resp. Med. Nagasaki Univ. Hosp.)、Yoshimasa Tanaka (Nagasaki Univ. Ctr. for Med. Innovation)、Hiroshi Mukae (Dept. Resp. Med. Nagasaki Univ. Hosp.)

Adoptive transfer of immune effector cells, especially allogeneic cells, has gained attention due to the immunosuppressed status of cancer patients. We focused on Vγ9Vδ2 T and NK cells, optimizing their large-scale expansion. These innate effectors exert tumor cytotoxicity independent of MHC. We evaluated their cytotoxic properties, including innate and antigen-specific cytotoxicity, ADCC, and cytokine production.PBMCs from healthy donors and patients were stimulated with PTA/IL-2 for Vγ9Vδ2 T cell expansion and IL-2/IL-18 for NK cell expansion. Expanded cells were co-cultured with lung cancer or pleural mesothelioma cells for in vitro assays. PTA served as the antigen, and anti-EGFR antibody was used for ADCC.Vγ9Vδ2 T cells exhibited PTA-dependent cytotoxicity against lung cancer and CD16-mediated ADCC. NK cells displayed innate cytotoxicity against mesothelioma, enhanced in an antibody-dependent manner.[Conclusion]PTA/IL-2 enabled high-purity Vγ9Vδ2 T cell expansion with robust tumor-killing functions, while IL-2/IL-18 expanded NK cells with strong innate cytotoxicity and ADCC. Future in vivo studies will explore their therapeutic potential.

[Disclosure of COI by Responsible researcher] Lead presenter is not the Responsible researcher.
[Lead presenter: Hiromi Tomono] We have no financial relationships to disclose.
[Principal Researcher: Yoshimasa Tanaka] ■employment by a company related to the presentation: 株式会社SCMbiomedica ■Advisor of the company and/or profit-making organization: 株式会社SCMbiomedica ■Profit of stock: 株式会社SCMbiomedica ■Fees of testimony judgement comment etc.: 株式会社SCMbiomedica ■Presents or other payment: 株式会社SCMbiomedica

### [P-3233] I-P11-16
### Immune cell therapy (CAR-T, T cell therapy) (1)

2025 / 9 / 27 (Sat) 15:30-16:15 Room P/(Motenashi Dome) Room P

### P-3233
### Withdrawn

No Abstract

### [P-3234] I-P11-16
### Immune cell therapy (CAR-T, T cell therapy) (1)

2025 / 9 / 27 (Sat) 15:30-16:15 Room P/(Motenashi Dome) Room P

---

### P-3234
### Acquisition of glioblastoma-specific T cell receptors for TCR-T cell therapy

[Speaker]Sora Yada (Fac. Med., Acad. Assem., Univ of Toyama) [Co-author]Kanami Tanaka (Fac. Med., Acad. Assem., Univ of Toyama)、Hiroyuki Kishi (Fac. Med., Acad. Assem., Univ of Toyama)、Eiji Kobayashi (Fac. Med., Acad. Assem., Univ of Toyama)

Glioblastoma is a disease with an extremely poor prognosis. Also, inhibition of apoptosis by deubiquitination is thought to be one of the main causes of glioblastoma. In addition, it is difficult to obtain cancer antigen-specific TCRs because of the limited use of tumor-infiltrating lymphocytes from cancer patients. Therefore, we attempted to clone deubiquitinase X-specific TCR genes from healthy human peripheral blood mononuclear cells (PBMCs) using our originally developed hTEC10 method. To this end, HLA-A24:02 binding peptides of deubiquitinase X were firstly predicted by using two algorithms and five predicted peptides with HLA-A24:02 binding capacity were synthesized. We next stimulated healthy PBMCs with the five peptides together with IL-2 for 2 weeks. We then performed single-cell sorting of peptide-specific T cells from the stimulated cells using the five peptide/HLA-A24 tetramer mixture and amplified TCR $\alpha$- and $\beta$-chains. As a result, three clonally-expanded TCRs were obtained. The TCRs were then expressed using our TAP method and peptide specificity of the TCRs were evaluated. As a result, one of the three TCRs was specific to deubiquitinase X peptide.

[Disclosure of COI by Responsible researcher] Lead presenter is not the Responsible researcher.
[Lead presenter: Sora Yada] We have no financial relationships to disclose.
[Principal Researcher: Eiji Kobayashi] We have no financial relationships to disclose.

### [P-3235] I-P11-16
### Immune cell therapy (CAR-T, T cell therapy) (1)

2025 / 9 / 27 (Sat) 15:30-16:15 Room P/(Motenashi Dome) Room P

---

### P-3235
### Short-term production of CAR-T cells using RetroNectin and G-Rex.

[Speaker]Seina Inui (Takara Bio Inc. CDM Center 3) [Co-author]Yasunori Amaishi (Takara Bio Inc. CDM Center 3)、Izumi Maki (Takara Bio Inc. CDM Center 3)、Sachiko Okamoto (Takara Bio Inc. CDM Center 3)

CAR-T cell therapies have shown remarkable efficacy in the treatment of hematologic malignancies. However, there are still many challenges in the cell manufacturing process. Most CAR-T cell manufacturing protocols expand T cells for 9 to 14 days. It is known that extended ***ex vivo*** culture period results in T cell differentiation and exhaustion, which reduces the potency of CAR-T cells after adoptive transfer. Therefore, there is a need to reduce manufacturing time to improve treatment efficacy. Then, we have already developed a short-term production method for CAR-T cell, Spo-T® (Short Period Operation for T-cell production), and reported its higher cytotoxic activity, greater proliferative ability and lower exhaustion compared to the conventional method. In this study, we established a simple and efficient method for CAR-T cell production by combining RetroNectin® and G-Rex (gas-permeable membrane bioreactor for high-density culture). Specifically, RetroNectin and anti-CD3 antibody are pre-coated on G-Rex bioreactor, and T-cell activation and transduction are performed simultaneously. This method can produce CAR-T cells in just 2 processing days, within a total of 3 days.

[Disclosure of COI by Responsible researcher] Lead presenter is not the Responsible researcher.
[Lead presenter: Seina Inui] ■Research funds from company: タカラバイオ株式会社　■ employment by a company related to the presentation: タカラバイオ株式会社
[Principal Researcher: Sachiko Okamoto] ■Research funds from company: タカラバイオ株式会社　■employment by a company related to the presentation: タカラバイオ株式会社

Poster Session

### [P-3236] I-P11-16
### Immune cell therapy (CAR-T, T cell therapy) (1)

2025 / 9 / 27 (Sat) 15:30-16:15 Room P/(Motenashi Dome) Room P

---

### P-3236
### The Simple CAR-T Cell Manufacture: Simultaneous T cell activation and transduction in RetroNectin-coated G-Rex

[Speaker]Yasunori Amaishi (Takara Bio Inc. CDM Center 3) [Co-author]Izumi Maki (Takara Bio Inc. CDM Center 3)、Seina Inui (Takara Bio Inc. CDM Center 3)、Sachiko Okamoto (Takara Bio Inc. CDM Center 3)

CAR-T cell therapy has shown high anti-tumor activity against hematologic malignancies, and several therapeutic agents have been marketed. However, the manufacturing process for CAR-T cells is complex and costly, presenting challenges that require improvements in manufacturing methods. RetroNectin® is a recombinant protein containing functional domains of human fibronectin, which promotes gene transfer to cells expressing integrins. Additionally, co-stimulation with RetroNectin and anti-CD3 antibodies can efficiently proliferate undifferentiated T cells. The G-Rex bioreactor, with its gas-permeable membrane, allows high-density culture of cells without the need for agitation or medium exchange. This study developed a method to produce high-quality CAR-T cells using a simple process by combining RetroNectin and G-Rex. By coating the G-Rex with RetroNectin, simultaneous T cell activation and gene transfer were achieved, eliminating the need for medium exchange and reducing the process to three operation days. This method simplifies the manufacturing process and enhances the quality and efficiency of CAR-T cells, providing a promising platform for cell therapy manufacturing.

[Disclosure of COI by Responsible researcher] Lead presenter is not the Responsible researcher.
[Lead presenter: Yasunori Amaishi] ■Research funds from company: タカラバイオ株式会社　■ employment by a company related to the presentation: タカラバイオ株式会社
[Principal Researcher: Sachiko Okamoto] ■Research funds from company: タカラバイオ株式会社　■employment by a company related to the presentation: タカラバイオ株式会社

### [P-3237] I-P11-16
### Immune cell therapy (CAR-T, T cell therapy) (1)

2025 / 9 / 27 (Sat) 15:30-16:15 Room P/(Motenashi Dome) Room P

---

### P-3237
### Harnessing Antigen-Independent TNF-$\alpha$ Mechanisms to Overcome Tumor Heterogeneity in Adoptive T Cell Therapy

[Speaker]Situo Deng (Dept. Oncology., Nagasaki Univ. Grad. Sch. of Biomed. Sci.) [Co-author]Daisuke Muraoka (Dept. Oncology., Nagasaki Univ. Grad. Sch. of Biomed. Sci. ／Aichi Cancer Center, Tumor Immunology and Translational Research Division.)、Takaaki Nakatsukasa (Dept. Oncology., Nagasaki Univ. Grad. Sch. of Biomed. Sci. ／Nagasaki Univ. Grad. Sch. of Biomed. Sci., Dept. of Surgery.)、Kiyoshi Yasui (Dept. Oncology., Nagasaki Univ. Grad. Sch. of Biomed. Sci.)、Mitsuhiro Yoneda (Dept. Oncology., Nagasaki Univ. Grad. Sch. of Biomed. Sci.)、Naozumi Harada (United Immunity Co., Ltd.)、Shinichi Sawada (cSIMVa, Chiba Univ.,)、Kazunari Akiyoshi (Department of Immunology Kyoto Univ. Grad)、Hiroaki Ikeda (Dept. Oncology., Nagasaki Univ. Grad. Sch. of Biomed. Sci.)

Recent advancements in adoptive cell therapies (ACTs) have ushered in a new era in cancer treatment. However, despite their potential, the efficacy of ACTs is often compromised by the emergence of antigen loss variants (ALVs) that evade recognition and elimination by antigen-specific T cells. To address this challenge, we developed an effective ACT strategy targeting ALV-containing tumors using our unique heterogeneous tumor mouse model. The combination of ACT and vaccination enables infused T cells to eradicate cancer cells, regardless of their antigen expression status. TNF-$\alpha$ mediates MHC-I-independent killing of antigen-negative cells, being produced not only by the infused ACT T cells but also by tumor-associated macrophages (TAMs) upon ACT activation. TNFR1 expression determines tumor susceptibility; its loss abolishes sensitivity. RNA-seq revealed NF-$\kappa$B mediates antigen-independent cytotoxicity. These findings demonstrate ACT stimulates innate immunity to secrete TNF-$\alpha$, overcoming heterogeneity. Targeting this pathway could enhance ACT efficacy against heterogeneous tumors.

[Disclosure of COI by Responsible researcher] Lead presenter is the Responsible researcher.
[Lead presenter: Ssituo Deng] We have no financial relationships to disclose.

▲TOP

[P-3238] I-P11-16
Immune cell therapy (CAR-T, T cell therapy) (1)

2025 / 9 / 27 (Sat) 15:30-16:15 Room P/(Motenashi Dome) Room P

P-3238
### Development of a Novel T cell Activator using Rabbit anti-CD28 antibody to prevent T cell Exhaustion

[Speaker]Yusuke Totsuka (FUJIFILM Corporation) [Co-author]Mayu Yamaguchi (FUJIFILM Corporation)、Yuki Machida (FUJIFILM Corporation)、Koki Endo (FUJIFILM Corporation／FUJIFILM Wako Pure Chemical Corporation)、Nao Yamazaki (FUJIFILM Corporation)、Yuta Murakami (FUJIFILM Corporation)、Takahiro Nishibu (FUJIFILM Corporation／FUJIFILM Wako Pure Chemical Corporation)

[Background]A key issue in CAR–T cell production is reduced efficacy caused by cell exhaustion and differentiation during growth. Focusing on the CD28 co–stimulatory signal tied to exhaustion, we developed a novel T cell activator with new anti–CD28 rabbit monoclonal antibodies (mAb) to preserve T cell proliferation while preventing exhaustion.[Methods and Results]Anti–CD28 rabbit mAb clones were obtained via our rabbit DNA immunization and B cell cloning method. Our T cell activators were developed by conjugating these mAbs with a commercially available mouse anti–CD3 antibody on magnetic beads. Human T cells were used to evaluate the activator's ability to promote proliferation and prevent exhaustion. The results showed these activators maintained undifferentiated T cells such as T memory stem cells and central memory T cells, while preserving or exceeding the proliferation rates of existing activators.[Conclusion]Our novel T cell activators strongly support proliferation and prevent exhaustion compared to conventional activators, showing potential for CAR–T cell manufacturing to address T cell exhaustion issues.

[Disclosure of COI by Responsible researcher] Lead presenter is the Responsible researcher.
[Lead presenter: Yusuke Totsuka] We have no financial relationships to disclose.

### [P-3239] I-P11-16
### Immune cell therapy (CAR-T, T cell therapy) (1)

2025 / 9 / 27 (Sat) 15:30-16:15 Room P/(Motenashi Dome) Room P

### P-3239
### Withdrawn

No Abstract

### [P-3245] I-P12-6
### Cancer diagnostic technologies (1)

2025 / 9 / 27 (Sat) 15:30-16:15 Room P/(Motenashi Dome) Room P

### P-3245
### The Impact of Peroxiredoxin 3 on Diagnosis and Prognosis in Human Pancreatic Ductal Adenocarcinoma

[Speaker]Anna Kakehashi (Dept. Mol. Pathol., Osaka Metropol. Univ. Grad. Sch. Med.) [Co-author]Yusaku Nishidoi (Dept. Mol. Pathol., Osaka Metropol. Univ. Grad. Sch. Med.)、Ikue Noura (Dept. Mol. Pathol., Osaka Metropol. Univ. Grad. Sch. Med.)、Guiyu Qiu (Dept. Mol. Pathol., Osaka Metropol. Univ. Grad. Sch. Med.)、Arpamas Vachiraarunwong (Dept. Mol. Pathol., Osaka Metropol. Univ. Grad. Sch. Med.／Dept. Environ. Risk Assessment, Osaka Metropol. Univ. Grad. Sch. Med.)、Masaki Fujioka (Dept. Mol. Pathol., Osaka Metropol. Univ. Grad. Sch. Med.)、Min Gi (Dept. Mol. Pathol., Osaka Metropol. Univ. Grad. Sch. Med.／Dept. Environ. Risk Assessment, Osaka Metropol. Univ. Grad. Sch. Med.)、Hideki Wanibuchi (Dept. Mol. Pathol., Osaka Metropol. Univ. Grad. Sch. Med.／Dept. Environ. Risk Assessment, Osaka Metropol. Univ. Grad. Sch. Med.)、Shugo Suzuki (Dept. Mol. Pathol., Osaka Metropol. Univ. Grad. Sch. Med.)

In the present study, novel biomarker candidates useful for early diagnosis and prognosis of human invasive PDAC were investigated. From proteome analysis results, peroxiredoxin 3 (PRX3) was chosen as a potential biomarker candidate. PRX3 protein and extracellular vesicle (EV) mRNA were found secreted from PANC-1, MIAPaCa-2, and SW1990 cells into the blood of Balb/c nude mice bearing tumors. PRX3 protein expression was significantly increased in 100 PDAC patients operated at Osaka Metropolitan University Hospital, predicted prognosis outcome in short-term postoperative period and improved prognosis when combined with blood SPan-1 and DUPAN-2 markers. A significant correlation was observed between expression of PRX3, CD44 variant 9, P-Nrf2, and FOXO3a. Next, PRX3 protein and EVs mRNA levels were measured in the blood of 36 PDAC and 10 IPMN patients, and a significant increase of PRX3 protein and EV mRNA was found in the blood serum of PDAC subjects as compared to IPMN and control. PRX3 EV mRNA elevation was significantly associated with patients poor survival. In conclusion, PRX3 protein and EVs mRNA may become the novel potential markers for PDAC diagnosis and prognosis.

[Disclosure of COI by Responsible researcher] Lead presenter is not the Responsible researcher.
[Lead presenter: Anna Kakehashi] We have no financial relationships to disclose.
[Principal Researcher: Shugo Suzuki] We have no financial relationships to disclose.

▲TOP

### [P-3246] I-P12-6
### Cancer diagnostic technologies (1)

2025 / 9 / 27 (Sat) 15:30-16:15 Room P/(Motenashi Dome) Room P

---

### P-3246
### Identification of circulating miRNA markers using a rat pancreatic cancer model

[Speaker]Atsuko Mizoguchi (Toray Industries, Inc.) [Co-author]Takashi Serizawa (Toray Industries, Inc.)、Aiko Takayama (Toray Industries, Inc.)、Hiroko Sudo (Toray Industries, Inc.)、Katsumi Fukamachi (Dept. Neurotoxicol. Nagoya City Univ. Grad. Sch. Med. Sci.)、David B. Alexander (Dept. Nanotoxicol. Nagoya City Univ. Grad. Sch. Med. Sci.)、Hiroyuki Tsuda (Dept. Nanotoxicol. Nagoya City Univ. Grad. Sch. Med. Sci.)

Circulating miRNAs can provide crucial information about cancerous conditions in a minimally invasive manner. However, it has not been clarified if miRNAs fluctuate in precancerous states. In this study, we found useful early cancer biomarkers by analyzing an animal model for human pancreatic ductal adenocarcinoma (PDAC) using transgenic rats in which expression of a human K-ras(G12V) oncogene is regulated by the Cre/lox system.We analyzed miRNA expression in blood and tissue samples from rats with pancreatic intraepithelial neoplasia and rats with PDAC, comparing them to controls.
The results revealed that 24 miRNAs exhibited consistent differential expression in the tissues and serum of rats with PDAC. Importantly, similar differences were observed in rats with the precursor lesion pancreatic intraepithelial neoplasia.Three of these miRNAs showed an approximately two-fold difference in expression in tissues and a 1.4-fold difference in the serum of rats with pancreatic intraepithelial neoplasia.Our findings suggest the potential for early diagnosis and therapeutic targeting of pancreatic cancer using circulating miRNAs.

[Disclosure of COI by Responsible researcher] Lead presenter is the Responsible researcher.
[Lead presenter: Atsuko Mizoguchi] We have no financial relationships to disclose.

▲TOP

### [P-3247] I-P12-6
### Cancer diagnostic technologies (1)

2025 / 9 / 27 (Sat) 15:30-16:15 Room P/(Motenashi Dome) Room P

---

### P-3247
### Identification of genes involved in latent neck lymph node metastasis of early oral squamous cell carcinoma

[Speaker]Kaito Isshiki (Ehime Univ Grad Sch of Med) [Co-author]Kohichi Nakashiro (Ehime Univ Grad Sch of Med)、Hiroyuki Goda (Ehime Univ Grad Sch of Med)、Daisuke Uchida (Ehime Univ Grad Sch of Med)

The most important prognostic factor in oral squamous cell carcinoma (OSCC) is the neck lymph node metastasis (LNM). However, although elective neck dissection and sentinel node biopsy are recommended for the management of the neck in early OSCC (eOSCC), watchful wait is often also performed in Japan. In this study, we explored genes affecting latent LNM in eOSCC. We used primary tumor tissues from 100 cases of eOSCC that underwent radical resection at our department, extracted total RNA, and performed comprehensive gene expression analysis using microarrays. As a result, we found 1,596 genes that showed significantly more than 2-fold expression changes in tumor tissues compared to adjacent normal tissues. Among these genes, 44 genes were identified that were significantly associated with latent neck LNM and showed more than 2-fold expression changes. In the up-regulated 6 genes, extracellular matrix-related genes such as COL11A1, MMP13, COL10A1, FN1, and MMP1 were included. In the future, we aim to construct a model for predicting latent neck LNM in eOSCC based on the conventional histopathological findings and expression quantification of these genes.

[Disclosure of COI by Responsible researcher] Lead presenter is not the Responsible researcher.
[Lead presenter: Kaito Isshiki] We have no financial relationships to disclose.
[Principal Researcher: Kohichi Nakashiro] We have no financial relationships to disclose.

### [P-3248] I-P12-6
### Cancer diagnostic technologies (1)

2025 / 9 / 27 (Sat) 15:30-16:15 Room P/(Motenashi Dome) Room P

---

### P-3248
### Machine learning-integrated phenotyping predicts cisplatin sensitivity based on tumor gene expression patterns

[Speaker]Haruki Ujiie (Dept. Clin. Pharm., Div. Info., Iwate Med. Univ., Sch, Pharm.,／Dept. Pharm., Iwate Med. Univ., Hosp.,) [Co-author]Tomoko Sakyo (Dept. Clin. Pharm., Div. Info., Iwate Med. Univ., Sch, Pharm.,)、Honami Yonezawa (Div. Health Chem., Iwate Med. Univ., Sch. Pharm.)、Naoyuki Nishiya (Dept. Clin. Pharm., Div. Info., Iwate Med. Univ., Sch, Pharm.,)

Although precision medicine advances personalized therapy with molecularly targeted drugs, its application to predict the efficacy of conventional anticancer drugs faces significant challenges. Here, we report a novel concept of phenotype-based classification leveraging machine learning (ML) of gene expression patterns to predict anticancer drug response. Hierarchical clustering using $IC_{50}$ values separated cisplatin (CP) -sensitive and resistant cell lines, and DEG analysis with SHAP-based ML identified 26 key genes to develop the Cisplatin Sensitivity Predictor 26 Genes (CSP26G) model. CP-resistant A549CR cells experimentally confirmed the CSP26G's external validity. The CSP26G also classified NSCLC patients in the TCGA into CP-sensitive and resistant groups, and the predicted sensitive group showed significantly longer survival than the resistant group. Furthermore, CSP26G predicts responsiveness to other DNA-damaging agents as well as the efficacy of CP. These findings indicate that DEG analysis integrated by ML is capable of forecasting drug sensitivity, thereby contributing to the advancement of more effective and personalized precision medicine in classical anticancer drugs.

[Disclosure of COI by Responsible researcher] Lead presenter is not the Responsible researcher.
[Lead presenter: Haruki Ujiie] We have no financial relationships to disclose.
[Principal Researcher: Naoyuki Nishiya] We have no financial relationships to disclose.

▲TOP

### [P-3249] I-P12-6
### Cancer diagnostic technologies (1)

2025 / 9 / 27 (Sat) 15:30-16:15 Room P/(Motenashi Dome) Room P

---

### P-3249
### Search for breast cancer risk predictors in HBOC using comprehensive gene expression analysis

[Speaker]Hirotsugu Yanai (Dept. Breast Surg., Kansai Med. Univ.／Kansai Med. Univ. Hosp., Clin. Genet. Ctr.) [Co-author]Naoko Yoshida (Dept. Pathol., Kansai Med. Univ.)、Chika Sato (Kansai Med. Univ. Hosp., Clin. Genet. Ctr.)、Saki Shimada (Kansai Med. Univ. Hosp., Clin. Genet. Ctr.)、Fumika Kanamori (Dept. Breast Surg., Kansai Med. Univ.)、Satsuki Sakaguchi (Dept. Breast Surg., Kansai Med. Univ.)、Tomoe Taji (Dept. Breast Surg., Kansai Med. Univ.)、Kayoko Endo (Dept. Breast Surg., Kansai Med. Univ.)、Yuichiro Kikawa (Dept. Breast Surg., Kansai Med. Univ.)、Tokiko Nakai (Dept. Pathol., Kansai Med. Univ.)、Koji Tsuta (Dept. Pathol., Kansai Med. Univ.)、Masahiro Takada (Dept. Breast Surg., Kansai Med. Univ.)

Background: For women with hereditary breast and ovarian cancer syndrome (HBOC), risk-reducing mastectomy (RRM) is recommended as a preventive measure before breast cancer (BC) develops. However, there is no objective indicator to determine the risk of developing BC. Therefore, we set out to find molecules that might increase the risk of BC in women with HBOC.
Materials and Methods: Total RNA was extracted from FFPE samples of BC and adjacent normal mammary gland (MG) from 29 women with HBOC and 18 women without HBOC ( ***BRCA1/2*** pathogenic variant-negative ). We performed a comprehensive gene expression analysis using the nCounter Analysis System to search for molecules associated with BC risk and compared the candidate molecules identified by RT-qPCR and other methods between BC and MG samples of women with or without HBOC.
Results and discussion: The three molecules that were commonly upregulated in BC of women with HBOC were molecules that have been reported to correlate with poor prognosis. We believe that in the future it will be possible to predict BC risk in HBOC women with high accuracy by combining several such markers.

[Disclosure of COI by Responsible researcher] Lead presenter is the Responsible researcher.
[Lead presenter: Hirotsugu Yanai] ■Research funds from company: 2023年度　科研費　基盤研究（C）（一般）

Poster Session

### [P-3250] I-P12-6
### Cancer diagnostic technologies (1)

2025 / 9 / 27 (Sat) 15:30-16:15 Room P/(Motenashi Dome) Room P

### P-3250
### Clinical significance of exosome-based microRNAs in urine of patients with gastric cancer and pancreatic cancer

[Speaker]Masateru Yamauchi (Div. Digestive Surg., Dept. of Surg., Kyoto Pref. Univ. Med.) [Co-author]Shuhei Komatsu (Div. Digestive Surg., Dept. of Surg., Kyoto Pref. Univ. Med.)、Taisuke Imamura (Div. Digestive Surg., Dept. of Surg., Kyoto Pref. Univ. Med.)、Yusuke Takashima (Div. Digestive Surg., Dept. of Surg., Kyoto Pref. Univ. Med.)、Ryo Ishida (Div. Digestive Surg., Dept. of Surg., Kyoto Pref. Univ. Med.)、Hiroshi Arakawa (Div. Digestive Surg., Dept. of Surg., Kyoto Pref. Univ. Med.)、Keiji Nishibeppu (Div. Digestive Surg., Dept. of Surg., Kyoto Pref. Univ. Med.)、Jun Kiuchi (Div. Digestive Surg., Dept. of Surg., Kyoto Pref. Univ. Med.)、Hiroki Shimizu (Div. Digestive Surg., Dept. of Surg., Kyoto Pref. Univ. Med.)、Tomohiro Arita (Div. Digestive Surg., Dept. of Surg., Kyoto Pref. Univ. Med.)、Toshiyuki Kosuga (Div. Digestive Surg., Dept. of Surg., Kyoto Pref. Univ. Med.)、Hirotaka Konishi (Div. Digestive Surg., Dept. of Surg., Kyoto Pref. Univ. Med.)、Yusuke Yamamoto (Div. Digestive Surg., Dept. of Surg., Kyoto Pref. Univ. Med.)、Ryo Morimura (Div. Digestive Surg., Dept. of Surg., Kyoto Pref. Univ. Med.)、Hitoshi Fujiwara (Div. Digestive Surg., Dept. of Surg., Kyoto Pref. Univ. Med.)、Atsushi Shiozaki (Div. Digestive Surg., Dept. of Surg., Kyoto Pref. Univ. Med.)

Although some urinary extracellular vesicle microRNAs(miRNA) as a useful biomarker for urological cancer had been reported in recent years, there was still few reports regarding digestive cancers. This study aims to investigate the clinical utility of urinary exosome-based miRNAs for non-invasive screening of gastric cancer (GC) and pancreatic cancer (PC). (1) PC (a) We selected four oncogenic miRNAs by comprehensive miRNA array analysis. (b) Small- and large-scale analysis showed that miR-210-3p was significantly upregulated in the urine of PC patients. (c) We examined a moderate positive correlation between miR-210-3p expression in plasma and urine. (2) GC (a) We selected five tumor suppressor miRNAs with the NCBI database. (b) Small- and large-scale analysis showed that urinary let-7e was significantly down-regulated in GC patients. (c) There were significant positive correlations of let-7e levels between in plasma and urine. (d) Overexpression of let-7e in GC cells inhibited cell proliferation, migration, invasion and suppressed the expression level of HMGA2. Urinary microRNAs might be useful for cancer detection and monitoring biomarkers in digestive cancers.

[Disclosure of COI by Responsible researcher] Lead presenter is not the Responsible researcher.
[Lead presenter: Masateru Yamauchi] We have no financial relationships to disclose.
[Principal Researcher: Shuhei Komatsu] We have no financial relationships to disclose.

▲TOP

### [P-3251] I-P12-6
### Cancer diagnostic technologies (1)

2025 / 9 / 27 (Sat) 15:30-16:15 Room P/(Motenashi Dome) Room P

---

### P-3251
### Investigation of the factors associated with high-grade fetal lung adenocarcinoma identified by gene network analysis

[Speaker]Rika Kasajima (Div. Mol. Path. and Genet, Kanagawa Cancer Ctr. Res. Inst.) [Co-author]Masaki Suzuki (Dept. Pathol., Kanagawa Cancer Ctr.／Dept. Pathol., Univ. Tokyo Hosp.)、Eigo Shimizu (Hum. Genome Ctr., Inst. Med. Sci., Univ. Tokyo)、Yoshinori Tamada (Res. Ctr. Health-Medical Data Sci. Grad. Sch. Med.,Hirosaki Univ.)、Atsushi Niida (Hum. Genome Ctr., Inst. Med. Sci., Univ. Tokyo)、Yukihiko Hiroshima (Div. Adv. Cancer Therap. Kanagawa Cancer Ctr. Res. Inst.)、Kotoe Katayama (Hum. Genome Ctr., Inst. Med. Sci., Univ. Tokyo)、Rui Yamaguchi (Hum. Genome Ctr., Inst. Med. Sci., Univ. Tokyo／Div. Cancer Sys. Bio., Aichi Cancer Ctr. Res. Inst.)、Kiyoshi Yamaguchi (Div. Clin. Genome Res., Inst. Med. Sci., Univ. Tokyo)、Yoichi Furukawa (Div. Clin. Genome Res., Inst. Med. Sci., Univ. Tokyo)、Satoru Miyano (Hum. Genome Ctr., Inst. Med. Sci., Univ. Tokyo／M&D Data Science Center, IIR, Institute of Science Tokyo)、Seiya Imoto (Hum. Genome Ctr., Inst. Med. Sci., Univ. Tokyo)、Tomoyuki Yokose (Dept. Pathol., Kanagawa Cancer Ctr.)、Yohei Miyagi (Div. Mol. Path. and Genet, Kanagawa Cancer Ctr. Res. Inst.)

Fetal adenocarcinoma of the lung (FLAC) is a rare variant of lung adenocarcinoma. Our previous study showed that lung adenocarcinoma with high-grade variant of FLAC (H-FLAC) has a poor prognosis, but the mechanism related to its characteristics and treatment targets have not been clearly appreciated yet. We conducted whole-exome and RNA sequencing in 16 surgically resected H-FLAC cases, and 16 common lung adenocarcinoma (CLAC) cases as controls. In order to extract networks related to H-FLAC specific features, we predicted gene regulatory networks from gene expression profiles by Bayesian networks (INGOR software). To integrate somatic mutations into these networks, we mapped genes harboring mutations identified via WES onto the respective regulatory networks. A core network for H-FLAC was then extracted by identifying the regulatory relationships involving the two genes upstream and downstream of each mutated gene. Interestingly, the core network of H-FLAC included not only mutated genes but also long non-coding RNAs and microRNAs. Regulation of oncogene and tumor suppressor gene expression by non-coding RNAs may represent one of the characteristic features of H-FLAC.

[Disclosure of COI by Responsible researcher] Lead presenter is the Responsible researcher.
[Lead presenter: Kasajima Rika] We have no financial relationships to disclose.

### [P-3252] I-P12-6
### Cancer diagnostic technologies (1)

2025 / 9 / 27 (Sat) 15:30-16:15 Room P/(Motenashi Dome) Room P

---

### P-3252
### Relationship between Desmoplastic Reaction (DR) Classification and Prognosis in Colorectal Cancer

[Speaker]Keigo Murakami (Tohoku University, Investigative Pathology) [Co-author]Yota Akamori (Tohoku University, Investigative Pathology／Tohoku University, Department of Surgery)、Hideaki Karasawa (Tohoku University, Department of Surgery)、Toru Furukawa (Tohoku University, Investigative Pathology)

While cancer-associated fibroblasts have been gaining attention, clinical utility of fibrotic cancer stroma classification by the Japanese cancer classifications guidelines has been questioned, leading to the removal of the quantitative fibrosis classification. The discrepancy highlights a significant gap between basic research and clinical practice in the evaluation of cancer stroma. DR classification in colorectal cancer has been proposed as a novel prognostic marker, where tumor stroma is classified into three types based on the morphological characteristics: Mature, Intermediate, and Immature. This study aimed to analyze the relationships between DR classification and prognostic clinicopathological factors in 229 patients with pStage II-IV colorectal cancer in out institution from 2013 to 2017. Among the three categories, significant differences were identified in the factors such as tumor depth, lymph node metastasis, and lymphovascular/perineural invasion. Disease-specific survival and recurrence-free survival were significantly worse in Intermediate/Immature group. These results suggest the potential utility of DR classification as a prognostic indicator.

[Disclosure of COI by Responsible researcher] Lead presenter is the Responsible researcher.
[Lead presenter: Keigo Murakami] We have no financial relationships to disclose.

### [P-3253] I-P12-6
### Cancer diagnostic technologies (1)

2025 / 9 / 27 (Sat) 15:30-16:15 Room P/(Motenashi Dome) Room P

---

### P-3253
### HR eye & MMR eye: One-Day Assessment of DNA Repair-Defective Tumors Eligible for Targeted Therapy

[Speaker]Shinta Saito (Grad. Sch. Nanobiosci., Yokohama City Univ.) [Co-author]Shingo Kato (Dept. Clin. Cancer Genomics, Yokohama City Univ. Hosp.)、Usaki Arai (Grad. Sch. Nanobiosci., Yokohama City Univ.)、Taichi Mizushima (Dept. Obstetrics and Gynecology, Yokohama City Univ. Grad. Sch. Med.)、Kensuke Tateishi (Dept. Neurosurgery, Yokohama City Univ. Grad. Sch. Med.)、Noritaka Adachi (Grad. Sch. Nanobiosci., Yokohama City Univ.)

Homologous recombination (HR) and mismatch repair (MMR) act as guardians of the human genome, and defects in HR or MMR are causative of at least a quarter of all malignant tumors. Although these DNA repair-deficient tumors are eligible for effective targeted therapies, fully reliable diagnostic strategies based on functional assay have yet to be established, potentially limiting safe and proper application of the molecular targeted drugs. Here we show that transient transfection of artificial DNA substrates enables ultrarapid detection of HR and MMR. This finding led us to develop a novel diagnostic strategy, in which the cellular HR/MMR status can be determined within just one day without the need for control cells or tissues. Notably, accuracy of this method triggered the discovery of a new pathogenic mutation, which has been missed by existing companion diagnostic tests. Our methods, termed HR eye and MMR eye, are applicable to frozen tumor tissues and roughly predicts response to therapy. Overall, the findings presented here will pave the way for accurately assessing malignant tumors with functional defects in HR or MMR, a major step forward in accelerating precision medicine.

[Disclosure of COI by Responsible researcher] Lead presenter is not the Responsible researcher.
[Lead presenter: Shinta Saito] We have no financial relationships to disclose.
[Principal Researcher: Noritaka Adachi] ■employment by a company related to the presentation: DnaQua, Inc. ■Profit of stock: DnaQua, Inc.

### [P-3263] I-P13-6
### Engineered antibody

2025 / 9 / 27 (Sat) 15:30-16:15 Room P/(Motenashi Dome) Room P

---

### P-3263
### Understanding ADC Resistance using Preclinical Enhertu-induced Resistant Models

[Speaker]Yuan Fang (GemPharmatech Co., Ltd.) [Co-author]Fang Zhu (GemPharmatech Co., Ltd.)、Hongyan Sun (GemPharmatech Co., Ltd.)、Jianming Xu (GemPharmatech Co., Ltd.)、Xiang Gao (GemPharmatech Co., Ltd.)

Antibody-drug conjugates (ADCs), such as HER2-targeting Enhertu (DS-8201), combine monoclonal antibody specificity with cytotoxic payloads, revolutionizing targeted cancer therapy. Despite its clinical success, drug resistance remains a major hurdle. To investigate resistance mechanisms, we developed a preclinical platform using HER2$^{+}$ JIMT-1 xenografts through iterative ***in vivo*** passaging and drug induction.
These preclinical Enhertu-resistant models offers a unique opportunity to investigate resistance mechanisms that may not yet be observed in clinics. Furthermore, these models provide a foundation for testing innovative therapeutic strategies, such as combination treatments targeting multiple pathways or the development of next-generation ADCs with improved payload efficacy. By enhancing our understanding of ADC resistance, these findings contribute to the advancement of personalized cancer therapies and may ultimately improve outcomes for patients with HER2$^{+}$ malignancies.
Note: This is an encore submission. The original abstract will be presented at AACR 2025, published abstract number: 1714. Permission has been obtained from AACR, and the copyright remains with AACR.

[Disclosure of COI by Responsible researcher] Lead presenter is the Responsible researcher.
[Lead presenter: Yuan Fang] We have no financial relationships to disclose.

### [P-3264] I-P13-6
### Engineered antibody

2025 / 9 / 27 (Sat) 15:30-16:15 Room P/(Motenashi Dome) Room P

---

### P-3264
### CD44-Targeting Near-Infrared Photoimmunotherapy as a Potential Treatment for Mesothelioma

[Speaker]Yumeta Shimazu (Kyoto University Hospital) [Co-author]Shigeto Nishikawa (Kyoto University Hospital)、Ryota Sumitomo (Kyoto University Hospital)、Daisuke Nakajima (Kyoto University Hospital)、Yojiro Yutaka (Kyoto University Hospital)、Satona Tanaka (Kyoto University Hospital)、Hidenao Kayawake (Kyoto University Hospital)、Ichiro Sakanoue (Kyoto University Hospital)、Toshi Menju (Kyoto University Hospital)

BackgroundCD44 is frequently observed at elevated levels in mesothelioma. Near-infrared photoimmunotherapy (NIR-PIT) is a novel therapeutic approach that utilizes an antibody-photoabsorber conjugate (APC) activated by near-infrared light. We hypothesized that CD44-targeting NIR-PIT could be an effective treatment for mesothelioma.MethodsThe expression of CD44 in murine mesothelioma AB12 cells was measured by immunofluorescence staining and Western blotting. AB12 cells were treated with an APC complex comprising CD44 antibody conjugated to IR700. After the incubation, the cells were exposed to near-infrared light, and the efficacy of NIR-PIT was evaluated.ResultsImmunofluorescence staining and Western blotting revealed CD44 expression in AB12 cells. Time-lapse imaging following NIR exposure revealed that treated cells exhibited swelling and ceased movement, followed by cytoplasmic leakage and cell death within a few hours.ConclusionThe results demonstrate the potential efficacy of CD44-targeted NIR-PIT for mesothelioma treatment. Further studies involving orthotopic transplantation models in mice are necessary to evaluate the safety and therapeutic benefits of this approach.

[Disclosure of COI by Responsible researcher] Lead presenter is the Responsible researcher.
[Lead presenter: Yumeta Shimazu] We have no financial relationships to disclose.

▲TOP

### [P-3265] I-P13-6
### Engineered antibody

2025 / 9 / 27 (Sat) 15:30-16:15 Room P/(Motenashi Dome) Room P

---

### P-3265
### Anti-CADM1/TSLC1 antibodies are potential therapeutic agents for cancer and pain

[Speaker]Azusa Yoneshige (KINDAI Univ., Sch. Med., Dept. Path.) [Co-author]Man Hagiyama (KINDAI Univ., Sch. Med., Dept. Path.)、Fuka Takeuchi (KINDAI Univ., Sch. Med., Dept. Path.)、Akihiko Ito (KINDAI Univ., Sch. Med., Dept. Path.)

Cell adhesion molecule 1 (CADM1), a single-pass transmembrane protein, is known to be expressed abundantly in central and peripheral neurons, respiratory and renal epithelial cells, and spermatogenic cell. Since our discovery of CADM1 as a novel adhesion molecule in mast cell in 2003, we have been investigating its pathogenic roles.
Recently, we demonstrated that two anti-CADM1 ectodomain antibodies, 3E1 and 9D2, are efficiently internalized into tumor cells via lipid raft-dependent endocytosis. Using 3E1 conjugated with monomethyl auristatin E (3E1-MMAE), we observed anti-tumor effects in cell lines of mesothelioma, small cell lung carcinoma, adult T-cell leukemia, and endometrial adenocarcinoma–all of which express CADM1. Furthermore, 3E1-MMAE treatment suppressed tumor growth in mouse models of melanoma and gastrointestinal stromal tumor. Separately, we found that 3E1 is also effective in blocking neurotransmission. Treatment with 3E1 reduced pain-related responses in mice, suggesting its utility as a long-acting analgesic.
In this session, we will provide an overview of our recent findings on anti-CADM1 antibodies and their potential applications in cancer and neural modulation.

[Disclosure of COI by Responsible researcher] Lead presenter is not the Responsible researcher.
[Lead presenter: Azusa Yoneshige] We have no financial relationships to disclose.
[Principal Researcher: Akihiko Ito] We have no financial relationships to disclose.

▲TOP

### [P-3266] I-P13-6
### Engineered antibody

2025 / 9 / 27 (Sat) 15:30-16:15 Room P/(Motenashi Dome) Room P

---

### P-3266
### Anti-tumor effect of nuclear transportable antibody-drug conjugate with triptolide via inhibition of RNA polymerase II

[Speaker]Taketo Yamada (Saitama Med Univ, Dept Path.) [Co-author]Mutsumi Hayashi (Saitama Med Univ, Dept Path.)

CD26 is a transmembrane glycoprotein overexpressed in many types of malignancies. We developed humanized anti-CD26 monoclonal antibody YS110 which has direct anti-tumor effect via nuclear transport of CD26 and transcriptional repression of POLR2A gene, a subunit of RNA polymerase II (Pol II). Subsequently we developed an antibody-drug conjugate Y-TR-1 with YS110 and a Pol II inhibitor triptolide. 5 triptolide molecules on average were crosslinked to YS110 by heterobifunctional linker SMCC. In this report, we present the pre-clinical studies and mechanism of action studies of Y-TR1. In pharmacological test, Y-TR-1 showed in vitro and in vivo cytotoxicity against CD26 positive cancer cells while non-cancer cells were less susceptible to Y-TR-1. Toxicity studies conducted in cynomolgus monkeys with cross-reactivity to Y-TR-1 showed no severe toxicity. Next, we investigated molecular mechanism of intracellular transport of Y-TR-1 by cell fractionation and immunofluorescent staining. Furthermore, we analyzed POL II inhibition by Y-TR-1 quantitatively with time lapse live cell imaging using an elongating POL II specific fluorescent probe RNAP2 Ser2-ph-mintbody.

[Disclosure of COI by Responsible researcher] Lead presenter is the Responsible researcher.
[Lead presenter: Taketo Yamada] ■Research funds from company: ヒト化抗体の供与

▲TOP

### [P-3267] I-P13-6
### Engineered antibody

2025 / 9 / 27 (Sat) 15:30-16:15 Room P/(Motenashi Dome) Room P

---

### P-3267
### Safety assessment of trastuzumab emtansine (T-DM1) for use in companion dog patients

[Speaker]Miki Ando (Lab. of Veterinary Surgery, Univ. of Tokyo) [Co-author]Shiyu Qin (Lab. of Veterinary Surgery, Univ. of Tokyo)、Hiroto Toyoda (Lab. of Veterinary Surgery, Univ. of Tokyo／Japan Small Animal Cancer Center)、Daiki Kato (Lab. of Veterinary Surgery, Univ. of Tokyo)、Kosei Sakai (Lab. of Small Animal Internal Medicine 1, Kitasato Univ.)、Namiko Ikeda (Lab. of Veterinary Surgery, Univ. of Tokyo)、Takaaki Iguchi (Lab. of Veterinary Surgery, Univ. of Tokyo)、Hayato Shibahara (Lab. of Veterinary Surgery, Univ. of Tokyo)、Shoma Koseki (Lab. of Veterinary Surgery, Univ. of Tokyo)、Takahiro Kako (Lab. of Veterinary Surgery, Univ. of Tokyo)、Haruka Motoi (Lab. of Veterinary Surgery, Univ. of Tokyo)、Takayuki Nakagawa (Lab. of Veterinary Surgery, Univ. of Tokyo)

Antibody-drug conjugates (ADCs) are promising cancer therapies, but their clinical application in veterinary medicine remains unexplored. Trastuzumab emtansine (T-DM1), an ADC targeting HER2, has demonstrated strong efficacy in HER2-positive human breast cancer. HER2 overexpression has been reported in various canine malignancies, suggesting the potential use of T-DM1 in veterinary oncology. This study aimed to assess the tolerability and adverse events of T-DM1 in a healthy experimental dog. The dog received premedication with diphenhydramine, famotidine, and prednisolone. T-DM1 was administered at 1.8 mg/kg (Dose1) and escalated to 3.6 mg/kg (Dose2). Adverse events were evaluated using VCOG-CTCAE v2.0.At 1.8 mg/kg, no severe adverse events were observed. However, Dose2 induced grade 4 thrombocytopenia, similar to findings in 28% of human patients, attributed to Fc receptor-mediated uptake into megakaryocytic progenitors. These results suggest that 1.8 mg/kg of T-DM1 might be tolerable in dogs, but further studies are required to determine the maximum tolerated dose and long-term safety.

[Disclosure of COI by Responsible researcher] Lead presenter is not the Responsible researcher.
[Lead presenter: Miki Ando] We have no financial relationships to disclose.
[Principal Researcher: Daiki Kato] We have no financial relationships to disclose.

### [P-3268] I-P13-6
### Engineered antibody

2025 / 9 / 27 (Sat) 15:30-16:15 Room P/(Motenashi Dome) Room P

---

P-3268

### Efficacy and Comparison of PPMX-T004b-PGAP-1, a New anti-CDH3 Antibody-Drug Conjugate (ADC), with Preceding ADC in Tumor

[Speaker]Sayuri Kaneko (Perseus Proteomics Inc, Research and Development Division) [Co-author]Hiroko Akiyoshi (Perseus Proteomics Inc, Research and Development Division)、Shimpei Nonouchi (UBE Corporation, Pharmaceutical Division, Pharmaceutical Research Laboratory)、Takashi Matsushita (UBE Corporation, Pharmaceutical Division, Pharmaceutical Research Laboratory)、Takamasa Kashiwagi (UBE Corporation, Pharmaceutical Division, Pharmaceutical Research Laboratory)、Gen Mizuno (UBE Corporation, Pharmaceutical Division, Pharmaceutical Research Laboratory)、Yukinori Wada (UBE Corporation, Pharmaceutical Division, Pharmaceutical Research Laboratory)、Megumi Hikichi (Perseus Proteomics Inc, Research and Development Division)、Yoichi Aikawa (Perseus Proteomics Inc, Research and Development Division)、Shinji Hagiwara (Perseus Proteomics Inc, Research and Development Division)

CDH3, a member of the cadherin family of proteins involved in cell-cell adhesion, is of particular clinical interest because it is highly expressed in many types of tumor while exhibiting minimal expression in normal tissues.We have developed a novel ADC targeting CDH3, named PPMX-T004b-PGAP-1. PPMX-T004b is a humanized antibody (Ab) that binds to the EC1 domain of CDH3, thereby achieving high internalization efficiency. PGAP-1 is a hydrophilic, peptide-based cleavable linker conjugated with Auristatin E, which has been improved through a 'two-step cleavage' to reduce off-target toxicity. PPMX-T004b-PGAP-1 demonstrated superior potency in vitro cell growth assay and in vivo xenograft mice models, compared to earlier developed anti-CDH3 ADC, PCA062 when evaluated with drug-Ab ratio (DAR) of 4 or higher, within our testing range of DAR 2-16. It exhibited high serum stability and comparable pharmacokinetic profile in mice to that of the naked Ab. Furthermore, it showed consistent bystander effects in the co-culture system. These preclinical results suggest that PPMX-T004b-PGAP-1 represents a promising therapeutic candidate for the treatment of solid tumors overexpressing CDH3.

[Disclosure of COI by Responsible researcher] Lead presenter is not the Responsible researcher.
[Lead presenter: Sayuri Kaneko] We have no financial relationships to disclose.
[Principal Researcher: Shinji Hagiwara] We have no financial relationships to disclose.

### [P-3269] I-P13-6
### Engineered antibody

2025 / 9 / 27 (Sat) 15:30-16:15 Room P/(Motenashi Dome) Room P

---

P-3269
### Development of astatine-211 labeled anti-interleukin-7 receptor antibody for treating acute lymphoblastic leukemia

[Speaker]Ryo Tsumura (Div. of Developmental Therap., EPOC., Natl. Cancer Ctr.) [Co-author]Hiroki Takashima (Div. of Developmental Therap., EPOC., Natl. Cancer Ctr.)、Motochika Hamada (Div. of Developmental Therap., EPOC., Natl. Cancer Ctr.)、Takahiro Anzai (Div. of Developmental Therap., EPOC., Natl. Cancer Ctr.)、Shino Manabe (Syn. BioChem., Sch. Pharm, Pharm. Sci., Inst. MedChem., Hoshi Uni.／Res. Ctr., Pharm. Development, Grad. Sch. Pharm. Sci., Tohoku Uni.／Glycometabolic Biochem. Lab., RIKEN)、Hiromitsu Haba (Nishina Ctrl. Accelerator-Based Sci., RIKEN)、Masahiro Yasunaga (Div. of Developmental Therap., EPOC., Natl. Cancer Ctr.)

Interleukin-7 receptor (IL-7R) has been reported to play a role in the proliferation and survival of acute lymphoblastic leukemia (ALL) cells. Also, IL-7R is expressed on the surface of leukemia cells in most ALL patients. Here, we investigated the feasibility of astatine-211 (211At) labeled anti-IL-7R antibody.
The results of the in vitro cell killing activity suggested that 211At labeled anti-IL-7R antibody exerted cytotoxicity depending on the expression of IL-7R. Also, we evaluated the in vivo efficacy of 211At labeled anti-IL-7R antibody in an aggressive leukemia mouse model, in which BALB/c mice were intravenously transplanted with NALM-6 cells. The results showed that almost all mice reached the humane endpoint due to weight loss by tumor burden in the DPBS-treated group and 211At labeled control antibody-treated groups until one month after initial treatment. On the other hand, weight loss and NALM-6 cell proliferation were not observed in all mice until three months after initial treatment in the 211At labeled anti-IL-7R antibody-treated group.
We concluded that 211At labeled anti-IL-7R antibody has the potential to be a novel therapeutic for treatments of ALL patients.

[Disclosure of COI by Responsible researcher] Lead presenter is not the Responsible researcher.
[Lead presenter: Ryo Tsumura] We have no financial relationships to disclose.
[Principal Researcher: Masahiro Yasunaga] ■Research funds from company: 国立がん研究センター研究開発費　■Profit of stock: 株式会社凛研究所　■Research expenses: 第一三共株式会社、味の素株式会社、シスメックス株式会社、H.U.グループホールディングス、株式会社同仁グループ

### [P-3270] I-P13-6
### Engineered antibody

2025 / 9 / 27 (Sat) 15:30-16:15 Room P/(Motenashi Dome) Room P

---

### P-3270
### Efficient internalization of EGFR-ADC by non-canonical endocytosis of EGFR

[Speaker]Kyoko Otsuyama (Dept. Cancer Cell Biol. Univ. Toyama／Dept. Med. Oncology and Palliative Care. Univ. Toyama／Dept. Med. Sch Med, 6 Grad. Univ. Toyama) [Co-author]Shinya Morita (Dept. Cancer Cell Biol. Univ. Toyama)、Maki Sato (Dept. Cancer Cell Biol. Univ. Toyama)、Yue Zhou (Dept. Cancer Cell Biol. Univ. Toyama)、Satoru Yokoyama (Dept. Cancer Cell Biol. Univ. Toyama)、Ryuji Hayashi (Dept. Med. Oncology and Palliative Care. Univ. Toyama)、Hiroaki Sakurai (Dept. Cancer Cell Biol. Univ. Toyama)

In recent years, antibody-drug conjugate (ADC) have been one of the most highly promising anticancer drugs. For example, Trastuzumab deruxtecan is HER2-ADC that has shown a high response rate against breast cancer. On the other hand, ADC targeting the EGFR, which is also a member of the ErbB family, is not yet in clinical use. One of the reasons is thought to be the low internalization efficiency of EGFR-ADC. Therefore, we try to solve this problem by inducing efficient endocytosis of EGFR. In this study, we focused on non-canonical endocytosis of EGFR induced by p38-mediated phosphorylation at Ser-1015 and investigated whether this property of EGFR could be exploited to efficiently induce endocytosis of EGFR-ADC in lung cancer cells with EGFR exon 19 deletion. We detected Ser-1015 phosphorylation when p38 is activated by TNF-$\alpha$ or cisplatin. Similar results were also obtained in EGFR-TKI resistant PC-9 cells. Currently, the efficacy of ABT-414, an EGFR-ADC, is under investigation. We would like to discuss the usefulness of this approach based on the results.

[Disclosure of COI by Responsible researcher] Lead presenter is the Responsible researcher.
[Lead presenter: Kyoko Otsuyama] We have no financial relationships to disclose.

### [P-3271] I-P13-6
### Engineered antibody

2025 / 9 / 27 (Sat) 15:30-16:15 Room P/(Motenashi Dome) Room P

---

### P-3271
### Antitumor effect of anti-EpCAM antibody-drug conjugate against adenocarcinoma

[Speaker]Asuka Hattori (Grad. Sch. Heal & Sports Sci., Toyo Univ.) [Co-author]Takumi Iwasawa (Inst. of Life Innova. Stu., Toyo Univ.)、Takumu Yamada (Grad. Sch. Sci. Eng., Toyo Univ.)、Tomoya Masuoka (Grad. Sch. Heal & Sports Sci., Toyo Univ.)、Kazunori Kato (Grad. Sch. Heal & Sports Sci., Toyo Univ.)

Objective: Epithelial cell adhesion molecule (EpCAM) a promising therapeutic target for cancer treatment due to its significantly elevated expression (100-1000-fold) in various carcinoma cells compared to normal tissues. In this study, we synthesized an antibody-drug conjugate (ADC), 1B7-DM1, consisting of an anti-EpCAM antibody established in our laboratory linked to the antimicrotubule agent mertansine (DM1), and evaluated its therapeutic efficacy against adenocarcinoma. Method: The cytotoxicity of 1B7-DM1 was evaluated by the sulforhodamine B assay. In addition, 1B7-DM1 was administered to tumor-bearing mice to evaluate its anti-tumor effect and safety. Results: 1B7-DM1 demonstrated significant cytotoxicity specifically against EpCAM-high expressing cancer cell lines. In tumor-bearing mice, treatment with 1B7-DM1 resulted in dose-dependent tumor regression. Importantly, no significant body weight loss or adverse changes in hepatic and renal function markers were observed, confirming the safety profile of 1B7-DM1. Conclusion: These findings demonstrate that 1B7-DM1 exhibits potent antitumor activity with a favorable safety profile.

[Disclosure of COI by Responsible researcher] Lead presenter is not the Responsible researcher.
[Lead presenter: Asuka Hattori] ■ employment by a company related to the presentation: 一般財団法人阪大微生物病研究会
[Principal Researcher: Kazunori Kato] We have no financial relationships to disclose.

### [P-3280] I-P13-8
### New molecular targeted therapy (2)

2025 / 9 / 27 (Sat) 15:30-16:15 Room P/(Motenashi Dome) Room P

---

### P-3280
### SLFN11 serves as a sensitivity biomarker for a novel ribonucleotide reductase inhibitor TAS1553

[Speaker]Junko Murai (Proteo-Science Center, Ehime University)

Ribonucleotide reductase (RNR) inhibitors deplete a pool of dNTPs and inhibit replication. TAS1553 is a novel RNR inhibitor, and its robust antitumor efficacy was reported using hematological and solid cancer xenograft model (Ueno et al., Communication Biology, 2022). A nuclear protein SLFN11 enhances antitumor activity of DNA damaging agents through activating apoptosis and blocking DNA replication (Murai et al., Mol Cell 2018, Ogawa et al., Mol Cell 2025). However, functions of SLFN11 in response to RNR inhibitors are mostly unknown. Using a couple of isogenic human cell lines with or without SLFN11, we show that TAS1553 blocked replication regardless of SLFN11. Nevertheless, SLFN11-positive cells exclusively activated apoptosis after 4 hours in response to TAS1553 treatment, concomitantly SLFN11 was recruited to chromatin and blocked rRNA synthesis. SLFN11-positive cells did not progress beyond mid-S phase under TAS1553 treatment, whose blockage was not rescued by the supply of excess dNTP. We conclude that the dNTP depletion by TAS1553 initiates the function of SLFN11 on chromatin. The expression of SLFN11 can be a potential biomarker to predict a superior response to TAS1553.

[Disclosure of COI by Responsible researcher] Lead presenter is the Responsible researcher.
[Lead presenter: Junko Murai] ■Research funds from company: 大鵬薬品工業株式会社　■Lecture fee: 武田薬品工業株式会社、ファイザー製薬株式会社

### [P-3281] I-P13-8
### New molecular targeted therapy (2)

2025 / 9 / 27 (Sat) 15:30-16:15 Room P/(Motenashi Dome) Room P

---

### P-3281
### A novel PARG inhibitor MOD000582 exerts replication-independent anticancer effects by accumulating PARylated proteins

[Speaker]Erina Kusakabe (Breast Center, Ehime Univ. Hosp.／Dept. Biochem. & Mol. Genet., Grad. Sch. Med., Ehime Univ.) [Co-author]Masahiro Matsuki (Alivexis, Inc.)、Yoh Terada (Alivexis, Inc.)、Haruna Noda (Breast Center, Ehime Univ. Hosp.)、Kana Taguchi (Breast Center, Ehime Univ. Hosp.)、Akari Murakami (Breast Center, Ehime Univ. Hosp.)、Yoshiaki Kamei (Breast Center, Ehime Univ. Hosp.)、Yuzo Umeda (Hepato-Biliary-Pancreatic and Breast Surg., Grad. Sch. Med., Ehime Univ.)、Junko Murai (Dept. Biochem. & Mol. Genet., Grad. Sch. Med., Ehime Univ.／Proteo-Science Center, Ehime Univ.)

Poly(ADP-ribose) (PAR) glycohydrolase (PARG) is an enzyme that hydrolyzes PAR chains formed primarily by PAR polymerase (PARP) 1/2. PARG inhibitors exert anticancer effects, but their detailed mechanism of action (MOA) remains unclear. Here, we investigate the MOA of a novel PARG inhibitor, MOD000582. A loss-of-function siRNA screen revealed XRCC1 is one of the genes that define sensitivity to PARG inhibition. Indeed, human lymphoma TK6 cells with XRCC1 knock-out (KO) exhibited significant sensitivity to the compound compared to the wild-type cells. Furthermore, XRCC1/POLB, both in base excision repair pathway, double KO cells showed enhanced sensitivity to the compound. The sensitivity was well correlated to the levels of PARylated proteins, which were further increased by adding a reduced form of nicotinamide riboside, an NAD+ precursor. MOD000582 showed anticancer effect without always inducing replication stress. In line with this finding, this compound killed G1-arrested cancer cells, suggesting that it has a replication-independent MOA in certain contexts. These observations may suggest that MOD000582 have potential to target slowly growing tumors and cancer stem cells.

[Disclosure of COI by Responsible researcher] Lead presenter is not the Responsible researcher.
[Lead presenter: Erina Kusakabe] We have no financial relationships to disclose.
[Principal Researcher: Junko Murai] ■Research funds from company: アリヴェクシス株式会社　■Lecture fee: ファイザー、武田薬品

▲TOP

[P-3282] I-P13-8
New molecular targeted therapy (2)

2025 / 9 / 27 (Sat) 15:30-16:15 Room P/(Motenashi Dome) Room P

P-3282
### Decreased sensitivity of tumor cells to topoisomerase I inhibitor irinotecan under PARG deficiency

[Speaker]Yongkang Shao (Dept. Molecular Genomic Biomedicine, CBMM, GSBS, Nagasaki Univ.) [Co-author]Goutham Sivanandan (Dept. Molecular Genomic Biomedicine, CBMM, GSBS, Nagasaki Univ.)、Mengting Xu (Dept. Molecular Genomic Biomedicine, CBMM, GSBS, Nagasaki Univ.)、Ankitha Vadi Velu (Dept. Molecular Genomic Biomedicine, CBMM, GSBS, Nagasaki Univ.)、Zongxiang Zhang (Dept. Molecular Genomic Biomedicine, CBMM, GSBS, Nagasaki Univ.)、Barkha Saraswat (Dept. Molecular Genomic Biomedicine, CBMM, GSBS, Nagasaki Univ.)、Ying Tong (Dept. Molecular Genomic Biomedicine, CBMM, GSBS, Nagasaki Univ.)、Mitsuko Masutani (Dept. Molecular Genomic Biomedicine, CBMM, GSBS, Nagasaki Univ.)

Poly(ADP-ribose) glycohydrolase (PARG) is the main enzyme for degradation of poly(ADP-ribose). PARG deficiency causes sensitization to alkylating agents and $\gamma$-irradiation. In several types of cancer a loss or decreased expression of PARG has been reported. The impact of PARG deficiency in sensitivities to chemotherapeutic agents has been analyzed. Here we report a reduced sensitivity of tumor cells under PARG deficiency to topoisomerase I inhibitor irinotecan (CPT-11).An inducible PARG shRNA expressing T-REx HeLa cells was previously established. It showed a reduced PARG level and accumulation of PARylation when PARG knockdown was induced and showed a higher IC50 value for CPT-11 treatment. PARG knockdown caused an increased G1 arrest with a decrease in S/G2/M phase arrest after CPT-11 treatment at 24 and 48 hrs. It caused a decreased level of phosphorylated CHK1, suggesting that a disturbed CHK1 activation occurs under PARG deficiency and could induce the alteration of cell cycle progression, leading to resistance to CPT-11. PARG deficiency may serve as a possible biomarker for CPT-11 resistance. The response in other cell types and mechanistic analysis are ongoing.

[Disclosure of COI by Responsible researcher] Lead presenter is not the Responsible researcher.
[Lead presenter: Yongkang Shao] We have no financial relationships to disclose.
[Principal Researcher: Mitsuko Masutani] We have no financial relationships to disclose.

### [P-3283] I-P13-8
### New molecular targeted therapy (2)

2025 / 9 / 27 (Sat) 15:30-16:15 Room P/(Motenashi Dome) Room P

---

### P-3283
### Anti-gastric cancer potential of the novel pyrrole-imidazole polyamide against SOX9 transcription factor

[Speaker]Osamu Shimozato (Lab. Precision Tumor Model Systems, Chiba Cancer Ctr. Res. Inst.) [Co-author]Natsue Akao (Lab. Precision Tumor Model Systems, Chiba Cancer Ctr. Res. Inst.)、Yoko Yanagisawa (Lab. Precision Tumor Model Systems, Chiba Cancer Ctr. Res. Inst.)、Yuki Kida (Div. Innovative Cancer Therapeutics, Chiba Cancer Ctr. Res. Inst.)、Takayoshi Watanabe (Div. Mol. Carcinogenesis, Chiba Cancer Ctr. Res. Inst.)、Atsushi Takatori (Div. Innovative Cancer Therapeutics, Chiba Cancer Ctr. Res. Inst.)

SOX9 is considered to be one of the critical transcription factors required for the development of colorectal and gastric cancers. Since its depletion reduced the tumorigenic activity in gastric cancer cells, SOX9 might be a promising therapeutic target against gastric cancer. Recently, we have demonstrated that the novel pyrrole imidazole polyamide (WNT-Chb) selectively binds to the Wnt effector TCF-responsive element and induces cell death in Wnt/β-catenin pathway-dependent colon cancer cells. Given that SOX9 binds to the TCF-responsive element, we sought to ask whether WNT-Chb could also suppress SOX9-dependent gastric cancer cells. To this end, we have employed human gastric cancer MKN74 and MKN45 cells and exposed them to WNT-Chb. Indeed, WNT-Chb strongly induced cell death in both cells. The knockdown experiments showed that MKN74 and MKN45 cells are dependent on ***SOX9*** and ***β-catenin***, respectively. In contrast, depletion of SOX9 or β-catenin had a negligible effect on WNT-Chb resistant human colon cancer HT-29 cells. Taken together, these results suggest that WNT-Chb exerts an anti-cancer activity against not only β-catenin-dependent but also SOX9-dependent gastric cancer.

[Disclosure of COI by Responsible researcher] Lead presenter is the Responsible researcher.
[Lead presenter: Osamu Shimozato] We have no financial relationships to disclose.

### [P-3284] I-P13-8
### New molecular targeted therapy (2)

2025 / 9 / 27 (Sat) 15:30-16:15 Room P/(Motenashi Dome) Room P

---

### P-3284
### Targeting of cyclin D3 by niclosamide in suppression of stomach cancer proliferation

[Speaker]Yu-Hsuan Hung (Ctr. for Cancer Res., Kaohsiung Med. Univ.) [Co-author]Li-Tzong Chen (Ctr. for Cancer Res., Kaohsiung Med. Univ.)

Niclosamide is an anti-parasite therapeutic with anti-cancer potential via modulations on gene expression and signal transduction. Our team has focused on this potential for years and validated it in lymphoma. We further explored this potential with systematic bioinformatic analysis and found niclosamide inhibited cancers of liver and ovary. While niclosamide was found to inhibit tumorigenesis of these two cancers, further analysis on related microarrays revealed that niclosamide affected cell cycle. A cell cycle regulator commonly downregulated in these two cancer types is cyclin D3 (CCND3). To validate the potential of niclosamide as a pan-cancer inhibitor, we applied the less studied stomach cancer model and found niclosamide similarly inhibited its proliferation. Niclosamide further decreased STAT3 phosphorylation and CCND3 expression, as this STAT3-CCND3 axis being previously reported. On the other hand, CCND3 knockdown inhibited stomach cancer proliferation and desensitized it to niclosamide. 3D culture and animal experiment will be applied to further solidify the targeting of CCND3 by niclosamide and this drug repurposing in stomach cancer therapy.

[Disclosure of COI by Responsible researcher] Lead presenter is the Responsible researcher.
[Lead presenter: Yu-Hsuan Hung] We have no financial relationships to disclose.

### [P-3285] I-P13-8
### New molecular targeted therapy (2)

2025 / 9 / 27 (Sat) 15:30-16:15 Room P/(Motenashi Dome) Room P

---

### P-3285
### Prognostic significance of Claudin18.2 expression in patients with gastric cancer

[Speaker]Yuichiro Miki (Osaka Metropolitan Universirty Department of Gastroenterological Surgery) [Co-author]Mami Yoshii (Osaka Metropolitan Universirty Department of Gastroenterological Surgery)、Yuki Seki (Osaka Metropolitan Universirty Department of Gastroenterological Surgery)、Kenji Kuroda (Osaka Metropolitan Universirty Department of Gastroenterological Surgery)、Hiroaki Kasashima (Osaka Metropolitan Universirty Department of Gastroenterological Surgery)、Tatsuro Tamura (Osaka Metropolitan Universirty Department of Gastroenterological Surgery)、Masatsune Shibutani (Osaka Metropolitan Universirty Department of Gastroenterological Surgery)、Takahiro Toyokawa (Osaka Metropolitan Universirty Department of Gastroenterological Surgery)、Masakazu Yashiro (Osaka Metropolitan Universirty Department of Gastroenterological Surgery)、Kiyoshi Maeda (Osaka Metropolitan Universirty Department of Gastroenterological Surgery)

Background: Claudin 18.2 (CLDN18.2) is a novel treatment target for patients with gastric cancer. However, it remains unclear whether the expression of CLDN18.2 affects survival outcomes.
Methods: In total, 586 patients with GC were enrolled in this study. CLDN18.2 expression in cancer cells was analyzed by immunohistochemistry. Correlations between CLDN18.2 expression and several clinicopathological factors and survival outcomes were investigated. We also performed a systematic review and a meta-analysis.
Results: The CLDN18.2 expression was not significantly correlated with any clinicopathological factor. In all patients, CLDN18.2 did not significantly affect OS. In patients with the diffuse type, the overall survival of patients with CLDN18.2-high expression was worse than that of patients with CLDN18.2-low expression, although the difference was not significant (p=0.092). Meta-analyses revealed that CLDN18.2 was not significant prognostic factor in resected cases.
Conclusion: CLDN18.2 was not a significant prognostic factor in general, whereas patients with CLDN18.2 high expression showed worse survival outcomes especially in the diffuse type.

[Disclosure of COI by Responsible researcher] Lead presenter is the Responsible researcher.
[Lead presenter: Yuichiro Miki] We have no financial relationships to disclose.

### [P-3296] I-P14-10
### Anticancer drug

2025 / 9 / 27 (Sat) 15:30-16:15 Room P/(Motenashi Dome) Room P

---

### P-3296
### Effect of olaparib, a PARP inhibitor, on cell death induced by daunorubicin, an anthracycline antibiotic

[Speaker]Hideki Mizutani (Coll. Pharm., Kinjo Gakuin Univ.) [Co-author]Noriko Yamamoto (Coll. Pharm., Kinjo Gakuin Univ.)、Maki Hasebe (Coll. Pharm., Kinjo Gakuin Univ.)、Sae Furuike (Coll. Pharm., Kinjo Gakuin Univ.)、Nako Matsuda (Coll. Pharm., Kinjo Gakuin Univ.)、Kinya Ohta (Coll. Pharm., Kinjo Gakuin Univ.)、Disuke Miyazawa (Coll. Pharm., Kinjo Gakuin Univ.)、Tohru Maeda (Coll. Pharm., Kinjo Gakuin Univ.)、Shosuke Kawanishi (Fac. Pharm. Sci., Suzuka Univ. Med. Sci.)

PARP inhibitors are molecularly targeted drugs with fewer side effects than conventional cancer drugs. PARP inhibitors tend to be effective against cancers with specific DNA repair abnormalities, and the combination of PARP inhibitors with conventional anticancer drugs is also thought to be effective. In this study, we investigated the effect of OLA on daunorubicin (DNR) cytotoxicity in human myeloid leukemia HL-60 cells. The DNR group was compared with the OLA group (DNR+OLA group), which was treated with OLA for 30 min prior to DNR administration. In cellular experiments, DNR-induced cytotoxicity, changes in cell morphology and changes in mitochondrial membrane potential were observed, with the degree of cytotoxicity in the DNR group > DNR+OLA group. No toxicity was observed in OLA itself at the concentrations used in the experiments. On the other hand, intracellular ROS increased in the DNR group < DNR+OLA group. In conclusion, the cytotoxicity of DNR was reduced by the combination of OLA. This suggests that caution should be exercised when using DNR in combination with OLA.

[Disclosure of COI by Responsible researcher] Lead presenter is the Responsible researcher.
[Lead presenter: Hideki Mizutani] ■Research funds from company: 日本学術振興会（科研費）

### [P-3297] I-P14-10
### Anticancer drug

2025 / 9 / 27 (Sat) 15:30-16:15 Room P/(Motenashi Dome) Room P

### P-3297
### Cell-death inducing mechanisms of the potential anti-cancer compound MO2455 in leukemia and lymphoma cells.

[Speaker]Ankitha Vadi Velu (Dept. Molecular Genomic Biomedicine, CBMM, GSBS, Nagasaki Univ.) [Co-author]Barkha Saraswat (Dept. Molecular Genomic Biomedicine, CBMM, GSBS, Nagasaki Univ.)、Ying Tong (Dept. Molecular Genomic Biomedicine, CBMM, GSBS, Nagasaki Univ.)、Kenji Matsuno (Dept. Pharmacy, Yasuda Woman's Univ. Hiroshima.)、Takeji Takamura (Fac. Engineering, Kanagawa Inst. Tech. Kanagawa.)、Fumiaki Koizumi (Dept. Molecular Genomic Biomedicine, CBMM, GSBS, Nagasaki Univ.)、Mitsuko Masutani (Dept. Molecular Genomic Biomedicine, CBMM, GSBS, Nagasaki Univ.)

Poly(ADP-ribose) (PAR) glycohydrolase (PARG) is a key enzyme involved in the hydrolysis of PAR. Deficiency in PARG, enhances cell sensitivity to $\gamma$-irradiation and alkylating agents, and this makes PARG a potentially attractive therapeutic target. We previously demonstrated that MO2455 induces PAR accumulation and exerts cytotoxicity on B cell lymphoma cells by disrupting the B cell pathway. In this study, the cellular mechanisms of MO2455-induced cell death was investigated in monocytic leukemia, B cell lymphoma and T cell leukemia cells. In U937 cells, apoptosis was confirmed by the increase of subG1 population, followed by the appearance of annexin-V+/propidium iodide (PI)- and annexin-V+/PI+ fractions. U937 cells showed a decreased mitochondrial membrane potential (MMP) and an early increase in $\gamma$-H2AX levels, indicating rapid DNA damage and induction of apoptosis. THP-1 and CCRF-CEM cells showed a rapid increase in the appearance of annexin-V+/PI+ fraction. Cell-death inducing cascade was also analyzed by western blot analysis. The results suggest that MO2455 effectively induces cell death through some common and distinct mechanisms in lymphoma and leukemia cells.

[Disclosure of COI by Responsible researcher] Lead presenter is not the Responsible researcher.
[Lead presenter: Ankitha Vadi Velu] We have no financial relationships to disclose.
[Principal Researcher: Mitsuko Masutani] We have no financial relationships to disclose.

### [P-3298] I-P14-10
### Anticancer drug

2025 / 9 / 27 (Sat) 15:30-16:15 Room P/(Motenashi Dome) Room P

---

### P-3298
### Synergistic Effects of Microtubule Inhibitors and MK2 Inhibition in Cancer Treatment with Reduced Toxicity

[Speaker]Mamoru Takada (Chiba Univ. Dept. of General Surgery) [Co-author]Aussie Suzuki (Univ. of Wisconsin, Madison, Carbone Cancer Center)、Takeshi Nagashima (Chiba Univ. Dept. of General Surgery)、Hiroshi Fujimoto (Chiba Univ. Dept. of General Surgery)、Junta Sakakibara (Chiba Univ. Dept. of General Surgery)、Ryotaro Teranaka (Chiba Univ. Dept. of General Surgery)、Masayuki Otsuka (Chiba Univ. Dept. of General Surgery)

CMPD1, a dual-target inhibitor of the p38-MK2 pathway and microtubule dynamics, shows increased sensitivity in cancer cells compared to normal cells, with significantly fewer side effects. This study demonstrates that CMPD1 induces irreversible mitotic defects in breast cancer cells, while non-transformed cells are largely unaffected. The key mechanism behind this selectivity is the distinct washout efficiency of CMPD1, following treatment, cancer cells struggle to recover from mitotic arrest, while normal cells resume mitosis quickly. CMPD1-treated mice showed no weight loss in animal models, unlike Taxol-treated mice, and displayed less liver damage and no renal toxicity. CMPD1 induces a robust prometaphase arrest in breast cancer cells via a p53-independent mechanism. Moreover, the combination of microtubule destabilizers, like vinblastine, with MK2 inhibitors, such as MK2i, significantly enhances mitotic defects and cell death, providing a synergistic therapeutic approach. These findings suggest that CMPD1's preferential action in cancer cells, combined with its reduced systemic toxicity, makes it a promising therapeutic candidate for cancer treatment.

[Disclosure of COI by Responsible researcher] Lead presenter is the Responsible researcher.
[Lead presenter: Mamoru Takada] ■Lecture fee: 日本イーライリリー株式会社

### [P-3299] I-P14-10
### Anticancer drug

2025 / 9 / 27 (Sat) 15:30-16:15 Room P/(Motenashi Dome) Room P

---

### P-3299
### Synergistic effect on gastric cancer cells of combination treatment with platinum agents

[Speaker]Kohei Hayashi (Dept. Molecular & Genomic Biomed., Grad. Sch. Biomed. Sci., Nagasaki Univ. ／Department of Gastroenterology and Hepatology, Nagasaki Univ. Hospital) [Co-author]Saraswat Barkha (Dept. Molecular & Genomic Biomed., Grad. Sch. Biomed. Sci., Nagasaki Univ.)、Vadivelu Ankitha (Dept. Molecular & Genomic Biomed., Grad. Sch. Biomed. Sci., Nagasaki Univ.)、Tong Ying (Dept. Molecular & Genomic Biomed., Grad. Sch. Biomed. Sci., Nagasaki Univ.)、Kazuhiko Nakao (Department of Gastroenterology and Hepatology, Nagasaki Univ. Hospital)、Hisamitsu Miyaaki (Department of Gastroenterology and Hepatology, Nagasaki Univ. Hospital)、Mitsuko Masutani (Dept. Molecular & Genomic Biomed., Grad. Sch. Biomed. Sci., Nagasaki Univ.)

Platinum-based agents, such as cisplatin (CDDP) and oxaliplatin (LOHP), are essential components of first-line systemic chemotherapy for unresectable gastric cancer. Despite both being platinum compounds, CDDP and LOHP differ in their mechanisms of action, indications, and toxicity profiles. These distinctions suggest the potential for additive or synergistic effects without cross-resistance when used in combination. This study investigated whether combining CDDP and LOHP enhances cytotoxicity in human gastric cancer cell lines. Eight cell lines were treated with CDDP, LOHP, or both, and cytotoxicity was evaluated using the MTT assay. Combination index (CI) values were calculated with CalcuSyn. Results showed a different range of IC50 values, indicating distinct sensitivities across cell lines. In MKN45 cells, combination treatment exhibited synergistic cytotoxicity with cell cycle arrest patterns and mitochondrial membrane potential similar to those of LOHP treatment. These findings support the therapeutic potential of combining CDDP and LOHP in gastric cancer treatment. Further mechanistic studies are ongoing.

[Disclosure of COI by Responsible researcher] Lead presenter is not the Responsible researcher.
[Lead presenter: Kohei Hayashi] We have no financial relationships to disclose.
[Principal Researcher: Mitsuko Masutani] We have no financial relationships to disclose.

### [P-3300] I-P14-10
### Anticancer drug

2025 / 9 / 27 (Sat) 15:30-16:15 Room P/(Motenashi Dome) Room P

### P-3300
### Novel prodrug targeting sulfotransferase for mesothelioma

[Speaker]Shigeaki Sunada (Juntendo Univ., JARIHES／Juntendo Univ., Grad. Sch. Med.) [Co-author]Ken (RIKEN) Matsumoto (RIKEN, CSRS)、Shingo Dan (JFCR, Cancer Chemother. Ctr.)、Saaya Suzuki (QST, Inst. Quantum Med. Sci.)、Qianqian Guo (Juntendo Univ., Grad. Sch. Med.)、Takashi(RIKEN) Watanabe (RIKEN, IMS)、Atsuo Kobayashi (RIKEN, IMS)、Rumi Kurokawa (RIKEN, CSRS)、Minoru Yoshida (RIKEN, CSRS)、Yukihide Momozawa (RIKEN, IMS)、Takashi Shimokawa (QST, Inst. Quantum Med. Sci.)、Yoshio Miki (Univ. Tsukuba, PMC)、Shunsuke Kato (Juntendo Univ., Grad. Sch. Med.)

Through a phenotypic analysis of compounds-formed DNA double-strand breaks (DSBs), we identified a seed compound S3, that induces DSBs and antitumor effects in specific cell lines. The unique human cancer cell line panel JFCR39 demonstrated that S3 caused the sulfotransferase ***SULT1A1/2*** expression-dependent cell lethality. In general, sulfonation plays an important role in chemical detoxification, but interestingly, S3 is converted to a DNA-damaging active form via the metabolism. Therefore, we have focused on malignant mesothelioma with higher expression of sulfotransferases, to develop S3 as a novel therapeutic agent. We have found that S3 would be converted to the active form via SULT1A1-specific metabolism and induce DNA adducts such as DNA-protein crosslinks, which contribute to the formation of lethal DSBs. Analysis of mesothelioma cell lines showed that S3 had highly target-specific antitumor effects compared to a conventional standard therapy. Profiling of mesothelioma features with high ***SULT1A1*** expression in the TCGA database suggested a high association with hormone response. Further data demonstrating the utility of S3 will be reported in this presentation.

[Disclosure of COI by Responsible researcher] Lead presenter is the Responsible researcher.
[Lead presenter: Shigeaki Sunada] ■Research funds from company: 国立研究開発法人 日本医療研究開発機構(AMED)、文部科学省 卓越研究員事業

### [P-3301] I-P14-10
### Anticancer drug

2025 / 9 / 27 (Sat) 15:30-16:15 Room P/(Motenashi Dome) Room P

---

### P-3301
### Effects of Anticancer Drugs on Mitochondrial Dynamics in Oral Cancer

[Speaker]Yusei Todoroki (Grad. Sch. Med. Sci., Kumamoto Univ.／Dept. Oral & Maxillofac. Surg., Fac. Life Sci., Kumamoto Univ.) [Co-author]Kenta Kawahara (Dept. Oral & Maxillofac. Surg., Fac. Life Sci., Kumamoto Univ.)、Kazumasa Ohyumi (Grad. Sch. Med. Sci., Kumamoto Univ.／Dept. Oral & Maxillofac. Surg., Fac. Life Sci., Kumamoto Univ.)、Kosuke Shinohara (Dept. Oral & Maxillofac. Surg., Fac. Life Sci., Kumamoto Univ.)、Mayuko Yamashita (Grad. Sch. Med. Sci., Kumamoto Univ.／Dept. Oral & Maxillofac. Surg., Fac. Life Sci., Kumamoto Univ.)、Yuki Seki (Dept. Oral & Maxillofac. Surg., Fac. Life Sci., Kumamoto Univ.)、Masatoshi Hirayama (Dept. Oral & Maxillofac. Surg., Fac. Life Sci., Kumamoto Univ.)、Akiyuki Hirosue (Dept. Oral & Maxillofac. Surg., Fac. Life Sci., Kumamoto Univ.)、Ryoji Yoshida (Dept. Oral & Maxillofac. Surg., Fac. Life Sci., Kumamoto Univ.)、Hideki Nakayama (Dept. Oral & Maxillofac. Surg., Fac. Life Sci., Kumamoto Univ.)

Background: Recently, mitochondrial networks have attracted attention in cancer research. This study aims to elucidate the mechanisms of treatment resistance based on morphological and functional changes in mitochondria induced by cisplatin and paclitaxel.Materials and Methods: Human oral cancer cell lines were treated with cisplatin and paclitaxel, and classified as sensitive or resistant. Image analysis was performed to evaluate mitochondrial morphological changes, and mitochondrial functions were also assessed.Results: Cisplatin induced mitochondrial fission in sensitive cell lines, while mitochondrial fusion was observed in resistant lines. In contrast, paclitaxel induced mitochondrial fission in both sensitive and resistant cell lines. Both drugs influenced oxidative phosphorylation and ATP production.Conclusion: Mitochondrial fusion appears to be associated with cisplatin resistance. These findings suggest that inducing mitochondrial fission with paclitaxel prior to cisplatin treatment may help overcome cisplatin resistance in resistant cancer cells.

[Disclosure of COI by Responsible researcher] Lead presenter is not the Responsible researcher.
[Lead presenter: Yusei Todoroki] We have no financial relationships to disclose.
[Principal Researcher: Hideki Nakayama] We have no financial relationships to disclose.

### [P-3307] I-P15
### Evaluation & Prediction of Pharmacological Effects

2025 / 9 / 27 (Sat) 15:30-16:15 Room P/(Motenashi Dome) Room P

### P-3307
### Investigation of extraction methods suitable for quantification of antibody concentrations in tumor tissue

[Speaker]Hirobumi Fuchigami (Natl. Canc. Ctr. EPOC Div. of Dev. Therap.) [Co-author]Shigehiro Koganemaru (Natl. Canc. Ctr. Hosp. E. Dept. of Exp. Therap.)、Masahiro Yasunaga (Natl. Canc. Ctr. EPOC Div. of Dev. Therap.)

Antibody-based therapeutics have become important drug therapies for various diseases including cancer. Although the concentration of antibodies in tumor tissue is crucial information for understanding their efficacy and safety, to our knowledge, no method exists that can directly quantify tissue-bound antibody concentrations. For this reason, we aimed to establish a method for direct quantification of immunoglobulin G (IgG) in tumor tissue, the major isotype of antibody-based therapeutics. In this study, we examined the possibility of quantifying tissue-bound antibodies using the ACES, a newly developed antibody extraction method. Cetuximab, one of the major antibody drugs, was used as a model antibody. ACES successfully evaluated tissue-bound cetuximab in model tumors. In addition, ACES allowed the measurement of Trastuzumab and Trastuzumab-deruxtecan (T-Dxd). We are further attempting to quantify smaller amounts of antibodies in tumor tissues using micro LC. We report on the progress of this study.

[Disclosure of COI by Responsible researcher] Lead presenter is not the Responsible researcher.
[Lead presenter: Hirobumi Fuchigami] ■Research funds from company: エーザイ株式会社
[Principal Researcher: Masahiro Yasunaga] ■Research funds from company: 第一三共株式会社、味の素株式会社、シスメックス株式会社、H.U.グループホールディングス、株式会社同仁グループ　■Profit of stock: 株式会社凜研究所

▲TOP

### [P-3308] I-P15
### Evaluation & Prediction of Pharmacological Effects

2025 / 9 / 27 (Sat) 15:30-16:15 Room P/(Motenashi Dome) Room P

### P-3308
### Automation and Optimization of a Three-Dimensional Blood Brain Barrier Model

[Speaker]Kanako Eto (Corning International K. K.) [Co-author]Hilary Sherman (Corning Life Sciences)、Ann Ferrie (Corning Life Sciences)

The blood-brain barrier (BBB) is a network of cells and blood vessels that filters and protects the brain, allowing essential substances in while blocking toxins. This protective feature complicates the study of CNS-targeted therapies. Existing in vitro models fail to accurately replicate the BBB's complex cellular environment necessary for researching neuroinflammation, toxicity, brain cancer, and other neurological conditions. We present a novel high-throughput screening method to optimize a BBB model by combining primary astrocytes, a neuroblastoma cell line, and a human brain endothelial cell line on Corning Transwell supports. Automation using World Precision Instruments´ EVOM Auto measured Transepithelial Electrical Resistance (TEER) for rapid, reproducible assessments of barrier integrity. The study evaluated how different cell type combinations, shear stress, and permeability-increasing compounds affect the BBB, using TEER measurements to develop and refine multiple models.

[Disclosure of COI by Responsible researcher] Lead presenter is not the Responsible researcher.
[Lead presenter: Kanako Eto] ■ employment by a company related to the presentation: コーニングインターナショナル株式会社
[Principal Researcher: Ann Ferrie] ■employment by a company related to the presentation: Corning Inc.

Poster Session

### [P-3309] I-P15
### Evaluation & Prediction of Pharmacological Effects

2025 / 9 / 27 (Sat) 15:30-16:15 Room P/(Motenashi Dome) Room P

---

### P-3309
### Integrated PK-PD/TD model-based prognostic prediction framework for FOLFIRINOX therapy in pancreatic cancer model mouse

[Speaker]Shinji Kobuchi (Kyoto Pharm. Univ. Dept. Pharmacokinetics) [Co-author]Kazuki Tosaka (Kyoto Pharm. Univ. Dept. Pharmacokinetics)、Kikuko Amagase (Ritsumeikan Univ. College of Pharm. Sci. Dept. Pharm.)、Yukako Ito (Kyoto Pharm. Univ. Dept. Pharmacokinetics)

Background: FOLFIRINOX therapy for pancreatic cancer lacks accurate prognostic indicators for personalized chemotherapy. This study aimed to develop a pharmacokinetic-pharmacodynamic/toxicodynamic (PK-PD/TD) model-based prognostic framework to quantify the relationship between prognostic markers and survival.
Methods: Pancreatic cancer xenograft mice were treated with FOLFIRINOX. Tumor volume, plasma drug levels, body weight, and survival time were monitored. A PK-PD/TD model with a parametric time-to-event (TTE) component was developed to predict survival considering efficacy and toxicity.
Results: The integrated PK-PD/TD model successfully described the observed data, including survival time. Mice with good tumor growth inhibition and stable body weight showed improved survival. TTE simulations indicated tumor inhibition had a greater impact on survival than body weight. The model might help optimize treatment by simulating dosing frequency and washout periods.
Conclusion: The PK-PD/TD model integrating a parametric TTE approach establishes a quantitative framework for survival prediction and may optimize individualized FOLFIRINOX therapy in pancreatic cancer.

[Disclosure of COI by Responsible researcher] Lead presenter is the Responsible researcher.
[Lead presenter: Shinji Kobuchi] We have no financial relationships to disclose.

### [P-3310] I-P15
### Evaluation & Prediction of Pharmacological Effects

2025 / 9 / 27 (Sat) 15:30-16:15 Room P/(Motenashi Dome) Room P

### P-3310
### Withdrawn

No Abstract

### [P-3311] I-P15
### Evaluation & Prediction of Pharmacological Effects

2025 / 9 / 27 (Sat) 15:30-16:15 Room P/(Motenashi Dome) Room P

---

### P-3311
### Withdrawn

No Abstract

### [P-3312] I-P15
### Evaluation & Prediction of Pharmacological Effects

2025 / 9 / 27 (Sat) 15:30-16:15 Room P/(Motenashi Dome) Room P

---

### P-3312
### Phenotypic Classification and Drug Sensitivity Profiling of Pancreatic Cancer Cell Lines Based on Public Data

[Speaker]Masakazu Fujii (Dept. of Gastroenterological Surg. II, Faculty of Med., Hokkaido Univ.) [Co-author]Shintaro Takeuchi (Dept. of Gastroenterological Surg. II, Faculty of Med., Hokkaido Univ.)、Takuro Noguchi (Shinshu Cancer Ctr., Shinshu Univ. Hosp.)、Satoshi Hirano (Dept. of Gastroenterological Surg. II, Faculty of Med., Hokkaido Univ.)

[Background] While molecular subtypes such as classical and basal-like have been proposed for pancreatic cancer, their relationship with drug sensitivity remains poorly defined. [Objective] To define transcriptome-based phenotypes of pancreatic cancer cell lines and examine their associations with drug sensitivity using public datasets. [Methods] We analyzed log2(TPM+1) gene expression data from 51 pancreatic cancer cell lines in the DepMap database. Consensus clustering based on phenotype-related genes identified distinct phenotypes. Drug sensitivity data from the CTD2 and GDSC2 datasets were compared across phenotypes using the Kruskal-Wallis test. [Results] Five phenotypes-Classical-A/B, Basal-like A/B, and Hybrid-were identified. GSEA revealed distinct enrichment patterns such as EMT and inflammatory signaling in Basal-like subtypes. Basal-like cell lines exhibited higher sensitivity to fluorouracil, vincristine, doxorubicin, etoposide, and gefitinib. [Conclusions] Molecular subtyping of pancreatic cancer cell lines reveals that certain agents may retain efficacy even in Basal-like phenotypes. These findings suggest the potential utility of phenotype-guided therapy selection.

[Disclosure of COI by Responsible researcher] Lead presenter is not the Responsible researcher.
[Lead presenter: Masakazu Fujii] We have no financial relationships to disclose.
[Principal Researcher: Shintaro Takeuchi] We have no financial relationships to disclose.

▲TOP

### [P-3322] II-P5-2
### Lung cancer (2)

2025 / 9 / 27 (Sat) 15:30-16:15 Room P/(Motenashi Dome) Room P

---

P-3322
### Design, synthesis, and biological evaluation of a novel 9-aminoacridine for lung cancer therapy

[Speaker]Ming-Chen Chiang (Grad. Inst. of Medical Sci., College of Med.,TMU／Dept. of Biochem. and Mol. Cell Biology, College of Med.,TMU)
[Co-author]Cheng-Wei Lin (Grad. Inst. of Medical Sci., College of Med.,TMU／Dept. of Biochem. and Mol. Cell Biology, College of Med.,TMU／Dept. of Biomed. Sci. and Environmental Biology, KMU)、Wen-Jing Hsu (Grad. Inst. of Medical Sci., College of Med.,TMU／Dept. of Biochem. and Mol. Cell Biology, College of Med.,TMU)、Cheng-Jui Huang (Dept. of Biochem. and Mol. Cell Biology, College of Med.,TMU)、Yang-Sen Lin (Dept. of Biochem. and Mol. Cell Biology, College of Med.,TMU)、Chu-Hung Chung (Grad. Inst. of Medical Sci., College of Med.,TMU／Dept. of Biochem. and Mol. Cell Biology, College of Med.,TMU)、Mei-Hsiang Lin (Sch. of Pharm., College of Pharmacy, TMU)

Lung cancer is the most commonly diagnosed malignancy and remains associated with poor prognosis. Despite advancements in treatments, including surgery, chemotherapy, radiotherapy, or their combinations, the overall survival rate is still unsatisfactory. About 50% of lung cancer patients harbor oncogenic mutations in KRAS or EGFR, making them key therapeutic targets. In this study, a series of 9-aminoacridine derivatives were synthesized using matrine as the lead compound. Among them, Compound M2 showed strong antitumor activity against EGFR- or KRAS-mutant lung cancer cell lines, such as PC9 and A549. Biological evaluation indicated that M2 significantly reduced cell viability by inducing apoptosis. Mechanistically, M2 increased intracellular ROS levels and upregulated ER stress markers. Additionally, it inhibited phosphorylation of AKT and EGFR and suppressed YAP signaling, leading to reduced EGFR protein expression. These findings identify Compound M2 as a potent dual EGFR-YAP pathway inhibitor with promising potential for lung cancer therapy.

[Disclosure of COI by Responsible researcher] Lead presenter is the Responsible researcher.
[Lead presenter: Ming-Chen Chiang ] We have no financial relationships to disclose.

### [P-3323] II-P5-2
### Lung cancer (2)

2025 / 9 / 27 (Sat) 15:30-16:15 Room P/(Motenashi Dome) Room P

P-3323
### Characteristics of CGP and RNA sequencing in EGFR-mutant lung cancer including cases of SCLC transformation

[Speaker]Shinsuke Ogusu (Division of Respiratory Medicine, Department of Internal Medicine, Saga University) [Co-author]Yuta Kishikawa (Division of Respiratory Medicine, Department of Internal Medicine, Saga University)、Mizuki Nanri (Division of Respiratory Medicine, Department of Internal Medicine, Saga University)、Yuki Kurihara (Division of Respiratory Medicine, Department of Internal Medicine, Saga University)、Natsuko Komiya (Division of Respiratory Medicine, Department of Internal Medicine, Saga University)、Hiroki Tashiro (Division of Respiratory Medicine, Department of Internal Medicine, Saga University)、Akemi Sato (Clinical laboratory Medicine, Saga University)、Chiho Nakashima (Division of Respiratory Medicine, Department of Internal Medicine, Saga University)、Koichiro Takahashi (Division of Respiratory Medicine, Department of Internal Medicine, Saga University)、Naoko Aragane (Department of Cancer Center, Takagi Hospital)

[Background]Resistance to EGFR-TKIs for EGFR-mutant lung cancer, including SCLC transformation, remains a problem to be solved. [Method] We conducted comprehensive genomic profiling (CGP) and RNA sequencing (RNA-Seq) using pre- and post-EGFR-TKIs treatment samples at our hospital between January 2015 and December 2023. Overall, six cases were included: three patients who developed SCLC (t-SCLC group) and three patients who did not (Ad group). Tumor microenvironment (TME) characterization was conducted using BostonGene portrait test (BG) and EcoTyper platforms. [Result] Concurrent truncating mutations in RB1 and TP53 were identified in one of three cases in the t-SCLC group. Differential gene expression analysis before and after EGFR-TKIs in the t-SCLC group revealed significantly increased expression of ASCL1, NEUROD1, and INSM1, and EGFR, REST, and YAP1 were downregulated. Notably, prolonged survival was observed in one patient whose tumor was classified as "Immune-Enriched" based on TME evaluation using BG and EcoTyper. [Conclusion] Integrating CGP and RNA sequencing analyses enables the identification of optimal, case-specific treatment strategies for EGFR-mutant lung cancer.

[Disclosure of COI by Responsible researcher] Lead presenter is the Responsible researcher.
[Lead presenter: Shinsuke Ogusu] ■Research funds from company: 文科省科学研究費補助金

### [P-3324] II-P5-2
### Lung cancer (2)

2025 / 9 / 27 (Sat) 15:30-16:15 Room P/(Motenashi Dome) Room P

---

### P-3324
### Spatiotemporal analysis identifies EGFR-TKI-resistant tumor epithelial clusters.

[Speaker]Shuichi Watanabe (Division of Cellular Signaling, National Cancer Center Research Institute.) [Co-author]Yoshiyuki Suehara (Division of Cellular Signaling, National Cancer Center Research Institute.)、Masachika Ikegami (Division of Cellular Signaling, National Cancer Center Research Institute.)、Toshihide Ueno (Division of Cellular Signaling, National Cancer Center Research Institute.)、Soonhwan Park (Division of Cellular Signaling, National Cancer Center Research Institute.／Department of General Thoracic Surgery, Juntendo University School of Medicine.)、Takuo Hayashi (Department of Human Pathology, Juntendo University School of Medicine.)、Kazuya Takamochi (Department of General Thoracic Surgery, Juntendo University School of Medicine.)、Shinji Kohsaka (Division of Cellular Signaling, National Cancer Center Research Institute.)

[Introduction]EGFR-TKI therapy in NSCLC shows high response rates, but relapse after MRD is inevitable. Our previous in vivo study demonstrated that drug-tolerant persister (DTP) cells contribute to MRD based on mutational heterogeneity (Ikeuchi H, Kohsaka S, et al. Oncogene. 2022). However, the mechanisms and molecular profiles of DTPs remain unclear.[Methods]FFPE samples from two NSCLC patients before and after therapy were analyzed using the Visium HD platform. 2$\mu$m tiles were integrated into 16$\mu$m tiles.[Results]After filtering, 322,604 (Case 1) and 399,489 (Case 2) tiles were obtained. UMAP identified 14 clusters. Tumor epithelial clusters were defined, and clusters with reduced EGFR expression ($p<2.2e-16$). These clusters expanded from focal to diffuse distributions after therapy. Enrichment of EMT-related genes in Case 1 and TNF$\alpha$-NF $\kappa$B signaling in Case 2 was shown.[Discussion]Single-cell spatial analysis of brain-metastasized NSCLC also reported EMT-related poor prognosis clusters (Tagore S, et al. Nat Med. 2025), consistent with our findings.[Conclusion]We identified spatial expansion and molecular features of EGFR-TKI-resistant tumor epithelial clusters.

[Disclosure of COI by Responsible researcher] Lead presenter is not the Responsible researcher.
[Lead presenter: Shuichi Watanabe] We have no financial relationships to disclose.
[Principal Researcher: Shinji Kohsaka] We have no financial relationships to disclose.

Poster Session

### [P-3325] II-P5-2
### Lung cancer (2)

2025 / 9 / 27 (Sat) 15:30-16:15 Room P/(Motenashi Dome) Room P

---

### P-3325
### Clinical outcomes of osimertinib for advanced EGFR-mutated LUAD based on the 9th TNM classification: a multicenter study

[Speaker]Ichidai Tanaka (Dept. of Respiratory Medicine, Nagoya University Graduate School of Medicine) [Co-author]Mika Sato (Dept. of Respiratory Medicine, Nagoya University Graduate School of Medicine)、Kazumi Hori (Dept. of Respiratory Medicine, Nagoya University Graduate School of Medicine)、Junji Koyama (Dept. of Respiratory Medicine, Nagoya University Graduate School of Medicine)、Yuta Kodama (Department of Respiratory Medicine, Japanese Red Cross Nagoya Daiichi Hospital)、Akira Matsui (Department of Respiratory Medicine, Kariya Toyota General Hospital)、Ayako Miyazawa (Department of Respiratory Medicine, Konan Kosei Hospital)、Shoko Miyamatsu (Department of Respiratory Medicine, Chukyo Hospital)、Shuichi Asano (Department of Respiratory Medicine, Chukyo Hospital)、Yoshitaka Hibino (Department of Respiratory Medicine, Konan Kosei Hospital)、Masahiro Morise (Dept. of Respiratory Medicine, Nagoya University Graduate School of Medicine)、Toshihiko Yokoyama (Department of Respiratory Medicine, Japanese Red Cross Nagoya Daiichi Hospital)、Tomoki Kimura (Department of Respiratory Medicine and Allergy, Tosei General Hospital)、Norio Yoshida (Department of Respiratory Medicine, Kariya Toyota General Hospital)、Mitsuo Sato (Dept. of Integrated Health Sciences, Nagoya University Graduate School Medicine)、Makoto Ishii (Dept. of Respiratory Medicine, Nagoya University Graduate School of Medicine)

The 9th edition of the TNM classification for lung cancer introduced M1c subdivisions (M1c1 and M1c2), but their clinical implications, particularly regarding molecular targeted therapies, are unclear. This study analyzed the efficacy of the 3rd generation EGFR-TKI, Osimertinib, in patients with recurrent EGFR-mutant lung adenocarcinoma (LUAD) versus those with de novo stage IV. We retrospectively reviewed clinical data from 275 patients treated with first-line osimertinib. Patients with postoperative recurrence were classified as r-M1a, r-M1b, r-M1c1, and r-M1c2 based on recurrence patterns. Among 183 de novo stage IV patients, distributions were M1a (26.8%), M1b (13.1%), M1c1 (28.4%), and M1c2 (31.7%). In 92 patients with recurrence, patterns were r-M1a (63.0%), r-M1b (12.0%), r-M1c1 (12.0%), and r-M1c2 (13.0%). Postoperative recurrence showed better PFS and OS than de novo stage IV (PFS: 34.6 vs. 17.9 months; OS: 53.4 vs. 35.2 months). In the both groups, M1c2 had the poorest outcomes. Thus, postoperative recurrence EGFR-mutant LUAD is associated with better survival, and M1c subclassification reflects significant differences in treatment response and prognosis.

[Disclosure of COI by Responsible researcher] Lead presenter is the Responsible researcher.
[Lead presenter: Ichidai Tanaka] ■Lecture fee: 中外製薬、アストラゼネカ社

### [P-3326] II-P5-2
### Lung cancer (2)

2025 / 9 / 27 (Sat) 15:30-16:15 Room P/(Motenashi Dome) Room P

---

### P-3326
### Impact of PD-L1 Expression on Outcomes in Advanced or Recurrent ALK-Positive NSCLC: A Multicenter Retrospective Study

[Speaker]Nobutaka Kataoka (Dept. of Respiratory Med., Kyoto Prefectural Univ. of Med.) [Co-author]Tadaaki Yamada (Dept. of Respiratory Med., Kyoto Prefectural Univ. of Med.)、Yoshiki Negi (Dept. of Respiratory and Hematol. Med., Hyogo Med. Univ.)、Akihiro Yoshimura (Dept. of Clin. Oncol., Kyoto Daiichi Red Cross Hosp.)、Hirokazu Taniguchi (Cli. Oncol. Ctr. Nagasaki Univ. Hosp.)、Satoshi Watanabe (Dept. of Respiratory and Infectious Diseases, Niigata Univ.)、Yusuke Chihara (Dept. of Respiratory Med., Uji Tokushukai Hosp.)、Takashi Kijima (Dept. of Respiratory and Hematol. Med., Hyogo Med. Univ.)、Koichi Takayama (Dept. of Respiratory Med., Kyoto Prefectural Univ. of Med.)

Background: In epidermal growth factor receptor (EGFR)-mutant non-small cell lung cancer (NSCLC), high intratumoral programmed death-ligand 1 (PD-L1) expression has been linked to reduced efficacy of EGFR tyrosine kinase inhibitors (EGFR-TKIs). However, its clinical relevance in anaplastic lymphoma kinase (ALK)-rearranged NSCLC remains unclear. Methods: We retrospectively analyzed data from patients with ALK-positive NSCLC who received first-line ALK-TKI therapy at 13 institutions in Japan between July 2014 and December 2022. Tumor PD-L1 expression was assessed at diagnosis. Results: A total of 129 patients were included in the analysis. Among them, 74 had low PD-L1 expression (0-49%), while 55 had high expression (>50%). There were no significant differences between the groups in overall survival (median: 119.9 vs. 76.6 months; hazard ratio [HR], 1.74; 95% confidence interval [CI], 0.91-3.32; P = .095) or progression-free survival with first-line ALK-TKI (median: 29.7 vs. 35.0 months; HR, 1.01; 95% CI, 0.63-1.63; P = .961). Conclusion: In ALK-rearranged NSCLC, PD-L1 expression levels at diagnosis were not significantly associated with the efficacy of first-line ALK-TKI treatment.

[Disclosure of COI by Responsible researcher] Lead presenter is not the Responsible researcher.
[Lead presenter: Nobutaka Kataoka] We have no financial relationships to disclose.
[Principal Researcher: Tadaaki Yamada] ■Lecture fee: 中外製薬、日本イーライリリー　■Research expenses: 小野薬品工業、武田薬品工業

▲TOP

### [P-3327] II-P5-2
### Lung cancer (2)

2025 / 9 / 27 (Sat) 15:30-16:15 Room P/(Motenashi Dome) Room P

### P-3327
### Multi-ion beam irradiation combined with KRASG12C inhibitors effectively destroys KRAS mutant lung cancer cells

[Speaker]Sei Sai (Dept Charg Part Ther Res, QST Hospt, QST) [Co-author]Yumei Kang (QST Hospital, QST／Div Radiat Oncol, Taipei Veter Gener, Taiwan)、Hyuncheol Kang (QST Hospital, QST／Div Radiat Oncol, Taipei Veter Gener, Taiwan／Dept Radiat Oncol, Seoul Nat Univ Colleg Med, Korea)、Masao Suzuki (Dept Charg Part Ther Res, QST Hospt, QST)、Hitoshi Ishikawa (QST Hospital, QST)

KRAS mutations occur primarily in the non-small cell lung cancer (NSCLC), and the presence of KRAS mutations is associated with poor prognosis. In this study we investigate the effects of a multi-ion (He-, C-, O-, Ne-) beam irradiation (IR) alone or in combination with KRASG12C inhibitors (sotorasib, adagrasib) on NSCLC cells. We found that LU99 (KRASG12C mutant) and A549 (KRASG12S mutant) cells were resistant to He and C-ion beam IR, but sensitive to Ne-ion beam IR, which was ineffective when combined with sotrasib or adagrasib. In contrast, LU65 (KRASG12C mutant) cells were sensitive to treatment with C-, Ne- and O-ion beam IR alone, and was further enhanced when combined with sotorasib or adagrasib, whereas LU99 cells were killed by C-, Ne-ion beam combined with relatively high dose of sotorasib or adagrasib. Furthermore, C-, and Ne-ion beam IR in combination with sotorasib significantly enhanced the expression of the apoptosis- and autophagy-related genes such as cleaved-caspase3 and ATG7. Taken together, combination of heavier ions such as Ne-ion IR, with KRAS12C inhibitors likely induces KRASG12C mutant NSCLC cell death more significantly.

[Disclosure of COI by Responsible researcher] Lead presenter is the Responsible researcher.
[Lead presenter: Sei Sai] We have no financial relationships to disclose.

### [P-3328] II-P5-2
### Lung cancer (2)

2025 / 9 / 27 (Sat) 15:30-16:15 Room P/(Motenashi Dome) Room P

### P-3328
### Clinical and prognostic significance of M2-like tumor-associated macrophages in malignant pleural mesothelioma

[Speaker]Ryota Sumitomo (Department of Thoracic Surgery, Graduate School of Medicine, Kyoto University) [Co-author]Tetsuya Fukui (Department of Thoracic Surgery, Graduate School of Medicine, Kyoto University)、Masashi Kobayashi (Department of Thoracic Surgery, Kurashiki Central Hospital)、Hiroaki Sakai (Department of Thoracic Surgery, Hyogo Prefectural Amagasaki General Medical Center)、Toshi Menju (Department of Thoracic Surgery, Graduate School of Medicine, Kyoto University)

Background: Malignant pleural mesothelioma (MPM) is an aggressive malignancy with a poor prognosis. Asbestos-exposed macrophages may contribute to both tumor initiation and progression.
Methods: A total of 101 patients diagnosed with MPM and treated between 1998 and 2010 were retrospectively analyzed. M2-like TAMs were evaluated in intratumoral and peritumoral regions by CD163 immunohistochemistry. Tumor cell proliferation was assessed using Ki-67 staining.
Results: The densities of M2-like TAMs in intratumoral and peritumoral regions were significantly correlated with pretreatment C-reactive protein levels ($r = 0.283$ and 0.255, respectively; both $P < 0.01$) and Ki-67 proliferation index ($r = 0.498$ and 0.435, respectively; both $P < 0.01$). High TAM density in intratumoral and peritumoral regions was associated with worse overall survival ($P = 0.044$ and 0.046, respectively). In multivariable analysis, peritumoral TAM status, clinical stage, and histology were independent prognostic factors.
Conclusion: M2-like TAMs may enhance tumor proliferation and aggressiveness in MPM, thereby contributing to poor clinical outcomes. Targeting these macrophages may offer a novel therapeutic strategy.

[Disclosure of COI by Responsible researcher] Lead presenter is the Responsible researcher.
[Lead presenter: Ryota Sumitomo] ■Research funds from company: 一般財団法人　藤原記念財団

▲TOP

### [P-3329] II-P5-2
### Lung cancer (2)

2025 / 9 / 27 (Sat) 15:30-16:15 Room P/(Motenashi Dome) Room P

---

### P-3329
### Lung cancer secreting activin A recruited tumor associated macrophages to promote tumor growth

[Speaker]Yo Kawaguchi (Div. of Gen Thorac Surg, Shiga Univ. of Med Sci) [Co-author]Makoto Yoden (Div. of Gen Thorac Surg, Shiga Univ. of Med Sci)、Keiko Ueda (Div. of Gen Thorac Surg, Shiga Univ. of Med Sci)、Yoko Kataoka (Div. of Gen Thorac Surg, Shiga Univ. of Med Sci)、Jun Hanaoka (Div. of Gen Thorac Surg, Shiga Univ. of Med Sci)

We have reported that lung cancer secreting activin A (Act A) induced skeletal muscle atrophy to lead sarcopenia, using knock-down experiments of Act A in Lewis lung cancer (LLC) cell line. In the Act A knock-down LLC bearing mice, tumor growth was suppressed and survival was prolonged. However, the mechanism of tumor growth via the Act A is not clarified. In vitro, we checked that Act A knock-down LLC itself did not slow the tumor growth and recombinant Act A also did not promote the growth of LLC cells. So, we suspected that Act A changed the tumor microenvironment (TME) into a tumor promoting environment. We demonstrated that LLC secreted Act A and Act A knock-down LLC reduced the secretion of Act A. Furthermore, tumor secreting Act A recruited tumor associated macrophages (TAM) in the TME and differentiated it into tumor promoting CD206 TAMs. In vivo, TAM in the tumor tissues decreased in Act A knock-down LLC bearing mice.This study indicated that interfering with the effect of Act A can be a therapeutic target for not only sarcopenia but also lung cancer progression.

[Disclosure of COI by Responsible researcher] Lead presenter is the Responsible researcher.
[Lead presenter: Yo Kawaguchi] We have no financial relationships to disclose.

### [P-3330] II-P5-2
### Lung cancer (2)

2025 / 9 / 27 (Sat) 15:30-16:15 Room P/(Motenashi Dome) Room P

---

### P-3330
### SLC7A11 overexpression as a histological and prognostic marker in non-small cell lung cancer

[Speaker]Dage Liu (Dept. of Thoracic Surg. , Faculty of Med., Kagawa Univ.) [Co-author]Nakashima Nariyasu (Dept. of Thoracic Surg. , Faculty of Med., Kagawa Univ.)、Yoshimasa Tokunaga (Dept. of Thoracic Surg. , Faculty of Med., Kagawa Univ.)、Xia Zhang (Dept.of Urology, Faculty of Med., Kagawa Univ.)、Toshiki Yajima (Dept. of Thoracic Surg. , Faculty of Med., Kagawa Univ.)

Background: SLC7A11 maintains redox homeostasis by importing cystine for glutathione synthesis. Despite its emerging role in ferroptosis resistance, its clinical relevance in NSCLC subtypes remains controversial. Methods: We analyzed SLC7A11 expression and p53 mutations status immunohistochemically, and survival outcomes in 104 NSCLC patients (58 adenocarcinomas [ADC], 46 squamous cell carcinomas [SCC]). Results: SCC exhibited 1.6-fold higher SLC7A11 expression than ADC (56.6% vs. 35.1%, $p=0.003$). Smokers showed elevated SLC7A11 positivity (47.0% vs. 26.3%, $p=0.038$). p53 mutations (58.8% in SCC) strongly correlated with SLC7A11 overexpression (77.3% vs. 29.3%, $p < 0.001$). SLC7A11-positive patients had reduced 5-year survival (26.3% vs. 47.0%, $p=0.038$), independent of Ki67 index. Conclusion: SLC7A11 defines a clinically aggressive NSCLC subset enriched in SCC and smokers, driven by p53 mutations.

[Disclosure of COI by Responsible researcher] Lead presenter is the Responsible researcher.
[Lead presenter: Liu Dage] We have no financial relationships to disclose.

### [P-3339] II-P6-2
### Tumor Immunity & Molecular Basis of Treatment Response in Breast Cancer

2025 / 9 / 27 (Sat) 15:30-16:15 Room P/(Motenashi Dome) Room P

---

### P-3339
### Modeling the Role of Extracellular Matrix Stiffness and Alignment in Regulating Tumor Immune Interactions

[Speaker]Hung-chi Hsu (Institute of Biophotonics, National Yang Ming Chiao Tung University) [Co-author]Chia-Yi Su (Institute of Biophotonics, National Yang Ming Chiao Tung University)

During tumor progression, the extracellular matrix (ECM) surrounding the tumor undergoes significant remodeling, resulting in radially aligned fibers and increased matrix stiffness in proximity to the tumor, contributing to tumor invasion and acting as physical barriers impairing immune cell infiltration. Biomimetic 3D models offer a powerful tool for studying tumor immune interactions, enabling precise control over the ECM physical properties. In this study, we have developed a 3D tumor model consisting of a collagen hydrogel scaffold with tunable stiffness and aligned fibers, mimicking in vivo tumor structure. By adjusting collagen concentration and polymerization conditions, we achieved controlled modulation of matrix stiffness while maintaining fiber alignment. A tumor spheroid and immune cells were embedded at opposite ends of the aligned collagen fiber track to study how defined ECM characteristics regulate the bidirectional interactions between immune and tumor cells. Our model serves as a physiologically relevant platform to study tumor immune interactions and may provide new insights for improving the efficacy of immunotherapies.

[Disclosure of COI by Responsible researcher] Lead presenter is not the Responsible researcher.
[Lead presenter: Hung-chi Hsu] We have no financial relationships to disclose.
[Principal Researcher: Hung-chi Hsu] We have no financial relationships to disclose.

### [P-3340] II-P6-2
### Tumor Immunity & Molecular Basis of Treatment Response in Breast Cancer

2025 / 9 / 27 (Sat) 15:30-16:15 Room P/(Motenashi Dome) Room P

---

### P-3340
### Therapeutic strategy targeting FXYD3-positive ancestor-like cancer stem cells in triple-negative breast cancer

[Speaker]Sohei Kawagoe (Kanazawa univ. Cancer Reserch Inst. CCB) [Co-author]Yasuto Takeuchi (Kanazawa univ. Cancer Reserch Inst. CCB)、Huazi Zhang (Kanazawa univ. Cancer Reserch Inst. CCB)、Noriko Gotoh (Kanazawa univ. Cancer Reserch Inst. CCB)

Patients with triple-negative breast cancer (TNBC) face recurrence or metastasis and have a poor prognosis. A population of drug-resistant cancer cells that survive chemotherapy are termed drug-tolerant persisters (DTPs). Targeting DTPs is expected to prevent recurrent and metastatic breast cancer. Recently, we identified a subpopulation of CSCs enriched in the plasma membrane protein FXYD3. FXYD3 is a subunit of the $Na^+/K^+$ pump and protects its activity (Li, Takeuchi, et al., J Clin Invest, 2023). Since this CSC subpopulation retains cell-of-origin traits, we refer to them as ancestor-like CSCs. Furthermore, we found that they possess drug-resistant potential, suggesting that they are indeed DTPs. This study aims to develop a TNBC therapy by targeting FXYD3. We have evaluated the use of digoxin, an old drug for heart failure, as it inhibits $Na^+/K^+$ pump activity and reduces drug resistance in FXYD3-positive ancestor-like CSCs. To establish an effective therapeutic strategy against DTPs, we are currently assessing the efficacy of digoxin in patient-derived cancer cells.

[Disclosure of COI by Responsible researcher] Lead presenter is not the Responsible researcher.
[Lead presenter: Sohei Kawagoe] We have no financial relationships to disclose.
[Principal Researcher: Noriko Gotoh] We have no financial relationships to disclose.

### [P-3341] II-P6-2
### Tumor Immunity & Molecular Basis of Treatment Response in Breast Cancer

2025 / 9 / 27 (Sat) 15:30-16:15 Room P/(Motenashi Dome) Room P

---

### P-3341
### Comparing Molecular Profile of Classical and Pleomorphic Invasive Lobular Carcinoma Using Spatial Transcriptome Analysis

[Speaker]Momoko Tokura (Lab. of Integrative Oncology, Natl. Cancer Ctr. Res. Inst.) [Co-author]Jun(NCC) Nakayama (Lab. of Integrative Oncology, Natl. Cancer Ctr. Res. Inst.／Dept. of Oncogenesis Growth Regulation, Osaka Intl. Cancer Inst.)、Hironori Suzuki (Lab. of Integrative Oncology, Natl. Cancer Ctr. Res. Inst.／Dept. Obst. & Gynecol., Nagoya Univ. Grad. Sch. of Med.)、Shin Takayama (Dept. Breast Surg., Natl. Cancer Ctr. Hosp.)、Masayuki Yoshida (Dept. Path., Natl. Cancer Ctr. Hosp.)、Yusuke Yamamoto (Lab. of Integrative Oncology, Natl. Cancer Ctr. Res. Inst.)

Invasive lobular carcinoma (ILC) is the most common of the special types of breast cancer accounting for approximately 10-15% of all cases. ILC is characterized by a lack of E-cadherin (CDH1) expression, leading to the loss of cell-cell adhesion and the dispersed spread of individual cancer cells. Histological subtypes of ILC exhibit diverse morphological features. Pleomorphic ILC (P-ILC) is known to have a worse prognosis than classic ILC (C-ILC) due to the appearance of high-grade nuclear atypia and mitotic cells. However, the molecular differences between C-ILC and P-ILC remain unclear. To address this deficiency, we performed spatial transcriptome profiling of four C-ILC samples and four P-ILC samples. Comparing C-ILC and P-ILC, several transcripts were highly expressed in P-ILC. Cell cycle-related genes were more enriched in cancer-dominant spots of P-ILC than in C-ILC. Moreover, expression levels of genes related to extracellular matrix (ECM) remodeling and angiogenesis tended to be higher in stroma-dominant spots of P-ILC than in C-ILC. These differences may be related to the aggressive phenotype of P-ILC.

[Disclosure of COI by Responsible researcher] Lead presenter is not the Responsible researcher.
[Lead presenter: Momoko Tokura] ■Research funds from company: 東芝、Nikon
[Principal Researcher: Yusuke Yamamoto] ■Research funds from company: 東芝、Nikon

### [P-3342] II-P6-2
### Tumor Immunity & Molecular Basis of Treatment Response in Breast Cancer

2025 / 9 / 27 (Sat) 15:30-16:15 Room P/(Motenashi Dome) Room P

### P-3342
### Low albumin-correlated systemic immunosuppression in metastatic breast cancer patients

[Speaker]Yuki Nakamura (Inter-Organ Communication Research Team, LiMe, Kyoto Univ.／Dept. of Breast Surg., Kyoto Univ.) [Co-author]Mayuko Yoda (Dept. of Integrative Bioanalytics, IDAC, Tohoku Univ.)、Yoshihiro Izumi (Div. of Metabolomics, Inst. of Bioregulation, Kyushu Univ.)、Atsushi Hatano (Dept. of Omics & Systems Biol., Niigata Univ.)、Riyo Konishi (Inter-Organ Communication Research Team, LiMe, Kyoto Univ.)、Daiya Ohara (Lab. of Integrative Biol. Sci., LiMe, Kyoto Univ.)、Yukie Kashima (Grad. Sch. of Frontier Sci., Tokyo Univ.)、Masatomo Takahashi (Div. of Metabolomics, Inst. of Bioregulation, Kyushu Univ.)、Kohta Nakatani (Div. of Metabolomics, Inst. of Bioregulation, Kyushu Univ.)、Takeshi Bamba (Div. of Metabolomics, Inst. of Bioregulation, Kyushu Univ.)、Alexis Vandenbon (Lab. of Tissue Homeostasis, LiMe, Kyoto Univ.／Inst. for Liberal Arts & Sci., Kyoto Univ.)、Yutaka Suzuki (Grad. Sch. of Frontier Sci., Tokyo Univ.)、Keiji Hirota (Lab. of Integrative Biol. Sci., LiMe, Kyoto Univ.)、Masaki Matsumoto (Dept. of Omics & Systems Biol., Niigata Univ.)、Masakazu Toi (Dept. of Breast Surg., Kyoto Univ.／Tokyo Metropolitan Ctr. & Infectious Disease Ctr, Komagome Hosp.)、Norikazu Masuda (Dept. of Breast Surg., Kyoto Univ.)、Kosuke Kawaguchi (Dept. of Breast Surg., Kyoto Univ.／Breast Ctr., Mie Univ. Hosp.)、Shinpei Kawaoka (Inter-Organ Communication Research Team, LiMe, Kyoto Univ.／Dept. of Integrative Bioanalytics, IDAC, Tohoku Univ.)

Cancer induces various adverse effects on the host, with cancer cachexia being the most well-known syndrome. Clinically, cancer cachexia is defined as an unintentional loss of weight, indicating a disruption in host homeostasis.Our research focuses on the early pathophysiological changes that occur before cancer cachexia. Specifically, we investigated low albumin levels, a common characteristic in advanced cancer patients, whose underlying mechanisms remain largely unexplored.In this study, we aimed to elucidate the pathophysiology of metastatic breast cancer patients from the perspective of low albumin status. Our transcriptome analysis of peripheral blood mononuclear cells revealed that low albumin levels correlate with transcriptome signatures indicative of an increase in neutrophils and a decrease in $CD8^+$ T cells. We also observed a reduction in immunoregulatory metabolites, such as arginine, in plasma correlating with albumin levels. These findings suggest that systemic immunosuppression is associated with low albumin levels.In this presentation, we will discuss our findings and explore the potential causal relationship between albumin levels and immunosuppressive phenotypes.

[Disclosure of COI by Responsible researcher] Lead presenter is not the Responsible researcher.
[Lead presenter: Yuki Nakamura] We have no financial relationships to disclose.
[Principal Researcher: Shinpei Kawaoka] We have no financial relationships to disclose.

▲TOP

### [P-3343] II-P6-2

### Tumor Immunity & Molecular Basis of Treatment Response in Breast Cancer

2025 / 9 / 27 (Sat) 15:30-16:15 Room P/(Motenashi Dome) Room P

---

### P-3343

### Mitochondrial BIG3-PHB2 complex as a potential therapeutic target in Triple-Negative Breast Cancer.

[Speaker]Ulziisaikhan Batmunkh (Natl. Inst. of Biomed. Innovation, Health & Nutrition／Dept. of Respiratory Med. & Rheumatology, Tokushima Univ.) [Co-author]Tetsuro Yoshimaru (Natl. Inst. of Biomed. Innovation, Health & Nutrition／Inst. of Advanced Med. Sci, Tokushima Univ.)、Yosuke Matsushita (Natl. Inst. of Biomed. Innovation, Health & Nutrition)、Keiji Uchiyama (Natl. Inst. of Biomed. Innovation, Health & Nutrition)、Yasuhiko Nishioka (Dept. of Respiratory Med. & Rheumatology, Tokushima Univ.)、Yasuo Miyoshi (Dept. of Surg., Hyogo College of Med.)、Mitsunori Sasa (Dept. of Surg., Tokushima Breast Care Clin.)、Toyomasa Katagiri (Natl. Inst. of Biomed. Innovation, Health & Nutrition)

Triple-negative breast cancer (TNBC), lacking hormone receptors and HER2, remains a major clinical challenge due to its aggressive nature, high recurrence rates, and limited treatment options. Our previous studies identified the BIG3-PHB2 complex as a key modulator of estrogen/ER$\alpha$ signaling in breast cancer cells. To target this pathway, we developed stERAP, a stapled cell-permeable peptide inhibitor that specifically disrupts the BIG3-PHB2 interaction, exhibiting potent anti-tumor effects. Analysis of TCGA data revealed frequent BIG3 upregulation in TNBC, correlating with poor prognosis. Inhibition of the BIG3-PHB2 complex by stERAP induced apoptosis and significantly suppressed TNBC cell proliferation in vitro and in vivo. More importantly, we found that the BIG3-PHB2 complex is localized in mitochondria in all TNBC cell lines examined. Treatment with stERAP led to elongation of mitochondrial morphology, suggesting that the BIG3-PHB2 complex plays a role in mitochondrial dynamics and maintenance in TNBC cells. These findings indicate that the mitochondrial BIG3-PHB2 complex functions as a critical modulator in TNBC and represents a promising therapeutic target.

[Disclosure of COI by Responsible researcher] Lead presenter is not the Responsible researcher.
[Lead presenter: Ulziisaikhan Batmunkh] We have no financial relationships to disclose.
[Principal Researcher: Ulziisaikhan Batmunkh, Yosuke Matsushita] ■Research funds from company: Oncotherapy Science Co., Ltd. ■Advisor of the company and/or profit-making organization: Oncotherapy Science Co., Ltd. ■Contributions: Tokushima Breast Care Clinic

▲TOP

Poster Session

### [P-3344] II-P6-2
### Tumor Immunity & Molecular Basis of Treatment Response in Breast Cancer

2025 / 9 / 27 (Sat) 15:30-16:15 Room P/(Motenashi Dome) Room P

---

### P-3344
### The role of splicing variant Rac1/Rac1b in triple negative breast cancer

[Speaker]Akifumi Mayama (Dept. Pathol. & Histotech. Tohoku Univ. Grad Sch. Med.／Dept. Clin.Lab.Med. Tohoku Univ. Hosp.) [Co-author]Kiyoshi Takagi (Dept. Pathol. & Histotech. Tohoku Univ. Grad Sch. Med.)、Mio Tanaka (Dept. Pathol. & Histotech. Tohoku Univ. Grad Sch. Med.)、Ai Sato (Dept. Pathol. & Histotech. Tohoku Univ. Grad Sch. Med.)、Yasuhiro Miki (Dept. Anatomic Pathol. Tohoku Univ. Grad Sch. Med.)、Shingo Sugawara (Dept. Clin.Lab.Med. Tohoku Univ. Hosp.)、Minoru Miyashita (Dept. Breast & Endocrine Surg. Oncol. Tohoku Univ. Grad Sch. Med.)、Takashi Suzuki (Dept. Pathol. Tohoku Univ. Hosp.)

RAS-related C3 botulinus toxin substrate 1 (Rac1) is a Rho subfamily small GTPase and Rac1b is one of the selective splice variants of Rac1, in which 19 amino acids (exon3b) are inserted. Rac1b has been considered constitutively active and .to promote tumor growth, invasion, and metastasis in vitro, but its association with clinicopathological factors in breast cancer remains unclear. In this study, we immunolocalized Rac1b in triple-negative breast cancer (TNBC) tissues and correlated them with clinicopathological characteristics. Rac1 immunoreactivity was detected in cytoplasm in TNBC cells and 43 cases (37%) were positive. Rac1b immunoreactivity was detected in cell membrane or cytoplasm in TNBC cells and 24 cases (21%) were positive. Rac1b immunoreactivity was positively correlated with Rac1 immunoreactivity, and it was significantly associated with shorter disease-free survival. These data suggested that Rac1b could be a potent poor prognostic factor in TNBC. Additionally, we established droplet digital PCR to specifically detect Rac1b from formalin-fixed paraffin-embedded samples.

[Disclosure of COI by Responsible researcher] Lead presenter is not the Responsible researcher.
[Lead presenter: Akifumi Mayama] We have no financial relationships to disclose.
[Principal Researcher: Takashi Suzuki] We have no financial relationships to disclose.

▲TOP

### [P-3345] II-P6-2
### Tumor Immunity & Molecular Basis of Treatment Response in Breast Cancer

2025 / 9 / 27 (Sat) 15:30-16:15 Room P/(Motenashi Dome) Room P

---

### P-3345
### The Relationship Between Hormone Levels, Lymphocyte Subsets, and Pathological Features in Triple-Negative Breast Cancer

[Speaker]Asumi Yamazaki (Dept. of Breast & Endocrine Surg.Oncology, Grad. Sch. of Med.,Tohoku Univ.／Dept. of Anatomic Pathol., Grad. Sch. of Med.,Tohoku Univ.) [Co-author]Yasuhiro Miki (Dept. of Anatomic Pathol., Grad. Sch. of Med.,Tohoku Univ.)、Erina Iwabuchi (Tokyo Univ. of Science, Dept. of Morphol. & Info. Analysis)、Chihiro Inoue (Dept. of Anatomic Pathol., Grad. Sch. of Med.,Tohoku Univ.)、Akiko Ebata (Dept. of Breast & Endocrine Surg.Oncology, Grad. Sch. of Med.,Tohoku Univ.)、Narumi(Tohoku Univ.) Harada (Dept. of Breast & Endocrine Surg.Oncology, Grad. Sch. of Med.,Tohoku Univ.)、Yohei Hamanaka (Dept. of Breast & Endocrine Surg.Oncology, Grad. Sch. of Med.,Tohoku Univ.)、Minoru Miyashita (Dept. of Breast & Endocrine Surg.Oncology, Grad. Sch. of Med.,Tohoku Univ.)、Hiroshi Tada (Dept. of Breast & Endocrine Surg.Oncology, Grad. Sch. of Med.,Tohoku Univ.)、Miku Sato (Dept. of Breast & Endocrine Surg.Oncology, Grad. Sch. of Med.,Tohoku Univ.)、Takashi Suzuki (Dept. of Anatomic Pathol., Grad. Sch. of Med.,Tohoku Univ.)

Background: Triple-negative breast cancer (TNBC) has a poor prognosis. Expression of estrogen receptor β (ERβ), androgen receptor (AR), and glucocorticoid receptor (GR) has been reported, suggesting steroid hormone involvement. However, the relationship between intratumoral hormone levels and immune cells remains unclear.Objective: To examine associations between concentrations of steroid hormones (estradiol, estrone, testosterone, dihydrotestosterone, cortisol, progesterone), T lymphocyte subsets (CD3, CD4, CD8), and pathological features in TNBC.Methods: We analyzed 62 TNBC patients who underwent surgery at Tohoku University Hospital from 2016 to 2023. Hormone levels were measured using LC-MS/MS. CD3, CD4, and CD8-positive lymphocytes were detected by immunohistochemistry and quantified via QuPath.Results: Estradiol and estrone showed negative correlations with CD4 and CD8-positive T cells and were elevated in early-stage tumors. Cortisol was increased in larger and more aggressive tumors.Conclusion: Estrogen may suppress T cell infiltration via ERβ signaling in TNBC. Cortisol could promote immune evasion through GR in advanced cases. Further in vitro studies are underway.

[Disclosure of COI by Responsible researcher] Lead presenter is the Responsible researcher.
[Lead presenter: Asumi Yamazaki] We have no financial relationships to disclose.

### [P-3346] II-P6-2
### Tumor Immunity & Molecular Basis of Treatment Response in Breast Cancer

2025 / 9 / 27 (Sat) 15:30-16:15 Room P/(Motenashi Dome) Room P

### P-3346
### Functional analysis and drug discovery on RHBDL2, an intramembrane serine protease in TNBC

[Speaker]Yosuke Matsushita (Lab. Biofunc. Mol. Med., NIBN／Div. Genome Med., Inst. Advanced Med. Sci., Tokushima Univ.) [Co-author]Kazumasa Okumura (Div. Genome Med., Inst. Advanced Med. Sci., Tokushima Univ.)、Masato Komatsu (Div. Genome Med., Inst. Advanced Med. Sci., Tokushima Univ.)、Tetsuro Yoshimaru (Lab. Biofunc. Mol. Med., NIBN／Div. Genome Med., Inst. Advanced Med. Sci., Tokushima Univ.)、Masaya Ono (Dept. Clin. Proteomics, Natl. Cancer Ctr. Res. Inst.)、Yasuo Miyoshi (Dept. Breast and Endocrine Surg., Hyogo College Med.)、Mitsunori Sasa (Dept. Surg., Tokushima Breast Care Clinic.)、Toyomasa Katagiri (Lab. Biofunc. Mol. Med., NIBN／Div. Genome Med., Inst. Advanced Med. Sci., Tokushima Univ.)

Cancer cells exploit distinct metabolic pathways to support abnormal proliferation and therapeutic resistance. In particular, glutaminolysis-driven anabolism plays a crucial role in cancer malignancy. Elucidating the molecular mechanisms underlying this process is essential for innovative drug discovery. We have identified RHBDL2 as a key regulator of glutamine metabolism in TNBC. RHBDL2 controls the stability and plasma membrane localization of glutamine transporter SLC1A5 (ASCT2), thereby promoting cell proliferation. Moreover, RHBDL2 localized in ER contributes to the maintenance of ASCT2 N-glycosylation through regulation of specific glycosyltransferases. RHBDL2 also forms complexes with other transporters involved in glutaminolysis. These findings highlight RHBDL2 as a central node in the metabolic network driving malignancy and drug resistance. To translate these insights into therapeutic strategies, we are currently conducting screening to identify lead monoclonal antibodies and antisense oligonucleotides targeting RHBDL2. Our findings strongly suggest that RHBDL2 represents a promising drug target for overcoming chemoresistance through modulation of glutamine metabolism.

[Disclosure of COI by Responsible researcher] Lead presenter is not the Responsible researcher.
[Lead presenter: Yosuke Matsushita] ■Research funds from company: 橋渡しシーズA、基盤研究C
[Principal Researcher: Toyomasa Katagiri] ■Research funds from company: ムーンショット型研究開発制度　■Advisor of the company and/or profit-making organization: オンコセラピー・サイエンス株式会社、株式会社Cancer Precision Medicine　■Research expenses: オンコセラピー・サイエンス株式会社　■Contributions: とくしまブレストケアクリニック

### [P-3354] II-P6-4
### Molecular Mechanisms of Breast Cancer Progression & Emerging Therapeutic Targets

2025 / 9 / 27 (Sat) 15:30-16:15 Room P/(Motenashi Dome) Room P

---

### P-3354
### Biological characteristics of breast cancer subtypes based on tumor-infiltrating lymphocyte subclass and RNA Analysis

[Speaker]Kyoko Asai (Saitama Med. Univ. Intl. Med. Ctr., Dept. Breast Oncology) [Co-author]Masataka Hirasaki (Saitama Med. Univ. Intl. Med. Ctr., Dept. Clinical Cancer Genomics)、Masahiro Ohara (Saitama Med. Univ. Intl. Med. Ctr., Dept. Breast Oncology)、Taeko Kurosaki (Saitama Med. Univ. Intl. Med. Ctr., Dept. Breast Oncology)、Ayako Nakame (Saitama Med. Univ. Intl. Med. Ctr., Dept. Breast Oncology)、Akihiro Fujimoto (Saitama Med. Univ. Intl. Med. Ctr., Dept. Breast Oncology)、Yuki Ichinose (Saitama Med. Univ. Intl. Med. Ctr., Dept. Breast Oncology)、Asami Nukui (Saitama Med. Univ. Intl. Med. Ctr., Dept. Breast Oncology)、Aya Asano (Saitama Med. Univ. Hosp., Dept. Breast Oncology)、Hiroko Shimada (Saitama Med. Univ. Intl. Med. Ctr., Dept. Breast Oncology)、Kazuo Matsuura (Saitama Med. Univ. Intl. Med. Ctr., Dept. Breast Oncology)、Hiroshi Ishiguro (Saitama Med. Univ. Intl. Med. Ctr., Dept. Breast Oncology)、Takahiro Hasebe (Saitama Med. Univ. Intl. Med. Ctr., Dept. Breast Oncology)、Akihiko Osaki (Saitama Med. Univ. Intl. Med. Ctr., Dept. Breast Oncology)、Toshiaki Saeki (Saitama Med. Univ. Intl. Med. Ctr., Dept. Breast Oncology)

Breast cancer is biologically classified based on hormone receptor (HR) and epidermal growth factor receptor type 2 (HER2) as HR+HER2-, HR+HER2+, HR-HER2+, or HR-HER2-. Tumor-infiltrating lymphocytes (TILs), central to tumor-immune interactions, include $CD8^+$ T cells, $CD4^+$ T cells and $CD20^+$ B cells etc. TILs were evaluated in 855 invasive ductal breast cancer cases using hematoxylin and eosin-stained sections. Additionally, 44 samples were analyzed for TIL subpopulations by immunohistochemistry, and 20 underwent RNA-Seq for gene expression analysis. In HR-HER2+ cases, a higher percentage of TILs was associated with better prognosis, while HR+HER2+ cases showed a poorer prognosis with more TILs. The HR+HER2+ group also had poorer prognosis with higher $CD8^+$ T cell percentages and better prognosis with more B cells. RNA-seq analysis revealed that the most significant expression changes in the poor prognosis HR+HER2+ group were immune-related, with EBV infection most strongly associated in KEGG pathway analysis. This suggests that in the less immunogenic HR+HER2+ subtype, $CD8^+$ T cells target infected cells, while B cells target viral antigens, not neoantigens.

[Disclosure of COI by Responsible researcher] Lead presenter is not the Responsible researcher.
[Lead presenter: Kyoko Asai] We have no financial relationships to disclose.
[Principal Researcher: Takahiro Hasebe] We have no financial relationships to disclose.

▲TOP

Poster Session

### [P-3355] II-P6-4
### Molecular Mechanisms of Breast Cancer Progression & Emerging Therapeutic Targets

2025 / 9 / 27 (Sat) 15:30-16:15 Room P/(Motenashi Dome) Room P

---

### P-3355
### Lysophosphatidic acid receptor 1 (LPAR1) is a poor prognostic factor associated with breast cancer progression

[Speaker]Yuka Koga (Dept. Pathol. & Histotech. Tohoku Univ. Grad Sch. Med.) [Co-author]Ai Sato (Dept. Pathol. & Histotech. Tohoku Univ. Grad Sch. Med.)、Kiyoshi Takagi (Dept. Pathol. & Histotech. Tohoku Univ. Grad Sch. Med.)、Mio Tanaka (Dept. Pathol. & Histotech. Tohoku Univ. Grad Sch. Med.)、Yasuhiro Miki (Dept. Anatomic Pathol. Tohoku Univ. Grad Sch. Med.)、Minoru Miyashita (Dept. Breast & Endocrine Surg. Oncol. Tohoku Univ. Grad Sch. Med.)、Takashi Suzuki (Dept. Pathol. & Histotech. Tohoku Univ. Grad Sch. Med.／Dept. Anatomic Pathol. Tohoku Univ. Grad Sch. Med.／Dept. Pathol. Tohoku Univ. Hosp.)

Lysophosphatidic acid (LPA) is a simple lipid consisting of a single fatty acyl chain attached to a glycerophosphate backbone. LPA has been reported to promote proliferation and invasion in ovarian cancer. By interacting with six G protein-coupled receptors (LPAR1-6), it exerts multifaceted biological activities. Among these receptors, LPAR1 has been postulated to be associated with cancer, inflammation, and tissue fibrosis. However, the clinical relevance of LPAR1 in breast cancer (BC) remains largely unknown. Therefore, we immunolocalized LPAR1 in 116 BC tissues to assess the role of LPAR1 in BC progression.LPAR1 immunoreactivity was detected in the plasma membrane of BC cells, with 28% of patients classified as positive. Immunohistochemical analysis showed that LPAR1 was positively correlated with menopause, pathological T factor, lymph node metastasis, and stage, while negatively correlated with estrogen receptor. LPAR1 was significantly associated with shorter disease-free survival. Knockdown of LPAR1 significantly inhibited cell proliferation in human BC cell lines. These data suggest that LPAR1 could be a poor prognostic factor associated cell proliferation in BC.

[Disclosure of COI by Responsible researcher] Lead presenter is not the Responsible researcher.
[Lead presenter: Yuka Koga] We have no financial relationships to disclose.
[Principal Researcher: Takashi Suzuki] We have no financial relationships to disclose.

### [P-3356] II-P6-4
### Molecular Mechanisms of Breast Cancer Progression & Emerging Therapeutic Targets

2025 / 9 / 27 (Sat) 15:30-16:15 Room P/(Motenashi Dome) Room P

---

### P-3356
### Kallikrein Related Peptidase 11 as a potent prognostic factor in human breast cancer related to chemotherapy resistance

[Speaker]Ai Sato (Dept. Pathol. & Histotech. Tohoku Univ. Grad Sch. Med.) [Co-author]Kiyoshi Takagi (Dept. Pathol. & Histotech. Tohoku Univ. Grad Sch. Med.)、Mio Tanaka (Dept. Pathol. & Histotech. Tohoku Univ. Grad Sch. Med.)、Hana Yamanaka (Dept. Pathol. & Histotech. Tohoku Univ. Grad Sch. Med.)、Yasuhiro Miki (Dept. Anatomic Pathol. Tohoku Univ. Grad Sch. Med.)、Minoru Miyashita (Dept. Breast & Endocrine Surg. Oncol. Tohoku Univ. Grad Sch. Med.)、Takashi Suzuki (Dept. Pathol. & Histotech. Tohoku Univ. Grad Sch. Med.／Dept. Anatomic Pathol. Tohoku Univ. Grad Sch. Med.／Dept. Pathol. Tohoku Univ. Hosp.)

The human kallikrein (KLK) family, a group of secreted serine endopeptidases consisting of 15 members, is involved in a variety of physiological processes and plays important roles in various cancers both positively and negatively. KLK11 has been reported to be involved in the progression of ovarian cancer, non-small cell lung cancer, etc. In breast cancer, KLK11 is reported to contribute to the cancer progression through IGFBP-3 degradation. However, the clinical relevance of KLK11 in breast cancer remains largely unknown. Therefore, we performed immunohistochemistry on 116 breast cancer tissues to assess the relationship between KLK11 expression and clinicopathological factors.KLK11 was detected in the cytoplasm of cancer cells, and its immunoreactivity was positively correlated with pT, lymph node metastasis, stage and Ki67. It was significantly associated with shorter disease-free survival (DFS) and breast cancer-specific survival. Interestingly, it was also significantly associated with shorter DFS in the chemotherapy received group.These data suggested that KLK11 could be a potent poor prognostic factor in breast cancer partly associated with chemotherapy resistance.

[Disclosure of COI by Responsible researcher] Lead presenter is not the Responsible researcher.
[Lead presenter: Ai Sato] We have no financial relationships to disclose.
[Principal Researcher: Takashi Suzuki] We have no financial relationships to disclose.

▲TOP

### [P-3357] II-P6-4
### Molecular Mechanisms of Breast Cancer Progression & Emerging Therapeutic Targets

2025 / 9 / 27 (Sat) 15:30-16:15 Room P/(Motenashi Dome) Room P

---

### P-3357
### Spatial Transcriptomic Characterization of the Tumor Microenvironment in Clinically Sequenced Breast Cancer Cases

[Speaker]Mariko Kikuchi (Dept. Prev. Med., Grad. Sch. of Med., The Univ. Tokyo／Dept. Breast Surg., Grad. Sch. Med., The Univ. Tokyo) [Co-author]Miwako Kakiuchi (Dept. Prev. Med., Grad. Sch. of Med., The Univ. Tokyo)、Hirotaka Inoue (Dept. Prev. Med., Grad. Sch. of Med., The Univ. Tokyo／Dept. Neurosurg., Kumamoto Univ.)、Ayumu Tsubosaka (Dept. Prev. Med., Grad. Sch. of Med., The Univ. Tokyo)、Kyohei Sano (Dept. Prev. Med., Grad. Sch. of Med., The Univ. Tokyo)、Daisuke Komura (Dept. Prev. Med., Grad. Sch. of Med., The Univ. Tokyo)、Hiroto Katoh (Div. Path., NCC Exploratory Oncol. Res. & Clin. Trial Ctr.)、Arisa Morizono (Dept. Breast Surg., Grad. Sch. Med., The Univ. Tokyo)、Katsutoshi Oda (Dept. Clin. Genomics, The Univ. Tokyo Hosp.)、Tanabe Masahiko (Dept. Breast Surg., Grad. Sch. Med., The Univ. Tokyo)、Yoshifumi Baba (Dept. Gastroesophageal Surg., Grad. Sch. Med., The Univ. Tokyo)、Shumpei Ishikawa (Dept. Prev. Med., Grad. Sch. of Med., The Univ. Tokyo／ Div. Path., NCC Exploratory Oncol. Res. & Clin. Trial Ctr.)

Breast cancer is a genetically heterogeneous disease, defined by diverse genetic alterations and molecular subtypes that differ in prognosis and treatment response. Germline ***BRCA1/2*** mutations are found in approximately 7–8% of cases. However, their associations with tumor histology, molecular subtype, and incomplete penetrance remain unclear and may be influenced by the tumor microenvironment (TME). To gain a more comprehensive understanding of the tumor ecosystem, it is essential to integrate genetic mutation data with spatial transcriptomic analysis of the TME.We performed spatial transcriptomic profiling on FFPE breast cancer tissues from eight cases harboring pathogenic ***BRCA1/2*** variants or relevant family histories. These samples were submitted to GenMineTOP (Todai OncoPanel; TOP), a dual DNA/RNA panel targeting 1,389 genes. Spatial analysis was conducted using the CosMx SMI platform with a 1000-gene panel. Remarkably, even small DCIS lesions exhibited transcriptional heterogeneity, including variation in the expression of known prognostic genes. These findings prompted further investigation into TME features that may drive the emergence of distinct tumor cell populations.

[Disclosure of COI by Responsible researcher] Lead presenter is not the Responsible researcher.
[Lead presenter: Mariko Kikuchi] We have no financial relationships to disclose.
[Principal Researcher: Yoshifumi Baba] ■Research funds from company: 日本医療研究開発機構(AMED)　■Advisor of the company and/or profit-making organization: 新日本科学株式会社　■Research expenses: Chordia therapeutics株式会社、大鵬薬品工業株式会社、ロート製薬株式会社、アステラス製薬株式会社

### [P-3358] II-P6-4
### Molecular Mechanisms of Breast Cancer Progression & Emerging Therapeutic Targets

2025 / 9 / 27 (Sat) 15:30-16:15 Room P/(Motenashi Dome) Room P

---

### P-3358
### Analyses of the reactive oxygen species reduction abilities of gamma-glutamylcyclotransferase (GGCT) variants.

[Speaker]Raito Sugiura (Dep.Biochem.,Fujita Haelth Univ.Sch.Med) [Co-author]Kenei Yamasaki (Dep.Biochem.,Fujita Haelth Univ.Sch.Med)、Fujiko Sueishi (Dep.Biochem.,Fujita Haelth Univ.Sch.Med)、Takanori Hayashi (Dep.Biochem.,Fujita Haelth Univ.Sch.Med)、Yohei Shimono (Dep.Biochem.,Fujita Haelth Univ.Sch.Med)

Gamma-glutamylcyclotransferase (GGCT) is an enzyme involved in the gamma-glutamyl cycle, that produces glutathione and reduces reactive oxygen species (ROS) level. We have previously shown that GGCT is a cancer stem cell gene in breast cancers. Although higher ability to remove ROS has been associated with cancer stemness, GGCT variant 1 (GGCTv1) is the only GGCT variant with a catalytic domain for glutathione production and ROS removal. In this study, we measured the ROS removal activities of GGCT variants and its mutant. DCFDA assay showed that overexpression of GGCTv1 reduced, and its knockdown increased intracellular ROS levels in breast cancer cells. Accordingly, the mutation of the catalytic domain of GGCTv1 abolished its ability to remove intracellular ROS. In contrast, the overexpression and knockdown of GGCTv2, which lacks the catalytic domain for glutathione production, did not affect intracellular ROS levels. These results suggest that GGCTv1 is the only variant of GGCT that promotes cancer stemness through its ROS removal ability.

[Disclosure of COI by Responsible researcher] Lead presenter is not the Responsible researcher.
[Lead presenter: Raito Sugiura] We have no financial relationships to disclose.
[Principal Researcher: Yohei Shimono] We have no financial relationships to disclose.

### [P-3359] II-P6-4
### Molecular Mechanisms of Breast Cancer Progression & Emerging Therapeutic Targets

2025 / 9 / 27 (Sat) 15:30-16:15 Room P/(Motenashi Dome) Room P

---

### P-3359
### Aging and obesity contribute to breast cancer progression

[Speaker]Yuna Ogawa (Dept. of Med. Sci., Med. and Health Sci., Kanazawa Univ.／Div. of Cancer Cell Biol., Cancer Res. Inst., Kanazawa Univ.)
[Co-author]Tsunaki Hongu (Div. of Cancer Cell Biol., Cancer Res. Inst., Kanazawa Univ.)、Haruna Tani (Ctr. for Environmental Response and Aging, IDAC, Tohoku Univ.)、Shohei Murakami (Ctr. for Environmental Response and Aging, IDAC, Tohoku Univ.)、Wei Fanyan (Ctr. for Environmental Response and Aging, IDAC, Tohoku Univ.)、Kozo Tanaka (Dept. of Mol. Oncology, IDAC, Tohoku Univ.)、Noriko Goto (Div. of Cancer Cell Biol., Cancer Res. Inst., Kanazawa Univ.)

Accumulating evidence suggests that aging and/or obesity influence tumorigenesis in various tissues, including the breast. However, how the tissue microenvironment altered by aging or obesity affects tumorigenesis remains largely unknown. In this study, we examined the effects of aging and obesity on the tumorigenesis and metastasis of breast cancer cells ***in vivo***. EO771 mouse breast carcinoma cells were transplanted into the mammary fat pad of aged mice or mice fed a high-fat diet, and tumor growth and lung metastasis were evaluated. The results showed that aged mice exhibited a marked increase in both tumorigenesis and lung metastasis, whereas obese mice showed a moderate increase in tumor formation and metastasis. These findings suggest that both aging and obesity contribute to the progression of breast cancer.

[Disclosure of COI by Responsible researcher] Lead presenter is not the Responsible researcher.
[Lead presenter: Yuna Ogawa] We have no financial relationships to disclose.
[Principal Researcher: Noriko Goto] We have no financial relationships to disclose.

▲TOP

### [P-3360] II-P6-4
### Molecular Mechanisms of Breast Cancer Progression & Emerging Therapeutic Targets

2025 / 9 / 27 (Sat) 15:30-16:15 Room P/(Motenashi Dome) Room P

---

### P-3360
### Analyses of the catalytic activity-independent promotion of cancer stemness by gamma-glutamylcyclotransferase

[Speaker]Fujiko Sueishi (Dep.Biochem.,Fujita Health Univ.Sch.Med) [Co-author]Takanori Hayashi (Dep.Biochem.,Fujita Health Univ.Sch.Med)、Yasuyoshi Mizutani (Dep.Mol.Oncology, Fujita Health Univ.Sch.Med.)、Motoshi Suzuki (Dep.Mol.Oncology, Fujita Health Univ.Sch.Med.)、Yohei Shimono (Dep.Biochem.,Fujita Health Univ.Sch.Med)

Gamma-glutamylcyclotransferase (GGCT) is an enzyme involved in the gamma-glutamyl cycle, that produces glutathione and reduces reactive oxygen species (ROS), and its higher expression is associated with poorer prognosis in breast cancer patients. We have previously shown that GGCT promotes tumor sphere formation ability of breast cancer cells and the patient-derived tumor xenograft (PDX) cells. However,, most of the variants of GGCT lack the catalytic domain for gamma-glutamylcyclotransferase. In this study, we investigated whether GGCTv2, a second variant of GGCT, is able to enhance cancer stem cell properties of breast cancer cells. Overexpression of GGCTv2 enhanced the tumor sphere formation of MCF7 and T47D breast cancer cells; however, its knockdown did not suppress that of MCF7 and breast cancer PDX cells. These results suggest that the catalytic activity-independent promotion of cancer stemness by cancer stem cell gene GGCT may exist, but it is highly cell-type dependent in breast cancer cells.

[Disclosure of COI by Responsible researcher] Lead presenter is not the Responsible researcher.
[Lead presenter: Fujiko Sueishi] We have no financial relationships to disclose.
[Principal Researcher: Yohei Shimono] We have no financial relationships to disclose.

### [P-3369] II-P7-2
### Ovarian Cancer: Molecular Mechanisms & Therapy

2025 / 9 / 27 (Sat) 15:30-16:15 Room P/(Motenashi Dome) Room P

---

### P-3369
### Clinical impact and the potential therapeutic target of HIF2A in Ovarian clear cell carcinoma

[Speaker]Mengxin Jiang (Dept. of Gynecol. & Obstetrics, Grad. of Medicine,Kyoto University) [Co-author]Ken(Kyoto Univ.) Yamaguchi (Dept. of Gynecol. & Obstetrics, Grad. of Medicine,Kyoto University)、Taito Miyamoto (Dept. of Gynecol. & Obstetrics, Grad. of Medicine,Kyoto University)、Rin Mizuno (Dept. of Gynecol. & Obstetrics, Grad. of Medicine,Kyoto University)、Mana Taki (Dept. of Gynecol. & Obstetrics, Grad. of Medicine,Kyoto University)、Koji Yamanoi (Dept. of Gynecol. & Obstetrics, Grad. of Medicine,Kyoto University)、Ryusuke Murakami (Dept. of Gynecol. & Obstetrics, Grad. of Medicine,Kyoto University)、Junzo Hamanishi (Dept. of Gynecol. & Obstetrics, Grad. of Medicine,Kyoto University)、Masaki Mandai (Dept. of Gynecol. & Obstetrics, Grad. of Medicine,Kyoto University)

Background: Ovarian clear cell carcinoma (OCCC) shows poor prognosis and is associated with hypoxia, characterized by high expression of HIF1A and HIF2A. This study aimed to clarify the role of HIF2A in OCCC. Methods: HIF2A nuclear expression was assessed by immunohistochemistry in OCCC cases from our institution, and its correlation with patient survival was analyzed. Functional analysis was performed by inhibiting HIF2A, including in vitro assessment of mitochondrial respiration and ROS production. In vivo therapeutic experiments were performed using cell line-derived xenograft (CDX) and patient-derived xenograft (PDX) models. Results: Log Rank test identified strong HIF2A nuclear expression as a poor prognostic factor for OCCC. In vitro functional analyses demonstrated that HIF2A suppression increased ATP production and ROS production, suggesting that suppression of HIF2A enhanced the mitochondrial activity. Treatment with the HIF2A inhibitor significantly suppressed tumor growth in both CDX and PDX models. Conclusion: High HIF2A nuclear expression is associated with poor prognosis in OCCC. Inhibition of HIF2A could represent a potential targeted therapeutic strategy for OCCC.

[Disclosure of COI by Responsible researcher] Lead presenter is not the Responsible researcher.
[Lead presenter: Mengxin Jiang] We have no financial relationships to disclose.
[Principal Researcher: Ken Yamaguchi] We have no financial relationships to disclose.

### [P-3370] II-P7-2
### Ovarian Cancer: Molecular Mechanisms & Therapy

2025 / 9 / 27 (Sat) 15:30-16:15 Room P/(Motenashi Dome) Room P

---

### P-3370
### Association of cell adhesion related gene expression with chemoresistance in ascites derived spheroids of ovarian cancer

[Speaker]Sae Hayakawa (Obstetrics and Gynecology, Nagoya University Graduate School of Medicine) [Co-author]Masato Yoshihara (Obstetrics and Gynecology, Nagoya University Graduate School of Medicine)、Shohei Iyoshi (Obstetrics and Gynecology, Nagoya University Graduate School of Medicine)、Kazumasa Mogi (Obstetrics and Gynecology, Nagoya University Graduate School of Medicine)、Emiri Miyamoto (Obstetrics and Gynecology, Nagoya University Graduate School of Medicine)、Hiroki Fujimoto (Obstetrics and Gynecology, Nagoya University Graduate School of Medicine)、Kaname Uno (Obstetrics and Gynecology, Nagoya University Graduate School of Medicine)、Kazuhisa Kitami (Obstetrics and Gynecology, Nagoya University Graduate School of Medicine)、Satoshi Nomura (Obstetrics and Gynecology, Nagoya University Graduate School of Medicine)、Yoshihiro Koya (Obstetrics and Gynecology, Nagoya University Graduate School of Medicine)、Marina Yoshikawa (Obstetrics and Gynecology, Nagoya University Graduate School of Medicine)、Atsushi Kunishima (Obstetrics and Gynecology, Nagoya University Graduate School of Medicine)、Maia Hayashi (Obstetrics and Gynecology, Nagoya University Graduate School of Medicine)、Hiroaki Kajiyama (Obstetrics and Gynecology, Nagoya University Graduate School of Medicine)

Ovarian cancer has the highest mortality among gynecologic cancers. In advanced stages, positive ascitic cytology is an independent prognostic factor. Most tumor cells in ascites form heterogeneous metastatic spheroids, not single cells. These spheroids often persist after chemotherapy and are linked to poor prognosis. This study aimed to examine the impact of chemotherapy on spheroids using two ovarian cancer cell lines (OV90 and A2780) and their cisplatin-resistant variants (OV90 CR and A2780 CR). Cell survival after cisplatin exposure confirmed drug resistance in CR lines. RNA sequencing revealed upregulation of extracellular and cell adhesion-related genes, including COL2A1 and COL5A1, in resistant cells. Time-lapse imaging showed denser aggregation in CR spheroids. These findings suggest that collagen gene expression may contribute to spheroid cohesion and chemoresistance and may serve as future therapeutic targets.

[Disclosure of COI by Responsible researcher] Lead presenter is not the Responsible researcher.
[Lead presenter: Sae Hayakawa] ■Research funds from company: 科研費
[Principal Researcher: Masato Yoshihara] ■Research funds from company: 科研費

### [P-3371] II-P7-2
### Ovarian Cancer: Molecular Mechanisms & Therapy

2025 / 9 / 27 (Sat) 15:30-16:15 Room P/(Motenashi Dome) Room P

---

### P-3371
### CRISPR-Cas9 screen identifies DHX38/PRP16 as an essential splicing factor for ovarian clear cell carcinoma tumorigenesis

[Speaker]Tomoatsu Hayashi (Inst. for Quant. Biosci., The Univ. of Tokyo) [Co-author]Brandon Cona (Inst. for Quant. Biosci., The Univ. of Tokyo)、Yoko Akasu (Dept. of ObGyn, Jikei Univ. Sch. of Med.)、Ayako Kawabata (Dept. of ObGyn, Jikei Univ. Sch. of Med.)、Aikou Okamoto (Dept. of ObGyn, Jikei Univ. Sch. of Med.)、Tetsu Akiyama (Inst. for Quant. Biosci., The Univ. of Tokyo)

Certain cancers, such as ovarian clear cell carcinoma (OCCC), exhibit high levels of interpatient genetic heterogeneity, posing a major challenge for the development of effective therapies. To identify novel genes essential for OCCC growth, we performed a comprehensive CRISPR-Cas9 knockout screen targeting cell proliferation, using both an OCCC cell line and a normal ovarian surface epithelium cell line. This screen identified DHX38/PRP16, an ATP-dependent RNA helicase involved in pre-mRNA splicing, as critical for OCCC cell growth and tumorigenesis. Knockdown of DHX38/PRP16 in OCCC cells, but not in normal cells, induced apoptosis and impaired tumor formation in a mouse model. These findings suggest that DHX38/PRP16 plays an important role in OCCC tumorigenesis and may represent a promising therapeutic target.

[Disclosure of COI by Responsible researcher] Lead presenter is not the Responsible researcher.
[Lead presenter: Tomoatsu Hayashi] We have no financial relationships to disclose.
[Principal Researcher: Tetsu Akiyama] We have no financial relationships to disclose.

### [P-3372] II-P7-2
### Ovarian Cancer: Molecular Mechanisms & Therapy

2025 / 9 / 27 (Sat) 15:30-16:15 Room P/(Motenashi Dome) Room P

---

### P-3372
### Exploring genes involved in anoikis resistancein ovarian cancer using the CRISPR/Cas9 libraries

[Speaker]Shiori Mori (Dept. Can. Biol., Inst. Biomed. Sci., Kansai Med. Univ.) [Co-author]Anh Vuduc (Dept. Can. Biol., Inst. Biomed. Sci., Kansai Med. Univ.)、Kanako Akamatsu (Dept. Can. Biol., Inst. Biomed. Sci., Kansai Med. Univ.)、Yoshiki Yasukochi (Dept. Genome. Analysis., Inst. Biomed. Sci., Kansai Med. Univ.)、Kouichiro Higasa (Dept. Genome. Analysis., Inst. Biomed. Sci., Kansai Med. Univ.)、Takeharu Sakamoto (Dept. Can. Biol., Inst. Biomed. Sci., Kansai Med. Univ.)

While advances in treatment have improved the overall long-term survival of ovarian cancer (OS) patients, the 5-year survival rate remains around 40% for those with advanced disease, such as peritoneal metastasis or recurrence. Therefore, elucidating the mechanisms of metastasis and developing new therapeutic strategies is urgently needed. Our previous studies demonstrated that KO of the anoikis-resistance gene X dramatically decreased peritoneal dissemination in human OC cells, suggesting that genes involved in anoikis-resistance may serve as promising therapeutic targets in OS. Based on these findings, we conducted a genome-wide CRISPR/Cas9 KO screen. Library was introduced into Cas9-expressing SKOV3 OS cells. Cells were divided into 2 experimental groups: floating and control conditions, and cultured for 2 days. Using the MAGeCK algorithm and RRA scoring, we identified ten negatively selected target genes. Consistent with these results, siRNA-mediated knockdown of the identified genes confirmed decreased anoikis resistance. We are currently validating these findings in both in vitro and in vivo models. Subsequently, we will explore additional targeted therapeutic approaches.

[Disclosure of COI by Responsible researcher] Lead presenter is not the Responsible researcher.
[Lead presenter: Shiori Mori] We have no financial relationships to disclose.
[Principal Researcher: Takeharu Sakamoto] We have no financial relationships to disclose.

### [P-3373] II-P7-2
### Ovarian Cancer: Molecular Mechanisms & Therapy

2025 / 9 / 27 (Sat) 15:30-16:15 Room P/(Motenashi Dome) Room P

---

### P-3373
### The molecular processes associated with the immune dynamics of macrophages in ovarian cancer

[Speaker]Mayuko Yamamoto (Dept. of Cell Path., Kumamoto Univ.／Dept. of Obstetrics and Gynecol., Kumamoto Univ.) [Co-author]Yukio Fujiwara (Dept. of Cell Path., Kumamoto Univ.)、Takeshi Motohara (Dept. of Obstetrics and Gynecol., Kumamoto Univ.)、Akiho Nishimura (Dept. of Obstetrics and Gynecol., Kumamoto Univ.)、Cheng Pan (Dept. of Cell Path., Kumamoto Univ.)、Eiji Kondoh (Dept. of Obstetrics and Gynecol., Kumamoto Univ.)、Yoshihiro Komohara (Dept. of Cell Path., Kumamoto Univ.)

Malignant ascites, a hallmark of ovarian cancer, contain both cancer cells and immune cells, including macrophages, which are thought to play critical roles in tumor progression. This study investigated the interactions between ovarian cancer cells and macrophages. Through single-cell analysis, we observed a significant accumulation of CD163-positive macrophages in malignant ascites, a finding further supported by histopathological analysis. Co-culture experiments with ovarian cancer cells and human monocyte-derived macrophages (HMDMs) revealed that HMDMs significantly promoted cancer cell proliferation. Similar results were obtained using mouse ovarian cancer cells co-cultured with peritoneal macrophages, where proliferation was more pronounced with wild-type (WT) macrophages compared to CD163 knockout (KO) macrophages. Furthermore, intraperitoneal injection of ovarian cancer cells into WT and CD163 KO mice demonstrated significantly prolonged survival in CD163 KO mice. In conclusion, CD163-positive macrophages promote ovarian cancer progression and may represent a promising therapeutic target.

[Disclosure of COI by Responsible researcher] Lead presenter is the Responsible researcher.
[Lead presenter: Mayuko Yamamoto] We have no financial relationships to disclose.

[P-3374] II-P7-2
### Ovarian Cancer: Molecular Mechanisms & Therapy

2025 / 9 / 27 (Sat) 15:30-16:15 Room P/(Motenashi Dome) Room P

P-3374
### Omental Tumor Microenvironment Abundant in IL-29 Is Crucial for Regression in Advanced Ovarian Cancer

[Speaker]Chang-Ni Lin (Dept. of Obstetrics and Gynecol., NCKUH)

Over 90% of ovarian cancer (OC) patients death related to metastasis, particularly to the omentum. The omental tumor microenvironment (TME) is a unique niche composed of tumor cells, immune cells, and adipocytes, which secrete cytokines and chemokines that modulate tumor-associated immunity. Among these, Interferons (IFNs) have been implicated in enhancing immunotherapy and in contributing to chemoresistance in OC. This study focuses on the role of IFN-λ1 (IL-29) within the omental TME of advanced OC. Analysis of 323 OC cases from TCGA showed that low IL-29 expression was significantly associated with poor prognosis. Our previous results showed that increased numbers of omental crown-like structure (CLS) correlate with worse outcome in advanced OC. The IL-29 level in ascites from patients with high CLS (N=4) were significantly lower than those in patients with low CLS (N=6; ***p***=0.0245). Similarly, IL-29 concentration in SKOV3/OA/THP-1 CM was markedly lower compared to IOSE398/OA/THP-1 CM (54±25 pg/ml vs. 191±28 pg/ml; ***p***=0.0659). Therefore, enhancing IL-29 in the omental TME may be a promising therapeutic strategy to promote regression in advanced OC.

[Disclosure of COI by Responsible researcher] Lead presenter is not the Responsible researcher.
[Lead presenter: Chang-Ni Lin] We have no financial relationships to disclose.
[Principal Researcher: ] We have no financial relationships to disclose.

### [P-3375] II-P7-2
### Ovarian Cancer: Molecular Mechanisms & Therapy

2025 / 9 / 27 (Sat) 15:30-16:15 Room P/(Motenashi Dome) Room P

---

### P-3375
### NGFR contributes to progression of ovarian cancer via promoting proliferation and invasion

[Speaker]Yoshihiro Koya (Dept. Ob. & Gynecol., Nagoya Univ., Grad. Sch. Med. ／R&D Section, Kishokai Med. Co.) [Co-author]Emiri Miyamoto (Dept. Ob. & Gynecol., Nagoya Univ., Grad. Sch. Med.)、Masato Yoshihara (Dept. Ob. & Gynecol., Nagoya Univ., Grad. Sch. Med.)、Shohei Iyoshi (Dept. Ob. & Gynecol., Nagoya Univ., Grad. Sch. Med.)、Kazumasa Mogi (Dept. Ob. & Gynecol., Nagoya Univ., Grad. Sch. Med. ／Dept. of Med. Genomics Center, Nagoya Univ. Hosp.)、Kaname Uno (Div. of Oncol, Dep. of Clinical Sciences, Lund Univ.)、Hiroki Fujimoto (Dept. Ob. & Gynecol., Aichi Med. Univ.)、Atsushi Kunishima (Dept. Ob. & Gynecol., Nagoya Univ., Grad. Sch. Med.)、Kazuhisa Kitami (Dept. of Gynecol. Oncol., Aichi Cancer Center)、Akihiro Nawa (R&D Section, Kishokai Med. Co.)、Hiroaki Kajiyama (Dept. Ob. & Gynecol., Nagoya Univ., Grad. Sch. Med.)

Nerve growth factor receptor (NGFR) is a cell surface molecule implicated in normal tissue function and various cancers, but its role in ovarian cancer remains unclear. This study investigated NGFR expression in ovarian cancer, its correlation with prognosis, and its involvement in migration, invasion, and proliferative potential. NGFR expression was evaluated in some clinical specimens and ovarian cancer cell lines, correlating with poor patient prognosis. High NGFR expression was associated with increased migration, invasion, and proliferation. siRNA-mediated NGFR knockdown significantly reduced these capabilities in vitro. In vivo, NGFR inhibition reduced peritoneal dissemination. RNA sequencing revealed several molecules potentially mediating the role of NGFR in ovarian cancer progression. NGFR contributes to the malignancy of ovarian cancer through its effects on migration, invasion, and proliferation, correlating with poor prognosis. These findings highlight NGFR as a potential therapeutic target, paving the way for future studies to explore its role in therapeutic targets for ovarian cancer.

[Disclosure of COI by Responsible researcher] Lead presenter is the Responsible researcher.
[Lead presenter: Yoshihiro Koya] ■ employment by a company related to the presentation: 医療法人葵鍾会

### [P-3376] II-P7-2
### Ovarian Cancer: Molecular Mechanisms & Therapy

2025 / 9 / 27 (Sat) 15:30-16:15 Room P/(Motenashi Dome) Room P

---

### P-3376
### Intraperitoneal Administration of DNase1-expressing AAV Vector and Its Effect on Ovarian Cancer Dissemination

[Speaker]Eri Suizu (Dept. of Obstetrics & Gynecology, Jichi Medical Univ.) [Co-author]Hideyuki Ohzawa (Dept. of Clin. Oncology, Jichi Medical Univ.)、Misaki Matsumiya (Dept. of Surg., Jichi Medical Univ.)、Rei Takahashi (Dept. of Surg., Jichi Medical Univ.)、Hirofumi Sonoda (Dept. of Surg., Jichi Medical Univ.)、Yasushi Saga (Dept. of Obstetrics & Gynecology, Jichi Medical Univ.)、Yuji Takei (Dept. of Obstetrics & Gynecology, Jichi Medical Univ.)、Hideyo Miyato (Dept. of Surg., Jichi Medical Univ.)、Hiroyuki Fujiwara (Dept. of Obstetrics & Gynecology, Jichi Medical Univ.)、Joji Kitayama (Dept. of Surg., Jichi Medical Univ.)

Objective: Neutrophil extracellular traps (NETs) are suggested to promote peritoneal dissemination in ovarian cancer. We examined whether DNase1 gene delivery to peritoneal tissues using an AAV vector can suppress dissemination. Methods: AAV-DNase1 was used to transduce ovarian cancer cells, and DNase1 expression and activity were assessed in culture supernatants after 72 hours. A syngeneic ovarian cancer model was created by injecting 5×10^6 ID8 cells intraperitoneally into C57BL/6 mice. AAV-DNase1 was then administered intraperitoneally, and dissemination was evaluated at 14 weeks. Results: DNase1 was secreted into the supernatant of transduced cells and effectively degraded NETs in a dose-dependent manner. At 14 weeks following injection, control mice developed numerous peritoneal nodules with a size of 1-2 mm on both sides of the peritoneum, while AAV-DNase1-treated mice tended to show a reduced number of nodules (control: 40.0±17.8, AAV-DNase: 16.5±23.3, p=0.23). Conclusion: AAV-mediated DNase1 delivery may inhibit ovarian cancer peritoneal dissemination via NET degradation, offering a potential new therapeutic strategy.

[Disclosure of COI by Responsible researcher] Lead presenter is the Responsible researcher.
[Lead presenter: Eri Suizu] We have no financial relationships to disclose.

▲TOP

[P-3010] I-P3-4
Inflammation & Cancer

2025 / 9 / 27 (Sat) 16:15-17:00 Room P/(Motenashi Dome) Room P

### P-3010
### Genetic dissection of tumor-suppressive tissue microenvironment in ***Drosophila***

[Speaker]Tomonori Nakanishi (Lab. of Genet, Grad. Sch. of Pharm. Sci., Kyoto Univ.) [Co-author]Masato Enomoto (Faculty of Med. Sci., Univ. of Fukui)、Tomoe Kobayashi (Shigei Med. Res. Inst. Div. of Mol. Genetics)、Makoto Matsuyama (Shigei Med. Res. Inst. Div. of Mol. Genetics)、Tatsushi Igaki (Lab. of Genetics, Grad. Sch. of Biostudies, Kyoto Univ.)

The frequency of cancer formation in a certain organ is largely different from region to region, depending on the cell type or tissue microenvironment. However, the molecular mechanism by which a certain cell type or tissue microenvironment makes differences in cancer sensitivity remains elusive. In ***Drosophila*** imaginal epithelium, oncogenic Ras-expressing cell clones with loss of tumor suppressor gene ***scrib*** (Ras$^{V12}$/***scrib***$^{-/-}$) massively overgrow and exhibit invasive behavior. Using this malignant tumor model, we analyzed the spatial pattern of tumor-proliferative activities in ***Drosophila*** wing imaginal epithelium. We found that tumor growth of Ras$^{V12}$/***scrib***$^{-/-}$ clones was significantly suppressed in a specific tissue region called "notum", while these mutant clones aggressively overgrew in other parts of the wing epithelium. Our genetic analyses showed that IL-6-JAK-STAT signaling pathway essential for driving overgrowth and invasion of Ras$^{V12}$/***scrib***$^{-/-}$ malignant tumors is downregulated specifically in the notum region. We will discuss the tumor-suppressive mechanism and its evolutionary conservation.

[Disclosure of COI by Responsible researcher] Lead presenter is the Responsible researcher.
[Lead presenter: Tomonori Nakanishi] We have no financial relationships to disclose.

### [P-3011] I-P3-4
### Inflammation & Cancer

2025 / 9 / 27 (Sat) 16:15-17:00 Room P/(Motenashi Dome) Room P

---

### P-3011
### Impact of systemic inflammation on immunotherapy efficacy in PD-L1 high advanced non-small cell lung cancer

[Speaker]Yusuke Kunimatsu (Kyoto Pref. Univ. of Med.) [Co-author]Tadaaki Yamada (Kyoto Pref. Univ. of Med.)、Yoshiki Negi (Hyogo Med. Univ.)、Motohiro Tamiya (Osaka International Cancer Inst.)、Keiko Tanimura (Kyoto Daini Hosp.)、Takayuki Takeda (Kyoto Daini Hosp.)、Yusuke Chihara (Uji Tokushukai Med. Ctr.)、Takashi Kijima (Hyogo Med. Univ.)、Koichi Takayama (Kyoto Pref. Univ. of Med.)

[Background] The standard treatment for advanced non-small cell lung cancer (NSCLC) with PD-L1 high is immune checkpoint inhibitor (ICI) monotherapy or combination immunotherapy. However, there are no clear criteria for treatment selection. We investigated relationship between systemic inflammation and the efficacy of ICI. [Methods] We used the systemic immune-inflammatory index (SII), which was calculated from blood data before treatment. We divided the patients into high and low inflammation population using SII, and retrospectively examined the treatment effects of pembrolizumab and combination immunotherapy. [Results] A total of 444 patients were enrolled, with 279 patients in the pembrolizumab group and 165 patients in the combination immunotherapy group. In the low inflammation population, progression-free survival (PFS) and overall survival (OS) were significantly prolonged in the combined immunotherapy group (median PFS: 9.8 months vs 16.0 months, p=0.03; median OS: 35.8 months vs Not reached, p=0.002). [Conclusion] Combination immunotherapy is promising for first-line treatment of PD-L1 high NSCLC with low systemic inflammation compared to pembrolizumab.

[Disclosure of COI by Responsible researcher] Lead presenter is not the Responsible researcher.
[Lead presenter: Yusuke Kunimatsu] We have no financial relationships to disclose.
[Principal Researcher: Tadaaki Yamada] ■Lecture fee: 中外製薬、日本イーライリリー ■Research expenses: 小野薬品工業、武田薬品工業

▲TOP

### [P-3012] I-P3-4
### Inflammation & Cancer

2025 / 9 / 27 (Sat) 16:15-17:00 Room P/(Motenashi Dome) Room P

---

P-3012
### Red Blood Cell-Bound cell-free DNA Triggers Pro-Inflammatory Macrophage Responses in Cancer Cachexia

[Speaker]Kaho Maehana (Dept. Mol. Pathol., Nara Med. Univ.) [Co-author]Ruiko Ogata (Dept. Mol. Pathol., Nara Med. Univ.)、Rina Tani (Dept. Mol. Pathol., Nara Med. Univ.)、Hiroki Kuniyasu (Dept. Mol. Pathol., Nara Med. Univ.)

Cancer cachexia affects 80% of patients with advanced cancer, reducing treatment tolerance and quality of life. However, its mechanisms are not fully understood. Damage-associated molecular patterns (DAMPs), including cell-free DNA (cfDNA), have been linked to cachexia and various chronic inflammatory diseases. We focused on red blood cell (RBC)-bound DNA, a specific cfDNA subtype. In a mouse cachexia model (Cx) using intraperitoneal CT26 colon cancer cell injection, peripheral RBCs carried DNA, unlike controls. In Cx mouse spleens, TLR9+/CD68+ macrophages phagocytosed DNA-bound RBCs. Co-culture of Cx RBCs with macrophage cell lines increased TLR9 and M1 markers (BatF2, CD68) and decreased Neudesin, a phagocytosis inhibitor. DNA-bound RBCs promote M1 polarization and enhance phagocytosis. We will further explore their role in cancer cachexia.

[Disclosure of COI by Responsible researcher] Lead presenter is not the Responsible researcher.
[Lead presenter: Kaho Maehana] We have no financial relationships to disclose.
[Principal Researcher: Hiroki Kuniyasu] We have no financial relationships to disclose.

[P-3013] I-P3-4
Inflammation & Cancer

2025 / 9 / 27 (Sat) 16:15-17:00 Room P/(Motenashi Dome) Room P

### P-3013
### Novel bioactive compounds and nanocarriers regulating macrophage diversity and malignant melanoma therapeutic approach

[Speaker]Haruka Saito (IVY Cosmetics Corporation) [Co-author]Yuuka Fukui (Graduate School of Science and Technology, Keio Univ.)、Nao Kuramochi (School of Advanced Engineering, Kogakuin Univ.)、Rino Yasuike (School of Advanced Engineering, Kogakuin Univ.)、Kyara Ohkawa (School of Advanced Engineering, Kogakuin Univ.)、Kenji Matsuno (Department of Pharmacy, Faculty of Pharmacy, Yasuda Women's Univ.)、Osamu Ohono (School of Advanced Engineering, Kogakuin Univ.)、Masatoshi Takeiri (IVY Cosmetics Corporation)、Keiko Arai (IVY Cosmetics Corporation)、Keiji Fujimoto (Graduate School of Science and Technology, Keio Univ.)、Yoshihide Kimura (IVY Cosmetics Corporation)

Macrophages are classified into some types by their activation state, function, and phenotype: M1, M2, and M0. M1 macrophages overactivation results in compromised barrier function of skin. In this skin, NF-κB activation was induced by excessive invasion of PAMPs (pathogen-associated molecular patterns), TLR4 ligands derived from bacteria, viruses, and fungi. M2 macrophages are closely associated with the tumor microenvironment (TME). Tumor-associated macrophages (TAMs), which are predominantly M2-like, are the most abundant innate immune cells in the TME. In malignant melanoma, TAMs promote carcinogenesis, angiogenesis, proliferation, invasion, and metastasis. Therefore, M1 and M2 macrophages represent a promising therapeutic target for the treatment of compromised barrier function of skin and malignant melanoma.In this study, we investigated the effects of plant extracts on M1-M2 polarization. Furthermore, we developed a nanocarrier system designed to effectively target skin with barrier dysfunction.Macrophages play essential roles in immune function; hence, our findings contribute to the advancement of novel therapeutic strategies aimed at regulating macrophage activity.

[Disclosure of COI by Responsible researcher] Lead presenter is the Responsible researcher.
[Lead presenter: Haruka Saito] We have no financial relationships to disclose.

### [P-3014] I-P3-4
### Inflammation & Cancer

2025 / 9 / 27 (Sat) 16:15-17:00 Room P/(Motenashi Dome) Room P

---

### P-3014
### Over expression of SOX9 with inflammation-related DNA damage in cholangiocarcinoma induced by liver fluke

[Speaker]Shiho Ohnishi (Grad. Sch. Pharm. Sci., Suzuka Univ. Med. Sci.／Dept. Pharm. Sci., Suzuka Univ. Med. Sci.) [Co-author]Shitao Li (Sch. Basic Med. Sci., Guangxi Med. Univ., China)、Yiqi Jiang (Sch. Basic Med. Sci., Guangxi Med. Univ., China)、Jun Kawanokuchi (Grad. Sch. Health Sci., Suzuka Univ. Med.)、Guifeng Wang (Dept. Acup-Mox., Suzuka Univ. Med. Sci.)、Zeli Tang (Sch. Basic Med. Sci., Guangxi Med. Univ., China)、Yasushi Arima (Grad. Sch. Health Sci., Suzuka Univ. Med.)

Cholangiocarcinoma (CCA) is a global health problem, which reveals high incidence and cause of death, particularly infected with liver fluke ***Clonorchis sinensis*** (Cs), in east China. In this study, we established a rat model of CCA by treating with a combination of Cs-infection and NDMA, and analyzed liver specimens by immunohistochemical staining. All of CCA samples showed strong staining of SOX9, an SRY-related HMG box transcription factor, in the proliferating epithelial cells of bile duct. Sox9 is a progenitor/precursor cell marker of the liver expressed during embryogenesis and following liver injury. Bile duct tissues from negative control rat showed little staining. Several reports have shown that over-expression of SOX9 was associated with poor prognosis and progression in inflammation-related cancer. Interestingly, HMGB1, COX-2, and 8-nitroguanine, an inflammation-related DNA damage, were strongly observed in SOX9-positive cells. These findings suggest that the combination of SOX9 and 8-nitroguanine might be a potential biomarker for CCA and a therapeutic target for CCA.

[Disclosure of COI by Responsible researcher] Lead presenter is not the Responsible researcher.
[Lead presenter: Ohnishi Shiho] ■Research funds from company: 科研費　国際共同研究加速基金(国際共同研究強化(B))
[Principal Researcher: Ning Ma] ■Research funds from company: 科研費　国際共同研究加速基金(国際共同研究強化(B))

[P-3015] I-P3-4
Inflammation & Cancer

2025 / 9 / 27 (Sat) 16:15-17:00 Room P/(Motenashi Dome) Room P

P-3015
### Inhibition of NLRP3 inflammasome suppresses inflammation-related colon carcinogenesis

[Speaker]Yohei Shirakami (Dpt. Gastroenterology., Gifu Univ. Grad. Sch. Med.) [Co-author]Hiroyasu Sakai (Dpt. Gastroenterology., Gifu Univ. Grad. Sch. Med.)、Hiroyuki Tomita (Dpt. Tumor Pathology., Gifu Univ. Grad. Sch. Med.)、Masahito Shimizu (Dpt. Gastroenterology., Gifu Univ. Grad. Sch. Med.)

NLRP3 inflammasome is up-regulated in patients with inflammatory bowel disease (IBD) and its expression in colorectal cancer tissue is a poor prognostic factor. In this study, we investigated the effects of NLRP3 inhibition on colitis and inflammation-related colon carcinogenesis. NLRP3-KO and control mice were employed and a sulfonylurea glibenclamide was used as an NLRP3 inhibitor. Mice were divided into three groups: control, glibenclamide-treated (GLB), and NLRP3-KO (KO). In colitis model, mice were treated with dextran sodium sulphate (DSS) in drinking water. In colorectal cancer model, azoxymethane and DSS were administered. Gene expressions of inflammatory cytokines and COX2 in the colonic mucosa decreased in GLB and KO groups in both models. In the colitis model, colonic shortening and increased histological inflammation scores were observed in GLB and KO groups. The carcinogenesis model showed decreases in the number and size of colorectal tumors in GLB and KO groups. The findings suggest that NLRP3 inhibition may exacerbate acute colonic inflammation, but have suppressive effects on inflammation-related colon tumorigenesis.

[Disclosure of COI by Responsible researcher] Lead presenter is the Responsible researcher.
[Lead presenter: Yohei Shirakami] We have no financial relationships to disclose.

### [P-3016] I-P3-4
### Inflammation & Cancer

2025 / 9 / 27 (Sat) 16:15-17:00 Room P/(Motenashi Dome) Room P

### P-3016
### Withdrawn

No Abstract

### [P-3017] I-P3-4
### Inflammation & Cancer

2025 / 9 / 27 (Sat) 16:15-17:00 Room P/(Motenashi Dome) Room P

---

### P-3017
### Pentraxin 3 contributes to stromal cell senescence and hepatocellular carcinoma cell sorafenib resistance

[Speaker]Ju-Ming Wang (Dept. of Biotechnology & Bioindustry Sci., National Cheng Kung University) [Co-author]Ping-Wen Chen (Inst. of Basic Med. Sci., National Cheng Kung University)

In hepatocellular carcinoma (HCC), sorafenib is the first-line targeted therapy for advanced HCC patients. The pro-inflammatory senescence-associated secretory phenotype (SASP) factors have been observed in tumor microenvironment (TME) upon anti-cancer treatment and suggested to promote HCC tumorigenesis. However, the details and regulations between cellular senescence in TME and its association with drug resistance development remain largely unknown. Pentraxin 3 (PTX3) is mainly elevated from tumor stroma following HCC progression and in the serum of sorafenib-received HCC patients, suggesting serving a poor prognostic factor. We found that PTX3 is responsive to sorafenib and further found that CD44 mediates the NF-κB-triggered cellular senescence in hepatic stellate cells and the sorafenib tolerance through Akt or ERK1/2 provoking CREB-involved Mcl-1 transcription in liver cancer cells. With the disruption of PTX3 and CD44 interaction by a neutralized αPTX3 antibody WHC001, the restricted sorafenib efficacy can be alleviated in vitro and in vivo via enhancing the sorafenib-induced apoptosis, suggesting an improved therapeutic efficacy in sorafenib-received HCC patients.

[Disclosure of COI by Responsible researcher] Lead presenter is the Responsible researcher.
[Lead presenter: Ju-Ming Wang] We have no financial relationships to disclose.

▲TOP

### [P-3027] I-P5-5
### Intracellular signal transduction (1)

2025 / 9 / 27 (Sat) 16:15-17:00 Room P/(Motenashi Dome) Room P

---

### P-3027
### p38-mediated non-canonical activation of Src facilitates tyrosine phosphorylation of TAB1

[Speaker]Iimi Onuma (Dept. Cancer Cell Biol., Grad. Sch. Pharm. Sci., Univ. Toyama) [Co-author]Yue Zhou (Dept. Cancer Cell Biol., Grad. Sch. Pharm. Sci., Univ. Toyama)、Satoru Yokoyama (Dept. Cancer Cell Biol., Grad. Sch. Pharm. Sci., Univ. Toyama)、Kazuyasu Chihara (Dept. Genome Sci and Microbiology, Med. Sci., Univ. Fukui)、Kenji Takeuchi (Dept. Genome Sci and Microbiology, Med. Sci., Univ. Fukui)、Kiyonao Sada (Dept. Genome Sci and Microbiology, Med. Sci., Univ. Fukui)、Hiroaki Sakurai (Dept. Cancer Cell Biol., Grad. Sch. Pharm. Sci., Univ. Toyama)

Src is a non-receptor tyrosine kinase that is highly activated in many cancers and contributes to malignancy. In current concepts, Src activity is controlled by tyrosine phosphorylation, and phospho (p) Y419 is often used as a marker of its activation. However, recent studies have shown that pY419 may contribute to substrate selection rather than Src activation. Therefore, the mechanisms underlying Src activation other than classical tyrosine phosphorylation warrant further study. We herein demonstrated that Src phosphorylates a novel substrate, TAB1, directly at Y481. p38 MAPK enhanced Src-mediated TAB1 phosphorylation through the direct phosphorylation of Src at S75. Moreover, the mode of substrate recognition by the SH2 domain of Src was different in TAB1 than in FAK, a known Src substrate. We identified a novel non-canonical Src activation mechanism based on serine phosphorylation and suggest pY481-TAB1 and pS75-Src as improved markers of Src activation, thereby offering alternative modes for assessing the Src activation status of Src-dependent cancers before and during targeted therapy.

[Disclosure of COI by Responsible researcher] Lead presenter is not the Responsible researcher.
[Lead presenter: Iimi Onuma] We have no financial relationships to disclose.
[Principal Researcher: Hiroaki Sakurai] We have no financial relationships to disclose.

### [P-3028] I-P5-5
### Intracellular signal transduction (1)

2025 / 9 / 27 (Sat) 16:15-17:00 Room P/(Motenashi Dome) Room P

---

### P-3028
### Analysis of Novel Genes Regulated by PRMT5 in Pancreatic Cancer Cells

[Speaker]Yoshinori Abe (Lab. Mol. Anal., Nippon Med. Sch.／Dept. Mol. Oncl., Inst. Adv. Med. Sci., Nippon Med. Sch.) [Co-author]Masashi Ogawa (Lab. Mol. Anal., Nippon Med. Sch.)、Tatsuki Kogure (Lab. Mol. Anal., Nippon Med. Sch.)、Kazuki Tsuchiya (Lab. Mol. Anal., Nippon Med. Sch.)、Nobuyuki Tanaka (Lab. Mol. Anal., Nippon Med. Sch.／Grad. Sch., Kobe University.)

An arginine methyltransferase, PRMT5, regulates epigenetic gene expression, mRNA splicing, DNA repair, and immune surveillance. The deregulation of these roles is also associated with oncogenesis. Aberrant expression of PRMT5 was observed in various cancers, including pancreatic cancer, suggesting PRMT5 is involved in tumor formation. However, the molecular mechanism of PRMT5 in oncogenesis has not been fully understood. In this study, we focused on the regulatory mechanism of PRMT5-mediated epigenetic gene expression and examined PRMT5-mediated regulatory genes in pancreatic cancer cells by comprehensive RNA-seq. RNA-seq analysis revealed a significant finding that regulates gene expression in the wound-healing signaling pathway. In this pathway, we found that PRMT5 upregulated a receptor tyrosine kinase, RON/MST1R. This finding was further validated by qPCR and western blot analysis. PRMT5-mediated gene expression is regulated by the methylation of Histone H3 or H4. Therefore, we examined whether PRMT5 directly upregulates RON expression. ChIP analysis revealed PRMT5 bound to the promoter region in the RON gene, suggesting PRMT5 epigenetically regulates RON expression.

[Disclosure of COI by Responsible researcher] Lead presenter is the Responsible researcher.
[Lead presenter: Yoshinori Abe] We have no financial relationships to disclose.

▲TOP

### [P-3029] I-P5-5
### Intracellular signal transduction (1)

2025 / 9 / 27 (Sat) 16:15-17:00 Room P/(Motenashi Dome) Room P

---

### P-3029
### Traffic communications between the organelles by RNF213

[Speaker]Toshiyuki Habu (Mukogawa Women's Univ., Dept. Food Sci. & Nut.) [Co-author]Hatasu Kobayashi (Mie Univ., Grad. Sch. of Med., Dept. Env. & Mol. Med.)

RNF213 is a susceptibility gene for moyamoya disease (MMD), a vascular disorder of intracranial arteries. It encodes RING finger type and RZ-type zinc-finger E3 ubiquitin ligase. This gene is also a translocation partner in anaplastic large cell lymphoma and inflammatory myofibroblastic tumor cases, with the ALK and MYC genes. The controlling RNF213 by PTP1B function promotes tumor survival under hypoxia. However, the cellular functions of RNF213 have been unclear. We believe that finding the substrate of ubiquitination by RNF213 is the first step to solving the MMD mechanisms. To investigate RNF213 function, we developed the RNF213 KO and KI mutants of mouse embryonic fibroblast cells. RNF213 KO cells show the translocation of lysosomal TFEB transcriptional factor into the nucleus under normal conditions and lower accumulations of lysosomal proteins. Furthermore, dysregulation of growth factors from the outside is also observed in RNF213 KO cells. These indicated that RNF213 plays a role in cellular trafficking communications between the Golgi, Lysosome, and/or plasma membranes. We will present updates on RNF213 cellular function in trafficking.

[Disclosure of COI by Responsible researcher] Lead presenter is the Responsible researcher.
[Lead presenter: Toshiyuki Habu] We have no financial relationships to disclose.

### [P-3030] I-P5-5
### Intracellular signal transduction (1)

2025 / 9 / 27 (Sat) 16:15-17:00 Room P/(Motenashi Dome) Room P

---

### P-3030
### Gallein, G protein $\beta\gamma$ subunits inhibitor, suppresses the TGF-$\alpha$-induced migration of HCC cells via inhibition of the JNK

[Speaker]Rie M. Nishiwaki (Dept. Pharmacol., Gifu Univ. Sch. Med.) [Co-author]Osamu Kozawa (Dept. Pharmacol., Gifu Univ. Sch. Med.)

G protein-coupled receptor (GPCR) signaling regulates a wide range of pathophysiological cell functions via G protein $\alpha$ and $\beta\gamma$ subunits. Small molecules targeting the subunits of G$\alpha$ and G$\beta\gamma$ have been developed as cancer therapeutics. We have previously reported that TGF-$\alpha$ induces the migration of human HCC cells (HuH7) through the activation of AKT, p38 MAPK, Rho-kinase, and JNK. The STAT3 signaling pathway was also involved in the regulation of the migration. This study aims to determine whether G$\beta\gamma$ subunits regulate the TGF-$\alpha$-induced migration of HuH7 cells using gallein, a G$\beta\gamma$ subunits inhibitor. Gallein significantly reduced the TGF-$\alpha$-induced cell migration. In contrast, fluorescein, a gallein-related compound that does not affect G$\beta\gamma$ subunits, failed to affect the cell migration. Gallein suppressed the TGF-$\alpha$-stimulated phosphorylation of JNK without affecting the phosphorylation of EGFR, AKT, p38 MAPK, target protein of Rho-kinase, and STAT3. Conversely, fluorescein did not attenuate the phosphorylation of JNK. These results strongly suggest that G$\beta\gamma$ subunits function as positive regulators in TGF-$\alpha$-induced migration of HCC cells via the JNK signaling pathway.

[Disclosure of COI by Responsible researcher] Lead presenter is not the Responsible researcher.
[Lead presenter: Rie M. Nishiwaki] We have no financial relationships to disclose.
[Principal Researcher: Osamu Kozawa] We have no financial relationships to disclose.

### [P-3031] I-P5-5
### Intracellular signal transduction (1)

2025 / 9 / 27 (Sat) 16:15-17:00 Room P/(Motenashi Dome) Room P

---

### P-3031
### The role of GPRC6A, a nutrient-sensing receptor, in pancreatic cancer and cancer-associated adipocytes

[Speaker]Yuki Nakamura (Div. Funct. Struct., Fukuoka Dent. Coll.) [Co-author]Takahito Otani (Div. Funct. Struct., Fukuoka Dent. Coll.)

Pancreatic Ductal Adenocarcinoma (PDAC) has the highest mortality rate of all cancers in Japan. PDAC is one of the poor prognosis cancers because it spreads easily and early. It has been reported that the adipocytes adjacent to pancreas are implicated in PDAC exacerbation. PDAC downregulates PPAR$\gamma$ expression and upregulates inflammatory cytokines and growth factors expression in the adipocytes, which is called cancer-associated adipocytes (CAA) exhibiting fibroblast-like cells. It is known that CAA aggravates the proliferation and invasion ability of PDAC. We have researched the effects of osteocalcin (OC), a non-collagenous bone matrix protein, on adipocyte function so far. In this process, we have revealed that OC binds to GPRC6A, a putative OC receptor, and promotes glucose and lipid metabolism accompanied with promotion of PPAR$\gamma$ expression in adipocytes. Then, we have verified the effects of GPRC6A signaling on CAA and PDAC. In this study, we have revealed that the activation of GPRC6A signaling suppresses both NF-$\kappa$B and Wnt/$\beta$-catenin signaling via promotion of PPAR$\gamma$ expression in CAA and PDAC, resulting in inhibition of PDAC growth and migration as well as CAA transformation.

[Disclosure of COI by Responsible researcher] Lead presenter is not the Responsible researcher.
[Lead presenter: Yuki Nakamura] We have no financial relationships to disclose.
[Principal Researcher: Takahito Otani] We have no financial relationships to disclose.

▲TOP

### [P-3032] I-P5-5
### Intracellular signal transduction (1)

2025 / 9 / 27 (Sat) 16:15-17:00 Room P/(Motenashi Dome) Room P

### P-3032
### Withdrawn

No Abstract

### [P-3033] I-P5-5
### Intracellular signal transduction (1)

2025 / 9 / 27 (Sat) 16:15-17:00 Room P/(Motenashi Dome) Room P

---

### P-3033
### cGAS STING Pathway Related Proteins as Prognostic Marker in Cholangiocarcinoma Patients

[Speaker]Parawee Artbua (Department of Pathology, Faculty of Medicine, Khon Kaen University, Thailand) [Co-author]Naruemon Kentachalee (Department of Pathology, Faculty of Medicine, Khon Kaen University, Thailand)、Prakasit Sa-Ngiamwibool (Department of Pathology, Faculty of Medicine, Khon Kaen University, Thailand／Cholangiocarcinoma Research Institute (CARI), Khon Kaen University, Thailand)、Phongsathorn Wichian (Department of Pathology, Faculty of Medicine, Khon Kaen University, Thailand)、Raksawan Deenonpoe (Department of Pathology, Faculty of Medicine, Khon Kaen University, Thailand／Cholangiocarcinoma Research Institute (CARI), Khon Kaen University, Thailand)

Cholangiocarcinoma (CCA) is an aggressive malignancy poor prognosis. Chromosome instability (CIN) promotes cancer progression and activates inflammatory signaling through the cGAS-STING pathway. $\gamma$H2AX, a marker of DNA damage, plays a central role in CIN and immune response regulations. This study aims to examine the cGAS-STING pathway-related proteins in CCA and their correlation with clinicopathological parameters. Immunohistochemistry (IHC) was performed on tissue microarrays (TMA) from formalin-fixed paraffin-embedded (FFPE) CCA samples. Statistical analysis assessed correlations between proteins expression and clinicopathological features. High $\gamma$H2AX was significantly associated with intraductal (ID) morphology ($P = 0.018$), and papillary tumors showed elevated cGAS ($P = 0.017$). Strong STING was more frequent in small tumors ($\leq$5 cm, $P < 0.001$) and remained significant in multivariate analysis. IFN$\alpha$ was higher in moderately differentiated tumors ($P = 0.001$). Periductal (PI) and poorly differentiated tumors were linked to shorter survival. These findings suggest the cGAS-STING pathway components may serve as prognostic markers in CCA.

[Disclosure of COI by Responsible researcher] Lead presenter is not the Responsible researcher.
[Lead presenter: Parawee Artbua] We have no financial relationships to disclose.
[Principal Researcher: Naruemon Kentachalee ] We have no financial relationships to disclose.

### [P-3034] I-P5-5
### Intracellular signal transduction (1)

2025 / 9 / 27 (Sat) 16:15-17:00 Room P/(Motenashi Dome) Room P

---

### P-3034
### CULLIN3, as a master regulator of intestinal mucosa regeneration

[Speaker]Yosuke Matsuoka (Dept. of Oncogenesis Growth Regulation, Osaka Intl. Cancer Inst.) [Co-author]Jun(NCC) Nakayama (Dept. of Oncogenesis Growth Regulation, Osaka Intl. Cancer Inst.)、Shigeki Higashiyama (Dept. of Oncogenesis Growth Regulation, Osaka Intl. Cancer Inst.)

CULLIN3 (CUL3) functions a scaffold to form CUL3-RING-ubiquitin ligases (CRL3s), which are involved in protein degradation process by the ubiquitin-proteasome system. Although it was revealed that CUL3 is involved in a variety of cellular processes and numerous human diseases including cancer, its function in intestinal epithelial cells had not been reported. We examined small intestine of intestinal epithelial cell-specific CUL3 knockout mice (CUL3 CKO) and found that the crypt region was expanded. Further investigation revealed that mucosa regeneration programs were activated in CUL3 CKO. Moreover, it was revealed that CUL3 activity was suppressed during the regenerative period after irradiation. Therefore, we speculate that CUL3 could function as a master regulator of shifting from homeostatic to regenerative state of intestinal mucosa after they are injured.

[Disclosure of COI by Responsible researcher] Lead presenter is the Responsible researcher.
[Lead presenter: Yosuke Matsuoka] We have no financial relationships to disclose.

### [P-3035] I-P5-5
### Intracellular signal transduction (1)

2025 / 9 / 27 (Sat) 16:15-17:00 Room P/(Motenashi Dome) Room P

---

### P-3035
### Analysis of tumor development through the increase of phosphorylated PKM2 in Adult T-cell leukemia/lymphoma

[Speaker]Tomonaga Ichikawa (Department of Microbiology, Saitama Medical University) [Co-author]Kazuhiro Morishita (Division of Pediatrics, Faculty of Medicine, University of Miyazaki)、Takashi Murakami (Department of Microbiology, Saitama Medical University)

We previously reported that downregulation of N-myc downstream-regulated gene 2 (NDRG2) plays an important role in the development of Adult T-cell leukemia/lymphoma (ATL). NDRG2 functions as a novel tumor suppressor to dephosphorylate important signaling molecules through the recruitment of PP2A, resulting in the suppression of signaling transduction pathways. We identified glycolytic enzyme Pyruvate Kinase M (PKM) as a NDRG2 binding partner. Furthermore, the phosphorylation of M2 isoform of Pyruvate Kinase (PKM2) at Serine 37 was remarkably increased in NDRG2 deficient ATL. Overexpression of NDRG2 in ATL cell lines suppressed the phosphorylated PKM2 at Serine 37 through the binding to PKM2 with NDRG2 followed by the recruitment of PP2A. We found differences in the metabolism of NDRG2-deficient ATL compared to T-ALL. The loss of PKM2 expression suppressed cell proliferation in ATL cell lines, suggesting that abnormal post-translational modification of PKM2 through the loss of NDRG2 expression induced cancer metabolism and development. Identifying function of PKM2 becomes a feasible and effective strategy for developing a novel treatment in NDRG2 deficient ATL.

[Disclosure of COI by Responsible researcher] Lead presenter is the Responsible researcher.
[Lead presenter: Tomonaga Ichikawa] We have no financial relationships to disclose.

### [P-3044] I-P5-7
### Intracellular signal transduction (3)

2025 / 9 / 27 (Sat) 16:15-17:00 Room P/(Motenashi Dome) Room P

---

### P-3044
### Non-canonical phosphorylation of EphA1 receptor tyrosine kinase

[Speaker]Yaling Zhou (Dept. Cancer Cell Biol., Univ. Toyama) [Co-author]Yue Zhou (Dept. Cancer Cell Biol., Univ. Toyama)、Naoki Yamada (Dept. Cancer Cell Biol., Univ. Toyama)、Satoru Yokoyama (Dept. Cancer Cell Biol., Univ. Toyama)、Yukinari Kato (Dept. Antibody Drug Dev., Tohoku Univ. Grad. Sch. Med.)、Hiroaki Sakurai (Dept. Cancer Cell Biol., Univ. Toyama)

Eph receptor family, a member of receptor tyrosine kinases, is often overexpressed in malignant tumors. We have reported that EphA2 is non-canonically phosphorylated at Ser-897 independent of its tyrosine kinase to promote cancer malignancy. Here, we focused on EphA1 that shows relatively high homology to EphA2. EphA1 phosphorylation was induced by growth factor EGF, phorbol ester 12-o-tetradecanoylphorbol-13-acetate (TPA), chemotherapeutic agent cisplatin, and protein synthesis inhibitor anisomycin, indicating that EphA1 is also non-canonically phosphorylated. EphA2 phosphorylation is catalyzed by p90 ribosomal S6 kinase (RSK). However, cisplatin or anisomycin induced EphA1 phosphorylation was blocked by a MAPK-activated protein kinase 2 (MK2) inhibitor, instead of an RSK inhibitor. These results were confirmed by MK2- or RSK-knockout cells. Interestingly, both RSK and MK2 were not involved in TPA-induced EphA1 phosphorylation, indicating that EphA1 phosphorylation is regulated in the multi-mechanisms under different stimulations. Overall, our findings suggest that non-canonical phosphorylation of EphA1 is induced in tumor microenvironment and regulates cancer progression.

[Disclosure of COI by Responsible researcher] Lead presenter is not the Responsible researcher.
[Lead presenter: Yaling Zhou] We have no financial relationships to disclose.
[Principal Researcher: Hiroaki Sakurai] We have no financial relationships to disclose.

### [P-3045] I-P5-7
### Intracellular signal transduction (3)

2025 / 9 / 27 (Sat) 16:15-17:00 Room P/(Motenashi Dome) Room P

---

### P-3045
### Characterization of pseudogene-like EP400NL in gene expression

[Speaker]Kiyoshi Yamaguchi (Div. Clin. Genome Res., Inst. Med. Sci., Univ. Tokyo) [Co-author]Saya Nakagawa (Div. Clin. Genome Res., Inst. Med. Sci., Univ. Tokyo)、Meihui Zuo (Div. Clin. Genome Res., Inst. Med. Sci., Univ. Tokyo)、Yumiko Isobe (Div. Clin. Genome Res., Inst. Med. Sci., Univ. Tokyo)、Kiyoko Takane (Div. Clin. Genome Res., Inst. Med. Sci., Univ. Tokyo)、Yoichi Furukawa (Div. Clin. Genome Res., Inst. Med. Sci., Univ. Tokyo)

The EP400 gene encodes an ATP-dependent chromatin remodeling enzyme involved in DNA double-strand break repair and transcription. The EP400 N-terminal-Like (EP400NL) gene, located adjacent to EP400 on chromosome 12q, has high similarity to the N-terminal 131-490 amino acid residues of EP400 and has been annotated as a pseudogene. However, it was recently reported that the EP400NL protein is expressed in certain types of cancer. To investigate the function of EP400NL in cancer cells, we examined whether EP400NL associates with the TIP60 complex because EP400 is a subunit of this complex. Immunoprecipitation experiments showed that TIP60 was co-precipitated with EP400 but not with EP400NL. On the other hand, EP400NL co-precipitated with BRD8, another subunit of this complex. BRD8 plays an important role in transcription in a TIP60-independent manner. Therefore, we compared gene expression profiles between EP400NL-depleted and BRD8-depleted HCT116 cells. Substantial overlap of differentially expressed genes was found between these cells. These data suggest that EP400NL may regulate gene expression partially in concert with BRD8, but independently of TIP60.

[Disclosure of COI by Responsible researcher] Lead presenter is the Responsible researcher.
[Lead presenter: Kiyoshi Yamaguchi] We have no financial relationships to disclose.

### [P-3046] I-P5-7
### Intracellular signal transduction (3)

2025 / 9 / 27 (Sat) 16:15-17:00 Room P/(Motenashi Dome) Room P

---

### P-3046
### Non-canonical phosphorylation of EphA2 in lung adenocarcinoma cells and lung squamous cell carcinoma cells

[Speaker]Saya Shimizu (Dept. Cancer Cell Biol., Univ. Toyama) [Co-author]Yue Zhou (Dept. Cancer Cell Biol., Univ. Toyama)、Satoru Yokoyama (Dept. Cancer Cell Biol., Univ. Toyama)、Seiji Yano (Dept. Respiratory Med., Kanazawa Univ.)、Hiroaki Sakurai (Dept. Cancer Cell Biol., Univ. Toyama)

EphA2 is a member of the receptor tyrosine kinase family that is overexpressed in malignant tumors. We previously reported that EphA2 phosphorylation at Ser-897 (pS-EphA2) induces cancer cell migration. pS-EphA2 does not depend on its tyrosine kinase activity and has unique functions in cancer malignancy; therefore, we define it as non-canonical activation and investigate its regulatory mechanisms and functions. In the present research, we determine pS-EphA2 in lung adenocarcinoma cells and lung squamous cell carcinoma cells. It has been reported that pS-EphA2 is catalyzed by p90 ribosomal S6 kinase (RSK), Akt, or PKA. In the most of tested cell lines, pS-EphA2 was regulated by RSK. In addition, pS-EphA2 and RSK phosphorylation were co-localized in the patient specimens. Although RSK is a kinase downstream of ERK, we recently reported that cellular stress induces the RSK-EphA2 pathway via MAPK-activated protein kinase 2 (MK2) independently of ERK. Interestingly, MK2 partially regulates the RSK-EphA2 pathway to promote cell migration in ERK-activated EML4-ALK lung cancer cells, suggesting that the importance of MK2 in the ERK-activated pS-EphA2 in lung cancer cells.

[Disclosure of COI by Responsible researcher] Lead presenter is not the Responsible researcher.
[Lead presenter: Saya Shimizu] We have no financial relationships to disclose.
[Principal Researcher: Hiroaki Sakurai] We have no financial relationships to disclose.

▲TOP

### [P-3047] I-P5-7
### Intracellular signal transduction (3)

2025 / 9 / 27 (Sat) 16:15-17:00 Room P/(Motenashi Dome) Room P

---

### P-3047
### Differences in the balance of canonical/non-canonical EGFR intracellular trafficking by different ligands

[Speaker]Maki Sato (Dept. Cancer Cell Biol., Univ. Toyama) [Co-author]Shinya Morita (Dept. Cancer Cell Biol., Univ. Toyama)、Kyoko Otsuyama (Dept. Cancer Cell Biol., Univ. Toyama)、Yue Zhou (Dept. Cancer Cell Biol., Univ. Toyama)、Satoru Yokoyama (Dept. Cancer Cell Biol., Univ. Toyama)、Hiroaki Sakurai (Dept. Cancer Cell Biol., Univ. Toyama)

Epidermal growth factor receptor (EGFR) has seven different ligands, which are classified into two categories, high affinity (EGF, TGF-$\alpha$, HB-EGF, BTC) and low affinity (AREG, EREG, EPGN). We have demonstrated that EGFR is regulated by canonical and non-canonical endocytosis. In the canonical mechanism, ligand induces EGFR dimerization and subsequent tyrosine autophosphorylation, which undergoes endocytic trafficking to lysosomal degradation. In contrast, in the non-canonical mechanism, ligand-activated p38 MAPK phosphorylates the C-terminal Ser-1015 of the ligand-unbound EGFR monomer, which triggers clathrin-mediated endocytoses and recycling to the cell surface. Using the high-affinity ligand EGF, we proposed a "dual-mode model" in which the ratio of these two endocytosis mechanisms varies with ligand concentrations. In this study, we investigated the differences between canonical and non-canonical phosphorylation and endocytic trafficking by seven different ligands. Interestingly, the pattern of EGFR phosphorylation and endocytosis differed depending on the affinity and concentrations of the ligands. These results support our proposed "dual-mode model".

[Disclosure of COI by Responsible researcher] Lead presenter is not the Responsible researcher.
[Lead presenter: Maki Sato] We have no financial relationships to disclose.
[Principal Researcher: Hiroaki Sakurai] We have no financial relationships to disclose.

### [P-3048] I-P5-7
### Intracellular signal transduction (3)

2025 / 9 / 27 (Sat) 16:15-17:00 Room P/(Motenashi Dome) Room P

---

### P-3048
### Activation mechanism of RSK1 via the p38-MK2 stress response signaling pathway

[Speaker]Takahiro Yokota (Dept. Cancer Cell Biol., Univ. Toyama) [Co-author]Yue Zhou (Dept. Cancer Cell Biol., Univ. Toyama)、Akihiro Tanaka (Dept. Cancer Cell Biol., Univ. Toyama)、Fang Zhang (Dept. Cancer Cell Biol., Univ. Toyama)、Satoru Yokoyama (Dept. Cancer Cell Biol., Univ. Toyama)、Hiroaki Sakurai (Dept. Cancer Cell Biol., Univ. Toyama)

p90 ribosomal S6 kinase1 (RSK1), a downstream kinase of ERK, regulates various cellular events involved in cell growth and survival. Its abnormal activity has been observed in various carcinomas. We have recently reported that the stress response kinases p38 and MAPK-activated protein kinase 2 (MK2) also regulated RSK1 activation; however, the detailed molecular mechanisms were still unknown. In the present study, we elucidated the activation mechanism of RSK1 by the p38-MK2 pathway. RSK1 phosphorylation at Ser-221 via ERK is crucial for RSK1 activity. In contrast, Ser-221 phosphorylation was not induced by the p38-MK2 pathway. We found that Ser-221 was weakly phosphorylated at the basal level and this phosphorylation was essential for RSK1 activation in a Ser-380 phosphorylation-dependent manner. In addition, EphA2, one of RSK substrates, regulated cell migration through the p38-MK2-RSK1 pathway. Besides, we demonstrated that Thr-359/Ser-363 phosphorylation was also involved in RSK1 activation through the p38-MK2 pathway. These results indicated that RSK1 is differently regulated by ERK and p38-MK2 to promote cancer malignancy.

[Disclosure of COI by Responsible researcher] Lead presenter is not the Responsible researcher.
[Lead presenter: Takahiro Yokota] We have no financial relationships to disclose.
[Principal Researcher: Hiroaki Sakurai] We have no financial relationships to disclose.

### [P-3049] I-P5-7
### Intracellular signal transduction (3)

2025 / 9 / 27 (Sat) 16:15-17:00 Room P/(Motenashi Dome) Room P

---

### P-3049
### Dynamic movement of FGFR1-FRS2 $\alpha$ complex observed at single-molecule level by High-Speed Atomic Force Microscopy

[Speaker]Yuma Myokan (Div cancer Cell Biol, Can Res Inst, Kanazawa Univ) [Co-author]Qiqige Saren (Div cancer Cell Biol, Can Res Inst, Kanazawa Univ)、Tsunaki Hongu (Div cancer Cell Biol, Can Res Inst, Kanazawa Univ)、Katsuya Sakai (Div Tumor Dynamics Reg, Can Res Inst, Kanazawa Univ／WPI-NanoLSI, Kanazawa Univ)、Neval Yilmaz (Div Tumor Dynamics Reg, Can Res Inst, Kanazawa Univ／WPI-NanoLSI, Kanazawa Univ)、Kunio Matsumoto (Div Tumor Dynamics Reg, Can Res Inst, Kanazawa Univ／WPI-NanoLSI, Kanazawa Univ)、Mikihiro Shibata (WPI-NanoLSI, Kanazawa Univ／High-Speed Bio-AFM Res Unit, InFiniti, Kanazawa Univ)、Noriko Gotoh (Div cancer Cell Biol, Can Res Inst, Kanazawa Univ)

Fibroblast growth factor (FGFR)1 is a receptor tyrosine kinase located on the plasma membrane, and plays roles in development, homeostasis, and tissue repair. Dysregulation of FGFR1 signaling leads to cancers. It is believed that the receptor dimerization activates tyrosine kinase activity in the cytoplasmic domain, resulting in autophosphorylation. FGFR1 then phosphorylates FRS2 adaptor protein (FRS2 $\alpha$), which, in turn, binds to Grb2. These are believed based on the results of the biochemical experiments. However, it has never been observed in dynamic state. To observe the dynamics of FGFR1 and its signal transduction at single-molecule level, we used cutting-edge technology High-Speed Atomic Force Microscopy (HS-AFM). HS-AFM imaging revealed that the FGFR1-FRS2 $\alpha$ complex, which is formed in both activated and inactivated states of FGF signaling, was observed to bind the phosphotyrosine-binding domain of FRS2 $\alpha$ to the Jaxta-membrane of FGFR1. Also, we observed a repeated behavior in which the complex is formed for a while and then the binding of these two proteins are uncoupled.The results suggest that the hypothesis that the FGFR1-FRS2 $\alpha$ complex is constantly formed is overturned.

[Disclosure of COI by Responsible researcher] Lead presenter is not the Responsible researcher.
[Lead presenter: Yuma Myokan] We have no financial relationships to disclose.
[Principal Researcher: Noriko Gotoh] We have no financial relationships to disclose.

▲TOP

### [P-3050] I-P5-7
### Intracellular signal transduction (3)

2025 / 9 / 27 (Sat) 16:15-17:00 Room P/(Motenashi Dome) Room P

---

### P-3050
### Analysis of the rapamycin and insulin-responsive mLST8 interactome using proximity biotinylated AirID

[Speaker]Suzuno Hirano (Proteo-Science Center, Ehime University) [Co-author]Kohdai Yamada (Proteo-Science Center, Ehime University)、Tatsuya Sawasaki (Proteo-Science Center, Ehime University)

mTOR is involved in cell proliferation and metabolism and functions by forming two complexes, mTORC1 and mTORC2. These complexes are activated by insulin, and the activity of mTORC1 is known to be selectively inhibited by rapamycin. mLST8 is a factor common to both complexes and is believed to contribute to their activity and stability, however the functional role and interactions of mLST8 protein require further analysis. In this study, we performed the proximity rapamycin- and insulin-dependent interactome of mLST8 using proximity biotinylation enzyme AirID. LC-MS/MS analysis of biotinylated proteins showed that mLST8 interacted with approximately 200 different proteins, of which 20-30 proteins showed changes in interaction by treatment with rapamycin or insulin. Biochemical and cell biological analyses of these biotinylated proteins suggest that at least four proteins may directly interact with mLST8 and be involved in mTOR signal. Functional analysis of the remaining proteins is ongoing.

[Disclosure of COI by Responsible researcher] Lead presenter is not the Responsible researcher.
[Lead presenter: Suzuno Hirano] We have no financial relationships to disclose.
[Principal Researcher: Tatsuya Sawasaki] ■Advisor of the company and/or profit-making organization: 株式会社セルフリーサイエンス

### [P-3051] I-P5-7
### Intracellular signal transduction (3)

2025 / 9 / 27 (Sat) 16:15-17:00 Room P/(Motenashi Dome) Room P

---

### P-3051
### Single-molecule imaging enables high-resolution quantification of oncogenic KRAS activation and therapeutic efficacy

[Speaker]Ryoma Yokoi (Dept. of Gastroenterological Surg., Gifu Univ. Sch. of Med.／iGCORE, Gifu Univ.) [Co-author]Jesse Y. Tajima (Dept. of Gastroenterological Surg., Gifu Univ. Sch. of Med.)、Yoshihiro Tanaka (Dept. of Gastroenterological Surg., Gifu Univ. Sch. of Med.)、Manabu Futamura (Dept. of Breast Surg., Gifu Univ. Sch. of Med.)、Nobuhisa Matsuhashi (Dept. of Gastroenterological Surg., Gifu Univ. Sch. of Med.)、Kenichi Suzuki (iGCORE, Gifu Univ.／Div. of Adv. Bioimaging., Natl. Cancer Ctr. Res. Inst.)

Introduction: Single-molecule imaging revealed that activated KRAS undergoes transient trapping. Quantifying the temporal fraction and zone size of these events enables precise assessment of KRAS activation.
Method: We performed single-molecule imaging and Western blot analyses of KRAS (WT, G13D, G12D/C/V) in living colon cancer cells before and after EGF stimulation, with or without molecular-targeted drug treatments.
Result: Western blot showed only slight or no increases in KRAS mutant activation upon EGF stimulation. In contrast, single-molecule imaging sensitively detected mutation-specific KRAS activation. While Western blot showed that 20-40% of active KRAS mutants remained after cetuximab treatment, KRAS inhibitors alone effectively eliminated them. Trapping events of KRAS mutants were reduced by either cetuximab or KRAS inhibitors. Notably, combination therapy further suppressed KRAS trapping in an additive manner, which was not detectable by Western blotting, although some residual trapping persisted.
Conclusion: Single-molecule imaging provides a more sensitive and precise evaluation of oncogenic KRAS activation and therapeutic efficacy than the Western blotting.

[Disclosure of COI by Responsible researcher] Lead presenter is not the Responsible researcher.
[Lead presenter: Ryoma Yokoi] We have no financial relationships to disclose.
[Principal Researcher: Kenichi Suzuki] We have no financial relationships to disclose.

[P-3059] I-P6-1
Cell cycle/Checkpoint

2025 / 9 / 27 (Sat) 16:15-17:00 Room P/(Motenashi Dome) Room P

P-3059
### Conflicting cell cycle signal-mediated cytotoxicity in acute myeloid leukemia cells

[Speaker]Tomohisa Baba (Cancer Research Institute, Kanazawa University) [Co-author]Keqi Gao (Cancer Research Institute, Kanazawa University)、Yoshikazu Johmura (Cancer Research Institute, Kanazawa University)

Accumulating evidence suggests that mitogenic signaling during cell cycle arrest can lead to severe cytotoxic outcomes, such as senescence, though the underlying mechanisms remain poorly understood. Here, we explored the link between cell cycle dynamics and the formation of PML-nuclear bodies (PML-NBs), intranuclear structures known to mediate cellular stress responses. Our findings demonstrate that PML-NBs increase during interphase arrest. Moreover, the activation of mitogenic ERK signaling by ATRA during CDK4/6 inhibitor-induced cell cycle arrest synergistically enhances the formation of larger PML-NBs through PML SUMOylation. This overconstruction, triggered by the simultaneous engagement of opposing cellular signals–a process we term "cell cycle burst"–results in potent cytotoxicity accompanied by terminal differentiation or apoptosis, depending on the cell type across multiple acute myeloid leukemia (AML) cell lines. Importantly, in an AML mouse model, this combination treatment significantly improved therapeutic efficacy with minimal effects on normal hematopoiesis. Our results introduce "cell cycle burst"-induced cytotoxicity as a promising therapeutic strategy for AML.

[Disclosure of COI by Responsible researcher] Lead presenter is the Responsible researcher.
[Lead presenter: Tomohisa Baba] We have no financial relationships to disclose.

### [P-3060] I-P6-1
### Cell cycle/Checkpoint

2025 / 9 / 27 (Sat) 16:15-17:00 Room P/(Motenashi Dome) Room P

---

### P-3060
### Ubiquitin-proteasome system-linked gene signature in prostate cancer

[Speaker]Yasuo Takashima (Kyoto Pref Univ Med, Genomic Med Sci) [Co-author]Kengo Yoshii (Kyoto Pref Univ Med, Math Stat Med Sci)、Masami Tanaka (Kyoto Pref Univ Med, Genomic Med Sci)、Kei Tashiro (Kyoto Pref Univ Med, Genomic Med Sci)

Prostate cancer (PCa) is the most frequently diagnosed cancer in men and a leading cause of cancer-related death. While prostate-specific antigen is a widely used biomarker, its specificity is limited. This study investigated the prognostic significance of gene subsets associated with the ubiquitin-proteasome pathway in PCa. We analyzed transcriptomic and clinical data of 94 early-onset patients with prostate cancer using public dataset. Differential expressions of IKBKB, UBQLN3, TMUB2, UBE2S, and BRCA1 were observed at relative-early stages of pT3a and Gleason 3+4. Similarly, HERPUD1, CDC20, UHRF1, PSMD7, PIAS3, MALT1, TNF, UBD, CD3E, CD247, SOCS1, UBE2C, CARD16, ZAP70, UBA7, and UBE3C expression levels also changed at pT3b and Gleason 4+3. At metastatic stages, DDB1, RPN1, UBE3B, UBE2H, PPIL2, WWP2, and CDH1 were downregulated. In addition, higher expressions of PSMD2, CDC20, NFKB1, and STIP1 or lower expressions of HERPUD2, NEDD4, ANAPC16, LNX1, and HERPUD1 were associated with poor prognoses for biochemical recurrence-free survival. A LASSO-Cox model identified six gene candidates including LNX1, PSMD2, SUMO4, UBE2C, UBR5, and UHRF1.

[Disclosure of COI by Responsible researcher] Lead presenter is the Responsible researcher.
[Lead presenter: Yasuo Takashima] We have no financial relationships to disclose.

### [P-3061] I-P6-1
### Cell cycle/Checkpoint

2025 / 9 / 27 (Sat) 16:15-17:00 Room P/(Motenashi Dome) Room P

---

### P-3061
### DNA damage and cell cycle delay induced by 5-aminolevulinic acid in cancer cells: a potential therapeutic strategy

[Speaker]Seiya Watanabe (Dept. Biosci, Grad. Sch. Life. Sci, Tokyo Univ. Agric.) [Co-author]Taku Chibazakura (Dept. Biosci, Grad. Sch. Life. Sci, Tokyo Univ. Agric.)

5-Aminolevulinic acid (5-ALA) is a native amino acid and a starting substrate for heme synthesis in animals. In cancer cells, the terminal metabolite of 5-ALA, protoporphyrin IX (PpIX), accumulates excessively due to inefficient conversion to heme. With excitation, PpIX emits red fluorescence and generates reactive oxygen species (ROS), leading to cell death. Using this property, 5-ALA has been clinically applied to photodynamic diagnosis and therapy of cancer. Meanwhile, we have found a novel effect of 5-ALA: it suppresses cancer cell proliferation and induces a cell cycle delay in the S/G2-phase progression without excitation. In addition, 5-ALA enhanced γ-H2AX foci formation, a DNA double strand break marker, suggesting that DNA replication stress contributes to the S/G2-phase delay. This effect did not depend on PpIX or ROS accumulation but likely on porphobilinogen (PBG), an immediate metabolite of 5-ALA. Furthermore, we have observed that 5-ALA suppresses the tumor growth in xenograft model. We are currently investigating the molecular mechanisms underlying the suppressive effect of 5-ALA on cancer growth and will discuss its implications for future anti-cancer strategies.

[Disclosure of COI by Responsible researcher] Lead presenter is not the Responsible researcher.
[Lead presenter: Seiya Watanabe] We have no financial relationships to disclose.
[Principal Researcher: Taku Chibazakura] We have no financial relationships to disclose.

### [P-3062] I-P6-1
### Cell cycle/Checkpoint

2025 / 9 / 27 (Sat) 16:15-17:00 Room P/(Motenashi Dome) Room P

### P-3062
### Novel Therapeutic Strategy for Neuroblastoma by Dual Chk1/Chk2 Inhibition

[Speaker]Ayumi Amano (Dept. Pathobiology, Nagoya City Univ., Sch. Phar.) [Co-author]Rina Kato (Dept. Pathobiology, Nagoya City Univ., Sch. Phar.)、Hiromasa Aoki (Dept. Pathobiology, Nagoya City Univ., Sch. Phar.)、Kohki Toriuchi (Dept. Pathobiology, Nagoya City Univ., Sch. Phar.)、Kazuya Izumi (Dept. Pathobiology, Nagoya City Univ., Sch. Phar.)、Mai Nagasaka (Dept. Cell Signaling, Nagoya City Univ., Sch. Phar.)、Yasumichi Inoue (Dept. Cell Signaling, Nagoya City Univ., Sch. Phar.)、Hidetoshi(Nagoya City Univ.) Hayashi (Dept. Cell Signaling, Nagoya City Univ., Sch. Phar.)、Mineyoshi Aoyama (Dept. Pathobiology, Nagoya City Univ., Sch. Phar.)

Neuroblastoma is one of the most common pediatric solid tumors. Due to treatment resistance and severe late effects, novel therapies with improved efficacy and reduced toxicity are urgently needed. Recent studies have identified CHK1 and CHK2 as potential therapeutic targets due to their role in DNA damage response. This study aimed to investigate the combined effects of CHK1 inhibitor (CHK1i; LY2606368) and CHK2 inhibitor (CHK2i; PV1019) on neuroblastoma cells and elucidate the underlying mechanisms. The dual inhibition of the checkpoint mechanism by CHK1i and CHK2i significantly increased DNA damage and replication stress, promoted cell cycle progression, and led to apoptosis more effectively than either inhibitor alone. These results demonstrated that the dual inhibition of Chk1 and Chk2 disrupted critical survival pathways in neuroblastoma cells. These findings support the dual inhibition of Chk1/Chk2 may be a promising therapeutic strategy for neuroblastoma with reduced side effects.

[Disclosure of COI by Responsible researcher] Lead presenter is not the Responsible researcher.
[Lead presenter: Ayumi Amano] We have no financial relationships to disclose.
[Principal Researcher: Mineyoshi Aoyama] We have no financial relationships to disclose.

▲TOP

### [P-3063] I-P6-1
### Cell cycle/Checkpoint

2025 / 9 / 27 (Sat) 16:15-17:00 Room P/(Motenashi Dome) Room P

---

### P-3063
### LAT1 Inhibition-Induced Cell Cycle Arrest via p38 MAPK Mediated Cyclin D1 Downregulation in Cancer Cells

[Speaker]Xinyu Zhou (Grad. Sch. of Med., The Univ. of Osaka) [Co-author]Ryuichi Ohgaki (Grad. Sch. of Med., The Univ. of Osaka／OTRI, The Univ. of Osaka)、Chunhuan Jin (Grad. Sch. of Med., The Univ. of Osaka)、Minhui Xu (Grad. Sch. of Med., The Univ. of Osaka)、Hiroki Okanishi (Grad. Sch. of Med., The Univ. of Osaka)、Yoshikatsu Kanai (Grad. Sch. of Med., The Univ. of Osaka／OTRI, The Univ. of Osaka／WPI-PRIMe, The Univ. of Osaka／Ctr. for Society-Academia Collaboration, Fujita Health Univ.)

L-type amino acid transporter 1 (LAT1/SLC7A5) is highly expressed in cancers and promotes tumor growth by mediating amino acid uptake. Nanvuranlat (Nanv; JPH203), a selective LAT1 inhibitor, shows antitumor effects, but its role in cell cycle regulation remains unclear. In this study, Nanv inhibited the G0/G1 to S phase transition in serum-synchronized MIA PaCa-2 pancreatic cancer cells. Mechanistically, Nanv activated p38 MAPK, leading to Cyclin D1 phosphorylation and proteasome-dependent degradation, blocking S phase entry. Proteasome inhibition restored Cyclin D1 and reversed cell cycle arrest. p38α was identified as the key responsible isoform. Similar effects were observed in AsPC-1 and PANC-1 cells, and in a MIA PaCa-2 xenograft model. These findings show that LAT1 inhibition induces G1 arrest via p38α-mediated Cyclin D1 downregulation, revealing a novel amino acid-dependent cell cycle checkpoint and a key mechanism behind Nanv's antitumor activity.

[Disclosure of COI by Responsible researcher] Lead presenter is not the Responsible researcher.
[Lead presenter: Xinyu Zhou] We have no financial relationships to disclose.
[Principal Researcher: Yoshikatsu Kanai] We have no financial relationships to disclose.

▲TOP

### [P-3064] I-P6-1
### Cell cycle/Checkpoint

2025 / 9 / 27 (Sat) 16:15-17:00 Room P/(Motenashi Dome) Room P

---

### P-3064
### Unveiling radiosensitization properties of PARP inhibitor talazoparib in cancer cells

[Speaker]Barkha Saraswat (Dept. of Mol. & Genomic Biomed., Sch. Med., CBMM, Nagasaki Univ.) [Co-author]Ankitha Vadi Velu (Dept. of Mol. & Genomic Biomed., Sch. Med., CBMM, Nagasaki Univ.)、Zhongming Gao (Dept. of Mol. & Genomic Biomed., Sch. Med., CBMM, Nagasaki Univ.)、Zhu Haoyang (Dept. of Mol. & Genomic Biomed., Sch. Med., CBMM, Nagasaki Univ.)、Ying Tong (Dept. of Mol. & Genomic Biomed., Sch. Med., CBMM, Nagasaki Univ.)、Mitsuko Masutani (Dept. of Mol. & Genomic Biomed., Sch. Med., CBMM, Nagasaki Univ.)

Poly(ADP-ribose) polymerases (PARP-1 and PARP-2) are crucial for DNA repair processes. Their inhibition impairs DNA repair mechanisms and makes them promising targets for radiosensitization strategies. We found that most of the clinically approved PARP inhibitors showed radiosensitization effects on lung adenocarcinoma A549 cells. Talazoparib showed radiosensitization at the lowest concentration. Here, we further studied the mechanistic action of radiosensitization by talazoparib and analyzed their impact on clonogenic survival, signal transduction, mitochondrial membrane potential, and cellular senescence, using SPiDER-β-gal assay for senescence analysis. We observed an increased level of cellular senescence, accompanied by a decrease in the mitochondrial membrane potential, by a combination of talazoparib and γ-irradiation. The senescence induction was further suggested to involve p21 action by knockdown experiments. Our study provides insights into potential therapeutic strategies for enhancing cancer cell sensitivity to radiation. The effect of PARP inhibitors in combination with γ-irradiation on different types of cancer cell lines will also be discussed.

[Disclosure of COI by Responsible researcher] Lead presenter is not the Responsible researcher.
[Lead presenter: Barkha Saraswat] We have no financial relationships to disclose.
[Principal Researcher: Mitsuko Masutani] We have no financial relationships to disclose.

### [P-3074] I-P6-3
### Genomic instability

2025 / 9 / 27 (Sat) 16:15-17:00 Room P/(Motenashi Dome) Room P

### P-3074
### Chromosomal Instability in Patient-Derived Gliomas: Implications for Aneuploidy and Tumor Evolution

[Speaker]Tetsuya Negoto (Div. Exp. Path., Cancer Inst., JFCR) [Co-author]Minji Jo (Div. Exp. Path., Cancer Inst., JFCR)、Hidenobu Yoshitake (Div. Exp. Path., Cancer Inst., JFCR／Dept. Neurosurg., Kurume Univ., Sch. of Med.)、Hideo Nakamura (Dept. Neurosurg., Kurume Univ., Sch. of Med.)、Motohiro Morioka (Dept. Neurosurg., Kurume Univ., Sch. of Med.)、Toru Hirota (Div. Exp. Path., Cancer Inst., JFCR)

Gliomas are malignant tumors driven by chromosomal instability, which promotes progression, resistance, and clonal evolution. We analyzed numerical and structural chromosomal abnormalities of 41 patient-derived gliomas (35 patients, six paired) via primary culture and spectral karyotyping (SKY). Numeric changes were more often in TP53-mutant tumors, whereas structural aberrations were observed in high-grade, TERT wild-type, MGMT-methylated, and recurrent cases, suggesting they may be therapy-driven rearrangements. High numeric changes were linked to poorer progression-free and overall survival in newly diagnosed Glioblastomas. Paired primary and recurrent tumors exhibited marked increases in both the frequency and variety of chromosomal abnormalities after therapy, reflecting therapy-driven genomic stress, while untreated cases showed gradual chromosomal changes consistent with intrinsic genomic instability. Our observations suggest that patient-derived gliomas retain both baseline and treatment-driven genomic diversity, and integrating SKY-based single-cell analysis well refines risk assessment and clarifies glioma evolution.

[Disclosure of COI by Responsible researcher] Lead presenter is not the Responsible researcher.
[Lead presenter: Tetsuya Negoto] We have no financial relationships to disclose.
[Principal Researcher: Toru Hirota] We have no financial relationships to disclose.

### [P-3075] I-P6-3
### Genomic instability

2025 / 9 / 27 (Sat) 16:15-17:00 Room P/(Motenashi Dome) Room P

---

P-3075
### Cell fate of aneuploidy in cancer cells to facilitate adaptation overcoming aneuploid-mediated intracellular stress.

[Speaker]Shicheng Zhang (Tokyo Univ. Sci., Res. Inst. for Biomed. Sci.) [Co-author]Tomoko Yamamori (Natl. Cancer Ctr., Exploratory Oncol. Res. & Clin. Trial Ctr.)、Kazuki Nakai (Natl. Cancer Ctr., Exploratory Oncol. Res. & Clin. Trial Ctr.)、Ryo Kamata (Natl. Cancer Ctr., Exploratory Oncol. Res. & Clin. Trial Ctr.)、Chiaki Mashima (Natl. Cancer Ctr., Exploratory Oncol. Res. & Clin. Trial Ctr.)、Akihiro Ohashi (Natl. Cancer Ctr., Exploratory Oncol. Res. & Clin. Trial Ctr.)、Hiroshi Haeno (Tokyo Univ. Sci., Res. Inst. for Biomed. Sci.)

Most solid tumors exhibit aneuploidy. The aim of this study was to identify key pathways that govern cell survival under aneuploid stress. To this end, a unique aneuploid model was established using pseudo-diploid HCT116 cells treated with an Aurora-B inhibitor. It has been observed that about 10% of treated cells exhibited comparable proliferative activity to parental cells, suggesting that these cells could overcome stress and facilitate the adaptation. In scRNA-seq analyses, the appearance of G1-phase-arrested cells corresponded to PI-staining results. Subsequently, the expression of proteasome degradation related genes in a subpopulation was upregulated which led to the termination of G1-phase-arrest. Heterogeneous activation of the p53 pathway was also demonstrated: in cells suffering proteotoxic stress it triggered apoptosis; in surviving cells, it engaged DNA repair. Further analysis of surviving cells revealed a temporal bottleneck distribution on UMAP, and the trajectory of gene expression dynamic transition was consistent with the actual time. Additionally, increased pro-inflammatory pathways and partial EMT were observed to be beneficial in overcoming the bottleneck.

[Disclosure of COI by Responsible researcher] Lead presenter is not the Responsible researcher.
[Lead presenter: Shicheng Zhang] We have no financial relationships to disclose.
[Principal Researcher: Hiroshi Haeno] ■Research funds from company: イミュノジェネティクス

### [P-3076] I-P6-3
### Genomic instability

2025 / 9 / 27 (Sat) 16:15-17:00 Room P/(Motenashi Dome) Room P

---

### P-3076
### PLK4 inhibition induces irreversible centrosome defect and DNA breakage in RB1-negative osteosarcoma

[Speaker]Hongxin Tu (Div. Oncol. Mol. Biol., Cancer Res. Inst., Kanazawa Univ) [Co-author]Richard Wong (NanoLSI., Kanazawa Univ)、Chiaki Takahashi (Div. Oncol. Mol. Biol., Cancer Res. Inst., Kanazawa Univ)

RB1 deficiency is known to be synthetic lethal with inhibition of several mitotic kinases. However, the mechanism has been only poorly explained by enhanced chromosomal instability. We noticed many of these kinases localize to centrosome. RB1 deletion induced polyploid centrosomes in an osteosarcoma cell line U2OS cells. A PLK4 inhibitor, centrinone, suppressed cell growth in RB1-positive U2OS cells, which was fully reversible when the agent was washed out. We, however, found that centrinone induced irreversible growth arrest and cellular senescence in RB1-negative U2OS cells. Inhibitors of other mitotic kinases residing in centrosomes did not exhibit such an RB1-loss specificity. Centrinone-induced downregulation of SAS-6 and pericentrin 1, which are required for proper centrosome duplication, continued after being washed out. We, however, observed that centrin-2 was upregulated much more robustly compared to p53 or CHK2, even after centrinone was washed out. These findings implicate that the PLK4 inhibitor might exhibit specific and safer cytotoxicity to treat RB1-lost cancer cells without frequent administration.

[Disclosure of COI by Responsible researcher] Lead presenter is not the Responsible researcher.
[Lead presenter: Hongxin Tu] We have no financial relationships to disclose.
[Principal Researcher: Richard Wong, Chiaki Takahashi] We have no financial relationships to disclose.

### [P-3077] I-P6-3
### Genomic instability

2025 / 9 / 27 (Sat) 16:15-17:00 Room P/(Motenashi Dome) Room P

---

### P-3077
### SMARCA4 regulates SMARCAD1 expression for toleration of replication stress i n non-small cell lung cancer

[Speaker]Siripan Limsirichaikul (Dept. Mol. Oncol., Fujita Health Univ. Sch. Med.) [Co-author]Yasuyoshi Mizutani (Dept. Mol. Oncol., Fujita Health Univ. Sch. Med.)、Toshiyuki Takeuchi (Dept. Mol. Oncol., Fujita Health Univ. Sch. Med.)、Dat Q. Tran (Dept. Mol. Oncol., Fujita Health Univ. Sch. Med.)、Taisuke Kajino (Div. Mol. Diagn., Aichi Cancer Ctr. Res. Inst.)、Motoshi Suzuki (Dept. Mol. Oncol., Fujita Health Univ. Sch. Med.)、Atsuko Niimi (Dept. Mol. Oncol., Fujita Health Univ. Sch. Med.)

SMARCA4, a core component of the SWI/SNF chromatin remodeling complex, is fr equently mutated in non-small cell lung cancer cases. SMARCA4-deficient canc er cells are known to be associated with increased replication stress, one o f the major causes of genomic instability, which may lead to cancer. Reporte d here are findings indicating that SMARCAD1, a chromatin remodeler and repl ication fork progressor, is highly expressed in SMARCA4-depleted cells under replication stress. In the presence of replication stress, immunofluorescen t analysis revealed significant accumulation of SMARCAD1 in stalled replicat ion forks of SMARCA4-depleted cells. In addition, chromatin immuno-precipita tion (ChIP) assay findings showed binding of SMARCA4 to the transcriptional regulatory region of SMARCAD1 and its decrease under a replication stress co ndition, suggesting that SMARCA4 is a transcriptional suppressor of SMARCAD1 . Clonogenic analysis also indicated that either SMARCA4 or SMARCAD1 was req uired to overcome replication fork stalling. Based on these results, the SMARCA4-SMARCAD1 axis is proposed as a novel mechanism for tolerating replicati on stress.

[Disclosure of COI by Responsible researcher] Lead presenter is not the Responsible researcher.
[Lead presenter: Siripan Limsirichaikul] We have no financial relationships to disclose.
[Principal Researcher: Atsuko Niimi] We have no financial relationships to disclose.

### [P-3078] I-P6-3
### Genomic instability

2025 / 9 / 27 (Sat) 16:15-17:00 Room P/(Motenashi Dome) Room P

### P-3078
### Molecular Basis of HP1-Dependent Activation of the Chromosomal Passenger Complex

[Speaker]Ishin Mori (Div. Exp. Pathol., Cancer Inst., JFCR／Tokyo Univ of Sci. Faculty Sci. Tech) [Co-author]Toru Hirota (Div. Exp. Pathol., Cancer Inst., JFCR)

Chromosomal instability (CIN) is a prominent feature of many cancer cells and can result from incorrect kinetochore–microtubule attachments during mitosis. The chromosomal passenger complex (CPC), including the mitotic kinase Aurora B, plays a crucial role in correcting these attachments by phosphorylating outer kinetochore proteins. We previously found that HP1 interacts with INCENP, another CPC component, enhances Aurora B activity, and that reduced HP1–INCENP binding in cancer cells is associated with CIN. How HP1 facilitates Aurora B activation is therefore an outstanding question. To investigate how HP1 mediates CPC assembly and promotes activation, we are taking both microscopic and reconstitution approaches. First, we use a super-resolution method called expansion microscopy to define CPC localization at centromere domains dependent on HP1. Second, to set up a reconstitution system, we have purified CPC components and HP1α. We will further purify the Aurora B–INCENP–HP1α complex to elucidate conformational changes of the CPC upon HP1 binding using cryo–electron microscopy. These experiments should provide insights into the molecular basis of Aurora B regulation by HP1.

[Disclosure of COI by Responsible researcher] Lead presenter is not the Responsible researcher.
[Lead presenter: Ishin Mori] We have no financial relationships to disclose.
[Principal Researcher: Toru Hirota] We have no financial relationships to disclose.

▲TOP

### [P-3079] I-P6-3
### Genomic instability

2025 / 9 / 27 (Sat) 16:15-17:00 Room P/(Motenashi Dome) Room P

---

### P-3079
### Effects of two active X chromosome on homologous recombination

[Speaker]Masatoshi Kitagawa (Hamamatsu Univ.. Sch. Med.) [Co-author]Yuka Tamura (Hamamatsu Univ.. Sch. Med.)、Chiharu Uchida (Hamamatsu Univ.. Sch. Med.)、Satoshi Sakai (Hamamatsu Univ.. Sch. Med.)、Hiroyuki Niida (Hamamatsu Univ.. Sch. Med.)、Tatsuya Ohhata (Hamamatsu Univ.. Sch. Med.)

In female mammalian somatic cells, one X chromosome is active and the other is inactive (XaXi). In contrast, ES cells and iPS cells have two active X chromosomes (XaXa). We discovered that female mouse ES cells (XaXa) shown lower homologous recombination (HR) activity comparing with male ES cells (XaYa). The reduced HR activity were recovered by Dox-mediated induction of Xist, which is a lncRNA promoting X chromosome inactivation. Moreover, we found that high expression of BRCC3, the X-linked HR-suppressor gene, in female ES cells (XaXa). It was suppressed by Dox-induced expression of Xist in one X chromosome. Interestingly, some breast cancer cases in women showed active two X chromosomes (XaXa). Using in silico analysis, we found that low expression of Xist and high expression of BRCC3 correlate with poor prognosis of breast cancers. To demonstrate this hypothesis, we generated Xist/p53 double knockout mice specifically in mammary gland cells to investigate the effects of XaXa on HR activity, chromosomal instabilities and cancer prognosis.

[Disclosure of COI by Responsible researcher] Lead presenter is the Responsible researcher.
[Lead presenter: Masatoshi Kitagawa] ■Research funds from company: 科研費挑戦的研究(開拓)

▲TOP

### [P-3080] I-P6-3
### Genomic instability

2025 / 9 / 27 (Sat) 16:15-17:00 Room P/(Motenashi Dome) Room P

---

### P-3080
### Inducing genomic instability: A novel therapeutic strategy for basal-like pancreatic cancer

[Speaker]Dosuke Iwadate (Dept. of Gastroenterology, Inst. Med. Sci., Asahi life Foundation／Grad. Sch. Med., Dept. of Gastroenterology, The Univ. of Tokyo) [Co-author]Keisuke Yamamoto (Grad. Sch. Med., Dept. of Gastroenterology, The Univ. of Tokyo)、Hiroaki Fujiwara (Dept. of Gastroenterology, Inst. Med. Sci., Asahi life Foundation／Grad. Sch. Med., Dept. of Gastroenterology, The Univ. of Tokyo)、Mitsuhiro Fujishiro (Grad. Sch. Med., Dept. of Gastroenterology, The Univ. of Tokyo)

Pancreatic ductal adenocarcinoma (PDAC) is a lethal disease with limited therapies. In our screening using patient-derived organoids, we identified cyclin-dependent kinase 12 (CDK12) as a promising target in basal-like PDAC. Both pharmacological and genetic inhibition of CDK12 exerted potent antitumor activity in vitro and in vivo, inducing double-strand DNA breaks and apoptosis. To elucidate the mechanisms underlying its antitumor activity, we used CRISPR-mediated genome editing to generate FKBP12$^{F36V}$ (dTAG) knock-in PDAC cell lines, enabling rapid elimination of endogenous CDK12 using a degrader against dTAG. Quantitative-image-based cytometry and time-lapse imaging showed that CDK12 ablation induces mitotic catastrophe. Specifically, loss of CDK12 kinase activity during S phase was sufficient to slow DNA replication speed, leading to spindle-assembly checkpoint activation and mitotic catastrophe. Importantly, we found that one of the features defining the basal-like lineage conferred pronounced sensitivity to CDK12 inhibition. Together, our results demonstrate that CDK12 safeguards genomic stability and is an attractive therapeutic target in basal-like PDAC.

[Disclosure of COI by Responsible researcher] Lead presenter is not the Responsible researcher.
[Lead presenter: Dosuke Iwadate] We have no financial relationships to disclose.
[Principal Researcher: Keisuke Yamamoto] We have no financial relationships to disclose.

▲TOP

### [P-3090] I-P7-2
### Insights into cancer pathogenesis based on genomic analysis

2025 / 9 / 27 (Sat) 16:15-17:00 Room P/(Motenashi Dome) Room P

---

### P-3090
### Molecular characteristics of polyp lesions in Gastric Adenocarcinoma and Proximal Polyposis of the Stomach (GAPPS)

[Speaker]Chihiro Matsumoto (Department of Gastroenterological Surgery, Kumamoto University) [Co-author]Kohei Yamashita (Department of Gastroenterological Surgery, Kumamoto University)、Kojiro Eto (Department of Gastroenterological Surgery, Kumamoto University)、Satoshi Ida (Department of Gastroenterological Surgery, Kumamoto University)、Yuji Miyamoto (Department of Gastroenterological Surgery, Kumamoto University)、Masaaki Iwatsuki (Department of Gastroenterological Surgery, Kumamoto University)

Background: GAPPS is characterized by polyposis localized in the gastric body and fundus with a strong tendency for adenocarcinoma. While polyp are numerous in GAPPS, the exact precursor lesion in this condition is not completely defined.Objective: We investigated the evolutionary process from polyp to carcinoma in GAPPS. Design: The polyps of seven patients with GAPPS (n=13) were classified into two groups; fundic gland polyp (FGP) and hyperproliferative aberrant pits (HPAP), based on pathological evaluation. Whole-exome and RNA sequencing were performed to investigate genomic alterations and transcriptome dynamics during carcinogenesis.Results: Somatic mutations in the APC gene were significantly more common in HPAPs compared to FGPs ($p = 0.02$). Furthermore, RNA expression analysis revealed that MKI67 expression was significantly higher in the HPAP group ($p = 0.02$), and pathways related to MYC targets and E2F targets were also upregulated in this group.Conclusion: Genetic differences between FGP and HPAP in GAPPS were elucidated. HPAPs exhibited higher proliferative activity and may represent precancerous lesions in GAPPS.

[Disclosure of COI by Responsible researcher] Lead presenter is not the Responsible researcher.
[Lead presenter: Chihiro Matsumoto] We have no financial relationships to disclose.
[Principal Researcher: Msaaki Iwatsuki] We have no financial relationships to disclose.

### [P-3091] I-P7-2
### Insights into cancer pathogenesis based on genomic analysis

2025 / 9 / 27 (Sat) 16:15-17:00 Room P/(Motenashi Dome) Room P

---

### P-3091
### Whole-genome sequencing of osteosarcoma

[Speaker]Masataka Hasegawa (Division of Cancer Evolution, National Cancer Center Research Institute／Department of Pediatrics, Hokkaido University Hospital) [Co-author]Kaito Mimura (Division of Cancer Evolution, National Cancer Center Research Institute)、Kentaro Watanabe (Department of Pediatrics, The University of Tokyo)、Ai Okada (Division of Genome Analysis Platform Development, National Cancer Center)、Kenichi Chiba (Division of Genome Analysis Platform Development, National Cancer Center)、Kotoe Katayama (Laboratory of Sequence Analysis, The University of Tokyo)、Miho Kato (Department of Childhood Cancer Data Management, Childhood Cancer Center, NCCHD)、Takako Yoshioka (Department of Pathology, NCCHD)、Hajime Okita (Division of Diagnostic Pathology, Keio University Hospital)、Akihiro Yoneda (Division of Surgery, NCCHD)、Seiya Imoto (Laboratory of Sequence Analysis, The University of Tokyo／Division of Health Medical Intelligence, The University of Tokyo)、Yuichi Shiraishi (Division of Genome Analysis Platform Development, National Cancer Center)、Atsushi Manabe (Department of Pediatrics, Hokkaido University Hospital)、Motohiro Kato (Department of Pediatrics, The University of Tokyo)、Kenichi Yoshida (Division of Cancer Evolution, National Cancer Center Research Institute)

Osteosarcoma is the most common malignant bone tumor in children and young adults. To clarify the genomic features of osteosarcoma, we studied whole-genome sequencing data of paired tumor and normal tissues from Japanese patients (N = 8), collected through the Observational Study of Childhood, Adolescent, and Young Adult Solid Tumors by the Japan Children's Cancer Group, along with publicly available data (N = 88). Pathogenic germline mutations in TP53 or RB1 were identified in 11% of the cases. Somatic variants or copy number alterations (CNAs) were most frequently observed in TP53 (90%), followed by ATRX and RB1. Tumor suppressor gene alterations were mainly caused by structural variants (SVs) disrupting exons or introns. Copy number alteration analysis revealed amplifications of CCNE1, MYC, and CDK4, and deletions of TP53, PTEN, and CDKN2A. Chromothripsis and extrachromosomal DNA (ecDNA) occurred in approximately half of the cases. EcDNA frequently involved oncogenes such as CCNE1 and CDK4. Complex CNAs and SVs contributed to the pathogenesis of osteosarcoma through the amplification of oncogenes and the loss of tumor suppressors.

[Disclosure of COI by Responsible researcher] Lead presenter is not the Responsible researcher.
[Lead presenter: Masataka Hasegawa] We have no financial relationships to disclose.
[Principal Researcher: Akihiro Yoneda] ■Contributions: 公益財団法人持田記念医学薬学振興財団, 公益財団法人内藤記念科学振興財団, 公益財団法人第一三共生命科学研究振興財団, 公益財団法人住友財団, 公益財団法人安田記念医学財団, 公益財団法人アステラス病態代謝研究会, 公益財団法人MSD生命科学財団, 一般社団法人日本血液学会, 公益財団法人小児医学研究振興財団, 公益財団法人上原記念生命科学財団, 公益財団法人高松宮妃癌研究基金, 公益信託日本白血病研究基金, 公益財団法人小林がん学術振興会, 公益財団法人武田科学振興財団, 公益財団法人大和証券財団, 公益財団法人ＳＧＨ財団, 公益財団法人川野小児医学奨学財団, 認定NPO法人ゴールドリボン・ネットワーク, 公益財団法人先進医薬研究振興財団, 公益財団法人テルモ生命科学振興財団, 公益財団法人小野薬品がん・免疫・神経研究財団, 公益財団法人細胞科学研究財団

### [P-3092] I-P7-2
### Insights into cancer pathogenesis based on genomic analysis

2025 / 9 / 27 (Sat) 16:15-17:00 Room P/(Motenashi Dome) Room P

---

### P-3092
### Molecular features of gastric metaplastic glands associated with carcinogenesis revealed by spatial transcriptomics

[Speaker]Haruka Amino (Kyoto Univ. Gastroenterology) [Co-author]Takahiro Shimizu (Kobe City Medical Center West Hosp. Gastroenterology)、Takahiko Ito (Japan Baptist Hosp. Gastroenterology)、Mari Teramura (Kyoto Katsura Hosp. Gastroenterology)、Atsushi Takai (Kyoto Univ. Gastroenterology)、Hiroshi Seno (Kyoto Univ. Gastroenterology)

Gastric cancer has high incidence and mortality rates globally. Metaplastic lesions, including intestinal metaplasia (IM) and spasmolytic polypeptide-expressing metaplasia (SPEM), are thought to be precancerous lesions. Despite various experiments using mouse models, the relationship between these metaplastic lesions and carcinogenesis in humans is still unknown. In this study, we investigated the association between gastric glands with metaplastic and non-metaplastic structures and carcinogenesis in humans using spatial transcriptomics. We performed Visium spatial transcriptomics analysis on four formalin-fixed paraffin-embedded specimens of intramucosal gastric carcinoma (IGC) and its surrounding glands resected by endoscopic submucosal dissection. We elucidated the overall gene expression profiles in IGC as well as in non-cancerous metaplastic and non-metaplastic lesions. Interestingly, we discovered that some parts of IM glands exhibited expression profiles closely resembling those of tumors, suggesting a potential involvement in carcinogenesis. Furthermore, it was considered that epithelial-mesenchymal transition might be involved in gastric carcinogenesis from metaplasia.

[Disclosure of COI by Responsible researcher] Lead presenter is the Responsible researcher.
[Lead presenter: Haruka Amino] We have no financial relationships to disclose.

▲TOP

### [P-3093] I-P7-2
### Insights into cancer pathogenesis based on genomic analysis

2025 / 9 / 27 (Sat) 16:15-17:00 Room P/(Motenashi Dome) Room P

### P-3093
### Impact of TP53, KRAS, and APC Mutations on Neoadjuvant Chemotherapy Outcomes in Locally Advanced Rectal Cancer

[Speaker]Tomohiro Takeda (Department of Surgery, Asahikawa Medical University) [Co-author]Tatsuya Shonaka (Department of Surgery, Asahikawa Medical University)、Mishie Tanino (Department of Diagnostic Pathology, Asahikawa Medical University Hospital)、Katsuro Enomoto (Department of Surgery, Asahikawa Medical University)、Shoichiro Mizukami (Department of Surgery, Asahikawa Medical University)、Masahide Otani (Department of Surgery, Asahikawa Medical University)、Mizuho Ohara (Department of Surgery, Asahikawa Medical University)、Chikayoshi Tani (Department of Surgery, Asahikawa Medical University)、Kimiharu Hasegawa (Department of Surgery, Asahikawa Medical University)、Yusuke Ono (Institute of Biomedical Research, Sapporo Higashi Tokushukai Hospital)、Yusuke Mizukami (Institute of Biomedical Research, Sapporo Higashi Tokushukai Hospital／Department of Medicine, Asahikawa Medical University／National Institute of Biomedical Innovation, Health and Nutrient)、Hideki Yokoo (Department of Surgery, Asahikawa Medical University)

Introduction: Locally advanced rectal cancer (LARC) often has a poor prognosis when it comes to local recurrence and distant metastasis. Neoadjuvant chemotherapy (NACT) offers a radiation-sparing approach may help to control locally and reduce distant recurrence; however, it can be challenging to determine which patients will benefit from NACT.Methods: In this study, 43 patients with LARC (cT3/4 or cN3) underwent NACT followed by primary tumor resection between January 2013 and June 2021. Targeted amplicon sequencing on the Ion S5 GeneStudio system assessed mutations in TP53, KRAS, APC, and other LARC-related genes.Results: TP53 wild type (25.6%) and KRAS+APC co-mutations (11.6%) were linked to poorer OS (HR = 0.124, p = 0.007/ HR = 4.60, p = 0.046).Conclusion: Genetic profiling of TP53, KRAS, and APC mutations offers crucial insights into NACT efficacy in LARC. These biomarkers could guide personalized therapeutic strategies to enhance patient outcomes.

[Disclosure of COI by Responsible researcher] Lead presenter is not the Responsible researcher.
[Lead presenter: Tomohiro Takeda] We have no financial relationships to disclose.
[Principal Researcher: Yusuke Ono] ■Research funds from company: 日立ハイテク

### [P-3094] I-P7-2
### Insights into cancer pathogenesis based on genomic analysis

2025 / 9 / 27 (Sat) 16:15-17:00 Room P/(Motenashi Dome) Room P

### P-3094
### Molecular and cellular heterogeneities in CN-AML are driven by cell-type specific effects of somatic mutations

[Speaker]MANLI YANG (Lab. for Regulatory Genomics, RIKEN IMS) [Co-author]Chung-Chau Hon (Lab. for Regulatory Genomics, RIKEN IMS)、Jonathan Moody (Lab. for Regulatory Genomics, RIKEN IMS)、Andreas Lennartsson (Dept. of Med., Karolinska Inst.)、Soren Lehmann (Dept. of Med., Karolinska Inst.)、Erik Arner (Dept. of Med., Karolinska Inst.)、Xiangfu Zhong (Dept. of Med., Karolinska Inst.)、Sofia Bengtzen (Dept. of Med., Karolinska Inst.)、Anna Bohlin (Dept. of Med., Karolinska Inst.)、Cheng-Tsung Pan (Grad. Sch. of Integrated Sci. for Life, Hiroshima Univ.)、Chi Wai Yip (Lab. for Transcriptome Tech., RIKEN IMS)、Miki Kojima (Lab. for Transcriptome Tech., RIKEN IMS)

Cytogenetically normal AML (CN-AML) lacks recurrent chromosomal abnormalities but is driven by somatic mutations (SMs), shaping disease and prognosis heterogeneity. We investigated the impact of these SMs on gene expression and their associations to molecular, cellular and phenotypic heterogeneity using 5'-based RNA-seq on bone marrow mononuclear cells from CN-AML patients, at both bulk (n=88) and single-cell (n=16) resolutions. Based on the bulk tCRE profiles, we classified four molecular subtypes differing in cell composition, clinical outcomes, and SMs. Using single-cell data, we compared SMs-affected tCREs to validate bulk deconvolution approach, identifying differential active tCREs in multiple cell types for key SMs. To understand the regulatory roles of these tCREs, we defined tCRE co-activity modules with biological functions and found several modules significantly associated with SMs and patient survival. Regulatory network analysis identified key transcription factors, super-enhancer-like loci as potential module regulators, revealing alternative tCRE-driven regulatory programs impacting survival-often missed by gene-centric RNA-seq analysis.

[Disclosure of COI by Responsible researcher] Lead presenter is the Responsible researcher.
[Lead presenter: MANLI YANG] We have no financial relationships to disclose.

### [P-3095] I-P7-2

### Insights into cancer pathogenesis based on genomic analysis

2025 / 9 / 27 (Sat) 16:15-17:00 Room P/(Motenashi Dome) Room P

---

### P-3095

### Highly chromosomal heterogeneity in parental sarcoma cells generating three phenotypically different tumor cell lines

[Speaker]Hideyuki Tanabe (Res. Center Integ. Evol. Sci., Grad. Univ. Adv. Studies, SOKENDAI) [Co-author]Masahiro Masuya (Dept. Hematol. & Oncol., Mie Univ. Grad. Sch. Med.)、Naoyuki Katayama (Dept. Hematol. & Oncol., Mie Univ. Grad. Sch. Med.)、Isao Tawara (Dept. Hematol. & Oncol., Mie Univ. Grad. Sch. Med.)

Previously we have reported molecular cytogenetic analysis of the 3 cell clones, named B12, D2 and D11, derived from a spontaneous sarcoma in an old female C57BL/6-EGFP mouse. All 3 clones represented similar morphology but tumorigenicities were quite different. To understand how these phenotypically differences have occurred during the process of establishment of cell clones, we have perfomed multi-color FISH techniques using the mouse chromosome specific painting probes. Results showed that several unique characteristics with numerical changes such as chromosomes 13, 17, and X, and structural rearrangements involving chromosomes 8, 14, 15, and 19 occurred in all 3 clones. Here we have examined the parental sarcoma bulk cells of 3 clones by M-FISH technique showing that highly chromosomal heterogeneity with numerical changes. Modal number of chromosomes were 62 (85.7%) for B12, 64 (57.1%) for D2, 66 (28.6%) for D11, but the parental cells showed 71 (15.8%) and distributed from 53 to 154. Therefore, 3 clones were generated through highly genomic heterogeneity and established phenotypically differences. Thus these cytogenetic data will be important for further tumorigenesis studies.

[Disclosure of COI by Responsible researcher] Lead presenter is the Responsible researcher.
[Lead presenter: Hideyuki Tanabe] We have no financial relationships to disclose.

### [P-3105] I-P7-4
### Pathophysiology based on clonal evolution

2025 / 9 / 27 (Sat) 16:15-17:00 Room P/(Motenashi Dome) Room P

---

### P-3105
### Characterization of Clonal Hematopoiesis in Liquid Biopsy Comprehensive Genomic Profiling Tests by C-CAT Repository Data

[Speaker]Manami Matsukawa (Dept. of Genet. Med. & Services., Natl. Cancer Ctr. Hosp.) [Co-author]Masachika Ikegami (Dept. of Msk. Oncol., Tokyo Met. Komagome Hosp.／Div. of Cellular Signaling, Natl. Cancer Ctr. Research Inst.／Ctr. for Cancer Genom. and Adv. Therap., Natl. Cancer Ctr.)、Chikako Tomozawa (Dept. of Genet. Med. & Services., Natl. Cancer Ctr. Hosp.)、Tomoko Watanabe (Dept. of Genet. Med. & Services., Natl. Cancer Ctr. Hosp.)、Satoyo Oda (Dept. of Genet. Med. & Services., Natl. Cancer Ctr. Hosp.／Dept. of Lab. Med., Natl. Cancer Ctr. Hosp.)、Fumito Yamazaki (Dept. of Genet. Med. & Services., Natl. Cancer Ctr. Hosp.)、Mizuki Watanabe (Dept. of Genet. Med. & Services., Natl. Cancer Ctr. Hosp.／Dept. of Hem. Stem Cell Transp., Natl. Cancer Ctr. Hosp.)、Ayaka Sato (Dept. of Genet. Med. & Services., Natl. Cancer Ctr. Hosp.／Dept. of Breast & Endo. Surg., The Univ. of Tokyo)、Noriko Tanabe (Dept. of Genet. Med. & Services., Natl. Cancer Ctr. Hosp.／Dept. of Clin. Genet., Saitama Med. Ctr.)、Takafumi Koyama (Dept. of Exp. Therap., Natl. Cancer Ctr. Hosp.)、Shinji Kohsaka (Div. of Cellular Signaling, Natl. Cancer Ctr. Research Inst.)、Shinji Kosugi (Dept. of Genom. Med., Kyoto Univ Sch. of Public Health)、Teruhiko Yoshida (Dept. of Genet. Med. & Services., Natl. Cancer Ctr. Hosp.)、Hiroyuki Mano (Div. of Cellular Signaling, Natl. Cancer Ctr. Research Inst.)、Makoto Hirata (Dept. of Genet. Med. & Services., Natl. Cancer Ctr. Hosp.)

Background: Clonal hematopoiesis (CH) is associated with aging, chemotherapy, and smoking. Identifying CH-derived variants (CHdv) in comprehensive genomic profiling (CGP) tests remains challenging. Here, we characterized putative CHdv in liquid biopsy CGP. Methods: This study used the C-CAT database including FoundationOne Liquid CDx (F1L) (n = 13,474) and FoundationOne CDx (F1) (62,104). We hypothesized that CHdv are more frequently detected in F1L than in F1, and compared the frequency of variant detection. CH hotspots (CHhs) were defined as variants detected in ≥5 cases in F1L with an odds ratio >4. Results and Discussion: Among 199,967 F1L variants, 15,344 were detected as CHhs in 8,044 cases (60%). A positive correlation between CHhs detection frequency in certain genes and known predisposing factors was observed. Splice site mutations were mainly seen in the second hit of CHhs, indicating that the first and second hits were caused by different mechanisms. A CHhs-prediction nomogram showed a strong predictive value with a C-index of 0.98. These results suggested that cancer type, mutation pattern, and the VAF-age correlation may help distinguish CHdv from other mutations.

[Disclosure of COI by Responsible researcher] Lead presenter is not the Responsible researcher.
[Lead presenter: Matsukawa Manami] We have no financial relationships to disclose.
[Principal Researcher: Makoto Hirata] We have no financial relationships to disclose.

### [P-3106] I-P7-4
### Pathophysiology based on clonal evolution

2025 / 9 / 27 (Sat) 16:15-17:00 Room P/(Motenashi Dome) Room P

---

### P-3106
### Analysis of clonal evolution in endometrioid endometrial cancer

[Speaker]Suguru Miyata (Div. of Cell Therap., Chiba Cancer Ctr. Res. Inst.) [Co-author]Junsuke Muraoka (Dept. of Genecology, Chiba Cancer Ctr.)、Yoshiaki Maru (Lab. Precis. Tumor Model. Syst., Chiba Cancer Ctr. Res. Inst.)、Takao Morinaga (Div. of Cell Therap., Chiba Cancer Ctr. Res. Inst.)、Takahiro Sugiyama (Dept. of Surgical Pathology, Chiba Cancer Ctr.)、Akinobu Araki (Dept. of Surgical Pathology, Chiba Cancer Ctr.)、Toyoyuki Hanazawa (Dept. of Otorhinolaryngology, Graduate School of Medicine, Chiba Univ.)、Makiko Itami (Dept. of Surgical Pathology, Chiba Cancer Ctr.)、Naotake Tanaka (Dept. of Genecology, Chiba Cancer Ctr.)、Masahito Kawazu (Div. of Cell Therap., Chiba Cancer Ctr. Res. Inst.)

Individual glands in the endometrium are often formed by clones harboring driver mutations; however, the detailed processes by which these clones contribute to cancer development remain unclear.We performed exome sequencing on multiple samples from the uterine cavity of cases with early-stage endometrioid endometrial cancers (EEC) to investigate the anatomical distribution of cancer clones. It was revealed that, even at an early stage of EEC, genetically diverse clones derived from a common origin can spread across the uterine cavity.To examine clonal diversity at the cellular level, we performed single-cell analysis on the selected cases with several molecular subtypes. Cancer cells exhibited diverse epithelial phenotypes, from differentiated types such as ciliated and lumenal cells, to undifferentiated progenitor-like cells, and this diversity in cellular composition was observed both across and within cases. Analyses of progenitor-like cells and organoid cultures suggested two distinct progenitor cell types.Our findings highlight the heterogeneity of early-stage EEC across multiple levels-from cellular to anatomical-and provide insights into the clonal evolution of the disease.

[Disclosure of COI by Responsible researcher] Lead presenter is not the Responsible researcher.
[Lead presenter: Suguru Miyata] We have no financial relationships to disclose.
[Principal Researcher: Masahito Kawazu] We have no financial relationships to disclose.

▲TOP

### [P-3107] I-P7-4
### Pathophysiology based on clonal evolution

2025 / 9 / 27 (Sat) 16:15-17:00 Room P/(Motenashi Dome) Room P

---

### P-3107
### Study on Acquisition Timing of KRAS Mutations in the Pancreatic Ductal Epithelium

[Speaker]Tomonori Hirano (Dept. of Path. & Tumor Biol., Kyoto Univ.) [Co-author]Nobuyuki Kakiuchi (Dept. of Path. & Tumor Biol., Kyoto Univ.／Dept. of Gastroenterology & Hepatology, Grad. Sch. of Med., Kyoto Univ.／Hakubi center, Kyoto Univ.)、Koichi Watanabe (Dept. of Path. & Tumor Biol., Kyoto Univ.)、Yasuhide Takeuchi (Dept. of Diagnostic Path., Grad. Sch. of Med., Kyoto Univ.)、Sachiko Minamiguchi (Fujita Health Univ. Hosp.)、Toshihiko Masui (Kurashiki Central Hosp.)、Kazuyuki Nagai (Dept. of Surgery. ,Kyoto Univ.)、Takayuki Anazawa (Dept. of Surgery. ,Kyoto Univ.)、Hiroko Tanaka (M&D Data Sci. Ctr., IIR, Inst. Sci. Tokyo)、Satoru Miyano (M&D Data Sci. Ctr., IIR, Inst. Sci. Tokyo)、Toshiro Sato (Dept. Integrated Med. and Biochem., Kei Univ Sch. of Med.)、Norimitsu Uza (Dept. of Gastroenterology, Kobe Univ. Grad. Sch. of Med.)、Hiroshi Seno (Dept. of Gastroenterology & Hepatology, Grad. Sch. of Med., Kyoto Univ.)、Seishi Ogawa (Dept. of Path. & Tumor Biol., Kyoto Univ.)

[Introduction] Pancreatic cancer is an aggressive malignancy with a poor prognosis. KRAS mutations are among the earliest events in pancreatic carcinogenesis, but the exact timing remains unclear.[Methods] Non-cancerous pancreatic tissues from surgical specimens were used to isolate the main pancreatic ducts and generate ductal organoids. DNA was extracted for whole-exome sequencing, and phylogenetic trees were reconstructed. Single-cell-derived organoids were established and analyzed to determine mutation rates. Then, the timing of KRAS mutation acquisition was estimated.[Results] A total of 430 organoids were analyzed. Phylogenetic analysis revealed frequent clonal expansion of KRAS-mutant clones in pancreatic cancer patients. The mutation rate in pancreatic duct cells was estimated to be 0.25 mutations per exome per year. KRAS mutations were acquired at various ages, with some arising as early as their early 20s.[Conclusions] This study provides insights into the evolution of early pancreatic cancer development. The results suggest that KRAS mutations occur early in life, which may contribute to the development of early detection strategies for pancreatic cancer.

[Disclosure of COI by Responsible researcher] Lead presenter is not the Responsible researcher.
[Lead presenter: Tomonori Hirano] We have no financial relationships to disclose.
[Principal Researcher: Seishi Ogawa] ■Advisor of the company and/or profit-making organization: エーザイ株式会社 Chordia Therapeutics Inc. Montage Bio, INC. ■Profit of stock: 朝日ゲノミクス株式会社 ■Lecture fee: 公益財団法人三菱財団 公益財団法人中谷医工計測技術振興財団 ■Research expenses: エーザイ株式会社 Chordia Therapeutics Inc. ■Contributions: 日本新薬株式会社 ■Endowed chair: Chordia Therapeutics Inc. 公益社団法人鹿児島共済会 南風病院

▲TOP

### [P-3108] I-P7-4
### Pathophysiology based on clonal evolution

2025 / 9 / 27 (Sat) 16:15-17:00 Room P/(Motenashi Dome) Room P

### P-3108
### Genetic Insights into Clonal Evolution from Normal Prostate Epithelium to Prostate Cancer via HGPIN

[Speaker]Kohsuke Hishiki (Dept. of Urology, Grad.Sch.of Med. & Faculty of Med. , Kyoto Univ.) [Co-author]Nobuyuki Kakiuchi (Dept. of Path. & Tumor Biol., Kyoto Univ.／Dept. of Gastroenterology & Hepatology, Kyoto Univ.／The Hakubi Center for Advanced Res., Kyoto Univ.)、Yuki Teramoto (Dept. of Diagnostic Path., Kyoto Univ.)、Koichi Watanabe (Dept. of Path. & Tumor Biol., Kyoto Univ.／Dept. of Gynecol., Grad.Sch.of Med. & Faculty of Med. , Kyoto Univ.)、Shun Kawaguchi (Dept. of Path. & Tumor Biol., Kyoto Univ.)、Kosuke Ieiri (Dept. of Path. & Tumor Biol., Kyoto Univ.／Dept. of Urology, Kyoto Univ.)、Hirona Maeda (Dept. of Path. & Tumor Biol., Kyoto Univ.／Dept. of Diagnostic Path., Kyoto Univ.)、Tomonori Hirano (Dept. of Path. & Tumor Biol., Kyoto Univ.／Dept. of Gastroenterology & Hepatology, Kyoto Univ.)、Yuki Kita (Dept. of Urology, Grad.Sch.of Med. & Faculty of Med. , Kyoto Univ.)、Takashi Kobayashi (Dept. of Urology, Grad.Sch.of Med. & Faculty of Med. , Kyoto Univ.)、Seishi Ogawa (Dept. of Path. & Tumor Biol., Kyoto Univ.／Inst. for the Advanced Study of Human Biol., Kyoto Univ.／Dept.of Med., Center for Hematology and Regenerative Med., KarolinskaInst., Sweden)

Prostate cancer (PC) is a significant health concern in aging populations and is believed to develop from high-grade prostatic intraepithelial neoplasia (HGPIN). However, the genetic relationship between them remains unclear. We collected 3-mm-thick prostate tissues from eight patients who underwent radical prostatectomy and prepared serial sections for laser microdissection. In total, 48 PC, 75 HGPIN, and 380 normal prostate epithelium (NPE) samples were subjected to whole-exome sequencing (WES), identifying 13,494 SNVs and indels. Known driver mutations, including ***FOXA1*** and ***KMT2C***, were detected in 11% of NPEs, 47% of HGPINs, and 67% of PCs. Clonal expansions spanning more than 2 mm were frequently observed in aged prostate glands. Phylogenetic analysis revealed that HGPIN and PC lesions often arose from a common ancestral clone that emerged around the age of 50. In one 75-year-old patient, PC, HGPIN, and NPE shared 12 mutations, suggesting that PC and HGPIN diverged from a common normal ancestor approximately 45 years prior. These findings support a model in which PC arises from adjacent, clonally expanded HGPIN lesions.

[Disclosure of COI by Responsible researcher] Lead presenter is not the Responsible researcher.
[Lead presenter: Kohsuke Hishiki] We have no financial relationships to disclose.
[Principal Researcher: Seishi Ogawa] ■Advisor of the company and/or profit-making organization: エーザイ株式会社、Chordia Therapeutics Inc.、Montage Bio, INC.　■Profit of stock: 朝日ゲノミクス株式会社　■Lecture fee: 公益財団法人三菱財団、公益財団法人中谷医工計測技術振興財団　■Research expenses: エーザイ株式会社、Chordia Therapeutics Inc.　■Contributions: 日本新薬株式会社　■Endowed chair: Chordia Therapeutics Inc.、公益社団法人鹿児島共済会 南風病院

### [P-3109] I-P7-4
### Pathophysiology based on clonal evolution

2025 / 9 / 27 (Sat) 16:15-17:00 Room P/(Motenashi Dome) Room P

---

### P-3109
### Splicing Junction Classifier for the Detection of Abnormal KEAP1-NRF2 System Activation

[Speaker]Raul N. Mateos (Div. Genome Analysis Platform Development, National Cancer Center Research Center) [Co-author]Wira Winardi (Department of Respiratory Medicine, Juntendo University Graduate School of Medicine)、Kenichi Chiba (Div. Genome Analysis Platform Development, National Cancer Center Research Center)、Ai Okada (Div. Genome Analysis Platform Development, National Cancer Center Research Center)、Ayako Suzuki (Department of Computational Biology and Medical Sciences, University of Tokyo)、Yoichiro Mitsuishi (Department of Respiratory Medicine, Juntendo University Graduate School of Medicine)、Yuichi Shiraishi (Div. Genome Analysis Platform Development, National Cancer Center Research Center)

Abnormal persistent activation of the KEAP1-NRF2 system in cancer has been shown to drive overexpression of NRF2-target genes, resulting in not only increased resistance to oxidative stress but also the appearance of abnormal splicing junctions throughout the genome. This study proposes a novel approach that integrates abnormal splicing patterns in a naive Bayes-based classifier to detect persistent activation of the KEAP1-NRF2 system. The classifier was applied to the large-scale dataset of The Cancer Genome Atlas (TCGA) and the Sequence Read Archive (SRA), demonstrating comparable precision against naive gene expression-based methods while identifying mutations previously missed on both datasets. Our results suggest that abnormal splicing junctions caused by overexpression can serve as a powerful biomarker for detecting abnormal pathway activations in cancer, offering a promising path for advancing cancer diagnosis and treatment.

[Disclosure of COI by Responsible researcher] Lead presenter is the Responsible researcher.
[Lead presenter: Raul N. Mateos] We have no financial relationships to disclose.

▲TOP

### [P-3118] I-P8-5
### Chromatin structure & others

2025 / 9 / 27 (Sat) 16:15-17:00 Room P/(Motenashi Dome) Room P

---

### P-3118
### Integrated Analysis of Epigenome and Chromatin Structure in CAEBV

[Speaker]Shuntaro Isogai (Dep. of Mol. Oncol., Chiba Univ. Grad. Sch. of Med.／Dep. of Endocrinology, Hematology and Gerontology, Chiba Univ. Grad. Sch.) [Co-author]Atsushi Okabe (Dep. of Mol. Oncol., Chiba Univ. Grad. Sch. of Med.／Health and Disease Omics Center, Chiba Univ.)、Shintaro Izumi (Dep. of Endocrinology, Hematology and Gerontology, Chiba Univ. Grad. Sch.／Dep. of Hematology, Chiba Univ. Hosp.)、Bahityar R. Nawai (Dep. of Mol. Oncol., Chiba Univ. Grad. Sch. of Med.)、Masaki Fukuyo (Dep. of Mol. Oncol., Chiba Univ. Grad. Sch. of Med.)、Emiko Sakaida (Dep. of Endocrinology, Hematology and Gerontology, Chiba Univ. Grad. Sch.／Dep. of Hematology, Chiba Univ. Hosp.)、Atsushi Kaneda (Dep. of Mol. Oncol., Chiba Univ. Grad. Sch. of Med.／Health and Disease Omics Center, Chiba Univ.)

Chronic active EBV disease (CAEBV) is a lymphoproliferative disorder with a poor prognosis involving the clonal proliferation of EBV-infected T/NK cells. Previously, we showed that EBV infection alters host epigenomes and contributes to the development of EBV-associated gastric and nasopharyngeal carcinomas. However, the epigenomic landscape of CAEBV remains largely unexplored. Here, we comprehensively analyzed EBV-host 3D interactions using CAEBV cell lines (SNK10, SNT15, and SNT16) by Hi-C and 4C-seq, combined with ChIP-seq to analyze histone marks (H3K27ac, H3K4me1, and H3K4me3) in CAEBV cell lines and control T cells (CD3+ from healthy donors). Hi-C data revealed distinct compartment profiles, and clustering analysis clearly separated CAEBV and control cells. Active histone marks also differed significantly, stratifying the cells into distinct subclusters. Furthermore, 4C-seq analysis revealed that EBV-host genome interaction regions were significantly enriched in areas that switched from B to A compartments in CAEBV cells. These results suggest that physical interactions between EBV and the host genomes in CAEBV may contribute to oncogene activation.

[Disclosure of COI by Responsible researcher] Lead presenter is not the Responsible researcher.
[Lead presenter: Shuntaro Isogai] We have no financial relationships to disclose.
[Principal Researcher: Atsushi Kaneda] ■Research expenses: SRL Inc., Scrum Inc.　■Contributions: Takeda Science Foundation., Uehara Memorial Foundation.

### [P-3119] I-P8-5
### Chromatin structure & others

2025 / 9 / 27 (Sat) 16:15-17:00 Room P/(Motenashi Dome) Room P

### P-3119
### Analysis of 3D chromatin architecture in PDAC

[Speaker]Wataru Kawase (Div. of Advanced Cancer Therap., Kanagawa Cancer Ctr. Res. Inst.／Ctr. for Cancer Genome Med., Kanagawa Cancer Ctr.) [Co-author]Yuri Demachi (Div. of Advanced Cancer Therap., Kanagawa Cancer Ctr. Res. Inst.／Ctr. for Cancer Genome Med., Kanagawa Cancer Ctr.)、Rika Kasajima (Ctr. for Cancer Genome Med., Kanagawa Cancer Ctr.／Mol. Pathol. & Genetics Div., Kanagawa Cancer Ctr. Res. Inst.)、Seira Hatakeyama (Div. of Advanced Cancer Therap., Kanagawa Cancer Ctr. Res. Inst.／Ctr. for Cancer Genome Med., Kanagawa Cancer Ctr.)、Yukihiko Hiroshima (Div. of Advanced Cancer Therap., Kanagawa Cancer Ctr. Res. Inst.／Ctr. for Cancer Genome Med., Kanagawa Cancer Ctr.)

The genomic DNA of eukaryotic cells is organized into a three-dimensional (3D) structure within the nucleus, playing a key role in gene regulation. Hi-C, a genome-wide method for analyzing 3D chromatin structure, has shown that DNA is folded into higher-order structures such as nuclear compartments, topologically associating domains and promoter-enhancer interactions. Recent studies suggest that disruption of these structures has been implicated in cancer development. Thus, combining Hi-C with multi-omics data can provide valuable insights into cancer diagnosis and treatment. Pancreatic ductal adenocarcinoma (PDAC) is one of the most lethal cancers, with a rising global incidence. The 5-year survival rate remains low, as most patients are diagnosed at an advanced or metastatic stage. Chemotherapy regimens such as FOLFIRINOX and gemcitabine/nab-paclitaxel have slightly improved outcomes, but overall prognosis remains poor. Therefore, new therapeutic targets and strategies are urgently needed. In this study, we analyzed 3D chromatin structure using normal pancreatic and pancreatic cancer cell lines. We identified PDAC-specific changes in chromatin structure, which will be presented.

[Disclosure of COI by Responsible researcher] Lead presenter is not the Responsible researcher.
[Lead presenter: Wataru Kawase] We have no financial relationships to disclose.
[Principal Researcher: Yukihiko Hiroshima] We have no financial relationships to disclose.

### [P-3120] I-P8-5
### Chromatin structure & others

2025 / 9 / 27 (Sat) 16:15-17:00 Room P/(Motenashi Dome) Room P

### P-3120
### Organization of the centromeric chromatin in cells entering mitosis

[Speaker]Hideki Wada (Div. Exp. Pathol., Cancer Inst., JFCR／Dept. JFCR., Institute of Science Tokyo) [Co-author]Ryusuke Nozawa (Div. Exp. Pathol., Cancer Inst., JFCR)、Toru Hirota (Div. Exp. Pathol., Cancer Inst., JFCR／Dept. JFCR., Institute of Science Tokyo)

One of the hallmarks of cancer is chromosomal instability, where chromosomes fail to be evenly transmitted to daughter cells. Fail-free chromosome segregation requires the correct attachment of microtubules to kinetochore pairs assembled at centromeres in mitosis. Centromere is therefore a key chromosomal domain defined by alpha-satellite DNA, but how centromeric chromatin structure is organized is not well understood. In this study, we focused to a centromere protein called CENP-B that specifically bind to alpha-satellite DNA and is thought to compact centromeric chromatin. To map CENP-B along the centromeres, we conducted ChIP-sequencing analysis. The resulting genomic distribution across the entire centromere was then correlated with its centromeric localization defined by Expansion Microscopy, a high-resolution imaging technique. Next, to investigate how CENP-B might contribute to assembly of chromatin architecture, we monitored the distance between sister kinetochores and found that centromeric compaction in G2 phase was specifically impaired without CENP-B. These results imply how centromeric DNA is organized in which CENP-B plays an essential role during the G2/M transition.

[Disclosure of COI by Responsible researcher] Lead presenter is not the Responsible researcher.
[Lead presenter: Hideki Wada] We have no financial relationships to disclose.
[Principal Researcher: Toru Hirota] We have no financial relationships to disclose.

### [P-3121] I-P8-5
### Chromatin structure & others

2025 / 9 / 27 (Sat) 16:15-17:00 Room P/(Motenashi Dome) Room P

---

### P-3121
### Spatial Elucidation of the Transcriptional Regulatory Mechanisms of Differentiation Abnormalities in Lung Adenocarcinoma

[Speaker]Dongchen He (Grad. Sch. of Front. Sci., Univ. Tokyo) [Co-author]Ayako Suzuki (Grad. Sch. of Front. Sci., Univ. Tokyo)、Yutaka Suzuki (Grad. Sch. of Front. Sci., Univ. Tokyo)、Junko Zenkoh (Grad. Sch. of Front. Sci., Univ. Tokyo)、Kotaro Nomura (Dept. Thoracic Surg., Natl. Cancer Ctr. Hosp. East)、Hiroto Katoh (Div. Pathol., EPOC, Natl. Cancer Ctr.)、Shumpei Ishikawa (Div. Pathol., EPOC, Natl. Cancer Ctr.)、Masahiro Tsuboi (Dept. Thoracic Surg., Natl. Cancer Ctr. Hosp. East)、Genichiro Ishii (Dept. Pathol. Clin. Lab., Natl. Cancer Ctr. Hosp. East)

Differentiation abnormalities in tumor cells drive tumor microenvironment formation and therapy resistance. Using spatial omics, we investigated transcriptional regulatory mechanisms underlying these abnormalities in lung adenocarcinoma. The study integrates Xenium for subcellular gene expression visualization and PhenoCycler for multiplex immunostaining analysis at single-cell resolution in hundreds of thousands of cells. Further, we conducted Curio Trekker for simultaneous transcriptomic and epigenomic (ATAC) profiling. Optimized protocols yielded spatially-resolved single-cell RNA and open chromatin profiles in over 2000 cells at the first experiment. Curio Trekker identified over 30000 high-confidence ATAC peaks, enabling precise chromatin accessibility mapping. The detected omics patterns changed from well-differentiated to hypoxic/de-differentiated regions. This integrative approach spatially maps epigenetic regulators, gene expression, and protein profiles linked to differentiation states, offering insights beyond conventional methods. Our findings deepen the understanding of molecular mechanism of tumor progression and support the development of next-generation therapies.

[Disclosure of COI by Responsible researcher] Lead presenter is not the Responsible researcher.
[Lead presenter: Dongchen He] We have no financial relationships to disclose.
[Principal Researcher: Ayako Suzuki] We have no financial relationships to disclose.

### [P-3122] I-P8-5
### Chromatin structure & others

2025 / 9 / 27 (Sat) 16:15-17:00 Room P/(Motenashi Dome) Room P

---

### P-3122
### Elucidation of the Molecular Mechanisms of Key Epigenomic regulator in Hepatoblastoma Cells

[Speaker]Ryoya Furugane (Dept. Mol. Oncol., Grad. Sch. Med., Chiba Univ.／Dept. Ped. Surg., Grad. Sch. Med., Chiba Univ.) [Co-author]Wataru Kudo (Dept. Mol. Oncol., Grad. Sch. Med., Chiba Univ.／Dept. Ped. Surg., Grad. Sch. Med., Chiba Univ.／Health and Disease Omics Center, Chiba Univ)、Takayuki Hoshii (Dept. Mol. Oncol., Grad. Sch. Med., Chiba Univ.)、Motoaki Seki (Dept. Mol. Oncol., Grad. Sch. Med., Chiba Univ.)、Atsushi Okabe (Dept. Mol. Oncol., Grad. Sch. Med., Chiba Univ.／Health and Disease Omics Center, Chiba Univ)、Masaki Fukuyo (Dept. Mol. Oncol., Grad. Sch. Med., Chiba Univ.)、Rahmutulla Bahityar (Dept. Mol. Oncol., Grad. Sch. Med., Chiba Univ.)、Tomoro Hishiki (Dept. Ped. Surg., Grad. Sch. Med., Chiba Univ.)、Atsushi Kaneda (Dept. Mol. Oncol., Grad. Sch. Med., Chiba Univ.／Health and Disease Omics Center, Chiba Univ)

In hepatoblastoma (HB), epigenomic alterations reportedly contribute to HB development along with ***CTNNB1*** mutations. However, its mechanisms are not fully clarified. Here, we conducted an integrative analysis of multiple public databases, including DepMap, Epifactors, and KEGG, and identified ***EP300*** as a key epigenetic regulator involved in HB cell proliferation. siRNA knockdown of ***EP300*** significantly suppressed HB cell proliferation. ChIP-seq analysis for p300 revealed 3421 and 3307 peaks at promoter and enhancer regions, overlapping with H3K27ac (91.9%) and ATAC-seq peaks (81.9%). Among the p300 -bound genes, 3092 and 1443 genes possessed active promoter and enhancers. Genes with p300 binding at active promoters were significantly enriched in the cell cycle pathway. Among ***EP300*** target genes, several oncogenes were listed in public databases. In addition, 22 genes were classified as essential in DepMap data, and half of them showed > 0.5-fold upregulation in HB clinical samples compared to background liver. These findings suggest a critical role of ***EP300*** in HB development. Functional analyses on these downstream targets are underway.

[Disclosure of COI by Responsible researcher] Lead presenter is not the Responsible researcher.
[Lead presenter: Ryoya Furugane] We have no financial relationships to disclose.
[Principal Researcher: Atsushi Kaneda] ■Research expenses: SRL Inc., Scrum Inc. ■Contributions: Takeda Science Foundation., Uehara Memorial Foundation.

▲TOP

### [P-3123] I-P8-5
### Chromatin structure & others

2025 / 9 / 27 (Sat) 16:15-17:00 Room P/(Motenashi Dome) Room P

---

### P-3123
### Effects of transient expression of Yamanaka factors to reprogram ARID1A-knockout gastric cells

[Speaker]Daichi Komiyama (Dept. Mol Oncol, Grad. Sch. Med., Chiba Univ.) [Co-author]Ryoji Fujiki (Dept. Mol Oncol, Grad. Sch. Med., Chiba Univ.)、Atsushi Okabe (Dept. Mol Oncol, Grad. Sch. Med., Chiba Univ.／Health and Disease Omics Ctr., Chiba Univ.)、Motoaki Seki (Dept. Mol Oncol, Grad. Sch. Med., Chiba Univ.)、Tomoka Okada (Dept. Mol Oncol, Grad. Sch. Med., Chiba Univ.)、Masaki Fukuyo (Dept. Mol Oncol, Grad. Sch. Med., Chiba Univ.)、Takayuki Hoshii (Dept. Mol Oncol, Grad. Sch. Med., Chiba Univ.)、Yasuhiro Yamada (Dept. Mol Path., Grad. Sch. Med., The Univ. Tokyo)、Atsushi Kaneda (Dept. Mol Oncol, Grad. Sch. Med., Chiba Univ.／Health and Disease Omics Ctr., Chiba Univ.)

ARID1A is a subunit of SWI/SNF chromatin remodeling complexes. While ARID1A is frequently mutated in gastric cancer, we and others previously showed that ARID1A knockout in gastric cells exhibited aberrant accumulation of DNA methylation over time. While Yamanaka 4 factors (Oct4, Sox2, Klf4, c-Myc; OSKM) are known to induce cell-fate reprogramming, we here knocked out ARID1A in gastric cells GES1 and cultured for 0 or 20 weeks (KO_0wk and KO_20wk), and examined the effect of transient OSKM expression using doxycycline-inducible system with 3 days on and 4 days off cycles. GES1_WT cells treated with OSKM for 0, 1, 2, and 4 cycles were clustered together in principal component analysis on RNA-seq, suggesting limited effect of transient OSKM induction on GES1_WT. Genes upregulated in ARID1A_KO_20wk compared to ARID1A_KO_0wk, e.g. inflammatory signature genes, were suppressed by transient OSKM expression, and KO_20wk cells treated with OSKM for 4 cycles were clustered together with KO_0wk cells with/without OSKM, but not with GES1_WT. OSKM induction may hardly affect cell-fate alteration in KO_0wk, but could intervene additional alteration in KO_20wk and reverse to KO_0wk state.

[Disclosure of COI by Responsible researcher] Lead presenter is not the Responsible researcher.
[Lead presenter: Daichi Komiyama] We have no financial relationships to disclose.
[Principal Researcher: Atsushi Kaneda] ■Research funds from company: SRL Inc., Scrum Inc., Tsubakimoto Chain Co.
■Contributions: Wedge Co.

### [P-3124] I-P8-5
### Chromatin structure & others

2025 / 9 / 27 (Sat) 16:15-17:00 Room P/(Motenashi Dome) Room P

---

### P-3124
### Breast CAF-derived exosomal miR-1290 promotes TNBC cell proliferation by targeting the TBC1D4-TSC1 axis

[Speaker]Jinkwon Lee (Korea Research Institute of Bioscience and Biotechnology) [Co-author]In Hwan Tae (Korea Research Institute of Bioscience and Biotechnology)、Yunsang Kang (Korea Research Institute of Bioscience and Biotechnology／Korea University of Science and Technology)、Jeongmin Lee (Korea Research Institute of Bioscience and Biotechnology／Korea University of Science and Technology)、Jinsan Kim (Korea Research Institute of Bioscience and Biotechnology／Korea University of Science and Technology)、Dae-Soo Kim (Korea Research Institute of Bioscience and Biotechnology／Korea University of Science and Technology)、Mi-Young Son (Korea Research Institute of Bioscience and Biotechnology／Korea University of Science and Technology)、Hyun-Soo Cho (Korea Research Institute of Bioscience and Biotechnology／Korea University of Science and Technology)

Breast cancer remains a leading cause of cancer mortality in women, with triple-negative breast cancer (TNBC) lacking effective targeted therapies. This study investigated the role of cancer-associated fibroblast (CAF) exosomes in TNBC progression. We identified exosomal miR-1290 as a key component derived from CAFs. When delivered to TNBC cells, miR-1290 suppressed TBC1D4 expression, leading to TSC1 downregulation and mTOR pathway activation. The miR-1290-TBC1D4-TSC1 axis promoted TNBC cell growth, as validated in 2D and 3D models and in vivo xenografts. Treatment with mTOR inhibitors rapamycin and everolimus effectively suppressed miR-1290-induced TNBC cell proliferation. In patient cohorts, elevated circulating miR-1290 distinguished breast cancer patients from healthy individuals and correlated with poor survival. The miR-1290-TBC1D4-TSC1 axis has potential value for both targeted treatment and diagnosis of TNBC.

[Disclosure of COI by Responsible researcher] Lead presenter is the Responsible researcher.
[Lead presenter: Jinkwon Lee] We have no financial relationships to disclose.

### [P-3125] I-P8-5
### Chromatin structure & others

2025 / 9 / 27 (Sat) 16:15-17:00 Room P/(Motenashi Dome) Room P

---

### P-3125
### Increased m5C RNA Methylation of HDGF Induces Cell Growth and Proliferation in Human Colorectal Cancer

[Speaker]Sanghun Jeong (Dept. of Biochem., Sch. of Med., Kyungpook Natl. Univ.／BK21 Plus KNU, Dep. of Biomed. Sci., Kyungpook Natl. Univ.) [Co-author]Eun Hwangbo (Dept. of Biochem., Sch. of Med., Kyungpook Natl. Univ.／BK21 Plus KNU, Dep. of Biomed. Sci., Kyungpook Natl. Univ.)、Gyeonghwa Kim (Dept. of Biochem., Sch. of Med., Kyungpook Natl. Univ.／Cell & Matrix Res. Inst., Sch. of Med., Kyungpook Natl. Univ.)、Hye Won Lee (Dep. of Pathol., Dongsan Med. Center, Keimyung Univ.)、Soo Young Park (Dep. of Internal Med., Kyungpook Natl. Univ.)、Won Young Tak (Dep. of Internal Med., Kyungpook Natl. Univ.)、Keun Hur (Dept. of Biochem., Sch. of Med., Kyungpook Natl. Univ.／BK21 Plus KNU, Dep. of Biomed. Sci., Kyungpook Natl. Univ.／Cell & Matrix Res. Inst., Sch. of Med., Kyungpook Natl. Univ.)

Recently, RNA methylations such as 5–methylcytosine (m5C) have emerged as key regulators of RNA stability and protein synthesis, although their role in colorectal cancer (CRC) remains unclear. This study investigated the effects of m5C methylation on the 3´UTR of HDGF mRNA in colorectal cancer cells. We examined both CRC cell lines and clinical samples obtained from CRC patients, including matched primary tumor tissues and adjacent normal colon tissues. CRC cells were cultured under varying conditions with different concentrations of FBS, glucose, and insulin. The levels of RNA methylation were assessed using quantitative bisulfite pyrosequencing with the PyroMark Q48 Autoprep system (Qiagen). Elevated m5C methylation on the 3´UTR of HDGF mRNA was observed in CRC cells cultured with high FBS, glucose, or insulin, along with increased HDGF protein expression. Clinical CRC tissues also showed higher m5C levels and HDGF protein compared to adjacent normal tissues. These results suggest that m5C methylation of HDGF mRNA is a distinct feature of CRC and may enhance mRNA stability, promoting cancer cell growth and proliferation.

[Disclosure of COI by Responsible researcher] Lead presenter is not the Responsible researcher.
[Lead presenter: Sanghun Jeong] We have no financial relationships to disclose.
[Principal Researcher: Keun Hur] We have no financial relationships to disclose.

▲TOP

### [P-3135] I-P9-6
### Metastasis & extracellular matrix

2025 / 9 / 27 (Sat) 16:15-17:00 Room P/(Motenashi Dome) Room P

---

### P-3135
### Matrix Stiffness Contributes to Tumor Progression in Intrahepatic Cholangiocarcinoma

[Speaker]Kei Naito (Dept. of General Surgery, Graduate School of Medicine, Chiba Univ.) [Co-author]Shigetsugu Takano (Dept. of General Surgery, Graduate School of Medicine, Chiba Univ.)、Kensuke Suzuki (Dept. of General Surgery, Graduate School of Medicine, Chiba Univ.)、Takashi Mishima (Dept. of General Surgery, Graduate School of Medicine, Chiba Univ.)、Takanori Konishi (Dept. of General Surgery, Graduate School of Medicine, Chiba Univ.)、Masayuki Otsuka (Dept. of General Surgery, Graduate School of Medicine, Chiba Univ.)

We investigated the functional roles of extracellular matrix (ECM) stiffness in tumor progression of intrahepatic cholangiocarcinoma (ICC). Tumor stiffness was quantitatively assessed preoperatively using shear wave elastography (SWE) in patients undergoing surgical resection. The stiffness ranged from 13.6 to 74.9 kPa. Higher stiffness was associated with larger tumor size (p=0.074), lymph node metastasis (p=0.134), and large duct subtype based on WHO classification(p=0.061). For in vitro analysis, we developed a 3D culture model of ICC using hydrogels with adjustable stiffness: soft (0.9-1.5 kPa), medium (14-20 kPa), and hard (35-47 kPa). Cells cultured under each condition exhibited distinct morphological phenotypes and differences in proliferation. These findings suggest that ECM stiffness contributes to ICC progression through mechanobiological regulation. Ongoing studies involve comprehensive pathological evaluation of 102 archived ICC specimens, along with tandem mass tag labelling-based quantitative proteomics with LC-MS/MS analysis to identify matrix stiffness-responsive molecular pathways with potential clinical relevance.

[Disclosure of COI by Responsible researcher] Lead presenter is not the Responsible researcher.
[Lead presenter: Kei Naito] We have no financial relationships to disclose.
[Principal Researcher: Masayuki Ohtsuka] We have no financial relationships to disclose.

▲TOP

### [P-3136] I-P9-6
### Metastasis & extracellular matrix

2025 / 9 / 27 (Sat) 16:15-17:00 Room P/(Motenashi Dome) Room P

---

### P-3136
### Elucidating the Mechanisms of Intercellular Interactions of Perineural Invasion in Pancreatic Cancer Microenvironment

[Speaker]Satoru Furuhashi (Department of Surgery, Hamamatsu University School of Medicine) [Co-author]Yoshifumi Morita (Department of Surgery, Hamamatsu University School of Medicine)、Koki Oda (Department of Surgery, Hamamatsu University School of Medicine)、Akio Matsumoto (Department of Surgery, Hamamatsu University School of Medicine)、Shinya Ida (Department of Surgery, Hamamatsu University School of Medicine)、Ryuta Muraki (Department of Surgery, Iwata City Hospital)、Makoto Takeda (Department of Surgery, Hamamatsu University School of Medicine)、Hirotoshi Kikuchi (Department of Surgery, Hamamatsu University School of Medicine)、Yoshihiro Hiramatsu (Department of Surgery, Hamamatsu University School of Medicine／Department of Perioperative Functioning Care and Support)、Hiroya Takeuchi (Department of Surgery, Hamamatsu University School of Medicine)

Background. PDAC is highly invasive and treatment-resistant. Perineural invasion (PNI), where cancer cells infiltrate perineural spaces, worsens prognosis. The tumor microenvironment (TME), including PSCs, immune cells, and neurons, plays a key role, however its mechanisms remain unclear.Methods.We established in vitro co-culture models of PDAC cells with dorsal root ganglion (DRG) neurons and a multi-co-culture system with PSCs. Migration assays, proteomic analysis, and immunohistochemistry identified PNI-related factors. TCGA and scRNA-seq validated the findings and evaluated the clinical relevance.Results.PDAC cells showed enhanced migration when co-cultured with PSCs. Proteomic analysis identified 51 upregulated proteins, with SPARC linked to poor prognosis. In silico analysis confirmed SPARC expression in PSCs and its correlation with shorter survival. Immunohistochemistry showed a strong correlation between SPARC and positive PNI.Conclusions.This study highlights roles of PSC in PNI via the cancer-neuron-immune axis. SPARC is a key regulator and potential therapeutic target, offering insights for TME-targeted PDAC treatments.

[Disclosure of COI by Responsible researcher] Lead presenter is not the Responsible researcher.
[Lead presenter: Satoru Furuhashi] We have no financial relationships to disclose.
[Principal Researcher: Hiroya Takeuchi] We have no financial relationships to disclose.

### [P-3137] I-P9-6
### Metastasis & extracellular matrix

2025 / 9 / 27 (Sat) 16:15-17:00 Room P/(Motenashi Dome) Room P

---

### P-3137
### The ability of proliferation in a soft environment promotes lung metastasis of osteosarcoma

[Speaker]Yoshihiro Yui (Research Institute, Nozaki Tokushukai Hospital) [Co-author]Jun Kumai (Research Institute, Nozaki Tokushukai Hospital)

<Background> Osteosarcoma often causes lung metastasis after primary tumor resection; however, effective treatments remain limited. The 5-year survival rate has stagnated at 60 - 70% over the past 30 years. We previously showed that lung fibrosis promotes osteosarcoma metastasis and that anti-fibrotic agents can suppress it. Osteosarcoma cells can also proliferate in the early soft, poorly adhesive lung microenvironment before fibrosis. This study investigates mechanisms enabling their growth under such conditions. <Results> RNA-seq comparing highly metastatic LM8 and low-metastatic Dunn cells cultured on soft substrates revealed upregulation of ribosome-related genes in LM8 cells. In vivo CRISPR activation library screening also showed enrichment of ribosome-related genes in lung metastases. LM8 cells exhibited higher ribosome activity under soft conditions, confirmed by increased pre-rRNA levels and FUrd incorporation. RNA polymerase I inhibition significantly suppressed lung metastasis in both tail vein injection and subcutaneous models. <Conclusion> Ribosome activity supports osteosarcoma proliferation in soft environments and may play a key role in lung metastasis.

[Disclosure of COI by Responsible researcher] Lead presenter is the Responsible researcher.
[Lead presenter: Yoshihiro Yui] We have no financial relationships to disclose.

[P-3138] I-P9-6
Metastasis & extracellular matrix

2025 / 9 / 27 (Sat) 16:15-17:00 Room P/(Motenashi Dome) Room P

P-3138

### Alternative splicing of ***FBLN2*** generates a prometastatic tumor microenvironment in gastrointestinal cancers

[Speaker]Ryo Funayama (Anticancer Strategies Lab., Advanced Res. Initiative, Inst. Sci. Tokyo) [Co-author]Yujue Wang (Dept. of Cell Proliferation, Grad. Sch. of Med., Tohoku Univ.／Dept. of Medical Biochemistry, Grad. Sch. of Med., Tohoku Univ.)、Masaki Hosogane (Dept. of Cell Proliferation, Grad. Sch. of Med., Tohoku Univ.)、Wei C. Kao (Dept. of Cell Proliferation, Grad. Sch. of Med., Tohoku Univ.)、Shingo Toyama (Dept. of Surgery, Grad. Sch. of Med., Tohoku Univ.)、Masaki Matsumoto (Grad. Sch. of Med. Dent. Sci., Niigata Univ.)、Keiko Nakayama (Dept. of Cell Proliferation, Grad. Sch. of Med., Tohoku Univ.／Biosci. Ctr., Inst. Sci. Tokyo)、Keiichi I. Nakayama (Anticancer Strategies Lab., Advanced Res. Initiative, Inst. Sci. Tokyo)

Most extracellular matrix (ECM)-related genes undergo extensive alternative splicing that generates multiple protein isoforms. Fibulin 2 (FBLN2) is an ECM glycoprotein that exists as two alternative splice variants - variant 1 and variant 2 - with only variant 1 including the amino acid sequence encoded by exon 9. Exclusion of exon 9 of ***FBLN2*** is one of the most recurrent splicing events across multiple types of solid cancer, but its functional relevance in cancer has remained unexplored. We here reveal that the exclusion of exon 9 of ***FBLN2*** results in the loss of a single N-glycosylation site that leads to misfolding of the FBLN2 protein as well as to a reduction in both its stability and secretion efficiency. Indeed, the ECM of human colorectal cancer tissue exhibits a reduced abundance of FBLN2. This deficiency of FBLN2 together with a concomitant increase in the abundance of fibronectin 1 in the tumor microenvironment promotes the adhesion and migration of colorectal cancer cells. Our data thus suggest that the alternative splicing of ***FBLN2*** exon 9 generates a prometastatic extracellular environment in cancer tissue by determining FBLN2 glycosylation.

[Disclosure of COI by Responsible researcher] Lead presenter is not the Responsible researcher.
[Lead presenter: Ryo Funayama] ■Research funds from company: 公的研究費（文部科学省 科学研究費助成事業）
[Principal Researcher: Keiichi I. Nakayama] We have no financial relationships to disclose.

### [P-3139] I-P9-6
### Metastasis & extracellular matrix

2025 / 9 / 27 (Sat) 16:15-17:00 Room P/(Motenashi Dome) Room P

---

### P-3139
### Hypoxia increased the hCXCL1 production from gastric cancer cells

[Speaker]Daiki Imanishi (Mol. Oncology & Therap., Osaka Metropolitan Univ.) [Co-author]Masakazu Yashiro (Mol. Oncology & Therap., Osaka Metropolitan Univ. ／Gastroenterological Surg., Osaka Metropolitan Univ.)、Hinano Nishikubo (Mol. Oncology & Therap., Osaka Metropolitan Univ.)、Dongheng Ma (Mol. Oncology & Therap., Osaka Metropolitan Univ.)、Yurie Yamamoto (Mol. Oncology & Therap., Osaka Metropolitan Univ.)、Canfeng Fan (Mol. Oncology & Therap., Osaka Metropolitan Univ.)、Tomoya Sano (Mol. Oncology & Therap., Osaka Metropolitan Univ. ／Gastroenterological Surg., Osaka Metropolitan Univ.)、Takashi Sakuma (Mol. Oncology & Therap., Osaka Metropolitan Univ. ／Gastroenterological Surg., Osaka Metropolitan Univ.)、Koji Maruo (Mol. Oncology & Therap., Osaka Metropolitan Univ. ／Gastroenterological Surg., Osaka Metropolitan Univ.)、Kiyoshi Maeda (Gastroenterological Surg., Osaka Metropolitan Univ.)

[Background] It has been reported that cancer-associated fibroblasts (CAFs) might originate from bone marrow mesenchymal stem cells via hCXCL1/CXCR2 signaling. Since solid tumors show hypoxia in the tumor tissue, hypoxic conditions might regulate hCXCL1 production from cancer cells. This study was conducted to clarify the relationship between hypoxia and hCXCL1 production from cancer cells.[Methods] Five cancer cell lines, OCUM-12, OCUG-1, PANC-1, MIA PaCa-2, RWP-1, and a CAF were cultured under various oxygen conditions, 20% O2, 5% O2, and 1% O2. hCXCL1 in each conditioned medium was measured by ELISA. [Results] hCXCL1/cell counts were maintained even in hypoxia for OCUM-12. OCUG-1 showed similar results to OCUM-12, but the other cell lines and CAF decreased with hypoxia. [Discussion] hCXCL1 production from cancer cells were different in cell types and O2 conditions. The hypoxic environment caused by the tumor may promote hCXCL1 production by tumor cells, which may facilitate the induction of CAF by increasing hCXCL1 levels.[Conclusion] Hypoxia in tumor environment may increase hCXCL1 production from cancer cells, resulting in CAF induction in the cancer stroma.

[Disclosure of COI by Responsible researcher] Lead presenter is not the Responsible researcher.
[Lead presenter: Daiki Imanishi] We have no financial relationships to disclose.
[Principal Researcher: Masakazu Yashiro] We have no financial relationships to disclose.

### [P-3140] I-P9-6
### Metastasis & extracellular matrix

2025 / 9 / 27 (Sat) 16:15-17:00 Room P/(Motenashi Dome) Room P

---

### P-3140
### Shared morphological features between migrating neurons and invading glioblastoma in the adult brain

[Speaker]Masato Sawada (Dept Dev Regen Neurobiol, IBS, Nagoya City Univ, Japan／Div Neural Dev Regen, NIPS, Japan) [Co-author]Yuzuki Hara (Dept Dev Regen Neurobiol, IBS, Nagoya City Univ, Japan)、Kazunobu Sawamoto (Dept Dev Regen Neurobiol, IBS, Nagoya City Univ, Japan／Div Neural Dev Regen, NIPS, Japan)

The adult mammalian brain consists of functional neuronal circuits, mature glial cells, and established vascular network. Even such a non-permissive environment, adult-born immature neurons, which are generated from neural stem cells in the ventricular-subventricular zone (V-SVZ) lining the walls of the lateral ventricles, could actively migrate toward their destinations. The active cellular migration in the adult brain could be also observed in the process of glioblastoma progression, in which malignant cells invade into the brain parenchyma. However, the morphological commonality between migrating neurons and invading glioblastoma cells remains to be fully addressed.
Here we analyzed the morphological features, migratory scaffolds, and interaction with surrounding environment in migrating neurons and invading glioblastoma cells in the adult rodent brain. We found that both cells form elongated cell clusters and interact with surrounding brain parenchymal cells during their migration. These results show the shared morphological features between migrating neurons and invading glioblastoma cells, which could be a strategy for active migration and invasion in the adult brain.

[Disclosure of COI by Responsible researcher] Lead presenter is the Responsible researcher.
[Lead presenter: Masato Sawada] ■Research funds from company: 科研費 基盤研究C

### [P-3141] I-P9-6
### Metastasis & extracellular matrix

2025 / 9 / 27 (Sat) 16:15-17:00 Room P/(Motenashi Dome) Room P

---

### P-3141
### The Role of Aligned Stromal Fibers-Induced Infiltrating Tumor Cells in Tumor Angiogenesis and Intravasation

[Speaker]Kuan-Cheng Chiu (School of Medicine, National Yang Ming Chiao Tung University, Taiwan) [Co-author]Chia-Yi Su (Institute of Biophotonics, National Yang Ming Chiao Tung University, Taiwan)

Infiltrating tumor cells (ITCs) invading along aligned tumor stromal fibers promote metastasis, yet the underlying mechanisms remain largely unclear. Our previous study suggested the potential role of ITCs in inducing angiogenesis through upregulating ID3. However, existing in vitro 3D tumor models rarely recapitulate the interaction between ITCs and blood vessels guided by aligned fibers, a key feature of tumor microenvironment. Therefore, this study has developed a 3D ITC-induced angiogenesis model to test whether ITCs invading along aligned fibers induce angiogenesis and intravasation. The 3D tumor model features a tumor spheroid and an endothelial-lined vascular channel on the opposite side, connected by aligned collagen fibers. Then, the model is applied to determine whether and how aligned collagen fibers enhance ITC infiltration and ID3 upregulation and whether the subsequent angiogenesis and intravasation are induced by ID3-highly expressed ITCs. Overall, the 3D ITC-induced angiogenesis model and the mechanism of ITC-induced angiogenesis established in this study may offer insights into metastatic mechanisms and potential therapeutic strategies.

[Disclosure of COI by Responsible researcher] Lead presenter is not the Responsible researcher.
[Lead presenter: Kuan-Cheng Chiu] We have no financial relationships to disclose.
[Principal Researcher: Kuan-Cheng Chiu] We have no financial relationships to disclose.

▲TOP

### [P-3152] I-P10-7
### Cell culture system/Organoid (1)

2025 / 9 / 27 (Sat) 16:15-17:00 Room P/(Motenashi Dome) Room P

---

### P-3152
### Spheroid Morphology of Lung Cancer Cell Lines Correlates with Oncological Profiles

[Speaker]Ryoichiro Doi (Dept. of Surgical Oncology, Nagasaki Univ.) [Co-author]Kaido Oishi (Dept. of Surgical Oncology, Nagasaki Univ.)、Shintaro Hashimoto (Dept. of Surgical Oncology, Nagasaki Univ.)、Fumitake Uchida (Dept. of Surgical Oncology, Nagasaki Univ.)、Ryosuke Hara (Dept. of Surgical Oncology, Nagasaki Univ.)、Masato Nishimuta (Dept. of Surgical Oncology, Nagasaki Univ.)、Mayumi Itawake (Inst. of Innovation for Future Society, Nagoya Univ.)、Takuro Miyazaki (Dept. of Surgical Oncology, Nagasaki Univ.)、Koichiro Shimoyama (Dept. of Surgical Oncology, Nagasaki Univ.)、Daisuke Taniguchi (Dept. of Surgical Oncology, Nagasaki Univ.)、Tomohiro Obata (Dept. of Surgical Oncology, Nagasaki Univ.)、Satoshi Mizoguchi (Dept. of Surgical Oncology, Nagasaki Univ.)、Tetsuro Tominaga (Dept. of Surgical Oncology, Nagasaki Univ.)、Ryota Otsubo (Dept. of Surgical Oncology, Nagasaki Univ.)、Takashi Nonaka (Dept. of Surgical Oncology, Nagasaki Univ.)、Keitaro Matsumoto (Dept. of Surgical Oncology, Nagasaki Univ.)

Multicellular tumor spheroids (MCTS) are recognized as a three–dimensional (3D) culture model that mimics cell–cell interactions and the tumor microenvironment (TME). However, the link between MCTS morphology and tumor characteristics remains unclear. In this study, we generated MCTS from five lung cancer cell lines (H1975, A549, H1299, PC6, and PC9) and categorized them based on aggregation strength: Type–A (tightly aggregated) and Type–B (loosely aggregated). Type–A MCTS were small, compact, and resistant to mechanical stress, while Type–B were larger and more fragile. Type–B cells had significantly higher migration and invasive capacity than Type–A cells, and this trend was even more pronounced in the 3D environment. Furthermore, the expression of EMT/CSC-related markers (VIM, FN1, CD44, SOX2, FOXC2, etc.) and CDH2 was enhanced in migrating and invading cells. Conversely, drug sensitivity assays indicated that Type–A MCTS were more resistant to cisplatin, paclitaxel, and gemcitabine. These results suggest that MCTS morphology is closely related to tumor invasiveness and drug response, and may provide a rapid, simple indicator for assessing malignancy in cancer models.

[Disclosure of COI by Responsible researcher] Lead presenter is the Responsible researcher.
[Lead presenter: Ryoichiro Doi] We have no financial relationships to disclose.

### [P-3153] I-P10-7
### Cell culture system/Organoid (1)

2025 / 9 / 27 (Sat) 16:15-17:00 Room P/(Motenashi Dome) Room P

### P-3153
### Glycolytic Metabolism of Pancreatic Cancer Cells in a Three-Dimensional Culture System

[Speaker]Chikako Yokoyama (Osaka Met. Univ., Grad. Eng., Chem. & Bioeng.) [Co-author]Hinami Ota (Osaka Met. Univ., Grad. Eng., Chem. & Bioeng.)

Solid tumors develop within three-dimensional (3D) microenvironments composed of tumor cells, non-tumor cells, and extracellular matrix components. This study investigated the glycolytic metabolism of pancreatic cancer cells using a 3D culture system, which better mimics the tumor microenvironment than conventional two-dimensional cultures. To assess glycolysis-related gene expression, three pancreatic cancer cell lines were analyzed via real-time PCR, revealing increased mRNA levels in 3D culture. Immunoblotting confirmed the upregulation of GPI and PGK1. Additionally, glucose consumption and lactate production were enhanced, consistent with the Warburg effect, despite reduced cell proliferation. Furthermore, intracellular ATP levels increased in MIA PaCa2 cells but remained unchanged in PANC-1 and SUIT-2 cells, potentially due to differences in aggregate morphology. We also examined the relationship between drug resistance and glycolytic inhibition in 3D-cultured cells and observed distinct responses across different cell lines. These findings indicate that our 3D pancreatic cancer models constitute a valuable platform for investigating drug metabolism.

[Disclosure of COI by Responsible researcher] Lead presenter is the Responsible researcher.
[Lead presenter: Chikako Yokoyama] We have no financial relationships to disclose.

### [P-3154] I-P10-7
### Cell culture system/Organoid (1)

2025 / 9 / 27 (Sat) 16:15-17:00 Room P/(Motenashi Dome) Room P

---

### P-3154
### Development of an Optimized Culture Medium Screening Method for Efficient Cancer Organoid (S-PDO) Establishment

[Speaker]Ayaka Nishio (Fukushima Cell Factory Inc.) [Co-author]Hirosumi Tamura (Translational Research Center, Fukushima Med. Univ.)、Honoka Suzuki (Fukushima Cell Factory Inc.)、Shinya Watanabe (Translational Research Center, Fukushima Med. Univ.)、Hirotaka Hoshi (Fukushima Cell Factory Inc.)

Patient-derived organoids (PDOs) have recently emerged as superior models for replicating clinical conditions compared to conventional cell lines. However, their culture requires specialized expertise and costly supplements, posing financial and technical challenges. This study aimed to develop a cost-effective and user-friendly method for establishing PDOs, enabling researchers with general skills to culture PDOs while preserving cancer-like tissue morphology and gene expression profiles over time. We selected 25 culture conditions by combining a basal medium with cytokines and other factors and evaluated 19 PDO lines (4 colorectal, 1 fallopian tube, 8 lung, 3 ovarian, and 3 peritoneal cancer lines). Primary cultured PDOs were enzymatically dissociated into single cells, seeded at 1,000 cells per well in 96-well plates, and cultured for 1-8 weeks. Cell proliferation was assessed based on cell morphology and ATP measurements. This method allowed for cost-effective and high-throughput screening of optimal culture conditions, significantly enhancing PDO establishment efficiency. To date, we have successfully established over 90 S-PDO lines (suspended patient-derived organoids).

[Disclosure of COI by Responsible researcher] Lead presenter is the Responsible researcher.
[Lead presenter: Ayaka Nishio] We have no financial relationships to disclose.

### [P-3155] I-P10-7
### Cell culture system/Organoid (1)

2025 / 9 / 27 (Sat) 16:15-17:00 Room P/(Motenashi Dome) Room P

---

### P-3155
### Establishment of a TME-Resembling In Vitro Assay System Using Freshly Isolated Human Tumoroids

[Speaker]Ryotaro Tabata (Axcelead Drug Discovery Partners, Inc.) [Co-author]Satoko Unno (Axcelead Drug Discovery Partners, Inc.)、Masahiro Ogawa (Axcelead Drug Discovery Partners, Inc.)、Masashi Yamasaki (Axcelead Drug Discovery Partners, Inc.)、Yasushi Fujitani (Axcelead Drug Discovery Partners, Inc.)、Masayuki Goto (Axcelead Drug Discovery Partners, Inc.)

Conventional 2D and single-cancer cell culture systems are often used for development of novel anti-cancer treatments, but the assay systems fail to mimic complex and heterogeneous tumor microenvironment (TME) and give a limitation to adequate prediction of efficacy of the new treatment options in human cancer. Thus, there is a growing need for in vitro preclinical assay platforms that mimic the patient-specific TME. To address this, we have established an assay system using freshly isolated tumoroids, which were prepared within 24 hours of surgery to remove a tumor, and evaluated efficacy of current standard of cares. In the system we mimic patient-specific TME with intercellular communication by optimizing size and seeding volume. Morphological analyses also suggest that the tumoroids closely mimic the native TME. Furthermore, currently existing anti-cancer drugs revealed marked different effects among donor patients in the system. These findings suggest that our in vitro assay system, in which tumoroids freshly isolated from human tumor tissues were used, may be powerful tool for the development of novel anti-cancer treatment options.

[Disclosure of COI by Responsible researcher] Lead presenter is not the Responsible researcher.
[Lead presenter: Ryotaro Tabata] We have no financial relationships to disclose.
[Principal Researcher: Masayuki Goto] We have no financial relationships to disclose.

### [P-3156] I-P10-7
### Cell culture system/Organoid (1)

2025 / 9 / 27 (Sat) 16:15-17:00 Room P/(Motenashi Dome) Room P

---

### P-3156
### Immune Cell function and Effect of Immune Checkpoint Inhibitor in Freshly Dispersed Human Tumors

[Speaker]Satoko Unno (Axcelead Drug Discovery Partners, Inc.) [Co-author]Ryotaro Tabata (Axcelead Drug Discovery Partners, Inc.)、Masahiro Ogawa (Axcelead Drug Discovery Partners, Inc.)、Masashi Yamasaki (Axcelead Drug Discovery Partners, Inc.)、Yasushi Fujitani (Axcelead Drug Discovery Partners, Inc.)、Masayuki Goto (Axcelead Drug Discovery Partners, Inc.)

In human cancer tissues, tumor cells coexist with various immune cells, and their interaction creates a complex tumor microenvironment (TME). Understanding immune response in the TME is important to elucidate immunosuppressive mechanisms by cancer cells. In this study, we were developing an in vitro assay system with human TME using freshly isolated human cancer tissues. Namely, the fresh tissues were enzymatically dissociated into singe-cell suspensions and tumor-infiltrating immune cells (TILs) composition was analyzed by flow cytometry. T cell response to various stimuli was evaluated, and various immune cells, including PD-$1^{high}$, $CD8^+$ /$CD4^+$ T cells and tumor-associated macrophages were found to infiltrate into the tumor tissues. Anti-CD3 antibody clearly induced IFN$\gamma$ production, but not cell proliferation, suggesting that IFN$\gamma$ production would be a reliable marker for T cell activation in the TME. Furthermore, the effect of immune checkpoint inhibitors on IFN$\gamma$ production will be shown. These findings suggest that IFN$\gamma$ production would be a functional marker of TIL activation and that the in vitro assay system may have some characteristic features of the human TME.

[Disclosure of COI by Responsible researcher] Lead presenter is not the Responsible researcher.
[Lead presenter: Satoko Unno] We have no financial relationships to disclose.
[Principal Researcher: Masayuki Goto] We have no financial relationships to disclose.

### [P-3157] I-P10-7
### Cell culture system/Organoid (1)

2025 / 9 / 27 (Sat) 16:15-17:00 Room P/(Motenashi Dome) Room P

---

### P-3157
### Withdrawn

No Abstract

### [P-3158] I-P10-7
### Cell culture system/Organoid (1)

2025 / 9 / 27 (Sat) 16:15-17:00 Room P/(Motenashi Dome) Room P

---

### P-3158
### Targeting BLU554-induced senescent cells to suppress pancreatic ductal adenocarcinoma

[Speaker]Masakazu Fujiwara (Res. Team for Geriatr. Pathol., Tokyo Met. Inst. Geriatr. Gerontol.) [Co-author]Yuuki Shichi (Res. Team for Geriatr. Pathol., Tokyo Met. Inst. Geriatr. Gerontol.)、Yasuko Hasegawa (Res. Team for Geriatr. Pathol., Tokyo Met. Inst. Geriatr. Gerontol.)、Seiichi Shinji (Dep. Gastroenterol. Surg., Nippon Med. Sch.)、Kimimasa Takahashi (Dep. Veterinary Pathol., Nippon Veterinary & Life Science Univ.)、Toshiyuki Ishiwata (Res. Team for Geriatr. Pathol., Tokyo Met. Inst. Geriatr. Gerontol.)

Fibroblast growth factor receptor 4 (FGFR4) is upregulated in approximately 50% of pancreatic ductal adenocarcinoma (PDAC) cases. BLU9931, an FGFR4 inhibitor, suppresses PDAC cell growth and induces cellular senescence. Here, we examined the senescence-inducing effects of BLU554, another FGFR4 inhibitor under clinical trials for hepatocellular carcinoma. We also evaluated whether dasatinib, a senolytic agent that eliminates senescent cells, could reduce the viability of BLU554-treated cells. PDAC cell lines PK-1 and T3M-4 were treated with BLU554. Senescent cells were detected using SPiDER-$\beta$-Gal (Dojindo Lab.), a fluorescent probe for senescence-associated $\beta$-galactosidase activity. Increased $\beta$-galactosidase activity was observed in both cell lines. BLU554 also increased p21 expression and decreased lamin B1 expression, indicating senescence induction. Subsequent dasatinib treatment reduced the viability of BLU554-treated PDAC cells, suggesting effective clearance of senescent cells. Future studies will investigate the combination of BLU554 with other senolytic agents and explore optimal dosing conditions to enhance therapeutic efficacy.

[Disclosure of COI by Responsible researcher] Lead presenter is not the Responsible researcher.
[Lead presenter: Masakazu Fujiwara] We have no financial relationships to disclose.
[Principal Researcher: Toshiyuki Ishiwata] We have no financial relationships to disclose.

[P-3159] I-P10-7
Cell culture system/Organoid (1)

2025 / 9 / 27 (Sat) 16:15-17:00 Room P/(Motenashi Dome) Room P

P-3159
### Analysis of the interaction between tissue-resident macrophages and HrasG12V-expressing cells on mammary organoids

[Speaker]Mihoko Kajita (RItsumeikan Univ. College of Life Sci.) [Co-author]Kazuki Motomura (RItsumeikan Univ. Res. Org. of Sci. & Tech.)、Toshiaki Ohteki (Inst. of Sci. Tokyo, Inst. of Integrated Res.)、Kyoko Shirakabe (RItsumeikan Univ. College of Life Sci.)

It has been not well studied how the immune system is triggered in the early stage of carcinogenesis. We focused on tissue-resident macrophages (tissue macrophages) as immune cells that initiate cancer immunity. In mammary tissues, CX3CR1+ tissue macrophages are attached to the two-layered mammary epithelial tissue of luminal and myoepithelial cells and can be visualized as GFP+ cells in mammary organoids derived from ***Cx3cr1**$^{gfp/+}$* mice. Using lentiviral transduction of "tdTomato-P2A-oncogene" into epithelial cells in mammary organoids, we established the system to visualize the interaction between oncogene-expressing cells (tdTomato+) and tissue macrophages (GFP+). Timelapse analysis of the mammary organoids showed that tissue macrophages recognized and phagocytosed mammary epithelial cells expressing HrasG12V (HrasG12V cells) and that this recognition required the direct interaction and did not involve cell death of HrasG12V cells. Moreover, phosphatidylserine (PS), known as eat me signal, was upregulated at the surface of the plasma membrane of HrasG12V cells, suggesting that tissue macrophages recognized PS and phagocytosed HrasG12V cells.

[Disclosure of COI by Responsible researcher] Lead presenter is the Responsible researcher.
[Lead presenter: Mihoko Kajita] We have no financial relationships to disclose.

### [P-3168] I-P10-9
### Cancer stem cells (1)

2025 / 9 / 27 (Sat) 16:15-17:00 Room P/(Motenashi Dome) Room P

---

### P-3168
### Elucidation of the mechanism of breast cancer progression by C1orf50, a gene of unknown function

[Speaker]Atsushi Fujimura (Kagawa University Faculty of Medicine) [Co-author]Yusuke Otani (Harvard Medical University BIDMC)、Atsushi Tanaka (Harvard Medical University BIDMC)

In breast cancer treatment strategy, it is important to develop methods to identify patients with poor prognosis in the early stages of the disease. We have discovered that the expression level of the previously functionally unknown gene C1orf50 defines the prognosis of breast cancer patients. Histopathological analysis revealed that C1orf50 expression is upregulated in breast cancer cells compared to normal mammary epithelium. Bioinformatics analysis and cell-based experiments also revealed that C1orf50 is required for the maintenance of stemness and cell proliferation in breast cancer. As a molecular mechanism, we found that C1orf50 maintains the activity of YAP/TAZ, a core factor in breast cancer stem cell maintenance. We also found that the expression level of C1orf50 correlates with a group of genes associated with immune evasion. Importantly, the expression level of C1orf50 was found to influence sensitivity to CDK4/6 inhibitors, an important therapeutic target for luminal breast cancer. This study suggests that C1orf50 may be useful in predicting prognosis and determining treatment response in breast cancer.

[Disclosure of COI by Responsible researcher] Lead presenter is the Responsible researcher.
[Lead presenter: Atsushi Fujimura] We have no financial relationships to disclose.

### [P-3169] I-P10-9
### Cancer stem cells (1)

2025 / 9 / 27 (Sat) 16:15-17:00 Room P/(Motenashi Dome) Room P

---

### P-3169
### Fatty acid-binding protein 5 in the intestinal tumorigenesis accelerated by dietary stearic acid

[Speaker]Kazuaki Nakata (National Institute of Global Health and Medicine, JIHS) [Co-author]Keisuke Sekine (Research Institute, National Cancer Center)、Kazuhiko Yamada (National Center for Global Health and Medicine, JIHS)、Norihiro Kokudo (National Center for Global Health and Medicine, JIHS)、Yuki I. Kawamura (National Institute of Global Health and Medicine, JIHS)

Dietary fat increases the risk of intestinal cancer; however, the effect of the fatty acid (FA) composition on tumorigenesis remains obscure. We previously demonstrated that dietary stearic acid (SA) more strongly increased the number of $Ki67^{+}$ proliferative cells expressing FA-binding protein 5 (FABP5) and accelerated tumorigenesis in the intestine than linoleic acid using murine models. In this study, we found that FABP5 inhibition suppressed SA-induced epithelial cell proliferation and tumorigenesis. Intestinal crypts from mice fed with the SA-rich high fat diet (HFD) formed more organoids than those from mice fed with control diet (CD) in the absence of SA. Moreover, the polyp development in $Apc^{Min/+}$ mice remained elevated after the cessation of SA-rich HFD intake. Spatial transcriptomics analysis using the Visium platform revealed that cancer-related genes such as DNA replication and cell cycle were upregulated in the Crypt cluster of SA-rich HFD-fed mice compared with that of CD-fed mice. These data indicate that dietary SA promotes intestinal epithelial cell proliferation and tumorigenesis via an FABP5 and the effect of dietary SA might be maintained in epithelial cells.

[Disclosure of COI by Responsible researcher] Lead presenter is not the Responsible researcher.
[Lead presenter: Kazuaki Nakata] We have no financial relationships to disclose.
[Principal Researcher: Yuki I. Kawamura] We have no financial relationships to disclose.

▲TOP

### [P-3170] I-P10-9
### Cancer stem cells (1)

2025 / 9 / 27 (Sat) 16:15-17:00 Room P/(Motenashi Dome) Room P

---

### P-3170
### The stemness and cancer stemness of Jag2 in the small intestine

[Speaker]Shinichiro Hasegawa (Department of gastroenterological surgery, The university of Osaka) [Co-author]Hisataka Ogawa (Osaka International Cancer Institute)

[Background]The Notch ligand Jag2 has recently been linked to human pancreatic and breast carcinoma. The purpose of this study is to evaluate the role of Jag2 in intestinal carcinogenesis, which is currently unknown. [Results]We have generated a new line of Jag2 knock out (KO) mice and performed phenotypic analysis of these mice. Using x-gal staining, we focused on the intestines due to the selective presence of Jag2 in the intestinal crypts. To evaluate Jag2 function in the "stem cell niche", we have established organoid cultures. We found that organoids derived from Jag2 heterozygous mice could not be maintained beyond one passage. This suggests reduced stemness of Jag2 heterozygous organoids. Additionally, we used dextran sulfate sodium (DSS) to induce intestinal mucosa inflammation in Jag2 heterozygous and wild type mice. We found that the weight of Jag2 heterozygous mice at day 8 was significantly lower compared to wild type mice. Histology showed that Jag2 heterozygous intestines have more severe inflammation than wild type at day8. In sum, these results are consistent with a functional role of Jag2 in the intestine, particularly in the maintenance of crypt stemness.

[Disclosure of COI by Responsible researcher] Lead presenter is the Responsible researcher.
[Lead presenter: Shinichiro Hasegawa] We have no financial relationships to disclose.

▲TOP

### [P-3171] I-P10-9
### Cancer stem cells (1)

2025 / 9 / 27 (Sat) 16:15-17:00 Room P/(Motenashi Dome) Room P

---

### P-3171
### Ovarian cancer-derived PDO models show different distribution patterns of cancer stem cell markers

[Speaker]Hirosumi Tamura (Translational Research Center, Fukushima Medical University) [Co-author]Ayaka Nishio (Fukushima Cell Factory Inc.)、Hirotaka Hoshi (Fukushima Cell Factory Inc.)、Shinya Watanabe (Translational Research Center, Fukushima Medical University)

Cancer stem cells are thought to be involved in various phenomena, such as cancer tissue metastasis and anticancer drug resistance, and are therefore promising therapeutic targets. We have established a number of patient-derived cancer cell models, which we named Fukushima PDO (F-PDO). Among many F-PDOs, 3D cultured cell models form cell clusters of various shapes and sizes. Analysis of cancer stem cells in such 3D culture models has not yet been performed. Therefore, we attempted to analyze the distribution of cancer stem cells in F-PDO derived from ovarian cancer. First, we analyzed the expression patterns of cancer stem cell markers in ovarian cancer-derived F-PDOs by immunohistochemistry. The results showed that the expression patterns of each cancer stem cell marker were not consistent. In some F-PDOs, cancer stem cell markers were positively or negatively expressed in cell clusters rather than in individual cells. These results suggest that different cancer stem cell subsets may be present in F-PDOs derived from ovarian cancer.

[Disclosure of COI by Responsible researcher] Lead presenter is the Responsible researcher.
[Lead presenter: Hirosumi Tamura] ■Research funds from company: 福島医薬品関連産業支援拠点化事業

### [P-3172] I-P10-9
### Cancer stem cells (1)

2025 / 9 / 27 (Sat) 16:15-17:00 Room P/(Motenashi Dome) Room P

---

### P-3172
### Tumor endothelial cell-derived Sfrp1 supports the maintenance of cancer stem cells via Wnt signaling

[Speaker]Yumiko Hayashi (Dept. Integrative Vascular Biol, Univ. of Fukui) [Co-author]Hiroyasu Kidoya (Dept. Integrative Vascular Biol, Univ. of Fukui)

Cancer stem cells (CSCs), which are critical targets for cancer therapy as they are involved in drug resistance to anticancer drugs, and metastasis, are maintained by angiocrine factors produced by particular niches that form within tumor tissue. Secreted frizzled-related protein 1 (Sfrp1) is an extracellular protein that modulates Wnt signaling. We aimed to elucidate angiocrine factors related to CSC maintenance, focusing on Sfrp1. We investigated the localization of Sfrp1 in tumors and found that it is expressed in some tumor vessels. Analysis of mice lacking Sfrp1 showed that tumor growth was suppressed in Sfrp1-deficient tumor tissues. Flow cytometry analysis indicated that CSCs were maintained in the early tumor growth phase in the Sfrp1 knockout (KO) mouse model of tumor-bearing cancer. However, tumor growth was inhibited in the late tumor growth phase because of the inability to maintain CSCs. Real-time PCR results from tumors of Sfrp1 KO mice showed that the expression of Wnt signaling target genes significantly decreased in the late stage of tumor growth. This suggests that Sfrp1 is involved in Wnt signaling-mediated mechanisms in tumor tissues.

[Disclosure of COI by Responsible researcher] Lead presenter is not the Responsible researcher.
[Lead presenter: Hayashi Yumiko] We have no financial relationships to disclose.
[Principal Researcher: Hiroyasu Kidoya] We have no financial relationships to disclose.

▲TOP

[P-3173] I-P10-9
Cancer stem cells (1)

2025 / 9 / 27 (Sat) 16:15-17:00 Room P/(Motenashi Dome) Room P

### P-3173
### p53-Phlda3 Axis Restrains Gastric Cancer Cell Plasticity by Blocking the PI3K-Akt Pathway

[Speaker]Kazuhiro Murakami (Kanazawa Univ., CRI, Div. of Epithelial Stem Cell Biology)

Gastric cancer is a malignant tumor that represents a significant global health problem. Cancer-initiating cells make up a subset within a tumor and are capable of self-renewal and differentiating into various cancer cell types. The ***leucine-rich repeat-containing G protein-coupled receptor 5*** (***Lgr5***) gene encodes the R-spondin receptor, which serves as a marker for cancer-initiating cells, particularly in epithelial tumors such as gastric cancer. In this study, we established gastric cancer organoids from a mouse model of chronic inflammation-driven gastric cancer. Using these organoids, we discovered that the plasticity between Lgr5+/- gastric cancer cells is regulated by transient activation of the PI3K-Akt signaling pathway, and that this transition is suppressed by the induction of ***Phlda3***. Additionally, we identified a novel cell population within the Lgr5- fraction that exhibits stem cell-like behavior, characterized by the expression of specific genes and the ability to generate Lgr5+ cells. Understanding these cell populations provides valuable insights into the biology of cancer-initiating cells and offers potential therapeutic targets for malignant gastric cancer.

[Disclosure of COI by Responsible researcher] Lead presenter is the Responsible researcher.
[Lead presenter: Kazuhiro Murakami] We have no financial relationships to disclose.

▲TOP

### [P-3174] I-P10-9
### Cancer stem cells (1)

2025 / 9 / 27 (Sat) 16:15-17:00 Room P/(Motenashi Dome) Room P

---

### P-3174
### Functional analysis of one-carbon metabolic enzyme SHMT2 in breast cancer

[Speaker]Itsuki Toriba (Kanazawa univ. Cancer Research Inst. CCB) [Co-author]Tsunaki Hongu (Kanazawa univ. Cancer Research Inst. CCB)、Hirokazu Kusunoki (Kanazawa univ. Cancer Research Inst. CCB)、Yasuto Takeuchi (Kanazawa univ. Cancer Research Inst. CCB)、Noriko Gotoh (Kanazawa univ. Cancer Research Inst. CCB)

One-carbon metabolism fuels the synthesis of nucleic acids and plays a pivotal role in cell growth. Although this metabolic pathway occurs both in the cytoplasm and mitochondria, mitochondrial one-carbon metabolism is known to be aberrantly activated in several types of cancers. Therefore, a group of enzymes responsible for mitochondrial one-carbon metabolism may be promising candidates for anticancer drugs. However, it is still unclear how each enzyme is functional for tumorigenesis and metastasis in a differential manner. In this study, we focused on a mitochondrial one-carbon metabolic enzyme SHMT2. SHMT2 knock down in human breast cancer cells results in significant reduction in cell proliferation and stemness compared to control cells. Analysis using a mouse lung metastasis model showed that suppression of SHMT2 expression significantly inhibited breast cancer lung metastasis. These compelling findings underscore the potential of targeting SHMT2 as a promising strategy to halt breast cancer progression and metastasis.

[Disclosure of COI by Responsible researcher] Lead presenter is not the Responsible researcher.
[Lead presenter: Itsuki Toriba] We have no financial relationships to disclose.
[Principal Researcher: Noriko Gotoh] We have no financial relationships to disclose.

### [P-3175] I-P10-9
### Cancer stem cells (1)

2025 / 9 / 27 (Sat) 16:15-17:00 Room P/(Motenashi Dome) Room P

---

### P-3175
### Molecular characterization for a neuroblastoma-like tumorigenesis model from cranial neural crest cells

[Speaker]Maya Shindo (Dept. Graduate School of Science and Engineering., Saitama Univ.／Res. Inst. for Clin. Oncol., Saitama Cancer Ctr.) [Co-author]Kyosuke Mukae (Res. Inst. for Clin. Oncol., Saitama Cancer Ctr.)、Tianyuan Shi (Dept. Graduate School of Science and Engineering., Saitama Univ.／Res. Inst. for Clin. Oncol., Saitama Cancer Ctr.)、Ritsuko Onuki (Res. Inst. for Clin. Oncol., Saitama Cancer Ctr.)、Atsuko Nakazawa (Dep. of Clin. Res., Saitama Children's Medical Ctr.)、Miki Ohira (Res. Inst. for Clin. Oncol., Saitama Cancer Ctr.)、Takehiko Kamijo (Res. Inst. for Clin. Oncol., Saitama Cancer Ctr.)

Neuroblastoma (NB), a pediatric cancer, is thought to originate from neural crest cells (NCCs), which are classified into cranial (cNCCs) and trunk NCCs (tNCCs) with distinct differentiation processes. A hiPSC-derived tNCC NB model was reported (Cell Reports, 2024), suggesting tNCCs as NB origin, but the role of cNCCs remains unclear. We have introduced MYCN and ALK overexpression into hiPSC-derived cNCCs and pathologically confirmed NB-like tumor formation. Here, microarray analysis was performed to study the molecular character of NB-like tumor. Comparison of gene expression profiles with 24 NB cell lines classified them as adrenergic type. We further performed integrated gene expression analysis using multiple tumor samples from Agilent/Affymetrix microarrays and RNA-seq. NB-like tumor clustered near NB samples. These results indicate that NB-like tumor resembles NB in pathology and gene expression, suggesting cNCCs, like tNCCs, may contribute to NB development. But large cluster dispersion limited accuracy. To address this, we are attempting RNA-seq analysis on NB-like tumors. We would like to discuss the analysis at this conference.

[Disclosure of COI by Responsible researcher] Lead presenter is not the Responsible researcher.
[Lead presenter: Maya Shindo] We have no financial relationships to disclose.
[Principal Researcher: Takehiko Kamijo] We have no financial relationships to disclose.

▲TOP

### [P-3185] I-P10-11
### Cell-to-cell interaction

2025 / 9 / 27 (Sat) 16:15-17:00 Room P/(Motenashi Dome) Room P

---

### P-3185
### Biological significance of thrombospondin-1 in pancreatic cancer progression

[Speaker]Hiromitsu Hayashi (Department of gastroenterological surgery, Kumamoto university) [Co-author]Kazuki Matsumura (Department of gastroenterological surgery, Kumamoto university)、Masaaki Iwatsuki (Department of gastroenterological surgery, Kumamoto university)

Pancreatic ductal adenocarcinoma (PDAC) is characterized by abundant stroma. Cancer-associated fibroblasts (CAFs) provide a favorable tumor microenvironment for cancer cells. SMAD4 acts as a tumor suppressor in PDAC. Dysregulation of SMAD4 is an important step in tumor progression. The matricellular protein thrombospondin (TSP) -1 is a major activator of TGF-beta, but its biological roles and those of TSP-1/TGF-beta interdependence during PDAC progression remain unclear. High substrate stiffness induced TSP-1 expression in CAFs, and TSP1 knockdown inhibited cell proliferation in CAFs. Paracrine TSP-1 treatment for PDAC cells promoted cell survival and EMT, and TSP1 knockdown in cancer cells abolished these phenotypes. Knockdown of SMAD4 induced TSP-1 overexpression with aberrant TGF-beta signaling in PDAC cells. Interestingly, TSP-1 overexpression induced loss of SMAD4 expression and enhanced cell proliferation. Treatment with LSKL peptide, which antagonizes TSP-1-mediated latent TGF-beta activation, attenuated cell survival, invasiveness, and chemoresistance with enhanced apoptosis in PDAC cells. Thus, TSP-1 and loss of SMAD4 coordinately accelerated malignant behavior in PDAC.

[Disclosure of COI by Responsible researcher] Lead presenter is the Responsible researcher.
[Lead presenter: Hiromitsu Hayashi] ■Lecture fee: アストラゼネカ社

### [P-3186] I-P10-11
### Cell-to-cell interaction

2025 / 9 / 27 (Sat) 16:15-17:00 Room P/(Motenashi Dome) Room P

---

### P-3186
### Identification of compounds that inhibit proliferation of tumor-promoting human breast cancer-associated fibroblasts

[Speaker]Zixu Wang (Juntendo University Graduate School of Medicine, Department of Molecular Pathogenesis) [Co-author]Kazunari Yamashita (Juntendo University Graduate School of Medicine, Department of Molecular Pathogenesis／Juntendo University Faculty of Medicine, Department of Pathology and Oncology)、Ritsuko Sasaki (Juntendo University Faculty of Medicine, Department of Breast Oncology／Juntendo University Graduate School of Medicine, Department of Breast Oncology)、Hiroko Onagi (Juntendo University Faculty of Medicine, Department of Human Pathology)、Yuwei Zhai (Juntendo University Graduate School of Medicine, Department of Molecular Pathogenesis)、Goro Kutomi (Juntendo University Faculty of Medicine, Department of Breast Oncology／Juntendo University Graduate School of Medicine, Department of Breast Oncology)、Akira Orimo (Juntendo University Graduate School of Medicine, Department of Molecular Pathogenesis／Juntendo University Faculty of Medicine, Department of Pathology and Oncology)

Cancer-associated fibroblasts (CAFs) influence tumor growth and progression. Discovery of new modality drugs targeting CAFs would therefore be useful for the development of cancer therapy. Using experimentally generated human breast CAFs (exp-CAFs), we screened 3,515 chemical compounds and identified zardaverine that could specifically inhibit proliferation of exp-CAFs but not control normal mammary fibroblasts. Zardaverine is velcrin, a novel class of molecular glue that stabilizes a PDE3A-SLFN12 complex, resulting in promotion of SLFN12 RNase activity to induce cytotoxicity. Knock-down of PDE3A expression by siRNA abrogated the growth-inhibitory effect by velcrin in exp-CAFs, indicating PDE3A expression to be required for velcrin sensitivity of these fibroblasts. PDE3A expression was also induced in normal breast fibroblasts by co-culture with breast carcinoma cells. When breast cancer cells and exp-CAFs were injected subcutaneously into immunodeficient mice, intratumor injection of BAY 2666605, another velcrin, attenuated growth of tumors presumably by targeting exp-CAFs within tumors. These findings suggest that PDE3A-positve CAFs may be a therapeutic target.

[Disclosure of COI by Responsible researcher] Lead presenter is not the Responsible researcher.
[Lead presenter: Zixu Wang] We have no financial relationships to disclose.
[Principal Researcher: Goro Kutomi] We have no financial relationships to disclose.

### [P-3187] I-P10-11
### Cell-to-cell interaction

2025 / 9 / 27 (Sat) 16:15-17:00 Room P/(Motenashi Dome) Room P

---

### P-3187
### PRSS3 from scirrhous gastric cancer, interact with cancer associated fibroblasts via PAR2/STC-1 axis

[Speaker]Takashi Sakuma (Department of Molecular Oncology and Therapeutics, Osaka Metropolitan University／Department of Gastroenterological Surgery, Osaka Metropolitan University) [Co-author]Masakazu Yashiro (Department of Molecular Oncology and Therapeutics, Osaka Metropolitan University／Department of Gastroenterological Surgery, Osaka Metropolitan University)、Dongheng Ma (Department of Molecular Oncology and Therapeutics, Osaka Metropolitan University)、Tomoya Sano (Department of Molecular Oncology and Therapeutics, Osaka Metropolitan University／Department of Gastroenterological Surgery, Osaka Metropolitan University)、Daiki Imanishi (Department of Molecular Oncology and Therapeutics, Osaka Metropolitan University／Department of Gastroenterological Surgery, Osaka Metropolitan University)、Hinano Nishikubo (Department of Molecular Oncology and Therapeutics, Osaka Metropolitan University)、Kyoka Kawabata (Department of Molecular Oncology and Therapeutics, Osaka Metropolitan University)、Canfeng Fan (Department of Molecular Oncology and Therapeutics, Osaka Metropolitan University)、Koji Maruo (Department of Molecular Oncology and Therapeutics, Osaka Metropolitan University／Department of Gastroenterological Surgery, Osaka Metropolitan University)、Yurie Yamamoto (Department of Molecular Oncology and Therapeutics, Osaka Metropolitan University)、Tatsunari Fukuoka (Department of Molecular Oncology and Therapeutics, Osaka Metropolitan University／Department of Gastroenterological Surgery, Osaka Metropolitan University)、Kiyoshi Maeda (Department of Gastroenterological Surgery, Osaka Metropolitan University)

In the microenvironment of scirrhous-type gastric cancer cells (SGCs), cancer associated fibroblasts (CAFs) in the stroma play critical roles in the regulation of tumor growth. However, the interaction between cancer cells and CAFs still remain unclear. To identify the interaction, we investigated RNA sequencing for each of three unique types of CAFs and normal fibroblasts (NFs) established in our department. As the result, we revealed that 55 genes were significantly expressed in CAFs compared to NFs, and we revealed stanniocalcin-1 (STC-1) with pathway analysis. The expression level of STC-1 in CAFs was significantly increased with the supernatant of SGCs, compared to non-SGCs. STC-1 stimulated the invasion activity and epithelial-mesenchymal transition of SGCs, and upregulated PRSS3, one of serine protease highly expressed in SGCs. On the other hand, PRSS3 increased the expression level of STC-1 in CAFs through the activation of receptor, PAR2. These results suggested that PRSS3 derived from SGCs stimulated PAR2/STC-1 signaling in CAFs, and might be associated with the interaction between cancer cells and CAFs, for promoting the invasion and metastasis of SGCs.

[Disclosure of COI by Responsible researcher] Lead presenter is not the Responsible researcher.
[Lead presenter: Takashi Sakuma] We have no financial relationships to disclose.
[Principal Researcher: Masakazu Yashiro] We have no financial relationships to disclose.

▲TOP

[P-3188] I-P10-11
Cell-to-cell interaction

2025 / 9 / 27 (Sat) 16:15-17:00 Room P/(Motenashi Dome) Room P

---

### P-3188
### Clinico-pathological significance of cancer-associated fibroblasts subtype, myCAF and apCAF, in gastric carcinomas

[Speaker]Dongheng Ma (Dept Mol Oncol & Therapeutics, Osaka Metro Univ) [Co-author]Hinano Nishikubo (Dept Mol Oncol & Therapeutics, Osaka Metro Univ)、Tomoya Sano (Dept Mol Oncol & Therapeutics, Osaka Metro Univ)、Daiki Imanishi (Dept Mol Oncol & Therapeutics, Osaka Metro Univ)、Takashi Sakuma (Dept Mol Oncol & Therapeutics, Osaka Metro Univ)、Koji Maruo (Dept Mol Oncol & Therapeutics, Osaka Metro Univ)、Canfeng Fan (Dept Mol Oncol & Therapeutics, Osaka Metro Univ)、Yurie Yamamoto (Dept Mol Oncol & Therapeutics, Osaka Metro Univ)、Masakazu Yashiro (Dept Mol Oncol & Therapeutics, Osaka Metro Univ)

Background: Cancer-associated fibroblasts (CAFs) has been reported to be closely associated with tumor development. Recent studies have identified three CAF subtypes: (i) myofibroblastic CAFs (myCAF), (ii) inflammatory CAFs (iCAF) and (iii) antigen-presenting CAFs (apCAF). The clinical significance of these subsets in GC remains unclear. We performed a clinicopathological analysis of CAF expression of myCAF and apCAF in GC. Methods: We retrospectively reviewed 1,442 patients with GC. Immunohistochemical staining was performed using anti-ACTIN2 and anti-HLA-DR $\alpha$ as primary antibodies. A cutoff was used to classify samples as " positive " or " negative " for myCAF and apCAF expression. Results: Among the 1,442 resected cases, myCAF positivity was correlated with worse OS and RFS ($p < 0.05$), associated with advanced T stage, N stage, lymphovascular invasion. Positivity of apCAF also showed poorer OS and RFS ($p < 0.05$), relating to advanced T stage and higher recurrence rates. Notably, positivity for both myCAF and apCAF indicated a poor prognosis ($p < 0.05$) in OS and RFS. Conclusion: Both myCAF and apCAF might play an important role for the development of GC.

[Disclosure of COI by Responsible researcher] Lead presenter is not the Responsible researcher.
[Lead presenter: Dongheng Ma] We have no financial relationships to disclose.
[Principal Researcher: Masakazu Yashiro] We have no financial relationships to disclose.

### [P-3189] I-P10-11
### Cell-to-cell interaction

2025 / 9 / 27 (Sat) 16:15-17:00 Room P/(Motenashi Dome) Room P

---

### P-3189
### Benchtop MALDI MS Imaging for Cancer Research: A New Approach Using HiPLEX-IHC Probes

[Speaker]Yumi Unno (Shimadzu, Analytical & Measuring Instruments Division, Solutions COE) [Co-author]Caroline J. Jones (KRATOS GROUP PLC. KRATOS ANALYTICAL LTD.)

We tested a novel technique called "MALDI-IHC," which combines benchtop MALDI-TOF mass spectrometry imaging (MSI ) with immunohistochemistry (IHC) to visualize protein distribution in human tonsil tissue sections. Using 6-plex and 20-plex antibody probes, this approach enables the localization of a variety of proteins, overcoming the protein detection sensitivity limitations typically encountered in conventional MSI techniques. MALDI-IHC uses photocleavable mass tags (PC-MT) that are conjugated to antibodies, allowing precise spatial protein information to be captured by mass spectrometry. This advancement enhances the understanding of cellular interactions and the behavior of target proteins, aiding cancer research and therapeutic development. By analyzing FFPE human tonsil tissue sections using MALDI-8020 and 8030 EasyCare with a 30 μm resolution, we visualized key cancer-related proteins such as CD68, VIM, and Ki67. This method, which enables the simultaneous analysis of multiple proteins, provides a low-cost and efficient solution for cancer biomarker discovery and pathogenesis research.

[Disclosure of COI by Responsible researcher] Lead presenter is the Responsible researcher.
[Lead presenter: Yumi Unno] We have no financial relationships to disclose.

### [P-3190] I-P10-11
### Cell-to-cell interaction

2025 / 9 / 27 (Sat) 16:15-17:00 Room P/(Motenashi Dome) Room P

---

### P-3190
### Mechanistic Elucidation of Connexin 43-Mediated Cancer Cell Extrusion from the Epithelial Monolayer

[Speaker]Takuya Kato (Dept. of Mol. Path., Fujita Health Univ. Sch. of Med.) [Co-author]Naoya Asai (Dept. of Mol. Path., Fujita Health Univ. Sch. of Med.)、Yoshiki Murakumo (Dept. of Path., Kitasato Univ. Sch. of Med.)、Sahai Erik (Tumour Cell Biol., The Francis Crick Inst.)

In epithelial tissues, it has been reported that surrounding normal cells extrude mutant cells from the epithelial-stromal interface. For mutant cells to overcome this elimination mechanism and progress to invasive cancer, it is presumed that they must acquire the ability to remain at the epithelial-stromal boundary; however, the molecular mechanisms underlying such phenomena remain largely unknown. We have identified a role for Connexin 43 (CX43) in regulating the behavior of cancer cells at the epithelial-stromal interface. Using an organotypic co-culture system of normal epithelial cells and cancer cells, we found that secretion of ATP from cancer cells, which is mediated by CX43, regulates the fate of cancer cells within epithelial cell layer. Intracellular signaling analysis revealed that in cancer cells, AKT activation occurred upon ATP stimulation, which was not observed in normal epithelial cells. This differential activation of AKT may underlie the selective elimination of cancer cells at the epithelial-stromal interface.

[Disclosure of COI by Responsible researcher] Lead presenter is not the Responsible researcher.
[Lead presenter: Takuya Kato] We have no financial relationships to disclose.
[Principal Researcher: Sahai Erik] We have no financial relationships to disclose.

▲TOP

### [P-3191] I-P10-11
### Cell-to-cell interaction

2025 / 9 / 27 (Sat) 16:15-17:00 Room P/(Motenashi Dome) Room P

---

### P-3191
### Spatial transcriptomics uncovers the tumor microenvironment in a mouse model of diffuse gastric cancer

[Speaker]Shin Suzuki (Dept. Prev. Med., Grad. Sch. Med., The Univ. of Tokyo) [Co-author]Ayumu Tsubosaka (Dept. Prev. Med., Grad. Sch. Med., The Univ. of Tokyo)、Kyohei Sano (Dept. Prev. Med., Grad. Sch. Med., The Univ. of Tokyo)、Miwako Kakiuchi (Dept. Prev. Med., Grad. Sch. Med., The Univ. of Tokyo)、Daisuke Komura (Dept. Prev. Med., Grad. Sch. Med., The Univ. of Tokyo)、Hiroto Katoh (Div. Path., NCC Exploratory Oncol. Res. & Clin. Trial Ctr.)、Shumpei Ishikawa (Dept. Prev. Med., Grad. Sch. Med., The Univ. of Tokyo／Div. Path., NCC Exploratory Oncol. Res. & Clin. Trial Ctr.)

Gastric cancer remains one of the most common and lethal malignancies worldwide, characterized by its histological and molecular heterogeneity. Diffuse-type gastric cancer (DGC) is often associated with a poorer prognosis compared to the intestinal subtype. A mouse model of DGC (an E-cadherin/p53 double conditional knockout (DCKO) mouse) exhibits histopathological features and gene expression profiles similar to those of human DGC. This model facilitates a deeper understanding of the tumor microenvironment (TME), which comprises non-cancerous cells and an altered extracellular matrix.
In recent years, spatial transcriptomics has emerged as a transformative technology that bridges gene expression profiles with tissue architecture. We performed spatial transcriptomic analysis on primary tumors and lymph node metastases from DCKO mice. We uncovered molecular crosstalk in the TME associated with DGC progression, invasion, and metastasis at single-cell resolution. This study contributes to our understanding of how the TME influences tumor malignancy at the molecular level.

[Disclosure of COI by Responsible researcher] Lead presenter is not the Responsible researcher.
[Lead presenter: Shin Suzuki] We have no financial relationships to disclose.
[Principal Researcher: Shumpei Ishikawa] ■Research funds from company: 日本学術振興会　■Advisor of the company and/or profit-making organization: 新日本科学株式会社　■Research expenses: Chordia theraputics株式会社、大鵬薬品工業株式会社、ロート製薬株式会社、アステラス製薬株式会社

▲TOP

[P-3192] I-P10-11
Cell-to-cell interaction

2025 / 9 / 27 (Sat) 16:15-17:00 Room P/(Motenashi Dome) Room P

### P-3192
### Uncovering Gastric Cancer Heterogeneity with Histopathology and Spatial Transcriptomics

[Speaker]Kyohei Sano (Dept. Prev. Med., Grad. Sch. Med., The Univ. of Tokyo) [Co-author]Takumi Onoyama (Div. Gastro. & Neph, Med., Tottori Univ.)、Ayumu Tsubosaka (Dept. Prev. Med., Grad. Sch. Med., The Univ. of Tokyo)、Miwako Kakiuchi (Dept. Prev. Med., Grad. Sch. Med., The Univ. of Tokyo)、Daisuke Komura (Dept. Prev. Med., Grad. Sch. Med., The Univ. of Tokyo)、Hiroto Katoh (Dept. Prev. Med., Grad. Sch. Med., The Univ. of Tokyo)、Tetsuo Ushiku (Dept. Pathol., Grad. Sch. Med., The Univ. of Tokyo)、Shumpei Ishikawa (Dept. Prev. Med., Grad. Sch. Med., The Univ. of Tokyo)

Gastric cancer (GC) demonstrates marked inter-tumoral heterogeneity, reflected in diverse histopathological features as well as distinct clinical and molecular characteristics. Despite previous classification efforts, mainly based on hematoxylin and eosin (H&E) staining or bulk sequencing, such approaches remain insufficient to capture the full complexity of the disease. In this study, we established a large-scale, multi-modal GC atlas by integrating single-cell spatial transcriptomic data, genomic profiles, and H&E-stained images from over 100 surgically resected cases. To overcome the limitations of discrete histological classifications, we used a deep neural network to extract continuous features from H&E images, capturing intermediate status beyond conventional categories. These features were integrated with spatial and genomic data to reveal how histological phenotypes are shaped by (1) genomic alterations, (2) cellular composition, and (3) intercellular interactions within the tumor microenvironment. This study provides a deeper understanding of gastric cancer heterogeneity, paving the way for advanced precision medicine in GC.

[Disclosure of COI by Responsible researcher] Lead presenter is not the Responsible researcher.
[Lead presenter: Kyohei Sano] ■Research funds from company: Eureka財団
[Principal Researcher: Shumpei Ishikawa] ■Research funds from company: 日本学術振興会　■Advisor of the company and/or profit-making organization: 新日本科学株式会社　■Research expenses: Chordia therapeutics株式会社、大鵬薬品工業株式会社、ロート製薬株式会社、アステラス製薬株式会社

### [P-3193] I-P10-11
### Cell-to-cell interaction

2025 / 9 / 27 (Sat) 16:15-17:00 Room P/(Motenashi Dome) Room P

---

P-3193

### $SOX10^{low}$ melanoma cells regulate the malignancies of $SOX10^{high}$ melanoma cells through secretion of IL-6.

[Speaker]Nana Takahashi (Dept. Cancer Cell Biol., Univ. Toyama) [Co-author]Yue Zhou (Dept. Cancer Cell Biol., Univ. Toyama)、Hiroaki Sakurai (Dept. Cancer Cell Biol., Univ. Toyama)、Satoru Yokoyama (Dept. Cancer Cell Biol., Univ. Toyama)

The transcription factor SOX10 is known to directly regulate the expression of MITF, a melanoma-specific oncogene, and regulates melanoma growth through MITF downstream targets. However, the MITF-independent functions of SOX10 in melanoma remain poorly understood. Moreover, both $SOX10^{high}$ and $SOX10^{low}$ melanoma cells coexist within melanoma tissues; however, their interactions are still unclear. Therefore, we investigated the interaction between $SOX10^{high}$ and $SOX10^{low}$ melanoma cells. By bulk RNA-seq analysis comparing gene expression between $SOX10^{high}$ and $SOX10^{low}$ melanoma cells, we identified the elevated expression of IL-6 in $SOX10^{low}$ cells. Furthermore, the conditioned medium from $SOX10^{low}$ cells led to enhanced cell motility in $SOX10^{high}$ cells. In future studies, we will investigate the mechanism in which $SOX10^{low}$ cells increase the induction of IL-6 secretion, and the involvement of IL-6 secreted by $SOX10^{low}$ cells in promoting melanoma metastasis and potentially contributing to melanoma progression and malignancy.

[Disclosure of COI by Responsible researcher] Lead presenter is not the Responsible researcher.
[Lead presenter: Nana Takahashi] We have no financial relationships to disclose.
[Principal Researcher: Satoru Yokoyama] We have no financial relationships to disclose.

### [P-3194] I-P10-11
### Cell-to-cell interaction

2025 / 9 / 27 (Sat) 16:15-17:00 Room P/(Motenashi Dome) Room P

---

### P-3194
### Effects of point mutations and nuclear localization of E-cadherin on entosis.

[Speaker]Yuki Takei (Dept. of Life Sci. & Med.Biosci., Waseda Univ.／Lab. of Integ. Onco., Natl. Cancer Ctr. Res. Inst.) [Co-author]Yusuke Hayashi (Dept. of Life Sci. & Med.Biosci., Waseda Univ.／Lab. of Integ. Onco., Natl. Cancer Ctr. Res. Inst.)、Kazushi Azuma (Dept. of Life Sci. & Med.Biosci., Waseda Univ.)、Kentaro Semba (Dept. of Life Sci. & Med.Biosci., Waseda Univ.／TR Ctr., Fukushima Med Univ.)、Yusuke Yamamoto (Lab. of Integ. Onco., Natl. Cancer Ctr. Res. Inst.)

Entosis is a process in which a live cell invades another and forms a Cell-in-Cell (CIC) structure. It contributes to nutrient acquisition, cell competition, and the evasion of therapeutic or immune-mediated attack. We previously evaluated entotic activity in 17 breast cancer cell lines and observed that those expressing E-cadherin, such as UACC-893 and UACC-812, showed high entotic levels. Knockdown of ***CDH1*** diminished entosis, while its overexpression enhanced it. Notably, CIC outcomes, including escape and degradation, varied by cell line. We also found that **in UACC-893 and UACC-812, E-cadherin and its binding partners, β-catenin and p120-catenin, localized to the nucleus rather than the membrane**, deviating from their canonical membrane-associated distribution. **These lines harbor point mutations in E-cadherin**, implying a potential link to altered entotic behavior. In this study, **we employed CRISPR/Cas9 to revert the mutant E-cadherin to the wild-type and examined how this affected entosis frequency and CIC-associated outcomes**. We also discuss the mechanistic basis by which E-cadherin mutations may drive nuclear localization and modulate entotic activity.

[Disclosure of COI by Responsible researcher] Lead presenter is not the Responsible researcher.
[Lead presenter: Yuki Takei] We have no financial relationships to disclose.
[Principal Researcher: Yusuke Yamamoto] ■Research funds from company: 東芝とNikonから共同研究費をいただいている

▲TOP

Poster Session

### [P-3195] I-P10-11
### Cell-to-cell interaction

2025 / 9 / 27 (Sat) 16:15-17:00 Room P/(Motenashi Dome) Room P

---

### P-3195
### Validation of a cancer-associated thrombosis model and early thrombosis detection in 4T1 breast cancer-bearing mice

[Speaker]Riyo Kamata (Lab. Host Defense, Fac. Pharm. Sci., Teikyo Univ.) [Co-author]Yuichi Kamikubo (Thrombo Translational Research Lab Inc.)、Lisa Munakata (Lab. Drug & Gene Delivery Research, Fac. Pharm. Sci., Teikyo Univ.)、Ryo Suzuki (Lab. Drug & Gene Delivery Research, Fac. Pharm. Sci., Teikyo Univ.)、Saori Kageyama (Lab. Theranostics, Fac. Pharm. Sci., Teikyo Univ.)、Kazuo Maruyama (Lab. Theranostics, Fac. Pharm. Sci., Teikyo Univ.)、Naoki Ohkura (Lab. Host Defense, Fac. Pharm. Sci., Teikyo Univ.)

Cancer-associated thrombosis (CAT) is the second leading cause of death in cancer patients and is induced by various factors released from cancer cells. However, methods for the early detection of CAT have not yet been established. To assess thrombotic tendency in an in vivo model, we monitored coagulation-associated factors and tissue factor (TF) pathway-driven initial thrombin generation (TF-TG) in plasma using 4T1 breast cancer cell-transplanted mice, a model known to exhibit neutrophil extracellular traps-dependent thrombotic tendencies. Compared to non-transplanted mice, plasma levels of TF, an initiator of the extrinsic coagulation pathway, and plasminogen activator inhibitor (PAI)-1, a fibrinolytic inhibitor, were elevated, whereas thrombin-antithrombin complex levels remained unchanged. Notably, TF-TG was significantly reduced in tumor-bearing mice, possibly due to consumptive depletion of coagulation factors such as Factor V and X. These findings suggest that 4T1-bearing mice develop a hypercoagulable and hypofibrinolytic state without clear evidence of thrombus formation and that reduced TF-TG may serve as a potential marker for early-stage thrombophilia.

[Disclosure of COI by Responsible researcher] Lead presenter is the Responsible researcher.
[Lead presenter: Riyo Kamata] We have no financial relationships to disclose.

### [P-3205] I-P10-13
### Cell death (2)

2025 / 9 / 27 (Sat) 16:15-17:00 Room P/(Motenashi Dome) Room P

---

### P-3205
### The transcription factor SOX10 regulates melanoma growth through modulation of nuclear receptor RXR $\gamma$ expression

[Speaker]Hana Ishizuka (Dept. Cancer Cell Biol., Univ. Toyama) [Co-author]Hiyori Ito (Dept. Cancer Cell Biol., Univ. Toyama)、Yue Zhou (Dept. Cancer Cell Biol., Univ. Toyama)、Hiroaki Sakurai (Dept. Cancer Cell Biol., Univ. Toyama)、Satoru Yokoyama (Dept. Cancer Cell Biol., Univ. Toyama)

The transcription factor SOX10 is highly expressed in melanomas and is involved in melanoma survival and proliferation through the regulation of gene expression. We investigated SOX10 to further elucidate its function. RNA-seq analysis identified the nuclear receptor ***RXRG*** as a downstream gene of SOX10. SOX10 knockdown (KD) reduced mRNA and protein expression of RXR $\gamma$ in five human malignant melanoma cells. An CUT&RUN assay revealed that SOX10 binds to the regulatory region of ***RXRG*** gene. RXR $\gamma$ forms heterodimers with various nuclear receptors such as retinoic acid receptor (RAR) and is involved in various cellular functions such as cell proliferation. In this study, we focused on ferroptosis as a function of RAR/RXR $\gamma$. The ferroptosis induced by ML162 was significantly inhibited by retinoic acid pretreatment. On the other hand, SOX10 KD increased ferroptosis sensitivity. These results indicate that SOX10 directly regulates the expression of RXR $\gamma$ and suppresses ferroptosis via RXR $\gamma$. Therefore, induction of ferroptosis via regulation of RXR $\gamma$ expression by SOX10 is expected to improve the prognosis of melanoma patients.

[Disclosure of COI by Responsible researcher] Lead presenter is not the Responsible researcher.
[Lead presenter: Hana Ishizuka] We have no financial relationships to disclose.
[Principal Researcher: Satoru Yokoyama] We have no financial relationships to disclose.

### [P-3206] I-P10-13
### Cell death (2)

2025 / 9 / 27 (Sat) 16:15-17:00 Room P/(Motenashi Dome) Room P

---

### P-3206
### Analysis of ferroptotic cell death in mesothelioma cells

[Speaker]Tatsuhiro Sato (Aichi Cancer Center, Div. Cancer Biol.) [Co-author]Lisa Ida (Aichi Cancer Center, Div. Cancer Biol.)、Emi Mishiro (Nagoya Univ., ITbM)、Satomi Mukai (Aichi Cancer Center, Div. Cancer Biol.)、Yoshitaka Sekido (Aichi Cancer Center, Div. Cancer Biol.)

Mesothelioma is an aggressive cancer that often develops after asbestos exposure. The histopathological subtypes of mesothelioma are usually classified into three major subtypes: epithelioid type, sarcomatoid type, and biphasic type that contains both components. In all cases, the patient prognosis is usually quite poor, with a five-year overall survival rate after diagnosis of less than 10%. Since the tumor cells diffusely spread in the thoracic or abdominal cavities from the early stage and thus cannot be surgically removed completely, systemic chemotherapy is regarded as the main therapeutic modality for MM patients. We found that approximately half of mesothelioma cell lines were vulnerable to RSL3, a ferroptosis inducer. Furthermore, sensitivity to RSL3 was significantly higher in cell lines derived from epithelial mesothelioma. We are currently performing gene expression and lipidome analyses to investigate the factors contributing to the vulnerability of mesothelioma cells to ferroptosis cell death, and report these results.

[Disclosure of COI by Responsible researcher] Lead presenter is the Responsible researcher.
[Lead presenter: Tatsuhiro Sato] We have no financial relationships to disclose.

[P-3207] I-P10-13
Cell death (2)

2025 / 9 / 27 (Sat) 16:15-17:00 Room P/(Motenashi Dome) Room P

### P-3207
### Exploration of factor involved in vacuolar formation associated with cell death induced by CDK4/6 inhibitor abemaciclib.

[Speaker]Hirotsugu Hino (Div Anat Sci, Dept Funct Morpho, Nihon Univ Sch Med) [Co-author]Yoshinori Tanaka (Biochem Unit, Veterinary Med, Okayama Univ Sci)、Tomoya Okimoto (Div Anat Sci, Dept Funct Morpho, Nihon Univ Sch Med)、Toshikatsu Ikeda (Div Anat Sci, Dept Funct Morpho, Nihon Univ Sch Med)、Tomoyo Hara (Div Anat Sci, Dept Funct Morpho, Nihon Univ Sch Med)、Noa Mizukami (Dept Biochem, Tokyo Med Univ)、Kosuke Takeya (Biochem Unit, Veterinary Med, Okayama Univ Sci)、Naoharu Takano (Dept Biochem, Tokyo Med Univ)、Masaki Hiramoto (Dept Biochem, Tokyo Med Univ)、Shin Aizawa (Div Anat Sci, Dept Funct Morpho, Nihon Univ Sch Med)、Keisuke Miyazawa (Dept Biochem, Tokyo Med Univ)、Masumi Eto (Biochem Unit, Veterinary Med, Okayama Univ Sci)、Shuichi Hirai (Div Anat Sci, Dept Funct Morpho, Nihon Univ Sch Med)

Sustained activation of CDK4/6, which controls G1/S transition in cell cycle induces oncogenesis. Recently, we reported that abemaciclib (Abe), a CDK4/6 inhibitor approved for breast cancer therapy, induced not only cell cycle arrest but also cell death in cancer cell lines. Abe also induced formation of many large vacuoles derived from lysosomes in the cytoplasm. Furthermore, bafilomycin A1, an autophagy inhibitor, inhibited both vacuolar formation and cell death induced by Abe, indicating a novel mechanism of cell death involving vacuolar formation. To elucidate the mechanism of the vacuolar formation, we analyzed the factors contained in these vacuoles using the lysosomal immunoprecipitation assay. The lysosomal fraction in Abe-treated cells contained autophagosomal membrane component LC3B as well as a late endosomal- and lysosomal-protein Rab7A and a lysosomal-protein LAMP2. The results support the hypothesis that these Abe-induced vacuoles are excessive fusion products of autophagosomes and lysosomes. We are currently analyzing the isolated lysosomal fractions by mass spectrometry with the aim of identifying new vacuolar-formation factors.

[Disclosure of COI by Responsible researcher] Lead presenter is the Responsible researcher.
[Lead presenter: Hirotsugu Hino] We have no financial relationships to disclose.

▲TOP

### [P-3208] I-P10-13
### Cell death (2)

2025 / 9 / 27 (Sat) 16:15-17:00 Room P/(Motenashi Dome) Room P

---

### P-3208
### Elucidating compensatory metabolic changes induced by glycolytic inhibition in pancreatic cancer cell lines

[Speaker]Kousuke Ishino (Dept. Integr. Diagn. Pathol., Nippon Med. Sch.) [Co-author]Wataru Nakajima (Dept. Mol. Oncol., Inst. Adv. Med. Sci., Nippon Med. Sch.)、Yui Takagi (Dept. Integr. Diagn. Pathol., Nippon Med. Sch.)、Kiyoshi Teduka (Dept. Integr. Diagn. Pathol., Nippon Med. Sch.)、Taeko Kitamura (Dept. Integr. Diagn. Pathol., Nippon Med. Sch.)、Yoko Kawamoto (Dept. Integr. Diagn. Pathol., Nippon Med. Sch.)、Takenori Fujii (Dept. Integr. Diagn. Pathol., Nippon Med. Sch.)、Akira Matsushita (Dept. Gastrointestinal & Hepato-Biliary-Pancreatic Surg., Nippon. Med. Sch.)、Ryuji Ohashi (Dept. Integr. Diagn. Pathol., Nippon Med. Sch.)

2-Deoxyglucose (2DG) inhibits glycolysis, reducing cancer cell viability by promoting misfolded proteins through abnormal N-glycosylation, which induces ER stress. However, the role of 2DG in pancreatic cancer (PDAC) and its relationship with protein N-glycosylation remains unclear. This study used mannose supplementation to enhance N-glycosylation, assess its effects on ER stress, and analyze protein expression in 2DG-treated PDAC cells. 2DG inhibited PDAC cell growth, and mannose partially restored it, with varying effects across cell lines. Mannose also suppressed the 2DG-induced reduction in N-glycosylation and GRP78 upregulation, suggesting that changes in protein N-glycosylation contribute to growth inhibition. Omics analysis revealed expression changes in pathways related to hexosamine biosynthesis, serine synthesis, ER stress, and replication. These findings suggest that 2DG-induced N-glycosylation disruption and ER stress contribute to apoptosis in PDAC cells. Mannose supplementation recovered N-glycosylation synthesis, indicating that targeting this pathway could be a potential therapeutic strategy for pancreatic cancer.

[Disclosure of COI by Responsible researcher] Lead presenter is the Responsible researcher.
[Lead presenter: Kousuke Ishino] We have no financial relationships to disclose.

### [P-3209] I-P10-13
### Cell death (2)

2025 / 9 / 27 (Sat) 16:15-17:00 Room P/(Motenashi Dome) Room P

---

### P-3209
### Role of Tubulin Acetylation in Paclitaxel Resistance in Lung Cancer

[Speaker]Varisa Pongrakhananon (Dept. of Pharmacol. & Physiol., Faculty of Pharm. Sci., Chulalongkorn Univ.)

Posttranslational modifications (PTMs) of microtubules play a crucial role in cancer aggressiveness, including resistance to apoptosis. This study explores how microtubule PTMs influence responsiveness to paclitaxel. We found that acetylated tubulin (Ace-tub) levels strongly correlate with paclitaxel sensitivity in xenografted immunodeficient mice and patient-derived primary lung cancer cells. Enhancing acetylation via $\alpha$TAT1 overexpression further suppressed paclitaxel-induced lung cancer cell death, while $\alpha$TAT1 knockdown decreased resistance. Paclitaxel-resistant lung cancer cells, generated through stepwise drug exposure, exhibited significantly upregulated tubulin acetylation, leading to enhance resistance. A microtubule sedimentation assay revealed that anti-apoptotic Mcl-1 preferentially binds to ace-tub, by preventing Mcl-1 degradation and prolonging its half-life. These findings indicate that tubulin acetylation enhances paclitaxel resistance by stabilizing Mcl-1, highlighting its potential as a therapeutic target in lung cancer treatment.

[Disclosure of COI by Responsible researcher] Lead presenter is the Responsible researcher.
[Lead presenter: Varisa Pongrakhananon] We have no financial relationships to disclose.

### [P-3210] I-P10-13
### Cell death (2)

2025 / 9 / 27 (Sat) 16:15-17:00 Room P/(Motenashi Dome) Room P

---

### P-3210
### Dual Targeting of mTOR and WNT Pathways Disrupts Mitosis and Induces Autophagic Cell Death in Neuroblastoma

[Speaker]Pei-Chen YU (Institute of Molecular and Cellular Biology, National Taiwan University) [Co-author]Yi-Chun Kao (Department of Life Science, National Taiwan University)、Hsin-Yi Chang (Graduate Institute of Medical Sciences, National Defense Medical Center)、Chen-Hao Huang (Graduate Institute of Biomedical Electronics and Bioinformatics, National Taiwan University)、Wen-Ming Hsu (Department of Surgery, National Taiwan University Hospital)、Hsuan-Cheng Huang (Institute of Biomedical Informatics, National Yang Ming Chiao Tung University)、Hsueh-Fen Juan (Institute of Molecular and Cellular Biology, National Taiwan University／Department of Life Science, National Taiwan University／Graduate Institute of Biomedical Electronics and Bioinformatics, National Taiwan University／Center for Computational and Systems Biology, National Taiwan University)

Neuroblastoma, an aggressive pediatric cancer, often resists standard therapies. We identified a novel drug combination-sirolimus (mTOR inhibitor) and pyrvinium pamoate (WNT inhibitor)-that targets mitotic vulnerabilities in neuroblastoma. Through transcriptomic screening and TMT-based quantitative proteomics, we demonstrated that the combination therapy significantly reduced cell proliferation and colony formation compared to monotherapies. Mechanistically, this dual treatment disrupted mitotic progression, downregulated key cell cycle proteins (e.g., CCNA2, MCM10, ERCC6L), and impaired cytoskeleton integrity, thereby suppressing cell migration. Notably, we observed increased autophagic flux without marked apoptosis, suggesting autophagy-mediated cell death as a dominant mechanism. Patient-derived organoids confirmed the in vitro findings, supporting translational potential. These results unveil a promising combinatorial strategy that leverages mitotic and metabolic vulnerabilities in neuroblastoma cells, offering a low-toxicity therapeutic avenue for future clinical applications.

[Disclosure of COI by Responsible researcher] Lead presenter is the Responsible researcher.
[Lead presenter: Pei-Chen YU] We have no financial relationships to disclose.

▲TOP

### [P-3211] I-P10-13
### Cell death (2)

2025 / 9 / 27 (Sat) 16:15-17:00 Room P/(Motenashi Dome) Room P

---

### P-3211
### Withdrawn

No Abstract

### [P-3212] I-P10-13
### Cell death (2)

2025 / 9 / 27 (Sat) 16:15-17:00 Room P/(Motenashi Dome) Room P

---

### P-3212
### ACSL3 is an unfavorable prognostic marker and confers ferroptosis resistance in cholangiocarcinoma

[Speaker]Apiwit Sae-Fung (Faculty of Allied Health Sciences, Chulalongkorn University, Bangkok, Thailand／Institute of Environmental Medicine, Karolinska Institutet, Stockholm, Sweden) [Co-author]Nawaporn Vinayavekhin (Faculty of Science, Chulalongkorn University, Bangkok, Thailand)、Bengt Fadeel (Institute of Environmental Medicine, Karolinska Institutet, Stockholm, Sweden)、Siriporn Jitkaew (Faculty of Allied Health Sciences, Chulalongkorn University, Bangkok, Thailand)

Cholangiocarcinoma (CCA) is a heterogeneous malignancy arising from the bile duct. Our previous analysis of ferroptosis-related genes (FRGs) found that FRG expression could be used to divide CCA patients into low- and high-risk groups based on survival. Here, we explored the role of ferroptosis in CCA. Based on mRNA expression and clinical data from public databases, we identified acyl-CoA synthetase long chain family member 3 (ACSL3) as a potential ferroptosis suppressor in high-risk CCA patients. Consistently, ACSL3 expression was upregulated in high-risk CCA cell lines and correlated with resistance to the ferroptosis inducer RSL3. Lipidomic analysis revealed that ferroptosis-resistant CCA cells had higher levels of monounsaturated fatty acid (MUFA)-containing phospholipids. Silencing ACSL3 sensitized the resistant cells to RSL3. Moreover, ferroptosis suppression was found to depend on exogenous MUFAs. We also found that resistance to RSL3 was enhanced upon inhibition of lipid droplet biogenesis. These findings highlight ACSL3 as a promising target to overcome ferroptosis resistance in CCA.

[Disclosure of COI by Responsible researcher] Lead presenter is not the Responsible researcher.
[Lead presenter: Apiwit Sae-Fung] We have no financial relationships to disclose.
[Principal Researcher: Siriporn Jitkaew] We have no financial relationships to disclose.

### [P-3213] I-P10-13
### Cell death (2)

2025 / 9 / 27 (Sat) 16:15-17:00 Room P/(Motenashi Dome) Room P

---

### P-3213
### Yes associated protine dictates copper homeostasis through ATOX1 and confers to cuproptosis sensitivity

[Speaker]Yu-Ling Chen (Grad. Inst. of Med. Sci. College of Med. TMU.／Dept. of Biochem. and Mol. Cell Biology, College of Med.,TMU) [Co-author]Zei-Wei Liu (Grad. Inst. of Med. Sci. College of Med. TMU.／Dept. of Biochem. and Mol. Cell Biology, College of Med.,TMU)、Cheng-Wei Lin (Grad. Inst. of Med. Sci. College of Med. TMU.／Dept. of Biochem. and Mol. Cell Biology, College of Med.,TMU)

Cuproptosis is a novel form of cell death triggered by copper ion accumulation, marked by lipoylated protein aggregation, TCA cycle disruption, and mitochondrial dysfunction. In our study, YAP expression was found closely associated with the cuproptosis signature in breast cancer and enhanced sensitivity to the cuproptosis inducer Es:Cu. YAP Inhibition attenuated Es:Cu-induced DLAT aggregation, preserved mitochondrial membrane potential, and reduced metabolic decline, indicating YAP as a critical mediator of cuproptosis. Mechanistically, YAP was found to directly upregulate the expression of the copper chaperone ATOX1, a key regulator of intracellular copper transport and homeostasis. In addition to its role in copper homeostasis, ATOX1 expression in breast cancer was found positively correlated with important clinical factors in breast cancer such as prognosis, BMI and T stage. These findings suggest the YAP ATOX1 axis plays a key role in cuproptosis sensitivity and may serve as a prognostic marker. In summary, YAP enhances sensitivity to cuproptosis and may offer a promising target for precision copper-based cancer therapy.

[Disclosure of COI by Responsible researcher] Lead presenter is the Responsible researcher.
[Lead presenter: Yu-Ling Chen] We have no financial relationships to disclose.

▲TOP

### [P-3223] I-P11-15
### Antibody therapy/Cancer vaccines (2)

2025 / 9 / 27 (Sat) 16:15-17:00 Room P/(Motenashi Dome) Room P

---

### P-3223
### Elucidating the pathophysiological role of monocyte and macrophage in antibody cancer therapeutics

[Speaker]Hao Shi (Division of Developmental Therapeutics, EOR&CT Center, National Cancer Center／Graduate School of Frontier Sciences, Tokyo University) [Co-author]Masahiro Yasunaga (Division of Developmental Therapeutics, EOR&CT Center, National Cancer Center／Graduate School of Frontier Sciences, Tokyo University)、Yoshihiro Komohara (Graduate school of Medical Sciences, Kumamoto University)

The tumor microenvironment is composed of cancer cells, non-cancer cells and surrounding stroma. Tumor-associated macrophages are one of the most important components of the TME, associated with therapeutic resistance and poor prognosis. Tumor-associated macrophages can be categorized into two types based on their functions: M1-like macrophages and M2-like macrophages. In antibody therapeutics, some antibodies in the TME may bind to monocytes or be phagocytosed by macrophages, limiting the antibodies' ability to kill the cancer cells. Therefore, evaluation systems to assess the binding and phagocytosis of therapeutic antibodies by monocytes and macrophages has been required. In this study, human THP-1 cells were differentiated into macrophages. To investigate the binding pathways of antibodies to monocytes and macrophages, Fc receptor blocker reagent and phagocytosis inhibitors were used to evaluate their inhibitory effects on antibody uptake. The cytotoxic efficacy of the antibodies was further assessed through in vitro co-culture of cancer cells and monocytes/macrophages. Finally, the animal model was established to study antibody delivery and cancer cell elimination in vivo.

[Disclosure of COI by Responsible researcher] Lead presenter is not the Responsible researcher.
[Lead presenter: Hao Shi] We have no financial relationships to disclose.
[Principal Researcher: Masahiro Yasunaga] We have no financial relationships to disclose.

### [P-3224] I-P11-15
### Antibody therapy/Cancer vaccines (2)

2025 / 9 / 27 (Sat) 16:15-17:00 Room P/(Motenashi Dome) Room P

---

### P-3224
### Expanding the Application of Shark IgNAR Antibodies for Next-next-generation Cancer Antibody Therapeutics

[Speaker]Yuki Nitta (The Univ. of Tokyo ／Natl. Cancer Ctr.) [Co-author]Wataru Takagi (The Univ. of Tokyo)、Hyodo Susumu (The Univ. of Tokyo)、Masahiro Yasunaga (The Univ. of Tokyo ／Natl. Cancer Ctr.)

Sharks produce a unique IgNAR antibody with exceptional structural stability and resistance to extreme conditions. These properties suggest its potential to penetrate the dense collagenous tumor stroma, overcoming the "stromal barrier." The IgNAR variable domain is the smallest among known antibodies and may bind tumor antigens inaccessible to human IgG. Moreover, its tolerance to acid, base, and denaturants makes it suitable for α-particle radioimmunotherapy (α-RIT), where IgG often fails due to instability. We established a shark-specific immunization method, distinct from murine protocols, and successfully generated tumor antigen-specific IgNAR antibodies using Schrylihinous torazame and phage display. Long-read RNA sequencing revealed unique IgNAR splice variants that may enhance antigen binding or stability.These findings highlight the promise of IgNAR as a next-next generation antibody platform. It offers advantages over mammalian single-domain antibodies like VHH, particularly in challenging therapeutic contexts such as α-RIT. This approach opens new avenues for the development of highly durable, precision-targeted therapeutics with clinical translational potential.

[Disclosure of COI by Responsible researcher] Lead presenter is not the Responsible researcher.
[Lead presenter: Yuki Nitta] We have no financial relationships to disclose.
[Principal Researcher: Masahiro Yasunaga] We have no financial relationships to disclose.

### [P-3225] I-P11-15
### Antibody therapy/Cancer vaccines (2)

2025 / 9 / 27 (Sat) 16:15-17:00 Room P/(Motenashi Dome) Room P

---

### P-3225
### Research of a novel treatment for malignant pleural mesothelioma targeting Annexin A2

[Speaker]Takumu Yamada (Grad. sch. Sci. & Eng. Dept. Biomed. Eng. TOYO Univ.) [Co-author]Tomoya Masuoka (Grad. Sch. of Health & Spo. Sci. TOYO Univ.)、Kenshin Oba (Grad. Sch. of Health & Spo. Sci. TOYO Univ.)、Takumi Iwasawa (Inst. of Life Innov. Stu. TOYO Univ.)、Kazunori Kato (Grad. sch. Sci. & Eng. Dept. Biomed. Eng. TOYO Univ.／Grad. Sch. of Health & Spo. Sci. TOYO Univ. ／Inst. of Life Innov. Stu. TOYO Univ.)

Malignant pleural mesothelioma (MPM) is a rare and aggressive cancer caused by asbestos exposure. Current treatments, including chemotherapy and immunotherapy, have limited efficacy, requiring new therapeutic strategies. Annexin A2, which is overexpressed in MPM, plays a role in tumor progression and therapy resistance. To develop a novel treatment, we generated anti-Annexin A2 antibodies by hybridoma technology and screened them using flow cytometry. Among the established clones, MS7F5 exhibited high internalization efficiency. A cytotoxicity assay using saporin-conjugated secondary antibodies demonstrated significant cancer cell death when combined with MS7F5, suggesting its potential for antibody-drug conjugate (ADC) therapy. In addition, several anti-Annexin A2 antibodies were established and investigated. Based on these findings, we aim to optimize ADC construction by evaluating differences in epitope binding and internalization efficiency to develop an effective treatment for MPM.

[Disclosure of COI by Responsible researcher] Lead presenter is not the Responsible researcher.
[Lead presenter: Takumu Yamada] We have no financial relationships to disclose.
[Principal Researcher: Kazunori Kato] We have no financial relationships to disclose.

### [P-3226] I-P11-15
### Antibody therapy/Cancer vaccines (2)

2025 / 9 / 27 (Sat) 16:15-17:00 Room P/(Motenashi Dome) Room P

---

### P-3226
### Targeting EphA2 and EGFR with Dual-Specific CrossMab Antibodies for Cholangiocarcinoma Therapy

[Speaker]Tsai Yu Lin (Ph.D. Program for Cancer Mol. Biol. & Drug Discovery, TMU) [Co-author]Fu Ling Chang (Ph.D. Program for Cancer Mol. Biol. & Drug Discovery, TMU)、Jia Huei Zheng (Taiwan Autoantibody Biobank Initiative, Hualien Tzu Chi Hosp.)、Yaw Dong Lang (Dept. of Med. Res., TMU Hosp.／Precision Med. Res. Ctr., TMU Hosp.／Grad. Inst. of Clin. Med., TMU)、Keng Chang Tsai (Natl.l Res. Inst. of Chinese Med., MOHW／Med. Biotechnology, TMU)、Wei Chun HuangFu (Ph.D. Program for Cancer Mol. Biol. & Drug Discovery, TMU／Drug Discovery & Development Industry, TMU)、Yu Ching Lee (TMU Res. Ctr. of Cancer Translational Med., TMU／Med. Biotechnology, TMU／Drug Discovery & Development Industry, TMU)

Cholangiocarcinoma (CCA) is an aggressive bile duct cancer with limited therapeutic options. Erythropoietin-producing hepatocellular receptor A2 (EphA2), a receptor tyrosine kinase overexpressed in CCA, promotes tumor progression but remains underexplored as a therapeutic target. Epidermal growth factor receptor (EGFR) is another candidate in CCA; however, monotherapies often fail due to resistance and compensatory signaling. Co-activation of oncogenic pathways by EphA2 and EGFR suggests that dual targeting may enhance therapeutic efficacy.We show that EphA2 is overexpressed in CCA and co-expressed with EGFR in tumor cells and extracellular vesicles. Our anti-EphA2 antibody, hSD5, induces EphA2 degradation and suppresses CCA cell proliferation, migration, and tumorsphere formation. Additionally, hSD5 regulates the cell cycle by downregulating E2F2. We further developed EE-CMab, a bispecific antibody integrating hSD5 and cetuximab via CrossMab technology. EE-CMab simultaneously targets EphA2 and EGFR and effectively suppresses tumor growth in the CCA xenograft model. These findings support dual EphA2/EGFR targeting and highlight EE-CMab as a promising therapeutic strategy for CCA.

[Disclosure of COI by Responsible researcher] Lead presenter is the Responsible researcher.
[Lead presenter: Tsai Yu Lin] We have no financial relationships to disclose.

### [P-3227] I-P11-15
### Antibody therapy/Cancer vaccines (2)

2025 / 9 / 27 (Sat) 16:15-17:00 Room P/(Motenashi Dome) Room P

---

### P-3227
### Inhibiting EndoMT-Promoted Tumor Growth in Pancreatic Adenocarcinoma with a Humanized Anti-HSP90 alpha Antibody

[Speaker]Chi-Shuan Fan (National Institute of Cancer Research, National Health Research) [Co-author]Chia-Chi Chen (National Institute of Cancer Research, National Health Research)、Li-Li Chen (National Institute of Cancer Research, National Health Research)、Tze-Sing Huang (National Institute of Cancer Research, National Health Research)、Tsu-An Hsu (National Institute of Cancer Research, National Health Research)、Hui-Chen Hung (National Institute of Cancer Research, National Health Research)

Extracellular HSP90a (eHSP90a) is markedly elevated in patients with colorectal cancer and pancreatic ductal adenocarcinoma (PDAC), making it a potential biomarker and therapeutic target. We developed HH01, a humanized monoclonal antibody targeting eHSP90a, and evaluated its anti-cancer efficacy. HH01 features unique complementarity-determining regions (CDRs) that bind with high affinity to two critical linear epitopes located within the HSP90a sequence, spanning amino acid residues 235 to 244 and 251 to 260, as identified by epitope mapping. In vitro, HH01 inhibited eHSP90a-induced invasion and spheroid formation by blocking CD91 interaction in colorectal and PDAC cells. In PDAC xenografts enriched with EndoMT-derived fibroblasts, HH01 suppressed tumor growth and reduced serum eHSP90a levels. HH01 also showed immunomodulatory effects by decreasing M2 macrophages and increasing T-cell activation. Pharmacologically, HH01 exhibited low aggregation, high solubility, and an extended half-life with no observed toxicity in mice. These findings support HH01 as a promising therapeutic candidate for PDAC through dual mechanisms: direct tumor inhibition and immune modulation.

[Disclosure of COI by Responsible researcher] Lead presenter is the Responsible researcher.
[Lead presenter: Chi-Shuan Fan] We have no financial relationships to disclose.

▲TOP

[P-3228] I-P11-15
Antibody therapy/Cancer vaccines (2)

2025 / 9 / 27 (Sat) 16:15-17:00 Room P/(Motenashi Dome) Room P

P-3228
### Trastuzumab Deruxtecan, HER2 targeting antibody-drug conjugates, exerted multiple anti-cancer activities against CCA.

[Speaker]Prin Sungwan (Div. of Hematopoiesis, Joint Res., Kumamoto Univ.) [Co-author]Jutatip Panaampon (Div. of Hematopoiesis, Joint Res., Kumamoto Univ.／Div. of Hematologic Neoplasia., Dana-Farber Cancer Inst., Harvard Med. Sch.)、Seiji Okada (Div. of Hematopoiesis, Joint Res., Kumamoto Univ.／Inst. of Industrial Nanomaterials., Kumamoto Univ.)

Cholangiocarcinoma (CCA) is a deadly cancer that arises from bile duct epithelial cells via multiple mechanisms, resulting in high tumor heterogeneity and very poor prognosis with very low 5-year survival, <40% response rate to standard chemotherapeutic regimens. R0 resection is the most effective treatment for CCA, whilst only < 3% of patients can be operated on resection due to lacking signs and asymptomatic. Effective treatment regimens for CCA are urgently needed. Cancer immunotherapy focusing on human epidermal growth factor receptor 2 (HER2) significantly increases clinical benefits in several HER2+ cancers. Here, we elucidated the anti-tumor activity of trastuzumab deruxtecan (T-DXd), the novel antibody-conjugated drugs against HER2+ CCA via multiple mechanisms. Including induction of cell cycle arrest, substantially induced cancer apoptosis as well as bystander killing in in-vitro, in-vivo CCA inoculated mice and CCA PDXs models. T-DXd demonstrated a greater anti-tumor effect than trastuzumab, the specific HER2 humanized monoclonal antibody. In sum, targeting HER2+ CCA cluster using T-DXd might be one of the promising therapeutic strategies for CCA treatment.

[Disclosure of COI by Responsible researcher] Lead presenter is the Responsible researcher.
[Lead presenter: Prin Sungwan] We have no financial relationships to disclose.

### [P-3229] I-P11-15
### Antibody therapy/Cancer vaccines (2)

2025 / 9 / 27 (Sat) 16:15-17:00 Room P/(Motenashi Dome) Room P

---

### P-3229
### Possibility of different functional roles among the Fab arms in monoclonal antibody found by electron density topography

[Speaker]Takashi Matsumoto (Rigaku Corp.) [Co-author]Akimi Sato (Tokyo University of Agriculture and Technology)、Takuma Kozono (Tokyo University of Agriculture and Technology)、Ryo Kitamura (Rigaku Corp.)、Takashi Sato (Rigaku Corp.)、Tomokazu Hasegawa (Rigaku Corp.)、Hiroyuki Kanda (Rigaku Corp.)、Takashi Tonozuka (Tokyo University of Agriculture and Technology)、Atsushi Nishikawa (Tokyo University of Agriculture and Technology)

In pharmaceutical development, three-dimensional structural information is crucial for molecular design and optimization. However, in the case of flexible molecules such as antibody, both crystallization and single-particle analysis are challenging, making use of higher-order structural information for design and quality control extremely limited. Should direct observation of antibody molecules become achievable, it could lead to a better understanding of their structural differences and conformational changes under different conditions, potentially opening up new industrial applications in the design, development, quality control, and production of antibody drugs.The molecular projection using electron density topography (EDT), which will be introduced in this presentation, does not require crystallization, freezing, fixation, or labeling, and allows for the direct observation of the shape and movement of antibody molecules.In this study, as a result of investigating mouse monoclonal antibody (IgG1) and its antigen protein complex with EDT, we report here the discovery of differences in Fab mobility and antigen interactions that have not been reported previously.

[Disclosure of COI by Responsible researcher] Lead presenter is the Responsible researcher.
[Lead presenter: Takashi Matsumoto] ■ employment by a company related to the presentation: 株式会社リガク　■Profit of stock: 株式会社リガク

### [P-3230] I-P11-15
### Antibody therapy/Cancer vaccines (2)

2025 / 9 / 27 (Sat) 16:15-17:00 Room P/(Motenashi Dome) Room P

---

### P-3230
### Establishment of the basis for the development of vaccine therapy for osteosarcoma

[Speaker]Takatsune Shimizu (Hoshi Univ, Pathophysiology) [Co-author]Tetsuya(Fujita Health Univ.) Takimoto (Fujita Health Univ, Oncology Innovation Center)、Nobusue Hiroyuki (Fujita Health Univ, Oncology Innovation Center)、Seiji Okada (Kumamoto Univ, Human Retrovirus Infection)、Akihiro Muto (Hoshi Univ, Pathophysiology)、Hideyuki Saya (Fujita Health Univ, Oncology Innovation Center)

Current immunotherapies have been largely ineffective for osteosarcoma (OS). We previously developed mouse OS cells. Inoculation of AXT cells into immune-competent B6 mice rapidly induces lethal OS with metastatic lesions that mimics human pathophysiology. Notably, injection of cell lysates from the MSC-like cells, that also form OS similar to AXT cells, into B6 mice prior to transplantation of AXT cells dramatically attenuated tumorigenesis of primary and metastatic lesions. This inhibitory effect was not observed in B6SCID mice, suggesting that immunity is involved in the anti-tumor process. Plasma membrane fraction was most effective, while the cytosol fraction showed no effect, indicating that the membrane peptide may have immunogenicity. IHC analysis revealed that T cells abundantly infiltrated into tumors with vaccination. In addition, tumor infiltrating CD8+ T cells sorted from OS in mice with vaccination highly expressed ***Gzmb***, ***Ifng***, and ***Fas*** compared to those from unvaccinated mice. Collectively, lysates from OS cells contain peptides that activate tumor immunity and exert anti-tumor effect. These findings provide a basis for the development of vaccine therapy for OS.

[Disclosure of COI by Responsible researcher] Lead presenter is the Responsible researcher.
[Lead presenter: Takatsune Shimizu] We have no financial relationships to disclose.

### [P-3231] I-P11-15
### Antibody therapy/Cancer vaccines (2)

2025 / 9 / 27 (Sat) 16:15-17:00 Room P/(Motenashi Dome) Room P

---

### P-3231
### Activation of Tumor-Specific CD8$^+$ T Cells by Engineered Extracellular Vesicles

[Speaker]Kanto Nagamori (Department of Immunology, Graduate School of Medicine, Kanazawa University) [Co-author]Tomoyoshi Yamano (Department of Immunology, Graduate School of Medicine, Kanazawa University／WPI Nano Life Science Institute (NanoLSI), Kanazawa University)

Extracellular vesicles (EVs), including exosomes, are nanoscale, membrane-enclosed particles that transport proteins RNAs, and lipids for intercellular communication. Their biocompatibility and low immunogenicity make EVs attractive drug delivery platforms with reduced risk of adverse effects. In this study, we engineered antigen-presenting EVs (AP-EVs) that express three critical signals for CD8$^+$ T cell activation: peptide-MHC class I complexes, CD80, and IL-2. Unlike conventional immunotherapies, which deliver these signals separately, AP-EVs present them in a single, integrated format. This avoids signal dispersion and enables precise, antigen-specific activation and expansion of CD8$^+$ T cells in vivo. In murine tumor models, AP-EVs accumulated at tumor sites, enhanced IFN-$\gamma^+$ CD8$^+$ T cell responses, and reduced exhausted T cell populations, thereby transforming the tumor microenvironment from "cold" to "hot". Importantly, AP-EVs alone suppressed tumor growth, demonstrating their potential as a powerful and targeted cancer immunotherapy. These findings support the development of AP-EVs as a novel, low-toxicity, high-specificity platform for next-generation cancer immunotherapy.

[Disclosure of COI by Responsible researcher] Lead presenter is not the Responsible researcher.
[Lead presenter: Kanto Nagamori] We have no financial relationships to disclose.
[Principal Researcher: Tomoyoshi Yamano] We have no financial relationships to disclose.

### [P-3240] I-P11-17
### Immune cell therapy (CAR-T, T cell therapy) (2)

2025 / 9 / 27 (Sat) 16:15-17:00 Room P/(Motenashi Dome) Room P

---

### P-3240
### Withdrawn

No Abstract

### [P-3241] I-P11-17
### Immune cell therapy (CAR-T, T cell therapy) (2)

2025 / 9 / 27 (Sat) 16:15-17:00 Room P/(Motenashi Dome) Room P

### P-3241
### Withdrawn

No Abstract

[P-3242] I-P11-17

Immune cell therapy (CAR-T, T cell therapy) (2)

2025 / 9 / 27 (Sat) 16:15-17:00 Room P/(Motenashi Dome) Room P

P-3242

### Oncolytic HSV enhances CAR T cell therapy by remodeling the tumor microenvironment in pancreatic cancer

[Speaker]Mona A. Aboalela (Cancer Immunetherapy Research Center, Grad. school of Med., Nagoya Univ.／Department of Gastroenterological surgery, Grad. school of Med., Nagoya Univ.／Department of Medical Microbiology & Immunology, Faculty of Med., Zagazig Univ.) [Co-author]Mohamed Abdelmoneim (Cancer Immunetherapy Research Center, Grad. school of Med., Nagoya Univ.／Department of Microbiology, Faculty of Veterinary Med., Zagazig Univ.)、Shigerua Matsumura (Cancer Immunetherapy Research Center, Grad. school of Med., Nagoya Univ.)、Ibrahim R. Eissa (Cancer Immunetherapy Research Center, Grad. school of Med., Nagoya Univ.／Surgical Oncology Division, Dept. of Surgery, Harvard Med. School.)、Yoshinori Naoe (Cancer Immunetherapy Research Center, Grad. school of Med., Nagoya Univ.)、Hideki Kasuya (Cancer Immunetherapy Research Center, Grad. school of Med., Nagoya Univ.)

Mesothelin (MSLN) has emerged as a promising target for CAR T cell therapy in pancreatic cancer; however, clinical outcomes remain suboptimal due to variable antigen expression, antigen shedding, and an immunosuppressive tumor microenvironment (TME). To overcome these challenges, we developed an oncolytic herpes simplex virus type 1 (HSV-1) engineered to express MSLN (HSV-MSLN) and tested its efficacy in combination with MSLN-targeted CAR T cells in a murine pancreatic tumor model. HSV-MSLN boosted MSLN surface presentation and lysed tumor cells in vitro. When co-cultured with CAR T cells, HSV-MSLN-infected cancer cells enhanced CAR T cell activation and cytotoxicity. In vivo, HSV-MSLN reprogrammed the TME to favor immune activation. The combined treatment led to stronger tumor suppression by promoting CAR T cell expansion and function, reducing T cell exhaustion and regulatory populations, increasing infiltration of migratory dendritic cells in tumors and tumor-draining lymph nodes, and enriching memory T cell subsets. These findings highlight the potential of HSV-MSLN to boost tumor-associated antigen expression and reshape the TME, thereby enhancing CAR T cell efficacy.

[Disclosure of COI by Responsible researcher] Lead presenter is the Responsible researcher.
[Lead presenter: Mona A. Aboalela] We have no financial relationships to disclose.

▲TOP

[P-3243] I-P11-17
Immune cell therapy (CAR-T, T cell therapy) (2)

2025 / 9 / 27 (Sat) 16:15-17:00 Room P/(Motenashi Dome) Room P

P-3243
### Combination therapy of T cell therapy and compound 433 overcomes tumor heterogeneity

[Speaker]Pengyu Miao (Dept. of Oncology, Nagasaki Univ. Biomed. Sci.) [Co-author]Situo Deng (Dept. of Oncology, Nagasaki Univ. Biomed. Sci.)、Daisuke Ehara (Dept. of Oncology, Nagasaki Univ. Biomed. Sci.／Dept. of Dermatology, Nagasaki Univ. Biomed. Sci.)、Daisuke Muraoka (Oncoimmunology Div., Aichi Cancer Center Res. Inst.)、Naohisa Ogo (Drug Discovery Ctr., Univ. of Shizuoka)、Mitsuhiro Yoneda (Dept. of Oncology, Nagasaki Univ. Biomed. Sci.)、Kiyoshi Yasui (Dept. of Oncology, Nagasaki Univ. Biomed. Sci.)、Akira Asai (Drug Discovery Ctr., Univ. of Shizuoka)、Hiroaki Ikeda (Dept. of Oncology, Nagasaki Univ. Biomed. Sci.)

Tumor heterogeneity is a major obstacle in solid tumor treatment because antigen-negative cells can escape T cell-mediated killing. This study investigated how T cell-derived cytokines sensitize tumors to the small-molecule compound 433. We used CT26 and CT26-9m colorectal cancer cells, the latter expressing antigen 9m, which is specifically recognized by CD8+ T cells derived from TCR-transgenic mice (DUC.18). Treatment with 433 in combination with IFN-γ or TNF-α induced significant apoptosis in tumor cells. In a heterogeneous tumor model using a mixture of CT26 and CT26-9m cells, combination therapy with antigen-specific T cells and 433 efficiently eliminated not only the antigen-positive CT26-9m cells but also the antigen-negative CT26 tumor cells. The adoptive transfer of DUC.18 T cells combined with 433 efficiently suppressed the growth of the heterogeneous tumor model. Mechanistically, tumor cells underwent caspase-dependent apoptosis, with TNF-α acting as the primary effector and IFN-γ serving as a sensitizer.These findings suggest that T cell-derived cytokines can reprogram tumor drug sensitivity, resulting in efficient eradication of tumors exhibiting antigen heterogeneity.

[Disclosure of COI by Responsible researcher] Lead presenter is not the Responsible researcher.
[Lead presenter: Pengyu Miao] We have no financial relationships to disclose.
[Principal Researcher: Situo Deng] We have no financial relationships to disclose.

[P-3244] I-P11-17
Immune cell therapy (CAR-T, T cell therapy) (2)

2025 / 9 / 27 (Sat) 16:15-17:00 Room P/(Motenashi Dome) Room P

P-3244
### Replacement of the linker sequence of the self-activating CAR enhances in vitro cytotoxic activity.

[Speaker]Taku Kouro (Div. Cancer Immunotherapy, Kanagawa Cancer Ctr. Res. Inst.／Cancer Vaccine Immunotherapy Ctr., Kanagawa Cancer Ctr.) [Co-author]Daisuke Hoshino (Cancer Biol. Div. Kanagawa Cancer Ctr. Res. Inst.)、Tetsuro Sasada (Div. Cancer Immunotherapy, Kanagawa Cancer Ctr. Res. Inst.／Cancer Vaccine Immunotherapy Ctr., Kanagawa Cancer Ctr.)

We have reported that the CAR targeting a modified HEG1 molecule specific to malignant mesothelioma induced tonic signaling through self-aggregation and elicited an exhaustive phenotype in transduced T cells. In this study, we sought to reduce the tonic signal by replacing its scFv linker. Unexpectedly, the substitution of the (G4S)4 linker (G4S-28z CAR) to the Whitlow/218 linker (W218-28z CAR) did not affect the extent of CAR self-aggregation, expression of exhaustion markers, and transcriptome profiles. However, W218-28z CAR T cells showed increased cytokine secretion upon target engagement and enhanced ***in vitro*** killing activity against target cells. Notably, despite the enhanced ***in vitro*** killing activity, W218-28z CAR-T cells did not demonstrate ***in vivo*** tumor control in the xenograft model. This may be attributable to the fact that ***in vivo*** tumor control may largely depend on the persistence of CAR-T cells, which is compromised by tonic signal-induced exhaustion of CAR-T cells.

[Disclosure of COI by Responsible researcher] Lead presenter is not the Responsible researcher.
[Lead presenter: Taku Kouro] We have no financial relationships to disclose.
[Principal Researcher: Tetsuro Sasada] ■Research expenses: 大鵬薬品工業株式会社

▲TOP

[P-3254] I-P12-7
Cancer diagnostic technologies (2)

2025 / 9 / 27 (Sat) 16:15-17:00 Room P/(Motenashi Dome) Room P

P-3254

### Development of $^{67}$Ga-labeled PEGylated heterodivalent probes for imaging negative charges and integrins on cancer cells

[Speaker]Toya Hiwatashi (Grad. Sch. Pharm., Kanazawa Univ.) [Co-author]Takeshi Fuchigami (Grad. Sch. Pharm., Kanazawa Univ.)、Hiroaki Echigo (Grad. Sch. Pharm., Kanazawa Univ.)、Masayuki Munekane (Grad. Sch. Pharm., Kanazawa Univ.)、Kenji Mishiro (Grad. Sch. Pharm., Kanazawa Univ.)、Hiroshi Wakabayashi (Dept. of Nuclear Med., Kanazawa Univ. Hosp.)、Seigo Kinuya (Dept. of Nuclear Med., Kanazawa Univ. Hosp.)、Kazuma Ogawa (Grad. Sch. Pharm., Kanazawa Univ.)

Previously, we developed a heterobivalent tumor imaging probe, [$^{67}$Ga]Ga-DOTA-KV6-Mal-c(RGDyK), fusing the KV6 peptide targeting negatively charged sites and a cyclic RGD peptide targeting integrin $\alpha_v\beta_3$ on the cancer cell membrane. This probe demonstrated higher tumor accumulation than those targeting a single target, though high liver and kidney uptake remained a concern. To address this, the present study introduced a PEG-based spacer between KV6 and c(RGDyK) to improve pharmacokinetics and dual-target accessibility, aiming for enhanced tumor selectivity. Two PEG-modified probes, [$^{67}$Ga]Ga-DOTA-KV6-PEG$_{12}$-c(RGDyK) and [$^{67}$Ga]Ga-DOTA-KV6-PEG$_{24}$-c(RGDyK), were synthesized and evaluated. Both PEG-modified probes showed significantly higher uptake in U87MG cells compared with [$^{67}$Ga]Ga-DOTA-KV6-Mal-c(RGDyK). Moreover, biodistribution studies in U87MG tumor-bearing mice revealed significantly higher tumor accumulation for these PEG-modified probes, with [$^{67}$Ga]Ga-DOTA-KV6-PEG$_{12}$-c(RGDyK) demonstrating the highest tumor-to-normal tissue ratio. These results indicated that PEG modification of the KV6-RGD peptide improves in vivo pharmacokinetics, including enhanced tumor accumulation.

[Disclosure of COI by Responsible researcher] Lead presenter is not the Responsible researcher.
[Lead presenter: Toya Hiwatashi] ■Research funds from company: JST創発 (2021-2028年度)、科研費基盤B (2022-2024年度)
[Principal Researcher: Takeshi Fuchigami] ■Research funds from company: JST創発 (2021-2028年度)、科研費基盤B (2022-2024年度)

### [P-3255] I-P12-7
### Cancer diagnostic technologies (2)

2025 / 9 / 27 (Sat) 16:15-17:00 Room P/(Motenashi Dome) Room P

### P-3255
### Withdrawn

No Abstract

### [P-3256] I-P12-7
### Cancer diagnostic technologies (2)

2025 / 9 / 27 (Sat) 16:15-17:00 Room P/(Motenashi Dome) Room P

---

### P-3256
### First-in-Human Phase 0 Study of Cu-PD32766, a CAIX-Targeting Peptide, in Patients with Clear Cell Renal Cell Carcinoma

[Speaker]Anri Inaki (Division of Functional Imaging, NCC-EPOC／Department of Diagnostic Radiology, NCCHE) [Co-author]Toshihiro Horii (Department of Diagnostic Radiology, NCCHE)、Mitsuyoshi Yoshimoto (Division of Functional Imaging, NCC-EPOC)、Akinori Kobayashi (Department of Gastrointestinal Oncology, NCCHE)、Chihiro Kondo (Department of Medical Oncology, NCCHE)、Nobuaki Matsubara (Department of Medical Oncology, NCCHE)、Chikako Funasaka (Department of Medical Oncology, NCCHE)、Hiromichi Nakajima (Department of Medical Oncology, NCCHE)、Yasutoshi Kuboki (Department of Experimental Therapeutics, NCCHE)、Kimiteru Ito (Department of Diagnostic Radiology, NCCH)、Masato Murakami (PeptiDream Inc.)、Naoki Kanazawa (PDR Pharma Inc.)、Akira Inagaki (Department of Radiological Technology, NCC)、Daisuke Kano (Department of Pharmacy, NCCHE)、Tatsushi Kobayashi (Department of Diagnostic Radiology, NCCHE)

This is an encore presentation from ASCO GU 2025.

**Background:** Carbonic anhydrase IX (CAIX) is a potential target for detecting tumors with poor prognosis. High CAIX expression is often seen in clear cell renal cell carcinoma (ccRCC). We developed a CAIX-targeted radioligand, PD-32766, and evaluated it in recurrent ccRCC cases for safety, imaging, pharmacokinetics, and dosimetry.

**Methods:** Between May-Sep 2024, five ccRCC patients with metastasis or recurrence received $^{64}$Cu-PD-32766 (148 MBq ±20%). PET/CT was done at 5, 60, 120, 240 min, 24, and 48 h. Vitals, labs, and urinalysis were assessed. Endpoints: PET positivity rate, lesion count, pharmacokinetics, dosimetry, AEs. Trial ID: jRCTs031240046.

**Results:** $^{64}$Cu-PD-32766 was well tolerated. Patient-level PET positivity at 120 min was 75%; lesion-level 96.2%. Uptake peaked at 60-120 min. Effective dose: 0.102±0.036 mSv/MBq. Highest organ doses: stomach (0.712±0.287) and small intestine (0.304±0.067 mGy/MBq).

**Conclusion:** $^{64}$Cu-PD-32766 showed high tumor uptake and good safety, supporting its diagnostic and future therapeutic use.

[Disclosure of COI by Responsible researcher] Lead presenter is the Responsible researcher.
[Lead presenter: Anri Inaki] ■Research expenses: ペプチドリーム株式会社

### [P-3257] I-P12-7
### Cancer diagnostic technologies (2)

2025 / 9 / 27 (Sat) 16:15-17:00 Room P/(Motenashi Dome) Room P

---

### P-3257
### Validation of a Semi-automatic Artificial Intelligence Model for Pan-cancer Tumor Segmentation

[Speaker]Ryota Shibaki (National Cancer Center Reserch Institute) [Co-author]Kazuma Kobayashi (National Cancer Center Reserch Institute／RIKEN Center for Advanced Intelligence Project)、Jun Sato (National Cancer Center Hospital)、Hiromi Matsumoto (National Cancer Center Reserch Institute／RIKEN Center for Advanced Intelligence Project)、Taro Hatsutani (FUJIFILM Corporation)、Yasuo Kawata (FUJIFILM Corporation)、Mao Okada (National Cancer Center Hospital)、Yuki Katsuya (National Cancer Center Hospital)、Takafumi Koyama (National Cancer Center Hospital)、Noboru Yamamoto (National Cancer Center Hospital)、Ryuji Hamamoto (National Cancer Center Reserch Institute／RIKEN Center for Advanced Intelligence Project)

3D assessment of tumor response evaluation can benefit precision oncology. We applied an off-the-shelf artificial intelligence (AI) tool to initiate 3D segmentation of pan-cancer lesions based on the Response Evaluation Criteria In Solid Tumors (RECIST) marks.Fifty-three patients with 17 cancer types were selected from participants in phase I trials at the National Cancer Center Hospital. A total of 1,203 multiple organ lesions, either primary or metastatic tumors, were identified. For each lesion, a RECIST mark was annotated for the input of the AI tool to output lesion-wise 3D segmentation. The endpoint was the Dice score, indicating the proportionate overlap between predicted and ground-truth 3D segmentation.The median Dice score was 0.720 (interquartile range 0.616-0.802). Stratified by organ, the median Dice scores were: lung (N=753), 0.714; lymph nodes (N=143), 0.719; liver (N=143), 0.748; peritoneum membrane (N=49), 0.732; and pleural membrane (N=47), 0.608. RECIST marks can be robustly propagated to lesion-wise 3D segmentation across various organs, which will help volumetric assessment of tumor evaluation.

[Disclosure of COI by Responsible researcher] Lead presenter is not the Responsible researcher.
[Lead presenter: Ryota Shibaki] We have no financial relationships to disclose.
[Principal Researcher: Ryuji Hmamoto] ■Research expenses: 富士フイルム株式会社、富士通株式会社

### [P-3258] I-P12-7
### Cancer diagnostic technologies (2)

2025 / 9 / 27 (Sat) 16:15-17:00 Room P/(Motenashi Dome) Room P

---

### P-3258
### Assessment of Metabolic Features and Development of Inhibitory Therapy for GI Cancer via Hyperpolarized 13C-MRI

[Speaker]Nobu Oshima (Dept. of GI Surgery, Kobe City Medical Center General Hospital／Dept. of Sugical Research, Kobe City Medical Center General Hospital／Dept. of Surgery, Kyoto Univ.) [Co-author]Akihiko Masuo (Dept. of Surgery, Kyoto Univ.)、Shigeru Tsunoda (Dept. of Surgery, Kyoto Univ.)、Shigeo Hisamori (Dept. of Surgery, Kyoto Univ.)、Masayuki Matsuo (Dept. of Radiology, Gifu Univ.)、Kazutaka Obama (Dept. of Surgery, Kyoto Univ.)

[Introduction]Hyperpolarized-13C-MR Imaging(HP-MRI) is a clinically used worldwide, allowing in vivo visualization of cancer-related pyruvate metabolism. [Objective]To define metabolic profiles and evaluate metabolism-targeted therapies using HP-MRI. [Methods] Three cancer cell lines, MiaPaCa2 (MP2, pancreatic), HCT116 (colorectal), and MKN45 (gastric), were analyzed for glycolytic(P-L) and oxidative(OXPHOS) pyruvate metabolism in vitro and in vivo. LDH and mitochondrial complex I(MC1) inhibitors were used to assess metabolic change. Antitumor effects of single and combined treatments were tested in mouse models. [Results] With HP-MRI, MP2 showed high P-L activity, reduced by ~80% with LDH inhibition; HCT116 and MKN45 showed ~50% reduction. HP-MRI also revealed 13C-Bicarbonate MR signal increased by LDH inhibition in MP2, indicating pseudglycolysis. In addition, MC1 inhibition increased P-L activity. These results indicate rewiring pyruvate metabolism. Combination therapy yielded stronger antitumor effects than a single treatment. [Conclusion] HP-MRI enables noninvasive assessment of tumor metabolism and prediction of therapeutic response, supporting its clinical potential.

[Disclosure of COI by Responsible researcher] Lead presenter is the Responsible researcher.
[Lead presenter: Nobu Oshima] We have no financial relationships to disclose.

### [P-3259] I-P12-7
### Cancer diagnostic technologies (2)

2025 / 9 / 27 (Sat) 16:15-17:00 Room P/(Motenashi Dome) Room P

---

### P-3259
### AI-Driven Uterine Sarcoma Diagnosis: An Ensemble Approach with Radiomics Integration

[Speaker]Boram Choi (Dept.of OBGY., SSMH) [Co-author]Youn Jin Choi (Dept.of OBGY., SSMH)、Sung Jong Lee (Dept.of OBGY., SSMH)、Keun Ho Lee (Dept.of OBGY., SSMH)、Soo Young Hur (Dept.of OBGY., SSMH)

This study develops an AI-driven MRI-based classification and segmentation pipeline to address diagnostic challenges in uterine sarcoma, often misclassified as leiomyomas. Using a multi-institutional dataset (63 sarcomas, 200 leiomyomas), the model integrates ensemble learning and advanced data augmentation (15 MRI sequences, 20 expansion) to tackle data scarcity and class imbalance. Optimized MRI protocols identified axial/sagittal T2WI and DWI as the most discriminative. Fine-tuned via cross-institutional federated learning, an ensemble of MobileNet-V2, UNet++, and DenseNet-121 reduced false positives from degenerated leiomyomas by 63.2%. The model achieved 92.44% accuracy (sensitivity: 92.25%, specificity: 92.50%), surpassing radiologists (88.3%) and single models (max: 89.1%), while tumor segmentation reached a Dice coefficient of 0.91. Bridging the gap between practitioners and specialists, this AI pipeline offers a scalable solution, with PET/CT and clinical data integration poised to enhance workflows and management strategies.

[Disclosure of COI by Responsible researcher] Lead presenter is the Responsible researcher.
[Lead presenter: Boram Choi] We have no financial relationships to disclose.

▲TOP

### [P-3260] I-P12-7
### Cancer diagnostic technologies (2)

2025 / 9 / 27 (Sat) 16:15-17:00 Room P/(Motenashi Dome) Room P

---

### P-3260
### Development of EBUS image classification model in pulmonary lymph nodes using deep learning

[Speaker]Naoki Teraya (Div. Med. AI Res. Dev., Natl. Cancer Ctr. Res. Inst.／Dept. Obstet. Gynecol., Showa Med. Univ. Sch. Med.) [Co-author]Yuji Matsumoto (Dept. Endoscopy, Respiratory Endoscopy Div., Natl. Cancer Ctr. Hosp.)、Masaaki Komatsu (Div. Med. AI Res. Dev., Natl. Cancer Ctr. Res. Inst.／Dept. Obstet. Gynecol., Showa Med. Univ. Sch. Med.／Cancer Transl. Res. Team, RIKEN Ctr. for AIP project)、Katsuji Takeda (Cancer Transl. Res. Team, RIKEN Ctr. for AIP project)、Naoaki Harada (Div. Med. AI Res. Dev., Natl. Cancer Ctr. Res. Inst.／Dept. NCC Cancer Sci., Grad. Sch., Inst. Sci. Tokyo)、Hideaki Furuse (Dept. Endoscopy, Respiratory Endoscopy Div., Natl. Cancer Ctr. Hosp.)、Ken Asada (Div. Med. AI Res. Dev., Natl. Cancer Ctr. Res. Inst.／Cancer Transl. Res. Team, RIKEN Ctr. for AIP project)、Syuzo Kaneko (Div. Med. AI Res. Dev., Natl. Cancer Ctr. Res. Inst.／Cancer Transl. Res. Team, RIKEN Ctr. for AIP project)、Takaaki Tsuchida (Dept. Endoscopy, Respiratory Endoscopy Div., Natl. Cancer Ctr. Hosp.)、Ryuji Hamamoto (Div. Med. AI Res. Dev., Natl. Cancer Ctr. Res. Inst.／Cancer Transl. Res. Team, RIKEN Ctr. for AIP project／Dept. NCC Cancer Sci., Grad. Sch., Inst. Sci. Tokyo)

Lung cancer is one of the most mortal tumors in the world, and appropriate staging and corresponding treatment are crucial. The stage is determined mainly based on contrast-enhanced CT and FDG-PET, and in the absence of distant metastasis, it largely depends on nodal factors. However, the diagnostic accuracy of these imaging modalities for nodal factors is insufficient, and in cases of suspected mediastinal lymph node (LN) metastasis, pathological confirmation using endobronchial ultrasound-guided transbronchial needle aspiration (EBUS-TBNA) is recommended. In this systematic staging, it is necessary to puncture multiple LNs, but there is also the burden of puncturing even those with an extremely low possibility of metastasis. Therefore, we aimed to develop a deep-learning model that evaluates whether target LNs are metastatic on EBUS images to reduce the frequency of LNs puncture. We attempted efficient feature extraction by using the difference between the B-mode and elastography images for each malignant and benign LN as input. This is expected to find malignant LNs with efficient workflow and stable accuracy, regardless of the skill levels of bronchoscopists.

[Disclosure of COI by Responsible researcher] Lead presenter is not the Responsible researcher.
[Lead presenter: Naoki Teraya] We have no financial relationships to disclose.
[Principal Researcher: Ryuji Hamamoto] We have no financial relationships to disclose.

### [P-3261] I-P12-7
### Cancer diagnostic technologies (2)

2025 / 9 / 27 (Sat) 16:15-17:00 Room P/(Motenashi Dome) Room P

---

### P-3261
### Withdrawn

No Abstract

### [P-3262] I-P12-7
### Cancer diagnostic technologies (2)

2025 / 9 / 27 (Sat) 16:15-17:00 Room P/(Motenashi Dome) Room P

---

### P-3262
### Withdrawn

No Abstract

### [P-3272] I-P13-7
### New molecular targeted therapy (1)

2025 / 9 / 27 (Sat) 16:15-17:00 Room P/(Motenashi Dome) Room P

---

### P-3272
### Development of an Anti-IL-7R Antibody-PROTAC Conjugate for the Treatment of Acute Lymphoblastic Leukemia

[Speaker]Huiqing Liang (Department of Integrated Biosciences, The University of Tokyo／Division of Developmental Therapeutics, National Cancer Center Japan) [Co-author]Masahiro Yasunaga (Department of Integrated Biosciences, The University of Tokyo／Division of Developmental Therapeutics, National Cancer Center Japan)、Yosuke Demizu (Division of Organic Chemistry, National Institute of Health Sciences)

The IL-7/IL-7R axis is critical in T-cell acute lymphoblastic leukemia, promoting leukemic cell survival, proliferation, and steroid resistance via oncogenic JAK/STAT signaling. These effects underscore the need for novel therapies targeting this pathway.PROTAC is a promising technology that induces degradation of intracellular proteins via the ubiquitin-proteasome system. Unlike conventional drugs, PROTAC eliminate target proteins, offering sustained suppression and the potential to overcome drug resistance. Moreover, it may achieve efficacy at lower doses, significantly reducing IC50.We are developing an anti-IL-7R antibody-drug conjugate (ADC) that delivers a PROTAC-based molecular targeting agent (MTA) to inhibit JAK-STAT signaling. Upon IL-7R binding and internalization, the ADC releases the MTA intracellularly, resulting in degradation of JAK proteins.This strategy may enable durable suppression of IL-7-driven oncogenic signaling and help overcome glucocorticoid resistance. The therapeutic advantages including dose reduction via PROTAC-mediated degradation, are under investigation. We report our current strategy and progress in the development of this novel targeted therapy.

[Disclosure of COI by Responsible researcher] Lead presenter is not the Responsible researcher.
[Lead presenter: Huiqing Liang] We have no financial relationships to disclose.
[Principal Researcher: Masahiro Yasunaga] ■Profit of stock: Rin Institute Inc.　■Research expenses: Daiichi Sankyo Co., Ltd., Ajinomoto Co., Inc., Sysmex Corporation, H.U. Group Holdings, Inc., and Dojin Group Co., Ltd.

Poster Session

### [P-3273] I-P13-7
### New molecular targeted therapy (1)

2025 / 9 / 27 (Sat) 16:15-17:00 Room P/(Motenashi Dome) Room P

---

### P-3273
### TRIP13, a potential drug target for ATL pharmacotherapy

[Speaker]Naoki Mori (Dept. Microbiol. Oncol., Grad. Sch. Med., Univ. Ryukyus) [Co-author]Chie Ishikawa (Dept. Microbiol. Oncol., Grad. Sch. Med., Univ. Ryukyus／Transdisciplinary Res. Organ. Subtrop. Isl. Stud., Univ. Ryukyus)

AAA-ATPase TRIP13 plays a crucial role in regulating DNA repair pathways and spindle assembly checkpoints. TRIP13 overexpression was observed in PHA-stimulated PBMCs and in HTLV-1-infected T-cell lines, including those derived from ATL. TRIP13 was upregulated during the immortalization of lymphocytes following HTLV-1 infection. TRIP13 knockdown decreased proliferation of HTLV-1-infected T-cell lines. Furthermore, the anti-ATL activity of the TRIP13 inhibitor DCZ0415 was examined. DCZ0415 inhibited proliferation and induced DNA damage, G1 cell cycle arrest, and caspase-mediated apoptosis in HTLV-1-infected T-cell lines. The expression of cell cycle-related proteins, including CDK4/6, cyclin D2/E, c-Myc, and phosphorylated pRb, was reduced following DCZ0415 treatment. Additionally, DCZ0415 increased the expression of the pro-apoptotic Bak while decreasing the expression of the anti-apoptotic Mcl-1, c-IAP2, and survivin. DCZ0415 also reduced JunB expression and the phosphorylation of IκBα and STAT3/5. These findings indicate that TRIP13 is involved in the leukemogenesis associated with HTLV-1 and that targeting TRIP13 exerts anti-ATL effects by inhibiting NF-κB, AP-1, and STAT3/5.

[Disclosure of COI by Responsible researcher] Lead presenter is the Responsible researcher.
[Lead presenter: Naoki Mori] We have no financial relationships to disclose.

▲TOP

### [P-3274] I-P13-7
### New molecular targeted therapy (1)

2025 / 9 / 27 (Sat) 16:15-17:00 Room P/(Motenashi Dome) Room P

---

### P-3274
### Development of a cancer-specific cell death induction method using new synthetic mRNA

[Speaker]Katsuya Fukino (Gunma Univ. GIAR) [Co-author]Noriko Ohta (Gunma Univ. GIAR)、Takashi Matsuzaki (Center of Medical Innovation and Translational Research)、Keisuke Nimura (Gunma Univ. GIAR)

Inducing cancer cell-specific cell death represent a promising therapeutic strategy for patients with tumor. We focused on diphtheria toxin, which acts on ANTXR1, known as tumor endothelial marker 8. We developed an anti-tumor therapy that induces cancer cell specific cell death by introducing PA-DT mRNA, a synthetic therapeutic gene fusing the protective antigen of anthrax with the diphtheria toxin. 22Rv1, human cancer prostate epithelial cells, cause cell death after PA-DT mRNA transfection. While PNT2, human normal prostate epithelial cells, does not cause cell death after PA-DT transfection. The tumor specific delivery of PA-DT mRNA using lipid-nano particles with a cell-surface-specific cationic property represses tumor growth in vivo without obvious adverse effect, including a decrease in body weight. Our findings indicate that PA-DT has the potential to be an anti-tumor therapeutic method.

[Disclosure of COI by Responsible researcher] Lead presenter is not the Responsible researcher.
[Lead presenter: Katsuya Fukino] We have no financial relationships to disclose.
[Principal Researcher: Keisuke Nimura] We have no financial relationships to disclose.

▲TOP

### [P-3275] I-P13-7
### New molecular targeted therapy (1)

2025 / 9 / 27 (Sat) 16:15-17:00 Room P/(Motenashi Dome) Room P

---

### P-3275
### Therapeutic efficacy and immune response of near-infrared photoimmunotherapy for lung cancer in orthotopic murine model

[Speaker]Hiroshi Yamasaki (Dept of Thoracic Surgery, Hokkaido University Faculty of Medicine) [Co-author]Kohei Nakajima (Lab. for Bioanalysis and Molecular Imaging, Graduate School of Pharmaceutical)、Ryohei Chiba (Dept of Thoracic Surgery, Hokkaido University Faculty of Medicine)、Haruhiko Shiiya (Dept of Thoracic Surgery, Hokkaido University Faculty of Medicine)、Kazuto Ohtaka (Dept of Thoracic Surgery, Hokkaido University Faculty of Medicine)、Hideki Ujiie (Dept of Thoracic Surgery, Hokkaido University Faculty of Medicine)、Aki Fujiwara (Dept of Thoracic Surgery, Hokkaido University Faculty of Medicine)、Masato Aragaki (Dept of Thoracic Surgery, Hokkaido University Faculty of Medicine)、Kanako Hatanaka (Ctr. for Development of Advanced Diagnostics, Hokkaido University Hospital)、Yutaka Hatanaka (Ctr. for Development of Advanced Diagnostics, Hokkaido University Hospital)、Mikako Ogawa (Lab. for Bioanalysis and Molecular Imaging, Graduate School of Pharmaceutical)、Tatsuya Kato (Dept of Thoracic Surgery, Hokkaido University Faculty of Medicine)

Despite advances in therapeutic strategies, lung cancer continues to have a poor prognosis, highlighting the need for novel treatment. Near-infrared photoimmunotherapy (NIR-PIT) is a novel cancer therapy that combines the specificity of antibodies with toxicity induced by photosensitizer activated by NIR light. In this study, we evaluated the therapeutic efficacy and immune response of NIR-PIT targeting CD44, a cell surface glycoprotein involved in cancer invasion and metastasis. Experiments were conducted with CD44 expressing cell line. An antibody-photosensitizer conjugate consisting of IM7 and a silicaphthalocyanine dye, IRDye700DX, was synthesized. In vitro NIR-PIT cytotoxicity was light dose dependent. For in vivo analysis, orthotopic mouse lung cancer model was established by transtracheal injection. After NIR-PIT, bio luminescence image demonstrated a significant reduction in lung cancer volume and survival time was prolonged. Moreover, immunohistochemical analysis revealed increased CD8+ T cells in the tumor microenvironment, suggesting an enhanced antitumor immune response. These results suggested that NIR-PIT for CD44 may represent a therapeutic option for lung cancer.

[Disclosure of COI by Responsible researcher] Lead presenter is not the Responsible researcher.
[Lead presenter: Hiroshi Yamasaki] ■Research funds from company: 科研費
[Principal Researcher: Tatsuya Kato] ■Research funds from company: 科研費

### [P-3276] I-P13-7
### New molecular targeted therapy (1)

2025 / 9 / 27 (Sat) 16:15-17:00 Room P/(Motenashi Dome) Room P

---

### P-3276
### Near-Infrared Photoimmunotherapy Targeting CD98 for Cancer Therapy

[Speaker]Citra R.A.P Palangka (Near InfraRed Photo-ImmunoTherapy Research Inst., Kansai Medical Univ.) [Co-author]Hirofumi Hanaoka (Near InfraRed Photo-ImmunoTherapy Research Inst., Kansai Medical Univ.)

Objectives: Near-infrared photoimmunotherapy (NIR-PIT) is a cancer treatment using antibody-photoabsorber (IR700) conjugates which bind to target cells and are activated by light inducing rapid necrotic cell death. CD98, a subunit of the LAT1/CD98 amino acid transporter complex, is overexpressed in many tumors, highlighting its potential as a cancer therapy target. The aim of this study was to evaluate the therapeutic effect of PIT with mAb CD98-IR700. Methods: IR700 conjugated to mAb CD98. It was evaluated in A549 cell line, a CD98-positive non-small cell lung cancer. PIT was conducted with irradiation of NIR light after the samples were incubated for 1 h with 10 μg/mL of mAb CD98-IR700. The morphology of samples was directly monitored under a microscope. Meanwhile, the viability samples were evaluated after 24 h. Results: Microscopic observation of the treated cell showed morphological changes typical of PIT. Cell viability was significantly reduced in vitro in a light-dose-dependent manner. Conclusion: These results suggest that PIT with mAb CD98-IR700 is a promising approach for cancer therapy.

[Disclosure of COI by Responsible researcher] Lead presenter is the Responsible researcher.
[Lead presenter: Citra R.A.P Palangka] We have no financial relationships to disclose.

### [P-3277] I-P13-7
### New molecular targeted therapy (1)

2025 / 9 / 27 (Sat) 16:15-17:00 Room P/(Motenashi Dome) Room P

### P-3277
### LPAR1 antagonist as a potential therapeutic agent for glioblastoma

[Speaker]Sumie Koike (Div. Experiment. Chemother., Cancer Chemother. Ctr., JFCR) [Co-author]Ai Takemoto (Div. Experiment. Chemother., Cancer Chemother. Ctr., JFCR)、Ryohei Katayama (Div. Experiment. Chemother., Cancer Chemother. Ctr., JFCR／Dept. CBMS, Grad. Sch. Front. Sci., The Univ. of Tokyo)、Satoshi Takagi (Div. Experiment. Chemother., Cancer Chemother. Ctr., JFCR)

Glioblastoma (GBM) is the most malignant brain tumor and has a poor prognosis with 5-year survival rate of lower than 10% although multimodality treatment combining surgery, radiotherapy, and chemotherapy has been developed. To identify novel therapeutic targets for GBM, we investigated the effect of a siRNA library targeting GPCRs, which are poor prognostic factors in patients with primary brain tumors, on the survival of GBM cells and identified lysophosphatidic acid receptor 1 (LPAR1) as a candidate molecular target. We found that LPA treatment accelerate LPAR1-positive GBM cell proliferation dose dependently and that genetic or pharmacological inhibition of LPAR1 suppresses proliferation and induces apoptosis in GBM cell lines. Furthermore, LPA-induced enhancement of GBM cell motility was cancelled by LPAR1 antagonist treatment.Moreover, oral administration of LPAR1 antagonist suppressed tumor growth in a GBM orthotopic xenograft mouse model. We have previously reported the possibility of LPAR1 antagonists as novel therapeutic agents for osteosarcoma (Oncogene 2021); these results suggest that LPAR1-targeted therapy would be also effective for GBM.

[Disclosure of COI by Responsible researcher] Lead presenter is not the Responsible researcher.
[Lead presenter: Sumie Koike] We have no financial relationships to disclose.
[Principal Researcher: Ai Takemoto] We have no financial relationships to disclose.

### [P-3278] I-P13-7
### New molecular targeted therapy (1)

2025 / 9 / 27 (Sat) 16:15-17:00 Room P/(Motenashi Dome) Room P

---

### P-3278
### PI polyamide-TPP conjugate targeting m.A3243G inhibits the tumor growth of teratoma harboring the mutation

[Speaker]Zhan Li (Div. Innov. Cancer Ther., Chiba Cancer Ctr. Res. Inst.) [Co-author]Nobuko Koshikawa (Div. Innov. Cancer Ther., Chiba Cancer Ctr. Res. Inst.)、Takumi Era (Dept. Cell Modula., Inst. Mol. Embry. Genet., Kumamoto Univ.)、Hiroki Nagase (Intract. Dis. Res. Ctr., Grad. Schl. Med, Juntendo Univ.)、Atsushi Takatori (Div. Innov. Cancer Ther., Chiba Cancer Ctr. Res. Inst.)、Keizo Takenaga (Div. Innov. Cancer Ther., Chiba Cancer Ctr. Res. Inst.)

[Purpose] We previously showed that triphenylphosphonium-conjugated pyrrole-imidazole polyamides (PIP-TPPs) targeting the mtDNA mutations of interest induce apoptosis of the cancer cells harboring the mutation through mtROS overproduction and mitophagy. Here we examined the effect of a PIP-TPP targeting m.A3243G (named CCC-018-TPP) on the tumor growth of teratoma carrying the mutation. [Methods] An iPS cell line (iMELA2) with m.A3243G mutation established from a MELAS patient was used. Teratoma was established by transplantation of iMELA2 cells in the gastrocnemial muscle of NOG mice. Delivery of CCC-018-TPP to teratoma tissues after intravenous administration was assessed by IHC using anti-TPP antibody. Apoptosis was detected by IHC using anti-cleaved caspase 3 antibody. [Results] CCC-018-TPP was well delivered to teratoma tissues after administration without any drug delivery systems, resulting in a significant retardation of teratoma growth and induction of apoptosis in the tumor tissues without obvious adverse effects. [Conclusion] These results further support that PIP-TPPs designed for mtDNA mutations of interest could be used to treat tumors harboring the target mutation.

[Disclosure of COI by Responsible researcher] Lead presenter is not the Responsible researcher.
[Lead presenter: Zhan Li] We have no financial relationships to disclose.
[Principal Researcher: Keizo Takenaga] ■Research funds from company: AMED

▲TOP

### [P-3279] I-P13-7
### New molecular targeted therapy (1)

2025 / 9 / 27 (Sat) 16:15-17:00 Room P/(Motenashi Dome) Room P

### P-3279
### Development of surviving gene siRNA in the therapy of fibrotic tumors

[Speaker]Saori Nishijima (Okinawa Institute of Science and Technology) [Co-author]Nao Ohmine (Okinawa Institute of Science and Technology)、Akiko Nishiyama (Okinawa Institute of Science and Technology)、Risa Ishida (Okinawa Institute of Science and Technology)、Kensuke Osada (National Institutes for Quantum Science and Technology)、Tadashi Yamamoto (Okinawa Institute of Science and Technology)

The TOB1 gene is known for its anti-proliferative effects; however, it also exhibits anti-apoptotic functions under certain conditions. This anti-apoptotic property of TOB1 presents a novel paradigm in cancer therapy. Expression of TOB confers resistance to cell death induced by genotoxic stress. We treated BxPC3 pancreatic cancer cells in vitro with genotoxic gemcitabine, either with or without TOB1 siRNA, and found that the presence of TOB1 siRNA significantly enhanced gemcitabine-induced cancer cell death. This combination therapy was further evaluated in mice xenografted with pancreatic cancer cells, using uPIC nanoparticles approximately 18 nm in diameter to effectively deliver TOB1 siRNA. Tumor growth suppression in xenografted mice was greater with the combination of gemcitabine and TOB1 siRNA than with gemcitabine alone. RNA-seq analysis of BxPC3 cells following the combination treatment revealed elevated expression levels of the pro-apoptotic gene BMF and several pro-inflammatory chemokine genes. These findings suggest that TOB regulates these pro-apoptotic signaling pathways.

[Disclosure of COI by Responsible researcher] Lead presenter is not the Responsible researcher.
[Lead presenter: Saori Nishijima] We have no financial relationships to disclose.
[Principal Researcher: Tadashi Yamamoto] We have no financial relationships to disclose.

### [P-3286] I-P14-9
### Drug delivery system

2025 / 9 / 27 (Sat) 16:15-17:00 Room P/(Motenashi Dome) Room P

---

### P-3286
### Size-dependent malignant brain tumor targeting by polymeric nanoruler

[Speaker]Yukine Ishibashi (The Univ. of Tokyo, Grad. Sch. of Eng.) [Co-author]Mitsuru Naito (The Univ. of Tokyo, Grad. Sch. of Eng.／Tokyo Univ. of Sci., Faculty of Adv. Eng.)、Yusuke Watanuki (The Univ. of Tokyo, Grad. Sch. of Eng.)、Masaru Cho (The Univ. of Tokyo, Grad. Sch. of Eng.)、Naoki Yamada (The Univ. of Tokyo, Grad. Sch. of Eng.)、Kanjiro Miyata (The Univ. of Tokyo, Grad. Sch. of Eng.)

There is currently no effective medication for the treatment of malignant brain tumors. They have a unique blood vessel structure called blood-brain tumor barrier (BBTB). Whereas low molecular weight antitumor agents can pass through BBTB because of their small size, they are rapidly excreted from the kidney due to the renal excretion threshold of ~8 nm in size. On the other hand, conventional nanomedicines (30-100 nm), cannot efficiently penetrate BBTB due to their substantially large size while avoiding renal excretion.
This study aimed to explore the optimal size of nanomedicines for brain tumor targeting. To this end, size-tunable PEG-grafted copolymers (gPEGs) were developed as a polymeric "nanoruler" with varying hydrodynamic diameters (8-30 nm) by grafting poly(ethylene glycol)s (PEGs) with different molecular weights (1-20 kDa) to poly(aspartic acid) (PAsp) backbone. gPEGs were intravenously injected into orthotopic brain tumor-bearing mice. 10-nm gPEG showed the maximum brain tumor accumulation level and deeper penetration into the brain tumor tissue compared with 20-30 nm gPEGs. These results demonstrate that nanomedicine size is critical for brain tumor targeting.[1]

[Disclosure of COI by Responsible researcher] Lead presenter is not the Responsible researcher.
[Lead presenter: Yukine Ishibashi] We have no financial relationships to disclose.
[Principal Researcher: Mitsuru Naito, Kanjiro Miyata] We have no financial relationships to disclose.

### [P-3287] I-P14-9
### Drug delivery system

2025 / 9 / 27 (Sat) 16:15-17:00 Room P/(Motenashi Dome) Room P

---

### P-3287
### TXB-001: A novel DDS anthracycline with high tumor stromal penetration as a potential treatment for refractory cancer

[Speaker]Ryotaro Yagi (Toray Industries, Inc.) [Co-author]Emi Tomikawa (Toray Industries, Inc.)、Chizuka Sakai (Toray Industries, Inc.)、Rieko Shimozono (Toray Industries, Inc.)、Yoko Koga (Toray Industries, Inc.)、Tsubasa Okano (Toray Industries, Inc.)、Yuki Takahashi (TOPPAN Holdings Inc.)、Tomoyuki Takaya (TOPPAN Holdings Inc.)、Rii Morimura (TOPPAN Holdings Inc.)、Shiro Kitano (TOPPAN Holdings Inc.)、Lisa Munakata (Faculty of Pharm. Sci., Teikyo Univ.)、Ryo Suzuki (Faculty of Pharm. Sci., Teikyo Univ.)、Masashi Uchida (Toray Industries, Inc.)

TXB-001, a novel anticancer drug, is an optimized N-(2-hydroxypropyl)methacrylamide copolymer-based conjugated pirarubicin (THP) that selectively accumulates in tumors and releases THP rapidly in acidic tumor environments, promising high efficacy. This study evaluated the stromal penetration and efficacy of TXB-001 in stroma-rich cancers. In a triple-negative breast cancer (TNBC) mouse model, intravenous doses of fluorescently labeled TXB-001 polymer (~10 nm in diameter) and DOXIL® like liposomes (~100 nm) showed extensive distribution of the polymer in the tumor, while the liposomes remained close to blood vessels. TXB-001 showed higher antiproliferative activity than DOXIL® in ***in vitro*** 3D cancer/stromal co-culture models by invivoid™ technology. TXB-001 exhibited a tumor/plasma concentration ratio 16-40 times higher than DOXIL® and high antitumor effect in TNBC and human pancreatic cancer models. These results suggest TXB-001 can reach the core of hypovascular and stroma-rich tumors, where tissue diffusion is poor with liposomal formulations, due to its about 1/10 size of DOXIL® and linear polymer. Thus, TXB-001 should be effective in treating stroma-rich refractory cancers.

[Disclosure of COI by Responsible researcher] Lead presenter is not the Responsible researcher.
[Lead presenter: Ryotaro Yagi] We have no financial relationships to disclose.
[Principal Researcher: Masashi Uchida] We have no financial relationships to disclose.

▲TOP

### [P-3288] I-P14-9
### Drug delivery system

2025 / 9 / 27 (Sat) 16:15-17:00 Room P/(Motenashi Dome) Room P

---

P-3288
### Enhanced Anticancer Activity of LLAA peptide using self-assemble nanoparticles in high-metastasis cancer cells

[Speaker]Mai Hazekawa (Dept. Drug Deliv., Fac. Pharm., Fukuoka Univ) [Co-author]Daisuke Watase (Dept. Drug Deliv., Fac. Pharm., Fukuoka Univ)、Takuya Nishinakagawa (Dept. Immuno. Mol. Pharm., Fac. Pharm., Fukuoka Univ)、Daisuke Ishibashi (Dept. Immuno. Mol. Pharm., Fac. Pharm., Fukuoka Univ)

LLAA, which is a peptide synthesized from LL-37, has demonstrated enhanced anticancer activity compared to its parent peptide. However, despite its promising anticancer potential, the activity of LLAA remains relatively weak due to its low-cell permeability. To improve the anticancer efficacy of LLAA, this study aimed to enhance its cell permeability through the development of a self-assemble nanoparticle (micelles) using biodegradable copolymer poly (lactic-co-glycolic acid). The efficacy of the LLAA-loaded micelles was evaluated in vitro using cell proliferation, migration, and invasion assays across four high-metastasis cancer cell lines: HM-1, B16/BL6, HeLa, and HepG2. Results showed that the LLAA-loaded micelles significantly inhibited cell proliferation, migration, and invasion, while also demonstrating enhanced cell permeability compared to LLAA alone. These findings suggest that the LLAA-loaded micelles represent a promising approach for improving the anticancer efficacy of LLAA and could be a valuable candidate for further development in cancer therapy.

[Disclosure of COI by Responsible researcher] Lead presenter is the Responsible researcher.
[Lead presenter: Mai Hazekawa] We have no financial relationships to disclose.

### [P-3289] I-P14-9
### Drug delivery system

2025 / 9 / 27 (Sat) 16:15-17:00 Room P/(Motenashi Dome) Room P

---

### P-3289
### Intra-tumor Administration of Doxorubicin with a Guide-equipe Jet Injector, Actranza®, in K562-xenografted Mice

[Speaker]Kazuhiro Terai (Kumamoto University, Institute of Industrial Nanomaterials／Kumamoto University, Research Center for Human Retrovirus Infection／Daicel Corporation, R&D, Research Center／Daicel Corporation, Life Sciences SBU) [Co-author]Ryusho Kariya (Kumamoto University, Research Center for Human Retrovirus Infection／Kobe Gakuin University, Pharmaceutical Sciences, Molecular Cellular Biology)、Hiromi Aoki (Kumamoto University, Research Center for Human Retrovirus Infection)、Hiromitsu Iga (Daicel Corporation, Life Sciences SBU)、Seiji Sakano (Daicel Corporation, Life Sciences SBU)、Satoshi Sakamoto (Kumamoto University, Institute of Industrial Nanomaterials)、Seiji Okada (Kumamoto University, Research Center for Human Retrovirus Infection)

Injection of therapeutic agents into organs is often hindered by high interstitial pressure, leading to leakage or backflow. We have shown that the needle-free jet injector Actranza® enables broad distribution of injected substances. Additionally, jet injection significantly enhances gene expression of naked plasmid DNA in the epidermis/dermis after transdermal delivery and in the myocardium after direct cardiac injection, compared to conventional needle-syringe (NS) methods.
In this study, we equipped a prototype of Actranza with a guide (30G needle) and administered the classical anticancer drug doxorubicin (DOX) intratumorally in tumor-bearing mice. When used for intra-tumor delivery of DOX (total dose: 4 mg/kg, on days 0 and 6), the device achieved a significant antitumor effect at a lower dose than previously reported (6-12 mg/kg), with the specific features of the device compared to syringe injection.
Jet injection using the guide-equipped Actranza effectively minimizes leakage and may be applicable to encapsulated tissues or organs, including tumors. This approach also holds promise for future applications in gene therapy, genome editing, and regenerative medicine.

[Disclosure of COI by Responsible researcher] Lead presenter is the Responsible researcher.
[Lead presenter: Kazuhiro Terai] ■ employment by a company related to the presentation: 株式会社ダイセル

▲TOP

### [P-3290] I-P14-9
### Drug delivery system

2025 / 9 / 27 (Sat) 16:15-17:00 Room P/(Motenashi Dome) Room P

### P-3290
### Development of NK cell-targeting technology using antibody-modified lipid nanoparticles

[Speaker]Natsumi Shima (Fac. Pharm. Sci., Hokkaido Univ.) [Co-author]Tokikazu Ikenaga (Fac. Pharm. Sci., Hokkaido Univ.)、Tomomi Matsunaga (Fac. Pharm. Sci., Hokkaido Univ.)、Yusuke Sato (Fac. Pharm. Sci., Hokkaido Univ.)、Yuma Yamada (Fac. Pharm. Sci., Hokkaido Univ.)、Hideyoshi Harashima (Fac. Pharm. Sci., Hokkaido Univ.)、Takashi Nakamura (Fac. Pharm. Sci., Hokkaido Univ.／Inst. Med. Pharm. Health Sci., Kanazawa Univ.)

The most advanced cancer immunotherapy using natural killer (NK) cells is ***ex vivo*** NK cell therapy, in which NK cells are expanded and functionally engineered ***ex vivo*** before being injected into patients. However, this therapy faces challenges related to cost and complexity. One potential strategy is to engineer NK cells directly ***in vivo***. However, no technologies have been reported to achieve this.
Lipid nanoparticles (LNPs) are useful drug delivery systems to deliver functional nucleic acids. In this study, we developed NK cell-targeting LNPs. LNPs were prepared by alcohol dilution method and modified with Fab' fragment targeting NK1.1. The Fab'-modified LNPs (NK1.1-LNPs) were added to mouse spleen cells or injected intravenously into mice, and the cellular uptake was analyzed. The NK1.1-LNPs exhibited the high affinity for NK1.1$^{+}$cells in a modification-dependent manner. Compared to unmodified LNPs, the NK1.1-LNPs showed higher NK cell selectivity with an increased percentage of NK cells among all cells bound to the LNPs.
Collectively, we successfully constructed NK cell-targeting LNPs. The NK1.1-LNPs may contribute to the development of innovative cancer immunotherapy.

[Disclosure of COI by Responsible researcher] Lead presenter is not the Responsible researcher.
[Lead presenter: Natsumi Shima] We have no financial relationships to disclose.
[Principal Researcher: Takashi Nakamura] ■Research funds from company: JSPS (JP24H00785) and AMED (JP21ak0101172)
■Contributions: Mochida Memorial Foundation

### [P-3291] I-P14-9
### Drug delivery system

2025 / 9 / 27 (Sat) 16:15-17:00 Room P/(Motenashi Dome) Room P

---

### P-3291
### Transient transfection using 222nm Far UV-C irradiation

[Speaker]Mane Nishimura (Faculty of Laboratory Science, Yamaguchi University Graduate School of Medicine) [Co-author]Jun Nishikawa (Faculty of Laboratory Science, Yamaguchi University Graduate School of Medicine)、Yutaka Suehiro (Department of Oncology and Laboratory Medicine, Yamaguchi University)、Takahiro Yamasaki (Department of Oncology and Laboratory Medicine, Yamaguchi University)

Ultraviolet (UV) light is avoided in gene transfer due to its DNA-damaging effects. Far UV-C (F-UV) light, at 222 nm, causes less DNA damage. We investigated plasmid introduction into cells using F-UV light. COS-7 and CHO-K1 cells were irradiated with 222 nm F-UV light. Then, DNA solution containing green fluorescent protein (EGFP) plasmid was added to the culture and incubated (37°C, 5% $CO_2$, 24 hours) for fluorescence microscopy. Cytotoxicity of cells irradiated with the same energy as for gene transfer was evaluated. Characteristics of EGFP-positive cells were compared with non-transfected cells by propidium iodide (PI) and Hoechst staining. Cells with distinct green fluorescence were observed after F-UV light irradiation and addition of 200 ng of EGFP plasmid. COS-7 cells showed the highest number of EGFP-positive cells at 0.5 mJ/cm2 irradiation, whereas that for CHO-K1 cells was at 1 mJ/cm2 irradiation. Cytotoxicity was low in COS-7 at ≤1 mJ/cm2 irradiation and CHO-K1 at ≤0.5 mJ/cm2. Transfected cells did not incorporate PI, and their nuclei did not differ from those of non-transfected cells. We successfully transfected two cell lines with EGFP plasmids by F-UV irradiation.

[Disclosure of COI by Responsible researcher] Lead presenter is not the Responsible researcher.
[Lead presenter: Mane Nishimura] We have no financial relationships to disclose.
[Principal Researcher: Jun Nishikawa] We have no financial relationships to disclose.

[P-3292] I-P14-9
Drug delivery system

2025 / 9 / 27 (Sat) 16:15-17:00 Room P/(Motenashi Dome) Room P

### P-3292
### Tumor-Type Specific Stromal Barriers in Antibody Delivery: Comparative 3D Spheroid Model

[Speaker]Yuki Tsuji (Grad. Sch. of Life Sci., Univ. of Tokyo／Center for Advanced Med. Development, National Cancer Center)

In pancreatic cancer, the tumor microenvironment (TME) contains abundant cancer-associated fibroblasts (CAFs), forming dense stroma that hinders antibody delivery. In contrast, colorectal cancer is less affected by CAF-mediated barriers and is a useful comparative model to study TME-dependent delivery. We developed 3D co-culture spheroids using pancreatic or colorectal cancer cells with CAFs. A transporter protein identified via in-house proteomic analysis was validated in both cancer types for future antibody delivery evaluation. Pancreatic cancer spheroids showed structural heterogeneity and compaction, whereas colorectal cancer spheroids were more expansive and uniform. These differences suggest tumor-type-specific CAF interactions and distinct stromal remodeling properties. We investigate whether impaired antibody delivery in pancreatic cancer is due to qualitative CAF differences or matrix components such as collagen and hyaluronic acid. This model enables systematic assessment of CAF composition, matrix complexity, and delivery potential, offering a preclinical platform to optimize antibody therapy in desmoplastic tumors with dense stromal architecture.

[Disclosure of COI by Responsible researcher] Lead presenter is the Responsible researcher.
[Lead presenter: Yuki Tsuji] ■Profit of stock: 株式会社凜研究所　■Research expenses: 第一三共株式会社、味の素株式会社、シスメックス株式会社、H.U.グループホールディングス、株式会社同仁グループ

### [P-3293] I-P14-9
### Drug delivery system

2025 / 9 / 27 (Sat) 16:15-17:00 Room P/(Motenashi Dome) Room P

---

### P-3293
### Anti-tumor effects of folate receptor targeted paclitaxel releasing mesoporous silica nanoparticles

[Speaker]Tetsuya Nakamura (Fac. Pharm., Pharm. Sci., Fukuyama Univ.) [Co-author]Tomoya Kubo (Fac. Pharm., Pharm. Sci., Fukuyama Univ.)、Rina Miyachi (Fac. Pharm., Pharm. Sci., Fukuyama Univ.)、Miyu Yasufuku (Fac. Pharm., Pharm. Sci., Fukuyama Univ.)、Ai Fujiwara (Fac. Pharm., Pharm. Sci., Fukuyama Univ.)、Yuina Inoue (Fac. Pharm., Pharm. Sci., Fukuyama Univ.)、Hazuki Iwamoto (Fac. Pharm., Pharm. Sci., Fukuyama Univ.)、Tetsuo Morita (Fac. Pharm., Pharm. Sci., Fukuyama Univ.)、Toshio Motoyashiki (Fac. Pharm., Pharm. Sci., Fukuyama Univ.)

The development of particles that selectively transport to cancer cells is expected to reduce side effects and maximize therapeutic effects. Paclitaxel is widely used in chemotherapy, but it has problems such as hypersensitivity symptoms and precipitation after dilution. Many studies have been conducted on improved formulations to solve this problem has been put on the market. However, these drugs have passive-targeting do not target overexpressed receptors or specific antigens and have an impact on normal tissues. Therefore, we created an active-targeting drug that targets the folate receptor which is overexpressed in many cancer cells. We used mesoporous silica nanoparticles with pores as a drug transport carrier adsorbed the drug and controlled the release. The particles were characterized to evaluate the particle surface area, pore size, particle diameter, and zeta potential. Drug release was quantified by HPLC. The intracellular transport of the fluorescent reagent bound particles, and the apoptosis of various cancer cells was evaluated. As a result, it became possible to prepare particles that induce apoptosis in cancer cells by targeting the folate receptor.

[Disclosure of COI by Responsible researcher] Lead presenter is the Responsible researcher.
[Lead presenter: Nakamura Tetsuya] We have no financial relationships to disclose.

### [P-3294] I-P14-9
### Drug delivery system

2025 / 9 / 27 (Sat) 16:15-17:00 Room P/(Motenashi Dome) Room P

---

### P-3294
### Targeted co-delivery of Doxorubicin and H. perforata via aptamer-modified nanovesicles in colorectal cancer cells

[Speaker]Watcharida Jindapong (Dept. of Biochem. & Mol. Biol., Thammasat Univ.) [Co-author]Boonchoy Soontornworajit (Dept. of Chemistry., Thammasat Univ.)、Pichayanoot Rotkrua (Dept. of Preclinical, Sci., Thammasat Univ.)

Targeted drug delivery enhances cancer treatment efficacy while reducing harm to normal cells. This study develops and evaluates niosomes made from cholesterol and Tween 20 for co-delivering crude Harrisonia perforata (Blanco) Merr. (HP-C) extract and doxorubicin to colorectal cancer cells (SW480), compared with normal intestinal cells (CCD841 CoN). To enhance delivery specificity, AS1411 aptamer was conjugated to the niosome surface due to its high affinity for nucleolin, a protein overexpressed on cancer cell membranes, including colorectal cancer. Niosome stability was assessed by dynamic light scattering, showing a particle size of approximately 250 nm and a zeta potential between -6 and -4 mV. Cellular uptake was confirmed by tracking fluorescence from FAM-tagged AS1411, and drug release efficiency was 40%. The cytotoxicity of HP-C and doxorubicin-loaded niosomes on cancerous and normal cells was assessed using MTS assays. Results showed that AS1411-functionalized niosomes with active compounds significantly inhibited cancer cell growth, highlighting their potential as an effective targeted drug delivery system.

[Disclosure of COI by Responsible researcher] Lead presenter is the Responsible researcher.
[Lead presenter: Watcharida Jindapong ] We have no financial relationships to disclose.

### [P-3295] I-P14-9
### Drug delivery system

2025 / 9 / 27 (Sat) 16:15-17:00 Room P/(Motenashi Dome) Room P

---

P-3295

### PLGA nanoparticles separately encapsulating ursolic acid and doxorubicin for improved cholangiocarcinoma therapy

[Speaker]Chadamas Sakonsinsiri (Dept. Biochem., Faculty of Med., Khon Kaen Univ., Thailand) [Co-author]Pornpattra Rattanaseth (Dept. Biochem., Faculty of Med., Khon Kaen Univ., Thailand)、Theerapong Puangmali (Dept. Physics, Faculty of Sci., Khon Kaen Univ., Thailand)、Kanlaya Katewongsa (Dept. Biochem., Faculty of Sci., Mahidol Uni., Thailand)、Somchai Pinlaor (Dept. Parasitol., Faculty of Med., Mahidol Uni., Thailand)、Raynoo Thanan (Dept. Biochem., Faculty of Med., Khon Kaen Univ., Thailand)

Cholangiocarcinoma (CCA), a fatal cancer prevalent in Thailand, often resists chemotherapy, necessitating improved treatment. Two different poly(lactic-co-glycolic acid) (PLGA) nanoparticle (NP) formulations were developed to enhance the delivery of ursolic acid (UA) and doxorubicin (DOX), addressing their solubility, toxicity, and cellular uptake limitations. UA-PLGA NPs (240 nm), synthesized via nanoprecipitation, showed high encapsulation efficiency, stability, and biocompatibility. Despite higher IC50 values (60-88 μg/mL) compared to free UA (8-12 μg/mL), the NPs enabled solvent-free delivery and dose-dependent cytotoxicity via diffusion in KKU-213A and KKU-055 cells. DOX-PLGA NPs (341 nm), prepared using oil-in-water emulsion, demonstrated biocompatible, sustained, pH-responsive release. DOX-PLGA NPs showed greater cytotoxicity than free DOX in KKU-213A cells (IC50: 0.36 vs. 0.66 μg/mL). In KKU-055 cells, both treatments had similar effects, though KKU-055 cells showed higher sensitivity to DOX-PLGA NPs than KKU-213A. These findings highlight UA- and DOX-loaded PLGA NPs as promising platforms for improved CCA therapy.

[Disclosure of COI by Responsible researcher] Lead presenter is the Responsible researcher.
[Lead presenter: Chadamas Sakonsinsiri] We have no financial relationships to disclose.

### [P-3302] I-P14-11
### Others

2025 / 9 / 27 (Sat) 16:15-17:00 Room P/(Motenashi Dome) Room P

---

### P-3302
### Characterization of tumors of older breast cancer patients that show ***de novo*** resistance to endocrine therapy

[Speaker]Yumiko Ishizuka (Dept. Breast Oncology, Juntendo Univ. Faculty Med.) [Co-author]Yoshiya Horimoto (Dept. Breast Oncology, Juntendo Univ. Faculty Med.／Dept. Human Path., Juntendo Univ. Faculty Med.／Dept. Breast Surg. & Oncology, Tokyo Med Univ.)、Takuo Hayashi (Dept. Human Path., Juntendo Univ. Faculty Med.)、Tsuyoshi Saito (Dept. Human Path., Juntendo Univ. Faculty Med.)、Hidetaka Eguchi (Intractable Disease Res. Ctr., Juntendo Univ. Grad. Sch. Med.)、Junichiro Watanabe (Dept. Breast Oncology, Juntendo Univ. Faculty Med.)、Goro Kutomi (Dept. Breast Oncology, Juntendo Univ. Faculty Med.)

Curative surgery followed by endocrine therapy is a standard for hormone receptor-positive breast cancer. However, in clinical practice, older patients may receive endocrine therapy alone for various reasons. To establish this approach as a standard treatment, accurately identifying patients who will benefit is essential. Fifty-one patients (over 70 years old) with ER-positive, HER2-negative invasive breast cancer who received endocrine therapy instead of surgery were retrospectively analyzed using a gene expression panel. Of the 51 patients, three developed progressive disease (PD) within 6 months of endocrine therapy. Gene expression analysis showed higher expression of cell cycle-related genes, including ***CDKN3***, in the PD group. ***CDKN3*** remained significantly overexpressed even after expanding the sample set (log2 fold change: 1.99; P=0.005). Public mRNA data confirmed worse outcomes in patients with high ***CDKN3*** expression. ***CDKN3*** emerged as a predictive marker in older breast cancer patients. Collectively, our findings may aid in developing tailored treatments for elderly patients.

[Disclosure of COI by Responsible researcher] Lead presenter is not the Responsible researcher.
[Lead presenter: Yumiko Ishizuka] We have no financial relationships to disclose.
[Principal Researcher: Yoshiya Horimoto] We have no financial relationships to disclose.

▲TOP

[P-3303] I-P14-11
Others

2025 / 9 / 27 (Sat) 16:15-17:00 Room P/(Motenashi Dome) Room P

P-3303

### The *C11orf24* gene as a useful biomarker for predicting severe neutropenia in modified FOLFIRINOX for pancreatic cancer

[Speaker]Gen Kanesada (Dept. of Gastroenterological, Breast & Endocrine Surg., Yamaguchi Univ.) [Co-author]Ryouichi Tsunedomi (Dept. of Gastroenterological, Breast & Endocrine Surg., Yamaguchi Univ.／Res. Inst. for Cell Design Medical Sci., Yamaguchi Unive.)、Yuki Nakagami (Health Data Sci. Lab., Shimonoseki City Unive.)、Masao Nakajima (Dept. of Gastroenterological, Breast & Endocrine Surg., Yamaguchi Univ.)、Mitsuo Nishiyama (Dept. of Gastroenterological, Breast & Endocrine Surg., Yamaguchi Univ.)、Hiroto Matsui (Dept. of Gastroenterological, Breast & Endocrine Surg., Yamaguchi Univ.)、Yukio Tokumitsu (Dept. of Gastroenterological, Breast & Endocrine Surg., Yamaguchi Univ.)、Yoshitaro Shindo (Dept. of Gastroenterological, Breast & Endocrine Surg., Yamaguchi Univ.)、Yusaku Watanabe (Dept. of Gastroenterological, Breast & Endocrine Surg., Yamaguchi Univ.)、Shinobu Tomochika (Dept. of Gastroenterological, Breast & Endocrine Surg., Yamaguchi Univ.)、Noriko Maeda (Dept. of Gastroenterological, Breast & Endocrine Surg., Yamaguchi Univ.)、Michihisa Iida (Dept. of Gastroenterological, Breast & Endocrine Surg., Yamaguchi Univ.)、Hirofumi Akita (Depart. of Digestive Surg., Osaka International Cancer Inst.)、Tatsuya Ioka (Oncology Center, Yamaguchi Unive. Hosp.)、Hidenori Takahashi (Dept. of Gastroenterological, Breast & Endocrine Surg., Yamaguchi Univ.)、Hiroaki Nagano (Dept. of Gastroenterological, Breast & Endocrine Surg., Yamaguchi Univ.／Res. Inst. for Cell Design Medical Sci., Yamaguchi Unive.)

Background: Pancreatic cancer (PC) has a poor prognosis. FOLFIRINOX improves outcomes, but severe neutropenia remains frequent despite UGT1A1 screening. This study aimed to identify biomarkers of severe neutropenia in modified FOLFIRINOX (mFFX) for PC.
Methods: We included 71 PC patients treated with mFFX, 92 with gemcitabine plus nab-paclitaxel (GnP), and 50 colorectal cancer patients treated with FOLFOXIRI. Whole-exome sequencing was used for screening. Validation analysis was performed using PCR genotyping, Cochran-Armitage trend test, and multivariate analysis. Predictive performance was assessed using logistic regression with leave-one-out cross-validation.
Results: Three SNPs were identified in screening. In validation, ***C11orf24*** (rs901827) was significantly associated with ≥grade 3 neutropenia in mFFX and FOLFOXIRI, but not in GnP. Multivariate analysis revealed ***C11orf24*** and baseline neutrophil count as independent risk factors. AUCs for the prediction model were 0.754 (≥grade 3) and 0.856 (≥grade 4).
Conclusion: ***C11orf24*** and baseline neutrophil count may be useful biomarkers for predicting severe neutropenia during irinotecan-containing triplet chemotherapy.

[Disclosure of COI by Responsible researcher] Lead presenter is not the Responsible researcher.
[Lead presenter: Gen Kanesada] ■Research expenses: CYTLIMIC Inc., Toyo Kohan Co., Ltd., NEC Corporation, Eisai, Incyte, Astellas Pharma Inc. ■Contributions: TAIHO PHARMACEUTICAL CO., LTD., Shionogi & Co., Ltd., MSD K.K, Otsuka Pharmaceutical Factory, Inc., Otsuka Pharmaceutical Co., Ltd., TSUMURA & CO., Kyowa Hakko Kirin Co.,Ltd., Kaken Pharmaceutical Co., Ltd., NIPPON KAYAKU Co., Ltd., DAIICHI SANKYO COMPANY, LIMITED., Japan Blood Products Organization, Chugai Pharmaceutical Co., Ltd.,
[Principal Researcher: Hiroaki Nagano] ■Research expenses: CYTLIMIC Inc., Toyo Kohan Co., Ltd., NEC Corporation, Eisai, Incyte, Astellas Pharma Inc. ■Contributions: TAIHO PHARMACEUTICAL CO., LTD., Shionogi & Co., Ltd., MSD K.K, Otsuka Pharmaceutical Factory, Inc., Otsuka Pharmaceutical Co., Ltd., TSUMURA & CO., Kyowa Hakko Kirin Co.,Ltd., Kaken Pharmaceutical Co., Ltd., NIPPON KAYAKU Co., Ltd., DAIICHI SANKYO COMPANY, LIMITED., Japan Blood Products Organization, Chugai Pharmaceutical Co., Ltd.,

▲TOP

### [P-3304] I-P14-11
### Others

2025 / 9 / 27 (Sat) 16:15-17:00 Room P/(Motenashi Dome) Room P

### P-3304
### Evaluation of Subclavian Vein Catheterization Under Ultrasound Guidance

[Speaker]Hiroki Miyachi (Dept. of Hematology, Itami City Hosp.) [Co-author]Masaaki Takahshi (Dept. of Hematology, Itami City Hosp.)、Yuka Umeki (Dept. of Hematology, Itami City Hosp.)、Kodai Ueshima (Dept. of Hematology, Itami City Hosp.)、Naoko Ishida (Dept. of Hematology, Itami City Hosp.)、Yukihiro Tokumine (Dept. of Hematology, Itami City Hosp.)、Megumu Inoue (Dept. of Hematology, Itami City Hosp.)

**BACHGROUND:**Central venous catheterization(CVC) has expanded its indications, including for chemotherapy. Because inserted catheter in the internal jugular vein or femoral vein leads to restrictions on patients physical activity, subclavian vein is the first choice in our department. **METHODS:**The assistant obtains a long-axis view of the subclavian vein by using ultrasound, and the operator punctured a vein using a needle guide attached to the probe. We retrospectively analyzed 520 cases who had CVC at the departments of hematology in our hospital between January 2014 and March 2025. **RESULTS:**514 cases had subclavian vein catheter without adverse events. There were 1 case of pneumothorax, and 1 case of mediastinal penetration. In 4 cases, the procedure was aborted due to resistance preventing guidewire advancement. In the successful cases, there were 16 cases of inadvertent insertion into the internal jugular vein, 1 case into the opposite subclavian vein, and 1 case of catheter inversion within the subclavian vein. In all these cases, the position of the catheter was adjusted under X-ray fluoroscopy.
**CONCLUSION:**Subclavian vein catheterization under ultrasound guidance is useful.

[Disclosure of COI by Responsible researcher] Lead presenter is not the Responsible researcher.
[Lead presenter: Hiroki Miyachi] We have no financial relationships to disclose.
[Principal Researcher: Masaaki Takahshi] We have no financial relationships to disclose.

▲TOP

### [P-3305] I-P14-11
### Others

2025 / 9 / 27 (Sat) 16:15-17:00 Room P/(Motenashi Dome) Room P

---

### P-3305
### Photodynamic therapy with hybrid liposomes containing indocyanine green in mouse models of breast cancer

[Speaker]Masaki Okumura (Div. Appl. Life Sci., Grad. Sch. Eng., Sojo Univ.) [Co-author]Junna Takai (Div. Appl. Life Sci., Grad. Sch. Eng., Sojo Univ.)、Koichi Goto (Div. Appl. Life Sci., Grad. Sch. Eng., Sojo Univ.)、Yoko Matsumoto (Div. Appl. Life Sci., Grad. Sch. Eng., Sojo Univ.)、Hideaki Ichihara (Div. Appl. Life Sci., Grad. Sch. Eng., Sojo Univ.)

Hybrid liposomes (HL) can be prepared by simply ultrasonicating a mixture of vesicular and micellar molecules. Inhibitory effects of HL on the growth of various tumor cells due to apoptosis have been achieved without using drugs. In this study, we examined the therapeutic effects of photodynamic therapy (PDT) with HL composed of DMPC, $C_{12}(EO)_{25}$ and indocyanine green in mice model subcutaneously inoculated with breast cancer (4T1-Luc) cells. PDT with HL/ICG inhibited proliferation of 4T1-Luc cells with generation of reactive oxygen species (ROS). A marked suppression of tumor growth based on tumor weight was obtained in model mice treated with PDT after intravenous administration of HL/ICG. Histological analysis revealed an increased apoptotic area in the tumor section of model mice treated intravenously with PDT-HL/ICG. Oxidative stress-positive cells were observed in tumor sections in tumor of model mice subcutaneously transplanted 4T1-Luc cells after the treatment with PDT-HL/ICG. Therapeutic effect of PDT with HL/ICG on a mouse model of breast cancer subcutaneously implanted 4T1-Luc cells was revealed due to ROS induction for the first time.

[Disclosure of COI by Responsible researcher] Lead presenter is not the Responsible researcher.
[Lead presenter: Masaki Okumura] We have no financial relationships to disclose.
[Principal Researcher: Hideaki Ichihara] We have no financial relationships to disclose.

### [P-3306] I-P14-11
### Others

2025 / 9 / 27 (Sat) 16:15-17:00 Room P/(Motenashi Dome) Room P

---

### P-3306
### Advanced mouse ascites carcinoma chemotherapy attempts to chase the localization of cancer stem cells

[Speaker]Jiro Fujimoto (Global Medical Science Laboratories)

Following intraperitoneal (ip) implantation, a single side-population (SP) cell of mouse ascites tumor (FAT-fu) cells selected as Hoechst 33342-effluxing cells developed into a tumor and killed 3 of the 17 male C3H/He mice into which it was implanted. The SP cells seemed to be cancer stem-like cells. The average survival period (ASP) of the FAT-fu bearing mice was 14.5 days, still remaining as low as 14.1 days following a single ip dose of 5-fluorouracil (5-FU) at 60 mg/kg. 5-FU was injected into the greater sac (GS), which comprises the majority of the peritoneal cavity, and eliminated the SP cells of the ascites tumor in the GS. On the other hand, ascites tumor cells containing the SP cells could still be detected in the lesser sac (LS) connected to the GS through the foramen of Winslow following administration of 5-FU; thus, the 5-FU injected into the GS hardly appeared to reach the LS. Administration of 40 mg/kg 5-FU into the GS as well as 20 mg/kg 5-FU into the LS extended the ASP of the mice up to 18.6 days. The results suggest that chemotherapy directed at chasing the localization of cancer stem cells might be more effective.

[Disclosure of COI by Responsible researcher] Lead presenter is the Responsible researcher.
[Lead presenter: Jiro Fujimoto] We have no financial relationships to disclose.

### [P-3313] II-P5-1
### Lung cancer (1)

2025 / 9 / 27 (Sat) 16:15-17:00 Room P/(Motenashi Dome) Room P

---

### P-3313
### RBM10 Loss Modulates Splicing and Defines a Unique Transcriptomic Signature in EGFR-Mutant LUAD

[Speaker]Satoshi Endo (Div. of Genome Biol., Natl. Cancer Ctr.／Dept. of Respiratory Med., Inst. of Sci. Tokyo) [Co-author]Shigenari Nukaga (Div. of Genome Biol., Natl. Cancer Ctr.／Div. of Pulmonary Med., Keio Univ. Sch. of Med.)、Kouya Shiraishi (Div. of Genome Biol., Natl. Cancer Ctr.)、Yasushi Yatabe (Dept. of Diagnostic Path., Natl. Cancer Ctr. Hosp.／Div. of Mol. Path., Natl. Cancer Ctr.)、Ryuji Hamamoto (Div. of Med. AI Res. and Development, Natl. Cancer Ctr.)、Takashi Kohno (Div. of Genome Biol., Natl. Cancer Ctr.)、Takashi Nakaoku (Div. of Genome Biol., Natl. Cancer Ctr.)

Background: RNA binding motif 10 (***RBM10***) is a key regulator of alternative splicing, particularly skipped exon (SE) events. ***RBM10*** mutations are reported in approximately 10% of lung adenocarcinoma (LUAD) cases and frequently co-occur with ***EGFR*** mutations. However, the biological consequences of ***RBM10*** loss in LUAD remain poorly understood. Methods: Whole-exome and RNA sequencing were performed on 727 LUAD samples obtained from patients who underwent surgical resection at the National Cancer Center Hospital between 1997 and 2018. SE events were analyzed using the percent spliced-in (PSI) metric calculated with the rMATS-turbo. Results: ***RBM10*** mutations were identified in 74 samples, the majority of which were truncating. Seventy-seven percent of ***RBM10***-mutated cases also harbored ***EGFR*** mutations and were mutually exclusive with ***TP53*** mutations. In ***EGFR***-mutated LUAD, co-mutation with ***RBM10*** was associated with reduced SE events. Moreover, UMAP analysis based on PSI revealed a distinct SE profile in LUAD with truncating ***RBM10*** mutations. Conclusion: Loss of ***RBM10*** may alter SE profiles in ***EGFR***-mutated LUAD, suggesting a potential role in modulating splicing patterns in this subset.

[Disclosure of COI by Responsible researcher] Lead presenter is not the Responsible researcher.
[Lead presenter: Satoshi Endo] We have no financial relationships to disclose.
[Principal Researcher: Takashi Nakaoku] We have no financial relationships to disclose.

### [P-3314] II-P5-1
### Lung cancer (1)

2025 / 9 / 27 (Sat) 16:15-17:00 Room P/(Motenashi Dome) Room P

---

### P-3314
### Investigating the impact of solid component-associated transcription factors on the lung adenocarcinoma malignancy.

[Speaker]Tomohiko Sakabe (Dept. of Path., Faculty of Med., Tottori Univ.) [Co-author]Karen Makishima (Dept. of Path., Faculty of Med., Tottori Univ.)、Tatsuya Miyamoto (Dept. General Thoracic Surg., Tottori Univ. Hosp.)、Kanae Ozaki (Dept. of Path., Faculty of Med., Tottori Univ.)、Yoshihisa Umekita (Dept. of Path., Faculty of Med., Tottori Univ.)

[Background] Solid component (SC) of lung adenocarcinoma (LUAD) has been associated with an unfavorable prognosis; however, the biological significance of the SC remains to be elucidated. The present study investigated the role of SC-associated transcription factors (SCATs) in the malignant phenotype of LUAD. [Methods] ***In silico*** analysis was conducted using our published RNA-seq data and public databases. SCATs expression in LUAD histologic subtypes was evaluated by immunohistochemistry (IHC). The impact of SCATs on LUAD malignancy was evaluated by examining the proliferation, invasion, and migration of A549 cells. [Results] ***In silico*** analysis and IHC revealed increased expression of six SCATs (TF-1, EZH2, HIF1A, NCAPG, TF-2, WDR5) in SC compared to other subtypes in LUAD patients. TF-1 overexpression in A549 cells resulted in increased proliferation and invasion. Overexpressing TF-2 in A549 cells enhanced proliferation, migration, and invasion. Additionally, IFN-γ-induced CD274 expression was also increased with TF-2 overexpression. [Conclusion] SCATs, particularly TF-1 and TF-2, play a pivotal role in the biological malignancy of SC in LUAD and may serve as therapeutic targets.

[Disclosure of COI by Responsible researcher] Lead presenter is the Responsible researcher.
[Lead presenter: Tomohiko Sakabe] ■Research expenses: 科研費 (基盤C)

### [P-3315] II-P5-1
### Lung cancer (1)

2025 / 9 / 27 (Sat) 16:15-17:00 Room P/(Motenashi Dome) Room P

### P-3315
### PKM1 Expression in Neuroendocrine-Type SCLC Is Associated with Reduced T Cell Infiltration

[Speaker]Naoki Shijubou (Department of Pathology, Sapporo Medical University School of Medicine (SMU)／Department of Respiratory Medicine and Allergology, SMU／Department of Pathology, Hakodate Goryoukaku Hospital) [Co-author]Terufumi Kubo (Department of Pathology, Sapporo Medical University School of Medicine (SMU))、Kenta Sasaki (Department of Pathology, Sapporo Medical University School of Medicine (SMU))、Tatsuru Ikeda (Department of Respiratory Medicine, Hakodate Goryoukaku Hospital)、Toshiyuki Sumi (Department of Pathology, Hakodate Goryoukaku Hospital)、Yoshihiko Hirohashi (Department of Pathology, Sapporo Medical University School of Medicine (SMU))、Hirofumi Chiba (Department of Respiratory Medicine and Allergology, SMU)、Toshihiko Torigoe (Department of Pathology, Sapporo Medical University School of Medicine (SMU))

Background:SCLC is a highly aggressive lung cancer comprising ~15% of lung cacer cases. Although chemotherapy plus ICIs is standard for ED-SCLC, the benefit of ICIs remains limited. Molecular subtyping based on transcription factor expression has revealed distinct immune landscapes, with NE and non-NE types differing in immune gene expression and CD8+ T cell infiltration. PKM, a glycolytic enzyme, has two isoforms: PKM1 and PKM2. Unlike most cancers that express PKM2, NE tumors like SCLC often express PKM1, potentially linking it to NE features and immune modulation.Methods:We analyzed surgical SCLC samples and classified them into molecular subtypes. IHC was performed for PKM1, NSE, CD8, and HLA class I.Results:NE-type tumors showed significantly lower CD8+ T cell infiltration than non-NE types. Within NE tumors, regions co-expressing PKM1 and NSE had especially low CD8+ T cells.Conclusion:PKM1 and NSE expression in NE-type SCLC correlates with reduced T cell infiltration. PKM1 may contribute to immune evasion and could be a potential target to enhance immune responses in SCLC.

[Disclosure of COI by Responsible researcher] Lead presenter is the Responsible researcher.
[Lead presenter: Naoki Shijubou] We have no financial relationships to disclose.

Poster Session

### [P-3316] II-P5-1
### Lung cancer (1)

2025 / 9 / 27 (Sat) 16:15-17:00 Room P/(Motenashi Dome) Room P

### P-3316
### Role of brain abundant membrane attached signal protein 1 (BASP1) in large cell neuroendocrine carcinoma of the lung

[Speaker]Ikue Noura (Dept. Mol. Path., Osaka Metropolitan. Univ. Grad. Sch. Med.) [Co-author]Shugo Suzuki (Dept. Mol. Path., Osaka Metropolitan. Univ. Grad. Sch. Med.)、Takeshi Inoue (Dept. Path., Osaka City Gen. Hosp.)、Anna Kakehashi (Dept. Mol. Path., Osaka Metropolitan. Univ. Grad. Sch. Med.)、Hideki Wanibuchi (Dept. Env. Risk Assess., Osaka Metropolitan. Univ. Grad. Sch. Med.)

Objective: Lung cancer is the second most common type of cancer in Japan, with a poor prognosis and the highest mortality rate. Large cell neuroendocrine carcinoma (LCNEC) accounts for a small proportion of lung cancers, but is known to have a worse prognosis than other histological types. The present study investigated the expression and function of brain abundant membrane attached signal protein 1 (BASP1) as a novel biomarker for LCNEC. Materials and Methods: BASP1 expression was evaluated in FFPE lung specimens diagnosed as LCNEC cases by immunohistochemical staining. The relationship between clinical information and BASP1 expression was examined. Furthermore, BASP1 was knocked down in two cell lines, NCI-H460 and NCI-H810, and cell invasion assays were conducted. Results: No correlation was found between BASP1 expression and overall survival, but a negative correlation was identified between BASP1 expression and clinical stage. No association was found with other clinical information, such as age or gender. In addition, reduction of BASP1 expression in cell lines promoted cell invasion. Conclusion: BASP1 expression is associated with the progression of LCNEC.

[Disclosure of COI by Responsible researcher] Lead presenter is not the Responsible researcher.
[Lead presenter: Ikue Noura] We have no financial relationships to disclose.
[Principal Researcher: Shugo Suzuki] We have no financial relationships to disclose.

▲TOP

### [P-3317] II-P5-1
### Lung cancer (1)

2025 / 9 / 27 (Sat) 16:15-17:00 Room P/(Motenashi Dome) Room P

---

### P-3317
### Establishment of intratumor heterogeneity cell model using lung cancer cell lines

[Speaker]Tomoko Funazo (Department of Respiratory Medicine, Graduate School of Medicine Kyoto University) [Co-author]Takahiro Tsuji (Fred Hutchinson Cancer Center)、Kazutaka Hosoya (Department of Respiratory Medicine, Graduate School of Medicine Kyoto University)、Yusuke Shima (Department of Respiratory Medicine, Graduate School of Medicine Kyoto University)、Masahiro Oi (Department of Respiratory Medicine, Graduate School of Medicine Kyoto University)、Keiichiro Suminaga (Department of Respiratory Medicine, Graduate School of Medicine Kyoto University)、Kentaro Hashimoto (Department of Respiratory Medicine, Graduate School of Medicine Kyoto University)、Hiroshi(Kyoto Univ.) Yoshida (Department of Respiratory Medicine, Graduate School of Medicine Kyoto University)、Hitomi Ajimizu (Department of Respiratory Medicine, Graduate School of Medicine Kyoto University)、Takashi Nomizo (Department of Respiratory Medicine, Graduate School of Medicine Kyoto University)、Hironori Yoshida (Department of Respiratory Medicine, Graduate School of Medicine Kyoto University)、Hiroaki Ozasa (Department of Respiratory Medicine, Graduate School of Medicine Kyoto University)

Intratumor heterogeneity is reported to be involved in cancer resistance to treatment. Previous studies have mainly involved collecting cancer cells from patients and classifying clusters with different properties. However, there have been few studies focusing on the early phase of intratumor heterogeneity. We hypothesized that exposure to anticancer drugs could reproduce intratumor heterogeneity in vitro.
As a result, bimodal CD47 expression was observed in H2228-AR1S, a commercially available ALK-positive lung cancer cell line (H2228) exposed to alectinib. The CD47high subpopulation had high tumorigenicity, while the CD47low subpopulation had high migration ability ***in vitro***. Next, bimodal EpCAM expression was observed in KTOR83-Dab, a patient-derived BRAF-positive lung cancer cell line (KTOR83) exposed to dabrafenib. The EpCAMlow subpopulation showed high migration ability.Enrichment analysis showed that the CD47low subpopulation of H2228-AR1S and the EpCAMlow subpopulation of KTOR83-Dab both showed enhanced pathways involved in cell-cell adhesion.
In conclusion, intratumor heterogeneity cell models having two subpopulations with different properties was established.

[Disclosure of COI by Responsible researcher] Lead presenter is not the Responsible researcher.
[Lead presenter: Tomoko Funazo] ■Contributions: 上原記念生命科学財団、鈴木謙三記念医科学応用研究財団、SGH財団、横山薬理研究
[Principal Researcher: Hiroaki Ozasa] ■Contributions: ベーリンガーインゲルハイム

### [P-3318] II-P5-1
### Lung cancer (1)

2025 / 9 / 27 (Sat) 16:15-17:00 Room P/(Motenashi Dome) Room P

### P-3318
### The role of phospholipase A2 in characteristics of pleural mesothelioma cells

[Speaker]Ayuko Sato (Dept. Mol. Pathol., Hyogo Med. Univ., Sch. Med.) [Co-author]Michiko Yuki (Dept. Mol. Pathol., Hyogo Med. Univ., Sch. Med.)、Tohru Tsujimura (Dept. Pathol., JCHO Osaka Minato Central Hosp.)、Kenji Ohshima (Dept. Mol. Pathol., Hyogo Med. Univ., Sch. Med.)

Pleural mesothelioma is a refractory tumor with a poor prognosis, and the most frequent initial symptom in patients with pleural mesothelioma is pleural effusion. The high anoikis resistance of mesothelioma cells in pleural effusion may contribute to the development and progression of mesothelioma, but the detailed mechanism remains to be elucidated. We previously established a mesothelioma cell line MM56-sph that acquired high anoikis resistance by long-term culture under ultra-low attachment condition. Here, we found that expression of members of the phospholipase A2 ($PLA_2$) family, which have important roles in lipid metabolism, was significantly upregulated in MM56-sph cells compared with parental MM56 cells. $PLA_2$ inhibitors suppressed cell growth and spheroid formation of MM56-sph cells. The siRNA-mediated knockdown of $PLA_2$ expression in MM56-sph cells also showed similar effects. These results suggest that $PLA_2$ may have an important role in the high anoikis resistance of mesothelioma cells.

[Disclosure of COI by Responsible researcher] Lead presenter is not the Responsible researcher.
[Lead presenter: Ayuko Sato] We have no financial relationships to disclose.
[Principal Researcher: Kenji Ohshima] We have no financial relationships to disclose.

[P-3319] II-P5-1
Lung cancer (1)

2025 / 9 / 27 (Sat) 16:15-17:00 Room P/(Motenashi Dome) Room P

P-3319

### Promotion of cellular functions via LPA receptor-mediated signaling in lung cancer cells co-cultured with stromal cells

[Speaker]Yuka Kusumoto (Mol Oncol. Life Science, Kindai Univ.) [Co-author]Mao Yamamoto (Mol Oncol. Life Science, Kindai Univ.)、Narumi Yashiro (Mol Oncol. Life Science, Kindai Univ.)、Moemi Tamura (Mol Oncol. Life Science, Kindai Univ.)、Shion Nagano (Mol Oncol. Life Science, Kindai Univ.)、Hiroko Ikeda (Mol Oncol. Life Science, Kindai Univ.)、Toshifumi Tsujiuchi (Mol Oncol. Life Science, Kindai Univ.)

Stromal cells enhance cancer progression in the tumor environment. The present study investigated the roles of lysophosphatidic acid (LPA) receptor-mediated signaling in modulating the biological process of lung cancer LL/2 cells co-cultured with fibroblast 3T3 cells and lymphatic endothelial cells SVEC4-10 cells. ***LPAR1***, ***LPAR2*** and ***LPAR5*** expression levels changed in LL/2 cells co-cultured with these cells. LPA treatment enhanced LL/2 cell growth in the supernatants from both cell types, exceeding the effects observed in the cell supernatants from 3T3 or SVEC4-10 cells alone. LL/2 cell motility increased in co-cultures with both 3T3 and SVEC4-10 cells, compared to either cell type alone. AM966 and TC LPA5 4 inhibited LL/2 cell motility, while GRI-977143 stimulated growth but reduced motility. ***Atx*** expression increased in LL/2 cells co-cultured with 3T3 and/or SVEC4-10 cells. Lysophosphatidylcholine (LPC) treatment enhanced LL/2 cell growth in the supernatants from both cell types. These results suggest that LPA receptor signaling mediates the cooperative effects of 3T3 and SVEC4-10 cells, which is involved in enhancing LL/2 cell proliferation and motility.

[Disclosure of COI by Responsible researcher] Lead presenter is not the Responsible researcher.
[Lead presenter: Yuka Kusumoto] We have no financial relationships to disclose.
[Principal Researcher: Toshifumi Tsujiuchi] We have no financial relationships to disclose.

### [P-3320] II-P5-1
### Lung cancer (1)

2025 / 9 / 27 (Sat) 16:15-17:00 Room P/(Motenashi Dome) Room P

---

### P-3320
### Regulation of cellular functions through activation of free fatty acid receptors (FFARs) in lung cancer cells

[Speaker]Shion Nagano (Mol Oncol. Life Science, Kindai Univ.) [Co-author]Mao Yamamoto (Mol Oncol. Life Science, Kindai Univ.)、Narumi Yashiro (Mol Oncol. Life Science, Kindai Univ.)、Yuka Kusumoto (Mol Oncol. Life Science, Kindai Univ.)、Moemi Tamura (Mol Oncol. Life Science, Kindai Univ.)、Hiroko Ikeda (Mol Oncol. Life Science, Kindai Univ.)、Toshifumi Tsujiuchi (Mol Oncol. Life Science, Kindai Univ.)

Free fatty acid receptors (FFARs) contributes to regulating cancer cell functions. This study investigated the roles of FFARs in promoting tumor progression using highly invasive A549-M8 cells derived from lung cancer A549 cells. A549-M8 cells indicated approximately eightfold higher invasive activity compared to parental A549 cells. ***FFAR1*** and ***FFAR4*** expressions were lower in A549-M8 cells than in A549 cells. TUG-770 (FFAR1 agonist) stimulated, while TUG-891 (FFAR4 agonist) inhibited A549-M8 cell invasion. A549-M8 cells demonstrated higher viability to cisplatin (CDDP) compared to A549 cells. TUG-770 enhanced, whereas TUG-891 reduced A549-M8 cell viability in response to CDDP. Under hypoxic conditions (1% $O_2$). ***FFAR1*** and ***FFAR4*** expressions were reduced in A549 cells under 1% $O_2$, while those levels were elevated in A549-M8 cells. A549-M8 cells showed markedly enhanced invasive activity compared to A549 cells. Under 1% $O_2$, A549 cell invasion was inhibited by TUG-770 and stimulated by TUG-891. Conversely, TUG-770 increased and TUG-891 suppressed A549-M8 cell invasion. These results suggest that FFAR-mediated signaling is involved in modulating malignant properties in lung cancer cells.

[Disclosure of COI by Responsible researcher] Lead presenter is not the Responsible researcher.
[Lead presenter: Shion Nagano] We have no financial relationships to disclose.
[Principal Researcher: Toshifumi Tsujiuchi] We have no financial relationships to disclose.

[P-3321] II-P5-1
Lung cancer (1)

2025 / 9 / 27 (Sat) 16:15-17:00 Room P/(Motenashi Dome) Room P

P-3321
### Roles of LPA receptor signaling in enhancing malignant potential of lung cancer cells under hypoxic conditions

[Speaker]Moemi Tamura (Mol Oncol. Life Science, Kindai Univ.) [Co-author]Narumi Yashiro (Mol Oncol. Life Science, Kindai Univ.)、Mao Yamamoto (Mol Oncol. Life Science, Kindai Univ.)、Yuka Kusumoto (Mol Oncol. Life Science, Kindai Univ.)、Shion Nagano (Mol Oncol. Life Science, Kindai Univ.)、Hiroko Ikeda (Mol Oncol. Life Science, Kindai Univ.)、Toshifumi Tsujiuchi (Mol Oncol. Life Science, Kindai Univ.)

Hypoxia promotes tumor progression by enhancing cancer cell motility, invasion, and metastasis. This study examined the roles of lysophosphatidic acid (LPA) receptor signaling in modulating malignant behavior under hypoxia (1% $O_2$) in lung cancer A549 cells. Highly migratory A549-R12 cells derived from A549 cells were used. ***LPAR1*** and ***LPAR2*** expression levels decreased under 1% $O_2$, while ***LPAR3*** expression remained unchanged. Cell motility increased under 1% $O_2$, with A549-R12 cells exhibiting higher motility. AM966 ($LPA_1$ antagonist) and (2S)-OMPT ($LPA_3$ agonist) increased A549-R12 cell motility, whereas GRI-977143 ($LPA_2$ agonist) reduced it. Moreover, the invasion activity of A549-R12 cells was higher than that of A549 cells, with 1% $O_2$ conditions significantly enhancing A549-R12 cell invasion. AM966 and (2S)-OMPT stimulated, whereas GRI-977143 inhibited, the invasion of A549-R12 cells. In the presence of LPA, cell viability to CDDP was higher in A549-R12 cells cultured at both 21% and 1% $O_2$ compared to A549 cells. These results suggest that LPA receptor signaling is involved in enhancing malignant progression in lung cancer cells under hypoxic conditions.

[Disclosure of COI by Responsible researcher] Lead presenter is not the Responsible researcher.
[Lead presenter: Moemi Tamura] We have no financial relationships to disclose.
[Principal Researcher: Toshifumi Tsujiuchi] We have no financial relationships to disclose.

### [P-3331] II-P6-1
### Molecular Pathology of Prognostic & Progression Markers in Breast Cancer

2025 / 9 / 27 (Sat) 16:15-17:00 Room P/(Motenashi Dome) Room P

---

### P-3331
### Validation of PREDICT and development of postoperative prognostic models for Japanese breast cancer patients

[Speaker]Nobuji Kouno (Div. Med. AI Res. Dev., Natl. Cancer Ctr. Res. Inst.／Cancer Transl. Res. Team, RIKEN Ctr. for AIP project／Dept. GI Surg., Kyoto Univ., Sch. Med.) [Co-author]Hiromi Hashiguchi (Dept. Breast Surg., Natl. Cancer Ctr. Hosp.)、Katsuji Takeda (Cancer Transl. Res. Team, RIKEN Ctr. for AIP project)、Satoshi Takahashi (Div. Med. AI Res. Dev., Natl. Cancer Ctr. Res. Inst.／Cancer Transl. Res. Team, RIKEN Ctr. for AIP project)、Masaaki Komatsu (Div. Med. AI Res. Dev., Natl. Cancer Ctr. Res. Inst.／Cancer Transl. Res. Team, RIKEN Ctr. for AIP project)、Takeshi Murata (Dept. Breast Surg., Natl. Cancer Ctr. Hosp.)、Shin Takayama (Dept. Breast Surg., Natl. Cancer Ctr. Hosp.)、Kazutaka Obama (Dept. GI Surg., Kyoto Univ., Sch. Med.)、Ryuji Hamamoto (Div. Med. AI Res. Dev., Natl. Cancer Ctr. Res. Inst.／Cancer Transl. Res. Team, RIKEN Ctr. for AIP project)

The Japanese breast cancer treatment guidelines refer to PREDICT, a UK–based tool for postoperative prognostic prediction; however, its generalizability to Japanese patients has not been thoroughly validated. In this study, we aimed to assess the applicability of PREDICT to Japanese patients and explore the potential for developing a more tailored prognostic model. We retrospectively analyzed clinical, pathological, and treatment data from 2,997 Japanese patients with primary breast cancer who underwent surgery at the National Cancer Center Hospital between 2006 and 2016. We evaluated the prognostic performance of PREDICT versions 2.2 and 3.0. Additionally, we developed prognostic models using Cox proportional hazards and other machine learning methods. While both PREDICT v2.2 and v3.0 demonstrated similar performance up to five years, the performance of v3.0 declined more significantly than that of v2.2 for longer–term predictions. Our machine learning–based models showed comparable or superior predictive accuracy to PREDICT, highlighting the potential for a more suitable prognostic model for Japanese breast cancer patients.

[Disclosure of COI by Responsible researcher] Lead presenter is not the Responsible researcher.
[Lead presenter: Nobuji Kouno] We have no financial relationships to disclose.
[Principal Researcher: Ryuji Hamamoto] We have no financial relationships to disclose.

### [P-3332] II-P6-1
### Molecular Pathology of Prognostic & Progression Markers in Breast Cancer

2025 / 9 / 27 (Sat) 16:15-17:00 Room P/(Motenashi Dome) Room P

---

### P-3332
### Relationships between RS of Oncotype Dx and clinicopathological features in HR+/HER2- early breast cancer

[Speaker]Eriko Tokunaga (Department of Breast Oncology, NHO Kyushu Cancer Center) [Co-author]Yumiko Koi (Department of Breast Oncology, NHO Kyushu Cancer Center)、Wakako Tajiri (Department of Breast Oncology, NHO Kyushu Cancer Center)、Hideki Ijichi (Department of Breast Oncology, NHO Kyushu Cancer Center)、Sayuri Akiyoshi (Department of Breast Oncology, NHO Kyushu Cancer Center)、Chinami Koga (Department of Breast Oncology, NHO Kyushu Cancer Center)、Yoshiaki Nakamura (Department of Breast Oncology, NHO Kyushu Cancer Center)、Kenichi Taguchi (Department of Pathology, NHO Kyushu Cancer Center)

Backgrounds: Oncotype Dx has been recently approved in Japan for hormone receptor positive (HR+)/HER2 negative (HER2-) early breast cancer (EBC). In this study, relationships between recurrence score (RS) of Oncotype Dx and the clinicopathological features were analyzed. Patients and Methods: A total of 199 HR+/HER2- EBC patients at pStage I-IIIA who underwent breast surgery between 2012 and 2024 were included. Results: RS was significantly correlated with histological grade (HG) and Ki67. RS was inversely correlated with PgR expression, Oncotype DX ER and PgR score. There were no relationships between RS and age, menopausal status, tumor size, nodal status, histology, HER2 and lymphovessel invasion (LVI). The positive relationships between RS and HG were observed in both pre and postmenopausal women, however, positive relationships between RS and Ki67 were observed in only premenopausal women. The fact that there was no relationship between RS and LVI, a classic poor prognostic factor, should be taken with caution when assessing the need for adjuvant chemotherapy (aCT). Conclusion: The necessity of aCT should be evaluated in light of both clinical risk and RS in HR+/HER2- EBC.

[Disclosure of COI by Responsible researcher] Lead presenter is the Responsible researcher.
[Lead presenter: Eriko Tokunaga] ■Lecture fee: 第一三共、アストラゼネカ、イーライリリー、中外

### [P-3333] II-P6-1
### Molecular Pathology of Prognostic & Progression Markers in Breast Cancer

2025 / 9 / 27 (Sat) 16:15-17:00 Room P/(Motenashi Dome) Room P

Poster Session

---

### P-3333
### RUNX3 synergizes with RUNX1 to suppress MYC in breast cancer

[Speaker]Nawaphat Jangphattananont (Cancer Sci. Inst. of Singapore, Natl. Univ. of Singapore, Singapore) [Co-author]Junichi Matsuo (Cancer Sci. Inst. of Singapore, Natl. Univ. of Singapore, Singapore)、Yong Wei Peng (Cancer Sci. Inst. of Singapore, Natl. Univ. of Singapore, Singapore／Dept. of Hematology-Oncology, Natl. Univ. Cancer Inst., Singapore)、Linda Shyue Huey Chuang (Cancer Sci. Inst. of Singapore, Natl. Univ. of Singapore, Singapore)、Yoshiaki Ito (Cancer Sci. Inst. of Singapore, Natl. Univ. of Singapore, Singapore)

The role of RUNX family genes in cancer is bifunctional, either oncogenic or tumor suppressive. However, the switch regulating this dual function remains unclear. In breast cancer, RUNX1 was listed within the top 10 highly mutated genes, and RUNX3 is frequently silenced by hypermethylation. To investigate RUNX function in breast cancer, we introduced RUNX3 expression into RUNX3-deficient, KRT8-expressing cells of the MMTV-PyMT breast cancer mouse model. RUNX3 introduction in the breast tumor resulted in upregulation of MYC activity and MYC-target gene CDK1. Intriguingly, RUNX3 attenuated RUNX1 expression. Histology and spatial transcriptomics analysis of the RUNX3-positive tumors revealed heterogeneity of RUNX1 expression. RUNX1 expression was also inversely correlated to MYC expression and MYC-pathway activity. We observed interaction between RUNX1 and RUNX3 proteins, indicating that the RUNX1-RUNX3 complex promotes MYC protein degradation. Additionally, overexpression of RUNX1 and RUNX3 strongly inhibited breast cancer growth. This study reveals an unappreciated synergy between the two RUNX paralogs in regulating breast homeostasis and suppressing breast cancer.

[Disclosure of COI by Responsible researcher] Lead presenter is the Responsible researcher.
[Lead presenter: Nawaphat Jangphattananont] We have no financial relationships to disclose.

▲TOP

### [P-3334] II-P6-1
### Molecular Pathology of Prognostic & Progression Markers in Breast Cancer

2025 / 9 / 27 (Sat) 16:15-17:00 Room P/(Motenashi Dome) Room P

---

### P-3334
### The clinical significance of MTX1 with genomic copy number amplification in breast cancer.

[Speaker]Yuki Ando (Department of Surgery, Kyusyu University Beppu Hospital) [Co-author]Shohei Shibuta (Department of Surgery, Kyusyu University Beppu Hospital)、Qingjiang Hu (Department of Surgery, Kyusyu University Beppu Hospital)、Hajime Otsu (Department of Surgery, Kyusyu University Beppu Hospital)、Yusuke Yonemura (Department of Surgery, Kyusyu University Beppu Hospital)、Koshi Mimori (Department of Surgery, Kyusyu University Beppu Hospital)

Background: Chromosome 1q amplification in breast cancer (BC) (Nature 2012) likely harbors key driver genes for BC progression. Materials and Methods: We identified MTX1 as a candidate driver gene on 1q using The Cancer Genome Atlas (TCGA) datasets. MTX1 mRNA expression was measured via RT-qPCR and correlated with clinical factors and prognosis. Its localization was observed by immunohistochemical staining (IHC). The biological role was examined by Gene Set Enrichment Analysis (GSEA). Results: MTX1 mRNA expression was significantly higher in BC tissues, and the higher expression group showed poor prognosis. IHC showed that MTX1 was overexpressed in BC cells compared with normal ductal epithelial cells. GSEA revealed that high COX19 expression tends to negatively correlate with MYC pathway. Conclusion: MTX1 could be a prognostic biomarker in BC. MTX1 belongs to Metaxins family. As the outer membrane proteins of mitochondria, the Metaxins family participates in the import of nuclear-encoded proteins into mitochondria and is thought to be the member of Sorting and Assembly Machinery (Cell Signal, 2014). We now investigate how MTX1 expression accelerates BC progression.

[Disclosure of COI by Responsible researcher] Lead presenter is not the Responsible researcher.
[Lead presenter: Yuki Ando] We have no financial relationships to disclose.
[Principal Researcher: Koshi Mimori] We have no financial relationships to disclose.

### [P-3335] II-P6-1
### Molecular Pathology of Prognostic & Progression Markers in Breast Cancer

2025 / 9 / 27 (Sat) 16:15-17:00 Room P/(Motenashi Dome) Room P

---

### P-3335
### The role of AMIGO2 in human breast cancer progression

[Speaker]Kanoko Nakamura (Dept. Pathol. & Histotech. Tohoku Univ. Grad Sch. Med.) [Co-author]Mio Tanaka (Dept. Pathol. & Histotech. Tohoku Univ. Grad Sch. Med.)、Kiyoshi Takagi (Dept. Pathol. & Histotech. Tohoku Univ. Grad Sch. Med.)、Ai Sato (Dept. Pathol. & Histotech. Tohoku Univ. Grad Sch. Med.)、Yasuhiro Miki (Dept. Anatomic Pathol. Tohoku Univ. Grad Sch. Med)、Minoru Miyashita (Dept. Breast & Endocrine Surg. Oncol. Tohoku Univ. Grad Sch. Med.)、Takashi Suzuki (Dept. Pathol. & Histotech. Tohoku Univ. Grad Sch. Med.／Dept. Anatomic Pathol. Tohoku Univ. Grad Sch. Med／Dept. Pathol. Tohoku Univ. Hosp.)

Adhesion molecule with immunoglobulin like domain 2 (AMIGO2) is a transmembrane protein that acts as an adhesion molecule. It has been reported to be expressed in various types of cancer cells and to contribute to tumor progression, particularly metastasis. However, its role in breast cancer (BC) remains unclear. In this study, we immunolocalized AMIGO2 in 116 BC tissues and investigated its function using human BC cell lines MCF-7, MDA-MB-231, T-47D, and SKBR-3.AMIGO2 immunoreactivity was detected in the membranes and cytoplasm of BC cells, with 22% of patients classified as AMIGO2-positive. Immunohistochemical analysis revealed that AMIGO2 was positively correlated with aggressive phenotype of breast cancers and risk of recurrence, metastasis and BC-specific death, especially in ER (+) and HER2 (-) cases. Knockdown of AMIGO2 significantly suppressed cell proliferation and migration in human BC cell lines. Moreover, suppression of AMIGO2 reduced the adhesion ability of BC cells to each other. In conclusion, AMIGO2 expression serves as a prognostic marker for poor outcomes in breast cancer, particularly in ER (+) and HER2 (-) cases, and plays a role in BC progression.

[Disclosure of COI by Responsible researcher] Lead presenter is not the Responsible researcher.
[Lead presenter: Kanoko Nakamura] We have no financial relationships to disclose.
[Principal Researcher: Takashi Suzuki] We have no financial relationships to disclose.

### [P-3336] II-P6-1
### Molecular Pathology of Prognostic & Progression Markers in Breast Cancer

2025 / 9 / 27 (Sat) 16:15-17:00 Room P/(Motenashi Dome) Room P

---

### P-3336
### Tim1 is a poor prognostic factor associated with proliferation and migration in breast cancer.

[Speaker]Mio Tanaka (Dept. Pathol. & Histotech. Tohoku Univ. Grad Sch. Med.) [Co-author]Kiyoshi Takagi (Dept. Pathol. & Histotech. Tohoku Univ. Grad Sch. Med.)、Mai Sawafuji (Dept. Pathol. & Histotech. Tohoku Univ. Grad Sch. Med.)、Ai Sato (Dept. Pathol. & Histotech. Tohoku Univ. Grad Sch. Med.)、Yasuhiro Miki (Dept. Anatomic Pathol. Tohoku Univ. Grad Sch. Med.)、Minoru Miyashita (Dept. Breast & Endocrine Surg. Oncol. Tohoku Univ. Grad Sch. Med.)、Takashi Suzuki (Dept. Pathol. & Histotech. Tohoku Univ. Grad Sch. Med.／Dept. Anatomic Pathol. Tohoku Univ. Grad Sch. Med.／Dept. Pathol. Tohoku Univ. Hosp.)

T-cell immunoglobulin and mucin domain (Tim) 1 is a phosphatidylserine receptor with a tyrosine kinase phosphorylation motif involved in immune cell activation. Although Tim1 expression has recently been reported in various types of cancer cells, its significance in breast cancer (BC) remains largely unknown. Here, we investigated the role of Tim1 in BC using immunohistochemistry and in vitro experiments.Tim1 immunoreactivity was detected in the cytoplasm of BC cells, and 53% of patients were considered positive for Tim1 (n = 116). Tim1 expression was positively correlated with aggressive phenotype and recurrence of breast cancers. Overexpression of Tim1 significantly promoted cell proliferation and migration in the human BC cell lines MDA-MB-231 and SKBR-3 but suppressed these effects in MCF-7. Deletion of the tyrosine kinase phosphorylation motif in Tim1 abolished these effects. Tim1 overexpression enhanced AKT phosphorylation in MDA-MB-231 cells, whereas no significant change was observed in MCF-7 cells. In summary, Tim1 may serve as a prognostic factor associated with cell proliferation and migration in BC, although its effects may vary depending on the cell type.

[Disclosure of COI by Responsible researcher] Lead presenter is not the Responsible researcher.
[Lead presenter: Mio Tanaka] We have no financial relationships to disclose.
[Principal Researcher: Takashi Suzuki] We have no financial relationships to disclose.

### [P-3337] II-P6-1
### Molecular Pathology of Prognostic & Progression Markers in Breast Cancer

2025 / 9 / 27 (Sat) 16:15-17:00 Room P/(Motenashi Dome) Room P

---

### P-3337
### Does ABO blood type affect recurrence and prognosis in resected breast cancer?

[Speaker]Koji Tezuka (JCHO Mishima General Hosp. Div. of Breast & General Surg.) [Co-author]Tomomi Hayashi (Shizuoka Cancer Center Div. of Breast Surg.)、Emima Bekku (Shizuoka Cancer Center Div. of Breast Surg.)、Yukiko Tadokoro (Shizuoka Cancer Center Div. of Breast Surg.)、Takayoshi Uematsu (Shizuoka Cancer Center Div. of Breast Imaging & Breast Intervention Radiology)、Kazuaki Nakajima (Shizuoka Cancer Center Div. of Breast Imaging & Breast Intervention Radiology)、Nahomi Tokudome (Shizuoka Cancer Center Div. of Breast Oncology)、Takashi Sugino (Shizuoka Cancer Center Div. of Path.)、Kaoru Takahashi (Shizuoka Cancer Center Div. of Breast Surg.)、Seiichiro Nishimura (Shizuoka Cancer Center Div. of Breast Surg.)

Introduction: The relationship between prognosis of breast cancer (BC) and ABO blood type is controversial. The aim of this study was to determine the relationship between ABO blood type and recurrence and prognosis in resected BC. Methods: 2928 patients who underwent radical surgery for BC of Stage 1-3 between 2010 and 2019 were included in this study. Recurrence was defined as recurrence at any site, and prognosis was evaluated as BC-specific survival (BCSS). Cumulative BCSS and recurrence rate were analyzed using the Kaplan-Meier method and compared using a log-rank test. A Cox proportional hazards model was used for multivariate analysis. Results: No clear relationship was found between ABO blood type and BCSS. However, blood type A/B was associated with a higher cumulative recurrence rate than blood type O/AB (10-year recurrence rate: 12.1% vs. 8.8%, P=0.035). Multivariate analysis showed that blood type A/B was an independent predictive factor for recurrence (hazard ratio 1.30, 95% confidence interval 1.01-1.67, P=0.040). Conclusion: No relationship was identified between ABO blood type and BCSS, but our analysis demonstrated that ABO blood type might influence recurrence.

[Disclosure of COI by Responsible researcher] Lead presenter is the Responsible researcher.
[Lead presenter: Koji Tezuka] We have no financial relationships to disclose.

### [P-3338] II-P6-1
### Molecular Pathology of Prognostic & Progression Markers in Breast Cancer

2025 / 9 / 27 (Sat) 16:15-17:00 Room P/(Motenashi Dome) Room P

---

### P-3338
### Analysis of gene expression and prognostic factors single-cell RNA sequencing (scRNA-seq) using in breast cancer(BC)

[Speaker]Kanako Naito (Kitasato University Graduate School of Medical Sciences) [Co-author]Shunpei Shibaki (Kitasato University Graduate School of Medical Sciences)、Hiroyuki Minoura (Kitasato University Graduate School of Medical Sciences)、Kyonosuke Ikemura (Kitasato University Graduate School of Medical Sciences)、Riku Okamoto (Kitasato University Graduate School of Medical Sciences)、Kota Okuno (Kitasato University Graduate School of Medical Sciences)、Yu Kuroda (Kitasato University Graduate School of Medical Sciences)、Keiko Oki (Kitasato University Graduate School of Medical Sciences)、Akiko Watanabe (Kitasato University Graduate School of Medical Sciences)、Shiori Fujino (Kitasato University Graduate School of Medical Sciences)、Takaaki Tokito (Kitasato University Department of Breast and Thyroid Surgery)、Mariko Kikuchi (Kitasato University Department of Breast and Thyroid Surgery)、Hiroshi Katoh (Kitasato University Department of Breast and Thyroid Surgery)、Naoki Hiki (Kitasato University Department of Upper Gastrointestinal Surgery)、Takafumi Sangai (Kitasato University Department of Breast and Thyroid Surgery)、Keishi Yamashita (Kitasato University , Center for New Century Medical Development)

Background: Single-cell RNA sequencing (scRNA-seq) enables gene expression profiling at single-cell resolution, aiding understanding of tumor heterogeneity. Objective: To identify gene expression features and prognostic markers in breast cancer (BC). Methods: scRNA-seq data from 26 BC cases (GSE176078) were analyzed using Seurat in R. Prognostic analysis used 637 BC cases (GSE199633) via JMP. Results: ***CXCL12*** was highly expressed in cancer-associated fibroblasts (CAFs), indicating dominance of inflammatory CAFs. ***KIT***-positive mast cells and ***CXCR2***-positive neutrophils were rare. ***MKI67*** formed clusters in T cells. Prognostic analysis showed low ***CD8A*** expression was linked to poor outcomes in triple-negative BC. High ***COL8A1*** expression, a myCAF marker, was associated with worse prognosis in luminal HER2 BC. In luminal BC, high ***MKI67*** expression also indicated poor prognosis. CellChat analysis revealed strong signaling from ***MKI67*** T cells to endothelial cells via ***CXCL13*** and ***CCL5***, suggesting enhanced angiogenesis. Conclusion: Distinct gene expression patterns in BC, especially involving CAFs and T cell interactions, may inform prognosis and therapeutic targets.

[Disclosure of COI by Responsible researcher] Lead presenter is not the Responsible researcher.
[Lead presenter: Kanako Naito] We have no financial relationships to disclose.
[Principal Researcher: Hiroyuki Minoura] We have no financial relationships to disclose.

### [P-3347] II-P6-3
### Molecular Profiling & Immune microenviroment in breast cancer

2025 / 9 / 27 (Sat) 16:15-17:00 Room P/(Motenashi Dome) Room P

---

### P-3347
### Association Between E-cadherin Expression and Tumor-Infiltrating Lymphocytes in Invasive Lobular Carcinoma

[Speaker]Yumiko Koi (Dept. Breast Surgery, NHO Kyushu Cancer Center) [Co-author]Katsumi Takizawa (Dept. Pathol, NHO Kyushu Cancer Center)、Wakako Tajiri (Dept. Breast Surgery, NHO Kyushu Cancer Center)、Sayuri Akiyoshi (Dept. Breast Surgery, NHO Kyushu Cancer Center)、Hideki Ijichi (Dept. Breast Surgery, NHO Kyushu Cancer Center)、Yoshiaki Nakamura (Dept. Breast Surgery, NHO Kyushu Cancer Center)、Chinami Koga (Dept. Breast Surgery, NHO Kyushu Cancer Center)、Kenichi Taguchi (Dept. Pathol, NHO Kyushu Cancer Center)、Eriko Tokunaga (Dept. Breast Surgery, NHO Kyushu Cancer Center)

E-cadherin is a cell adhesion molecule essential for epithelial structure, and its loss is a hallmark of invasive lobular carcinoma (ILC). Tumor-infiltrating lymphocytes (TILs) reflect host antitumor immunity; however, their relationship with E-cadherin in ILC is unclear. This study examined the association between E-cadherin and TILs in ILC. We retrospectively analyzed 201 patients with pStage I-III ILC who underwent curative surgery without neoadjuvant chemotherapy from 2000 to 2024. E-cadherin was evaluated by immunohistochemistry and categorized into four levels (0 to 3+) per ASCO/CAP HER2 scoring. TILs were assessed on H&E-stained sections per International TILs Working Group guidelines. Median age was 57, and most were hormone receptor (HR)-positive/HER2-negative (n=176). E-cadherin was 0 in 131 cases, 1+ in 26, 2+ in 41, and 3+ in 3 cases. Median TIL score was 4%, with no difference across subtypes. Multivariate analysis identified E-cadherin as the only independent factor related to TILs. Reduced TILs in E-cadherin-negative tumors might reflect immune suppression via epithelial-mesenchymal transition.

[Disclosure of COI by Responsible researcher] Lead presenter is not the Responsible researcher.
[Lead presenter: Yumiko Koi] We have no financial relationships to disclose.
[Principal Researcher: Eriko Tokunaga] We have no financial relationships to disclose.

### [P-3348] II-P6-3
### Molecular Profiling & Immune microenviroment in breast cancer

2025 / 9 / 27 (Sat) 16:15-17:00 Room P/(Motenashi Dome) Room P

---

### P-3348
### The influence of BRCA1/2 gene status on treatment strategies in patients with breast cancer

[Speaker]Rie Sugihara (Kurume Univ.Surg) [Co-author]Shuntaro Matsushima (Kurume Univ.Surg)、Ayano Nakamura (Kurume Univ.Surg)、Shuko Saku (Kurume Univ.Surg)、Sanae Numata (Kurume Univ.Cancer center)、Tomoya Sudo (Kurume Univ.Surg)、Uhi Toh (Kurume Univ.Surg)

Background:Since 2020 BRCA analysis testing and risk reduction mastectomy or risk reduction salphinco-oophorectomy were available in Japan. We investigated how the BRCA 1/2 status affects treatment strategies, particular impact in surgical procedures in pts with BC. Patients: 233 pts underwent BRCA analysis in Apr.2018- Aug.2024, 175 pts for HBOC diagnosis and 58 pts for diagnosing targeted therapy options(54 pts with recurrent metastatic(rm)BC, 4 pts with postoperative high recurrence risk). Result:31 pts were diagnosed as HBOC, the causative genes were BRCA1: 12 pts(38.7%) and BRCA2: 19 pts(61.2%). The most common BRCA1 /2mutation were c.188T>A(p.Leu63*) in 6 pts, and c.5576_5579del in 4 pts. The BRCA analysis performed for 5 pts with rmBC and for 26 pts with high recurrence risk perioperatively. Among these HBOC pts, 22 of 29(75.9%) pts changed their treatment strategies, including 8 pts switched the scheduled conserving surgery to total mastectomy, 9 and 21 pts underwent RRM and RRSO, respectively.Summary:BRCA1/2 gene testing has significant implications for surgical decisions in both the treatment of primary cancer and the prevention of future cancers in HBOC pts with BC.

[Disclosure of COI by Responsible researcher] Lead presenter is not the Responsible researcher.
[Lead presenter: Rie Sugihara] We have no financial relationships to disclose.
[Principal Researcher: Uhi Toh] We have no financial relationships to disclose.

▲TOP

### [P-3349] II-P6-3
### Molecular Profiling & Immune microenviroment in breast cancer

2025 / 9 / 27 (Sat) 16:15-17:00 Room P/(Motenashi Dome) Room P

---

### P-3349
### Clinical Significance of FOXA1 Expression in Breast Cancer: A Retrospective Analysis of Cases Without Systemic Therapy

[Speaker]Yuka Inoue (Breast Surgical Oncology, Cancer Institute Hospital of JFCR) [Co-author]Tomo Osako (Division of Pathology, Cancer Institute of JFCR)、Masashi Akiya (Division of Pathology, Cancer Institute of JFCR)、Tomohiro Chiba (Division of Pathology, Cancer Institute of JFCR)、Asumi Iesato (Breast Surgical Oncology, Cancer Institute Hospital of JFCR)、Meiko Nishimura (Breast Medical Oncology, Cancer Institute Hospital of JFCR)、Yukinori Ozaki (Breast Medical Oncology, Cancer Institute Hospital of JFCR)、Tetsuyo Maeda (Breast Surgical Oncology, Cancer Institute Hospital of JFCR)、Natsue Uehiro (Breast Surgical Oncology, Cancer Institute Hospital of JFCR)、Nami Yamashita (Breast Surgical Oncology, Cancer Institute Hospital of JFCR)、Takayuki Kobayashi (Breast Medical Oncology, Cancer Institute Hospital of JFCR)、Takehiko Sakai (Breast Surgical Oncology, Cancer Institute Hospital of JFCR)、Toshimi Takano (Breast Medical Oncology, Cancer Institute Hospital of JFCR)、Takayuki Ueno (Breast Surgical Oncology, Cancer Institute Hospital of JFCR)

FOXA1 is a key transcription factor associated with ER signaling and favorable prognosis in ER+HER2- breast cancer. Its role in other subtypes remains unclear, and studies excluding adjuvant therapy cases are limited. We analyzed FOXA1 expression in 758 cases treated with surgery alone between 1979-1982. Tissue microarrays were constructed, and all immunohistochemical and pathological features re-evaluated. Subtypes were ER+HER2- (71.3%), ER+HER2+ (6.2%), ER-HER2+ (9.8%), and ER-HER2- (12.7%). FOXA1 expression (cutoff 10%) was found in 96.35%, 100%, 92.86%, and 23.16% of these subtypes, respectively. No significant correlation between FOXA1 expression and overall survival was observed, including within ER+ tumors. However, in ER-HER2- cases, FOXA1 positivity suggested possible involvement in late recurrence. Our findings indicate FOXA1 is expressed across all subtypes. Although previously regarded as a favorable prognostic marker in ER+ tumors, analysis of historical cases without adjuvant therapy revealed no significant survival differences by FOXA1 status. These results suggest FOXA1 may reflect endocrine responsiveness rather than intrinsic tumor aggressiveness.

[Disclosure of COI by Responsible researcher] Lead presenter is the Responsible researcher.
[Lead presenter: Yuka Inoue] We have no financial relationships to disclose.

### [P-3350] II-P6-3
### Molecular Profiling & Immune microenviroment in breast cancer

2025 / 9 / 27 (Sat) 16:15-17:00 Room P/(Motenashi Dome) Room P

---

### P-3350
### Adipocytes induce CSC phenotype and tamoxifen resistance by activating PI3K/Akt/mTOR pathway in ER+ breast cancer cells

[Speaker]Masatoshi Nakatsuji (Faculty of Pharmacy, Osaka Medical and Pharmaceutical University) [Co-author]Ko Fujimori (Faculty of Pharmacy, Osaka Medical and Pharmaceutical University)

Resistance to endocrine therapy remains a major challenge in ER-positive breast cancer therapy. Obesity is associated with the clinical response in ER-positive breast cancer. In this study, we investigated the molecular mechanisms underlying obesity-induced resistance to tamoxifen (TAM) in the ER-positive breast cancer cell line MCF-7 using differentiated adipocyte-conditioned medium (D-CM). D-CM treatment promoted TAM resistance by decreasing TAM-induced apoptosis in MCF-7 cells. The expression of the markers for cancer stem-like cells (CSCs) and mammosphere formation was enhanced by co-treating with D-CM and TAM, compared with TAM alone. Moreover, the PI3K/Akt/mTOR pathway was activated by D-CM treatment. The combination of TAM with Akt or mTOR inhibitor decreased the expression levels of the CSC markers, suppressed mammosphere formation, and resensitized to TAM via inducing apoptosis in D-CM-treated MCF-7 cells. These results indicate that the conditioned medium of differentiated adipocytes promoted TAM resistance by elevating TAM-induced CSC phenotype through activation of the PI3K/Akt/mTOR pathway in ER-positive breast cancer cells.

[Disclosure of COI by Responsible researcher] Lead presenter is not the Responsible researcher.
[Lead presenter: Masatoshi Nakatsuji] We have no financial relationships to disclose.
[Principal Researcher: Ko Fujimori] ■Research expenses: 株式会社美山

### [P-3351] II-P6-3
### Molecular Profiling & Immune microenviroment in breast cancer

2025 / 9 / 27 (Sat) 16:15-17:00 Room P/(Motenashi Dome) Room P

### P-3351
### EGFR/SRC/STAT3 signaling promotes radioresistance and cancer stemness by regulating hexokinase2 expression in TNBC cells

[Speaker]YU HAO HUANG (Department of Biomedical Sciences, Chung Shan Medical University, Taichung, Taiwan) [Co-author]Yu-Ci Tu (Department of Biomedical Sciences, Chung Shan Medical University, Taichung, Taiwan)、Wen-Wei Chang (Department of Biomedical Sciences, Chung Shan Medical University, Taichung, Taiwan)

Triple-negative breast cancer (TNBC) is an aggressive subtype with high recurrence, metastasis, and radioresistance, partly driven by cancer stem cells (CSCs). In this study, a radioresistant MDA-MB-231 cell line (231-RR) was established, exhibiting enhanced CSC activity. EGFR/SRC/STAT3 signaling was significantly upregulated in 231-RR cells, increasing their sensitivity to SRC/STAT3 inhibitors. Overexpression of SRCY530F in parental 231 cells further enhanced CSC activity and STAT3 activation. RNA sequencing analysis from the Cancer Genome Atlas (TCGA) revealed glycolysis as a significantly upregulated pathway in high-SRC expression patients. Notably, inhibition of SRC/STAT3 signaling downregulated hexokinase-2 (HK-2), reducing CSC activity and the expression of pluripotency-associated transcription factors, including c-MYC and OCT4. Future studies will evaluate radiation efficacy in relation to EGFR/SRC/STAT3 activation and HK-2 expression in clinical specimens. These findings establish EGFR/SRC/STAT3 as a key driver of TNBC radioresistance, offering a foundation for novel therapies targeting metabolic vulnerabilities to improve treatment outcomes.

[Disclosure of COI by Responsible researcher] Lead presenter is not the Responsible researcher.
[Lead presenter: YU HAO HUANG] We have no financial relationships to disclose.
[Principal Researcher: Wen-Wei Chang] We have no financial relationships to disclose.

### [P-3352] II-P6-3
### Molecular Profiling & Immune microenviroment in breast cancer

2025 / 9 / 27 (Sat) 16:15-17:00 Room P/(Motenashi Dome) Room P

### P-3352
### Investigating the anticancer mechanism of Piperlongumine in Triple-negative breast cancer

[Speaker]Ming Yi Hsieh (Grad. Inst. of Med. Sci., College of Med.,TMU.／Dept. of Biochem. and Mol. Cell Biol.,College of Med., TMU.) [Co-author]Cheng Jui Huang (Dept. of Biochem. and Mol. Cell Biol.,College of Med., TMU.)、Cheng Wei Lin (Grad. Inst. of Med. Sci., College of Med.,TMU.／Dept. of Biochem. and Mol. Cell Biol.,College of Med., TMU.)

Triple-negative breast cancer (TNBC) is an aggressive subtype with high proliferation, invasion, metastasis, and drug resistance, yet lacks effective therapies. We investigated the anticancer effects of Piperlongumine (PL), a natural small-molecule compound from Piper longum. Our study showed that PL inhibits TNBC cell proliferation, induces apoptosis, and reduces migration, invasion, and EMT-related gene expression, including Twist, Snail, and Vimentin. Mechanistically, PL activates the LATS and AMPK signaling pathways, leading to phosphorylation and degradation of YAP, thereby inhibiting its nuclear translocation. Since YAP is a key regulator of cancer progression and metastasis, its inactivation by PL contributes to reduced tumor aggressiveness. We also identified Endothelin-1 (EDN1), a gene associated with poor TNBC prognosis and regulated by nuclear YAP, as a downstream target of PL. PL treatment significantly suppresses EDN1 expression via LATS/AMPK-mediated YAP inactivation. These findings suggest that PL disrupts the YAP/EDN1 axis and offers a novel therapeutic strategy for TNBC.

[Disclosure of COI by Responsible researcher] Lead presenter is the Responsible researcher.
[Lead presenter: Ming Yi Hsieh] We have no financial relationships to disclose.

### [P-3353] II-P6-3
### Molecular Profiling & Immune microenviroment in breast cancer

2025 / 9 / 27 (Sat) 16:15-17:00 Room P/(Motenashi Dome) Room P

---

### P-3353
### CDK4/6 Inhibitors retrial and therapy sequences after initial CDK4/6 Inhibitors in HR+, HER2- Advanced Breast Cancer

[Speaker]Muhan Yu (Chiba Univ. Grad. Sch. of Med. Dept. of General Surg.) [Co-author]Mamoru Takada (Chiba Univ. Grad. Sch. of Med. Dept. of General Surg.)、Takeshi Nagashima (Chiba Univ. Grad. Sch. of Med. Dept. of General Surg.)、Hiroshi Fujimoto (Chiba Univ. Grad. Sch. of Med. Dept. of General Surg.)、Junta Sakakibara (Chiba Univ. Grad. Sch. of Med. Dept. of General Surg.)、Ryotaro Teranaka (Chiba Univ. Grad. Sch. of Med. Dept. of General Surg.)、Hiroto Yamamoto (Chiba Univ. Grad. Sch. of Med. Dept. of General Surg.)、Masayuki Otsuka (Chiba Univ. Grad. Sch. of Med. Dept. of General Surg.)

This study explores factors influencing the effectiveness of CDK4/6 inhibitor (CDK4/6i) re-treatment in patients with HR+, HER2- advanced breast cancer (ABC) following disease progression. The analysis of clinicopathological factors and treatment history revealed that CDK4/6i re-treatment is more beneficial for patients with bone-only disease, prior fulvestrant treatment, and those who did not receive chemotherapy between CDK4/6i treatments. In contrast, patients with visceral disease, de novo metastases, or prior chemotherapy experienced shorter treatment durations, suggesting the need for alternative therapies. Tailoring treatment based on individual factors, such as disease type and previous treatment responses, may improve outcomes for patients undergoing CDK4/6i re-treatment. However, in patients who didn't undergo CDK4/6i retrial, once they started chemotherapy after CDK4/6i failed, their treatment will be chemotherapy centered. Chemotherapy becomes the primary treatment after CDK4/6i failure, limiting the potential for returning to hormone therapy. This study highlights the importance of personalized treatment strategies for patients with HR+, HER2- ABC.

[Disclosure of COI by Responsible researcher] Lead presenter is not the Responsible researcher.
[Lead presenter: Muhan Yu] We have no financial relationships to disclose.
[Principal Researcher: Takeshi Nagashima] We have no financial relationships to disclose.

### [P-3361] II-P7-1
### Omics Analyses in Gynecologic Cancers

2025 / 9 / 27 (Sat) 16:15-17:00 Room P/(Motenashi Dome) Room P

---

### P-3361
### Investigation of Choriocarcinoma-Associated miRNAs Through Small RNA Sequencing

[Speaker]Kosuke Yoshida (Dept. of Obstetrics and Gynecology, Nagoya University) [Co-author]Shohei Iyoshi (Dept. of Obstetrics and Gynecology, Nagoya University)、Kazumasa Mogi (Dept. of Obstetrics and Gynecology, Nagoya University)、Masato Yoshihara (Dept. of Obstetrics and Gynecology, Nagoya University)、Yukari Nagao (Dept. of Obstetrics and Gynecology, Nagoya University)、Satoshi Tamauchi (Dept. of Obstetrics and Gynecology, Nagoya University)、Akira Yokoi (Dept. of Obstetrics and Gynecology, Nagoya University)、Kaoru Niimi (Dept. of Obstetrics and Gynecology, Nagoya University)、Hiroaki Kajiyama (Dept. of Obstetrics and Gynecology, Nagoya University)

Despite the crucial role of miRNAs in cancer development, their functions in choriocarcinoma (CC) remain poorly understood. In this study, we aimed to identify CC-specific miRNAs and investigate their functional roles.We included 11 CC patients and 5 complete hydatidiform mole (CHM) patients. Total RNA was extracted from trophoblasts in FFPE specimens using laser capture microdissection. miRNA sequencing revealed 87 significantly upregulated and 28 downregulated miRNAs in CC compared to CHM. Notably, 13 of the 28 downregulated miRNAs were members of the Chromosome 19 miRNA cluster (C19MC). In vitro analyses showed that overexpression of miR-517a-3p, a miRNA from C19MC, significantly inhibited proliferation, migration, and invasion in CC cell lines. Furthermore, RNA sequencing and in silico analysis identified SRSF1 as a target gene. The downregulation of SRSF1 also resulted in significant reductions in proliferation, migration, and invasion. Immunohistochemistry confirmed that SRSF1 was overexpressed in CC compared to CHM.In conclusion, the downregulation of C19MC is a distinctive feature of CC, and miR-517a-3p acts as a tumor suppressor by targeting SRSF1.

[Disclosure of COI by Responsible researcher] Lead presenter is the Responsible researcher.
[Lead presenter: Kosuke Yoshida] We have no financial relationships to disclose.

### [P-3362] II-P7-1
### Omics Analyses in Gynecologic Cancers

2025 / 9 / 27 (Sat) 16:15-17:00 Room P/(Motenashi Dome) Room P

---

### P-3362
### Elucidation of Choriocarcinoma-Specific Genomic Profiles by Whole Exome Sequencing

[Speaker]Kaoru Niimi (Dept. Obst. & Gynecol., Nagoya Univ.) [Co-author]Yukari Oda (Dept. Obst. & Gynecol., Nagoya Univ.)、Yusuke Okuno (Dept. Virology, Nagoya City Univ.)、Yuko Yasui (Dept. Obst. & Gynecol., Nagoya Univ.)、Kosuke Yoshida (Dept. Obst. & Gynecol., Nagoya Univ.)、Masato Yoshihara (Dept. Obst. & Gynecol., Nagoya Univ.)、Satoshi Tamauchi (Dept. Obst. & Gynecol., Nagoya Univ.)、Akira Yokoi (Dept. Obst. & Gynecol., Nagoya Univ.)、Nobuhisa Yoshikawa (Dept. Obst. & Gynecol., Nagoya Univ.)、Eiko Yamamoto (Dept. Healthcare Administration, Nagoya Univ.)、Hiroaki Kajiyama (Dept. Obst. & Gynecol., Nagoya Univ.)

Objective: Genome analysis is increasingly used to reveal disease mechanisms and develop new treatments. However, studies on choriocarcinoma, a rare malignancy, are limited. This study aimed to investigate genetic alterations and drug resistance in choriocarcinoma using whole exome sequencing (WES).Methods: DNA was extracted from 14 choriocarcinoma samples (8 gestational, 6 non-gestational) and matched normal tissues. For gestational cases, normal DNA came from both patient and partner; for non-gestational, from the patient alone. WES was used to compare tumor and normal sequences. Ethical approval and informed consent were obtained.Results: In gestational choriocarcinoma, mutation frequency was higher in chemotherapy-treated cases than untreated ones. No such trend was found in non-gestational cases. No common mutations were shared across all samples. Six gestational cases had two novel mutations; all non-gestational cases shared two others. Copy number alterations (CNAs) were common, especially post-chemotherapy.Conclusion: Choriocarcinoma showed frequent CNAs but no consistent mutations, suggesting CNAs may drive tumorigenesis and drug resistance.

[Disclosure of COI by Responsible researcher] Lead presenter is the Responsible researcher.
[Lead presenter: Kaoru Niimi] We have no financial relationships to disclose.

### [P-3363] II-P7-1
### Omics Analyses in Gynecologic Cancers

2025 / 9 / 27 (Sat) 16:15-17:00 Room P/(Motenashi Dome) Room P

---

### P-3363
### Identification of choriocarcinoma specific molecular profile by proteome analysis

[Speaker]Yuko Yasui (Dept. Obst. & Gynecol. , Nagoya Univ. Grad. Sch. of Med.) [Co-author]Kaoru Niimi (Dept. Obst. & Gynecol. , Nagoya Univ. Grad. Sch. of Med.)、Shohei Iyoshi (Dept. Obst. & Gynecol. , Nagoya Univ. Grad. Sch. of Med.)、Kosuke Yoshida (Dept. Obst. & Gynecol. , Nagoya Univ. Grad. Sch. of Med.)、Mari Shirasaki (Dept. Obst. & Gynecol. , Nagoya Univ. Grad. Sch. of Med.)、Kimihiro Nishino (Dept. Health. Administration, Nagoya Univ. Grad. Sch. of Med.)、Eiko Yamamoto (Dept. Health. Administration, Nagoya Univ. Grad. Sch. of Med.)、Hiroaki Kajiyama (Dept. Obst. & Gynecol. , Nagoya Univ. Grad. Sch. of Med.)

[Objective] We aimed to identify the choriocarcinoma-specific protein profile through proteome analysis.[Methods] We used 5 samples of choriocarcinoma and 10 samples of complete hydatidiform moles. Of the cases of complete hydatidiform moles, 5 cases were in remission and 5 cases developed invasive moles after curettage. Proteins were extracted from the tissue samples of these cases, and shotgun proteomics using liquid chromatography-tandem mass spectrometry (LC-MS/MS) was performed.[Results] Proteome analysis identified 787 proteins. Volcano plot showed that 81 proteins were significantly overexpressed and 99 proteins were significantly downregulated in choriocarcinoma compared with complete hydatidiform moles. Moreover, we referred RNA-seq data of our previous study and narrow down the candidate proteins, FTL, SOD2, and GPX1.[Conclusion] Proteome and RNA-seq analysis identified five proteins, FTL, SOD2, and GPX1, were overexpressed in choriocarcinoma. These proteins may be involved in the progression of choriocarcinoma, and are expected to be applied to novel biomarkers and therapeutic targets.

[Disclosure of COI by Responsible researcher] Lead presenter is not the Responsible researcher.
[Lead presenter: Yuko Yasui] We have no financial relationships to disclose.
[Principal Researcher: Kaoru Niimi] We have no financial relationships to disclose.

### [P-3364] II-P7-1
### Omics Analyses in Gynecologic Cancers

2025 / 9 / 27 (Sat) 16:15-17:00 Room P/(Motenashi Dome) Room P

---

### P-3364
### Impact of Copy Number Variations on EV-miRNA Profiles in Ovarian Cancer

[Speaker]Hajime Araki (Nagoya University Graduate School of Medicine, Obstetrics and Gynecology) [Co-author]Akira Yokoi (Nagoya University Graduate School of Medicine, Obstetrics and Gynecology)、Kosuke Yoshida (Nagoya University Graduate School of Medicine, Obstetrics and Gynecology)、Eri Inami (Nagoya University Graduate School of Medicine, Obstetrics and Gynecology)、Masami Kitagawa (Nagoya University Graduate School of Medicine, Obstetrics and Gynecology)、Kazuhiro Suzuki (Nagoya University Graduate School of Medicine, Obstetrics and Gynecology)、Hironori Suzuki (Nagoya University Graduate School of Medicine, Obstetrics and Gynecology)、Hiroaki Kajiyama (Nagoya University Graduate School of Medicine, Obstetrics and Gynecology)

***TP53*** mutations with various copy number variations (CNVs) are major genomic alterations in ovarian cancer, yet the functional impact of CNVs remains unclear. This study aimed to explore how CNVs affect extracellular vesicle (EV)-derived miRNA profiles and their clinical relevance. ***TP53***-mutant ovarian cancer cases (n=247) from TCGA were classified into CNV-high (n=21) and CNV-low (n=62) groups based on the CNV calls, and the CNV-high group showed poorer overall survival. To examine CNV-associated miRNA alternation, we analyzed 23 ***TP53***-mutant ovarian cancer cell lines (CNV-high: n=17, CNV-low: n=6), and identified seven upregulated and nine downregulated miRNAs in the CNV-high group. Among them, miR-203a was more highly expressed in CNV-high cell lines and linked to poor prognosis. Its expression was also elevated in tumor tissues, patient serum, and EVs from some CNV-high cell lines. This elevated expression was also associated with increased cisplatin resistance. Furthermore, overexpression of miR-203a in CNV-low cell lines significantly enhanced cell proliferation. These findings suggest that CNV-associated miR-203a promotes malignancy and could be a therapeutic target.

[Disclosure of COI by Responsible researcher] Lead presenter is not the Responsible researcher.
[Lead presenter: Hajime Araki] We have no financial relationships to disclose.
[Principal Researcher: Kosuke Yoshida] We have no financial relationships to disclose.

### [P-3365] II-P7-1
### Omics Analyses in Gynecologic Cancers

2025 / 9 / 27 (Sat) 16:15-17:00 Room P/(Motenashi Dome) Room P

---

### P-3365
### Investigating the malignant mechanism of gastric-type endocervical adenocarcinoma

[Speaker]Hiroaki Yamada (Department of Obstetrics and Gynecology, Nagoya University School of Medicine) [Co-author]Akira Yokoi (Department of Obstetrics and Gynecology, Nagoya University School of Medicine)、Kosuke Yoshida (Department of Obstetrics and Gynecology, Nagoya University School of Medicine)、Kazuhiro Suzuki (Department of Obstetrics and Gynecology, Nagoya University School of Medicine)、Ryosuke Uekusa (Department of Obstetrics and Gynecology, Nagoya University School of Medicine)、Masami Kitagawa (Department of Obstetrics and Gynecology, Nagoya University School of Medicine)、Eri Inami (Department of Obstetrics and Gynecology, Nagoya University School of Medicine)、Naotake Tsuda (Department of Obstetrics and Gynecology, Kurume University School of Medicine)、Hiroaki Kajiyama (Department of Obstetrics and Gynecology, Nagoya University School of Medicine)

Gastric-type endocervical adenocarcinoma (GAS) is an HPV-independent cervical cancer with a worse prognosis than usual-type endocervical adenocarcinoma (UEA). This study investigated the function of GAS to identify disease mechanisms and therapeutic targets. mRNA-seq was performed in GAS, UEA, LEGH, and atypical LEGH (total n=18), and clustering analyses clearly distinguished each group. DESeq2 analysis revealed unique gene sets in GAS compared to LEGH or UEA, and the similarity of GAS gene profiles was closer to LEGH than UEA, suggesting GAS shared cell lineage with LEGH. ***PGC*** was identified as a GAS-unique gene, and RT-qPCR in GAS cell lines (KGAS) was also highly expressed than other cells. ***PGC*** are also highly expressed in GAS tissues by IHC staining. However, suppression of ***PGC*** did not affect cell proliferation and drug sensitivities in vitro. IPA analysis detected multiple activated upstream kinase activities in GAS, such as DYRK1A, RET, BRD4, and RAF1, and their inhibitors were effective KGAS cells in vitro. In conclusion, transcriptome analyses for GAS revealed novel GAS-specific biomarkers and therapeutic targets, contributing to improving the GAS patient prognosis.

[Disclosure of COI by Responsible researcher] Lead presenter is not the Responsible researcher.
[Lead presenter: Hiroaki Yamada] We have no financial relationships to disclose.
[Principal Researcher: Kosuke Yoshida] We have no financial relationships to disclose.

### [P-3366] II-P7-1
### Omics Analyses in Gynecologic Cancers

2025 / 9 / 27 (Sat) 16:15-17:00 Room P/(Motenashi Dome) Room P

---

### P-3366
### Genomic and molecular profiles of undifferentiated/dedifferentiated endometrial carcinomas

[Speaker]Tetsuro Shiraishi (Dept. Gynecology., Natl. Cancer Ctr. Hosp.／Dept. Obstet.&Gynecol., Keio Univ. School of Medicine.) [Co-author]Mayumi Kato (Dept. Gynecology., Natl. Cancer Ctr. Hosp.)、Ai Ikki (Dept. Clin. Genomics., Natl. Cancer Ctr. Res. Inst.)、Daiki Higuchi (Div. Genome Biol., Natl. Cancer Ctr. Res. Inst.)、Maiko Yamaguchi (Div. Genome Biol., Natl. Cancer Ctr. Res. Inst.)、Takayuki Kawai (Div. Genome Biol., Natl. Cancer Ctr. Res. Inst.)、Takafumi Koyama (Dept. Experimental Therapeutics., Natl. Cancer Ctr. Hosp.)、Masaaki Komatsu (Dept. AI Research and Development., Natl. Cancer Ctr. Res. Inst.)、Ryuji Hamamoto (Dept. AI Research and Development., Natl. Cancer Ctr. Res. Inst.)、Akihide Yoshimi (Dept. Cancer RNA Research., Natl. Cancer Ctr. Res. Inst.)、Wataru Yamagami (Dept. Obstet.&Gynecol., Keio Univ. School of Medicine.)、Takashi Kohno (Div. Genome Biol., Natl. Cancer Ctr. Res. Inst.)、Mitsuya Ishikawa (Dept. Gynecology., Natl. Cancer Ctr. Hosp.)、Hiroshi Yoshida (Dept. Diagnostic Path., Natl. Cancer Ctr. Hosp.)、Kouya Shiraishi (Dept. Clin. Genomics., Natl. Cancer Ctr. Res. Inst.／Div. Genome Biol., Natl. Cancer Ctr. Res. Inst.)

Undifferentiated/dedifferentiated endometrial carcinoma of uteri (UDEC) is rare, has an aggressive growth pattern, tends to be diagnosed at an advanced stage, and is resistant to conventional chemotherapies. Genome analysis focusing on Japanese UDEC has seldom been reported. We investigated the distribution of genomic alterations in UDEC registered in Japan's Center for Cancer Genomics and Advanced Therapeutics (C-CAT) database (IRB number 2017-331 and 2020-067). Forty patients with UDEC were included in the C-CAT database. According to the ProMiSE classifications, ***POLE*** mutations (5%), high-frequency microsatellite instability (30%), p53-abnormality (32.5%), and no specific molecular profile (32.5%) were detected. In addition, 60% of patients with UDEC had oncogenic mutations in SWI/SNF chromatin remodeling complex members, including ***ARID1A/B***, ***SMARCA4***, and ***SMARCB1***, higher than those in endometrioid carcinoma. Furthermore, we are currently preparing and analyzing WES for UDEC cases diagnosed at our hospital. About half of UDEC are deficient in members of the SWI/SNF chromatin remodeling complex and may be therapeutic targets.

[Disclosure of COI by Responsible researcher] Lead presenter is not the Responsible researcher.
[Lead presenter: Tetsuro Shiraishi] We have no financial relationships to disclose.
[Principal Researcher: Kouya Shiraishi] We have no financial relationships to disclose.

### [P-3367] II-P7-1
### Omics Analyses in Gynecologic Cancers

2025 / 9 / 27 (Sat) 16:15-17:00 Room P/(Motenashi Dome) Room P

---

### P-3367
### Metabolomic and transcriptomic analyses identify metabolic alterations and immune suppression in ovarian cancer

[Speaker]Maiko Yamaguchi (Division of Genome Biology, National Cancer Center Research Institute／Department of Obstetrics and Gynecology, Juntendo University Faculty of Medicine) [Co-author]Daiki Higuchi (Division of Genome Biology, National Cancer Center Research Institute／Department of Obstetrics and Gynecology, Showa University School of Medicine)、Erisa Fujii (Department of Gynecology, National Cancer Center Hospital)、Kengo Hiranuma (Department of Obstetrics and Gynecology, Juntendo University Faculty of Medicine)、Yuka Asami (Department of Obstetrics and Gynecology, Showa University School of Medicine)、Hanako Ono (Department of Clinical Genomics, National Cancer Center Research Institute)、Masaaki Komatsu (Division of Medical AI Research and Development, NCC Research Institute／Cancer Translational Research Team, RIKEN Center for Advanced Intelligence Project)、Ryuji Hamamoto (Division of Medical AI Research and Development, NCC Research Institute／Cancer Translational Research Team, RIKEN Center for Advanced Intelligence Project)、Yasuhisa Terao (Department of Obstetrics and Gynecology, Juntendo University Faculty of Medicine)、Koji Matsumoto (Department of Obstetrics and Gynecology, Showa University School of Medicine)、Mitsuya Ishikawa (Department of Gynecology, National Cancer Center Hospital)、Takashi Kohno (Division of Genome Biology, National Cancer Center Research Institute)、Hiroshi Yoshida (Department of Diagnostic Pathology, National Cancer Center Hospital)、Hideki Makinoshima (Tsuruoka Metabolomics Laboratory, National Cancer Center Tsuruoka)、Tomoyasu Kato (Department of Gynecology, National Cancer Center Hospital)、Kouya Shiraishi (Division of Genome Biology, National Cancer Center Research Institute／Department of Clinical Genomics, National Cancer Center Research Institute)

To identify metabolic alterations and related phenotypes specific to ovarian cancer (OC), we performed metabolomic analyses of OC tissues, non-cancerous ovarian tissues, and plasma from patients with OC or benign ovarian tumors. The relationship with transcriptomics was investigated using transcriptome analysis. Fifty-one patients with OC and three with benign tumors, diagnosed between 2011 and 2014, were enrolled. Gas chromatography-mass spectrometry was used for metabolomic analysis of plasma (n=54) and tissues (n=44). Transcriptomic analysis was conducted using cancerous tissues from 39 OC patients. As plasma data could not classify patients, OC patients were classified into two groups, hypermetabolic and hypometabolic, based on tissue analysis alone. The hypermetabolic group had more advanced-stage tumors (P = 0.02). Transcriptome pathway analysis revealed the suppression of immunity-related pathways. Furthermore, the percentage of NK cells in the hypermetabolic group was lower than that in the hypometabolic group (P = 0.04). Thus, these findings suggest that hypermetabolism may be directly or indirectly involved in suppression of immune activity.

[Disclosure of COI by Responsible researcher] Lead presenter is not the Responsible researcher.
[Lead presenter: Maiko Yamaguchi] We have no financial relationships to disclose.
[Principal Researcher: Kouya Shiraishi] We have no financial relationships to disclose.

[P-3368] II-P7-1
Omics Analyses in Gynecologic Cancers

2025 / 9 / 27 (Sat) 16:15-17:00 Room P/(Motenashi Dome) Room P

P-3368
### Landscape of genetic alterations associated with early-onset ovarian cancer development

[Speaker]Daiki Higuchi (Division of Genome Biology, National Cancer Center Research Institute／Department of Obstetrics and Gynecology, Showa Medical University) [Co-author]Ai Ikki (Department of Clinical Genomics, National Cancer Center Research Institute／Department of Obstetrics and Gynecology, Nippon Medical School)、Maiko Yamaguchi (Division of Genome Biology, National Cancer Center Research Institute／Department of Obstetrics and Gynecology, Juntendo University Faculty of Medicine)、Takayuki Kawai (Division of Genome Biology, National Cancer Center Research Institute／Department of Obstetrics and Gynecology, Juntendo University Faculty of Medicine)、Tetsuro Shiraishi (Department of Gynecology, National Cancer Center Hospital)、Mayumi Kato (Department of Gynecology, National Cancer Center Hospital)、Koji Matsumoto (Department of Obstetrics and Gynecology, Showa Medical University)、Kosei Hasegawa (Gynecologic Oncology, Saitama Medical University International Medical Center)、Haruya Saji (Department of Gynecology, Kanagawa Cancer Center Research Institute)、Shu Soeda (Department of OG, Fukushima Medical University School of Medicine)、Koichi Matsuda (Computational Biology and Medical Sciences, The University of Tokyo)、Aikou Okamoto (Department of OG, The Jikei University School of Medicine)、Takashi Kohno (Division of Genome Biology, National Cancer Center Research Institute)、Mitsuya Ishikawa (Department of Gynecology, National Cancer Center Hospital)、Hiroshi Yoshida (Department of Diagnostic Pathology, National Cancer Center Hospital)、Kouya Shiraishi (Division of Genome Biology, National Cancer Center Research Institute／Department of Clinical Genomics, National Cancer Center Research Institute)

Pathogenic germline variants (PGVs) in BRCA1 and BRCA2 are well-established risk factors for ovarian cancer, particularly in women aged > 50 years. However, the genetic background of adolescents and young adults (AYA; <40 years) with ovarian cancer remains poorly defined. Moreover, large-scale analyses of structural variants (SVs) in ovarian cancer are limited.In this study, we analyzed 230 cases of ovarian and borderline tumors using next-generation sequencing to identify SNVs/INDELs and SVs. BRCA1 and BRCA2 variants were found in six (2.6%) and two (0.9%) cases, respectively (total: 3.5%), significantly lower than previous reports across all age groups (15%; p < 0.001), especially among AYA cases.POLH variants were observed in 8 cases (3.5%), most frequently in this cohort. Additionally, PGVs in Lynch syndrome-related genes were found in four cases (1.7%), and PGVs in PRSS1 and SPINK1, previously unlinked to ovarian cancer, were also detected.These findings suggest that early-onset ovarian cancer may have a distinct genetic profile. SVs analysis is underway and will be presented at a conference.

[Disclosure of COI by Responsible researcher] Lead presenter is not the Responsible researcher.
[Lead presenter: Daiki Higuchi] We have no financial relationships to disclose.
[Principal Researcher: Kouya Shiraishi] We have no financial relationships to disclose.

### [P-3377] II-P7-3
### Molecular Mechanisms & Therapy in Gynecologic cancer

2025 / 9 / 27 (Sat) 16:15-17:00 Room P/(Motenashi Dome) Room P

### P-3377
### Expression of iron exporter ferroportin and its role in ovarian cancer

[Speaker]Yuki Minamikawa (Dept. Anatomic Pathol., Grad. Sch. Med., Tohoku. univ.) [Co-author]Erina Iwabuchi (Dept. Morphological Analysis., Grad. Med. & Dent., Science Tokyo. univ.)、Yasuhiro Miki (Dept. Anatomic Pathol., Grad. Sch. Med., Tohoku. univ.)、Yoshiaki Onodera (Dept. Anatomic Pathol., Grad. Sch. Med., Tohoku. univ.)、Yusuke Shibuya (Dept. Obstetrics & Gynecology., Grad. Sch. Med., Tohoku. univ.／Dept. Obstetrics & Gynecology., Tohoku. Hosp.)、Muneaki Shimada (Dept. Obstetrics & Gynecology., Grad. Sch. Med., Tohoku. univ.／Dept. Obstetrics & Gynecology., Tohoku. Hosp.／Advanced Research Center for Innovations in Next-Generation Medicine, Tohoku. univ.)、Takashi Suzuki (Dept. Anatomic Pathol., Grad. Sch. Med., Tohoku. univ.／Dept. Pathol & Histotechnology., Grad. Sch. Med., Tohoku. univ.／Dept. Pathol., Tohoku. Hosp.)

It has been revealed that iron is involved in the metastasis and recurrence of ovarian cancer. Ferroportin acts as an iron efflux transporter and plays a role in regulating intracellular iron concentration. It is known that ferroportin expression is decreased in ovarian cancer compared to normal ovarian tissue. The purpose of this study was to clarify the significance of ferroportin expression in ovarian cancer. The expression of ferroportin was evaluated by immunohistochemical staining in 89 cases of ovarian cancer (56 cases of high-grade serous carcinoma, 33 cases of clear cell carcinoma). The ferroportin-positive group had significantly fewer distant metastatic cases ($p = 0.023$). Furthermore, survival analysis showed a tendency for overall survival to be longer in ferroportin-positive ovarian cancer ($p = 0.1323$). These results suggest that ferroportin is involved in suppressing metastasis in ovarian cancer and may contribute to prolonging prognosis. The effects of ferroportin knockdown on proliferation and invasion will be examined using cell lines. Furthermore, it is necessary to clarify the relationship between ferroportin expression and intracellular iron concentration.

[Disclosure of COI by Responsible researcher] Lead presenter is not the Responsible researcher.
[Lead presenter: Yuki Minamikawa] We have no financial relationships to disclose.
[Principal Researcher: Yasuhiro Miki] We have no financial relationships to disclose.

### [P-3378] II-P7-3
### Molecular Mechanisms & Therapy in Gynecologic cancer

2025 / 9 / 27 (Sat) 16:15-17:00 Room P/(Motenashi Dome) Room P

---

### P-3378
### Effect of p53 aggregation on the expression of 6-sulfo sialyl glycans in p53-mutated cancer

[Speaker]Kazuchika Nishitsuji (Wakayama Med. Univ., Dept. Biochem.／CNRS, Univ. Lille, UMR 8576-UGSF) [Co-author]Midori Ikezaki (Wakayama Med. Univ., Dept. Biochem.)、Naoyuki Iwahashi (Wakayama Med. Univ., Dept. Obstet. Gynecol.)、Megumi Fujino (Wakayama Med. Univ., Dept. Obstet. Gynecol.)、Kaho Nishioka (Wakayama Med. Univ., Dept. Obstet. Gynecol.)、Masakazu Fujimoto (Kyoto Univ., Dept. Diagn. Pathol.)、Noa Mori (Wakayama Med. Univ., Dept. Biochem.)、Yoshiko Uchimura (CNRS, Univ. Lille, UMR 8576-UGSF)、Yoshito Ihara (Wakayama Med. Univ., Dept. Biochem.)、Yukio Fujiwara (Kumamoto Univ., Dept. Cell Pathol.)、Yoshihiro Komohara (Kumamoto Univ., Dept. Cell Pathol.)、Kazuhiko Ino (Wakayama Med. Univ., Dept. Obstet. Gynecol.)、Kenji Uchimura (CNRS, Univ. Lille, UMR 8576-UGSF)

Mutant p53 proteins form protein aggregates and the presence of cytoplasmic p53 aggregates correlates with poor prognosis in several cancers. Currently, the relationship between p53 aggregation and the expression of aberrant glycan structures, another characteristic of many cancers, is unknown. Here, we investigated the existence of sulfated and sialylated glycans in ovarian cancer by using the CL40 antibody. CL40 recognizes 6-sulfo sialyl Lewis X and 6-sulfo sialyl LacNAc with an absolute dependency on GlcNAc 6-***O***-sulfation. CL40-positive cancer cells were present in primary or peritoneal- or omentum-disseminated ovarian cancer tissues. We further showed that the expression of CL40-reactive glycans and the mRNA expression of GlcNAc-6-***O***-sulfotransferase genes were reduced by preventing p53 aggregation by using ovarian cancer-derived OVCAR-3 cells that harbor R248Q mutant p53 aggregates. Overall, our results suggest a novel function for p53 aggregation in regulating the synthesis of CL40-reactive 6-***O***-sulfated sialyl glycans. Future work that clarifies the role of CL40-reactive glycans in cancer pathology is necessary.

[Disclosure of COI by Responsible researcher] Lead presenter is the Responsible researcher.
[Lead presenter: Kazuchika Nishitsuji] We have no financial relationships to disclose.

### [P-3379] II-P7-3
### Molecular Mechanisms & Therapy in Gynecologic cancer

2025 / 9 / 27 (Sat) 16:15-17:00 Room P/(Motenashi Dome) Room P

---

### P-3379
### Exploration of LRRN4 as a Marker for Normal Mesothelial Cells

[Speaker]Maia Hayashi (Obstetrics and Gynecology, Nagoya University Graduate School of Medicine) [Co-author]Masato Yoshihara (Obstetrics and Gynecology, Nagoya University Graduate School of Medicine)、Shohei Iyoshi (Obstetrics and Gynecology, Nagoya University Graduate School of Medicine)、Kazumasa Mogi (Obstetrics and Gynecology, Nagoya University Graduate School of Medicine)、Emiri Miyamoto (Obstetrics and Gynecology, Nagoya University Graduate School of Medicine)、Hiroki Fujimoto (Obstetrics and Gynecology, Nagoya University Graduate School of Medicine)、Kaname Uno (Obstetrics and Gynecology, Nagoya University Graduate School of Medicine)、Yoshihiro Koya (Obstetrics and Gynecology, Nagoya University Graduate School of Medicine)、Atsushi Kunishima (Obstetrics and Gynecology, Nagoya University Graduate School of Medicine)、Marina Yoshikawa (Obstetrics and Gynecology, Nagoya University Graduate School of Medicine)、Sae Hayakawa (Obstetrics and Gynecology, Nagoya University Graduate School of Medicine)、Hiroaki Kajiyama (Obstetrics and Gynecology, Nagoya University Graduate School of Medicine)

Ovarian cancer is the most prognostically unfavorable gynecologic malignancy, largely due to its late-stage diagnosis following extensive intraperitoneal metastasis. Previous studies have shown that peritoneal fibrosis and mesothelial-to-mesenchymal transition (MMT) of peritoneal mesothelial cells, triggered by soluble factors secreted by ovarian cancer cells, contribute to peritoneal dissemination and disease progression. Based on this background, the present study aimed to identify specific markers distinguishing normal from mesenchymally transformed mesothelial cells and to investigate their biological roles. Comparative analyses revealed that LRRN4 is specifically expressed in normal peritoneal mesothelial cells. Its expression was downregulated under stress conditions and absent in other peritoneal cell types or cancer cells. Functional analyses using LRRN4 knockdown and overexpression cell models identified signaling pathways and interacting proteins regulated by LRRN4. These findings suggest that LRRN4 is a reliable marker for normal peritoneal mesothelial cells and a potential therapeutic target in ovarian cancer by modulating mesothelial cell function.

[Disclosure of COI by Responsible researcher] Lead presenter is not the Responsible researcher.
[Lead presenter: Maia Hayashi] We have no financial relationships to disclose.
[Principal Researcher: Masato Yoshihara] ■Research funds from company: 科研費

### [P-3380] II-P7-3
### Molecular Mechanisms & Therapy in Gynecologic cancer

2025 / 9 / 27 (Sat) 16:15-17:00 Room P/(Motenashi Dome) Room P

---

### P-3380
### Immune microenvironment in uterine carcinosarcoma

[Speaker]Haruko Okamoto (Kyoto university Medical Gynecol.) [Co-author]Mana Taki (Kyoto university Medical Gynecol.)、Taito Miyamoto (Kyoto university Medical Gynecol.)、Rin Mizuno (Kyoto university Medical Gynecol.)、Koji Yamanoi (Kyoto university Medical Gynecol.)、Ryusuke Murakami (Kyoto university Medical Gynecol.)、Ken(Kyoto Univ.) Yamaguchi (Kyoto university Medical Gynecol.)、Junzo Hamanishi (Kyoto university Medical Gynecol.)、Masaki Mandai (Kyoto university Medical Gynecol.)

[Objective] Uterine carcinosarcoma (UCS) is a rare endometrial cancer with the poorest prognosis, characterized by strong "tumor heterogeneity" comprising carcinoma and sarcoma components. We hypothesize that "immune heterogeneity" also affects prognosis and aimed to clarify the immunosuppressive mechanism. [Method] To compare the tumor immune environment with uterine serous carcinoma (USC), another aggressive endometrial cancer, we performed immunohistochemistry on UCS (55 cases) and USC (49 cases) specimens from our hospital. We also focused on SPARC, predominantly expressed in UCS sarcoma component, and generated SPARC-knockdown cell lines. A mouse immune model assessed tumor immunity. [Results] UCS had fewer $CD8^{+}$T cells and more $CD206^{+}$M2 macrophages than USC, especially in the sarcoma component. In mice, SPARC-knockdown tumors showed suppressed growth, more $CD8^{+}$T cells, and fewer M2 macrophages. A cytokine array revealed lower C5a levels in tumors and blood from the SPARC-knockdown group. [Conclusion] These findings suggest that SPARC, highly expressed in sarcoma, promotes M2 macrophage accumulation via C5a, contributing to UCS immunosuppression.

[Disclosure of COI by Responsible researcher] Lead presenter is the Responsible researcher.
[Lead presenter: Haruko Okamoto] We have no financial relationships to disclose.

### [P-3381] II-P7-3
### Molecular Mechanisms & Therapy in Gynecologic cancer

2025 / 9 / 27 (Sat) 16:15-17:00 Room P/(Motenashi Dome) Room P

---

### P-3381
### Histone methyltransferase SUV39H2 is associated with tumor growth and metastasis in uterine endometrial serous carcinoma

[Speaker]Yuri Jonouchi (The University of Tokyo Hospital Obstetrics and Gynecology) [Co-author]Kenbun Sone (The University of Tokyo Hospital Obstetrics and Gynecology)、Saki Aota (The University of Tokyo Hospital Obstetrics and Gynecology)、Ryuta Hachijo (The University of Tokyo Hospital Obstetrics and Gynecology)、Eri Suzuki (The University of Tokyo Hospital Obstetrics and Gynecology)、Aya Ishizaka (The University of Tokyo Hospital Obstetrics and Gynecology)、Minami Hayashi (The University of Tokyo Hospital Obstetrics and Gynecology)、Natsumi Tsuboyama (The University of Tokyo Hospital Obstetrics and Gynecology)、Saki Tanimoto (The University of Tokyo Hospital Obstetrics and Gynecology)、Saki Tsuchimochi (The University of Tokyo Hospital Obstetrics and Gynecology)、Katsutoshi Oda (The University of Tokyo Hospital Department of clinical genomics)、Yasushi Hirota (The University of Tokyo Hospital Obstetrics and Gynecology)、Yutaka Osuga (The University of Tokyo Hospital Obstetrics and Gynecology)

<Background>Among uterine endometrial cancers, serous carcinoma has a high risk of metastatic recurrence and a poor prognosis. The Histone methyltransferase SUV39H2 has been reported to be related in tumor growth and metastasis in many cancers. We investigated whether SUV39H2 is a therapeutic target in serous carcinoma.<Method>We targeted SUV39H2, which is highly expressed in serous carcinoma in the database, and analyzed its expression in clinical samples by qRT-PCR and immunohistochemistry. Next, we examined the antitumor effects of SUV39H2 inhibitor and knockdown of SUV39H2 by siRNA and EMT markers. And we used RNA-seq to search for downstream genes whose expression is regulated by SUV39H2.<Results>SUV39H2 was significantly expressed in serous carcinoma compared with normal endometrium. In serous carcinoma cell lines, SUV39H2 inhibitor and knockdown of SUV39H2 by siRNA inhibited cell growth and induced apoptosis. Western blot, Immunoblotting, scratch assays and invasion assays confirmed that SUV39H2 induces EMT involved in metastasis. RNA- seq showed changes in genes related to apoptosis and EMT.<Conclusion>SUV39H2 is a potential new therapeutic target in serous carcinoma.

[Disclosure of COI by Responsible researcher] Lead presenter is not the Responsible researcher.
[Lead presenter: Yuri Jonouchi] We have no financial relationships to disclose.
[Principal Researcher: Kenbun Sone] ■Research funds from company: 文科省科研費、政府系委託費

▲TOP

### [P-3382] II-P7-3
### Molecular Mechanisms & Therapy in Gynecologic cancer

2025 / 9 / 27 (Sat) 16:15-17:00 Room P/(Motenashi Dome) Room P

---

### P-3382
### Elucidation of the role of L1CAM-NF-κB pathway in human endometrial cancer

[Speaker]Hiroyuki Kurosu (Dept. of Obstet. & Gynecol. Hokkaido Univ. Grad. Sch. of Med.／Dept. of Path. Hokkaido Univ. Grad. Sch. of Med.)
[Co-author]Hiroshi Asano (Dept. of Obstet. & Gynecol. Hokkaido Univ. Grad. Sch. of Med.)、Shugo Tanaka (Dept. of Path. Hokkaido Univ. Grad. Sch. of Med.)、Issei Kawakita (Dept. of Path. Hokkaido Univ. Grad. Sch. of Med.)、Kentaro Kumagai (Dept. of Path. Hokkaido Univ. Grad. Sch. of Med.)、Kensuke Nakazono (Dept. of Path. Hokkaido Univ. Grad. Sch. of Med.)、Rino Saito (Dept. of Path. Hokkaido Univ. Grad. Sch. of Med.)、Chihiro Terasaka (Dept. of Path. Hokkaido Univ. Grad. Sch. of Med.)、Kazuya Hamada (Dept. of Path. Hokkaido Univ. Grad. Sch. of Med.)、Sari Iwasaki (Dept. of Path. Hokkaido Univ. Grad. Sch. of Med.)、Satoshi Tanaka (Dept. of Path. Hokkaido Univ. Grad. Sch. of Med.)、Saki Yamamoto (Dept. of Obstet. & Gynecol. Hokkaido Univ. Grad. Sch. of Med.)、Hidemichi Watari (Dept. of Obstet. & Gynecol. Hokkaido Univ. Grad. Sch. of Med.)、Koji Taniguchi (Dept. of Path. Hokkaido Univ. Grad. Sch. of Med.)

In 2023, FIGO staging of endometrial cancer was revised based on TCGA classification. L1CAM is not included in this classification, but in endometrial cancer, there have been reports that high expression of L1CAM is associated with metastasis, advanced tumor stage, and resistance to chemotherapy. L1CAM is a transmembrane glycoprotein belonging to the immunoglobulin superfamily, and is involved in various cellular functions such as cell adhesion, migration, and signal transduction. The aim of our study is to investigate the mechanisms by which L1CAM acts as a poor prognostic factor in endometrial cancer. We generated L1CAM knocked-down cell lines and L1CAM overexpression cell lines using the endometrial cancer cell lines (HHUA, SPAC-1-L, Ishikawa), and several assays evaluated these cancer cell lines. We found that L1CAM knockdown decreases cancer cell proliferation without affecting cell survival. Conversely, overexpressing L1CAM increased the proliferation of cancer cells. RNA-seq signal pathway analysis suggests that L1CAM knockdown may affect the NF-κB pathway. We are conducting additional experiments to investigate the role of L1CAM in this pathway.

[Disclosure of COI by Responsible researcher] Lead presenter is not the Responsible researcher.
[Lead presenter: Hiroyuki Kurosu] We have no financial relationships to disclose.
[Principal Researcher: Koji Taniguchi] We have no financial relationships to disclose.

▲TOP

### [P-3383] II-P7-3
### Molecular Mechanisms & Therapy in Gynecologic cancer

2025 / 9 / 27 (Sat) 16:15-17:00 Room P/(Motenashi Dome) Room P

---

### P-3383
### Expression and Function of Malic Enzyme 1 in Endometrial Cancer

[Speaker]Natsuki Uchiyama (Dept. of Obstetrics and Gynecology, Shinshu Univ.) [Co-author]Tsutomu Miyamoto (Dept. of Obstetrics and Gynecology, Shinshu Univ.)、Ryoichi Asaka (Dept. of Obstetrics and Gynecology, Shinshu Univ.)、Manaka Shinagawa (Dept. of Obstetrics and Gynecology, Shinshu Univ.)、Yusuke Yokokawa (Dept. of Obstetrics and Gynecology, Shinshu Univ.)、Kyosuke Kamijo (Dept. of Obstetrics and Gynecology, Shinshu Univ.)、Marina Fujioka (Dept. of Obstetrics and Gynecology, Shinshu Univ.)

**Objective** : Malic Enzyme 1 (ME1) catalyzes malate to pyruvate, playing a key role in energy metabolism. This study examined the expression and function of ME1 in endometrial carcinoma (EC). **Methods**: We analyzed ME1 expression by immunohistochemistry (IHC) in 172 EC and 6 normal endometria (NE) as control with ethics approval. We assessed proliferation and medroxyprogesterone acetate (MPA) sensitivity via WST-1 assay in HEC1A, Ishikawa, and HEC265 cells with or without ME1 knockdown. Furthermore, the ME1 inhibitor was used with MPA in Ishikawa cells. **Results**: IHC revealed that ME1 H-score is higher in EC than NE (75.7 vs. 2.0; $p<0.05$) and high ME1 expression correlated with shorter progression-free survival ($p<0.05$). ME1 knockdown reduced proliferation in HEC1A, Ishikawa, and HEC265 (29.9%, 53.4%, and 18.7% down, respectively) ($p<0.05$) and enhanced MPA-associated growth inhibition (49.5%, 50.7%, and 72.8% down, respectively) ($p<0.05$). In Ishikawa cells, an ME1 inhibitor also enhanced MPA-associated growth inhibition ($p<0.05$). **Conclusion**: High ME1 expression is associated with poor prognosis, and ME1 inhibition reduced cell growth, suggesting ME1 as a therapeutic target in EC.

[Disclosure of COI by Responsible researcher] Lead presenter is not the Responsible researcher.
[Lead presenter: Natsuki Uchiyama] We have no financial relationships to disclose.
[Principal Researcher: Tsutomu Miyamoto] We have no financial relationships to disclose.

Poster Session

### [P-3384] II-P7-3
### Molecular Mechanisms & Therapy in Gynecologic cancer

2025 / 9 / 27 (Sat) 16:15-17:00 Room P/(Motenashi Dome) Room P

---

### P-3384
### Impact of Histological Type on MRI-Based Evaluation of Tumor Size and LVSI in Early-Stage Cervical Cancer

[Speaker]Kazuhiro Kou (Dept. Gynecology and Obstetrics. Kyoto University Graduate School of Medicine.) [Co-author]Koji Yamanoi (Dept. Gynecology and Obstetrics. Kyoto University Graduate School of Medicine.)、Nozomi Higashiyama (Dept. Gynecology and Obstetrics. Kyoto University Graduate School of Medicine.)、Taito Miyamoto (Dept. Gynecology and Obstetrics. Kyoto University Graduate School of Medicine.)、Rin Mizuno (Dept. Gynecology and Obstetrics. Kyoto University Graduate School of Medicine.)、Mana Taki (Dept. Gynecology and Obstetrics. Kyoto University Graduate School of Medicine.)、Ryusuke Murakami (Dept. Gynecology and Obstetrics. Kyoto University Graduate School of Medicine.)、Ken(Kyoto Univ.) Yamaguchi (Dept. Gynecology and Obstetrics. Kyoto University Graduate School of Medicine.)、Junzo Hamanishi (Dept. Gynecology and Obstetrics. Kyoto University Graduate School of Medicine.)、Masaki Mandai (Dept. Gynecology and Obstetrics. Kyoto University Graduate School of Medicine.)

Purpose: This study examined whether detailed MRI interpretation can predict pathological factors relevant to treatment choice.Methods: We retrospectively analyzed 290 cervical cancer cases (FIGO 2008 stage ≤IB1) treated at two centers from 2006–2020. Tumor size and LVSI were extracted from pathology reports, and MRI was reviewed for tumor visibility and size. Sensitivity to tumor diameter <2cm on specimen and LVSI status were compared between SCC and non SCC across four MRI based tumor size groups: invisible, <1cm, 1–2cm, and >2cm.Results: In SCC, sensitivity markedly declined with tumor size, from 9/10, 90% at <1cm to 9/56, 16% at >2cm. In non SCC, it remained stable at <2cm (<1cm; 7/9, 78%, 1–2cm; 10/15, 67%), but decreased to 5/30, 17% at >2cm. In SCC, LVSI increased from 1/10, 10% at <1cm to 22/33, 67% at 1–2cm, remaining high at 29/56, 52% at >2cm. Non SCC showed low LVSI across sizes (<1cm; 2/9, 22%, 1–2cm; 3/15, 20%, >2cm; 10/30, 33%). Tumor size and histology may affect sensitivity and LVSI risk.Conclusion: Detailed MRI interpretation can estimate maximum tumor size and LVSI extent on pathology. Histological type should be considered in evaluation.

[Disclosure of COI by Responsible researcher] Lead presenter is the Responsible researcher.
[Lead presenter: Kazuhiro Kou] We have no financial relationships to disclose.

▲TOP

### [P-3385] II-P7-3
### Molecular Mechanisms & Therapy in Gynecologic cancer

2025 / 9 / 27 (Sat) 16:15-17:00 Room P/(Motenashi Dome) Room P

---

### P-3385
### Evaluation of zolbetuximab efficacy in gastric-type cervical adenocarcinoma using patient-derived organoid models

[Speaker]Yusuke Yokokawa (Dept. of Obstet. Gynecol., Shinshu Univ. Sch. Med.) [Co-author]Ryoichi Asaka (Dept. of Obstet. Gynecol., Shinshu Univ. Sch. Med.)、Manaka Shinagawa (Dept. of Obstet. Gynecol., Shinshu Univ. Sch. Med.)、Marina Fujioka (Dept. of Obstet. Gynecol., Shinshu Univ. Sch. Med.)、Natsuki Uchiyama (Dept. of Obstet. Gynecol., Shinshu Univ. Sch. Med.)、Hirofumi Ando (Dept. of Obstet. Gynecol., Shinshu Univ. Sch. Med.)、Tomoyuki Nakajima (Shinshu Univ. Hosp. Dept. of Lab. Med.)、Yoshiaki Maru (Lab. Precis. Tumor Model. Syst., Chiba Cancer Ctr. Res. Inst.)、Tsutomu Miyamoto (Dept. of Obstet. Gynecol., Shinshu Univ. Sch. Med.)

Gastric-type cervical adenocarcinoma (GAS) is a refractory disease with chemotherapy and radiation resistance. Zolbetuximab (ZOL) is a monoclonal antibody against CLDN18.2, a specific marker of gastric differentiation, and is available as a therapeutic against gastric cancer. In this study, we evaluated the sensitivity of ZOL to GAS using patient-derived organoids (GPDOs) and organoid-derived xenografts (GODXs), which were confirmed CLDN18-positive by immunohistochemistry. First, we evaluated the antibody-dependent cellular cytotoxicity (ADCC) activity of ZOL for GPDOs with peripheral blood mononuclear cells (PBMC) and confirmed it. In experiments using GODXs, which were transplanted and passaged subcutaneously in BALB/c nude mice, cisplatin alone (6 mg/kg/week) or ZOL alone (60 mg/kg/week) intraperitoneal administrations showed no growth inhibition effect for GODXs. However, a combination of cisplatin and ZOL administration demonstrated significant growth suppression compared with the placebo-administered control group (50.0 % reduced, $P < 0.05$). These results suggest that ZOL can be a candidate therapeutic for GAS.

[Disclosure of COI by Responsible researcher] Lead presenter is the Responsible researcher.
[Lead presenter: Yusuke Yokokawa] We have no financial relationships to disclose.
